# Loss of RNA–Dependent RNA Polymerase 2 (RDR2) Function Causes Widespread and Unexpected Changes in the Expression of Transposons, Genes, and 24-nt Small RNAs

**DOI:** 10.1371/journal.pgen.1000737

**Published:** 2009-11-20

**Authors:** Yi Jia, Damon R. Lisch, Kazuhiro Ohtsu, Michael J. Scanlon, Dan Nettleton, Patrick S. Schnable

**Affiliations:** 1 Interdepartmental Plant Biology Program, Iowa State University, Ames, Iowa, United States of America; 2 Department of Genetics, Development, and Cell Biology, Iowa State University, Ames, Iowa, United States of America; 3 Department of Plant and Microbial Biology, University of California Berkeley, Berkeley, California, United States of America; 4 Department of Agronomy, Iowa State University, Ames, Iowa, United States of America; 5 Department of Plant Biology, Cornell University, Ithaca, New York, United States of America; 6 Department of Statistics, Iowa State University, Ames, Iowa, United States of America; 7 Center for Plant Genomics, Iowa State University, Ames, Iowa, United States of America; The Salk Institute for Biological Studies, United States of America

## Abstract

Transposable elements (TEs) comprise a substantial portion of many eukaryotic genomes and are typically transcriptionally silenced. RNA–dependent RNA polymerase 2 (RDR2) is a component of the RNA–directed DNA methylation (RdDM) silencing pathway. In maize, loss of *mediator of paramutation1* (*mop1*) encoded RDR2 function results in reactivation of transcriptionally silenced *Mu* transposons and a substantial reduction in the accumulation of 24 nt short-interfering RNAs (siRNAs) that recruit RNA silencing components. An RNA–seq experiment conducted on shoot apical meristems (SAMs) revealed that, as expected based on a model in which RDR2 generates 24 nt siRNAs that suppress expression, most differentially expressed DNA TEs (78%) were up-regulated in the *mop1* mutant. In contrast, most differentially expressed retrotransposons (68%) were down-regulated. This striking difference suggests that distinct silencing mechanisms are applied to different silencing templates. In addition, >6,000 genes (24% of analyzed genes), including nearly 80% (286/361) of genes in chromatin modification pathways, were differentially expressed. Overall, two-thirds of differentially regulated genes were down-regulated in the *mop1* mutant. This finding suggests that RDR2 plays a significant role in regulating the expression of not only transposons, but also of genes. A re-analysis of existing small RNA data identified both RDR2–sensitive and RDR2–resistant species of 24 nt siRNAs that we hypothesize may at least partially explain the complex changes in the expression of genes and transposons observed in the *mop1* mutant.

## Author Summary

Shoot apical meristems (SAMs) are ultimately responsible for generating all above-ground plant tissues. Recent studies highlighted the effects of chromatin remodeling on the expression of various genes important to SAM development. The transposons that comprise a substantial portion of many eukaryotic genomes are typically transcriptionally silenced, presumably to promote genome stability. We demonstrate that a loss of a key component of the RNA–dependent DNA Methylation (RdDM) silencing pathway affects the expression of not only transposons but also thousands of genes, including nearly 80% of the chromatin-associated genes. Surprisingly, the expression of many transposons and genes is down-regulated via the loss of this component of the silencing pathway. In this study, we have shown that a maize mutation of RDR2 causes significant changes in SAM morphology. In combination, these observations indicate the complexity of transcriptome regulation and the crucial roles of RDR2 on transcriptome regulation, chromatin modification, and SAM development.

## Introduction

Repetitive sequences, including transposable elements (TEs) and tandem repeats, comprise a substantial fraction of many eukaryotic genomes. To protect genome integrity TEs are typically transcriptionally silenced via epigenetic mechanisms [1]–[3]. At the core of many of these mechanisms are a variety of small RNAs. Diverse small RNA pathways exist in most eukaroytes producing microRNAs and short-interfering RNAs (siRNAs) that function to negatively regulate gene expression and/or to suppress the activity of transposons. siRNAs derived from double-stranded RNAs via mechanisms that are either RNA-dependent RNA polymerase (RDR) dependent or independent. Various RDRs (e.g. RDR1, RDR2 and RDR6) are functional in different siRNA pathways and most siRNAs are biosynthesized from the heterochromatic loci, a process that generally requires RDR2 and DICER-LIKE3 (DCL3)[4]. In Arabidopsis, these heterochromatic 24 nt siRNAs, predominate the small RNA population. These siRNAs recruit chromatin-targeted RNA silencing components to form transcriptionally silent heterochromatin, which is derived mainly from TEs and tandem repeats [5], by cytosine methylation and various histone modifications, such as histone deacetylation and histone H3 lysine 9 dimethylation. RDR2 is required for the biogenesis of most of these 24 nt siRNAs [5],[6]. Thus, RDR2 is a key component of the chromatin-targeted RNA silencing process, which is also called the RNA dependent DNA Methylation (RdDM) pathway.

The maize homolog of AtRDR2, *mediator of paramutation1* (*mop1*) [7], is required to establish and maintain paramutation at multiple genetic loci [8]. Paramutation is an epigenetic process in which the interaction of two alleles of a single locus causes a heritable change of one allele that is induced by the other allele. Paramutation was first observed on the *red1* (*r1*) locus in maize by R.A. Brink in the 1950s in which specific weakly expressed alleles can heritably change other strongly expressed alleles to weakly expressed alleles [9]. In maize, paramutation is also observed in *b1* and *pl1* loci. The *mop1* gene, which encodes the maize version of RDR2, is required for paramutation in *b1*, *r1and pl1* loci, demonstrating that an RNA-dependent mechanism is important for paramutation in maize [7]. A tandem repeat 100 kb upstream of *b1* locus is required for paramutation in *b1* locus. This repeat does not share sequence similarity with the *b1* coding sequence, but has been demonstrated to physically interact with the *b1* transcription start site [10]. Mutations in *mop1* can also result in reactivation of transcriptionally silenced *Mutator* transposons and can substantially reduce the overall levels of 24 nt siRNAs, demonstrating that in maize RDR2 contributes to the silencing of repetitive elements and plays an important role in the biogenesis of 24 nt siRNAs [11],[12].

Shoot apical meristems (SAMs) are responsible for the elaboration of all above-ground plant organs [13]. Maintenance of SAM identity and organogenesis are precisely regulated by a complex regulatory network involving various transcriptional factors and signal transduction proteins, as well as epigenetic factors [14]. In a previously conducted global gene expression analysis of maize SAMs and seedlings we identified 2,700+ genes as being preferentially expressed in SAMs [15]. This included ∼60 retrotransposons that were substantially up-regulated in SAMs as compared to seedlings despite the fact that *mop1* was expressed >100 fold higher in SAMs than in seedlings. It was not clear at that time why repetitive retrotransposons and *mop1*, which contributes to the silencing of repetitive elements, would be both significantly up-regulated in SAMs. Given that retrotransposon-derived transcripts represent ∼9% of all SAM transcripts (as opposed to ∼0.3% of seedling transcripts)[15], the SAM is an ideal model for studying the effects of *mop1* on the accumulation of retrotransposon-derived transcripts.

Here we report an analysis of an RNA-seq experiment that detected hundreds of transposons and thousands of the genes that are differentially expressed in *mop1* mutant and non-mutant SAMs. This finding suggests that RDR2 plays a role in regulating the expression of not only transposons, but also of genes. Consistent with this observation, RDR2 mutants also exhibited a distinct SAM morphology relative to their wild type siblings, suggesting a role for RDR2 in normal SAM development. As expected based on its role in the RdDM pathway, loss of RDR2 function resulted in the up-regulation of many DNA TEs, retrotransposons and genes. However, some DNA TEs and many retrotransposons and genes were down-regulated in the *mop1* mutant.

## Results

### Collection of SAMs via Laser Capture Microdissection (LCM) and RNA–seq

**Figure 1:**
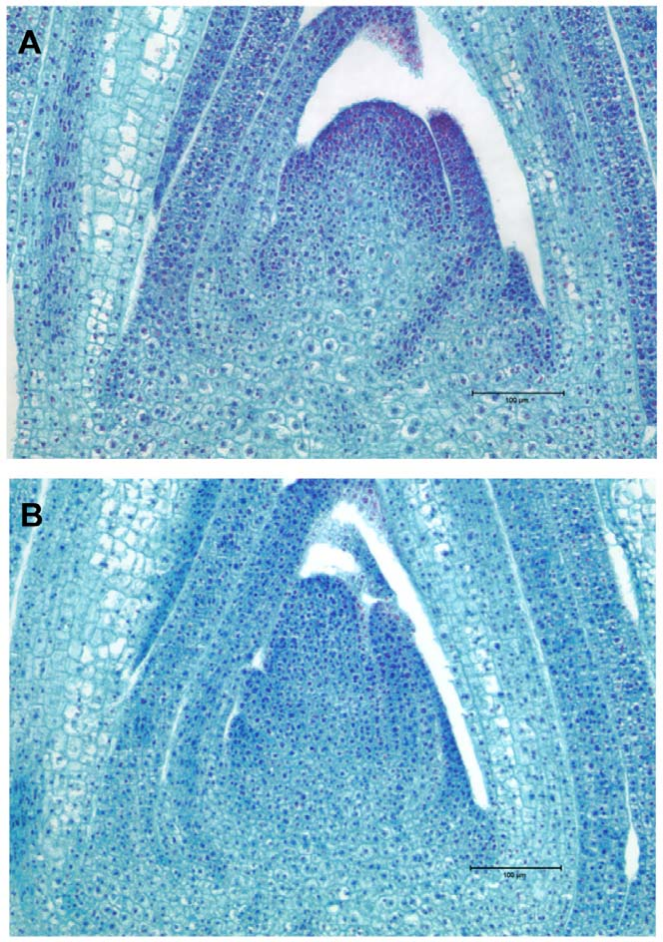
Comparison of SAM morphologies between *mop1* mutants and non-mutants. Safranin O/FastGreen stained image of SAMs from (A) a *mop1/mop1* mutant and (B) a non-mutant sibling. See Table S1 for a quantitative analysis.

A family segregating 1∶1 for *mop1* homozygotes and heterozygotes was planted in a growth chamber and harvested 14-days after planting. Individual seedlings were genotyped to identify *mop1* homozygotes and heterozygotes (see Materials and Methods). To determine the effects of *mop1* on SAM development the ratios of height versus width of mutants (homozygous) and non-mutants (heterozygous) SAMs were compared (Figure 1); mutant ratios were significantly smaller than non-mutant ratios (p-value = 0.006; Table S1). SAMs plus leaves at plastochron 0 (P0) and P1 stages were collected via LCM followed by RNA extraction and amplification according to our previously published procedures [15]. A pooled RNA sample from twelve mutant SAMs and a pooled RNA sample from ten non-mutant SAMs were subjected to Illumina/Solexa sequencing (see Materials and Methods). 2.8 million reads from *mop1* mutants and 4.1 million reads from non-mutants could be uniquely mapped to the Maize Genome Sequencing Project's (MGSP's) B73 reference genome [16] (see Materials and Methods).

### Expression of various TEs is differentially affected by loss of RDR2 function

More than 80% of the B73 reference genome is composed of TEs [16]. To ensure genome stability these elements are mostly suppressed via genome defense systems such as chromatin-based silencing, which is guided and reinforced by 24 nt siRNAs [17]. RDR2 plays an important role in the biogenesis of 24 nt siRNAs [18]. To test the hypothesis that the loss of RDR2 function in the *mop1* mutant would result in the activation of TEs (i.e, an increased accumulation of TE-derived transcripts), the Illumina/Solexa reads obtained from the SAM were aligned to all annotated TEs in the B73 genome (see Materials and Methods). As expected based on the mechanism associated with the RdDM silencing pathway, many DNA TEs (class II) and retrotransposons (class I) were up-regulated in the *mop1* mutant (Table 1). However, a significant fraction of DNA TEs and retrotransposons were down-regulated (Table 1). The most strongly up-regulated DNA TE super-families were *Stowaway* and *Tourist* and the most strongly down-regulated DNA TE family was *CACTA* (Figure S1). Both the Ty1/*Copia*-like and Ty3/*Gypsy*-like super-families of retrotransposons include some families that were up-regulated and others that were down-regulated (Figure S2).

**Table 1:** Expression patterns from diverse super-families of TEs.

TE class	Superfamily^a^	Total	Up^b^	Down^b^	Up+Down^b^
I	RIL	3	0	2	2
	RIX	18	1	12	13
	RLC	153	44	62	106
	RLG	188	49	105	154
	RLX	246	45	112	157
	**Total**	**608**	**139 (32%)**	**293 (68%)**	**432**
II	*CACTA*	151	37	21	58
	*hAT*	228	23	8	31
	*Helitron*	63	12	4	16
	*Mariner*	9	0	0	0
	*MULE*	140	29	3	32
	*PIF/Harbinger*	38	10	3	13
	*Stowaway*	101	14	0	14
	*Tourist*	67	11	0	11
	**Total**	**797**	**136 (78%)**	**39 (22%)**	**175**

**a **RIL, LINE (L1) retrotransposons; RIX, unknown LINE retrotransposons; RLC, Ty1/*Copia* LTR retrotransposons; RLG, Ty3/*Gypsy* LTR retrotransposons; RLX, unknown LTR retrotransposons.

**b **Up, number of up-regulated sub-families/families; Down, number of down-regulated sub-families/families; sub-families for class I TEs; families for class II TEs; Up+down, total number of differentially expressed sub-families/families.

**Figure 2:**
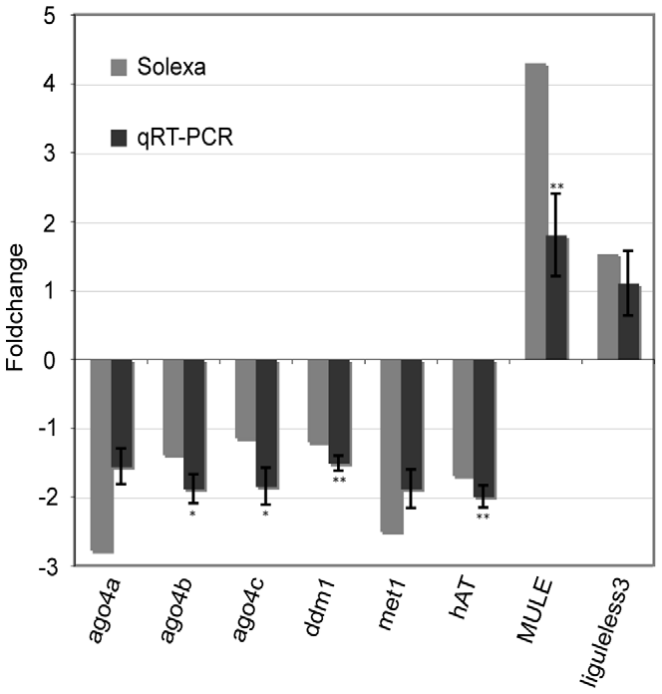
Validation of eight differentially expressed genes via qRT–PCR. Eight differentially expressed genes were chosen from the RdDM pathway (*ago4a* [Chromdb ID: AGO104], *ago4b* [Chromdb ID: AGO105], *ago4c* [Chromdb ID: AGO119], *ddm1* [Chromdb ID: CHR101], *met1* [Chromdb ID: DMT101]), transposons (*hAT* [a member of the ZM_hAT_8 sub-family; http://www.maizesequence.org]) and *MULE* [a member of the *MULE* sub-family DTM_Zm33205; http://www.maizesequence.org]) and a regulator of SAM development (*liguleless3* [Gene model ID: GRMZM2G087741; http://www.maizesequence.org]) for qRT–PCR validation. Primers used for qRT–PCR are presented in 8. Fold change was presented as the relative abundance of transcript in the *mop1* mutant/non-mutant. The quantitative fold changes obtained from between RNA–seq and qRT–PCR experiments were significantly correlated (Pearson correlation coefficient was 0.94, r^2^ = 0.88, p-value = 0.0005). A t-test of equal expression between the mutant and non-mutant using the data from four biological replications of qRT–PCR were conducted (p-value ≤0.05, *; p-value ≤0.01, **).

To assess the effects of *mop1* on specific groups of DNA TEs, each DNA TE super-family was divided into families based on a phylogenetic analysis conducted by the Maize Transposon Consortium that defined 797 unique families (i.e., monophyletic clades) [16]. Of these families, 22% (175/797) were differentially expressed in the *mop1* mutant (Table 1; Table S2). Consistent with our hypothesis, most (78%; 136/175) of the differentially expressed DNA TEs were up-regulated in the *mop1* mutant. In particular, of the 32/140 TE families annotated as *Mutator*-like elements (MULEs) that were differentially expressed, 29 (91%) were up-regulated. This is consistent with the report that silenced *Mutator* TEs can be reactivated in *mop1* mutants [11]. Similarly, of the 31/228 *hAT* families that were differentially expressed, 74% (23/31) were up-regulated. Among the 58/151 differentially expressed *CACTA* families many (37/58) individual *CACTA* families were up-regulated. Even so, *CACTA* DNA TEs as a group were down-regulated by loss of RDR2 function (Figure S1). The up-regulation of one MULE element and the down-regulation of one *hAT* element (viz., an *Ac*-like element) were confirmed by quantitative real-time PCR (qRT-PCR) (Figure 2).

A similar analysis was performed on retrotransposons, which have been categorized by the Maize Transposon Consortium into super-families (e.g., RLG, retrotransposon LTR *Gypsy*), families (e.g., *Huck*) and sub-families (e.g., Ac186577_1525). 71% of the 608 unique retrotransposon sub-families were differentially expressed to various degrees in the *mop1* mutant (Table S3). Consistent with current understanding of the RdDM silencing pathway, approximately one-third (32%) of the differentially expressed retrotransposon sub-families were up-regulated in *mop1* mutants as compared to non-mutants. But inconsistent with expectations, the majority (68%) of the differentially regulated retrotransposons were down-regulated in the mutant (Table 1; Table S3). Some specific sub-families of retrotransposons were mostly up- or mostly down-regulated. For example, within the Ty1/*Copia*-like super-family, 12/16 *Ji* and 9/17 *Opie* sub-families were up-regulated. Within the Ty3/*Gypsy*-like super-family, 6/7 *Flip* and 19/20 *Huck* sub-families were up-regulated. In contrast, 38/41 Ty3/*Gypsy*-like *Cinful-zeon* sub-families and 9/10 annotated Ty3/*Gypsy*-like *Prem1* sub-families were down-regulated in the *mop1* mutant (Table S3). These distinct responses to loss of RDR2 function suggest that the expressions of different retrotransposon families are regulated via different mechanisms.

### Many chromatin modification genes are down-regulated in the *mop1* mutant

To study the impacts of *mop1* on chromatin modification pathways, 386 maize genes annotated as being involved in chromatin modification were downloaded in Dec. 2008 from ChromDB [19]. In total, 361 of these genes can be uniquely mapped to the B73 reference genome (see Materials and Methods). RNA-seq reads that map to these chromatin-associated genes were used to conduct Fisher's exact tests. Nearly 80% (286/361) of these chromatin pathway genes were differentially expressed between the *mop1* mutants and non-mutant siblings using a 5% false discover rate (FDR) cutoff, a frequency far higher than observed when considering all genes (24%). Approximately ¾ (76%) of differentially expressed chromatin genes were down-regulated in the *mop1* mutant (Table S4). A wide variety of chromatin-associated genes exhibited differential expression between *mop1* mutants and non-mutants, including those affecting various histone modifications, such as histone ubiquitination, methylation, acetylation and deacetylation (Table S4). As the maize homolog of AtRDR2, *mop1* is expected to function in the RdDM pathway, which involves the biogenesis of 24 nt siRNAs, *de novo* methylation of DNA, maintenance of DNA methylation, and demethylation [20]. Almost all of the genes known to be implicated in the RdDM pathway were down-regulated in the *mop1* mutants (Table 2). The maize genome contains two *DCL3* paralogs, which are involved in the biogenesis of 24 nt small RNAs. One of these (*Zmdcl3b*) was down-regulated while the other (*Zmdcl3a*) was up-regulated (Table 2). These opposite responses suggest that the two *DCL3* paralogs may be functionally distinct. This type of divergent gene expression pattern was also observed for DRM protein in which one maize homolog was down-regulated and another was up-regulated. In addition to DRM protein, AGO4, DRD1, and MET1proteins function in the *de novo* methylation pathway [20]. All maize homologs of these genes were down-regulated (Table 2). CMT3, MET1, DDM1, HDA6, SUVH4 function in the maintenance methylation pathway [20]. All maize homologs of these genes were down-regulated (Table 2). DNA demethylation is thought to regulate epigenome dynamics in opposition to the RdDM pathway. In Arabidopsis, ROS1 and DME remove DNA methylation [20]. There are two homologs of DME gene in maize and both were down-regulated as well (Table 2). The expression levels of several of genes important for epigenetic silencing (viz., *met1*, *met3*, three *ago4* paralogs, and *ddm1*) were tested via qRT-PCR with results that were consistent with those obtained from RNA-seq (Figure 2). These observations demonstrate that there is widespread down-regulation of components in the RdDM pathway in the *mop1* mutant, suggesting either that MOP1 positively regulates the entire pathway, or that genes involved in chromatin modification and DNA methylation are co-regulated in maize.

**Table 2:** Differentially expressed genes in RdDM pathway.

Chromdb ID^a^	Gene Name^b^	log_2_(FC)^c^	FDR^d^
DCL102	*dcl3a*	1.2	8e-06
DCL104	*dcl3b*	−0.5	1e-02
AGO104	*ago4a*	−0.7	9e-21
AGO105	*ago4b*	−1.7	6e-17
AGO119	*ago4c*	−0.5	1e-06
CHR127	*drd1*	−1.3	1e-11
DMT101	*met1*	−1.0	3e-11
DMT106	*drm1/2*	−0.7	3e-13
DMT102	*cmt3a*	−1.0	3e-40
DMT105	*cmt3b*	−0.7	4e-22
SDG118	*suvh4/kyp*	−1.0	4e-16
HDA108	*hda6*	−0.7	2e-09
CHR101	*ddm1*	−0.5	8e-04
DNG101	*dme1*	−1.0	4e-12
DNG103	*dme2*	−1.7	9e-26

**a **ID used in Chromdb (http://www.chromdb.org).

**b **ID used in general RdDM pathway [20].

**c **log_2_ transformation of fold change as the relative abandunce of transcripts in mutants/non-mutants. Positive value indicates the up regulation and negative value indicates down regulation.

**d **The false discovery rate calculated using Benjamini and Hochberg's procedure [24] for the p-value from Fisher's exact test.

In addition to *mop1*, mutations in two other maize genes are known to affect the accumulation of both 24–26 nt siRNAs and DNA methylation. *rmr1* (*required to maintain repression1*) encodes a SWI/SNF2 class chromatin remodeling protein. Mutations in *rmr1* have dramatic effects on accumulation of 24–26 nt siRNAs, maintenance of the repressed state of paramutant genes, and methylation of *Mu* transposons [21],[22]. Unlike the related DDM1 orthologs (Table 2), expression of *rmr1* was not significantly changed in the *mop1* mutant. This observation is consistent with the suggestion that RMR1 may act genetically upstream and sometimes independently of RDR2 [22]. The second cloned gene that affects 24–26 nt siRNA accumulation in maize is *rmr6*. This gene encodes the conserved Pol IV largest subunit (RPD1) and is required for paramutation [23]. Although expression of this gene was reduced in the *mop1* mutant, this change was not significant.

### Widespread changes in gene expression following loss of RDR2 activity

The finding that SAM morphology differs between *mop1* mutant and non-mutants led us to hypothesize that *mop1* affects not only the expression of TEs and components in RdDM pathway but also genes important to the development of the SAM. To test this hypothesis the Illumina/Solexa reads were mapped to the “filtered gene set” of maize generated by the MGSP (see Materials and Methods). Among reads that could be uniquely mapped to the genome, 2.2 million (78%) from *mop1* mutants and 3.2 million (79%) from non-mutants aligned to gene models (Figure S3; Table S5). At least one Illumina/Solexa read from at least one of the two genotypes aligned to 24,743 of the 32,540 genes in the MGSP's filtered gene set (Table S5). Of these genes, 6,016 (24% of 24,743) could be declared to be differentially expressed between the mutant and non-mutant pools at an estimated 5% FDR [24]. The ratio of number of genes that were up-regulated in the *mop1* mutant to those that were down-regulated was ∼1∶2 (Table S6). Consistent with our finding that *mop1* mutant SAMs differ morphologically from non-mutant siblings (Figure 1; Table S1), several key regulators of SAM development, including *fasciated ear2* [25], *terminal ear1-like 2* [26], *outer cell layer4* [27] and *liguleless3* (encoding a *Knotted* class 1 homeodomain protein) [28] were differentially expressed (Table 3). The differential expression of one of these genes, *liguleless3*, was validated via quantitative real-time PCR (qRT-PCR) (Figure 2). This finding suggests that 24 nt siRNAs play a role in regulating (directly or indirectly) the expression of not only transposons, but also of genes.

**Table 3:** Key regulators of SAM developments showing differential expression.

Gene ID^a^	log2(FC)^b^	FDR^c^	SwissProt ID^d^	Protein Name	E-value^e^	Ref
GRMZM2G104925	−0.76	5.00e-04	Q940E8	Fasciated ear2	0	[19]
GRMZM2G085113	−0.86	1.62e-09	A9XIW7	Terminal ear1-like 2 protein	6e-138	[20]
GRMZM2G123140	−0.50	1.05e-02	B3GW90	Putative HD-ZIP IV family transcription factor OCL4	0	[21]
GRMZM2G087741	0.50	6.09e-06	Q9SYT6	Knotted class 1 homeodomain protein liguleless3	0	[22]

**a **Refer to http://www.maizesequence.org.

**b **log_2_ transformation of fold change as the relative abandunce of transcripts in mutants/non-mutants. Postive values indicate up regulation and negative values indicate down regulation.

**c **False discovery rate calculated using Benjamini and Hochberg's procedure [24] for the p-value from Fisher's exact test.

**d **Protein ID retrieved from SwissProt_Trembl database via blastp (1e-10 as cutoff).

**e **E-value from blastp search.

### Re-analysis of existing 24 nt siRNA data

The analyses described above demonstrate that loss of RDR2 function results in widespread changes in the accumulation of transcripts from DNA TEs, retrotransposons and genes. As expected based on a model in which RDR2 generates 24 nt siRNAs that suppress expression, many TEs and genes were up-regulated in the *mop1* mutant. Interestingly, some DNA TEs and many retrotransposons and genes were actually down-regulated in the *mop1* mutant, demonstrating new complexities in the regulation of the expression of transposons and genes.

To explore this complexity we undertook a re-analysis of 24 nt siRNAs isolated from immature ears of *mop1* and non-mutant plants [18]. Nobuta et al. reported that *mop1* mutants accumulated many fewer 24 nt siRNAs (as a proportion of all small RNAs) than do wild-type [18]. Their analysis treated all 24 nt siRNAs as a group. We extended their analysis by considering the effects of the *mop1* mutation on the accumulation of each individual species of 24 nt siRNAs in their data set. As such, our analysis enabled us to identify specific RNA species that make up a significantly greater proportion of the observed reads from one genotype than from the other (see Materials and Methods).

Considering the union of *mop1* mutant and non-mutant reads, the Nobuta et al. data set contains >2.3M distinct 24 nt siRNA species. Many of these are present at very low abundance and as such it would not be possible to detect statistically significant differences between the two genotypes even if such differences exist. Of the 5% of RNA species for which 5 or more counts were recorded in the union of the two genotypes (125,344/2.3M), we found that 30% (38,564/125,344) of the 24 nt siRNAs were differentially expressed between the two genotypes. Consistent with the report of Nobuta et al., most of the 38,564 differentially regulated species of 24 nt siRNAs (33,614) were down-regulated in the *mop1* mutant (Figure S4; Table S7). We term these “RDR2-sensitive 24 nt siRNAs”. Quite unexpectedly, 4,950 distinct species of 24 nt siRNAs were significantly “up-regulated” in the *mop1* mutant (Figure S4; Table S7). Although some of these may be actually up-regulated, others may simply be less down-regulated in the mutant than are other species of 24 nt siRNAs (see Materials and Methods). We have therefore termed these “up-regulated” species “RDR2-resistant 24 nt siRNAs”.

## Discussion

RDR2 is an essential component of the heterochromatin silencing pathway in multiple species [5],[29] and functions in DNA and histone methylation, the biogenesis of 24 nt siRNAs and the silencing of repetitive DNAs [30]. The maize homolog of RDR2, *mop1*, was originally identified as a mutant that functions as an epigenetic regulator of a target gene via interactions with upstream tandem repeats [7]. *mop1* is also required for the methylation of the terminal inverted repeats of *Mu* TEs and for the maintenance of silencing of *MuDR* transposons [31]. Based on its mutant phenotypes, it has been hypothesized that the *mop1* gene regulates many loci [8]. To test this hypothesis and to examine the effect of RDR2 on the silencing of TEs in a large, complex genome, we conducted RNA-seq experiments on SAMs of *mop1* mutant and non-mutant seedlings. SAMs were selected for analysis because they are responsible for the elaboration of all aerial organs [13], they have a complex transcriptome [32], and our prior analyses had revealed that multiple retrotransposons and *mop1* are all substantially up-regulated in SAMs as compared to seedlings [15].

### Effect of RDR2 on gene expression

We identified more than 6,000 genes whose expression differed between *mop1* mutant and non-mutant SAMs. These widespread differences in gene expression are consistent with the multiple developmental defects associated with the loss of *mop1* function in mutants [8].

Over several generations, maize lines that carry the *mop1* mutation can accumulate a variety of epimutant phenotypes (Lisch, unpublished data). In this study we controlled for the effects of any segregating epi-alleles by analyzing RNA from pools of *mop1* and non-mutant SAMs. However, our discovery that genes involved in a variety of silencing pathways including DNA methylation, histone modification and RNA-mediated silencing, are differentially regulated in the *mop1* mutant complicates any facile explanation for the origins of these phenotypes. Unlike *rdr2* mutants in Arabidopsis, *ddm1* and *met1* mutants can have severe effects on plant morphology [33], and the maize homologs of both of these genes are down-regulated in the *mop1* SAMs. It is not clear whether or not the down-regulation in the maize *mop1* mutants of so many genes involved in epigenetic regulation is consequential, but it does suggest that many of the phenotypes that arise in *mop1*-containing lines over multiple generations may not be the direct result of the loss of RDR2 activity. The same may well be true for at least some of the differences in gene expression that we observed.

### Effect of RDR2 on the accumulation of TE-derived transcripts

RNA-seq data identified hundreds of DNA TEs and retrotransposons that are differentially regulated in SAMs. Based on a model in which RDR2 generates 24 nt siRNAs that silence DNA TEs and retrotransposons, our expectation was that loss of RDR2 function in *mop1* SAMs would result in the up-regulation of DNA TEs and retrotransposons. Although we did observe that the majority of differentially expressed DNA TEs (78%) were up-regulated in the *mop1* mutant, less than half of all differentially regulated retrotransposons (32%) were up-regulated. This suggests that at least some DNA TEs and retrotransposons are silenced via distinct mechanisms.

### RDR2–dependent silencing of pericentromeric TEs

Pericentric heterochromatin is rich in TEs in many species, and these sequences are typically heavily methylated and associated with large numbers of 24 nt siRNAs [2]. Consistent with its role in the RdDM pathway loss of RDR2 function results in the up-regulation of certain TEs, including *Huck* elements which are members of the Ty3/*Gypsy*-like super-family of retrotransposons. Fluorescence *in situ* hybridization (FISH) experiments reveal that although *Huck* elements are dispersed along all chromosomes they are significantly enriched in the vicinity of, but not in, centromeres [34]. Indeed, in general Ty3/*Gypsy*-like sequences cluster in pericentromeric regions across all grass species [35]. Our observations provide evidence that at least one pericentromeric repeat (i.e., *Huck*) is transcriptionally silenced via the RdDM pathway.

### RDR2–sensitive and RDR2–resistant 24 nt siRNAs

In Arabidopsis RNA gel blot experiments, the population of 24 nt siRNAs is almost entirely eliminated in the *rdr2* mutant [5], indicating RDR2 is required for the biogenesis of nearly all 24 nt siRNA. In the maize *mop1* mutant, the population of 24 nt siRNAs is dramatically reduced, but not eliminated [18]. Via a re-analysis of an existing small-RNA data set we identified >33,000 unique “RDR2-sensitive” and ∼5,000 unique “RDR2-resistant” 24 nt siRNAs that are “down-regulated and “up-regulated” in the *mop1* mutant, respectively.

### RDR2–independent silencing of DNA TEs and retrotransposons

In contrast to elements such as *Huck*, the silencing of some types of DNA TEs and retrotransposons (e.g., most *Prem1* elements) does not appear to require RDR2, as evidenced by the fact that they are down-regulated in the *mop1* mutant. The hypothesis that an RDR2-independent heterochromatin silencing pathway exists in maize is consistent with our previous observation that many retrotransposon are significantly up-regulated (some >1,000×) in SAMs as compared to seedlings even though *mop1* transcripts accumulate in SAMs to a level 100× higher than in seedlings. On the other hand, because new retrotransposon insertions are quite rare in maize [36], we considered the possibility that a significant proportion of the retrotransposon-derived transcripts we detected in SAMs are generated via RDR2 activity itself [37], which can produce aberrant non-polyadenylated RNAs. If this were the case, these species would indeed be lost in the RDR2 mutant (along with associated siRNAs). However, because the procedures we used to construct our RNA-seq libraries preferentially target mRNA species this possibility seems unlikely.

We therefore considered other RDR2-independent mechanisms for silencing DNA transposons and retrotransposons in a complex genome such as that of maize. Because the expression of many genes is affected by the *mop1* mutant, it is possible, for example, that some of these effects could be antagonistic to the direct effects of *mop1* on gene silencing. In addition, Lippman et al. [38] reported that the epigenetic inheritance of different TEs differed from mutant to mutant in Arabidopsis and proposed the existence of distinct but interacting pathways responsible for transposon silencing via siRNAs and histone modifications. Observations from fission yeast offer a plausible possibility for an RDR2-independent pathway. In this yeast, inhibition of histone deacetyltransferases causes an inherited loss of heterochromatin [39]. Several genes encoding histone deacetyltransferases (HDACs) were up-regulated in the *mop1* mutant. It is possible that enhanced expression of these HDACs could enhance silencing of some TEs. Similarly, the reduction in expression of the maize orthologs of ROS1 and DME1, both of which are required for demethylation of a variety of target genes in Arabidopsis [40], could result in the silencing of a variety of genes in *mop1* mutants. Hence, our observations in the *mop1* mutant of the down-regulation of some DNA TEs and many retrotransposons, enhanced expression of genes in the HDAC silencing pathway, and decreased expression of genes in the demethylation pathway are consistent with the existence of multiple silencing mechanisms, but suggest that these mechanisms can potentially interact antagonistically.

Nobuta et al. reported that 22 nt small RNAs are highly abundant in the *mop1* mutant [18] and suggested that these small RNAs may be the result of an alternative mode of heterochromatic siRNA production that is independent of, and may even be enhanced by, the loss of RDR2. Alternatively, or in addition, the RDR2-independent silencing we observed could be the result of the RDR2-resistant 24 nt siRNAs we identified. As discussed in the Materials and Methods section, these RDR2-resistant 24 nt siRNAs may actually be produced at higher levels in the *mop1* mutant. If this were the case, then these RDR2-resistant siRNAs could be responsible for the enhanced silencing of some of the DNA TEs and retrotransposons we observed in the *mop1* mutant. If, on the other hand, the RDR2-resistant siRNAs are simply less susceptible to loss of RDR2 function, they would need to be more effective at silencing in the *mop1* background to explain the enhanced silencing of DNA TEs and retrotransposons we observed. RDR2-resistant 24 nt siRNAs might, for example, exhibit enhanced repressive activity in response to changes in chromatin structure resulting from loss of RDR2 activity.

Potential sources of RDR2-resistant siRNAs include novel combinations of sense/anti-sense transcripts and transcribed inverted repeats. In maize retrotransposons are often present in vast nested arrays [36]. Enhanced transcription of these nested retrotransposons (due perhaps to loss of RDR2-dependent silencing) could result in the production of novel combinations of sense and antisense RNAs that could be processed into biologically active siRNAs even in the absence of RDR2. Thus, the effects of the *mop1* mutant on a given transposon family may be a reflection not just of its sequence, but of the physical distribution of that family within the genome. With that in mind, it is interesting to note that the one family of DNA TEs with a high proportion of up-regulated members (*CACTA* elements) exhibits a distinct chromosomal distribution relative to the other families of DNA transposons. *CACTA* elements are significantly more likely to be found in gene-poor, heterochromatic regions of the genome than are all other DNA TEs [16].

In addition, dosage effect has been reported in the maize *Activator/Dissociation* (*Ac/Ds*) and *Mutator* transposon families [41]–[44], demonstrating that the regulation of the TE transposition is complex; similar complexity likely contributes to our observation of the down-regulation of TEs in the *mop1* mutant. For example, some of the changes in expression of TEs and genes observed in this study could be due to epigenetic interactions between TEs and genes. In several cases it has been demonstrated that expression of TEs can reduce expression of nearby genes [45]–[49]. For example, de-repression of an LTR retrotransposon flanking the *BONSAI* gene in Arabidopsis in a *ddm1* mutant background results in epigenetic silencing of *BONSAI* [50]. This process involves the production of *BONSAI*-specific siRNAs. Given our observation that DNA TEs, which tend to be preferentially located in gene-rich regions of the genome are likely to be up-regulated in the *mop1* mutants, it is possible that many of the negative effects on gene expression are due to similar interactions between genes and nearby TEs. To more comprehensively analyze the relationship between levels of gene expression, siRNA production, and DNA methylation, it will be necessary to analyze all of these variables in a single tissue. Further, given the number of variables involved, a clear understanding of cause and effect relationships between RDR2 activity and expression will require detailed analyses of individual transposons, retrotransposons and genes.

## Materials and Methods

### Genetic stocks, plant growth conditions, genotyping, and RNA–seq

The *mop1-1* allele used in this study has been described previously [12]. This mutant is within a mixed genetic background, including both the highly inbred *a1-mum2* minimal *Mutator* line [51] and the *Mutator* line from which *mop1-1* was first derived [8]. The *mop1-1* mutation in this background was maintained through several generations via sib crosses, self fertilizations, or back-crosses with the *a1-mum2* stock. Although the progenitors of this line contained active *MuDR* elements, these elements were no longer present in the line used for this study, which lacked detectable *Mutator* activity.

This genetic background is distinct from that analyzed by Nobuta et al. [18]. Importantly, the family used in these experiments is closely related to a *mop1* mutant lineage that gave rise to a large number of unique morphological phenotypes not previously observed in *mop1* mutant plants (Lisch, unpublished observation). Given this, and given the dramatic differences in TE composition between maize inbred lines, direct comparisons of transcript data between the current data set and that of Nobuta et al. should be treated with caution.

A plant having the genotype *mop1-1/mop1-1* was crossed to *Mop1*/*mop1-1* heterozygote and the resulting progeny kernels planted in growth chambers (PGW-40, Percival Scientific, http://www.percival-scientific.com). Temperature and light cycles were set as 25 degrees for 15 hours of light and 20 degrees for 9 hours of dark. During the light period the light intensity at the surface of the growth medium was maintained between 650 and 800 umol m^−2^ sec^−1^.

At 14-days after planting SAMs were collected using the PALM MicroBeam System (115V Z, P.A.L.M. Microlaser Technologies, http://www.palm-microlaser.com). Plants homozygous and heterozygous for the *mop1-1* mutant allele were distinguished using two pairs of primers: a pair of *mop1*-specific primers consisting of RDRF3 (sequence: 5′-TCTCCACCGCCCACTTGAT-3′) and RDRR2 (sequence: 5′-ATGGCCAGCAGGGTGTCGCAGAT-3′) and a primer pair consisting of the *Mutator* TIR primer *Mu*-TIR (5′-AGAGAAGCCAACGCCAWCGCCTCYATTTCGTC-3′) and the *mop1*-specific primer RDRF3. Twelve *mop1-1/mop1-1* and ten *Mop1/mop1-1* SAMs were used to form *mop1* mutant and non-mutant pools. Collected SAM tissues were used for RNA extraction, RNA amplification and synthesis of double stranded cDNAs according to our previous published procedures [15]. These procedures preferentially target polyadenylated transcripts. Illumina/Solexa libraries were constructed using these double stranded cDNAs following Illumina/Solexa's standard protocol for genomic library preparation. The resulting libraries were sequenced on the Solexa 1G Genome Analyzer at the Michael Smith Genome Sciences Centre (Vancouver, BC, Canada). Each library was sequenced using 2 lanes on a Solexa flow cell. The Gene Expression Omnibus (GEO) database (http://www.ncbi.nlm.nih.gov/geo) accession number for the data used in the paper is GSE16789.

### Alignments of RNA–seq to the maize reference genome and TEs

The resulting Solexa reads were aligned to the maize B73 reference genome (Release 4a.53) (http://www.maizesequence.org) with the short read aligner NOVOALIGN (http://www.novocraft.com) using 32 base sequences. Low quality bases located at the end of reads were trimmed and only reads that mapped uniquely to the genome with a maximum of two mismatches including insertion/deletion (indel) across 32 bases were used for subsequent analyses. The “filtered gene set”, a collection of high-quality gene models developed by the MGSP, was projected onto the B73 reference genome.

In addition, the Illumina/Solexa reads were also aligned directly to the DNA TE families and retrotransposon subfamilies. Due to the repetitive property of the TEs, each read is allowed to be mapped to multiple DNA TE families or retrotransposon subfamilies but each read is only counted once within each family or sub-family with same alignment criteria as used for alignments to the reference genome.

The 386 chromatin-associated genes were mapped to the maize B73 reference genome using criteria of 95% identity and 90% coverage. Reads that uniquely mapped to the reference genome were projected onto each of these chromatin-associated genes allowing us to detect differential expression.

### Identifying differential expression via a likelihood ratio test and Fisher's exact test

Two statistical procedures to identify differentially expressed genes were compared and evaluated: a likelihood ratio test based on a Poisson model (below) [52] and Fisher's exact test. Although the two procedures produced similar p-values (R = 0.9; Figure S5), the Fisher's exact test was more conservative. It was therefore selected for use in this study.

The likelihood ratio test analysis generally followed the procedure described in Marioni et al. [52]. For each gene, the number of reads from the *mop1-1* mutant sample and the non-mutant sample were modeled as independent Poisson random variables with mean λ_m_C_m_ for mutant and mean λ_n_C_n_ for non-mutant, where C_m_ and C_n_ denote counts of the total number of mapped reads for the mutant (2,156,241) and non-mutant (3,248,869) samples, respectively. It is straightforward to show that the likelihood ratio statistic for testing the null hypothesis of H_0_: λ_m_ = λ_n_ is T = 2{k_m_ log (k_m_/C_m_)+k_n_ log(k_n_/C_n_)−k log(k/C)}, where k_m_ is the number of mutant reads for the gene in question, k_n_ is the number of non-mutant reads for the gene in question, k = k_m_+k_n_, and C = C_m_+C_n_. This statistic is distributed approximately as a chi-square random variable with 1 degree of freedom when the null hypothesis of no differential expression is true. Thus, p-values were obtained by comparing the observed statistic for each gene to the chi-square distribution with 1 degree of freedom. Ideally, sequencing would have been carried our separately for multiple independent biological replications of each genotype so that over dispersion relative to the Poisson distribution could have been assessed and accounted for using an analysis like that proposed by Robinson and Smyth [53]. Note that our qRT-PCR validation and analysis (discussed below) was based on separate measurements of independent biological replications.

### Detection of RDR2–sensitive and –resistant 24 nt siRNAs

Each species of 24 nt siRNAs was tested whether the proportions in the library between *mop1* mutant and non-mutant were significantly different via Fisher's exact test. Because we are able to measure only the abundance of each species in a genotype relative to the total number of reads for that genotype, it is difficult to formally distinguish 24 nt siRNAs that are up-regulated in the mutant from those that make up a significantly greater proportion of the observed reads only because of the absence of many other 24 nt siRNA species in the *mop1* mutant, thereby making them proportionately more abundant. For the purposes of this study we therefore carefully define up-regulation to mean that a particular species is significantly more abundant in one sample of reads than in another. It is important to note that this does not necessarily mean that the number of RNA molecules of that particular species increases on a per cell basis.

### qRT–PCR validation and data analysis

Primer design for qRT-PCR was conducted as described [54]. RNA samples independent from those used in the RNA-seq experiment were extracted from four biological replications from *mop1-1/mop1-1* and *Mop1/mop1-1* (3 SAMs pooled within each replicate per genotype) by using the same procedure as the RNA-seq experiment. To prepare the cDNA template, combined oligodT and random hexamers was used to perform reverse transcription reactions at 55°C for 1 hour with SuperScript III. A reverse transcription without SuperScript III was conducted to control for genomic DNA contamination. qRT-PCR was conducted on an Mx4000 multiplex quantitative PCR system (Stratagene). RNA from a human gene (GenBank accession no. AA418251) was spiked into each reaction as an external reference for data normalization. Genotype-specific Ct values for each gene and control were calculated and then the ΔΔCt was computed. For each gene, ΔΔCt across 4 biological replications was used to conduct a t-test in R (www.r-project.org) [55].

## Supporting Information

Figure S1 Overall expression fold changes of mutant versus non-mutant for differentially expressed DNA TEs. In this analysis all members of each differentially expressed super-family were treated as a group. The percentage of reads that match each super-family among all mapped reads in each genotype was calculated and the fold change was computed as the ratio of the percentage of mutant versus non-mutant for each super-family.
(0.22 MB TIF)

Figure S2 Overall fold changes of mutant versus non-mutant for differentially expressed retrotransposons. In this analysis all members of each differentially expressed family were treated as a group. The percentage of reads that match each family among all mapped reads in each genotype was calculated and the fold change was computed as the ratio of the percentage of mutant versus non-mutant for each family.
(0.24 MB TIF)

Figure S3 Distribution of numbers of mapped reads across tested genes.
(0.29 MB TIF)

Figure S4 RDR2-sensitive and RDR2-resistant 24 nt siRNAs in wild-type and *mop1* mutants. The log_2_ transformation of read counts in non-mutant (x-axis) versus *mop1* mutant (y-axis) for each species of the 4,950 RDR2-resistant 24 nt siRNAs (green dots) and the 33,614 RDR2-sensitive 24 nt siRNAs (red dots) were plotted.
(0.36 MB TIF)

Figure S5 p-value comparison between likelihood ratio test and Fisher's exact test.
(0.27 MB TIF)

Table S1 *mop1* mutants and non-mutants have distinct SAM morphologies.
(0.01 MB PDF)

Table S2 List of differentially expressed DNA transposon families.
(0.03 MB PDF)

Table S3 List of differentially expressed retrotransposon sub-families.
(0.04 MB PDF)

Table S4 List of differentially expressed chromatin-associated genes.
(0.66 MB DOC)

Table S5 Alignment of RNA-seq reads to genome and genes.
(0.01 MB PDF)

Table S6 List of differentially expressed genes and related annotation.
(7.02 MB DOC)

Table S7 List of RDR2-sensitive and RDR2-resistant 24 nt siRNA.
(1.63 MB PDF)

Table S8 Primer sequences used for qRT-PCR experiment.
(0.01 MB PDF)
