# Supplementary figures and images for: Loss of RNA–Dependent RNA Polymerase 2 (RDR2) Function Causes Widespread and Unexpected Changes in the Expression of Transposons, Genes, and 24-nt Small RNAs
Source: PLoS Genet. 2009 Nov 20;5(11):e1000737. doi: 10.1371/journal.pgen.1000737 (PMC2774947; doi:10.1371/journal.pgen.1000737)

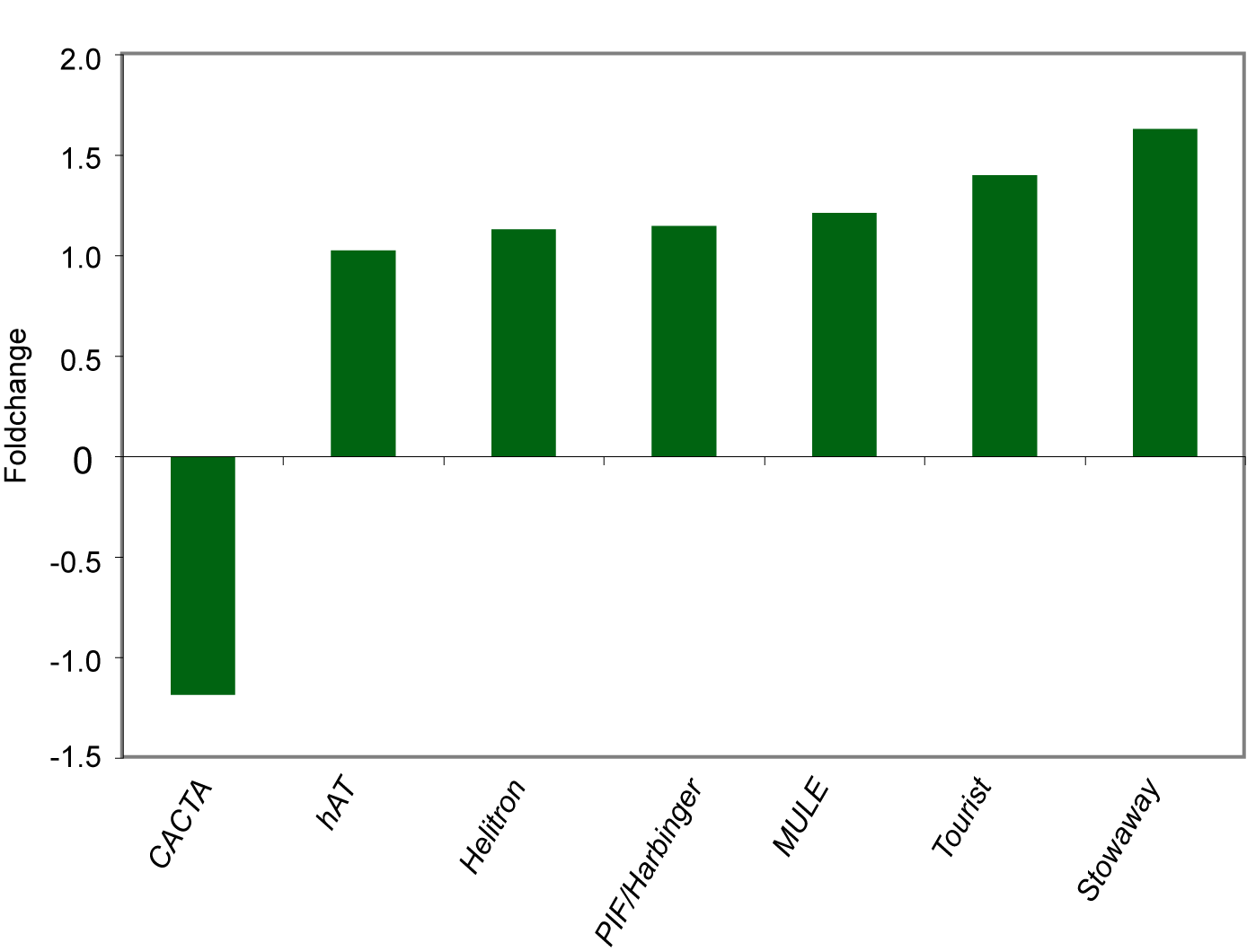

Supplement: Figure S1 — Overall expression fold changes of mutant versus non-mutant for differentially expressed DNA TEs. In this analysis all members of each differentially expressed super-family were treated as a group. The percentage of reads that match each super-family among all mapped reads in each genotype was calculated and the fold change was computed as the ratio of the percentage of mutant versus non-mutant for each super-family. (0.22 MB TIF) [file pgen.1000737.s001.tif]

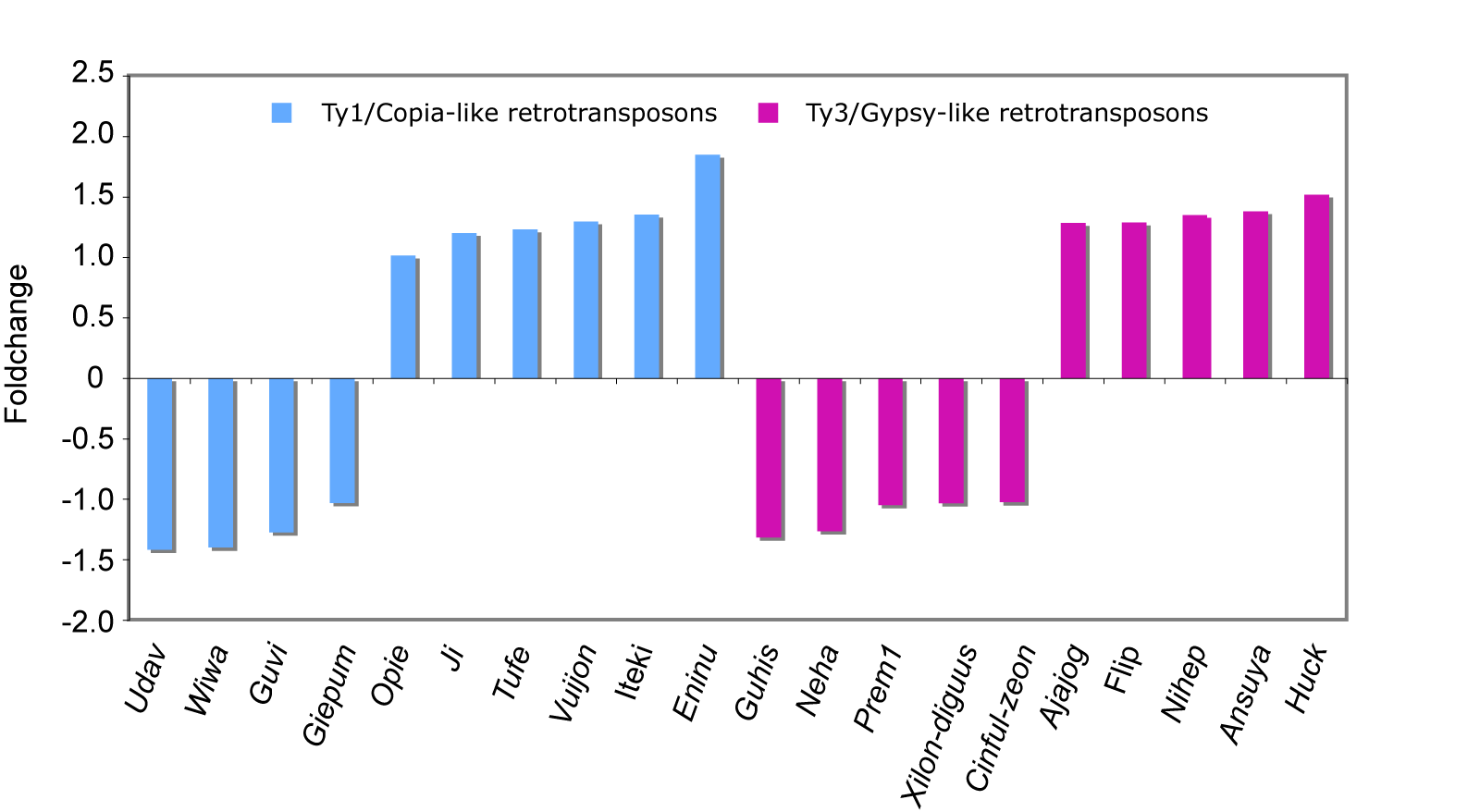

Supplement: Figure S2 — Overall fold changes of mutant versus non-mutant for differentially expressed retrotransposons. In this analysis all members of each differentially expressed family were treated as a group. The percentage of reads that match each family among all mapped reads in each genotype was calculated and the fold change was computed as the ratio of the percentage of mutant versus non-mutant for each family. (0.24 MB TIF) [file pgen.1000737.s002.tif]

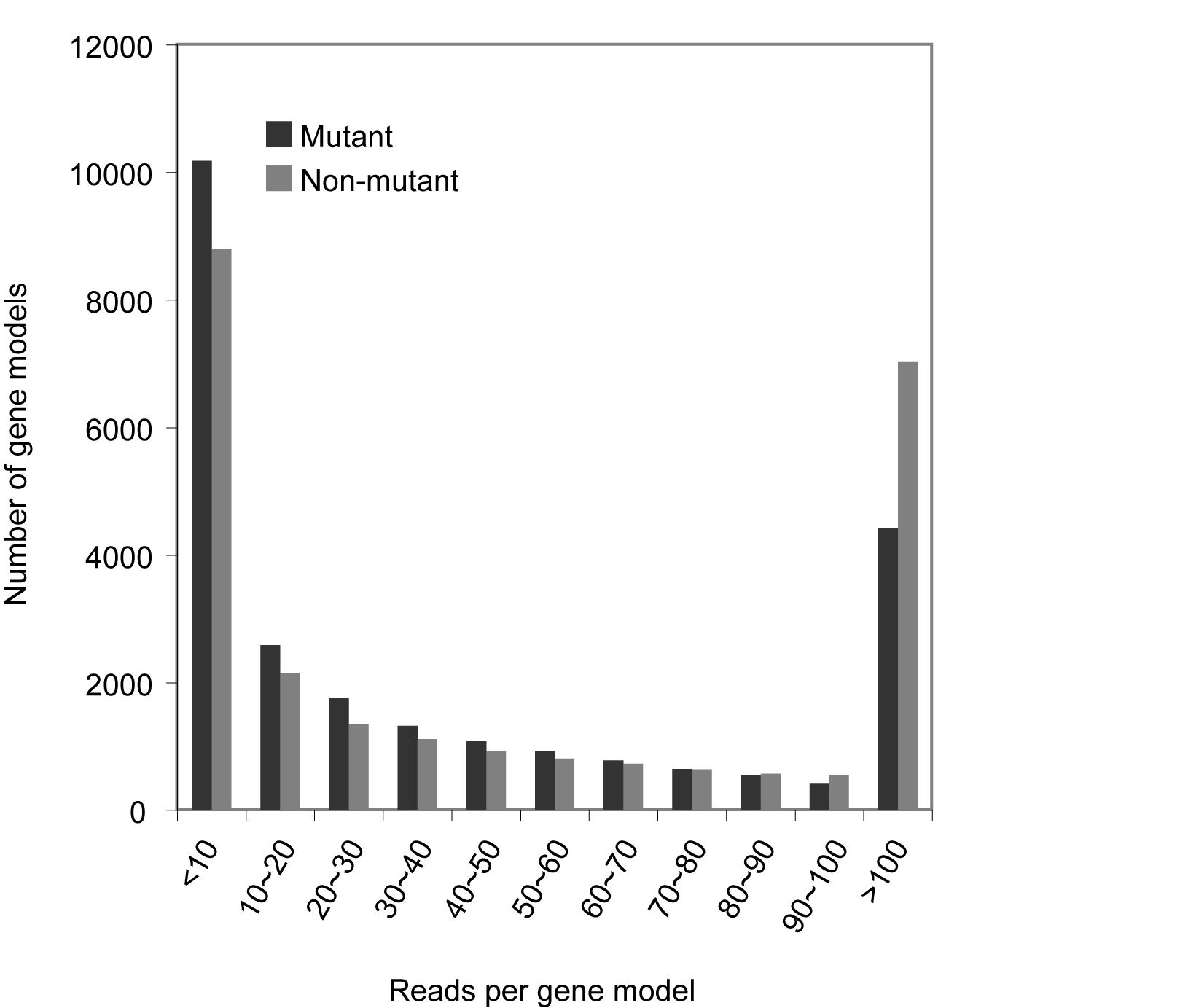

Supplement: Figure S3 — Distribution of numbers of mapped reads across tested genes. (0.29 MB TIF) [file pgen.1000737.s003.tif]

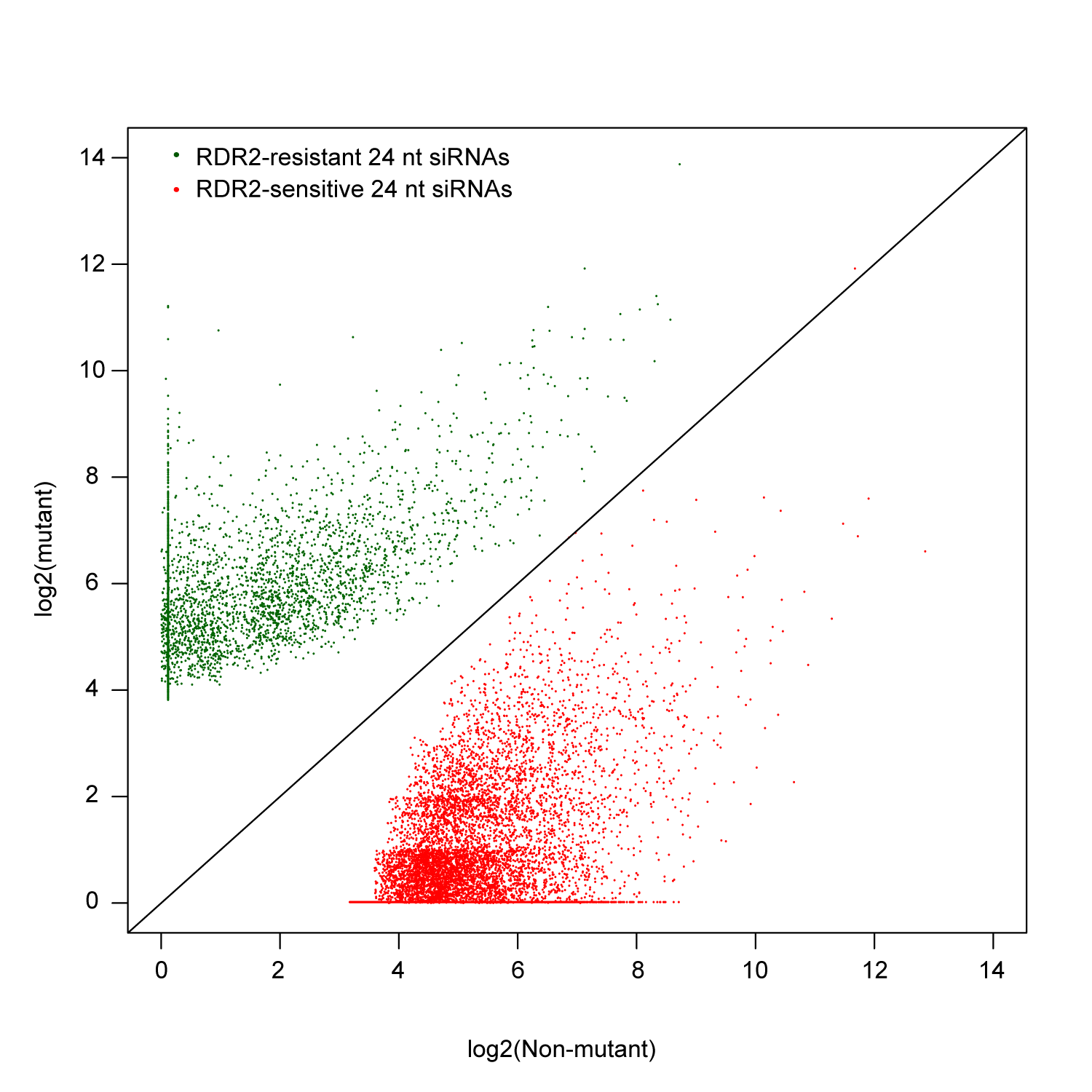

Supplement: Figure S4 — RDR2-sensitive and RDR2-resistant 24 nt siRNAs in wild-type and mop1 mutants. The log2 transformation of read counts in non-mutant (x-axis) versus mop1 mutant (y-axis) for each species of the 4,950 RDR2-resistant 24 nt siRNAs (green dots) and the 33,614 RDR2-sensitive 24 nt siRNAs (red dots) were plotted. (0.36 MB TIF) [file pgen.1000737.s004.tif]

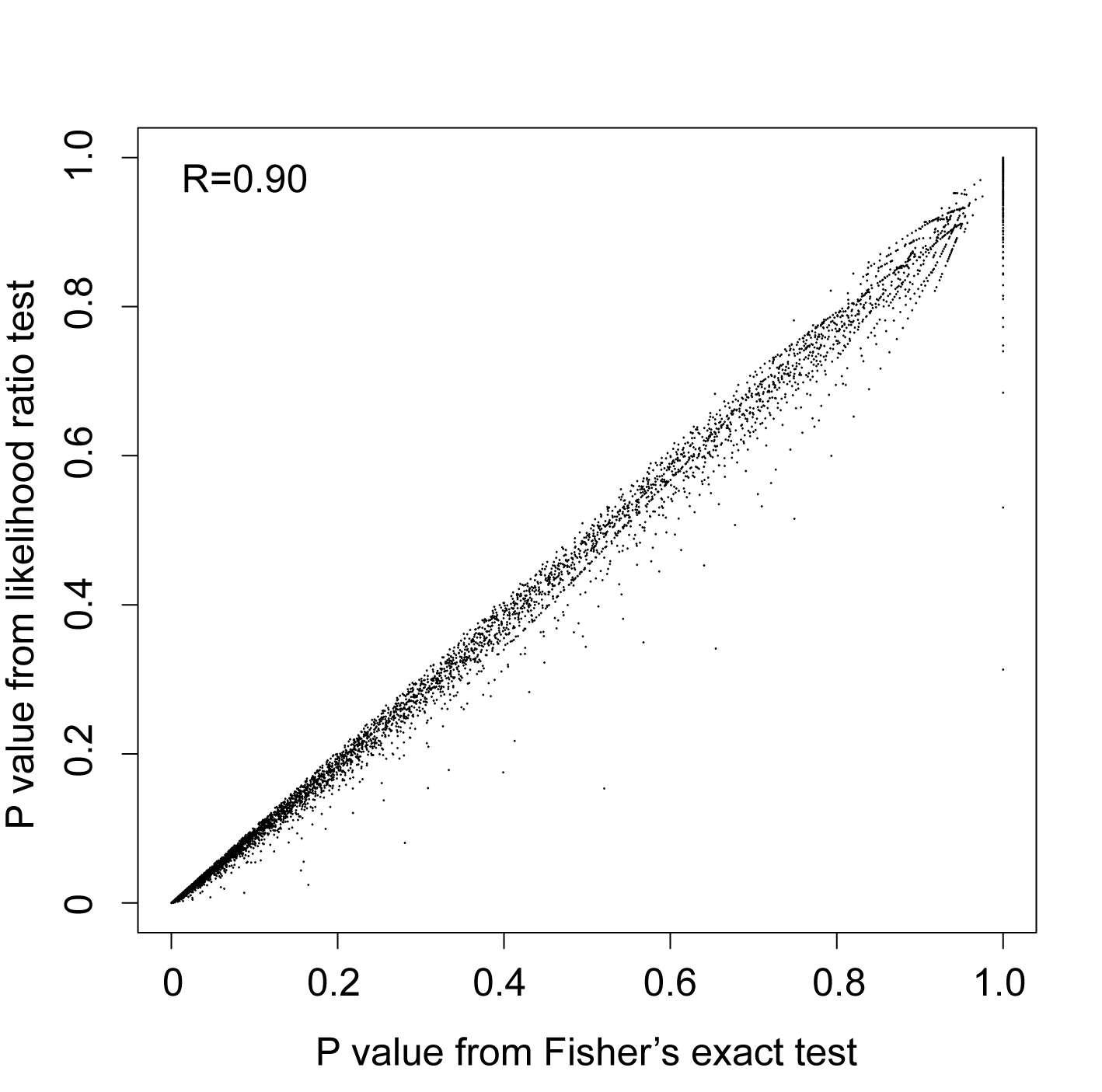

Supplement: Figure S5 — p-value comparison between likelihood ratio test and Fisher's exact test. (0.27 MB TIF) [file pgen.1000737.s005.tif]
