# Supplementary material for: Loss of RNA–Dependent RNA Polymerase 2 (RDR2) Function Causes Widespread and Unexpected Changes in the Expression of Transposons, Genes, and 24-nt Small RNAs
Source: PLoS Genet. 2009 Nov 20;5(11):e1000737. doi: 10.1371/journal.pgen.1000737 (PMC2774947; doi:10.1371/journal.pgen.1000737)
Supplement: Table S1 — mop1 mutants and non-mutants have distinct SAM morphologies. (0.01 MB PDF) [file pgen.1000737.s006.pdf]

Table S1. *mop1* mutants and non-mutants have distinct SAM morphologies

Genotype	SAM ID	Width (μM) [a]	Height (μM) [b]	Ratio(Height/Width)
mop1/mop1	1	171.0	118.4	0.69
	2	180.1	162.6	0.90
	3	170.7	145.2	0.85
	4	161.3	142.7	0.88
	**Mean**	**170.8**	**142.2**	**0.83**
Mop1/mop1	1	221.3	249.3	1.13
	2	156.8	171.6	1.09
	3	157.3	181.3	1.15
	4	151.4	160.8	1.06
	**Mean**	**171.7**	**190.8**	**1.11**

[a] The length of a horizontal line drawn between the initiation points of P0 and P1 was defined as the width of the SAM. [b] The length of a vertical line drawn from the top of the SAM to the horizontal line was defined as the height of the SAM. The averaged width and height were calculated for mutant and non-mutants across four different SAMs, respectively. A Welch two-sample t-test demonstrated that the mean ratio (height/width) of mutants (0.83) is significantly smaller than that of non-mutants (1.11) with p-value 0.006.
