# Supplementary material for: Loss of RNA–Dependent RNA Polymerase 2 (RDR2) Function Causes Widespread and Unexpected Changes in the Expression of Transposons, Genes, and 24-nt Small RNAs
Source: PLoS Genet. 2009 Nov 20;5(11):e1000737. doi: 10.1371/journal.pgen.1000737 (PMC2774947; doi:10.1371/journal.pgen.1000737)
Supplement: Table S2 — List of differentially expressed DNA transposon families. (0.03 MB PDF) [file pgen.1000737.s007.pdf]

Table S2. List of differentially expressed DNA transposons

DNA transposon ID	No. Illumina/Solexa Reads [a]		log2(FC) [b]	BH.FDR [c]
	Mutant	Non-mutant		
ZM_CACTA_105	14,280	35,351	-0.6	0.00E+01
ZM_CACTA_85	10,276	7,755	1.1	0.00E+01
ZM_CACTA_89	13,242	29,148	-0.4	2.19E-190
ZM_CACTA_93	1,856	6,206	-1.0	4.86E-179
ZM_CACTA_18	2,264	6,205	-0.7	1.14E-103
ZM_CACTA_16	2,585	6,806	-0.7	5.62E-99
ZM_CACTA_102	4,857	5,614	0.5	3.95E-69
ZM_CACTA_21	3,436	3,931	0.5	2.54E-51
ZM_PIF/Harbinger_17	1,279	1,137	0.9	6.60E-49
ZM_Stowaway_70	242	62	2.7	7.53E-48
ZM_CACTA_32	1,365	1,366	0.7	7.12E-36
ZM_CACTA_2	8,113	15,692	-0.2	7.38E-34
DTM_Zm00665_AC184764-1	2,334	2,771	0.5	1.58E-28
ZM_CACTA_73	775	1,993	-0.7	2.65E-26
ZM_CACTA_1	1,459	3,341	-0.5	5.20E-26
ZM_Stowaway_5	694	635	0.8	1.77E-24
ZM_PIF/Harbinger_16	821	800	0.7	9.24E-24
ZM_CACTA_63	1,291	1,465	0.5	4.44E-20
DTM_TAFT1_DQ493649	983	1,060	0.6	2.17E-19
DTM_Zm00473_AC191070-1	1,190	1,401	0.5	2.81E-15
ZM_CACTA_77	1,690	3,529	-0.3	2.81E-15
DTM_Zm00257_consensus	286	233	1.0	8.52E-14
DTM_Zm00268_AC191380-1	715	788	0.6	7.22E-13
ZM_CACTA_26	1,186	2,522	-0.4	1.59E-12
DTM_Zm09546_AC191082-1	502	513	0.7	2.20E-12
DTM_Zm00850_AC186314-1	298	262	0.9	6.33E-12
DTM_Zm32512_AC187775-1	459	474	0.7	6.19E-11
ZM_hAT_2	611	682	0.6	1.97E-10
DTM_Zm09664_AC195786-1	480	509	0.6	3.00E-10
ZM_CACTA_noncoding_39	602	677	0.5	5.84E-10
ZM_CACTA_20	3,902	5,564	0.2	6.02E-10
ZM_Tourist_34	816	978	0.5	1.39E-09
ZM_CACTA_22	1,252	1,603	0.4	1.76E-09
ZM_CACTA_4	1,947	3,808	-0.3	2.75E-09
ZM_hAT_36	525	1,196	-0.5	3.16E-09
ZM_hAT_noncoding_31	245	225	0.8	9.82E-09
DTM_Zm00673_AC187563-1	354	364	0.7	1.24E-08
ZM_hAT_noncoding_15	93	54	1.5	1.29E-08
ZM_CACTA_79	986	1,244	0.4	2.19E-08
ZM_CACTA_31	1,498	2,967	-0.3	2.59E-08
ZM_hAT_noncoding_45	553	640	0.5	4.66E-08
DTM_Zm33205_AC203276-1	92	57	1.4	8.46E-08
ZM_CACTA_90	1,431	1,926	0.3	3.26E-07
DTM_Zm02117_AC177838-1	1,781	2,449	0.3	3.30E-07

ZM_CACTA_35	781	1,628	-0.3	3.49E-07
DTM_Zm00364_AC196271-1	444	505	0.5	4.05E-07
ZM_Tourist_2	96	65	1.3	4.97E-07
ZM_CACTA_107	1,500	2,919	-0.2	7.19E-07
ZM_Tourist_35	72	42	1.5	8.88E-07
ZM_PIF/Harbinger_19	1,222	1,649	0.3	4.71E-06
ZM_PIF/Harbinger_25	904	1,181	0.3	5.14E-06
ZM_hAT_4	461	546	0.5	5.23E-06
ZM_PIF/Harbinger_24	220	221	0.7	5.23E-06
ZM_CACTA_19	582	1,224	-0.4	7.51E-06
ZM_Stowaway_13	55	30	1.6	1.11E-05
ZM_Tourist_18	190	187	0.7	1.20E-05
ZM_CACTA_45	707	910	0.3	2.29E-05
ZM_hAT_noncoding_30	102	83	1.0	2.71E-05
ZM_PIF/Harbinger_32	208	214	0.7	2.71E-05
ZM_CACTA_55	672	863	0.4	3.31E-05
DTM_Zm17860_AC184871-1	137	125	0.8	3.44E-05
ZM_CACTA_28	859	1,145	0.3	6.52E-05
ZM_CACTA_53	1,001	1,357	0.3	6.88E-05
ZM_Stowaway_64	71	51	1.2	6.91E-05
ZM_PIF/Harbinger_35	348	411	0.5	9.22E-05
ZM_CACTA_56	1,538	2,174	0.2	1.14E-04
ZM_hAT_8	657	1,323	-0.3	1.41E-04
DTM_Zm09941_consensus	97	82	1.0	1.46E-04
ZM_Tourist_61	146	143	0.7	1.61E-04
ZM_PIF/Harbinger_37	849	1,144	0.3	1.78E-04
DTM_Zm20615_AC216184-1	96	82	0.9	1.89E-04
ZM_hAT_18	29	110	-1.2	2.30E-04
ZM_CACTA_109	1,280	1,796	0.2	2.36E-04
ZM_Stowaway_6	107	96	0.9	2.36E-04
ZM_CACTA_noncoding_25	289	339	0.5	3.68E-04
ZM_hAT_noncoding_183	118	112	0.8	4.00E-04
ZM_CACTA_110	589	1,183	-0.3	4.47E-04
ZM_PIF/Harbinger_18	348	739	-0.4	5.09E-04
ZM_hAT_noncoding_42	139	140	0.7	5.26E-04
ZM_Tourist_49	25	10	2.0	6.77E-04
ZM_CACTA_62	896	1,234	0.3	7.48E-04
ZM_CACTA_91	626	833	0.3	8.03E-04
ZM_PIF/Harbinger_34	287	620	-0.4	8.27E-04
ZM_hAT_33	109	274	-0.6	1.02E-03
ZM_Tourist_50	112	110	0.7	1.51E-03
ZM_CACTA_noncoding_7	200	227	0.5	1.52E-03
DTM_Zm01468_AC186160-1	98	93	0.8	1.52E-03
ZM_Stowaway_21	164	179	0.6	1.56E-03
ZM_PIF/Harbinger_12	96	91	0.8	1.71E-03
ZM_CACTA_noncoding_1	153	165	0.6	1.72E-03
ZM_hAT_34	251	299	0.5	1.74E-03

ZM_Tourist_66	204	234	0.5	1.74E-03
DTM_Zm04817_AC194407-1	819	1,131	0.2	1.75E-03
ZM_CACTA_75	585	783	0.3	1.81E-03
DTM_Zm10271_AC186904-1	50	37	1.1	1.82E-03
ZM_CACTA_101	790	1,089	0.2	1.88E-03
ZM_CACTA_83	587	790	0.3	2.53E-03
ZM_CACTA_33	548	732	0.3	2.61E-03
ZM_CACTA_72	978	1,379	0.2	2.61E-03
ZM_hAT_noncoding_174	40	27	1.3	2.61E-03
ZM_PIF/Harbinger_33	360	740	-0.3	2.84E-03
DTM_Zm02703_AC177838-1	217	256	0.5	3.19E-03
ZM_CACTA_59	558	752	0.3	3.61E-03
ZM_CACTA_67	1,658	3,016	-0.2	4.21E-03
ZM_CACTA_71	1,118	1,602	0.2	4.30E-03
ZM_CACTA_103	1,035	1,475	0.2	4.33E-03
ZM_Stowaway_8	67	60	0.9	4.73E-03
DTM_Zm33207_AC210869-1	68	61	0.9	4.98E-03
ZM_CACTA_9	2,002	2,976	0.1	5.32E-03
ZM_hAT_38	203	242	0.5	5.92E-03
ZM_hAT_39	249	307	0.4	6.81E-03
ZM_hAT_11	287	593	-0.3	8.01E-03
DTM_Zm00884_AC214130-1	106	111	0.6	8.07E-03
ZM_CACTA_41	670	931	0.2	8.17E-03
ZM_CACTA_14	1,692	3,053	-0.1	8.87E-03
ZM_hAT_noncoding_4	77	75	0.8	9.17E-03
ZM_Stowaway_12	187	223	0.5	9.89E-03
ZM_CACTA_61	2,810	4,265	0.1	1.00E-02
ZM_PIF/Harbinger_36	366	481	0.3	1.00E-02
DTM_Zm06136_AC196794-1	307	398	0.3	1.32E-02
ZM_hAT_19	98	104	0.6	1.38E-02
ZM_hAT_noncoding_22	83	85	0.7	1.49E-02
ZM_CACTA_99	2,106	3,170	0.1	1.52E-02
ZM_CACTA_88	419	823	-0.3	1.52E-02
ZM_hAT_noncoding_7	154	181	0.5	1.65E-02
ZM_Stowaway_82	125	141	0.5	1.70E-02
ZM_hAT_noncoding_2	172	206	0.5	1.71E-02
ZM_Tourist_27	6	0	#VALUE!	1.71E-02
DTM_Zm00851_AC186342-1	170	205	0.4	1.94E-02
DTM_Zm00743_AC177943-1	1,266	1,866	0.2	2.11E-02
ZM_hAT_15	64	160	-0.6	2.11E-02
ZM_hAT_noncoding_58	125	143	0.5	2.11E-02
DTM_Zm20301_AC191081-1	63	61	0.8	2.19E-02
DTM_Zm02661_AC177816-1	697	991	0.2	2.31E-02
ZM_Stowaway_30	236	301	0.4	2.39E-02
ZM_CACTA_94	794	1,140	0.2	2.45E-02
ZM_CACTA_44	743	1,063	0.2	2.61E-02
DTM_Zm11868_AC202888-1	419	573	0.3	2.85E-02

ZM_hAT_noncoding_37	16	8	1.7	2.85E-02
ZM_hAT_noncoding_86	49	45	0.8	2.91E-02
ZM_CACTA_74	13,198	22,273	-0.0	2.93E-02
ZM_Stowaway_89	27	19	1.2	2.93E-02
ZM_Stowaway_55	20	12	1.4	2.93E-02
DTM_Zm22051IC_AC201818-1	173	366	-0.4	2.98E-02
ZM_CACTA_noncoding_2	182	226	0.4	2.98E-02
ZM_hAT_noncoding_98	35	29	1.0	3.38E-02
ZM_Stowaway_95	27	20	1.1	3.39E-02
ZM_CACTA_87	289	575	-0.3	3.44E-02
DTM_Zm33202_AC177945-1	28	21	1.1	3.69E-02
ZM_hAT_35	179	374	-0.4	3.69E-02
ZM_hAT_noncoding_117	5	0	#VALUE!	3.83E-02
ZM_Tourist_40	84	92	0.6	3.97E-02
DTM_Zm33222_AC190995-1	95	215	-0.5	4.04E-02
ZM_CACTA_noncoding_18	8	2	2.7	4.06E-02
ZM_Tourist_13	31	25	1.0	4.11E-02
DTM_Zm03788_AC184173-1	41	108	-0.7	4.15E-02
ZM_Stowaway_47	62	63	0.7	4.49E-02
ZM_hAT_noncoding_70	23	69	-0.9	4.63E-02
ZM_hAT_noncoding_8	59	60	0.7	4.88E-02

[a] The number of reads mapped to each gene model from mutant and non-mutant RNA-seq

[b] log_2 transformation of foldchange as the relative abandunce of transcripts in mutants/non-mutants

[c] The fasle discovery rate calculated using Benjamini and Hochberg's procedure for the p value from Fisher's exact test
