# Supplementary material for: Loss of RNA–Dependent RNA Polymerase 2 (RDR2) Function Causes Widespread and Unexpected Changes in the Expression of Transposons, Genes, and 24-nt Small RNAs
Source: PLoS Genet. 2009 Nov 20;5(11):e1000737. doi: 10.1371/journal.pgen.1000737 (PMC2774947; doi:10.1371/journal.pgen.1000737)
Supplement: Table S3 — List of differentially expressed retrotransposon sub-families. (0.04 MB PDF) [file pgen.1000737.s008.pdf]

Table S3. List of differentially expressed retrotransposons

Retrotransposon ID	No. Illumina/Solexa reads [a]		log$_2$(FC) [b]	BH.FDR [c]
	Mutant	Non-Mutant		
RLC_opie_AC198924_6206	12,344	29,617	-0.8	0.00E+01
RLG_flip_AC213672_12280	16,317	14,378	0.7	0.00E+01
RLX_ipiki_AC212468_11834	15,260	11,162	0.9	0.00E+01
RLX_iwik_AC203371_7824	19,200	18,967	0.5	1.02E-242
RLC_iteki_AC214131_12504	25,079	27,446	0.3	1.17E-163
RLG_ansuya_AC207803_9728	13,053	13,682	0.4	1.90E-115
RLG_flip_AC203163_7675	15,353	16,674	0.4	1.13E-105
RLG_prem1_AC196065_4927	18,592	31,379	-0.3	9.63E-100
RLG_prem1_AC200105_6751	22,071	36,643	-0.3	7.28E-99
RLG_xilon-diguus_AC185317_1058	8,677	15,934	-0.4	1.08E-98
RLG_xilon-diguus_AC195486_4629	5,650	10,894	-0.5	1.57E-90
RLG_nihep_AC194441_4115	13,881	15,234	0.3	3.48E-88
RLG_ansuya_AC208678_9984	9,479	9,919	0.4	7.30E-85
RLX_sari_AC184117_11	10,400	11,319	0.4	1.90E-70
RLX_odoj_AC194387_4072	8,566	15,084	-0.3	3.66E-69
RLX_egisu_AC206234_9137	15,262	25,262	-0.3	7.72E-66
RLC_ji_AC192600_3526	7,933	8,490	0.4	5.95E-61
RIX_ekeje_AC197537-0	932	2,312	-0.8	1.59E-52
RLX_tekay_AC200856_6996	5,032	9,106	-0.4	3.73E-51
RLG_cinful-zeon_AC198933_6214	5,007	9,065	-0.4	3.98E-51
RLX_tuteh_AC183372_584	995	650	1.1	1.45E-50
RLG_flip_AC211750_11412	12,637	14,670	0.3	4.56E-48
RLG_prem1_AC212325_11702	22,480	35,258	-0.2	6.74E-46
RLG_huck_AC190900_2713	6,371	6,882	0.4	7.68E-46
RLX_gunu_AC200292_6838	5,000	5,231	0.4	4.81E-45
RLX_hutu_AC210780_10851	3,822	3,852	0.5	2.59E-43
RLG_flip_AC194904_4319	10,111	11,684	0.3	1.97E-40
RLG_huck_AC194973_4393	1,416	1,167	0.8	1.39E-38
RLC_ji_AC200613_6936	8,746	10,054	0.3	6.04E-37
RLX_gunu_AC204937_8509	8,659	14,315	-0.3	6.18E-37
RLG_huck_AC186656_1609	926	673	0.9	1.00E-36
RIX_nugimu_AC203843-0	620	1,546	-0.8	5.31E-36
RLG_flip_AC214266_12595	13,577	16,289	0.2	3.03E-35
RLG_prem1_AC184142_802	12,769	20,390	-0.2	1.37E-34
RLC_vuijon_AC194895_4312	7,687	8,787	0.3	2.01E-34
RLC_ji_AC182107_448	7,543	8,634	0.3	2.54E-33
RLG_cinful-zeon_AC203004_7602	14,357	22,672	-0.2	7.56E-33
RLG_prem1_AC200740_6986	10,332	16,655	-0.2	4.65E-32
RLC_ji_AC197052_5349	7,271	8,330	0.3	6.63E-32
RLC_ji_AC210731_10832	7,582	8,725	0.3	7.70E-32
RLC_giepum_AC197531_5634	2,734	5,013	-0.4	3.76E-31
RLG_guhis_AC198413_5999	1,805	3,510	-0.5	4.94E-31
RLX_hiimam_AC207734_9687	650	1,533	-0.8	3.27E-30
RLG_huck_AC199418_6452	797	593	0.9	6.16E-30

RLG_flip_AC208040_9765	12,426	15,002	0.2	7.95E-30
RLC_ji_AC215728_13156	10,095	12,003	0.2	1.56E-29
RLG_huck_AC216048_13250	1,426	1,271	0.6	1.94E-29
RLG_huck_AC191259_3001	995	803	0.8	2.22E-29
RIX_afeda_AC208414-0	1,193	2,464	-0.6	2.26E-29
RLC_ji_AC186528_1508	1,230	2,514	-0.6	1.28E-28
RLG_huck_AC210079_10574	1,239	1,081	0.7	7.19E-28
RLG_huck_AC186603_1556	1,403	1,266	0.6	1.27E-27
RLX_ilyl_AC196209_163	457	281	1.2	9.03E-27
RLG_cinful-zeon_AC211573_11290	5,858	9,710	-0.3	4.33E-26
RLG_huck_AC195575_4652	461	296	1.1	7.97E-25
RLX_defub_AC191393_106	1,867	1,844	0.5	3.97E-24
RLG_huck_AC213612_12218	766	607	0.8	4.22E-24
RLG_neha_AC215285_13046	1,804	3,375	-0.4	4.38E-24
RLG_prem1_AC201801_7095	11,957	18,713	-0.2	7.72E-24
RLX_crm_AC206920_9397	1,867	3,464	-0.4	1.73E-23
RLX_ebeg_AC213788_12351	857	1,797	-0.6	4.08E-23
RLG_ajajog_AC191578_3186	5,539	6,391	0.3	6.25E-23
RLG_huck_AC212331_11708	1,212	1,106	0.6	6.37E-23
RLC_opie_AC202033_7274	3,336	3,643	0.3	1.33E-22
RLG_dagaf_AC182835_556	6,110	7,137	0.2	3.27E-22
RIX_leijoh_AC212369-0	646	1,420	-0.7	3.88E-22
RLX_ubow_AC194933_4355	1,259	2,446	-0.5	7.30E-22
RLX_votaed_AC215881_13209	670	1,457	-0.6	8.23E-22
RLC_ji_AC190978_2799	7,288	8,665	0.2	1.30E-21
RLG_huck_AC193313_3542	388	257	1.1	9.76E-20
RLG_cinful-zeon_AC191523_3148	1,323	2,509	-0.4	1.32E-19
RIX_koajav_AC205853-0	351	857	-0.8	3.53E-19
RIX_ejoet_AC212216-0	298	758	-0.9	3.95E-19
RLC_eninu_AC191055_2893	598	479	0.8	1.68E-18
RLC_ji_AC193479_3665	10,661	13,171	0.2	1.94E-18
RLC_udav_AC196188_5022	670	1,404	-0.6	3.30E-18
RLC_ji_AC213834_12382	16,754	21,245	0.1	7.56E-18
RLC_guvi_AC185473_1128	1,210	2,280	-0.4	2.96E-17
RLC_opie_AC217577_13524	3,658	4,177	0.3	4.78E-17
RLX_eweko_AC200180_6781	489	378	0.8	6.24E-17
RIX_edaej_AC215611-0	461	1,019	-0.7	1.89E-16
RLG_cinful-zeon_AC202991_7588	3,002	5,052	-0.3	3.39E-16
RLG_cinful-zeon_AC212696_11896	15,229	23,065	-0.1	4.10E-16
RLG_wuge_AC190810_2615	607	512	0.7	6.62E-16
RLG_cinful-zeon_AC215255_13029	3,134	5,239	-0.3	9.66E-16
RLG_dagaf_AC195302_4533	6,828	10,734	-0.2	5.54E-15
RLX_wihov_AC205351_8739	3,401	5,608	-0.2	1.30E-14
RLG_cinful-zeon_AC194954_4372	4,537	7,305	-0.2	2.44E-14
RLC_opie_AC197201_5474	5,068	6,081	0.2	1.11E-13
RLG_cinful-zeon_AC205031_8560	3,475	5,686	-0.2	1.15E-13
RLC_tufe_AC211502_11228	5,232	6,294	0.2	1.20E-13

RLC_opie_AC187149_1780	5,747	6,965	0.2	1.51E-13
RLC_opie_AC210610_10772	1,702	2,970	-0.3	2.33E-13
RLG_cinful-zeon_AC210140_10614	9,294	14,263	-0.1	3.05E-13
RLC_ji_AC207234_9488	11,265	14,240	0.1	4.84E-13
RLG_doke_AC186158_1307	959	945	0.5	4.90E-13
RLG_huck_AC208842_10038	1,130	1,152	0.4	1.17E-12
RLG_cinful-zeon_AC186614_1565	3,470	5,641	-0.2	1.31E-12
RLC_ji_AC202456_7443	9,196	11,540	0.1	2.34E-12
RLX_nisow_AC208616_9947	58	213	-1.4	9.78E-12
RIX_loneok_AC193379-0	812	1,522	-0.4	1.78E-11
RLG_muojo_AC186807_1674	450	924	-0.6	2.85E-11
RLX_bori_AC190960_100	244	172	1.0	3.80E-11
RLX_naadira_AC201761_7053	1,903	2,132	0.3	6.47E-11
RLG_prem1_AC191715_3282	17,932	26,569	-0.1	8.07E-11
RLC_wiwa_AC191531_3153	405	839	-0.6	1.06E-10
RLG_huck_AC199444_6460	633	603	0.5	2.44E-10
RLG_cinful-zeon_AC206171_9091	5,357	8,329	-0.2	5.18E-10
RLG_cinful-zeon_AC199960_6694	2,875	4,648	-0.2	6.76E-10
RLG_gyte_AC207411_9568	487	954	-0.5	2.09E-09
RLX_kahoba_AC186618_29	340	288	0.7	3.58E-09
RLX_peeve_AC209948_10499	3,164	5,051	-0.2	3.65E-09
RLC_opie_AC188002_2029	3,549	4,289	0.2	5.29E-09
RLC_sawujo_AC193398_3610	1,133	1,222	0.4	5.37E-09
RLG_cinful-zeon_AC186530_1513	12,546	18,686	-0.1	5.59E-09
RLG_cinful-zeon_AC208661_9976	2,102	3,445	-0.2	9.72E-09
RLG_gyma_AC212146_11616	917	1,628	-0.4	1.05E-08
RLG_cinful-zeon_AC208228_9805	6,851	10,432	-0.1	1.41E-08
RLC_ji_AC211489_11215	10,988	14,153	0.1	1.47E-08
RLX_oweiw_AC190860_2662	45	159	-1.3	1.83E-08
RLG_cinful-zeon_AC199396_6426	2,658	4,252	-0.2	4.77E-08
RLX_uwum_AC213069_12092	4,232	5,219	0.2	4.88E-08
RLX_leso_AC215500_13080	568	560	0.5	5.32E-08
RLG_xilon-diguus_AC203313_7774	52,621	75,427	-0.0	6.12E-08
RLG_huck_AC214833_12913	447	425	0.5	1.20E-07
RLX_hoda_AC193645_3730	53	169	-1.2	1.22E-07
RLX_alaw_AC197914_166	315	276	0.7	1.23E-07
RLX_ubel_AC183941_731	38	136	-1.4	1.55E-07
RLX_ojah_AC184792_875	1,664	1,924	0.3	2.31E-07
RLG_ansuya_AC191576_3184	853	1,491	-0.3	3.34E-07
RLX_mada_AC208456_9876	574	580	0.5	3.43E-07
RLG_huck_AC203007_7610	777	824	0.4	3.47E-07
RLG_cinful-zeon_AC195790_4706	1,867	3,031	-0.2	4.35E-07
RIX_pyelum_AC203928-0	813	1,424	-0.3	4.68E-07
RLG_cinful-zeon_AC205768_9013	11,932	17,619	-0.1	8.83E-07
RLG_huck_AC210804_10865	1,232	1,398	0.3	1.20E-06
RLX_kawivo_AC186793_30	791	851	0.4	1.25E-06
RLC_lusi_AC198175_5903	9	61	-2.3	1.25E-06

RLX_teki_AC202867_7492	34	8	2.6	1.38E-06
RLG_cinful-zeon_AC200200_6794	3,016	4,715	-0.2	1.54E-06
RLX_dala_AC216254_13336	259	226	0.7	1.59E-06
RLG_cinful-zeon_AC193672_3742	11,721	17,284	-0.1	1.97E-06
RLX_vafim_AC196676_5176	38	127	-1.3	2.10E-06
RLG_riiryl_AC193745_3743	1,688	1,988	0.2	2.83E-06
RLC_kameer_AC194009_3819	84	216	-0.9	3.19E-06
RLG_huck_AC186577_1525	2,020	2,417	0.2	3.60E-06
RLG_cinful-zeon_AC191398_3106	1,963	3,139	-0.2	3.66E-06
RIL_etiti_AC211734-0	419	778	-0.4	5.30E-06
RLG_prem1_AC215184_12949	23,209	33,573	-0.1	5.77E-06
RLG_cinful-zeon_AC177813_189	13,225	19,382	-0.1	6.33E-06
RLG_cinful-zeon_AC216587_13387	2,770	4,314	-0.2	9.18E-06
RLX_bobeg_AC193485_3670	216	187	0.7	9.64E-06
RLG_apil_AC198662_6085	8	52	-2.2	9.88E-06
RLC_tata_AC205418_8792	49	143	-1.1	1.30E-05
RLG_grande_AC214148_12520	2,647	3,254	0.2	1.30E-05
RLC_stonor_AC212476_11849	269	527	-0.5	1.33E-05
RLC_ulyg_AC196397_5082	8,605	12,746	-0.1	1.37E-05
RLG_doke_AC197224_5479	6,271	8,063	0.1	1.84E-05
RLX_vusu_AC187081_1751	119	270	-0.7	2.14E-05
RLX_mibaab_AC205139_8652	9	53	-2.1	2.57E-05
RLG_gyma_AC189750_2238	3,972	6,041	-0.1	3.03E-05
RLG_cinful-zeon_AC194588_4160	7,182	10,665	-0.1	3.95E-05
RLG_cinful-zeon_AC207332_9527	3,646	5,558	-0.1	4.40E-05
RLC_opie_AC202020_7258	4,200	5,335	0.1	5.65E-05
RLC_opie_AC196469_5133	12,715	18,546	-0.1	6.79E-05
RLX_demo_AC202036_173	203	406	-0.5	6.79E-05
RLG_cinful-zeon_AC194967_4388	3,078	4,719	-0.1	6.92E-05
RLG_cinful-zeon_AC177930_407	3,587	5,459	-0.1	7.09E-05
RLG_lata_AC191117_2960	944	1,559	-0.2	8.88E-05
RLX_uwum_AC177933_415	7,207	10,668	-0.1	9.65E-05
RLC_ekoj_AC194603_4173	742	834	0.3	1.10E-04
RLG_cinful-zeon_AC203825_7980	2,490	3,831	-0.1	2.41E-04
RLG_huck_AC208546_9913	543	595	0.3	2.52E-04
RLG_lyruom_AC185669_1267	20	72	-1.4	2.58E-04
RLC_anar_AC206985_9422	202	393	-0.5	3.15E-04
RLX_milt_AC211742_11402	2,298	3,537	-0.1	4.27E-04
RLX_ywely_AC190897_98	97	212	-0.7	5.87E-04
RLG_gyma_AC197250_5506	5,324	6,909	0.1	6.83E-04
RLG_cinful-zeon_AC199790_6582	6,646	9,785	-0.1	7.71E-04
RLG_cinful-zeon_AC207755_9705	7,239	10,629	-0.1	8.28E-04
RLG_feki_AC195127_4412	30	88	-1.1	8.28E-04
RLX_daju_AC190492_79	18	63	-1.3	9.00E-04
RLX_ebel_AC210216_10670	72	165	-0.7	1.18E-03
RLG_cinful-zeon_AC211144_11003	3,624	5,427	-0.1	1.42E-03
RLX_ebel_AC188777_2128	86	187	-0.6	1.51E-03

RLX_liove_AC207121_9462	3,335	5,005	-0.1	1.65E-03
RLX_bida_AC205396_8768	1,182	1,867	-0.2	1.68E-03
RLG_apil_AC204354_8209	6	33	-2.0	1.73E-03
RLG_cinful-zeon_AC209373_10201	6,418	9,417	-0.1	2.10E-03
RLX_ebel_AC211737_11397	82	178	-0.6	2.15E-03
RLX_lamyab_AC208713_10008	9	40	-1.7	2.27E-03
RLC_nida_AC206942_9401	3,749	4,837	0.1	2.34E-03
RLC_victim_AC183319_577	137	270	-0.5	2.36E-03
RLX_petopi_AC195376_4582	15	53	-1.3	2.50E-03
RLC_opie_AC187207_1792	6,480	8,517	0.1	2.58E-03
RLG_prem1_AC186287_1362	9,183	13,341	-0.1	2.64E-03
RLX_naseup_AC196428_5108	3,197	4,108	0.1	3.10E-03
RIX_totyru_AC203014-0	151	291	-0.5	3.15E-03
RLX_jelat_AC194217_3960	26	74	-1.0	3.43E-03
RLG_cinful-zeon_AC213887_12415	4,325	6,403	-0.1	3.45E-03
RLX_gufa_AC194066_3854	42	104	-0.8	4.11E-03
RLX_baso_AC192251_3423	649	759	0.2	4.29E-03
RLG_boja_AC200053_6723	9	38	-1.6	4.80E-03
RLX_mako_AC200748_6991	54	124	-0.7	5.30E-03
RLX_fanuab_AC193594_3712	11	42	-1.5	5.64E-03
RLX_nuhan_AC206272_9161	2,182	3,299	-0.1	6.04E-03
RLC_opie_AC214122_12495	5,466	7,183	0.1	6.09E-03
RLX_osed_AC191084_2931	595	695	0.2	6.09E-03
RLG_cinful-zeon_AC183943_734	2,111	3,192	-0.1	6.95E-03
RLX_mafigi_AC216705_13396	12	43	-1.4	6.95E-03
RLX_milt_AC194936_4356	763	1,219	-0.2	7.05E-03
RLG_cinful-zeon_AC208420_9852	3,383	5,024	-0.1	7.18E-03
RLX_pute_AC197188_5467	140	266	-0.5	7.18E-03
RLC_gudyeg_AC206942_9404	510	837	-0.2	8.02E-03
RLX_tekay_AC211245_11065	5,429	7,946	-0.1	8.02E-03
RLX_yreud_AC198385_5976	550	898	-0.2	8.02E-03
RLC_bote_AC211535_11252	10	38	-1.5	8.62E-03
RLG_aneas_AC203312_7773	15	49	-1.2	8.99E-03
RLX_dugiab_AC207724_9683	8,602	11,456	0.1	8.99E-03
RLX_lamyab_AC215655_13126	32	81	-0.9	1.11E-02
RLX_pibo_AC201915_172	35	86	-0.8	1.13E-02
RLC_ruda_AC206281_9164	1,466	1,841	0.1	1.17E-02
RLX_japov_AC213985_12447	320	356	0.3	1.20E-02
RLX_etug_AC187099_1770	77	159	-0.6	1.21E-02
RIX_keneat_AC209697-0	145	268	-0.4	1.41E-02
RLX_bumy_AC216354_13379	281	481	-0.3	1.41E-02
RLG_ywyt_AC209975_10517	494	579	0.2	1.57E-02
RLX_milt_AC209648_10275	379	629	-0.3	1.61E-02
RLG_grande_AC190611_2432	2,250	3,365	-0.1	1.71E-02
RLX_hesa_AC204349_8207	10	36	-1.4	1.71E-02
RLX_hopscotch_AC209396_10229	29	73	-0.9	1.71E-02
RLC_leviathan_AC208826_10024	11,307	16,226	-0.0	1.88E-02

RLC_wamenu_AC191287_3028	80	160	-0.5	1.89E-02
RLX_vedi_AC198992_6258	227	394	-0.3	1.89E-02
RLG_grande_AC214497_12729	1,753	2,644	-0.1	1.92E-02
RLX_wiolus_AC210058_10567	5	24	-1.8	1.93E-02
RLX_halo_AC205330_8725	3	19	-2.2	2.06E-02
RLC_bipide_AC205969_9058	128	236	-0.4	2.73E-02
RLG_ivuk_AC194103_3869	1	12	-3.1	2.81E-02
RLC_ruda_AC195952_4839	1,905	2,448	0.1	2.84E-02
RLC_vuna_AC193505_121	1	13	-3.2	2.90E-02
RLG_yfages_AC197085_5383	5	23	-1.7	2.90E-02
RLG_boha_AC205574_8933	4	20	-1.8	3.08E-02
RLX_mada_AC215312_13067	2,779	4,103	-0.1	3.14E-02
RLX_uwub_AC195372_161	693	1,086	-0.2	3.29E-02
RLC_raider_AC209705_10304	103	194	-0.4	3.30E-02
RLX_arar_AC208428_9858	12	37	-1.2	3.35E-02
RLX_panen_AC192606_115	294	490	-0.3	3.38E-02
RLC_nida_AC201982_7215	3,850	5,076	0.1	3.71E-02
RLX_lyna_AC194093_3868	146	260	-0.4	3.83E-02
RLG_cinful-zeon_AC201757_7049	10,158	13,673	0.0	4.03E-02
RLG_cinful-zeon_AC206615_9266	7,913	11,381	-0.1	4.03E-02
RLG_fourf_AC202975_7570	1,285	1,945	-0.1	4.04E-02
RLX_toro_AC197199_5471	124	225	-0.4	4.05E-02
RLG_cinful-zeon_AC205118_8623	2,142	3,178	-0.1	4.31E-02
RLX_eugene_AC200048_6717	2,036	2,637	0.1	4.35E-02
RLC_opie_AC211653_11340	7,072	9,466	0.1	4.47E-02
RLG_laiwa_AC214288_12602	4	19	-1.8	4.47E-02
RLC_dadeir_AC201957_7187	2	14	-2.3	4.77E-02
RLX_ovev_AC199878_6641	7	26	-1.4	4.77E-02
RLC_tiwe_AC187032_1709	17	45	-0.9	4.82E-02
RLC_dolovu_AC211740_11400	3	17	-2.0	4.84E-02

[a] The number of reads mapped to each gene model from mutant and non-mutant RNA-seq
[b] $\log_2$ transformation of foldchange as the relative abandunce of transcripts in mutants/non-mutants
[c] The fasle discovery rate calculated using Benjamini and Hochberg's procedure for the p value from Fisher's exact test
