# Supplementary material for: Loss of RNA–Dependent RNA Polymerase 2 (RDR2) Function Causes Widespread and Unexpected Changes in the Expression of Transposons, Genes, and 24-nt Small RNAs
Source: PLoS Genet. 2009 Nov 20;5(11):e1000737. doi: 10.1371/journal.pgen.1000737 (PMC2774947; doi:10.1371/journal.pgen.1000737)
Supplement: Table S4 — List of differentially expressed chromatin-associated genes. (0.66 MB DOC) [file pgen.1000737.s009.doc]

**Table S4.** List of differentially expressed chromatin-associated genes

| **Chromdb.ID** | No. Illumina/Solexa ^a^ | | **log2(FC) ^b^** | **BH.FDR ^c^** | **Protein.group** | **Protein.description** |
| --- | --- | --- | --- | --- | --- | --- |
|  | **Mutant** | **Non-mutant** |  |  |  |  |
| AGO101 | 81 | 201 | -1.32 | 7.25E-13 | Pinhead_Group | Argonaute superfamily member; Pinhead subfamily group; predicted homolog of Arabidopsis Pinhead (AGO10) |
| AGO104 | 707 | 1,102 | -0.65 | 8.36E-21 | Plant AGO4/6/9 class | A PIWI/PAZ domain containing member of the Argonuate gene family. |
| AGO105 | 77 | 220 | -1.53 | 5.36E-17 | Plant AGO4/6/9 class | A PIWI/PAZ domain containing member of the Argonuate gene family. |
| AGO106 | 3 | 11 | -1.89 | 4.37E-02 | AGO1 Group (Dicots and Monocots) | A PIWI/PAZ domain containing member of the Argonuate gene family. |
| AGO108 | 111 | 201 | -0.87 | 4.28E-07 | ARATH_AGO5 Group | A PIWI/PAZ domain containing member of the Argonuate gene family. |
| AGO110 | 147 | 312 | -1.10 | 1.32E-14 | AGO1 Group (Dicots and Monocots) | A PIWI/PAZ domain containing member of the Argonuate gene family. |
| AGO112 | 47 | 74 | -0.67 | 1.85E-02 | AGO2_AGO3 Group | A PIWI/PAZ domain containing member of the Argonuate gene family. |
| AGO113 | 465 | 545 | -0.24 | 1.22E-02 | AGO1 Group (Dicots and Monocots) | A PIWI/PAZ domain containing member of the Argonuate gene family. |
| AGO117 | 1,775 | 1,389 | 0.34 | 6.95E-11 | AGO1 Group (Dicots and Monocots) | A PIWI/PAZ domain containing member of the Argonuate gene family. |
| AGO119 | 419 | 558 | -0.42 | 9.24E-06 | Plant AGO4/6/9 class | A PIWI/PAZ domain containing member of the Argonuate gene family. |
| AGO120 | 262 | 102 | 1.35 | 1.23E-16 | Zippy (AGO7) Group | A PIWI/PAZ domain containing member of the Argonuate gene family. |
| AGO121 | 101 | 208 | -1.05 | 1.27E-09 | Plant AGO4/6/9 class | A PIWI/PAZ domain containing member of the Argonuate gene family. |
| ARID101 | 90 | 205 | -1.20 | 2.08E-11 | ARID/BRIGHT DNA binding domain group | NA |
| ARID102 | 18 | 59 | -1.72 | 3.39E-06 | ARID/BRIGHT DNA binding domain group | NA |
| ARID103 | 90 | 199 | -1.16 | 1.46E-10 | ARID/BRIGHT DNA binding domain group | NA |
| ARID104 | 181 | 97 | 0.89 | 1.39E-06 | ARID/BRIGHT DNA binding domain group | NA |
| ARID105 | 57 | 148 | -1.39 | 2.36E-10 | ARID/BRIGHT DNA binding domain group | NA |
| ARP101 | 1,569 | 737 | 1.08 | 2.01E-66 | Actin superfamily; actin-related proteins | Nuclear actin-related protein involved in chromatin remodeling, component of chromatin-remodeling enzyme complexes; putative homolog of Saccharomyces cerevisiae ARP4. |
| ARP102 | 161 | 224 | -0.49 | 1.62E-03 | Actin superfamily; actin-related proteins | Nuclear actin-related protein involved in chromatin remodeling; homolog of Arabidopsis thaliana ARP7 |
| ARP103 | 46 | 124 | -1.44 | 2.53E-09 | Actin superfamily; actin-related proteins | Nuclear actin-related protein involved in chromatin remodeling, component of chromatin-remodeling enzyme complexes; putative homolog of Arabidopsis thaliana ARP8 |
| ARP104 | 41 | 85 | -1.06 | 1.25E-04 | Actin superfamily; actin-related proteins | Nuclear actin-related protein involved in chromatin remodeling, component of chromatin-remodeling enzyme complexes; putative homolog of Arabidopsis thaliana ARP5 |
| ARP106 | 123 | 298 | -1.29 | 1.05E-17 | Actin superfamily; actin-related proteins | Nuclear actin-related protein involved in chromatin remodeling, component of chromatin-remodeling enzyme complexes; putative homolog of Arabidopsis thaliana ARP9 and Saccharomyces cerevisiae ARP8 |
| ARP107 | 92 | 243 | -1.41 | 9.64E-17 | Actin superfamily; actin-related proteins | NA |
| BRD101 | 48 | 73 | -0.62 | 2.94E-02 | Diverse bromodomain-containing proteins (polybromo1, yeast RSC, and human BRD homologs) | Single bromodomain-containing protein similar to Homo sapiens BRD7; bromodomains are found in many chromatin associated proteins and can interact specifically with acetylated lysine |
| BRD102 | 139 | 81 | 0.77 | 1.84E-04 | Diverse bromodomain-containing proteins (polybromo1, yeast RSC, and human BRD homologs) | Single bromodomain-containing protein similar to Homo sapiens BRD7; bromodomains are found in many chromatin associated proteins and can interact specifically with acetylated lysine |
| BRD104 | 10 | 44 | -2.15 | 3.49E-06 | Diverse bromodomain-containing proteins (polybromo1, yeast RSC, and human BRD homologs) | Single bromodomain-containing protein similar to Homo sapiens BRD7; bromodomains are found in many chromatin associated proteins and can interact specifically with acetylated lysine |
| BRD105 | 47 | 116 | -1.31 | 8.43E-08 | Diverse bromodomain-containing proteins (polybromo1, yeast RSC, and human BRD homologs) | NA |
| CHB101 | 54 | 84 | -0.65 | 1.42E-02 | ARATH SWI3D Group | Putative homolog of the Saccharomyces cerevisiae SWI3 and RSC8 proteins. Yeast SWI3 and RSC8 are subunits of the SWI/SNF and RSC8 chromatin remodeling complexes which regulate transcription by remodeling chromatin. |
| CHC101 | 228 | 430 | -0.93 | 3.15E-15 | SMARD/SWP73/RSC6 group | SWIB domain-containing protein; sequence similarity indicates the protein is a likely homolog of Homo sapiens SMARD proteins and Saccharomyces cerevisiae SWP73 and RSC6 proteins; potential SWI/SNF complex component |
| CHC102 | 228 | 430 | -0.93 | 3.15E-15 | SMARD/SWP73/RSC6 group | SWIB domain-containing protein; sequence similarity indicates the protein is a likely homolog of Homo sapiens SMARD proteins and Saccharomyces cerevisiae SWP73 and RSC6 proteins; potential SWI/SNF complex component |
| CHE101 | 115 | 161 | -0.50 | 6.53E-03 | SWI/SNF chromatin remodeling complex proteins (Snf5 homologs) | Chromatin remodeling complex subunit E; probable ortholog of yeast SNF5, a component of the yeast SWI/SNF chromatin remodeling complex |
| CHR101 | 187 | 273 | -0.56 | 7.87E-05 | SNF2 super family (Snf2, Ris1, Rad26 superclasses) | Putative ortholog of Arabidopsis thalian DDM1, a SWI2/SNF2 chromatin remodeling protein involved in the maintenance of DNA methylation |
| CHR102 | 126 | 201 | -0.69 | 4.18E-05 | SNF2 super family (Snf2, Ris1, Rad26 superclasses) | SNF2 Superfamily; RIS1 Superclass; SMARCA3 class |
| CHR104 | 69 | 128 | -0.90 | 3.94E-05 | SNF2 super family (Snf2, Ris1, Rad26 superclasses) | SNF2 Superfamily; RIS1 Superclass; RAD16 class |
| CHR106 | 2,211 | 1,131 | 0.96 | 1.13E-76 | SNF2 super family (Snf2, Ris1, Rad26 superclasses) | Putative ortholog of Arabidopsis thalian DDM1, a SWI2/SNF2 chromatin remodeling protein involved in the maintenance of DNA methylation |
| CHR110 | 253 | 541 | -1.11 | 9.57E-25 | SNF2 super family (Snf2, Ris1, Rad26 superclasses) | SNF2 Superfamily; SNF2 Superclass; ISWI class |
| CHR112 | 292 | 771 | -1.41 | 2.45E-50 | SNF2 super family (Snf2, Ris1, Rad26 superclasses) | SNF2 Superfamily; RIS1 Superclass; RAD5 class |
| CHR113 | 637 | 525 | 0.27 | 2.44E-03 | SNF2 super family (Snf2, Ris1, Rad26 superclasses) | Similar to Arabidopsis thaliana PKL, a SWI/SWF nuclear-localized chromatin remodeling factor of the CHD (chomodomain helicase domain) group; involved in post-germination repression of embryonic development |
| CHR115 | 32 | 86 | -1.44 | 8.42E-07 | SNF2 super family (Snf2, Ris1, Rad26 superclasses) | SNF2 family DNA-dependent ATPase similar to transcription-coupled repair nucleotide excision repair proteins like Saccharomyces cerevisiae RAD26 |
| CHR118 | 98 | 205 | -1.08 | 8.60E-10 | SNF2 super family (Snf2, Ris1, Rad26 superclasses) | SNF2 superfamily chromatin remodeling protein; putative homolog of Saccharomyces cerevisiae MOT1, an essential abundant protein involved in regulation of transcription |
| CHR119 | 128 | 315 | -1.31 | 4.23E-19 | SNF2 super family (Snf2, Ris1, Rad26 superclasses) | Similar to Arabidopsis thaliana PKL, a SWI/SWF nuclear-localized chromatin remodeling factor of the CHD (chomodomain helicase domain) group; involved in post-germination repression of embryonic development |
| CHR120 | 226 | 421 | -0.91 | 1.66E-14 | SNF2 super family (Snf2, Ris1, Rad26 superclasses) | Chromatin remodeling complex subunit R; SWI2/SNF2 superfamily |
| CHR122 | 54 | 222 | -2.05 | 5.32E-25 | SNF2 super family (Snf2, Ris1, Rad26 superclasses) | SWI/SNF class chromatin remodeling complex protein involved in transcriptional regulation; putative homolog of Saccharomyces cerevisiae SNF2 |
| CHR125 | 28 | 57 | -1.04 | 2.28E-03 | SNF2 super family (Snf2, Ris1, Rad26 superclasses) | SNF2 superfamily chromatin remodeling protein; putative homolog of Saccharomyces cerevisiae INO80, an ATPase that forms a large complex, containing actin and several actin-related proteins, that has chromatin remodeling activity |
| CHR127 | 64 | 168 | -1.40 | 9.48E-12 | SNF2 super family (Snf2, Ris1, Rad26 superclasses) | SNF2 Superfamily; RAD26 Superclass; DRD1 class |
| CHR131 | 79 | 294 | -1.91 | 5.24E-30 | SNF2 super family (Snf2, Ris1, Rad26 superclasses) | SWI/SNF class chromatin remodeling complex protein involved in transcriptional regulation; putative homolog of Saccharomyces cerevisiae SNF2 |
| CHR132 | 176 | 88 | 0.99 | 1.84E-07 | SNF2 super family (Snf2, Ris1, Rad26 superclasses) | SNF2 superfamily chromatin remodeling protein; putative homolog of Saccharomyces cerevisiae INO80, an ATPase that forms a large complex, containing actin and several actin-related proteins, that has chromatin remodeling activity |
| CHR135 | 86 | 171 | -1.00 | 1.76E-07 | SNF2 super family (Snf2, Ris1, Rad26 superclasses) | Putative ortholog of Arabidopsis thaliana PIE1 (PHOTOPERIOD-INDEPENDENT EARLY FLOWERING 1); Swi2/Snf2-related ATPase, component of the SWR1 complex; homolog of Saccharomyces cerevisiae SWR1, a protein required for the incorporation of Htz1p into chromatin |
| CHR136 | 113 | 265 | -1.24 | 4.30E-15 | SNF2 super family (Snf2, Ris1, Rad26 superclasses) | SNF2 Superfamily; RIS1 class |
| CHR139 | 156 | 264 | -0.77 | 1.78E-07 | SNF2 super family (Snf2, Ris1, Rad26 superclasses) | SNF2 family DNA-dependent ATPase similar to transcription-coupled repair nucleotide excision repair proteins like Saccharomyces cerevisiae RAD26 |
| CHR140 | 72 | 107 | -0.58 | 1.21E-02 | SNF2 super family (Snf2, Ris1, Rad26 superclasses) | SNF2 Superfamily; RAD26 Superclass; HARP class |
| CHR143 | 78 | 213 | -1.46 | 1.48E-15 | SNF2 super family (Snf2, Ris1, Rad26 superclasses) | SWI/SNF class chromatin remodeling complex protein involved in transcriptional regulation; putative homolog of Saccharomyces cerevisiae SNF2 |
| CHR153 | 72 | 151 | -1.08 | 1.79E-07 | SNF2 super family (Snf2, Ris1, Rad26 superclasses) | Simlar to Homo Sapiens CHDL (chromodomain helicase DNA binding protein 1-like), a subgroup within the SNF2 superfamily of ATP dependent helicases |
| CHR154 | 7 | 25 | -1.85 | 1.89E-03 | SNF2 super family (Snf2, Ris1, Rad26 superclasses) | SNF2 superfamily chromatin remodeling protein; putative homolog of Saccharomyces cerevisiae FUN30, a SWI/SNF2 family protein, over expression of the protein affects chromosome stability |
| CHR155 | 10 | 26 | -1.39 | 1.03E-02 | SNF2 super family (Snf2, Ris1, Rad26 superclasses) | Chromatin remodeling complex subunit R; probable SWI2/SNF2-like protein based on similarity to CHR proteins in rice and Arabidopsis, but not enough sequence information exists to firmly establish subclass |
| CHR156 | 28 | 55 | -0.99 | 4.14E-03 | SNF2 super family (Snf2, Ris1, Rad26 superclasses) | SNF2 Superfamily; RAD26 Superclass; DRD1 class |
| CHR159 | 49 | 122 | -1.33 | 3.05E-08 | SNF2 super family (Snf2, Ris1, Rad26 superclasses) | SNF2 Superfamily; RIS1 Superclass; CHR36/39 class |
| CHR160 | 32 | 136 | -2.10 | 2.92E-16 | SNF2 super family (Snf2, Ris1, Rad26 superclasses) | SNF2 Superfamily; RAD26 Superclass; HARP class |
| CPC102 | 220 | 479 | -1.13 | 6.92E-23 | Condensin complex component; SMC protein group | A member of the Structural Maintenance of Chromosome (SMC) superfamily and a member of the condensin sub-family; homolog of Saccharomyces cerevisiae SMC2. Condensins regulate chromsome condensation during cell division. |
| CPC103 | 1,783 | 833 | 1.09 | 2.58E-76 | Condensin complex component; SMC protein group | NA |
| CPD101 | 112 | 352 | -1.66 | 1.08E-29 | Condensin complex component, non-SMC subunit (Cnd1 homologs) | Condensin complex components subunit D; one of the non-SMC subunits, known as Cnd1 in Schizosaccharomyces pombe, and XCAP-D2 in Xenopus laevis |
| CPG101 | 183 | 265 | -0.55 | 1.36E-04 | Condensin complex component, non-SMC subunit | Condensin complex components subunit G; one of the non-SMC subunits |
| CPH101 | 159 | 251 | -0.67 | 7.06E-06 | Condensin complex component; Barren domain-containing, non-SMC subunit | Condensin complex components subunit H; one of the non-SMC subunits, similar to the Drosophila Barren (barr) gene which is required for sister-chromatid segregation in mitosis |
| CRD101 | 115 | 236 | -1.05 | 1.19E-10 | Diverse group of chromodomain-containing proteins | Chromodomain and a chromo shadow domain containing protein |
| DCL101 | 573 | 369 | 0.62 | 1.45E-10 | ARATH_DCL1 Group | Dicer-Like; Dicer-like; similar to the Drosophila melanogaster gene Dicer, a multidomain ribonuclease essential for RNA silencing |
| DCL102 | 111 | 47 | 1.23 | 7.62E-07 | ARATH_DCL3 Group | Dicer-like; similar to the Drosophila melanogaster gene Dicer, a multidomain ribonuclease essential for RNA silencing. |
| DCL103 | 47 | 106 | -1.18 | 2.62E-06 | ARATH_DCL4 Group | Dicer-Like; Dicer-like; similar to the Drosophila melanogaster gene Dicer, a multidomain ribonuclease essential for RNA silencing |
| DCL104 | 109 | 149 | -0.46 | 1.44E-02 | ARATH_DCL3 Group | Dicer-Like; similar to the Drosophila melanogaster gene Dicer, a multidomain ribonuclease essential for RNA silencing |
| DEK101 | 418 | 1,058 | -1.35 | 1.85E-64 | DEK_C domain proteins | Predicted homolog of the human DEK protein, an abundant chromatin protein that is linked with cancers and autoimmune disease. |
| DEK102 | 182 | 312 | -0.79 | 6.62E-09 | DEK_C domain proteins | Predicted homolog of the human DEK protein, an abundant chromatin protein that is linked with cancers and autoimmune disease. |
| DEK103 | 1,242 | 782 | 0.66 | 2.34E-23 | DEK_C domain proteins | Predicted homolog of the human DEK protein, an abundant chromatin protein that is linked with cancers and autoimmune disease. |
| DMT101 | 106 | 235 | -1.16 | 2.79E-12 | DNA methyltransferases | Class I DNA methyltransferase-A putative DNA methyltransferase related to the mammalian DNMT1 methyltransferases |
| DMT102 | 619 | 1,186 | -0.95 | 2.23E-41 | DNA methyltransferases | Class II DNA methyltransferase; a DNA methyltransferase containing a chromodomain (chromomethylase) |
| DMT104 | 78 | 184 | -1.25 | 7.64E-11 | DNA methyltransferases | Class IV DNA methyltransferase- A putative DNA methyltransferase similar to the mammalian DNMT2 methyltransferases |
| DMT105 | 828 | 1,279 | -0.64 | 3.80E-23 | DNA methyltransferases | Class II DNA methyltransferase; a DNA methyltransferase containing a chromodomain (chromomethylase) |
| DMT106 | 253 | 446 | -0.83 | 3.06E-13 | DNA methyltransferases | Class III DNA methyltransferase; a putative DNA methyltransferase with rearranged catalytic domains; similar to mammalian DNMT3 methyltransferases |
| DMT107 | 294 | 126 | 1.21 | 6.95E-16 | DNA methyltransferases | Putative DNA methyltransferase |
| DNG101 | 109 | 238 | -1.14 | 4.38E-12 | Superfamily of DNA glycosylases | DEMETER (DME) family of bifunctional DNA glycocylases; DEMETER-LIKE (DML) protein |
| DNG103 | 97 | 304 | -1.66 | 9.07E-26 | Superfamily of DNA glycosylases | DEMETER (DME) family of bifunctional DNA glycocylases; DEMETER-LIKE (DML) protein |
| DRB101 | 85 | 118 | -0.48 | 2.65E-02 | Double stranded RNA-Binding protein group | NA |
| DRB103 | 145 | 276 | -0.94 | 2.05E-10 | Double stranded RNA-Binding protein group | NA |
| DRB104 | 152 | 233 | -0.63 | 4.62E-05 | Double stranded RNA-Binding protein group | NA |
| DRB105 | 37 | 57 | -0.63 | 4.92E-02 | Double stranded RNA-Binding protein group | NA |
| EBP101 | 99 | 259 | -1.40 | 1.64E-17 | BAH-PHD domain-containing protein group | NA |
| EBP103 | 92 | 249 | -1.45 | 1.02E-17 | BAH-PHD domain-containing protein group | NA |
| EBP104 | 31 | 81 | -1.40 | 3.01E-06 | BAH-PHD domain-containing protein group | NA |
| EPL101 | 86 | 244 | -1.52 | 1.57E-18 | Enhancer of Polycomb-like protein group [E(Pc) homologs] | Enhancer of Polycomb-like protein, related to the Drosophila PcG protein Enhancer of PolycombEnhancer of Polycomb-like protein, related to the Drosophila PcG protein Enhancer of Polycomb |
| FGP101 | 57 | 33 | 0.78 | 1.98E-02 | Flowering control-associated proteins; FRIGIDA group | NA |
| FGP102 | 57 | 21 | 1.43 | 9.31E-05 | Flowering control-associated proteins; FRIGIDA group | NA |
| FGP103 | 481 | 279 | 0.77 | 1.04E-12 | Flowering control-associated proteins; FRIGIDA group | NA |
| FLCP102 | 26 | 44 | -0.77 | 3.98E-02 | Flowering control-associated proteins | FLC homologs |
| FLCP103 | 45 | 191 | -2.10 | 2.76E-22 | Flowering control-associated proteins | FLC homologs |
| FLCP104 | 36 | 76 | -1.09 | 2.19E-04 | Flowering control-associated proteins | FLC homologs |
| FLCP105 | 874 | 372 | 1.22 | 2.14E-45 | Flowering control-associated proteins | FLC homologs |
| FLCP106 | 247 | 488 | -0.99 | 5.12E-19 | Flowering control-associated proteins | FLC homologs |
| FLCP112 | 105 | 156 | -0.58 | 1.89E-03 | Flowering control-associated proteins | NA |
| FLCP113 | 210 | 405 | -0.96 | 3.29E-15 | Flowering control-associated proteins | NA |
| FLCP115 | 46 | 26 | 0.81 | 3.15E-02 | Flowering control-associated proteins | FLC homologs |
| FLCP118 | 13,467 | 5,347 | 1.32 | 0.00E+01 | Flowering control-associated proteins | FLC homologs |
| FLCP127 | 19 | 71 | -1.91 | 3.70E-08 | Flowering control-associated proteins | FLC homologs |
| FLCP131 | 346 | 136 | 1.34 | 2.56E-21 | Flowering control-associated proteins | NA |
| GTA107 | 137 | 245 | -0.85 | 4.07E-08 | Transcription elongation-nucleosome displacement proteins (Spt5 homologs) | Putatiave homolog of Saccharomyces cerevisiae SPT5. The yeast protein forms a complex with Spt4p and mediates both activation and inhibition of transcription elongation, and plays a role in pre-mRNA processing. Signature Pfam motifs are two tandem Supt5 domains with unknown function and several KOW (Kyprides, Ouzounis, Woese) motifs that are found in a variety of ribosomal proteins and in some proteins involved in transcriptional and translational regulation. |
| GTB101 | 489 | 894 | -0.88 | 5.26E-28 | Transcription elongation-nucleosome displacement proteins (Spt6 homologs) | Putative homolog Saccharomyces cerevisiae Spt6 protein, which functions as part of a protein complex in transcription initiation and also plays a role in chromatin structure/assembly. |
| GTC102 | 466 | 758 | -0.71 | 5.79E-17 | Global transcription factor group C (Spt16 homologs) | Putative homolog Saccharomyces cerevisiae Spt16 protein, a subunit of the heterodimeric FACT complex (Spt16p-Pob3p), facilitates RNA Polymerase II transcription elongation through nucleosomes by destabilizing and then reassembling nucleosome structure |
| GTE102 | 120 | 204 | -0.78 | 3.76E-06 | Global transcription factor group E (Bdf1, BRD4 and BRD2 (RING3) homologs) | Global transcription factor group E (BDF1-FSH-RING3 homologs) |
| GTE103 | 593 | 911 | -0.63 | 1.82E-16 | Global transcription factor group E (Bdf1, BRD4 and BRD2 (RING3) homologs) | Global transcription factor group E (BDF1-FSH-RING3 homologs) |
| GTE108 | 106 | 181 | -0.78 | 1.15E-05 | Global transcription factor group E (Bdf1, BRD4 and BRD2 (RING3) homologs) | Global transcription factor group E (BDF1-FSH-RING3 homologs) |
| GTE110 | 74 | 125 | -0.77 | 4.38E-04 | Global transcription factor group E (Bdf1, BRD4 and BRD2 (RING3) homologs) | Global transcription factor group E (BDF1-FSH-RING3 homologs) |
| GTE113 | 5 | 53 | -3.42 | 4.13E-11 | Global transcription factor group E (Bdf1, BRD4 and BRD2 (RING3) homologs) | Global transcription factor group E (BDF1-FSH-RING3 homologs) |
| GTE114 | 61 | 146 | -1.27 | 4.53E-09 | Global transcription factor group E (Bdf1, BRD4 and BRD2 (RING3) homologs) | NA |
| GTE115 | 83 | 143 | -0.80 | 9.82E-05 | Global transcription factor group E (Bdf1, BRD4 and BRD2 (RING3) homologs) | NA |
| GTI101 | 49 | 89 | -0.87 | 9.44E-04 | Global transcription factor group I (Spt2 homologs) | Putative homolog Saccharomyces cerevisiae Spt2, a protein involved in negative regulation of transcription, exhibits regulated interactions with both histones and SWI-SNF component |
| HAF101 | 177 | 370 | -1.08 | 1.18E-16 | Histone acetyltransferases (TafI homologs) | Member of the histone acetyltransferase (TafII-250 family homology group). The 250 kD subunit of the TFIID transcription initiation factor complex has histone acetyl transferase activity. |
| HAG102 | 134 | 509 | -1.94 | 3.25E-52 | Histone acetyltransferases (GNAT superfamily) | Histone acetyltransferases (GNAT/MYST superfamily); probable ortholog of yeast HAT1 |
| HAG103 | 85 | 161 | -0.93 | 1.98E-06 | Histone acetyltransferases (GNAT superfamily) | Histone acetyltransferases (GNAT/MYST superfamily) |
| HAG107 | 154 | 248 | -0.70 | 3.54E-06 | Histone acetyltransferases (GNAT superfamily) | Histone acetyltransferases (GNAT/MYST superfamily); probable ortholog of ELP3 |
| HAM101 | 26 | 78 | -1.60 | 3.92E-07 | Histone acetyltransferases (MYST family) | Histone acetyltransferase in the MYST family; probable ortholog of yeast ESA1 |
| HAM102 | 13 | 51 | -1.98 | 2.04E-06 | Histone acetyltransferases (MYST family) | Histone acetyltransferases (GNAT/MYST superfamily) |
| HCP101 | 536 | 409 | 0.38 | 1.00E-04 | Sin3 complex components (SAP18 homologs) | Histone deacetylase protein; possible SAP18 homolog; Sin3 complex component |
| HCP102 | 113 | 238 | -1.09 | 2.71E-11 | Sin3 complex components (SAP18 homologs) | Histone deacetylase protein; possible SAP18 homolog; Sin3 complex component |
| HDA101 | 143 | 287 | -1.02 | 4.36E-12 | Histone deacetylases (Rpd3/HDA1 superfamily) | Class I RPD3 type histone deacetylase protein |
| HDA102 | 142 | 209 | -0.57 | 4.31E-04 | Histone deacetylases (Rpd3/HDA1 superfamily) | Class I RPD3 type histone deacetylase protein |
| HDA108 | 335 | 522 | -0.65 | 1.68E-10 | Histone deacetylases (Rpd3/HDA1 superfamily) | Class I RPD3 type histone deacetylase protein |
| HDA109 | 179 | 238 | -0.42 | 4.63E-03 | Histone deacetylases (Rpd3/HDA1 superfamily) | Class II RPD3 type histone deacetylase protein |
| HDA110 | 212 | 266 | -0.34 | 1.58E-02 | Histone deacetylases (Rpd3/HDA1 superfamily) | Class II RPD3 type histone deacetylase protein |
| HDA117 | 72 | 126 | -0.82 | 1.82E-04 | Histone deacetylases (Rpd3/HDA1 superfamily) | Unclassified RPD3 type histone deacetylase protein with similarity to acetylpolyamine aminohydrolase (aphA) proteins of Archae. |
| HDMA101 | 29 | 95 | -1.72 | 2.85E-09 | Histone demethylases (AOF2/ LSD1 homologs) | Predictecd homolog of Arabidopsis LDL1; a predicted homolog of human AOF2/LDL2; LDL1 acts in partial redundancy with FLD (FLOWERING LOCUS D), an additional AOF2/LSD1 homolog, to repress FLC (FLOWERING LOCUS C) expression in Arabidopsis. |
| HDMA102 | 31 | 63 | -1.03 | 1.35E-03 | Histone demethylases (AOF2/ LSD1 homologs) | Predicted homolog of the Arabidopsis thaliana FLD (Flowering Locus D). FLD mutants dmeonstrate hyperacetylation of histones in FLC chromatin, up-regulation of FLC expression and extremely delayed flowering. |
| HDMA104 | 39 | 21 | 0.88 | 3.50E-02 | Histone demethylases (AOF2/ LSD1 homologs) | Predicted homolog of human AOF2/LSD1 histone demethylase; Arabidopsis LDL3 family. |
| HDT101 | 64 | 120 | -0.92 | 5.36E-05 | Histone deacetylases (plant-specific HD2 family) | Plant specific HD2 type histone deacetylase |
| HDT102 | 990 | 1,762 | -0.84 | 3.41E-50 | Histone deacetylases (plant-specific HD2 family) | Plant specific HD2 type histone deacetylase |
| HDT103 | 2,433 | 1,643 | 0.56 | 1.85E-33 | Histone deacetylases (plant-specific HD2 family) | Plant specific HD2 type histone deacetylase |
| HDT104 | 442 | 953 | -1.12 | 1.37E-43 | Histone deacetylases (plant-specific HD2 family) | Plant specific HD2 type histone deacetylase |
| HEN101 | 25 | 74 | -1.58 | 1.00E-06 | HUA Enhancer | Probable ortholog of the Arabidopsis protein HUA Enhancer |
| HFO101 | 4,389 | 2,455 | 0.83 | 2.25E-119 | Histone H4 | Histone H4, core histone required for chromatin assembly and chromosome function |
| HFO102 | 5,701 | 1,161 | 2.28 | 0.00E+01 | Histone H4 | Histone H4, core histone required for chromatin assembly and chromosome function |
| HFO103 | 5,365 | 4,069 | 0.39 | 2.54E-38 | Histone H4 | Histone H4, core histone required for chromatin assembly and chromosome function |
| HFO104 | 4,441 | 4,022 | 0.13 | 4.18E-05 | Histone H4 | Histone H4, core histone required for chromatin assembly and chromosome function |
| HFO105 | 2,622 | 1,592 | 0.71 | 7.94E-55 | Histone H4 | Histone H4, core histone required for chromatin assembly and chromosome function |
| HFO106 | 1,624 | 1,880 | -0.22 | 8.99E-06 | Histone H4 | Histone H4, core histone required for chromatin assembly and chromosome function |
| HFO107 | 2,168 | 2,371 | -0.14 | 1.57E-03 | Histone H4 | Histone H4, core histone required for chromatin assembly and chromosome function |
| HFO109 | 10,896 | 12,945 | -0.26 | 7.55E-45 | Histone H4 | Histone H4, core histone required for chromatin assembly and chromosome function |
| HFO112 | 2,459 | 4,941 | -1.02 | 4.53E-192 | Histone H4 | Histone H4, core histone required for chromatin assembly and chromosome function |
| HFO113 | 2,827 | 547 | 2.36 | 0.00E+01 | Histone H4 | Histone H4, core histone required for chromatin assembly and chromosome function |
| HFO115 | 514 | 927 | -0.86 | 6.16E-28 | Histone H4 | Histone H4, core histone required for chromatin assembly and chromosome function |
| HFO116 | 597 | 117 | 2.34 | 1.38E-76 | Histone H4 | Histone H4, core histone required for chromatin assembly and chromosome function |
| HFO117 | 2,117 | 855 | 1.30 | 1.62E-119 | Histone H4 | Histone H4, core histone required for chromatin assembly and chromosome function |
| HFO118 | 6,055 | 4,632 | 0.38 | 1.04E-40 | Histone H4 | Histone H4, core histone required for chromatin assembly and chromosome function |
| HIRA101 | 106 | 157 | -0.58 | 1.97E-03 | HIRA protein group | HIRA proteins are histone-interacting proteins which function in the nucleosome assembly pathway to facilitate the deposition of histones onto the DNA |
| HMGA102 | 8,422 | 4,528 | 0.88 | 1.94E-258 | High Mobility Group Family A | HMGA protein containing a linker histone and multiple AT hook domains. |
| HMGA103 | 1,254 | 1,746 | -0.49 | 1.08E-19 | High Mobility Group Family A | HMGA protein containing a linker histone domain and multiple AT hook domains. |
| HMGB101 | 3,049 | 4,494 | -0.57 | 8.33E-65 | High Mobility Group Family B | Single HMG domain chromatin protein |
| HMGB102 | 253 | 499 | -0.99 | 2.53E-19 | High Mobility Group Family B | Single HMG domain chromatin protein |
| HMGB103 | 5,297 | 6,541 | -0.32 | 2.11E-32 | High Mobility Group Family B | Single HMG domain chromatin protein |
| HMGB104 | 768 | 1,330 | -0.80 | 2.91E-35 | High Mobility Group Family B | nfd104 |
| HMGB105 | 937 | 2,329 | -1.32 | 3.92E-137 | High Mobility Group Family B | Single HMG domain chromatin protein |
| HMGB106 | 929 | 1,509 | -0.71 | 1.83E-32 | High Mobility Group Family B | Single HMG domain chromatin protein |
| HMGB108 | 274 | 598 | -1.14 | 1.83E-28 | High Mobility Group Family B | Triple HMG domain chromatin protein |
| HMTA101 | 64 | 103 | -0.70 | 3.52E-03 | Set1 complex component (Ash2/Bre2 homologs) | NA |
| HON101 | 2,955 | 3,153 | -0.10 | 6.05E-03 | Histone H1 linker protein | Linker histone family protein: Histones in the linker histone family (H1 and H5) contain globular winged helix domains; they interact with the linker DNA between nucleosomes, as well as having other proposed nucleosome functions relating to chromatin condensation and transcriptional repression. Linker histone proteins are less conserved than the core histone proteins and can be regulated developmentally in some organisms, e.g. H5. |
| HON102 | 3,094 | 4,701 | -0.61 | 3.15E-77 | Histone H1 linker protein | Linker histone family protein: Histones in the linker histone family (H1 and H5) contain globular winged helix domains; they interact with the linker DNA between nucleosomes, as well as having other proposed nucleosome functions relating to chromatin condensation and transcriptional repression. Linker histone proteins are less conserved than the core histone proteins and can be regulated developmentally in some organisms, e.g. H5. |
| HON103 | 55 | 30 | 0.86 | 1.22E-02 | Histone H1 linker protein | Linker histone family protein: Histones in the linker histone family (H1 and H5) contain globular winged helix domains; they interact with the linker DNA between nucleosomes, as well as having other proposed nucleosome functions relating to chromatin condensation and transcriptional repression. Linker histone proteins are less conserved than the core histone proteins and can be regulated developmentally in some organisms, e.g. H5. |
| HON106 | 1,822 | 2,166 | -0.26 | 2.43E-08 | Histone H1 linker protein | Linker histone family protein: Histones in the linker histone family (H1 and H5) contain globular winged helix domains; they interact with the linker DNA between nucleosomes, as well as having other proposed nucleosome functions relating to chromatin condensation and transcriptional repression. Linker histone proteins are less conserved than the core histone proteins and can be regulated developmentally in some organisms, e.g. H5. |
| HON110 | 4,361 | 1,817 | 1.25 | 1.98E-233 | Histone H1 linker protein | Linker histone family protein: Histones in the linker histone family (H1 and H5) contain globular winged helix domains; they interact with the linker DNA between nucleosomes, as well as having other proposed nucleosome functions relating to chromatin condensation and transcriptional repression. Linker histone proteins are less conserved than the core histone proteins and can be regulated developmentally in some organisms, e.g. H5. |
| HTA101 | 759 | 1,164 | -0.63 | 1.42E-20 | Histone Variants | Histone 2A family protein; histone variant H2AZ group |
| HTA103 | 6,715 | 5,433 | 0.29 | 4.92E-29 | Core | Histone H2A, core histone required for chromatin assembly and chromosome function |
| HTA104 | 10,878 | 17,382 | -0.69 | 0.00E+01 | Core | Histone H2A, core histone required for chromatin assembly and chromosome function |
| HTA105 | 7,829 | 9,328 | -0.26 | 3.16E-33 | Core | Histone H2A, core histone required for chromatin assembly and chromosome function |
| HTA108 | 3,112 | 5,538 | -0.84 | 2.59E-157 | H2A variant | Histone H2A, core histone required for chromatin assembly and chromosome function |
| HTA109 | 2,644 | 1,058 | 1.31 | 1.39E-151 | Histone Variants | Histone 2A family protein; histone variant H2AZ group |
| HTA110 | 1,066 | 1,967 | -0.90 | 7.99E-62 | Core | Histone H2A, core histone required for chromatin assembly and chromosome function |
| HTA111 | 1,428 | 1,868 | -0.40 | 7.57E-15 | Core | Histone H2A, core histone required for chromatin assembly and chromosome function |
| HTA112 | 2,267 | 5,670 | -1.33 | 0.00E+01 | Core | Histone H2A, core histone required for chromatin assembly and chromosome function |
| HTA115 | 397 | 834 | -1.08 | 3.05E-36 | Core | Histone H2A, core histone required for chromatin assembly and chromosome function |
| HTB101 | 959 | 1,256 | -0.40 | 1.86E-10 | Histone H2B | Histone H2B, core histone required for chromatin assembly and chromosome function |
| HTB102 | 2,365 | 2,766 | -0.24 | 7.55E-09 | Histone H2B | Histone H2B, core histone required for chromatin assembly and chromosome function |
| HTB103 | 1,492 | 791 | 0.90 | 1.77E-47 | Histone H2B | Histone H2B, core histone required for chromatin assembly and chromosome function |
| HTB105 | 4,341 | 1,433 | 1.59 | 0.00E+01 | Histone H2B | Histone H2B, core histone required for chromatin assembly and chromosome function |
| HTB108 | 3,355 | 2,331 | 0.51 | 6.06E-40 | Histone H2B | Histone H2B, core histone required for chromatin assembly and chromosome function |
| HTB109 | 3,801 | 1,087 | 1.79 | 0.00E+01 | Histone H2B | Histone H2B, core histone required for chromatin assembly and chromosome function |
| HTB113 | 550 | 362 | 0.59 | 2.16E-09 | Histone H2B | Histone H2B, core histone required for chromatin assembly and chromosome function |
| HTB114 | 4,472 | 5,658 | -0.35 | 5.65E-34 | Histone H2B | Histone H2B, core histone required for chromatin assembly and chromosome function |
| HTB115 | 712 | 368 | 0.94 | 1.00E-24 | Histone H2B | Histone H2B, core histone required for chromatin assembly and chromosome function |
| HTB116 | 1,731 | 1,515 | 0.18 | 5.48E-04 | Histone H2B | Histone H2B, core histone required for chromatin assembly and chromosome function |
| HTR101 | 1,085 | 575 | 0.90 | 7.96E-35 | Core | Histone H3, core histone required for chromatin assembly and chromosome function |
| HTR103 | 3,981 | 2,389 | 0.73 | 1.76E-86 | Core | Histone H3, core histone required for chromatin assembly and chromosome function |
| HTR104 | 69 | 191 | -1.48 | 3.40E-14 | Core | Histone H3, core histone required for chromatin assembly and chromosome function |
| HTR105 | 3,215 | 1,357 | 1.23 | 9.61E-168 | Core | Histone H3, core histone required for chromatin assembly and chromosome function |
| HTR106 | 364 | 1,672 | -2.21 | 2.74E-201 | Core | Histone H3, core histone required for chromatin assembly and chromosome function |
| HTR107 | 1,785 | 4,309 | -1.28 | 1.18E-241 | Core | Histone H3, core histone required for chromatin assembly and chromosome function |
| HTR108 | 2,504 | 2,657 | -0.10 | 2.05E-02 | Core | Histone H3, core histone required for chromatin assembly and chromosome function |
| HTR109 | 1,732 | 1,122 | 0.61 | 1.07E-28 | Core | Histone H3, core histone required for chromatin assembly and chromosome function |
| HTR110 | 11,177 | 4,235 | 1.39 | 0.00E+01 | Core | Histone H3, core histone required for chromatin assembly and chromosome function |
| HTR111 | 4,273 | 3,257 | 0.38 | 1.19E-29 | Core | Histone H3, core histone required for chromatin assembly and chromosome function |
| HTR112 | 8,908 | 6,451 | 0.45 | 8.11E-85 | Core | Histone H3, core histone required for chromatin assembly and chromosome function |
| HTR113 | 42 | 210 | -2.33 | 1.06E-27 | Core | Histone H3, core histone required for chromatin assembly and chromosome function |
| HTR114 | 310 | 595 | -0.95 | 1.84E-21 | Centromeric Histone H3 | Centromeric histone H3 |
| HUPA101 | 61 | 258 | -2.09 | 9.06E-30 | Histone ubiquitination proteins group A (Bre1 homologs) | NA |
| HUPB101 | 169 | 321 | -0.94 | 8.18E-12 | Histone ubiquitination proteins group B (Rad6 homologs) | NA |
| HUPB102 | 101 | 197 | -0.98 | 3.14E-08 | Histone ubiquitination proteins group B (Rad6 homologs) | NA |
| HXA102 | 1,098 | 434 | 1.33 | 1.54E-64 | Histone acetyltransferase complex component (Ada2 homologs) | Homologous to yeast ADA2, a transcriptional adaptor protein that is part of the ADA and SAGA complexes |
| INGF101 | 70 | 44 | 0.66 | 2.48E-02 | Inhibitor of Growth protein group (ING1-5 homologs) | ING Family of PHD Finger Proteins |
| JMJ103 | 62 | 200 | -1.70 | 7.90E-18 | Jumonji domain group | NA |
| JMJ105 | 67 | 195 | -1.55 | 1.99E-15 | Jumonji domain group | NA |
| JMJ106 | 38 | 61 | -0.69 | 2.67E-02 | Jumonji domain group | NA |
| JMJ107 | 1,520 | 722 | 1.06 | 7.54E-63 | Jumonji domain group | NA |
| JMJ108 | 277 | 492 | -0.84 | 9.27E-15 | Jumonji domain group | NA |
| JMJ109 | 22 | 128 | -2.55 | 3.82E-19 | Jumonji domain group | NA |
| LFY102 | 48 | 27 | 0.82 | 2.65E-02 | Flowering control-associated proteins; LFY group | NA |
| MBD101 | 88 | 230 | -1.40 | 1.08E-15 | Methyl binding domain proteins | Protein containing a putative methyl-CpG-binding domain; genomic sequence from BAC may have sequence errors; predicted translational start site different from cDNA and ESTs |
| MBD105 | 299 | 571 | -0.94 | 2.05E-20 | Methyl binding domain proteins | Protein containing a putative methyl-CpG-binding domain |
| MBD106 | 932 | 444 | 1.06 | 1.30E-38 | Methyl binding domain proteins | Protein containing a putative methyl-CpG-binding domain |
| MBD108 | 95 | 192 | -1.03 | 1.17E-08 | Methyl binding domain proteins | Protein containing a putative methyl-CpG-binding domain |
| MBD113 | 325 | 481 | -0.58 | 3.96E-08 | Methyl binding domain proteins | Protein containing a putative methyl-CpG-binding domain |
| MBD116 | 85 | 199 | -1.24 | 1.24E-11 | Methyl binding domain proteins | Protein containing a putative methyl-CpG-binding domain |
| MBD117 | 14 | 31 | -1.16 | 1.49E-02 | Methyl binding domain proteins | Protein containing a putative methyl-CpG-binding domain |
| MBD119 | 260 | 130 | 0.99 | 1.47E-10 | Methyl binding domain proteins | Protein containing a putative methyl-CpG-binding domain |
| MRG101 | 171 | 417 | -1.30 | 1.29E-24 | MRG domain-containing proteins | NA |
| NFA101 | 580 | 2,101 | -1.87 | 2.69E-203 | NAP1 Class | Nucleosome assembly protein; putative homolog of Saccharomyces cerevisiae NAP1, a protein involved in the transport of H2A and H2B histones to the nucleus |
| NFA104 | 936 | 1,197 | -0.37 | 1.11E-08 | SET translocation (myeloid leukemia-associated) Cl | A nucleosome assembly-like protein with more similarity to Homo sapiens TSPY, TSPLY and SET translocation (myeloid leukemia-associated)proteins than Saccharomyces cerevisiae NAP1; required for establishing siRNA-mediated, heritable silencing of a maize transposon |
| NFA105 | 580 | 2,101 | -1.87 | 2.69E-203 | NAP1 Class | Nucleosome assembly protein; putative homolog of Saccharomyces cerevisiae NAP1, a protein involved in the transport of H2A and H2B histones to the nucleus |
| NFB102 | 159 | 247 | -0.65 | 1.61E-05 | Nucleosome/chromatin assembly complex proteins (Cac2 homologs) | Putative homolog of human CAF1 subunit B or yeast CAC2, the p60 subunit s of human and yeast chromatin assembly factor I , respectively. Human CAF1 is composed of three subunits that are 150 kd, 60 kd and 48 kd in size. The smallest subunit dissociates during G1 phase of the cell cycle. CAF1 is thought to deposit histones H3 and H4 onto newly replicated DNA such that histone H2A and H2B can then bind to form nucleosome core particles. |
| NFC101 | 1,284 | 1,436 | -0.17 | 2.67E-03 | NURF complex component (RBBP4/Caf1 homologs) | Member of the nucleosome/chromatin assembly factor group C (RBP4/7 homolog); highly similar to human RbBp48 (Retinoblastoma binding protein) - a WD-40 protein found in several chromatin regulatory complexes |
| NFC102 | 557 | 1,059 | -0.94 | 1.97E-36 | NURF complex component (RBBP4/Caf1 homologs) | Member of the nucleosome/chromatin assembly factor group C (RBP4/7 homolog); highly similar to human RbBp48 (Retinoblastoma binding protein) - a WD-40 protein found in several chromatin regulatory complexes |
| NFC103 | 674 | 212 | 1.66 | 4.65E-55 | NURF complex component (RBBP4/Caf1 homologs) | Member of the nucleosome/chromatin assembly factor group C (RBP4/7 homolog); highly similar to human RbBp48 (Retinoblastoma binding protein) - a WD-40 protein found in several chromatin regulatory complexes |
| NFC104 | 173 | 437 | -1.35 | 3.19E-27 | NURF complex component (RBBP4/Caf1 homologs) | Member of the nucleosome/chromatin assembly factor group C (RBP4/7 homolog); highly similar to human RbBp48 (Retinoblastoma binding protein) - a WD-40 protein found in several chromatin regulatory complexes |
| NFE101 | 287 | 341 | -0.26 | 3.24E-02 | Nucleosome positioning into regularly spaced arrays (Acf1 homologs) | Putative homolog of the ACF (ATP-dependent chromatin assembly factor) proteins BAZ1A (Homo sapiens) and Acf1 (Drosophila). Invertebrate and vertebrate ACF proteins contain bromodomain and PHD finger motifs, but putative plant and fungal homologs lack these conserved domains. In Drosophila Acf1, along with Iswi and Nap-1, functions in the assembly of core histones and DNA into nucleosomes; interaction of Acf1 with Iswi enhances the efficiency of nucleosome sliding. |
| NFF102 | 513 | 391 | 0.38 | 1.36E-04 | Nucleosome/chromatin assembly complex proteins (Cac1 homologs) | Putative homolog of Chromatin assembly factor 1 (Caf1), subunit A; Caf1 is a multi-protein complex originally isolated from human cells and named for its ability to assemble nucleosomes onto newly replicated DNA. The human complex is comprised of the three subunits, p150 (CHAF1A), p60 (CHAF1B) and p48 subunits. |
| NFF103 | 18 | 46 | -1.37 | 6.27E-04 | Nucleosome/chromatin assembly complex proteins (Cac1 homologs) | Putative homolog of Chromatin assembly factor 1 (Caf1), subunit A; Caf1 is a multi-protein complex originally isolated from human cells and named for its ability to assemble nucleosomes onto newly replicated DNA. The human complex is comprised of the three subunits, p150 (CHAF1A), p60 (CHAF1B) and p48 subunits. |
| NRPDB101 | 509 | 364 | 0.47 | 3.19E-06 | RNA Polymerase IV Small Subunit | NA |
| NRPDB102 | 30 | 64 | -1.10 | 6.30E-04 | RNA Polymerase IV Small Subunit | NA |
| PAFB101 | 173 | 217 | -0.34 | 2.92E-02 | PAF1 complex protein (Leo1 homologs) | Putatiave homolog of Saccharomyces cerevisiae LEO1, a RNA polymerase II-associated Paf1p complex protein. The Saccharomyces cerevisiae Paf1 complex consists of five proteins (Paf1, Rtf1, Cdc73, Leo1, Ctr9) and is associated with the elongating RNA polymerase II complex. |
| PAFD101 | 27 | 74 | -1.47 | 3.63E-06 | PAF1 complex protein (Rtf1 homologs) | Putatiave homolog of Saccharomyces cerevisiae RTF1, a RNA polymerase II-associated Paf1p complex protein. The Saccharomyces cerevisiae Paf1 complex consists of five proteins (Paf1, Rtf1, Cdc73, Leo1, Ctr9) and is associated with the elongating RNA polymerase II complex. |
| PATPB101 | 506 | 837 | -0.74 | 1.11E-19 | Proteasomal ATPases; Group B | Predicted homolog of Saccharomyces cerevisiae RPT6, Proteasomal ATPases are proposed to link Ubiquitylation of Histone H2B to Methylation of Histone H3. |
| PGE102 | 119 | 394 | -1.74 | 3.94E-35 | Polycomb group (esc homologs) | Polycomb group; one of two closely related maize genes that encode a protein related to the Drosophila PcG protein ESC. The related Arabidopsis protein, FIE1, is required for proper regulation of endosperm development. |
| PRMT102 | 868 | 1,019 | -0.24 | 4.23E-04 | Protein arginine methyltransferases | Protein arginine N-methyltransferase, ChromDB Class A; homologous to human PRMT1 |
| PRMT103 | 82 | 136 | -0.74 | 3.77E-04 | Protein arginine methyltransferases | Protein arginine N-methyltransferase, ChromDB Class C; homologous to human PRMT7 |
| PRMT104 | 81 | 130 | -0.69 | 1.08E-03 | Protein arginine methyltransferases | Protein arginine N-methyltransferase, ChromDB Class IV; orthologous to human PRMT5 |
| PRMT105 | 98 | 225 | -1.21 | 1.47E-12 | Protein arginine methyltransferases | Protein arginine N-methyltransferase, ChromDB ClassV; orthologous to human PRMT3 |
| RDR101 | 12 | 83 | -2.80 | 4.25E-14 | RNA-dependent RNA polymerases | NA |
| RHEL101 | 79 | 229 | -1.55 | 5.79E-18 | RNA helicases | Silencing defective; a probably ortholog of the Arabidopsis gene that encodes a protein with similarity to RNA helicases |
| RUVBL102 | 187 | 457 | -1.30 | 6.25E-27 | RVB1 like proteins | Member of the RUVB-like protein family; homologous to Saccharomyces cerevisiae RVB proteins which are involved in transcription regulation and are components of chromatin remodeling complexes. |
| RUVBL103 | 183 | 462 | -1.35 | 1.19E-28 | RVB2 like proteins | Member of the RUVB-like protein family; homologous to Saccharomyces cerevisiae RVB proteins which are involved in transcription regulation and are components of chromatin remodeling complexes. |
| SDG101 | 34 | 103 | -1.61 | 3.81E-09 | SUVH1/SUVH3 Group | Su(var)3-9 group; plant specific sub-group with YDG_SRA, Pre-SET, and SET domains |
| SDG102 | 74 | 110 | -0.58 | 1.08E-02 | ARATH_ASHH1 Group | SET domain protein; predicted histone H3 lysine 36 histone methyltransferase; ASH1 group |
| SDG103 | 103 | 71 | 0.53 | 2.46E-02 | ARATH_SUVH5/SUVH6 Group | Su(var)3-9 group; plant specific sub-group with YDG_SRA, Pre-SET, and SET domains |
| SDG104 | 73 | 117 | -0.69 | 2.00E-03 | ARATH_SUVH5/SUVH6 Group | Su(var)3-9 group; plant specific sub-group with YDG_SRA, Pre-SET, and SET domains |
| SDG105 | 66 | 299 | -2.19 | 2.68E-36 | SUVH1/SUVH3 Group | Su(var)3-9 group; plant specific sub-group with YDG_SRA, Pre-SET, and SET domains |
| SDG106 | 260 | 511 | -0.99 | 1.38E-19 | SDG732 Group | SET domain protein |
| SDG111 | 127 | 365 | -1.53 | 1.72E-27 | SUVH1/SUVH3 Group | Su(var)3-9 group; plant specific sub-group with YDG_SRA, Pre-SET, and SET domains |
| SDG113 | 72 | 140 | -0.97 | 4.46E-06 | SUVH1/SUVH3 Group | Su(var)3-9 group; plant specific sub-group with YDG_SRA, Pre-SET, and SET domains |
| SDG116 | 72 | 42 | 0.77 | 8.98E-03 | ARATH_SUVR3 Group | Su(var)3-9 group of confirmed and predicted histone H3 lysine 9 methyltransferases |
| SDG117 | 98 | 267 | -1.46 | 4.51E-19 | ARATH_SUVR5 Group | SET domain protein |
| SDG118 | 164 | 348 | -1.10 | 4.18E-16 | ARATH_Kryptonite Group | Su(var)3-9 group; plant specific sub-group with YDG_SRA, Pre-SET, and SET domains |
| SDG119 | 8 | 29 | -1.87 | 6.94E-04 | ARATH_SUVH5/SUVH6 Group | Su(var)3-9 group; plant specific sub-group with YDG_SRA, Pre-SET, and SET domains |
| SDG122 | 217 | 127 | 0.76 | 3.40E-06 | S-ET interrupted and unclassified | SET domain protein |
| SDG123 | 129 | 91 | 0.49 | 1.68E-02 | S-ET interrupted and unclassified | SET domain protein |
| SDG124 | 221 | 478 | -1.12 | 1.51E-22 | ARATH_CLF Group | Enhancer of zeste family of predicted histone H3 lysine 27 histone methyltransferases |
| SDG126 | 38 | 18 | 1.07 | 1.42E-02 | ARATH_EZA1 Group | Enhancer of zeste family of predicted histone H3 lysine 27 histone methyltransferases |
| SDG130 | 33 | 83 | -1.34 | 4.46E-06 | S-ET interrupted and unclassified | SET domain protein |
| SDG131 | 18 | 37 | -1.05 | 1.39E-02 | ARATH_SUVR5 Group | SET domain protein |
| SDG135 | 213 | 67 | 1.66 | 4.29E-18 | ARATH_SUVH2/SUVH9 Group | Su(var)3-9 group; plant specific sub-group with YDG_SRA, Pre-SET, and SET domains |
| SDG136 | 16 | 74 | -2.22 | 5.39E-10 | ARATH_SUVH2/SUVH9 Group | Su(var)3-9 group; plant specific sub-group with YDG_SRA, Pre-SET, and SET domains |
| SDG138 | 58 | 115 | -1.00 | 2.14E-05 | TRR_TRX | Member of the TRR (Trithorax-related protein) family of predicted histone H3 lysine 4 methyltransferases |
| SDG145 | 116 | 160 | -0.48 | 9.40E-03 | ARATH_EFS Group | ARATH EFS histone H3 lysine 36 histone methyltransferase family |
| SDG146 | 40 | 73 | -0.88 | 2.63E-03 | ARATH_ATX4/5 Group | Member of the ARATH_ATX4/5 family of predicted histone H3 lysine 4 methyltransferases |
| SGA101 | 128 | 177 | -0.48 | 5.90E-03 | Nucleosome assembly factor (ASF1 homologs) | Putative homolog of Saccharomyces cerevisiae ASF1 (Anti-Silencing Function), a histone H3/H4 chaperone that functions in nucleosome assembly as a histone donor. |
| SGA102 | 385 | 474 | -0.31 | 2.44E-03 | Nucleosome assembly factor (ASF1 homologs) | Putative homolog of Saccharomyces cerevisiae ASF1 (Anti-Silencing Function), a histone H3/H4 chaperone that functions in nucleosome assembly as a histone donor. |
| SGS101 | 505 | 833 | -0.73 | 2.01E-19 | Suppressor of gene silencing | NA |
| SMH103 | 53 | 103 | -0.97 | 9.08E-05 | Single myb histone protein group | Single myb histone gene containing an N-terminal MYB-like domain, an internal globular linker histone (H1/H5) domain, and a C-terminal coiled coil domain. |
| SMH104 | 125 | 229 | -0.88 | 3.96E-08 | Single myb histone protein group | Single myb histone gene containing an N-terminal MYB-like domain, an internal globular linker histone (H1/H5) domain, and a C-terminal coiled coil domain. |
| SMH105 | 57 | 184 | -1.70 | 1.57E-16 | Single myb histone protein group | Single myb histone gene containing an N-terminal MYB-like domain, an internal globular linker histone (H1/H5) domain, and a C-terminal coiled coil domain. |
| SMH106 | 47 | 82 | -0.81 | 2.83E-03 | Single myb histone protein group | Single myb histone gene containing an N-terminal MYB-like domain, an internal globular linker histone (H1/H5) domain, and a C-terminal coiled coil domain. |
| SNT101 | 308 | 514 | -0.75 | 6.26E-13 | Histone deacetylase complex protein (Sin3 homologs) | NA |
| SRT101 | 23 | 61 | -1.42 | 4.41E-05 | Histone deacetylases (SIR2 family) | Class IV Sirtuin - Homolog of scSIR2, an NADH dependent Histone Deacetylase; probable maize ortholog of Arabidopsis SRT1 |
| SSRP101 | 341 | 531 | -0.65 | 1.27E-10 | Histone chaperone (Pob3 and SSRP homologs; FACT complex proteins) | Putative homolog of yeast Pob3 and human SSRP1, two subunits comprising the FACT complex (Facilitates Chromatin Transcription); the FACT complex regulates important chromatin-related processes such as transcription, DNA replication and DNA repair. |
| SWDB101 | 34 | 98 | -1.54 | 2.87E-08 | COMPASS (Set1C) complex protein (SWD2 homologs) | NA |
| SWDB102 | 135 | 256 | -0.93 | 1.14E-09 | COMPASS (Set1C) complex protein (SWD2 homologs) | NA |
| SWDB103 | 41 | 94 | -1.21 | 6.96E-06 | COMPASS (Set1C) complex protein (SWD2 homologs) | NA |
| SWDC101 | 96 | 38 | 1.33 | 1.13E-06 | COMPASS (Set1C) complex protein (SWD3 homologs) | NA |
| VEF102 | 540 | 164 | 1.71 | 3.02E-46 | VEF Family (VRN2, EMF2, FIS2) | Similar to Arabidopsis VEF2 (EMF2) |
| VEF103 | 538 | 164 | 1.70 | 6.92E-46 | VEF Family (VRN2, EMF2, FIS2) | Similar to Arabidopsis VEF2 (EMF2) |
| VPGA102 | 155 | 87 | 0.82 | 2.61E-05 | Flowering control-associated proteins (VRN1 homologs) | NA |
| VPGB101 | 112 | 253 | -1.19 | 1.44E-13 | Flowering control-associated proteins (VIN3 homologs) | NA |
| VPGB102 | 155 | 217 | -0.50 | 1.61E-03 | Flowering control-associated proteins (VIN3 homologs) | NA |
| VPGB104 | 66 | 144 | -1.14 | 1.05E-07 | Flowering control-associated proteins (VIN3 homologs) | NA |

^a^ The number of reads mapped to each gene model from mutant and non-mutant RNA-seq

^b^ log_2_ transformation of foldchange as the relative abandunce of transcripts in mutants/non-mutants

^c^ The fasle discovery rate calculated using Benjamini and Hochberg’s procedure for the p value from Fisher’s exact test
