# Supplementary material for: Loss of RNA–Dependent RNA Polymerase 2 (RDR2) Function Causes Widespread and Unexpected Changes in the Expression of Transposons, Genes, and 24-nt Small RNAs
Source: PLoS Genet. 2009 Nov 20;5(11):e1000737. doi: 10.1371/journal.pgen.1000737 (PMC2774947; doi:10.1371/journal.pgen.1000737)
Supplement: Table S5 — Alignment of RNA-seq reads to genome and genes. (0.01 MB PDF) [file pgen.1000737.s010.pdf]

Table S5. Alignment of RNA seq reads to genome and genes

Genotype	Total Reads [a]	No. Non-uniquely mapped [b]	No. Uniquely Mapped Reads		Total genes [e]
			To Genome [c]	To Genes [d]	
mop1/mop1	4,474,243	950,295 (21%)	2,775,266 (62%)	2,156,241 (78%)	24,743 (76%)
Mop1/mop1	5,982,599	1,350,921 (23%)	4,113,045 (69%)	3,248,869 (79%)	

[a] The number of reads after trimming low quality bases at the 3' end and removing the reads shorter than 32 bp ; [b] The number of reads (percentage) that aligned to more than 1 genomic locations on B73 reference genome allowing a maximum of 2 mismatches including insertion/deletions; [c] The number of reads (percentage of total reads) that aligned to a unique location of the B73 reference genome; [d] The number of uniquely mapped reads (percentage of uniquely mapped to genome) that can be projected onto gene models; [e] The union of genes (percentage of all 32,540 all genes) captured from two genotypes.
