# Supplementary material for: Loss of RNA–Dependent RNA Polymerase 2 (RDR2) Function Causes Widespread and Unexpected Changes in the Expression of Transposons, Genes, and 24-nt Small RNAs
Source: PLoS Genet. 2009 Nov 20;5(11):e1000737. doi: 10.1371/journal.pgen.1000737 (PMC2774947; doi:10.1371/journal.pgen.1000737)
Supplement: Table S6 — List of differentially expressed genes and related annotation. (7.02 MB DOC) [file pgen.1000737.s011.doc]

**Table S6.** List of differentially expressed genes

| **Gene.ID** | **No. Illumina/Solexa Reads ^a^** | | **log2(FC) ^b^** | **BH.FDR ^c^** | **Protein Name** |
| --- | --- | --- | --- | --- | --- |
|  | **Mutant** | **Non-Mutant** |  |  |  |
| AC149475.2_FG003 | 40 | 120 | -0.99 | 7.41E-04 | Proteasome subunit alpha type (EC 3.4.25.1) |
| AC149828.2_FG002 | 51 | 199 | -1.37 | 1.30E-09 | Putative uncharacterized protein |
| AC155377.1_FG002 | 17 | 61 | -1.25 | 6.25E-03 | Putative uncharacterized protein |
| AC155610.2_FG004 | 30 | 82 | -0.86 | 2.34E-02 | Putative uncharacterized protein |
| AC155622.2_FG001 | 30 | 86 | -0.93 | 9.46E-03 | cDNA clone:J023075G08, full insert sequence |
| AC155624.2_FG006 | 1 | 13 | -3.11 | 4.97E-02 | Putative uncharacterized protein |
| AC155624.2_FG011 | 1 | 13 | -3.11 | 4.97E-02 | Putative uncharacterized protein |
| AC159612.1_FG008 | 34 | 108 | -1.08 | 5.80E-04 | Replication factor C subunit 5 |
| AC165171.2_FG003 | 21 | 64 | -1.02 | 1.90E-02 | Putative uncharacterized protein |
| AC177924.2_FG001 | 0 | 15 | #NUM! ^d^ | 4.61E-03 | Putative uncharacterized protein OJ1369_G08.15 |
| AC182418.6_FG009 | 271 | 251 | 0.70 | 5.06E-07 | 40S ribosomal protein S26 (Putative uncharacterized protein) |
| AC183950.2_FG001 | 68 | 269 | -1.39 | 4.94E-13 | Histone deacetylase 2b (HD2 type histone deacetylase HDA106) |
| AC185415.3_FG005 | 33 | 24 | 1.05 | 3.01E-02 | Os07g0296200 protein (Putative uncharacterized protein) (cDNA clone:002-108-B11, full insert sequence) (RRM-containing RNA-binding protein-like) |
| AC185467.3_FG002 | 11 | 54 | -1.70 | 8.29E-04 | Putative uncharacterized protein |
| AC185532.4_FG006 | 21 | 93 | -1.56 | 1.38E-05 | Putative uncharacterized protein |
| AC186299.3_FG004 | 10 | 38 | -1.33 | 3.31E-02 | Putative uncharacterized protein |
| AC186319.4_FG002 | 57 | 312 | -1.86 | 1.40E-22 | Os08g0320100 protein (cDNA clone:J033097J11, full insert sequence) (RNA recognition motif (RRM)-containing protein-like) |
| AC186433.4_FG002 | 22 | 104 | -1.65 | 1.13E-06 | CUE domain containing protein (Putative uncharacterized protein) |
| AC187098.4_FG011 | 4 | 34 | -2.50 | 7.24E-04 | PWWP domain containing protein |
| AC188759.3_FG005 | 163 | 162 | 0.60 | 1.57E-03 | Putative uncharacterized protein |
| AC189879.3_FG003 | 54 | 138 | -0.76 | 5.21E-03 | ZLL/PNH homologous protein |
| AC190609.3_FG002 | 43 | 113 | -0.80 | 9.86E-03 | 60S ribosomal protein L38 (Putative uncharacterized protein) |
| AC190609.3_FG004 | 18 | 55 | -1.02 | 3.61E-02 | Putative uncharacterized protein |
| AC190750.2_FG014 | 12 | 54 | -1.58 | 1.55E-03 | F-box domain containing protein |
| AC190801.3_FG007 | 19 | 57 | -0.99 | 4.06E-02 | Putative uncharacterized protein |
| AC190982.3_FG008 | 38 | 107 | -0.90 | 4.09E-03 | Putative uncharacterized protein |
| AC190999.2_FG004 | 22 | 72 | -1.12 | 5.90E-03 | Putative SNM1 |
| AC191387.3_FG009 | 18 | 67 | -1.30 | 2.32E-03 | Dihydrolipoyl dehydrogenase (EC 1.8.1.4) |
| AC191628.3_FG002 | 21 | 81 | -1.36 | 3.64E-04 | Putative uncharacterized protein (Ras-related protein RHN1) |
| AC192244.3_FG007 | 125 | 513 | -1.45 | 4.24E-26 | Bifunctional coenzyme A synthase |
| AC192244.3_FG008 | 117 | 59 | 1.58 | 3.50E-11 | Putative uncharacterized protein |
| AC194015.3_FG003 | 32 | 100 | -1.05 | 1.27E-03 | Putative uncharacterized protein |
| AC194264.3_FG006 | 21 | 11 | 1.52 | 1.81E-02 | Os08g0556700 protein (Fragment) |
| AC194341.4_FG002 | 31 | 124 | -1.41 | 2.20E-06 | Hydrogen-transporting ATP synthase, rotational mechanism |
| AC194409.3_FG005 | 35 | 110 | -1.06 | 6.62E-04 | Putative uncharacterized protein |
| AC194671.1_FG004 | 36 | 158 | -1.54 | 4.78E-09 | Dihydroflavonol-4-reductase |
| AC195340.3_FG001 | 235 | 275 | 0.36 | 2.38E-02 | Alpha tubulin |
| AC195340.3_FG002 | 36 | 153 | -1.50 | 1.90E-08 | NADH-ubiquinone oxidoreductase 23 kDa subunit |
| AC195343.3_FG002 | 13 | 74 | -1.92 | 9.62E-06 | Chaperonin |
| AC196125.3_FG002 | 306 | 722 | -0.65 | 3.67E-10 | Glycine-rich RNA-binding protein 8 |
| AC196961.2_FG003 | 30 | 22 | 1.04 | 4.41E-02 | Histone H4 |
| AC196971.3_FG002 | 27 | 85 | -1.06 | 4.12E-03 | Putative uncharacterized protein |
| AC196978.5_FG001 | 74 | 163 | -0.55 | 2.95E-02 | Putative uncharacterized protein |
| AC197118.3_FG005 | 9 | 53 | -1.97 | 1.96E-04 | Antigenic determinant of rec-A protein (Putative uncharacterized protein) |
| AC197122.3_FG003 | 21 | 93 | -1.56 | 1.38E-05 | Os03g0652100 protein (Not1 N-terminal domain, CCR4-Not complex component family protein, expressed) (Putative uncharacterized protein OSJNBa0093M23.12) |
| AC197146.3_FG001 | 38 | 158 | -1.46 | 2.26E-08 | Putative uncharacterized protein |
| AC197246.3_FG001 | 16 | 7 | 1.78 | 2.33E-02 | Putative uncharacterized protein (Ras-related protein ARA-4) |
| AC197555.3_FG008 | 19 | 58 | -1.02 | 3.18E-02 | DNA-damage-repair/toleration protein DRT102 |
| AC197578.4_FG003 | 6 | 37 | -2.03 | 1.94E-03 | Putative uncharacterized protein |
| AC197672.3_FG002 | 33 | 126 | -1.34 | 4.72E-06 | Acetyl-coenzyme A carboxylase ACC1A |
| AC197717.3_FG002 | 7 | 34 | -1.69 | 1.19E-02 | Putative uncharacterized protein |
| AC197779.3_FG001 | 9 | 64 | -2.24 | 5.53E-06 | Putative uncharacterized protein |
| AC198169.4_FG004 | 31 | 107 | -1.20 | 1.60E-04 | EMB1586 (Putative uncharacterized protein) |
| AC198361.3_FG004 | 42 | 124 | -0.97 | 7.29E-04 | Putative uncharacterized protein |
| AC198518.3_FG003 | 31 | 94 | -1.01 | 3.13E-03 | Putative uncharacterized protein |
| AC198937.4_FG004 | 62 | 158 | -0.76 | 2.25E-03 | 60S ribosomal protein L34 |
| AC198937.4_FG006 | 24 | 83 | -1.20 | 1.16E-03 | Malate dehydrogenase (EC 1.1.1.37) |
| AC199173.3_FG006 | 22 | 87 | -1.39 | 1.30E-04 | Putative uncharacterized protein (Chromdomain-containing protein CRD101) |
| AC199193.3_FG003 | 45 | 160 | -1.24 | 9.31E-07 | ABI3-interacting protein 2 (Putative uncharacterized protein) |
| AC199315.4_FG001 | 9 | 49 | -1.85 | 6.14E-04 | Protein transport protein Sec61 beta subunit (Putative uncharacterized protein) |
| AC199371.3_FG001 | 2 | 17 | -2.50 | 3.74E-02 | Putative uncharacterized protein |
| AC199768.4_FG006 | 49 | 119 | -0.69 | 2.15E-02 | Expressed protein |
| AC199782.5_FG002 | 42 | 119 | -0.91 | 1.91E-03 | Hydroxymethylglutaryl-CoA synthase |
| AC199858.4_FG002 | 9 | 35 | -1.37 | 3.67E-02 | OSIGBa0139P06.9 protein |
| AC199922.3_FG003 | 90 | 291 | -1.10 | 6.39E-10 | Os03g0347200 protein (MIF4G domain containing protein, expressed) |
| AC200881.4_FG004 | 18 | 60 | -1.15 | 1.28E-02 | Putative uncharacterized protein |
| AC202439.3_FG005 | 16 | 55 | -1.19 | 1.25E-02 | Putative uncharacterized protein |
| AC202930.4_FG003 | 307 | 585 | -0.34 | 4.86E-03 | 40S ribosomal protein S14 |
| AC203173.3_FG004 | 97 | 372 | -1.35 | 3.96E-17 | Elongation factor 2 |
| AC203535.4_FG001 | 99 | 230 | -0.62 | 1.72E-03 | CDC5 protein |
| AC203812.3_FG004 | 15 | 63 | -1.48 | 8.85E-04 | Putative uncharacterized protein (Splicing factor, arginine/serine-rich 2) |
| AC203841.3_FG001 | 33 | 105 | -1.08 | 8.66E-04 | Grx_S15.2-glutaredoxin subgroup II (Putative uncharacterized protein) |
| AC203841.3_FG009 | 9 | 35 | -1.37 | 3.67E-02 | Putative uncharacterized protein |
| AC203843.4_FG004 | 10 | 39 | -1.37 | 2.44E-02 | Putative uncharacterized protein |
| AC203862.4_FG002 | 7 | 30 | -1.51 | 4.50E-02 | Putative uncharacterized protein |
| AC203862.4_FG003 | 1,965 | 1,481 | 1.00 | 8.73E-89 | Putative uncharacterized protein |
| AC203957.3_FG004 | 16 | 2 | 3.59 | 2.18E-04 | Putative uncharacterized protein |
| AC203972.3_FG001 | 5 | 0 | #VALUE! ^e^ | 4.27E-02 | Putative uncharacterized protein |
| AC204418.3_FG006 | 39 | 28 | 1.07 | 1.37E-02 | Putative STH1 protein |
| AC204530.4_FG002 | 39 | 118 | -1.01 | 6.60E-04 | Putative uncharacterized protein |
| AC204868.3_FG004 | 79 | 72 | 0.73 | 1.12E-02 | Kelch motif family protein (Putative uncharacterized protein OSJNBb0059G13.9) |
| AC204921.4_FG011 | 34 | 120 | -1.23 | 3.53E-05 | 1-(5-phosphoribosyl)-5-[(5-phosphoribosylamino)methylideneamino]imi dazole-4-carboxamide isomerase |
| AC205122.4_FG004 | 21 | 60 | -0.92 | 4.97E-02 | Protein kinase CK2 catalytic subunit CK2 alpha-3 |
| AC205521.3_FG003 | 156 | 472 | -1.01 | 7.58E-14 | Putative uncharacterized protein |
| AC205568.3_FG007 | 6 | 29 | -1.68 | 2.50E-02 | Putative gag-pol polyprotein |
| AC205608.4_FG004 | 15 | 67 | -1.57 | 3.22E-04 | Peptide transporter PTR2 |
| AC205677.3_FG001 | 9 | 59 | -2.12 | 2.75E-05 | Putative uncharacterized protein |
| AC205677.3_FG002 | 77 | 168 | -0.53 | 3.29E-02 | 40S ribosomal protein S13 (Putative uncharacterized protein) |
| AC206259.3_FG003 | 104 | 214 | -0.45 | 4.19E-02 | Putative uncharacterized protein |
| AC206586.2_FG007 | 64 | 182 | -0.92 | 6.22E-05 | Putative uncharacterized protein |
| AC206642.4_FG001 | 247 | 469 | -0.33 | 1.66E-02 | 60S ribosomal protein L35 |
| AC206840.3_FG002 | 4 | 26 | -2.11 | 1.27E-02 | Putative uncharacterized protein |
| AC207342.3_FG007 | 11 | 40 | -1.27 | 4.00E-02 | Putative uncharacterized protein |
| AC207652.3_FG002 | 12 | 50 | -1.47 | 4.22E-03 | ATP-dependent Clp protease proteolytic subunit |
| AC207890.3_FG002 | 39 | 119 | -1.02 | 6.65E-04 | Putative uncharacterized protein |
| AC208201.3_FG001 | 6 | 28 | -1.63 | 3.52E-02 | Putative uncharacterized protein |
| AC208327.4_FG003 | 20 | 62 | -1.04 | 2.17E-02 | Glutamate-rich WD repeat-containing protein 1 (Putative uncharacterized protein) |
| AC208346.3_FG004 | 62 | 199 | -1.09 | 6.38E-07 | Putative uncharacterized protein (cDNA clone:J013133L14, full insert sequence) (Leucine zipper protein-like) (Os01g0827500 protein) |
| AC208348.3_FG005 | 85 | 237 | -0.89 | 5.93E-06 | Putative uncharacterized protein (Putative RH2 protein) (RNA helicase 2) |
| AC208415.3_FG003 | 4 | 24 | -1.99 | 2.73E-02 | Putative uncharacterized protein |
| AC208436.3_FG007 | 15 | 52 | -1.20 | 1.87E-02 | Putative uncharacterized protein |
| AC208440.3_FG002 | 8 | 48 | -1.99 | 4.21E-04 | Putative uncharacterized protein |
| AC208833.3_FG005 | 44 | 122 | -0.88 | 2.34E-03 | CAAX prenyl protease 1 (Putative uncharacterized protein) |
| AC209206.3_FG011 | 40 | 114 | -0.92 | 2.51E-03 | Putative uncharacterized protein |
| AC209374.4_FG001 | 19 | 72 | -1.33 | 1.12E-03 | Putative uncharacterized protein |
| AC209462.3_FG003 | 23 | 77 | -1.15 | 3.06E-03 | Peroxisomal membrane carrier protein (Putative uncharacterized protein) |
| AC209718.1_FG009 | 17 | 6 | 2.09 | 6.53E-03 | Os01g0197500 protein |
| AC209755.3_FG002 | 38 | 126 | -1.14 | 6.84E-05 | Vacuolar protein sorting protein 25 |
| AC209858.4_FG002 | 187 | 644 | -1.19 | 1.91E-24 | Putative uncharacterized protein |
| AC209898.2_FG004 | 45 | 38 | 0.84 | 4.09E-02 | Putative uncharacterized protein |
| AC209974.4_FG002 | 17 | 58 | -1.18 | 1.10E-02 | Putative uncharacterized protein |
| AC210003.2_FG004 | 112 | 71 | 1.25 | 1.48E-07 | Peroxidase 16 (Putative uncharacterized protein) |
| AC210013.4_FG019 | 198 | 213 | 0.49 | 4.45E-03 | Putative uncharacterized protein |
| AC210050.3_FG007 | 18 | 115 | -2.08 | 1.18E-09 | Putative uncharacterized protein |
| AC210691.2_FG003 | 8 | 72 | -2.58 | 6.19E-08 | Putative uncharacterized protein |
| AC210780.3_FG006 | 170 | 400 | -0.64 | 7.91E-06 | Putative beta 1,3 glucan synthase |
| AC210993.3_FG005 | 17 | 52 | -1.02 | 4.08E-02 | Putative uncharacterized protein |
| AC211394.4_FG004 | 21 | 67 | -1.08 | 1.15E-02 | Putative uncharacterized protein |
| AC211401.4_FG003 | 6 | 27 | -1.58 | 4.89E-02 | Putative uncharacterized protein |
| AC211762.4_FG005 | 16 | 81 | -1.75 | 1.30E-05 | SET domain-containing protein SET118 |
| AC211955.4_FG013 | 329 | 895 | -0.85 | 9.52E-20 | 60S ribosomal protein L7-2 (Putative uncharacterized protein) |
| AC212112.4_FG002 | 49 | 119 | -0.69 | 2.15E-02 | Os02g0742500 protein (Putative DegP2 protease) |
| AC212390.3_FG005 | 58 | 150 | -0.78 | 2.54E-03 | Putative uncharacterized protein |
| AC212565.3_FG001 | 124 | 86 | 1.12 | 4.12E-07 | Histone H4 |
| AC212570.3_FG006 | 111 | 356 | -1.09 | 6.04E-12 | Small nucleolar ribonucleoprotein complex subunit |
| AC212668.2_FG010 | 24 | 69 | -0.93 | 2.64E-02 | Embryonic flower 2 (VEF family protein) |
| AC212835.3_FG001 | 2 | 20 | -2.73 | 1.09E-02 | Putative uncharacterized protein |
| AC212835.3_FG007 | 0 | 12 | #NUM! | 1.33E-02 | Putative uncharacterized protein |
| AC212859.3_FG007 | 27 | 130 | -1.68 | 2.38E-08 | Putative uncharacterized protein |
| AC213521.3_FG003 | 15 | 52 | -1.20 | 1.87E-02 | Putative uncharacterized protein |
| AC213521.3_FG004 | 217 | 543 | -0.73 | 1.64E-09 | Membrane steroid-binding protein 1 |
| AC213600.3_FG002 | 32 | 84 | -0.80 | 3.41E-02 | Putative uncharacterized protein |
| AC213621.5_FG005 | 1,192 | 2,417 | -0.43 | 6.65E-16 | 60S ribosomal protein L36 |
| AC213654.3_FG005 | 14 | 47 | -1.16 | 3.67E-02 | Putative uncharacterized protein |
| AC214244.4_FG002 | 91 | 211 | -0.62 | 3.29E-03 | CUE domain containing protein |
| AC214266.3_FG002 | 18 | 66 | -1.28 | 3.13E-03 | Putative uncharacterized protein (UDP-galactose translocator) |
| AC214350.3_FG007 | 32 | 99 | -1.04 | 1.65E-03 | Ubiquinol-cytochrome c reductase iron-sulfur subunit (EC 1.10.2.2) |
| AC214437.4_FG004 | 28 | 75 | -0.83 | 3.77E-02 | Putative uncharacterized protein |
| AC214479.2_FG001 | 78 | 191 | -0.70 | 1.62E-03 | Os02g0832500 protein (Callose synthase-like protein) (Fragment) |
| AC214507.3_FG001 | 6 | 31 | -1.78 | 1.82E-02 | CFM6 |
| AC215201.3_FG008 | 11 | 47 | -1.50 | 6.39E-03 | Putative uncharacterized protein |
| AC216010.3_FG001 | 59 | 206 | -1.21 | 3.03E-08 | Pseudouridylate synthase (EC 5.4.99.-) |
| AC216855.2_FG002 | 52 | 21 | 1.90 | 1.46E-06 | Wax synthase isoform 1 |
| AC217121.3_FG007 | 7 | 43 | -2.03 | 9.03E-04 | Putative uncharacterized protein |
| AC217271.3_FG002 | 106 | 218 | -0.45 | 3.88E-02 | Os02g0280100 protein (Putative uncharacterized protein) (Putative MFAP1 protein) |
| AC217358.3_FG004 | 53 | 169 | -1.08 | 7.82E-06 | Putative uncharacterized protein |
| AC217358.3_FG005 | 78 | 218 | -0.89 | 1.52E-05 | Mitochondrial prohibitin complex protein 2 (Putative uncharacterized protein) |
| AC217358.3_FG011 | 64 | 188 | -0.96 | 1.87E-05 | Putative uncharacterized protein |
| AC217665.3_FG005 | 18 | 62 | -1.19 | 7.31E-03 | Putative uncharacterized protein |
| AC217908.2_FG003 | 19 | 65 | -1.18 | 6.50E-03 | Putative uncharacterized protein |
| AC217977.3_FG004 | 57 | 149 | -0.79 | 1.90E-03 | Endothelial differentiation-related factor 1 (Putative uncharacterized protein) |
| AC218148.2_FG008 | 22 | 103 | -1.64 | 1.61E-06 | Putative uncharacterized protein |
| AC218457.2_FG013 | 30 | 90 | -0.99 | 4.54E-03 | Expressed protein |
| AC218998.2_FG003 | 1 | 13 | -3.11 | 4.97E-02 | Os11g0607100 protein (Pentatricopeptide, putative, expressed) |
| AC220927.3_FG004 | 170 | 524 | -1.03 | 6.10E-16 | Polyadenylate-binding protein 2 (Putative uncharacterized protein) |
| AC220927.3_FG011 | 18 | 54 | -0.99 | 4.59E-02 | Os02g0758100 protein (LMBR1 integral membrane protein-like) |
| AC225147.4_FG002 | 94 | 225 | -0.67 | 9.20E-04 | 40S ribosomal protein S23 (Putative uncharacterized protein) |
| AC225185.3_FG004 | 20 | 63 | -1.06 | 1.68E-02 | Os12g0271600 protein (cDNA clone:J023050F09, full insert sequence) (Myosin heavy chain, putative, expressed) |
| AC226235.2_FG002 | 104 | 332 | -1.08 | 4.55E-11 | Putative uncharacterized protein |
| AC226373.2_FG010 | 17 | 74 | -1.53 | 1.99E-04 | Zinc finger C-x8-C-x5-C-x3-H type family protein |
| AC229673.2_FG006 | 49 | 129 | -0.81 | 4.42E-03 | Putative uncharacterized protein |
| AC230020.1_FG009 | 9 | 36 | -1.41 | 2.71E-02 | Putative uncharacterized protein |
| AC231180.2_FG001 | 10 | 38 | -1.33 | 3.31E-02 | Putative uncharacterized protein |
| AC233851.1_FG014 | 16 | 78 | -1.69 | 2.64E-05 | Putative uncharacterized protein |
| AC233856.1_FG003 | 21 | 59 | -0.90 | 4.95E-02 | Folylpolyglutamate synthase |
| AC233859.1_FG003 | 13 | 52 | -1.41 | 5.23E-03 | NHP2-like protein 1 (Putative uncharacterized protein) |
| AC233859.1_FG005 | 37 | 92 | -0.72 | 3.90E-02 | Putative uncharacterized protein |
| AC233865.1_FG002 | 12 | 4 | 2.18 | 3.60E-02 | Os09g0552900 protein |
| AC233866.1_FG006 | 772 | 1,497 | -0.36 | 1.46E-07 | Elongation factor 1-alpha |
| AC233869.1_FG002 | 2 | 24 | -2.99 | 2.83E-03 | Putative uncharacterized protein |
| AC233869.1_FG004 | 11 | 55 | -1.73 | 5.77E-04 | Putative uncharacterized protein |
| AC233870.1_FG005 | 57 | 171 | -0.99 | 2.88E-05 | Adhesion regulating molecule conserved region family protein (Putative uncharacterized protein) |
| AC233872.1_FG003 | 83 | 216 | -0.79 | 1.39E-04 | Mitochondrial glycoprotein (Putative uncharacterized protein) |
| AC233878.1_FG004 | 17 | 52 | -1.02 | 4.08E-02 | Calcium-transporting ATPase 3, endoplasmic reticulum-type, putative, expressed |
| AC233882.1_FG005 | 51 | 41 | 0.91 | 1.44E-02 | Putative outwardly rectifying potassium channel |
| AC233883.1_FG003 | 24 | 74 | -1.03 | 1.00E-02 | Putative uncharacterized protein |
| AC233893.1_FG001 | 21 | 77 | -1.28 | 9.16E-04 | Homocysteine S-methyltransferase 1 (Putative uncharacterized protein) |
| AC233893.1_FG002 | 31 | 139 | -1.57 | 3.98E-08 | Putative uncharacterized protein (No apical meristem protein, expressed) (Putative uncharacterized protein OJ1743A09.3) |
| AC233895.1_FG001 | 286 | 780 | -0.86 | 2.34E-17 | Proliferation-associated protein 2G4 |
| AC233901.1_FG001 | 33 | 114 | -1.20 | 9.41E-05 | Putative uncharacterized protein |
| AC233939.1_FG002 | 20 | 65 | -1.11 | 9.93E-03 | Os03g0280000 protein (ABC transporter family protein, putative, expressed) |
| AC233942.1_FG001 | 53 | 47 | 0.76 | 4.34E-02 | Putative uncharacterized protein |
| AC233949.1_FG004 | 259 | 474 | -0.28 | 4.77E-02 | Putative uncharacterized protein (Cell division cycle protein 48, putative, expressed) |
| AC233955.1_FG008 | 28 | 6 | 2.81 | 6.50E-06 | Putative uncharacterized protein |
| AC233958.1_FG002 | 32 | 89 | -0.88 | 1.45E-02 | RNA-binding protein 8A |
| AC233959.1_FG002 | 64 | 142 | -0.56 | 4.34E-02 | Protein phosphatase 2C isoform epsilon (Putative uncharacterized protein) |
| AC233961.1_FG001 | 30 | 102 | -1.17 | 3.18E-04 | Putative uncharacterized protein |
| AC233979.1_FG009 | 21 | 90 | -1.51 | 3.87E-05 | Putative uncharacterized protein |
| AC234154.1_FG007 | 13 | 74 | -1.92 | 9.62E-06 | CHY1 |
| AC234156.1_FG005 | 25 | 13 | 1.53 | 8.11E-03 | Fasciclin-like arabinogalactan protein 7 (Putative uncharacterized protein) |
| AC234164.1_FG002 | 30 | 102 | -1.17 | 3.18E-04 | Putative uncharacterized protein |
| AC234175.1_FG010 | 14 | 45 | -1.09 | 4.67E-02 | Putative fertility restorer homologue |
| AC234180.1_FG002 | 4 | 22 | -1.87 | 3.96E-02 | Putative uncharacterized protein |
| AC234201.1_FG001 | 142 | 95 | 1.17 | 1.21E-08 | Putative uncharacterized protein |
| AC234201.1_FG002 | 10 | 41 | -1.44 | 1.32E-02 | Putative uncharacterized protein |
| AC234201.1_FG003 | 84 | 85 | 0.57 | 4.85E-02 | Putative uncharacterized protein |
| AC234520.1_FG004 | 25 | 76 | -1.01 | 8.80E-03 | Putative uncharacterized protein |
| AC234521.1_FG005 | 3 | 27 | -2.58 | 3.47E-03 | Putative uncharacterized protein |
| AC234528.1_FG005 | 22 | 72 | -1.12 | 5.90E-03 | Putative uncharacterized protein |
| AC234575.1_FG006 | 10 | 44 | -1.55 | 6.84E-03 | Os05g0196200 protein |
| AC235535.1_FG001 | 312 | 658 | -0.49 | 8.55E-06 | Putative uncharacterized protein (cDNA clone:001-115-D05, full insert sequence) |
| AC235540.1_FG001 | 2 | 34 | -3.50 | 4.26E-05 | Aminoacylase-1 |
| AF546187.1_FG009 | 22 | 10 | 1.73 | 9.10E-03 | NA |
| EF517601.1_FG012 | 202 | 572 | -0.91 | 3.62E-14 | NA |
| EF517601.1_FG016 | 25 | 11 | 1.78 | 3.09E-03 | NA |
| GRMZM2G000039 | 79 | 183 | -0.62 | 7.18E-03 | Putative uncharacterized protein |
| GRMZM2G000042 | 71 | 169 | -0.66 | 5.74E-03 | NA |
| GRMZM2G000093 | 97 | 204 | -0.48 | 3.05E-02 | Putative uncharacterized protein |
| GRMZM2G000171 | 80 | 200 | -0.73 | 6.96E-04 | Putative uncharacterized protein |
| GRMZM2G000219 | 46 | 20 | 1.79 | 1.85E-05 | Copper-transporting ATPase PAA1 |
| GRMZM2G000245 | 135 | 290 | -0.51 | 3.88E-03 | H0311C03.6 protein |
| GRMZM2G000278 | 94 | 77 | 0.88 | 6.62E-04 | Putative uncharacterized protein |
| GRMZM2G000371 | 72 | 166 | -0.61 | 1.22E-02 | Putative uncharacterized protein |
| GRMZM2G000481 | 74 | 66 | 0.76 | 1.27E-02 | Tyrosyl-tRNA synthetase |
| GRMZM2G000608 | 124 | 304 | -0.70 | 3.42E-05 | Putative uncharacterized protein |
| GRMZM2G000614 | 17 | 54 | -1.08 | 3.23E-02 | Putative uncharacterized protein |
| GRMZM2G000622 | 67 | 246 | -1.29 | 1.29E-10 | Putative uncharacterized protein (Putative formylglycineamide ribotide amidotransferase) |
| GRMZM2G000623 | 15 | 50 | -1.15 | 2.43E-02 | Elongin C |
| GRMZM2G000686 | 47 | 146 | -1.04 | 6.37E-05 | Putative uncharacterized protein |
| GRMZM2G000710 | 133 | 139 | 0.53 | 1.49E-02 | Putative uncharacterized protein |
| GRMZM2G000739 | 24 | 83 | -1.20 | 1.16E-03 | Putative uncharacterized protein |
| GRMZM2G000749 | 49 | 158 | -1.10 | 1.30E-05 | Putative uncharacterized protein |
| GRMZM2G000777 | 51 | 164 | -1.09 | 7.75E-06 | Late embryogenesis abundant protein |
| GRMZM2G000923 | 614 | 1,319 | -0.51 | 4.54E-12 | Nascent polypeptide-associated complex alpha subunit-like protein |
| GRMZM2G000936 | 81 | 81 | 0.59 | 4.27E-02 | ETO1-like protein 1, putative, expressed (Putative uncharacterized protein) |
| GRMZM2G000937 | 48 | 147 | -1.02 | 9.41E-05 | Putative uncharacterized protein |
| GRMZM2G000976 | 22 | 96 | -1.53 | 1.28E-05 | Exostosin-like |
| GRMZM2G000980 | 247 | 193 | 0.95 | 2.08E-10 | Putative uncharacterized protein |
| GRMZM2G001084 | 62 | 156 | -0.74 | 3.35E-03 | OSJNBa0039C07.4 protein |
| GRMZM2G001160 | 121 | 105 | 0.80 | 3.51E-04 | ATP-dependent DNA helicase, RecQ family protein, expressed (Os11g0672700 protein) (Putative uncharacterized protein) |
| GRMZM2G001169 | 91 | 78 | 0.81 | 1.94E-03 | Calmodulin binding protein |
| GRMZM2G001180 | 51 | 140 | -0.87 | 1.14E-03 | Leucine Rich Repeat family protein, expressed |
| GRMZM2G001184 | 99 | 257 | -0.78 | 2.48E-05 | Os02g0480900 protein (cDNA clone:J033068A12, full insert sequence) (Putative Poly(A)-binding protein binding protein) |
| GRMZM2G001200 | 47 | 16 | 2.15 | 5.45E-07 | Putative uncharacterized protein |
| GRMZM2G001247 | 5 | 0 | #VALUE! | 4.27E-02 | Putative uncharacterized protein |
| GRMZM2G001255 | 101 | 101 | 0.59 | 1.95E-02 | Putative uncharacterized protein P0410E01.26 |
| GRMZM2G001272 | 169 | 407 | -0.68 | 2.07E-06 | Tubby-like protein |
| GRMZM2G001327 | 4,351 | 3,841 | 0.77 | 3.51E-126 | Elongation factor 1-alpha |
| GRMZM2G001334 | 66 | 177 | -0.83 | 3.72E-04 | Os04g0626900 protein |
| GRMZM2G001415 | 54 | 132 | -0.70 | 1.38E-02 | Putative uncharacterized protein |
| GRMZM2G001500 | 85 | 291 | -1.18 | 3.86E-11 | Stromal 70 kDa heat shock-related protein |
| GRMZM2G001541 | 47 | 114 | -0.69 | 2.79E-02 | Putative uncharacterized protein |
| GRMZM2G001639 | 30 | 18 | 1.33 | 9.74E-03 | Putative uncharacterized protein |
| GRMZM2G001652 | 66 | 145 | -0.54 | 4.67E-02 | Os09g0346700 protein (Putative uncharacterized protein) (cDNA clone:J023062D09, full insert sequence) (Putative uncharacterized protein P0512H04.9-1) |
| GRMZM2G001661 | 38 | 6 | 3.25 | 8.72E-09 | Wiscott-Aldrich syndrome, C-terminal |
| GRMZM2G001748 | 123 | 309 | -0.74 | 9.81E-06 | Putative uncharacterized protein |
| GRMZM2G001755 | 50 | 140 | -0.89 | 8.22E-04 | Putative uncharacterized protein |
| GRMZM2G001816 | 90 | 189 | -0.48 | 4.28E-02 | 60S ribosomal protein L11-1 |
| GRMZM2G001887 | 1,855 | 1,808 | 0.63 | 2.36E-37 | Putative uncharacterized protein (Transcription factor BTF3) |
| GRMZM2G001918 | 217 | 472 | -0.53 | 6.15E-05 | Putative uncharacterized protein |
| GRMZM2G001934 | 56 | 43 | 0.97 | 5.60E-03 | Putative uncharacterized protein (Receptor protein kinase TMK1) |
| GRMZM2G002002 | 105 | 237 | -0.58 | 3.18E-03 | Nucleotidyltransferase domain containing protein, expressed |
| GRMZM2G002104 | 57 | 15 | 2.52 | 3.37E-10 | Putative uncharacterized protein |
| GRMZM2G002130 | 78 | 184 | -0.65 | 4.84E-03 | Putative uncharacterized protein |
| GRMZM2G002147 | 235 | 210 | 0.75 | 5.85E-07 | Putative uncharacterized protein |
| GRMZM2G002220 | 56 | 147 | -0.80 | 2.17E-03 | Os08g0487800 protein (Putative heat-shock protein) |
| GRMZM2G002361 | 63 | 193 | -1.02 | 4.73E-06 | Os01g0101300 protein |
| GRMZM2G002416 | 286 | 266 | 0.70 | 2.82E-07 | Putative uncharacterized protein (Os01g0300200 protein) (Putative ATP citrate lyase a-subunit) |
| GRMZM2G002420 | 183 | 176 | 0.65 | 2.17E-04 | NA |
| GRMZM2G002427 | 88 | 209 | -0.66 | 1.98E-03 | Putative uncharacterized protein |
| GRMZM2G002520 | 145 | 444 | -1.02 | 2.55E-13 | Putative uncharacterized protein |
| GRMZM2G002558 | 270 | 695 | -0.77 | 4.29E-13 | Pinin/SDK/memA/ protein conserved region containing protein |
| GRMZM2G002603 | 129 | 500 | -1.36 | 2.37E-23 | Putative uncharacterized protein |
| GRMZM2G002616 | 1,136 | 867 | 0.98 | 9.12E-50 | Putative uncharacterized protein |
| GRMZM2G002617 | 94 | 206 | -0.54 | 1.35E-02 | Putative uncharacterized protein (TMV-MP30 binding protein 2C) |
| GRMZM2G002765 | 15 | 62 | -1.46 | 1.22E-03 | Putative uncharacterized protein |
| GRMZM2G002786 | 206 | 590 | -0.93 | 4.70E-15 | Putative uncharacterized protein (Seed maturation protein) |
| GRMZM2G002825 | 248 | 760 | -1.02 | 1.03E-22 | Actin-depolymerizing factor 3 (Putative uncharacterized protein) |
| GRMZM2G002828 | 43 | 162 | -1.32 | 1.87E-07 | Transcription initiation factor IIB |
| GRMZM2G002830 | 328 | 618 | -0.32 | 5.94E-03 | Putative uncharacterized protein |
| GRMZM2G002859 | 37 | 115 | -1.04 | 5.13E-04 | Putative uncharacterized protein |
| GRMZM2G002879 | 44 | 124 | -0.90 | 1.85E-03 | Methionine aminopeptidase (EC 3.4.11.18) |
| GRMZM2G002903 | 227 | 476 | -0.48 | 3.11E-04 | Putative uncharacterized protein |
| GRMZM2G002948 | 344 | 834 | -0.69 | 7.84E-13 | Zinc finger protein hangover |
| GRMZM2G002978 | 50 | 124 | -0.72 | 1.30E-02 | Putative uncharacterized protein |
| GRMZM2G003002 | 53 | 32 | 1.32 | 2.85E-04 | Putative uncharacterized protein |
| GRMZM2G003022 | 183 | 207 | 0.41 | 2.44E-02 | Putative uncharacterized protein |
| GRMZM2G003028 | 63 | 145 | -0.61 | 2.19E-02 | Putative uncharacterized protein |
| GRMZM2G003033 | 21 | 59 | -0.90 | 4.95E-02 | Putative uncharacterized protein |
| GRMZM2G003038 | 873 | 1,042 | 0.34 | 5.71E-06 | Protein arginine N-methyltransferase 1 (Putative uncharacterized protein) |
| GRMZM2G003043 | 55 | 147 | -0.83 | 1.63E-03 | Putative uncharacterized protein |
| GRMZM2G003064 | 114 | 64 | 1.42 | 2.03E-09 | Erg28 like protein |
| GRMZM2G003108 | 9 | 41 | -1.60 | 7.36E-03 | Putative uncharacterized protein |
| GRMZM2G003130 | 176 | 426 | -0.68 | 8.38E-07 | Chromatin modification-related protein EAF3 |
| GRMZM2G003306 | 6,585 | 5,735 | 0.79 | 7.69E-200 | Histone H2A |
| GRMZM2G003318 | 86 | 67 | 0.95 | 3.96E-04 | Plastid-lipid associated protein 3 |
| GRMZM2G003379 | 22 | 98 | -1.56 | 6.52E-06 | Endo-1,4-beta-glucanase |
| GRMZM2G003384 | 1,305 | 2,839 | -0.53 | 3.36E-27 | Putative uncharacterized protein |
| GRMZM2G003385 | 937 | 1,121 | 0.33 | 2.92E-06 | Putative uncharacterized protein |
| GRMZM2G003389 | 5 | 25 | -1.73 | 3.74E-02 | Putative uncharacterized protein |
| GRMZM2G003417 | 71 | 51 | 1.07 | 4.79E-04 | cDNA clone:001-129-A10, full insert sequence (Os01g0318700 protein) (Putative ABC1 protein) |
| GRMZM2G003452 | 184 | 116 | 1.26 | 3.24E-12 | B0222C05.5 protein |
| GRMZM2G003530 | 42 | 140 | -1.15 | 2.39E-05 | Putative uncharacterized protein |
| GRMZM2G003563 | 246 | 288 | 0.36 | 1.98E-02 | Putative uncharacterized protein |
| GRMZM2G003642 | 109 | 112 | 0.55 | 2.29E-02 | Putative uncharacterized protein |
| GRMZM2G003699 | 593 | 610 | 0.55 | 1.02E-09 | Predicted CDS Pa_7_9840 |
| GRMZM2G003715 | 16 | 64 | -1.41 | 1.54E-03 | Putative uncharacterized protein |
| GRMZM2G003718 | 10 | 65 | -2.11 | 8.55E-06 | Os06g0633900 protein (Cgi67 serine protease-like) |
| GRMZM2G003725 | 66 | 197 | -0.99 | 6.78E-06 | RING-H2 finger protein ATL5I |
| GRMZM2G003732 | 133 | 323 | -0.69 | 2.28E-05 | Os05g0295100 protein |
| GRMZM2G003752 | 410 | 1,055 | -0.77 | 1.17E-19 | Fasciclin-like arabinogalactan protein 10 |
| GRMZM2G003835 | 123 | 80 | 1.21 | 6.70E-08 | EMB2752 |
| GRMZM2G003852 | 275 | 258 | 0.68 | 8.72E-07 | Putative uncharacterized protein |
| GRMZM2G003853 | 108 | 302 | -0.89 | 1.64E-07 | 3-beta-hydroxysteroid-delta-isomerase |
| GRMZM2G003861 | 237 | 232 | 0.62 | 3.75E-05 | SLL2 |
| GRMZM2G003883 | 228 | 901 | -1.39 | 2.97E-43 | Putative uncharacterized protein |
| GRMZM2G003897 | 354 | 1,000 | -0.91 | 2.22E-24 | Nucleolin (Putative uncharacterized protein) |
| GRMZM2G003947 | 92 | 89 | 0.64 | 1.55E-02 | Putative uncharacterized protein |
| GRMZM2G004012 | 297 | 258 | 0.79 | 2.30E-09 | Basic blue protein (Putative uncharacterized protein) |
| GRMZM2G004040 | 13 | 49 | -1.32 | 1.34E-02 | Putative uncharacterized protein |
| GRMZM2G004057 | 1,332 | 1,371 | 0.55 | 3.26E-21 | 60S ribosomal protein L21 |
| GRMZM2G004083 | 168 | 159 | 0.67 | 2.43E-04 | Putative uncharacterized protein B1046G12.6 (Putative uncharacterized protein P0419B01.18) |
| GRMZM2G004111 | 1,390 | 1,227 | 0.77 | 2.75E-40 | Putative uncharacterized protein |
| GRMZM2G004140 | 170 | 354 | -0.47 | 3.20E-03 | Putative uncharacterized protein |
| GRMZM2G004157 | 139 | 127 | 0.72 | 4.63E-04 | Putative uncharacterized protein |
| GRMZM2G004172 | 180 | 429 | -0.66 | 1.85E-06 | NADH-ubiquinone oxidoreductase B16.6 subunit |
| GRMZM2G004259 | 115 | 235 | -0.44 | 3.32E-02 | Mitochondrial ribosomal protein L43 |
| GRMZM2G004320 | 109 | 297 | -0.85 | 7.26E-07 | Protein-S-isoprenylcysteine O-methyltransferase |
| GRMZM2G004349 | 463 | 906 | -0.38 | 4.14E-05 | Putative uncharacterized protein (Seed maturation protein) |
| GRMZM2G004377 | 18 | 57 | -1.07 | 2.17E-02 | Putative uncharacterized protein |
| GRMZM2G004468 | 65 | 152 | -0.63 | 1.45E-02 | Putative uncharacterized protein |
| GRMZM2G004528 | 232 | 500 | -0.52 | 4.92E-05 | Putative uncharacterized protein (Putative inositol-3-phosphate synthase) (EC 5.5.1.4) |
| GRMZM2G004534 | 423 | 495 | 0.36 | 1.16E-03 | Pyruvate kinase (EC 2.7.1.40) |
| GRMZM2G004679 | 59 | 149 | -0.75 | 4.03E-03 | AT hook-containing MAR binding 1-like protein |
| GRMZM2G004690 | 15 | 5 | 2.18 | 1.13E-02 | Putative uncharacterized protein |
| GRMZM2G004699 | 120 | 351 | -0.96 | 1.34E-09 | Putative uncharacterized protein |
| GRMZM2G004736 | 38 | 97 | -0.76 | 2.33E-02 | Putative uncharacterized protein (Fragment) |
| GRMZM2G004835 | 10 | 37 | -1.30 | 4.43E-02 | Esterase (Putative uncharacterized protein) |
| GRMZM2G004878 | 1,313 | 1,795 | 0.14 | 3.50E-02 | Putative uncharacterized protein |
| GRMZM2G004888 | 8 | 35 | -1.54 | 2.17E-02 | Putative pentatricopeptide (PPR) repeat-containing protein |
| GRMZM2G004932 | 303 | 226 | 1.01 | 3.36E-14 | Os05g0524400 protein (cDNA clone:J023139C03, full insert sequence) (Putative diphosphate-fructose-6-phosphate 1-phosphotransferase) |
| GRMZM2G004949 | 60 | 143 | -0.66 | 1.34E-02 | Putative uncharacterized protein |
| GRMZM2G004955 | 44 | 11 | 2.59 | 3.49E-08 | Os06g0725300 protein |
| GRMZM2G004959 | 254 | 230 | 0.73 | 4.34E-07 | NA |
| GRMZM2G004988 | 95 | 310 | -1.11 | 8.22E-11 | Putative uncharacterized protein |
| GRMZM2G004996 | 12 | 46 | -1.35 | 1.47E-02 | Charged multivesicular body protein 2b (Putative uncharacterized protein) |
| GRMZM2G005024 | 73 | 65 | 0.76 | 1.20E-02 | Tryptophan synthase beta-subunit |
| GRMZM2G005061 | 105 | 373 | -1.24 | 4.70E-15 | SnRK1-interacting protein 1 |
| GRMZM2G005080 | 574 | 510 | 0.76 | 1.78E-16 | Proteasome subunit alpha type (EC 3.4.25.1) |
| GRMZM2G005107 | 60 | 152 | -0.75 | 3.57E-03 | Peroxisomal 2,4-dienoyl-CoA reductase |
| GRMZM2G005229 | 69 | 61 | 0.77 | 1.52E-02 | Putative uncharacterized protein |
| GRMZM2G005236 | 43 | 14 | 2.21 | 1.09E-06 | Putative uncharacterized protein (Os01g0876800 protein) (Putative RRM-containing protein SEB-4) |
| GRMZM2G005256 | 379 | 264 | 1.11 | 1.58E-20 | Putative uncharacterized protein |
| GRMZM2G005308 | 130 | 268 | -0.45 | 1.75E-02 | Putative uncharacterized protein |
| GRMZM2G005339 | 26 | 125 | -1.67 | 5.24E-08 | Putative uncharacterized protein |
| GRMZM2G005346 | 128 | 330 | -0.77 | 1.47E-06 | Protein phosphatase methylesterase 1 |
| GRMZM2G005433 | 18 | 10 | 1.44 | 4.65E-02 | Photosystem II reaction center psb28 protein |
| GRMZM2G005435 | 96 | 248 | -0.78 | 4.56E-05 | Putative uncharacterized protein |
| GRMZM2G005483 | 31 | 18 | 1.38 | 6.63E-03 | Putative uncharacterized protein |
| GRMZM2G005592 | 94 | 422 | -1.58 | 2.60E-24 | Putative uncharacterized protein |
| GRMZM2G005622 | 22 | 134 | -2.02 | 7.84E-11 | Putative uncharacterized protein |
| GRMZM2G005624 | 61 | 185 | -1.01 | 1.02E-05 | Putative uncharacterized protein |
| GRMZM2G005640 | 10 | 97 | -2.69 | 6.73E-11 | Putative uncharacterized protein |
| GRMZM2G005641 | 39 | 23 | 1.35 | 2.50E-03 | Vesicle-associated membrane protein 724 |
| GRMZM2G005715 | 132 | 110 | 0.85 | 5.25E-05 | Putative uncharacterized protein |
| GRMZM2G005732 | 113 | 239 | -0.49 | 1.42E-02 | Putative uncharacterized protein |
| GRMZM2G005743 | 244 | 661 | -0.85 | 1.56E-14 | Putative uncharacterized protein |
| GRMZM2G005849 | 144 | 118 | 0.88 | 1.17E-05 | Putative uncharacterized protein |
| GRMZM2G005886 | 38 | 103 | -0.85 | 1.01E-02 | Putative uncharacterized protein |
| GRMZM2G005887 | 292 | 336 | 0.39 | 4.98E-03 | Cysteine synthase (EC 2.5.1.47) |
| GRMZM2G005939 | 40 | 97 | -0.69 | 4.66E-02 | Putative uncharacterized protein |
| GRMZM2G006071 | 134 | 274 | -0.44 | 1.92E-02 | Putative uncharacterized protein (Ribonucleoprotein like protein) |
| GRMZM2G006080 | 89 | 192 | -0.52 | 2.38E-02 | Putative uncharacterized protein (Os01g0769700 protein) (Putative uncharacterized protein B1143G03.32) (Putative uncharacterized protein P0665A11.8) |
| GRMZM2G006083 | 54 | 140 | -0.78 | 3.49E-03 | Putative uncharacterized protein |
| GRMZM2G006178 | 103 | 220 | -0.50 | 1.59E-02 | ATP-binding cassette sub-family E member 1, putative, expressed (Os11g0546000 protein) |
| GRMZM2G006229 | 11 | 45 | -1.44 | 8.73E-03 | Thioredoxin family protein |
| GRMZM2G006246 | 86 | 238 | -0.88 | 6.56E-06 | DNA damage signaling and repair protein |
| GRMZM2G006293 | 3,055 | 2,267 | 1.02 | 2.77E-143 | 40S ribosomal protein S27a |
| GRMZM2G006297 | 118 | 239 | -0.43 | 3.57E-02 | Putative uncharacterized protein |
| GRMZM2G006363 | 45 | 122 | -0.85 | 3.98E-03 | Putative uncharacterized protein |
| GRMZM2G006377 | 40 | 113 | -0.91 | 3.17E-03 | Putative uncharacterized protein |
| GRMZM2G006416 | 126 | 124 | 0.61 | 5.66E-03 | Putative uncharacterized protein |
| GRMZM2G006429 | 144 | 310 | -0.51 | 2.55E-03 | Putative uncharacterized protein |
| GRMZM2G006450 | 0 | 11 | #NUM! | 2.14E-02 | Putative uncharacterized protein |
| GRMZM2G006452 | 217 | 494 | -0.60 | 3.48E-06 | Os01g0904400 protein (SMC2 protein) |
| GRMZM2G006463 | 49 | 40 | 0.88 | 2.22E-02 | Putative uncharacterized protein |
| GRMZM2G006468 | 5 | 25 | -1.73 | 3.74E-02 | Putative wound responsive protein |
| GRMZM2G006474 | 205 | 410 | -0.41 | 5.09E-03 | Putative uncharacterized protein |
| GRMZM2G006493 | 282 | 331 | 0.36 | 1.20E-02 | Zinc finger CCCH type domain-containing protein ZFN-like 6 |
| GRMZM2G006505 | 115 | 67 | 1.37 | 5.47E-09 | Receptor-kinase isolog |
| GRMZM2G006631 | 40 | 17 | 1.83 | 4.72E-05 | Putative uncharacterized protein |
| GRMZM2G006661 | 46 | 129 | -0.90 | 1.41E-03 | Putative uncharacterized protein |
| GRMZM2G006673 | 127 | 126 | 0.60 | 6.02E-03 | Putative uncharacterized protein |
| GRMZM2G006676 | 298 | 1,203 | -1.42 | 1.16E-59 | NA |
| GRMZM2G006752 | 37 | 105 | -0.91 | 4.71E-03 | NA |
| GRMZM2G006763 | 962 | 1,276 | 0.18 | 1.49E-02 | Putative uncharacterized protein |
| GRMZM2G006765 | 85 | 186 | -0.54 | 2.07E-02 | Putative uncharacterized protein |
| GRMZM2G006781 | 21 | 59 | -0.90 | 4.95E-02 | Putative uncharacterized protein |
| GRMZM2G006790 | 45 | 107 | -0.66 | 4.27E-02 | Calcium ion binding protein (Putative uncharacterized protein) |
| GRMZM2G006806 | 88 | 209 | -0.66 | 1.98E-03 | Protein SSU72 (Putative uncharacterized protein) |
| GRMZM2G006871 | 43 | 30 | 1.11 | 6.67E-03 | Putative uncharacterized protein |
| GRMZM2G006958 | 42 | 101 | -0.67 | 4.33E-02 | Os03g0205000 protein (cDNA clone:J023025N13, full insert sequence) (CUE domain containing protein, expressed) |
| GRMZM2G006977 | 103 | 98 | 0.66 | 6.72E-03 | Os03g0203700 protein (cDNA clone:J023087M18, full insert sequence) (cDNA clone:J023101G05, full insert sequence) (Calcium-transporting ATPase 2, plasma membrane-type, putative, expressed) |
| GRMZM2G006981 | 22 | 4 | 3.05 | 5.56E-05 | Putative uncharacterized protein |
| GRMZM2G007063 | 131 | 94 | 1.07 | 5.53E-07 | Putative uncharacterized protein |
| GRMZM2G007080 | 28 | 94 | -1.16 | 7.15E-04 | Mitochondrial import inner membrane translocase subunit tim22 |
| GRMZM2G007130 | 12 | 48 | -1.41 | 7.87E-03 | Putative uncharacterized protein |
| GRMZM2G007146 | 74 | 63 | 0.82 | 5.27E-03 | Putative uncharacterized protein |
| GRMZM2G007160 | 40 | 103 | -0.77 | 1.79E-02 | NA |
| GRMZM2G007229 | 46 | 115 | -0.73 | 1.81E-02 | Os02g0290300 protein (Transducin-like) |
| GRMZM2G007277 | 18 | 66 | -1.28 | 3.13E-03 | Putative uncharacterized protein |
| GRMZM2G007288 | 107 | 105 | 0.62 | 1.07E-02 | Putative uncharacterized protein |
| GRMZM2G007339 | 56 | 142 | -0.75 | 4.88E-03 | Putative uncharacterized protein |
| GRMZM2G007399 | 32 | 96 | -0.99 | 3.54E-03 | Putative uncharacterized protein |
| GRMZM2G007486 | 119 | 241 | -0.43 | 3.65E-02 | Peptidylprolyl isomerase, putative, expressed |
| GRMZM2G007647 | 197 | 224 | 0.41 | 2.17E-02 | Acetolactate synthase/ amino acid binding protein |
| GRMZM2G007695 | 256 | 592 | -0.62 | 8.89E-08 | Putative uncharacterized protein |
| GRMZM2G007721 | 47 | 122 | -0.78 | 7.05E-03 | Os04g0131900 protein (OSJNBb0080H08.21 protein) |
| GRMZM2G007899 | 6 | 30 | -1.73 | 1.79E-02 | Os04g0549400 protein (cDNA clone:002-175-G09, full insert sequence) |
| GRMZM2G007933 | 52 | 136 | -0.80 | 3.69E-03 | Putative uncharacterized protein |
| GRMZM2G007939 | 36 | 18 | 1.59 | 6.18E-04 | Putative chloroplast-targeted beta-amylase |
| GRMZM2G007953 | 67 | 49 | 1.04 | 9.61E-04 | Putative uncharacterized protein |
| GRMZM2G008058 | 118 | 285 | -0.68 | 1.08E-04 | Putative uncharacterized protein |
| GRMZM2G008072 | 16 | 7 | 1.78 | 2.33E-02 | Putative uncharacterized protein |
| GRMZM2G008095 | 85 | 208 | -0.70 | 9.54E-04 | Protein SFT2 |
| GRMZM2G008226 | 150 | 150 | 0.59 | 3.16E-03 | Os08g0414700 protein (Putative trehalose-6-phosphate synthase) |
| GRMZM2G008242 | 17 | 80 | -1.64 | 3.59E-05 | Putative uncharacterized protein |
| GRMZM2G008250 | 270 | 251 | 0.70 | 6.27E-07 | Nuclear transcription factor Y subunit A-2 |
| GRMZM2G008252 | 1 | 13 | -3.11 | 4.97E-02 | Putative uncharacterized protein |
| GRMZM2G008273 | 57 | 196 | -1.19 | 1.26E-07 | COP9 signalosome complex subunit 3 |
| GRMZM2G008287 | 81 | 188 | -0.62 | 5.65E-03 | Protein arginine N-methyltransferase 6 (Putative uncharacterized protein) |
| GRMZM2G008309 | 187 | 127 | 1.15 | 6.42E-11 | Fiber expressed protein |
| GRMZM2G008410 | 287 | 884 | -1.03 | 1.24E-26 | Putative uncharacterized protein |
| GRMZM2G008456 | 65 | 190 | -0.96 | 2.11E-05 | DNA-binding protein |
| GRMZM2G008464 | 82 | 344 | -1.48 | 4.13E-18 | NADH-ubiquinone oxidoreductase 10.5 kDa subunit |
| GRMZM2G008478 | 52 | 179 | -1.19 | 4.08E-07 | Putative uncharacterized protein (Serine/threonine-protein kinase NAK) |
| GRMZM2G008497 | 178 | 380 | -0.50 | 7.58E-04 | Os02g0474700 protein (Putative Importin 7<Ran-binding protein 7) |
| GRMZM2G008501 | 45 | 136 | -1.00 | 2.32E-04 | Transferase, transferring glycosyl groups |
| GRMZM2G008513 | 118 | 242 | -0.44 | 2.77E-02 | Serine/threonine-protein kinase 38 |
| GRMZM2G008607 | 64 | 55 | 0.81 | 1.36E-02 | Putative uncharacterized protein |
| GRMZM2G008643 | 177 | 338 | -0.34 | 4.34E-02 | Putative uncharacterized protein |
| GRMZM2G008657 | 255 | 295 | 0.38 | 1.21E-02 | Putative uncharacterized protein |
| GRMZM2G008715 | 12 | 44 | -1.28 | 1.99E-02 | Putative uncharacterized protein |
| GRMZM2G008728 | 20 | 63 | -1.06 | 1.68E-02 | Putative uncharacterized protein |
| GRMZM2G008748 | 1,042 | 780 | 1.01 | 7.36E-48 | 60S ribosomal protein L6 |
| GRMZM2G008773 | 76 | 39 | 1.55 | 2.68E-07 | LysM domain containing protein |
| GRMZM2G008919 | 197 | 198 | 0.58 | 5.77E-04 | Vesicle-associated membrane protein 725 |
| GRMZM2G009025 | 205 | 225 | 0.46 | 6.59E-03 | Putative uncharacterized protein |
| GRMZM2G009091 | 184 | 424 | -0.61 | 1.13E-05 | Queuine tRNA-ribosyltransferase (EC 2.4.2.29) |
| GRMZM2G009136 | 29 | 80 | -0.87 | 2.14E-02 | Annexin A4 (Putative uncharacterized protein) |
| GRMZM2G009163 | 107 | 87 | 0.89 | 1.73E-04 | Ca2+/calmodulin-dependent protein kinase phosphatase (Putative uncharacterized protein) |
| GRMZM2G009253 | 136 | 350 | -0.77 | 7.65E-07 | Putative uncharacterized protein |
| GRMZM2G009265 | 98 | 217 | -0.56 | 8.48E-03 | Putative uncharacterized protein (Ubiquitin ligase SINAT5) |
| GRMZM2G009282 | 215 | 529 | -0.71 | 8.77E-09 | Putative alpha-glucosidase |
| GRMZM2G009326 | 111 | 270 | -0.69 | 1.26E-04 | Putative uncharacterized protein |
| GRMZM2G009335 | 115 | 234 | -0.43 | 3.75E-02 | Putative uncharacterized protein |
| GRMZM2G009365 | 701 | 266 | 1.99 | 4.46E-91 | Os02g0130600 protein (Putative uncharacterized protein) (cDNA clone:J023041I07, full insert sequence) (Putative uncharacterized protein OJ1007_D04.9) |
| GRMZM2G009387 | 46 | 188 | -1.44 | 1.08E-09 | Putative uncharacterized protein |
| GRMZM2G009412 | 1,768 | 2,269 | 0.23 | 6.03E-06 | 60S ribosomal protein L44 (Putative uncharacterized protein) |
| GRMZM2G009413 | 56 | 159 | -0.91 | 2.31E-04 | Putative uncharacterized protein |
| GRMZM2G009438 | 42 | 133 | -1.07 | 1.23E-04 | Putative uncharacterized protein |
| GRMZM2G009448 | 970 | 1,652 | -0.18 | 1.28E-02 | Glycine-rich RNA-binding protein 7 |
| GRMZM2G009538 | 102 | 218 | -0.50 | 1.78E-02 | Putative uncharacterized protein |
| GRMZM2G009571 | 36 | 98 | -0.85 | 1.05E-02 | Putative uncharacterized protein |
| GRMZM2G009593 | 56 | 158 | -0.91 | 2.93E-04 | Serine/threonine protein phosphatase (EC 3.1.3.16) |
| GRMZM2G009653 | 84 | 209 | -0.72 | 6.11E-04 | Putative uncharacterized protein |
| GRMZM2G009655 | 168 | 389 | -0.62 | 2.47E-05 | Ubiquitin carboxyl-terminal hydrolase (EC 3.1.2.15) |
| GRMZM2G009661 | 45 | 114 | -0.75 | 1.39E-02 | Putative uncharacterized protein |
| GRMZM2G009673 | 78 | 186 | -0.66 | 3.42E-03 | Putative RNA polymerase I subunit |
| GRMZM2G009724 | 190 | 132 | 1.12 | 1.25E-10 | Putative uncharacterized protein (Speckle-type POZ protein) |
| GRMZM2G009795 | 70 | 331 | -1.65 | 2.16E-20 | Putative uncharacterized protein |
| GRMZM2G009845 | 565 | 1,076 | -0.34 | 5.68E-05 | Putative uncharacterized protein |
| GRMZM2G009849 | 121 | 344 | -0.92 | 8.40E-09 | Putative uncharacterized protein (Os01g0816400 protein) (Putative microtubule bundling polypeptide TMBP200) |
| GRMZM2G009871 | 335 | 661 | -0.39 | 4.24E-04 | Putative uncharacterized protein |
| GRMZM2G009876 | 376 | 723 | -0.35 | 8.80E-04 | Putative uncharacterized protein (TGF-beta-inducible nuclear protein 1) |
| GRMZM2G009895 | 58 | 145 | -0.73 | 5.60E-03 | Putative uncharacterized protein |
| GRMZM2G009901 | 43 | 35 | 0.89 | 3.35E-02 | Putative uncharacterized protein |
| GRMZM2G009913 | 93 | 284 | -1.02 | 1.09E-08 | Mitotic spindle checkpoint component mad3 |
| GRMZM2G009936 | 1,394 | 1,528 | 0.46 | 5.40E-16 | 60S ribosomal protein L36 |
| GRMZM2G009940 | 96 | 326 | -1.17 | 3.09E-12 | Putative uncharacterized protein (Retinol dehydrogenase 14) |
| GRMZM2G010000 | 133 | 75 | 1.42 | 1.01E-10 | Putative uncharacterized protein |
| GRMZM2G010054 | 315 | 350 | 0.44 | 7.82E-04 | cDNA clone:J023075G08, full insert sequence |
| GRMZM2G010056 | 22 | 76 | -1.20 | 2.65E-03 | Putative uncharacterized protein |
| GRMZM2G010085 | 250 | 525 | -0.48 | 1.17E-04 | Putative uncharacterized protein (cDNA clone:001-115-D05, full insert sequence) |
| GRMZM2G010136 | 34 | 107 | -1.06 | 7.52E-04 | F-actin capping protein alpha subunit |
| GRMZM2G010238 | 16 | 89 | -1.88 | 9.37E-07 | Putative uncharacterized protein |
| GRMZM2G010321 | 131 | 352 | -0.83 | 8.87E-08 | Putative uncharacterized protein |
| GRMZM2G010323 | 100 | 266 | -0.82 | 7.43E-06 | Zinc finger C-x8-C-x5-C-x3-H type family protein |
| GRMZM2G010328 | 721 | 270 | 2.01 | 5.34E-95 | Putative uncharacterized protein |
| GRMZM2G010357 | 102 | 627 | -2.03 | 1.17E-50 | CCR4-NOT transcription complex subunit 2 |
| GRMZM2G010406 | 724 | 871 | 0.32 | 8.30E-05 | Argininosuccinate synthase (EC 6.3.4.5) |
| GRMZM2G010433 | 21 | 101 | -1.67 | 1.19E-06 | Os06g0228500 protein (Putative uncharacterized protein) (cDNA clone:002-129-B05, full insert sequence) (Putative amino acid transport protein) |
| GRMZM2G010452 | 268 | 655 | -0.70 | 1.78E-10 | Putative uncharacterized protein |
| GRMZM2G010466 | 7 | 0 | #VALUE! | 8.95E-03 | Putative uncharacterized protein P0022B05.128 |
| GRMZM2G010551 | 4 | 24 | -1.99 | 2.73E-02 | Putative uncharacterized protein |
| GRMZM2G010599 | 362 | 690 | -0.34 | 1.81E-03 | 60S ribosomal protein L35 |
| GRMZM2G010637 | 126 | 411 | -1.11 | 3.88E-14 | cDNA clone:J023074F05, full insert sequence (Putative Spo76 protein) |
| GRMZM2G010649 | 89 | 207 | -0.63 | 3.55E-03 | Putative uncharacterized protein |
| GRMZM2G010693 | 315 | 162 | 1.55 | 1.15E-28 | Putative uncharacterized protein |
| GRMZM2G010754 | 163 | 354 | -0.53 | 7.07E-04 | Splicing factor, arginine/serine-rich 12 |
| GRMZM2G010765 | 63 | 182 | -0.94 | 4.44E-05 | Putative uncharacterized protein (Transparent testa 12 protein) |
| GRMZM2G010797 | 46 | 118 | -0.77 | 9.93E-03 | Putative uncharacterized protein |
| GRMZM2G010823 | 81 | 259 | -1.09 | 1.06E-08 | Putative uncharacterized protein |
| GRMZM2G010834 | 69 | 66 | 0.66 | 3.67E-02 | Os08g0341700 protein (Phosphatidylinositol transfer-like) |
| GRMZM2G010836 | 180 | 179 | 0.60 | 7.64E-04 | Vesicle transport v-SNARE 13 |
| GRMZM2G010871 | 46 | 118 | -0.77 | 9.93E-03 | Heat shock factor protein HSF30 |
| GRMZM2G010960 | 54 | 48 | 0.76 | 3.67E-02 | Os06g0237300 protein (Putative pollen-specific LIM domain protein) |
| GRMZM2G010973 | 164 | 119 | 1.05 | 2.28E-08 | Putative uncharacterized protein |
| GRMZM2G010991 | 700 | 705 | 0.58 | 1.69E-12 | 60S ribosomal protein L22-2 |
| GRMZM2G011031 | 42 | 119 | -0.91 | 1.91E-03 | Putative uncharacterized protein |
| GRMZM2G011085 | 316 | 239 | 0.99 | 2.75E-14 | Putative uncharacterized protein |
| GRMZM2G011129 | 103 | 262 | -0.76 | 4.10E-05 | Putative uncharacterized protein (Ribonucleoprotein) (Nucleic acid-binding protein) |
| GRMZM2G011169 | 6 | 31 | -1.78 | 1.82E-02 | Os05g0432600 protein (Putative uncharacterized protein) (cDNA clone:J033088B21, full insert sequence) (Putative embryogenesis-associated protein) |
| GRMZM2G011355 | 869 | 644 | 1.02 | 6.55E-41 | Os03g0811900 protein (ADP-ribosylation factor) (ADP-ribosylation factor, putative, expressed) |
| GRMZM2G011404 | 39 | 100 | -0.77 | 2.03E-02 | EMB2756 |
| GRMZM2G011434 | 134 | 265 | -0.39 | 4.42E-02 | Putative uncharacterized protein |
| GRMZM2G011436 | 34 | 97 | -0.92 | 5.55E-03 | Putative uncharacterized protein |
| GRMZM2G011437 | 161 | 345 | -0.51 | 1.38E-03 | RanBP1 domain containing protein |
| GRMZM2G011469 | 116 | 253 | -0.53 | 5.69E-03 | H0322F07.2 protein |
| GRMZM2G011491 | 336 | 410 | 0.30 | 2.15E-02 | Putative uncharacterized protein |
| GRMZM2G011513 | 17 | 5 | 2.36 | 3.93E-03 | Novel protein |
| GRMZM2G011592 | 726 | 783 | 0.48 | 2.30E-09 | Os01g0557500 protein |
| GRMZM2G011631 | 284 | 331 | 0.37 | 9.54E-03 | DNA polymerase (EC 2.7.7.7) |
| GRMZM2G011655 | 44 | 24 | 1.47 | 4.35E-04 | Putative uncharacterized protein |
| GRMZM2G011777 | 49 | 169 | -1.19 | 1.05E-06 | Putative uncharacterized protein |
| GRMZM2G011862 | 28 | 0 | #VALUE! | 1.64E-10 | H0701F11.1 protein (H0723C07.14 protein) |
| GRMZM2G011912 | 34 | 109 | -1.09 | 4.47E-04 | Putative uncharacterized protein |
| GRMZM2G011919 | 1 | 13 | -3.11 | 4.97E-02 | CBL-interacting serine/threonine-protein kinase 15 (Putative uncharacterized protein) |
| GRMZM2G011968 | 32 | 6 | 3.01 | 4.46E-07 | NA |
| GRMZM2G011998 | 74 | 187 | -0.75 | 8.41E-04 | Putative uncharacterized protein |
| GRMZM2G012030 | 198 | 205 | 0.54 | 1.41E-03 | Putative uncharacterized protein |
| GRMZM2G012041 | 133 | 406 | -1.02 | 3.74E-12 | Putative uncharacterized protein |
| GRMZM2G012102 | 29 | 17 | 1.36 | 1.18E-02 | Putative uncharacterized protein |
| GRMZM2G012119 | 363 | 720 | -0.40 | 1.47E-04 | Putative uncharacterized protein |
| GRMZM2G012160 | 38 | 27 | 1.08 | 1.65E-02 | Cysteine proteinase inhibitor |
| GRMZM2G012209 | 204 | 456 | -0.57 | 2.34E-05 | Methylosome subunit pICln (Putative uncharacterized protein) |
| GRMZM2G012224 | 2,446 | 1,800 | 1.03 | 5.57E-117 | Putative uncharacterized protein |
| GRMZM2G012284 | 93 | 316 | -1.17 | 8.10E-12 | Putative uncharacterized protein |
| GRMZM2G012302 | 214 | 461 | -0.52 | 1.17E-04 | Putative uncharacterized protein (cDNA clone:J023099P19, full insert sequence) (MEI2-like RNA binding protein) (Putative AML1) |
| GRMZM2G012391 | 41 | 126 | -1.03 | 3.03E-04 | Putative uncharacterized protein |
| GRMZM2G012393 | 94 | 86 | 0.72 | 5.95E-03 | Putative uncharacterized protein |
| GRMZM2G012416 | 730 | 1,553 | -0.50 | 1.73E-13 | Eukaryotic initiation factor 5C CG2922-PF, isoform F (Putative uncharacterized protein) |
| GRMZM2G012434 | 147 | 148 | 0.58 | 3.55E-03 | Putative peptide transporter |
| GRMZM2G012498 | 7 | 0 | #VALUE! | 8.95E-03 | Putative uncharacterized protein |
| GRMZM2G012501 | 129 | 403 | -1.05 | 9.62E-13 | Putative uncharacterized protein |
| GRMZM2G012601 | 65 | 148 | -0.60 | 2.40E-02 | Putative uncharacterized protein |
| GRMZM2G012628 | 494 | 942 | -0.34 | 1.83E-04 | Putative uncharacterized protein |
| GRMZM2G012631 | 4,746 | 7,656 | -0.10 | 1.54E-03 | Putative uncharacterized protein |
| GRMZM2G012737 | 187 | 562 | -1.00 | 3.94E-16 | Acyl-protein thioesterase 2 (Putative uncharacterized protein) |
| GRMZM2G012758 | 14 | 3 | 2.81 | 3.86E-03 | Glucan endo-1,3-beta-glucosidase 7 (Putative uncharacterized protein) |
| GRMZM2G012761 | 45 | 129 | -0.93 | 1.01E-03 | Putative uncharacterized protein |
| GRMZM2G012841 | 161 | 359 | -0.57 | 2.59E-04 | Iron-stress related protein (Putative uncharacterized protein) |
| GRMZM2G012863 | 185 | 404 | -0.54 | 2.19E-04 | 3-oxoacyl-synthase I |
| GRMZM2G012874 | 126 | 131 | 0.54 | 1.68E-02 | Putative uncharacterized protein |
| GRMZM2G012964 | 193 | 791 | -1.44 | 4.55E-40 | Putative uncharacterized protein |
| GRMZM2G012970 | 227 | 600 | -0.81 | 2.52E-12 | Putative uncharacterized protein |
| GRMZM2G012992 | 52 | 139 | -0.83 | 1.94E-03 | Putative uncharacterized protein |
| GRMZM2G012999 | 89 | 192 | -0.52 | 2.38E-02 | Putative uncharacterized protein |
| GRMZM2G013152 | 250 | 240 | 0.65 | 8.74E-06 | Adenylate kinase |
| GRMZM2G013201 | 114 | 62 | 1.47 | 8.85E-10 | Putative uncharacterized protein |
| GRMZM2G013283 | 50 | 41 | 0.88 | 1.88E-02 | B0308C03.3 protein (OSJNBa0084A10.14 protein) |
| GRMZM2G013318 | 798 | 1,679 | -0.48 | 9.67E-14 | Putative GTP binding protein |
| GRMZM2G013324 | 310 | 305 | 0.61 | 2.10E-06 | Putative uncharacterized protein |
| GRMZM2G013357 | 65 | 33 | 1.57 | 1.97E-06 | OSJNBa0017P10.2 protein (OSJNBb0046P18.7 protein) |
| GRMZM2G013378 | 31 | 120 | -1.36 | 5.86E-06 | Os01g0273300 protein (Putative uncharacterized protein P0693B08.37) |
| GRMZM2G013430 | 7 | 1 | 3.40 | 3.67E-02 | Putative uncharacterized protein |
| GRMZM2G013448 | 58 | 35 | 1.32 | 1.54E-04 | Putative uncharacterized protein |
| GRMZM2G013463 | 449 | 204 | 1.73 | 6.87E-48 | Putative uncharacterized protein |
| GRMZM2G013471 | 14 | 48 | -1.19 | 2.77E-02 | Putative uncharacterized protein |
| GRMZM2G013478 | 60 | 147 | -0.70 | 7.66E-03 | Nucleoside diphosphate kinase (EC 2.7.4.6) |
| GRMZM2G013555 | 170 | 184 | 0.48 | 1.06E-02 | Putative uncharacterized protein |
| GRMZM2G013600 | 387 | 804 | -0.46 | 2.11E-06 | Putative uncharacterized protein |
| GRMZM2G013619 | 292 | 28 | 3.97 | 6.47E-81 | Polyadenylate-binding protein 2 |
| GRMZM2G013634 | 49 | 122 | -0.72 | 1.49E-02 | Os03g0119000 protein (Putative uncharacterized protein) (cDNA clone:006-210-A11, full insert sequence) (cDNA clone:006-306-G01, full insert sequence) (cDNA clone:J023091L16, full insert sequence) (ABR017Cp, putative, expressed) |
| GRMZM2G013639 | 145 | 100 | 1.13 | 2.35E-08 | Putative uncharacterized protein |
| GRMZM2G013695 | 75 | 51 | 1.15 | 9.73E-05 | Grx_S14-glutaredoxin subgroup II |
| GRMZM2G013728 | 21 | 78 | -1.30 | 9.16E-04 | Os03g0180000 protein (Cation exchanger, putative, expressed) (Putative potassium-dependent sodium-calcium exchanger-like protein) |
| GRMZM2G013750 | 212 | 499 | -0.64 | 3.95E-07 | Putative uncharacterized protein |
| GRMZM2G013767 | 26 | 14 | 1.48 | 9.82E-03 | Putative uncharacterized protein |
| GRMZM2G013798 | 398 | 1,313 | -1.13 | 8.11E-46 | ATP synthase subunit C (EC 3.6.3.14) |
| GRMZM2G013821 | 716 | 1,357 | -0.33 | 6.89E-06 | Putative uncharacterized protein (HMGc1 protein) |
| GRMZM2G013880 | 38 | 24 | 1.25 | 5.95E-03 | Putative uncharacterized protein |
| GRMZM2G013908 | 146 | 146 | 0.59 | 3.31E-03 | Putative uncharacterized protein |
| GRMZM2G013944 | 36 | 94 | -0.79 | 2.01E-02 | Senescence-associated-like protein |
| GRMZM2G014009 | 37 | 19 | 1.55 | 7.49E-04 | Putative uncharacterized protein |
| GRMZM2G014043 | 11 | 43 | -1.38 | 1.63E-02 | Putative uncharacterized protein |
| GRMZM2G014091 | 225 | 874 | -1.37 | 8.38E-41 | Small nuclear ribonucleoprotein E |
| GRMZM2G014106 | 57 | 154 | -0.84 | 7.96E-04 | Enhancer of polycomb-like protein |
| GRMZM2G014154 | 100 | 263 | -0.80 | 1.15E-05 | Histidine-containing phosphotransfer protein 1 (Putative uncharacterized protein) (Histidine-containing phosphotransfer protein) |
| GRMZM2G014170 | 55 | 134 | -0.69 | 1.21E-02 | Putative uncharacterized protein |
| GRMZM2G014193 | 21 | 59 | -0.90 | 4.95E-02 | Putative uncharacterized protein |
| GRMZM2G014240 | 468 | 304 | 1.21 | 3.69E-29 | Putative uncharacterized protein |
| GRMZM2G014341 | 113 | 282 | -0.73 | 3.65E-05 | Putative uncharacterized protein |
| GRMZM2G014376 | 152 | 152 | 0.59 | 2.81E-03 | Putative uncharacterized protein |
| GRMZM2G014387 | 124 | 275 | -0.56 | 1.93E-03 | Putative uncharacterized protein |
| GRMZM2G014397 | 84 | 267 | -1.08 | 7.20E-09 | Putative uncharacterized protein |
| GRMZM2G014400 | 21 | 62 | -0.97 | 3.11E-02 | Putative uncharacterized protein |
| GRMZM2G014419 | 136 | 148 | 0.47 | 2.92E-02 | Harpin-induced protein (Putative uncharacterized protein) |
| GRMZM2G014444 | 868 | 809 | 0.69 | 6.56E-21 | Putative uncharacterized protein |
| GRMZM2G014452 | 87 | 77 | 0.77 | 4.67E-03 | Putative uncharacterized protein |
| GRMZM2G014508 | 64 | 183 | -0.92 | 4.93E-05 | Decaprenyl-diphosphate synthase subunit 1 |
| GRMZM2G014676 | 184 | 176 | 0.66 | 1.78E-04 | Prefoldin subunit 5 (Putative uncharacterized protein) |
| GRMZM2G014709 | 24 | 70 | -0.95 | 2.08E-02 | Putative uncharacterized protein |
| GRMZM2G014750 | 539 | 1,109 | -0.45 | 3.15E-08 | Putative uncharacterized protein |
| GRMZM2G014805 | 137 | 359 | -0.80 | 2.31E-07 | Ubiquitin-specific protease 12, putative, expressed |
| GRMZM2G014872 | 26 | 93 | -1.25 | 3.90E-04 | Putative uncharacterized protein |
| GRMZM2G015011 | 65 | 170 | -0.80 | 8.04E-04 | Topoisomerase 6 subunit B-like protein |
| GRMZM2G015024 | 10 | 57 | -1.92 | 1.35E-04 | 50S ribosomal protein L22 |
| GRMZM2G015033 | 127 | 119 | 0.69 | 1.76E-03 | Putative uncharacterized protein |
| GRMZM2G015080 | 11 | 40 | -1.27 | 4.00E-02 | cDNA, clone: J100030A12, full insert sequence |
| GRMZM2G015090 | 72 | 156 | -0.52 | 4.29E-02 | Leucine-rich repeat-containing protein 40 |
| GRMZM2G015097 | 212 | 162 | 0.98 | 1.69E-09 | Putative uncharacterized protein (Tesmin/TSO1-like CXC domain containing protein) |
| GRMZM2G015100 | 129 | 94 | 1.05 | 1.04E-06 | Putative uncharacterized protein |
| GRMZM2G015126 | 116 | 477 | -1.45 | 2.77E-24 | Ankyrin like protein |
| GRMZM2G015132 | 305 | 318 | 0.53 | 5.41E-05 | Dihydrolipoamide S-acetyltransferase (EC 2.3.1.12) |
| GRMZM2G015159 | 135 | 312 | -0.62 | 2.21E-04 | Putative uncharacterized protein (Ras-related protein RHN1) |
| GRMZM2G015287 | 108 | 87 | 0.90 | 1.32E-04 | Ubiquitin carrier protein (EC 6.3.2.-) |
| GRMZM2G015289 | 29 | 86 | -0.98 | 6.62E-03 | Putative uncharacterized protein |
| GRMZM2G015291 | 93 | 266 | -0.92 | 4.83E-07 | OSJNBa0027O01.13 protein (OSJNBb0006L01.3 protein) |
| GRMZM2G015295 | 1,281 | 3,063 | -0.67 | 4.45E-44 | Putative uncharacterized protein |
| GRMZM2G015355 | 85 | 456 | -1.83 | 3.33E-32 | Chloroplast PSII K protein |
| GRMZM2G015368 | 7 | 1 | 3.40 | 3.67E-02 | Oral cancer overexpressed protein 1 |
| GRMZM2G015384 | 45 | 112 | -0.72 | 2.05E-02 | Putative uncharacterized protein |
| GRMZM2G015401 | 516 | 530 | 0.55 | 1.29E-08 | Putative uncharacterized protein |
| GRMZM2G015578 | 55 | 41 | 1.02 | 4.65E-03 | Putative uncharacterized protein |
| GRMZM2G015592 | 36 | 132 | -1.28 | 6.34E-06 | Putative splicing factor |
| GRMZM2G015642 | 65 | 191 | -0.96 | 1.63E-05 | Putative uncharacterized protein |
| GRMZM2G015727 | 9 | 43 | -1.66 | 3.77E-03 | Putative uncharacterized protein |
| GRMZM2G015735 | 292 | 606 | -0.46 | 5.72E-05 | Putative uncharacterized protein |
| GRMZM2G015739 | 17 | 4 | 2.68 | 1.25E-03 | Putative uncharacterized protein |
| GRMZM2G015784 | 1,113 | 947 | 0.82 | 2.98E-36 | Peptidyl-prolyl isomerase FKBP12 (Immunophilin) |
| GRMZM2G015869 | 104 | 252 | -0.69 | 2.57E-04 | Cystinosin (Putative uncharacterized protein) |
| GRMZM2G015880 | 70 | 181 | -0.78 | 6.31E-04 | ACI13 (Putative uncharacterized protein) |
| GRMZM2G015886 | 267 | 520 | -0.37 | 3.88E-03 | Cellulose synthase A catalytic subunit 3 [UDP-forming], putative (EC 2.4.1.12) |
| GRMZM2G015889 | 88 | 60 | 1.14 | 2.19E-05 | Putative uncharacterized protein (Serine/threonine-protein kinase NAK) |
| GRMZM2G015902 | 90 | 200 | -0.56 | 1.17E-02 | Hepatocellular carcinoma-associated antigen 59 family protein (Putative uncharacterized protein) |
| GRMZM2G015955 | 75 | 177 | -0.65 | 5.92E-03 | Putative uncharacterized protein |
| GRMZM2G015973 | 10 | 3 | 2.33 | 3.88E-02 | Putative uncharacterized protein |
| GRMZM2G015989 | 551 | 1,227 | -0.56 | 2.47E-13 | RuBisCO large subunit-binding protein subunit beta |
| GRMZM2G016184 | 37 | 147 | -1.40 | 2.52E-07 | Putative uncharacterized protein |
| GRMZM2G016232 | 570 | 122 | 2.82 | 6.44E-114 | Histone H4 |
| GRMZM2G016250 | 2,079 | 2,307 | 0.44 | 5.05E-22 | 60S ribosomal protein L27 |
| GRMZM2G016296 | 539 | 527 | 0.62 | 5.29E-11 | Putative uncharacterized protein |
| GRMZM2G016323 | 412 | 1,183 | -0.93 | 4.81E-30 | Ubiquitin carboxyl-terminal hydrolase (EC 3.1.2.15) |
| GRMZM2G016447 | 91 | 253 | -0.88 | 2.64E-06 | Putative uncharacterized protein |
| GRMZM2G016480 | 239 | 143 | 1.33 | 2.86E-17 | Putative uncharacterized protein |
| GRMZM2G016551 | 46 | 136 | -0.97 | 3.36E-04 | Putative uncharacterized protein |
| GRMZM2G016581 | 155 | 304 | -0.38 | 3.37E-02 | Putative uncharacterized protein |
| GRMZM2G016602 | 80 | 211 | -0.81 | 1.09E-04 | Protein recA |
| GRMZM2G016605 | 63 | 43 | 1.14 | 4.88E-04 | Putative GID1-like gibberellin receptor |
| GRMZM2G016644 | 24 | 67 | -0.89 | 4.16E-02 | Putative uncharacterized protein |
| GRMZM2G016660 | 47 | 150 | -1.08 | 2.89E-05 | Putative uncharacterized protein |
| GRMZM2G016671 | 77 | 172 | -0.57 | 1.79E-02 | Tousled-like kinase 1 (Fragment) |
| GRMZM2G016774 | 59 | 53 | 0.75 | 3.06E-02 | Os03g0845500 protein (Putative uncharacterized protein) (AMP-binding protein, putative, expressed) (Putative AMP-binding protein) |
| GRMZM2G016803 | 46 | 123 | -0.83 | 4.29E-03 | Outer mitochondrial membrane protein porin (Putative uncharacterized protein) (Voltage-dependent anion channel protein 2) |
| GRMZM2G016819 | 28 | 78 | -0.89 | 2.46E-02 | Putative uncharacterized protein |
| GRMZM2G016875 | 86 | 303 | -1.23 | 4.30E-12 | Putative uncharacterized protein |
| GRMZM2G016878 | 79 | 203 | -0.77 | 3.59E-04 | Putative uncharacterized protein |
| GRMZM2G016894 | 77 | 223 | -0.94 | 4.24E-06 | P0076O17.9 protein |
| GRMZM2G016923 | 73 | 170 | -0.63 | 9.04E-03 | Os02g0258300 protein (Putative uncharacterized protein) (Extra-large G-protein-like) |
| GRMZM2G016926 | 182 | 204 | 0.43 | 2.04E-02 | Putative uncharacterized protein |
| GRMZM2G016930 | 290 | 259 | 0.75 | 2.09E-08 | Serine/threonine protein phosphatase (EC 3.1.3.16) |
| GRMZM2G016939 | 189 | 154 | 0.89 | 2.38E-07 | Protein kinase G11A (Putative uncharacterized protein) |
| GRMZM2G016958 | 42 | 114 | -0.85 | 4.74E-03 | Putative uncharacterized protein |
| GRMZM2G017016 | 328 | 349 | 0.50 | 7.24E-05 | Putative uncharacterized protein |
| GRMZM2G017047 | 40 | 133 | -1.14 | 4.03E-05 | NA |
| GRMZM2G017086 | 570 | 595 | 0.53 | 8.77E-09 | Ubiquitin carboxyl-terminal hydrolase (EC 3.1.2.15) |
| GRMZM2G017087 | 1,010 | 1,750 | -0.20 | 2.68E-03 | Homeobox protein OSH1 (Homeobox transcription factor KNOTTED1) (KNOTTED1) |
| GRMZM2G017159 | 244 | 470 | -0.35 | 9.48E-03 | Putative uncharacterized protein |
| GRMZM2G017187 | 643 | 1,264 | -0.38 | 4.43E-07 | NA |
| GRMZM2G017257 | 74 | 284 | -1.35 | 3.75E-13 | Putative uncharacterized protein |
| GRMZM2G017266 | 86 | 45 | 1.53 | 5.62E-08 | Putative uncharacterized protein |
| GRMZM2G017269 | 30 | 87 | -0.94 | 9.47E-03 | HEAT repeat family protein (Putative uncharacterized protein) |
| GRMZM2G017305 | 66 | 187 | -0.91 | 4.79E-05 | Putative uncharacterized protein |
| GRMZM2G017329 | 64 | 228 | -1.24 | 2.07E-09 | Putative uncharacterized protein (Syntaxin 23) |
| GRMZM2G017334 | 26 | 74 | -0.92 | 2.02E-02 | Putative uncharacterized protein |
| GRMZM2G017368 | 157 | 55 | 2.10 | 6.04E-22 | Putative anther ethylene-upregulated protein ER1 |
| GRMZM2G017426 | 28 | 84 | -0.99 | 7.62E-03 | Putative uncharacterized protein |
| GRMZM2G017460 | 179 | 452 | -0.74 | 3.49E-08 | Os03g0353500 protein (Putative uncharacterized protein) (cDNA clone:J013014L03, full insert sequence) (Expressed protein) |
| GRMZM2G017525 | 260 | 524 | -0.42 | 8.29E-04 | Os12g0640900 protein (Putative uncharacterized protein) (cDNA clone:J013074D12, full insert sequence) (Expressed protein) |
| GRMZM2G017536 | 273 | 245 | 0.75 | 7.78E-08 | Os03g0302900 protein (Putative uncharacterized protein) (cDNA clone:J023014L15, full insert sequence) (Expressed protein) |
| GRMZM2G017606 | 23 | 9 | 1.95 | 2.63E-03 | SHI |
| GRMZM2G017616 | 46 | 28 | 1.31 | 9.20E-04 | Lipoxygenase (EC 1.13.11.12) |
| GRMZM2G017624 | 90 | 197 | -0.54 | 1.58E-02 | Putative uncharacterized protein |
| GRMZM2G017643 | 182 | 146 | 0.91 | 2.18E-07 | Putative uncharacterized protein |
| GRMZM2G017647 | 6 | 45 | -2.32 | 1.73E-04 | Putative uncharacterized protein |
| GRMZM2G017671 | 174 | 485 | -0.89 | 1.21E-11 | Putative uncharacterized protein |
| GRMZM2G017682 | 18 | 55 | -1.02 | 3.61E-02 | Expressed protein (Putative uncharacterized protein OJ1754_E06.15) |
| GRMZM2G017804 | 55 | 146 | -0.82 | 1.62E-03 | Putative uncharacterized protein |
| GRMZM2G017821 | 118 | 471 | -1.41 | 4.97E-23 | Putative uncharacterized protein |
| GRMZM2G017845 | 33 | 84 | -0.76 | 4.43E-02 | Putative uncharacterized protein |
| GRMZM2G017865 | 82 | 223 | -0.85 | 2.79E-05 | Putative uncharacterized protein DUPR11.33 |
| GRMZM2G017923 | 52 | 144 | -0.88 | 7.93E-04 | Putative uncharacterized protein |
| GRMZM2G017933 | 54 | 288 | -1.82 | 2.38E-20 | Putative uncharacterized protein |
| GRMZM2G017941 | 26 | 94 | -1.26 | 2.88E-04 | Putative uncharacterized protein |
| GRMZM2G018022 | 5 | 38 | -2.33 | 5.36E-04 | Putative uncharacterized protein |
| GRMZM2G018074 | 15 | 76 | -1.75 | 2.85E-05 | Putative uncharacterized protein |
| GRMZM2G018103 | 215 | 793 | -1.29 | 5.97E-34 | Putative uncharacterized protein |
| GRMZM2G018126 | 134 | 374 | -0.89 | 4.70E-09 | Transmembrane 9 superfamily protein member 4 |
| GRMZM2G018177 | 475 | 417 | 0.78 | 2.95E-14 | Triosephosphate isomerase (EC 5.3.1.1) |
| GRMZM2G018189 | 223 | 456 | -0.44 | 1.21E-03 | SLT1 protein |
| GRMZM2G018197 | 36 | 25 | 1.12 | 1.82E-02 | Putative uncharacterized protein |
| GRMZM2G018223 | 34 | 127 | -1.31 | 8.03E-06 | Os07g0563700 protein |
| GRMZM2G018228 | 1,971 | 2,535 | 0.23 | 2.07E-06 | 40S ribosomal protein S15a |
| GRMZM2G018251 | 280 | 306 | 0.46 | 9.08E-04 | Putative uncharacterized protein (WW domain-containing oxidoreductase) |
| GRMZM2G018341 | 30 | 13 | 1.80 | 8.97E-04 | Putative uncharacterized protein |
| GRMZM2G018356 | 23 | 93 | -1.42 | 6.20E-05 | Putative uncharacterized protein |
| GRMZM2G018369 | 33 | 134 | -1.43 | 5.02E-07 | Putative uncharacterized protein |
| GRMZM2G018398 | 116 | 315 | -0.85 | 3.53E-07 | Ethylene response element binding protein (Putative uncharacterized protein) |
| GRMZM2G018484 | 13 | 5 | 1.97 | 3.14E-02 | Putative uncharacterized protein |
| GRMZM2G018508 | 141 | 390 | -0.88 | 2.85E-09 | Putative uncharacterized protein |
| GRMZM2G018595 | 381 | 451 | 0.35 | 3.73E-03 | ATP binding protein |
| GRMZM2G018607 | 145 | 356 | -0.70 | 4.76E-06 | Putative uncharacterized protein |
| GRMZM2G018689 | 82 | 253 | -1.03 | 6.11E-08 | Ribosome recycling factor |
| GRMZM2G018697 | 192 | 485 | -0.75 | 9.22E-09 | Putative uncharacterized protein |
| GRMZM2G018716 | 19 | 8 | 1.84 | 1.28E-02 | Putative uncharacterized protein |
| GRMZM2G018770 | 989 | 736 | 1.02 | 4.54E-46 | Putative uncharacterized protein |
| GRMZM2G018775 | 115 | 236 | -0.45 | 2.92E-02 | Putative uncharacterized protein |
| GRMZM2G018901 | 211 | 525 | -0.72 | 5.50E-09 | Putative uncharacterized protein |
| GRMZM2G018941 | 520 | 964 | -0.30 | 9.96E-04 | NADH-ubiquinone oxidoreductase 13 kDa-B subunit |
| GRMZM2G018943 | 90 | 298 | -1.14 | 1.17E-10 | Translation initiation factor eIF-2B delta subunit |
| GRMZM2G018955 | 218 | 228 | 0.53 | 9.67E-04 | Putative uncharacterized protein |
| GRMZM2G018971 | 41 | 156 | -1.34 | 2.30E-07 | Putative uncharacterized protein |
| GRMZM2G019121 | 465 | 486 | 0.53 | 3.20E-07 | Putative uncharacterized protein |
| GRMZM2G019200 | 653 | 209 | 2.23 | 5.17E-99 | Putative uncharacterized protein |
| GRMZM2G019251 | 688 | 668 | 0.63 | 2.95E-14 | Putative uncharacterized protein |
| GRMZM2G019257 | 29 | 86 | -0.98 | 6.62E-03 | Putative Myb-like DNA-binding protein |
| GRMZM2G019267 | 127 | 263 | -0.46 | 1.61E-02 | Putative uncharacterized protein |
| GRMZM2G019291 | 29 | 76 | -0.80 | 4.08E-02 | Putative uncharacterized protein |
| GRMZM2G019317 | 221 | 168 | 0.99 | 5.11E-10 | Putative uncharacterized protein |
| GRMZM2G019325 | 758 | 1,416 | -0.31 | 1.80E-05 | 40S ribosomal protein S11 (Putative uncharacterized protein) |
| GRMZM2G019404 | 122 | 63 | 1.54 | 2.62E-11 | Putative uncharacterized protein |
| GRMZM2G019450 | 114 | 376 | -1.13 | 3.19E-13 | Putative uncharacterized protein |
| GRMZM2G019500 | 379 | 450 | 0.34 | 4.57E-03 | Putative uncharacterized protein |
| GRMZM2G019536 | 704 | 715 | 0.57 | 4.06E-12 | Putative uncharacterized protein |
| GRMZM2G019538 | 92 | 205 | -0.56 | 9.16E-03 | Putative uncharacterized protein |
| GRMZM2G019586 | 129 | 404 | -1.06 | 7.35E-13 | Putative uncharacterized protein |
| GRMZM2G019604 | 1,026 | 1,934 | -0.32 | 8.72E-08 | 40S ribosomal protein S6 (Ribosomal protein s6 RPS6-2) |
| GRMZM2G019621 | 234 | 445 | -0.34 | 1.85E-02 | Putative uncharacterized protein |
| GRMZM2G019673 | 135 | 120 | 0.76 | 2.57E-04 | Os10g0339600 protein (Putative uncharacterized protein) (Large secreted protein, putative, expressed) |
| GRMZM2G019686 | 157 | 110 | 1.10 | 1.11E-08 | FLP1 |
| GRMZM2G019838 | 28 | 85 | -1.01 | 5.93E-03 | Ankyrin repeat protein (Putative uncharacterized protein) |
| GRMZM2G019879 | 121 | 103 | 0.82 | 1.90E-04 | Putative uncharacterized protein (Putative VERNALIZATION INDEPENDENCE 4) |
| GRMZM2G019919 | 214 | 404 | -0.33 | 3.37E-02 | Os04g0543200 protein (cDNA clone:J033076A11, full insert sequence) (cDNA clone:J033085H02, full insert sequence) (OSJNBb0038F03.1 protein) (OSJNBb0103I08.18 protein) |
| GRMZM2G019991 | 30 | 86 | -0.93 | 9.46E-03 | Putative uncharacterized protein |
| GRMZM2G020016 | 239 | 91 | 1.98 | 7.44E-31 | Putative uncharacterized protein |
| GRMZM2G020040 | 42 | 116 | -0.87 | 3.81E-03 | Putative uncharacterized protein |
| GRMZM2G020096 | 173 | 506 | -0.96 | 1.28E-13 | Putative uncharacterized protein |
| GRMZM2G020098 | 5 | 28 | -1.89 | 1.86E-02 | Putative uncharacterized protein |
| GRMZM2G020126 | 11 | 41 | -1.31 | 2.99E-02 | Putative uncharacterized protein |
| GRMZM2G020142 | 118 | 267 | -0.59 | 1.51E-03 | Putative uncharacterized protein |
| GRMZM2G020201 | 115 | 250 | -0.53 | 6.35E-03 | Putative uncharacterized protein |
| GRMZM2G020255 | 228 | 429 | -0.32 | 3.05E-02 | Putative uncharacterized protein |
| GRMZM2G020281 | 116 | 255 | -0.54 | 4.19E-03 | Putative uncharacterized protein |
| GRMZM2G020291 | 29 | 131 | -1.58 | 9.02E-08 | Putative uncharacterized protein |
| GRMZM2G020366 | 75 | 172 | -0.61 | 1.18E-02 | Putative uncharacterized protein |
| GRMZM2G020401 | 69 | 164 | -0.66 | 7.33E-03 | Putative uncharacterized protein (cDNA clone:001-003-G05, full insert sequence) (Putative uncharacterized protein B1274F11.37) |
| GRMZM2G020461 | 46 | 111 | -0.68 | 3.16E-02 | Putative uncharacterized protein |
| GRMZM2G020484 | 39 | 104 | -0.82 | 1.09E-02 | Putative uncharacterized protein |
| GRMZM2G020500 | 47 | 16 | 2.15 | 5.45E-07 | Putative uncharacterized protein |
| GRMZM2G020544 | 105 | 221 | -0.48 | 2.22E-02 | Putative uncharacterized protein (Ras-related protein Rab11C) |
| GRMZM2G020620 | 41 | 123 | -0.99 | 6.52E-04 | Putative ankyrin protein |
| GRMZM2G020653 | 41 | 124 | -1.01 | 5.06E-04 | Putative uncharacterized protein |
| GRMZM2G020661 | 106 | 287 | -0.85 | 1.36E-06 | Putative uncharacterized protein (Ras-related protein Rab11B) |
| GRMZM2G020721 | 62 | 182 | -0.96 | 3.16E-05 | Os03g0314200 protein (cDNA clone:J023119N22, full insert sequence) (O-acetyltransferase, putative, expressed) |
| GRMZM2G020801 | 406 | 1,116 | -0.87 | 2.54E-25 | Putative uncharacterized protein |
| GRMZM2G020814 | 35 | 96 | -0.86 | 1.22E-02 | C-terminal zinc-finger |
| GRMZM2G020864 | 8 | 39 | -1.69 | 5.64E-03 | Pentatricopeptide repeat protein PPR868-14 |
| GRMZM2G020912 | 36 | 101 | -0.90 | 6.73E-03 | Putative uncharacterized protein OSJNBa0069C14.14 |
| GRMZM2G020920 | 148 | 321 | -0.53 | 1.62E-03 | Os08g0564800 protein (Putative uncharacterized protein) (cDNA clone:J023088K02, full insert sequence) (Putative CTV.22) |
| GRMZM2G020940 | 441 | 1,410 | -1.09 | 3.90E-46 | Putative uncharacterized protein |
| GRMZM2G020943 | 32 | 22 | 1.13 | 2.38E-02 | Putative uncharacterized protein |
| GRMZM2G021069 | 278 | 686 | -0.71 | 2.63E-11 | Minichromosome maintenance protein |
| GRMZM2G021170 | 297 | 223 | 1.00 | 8.60E-14 | Putative uncharacterized protein |
| GRMZM2G021219 | 214 | 563 | -0.80 | 1.89E-11 | ATP/GTP binding protein |
| GRMZM2G021270 | 284 | 688 | -0.69 | 1.24E-10 | DNA topoisomerase 2 (EC 5.99.1.3) |
| GRMZM2G021299 | 99 | 275 | -0.88 | 8.94E-07 | Putative ubiquitin-protein ligase 1 |
| GRMZM2G021331 | 473 | 1,017 | -0.51 | 1.77E-09 | ATP synthase subunit beta (EC 3.6.3.14) |
| GRMZM2G021339 | 11 | 0 | #VALUE! | 3.46E-04 | Putative uncharacterized protein |
| GRMZM2G021464 | 20 | 69 | -1.20 | 4.36E-03 | Small nuclear ribonucleoprotein LSM1 |
| GRMZM2G021470 | 142 | 109 | 0.97 | 1.43E-06 | Putative uncharacterized protein |
| GRMZM2G021498 | 27 | 101 | -1.31 | 7.67E-05 | Putative uncharacterized protein |
| GRMZM2G021567 | 105 | 294 | -0.89 | 2.47E-07 | Putative uncharacterized protein |
| GRMZM2G021621 | 84 | 196 | -0.63 | 4.65E-03 | Beta-expansin 1a (Putative uncharacterized protein) |
| GRMZM2G021704 | 13 | 55 | -1.49 | 2.86E-03 | Dihydroorotase |
| GRMZM2G021710 | 11 | 44 | -1.41 | 1.20E-02 | Os04g0488500 protein |
| GRMZM2G021742 | 489 | 518 | 0.51 | 4.44E-07 | U2 small nuclear ribonucleoprotein A |
| GRMZM2G021777 | 72 | 33 | 1.72 | 8.61E-08 | CONSTANS-like protein CO5 |
| GRMZM2G021816 | 406 | 334 | 0.87 | 8.28E-15 | Activator of 90 kDa heat shock protein ATPase |
| GRMZM2G021834 | 11 | 41 | -1.31 | 2.99E-02 | Putative uncharacterized protein |
| GRMZM2G021885 | 120 | 110 | 0.72 | 1.43E-03 | cDNA, clone: J090049M22, full insert sequence (OSJNBa0053K19.22 protein) |
| GRMZM2G021912 | 143 | 151 | 0.51 | 1.26E-02 | Putative uncharacterized protein |
| GRMZM2G022019 | 176 | 154 | 0.78 | 1.05E-05 | Putative uncharacterized protein |
| GRMZM2G022041 | 832 | 925 | 0.44 | 5.25E-09 | Putative uncharacterized protein (Small nuclear ribonucleoprotein-associated protein B) |
| GRMZM2G022061 | 74 | 331 | -1.57 | 6.60E-19 | Putative uncharacterized protein OSJNBb0004M10.11 |
| GRMZM2G022088 | 120 | 109 | 0.73 | 1.09E-03 | Putative uncharacterized protein |
| GRMZM2G022090 | 69 | 54 | 0.95 | 2.09E-03 | Putative uncharacterized protein (Cyanobacteria-specific protein-like) (Os01g0338600 protein) |
| GRMZM2G022107 | 30 | 84 | -0.89 | 1.50E-02 | Putative uncharacterized protein |
| GRMZM2G022120 | 40 | 99 | -0.72 | 3.22E-02 | Putative uncharacterized protein |
| GRMZM2G022180 | 488 | 1,293 | -0.81 | 2.30E-26 | Calnexin (Fragment) |
| GRMZM2G022206 | 105 | 249 | -0.65 | 5.68E-04 | Ubiquitin carrier protein (EC 6.3.2.-) |
| GRMZM2G022212 | 8 | 0 | #VALUE! | 4.02E-03 | Putative uncharacterized protein |
| GRMZM2G022253 | 626 | 666 | 0.50 | 1.04E-08 | Putative uncharacterized protein |
| GRMZM2G022258 | 116 | 308 | -0.82 | 1.08E-06 | Os03g0858100 protein (Putative chromosome region maintenance protein) |
| GRMZM2G022269 | 482 | 898 | -0.31 | 1.20E-03 | Elongation factor Tu |
| GRMZM2G022275 | 189 | 411 | -0.53 | 2.17E-04 | Putative uncharacterized protein |
| GRMZM2G022279 | 39 | 105 | -0.84 | 8.78E-03 | Putative uncharacterized protein |
| GRMZM2G022298 | 88 | 216 | -0.70 | 6.48E-04 | Putative uncharacterized protein |
| GRMZM2G022365 | 234 | 459 | -0.38 | 5.74E-03 | Putative uncharacterized protein (Putative methyl-binding domain protein MBD106) |
| GRMZM2G022413 | 34 | 9 | 2.51 | 4.15E-06 | Peptide methionine sulfoxide reductase |
| GRMZM2G022603 | 107 | 382 | -1.24 | 1.50E-15 | Os10g0577800 protein (Putative uncharacterized protein) (cDNA clone:J023139M21, full insert sequence) (Poly polymerase catalytic domain containing protein, expressed) (Putative CEO protein) |
| GRMZM2G022611 | 18 | 1 | 4.76 | 9.34E-06 | Os03g0303100 protein (cDNA clone:J023081F02, full insert sequence) (Expressed protein) |
| GRMZM2G022619 | 114 | 243 | -0.50 | 1.11E-02 | Putative uncharacterized protein |
| GRMZM2G022627 | 53 | 20 | 2.00 | 3.61E-07 | Nucleotide binding protein |
| GRMZM2G022645 | 16 | 7 | 1.78 | 2.33E-02 | Putative uncharacterized protein |
| GRMZM2G022694 | 67 | 199 | -0.98 | 7.63E-06 | Putative uncharacterized protein |
| GRMZM2G022763 | 69 | 204 | -0.97 | 5.86E-06 | Putative uncharacterized protein |
| GRMZM2G022768 | 103 | 84 | 0.89 | 3.01E-04 | Putative uncharacterized protein |
| GRMZM2G022793 | 45 | 109 | -0.68 | 2.99E-02 | Putative uncharacterized protein |
| GRMZM2G022861 | 24 | 72 | -0.99 | 1.29E-02 | Cyclic nucleotide-gated ion channel 9 |
| GRMZM2G022866 | 5 | 25 | -1.73 | 3.74E-02 | Putative uncharacterized protein |
| GRMZM2G022987 | 128 | 103 | 0.90 | 2.41E-05 | PHD finger protein-like |
| GRMZM2G023020 | 176 | 114 | 1.22 | 2.97E-11 | Putative uncharacterized protein |
| GRMZM2G023051 | 87 | 243 | -0.89 | 4.55E-06 | Putative uncharacterized protein |
| GRMZM2G023105 | 22 | 14 | 1.24 | 4.47E-02 | Putative uncharacterized protein |
| GRMZM2G023110 | 263 | 591 | -0.58 | 5.73E-07 | Atypical receptor-like kinase MARK |
| GRMZM2G023194 | 363 | 339 | 0.69 | 5.63E-09 | Ubiquinol-cytochrome c reductase iron-sulfur subunit (EC 1.10.2.2) |
| GRMZM2G023220 | 44 | 121 | -0.87 | 2.92E-03 | Putative uncharacterized protein |
| GRMZM2G023232 | 346 | 876 | -0.75 | 1.40E-15 | Heat shock 70 kDa protein 4 |
| GRMZM2G023242 | 280 | 579 | -0.46 | 1.05E-04 | Putative uncharacterized protein (Transcription factor BTF3) |
| GRMZM2G023275 | 57 | 153 | -0.83 | 9.88E-04 | cDNA clone:J033046B22, full insert sequence (Putative octicosapeptide/Phox/Bem1p (PB1) domain-/tetratricopeptide repeat (TPR)-containing protein) |
| GRMZM2G023313 | 27 | 86 | -1.08 | 3.18E-03 | Putative uncharacterized protein |
| GRMZM2G023392 | 29 | 104 | -1.25 | 1.13E-04 | Putative uncharacterized protein |
| GRMZM2G023475 | 23 | 72 | -1.05 | 8.80E-03 | Putative uncharacterized protein |
| GRMZM2G023563 | 175 | 186 | 0.50 | 6.02E-03 | Putative uncharacterized protein |
| GRMZM2G023575 | 70 | 207 | -0.97 | 5.12E-06 | Putative uncharacterized protein (Vacuolar protein sorting 37C) |
| GRMZM2G023585 | 183 | 580 | -1.07 | 1.06E-18 | NA |
| GRMZM2G023667 | 243 | 514 | -0.49 | 9.41E-05 | Putative uncharacterized protein (Single myb histone 4) (Single myb histone 3) |
| GRMZM2G023884 | 22 | 96 | -1.53 | 1.28E-05 | Putative uncharacterized protein |
| GRMZM2G023921 | 139 | 114 | 0.88 | 1.93E-05 | DNA-binding WRKY |
| GRMZM2G023973 | 84 | 241 | -0.93 | 1.64E-06 | Formin binding protein 3-like |
| GRMZM2G023988 | 72 | 171 | -0.66 | 6.02E-03 | Zinc finger, C2H2 type family protein |
| GRMZM2G024054 | 106 | 93 | 0.78 | 1.14E-03 | Putative uncharacterized protein |
| GRMZM2G024099 | 366 | 363 | 0.60 | 3.09E-07 | Putative uncharacterized protein |
| GRMZM2G024151 | 354 | 868 | -0.70 | 6.41E-14 | Glycogen synthase kinase-3 MsK-3 |
| GRMZM2G024159 | 98 | 302 | -1.03 | 2.54E-09 | Protein YIP1 |
| GRMZM2G024196 | 30 | 22 | 1.04 | 4.41E-02 | OSIGBa0153E02-OSIGBa0093I20.14 protein |
| GRMZM2G024267 | 120 | 60 | 1.59 | 1.87E-11 | Putative uncharacterized protein |
| GRMZM2G024293 | 288 | 623 | -0.52 | 3.37E-06 | XPA-binding protein 1 |
| GRMZM2G024354 | 1,926 | 1,429 | 1.02 | 2.49E-90 | Ribosomal protein L15 |
| GRMZM2G024389 | 162 | 465 | -0.93 | 5.57E-12 | Putative uncharacterized protein |
| GRMZM2G024395 | 53 | 139 | -0.80 | 3.25E-03 | Putative uncharacterized protein |
| GRMZM2G024435 | 1 | 14 | -3.22 | 3.22E-02 | Putative uncharacterized protein |
| GRMZM2G024451 | 107 | 258 | -0.68 | 2.53E-04 | Pre-mRNA-splicing factor ISY1 |
| GRMZM2G024477 | 195 | 417 | -0.51 | 3.59E-04 | Os09g0134500 protein (cDNA clone:J033074A15, full insert sequence) (Trithorax-like) |
| GRMZM2G024499 | 23 | 14 | 1.31 | 3.13E-02 | NA |
| GRMZM2G024576 | 197 | 140 | 1.08 | 1.69E-10 | Putative uncharacterized protein (cDNA clone:J013159H12, full insert sequence) (Putative kinesin) |
| GRMZM2G024612 | 51 | 158 | -1.04 | 2.95E-05 | Putative uncharacterized protein |
| GRMZM2G024626 | 55 | 134 | -0.69 | 1.21E-02 | Putative uncharacterized protein |
| GRMZM2G024647 | 921 | 916 | 0.60 | 3.22E-17 | 60S ribosomal protein L6 |
| GRMZM2G024657 | 96 | 99 | 0.55 | 3.69E-02 | OTU domain-containing protein 6B |
| GRMZM2G024668 | 1,894 | 2,567 | 0.15 | 3.18E-03 | Heat shock protein 90 |
| GRMZM2G024686 | 160 | 451 | -0.90 | 4.05E-11 | Aspartokinase (EC 2.7.2.4) |
| GRMZM2G024690 | 65 | 146 | -0.58 | 3.32E-02 | Protein binding protein |
| GRMZM2G024693 | 59 | 142 | -0.68 | 1.27E-02 | Putative uncharacterized protein |
| GRMZM2G024730 | 98 | 210 | -0.51 | 1.80E-02 | Putative uncharacterized protein |
| GRMZM2G024733 | 213 | 232 | 0.47 | 4.27E-03 | Putative uncharacterized protein |
| GRMZM2G024806 | 81 | 56 | 1.12 | 6.76E-05 | F-box domain containing protein |
| GRMZM2G024823 | 147 | 148 | 0.58 | 3.55E-03 | Putative uncharacterized protein |
| GRMZM2G024838 | 103 | 93 | 0.74 | 2.87E-03 | Structural constituent of ribosome |
| GRMZM2G024910 | 955 | 1,676 | -0.22 | 1.22E-03 | Ubiquitin carrier protein (EC 6.3.2.-) |
| GRMZM2G024992 | 30 | 80 | -0.82 | 2.91E-02 | P0432B10.2 protein |
| GRMZM2G025059 | 68 | 164 | -0.68 | 5.82E-03 | Putative uncharacterized protein |
| GRMZM2G025113 | 25 | 101 | -1.42 | 2.74E-05 | Os03g0340900 protein (Putative uncharacterized protein) (Chloroplast RelA homologue 1) (RelA/SpoT protein, putative, expressed) |
| GRMZM2G025154 | 76 | 69 | 0.73 | 1.47E-02 | Serine carboxypeptidase F13S12.6 |
| GRMZM2G025182 | 5 | 26 | -1.79 | 2.67E-02 | Pectinesterase (EC 3.1.1.11) |
| GRMZM2G025214 | 462 | 1,108 | -0.67 | 2.84E-16 | Putative uncharacterized protein |
| GRMZM2G025215 | 152 | 164 | 0.48 | 1.69E-02 | Proline-rich protein |
| GRMZM2G025281 | 190 | 376 | -0.39 | 1.08E-02 | Putative uncharacterized protein |
| GRMZM2G025303 | 19 | 70 | -1.29 | 2.08E-03 | NA |
| GRMZM2G025356 | 494 | 410 | 0.86 | 1.91E-17 | Putative uncharacterized protein |
| GRMZM2G025366 | 72 | 201 | -0.89 | 3.42E-05 | Putative uncharacterized protein |
| GRMZM2G025387 | 70 | 204 | -0.95 | 8.38E-06 | Calcium-dependent protein kinase, isoform 2 |
| GRMZM2G025531 | 44 | 117 | -0.82 | 6.97E-03 | Putative uncharacterized protein |
| GRMZM2G025592 | 652 | 1,241 | -0.34 | 1.34E-05 | Chromomethylase OsMET2a |
| GRMZM2G025594 | 54 | 124 | -0.61 | 3.95E-02 | F-box domain containing protein |
| GRMZM2G025598 | 246 | 576 | -0.64 | 5.37E-08 | Putative uncharacterized protein |
| GRMZM2G025648 | 22 | 75 | -1.18 | 2.63E-03 | Putative uncharacterized protein |
| GRMZM2G025685 | 63 | 18 | 2.40 | 1.16E-10 | Putative uncharacterized protein |
| GRMZM2G025742 | 0 | 11 | #NUM! | 2.14E-02 | Auxin efflux carrier component 6 |
| GRMZM2G025812 | 45 | 26 | 1.38 | 6.38E-04 | BZIP transcription factor protein |
| GRMZM2G025855 | 32 | 24 | 1.01 | 4.05E-02 | Putative uncharacterized protein |
| GRMZM2G025906 | 169 | 132 | 0.95 | 2.69E-07 | Putative uncharacterized protein |
| GRMZM2G025977 | 58 | 39 | 1.16 | 7.89E-04 | Putative uncharacterized protein |
| GRMZM2G025992 | 164 | 363 | -0.55 | 2.91E-04 | Superoxide dismutase [Cu-Zn] (EC 1.15.1.1) |
| GRMZM2G026043 | 28 | 110 | -1.38 | 1.46E-05 | Putative uncharacterized protein (cDNA clone:J023140C12, full insert sequence) (Cyclin G-associated kinase-like protein) |
| GRMZM2G026065 | 252 | 251 | 0.60 | 4.16E-05 | Putative uncharacterized protein |
| GRMZM2G026085 | 437 | 414 | 0.67 | 3.53E-10 | Putative uncharacterized protein |
| GRMZM2G026117 | 356 | 226 | 1.25 | 3.71E-23 | Putative uncharacterized protein |
| GRMZM2G026180 | 77 | 196 | -0.76 | 5.68E-04 | Putative uncharacterized protein |
| GRMZM2G026301 | 167 | 387 | -0.62 | 2.35E-05 | Putative uncharacterized protein pk8-b |
| GRMZM2G026346 | 229 | 571 | -0.73 | 7.43E-10 | Putative uncharacterized protein |
| GRMZM2G026417 | 27 | 16 | 1.35 | 1.45E-02 | Putative uncharacterized protein |
| GRMZM2G026490 | 79 | 79 | 0.59 | 4.79E-02 | Putative uncharacterized protein |
| GRMZM2G026558 | 96 | 97 | 0.58 | 2.93E-02 | OSIGBa0115K01-H0319F09.22 protein |
| GRMZM2G026576 | 115 | 121 | 0.52 | 2.91E-02 | Putative uncharacterized protein |
| GRMZM2G026702 | 60 | 50 | 0.85 | 1.26E-02 | Putative uncharacterized protein |
| GRMZM2G026758 | 137 | 90 | 1.20 | 1.21E-08 | Putative uncharacterized protein |
| GRMZM2G026767 | 12 | 44 | -1.28 | 1.99E-02 | Putative uncharacterized protein |
| GRMZM2G026802 | 219 | 157 | 1.07 | 2.56E-11 | Putative uncharacterized protein |
| GRMZM2G026839 | 239 | 225 | 0.68 | 5.75E-06 | Putative uncharacterized protein |
| GRMZM2G026868 | 225 | 494 | -0.54 | 2.43E-05 | Putative uncharacterized protein |
| GRMZM2G026892 | 281 | 728 | -0.78 | 5.01E-14 | Putative uncharacterized protein |
| GRMZM2G026918 | 78 | 200 | -0.77 | 4.07E-04 | Putative uncharacterized protein |
| GRMZM2G026952 | 29 | 83 | -0.93 | 1.37E-02 | Putative uncharacterized protein |
| GRMZM2G026991 | 358 | 1,160 | -1.10 | 4.97E-39 | Putative uncharacterized protein |
| GRMZM2G027019 | 268 | 767 | -0.93 | 2.09E-19 | Putative uncharacterized protein |
| GRMZM2G027043 | 13 | 66 | -1.75 | 8.78E-05 | Phosphatidate cytidylyltransferase (EC 2.7.7.41) |
| GRMZM2G027047 | 66 | 160 | -0.69 | 6.25E-03 | Putative uncharacterized protein |
| GRMZM2G027059 | 40 | 103 | -0.77 | 1.79E-02 | Putative uncharacterized protein |
| GRMZM2G027079 | 36 | 18 | 1.59 | 6.18E-04 | Putative uncharacterized protein |
| GRMZM2G027105 | 208 | 410 | -0.39 | 8.64E-03 | Putative uncharacterized protein |
| GRMZM2G027183 | 59 | 137 | -0.62 | 2.55E-02 | Putative uncharacterized protein |
| GRMZM2G027209 | 69 | 160 | -0.62 | 1.25E-02 | Putative uncharacterized protein |
| GRMZM2G027232 | 177 | 192 | 0.47 | 9.37E-03 | 50S ribosomal protein L11 (Putative uncharacterized protein) |
| GRMZM2G027282 | 313 | 929 | -0.98 | 1.23E-25 | Putative uncharacterized protein |
| GRMZM2G027441 | 1,989 | 4,255 | -0.51 | 1.80E-37 | 60S ribosomal protein L5-1 |
| GRMZM2G027451 | 4,876 | 6,679 | 0.14 | 5.60E-06 | 60S ribosomal protein L10-3 |
| GRMZM2G027603 | 28 | 80 | -0.92 | 1.56E-02 | Putative uncharacterized protein |
| GRMZM2G027663 | 82 | 220 | -0.83 | 4.32E-05 | Putative uncharacterized protein |
| GRMZM2G027673 | 207 | 142 | 1.14 | 8.07E-12 | Putative uncharacterized protein |
| GRMZM2G027695 | 52 | 22 | 1.83 | 2.07E-06 | Putative uncharacterized protein |
| GRMZM2G027756 | 65 | 171 | -0.80 | 6.52E-04 | GCK-like kinase MIK |
| GRMZM2G027932 | 100 | 211 | -0.49 | 2.52E-02 | Putative uncharacterized protein |
| GRMZM2G027995 | 1,095 | 2,459 | -0.58 | 2.61E-27 | Translational initiation factor eIF-4A |
| GRMZM2G028004 | 37 | 102 | -0.87 | 7.34E-03 | Putative uncharacterized protein |
| GRMZM2G028041 | 204 | 611 | -0.99 | 2.34E-17 | Putative uncharacterized protein |
| GRMZM2G028136 | 7 | 30 | -1.51 | 4.50E-02 | Putative uncharacterized protein |
| GRMZM2G028151 | 389 | 1,205 | -1.04 | 8.49E-37 | Putative uncharacterized protein |
| GRMZM2G028156 | 187 | 401 | -0.51 | 4.26E-04 | Putative uncharacterized protein |
| GRMZM2G028218 | 646 | 675 | 0.53 | 8.14E-10 | Putative uncharacterized protein |
| GRMZM2G028307 | 92 | 382 | -1.46 | 8.17E-20 | Putative uncharacterized protein |
| GRMZM2G028313 | 171 | 408 | -0.66 | 3.38E-06 | Putative uncharacterized protein |
| GRMZM2G028335 | 87 | 203 | -0.63 | 3.84E-03 | Putative uncharacterized protein |
| GRMZM2G028346 | 88 | 303 | -1.19 | 1.11E-11 | Proteasome subunit alpha type (EC 3.4.25.1) |
| GRMZM2G028353 | 16 | 68 | -1.50 | 5.95E-04 | Cellulose synthase-7 |
| GRMZM2G028379 | 458 | 1,920 | -1.48 | 8.15E-101 | p8MTCP1 |
| GRMZM2G028432 | 181 | 181 | 0.59 | 8.17E-04 | V-ATPase subunit c |
| GRMZM2G028500 | 31 | 89 | -0.93 | 8.22E-03 | Putative uncharacterized protein |
| GRMZM2G028501 | 39 | 99 | -0.75 | 2.48E-02 | Glycosyltransferase QUASIMODO1, putative, expressed |
| GRMZM2G028535 | 288 | 779 | -0.84 | 5.61E-17 | Delta 1-pyrroline-5-carboxylate synthetase |
| GRMZM2G028587 | 243 | 242 | 0.60 | 6.41E-05 | Cylicin-1 |
| GRMZM2G028690 | 134 | 325 | -0.69 | 2.41E-05 | Putative uncharacterized protein |
| GRMZM2G028718 | 43 | 146 | -1.17 | 9.05E-06 | Putative uncharacterized protein P0030H07.23 |
| GRMZM2G028730 | 38 | 232 | -2.02 | 1.01E-18 | Putative uncharacterized protein |
| GRMZM2G028766 | 253 | 206 | 0.89 | 1.08E-09 | Putative uncharacterized protein |
| GRMZM2G028813 | 58 | 131 | -0.58 | 4.00E-02 | Putative uncharacterized protein |
| GRMZM2G028834 | 263 | 243 | 0.71 | 6.63E-07 | Eukaryotic translation initiation factor 3 subunit 2 |
| GRMZM2G028852 | 9 | 35 | -1.37 | 3.67E-02 | Putative uncharacterized protein (Mitochondrial import inner membrane translocase subunit Tim17 family protein, expressed) |
| GRMZM2G028883 | 890 | 2,385 | -0.83 | 3.57E-50 | Putative uncharacterized protein |
| GRMZM2G028900 | 306 | 348 | 0.41 | 2.52E-03 | GTP binding protein |
| GRMZM2G028905 | 18 | 64 | -1.24 | 4.19E-03 | Putative uncharacterized protein |
| GRMZM2G028914 | 51 | 142 | -0.89 | 9.09E-04 | Putative mechanosensitive ion channel protein |
| GRMZM2G028924 | 15 | 57 | -1.33 | 4.32E-03 | Putative uncharacterized protein |
| GRMZM2G028929 | 471 | 506 | 0.49 | 2.19E-06 | Putative uncharacterized protein (Epsilon-COP) |
| GRMZM2G028955 | 904 | 997 | 0.45 | 3.30E-10 | Histone H2A |
| GRMZM2G028986 | 421 | 406 | 0.64 | 3.62E-09 | Cytochrome b5 (Putative uncharacterized protein) |
| GRMZM2G029001 | 63 | 198 | -1.06 | 1.67E-06 | Putative uncharacterized protein |
| GRMZM2G029027 | 12 | 41 | -1.18 | 4.59E-02 | Arginyl-tRNA synthetase (Putative uncharacterized protein) |
| GRMZM2G029096 | 130 | 103 | 0.93 | 1.39E-05 | DNA-binding protein |
| GRMZM2G029101 | 36 | 25 | 1.12 | 1.82E-02 | Expp1 protein |
| GRMZM2G029148 | 480 | 1,538 | -1.09 | 1.85E-50 | Profilin |
| GRMZM2G029184 | 207 | 541 | -0.79 | 9.51E-11 | Fiber protein Fb34 |
| GRMZM2G029242 | 17 | 8 | 1.68 | 3.02E-02 | Serine/threonine-protein kinase receptor |
| GRMZM2G029262 | 209 | 412 | -0.39 | 8.83E-03 | Os12g0608600 protein |
| GRMZM2G029307 | 41 | 148 | -1.26 | 1.98E-06 | Putative uncharacterized protein |
| GRMZM2G029314 | 12 | 44 | -1.28 | 1.99E-02 | Putative uncharacterized protein |
| GRMZM2G029345 | 13 | 1 | 4.29 | 4.67E-04 | Putative uncharacterized protein OSJNBa0029G06.14 |
| GRMZM2G029478 | 101 | 256 | -0.75 | 5.38E-05 | F-box domain containing protein |
| GRMZM2G029486 | 5 | 26 | -1.79 | 2.67E-02 | Putative uncharacterized protein (Ras-related protein Rab11A) |
| GRMZM2G029536 | 36 | 106 | -0.97 | 2.69E-03 | Nucleotide binding protein |
| GRMZM2G029543 | 107 | 261 | -0.70 | 1.74E-04 | Malonyl CoA-acyl carrier protein transacylase |
| GRMZM2G029559 | 1,265 | 1,247 | 0.61 | 2.18E-24 | Elongation factor 1-gamma 2 |
| GRMZM2G029566 | 86 | 184 | -0.51 | 3.32E-02 | Putative uncharacterized protein |
| GRMZM2G029573 | 328 | 400 | 0.31 | 2.40E-02 | Putative uncharacterized protein |
| GRMZM2G029583 | 431 | 1,045 | -0.69 | 5.36E-16 | 26S proteasome non-ATPase regulatory subunit 6 (Putative uncharacterized protein) |
| GRMZM2G029698 | 85 | 56 | 1.19 | 1.31E-05 | Putative uncharacterized protein |
| GRMZM2G029731 | 58 | 133 | -0.61 | 3.41E-02 | Inositol-1-monophosphatase (Putative uncharacterized protein) |
| GRMZM2G029785 | 96 | 220 | -0.60 | 3.52E-03 | Putative uncharacterized protein |
| GRMZM2G029845 | 71 | 69 | 0.63 | 4.09E-02 | Putative uncharacterized protein (Pyrimidine-specific ribonucleoside hydrolase rihA) |
| GRMZM2G029912 | 41 | 28 | 1.14 | 7.15E-03 | Putative uncharacterized protein |
| GRMZM2G029933 | 42 | 116 | -0.87 | 3.81E-03 | mTERF family protein |
| GRMZM2G030016 | 3,248 | 6,104 | -0.32 | 5.53E-23 | 40S ribosomal protein S7 |
| GRMZM2G030038 | 119 | 114 | 0.65 | 3.90E-03 | Putative uncharacterized protein |
| GRMZM2G030072 | 116 | 240 | -0.46 | 2.31E-02 | ATP-dependent Clp protease proteolytic subunit |
| GRMZM2G030125 | 40 | 169 | -1.49 | 3.52E-09 | Auxin Efflux Carrier family protein (Putative uncharacterized protein) |
| GRMZM2G030167 | 306 | 285 | 0.69 | 9.84E-08 | Putative uncharacterized protein |
| GRMZM2G030173 | 45 | 18 | 1.91 | 6.03E-06 | Xyloglucan endotransglucosylase/hydrolase protein 5 |
| GRMZM2G030223 | 81 | 50 | 1.29 | 7.53E-06 | Patatin-like protein 3 |
| GRMZM2G030228 | 814 | 564 | 1.12 | 1.99E-44 | Ribosomal protein S8 |
| GRMZM2G030235 | 81 | 178 | -0.54 | 2.43E-02 | Putative uncharacterized protein |
| GRMZM2G030272 | 60 | 51 | 0.83 | 1.36E-02 | WRKY55-superfamily of TFs having WRKY and zinc finger domains |
| GRMZM2G030293 | 401 | 751 | -0.31 | 2.83E-03 | S28 ribosomal protein (Fragment) |
| GRMZM2G030384 | 33 | 98 | -0.98 | 3.10E-03 | Putative uncharacterized protein |
| GRMZM2G030422 | 520 | 505 | 0.63 | 6.12E-11 | Putative uncharacterized protein |
| GRMZM2G030458 | 47 | 29 | 1.29 | 1.06E-03 | Putative uncharacterized protein |
| GRMZM2G030510 | 265 | 578 | -0.53 | 4.96E-06 | NADH-ubiquinone oxidoreductase 10.5 kDa subunit |
| GRMZM2G030529 | 78 | 221 | -0.91 | 7.53E-06 | Amine oxidase, flavin-containing family protein, expressed |
| GRMZM2G030557 | 93 | 194 | -0.47 | 4.06E-02 | Putative uncharacterized protein |
| GRMZM2G030628 | 45 | 16 | 2.08 | 1.58E-06 | Putative uncharacterized protein |
| GRMZM2G030659 | 161 | 377 | -0.64 | 2.08E-05 | Putative uncharacterized protein |
| GRMZM2G030673 | 183 | 428 | -0.63 | 4.49E-06 | Os05g0467000 protein (Putative calcium-dependent protein kinase) |
| GRMZM2G030692 | 56 | 160 | -0.92 | 1.82E-04 | Putative uncharacterized protein |
| GRMZM2G030710 | 181 | 438 | -0.68 | 6.35E-07 | Putative uncharacterized protein |
| GRMZM2G030731 | 1,479 | 3,029 | -0.44 | 4.83E-21 | 60S ribosomal protein L27a-3 (Putative uncharacterized protein) |
| GRMZM2G030768 | 313 | 811 | -0.78 | 1.46E-15 | Putative uncharacterized protein |
| GRMZM2G030858 | 1,064 | 1,337 | 0.26 | 1.04E-04 | Putative uncharacterized protein |
| GRMZM2G030873 | 65 | 164 | -0.74 | 2.26E-03 | cDNA clone:001-117-A11, full insert sequence |
| GRMZM2G030902 | 325 | 393 | 0.32 | 1.86E-02 | Putative uncharacterized protein |
| GRMZM2G030915 | 2,736 | 3,263 | 0.34 | 1.24E-17 | 40S ribosomal protein S3a |
| GRMZM2G031022 | 10 | 76 | -2.33 | 2.07E-07 | Putative uncharacterized protein |
| GRMZM2G031053 | 63 | 149 | -0.65 | 1.31E-02 | Putative uncharacterized protein |
| GRMZM2G031107 | 97 | 94 | 0.64 | 1.27E-02 | Putative uncharacterized protein |
| GRMZM2G031204 | 64 | 144 | -0.58 | 3.18E-02 | FK506-binding protein 2-1 |
| GRMZM2G031298 | 78 | 170 | -0.53 | 2.94E-02 | Putative uncharacterized protein (Tankyrase 2) |
| GRMZM2G031326 | 203 | 407 | -0.41 | 4.87E-03 | Nucleolar complex protein 4 |
| GRMZM2G031461 | 25 | 74 | -0.97 | 1.44E-02 | Putative uncharacterized protein |
| GRMZM2G031501 | 337 | 1,283 | -1.34 | 5.36E-58 | Putative uncharacterized protein |
| GRMZM2G031529 | 195 | 152 | 0.95 | 2.17E-08 | Putative uncharacterized protein |
| GRMZM2G031545 | 2,323 | 1,901 | 0.88 | 9.40E-85 | Elongation factor 1-delta 1 (Putative uncharacterized protein) |
| GRMZM2G031568 | 14 | 48 | -1.19 | 2.77E-02 | Putative uncharacterized protein |
| GRMZM2G031584 | 22 | 75 | -1.18 | 2.63E-03 | DNAJ heat shock N-terminal domain-containing protein-like (Os01g0927400 protein) |
| GRMZM2G031586 | 1,253 | 1,054 | 0.84 | 3.18E-42 | Putative uncharacterized protein |
| GRMZM2G031591 | 29 | 78 | -0.84 | 3.32E-02 | Putative uncharacterized protein |
| GRMZM2G031607 | 150 | 308 | -0.45 | 1.03E-02 | Calcyclin-binding protein (Putative uncharacterized protein) |
| GRMZM2G031656 | 166 | 354 | -0.50 | 1.41E-03 | Putative uncharacterized protein |
| GRMZM2G031724 | 50 | 20 | 1.91 | 1.65E-06 | cDNA clone:001-028-C12, full insert sequence (cDNA clone:001-031-D01, full insert sequence) (cDNA clone:J033060N07, full insert sequence) (Gibberellin 2-oxidase) (Os01g0757200 protein) |
| GRMZM2G031761 | 22 | 72 | -1.12 | 5.90E-03 | FLU (Putative uncharacterized protein) |
| GRMZM2G031802 | 60 | 135 | -0.58 | 4.34E-02 | ER lumen protein retaining receptor |
| GRMZM2G031825 | 109 | 233 | -0.50 | 1.25E-02 | Putative uncharacterized protein |
| GRMZM2G031827 | 215 | 560 | -0.79 | 4.91E-11 | Splicing factor U2af 38 kDa subunit |
| GRMZM2G031846 | 612 | 500 | 0.88 | 1.94E-22 | Putative uncharacterized protein |
| GRMZM2G032003 | 491 | 584 | 0.34 | 9.45E-04 | Putative uncharacterized protein |
| GRMZM2G032071 | 170 | 350 | -0.45 | 4.70E-03 | Putative uncharacterized protein |
| GRMZM2G032107 | 67 | 33 | 1.61 | 8.04E-07 | Putative uncharacterized protein |
| GRMZM2G032110 | 174 | 184 | 0.51 | 5.76E-03 | Putative uncharacterized protein |
| GRMZM2G032163 | 110 | 295 | -0.83 | 1.56E-06 | Putative uncharacterized protein |
| GRMZM2G032258 | 354 | 418 | 0.35 | 4.94E-03 | Nucleic acid binding protein |
| GRMZM2G032314 | 10 | 37 | -1.30 | 4.43E-02 | PRGR1 |
| GRMZM2G032315 | 3,708 | 4,925 | 0.18 | 1.20E-07 | 60S acidic ribosomal protein P1 |
| GRMZM2G032337 | 61 | 136 | -0.57 | 4.50E-02 | Putative uncharacterized protein |
| GRMZM2G032339 | 259 | 179 | 1.12 | 1.93E-14 | ZMM4 MADS-box protein (M4 protein) |
| GRMZM2G032348 | 349 | 197 | 1.42 | 1.27E-27 | Putative uncharacterized protein |
| GRMZM2G032367 | 378 | 363 | 0.65 | 1.97E-08 | Cytochrome c1, heme protein |
| GRMZM2G032409 | 169 | 515 | -1.02 | 2.63E-15 | Dolichyl-diphosphooligosaccharide--protein glycosyltransferase 67 kDasubunit |
| GRMZM2G032484 | 47 | 116 | -0.71 | 1.92E-02 | Putative uncharacterized protein |
| GRMZM2G032505 | 82 | 209 | -0.76 | 2.93E-04 | Putative uncharacterized protein |
| GRMZM2G032564 | 668 | 594 | 0.76 | 4.91E-19 | 60S ribosomal protein L34 |
| GRMZM2G032684 | 121 | 121 | 0.59 | 8.83E-03 | Putative uncharacterized protein |
| GRMZM2G032711 | 195 | 476 | -0.70 | 1.01E-07 | Putative uncharacterized protein (WD-40 repeat protein MSI1) |
| GRMZM2G032763 | 54 | 39 | 1.06 | 2.88E-03 | Putative uncharacterized protein |
| GRMZM2G032847 | 84 | 407 | -1.69 | 1.15E-25 | Putative uncharacterized protein |
| GRMZM2G032852 | 31 | 111 | -1.25 | 6.63E-05 | Putative uncharacterized protein |
| GRMZM2G033027 | 14 | 54 | -1.36 | 6.51E-03 | Putative uncharacterized protein |
| GRMZM2G033117 | 114 | 274 | -0.67 | 1.80E-04 | Putative uncharacterized protein |
| GRMZM2G033480 | 183 | 167 | 0.72 | 3.37E-05 | DNA-binding protein-like |
| GRMZM2G033526 | 135 | 358 | -0.82 | 1.25E-07 | Pyruvate kinase (EC 2.7.1.40) |
| GRMZM2G033576 | 286 | 279 | 0.63 | 3.77E-06 | C2 domain containing protein |
| GRMZM2G033592 | 31 | 95 | -1.02 | 2.44E-03 | Putative uncharacterized protein |
| GRMZM2G033619 | 241 | 267 | 0.44 | 3.63E-03 | Putative uncharacterized protein |
| GRMZM2G033626 | 410 | 472 | 0.39 | 5.99E-04 | 26S proteasome non-ATPase regulatory subunit 14 (Putative uncharacterized protein) |
| GRMZM2G033641 | 56 | 27 | 1.64 | 5.76E-06 | Putative uncharacterized protein |
| GRMZM2G033649 | 301 | 263 | 0.79 | 2.44E-09 | Putative uncharacterized protein |
| GRMZM2G033653 | 14 | 60 | -1.51 | 1.35E-03 | Putative uncharacterized protein |
| GRMZM2G033724 | 39 | 119 | -1.02 | 6.65E-04 | Putative uncharacterized protein |
| GRMZM2G033799 | 86 | 188 | -0.54 | 2.15E-02 | Putative uncharacterized protein |
| GRMZM2G033828 | 170 | 186 | 0.46 | 1.48E-02 | cDNA, clone: J065014C16, full insert sequence (cDNA, clone: J065187G05, full insert sequence) |
| GRMZM2G033829 | 25 | 78 | -1.05 | 6.78E-03 | PDIL1-4-Zea mays protein disulfide isomerase (Protein disulfide isomerase) |
| GRMZM2G033846 | 152 | 75 | 1.61 | 8.39E-15 | Caltractin |
| GRMZM2G033867 | 51 | 130 | -0.76 | 7.73E-03 | MARD1 |
| GRMZM2G033876 | 90 | 199 | -0.55 | 1.17E-02 | Os02g0742000 protein (Putative serine-threonine rich antigen) |
| GRMZM2G033894 | 290 | 567 | -0.38 | 1.95E-03 | Putative uncharacterized protein |
| GRMZM2G033971 | 56 | 38 | 1.15 | 9.72E-04 | Putative uncharacterized protein |
| GRMZM2G034069 | 430 | 414 | 0.65 | 2.24E-09 | Putative uncharacterized protein |
| GRMZM2G034143 | 21 | 84 | -1.41 | 1.96E-04 | Uridine kinase (EC 2.7.1.48) |
| GRMZM2G034225 | 557 | 372 | 1.17 | 8.93E-33 | Putative uncharacterized protein |
| GRMZM2G034260 | 59 | 132 | -0.57 | 4.89E-02 | Mediator complex subunit 10 CG5057-PA |
| GRMZM2G034276 | 138 | 323 | -0.64 | 1.08E-04 | Putative uncharacterized protein |
| GRMZM2G034288 | 87 | 203 | -0.63 | 3.84E-03 | Putative uncharacterized protein |
| GRMZM2G034383 | 40 | 104 | -0.79 | 1.45E-02 | TOM2B |
| GRMZM2G034410 | 175 | 120 | 1.14 | 5.34E-10 | Putative uncharacterized protein |
| GRMZM2G034417 | 263 | 255 | 0.64 | 7.20E-06 | Inositol monophosphatase 3 (Putative uncharacterized protein) |
| GRMZM2G034453 | 47 | 146 | -1.04 | 6.37E-05 | Putative uncharacterized protein |
| GRMZM2G034503 | 100 | 215 | -0.51 | 1.66E-02 | Putative uncharacterized protein |
| GRMZM2G034526 | 39 | 120 | -1.03 | 5.13E-04 | Putative uncharacterized protein |
| GRMZM2G034551 | 14 | 64 | -1.60 | 3.47E-04 | ZIP zinc/iron transport family protein |
| GRMZM2G034572 | 145 | 399 | -0.87 | 2.63E-09 | Os07g0145400 protein (Putative phytosulfokine receptor) |
| GRMZM2G034575 | 222 | 221 | 0.60 | 1.39E-04 | Putative uncharacterized protein |
| GRMZM2G034622 | 74 | 165 | -0.57 | 2.16E-02 | Os05g0159000 protein (Putative uncharacterized protein OSJNBa0017J22.6) |
| GRMZM2G034631 | 17 | 52 | -1.02 | 4.08E-02 | Putative uncharacterized protein |
| GRMZM2G034639 | 113 | 246 | -0.53 | 6.86E-03 | Putative uncharacterized protein |
| GRMZM2G034647 | 166 | 476 | -0.93 | 3.23E-12 | Cyclin IbZm |
| GRMZM2G034668 | 41 | 31 | 0.99 | 1.85E-02 | Cytochrome b561 (Putative uncharacterized protein) |
| GRMZM2G034684 | 52 | 161 | -1.04 | 2.57E-05 | Putative uncharacterized protein |
| GRMZM2G034764 | 72 | 17 | 2.67 | 1.19E-13 | C2 domain containing protein |
| GRMZM2G034794 | 1,527 | 1,561 | 0.56 | 5.25E-25 | Ribosomal protein L15 |
| GRMZM2G034804 | 406 | 485 | 0.33 | 3.89E-03 | Nuclear cap-binding protein subunit 2 (Putative uncharacterized protein) |
| GRMZM2G034828 | 177 | 377 | -0.50 | 9.76E-04 | Kinesin heavy chain (Fragment) |
| GRMZM2G034833 | 113 | 275 | -0.69 | 1.17E-04 | Palmitoyltransferase ZDHHC9 (Putative uncharacterized protein) |
| GRMZM2G034840 | 346 | 736 | -0.50 | 1.08E-06 | Putative uncharacterized protein |
| GRMZM2G034882 | 5 | 25 | -1.73 | 3.74E-02 | Putative uncharacterized protein |
| GRMZM2G034943 | 326 | 355 | 0.47 | 2.39E-04 | Putative uncharacterized protein |
| GRMZM2G034985 | 30 | 20 | 1.18 | 2.58E-02 | Putative uncharacterized protein |
| GRMZM2G035008 | 1 | 14 | -3.22 | 3.22E-02 | Putative uncharacterized protein |
| GRMZM2G035017 | 1,051 | 2,400 | -0.60 | 9.87E-29 | 40S ribosomal protein S4 (Putative uncharacterized protein) (Ribsomal protein S4) |
| GRMZM2G035063 | 199 | 401 | -0.42 | 4.46E-03 | Chaperonin |
| GRMZM2G035118 | 59 | 161 | -0.86 | 4.88E-04 | cDNA clone:J013001E01, full insert sequence (Putative pumilio-family RNA-binding domain-containing protein(PPD1)) |
| GRMZM2G035150 | 33 | 97 | -0.96 | 3.92E-03 | Putative uncharacterized protein |
| GRMZM2G035325 | 100 | 248 | -0.72 | 1.66E-04 | SWIB/MDM2 domain containing protein |
| GRMZM2G035341 | 253 | 500 | -0.39 | 2.68E-03 | Putative uncharacterized protein |
| GRMZM2G035356 | 240 | 147 | 1.30 | 8.43E-17 | Putative uncharacterized protein |
| GRMZM2G035430 | 12 | 44 | -1.28 | 1.99E-02 | Putative uncharacterized protein |
| GRMZM2G035445 | 161 | 173 | 0.49 | 1.13E-02 | Os07g0244300 protein (cDNA clone:J013149E04, full insert sequence) (Organic solute transporter-like) |
| GRMZM2G035520 | 419 | 438 | 0.53 | 1.50E-06 | Os12g0638700 protein (Plasma membrane ATPase 1, putative, expressed) (Plasma membrane H+ ATPase) (EC 3.6.3.6) |
| GRMZM2G035579 | 38 | 134 | -1.23 | 1.23E-05 | Os06g0603600 protein (Putative uncharacterized protein) (cDNA clone:001-117-D01, full insert sequence) (cDNA clone:J013114F06, full insert sequence) (Putative ids-4 protein) |
| GRMZM2G035632 | 15 | 7 | 1.69 | 3.69E-02 | Putative uncharacterized protein |
| GRMZM2G035665 | 207 | 226 | 0.46 | 5.85E-03 | OSIGBa0132G14.1 protein (OSIGBa0148J22.4 protein) |
| GRMZM2G035688 | 124 | 306 | -0.71 | 2.32E-05 | Response regulator |
| GRMZM2G035719 | 59 | 160 | -0.85 | 6.09E-04 | Nucleic acid binding protein (Putative uncharacterized protein) |
| GRMZM2G035732 | 196 | 100 | 1.56 | 3.64E-18 | NA |
| GRMZM2G035779 | 17 | 54 | -1.08 | 3.23E-02 | Ser/Thr protein phosphatase family |
| GRMZM2G035785 | 117 | 124 | 0.51 | 3.13E-02 | Protein binding protein (Putative uncharacterized protein) |
| GRMZM2G035807 | 445 | 437 | 0.62 | 5.44E-09 | Putative uncharacterized protein |
| GRMZM2G035843 | 168 | 329 | -0.38 | 2.75E-02 | Putative uncharacterized protein (Calcium-dependent protein kinase ZmCPK11) |
| GRMZM2G035849 | 87 | 211 | -0.69 | 1.08E-03 | Palmitoyltransferase PFA4 |
| GRMZM2G035933 | 55 | 29 | 1.51 | 3.27E-05 | Putative uncharacterized protein |
| GRMZM2G036007 | 38 | 110 | -0.94 | 2.60E-03 | Putative uncharacterized protein |
| GRMZM2G036034 | 513 | 368 | 1.07 | 4.52E-26 | Coatomer subunit gamma (Putative uncharacterized protein) |
| GRMZM2G036050 | 189 | 457 | -0.68 | 3.28E-07 | Putative uncharacterized protein |
| GRMZM2G036134 | 1,225 | 966 | 0.93 | 2.30E-49 | Putative uncharacterized protein |
| GRMZM2G036351 | 7 | 1 | 3.40 | 3.67E-02 | ZIM motif family protein |
| GRMZM2G036543 | 91 | 219 | -0.68 | 9.35E-04 | Putative uncharacterized protein |
| GRMZM2G036596 | 150 | 297 | -0.39 | 3.07E-02 | Putative uncharacterized protein |
| GRMZM2G036605 | 89 | 220 | -0.71 | 4.69E-04 | NA |
| GRMZM2G036720 | 66 | 147 | -0.56 | 3.46E-02 | Putative uncharacterized protein |
| GRMZM2G036765 | 996 | 784 | 0.94 | 2.29E-40 | Putative uncharacterized protein (Cell division cycle protein 48, putative, expressed) |
| GRMZM2G036829 | 58 | 131 | -0.58 | 4.00E-02 | Putative uncharacterized protein |
| GRMZM2G036837 | 47 | 114 | -0.69 | 2.79E-02 | Putative uncharacterized protein |
| GRMZM2G036872 | 14 | 70 | -1.73 | 5.88E-05 | Putative uncharacterized protein |
| GRMZM2G036908 | 180 | 83 | 1.71 | 5.30E-19 | Putative uncharacterized protein |
| GRMZM2G036976 | 37 | 99 | -0.83 | 1.44E-02 | NA |
| GRMZM2G036991 | 211 | 423 | -0.41 | 4.05E-03 | NA |
| GRMZM2G037012 | 14 | 5 | 2.08 | 1.90E-02 | Putative uncharacterized protein |
| GRMZM2G037104 | 100 | 40 | 1.91 | 1.75E-12 | 1-acyl-sn-glycerol-3-phosphate acyltransferase PLS1 |
| GRMZM2G037164 | 78 | 64 | 0.88 | 2.08E-03 | Putative uncharacterized protein |
| GRMZM2G037200 | 162 | 318 | -0.38 | 2.74E-02 | Nucleic acid binding protein (Putative uncharacterized protein) |
| GRMZM2G037226 | 184 | 630 | -1.18 | 1.24E-23 | RNA-binding protein AKIP1 |
| GRMZM2G037335 | 45 | 130 | -0.94 | 7.93E-04 | Chromosome chr12 scaffold_18, whole genome shotgun sequence |
| GRMZM2G037368 | 147 | 324 | -0.55 | 8.39E-04 | IAA5-auxin-responsive Aux/IAA family member |
| GRMZM2G037444 | 71 | 191 | -0.84 | 1.53E-04 | Putative uncharacterized protein |
| GRMZM2G037585 | 38 | 94 | -0.72 | 4.14E-02 | Putative uncharacterized protein |
| GRMZM2G037614 | 18 | 117 | -2.11 | 5.00E-10 | Putative uncharacterized protein |
| GRMZM2G037617 | 17 | 74 | -1.53 | 1.99E-04 | Serine/threonine-protein kinase 12 |
| GRMZM2G037650 | 15 | 56 | -1.31 | 5.82E-03 | Putative uncharacterized protein |
| GRMZM2G037685 | 26 | 13 | 1.59 | 5.28E-03 | Putative uncharacterized protein |
| GRMZM2G037698 | 536 | 1,110 | -0.46 | 1.68E-08 | Pre-mRNA-splicing factor 19 (Putative uncharacterized protein) |
| GRMZM2G037725 | 152 | 370 | -0.69 | 4.15E-06 | Putative uncharacterized protein |
| GRMZM2G037823 | 2 | 21 | -2.80 | 1.11E-02 | Putative uncharacterized protein |
| GRMZM2G038032 | 1,656 | 2,225 | 0.17 | 2.96E-03 | Guanine nucleotide-binding protein beta subunit-like protein |
| GRMZM2G038126 | 241 | 579 | -0.67 | 9.26E-09 | 26S protease regulatory subunit 6B (Putative uncharacterized protein) |
| GRMZM2G038183 | 12 | 52 | -1.52 | 3.07E-03 | Putative uncharacterized protein |
| GRMZM2G038279 | 67 | 56 | 0.85 | 6.90E-03 | Putative uncharacterized protein |
| GRMZM2G038313 | 180 | 380 | -0.49 | 1.24E-03 | Putative uncharacterized protein |
| GRMZM2G038375 | 464 | 497 | 0.49 | 2.09E-06 | Protein translocase/ protein transporter (Putative uncharacterized protein) |
| GRMZM2G038394 | 5 | 27 | -1.84 | 2.70E-02 | Putative uncharacterized protein |
| GRMZM2G038401 | 159 | 438 | -0.87 | 3.08E-10 | Putative uncharacterized protein |
| GRMZM2G038412 | 56 | 127 | -0.59 | 4.30E-02 | Putative uncharacterized protein |
| GRMZM2G038636 | 17 | 117 | -2.19 | 1.33E-10 | Putative uncharacterized protein |
| GRMZM2G038643 | 145 | 302 | -0.47 | 6.90E-03 | Putative uncharacterized protein |
| GRMZM2G038691 | 55 | 134 | -0.69 | 1.21E-02 | Putative uncharacterized protein |
| GRMZM2G038783 | 33 | 6 | 3.05 | 2.29E-07 | CONSTANS-like protein CO6 |
| GRMZM2G038791 | 256 | 228 | 0.76 | 1.55E-07 | Ribose-phosphate pyrophosphokinase (EC 2.7.6.1) |
| GRMZM2G038827 | 1,181 | 405 | 2.14 | 8.73E-169 | NA |
| GRMZM2G038882 | 24 | 68 | -0.91 | 3.33E-02 | Putative uncharacterized protein |
| GRMZM2G038988 | 320 | 276 | 0.80 | 2.93E-10 | Calmodulin binding protein |
| GRMZM2G039089 | 51 | 134 | -0.80 | 3.42E-03 | Putative uncharacterized protein |
| GRMZM2G039106 | 14 | 48 | -1.19 | 2.77E-02 | Putative uncharacterized protein |
| GRMZM2G039155 | 248 | 691 | -0.89 | 2.10E-16 | GTP binding protein |
| GRMZM2G039214 | 109 | 247 | -0.59 | 2.32E-03 | Putative uncharacterized protein |
| GRMZM2G039251 | 684 | 609 | 0.76 | 2.19E-19 | Succinyl-CoA ligase alpha-chain 2 |
| GRMZM2G039254 | 130 | 282 | -0.53 | 3.29E-03 | Os02g0127600 protein (Putative pentatricopeptide (PPR) repeat-containing protein) |
| GRMZM2G039280 | 123 | 120 | 0.63 | 4.87E-03 | Ethylene-overproduction protein 1 |
| GRMZM2G039365 | 108 | 235 | -0.53 | 7.73E-03 | Putative uncharacterized protein (Seven-transmembrane-domain protein 1) |
| GRMZM2G039373 | 43 | 106 | -0.71 | 2.65E-02 | Putative uncharacterized protein (Trafficking protein particle complex subunit 1) |
| GRMZM2G039385 | 252 | 267 | 0.51 | 5.23E-04 | Phosphatidylserine synthase 2 |
| GRMZM2G039396 | 66 | 159 | -0.68 | 6.24E-03 | Putative uncharacterized protein |
| GRMZM2G039454 | 387 | 790 | -0.44 | 9.03E-06 | Cellulose synthase BoCesA2 |
| GRMZM2G039455 | 1,838 | 1,432 | 0.95 | 1.66E-76 | Putative uncharacterized protein |
| GRMZM2G039542 | 144 | 149 | 0.54 | 8.54E-03 | Succinate dehydrogenase subunit 3 |
| GRMZM2G039583 | 4 | 24 | -1.99 | 2.73E-02 | Putative uncharacterized protein |
| GRMZM2G039588 | 78 | 279 | -1.25 | 1.48E-11 | Putative uncharacterized protein |
| GRMZM2G039600 | 134 | 305 | -0.60 | 4.17E-04 | H0805A05.2 protein |
| GRMZM2G039618 | 44 | 121 | -0.87 | 2.92E-03 | NA |
| GRMZM2G039648 | 3 | 20 | -2.15 | 3.95E-02 | NA |
| GRMZM2G039664 | 117 | 122 | 0.53 | 2.54E-02 | Putative uncharacterized protein |
| GRMZM2G039671 | 95 | 303 | -1.08 | 4.37E-10 | Putative uncharacterized protein |
| GRMZM2G039711 | 80 | 241 | -1.00 | 2.96E-07 | Putative uncharacterized protein |
| GRMZM2G039811 | 19 | 58 | -1.02 | 3.18E-02 | Putative uncharacterized protein |
| GRMZM2G039867 | 8 | 1 | 3.59 | 1.85E-02 | Putative uncharacterized protein |
| GRMZM2G039889 | 59 | 135 | -0.60 | 3.03E-02 | Putative uncharacterized protein |
| GRMZM2G039895 | 131 | 294 | -0.57 | 9.81E-04 | Putative uncharacterized protein |
| GRMZM2G039906 | 44 | 145 | -1.13 | 1.87E-05 | Putative uncharacterized protein |
| GRMZM2G039919 | 504 | 119 | 2.67 | 4.73E-95 | Putative uncharacterized protein |
| GRMZM2G040017 | 81 | 171 | -0.49 | 4.97E-02 | Putative uncharacterized protein |
| GRMZM2G040078 | 32 | 118 | -1.29 | 1.82E-05 | Putative uncharacterized protein |
| GRMZM2G040079 | 159 | 364 | -0.60 | 7.47E-05 | Bromodomain protein 103 |
| GRMZM2G040100 | 6 | 31 | -1.78 | 1.82E-02 | Putative uncharacterized protein |
| GRMZM2G040102 | 49 | 35 | 1.08 | 4.58E-03 | Putative uncharacterized protein |
| GRMZM2G040131 | 85 | 237 | -0.89 | 5.93E-06 | Putative uncharacterized protein (Methyl-binding domain protein MBD101) |
| GRMZM2G040145 | 47 | 120 | -0.76 | 1.07E-02 | Expressed protein |
| GRMZM2G040164 | 800 | 824 | 0.55 | 7.46E-13 | Proteasome subunit beta type 4 (Putative uncharacterized protein) |
| GRMZM2G040182 | 106 | 248 | -0.63 | 8.51E-04 | Putative F-box protein family |
| GRMZM2G040209 | 86 | 211 | -0.70 | 8.40E-04 | Putative uncharacterized protein |
| GRMZM2G040278 | 27 | 5 | 3.02 | 4.91E-06 | Putative uncharacterized protein |
| GRMZM2G040387 | 51 | 173 | -1.17 | 1.09E-06 | Putative uncharacterized protein |
| GRMZM2G040389 | 534 | 1,078 | -0.42 | 3.32E-07 | Putative uncharacterized protein |
| GRMZM2G040477 | 1,021 | 2,273 | -0.56 | 2.46E-24 | Guanine nucleotide-binding protein beta subunit-like protein (Putative uncharacterized protein) |
| GRMZM2G040561 | 41 | 123 | -0.99 | 6.52E-04 | Putative DNAJ protein |
| GRMZM2G040618 | 18 | 59 | -1.12 | 1.69E-02 | Putative uncharacterized protein |
| GRMZM2G040627 | 124 | 109 | 0.78 | 3.53E-04 | Uracil-DNA glycosylase (EC 3.2.2.-) |
| GRMZM2G040634 | 29 | 79 | -0.85 | 2.67E-02 | Putative uncharacterized protein |
| GRMZM2G040642 | 16 | 67 | -1.47 | 5.86E-04 | Putative uncharacterized protein |
| GRMZM2G040689 | 3,340 | 3,147 | 0.68 | 5.46E-77 | Putative uncharacterized protein |
| GRMZM2G040702 | 443 | 792 | -0.25 | 1.97E-02 | DNA-directed RNA polymerase II 19 kDa polypeptide (Putative uncharacterized protein) |
| GRMZM2G040736 | 14 | 5 | 2.08 | 1.90E-02 | Putative uncharacterized protein |
| GRMZM2G040762 | 103 | 351 | -1.18 | 2.95E-13 | Os03g0121800 protein (DEAD/DEAH box helicase carpel factory, putative, expressed) (Putative CAF protein) |
| GRMZM2G040843 | 19 | 59 | -1.04 | 2.46E-02 | Alkaline/neutral invertase (Putative uncharacterized protein) |
| GRMZM2G040947 | 7 | 31 | -1.56 | 3.26E-02 | Putative uncharacterized protein |
| GRMZM2G040965 | 144 | 143 | 0.60 | 3.00E-03 | AFH1 |
| GRMZM2G040995 | 139 | 381 | -0.86 | 7.62E-09 | Putative uncharacterized protein |
| GRMZM2G041058 | 98 | 315 | -1.09 | 1.29E-10 | Uracil phosphoribosyltransferase |
| GRMZM2G041181 | 82 | 216 | -0.81 | 1.04E-04 | Putative uncharacterized protein (SC3 protein) |
| GRMZM2G041223 | 77 | 305 | -1.39 | 7.61E-15 | Putative growth-regulating factor 6 |
| GRMZM2G041238 | 4,381 | 3,100 | 1.09 | 2.89E-229 | 60S ribosomal protein L26-1 |
| GRMZM2G041258 | 125 | 65 | 1.53 | 1.80E-11 | Putative uncharacterized protein |
| GRMZM2G041328 | 261 | 270 | 0.54 | 1.52E-04 | Putative uncharacterized protein |
| GRMZM2G041381 | 3,307 | 2,810 | 0.83 | 4.29E-108 | Histone H2A |
| GRMZM2G041472 | 444 | 377 | 0.83 | 1.00E-14 | Putative uncharacterized protein |
| GRMZM2G041549 | 9 | 2 | 2.76 | 4.06E-02 | Putative uncharacterized protein (Zinc finger, C3HC4 type family protein) |
| GRMZM2G041645 | 99 | 246 | -0.72 | 1.55E-04 | Os07g0557700 protein (Putative uncharacterized protein P0567H04.2) |
| GRMZM2G041697 | 427 | 886 | -0.46 | 5.57E-07 | Putative uncharacterized protein |
| GRMZM2G041699 | 25 | 5 | 2.91 | 1.80E-05 | Cytokinin-O-glucosyltransferase 2 |
| GRMZM2G041761 | 13 | 1 | 4.29 | 4.67E-04 | Putative uncharacterized protein |
| GRMZM2G041765 | 883 | 955 | 0.48 | 4.19E-11 | IQ calmodulin-binding motif family protein |
| GRMZM2G041799 | 135 | 135 | 0.59 | 4.74E-03 | Zinc finger C-x8-C-x5-C-x3-H type family protein |
| GRMZM2G041818 | 111 | 112 | 0.58 | 1.63E-02 | Putative uncharacterized protein |
| GRMZM2G041822 | 137 | 409 | -0.99 | 1.06E-11 | Serine/threonine protein phosphatase (EC 3.1.3.16) |
| GRMZM2G041831 | 46 | 129 | -0.90 | 1.41E-03 | Putative uncharacterized protein |
| GRMZM2G041842 | 428 | 216 | 1.58 | 6.00E-40 | Putative uncharacterized protein |
| GRMZM2G041876 | 89 | 221 | -0.72 | 3.88E-04 | H0311C03.6 protein |
| GRMZM2G041881 | 449 | 567 | 0.25 | 2.49E-02 | Transcription factor BTF3 |
| GRMZM2G041885 | 61 | 140 | -0.61 | 2.79E-02 | Signal peptide peptidase-like 3 |
| GRMZM2G041980 | 29 | 19 | 1.20 | 2.27E-02 | Aquaporin NIP1.2 (Putative uncharacterized protein) |
| GRMZM2G042008 | 318 | 583 | -0.28 | 2.27E-02 | Nucleoid DNA-binding protein cnd41-like (Os01g0598600 protein) |
| GRMZM2G042027 | 49 | 154 | -1.06 | 2.92E-05 | Putative uncharacterized protein |
| GRMZM2G042032 | 162 | 542 | -1.15 | 1.71E-19 | Putative uncharacterized protein |
| GRMZM2G042040 | 520 | 249 | 1.65 | 6.36E-52 | Microsomal glutathione S-transferase 3 (Putative uncharacterized protein) |
| GRMZM2G042047 | 406 | 846 | -0.47 | 8.06E-07 | Histone H2A |
| GRMZM2G042074 | 152 | 411 | -0.84 | 4.04E-09 | Putative uncharacterized protein |
| GRMZM2G042089 | 236 | 452 | -0.35 | 1.41E-02 | Putative uncharacterized protein |
| GRMZM2G042099 | 98 | 241 | -0.71 | 2.59E-04 | Putative uncharacterized protein |
| GRMZM2G042118 | 2,361 | 3,180 | 0.16 | 3.25E-04 | Glycine-rich RNA-binding protein 2 |
| GRMZM2G042136 | 23 | 12 | 1.53 | 1.44E-02 | Flavoprotein wrbA |
| GRMZM2G042143 | 69 | 64 | 0.70 | 2.72E-02 | Putative uncharacterized protein |
| GRMZM2G042146 | 199 | 218 | 0.46 | 7.64E-03 | Putative uncharacterized protein |
| GRMZM2G042171 | 18 | 76 | -1.49 | 1.84E-04 | Putative uncharacterized protein (Putative sulfate transporter) |
| GRMZM2G042231 | 164 | 340 | -0.46 | 4.58E-03 | Putative uncharacterized protein |
| GRMZM2G042292 | 90 | 308 | -1.18 | 1.19E-11 | Putative uncharacterized protein |
| GRMZM2G042343 | 14 | 68 | -1.69 | 1.21E-04 | Putative uncharacterized protein (Ribonucleoprotein like protein) |
| GRMZM2G042398 | 19 | 58 | -1.02 | 3.18E-02 | mRNA |
| GRMZM2G042443 | 14 | 87 | -2.04 | 2.77E-07 | RNA-dependent RNA polymerase |
| GRMZM2G042488 | 13 | 4 | 2.29 | 2.21E-02 | Putative uncharacterized protein |
| GRMZM2G042502 | 642 | 612 | 0.66 | 2.28E-14 | Putative uncharacterized protein (Pyrophosphate--fructose 6-phosphate 1-phosphotransferase alpha subunit) |
| GRMZM2G042552 | 59 | 208 | -1.23 | 1.65E-08 | Putative uncharacterized protein |
| GRMZM2G042604 | 340 | 693 | -0.44 | 4.15E-05 | Proteasome subunit alpha type (EC 3.4.25.1) |
| GRMZM2G042636 | 431 | 836 | -0.36 | 1.71E-04 | Putative uncharacterized protein |
| GRMZM2G042754 | 26 | 78 | -0.99 | 9.86E-03 | Putative uncharacterized protein |
| GRMZM2G042807 | 76 | 206 | -0.85 | 6.21E-05 | Pre-mRNA processing factor |
| GRMZM2G042897 | 14 | 48 | -1.19 | 2.77E-02 | Membrane-associated phospholipid phosphatase |
| GRMZM2G042992 | 531 | 975 | -0.29 | 1.62E-03 | Os08g0162100 protein |
| GRMZM2G043030 | 63 | 244 | -1.36 | 1.56E-11 | Putative uncharacterized protein |
| GRMZM2G043056 | 74 | 168 | -0.59 | 1.57E-02 | Putative uncharacterized protein |
| GRMZM2G043119 | 36 | 90 | -0.73 | 4.44E-02 | Mitochondrial glycoprotein |
| GRMZM2G043240 | 9 | 40 | -1.56 | 1.03E-02 | Putative uncharacterized protein |
| GRMZM2G043279 | 1,553 | 1,095 | 1.10 | 9.89E-82 | 60S ribosomal protein L35 (Putative uncharacterized protein) |
| GRMZM2G043295 | 13 | 3 | 2.71 | 6.90E-03 | Anthocyanidin 5,3-O-glucosyltransferase (Putative uncharacterized protein) |
| GRMZM2G043348 | 64 | 58 | 0.73 | 2.53E-02 | Putative uncharacterized protein |
| GRMZM2G043350 | 62 | 166 | -0.83 | 6.34E-04 | Putative uncharacterized protein |
| GRMZM2G043414 | 64 | 55 | 0.81 | 1.36E-02 | Diacylglycerol kinase |
| GRMZM2G043435 | 61 | 165 | -0.84 | 4.66E-04 | Respiratory burst oxidase-like protein C |
| GRMZM2G043456 | 207 | 407 | -0.38 | 9.50E-03 | DNA-directed RNA polymerase (EC 2.7.7.6) |
| GRMZM2G043464 | 30 | 87 | -0.94 | 9.47E-03 | EMB2756 |
| GRMZM2G043489 | 38 | 117 | -1.03 | 5.81E-04 | Putative uncharacterized protein |
| GRMZM2G043501 | 12 | 51 | -1.50 | 4.25E-03 | Os09g0394100 protein (Putative Malonyl-CoA decarboxylase, mitochondrial (MCD)) |
| GRMZM2G043509 | 51 | 124 | -0.69 | 1.68E-02 | Putative uncharacterized protein |
| GRMZM2G043584 | 248 | 236 | 0.66 | 6.09E-06 | Os03g0228800 protein (Putative uncharacterized protein) (Receptor protein kinase CLAVATA1, putative, expressed) |
| GRMZM2G043602 | 1,445 | 861 | 1.34 | 7.00E-105 | 3-oxoacyl-reductase |
| GRMZM2G043724 | 153 | 302 | -0.39 | 2.90E-02 | ATP-dependent RNA helicase DDX41 |
| GRMZM2G043764 | 192 | 376 | -0.38 | 1.58E-02 | Putative uncharacterized protein |
| GRMZM2G043773 | 66 | 198 | -0.99 | 5.28E-06 | Os07g0598900 protein (Putative uncharacterized protein) (cDNA clone:J033099K03, full insert sequence) (Putative cobW protein) |
| GRMZM2G043776 | 50 | 130 | -0.79 | 4.76E-03 | Dihydroorotate dehydrogenase |
| GRMZM2G043783 | 23 | 84 | -1.28 | 5.40E-04 | Putative uncharacterized protein |
| GRMZM2G043799 | 19 | 9 | 1.67 | 1.68E-02 | Putative uncharacterized protein |
| GRMZM2G043822 | 833 | 2,353 | -0.91 | 2.62E-57 | Putative uncharacterized protein (cDNA clone:J023010B07, full insert sequence) |
| GRMZM2G043983 | 162 | 314 | -0.36 | 3.81E-02 | Mitochondrial import receptor subunit TOM40 (Putative uncharacterized protein) |
| GRMZM2G044004 | 1,337 | 440 | 2.19 | 3.03E-198 | Nucleic acid binding protein |
| GRMZM2G044027 | 15 | 83 | -1.88 | 3.03E-06 | Putative uncharacterized protein |
| GRMZM2G044038 | 60 | 136 | -0.59 | 3.71E-02 | CBL-interacting serine/threonine-protein kinase 15 |
| GRMZM2G044062 | 20 | 11 | 1.45 | 3.89E-02 | Thioredoxin X |
| GRMZM2G044077 | 7 | 1 | 3.40 | 3.67E-02 | cDNA clone:002-107-B02, full insert sequence (cDNA clone:J033015K21, full insert sequence) (Os01g0224100 protein) (Pti6-like) |
| GRMZM2G044085 | 9 | 56 | -2.05 | 6.14E-05 | Oligosaccharyl transferase STT3 subunit |
| GRMZM2G044107 | 40 | 21 | 1.52 | 5.16E-04 | Glycosyl hydrolase family 3 C terminal domain containing protein, expressed (Os11g0297800 protein) (cDNA clone:002-149-D05, full insert sequence) (Beta-D-xylosidase) |
| GRMZM2G044126 | 27 | 72 | -0.82 | 4.29E-02 | Putative uncharacterized protein (Histone aceytl-transferase HAC108) |
| GRMZM2G044128 | 1,554 | 3,687 | -0.66 | 1.72E-51 | Os07g0636000 protein (Putative uncharacterized protein) (cDNA clone:J033094C11, full insert sequence) (Putative centromere/microtubule binding protein) |
| GRMZM2G044143 | 42 | 103 | -0.70 | 3.66E-02 | OSJNBb0012E24.4 protein |
| GRMZM2G044237 | 392 | 739 | -0.32 | 2.07E-03 | Putative uncharacterized protein |
| GRMZM2G044273 | 23 | 66 | -0.93 | 3.02E-02 | Putative uncharacterized protein |
| GRMZM2G044301 | 36 | 96 | -0.82 | 1.63E-02 | Os05g0302300 protein |
| GRMZM2G044343 | 50 | 131 | -0.80 | 3.90E-03 | Putative uncharacterized protein |
| GRMZM2G044354 | 26 | 86 | -1.13 | 1.60E-03 | Protein OS-9 (Putative uncharacterized protein) |
| GRMZM2G044382 | 86 | 385 | -1.57 | 4.70E-22 | Putative uncharacterized protein |
| GRMZM2G044398 | 506 | 596 | 0.36 | 4.49E-04 | Zinc finger C-x8-C-x5-C-x3-H type family protein |
| GRMZM2G044422 | 28 | 78 | -0.89 | 2.46E-02 | Putative uncharacterized protein |
| GRMZM2G044457 | 65 | 169 | -0.79 | 9.90E-04 | Putative uncharacterized protein |
| GRMZM2G044493 | 5 | 0 | #VALUE! | 4.27E-02 | Putative uncharacterized protein |
| GRMZM2G044495 | 69 | 175 | -0.75 | 1.33E-03 | Putative uncharacterized protein |
| GRMZM2G044501 | 70 | 184 | -0.80 | 4.14E-04 | Putative uncharacterized protein |
| GRMZM2G044527 | 74 | 168 | -0.59 | 1.57E-02 | Syntaxin 132 |
| GRMZM2G044552 | 80 | 185 | -0.62 | 7.58E-03 | PRPK |
| GRMZM2G044576 | 5 | 0 | #VALUE! | 4.27E-02 | Putative uncharacterized protein |
| GRMZM2G044681 | 44 | 106 | -0.68 | 4.06E-02 | H0525E10.11 protein |
| GRMZM2G044684 | 184 | 187 | 0.57 | 1.19E-03 | GrpE protein homolog |
| GRMZM2G044771 | 33 | 10 | 2.31 | 1.31E-05 | Putative uncharacterized protein |
| GRMZM2G044800 | 2,082 | 2,352 | 0.42 | 8.63E-20 | 40S ribosomal protein S11 (Putative uncharacterized protein) |
| GRMZM2G044851 | 16 | 7 | 1.78 | 2.33E-02 | Putative uncharacterized protein |
| GRMZM2G044963 | 541 | 1,280 | -0.65 | 7.89E-18 | Auxin-regulated calmodulin (Calmodulin) |
| GRMZM2G045070 | 150 | 292 | -0.37 | 4.75E-02 | Topoisomerase-like protein |
| GRMZM2G045084 | 49 | 33 | 1.16 | 2.68E-03 | Protein binding protein |
| GRMZM2G045090 | 49 | 139 | -0.91 | 7.52E-04 | cDNA clone:J013023K07, full insert sequence (Putative 5-oxoprolinase) |
| GRMZM2G045183 | 44 | 138 | -1.06 | 9.39E-05 | Ubiquinone biosynthesis protein ubiB |
| GRMZM2G045192 | 75 | 166 | -0.55 | 2.63E-02 | Putative uncharacterized protein |
| GRMZM2G045241 | 52 | 120 | -0.62 | 4.26E-02 | Putative uncharacterized protein (Serine/threonine-protein kinase MHK) |
| GRMZM2G045249 | 60 | 139 | -0.62 | 2.26E-02 | Putative uncharacterized protein |
| GRMZM2G045270 | 166 | 98 | 1.35 | 2.08E-12 | Putative uncharacterized protein (60S ribosomal protein L18A-like) (Os01g0667100 protein) |
| GRMZM2G045275 | 12 | 50 | -1.47 | 4.22E-03 | Putative uncharacterized protein |
| GRMZM2G045280 | 31 | 109 | -1.22 | 1.21E-04 | Putative uncharacterized protein |
| GRMZM2G045287 | 15 | 49 | -1.12 | 3.18E-02 | Putative uncharacterized protein |
| GRMZM2G045314 | 185 | 393 | -0.50 | 8.22E-04 | Putative uncharacterized protein |
| GRMZM2G045319 | 39 | 19 | 1.63 | 3.19E-04 | Cytochrome P450 monooxygenase (Putative cytochrome P450 monooxygenase) |
| GRMZM2G045330 | 277 | 298 | 0.49 | 4.93E-04 | Putative uncharacterized protein |
| GRMZM2G045430 | 123 | 123 | 0.59 | 7.87E-03 | KI domain interacting kinase 1 |
| GRMZM2G045467 | 2 | 19 | -2.66 | 1.65E-02 | Transferase, transferring glycosyl groups |
| GRMZM2G045503 | 199 | 612 | -1.03 | 1.92E-18 | Os08g0542900 protein (Putative uncharacterized protein) (cDNA clone:J033074I09, full insert sequence) (Putative RNA recognition motif (RRM)-containing protein) |
| GRMZM2G045544 | 19 | 59 | -1.04 | 2.46E-02 | Putative uncharacterized protein |
| GRMZM2G045686 | 32 | 24 | 1.01 | 4.05E-02 | Putative uncharacterized protein |
| GRMZM2G045714 | 44 | 190 | -1.52 | 1.69E-10 | Putative uncharacterized protein |
| GRMZM2G045732 | 31 | 23 | 1.02 | 4.84E-02 | NA |
| GRMZM2G045781 | 479 | 418 | 0.79 | 1.18E-14 | Permease I |
| GRMZM2G045854 | 77 | 193 | -0.73 | 8.43E-04 | Hydroxyproline-rich glycoprotein family protein-like |
| GRMZM2G045977 | 70 | 38 | 1.47 | 2.64E-06 | Growth-regulating factor |
| GRMZM2G045981 | 34 | 113 | -1.14 | 1.95E-04 | Os08g0117700 protein (Putative SERK1 protein) |
| GRMZM2G045987 | 206 | 387 | -0.32 | 4.41E-02 | Putative uncharacterized protein |
| GRMZM2G046011 | 3,600 | 2,849 | 0.93 | 7.22E-144 | 60S ribosomal protein L38 |
| GRMZM2G046024 | 8 | 1 | 3.59 | 1.85E-02 | ATPase, coupled to transmembrane movement of substances |
| GRMZM2G046037 | 21 | 63 | -0.99 | 2.43E-02 | NA |
| GRMZM2G046055 | 1,406 | 1,315 | 0.69 | 2.80E-33 | Histone H2A |
| GRMZM2G046143 | 44 | 107 | -0.69 | 3.40E-02 | Bzip-related transcription factor-like |
| GRMZM2G046274 | 48 | 28 | 1.37 | 4.30E-04 | Clathrin binding protein |
| GRMZM2G046293 | 36 | 93 | -0.78 | 2.46E-02 | Putative uncharacterized protein |
| GRMZM2G046313 | 135 | 393 | -0.95 | 1.35E-10 | RNA recognition motif 2 family protein |
| GRMZM2G046337 | 36 | 25 | 1.12 | 1.82E-02 | Exosome complex exonuclease RRP40 |
| GRMZM2G046353 | 262 | 52 | 2.92 | 1.87E-54 | Putative uncharacterized protein |
| GRMZM2G046402 | 127 | 274 | -0.52 | 4.58E-03 | Putative uncharacterized protein |
| GRMZM2G046459 | 362 | 685 | -0.33 | 2.99E-03 | Putative uncharacterized protein |
| GRMZM2G046474 | 23 | 9 | 1.95 | 2.63E-03 | Putative uncharacterized protein |
| GRMZM2G046558 | 376 | 408 | 0.47 | 5.11E-05 | Esterase D |
| GRMZM2G046574 | 123 | 105 | 0.82 | 1.71E-04 | Putative uncharacterized protein (TPR repeat region family protein) |
| GRMZM2G046576 | 102 | 224 | -0.54 | 8.44E-03 | Signal recognition particle 9 kDa protein |
| GRMZM2G046681 | 24 | 101 | -1.48 | 1.10E-05 | Putative uncharacterized protein |
| GRMZM2G046776 | 14 | 51 | -1.27 | 1.18E-02 | Putative uncharacterized protein |
| GRMZM2G046841 | 4,826 | 6,003 | 0.28 | 3.02E-21 | Histone H2B |
| GRMZM2G046885 | 48 | 195 | -1.43 | 6.54E-10 | MADS-box transcription factor 22 (Putative uncharacterized protein) |
| GRMZM2G046900 | 43 | 117 | -0.85 | 4.14E-03 | Putative uncharacterized protein |
| GRMZM2G046909 | 37 | 26 | 1.10 | 1.48E-02 | Putative uncharacterized protein |
| GRMZM2G046932 | 208 | 663 | -1.08 | 1.40E-21 | Glutamyl-tRNA synthetase, cytoplasmic |
| GRMZM2G046968 | 86 | 58 | 1.16 | 2.36E-05 | Putative uncharacterized protein |
| GRMZM2G047018 | 418 | 502 | 0.33 | 4.15E-03 | PHD finger-like domain-containing protein 5A (Putative uncharacterized protein) |
| GRMZM2G047042 | 22 | 95 | -1.52 | 1.82E-05 | Putative uncharacterized protein |
| GRMZM2G047055 | 685 | 1,491 | -0.53 | 1.64E-14 | NA |
| GRMZM2G047093 | 612 | 1,052 | -0.19 | 4.20E-02 | Putative uncharacterized protein |
| GRMZM2G047143 | 181 | 522 | -0.94 | 1.50E-13 | Mitotic spindle checkpoint protein MAD2 (Putative uncharacterized protein) |
| GRMZM2G047161 | 1,495 | 2,612 | -0.21 | 4.90E-05 | Craniofacial development protein 1 (Putative uncharacterized protein) |
| GRMZM2G047167 | 42 | 111 | -0.81 | 9.05E-03 | Protein binding protein |
| GRMZM2G047178 | 18 | 60 | -1.15 | 1.28E-02 | Putative uncharacterized protein |
| GRMZM2G047204 | 199 | 156 | 0.94 | 1.75E-08 | Putative uncharacterized protein |
| GRMZM2G047223 | 32 | 97 | -1.01 | 2.76E-03 | USP6 N-terminal-like protein |
| GRMZM2G047274 | 91 | 212 | -0.63 | 2.77E-03 | Putative uncharacterized protein |
| GRMZM2G047365 | 41 | 121 | -0.97 | 8.33E-04 | Putative uncharacterized protein |
| GRMZM2G047370 | 182 | 546 | -0.99 | 1.31E-15 | Putative uncharacterized protein |
| GRMZM2G047372 | 57 | 130 | -0.60 | 3.83E-02 | Putative uncharacterized protein |
| GRMZM2G047419 | 70 | 156 | -0.56 | 2.96E-02 | Putative uncharacterized protein |
| GRMZM2G047474 | 51 | 119 | -0.63 | 4.08E-02 | Putative uncharacterized protein (TLD family protein) |
| GRMZM2G047486 | 80 | 291 | -1.27 | 2.50E-12 | Putative uncharacterized protein (Calcium-dependent protein kinase ZmCPK11) |
| GRMZM2G047512 | 480 | 619 | 0.22 | 4.77E-02 | Putative uncharacterized protein |
| GRMZM2G047572 | 30 | 89 | -0.98 | 5.82E-03 | Putative uncharacterized protein |
| GRMZM2G047607 | 86 | 198 | -0.61 | 5.09E-03 | Putative uncharacterized protein |
| GRMZM2G047727 | 1,163 | 1,449 | 0.27 | 1.82E-05 | Putative uncharacterized protein (Ubiquitin fusion protein) |
| GRMZM2G047732 | 1,469 | 2,538 | -0.20 | 2.54E-04 | Putative uncharacterized protein (Ubiquitin fusion protein) |
| GRMZM2G047759 | 10 | 1 | 3.91 | 4.41E-03 | Putative uncharacterized protein |
| GRMZM2G047774 | 193 | 216 | 0.43 | 1.47E-02 | Putative uncharacterized protein |
| GRMZM2G047781 | 77 | 208 | -0.84 | 6.76E-05 | Putative uncharacterized protein |
| GRMZM2G047813 | 3,308 | 5,742 | -0.20 | 1.50E-09 | Histone H2A |
| GRMZM2G047855 | 374 | 451 | 0.32 | 8.64E-03 | Casein kinase II subunit alpha-2 (Putative uncharacterized protein) |
| GRMZM2G047860 | 138 | 292 | -0.49 | 5.65E-03 | Putative uncharacterized protein |
| GRMZM2G047894 | 85 | 199 | -0.64 | 3.47E-03 | Os03g0290500 protein (cDNA clone:J023008J23, full insert sequence) |
| GRMZM2G047949 | 28 | 111 | -1.40 | 1.06E-05 | DEAD/DEAH box helicase family protein, expressed (Os11g0176200 protein) (Putative uncharacterized protein) (HUA enhancer 2) |
| GRMZM2G047971 | 471 | 540 | 0.39 | 1.52E-04 | Putative uncharacterized protein |
| GRMZM2G048006 | 115 | 353 | -1.03 | 9.18E-11 | Phosphatidate cytidylyltransferase (EC 2.7.7.41) |
| GRMZM2G048008 | 49 | 129 | -0.81 | 4.42E-03 | Glycosyl transferase-like protein |
| GRMZM2G048045 | 69 | 153 | -0.56 | 3.32E-02 | Putative uncharacterized protein |
| GRMZM2G048117 | 274 | 140 | 1.56 | 4.13E-25 | Putative uncharacterized protein |
| GRMZM2G048194 | 78 | 204 | -0.80 | 1.76E-04 | Putative uncharacterized protein |
| GRMZM2G048205 | 36 | 102 | -0.91 | 5.35E-03 | Putative uncharacterized protein |
| GRMZM2G048274 | 80 | 208 | -0.79 | 2.06E-04 | Putative uncharacterized protein |
| GRMZM2G048276 | 190 | 156 | 0.88 | 3.62E-07 | Putative uncharacterized protein |
| GRMZM2G048277 | 263 | 263 | 0.59 | 3.02E-05 | Peptidyl-prolyl cis-trans isomerase NIMA-interacting 4 |
| GRMZM2G048287 | 80 | 171 | -0.50 | 4.17E-02 | Putative uncharacterized protein |
| GRMZM2G048324 | 74 | 40 | 1.48 | 1.18E-06 | PDI-like protein |
| GRMZM2G048335 | 13 | 2 | 3.29 | 1.88E-03 | Putative uncharacterized protein |
| GRMZM2G048366 | 148 | 296 | -0.41 | 2.33E-02 | Putative uncharacterized protein |
| GRMZM2G048371 | 1,712 | 2,231 | 0.21 | 7.04E-05 | Putative uncharacterized protein |
| GRMZM2G048482 | 97 | 256 | -0.81 | 1.38E-05 | Putative uncharacterized protein (SLT1 protein) |
| GRMZM2G048497 | 44 | 25 | 1.41 | 5.48E-04 | Putative uncharacterized protein |
| GRMZM2G048611 | 49 | 122 | -0.72 | 1.49E-02 | Putative uncharacterized protein |
| GRMZM2G048635 | 14 | 3 | 2.81 | 3.86E-03 | Putative uncharacterized protein |
| GRMZM2G048644 | 172 | 91 | 1.51 | 3.53E-15 | Os06g0712400 protein (cDNA clone:J013108E08, full insert sequence) (Putative NF-E2 inducible protein) |
| GRMZM2G048703 | 123 | 292 | -0.66 | 1.43E-04 | Putative uncharacterized protein |
| GRMZM2G048733 | 28 | 102 | -1.27 | 1.28E-04 | Putative uncharacterized protein |
| GRMZM2G048762 | 49 | 114 | -0.63 | 4.37E-02 | Putative uncharacterized protein |
| GRMZM2G048800 | 16 | 8 | 1.59 | 4.55E-02 | Os02g0539900 protein (Putative uncharacterized protein) (Rab11 binding protein-like) |
| GRMZM2G048819 | 70 | 249 | -1.24 | 3.09E-10 | Chromosome chr12 scaffold_36, whole genome shotgun sequence |
| GRMZM2G048821 | 86 | 226 | -0.80 | 6.05E-05 | Putative uncharacterized protein (Putative thymidine kinase 1) (EC 2.7.1.21) (Fragment) |
| GRMZM2G048843 | 108 | 259 | -0.67 | 3.23E-04 | Putative uncharacterized protein (Os01g0764300 protein) (Putative uncharacterized protein P0403C05.7) |
| GRMZM2G048846 | 52 | 177 | -1.18 | 7.26E-07 | Caltractin (Putative uncharacterized protein) |
| GRMZM2G048907 | 28 | 18 | 1.23 | 2.79E-02 | ATP synthase gamma chain |
| GRMZM2G048912 | 31 | 82 | -0.81 | 3.14E-02 | Putative uncharacterized protein OJ1138_B05.118 |
| GRMZM2G049031 | 48 | 123 | -0.77 | 9.42E-03 | Cell division control protein 50 (Putative uncharacterized protein) |
| GRMZM2G049057 | 212 | 141 | 1.18 | 8.64E-13 | NA |
| GRMZM2G049076 | 60 | 144 | -0.67 | 1.12E-02 | Putative sucrose-phosphate synthase |
| GRMZM2G049088 | 137 | 149 | 0.47 | 2.97E-02 | Putative uncharacterized protein |
| GRMZM2G049141 | 115 | 279 | -0.69 | 1.09E-04 | Putative HECT ubiquitin-protein ligase 3 |
| GRMZM2G049190 | 22 | 66 | -0.99 | 2.14E-02 | Putative uncharacterized protein |
| GRMZM2G049201 | 161 | 368 | -0.60 | 6.91E-05 | Os12g0609800 protein (Transducin family protein, putative, expressed) |
| GRMZM2G049269 | 576 | 521 | 0.74 | 1.35E-15 | Putative uncharacterized protein |
| GRMZM2G049329 | 80 | 303 | -1.33 | 1.07E-13 | Putative uncharacterized protein |
| GRMZM2G049342 | 160 | 313 | -0.38 | 3.31E-02 | Target of rapamycin |
| GRMZM2G049382 | 39 | 97 | -0.72 | 3.67E-02 | Putative uncharacterized protein |
| GRMZM2G049416 | 149 | 445 | -0.99 | 1.22E-12 | 50S ribosomal protein L3-2 |
| GRMZM2G049487 | 282 | 112 | 1.92 | 5.63E-35 | Putative uncharacterized protein |
| GRMZM2G049525 | 123 | 316 | -0.77 | 3.55E-06 | Putative uncharacterized protein |
| GRMZM2G049534 | 1 | 19 | -3.66 | 5.11E-03 | Putative uncharacterized protein |
| GRMZM2G049536 | 169 | 367 | -0.53 | 5.58E-04 | DNA replication complex GINS protein PSF1 (Putative uncharacterized protein) |
| GRMZM2G049641 | 105 | 249 | -0.65 | 5.68E-04 | Putative uncharacterized protein |
| GRMZM2G049675 | 2 | 18 | -2.58 | 2.48E-02 | Gibberellin receptor GID1L2 |
| GRMZM2G049693 | 271 | 830 | -1.02 | 1.22E-24 | Putative uncharacterized protein |
| GRMZM2G049695 | 5 | 0 | #VALUE! | 4.27E-02 | Putative uncharacterized protein |
| GRMZM2G049823 | 29 | 80 | -0.87 | 2.14E-02 | Embryonic flower 2 (VEF family protein) |
| GRMZM2G049839 | 40 | 125 | -1.05 | 2.67E-04 | Os02g0198600 protein (Putative uncharacterized protein) (cDNA clone:J013126L13, full insert sequence) (Putative DNA-damage inducible protein) |
| GRMZM2G049866 | 272 | 549 | -0.42 | 5.92E-04 | Heterogeneous nuclear ribonucleoprotein A3-like protein 2 |
| GRMZM2G049902 | 58 | 201 | -1.20 | 6.20E-08 | Putative pre-mRNA splicing factor (U5 snRNP-associated 102 kDa protein, putative, expressed) |
| GRMZM2G050137 | 36 | 11 | 2.30 | 6.90E-06 | Putative uncharacterized protein |
| GRMZM2G050159 | 0 | 10 | #NUM! | 3.46E-02 | Putative uncharacterized protein |
| GRMZM2G050193 | 82 | 231 | -0.90 | 5.57E-06 | Calcium lipid binding protein-like |
| GRMZM2G050218 | 187 | 565 | -1.00 | 2.37E-16 | Putative uncharacterized protein |
| GRMZM2G050270 | 186 | 181 | 0.63 | 2.65E-04 | 3-N-debenzoyl-2-deoxytaxol N-benzoyltransferase (Putative uncharacterized protein) |
| GRMZM2G050309 | 16 | 58 | -1.27 | 6.93E-03 | Putative uncharacterized protein |
| GRMZM2G050325 | 21 | 92 | -1.54 | 1.95E-05 | Phenazine biosynthesis protein |
| GRMZM2G050371 | 54 | 40 | 1.02 | 4.22E-03 | Ornithine carbamoyltransferase |
| GRMZM2G050375 | 318 | 679 | -0.50 | 2.47E-06 | BRASSINOSTEROID INSENSITIVE 1-associated receptor kinase 1 (Putative uncharacterized protein) |
| GRMZM2G050460 | 759 | 421 | 1.44 | 1.05E-61 | 60S ribosomal protein L30 |
| GRMZM2G050484 | 27 | 100 | -1.30 | 1.04E-04 | ZIP-like protein 1 |
| GRMZM2G050501 | 123 | 377 | -1.02 | 1.87E-11 | Putative uncharacterized protein |
| GRMZM2G050590 | 15 | 76 | -1.75 | 2.85E-05 | Putative uncharacterized protein |
| GRMZM2G050628 | 40 | 117 | -0.96 | 1.22E-03 | Putative uncharacterized protein |
| GRMZM2G050645 | 78 | 53 | 1.15 | 6.50E-05 | Putative uncharacterized protein |
| GRMZM2G050647 | 59 | 201 | -1.18 | 9.71E-08 | H0525E10.11 protein |
| GRMZM2G050649 | 117 | 256 | -0.54 | 4.34E-03 | Growth regulator like protein (Putative uncharacterized protein) |
| GRMZM2G050714 | 44 | 108 | -0.70 | 2.81E-02 | Putative uncharacterized protein |
| GRMZM2G050803 | 44 | 37 | 0.84 | 3.88E-02 | Putative uncharacterized protein |
| GRMZM2G050882 | 52 | 29 | 1.43 | 1.12E-04 | OSJNBb0065L13.5 protein |
| GRMZM2G050914 | 168 | 327 | -0.37 | 3.06E-02 | Os03g0731500 protein (cDNA clone:J013116D21, full insert sequence) (cDNA clone:J013123C23, full insert sequence) (Major surface like glycoprotein, putative, expressed) (Putative leishmanolysin-like protein) |
| GRMZM2G050925 | 1,903 | 1,120 | 1.36 | 3.79E-141 | Putative uncharacterized protein |
| GRMZM2G051043 | 32 | 96 | -0.99 | 3.54E-03 | CID11 |
| GRMZM2G051129 | 48 | 171 | -1.24 | 3.63E-07 | Putative uncharacterized protein |
| GRMZM2G051153 | 165 | 173 | 0.52 | 6.01E-03 | cDNA clone:002-175-B04, full insert sequence (Os01g0306100 protein) |
| GRMZM2G051228 | 44 | 140 | -1.08 | 5.58E-05 | Putative uncharacterized protein |
| GRMZM2G051247 | 25 | 99 | -1.39 | 3.69E-05 | Putative uncharacterized protein |
| GRMZM2G051262 | 135 | 269 | -0.40 | 3.61E-02 | Alpha-1,2-mannosyltransferase ALG9 (Putative uncharacterized protein) |
| GRMZM2G051270 | 105 | 98 | 0.69 | 4.45E-03 | Putative uncharacterized protein |
| GRMZM2G051330 | 5 | 25 | -1.73 | 3.74E-02 | Putative uncharacterized protein |
| GRMZM2G051367 | 417 | 418 | 0.59 | 8.41E-08 | Putative uncharacterized protein |
| GRMZM2G051403 | 8 | 1 | 3.59 | 1.85E-02 | Putative uncharacterized protein |
| GRMZM2G051458 | 90 | 233 | -0.78 | 8.26E-05 | Putative uncharacterized protein |
| GRMZM2G051622 | 95 | 294 | -1.04 | 3.83E-09 | Os01g0111200 protein (Putative uncharacterized protein P0439B06.21) |
| GRMZM2G051630 | 124 | 133 | 0.49 | 3.29E-02 | Putative uncharacterized protein |
| GRMZM2G051677 | 506 | 400 | 0.93 | 2.64E-20 | Fructokinase-2 |
| GRMZM2G051689 | 29 | 15 | 1.54 | 3.69E-03 | Putative uncharacterized protein |
| GRMZM2G051724 | 22 | 84 | -1.34 | 3.27E-04 | Methyl-binding domain protein MBD111 (Putative uncharacterized protein) (Putative methyl-binding domain protein MBD111) |
| GRMZM2G051750 | 41 | 31 | 0.99 | 1.85E-02 | Putative uncharacterized protein |
| GRMZM2G051753 | 9 | 37 | -1.45 | 1.99E-02 | Boron transporter-like protein 2 |
| GRMZM2G051764 | 313 | 588 | -0.32 | 8.65E-03 | AKIN gamma (Putative uncharacterized protein) |
| GRMZM2G051767 | 32 | 103 | -1.10 | 7.47E-04 | Putative uncharacterized protein |
| GRMZM2G051769 | 193 | 224 | 0.38 | 3.56E-02 | IAA16-auxin-responsive Aux/IAA family member |
| GRMZM2G051792 | 61 | 140 | -0.61 | 2.79E-02 | Putative uncharacterized protein |
| GRMZM2G051808 | 7 | 32 | -1.60 | 2.34E-02 | NA |
| GRMZM2G051848 | 495 | 500 | 0.58 | 7.30E-09 | Ribosomal protein S8 |
| GRMZM2G051879 | 3,641 | 4,409 | 0.32 | 1.34E-20 | Histone H3 |
| GRMZM2G051883 | 79 | 76 | 0.65 | 2.48E-02 | Os05g0187500 protein (Putative uncharacterized protein OJ1097_A12.6) |
| GRMZM2G051917 | 107 | 272 | -0.75 | 2.98E-05 | H0901F07.12 protein |
| GRMZM2G051955 | 72 | 51 | 1.09 | 3.54E-04 | ZF-HD homeobox protein |
| GRMZM2G052078 | 68 | 214 | -1.06 | 5.08E-07 | Os04g0507500 protein (OSJNBa0043L24.9 protein) |
| GRMZM2G052148 | 118 | 358 | -1.01 | 1.36E-10 | Os03g0349000 protein (Putative uncharacterized protein) (cDNA clone:J013134K18, full insert sequence) (Nucleoporin interacting component, putative, expressed) |
| GRMZM2G052178 | 367 | 439 | 0.33 | 6.89E-03 | Putative uncharacterized protein |
| GRMZM2G052200 | 31 | 91 | -0.96 | 6.53E-03 | Putative uncharacterized protein |
| GRMZM2G052328 | 45 | 128 | -0.92 | 1.28E-03 | Putative uncharacterized protein |
| GRMZM2G052344 | 140 | 55 | 1.94 | 1.48E-17 | RING-H2 finger protein ATL2B |
| GRMZM2G052389 | 21 | 61 | -0.95 | 3.93E-02 | Putative uncharacterized protein |
| GRMZM2G052422 | 24 | 14 | 1.37 | 2.14E-02 | 1-aminocyclopropane-1-carboxylate oxidase 1 (Putative uncharacterized protein) (Acc oxidase) |
| GRMZM2G052435 | 392 | 432 | 0.45 | 8.25E-05 | Os02g0474700 protein (Putative Importin 7<Ran-binding protein 7) |
| GRMZM2G052476 | 150 | 43 | 2.39 | 1.22E-24 | Ternary complex factor MIP1 |
| GRMZM2G052515 | 70 | 179 | -0.76 | 9.48E-04 | Putative uncharacterized protein (Transcription factor E2F2) |
| GRMZM2G052569 | 143 | 454 | -1.08 | 9.73E-15 | Putative uncharacterized protein |
| GRMZM2G052586 | 42 | 103 | -0.70 | 3.66E-02 | 50S ribosomal protein L35 (Fragment) |
| GRMZM2G052650 | 210 | 188 | 0.75 | 3.49E-06 | cDNA clone:J023075D08, full insert sequence |
| GRMZM2G052658 | 9 | 38 | -1.49 | 2.01E-02 | Putative uncharacterized protein |
| GRMZM2G052671 | 4 | 23 | -1.93 | 3.97E-02 | Putative uncharacterized protein |
| GRMZM2G052699 | 52 | 130 | -0.73 | 1.00E-02 | Putative uncharacterized protein |
| GRMZM2G052855 | 69 | 157 | -0.59 | 2.06E-02 | Putative uncharacterized protein |
| GRMZM2G052869 | 438 | 295 | 1.16 | 2.04E-25 | Metallothionein-like protein type 2 |
| GRMZM2G052926 | 58 | 160 | -0.87 | 4.48E-04 | Putative uncharacterized protein |
| GRMZM2G052935 | 36 | 166 | -1.61 | 4.10E-10 | Aspartokinase (EC 2.7.2.4) |
| GRMZM2G053019 | 44 | 130 | -0.97 | 5.61E-04 | Translocon Tic40 |
| GRMZM2G053027 | 83 | 52 | 1.27 | 7.14E-06 | Putative uncharacterized protein |
| GRMZM2G053079 | 90 | 83 | 0.71 | 7.89E-03 | Putative uncharacterized protein OSJNBa0027L23.9 |
| GRMZM2G053150 | 12 | 43 | -1.25 | 2.64E-02 | Putative uncharacterized protein |
| GRMZM2G053236 | 68 | 175 | -0.77 | 1.01E-03 | ATP-dependent Clp protease proteolytic subunit |
| GRMZM2G053261 | 118 | 64 | 1.47 | 3.98E-10 | Os05g0593800 protein (Putative uncharacterized protein) (cDNA clone:001-033-D10, full insert sequence) (Putative uncharacterized protein P0663C08.15) |
| GRMZM2G053299 | 174 | 414 | -0.66 | 3.33E-06 | Putative uncharacterized protein |
| GRMZM2G053319 | 228 | 227 | 0.60 | 1.18E-04 | Putative uncharacterized protein |
| GRMZM2G053338 | 194 | 167 | 0.81 | 1.64E-06 | Putative uncharacterized protein |
| GRMZM2G053397 | 85 | 216 | -0.75 | 2.41E-04 | Putative uncharacterized protein |
| GRMZM2G053503 | 159 | 41 | 2.55 | 4.49E-28 | Ethylene-responsive factor-like protein 1 |
| GRMZM2G053511 | 21 | 88 | -1.48 | 5.32E-05 | Ubiquitin-protein ligase/ zinc ion binding protein |
| GRMZM2G053557 | 27 | 93 | -1.19 | 6.08E-04 | Myb-like DNA-binding domain containing protein (Putative uncharacterized protein) |
| GRMZM2G053627 | 9 | 2 | 2.76 | 4.06E-02 | Putative uncharacterized protein |
| GRMZM2G053652 | 2,187 | 3,985 | -0.27 | 1.70E-11 | 40S ribosomal protein S7 (Putative uncharacterized protein) |
| GRMZM2G053766 | 186 | 216 | 0.38 | 4.03E-02 | Os02g0785900 protein |
| GRMZM2G053767 | 591 | 620 | 0.52 | 7.39E-09 | 40S ribosomal protein S4-like (Putative uncharacterized protein) |
| GRMZM2G053790 | 18 | 57 | -1.07 | 2.17E-02 | Putative uncharacterized protein |
| GRMZM2G053803 | 108 | 222 | -0.45 | 3.60E-02 | Acyl-CoA binding protein |
| GRMZM2G053908 | 24 | 79 | -1.13 | 3.56E-03 | Putative uncharacterized protein |
| GRMZM2G053916 | 39 | 119 | -1.02 | 6.65E-04 | Putative uncharacterized protein |
| GRMZM2G054012 | 184 | 154 | 0.85 | 1.14E-06 | 40S ribosomal protein S30 |
| GRMZM2G054013 | 56 | 171 | -1.02 | 1.97E-05 | Putative uncharacterized protein |
| GRMZM2G054093 | 25 | 71 | -0.91 | 2.94E-02 | Putative uncharacterized protein |
| GRMZM2G054136 | 2,728 | 2,549 | 0.69 | 8.08E-65 | 40S ribosomal protein S6 |
| GRMZM2G054162 | 141 | 456 | -1.10 | 2.38E-15 | Putative uncharacterized protein |
| GRMZM2G054201 | 211 | 222 | 0.52 | 1.60E-03 | Putative uncharacterized protein |
| GRMZM2G054225 | 512 | 372 | 1.05 | 3.51E-25 | DNA-directed RNA polymerase (EC 2.7.7.6) (Fragment) |
| GRMZM2G054247 | 44 | 129 | -0.96 | 7.20E-04 | Putative uncharacterized protein |
| GRMZM2G054300 | 1,331 | 2,392 | -0.25 | 2.79E-06 | APx1-Cytosolic Ascorbate Peroxidase |
| GRMZM2G054350 | 27 | 86 | -1.08 | 3.18E-03 | Putative uncharacterized protein |
| GRMZM2G054354 | 59 | 198 | -1.16 | 1.75E-07 | Protein transporter (Putative uncharacterized protein) |
| GRMZM2G054380 | 174 | 362 | -0.47 | 2.73E-03 | SET domain-containing protein SET118 |
| GRMZM2G054415 | 28 | 83 | -0.98 | 7.59E-03 | Putative uncharacterized protein |
| GRMZM2G054468 | 32 | 82 | -0.77 | 4.13E-02 | Putative uncharacterized protein |
| GRMZM2G054470 | 66 | 191 | -0.94 | 2.33E-05 | Putative uncharacterized protein |
| GRMZM2G054537 | 49 | 120 | -0.70 | 1.80E-02 | DAG protein |
| GRMZM2G054564 | 9 | 2 | 2.76 | 4.06E-02 | Putative uncharacterized protein |
| GRMZM2G054706 | 114 | 303 | -0.82 | 1.40E-06 | Putative uncharacterized protein |
| GRMZM2G054765 | 149 | 302 | -0.43 | 1.47E-02 | Os02g0135700 protein (Putative DNA polymerase V) |
| GRMZM2G054827 | 15 | 65 | -1.52 | 6.36E-04 | Os09g0573100 protein (Putative uncharacterized protein) (cDNA clone:J023093B21, full insert sequence) (Putative uncharacterized protein B1130E07.15) |
| GRMZM2G054830 | 1,217 | 1,504 | 0.29 | 4.11E-06 | 40S ribosomal protein S9 |
| GRMZM2G054860 | 207 | 224 | 0.48 | 4.19E-03 | NA |
| GRMZM2G054896 | 16 | 6 | 2.01 | 1.74E-02 | Plastid-specific ribosomal protein 6 |
| GRMZM2G054916 | 7 | 1 | 3.40 | 3.67E-02 | Cupin family protein |
| GRMZM2G055000 | 61 | 53 | 0.79 | 1.93E-02 | SR protein related family member |
| GRMZM2G055020 | 75 | 259 | -1.20 | 4.19E-10 | Putative uncharacterized protein |
| GRMZM2G055025 | 118 | 263 | -0.56 | 2.45E-03 | Outer mitochondrial membrane protein porin |
| GRMZM2G055116 | 230 | 111 | 1.64 | 9.12E-23 | Putative uncharacterized protein |
| GRMZM2G055135 | 101 | 80 | 0.93 | 1.68E-04 | LOL1 |
| GRMZM2G055172 | 60 | 176 | -0.96 | 4.06E-05 | Putative uncharacterized protein |
| GRMZM2G055217 | 21 | 67 | -1.08 | 1.15E-02 | Putative uncharacterized protein |
| GRMZM2G055273 | 50 | 42 | 0.84 | 2.56E-02 | Membrane protein |
| GRMZM2G055276 | 2,726 | 2,249 | 0.87 | 3.24E-97 | Putative uncharacterized protein |
| GRMZM2G055320 | 114 | 48 | 1.84 | 2.01E-13 | 4-coumarate:CoA ligase |
| GRMZM2G055331 | 79 | 277 | -1.22 | 4.38E-11 | Sucrose phosphate synthase II |
| GRMZM2G055404 | 70 | 36 | 1.55 | 1.08E-06 | DNA-binding WRKY |
| GRMZM2G055435 | 114 | 37 | 2.21 | 3.27E-17 | Os02g0667600 protein (cDNA clone:J033044J15, full insert sequence) (Putative uncharacterized protein OJ1486_E07.31-1) (Putative uncharacterized protein OJ1725_H08.8-1) |
| GRMZM2G055450 | 76 | 63 | 0.86 | 3.20E-03 | Putative uncharacterized protein |
| GRMZM2G055489 | 82 | 276 | -1.16 | 3.19E-10 | Putative uncharacterized protein |
| GRMZM2G055520 | 57 | 147 | -0.78 | 2.89E-03 | Os08g0157100 protein (Proline-rich protein-like) |
| GRMZM2G055567 | 26 | 76 | -0.96 | 1.61E-02 | DNA-directed RNA polymerase (EC 2.7.7.6) |
| GRMZM2G055619 | 233 | 434 | -0.31 | 3.90E-02 | Putative uncharacterized protein (Putative uncharacterized protein P0452F04.29) |
| GRMZM2G055678 | 81 | 60 | 1.02 | 2.68E-04 | Putative uncharacterized protein |
| GRMZM2G055682 | 313 | 365 | 0.37 | 5.80E-03 | Putative uncharacterized protein |
| GRMZM2G055705 | 4 | 26 | -2.11 | 1.27E-02 | Leucine Rich Repeat family protein |
| GRMZM2G055713 | 11 | 47 | -1.50 | 6.39E-03 | Putative uncharacterized protein |
| GRMZM2G055724 | 142 | 103 | 1.05 | 2.30E-07 | Putative uncharacterized protein |
| GRMZM2G055752 | 113 | 507 | -1.57 | 4.63E-29 | Putative uncharacterized protein |
| GRMZM2G055754 | 121 | 322 | -0.82 | 5.43E-07 | F-box domain containing protein |
| GRMZM2G055809 | 90 | 47 | 1.53 | 2.46E-08 | Putative uncharacterized protein |
| GRMZM2G055880 | 263 | 206 | 0.94 | 4.99E-11 | 60Kd inner membrane protein, expressed |
| GRMZM2G055899 | 182 | 368 | -0.42 | 6.31E-03 | Branched-chain-amino-acid aminotransferase (EC 2.6.1.42) |
| GRMZM2G055905 | 120 | 320 | -0.82 | 6.26E-07 | Serine/threonine protein phosphatase (EC 3.1.3.16) |
| GRMZM2G055970 | 195 | 609 | -1.05 | 4.91E-19 | Putative uncharacterized protein |
| GRMZM2G055999 | 361 | 782 | -0.52 | 1.05E-07 | Heavy metal-associated domain containing protein |
| GRMZM2G056014 | 48 | 129 | -0.83 | 3.34E-03 | Similar to rice Ca+2-ATPase |
| GRMZM2G056039 | 2,935 | 2,930 | 0.59 | 1.92E-53 | Putative uncharacterized protein |
| GRMZM2G056075 | 146 | 367 | -0.74 | 1.05E-06 | Os05g0274200 protein (Putative uncharacterized protein) |
| GRMZM2G056081 | 79 | 69 | 0.79 | 5.95E-03 | Saccharopine dehydrogenase |
| GRMZM2G056093 | 285 | 341 | 0.33 | 2.07E-02 | Putative uncharacterized protein |
| GRMZM2G056120 | 104 | 307 | -0.97 | 1.17E-08 | Putative uncharacterized protein |
| GRMZM2G056143 | 14 | 47 | -1.16 | 3.67E-02 | Putative uncharacterized protein |
| GRMZM2G056145 | 96 | 270 | -0.90 | 8.43E-07 | Putative uncharacterized protein |
| GRMZM2G056231 | 829 | 1,070 | 0.22 | 5.24E-03 | Histone H2A |
| GRMZM2G056252 | 112 | 108 | 0.64 | 6.72E-03 | Putative uncharacterized protein |
| GRMZM2G056270 | 146 | 312 | -0.50 | 2.72E-03 | Putative uncharacterized protein (RING finger and CHY zinc finger domain-containing protein 1) |
| GRMZM2G056350 | 513 | 1,024 | -0.41 | 2.16E-06 | MKI67 FHA domain-interacting nucleolar phosphoprotein-like (Putative uncharacterized protein) |
| GRMZM2G056357 | 37 | 146 | -1.39 | 2.52E-07 | Os10g0506800 protein (cDNA clone:J013122F06, full insert sequence) (TPR Domain containing protein, expressed) (Tetratricopeptide repeat, putative) |
| GRMZM2G056407 | 55 | 30 | 1.47 | 6.18E-05 | Putative uncharacterized protein |
| GRMZM2G056419 | 38 | 105 | -0.87 | 6.45E-03 | Actin-related protein 2/3 complex subunit 1B |
| GRMZM2G056501 | 306 | 374 | 0.30 | 3.12E-02 | Putative uncharacterized protein |
| GRMZM2G056564 | 20 | 69 | -1.20 | 4.36E-03 | Putative uncharacterized protein |
| GRMZM2G056569 | 213 | 771 | -1.26 | 4.65E-32 | Os07g0691800 protein (cDNA clone:001-013-H11, full insert sequence) (cDNA clone:001-204-A02, full insert sequence) (cDNA clone:J023018C04, full insert sequence) |
| GRMZM2G056572 | 183 | 408 | -0.57 | 7.91E-05 | Protein phosphatase 2C isoform epsilon (Putative uncharacterized protein) |
| GRMZM2G056573 | 676 | 538 | 0.92 | 1.19E-26 | Putative uncharacterized protein (YT521-B-like family protein) |
| GRMZM2G056600 | 48 | 21 | 1.78 | 9.73E-06 | Putative uncharacterized protein |
| GRMZM2G056612 | 49 | 140 | -0.92 | 5.92E-04 | Putative CDPK-related protein kinase |
| GRMZM2G056632 | 30 | 123 | -1.44 | 1.78E-06 | Putative uncharacterized protein |
| GRMZM2G056668 | 8 | 62 | -2.36 | 3.16E-06 | SR protein related family member |
| GRMZM2G056702 | 336 | 164 | 1.63 | 9.83E-33 | Putative uncharacterized protein |
| GRMZM2G056732 | 193 | 196 | 0.57 | 9.17E-04 | Putative uncharacterized protein (Serine/threonine-protein kinase SAPK2) |
| GRMZM2G056756 | 54 | 153 | -0.91 | 3.02E-04 | Putative uncharacterized protein |
| GRMZM2G056762 | 44 | 141 | -1.09 | 5.59E-05 | Putative uncharacterized protein |
| GRMZM2G056929 | 160 | 106 | 1.19 | 8.97E-10 | DNA-binding protein S1FA2 |
| GRMZM2G056988 | 10 | 2 | 2.91 | 2.36E-02 | Putative uncharacterized protein |
| GRMZM2G057000 | 350 | 923 | -0.81 | 1.23E-18 | Brassinosteroid biosynthesis-like protein |
| GRMZM2G057026 | 104 | 234 | -0.58 | 3.58E-03 | Putative uncharacterized protein (cDNA clone:001-026-C09, full insert sequence) (cDNA clone:J013116B18, full insert sequence) (cDNA clone:J023121K11, full insert sequence) |
| GRMZM2G057075 | 39 | 202 | -1.78 | 6.97E-14 | Putative uncharacterized protein (Ras association and pleckstrin homology domains 1 isoform 2) |
| GRMZM2G057078 | 134 | 140 | 0.53 | 1.54E-02 | Os11g0160100 protein (PHD-finger family protein, expressed) |
| GRMZM2G057091 | 72 | 205 | -0.92 | 1.68E-05 | Putative uncharacterized protein |
| GRMZM2G057150 | 220 | 477 | -0.53 | 5.87E-05 | Putative uncharacterized protein |
| GRMZM2G057186 | 274 | 897 | -1.12 | 9.41E-31 | Putative uncharacterized protein |
| GRMZM2G057251 | 246 | 512 | -0.47 | 2.36E-04 | Syntaxin 72 |
| GRMZM2G057329 | 600 | 1,224 | -0.44 | 1.43E-08 | Peptidyl-prolyl cis-trans isomerase (EC 5.2.1.8) |
| GRMZM2G057352 | 165 | 366 | -0.56 | 2.58E-04 | Early nodulin-like protein 1 |
| GRMZM2G057369 | 222 | 187 | 0.84 | 8.62E-08 | Putative uncharacterized protein |
| GRMZM2G057437 | 114 | 263 | -0.61 | 8.93E-04 | Putative uncharacterized protein (SAM domain family protein) |
| GRMZM2G057441 | 144 | 283 | -0.38 | 4.27E-02 | Ubiquitin-activating enzyme E1 |
| GRMZM2G057448 | 136 | 317 | -0.63 | 1.39E-04 | Histone-arginine methyltransferase CARM1 |
| GRMZM2G057450 | 478 | 1,100 | -0.61 | 8.23E-14 | Putative uncharacterized protein (Splicing factor U2AF 65 kDa subunit) |
| GRMZM2G057466 | 13 | 48 | -1.29 | 1.32E-02 | Putative uncharacterized protein |
| GRMZM2G057525 | 92 | 204 | -0.56 | 1.08E-02 | Putative uncharacterized protein |
| GRMZM2G057535 | 154 | 161 | 0.53 | 7.17E-03 | Elongation factor 1-alpha |
| GRMZM2G057623 | 114 | 257 | -0.58 | 2.07E-03 | Putative uncharacterized protein (Putative uncharacterized protein OSJNBa0040E01.12) |
| GRMZM2G057642 | 164 | 437 | -0.82 | 2.40E-09 | RNA-binding protein 25 |
| GRMZM2G057753 | 25 | 8 | 2.24 | 3.70E-04 | Os12g0158300 protein (Putative uncharacterized protein) (cDNA, clone: J080307E12, full insert sequence) (Uncharacterized plant-specific domain TIGR01568 family protein, expressed) |
| GRMZM2G057779 | 84 | 255 | -1.01 | 1.02E-07 | Putative uncharacterized protein |
| GRMZM2G057823 | 1,596 | 2,019 | 0.25 | 2.66E-06 | Fructose-bisphosphate aldolase (EC 4.1.2.13) |
| GRMZM2G057852 | 757 | 365 | 1.64 | 5.01E-75 | Histone H2B |
| GRMZM2G057853 | 154 | 315 | -0.44 | 1.00E-02 | Seh1-like protein |
| GRMZM2G057973 | 158 | 163 | 0.55 | 4.61E-03 | Putative uncharacterized protein |
| GRMZM2G058039 | 68 | 149 | -0.54 | 4.31E-02 | Putative uncharacterized protein (cDNA clone:001-044-B01, full insert sequence) (Polypyrimidine tract-binding protein 1-like) |
| GRMZM2G058057 | 289 | 704 | -0.69 | 4.36E-11 | Putative uncharacterized protein |
| GRMZM2G058158 | 235 | 175 | 1.02 | 3.75E-11 | EMF2 |
| GRMZM2G058250 | 96 | 97 | 0.58 | 2.93E-02 | PSRP4 |
| GRMZM2G058252 | 40 | 123 | -1.03 | 4.54E-04 | Putative uncharacterized protein (Small nuclear ribonucleoprotein F) |
| GRMZM2G058261 | 44 | 29 | 1.19 | 3.12E-03 | Deoxyribodipyrimidine photolyase |
| GRMZM2G058276 | 258 | 500 | -0.36 | 5.68E-03 | T-complex protein 1 subunit beta |
| GRMZM2G058292 | 46 | 115 | -0.73 | 1.81E-02 | Anthocyanin biosynthetic gene regulator PAC1 |
| GRMZM2G058310 | 11 | 45 | -1.44 | 8.73E-03 | Beta-amylase (EC 3.2.1.2) |
| GRMZM2G058407 | 36 | 111 | -1.03 | 7.56E-04 | Putative uncharacterized protein (Signal recognition particle 9 kDa protein) |
| GRMZM2G058432 | 433 | 498 | 0.39 | 3.67E-04 | Mitochondrial import inner membrane translocase subunit Tim13 (Putative uncharacterized protein) |
| GRMZM2G058444 | 77 | 48 | 1.27 | 1.61E-05 | Os02g0809800 protein (Putative uncharacterized protein) (cDNA clone:J023079I02, full insert sequence) (Putative phosphate transporter) |
| GRMZM2G058451 | 40 | 101 | -0.74 | 2.67E-02 | BHLH transcription factor (Os07g0543000 protein) (cDNA clone:001-007-D03, full insert sequence) (BHLH-HALZ myc like protein) (Putative bHLH protein) (Putative uncharacterized protein) |
| GRMZM2G058479 | 144 | 321 | -0.57 | 6.41E-04 | Putative uncharacterized protein |
| GRMZM2G058481 | 48 | 36 | 1.01 | 9.23E-03 | Putative uncharacterized protein 4A |
| GRMZM2G058522 | 52 | 170 | -1.12 | 3.96E-06 | Superoxide dismutase [Cu-Zn] (EC 1.15.1.1) |
| GRMZM2G058560 | 154 | 371 | -0.68 | 6.80E-06 | Serine/threonine-protein phosphatase 2A activator 2 |
| GRMZM2G058573 | 23 | 66 | -0.93 | 3.02E-02 | Putative uncharacterized protein (SIR2-like histone deacetylase) |
| GRMZM2G058588 | 0 | 10 | #NUM! | 3.46E-02 | LIGULELESS1 |
| GRMZM2G058612 | 8 | 63 | -2.39 | 2.05E-06 | Putative uncharacterized protein |
| GRMZM2G058659 | 97 | 284 | -0.96 | 6.40E-08 | 3-phosphoshikimate 1-carboxyvinyltransferase (Epsp-synthase) (EC 2.5.1.19) (Fragment) |
| GRMZM2G058675 | 76 | 66 | 0.79 | 6.34E-03 | Aldehyde dehydrogenase (Putative uncharacterized protein) (RF2) (EC 1.2.1.3) (T cytoplasm male sterility restorer factor 2) |
| GRMZM2G058690 | 44 | 30 | 1.14 | 4.81E-03 | IQ calmodulin-binding motif family protein |
| GRMZM2G058702 | 77 | 188 | -0.70 | 1.85E-03 | Putative uncharacterized protein |
| GRMZM2G058760 | 124 | 114 | 0.71 | 1.12E-03 | Ferredoxin--NADP reductase (EC 1.18.1.2) |
| GRMZM2G058870 | 263 | 557 | -0.49 | 4.24E-05 | Putative uncharacterized protein |
| GRMZM2G058872 | 165 | 116 | 1.10 | 4.55E-09 | Putative uncharacterized protein |
| GRMZM2G058954 | 186 | 215 | 0.38 | 3.97E-02 | Putative uncharacterized protein |
| GRMZM2G059013 | 62 | 144 | -0.62 | 2.09E-02 | Fringe protein, putative, expressed |
| GRMZM2G059015 | 182 | 420 | -0.62 | 1.22E-05 | Putative uncharacterized protein |
| GRMZM2G059033 | 17 | 7 | 1.87 | 1.45E-02 | Putative uncharacterized protein |
| GRMZM2G059037 | 47 | 114 | -0.69 | 2.79E-02 | Cohesion protein |
| GRMZM2G059117 | 39 | 29 | 1.02 | 2.02E-02 | Putative uncharacterized protein |
| GRMZM2G059225 | 144 | 325 | -0.58 | 3.35E-04 | Os09g0510700 protein |
| GRMZM2G059266 | 80 | 254 | -1.08 | 2.09E-08 | PHD-finger family protein (Putative uncharacterized protein) |
| GRMZM2G059282 | 214 | 114 | 1.50 | 1.34E-18 | Elongin C |
| GRMZM2G059308 | 43 | 36 | 0.85 | 4.60E-02 | Ent-kaurene oxidase (Putative uncharacterized protein) |
| GRMZM2G059325 | 148 | 322 | -0.53 | 1.38E-03 | Transmembrane protein 18 |
| GRMZM2G059381 | 47 | 112 | -0.66 | 3.33E-02 | AMP-binding protein |
| GRMZM2G059428 | 46 | 31 | 1.16 | 3.98E-03 | Os03g0133000 protein (Putative uncharacterized protein) (No apical meristem protein, expressed) |
| GRMZM2G059432 | 51 | 119 | -0.63 | 4.08E-02 | Os08g0109500 protein (DNA-binding protein family-like) |
| GRMZM2G059449 | 45 | 114 | -0.75 | 1.39E-02 | Putative uncharacterized protein |
| GRMZM2G059496 | 28 | 87 | -1.04 | 3.58E-03 | Putative uncharacterized protein |
| GRMZM2G059556 | 22 | 64 | -0.95 | 3.48E-02 | Putative uncharacterized protein |
| GRMZM2G059565 | 9 | 2 | 2.76 | 4.06E-02 | NA |
| GRMZM2G059580 | 303 | 900 | -0.98 | 6.18E-25 | Elongation factor 1-gamma 3 |
| GRMZM2G059618 | 46 | 166 | -1.26 | 3.39E-07 | Putative uncharacterized protein |
| GRMZM2G059702 | 149 | 122 | 0.88 | 7.24E-06 | Putative uncharacterized protein |
| GRMZM2G059706 | 36 | 15 | 1.85 | 1.04E-04 | Disease resistance response protein 206 |
| GRMZM2G059801 | 52 | 42 | 0.90 | 1.54E-02 | Putative uncharacterized protein |
| GRMZM2G059865 | 312 | 601 | -0.35 | 2.59E-03 | Putative uncharacterized protein |
| GRMZM2G059887 | 95 | 266 | -0.89 | 1.22E-06 | AGP16 (Putative uncharacterized protein) |
| GRMZM2G059922 | 35 | 124 | -1.23 | 2.35E-05 | Cyclin-A2 |
| GRMZM2G059937 | 182 | 92 | 1.58 | 3.98E-17 | Outer mitochondrial membrane protein porin (Putative uncharacterized protein) |
| GRMZM2G059958 | 27 | 94 | -1.21 | 4.55E-04 | Putative uncharacterized protein |
| GRMZM2G059974 | 128 | 454 | -1.24 | 3.34E-18 | Putative uncharacterized protein |
| GRMZM2G059985 | 24 | 15 | 1.27 | 3.59E-02 | Putative uncharacterized protein |
| GRMZM2G060163 | 1,040 | 481 | 1.70 | 9.33E-109 | Ribonucleoside-diphosphate reductase small chain |
| GRMZM2G060167 | 285 | 349 | 0.30 | 4.43E-02 | Os02g0258300 protein (Putative uncharacterized protein) (Extra-large G-protein-like) |
| GRMZM2G060185 | 46 | 26 | 1.41 | 4.33E-04 | Putative uncharacterized protein |
| GRMZM2G060194 | 78 | 238 | -1.02 | 2.30E-07 | Putative uncharacterized protein |
| GRMZM2G060213 | 113 | 121 | 0.49 | 3.97E-02 | Putative uncharacterized protein |
| GRMZM2G060216 | 30 | 21 | 1.11 | 2.99E-02 | Basic leucine zipper protein (Liguleless2) |
| GRMZM2G060253 | 289 | 636 | -0.55 | 8.77E-07 | Putative uncharacterized protein |
| GRMZM2G060276 | 12 | 45 | -1.32 | 1.99E-02 | Putative uncharacterized protein |
| GRMZM2G060296 | 165 | 466 | -0.91 | 1.61E-11 | Putative uncharacterized protein |
| GRMZM2G060349 | 31 | 125 | -1.42 | 1.61E-06 | Putative uncharacterized protein |
| GRMZM2G060355 | 51 | 145 | -0.92 | 4.52E-04 | Putative uncharacterized protein |
| GRMZM2G060357 | 36 | 96 | -0.82 | 1.63E-02 | Putative uncharacterized protein (Cytochrome b561) |
| GRMZM2G060373 | 3,537 | 3,989 | 0.42 | 8.15E-34 | 40S ribosomal protein S29 |
| GRMZM2G060485 | 245 | 137 | 1.43 | 7.63E-20 | Response regulator 9 |
| GRMZM2G060554 | 75 | 226 | -1.00 | 9.74E-07 | Putative uncharacterized protein |
| GRMZM2G060561 | 59 | 194 | -1.13 | 5.40E-07 | Putative uncharacterized protein |
| GRMZM2G060567 | 424 | 420 | 0.61 | 2.42E-08 | Eukaryotic translation initiation factor 3 subunit 1 alpha, 35kDa |
| GRMZM2G060611 | 191 | 400 | -0.48 | 1.13E-03 | Os05g0519900 protein (Putative uncharacterized protein) (cDNA clone:J013099L06, full insert sequence) (Putative oligosaccharyl transferase STT3) (Putative oligosaccharyl transferase STT3 subunit) |
| GRMZM2G060669 | 505 | 402 | 0.92 | 5.54E-20 | Histone deacetylase complex subunit SAP18 |
| GRMZM2G060690 | 122 | 315 | -0.78 | 2.66E-06 | Cyclin-A2 |
| GRMZM2G060702 | 675 | 1,446 | -0.51 | 4.62E-13 | NA |
| GRMZM2G060726 | 65 | 163 | -0.73 | 2.76E-03 | Putative uncharacterized protein |
| GRMZM2G060742 | 18 | 10 | 1.44 | 4.65E-02 | Citrate transporter family protein |
| GRMZM2G060765 | 121 | 294 | -0.69 | 6.07E-05 | Putative uncharacterized protein |
| GRMZM2G060842 | 11 | 51 | -1.62 | 1.67E-03 | Putative uncharacterized protein |
| GRMZM2G060856 | 107 | 367 | -1.19 | 5.56E-14 | Membrane protein |
| GRMZM2G060857 | 112 | 103 | 0.71 | 2.43E-03 | Tubulin-specific chaperone E |
| GRMZM2G060868 | 186 | 509 | -0.86 | 1.53E-11 | Putative uncharacterized protein |
| GRMZM2G060872 | 56 | 127 | -0.59 | 4.30E-02 | Putative uncharacterized protein (Serine/threonine-protein kinase 16) |
| GRMZM2G060886 | 27 | 96 | -1.24 | 2.56E-04 | Phosphoethanolamine N-methyltransferase |
| GRMZM2G060906 | 1 | 20 | -3.73 | 3.19E-03 | OSJNBa0022H21.11 protein |
| GRMZM2G060924 | 129 | 290 | -0.58 | 9.00E-04 | Putative uncharacterized protein |
| GRMZM2G060952 | 194 | 130 | 1.17 | 1.26E-11 | Putative uncharacterized protein |
| GRMZM2G061023 | 36 | 103 | -0.93 | 4.23E-03 | Putative uncharacterized protein |
| GRMZM2G061043 | 221 | 555 | -0.74 | 9.67E-10 | Putative uncharacterized protein |
| GRMZM2G061127 | 26 | 75 | -0.94 | 2.04E-02 | Putative uncharacterized protein (Os01g0967900 protein) (Putative aspartyl aminopeptidase) |
| GRMZM2G061135 | 1,227 | 1,657 | 0.16 | 1.90E-02 | S-adenosylmethionine synthetase (EC 2.5.1.6) |
| GRMZM2G061206 | 97 | 278 | -0.93 | 2.22E-07 | Antiporter/ drug transporter/ transporter |
| GRMZM2G061446 | 48 | 119 | -0.72 | 1.69E-02 | Putative uncharacterized protein |
| GRMZM2G061487 | 56 | 36 | 1.23 | 5.24E-04 | DRE binding factor 1 |
| GRMZM2G061620 | 54 | 162 | -0.99 | 5.50E-05 | DNA repair protein RAD23 (Putative uncharacterized protein) |
| GRMZM2G061624 | 213 | 513 | -0.68 | 6.49E-08 | Putative uncharacterized protein (Ubiquitin ligase SINAT4) (SINA4) |
| GRMZM2G061634 | 32 | 108 | -1.16 | 2.50E-04 | Putative uncharacterized protein |
| GRMZM2G061662 | 71 | 233 | -1.12 | 2.92E-08 | Putative uncharacterized protein |
| GRMZM2G061663 | 4 | 26 | -2.11 | 1.27E-02 | Os05g0550000 protein (Putative uncharacterized protein P0560C03.4) |
| GRMZM2G061672 | 143 | 154 | 0.48 | 2.12E-02 | Putative uncharacterized protein |
| GRMZM2G061684 | 18 | 61 | -1.17 | 9.71E-03 | Putative uncharacterized protein |
| GRMZM2G061728 | 41 | 153 | -1.31 | 5.85E-07 | Putative uncharacterized protein |
| GRMZM2G061734 | 66 | 43 | 1.21 | 1.81E-04 | Putative uncharacterized protein |
| GRMZM2G061735 | 116 | 343 | -0.97 | 1.41E-09 | Putative uncharacterized protein |
| GRMZM2G061745 | 212 | 447 | -0.48 | 3.99E-04 | Putative uncharacterized protein |
| GRMZM2G061751 | 59 | 53 | 0.75 | 3.06E-02 | Putative uncharacterized protein |
| GRMZM2G061758 | 695 | 456 | 1.20 | 2.19E-42 | Putative uncharacterized protein (Ubiquitin-protein ligase/ zinc ion binding protein) |
| GRMZM2G061783 | 22 | 63 | -0.93 | 3.46E-02 | Putative uncharacterized protein |
| GRMZM2G061900 | 95 | 274 | -0.94 | 2.27E-07 | Putative uncharacterized protein (Ras-related protein ARA-3) |
| GRMZM2G061928 | 153 | 379 | -0.72 | 1.37E-06 | Putative uncharacterized protein (Soluble inorganic pyrophosphatase) |
| GRMZM2G061932 | 35 | 21 | 1.33 | 5.28E-03 | Putative uncharacterized protein |
| GRMZM2G061938 | 173 | 343 | -0.40 | 1.54E-02 | Putative uncharacterized protein |
| GRMZM2G061988 | 616 | 439 | 1.08 | 1.13E-31 | Os10g0377800 protein (Pyridoxamine 5'-phosphate oxidase, putative, expressed) |
| GRMZM2G061996 | 75 | 223 | -0.98 | 1.59E-06 | Protein arginine n-methyltransferase, putative (EC 2.1.1.125) |
| GRMZM2G062019 | 16 | 0 | #VALUE! | 5.24E-06 | Gibberellin receptor GID1L2 |
| GRMZM2G062024 | 49 | 115 | -0.64 | 3.70E-02 | Carbohydrate transporter/ sugar porter/ transporter |
| GRMZM2G062056 | 22 | 10 | 1.73 | 9.10E-03 | Lipoxygenase (EC 1.13.11.12) |
| GRMZM2G062084 | 32 | 91 | -0.92 | 9.11E-03 | Putative phragmoplast-associated kinesin |
| GRMZM2G062179 | 13 | 44 | -1.17 | 4.08E-02 | OSIGBa0153E02-OSIGBa0093I20.21 protein |
| GRMZM2G062262 | 44 | 161 | -1.28 | 4.21E-07 | Gigantea 1B |
| GRMZM2G062333 | 55 | 129 | -0.64 | 2.49E-02 | Minichromosome maintenance protein |
| GRMZM2G062373 | 358 | 832 | -0.63 | 6.13E-11 | Putative uncharacterized protein (USP family protein) |
| GRMZM2G062391 | 37 | 91 | -0.71 | 4.71E-02 | Putative uncharacterized protein |
| GRMZM2G062416 | 57 | 137 | -0.67 | 1.36E-02 | Phosphatidate cytidylyltransferase (EC 2.7.7.41) |
| GRMZM2G062420 | 37 | 132 | -1.24 | 1.03E-05 | GTP cyclohydrolase I 1 |
| GRMZM2G062481 | 126 | 250 | -0.40 | 4.75E-02 | Acyl carrier protein |
| GRMZM2G062504 | 171 | 97 | 1.41 | 1.09E-13 | Putative uncharacterized protein |
| GRMZM2G062527 | 115 | 58 | 1.58 | 6.38E-11 | GAST1 protein |
| GRMZM2G062552 | 122 | 82 | 1.16 | 1.79E-07 | Putative uncharacterized protein |
| GRMZM2G062577 | 46 | 108 | -0.64 | 4.48E-02 | Anther-specific proline-rich protein APG |
| GRMZM2G062591 | 155 | 338 | -0.53 | 8.32E-04 | DNA-binding protein (Putative uncharacterized protein) |
| GRMZM2G062657 | 44 | 114 | -0.78 | 1.06E-02 | Putative uncharacterized protein |
| GRMZM2G062683 | 13 | 47 | -1.26 | 1.77E-02 | Putative uncharacterized protein (STE20/SPS1-related proline-alanine-rich protein kinase) |
| GRMZM2G062854 | 41 | 109 | -0.82 | 8.35E-03 | ATP synthase subunit beta (EC 3.6.3.14) |
| GRMZM2G062860 | 8 | 39 | -1.69 | 5.64E-03 | Os03g0213300 protein (Putative uncharacterized protein) (Expressed protein) |
| GRMZM2G062910 | 31 | 96 | -1.04 | 1.89E-03 | mTERF family protein |
| GRMZM2G062953 | 167 | 192 | 0.39 | 4.68E-02 | Putative uncharacterized protein |
| GRMZM2G063060 | 86 | 75 | 0.79 | 4.20E-03 | Os03g0151800 protein (Putative uncharacterized protein) (cDNA clone:J013001D04, full insert sequence) (cDNA clone:J013082G14, full insert sequence) (cDNA clone:J013093N05, full insert sequence) (cDNA clone:J013112O07, full insert sequence) (Cell division cycle protein 48, putative, expressed) |
| GRMZM2G063156 | 22 | 9 | 1.88 | 4.18E-03 | NA |
| GRMZM2G063188 | 159 | 389 | -0.70 | 2.02E-06 | Putative uncharacterized protein |
| GRMZM2G063192 | 972 | 823 | 0.83 | 4.77E-32 | Cell division cycle protein 20 |
| GRMZM2G063216 | 66 | 41 | 1.28 | 6.59E-05 | Putative uncharacterized protein (WRKY46-superfamily of TFs having WRKY and zinc finger domains) |
| GRMZM2G063244 | 80 | 53 | 1.19 | 3.31E-05 | Peptidyl-prolyl cis-trans isomerase (EC 5.2.1.8) |
| GRMZM2G063253 | 109 | 311 | -0.92 | 4.30E-08 | Putative glucose-6-phosphate/phosphate-tranlocat or |
| GRMZM2G063298 | 28 | 18 | 1.23 | 2.79E-02 | NA |
| GRMZM2G063340 | 1,494 | 3,702 | -0.72 | 1.21E-60 | 40S ribosomal protein S12 |
| GRMZM2G063369 | 75 | 216 | -0.93 | 6.99E-06 | Putative uncharacterized protein |
| GRMZM2G063394 | 55 | 137 | -0.73 | 8.25E-03 | Putative uncharacterized protein (Armadillo/beta-catenin repeat family protein, putative, expressed) |
| GRMZM2G063431 | 10 | 2 | 2.91 | 2.36E-02 | Putative uncharacterized protein |
| GRMZM2G063468 | 11 | 54 | -1.70 | 8.29E-04 | Putative uncharacterized protein |
| GRMZM2G063473 | 12 | 44 | -1.28 | 1.99E-02 | Putative uncharacterized protein |
| GRMZM2G063517 | 136 | 382 | -0.90 | 2.23E-09 | Putative uncharacterized protein |
| GRMZM2G063617 | 4,164 | 2,765 | 1.18 | 1.40E-248 | 60S ribosomal protein L22-2 (Putative uncharacterized protein) |
| GRMZM2G063643 | 53 | 176 | -1.14 | 1.51E-06 | Putative uncharacterized protein |
| GRMZM2G063676 | 331 | 396 | 0.33 | 1.15E-02 | Heat shock 70 kDa protein 4 (Putative uncharacterized protein) |
| GRMZM2G063688 | 82 | 177 | -0.52 | 3.38E-02 | Putative uncharacterized protein |
| GRMZM2G063700 | 155 | 77 | 1.60 | 5.79E-15 | Putative uncharacterized protein |
| GRMZM2G063723 | 65 | 56 | 0.81 | 1.15E-02 | Putative uncharacterized protein |
| GRMZM2G063737 | 55 | 195 | -1.23 | 4.87E-08 | Putative uncharacterized protein |
| GRMZM2G063756 | 275 | 38 | 3.45 | 3.47E-67 | Cytochrome P450 CYP71C3v2 (Cytochrome P450 monooxygenase CYP71C3v2) |
| GRMZM2G063792 | 142 | 384 | -0.84 | 1.26E-08 | Protein kinase AFC1, putative, expressed |
| GRMZM2G063802 | 34 | 25 | 1.04 | 3.29E-02 | Nicotiana lesion-inducing like |
| GRMZM2G063850 | 354 | 695 | -0.38 | 3.90E-04 | Heat shock protein STI |
| GRMZM2G063880 | 23 | 9 | 1.95 | 2.63E-03 | Os05g0571200 protein (Putative uncharacterized protein) (cDNA clone:002-142-F03, full insert sequence) |
| GRMZM2G063896 | 4,525 | 4,109 | 0.73 | 2.48E-119 | Histone H4 |
| GRMZM2G063931 | 51 | 154 | -1.00 | 8.19E-05 | Ubiquitin carrier protein (EC 6.3.2.-) |
| GRMZM2G063961 | 24 | 78 | -1.11 | 4.65E-03 | Putative uncharacterized protein |
| GRMZM2G063972 | 40 | 120 | -0.99 | 7.41E-04 | Heat shock protein binding protein |
| GRMZM2G064031 | 65 | 149 | -0.61 | 2.03E-02 | Putative uncharacterized protein |
| GRMZM2G064042 | 97 | 68 | 1.10 | 1.60E-05 | Putative uncharacterized protein |
| GRMZM2G064050 | 13 | 61 | -1.64 | 5.36E-04 | Putative uncharacterized protein |
| GRMZM2G064056 | 176 | 164 | 0.69 | 1.06E-04 | Putative uncharacterized protein |
| GRMZM2G064111 | 32 | 131 | -1.44 | 7.78E-07 | Putative uncharacterized protein |
| GRMZM2G064136 | 577 | 394 | 1.14 | 1.61E-32 | Cation transport protein chaC (Putative uncharacterized protein) |
| GRMZM2G064145 | 297 | 345 | 0.38 | 6.35E-03 | Putative uncharacterized protein |
| GRMZM2G064168 | 194 | 149 | 0.97 | 1.25E-08 | Putative uncharacterized protein |
| GRMZM2G064212 | 56 | 146 | -0.79 | 2.69E-03 | Autophagy-related 4 variant 1 (Cysteine protease ATG4B) (Putative uncharacterized protein) |
| GRMZM2G064296 | 186 | 140 | 1.00 | 9.53E-09 | Endoplasmic oxidoreductin-1 |
| GRMZM2G064302 | 790 | 2,169 | -0.87 | 5.69E-49 | Enolase (EC 4.2.1.11) |
| GRMZM2G064330 | 165 | 337 | -0.44 | 6.92E-03 | Putative uncharacterized protein |
| GRMZM2G064336 | 160 | 435 | -0.85 | 8.57E-10 | NifU-like N-terminal domain-containing protein (Putative uncharacterized protein) |
| GRMZM2G064366 | 65 | 170 | -0.80 | 8.04E-04 | Lung seven transmembrane receptor family protein, expressed (Os11g0546100 protein) |
| GRMZM2G064382 | 284 | 803 | -0.91 | 9.94E-20 | Putative uncharacterized protein |
| GRMZM2G064426 | 237 | 457 | -0.36 | 1.04E-02 | Putative uncharacterized protein |
| GRMZM2G064630 | 39 | 95 | -0.69 | 4.40E-02 | Os07g0627300 protein (Putative uncharacterized protein) (cDNA clone:001-115-D02, full insert sequence) (Myb-related protein-like) |
| GRMZM2G064640 | 702 | 1,451 | -0.46 | 8.13E-11 | 40S ribosomal protein S9 |
| GRMZM2G064732 | 109 | 233 | -0.50 | 1.25E-02 | Guanine nucleotide-binding protein alpha-1 subunit (Putative uncharacterized protein) |
| GRMZM2G064753 | 61 | 143 | -0.64 | 1.68E-02 | Cytochrome c oxidase polypeptide Vb |
| GRMZM2G064767 | 42 | 131 | -1.05 | 1.60E-04 | Putative uncharacterized protein |
| GRMZM2G064799 | 323 | 238 | 1.03 | 1.56E-15 | Putative uncharacterized protein |
| GRMZM2G064831 | 23 | 66 | -0.93 | 3.02E-02 | Vacuolar membrane protein |
| GRMZM2G064852 | 38 | 100 | -0.80 | 1.54E-02 | C2 domain-containing protein, putative, expressed (Putative C2 protein) (Putative phosphoribosyltransferase) |
| GRMZM2G064853 | 23 | 65 | -0.91 | 3.78E-02 | cDNA clone:J023109M13, full insert sequence (Os01g0844400 protein) (Zinc finger DHHC domain containing protein 2-like) |
| GRMZM2G064868 | 124 | 281 | -0.59 | 1.01E-03 | Putative uncharacterized protein |
| GRMZM2G064967 | 17 | 52 | -1.02 | 4.08E-02 | Putative uncharacterized protein |
| GRMZM2G064984 | 91 | 211 | -0.62 | 3.29E-03 | Putative uncharacterized protein |
| GRMZM2G064988 | 112 | 233 | -0.47 | 2.37E-02 | Histone deacetylase complex subunit SAP18 (Putative uncharacterized protein) |
| GRMZM2G064993 | 12 | 43 | -1.25 | 2.64E-02 | Annexin-like protein RJ4 (Putative uncharacterized protein) (Annexin p33) |
| GRMZM2G065076 | 42 | 102 | -0.69 | 3.64E-02 | Putative uncharacterized protein |
| GRMZM2G065105 | 100 | 266 | -0.82 | 7.43E-06 | Putative uncharacterized protein |
| GRMZM2G065154 | 4 | 27 | -2.16 | 8.55E-03 | Putative uncharacterized protein |
| GRMZM2G065276 | 173 | 332 | -0.35 | 4.08E-02 | Putative uncharacterized protein |
| GRMZM2G065423 | 31 | 144 | -1.62 | 6.66E-09 | Putative uncharacterized protein |
| GRMZM2G065451 | 158 | 123 | 0.95 | 6.71E-07 | Putative uncharacterized protein |
| GRMZM2G065494 | 177 | 166 | 0.68 | 1.17E-04 | Putative uncharacterized protein |
| GRMZM2G065496 | 28 | 81 | -0.94 | 1.23E-02 | B3 DNA binding domain containing protein (Putative uncharacterized protein) |
| GRMZM2G065498 | 166 | 424 | -0.76 | 5.86E-08 | Os03g0648500 protein (Putative uncharacterized protein) (cDNA clone:002-133-B02, full insert sequence) (Phytocyanin-related) (Plastocyanin-like domain containing protein, expressed) |
| GRMZM2G065532 | 123 | 292 | -0.66 | 1.43E-04 | Os08g0113200 protein (Putative RNA recognition motif (RRM)-containing protein) |
| GRMZM2G065566 | 246 | 574 | -0.63 | 7.66E-08 | Protein binding protein |
| GRMZM2G065596 | 229 | 426 | -0.30 | 4.45E-02 | Putative uncharacterized protein orf114-a |
| GRMZM2G065599 | 112 | 267 | -0.66 | 2.79E-04 | Putative uncharacterized protein (DNA methyltransferase DMT106) |
| GRMZM2G065665 | 28 | 75 | -0.83 | 3.77E-02 | Putative oligopeptidase |
| GRMZM2G065669 | 154 | 307 | -0.40 | 2.09E-02 | Putative uncharacterized protein |
| GRMZM2G065734 | 1,837 | 3,581 | -0.37 | 4.77E-18 | 40S ribosomal protein S3a (Putative uncharacterized protein) |
| GRMZM2G065757 | 140 | 348 | -0.72 | 4.24E-06 | Putative uncharacterized protein |
| GRMZM2G065804 | 89 | 375 | -1.48 | 7.45E-20 | Ribonuclease (EC 3.1.26.-) |
| GRMZM2G065806 | 70 | 193 | -0.87 | 7.08E-05 | NA |
| GRMZM2G065822 | 234 | 481 | -0.45 | 6.45E-04 | Putative uncharacterized protein |
| GRMZM2G065839 | 5 | 33 | -2.13 | 4.21E-03 | Putative uncharacterized protein |
| GRMZM2G065913 | 157 | 157 | 0.59 | 2.30E-03 | Putative uncharacterized protein |
| GRMZM2G065950 | 278 | 596 | -0.51 | 1.05E-05 | Putative uncharacterized protein |
| GRMZM2G065956 | 176 | 160 | 0.73 | 4.59E-05 | 50S ribosomal protein L12-2 |
| GRMZM2G066024 | 3,556 | 1,258 | 2.09 | 0.00E+01 | Fructose-bisphosphate aldolase (EC 4.1.2.13) |
| GRMZM2G066051 | 40 | 102 | -0.76 | 2.18E-02 | Putative uncharacterized protein |
| GRMZM2G066059 | 18 | 61 | -1.17 | 9.71E-03 | Putative uncharacterized protein |
| GRMZM2G066080 | 146 | 362 | -0.72 | 2.85E-06 | Acetyl-coenzyme A carboxylase |
| GRMZM2G066101 | 452 | 988 | -0.54 | 6.12E-10 | Putative uncharacterized protein |
| GRMZM2G066111 | 469 | 601 | 0.23 | 3.80E-02 | ATPase subunit 4 |
| GRMZM2G066158 | 139 | 71 | 1.56 | 6.08E-13 | Putative uncharacterized protein |
| GRMZM2G066222 | 1,817 | 3,315 | -0.28 | 8.85E-10 | Ribosomal protein S27 |
| GRMZM2G066225 | 67 | 49 | 1.04 | 9.61E-04 | Putative uncharacterized protein |
| GRMZM2G066274 | 87 | 190 | -0.54 | 1.91E-02 | Putative uncharacterized protein |
| GRMZM2G066293 | 23 | 104 | -1.59 | 2.18E-06 | ATPase |
| GRMZM2G066329 | 51 | 184 | -1.26 | 7.69E-08 | Coatomer subunit zeta-1 |
| GRMZM2G066341 | 77 | 218 | -0.91 | 1.09E-05 | Putative uncharacterized protein |
| GRMZM2G066362 | 54 | 151 | -0.89 | 4.82E-04 | Putative uncharacterized protein |
| GRMZM2G066400 | 113 | 233 | -0.45 | 2.77E-02 | Putative uncharacterized protein |
| GRMZM2G066440 | 136 | 514 | -1.33 | 5.77E-23 | Putative uncharacterized protein |
| GRMZM2G066460 | 3,947 | 4,290 | 0.47 | 4.35E-47 | Putative uncharacterized protein |
| GRMZM2G066469 | 82 | 69 | 0.84 | 2.92E-03 | Mitochondrial inner membrane protease subunit 1 |
| GRMZM2G066496 | 49 | 182 | -1.30 | 3.82E-08 | Putative uncharacterized protein |
| GRMZM2G066528 | 2,781 | 4,565 | -0.12 | 2.46E-03 | Putative uncharacterized protein (HMGd1 protein) (Nucleasome/chromatin assembly factor D protein NFD101) |
| GRMZM2G066561 | 4 | 23 | -1.93 | 3.97E-02 | Os03g0665800 protein (Expressed protein) |
| GRMZM2G066650 | 114 | 240 | -0.48 | 1.68E-02 | Os12g0568800 protein (Putative uncharacterized protein) (Importin-beta N-terminal domain containing protein, expressed) (Importin-beta2) (Fragment) |
| GRMZM2G066851 | 54 | 148 | -0.86 | 9.60E-04 | Putative uncharacterized protein |
| GRMZM2G066860 | 17 | 86 | -1.75 | 6.03E-06 | Putative uncharacterized protein |
| GRMZM2G066981 | 19 | 61 | -1.09 | 1.47E-02 | Putative uncharacterized protein |
| GRMZM2G067019 | 68 | 168 | -0.71 | 3.36E-03 | Putative uncharacterized protein |
| GRMZM2G067036 | 105 | 87 | 0.86 | 3.75E-04 | Putative uncharacterized protein |
| GRMZM2G067063 | 119 | 358 | -1.00 | 1.99E-10 | Protein disulfide isomerase |
| GRMZM2G067122 | 35 | 6 | 3.14 | 6.02E-08 | Putative uncharacterized protein |
| GRMZM2G067156 | 220 | 457 | -0.46 | 5.79E-04 | Putative uncharacterized protein |
| GRMZM2G067205 | 51 | 38 | 1.02 | 6.24E-03 | JAG |
| GRMZM2G067223 | 3 | 27 | -2.58 | 3.47E-03 | Putative uncharacterized protein |
| GRMZM2G067265 | 118 | 110 | 0.69 | 2.20E-03 | Asparate aminotransferase |
| GRMZM2G067277 | 63 | 225 | -1.25 | 2.31E-09 | Putative uncharacterized protein |
| GRMZM2G067299 | 21 | 72 | -1.19 | 3.91E-03 | Inositol 1,3,4,5,6-pentakisphosphate 2-kinase |
| GRMZM2G067303 | 753 | 738 | 0.62 | 5.45E-15 | 40S ribosomal protein S20 |
| GRMZM2G067371 | 19 | 10 | 1.52 | 3.14E-02 | Putative uncharacterized protein |
| GRMZM2G067417 | 227 | 451 | -0.40 | 3.99E-03 | Cytochrome c oxidase polypeptide VIb |
| GRMZM2G067426 | 73 | 57 | 0.95 | 1.54E-03 | Ternary complex factor MIP1 |
| GRMZM2G067453 | 35 | 113 | -1.10 | 2.99E-04 | Putative uncharacterized protein (Uridylate kinase) |
| GRMZM2G067460 | 62 | 166 | -0.83 | 6.34E-04 | Putative Avr9/Cf-9 rapidly elicited protein |
| GRMZM2G067476 | 822 | 896 | 0.47 | 6.03E-10 | Putative uncharacterized protein |
| GRMZM2G067511 | 191 | 493 | -0.78 | 1.59E-09 | Calmodulin |
| GRMZM2G067520 | 17 | 56 | -1.13 | 1.90E-02 | NA |
| GRMZM2G067555 | 11 | 43 | -1.38 | 1.63E-02 | Putative uncharacterized protein |
| GRMZM2G067581 | 252 | 289 | 0.39 | 9.87E-03 | Putative uncharacterized protein |
| GRMZM2G067601 | 172 | 357 | -0.46 | 3.38E-03 | Cytochrome b5 |
| GRMZM2G067624 | 983 | 1,925 | -0.38 | 3.08E-10 | SBP domain containing protein |
| GRMZM2G067646 | 288 | 351 | 0.31 | 3.74E-02 | Os06g0183900 protein (cDNA clone:006-207-D12, full insert sequence) (Putative uncharacterized protein OSJNBb0036B04.23) (Putative uncharacterized protein P0554A06.7) |
| GRMZM2G067707 | 161 | 357 | -0.56 | 3.02E-04 | Putative uncharacterized protein (Ubiquinol-cytochrome c reductase complex ubiquinone-binding protein QP-C) |
| GRMZM2G067747 | 61 | 245 | -1.41 | 3.70E-12 | Sodium/hydrogen exchanger |
| GRMZM2G067752 | 73 | 375 | -1.77 | 2.34E-25 | Annexin A4 (Putative uncharacterized protein) |
| GRMZM2G067756 | 37 | 108 | -0.95 | 2.32E-03 | Putative uncharacterized protein |
| GRMZM2G067789 | 105 | 320 | -1.02 | 1.31E-09 | Putative sugar transporter protein |
| GRMZM2G067830 | 78 | 44 | 1.42 | 1.79E-06 | Expressed protein |
| GRMZM2G067877 | 213 | 596 | -0.89 | 2.65E-14 | Grave disease carrier protein (Putative uncharacterized protein) |
| GRMZM2G067883 | 70 | 336 | -1.67 | 4.86E-21 | Chloroplast PSII K protein |
| GRMZM2G067921 | 866 | 1,000 | 0.38 | 1.91E-07 | Delayed flowering1 |
| GRMZM2G067985 | 391 | 1,317 | -1.16 | 7.36E-48 | Actin |
| GRMZM2G068028 | 70 | 211 | -1.00 | 2.42E-06 | Putative uncharacterized protein |
| GRMZM2G068220 | 56 | 36 | 1.23 | 5.24E-04 | Putative uncharacterized protein |
| GRMZM2G068244 | 27 | 107 | -1.40 | 1.61E-05 | NAD-dependent epimerase/dehydratase |
| GRMZM2G068255 | 101 | 211 | -0.47 | 2.99E-02 | Putative uncharacterized protein |
| GRMZM2G068294 | 24 | 15 | 1.27 | 3.59E-02 | Putative uncharacterized protein |
| GRMZM2G068316 | 15 | 59 | -1.38 | 3.19E-03 | DnaJ domain containing protein, expressed |
| GRMZM2G068465 | 97 | 280 | -0.94 | 1.74E-07 | Putative uncharacterized protein |
| GRMZM2G068471 | 687 | 871 | 0.25 | 4.68E-03 | Putative uncharacterized protein |
| GRMZM2G068476 | 137 | 357 | -0.79 | 2.86E-07 | Putative uncharacterized protein |
| GRMZM2G068479 | 114 | 294 | -0.78 | 6.32E-06 | Pre-mRNA-splicing factor ATP-dependent RNA helicase |
| GRMZM2G068489 | 350 | 210 | 1.33 | 3.75E-25 | Vacuolar protein sorting 29 |
| GRMZM2G068496 | 2,017 | 2,640 | 0.20 | 2.31E-05 | 60S ribosomal protein L29 |
| GRMZM2G068590 | 121 | 104 | 0.81 | 2.58E-04 | Inhibitor of apoptosis-like protein |
| GRMZM2G068657 | 381 | 208 | 1.46 | 7.80E-32 | Putative uncharacterized protein |
| GRMZM2G068665 | 112 | 228 | -0.43 | 3.95E-02 | Pyrroline-5-carboxylate reductase (EC 1.5.1.2) |
| GRMZM2G068690 | 49 | 38 | 0.96 | 1.13E-02 | Putative uncharacterized protein |
| GRMZM2G068703 | 13 | 69 | -1.82 | 4.23E-05 | Acid phosphatase/vanadium-dependent haloperoxidase related |
| GRMZM2G068862 | 102 | 258 | -0.75 | 5.73E-05 | Os03g0134300 protein (ATP phosphoribosyltransferase family protein, expressed) |
| GRMZM2G068952 | 548 | 707 | 0.22 | 3.05E-02 | Putative uncharacterized protein |
| GRMZM2G068984 | 37 | 108 | -0.95 | 2.32E-03 | Putative uncharacterized protein |
| GRMZM2G069018 | 83 | 69 | 0.86 | 1.78E-03 | Fiber protein Fb2 (Putative uncharacterized protein) |
| GRMZM2G069024 | 71 | 66 | 0.70 | 2.42E-02 | Putative uncharacterized protein |
| GRMZM2G069061 | 135 | 270 | -0.41 | 3.20E-02 | NADH-ubiquinone oxidoreductase 20 kDa subunit (Putative uncharacterized protein) |
| GRMZM2G069078 | 36 | 21 | 1.37 | 3.71E-03 | Putative uncharacterized protein |
| GRMZM2G069092 | 58 | 51 | 0.78 | 2.74E-02 | Expressed protein (Putative uncharacterized protein) |
| GRMZM2G069095 | 79 | 218 | -0.87 | 2.11E-05 | Vacuolar H+-translocating inorganic pyrophosphatase (EC 3.6.1.1) |
| GRMZM2G069102 | 60 | 40 | 1.18 | 4.65E-04 | CID11 |
| GRMZM2G069174 | 143 | 446 | -1.05 | 5.11E-14 | RNA-binding protein-like |
| GRMZM2G069177 | 99 | 69 | 1.11 | 9.38E-06 | cDNA clone:J023134M09, full insert sequence (Os01g0168500 protein) (Putative uncharacterized protein OJ1276_B06.19) |
| GRMZM2G069262 | 107 | 239 | -0.57 | 4.03E-03 | Putative uncharacterized protein |
| GRMZM2G069316 | 172 | 428 | -0.72 | 2.02E-07 | Nucleolin |
| GRMZM2G069365 | 119 | 30 | 2.58 | 2.52E-21 | Zinc finger homeodomain protein 1 |
| GRMZM2G069389 | 116 | 97 | 0.85 | 1.65E-04 | Putative uncharacterized protein |
| GRMZM2G069405 | 212 | 479 | -0.58 | 7.53E-06 | Putative uncharacterized protein |
| GRMZM2G069444 | 108 | 78 | 1.06 | 7.56E-06 | Putative uncharacterized protein |
| GRMZM2G069486 | 14 | 45 | -1.09 | 4.67E-02 | NA |
| GRMZM2G069518 | 289 | 745 | -0.77 | 3.95E-14 | Brix domain-containing protein 1 |
| GRMZM2G069542 | 446 | 335 | 1.00 | 2.18E-20 | Phosphoenolpyruvate carboxylase (EC 4.1.1.31) |
| GRMZM2G069594 | 1,559 | 1,233 | 0.93 | 2.77E-62 | 60S ribosomal protein L38 (Putative uncharacterized protein) |
| GRMZM2G069618 | 37 | 98 | -0.81 | 1.43E-02 | Putative uncharacterized protein (TPR domain containing protein) |
| GRMZM2G069649 | 171 | 665 | -1.37 | 4.54E-31 | Putative uncharacterized protein (Putative F-box protein) |
| GRMZM2G069651 | 1,051 | 828 | 0.94 | 1.88E-42 | Os09g0482100 protein |
| GRMZM2G069687 | 66 | 151 | -0.60 | 2.13E-02 | Putative uncharacterized protein |
| GRMZM2G069726 | 117 | 117 | 0.59 | 1.12E-02 | GTP-binding protein (Putative uncharacterized protein) |
| GRMZM2G069758 | 46 | 129 | -0.90 | 1.41E-03 | Pyridoxal kinase |
| GRMZM2G069762 | 454 | 372 | 0.88 | 1.37E-16 | Putative uncharacterized protein |
| GRMZM2G069772 | 148 | 313 | -0.49 | 3.83E-03 | Inosine-5-monophosphate dehydrogenase 2 |
| GRMZM2G069773 | 18 | 55 | -1.02 | 3.61E-02 | Putative uncharacterized protein |
| GRMZM2G069841 | 0 | 20 | #NUM! | 3.61E-04 | Putative uncharacterized protein |
| GRMZM2G069856 | 50 | 142 | -0.91 | 5.19E-04 | Putative uncharacterized protein |
| GRMZM2G069928 | 74 | 37 | 1.59 | 2.49E-07 | Putative uncharacterized protein OSJNBb0055I24.117 |
| GRMZM2G069970 | 29 | 86 | -0.98 | 6.62E-03 | Putative uncharacterized protein |
| GRMZM2G070045 | 75 | 180 | -0.67 | 3.48E-03 | Os02g0749500 protein (cDNA clone:001-133-E02, full insert sequence) (Putative SEC6) |
| GRMZM2G070047 | 378 | 436 | 0.39 | 1.23E-03 | Ubiquitin carrier protein (EC 6.3.2.-) |
| GRMZM2G070068 | 199 | 159 | 0.92 | 4.01E-08 | Putative uncharacterized protein |
| GRMZM2G070075 | 166 | 167 | 0.58 | 1.85E-03 | Putative uncharacterized protein |
| GRMZM2G070199 | 135 | 369 | -0.86 | 1.65E-08 | Putative uncharacterized protein |
| GRMZM2G070211 | 30 | 89 | -0.98 | 5.82E-03 | Putative uncharacterized protein |
| GRMZM2G070218 | 27 | 100 | -1.30 | 1.04E-04 | Shikimate kinase |
| GRMZM2G070239 | 155 | 159 | 0.55 | 4.87E-03 | Splicing factor, arginine/serine-rich 7 |
| GRMZM2G070255 | 403 | 357 | 0.77 | 8.14E-12 | Putative uncharacterized protein |
| GRMZM2G070279 | 47 | 41 | 0.79 | 4.93E-02 | Putative uncharacterized protein |
| GRMZM2G070284 | 257 | 279 | 0.47 | 1.26E-03 | Putative uncharacterized protein |
| GRMZM2G070292 | 54 | 29 | 1.49 | 4.93E-05 | ATPase, coupled to transmembrane movement of substances |
| GRMZM2G070305 | 200 | 215 | 0.49 | 4.01E-03 | Putative peroxisome biogenesis protein PEX1 |
| GRMZM2G070322 | 1 | 13 | -3.11 | 4.97E-02 | Systemin receptor SR160 |
| GRMZM2G070360 | 165 | 111 | 1.16 | 8.95E-10 | Putative uncharacterized protein (Vacuolar ATP synthase subunit E) |
| GRMZM2G070375 | 68 | 24 | 2.09 | 1.24E-09 | FIP1 |
| GRMZM2G070389 | 274 | 560 | -0.44 | 2.56E-04 | Putative uncharacterized protein (Trafficking protein particle complex subunit 3) |
| GRMZM2G070422 | 1,532 | 1,226 | 0.91 | 2.01E-59 | Putative uncharacterized protein |
| GRMZM2G070429 | 12 | 48 | -1.41 | 7.87E-03 | Lipase |
| GRMZM2G070446 | 14 | 49 | -1.22 | 2.08E-02 | Putative uncharacterized protein |
| GRMZM2G070487 | 419 | 795 | -0.33 | 9.18E-04 | Translation initiation factor 3 |
| GRMZM2G070500 | 13 | 0 | #VALUE! | 6.50E-05 | Putative uncharacterized protein |
| GRMZM2G070542 | 193 | 596 | -1.04 | 4.37E-18 | Putative uncharacterized protein (T-complex protein 1 subunit epsilon) |
| GRMZM2G070555 | 89 | 45 | 1.58 | 1.51E-08 | Putative uncharacterized protein |
| GRMZM2G070605 | 34 | 118 | -1.20 | 6.27E-05 | Os01g0239200 protein (Putative triose phosphate/phosphate translocator) |
| GRMZM2G070633 | 49 | 145 | -0.97 | 2.26E-04 | Putative uncharacterized protein |
| GRMZM2G070649 | 14 | 55 | -1.38 | 4.75E-03 | Putative uncharacterized protein (Ribosomal protein L2) |
| GRMZM2G070685 | 47 | 17 | 2.06 | 9.16E-07 | Chloroplast NADH dehydrogenase subunit J (NADH-plastoquinone oxidoreductase subunit J) |
| GRMZM2G070708 | 133 | 336 | -0.75 | 3.22E-06 | Putative uncharacterized protein |
| GRMZM2G070804 | 123 | 127 | 0.55 | 1.51E-02 | Salt tolerant protein |
| GRMZM2G070807 | 400 | 271 | 1.15 | 6.18E-23 | Putative uncharacterized protein |
| GRMZM2G070863 | 58 | 159 | -0.86 | 4.47E-04 | Putative uncharacterized protein |
| GRMZM2G070881 | 11 | 41 | -1.31 | 2.99E-02 | Putative uncharacterized protein (OSJNBa0033G05.14 protein) |
| GRMZM2G071010 | 75 | 193 | -0.77 | 4.93E-04 | Ubiquitin-conjugating enzyme spm2 |
| GRMZM2G071025 | 569 | 1,155 | -0.43 | 6.82E-08 | Putative uncharacterized protein |
| GRMZM2G071034 | 64 | 187 | -0.96 | 2.39E-05 | Putative uncharacterized protein (Zinc finger C-x8-C-x5-C-x3-H type family protein) |
| GRMZM2G071042 | 4 | 30 | -2.32 | 3.91E-03 | Zinc finger A20 and AN1 domains-containing protein (AN12) |
| GRMZM2G071059 | 49 | 210 | -1.51 | 1.47E-11 | CCR4-NOT transcription complex subunit 7 (Putative uncharacterized protein) |
| GRMZM2G071071 | 329 | 671 | -0.44 | 5.30E-05 | Putative uncharacterized protein |
| GRMZM2G071100 | 28 | 78 | -0.89 | 2.46E-02 | Putative uncharacterized protein |
| GRMZM2G071113 | 30 | 83 | -0.88 | 1.88E-02 | F-box domain containing protein |
| GRMZM2G071163 | 52 | 124 | -0.66 | 2.55E-02 | Putative uncharacterized protein (OSJNBa0033G05.21 protein) (OSJNBa0070O11.1 protein) |
| GRMZM2G071172 | 19 | 74 | -1.37 | 6.11E-04 | Putative uncharacterized protein |
| GRMZM2G071208 | 93 | 252 | -0.85 | 7.90E-06 | Branched-chain-amino-acid aminotransferase (EC 2.6.1.42) |
| GRMZM2G071272 | 1,225 | 570 | 1.70 | 4.67E-127 | Putative uncharacterized protein |
| GRMZM2G071288 | 181 | 358 | -0.39 | 1.43E-02 | Putative uncharacterized protein |
| GRMZM2G071304 | 96 | 235 | -0.70 | 3.39E-04 | DNA ligase (EC 6.5.1.1) |
| GRMZM2G071441 | 86 | 216 | -0.74 | 3.90E-04 | Ubiquitin carboxyl-terminal hydrolase (EC 3.1.2.15) |
| GRMZM2G071491 | 302 | 317 | 0.52 | 8.61E-05 | Putative uncharacterized protein |
| GRMZM2G071518 | 186 | 439 | -0.65 | 2.13E-06 | Putative uncharacterized protein |
| GRMZM2G071582 | 29 | 116 | -1.41 | 5.11E-06 | ZAC |
| GRMZM2G071602 | 307 | 316 | 0.55 | 2.62E-05 | Putative uncharacterized protein |
| GRMZM2G071626 | 32 | 122 | -1.34 | 7.14E-06 | Putative uncharacterized protein |
| GRMZM2G071714 | 13 | 58 | -1.57 | 1.02E-03 | Putative uncharacterized protein |
| GRMZM2G071739 | 63 | 58 | 0.71 | 3.16E-02 | Putative uncharacterized protein (UBA/TS-N domain) |
| GRMZM2G071768 | 75 | 175 | -0.63 | 8.43E-03 | Putative uncharacterized protein |
| GRMZM2G071846 | 291 | 282 | 0.64 | 1.94E-06 | Adenine phosphoribosyltransferase 2 |
| GRMZM2G071877 | 66 | 145 | -0.54 | 4.67E-02 | Protein kinase Pti1 (Putative uncharacterized protein) |
| GRMZM2G071907 | 183 | 35 | 2.98 | 9.52E-39 | WRKY68-superfamily of TFs having WRKY and zinc finger domains |
| GRMZM2G071959 | 3,915 | 1,094 | 2.43 | 0.00E+01 | Histone H2B |
| GRMZM2G072117 | 118 | 319 | -0.84 | 3.35E-07 | AT-hook protein 1 (Putative uncharacterized protein) |
| GRMZM2G072156 | 86 | 193 | -0.57 | 9.88E-03 | Putative uncharacterized protein |
| GRMZM2G072171 | 22 | 62 | -0.90 | 4.30E-02 | Os03g0775600 protein (Putative uncharacterized protein) (cDNA clone:J013074F23, full insert sequence) (Dehydrogenase, putative, expressed) |
| GRMZM2G072210 | 165 | 150 | 0.73 | 8.35E-05 | Os05g0295900 protein (cDNA clone:J023037D24, full insert sequence) |
| GRMZM2G072231 | 72 | 155 | -0.51 | 4.97E-02 | Putative uncharacterized protein 170F8.6 |
| GRMZM2G072238 | 249 | 249 | 0.59 | 5.63E-05 | Putative uncharacterized protein |
| GRMZM2G072315 | 1,226 | 1,122 | 0.72 | 2.20E-31 | Putative uncharacterized protein |
| GRMZM2G072339 | 159 | 150 | 0.68 | 3.74E-04 | Putative uncharacterized protein |
| GRMZM2G072415 | 314 | 273 | 0.79 | 8.31E-10 | Protein translocase/ protein transporter |
| GRMZM2G072462 | 83 | 76 | 0.72 | 1.08E-02 | Putative uncharacterized protein |
| GRMZM2G072501 | 25 | 102 | -1.44 | 1.96E-05 | Putative uncharacterized protein |
| GRMZM2G072569 | 318 | 322 | 0.57 | 7.76E-06 | Putative uncharacterized protein |
| GRMZM2G072578 | 76 | 66 | 0.79 | 6.34E-03 | Phospholipase A2 |
| GRMZM2G072582 | 22 | 74 | -1.16 | 3.46E-03 | Putative uncharacterized protein |
| GRMZM2G072584 | 47 | 112 | -0.66 | 3.33E-02 | Putative uncharacterized protein (Protein kinase domain containing protein, expressed) (Putative uncharacterized protein OSJNBa0094J08.29) |
| GRMZM2G072612 | 52 | 121 | -0.63 | 3.62E-02 | Putative uncharacterized protein |
| GRMZM2G072658 | 12 | 48 | -1.41 | 7.87E-03 | Putative uncharacterized protein 170F8.8 |
| GRMZM2G072690 | 63 | 176 | -0.89 | 1.45E-04 | Golgi transport 1 protein B (Putative uncharacterized protein) |
| GRMZM2G072700 | 105 | 220 | -0.48 | 2.55E-02 | Copper-translocating P-type ATPase family protein, expressed |
| GRMZM2G072729 | 1,402 | 699 | 1.60 | 3.26E-133 | 60S ribosomal protein L2 (Putative uncharacterized protein) |
| GRMZM2G072755 | 16 | 6 | 2.01 | 1.74E-02 | T-cytoplasm male sterility restorer factor 2 |
| GRMZM2G072850 | 188 | 71 | 2.00 | 1.52E-24 | H0423H10.7 protein |
| GRMZM2G072855 | 5,818 | 1,251 | 2.81 | 0.00E+01 | Histone H4 |
| GRMZM2G072892 | 15 | 49 | -1.12 | 3.18E-02 | Os03g0334800 protein (Lung seven transmembrane receptor family protein, expressed) |
| GRMZM2G072894 | 83 | 193 | -0.63 | 5.24E-03 | Poly polymerase catalytic domain containing protein, expressed (Putative CEO protein (Alternative splicing products)) |
| GRMZM2G073003 | 318 | 821 | -0.78 | 1.40E-15 | Putative uncharacterized protein |
| GRMZM2G073023 | 98 | 100 | 0.56 | 3.20E-02 | ER-derived vesicles protein ERV14 |
| GRMZM2G073045 | 155 | 378 | -0.69 | 3.41E-06 | Putative uncharacterized protein |
| GRMZM2G073054 | 29 | 18 | 1.28 | 1.42E-02 | Starch branching enzyme IIa (EC 2.4.1.18) (Fragment) |
| GRMZM2G073123 | 80 | 172 | -0.51 | 3.65E-02 | Putative uncharacterized protein |
| GRMZM2G073150 | 1,432 | 1,806 | 0.26 | 6.77E-06 | Putative uncharacterized protein |
| GRMZM2G073192 | 85 | 80 | 0.68 | 1.60E-02 | Putative uncharacterized protein |
| GRMZM2G073199 | 96 | 264 | -0.87 | 2.69E-06 | AT-hook protein 1 (Putative uncharacterized protein) |
| GRMZM2G073275 | 2,761 | 1,643 | 1.34 | 3.66E-201 | Histone H4 |
| GRMZM2G073312 | 49 | 127 | -0.78 | 6.72E-03 | Putative uncharacterized protein |
| GRMZM2G073377 | 36 | 101 | -0.90 | 6.73E-03 | Putative uncharacterized protein (YY1 protein) |
| GRMZM2G073396 | 7 | 0 | #VALUE! | 8.95E-03 | Os02g0116700 protein (Putative Phytosulfokine receptor) |
| GRMZM2G073401 | 333 | 243 | 1.05 | 2.20E-16 | Chaperonin |
| GRMZM2G073465 | 54 | 155 | -0.93 | 2.39E-04 | Cysteine protease 1 |
| GRMZM2G073495 | 116 | 308 | -0.82 | 1.08E-06 | Putative uncharacterized protein |
| GRMZM2G073498 | 305 | 646 | -0.49 | 7.93E-06 | Putative uncharacterized protein |
| GRMZM2G073504 | 54 | 137 | -0.75 | 6.39E-03 | Putative uncharacterized protein |
| GRMZM2G073511 | 23 | 64 | -0.89 | 4.73E-02 | Putative uncharacterized protein |
| GRMZM2G073521 | 60 | 194 | -1.10 | 8.25E-07 | Putative uncharacterized protein |
| GRMZM2G073535 | 298 | 552 | -0.30 | 1.99E-02 | Protein translation factor SUI1 (Putative uncharacterized protein) |
| GRMZM2G073551 | 58 | 164 | -0.91 | 1.77E-04 | Putative uncharacterized protein |
| GRMZM2G073555 | 66 | 42 | 1.24 | 1.10E-04 | Putative uncharacterized protein |
| GRMZM2G073567 | 187 | 60 | 2.23 | 3.52E-28 | Putative uncharacterized protein |
| GRMZM2G073591 | 39 | 100 | -0.77 | 2.03E-02 | Putative uncharacterized protein OJ1041F02.2 |
| GRMZM2G073609 | 291 | 257 | 0.77 | 9.28E-09 | Putative uncharacterized protein |
| GRMZM2G073628 | 12 | 44 | -1.28 | 1.99E-02 | Putative uncharacterized protein (Protein disulfide isomerase) |
| GRMZM2G073630 | 81 | 199 | -0.71 | 1.09E-03 | Putative uncharacterized protein |
| GRMZM2G073668 | 162 | 126 | 0.95 | 3.72E-07 | Ethylene receptor |
| GRMZM2G073671 | 143 | 347 | -0.69 | 1.10E-05 | Cyclin IIIZm |
| GRMZM2G073725 | 769 | 553 | 1.07 | 8.68E-39 | Putative uncharacterized protein |
| GRMZM2G073741 | 8 | 37 | -1.62 | 1.12E-02 | Putative uncharacterized protein |
| GRMZM2G073750 | 39 | 113 | -0.94 | 2.29E-03 | Putative uncharacterized protein |
| GRMZM2G073755 | 72 | 54 | 1.01 | 8.74E-04 | Auxin-repressed protein (Putative uncharacterized protein) |
| GRMZM2G073770 | 33 | 16 | 1.64 | 8.79E-04 | GSDL-motif lipase |
| GRMZM2G073788 | 121 | 246 | -0.43 | 2.99E-02 | Catalytic/ protein phosphatase type 2C/ protein serine/threonine phosphatase |
| GRMZM2G073860 | 13 | 5 | 1.97 | 3.14E-02 | Putative uncharacterized protein |
| GRMZM2G073861 | 38 | 99 | -0.79 | 1.90E-02 | Putative uncharacterized protein |
| GRMZM2G073892 | 21 | 77 | -1.28 | 9.16E-04 | BZIP transcription factor |
| GRMZM2G073912 | 118 | 286 | -0.69 | 8.94E-05 | Putative uncharacterized protein |
| GRMZM2G073934 | 120 | 373 | -1.04 | 1.26E-11 | Putative uncharacterized protein |
| GRMZM2G073959 | 64 | 176 | -0.87 | 1.99E-04 | Putative uncharacterized protein |
| GRMZM2G073979 | 53 | 171 | -1.10 | 4.55E-06 | EREBP-4 like protein (Putative uncharacterized protein) |
| GRMZM2G074028 | 134 | 126 | 0.68 | 1.27E-03 | Putative uncharacterized protein |
| GRMZM2G074037 | 228 | 446 | -0.38 | 7.15E-03 | 26S protease regulatory subunit S10B |
| GRMZM2G074082 | 87 | 272 | -1.05 | 1.10E-08 | Putative uncharacterized protein |
| GRMZM2G074085 | 653 | 1,567 | -0.67 | 6.14E-23 | 60S ribosomal protein L31 |
| GRMZM2G074087 | 221 | 228 | 0.55 | 5.91E-04 | Putative uncharacterized protein |
| GRMZM2G074094 | 17 | 65 | -1.34 | 1.90E-03 | NA |
| GRMZM2G074097 | 65 | 54 | 0.86 | 7.73E-03 | Putative uncharacterized protein |
| GRMZM2G074099 | 4 | 23 | -1.93 | 3.97E-02 | Bile acid sodium symporter |
| GRMZM2G074114 | 75 | 172 | -0.61 | 1.18E-02 | Putative uncharacterized protein |
| GRMZM2G074122 | 191 | 190 | 0.60 | 4.45E-04 | Phosphoenolpyruvate carboxylase (EC 4.1.1.31) |
| GRMZM2G074193 | 23 | 78 | -1.17 | 2.32E-03 | Putative uncharacterized protein |
| GRMZM2G074254 | 14 | 58 | -1.46 | 1.85E-03 | Protein binding protein |
| GRMZM2G074267 | 100 | 229 | -0.60 | 2.56E-03 | Putative auxin efflux carrier |
| GRMZM2G074280 | 228 | 265 | 0.37 | 2.10E-02 | Putative RNA polymerase II complex component SRB7 |
| GRMZM2G074282 | 209 | 239 | 0.40 | 1.87E-02 | Putative uncharacterized protein |
| GRMZM2G074300 | 3,142 | 2,192 | 1.11 | 2.91E-169 | 40S ribosomal protein S3 |
| GRMZM2G074306 | 52 | 25 | 1.65 | 1.28E-05 | Putative uncharacterized protein |
| GRMZM2G074314 | 100 | 208 | -0.47 | 3.32E-02 | Putative GTPase activating protein |
| GRMZM2G074351 | 54 | 220 | -1.44 | 3.76E-11 | Putative uncharacterized protein |
| GRMZM2G074377 | 182 | 532 | -0.96 | 2.90E-14 | Putative uncharacterized protein |
| GRMZM2G074404 | 33 | 105 | -1.08 | 8.66E-04 | Transmembrane BAX inhibitor motif-containing protein 4 |
| GRMZM2G074436 | 44 | 151 | -1.19 | 4.55E-06 | Putative uncharacterized protein |
| GRMZM2G074466 | 117 | 471 | -1.42 | 2.74E-23 | Putative uncharacterized protein |
| GRMZM2G074479 | 227 | 442 | -0.37 | 8.79E-03 | Ubiquitin carrier protein (EC 6.3.2.-) |
| GRMZM2G074489 | 62 | 51 | 0.87 | 8.11E-03 | H0702G05.9 protein |
| GRMZM2G074501 | 13 | 5 | 1.97 | 3.14E-02 | Putative uncharacterized protein |
| GRMZM2G074567 | 152 | 354 | -0.63 | 5.39E-05 | Putative uncharacterized protein |
| GRMZM2G074569 | 1 | 14 | -3.22 | 3.22E-02 | Putative uncharacterized protein |
| GRMZM2G074599 | 167 | 343 | -0.45 | 5.65E-03 | Putative uncharacterized protein |
| GRMZM2G074604 | 13 | 46 | -1.23 | 2.34E-02 | Phenylalanine ammonia-lyase (EC 4.3.1.24) |
| GRMZM2G074645 | 125 | 112 | 0.75 | 5.27E-04 | Putative uncharacterized protein |
| GRMZM2G074689 | 40 | 143 | -1.25 | 4.05E-06 | Putative uncharacterized protein |
| GRMZM2G074787 | 183 | 348 | -0.34 | 4.76E-02 | Cleavage and polyadenylation specificity factor 5 (Putative uncharacterized protein) |
| GRMZM2G074790 | 143 | 363 | -0.75 | 8.44E-07 | Putative uncharacterized protein |
| GRMZM2G074805 | 250 | 502 | -0.41 | 1.39E-03 | Putative uncharacterized protein |
| GRMZM2G074818 | 187 | 386 | -0.45 | 2.62E-03 | Putative uncharacterized protein |
| GRMZM2G074898 | 679 | 1,264 | -0.31 | 7.57E-05 | 60S ribosomal protein L24 |
| GRMZM2G074914 | 13 | 3 | 2.71 | 6.90E-03 | Putative uncharacterized protein |
| GRMZM2G074957 | 77 | 181 | -0.64 | 5.50E-03 | Putative uncharacterized protein |
| GRMZM2G075003 | 110 | 101 | 0.71 | 2.75E-03 | Mitochondrial import inner membrane translocase subunit Tim8 |
| GRMZM2G075027 | 73 | 273 | -1.31 | 3.97E-12 | Putative uncharacterized protein |
| GRMZM2G075074 | 30 | 103 | -1.19 | 2.40E-04 | Threonine endopeptidase |
| GRMZM2G075092 | 28 | 19 | 1.15 | 3.18E-02 | Putative uncharacterized protein (cDNA clone:J033102P10, full insert sequence) (Cytochrome B561-like) (Os01g0666500 protein) |
| GRMZM2G075104 | 84 | 186 | -0.56 | 1.72E-02 | Putative uncharacterized protein |
| GRMZM2G075117 | 236 | 258 | 0.46 | 2.65E-03 | D-type cyclin |
| GRMZM2G075150 | 85 | 265 | -1.05 | 1.85E-08 | Putative uncharacterized protein |
| GRMZM2G075153 | 155 | 410 | -0.81 | 1.28E-08 | Cystathionine gamma-synthase (EC 4.2.99.9) |
| GRMZM2G075196 | 235 | 516 | -0.54 | 1.39E-05 | Putative uncharacterized protein |
| GRMZM2G075227 | 67 | 269 | -1.41 | 2.01E-13 | Senescence-associated protein |
| GRMZM2G075255 | 105 | 279 | -0.82 | 4.76E-06 | Putative uncharacterized protein |
| GRMZM2G075294 | 113 | 91 | 0.90 | 8.10E-05 | NA |
| GRMZM2G075485 | 130 | 319 | -0.70 | 1.89E-05 | Putative uncharacterized protein |
| GRMZM2G075488 | 88 | 205 | -0.63 | 3.38E-03 | Putative uncharacterized protein |
| GRMZM2G075505 | 64 | 213 | -1.14 | 7.00E-08 | Putative uncharacterized protein |
| GRMZM2G075528 | 92 | 208 | -0.59 | 6.66E-03 | Yip1 domain family member 1 |
| GRMZM2G075551 | 68 | 153 | -0.58 | 2.73E-02 | Putative uncharacterized protein |
| GRMZM2G075582 | 54 | 175 | -1.10 | 3.06E-06 | Os06g0164000 protein (Putative uncharacterized protein) (Putative transcription factor) |
| GRMZM2G075624 | 1,252 | 1,372 | 0.46 | 1.78E-14 | Putative uncharacterized protein (Translationally-controlled tumor protein) |
| GRMZM2G075637 | 946 | 1,227 | 0.22 | 3.69E-03 | Putative uncharacterized protein (Nucleosome/chromatin assembly factor A) |
| GRMZM2G075676 | 24 | 92 | -1.35 | 1.44E-04 | Putative uncharacterized protein |
| GRMZM2G075690 | 52 | 144 | -0.88 | 7.93E-04 | Mov34/MPN/PAD-1 family protein (Putative uncharacterized protein) |
| GRMZM2G075744 | 112 | 228 | -0.43 | 3.95E-02 | Putative uncharacterized protein |
| GRMZM2G075767 | 192 | 527 | -0.87 | 4.15E-12 | Putative uncharacterized protein |
| GRMZM2G075775 | 123 | 132 | 0.49 | 3.22E-02 | Putative uncharacterized protein |
| GRMZM2G075839 | 111 | 275 | -0.72 | 5.80E-05 | Programmed cell death protein 5 (Putative uncharacterized protein) |
| GRMZM2G075844 | 372 | 271 | 1.05 | 2.47E-18 | Putative uncharacterized protein |
| GRMZM2G075942 | 128 | 131 | 0.56 | 1.21E-02 | Heterogeneous nuclear ribonucleoprotein R |
| GRMZM2G075956 | 84 | 199 | -0.65 | 2.76E-03 | Putative uncharacterized protein |
| GRMZM2G076049 | 1,045 | 529 | 1.57 | 2.81E-97 | Endoglucanase 1 |
| GRMZM2G076075 | 18 | 55 | -1.02 | 3.61E-02 | Putative uncharacterized protein |
| GRMZM2G076128 | 91 | 225 | -0.71 | 3.64E-04 | TTN10 |
| GRMZM2G076145 | 150 | 112 | 1.01 | 3.23E-07 | Putative uncharacterized protein |
| GRMZM2G076276 | 38 | 105 | -0.87 | 6.45E-03 | Putative uncharacterized protein |
| GRMZM2G076387 | 30 | 83 | -0.88 | 1.88E-02 | Os07g0511000 protein (Putative uncharacterized protein) (cDNA clone:J023050A04, full insert sequence) (Putative uncharacterized protein P0409B11.37) |
| GRMZM2G076399 | 436 | 531 | 0.31 | 6.62E-03 | Small nuclear ribonucleoprotein Sm D2 |
| GRMZM2G076423 | 12 | 70 | -1.95 | 1.39E-05 | Putative uncharacterized protein |
| GRMZM2G076450 | 100 | 71 | 1.09 | 1.20E-05 | Regulatory protein NPR1 |
| GRMZM2G076539 | 365 | 384 | 0.52 | 1.35E-05 | Putative uncharacterized protein |
| GRMZM2G076631 | 5 | 0 | #VALUE! | 4.27E-02 | Putative uncharacterized protein |
| GRMZM2G076705 | 288 | 538 | -0.31 | 1.61E-02 | Nucleic acid binding protein (Putative uncharacterized protein) |
| GRMZM2G076796 | 7 | 57 | -2.43 | 6.54E-06 | Putative uncharacterized protein |
| GRMZM2G076827 | 101 | 247 | -0.70 | 2.58E-04 | Putative uncharacterized protein |
| GRMZM2G076841 | 80 | 232 | -0.94 | 2.19E-06 | OSIGBa0139P06.9 protein |
| GRMZM2G076885 | 135 | 351 | -0.79 | 4.64E-07 | Putative uncharacterized protein |
| GRMZM2G076985 | 53 | 125 | -0.65 | 2.68E-02 | L-ascorbate oxidase (Putative uncharacterized protein) |
| GRMZM2G076987 | 30 | 79 | -0.81 | 3.60E-02 | Receptor-like serine-threonine protein kinase |
| GRMZM2G077002 | 71 | 57 | 0.91 | 2.68E-03 | GATA transcription factor 29 (Putative uncharacterized protein) |
| GRMZM2G077036 | 11 | 40 | -1.27 | 4.00E-02 | Os04g0570800 protein (cDNA clone:J023031N01, full insert sequence) (OSJNBa0088I22.11 protein) |
| GRMZM2G077068 | 40 | 126 | -1.06 | 2.05E-04 | Putative Mla1 |
| GRMZM2G077114 | 23 | 13 | 1.41 | 2.62E-02 | Putative uncharacterized protein |
| GRMZM2G077124 | 32 | 10 | 2.27 | 2.35E-05 | Putative uncharacterized protein |
| GRMZM2G077150 | 21 | 74 | -1.23 | 2.21E-03 | Putative uncharacterized protein |
| GRMZM2G077197 | 16 | 59 | -1.29 | 5.18E-03 | Regulatory protein NPR1 |
| GRMZM2G077208 | 5,523 | 6,434 | 0.37 | 2.69E-42 | 60S acidic ribosomal protein P3 (Putative uncharacterized protein) |
| GRMZM2G077219 | 7 | 1 | 3.40 | 3.67E-02 | Putative uncharacterized protein |
| GRMZM2G077227 | 270 | 626 | -0.62 | 2.56E-08 | Putative uncharacterized protein |
| GRMZM2G077253 | 268 | 227 | 0.83 | 3.75E-09 | Ubiquitin family protein |
| GRMZM2G077256 | 115 | 36 | 2.27 | 1.02E-17 | Putative uncharacterized protein |
| GRMZM2G077258 | 2 | 17 | -2.50 | 3.74E-02 | Putative uncharacterized protein |
| GRMZM2G077295 | 37 | 107 | -0.94 | 2.95E-03 | Putative uncharacterized protein |
| GRMZM2G077307 | 33 | 101 | -1.02 | 1.87E-03 | Putative uncharacterized protein (RING finger and CHY zinc finger domain-containing protein 1) |
| GRMZM2G077316 | 79 | 74 | 0.69 | 1.86E-02 | Putative uncharacterized protein (Allene oxide cyclase) |
| GRMZM2G077420 | 47 | 111 | -0.65 | 3.97E-02 | Os04g0514500 protein (cDNA clone:J033104I01, full insert sequence) (OSJNBb0070J16.5 protein) (OSJNBb0072M01.1 protein) |
| GRMZM2G077460 | 99 | 292 | -0.97 | 2.95E-08 | Os02g0816000 protein (Putative uncharacterized protein) (cDNA, clone: J065196G22, full insert sequence) (Putative anthranilate phosphoribosyltransferase) |
| GRMZM2G077463 | 89 | 225 | -0.75 | 2.13E-04 | Putative uncharacterized protein (Signal peptidase complex subunit 3) |
| GRMZM2G077503 | 55 | 141 | -0.77 | 4.60E-03 | Putative uncharacterized protein |
| GRMZM2G077673 | 24 | 83 | -1.20 | 1.16E-03 | Beta-ureidopropionase (Putative uncharacterized protein) |
| GRMZM2G077718 | 480 | 459 | 0.66 | 1.07E-10 | NA |
| GRMZM2G077744 | 411 | 276 | 1.17 | 5.41E-24 | Putative uncharacterized protein (Signal recognition particle 19 kDa protein) |
| GRMZM2G077757 | 60 | 183 | -1.02 | 8.99E-06 | G-patch domain containing protein |
| GRMZM2G077797 | 58 | 176 | -1.01 | 1.52E-05 | RNA binding protein |
| GRMZM2G077809 | 27 | 73 | -0.84 | 3.50E-02 | Putative uncharacterized protein |
| GRMZM2G077811 | 37 | 103 | -0.89 | 5.88E-03 | Putative uncharacterized protein (OSJNBa0010H02.15 protein) |
| GRMZM2G077837 | 20 | 60 | -0.99 | 2.78E-02 | Putative uncharacterized protein |
| GRMZM2G077851 | 5,925 | 5,708 | 0.65 | 2.37E-125 | 60S ribosomal protein L2 |
| GRMZM2G077942 | 211 | 214 | 0.57 | 4.67E-04 | Actin-depolymerizing factor 5 |
| GRMZM2G077991 | 1,949 | 1,649 | 0.83 | 1.64E-64 | Ribosomal protein L37 |
| GRMZM2G078022 | 592 | 1,688 | -0.92 | 3.32E-42 | Co-chaperone protein SBA1 |
| GRMZM2G078174 | 226 | 223 | 0.61 | 8.45E-05 | Putative uncharacterized protein |
| GRMZM2G078178 | 96 | 92 | 0.65 | 1.17E-02 | Putative uncharacterized protein |
| GRMZM2G078272 | 23 | 81 | -1.22 | 1.31E-03 | Putative uncharacterized protein |
| GRMZM2G078283 | 69 | 157 | -0.59 | 2.06E-02 | Putative uncharacterized protein |
| GRMZM2G078314 | 4,038 | 2,543 | 1.26 | 1.78E-266 | Histone H3 |
| GRMZM2G078360 | 117 | 254 | -0.53 | 5.87E-03 | P0044F08.17 protein |
| GRMZM2G078373 | 49 | 37 | 1.00 | 1.02E-02 | Putative uncharacterized protein |
| GRMZM2G078440 | 50 | 117 | -0.64 | 3.88E-02 | Putative uncharacterized protein |
| GRMZM2G078541 | 200 | 452 | -0.58 | 1.41E-05 | Putative uncharacterized protein |
| GRMZM2G078569 | 811 | 1,468 | -0.26 | 2.20E-04 | Putative uncharacterized protein |
| GRMZM2G078636 | 7 | 1 | 3.40 | 3.67E-02 | Putative uncharacterized protein |
| GRMZM2G078691 | 541 | 1,216 | -0.58 | 9.87E-14 | Nuclear transcription factor Y subunit C-2 |
| GRMZM2G078725 | 207 | 239 | 0.38 | 2.69E-02 | Microtubule-associated protein TORTIFOLIA1 |
| GRMZM2G078754 | 82 | 267 | -1.11 | 2.33E-09 | Putative uncharacterized protein |
| GRMZM2G078756 | 71 | 156 | -0.54 | 3.59E-02 | ATP binding protein |
| GRMZM2G078826 | 341 | 779 | -0.60 | 1.27E-09 | Putative uncharacterized protein |
| GRMZM2G078832 | 115 | 361 | -1.06 | 1.42E-11 | Putative uncharacterized protein |
| GRMZM2G078876 | 360 | 373 | 0.54 | 6.14E-06 | Putative uncharacterized protein (Xylogen protein 1) |
| GRMZM2G078890 | 3 | 22 | -2.28 | 1.80E-02 | Putative uncharacterized protein (Transferase, transferring glycosyl groups) |
| GRMZM2G078894 | 147 | 288 | -0.38 | 4.04E-02 | Phosphoribosylaminoimidazole-succinocarboxamide synthase (EC 6.3.2.6) |
| GRMZM2G078895 | 106 | 252 | -0.66 | 5.00E-04 | Putative uncharacterized protein |
| GRMZM2G078941 | 118 | 100 | 0.83 | 2.02E-04 | Putative uncharacterized protein (RER1A protein) |
| GRMZM2G078985 | 2,777 | 2,195 | 0.93 | 2.43E-111 | Putative uncharacterized protein |
| GRMZM2G079031 | 197 | 168 | 0.82 | 1.08E-06 | Putative arm repeat protein |
| GRMZM2G079066 | 80 | 287 | -1.25 | 6.29E-12 | Putative uncharacterized protein |
| GRMZM2G079080 | 228 | 234 | 0.55 | 3.55E-04 | ZLL/PNH homologous protein |
| GRMZM2G079083 | 186 | 480 | -0.78 | 3.16E-09 | Pre-rRNA-processing protein TSR2 |
| GRMZM2G079089 | 1,480 | 765 | 1.54 | 7.93E-134 | Histone H2B |
| GRMZM2G079127 | 9 | 49 | -1.85 | 6.14E-04 | Putative uncharacterized protein |
| GRMZM2G079143 | 164 | 332 | -0.43 | 9.90E-03 | Putative uncharacterized protein |
| GRMZM2G079196 | 147 | 317 | -0.52 | 2.10E-03 | Putative uncharacterized protein |
| GRMZM2G079206 | 46 | 110 | -0.67 | 3.78E-02 | Putative uncharacterized protein |
| GRMZM2G079226 | 39 | 127 | -1.11 | 1.04E-04 | Putative uncharacterized protein |
| GRMZM2G079231 | 18 | 58 | -1.10 | 1.68E-02 | Putative uncharacterized protein P0669G04.8 |
| GRMZM2G079256 | 31 | 93 | -0.99 | 3.99E-03 | Enoyl-[acyl-carrier-protein] reductase [NADH] |
| GRMZM2G079257 | 144 | 398 | -0.88 | 1.90E-09 | Putative uncharacterized protein |
| GRMZM2G079293 | 611 | 635 | 0.54 | 1.47E-09 | Putative uncharacterized protein |
| GRMZM2G079308 | 28 | 126 | -1.58 | 1.98E-07 | Myristoyl-acyl carrier protein thioesterase (Putative uncharacterized protein) |
| GRMZM2G079381 | 23 | 65 | -0.91 | 3.78E-02 | Ferredoxin--nitrite reductase |
| GRMZM2G079397 | 496 | 484 | 0.63 | 2.94E-10 | Putative uncharacterized protein |
| GRMZM2G079468 | 33 | 136 | -1.45 | 3.64E-07 | Putative uncharacterized protein |
| GRMZM2G079477 | 10 | 44 | -1.55 | 6.84E-03 | Phosphoribosylformylglycinamidine cyclo-ligase (EC 6.3.3.1) |
| GRMZM2G079545 | 8 | 42 | -1.80 | 2.76E-03 | Putative uncharacterized protein |
| GRMZM2G079583 | 78 | 50 | 1.23 | 2.18E-05 | Putative uncharacterized protein |
| GRMZM2G079613 | 25 | 74 | -0.97 | 1.44E-02 | Pollenless3 (Putative uncharacterized protein) |
| GRMZM2G079617 | 29 | 92 | -1.07 | 1.87E-03 | Polygalacturonase |
| GRMZM2G079625 | 84 | 248 | -0.97 | 4.79E-07 | Putative uncharacterized protein |
| GRMZM2G079653 | 6 | 41 | -2.18 | 5.78E-04 | Ethylene-responsive transcription factor 3 |
| GRMZM2G079727 | 76 | 58 | 0.98 | 7.21E-04 | Putative uncharacterized protein |
| GRMZM2G079746 | 248 | 287 | 0.38 | 1.38E-02 | Fiber protein Fb11 |
| GRMZM2G079772 | 45 | 166 | -1.29 | 2.05E-07 | Low phytic acid 1 |
| GRMZM2G079805 | 238 | 498 | -0.47 | 2.15E-04 | Putative uncharacterized protein |
| GRMZM2G079817 | 199 | 465 | -0.63 | 1.73E-06 | Putative uncharacterized protein |
| GRMZM2G079823 | 93 | 260 | -0.89 | 1.59E-06 | Putative uncharacterized protein |
| GRMZM2G079832 | 42 | 221 | -1.80 | 2.05E-15 | Putative uncharacterized protein |
| GRMZM2G079873 | 19 | 73 | -1.35 | 8.29E-04 | Putative uncharacterized protein |
| GRMZM2G079908 | 178 | 620 | -1.21 | 4.23E-24 | Acyl-CoA-binding protein (Putative uncharacterized protein) |
| GRMZM2G079938 | 31 | 90 | -0.95 | 6.51E-03 | Putative uncharacterized protein |
| GRMZM2G079944 | 11 | 55 | -1.73 | 5.77E-04 | Putative uncharacterized protein |
| GRMZM2G080048 | 421 | 971 | -0.61 | 2.42E-12 | Eukaryotic translation initiation factor 2 beta subunit |
| GRMZM2G080222 | 1,034 | 1,039 | 0.58 | 2.03E-18 | 40S ribosomal protein S15 (Putative uncharacterized protein) |
| GRMZM2G080231 | 7 | 1 | 3.40 | 3.67E-02 | Putative uncharacterized protein |
| GRMZM2G080274 | 3,014 | 3,166 | 0.52 | 3.35E-43 | Histone H1 (Putative uncharacterized protein) (Histone H1-like protein HON101) |
| GRMZM2G080307 | 93 | 209 | -0.58 | 6.90E-03 | Os02g0772500 protein (Putative uncharacterized protein) (Putative uncharacterized protein OJ1611_C08.15) |
| GRMZM2G080375 | 5 | 27 | -1.84 | 2.70E-02 | 6-phosphofructokinase 2 |
| GRMZM2G080462 | 15 | 91 | -2.01 | 1.94E-07 | Putative uncharacterized protein (SET domain protein 123) |
| GRMZM2G080466 | 30 | 17 | 1.41 | 8.19E-03 | Induced stolen tip protein TUB8 |
| GRMZM2G080521 | 55 | 190 | -1.20 | 1.58E-07 | Putative uncharacterized protein |
| GRMZM2G080524 | 30 | 16 | 1.50 | 4.49E-03 | Catalytic/ hydrolase |
| GRMZM2G080583 | 29 | 101 | -1.21 | 2.71E-04 | 6b-interacting protein 1 |
| GRMZM2G080603 | 3,941 | 7,656 | -0.37 | 2.32E-37 | Glycine-rich RNA-binding protein 2 |
| GRMZM2G080644 | 60 | 157 | -0.80 | 1.58E-03 | Putative uncharacterized protein |
| GRMZM2G080663 | 250 | 247 | 0.61 | 3.03E-05 | Putative uncharacterized protein |
| GRMZM2G080670 | 37 | 22 | 1.34 | 3.06E-03 | Putative uncharacterized protein ZMRS072.10 |
| GRMZM2G080698 | 23 | 3 | 3.53 | 5.18E-06 | NA |
| GRMZM2G080775 | 50 | 124 | -0.72 | 1.30E-02 | Putative uncharacterized protein |
| GRMZM2G080816 | 19 | 84 | -1.55 | 4.42E-05 | Putative uncharacterized protein (Rhodanese domain-containing protein-like) |
| GRMZM2G080842 | 45 | 138 | -1.03 | 1.40E-04 | Os02g0665200 protein (Mitochondrial substrate carrier protein-like) |
| GRMZM2G080889 | 5 | 27 | -1.84 | 2.70E-02 | Putative uncharacterized protein |
| GRMZM2G080912 | 195 | 194 | 0.60 | 4.21E-04 | Putative uncharacterized protein |
| GRMZM2G081012 | 59 | 49 | 0.86 | 1.18E-02 | Transcription initiation factor IID, 18kD subunit family protein |
| GRMZM2G081013 | 482 | 449 | 0.69 | 8.30E-12 | Periodic tryptophan protein 1 |
| GRMZM2G081037 | 302 | 610 | -0.42 | 2.36E-04 | Putative uncharacterized protein |
| GRMZM2G081077 | 10 | 45 | -1.58 | 4.89E-03 | Putative uncharacterized protein |
| GRMZM2G081102 | 2,517 | 1,912 | 0.99 | 1.30E-111 | 60S ribosomal protein L13 |
| GRMZM2G081127 | 11 | 41 | -1.31 | 2.99E-02 | Putative uncharacterized protein |
| GRMZM2G081144 | 71 | 235 | -1.14 | 1.64E-08 | HDDC2 protein (Putative uncharacterized protein) |
| GRMZM2G081155 | 138 | 299 | -0.52 | 2.40E-03 | Putative uncharacterized protein |
| GRMZM2G081221 | 25 | 84 | -1.16 | 1.81E-03 | Putative uncharacterized protein |
| GRMZM2G081445 | 83 | 82 | 0.61 | 3.11E-02 | Putative uncharacterized protein |
| GRMZM2G081474 | 30 | 95 | -1.07 | 1.64E-03 | Putative uncharacterized protein |
| GRMZM2G081511 | 17 | 63 | -1.30 | 3.48E-03 | Putative uncharacterized protein |
| GRMZM2G081521 | 237 | 473 | -0.41 | 2.44E-03 | Putative uncharacterized protein |
| GRMZM2G081529 | 137 | 119 | 0.79 | 1.21E-04 | Putative uncharacterized protein |
| GRMZM2G081541 | 33 | 98 | -0.98 | 3.10E-03 | Os08g0564100 protein (Putative uncharacterized protein) (cDNA clone:001-113-A02, full insert sequence) (Putative iron inhibited ABC transporter 2) |
| GRMZM2G081580 | 5,494 | 6,773 | 0.29 | 2.63E-26 | DNA-binding protein MNB1B (Putative uncharacterized protein) (HMGa protein) (Nucleosome/chromatin assembly factor group D protein) |
| GRMZM2G081644 | 57 | 41 | 1.07 | 1.93E-03 | Putative uncharacterized protein |
| GRMZM2G081653 | 202 | 96 | 1.66 | 2.16E-20 | Putative uncharacterized protein |
| GRMZM2G081745 | 124 | 303 | -0.70 | 4.14E-05 | Os04g0129500 protein |
| GRMZM2G081886 | 290 | 529 | -0.28 | 3.95E-02 | Putative uncharacterized protein |
| GRMZM2G081919 | 51 | 129 | -0.75 | 7.69E-03 | Myb-like DNA-binding domain containing protein (Putative uncharacterized protein) |
| GRMZM2G081924 | 98 | 291 | -0.98 | 2.61E-08 | Putative uncharacterized protein |
| GRMZM2G082007 | 62 | 236 | -1.34 | 8.69E-11 | Putative uncharacterized protein |
| GRMZM2G082037 | 7 | 1 | 3.40 | 3.67E-02 | Putative uncharacterized protein |
| GRMZM2G082181 | 27 | 101 | -1.31 | 7.67E-05 | Putative uncharacterized protein |
| GRMZM2G082185 | 64 | 233 | -1.27 | 6.21E-10 | Cysteine synthase (EC 2.5.1.47) |
| GRMZM2G082191 | 44 | 104 | -0.65 | 4.84E-02 | OSIGBa0145C12.4 protein (OSJNBb0002J11.4 protein) |
| GRMZM2G082222 | 65 | 238 | -1.28 | 2.99E-10 | Putative uncharacterized protein |
| GRMZM2G082271 | 704 | 1,499 | -0.50 | 4.33E-13 | Os10g0182000 protein (cDNA clone:J013059M16, full insert sequence) (Alanyl-tRNA synthetase, mitochondrial, putative, expressed) |
| GRMZM2G082322 | 329 | 183 | 1.44 | 9.80E-27 | Putative uncharacterized protein |
| GRMZM2G082384 | 202 | 599 | -0.98 | 1.12E-16 | Putative uncharacterized protein |
| GRMZM2G082390 | 477 | 602 | 0.26 | 2.05E-02 | Putative uncharacterized protein (SUMO1a protein) (Ubiquitin-like protein SMT3) |
| GRMZM2G082468 | 161 | 123 | 0.98 | 2.12E-07 | Putative uncharacterized protein |
| GRMZM2G082522 | 29 | 90 | -1.04 | 3.16E-03 | Putative uncharacterized protein |
| GRMZM2G082529 | 99 | 97 | 0.62 | 1.42E-02 | Putative uncharacterized protein |
| GRMZM2G082581 | 84 | 181 | -0.52 | 3.12E-02 | Putative uncharacterized protein |
| GRMZM2G082586 | 80 | 32 | 1.91 | 4.85E-10 | Putative uncharacterized protein |
| GRMZM2G082608 | 10 | 3 | 2.33 | 3.88E-02 | Putative uncharacterized protein |
| GRMZM2G082640 | 129 | 286 | -0.56 | 1.72E-03 | Os08g0477800 protein (Putative uncharacterized protein) (cDNA clone:J013060B01, full insert sequence) (Putative uncharacterized protein OJ1666_A04.14) |
| GRMZM2G082664 | 65 | 240 | -1.29 | 1.59E-10 | Os06g0499900 protein (cDNA clone:J023128I09, full insert sequence) (Putative dihydrolipoamide S-acetyltransferase) |
| GRMZM2G082745 | 59 | 149 | -0.75 | 4.03E-03 | Putative uncharacterized protein |
| GRMZM2G082823 | 73 | 163 | -0.57 | 2.43E-02 | Os02g0138000 protein (Putative uncharacterized protein) (Putative extensin) |
| GRMZM2G082855 | 37 | 28 | 0.99 | 3.28E-02 | Os06g0130100 protein (Transmembrane protein kinase) |
| GRMZM2G082874 | 82 | 184 | -0.57 | 1.36E-02 | Plant-specific domain TIGR01589 family protein (Putative uncharacterized protein) |
| GRMZM2G082931 | 84 | 216 | -0.77 | 1.82E-04 | Glycine-rich RNA-binding protein 7 |
| GRMZM2G082962 | 152 | 356 | -0.64 | 3.76E-05 | Putative uncharacterized protein |
| GRMZM2G082974 | 218 | 615 | -0.90 | 3.92E-15 | Eukaryotic translation initiation factor 5A-1/2 (Putative uncharacterized protein) |
| GRMZM2G082976 | 72 | 202 | -0.90 | 3.44E-05 | Putative uncharacterized protein |
| GRMZM2G083012 | 74 | 168 | -0.59 | 1.57E-02 | Putative uncharacterized protein |
| GRMZM2G083058 | 217 | 533 | -0.71 | 9.99E-09 | Os09g0344800 protein (cDNA clone:001-044-G03, full insert sequence) (Membrane protein-like) |
| GRMZM2G083068 | 55 | 128 | -0.63 | 2.97E-02 | Os05g0185700 protein (cDNA clone:002-103-F05, full insert sequence) (Putative uncharacterized protein OJ1111_A10.13) |
| GRMZM2G083095 | 356 | 1,008 | -0.91 | 1.04E-24 | Putative uncharacterized protein |
| GRMZM2G083195 | 119 | 125 | 0.52 | 2.74E-02 | Putative uncharacterized protein |
| GRMZM2G083243 | 441 | 897 | -0.43 | 2.32E-06 | Putative uncharacterized protein |
| GRMZM2G083253 | 1,271 | 2,341 | -0.29 | 1.12E-07 | 60S ribosomal protein L23a (Putative uncharacterized protein) |
| GRMZM2G083346 | 85 | 213 | -0.73 | 4.42E-04 | Cleavage and polyadenylation specificity factor, 73 kDa subunit (Putative uncharacterized protein) |
| GRMZM2G083347 | 190 | 64 | 2.16 | 1.52E-27 | Os12g0477400 protein (cDNA, clone: J100046N20, full insert sequence) (No apical meristem protein, expressed) |
| GRMZM2G083374 | 49 | 123 | -0.74 | 1.23E-02 | Putative uncharacterized protein |
| GRMZM2G083402 | 27 | 10 | 2.02 | 5.16E-04 | Putative uncharacterized protein |
| GRMZM2G083408 | 17 | 4 | 2.68 | 1.25E-03 | NA |
| GRMZM2G083410 | 146 | 297 | -0.43 | 1.56E-02 | Putative uncharacterized protein |
| GRMZM2G083459 | 147 | 374 | -0.76 | 4.93E-07 | Putative uncharacterized protein At5g18580 (Tonneau 2) |
| GRMZM2G083475 | 158 | 346 | -0.54 | 6.89E-04 | Chromosome chr6 scaffold_3, whole genome shotgun sequence |
| GRMZM2G083497 | 993 | 1,175 | 0.35 | 3.81E-07 | 40S ribosomal protein S15a |
| GRMZM2G083580 | 94 | 301 | -1.09 | 3.81E-10 | Putative uncharacterized protein |
| GRMZM2G083642 | 592 | 1,116 | -0.32 | 9.26E-05 | Small nuclear ribonucleoprotein G |
| GRMZM2G083670 | 81 | 48 | 1.35 | 2.54E-06 | Putative uncharacterized protein |
| GRMZM2G083711 | 71 | 44 | 1.28 | 3.64E-05 | Formiminotransferase-like |
| GRMZM2G083783 | 162 | 315 | -0.37 | 3.81E-02 | Putative uncharacterized protein |
| GRMZM2G083810 | 45 | 174 | -1.36 | 2.26E-08 | Putative uncharacterized protein |
| GRMZM2G083836 | 35 | 123 | -1.22 | 3.14E-05 | Asparaginyl-tRNA synthetase, cytoplasmic 3 |
| GRMZM2G084005 | 24 | 7 | 2.37 | 2.38E-04 | Putative uncharacterized protein |
| GRMZM2G084021 | 37 | 13 | 2.10 | 1.71E-05 | Putative uncharacterized protein |
| GRMZM2G084046 | 86 | 222 | -0.78 | 1.41E-04 | Putative uncharacterized protein |
| GRMZM2G084063 | 204 | 475 | -0.63 | 1.56E-06 | Putative uncharacterized protein |
| GRMZM2G084134 | 61 | 187 | -1.02 | 6.09E-06 | Putative uncharacterized protein |
| GRMZM2G084181 | 143 | 386 | -0.84 | 1.38E-08 | H0714H04.5 protein |
| GRMZM2G084195 | 1,859 | 2,108 | 0.41 | 2.83E-17 | Histone H4 |
| GRMZM2G084347 | 27 | 109 | -1.42 | 1.20E-05 | Putative uncharacterized protein |
| GRMZM2G084406 | 76 | 210 | -0.87 | 3.16E-05 | Putative uncharacterized protein |
| GRMZM2G084429 | 743 | 622 | 0.85 | 1.86E-25 | Putative uncharacterized protein |
| GRMZM2G084463 | 165 | 100 | 1.31 | 6.59E-12 | Putative uncharacterized protein |
| GRMZM2G084465 | 2,427 | 6,166 | -0.75 | 4.23E-110 | 40S ribosomal protein S23 (Putative uncharacterized protein) |
| GRMZM2G084498 | 37 | 16 | 1.80 | 1.38E-04 | Putative uncharacterized protein |
| GRMZM2G084515 | 7 | 37 | -1.81 | 5.85E-03 | Putative uncharacterized protein |
| GRMZM2G084521 | 118 | 242 | -0.44 | 2.77E-02 | Peptidyl-prolyl cis-trans isomerase (EC 5.2.1.8) |
| GRMZM2G084540 | 182 | 421 | -0.62 | 1.02E-05 | Putative uncharacterized protein |
| GRMZM2G084586 | 103 | 246 | -0.66 | 5.09E-04 | Os04g0660500 protein (cDNA clone:J013107K18, full insert sequence) (OSJNBa0015K02.14 protein) |
| GRMZM2G084647 | 141 | 288 | -0.44 | 1.56E-02 | Translation initiation factor eIF-2B alpha subunit |
| GRMZM2G084729 | 44 | 112 | -0.76 | 1.59E-02 | Putative uncharacterized protein |
| GRMZM2G084739 | 1,174 | 1,151 | 0.62 | 4.02E-23 | 60S ribosomal protein L9 |
| GRMZM2G084762 | 98 | 64 | 1.21 | 1.84E-06 | Putative uncharacterized protein |
| GRMZM2G084767 | 931 | 1,140 | 0.30 | 3.23E-05 | Putative uncharacterized protein |
| GRMZM2G084791 | 111 | 318 | -0.93 | 2.55E-08 | WNK6 |
| GRMZM2G084812 | 435 | 322 | 1.03 | 1.43E-20 | Fasciclin-like arabinogalactan protein 8 |
| GRMZM2G084821 | 101 | 389 | -1.35 | 5.21E-18 | NA |
| GRMZM2G084859 | 5 | 0 | #VALUE! | 4.27E-02 | Putative phytosulfokine peptide |
| GRMZM2G084881 | 155 | 112 | 1.06 | 5.07E-08 | Os09g0567300 protein (Putative monodehydroascorbate reductase) |
| GRMZM2G084968 | 30 | 94 | -1.06 | 2.14E-03 | Os06g0107000 protein (Fragment) |
| GRMZM2G085005 | 138 | 418 | -1.01 | 2.49E-12 | Partner of Nob1 (Putative uncharacterized protein) |
| GRMZM2G085019 | 111 | 94 | 0.83 | 3.64E-04 | Malic enzyme |
| GRMZM2G085049 | 172 | 170 | 0.61 | 8.13E-04 | Putative uncharacterized protein |
| GRMZM2G085054 | 974 | 302 | 2.28 | 1.34E-151 | UDP-glucosyltransferase BX8 |
| GRMZM2G085111 | 22 | 71 | -1.10 | 7.70E-03 | MDR-like ABC transporter (Putative CjMDR1) |
| GRMZM2G085113 | 152 | 415 | -0.86 | 1.62E-09 | Terminal ear1-like 2 protein |
| GRMZM2G085195 | 114 | 301 | -0.81 | 2.17E-06 | Putative uncharacterized protein |
| GRMZM2G085236 | 298 | 190 | 1.24 | 3.01E-19 | H0423H10.7 protein |
| GRMZM2G085248 | 245 | 520 | -0.49 | 7.52E-05 | Putative uncharacterized protein |
| GRMZM2G085249 | 126 | 296 | -0.64 | 2.01E-04 | Putative uncharacterized protein (Adenosine 5'-phosphosulfate reductase 5) |
| GRMZM2G085301 | 29 | 117 | -1.42 | 3.72E-06 | Putative sugar transporter protein |
| GRMZM2G085530 | 27 | 75 | -0.88 | 2.79E-02 | Os06g0195600 protein (Putative uncharacterized protein) (Putative Sac domain-containing inositol phosphatase 3) |
| GRMZM2G085547 | 188 | 191 | 0.57 | 1.12E-03 | Putative uncharacterized protein |
| GRMZM2G085630 | 76 | 66 | 0.79 | 6.34E-03 | Holocarboxylase synthetase |
| GRMZM2G085713 | 166 | 172 | 0.54 | 4.23E-03 | Putative uncharacterized protein |
| GRMZM2G085750 | 386 | 283 | 1.04 | 1.01E-18 | Early nodulin 93 (Putative uncharacterized protein) |
| GRMZM2G085827 | 47 | 119 | -0.75 | 1.31E-02 | Putative uncharacterized protein |
| GRMZM2G085833 | 91 | 205 | -0.58 | 7.49E-03 | Putative uncharacterized protein |
| GRMZM2G085856 | 174 | 133 | 0.98 | 6.97E-08 | Putative uncharacterized protein |
| GRMZM2G085872 | 6 | 36 | -1.99 | 2.84E-03 | Putative uncharacterized protein |
| GRMZM2G085892 | 41 | 14 | 2.14 | 3.25E-06 | Os04g0385600 protein (OSJNBa0044M19.1 protein) (OSJNBa0084N21.16 protein) |
| GRMZM2G085909 | 700 | 1,221 | -0.21 | 1.05E-02 | Putative uncharacterized protein |
| GRMZM2G085926 | 127 | 319 | -0.74 | 7.08E-06 | Choline-phosphate cytidylyltransferase B (Putative uncharacterized protein) |
| GRMZM2G085932 | 428 | 881 | -0.45 | 1.13E-06 | Mitochondrial glycoprotein (Putative uncharacterized protein) |
| GRMZM2G085948 | 82 | 240 | -0.96 | 1.03E-06 | Protein binding protein |
| GRMZM2G086030 | 40 | 104 | -0.79 | 1.45E-02 | H0811D08.1 protein |
| GRMZM2G086032 | 77 | 57 | 1.03 | 3.67E-04 | Putative uncharacterized protein |
| GRMZM2G086069 | 218 | 521 | -0.67 | 8.69E-08 | Calmodulin-like |
| GRMZM2G086072 | 24 | 90 | -1.32 | 2.70E-04 | Putative uncharacterized protein |
| GRMZM2G086088 | 194 | 203 | 0.53 | 2.21E-03 | Ubiquitin carrier protein (EC 6.3.2.-) |
| GRMZM2G086116 | 156 | 515 | -1.13 | 4.95E-18 | ATP-dependent RNA helicase dhh1 (Putative uncharacterized protein) |
| GRMZM2G086123 | 44 | 30 | 1.14 | 4.81E-03 | Putative uncharacterized protein |
| GRMZM2G086138 | 58 | 146 | -0.74 | 4.59E-03 | Putative uncharacterized protein |
| GRMZM2G086191 | 15 | 60 | -1.41 | 2.33E-03 | Microtubule motor |
| GRMZM2G086277 | 18 | 60 | -1.15 | 1.28E-02 | Zinc finger, C2H2 type family protein |
| GRMZM2G086371 | 64 | 149 | -0.63 | 1.64E-02 | Os04g0502200 protein |
| GRMZM2G086430 | 7 | 35 | -1.73 | 8.45E-03 | Putative uncharacterized protein (OSJNBa0019K04.6 protein) |
| GRMZM2G086474 | 139 | 56 | 1.90 | 3.76E-17 | Putative uncharacterized protein |
| GRMZM2G086489 | 110 | 251 | -0.60 | 1.74E-03 | Putative uncharacterized protein |
| GRMZM2G086497 | 64 | 276 | -1.52 | 4.35E-15 | Putative uncharacterized protein |
| GRMZM2G086577 | 72 | 52 | 1.06 | 3.97E-04 | Putative uncharacterized protein |
| GRMZM2G086636 | 306 | 369 | 0.32 | 2.01E-02 | Putative uncharacterized protein |
| GRMZM2G086648 | 35 | 93 | -0.82 | 1.86E-02 | NA |
| GRMZM2G086669 | 190 | 410 | -0.52 | 3.02E-04 | Putative uncharacterized protein (Ribosomal protein L35 containing protein) |
| GRMZM2G086733 | 97 | 75 | 0.96 | 1.45E-04 | Putative uncharacterized protein |
| GRMZM2G086757 | 23 | 72 | -1.05 | 8.80E-03 | Os07g0249100 protein (cDNA clone:J013092H05, full insert sequence) (Putative pentatricopeptide (PPR) repeat-containing protein) |
| GRMZM2G086788 | 30 | 16 | 1.50 | 4.49E-03 | 50S ribosomal protein L6 |
| GRMZM2G086789 | 53 | 148 | -0.89 | 5.52E-04 | Putative uncharacterized protein |
| GRMZM2G086801 | 101 | 313 | -1.04 | 7.75E-10 | COP9 signalosome complex subunit 5b (Putative uncharacterized protein) |
| GRMZM2G086882 | 277 | 338 | 0.30 | 4.06E-02 | Putative uncharacterized protein |
| GRMZM2G086887 | 106 | 314 | -0.98 | 6.94E-09 | Putative uncharacterized protein |
| GRMZM2G086906 | 1,913 | 2,155 | 0.42 | 1.61E-18 | 40S ribosomal protein S17-4 |
| GRMZM2G086934 | 631 | 537 | 0.82 | 1.41E-20 | Putative uncharacterized protein (Replication protein A 70 kDa DNA-binding subunit) |
| GRMZM2G086943 | 137 | 270 | -0.39 | 4.69E-02 | PRA1 family protein (Putative uncharacterized protein) |
| GRMZM2G086949 | 77 | 165 | -0.51 | 4.37E-02 | Putative uncharacterized protein |
| GRMZM2G086964 | 191 | 223 | 0.37 | 4.39E-02 | Putative uncharacterized protein |
| GRMZM2G087032 | 100 | 78 | 0.95 | 1.43E-04 | Putative uncharacterized protein |
| GRMZM2G087063 | 49 | 115 | -0.64 | 3.70E-02 | Putative uncharacterized protein |
| GRMZM2G087103 | 277 | 235 | 0.83 | 2.33E-09 | OB-fold nucleic acid binding domain containing protein |
| GRMZM2G087105 | 226 | 213 | 0.68 | 1.17E-05 | Putative uncharacterized protein |
| GRMZM2G087137 | 516 | 430 | 0.85 | 5.85E-18 | Putative uncharacterized protein |
| GRMZM2G087161 | 27 | 80 | -0.98 | 1.12E-02 | Putative uncharacterized protein |
| GRMZM2G087196 | 187 | 365 | -0.37 | 2.00E-02 | Nucleolar protein 10 |
| GRMZM2G087212 | 118 | 371 | -1.06 | 7.23E-12 | Transmembrane 9 superfamily protein member 3 |
| GRMZM2G087233 | 1,890 | 3,307 | -0.22 | 2.47E-06 | 60S ribosomal protein L10-3 (Putative uncharacterized protein) |
| GRMZM2G087254 | 60 | 232 | -1.36 | 5.56E-11 | Adenosine 5'-phosphosulfate reductase 2 |
| GRMZM2G087259 | 21 | 10 | 1.66 | 1.39E-02 | Carbonic anhydrase (Putative uncharacterized protein) |
| GRMZM2G087312 | 64 | 61 | 0.66 | 4.43E-02 | Oxidoreductase |
| GRMZM2G087326 | 707 | 899 | 0.24 | 4.68E-03 | Putative uncharacterized protein |
| GRMZM2G087426 | 16 | 7 | 1.78 | 2.33E-02 | Expressed protein (Putative uncharacterized protein) |
| GRMZM2G087431 | 128 | 348 | -0.85 | 6.87E-08 | Putative uncharacterized protein |
| GRMZM2G087531 | 52 | 138 | -0.82 | 2.42E-03 | Os12g0239200 protein (Putative uncharacterized protein) (Expressed protein) |
| GRMZM2G087549 | 241 | 459 | -0.34 | 1.68E-02 | Putative uncharacterized protein |
| GRMZM2G087570 | 252 | 487 | -0.36 | 7.04E-03 | Peptidyl-prolyl cis-trans isomerase (EC 5.2.1.8) |
| GRMZM2G087600 | 286 | 532 | -0.30 | 1.89E-02 | Putative uncharacterized protein |
| GRMZM2G087612 | 34 | 110 | -1.10 | 4.47E-04 | Putative uncharacterized protein |
| GRMZM2G087712 | 158 | 472 | -0.99 | 2.11E-13 | Putative uncharacterized protein |
| GRMZM2G087714 | 140 | 63 | 1.74 | 2.72E-15 | Putative uncharacterized protein |
| GRMZM2G087741 | 410 | 436 | 0.50 | 6.09E-06 | Knotted class 1 homeodomain protein liguleless3 |
| GRMZM2G087806 | 45 | 181 | -1.42 | 4.54E-09 | Os06g0644500 protein (Putative uncharacterized protein) (Ankyrin repeat-containing protein-like) |
| GRMZM2G087817 | 104 | 82 | 0.93 | 1.15E-04 | Putative uncharacterized protein (Single myb histone 1) |
| GRMZM2G087850 | 64 | 233 | -1.27 | 6.21E-10 | Putative uncharacterized protein |
| GRMZM2G087896 | 49 | 23 | 1.68 | 1.82E-05 | Putative uncharacterized protein |
| GRMZM2G087901 | 18 | 7 | 1.95 | 8.95E-03 | Sucrose transporter |
| GRMZM2G087918 | 49 | 116 | -0.65 | 3.71E-02 | Putative uncharacterized protein |
| GRMZM2G087920 | 260 | 484 | -0.31 | 2.87E-02 | Putative uncharacterized protein |
| GRMZM2G087924 | 118 | 103 | 0.79 | 5.00E-04 | Putative uncharacterized protein |
| GRMZM2G088014 | 141 | 345 | -0.70 | 8.01E-06 | Os09g0101800 protein (Putative uncharacterized protein OSJNBa0066B16.37) |
| GRMZM2G088060 | 872 | 1,599 | -0.28 | 3.01E-05 | Putative uncharacterized protein |
| GRMZM2G088083 | 124 | 340 | -0.86 | 5.92E-08 | Putative uncharacterized protein |
| GRMZM2G088088 | 530 | 1,172 | -0.55 | 2.37E-12 | Putative uncharacterized protein |
| GRMZM2G088114 | 50 | 180 | -1.26 | 1.16E-07 | Os02g0115600 protein (Putative uncharacterized protein) (cDNA clone:J013106A17, full insert sequence) (Heat shock factor protein hsf8-like) |
| GRMZM2G088212 | 209 | 618 | -0.97 | 5.62E-17 | Catalase (EC 1.11.1.6) |
| GRMZM2G088242 | 57 | 40 | 1.10 | 1.71E-03 | AT-HSFB4 |
| GRMZM2G088261 | 81 | 180 | -0.56 | 1.79E-02 | Os09g0267500 protein (Putative NEDD1 protein) |
| GRMZM2G088309 | 18 | 64 | -1.24 | 4.19E-03 | Protein DROOPING LEAF (Putative uncharacterized protein) |
| GRMZM2G088361 | 29 | 130 | -1.57 | 9.01E-08 | Os05g0467000 protein (Putative calcium-dependent protein kinase) |
| GRMZM2G088397 | 62 | 146 | -0.64 | 1.48E-02 | HD domain containing protein |
| GRMZM2G088511 | 2,220 | 4,943 | -0.56 | 7.32E-53 | NA |
| GRMZM2G088565 | 298 | 317 | 0.50 | 1.63E-04 | Pyruvate dehydrogenase E1 component subunit beta |
| GRMZM2G088590 | 113 | 241 | -0.50 | 1.24E-02 | Zn-finger, RanBP-type, containing protein |
| GRMZM2G088669 | 85 | 72 | 0.83 | 2.19E-03 | Putative uncharacterized protein |
| GRMZM2G088737 | 33 | 88 | -0.82 | 2.43E-02 | Putative uncharacterized protein (MAP kinase activating protein-like) |
| GRMZM2G088849 | 93 | 60 | 1.22 | 2.87E-06 | Putative uncharacterized protein |
| GRMZM2G088874 | 10 | 1 | 3.91 | 4.41E-03 | Putative uncharacterized protein |
| GRMZM2G088880 | 1,011 | 1,871 | -0.30 | 1.53E-06 | Putative uncharacterized protein |
| GRMZM2G088961 | 82 | 78 | 0.66 | 2.19E-02 | Putative uncharacterized protein |
| GRMZM2G088974 | 107 | 316 | -0.97 | 7.92E-09 | Putative uncharacterized protein |
| GRMZM2G089050 | 35 | 91 | -0.79 | 2.81E-02 | Nucleic acid binding protein |
| GRMZM2G089193 | 148 | 307 | -0.46 | 7.51E-03 | Putative uncharacterized protein |
| GRMZM2G089231 | 17 | 8 | 1.68 | 3.02E-02 | Putative uncharacterized protein |
| GRMZM2G089248 | 33 | 23 | 1.11 | 2.70E-02 | Putative uncharacterized protein |
| GRMZM2G089259 | 43 | 133 | -1.04 | 1.80E-04 | Putative uncharacterized protein (Seven transmembrane protein Mlo4) |
| GRMZM2G089355 | 29 | 78 | -0.84 | 3.32E-02 | Putative uncharacterized protein (Ribosomal protein L11 methyltransferase containing protein) |
| GRMZM2G089365 | 197 | 223 | 0.41 | 1.89E-02 | Fructose-bisphosphate aldolase (EC 4.1.2.13) |
| GRMZM2G089406 | 126 | 371 | -0.97 | 2.79E-10 | Putative uncharacterized protein |
| GRMZM2G089484 | 114 | 118 | 0.54 | 2.28E-02 | Putative uncharacterized protein (Mitogen activated protein kinase 6) |
| GRMZM2G089491 | 950 | 488 | 1.55 | 1.10E-86 | Protein transport protein SEC61 gamma subunit |
| GRMZM2G089562 | 305 | 155 | 1.57 | 2.93E-28 | Putative uncharacterized protein |
| GRMZM2G089630 | 45 | 150 | -1.15 | 9.47E-06 | Putative uncharacterized protein |
| GRMZM2G089676 | 200 | 498 | -0.72 | 1.30E-08 | Putative uncharacterized protein |
| GRMZM2G089698 | 95 | 62 | 1.21 | 2.74E-06 | Anthranilate N-benzoyltransferase protein 1 |
| GRMZM2G089713 | 292 | 338 | 0.38 | 6.51E-03 | Sucrose synthase 1 |
| GRMZM2G089819 | 76 | 214 | -0.90 | 1.58E-05 | Brassinosteroid LRR receptor kinase (Putative uncharacterized protein) |
| GRMZM2G089857 | 63 | 146 | -0.62 | 1.86E-02 | Putative uncharacterized protein |
| GRMZM2G089976 | 23 | 86 | -1.31 | 4.07E-04 | Putative uncharacterized protein |
| GRMZM2G089992 | 181 | 701 | -1.36 | 1.65E-32 | Mitotic checkpoint protein BUB3 (Putative uncharacterized protein) |
| GRMZM2G090037 | 60 | 199 | -1.14 | 2.70E-07 | Chaperone protein dnaJ 15 (Putative uncharacterized protein) |
| GRMZM2G090061 | 70 | 157 | -0.57 | 2.52E-02 | Myb family transcription factor-related protein (Putative uncharacterized protein) |
| GRMZM2G090168 | 35 | 23 | 1.20 | 1.02E-02 | Pre-mRNA-splicing factor PRP17 (Putative uncharacterized protein) |
| GRMZM2G090190 | 19 | 67 | -1.23 | 3.72E-03 | Putative uncharacterized protein |
| GRMZM2G090213 | 297 | 581 | -0.38 | 1.59E-03 | RNA polymerase II transcriptional coactivator KIWI |
| GRMZM2G090217 | 228 | 212 | 0.70 | 5.98E-06 | Putative uncharacterized protein (MSI type nucleosome/chromatin assembly factor C) |
| GRMZM2G090241 | 166 | 371 | -0.57 | 1.67E-04 | Putative uncharacterized protein |
| GRMZM2G090300 | 170 | 379 | -0.57 | 1.43E-04 | Plastidic phosphate translocator-like protein1 (Putative uncharacterized protein) |
| GRMZM2G090338 | 283 | 560 | -0.39 | 1.21E-03 | Ferredoxin-sulfite reductase (EC 1.8.7.1) |
| GRMZM2G090480 | 52 | 133 | -0.76 | 5.55E-03 | Putative uncharacterized protein |
| GRMZM2G090500 | 112 | 260 | -0.62 | 8.12E-04 | Putative uncharacterized protein |
| GRMZM2G090542 | 157 | 328 | -0.47 | 4.32E-03 | Os08g0398700 protein (cDNA clone:J013002N11, full insert sequence) |
| GRMZM2G090594 | 27 | 14 | 1.54 | 6.57E-03 | WRKY25-superfamily of TFs having WRKY and zinc finger domains |
| GRMZM2G090619 | 21 | 73 | -1.21 | 2.95E-03 | Putative uncharacterized protein |
| GRMZM2G090669 | 294 | 89 | 2.32 | 2.21E-46 | Putative uncharacterized protein |
| GRMZM2G090723 | 52 | 44 | 0.83 | 2.30E-02 | NA |
| GRMZM2G090736 | 108 | 102 | 0.67 | 5.29E-03 | Putative uncharacterized protein |
| GRMZM2G090738 | 366 | 986 | -0.84 | 3.56E-21 | 60S ribosomal protein L5-1 |
| GRMZM2G090747 | 77 | 258 | -1.15 | 1.90E-09 | Putative uncharacterized protein |
| GRMZM2G090779 | 184 | 198 | 0.49 | 6.76E-03 | Putative uncharacterized protein |
| GRMZM2G090868 | 18 | 81 | -1.58 | 4.76E-05 | Putative uncharacterized protein |
| GRMZM2G090887 | 94 | 285 | -1.01 | 1.61E-08 | Putative uncharacterized protein |
| GRMZM2G090904 | 201 | 529 | -0.80 | 8.95E-11 | 26S protease regulatory subunit 6B |
| GRMZM2G090935 | 139 | 82 | 1.35 | 1.73E-10 | Putative uncharacterized protein (Seed specific protein Bn15D14A) |
| GRMZM2G091058 | 167 | 375 | -0.58 | 1.27E-04 | Putative uncharacterized protein |
| GRMZM2G091069 | 203 | 221 | 0.47 | 5.28E-03 | Ubiquitin carboxyl-terminal hydrolase (EC 3.1.2.15) |
| GRMZM2G091119 | 649 | 1,558 | -0.67 | 8.62E-23 | Importin alpha-1b subunit |
| GRMZM2G091151 | 32 | 18 | 1.42 | 4.51E-03 | USP39 protein |
| GRMZM2G091155 | 732 | 2,053 | -0.90 | 4.09E-49 | 14-3-3-like protein |
| GRMZM2G091226 | 61 | 44 | 1.06 | 1.42E-03 | Putative uncharacterized protein |
| GRMZM2G091228 | 22 | 79 | -1.25 | 1.09E-03 | Putative uncharacterized protein |
| GRMZM2G091258 | 98 | 261 | -0.82 | 9.79E-06 | Putative uncharacterized protein |
| GRMZM2G091265 | 109 | 265 | -0.69 | 1.62E-04 | DNA binding protein |
| GRMZM2G091302 | 118 | 237 | -0.41 | 4.51E-02 | Oxidoreductase/ zinc ion binding protein |
| GRMZM2G091331 | 66 | 20 | 2.31 | 1.10E-10 | WRKY68-superfamily of TFs having WRKY and zinc finger domains |
| GRMZM2G091362 | 156 | 79 | 1.57 | 1.01E-14 | Putative uncharacterized protein |
| GRMZM2G091383 | 1,185 | 596 | 1.58 | 3.27E-111 | 40S ribosomal protein S24 |
| GRMZM2G091433 | 54 | 139 | -0.77 | 4.25E-03 | Putative uncharacterized protein (Repressor protein) |
| GRMZM2G091449 | 122 | 128 | 0.52 | 2.15E-02 | Putative uncharacterized protein (Putative Splicing factor 3B subunit 3) |
| GRMZM2G091481 | 461 | 286 | 1.28 | 3.18E-31 | Protein disulfide isomerase |
| GRMZM2G091503 | 89 | 282 | -1.07 | 2.83E-09 | Signal recognition particle receptor beta subunit |
| GRMZM2G091578 | 945 | 1,058 | 0.43 | 8.90E-10 | Subtilisin-like protease |
| GRMZM2G091586 | 38 | 107 | -0.90 | 4.09E-03 | Putative uncharacterized protein |
| GRMZM2G091643 | 26 | 88 | -1.17 | 1.21E-03 | Putative uncharacterized protein |
| GRMZM2G091652 | 21 | 85 | -1.43 | 1.42E-04 | Putative uncharacterized protein |
| GRMZM2G091715 | 729 | 969 | 0.18 | 4.48E-02 | Acyl carrier protein |
| GRMZM2G091845 | 114 | 105 | 0.71 | 2.15E-03 | Putative uncharacterized protein |
| GRMZM2G091916 | 47 | 116 | -0.71 | 1.92E-02 | Putative uncharacterized protein |
| GRMZM2G091921 | 88 | 76 | 0.80 | 2.79E-03 | Putative uncharacterized protein (Ribosomal protein L32 containing protein) |
| GRMZM2G092101 | 31 | 82 | -0.81 | 3.14E-02 | Putative uncharacterized protein |
| GRMZM2G092107 | 105 | 269 | -0.77 | 2.58E-05 | Putative uncharacterized protein |
| GRMZM2G092109 | 55 | 137 | -0.73 | 8.25E-03 | Putative uncharacterized protein |
| GRMZM2G092120 | 37 | 169 | -1.60 | 3.83E-10 | Ankyrin-like protein |
| GRMZM2G092123 | 80 | 65 | 0.89 | 1.70E-03 | Os03g0284900 protein (cDNA clone:J013135M09, full insert sequence) (Pentatricopeptide, putative, expressed) |
| GRMZM2G092125 | 63 | 32 | 1.57 | 3.49E-06 | Aquaporin PIP2.1 (Putative uncharacterized protein) |
| GRMZM2G092129 | 117 | 348 | -0.98 | 7.37E-10 | Putative uncharacterized protein |
| GRMZM2G092131 | 24 | 119 | -1.72 | 5.64E-08 | Os07g0471100 protein (SET-domain transcriptional regulator family-like protein) |
| GRMZM2G092146 | 19 | 10 | 1.52 | 3.14E-02 | Putative uncharacterized protein |
| GRMZM2G092147 | 144 | 284 | -0.39 | 3.83E-02 | Putative uncharacterized protein |
| GRMZM2G092154 | 58 | 47 | 0.89 | 1.02E-02 | Putative uncharacterized protein |
| GRMZM2G092167 | 6 | 31 | -1.78 | 1.82E-02 | Os11g0106800 protein |
| GRMZM2G092190 | 27 | 89 | -1.13 | 1.40E-03 | Os04g0589600 protein (Putative uncharacterized protein) (cDNA clone:J013071F09, full insert sequence) (cDNA clone:J013157H01, full insert sequence) (OSJNBa0086O06.10 protein) |
| GRMZM2G092198 | 11 | 42 | -1.34 | 2.20E-02 | Os03g0728200 protein (Pentatricopeptide, putative) (Putative PPR repeat containing protein) |
| GRMZM2G092214 | 57 | 53 | 0.70 | 4.73E-02 | Putative uncharacterized protein |
| GRMZM2G092232 | 111 | 100 | 0.74 | 1.68E-03 | Putative uncharacterized protein |
| GRMZM2G092258 | 184 | 381 | -0.46 | 2.41E-03 | Putative uncharacterized protein |
| GRMZM2G092284 | 151 | 351 | -0.63 | 6.07E-05 | Putative uncharacterized protein |
| GRMZM2G092325 | 30 | 191 | -2.08 | 4.97E-16 | Putative uncharacterized protein |
| GRMZM2G092327 | 415 | 483 | 0.37 | 9.92E-04 | Putative uncharacterized protein |
| GRMZM2G092371 | 72 | 167 | -0.62 | 1.02E-02 | Putative uncharacterized protein |
| GRMZM2G092447 | 139 | 574 | -1.45 | 1.84E-29 | Putative uncharacterized protein |
| GRMZM2G092497 | 106 | 112 | 0.51 | 4.35E-02 | Putative uncharacterized protein |
| GRMZM2G092535 | 346 | 421 | 0.31 | 1.78E-02 | cDNA, clone: J100009E11, full insert sequence |
| GRMZM2G092581 | 98 | 73 | 1.02 | 5.05E-05 | Putative uncharacterized protein |
| GRMZM2G092632 | 2 | 22 | -2.87 | 6.92E-03 | ATP-dependent Clp protease proteolytic subunit |
| GRMZM2G092648 | 30 | 81 | -0.84 | 2.92E-02 | Putative uncharacterized protein |
| GRMZM2G092669 | 219 | 574 | -0.80 | 1.49E-11 | Putative uncharacterized protein |
| GRMZM2G092719 | 1,234 | 1,184 | 0.65 | 1.64E-26 | Putative uncharacterized protein |
| GRMZM2G092741 | 138 | 275 | -0.40 | 3.41E-02 | Putative uncharacterized protein |
| GRMZM2G092743 | 144 | 313 | -0.53 | 1.62E-03 | Putative uncharacterized protein |
| GRMZM2G092817 | 10 | 3 | 2.33 | 3.88E-02 | 4,5-DOPA dioxygenase extradiol (Putative uncharacterized protein) |
| GRMZM2G092910 | 164 | 442 | -0.84 | 9.80E-10 | Putative uncharacterized protein |
| GRMZM2G092959 | 176 | 348 | -0.39 | 1.63E-02 | Putative uncharacterized protein |
| GRMZM2G092975 | 397 | 886 | -0.57 | 7.49E-10 | Putative uncharacterized protein |
| GRMZM2G093006 | 4 | 37 | -2.62 | 3.08E-04 | Protein held out wings (Putative uncharacterized protein) |
| GRMZM2G093050 | 915 | 1,839 | -0.42 | 1.53E-11 | Putative uncharacterized protein |
| GRMZM2G093092 | 509 | 453 | 0.76 | 1.36E-14 | Putative uncharacterized protein |
| GRMZM2G093217 | 133 | 294 | -0.55 | 1.46E-03 | Chromosome transmission fidelity factor, putative |
| GRMZM2G093239 | 39 | 97 | -0.72 | 3.67E-02 | Putative uncharacterized protein |
| GRMZM2G093254 | 33 | 93 | -0.90 | 7.98E-03 | Putative uncharacterized protein |
| GRMZM2G093256 | 182 | 544 | -0.99 | 2.19E-15 | NA |
| GRMZM2G093347 | 582 | 626 | 0.49 | 9.47E-08 | Putative uncharacterized protein |
| GRMZM2G093574 | 4 | 35 | -2.54 | 4.79E-04 | 40S ribosomal protein S21 |
| GRMZM2G093598 | 61 | 159 | -0.79 | 1.37E-03 | Putative uncharacterized protein |
| GRMZM2G093603 | 108 | 273 | -0.75 | 3.18E-05 | Ent-kaurene synthase-like protein 1 |
| GRMZM2G093623 | 89 | 304 | -1.18 | 1.79E-11 | OSJNBa0032F06.21 protein |
| GRMZM2G093666 | 62 | 140 | -0.58 | 3.44E-02 | S-adenosylmethionine-dependent methyltransferase |
| GRMZM2G093731 | 126 | 117 | 0.70 | 1.29E-03 | Putative uncharacterized protein |
| GRMZM2G093744 | 4 | 41 | -2.77 | 5.20E-05 | BHLH transcription factor |
| GRMZM2G093789 | 32 | 86 | -0.83 | 2.22E-02 | Putative uncharacterized protein |
| GRMZM2G093858 | 67 | 58 | 0.80 | 1.29E-02 | Putative uncharacterized protein |
| GRMZM2G093900 | 27 | 72 | -0.82 | 4.29E-02 | Aminotransferase y4uB |
| GRMZM2G093945 | 95 | 256 | -0.84 | 7.52E-06 | Putative uncharacterized protein |
| GRMZM2G093997 | 120 | 395 | -1.13 | 6.34E-14 | Nonspecific lipid-transfer protein 4 |
| GRMZM2G094039 | 110 | 330 | -0.99 | 1.46E-09 | Putative uncharacterized protein |
| GRMZM2G094047 | 2 | 28 | -3.22 | 7.48E-04 | NA |
| GRMZM2G094050 | 34 | 105 | -1.04 | 1.27E-03 | Putative uncharacterized protein |
| GRMZM2G094051 | 794 | 687 | 0.80 | 1.50E-24 | 60S ribosomal protein L7-2 (Putative uncharacterized protein) |
| GRMZM2G094074 | 133 | 351 | -0.81 | 2.07E-07 | 40S ribosomal protein S14 (Putative uncharacterized protein) |
| GRMZM2G094083 | 16 | 50 | -1.05 | 4.71E-02 | Putative uncharacterized protein |
| GRMZM2G094273 | 51 | 21 | 1.87 | 2.33E-06 | Cis,cis-muconate cycloisomerase-like |
| GRMZM2G094428 | 32 | 96 | -0.99 | 3.54E-03 | 3-N-debenzoyl-2-deoxytaxol N-benzoyltransferase (Putative uncharacterized protein) |
| GRMZM2G094497 | 118 | 317 | -0.83 | 5.28E-07 | Vacuolar ATP synthase subunit B isoform 1 |
| GRMZM2G094500 | 24 | 95 | -1.39 | 5.61E-05 | Putative uncharacterized protein |
| GRMZM2G094541 | 46 | 34 | 1.03 | 1.02E-02 | Putative uncharacterized protein |
| GRMZM2G094543 | 148 | 127 | 0.81 | 3.63E-05 | Putative uncharacterized protein |
| GRMZM2G094579 | 43 | 22 | 1.56 | 1.92E-04 | Putative uncharacterized protein |
| GRMZM2G094616 | 85 | 267 | -1.06 | 1.08E-08 | Protein binding protein |
| GRMZM2G094639 | 629 | 2,037 | -1.10 | 2.02E-68 | Putative uncharacterized protein (Protease inhibitor/seed storage/LTP family protein, expressed) |
| GRMZM2G094712 | 328 | 602 | -0.28 | 1.95E-02 | Aspartate aminotransferase (EC 2.6.1.1) |
| GRMZM2G094867 | 106 | 61 | 1.39 | 1.81E-08 | Putative uncharacterized protein |
| GRMZM2G094898 | 17 | 61 | -1.25 | 6.25E-03 | Putative uncharacterized protein |
| GRMZM2G094928 | 43 | 282 | -2.12 | 5.10E-24 | Putative uncharacterized protein (Vacuolar ATP synthase subunit G) |
| GRMZM2G094951 | 32 | 85 | -0.82 | 2.76E-02 | Putative uncharacterized protein |
| GRMZM2G094959 | 6 | 27 | -1.58 | 4.89E-02 | Putative uncharacterized protein |
| GRMZM2G095039 | 252 | 222 | 0.77 | 1.09E-07 | Putative uncharacterized protein (Rapid alkalinization factor 1) |
| GRMZM2G095043 | 156 | 329 | -0.49 | 3.21E-03 | Os03g0300300 protein (Putative uncharacterized protein) (cDNA clone:J013074C22, full insert sequence) (Transducin family protein, putative, expressed) |
| GRMZM2G095082 | 4 | 28 | -2.22 | 5.82E-03 | Putative uncharacterized protein |
| GRMZM2G095124 | 258 | 262 | 0.57 | 8.51E-05 | Adapter-related protein complex 1 beta 1 subunit, putative, expressed |
| GRMZM2G095141 | 141 | 133 | 0.68 | 9.25E-04 | Putative uncharacterized protein |
| GRMZM2G095185 | 268 | 498 | -0.30 | 2.63E-02 | Putative uncharacterized protein |
| GRMZM2G095211 | 150 | 132 | 0.78 | 7.90E-05 | Putative uncharacterized protein OSJNBa0019F11.13 (Putative uncharacterized protein P0541H01.35) |
| GRMZM2G095219 | 24 | 68 | -0.91 | 3.33E-02 | Os10g0565300 protein (C2 domain-containing protein, putative, expressed) |
| GRMZM2G095299 | 51 | 14 | 2.46 | 6.49E-09 | Putative uncharacterized protein |
| GRMZM2G095302 | 13 | 45 | -1.20 | 3.11E-02 | ATP binding protein |
| GRMZM2G095308 | 24 | 72 | -0.99 | 1.29E-02 | Putative uncharacterized protein |
| GRMZM2G095348 | 95 | 290 | -1.02 | 8.40E-09 | Coatomer complex subunit |
| GRMZM2G095541 | 7 | 1 | 3.40 | 3.67E-02 | Putative uncharacterized protein (Putative uncharacterized protein P0506E04.21) |
| GRMZM2G095552 | 56 | 189 | -1.16 | 3.35E-07 | Putative uncharacterized protein |
| GRMZM2G095579 | 15 | 58 | -1.36 | 4.34E-03 | Dihydroneopterin aldolase (Putative uncharacterized protein) |
| GRMZM2G095631 | 70 | 60 | 0.81 | 8.99E-03 | HEAT repeat family protein |
| GRMZM2G095670 | 121 | 113 | 0.69 | 2.03E-03 | G10-like protein (Putative uncharacterized protein) |
| GRMZM2G095786 | 64 | 144 | -0.58 | 3.18E-02 | Putative uncharacterized protein |
| GRMZM2G095826 | 42 | 25 | 1.34 | 1.66E-03 | Ferredoxin-3 (Putative uncharacterized protein) |
| GRMZM2G095892 | 295 | 349 | 0.35 | 1.32E-02 | NA |
| GRMZM2G095931 | 34 | 102 | -0.99 | 2.11E-03 | Os08g0427500 protein (Putative xeroderma pigmentosum group C protein) |
| GRMZM2G095964 | 51 | 28 | 1.46 | 1.35E-04 | Putative uncharacterized protein |
| GRMZM2G096020 | 145 | 58 | 1.91 | 5.87E-18 | Putative uncharacterized protein |
| GRMZM2G096051 | 108 | 240 | -0.56 | 4.19E-03 | Putative uncharacterized protein |
| GRMZM2G096106 | 21 | 63 | -0.99 | 2.43E-02 | Os07g0613300 protein (cDNA clone:J013106D10, full insert sequence) (Putative exportin, tRNA) (Putative exportin, tRNA (Nuclear export receptor for tRNAs)) |
| GRMZM2G096107 | 167 | 410 | -0.70 | 7.04E-07 | Putative uncharacterized protein |
| GRMZM2G096153 | 101 | 271 | -0.83 | 4.19E-06 | Glutathione S-transferase (Putative uncharacterized protein) |
| GRMZM2G096240 | 45 | 114 | -0.75 | 1.39E-02 | Putative uncharacterized protein |
| GRMZM2G096261 | 268 | 203 | 0.99 | 3.63E-12 | Putative uncharacterized protein |
| GRMZM2G096355 | 43 | 116 | -0.84 | 5.14E-03 | Putative uncharacterized protein |
| GRMZM2G096389 | 33 | 99 | -0.99 | 3.13E-03 | Putative uncharacterized protein |
| GRMZM2G096458 | 478 | 466 | 0.63 | 5.90E-10 | Faciata 1-like protein (Fragment) |
| GRMZM2G096546 | 38 | 15 | 1.93 | 3.78E-05 | Plant-specific domain TIGR01627 family protein (Putative uncharacterized protein) |
| GRMZM2G096548 | 428 | 463 | 0.48 | 1.12E-05 | Putative uncharacterized protein |
| GRMZM2G096585 | 1,177 | 3,706 | -1.06 | 2.34E-117 | Peptidyl-prolyl isomerase |
| GRMZM2G096591 | 45 | 167 | -1.30 | 1.50E-07 | Glucan endo-1,3-beta-glucosidase 7 |
| GRMZM2G096596 | 59 | 208 | -1.23 | 1.65E-08 | Adenylyl cyclase-associated protein |
| GRMZM2G096690 | 2,293 | 2,053 | 0.75 | 2.36E-63 | Os06g0172600 protein (40S ribosomal protein S30-like) |
| GRMZM2G096693 | 316 | 288 | 0.73 | 1.57E-08 | Putative uncharacterized protein |
| GRMZM2G096695 | 73 | 37 | 1.57 | 3.87E-07 | Enzyme of the cupin superfamily (Putative uncharacterized protein) |
| GRMZM2G096705 | 238 | 271 | 0.40 | 9.73E-03 | Putative uncharacterized protein |
| GRMZM2G096759 | 54 | 137 | -0.75 | 6.39E-03 | Putative uncharacterized protein |
| GRMZM2G096802 | 35 | 89 | -0.76 | 3.44E-02 | Putative uncharacterized protein |
| GRMZM2G096815 | 2,453 | 2,290 | 0.69 | 1.79E-58 | Putative uncharacterized protein |
| GRMZM2G096824 | 26 | 69 | -0.82 | 4.93E-02 | Putative uncharacterized protein |
| GRMZM2G096877 | 46 | 119 | -0.78 | 8.09E-03 | Putative uncharacterized protein |
| GRMZM2G096909 | 48 | 130 | -0.85 | 2.68E-03 | Putative uncharacterized protein |
| GRMZM2G096962 | 171 | 186 | 0.47 | 1.28E-02 | 50S ribosomal protein L9 (Putative uncharacterized protein) |
| GRMZM2G096972 | 390 | 693 | -0.24 | 3.94E-02 | Putative splicing factor 3b, subunit 3, 130kDa |
| GRMZM2G097021 | 140 | 155 | 0.44 | 3.83E-02 | Putative uncharacterized protein |
| GRMZM2G097043 | 42 | 131 | -1.05 | 1.60E-04 | Putative uncharacterized protein |
| GRMZM2G097129 | 26 | 16 | 1.29 | 2.10E-02 | Putative uncharacterized protein |
| GRMZM2G097226 | 321 | 348 | 0.47 | 2.07E-04 | Pyruvate dehydrogenase E1 beta subunit isoform 2 (EC 1.2.4.1) |
| GRMZM2G097277 | 23 | 68 | -0.97 | 2.41E-02 | Putative uncharacterized protein (Putative arabinoxylan narabinofuranohydrolase isoenzyme AXAH-I) |
| GRMZM2G097289 | 56 | 223 | -1.40 | 5.99E-11 | Os05g0144300 protein (Putative transcriptional regulator) |
| GRMZM2G097313 | 187 | 443 | -0.65 | 1.57E-06 | Putative uncharacterized protein |
| GRMZM2G097395 | 30 | 22 | 1.04 | 4.41E-02 | Putative uncharacterized protein |
| GRMZM2G097499 | 246 | 162 | 1.19 | 5.40E-15 | Putative uncharacterized protein |
| GRMZM2G097502 | 37 | 120 | -1.11 | 1.76E-04 | Os05g0430300 protein (cDNA clone:J033067N19, full insert sequence) (Putative uncharacterized protein OSJNBb0048I21.11) |
| GRMZM2G097573 | 29 | 95 | -1.12 | 1.09E-03 | Putative uncharacterized protein (cDNA clone:J023004E10, full insert sequence) |
| GRMZM2G097593 | 288 | 190 | 1.19 | 1.57E-17 | Splicing factor, arginine/serine-rich 7 |
| GRMZM2G097605 | 87 | 185 | -0.50 | 3.43E-02 | DNA repair helicase UVH6 |
| GRMZM2G097726 | 99 | 256 | -0.78 | 3.07E-05 | SHL1 |
| GRMZM2G097802 | 45 | 116 | -0.77 | 1.14E-02 | Putative uncharacterized protein |
| GRMZM2G097805 | 158 | 363 | -0.61 | 7.15E-05 | Os01g0201000 protein |
| GRMZM2G097812 | 37 | 95 | -0.77 | 2.65E-02 | Putative uncharacterized protein |
| GRMZM2G097898 | 102 | 221 | -0.52 | 1.15E-02 | DNA polymerase I (Putative uncharacterized protein) |
| GRMZM2G097935 | 8 | 32 | -1.41 | 4.06E-02 | Putative uncharacterized protein |
| GRMZM2G097959 | 56 | 131 | -0.63 | 2.62E-02 | Putative uncharacterized protein |
| GRMZM2G097989 | 208 | 212 | 0.56 | 5.26E-04 | Glutathione S-transferase GSTU6 |
| GRMZM2G098039 | 90 | 215 | -0.66 | 1.28E-03 | Putative uncharacterized protein |
| GRMZM2G098046 | 205 | 458 | -0.57 | 2.07E-05 | Putative uncharacterized protein |
| GRMZM2G098076 | 495 | 557 | 0.42 | 2.87E-05 | Putative uncharacterized protein |
| GRMZM2G098079 | 67 | 211 | -1.06 | 5.73E-07 | Putative uncharacterized protein |
| GRMZM2G098084 | 269 | 300 | 0.43 | 2.52E-03 | Putative uncharacterized protein |
| GRMZM2G098167 | 52 | 23 | 1.77 | 4.43E-06 | Putative uncharacterized protein |
| GRMZM2G098298 | 154 | 500 | -1.11 | 6.21E-17 | Putative uncharacterized protein |
| GRMZM2G098301 | 35 | 123 | -1.22 | 3.14E-05 | Os02g0117500 protein (Putative glutamate receptor) |
| GRMZM2G098335 | 128 | 83 | 1.22 | 2.53E-08 | Putative uncharacterized protein |
| GRMZM2G098391 | 960 | 957 | 0.60 | 9.42E-18 | NA |
| GRMZM2G098397 | 57 | 44 | 0.96 | 6.12E-03 | Putative uncharacterized protein |
| GRMZM2G098427 | 232 | 213 | 0.71 | 2.64E-06 | Myb-binding protein (Os06g0116100 protein) (Putative uncharacterized protein) (cDNA clone:J023025B11, full insert sequence) (Putative GAMYB-binding protein) |
| GRMZM2G098434 | 26 | 74 | -0.92 | 2.02E-02 | Putative uncharacterized protein |
| GRMZM2G098494 | 102 | 67 | 1.20 | 1.37E-06 | Na+/H+ antiporter |
| GRMZM2G098496 | 181 | 418 | -0.62 | 1.16E-05 | Alpha-soluble NSF attachment protein (Putative uncharacterized protein) |
| GRMZM2G098569 | 88 | 86 | 0.62 | 2.44E-02 | Putative uncharacterized protein |
| GRMZM2G098577 | 597 | 285 | 1.66 | 5.45E-60 | Putative eukaryotic translation initiation factor 4 gamma |
| GRMZM2G098594 | 82 | 72 | 0.78 | 5.61E-03 | Putative growth-regulating factor 14 |
| GRMZM2G098603 | 343 | 308 | 0.75 | 1.19E-09 | Putative uncharacterized protein |
| GRMZM2G098643 | 685 | 681 | 0.60 | 6.08E-13 | Putative auxin efflux carrier |
| GRMZM2G098674 | 8 | 41 | -1.77 | 3.95E-03 | Putative uncharacterized protein |
| GRMZM2G098731 | 73 | 57 | 0.95 | 1.54E-03 | NA |
| GRMZM2G098747 | 194 | 404 | -0.47 | 1.41E-03 | Putative uncharacterized protein |
| GRMZM2G098754 | 39 | 98 | -0.74 | 3.03E-02 | Putative uncharacterized protein |
| GRMZM2G098800 | 194 | 173 | 0.76 | 7.62E-06 | Putative uncharacterized protein |
| GRMZM2G098813 | 47 | 23 | 1.62 | 6.62E-05 | Floricaula/leafy-like 1 |
| GRMZM2G098815 | 94 | 199 | -0.49 | 2.79E-02 | Putative uncharacterized protein |
| GRMZM2G098819 | 156 | 152 | 0.63 | 1.09E-03 | Mps one binder kinase activator-like 1A (Putative uncharacterized protein) |
| GRMZM2G098828 | 203 | 153 | 1.00 | 1.72E-09 | Putative uncharacterized protein |
| GRMZM2G098859 | 46 | 115 | -0.73 | 1.81E-02 | Putative uncharacterized protein |
| GRMZM2G098884 | 273 | 510 | -0.31 | 1.93E-02 | Putative uncharacterized protein |
| GRMZM2G098900 | 71 | 155 | -0.53 | 4.14E-02 | Os06g0702500 protein (cDNA clone:J013108L09, full insert sequence) (GHMP kinase-like) |
| GRMZM2G098957 | 103 | 56 | 1.47 | 6.55E-09 | 50S ribosomal protein L14 |
| GRMZM2G098988 | 92 | 60 | 1.21 | 4.12E-06 | Putative uncharacterized protein |
| GRMZM2G099007 | 389 | 305 | 0.94 | 4.37E-16 | G10-like protein (Putative uncharacterized protein) |
| GRMZM2G099036 | 17 | 52 | -1.02 | 4.08E-02 | Putative uncharacterized protein |
| GRMZM2G099049 | 102 | 89 | 0.79 | 1.45E-03 | Auxin-repressed protein |
| GRMZM2G099066 | 2,367 | 6,013 | -0.75 | 2.50E-107 | Histone H2A |
| GRMZM2G099074 | 74 | 170 | -0.61 | 1.13E-02 | Putative uncharacterized protein |
| GRMZM2G099097 | 858 | 246 | 2.39 | 7.96E-142 | Putative uncharacterized protein |
| GRMZM2G099101 | 408 | 293 | 1.07 | 1.01E-20 | Endo-1,4-beta-glucanase |
| GRMZM2G099136 | 164 | 120 | 1.04 | 2.56E-08 | Putative uncharacterized protein |
| GRMZM2G099167 | 265 | 616 | -0.63 | 2.84E-08 | Alpha tubulin |
| GRMZM2G099183 | 378 | 426 | 0.42 | 3.85E-04 | Putative uncharacterized protein |
| GRMZM2G099191 | 27 | 72 | -0.82 | 4.29E-02 | Putative uncharacterized protein |
| GRMZM2G099253 | 93 | 285 | -1.02 | 8.46E-09 | Putative uncharacterized protein |
| GRMZM2G099319 | 15 | 4 | 2.50 | 4.23E-03 | Putative uncharacterized protein |
| GRMZM2G099328 | 21 | 70 | -1.15 | 5.10E-03 | Zinc ion binding protein |
| GRMZM2G099334 | 133 | 404 | -1.01 | 6.41E-12 | Putative uncharacterized protein (WD40 repeat protein) |
| GRMZM2G099337 | 56 | 153 | -0.86 | 7.29E-04 | Expressed protein |
| GRMZM2G099352 | 3,114 | 3,236 | 0.54 | 4.36E-47 | Putative uncharacterized protein |
| GRMZM2G099355 | 834 | 732 | 0.78 | 1.01E-24 | Os05g0163200 protein (Putative PRP8 protein) |
| GRMZM2G099382 | 4 | 28 | -2.22 | 5.82E-03 | Tonoplast dicarboxylate transporter |
| GRMZM2G099390 | 86 | 77 | 0.75 | 5.88E-03 | OSIGBa0153E02-OSIGBa0093I20.15 protein |
| GRMZM2G099449 | 34 | 103 | -1.01 | 1.65E-03 | Transmembrane BAX inhibitor motif-containing protein 4 |
| GRMZM2G099461 | 43 | 27 | 1.26 | 2.29E-03 | Histone-like transcription factor and archaeal histone family protein |
| GRMZM2G099474 | 32 | 107 | -1.15 | 3.34E-04 | Thioredoxin-like protein 1 |
| GRMZM2G099628 | 63 | 140 | -0.56 | 4.17E-02 | Putative uncharacterized protein |
| GRMZM2G099657 | 668 | 1,192 | -0.24 | 2.99E-03 | 40S ribosomal protein SA (Putative uncharacterized protein) |
| GRMZM2G099696 | 223 | 414 | -0.30 | 4.97E-02 | Putative uncharacterized protein |
| GRMZM2G099754 | 175 | 99 | 1.41 | 4.81E-14 | Putative uncharacterized protein (Serine/threonine-protein kinase NAK) |
| GRMZM2G099758 | 60 | 142 | -0.65 | 1.60E-02 | Putative uncharacterized protein |
| GRMZM2G099862 | 85 | 263 | -1.04 | 3.19E-08 | Putative uncharacterized protein |
| GRMZM2G099914 | 674 | 703 | 0.53 | 2.73E-10 | Putative uncharacterized protein |
| GRMZM2G099981 | 140 | 115 | 0.88 | 1.61E-05 | Putative uncharacterized protein |
| GRMZM2G100020 | 59 | 39 | 1.19 | 5.70E-04 | Putative uncharacterized protein |
| GRMZM2G100090 | 134 | 80 | 1.34 | 5.71E-10 | Ubiquitin-protein ligase |
| GRMZM2G100107 | 12 | 41 | -1.18 | 4.59E-02 | Putative uncharacterized protein |
| GRMZM2G100120 | 302 | 556 | -0.29 | 2.47E-02 | Putative uncharacterized protein |
| GRMZM2G100121 | 296 | 247 | 0.85 | 1.89E-10 | Os12g0601000 protein (Leucine Rich Repeat family protein, expressed) |
| GRMZM2G100133 | 52 | 35 | 1.16 | 1.78E-03 | Putative uncharacterized protein |
| GRMZM2G100225 | 994 | 1,266 | 0.24 | 6.09E-04 | 60S ribosomal protein L7-1 (Putative uncharacterized protein) |
| GRMZM2G100229 | 146 | 355 | -0.69 | 7.44E-06 | Putative uncharacterized protein |
| GRMZM2G100402 | 168 | 410 | -0.70 | 1.11E-06 | Putative uncharacterized protein |
| GRMZM2G100431 | 74 | 163 | -0.55 | 2.95E-02 | Chromosome chr17 scaffold_12, whole genome shotgun sequence |
| GRMZM2G100448 | 163 | 393 | -0.68 | 3.10E-06 | Os05g0289400 protein (Putative crn) (cDNA clone:J023095B21, full insert sequence) (Putative crooked neck protein) |
| GRMZM2G100467 | 820 | 1,420 | -0.20 | 8.46E-03 | Putative 60S ribosomal protein L39 |
| GRMZM2G100473 | 36 | 108 | -0.99 | 1.62E-03 | Putative uncharacterized protein |
| GRMZM2G100478 | 262 | 941 | -1.25 | 1.70E-38 | Syntaxin-related protein KNOLLE |
| GRMZM2G100484 | 81 | 229 | -0.91 | 6.41E-06 | Putative uncharacterized protein |
| GRMZM2G100505 | 322 | 290 | 0.74 | 4.51E-09 | Putative uncharacterized protein |
| GRMZM2G100511 | 237 | 453 | -0.34 | 1.59E-02 | Mitochondrial-processing peptidase beta subunit |
| GRMZM2G100579 | 25 | 106 | -1.49 | 5.26E-06 | Chloroplast outer envelope protein 86 |
| GRMZM2G100620 | 191 | 105 | 1.45 | 8.20E-16 | Putative uncharacterized protein (Splicing factor 3A subunit 2) |
| GRMZM2G100639 | 302 | 608 | -0.42 | 2.67E-04 | Putative uncharacterized protein |
| GRMZM2G100732 | 118 | 252 | -0.50 | 9.41E-03 | Putative uncharacterized protein |
| GRMZM2G100741 | 19 | 62 | -1.11 | 1.14E-02 | Putative uncharacterized protein |
| GRMZM2G100815 | 138 | 278 | -0.42 | 2.38E-02 | Putative uncharacterized protein |
| GRMZM2G100819 | 104 | 93 | 0.75 | 1.83E-03 | Sm protein |
| GRMZM2G100858 | 35 | 95 | -0.85 | 1.21E-02 | Rab geranylgeranyl transferase like protein |
| GRMZM2G100864 | 380 | 424 | 0.43 | 2.17E-04 | Os05g0370600 protein (Putative uncharacterized protein) (Putative anthranilate phosphoribosyltransferase) |
| GRMZM2G100872 | 79 | 171 | -0.52 | 3.53E-02 | Putative uncharacterized protein |
| GRMZM2G100881 | 41 | 129 | -1.06 | 1.80E-04 | Putative uncharacterized protein |
| GRMZM2G101020 | 252 | 292 | 0.38 | 1.31E-02 | Putative uncharacterized protein |
| GRMZM2G101036 | 34 | 97 | -0.92 | 5.55E-03 | F-box domain containing protein |
| GRMZM2G101060 | 93 | 57 | 1.30 | 8.69E-07 | Putative uncharacterized protein |
| GRMZM2G101186 | 9 | 56 | -2.05 | 6.14E-05 | Putative uncharacterized protein |
| GRMZM2G101271 | 141 | 328 | -0.63 | 1.05E-04 | Putative uncharacterized protein |
| GRMZM2G101287 | 16 | 50 | -1.05 | 4.71E-02 | Pathogen induced protein 2-4 (Putative uncharacterized protein) |
| GRMZM2G101390 | 47 | 145 | -1.03 | 8.26E-05 | Putative uncharacterized protein |
| GRMZM2G101408 | 49 | 146 | -0.98 | 1.76E-04 | Os04g0561600 protein |
| GRMZM2G101412 | 19 | 68 | -1.25 | 2.81E-03 | Putative uncharacterized protein |
| GRMZM2G101446 | 1,937 | 3,448 | -0.24 | 6.16E-08 | Putative uncharacterized protein (SKP1-like protein 1A) |
| GRMZM2G101460 | 49 | 134 | -0.86 | 1.87E-03 | Pentatricopeptide, putative |
| GRMZM2G101463 | 594 | 480 | 0.90 | 1.56E-22 | Putative uncharacterized protein |
| GRMZM2G101480 | 809 | 618 | 0.98 | 2.31E-35 | Putative uncharacterized protein |
| GRMZM2G101502 | 136 | 111 | 0.88 | 2.00E-05 | Putative uncharacterized protein |
| GRMZM2G101515 | 357 | 333 | 0.69 | 6.64E-09 | Putative uncharacterized protein |
| GRMZM2G101635 | 206 | 173 | 0.84 | 2.55E-07 | Cyclin-dependent protein kinase |
| GRMZM2G101682 | 737 | 1,487 | -0.42 | 1.11E-09 | Putative uncharacterized protein |
| GRMZM2G101689 | 106 | 223 | -0.48 | 2.30E-02 | Putative uncharacterized protein |
| GRMZM2G101711 | 48 | 139 | -0.94 | 4.20E-04 | NA |
| GRMZM2G101760 | 168 | 414 | -0.71 | 5.06E-07 | Putative uncharacterized protein |
| GRMZM2G101769 | 12 | 2 | 3.18 | 3.76E-03 | Putative uncharacterized protein |
| GRMZM2G101791 | 32 | 95 | -0.98 | 4.51E-03 | Putative uncharacterized protein |
| GRMZM2G101852 | 76 | 176 | -0.62 | 8.80E-03 | SINA5 |
| GRMZM2G101859 | 1,738 | 1,506 | 0.80 | 1.83E-53 | Putative uncharacterized protein |
| GRMZM2G101872 | 77 | 67 | 0.79 | 6.67E-03 | Putative uncharacterized protein (Phytocyanin protein-like) |
| GRMZM2G101874 | 200 | 415 | -0.46 | 1.27E-03 | Microtubule-associated protein MAP65-1a (Putative uncharacterized protein) |
| GRMZM2G101900 | 123 | 258 | -0.48 | 1.26E-02 | Aspartate carbamoyltransferase 1 |
| GRMZM2G101920 | 10 | 1 | 3.91 | 4.41E-03 | Putative uncharacterized protein |
| GRMZM2G101938 | 190 | 377 | -0.40 | 1.08E-02 | Putative uncharacterized protein (Ras-related protein RIC2) |
| GRMZM2G102021 | 348 | 371 | 0.50 | 4.11E-05 | Gamma-secretase subunit APH-1B (Putative uncharacterized protein) |
| GRMZM2G102075 | 526 | 963 | -0.28 | 2.17E-03 | 40S ribosomal protein S15 |
| GRMZM2G102161 | 10 | 0 | #VALUE! | 7.87E-04 | Putative MADS-domain transcription factor (Fragment) |
| GRMZM2G102174 | 116 | 104 | 0.75 | 1.05E-03 | PWWP domain containing protein, expressed (PWWP domain, putative) |
| GRMZM2G102196 | 17 | 57 | -1.15 | 1.45E-02 | Putative uncharacterized protein |
| GRMZM2G102271 | 40 | 158 | -1.39 | 7.05E-08 | Polymerase (DNA directed) sigma-like |
| GRMZM2G102346 | 202 | 227 | 0.42 | 1.36E-02 | Putative uncharacterized protein |
| GRMZM2G102347 | 230 | 155 | 1.16 | 1.88E-13 | Putative uncharacterized protein |
| GRMZM2G102421 | 914 | 398 | 1.79 | 9.62E-103 | Ubiquitin carrier protein (EC 6.3.2.-) |
| GRMZM2G102471 | 977 | 1,969 | -0.42 | 1.44E-12 | Ubiquitin carrier protein (EC 6.3.2.-) |
| GRMZM2G102475 | 78 | 197 | -0.75 | 6.08E-04 | Putative uncharacterized protein |
| GRMZM2G102497 | 41 | 30 | 1.04 | 1.68E-02 | Os03g0355700 protein (Putative uncharacterized protein) (IQ calmodulin-binding motif family protein, expressed) |
| GRMZM2G102514 | 31 | 81 | -0.79 | 3.86E-02 | BES1/BZR1 protein |
| GRMZM2G102521 | 30 | 78 | -0.79 | 4.38E-02 | Putative uncharacterized protein (Solute carrier family 35 member C2) |
| GRMZM2G102548 | 113 | 253 | -0.57 | 2.75E-03 | Putative uncharacterized protein (Os01g0815900 protein) |
| GRMZM2G102560 | 78 | 184 | -0.65 | 4.84E-03 | Putative uncharacterized protein |
| GRMZM2G102580 | 119 | 244 | -0.44 | 2.84E-02 | Prefoldin subunit 3 |
| GRMZM2G102745 | 55 | 133 | -0.68 | 1.45E-02 | Putative uncharacterized protein |
| GRMZM2G102754 | 39 | 97 | -0.72 | 3.67E-02 | Ethylene insensitive 2 |
| GRMZM2G102779 | 226 | 474 | -0.48 | 3.01E-04 | cDNA clone:001-031-F02, full insert sequence (Putative eukaryotic translation initiation factor (EIF3d)) |
| GRMZM2G102802 | 23 | 11 | 1.66 | 7.53E-03 | Putative uncharacterized protein |
| GRMZM2G102815 | 11 | 61 | -1.88 | 9.17E-05 | Putative uncharacterized protein |
| GRMZM2G102829 | 1,399 | 2,684 | -0.35 | 3.73E-12 | Os09g0115400 protein (Poly(A)-binding protein) (Putative uncharacterized protein) (cDNA clone:J013116F07, full insert sequence) (Putative poly(A)-binding protein) |
| GRMZM2G102838 | 9 | 46 | -1.76 | 1.85E-03 | Plastid high chlorophyll fluorescence 136 |
| GRMZM2G102845 | 114 | 283 | -0.72 | 4.80E-05 | Putative uncharacterized protein |
| GRMZM2G102944 | 87 | 221 | -0.75 | 2.27E-04 | Putative uncharacterized protein (Serine/threonine-protein kinase Haspin) |
| GRMZM2G102946 | 414 | 295 | 1.08 | 2.52E-21 | Putative Rop family GTPase ROP5 |
| GRMZM2G102964 | 77 | 189 | -0.70 | 1.86E-03 | Putative uncharacterized protein |
| GRMZM2G103116 | 73 | 72 | 0.61 | 4.57E-02 | Microtubule motor |
| GRMZM2G103216 | 73 | 62 | 0.83 | 6.26E-03 | Putative uncharacterized protein |
| GRMZM2G103230 | 111 | 266 | -0.67 | 2.65E-04 | Putative uncharacterized protein |
| GRMZM2G103258 | 64 | 179 | -0.89 | 1.28E-04 | Signal peptide peptidase-like 3 |
| GRMZM2G103266 | 122 | 324 | -0.82 | 5.90E-07 | Putative uncharacterized protein |
| GRMZM2G103276 | 76 | 33 | 1.79 | 8.01E-09 | Putative uncharacterized protein |
| GRMZM2G103345 | 217 | 432 | -0.40 | 4.60E-03 | Putative uncharacterized protein (cDNA clone:J013160I10, full insert sequence) (Putative uncharacterized protein OJ1003_C09.7-1) |
| GRMZM2G103430 | 286 | 784 | -0.86 | 9.20E-18 | Putative uncharacterized protein |
| GRMZM2G103465 | 33 | 111 | -1.16 | 2.24E-04 | DHHC zinc finger domain containing protein |
| GRMZM2G103526 | 173 | 163 | 0.68 | 1.90E-04 | Acid phosphatase 1 (Putative uncharacterized protein) |
| GRMZM2G103595 | 26 | 69 | -0.82 | 4.93E-02 | Putative uncharacterized protein |
| GRMZM2G103672 | 90 | 223 | -0.72 | 4.13E-04 | Beta-expansin 4 |
| GRMZM2G103721 | 68 | 167 | -0.70 | 3.35E-03 | Os05g0180600 protein (Putative uncharacterized protein) (Putative phosphatidylinositol 3-kinase) |
| GRMZM2G103740 | 361 | 399 | 0.45 | 2.08E-04 | Putative uncharacterized protein |
| GRMZM2G103843 | 4 | 22 | -1.87 | 3.96E-02 | Putative kinase |
| GRMZM2G103864 | 92 | 70 | 0.99 | 1.63E-04 | Putative uncharacterized protein |
| GRMZM2G103873 | 12 | 2 | 3.18 | 3.76E-03 | Putative uncharacterized protein |
| GRMZM2G103884 | 72 | 191 | -0.82 | 2.57E-04 | Putative uncharacterized protein |
| GRMZM2G103896 | 212 | 235 | 0.44 | 7.94E-03 | Putative uncharacterized protein |
| GRMZM2G103900 | 10 | 42 | -1.48 | 1.35E-02 | Putative uncharacterized protein |
| GRMZM2G103909 | 94 | 321 | -1.18 | 3.95E-12 | Putative uncharacterized protein |
| GRMZM2G104017 | 388 | 868 | -0.57 | 9.91E-10 | Actin |
| GRMZM2G104045 | 51 | 170 | -1.15 | 1.95E-06 | Os12g0612400 protein (DNAJ heat shock N-terminal domain-containing protein, putative, expressed) |
| GRMZM2G104118 | 131 | 107 | 0.88 | 3.27E-05 | N-acetylglucosaminyltransferase III |
| GRMZM2G104179 | 18 | 69 | -1.35 | 1.25E-03 | Putative uncharacterized protein (Tetratricopeptide repeat protein 1) |
| GRMZM2G104258 | 172 | 402 | -0.63 | 1.05E-05 | Putative uncharacterized protein |
| GRMZM2G104269 | 17 | 61 | -1.25 | 6.25E-03 | Putative uncharacterized protein |
| GRMZM2G104288 | 15 | 81 | -1.84 | 4.32E-06 | Os07g0495300 protein (Putative uncharacterized protein) (Pentatricopeptide repeat-containing protein-like protein) |
| GRMZM2G104294 | 190 | 142 | 1.01 | 4.70E-09 | Putative uncharacterized protein |
| GRMZM2G104342 | 87 | 78 | 0.75 | 6.19E-03 | Os03g0723000 protein (cDNA clone:001-125-A04, full insert sequence) (cDNA clone:J033051K20, full insert sequence) (cDNA clone:J033110P03, full insert sequence) (GRAS family transcription factor containing protein, expressed) (GRAS family transcription factor, putative) |
| GRMZM2G104357 | 34 | 105 | -1.04 | 1.27E-03 | Putative uncharacterized protein |
| GRMZM2G104373 | 236 | 726 | -1.03 | 6.78E-22 | Putative uncharacterized protein |
| GRMZM2G104375 | 144 | 288 | -0.41 | 2.72E-02 | THUMP domain containing protein |
| GRMZM2G104394 | 61 | 16 | 2.52 | 6.12E-11 | Peroxidase 1 |
| GRMZM2G104400 | 43 | 160 | -1.30 | 3.53E-07 | Os05g0426200 protein (Putative uncharacterized protein) (cDNA clone:J013032G17, full insert sequence) (Transcription factor) (Unknow protein) |
| GRMZM2G104430 | 101 | 216 | -0.51 | 1.72E-02 | cDNA clone:J033023O17, full insert sequence (OSJNBa0043A12.36 protein) |
| GRMZM2G104456 | 592 | 432 | 1.05 | 6.41E-29 | Nucleolin |
| GRMZM2G104464 | 50 | 32 | 1.24 | 1.18E-03 | Putative uncharacterized protein |
| GRMZM2G104516 | 35 | 5 | 3.40 | 1.19E-08 | Putative uncharacterized protein |
| GRMZM2G104538 | 103 | 269 | -0.79 | 1.17E-05 | Phytol kinase 2 |
| GRMZM2G104613 | 163 | 478 | -0.96 | 4.96E-13 | 3-isopropylmalate dehydrogenase (EC 1.1.1.85) |
| GRMZM2G104632 | 479 | 876 | -0.28 | 4.01E-03 | Putative uncharacterized protein |
| GRMZM2G104649 | 606 | 574 | 0.67 | 6.71E-14 | Eukaryotic translation initiation factor 3 subunit 3 (Putative uncharacterized protein) |
| GRMZM2G104658 | 215 | 490 | -0.60 | 3.17E-06 | Putative uncharacterized protein |
| GRMZM2G104676 | 39 | 116 | -0.98 | 1.08E-03 | Os03g0397300 protein (Putative uncharacterized protein) (cDNA clone:J023089F18, full insert sequence) (Putative cell division control protein) (Ser/Thr protein phosphatase family protein, expressed) |
| GRMZM2G104769 | 96 | 34 | 2.09 | 1.61E-13 | Putative uncharacterized protein (STIP1 homology and U box-containing protein 1) |
| GRMZM2G104918 | 217 | 473 | -0.53 | 5.29E-05 | Soluble inorganic pyrophosphatase |
| GRMZM2G104925 | 78 | 199 | -0.76 | 5.00E-04 | Fasciated ear2 |
| GRMZM2G104942 | 60 | 232 | -1.36 | 5.56E-11 | Putative uncharacterized protein |
| GRMZM2G104958 | 146 | 151 | 0.54 | 7.62E-03 | Putative uncharacterized protein |
| GRMZM2G104999 | 12 | 65 | -1.85 | 6.23E-05 | Pyrimidine-specific ribonucleoside hydrolase rihB |
| GRMZM2G105019 | 359 | 833 | -0.62 | 6.44E-11 | Putative uncharacterized protein |
| GRMZM2G105025 | 99 | 83 | 0.85 | 7.84E-04 | Os03g0762900 protein (Expressed protein) |
| GRMZM2G105167 | 7 | 45 | -2.09 | 4.07E-04 | Putative uncharacterized protein |
| GRMZM2G105184 | 24 | 88 | -1.28 | 3.59E-04 | Putative uncharacterized protein |
| GRMZM2G105224 | 31 | 18 | 1.38 | 6.63E-03 | C2H2 zinc finger protein (Nucleic acid binding protein) |
| GRMZM2G105250 | 27 | 79 | -0.96 | 1.11E-02 | Putative leaf development protein Argonaute |
| GRMZM2G105253 | 128 | 257 | -0.41 | 3.50E-02 | Putative uncharacterized protein |
| GRMZM2G105266 | 160 | 167 | 0.53 | 6.05E-03 | AP2 domain containing protein |
| GRMZM2G105283 | 70 | 39 | 1.44 | 4.75E-06 | Putative uncharacterized protein |
| GRMZM2G105297 | 1 | 18 | -3.58 | 8.30E-03 | Putative uncharacterized protein |
| GRMZM2G105302 | 255 | 298 | 0.37 | 1.59E-02 | Putative uncharacterized protein |
| GRMZM2G105364 | 127 | 292 | -0.61 | 4.23E-04 | Putative uncharacterized protein |
| GRMZM2G105523 | 24 | 2 | 4.18 | 4.99E-07 | Putative uncharacterized protein |
| GRMZM2G105531 | 217 | 437 | -0.42 | 2.88E-03 | Mitochondrial import receptor subunit TOM20 (Putative uncharacterized protein) |
| GRMZM2G105570 | 12 | 42 | -1.22 | 3.50E-02 | Putative uncharacterized protein |
| GRMZM2G105571 | 1,088 | 1,874 | -0.19 | 2.95E-03 | ADP,ATP carrier protein (Putative uncharacterized protein) |
| GRMZM2G105579 | 26 | 81 | -1.05 | 6.03E-03 | Putative uncharacterized protein |
| GRMZM2G105587 | 239 | 205 | 0.81 | 6.30E-08 | MFS18 protein |
| GRMZM2G105712 | 1,080 | 900 | 0.85 | 2.31E-37 | 60S acidic ribosomal protein P2A (Putative uncharacterized protein) |
| GRMZM2G105770 | 65 | 204 | -1.06 | 9.74E-07 | Kinesin heavy chain, putative, expressed |
| GRMZM2G105772 | 129 | 80 | 1.28 | 5.39E-09 | DNA repair protein RAD23 (Putative uncharacterized protein) |
| GRMZM2G105801 | 105 | 272 | -0.78 | 1.37E-05 | Putative uncharacterized protein |
| GRMZM2G105807 | 66 | 185 | -0.90 | 7.61E-05 | Os06g0622300 protein (Putative uncharacterized protein) (cDNA clone:J013170P10, full insert sequence) (cDNA clone:J033020C22, full insert sequence) (DNA-binding protein-like) |
| GRMZM2G105844 | 560 | 1,248 | -0.56 | 1.35E-13 | Alpha-expansin 15 |
| GRMZM2G105863 | 535 | 626 | 0.36 | 1.84E-04 | Putative uncharacterized protein |
| GRMZM2G105869 | 65 | 38 | 1.37 | 2.75E-05 | Histone-lysine N-methyltransferase SUVR3 |
| GRMZM2G105901 | 15 | 7 | 1.69 | 3.69E-02 | KID-containing protein |
| GRMZM2G105996 | 935 | 1,031 | 0.45 | 1.50E-10 | Os07g0223400 protein (cDNA, clone: J075171N24, full insert sequence) (cDNA, clone: J100075G09, full insert sequence) (ADP-ribosylation factor 1) |
| GRMZM2G106039 | 98 | 53 | 1.48 | 1.22E-08 | Ribosomal protein S2 |
| GRMZM2G106042 | 55 | 139 | -0.75 | 5.58E-03 | Putative uncharacterized protein |
| GRMZM2G106056 | 87 | 290 | -1.15 | 1.72E-10 | Double-strand break repair protein MRE11 |
| GRMZM2G106061 | 192 | 213 | 0.44 | 1.22E-02 | Elongation factor Tu |
| GRMZM2G106133 | 2,162 | 4,698 | -0.53 | 8.79E-45 | Putative uncharacterized protein (High mobility group I/Y-2) |
| GRMZM2G106140 | 43 | 140 | -1.11 | 3.65E-05 | Putative uncharacterized protein |
| GRMZM2G106213 | 120 | 293 | -0.70 | 5.72E-05 | Glucose-1-phosphate adenylyltransferase (EC 2.7.7.27) |
| GRMZM2G106218 | 23 | 71 | -1.03 | 1.14E-02 | Putative uncharacterized protein |
| GRMZM2G106233 | 22 | 67 | -1.02 | 1.67E-02 | Putative MAR binding protein |
| GRMZM2G106245 | 646 | 563 | 0.79 | 1.25E-19 | Silencing group B protein |
| GRMZM2G106263 | 44 | 31 | 1.10 | 7.41E-03 | Hydroxymethylglutaryl-CoA synthase |
| GRMZM2G106308 | 106 | 385 | -1.27 | 3.56E-16 | Putative uncharacterized protein |
| GRMZM2G106331 | 61 | 191 | -1.06 | 2.80E-06 | Putative uncharacterized protein |
| GRMZM2G106344 | 63 | 54 | 0.81 | 1.28E-02 | Putative uncharacterized protein |
| GRMZM2G106389 | 42 | 132 | -1.06 | 1.61E-04 | Putative uncharacterized protein |
| GRMZM2G106393 | 21 | 9 | 1.81 | 6.66E-03 | Putative uncharacterized protein (Wound induced protein) |
| GRMZM2G106401 | 133 | 64 | 1.65 | 2.41E-13 | Non-ribosomal peptide synthetase |
| GRMZM2G106412 | 204 | 442 | -0.52 | 1.29E-04 | Putative uncharacterized protein |
| GRMZM2G106424 | 1,123 | 2,008 | -0.25 | 4.04E-05 | Putative uncharacterized protein |
| GRMZM2G106427 | 32 | 116 | -1.27 | 3.28E-05 | Putative uncharacterized protein |
| GRMZM2G106445 | 13 | 4 | 2.29 | 2.21E-02 | Putative uncharacterized protein (Wound induced protein) |
| GRMZM2G106526 | 41 | 112 | -0.86 | 5.41E-03 | ATP10 protein |
| GRMZM2G106647 | 170 | 434 | -0.76 | 3.38E-08 | CsAtPR5 (Putative uncharacterized protein) |
| GRMZM2G106766 | 109 | 255 | -0.63 | 7.00E-04 | Putative uncharacterized protein |
| GRMZM2G106792 | 20 | 58 | -0.94 | 4.45E-02 | Harpin inducing protein (Putative uncharacterized protein) |
| GRMZM2G106881 | 72 | 213 | -0.97 | 3.08E-06 | Os04g0574600 protein (Putative uncharacterized protein) (cDNA clone:J023132O07, full insert sequence) |
| GRMZM2G106928 | 219 | 476 | -0.53 | 5.67E-05 | Superoxide dismutase [Cu-Zn] (EC 1.15.1.1) |
| GRMZM2G107003 | 917 | 1,158 | 0.25 | 5.74E-04 | Gibberellin-regulated protein 1 (Putative uncharacterized protein) (GASA-like protein) |
| GRMZM2G107073 | 111 | 113 | 0.57 | 1.99E-02 | Putative uncharacterized protein |
| GRMZM2G107082 | 98 | 242 | -0.71 | 2.14E-04 | Putative uncharacterized protein |
| GRMZM2G107089 | 90 | 200 | -0.56 | 1.17E-02 | Prolyl-tRNA synthetase |
| GRMZM2G107092 | 180 | 173 | 0.65 | 2.36E-04 | Os06g0244700 protein |
| GRMZM2G107106 | 193 | 448 | -0.62 | 3.69E-06 | Putative uncharacterized protein |
| GRMZM2G107111 | 30 | 91 | -1.01 | 3.57E-03 | Putative uncharacterized protein |
| GRMZM2G107114 | 1,476 | 1,555 | 0.52 | 6.30E-21 | Prohibitin |
| GRMZM2G107199 | 43 | 147 | -1.18 | 6.85E-06 | Putative uncharacterized protein |
| GRMZM2G107205 | 48 | 32 | 1.18 | 2.34E-03 | Putative uncharacterized protein |
| GRMZM2G107289 | 619 | 536 | 0.80 | 3.50E-19 | Putative uncharacterized protein (Ubiquitin thioesterase otubain-like protein) |
| GRMZM2G107309 | 226 | 263 | 0.37 | 2.30E-02 | Putative uncharacterized protein (Histone deacetylase HDA110 isoform 1) |
| GRMZM2G107336 | 777 | 862 | 0.44 | 1.56E-08 | Putative uncharacterized protein |
| GRMZM2G107377 | 792 | 236 | 2.34 | 3.70E-127 | Cyclin delta-3 |
| GRMZM2G107437 | 34 | 89 | -0.80 | 2.61E-02 | Putative uncharacterized protein |
| GRMZM2G107444 | 558 | 333 | 1.34 | 2.12E-40 | Membrane steroid-binding protein 1 |
| GRMZM2G107463 | 78 | 178 | -0.60 | 1.15E-02 | Putative uncharacterized protein |
| GRMZM2G107499 | 143 | 341 | -0.66 | 2.79E-05 | Putative uncharacterized protein |
| GRMZM2G107532 | 200 | 112 | 1.43 | 3.04E-16 | Putative uncharacterized protein |
| GRMZM2G107540 | 471 | 386 | 0.88 | 2.72E-17 | Histone H2A |
| GRMZM2G107562 | 17 | 65 | -1.34 | 1.90E-03 | Copper ion binding protein |
| GRMZM2G107565 | 41 | 15 | 2.04 | 8.29E-06 | Putative uncharacterized protein |
| GRMZM2G107571 | 112 | 278 | -0.72 | 5.09E-05 | Os10g0477000 protein (Expressed protein) |
| GRMZM2G107588 | 99 | 214 | -0.52 | 1.39E-02 | Putative uncharacterized protein |
| GRMZM2G107629 | 83 | 70 | 0.84 | 2.44E-03 | Putative uncharacterized protein |
| GRMZM2G107696 | 674 | 647 | 0.65 | 1.25E-14 | Putative uncharacterized protein |
| GRMZM2G107737 | 192 | 190 | 0.61 | 3.68E-04 | Putative uncharacterized protein |
| GRMZM2G107798 | 39 | 18 | 1.71 | 1.58E-04 | Peptidase/ serine-type peptidase |
| GRMZM2G107807 | 491 | 469 | 0.66 | 5.84E-11 | PHD finger protein |
| GRMZM2G107838 | 79 | 67 | 0.83 | 4.17E-03 | Dolichyldiphosphatase 1 |
| GRMZM2G107854 | 67 | 173 | -0.78 | 9.35E-04 | Putative uncharacterized protein (cDNA, clone: J100048D21, full insert sequence) (Glycosyl transferase family 8 protein, expressed) |
| GRMZM2G107985 | 35 | 105 | -0.99 | 1.85E-03 | Putative uncharacterized protein |
| GRMZM2G108032 | 619 | 558 | 0.74 | 7.61E-17 | Putative uncharacterized protein |
| GRMZM2G108085 | 144 | 116 | 0.90 | 6.03E-06 | Putative uncharacterized protein |
| GRMZM2G108115 | 26 | 70 | -0.84 | 4.94E-02 | Endoplasmic oxidoreductin-1 |
| GRMZM2G108133 | 5 | 0 | #VALUE! | 4.27E-02 | Putative uncharacterized protein |
| GRMZM2G108138 | 12 | 52 | -1.52 | 3.07E-03 | Putative uncharacterized protein |
| GRMZM2G108228 | 97 | 90 | 0.70 | 5.82E-03 | Tubby-like protein |
| GRMZM2G108255 | 21 | 106 | -1.74 | 2.74E-07 | Putative uncharacterized protein |
| GRMZM2G108267 | 81 | 44 | 1.47 | 3.59E-07 | Putative uncharacterized protein |
| GRMZM2G108277 | 441 | 541 | 0.30 | 7.91E-03 | Putative thioredoxin-like U5 small ribonucleoprotein particle protein |
| GRMZM2G108348 | 3,698 | 6,371 | -0.19 | 1.53E-09 | 40S ribosomal protein S9 (Putative uncharacterized protein) |
| GRMZM2G108384 | 50 | 122 | -0.70 | 1.90E-02 | Palmitoyltransferase swf1 (Putative uncharacterized protein) |
| GRMZM2G108474 | 686 | 1,944 | -0.91 | 8.04E-48 | Putative uncharacterized protein (Translationally-controlled tumor protein) |
| GRMZM2G108537 | 9 | 1 | 3.76 | 9.10E-03 | Nodulin protein |
| GRMZM2G108546 | 66 | 152 | -0.61 | 1.81E-02 | Putative uncharacterized protein |
| GRMZM2G108686 | 10 | 45 | -1.58 | 4.89E-03 | Putative uncharacterized protein |
| GRMZM2G108712 | 962 | 2,717 | -0.91 | 3.85E-66 | Proliferating cell nuclear antigen |
| GRMZM2G108737 | 122 | 284 | -0.63 | 3.96E-04 | Putative uncharacterized protein P0592C05.16 |
| GRMZM2G108741 | 20 | 58 | -0.94 | 4.45E-02 | Os04g0690400 protein |
| GRMZM2G108775 | 25 | 89 | -1.24 | 5.84E-04 | Putative uncharacterized protein |
| GRMZM2G108780 | 910 | 1,542 | -0.17 | 2.38E-02 | Profilin |
| GRMZM2G108829 | 72 | 218 | -1.01 | 1.11E-06 | Putative uncharacterized protein |
| GRMZM2G108849 | 84 | 202 | -0.67 | 1.92E-03 | Cathepsin B-like cysteine proteinase 3 |
| GRMZM2G108861 | 12 | 1 | 4.18 | 9.96E-04 | Putative uncharacterized protein |
| GRMZM2G108871 | 186 | 26 | 3.43 | 4.13E-45 | 60S ribosomal protein L26-1 |
| GRMZM2G108874 | 89 | 238 | -0.83 | 2.11E-05 | Putative uncharacterized protein |
| GRMZM2G108919 | 172 | 155 | 0.74 | 4.44E-05 | Polyadenylate-binding protein 2 |
| GRMZM2G108991 | 58 | 35 | 1.32 | 1.54E-04 | Os09g0542700 protein (Putative uncharacterized protein B1274F11.50) (Serine/threonine protein phosphatase 2A-like protein) |
| GRMZM2G108996 | 142 | 290 | -0.44 | 1.41E-02 | Putative uncharacterized protein |
| GRMZM2G109009 | 97 | 358 | -1.29 | 1.81E-15 | Putative uncharacterized protein |
| GRMZM2G109032 | 52 | 135 | -0.78 | 4.52E-03 | S-adenosylmethionine-dependent methyltransferase/ catalytic |
| GRMZM2G109039 | 89 | 279 | -1.06 | 4.90E-09 | WD-repeat protein-like |
| GRMZM2G109121 | 207 | 615 | -0.98 | 4.42E-17 | ATP-binding cassette sub-family E member 1, putative, expressed (Os11g0546000 protein) |
| GRMZM2G109130 | 95 | 87 | 0.72 | 5.04E-03 | Lipoxygenase (EC 1.13.11.12) |
| GRMZM2G109165 | 115 | 90 | 0.95 | 3.27E-05 | Putative uncharacterized protein |
| GRMZM2G109225 | 27 | 139 | -1.77 | 1.24E-09 | Os04g0221000 protein (Putative uncharacterized protein) (cDNA clone:J033095J08, full insert sequence) (OSJNBa0055H05.9 protein) |
| GRMZM2G109244 | 14 | 99 | -2.23 | 4.54E-09 | Putative uncharacterized protein |
| GRMZM2G109268 | 24 | 76 | -1.07 | 5.95E-03 | Putative uncharacterized protein (Putative uncharacterized protein OSJNBa0026E05.3) (Putative uncharacterized protein OSJNBa0081C13.27) |
| GRMZM2G109284 | 476 | 399 | 0.85 | 2.66E-16 | Putative uncharacterized protein |
| GRMZM2G109352 | 27 | 74 | -0.86 | 3.51E-02 | Putative uncharacterized protein |
| GRMZM2G109425 | 651 | 1,491 | -0.60 | 3.58E-18 | T-complex protein 1 subunit zeta |
| GRMZM2G109550 | 113 | 361 | -1.08 | 6.12E-12 | Ribosomal protein L16-like |
| GRMZM2G109595 | 8 | 41 | -1.77 | 3.95E-03 | Putative uncharacterized protein |
| GRMZM2G109639 | 20 | 60 | -0.99 | 2.78E-02 | Zinc finger protein 511 |
| GRMZM2G109680 | 54 | 220 | -1.44 | 3.76E-11 | Putative uncharacterized protein |
| GRMZM2G109725 | 148 | 148 | 0.59 | 2.96E-03 | Putative uncharacterized protein |
| GRMZM2G109731 | 58 | 163 | -0.90 | 2.23E-04 | Putative uncharacterized protein |
| GRMZM2G109743 | 53 | 183 | -1.20 | 2.70E-07 | Putative uncharacterized protein |
| GRMZM2G109783 | 25 | 81 | -1.10 | 3.13E-03 | Putative uncharacterized protein |
| GRMZM2G109818 | 64 | 177 | -0.88 | 1.59E-04 | Sad1-unc84-like protein |
| GRMZM2G109879 | 22 | 73 | -1.14 | 4.50E-03 | Putative uncharacterized protein |
| GRMZM2G109966 | 66 | 241 | -1.28 | 2.71E-10 | Panicle organization related protein (Putative stamina pistilloidia) |
| GRMZM2G110004 | 31 | 87 | -0.90 | 1.31E-02 | Putative uncharacterized protein |
| GRMZM2G110143 | 177 | 444 | -0.74 | 6.87E-08 | Putative uncharacterized protein |
| GRMZM2G110175 | 48 | 41 | 0.82 | 3.89E-02 | Cinnamyl alcohol dehydrogenase |
| GRMZM2G110198 | 112 | 248 | -0.56 | 4.20E-03 | Putative uncharacterized protein |
| GRMZM2G110233 | 686 | 1,511 | -0.55 | 1.56E-15 | Nucleolar protein Nop56 |
| GRMZM2G110258 | 23 | 64 | -0.89 | 4.73E-02 | Putative uncharacterized protein |
| GRMZM2G110279 | 78 | 75 | 0.65 | 2.88E-02 | Putative uncharacterized protein |
| GRMZM2G110289 | 34 | 86 | -0.75 | 3.91E-02 | DeliriumA-like |
| GRMZM2G110345 | 69 | 31 | 1.75 | 7.81E-08 | Expressed protein (Putative uncharacterized protein OJ1006F06.20) |
| GRMZM2G110378 | 536 | 1,205 | -0.58 | 1.10E-13 | Actin |
| GRMZM2G110406 | 79 | 202 | -0.76 | 3.57E-04 | Guanine nucleotide exchange factor, putative, expressed |
| GRMZM2G110483 | 16 | 55 | -1.19 | 1.25E-02 | Chromosome chr14 scaffold_21, whole genome shotgun sequence |
| GRMZM2G110509 | 178 | 380 | -0.50 | 7.58E-04 | Elongation factor 1-alpha |
| GRMZM2G110548 | 90 | 245 | -0.85 | 9.47E-06 | Enhancer of polycomb-like protein |
| GRMZM2G110646 | 314 | 306 | 0.63 | 1.01E-06 | CDP-diacylglycerol--inositol 3-phosphatidyltransferase 1 (Putative uncharacterized protein) |
| GRMZM2G110714 | 689 | 222 | 2.23 | 7.73E-104 | Putative uncharacterized protein |
| GRMZM2G110834 | 52 | 121 | -0.63 | 3.62E-02 | Putative uncharacterized protein |
| GRMZM2G110843 | 282 | 196 | 1.12 | 2.13E-15 | Putative uncharacterized protein |
| GRMZM2G110851 | 31 | 100 | -1.10 | 8.44E-04 | Putative uncharacterized protein (Putative fertility restorer) |
| GRMZM2G111014 | 221 | 553 | -0.73 | 1.18E-09 | Putative uncharacterized protein |
| GRMZM2G111045 | 80 | 36 | 1.74 | 8.03E-09 | Putative uncharacterized protein |
| GRMZM2G111123 | 79 | 224 | -0.91 | 6.58E-06 | Putative uncharacterized protein |
| GRMZM2G111172 | 342 | 947 | -0.88 | 5.71E-22 | 40S ribosomal protein S15 (Putative uncharacterized protein) |
| GRMZM2G111191 | 94 | 206 | -0.54 | 1.35E-02 | Os03g0209500 protein (Putative uncharacterized protein) (cDNA clone:J033105H03, full insert sequence) (Zinc finger family protein, putative, expressed) |
| GRMZM2G111214 | 74 | 165 | -0.57 | 2.16E-02 | Golgi transport 1 protein B (Putative uncharacterized protein) |
| GRMZM2G111269 | 161 | 173 | 0.49 | 1.13E-02 | ATP binding protein (Putative uncharacterized protein) |
| GRMZM2G111324 | 90 | 91 | 0.58 | 3.46E-02 | Putative uncharacterized protein |
| GRMZM2G111411 | 446 | 461 | 0.54 | 2.66E-07 | Ran-binding protein 1 |
| GRMZM2G111436 | 303 | 279 | 0.71 | 5.96E-08 | Werner syndrome ATP-dependent helicase |
| GRMZM2G111451 | 98 | 230 | -0.64 | 1.37E-03 | Putative uncharacterized protein |
| GRMZM2G111472 | 38 | 126 | -1.14 | 6.84E-05 | Putative uncharacterized protein |
| GRMZM2G111510 | 184 | 586 | -1.08 | 4.04E-19 | Putative uncharacterized protein |
| GRMZM2G111566 | 286 | 281 | 0.62 | 4.95E-06 | Proteasome subunit beta type 2 (Putative uncharacterized protein) |
| GRMZM2G111579 | 96 | 278 | -0.94 | 1.54E-07 | Putative uncharacterized protein |
| GRMZM2G111593 | 79 | 64 | 0.90 | 1.59E-03 | Putative uncharacterized protein |
| GRMZM2G111632 | 97 | 88 | 0.73 | 4.25E-03 | Phytol kinase 2 |
| GRMZM2G111672 | 38 | 118 | -1.04 | 4.48E-04 | Putative uncharacterized protein |
| GRMZM2G111679 | 33 | 97 | -0.96 | 3.92E-03 | Putative uncharacterized protein |
| GRMZM2G111713 | 92 | 269 | -0.96 | 1.62E-07 | Putative uncharacterized protein (Tim44-like domain containing protein) |
| GRMZM2G111720 | 322 | 352 | 0.46 | 3.02E-04 | Putative uncharacterized protein |
| GRMZM2G111756 | 67 | 151 | -0.58 | 2.62E-02 | ATP-dependent Clp protease proteolytic subunit |
| GRMZM2G111760 | 143 | 104 | 1.05 | 2.58E-07 | Putative uncharacterized protein |
| GRMZM2G111780 | 5 | 31 | -2.04 | 5.95E-03 | Putative uncharacterized protein |
| GRMZM2G111782 | 72 | 62 | 0.81 | 7.95E-03 | Protein binding protein |
| GRMZM2G111818 | 332 | 648 | -0.37 | 8.82E-04 | Ubiquitin-activating enzyme E1 |
| GRMZM2G111872 | 146 | 493 | -1.16 | 4.07E-18 | Putative uncharacterized protein |
| GRMZM2G111923 | 134 | 100 | 1.01 | 1.42E-06 | Putative uncharacterized protein |
| GRMZM2G111954 | 22 | 10 | 1.73 | 9.10E-03 | Putative uncharacterized protein |
| GRMZM2G112072 | 66 | 158 | -0.67 | 7.47E-03 | Mitotic spindle checkpoint protein MAD2 (Putative uncharacterized protein) |
| GRMZM2G112074 | 51 | 124 | -0.69 | 1.68E-02 | Putative uncharacterized protein |
| GRMZM2G112149 | 259 | 245 | 0.67 | 2.61E-06 | Putative uncharacterized protein |
| GRMZM2G112165 | 3,082 | 6,023 | -0.38 | 1.18E-30 | Os09g0482100 protein |
| GRMZM2G112176 | 4 | 25 | -2.05 | 1.86E-02 | Putative uncharacterized protein |
| GRMZM2G112204 | 64 | 41 | 1.23 | 1.38E-04 | Putative uncharacterized protein orf105-e |
| GRMZM2G112240 | 141 | 306 | -0.53 | 1.97E-03 | Putative uncharacterized protein |
| GRMZM2G112285 | 8 | 39 | -1.69 | 5.64E-03 | Putative uncharacterized protein |
| GRMZM2G112337 | 171 | 140 | 0.88 | 1.18E-06 | Putative uncharacterized protein |
| GRMZM2G112352 | 102 | 315 | -1.04 | 8.84E-10 | Putative uncharacterized protein |
| GRMZM2G112366 | 262 | 115 | 1.78 | 5.14E-29 | Putative uncharacterized protein (Signal peptidase complex subunit 1) |
| GRMZM2G112392 | 291 | 200 | 1.13 | 2.44E-16 | Putative uncharacterized protein |
| GRMZM2G112425 | 78 | 205 | -0.80 | 1.77E-04 | Putative uncharacterized protein |
| GRMZM2G112429 | 45 | 120 | -0.82 | 4.89E-03 | Esterase/lipase/thioesterase |
| GRMZM2G112470 | 100 | 253 | -0.75 | 7.49E-05 | Putative uncharacterized protein |
| GRMZM2G112535 | 99 | 239 | -0.68 | 4.88E-04 | cDNA clone:006-210-D10, full insert sequence (cDNA clone:006-303-D08, full insert sequence) (cDNA clone:J023055M24, full insert sequence) (Os01g0706000 protein) (Transcriptional coactivator-like) |
| GRMZM2G112609 | 4 | 29 | -2.27 | 5.95E-03 | Os03g0852200 protein (cDNA clone:J013132M16, full insert sequence) |
| GRMZM2G112640 | 65 | 174 | -0.83 | 4.23E-04 | Protein translocase/ protein transporter |
| GRMZM2G112686 | 139 | 70 | 1.58 | 3.11E-13 | Esterase |
| GRMZM2G112764 | 144 | 134 | 0.70 | 5.22E-04 | DNA binding protein (Putative uncharacterized protein) |
| GRMZM2G112782 | 26 | 96 | -1.29 | 1.58E-04 | Putative uncharacterized protein |
| GRMZM2G112805 | 148 | 319 | -0.52 | 1.86E-03 | Putative uncharacterized protein |
| GRMZM2G112912 | 550 | 348 | 1.25 | 6.13E-36 | Histone H2B |
| GRMZM2G112956 | 565 | 543 | 0.65 | 2.67E-12 | NADH dehydrogenase subunit 2 |
| GRMZM2G112965 | 13 | 3 | 2.71 | 6.90E-03 | Putative uncharacterized protein |
| GRMZM2G113016 | 44 | 106 | -0.68 | 4.06E-02 | Putative uncharacterized protein |
| GRMZM2G113062 | 59 | 212 | -1.25 | 6.81E-09 | Methionine aminopeptidase (EC 3.4.11.18) |
| GRMZM2G113098 | 14 | 6 | 1.81 | 4.24E-02 | GATA transcription factor 9 (Putative uncharacterized protein) |
| GRMZM2G113156 | 147 | 359 | -0.70 | 5.41E-06 | Putative uncharacterized protein |
| GRMZM2G113159 | 282 | 189 | 1.17 | 1.12E-16 | Cp protein |
| GRMZM2G113196 | 137 | 98 | 1.07 | 2.47E-07 | Putative uncharacterized protein |
| GRMZM2G113202 | 28 | 108 | -1.36 | 2.79E-05 | Myosin VIII ZMM3 (Fragment) |
| GRMZM2G113228 | 35 | 161 | -1.61 | 8.85E-10 | Putative uncharacterized protein |
| GRMZM2G113253 | 138 | 357 | -0.78 | 4.75E-07 | Putative uncharacterized protein |
| GRMZM2G113257 | 24 | 4 | 3.18 | 1.64E-05 | DNA binding protein |
| GRMZM2G113332 | 1,617 | 1,409 | 0.79 | 7.43E-49 | Putative uncharacterized protein |
| GRMZM2G113351 | 23 | 89 | -1.36 | 1.59E-04 | Putative uncharacterized protein |
| GRMZM2G113364 | 23 | 64 | -0.89 | 4.73E-02 | Programmed cell death protein 2 |
| GRMZM2G113372 | 33 | 25 | 0.99 | 4.37E-02 | NA |
| GRMZM2G113373 | 93 | 249 | -0.83 | 1.22E-05 | Putative uncharacterized protein |
| GRMZM2G113408 | 786 | 1,341 | -0.18 | 2.63E-02 | Putative uncharacterized protein |
| GRMZM2G113414 | 180 | 357 | -0.40 | 1.40E-02 | Protein translation factor SUI1 |
| GRMZM2G113506 | 68 | 269 | -1.39 | 4.94E-13 | Putative uncharacterized protein (cDNA, clone: J100048D21, full insert sequence) (Glycosyl transferase family 8 protein, expressed) |
| GRMZM2G113592 | 316 | 924 | -0.96 | 1.37E-24 | U1 snRNP |
| GRMZM2G113613 | 137 | 303 | -0.55 | 1.25E-03 | Os06g0687800 protein (Putative uncharacterized protein) (cDNA clone:J023133F22, full insert sequence) (Putative receptor-mediated endocytosis 1 isoform I) |
| GRMZM2G113619 | 128 | 121 | 0.67 | 1.90E-03 | Putative uncharacterized protein |
| GRMZM2G113626 | 54 | 134 | -0.72 | 9.41E-03 | Putative uncharacterized protein |
| GRMZM2G113629 | 26 | 124 | -1.66 | 7.62E-08 | Protein disulfide isomerase |
| GRMZM2G113652 | 219 | 450 | -0.45 | 1.09E-03 | Kinesin-4 |
| GRMZM2G113668 | 41 | 22 | 1.49 | 6.42E-04 | Os03g0857600 protein (cDNA clone:006-206-D05, full insert sequence) (cDNA clone:J023117O12, full insert sequence) (Expressed protein) (Putative uncharacterized protein OSJNBa0059G06.14) |
| GRMZM2G113696 | 2,157 | 3,743 | -0.20 | 2.17E-06 | Eukaryotic translation initiation factor 5A |
| GRMZM2G113720 | 1,114 | 1,892 | -0.17 | 8.60E-03 | 60S ribosomal protein L18a |
| GRMZM2G113742 | 98 | 53 | 1.48 | 1.22E-08 | Myb family transcription factor-related protein (Putative uncharacterized protein) |
| GRMZM2G113775 | 35 | 99 | -0.91 | 6.09E-03 | Putative uncharacterized protein |
| GRMZM2G113815 | 34 | 22 | 1.22 | 1.25E-02 | Polygalacturonase |
| GRMZM2G113863 | 647 | 502 | 0.96 | 3.64E-27 | Copper chaperone |
| GRMZM2G113873 | 165 | 504 | -1.02 | 4.51E-15 | Putative uncharacterized protein |
| GRMZM2G113899 | 196 | 508 | -0.78 | 6.54E-10 | Os08g0496900 protein (Putative transmembrane 9 superfamily protein member 2) |
| GRMZM2G113964 | 245 | 213 | 0.79 | 8.15E-08 | Putative uncharacterized protein |
| GRMZM2G113967 | 57 | 206 | -1.26 | 8.49E-09 | CBL-interacting serine/threonine-protein kinase 15 (Putative uncharacterized protein) |
| GRMZM2G114008 | 19 | 65 | -1.18 | 6.50E-03 | Putative uncharacterized protein |
| GRMZM2G114057 | 83 | 204 | -0.71 | 1.02E-03 | Putative uncharacterized protein |
| GRMZM2G114071 | 502 | 410 | 0.88 | 1.70E-18 | Transposon protein Mutator sub-class |
| GRMZM2G114098 | 50 | 120 | -0.67 | 2.75E-02 | Os06g0138200 protein (Oxidoreductase, 2OG-Fe(II) oxygenase family protein-like) |
| GRMZM2G114107 | 50 | 135 | -0.84 | 2.04E-03 | Putative uncharacterized protein P0478E02.2 |
| GRMZM2G114172 | 96 | 100 | 0.53 | 4.44E-02 | Putative uncharacterized protein |
| GRMZM2G114220 | 16 | 75 | -1.64 | 7.71E-05 | Ubiquitin fusion degradation protein 1 |
| GRMZM2G114557 | 144 | 155 | 0.49 | 1.88E-02 | Peptidase/ serine-type peptidase |
| GRMZM2G114570 | 58 | 48 | 0.86 | 1.42E-02 | Putative uncharacterized protein |
| GRMZM2G114584 | 238 | 229 | 0.65 | 1.54E-05 | Putative uncharacterized protein |
| GRMZM2G114650 | 6 | 31 | -1.78 | 1.82E-02 | Putative uncharacterized protein |
| GRMZM2G114688 | 20 | 58 | -0.94 | 4.45E-02 | Putative uncharacterized protein |
| GRMZM2G114692 | 123 | 431 | -1.22 | 4.95E-17 | Putative uncharacterized protein |
| GRMZM2G114702 | 55 | 142 | -0.78 | 3.76E-03 | Putative uncharacterized protein |
| GRMZM2G114706 | 274 | 299 | 0.47 | 9.15E-04 | Ankyrin protein kinase-like |
| GRMZM2G114739 | 60 | 146 | -0.69 | 9.30E-03 | Sulfite oxidase |
| GRMZM2G114748 | 26 | 78 | -0.99 | 9.86E-03 | Putative uncharacterized protein |
| GRMZM2G114758 | 70 | 170 | -0.69 | 3.77E-03 | Putative uncharacterized protein |
| GRMZM2G114816 | 50 | 130 | -0.79 | 4.76E-03 | Putative uncharacterized protein |
| GRMZM2G114861 | 162 | 372 | -0.61 | 5.19E-05 | Kinesin-1 |
| GRMZM2G114888 | 206 | 477 | -0.62 | 2.06E-06 | Structural constituent of ribosome |
| GRMZM2G114930 | 39 | 98 | -0.74 | 3.03E-02 | Putative uncharacterized protein |
| GRMZM2G114954 | 88 | 274 | -1.05 | 9.57E-09 | Putative uncharacterized protein |
| GRMZM2G115047 | 39 | 162 | -1.46 | 1.49E-08 | Ankyrin-3 (Putative uncharacterized protein) |
| GRMZM2G115152 | 12 | 58 | -1.68 | 5.57E-04 | Putative uncharacterized protein |
| GRMZM2G115182 | 71 | 223 | -1.06 | 2.61E-07 | Putative uncharacterized protein |
| GRMZM2G115209 | 150 | 170 | 0.41 | 4.93E-02 | Putative uncharacterized protein |
| GRMZM2G115243 | 259 | 600 | -0.62 | 6.11E-08 | 14-3-3-like protein (Putative uncharacterized protein) |
| GRMZM2G115304 | 67 | 148 | -0.55 | 4.17E-02 | Putative uncharacterized protein |
| GRMZM2G115329 | 81 | 50 | 1.29 | 7.53E-06 | Putative uncharacterized protein |
| GRMZM2G115342 | 56 | 172 | -1.03 | 1.52E-05 | Putative uncharacterized protein |
| GRMZM2G115400 | 102 | 263 | -0.78 | 2.53E-05 | Putative UDP-glucose:glycoprotein glucosyltransferase |
| GRMZM2G115420 | 78 | 189 | -0.69 | 2.38E-03 | Putative uncharacterized protein |
| GRMZM2G115424 | 151 | 316 | -0.47 | 4.77E-03 | PHD finger protein (Putative uncharacterized protein) |
| GRMZM2G115437 | 65 | 268 | -1.45 | 8.93E-14 | Protein binding protein |
| GRMZM2G115456 | 171 | 364 | -0.50 | 1.24E-03 | Putative uncharacterized protein |
| GRMZM2G115579 | 73 | 183 | -0.73 | 1.18E-03 | Putative uncharacterized protein |
| GRMZM2G115621 | 246 | 257 | 0.53 | 4.04E-04 | NADH ubiquinone oxidoreductase B22-like subunit |
| GRMZM2G115633 | 84 | 230 | -0.86 | 1.71E-05 | NA |
| GRMZM2G115635 | 927 | 398 | 1.81 | 8.77E-106 | Permease (Putative uncharacterized protein) |
| GRMZM2G115674 | 113 | 408 | -1.26 | 6.36E-17 | Putative uncharacterized protein |
| GRMZM2G115750 | 740 | 710 | 0.65 | 4.40E-16 | Putative uncharacterized protein |
| GRMZM2G115755 | 65 | 176 | -0.85 | 2.72E-04 | cDNA clone:J013093F08, full insert sequence |
| GRMZM2G115757 | 151 | 115 | 0.98 | 5.74E-07 | FK506-binding protein 2-1 |
| GRMZM2G115766 | 162 | 143 | 0.77 | 3.65E-05 | Putative uncharacterized protein |
| GRMZM2G115773 | 6 | 0 | #VALUE! | 1.98E-02 | Os04g0559000 protein (Putative uncharacterized protein) (OSJNBa0065O17.13 protein) |
| GRMZM2G115775 | 94 | 224 | -0.66 | 1.11E-03 | Putative uncharacterized protein |
| GRMZM2G115812 | 215 | 679 | -1.07 | 1.29E-21 | Putative uncharacterized protein |
| GRMZM2G115817 | 72 | 163 | -0.59 | 2.00E-02 | Circadian clock coupling factor ZGT (Putative uncharacterized protein) |
| GRMZM2G115828 | 281 | 653 | -0.63 | 1.08E-08 | Ubiquitin carrier protein (EC 6.3.2.-) |
| GRMZM2G115834 | 87 | 211 | -0.69 | 1.08E-03 | Putative uncharacterized protein |
| GRMZM2G115875 | 35 | 92 | -0.80 | 2.29E-02 | Putative uncharacterized protein |
| GRMZM2G115895 | 13 | 5 | 1.97 | 3.14E-02 | NA |
| GRMZM2G115901 | 206 | 938 | -1.60 | 5.35E-55 | 60S ribosomal protein L32 (Putative uncharacterized protein) |
| GRMZM2G115925 | 208 | 434 | -0.47 | 7.09E-04 | Putative uncharacterized protein (Small nuclear ribonucleoprotein Sm D3) |
| GRMZM2G115939 | 207 | 170 | 0.88 | 7.30E-08 | Ubiquitin carrier protein (EC 6.3.2.-) |
| GRMZM2G115975 | 98 | 231 | -0.65 | 1.15E-03 | Rho GTPase activator |
| GRMZM2G116086 | 182 | 692 | -1.34 | 2.86E-31 | Putative uncharacterized protein (cDNA clone:J023029O03, full insert sequence) (Os01g0772600 protein) (Putative serine/threonine protein kinase) |
| GRMZM2G116126 | 151 | 356 | -0.65 | 3.01E-05 | Putative uncharacterized protein |
| GRMZM2G116133 | 12 | 76 | -2.07 | 1.30E-06 | Exosome complex exonuclease RRP42 |
| GRMZM2G116135 | 4,642 | 3,264 | 1.10 | 2.98E-246 | Ribosomal protein L19 |
| GRMZM2G116140 | 52 | 128 | -0.71 | 1.22E-02 | Ripening-related protein |
| GRMZM2G116196 | 52 | 175 | -1.16 | 1.30E-06 | Putative uncharacterized protein |
| GRMZM2G116282 | 2,167 | 1,672 | 0.97 | 1.69E-92 | Plasminogen activator inhibitor 1 RNA-binding protein |
| GRMZM2G116327 | 38 | 114 | -0.99 | 9.66E-04 | CrcB-like protein (Putative uncharacterized protein) |
| GRMZM2G116362 | 6 | 49 | -2.44 | 3.14E-05 | Zinc finger, C3HC4 type family protein |
| GRMZM2G116614 | 91 | 38 | 1.85 | 7.73E-11 | Putative uncharacterized protein |
| GRMZM2G116634 | 53 | 153 | -0.94 | 2.14E-04 | ABC transporter family protein, expressed |
| GRMZM2G116638 | 39 | 124 | -1.08 | 1.80E-04 | Chitin-inducible gibberellin-responsive protein 2, putative, expressed |
| GRMZM2G116689 | 347 | 798 | -0.61 | 4.22E-10 | Polyubiquitin 2 (Putative uncharacterized protein) |
| GRMZM2G116752 | 9 | 43 | -1.66 | 3.77E-03 | Putative uncharacterized protein |
| GRMZM2G116774 | 759 | 623 | 0.88 | 2.11E-27 | 60S ribosomal protein L33-B |
| GRMZM2G116812 | 4 | 23 | -1.93 | 3.97E-02 | Putative uncharacterized protein |
| GRMZM2G116885 | 78 | 171 | -0.54 | 2.95E-02 | Putative uncharacterized protein |
| GRMZM2G117064 | 55 | 145 | -0.81 | 2.00E-03 | Putative uncharacterized protein |
| GRMZM2G117198 | 1,003 | 2,040 | -0.43 | 1.07E-13 | S-adenosylmethionine synthetase (EC 2.5.1.6) |
| GRMZM2G117207 | 87 | 185 | -0.50 | 3.43E-02 | Protein YIP1 |
| GRMZM2G117238 | 71 | 278 | -1.38 | 2.63E-13 | Origin recognition complex subunit 2 |
| GRMZM2G117240 | 214 | 454 | -0.49 | 2.79E-04 | Homocysteine S-methyltransferase 3 (Putative uncharacterized protein) |
| GRMZM2G117298 | 98 | 284 | -0.94 | 1.18E-07 | Putative uncharacterized protein |
| GRMZM2G117401 | 228 | 483 | -0.49 | 1.60E-04 | Putative uncharacterized protein |
| GRMZM2G117439 | 62 | 171 | -0.87 | 2.62E-04 | Putative uncharacterized protein |
| GRMZM2G117458 | 135 | 381 | -0.91 | 1.57E-09 | Putative uncharacterized protein |
| GRMZM2G117459 | 62 | 142 | -0.60 | 2.48E-02 | Putative uncharacterized protein |
| GRMZM2G117507 | 458 | 420 | 0.72 | 6.12E-12 | Os05g0304400 protein (Putative uncharacterized protein) (cDNA clone:J033023F13, full insert sequence) |
| GRMZM2G117544 | 139 | 309 | -0.56 | 8.39E-04 | Putative uncharacterized protein |
| GRMZM2G117615 | 46 | 121 | -0.80 | 6.58E-03 | Putative uncharacterized protein |
| GRMZM2G117619 | 30 | 92 | -1.03 | 3.61E-03 | Putative uncharacterized protein |
| GRMZM2G117642 | 2,136 | 1,540 | 1.06 | 4.43E-107 | Putative uncharacterized protein |
| GRMZM2G117700 | 162 | 323 | -0.40 | 1.74E-02 | Putative uncharacterized protein |
| GRMZM2G117715 | 350 | 820 | -0.64 | 4.06E-11 | Putative uncharacterized protein |
| GRMZM2G117742 | 19 | 68 | -1.25 | 2.81E-03 | Putative uncharacterized protein |
| GRMZM2G117746 | 225 | 133 | 1.35 | 1.02E-16 | Peptidyl-prolyl isomerase PASTICCINO1 |
| GRMZM2G117755 | 122 | 85 | 1.11 | 7.07E-07 | Putative uncharacterized protein (Hypersensitive-induced response protein) |
| GRMZM2G117811 | 867 | 410 | 1.67 | 3.98E-88 | Putative uncharacterized protein |
| GRMZM2G117854 | 217 | 158 | 1.05 | 7.30E-11 | Putative uncharacterized protein |
| GRMZM2G117870 | 651 | 437 | 1.17 | 5.36E-38 | Os03g0694900 protein (Putative valyl tRNA synthetase) (Valyl-tRNA synthetase, putative, expressed) |
| GRMZM2G117935 | 85 | 205 | -0.68 | 1.40E-03 | Putative uncharacterized protein (Superal1) |
| GRMZM2G117961 | 41 | 219 | -1.83 | 1.43E-15 | MADS-box transcription factor 26 |
| GRMZM2G117978 | 19 | 82 | -1.52 | 8.77E-05 | NA |
| GRMZM2G117984 | 47 | 221 | -1.64 | 1.62E-13 | Putative uncharacterized protein |
| GRMZM2G117993 | 52 | 136 | -0.80 | 3.69E-03 | Putative uncharacterized protein |
| GRMZM2G118014 | 357 | 642 | -0.26 | 3.28E-02 | Putative uncharacterized protein |
| GRMZM2G118063 | 110 | 268 | -0.69 | 1.43E-04 | Putative uncharacterized protein |
| GRMZM2G118098 | 119 | 309 | -0.79 | 2.61E-06 | Putative uncharacterized protein |
| GRMZM2G118106 | 14 | 4 | 2.40 | 7.64E-03 | Os12g0179800 protein (Transcriptional regulator, Sir2 family protein, expressed) |
| GRMZM2G118113 | 202 | 506 | -0.73 | 6.69E-09 | Putative uncharacterized protein |
| GRMZM2G118208 | 67 | 174 | -0.79 | 7.62E-04 | Putative uncharacterized protein (Phosphate carrier protein, mitochondrial, putative, expressed) |
| GRMZM2G118241 | 3 | 34 | -2.91 | 2.44E-04 | Putative uncharacterized protein |
| GRMZM2G118265 | 179 | 76 | 1.83 | 8.59E-21 | cDNA clone:J013020F11, full insert sequence (Copine I-like) (Os01g0960500 protein) |
| GRMZM2G118316 | 75 | 187 | -0.73 | 1.34E-03 | Putative uncharacterized protein |
| GRMZM2G118344 | 151 | 156 | 0.54 | 6.27E-03 | Putative uncharacterized protein |
| GRMZM2G118355 | 871 | 901 | 0.54 | 1.07E-13 | Histone H3 |
| GRMZM2G118362 | 187 | 362 | -0.36 | 2.47E-02 | Putative uncharacterized protein |
| GRMZM2G118363 | 82 | 235 | -0.93 | 2.72E-06 | Putative uncharacterized protein (Os01g0825700 protein) (Putative VHS2 protein) |
| GRMZM2G118403 | 115 | 245 | -0.50 | 1.14E-02 | Putative uncharacterized protein |
| GRMZM2G118453 | 1 | 16 | -3.41 | 1.35E-02 | Heat shock transcription factor |
| GRMZM2G118459 | 215 | 573 | -0.82 | 4.30E-12 | Putative uncharacterized protein |
| GRMZM2G118462 | 55 | 138 | -0.74 | 6.76E-03 | Putative uncharacterized protein |
| GRMZM2G118571 | 70 | 226 | -1.10 | 7.58E-08 | Protein Z |
| GRMZM2G118579 | 112 | 30 | 2.49 | 1.78E-19 | Putative uncharacterized protein |
| GRMZM2G118590 | 226 | 494 | -0.54 | 2.94E-05 | Putative uncharacterized protein |
| GRMZM2G118637 | 568 | 1,901 | -1.15 | 2.72E-68 | Polyubiquitin containing 7 ubiquitin monomers (Ubiquitin) |
| GRMZM2G118644 | 69 | 174 | -0.74 | 1.63E-03 | Putative uncharacterized protein |
| GRMZM2G118687 | 140 | 134 | 0.65 | 1.45E-03 | Peptidyl-prolyl cis-trans isomerase (EC 5.2.1.8) |
| GRMZM2G118743 | 111 | 252 | -0.59 | 1.81E-03 | Putative uncharacterized protein |
| GRMZM2G118825 | 31 | 18 | 1.38 | 6.63E-03 | Tetratricopeptide repeat protein 15 |
| GRMZM2G118939 | 23 | 73 | -1.07 | 6.77E-03 | BRASSINOSTEROID INSENSITIVE 1-associated receptor kinase 1 |
| GRMZM2G119071 | 2,607 | 2,560 | 0.62 | 6.77E-51 | Histone H2B |
| GRMZM2G119079 | 185 | 164 | 0.77 | 9.34E-06 | Protein-tyrosine phosphatase mitochondrial 1 |
| GRMZM2G119146 | 170 | 555 | -1.12 | 4.60E-19 | Nucleic acid binding protein |
| GRMZM2G119169 | 330 | 845 | -0.77 | 1.23E-15 | Putative uncharacterized protein |
| GRMZM2G119175 | 276 | 759 | -0.87 | 2.92E-17 | Pyruvate kinase (EC 2.7.1.40) |
| GRMZM2G119256 | 85 | 248 | -0.95 | 6.83E-07 | CMP-KDO synthetase (EC 2.7.7.38) |
| GRMZM2G119258 | 264 | 219 | 0.86 | 1.66E-09 | Ethylene response protein |
| GRMZM2G119287 | 342 | 132 | 1.96 | 1.31E-43 | Putative uncharacterized protein |
| GRMZM2G119300 | 109 | 241 | -0.55 | 5.10E-03 | Putative uncharacterized protein |
| GRMZM2G119303 | 43 | 120 | -0.89 | 2.68E-03 | Putative uncharacterized protein |
| GRMZM2G119311 | 254 | 172 | 1.15 | 1.07E-14 | Putative uncharacterized protein |
| GRMZM2G119345 | 22 | 79 | -1.25 | 1.09E-03 | ABC-2 type transporter family protein |
| GRMZM2G119357 | 12 | 53 | -1.55 | 2.18E-03 | DNA binding protein (Putative uncharacterized protein) |
| GRMZM2G119361 | 33 | 17 | 1.55 | 1.66E-03 | Putative uncharacterized protein |
| GRMZM2G119383 | 133 | 104 | 0.95 | 6.60E-06 | Putative uncharacterized protein |
| GRMZM2G119393 | 196 | 162 | 0.87 | 2.54E-07 | DNA-directed RNA polymerase II 16 kDa polypeptide (Putative uncharacterized protein) |
| GRMZM2G119426 | 141 | 82 | 1.37 | 8.08E-11 | Putative uncharacterized protein |
| GRMZM2G119471 | 190 | 144 | 0.99 | 7.92E-09 | 50S ribosomal protein L5 |
| GRMZM2G119482 | 48 | 117 | -0.69 | 2.46E-02 | Putative uncharacterized protein |
| GRMZM2G119485 | 25 | 80 | -1.09 | 4.04E-03 | Putative uncharacterized protein |
| GRMZM2G119494 | 191 | 146 | 0.98 | 1.26E-08 | Glycoside hydrolase, family 28 (Putative uncharacterized protein) |
| GRMZM2G119536 | 74 | 234 | -1.07 | 1.03E-07 | Os06g0694000 protein (Putative uncharacterized protein) |
| GRMZM2G119546 | 102 | 89 | 0.79 | 1.45E-03 | Serine/threonine protein phosphatase (EC 3.1.3.16) |
| GRMZM2G119583 | 81 | 180 | -0.56 | 1.79E-02 | Os05g0129100 protein (Putative acetylornithine aminotransferase) |
| GRMZM2G119640 | 240 | 272 | 0.41 | 7.64E-03 | Zinc finger C-x8-C-x5-C-x3-H type family protein |
| GRMZM2G119650 | 82 | 298 | -1.27 | 1.47E-12 | F-box domain containing protein |
| GRMZM2G119725 | 50 | 168 | -1.16 | 2.18E-06 | Lysine ketoglutarate reductase trans-splicing related 1 (Putative uncharacterized protein) |
| GRMZM2G119745 | 186 | 189 | 0.57 | 1.06E-03 | Os07g0632600 protein (Putative uncharacterized protein P0519E12.120) |
| GRMZM2G119761 | 135 | 391 | -0.94 | 2.23E-10 | Putative uncharacterized protein |
| GRMZM2G119773 | 121 | 470 | -1.37 | 5.18E-22 | Putative uncharacterized protein |
| GRMZM2G119778 | 66 | 168 | -0.76 | 1.62E-03 | Putative uncharacterized protein |
| GRMZM2G119782 | 573 | 1,086 | -0.33 | 7.82E-05 | Membrane steroid-binding protein 1 (Putative uncharacterized protein) |
| GRMZM2G119791 | 36 | 122 | -1.17 | 6.57E-05 | Putative uncharacterized protein |
| GRMZM2G119809 | 641 | 1,274 | -0.40 | 1.30E-07 | 60S acidic ribosomal protein P2A |
| GRMZM2G119930 | 69 | 170 | -0.71 | 2.94E-03 | Putative uncharacterized protein (Zinc finger, RING-type) |
| GRMZM2G120035 | 91 | 69 | 0.99 | 1.50E-04 | Gtk16 protein |
| GRMZM2G120047 | 215 | 217 | 0.58 | 3.00E-04 | Proteasome subunit alpha type (EC 3.4.25.1) |
| GRMZM2G120085 | 184 | 382 | -0.46 | 2.10E-03 | Putative uncharacterized protein |
| GRMZM2G120115 | 96 | 232 | -0.68 | 5.95E-04 | Syntaxin 23 |
| GRMZM2G120225 | 71 | 207 | -0.95 | 7.32E-06 | Os01g0243100 protein (Fragment) |
| GRMZM2G120271 | 121 | 276 | -0.60 | 8.80E-04 | Putative uncharacterized protein |
| GRMZM2G120304 | 27 | 101 | -1.31 | 7.67E-05 | Putative uncharacterized protein |
| GRMZM2G120371 | 21 | 67 | -1.08 | 1.15E-02 | G protein-coupled receptor |
| GRMZM2G120432 | 303 | 601 | -0.40 | 6.35E-04 | 40S ribosomal protein S14 (Putative uncharacterized protein) |
| GRMZM2G120539 | 5 | 0 | #VALUE! | 4.27E-02 | AMP binding protein |
| GRMZM2G120572 | 45 | 152 | -1.16 | 7.14E-06 | Putative uncharacterized protein |
| GRMZM2G120652 | 283 | 332 | 0.36 | 1.22E-02 | Putative uncharacterized protein |
| GRMZM2G120657 | 107 | 82 | 0.98 | 4.97E-05 | Putative uncharacterized protein |
| GRMZM2G120750 | 443 | 475 | 0.49 | 4.36E-06 | Os08g0295300 protein (cDNA clone:J023052L01, full insert sequence) (Putative threonyl-tRNA synthetase) |
| GRMZM2G120814 | 144 | 125 | 0.80 | 6.63E-05 | SnRK1-interacting protein 1 |
| GRMZM2G120816 | 42 | 143 | -1.18 | 1.03E-05 | RING-finger protein like |
| GRMZM2G120870 | 41 | 125 | -1.02 | 3.93E-04 | Sentrin-specific protease 2 |
| GRMZM2G120938 | 12 | 1 | 4.18 | 9.96E-04 | Male sterility protein 2 |
| GRMZM2G120954 | 80 | 72 | 0.74 | 8.87E-03 | Putative uncharacterized protein |
| GRMZM2G121022 | 349 | 710 | -0.43 | 3.54E-05 | Putative uncharacterized protein |
| GRMZM2G121063 | 34 | 102 | -0.99 | 2.11E-03 | Putative uncharacterized protein |
| GRMZM2G121075 | 1,413 | 2,743 | -0.37 | 1.71E-13 | 60S ribosomal protein L12 (Putative uncharacterized protein) |
| GRMZM2G121117 | 35 | 26 | 1.02 | 2.76E-02 | Putative uncharacterized protein OJ1014_C08.6 |
| GRMZM2G121123 | 249 | 295 | 0.35 | 2.68E-02 | Os04g0112300 protein (cDNA clone:J033005K19, full insert sequence) (cDNA clone:J033099O19, full insert sequence) (OSJNBb0021I10.2 protein) (OSJNBb0060M15.9 protein) |
| GRMZM2G121150 | 65 | 165 | -0.75 | 1.85E-03 | Putative uncharacterized protein |
| GRMZM2G121166 | 52 | 134 | -0.77 | 5.58E-03 | Putative uncharacterized protein |
| GRMZM2G121186 | 309 | 317 | 0.55 | 1.92E-05 | Nucleosome/chromatin assembly factor group A |
| GRMZM2G121210 | 810 | 860 | 0.51 | 3.02E-11 | DNA topoisomerase 2 (EC 5.99.1.3) |
| GRMZM2G121221 | 1,952 | 2,233 | 0.40 | 4.17E-17 | Histone H1 |
| GRMZM2G121223 | 90 | 268 | -0.98 | 9.95E-08 | Putative uncharacterized protein |
| GRMZM2G121237 | 33 | 96 | -0.95 | 4.96E-03 | Putative uncharacterized protein |
| GRMZM2G121262 | 291 | 545 | -0.31 | 1.39E-02 | Putative uncharacterized protein |
| GRMZM2G121303 | 44 | 143 | -1.11 | 3.23E-05 | Ubiquitinating enzyme |
| GRMZM2G121309 | 567 | 1,132 | -0.41 | 4.83E-07 | Putative uncharacterized protein |
| GRMZM2G121404 | 42 | 115 | -0.86 | 4.75E-03 | Putative 5-3 exoribonuclease |
| GRMZM2G121456 | 66 | 200 | -1.01 | 4.12E-06 | ATP-dependent Clp protease proteolytic subunit |
| GRMZM2G121495 | 161 | 124 | 0.97 | 3.10E-07 | Beta-galactosidase (EC 3.2.1.23) |
| GRMZM2G121776 | 65 | 152 | -0.63 | 1.45E-02 | Putative uncharacterized protein |
| GRMZM2G121790 | 171 | 400 | -0.63 | 10.00E-06 | Putative uncharacterized protein (Shaggy-related protein kinase eta) |
| GRMZM2G121820 | 589 | 705 | 0.33 | 3.61E-04 | Putative uncharacterized protein |
| GRMZM2G121826 | 77 | 251 | -1.11 | 7.76E-09 | Putative uncharacterized protein |
| GRMZM2G121840 | 63 | 155 | -0.71 | 5.21E-03 | Putative uncharacterized protein |
| GRMZM2G121942 | 159 | 309 | -0.37 | 4.02E-02 | Putative uncharacterized protein (Splicing factor 3B subunit 5) |
| GRMZM2G122108 | 45 | 32 | 1.08 | 6.23E-03 | Putative uncharacterized protein |
| GRMZM2G122116 | 82 | 228 | -0.88 | 1.12E-05 | Pentatricopeptide, putative, expressed (Putative chloroplast RNA processing protein) |
| GRMZM2G122135 | 247 | 597 | -0.68 | 3.49E-09 | Putative uncharacterized protein |
| GRMZM2G122139 | 7 | 64 | -2.60 | 4.71E-07 | Cytosolic purine 5-nucleotidase |
| GRMZM2G122145 | 304 | 879 | -0.94 | 9.10E-23 | PHD finger protein (Putative uncharacterized protein) |
| GRMZM2G122231 | 33 | 21 | 1.24 | 1.08E-02 | Trehalose-6-phosphate synthase |
| GRMZM2G122290 | 192 | 417 | -0.53 | 2.08E-04 | 50S ribosomal protein L1 |
| GRMZM2G122344 | 114 | 236 | -0.46 | 2.49E-02 | Putative uncharacterized protein (cDNA clone:J023013K01, full insert sequence) (Fertility restorer-like protein) (Os01g0546500 protein) |
| GRMZM2G122476 | 151 | 126 | 0.85 | 1.24E-05 | Putative uncharacterized protein |
| GRMZM2G122479 | 305 | 593 | -0.37 | 1.84E-03 | Malic enzyme |
| GRMZM2G122481 | 438 | 321 | 1.04 | 3.71E-21 | Cytochrome c oxidase polypeptide Vc (Putative uncharacterized protein) |
| GRMZM2G122563 | 16 | 1 | 4.59 | 4.55E-05 | Ribulose-1,5-bisphosphate carboxylase/oxygenase large subunit (Fragment) |
| GRMZM2G122607 | 12 | 45 | -1.32 | 1.99E-02 | WD-repeat domain phosphoinositide-interacting protein 3 |
| GRMZM2G122666 | 368 | 793 | -0.52 | 1.34E-07 | GTP-binding protein SAR1A (Putative uncharacterized protein) |
| GRMZM2G122715 | 39 | 26 | 1.18 | 7.69E-03 | Putative uncharacterized protein |
| GRMZM2G122750 | 92 | 286 | -1.04 | 4.33E-09 | Putative uncharacterized protein |
| GRMZM2G122767 | 312 | 1,068 | -1.18 | 4.70E-40 | T-complex protein 1, delta subunit |
| GRMZM2G122780 | 168 | 179 | 0.50 | 8.12E-03 | Putative uncharacterized protein |
| GRMZM2G122805 | 40 | 126 | -1.06 | 2.05E-04 | Putative uncharacterized protein (Ras-related protein Rab11D) |
| GRMZM2G122810 | 323 | 620 | -0.35 | 2.50E-03 | Cycloartenol-C-24-methyltransferase 1 (Putative uncharacterized protein) (Endosperm C-24 sterol methyltransferase) |
| GRMZM2G122863 | 48 | 122 | -0.75 | 9.38E-03 | Putative uncharacterized protein |
| GRMZM2G122869 | 93 | 213 | -0.60 | 4.25E-03 | Putative uncharacterized protein |
| GRMZM2G122871 | 1,452 | 2,805 | -0.36 | 2.43E-13 | Elongation factor 1-gamma 3 |
| GRMZM2G122937 | 26 | 69 | -0.82 | 4.93E-02 | Putative uncharacterized protein (Remorin) |
| GRMZM2G122999 | 312 | 346 | 0.44 | 7.29E-04 | Putative uncharacterized protein |
| GRMZM2G123140 | 117 | 249 | -0.50 | 1.05E-02 | Putative HD-ZIP IV family transcription factor OCL4 |
| GRMZM2G123159 | 322 | 317 | 0.61 | 1.25E-06 | N-acetyltransferase 5 (Silencing group B protein) |
| GRMZM2G123202 | 77 | 22 | 2.40 | 9.62E-13 | OSJNBb0039L24.12 protein |
| GRMZM2G123227 | 142 | 102 | 1.07 | 1.51E-07 | Putative uncharacterized protein |
| GRMZM2G123308 | 73 | 159 | -0.53 | 3.84E-02 | KANADI-like transcription factor FEATHERED |
| GRMZM2G123328 | 97 | 79 | 0.89 | 4.54E-04 | CCR4-NOT transcription complex subunit 8 (Putative uncharacterized protein) |
| GRMZM2G123355 | 71 | 41 | 1.38 | 7.20E-06 | Glycosyltransferase 6 |
| GRMZM2G123371 | 584 | 1,019 | -0.21 | 2.22E-02 | Pyrophosphate--fructose 6-phosphate 1-phosphotransferase alpha subunit |
| GRMZM2G123440 | 183 | 374 | -0.44 | 4.46E-03 | Putative uncharacterized protein |
| GRMZM2G123459 | 28 | 80 | -0.92 | 1.56E-02 | Putative uncharacterized protein |
| GRMZM2G123482 | 179 | 82 | 1.72 | 6.24E-19 | Os06g0639300 protein (cDNA clone:002-139-H06, full insert sequence) (Putative uncharacterized protein P0458E02.9) (Putative uncharacterized protein P0523F01.38) |
| GRMZM2G123495 | 794 | 644 | 0.89 | 1.11E-29 | 60S ribosomal protein L23 (Putative uncharacterized protein) |
| GRMZM2G123519 | 78 | 222 | -0.92 | 7.55E-06 | Os01g0232500 protein (Ubiquitin-conjugating enzyme-like) |
| GRMZM2G123527 | 502 | 546 | 0.47 | 2.13E-06 | Putative uncharacterized protein |
| GRMZM2G123534 | 45 | 134 | -0.98 | 3.85E-04 | NA |
| GRMZM2G123540 | 116 | 307 | -0.81 | 1.33E-06 | Putative uncharacterized protein |
| GRMZM2G123714 | 712 | 574 | 0.90 | 4.62E-27 | Glucan endo-1,3-beta-glucosidase 3 (Putative uncharacterized protein) |
| GRMZM2G123762 | 3 | 20 | -2.15 | 3.95E-02 | Os04g0166000 protein |
| GRMZM2G123796 | 65 | 59 | 0.73 | 2.67E-02 | RNA-binding region-containing protein 1 |
| GRMZM2G123807 | 129 | 132 | 0.56 | 1.04E-02 | Transport protein particle component, Bet3 containing protein |
| GRMZM2G123876 | 64 | 144 | -0.58 | 3.18E-02 | Putative uncharacterized protein |
| GRMZM2G123887 | 179 | 397 | -0.56 | 1.28E-04 | Putative uncharacterized protein |
| GRMZM2G123901 | 91 | 82 | 0.74 | 4.87E-03 | B-cell receptor-associated protein 31-like containing protein |
| GRMZM2G123922 | 345 | 646 | -0.31 | 6.15E-03 | Putative uncharacterized protein |
| GRMZM2G123940 | 89 | 58 | 1.21 | 6.14E-06 | Putative uncharacterized protein |
| GRMZM2G123972 | 90 | 272 | -1.00 | 3.57E-08 | Putative uncharacterized protein |
| GRMZM2G124026 | 10 | 60 | -1.99 | 4.24E-05 | Putative uncharacterized protein |
| GRMZM2G124051 | 630 | 1,332 | -0.49 | 2.98E-11 | CCR4-NOT transcription complex subunit 6 |
| GRMZM2G124070 | 56 | 180 | -1.09 | 3.16E-06 | Putative uncharacterized protein |
| GRMZM2G124143 | 1,004 | 1,341 | 0.17 | 2.01E-02 | S28 ribosomal protein (Fragment) |
| GRMZM2G124151 | 55 | 125 | -0.59 | 4.88E-02 | Putative uncharacterized protein |
| GRMZM2G124179 | 305 | 111 | 2.05 | 3.58E-41 | Os05g0205100 protein (cDNA clone:002-181-E05, full insert sequence) (Putative uncharacterized protein OSJNBb0115F21.3) |
| GRMZM2G124254 | 119 | 107 | 0.74 | 9.84E-04 | Putative uncharacterized protein |
| GRMZM2G124281 | 45 | 130 | -0.94 | 7.93E-04 | Mitochondrial glycoprotein (Putative uncharacterized protein) |
| GRMZM2G124288 | 116 | 313 | -0.84 | 4.40E-07 | Putative uncharacterized protein |
| GRMZM2G124301 | 28 | 95 | -1.17 | 5.43E-04 | Putative uncharacterized protein |
| GRMZM2G124307 | 160 | 118 | 1.03 | 7.12E-08 | Putative uncharacterized protein (TIL-2-Zea mays Temperature-induced lipocalin-2) (Temperature-induced lipocalin-2) |
| GRMZM2G124321 | 65 | 178 | -0.86 | 2.19E-04 | Os08g0172200 protein (Putative uncharacterized protein OJ1590_E05.35-1) |
| GRMZM2G124335 | 163 | 377 | -0.62 | 3.25E-05 | Putative uncharacterized protein |
| GRMZM2G124371 | 86 | 182 | -0.49 | 3.81E-02 | Os04g0686200 protein (Putative uncharacterized protein) (OSJNBa0070M12.5 protein) |
| GRMZM2G124377 | 70 | 234 | -1.15 | 1.43E-08 | Putative uncharacterized protein |
| GRMZM2G124411 | 751 | 959 | 0.24 | 4.41E-03 | Putative uncharacterized protein |
| GRMZM2G124434 | 267 | 219 | 0.88 | 5.79E-10 | Putative uncharacterized protein |
| GRMZM2G124441 | 132 | 300 | -0.59 | 5.33E-04 | Putative uncharacterized protein |
| GRMZM2G124530 | 179 | 93 | 1.54 | 2.98E-16 | Putative uncharacterized protein |
| GRMZM2G124576 | 974 | 2,457 | -0.74 | 9.18E-43 | cDNA clone:J013092O17, full insert sequence (Nucleolar protein NOP5, putative, expressed) |
| GRMZM2G124644 | 94 | 68 | 1.06 | 4.05E-05 | Heat shock protein binding protein |
| GRMZM2G124671 | 21 | 59 | -0.90 | 4.95E-02 | Putative uncharacterized protein (UDP-N-acetylglucosamine transferase subunit alg13) |
| GRMZM2G124718 | 79 | 205 | -0.78 | 2.35E-04 | Putative uncharacterized protein |
| GRMZM2G124732 | 35 | 95 | -0.85 | 1.21E-02 | Os09g0327100 protein (Fragment) |
| GRMZM2G124809 | 111 | 250 | -0.58 | 2.53E-03 | Putative uncharacterized protein |
| GRMZM2G124886 | 138 | 281 | -0.43 | 1.87E-02 | Putative uncharacterized protein |
| GRMZM2G124887 | 63 | 26 | 1.87 | 9.83E-08 | Putative uncharacterized protein |
| GRMZM2G124965 | 76 | 214 | -0.90 | 1.58E-05 | Putative uncharacterized protein |
| GRMZM2G125004 | 26 | 6 | 2.71 | 4.16E-05 | F-box protein |
| GRMZM2G125044 | 84 | 72 | 0.81 | 3.54E-03 | Putative uncharacterized protein (Os01g0663800 protein) (TA9 protein-like) |
| GRMZM2G125054 | 28 | 95 | -1.17 | 5.43E-04 | Putative uncharacterized protein |
| GRMZM2G125138 | 127 | 96 | 1.00 | 4.24E-06 | Putative uncharacterized protein |
| GRMZM2G125141 | 23 | 67 | -0.95 | 2.39E-02 | Postsynaptic protein CRIPT |
| GRMZM2G125148 | 1,733 | 6,098 | -1.22 | 1.44E-241 | Os03g0350300 protein (Putative uncharacterized protein) (cDNA clone:J033023B01, full insert sequence) (Nucleolar protein NOP5, putative, expressed) |
| GRMZM2G125149 | 75 | 282 | -1.32 | 1.22E-12 | Putative uncharacterized protein |
| GRMZM2G125193 | 236 | 283 | 0.33 | 4.43E-02 | Putative uncharacterized protein |
| GRMZM2G125239 | 156 | 626 | -1.41 | 9.89E-31 | Putative uncharacterized protein |
| GRMZM2G125268 | 100 | 61 | 1.30 | 2.65E-07 | Mitochondrial aldehyde dehydrogenase RF2B |
| GRMZM2G125271 | 2,223 | 2,875 | 0.22 | 1.07E-06 | 40S ribosomal protein S4 (Putative uncharacterized protein) |
| GRMZM2G125294 | 71 | 189 | -0.82 | 2.35E-04 | Putative uncharacterized protein |
| GRMZM2G125300 | 48 | 120 | -0.73 | 1.39E-02 | 40S ribosomal protein S21 |
| GRMZM2G125310 | 117 | 352 | -1.00 | 2.63E-10 | Lactoylglutathione lyase |
| GRMZM2G125424 | 1,906 | 880 | 1.71 | 6.89E-200 | Putative uncharacterized protein |
| GRMZM2G125494 | 20 | 86 | -1.51 | 5.82E-05 | Putative uncharacterized protein |
| GRMZM2G125512 | 276 | 101 | 2.04 | 3.71E-37 | Putative uncharacterized protein |
| GRMZM2G125513 | 31 | 105 | -1.17 | 2.81E-04 | Putative uncharacterized protein |
| GRMZM2G125531 | 179 | 72 | 1.91 | 6.86E-22 | Putative uncharacterized protein |
| GRMZM2G125556 | 21 | 75 | -1.25 | 1.65E-03 | Pleckstrin homology domain containing, family A |
| GRMZM2G125617 | 111 | 262 | -0.65 | 4.55E-04 | Putative uncharacterized protein |
| GRMZM2G125635 | 371 | 1,000 | -0.84 | 1.77E-21 | Putative uncharacterized protein (S-adenosylmethionine decarboxylase proenzyme) |
| GRMZM2G125642 | 36 | 117 | -1.11 | 2.01E-04 | Putative uncharacterized protein |
| GRMZM2G125777 | 444 | 344 | 0.96 | 7.68E-19 | Putative uncharacterized protein |
| GRMZM2G125784 | 202 | 178 | 0.77 | 2.52E-06 | Putative uncharacterized protein |
| GRMZM2G125893 | 400 | 363 | 0.73 | 7.30E-11 | Putative uncharacterized protein |
| GRMZM2G125943 | 31 | 82 | -0.81 | 3.14E-02 | Histidine kinase |
| GRMZM2G125969 | 24 | 70 | -0.95 | 2.08E-02 | Heat shock factor protein HSF30 (Putative uncharacterized protein) |
| GRMZM2G126026 | 43 | 28 | 1.21 | 3.73E-03 | CCT motif family protein |
| GRMZM2G126106 | 12 | 1 | 4.18 | 9.96E-04 | Putative uncharacterized protein |
| GRMZM2G126128 | 17 | 55 | -1.10 | 2.48E-02 | Putative uncharacterized protein |
| GRMZM2G126131 | 59 | 137 | -0.62 | 2.55E-02 | Putative uncharacterized protein |
| GRMZM2G126190 | 447 | 1,121 | -0.74 | 3.34E-19 | Actin-7 (Putative uncharacterized protein) |
| GRMZM2G126266 | 95 | 209 | -0.55 | 1.21E-02 | cDNA clone:J023002A11, full insert sequence (Os01g0928100 protein) (Putative uncharacterized protein OSJNBa0052O12.5) |
| GRMZM2G126338 | 319 | 284 | 0.76 | 2.54E-09 | Putative uncharacterized protein |
| GRMZM2G126361 | 26 | 91 | -1.22 | 5.15E-04 | cDNA clone:J013001F19, full insert sequence |
| GRMZM2G126397 | 876 | 2,063 | -0.64 | 5.23E-28 | Nonspecific lipid-transfer protein (Putative uncharacterized protein) |
| GRMZM2G126447 | 67 | 149 | -0.56 | 3.60E-02 | Os02g0244300 protein |
| GRMZM2G126453 | 161 | 169 | 0.52 | 6.39E-03 | Putative uncharacterized protein |
| GRMZM2G126471 | 98 | 103 | 0.52 | 4.80E-02 | Putative uncharacterized protein |
| GRMZM2G126484 | 97 | 238 | -0.70 | 2.96E-04 | Putative uncharacterized protein |
| GRMZM2G126517 | 53 | 187 | -1.23 | 1.11E-07 | Putative uncharacterized protein |
| GRMZM2G126601 | 12 | 42 | -1.22 | 3.50E-02 | Putative uncharacterized protein |
| GRMZM2G126603 | 141 | 136 | 0.64 | 1.58E-03 | 50S ribosomal protein L34 |
| GRMZM2G126646 | 19 | 68 | -1.25 | 2.81E-03 | Putative uncharacterized protein |
| GRMZM2G126682 | 346 | 242 | 1.11 | 1.11E-18 | Putative uncharacterized protein |
| GRMZM2G126691 | 111 | 232 | -0.47 | 2.01E-02 | Putative uncharacterized protein |
| GRMZM2G126742 | 77 | 254 | -1.13 | 4.41E-09 | OSJNBb0038F03.3 protein (OSJNBb0103I08.20 protein) |
| GRMZM2G126821 | 1,064 | 2,053 | -0.36 | 8.57E-10 | Putative uncharacterized protein |
| GRMZM2G126832 | 47 | 167 | -1.24 | 5.48E-07 | Putative uncharacterized protein |
| GRMZM2G126835 | 124 | 260 | -0.48 | 1.30E-02 | Putative glycosyltransferase |
| GRMZM2G126839 | 119 | 247 | -0.46 | 1.92E-02 | Putative uncharacterized protein |
| GRMZM2G126860 | 209 | 198 | 0.67 | 3.94E-05 | Putative uncharacterized protein |
| GRMZM2G126928 | 289 | 318 | 0.45 | 9.95E-04 | 60S ribosomal protein L18a |
| GRMZM2G127034 | 35 | 98 | -0.89 | 7.66E-03 | AT hook motif family protein |
| GRMZM2G127053 | 11 | 52 | -1.65 | 1.18E-03 | Cardiolipin synthetase |
| GRMZM2G127067 | 132 | 426 | -1.10 | 2.35E-14 | C-4 methylsterol oxidase (Putative uncharacterized protein) |
| GRMZM2G127154 | 66 | 40 | 1.31 | 3.97E-05 | Putative uncharacterized protein |
| GRMZM2G127308 | 29 | 15 | 1.54 | 3.69E-03 | Putative alliinase |
| GRMZM2G127312 | 282 | 336 | 0.34 | 1.97E-02 | Putative uncharacterized protein |
| GRMZM2G127386 | 291 | 850 | -0.96 | 1.19E-22 | Nitrilase-associated protein |
| GRMZM2G127393 | 186 | 374 | -0.42 | 6.89E-03 | Os06g0308000 protein (Putative uncharacterized protein) (cDNA clone:J013071B03, full insert sequence) (Trigger factor-like) |
| GRMZM2G127404 | 411 | 378 | 0.71 | 1.25E-10 | Putative uncharacterized protein |
| GRMZM2G127426 | 30 | 14 | 1.69 | 1.24E-03 | Putative uncharacterized protein |
| GRMZM2G127521 | 121 | 402 | -1.14 | 2.33E-14 | 60S ribosomal protein L17 |
| GRMZM2G127537 | 140 | 131 | 0.69 | 8.52E-04 | Putative uncharacterized protein |
| GRMZM2G127548 | 35 | 101 | -0.94 | 3.85E-03 | BSD domain containing protein (Putative uncharacterized protein) |
| GRMZM2G127581 | 181 | 418 | -0.62 | 1.16E-05 | Tubulin-specific chaperone B |
| GRMZM2G127648 | 135 | 314 | -0.63 | 1.57E-04 | Ras-related protein RIC2 |
| GRMZM2G127656 | 158 | 395 | -0.73 | 4.75E-07 | Putative uncharacterized protein |
| GRMZM2G127665 | 215 | 238 | 0.44 | 6.39E-03 | Putative uncharacterized protein |
| GRMZM2G127668 | 0 | 10 | #NUM! | 3.46E-02 | H0306F03.8 protein |
| GRMZM2G127687 | 105 | 252 | -0.67 | 3.96E-04 | Putative uncharacterized protein |
| GRMZM2G127739 | 160 | 172 | 0.49 | 1.28E-02 | Extra-large guanine nucleotide binding protein, putative, expressed |
| GRMZM2G127780 | 302 | 352 | 0.37 | 6.91E-03 | cDNA clone:J013046L18, full insert sequence (Os01g0679900 protein) (Rubisco subunit binding-protein beta subunit-like) |
| GRMZM2G127798 | 31 | 22 | 1.09 | 3.26E-02 | 6-phosphogluconate dehydrogenase, decarboxylating (EC 1.1.1.44) |
| GRMZM2G127844 | 46 | 115 | -0.73 | 1.81E-02 | Os05g0520200 protein (Putative uncharacterized protein) (Putative uncharacterized protein P0483D07.6) (Putative uncharacterized protein P0599F04.16) |
| GRMZM2G127911 | 65 | 58 | 0.76 | 2.03E-02 | Phosphopantothenoylcysteine decarboxylase (Putative uncharacterized protein) |
| GRMZM2G127949 | 209 | 248 | 0.34 | 4.71E-02 | Auxin transporter-like protein 1 |
| GRMZM2G128012 | 14 | 69 | -1.71 | 8.39E-05 | Putative uncharacterized protein |
| GRMZM2G128057 | 53 | 48 | 0.73 | 4.58E-02 | Putative uncharacterized protein |
| GRMZM2G128080 | 98 | 205 | -0.47 | 3.14E-02 | Putative uncharacterized protein |
| GRMZM2G128092 | 124 | 303 | -0.70 | 4.14E-05 | OSJNBa0009P12.21 protein |
| GRMZM2G128171 | 167 | 331 | -0.40 | 1.92E-02 | Putative uncharacterized protein |
| GRMZM2G128176 | 104 | 223 | -0.51 | 1.42E-02 | PHD finger transcription factor-like protein |
| GRMZM2G128315 | 193 | 485 | -0.74 | 1.21E-08 | Putative uncharacterized protein OSJNBa0018K15.10 |
| GRMZM2G128319 | 108 | 441 | -1.44 | 3.43E-22 | Putative uncharacterized protein |
| GRMZM2G128358 | 20 | 8 | 1.91 | 5.11E-03 | Putative uncharacterized protein |
| GRMZM2G128518 | 150 | 122 | 0.89 | 5.56E-06 | Putative uncharacterized protein |
| GRMZM2G128560 | 28 | 17 | 1.31 | 1.69E-02 | Putative uncharacterized protein |
| GRMZM2G128579 | 500 | 1,173 | -0.64 | 7.90E-16 | Os05g0567400 protein |
| GRMZM2G128613 | 32 | 108 | -1.16 | 2.50E-04 | Putative uncharacterized protein OJ1743A09.20 |
| GRMZM2G128658 | 78 | 205 | -0.80 | 1.77E-04 | Electron transporter |
| GRMZM2G128663 | 120 | 102 | 0.83 | 2.28E-04 | PAPA-1-like conserved region family protein |
| GRMZM2G128737 | 99 | 286 | -0.94 | 1.02E-07 | Putative uncharacterized protein |
| GRMZM2G128744 | 51 | 184 | -1.26 | 7.69E-08 | Cysteine synthase (EC 2.5.1.47) |
| GRMZM2G128807 | 739 | 1,463 | -0.39 | 1.73E-08 | Putative uncharacterized protein |
| GRMZM2G128809 | 32 | 91 | -0.92 | 9.11E-03 | Os05g0373400 protein (Fragment) |
| GRMZM2G128929 | 202 | 189 | 0.69 | 3.27E-05 | L-lactate dehydrogenase (EC 1.1.1.27) |
| GRMZM2G128934 | 84 | 240 | -0.92 | 2.08E-06 | Putative uncharacterized protein |
| GRMZM2G129007 | 126 | 446 | -1.23 | 7.69E-18 | ATP-dependent RNA helicase dhh1 |
| GRMZM2G129015 | 1,349 | 1,662 | 0.29 | 7.16E-07 | 40S ribosomal protein S26 |
| GRMZM2G129147 | 176 | 193 | 0.46 | 1.28E-02 | Growth-regulating factor |
| GRMZM2G129150 | 58 | 35 | 1.32 | 1.54E-04 | Putative uncharacterized protein |
| GRMZM2G129169 | 38 | 97 | -0.76 | 2.33E-02 | B0403H10-OSIGBa0105A11.8 protein |
| GRMZM2G129238 | 23 | 12 | 1.53 | 1.44E-02 | Phosphoinositide-specific phospholipase C |
| GRMZM2G129268 | 68 | 187 | -0.87 | 1.17E-04 | Putative uncharacterized protein |
| GRMZM2G129278 | 104 | 237 | -0.60 | 2.60E-03 | DNA binding protein |
| GRMZM2G129354 | 94 | 209 | -0.56 | 8.47E-03 | ARF GAP-like zinc finger-containing protein ZIGA3 |
| GRMZM2G129428 | 63 | 183 | -0.95 | 3.48E-05 | Nucleic acid binding protein |
| GRMZM2G129431 | 209 | 415 | -0.40 | 6.23E-03 | Putative uncharacterized protein |
| GRMZM2G129444 | 206 | 199 | 0.64 | 9.10E-05 | Mitotic checkpoint protein |
| GRMZM2G129554 | 160 | 348 | -0.53 | 7.39E-04 | Putative uncharacterized protein |
| GRMZM2G129585 | 13 | 5 | 1.97 | 3.14E-02 | Putative uncharacterized protein |
| GRMZM2G129620 | 51 | 176 | -1.20 | 4.64E-07 | Putative uncharacterized protein |
| GRMZM2G129817 | 242 | 285 | 0.36 | 2.40E-02 | Putative uncharacterized protein (Histone acetyltransferase complex component) |
| GRMZM2G129874 | 2,508 | 3,303 | 0.19 | 5.14E-06 | 60S ribosomal protein L7a (Putative uncharacterized protein) |
| GRMZM2G129987 | 374 | 1,037 | -0.88 | 4.09E-24 | Putative uncharacterized protein |
| GRMZM2G130010 | 81 | 190 | -0.64 | 4.75E-03 | Putative uncharacterized protein |
| GRMZM2G130046 | 52 | 153 | -0.97 | 1.52E-04 | Os02g0498700 protein (Fragment) |
| GRMZM2G130062 | 52 | 173 | -1.14 | 1.71E-06 | Putative uncharacterized protein |
| GRMZM2G130079 | 3,313 | 1,406 | 1.83 | 0.00E+01 | Histone H3 |
| GRMZM2G130095 | 877 | 1,510 | -0.19 | 9.45E-03 | Putative uncharacterized protein |
| GRMZM2G130101 | 8 | 33 | -1.45 | 3.02E-02 | Putative uncharacterized protein |
| GRMZM2G130109 | 61 | 153 | -0.74 | 3.81E-03 | Putative uncharacterized protein |
| GRMZM2G130127 | 77 | 52 | 1.16 | 7.97E-05 | Putative uncharacterized protein |
| GRMZM2G130169 | 43 | 155 | -1.26 | 1.18E-06 | Ubiquitin carrier protein (EC 6.3.2.-) |
| GRMZM2G130207 | 344 | 307 | 0.76 | 7.08E-10 | DNA-directed RNA polymerase II subunit J (Putative uncharacterized protein) |
| GRMZM2G130287 | 78 | 177 | -0.59 | 1.35E-02 | Putative uncharacterized protein |
| GRMZM2G130339 | 306 | 346 | 0.41 | 1.88E-03 | Putative uncharacterized protein (Ribosome biogenesis protein NEP1) |
| GRMZM2G130351 | 41 | 28 | 1.14 | 7.15E-03 | Putative uncharacterized protein |
| GRMZM2G130354 | 54 | 34 | 1.26 | 5.58E-04 | Putative uncharacterized protein |
| GRMZM2G130358 | 61 | 55 | 0.74 | 2.73E-02 | 50S ribosomal protein L31 |
| GRMZM2G130366 | 185 | 401 | -0.52 | 2.95E-04 | DNA-binding protein |
| GRMZM2G130404 | 191 | 547 | -0.93 | 6.27E-14 | Putative GTP-binding protein ara-3 (Rab-type small GTP-binding protein-like) |
| GRMZM2G130440 | 441 | 352 | 0.92 | 2.10E-17 | Putative uncharacterized protein |
| GRMZM2G130442 | 112 | 271 | -0.68 | 1.61E-04 | Putative uncharacterized protein |
| GRMZM2G130449 | 37 | 106 | -0.93 | 3.74E-03 | Beta-1,3-galactosyltransferase 6 |
| GRMZM2G130544 | 811 | 1,444 | -0.24 | 1.04E-03 | 40S ribosomal protein S18 |
| GRMZM2G130558 | 46 | 37 | 0.91 | 2.31E-02 | Plastidic phosphate translocator-like protein1 |
| GRMZM2G130586 | 285 | 283 | 0.60 | 9.29E-06 | Putative uncharacterized protein (Os01g0663800 protein) (TA9 protein-like) |
| GRMZM2G130625 | 148 | 142 | 0.65 | 1.10E-03 | Putative uncharacterized protein |
| GRMZM2G130724 | 72 | 169 | -0.64 | 8.62E-03 | Putative uncharacterized protein |
| GRMZM2G130746 | 1,185 | 2,192 | -0.30 | 1.62E-07 | Histone H2A |
| GRMZM2G130790 | 63 | 143 | -0.59 | 3.06E-02 | FolD bifunctional protein |
| GRMZM2G130953 | 74 | 72 | 0.63 | 3.78E-02 | Putative uncharacterized protein |
| GRMZM2G130987 | 506 | 1,394 | -0.87 | 1.03E-31 | Putative uncharacterized protein |
| GRMZM2G131020 | 100 | 210 | -0.48 | 2.90E-02 | Putative uncharacterized protein |
| GRMZM2G131106 | 678 | 810 | 0.33 | 9.32E-05 | Putative uncharacterized protein |
| GRMZM2G131155 | 48 | 27 | 1.42 | 2.47E-04 | Putative uncharacterized protein |
| GRMZM2G131167 | 80 | 216 | -0.84 | 4.52E-05 | Putative uncharacterized protein |
| GRMZM2G131221 | 6 | 31 | -1.78 | 1.82E-02 | Putative uncharacterized protein |
| GRMZM2G131249 | 93 | 197 | -0.49 | 3.12E-02 | Putative uncharacterized protein |
| GRMZM2G131305 | 35 | 110 | -1.06 | 6.62E-04 | Putative uncharacterized protein |
| GRMZM2G131321 | 104 | 415 | -1.41 | 2.76E-20 | Putative uncharacterized protein (Signal peptidase complex subunit 2) |
| GRMZM2G131329 | 187 | 188 | 0.58 | 8.56E-04 | Putative uncharacterized protein |
| GRMZM2G131431 | 72 | 48 | 1.18 | 9.30E-05 | Putative uncharacterized protein |
| GRMZM2G131434 | 277 | 247 | 0.76 | 4.35E-08 | Putative uncharacterized protein |
| GRMZM2G131467 | 206 | 187 | 0.73 | 8.12E-06 | Activator of basal transcription 1 (Putative uncharacterized protein) |
| GRMZM2G131473 | 606 | 649 | 0.49 | 3.38E-08 | Methionine aminopeptidase (EC 3.4.11.18) |
| GRMZM2G131482 | 75 | 205 | -0.86 | 5.68E-05 | Os06g0489200 protein (Putative uncharacterized protein) (cDNA clone:J033132P04, full insert sequence) (Putative SWAP (Suppressor-of-White-APricot)/surp domain-containing protein) |
| GRMZM2G131516 | 465 | 235 | 1.58 | 2.93E-43 | cDNA, clone: J100030A12, full insert sequence |
| GRMZM2G131525 | 70 | 177 | -0.75 | 1.43E-03 | Syntaxin-related protein KNOLLE |
| GRMZM2G131554 | 248 | 162 | 1.21 | 1.86E-15 | Putative uncharacterized protein |
| GRMZM2G131577 | 521 | 920 | -0.23 | 1.83E-02 | Putative uncharacterized protein (Transcription factor BTF3) |
| GRMZM2G131638 | 222 | 260 | 0.36 | 2.79E-02 | Protein CYPRO4 |
| GRMZM2G131715 | 195 | 223 | 0.40 | 2.43E-02 | NA |
| GRMZM2G131733 | 39 | 122 | -1.05 | 3.02E-04 | Putative uncharacterized protein |
| GRMZM2G131756 | 44 | 172 | -1.38 | 2.49E-08 | Putative uncharacterized protein |
| GRMZM2G131793 | 61 | 142 | -0.63 | 1.99E-02 | Putative uncharacterized protein |
| GRMZM2G131822 | 40 | 30 | 1.01 | 2.22E-02 | Putative uncharacterized protein |
| GRMZM2G131907 | 357 | 928 | -0.79 | 6.63E-18 | Putative uncharacterized protein |
| GRMZM2G131943 | 2,520 | 2,389 | 0.67 | 1.14E-56 | Putative uncharacterized protein |
| GRMZM2G131957 | 326 | 602 | -0.29 | 1.61E-02 | Os08g0490300 protein (Putative uncharacterized protein) (cDNA clone:J013090I22, full insert sequence) (Putative cleavage and polyadenylation specific factor) |
| GRMZM2G131988 | 6 | 32 | -1.82 | 1.26E-02 | Lysine ketoglutarate reductase trans-splicing related 1 (Putative uncharacterized protein) |
| GRMZM2G132000 | 83 | 185 | -0.56 | 1.41E-02 | Os11g0145400 protein (Putative uncharacterized protein) (Ubiquitin-like protein 5, putative, expressed) (cDNA, clone: J080308D02, full insert sequence) |
| GRMZM2G132019 | 63 | 139 | -0.55 | 4.88E-02 | Putative uncharacterized protein |
| GRMZM2G132060 | 182 | 545 | -0.99 | 1.69E-15 | Putative uncharacterized protein |
| GRMZM2G132069 | 31 | 23 | 1.02 | 4.84E-02 | Putative uncharacterized protein |
| GRMZM2G132077 | 5,944 | 10,355 | -0.21 | 1.21E-17 | NA |
| GRMZM2G132093 | 6 | 0 | #VALUE! | 1.98E-02 | Putative uncharacterized protein |
| GRMZM2G132116 | 264 | 229 | 0.80 | 1.91E-08 | Putative uncharacterized protein (Serine/threonine-protein kinase Eg2-like) |
| GRMZM2G132121 | 1,895 | 1,868 | 0.61 | 1.93E-36 | Putative uncharacterized protein |
| GRMZM2G132130 | 2 | 19 | -2.66 | 1.65E-02 | Os05g0358400 protein (cDNA clone:006-202-A08, full insert sequence) (cDNA clone:J023108P17, full insert sequence) (Putative uncharacterized protein OJ1045_C06.12) (Putative uncharacterized protein OSJNBa0009L15.2) |
| GRMZM2G132184 | 54 | 126 | -0.63 | 3.36E-02 | Putative uncharacterized protein |
| GRMZM2G132218 | 44 | 125 | -0.91 | 1.46E-03 | Putative uncharacterized protein |
| GRMZM2G132371 | 325 | 388 | 0.34 | 1.18E-02 | Kinesin heavy chain (Fragment) |
| GRMZM2G132450 | 26 | 134 | -1.77 | 2.65E-09 | Cytochrome P450 CYP81N5 |
| GRMZM2G132465 | 349 | 891 | -0.76 | 2.63E-16 | RNA binding protein |
| GRMZM2G132486 | 123 | 289 | -0.64 | 2.45E-04 | AFH1 |
| GRMZM2G132547 | 181 | 211 | 0.37 | 4.77E-02 | CTP synthase (Putative uncharacterized protein) |
| GRMZM2G132623 | 249 | 610 | -0.70 | 6.89E-10 | 60S ribosomal protein L31 |
| GRMZM2G132636 | 16 | 51 | -1.08 | 3.66E-02 | Putative uncharacterized protein |
| GRMZM2G132644 | 83 | 239 | -0.93 | 1.87E-06 | Putative uncharacterized protein |
| GRMZM2G132682 | 14 | 3 | 2.81 | 3.86E-03 | BCL-2 binding anthanogene-1 |
| GRMZM2G132748 | 170 | 114 | 1.17 | 3.38E-10 | Putative uncharacterized protein |
| GRMZM2G132749 | 341 | 361 | 0.51 | 3.76E-05 | Putative uncharacterized protein |
| GRMZM2G132759 | 301 | 1,159 | -1.35 | 2.30E-53 | Ubiquitin carrier protein (EC 6.3.2.-) |
| GRMZM2G132780 | 58 | 135 | -0.63 | 2.43E-02 | C2H2 zinc-finger protein (Fragment) |
| GRMZM2G132847 | 50 | 142 | -0.91 | 5.19E-04 | Os06g0489500 protein (Putative cytosine deaminase) |
| GRMZM2G132857 | 161 | 480 | -0.98 | 1.41E-13 | Putative uncharacterized protein |
| GRMZM2G132862 | 98 | 60 | 1.30 | 4.79E-07 | Putative uncharacterized protein |
| GRMZM2G132880 | 7 | 0 | #VALUE! | 8.95E-03 | RCN1-Corn Centroradialis/TFL1-like protein (ZCN6) |
| GRMZM2G132929 | 889 | 1,806 | -0.43 | 4.26E-12 | 40S ribosomal protein S12 |
| GRMZM2G132936 | 69 | 165 | -0.67 | 6.14E-03 | Putative uncharacterized protein |
| GRMZM2G132968 | 1,815 | 1,875 | 0.54 | 2.90E-28 | 60S ribosomal protein L3 (Putative uncharacterized protein) |
| GRMZM2G133012 | 82 | 224 | -0.86 | 2.22E-05 | Putative uncharacterized protein |
| GRMZM2G133023 | 10 | 2 | 2.91 | 2.36E-02 | Expressed protein (Stem-specific protein TSJT1, putative, expressed) |
| GRMZM2G133028 | 8 | 39 | -1.69 | 5.64E-03 | Putative uncharacterized protein |
| GRMZM2G133050 | 162 | 414 | -0.76 | 8.07E-08 | Putative uncharacterized protein |
| GRMZM2G133121 | 142 | 157 | 0.45 | 3.48E-02 | Putative uncharacterized protein |
| GRMZM2G133173 | 839 | 1,030 | 0.30 | 1.17E-04 | Putative uncharacterized protein |
| GRMZM2G133203 | 4 | 30 | -2.32 | 3.91E-03 | Putative uncharacterized protein |
| GRMZM2G133302 | 5 | 26 | -1.79 | 2.67E-02 | Putative uncharacterized protein |
| GRMZM2G133314 | 47 | 160 | -1.18 | 3.19E-06 | cDNA clone:J033109B05, full insert sequence (Os01g0205100 protein) (Putative G-protein beta) |
| GRMZM2G133331 | 312 | 146 | 1.69 | 3.94E-32 | Putative uncharacterized protein |
| GRMZM2G133359 | 16 | 51 | -1.08 | 3.66E-02 | Cysteine synthase (EC 2.5.1.47) |
| GRMZM2G133394 | 45 | 18 | 1.91 | 6.03E-06 | Putative uncharacterized protein |
| GRMZM2G133396 | 29 | 82 | -0.91 | 1.73E-02 | Protein binding protein (Putative uncharacterized protein) |
| GRMZM2G133413 | 27 | 83 | -1.03 | 5.23E-03 | Putative uncharacterized protein |
| GRMZM2G133428 | 117 | 120 | 0.55 | 1.73E-02 | Putative uncharacterized protein |
| GRMZM2G133464 | 82 | 192 | -0.64 | 4.19E-03 | Serine/threonine protein phosphatase (EC 3.1.3.16) |
| GRMZM2G133483 | 117 | 120 | 0.55 | 1.73E-02 | NA |
| GRMZM2G133613 | 10 | 2 | 2.91 | 2.36E-02 | Putative uncharacterized protein |
| GRMZM2G133631 | 151 | 112 | 1.02 | 1.77E-07 | CAPIP1 (Putative uncharacterized protein) |
| GRMZM2G133652 | 108 | 247 | -0.60 | 1.88E-03 | Putative uncharacterized protein |
| GRMZM2G133749 | 49 | 135 | -0.87 | 1.49E-03 | Putative uncharacterized protein |
| GRMZM2G133756 | 165 | 343 | -0.46 | 4.14E-03 | Putative uncharacterized protein |
| GRMZM2G133764 | 443 | 1,038 | -0.64 | 5.74E-14 | Putative uncharacterized protein (Putative RH2 protein) (RNA helicase 2) |
| GRMZM2G133802 | 400 | 2,185 | -1.86 | 2.54E-158 | cDNA clone:J033132F08, full insert sequence (Os01g0282800 protein) |
| GRMZM2G133838 | 257 | 122 | 1.67 | 5.08E-26 | H/ACA ribonucleoprotein complex subunit 3-like protein |
| GRMZM2G133919 | 207 | 511 | -0.71 | 1.39E-08 | Protein BRICK1 |
| GRMZM2G133986 | 43 | 129 | -0.99 | 3.90E-04 | Putative uncharacterized protein |
| GRMZM2G134054 | 80 | 11 | 3.45 | 1.19E-19 | Putative uncharacterized protein |
| GRMZM2G134062 | 49 | 164 | -1.15 | 3.28E-06 | Putative uncharacterized protein (SPX (SYG1/Pho81/XPR1) domain-containing protein-like) |
| GRMZM2G134064 | 17 | 5 | 2.36 | 3.93E-03 | Putative uncharacterized protein |
| GRMZM2G134104 | 183 | 393 | -0.51 | 5.00E-04 | Putative uncharacterized protein |
| GRMZM2G134107 | 413 | 436 | 0.51 | 3.24E-06 | 60S ribosomal protein L22-2 |
| GRMZM2G134134 | 487 | 542 | 0.44 | 1.68E-05 | Putative uncharacterized protein |
| GRMZM2G134176 | 35 | 122 | -1.21 | 4.18E-05 | Ubiquitin carrier protein (EC 6.3.2.-) |
| GRMZM2G134230 | 188 | 408 | -0.53 | 2.43E-04 | Putative uncharacterized protein |
| GRMZM2G134234 | 203 | 128 | 1.26 | 1.80E-13 | Putative uncharacterized protein |
| GRMZM2G134270 | 52 | 139 | -0.83 | 1.94E-03 | Putative uncharacterized protein (Putative oxygenase) (Ubiquinone biosynthesis mono0xygenase COQ6 family protein, expressed) |
| GRMZM2G134329 | 20 | 146 | -2.28 | 1.22E-13 | Rhodopsin-like receptor |
| GRMZM2G134341 | 77 | 43 | 1.43 | 1.45E-06 | Putative uncharacterized protein |
| GRMZM2G134367 | 24 | 80 | -1.15 | 2.71E-03 | Putative uncharacterized protein |
| GRMZM2G134389 | 71 | 67 | 0.68 | 3.11E-02 | Tyrosine specific protein phosphatase family protein |
| GRMZM2G134439 | 47 | 113 | -0.67 | 3.35E-02 | Putative uncharacterized protein |
| GRMZM2G134480 | 126 | 296 | -0.64 | 2.01E-04 | Ubiquitin-activating enzyme E1 |
| GRMZM2G134508 | 188 | 176 | 0.69 | 6.38E-05 | Nucleoporin p58/p45 (Putative uncharacterized protein) |
| GRMZM2G134544 | 106 | 274 | -0.78 | 1.48E-05 | Putative uncharacterized protein (Plastidic general dicarboxylate transporter) |
| GRMZM2G134613 | 52 | 38 | 1.04 | 4.63E-03 | Autophagy-related 8d variant 1 (Autophagy-related protein 8) |
| GRMZM2G134711 | 23 | 116 | -1.74 | 5.89E-08 | Putative uncharacterized protein |
| GRMZM2G134731 | 261 | 512 | -0.38 | 3.13E-03 | Cyclase/dehydrase family protein |
| GRMZM2G134738 | 259 | 496 | -0.35 | 9.78E-03 | Cytochrome c oxidase polypeptide Vb |
| GRMZM2G134747 | 100 | 210 | -0.48 | 2.90E-02 | Cyanate hydratase (Putative uncharacterized protein) |
| GRMZM2G134756 | 242 | 568 | -0.64 | 6.20E-08 | Tyrosyl-tRNA synthetase |
| GRMZM2G134761 | 28 | 87 | -1.04 | 3.58E-03 | Putative uncharacterized protein |
| GRMZM2G134770 | 197 | 547 | -0.88 | 6.25E-13 | Zn-finger, RanBP-type, containing protein |
| GRMZM2G134797 | 109 | 96 | 0.77 | 1.07E-03 | Putative uncharacterized protein |
| GRMZM2G134889 | 88 | 308 | -1.22 | 3.34E-12 | Protein disulfide isomerase |
| GRMZM2G134901 | 103 | 106 | 0.55 | 3.20E-02 | DNA binding protein (Putative uncharacterized protein) |
| GRMZM2G134917 | 231 | 241 | 0.53 | 6.01E-04 | Putative uncharacterized protein |
| GRMZM2G134982 | 47 | 151 | -1.09 | 2.21E-05 | Os03g0650700 protein (Putative uncharacterized protein) (Elongation factor Tu family protein, putative, expressed) (Putative Translation Elongation factor protein) |
| GRMZM2G135091 | 118 | 58 | 1.62 | 1.14E-11 | Phosphoenolpyruvate carboxylase kinase 2 |
| GRMZM2G135095 | 38 | 94 | -0.72 | 4.14E-02 | Ribosomal protein S18 containing protein |
| GRMZM2G135186 | 770 | 2,055 | -0.82 | 9.33E-43 | Putative uncharacterized protein |
| GRMZM2G135199 | 30 | 99 | -1.13 | 7.39E-04 | Putative uncharacterized protein |
| GRMZM2G135236 | 64 | 150 | -0.64 | 1.38E-02 | Putative ATPase, aminophospholipid transporter (APLT), class I, type 8A, member 1 |
| GRMZM2G135256 | 32 | 89 | -0.88 | 1.45E-02 | Putative uncharacterized protein |
| GRMZM2G135320 | 75 | 178 | -0.66 | 4.94E-03 | Putative uncharacterized protein OJ1504_G04.6 |
| GRMZM2G135322 | 101 | 213 | -0.49 | 2.61E-02 | Putative uncharacterized protein |
| GRMZM2G135337 | 28 | 84 | -0.99 | 7.62E-03 | Ubiquitin-activating enzyme E1 |
| GRMZM2G135341 | 134 | 281 | -0.48 | 8.73E-03 | BADH-like protein |
| GRMZM2G135367 | 43 | 26 | 1.32 | 1.38E-03 | Electron transporter |
| GRMZM2G135470 | 56 | 170 | -1.01 | 2.55E-05 | Putative uncharacterized protein |
| GRMZM2G135476 | 28 | 78 | -0.89 | 2.46E-02 | Putative uncharacterized protein |
| GRMZM2G135654 | 932 | 1,179 | 0.25 | 5.99E-04 | Putative uncharacterized protein |
| GRMZM2G135688 | 126 | 126 | 0.59 | 7.21E-03 | Putative uncharacterized protein |
| GRMZM2G135727 | 1,624 | 1,449 | 0.76 | 1.83E-45 | 60S ribosomal protein L3 (Putative uncharacterized protein) |
| GRMZM2G135756 | 145 | 290 | -0.41 | 2.46E-02 | RER1A protein |
| GRMZM2G135778 | 257 | 472 | -0.29 | 4.67E-02 | Splicing factor, arginine/serine-rich 7 |
| GRMZM2G135782 | 90 | 234 | -0.79 | 6.66E-05 | Putative uncharacterized protein |
| GRMZM2G135839 | 64 | 161 | -0.74 | 2.59E-03 | Os07g0106000 protein (Putative uncharacterized protein) (cDNA clone:J033024P06, full insert sequence) (Putative uncharacterized protein B1317D11.117-1) (Putative uncharacterized protein P0617C02.122-1) |
| GRMZM2G135968 | 41 | 232 | -1.91 | 1.89E-17 | Putative uncharacterized protein |
| GRMZM2G135970 | 52 | 33 | 1.25 | 6.81E-04 | Putative uncharacterized protein |
| GRMZM2G135978 | 592 | 711 | 0.33 | 4.25E-04 | Putative uncharacterized protein (Transport inhibitor response 1) |
| GRMZM2G136058 | 225 | 250 | 0.44 | 6.02E-03 | Putative uncharacterized protein (Transmembrane emp24 domain-containing protein 10) |
| GRMZM2G136178 | 63 | 55 | 0.79 | 1.72E-02 | Protein AIG1 |
| GRMZM2G136237 | 79 | 176 | -0.56 | 1.93E-02 | Putative uncharacterized protein |
| GRMZM2G136250 | 61 | 200 | -1.12 | 3.17E-07 | Cupin, RmlC-type |
| GRMZM2G136262 | 138 | 289 | -0.47 | 7.43E-03 | Acyl carrier protein |
| GRMZM2G136283 | 271 | 614 | -0.59 | 1.82E-07 | Putative uncharacterized protein |
| GRMZM2G136296 | 223 | 483 | -0.52 | 5.62E-05 | Putative uncharacterized protein |
| GRMZM2G136369 | 44 | 122 | -0.88 | 2.34E-03 | Homeodomain transcription factor |
| GRMZM2G136427 | 119 | 99 | 0.86 | 1.46E-04 | Transcription regulator |
| GRMZM2G136494 | 210 | 465 | -0.56 | 2.98E-05 | Os04g0690100 protein (Putative uncharacterized protein) (OSJNBa0039K24.23 protein) |
| GRMZM2G136513 | 73 | 50 | 1.14 | 1.65E-04 | Os03g0254000 protein (cDNA, clone: J090087F12, full insert sequence) (NB-ARC domain containing protein, expressed) |
| GRMZM2G136624 | 209 | 123 | 1.36 | 1.17E-15 | Putative uncharacterized protein |
| GRMZM2G136635 | 401 | 227 | 1.41 | 1.07E-31 | Putative uncharacterized protein |
| GRMZM2G136644 | 400 | 490 | 0.30 | 1.23E-02 | Putative uncharacterized protein |
| GRMZM2G136665 | 238 | 216 | 0.73 | 1.03E-06 | NA |
| GRMZM2G136712 | 87 | 233 | -0.83 | 2.77E-05 | Os09g0294000 protein (cDNA clone:J013146N15, full insert sequence) (Aspartate kinase-homoserine dehydrogenase) |
| GRMZM2G136765 | 11 | 44 | -1.41 | 1.20E-02 | Protein phosphatase 2C containing protein |
| GRMZM2G136889 | 117 | 108 | 0.71 | 2.00E-03 | Putative uncharacterized protein |
| GRMZM2G136895 | 170 | 385 | -0.59 | 7.47E-05 | Os04g0640700 protein (OSJNBb0079B02.3 protein) |
| GRMZM2G136918 | 54 | 219 | -1.43 | 3.73E-11 | Putative uncharacterized protein |
| GRMZM2G137064 | 28 | 80 | -0.92 | 1.56E-02 | Putative uncharacterized protein |
| GRMZM2G137288 | 76 | 69 | 0.73 | 1.47E-02 | Putative uncharacterized protein |
| GRMZM2G137312 | 180 | 383 | -0.50 | 8.08E-04 | NADH-ubiquinone oxidoreductase B18 subunit (Putative uncharacterized protein) |
| GRMZM2G137338 | 46 | 139 | -1.00 | 2.03E-04 | Unknow protein |
| GRMZM2G137352 | 43 | 120 | -0.89 | 2.68E-03 | Remorin |
| GRMZM2G137375 | 665 | 575 | 0.80 | 1.06E-20 | Putative uncharacterized protein |
| GRMZM2G137409 | 12 | 44 | -1.28 | 1.99E-02 | Putative uncharacterized protein |
| GRMZM2G137468 | 114 | 57 | 1.59 | 5.02E-11 | Protein kinase APK1A (Putative uncharacterized protein) |
| GRMZM2G137495 | 9 | 62 | -2.19 | 8.07E-06 | Putative uncharacterized protein |
| GRMZM2G137532 | 7 | 1 | 3.40 | 3.67E-02 | Os09g0474000 protein (Putative uncharacterized protein) (cDNA clone:002-141-G12, full insert sequence) (Putative uncharacterized protein OSJNBa0026C08.19) |
| GRMZM2G137558 | 100 | 281 | -0.90 | 4.90E-07 | Putative uncharacterized protein |
| GRMZM2G137596 | 103 | 107 | 0.54 | 3.30E-02 | Putative uncharacterized protein |
| GRMZM2G137704 | 24 | 83 | -1.20 | 1.16E-03 | Os05g0272900 protein (Putative uncharacterized protein) (cDNA clone:002-121-H04, full insert sequence) (Putative uncharacterized protein OSJNBb0061M13.14) |
| GRMZM2G137707 | 34 | 115 | -1.17 | 1.51E-04 | Putative uncharacterized protein |
| GRMZM2G137839 | 697 | 1,635 | -0.64 | 6.19E-22 | APx1-Cytosolic Ascorbate Peroxidase (Ascorbate peroxidase) |
| GRMZM2G137849 | 80 | 79 | 0.61 | 4.01E-02 | Protein brittle-1 (Nucleotide sugar translocator BT2B) |
| GRMZM2G137930 | 8 | 86 | -2.83 | 2.51E-10 | Putative uncharacterized protein (Soluble inorganic pyrophosphatase) |
| GRMZM2G137947 | 438 | 469 | 0.49 | 4.43E-06 | 26S proteasome non-ATPase regulatory subunit 13 (Putative uncharacterized protein) |
| GRMZM2G137965 | 1,274 | 1,461 | 0.39 | 3.80E-11 | Putative uncharacterized protein (Nucleosome/chromatin assembly factor group C) |
| GRMZM2G137968 | 99 | 256 | -0.78 | 3.07E-05 | KAP-2 |
| GRMZM2G138041 | 11 | 43 | -1.38 | 1.63E-02 | ATP-dependent RNA helicase DBP5 |
| GRMZM2G138053 | 155 | 118 | 0.98 | 3.19E-07 | Putative uncharacterized protein |
| GRMZM2G138067 | 24 | 8 | 2.18 | 6.31E-04 | Putative uncharacterized protein |
| GRMZM2G138152 | 42 | 114 | -0.85 | 4.74E-03 | Respiratory burst oxidase protein B |
| GRMZM2G138176 | 75 | 65 | 0.80 | 7.52E-03 | F-box/LRR-repeat protein 2 (Putative uncharacterized protein) |
| GRMZM2G138178 | 110 | 273 | -0.72 | 6.60E-05 | Os09g0306700 protein (cDNA, clone: J100045B11, full insert sequence) (Putative SMA-9 class B) |
| GRMZM2G138220 | 417 | 861 | -0.45 | 1.25E-06 | ATP synthase delta chain (Putative uncharacterized protein) |
| GRMZM2G138245 | 5 | 25 | -1.73 | 3.74E-02 | CDPK-related protein kinase, putative, expressed (Putative kinase) |
| GRMZM2G138258 | 80 | 65 | 0.89 | 1.70E-03 | Putative uncharacterized protein |
| GRMZM2G138342 | 55 | 43 | 0.95 | 7.41E-03 | NAD kinase 1 |
| GRMZM2G138410 | 23 | 73 | -1.07 | 6.77E-03 | Putative uncharacterized protein (UDP-glucose 4-epimerase GEPI48) |
| GRMZM2G138419 | 226 | 479 | -0.49 | 1.73E-04 | Os12g0568800 protein (Putative uncharacterized protein) (Importin-beta N-terminal domain containing protein, expressed) (Importin-beta2) (Fragment) |
| GRMZM2G138421 | 50 | 119 | -0.66 | 2.74E-02 | Putative uncharacterized protein |
| GRMZM2G138494 | 37 | 111 | -0.99 | 1.42E-03 | Putative uncharacterized protein |
| GRMZM2G138496 | 41 | 133 | -1.11 | 6.22E-05 | Putative uncharacterized protein |
| GRMZM2G138511 | 143 | 318 | -0.56 | 7.20E-04 | Putative uncharacterized protein (DnaJ domain containing protein, expressed) (Putative heat shock protein) |
| GRMZM2G138527 | 790 | 1,795 | -0.59 | 4.65E-21 | Fiber protein Fb15 |
| GRMZM2G138550 | 745 | 1,716 | -0.61 | 2.57E-21 | NA |
| GRMZM2G138566 | 2 | 19 | -2.66 | 1.65E-02 | Calcium binding atopy-related autoantigen 1 |
| GRMZM2G138583 | 82 | 222 | -0.85 | 3.49E-05 | Brain protein 16 (Putative uncharacterized protein) |
| GRMZM2G138659 | 43 | 142 | -1.13 | 2.83E-05 | Putative uncharacterized protein |
| GRMZM2G138676 | 120 | 284 | -0.65 | 2.09E-04 | Putative uncharacterized protein |
| GRMZM2G138819 | 213 | 203 | 0.66 | 3.95E-05 | Putative uncharacterized protein |
| GRMZM2G138881 | 74 | 174 | -0.64 | 6.67E-03 | ER lumen protein retaining receptor C28H8.4 (Putative uncharacterized protein) |
| GRMZM2G138943 | 32 | 92 | -0.93 | 7.17E-03 | Putative uncharacterized protein |
| GRMZM2G138964 | 21 | 84 | -1.41 | 1.96E-04 | Putative uncharacterized protein |
| GRMZM2G138987 | 796 | 843 | 0.51 | 3.23E-11 | Putative uncharacterized protein |
| GRMZM2G139031 | 53 | 136 | -0.77 | 4.84E-03 | Putative uncharacterized protein |
| GRMZM2G139035 | 63 | 44 | 1.11 | 7.60E-04 | Putative uncharacterized protein |
| GRMZM2G139047 | 46 | 121 | -0.80 | 6.58E-03 | NOL1/NOP2/sun family protein (Putative uncharacterized protein) |
| GRMZM2G139141 | 133 | 362 | -0.85 | 2.72E-08 | Putative uncharacterized protein (cDNA clone:J023120B17, full insert sequence) (cDNA clone:J033082K10, full insert sequence) (Drought-inducible protein 1OS) (Os01g0785700 protein) |
| GRMZM2G139160 | 40 | 30 | 1.01 | 2.22E-02 | C2H2 zinc finger protein |
| GRMZM2G139198 | 98 | 205 | -0.47 | 3.14E-02 | Partner of Nob1 (Putative uncharacterized protein) |
| GRMZM2G139210 | 55 | 220 | -1.41 | 6.48E-11 | Putative uncharacterized protein |
| GRMZM2G139250 | 80 | 50 | 1.27 | 1.07E-05 | Putative uncharacterized protein |
| GRMZM2G139336 | 113 | 272 | -0.68 | 1.71E-04 | Putative uncharacterized protein |
| GRMZM2G139341 | 158 | 358 | -0.59 | 1.41E-04 | Putative uncharacterized protein |
| GRMZM2G139349 | 1,321 | 2,265 | -0.19 | 1.35E-03 | 40S ribosomal protein S25-1 (Putative uncharacterized protein) |
| GRMZM2G139407 | 237 | 481 | -0.43 | 1.16E-03 | Putative uncharacterized protein |
| GRMZM2G139419 | 77 | 206 | -0.83 | 1.06E-04 | H0525C06.10 protein |
| GRMZM2G139434 | 8 | 1 | 3.59 | 1.85E-02 | Putative uncharacterized protein |
| GRMZM2G139441 | 408 | 409 | 0.59 | 1.09E-07 | DAG protein (Putative uncharacterized protein) |
| GRMZM2G139462 | 18 | 70 | -1.37 | 9.23E-04 | Putative uncharacterized protein (Uridylate kinase) |
| GRMZM2G139617 | 474 | 1,149 | -0.69 | 1.50E-17 | Dek protein |
| GRMZM2G139650 | 43 | 103 | -0.67 | 4.59E-02 | Phytoene dehydrogenase-like |
| GRMZM2G139657 | 134 | 92 | 1.13 | 8.63E-08 | Putative uncharacterized protein |
| GRMZM2G139680 | 381 | 266 | 1.11 | 1.45E-20 | 2-cys peroxiredoxin BAS1 |
| GRMZM2G139714 | 59 | 49 | 0.86 | 1.18E-02 | UL36 tegument protein |
| GRMZM2G139760 | 132 | 326 | -0.71 | 1.20E-05 | Putative uncharacterized protein |
| GRMZM2G139822 | 46 | 108 | -0.64 | 4.48E-02 | Putative uncharacterized protein |
| GRMZM2G139861 | 74 | 55 | 1.02 | 5.42E-04 | Os10g0196400 protein |
| GRMZM2G139874 | 77 | 62 | 0.90 | 1.76E-03 | Putative uncharacterized protein |
| GRMZM2G139880 | 93 | 195 | -0.48 | 3.58E-02 | Bifunctional dihydrofolate reductase-thymidylate synthase |
| GRMZM2G139882 | 53 | 198 | -1.31 | 7.21E-09 | Putative uncharacterized protein |
| GRMZM2G139892 | 73 | 197 | -0.84 | 1.17E-04 | Putative uncharacterized protein |
| GRMZM2G139894 | 620 | 1,322 | -0.50 | 1.20E-11 | Putative uncharacterized protein |
| GRMZM2G139900 | 948 | 2,645 | -0.89 | 2.07E-62 | 40S ribosomal protein S12 |
| GRMZM2G139931 | 48 | 117 | -0.69 | 2.46E-02 | Putative uncharacterized protein |
| GRMZM2G139952 | 507 | 545 | 0.49 | 8.41E-07 | Putative uncharacterized protein |
| GRMZM2G140051 | 544 | 2,162 | -1.40 | 6.71E-105 | Nucleosome/chromatin assembly factor group A |
| GRMZM2G140116 | 728 | 1,319 | -0.27 | 4.76E-04 | Putative uncharacterized protein |
| GRMZM2G140156 | 90 | 210 | -0.63 | 2.65E-03 | Putative uncharacterized protein |
| GRMZM2G140179 | 52 | 139 | -0.83 | 1.94E-03 | Putative uncharacterized protein |
| GRMZM2G140201 | 318 | 139 | 1.79 | 1.39E-35 | Putative uncharacterized protein |
| GRMZM2G140288 | 14 | 54 | -1.36 | 6.51E-03 | Putative uncharacterized protein |
| GRMZM2G140328 | 215 | 480 | -0.57 | 1.40E-05 | Os04g0605500 protein (Putative uncharacterized protein) (OSJNBa0035M09.2 protein) (OSJNBb0015N08.12 protein) |
| GRMZM2G140342 | 84 | 285 | -1.17 | 1.04E-10 | Putative uncharacterized protein (TMEM87A protein) |
| GRMZM2G140362 | 0 | 10 | #NUM! | 3.46E-02 | Putative uncharacterized protein |
| GRMZM2G140451 | 481 | 1,043 | -0.53 | 4.44E-10 | Putative uncharacterized protein |
| GRMZM2G140537 | 85 | 84 | 0.61 | 3.30E-02 | Protein kinase family protein, putative, expressed |
| GRMZM2G140545 | 399 | 250 | 1.27 | 1.86E-26 | 14-3-3-like protein A |
| GRMZM2G140577 | 150 | 553 | -1.29 | 9.11E-24 | SET domain protein 105 |
| GRMZM2G140590 | 68 | 188 | -0.88 | 9.24E-05 | Putative uncharacterized protein |
| GRMZM2G140609 | 355 | 996 | -0.90 | 6.55E-24 | 40S ribosomal protein S23 (Putative uncharacterized protein) |
| GRMZM2G140633 | 101 | 42 | 1.86 | 3.73E-12 | Cyclin delta-2 |
| GRMZM2G140635 | 4 | 41 | -2.77 | 5.20E-05 | Putative uncharacterized protein |
| GRMZM2G140674 | 11 | 54 | -1.70 | 8.29E-04 | Putative uncharacterized protein |
| GRMZM2G140694 | 22 | 14 | 1.24 | 4.47E-02 | Putative uncharacterized protein |
| GRMZM2G140737 | 193 | 191 | 0.61 | 3.81E-04 | Putative uncharacterized protein (Vacuolar protein sorting 26) |
| GRMZM2G140758 | 177 | 357 | -0.42 | 8.13E-03 | Putative uncharacterized protein |
| GRMZM2G140763 | 5 | 0 | #VALUE! | 4.27E-02 | Putative uncharacterized protein |
| GRMZM2G140809 | 232 | 233 | 0.59 | 1.43E-04 | Putative uncharacterized protein |
| GRMZM2G140837 | 64 | 52 | 0.89 | 6.74E-03 | Putative uncharacterized protein (Serologically defined breast cancer antigen NY-BR-84) |
| GRMZM2G140867 | 364 | 694 | -0.34 | 1.86E-03 | Proteasome subunit beta type 7-A (Putative uncharacterized protein) |
| GRMZM2G140970 | 174 | 368 | -0.49 | 1.37E-03 | PWWP domain containing protein |
| GRMZM2G141002 | 8 | 35 | -1.54 | 2.17E-02 | Os06g0171600 protein (cDNA clone:001-127-G09, full insert sequence) (Inner membrane ALBINO3-like protein) |
| GRMZM2G141216 | 20 | 68 | -1.17 | 5.81E-03 | Zinc finger, C3HC4 type family protein |
| GRMZM2G141222 | 84 | 217 | -0.78 | 1.49E-04 | NA |
| GRMZM2G141241 | 83 | 75 | 0.74 | 8.25E-03 | Putative uncharacterized protein |
| GRMZM2G141273 | 104 | 265 | -0.76 | 3.60E-05 | P-protein (Putative uncharacterized protein) |
| GRMZM2G141332 | 24 | 74 | -1.03 | 1.00E-02 | Leucine Rich Repeat family protein, expressed |
| GRMZM2G141399 | 14 | 49 | -1.22 | 2.08E-02 | Starch synthase DULL1 |
| GRMZM2G141432 | 1,745 | 1,514 | 0.80 | 1.83E-53 | Histone H2B |
| GRMZM2G141472 | 144 | 349 | -0.69 | 1.17E-05 | Putative uncharacterized protein |
| GRMZM2G141499 | 74 | 178 | -0.67 | 3.93E-03 | Putative uncharacterized protein |
| GRMZM2G141503 | 20 | 59 | -0.97 | 3.54E-02 | Putative uncharacterized protein |
| GRMZM2G141600 | 40 | 103 | -0.77 | 1.79E-02 | Putative uncharacterized protein |
| GRMZM2G141665 | 58 | 40 | 1.13 | 1.25E-03 | Putative uncharacterized protein (Syringomycin biosynthesis enzyme) |
| GRMZM2G141679 | 561 | 1,340 | -0.66 | 2.66E-19 | DRE binding factor 2 |
| GRMZM2G141723 | 14 | 47 | -1.16 | 3.67E-02 | Putative uncharacterized protein |
| GRMZM2G141735 | 14 | 49 | -1.22 | 2.08E-02 | Putative uncharacterized protein |
| GRMZM2G141784 | 65 | 179 | -0.87 | 1.74E-04 | Chaperone protein dnaJ 10 |
| GRMZM2G141799 | 155 | 122 | 0.94 | 1.08E-06 | Tropinone reductase |
| GRMZM2G141810 | 28 | 2 | 4.40 | 2.06E-08 | Putative uncharacterized protein |
| GRMZM2G141818 | 927 | 1,212 | 0.20 | 7.20E-03 | Os04g0151800 protein (Putative uncharacterized protein) |
| GRMZM2G141848 | 29 | 96 | -1.14 | 8.29E-04 | Putative uncharacterized protein (Adenosine 5'-phosphosulfate reductase 6) |
| GRMZM2G141856 | 117 | 420 | -1.25 | 2.96E-17 | Uridine kinase (EC 2.7.1.48) |
| GRMZM2G141858 | 32 | 124 | -1.36 | 3.86E-06 | Putative uncharacterized protein |
| GRMZM2G141873 | 10 | 43 | -1.51 | 9.62E-03 | Putative uncharacterized protein |
| GRMZM2G141903 | 338 | 272 | 0.90 | 3.93E-13 | Casein kinase II subunit alpha-2 (Putative uncharacterized protein) |
| GRMZM2G141922 | 107 | 70 | 1.20 | 7.47E-07 | Putative uncharacterized protein |
| GRMZM2G141925 | 702 | 626 | 0.76 | 8.67E-20 | Seed maturation protein |
| GRMZM2G141998 | 33 | 85 | -0.77 | 3.65E-02 | Os04g0686700 protein |
| GRMZM2G142057 | 60 | 31 | 1.54 | 9.89E-06 | Putative uncharacterized protein |
| GRMZM2G142072 | 80 | 225 | -0.90 | 9.25E-06 | Suppressor of ty, putative |
| GRMZM2G142111 | 39 | 141 | -1.26 | 3.36E-06 | Putative uncharacterized protein |
| GRMZM2G142119 | 42 | 168 | -1.41 | 2.22E-08 | MAGE (Putative uncharacterized protein) |
| GRMZM2G142168 | 79 | 180 | -0.60 | 1.01E-02 | Os09g0566100 protein |
| GRMZM2G142266 | 52 | 38 | 1.04 | 4.63E-03 | Putative uncharacterized protein |
| GRMZM2G142336 | 32 | 138 | -1.52 | 1.02E-07 | Putative uncharacterized protein |
| GRMZM2G142409 | 24 | 119 | -1.72 | 5.64E-08 | Reticulon |
| GRMZM2G142502 | 96 | 228 | -0.66 | 1.03E-03 | Putative uncharacterized protein |
| GRMZM2G142553 | 38 | 116 | -1.02 | 7.52E-04 | Putative uncharacterized protein |
| GRMZM2G142557 | 412 | 454 | 0.45 | 5.39E-05 | RNA-binding post-transcriptional regulator csx1 |
| GRMZM2G142620 | 307 | 361 | 0.36 | 8.71E-03 | Putative uncharacterized protein |
| GRMZM2G142640 | 1,186 | 2,699 | -0.59 | 8.33E-32 | 60S ribosomal protein L24 |
| GRMZM2G142667 | 22 | 62 | -0.90 | 4.30E-02 | Os04g0678300 protein (Putative uncharacterized protein) (OSJNBa0064G10.23 protein) |
| GRMZM2G142712 | 64 | 182 | -0.92 | 6.22E-05 | Putative uncharacterized protein |
| GRMZM2G142718 | 2 | 17 | -2.50 | 3.74E-02 | Putative uncharacterized protein |
| GRMZM2G142721 | 18 | 4 | 2.76 | 6.73E-04 | Putative uncharacterized protein |
| GRMZM2G142806 | 108 | 91 | 0.84 | 3.84E-04 | TBC1 domain family member 22A |
| GRMZM2G142825 | 67 | 158 | -0.65 | 1.14E-02 | Putative uncharacterized protein |
| GRMZM2G142850 | 36 | 134 | -1.30 | 3.45E-06 | Putative uncharacterized protein |
| GRMZM2G142870 | 63 | 158 | -0.74 | 3.59E-03 | cDNA, clone: J075142E09, full insert sequence (MRP-like ABC transporter) (Putative AtMRP4) |
| GRMZM2G142875 | 24 | 13 | 1.48 | 1.25E-02 | Putative uncharacterized protein |
| GRMZM2G142913 | 43 | 110 | -0.76 | 1.47E-02 | Putative uncharacterized protein |
| GRMZM2G142918 | 105 | 63 | 1.33 | 8.31E-08 | Putative uncharacterized protein |
| GRMZM2G142984 | 169 | 353 | -0.47 | 2.72E-03 | Putative uncharacterized protein |
| GRMZM2G143071 | 145 | 159 | 0.46 | 2.73E-02 | Putative uncharacterized protein |
| GRMZM2G143128 | 57 | 224 | -1.38 | 7.42E-11 | Putative uncharacterized protein (Vacuolar ATP synthase 21 kDa proteolipid subunit) |
| GRMZM2G143160 | 82 | 260 | -1.07 | 1.22E-08 | Putative uncharacterized protein |
| GRMZM2G143165 | 231 | 242 | 0.52 | 7.27E-04 | Ectonucleotide pyrophosphatase/phosphodiesterase 1 |
| GRMZM2G143205 | 85 | 211 | -0.72 | 5.38E-04 | Os04g0388500 protein |
| GRMZM2G143210 | 4 | 37 | -2.62 | 3.08E-04 | DNA binding protein (Putative uncharacterized protein) |
| GRMZM2G143234 | 409 | 354 | 0.80 | 7.37E-13 | ATP synthase epsilon chain, mitochondrial |
| GRMZM2G143242 | 70 | 152 | -0.53 | 4.64E-02 | Putative uncharacterized protein |
| GRMZM2G143246 | 204 | 445 | -0.53 | 9.53E-05 | Putative uncharacterized protein |
| GRMZM2G143330 | 49 | 132 | -0.84 | 2.34E-03 | Eukaryotic translation initiation factor 3 subunit 2 |
| GRMZM2G143357 | 11 | 48 | -1.53 | 4.57E-03 | Putative uncharacterized protein |
| GRMZM2G143377 | 12 | 58 | -1.68 | 5.57E-04 | Os06g0613100 protein (Selenium-binding protein-like) |
| GRMZM2G143392 | 73 | 69 | 0.67 | 2.76E-02 | Sigma factor SIG2A |
| GRMZM2G143402 | 366 | 418 | 0.40 | 9.45E-04 | ZIM motif family protein |
| GRMZM2G143403 | 7 | 46 | -2.12 | 2.72E-04 | Putative uncharacterized protein |
| GRMZM2G143443 | 64 | 151 | -0.65 | 1.39E-02 | Putative uncharacterized protein |
| GRMZM2G143445 | 88 | 37 | 1.84 | 1.52E-10 | Os02g0179800 protein (Putative uncharacterized protein P0544B02.28) |
| GRMZM2G143462 | 507 | 630 | 0.28 | 7.52E-03 | DNA repair protein RAD23-1 |
| GRMZM2G143469 | 10 | 3 | 2.33 | 3.88E-02 | OHP2 |
| GRMZM2G143499 | 146 | 289 | -0.39 | 3.18E-02 | Peptidyl-tRNA hydrolase 2 (Putative uncharacterized protein) |
| GRMZM2G143568 | 202 | 406 | -0.42 | 4.76E-03 | Os08g0200400 protein (cDNA clone:J013122M06, full insert sequence) (Putative KH domain protein) |
| GRMZM2G143602 | 90 | 218 | -0.68 | 8.82E-04 | Putative uncharacterized protein |
| GRMZM2G143646 | 10 | 57 | -1.92 | 1.35E-04 | Putative uncharacterized protein (Putative pentatricopeptide repeat containing protein) |
| GRMZM2G143655 | 65 | 193 | -0.98 | 9.92E-06 | PYM protein |
| GRMZM2G143703 | 52 | 126 | -0.69 | 1.78E-02 | Catalytic/ hydrolase |
| GRMZM2G143725 | 173 | 473 | -0.86 | 9.02E-11 | Os04g0129500 protein |
| GRMZM2G143767 | 89 | 198 | -0.56 | 1.12E-02 | Zinc-binding protein |
| GRMZM2G143780 | 2,297 | 2,453 | 0.50 | 3.62E-30 | Histone H4 |
| GRMZM2G143782 | 27 | 7 | 2.54 | 3.97E-05 | Speckle-type POZ protein |
| GRMZM2G143788 | 240 | 250 | 0.53 | 3.96E-04 | Putative uncharacterized protein |
| GRMZM2G143791 | 1,662 | 1,885 | 0.41 | 1.70E-15 | Putative uncharacterized protein |
| GRMZM2G143854 | 93 | 212 | -0.60 | 5.02E-03 | Putative uncharacterized protein |
| GRMZM2G143870 | 16 | 49 | -1.02 | 4.67E-02 | Putative uncharacterized protein |
| GRMZM2G143878 | 38 | 94 | -0.72 | 4.14E-02 | Putative uncharacterized protein |
| GRMZM2G143883 | 113 | 91 | 0.90 | 8.10E-05 | Putative uncharacterized protein |
| GRMZM2G143917 | 9 | 38 | -1.49 | 2.01E-02 | Putative uncharacterized protein |
| GRMZM2G144008 | 47 | 136 | -0.94 | 6.13E-04 | Putative uncharacterized protein (Ras-related protein Rab11A) |
| GRMZM2G144030 | 1,285 | 1,581 | 0.29 | 1.16E-06 | Eukaryotic translation initiation factor 5A |
| GRMZM2G144097 | 15 | 56 | -1.31 | 5.82E-03 | Putative uncharacterized protein |
| GRMZM2G144101 | 192 | 198 | 0.55 | 1.38E-03 | Mitochondrial import inner membrane translocase subunit Tim9 (Putative uncharacterized protein) |
| GRMZM2G144146 | 133 | 352 | -0.81 | 2.09E-07 | Putative uncharacterized protein |
| GRMZM2G144166 | 50 | 158 | -1.07 | 1.97E-05 | Putative uncharacterized protein OSJNBa0038P01.31 |
| GRMZM2G144254 | 52 | 165 | -1.07 | 1.16E-05 | Putative uncharacterized protein |
| GRMZM2G144387 | 2,434 | 3,301 | 0.15 | 6.76E-04 | Ribosomal protein L1 |
| GRMZM2G144615 | 30 | 143 | -1.66 | 4.85E-09 | F-box domain containing protein |
| GRMZM2G144618 | 112 | 96 | 0.81 | 4.17E-04 | Putative uncharacterized protein |
| GRMZM2G144635 | 126 | 122 | 0.64 | 3.61E-03 | Putative uncharacterized protein |
| GRMZM2G144665 | 51 | 133 | -0.79 | 4.18E-03 | Putative uncharacterized protein |
| GRMZM2G144674 | 12 | 1 | 4.18 | 9.96E-04 | NA |
| GRMZM2G144701 | 6 | 30 | -1.73 | 1.79E-02 | Putative uncharacterized protein |
| GRMZM2G144705 | 1,311 | 2,493 | -0.34 | 1.33E-10 | Putative uncharacterized protein |
| GRMZM2G144726 | 1,402 | 2,684 | -0.35 | 6.03E-12 | Putative uncharacterized protein |
| GRMZM2G144764 | 117 | 434 | -1.30 | 6.92E-19 | Putative uncharacterized protein |
| GRMZM2G144782 | 19 | 5 | 2.52 | 1.35E-03 | Putative uncharacterized protein (RING finger and CHY zinc finger domain-containing protein 1) |
| GRMZM2G144868 | 386 | 943 | -0.70 | 7.70E-15 | Putative uncharacterized protein |
| GRMZM2G144873 | 97 | 260 | -0.83 | 7.18E-06 | Putative uncharacterized protein (Transferase, transferring glycosyl groups) |
| GRMZM2G145008 | 10 | 46 | -1.61 | 3.53E-03 | Putative uncharacterized protein (GTPase activating protein, putative, expressed) |
| GRMZM2G145034 | 140 | 277 | -0.39 | 3.97E-02 | Putative uncharacterized protein |
| GRMZM2G145041 | 12 | 53 | -1.55 | 2.18E-03 | Putative uncharacterized protein |
| GRMZM2G145101 | 112 | 109 | 0.63 | 6.98E-03 | Putative uncharacterized protein |
| GRMZM2G145107 | 49 | 145 | -0.97 | 2.26E-04 | Putative uncharacterized protein (Sucrose transporter 2) |
| GRMZM2G145112 | 152 | 135 | 0.76 | 9.36E-05 | Os06g0728500 protein (Putative uncharacterized protein) (cDNA clone:J013118C12, full insert sequence) (cDNA clone:J033068A13, full insert sequence) (Putative uncharacterized protein P0017G10.33) |
| GRMZM2G145146 | 67 | 34 | 1.57 | 1.57E-06 | DNA binding protein |
| GRMZM2G145175 | 72 | 164 | -0.60 | 1.70E-02 | Putative uncharacterized protein |
| GRMZM2G145201 | 2 | 19 | -2.66 | 1.65E-02 | Putative RNA-dependent RNA polymerase (SHOOTLESS2) |
| GRMZM2G145213 | 132 | 141 | 0.50 | 2.50E-02 | Putative uncharacterized protein |
| GRMZM2G145236 | 29 | 145 | -1.73 | 1.17E-09 | Os10g0577800 protein (Putative uncharacterized protein) (cDNA clone:J023139M21, full insert sequence) (Poly polymerase catalytic domain containing protein, expressed) (Putative CEO protein) |
| GRMZM2G145258 | 1,147 | 1,148 | 0.59 | 9.92E-21 | Putative uncharacterized protein |
| GRMZM2G145280 | 2,815 | 2,383 | 0.83 | 4.56E-93 | 60S ribosomal protein L13 |
| GRMZM2G145308 | 70 | 61 | 0.79 | 9.66E-03 | Putative uncharacterized protein |
| GRMZM2G145360 | 27 | 18 | 1.18 | 3.84E-02 | Putative salt-inducible protein kinase |
| GRMZM2G145396 | 318 | 711 | -0.57 | 4.30E-08 | ATOZI1 |
| GRMZM2G145473 | 45 | 112 | -0.72 | 2.05E-02 | Os02g0829400 protein (Putative uncharacterized protein OJ1124_D06.10) |
| GRMZM2G145482 | 39 | 114 | -0.96 | 1.79E-03 | Putative uncharacterized protein |
| GRMZM2G145500 | 294 | 297 | 0.58 | 1.48E-05 | Putative uncharacterized protein |
| GRMZM2G145578 | 32 | 127 | -1.40 | 2.02E-06 | Putative uncharacterized protein |
| GRMZM2G145651 | 36 | 151 | -1.48 | 3.73E-08 | Putative uncharacterized protein |
| GRMZM2G145690 | 17 | 66 | -1.37 | 1.93E-03 | Putative uncharacterized protein |
| GRMZM2G145699 | 125 | 249 | -0.40 | 4.65E-02 | GPI transamidase subunit PIG-U family protein (Putative uncharacterized protein) |
| GRMZM2G145715 | 424 | 468 | 0.45 | 4.00E-05 | Putative uncharacterized protein |
| GRMZM2G145720 | 11 | 43 | -1.38 | 1.63E-02 | Putative uncharacterized protein |
| GRMZM2G145753 | 29 | 14 | 1.64 | 2.96E-03 | Putative receptor-like protein kinase |
| GRMZM2G145758 | 68 | 202 | -0.98 | 6.77E-06 | Histone H3 |
| GRMZM2G145775 | 13 | 61 | -1.64 | 5.36E-04 | Putative uncharacterized protein |
| GRMZM2G145788 | 22 | 1 | 5.05 | 3.68E-07 | NA |
| GRMZM2G145850 | 38 | 13 | 2.14 | 1.01E-05 | Os03g0713200 protein (Putative uncharacterized protein) (Expressed protein) (Putative uncharacterized protein OSJNBa0014G15.28) |
| GRMZM2G145854 | 372 | 455 | 0.30 | 1.62E-02 | NADH-ubiquinone oxidoreductase 75 kDa subunit (EC 1.6.5.3) |
| GRMZM2G145870 | 130 | 379 | -0.95 | 2.70E-10 | Putative uncharacterized protein |
| GRMZM2G145879 | 58 | 134 | -0.62 | 2.88E-02 | CDC2+/CDC28-related protein kinase R2 (Putative uncharacterized protein) |
| GRMZM2G145950 | 268 | 220 | 0.88 | 6.22E-10 | Putative uncharacterized protein |
| GRMZM2G145951 | 70 | 163 | -0.63 | 1.11E-02 | Putative uncharacterized protein |
| GRMZM2G145968 | 242 | 486 | -0.41 | 1.67E-03 | HMG1 protein |
| GRMZM2G146000 | 17 | 57 | -1.15 | 1.45E-02 | Tetratricopeptide repeat domain 4 |
| GRMZM2G146015 | 350 | 332 | 0.67 | 3.40E-08 | Blue copper protein |
| GRMZM2G146020 | 217 | 125 | 1.39 | 7.46E-17 | Transcription factor PosF21 |
| GRMZM2G146041 | 29 | 85 | -0.96 | 8.45E-03 | Os05g0247900 protein (cDNA clone:J023109O07, full insert sequence) (Uknown protein) |
| GRMZM2G146111 | 141 | 303 | -0.51 | 3.09E-03 | Putative uncharacterized protein |
| GRMZM2G146115 | 409 | 1,085 | -0.82 | 3.46E-22 | GTP-binding protein PTD004 (Putative uncharacterized protein) |
| GRMZM2G146118 | 80 | 77 | 0.65 | 2.56E-02 | Putative uncharacterized protein |
| GRMZM2G146143 | 98 | 95 | 0.64 | 1.31E-02 | Putative uncharacterized protein |
| GRMZM2G146173 | 323 | 676 | -0.47 | 1.03E-05 | Putative uncharacterized protein |
| GRMZM2G146192 | 92 | 93 | 0.58 | 3.10E-02 | Putative uncharacterized protein |
| GRMZM2G146225 | 194 | 151 | 0.95 | 1.99E-08 | Putative uncharacterized protein |
| GRMZM2G146267 | 89 | 255 | -0.93 | 8.33E-07 | Expressed protein |
| GRMZM2G146278 | 71 | 62 | 0.79 | 1.01E-02 | Putative uncharacterized protein |
| GRMZM2G146292 | 55 | 168 | -1.02 | 2.25E-05 | Putative uncharacterized protein |
| GRMZM2G146354 | 64 | 33 | 1.55 | 4.52E-06 | Ubiquitin ligase SINAT2 (SINA2) |
| GRMZM2G146358 | 715 | 1,293 | -0.26 | 6.86E-04 | Ribosomal protein L19 |
| GRMZM2G146374 | 167 | 125 | 1.01 | 5.83E-08 | Ubiquitin carrier protein (EC 6.3.2.-) |
| GRMZM2G146490 | 53 | 122 | -0.61 | 4.46E-02 | DNA repair protein XRCC1 (Putative uncharacterized protein) |
| GRMZM2G146553 | 27 | 76 | -0.90 | 2.22E-02 | Serine/threonine kinase |
| GRMZM2G146589 | 299 | 656 | -0.54 | 6.86E-07 | Lysyl-tRNA synthetase (EC 6.1.1.6) |
| GRMZM2G146670 | 568 | 1,045 | -0.29 | 9.19E-04 | Voltage-dependent anion channel protein 1b |
| GRMZM2G146761 | 141 | 339 | -0.67 | 2.08E-05 | Putative uncharacterized protein |
| GRMZM2G146819 | 71 | 198 | -0.89 | 4.95E-05 | Os03g0586700 protein (Expressed protein) |
| GRMZM2G146847 | 110 | 25 | 2.73 | 3.02E-21 | Putative uncharacterized protein |
| GRMZM2G146862 | 159 | 306 | -0.35 | 4.97E-02 | Os11g0266800 protein (TPR Domain containing protein, expressed) |
| GRMZM2G146951 | 168 | 413 | -0.71 | 6.14E-07 | Putative uncharacterized protein (Transmembrane 9 superfamily protein member 2) |
| GRMZM2G147046 | 208 | 222 | 0.50 | 2.58E-03 | Putative uncharacterized protein (Suppressor/enhancer of lin-12 protein 9) |
| GRMZM2G147056 | 111 | 119 | 0.49 | 4.40E-02 | Putative uncharacterized protein |
| GRMZM2G147145 | 146 | 426 | -0.95 | 1.85E-11 | Putative uncharacterized protein |
| GRMZM2G147221 | 19 | 59 | -1.04 | 2.46E-02 | Cellulase containing protein |
| GRMZM2G147243 | 331 | 945 | -0.92 | 9.60E-24 | Putative uncharacterized protein |
| GRMZM2G147266 | 48 | 26 | 1.48 | 1.99E-04 | Putative uncharacterized protein |
| GRMZM2G147268 | 5 | 26 | -1.79 | 2.67E-02 | Os04g0516600 protein (Putative uncharacterized protein) (cDNA clone:001-031-C08, full insert sequence) (cDNA clone:J023133K04, full insert sequence) (OSJNBb0072M01.18 protein) |
| GRMZM2G147346 | 14 | 5 | 2.08 | 1.90E-02 | Os10g0478300 protein (cDNA clone:002-153-H11, full insert sequence) (Myb-related protein Myb4, putative, expressed) |
| GRMZM2G147355 | 45 | 124 | -0.87 | 2.56E-03 | Putative uncharacterized protein |
| GRMZM2G147377 | 55 | 155 | -0.90 | 3.34E-04 | Kinesin light chain |
| GRMZM2G147402 | 61 | 211 | -1.20 | 2.36E-08 | Putative uncharacterized protein |
| GRMZM2G147450 | 121 | 258 | -0.50 | 8.98E-03 | Putative uncharacterized protein |
| GRMZM2G147459 | 152 | 364 | -0.67 | 1.06E-05 | ER lumen protein retaining receptor C28H8.4 (Putative uncharacterized protein) |
| GRMZM2G147500 | 130 | 353 | -0.85 | 5.19E-08 | Putative uncharacterized protein |
| GRMZM2G147579 | 113 | 112 | 0.60 | 9.48E-03 | Putative uncharacterized protein |
| GRMZM2G147587 | 267 | 190 | 1.08 | 5.38E-14 | Putative uncharacterized protein |
| GRMZM2G147603 | 57 | 146 | -0.77 | 3.54E-03 | Putative uncharacterized protein |
| GRMZM2G147687 | 305 | 294 | 0.64 | 7.73E-07 | Putative uncharacterized protein |
| GRMZM2G147709 | 41 | 100 | -0.69 | 4.11E-02 | Membrane protein |
| GRMZM2G147716 | 225 | 418 | -0.30 | 4.65E-02 | MADS-box transcription factor 18 (Putative uncharacterized protein) (Putative MADS-domain transcription factor) |
| GRMZM2G147800 | 150 | 330 | -0.55 | 8.08E-04 | cDNA, clone: J090091D10, full insert sequence |
| GRMZM2G147840 | 24 | 12 | 1.59 | 9.60E-03 | Putative uncharacterized protein (Os01g0190500 protein) (Putative uncharacterized protein P0710E05.21) |
| GRMZM2G147961 | 0 | 12 | #NUM! | 1.33E-02 | Putative uncharacterized protein |
| GRMZM2G148057 | 180 | 168 | 0.69 | 8.39E-05 | Protein Kinase interacting protein |
| GRMZM2G148106 | 109 | 224 | -0.45 | 3.69E-02 | ATP-dependent Clp protease proteolytic subunit |
| GRMZM2G148194 | 70 | 30 | 1.81 | 3.35E-08 | Putative uncharacterized protein |
| GRMZM2G148198 | 1 | 16 | -3.41 | 1.35E-02 | SWIb domain-containing protein |
| GRMZM2G148200 | 428 | 799 | -0.31 | 2.27E-03 | Putative uncharacterized protein |
| GRMZM2G148211 | 61 | 143 | -0.64 | 1.68E-02 | Putative uncharacterized protein |
| GRMZM2G148216 | 73 | 196 | -0.83 | 1.45E-04 | Putative uncharacterized protein |
| GRMZM2G148270 | 25 | 97 | -1.36 | 6.91E-05 | Putative uncharacterized protein |
| GRMZM2G148301 | 268 | 1,059 | -1.39 | 9.10E-51 | Ran GTPase activating protein |
| GRMZM2G148323 | 125 | 396 | -1.07 | 7.16E-13 | Os10g0476600 protein (Putative uncharacterized protein) (cDNA clone:J013116N14, full insert sequence) (cDNA clone:J033037A09, full insert sequence) (EF hand family protein, expressed) |
| GRMZM2G148333 | 31 | 17 | 1.46 | 3.85E-03 | Ethylene response factor (Putative uncharacterized protein) |
| GRMZM2G148374 | 48 | 19 | 1.93 | 3.11E-06 | Putative uncharacterized protein (Putative H+-exporting ATPase) |
| GRMZM2G148411 | 54 | 150 | -0.88 | 6.07E-04 | Putative uncharacterized protein |
| GRMZM2G148467 | 60 | 25 | 1.85 | 1.97E-07 | cDNA clone:J013158C23, full insert sequence |
| GRMZM2G148555 | 107 | 380 | -1.24 | 2.00E-15 | Os01g0621300 protein |
| GRMZM2G148561 | 22 | 10 | 1.73 | 9.10E-03 | WRKY25-superfamily of TFs having WRKY and zinc finger domains |
| GRMZM2G148633 | 121 | 307 | -0.75 | 8.58E-06 | Putative uncharacterized protein |
| GRMZM2G148709 | 774 | 593 | 0.98 | 1.60E-33 | Putative uncharacterized protein |
| GRMZM2G148723 | 70 | 154 | -0.55 | 4.02E-02 | Putative uncharacterized protein |
| GRMZM2G148744 | 2,684 | 4,489 | -0.15 | 1.60E-04 | Putative uncharacterized protein |
| GRMZM2G148751 | 45 | 123 | -0.86 | 3.20E-03 | Putative uncharacterized protein |
| GRMZM2G148769 | 612 | 626 | 0.56 | 2.83E-10 | 6-phosphogluconolactonase |
| GRMZM2G148810 | 81 | 67 | 0.87 | 1.97E-03 | Putative uncharacterized protein |
| GRMZM2G148811 | 64 | 176 | -0.87 | 1.99E-04 | Putative uncharacterized protein |
| GRMZM2G148867 | 248 | 172 | 1.12 | 1.15E-13 | Putative uncharacterized protein |
| GRMZM2G148896 | 210 | 155 | 1.03 | 3.42E-10 | Putative uncharacterized protein |
| GRMZM2G148924 | 128 | 429 | -1.15 | 1.28E-15 | Putative uncharacterized protein |
| GRMZM2G148937 | 46 | 32 | 1.11 | 4.50E-03 | Putative uncharacterized protein |
| GRMZM2G148962 | 79 | 207 | -0.80 | 1.54E-04 | Putative uncharacterized protein (Putative serine/threonine kinase) |
| GRMZM2G148985 | 234 | 463 | -0.39 | 4.09E-03 | Heat-and acid-stable phosphoprotein (Putative uncharacterized protein) |
| GRMZM2G149051 | 9 | 1 | 3.76 | 9.10E-03 | Putative Receptor-like protein kinase |
| GRMZM2G149073 | 88 | 71 | 0.90 | 7.15E-04 | Putative uncharacterized protein |
| GRMZM2G149105 | 492 | 553 | 0.42 | 3.07E-05 | Putative uncharacterized protein |
| GRMZM2G149108 | 83 | 207 | -0.73 | 5.73E-04 | Putative uncharacterized protein |
| GRMZM2G149115 | 130 | 113 | 0.79 | 1.73E-04 | Putative uncharacterized protein |
| GRMZM2G149135 | 19 | 60 | -1.07 | 1.91E-02 | Os03g0412900 protein (Expressed protein) |
| GRMZM2G149138 | 73 | 200 | -0.86 | 5.96E-05 | Nucleic acid binding protein |
| GRMZM2G149175 | 82 | 196 | -0.67 | 2.48E-03 | Putative uncharacterized protein |
| GRMZM2G149178 | 4,802 | 3,013 | 1.26 | 2.73E-319 | Histone H4 |
| GRMZM2G149211 | 148 | 149 | 0.58 | 3.64E-03 | Putative uncharacterized protein |
| GRMZM2G149238 | 75 | 173 | -0.61 | 9.94E-03 | TATA-binding protein 2 |
| GRMZM2G149257 | 830 | 1,044 | 0.26 | 8.30E-04 | 60S ribosomal protein L21 (Putative uncharacterized protein) |
| GRMZM2G149272 | 25 | 69 | -0.87 | 3.65E-02 | Putative uncharacterized protein |
| GRMZM2G149281 | 156 | 178 | 0.40 | 4.91E-02 | UMP-CMP kinase family protein |
| GRMZM2G149335 | 331 | 382 | 0.38 | 2.84E-03 | Putative uncharacterized protein |
| GRMZM2G149414 | 711 | 447 | 1.26 | 5.44E-47 | Fb14 |
| GRMZM2G149422 | 93 | 23 | 2.61 | 5.84E-17 | Phi-1-like phosphate-induced protein |
| GRMZM2G149446 | 8 | 0 | #VALUE! | 4.02E-03 | Glycine-rich cell wall structural protein 2 |
| GRMZM2G149480 | 151 | 319 | -0.49 | 3.65E-03 | Os08g0520000 protein (Putative uncharacterized protein) (cDNA clone:001-117-E10, full insert sequence) (Putative mitochondrial energy transfer protein) |
| GRMZM2G149543 | 54 | 133 | -0.71 | 1.14E-02 | Putative uncharacterized protein |
| GRMZM2G149556 | 182 | 460 | -0.75 | 2.32E-08 | Seed specific protein Bn15D1B |
| GRMZM2G149580 | 19 | 58 | -1.02 | 3.18E-02 | Acyl carrier protein |
| GRMZM2G149617 | 25 | 81 | -1.10 | 3.13E-03 | Putative uncharacterized protein |
| GRMZM2G149649 | 238 | 514 | -0.52 | 3.34E-05 | 60S ribosomal protein L12 (Putative uncharacterized protein) |
| GRMZM2G149704 | 61 | 147 | -0.68 | 9.80E-03 | SGT1 |
| GRMZM2G149751 | 1,235 | 1,108 | 0.75 | 6.03E-34 | Putative uncharacterized protein |
| GRMZM2G149761 | 237 | 729 | -1.03 | 5.95E-22 | Fiber protein Fb34 |
| GRMZM2G149768 | 5,059 | 2,955 | 1.37 | 0.00E+01 | Elongation factor 1-alpha |
| GRMZM2G149775 | 96 | 291 | -1.01 | 9.53E-09 | Histone H2A |
| GRMZM2G149802 | 188 | 523 | -0.88 | 2.23E-12 | DNA polymerase (EC 2.7.7.7) |
| GRMZM2G149837 | 56 | 35 | 1.27 | 3.18E-04 | Os01g0917200 protein (Putative uncharacterized protein P0413C03.17) |
| GRMZM2G149903 | 40 | 101 | -0.74 | 2.67E-02 | Inositol-tetrakisphosphate 1-kinase 3 (Putative uncharacterized protein) |
| GRMZM2G149943 | 38 | 20 | 1.52 | 9.42E-04 | ATP binding protein |
| GRMZM2G149946 | 174 | 377 | -0.52 | 4.93E-04 | Oligosaccharide transporter (Putative uncharacterized protein) |
| GRMZM2G149952 | 26 | 111 | -1.50 | 3.53E-06 | Putative uncharacterized protein |
| GRMZM2G149958 | 121 | 75 | 1.28 | 1.52E-08 | Putative uncharacterized protein |
| GRMZM2G149975 | 1,986 | 1,140 | 1.39 | 1.80E-153 | 60S ribosomal protein L29 |
| GRMZM2G149994 | 60 | 213 | -1.24 | 8.16E-09 | OSJNBa0029H02.6 protein |
| GRMZM2G150024 | 166 | 321 | -0.36 | 4.06E-02 | Putative uncharacterized protein |
| GRMZM2G150058 | 223 | 201 | 0.74 | 1.80E-06 | Os02g0814700 protein (Os03g0139100 protein) (Os10g0466700 protein) (Putative uncharacterized protein) (cDNA clone:J013057L24, full insert sequence) (60S ribosomal protein L17) (60S ribosomal protein L23, putative, expressed) |
| GRMZM2G150160 | 27 | 94 | -1.21 | 4.55E-04 | Putative uncharacterized protein |
| GRMZM2G150166 | 98 | 224 | -0.60 | 3.25E-03 | Adenylyl cyclase-associated protein |
| GRMZM2G150217 | 29 | 90 | -1.04 | 3.16E-03 | Os05g0238400 protein (cDNA clone:J033044N23, full insert sequence) (Putative uncharacterized protein OJ1122_B08.9) |
| GRMZM2G150248 | 140 | 121 | 0.80 | 8.37E-05 | Lysine-specific histone demethylase 1 |
| GRMZM2G150256 | 29 | 78 | -0.84 | 3.32E-02 | Cysteine proteinase Mir2 |
| GRMZM2G150262 | 133 | 314 | -0.65 | 9.95E-05 | Zinc finger C-x8-C-x5-C-x3-H type family protein |
| GRMZM2G150286 | 279 | 322 | 0.38 | 7.52E-03 | Putative uncharacterized protein |
| GRMZM2G150323 | 39 | 95 | -0.69 | 4.40E-02 | Acetylglucosaminyltransferase (Putative uncharacterized protein) |
| GRMZM2G150337 | 19 | 62 | -1.11 | 1.14E-02 | Putative uncharacterized protein |
| GRMZM2G150367 | 212 | 209 | 0.61 | 1.34E-04 | Stress-related protein |
| GRMZM2G150374 | 35 | 104 | -0.98 | 2.38E-03 | Os02g0703900 protein (cDNA clone:J033084H22, full insert sequence) (Nodulin-like protein) |
| GRMZM2G150383 | 10 | 39 | -1.37 | 2.44E-02 | Putative uncharacterized protein |
| GRMZM2G150406 | 14 | 61 | -1.53 | 9.55E-04 | Putative uncharacterized protein |
| GRMZM2G150408 | 196 | 177 | 0.74 | 1.19E-05 | Putative uncharacterized protein |
| GRMZM2G150448 | 105 | 216 | -0.45 | 3.78E-02 | Atypical receptor-like kinase MARK |
| GRMZM2G150484 | 376 | 445 | 0.35 | 3.89E-03 | Putative uncharacterized protein |
| GRMZM2G150503 | 104 | 229 | -0.55 | 6.67E-03 | Putative uncharacterized protein |
| GRMZM2G150541 | 51 | 217 | -1.50 | 8.74E-12 | Os09g0516500 protein (3-beta-hydroxysteroid dehydrogenase-like) |
| GRMZM2G150648 | 1,703 | 5,383 | -1.07 | 4.15E-172 | Putative uncharacterized protein |
| GRMZM2G150714 | 42 | 19 | 1.74 | 5.64E-05 | Putative uncharacterized protein |
| GRMZM2G150754 | 94 | 73 | 0.96 | 2.13E-04 | Putative uncharacterized protein |
| GRMZM2G150755 | 64 | 30 | 1.68 | 5.75E-07 | Putative uncharacterized protein |
| GRMZM2G150762 | 66 | 35 | 1.51 | 4.60E-06 | Hypersensitive-induced response protein |
| GRMZM2G150806 | 44 | 21 | 1.66 | 9.53E-05 | Putative uncharacterized protein |
| GRMZM2G150827 | 14 | 179 | -3.09 | 5.33E-23 | Os03g0260000 protein (Putative uncharacterized protein) (Dynamin family protein, putative, expressed) |
| GRMZM2G150867 | 34 | 91 | -0.83 | 1.72E-02 | Ubiquitin carrier protein (EC 6.3.2.-) |
| GRMZM2G150932 | 76 | 215 | -0.91 | 1.24E-05 | Putative uncharacterized protein |
| GRMZM2G150984 | 64 | 161 | -0.74 | 2.59E-03 | Putative phragmoplast-associated kinesin |
| GRMZM2G151009 | 96 | 84 | 0.78 | 2.20E-03 | ORM1-like protein 2 (Putative uncharacterized protein) |
| GRMZM2G151169 | 250 | 466 | -0.31 | 3.05E-02 | Os07g0695800 protein (cDNA clone:J023097A04, full insert sequence) (Putative 2-oxoglutarate dehydrogenase, E1 subunit) |
| GRMZM2G151223 | 155 | 412 | -0.82 | 1.03E-08 | Putative uncharacterized protein |
| GRMZM2G151236 | 85 | 215 | -0.75 | 2.95E-04 | Protein kinase domain containing protein, expressed |
| GRMZM2G151252 | 2,243 | 3,642 | -0.11 | 2.52E-02 | 40S ribosomal protein S24 |
| GRMZM2G151285 | 223 | 420 | -0.32 | 3.14E-02 | Putative uncharacterized protein |
| GRMZM2G151440 | 18 | 59 | -1.12 | 1.69E-02 | Putative uncharacterized protein |
| GRMZM2G151529 | 44 | 36 | 0.88 | 3.62E-02 | HCF106C protein |
| GRMZM2G151564 | 61 | 143 | -0.64 | 1.68E-02 | Calcium ion binding protein |
| GRMZM2G151651 | 161 | 355 | -0.55 | 4.15E-04 | Putative uncharacterized protein |
| GRMZM2G151656 | 25 | 5 | 2.91 | 1.80E-05 | SAUR52-auxin-responsive SAUR family member |
| GRMZM2G151689 | 314 | 375 | 0.34 | 1.41E-02 | Zinc finger C-x8-C-x5-C-x3-H type family protein |
| GRMZM2G151700 | 93 | 201 | -0.52 | 2.03E-02 | Actin associated protein |
| GRMZM2G151701 | 26 | 71 | -0.86 | 3.97E-02 | Putative uncharacterized protein |
| GRMZM2G151726 | 1,505 | 1,899 | 0.26 | 4.09E-06 | Histone H2A |
| GRMZM2G151734 | 156 | 325 | -0.47 | 4.80E-03 | Os06g0665100 protein (Putative uncharacterized protein) (cDNA clone:006-310-E08, full insert sequence) (cDNA clone:J033034G21, full insert sequence) (Dreg-2 like protein) |
| GRMZM2G151807 | 282 | 718 | -0.76 | 4.54E-13 | NA |
| GRMZM2G151826 | 2,705 | 2,824 | 0.53 | 5.67E-40 | Histone H2A |
| GRMZM2G151893 | 96 | 243 | -0.75 | 1.04E-04 | Putative uncharacterized protein |
| GRMZM2G151997 | 9 | 55 | -2.02 | 9.00E-05 | Putative uncharacterized protein |
| GRMZM2G152007 | 58 | 131 | -0.58 | 4.00E-02 | Os12g0125300 protein (Putative uncharacterized protein) (Protein kinase, putative, expressed) |
| GRMZM2G152059 | 57 | 51 | 0.75 | 3.44E-02 | Legume lectins beta domain containing protein |
| GRMZM2G152105 | 121 | 247 | -0.44 | 2.99E-02 | Putative uncharacterized protein |
| GRMZM2G152111 | 207 | 440 | -0.50 | 2.97E-04 | Putative uncharacterized protein (Transformer-2 protein) |
| GRMZM2G152175 | 2 | 17 | -2.50 | 3.74E-02 | Dihydroflavonol-4-reductase |
| GRMZM2G152328 | 345 | 723 | -0.48 | 4.32E-06 | Actin-1 |
| GRMZM2G152360 | 24 | 105 | -1.54 | 4.02E-06 | Putative uncharacterized protein |
| GRMZM2G152421 | 283 | 551 | -0.37 | 2.69E-03 | Putative uncharacterized protein |
| GRMZM2G152432 | 29 | 8 | 2.45 | 2.53E-05 | Calmodulin |
| GRMZM2G152438 | 66 | 158 | -0.67 | 7.47E-03 | Putative uncharacterized protein |
| GRMZM2G152466 | 3,701 | 7,619 | -0.45 | 2.67E-54 | Tubulin alpha-3 chain |
| GRMZM2G152470 | 566 | 645 | 0.40 | 1.65E-05 | Putative uncharacterized protein |
| GRMZM2G152485 | 27 | 100 | -1.30 | 1.04E-04 | Putative uncharacterized protein |
| GRMZM2G152548 | 38 | 108 | -0.92 | 3.28E-03 | Putative uncharacterized protein |
| GRMZM2G152549 | 213 | 493 | -0.62 | 1.21E-06 | Putative uncharacterized protein |
| GRMZM2G152552 | 501 | 1,005 | -0.41 | 1.65E-06 | 60S ribosomal protein L34 (Putative uncharacterized protein) |
| GRMZM2G152561 | 295 | 245 | 0.86 | 1.28E-10 | ATP-dependent RNA helicase |
| GRMZM2G152573 | 2,123 | 1,850 | 0.79 | 3.64E-64 | Putative uncharacterized protein |
| GRMZM2G152591 | 28 | 97 | -1.20 | 4.07E-04 | Putative uncharacterized protein |
| GRMZM2G152599 | 1,088 | 1,047 | 0.65 | 3.70E-23 | 30S ribosomal protein S16 (Putative uncharacterized protein) |
| GRMZM2G152686 | 51 | 161 | -1.07 | 1.73E-05 | Putative uncharacterized protein |
| GRMZM2G152703 | 297 | 198 | 1.18 | 1.20E-17 | Putative uncharacterized protein |
| GRMZM2G152732 | 38 | 121 | -1.08 | 2.67E-04 | Putative uncharacterized protein |
| GRMZM2G152764 | 4 | 26 | -2.11 | 1.27E-02 | Lipase, putative, expressed |
| GRMZM2G152774 | 57 | 49 | 0.81 | 1.95E-02 | 3-5 exonuclease/ nucleic acid binding protein |
| GRMZM2G152808 | 73 | 176 | -0.68 | 3.72E-03 | Putative uncharacterized protein |
| GRMZM2G152827 | 704 | 813 | 0.38 | 3.52E-06 | Mitochondrial phosphate transporter |
| GRMZM2G152908 | 464 | 517 | 0.44 | 2.83E-05 | Putative uncharacterized protein |
| GRMZM2G152921 | 227 | 561 | -0.71 | 2.15E-09 | Putative uncharacterized protein |
| GRMZM2G152925 | 206 | 482 | -0.63 | 1.02E-06 | Cytochrome c oxidase subunit (Putative uncharacterized protein) |
| GRMZM2G152955 | 62 | 165 | -0.82 | 7.91E-04 | Putative uncharacterized protein |
| GRMZM2G152963 | 257 | 241 | 0.68 | 2.18E-06 | Putative uncharacterized protein |
| GRMZM2G153058 | 226 | 425 | -0.32 | 3.27E-02 | Putative uncharacterized protein |
| GRMZM2G153119 | 71 | 161 | -0.59 | 1.91E-02 | Putative uncharacterized protein |
| GRMZM2G153181 | 241 | 484 | -0.41 | 1.64E-03 | Eukaryotic translation initiation factor 3 subunit 6-interacting protein (Putative uncharacterized protein) |
| GRMZM2G153206 | 206 | 187 | 0.73 | 8.12E-06 | RALF |
| GRMZM2G153212 | 22 | 8 | 2.05 | 1.83E-03 | Os02g0515200 protein (Putative uncharacterized protein) (cDNA clone:J023101N14, full insert sequence) (Hydroxyproline-rich glycoprotein-like) |
| GRMZM2G153227 | 198 | 226 | 0.40 | 2.23E-02 | H/ACA ribonucleoprotein complex subunit 3-like protein |
| GRMZM2G153274 | 85 | 243 | -0.92 | 1.82E-06 | Putative uncharacterized protein (cDNA clone:006-311-E04, full insert sequence) (cDNA clone:J013070K24, full insert sequence) (Os01g0763600 protein) (Putative uncharacterized protein P0512C01.48) |
| GRMZM2G153275 | 7 | 48 | -2.19 | 1.25E-04 | Pentatricopeptide, putative, expressed |
| GRMZM2G153292 | 3,009 | 5,792 | -0.35 | 2.25E-26 | Tubulin alpha-3 chain |
| GRMZM2G153409 | 63 | 184 | -0.95 | 2.74E-05 | Putative uncharacterized protein |
| GRMZM2G153450 | 414 | 267 | 1.22 | 4.24E-26 | Putative uncharacterized protein |
| GRMZM2G153454 | 33 | 87 | -0.81 | 2.42E-02 | DNA binding protein |
| GRMZM2G153488 | 2,291 | 849 | 2.02 | 2.39E-305 | Low-molecular-weight cysteine-rich protein LCR69 |
| GRMZM2G153594 | 94 | 217 | -0.62 | 3.21E-03 | Os10g0375600 protein (Anther ethylene-upregulated protein ER1, putative, expressed) |
| GRMZM2G153611 | 48 | 113 | -0.64 | 4.17E-02 | Os03g0233500 protein (Putative uncharacterized protein) (IBR domain containing protein, expressed) |
| GRMZM2G153648 | 201 | 443 | -0.55 | 5.38E-05 | Putative uncharacterized protein |
| GRMZM2G153675 | 115 | 98 | 0.82 | 2.91E-04 | Putative uncharacterized protein |
| GRMZM2G153704 | 254 | 509 | -0.41 | 1.34E-03 | Putative uncharacterized protein |
| GRMZM2G153754 | 14 | 5 | 2.08 | 1.90E-02 | Os07g0176200 protein (Putative CXC domain protein TSO1) |
| GRMZM2G153766 | 92 | 211 | -0.61 | 4.06E-03 | Surfeit locus protein 5 |
| GRMZM2G153769 | 117 | 482 | -1.45 | 1.29E-24 | COP9 signalosome complex subunit 4 (Putative uncharacterized protein) |
| GRMZM2G153792 | 85 | 188 | -0.55 | 1.53E-02 | Putative uncharacterized protein |
| GRMZM2G153863 | 108 | 249 | -0.61 | 1.33E-03 | 50S ribosomal protein L21 |
| GRMZM2G153877 | 94 | 66 | 1.10 | 2.40E-05 | Putative uncharacterized protein |
| GRMZM2G153899 | 95 | 201 | -0.49 | 2.87E-02 | Putative uncharacterized protein |
| GRMZM2G153924 | 217 | 469 | -0.52 | 8.26E-05 | Ubiquitin carrier protein (EC 6.3.2.-) |
| GRMZM2G153949 | 850 | 1,701 | -0.41 | 1.86E-10 | GTP-binding nuclear protein Ran-A1 (Putative uncharacterized protein) |
| GRMZM2G153969 | 676 | 1,164 | -0.19 | 2.67E-02 | OB-fold nucleic acid binding domain containing protein (Putative uncharacterized protein) |
| GRMZM2G153999 | 160 | 135 | 0.84 | 8.45E-06 | Putative uncharacterized protein |
| GRMZM2G154093 | 0 | 10 | #NUM! | 3.46E-02 | Homogentisate 1,2-dioxygenase |
| GRMZM2G154165 | 61 | 159 | -0.79 | 1.37E-03 | Os05g0519900 protein (Putative uncharacterized protein) (cDNA clone:J013099L06, full insert sequence) (Putative oligosaccharyl transferase STT3) (Putative oligosaccharyl transferase STT3 subunit) |
| GRMZM2G154169 | 530 | 612 | 0.38 | 8.47E-05 | GIF2 (Putative GRF-interacting factor 2) (Putative uncharacterized protein) |
| GRMZM2G154218 | 3,819 | 10,213 | -0.83 | 2.48E-214 | Elongation factor 1-alpha |
| GRMZM2G154267 | 86 | 267 | -1.04 | 1.63E-08 | DNA polymerase epsilon subunit 2 |
| GRMZM2G154312 | 928 | 2,169 | -0.63 | 1.49E-28 | Putative uncharacterized protein |
| GRMZM2G154316 | 453 | 574 | 0.25 | 2.81E-02 | Putative uncharacterized protein |
| GRMZM2G154344 | 345 | 185 | 1.49 | 1.75E-29 | Putative uncharacterized protein |
| GRMZM2G154394 | 63 | 41 | 1.21 | 2.72E-04 | Putative uncharacterized protein |
| GRMZM2G154397 | 782 | 2,712 | -1.20 | 2.00E-104 | Putative uncharacterized protein |
| GRMZM2G154426 | 128 | 336 | -0.80 | 5.05E-07 | Putative uncharacterized protein |
| GRMZM2G154487 | 126 | 136 | 0.48 | 3.52E-02 | Putative uncharacterized protein |
| GRMZM2G154499 | 22 | 14 | 1.24 | 4.47E-02 | HESB-like domain-containing protein 2 (Putative uncharacterized protein) |
| GRMZM2G154509 | 28 | 107 | -1.34 | 2.78E-05 | Putative uncharacterized protein |
| GRMZM2G154574 | 71 | 182 | -0.77 | 8.32E-04 | Putative uncharacterized protein |
| GRMZM2G154578 | 35 | 13 | 2.02 | 4.85E-05 | AIR12 |
| GRMZM2G154621 | 200 | 234 | 0.36 | 4.00E-02 | Putative uncharacterized protein |
| GRMZM2G154747 | 1 | 16 | -3.41 | 1.35E-02 | NA |
| GRMZM2G154752 | 16 | 2 | 3.59 | 2.18E-04 | Putative uncharacterized protein |
| GRMZM2G154864 | 286 | 841 | -0.96 | 8.57E-23 | Putative uncharacterized protein |
| GRMZM2G154883 | 68 | 156 | -0.61 | 1.68E-02 | Putative lyncein |
| GRMZM2G154890 | 95 | 252 | -0.82 | 1.45E-05 | Putative uncharacterized protein |
| GRMZM2G154896 | 172 | 373 | -0.53 | 5.35E-04 | Pollen-specific protein like |
| GRMZM2G154936 | 1,503 | 1,457 | 0.64 | 5.94E-31 | Putative uncharacterized protein |
| GRMZM2G154939 | 233 | 1,013 | -1.53 | 8.79E-56 | Protein RCC2 |
| GRMZM2G155123 | 111 | 256 | -0.61 | 1.09E-03 | Os05g0125000 protein (cDNA clone:001-116-F09, full insert sequence) (Putative DNA-binding protein) |
| GRMZM2G155232 | 9 | 34 | -1.33 | 4.94E-02 | Putative uncharacterized protein |
| GRMZM2G155242 | 274 | 795 | -0.95 | 7.42E-21 | Inositol-3-phosphate synthase |
| GRMZM2G155281 | 325 | 187 | 1.39 | 5.57E-25 | ATFP3 |
| GRMZM2G155312 | 113 | 254 | -0.58 | 2.34E-03 | Putative uncharacterized protein |
| GRMZM2G155314 | 177 | 394 | -0.56 | 1.18E-04 | Ankyrin-1 (Putative uncharacterized protein) |
| GRMZM2G155321 | 18 | 68 | -1.33 | 1.70E-03 | Putative uncharacterized protein |
| GRMZM2G155323 | 116 | 314 | -0.85 | 4.44E-07 | Os03g0713000 protein (Putative dehydratase/deaminase) |
| GRMZM2G155357 | 57 | 138 | -0.68 | 1.14E-02 | Putative uncharacterized protein |
| GRMZM2G155375 | 32 | 86 | -0.83 | 2.22E-02 | Putative uncharacterized protein |
| GRMZM2G155384 | 600 | 688 | 0.39 | 1.47E-05 | Os02g0146700 protein (cDNA clone:001-200-B03, full insert sequence) (cDNA clone:J033108C09, full insert sequence) (Putative 26S proteasome regulatory subunit S2) |
| GRMZM2G155437 | 488 | 411 | 0.84 | 1.68E-16 | mRNA turnover protein 4 |
| GRMZM2G155490 | 35 | 25 | 1.08 | 2.44E-02 | NA |
| GRMZM2G155543 | 205 | 434 | -0.49 | 4.27E-04 | Putative uncharacterized protein |
| GRMZM2G155546 | 174 | 88 | 1.57 | 3.14E-16 | Putative uncharacterized protein (Ribonucleoside-diphosphate reductase small chain) |
| GRMZM2G155580 | 469 | 492 | 0.52 | 3.73E-07 | Putative uncharacterized protein |
| GRMZM2G155593 | 400 | 255 | 1.24 | 8.54E-26 | Putative uncharacterized protein |
| GRMZM2G155642 | 187 | 157 | 0.84 | 1.09E-06 | Mitochondrial inner membrane protease subunit 1 |
| GRMZM2G155753 | 35 | 90 | -0.77 | 2.81E-02 | FK506 binding protein (Putative uncharacterized protein) |
| GRMZM2G155767 | 34 | 21 | 1.29 | 7.53E-03 | Histidine kinase |
| GRMZM2G155931 | 2,818 | 2,647 | 0.68 | 1.12E-65 | 60S ribosomal protein L32 (Putative uncharacterized protein) |
| GRMZM2G155935 | 80 | 208 | -0.79 | 2.06E-04 | Putative uncharacterized protein |
| GRMZM2G156013 | 111 | 231 | -0.47 | 2.31E-02 | Serine/threonine protein kinase |
| GRMZM2G156033 | 60 | 141 | -0.64 | 1.90E-02 | DnaJ |
| GRMZM2G156068 | 632 | 1,376 | -0.53 | 1.82E-13 | ATP synthase |
| GRMZM2G156099 | 35 | 99 | -0.91 | 6.09E-03 | Putative uncharacterized protein |
| GRMZM2G156105 | 44 | 129 | -0.96 | 7.20E-04 | Integrin beta-1-binding protein 2 |
| GRMZM2G156110 | 597 | 594 | 0.60 | 2.78E-11 | 40S ribosomal protein S15 (Putative uncharacterized protein) |
| GRMZM2G156156 | 87 | 183 | -0.48 | 4.48E-02 | Putative uncharacterized protein |
| GRMZM2G156174 | 95 | 222 | -0.63 | 2.00E-03 | B0402A04.2 protein |
| GRMZM2G156203 | 67 | 170 | -0.75 | 1.74E-03 | NA |
| GRMZM2G156255 | 108 | 63 | 1.37 | 1.81E-08 | Potassium outward rectifying channel (Fragment) |
| GRMZM2G156296 | 151 | 34 | 2.74 | 3.27E-29 | Anthranilate N-benzoyltransferase protein 1 |
| GRMZM2G156320 | 237 | 221 | 0.69 | 3.91E-06 | Putative uncharacterized protein |
| GRMZM2G156388 | 10 | 37 | -1.30 | 4.43E-02 | Putative uncharacterized protein |
| GRMZM2G156486 | 26 | 77 | -0.97 | 1.27E-02 | Putative uncharacterized protein |
| GRMZM2G156608 | 171 | 373 | -0.53 | 4.45E-04 | Putative uncharacterized protein |
| GRMZM2G156673 | 1,597 | 2,790 | -0.21 | 2.41E-05 | 40S ribosomal protein S5 (Putative uncharacterized protein) |
| GRMZM2G156756 | 94 | 292 | -1.04 | 3.31E-09 | Putative uncharacterized protein |
| GRMZM2G156785 | 938 | 2,375 | -0.75 | 6.72E-42 | Putative uncharacterized protein (HMGc2 protein) |
| GRMZM2G156818 | 64 | 161 | -0.74 | 2.59E-03 | Os06g0574400 protein |
| GRMZM2G156879 | 69 | 153 | -0.56 | 3.32E-02 | NA |
| GRMZM2G156956 | 79 | 251 | -1.08 | 2.36E-08 | Putative uncharacterized protein |
| GRMZM2G156960 | 37 | 126 | -1.18 | 4.39E-05 | Putative uncharacterized protein |
| GRMZM2G157007 | 64 | 33 | 1.55 | 4.52E-06 | Putative uncharacterized protein |
| GRMZM2G157018 | 793 | 1,512 | -0.34 | 9.30E-07 | ATP synthase D chain, mitochondrial (Putative uncharacterized protein) |
| GRMZM2G157019 | 57 | 274 | -1.67 | 3.02E-17 | Putative uncharacterized protein (Nucleosome/chromatin assembly factor A) |
| GRMZM2G157102 | 9 | 36 | -1.41 | 2.71E-02 | Putative uncharacterized protein |
| GRMZM2G157147 | 280 | 172 | 1.29 | 2.18E-19 | Putative phosphatidylinositol-4-phosphate-5-kinase |
| GRMZM2G157157 | 181 | 208 | 0.39 | 3.63E-02 | Putative uncharacterized protein |
[truncated: 150,849 more chars]
