# Supplementary material for: Loss of RNA–Dependent RNA Polymerase 2 (RDR2) Function Causes Widespread and Unexpected Changes in the Expression of Transposons, Genes, and 24-nt Small RNAs
Source: PLoS Genet. 2009 Nov 20;5(11):e1000737. doi: 10.1371/journal.pgen.1000737 (PMC2774947; doi:10.1371/journal.pgen.1000737)
Supplement: Table S7 — List of RDR2-sensitive and RDR2-resistant 24 nt siRNA. (1.63 MB PDF) [file pgen.1000737.s012.pdf]

Table S7. List of RDR2-sensitive and RDR2-resistant 24 nt siRNAs

	No. 24 nt siRNAs		
**siRNA sequence**	**Mutant**	**Non-mutant**	**Type**
AAAAAAAAAAAAAAAAAAAAAAAA	14	0	RDR2-resistant
AAAAAATCGAGAGGACGGTGGCAT	0	9	RDR2-sensitive
AAAAAGCTCGTAGTTGGACCTTGG	90	21	RDR2-resistant
AAAAATACTCGTTTGGCACGGCTC	0	9	RDR2-sensitive
AAAAATACTGTAGAAGCCGCAGCC	2	18	RDR2-sensitive
AAAAATTGTGTGCTTTGAGCCGGC	0	10	RDR2-sensitive
AAAAATTGTTTGGGCTTTCTTGGC	0	15	RDR2-sensitive
AAAACACTCGGCAAAGAATATGGC	0	17	RDR2-sensitive
AAAACCACAATCGTGATATCGGAG	51	6	RDR2-resistant
AAAACCCGGGCTGTGAGAGGAGGG	2	25	RDR2-sensitive
AAAACCGGATGCAGAAGATGTACG	49	0	RDR2-resistant
AAAACCGTCGAGCTAGGCTAGCAC	0	23	RDR2-sensitive
AAAACGCGACGGGGCATTGTAAGT	74	1	RDR2-resistant
AAAACGGCTGGCTCTTTGATGGCT	0	19	RDR2-sensitive
AAAACGGTCGGAAACGGTATTTAT	1	23	RDR2-sensitive
AAAACGTCACTGTAAATAGGTTTA	0	14	RDR2-sensitive
AAAACTAGGACAGAACGGCTCCGT	0	43	RDR2-sensitive
AAAACTCACCAGTTGATCCGTGGC	16	0	RDR2-resistant
AAAACTCGATTCGAATCGTCTGCT	46	0	RDR2-resistant
AAAACTCGGACAGAACGGCTCCAT	0	9	RDR2-sensitive
AAAACTGGGACAGAACGGCTCCAT	0	9	RDR2-sensitive
AAAACTGGGACAGAACGGCTCCGT	1	55	RDR2-sensitive
AAAACTGGGATAGAACGGCTCCGT	0	10	RDR2-sensitive
AAAACTGTGTTACCTCGGCGTCAT	0	10	RDR2-sensitive
AAAAGAGCTCAAAGCCTGCGGCAC	0	15	RDR2-sensitive
AAAAGAGGGTGGCATAGAACTGAG	31	2	RDR2-resistant
AAAAGGAACCAACGGACAGGGCAA	0	10	RDR2-sensitive
AAAAGGATGGTAGTGGTGGGGAAT	0	10	RDR2-sensitive
AAAAGGCGGCTCAGCTATGAAGAG	0	10	RDR2-sensitive
AAAAGGCTGGCTTGCGTTCGCTTC	1	12	RDR2-sensitive
AAAAGGGCGGGCCTGGTTTATTTT	14	0	RDR2-resistant
AAAAGGTTAAGAAGGTATGATAAC	36	1	RDR2-resistant
AAAAGTACTCGGCAAAGAGGCTGT	1	12	RDR2-sensitive
AAAAGTGTGTGACTCGTGCGGTGC	23	0	RDR2-resistant
AAAAGTTATGGACTGACGGTTGGT	0	9	RDR2-sensitive
AAAAGTTGTAGTTTTATTAGCAGA	15	0	RDR2-resistant
AAAATACAATGTTATTTTCGTCGG	1	14	RDR2-sensitive
AAAATACTGTAGAAGCCGCAGCCG	0	13	RDR2-sensitive
AAAATACTGTAGATGGCGTTGTTT	0	12	RDR2-sensitive
AAAATAGAAGGTTATTTTCGTCGG	0	11	RDR2-sensitive
AAAATAGGACACATTTTCGTCGGT	0	12	RDR2-sensitive
AAAATATCTGATCTGTTAGATGGT	50	0	RDR2-resistant
AAAATGGTTGTCCATATGTCGGAT	0	13	RDR2-sensitive
AAAATGTAGTTAGCGGAGTGGCTC	26	0	RDR2-resistant
AAAATGTGGCTGTACGGACGCGGA	0	9	RDR2-sensitive
AAAATTCACCGGACTGTACGGTAT	1	30	RDR2-sensitive

AAAATTCATCGGACTGTTCGGTGT	0	10	RDR2-sensitive
AAAATTGGAGGGACGAGAGAGGAC	1	21	RDR2-sensitive
AAAATTTACCGGACTGTTCGGTGT	0	11	RDR2-sensitive
AAAATTTAGTTTGGAAACGGAAGC	17	1	RDR2-resistant
AAACAAAAAACTGGACTTGGCTAT	14	0	RDR2-resistant
AAACAAATCTTGGTCGTCCCCTTC	74	2	RDR2-resistant
AAACAACCTAATTTGAGGGATCGT	2	15	RDR2-sensitive
AAACAACCTTATTTGAGGGATCGT	1	26	RDR2-sensitive
AAACAACGGGCTTCGTGTCGGGCT	0	9	RDR2-sensitive
AAACAACTGCAACAGTGTCGGCTT	1	13	RDR2-sensitive
AAACAATCTAAACTCTGGAGCAGA	0	14	RDR2-sensitive
AAACACAGAACAGAGCGGCTCCGT	1	18	RDR2-sensitive
AAACACGGACTCTGTCGGTCACCC	2	28	RDR2-sensitive
AAACACTGCGGAGAGACTAAGGCG	21	1	RDR2-resistant
AAACAGGACAAAGGACGGAGGCGT	0	11	RDR2-sensitive
AAACAGGAGCTGAGCTATGGTCAA	0	17	RDR2-sensitive
AAACATACTGCACTGTAGTAGGCT	0	20	RDR2-sensitive
AAACATGCTCGGGCTGGGTCGGGC	0	13	RDR2-sensitive
AAACATGGTCGTGAACTTGGCATC	4	62	RDR2-sensitive
AAACATTCGATGGGACAGGGGCTA	0	12	RDR2-sensitive
AAACATTGATCTAGGAGACCATGA	48	4	RDR2-resistant
AAACCAATGGTTTAGATTGTTGGA	0	18	RDR2-sensitive
AAACCAGCGACTGCAGCGAGAGAC	0	13	RDR2-sensitive
AAACCATTTAAGATCATGTTCGGT	1	32	RDR2-sensitive
AAACCCATCGGACGACACGAGCAT	0	10	RDR2-sensitive
AAACCCGGCAGATAGCGCGATCAC	1	12	RDR2-sensitive
AAACCCGGGCTGTGAGAGGAGGGG	0	16	RDR2-sensitive
AAACCGTTGGATCTAGATTGGATG	0	10	RDR2-sensitive
AAACCTAGGCTGATAGAGGAGGGA	0	11	RDR2-sensitive
AAACCTCGGGCAGGAGACAGTCAC	0	15	RDR2-sensitive
AAACCTGAGTATCGTTGGGATTAC	0	13	RDR2-sensitive
AAACCTGCTCTGCACCGGCTCTGC	0	10	RDR2-sensitive
AAACCTGGGAACCGGCAAAGACAT	1	19	RDR2-sensitive
AAACCTGGGAACCGGCAAAGATGT	0	9	RDR2-sensitive
AAACCTGTTTTTGAGCTCGTTGGC	0	23	RDR2-sensitive
AAACCTTTGGCATGGTTTGTCGGT	80	0	RDR2-resistant
AAACGAATTTTATAGAAGCTGGCT	2	17	RDR2-sensitive
AAACGACGAGATCAACGACGCGAT	0	9	RDR2-sensitive
AAACGACGGGCAGAACCGGTGCTG	0	13	RDR2-sensitive
AAACGACGGTATCTGACTTTGTGC	36	0	RDR2-resistant
AAACGACTTTGGCTCTTTGATGGC	0	15	RDR2-sensitive
AAACGAGACTGATTGATAGCGCAT	0	11	RDR2-sensitive
AAACGAGTGATAGAACTTGGCGGT	28	4	RDR2-resistant
AAACGCACGAACGGACAATGGCGA	0	9	RDR2-sensitive
AAACGCGACGGGGCATTGTAAGTG	22	2	RDR2-resistant
AAACGCGGAATAGAACGGCTCCAT	1	73	RDR2-sensitive
AAACGGACCCAGGAACGTGCGGAT	0	25	RDR2-sensitive
AAACGGACCGAGCTGAGTCGGGCT	0	40	RDR2-sensitive
AAACGGACCGGCACGGTAAGTCGT	1	21	RDR2-sensitive

AAACGGACGTATCTGGTCGGCTAG	0	15	RDR2-sensitive
AAACGGATCGGACCGAGTCGGTAA	0	11	RDR2-sensitive
AAACGGCGTGTAGACGTGCGGCTT	1	28	RDR2-sensitive
AAACGGCTCTGGCTCCTTGATGGC	0	25	RDR2-sensitive
AAACGGCTCTGGCTCTTTGATGGC	0	24	RDR2-sensitive
AAACGGCTGGCTCTTTGATGGCTC	0	9	RDR2-sensitive
AAACGGGCCGGGCTGTCTAGGCAC	0	9	RDR2-sensitive
AAACGGGCGGATACGGACGGATAA	0	22	RDR2-sensitive
AAACGGGCGGATACGGACGGATAG	0	65	RDR2-sensitive
AAACGGGCGGATACGGACGGATAT	0	84	RDR2-sensitive
AAACGGGCTAAACGGGCTAAACGG	0	9	RDR2-sensitive
AAACGGGCTATATGGGCTAAACGG	1	17	RDR2-sensitive
AAACGGTTTGTAGAAGACATCGGC	0	13	RDR2-sensitive
AAACGTAGGACGTAATGGCCCCAT	0	10	RDR2-sensitive
AAACGTAGGGCTCTTTGATGGGCT	0	9	RDR2-sensitive
AAACGTCACTGTAAATATGTTTAG	0	10	RDR2-sensitive
AAACGTGTCGTGCTGGTCCGGCTA	3	45	RDR2-sensitive
AAACGTTGAGATTGTTCCAGGAGA	82	6	RDR2-resistant
AAACTAACAGTTTAGATCGTTGGA	0	11	RDR2-sensitive
AAACTAAGTTGGATAGACAGGCGT	0	10	RDR2-sensitive
AAACTCTCGTAGACGTGCTGGTAT	0	12	RDR2-sensitive
AAACTCTGCTGCATATTGTTAAGT	14	0	RDR2-resistant
AAACTCTGGGTGGAGCAGCTCTGC	0	39	RDR2-sensitive
AAACTGACAAGTTCCTGATGTGCC	21	2	RDR2-resistant
AAACTGAGCACAGGATGGACGCAA	0	14	RDR2-sensitive
AAACTGGACCAGAACGGCTCTTGG	0	15	RDR2-sensitive
AAACTGGAGAAACCTAACGGGCAG	0	10	RDR2-sensitive
AAACTGTAGCCCCTATACGGTGAA	0	9	RDR2-sensitive
AAACTGTAGCTCCTAAACGGTGAA	0	13	RDR2-sensitive
AAACTGTAGCTGTATGGCCGCAAA	0	11	RDR2-sensitive
AAACTGTATCTTCACTAGGGCGGT	0	9	RDR2-sensitive
AAACTGTGAATACGTCATCTAGCC	22	0	RDR2-resistant
AAACTGTGGCTCTTGACCAGTGGC	0	18	RDR2-sensitive
AAACTGTTAGTCGGTATATGTTAG	0	21	RDR2-sensitive
AAACTTAGACACGATGGACGGTAT	0	13	RDR2-sensitive
AAACTTATCTGTGAGTTTGAGCGC	0	15	RDR2-sensitive
AAACTTCACTCTGCAAACGGTGTT	0	10	RDR2-sensitive
AAACTTTAGAGCACGGGCTGGCAT	0	9	RDR2-sensitive
AAACTTTGATTAGCCGGAACGATT	7	51	RDR2-sensitive
AAACTTTGATTAGCTGAAACGATT	2	16	RDR2-sensitive
AAACTTTGATTAGCTGGAACGATT	16	37	RDR2-sensitive
AAAGAACCCTAACCCTAGGGCTGC	0	14	RDR2-sensitive
AAAGAACCGGAACCGAGGAACCGA	1	16	RDR2-sensitive
AAAGAACCGGAACCGAGGAGTCGA	0	11	RDR2-sensitive
AAAGAACGTCGCTGACTGAGGCAT	0	16	RDR2-sensitive
AAAGAACTCTTGACAACTGGTATT	18	1	RDR2-resistant
AAAGAAGACTGTAAAGGACGGATA	0	22	RDR2-sensitive
AAAGAAGCAGGAGCTGGAGCTTTG	0	19	RDR2-sensitive
AAAGAAGGAAACCTGGGAACCGGC	1	42	RDR2-sensitive

AAAGAAGGCTTTGCCGAGTGCCGC	0	22	RDR2-sensitive
AAAGAATTTGAGCTCAGATGCGCG	17	1	RDR2-resistant
AAAGAATTTGTCAGCAGCTTTATG	14	0	RDR2-resistant
AAAGACAACTGGACCGGAGCACAT	0	10	RDR2-sensitive
AAAGACATGTTTGGATGGCTGCAC	0	9	RDR2-sensitive
AAAGACCTGTAGGTGAGAGACCGC	0	49	RDR2-sensitive
AAAGACTAAGATCATTATTGATGG	29	0	RDR2-resistant
AAAGACTAGAGCTGGAGTGGGTGT	14	0	RDR2-resistant
AAAGACTGGCTCGAAACGTTGGCT	0	11	RDR2-sensitive
AAAGAGAAGAACTACGAATAGATT	30	1	RDR2-resistant
AAAGAGACCTGGGCGTACCTGCGA	0	14	RDR2-sensitive
AAAGAGCACAGACCTGGGCACCGG	0	17	RDR2-sensitive
AAAGAGGGCTCGGCAACAGTACAT	1	43	RDR2-sensitive
AAAGAGGTTGAGAACGGCATCTAT	19	0	RDR2-resistant
AAAGATATCGGTCTAAATAATCTC	20	0	RDR2-resistant
AAAGATCACGGACCAAGCAGGCGT	0	10	RDR2-sensitive
AAAGATCTCAAAGCGTCGAATGTC	19	0	RDR2-resistant
AAAGATGTGTATGTTGCGTTGGAT	25	2	RDR2-resistant
AAAGATTAAGAGATCATATGTGCA	17	0	RDR2-resistant
AAAGATTTTCTCTTCAATCGTCAC	43	4	RDR2-resistant
AAAGCAATCTTTCGGATCGGCTGG	0	11	RDR2-sensitive
AAAGCACCAGTTTCAACGGTCGTC	0	9	RDR2-sensitive
AAAGCACCCTGAACCCGGCTCTGC	0	12	RDR2-sensitive
AAAGCACGGACTGTTCGGTGCGAC	0	31	RDR2-sensitive
AAAGCAGATGTAGGCCCACTCGCT	0	9	RDR2-sensitive
AAAGCAGCAGCTTTCTGTCGTCGG	4	25	RDR2-sensitive
AAAGCAGGACATCGGTTCACTCAC	0	9	RDR2-sensitive
AAAGCCTCGGATTCCGGTAGTGAT	0	10	RDR2-sensitive
AAAGCCTTCGTCGGAGAAGGATGG	0	16	RDR2-sensitive
AAAGCGAAGTCCGGACAGTCCGCC	17	0	RDR2-resistant
AAAGCGACTACAGAGGACAGTCAA	1	13	RDR2-sensitive
AAAGCGCTTCGTCTCGCAGTCGGC	0	9	RDR2-sensitive
AAAGCGGTCAGACGGTGGAGACAG	0	21	RDR2-sensitive
AAAGCGTGACTTCAACGGCCTCAT	0	21	RDR2-sensitive
AAAGCGTGTATCCGGATCGTTGGT	0	23	RDR2-sensitive
AAAGCTACCTCCGGTTGTCGCGAT	20	0	RDR2-resistant
AAAGCTAGAACATGCTACGCAGTG	14	0	RDR2-resistant
AAAGCTCAGCGGATAACGGGTCGT	0	11	RDR2-sensitive
AAAGCTCCGTCGGCTACGTATCAC	60	0	RDR2-resistant
AAAGCTCGACTGTGAAGGGGCCGG	0	10	RDR2-sensitive
AAAGCTCGCTTCAAGGTCGTGGAT	22	0	RDR2-resistant
AAAGCTCTGGACATGTCAGACTGC	0	9	RDR2-sensitive
AAAGCTGAGCGTTTGGCAGTCCGC	0	10	RDR2-sensitive
AAAGCTGCTGTAGCTGTAAGATGT	0	15	RDR2-sensitive
AAAGCTGTATCTAGTGGCACTTGT	14	0	RDR2-resistant
AAAGCTTAGACTCTAATTACGGTC	0	9	RDR2-sensitive
AAAGGAACTGGGACGGACGAGAAC	0	10	RDR2-sensitive
AAAGGACCCACAGCAGAACTGCTA	0	19	RDR2-sensitive
AAAGGACCGTTTAGAGGACGTTGC	6	55	RDR2-sensitive

AAAGGACGGATATAGAGGATGGAT	0	14	RDR2-sensitive
AAAGGACGGATATAGAGGATGTAT	1	17	RDR2-sensitive
AAAGGACGTTGCTGGAGATGACGA	0	10	RDR2-sensitive
AAAGGACTACAGAGGACAGTCAAA	1	18	RDR2-sensitive
AAAGGACTCCCAGCAGCAATCAGC	23	1	RDR2-resistant
AAAGGAGACTGTAAAGGACGGATA	0	15	RDR2-sensitive
AAAGGATGTAGATTTAGGCATTTG	0	10	RDR2-sensitive
AAAGGATTTCTCTGTTTTCGGTGC	0	11	RDR2-sensitive
AAAGGCAGAACGGTAGACTACCCT	0	15	RDR2-sensitive
AAAGGCCATGTAGACAGGCGATGC	0	9	RDR2-sensitive
AAAGGCCATGTAGACAGGCGATGT	0	10	RDR2-sensitive
AAAGGCTAGAACTGATCCGGCTCT	0	42	RDR2-sensitive
AAAGGCTCTGAGCGAACGGATCCT	0	11	RDR2-sensitive
AAAGGCTCTGTCACTTGACGTCGT	0	10	RDR2-sensitive
AAAGGCTGGTTATGTCTGACGGCT	0	13	RDR2-sensitive
AAAGGGAGGTGCTGTAGCCGGCTA	1	42	RDR2-sensitive
AAAGGGCTGGATCCAGGAAGCAGC	3	20	RDR2-sensitive
AAAGGGCTGTTAGCAGACGTTTAT	0	10	RDR2-sensitive
AAAGGGGTACGGCCGATTGAGAAC	0	12	RDR2-sensitive
AAAGGGTGAGGACAACAGTGGCAT	1	12	RDR2-sensitive
AAAGGTAGAACCAGCGTGAGCCGT	0	29	RDR2-sensitive
AAAGGTAGTCGTTTAGGACATGAT	1	67	RDR2-sensitive
AAAGGTTCTGTCGGGAGCGTGCGG	0	9	RDR2-sensitive
AAAGGTTGGATCGACGTAGAGGCA	0	32	RDR2-sensitive
AAAGTAATGATGGCTTAGAAACAT	57	5	RDR2-resistant
AAAGTACTCGGCAAAGAAGGCTTT	1	18	RDR2-sensitive
AAAGTAGACTGTAAAGGACGGATA	0	9	RDR2-sensitive
AAAGTAGAGCTACGCTACATGCGG	45	6	RDR2-resistant
AAAGTAGGATGCAGCACACGACGG	0	12	RDR2-sensitive
AAAGTAGGGACTAATTGGCTTTAG	0	10	RDR2-sensitive
AAAGTAGTGGATTGGGCATGTTCG	6	21	RDR2-sensitive
AAAGTCAGGAGACATCTGAGGCGT	1	20	RDR2-sensitive
AAAGTCAGTTATCGTCGTTTTGCC	63	6	RDR2-resistant
AAAGTCATTCTATCTTTAAGTCGG	2	31	RDR2-sensitive
AAAGTCCAGGACTGCAGGGGCGGT	2	14	RDR2-sensitive
AAAGTCCGGGCTGTGAGAGGAGGG	0	14	RDR2-sensitive
AAAGTCGACTTTGCCGAGGGCCGC	2	21	RDR2-sensitive
AAAGTCGGCATTGCAGCAGATCGG	0	9	RDR2-sensitive
AAAGTCTGGCCTGTTTTCGCGCGT	0	17	RDR2-sensitive
AAAGTGACTGCATGGACGGTCCAT	0	12	RDR2-sensitive
AAAGTGGAACCGGAGGCAGGGCGT	0	10	RDR2-sensitive
AAAGTGGATCCGGCGTTCTTGAGT	20	0	RDR2-resistant
AAAGTGTACTTTATGATCTGTGGA	0	9	RDR2-sensitive
AAAGTTCGGAGAGCCAGGAGACAC	0	11	RDR2-sensitive
AAAGTTGGCATTGCAGTAGACCGG	1	15	RDR2-sensitive
AAAGTTTGGATATGTCAGCGGCTC	31	2	RDR2-resistant
AAATAACACGGGCTGAGCTGGCAT	0	13	RDR2-sensitive
AAATAACTGTAGAATGGACGGTTG	0	20	RDR2-sensitive
AAATAAGTTGTTATTTTCGTCGGT	0	27	RDR2-sensitive

AAATAATATGGGTCGGGCTAGCAT	0	10	RDR2-sensitive
AAATAATCACTATTTTCGTCGGTC	0	9	RDR2-sensitive
AAATAATCTGTATTTTCGTCGGTC	0	20	RDR2-sensitive
AAATACAATGTTATTTTCGTCGGC	2	23	RDR2-sensitive
AAATACAGCTTATTTTCGTCGGCC	0	10	RDR2-sensitive
AAATACATTGTAGTTGTCTCGTCA	0	10	RDR2-sensitive
AAATACGAAAGTTATTTTCGTCGG	0	14	RDR2-sensitive
AAATACGGACTCTATCTAAGGCTG	0	9	RDR2-sensitive
AAATACGGAGTCTGCTGGAAACGG	0	12	RDR2-sensitive
AAATACGTCGGGTAGGGTACGGAT	0	31	RDR2-sensitive
AAATACGTGCTGGCAGCTGATGTG	0	11	RDR2-sensitive
AAATACTCGGCGAATGACGGTCCT	0	14	RDR2-sensitive
AAATACTGTAGAAGCCGCAGCCGC	13	231	RDR2-sensitive
AAATACTTCGGACCAGGCTAGCAC	0	13	RDR2-sensitive
AAATACTTCGGGCTGGGCTAGCAC	0	21	RDR2-sensitive
AAATAGACTGCTGAGGGTATTAAG	51	0	RDR2-resistant
AAATAGAGCTATTTTCGACGGTAC	0	11	RDR2-sensitive
AAATAGGACTTTGCCGAGAGCCGC	1	13	RDR2-sensitive
AAATAGGCTATTTTCGTCGGTTTA	0	9	RDR2-sensitive
AAATAGGTTTACGATCGTCACTAT	0	10	RDR2-sensitive
AAATAGTGTTCGGATGGCAGCTAG	0	11	RDR2-sensitive
AAATATAATTGTGTGTCGTCGTAA	34	1	RDR2-resistant
AAATATGACTGCGGAAGATTGTAT	1	25	RDR2-sensitive
AAATATGATTGTTTAAGTGATTTC	25	1	RDR2-resistant
AAATATGTCTAGATTGTATCGGTG	0	27	RDR2-sensitive
AAATATTCGGCATTTTCTATAGAT	164	0	RDR2-resistant
AAATATTGGGCTTTTTACCGGTTA	23	1	RDR2-resistant
AAATATTTCGGATCGTGTCGTGCC	0	9	RDR2-sensitive
AAATCATGGTCTGTATTTGGCGCA	0	10	RDR2-sensitive
AAATCGGTACGACTCATGACGGGC	1	13	RDR2-sensitive
AAATCGGTCGTGTATTTAGTCGGT	1	30	RDR2-sensitive
AAATCGTGCCTGGACCGGGTCGGC	0	12	RDR2-sensitive
AAATCGTTCGTCTGACTTGGGTAT	288	54	RDR2-resistant
AAATCTAATTAGGTCGTCTTGTGC	0	11	RDR2-sensitive
AAATCTCTGACTGCGAAGATCGAC	0	10	RDR2-sensitive
AAATGAACCCAAGCTGAGGCTCTT	0	31	RDR2-sensitive
AAATGAACGATGACGGTCTGTCGC	0	9	RDR2-sensitive
AAATGAACTCGGCCCCGATGGCGT	0	16	RDR2-sensitive
AAATGACGTGAGCTGAAGCCGGGT	0	10	RDR2-sensitive
AAATGACTCGTATCAACAGGGGCG	0	9	RDR2-sensitive
AAATGAGACTGTAAATGACGGATA	0	10	RDR2-sensitive
AAATGATCTACCTGTAGAGATGGC	1	14	RDR2-sensitive
AAATGCCTCGGTGCTGTTGGGCGC	0	9	RDR2-sensitive
AAATGCCTGTAGCACTAGCGGCAC	0	9	RDR2-sensitive
AAATGCGAGGCTGAGGTATATGGC	31	1	RDR2-resistant
AAATGCGGATGCGGAGTGGGATGG	0	9	RDR2-sensitive
AAATGCTACTTCATCTTGTCGGTC	25	0	RDR2-resistant
AAATGCTCCTGCGATTTGGTCGGT	2	18	RDR2-sensitive
AAATGGAACTCTCGAGAAGACTCT	0	11	RDR2-sensitive

AAATGGAACTGTAAAATAGCGGAT	0	9	RDR2-sensitive
AAATGGAAGGTGGAGGTTTTAGGT	14	0	RDR2-resistant
AAATGGACTGGGCTAGCACGGCAC	0	20	RDR2-sensitive
AAATGGACTGGGCTTGTCGGGCGG	0	11	RDR2-sensitive
AAATGGATTGTGTCTATCAGGCTG	0	14	RDR2-sensitive
AAATGGATTTGCAGATTTGATGGT	0	25	RDR2-sensitive
AAATGGGTCGTGCTTCGGACCGGC	1	28	RDR2-sensitive
AAATGGTTGTAACGTAGATGGTAA	0	14	RDR2-sensitive
AAATGTCTGATCGTTTGGAGGGAG	42	3	RDR2-resistant
AAATGTCTTCTCTAGAAACGGCTC	0	15	RDR2-sensitive
AAATGTGGCTGTACGGACGCGGAT	0	12	RDR2-sensitive
AAATGTTGGGCTTTTTACCGGTTA	55	17	RDR2-resistant
AAATGTTTGGACGGCTATAGCTAC	0	9	RDR2-sensitive
AAATTAGCGTCAGGAGGTCGGCCC	34	0	RDR2-resistant
AAATTAGTCTCTGTAGAGTGGCAG	24	0	RDR2-resistant
AAATTAGTGGACGGTGATGGTGAT	0	14	RDR2-sensitive
AAATTATCTATTTTCGTCGGTTTA	0	21	RDR2-sensitive
AAATTATCTGATACATTAGATCGT	0	11	RDR2-sensitive
AAATTATTTGGTTGCGTTGGCTCT	20	0	RDR2-resistant
AAATTCACTAGTCGGTTGACGGTC	0	9	RDR2-sensitive
AAATTCGAATGTTATTTTCGTCGG	0	16	RDR2-sensitive
AAATTCTAAGTGGGACGACATCAT	0	13	RDR2-sensitive
AAATTGAACTAACGGCATTAACTC	31	4	RDR2-resistant
AAATTGAACTCGGTTGAATTCGGT	51	2	RDR2-resistant
AAATTGACTTTGTTCTCGGTTCTT	143	0	RDR2-resistant
AAATTGCGGACTATTTTCGGCGGT	0	17	RDR2-sensitive
AAATTGGAGGGACGAGAGAGGACG	1	20	RDR2-sensitive
AAATTGTATTGTTTTATGATCCGG	0	9	RDR2-sensitive
AAATTGTTAGTCGGTATACATTGC	0	21	RDR2-sensitive
AAATTGTTCAGTTGCATACTCATT	26	0	RDR2-resistant
AAATTGTTGCGGATCTATCGGCTT	0	9	RDR2-sensitive
AAATTTACTTGTCGGAGCGAGGTT	3	23	RDR2-sensitive
AAATTTAGAGGACGAGGACGTCAT	0	40	RDR2-sensitive
AAATTTAGCGGACAGGACGGTTTA	1	13	RDR2-sensitive
AAATTTAGGGGACGCTGCTGGAGA	0	13	RDR2-sensitive
AAATTTAGTGGACGAGGACGTGCG	0	9	RDR2-sensitive
AAATTTATTGGTCGGAAATGGAGC	1	34	RDR2-sensitive
AAATTTCGTGGAGCTGGAGCGGTC	0	23	RDR2-sensitive
AAATTTCTACTCGTCGGGTTACCC	0	10	RDR2-sensitive
AAATTTGGACCATTTCTCGATTTG	17	0	RDR2-resistant
AAATTTGGGATTATTCGGATTTTC	109	5	RDR2-resistant
AAATTTGGTAGAGCAGGTCATTAG	20	0	RDR2-resistant
AAATTTGTACTAACGGACGACATA	0	33	RDR2-sensitive
AAATTTTATTTGCACGTTTGGACC	0	26	RDR2-sensitive
AAATTTTCGGTTCGGTAATTCGGT	0	20	RDR2-sensitive
AAATTTTGTAAATAATCGTTTGTT	15	0	RDR2-resistant
AACAAAACTAGAACAGAACGGCTC	1	23	RDR2-sensitive
AACAAAACTAGGACAGAACGGCTC	0	29	RDR2-sensitive
AACAAAACTGGAAAAGAACGGCTC	0	11	RDR2-sensitive

AACAAAACTGGGACAGAACGGCTC	3	86	RDR2-sensitive
AACAAAACTGGGACAGAACGGTTC	0	15	RDR2-sensitive
AACAAAACTGGGATAGAACGGCTC	0	9	RDR2-sensitive
AACAAAGACTGTCGCTGTCGGCGT	0	18	RDR2-sensitive
AACAAATCTTGGTCGTCCCCTTCT	19	0	RDR2-resistant
AACAACACTGTAGATCATTTTCGG	0	19	RDR2-sensitive
AACAACCTAATTTGAGGGATCGTC	0	29	RDR2-sensitive
AACAACCTTATTTGAGGGATCGTC	8	44	RDR2-sensitive
AACAACGACTGCGGATCGGCTAAA	25	0	RDR2-resistant
AACAACGGACGCAACGCACGGCTC	0	10	RDR2-sensitive
AACAACGTACGGGCATACAGCTAG	0	29	RDR2-sensitive
AACAACTCCGAATGGGATGCGCGT	0	9	RDR2-sensitive
AACAACTGTAGCAAGAAACTCGGA	0	11	RDR2-sensitive
AACAACTGTGACCAGACGAAGCGC	0	9	RDR2-sensitive
AACAACTTGTGCTGATTGGATGAC	761	21	RDR2-resistant
AACAACTTTCTGTCGATGGACGCA	0	12	RDR2-sensitive
AACAAGACGGTGCTGAACGTCGAC	0	17	RDR2-sensitive
AACAAGCTCTAAGGTGTGTTTGGT	15	0	RDR2-resistant
AACAATAGATTGATTCGGTTGCCT	0	14	RDR2-sensitive
AACAATATTATGTACGGCTATTGA	22	0	RDR2-resistant
AACAATCCGGTGACTGTCCGGCCG	0	23	RDR2-sensitive
AACAATCTCGTCGCGCGGCTTCGC	1	15	RDR2-sensitive
AACAATCTTATTTGAGGGATCGTC	0	10	RDR2-sensitive
AACACAAACAGCGGACTTCGTGGC	0	12	RDR2-sensitive
AACACAAGTGACGAGCAGCTCCGA	0	10	RDR2-sensitive
AACACAAGTTTGTCTAGTCGTTGG	1	33	RDR2-sensitive
AACACACGCAGCGAATCTGGGCAT	0	13	RDR2-sensitive
AACACATGCGTTCGGTCTAGATGG	0	11	RDR2-sensitive
AACACATGTTGGATAAAATGGCGT	166	10	RDR2-resistant
AACACCAGTTTCAACGGTCGTCGT	0	9	RDR2-sensitive
AACACCGGAGTAGTTTTTGATGGC	0	30	RDR2-sensitive
AACACCGTTGGATACTGGACGGAT	0	15	RDR2-sensitive
AACACCGTTGGATATGGAGGGTGC	0	9	RDR2-sensitive
AACACCGTTGGATGTGGAGGGTGC	1	65	RDR2-sensitive
AACACCGTTGGATGTGGAGGGTGT	0	41	RDR2-sensitive
AACACCTTCGGAAGACTGGCACAA	0	18	RDR2-sensitive
AACACCTTCTGCAGCGGAGACTGT	0	11	RDR2-sensitive
AACACGCCTGTGAAGCACGACGCC	0	9	RDR2-sensitive
AACACGCTACGGGCTTAAACGGAT	0	9	RDR2-sensitive
AACACGCTTGTTCGGACAACGCAT	0	55	RDR2-sensitive
AACACGGACCATAGGGGGGCCGGG	16	0	RDR2-resistant
AACACGTGACAGTGGAGTCGGCGC	0	39	RDR2-sensitive
AACACTCATGTTGGTCTGTGGGGA	0	12	RDR2-sensitive
AACACTCCTGAACTCGGCTGGTAA	0	14	RDR2-sensitive
AACACTCGGCAAAGAACACTCGGC	12	65	RDR2-sensitive
AACACTCGGCAAAGAACGCTCGGC	33	375	RDR2-sensitive
AACACTCGGCAAAGAAGGCTCGGC	8	114	RDR2-sensitive
AACACTCGGCAAAGCCTCGGATTC	0	14	RDR2-sensitive
AACACTCGGCACAGAACGGCACTC	0	15	RDR2-sensitive

AACACTCTCGTACTGTGCTTAGGC	9	65	RDR2-sensitive
AACACTCTCGTCGCGCGGCTTCGC	0	12	RDR2-sensitive
AACACTCTCGTCGTGCGGCTTCGC	0	9	RDR2-sensitive
AACACTGCTCGGAGGTACTGGCAT	0	26	RDR2-sensitive
AACACTGTAGCCGGCTAACCGTGT	0	14	RDR2-sensitive
AACACTGTAGCTTTAGGGGACCGA	1	28	RDR2-sensitive
AACACTGTAGCTTTAGGGGACGTT	0	10	RDR2-sensitive
AACACTGTAGCTTTAGGGGATCGA	0	9	RDR2-sensitive
AACACTGTATAGCATTTTCGACGG	0	9	RDR2-sensitive
AACACTGTTGGACTGGCCCACGGT	0	12	RDR2-sensitive
AACAGAACCCTAGCAGCGACTCAT	0	30	RDR2-sensitive
AACAGAAGGGCACAACTGAGCAAA	0	10	RDR2-sensitive
AACAGACGTAGACCACATGACCGG	0	10	RDR2-sensitive
AACAGACGTGGGGCAGGTGGCGCA	1	14	RDR2-sensitive
AACAGACTGGTCTGACACGGTCAA	0	9	RDR2-sensitive
AACAGAGATTGGTTTCATTCTGCA	0	10	RDR2-sensitive
AACAGAGCTCATGATCGTCGAGAC	0	10	RDR2-sensitive
AACAGATCAGTTATTTTCCAGGTT	26	0	RDR2-resistant
AACAGATCGGATTCGGATCGGATA	0	9	RDR2-sensitive
AACAGATGACTAGATAACATTGCC	15	0	RDR2-resistant
AACAGATTGATGGGCGGTACGGTA	18	0	RDR2-resistant
AACAGATTGCTGGTCGTGCTGGTC	0	10	RDR2-sensitive
AACAGATTGTTAGAGACAGACGTT	0	13	RDR2-sensitive
AACAGCGTAGACGGTTCTGTCCGA	0	31	RDR2-sensitive
AACAGCTACTGTGGAAACCGGCGT	2	36	RDR2-sensitive
AACAGCTCAGGACCTTGACGACGG	0	31	RDR2-sensitive
AACAGCTGCAACAGTGTCGGCTTG	1	21	RDR2-sensitive
AACAGGACGCTTTCAATATATAAT	19	0	RDR2-resistant
AACAGGACGGCTGAAAGCACACAA	0	41	RDR2-sensitive
AACAGGACTGCAGAAGAATGGGCT	0	14	RDR2-sensitive
AACAGGCAACGTAGCATAGCACTC	0	9	RDR2-sensitive
AACAGGCGTATGAGGCACTGAAGC	19	0	RDR2-resistant
AACAGGCTGTCGGGTGCGCCACCT	0	12	RDR2-sensitive
AACAGGGGACGACATGGGACGGTC	0	10	RDR2-sensitive
AACAGGTACCGCAGGATGAGGCGC	0	10	RDR2-sensitive
AACAGTAAGAACTCTGTTGGTGGC	0	11	RDR2-sensitive
AACAGTACGGATCGGAACGGTATT	0	9	RDR2-sensitive
AACAGTAGAACCGGACATTCGGCC	0	10	RDR2-sensitive
AACAGTAGTCCCGGACGGATTCAC	1	32	RDR2-sensitive
AACAGTCGGAAAGGCAGTAGTCGC	2	14	RDR2-sensitive
AACAGTCGGAGAGGCAGTAGTCAC	0	13	RDR2-sensitive
AACAGTCGGATAGGCAGTAGTCAC	0	15	RDR2-sensitive
AACAGTCGGCTCTCGGCAAAGATG	0	9	RDR2-sensitive
AACAGTCTTTGTTGGCTCGGTACA	0	23	RDR2-sensitive
AACAGTGGGCGTGACGTACTTGGG	1	13	RDR2-sensitive
AACAGTTGTGATTCGGTATGGCTT	40	0	RDR2-resistant
AACATAACTTATTTTACGTCGGTC	0	11	RDR2-sensitive
AACATAGTTTATGTGTAGTTCGGT	22	1	RDR2-resistant
AACATCAACTGTAGCTCGATGGTT	0	12	RDR2-sensitive

AACATCGGAGTAGTTTTTGGTGGC	0	14	RDR2-sensitive
AACATCTCGCTGTGAGCCAGGCTG	0	12	RDR2-sensitive
AACATCTGGATCCAGAACGCGCAC	0	9	RDR2-sensitive
AACATGAACTGTTGCTAATGTCGT	17	0	RDR2-resistant
AACATGACTGCAGAGGATTGTGGT	0	16	RDR2-sensitive
AACATGCCCGTTTATGTGTCGGAC	0	16	RDR2-sensitive
AACATGCTTGTTCGGACGACGCAT	0	12	RDR2-sensitive
AACATGGACGAGCAGACACGACAC	0	20	RDR2-sensitive
AACATGGACGGCGGGGAGGGTCGC	35	1	RDR2-resistant
AACATGTGAGGATTGTGACGCATC	0	19	RDR2-sensitive
AACATTGTAATTGGACTTTCGGTA	0	13	RDR2-sensitive
AACATTGTTGACTGTGAAGCCGGA	62	3	RDR2-resistant
AACCAACCGGGACACGTAGGGCGG	0	10	RDR2-sensitive
AACCAACTTTATATCGGGCGTCGG	0	9	RDR2-sensitive
AACCAAGACGGCTCTAGACGACAT	0	14	RDR2-sensitive
AACCAAGACGGCTCTAGACGACGT	0	9	RDR2-sensitive
AACCAAGCGGCCAGCGCGCTCTGT	19	1	RDR2-resistant
AACCAATCTTGGGCTGCCCAACGG	0	13	RDR2-sensitive
AACCAATGGTTTAGATTGTTGGAT	0	10	RDR2-sensitive
AACCACATTTCATCGTGCGGGCTG	28	5	RDR2-resistant
AACCACGGTCGCGTGCTGACACGG	0	14	RDR2-sensitive
AACCACTCGAGACTGTGTGGGCAG	5	57	RDR2-sensitive
AACCAGAAGGACGACCGAAGACAG	0	14	RDR2-sensitive
AACCAGAAGGACGACCGAAGACTT	0	21	RDR2-sensitive
AACCAGATCGATATCAGGACTCGA	0	11	RDR2-sensitive
AACCAGATTGTAGGACTGGTCGGC	0	9	RDR2-sensitive
AACCAGCGACTGCAGCGAGAGACG	0	9	RDR2-sensitive
AACCAGGACTACGTAGAAGAGGAA	0	9	RDR2-sensitive
AACCAGGCTCAAGAGAACACGCAT	0	41	RDR2-sensitive
AACCAGGCTGTCTAGAGAAGCGAA	0	9	RDR2-sensitive
AACCAGGTCGTGTCGGGCTCGGGC	0	16	RDR2-sensitive
AACCAGGTCGTGTCGGGCTTGCGG	0	34	RDR2-sensitive
AACCAGGTGTGGACGCTGACGGTA	1	57	RDR2-sensitive
AACCAGTTGCATGCATAGAGTCTG	0	11	RDR2-sensitive
AACCAGTTTGTAGGGATGGTCGGC	0	14	RDR2-sensitive
AACCAGTTTGTAGGGGTGGTCGGC	1	15	RDR2-sensitive
AACCATAAAGTTTGTAGCACGGGT	56	2	RDR2-resistant
AACCATACTGTAATTCGCGCGGAC	0	14	RDR2-sensitive
AACCATAGGGTATCGGATCGTGGG	0	16	RDR2-sensitive
AACCATAGTTGACTGGAAGACGAC	0	12	RDR2-sensitive
AACCATAGTTGACTGGGAGACGAC	1	96	RDR2-sensitive
AACCATCCTGGTTTGTAGTCGGGC	0	13	RDR2-sensitive
AACCATCGCGGGCTGTACGACGGT	0	13	RDR2-sensitive
AACCATCGGGTATCGGATCGTGGG	0	10	RDR2-sensitive
AACCATCGTCGTACTTATACGGTC	0	42	RDR2-sensitive
AACCATTAGTTCTTTATTGTCGGC	0	24	RDR2-sensitive
AACCATTTAAGATCATGTTCGGTT	0	16	RDR2-sensitive
AACCCAAAGTTGACTGGGAGACGA	0	15	RDR2-sensitive
AACCCAACGGCCTGGTGACGTGGC	0	9	RDR2-sensitive

AACCCATCTGGGCTGGGACGCGCA	0	11	RDR2-sensitive
AACCCCGGCTTGTAGTGTGTGCTT	0	12	RDR2-sensitive
AACCCGAGAGTCGTCGACATTGGC	0	11	RDR2-sensitive
AACCCGGACTTTGAGAAGAGGGAT	0	20	RDR2-sensitive
AACCCGGCACGGTAGAATAAACGG	1	15	RDR2-sensitive
AACCCGGCACGGTAGAATAAGCGG	2	331	RDR2-sensitive
AACCCGGCATGGTAGAATAAGCGG	0	13	RDR2-sensitive
AACCCGGGAGCAGGATGAACGAGC	25	3	RDR2-resistant
AACCCGGGCTGTGAGAGAAGAGGA	0	13	RDR2-sensitive
AACCCGGGCTGTGAGAGGAGGGGA	2	47	RDR2-sensitive
AACCCGTAGACCTGAACTCGACGG	0	35	RDR2-sensitive
AACCCGTCGTGACACGCCTTCGGC	0	13	RDR2-sensitive
AACCCTAATTCGTGGTGGTCTCGG	0	10	RDR2-sensitive
AACCCTGCGCCATCGCGGATCGTC	0	10	RDR2-sensitive
AACCCTGTGATGCGTAGTCGGATC	0	9	RDR2-sensitive
AACCCTTGTTCGTTGGGACACCAC	0	12	RDR2-sensitive
AACCGAACAAGGGGAACTAGGCAT	0	19	RDR2-sensitive
AACCGAACGCCTTTAGACGGACAT	0	15	RDR2-sensitive
AACCGAAGGATAGAGCAGACGAGA	0	11	RDR2-sensitive
AACCGACACGTAGGGGGCGCTGTA	0	10	RDR2-sensitive
AACCGACTCAGAACTGGTACGGAC	65	126	RDR2-sensitive
AACCGACTGGAGCAGGAGCACAAC	0	15	RDR2-sensitive
AACCGACTGTATCGCAGGCGCATT	1	15	RDR2-sensitive
AACCGACTGTTGATCCAAGGGCGC	0	16	RDR2-sensitive
AACCGAGGACGTAGACGCGGCCGT	0	16	RDR2-sensitive
AACCGATCTGTAGTGGAACAGGCT	0	15	RDR2-sensitive
AACCGGACCGCCTGTACAGAGGTT	0	28	RDR2-sensitive
AACCGGACCGGGTCGTGCCTGGAC	0	9	RDR2-sensitive
AACCGGACGACCCATACTGTTTGG	0	17	RDR2-sensitive
AACCGGACTGCCGACACACGGCGT	4	25	RDR2-sensitive
AACCGGATGCAGAAGATGTACGAT	29	0	RDR2-resistant
AACCGGCTAGTGTAGAGGACACAT	0	10	RDR2-sensitive
AACCGGGTCGTGCCGTGCTCGGAC	1	13	RDR2-sensitive
AACCGTAGGTCGCTGAAGGCCGTT	0	13	RDR2-sensitive
AACCGTCAGGTCGGATCGTGGGCC	1	18	RDR2-sensitive
AACCGTCCACTCTGTAACAACGGT	0	51	RDR2-sensitive
AACCGTGACTGTCGGGCCAGGCCC	0	11	RDR2-sensitive
AACCGTGCCGGGCTTAGGCTGCAT	0	10	RDR2-sensitive
AACCGTGCTGGGCTCAAACAGGCT	0	10	RDR2-sensitive
AACCGTGGATAGGAACATAGGCTG	0	11	RDR2-sensitive
AACCGTGTCTGATCGTGCCTGGGA	0	11	RDR2-sensitive
AACCGTTAGGGTTCGACCGTTGGA	8	69	RDR2-sensitive
AACCGTTCAATTTCGATCGTCGCC	0	27	RDR2-sensitive
AACCGTTGGATCTAGATTGGATGC	0	15	RDR2-sensitive
AACCGTTGGCTCTCGGCAAAGACT	1	12	RDR2-sensitive
AACCTAATTGTGGGACTGGGCCGG	2	31	RDR2-sensitive
AACCTAATTGTGGGCCTGGGCCGG	5	31	RDR2-sensitive
AACCTAATTGTGGGTCTAGGTCGG	0	9	RDR2-sensitive
AACCTAATTTGAGGGATCGTCCCA	5	128	RDR2-sensitive

AACCTAATTTGAGGGATCGTCTCA	0	17	RDR2-sensitive
AACCTACGGTGGCTTAGATCTCGG	0	10	RDR2-sensitive
AACCTACTCCTTGGACTACGGCGA	0	42	RDR2-sensitive
AACCTACTTAGGACTTATTCGGTT	0	9	RDR2-sensitive
AACCTAGACACTGCTGCACGGTAC	1	21	RDR2-sensitive
AACCTAGCGAGACGGTACTGTCGA	0	9	RDR2-sensitive
AACCTAGCGGATCGTGCCTAAGTC	0	16	RDR2-sensitive
AACCTAGGACTCAGCACGGTTCGT	0	33	RDR2-sensitive
AACCTAGGCTGAGAGAGTGTGCAT	0	10	RDR2-sensitive
AACCTAGTCGTCAGGAGCAGCAGA	24	0	RDR2-resistant
AACCTATTGAAAACTAGTCGGGCC	0	10	RDR2-sensitive
AACCTCACCAGTCGTCGGAGATAG	1	16	RDR2-sensitive
AACCTCGCCGGAGAAGACAGCTAT	7	88	RDR2-sensitive
AACCTCGGGTTGTAGTGTGTGCTT	0	9	RDR2-sensitive
AACCTCGTGGTACAGATCGTCCGT	0	10	RDR2-sensitive
AACCTGAACACACAAGAGCGGTGT	0	25	RDR2-sensitive
AACCTGACCCGGGTGTAGAGATGG	0	13	RDR2-sensitive
AACCTGACTCTTGGTAGACTGGCT	0	9	RDR2-sensitive
AACCTGGCTCTGATACCACTGAAG	52	0	RDR2-resistant
AACCTGTTAGAGGCATGTGGTCCA	45	0	RDR2-resistant
AACCTTAGGCAAAGTGTGGCACAT	0	13	RDR2-sensitive
AACCTTATTTAAGGGATCGTCTCA	1	26	RDR2-sensitive
AACCTTATTTGAGGGATCGTCACA	0	11	RDR2-sensitive
AACCTTATTTGAGGGATCGTCATA	0	16	RDR2-sensitive
AACCTTATTTGAGGGATCGTCATG	0	41	RDR2-sensitive
AACCTTATTTGAGGGATCGTCCAA	0	13	RDR2-sensitive
AACCTTATTTGAGGGATCGTCCCA	0	23	RDR2-sensitive
AACCTTATTTGAGGGATCGTCCCG	0	20	RDR2-sensitive
AACCTTATTTGAGGGATCGTCCTA	0	37	RDR2-sensitive
AACCTTATTTGAGGGATCGTCTAA	0	20	RDR2-sensitive
AACCTTATTTGAGGGATCGTCTCA	17	81	RDR2-sensitive
AACCTTATTTGAGGGATCGTCTCG	0	14	RDR2-sensitive
AACCTTATTTGAGGGATCGTTCTA	0	9	RDR2-sensitive
AACCTTCAGGACTGTCACGGGTGT	0	16	RDR2-sensitive
AACCTTGACGTGGACAGAGGCTGT	1	12	RDR2-sensitive
AACCTTGCGAGGCAGAACGGACGA	0	9	RDR2-sensitive
AACCTTGTAGTAAGATTGTCGGTC	1	12	RDR2-sensitive
AACCTTTGGCATGGTTTGTCGGTC	44	0	RDR2-resistant
AACCTTTTATATTTCATGTCGGGC	0	13	RDR2-sensitive
AACGAACCATCTGACGGTGCTATC	0	9	RDR2-sensitive
AACGAAGGCAGAACGGCAGAACGG	0	21	RDR2-sensitive
AACGAAGGCAGAACGGCAGGCTAC	0	10	RDR2-sensitive
AACGAAGGCAGAACGGTAGGCTAC	2	15	RDR2-sensitive
AACGAATCTTGAGATATTTGGCTT	51	8	RDR2-resistant
AACGAATTTTATAGAAGCTGGCTG	1	15	RDR2-sensitive
AACGACAAATTTACTCGTCGGTCC	0	9	RDR2-sensitive
AACGACAATTTTACTCGTCGGTCC	0	9	RDR2-sensitive
AACGACACCGTGGAGCCAAGGCAA	0	28	RDR2-sensitive
AACGACATTCAGAATATTTGGCAA	0	12	RDR2-sensitive

AACGACGATAGCTTACCTTGTACC	28	5	RDR2-resistant
AACGACGGCAGAACGGCAGAACGG	0	10	RDR2-sensitive
AACGACTAAGGACCCTAGCGACGG	0	9	RDR2-sensitive
AACGACTCAGGATCCTGACGACGG	0	9	RDR2-sensitive
AACGACTCTGACGATTTGGCGGCG	0	21	RDR2-sensitive
AACGACTGAGGACCCTAGCGACGG	1	48	RDR2-sensitive
AACGACTGATGATCAAACGGCTAA	0	10	RDR2-sensitive
AACGACTGCGGACCGAGCCAGCAT	0	9	RDR2-sensitive
AACGACTGTAGCAAAATGGTTCGT	10	88	RDR2-sensitive
AACGACTGTTAGACTAGCCGTCAT	0	16	RDR2-sensitive
AACGACTGTTGGGCGGAGGCGCAC	0	13	RDR2-sensitive
AACGACTTTGGCTCTTTGATGGCT	0	20	RDR2-sensitive
AACGAGAGACTGATATGGCACCAA	0	16	RDR2-sensitive
AACGAGCCAGCTCGAACTCGGACG	0	11	RDR2-sensitive
AACGAGCGTCACTGTAGCGTCGTC	0	12	RDR2-sensitive
AACGAGGATTAGTCGGGACCTTGC	1	12	RDR2-sensitive
AACGAGGCACGTAGCAGCGACGAC	0	13	RDR2-sensitive
AACGAGGCATGTGTTGGTCGCGGT	4	61	RDR2-sensitive
AACGAGTACTGAGAAGCGCGCTGT	0	24	RDR2-sensitive
AACGAGTGATAGAACTTGGCGGGT	52	0	RDR2-resistant
AACGAGTGATAGAACTTGGCGGTT	87	2	RDR2-resistant
AACGAGTGGAGTGATAGAGGACGC	0	11	RDR2-sensitive
AACGATTACATAGTTGGACATCAT	2	20	RDR2-sensitive
AACGATTATGGATATGTGGATGGC	0	10	RDR2-sensitive
AACGATTCGGTAGATTATAATGGC	3	27	RDR2-sensitive
AACGATTCGGTGGATTATAATGGC	2	26	RDR2-sensitive
AACGCAACACGACCTCGGCGCCAT	0	16	RDR2-sensitive
AACGCAAGCAGTAGATCACGGAAC	0	20	RDR2-sensitive
AACGCACGAACGGACAATGGCGAC	0	10	RDR2-sensitive
AACGCACGATAGGATACGGACGGT	0	10	RDR2-sensitive
AACGCACGTAGTGGTCTGGGACAA	0	15	RDR2-sensitive
AACGCATCGGATCATCCACAGCAT	0	20	RDR2-sensitive
AACGCATCTGATAGTAGACGAGCG	0	9	RDR2-sensitive
AACGCCATCGGGCCCAGAAGACAC	0	9	RDR2-sensitive
AACGCCGGCGCTCGGACGGAAGGC	0	11	RDR2-sensitive
AACGCGACGGGGCATTGTAAGTGG	89	3	RDR2-resistant
AACGCGACTCTGTCAGGAACGGAA	0	43	RDR2-sensitive
AACGCGAGACTGGGGGCGTCGGGC	0	10	RDR2-sensitive
AACGCGGAACAGGAGATGGAGCGA	0	9	RDR2-sensitive
AACGCGTCTGAGACTGTTCGGTGC	0	24	RDR2-sensitive
AACGCGTCTGAGACTGTTCGGTGT	3	38	RDR2-sensitive
AACGCTAACCCGGCTTGTAGTTGT	12	57	RDR2-sensitive
AACGCTAGCGTAGGAGATCGGCAC	0	9	RDR2-sensitive
AACGCTATACTGGACTAGGGGCGT	0	9	RDR2-sensitive
AACGCTCGGCAAAGAACACTCGGC	0	9	RDR2-sensitive
AACGCTCGGCGAACTGTACATCGG	7	141	RDR2-sensitive
AACGCTGATGGAGGATAGACTGGT	43	4	RDR2-resistant
AACGCTGGCAAGGAACACGGGCGC	27	0	RDR2-resistant
AACGCTGGTAGATGAGGACGACGA	0	10	RDR2-sensitive

AACGCTTAGTCCGGTGAACGTCTG	0	14	RDR2-sensitive
AACGCTTGATGTGACTCGGGCTAA	0	10	RDR2-sensitive
AACGCTTGGAACATCCGACAGCTA	0	9	RDR2-sensitive
AACGGAACAGAAGTGGAGCGGCTT	0	19	RDR2-sensitive
AACGGAACGTGTGATGGTTTAACA	63	0	RDR2-resistant
AACGGAAGACACCTGTGCACGCAT	0	13	RDR2-sensitive
AACGGAAGAGGAGTGGAACGGCTC	0	10	RDR2-sensitive
AACGGACAGAAACGGTCGCGCATT	0	28	RDR2-sensitive
AACGGACAGAGCCGGGTCGGGCAC	0	23	RDR2-sensitive
AACGGACAGGGGAGGGCAGAACAG	0	37	RDR2-sensitive
AACGGACCACAAGCAGGCTTTCGG	0	10	RDR2-sensitive
AACGGACCACTATTTTCGTCGGCC	0	11	RDR2-sensitive
AACGGACCAGCCGGAGGATGGCGC	0	10	RDR2-sensitive
AACGGACCAGCTGAAGGGTGACGC	0	45	RDR2-sensitive
AACGGACCCAGGAACGTGCGGATC	0	12	RDR2-sensitive
AACGGACCGAGCTGAGTCGGGCTC	0	28	RDR2-sensitive
AACGGACCGGCGTCAACTGTCCAC	1	19	RDR2-sensitive
AACGGACCGGCGTCAACTGTTCAC	0	24	RDR2-sensitive
AACGGACGACGACAACCTCATCAT	0	10	RDR2-sensitive
AACGGACGTCTCTAGCACTAGCAG	0	10	RDR2-sensitive
AACGGACTGATCTATTAGAAGGGC	22	0	RDR2-resistant
AACGGACTTCGGGCTAAACGGGTA	0	10	RDR2-sensitive
AACGGAGCGTGACTGTGAGAGCGG	0	18	RDR2-sensitive
AACGGAGGCAGAACGGCAGAACAC	0	15	RDR2-sensitive
AACGGAGGCGGCTTAGTAGTTCTC	0	13	RDR2-sensitive
AACGGAGTAGAGCAGAACGGTTCC	0	10	RDR2-sensitive
AACGGATACGGATACGGATAGTCT	0	9	RDR2-sensitive
AACGGATCAGATGGTTTGCTTGCA	5	62	RDR2-sensitive
AACGGATGACGCTGCCGCTCCCAC	0	10	RDR2-sensitive
AACGGATGGCACTTGTACACGCAT	0	21	RDR2-sensitive
AACGGATTCGGGATCGGATACGGA	0	11	RDR2-sensitive
AACGGCAGAACACACTGATGGCAG	0	49	RDR2-sensitive
AACGGCAGAACAGCAGACTACTCT	0	12	RDR2-sensitive
AACGGCAGAACGGCAGAACCACAA	0	9	RDR2-sensitive
AACGGCAGAACGGCAGAACGCAGG	0	12	RDR2-sensitive
AACGGCAGAACGGCAGAACGCGTC	0	14	RDR2-sensitive
AACGGCAGAACGGCAGACTACCCT	0	17	RDR2-sensitive
AACGGCAGAACGGCAGACTACTCT	0	31	RDR2-sensitive
AACGGCAGAACGTAAGGAAGGCAT	0	18	RDR2-sensitive
AACGGCAGAACGTAAGGGAGGCAG	0	11	RDR2-sensitive
AACGGCAGAATACGGGTAGGACAG	0	9	RDR2-sensitive
AACGGCAGCGACTCGGACAAGGAA	0	9	RDR2-sensitive
AACGGCAGTCAGGAGGACATCATC	14	0	RDR2-resistant
AACGGCATAGGATTCCTTCGGGTA	0	22	RDR2-sensitive
AACGGCATGTACTTAGGGCGCTAG	0	35	RDR2-sensitive
AACGGCCTTCAGCGACCTACGGTT	1	30	RDR2-sensitive
AACGGCCTTGTTACGCATACTCAC	0	19	RDR2-sensitive
AACGGCCTTTAGCGACCTACGGTT	0	15	RDR2-sensitive
AACGGCGCTTAACTCGGACGTCGG	1	24	RDR2-sensitive

AACGGCTAACTCGGTCTTCGACGG	0	13	RDR2-sensitive
AACGGCTACTGTTTTCGGCGGCGC	0	12	RDR2-sensitive
AACGGCTAGAAGGGGGCACCGGAC	0	9	RDR2-sensitive
AACGGCTCAGAGTCCAGACGATGT	0	15	RDR2-sensitive
AACGGCTCTGAATCTGCAACGGTC	0	12	RDR2-sensitive
AACGGCTCTGCCTAGCTCCGACGG	0	16	RDR2-sensitive
AACGGCTCTGGCTCCTTGATGGCT	0	15	RDR2-sensitive
AACGGCTCTGGCTCTTTGATGGCT	0	41	RDR2-sensitive
AACGGCTCTGTCTCTTTGATGGCT	0	18	RDR2-sensitive
AACGGCTGCAATTTTAGACGGTGA	0	9	RDR2-sensitive
AACGGCTGCAGACAGCGAGACCGA	0	12	RDR2-sensitive
AACGGCTGGCACTCGGCAAAGAGG	2	14	RDR2-sensitive
AACGGCTGGTCTCGGTAACCTTGA	117	11	RDR2-resistant
AACGGCTTGGACGGAACACGGGGC	0	13	RDR2-sensitive
AACGGCTTTCTACGCTACCGTCGG	0	9	RDR2-sensitive
AACGGCTTTGTAGGAAATTGTCGG	0	14	RDR2-sensitive
AACGGGAAAGTCGGAAACGGATAA	0	13	RDR2-sensitive
AACGGGAACGAGAATGTTGGACGG	0	21	RDR2-sensitive
AACGGGACTAACCGAACAAGGCAT	0	13	RDR2-sensitive
AACGGGCAACGGATGAGGTTACTT	19	1	RDR2-resistant
AACGGGCAGTGGAAGATGATGTGC	0	9	RDR2-sensitive
AACGGGCCGGCGTCAACTGTCCAC	4	56	RDR2-sensitive
AACGGGCCTTGCCTGGGTCGCGGC	0	11	RDR2-sensitive
AACGGGCGGATACGGACGGATAGT	0	31	RDR2-sensitive
AACGGGCGGCGTGCTGAATCCTTT	36	2	RDR2-resistant
AACGGGCTGAATAGTGTGCACGAG	0	16	RDR2-sensitive
AACGGGCTGGACCGACACGGCTAC	0	10	RDR2-sensitive
AACGGGCTTAGAGCTAAACGGGCC	0	15	RDR2-sensitive
AACGGGCTTAGAGGTAAACGGGCC	2	41	RDR2-sensitive
AACGGGCTTAGAGGTAAACGGGTC	0	26	RDR2-sensitive
AACGGGGGTGAACAATGGCGACAA	0	9	RDR2-sensitive
AACGGGTATGGGTGAAGGCGGAAC	0	12	RDR2-sensitive
AACGGGTATGTGTGAAGGCGGAAC	0	9	RDR2-sensitive
AACGGGTCGGATTCGGATCGGATA	0	13	RDR2-sensitive
AACGGGTCGGATTCGGGTCGGGTA	0	9	RDR2-sensitive
AACGGGTCTCTGTGGAGAACGGGA	2	39	RDR2-sensitive
AACGGGTCTGCGGCAATGTATCAT	0	24	RDR2-sensitive
AACGGGTTACTGTTATTGATCGGC	199	27	RDR2-resistant
AACGGGTTGATCGTGCCCCCGGAC	0	13	RDR2-sensitive
AACGGGTTGGTCTGTTACGGTCAT	0	14	RDR2-sensitive
AACGGTAATTTTACTCGTCGGTCC	0	10	RDR2-sensitive
AACGGTAGAATGGACGGCTGAGAT	0	32	RDR2-sensitive
AACGGTAGCTCTGATGACGACTGT	0	16	RDR2-sensitive
AACGGTCACCTGCTCTGACAGCGG	7	46	RDR2-sensitive
AACGGTCCGTAATGAACAGCGGTG	0	16	RDR2-sensitive
AACGGTCCTGACAGTCATGGACGG	0	18	RDR2-sensitive
AACGGTCGACTCTGACGGATGAAC	0	9	RDR2-sensitive
AACGGTCGACTGCAACGCATTAAA	5	34	RDR2-sensitive
AACGGTCGACTGCAACGCATTTAA	2	33	RDR2-sensitive

AACGGTCGACTGCAACGGCGCCTG	0	36	RDR2-sensitive
AACGGTCGACTGCTACAGACCCCA	0	9	RDR2-sensitive
AACGGTCGACTGCTACAGACGCCA	0	19	RDR2-sensitive
AACGGTCGACTGCTACAGGCGCCA	7	55	RDR2-sensitive
AACGGTCGAGATTGACGGCAGAAC	0	9	RDR2-sensitive
AACGGTCGGAAACGGTATTTATTC	1	14	RDR2-sensitive
AACGGTCGGCTGACGTGGCGTGCA	1	16	RDR2-sensitive
AACGGTCGGTCCAGCATAGCTACC	0	54	RDR2-sensitive
AACGGTCGGTCCGGCACAACCCGT	0	12	RDR2-sensitive
AACGGTCGGTTGGTCGCTACAAGT	1	12	RDR2-sensitive
AACGGTCGTCAGCTGCCGTCGGCT	0	13	RDR2-sensitive
AACGGTCGTCATCTATAGACGGTT	0	17	RDR2-sensitive
AACGGTCTATTTAGTAGGGCGGTA	0	9	RDR2-sensitive
AACGGTCTCGTAGACAGCCGTCGG	0	23	RDR2-sensitive
AACGGTCTCTAGCCGTCGGACATA	0	9	RDR2-sensitive
AACGGTCTGTGCTCTGGGCCGGAT	1	33	RDR2-sensitive
AACGGTGGCTGGAGCGGCTGGGAC	0	14	RDR2-sensitive
AACGGTTAAAGTGCATGCTCAATA	17	1	RDR2-resistant
AACGGTTAGACTGTTGCTGACGGC	0	9	RDR2-sensitive
AACGGTTCAGATCGTTGGATAACC	0	16	RDR2-sensitive
AACGGTTCAGATCGTTGGATAACT	0	14	RDR2-sensitive
AACGGTTCAGATCGTTGGATAATC	0	14	RDR2-sensitive
AACGGTTCGCGCTCTGGACCGGAC	0	28	RDR2-sensitive
AACGGTTGACTGCATGTGCTACGC	13	36	RDR2-sensitive
AACGGTTGGTCTGATAGTCGTCGG	0	14	RDR2-sensitive
AACGGTTGTAGAGCGAGTCGGCAG	0	10	RDR2-sensitive
AACGGTTGTCAACTGTAGATGGCT	0	101	RDR2-sensitive
AACGGTTTACGTTTGATTACTGGC	0	9	RDR2-sensitive
AACGGTTTAGATTGTTGGATAACA	0	9	RDR2-sensitive
AACGTACACGGCACCGCTGAGTCT	0	9	RDR2-sensitive
AACGTACGGGCATACAGCTAGTTA	0	9	RDR2-sensitive
AACGTACTGAACCGGTGTGTCATT	0	13	RDR2-sensitive
AACGTAGAACTCGACCTCGGGCGT	0	10	RDR2-sensitive
AACGTAGACTGTGGTGCACGGTTC	0	21	RDR2-sensitive
AACGTAGGCCTCTGAGCTGGACGA	0	10	RDR2-sensitive
AACGTATAGGACACTCTTCGGCTC	0	10	RDR2-sensitive
AACGTCAAAATGTAGTTAGCGGAG	14	0	RDR2-resistant
AACGTCAGAAGCGGCTCTGTGCCA	0	26	RDR2-sensitive
AACGTCATATCGGATTAAGGTCGG	0	12	RDR2-sensitive
AACGTCCCAGTCGGAGGGTGGCGC	0	30	RDR2-sensitive
AACGTCCGGAGTGTGTGATGGCTT	2	31	RDR2-sensitive
AACGTCCGGTAAATGATTCGGTAT	0	22	RDR2-sensitive
AACGTCTCCCGTCTGACCGAGGCT	0	9	RDR2-sensitive
AACGTCTGTAGGTATGTCGGCGGG	0	11	RDR2-sensitive
AACGTGACCTGTAGATGAGGTCTA	0	9	RDR2-sensitive
AACGTGAGCTGCATGAGAGGACGT	0	32	RDR2-sensitive
AACGTGAGGTGTAGAGGCGCGCGC	0	12	RDR2-sensitive
AACGTGGAACTGATGTGGCAGCAA	0	10	RDR2-sensitive
AACGTGGAAGTAGGCAAGTGTTAC	0	28	RDR2-sensitive

AACGTGGAGGTAGGCAAGTGTTAT	0	14	RDR2-sensitive
AACGTGGGGCTGAGAGGCGGCATG	0	9	RDR2-sensitive
AACGTGGGTGATAGAACGGACGGT	1	20	RDR2-sensitive
AACGTGTACCGGACTGTTCGGTGT	1	15	RDR2-sensitive
AACGTGTCGCTTAGGACGTCCGGA	0	16	RDR2-sensitive
AACGTGTCGTGCTGGTCCGGCTAT	0	30	RDR2-sensitive
AACGTGTCTGAGACTGTTCGGTGT	0	10	RDR2-sensitive
AACGTGTGACTGTTATGAAGGTAT	0	53	RDR2-sensitive
AACGTGTGTCTGCACGTGTCGGGC	0	50	RDR2-sensitive
AACGTTAGTGACAGGACGACGGGA	0	13	RDR2-sensitive
AACGTTCGACGGCTGTCCGCGCGT	0	9	RDR2-sensitive
AACGTTCGGAGTGTGTGATGGCTT	0	11	RDR2-sensitive
AACGTTCGGATCGGGGAGACGCAT	0	13	RDR2-sensitive
AACGTTGACACTGCGGACGGTGAG	1	14	RDR2-sensitive
AACGTTGAGCTGGTGTTTGAGGTT	0	9	RDR2-sensitive
AACGTTGCTGTAGCGAGTAGAGGA	0	35	RDR2-sensitive
AACGTTGGCAGCAGTCGGACATAA	0	10	RDR2-sensitive
AACGTTGGCTCTTGACGGACATGT	0	25	RDR2-sensitive
AACGTTGGGACTGTCCGGGCGAGC	0	10	RDR2-sensitive
AACGTTGGGCTGAGAGCAGGCATG	0	10	RDR2-sensitive
AACGTTGGTTGAATTTGAGACGAC	0	9	RDR2-sensitive
AACGTTGTACGGCGTGGTAGACGG	3	16	RDR2-sensitive
AACGTTTCGGACGTACGTGTGGAT	0	12	RDR2-sensitive
AACGTTTGTTTGGTTGCTCGGTAC	0	17	RDR2-sensitive
AACGTTTGTTTGGTTGCTGGATAC	0	16	RDR2-sensitive
AACGTTTGTTTGGTTGTTCGGTAC	0	14	RDR2-sensitive
AACTAAAACGTTATCTGCTGGACC	1	17	RDR2-sensitive
AACTAAATTTTGTAGTGGCTTGGA	30	0	RDR2-resistant
AACTAAGCTACCACCTCGTCGTTC	24	0	RDR2-resistant
AACTACAACGTTATCTGGTGGACC	0	9	RDR2-sensitive
AACTACACTTATGTGATCTTAGGG	0	11	RDR2-sensitive
AACTACATTTATGTGATCTTAGGG	0	20	RDR2-sensitive
AACTACTGGAGAACACGCCGGCAT	0	11	RDR2-sensitive
AACTAGAACTACGCTACATCTACT	1	17	RDR2-sensitive
AACTAGAGTGACGGGTACCCTGGA	0	9	RDR2-sensitive
AACTAGCCGGGCAGAAATGACAGA	0	21	RDR2-sensitive
AACTAGGGTGGACTCTTCGATGGC	1	17	RDR2-sensitive
AACTAGTCGTTGGACCCAGACGGT	0	11	RDR2-sensitive
AACTATAGACGGCTGCTGACGGTC	0	13	RDR2-sensitive
AACTATATCGTCGACTTATGGTGT	0	10	RDR2-sensitive
AACTATGCGACTGTCAGAAGGTGA	2	22	RDR2-sensitive
AACTATGGTTGCTGGATCGTTGTC	1	17	RDR2-sensitive
AACTATGTCGGAGTAGGTATGCAT	0	9	RDR2-sensitive
AACTCAGCAGCCGTCGGTCGTCGC	0	11	RDR2-sensitive
AACTCATCGGATACGGGACGGATA	0	19	RDR2-sensitive
AACTCATCGGCTACAAATGGGCAT	0	30	RDR2-sensitive
AACTCATGTTGGATTGTGATGGTG	1	39	RDR2-sensitive
AACTCCATCTGTTGCTTCTGAGGC	0	9	RDR2-sensitive
AACTCCCAATCTGTCGGGCACCAT	0	11	RDR2-sensitive

AACTCCCGCCGAAGACTGCTTCTT	0	13	RDR2-sensitive
AACTCCCGTCGGCTGCTAGTGGGC	0	9	RDR2-sensitive
AACTCGACTGGATTGGTACGGCTC	0	12	RDR2-sensitive
AACTCGCAGGCTTAGACGACCTAA	0	11	RDR2-sensitive
AACTCGCTCGACTCGGCTCGTTAG	0	19	RDR2-sensitive
AACTCGCTCGACTCGGCTTGTTAG	0	9	RDR2-sensitive
AACTCGGAGGCATCACGTAGGGAC	0	12	RDR2-sensitive
AACTCGGATCTGTGACGCACGGCC	0	9	RDR2-sensitive
AACTCGGCTCGACTCGGACCGGCT	0	11	RDR2-sensitive
AACTCGGCTCGGCTCGAAGCGGCT	91	0	RDR2-resistant
AACTCGGCTCGGCTCGGATCGGCT	0	17	RDR2-sensitive
AACTCGGCTTAACTCAGATCGGCT	0	10	RDR2-sensitive
AACTCGGGTGTCGTCTGATGGCAC	0	17	RDR2-sensitive
AACTCGGTCAGTCGGTGCGGCGAT	7	29	RDR2-sensitive
AACTCGGTCTCGGACGTCCGGCCT	0	16	RDR2-sensitive
AACTCGGTCTTCGACGGTCATCTC	0	10	RDR2-sensitive
AACTCGTCGGTTGGATTGAGGCAT	0	9	RDR2-sensitive
AACTCTAAACTCGTCGGTTGGCAT	0	9	RDR2-sensitive
AACTCTAAGCTCGTCGGTTGGCAT	0	34	RDR2-sensitive
AACTCTACATCGTCTAGTGGCATT	0	11	RDR2-sensitive
AACTCTACGTCGTCTGGAGACGTC	2	16	RDR2-sensitive
AACTCTATCAGACAGACTCAGGCT	0	13	RDR2-sensitive
AACTCTATCATCAGCTTGGGACAG	14	0	RDR2-resistant
AACTCTCAGCAGGTACGACGGACA	0	61	RDR2-sensitive
AACTCTCCCGTTGCAGTGGACATG	63	0	RDR2-resistant
AACTCTCGTAGACGTGCTGGTATT	0	19	RDR2-sensitive
AACTCTCTCTGCAGGATGACTCTC	178	5	RDR2-resistant
AACTCTCTGTAGCGGTTCGGACGG	0	17	RDR2-sensitive
AACTCTCTTGATCGTAACTCGGAT	28	4	RDR2-resistant
AACTCTGACACTGTTGCACGGTAC	0	10	RDR2-sensitive
AACTCTGCAGGTAGAGCTGTACGG	0	46	RDR2-sensitive
AACTCTGCCTCGCACTGTGACTGC	0	10	RDR2-sensitive
AACTCTGTAGAGAGCCGCGGGCAT	0	18	RDR2-sensitive
AACTCTGTAGGTGGCGATGTCTAT	0	11	RDR2-sensitive
AACTGAACGTAGGACATTCGGTCT	0	11	RDR2-sensitive
AACTGAACTGAAGAACGGAATGCC	0	10	RDR2-sensitive
AACTGACAAGTTCCTGATGTGCCT	14	0	RDR2-resistant
AACTGACACAGGCGGAACTGACAC	2	49	RDR2-sensitive
AACTGACACTGCAAGCAGCTGGAT	0	12	RDR2-sensitive
AACTGACGGACTCGGAACGGCGGA	0	17	RDR2-sensitive
AACTGACTCTGCTCATGCGTGGAC	0	14	RDR2-sensitive
AACTGAGGCGAGGACCATCATTAT	0	12	RDR2-sensitive
AACTGAGGCTCTGATACCAGATGA	9	37	RDR2-sensitive
AACTGAGGCTTTGATACCAGATGA	28	258	RDR2-sensitive
AACTGAGTAGAACTGACGGTCCAT	0	33	RDR2-sensitive
AACTGATGTAGCTTTGTTGTCGGT	0	18	RDR2-sensitive
AACTGCACCGTTGAGTTGTGGACA	0	153	RDR2-sensitive
AACTGCCGCTGTAGACAGAAGATG	0	45	RDR2-sensitive
AACTGCCTCTGAACCCTGTGCGGT	37	4	RDR2-resistant

AACTGCGACTGGACACGACGTGGC	0	9	RDR2-sensitive
AACTGCTAATGTAGATGTCGGCGT	0	9	RDR2-sensitive
AACTGCTAGGTTCGCGACGGCGGC	23	0	RDR2-resistant
AACTGCTGGGGACGGATGGAACAC	0	19	RDR2-sensitive
AACTGCTGTGCTGTATGACGGATC	0	23	RDR2-sensitive
AACTGGACAGATGGCGTGGACACT	22	0	RDR2-resistant
AACTGGACCAAGCCTAAATGGTTG	0	17	RDR2-sensitive
AACTGGACCATGCCAGGCTCGGAC	0	11	RDR2-sensitive
AACTGGAGGGACAAGAGAGGACGC	0	12	RDR2-sensitive
AACTGGATCCGTCGCTTAGGGCAT	0	13	RDR2-sensitive
AACTGGATTCGGGACCGGATACGG	1	25	RDR2-sensitive
AACTGGCTCGAACAGGCTGGATGC	0	12	RDR2-sensitive
AACTGGCTCTGTGCACGCGTGGAC	0	10	RDR2-sensitive
AACTGGGACAGAACGGCTCCGTTC	0	29	RDR2-sensitive
AACTGGGGTGAACTCTTCGATGGC	0	14	RDR2-sensitive
AACTGGTCTTAGGGTCGTCTGTGC	31	0	RDR2-resistant
AACTGTAAAATGGACGGTTGGATG	0	13	RDR2-sensitive
AACTGTAATATGGACGGTTGGATG	2	45	RDR2-sensitive
AACTGTACATCGGCAACAGCCTCT	1	19	RDR2-sensitive
AACTGTACCGATGACTGCACATGT	0	15	RDR2-sensitive
AACTGTACTGTTACTGACGGCCGC	0	12	RDR2-sensitive
AACTGTAGAAGGCTTTTTACTAGG	50	2	RDR2-resistant
AACTGTAGAAGGCTTTTTACTTGG	90	24	RDR2-resistant
AACTGTAGAATGGACGGTTGGATG	0	22	RDR2-sensitive
AACTGTAGAATGTACGGTTAGATG	0	14	RDR2-sensitive
AACTGTAGACGATACGGTGACTGT	0	16	RDR2-sensitive
AACTGTAGATAGGCCTAATGACTT	0	11	RDR2-sensitive
AACTGTAGCCTTTATTGCAGAGTG	0	9	RDR2-sensitive
AACTGTAGCGCGTAGGGGGCTCTC	0	13	RDR2-sensitive
AACTGTAGGATCAACGGTGGGATG	0	14	RDR2-sensitive
AACTGTAGGTCGCTGAAGACGCAT	0	13	RDR2-sensitive
AACTGTAGGTGGGCCTAATGACTT	2	27	RDR2-sensitive
AACTGTAGGTGGGCCTAATTACTT	2	29	RDR2-sensitive
AACTGTAGTCCCAGGCGAATTCAC	2	15	RDR2-sensitive
AACTGTAGTCTTTATTGCAGAGTG	0	18	RDR2-sensitive
AACTGTAGTCTTTGCCCGGCCCAC	2	21	RDR2-sensitive
AACTGTAGTTCCGGACGGATTCAC	0	14	RDR2-sensitive
AACTGTCATGTAGTGTAGTGGTAT	23	0	RDR2-resistant
AACTGTCCACTCTGCAACGGCCCA	0	11	RDR2-sensitive
AACTGTCCCAATCTGGCGCGGCTG	2	39	RDR2-sensitive
AACTGTCCTAATCTGACGCGGCTG	0	33	RDR2-sensitive
AACTGTCTGAGACGATACGGACGC	0	12	RDR2-sensitive
AACTGTGACTGTGGCAGACTGGCT	10	285	RDR2-sensitive
AACTGTGACTGTTGCAGACTGGCT	1	74	RDR2-sensitive
AACTGTGAGACAAAGTTGGCCATT	15	0	RDR2-resistant
AACTGTGATTGTAGCAGACCGGCT	0	9	RDR2-sensitive
AACTGTGCCAAGCCTGGGCCGCGT	0	9	RDR2-sensitive
AACTGTGCTGGACCTAGATCGCGT	0	12	RDR2-sensitive
AACTGTGGCGCGGCGTGAGGACAT	0	19	RDR2-sensitive

AACTGTGGTGTGGTTATAGATGAA	17	0	RDR2-resistant
AACTGTGTGTAAATCGAATGGTGC	16	0	RDR2-resistant
AACTGTTAGATGTGCATCGGTCGT	0	25	RDR2-sensitive
AACTGTTGTCGGCTCGATCGGTTT	0	12	RDR2-sensitive
AACTTAAGGGCTATTGACGTCGAC	138	13	RDR2-resistant
AACTTATCGAGCGGATCGACGTCA	0	19	RDR2-sensitive
AACTTATCGTATTAGAAACGTCGG	0	11	RDR2-sensitive
AACTTATGATGTTGGATATTAATT	16	0	RDR2-resistant
AACTTATGGTTGGTCGCTATAGAT	3	95	RDR2-sensitive
AACTTATGTAGAAATATGTGGTGT	0	9	RDR2-sensitive
AACTTATGTTGGTCTGTGAGGATA	0	12	RDR2-sensitive
AACTTCACTGACGTTGGATGGCTG	50	11	RDR2-resistant
AACTTCAGGCGTCAGGAAGGAGGC	0	11	RDR2-sensitive
AACTTCCGTTGTAGTCTAGTTGGT	76	0	RDR2-resistant
AACTTCGACTGTAATATATGGTGT	0	10	RDR2-sensitive
AACTTCGATCGGAGAACGGTCCAT	0	30	RDR2-sensitive
AACTTCGGCAGACTCTTTCAGAAG	0	11	RDR2-sensitive
AACTTCTGGGACTGCCTTGACGGT	0	16	RDR2-sensitive
AACTTGACGGAGCCTCGGACGCTA	57	5	RDR2-resistant
AACTTGGACTTTATTGGACGCGCC	0	9	RDR2-sensitive
AACTTGGGCAGAACGCACGGGCAC	1	17	RDR2-sensitive
AACTTGTAGGACGATTTTCGGCTC	0	13	RDR2-sensitive
AACTTGTGACTTGATGGCTGTCCT	18	1	RDR2-resistant
AACTTGTGCTGATTGGATGACTTG	65	7	RDR2-resistant
AACTTGTGGGTACTGTGTCCGCGG	0	14	RDR2-sensitive
AACTTTCAGTGGGACATTCGACGT	30	3	RDR2-resistant
AACTTTCTTGTTTTATTCGGACCA	42	0	RDR2-resistant
AACTTTGACGTGGACAGAGGCTGT	0	11	RDR2-sensitive
AACTTTGACTGTGGCAGATCGGCT	0	18	RDR2-sensitive
AACTTTGATTAGCCGGAACGATTC	0	18	RDR2-sensitive
AACTTTGCGTTGCTAGATCGTTGT	0	20	RDR2-sensitive
AACTTTGGGTTGCTGGATCGTTGT	5	26	RDR2-sensitive
AACTTTTGCATGAGCCAGAGCCGA	0	10	RDR2-sensitive
AACTTTTTGTAGTGTTGATCTCGG	0	24	RDR2-sensitive
AAGAAAACTTTGAGAGATTTTGGC	18	0	RDR2-resistant
AAGAAAATCGTCGACTGACGTCGA	0	10	RDR2-sensitive
AAGAAATGGACAAGGGCGTGCCAC	4	18	RDR2-sensitive
AAGAACACGTCTGATTGGGTGGCG	0	18	RDR2-sensitive
AAGAACCAAGTAGAACGGAGCCGT	0	10	RDR2-sensitive
AAGAACCAAGTAGAATGGAGCCGT	0	9	RDR2-sensitive
AAGAACGTCGCTGACTGAGGCATT	0	13	RDR2-sensitive
AAGAACTATCTGACCTGGGCTGAC	0	16	RDR2-sensitive
AAGAACTCGAGGAATTTCAGGCAT	42	9	RDR2-resistant
AAGAACTCTGTTGGTGGCTGAACT	116	6	RDR2-resistant
AAGAACTTGGTCTGCTAGATGACG	0	11	RDR2-sensitive
AAGAAGACGCATGCAACCGAACAT	0	9	RDR2-sensitive
AAGAAGACGGGCGGCTGAACTAGC	2	21	RDR2-sensitive
AAGAAGCAGGAGCTGGAGCTTTGC	0	9	RDR2-sensitive
AAGAAGCTGGTGCCCATGGGGTGA	39	1	RDR2-resistant

AAGAAGGACTGTGCGGCCATCCAC	0	13	RDR2-sensitive
AAGAAGGCTCGGCAAACAGTACAT	1	17	RDR2-sensitive
AAGAAGGCTTAGTGACGTGCTTGT	0	10	RDR2-sensitive
AAGAATAAACTCTGATAGTCTGAT	55	0	RDR2-resistant
AAGAATGATAATGATCGAGCGATG	17	0	RDR2-resistant
AAGACACACGCTAGGATCTCGGAG	0	19	RDR2-sensitive
AAGACAGGATGGACGGGATAGGAC	0	9	RDR2-sensitive
AAGACATGTATCTTGACGAAGACA	1	30	RDR2-sensitive
AAGACCGGGATTTGTAGTGGGCAG	0	33	RDR2-sensitive
AAGACCTAGCTATGACACTGGCAC	0	27	RDR2-sensitive
AAGACCTTAGACGACTATGCATGT	0	12	RDR2-sensitive
AAGACGAACGTAGAAGCGCAGTGC	0	9	RDR2-sensitive
AAGACGAAGATAGAATCATGTACA	0	13	RDR2-sensitive
AAGACGAAGCTAGCAGCGCGATAT	0	15	RDR2-sensitive
AAGACGATAGTGTAGACCAGGCAC	0	21	RDR2-sensitive
AAGACGCCTCTGAACGACGTAGAA	0	20	RDR2-sensitive
AAGACGGAGCCAGCAACGTGATAT	1	44	RDR2-sensitive
AAGACGGATCTAGCTAGACGACGT	0	9	RDR2-sensitive
AAGACGGCCTGAGTGGGACGGTTG	0	9	RDR2-sensitive
AAGACGGTAGTGTAGACCAGGCAC	1	35	RDR2-sensitive
AAGACGGTGCTGAACGTCGACGTC	0	11	RDR2-sensitive
AAGACGTGACTGCATATGTCTGAC	0	10	RDR2-sensitive
AAGACTAAGATCATTATTGATGGC	28	4	RDR2-resistant
AAGACTACAATCGAAGGTCGTCGT	0	9	RDR2-sensitive
AAGACTATGAGAGATGAGACGACA	0	12	RDR2-sensitive
AAGACTCATAAACTCGGCTGGTAA	0	32	RDR2-sensitive
AAGACTCATACAAGCGCGGAACAG	0	16	RDR2-sensitive
AAGACTCATCGGCAGCAGACCAAC	0	10	RDR2-sensitive
AAGACTCCTAAACTCGGCTGGTAA	0	78	RDR2-sensitive
AAGACTCGACTCTCCCCAAGGACG	26	2	RDR2-resistant
AAGACTCTTAAACTCGGCTGGTAA	0	35	RDR2-sensitive
AAGACTGCAGAGTGGAAGCAGCAT	0	32	RDR2-sensitive
AAGACTGCAGGAGCTACGGTGGGA	71	4	RDR2-resistant
AAGACTGCATGCGCTCGCGGCTGC	3	21	RDR2-sensitive
AAGACTGCATGCGCTCGCGGGTGC	0	16	RDR2-sensitive
AAGACTGTAAAGGACGGATATAGA	0	22	RDR2-sensitive
AAGACTGTAAGGGGTAGGCTGCCT	1	35	RDR2-sensitive
AAGACTGTCTGCAGCGTTTGACGT	0	9	RDR2-sensitive
AAGACTGTGTCCAGCAGACCGCTA	0	10	RDR2-sensitive
AAGACTGTGTGTAAGGCTATCTCC	0	19	RDR2-sensitive
AAGACTGTTATGTTGACGGGTCAT	0	25	RDR2-sensitive
AAGACTGTTGTCTTTATTGCAGAG	0	21	RDR2-sensitive
AAGACTTCAATCCAAGATCGTCGC	0	18	RDR2-sensitive
AAGACTTCAATCGAAGATCGTCGT	5	115	RDR2-sensitive
AAGACTTCAATCGAAGGTCGTCGT	15	116	RDR2-sensitive
AAGACTTCAATCGAGGGTCGTCGT	0	9	RDR2-sensitive
AAGACTTCTTAGTCGTGTCGGCAC	0	14	RDR2-sensitive
AAGACTTGGTTATGTCTGACGGCT	0	11	RDR2-sensitive
AAGACTTGTTGGACCAGGCGTTGT	0	9	RDR2-sensitive

AAGACTTGTTTGATCGGTTGTGGT	0	15	RDR2-sensitive
AAGACTTTAATCAAAGGTCGTCGT	0	12	RDR2-sensitive
AAGACTTTGATCGAAGGTCGTCAT	0	13	RDR2-sensitive
AAGAGACACAGTGGATGGAAGCTC	0	11	RDR2-sensitive
AAGAGACAGGCTAGCACGGAACTT	1	25	RDR2-sensitive
AAGAGACTGAAGCTGAGTCGCGAC	23	0	RDR2-resistant
AAGAGACTGCATAACACAATCCTG	0	12	RDR2-sensitive
AAGAGATATGGATGACTTACTTTT	17	0	RDR2-resistant
AAGAGCCAAGTAGAACAGAGCCGT	0	18	RDR2-sensitive
AAGAGCCAAGTAGAACGGAGCCAT	1	38	RDR2-sensitive
AAGAGCCAAGTAGAACGGAGCCGT	7	227	RDR2-sensitive
AAGAGCCAAGTAGAACGGAGTCAT	0	29	RDR2-sensitive
AAGAGCCAAGTAGAACGGAGTCGT	2	68	RDR2-sensitive
AAGAGCCAAGTAGAATGGAGCCGT	0	10	RDR2-sensitive
AAGAGCCAAGTGGAACGGAACCGT	1	15	RDR2-sensitive
AAGAGGAAGGAGGACGCAGGATAT	4	56	RDR2-sensitive
AAGAGGAAGGAGGACGCGGGATAT	0	12	RDR2-sensitive
AAGAGGATGGTTGGGGACGGAGTT	22	2	RDR2-resistant
AAGAGGCGTCTGGTATACCTGAGT	15	0	RDR2-resistant
AAGAGGGCTCCCTCAGGGTCTGGA	114	0	RDR2-resistant
AAGAGGGCTCGGCAAACAGTACAT	0	11	RDR2-sensitive
AAGAGGGCTCGGCACACAGTGCAT	0	28	RDR2-sensitive
AAGAGGTGGATACGTCAACCCTGA	0	9	RDR2-sensitive
AAGAGTAGAGGATGATGATAAGTA	17	1	RDR2-resistant
AAGAGTCGACGTCTACGTGGATCT	5	31	RDR2-sensitive
AAGAGTGGCATGATCCTGACGGAA	0	11	RDR2-sensitive
AAGATACTGTATCTCAGCTGGAGG	29	5	RDR2-resistant
AAGATAGAAGTCATCTGAGGCGTC	2	16	RDR2-sensitive
AAGATAGTATCCGGTGCTTACTCC	63	1	RDR2-resistant
AAGATATTGATGATGACCTCCCGT	14	0	RDR2-resistant
AAGATCGGAAAGACCAGCGCGCGC	0	17	RDR2-sensitive
AAGATCGGGGGTCGCGGACTCGGC	0	9	RDR2-sensitive
AAGATGACTTGTTAGCAAAGGGCG	15	0	RDR2-resistant
AAGATGCGACTGAACCAGCACAGA	0	21	RDR2-sensitive
AAGATGGCTTCACTCGGACTACAT	0	13	RDR2-sensitive
AAGATGGTATTGTGGACCAGGCAC	0	13	RDR2-sensitive
AAGATGGTGAACACTGATTCGGAT	0	9	RDR2-sensitive
AAGATGTTAGAAGATGTTGTGCTG	57	3	RDR2-resistant
AAGATTAAGAGATCATATGTGCAT	21	2	RDR2-resistant
AAGATTATAGGGATGTTTGGACTG	0	9	RDR2-sensitive
AAGATTCGGGACGGATACAGGACG	1	12	RDR2-sensitive
AAGATTGTCCTATGTCTGACGGCT	0	12	RDR2-sensitive
AAGATTTCAATCAAAGGTCGTCGT	0	10	RDR2-sensitive
AAGATTTGTAGAGTGTACATCAAG	1	19	RDR2-sensitive
AAGATTTGTGTTCGGCAGGGGCGT	0	14	RDR2-sensitive
AAGATTTTGAGTCTATTTCGTCGG	1	21	RDR2-sensitive
AAGCAAACGGCACATATGCTCTGA	1	14	RDR2-sensitive
AAGCAACCGAGCCAGAGAACCAGC	0	9	RDR2-sensitive
AAGCAACCTTGGCCAGATCGTCAC	0	30	RDR2-sensitive

AAGCAACGGACACACCTTCGGCTC	7	52	RDR2-sensitive
AAGCAACGGACGCACCTTCGACTC	0	14	RDR2-sensitive
AAGCAATCCGGCTCGGAATCGTTC	2	38	RDR2-sensitive
AAGCAATCCGGTTAGAAATCGTTC	1	12	RDR2-sensitive
AAGCAATCCGGTTAGGAATCGTTC	0	11	RDR2-sensitive
AAGCAATCTTTCGGATCGGCTGGC	0	13	RDR2-sensitive
AAGCAATTCGTTGGTTGTTGCATA	0	17	RDR2-sensitive
AAGCACAGATGTAGGTCGTCACTC	0	15	RDR2-sensitive
AAGCACAGTGGACTAGCTCGGCAT	3	35	RDR2-sensitive
AAGCACCGTCGGCACGATCTACAC	90	2	RDR2-resistant
AAGCACGACAGAGGAAACACACGA	0	15	RDR2-sensitive
AAGCACGACCTAGGCATGACTCGA	0	15	RDR2-sensitive
AAGCACGAGATTCTGATTGGTTGC	0	11	RDR2-sensitive
AAGCACGCGACAGACACGGAGCAC	0	10	RDR2-sensitive
AAGCACGCGTTTGGATGGCTGCAC	0	9	RDR2-sensitive
AAGCACGGCACGAATAACGGACAG	0	13	RDR2-sensitive
AAGCACGGGGGTCGCGGACTCGGC	2	50	RDR2-sensitive
AAGCACGGGGTCGCGGACTCGGCC	2	17	RDR2-sensitive
AAGCACGGTGTGTAGACGTCCATA	0	12	RDR2-sensitive
AAGCACGTGAGCTCTCGTATGGCC	0	17	RDR2-sensitive
AAGCACTCGGCAAACATGGCTCTC	3	23	RDR2-sensitive
AAGCACTGCTTGCAGCATCGTCGA	0	20	RDR2-sensitive
AAGCAGACCTGGCGATCTCTCTAC	0	9	RDR2-sensitive
AAGCAGACTGCAAACATGTTCTTT	0	10	RDR2-sensitive
AAGCAGACTGCTCACGGACTCCAA	0	9	RDR2-sensitive
AAGCAGACTGTCATGATAATGCTT	78	3	RDR2-resistant
AAGCAGACTGTCCATATTTTGTAG	35	0	RDR2-resistant
AAGCAGACTGTTGCGCGACCTCAA	1	77	RDR2-sensitive
AAGCAGACTGTTGCGCGACCTCGA	2	77	RDR2-sensitive
AAGCAGAGGACTAGATTTTTCTCT	22	1	RDR2-resistant
AAGCAGAGTCGAACGCAGAGACCA	0	14	RDR2-sensitive
AAGCAGCATCTGCTTGTTGGTTGT	0	10	RDR2-sensitive
AAGCAGCTGTAGGCTATAAGCTGT	0	33	RDR2-sensitive
AAGCAGGACGCGGGAACTCTCCAC	0	9	RDR2-sensitive
AAGCAGGACTGCCCATATGCGCCA	0	11	RDR2-sensitive
AAGCAGGAGCTAGAGCTTTGCCAA	0	9	RDR2-sensitive
AAGCAGGAGCTGGAGCTTTGCCAA	4	101	RDR2-sensitive
AAGCAGGAGTTGGAGCTTTGCCAA	0	9	RDR2-sensitive
AAGCAGGCTGATCGAATTTGGAAG	26	1	RDR2-resistant
AAGCAGGTCGCACGGGTTGCTTAG	0	27	RDR2-sensitive
AAGCAGTAGGACATAAGTTAATGT	0	12	RDR2-sensitive
AAGCAGTAGTTTGGACCGAGCCTC	1	17	RDR2-sensitive
AAGCAGTCGGTAGCAACTACTACT	0	23	RDR2-sensitive
AAGCAGTCTCGGATGAAGTGGTAT	46	9	RDR2-resistant
AAGCAGTTGAGAGTAGTGGAAGGC	28	4	RDR2-resistant
AAGCAGTTGTAGGCTGTAAGCTGT	0	9	RDR2-sensitive
AAGCATATAGGTTTGTGGCTCGGT	0	9	RDR2-sensitive
AAGCATCAAACTGTTAGTCGGTAT	0	37	RDR2-sensitive
AAGCATCGGCCTACACTCTCAGGC	1	13	RDR2-sensitive

AAGCATCGTGAACTGTCCGGCACC	0	10	RDR2-sensitive
AAGCATGGGCTGGTATATTGACAC	18	1	RDR2-resistant
AAGCATGTTTGACTGCCTCAGCAT	0	12	RDR2-sensitive
AAGCATTGATGGTGAAGTGTCTCT	31	3	RDR2-resistant
AAGCCACAGATCGTTCGTGTCGCC	0	19	RDR2-sensitive
AAGCCACGTTCGGACATACGTGCG	0	13	RDR2-sensitive
AAGCCCGAAGCACGATGAACCCGC	0	14	RDR2-sensitive
AAGCCCGCCCGCTTATTCTACCGT	0	10	RDR2-sensitive
AAGCCCGTTGGACCGGCAGCTCAG	0	10	RDR2-sensitive
AAGCCGAGCTCCCACGGTCGTCGC	0	22	RDR2-sensitive
AAGCCGCTGACGGTGTAGAATCAA	0	48	RDR2-sensitive
AAGCCGGGTTGGACTGCACGACGG	0	17	RDR2-sensitive
AAGCCGTGTCGGGTCTGGACTGGC	0	45	RDR2-sensitive
AAGCCTCTCAGGAAACGGTACTGT	1	14	RDR2-sensitive
AAGCCTCTGTACTAGGTCGCGGAC	1	17	RDR2-sensitive
AAGCCTGAACCAGACGAAGGTCGA	0	17	RDR2-sensitive
AAGCCTGCGGCACGTTCAACTTTT	0	16	RDR2-sensitive
AAGCGAAGTGAGCACACTGTACGA	0	29	RDR2-sensitive
AAGCGACGCTGAGCAGAGGACTTC	0	28	RDR2-sensitive
AAGCGAGACATGGTTGATTTATGA	31	0	RDR2-resistant
AAGCGCTTGGCTGATCTGTCGGGT	16	0	RDR2-resistant
AAGCGGACCTACTAGCCAGCCGGG	0	10	RDR2-sensitive
AAGCGGGCATTGCAATTGACGCAT	31	1	RDR2-resistant
AAGCGGGCGGGCTTGGACAGGAAA	1	12	RDR2-sensitive
AAGCGGGTCGGGCTCGGACAGAAA	0	9	RDR2-sensitive
AAGCGGGTGAGTAGCAGTAGGCTA	17	0	RDR2-resistant
AAGCGGTAGACTTTTTACATCACA	3	89	RDR2-sensitive
AAGCGGTAGACTTTTTACATCCCA	0	12	RDR2-sensitive
AAGCGGTAGGATTTTTACATCACA	1	27	RDR2-sensitive
AAGCGGTAGGCTTTTTACATCACA	4	87	RDR2-sensitive
AAGCGGTCAGACGGTGGAGACAGA	0	11	RDR2-sensitive
AAGCGGTCGCTGTAGACGGTGCGC	0	14	RDR2-sensitive
AAGCGGTTTCTCCAAAGATTAGTG	22	2	RDR2-resistant
AAGCGTCGAGACGGTCCGAGCCGA	0	9	RDR2-sensitive
AAGCGTCTAGACTTGAACCGGTAG	0	11	RDR2-sensitive
AAGCGTTGTACGGATGCAGTGCGC	2	20	RDR2-sensitive
AAGCTAACTTGTGAATTTGATGCA	14	0	RDR2-resistant
AAGCTAAGCTCTGGCAGACTTCAT	1	16	RDR2-sensitive
AAGCTAATTACACAGATGAGGACT	20	0	RDR2-resistant
AAGCTACAAGTTGGACGGTGTCTT	26	0	RDR2-resistant
AAGCTACATAATAGGACGATTGCT	33	2	RDR2-resistant
AAGCTACGGGACAGTGAGAAGGAC	0	14	RDR2-sensitive
AAGCTACTGTGCTACTGGGCGGAC	0	10	RDR2-sensitive
AAGCTACTGTTGCAGTTTGGACGG	1	20	RDR2-sensitive
AAGCTAGACTGTCGCAGGGCCGGA	0	9	RDR2-sensitive
AAGCTAGAGCTCATGGAGCTGGAC	18	0	RDR2-resistant
AAGCTAGATCTCGCTCGACGCAAC	0	28	RDR2-sensitive
AAGCTAGATCTCGCTCGTCGCAAC	0	12	RDR2-sensitive
AAGCTAGATCTCGTTCGTCGCAAC	0	12	RDR2-sensitive

AAGCTAGCAACCGGCGCCTCTGCT	94	5	RDR2-resistant
AAGCTAGCGACGGTGTCCTGACAA	0	10	RDR2-sensitive
AAGCTAGCTGTTTGGCAGAACTTC	1	31	RDR2-sensitive
AAGCTATGTTCGACGGTATCTGCT	0	44	RDR2-sensitive
AAGCTCCGTCGGCTACGTATCACC	95	2	RDR2-resistant
AAGCTCCTGCACCAGGTCGCGGAC	0	17	RDR2-sensitive
AAGCTCCTGTTTGATTGTCTGTAC	0	10	RDR2-sensitive
AAGCTCGAAGAAGGAACTCGGCTT	2	37	RDR2-sensitive
AAGCTCGGACAGGAAACTAGGCAC	0	10	RDR2-sensitive
AAGCTCGGCACTACAGATCACGGT	0	49	RDR2-sensitive
AAGCTCGGCGCCAATGATGACGGT	0	13	RDR2-sensitive
AAGCTCGGCTTGGGCTCGTTCGGA	0	11	RDR2-sensitive
AAGCTCTGATGAAGCTGCTGCATT	93	6	RDR2-resistant
AAGCTCTGGACATGTCAGACTGCA	1	14	RDR2-sensitive
AAGCTGACGGGCAGGATGAAGCAA	0	14	RDR2-sensitive
AAGCTGACGTGGCTATAGCCTGAG	0	10	RDR2-sensitive
AAGCTGACTGTGGGGAAAAGGTGG	0	10	RDR2-sensitive
AAGCTGACTGTGGGGAGAAGCTGG	1	38	RDR2-sensitive
AAGCTGACTTCGGACGGCTCCCTC	0	12	RDR2-sensitive
AAGCTGATGTGGCTATAGCCTGAG	1	15	RDR2-sensitive
AAGCTGCAAAGGCCTGATGGGCGC	0	9	RDR2-sensitive
AAGCTGCAAAGGCCTGATGGGTGC	1	14	RDR2-sensitive
AAGCTGCAGAAGTCTGATGGACGC	0	13	RDR2-sensitive
AAGCTGCAGAGGCCTGATGGGTGC	1	22	RDR2-sensitive
AAGCTGCTGTGTGATGGCGACTGT	0	13	RDR2-sensitive
AAGCTGCTTTTTCAACAAGCAGCA	0	12	RDR2-sensitive
AAGCTGGATGCATGGATGATCTCT	18	1	RDR2-resistant
AAGCTGGGCTGCACGCGCGGATAT	0	9	RDR2-sensitive
AAGCTGGTAGGGCCGAGCACACGG	0	22	RDR2-sensitive
AAGCTGTATCTAGTGGCACTTGTG	31	1	RDR2-resistant
AAGCTGTATGAGCGGATGTAAAAC	0	9	RDR2-sensitive
AAGCTGTATGAGCGGATGTAAGAT	0	16	RDR2-sensitive
AAGCTGTTGCAGGCTTGTCGGCGT	0	14	RDR2-sensitive
AAGCTGTTGTAGGCTGCAAGCTGT	9	34	RDR2-sensitive
AAGCTTATATTCGACGGTCTCTAT	1	14	RDR2-sensitive
AAGCTTATCTCGGACGGCTTGTCT	0	18	RDR2-sensitive
AAGCTTATGTCCGACGGCTGTCAG	1	25	RDR2-sensitive
AAGCTTATGTCCGACGGTCACTGA	0	37	RDR2-sensitive
AAGCTTATGTCCGACGGTCTCTGC	1	27	RDR2-sensitive
AAGCTTATGTCTGACGGCTTTGAT	0	9	RDR2-sensitive
AAGCTTATGTCTGACGGTCTCCTA	0	31	RDR2-sensitive
AAGCTTATGTTCGACGGCCGCTAG	0	9	RDR2-sensitive
AAGCTTATGTTTGGTTGTCTGCAC	0	9	RDR2-sensitive
AAGCTTGACTGGGACGGCGATCAC	1	17	RDR2-sensitive
AAGCTTGAGGCTCGCGAGTCGGCT	0	10	RDR2-sensitive
AAGCTTGAGTGAGTTTGTCTGAAA	47	0	RDR2-resistant
AAGCTTGTAGACATAATAATTTAA	78	3	RDR2-resistant
AAGCTTTGGATGGTCTGTCGACGC	0	12	RDR2-sensitive
AAGGAAACCTGGGAACCGGCAAAG	1	13	RDR2-sensitive

AAGGAACATGCAGGTGGGACCCAT	0	21	RDR2-sensitive
AAGGAACCCTGTGGTGCGGTCGGA	0	12	RDR2-sensitive
AAGGAACCGAGGCAGAGAACCAGC	0	9	RDR2-sensitive
AAGGAACGTCGCTGTCATGGCTAT	0	23	RDR2-sensitive
AAGGAACTAGAGTGCAGGGACATA	0	13	RDR2-sensitive
AAGGAACTGTAGCCCAACGGCTAG	0	13	RDR2-sensitive
AAGGAACTTAAGAACGCAGTGTAG	0	10	RDR2-sensitive
AAGGAAGAACTGTGGTGAGGACGC	0	10	RDR2-sensitive
AAGGAAGACGCAGGCTGCATGAGC	0	10	RDR2-sensitive
AAGGAAGTAGCAGCACGGTCACTA	15	0	RDR2-resistant
AAGGAATGTGGACGAGTGATGGTT	0	11	RDR2-sensitive
AAGGAATGTTGATTTTAAGACGGG	0	11	RDR2-sensitive
AAGGAATGTTGATTTTAAGACGGT	0	9	RDR2-sensitive
AAGGAATGTTGATTTTAAGGCGGG	0	10	RDR2-sensitive
AAGGACAAACAGGAGGAGGAGCGC	0	10	RDR2-sensitive
AAGGACAACTGTAGATAGGCCTAA	0	10	RDR2-sensitive
AAGGACAACTGTAGGTGGGCCTAA	10	522	RDR2-sensitive
AAGGACAACTGTAGGTGGGCCTCA	0	13	RDR2-sensitive
AAGGACAACTGTAGGTGGGTCTAA	0	20	RDR2-sensitive
AAGGACAACTGTAGTGGGCCTAAT	0	11	RDR2-sensitive
AAGGACAGGAACACAGTGGGCTGA	0	12	RDR2-sensitive
AAGGACAGTAGTCCCGGACGGTTT	0	14	RDR2-sensitive
AAGGACAGTTGGTTAGGAAGGCTT	0	10	RDR2-sensitive
AAGGACATAGTTGGCACGACGCAT	0	16	RDR2-sensitive
AAGGACATCTGTAGGTAGGCCTAA	0	20	RDR2-sensitive
AAGGACATCTGTAGGTGGGCCTAA	0	30	RDR2-sensitive
AAGGACATGTGCATGTGGGCCCAA	1	15	RDR2-sensitive
AAGGACCGTACCGAAAGTAGGCTT	0	9	RDR2-sensitive
AAGGACCGTACCGTTGACTGGCCT	2	17	RDR2-sensitive
AAGGACCGTTTAGAGGACGTTGCT	2	50	RDR2-sensitive
AAGGACCTTCGTCCCTCGAGGCAT	2	16	RDR2-sensitive
AAGGACGAACGACAGGAGAGCCAA	2	16	RDR2-sensitive
AAGGACGCCGACGACTGCACGGCA	75	0	RDR2-resistant
AAGGACGGGAGAACCAGTCGACGC	0	10	RDR2-sensitive
AAGGACGTGCTTTTCTAATATCTT	90	0	RDR2-resistant
AAGGACGTGGTCATGAGGCTTCAA	0	14	RDR2-sensitive
AAGGACTACCGTAACAGCGACCGC	0	14	RDR2-sensitive
AAGGACTCCCAGCAGCAATCAGCC	17	0	RDR2-resistant
AAGGACTCGACAGCCCCGACAGCT	6	22	RDR2-sensitive
AAGGACTCGCCCCTGGCGGCGGCC	25	1	RDR2-resistant
AAGGACTGACAACAACGTCGGTTG	69	6	RDR2-resistant
AAGGACTGCCACACGCGCGCGCGC	3	20	RDR2-sensitive
AAGGACTGCTTGCATAACCGTCGT	0	9	RDR2-sensitive
AAGGACTGCTTGCATAGCCGTTGT	0	11	RDR2-sensitive
AAGGACTTAGGGCTCTGGCGACGG	0	15	RDR2-sensitive
AAGGACTTGTTCGTTTGTGTCGGA	34	4	RDR2-resistant
AAGGAGACGTCAGCAAGGACCCGA	1	16	RDR2-sensitive
AAGGAGACGTCAGCAAGGATTCGA	0	9	RDR2-sensitive
AAGGAGATGGTGACCGCTGATTGA	0	30	RDR2-sensitive

AAGGAGCCTGCAGAAGCGCTGCTT	0	12	RDR2-sensitive
AAGGAGCGTAGGCGTGCACACCAA	0	14	RDR2-sensitive
AAGGAGCTCGTAGAGAAGGCGCCC	0	25	RDR2-sensitive
AAGGAGGAGCTGGCCAGAGCATGC	0	27	RDR2-sensitive
AAGGAGGCTAGTAGGAACGGTCCA	0	10	RDR2-sensitive
AAGGAGTCGATGGCTGGCGCGTGC	26	0	RDR2-resistant
AAGGAGTGATGGTTCATTAGCTTT	21	1	RDR2-resistant
AAGGATACGGGCAACACTTCTCAC	0	11	RDR2-sensitive
AAGGATAGCCGACGCGGACAGCAT	0	17	RDR2-sensitive
AAGGATCGACAGGCGCGAGACGAG	21	2	RDR2-resistant
AAGGATCGAGAAGAGGAGGAGCAA	0	16	RDR2-sensitive
AAGGATCTGACCAGCAAGATGCAT	0	16	RDR2-sensitive
AAGGATCTGGATCAGAGCGCATGG	0	9	RDR2-sensitive
AAGGATCTGGATCATCAGAGCGCA	1	68	RDR2-sensitive
AAGGATCTGGATCATCAGAGCGTA	0	26	RDR2-sensitive
AAGGATCTTCCCAAGAACTGGCAA	0	9	RDR2-sensitive
AAGGATGAAGACTGCTGTAGTTGA	0	12	RDR2-sensitive
AAGGATGAGATGTGGGTGATGCAT	0	12	RDR2-sensitive
AAGGATGAGCTGAACCGTGTGCAT	0	9	RDR2-sensitive
AAGGATGTGTAGTGTGATGGCTGC	0	10	RDR2-sensitive
AAGGATGTGTTTGGTTGGATGGTC	0	10	RDR2-sensitive
AAGGATGTTTGTTTATTTGGGTAT	1196	0	RDR2-resistant
AAGGATTCCCTTGGTCTCGAGTTT	24	1	RDR2-resistant
AAGGATTGCTTGCACAACAGTCGT	0	9	RDR2-sensitive
AAGGATTGGTATACGTGGGACCGA	0	9	RDR2-sensitive
AAGGATTGTGGTGTAAGATTGCAC	0	34	RDR2-sensitive
AAGGATTTAGCGACTGACCGTCGG	0	26	RDR2-sensitive
AAGGCAAGTAAACTTGTGATGGTT	21	0	RDR2-resistant
AAGGCAATAGTGTAGACTAGGCAC	0	27	RDR2-sensitive
AAGGCAATTAATGTATGGATATGG	14	0	RDR2-resistant
AAGGCACAGTGTCGGACGGTCCGC	0	10	RDR2-sensitive
AAGGCACGCAGGACTGTGCAGGCA	0	107	RDR2-sensitive
AAGGCACGCAGGACTGTGCAGGCC	0	10	RDR2-sensitive
AAGGCACGCAGGACTGTGCAGGCT	3	37	RDR2-sensitive
AAGGCACGTTAGAATGGACGGAGA	0	9	RDR2-sensitive
AAGGCACTCGACAAAGGGACTGGC	0	15	RDR2-sensitive
AAGGCACTCGGCAAAGATACTGGC	0	14	RDR2-sensitive
AAGGCACTCGGCAAAGGGACTGGC	2	14	RDR2-sensitive
AAGGCACTGTTGGGGTAGTTTTTC	0	9	RDR2-sensitive
AAGGCAGAACATGGGTGTGAACGC	0	9	RDR2-sensitive
AAGGCAGACTCACCACAATGACGC	2	22	RDR2-sensitive
AAGGCAGATAGACGAAGCCTCTGA	0	15	RDR2-sensitive
AAGGCAGCAAGCATGAACAAGCAT	0	9	RDR2-sensitive
AAGGCATCACGTAGGGACTGGCGT	0	23	RDR2-sensitive
AAGGCCAGGTAGAGCGGTTAGTTT	0	54	RDR2-sensitive
AAGGCCAGGTAGAGCGGTTGGTTT	0	173	RDR2-sensitive
AAGGCCCGTGGGCCGTCGGACGAC	59	0	RDR2-resistant
AAGGCCTATGTACGAGCGTCGGTT	0	31	RDR2-sensitive
AAGGCCTCGAGCGAAGGGTGGCGT	0	9	RDR2-sensitive

AAGGCCTGTGGCATGCACACTAGT	63	9	RDR2-resistant
AAGGCCTGTTTGGTTCGTGGCTAA	0	9	RDR2-sensitive
AAGGCGGCACTACAGTACACACAT	0	11	RDR2-sensitive
AAGGCGGGCAACATACGTGTAGAC	0	13	RDR2-sensitive
AAGGCGGGCTGGGCAGCAGCTAGG	22	1	RDR2-resistant
AAGGCTAGTAGCAAGGTCTCGGTC	0	10	RDR2-sensitive
AAGGCTATATACTCGTTGGATACA	22	2	RDR2-resistant
AAGGCTATGTCTGACGGTTGGTCC	0	18	RDR2-sensitive
AAGGCTCAGCTCTGGTCGTGGGTT	0	16	RDR2-sensitive
AAGGCTCGGCAAACAGTACATCGG	1	27	RDR2-sensitive
AAGGCTCGGCAAACAGTGCATCGG	0	13	RDR2-sensitive
AAGGCTCTTAACCTTGTGGTCGTG	12	65	RDR2-sensitive
AAGGCTGCAAGAGTAATCGGTCAT	0	17	RDR2-sensitive
AAGGCTGCAAGAGTAGTCGGCCAT	0	9	RDR2-sensitive
AAGGCTGCAAGAGTAGTTGGTCAT	0	20	RDR2-sensitive
AAGGCTGCAAGCATGAACAAGCAT	1	21	RDR2-sensitive
AAGGCTGCAGAGTTGCCGACGCTG	26	2	RDR2-resistant
AAGGCTGCCTCCAACAATAGACGA	0	14	RDR2-sensitive
AAGGCTGGGACTAACAGTGGCTGT	0	9	RDR2-sensitive
AAGGCTGGGAGACGCTACATGCAT	0	10	RDR2-sensitive
AAGGCTGGGATGAGCAACGCACAC	0	14	RDR2-sensitive
AAGGCTGTAAGAGTAGTCGGCCAT	2	23	RDR2-sensitive
AAGGCTGTAAGGGGTAGACTGCCT	0	13	RDR2-sensitive
AAGGCTGTAAGGGGTAGGCTGCCT	3	28	RDR2-sensitive
AAGGCTGTAGACAGGTCGTCAGGC	0	9	RDR2-sensitive
AAGGCTGTCAGATATAAAGGTATT	0	10	RDR2-sensitive
AAGGCTGTCTCTGTCGAGGACCAT	0	39	RDR2-sensitive
AAGGCTGTGGATGATCCGGTATGT	1	13	RDR2-sensitive
AAGGCTGTGTGTGTGAGATGACGC	0	11	RDR2-sensitive
AAGGCTTCTAACCTTATGGTCGTG	0	10	RDR2-sensitive
AAGGCTTCTAACCTTGTAGTCGTG	0	13	RDR2-sensitive
AAGGCTTCTAACCTTGTGGTCGTG	4	67	RDR2-sensitive
AAGGCTTGTCTGGTTGTTCGTCGC	0	13	RDR2-sensitive
AAGGGACAACAGGGGACAACACAG	0	21	RDR2-sensitive
AAGGGACCGAATTTAGGGGACGTT	0	11	RDR2-sensitive
AAGGGACCGACACGTAGAGAGCGC	0	14	RDR2-sensitive
AAGGGACCGGTATGCACGCCTTGG	0	9	RDR2-sensitive
AAGGGACCTGTGGTAGACGGGCAA	0	14	RDR2-sensitive
AAGGGACGGTGTAGTGGATCACGA	0	30	RDR2-sensitive
AAGGGACTGTAAACGAACCGGTAA	0	43	RDR2-sensitive
AAGGGAGCGCGACGGCAGAACCGT	0	11	RDR2-sensitive
AAGGGAGCTTCGGCATGATGACAT	0	10	RDR2-sensitive
AAGGGAGGACGGGTTCAACAGCTC	0	9	RDR2-sensitive
AAGGGAGGGATTAGCAACTCACAC	0	11	RDR2-sensitive
AAGGGAGGTGCTGTAGCCGGCTAG	0	12	RDR2-sensitive
AAGGGATGTTGACGAGTTGGAGGC	0	10	RDR2-sensitive
AAGGGATTGGGTGAAAGGCTTGGA	0	9	RDR2-sensitive
AAGGGCAGTAGGGGAGTGGTCAGT	2	19	RDR2-sensitive
AAGGGCATGTACAACGGTGTTTAA	0	9	RDR2-sensitive

AAGGGCATGTACAGTGGGTGTCTT	0	12	RDR2-sensitive
AAGGGCATGTACAGTGGTGTTTAA	2	19	RDR2-sensitive
AAGGGCGCATCACCGGTCGGATAG	0	9	RDR2-sensitive
AAGGGCTAGGACGACATGCTTCAC	0	9	RDR2-sensitive
AAGGGCTAGTTTGGATCGGCGCGC	0	11	RDR2-sensitive
AAGGGCTTGTTCGGTTACACCAAT	1	16	RDR2-sensitive
AAGGGCTTGTTCGGTTATTCCCAA	14	60	RDR2-sensitive
AAGGGCTTGTTCGGTTATTCCTAT	2	14	RDR2-sensitive
AAGGGCTTGTTCGGTTCTACCCCA	0	12	RDR2-sensitive
AAGGGGACAGATAGCATGATGCAT	0	12	RDR2-sensitive
AAGGGGACCGACACGTAGGGACGC	0	15	RDR2-sensitive
AAGGGGGTAACCACTAGATCTCGT	3	16	RDR2-sensitive
AAGGGGTAGACTGTCATTCCGTGT	0	9	RDR2-sensitive
AAGGGTAACTCACCACGTCGTCGT	1	13	RDR2-sensitive
AAGGGTAGTCTTTGTTCTGTCGTT	0	18	RDR2-sensitive
AAGGGTCAGCAGAGAACGACAGAG	0	14	RDR2-sensitive
AAGGGTCGGAAGGAGGAGGACGGA	19	0	RDR2-resistant
AAGGGTCTGACGCAGAAGAACTGT	0	10	RDR2-sensitive
AAGGGTTGTTTTCTTTGATTCGGA	0	10	RDR2-sensitive
AAGGGTTTAGGGGTGTACTGTAGC	0	9	RDR2-sensitive
AAGGGTTTTGTGAAGATATCGGCT	80	0	RDR2-resistant
AAGGTAACGACCTGATTGTTAGGC	0	10	RDR2-sensitive
AAGGTAAGCTTTAGGTCGTTCGGC	0	13	RDR2-sensitive
AAGGTACACTAGAGTAGATATCAT	14	0	RDR2-resistant
AAGGTAGACGGCTGCAGTAGTTTT	0	10	RDR2-sensitive
AAGGTAGACTGACTTAGACGGATT	0	11	RDR2-sensitive
AAGGTAGAGCAGTGCTGATGACGT	0	14	RDR2-sensitive
AAGGTAGGATTCTGGAATTGCATA	28	4	RDR2-resistant
AAGGTCAGGGTATGGATAGACGAC	0	10	RDR2-sensitive
AAGGTCATTCTATCTTTAAGTCGG	11	102	RDR2-sensitive
AAGGTCCGTAACTCTGGATCGGAT	0	14	RDR2-sensitive
AAGGTCGAGTCGTAGGTCGTTTGG	1	13	RDR2-sensitive
AAGGTCGGTGTAGTTGACGCGCCA	1	43	RDR2-sensitive
AAGGTCGTGCTGTTGTTATGGTGC	0	10	RDR2-sensitive
AAGGTCTCGAACGGCAAGGGCTTC	3	33	RDR2-sensitive
AAGGTCTCGAACGGCACCGGCTTC	1	19	RDR2-sensitive
AAGGTCTGATGAAAATTTTCGGCT	0	9	RDR2-sensitive
AAGGTCTTCGAGGAACGGCGACAG	0	10	RDR2-sensitive
AAGGTGACGGCCAGAGTAGAAGCA	0	23	RDR2-sensitive
AAGGTGACTTGCATCAACTGGACC	0	13	RDR2-sensitive
AAGGTGCAACTCGTGAGCCGGCAT	0	13	RDR2-sensitive
AAGGTGCTTTCAGGAGTAGGACGG	0	15	RDR2-sensitive
AAGGTGGTTGATGGATAAGTTGGA	23	0	RDR2-resistant
AAGGTGTTGTTGGAGGCAGTCTAA	0	18	RDR2-sensitive
AAGGTGTTTTAGGAGTAGGACGGT	9	107	RDR2-sensitive
AAGGTGTTTTCGGACCTTCGGCAT	0	10	RDR2-sensitive
AAGGTGTTTTTAGGAGTAGGACGG	4	34	RDR2-sensitive
AAGGTTAAGAAGGTATGATAACTA	228	7	RDR2-resistant
AAGGTTAGCCTCGGACGAGTCATG	0	11	RDR2-sensitive

AAGGTTAGGTCCGACGGCTTGTCT	0	22	RDR2-sensitive
AAGGTTAGTTATTCAATTCGGACG	0	9	RDR2-sensitive
AAGGTTAGTTCCTCGATTCGGATG	0	17	RDR2-sensitive
AAGGTTAGTTCTTCAATTCGGACG	0	20	RDR2-sensitive
AAGGTTATTCTTAGATCACTCGTT	32	3	RDR2-resistant
AAGGTTGAAGTAGATTACATCGGA	86	0	RDR2-resistant
AAGGTTGATAGTTGTATGGACGGT	0	17	RDR2-sensitive
AAGGTTGCTTCTCTGCGAAGACTG	0	29	RDR2-sensitive
AAGGTTGGCATGCTAGGTTTCGGT	15	0	RDR2-resistant
AAGGTTGGCCGCGCGGGAGAACGG	0	9	RDR2-sensitive
AAGGTTGGGGATGGTTCAAACACC	20	2	RDR2-resistant
AAGGTTGTACATGCTCTTAGAGGA	0	11	RDR2-sensitive
AAGGTTGTAGCTAGGACTGGGCAT	0	128	RDR2-sensitive
AAGGTTGTTCGTCGACGACGACAT	0	11	RDR2-sensitive
AAGGTTGTTCTGTGACTTGGTCGT	0	9	RDR2-sensitive
AAGGTTGTTTCTAGTAGACGGCGA	0	16	RDR2-sensitive
AAGGTTTACGAGAGCTGACGACAT	18	0	RDR2-resistant
AAGGTTTCGTAGAGCAGGAGGGGA	1	17	RDR2-sensitive
AAGGTTTTCTCCTGTTCCTCGGAC	63	14	RDR2-resistant
AAGGTTTTCTCTTGTTCTTCGGAT	40	0	RDR2-resistant
AAGGTTTTGTAGAGCAGAAGGTGA	0	11	RDR2-sensitive
AAGTAAAGTTTGGCGCTGTTAATC	138	2	RDR2-resistant
AAGTAACGGTGACGGATCCTTTGC	0	17	RDR2-sensitive
AAGTAATAGATGGACTGCTTTTTA	27	0	RDR2-resistant
AAGTAATGATGGCTTAGAAACATT	24	1	RDR2-resistant
AAGTACACGGACGCTCGCGCACGC	0	30	RDR2-sensitive
AAGTACCGGATGACTGAGCGGAGC	0	12	RDR2-sensitive
AAGTACTCGGTATGCAGGGGCGAA	0	9	RDR2-sensitive
AAGTACTCTGATTATATTATGGCA	0	12	RDR2-sensitive
AAGTACTGTGACGGAACCTCCCAA	0	85	RDR2-sensitive
AAGTACTGTTTCTCACGCGTGCAT	0	26	RDR2-sensitive
AAGTAGAACATTAAGAATGGCATT	38	3	RDR2-resistant
AAGTAGAACGGACGGATGGGACGC	0	12	RDR2-sensitive
AAGTAGAACGGTAGAACCACGGTG	0	10	RDR2-sensitive
AAGTAGAACTCCGTATAAACGGTG	0	15	RDR2-sensitive
AAGTAGACTGTAAATGACGGGTAT	0	13	RDR2-sensitive
AAGTAGAGTGGAGAGTAGACGCAT	0	10	RDR2-sensitive
AAGTAGATCTTGAAGATGGATGAC	22	2	RDR2-resistant
AAGTAGATTACATCGGATATGACT	18	0	RDR2-resistant
AAGTAGGGCTGATTCGGTGACCAG	0	37	RDR2-sensitive
AAGTAGTAGTAGAGATATGTACAT	0	23	RDR2-sensitive
AAGTAGTAGTGTGGCACTCAGCAT	0	21	RDR2-sensitive
AAGTAGTGGATTGGGCATGTTCGT	10	54	RDR2-sensitive
AAGTAGTTCTGCAGGATGGAGGCG	17	0	RDR2-resistant
AAGTATAGGGCAAATGACATAATA	16	0	RDR2-resistant
AAGTATATGGTGGCTTTATTACTC	160	0	RDR2-resistant
AAGTATCAGACTGTTCTTAGGCCG	0	21	RDR2-sensitive
AAGTATGAGGATGTGTATTGATTA	24	3	RDR2-resistant
AAGTATGATGGGCTGATTCGGTAC	0	12	RDR2-sensitive

AAGTATGTTATTGCGTCGTACATA	63	3	RDR2-resistant
AAGTATTAGACTATGTAACATATT	32	4	RDR2-resistant
AAGTATTAGGGTAGAGGCCGGACA	0	29	RDR2-sensitive
AAGTATTTGCTATATGTTCGGACG	49	0	RDR2-resistant
AAGTCAATAGTGTAGACCATGGCA	0	13	RDR2-sensitive
AAGTCAATTCATCTTTAAGTCGGT	6	24	RDR2-sensitive
AAGTCAGACTGAGGATGTGGTGTC	0	11	RDR2-sensitive
AAGTCATTCTATCTTTAAGTCGGT	2	15	RDR2-sensitive
AAGTCATTTCATCTTTAAGTCGGT	2	24	RDR2-sensitive
AAGTCCTCTGTCGGCGACGCGAAC	0	10	RDR2-sensitive
AAGTCGAAGACAGCATGTAGGGAC	0	18	RDR2-sensitive
AAGTCGTGGGGAATAATGTGGCAT	0	11	RDR2-sensitive
AAGTCGTTGAACTGCTATCGGCAC	0	22	RDR2-sensitive
AAGTCTACATCGGTACTTGCGGCT	21	0	RDR2-resistant
AAGTCTAGACGCTTCGAACAACTT	1	25	RDR2-sensitive
AAGTCTAGATTGTATCGATGCGTA	0	9	RDR2-sensitive
AAGTCTAGGCTGGAACGACCCGGC	0	11	RDR2-sensitive
AAGTCTCCTTTAGGCTAGTCGGAT	0	19	RDR2-sensitive
AAGTCTGCATGATACTGATGGTTG	1	21	RDR2-sensitive
AAGTCTGTATGATACTGATGGTTG	1	13	RDR2-sensitive
AAGTCTGTGAGCCAGGGTGGACGA	0	9	RDR2-sensitive
AAGTGAACATGTAGGCGCGGCTGA	0	20	RDR2-sensitive
AAGTGAAGACGGAGCCAGCAACGT	2	20	RDR2-sensitive
AAGTGACCTGACCCGACCTGGCAT	0	9	RDR2-sensitive
AAGTGACGGCACGGCACGGCTCGA	0	9	RDR2-sensitive
AAGTGACGGTGACAGAGCCTTTGC	0	18	RDR2-sensitive
AAGTGACGGTGACGGATCCTTTGC	2	58	RDR2-sensitive
AAGTGACGGTGACGGATGCTTTGC	0	11	RDR2-sensitive
AAGTGACTGAATATATGCAGAAAG	23	1	RDR2-resistant
AAGTGACTGTTTTAGGATGGCCTA	0	11	RDR2-sensitive
AAGTGAGCCTCTAAGTTCTGGCAT	25	0	RDR2-resistant
AAGTGAGTTTGTATGAGTGAGGTT	44	0	RDR2-resistant
AAGTGATAACTGTGGCAACATAAG	20	0	RDR2-resistant
AAGTGATCCTGTTGACCGTTGTTC	123	9	RDR2-resistant
AAGTGATGGTAGCGTACTAGACAT	0	10	RDR2-sensitive
AAGTGATTACTATGTGATGTGGAC	45	5	RDR2-resistant
AAGTGCGACGGATAGCGGTGGCGT	0	9	RDR2-sensitive
AAGTGCGCGGACGGCTTCCAGCGG	0	12	RDR2-sensitive
AAGTGCGCTGTAGGCCGAGAGGCT	0	12	RDR2-sensitive
AAGTGCGGCTGAACCGGATGGCAC	0	17	RDR2-sensitive
AAGTGCTAGACGCAGGACACTCAA	0	12	RDR2-sensitive
AAGTGCTCTGTGGCCTGAGGGGTT	0	11	RDR2-sensitive
AAGTGGACTCATCGCTGTCGTCGT	2	14	RDR2-sensitive
AAGTGGAGGACCCTAGACTAAAAT	26	0	RDR2-resistant
AAGTGGGAGTGGAGCTTTTGCGGC	0	14	RDR2-sensitive
AAGTGGGCAGTGAACGAGCGTCAC	0	13	RDR2-sensitive
AAGTGGGGGAGGAGTAGGAGCGCA	35	0	RDR2-resistant
AAGTGTAGACGTGGGCTTCGGCAT	0	17	RDR2-sensitive
AAGTGTAGCTCTACGAACGGCGAT	0	10	RDR2-sensitive

AAGTGTAGCTCTATGGACGGTGAT	0	11	RDR2-sensitive
AAGTGTAGCTCTGCGGACGGTGAT	0	40	RDR2-sensitive
AAGTGTATCTCTGCGGACGACGAT	2	45	RDR2-sensitive
AAGTGTATCTCTGCGGTCGGTGAT	5	19	RDR2-sensitive
AAGTGTTGGAACTGTATTAGGGTT	1	81	RDR2-sensitive
AAGTGTTGGACTAGCACGGCCAGA	0	9	RDR2-sensitive
AAGTGTTTGGTTAGATGGAATAGG	46	0	RDR2-resistant
AAGTGTTTTTTGGAGTAGGACGGT	0	12	RDR2-sensitive
AAGTTAATTGTTTTGGTTGTCTGG	3	22	RDR2-sensitive
AAGTTACGGAGAGCTGACGTCATG	36	0	RDR2-resistant
AAGTTAGCGCTGTGTGACGGTGAA	0	26	RDR2-sensitive
AAGTTATCTATGTTTCGATCGGGC	0	9	RDR2-sensitive
AAGTTATGGACTGACGGTTGGTCG	0	10	RDR2-sensitive
AAGTTCCGGCGGCTCTGGGACGGT	0	22	RDR2-sensitive
AAGTTCGACGGTACCCACGTTCTT	0	9	RDR2-sensitive
AAGTTCGCGACTGACAGACGGACG	0	19	RDR2-sensitive
AAGTTCGCTGACTGCAGAGGACAA	0	11	RDR2-sensitive
AAGTTCGTGGACCTAAGTGGCATT	18	0	RDR2-resistant
AAGTTCTAACAGACTGTCCGTCGG	0	9	RDR2-sensitive
AAGTTGAATTCGGTCTCGGTTGGT	0	10	RDR2-sensitive
AAGTTGACGTAGGCAAGAGGGTGC	119	4	RDR2-resistant
AAGTTGACTGGGAGACGACGGCCT	6	25	RDR2-sensitive
AAGTTGAGATACAGAATAGCGGAA	21	0	RDR2-resistant
AAGTTGCAGTAGCCGGAACAAGAT	0	9	RDR2-sensitive
AAGTTGCTCGGACAGCGTCGGCAA	75	22	RDR2-resistant
AAGTTGCTTAGACTGTACAACGGC	1	12	RDR2-sensitive
AAGTTGGAAGTATGCTTTTATGTC	50	0	RDR2-resistant
AAGTTGGAATGAACAGTTTGACGT	49	7	RDR2-resistant
AAGTTGGACGGTTTTATACAGTAC	0	11	RDR2-sensitive
AAGTTGGATCCAGACGACATGACA	128	0	RDR2-resistant
AAGTTGGCATTGCAGTAGACCGGA	0	10	RDR2-sensitive
AAGTTGGCTGCGAGCATACAGTTC	0	18	RDR2-sensitive
AAGTTGGTAACGTGTTGGAGGCTT	0	12	RDR2-sensitive
AAGTTGGTAAGGTGTTGGAGGCTT	22	0	RDR2-resistant
AAGTTGTATGTTTGGATGTGCATG	69	0	RDR2-resistant
AAGTTGTATTGAAGGGCAAGCCAC	32	1	RDR2-resistant
AAGTTTAGATCGTGCCGACGGTGC	249	27	RDR2-resistant
AAGTTTATCTCTGACGGCTTCCTA	0	9	RDR2-sensitive
AAGTTTCGAACTTGTCTTGTCTGT	27	0	RDR2-resistant
AAGTTTCTACTATCGTTGTGCGTC	0	9	RDR2-sensitive
AAGTTTGCGACTCGGACAGGCCTA	0	20	RDR2-sensitive
AAGTTTGTAGCACGGGTGACAAGG	23	1	RDR2-resistant
AAGTTTGTCTAGTCGTTGGTGTCG	1	29	RDR2-sensitive
AAGTTTGTTGGCTGAGTCGGCTTG	0	16	RDR2-sensitive
AAGTTTTGGAGAGCTGACGTCATG	23	0	RDR2-resistant
AAGTTTTGGAGCGCTGACGTCATG	38	0	RDR2-resistant
AATAAAAAGGTATTGTGTCGTTGT	16	0	RDR2-resistant
AATAAAACTAGGACAGAACGGCTC	0	10	RDR2-sensitive
AATAAAACTGGGACAGAACGGCTC	0	15	RDR2-sensitive

AATAAACCCGGGCTGTGTCGGCAC	1	20	RDR2-sensitive
AATAAACGGCTACTGTTTTCGGCG	0	10	RDR2-sensitive
AATAAATACTCTGATTATTGTGAC	2	14	RDR2-sensitive
AATAACAACTTGTGCTGATTGGAT	21	0	RDR2-resistant
AATAACACAACCGAGCTCGGCGCC	1	24	RDR2-sensitive
AATAACGTTAAAAGTAGTGGTATT	20	0	RDR2-resistant
AATAACTGTAGAATGTACGGTTAG	0	11	RDR2-sensitive
AATAACTGTATAAATGGACGGTTG	0	13	RDR2-sensitive
AATAAGCTGGACTGGGCACAGCAC	0	12	RDR2-sensitive
AATAAGGTCGATGTGGAGCGCAGC	14	0	RDR2-resistant
AATAAGTAGTAGAGACTTGTGCAT	0	10	RDR2-sensitive
AATAAGTTGTTATTTTCGTCGGTC	0	30	RDR2-sensitive
AATAATAAAGCAGAGGACTAGATT	19	0	RDR2-resistant
AATAATCATGTCGGACCGGCCCAC	0	9	RDR2-sensitive
AATAATCGTGTCGGATCGGCCCAC	0	16	RDR2-sensitive
AATAATCGTGTCGGGCCGGCCCAC	0	16	RDR2-sensitive
AATAATCTGTTATTTTCGGCGGCC	0	10	RDR2-sensitive
AATAATGACTTTGGACAGACGTTT	0	10	RDR2-sensitive
AATAATTTGGACTGGTGGGAGGGC	1	27	RDR2-sensitive
AATAATTTGTTGTGTCGATCGGAG	33	0	RDR2-resistant
AATACAAATTGTAAGTAATGCGTT	27	3	RDR2-resistant
AATACCAATTATGTCGGATGTAAG	27	0	RDR2-resistant
AATACCCACGGGTACGGTCGCGGA	1	36	RDR2-sensitive
AATACCCTTATTTACGACGTCGGC	0	12	RDR2-sensitive
AATACGAAAGGTATTTTCGTCGGC	0	12	RDR2-sensitive
AATACGAAATAGACTGGACTGCTA	0	10	RDR2-sensitive
AATACGGGCCTTTTCGGGCTTGGG	0	13	RDR2-sensitive
AATACGTACGTAGGACAGGCCGGT	0	9	RDR2-sensitive
AATACGTCGGGTAGGGTACGGATA	0	11	RDR2-sensitive
AATACTACGCAGATCACGGATGTC	0	9	RDR2-sensitive
AATACTGGGCTGCAGATGGGGCAT	0	9	RDR2-sensitive
AATACTGTAGAAGCCGCAGCCGCA	0	183	RDR2-sensitive
AATACTGTAGATGGCGTTGTTTGA	0	16	RDR2-sensitive
AATACTGTTCATGTTGTAGAGGCT	0	15	RDR2-sensitive
AATACTGTTTATTTTTCTTCGTCA	29	0	RDR2-resistant
AATACTTTTTCTCGGACAGTCGGT	0	10	RDR2-sensitive
AATAGAACAGTAGTAGCTTGTGCA	65	6	RDR2-resistant
AATAGACTCGGAACGGACGGGCTC	0	12	RDR2-sensitive
AATAGACTGGCTTCGAATTGGCTT	0	9	RDR2-sensitive
AATAGATGGCACAGTAGACGTCTC	0	12	RDR2-sensitive
AATAGCTTGTACTGTAGTTGGCAG	0	9	RDR2-sensitive
AATAGGGCACTGTGGCATAGATAA	0	12	RDR2-sensitive
AATAGGTAGGACGGCGCGGCTGCT	37	2	RDR2-resistant
AATAGGTAGTATAGATTCGATCGA	0	9	RDR2-sensitive
AATAGGTGGACTGGCTGAGGACGA	0	9	RDR2-sensitive
AATAGTACTCGGCAAAGAGGCTGT	0	12	RDR2-sensitive
AATAGTCCGGACACGTGGTGGCGC	0	13	RDR2-sensitive
AATAGTCTTTTCTGTAATGACGGT	0	24	RDR2-sensitive
AATAGTGCCGGGCTGGGCATGACA	0	16	RDR2-sensitive

AATAGTGTTGTGTTTGGACCAGTA	0	9	RDR2-sensitive
AATAGTTAGTAGAGATTGGACGGT	0	11	RDR2-sensitive
AATAGTTGTAGAGATGTGCCACTT	0	14	RDR2-sensitive
AATATAAGTGTTGGACTAGCACGG	0	30	RDR2-sensitive
AATATAGAGGACGTTGCTGGAGAC	0	9	RDR2-sensitive
AATATATTCAAATGGGTCGTCGTG	2	31	RDR2-sensitive
AATATATTCAAATGGGTCGTTGTG	0	21	RDR2-sensitive
AATATCCCTGCAGCGGTGGACTGT	1	36	RDR2-sensitive
AATATCCGAGGGCTGGGAGGCGGA	0	10	RDR2-sensitive
AATATCGCATCTGTTTCGTCGGTC	2	34	RDR2-sensitive
AATATCGGGAGGACGGTCTGTCTC	0	11	RDR2-sensitive
AATATCGGTTCCTCTGCGTCGGTT	0	14	RDR2-sensitive
AATATCGTGCTGTGTCCGCGTCAT	0	26	RDR2-sensitive
AATATCGTGTCGGGTTAGACTGGC	0	13	RDR2-sensitive
AATATCTGATCTGTTAGATGGTCT	1590	0	RDR2-resistant
AATATGATATTGATTGTGGCGATG	19	0	RDR2-resistant
AATATGCTGGAGCGGTATTACTCA	17	0	RDR2-resistant
AATATGGCTGCAGATTAGGTGCAA	0	9	RDR2-sensitive
AATATGTCTAGATTGTATCGGTGC	0	12	RDR2-sensitive
AATATGTCTTGTTTTGTGGATGCT	24	0	RDR2-resistant
AATATGTGGATACTCGGCTATAAG	30	0	RDR2-resistant
AATATTAGCAACGGACGGTCGGTC	1	18	RDR2-sensitive
AATATTAGTAAGCAGACTGTCATG	58	4	RDR2-resistant
AATATTAGTACTCGTTGGCTCGGC	0	11	RDR2-sensitive
AATATTATGTACGGCTATTGAACT	34	2	RDR2-resistant
AATATTCTGTCTGATTGGTTCGGT	0	26	RDR2-sensitive
AATATTGTGTGACTCGAGATTAAA	14	0	RDR2-resistant
AATATTTTACTGTCGGACCGGTAC	0	24	RDR2-sensitive
AATCAAACTCGTCTCGGCTCGGCT	0	12	RDR2-sensitive
AATCAACAGTGGTCGTCACGTGCA	0	24	RDR2-sensitive
AATCAACAGTGTCTGACGTGAGGC	18	1	RDR2-resistant
AATCACGGACGGTTATTATATGTA	0	10	RDR2-sensitive
AATCACGTCGATGCTTAGGAGGCT	17	0	RDR2-resistant
AATCACTTGAACATGGTCGACGGC	0	43	RDR2-sensitive
AATCACTTGAACATGGTCGACGGT	1	18	RDR2-sensitive
AATCAGACGAACACAGCACCACAC	0	12	RDR2-sensitive
AATCAGACTCTGCATATGTATTGA	0	21	RDR2-sensitive
AATCAGGAGCTGGAGCTGTGCCAA	0	10	RDR2-sensitive
AATCAGGAGTAGTGATGGTTAATG	0	10	RDR2-sensitive
AATCAGGGCATGGCACTACATACA	0	11	RDR2-sensitive
AATCATAGACACAGACGGTCCCAC	0	10	RDR2-sensitive
AATCATATTTGTGAGGATCTTCGG	0	10	RDR2-sensitive
AATCATCATCATCTGATCTGCGGT	19	1	RDR2-resistant
AATCATGTAGATGGTGTAAATGCT	94	2	RDR2-resistant
AATCATGTTTTTGAGGACCTTCGG	3	22	RDR2-sensitive
AATCATGTTTTTGAGGATCTTCGG	0	24	RDR2-sensitive
AATCATTGTTGGATCAAAGGGCAT	18	0	RDR2-resistant
AATCCACATGGATTGAGAGCGAAC	0	9	RDR2-sensitive
AATCCACATGGATTGAGAGCTAAC	6	416	RDR2-sensitive

AATCCACCGTAGTCAGGACGTCAT	0	9	RDR2-sensitive
AATCCACTGTAGTCAGACCGTCAC	0	13	RDR2-sensitive
AATCCACTGTATGGCTTCGGCGGC	119	8	RDR2-resistant
AATCCAGGGCTGCTGTGAAGACGA	0	12	RDR2-sensitive
AATCCATATGGATTGAGAGCTAAC	1	89	RDR2-sensitive
AATCCATATGGATTGAGAGCTGAC	0	16	RDR2-sensitive
AATCCATATGGATTGGGGTAGAAC	0	30	RDR2-sensitive
AATCCATTCTTGGATTCGTCACTG	31	3	RDR2-resistant
AATCCGACTGCGGCTGCGGCTTCT	7	60	RDR2-sensitive
AATCCGGACGGATAAGAAACGGAT	0	9	RDR2-sensitive
AATCCGGACGGGTAAGAAACGGAT	4	57	RDR2-sensitive
AATCCGGACGTCGGGTGGAAGCGG	0	13	RDR2-sensitive
AATCCGGACTGGGTTGACATGGCC	0	24	RDR2-sensitive
AATCCGGCTAGGAATCGTTCCGAC	1	44	RDR2-sensitive
AATCCGGCTATGAATCGTTCCGAC	0	9	RDR2-sensitive
AATCCGGCTGCGGCTGCGGCTTCT	1	15	RDR2-sensitive
AATCCGGCTGTGGCTTCTACGGTA	0	10	RDR2-sensitive
AATCCGGTTAGGAATCGTTCCGAC	0	18	RDR2-sensitive
AATCCGGTTAGGAATCGTTCGGAT	0	28	RDR2-sensitive
AATCCGGTTAGGAATCGTTCTGAC	1	67	RDR2-sensitive
AATCCGGTTCGAAATCGTTCTGAC	0	10	RDR2-sensitive
AATCCGTGTCTACTCTGTGATTGA	0	11	RDR2-sensitive
AATCCGTGTGATTGGACGTCTGCA	0	9	RDR2-sensitive
AATCGAACACGGACTGTCCGGCCA	0	9	RDR2-sensitive
AATCGAATCGGACAGGTGCGACAT	0	9	RDR2-sensitive
AATCGACGGATTTGGCTTCGGCTC	2	23	RDR2-sensitive
AATCGACTCAGACGGAGACACTAC	8	29	RDR2-sensitive
AATCGACTGTCCGGAATACCGTAG	0	16	RDR2-sensitive
AATCGATCTGGACCGTCCGTGGCA	0	9	RDR2-sensitive
AATCGATGTGTAGTGTGATGGTCT	161	0	RDR2-resistant
AATCGCAGGCGTGGAGAAGGCTAG	1	14	RDR2-sensitive
AATCGCCCCTGGACGGTCGCGGAC	59	3	RDR2-resistant
AATCGCGCGATGGCAGAACAGGGC	0	9	RDR2-sensitive
AATCGGACACGGATAGCGTCGGTA	0	9	RDR2-sensitive
AATCGGACCGTATCAAGGTCGGTG	1	13	RDR2-sensitive
AATCGGACCGTGCCCGTGTCGGCC	0	17	RDR2-sensitive
AATCGGACGGTCGAGATAGTGGCG	0	44	RDR2-sensitive
AATCGGACGGTCGGGATAGTGGCG	0	10	RDR2-sensitive
AATCGGAGTTCGGATACATTCTAG	0	13	RDR2-sensitive
AATCGGATCGTGTCGGGCCGGCAC	0	10	RDR2-sensitive
AATCGGCCAGGTCGACGACGCGAT	0	25	RDR2-sensitive
AATCGGCTCCTGAACGAGGCTGCA	1	15	RDR2-sensitive
AATCGGGCCAAGGCACAACAGGAT	1	12	RDR2-sensitive
AATCGGGCCACCAGTCACGGGCAT	0	14	RDR2-sensitive
AATCGGGCCGGGCTGTCTAGGCAC	0	10	RDR2-sensitive
AATCGGGCCGTTGACACATGGCAT	0	15	RDR2-sensitive
AATCGGGCGGGTAGAACGCGGATA	0	28	RDR2-sensitive
AATCGGGCGGGTAGTACACGGATA	0	23	RDR2-sensitive
AATCGGGTGGGTAGTACGCGGATA	0	17	RDR2-sensitive

AATCGGTACGACTCATGACGGGCT	0	11	RDR2-sensitive
AATCGGTCGTGTATTTAGTCGGTC	0	9	RDR2-sensitive
AATCGGTGCATGTGCGCGGTACTC	0	10	RDR2-sensitive
AATCGGTTATACGATAACGGTCGG	1	13	RDR2-sensitive
AATCGGTTGGAGCGCGTGGAGCGG	0	9	RDR2-sensitive
AATCGTAATCGGATACTGTCGGCG	0	10	RDR2-sensitive
AATCGTGCCAGGCCTGGACCGCAT	0	12	RDR2-sensitive
AATCGTGCTGTACCGACATTATAC	0	10	RDR2-sensitive
AATCGTGCTGTCTAGACGCTTCAG	0	9	RDR2-sensitive
AATCGTGGCTGAGTGAGGGCGGGC	0	18	RDR2-sensitive
AATCGTTCGTCTGACTTGGGTATA	113	34	RDR2-resistant
AATCGTTCGTTGGCAGAAGGCCAT	0	10	RDR2-sensitive
AATCTAAACTCTGGAGCAGAGCTG	1	48	RDR2-sensitive
AATCTAATCTCGGGACACGCGGAC	3	106	RDR2-sensitive
AATCTAATTGTGGGCCTGGGCCGG	0	19	RDR2-sensitive
AATCTAATTGTGGGTCTGGGCCGG	0	12	RDR2-sensitive
AATCTACTTTCTGTTCGTCGGTTC	0	44	RDR2-sensitive
AATCTAGATTTGAGGCTTTACTGT	1	17	RDR2-sensitive
AATCTATACAGTCGTCGGGAGGCT	0	15	RDR2-sensitive
AATCTATGGGACAGTAGTGGTATG	0	12	RDR2-sensitive
AATCTATTTGATGTTGAATACGGT	14	0	RDR2-resistant
AATCTCGGGGCTGGGTGGAGGGCA	0	10	RDR2-sensitive
AATCTCGGTGAACTGTACATTGGC	0	42	RDR2-sensitive
AATCTCTGAACCAGACGTGGTGAT	3	22	RDR2-sensitive
AATCTCTGACTGCGAAGATCGACA	0	22	RDR2-sensitive
AATCTGCACAAGTACTACAGGTAT	17	0	RDR2-resistant
AATCTGCATAGTCGTCGGTAGGCC	0	14	RDR2-sensitive
AATCTGCTAGCGGTATTTCTGAAT	25	2	RDR2-resistant
AATCTGGAACGGAGCGGCTCTGTT	6	46	RDR2-sensitive
AATCTGGACAAATAAGCTAGACTG	0	13	RDR2-sensitive
AATCTGGACCGTAGTACCGGCTGT	0	10	RDR2-sensitive
AATCTGGACGTGGCCCAACGGTAC	0	11	RDR2-sensitive
AATCTGGCTGTAGGGAGAATCCGG	0	57	RDR2-sensitive
AATCTGGGCGCAAGAACGGCTCCT	0	9	RDR2-sensitive
AATCTGTACAGTCGTCGAGAGGCC	0	15	RDR2-sensitive
AATCTGTACAGTCGTCGAGATGCT	0	12	RDR2-sensitive
AATCTGTACAGTCGTCGGGAGGCC	0	28	RDR2-sensitive
AATCTGTACAGTCGTCGGGAGGCT	0	38	RDR2-sensitive
AATCTGTACAGTCGTCGTGAGGCC	0	10	RDR2-sensitive
AATCTGTAGGATAGTATGGTCAGC	0	25	RDR2-sensitive
AATCTGTATCGACTCTTACCGGTG	1	12	RDR2-sensitive
AATCTGTTGGACGGCTACATCCGC	0	19	RDR2-sensitive
AATCTTAAGGATGGTGCGTCGTAG	33	3	RDR2-resistant
AATCTTATCTCGGGACACGCGGAC	0	11	RDR2-sensitive
AATCTTATTGGTTGGGTCTTCGGT	0	11	RDR2-sensitive
AATCTTATTTGAGAGATCGTCCTA	0	15	RDR2-sensitive
AATCTTATTTGAGGGATCGTCTCA	0	11	RDR2-sensitive
AATCTTATTTGATGGATCGTCTCA	0	15	RDR2-sensitive
AATCTTCTCTGTATCGTTCCAATT	25	0	RDR2-resistant

AATCTTGGCCGGCTGGAGATGGAG	2	19	RDR2-sensitive
AATCTTGGCCTTTGAACTTACGGT	0	14	RDR2-sensitive
AATCTTGTATTGTTTGAATTTCGG	0	9	RDR2-sensitive
AATCTTTGTCTTGCTGTCGTCGTT	0	11	RDR2-sensitive
AATCTTTTAGAGAAGACTGTAAAG	0	25	RDR2-sensitive
AATCTTTTAGAGGAGACTGTAAAG	0	10	RDR2-sensitive
AATGAACAGTTTGACGTTAGTATA	65	5	RDR2-resistant
AATGAACCCAAGCTGAGGCTCTTC	0	17	RDR2-sensitive
AATGAACTAGAGGGCATGAGCCAA	3	17	RDR2-sensitive
AATGAACTTCGGACGAAGGCCATG	0	10	RDR2-sensitive
AATGAAGACCGTTGGTGGGCATGT	20	0	RDR2-resistant
AATGAAGGACTGCCACACGCGCGC	0	10	RDR2-sensitive
AATGACAGATGTTGGAATTGTTCT	38	0	RDR2-resistant
AATGACAGGACGGGACGATACCTC	0	9	RDR2-sensitive
AATGACAGGACGGGATGGGACGGG	3	22	RDR2-sensitive
AATGACAGGACGGGATGGGATGGA	0	9	RDR2-sensitive
AATGACATGTAGGGTCCATGGTAT	0	14	RDR2-sensitive
AATGACATGTAGGGTTCATGGTAT	0	10	RDR2-sensitive
AATGACATGTGGGATCAGTGGCAA	0	21	RDR2-sensitive
AATGACATGTGGGATCCATGGTAT	0	9	RDR2-sensitive
AATGACATGTGGGATCTATGGTAT	0	19	RDR2-sensitive
AATGACATGTGGGGTCCATGGTAT	0	23	RDR2-sensitive
AATGACCACTCGCCATCGTCGGGG	12	47	RDR2-sensitive
AATGACCGTCAACTATAGAAGGCT	2	27	RDR2-sensitive
AATGACCTGCAGAATTGATGGTAT	0	9	RDR2-sensitive
AATGACGACGTACATCTCTTATGT	23	0	RDR2-resistant
AATGACGCTTGTCGTGGGACACTC	30	0	RDR2-resistant
AATGACTATAAAATCTGATGGCAT	0	17	RDR2-sensitive
AATGACTGTAACTCTTGTAGTGGC	0	15	RDR2-sensitive
AATGACTGTCAGCTAGTAACGGAC	0	9	RDR2-sensitive
AATGACTTGGACTTGAGGGGTCGG	0	10	RDR2-sensitive
AATGAGAAAGTTCTTCTTCCTAAG	18	0	RDR2-resistant
AATGAGAACTTTGAAGGCCGAAGA	20	1	RDR2-resistant
AATGAGACTCGGAGGCAGACCAAA	32	0	RDR2-resistant
AATGAGATATTGTTTGATTGTTGT	0	10	RDR2-sensitive
AATGAGCCAGTGACTGATTTTACT	19	1	RDR2-resistant
AATGAGCTATTGTTTGATTGTTGT	0	9	RDR2-sensitive
AATGAGTAGAGGACGTTCTGATCC	0	9	RDR2-sensitive
AATGAGTCTGGATTGGAATCGGTT	0	11	RDR2-sensitive
AATGAGTTGATTTGTAAGTTCGGT	20	0	RDR2-resistant
AATGAGTTTCGGACGAAGGTCATA	0	14	RDR2-sensitive
AATGATAAGTAGAATTGATTGGAT	0	9	RDR2-sensitive
AATGATAATGATCGAGCGATGACA	268	17	RDR2-resistant
AATGATAGATTGTAATTGCGGCCT	17	0	RDR2-resistant
AATGATATTGTGTTGGACATTCAA	0	9	RDR2-sensitive
AATGATCAGTTGTTTCCTGTTGGC	36	0	RDR2-resistant
AATGATCGAGCGATGACATTTATT	449	23	RDR2-resistant
AATGATCGGTTCGGCTTGGGTCGG	1	19	RDR2-sensitive
AATGATCTAGGATCTGCTTGCGGT	0	15	RDR2-sensitive

AATGATCTCCGGACGAAGGTCATG	0	9	RDR2-sensitive
AATGATGACTTTGAGTGGACGTTT	0	25	RDR2-sensitive
AATGATGTCTGTATCACTGTCGGT	0	21	RDR2-sensitive
AATGATGTGGCTCTCTAGCTCGGC	0	12	RDR2-sensitive
AATGATTGAGAACGAGATGACTGC	0	17	RDR2-sensitive
AATGATTTACACTCGAATGTCGGT	0	9	RDR2-sensitive
AATGATTTGTTATTATGGTCTGAT	20	0	RDR2-resistant
AATGCAAAATTTGTAGAATGGCAT	0	17	RDR2-sensitive
AATGCAAGGGTAGAAGTTGGGCGC	0	16	RDR2-sensitive
AATGCACGCAGAGGACCAGAATAA	0	15	RDR2-sensitive
AATGCAGCGGAGGAATGTTTTTAT	48	2	RDR2-resistant
AATGCAGGATTCTGGAAACGGATG	37	4	RDR2-resistant
AATGCAGGCTATAGTGAGGGCGAT	0	12	RDR2-sensitive
AATGCATGCGGAGGATCTGGCCAA	0	67	RDR2-sensitive
AATGCATTTTGTGGATCTGAGGCC	0	18	RDR2-sensitive
AATGCCCTGAATTCCTGCAGTTTC	19	0	RDR2-resistant
AATGCCGGACTGGGTTTGGCATGA	1	13	RDR2-sensitive
AATGCGAGGCTGAGGTATATGGCG	31	2	RDR2-resistant
AATGCGGGAGTTTGGCTACTGTAT	0	22	RDR2-sensitive
AATGCTAGCACACTGACGTCACGA	0	20	RDR2-sensitive
AATGCTCCTGCGATTCGGTCGGTC	1	19	RDR2-sensitive
AATGCTCCTGCGATTTGGTCGGTT	0	10	RDR2-sensitive
AATGCTCTAGACTGTTATTGGCAT	1	29	RDR2-sensitive
AATGCTCTCGCGGAAGTCGTCTGT	0	11	RDR2-sensitive
AATGCTCTGATTGGTACAATGAAT	0	15	RDR2-sensitive
AATGCTGGACTTTAGATAAACGGG	0	10	RDR2-sensitive
AATGCTGGTCTGCATACACTCATG	0	19	RDR2-sensitive
AATGCTTCAGCGGACGAAAGGCAT	0	30	RDR2-sensitive
AATGCTTCAGCGGACGAAGGGCAC	0	14	RDR2-sensitive
AATGCTTCAGCGGACGAAGGGCAT	2	43	RDR2-sensitive
AATGCTTCGAAGGACGAAGGGCAT	0	29	RDR2-sensitive
AATGCTTCGTAGGACGAAAGGCAT	0	12	RDR2-sensitive
AATGCTTGGCCGTTCGATCGTTGG	0	21	RDR2-sensitive
AATGGAAAACGAAGATGGAATCAT	19	0	RDR2-resistant
AATGGACAATGTTTACAGGTTCGG	0	11	RDR2-sensitive
AATGGACAGTGCTGGAGGATATGA	0	13	RDR2-sensitive
AATGGACAGTGTTTACAGGTTCGG	3	20	RDR2-sensitive
AATGGACATAGATATTTTTCACGG	0	66	RDR2-sensitive
AATGGACCACCTTAGATGATCATA	808	211	RDR2-resistant
AATGGACCAGATAGTTTGCTTGCA	0	61	RDR2-sensitive
AATGGACCCCAACCTCGGCGTCAT	0	10	RDR2-sensitive
AATGGACCCTATGCTCGGCGCCAT	0	11	RDR2-sensitive
AATGGACGACAGAATCGATCGGTT	0	17	RDR2-sensitive
AATGGACGAGCCGAGCCGAGCCGA	0	38	RDR2-sensitive
AATGGACGGCTGAGATCGCAGAAC	0	23	RDR2-sensitive
AATGGACGGCTGAGATCGCAGAAT	1	16	RDR2-sensitive
AATGGACTGATATGGCTTCGGCTC	0	9	RDR2-sensitive
AATGGACTGGACAGGCACGCCGCC	0	15	RDR2-sensitive
AATGGACTGTGTCTGACGGCCCGG	0	15	RDR2-sensitive

AATGGACTGTTACACTACAGATGG	0	19	RDR2-sensitive
AATGGACTTGGACTGGATTGGCCT	0	85	RDR2-sensitive
AATGGAGTTGGTGGAGGGCGAGCT	18	1	RDR2-resistant
AATGGATCAGATGGTTTGCTTGCA	4	25	RDR2-sensitive
AATGGATCCTATGTCTATATCGTC	31	0	RDR2-resistant
AATGGATCTGTTCTGCTTAGCCGG	0	14	RDR2-sensitive
AATGGATTGAGAGCCTATTCGGTT	0	17	RDR2-sensitive
AATGGATTTGCAGATTTGATGGTT	0	10	RDR2-sensitive
AATGGCAGAACACTGGCAGAACAC	0	13	RDR2-sensitive
AATGGCAGAGGTAGATTTGGGCAG	0	15	RDR2-sensitive
AATGGCCTCGACTGCTGCTTCAAT	22	0	RDR2-resistant
AATGGCGTGTCTGAGCGACGTAAT	30	0	RDR2-resistant
AATGGCTATAGATATTTTTCACGG	0	26	RDR2-sensitive
AATGGCTCAGGACCCTAGCGACGG	0	10	RDR2-sensitive
AATGGCTCTGGCTCTTTGATGGCT	0	11	RDR2-sensitive
AATGGCTGACATGACATCGACGTG	0	27	RDR2-sensitive
AATGGCTGTGATTATGGCTTCGGA	28	0	RDR2-resistant
AATGGCTTTGTAAAAATATCGGCT	15	0	RDR2-resistant
AATGGGAATGACAGAACACGGAAT	0	9	RDR2-sensitive
AATGGGAATGGCAGAACACGGAAT	0	61	RDR2-sensitive
AATGGGCATAGATATTTTTCACGG	2	86	RDR2-sensitive
AATGGGCGACCGAACTCTCCGGCT	19	1	RDR2-resistant
AATGGGCGCTGTAGGGGGCACCGC	2	23	RDR2-sensitive
AATGGGCTCCGACGCGATACACGG	0	15	RDR2-sensitive
AATGGGCTGGGCTGGAATGGGCTT	0	14	RDR2-sensitive
AATGGGCTGTCACCTGACCGGTGT	0	10	RDR2-sensitive
AATGGGCTGTCTGATAGGCTTCGG	3	113	RDR2-sensitive
AATGGGTTGTATATGGTTGACAGG	15	0	RDR2-resistant
AATGGTAATGGGTTGTATATGGTT	91	0	RDR2-resistant
AATGGTACTGCAGAGCATTTTCGG	0	10	RDR2-sensitive
AATGGTCGCGATTGTATTGCACGG	0	22	RDR2-sensitive
AATGGTCGGTCGCTATAGAGGTAC	0	19	RDR2-sensitive
AATGGTCGTCAGCTGCAGACGGTG	1	13	RDR2-sensitive
AATGGTCTTCGGACGAAGGTCATG	16	0	RDR2-resistant
AATGGTCTTTGGACGAAGGTCATG	24	2	RDR2-resistant
AATGGTTATGTGACTGATTATGGT	49	6	RDR2-resistant
AATGGTTCCGGATCTGAGGAGAAT	0	17	RDR2-sensitive
AATGGTTGAGGATCTAGACGACTA	333	0	RDR2-resistant
AATGGTTTACAATCGATTACGGAC	0	16	RDR2-sensitive
AATGGTTTACACTTGATTACGGAC	0	10	RDR2-sensitive
AATGGTTTGGCAAAACGGCTCCTC	0	9	RDR2-sensitive
AATGGTTTTGTATTGTTGTTGAGA	15	0	RDR2-resistant
AATGTAAACTTTGTAGCAGAGCTG	0	32	RDR2-sensitive
AATGTACCTGGTAGACACGTCGAC	0	16	RDR2-sensitive
AATGTAGAAATAGTAGAATGCGGT	0	20	RDR2-sensitive
AATGTAGACCACGAGTGGAACCGT	1	15	RDR2-sensitive
AATGTAGGACATTGTTACGGTGGC	58	7	RDR2-resistant
AATGTAGTGACTCTCCACGGCCAT	0	9	RDR2-sensitive
AATGTATGGATATGGGAGGGCAGA	29	5	RDR2-resistant

AATGTCAGATGACGGCTGTTAGCT	0	11	RDR2-sensitive
AATGTCGGTGGCGTCTACATGCTC	0	20	RDR2-sensitive
AATGTCTCGAAGGACGAAGGGCAT	0	13	RDR2-sensitive
AATGTCTCGCGTCGGTCTGTCGGC	0	9	RDR2-sensitive
AATGTCTGATCGTTTGGAGGGAGC	33	1	RDR2-resistant
AATGTCTGGGAAGCTCTCACGGTG	0	10	RDR2-sensitive
AATGTGAACTCATCTATTGGCATT	55	3	RDR2-resistant
AATGTGAACTCATTGTATTGCATA	0	11	RDR2-sensitive
AATGTGACAGAGGATAGAAGCATT	17	0	RDR2-resistant
AATGTGGAACTCTAGCGAGGGGCG	0	25	RDR2-sensitive
AATGTGGATCTCTTAGGTTTGTAC	25	0	RDR2-resistant
AATGTGGTCTAGTTCTGGCACGGT	0	16	RDR2-sensitive
AATGTGTAGTGTGATGGTCTGGGT	22	0	RDR2-resistant
AATGTGTCCAGGATGAAGATAGGT	19	0	RDR2-resistant
AATGTGTGAACAGGTATCTTCGGA	4	29	RDR2-sensitive
AATGTGTTTGACATGATCTGGGAA	164	5	RDR2-resistant
AATGTGTTTGGTTGTAGGTTGCAT	0	13	RDR2-sensitive
AATGTTATGGACTCGCATGTTGCT	62	0	RDR2-resistant
AATGTTATGGACTGTGTTGCAGAA	0	15	RDR2-sensitive
AATGTTCTGAACGAATTGTGGCAA	48	1	RDR2-resistant
AATGTTGCACAGGATTGTTGGCTA	44	6	RDR2-resistant
AATGTTGCATATGATTGTTGGCTA	23	0	RDR2-resistant
AATGTTGGACAGCAGATAGATTGG	27	3	RDR2-resistant
AATGTTGTCGCAGTAGACCACGGT	0	14	RDR2-sensitive
AATGTTGTCTGTAGACGGCGGCTT	0	13	RDR2-sensitive
AATGTTGTTGGACGCATTGCGCAT	0	14	RDR2-sensitive
AATGTTTCAGCGGACGAAGGGCAT	0	11	RDR2-sensitive
AATGTTTCGAAGGACGAAGGGCAT	0	18	RDR2-sensitive
AATGTTTGACTGTCGCGTGCCTTT	84	0	RDR2-resistant
AATGTTTGGACGGCTATAGCTACA	0	10	RDR2-sensitive
AATGTTTGGCACTGAATCGACGTC	0	37	RDR2-sensitive
AATTAAATATGTTGGACACTTAAT	0	9	RDR2-sensitive
AATTAATTTGCACCGTTGGTCTGA	105	0	RDR2-resistant
AATTACAGCTGTAGCTAGGATGGC	0	9	RDR2-sensitive
AATTACTCGCGGGCCCACGGCTAC	0	9	RDR2-sensitive
AATTACTCGTGGACCTACAGCATA	0	12	RDR2-sensitive
AATTACTTGAACATGGTCGACGGC	0	27	RDR2-sensitive
AATTAGCCTCTTCGGACGGTCGGA	0	18	RDR2-sensitive
AATTAGCGGCTCTGGCGACGTCTG	0	11	RDR2-sensitive
AATTAGGCCTTTCGGACGGTCGGA	0	16	RDR2-sensitive
AATTAGGTTGGGCCGTGCCGGATA	0	9	RDR2-sensitive
AATTAGTAAAATTGGTGCGTCGTT	14	0	RDR2-resistant
AATTAGTACTATTTTCGGCGGCAT	0	9	RDR2-sensitive
AATTAGTCTCTGTAGAGTGGCAGA	135	0	RDR2-resistant
AATTAGTGACGCGCATGAATGGAT	18	1	RDR2-resistant
AATTAGTTTGGCGTGTACATGTAA	0	12	RDR2-sensitive
AATTATAGACTGTAGTGTGGTGTC	0	21	RDR2-sensitive
AATTATTGTAGATCTTGAGAGGTT	20	2	RDR2-resistant
AATTATTTGGTTGCGTTGGCTCTT	20	0	RDR2-resistant

AATTATTTGTGTTGGACAATTCAA	0	12	RDR2-sensitive
AATTCAAACGGACTAGACTGGCTC	0	20	RDR2-sensitive
AATTCAAACGGGCTGGATCGGCCT	0	10	RDR2-sensitive
AATTCAACAATGTGATCTATGGCA	14	0	RDR2-resistant
AATTCACCGGACTGTACGGTGGTG	6	28	RDR2-sensitive
AATTCACCGGACTGTCTGGTGGTG	1	13	RDR2-sensitive
AATTCACTAGTCGGTTGACGGTCC	0	15	RDR2-sensitive
AATTCAGCCTGTAGTGTCGGCCCA	0	10	RDR2-sensitive
AATTCATCTGAGATTGATTGTAGC	56	6	RDR2-resistant
AATTCATGTCGGGCCGGCACGATC	0	15	RDR2-sensitive
AATTCGAACGACTAACCTTAGGCA	1	68	RDR2-sensitive
AATTCGACTGACGTGGATGGGCTA	0	10	RDR2-sensitive
AATTCGATCTATTCGGGTTCGGTT	0	11	RDR2-sensitive
AATTCGATTTTGGATAGACAGGCC	25	0	RDR2-resistant
AATTCGGACGACGGTGCGGCGCGC	0	12	RDR2-sensitive
AATTCGGACGACTAATCTTAGGCA	0	10	RDR2-sensitive
AATTCGGGCTGGCACGACACGGTT	0	11	RDR2-sensitive
AATTCGGTCTATTCAGGTTCGGTT	0	10	RDR2-sensitive
AATTCGGTCTATTCGGGTTCGGTT	5	378	RDR2-sensitive
AATTCGGTCTATTCGTGTTCGGTT	0	14	RDR2-sensitive
AATTCGGTCTATTTGGGTTCGGTT	0	17	RDR2-sensitive
AATTCGGTCTGTTCGGGTTCGGTT	0	39	RDR2-sensitive
AATTCGGTTCGGTCTATTCGGGTT	4	23	RDR2-sensitive
AATTCGGTTCGGTCTATTCGGTTT	0	28	RDR2-sensitive
AATTCGTTGGTTGTTGCATATTGG	0	14	RDR2-sensitive
AATTCTACACTTATGCAAGGACTG	0	12	RDR2-sensitive
AATTCTAGTGAATTGGACCGTCTG	0	14	RDR2-sensitive
AATTCTGTGCATGAGTGGACGGTC	0	12	RDR2-sensitive
AATTCTTAAGACGGTAGTGTAGAC	0	22	RDR2-sensitive
AATTCTTTCGGTCCGGCATGACAT	0	9	RDR2-sensitive
AATTGAACGGACCGAGTTGGCCAG	0	10	RDR2-sensitive
AATTGAACGGGCGGAGCTGGCTAG	0	10	RDR2-sensitive
AATTGAAGGCGGGCCCACAGGAAC	0	32	RDR2-sensitive
AATTGAAGGGACGAGAGAGGACGC	0	13	RDR2-sensitive
AATTGACTGTAGTTGGTACCTTGC	0	12	RDR2-sensitive
AATTGATGATCGTTCTTTTTGGCC	87	10	RDR2-resistant
AATTGCGCGGCGGACTAGCGGCTA	0	34	RDR2-sensitive
AATTGGAACGATACAGAGAAGATT	46	2	RDR2-resistant
AATTGGAACTCCAGCATAGCTGCA	0	16	RDR2-sensitive
AATTGGAAGGATGAGAGAGGACGC	0	9	RDR2-sensitive
AATTGGAAGTGGGCCCACGTGAAC	0	15	RDR2-sensitive
AATTGGACTTTCGGTATTATAACA	0	29	RDR2-sensitive
AATTGGAGCTCAAGCGAACGGACT	0	11	RDR2-sensitive
AATTGGAGGGACAAGAGAGGACGC	0	31	RDR2-sensitive
AATTGGAGGGACAAGAGAGGACGT	0	9	RDR2-sensitive
AATTGGAGGGACGAGAGAGGACGA	0	14	RDR2-sensitive
AATTGGAGGGACGAGAGAGGACGC	1	48	RDR2-sensitive
AATTGGAGGGACGAGAGAGGACGT	1	35	RDR2-sensitive
AATTGGAGTGACGAGAGAGGACGC	0	9	RDR2-sensitive

AATTGGATCGTGCCTGTACCCGGC	0	9	RDR2-sensitive
AATTGGATGGACGAGAGAGGACGT	0	12	RDR2-sensitive
AATTGGTTTCGGTACAAAAGGTTG	16	0	RDR2-resistant
AATTGTCAGCGACCTTCCCGGCCT	0	9	RDR2-sensitive
AATTGTCGTCAACTGTATATGGCT	1	63	RDR2-sensitive
AATTGTCTTTTTCGCTCGGTCACG	0	10	RDR2-sensitive
AATTGTGGACGGATGAACTGGCGT	0	14	RDR2-sensitive
AATTGTGGTATTGTAAACTGCGGT	3	45	RDR2-sensitive
AATTGTGGTTGTAGTAAAGGCAAT	1	21	RDR2-sensitive
AATTGTGTGGAACGTACAACGGCT	0	9	RDR2-sensitive
AATTGTGTGGAACTTACAACGGCA	0	10	RDR2-sensitive
AATTGTTAGTCGGTATACATTGCC	0	15	RDR2-sensitive
AATTGTTCAGATCGTTGGATAACT	0	12	RDR2-sensitive
AATTGTTCGGACTGCGGCAGCTAC	0	25	RDR2-sensitive
AATTGTTGATGTGGAACAATCGGG	19	0	RDR2-resistant
AATTGTTGTGCTACGGTCTAATTC	42	1	RDR2-resistant
AATTGTTTATTTAATCGGGCATTT	17	1	RDR2-resistant
AATTGTTTGGTATCTTAGGCACAA	18	0	RDR2-resistant
AATTTAAATTTCTGACGCGTCTGC	14	0	RDR2-resistant
AATTTAGAGGACCGTTTAGAGGAC	0	9	RDR2-sensitive
AATTTAGAGGACGCTGCTGGAGAC	0	16	RDR2-sensitive
AATTTAGAGGACGCTGCTGGATTC	1	15	RDR2-sensitive
AATTTAGAGGACGTTGCTGGAGAC	1	17	RDR2-sensitive
AATTTAGGGGACGCTGCTGGAGAC	5	21	RDR2-sensitive
AATTTAGGGGACGTTGCTGGAGAC	1	23	RDR2-sensitive
AATTTAGTGGACGAGGACGTGCGC	0	15	RDR2-sensitive
AATTTATGTGTTTGTTGGATGGTT	2	15	RDR2-sensitive
AATTTCCTGGTGAGATCTGTCGGT	0	12	RDR2-sensitive
AATTTCGCTGGTACTGTGGTGGGC	0	17	RDR2-sensitive
AATTTCGGTTCGGTCTATTCGGTC	13	111	RDR2-sensitive
AATTTCGGTTCGGTCTATTCGGTT	2	22	RDR2-sensitive
AATTTCGGTTCGGTTCGGTACGGT	1	26	RDR2-sensitive
AATTTCTAGTGGCATTGAACGGAT	16	0	RDR2-resistant
AATTTGACTGCGGTTTCTACGGTA	0	11	RDR2-sensitive
AATTTGAGCATCGTTGGACAATGG	34	4	RDR2-resistant
AATTTGCACCGTTGGTCTGAAAGA	26	0	RDR2-resistant
AATTTGCCTCTTCGGACGATCGGT	0	9	RDR2-sensitive
AATTTGGACGACTAACCTTAGGCA	3	18	RDR2-sensitive
AATTTGGACTGATGGGAGGGCTAT	0	15	RDR2-sensitive
AATTTGGACTGGTGGGAGGGCTAT	9	150	RDR2-sensitive
AATTTGGGATTATTCGGATTTTCT	16	0	RDR2-resistant
AATTTGGTATCAGAGGACGGTCGA	0	10	RDR2-sensitive
AATTTGGTCAGTCGGTGTGGCGAT	4	27	RDR2-sensitive
AATTTGTCAGCAGCTTTATGGATT	29	1	RDR2-resistant
AATTTGTGGTAAGATTTTTCGGAT	51	0	RDR2-resistant
AATTTGTTGATGATGAAGCGTGCT	17	0	RDR2-resistant
AATTTTAGAGGACGCCGGACGCAC	0	9	RDR2-sensitive
AATTTTATTTGCACGTTTGGACCC	0	12	RDR2-sensitive
AATTTTCAGATTGTGCAGTGTGTT	14	0	RDR2-resistant

AATTTTCGGCGGCCAGGGATGAGC	0	15	RDR2-sensitive
AATTTTCGGTTCGGTAATTCGGTT	1	20	RDR2-sensitive
AATTTTGAGGGACAACAGGGGACA	0	10	RDR2-sensitive
AATTTTGGACCCATTTCTCGGCGC	1	17	RDR2-sensitive
AATTTTGTAGTGGCTTGGATTTAA	20	0	RDR2-resistant
AATTTTGTTAAGAAGATACTGTAT	38	4	RDR2-resistant
AATTTTGTTAGTTTTGAATGCATG	14	0	RDR2-resistant
AATTTTTGTTCGGTCGGAATCGGT	2	54	RDR2-sensitive
ACAAAACTAGAACAGAACGGCTCC	0	10	RDR2-sensitive
ACAAAACTAGGACTGAACGGCTAC	0	9	RDR2-sensitive
ACAAAACTGGGACAGAACGGCTCC	0	49	RDR2-sensitive
ACAAAACTGGGACAGAACGGCTTC	0	9	RDR2-sensitive
ACAAAAGGCTGGCTTGCGTTCGCT	0	15	RDR2-sensitive
ACAAACTCGCAGGCTTAGACGACC	0	13	RDR2-sensitive
ACAAAGCTGACTGAGAGACGCCAC	0	14	RDR2-sensitive
ACAAAGCTGACTGGGAGACGCCAC	0	25	RDR2-sensitive
ACAAATATTTGTGATTGACGTGAC	0	10	RDR2-sensitive
ACAACACAGGACTGTAGGAGCATA	0	10	RDR2-sensitive
ACAACACCGGCTAGGGGACGACGT	0	10	RDR2-sensitive
ACAACACTCGGCAAAGCCTCGGAT	0	12	RDR2-sensitive
ACAACACTGTAGATCATTTTCGGC	0	11	RDR2-sensitive
ACAACACTGTAGCCGGCTAACCGT	0	26	RDR2-sensitive
ACAACAGATCTGGCTGCAAGTTTC	20	0	RDR2-resistant
ACAACAGTCTGAACAGTCGGCTTT	0	15	RDR2-sensitive
ACAACCGGCAGAGCAGAACGACAT	1	13	RDR2-sensitive
ACAACCTAATTTGAGGGATCGTCC	2	30	RDR2-sensitive
ACAACCTAATTTGAGGGATCGTCT	0	18	RDR2-sensitive
ACAACCTCCGTCGTCAGGCCACAT	14	0	RDR2-resistant
ACAACCTTATTTGAGGGATCGTCA	2	15	RDR2-sensitive
ACAACCTTATTTGAGGGATCGTCC	0	14	RDR2-sensitive
ACAACCTTATTTGAGGGATCGTCT	11	44	RDR2-sensitive
ACAACGACCGGACGGTCCGCGCAT	0	14	RDR2-sensitive
ACAACGACTGCGGATCGGCTAAAT	18	0	RDR2-resistant
ACAACGTACGGGCATACAGCTAGT	0	18	RDR2-sensitive
ACAACGTCAGCAACGGTCGTCGTC	0	10	RDR2-sensitive
ACAACGTGGAAGTAGGCAAGTGTT	0	24	RDR2-sensitive
ACAACTATTTATTGGACTGGGCAA	17	1	RDR2-resistant
ACAACTCCGTTGGACTACGGGTGG	44	5	RDR2-resistant
ACAACTCGGACTCCGCAAGACGGA	0	9	RDR2-sensitive
ACAACTCTGGAGTTAGGGTGTTTG	0	10	RDR2-sensitive
ACAACTCTGGCCCAGACACGTCAT	4	42	RDR2-sensitive
ACAACTCTGGCCCAGGCACGTCAT	4	73	RDR2-sensitive
ACAACTCTGGCCCATGCACGTCAT	1	18	RDR2-sensitive
ACAACTCTGGCCTAGGCACGTCAT	2	21	RDR2-sensitive
ACAACTCTGGTCCATGCACGTCAT	0	10	RDR2-sensitive
ACAACTGTAGGTGGGCCTAATGAC	2	22	RDR2-sensitive
ACAACTGTAGGTGGGCCTAATTAC	0	9	RDR2-sensitive
ACAACTGTCTTTAATGTCGTCGGT	0	9	RDR2-sensitive
ACAACTTATCAGACCTGACGAGCT	0	14	RDR2-sensitive

ACAACTTGTGCTGATTGGATGACT	289	9	RDR2-resistant
ACAACTTTCTGTCGATGGACGCAT	0	22	RDR2-sensitive
ACAAGACTAGTCGGCTAATTAATT	22	0	RDR2-resistant
ACAAGACTGCATAGGGTACACCAC	0	19	RDR2-sensitive
ACAAGACTGCATGCGCTCGCGGCT	0	15	RDR2-sensitive
ACAAGACTGCATGCGCTCGCGGGA	0	9	RDR2-sensitive
ACAAGACTGCATGCGCTCGCGGGT	0	9	RDR2-sensitive
ACAAGATCGTATCTGGCCCGGCAC	0	10	RDR2-sensitive
ACAAGATCTAAGGGCCCTTCGGTT	0	10	RDR2-sensitive
ACAAGCAGTCGGTAGCAACTACTA	0	28	RDR2-sensitive
ACAAGCCCGCGTGCTTAGTCGGTC	0	13	RDR2-sensitive
ACAAGCCCTGACGGAACTCGTCGT	0	9	RDR2-sensitive
ACAAGCGGTTTCTCCAAAGATTAG	17	1	RDR2-resistant
ACAAGCTATAAGAAGGGGTCGGAC	26	1	RDR2-resistant
ACAAGCTGGGCTGCACGCGCGGGT	0	10	RDR2-sensitive
ACAAGCTTATCTAGTCGTCGGTGC	0	9	RDR2-sensitive
ACAAGGACAACTGTAGGTGGGCCT	8	57	RDR2-sensitive
ACAAGGACGAGCAGGGTGGGGCAA	0	9	RDR2-sensitive
ACAAGGACGGGGGAACCCGACGTC	19	1	RDR2-resistant
ACAAGGACGTGCTTTTCTAATATC	15	0	RDR2-resistant
ACAAGGACTACACCGCGGCAGCAG	0	10	RDR2-sensitive
ACAAGGACTCGGCAGATTCGGTAT	0	16	RDR2-sensitive
ACAAGGATCTGGATCAGAGCGCAT	1	15	RDR2-sensitive
ACAAGGCCTCAGCCTGTGTCGGAC	0	12	RDR2-sensitive
ACAAGGCCTGATCTCGTTCGTCGT	0	9	RDR2-sensitive
ACAAGGCTAGGTAGGACTAGGCAT	0	17	RDR2-sensitive
ACAAGGCTAGTAGCAAGGTCTCGG	0	13	RDR2-sensitive
ACAAGGGACTGTAAACGAACCGGT	1	12	RDR2-sensitive
ACAAGTAATGTGCGGGCTGGGCAC	0	21	RDR2-sensitive
ACAAGTACCTCGGGAGCATCTACC	20	0	RDR2-resistant
ACAAGTATTGGGTGTGCTGATAAT	43	0	RDR2-resistant
ACAAGTCCGCGGGCTTAGACGGTC	1	15	RDR2-sensitive
ACAAGTCGGCGCACCTATCGGCGT	0	29	RDR2-sensitive
ACAAGTCGGCGTTGTGGGTTGGCC	18	0	RDR2-resistant
ACAAGTGGAGGACCCTAGACTAAA	27	2	RDR2-resistant
ACAAGTTCGTCTAGACATCGGTGT	0	16	RDR2-sensitive
ACAAGTTGAGATGTCTATGGCAAG	18	0	RDR2-resistant
ACAATACAAGATTTTTCGGGATTG	0	9	RDR2-sensitive
ACAATATTATGTACGGCTATTGAA	41	1	RDR2-resistant
ACAATCAGGTGTAGATCGTCTGGC	4	21	RDR2-sensitive
ACAATCCGGTGACTGTCCGGCCGA	0	16	RDR2-sensitive
ACAATCGACTTAGAATTGAGATGG	0	9	RDR2-sensitive
ACAATCGTGATATCGGAGAAAGGT	21	0	RDR2-resistant
ACAATCTACTTTCTGTTCGTCGGT	0	22	RDR2-sensitive
ACAATCTGGCTTATGAAACGGTGA	65	2	RDR2-resistant
ACAATCTTATTTGATGGATCGTCT	0	14	RDR2-sensitive
ACAATGACACGTAGACTGGTACGG	0	10	RDR2-sensitive
ACAATGGACTGTTCGGTGCACCAC	0	11	RDR2-sensitive
ACAATTACTCGTGGACCCACAGCA	0	12	RDR2-sensitive

ACAATTAGCGGCTCTGGCGACGTC	1	12	RDR2-sensitive
ACAATTGGTCATCGCGCTTGGTTG	29	3	RDR2-resistant
ACAATTTCGGTTCGGTCTATTCGG	1	15	RDR2-sensitive
ACAATTTCGTTGGATTAGATGTGG	18	0	RDR2-resistant
ACAATTTTTTCTGGTTGTCGGAGC	0	10	RDR2-sensitive
ACACAAGACGGATCTAGACGACAT	0	13	RDR2-sensitive
ACACAAGTTTGTCTAGTCGTTGGT	0	32	RDR2-sensitive
ACACAAGTTTGTTTAGTCGTTGGT	0	9	RDR2-sensitive
ACACAATCTGATCTGACGGAGTCT	15	0	RDR2-resistant
ACACAATCTGGCTTATGAAACGGT	66	6	RDR2-resistant
ACACAGAACTTGGTGTCGGCGGAA	1	19	RDR2-sensitive
ACACAGACTGGACCTGAGCGCGGC	0	12	RDR2-sensitive
ACACAGATGGATTGGACTTGGTAT	0	13	RDR2-sensitive
ACACAGATTGTTTGGTAAGAGCAT	0	11	RDR2-sensitive
ACACAGCTCGACGTCATAGACTAT	0	9	RDR2-sensitive
ACACAGCTCGGCGCCATAGGCTAT	2	16	RDR2-sensitive
ACACAGCTCGGCGTCATAGGCTAT	0	9	RDR2-sensitive
ACACAGGACTCAGGAAGAGAGACG	0	9	RDR2-sensitive
ACACAGGACTGTCCGGTGCGCCAA	0	16	RDR2-sensitive
ACACAGGACTGTGCAGGCGCAGCC	0	16	RDR2-sensitive
ACACAGGAGCGACATGAACACCGG	0	20	RDR2-sensitive
ACACAGGTCGAGACTGGACGGTTT	0	13	RDR2-sensitive
ACACAGTAGGATTGTCGGCACTAC	0	10	RDR2-sensitive
ACACATAACTAATTGTGGGTCGTG	0	9	RDR2-sensitive
ACACATAATCGGTTGAGTCGTTGT	1	16	RDR2-sensitive
ACACATGACTGCACCCAAGACCAC	0	9	RDR2-sensitive
ACACATGCAGAGGACACGGGAAAC	0	9	RDR2-sensitive
ACACATGCGTTCGGTCTAGATGGA	0	10	RDR2-sensitive
ACACATGGATTGGATGAGATTGGA	0	31	RDR2-sensitive
ACACATGGATTGGATGGGATTGGA	0	11	RDR2-sensitive
ACACATGTTGGATAAAATGGCGTG	62	14	RDR2-resistant
ACACCACTGTTGGGTTGTCGGCCC	0	14	RDR2-sensitive
ACACCAGGTCGCGGACCGTCCGGC	0	15	RDR2-sensitive
ACACCAGTCGGAGACGTCACGTAG	0	9	RDR2-sensitive
ACACCATAGACTATGACGACGAGC	0	12	RDR2-sensitive
ACACCCCCTGTAGACCCGGCTGGC	0	11	RDR2-sensitive
ACACCGATCCTGCTCTGGCGGCGC	0	14	RDR2-sensitive
ACACCGCGCACTGTAGACGACGGC	0	23	RDR2-sensitive
ACACCGCGGCTGGCATAGTGCACA	0	20	RDR2-sensitive
ACACCGCTGTAGAGGGAGGGGTAG	0	13	RDR2-sensitive
ACACCGGACTGTCCAGTGGTGCAC	3	34	RDR2-sensitive
ACACCGGACTGTCCGATGGTGCAC	0	12	RDR2-sensitive
ACACCGGAGTAGTTTTTGATGGCG	1	48	RDR2-sensitive
ACACCGTAGTAATTTTTGATGGCG	0	12	RDR2-sensitive
ACACCGTTGGATACTGGACGGATG	0	14	RDR2-sensitive
ACACCGTTGGATATTGGACGGATG	0	64	RDR2-sensitive
ACACCGTTGGATATTGGATGGATG	0	43	RDR2-sensitive
ACACCGTTGGATGTGGAGGGTGCG	0	19	RDR2-sensitive
ACACCGTTGGATGTGGAGGGTGTG	0	18	RDR2-sensitive

ACACCGTTGGCGCACTGTTGGCAC	0	10	RDR2-sensitive
ACACCTAGGTCGTGGACTGTCCGG	0	16	RDR2-sensitive
ACACCTCAAGCCAGTCTGTCGGAT	0	9	RDR2-sensitive
ACACCTCGTACATGTAGCTAGCGG	0	14	RDR2-sensitive
ACACCTCTGAGTCTGCCGGCACAA	0	9	RDR2-sensitive
ACACCTGGTGATTATGTTCGCGCC	0	11	RDR2-sensitive
ACACCTGTGTTTGCGTTGTCGGGC	0	11	RDR2-sensitive
ACACCTTCGGTCTAGCCACGGCTC	0	11	RDR2-sensitive
ACACCTTTGGACGTTTTCCGGCGC	0	9	RDR2-sensitive
ACACCTTTTCGTCGAATGTCGGTT	0	25	RDR2-sensitive
ACACGAAGGGCGACACGAAGATGT	0	10	RDR2-sensitive
ACACGAATAAGCTGGACTGGGCAC	0	15	RDR2-sensitive
ACACGACCTAGTAGCAGGAACGAC	0	47	RDR2-sensitive
ACACGACGGACACCAAGCGGGGAG	0	11	RDR2-sensitive
ACACGACGGATGCGGCTTCGACGA	0	13	RDR2-sensitive
ACACGACGGTTGATCTTTAGCGAC	4	32	RDR2-sensitive
ACACGACTCTCGGCAACGGATATC	548	36	RDR2-resistant
ACACGACTGAGGAGTGGACGTGAC	0	9	RDR2-sensitive
ACACGATGAGACTGGAGTAGAGGC	1	31	RDR2-sensitive
ACACGATTGAGGATGTATTTGGCA	0	18	RDR2-sensitive
ACACGCAGCCGTTGGACATTGCAC	0	10	RDR2-sensitive
ACACGCATGGACTGTCTAGGGCGC	0	15	RDR2-sensitive
ACACGCCTGCTAGACGAGTCGGAC	0	22	RDR2-sensitive
ACACGCCTGTAAGACGAGTCGGAC	0	9	RDR2-sensitive
ACACGCGGGACGGCGGACAGACAC	0	9	RDR2-sensitive
ACACGCTGGACGAACGAGATCGGC	0	11	RDR2-sensitive
ACACGCTGGAGGCAGAACACGTCA	0	11	RDR2-sensitive
ACACGCTGTGGCAGAACCGTGCAC	1	26	RDR2-sensitive
ACACGCTTGTTCGGACAACGCATA	0	134	RDR2-sensitive
ACACGCTTTGGTACATTTAGGCAT	19	0	RDR2-resistant
ACACGGAATGACAGCCTACCCCTT	0	9	RDR2-sensitive
ACACGGACCATAGGGGGGCCGGGG	40	1	RDR2-resistant
ACACGGACGGTCTAGATCGCCGGG	0	21	RDR2-sensitive
ACACGGACGGTGTAGATTAGCCGG	0	13	RDR2-sensitive
ACACGGATGTGAAGGCCTACACTA	0	29	RDR2-sensitive
ACACGGATTTGTGGACATAGGTAT	0	10	RDR2-sensitive
ACACGGCTGAGCACGTGGGCCGGC	0	9	RDR2-sensitive
ACACGGCTGGAGAATGTAGAGGAC	1	16	RDR2-sensitive
ACACGGCTTATAGTGATTCGTCGT	0	11	RDR2-sensitive
ACACGGTACTGTTAGTGAGGACGT	0	9	RDR2-sensitive
ACACGTAGACGGTCTGTGCTGCTA	0	9	RDR2-sensitive
ACACGTAGGAGGCGCTGTAGGGGC	0	20	RDR2-sensitive
ACACGTAGGGCGCTGTAGCAGGCA	0	15	RDR2-sensitive
ACACGTAGGGGCGCTGTAGAGGGC	0	91	RDR2-sensitive
ACACGTAGGGGCGCTGTAGGGGGC	0	31	RDR2-sensitive
ACACGTAGGGGGCGCTGTAGGGGC	3	35	RDR2-sensitive
ACACGTAGGGGTGCTGTAGGGGGC	0	14	RDR2-sensitive
ACACGTAGTGGGCGCTGTAGGGGC	1	25	RDR2-sensitive
ACACGTCTGCTGCTCCTGACGGCT	0	9	RDR2-sensitive

ACACGTGCTCGCGTTTGTCGTCGA	0	16	RDR2-sensitive
ACACGTGGACCAATCAGATCGCGA	0	58	RDR2-sensitive
ACACGTGGACCAATCAGATCGCGC	0	26	RDR2-sensitive
ACACGTGGGTCTGTTATTGGGCAA	0	9	RDR2-sensitive
ACACGTTACGTGCGAGATCTCGGG	0	27	RDR2-sensitive
ACACGTTTGGCTTATGAGCAGGCT	0	9	RDR2-sensitive
ACACTAAGACTGTGTTTGGTTGGA	0	12	RDR2-sensitive
ACACTACACATGGTCGGACGGTCC	0	18	RDR2-sensitive
ACACTACCATCTGATCTTTGGACA	0	12	RDR2-sensitive
ACACTAGTGCCTGCAGATTCGGTG	27	2	RDR2-resistant
ACACTCACTCGTCGGCTTAGGGAC	3	36	RDR2-sensitive
ACACTCATGTCTGTTGGATGGTTT	391	0	RDR2-resistant
ACACTCGACACTCGGCAAAGACGC	0	11	RDR2-sensitive
ACACTCGGCAAAGAACACTCGGCA	0	57	RDR2-sensitive
ACACTCGGCAAAGAACGCTCGGCG	1	41	RDR2-sensitive
ACACTCGGCAAAGAAGGCTCGGCA	0	22	RDR2-sensitive
ACACTCGGCAAAGACGGCTCTCGG	0	9	RDR2-sensitive
ACACTCGGCAAAGATGGCTCTCGG	0	12	RDR2-sensitive
ACACTCGGCAAAGCCTCGGATTCC	0	24	RDR2-sensitive
ACACTCGGCAAAGTCTCGGATTCC	0	11	RDR2-sensitive
ACACTCGGCACAGAATGATCGGCG	0	14	RDR2-sensitive
ACACTCGGGAGTAGAAAGACGGAT	0	15	RDR2-sensitive
ACACTCTATGGGCTGTCTGATGGC	0	10	RDR2-sensitive
ACACTCTCTCTCTCGGACTCGGTC	0	12	RDR2-sensitive
ACACTCTGCAGAGGCTGCACATCT	0	9	RDR2-sensitive
ACACTCTGCAGAGGTGGTACCCTT	2	15	RDR2-sensitive
ACACTCTGCAGAGGTTGTGCACTT	3	16	RDR2-sensitive
ACACTCTGGCCCATGAACACGCTT	0	34	RDR2-sensitive
ACACTCTGTAACTGCATGCATTAA	29	1	RDR2-resistant
ACACTCTGTATCTTAGAACGCGCA	0	39	RDR2-sensitive
ACACTCTTGAACCCCTCGGATGGT	30	0	RDR2-resistant
ACACTGAACAGGAGAGAGCTCCAG	0	13	RDR2-sensitive
ACACTGCTCGGAGGTACTGGCATG	0	13	RDR2-sensitive
ACACTGCTGAATCATGGATGGTGT	0	11	RDR2-sensitive
ACACTGGACGGACAGCGACGGTCG	0	14	RDR2-sensitive
ACACTGGCAGAACACGGAATGGCA	0	9	RDR2-sensitive
ACACTGGCCTTTAGAACTAGCGGC	0	28	RDR2-sensitive
ACACTGGGATGAGCATCGACACAT	0	10	RDR2-sensitive
ACACTGGGCTGTATAAACCGCGGC	0	15	RDR2-sensitive
ACACTGGTTTCGTCGATCTCGGAT	0	10	RDR2-sensitive
ACACTGTACGAATGTGTCGTCTGT	1	17	RDR2-sensitive
ACACTGTAGACGACGTTGTTTGAC	0	10	RDR2-sensitive
ACACTGTAGACGCCTACACGGTGT	0	10	RDR2-sensitive
ACACTGTAGACTGCATATAGTCAT	0	13	RDR2-sensitive
ACACTGTAGATCATTTTCGGCGGC	1	13	RDR2-sensitive
ACACTGTAGATCATTTTCGGCGGT	0	13	RDR2-sensitive
ACACTGTAGATTGCACGGTTCGTA	0	14	RDR2-sensitive
ACACTGTAGCCGCTATAGGATGCA	0	21	RDR2-sensitive
ACACTGTAGCTTTAATCCGACTGC	0	36	RDR2-sensitive

ACACTGTAGCTTTAGGGGACCGAA	0	27	RDR2-sensitive
ACACTGTAGCTTTAGGGGACGTTG	0	21	RDR2-sensitive
ACACTGTAGCTTTAGGGGATGGAA	0	11	RDR2-sensitive
ACACTGTAGTAATTGTTTGGTGGC	0	25	RDR2-sensitive
ACACTGTCGGGAGGGTCGCCGGGA	0	10	RDR2-sensitive
ACACTGTCTTGCGCAATTCGTCGT	0	9	RDR2-sensitive
ACACTGTGAGTTATATTGTCTGGG	0	13	RDR2-sensitive
ACACTGTGCTAGCTAGGTCGGGTC	0	10	RDR2-sensitive
ACACTGTGTGTGGCTTTCTGTCGT	0	11	RDR2-sensitive
ACACTGTTCACTCCTAGAGGCCGC	0	11	RDR2-sensitive
ACACTGTTGAAGCATGGACGGGGT	0	18	RDR2-sensitive
ACACTGTTGCAGCTGTTTGGACTG	1	141	RDR2-sensitive
ACACTGTTGCAGCTGTTTGGATTG	0	33	RDR2-sensitive
ACACTGTTGGACTGGCCCACGGTC	0	25	RDR2-sensitive
ACACTTCGGACTAGCACGACACGG	0	22	RDR2-sensitive
ACACTTCGGACTAGCACGGCACAA	0	18	RDR2-sensitive
ACACTTCGGACTAGCACGGCACGG	1	25	RDR2-sensitive
ACACTTCGGACTAGTACGGCATGA	0	14	RDR2-sensitive
ACACTTCGGACTCGCACGGCATGA	0	16	RDR2-sensitive
ACACTTCGGACTGGCACGGCACGA	0	16	RDR2-sensitive
ACACTTCGGACTGTCACGACACGG	0	13	RDR2-sensitive
ACACTTCGGACTGTCCGGTGCAAC	0	11	RDR2-sensitive
ACACTTCGGGCTGACACGACACGG	0	43	RDR2-sensitive
ACACTTCGGGCTGACACGTCACAG	0	31	RDR2-sensitive
ACACTTCGGGCTGGCACGACACGG	6	190	RDR2-sensitive
ACACTTCGGGCTGGCACGATACGG	1	26	RDR2-sensitive
ACACTTCGGGCTGGCACGGCACAA	0	10	RDR2-sensitive
ACACTTCGGGCTGGCACGGCACGG	0	72	RDR2-sensitive
ACACTTCGGGCTGGCACGGCATGT	0	12	RDR2-sensitive
ACACTTCGGGCTGGCACGGTACGG	0	30	RDR2-sensitive
ACACTTCGGGCTGGCACGGTATGA	0	41	RDR2-sensitive
ACACTTCGGGCTGGCACGGTGTGA	0	11	RDR2-sensitive
ACACTTCGGGCTGGCATGACACGG	0	12	RDR2-sensitive
ACACTTCGGGCTGGCATGGTATGA	0	9	RDR2-sensitive
ACACTTCGGGCTGGTACGACACGC	0	9	RDR2-sensitive
ACACTTCGGGCTGGTACGACACGG	0	31	RDR2-sensitive
ACACTTCGTCTGTATGTAAGCGGT	0	9	RDR2-sensitive
ACACTTTCAACTGGGCGAGGACGT	0	9	RDR2-sensitive
ACACTTTGCTTAAGGTTGGTCGTC	0	14	RDR2-sensitive
ACACTTTGGACTGGCACGACACGG	1	41	RDR2-sensitive
ACACTTTGGGCTGGCACGACACGG	2	57	RDR2-sensitive
ACACTTTGGGCTGGCACGGTACGT	0	11	RDR2-sensitive
ACACTTTGGGCTGGTATGGTACGG	1	56	RDR2-sensitive
ACAGAACACGGAATGGGGCAGCCT	0	10	RDR2-sensitive
ACAGAACAGAGCGGCTCCGTTCCT	4	77	RDR2-sensitive
ACAGAACATCGAACGGCAGGTCCT	0	22	RDR2-sensitive
ACAGAACGCCGACGGATGGCTCGC	0	9	RDR2-sensitive
ACAGAACGCTGACGTGGCACGTGA	0	20	RDR2-sensitive
ACAGAACGGATGGCTTGGATGCAT	0	12	RDR2-sensitive

ACAGAACGGATGGGCTGGATGCAT	0	13	RDR2-sensitive
ACAGAAGACTGTAAAGGACGGATA	0	48	RDR2-sensitive
ACAGAAGGACAGCGTCGCCTGCGC	0	10	RDR2-sensitive
ACAGAATGGCAGAACGGCTGGCTA	0	10	RDR2-sensitive
ACAGAATGGTAGAACGGATGGTTG	0	17	RDR2-sensitive
ACAGAATGTTGTCTGTAGACGGCG	1	23	RDR2-sensitive
ACAGACAGAAGCAGGACGTGACAC	0	12	RDR2-sensitive
ACAGACCAACAGTAGATCGGGCCC	0	10	RDR2-sensitive
ACAGACCAGCGAGAACGACGACAT	0	14	RDR2-sensitive
ACAGACGGCATGCAGGACAGACGG	0	9	RDR2-sensitive
ACAGACGGTCGTGCTCGTAAGCAG	0	10	RDR2-sensitive
ACAGACGTGGAACAGGTGGCGCAC	0	9	RDR2-sensitive
ACAGACGTGGGACAGGTGGCGCAC	0	15	RDR2-sensitive
ACAGACGTGGGGCAGGTGACGCAC	0	9	RDR2-sensitive
ACAGACGTGGGGCAGGTGGCGCAC	2	41	RDR2-sensitive
ACAGACGTTATAATGGATTAGTGA	21	1	RDR2-resistant
ACAGACTACGCTCGGCCGATGCAG	0	10	RDR2-sensitive
ACAGACTCAAAGCACGGCTCGACA	0	14	RDR2-sensitive
ACAGACTCGGACGTGAGCGACGGA	0	10	RDR2-sensitive
ACAGACTCTGCTCGGATCGGTCTA	0	23	RDR2-sensitive
ACAGACTGTAGCGGCCCACGTCAC	0	15	RDR2-sensitive
ACAGACTTTAGTACCGGTTTTCGT	1	12	RDR2-sensitive
ACAGAGCAGGACTGCAGGAGGGCG	0	21	RDR2-sensitive
ACAGAGCCGTTGGACTCATGGCGC	0	16	RDR2-sensitive
ACAGAGCTCGCACGCGCATAGCCT	1	34	RDR2-sensitive
ACAGAGTGGGCTAACGACGACGGA	84	3	RDR2-resistant
ACAGAGTGTCAGAGCAGAGGATAA	0	9	RDR2-sensitive
ACAGATCCTTAGACTGTTTGGCGT	0	12	RDR2-sensitive
ACAGATCGCTGAACGCTGACGGTG	1	22	RDR2-sensitive
ACAGATCGGACTAGCACGGTTCAA	0	29	RDR2-sensitive
ACAGATCTCAAGACGAAGGGTCGG	0	18	RDR2-sensitive
ACAGATCTGAACGCGTGGCGGCAC	0	10	RDR2-sensitive
ACAGATGAGGATGTGCCGGATTCG	79	2	RDR2-resistant
ACAGATGTGGTATAACTTGTTATC	15	0	RDR2-resistant
ACAGATTACGGGCCGGTCCAACAC	0	10	RDR2-sensitive
ACAGATTCCTGTGACTCGGCGAGC	0	21	RDR2-sensitive
ACAGATTGATGGGCGGTACGGTAT	26	0	RDR2-resistant
ACAGATTGCTGGTCGTGCTGGTCG	0	9	RDR2-sensitive
ACAGCACGGCAGAGGACAAAGCAG	0	9	RDR2-sensitive
ACAGCACTGTAGACACGGCTGCAG	0	15	RDR2-sensitive
ACAGCAGATGGATAAGCTTGTAGA	18	1	RDR2-resistant
ACAGCATGGACGAGCAGACACGAC	0	9	RDR2-sensitive
ACAGCCGACGGCTGTCAGACTGGC	0	14	RDR2-sensitive
ACAGCCGGCTGATAACGCGTGCAG	0	14	RDR2-sensitive
ACAGCCGGCTGTACGTAGACGTCG	0	12	RDR2-sensitive
ACAGCCTGAGCGCGCTGTCGGGCG	133	0	RDR2-resistant
ACAGCCTGCTTGTGCTCCGTCGTC	37	0	RDR2-resistant
ACAGCGAAGGTTTATGATTGTCGA	16	0	RDR2-resistant
ACAGCGACAATTTACTCGTCGGTC	0	17	RDR2-sensitive

ACAGCGCATCGGCTGGGAAGCATG	2	14	RDR2-sensitive
ACAGCGGCTGGGAGGAGGGCCGAG	183	21	RDR2-resistant
ACAGCGTTCGTTTGGTTGGCCGTA	0	9	RDR2-sensitive
ACAGCTACTGTGGAAACCGGCGTC	0	24	RDR2-sensitive
ACAGCTAGCCGTTGGACTGATGGC	0	23	RDR2-sensitive
ACAGCTCTGCTTGCTGTCGGCGTG	0	9	RDR2-sensitive
ACAGCTGAGCACGTGGACCGGCAC	0	11	RDR2-sensitive
ACAGCTGCACATGAACGTAGACGA	0	13	RDR2-sensitive
ACAGCTGCACGGTACTCCAAGTAT	0	19	RDR2-sensitive
ACAGCTGCGTGGGACTTGGCGATA	0	9	RDR2-sensitive
ACAGCTGCTGTGCTCGCCGGATAA	0	11	RDR2-sensitive
ACAGCTGTCAGACAGGTCGTTGGA	0	14	RDR2-sensitive
ACAGCTTCGGATTCGGGTCGGATA	0	17	RDR2-sensitive
ACAGGAACTGTAAATTTTTACGGC	0	16	RDR2-sensitive
ACAGGAACTTCAGGGACTTGGCGT	0	10	RDR2-sensitive
ACAGGACAAAGCCAGGTGAGACAT	0	10	RDR2-sensitive
ACAGGACAACCTGCTTGAGTCGGC	0	9	RDR2-sensitive
ACAGGACAACGAGCAGACTGGTTT	0	9	RDR2-sensitive
ACAGGACACCTAGGATCCGTCTTA	15	0	RDR2-resistant
ACAGGACACGGCACGGCTAGACAC	0	55	RDR2-sensitive
ACAGGACAGACGAAGGACAGACGA	0	11	RDR2-sensitive
ACAGGACAGGACGGGATGAGACAA	0	9	RDR2-sensitive
ACAGGACCCCACTCGGCAAAGACA	0	9	RDR2-sensitive
ACAGGACCCTGCTGGAGCCAGCCG	0	15	RDR2-sensitive
ACAGGACCCTGCTGGAGCCAGCCT	0	9	RDR2-sensitive
ACAGGACCTTCGGCGACAAAGCAG	0	16	RDR2-sensitive
ACAGGACGCACTCGGCAAAGACAA	0	12	RDR2-sensitive
ACAGGACGCGCGGTAAGACTGAGC	0	11	RDR2-sensitive
ACAGGACGGAAGCGAGCACGCCAA	0	9	RDR2-sensitive
ACAGGACGGCCGAGGGCGCGATGA	0	11	RDR2-sensitive
ACAGGACGGCGATTAGGCCTGCTA	47	4	RDR2-resistant
ACAGGACGGCTGAAAGCACACAAT	0	36	RDR2-sensitive
ACAGGACGGGACGGGACGAGACGA	0	15	RDR2-sensitive
ACAGGACGGTTCGCGCTTCGGTGC	2	29	RDR2-sensitive
ACAGGACGTACACGGGATATTCAG	0	11	RDR2-sensitive
ACAGGACGTGTTAGCGGTTGTTGC	0	11	RDR2-sensitive
ACAGGACTCAGGAAGAGAGACGAC	0	10	RDR2-sensitive
ACAGGACTCGCTCGCGCAGGGCTG	0	22	RDR2-sensitive
ACAGGACTGCAGAAGAATGGGCTA	0	47	RDR2-sensitive
ACAGGACTGTAGGAGCATAGGTTA	0	14	RDR2-sensitive
ACAGGAGCGGCTCCTTCTCTGCAC	0	15	RDR2-sensitive
ACAGGAGGCAGAACCACGGTGCAT	0	10	RDR2-sensitive
ACAGGAGGTTCATGTGGACCGCGG	18	0	RDR2-resistant
ACAGGAGTACTTTGGGATGAGCAT	0	12	RDR2-sensitive
ACAGGATATGGCTACATTGTTATT	18	0	RDR2-resistant
ACAGGATCTTCGGCGACGAAGCAT	0	13	RDR2-sensitive
ACAGGATGACGAACAAGGTGGCGA	0	16	RDR2-sensitive
ACAGGATGTAGCCGGGTTAGACGA	0	9	RDR2-sensitive
ACAGGCAACGTAGCATAGCACTCT	1	27	RDR2-sensitive

ACAGGCACGGCACGGATCTTCTAC	0	13	RDR2-sensitive
ACAGGCACTGTAGGCTGTCCGGTG	0	17	RDR2-sensitive
ACAGGCATGCTCGGCTACTCCAGT	38	0	RDR2-resistant
ACAGGCCTGAGACAGACGCGCTGG	0	10	RDR2-sensitive
ACAGGCCTTAGACTATATTCAACG	0	17	RDR2-sensitive
ACAGGCGACGGGACGGAAGAACAG	0	9	RDR2-sensitive
ACAGGCGAGTAGCGTGCGGACAAC	1	25	RDR2-sensitive
ACAGGCTAAGCGGACGGTAAGCAC	0	9	RDR2-sensitive
ACAGGCTCTGGACGACAGCCTGCA	0	10	RDR2-sensitive
ACAGGCTGCAGCAGGCTACAACCT	0	10	RDR2-sensitive
ACAGGGCAGTAGCTGAGAAAGCGG	0	13	RDR2-sensitive
ACAGGGCCTCGGATTTTACTGGCA	0	11	RDR2-sensitive
ACAGGGCTTGTAGAGAGGTTGTAA	0	29	RDR2-sensitive
ACAGGGGGCTCTCGGCAAAGACAC	0	10	RDR2-sensitive
ACAGGGGTCCTGTGTCTTCTGGGC	0	10	RDR2-sensitive
ACAGGGTCAGACTGTCCACGACAT	0	14	RDR2-sensitive
ACAGGGTTGTCTGAGGTCTGTTGG	0	27	RDR2-sensitive
ACAGGTACTGCCTAGAGGGGCTAA	0	10	RDR2-sensitive
ACAGGTACTGTAGACTGTCCGGTG	0	11	RDR2-sensitive
ACAGGTACTGTTCACTGTCCGGTG	0	10	RDR2-sensitive
ACAGGTACTGTTTGATGTCCGGTG	0	22	RDR2-sensitive
ACAGGTAGGCACTCGGCAAAGACA	1	18	RDR2-sensitive
ACAGGTCAAAGGCAGAACGGTCAA	1	41	RDR2-sensitive
ACAGGTCAGAACGGAAGCAACCAC	0	24	RDR2-sensitive
ACAGGTCCTGTAGACTGTCCGGTG	2	78	RDR2-sensitive
ACAGGTCGGTCTGAGCCCGCCGGA	3	17	RDR2-sensitive
ACAGGTCTGACGAGCTGACACGGT	0	32	RDR2-sensitive
ACAGGTCTGGCGAGCTGACGCGGT	0	23	RDR2-sensitive
ACAGGTGACTGCAGAGAGGAGGTT	0	21	RDR2-sensitive
ACAGGTGCACTGGTAGAACAACGG	0	9	RDR2-sensitive
ACAGGTGGTGGACTTCCGGATCGT	5	36	RDR2-sensitive
ACAGGTTCGTCGTGCCTCCGGCCC	0	9	RDR2-sensitive
ACAGGTTCTCTGGAGGACATGGTT	9	25	RDR2-sensitive
ACAGGTTGTTGATATGGCATGTGA	0	9	RDR2-sensitive
ACAGGTTTGTTGTCGGGATAAAAT	122	0	RDR2-resistant
ACAGTAAACTGTCACGGTCGTCCC	2335	2263	RDR2-sensitive
ACAGTAACTCTGCAAGGTGGGACC	0	11	RDR2-sensitive
ACAGTACATCGGCAAACAGGGGCG	0	11	RDR2-sensitive
ACAGTACCGTTGGATGTAGATGGT	0	15	RDR2-sensitive
ACAGTACTGTCTCAGGTCGCGGAC	1	14	RDR2-sensitive
ACAGTAGAACCGGACATTCGGCCA	0	11	RDR2-sensitive
ACAGTAGAATAAGCGGACGGGCTT	0	30	RDR2-sensitive
ACAGTAGGATTGTCGGCACTACAA	0	9	RDR2-sensitive
ACAGTATCGTACTTGGACAGGCAA	0	23	RDR2-sensitive
ACAGTATCTGGGTAATCGGACAAT	0	15	RDR2-sensitive
ACAGTATGGGACAGCGACCAGCAC	0	13	RDR2-sensitive
ACAGTCAAGACATGCGGAGGACAT	0	40	RDR2-sensitive
ACAGTCACTGTAGACGGTCCGGTG	4	21	RDR2-sensitive
ACAGTCACTGTAGCTGTCCGGTGC	0	12	RDR2-sensitive

ACAGTCAGTAGACCGGCATGACGT	0	9	RDR2-sensitive
ACAGTCCTGACTATAGATGGCCAA	0	10	RDR2-sensitive
ACAGTCGAATCGGCAACAAAGCGT	1	17	RDR2-sensitive
ACAGTCGACTGCTACAGACCCCAA	0	9	RDR2-sensitive
ACAGTCGACTTAGAATGAAGATGG	0	15	RDR2-sensitive
ACAGTCGGCTCTCGGCAAAGATGC	0	11	RDR2-sensitive
ACAGTCGGTGCACCACAAGCGGTT	1	25	RDR2-sensitive
ACAGTCTAGAACTCTCGCGGCTGT	0	16	RDR2-sensitive
ACAGTCTCCGCTGCAGAAGGTGTT	0	55	RDR2-sensitive
ACAGTCTGATTTGTAGTAGGGCTG	0	10	RDR2-sensitive
ACAGTCTGGTATAGTCGGTGCGCT	0	21	RDR2-sensitive
ACAGTGACACGCTAGATCTAGGGG	0	12	RDR2-sensitive
ACAGTGACACGCTAGATCTGGGGA	0	17	RDR2-sensitive
ACAGTGACACGCTAGATCTGGGGG	0	24	RDR2-sensitive
ACAGTGACTCTGTTCTTCTTGGCA	0	14	RDR2-sensitive
ACAGTGACTGTACGGTGCGCCACC	0	12	RDR2-sensitive
ACAGTGCGGACCAGTGTTGGGCAT	0	18	RDR2-sensitive
ACAGTGGTCTGGGTGGTCTTCGAA	14	0	RDR2-resistant
ACAGTGTATAGCGCATGTAGACGT	0	17	RDR2-sensitive
ACAGTGTCGCGACTGAATCGTCAT	0	10	RDR2-sensitive
ACAGTGTCGTGCTTGGGCAGTCAG	0	10	RDR2-sensitive
ACAGTGTTGGGAGGTGAGAGACGC	0	26	RDR2-sensitive
ACAGTTCAGATCGTTGGATAATAC	0	12	RDR2-sensitive
ACAGTTGTAGATCTTTATTGCAGA	0	10	RDR2-sensitive
ACAGTTGTTGGCTAGATTCGCGGA	0	13	RDR2-sensitive
ACAGTTTAGCTGTTTTTCGATCGT	0	12	RDR2-sensitive
ACAGTTTAGGCAGACATACTGAAG	38	3	RDR2-resistant
ACAGTTTGACGTTAGTATAATAGT	18	1	RDR2-resistant
ACAGTTTGACTCGATCGATACGGT	1	15	RDR2-sensitive
ACAGTTTTTAATGTAGAATCAGGA	35	3	RDR2-resistant
ACATAAGAAGCTAATGTAGACGTT	0	16	RDR2-sensitive
ACATAAGCCCGTTGGACCGGCAGC	0	15	RDR2-sensitive
ACATAAGCTTATGTCTGACGGTCT	1	21	RDR2-sensitive
ACATAAGGCTATGTCTGACGGTTG	0	9	RDR2-sensitive
ACATAAGTACACGGACGCAGGCAC	0	11	RDR2-sensitive
ACATAAGTCGAAGACAGCATGTAG	0	9	RDR2-sensitive
ACATACACGTTTGGACCACGCATA	1	60	RDR2-sensitive
ACATACAGACTACGCTCGGCCGAT	0	13	RDR2-sensitive
ACATACCGTCGTAATGTATGGGCA	0	52	RDR2-sensitive
ACATACGACAGGATCGGCTCGGCT	0	10	RDR2-sensitive
ACATACGACGCGGCGACGAACGAC	0	10	RDR2-sensitive
ACATACGGTGCTGTCAGCGACGCG	73	3	RDR2-resistant
ACATACGTTATTTTTTTCGTCGGT	1	31	RDR2-sensitive
ACATACTGCACTGTAGTAGGCTAC	0	15	RDR2-sensitive
ACATAGATCTGATTCCTGGAACGG	14	0	RDR2-resistant
ACATAGGGGCGCTGTAGGGGGCAC	0	25	RDR2-sensitive
ACATAGGTTCGGCCTGGGTGCATT	0	42	RDR2-sensitive
ACATAGTTTATGTGTAGTTCGGTA	31	5	RDR2-resistant
ACATATAATCGGTTGAGTCGTCGC	3	18	RDR2-sensitive

ACATATATCGCGTGATTGGACGTC	0	12	RDR2-sensitive
ACATATCCAAGATGGACTGGGCTT	0	14	RDR2-sensitive
ACATATCGGATTTCGTACTAGGCC	0	24	RDR2-sensitive
ACATATCGTCAGTAGGTCGTCGAC	0	15	RDR2-sensitive
ACATATCTAATTATGGGTCGTGCT	198	1	RDR2-resistant
ACATATTCGGGCTTCTTGCGGATC	41	8	RDR2-resistant
ACATATTCTGAACAATGTGTAGAA	0	11	RDR2-sensitive
ACATCACCCTTCAGGTCGTCTGGC	0	12	RDR2-sensitive
ACATCAGACCTGCGAGGACGTCGT	2	23	RDR2-sensitive
ACATCATCGGATGGTCCGGCCATA	0	12	RDR2-sensitive
ACATCCACGGGCTAGCAGGCGGGT	0	15	RDR2-sensitive
ACATCCAGAACTCTGCAGGTAGAG	0	10	RDR2-sensitive
ACATCGGGCGACGGATCCGGCTTC	3	22	RDR2-sensitive
ACATCGGGCGGGAGACGCAGACAT	0	23	RDR2-sensitive
ACATCGTGGACAGAGCATGGGCAT	0	11	RDR2-sensitive
ACATCGTTATATTTGTAGAGTGGC	0	73	RDR2-sensitive
ACATCTAGTAGAGCGTGATAGCCG	0	12	RDR2-sensitive
ACATCTATAGGGTTGTCTGATGTC	0	11	RDR2-sensitive
ACATCTCCTGTGAACTAGACTGGC	0	9	RDR2-sensitive
ACATCTCTTGTGAACCAGACTGGC	0	9	RDR2-sensitive
ACATCTGATGTGGATCGCGGTAGC	32	1	RDR2-resistant
ACATCTGTATCTAGGGCTGGGCAT	0	19	RDR2-sensitive
ACATCTTAGGACTTAGGGCGGGTG	0	20	RDR2-sensitive
ACATCTTTGTATGATTGGTGGACG	0	22	RDR2-sensitive
ACATCTTTGTTTGTTGTGGAGGCT	14	0	RDR2-resistant
ACATGACTGCAGAGGATTGTGGTA	0	18	RDR2-sensitive
ACATGACTGTAGAGGATTGTGGTA	0	13	RDR2-sensitive
ACATGAGAATGGACTGTGCGGGGA	0	12	RDR2-sensitive
ACATGAGGACTACCAGATCGTGGC	0	27	RDR2-sensitive
ACATGAGTTACTGGGCCGTCGGAT	0	16	RDR2-sensitive
ACATGATCGGACACACGTGGGTAT	0	9	RDR2-sensitive
ACATGCACTGTAGAGTGTCCGGTG	16	35	RDR2-sensitive
ACATGCAGAGGACACGGGAAACTC	1	12	RDR2-sensitive
ACATGCAGGTCGGTATCGTTACAT	0	20	RDR2-sensitive
ACATGCAGTTGAACTGGTCCTCGG	0	11	RDR2-sensitive
ACATGCCCGTTTATGTGTCGGACC	0	14	RDR2-sensitive
ACATGCGTCGCTGTGTTCGGCAAG	0	10	RDR2-sensitive
ACATGCGTTCGGTCTAGATGGATG	0	17	RDR2-sensitive
ACATGCTATATGTAGGATTCGGCA	0	10	RDR2-sensitive
ACATGCTCGGGCTGGGTCGGGCCG	0	32	RDR2-sensitive
ACATGCTTGTTCGGACGACGCATA	0	12	RDR2-sensitive
ACATGGACCGATGAGGCACGACAT	0	21	RDR2-sensitive
ACATGGACCTGATGGCTTTAATGG	16	0	RDR2-resistant
ACATGGACGTAGGATGGACGGTGG	0	10	RDR2-sensitive
ACATGGACTGAAGTTTTTATGGTA	39	3	RDR2-resistant
ACATGGACTGTAGGAGCGGGGTTC	0	11	RDR2-sensitive
ACATGGATTTGTTGATGGATTTAA	0	30	RDR2-sensitive
ACATGGCCAGAAGACGAACCTCTT	0	15	RDR2-sensitive
ACATGGCGTCGGATCTGCCGGTAC	0	11	RDR2-sensitive

ACATGGCTGAAGTAAGAGACAACA	43	5	RDR2-resistant
ACATGGCTGTTCGGATGATCGCAT	30	3	RDR2-resistant
ACATGGGAGATGCTGACATGACGT	31	1	RDR2-resistant
ACATGGTTGTTCGGATGATCGCAT	71	5	RDR2-resistant
ACATGGTTTTGTAGAGCATGGTAT	0	26	RDR2-sensitive
ACATGTACGTGCATGCCGCGTCGG	0	9	RDR2-sensitive
ACATGTACTGTAGAGTGTCCGGTG	8	90	RDR2-sensitive
ACATGTATGAACGTTTTTTTGGAT	15	0	RDR2-resistant
ACATGTCAGACACGTAGAGGTGGT	0	16	RDR2-sensitive
ACATGTCAGAGGCAGAACGGCAGA	0	20	RDR2-sensitive
ACATGTCGCGTAGGACAGTTCGGC	0	19	RDR2-sensitive
ACATGTCGTGTGTTTAGGTTTACC	46	0	RDR2-resistant
ACATGTCGTTGCTGGAGTTGGCAT	0	12	RDR2-sensitive
ACATGTCGTTGTAGTTGCAGGCAC	0	47	RDR2-sensitive
ACATGTCTGGCTAAAGCATCGGTT	0	15	RDR2-sensitive
ACATGTGTTTGTGTTGATCGTCAA	0	10	RDR2-sensitive
ACATGTTCGGTGCATACATAGATT	0	9	RDR2-sensitive
ACATGTTGAGGTGTTCGCCTGGAT	0	14	RDR2-sensitive
ACATGTTGGATAAAATGGCGTGAG	660	132	RDR2-resistant
ACATGTTGTGATTTGACTTGGCAT	0	33	RDR2-sensitive
ACATGTTTGGATGGCTGCACGCGA	0	16	RDR2-sensitive
ACATGTTTGGTTCATGGACGGTGT	0	9	RDR2-sensitive
ACATTAGGGTAGGATACGGGATGA	0	24	RDR2-sensitive
ACATTCGGCGAGAACACGGGTCAT	0	10	RDR2-sensitive
ACATTCTAACGGCTGGTCTCGGTA	71	3	RDR2-resistant
ACATTCTGCGTGATTGGTTGGCTC	0	9	RDR2-sensitive
ACATTCTGTGTTGTGCTCTATCGT	0	23	RDR2-sensitive
ACATTGAAGACGTGGGCTGCACAA	0	39	RDR2-sensitive
ACATTGGATACGTGCTGCCTCAGA	23	0	RDR2-resistant
ACATTGGATTATATACAGACTGCA	0	49	RDR2-sensitive
ACATTGGTCAGTTCGGATCGGGTG	0	9	RDR2-sensitive
ACATTGGTGGTACGCTGGACTAAC	0	14	RDR2-sensitive
ACATTGGTTATCATGTCGTTTCTG	32	0	RDR2-resistant
ACATTGTAATTGGACTTTCGGTAT	1	167	RDR2-sensitive
ACATTGTGGGCAGTGAAGATGGAG	124	3	RDR2-resistant
ACATTGTTGGATGGATACAGGGGC	1	21	RDR2-sensitive
ACATTTAAGACTGAGACATACCGG	0	9	RDR2-sensitive
ACATTTATGAGGGATCGTCCTACT	19	1	RDR2-resistant
ACATTTATGGCTAGGCTCGTTGGC	0	17	RDR2-sensitive
ACATTTATTTTTTGACGATCGGTG	41	5	RDR2-resistant
ACATTTCATTGTGCGAGCTGGATA	30	0	RDR2-resistant
ACATTTCGAGGACGACGAAGGCAC	0	9	RDR2-sensitive
ACATTTCGGGCTGACACGTCACGG	0	43	RDR2-sensitive
ACATTTGAAAGTGATTGTTGGTCA	44	10	RDR2-resistant
ACATTTGGAGGGTAGACGATGGCT	0	22	RDR2-sensitive
ACATTTTGTTTGGTTGTTGGATAA	0	15	RDR2-sensitive
ACATTTTTTGTACATGAGACTGCT	49	3	RDR2-resistant
ACCAAACAAGCGGCAAAGTAGCAC	0	15	RDR2-sensitive
ACCAAACACAGAACAGAGCGGCTC	3	97	RDR2-sensitive

ACCAAACACTCGGCGAATGACGAC	0	18	RDR2-sensitive
ACCAAACCGGGTCGGGCTAGGCTT	0	12	RDR2-sensitive
ACCAAACTTCACGCGTCGGGTCGA	0	12	RDR2-sensitive
ACCAAAGACTGGATGTAGGGACGT	0	12	RDR2-sensitive
ACCAAAGGCTGTGGCGATGAGGGA	0	17	RDR2-sensitive
ACCAAAGGCTGTGGCGATGAGGTA	0	14	RDR2-sensitive
ACCAAATGTGTCTCGCGGACGGTC	0	9	RDR2-sensitive
ACCAAATTGAGCGGGCGGACGGTC	0	14	RDR2-sensitive
ACCAAATTTTATGTGGGTCTGGAC	0	9	RDR2-sensitive
ACCAACGACTGCAGCGAAGGCCTC	0	10	RDR2-sensitive
ACCAACGAGGATTAGTCGGGACCT	0	33	RDR2-sensitive
ACCAACGGAGACAAGACGACGGAC	0	11	RDR2-sensitive
ACCAACGGAGACAAGACGACTGGC	0	31	RDR2-sensitive
ACCAACGTAGATCGCTGAAGGCAC	0	10	RDR2-sensitive
ACCAACTCTGGCCAGGCACACCGA	0	10	RDR2-sensitive
ACCAACTCTGGCCAGGCACACCGG	0	11	RDR2-sensitive
ACCAACTGGATCAGGCTGTCGGTT	0	16	RDR2-sensitive
ACCAAGACGGTTCTCCAAGCACAA	0	9	RDR2-sensitive
ACCAAGAGGGAGATCGTGACCGGC	0	10	RDR2-sensitive
ACCAAGCCTGATGACGTACTGAAC	0	16	RDR2-sensitive
ACCAAGCGTAGTACACTCGGCATA	0	9	RDR2-sensitive
ACCAAGGCTGGGACTAACAGTGGC	0	19	RDR2-sensitive
ACCAAGTAGCAACAGACGCCTCAC	1	18	RDR2-sensitive
ACCAAGTCGCAGTAGAGAACCGGT	0	14	RDR2-sensitive
ACCAAGTCTTCGGAACGGCTCCAG	0	9	RDR2-sensitive
ACCAAGTTGCTCGGACAGCGTCAG	0	12	RDR2-sensitive
ACCAATCTGGCGATGACGGGCTGC	0	9	RDR2-sensitive
ACCAATGACTGGAAGTAGGGACGT	0	47	RDR2-sensitive
ACCAATGACTGTAAGCAGGGACGT	0	22	RDR2-sensitive
ACCAATTGGACTGTTACACCGGAT	0	9	RDR2-sensitive
ACCAATTGGGCCTGTAGTGTCGGC	0	10	RDR2-sensitive
ACCAATTGTTAGAACCGGCCCCTG	0	20	RDR2-sensitive
ACCACACCAGGTTGTGAACGTCTA	0	10	RDR2-sensitive
ACCACACTCGGTGCATACTGGACA	0	12	RDR2-sensitive
ACCACACTGAACAGCAGAGAGCTC	0	18	RDR2-sensitive
ACCACACTGAACAGGAGAGAGCTC	0	17	RDR2-sensitive
ACCACAGTAGAGCGCATGATTGGC	0	10	RDR2-sensitive
ACCACATAGCGGTGTTCTCTGCGG	0	15	RDR2-sensitive
ACCACATGTCGGTCAGTTTTCGGT	0	9	RDR2-sensitive
ACCACATTGTGTCGGGCCATGCAT	0	23	RDR2-sensitive
ACCACCAATGACTGTAAGCAGGGA	0	9	RDR2-sensitive
ACCACCACGCTAGCTGAAGGACGA	0	10	RDR2-sensitive
ACCACCCAGAGTAGGTCGTCACGG	0	13	RDR2-sensitive
ACCACCCTAAGGGATTGTTCGGAT	0	11	RDR2-sensitive
ACCACCTGTAGCCTGACGGTGACC	0	24	RDR2-sensitive
ACCACCTGTTATGAACCTTGTCGG	25	3	RDR2-resistant
ACCACGACTGCATGCAACTACCAC	0	20	RDR2-sensitive
ACCACGCGACGTAGATCCAAGCGG	0	11	RDR2-sensitive
ACCACGCTAGCTGAAGGACGACGA	0	10	RDR2-sensitive

ACCACGCTGGTAAGACGGGAACAC	0	47	RDR2-sensitive
ACCACGGATGGGTGTGACGTCTAC	0	14	RDR2-sensitive
ACCACGGATTGTCCGGACCGCGGA	0	10	RDR2-sensitive
ACCACGTCGACAGAAGCGGAACAA	0	16	RDR2-sensitive
ACCACGTGACTGCTACGCCGGAAC	0	9	RDR2-sensitive
ACCACTAGACGTGTAGGGCAGCGG	0	9	RDR2-sensitive
ACCACTCGTCGCAGCTCTGGCCGG	0	9	RDR2-sensitive
ACCACTGCGGGAGATGAAAAAGTA	2	23	RDR2-sensitive
ACCACTGGACTCGGTTCTTCGGTT	33	0	RDR2-resistant
ACCACTGGGCTGTATAAACCGCGG	0	12	RDR2-sensitive
ACCACTGTAGCAGCTTTAGCGGTC	1	15	RDR2-sensitive
ACCACTGTAGCGCGTAGAGGATGA	0	20	RDR2-sensitive
ACCACTGTAGGATTACGGGGAGGC	0	10	RDR2-sensitive
ACCACTTCAGACAAGTGGGTCGGA	1	31	RDR2-sensitive
ACCACTTGTCAGCAGACTGGGCGT	0	9	RDR2-sensitive
ACCAGAAACCTGATTCGCCGGCAC	0	9	RDR2-sensitive
ACCAGAAATGAGCCGGCGGACTGT	0	10	RDR2-sensitive
ACCAGAACCAGATCGAGCGCGCGT	0	33	RDR2-sensitive
ACCAGACGAGTAGACGACGCGAGT	0	9	RDR2-sensitive
ACCAGACTGGCCACATACACACAA	0	34	RDR2-sensitive
ACCAGACTGGTAGAGAGGGCAGAG	0	9	RDR2-sensitive
ACCAGACTGTCCGGTGCGCCATGC	0	9	RDR2-sensitive
ACCAGAGACTGTTTCTTCCCCGGC	0	10	RDR2-sensitive
ACCAGAGATGGACTGTCTGGCGGT	0	27	RDR2-sensitive
ACCAGAGCTGGCATGTGGACCTAA	0	9	RDR2-sensitive
ACCAGAGGTGATTGTAGGGGCTAA	0	14	RDR2-sensitive
ACCAGATACGAGCAGATTGGTCGC	0	9	RDR2-sensitive
ACCAGATCGGGGTGATGAAGTCGG	0	20	RDR2-sensitive
ACCAGATCGTTTGATTGCATGGTT	0	21	RDR2-sensitive
ACCAGATTAGTAGGAGCCGCGTTC	0	17	RDR2-sensitive
ACCAGCACACGATCGGACGCGCGT	0	19	RDR2-sensitive
ACCAGCGACTGCAGCGAAAGGCGA	1	86	RDR2-sensitive
ACCAGCGACTGCAGCGAAATGCGA	0	10	RDR2-sensitive
ACCAGCGACTGCAGCGAAGACCTC	0	13	RDR2-sensitive
ACCAGCGACTGCAGCGAAGGCCTC	3	153	RDR2-sensitive
ACCAGCGACTGCAGCGAGAGACGA	0	167	RDR2-sensitive
ACCAGCGACTGCAGTGAAAGGCGA	0	22	RDR2-sensitive
ACCAGCGATGGTGTTCTCTGCGGC	1	12	RDR2-sensitive
ACCAGCTCGGCTCGTAGTGTCGGG	0	9	RDR2-sensitive
ACCAGCTCGGCTCGTAGTGTCGGT	1	48	RDR2-sensitive
ACCAGCTGAGAATTAGGCGTCTTG	3	93	RDR2-sensitive
ACCAGCTGTGACGGAACCTCCCAA	2	54	RDR2-sensitive
ACCAGGACCTGCTGCGCAACACGG	0	12	RDR2-sensitive
ACCAGGACGTAGGGTATAACGCAT	2	20	RDR2-sensitive
ACCAGGACGTAGGGTATTACGCAT	0	18	RDR2-sensitive
ACCAGGACGTAGGGTGTTACGCAC	0	14	RDR2-sensitive
ACCAGGACGTAGGGTGTTACGCAT	7	303	RDR2-sensitive
ACCAGGAGAGGGACGAGAGGGACC	39	1	RDR2-resistant
ACCAGGATGTAGGGTGTTACGCAT	0	13	RDR2-sensitive

ACCAGGCACGGCACGACAGGACAC	0	14	RDR2-sensitive
ACCAGGGAACAGTAGCACAACGGT	0	13	RDR2-sensitive
ACCAGGGACAGTAGGAGGGCCGAG	0	10	RDR2-sensitive
ACCAGGGTCCCACCACGTCGTCGC	0	10	RDR2-sensitive
ACCAGGTCGATAGGTGGTTGGGGC	0	9	RDR2-sensitive
ACCAGGTCGCGGACCGTCCGGCCC	20	103	RDR2-sensitive
ACCAGGTCGCGGACTGTCCGGCCC	5	26	RDR2-sensitive
ACCAGGTCGTGACTGAGCTGCCGG	0	10	RDR2-sensitive
ACCAGGTCGTGTCGGGCTTGCGGG	0	13	RDR2-sensitive
ACCAGGTGTGGACGCTGACGGTAC	1	129	RDR2-sensitive
ACCAGGTTGGCGAGAAACAGGCAA	0	22	RDR2-sensitive
ACCAGGTTGTAGAGGTAATCGGCC	0	24	RDR2-sensitive
ACCAGTAACGGTAGTAGATGGCAT	0	16	RDR2-sensitive
ACCAGTCGTGTAGGACAGTTCGGT	0	13	RDR2-sensitive
ACCAGTCTGTAGGGGTGGTCGGCC	0	20	RDR2-sensitive
ACCAGTTGGAACTGTTCGGGTAAC	0	20	RDR2-sensitive
ACCAGTTTGAAGGGGTGGTCGGCC	1	28	RDR2-sensitive
ACCAGTTTGCAGGGGTGGTCGGCC	0	33	RDR2-sensitive
ACCAGTTTGTAGGGATGGTCGGCC	0	33	RDR2-sensitive
ACCAGTTTGTAGGGATGGTCGGTC	0	9	RDR2-sensitive
ACCAGTTTGTAGGGGTGGTCGGCC	0	40	RDR2-sensitive
ACCATAAAGTTTGTAGCACGGGTG	49	1	RDR2-resistant
ACCATAAGCAGTAGGCACTCGCGT	0	18	RDR2-sensitive
ACCATACTGTAATTCGCGCGGACT	0	13	RDR2-sensitive
ACCATAGACTATGACGACGAGCTG	0	54	RDR2-sensitive
ACCATAGCTCATGGACGGTAACAG	0	12	RDR2-sensitive
ACCATAGGCTGGTCTGAACGGCAA	1	16	RDR2-sensitive
ACCATAGTTGACTGGGAGACGACG	0	9	RDR2-sensitive
ACCATATCGCGAGAGCTCCGGCAG	0	9	RDR2-sensitive
ACCATATCGCTTCGTCGGGGGCTT	0	19	RDR2-sensitive
ACCATCACATATCGGGGTCGTTGG	0	9	RDR2-sensitive
ACCATCAGCGTGGGCCGTCCGGAC	0	11	RDR2-sensitive
ACCATCAGTGCGGATTGTCCGGAC	0	9	RDR2-sensitive
ACCATCATTATCGTGCGGAGGTAT	3	22	RDR2-sensitive
ACCATCCGGATCTAGAACGCGCAT	0	57	RDR2-sensitive
ACCATCCGGCCTCTGACCACGGAC	0	9	RDR2-sensitive
ACCATCCGGTCTGTGGATAGAGGA	0	18	RDR2-sensitive
ACCATCGAGTTGCGATTGTCGGCG	0	10	RDR2-sensitive
ACCATCGCAGGACTGACCGGCTGC	72	0	RDR2-resistant
ACCATCGCGGGCTGTACGACGGTT	0	26	RDR2-sensitive
ACCATCGGACATAAGCCTTCGGCC	0	15	RDR2-sensitive
ACCATCGGGCTGGTTCGGTGCACC	0	21	RDR2-sensitive
ACCATCGGTGTCCGATCTGACGGC	0	15	RDR2-sensitive
ACCATCGTCGTACTTATACGGTCC	0	9	RDR2-sensitive
ACCATCGTGGATTGTTCGGCCCAG	0	12	RDR2-sensitive
ACCATGATGGTAGAAATTCGTCGC	0	13	RDR2-sensitive
ACCATGCTCTGTGAACTCGCGGCT	14	0	RDR2-resistant
ACCATGCTGCTTGGGACCGACGGA	0	10	RDR2-sensitive
ACCATGGACGCATGCGCGACTCGA	0	14	RDR2-sensitive

ACCATGTTTGGGAGAGGCTGACAG	0	10	RDR2-sensitive
ACCATTAATGGCATGGTCGGTCGG	0	15	RDR2-sensitive
ACCATTAGATCTAGATCGGATGCA	2	45	RDR2-sensitive
ACCATTAGTTTCACCGGTCGTCGT	0	21	RDR2-sensitive
ACCATTGGACTCGGTTCTTCGGTT	21	1	RDR2-resistant
ACCATTGGACTGTCCGGTGTGCCC	1	27	RDR2-sensitive
ACCATTGGGTCTTGATCGGATGTT	0	10	RDR2-sensitive
ACCATTGTAAGTACTGTGACGGAA	1	13	RDR2-sensitive
ACCATTTAAGATCATGTTCGGTTC	0	9	RDR2-sensitive
ACCATTTAGCTACAGTCGGCGACA	2	21	RDR2-sensitive
ACCATTTGTCTAGGTTGTTGGACC	59	4	RDR2-resistant
ACCATTTGTGGCACTGAGCACGGT	1	24	RDR2-sensitive
ACCATTTTATGGGTCAGAGGACTG	14	0	RDR2-resistant
ACCATTTTGCTACAGTCGGCGACA	0	17	RDR2-sensitive
ACCCAAAGTTGACTGGGAGACGAC	1	31	RDR2-sensitive
ACCCAAGACGGATCTAGACGACAT	0	29	RDR2-sensitive
ACCCAAGACGGATCTAGACGACGT	2	83	RDR2-sensitive
ACCCAAGTGGCTCTGACGTGGCAT	0	18	RDR2-sensitive
ACCCAATCTTGGTCGTTCGTTGGC	0	58	RDR2-sensitive
ACCCAATGGTCGGATTAGAAGGGC	0	9	RDR2-sensitive
ACCCACACCCATACAGGTCGTCGG	1	12	RDR2-sensitive
ACCCACACGACGGACACCAAGCGG	0	11	RDR2-sensitive
ACCCACAGCTGTAGCGGAGGCCAA	0	11	RDR2-sensitive
ACCCACAGGTATAATCACGGACGG	0	11	RDR2-sensitive
ACCCACAGGTATAGTCGCGGACGG	0	17	RDR2-sensitive
ACCCACAGGTATGGTCACGGACGA	0	9	RDR2-sensitive
ACCCACCCGTTGTATTGGTCGGAC	0	23	RDR2-sensitive
ACCCACGGATCTGAGGAAGAAGAC	0	9	RDR2-sensitive
ACCCACGTGTAGAATATTTAGGCC	0	9	RDR2-sensitive
ACCCACTCTGGACCCTTCAGACGA	0	30	RDR2-sensitive
ACCCACTGTGGTTATCATGTCAAT	17	0	RDR2-resistant
ACCCAGAGACTGCAGAAGTGTGGC	44	5	RDR2-resistant
ACCCAGAGGTTTAGATCGTTGGAT	0	13	RDR2-sensitive
ACCCAGATACGGCTGCACGGTACT	0	10	RDR2-sensitive
ACCCAGCATGCTGATCGGACGGGA	0	14	RDR2-sensitive
ACCCAGCCTGATCGACGAGGACGG	0	61	RDR2-sensitive
ACCCAGCTGGTGTGATGTCGTGGC	6	21	RDR2-sensitive
ACCCAGTTGGGCTGTAAGTGGCGT	0	13	RDR2-sensitive
ACCCATAAGGATGGTCGCTGTAAG	0	9	RDR2-sensitive
ACCCATCGGGTATCGGATCGCGGG	0	19	RDR2-sensitive
ACCCATCGGGTATCGGGCGGATAT	0	12	RDR2-sensitive
ACCCATCGGGTATCGGGTCGCGGG	0	13	RDR2-sensitive
ACCCATCTGGACTGGGGCACGCAG	1	12	RDR2-sensitive
ACCCATCTGGACTGGGGCACGCGG	0	30	RDR2-sensitive
ACCCATCTGGGCTGGAGCACGCGG	1	17	RDR2-sensitive
ACCCATCTGGGCTGGGACACGCAG	0	15	RDR2-sensitive
ACCCATCTGGGCTGGGACACGCAT	0	9	RDR2-sensitive
ACCCATCTGGGCTGGGACACGCGG	1	90	RDR2-sensitive
ACCCATCTGGGCTGGGACGCGCAA	0	10	RDR2-sensitive

ACCCATCTGGGCTGGGACGCGCAG	0	40	RDR2-sensitive
ACCCATCTGGGCTGGGGCACGCAG	9	184	RDR2-sensitive
ACCCATCTGGGCTGGGGCACGCGG	5	145	RDR2-sensitive
ACCCATGAACCCGTCGGGTATACA	0	11	RDR2-sensitive
ACCCATGTAATCGGGCTGAGGCAC	1	40	RDR2-sensitive
ACCCATTCCGAAAACGGGTGTCGG	0	29	RDR2-sensitive
ACCCCAACGGTCGGCTGACATGGC	0	12	RDR2-sensitive
ACCCCAACGGTCGGCTGACGTGGC	1	32	RDR2-sensitive
ACCCCAACGGTCTGCTGACGTGGC	0	15	RDR2-sensitive
ACCCCAACTGTCGGCTGACGTGGC	0	9	RDR2-sensitive
ACCCCAGGTTGTGGACTGTCCGGC	2	113	RDR2-sensitive
ACCCCATATGTAGAGTGCATGCAA	1	29	RDR2-sensitive
ACCCCCATGGGAAAGTGTCGGACC	0	10	RDR2-sensitive
ACCCCCGGACAGAAGGCAACTAGG	0	12	RDR2-sensitive
ACCCCCGGATGAGAAATGAAGGCA	0	10	RDR2-sensitive
ACCCCCTGACGCAGCACCGTTGGA	0	9	RDR2-sensitive
ACCCCCTGTAGACCCGGCTGGCGC	0	65	RDR2-sensitive
ACCCCGGACAGGAGACGCAGTCAT	0	11	RDR2-sensitive
ACCCCGGATCGTAGGCGCAGAGGC	0	14	RDR2-sensitive
ACCCCGTTAGCTGAATGAGGACGA	0	9	RDR2-sensitive
ACCCCTAGGCGTGGACTGTCCGGA	0	11	RDR2-sensitive
ACCCCTCAGGGGCAAAGTCGGTAT	0	13	RDR2-sensitive
ACCCCTCGGGCGTGGTAGCGTCGC	2	109	RDR2-sensitive
ACCCCTGATCAGTAGTGGGCGCGG	0	9	RDR2-sensitive
ACCCCTGGATCTAGCGGACGGCTG	0	10	RDR2-sensitive
ACCCCTGTAGATGGTATGGCTTGA	0	24	RDR2-sensitive
ACCCCTTGGTGAAGATGTCGGCGA	0	15	RDR2-sensitive
ACCCCTTGTTGGTTCTTTGGCATT	340	0	RDR2-resistant
ACCCGACAGGCTGGTTGGACGCGC	0	10	RDR2-sensitive
ACCCGACGCACGGAACGATGACGG	0	20	RDR2-sensitive
ACCCGAGGACGAAGACGCCGGTGC	0	13	RDR2-sensitive
ACCCGAGGCTTTAGAATAGGGCTG	0	18	RDR2-sensitive
ACCCGAGTCGTGAAGAAGATGCTG	0	18	RDR2-sensitive
ACCCGATAGACTGGTTAGGCGCGC	0	9	RDR2-sensitive
ACCCGATGCACAGAACGACGACGG	0	13	RDR2-sensitive
ACCCGCACACATCCAGATCGTCGT	0	37	RDR2-sensitive
ACCCGCAGAGGACCTGGGCGCAGC	0	16	RDR2-sensitive
ACCCGCATAATATGCAGAGGCTGG	0	9	RDR2-sensitive
ACCCGCATAGAACCGGACTCGGAC	0	10	RDR2-sensitive
ACCCGCCTCGGACGGAAACGGCCT	0	10	RDR2-sensitive
ACCCGCGGGTCTGTTATTGGGCAA	8	24	RDR2-sensitive
ACCCGCGGGTTTAGACAGTTCGGC	0	18	RDR2-sensitive
ACCCGCGTAGAACCAGACCGTGCT	0	13	RDR2-sensitive
ACCCGCGTTGATGCAGAGGATCGT	0	9	RDR2-sensitive
ACCCGGAACGATGCAAAGCAGCAC	0	11	RDR2-sensitive
ACCCGGACATGTGGTTGGTGGCCT	26	3	RDR2-resistant
ACCCGGACCGCTGAGAACACGGGA	0	17	RDR2-sensitive
ACCCGGACGCCGTGGCGTTGACGT	0	18	RDR2-sensitive
ACCCGGACGGTCTACACTAATGCC	0	17	RDR2-sensitive

ACCCGGACTGTTCGGCCCATAGGC	1	14	RDR2-sensitive
ACCCGGACTTTGAGAAGAGGGATG	1	28	RDR2-sensitive
ACCCGGAGACTGCAGAAGTATGGC	0	9	RDR2-sensitive
ACCCGGAGAGGATTGGAGGGGCTA	3	19	RDR2-sensitive
ACCCGGAGGCAGAACCAGGGGAGG	0	24	RDR2-sensitive
ACCCGGATAGAATCGTATCGGATA	0	10	RDR2-sensitive
ACCCGGATATACAGCAGTCGGATA	0	13	RDR2-sensitive
ACCCGGCACGGTAGAATAAACGGG	0	21	RDR2-sensitive
ACCCGGCACGGTAGAATAAGCGGA	0	64	RDR2-sensitive
ACCCGGCACGGTAGAATAAGCGGG	7	574	RDR2-sensitive
ACCCGGCATGGTAGAATAAGCGGG	0	15	RDR2-sensitive
ACCCGGCTTGTAGTTGTGCTTAAG	3	18	RDR2-sensitive
ACCCGGGAGCAGGATGAACGAGCG	33	4	RDR2-resistant
ACCCGGGCTGTGAGAGAAGAGGAA	0	63	RDR2-sensitive
ACCCGGGCTGTGAGAGGAGGGGAA	4	142	RDR2-sensitive
ACCCGGGTGTAGAGATGGATTCGG	0	9	RDR2-sensitive
ACCCGTAGACCTGAACTCGACGGG	1	13	RDR2-sensitive
ACCCGTAGATCTCGAGCAAGGCAG	0	31	RDR2-sensitive
ACCCGTAGGTCTGTTATTGGGCAA	1	20	RDR2-sensitive
ACCCGTCGCGGATAGTGGGTACGG	0	13	RDR2-sensitive
ACCCGTCGGGTATTCGGATTTCGG	0	23	RDR2-sensitive
ACCCGTCGTAGAGTTACTCGGCAT	0	10	RDR2-sensitive
ACCCGTCGTCTTGCTGGACGCGCT	0	16	RDR2-sensitive
ACCCGTCTTTCTGTAGGGTGGCCA	1	22	RDR2-sensitive
ACCCGTGCTGCAAGCTTTATGGTT	420	1	RDR2-resistant
ACCCGTGGATCTGTTATTGGGCAA	0	10	RDR2-sensitive
ACCCGTGGGTATAGTCATGGACGG	1	49	RDR2-sensitive
ACCCGTGGGTCTGTAATTGGGCAA	0	32	RDR2-sensitive
ACCCGTGGGTCTGTTATTAGGCAA	1	19	RDR2-sensitive
ACCCGTGGGTCTGTTATTGGCAAA	1	20	RDR2-sensitive
ACCCGTGGGTCTGTTATTGGGCAA	87	714	RDR2-sensitive
ACCCGTGGGTCTGTTATTGGGCAC	2	17	RDR2-sensitive
ACCCGTGGGTCTGTTATTGGGCAT	5	23	RDR2-sensitive
ACCCGTGGGTCTGTTATTTGGCAA	0	17	RDR2-sensitive
ACCCGTGGGTCTGTTGTTGGGCAA	0	27	RDR2-sensitive
ACCCGTTGCACGTCTGTAGATGGC	0	11	RDR2-sensitive
ACCCGTTGGTCTGTTATTGGGCAA	1	21	RDR2-sensitive
ACCCGTTTTAGAGTTTGTCGGTCG	0	15	RDR2-sensitive
ACCCGTTTTGATGCGGGGTCGGAT	0	19	RDR2-sensitive
ACCCTAACCCTAATGGGCCGGCAG	0	10	RDR2-sensitive
ACCCTAACCTGGACTGAGAAGGCG	0	9	RDR2-sensitive
ACCCTAACTGACTCTGCGCACGCG	0	9	RDR2-sensitive
ACCCTAAGAACCGTCGGAAACAGT	0	13	RDR2-sensitive
ACCCTAAGCCACAGATCGTTCGTG	0	9	RDR2-sensitive
ACCCTAATGAGTCGGGCGTCCGGA	0	10	RDR2-sensitive
ACCCTAATTCGTGGTGGTCTCGGA	0	42	RDR2-sensitive
ACCCTAATTCGTGGTGGTCTCGGG	0	18	RDR2-sensitive
ACCCTACTGTAGTCAGCGGAGCAT	0	10	RDR2-sensitive
ACCCTAGAACCAAGCCTGCGGCAG	0	36	RDR2-sensitive

ACCCTAGAACTTACTCCAGGAGAG	0	9	RDR2-sensitive
ACCCTAGAGTCGTCGGCTGCGGCT	0	10	RDR2-sensitive
ACCCTAGTGTTGTCGGCTGCGGTT	0	9	RDR2-sensitive
ACCCTATGGGCTGGCCCACCGGAC	1	16	RDR2-sensitive
ACCCTATGGGCTGGTCTACCGGAC	0	23	RDR2-sensitive
ACCCTATTTGAGGGATCGTCCCGT	0	10	RDR2-sensitive
ACCCTCAAGACGCCTAATTCTCAG	0	25	RDR2-sensitive
ACCCTCAGGGTCGGACGAGGCGGA	0	46	RDR2-sensitive
ACCCTCAGTAAAGGTTGTCGGTAT	0	10	RDR2-sensitive
ACCCTCCCATACGCTCTGATGATC	0	10	RDR2-sensitive
ACCCTCGGATTCCGCAGACGATGC	0	37	RDR2-sensitive
ACCCTCGGATTCGGCAGACGATGC	0	27	RDR2-sensitive
ACCCTCTACAGATAGCGGACGCAT	0	20	RDR2-sensitive
ACCCTCTGCGACGGTGTGGACGGT	0	12	RDR2-sensitive
ACCCTCTGGAGCCCAGAACGTGTA	0	15	RDR2-sensitive
ACCCTCTGTAGCAGCGCGGACGAT	0	15	RDR2-sensitive
ACCCTGAAAGAACTTGCTCGGTAT	0	10	RDR2-sensitive
ACCCTGAGAGTCACGTAGAGACGA	0	9	RDR2-sensitive
ACCCTGCTGGAGTTCGGACCTACC	0	11	RDR2-sensitive
ACCCTGGAACTGCGGAGGCCGAAT	14	0	RDR2-resistant
ACCCTGGACCAGGCTCTGCAGGCA	1	35	RDR2-sensitive
ACCCTGGAGGATGAGGTCGAGGAC	427	1	RDR2-resistant
ACCCTGGCCAGGCTCTGCAGGCGT	0	37	RDR2-sensitive
ACCCTGGGCCAGGCTCTGCAGGCG	0	12	RDR2-sensitive
ACCCTGGGCCAGGCTCTGTAGGCA	0	9	RDR2-sensitive
ACCCTGGTTGACCTGATACGGTAT	0	12	RDR2-sensitive
ACCCTGTAGAGTGAGGATTTGGGT	0	10	RDR2-sensitive
ACCCTGTAGGGCTGATTTGGTGAC	0	9	RDR2-sensitive
ACCCTGTGGATACCCGATCGGCAT	0	25	RDR2-sensitive
ACCCTGTTGGGACCTGACAAGCAG	0	12	RDR2-sensitive
ACCCTTAACCGTGTCGGGCCGGGC	0	13	RDR2-sensitive
ACCCTTACACTGGATTGTTCGGAT	2	46	RDR2-sensitive
ACCCTTCGCAGGACTCGGCGCTTT	0	9	RDR2-sensitive
ACCCTTCGGACTCGGAGCGAGCAT	0	20	RDR2-sensitive
ACCCTTGACTATTGGACTAGGCGA	30	0	RDR2-resistant
ACCCTTTACAGTAGTGCGTCTGAC	0	32	RDR2-sensitive
ACCGAAAATGGACGGTAGCCGGAC	0	10	RDR2-sensitive
ACCGAAACCGTAGAGAGGGCCACC	26	0	RDR2-resistant
ACCGAACAAGCCCTGACGGAACTC	0	9	RDR2-sensitive
ACCGAACACTGGACGTAGAGACGT	0	29	RDR2-sensitive
ACCGAACAGCGGACAGGAGGGCTA	0	15	RDR2-sensitive
ACCGAACAGGGCCTTAGACGGAAC	0	14	RDR2-sensitive
ACCGAACCGAACCGAACCGAAGAA	0	9	RDR2-sensitive
ACCGAACCGAACCGAAGAACCGAA	0	17	RDR2-sensitive
ACCGAACCGGAAGCACTGAGACGA	0	22	RDR2-sensitive
ACCGAACTATAGAGGAAGGGCAAA	0	13	RDR2-sensitive
ACCGAACTCGCTGTGGAACACGGG	0	9	RDR2-sensitive
ACCGAACTGGCGAAGCACGGCTGC	3	67	RDR2-sensitive
ACCGAACTGTACAGATGCGGAGCT	0	16	RDR2-sensitive

ACCGAACTGTTGTGTTGGCTTCAT	0	10	RDR2-sensitive
ACCGAAGACTAGATGTAGGGACGT	0	19	RDR2-sensitive
ACCGAAGACTGAAAGTAGGGACGT	0	11	RDR2-sensitive
ACCGAAGACTGGAAGTAGGGACGT	0	81	RDR2-sensitive
ACCGAAGACTGGATGTAGAGACGT	0	50	RDR2-sensitive
ACCGAAGGATAGAGCAGACGAGAC	0	53	RDR2-sensitive
ACCGAAGGCAGAACCAGGGGACAC	0	20	RDR2-sensitive
ACCGAAGGCAGAACCAGGGGAGGC	0	28	RDR2-sensitive
ACCGAAGGGGATTGTAGGGGCTAA	0	9	RDR2-sensitive
ACCGAATACTGGATGTAGGGACGT	0	55	RDR2-sensitive
ACCGAATGCAGAACCATGGTGCAC	0	11	RDR2-sensitive
ACCGAATTCGTAGAGACCGCGCAT	0	14	RDR2-sensitive
ACCGAATTCGTAGGGATCGCTCGA	0	10	RDR2-sensitive
ACCGACAAGGGCCGTCTGACGCAC	0	12	RDR2-sensitive
ACCGACACAGCTTGCAACGACGTT	0	17	RDR2-sensitive
ACCGACACGTAGAGGGCGCTGTAG	0	74	RDR2-sensitive
ACCGACACGTAGGAGGCGCTGTAG	0	65	RDR2-sensitive
ACCGACACGTAGGGGACGCTGTAA	0	23	RDR2-sensitive
ACCGACACGTAGGGGACGCTGTAG	0	56	RDR2-sensitive
ACCGACACGTAGGGGGCGCTGTAG	1	48	RDR2-sensitive
ACCGACACTGTGGTGATAACTGGT	0	18	RDR2-sensitive
ACCGACAGGCTGGACATTAGAAGA	0	49	RDR2-sensitive
ACCGACAGTTGACTTTATAGGCTA	0	36	RDR2-sensitive
ACCGACATGTAGGGGGCGCTGTAG	0	9	RDR2-sensitive
ACCGACCAGGTGCTCTGACCGGCG	0	55	RDR2-sensitive
ACCGACCCTGGCAGAACAGTGCAC	0	10	RDR2-sensitive
ACCGACGAGCTAGATCTGCGACGG	0	12	RDR2-sensitive
ACCGACGAGGACCCAGAGTTCGGA	0	9	RDR2-sensitive
ACCGACGGATGCTGTGATGGTATT	0	9	RDR2-sensitive
ACCGACGTCGAGCAGGAAGCGTGA	0	9	RDR2-sensitive
ACCGACGTGGAACCGAGCTCGGCG	0	12	RDR2-sensitive
ACCGACTGACCAAATTGCAGGGGC	0	15	RDR2-sensitive
ACCGACTGCGTACAACTACATCGA	0	50	RDR2-sensitive
ACCGACTGGACCAGGCTCACGGTT	2	32	RDR2-sensitive
ACCGACTGTAAAATTTTATCGGTG	0	13	RDR2-sensitive
ACCGACTGTAGCTCGATGGTTCGT	5	67	RDR2-sensitive
ACCGACTGTCCGAAGTACTCGGCA	0	15	RDR2-sensitive
ACCGACTGTGCTATTTCTAGTGGC	2	38	RDR2-sensitive
ACCGACTGTGGGGGCAGATATCAC	0	15	RDR2-sensitive
ACCGACTGTGTTATTTCTAGTGGC	21	106	RDR2-sensitive
ACCGACTGTGTTGGAGCCGGCTCT	0	16	RDR2-sensitive
ACCGACTGTTGATCCAAGGGCGCC	0	9	RDR2-sensitive
ACCGACTGTTTGGTTACGCGGCCG	0	24	RDR2-sensitive
ACCGACTTCGTACGGCTACATCGA	1	46	RDR2-sensitive
ACCGACTTGAGTCCGAAGATGCCT	0	12	RDR2-sensitive
ACCGAGACCAGGGTCGGACGTCGA	0	13	RDR2-sensitive
ACCGAGACTACATGTAGGGACGTT	0	9	RDR2-sensitive
ACCGAGACTGCATGCGTGGACGTG	2	14	RDR2-sensitive
ACCGAGAGTAGGACACTCGGCATA	0	12	RDR2-sensitive

ACCGAGAGTAGGACGGCGCTCGAC	0	14	RDR2-sensitive
ACCGAGATCGCGGTCCACGGACGG	0	20	RDR2-sensitive
ACCGAGATTTGTGGTGGACTGCAT	0	91	RDR2-sensitive
ACCGAGCAAGTTCCGTAGACGTAG	0	16	RDR2-sensitive
ACCGAGCGCAGGACGTTGCCGAGT	0	20	RDR2-sensitive
ACCGAGCTAGGATGTCAGCTCAGC	0	12	RDR2-sensitive
ACCGAGCTCGGCGCCATAGATCAT	2	18	RDR2-sensitive
ACCGAGCTCGGCGCCATAGATCTT	0	23	RDR2-sensitive
ACCGAGCTCGGCGTCATAGATCTT	0	14	RDR2-sensitive
ACCGAGCTTGGACTGCGGCGTAGG	1	49	RDR2-sensitive
ACCGAGGTCGCGGCCCATGGACGG	1	12	RDR2-sensitive
ACCGAGTGGACGAAGAGTGATGGC	0	11	RDR2-sensitive
ACCGAGTGTAGCACGTGGCAAAGA	0	10	RDR2-sensitive
ACCGATAGACTACAGAAATAGGCC	15	0	RDR2-resistant
ACCGATAGACTATAGAAATAGGCC	24	0	RDR2-resistant
ACCGATAGTAGAACACTCGGCATA	0	11	RDR2-sensitive
ACCGATATCAGGACACTCGGCATA	1	108	RDR2-sensitive
ACCGATCCGATCTGACGATAGGCA	0	22	RDR2-sensitive
ACCGATCCGGATCTGCTTGGAGGC	0	20	RDR2-sensitive
ACCGATCGTTTGAGTATGGGCATC	0	10	RDR2-sensitive
ACCGATCTCTCGGGACTGGCGTCA	0	9	RDR2-sensitive
ACCGATCTGTAGTGGAACAGGCTC	0	28	RDR2-sensitive
ACCGATGACTAGACACACGTGCTG	0	54	RDR2-sensitive
ACCGATGACTGAAAGTAGGGACGT	0	15	RDR2-sensitive
ACCGATGACTGGAAGTAGGGACGT	1	89	RDR2-sensitive
ACCGATGACTGGATGCAGGGACGT	0	16	RDR2-sensitive
ACCGATGACTGGATGTAGGGACGT	0	11	RDR2-sensitive
ACCGATGACTGGGCACACGTGCTG	0	16	RDR2-sensitive
ACCGATGACTGTAAGTAGGGATGT	0	22	RDR2-sensitive
ACCGATGAGGACCTAGAAGCCGGA	2	17	RDR2-sensitive
ACCGATGATTGGAAGTAGGGACGT	0	9	RDR2-sensitive
ACCGATGCTGCGATCCTGAAGACG	0	13	RDR2-sensitive
ACCGATGGCTTGACTCGGCTCGTT	0	14	RDR2-sensitive
ACCGATTGCTGGACTGGATGGTGC	0	27	RDR2-sensitive
ACCGCAAGCAGCAGACTCGAACAA	0	9	RDR2-sensitive
ACCGCACTGGTGTAGCTCGGCGTC	1	30	RDR2-sensitive
ACCGCAGACTGAAAGTAGGGACGT	0	9	RDR2-sensitive
ACCGCAGAGCGGATGAGGATGATT	33	2	RDR2-resistant
ACCGCAGGTAGATTTGGACGGCGC	0	20	RDR2-sensitive
ACCGCATCCGACGACGACGCCATC	1	14	RDR2-sensitive
ACCGCATGCGTCGCTGTGTTCGGC	0	15	RDR2-sensitive
ACCGCCAGATGCGTTGATCGTGGA	0	11	RDR2-sensitive
ACCGCCCACTCTGCAACGGCCCAG	0	24	RDR2-sensitive
ACCGCCTCGTAGCAACGCACGGAC	0	15	RDR2-sensitive
ACCGCCTGGGCTGTTGGATGGTAA	0	17	RDR2-sensitive
ACCGCGAACGTGGACCGTCCAGAC	0	12	RDR2-sensitive
ACCGCGATTGTAACGGCCTGCCAA	0	9	RDR2-sensitive
ACCGCGCACTGTAGACGACGGCCC	2	44	RDR2-sensitive
ACCGCGCACTGTAGACGACGGCTC	1	29	RDR2-sensitive

ACCGCGCCTGTAGACCGGACGGTC	0	11	RDR2-sensitive
ACCGCGCTCTGAACCAGACGACCC	0	9	RDR2-sensitive
ACCGCGCTCTGTCATGCTCGACGG	0	14	RDR2-sensitive
ACCGCGGAACAGGATTCGGCCCAC	1	14	RDR2-sensitive
ACCGCGGACCGTCCGGCCTCAGGG	0	23	RDR2-sensitive
ACCGCGGATTGTCTGCGCCGGCAC	0	16	RDR2-sensitive
ACCGCGGGACTGCGGCATCTTCAC	0	10	RDR2-sensitive
ACCGCGTACACGTATCGTTCGGAA	0	11	RDR2-sensitive
ACCGCTCGACTGCGTAGAAGAGGA	0	11	RDR2-sensitive
ACCGCTCGCTTTTGACTGGGACGG	0	16	RDR2-sensitive
ACCGCTCTGCAGCACGTATGACGT	0	37	RDR2-sensitive
ACCGCTCTGGCCTCTGGACGCGGC	0	52	RDR2-sensitive
ACCGCTGAAGACGGTCGGACTCTC	0	13	RDR2-sensitive
ACCGCTGTAGAGCGAGTCGGCGGC	1	15	RDR2-sensitive
ACCGCTTTCTGTCGACGGACGCAC	0	9	RDR2-sensitive
ACCGGAAACTCCTGACGTAGACGA	17	1	RDR2-resistant
ACCGGAACGAACCATCTGACGGTG	0	32	RDR2-sensitive
ACCGGAACTGTTTCGGCCAGGATT	1	31	RDR2-sensitive
ACCGGAATCGCGTTCTTTGCCGAG	0	10	RDR2-sensitive
ACCGGAATGGTGGCAGAACAAGGA	0	17	RDR2-sensitive
ACCGGACAAGCAGCGCATTTCCGA	0	11	RDR2-sensitive
ACCGGACACAACCCTGCAAGGACG	0	12	RDR2-sensitive
ACCGGACACTGCACAGTAGATGTC	1	17	RDR2-sensitive
ACCGGACACTGCATAGTGCAACGA	0	13	RDR2-sensitive
ACCGGACAGAACGGTTTACAGACC	0	9	RDR2-sensitive
ACCGGACAGGACGCTTTAGCGGTC	0	23	RDR2-sensitive
ACCGGACAGGATTAGAGGGGCTAA	0	15	RDR2-sensitive
ACCGGACAGGTCCTGTAGACGGTC	2	14	RDR2-sensitive
ACCGGACAGTCACTGTAGACGGTC	6	42	RDR2-sensitive
ACCGGACAGTCCAGCGTGTGGCAC	0	9	RDR2-sensitive
ACCGGACAGTCCGGTGCACACCGG	30	153	RDR2-sensitive
ACCGGACAGTCCGGTGTACACCGG	7	23	RDR2-sensitive
ACCGGACAGTCTGCGCAGAGAGGC	0	20	RDR2-sensitive
ACCGGACAGTCTGGCAACGGTCGA	0	9	RDR2-sensitive
ACCGGACAGTCTGGTGGTAGGCAC	0	17	RDR2-sensitive
ACCGGACAGTGAACAGTAACGGAT	1	194	RDR2-sensitive
ACCGGACAGTGAACAGTGCGCGGG	0	23	RDR2-sensitive
ACCGGACAGTGCAGTAACGACTAG	0	9	RDR2-sensitive
ACCGGACATCCAGGCGGGCCCAGA	0	12	RDR2-sensitive
ACCGGACCAGCTCGAGGTAGACGA	0	11	RDR2-sensitive
ACCGGACCATCAGGCGATATGCAG	0	11	RDR2-sensitive
ACCGGACCCAGGTGGTTGGCTGGA	0	17	RDR2-sensitive
ACCGGACCCTATAGCGGACGTCGT	0	11	RDR2-sensitive
ACCGGACCCTTTTTGGCTTATTGT	4	23	RDR2-sensitive
ACCGGACCGAGCACGCAGAGACGA	2	14	RDR2-sensitive
ACCGGACCGAGTACTGTTAGCGGT	0	10	RDR2-sensitive
ACCGGACCGATTATGCAGACGCCT	1	14	RDR2-sensitive
ACCGGACCGCCTGTACAGAGGATC	0	14	RDR2-sensitive
ACCGGACCGCCTGTATAGAGGATA	1	29	RDR2-sensitive

ACCGGACCGGACGCATGCCTGCAC	0	37	RDR2-sensitive
ACCGGACCGGCTCGACACGGCTAG	0	25	RDR2-sensitive
ACCGGACCTAGGTGGTTGGCTGGA	0	27	RDR2-sensitive
ACCGGACCTCTAGCATGGCGTAGA	0	18	RDR2-sensitive
ACCGGACCTGGCAGACACTAGGCG	0	15	RDR2-sensitive
ACCGGACGACCCATACTGTTTGGA	0	26	RDR2-sensitive
ACCGGACGCACCGATTTTCGGAAC	0	12	RDR2-sensitive
ACCGGACTATACAGTACACACCGG	0	10	RDR2-sensitive
ACCGGACTATCCGATGGGACGCAT	0	31	RDR2-sensitive
ACCGGACTGGCACGGTTTGAAGGT	0	14	RDR2-sensitive
ACCGGACTGTCCGGTGCACCATAC	1	15	RDR2-sensitive
ACCGGACTGTCCGGTGCGCCCATC	2	24	RDR2-sensitive
ACCGGACTGTCTGGTGTGCCCATC	0	9	RDR2-sensitive
ACCGGACTGTTAGGTGTACCACCC	0	25	RDR2-sensitive
ACCGGACTGTTCGGTGCGCCCATC	0	17	RDR2-sensitive
ACCGGACTGTTCGGTGTGCCACGT	0	12	RDR2-sensitive
ACCGGAGAGGATTGTAGAAGGGGA	0	11	RDR2-sensitive
ACCGGAGGACCTGTAGAGACGGAG	0	13	RDR2-sensitive
ACCGGAGGATGACGTACATGACGT	0	18	RDR2-sensitive
ACCGGAGGATGACGTGCAGGACAT	0	18	RDR2-sensitive
ACCGGAGGCAGAACACAAAAAGGC	0	20	RDR2-sensitive
ACCGGAGGGGATTGGAGGGGCTAA	1	13	RDR2-sensitive
ACCGGAGTAGACTAGAGAGGCTAA	0	43	RDR2-sensitive
ACCGGAGTCGGCATACCACTCGGA	0	9	RDR2-sensitive
ACCGGAGTCTGGATGCGAAGGCCT	24	1	RDR2-resistant
ACCGGAGTGAATTGGAGGGGCTAA	0	10	RDR2-sensitive
ACCGGATATCGACGTAGCATGCCA	0	10	RDR2-sensitive
ACCGGATCGAAGATCGGCATTCCC	0	12	RDR2-sensitive
ACCGGATCTGCGACAGAGCGGCAC	1	13	RDR2-sensitive
ACCGGATGAAGAAGCAGTCACGAC	1	21	RDR2-sensitive
ACCGGATGACTGAGCGGAGCGGCG	0	12	RDR2-sensitive
ACCGGATGTAGGGACGTGCAGCCC	0	9	RDR2-sensitive
ACCGGATGTCGACGTGGCGTGCCA	0	13	RDR2-sensitive
ACCGGATTAGTAGGCTAGATGGTT	23	3	RDR2-resistant
ACCGGATTGTTCAGTGCTACCCAG	0	22	RDR2-sensitive
ACCGGCAACAGAAGGTGGACAACA	0	11	RDR2-sensitive
ACCGGCACGACTAGACGAAGGCAC	0	16	RDR2-sensitive
ACCGGCACGGACCAACGAAGGCAC	0	33	RDR2-sensitive
ACCGGCAGAAACGAACAAGGCCTC	0	10	RDR2-sensitive
ACCGGCATACCCTGTAAAACCAAC	0	11	RDR2-sensitive
ACCGGCATGACGTGCATGAGCCAT	0	9	RDR2-sensitive
ACCGGCATGTAGATGTGGCCCAGC	5	20	RDR2-sensitive
ACCGGCCACTGAAAGAGACGGAAC	0	24	RDR2-sensitive
ACCGGCCACTGAACTCGAAGACGA	0	9	RDR2-sensitive
ACCGGCCTGACAGAAAGACAGCGC	0	9	RDR2-sensitive
ACCGGCCTGACAGAAGGACAGCAC	0	10	RDR2-sensitive
ACCGGCCTGACAGAAGGACAGCGC	1	45	RDR2-sensitive
ACCGGCCTGACAGAAGGACAGCGT	0	18	RDR2-sensitive
ACCGGCCTGGGTGGGACGGTTGGC	1	34	RDR2-sensitive

ACCGGCCTGGTTGCAGGAGATGTT	0	9	RDR2-sensitive
ACCGGCCTGTTAGTGATGGGGCTA	0	12	RDR2-sensitive
ACCGGCCTGTTGACCGTGTCGGAC	0	40	RDR2-sensitive
ACCGGCGGCAGGCTTGAGGCAGGA	0	14	RDR2-sensitive
ACCGGCTAGTGTAGAGGACACATC	0	9	RDR2-sensitive
ACCGGCTCGCTAGACACGGCTCAT	0	9	RDR2-sensitive
ACCGGCTCGGATTAGTTAGCAGGC	0	9	RDR2-sensitive
ACCGGCTCTGACCGCGTTTTGGCA	0	12	RDR2-sensitive
ACCGGCTCTGACTCCTGGAGAAGC	0	12	RDR2-sensitive
ACCGGCTGGAACAGTAGAAGTAGA	0	12	RDR2-sensitive
ACCGGCTGTGTAGAGGCTAACTAG	0	15	RDR2-sensitive
ACCGGGACGAGCCAGATGAGACGC	0	10	RDR2-sensitive
ACCGGGACTGGGACTCAGGAGACG	0	10	RDR2-sensitive
ACCGGGATTTCTACTGGGGTCGGA	0	18	RDR2-sensitive
ACCGGGATTTGTAGTGGGCAGTAG	0	14	RDR2-sensitive
ACCGGGCCATGACTGAGGACGTGA	0	20	RDR2-sensitive
ACCGGGCCGAATGCATGACGGGCT	0	71	RDR2-sensitive
ACCGGGCCGGGCTGTTCGACAGGC	0	9	RDR2-sensitive
ACCGGGCTAGCACGGCATGAAGGC	0	11	RDR2-sensitive
ACCGGGCTAGGCCTGACCCGCATA	0	12	RDR2-sensitive
ACCGGGCTCCAGAACGGTCTCCGC	0	14	RDR2-sensitive
ACCGGGCTGGACGGTCGAGTGGGA	0	10	RDR2-sensitive
ACCGGGCTGGGTTGGTACGGGCCT	0	9	RDR2-sensitive
ACCGGGCTGTCCGATGGGTAGCAC	0	12	RDR2-sensitive
ACCGGGCTGTGCCGAGGCACTAAT	0	23	RDR2-sensitive
ACCGGGGCGAGCCAGATGGGACGC	0	10	RDR2-sensitive
ACCGGGTGTAGGGTTCCTTACGGC	0	10	RDR2-sensitive
ACCGGGTTGGACGGTCGAGTGGGA	2	23	RDR2-sensitive
ACCGGGTTGGACTGTCGAGTGGGA	0	25	RDR2-sensitive
ACCGGTATCGTGATGTTGGGAAGC	0	9	RDR2-sensitive
ACCGGTCAGAGGCAGAACGGTCAA	0	49	RDR2-sensitive
ACCGGTCAGAGGTAGAACGGTCAA	0	10	RDR2-sensitive
ACCGGTCAGTGGAGCAGCGGAGTG	0	10	RDR2-sensitive
ACCGGTCAGTGGAGCGGCGAAGTG	0	58	RDR2-sensitive
ACCGGTCCGGACACGTAGCGTCAT	0	25	RDR2-sensitive
ACCGGTCCGGTATACGTTGCTGAT	0	11	RDR2-sensitive
ACCGGTCGAGGAACAAAAGGACGG	0	20	RDR2-sensitive
ACCGGTCGGAGAACGTGGATGGTA	0	10	RDR2-sensitive
ACCGGTCGGTCTGGTGCACGGTTC	0	13	RDR2-sensitive
ACCGGTCGTGAGCTCCTGCCCGGC	0	15	RDR2-sensitive
ACCGGTCGTTAGGACAGACGTCGT	0	12	RDR2-sensitive
ACCGGTCTGACAAGAGGACAGCGC	0	11	RDR2-sensitive
ACCGGTCTGACAGAAGGACAGCGC	1	32	RDR2-sensitive
ACCGGTCTGGGTGAGACGGTTGGC	0	19	RDR2-sensitive
ACCGGTCTGTCAAATGGAGGCGAT	0	13	RDR2-sensitive
ACCGGTGAATTGAGTGGTCGTCGC	0	10	RDR2-sensitive
ACCGGTGACGGTAGTCGACGACGG	0	13	RDR2-sensitive
ACCGGTGATCTAATGGGCTGCGGC	0	21	RDR2-sensitive
ACCGGTTCTTGTTGAGTGTTTGGC	0	31	RDR2-sensitive

ACCGGTTTTCTGTTTTCTGGCGGC	353	59	RDR2-resistant
ACCGTACAGATCGGTTCACGGACA	0	18	RDR2-sensitive
ACCGTACGAAGACGCAGTACTCAT	0	11	RDR2-sensitive
ACCGTACTGGTAGACCGGCCCAGG	0	14	RDR2-sensitive
ACCGTAGAATGCACTGGTCGCAGA	0	13	RDR2-sensitive
ACCGTAGAATGCCCCTAGACGCCA	0	10	RDR2-sensitive
ACCGTAGACCTCACCCTCGCGCGT	0	20	RDR2-sensitive
ACCGTAGGCTGACCGGTGGCCCAA	0	26	RDR2-sensitive
ACCGTAGGCTGGTCTGAACGGCAA	1	46	RDR2-sensitive
ACCGTAGTCGGTAAGATCTGTCGG	0	14	RDR2-sensitive
ACCGTATCGGGGGCTCGACGGACC	0	10	RDR2-sensitive
ACCGTCATCGCTTGGTCGTCGTGC	0	9	RDR2-sensitive
ACCGTCATTGCTTGGTCGTCGTGC	0	9	RDR2-sensitive
ACCGTCCACTCTGTAACAACGGTC	0	22	RDR2-sensitive
ACCGTCCCGGGCTGGAAAGGTTGC	1	13	RDR2-sensitive
ACCGTCCGACCTGTAGGCGCGGAT	0	29	RDR2-sensitive
ACCGTCCGCGACTGTGCAGAGACC	0	11	RDR2-sensitive
ACCGTCCGCGCCTGACCAGAGAGC	1	23	RDR2-sensitive
ACCGTCCGCGGTCGGCAGAATGGA	1	18	RDR2-sensitive
ACCGTCCGCGTCTGACCAGAGAGC	0	11	RDR2-sensitive
ACCGTCCGGACGTACGCAGAGGAG	0	16	RDR2-sensitive
ACCGTCCGGACGTCAGGCAGGAAA	0	11	RDR2-sensitive
ACCGTCCGGCTTCGGCGTAGACAA	0	14	RDR2-sensitive
ACCGTCCGGGCTATACACGCGCAG	0	12	RDR2-sensitive
ACCGTCTACTCTGCAACGGCCCAG	0	11	RDR2-sensitive
ACCGTCTCGGACAGGCAGTGGACA	0	10	RDR2-sensitive
ACCGTCTGTGTAGAGGCTAACCGG	1	12	RDR2-sensitive
ACCGTGACAGAAAGCTGGAGGCAC	0	14	RDR2-sensitive
ACCGTGACAGTAGCAAGTGATGGT	0	9	RDR2-sensitive
ACCGTGACCGAGCGAAAAAGACAA	0	13	RDR2-sensitive
ACCGTGACTGGGGAATACCGGCGT	0	45	RDR2-sensitive
ACCGTGACTTAGTTCGTTCGTCGT	0	9	RDR2-sensitive
ACCGTGCACCGTGTGATTGTGGAC	0	42	RDR2-sensitive
ACCGTGCCAGGTCACATGTCGGAC	0	14	RDR2-sensitive
ACCGTGCCTGGCTCTCGGACGCGA	1	25	RDR2-sensitive
ACCGTGCGCCATGCGGTGTTGGAC	0	9	RDR2-sensitive
ACCGTGCGCTGGGGGAAGAGAGCA	26	1	RDR2-resistant
ACCGTGCTAGTAGGCTCCGCCGGC	0	20	RDR2-sensitive
ACCGTGCTATGCAATATCGTCGGT	0	29	RDR2-sensitive
ACCGTGCTGGGCTCAAACAGGCTG	0	10	RDR2-sensitive
ACCGTGCTTTAATGATCTGTAGGT	0	16	RDR2-sensitive
ACCGTGGACTGGCCTCTCCGGCGA	1	24	RDR2-sensitive
ACCGTGGACTGGTGCTCGACGGCG	0	17	RDR2-sensitive
ACCGTGGATTCGTGATTGGCGATG	27	1	RDR2-resistant
ACCGTGGTCTCTGAGAGTCGGCAC	0	21	RDR2-sensitive
ACCGTGTACCGGAGGGAACGAGGC	1	35	RDR2-sensitive
ACCGTGTACTTGAGGGAACGAGGC	0	11	RDR2-sensitive
ACCGTGTAGAATAGAGGACGCGAC	0	78	RDR2-sensitive
ACCGTGTCAAGTAGGACTGGGCAT	0	9	RDR2-sensitive

ACCGTGTCGGTACGGTACGGCTAT	2	24	RDR2-sensitive
ACCGTGTCTGATCGTGCCTGGGAC	0	48	RDR2-sensitive
ACCGTGTCTGCCGGGTTAGACTGC	0	11	RDR2-sensitive
ACCGTGTCTTCACGTCGGATAGCG	0	9	RDR2-sensitive
ACCGTTAAAGGAACTGGGACGGAC	0	10	RDR2-sensitive
ACCGTTAGGGTTCGACCGTTGGAG	11	68	RDR2-sensitive
ACCGTTCAAGGGACTGCACGGCGT	0	26	RDR2-sensitive
ACCGTTCCATGTGTGTCGGACCGT	0	10	RDR2-sensitive
ACCGTTCGACTGACGAGAAGAGGA	0	10	RDR2-sensitive
ACCGTTCGAGAGACTGCACGGCGT	2	25	RDR2-sensitive
ACCGTTCGAGGGACTACATGGCGT	0	11	RDR2-sensitive
ACCGTTCGAGGGACTGCACAGCGT	1	29	RDR2-sensitive
ACCGTTCGAGGGACTGCATGGCGT	0	12	RDR2-sensitive
ACCGTTCGAGTGACTGCATGGCGT	2	14	RDR2-sensitive
ACCGTTCGGCACCTGACCGCAGAC	0	22	RDR2-sensitive
ACCGTTCGTTTAGCTTTGAACCGG	0	12	RDR2-sensitive
ACCGTTGGATACAGAACGGATGGC	0	19	RDR2-sensitive
ACCGTTGGATGTAGAGGGTGTGAT	0	13	RDR2-sensitive
ACCGTTGGATGTAGATGGTGTGGT	1	37	RDR2-sensitive
ACCGTTGGATGTGGAAGGTGTGGT	0	18	RDR2-sensitive
ACCGTTGGATGTGGAGGATGTGGT	0	28	RDR2-sensitive
ACCGTTGGATGTGGAGGGTGCGGT	0	19	RDR2-sensitive
ACCGTTGGATGTGGAGGGTGTGAT	0	10	RDR2-sensitive
ACCGTTGGATGTGGAGGGTGTGGC	0	20	RDR2-sensitive
ACCGTTGGATGTGGAGGGTGTGGT	7	130	RDR2-sensitive
ACCGTTGGCCCGGCCGACCGTTGG	2	16	RDR2-sensitive
ACCGTTGGCGCTCGGCAAAGACTG	0	10	RDR2-sensitive
ACCGTTGGCTCTCGGCAAAGACTG	1	14	RDR2-sensitive
ACCGTTGGCTGAGGCACGTGGCTA	0	10	RDR2-sensitive
ACCGTTGGGGTTCGACCGTTGGAG	2	24	RDR2-sensitive
ACCGTTGGTCTGAAAGATATCGGT	245	1	RDR2-resistant
ACCGTTGTAGAGCGAGTCGGCAGC	0	13	RDR2-sensitive
ACCGTTTAGAGGACGCTGCTGGAG	0	29	RDR2-sensitive
ACCGTTTAGAGGACGTTGCTGGAG	1	21	RDR2-sensitive
ACCGTTTCAAGCCGGGGTCGTCAA	0	87	RDR2-sensitive
ACCTAAAGTCGGGCGGGCACGCAT	0	9	RDR2-sensitive
ACCTAACCGATGGACGTACGGCCC	0	17	RDR2-sensitive
ACCTAACTCAGGAGCGGACGTGAC	0	9	RDR2-sensitive
ACCTAACTGTAGTATGGGCCGCAC	1	20	RDR2-sensitive
ACCTAAGACGGATCTAGACGACAT	0	9	RDR2-sensitive
ACCTAATTGTGGGACTGGGCCGGC	0	35	RDR2-sensitive
ACCTAATTGTGGGCCTGGGCCGGC	1	19	RDR2-sensitive
ACCTAATTTGAGGGATCGTCCCAC	0	9	RDR2-sensitive
ACCTAATTTGAGGGATCGTCCCAT	1	83	RDR2-sensitive
ACCTAATTTGAGGGATCGTCCCGT	0	10	RDR2-sensitive
ACCTAATTTGAGGGATCGTCTCAT	2	14	RDR2-sensitive
ACCTAATTTGAGGGATCGTTCCAT	0	13	RDR2-sensitive
ACCTACAACTCGGATGACGGGCCC	1	14	RDR2-sensitive
ACCTACAGGGTTGTCTGAGGTCTG	0	9	RDR2-sensitive

ACCTACCTGTCGGATTGGTCGGCT	0	58	RDR2-sensitive
ACCTACGAATCTCTCGGACTACGG	0	18	RDR2-sensitive
ACCTACTCCTTGGACTACGGCGAA	0	28	RDR2-sensitive
ACCTACTCTGGGCCGGACCGGCAC	1	17	RDR2-sensitive
ACCTAGAACACTCGGCAAAGGCAC	0	11	RDR2-sensitive
ACCTAGAACACTCGGCAAAGGCGC	0	11	RDR2-sensitive
ACCTAGACATGCCGATCGGACTAA	0	9	RDR2-sensitive
ACCTAGACTGAGAGAGGAGGGAGG	0	14	RDR2-sensitive
ACCTAGATCACGGACCGTTCAGGC	0	14	RDR2-sensitive
ACCTAGATCTGACCCTCAGGAGAG	0	12	RDR2-sensitive
ACCTAGCAAGGTGTACCGTTGGGC	0	15	RDR2-sensitive
ACCTAGCTGTAGGGGCGGCTCCTT	0	10	RDR2-sensitive
ACCTAGGACACTCGACACTCGGCA	0	11	RDR2-sensitive
ACCTAGGACACTCGGCAAAGACGC	1	13	RDR2-sensitive
ACCTAGGACTGGGCCAGTAGGGCA	0	11	RDR2-sensitive
ACCTAGGCCGTCGGAAATAAGCAT	0	13	RDR2-sensitive
ACCTAGGCTGATAGAGGAGGGAGG	0	14	RDR2-sensitive
ACCTAGGTCGTGGACTGTCCGGCC	3	49	RDR2-sensitive
ACCTAGTATTGTAGAAGCTTCGGC	0	15	RDR2-sensitive
ACCTATAGGTTGTTCGCTTCGGAT	0	12	RDR2-sensitive
ACCTATCGGCGTTTCTGTTTCGGG	0	16	RDR2-sensitive
ACCTATCGTAGACTGTCCGGCCGT	0	17	RDR2-sensitive
ACCTATCGTTGTATTGGTCGGGTC	0	16	RDR2-sensitive
ACCTATCTCGACGGCACAGATATT	0	9	RDR2-sensitive
ACCTATCTGGGCTGGGGTACGCGG	0	9	RDR2-sensitive
ACCTATCTTCAGGCTCATCGGACA	2	15	RDR2-sensitive
ACCTATGGATTGGACGGGATTGGA	0	17	RDR2-sensitive
ACCTATGGTAGACTGTACGGTGAC	0	15	RDR2-sensitive
ACCTATTAAGACTGTAAGGGGTAG	0	20	RDR2-sensitive
ACCTCAACGGTCGGCTGACGTGGC	0	9	RDR2-sensitive
ACCTCAATCTGACTGGATGGCTGT	0	39	RDR2-sensitive
ACCTCACGTGGTTCCTAGAACGGC	0	12	RDR2-sensitive
ACCTCACTCGGATCGTCATGTGTC	0	11	RDR2-sensitive
ACCTCCCGTCAGCAGTAGAGGCGC	0	20	RDR2-sensitive
ACCTCCCTGGAAGGACTCAGTCGG	0	15	RDR2-sensitive
ACCTCCTCCGTTGGATTGGTGGTA	0	19	RDR2-sensitive
ACCTCGCCGGAGAAGACAGCTATC	0	17	RDR2-sensitive
ACCTCGCGCTGGGCTGTGAGGCAG	54	7	RDR2-resistant
ACCTCGCGTCTGTCTGGTGTTCGA	0	11	RDR2-sensitive
ACCTCGGACGGGAGACGCAGTCAC	1	18	RDR2-sensitive
ACCTCGGACGGGAGACGTAGTCAC	0	18	RDR2-sensitive
ACCTCGGACGGTAGACGCAGTCAC	0	13	RDR2-sensitive
ACCTCGGATACAGGTAGGATACGG	0	14	RDR2-sensitive
ACCTCGGATACGGGTAGGATACGG	0	107	RDR2-sensitive
ACCTCGGATTAGTTTTTTGTGGCG	1	16	RDR2-sensitive
ACCTCGGGCGGGACACGCAGTCAC	0	10	RDR2-sensitive
ACCTCGGGCGGGAGACGCAGTCAC	1	19	RDR2-sensitive
ACCTCGGGGTTACTTGAGGGTCGG	0	17	RDR2-sensitive
ACCTCGGGTAGAAGATACAATCAT	0	10	RDR2-sensitive

ACCTCGGTTTGGCTCGGACAGGCT	0	9	RDR2-sensitive
ACCTCGTCACAGGTCTTCGTCCGG	0	11	RDR2-sensitive
ACCTCGTCACAGGTTTTCGTCCGG	0	12	RDR2-sensitive
ACCTCTCAGGACAAAGTCGGTATT	0	9	RDR2-sensitive
ACCTCTCCGGCCTGGGACGGTGCT	0	9	RDR2-sensitive
ACCTCTCCTTCTGGGAGCGTCGGC	0	21	RDR2-sensitive
ACCTCTCGCGGATAGACGGATGAT	0	13	RDR2-sensitive
ACCTCTCTCGGACTCTACGTCAGT	0	12	RDR2-sensitive
ACCTCTGCGGACGGCGATTTCGGA	0	14	RDR2-sensitive
ACCTCTGTAGTTCAATTGGGGACG	0	9	RDR2-sensitive
ACCTGAACACTTAGATGCTGGCCT	0	55	RDR2-sensitive
ACCTGAACAGCATAGGGCCTGATT	0	10	RDR2-sensitive
ACCTGAACCAGTGGGTACCTCTAA	0	9	RDR2-sensitive
ACCTGAACCCGAGTAGACTGACAA	1	12	RDR2-sensitive
ACCTGAACGAGCGGTGGCGCGCAT	0	12	RDR2-sensitive
ACCTGAACGCTTGGATGCCGGCAT	0	10	RDR2-sensitive
ACCTGAACGCTTGGATGCCGGCCT	0	14	RDR2-sensitive
ACCTGAACTGATTCTGTGCACCAT	0	21	RDR2-sensitive
ACCTGACAAGCTGACATCTGGTTC	0	13	RDR2-sensitive
ACCTGACAGCACTCGGCAAAGAAT	0	15	RDR2-sensitive
ACCTGACCTGGGCGCGGGACGAAC	0	13	RDR2-sensitive
ACCTGACGACTAGTGAAGAGGCAT	0	17	RDR2-sensitive
ACCTGAGCTCTGTGGAATGCACGT	152	36	RDR2-resistant
ACCTGAGTCGTTGTAATGTGGCAT	0	17	RDR2-sensitive
ACCTGATCATGTTGGACGGGCCGA	0	13	RDR2-sensitive
ACCTGATCCAATCTGATCGCGGAC	0	21	RDR2-sensitive
ACCTGATCTGACTGAAGAGGGCGT	0	28	RDR2-sensitive
ACCTGATCTGCGACTGACTGACGG	0	25	RDR2-sensitive
ACCTGATGCAAGATCTCGAAGGCG	0	11	RDR2-sensitive
ACCTGATGCTGTTCAGAGGTCGGA	0	16	RDR2-sensitive
ACCTGATGCTGTTTAGGGGTCGGA	11	35	RDR2-sensitive
ACCTGATTGTTAGGCGAGAGGTAC	0	16	RDR2-sensitive
ACCTGATTGTTCGTTGTAGGGGTA	0	15	RDR2-sensitive
ACCTGCAATATTGTGTGACTCGAG	471	2	RDR2-resistant
ACCTGCACGCGGACGGTTCGACGG	0	10	RDR2-sensitive
ACCTGCATGCGCGGAGACGGGACC	0	14	RDR2-sensitive
ACCTGCATGTCGTGACCAGGGCAA	22	0	RDR2-resistant
ACCTGCCAAGAAGCTGATCAGCGT	0	11	RDR2-sensitive
ACCTGCCGGTAGAAGTTCCGTCGT	1	29	RDR2-sensitive
ACCTGCGGAAGTGGTAGTTCGTGT	23	2	RDR2-resistant
ACCTGCGGCAGGTACGAGGACTAC	0	9	RDR2-sensitive
ACCTGCTAGGGTCGGATCGGTATG	0	12	RDR2-sensitive
ACCTGCTCGAGCTCGGACCAGAGG	0	13	RDR2-sensitive
ACCTGCTCGTTGTATTGGTCGGTC	1	28	RDR2-sensitive
ACCTGCTCTGACAAACTAACGGCT	17	1	RDR2-resistant
ACCTGCTCTGACAGGGCAACGGCT	0	9	RDR2-sensitive
ACCTGCTGGTACTGTATCGGCTAT	0	13	RDR2-sensitive
ACCTGCTGGTGCAGTCTGACATGT	0	11	RDR2-sensitive
ACCTGGAACTGAAGGCGCGCCTGG	0	18	RDR2-sensitive

ACCTGGAAGCGGAGCTCGGCGCGT	0	9	RDR2-sensitive
ACCTGGACACGGATGGCCTGGCAA	0	13	RDR2-sensitive
ACCTGGACCGTTGCAACGATGGCG	0	10	RDR2-sensitive
ACCTGGACCGTTGCAGCGATGGCG	3	27	RDR2-sensitive
ACCTGGACGCAGGAGCGGATCCTC	0	11	RDR2-sensitive
ACCTGGACGGGGTGCTACTCGGAA	0	18	RDR2-sensitive
ACCTGGACTGGAGAAGATGGTGCC	0	11	RDR2-sensitive
ACCTGGAGGAGACGCTGGAAACGC	1	14	RDR2-sensitive
ACCTGGATCATGTGGAAGACGACT	0	25	RDR2-sensitive
ACCTGGCACGGTCGTGACTCGGCA	0	9	RDR2-sensitive
ACCTGGCAGAAGGACCGTTGGACA	0	21	RDR2-sensitive
ACCTGGCCGTGGATTGTTACGGAT	0	11	RDR2-sensitive
ACCTGGCGCGTCAACTGTCGGCGT	2	14	RDR2-sensitive
ACCTGGCGGCTGGTGTACATAGAC	37	3	RDR2-resistant
ACCTGGCTCTGACGTGGCGGCGGC	0	28	RDR2-sensitive
ACCTGGCTCTGACGTGGTGGCGGC	1	15	RDR2-sensitive
ACCTGGCTCTGATACCAAGTGTTG	0	9	RDR2-sensitive
ACCTGGCTCTGATACCACTGAAGG	211	0	RDR2-resistant
ACCTGGGACACTCGACAAAGACGC	0	12	RDR2-sensitive
ACCTGGGACACTCGGCAAAGACGC	1	26	RDR2-sensitive
ACCTGGGACACTCGGTAAAGACGC	0	10	RDR2-sensitive
ACCTGGGCGCGGGACGAACACGAA	10	32	RDR2-sensitive
ACCTGGGGTGTAGAAGGCAGGCGC	0	23	RDR2-sensitive
ACCTGGTAGTGCAGAATCTCGGTG	0	16	RDR2-sensitive
ACCTGGTCGGTACGTGGTCGAGGC	0	27	RDR2-sensitive
ACCTGGTCGGTACGTGGTCGGGGC	7	154	RDR2-sensitive
ACCTGGTCGGTACGTGGTTGGGGC	0	13	RDR2-sensitive
ACCTGGTCGGTGGGTGGTCGGTGC	1	13	RDR2-sensitive
ACCTGGTCGGTGGGTGGTTGGGGC	0	9	RDR2-sensitive
ACCTGGTCGGTGTCTGGTCGGGGC	1	22	RDR2-sensitive
ACCTGGTCGGTTGCTGGTCGGGGC	0	13	RDR2-sensitive
ACCTGGTGCGGCTGAACCGGATGG	0	11	RDR2-sensitive
ACCTGGTTGGTCGGCCTGGTCGGT	0	11	RDR2-sensitive
ACCTGGTTTTGGAGATCGCTGGAG	0	10	RDR2-sensitive
ACCTGGTTTTTGTCGGCTACGGTT	26	3	RDR2-resistant
ACCTGTAAGAGCCTTTGACGGGAG	0	22	RDR2-sensitive
ACCTGTAAGAGGCTTTTGACGGAT	0	14	RDR2-sensitive
ACCTGTAGAAGGGCCGAGCGGCGT	0	9	RDR2-sensitive
ACCTGTAGAGCCCGAGAGGCGAGA	0	9	RDR2-sensitive
ACCTGTAGCGCTGACTCTTGGCAT	1154	149	RDR2-resistant
ACCTGTAGCGCTGACTCTTGGCCT	32	5	RDR2-resistant
ACCTGTAGGCGCGGATCGTCCGGC	0	13	RDR2-sensitive
ACCTGTAGGGGCACGTAGATGGCA	0	11	RDR2-sensitive
ACCTGTAGGTCCACCAGATAACGT	2	78	RDR2-sensitive
ACCTGTAGTACCTATGCATGTTGA	15	0	RDR2-resistant
ACCTGTAGTCCTATGCGCGTGCAT	0	34	RDR2-sensitive
ACCTGTAGTGCACCGACGCGCCCT	0	13	RDR2-sensitive
ACCTGTATGGCATACTCTCGTCCT	1	37	RDR2-sensitive
ACCTGTATGTGCCTGCCAGACGTG	0	24	RDR2-sensitive

ACCTGTCAGAGACCGAACCGGCAG	0	11	RDR2-sensitive
ACCTGTCATCGGAACGAGGGGAGA	0	9	RDR2-sensitive
ACCTGTCGCGTAGGACAGTTCGGC	0	30	RDR2-sensitive
ACCTGTCGGATTGGTCGGCTTGGG	0	18	RDR2-sensitive
ACCTGTCTGTTGCATTGGTCGGAT	0	12	RDR2-sensitive
ACCTGTGAAGCAAGAACCGACGCC	0	9	RDR2-sensitive
ACCTGTGACTGTAGCTTCGGCTAA	0	10	RDR2-sensitive
ACCTGTGCAAGTGTAGATCTGCGG	0	13	RDR2-sensitive
ACCTGTGGACGCCTACACTAACAT	0	13	RDR2-sensitive
ACCTGTGGCTGAAAAGTGTGGCGC	0	15	RDR2-sensitive
ACCTGTGGGTCTGTTATTGGGCAA	4	30	RDR2-sensitive
ACCTGTGGGTCTTGTCGAGTCGGA	0	13	RDR2-sensitive
ACCTGTGTGGTGCTCTGTCTGTTC	0	39	RDR2-sensitive
ACCTGTTAGACGGTGTAGGCGATG	0	10	RDR2-sensitive
ACCTGTTATGAACCTTGTCGGGAT	44	3	RDR2-resistant
ACCTGTTCAGGAGTAGGACGGAGC	0	11	RDR2-sensitive
ACCTGTTCGTTGCCGGAGAAGGAC	1	21	RDR2-sensitive
ACCTGTTCGTTGGTGGAACGACGG	300	40	RDR2-resistant
ACCTGTTCTTGACGTAGAGGATGT	0	11	RDR2-sensitive
ACCTGTTGACTACTGTGTCGGGCC	0	14	RDR2-sensitive
ACCTGTTTGGCACGAAGCACGATG	0	11	RDR2-sensitive
ACCTGTTTGGCACGGCTCCTCTAG	0	9	RDR2-sensitive
ACCTGTTTGGCACGGCTCCTTCAG	0	12	RDR2-sensitive
ACCTTAAAGGGATTCTTGAGACGG	24	1	RDR2-resistant
ACCTTAACGAGCCGAGCCGAGCTG	0	17	RDR2-sensitive
ACCTTAAGGCTGGAGCGAGGGCGT	0	78	RDR2-sensitive
ACCTTAAGTAGTGGACGAGGGCAG	0	40	RDR2-sensitive
ACCTTACTGGACGCCTAGAGGACG	0	103	RDR2-sensitive
ACCTTACTGGACGTGTAGAGGACG	0	42	RDR2-sensitive
ACCTTATGTGACGTAGGACCACAC	0	12	RDR2-sensitive
ACCTTATTTGAGGAATCGTCTCGT	0	10	RDR2-sensitive
ACCTTATTTGAGGGATCGTCATGT	0	13	RDR2-sensitive
ACCTTATTTGAGGGATCGTCCCGT	0	35	RDR2-sensitive
ACCTTATTTGAGGGATCGTCTCAT	5	27	RDR2-sensitive
ACCTTATTTGAGGGATCGTCTCGT	0	21	RDR2-sensitive
ACCTTATTTGAGGGATCGTTCCAT	0	15	RDR2-sensitive
ACCTTATTTGAGGGATCGTTCTAT	0	9	RDR2-sensitive
ACCTTCACGGGAGGGAGGACCATC	14	0	RDR2-resistant
ACCTTCATACTGTAGTAACGGATA	0	12	RDR2-sensitive
ACCTTCATACTGTAGTAACGGGTA	1	19	RDR2-sensitive
ACCTTCCATCTGTAGTATTCGGTT	0	11	RDR2-sensitive
ACCTTCGGGGCGTGTTAGACTGGC	0	10	RDR2-sensitive
ACCTTCGTCGTTCGCATACTTGGT	0	13	RDR2-sensitive
ACCTTCTAGTGGACTCGGGGATAT	0	14	RDR2-sensitive
ACCTTCTCTGGACACGTGGCGGCT	0	9	RDR2-sensitive
ACCTTGCAGTAGACCTTTCCGATA	0	11	RDR2-sensitive
ACCTTGCGGGACTGGTCATCGGGC	0	25	RDR2-sensitive
ACCTTGCTGAGCGAAGAACGACGA	0	9	RDR2-sensitive
ACCTTGCTGGGCGTGTAGAGGACG	0	140	RDR2-sensitive

ACCTTGGACGACGTGTTTTGGCAT	0	39	RDR2-sensitive
ACCTTGGACGGCGTGTTTTGGCAT	0	11	RDR2-sensitive
ACCTTGGGCGGGAGACGTAGTCAC	0	11	RDR2-sensitive
ACCTTGGTCGGGCCGAGCAGGGAG	0	11	RDR2-sensitive
ACCTTGTCAGTGTGGCTACTTAAA	17	1	RDR2-resistant
ACCTTGTCGAAGCTGTATTGCATC	25	2	RDR2-resistant
ACCTTGTGATGTTAACTTTGTGAT	21	2	RDR2-resistant
ACCTTTGGCATGGTTTGTCGGTCA	79	1	RDR2-resistant
ACCTTTGTTCGGCGGCTCGGCACC	0	15	RDR2-sensitive
ACCTTTGTTTGCTCGGTACTTCGG	22	0	RDR2-resistant
ACGAAAAAGTGGAACCGGAGGCAG	0	9	RDR2-sensitive
ACGAAACAGTGACTGGGACCGCAT	0	12	RDR2-sensitive
ACGAAAGTCGTCGACGGTGTTTAG	0	15	RDR2-sensitive
ACGAACACGGCTATAGTCAATGCA	0	12	RDR2-sensitive
ACGAACACGGTACACAATCTGATC	17	0	RDR2-resistant
ACGAACAGACATGGTACGGCTCAC	0	23	RDR2-sensitive
ACGAACAGGGCAACAGACGAACAG	0	19	RDR2-sensitive
ACGAACATGAAGTGCGGTCAGGAC	0	11	RDR2-sensitive
ACGAACCAGCGACGGTGCTCTCTG	0	9	RDR2-sensitive
ACGAACCATGAACGATCGGACGAA	0	11	RDR2-sensitive
ACGAACCGAGCGAGCCGACGAGCC	0	17	RDR2-sensitive
ACGAACCGCGGAACAGGATTCGGC	0	13	RDR2-sensitive
ACGAACGCACGGCAGGAGAGACGA	0	12	RDR2-sensitive
ACGAACGGACAATGGCGACGTGCA	0	31	RDR2-sensitive
ACGAACGGACGGCTTAGATTGGCT	0	10	RDR2-sensitive
ACGAACTAAGACTCTCCACGGCAC	0	14	RDR2-sensitive
ACGAACTGGGGGAGACGACCGAAC	0	27	RDR2-sensitive
ACGAAGACGCAGGCAACCGAACAC	0	10	RDR2-sensitive
ACGAAGAGGGACGGAAGAGGACAA	0	10	RDR2-sensitive
ACGAAGCACGATAGACTTGGGCCA	0	22	RDR2-sensitive
ACGAAGGCAGAACGGCAGAACGGC	0	10	RDR2-sensitive
ACGAAGGGGGCACGGTAGAGGGCT	0	10	RDR2-sensitive
ACGAAGTCGGTGCAGGAAGATGTA	1	31	RDR2-sensitive
ACGAATAAGCGGGTCGGGCTCGGA	0	24	RDR2-sensitive
ACGAATAAGCGGGTTGGCTCGGAC	0	11	RDR2-sensitive
ACGAATACGGATATTTCGGATCTC	0	11	RDR2-sensitive
ACGAATATTTTTGTCGGATATCGT	0	14	RDR2-sensitive
ACGAATCCTGGATGAGACGTGACA	0	16	RDR2-sensitive
ACGAATTTCTGGGGCATCGTCACT	53	0	RDR2-resistant
ACGAATTTTATAGAAGCTGGCTGA	1	64	RDR2-sensitive
ACGACAACCCTGTAGAGCGCGGCG	0	19	RDR2-sensitive
ACGACAAGAGAACCGGCTCAACAA	0	16	RDR2-sensitive
ACGACACGAGGCTGGACGGTCCGC	0	15	RDR2-sensitive
ACGACACGATTAACTGGCCGGCAC	0	12	RDR2-sensitive
ACGACACGCGGAACGACGACGAGA	0	24	RDR2-sensitive
ACGACACGGAAGAGGCTTAAACAG	0	22	RDR2-sensitive
ACGACACGGCACGATTGGAACCAG	0	11	RDR2-sensitive
ACGACACGTCCATGTCTAGATCGT	0	24	RDR2-sensitive
ACGACACTGTACAACATTTTCGGC	0	12	RDR2-sensitive

ACGACACTGTATCGGCCCGATCGT	0	15	RDR2-sensitive
ACGACAGACGAACTAAGTCGCGGC	0	25	RDR2-sensitive
ACGACAGACGAACTACGTCACGGC	0	9	RDR2-sensitive
ACGACAGAGGAACACACGAGACAC	0	30	RDR2-sensitive
ACGACAGTCGAATCGGCAACAAAG	0	12	RDR2-sensitive
ACGACAGTCGAATCGGCAATAGAG	0	11	RDR2-sensitive
ACGACATGATTAGGACACGGCCTG	0	13	RDR2-sensitive
ACGACCAAGGGCTGGACGGTCCGC	0	14	RDR2-sensitive
ACGACCAGATGCGAGCGCTCTCAT	0	9	RDR2-sensitive
ACGACCAGTGAAGCAGATCGACAT	1	18	RDR2-sensitive
ACGACCCAGTAGAGCAGGCAGTGC	0	29	RDR2-sensitive
ACGACCCGGAATAGCTCAGAGGCT	0	15	RDR2-sensitive
ACGACCTAGAACCGGACGGTCCAC	0	44	RDR2-sensitive
ACGACCTAGTAGCAGGAACGACGT	2	139	RDR2-sensitive
ACGACCTGACTTGGCATACTCCGT	23	3	RDR2-resistant
ACGACCTTCTATGTCATCGCGGAC	0	19	RDR2-sensitive
ACGACGACGCTGTAGTGAGGTTGC	0	12	RDR2-sensitive
ACGACGACTGTACAGATTACGCCG	0	10	RDR2-sensitive
ACGACGATCAGATCGAGTCGGCAC	0	18	RDR2-sensitive
ACGACGGACATTGTGGTACGGTGT	1	26	RDR2-sensitive
ACGACGGATGCGGCTTCGACGAAA	0	9	RDR2-sensitive
ACGACGGCAGAACGGCAGAACGGC	0	17	RDR2-sensitive
ACGACGGCCGTTAGATGGGAGACG	0	9	RDR2-sensitive
ACGACGGCCGTTGGATGGAAGACG	0	15	RDR2-sensitive
ACGACGGTATCTGACTTTGTGCCA	40	0	RDR2-resistant
ACGACGTGGTTTAGAGCTAGGCTG	0	19	RDR2-sensitive
ACGACGTGGTTTAGGACTAGGCTG	0	9	RDR2-sensitive
ACGACTAACAGACTCACGGGCCGT	0	16	RDR2-sensitive
ACGACTAAGGGTATACGTGAGCAC	0	26	RDR2-sensitive
ACGACTACTAGACTGGCGTGACGT	0	17	RDR2-sensitive
ACGACTAGCAAACTGACGGACCGT	0	14	RDR2-sensitive
ACGACTAGCACACTGACGGGCCGT	1	32	RDR2-sensitive
ACGACTAGCAGACTGACGGGCCGT	0	9	RDR2-sensitive
ACGACTAGCAGGCTGACGGGCCGT	2	16	RDR2-sensitive
ACGACTAGTGTATTCATACGGTTA	32	0	RDR2-resistant
ACGACTATTGGACCGGGCTGGCCT	0	12	RDR2-sensitive
ACGACTCCCGATCTTTCGGCTCCA	0	12	RDR2-sensitive
ACGACTCCTGATCTTTCGGCTCCA	0	24	RDR2-sensitive
ACGACTCGACTGTGAACGGCGCAA	1	12	RDR2-sensitive
ACGACTCGAGCATGATGTAGGCAC	0	9	RDR2-sensitive
ACGACTCGCTCTAGGGGTCGTCGG	0	11	RDR2-sensitive
ACGACTCTAGCTCCTTGATGGCTC	0	11	RDR2-sensitive
ACGACTCTCGGCAACGGATATCTC	164	37	RDR2-resistant
ACGACTCTGACGATTTGACGGCCA	0	26	RDR2-sensitive
ACGACTCTGACGATTTGGCGGCGA	0	9	RDR2-sensitive
ACGACTCTGAGTCAAGGACGACAC	44	0	RDR2-resistant
ACGACTCTGGCAGCCGTTAGATGT	0	37	RDR2-sensitive
ACGACTGACGATTTGACAGCGGCC	0	12	RDR2-sensitive
ACGACTGACGATTTGACGGCGGCC	2	45	RDR2-sensitive

ACGACTGAGGGAGGCCGCCAGTGC	0	10	RDR2-sensitive
ACGACTGATAGGTAGATTGGCCGC	0	10	RDR2-sensitive
ACGACTGCACGGCACTGCTCGACG	25	2	RDR2-resistant
ACGACTGCAGGCAGAACCGTGCAC	0	11	RDR2-sensitive
ACGACTGGAGTTTGAAACGGTCTA	0	9	RDR2-sensitive
ACGACTGGGAGTAGAGCAGGGCAC	0	14	RDR2-sensitive
ACGACTGTACAGATCGCGCCGCAT	0	12	RDR2-sensitive
ACGACTGTACAGATTACGCCACAT	0	9	RDR2-sensitive
ACGACTGTACAGATTACGCCGCAT	1	32	RDR2-sensitive
ACGACTGTACAGATTGCGCCACAT	2	45	RDR2-sensitive
ACGACTGTACAGATTGCGTCACAT	0	10	RDR2-sensitive
ACGACTGTAGGAAGCCGTCGGATA	1	29	RDR2-sensitive
ACGACTGTATAGATTGCGTCACAT	0	17	RDR2-sensitive
ACGACTGTCAGAGCAGGCCGTCAG	0	9	RDR2-sensitive
ACGACTGTCTCCAGTAGAGCGCGT	0	11	RDR2-sensitive
ACGACTGTCTGACGTAGAGATGGC	0	13	RDR2-sensitive
ACGACTGTGAGCTACAACGGTGTC	0	9	RDR2-sensitive
ACGACTGTGATCTAGGGAGGCCAT	1	13	RDR2-sensitive
ACGACTGTGCTTGAACACGTGCAT	0	34	RDR2-sensitive
ACGACTGTGGTTAGGCTAGTGCTC	0	9	RDR2-sensitive
ACGACTTAAAACGCGACGGGGCAT	35	6	RDR2-resistant
ACGACTTCTTAGGACGGCAGTAGA	0	16	RDR2-sensitive
ACGACTTGACGGTAAACGCGAGCA	0	10	RDR2-sensitive
ACGACTTTGGCTCTTTGATGGCTC	0	10	RDR2-sensitive
ACGAGACACAGGCGCTGATGCATA	0	10	RDR2-sensitive
ACGAGACGGATACGGGACGAGCAG	0	10	RDR2-sensitive
ACGAGACGTAGTGAACCGGACCGA	0	9	RDR2-sensitive
ACGAGACTGTGCATCCGTCCCCGT	0	9	RDR2-sensitive
ACGAGAGAGGACGACAAGGGACAT	0	17	RDR2-sensitive
ACGAGAGCTGTAGCCCAGGAGCGC	0	12	RDR2-sensitive
ACGAGATCAGGCCTTGTTGGACGG	0	10	RDR2-sensitive
ACGAGATGAAGACGCCAGGAACCA	0	9	RDR2-sensitive
ACGAGATGCAGAGCTGCTTGTCGG	0	24	RDR2-sensitive
ACGAGCAACTGCTAGAGTGGGCAA	0	10	RDR2-sensitive
ACGAGCACACGGACTTAGATGGCC	0	9	RDR2-sensitive
ACGAGCAGATTGGTCGCATCTCTA	1	21	RDR2-sensitive
ACGAGCAGCTGGGTTGGAGGAATC	29	4	RDR2-resistant
ACGAGCCAGCTCGGATCGACTCGT	0	11	RDR2-sensitive
ACGAGCCCACGGACTTAGATGGCC	0	23	RDR2-sensitive
ACGAGCCCGTGGACTTAGACGGCT	0	14	RDR2-sensitive
ACGAGCTAACAGTCGGCGTGTCGT	0	14	RDR2-sensitive
ACGAGCTATAGAGCGTGACGGCAC	0	10	RDR2-sensitive
ACGAGCTCGATTAGGACACGGCAC	0	21	RDR2-sensitive
ACGAGGACGCCACCAACATCGCCG	60	1	RDR2-resistant
ACGAGGACTCTACGCGGACCACAA	0	15	RDR2-sensitive
ACGAGGAGGGCAGAACGGCAGAAC	0	14	RDR2-sensitive
ACGAGGATCGGTAACACTGCGAAG	0	9	RDR2-sensitive
ACGAGTCTGAACACGTAGCAACGG	0	9	RDR2-sensitive
ACGATAATCGGTCTAGACTGGCAT	0	9	RDR2-sensitive

ACGATACCCGTCGTCTCGCTGGAC	0	13	RDR2-sensitive
ACGATACTGAACCATACCGGGCTT	0	9	RDR2-sensitive
ACGATAGGACACCTGATCACGGTC	0	19	RDR2-sensitive
ACGATAGGCTTAGACCAGACTGGC	0	13	RDR2-sensitive
ACGATAGGTCTGGACCGTGCCGGC	0	10	RDR2-sensitive
ACGATATAGGGCTGGACGGTTCAC	2	37	RDR2-sensitive
ACGATATGGTTGAAGCTCGACGGT	0	19	RDR2-sensitive
ACGATCATGCCGTGACGATCTGAT	0	12	RDR2-sensitive
ACGATCGGATGCACGGCTACCTGC	0	14	RDR2-sensitive
ACGATCTAGACATGGACGTGCCGT	0	24	RDR2-sensitive
ACGATCTGAGCTAGCGCGGGCGTA	0	24	RDR2-sensitive
ACGATGAGACTGGAGTAGAGGCCG	0	16	RDR2-sensitive
ACGATGAGTATTTCTGATCGTTGG	1	99	RDR2-sensitive
ACGATGCGCGGGGTAGACAAACGG	1	35	RDR2-sensitive
ACGATGGACGAACTATGTCGCGGC	0	19	RDR2-sensitive
ACGATGGACGTATGGATGGCCAGC	0	20	RDR2-sensitive
ACGATGTTCTGTTTGTAACGGTTG	51	6	RDR2-resistant
ACGATTCGGTAGATTATAATGGCA	0	62	RDR2-sensitive
ACGATTCGGTGGATTATAATGGCA	2	22	RDR2-sensitive
ACGATTCGTATAGCACTTCGGCAC	0	12	RDR2-sensitive
ACGATTGACGCGGAATTTCGGTTG	1	12	RDR2-sensitive
ACGATTGGACACGGCCTGACTCAG	0	14	RDR2-sensitive
ACGATTTCTAACCGGATTGTCTGT	0	19	RDR2-sensitive
ACGATTTGTAGAGTATACATCAAG	0	13	RDR2-sensitive
ACGATTTTCGGACCGTATCGGCAT	0	9	RDR2-sensitive
ACGCAATTGTCTTTTTCGCTCGGT	0	20	RDR2-sensitive
ACGCACATATAGTGACGCTGGACG	0	9	RDR2-sensitive
ACGCACATTGGTGGTACGCTGGAC	0	10	RDR2-sensitive
ACGCACCGGACTGTCCGATGTGCA	0	12	RDR2-sensitive
ACGCAGAACTGTGCAGGCCCGACC	0	15	RDR2-sensitive
ACGCAGAACTGTGCTGGCCCGGCC	0	13	RDR2-sensitive
ACGCAGAATCGGTCGAGACGTCAC	0	9	RDR2-sensitive
ACGCAGATCGAGCATAGAACCGGC	0	9	RDR2-sensitive
ACGCAGCACACTCACTCGTCGGCT	0	20	RDR2-sensitive
ACGCAGCACGTACCTCGGCGCCAT	0	21	RDR2-sensitive
ACGCAGCAGGCCCCTACCTGGCGC	0	9	RDR2-sensitive
ACGCAGCGACAATTTACTCGTCGG	0	9	RDR2-sensitive
ACGCAGCTGCAGAAGGGACGACAC	0	12	RDR2-sensitive
ACGCAGGACGTAACATGAGAGCGG	0	26	RDR2-sensitive
ACGCAGGACTGTGCAGGCACGACT	0	18	RDR2-sensitive
ACGCAGGACTGTGCAGGCACGGCA	1	47	RDR2-sensitive
ACGCAGGACTGTGCAGGCACGGCC	1	21	RDR2-sensitive
ACGCAGGACTGTGCAGGCCCGGCC	2	99	RDR2-sensitive
ACGCAGGACTGTGTAGGCCCGGCC	1	71	RDR2-sensitive
ACGCAGGCGACAGACAAACTCCAA	0	17	RDR2-sensitive
ACGCAGGGGCGGGCCCAGAATTTG	29	0	RDR2-resistant
ACGCATAGACGGTAGGGCAGGTAA	0	13	RDR2-sensitive
ACGCATAGGAAGACGAACGGACAG	0	10	RDR2-sensitive
ACGCATCTGTGGAGCCAGGCTCAC	0	9	RDR2-sensitive

ACGCCAAAATGGACATGAGACGGT	0	10	RDR2-sensitive
ACGCCAACGCCTCCATTTCGTCGA	0	9	RDR2-sensitive
ACGCCAACTGTTCAACACGTGCAC	0	13	RDR2-sensitive
ACGCCAGAAGTTGGACGTTTGGTT	0	13	RDR2-sensitive
ACGCCAGACTGCTGACTGTACGGC	0	17	RDR2-sensitive
ACGCCAGATGCAGGGGACGACTGT	0	10	RDR2-sensitive
ACGCCAGGTCGCGGACCGTCCGGC	2	85	RDR2-sensitive
ACGCCATCGTAGGTCGTTCTCGGG	0	17	RDR2-sensitive
ACGCCATTGCTGTAGAAGTGTGCA	0	16	RDR2-sensitive
ACGCCCAGCGCACGATCTGTTGAC	0	13	RDR2-sensitive
ACGCCCATTCACCGTCGGTACGTT	0	12	RDR2-sensitive
ACGCCCCCCGTTGTACGTCTGGAC	0	17	RDR2-sensitive
ACGCCGGCGCTCGGACGGAAGGCG	0	19	RDR2-sensitive
ACGCCGGTGACGAACTCCAAGCGG	0	75	RDR2-sensitive
ACGCCGTCTCTCACTGATCGTCTT	0	10	RDR2-sensitive
ACGCCGTGCGACTGGGTAGGCAGC	0	70	RDR2-sensitive
ACGCCGTTGGATTTGAGGGGAAGG	17	1	RDR2-resistant
ACGCCTCCAAATCGTCGAAGCCGC	0	76	RDR2-sensitive
ACGCCTGAGCGCTCGGACTATGGC	0	10	RDR2-sensitive
ACGCCTGCTAGACGAGTCGGACAC	0	50	RDR2-sensitive
ACGCCTGGTGGGCTGTGGACCGGC	1	24	RDR2-sensitive
ACGCCTGTAAGACGAGTCGGACAC	0	17	RDR2-sensitive
ACGCCTTCGTTGGATGTGGACGGC	0	13	RDR2-sensitive
ACGCCTTCTTTGGAGGCCGTCGGA	0	21	RDR2-sensitive
ACGCCTTGGGGAAATCATCGTCGC	0	9	RDR2-sensitive
ACGCGACAAATGGGACACACGGAG	0	13	RDR2-sensitive
ACGCGACATGACGAGTACCGATGA	0	9	RDR2-sensitive
ACGCGACGGCGGATCTGGCGGCGC	0	16	RDR2-sensitive
ACGCGACGGGGCATTGTAAGTGGC	31	3	RDR2-resistant
ACGCGACGTCGTACGCCTCGGCTA	0	15	RDR2-sensitive
ACGCGACTCAGCGATGGCGGTCTG	2	14	RDR2-sensitive
ACGCGACTCTGTCAGGAACGGAAC	0	37	RDR2-sensitive
ACGCGACTGCTCGTGATCTGCTGA	5	21	RDR2-sensitive
ACGCGACTGCTCGTGATCTTCTGA	3	27	RDR2-sensitive
ACGCGATATGGCAGAACCGTGCGC	0	12	RDR2-sensitive
ACGCGATCGGCTGAAGATGCGCCG	0	12	RDR2-sensitive
ACGCGATGGTAGAACGTGGCGCAC	0	10	RDR2-sensitive
ACGCGCAGCGCGTCGGCAGGGGCG	17	1	RDR2-resistant
ACGCGCAGGATGACTTCTTTTACT	1	20	RDR2-sensitive
ACGCGCCGGAATCAGCAGGGTCGA	4	82	RDR2-sensitive
ACGCGCCTGTAGGGCGGACGGTCC	0	9	RDR2-sensitive
ACGCGCGACACTTGCAAACGACAT	0	9	RDR2-sensitive
ACGCGCGACATAGCATGCTGACGG	0	31	RDR2-sensitive
ACGCGCTAGCTGAAGGTCGTCCGT	0	9	RDR2-sensitive
ACGCGCTCGACCCATCTGGGCTGG	0	9	RDR2-sensitive
ACGCGCTGTGGCAGAACCGTGCAC	0	15	RDR2-sensitive
ACGCGGAAACGAGCATGCTGGCAC	0	9	RDR2-sensitive
ACGCGGAAGGCGGTCTGAACTCTA	0	11	RDR2-sensitive
ACGCGGAATAGAACGGCTCCATTC	0	12	RDR2-sensitive

ACGCGGACGGCTGACGGACGACGC	0	14	RDR2-sensitive
ACGCGGATCGGCAACACTGCGAAG	0	9	RDR2-sensitive
ACGCGGATGAACGCACAGTAACGG	0	17	RDR2-sensitive
ACGCGGCAAGAACACGTACTCGGC	0	12	RDR2-sensitive
ACGCGGCACACTCACTCGTCGGCT	0	16	RDR2-sensitive
ACGCGGCACTCACTCGTCGGCCTG	0	11	RDR2-sensitive
ACGCGGCGACAATTTACTCGTCGG	0	9	RDR2-sensitive
ACGCGGCTCTAGTCGGATGGAAGG	19	0	RDR2-resistant
ACGCGGGACGGCGGACAGACACAA	0	11	RDR2-sensitive
ACGCGGGACTGTACGACCGGAGGC	0	26	RDR2-sensitive
ACGCGGGCTGCCACGTCATGAAGC	0	13	RDR2-sensitive
ACGCGGTACTGTTGGACTACGGGT	0	10	RDR2-sensitive
ACGCGGTGCGATACTCACGACGGT	0	15	RDR2-sensitive
ACGCGGTTGTCACGGTCTCGCAAG	0	9	RDR2-sensitive
ACGCGTAGAGGGCGCTGTAGGGGA	0	9	RDR2-sensitive
ACGCGTAGGGGACGCTGTAGGGGC	1	21	RDR2-sensitive
ACGCGTAGGGGGCGCTGTAGGGGC	0	11	RDR2-sensitive
ACGCGTCAGAGGCAGAACACGTCA	0	21	RDR2-sensitive
ACGCGTCAGAGGCAGAACGGCAGA	0	38	RDR2-sensitive
ACGCGTCAGATGCGCGATGGTCGG	1	14	RDR2-sensitive
ACGCGTCGACACGGTGATGGCTGT	0	12	RDR2-sensitive
ACGCGTCGGCTGGGCGGATAGCGT	0	20	RDR2-sensitive
ACGCGTCTGAGACTGTGCGTGCGT	1	14	RDR2-sensitive
ACGCGTCTGAGACTGTTCGGTGCA	0	10	RDR2-sensitive
ACGCGTCTGAGACTGTTCGGTGTT	0	15	RDR2-sensitive
ACGCGTCTTAGACTGTTCGGTGTT	0	10	RDR2-sensitive
ACGCGTGACGGCAGCAACCTCTCT	0	11	RDR2-sensitive
ACGCGTGCGGGCTGTTCGCGGCGT	0	56	RDR2-sensitive
ACGCGTGCGGGTAGGAAGCGAGGA	0	19	RDR2-sensitive
ACGCGTGCTCGCGTTTGTCGTCGA	4	28	RDR2-sensitive
ACGCGTGTCTGAAGCCGGACCCAA	0	11	RDR2-sensitive
ACGCGTTGGACGGATCACGACGTC	51	9	RDR2-resistant
ACGCTAAAGCTAGCGGATCTCGGG	0	13	RDR2-sensitive
ACGCTAAGACTGTGTTCACCGCAT	0	21	RDR2-sensitive
ACGCTACGGGCTTAAACGGATCGG	0	10	RDR2-sensitive
ACGCTACGGGCTTAAACGGGCTAG	0	13	RDR2-sensitive
ACGCTACTGGAGAACGTAGAGGAC	0	35	RDR2-sensitive
ACGCTACTGGTGTTCTCGGCTTCT	0	10	RDR2-sensitive
ACGCTAGGATGAGCATCGACACAT	0	28	RDR2-sensitive
ACGCTAGTGGGTTGTAATTCGGCT	0	11	RDR2-sensitive
ACGCTATAAGTGCCGTCGGGATAA	0	9	RDR2-sensitive
ACGCTATACTGCATGCACTCAGCC	0	38	RDR2-sensitive
ACGCTATCGCAACGCACGGACACA	0	25	RDR2-sensitive
ACGCTCACCGGAACTACGAGGACG	0	17	RDR2-sensitive
ACGCTCACGTATGATCCTGTCGCT	0	33	RDR2-sensitive
ACGCTCATCTCCTGCGGATCTCGG	0	13	RDR2-sensitive
ACGCTCCAGAGTTCTGACTGACGG	0	10	RDR2-sensitive
ACGCTCCAGATTGAACGAAGGCAG	0	13	RDR2-sensitive
ACGCTCGACGAACTGTACATCGGC	0	14	RDR2-sensitive

ACGCTCGACGACTGGCAGAAGGAC	0	12	RDR2-sensitive
ACGCTCGCAGAGCCCGGACGTCGT	0	13	RDR2-sensitive
ACGCTCGGCAAACTGTACATCGGC	0	10	RDR2-sensitive
ACGCTCGGCGAACTGTACATCGGC	26	337	RDR2-sensitive
ACGCTCGGCGAACTGTATATCGGC	0	9	RDR2-sensitive
ACGCTCGGTCACGCGCAGGATGAC	0	14	RDR2-sensitive
ACGCTCGTCGAGCCAAGCTGAGAC	0	19	RDR2-sensitive
ACGCTCTCGGGCAACCGTCGTCGT	0	16	RDR2-sensitive
ACGCTCTGGATGCGGCAGAACCGT	2	37	RDR2-sensitive
ACGCTCTGTACCATCATGGACCGT	0	10	RDR2-sensitive
ACGCTGAGATGAGCATCAACACAT	0	11	RDR2-sensitive
ACGCTGAGCGTGACTGCATGAGGA	0	9	RDR2-sensitive
ACGCTGATGGAGGATAGACTGGTG	43	3	RDR2-resistant
ACGCTGCTGGAGAGCATAGAAGAC	0	17	RDR2-sensitive
ACGCTGCTGGAGAGCGTAGAGGAC	0	9	RDR2-sensitive
ACGCTGCTGGAGGACGTAGAGGAC	0	11	RDR2-sensitive
ACGCTGCTGTAATGGACAGGGGTA	0	38	RDR2-sensitive
ACGCTGCTGTAGAGCGTAGACGAC	0	26	RDR2-sensitive
ACGCTGGAACACTGGGACGAGCGC	0	15	RDR2-sensitive
ACGCTGGAAGCTGTGAACCGTCAT	0	10	RDR2-sensitive
ACGCTGGACGCAGGAAGCAGAACG	0	11	RDR2-sensitive
ACGCTGGAGGCAGAACACGTCAGA	0	12	RDR2-sensitive
ACGCTGGATGTAGCGCAACGGTAG	0	9	RDR2-sensitive
ACGCTGGCAAGGAACACGGGCGCG	44	8	RDR2-resistant
ACGCTGGCACAACCCGGACGTCGT	0	11	RDR2-sensitive
ACGCTGGCACAACCTGAACGACGT	2	22	RDR2-sensitive
ACGCTGGCAGATTAAGACTCGCAT	0	9	RDR2-sensitive
ACGCTGGCCAGTGCGACGTGACAT	0	12	RDR2-sensitive
ACGCTGGCCCAACCTGAACGACGT	1	22	RDR2-sensitive
ACGCTGGGATGAGCATCGACACAT	0	80	RDR2-sensitive
ACGCTGGGATGAGCATCGATACAT	0	30	RDR2-sensitive
ACGCTGGTAAGACGGGAACACTAG	0	27	RDR2-sensitive
ACGCTGGTGCTGTGAACACGTCGA	0	17	RDR2-sensitive
ACGCTGTAGAAGAGGGTACCCCTT	0	10	RDR2-sensitive
ACGCTGTAGGGGCACCGCTGCGGC	0	27	RDR2-sensitive
ACGCTGTATATGAGGTTTTTCCGT	0	10	RDR2-sensitive
ACGCTGTCGTGCTGTTTCGTGCAT	3	27	RDR2-sensitive
ACGCTGTGGATGACGACCAGTTGA	0	13	RDR2-sensitive
ACGCTGTTGTAGAATGTAGAGGAC	0	15	RDR2-sensitive
ACGCTTAGTCCGGTGAACGTCTGC	0	10	RDR2-sensitive
ACGCTTCGGACTGGCACGACACGG	0	68	RDR2-sensitive
ACGCTTCGGGCTGGCACAACACGG	0	67	RDR2-sensitive
ACGCTTCTGGAGCGAGGTCGTGGA	0	9	RDR2-sensitive
ACGCTTGATGTGACCCGGACTAAA	0	28	RDR2-sensitive
ACGCTTGATGTGACCCGGGCTAAA	0	19	RDR2-sensitive
ACGCTTGATGTGACTCGGGCTAAA	0	10	RDR2-sensitive
ACGCTTGATGTGATCCGGGCTAAA	0	19	RDR2-sensitive
ACGGAAAAAAGCACGGCACGACAC	0	14	RDR2-sensitive
ACGGAAAAGCAGCAGAGCACTCAC	0	9	RDR2-sensitive

ACGGAAATCTCGGGTCCGAGGACA	0	9	RDR2-sensitive
ACGGAACAAGAACCACAAGGGCAT	0	35	RDR2-sensitive
ACGGAACAGGAGAGGGCAGAACAG	0	22	RDR2-sensitive
ACGGAACGACGACGACACAGGAGG	0	23	RDR2-sensitive
ACGGAACGAGAACTACCCGGTAAA	0	11	RDR2-sensitive
ACGGAACGGGGACGGCAGAACGGC	0	20	RDR2-sensitive
ACGGAACGTGTGATGGTTTAACAG	16	0	RDR2-resistant
ACGGAACTAGTCGGACACGGATAT	0	15	RDR2-sensitive
ACGGAACTGTACGCGGGGACCCAT	0	37	RDR2-sensitive
ACGGAAGCAAGAGGACGTCAGCAA	5	41	RDR2-sensitive
ACGGAAGCACGCGTTGGACGGATC	84	7	RDR2-resistant
ACGGAAGCAGACAGAGCTGGGCAT	0	28	RDR2-sensitive
ACGGAAGCAGGACAACGTCAGCAG	1	17	RDR2-sensitive
ACGGAAGCAGGACGACGTCAGCAG	0	14	RDR2-sensitive
ACGGAAGCAGGAGGACGTCAGCAA	1	16	RDR2-sensitive
ACGGAAGGGAATAAGACGGGCCAG	0	30	RDR2-sensitive
ACGGAAGTACGATGAGATGAGCAT	0	33	RDR2-sensitive
ACGGAAGTACGATGGGATGAGCAT	1	17	RDR2-sensitive
ACGGAAGTACGCTAAGATGAGCAT	0	10	RDR2-sensitive
ACGGAAGTACGCTGGGATGAGCAT	0	36	RDR2-sensitive
ACGGAATACGGCTCGAACTTCCAG	0	12	RDR2-sensitive
ACGGAATGAATCACCTGACGGTGC	0	10	RDR2-sensitive
ACGGAATGACAGAGCGGAATGGGA	0	10	RDR2-sensitive
ACGGAATGAGACAGCCTACCCCTT	0	27	RDR2-sensitive
ACGGAATGAGGCAGCCTACCCCTT	0	14	RDR2-sensitive
ACGGAATGTGGAGAGCCCAGACGC	0	17	RDR2-sensitive
ACGGACAATGGCGACGTGCAAACA	0	10	RDR2-sensitive
ACGGACACACCGCGGATACTTCTG	0	9	RDR2-sensitive
ACGGACACCTAACTAGATGGGCTT	0	13	RDR2-sensitive
ACGGACACGGTATGTTAGTGCCAT	0	22	RDR2-sensitive
ACGGACACGGTCATATATAGGGTT	0	10	RDR2-sensitive
ACGGACAGGATCCCTACAAGACGA	0	10	RDR2-sensitive
ACGGACAGGGGAGGGCAGAACAGA	0	9	RDR2-sensitive
ACGGACAGTACGAAGACGCTGCAC	0	10	RDR2-sensitive
ACGGACCAAACGGACGGCCCACAC	0	19	RDR2-sensitive
ACGGACCACTATTTTCGTCGGCCC	0	19	RDR2-sensitive
ACGGACCAGACGCACGCGGGCCAC	0	15	RDR2-sensitive
ACGGACCAGGATATTACCGTGCAG	0	9	RDR2-sensitive
ACGGACCATCCGGCTACTAGGCGC	0	32	RDR2-sensitive
ACGGACCATCTGTAGGGAAATGGC	0	9	RDR2-sensitive
ACGGACCATGCCCGGCACTGGCAC	0	12	RDR2-sensitive
ACGGACCATTGGGGCGGCTGGGGC	0	17	RDR2-sensitive
ACGGACCCGATGAATGACGGCTAG	0	12	RDR2-sensitive
ACGGACCGAGATAGCACGGCTCGA	0	15	RDR2-sensitive
ACGGACCGAGCTGGCCAGGGACAG	0	13	RDR2-sensitive
ACGGACCGGGCTAGCGCGGCTTGA	0	9	RDR2-sensitive
ACGGACCGTCCAAGCCCTAGGCGC	0	11	RDR2-sensitive
ACGGACCGTCCATACCACAGGTGC	0	11	RDR2-sensitive
ACGGACCGTCCGACCATCAGACGC	0	13	RDR2-sensitive

ACGGACCGTCCGGCATTAGGCCAT	0	9	RDR2-sensitive
ACGGACCGTTAGACCCACAGGCGC	0	12	RDR2-sensitive
ACGGACCGTTCAAGCTACGCCCGC	0	23	RDR2-sensitive
ACGGACCTCAGTGGACAGAGGCAT	0	17	RDR2-sensitive
ACGGACCTGGATGTGCAGAGATAC	0	41	RDR2-sensitive
ACGGACGACGACTGATTGGGACGG	0	10	RDR2-sensitive
ACGGACGACGTAGATAGTGTGGCC	0	11	RDR2-sensitive
ACGGACGAGCAGGGCAAACCGAAG	0	9	RDR2-sensitive
ACGGACGATTACAGATGACGGGAC	0	15	RDR2-sensitive
ACGGACGCTGGACGCAGGAAGCAG	1	59	RDR2-sensitive
ACGGACGGATGCAGACGGATGCAG	0	12	RDR2-sensitive
ACGGACGGCGTATGGTCGAAGGCA	0	14	RDR2-sensitive
ACGGACGGTCCAGATTACCCGGAG	0	9	RDR2-sensitive
ACGGACGGTCTAGATCGCCGGGAG	0	19	RDR2-sensitive
ACGGACGGTGTAGATTAGCCGGAG	0	67	RDR2-sensitive
ACGGACGGTGTCCAACGACGTCAT	0	10	RDR2-sensitive
ACGGACGGTTATCATGACGGTCAT	0	51	RDR2-sensitive
ACGGACGTGGACGCCTACACTACT	1	17	RDR2-sensitive
ACGGACTAACAGGACGGGGTGAGG	0	9	RDR2-sensitive
ACGGACTGATCTATTAGAAGGGCC	186	3	RDR2-resistant
ACGGACTGGACAGGTGCGCCGTCT	0	9	RDR2-sensitive
ACGGACTGTGAACGCCTACGGTAC	0	59	RDR2-sensitive
ACGGACTTAGACGGTACCCTATTT	19	1	RDR2-resistant
ACGGACTTATTTCGGGCGGTCCGA	0	12	RDR2-sensitive
ACGGAGACGAGCCCATAAGGACGA	0	10	RDR2-sensitive
ACGGAGAGACGGCTCTAAGATTAT	0	18	RDR2-sensitive
ACGGAGAGACGGCTCTAAGATTTT	2	47	RDR2-sensitive
ACGGAGCCGCTCTGTTCTGTGTTT	0	20	RDR2-sensitive
ACGGAGCCGGATACGGAGACTGCT	0	23	RDR2-sensitive
ACGGAGCGAGGAGACAGACACCAT	0	16	RDR2-sensitive
ACGGAGCGGCAGAACACGATTGTG	0	11	RDR2-sensitive
ACGGAGCTCTGACATGACGACGTC	0	35	RDR2-sensitive
ACGGAGGATAGAGGAGTATAGCAT	0	17	RDR2-sensitive
ACGGAGGATATGATGTGTAGTCGG	177	0	RDR2-resistant
ACGGAGGATTGGGAGTAGAAGCGA	0	30	RDR2-sensitive
ACGGAGGCAGAACAGCCGTTGGCA	0	11	RDR2-sensitive
ACGGAGGCAGAACGCGGCTCGGGT	0	12	RDR2-sensitive
ACGGAGGGCTGGTTTGGACGTCAT	0	11	RDR2-sensitive
ACGGAGGTGTGGGCATAAGAGGAA	0	18	RDR2-sensitive
ACGGAGTAGAGCAGAACGGTTCCT	1	24	RDR2-sensitive
ACGGAGTCCGAACGGGTTGACGGT	0	9	RDR2-sensitive
ACGGAGTCTGCTGGAACGCACGGT	0	15	RDR2-sensitive
ACGGAGTCTGCTGGAGCGCGCAAC	1	13	RDR2-sensitive
ACGGAGTGACGCAAGCAAGGACCC	0	10	RDR2-sensitive
ACGGAGTGGTAGTGGAGGGGATTT	19	0	RDR2-resistant
ACGGAGTTCTCCTGTGTCGTTGGT	0	20	RDR2-sensitive
ACGGAGTTGGGAATGGGAATGGCA	0	12	RDR2-sensitive
ACGGAGTTTTTAGATCTACGGTAT	0	27	RDR2-sensitive
ACGGATAACCCGCGGGTAGAAGGT	0	9	RDR2-sensitive

ACGGATACAGGACGGGACCGGGTA	0	9	RDR2-sensitive
ACGGATACGAGTATTTTTTTGACA	2	15	RDR2-sensitive
ACGGATACGGAGAGGAGAGAGAAT	0	21	RDR2-sensitive
ACGGATACGGATACAGGGACCGGA	0	31	RDR2-sensitive
ACGGATACGGATACGGATAGTCTG	0	12	RDR2-sensitive
ACGGATACGGATAGTCTGTTCGGA	0	12	RDR2-sensitive
ACGGATACGGGTTTGGGATAGCAA	0	10	RDR2-sensitive
ACGGATACTTTTGGGGCACCTGGC	22	0	RDR2-resistant
ACGGATAGGGGCATTCGAGGCGGA	87	8	RDR2-resistant
ACGGATAGTTAGAAACAGGACGGA	0	26	RDR2-sensitive
ACGGATAGTTAGAAACGGGACGGA	1	76	RDR2-sensitive
ACGGATAGTTAGAAACGGGGCGGA	0	13	RDR2-sensitive
ACGGATATAGAGGATGTATATAGA	0	12	RDR2-sensitive
ACGGATATGACTCGGCGAGCCCGG	0	14	RDR2-sensitive
ACGGATCACTACAGTATGTGGCAC	0	11	RDR2-sensitive
ACGGATCAGAGCAGACCTTGGCGT	0	12	RDR2-sensitive
ACGGATCAGATGATTTGCTTGTAC	0	9	RDR2-sensitive
ACGGATCAGATGGTTTGCTTGCAC	0	20	RDR2-sensitive
ACGGATCATACCCGGATCGAGCTC	0	27	RDR2-sensitive
ACGGATCTAGACTGCGAAGACCAT	0	9	RDR2-sensitive
ACGGATGACCACATTGAAGAGCGG	0	10	RDR2-sensitive
ACGGATGACGTGGTCTAGCTCGGC	1	15	RDR2-sensitive
ACGGATGAGCTCAACGACGTCATT	1	18	RDR2-sensitive
ACGGATGCTGCAGGAGGGTTCATC	0	9	RDR2-sensitive
ACGGATGTGAAGGCCTACACTACT	0	19	RDR2-sensitive
ACGGATGTGGATTGGGAGACCGTT	0	10	RDR2-sensitive
ACGGATTAGGACCATGCCGGCCCG	0	14	RDR2-sensitive
ACGGATTCAGAATCGGATACGGAT	0	14	RDR2-sensitive
ACGGATTCAGGATCGGATACGGAT	0	15	RDR2-sensitive
ACGGATTCGAGATCGGATACGGAT	0	16	RDR2-sensitive
ACGGATTCGGATACTCGTTTCGGA	0	11	RDR2-sensitive
ACGGATTCGGGATCGGATACGGAT	0	137	RDR2-sensitive
ACGGATTGAATACCTGGAACGGTT	0	10	RDR2-sensitive
ACGGATTGAGTAGTAGCAGCCGAC	0	89	RDR2-sensitive
ACGGATTGTTGTTACGGTGCTCAC	0	18	RDR2-sensitive
ACGGATTTCCTTTTATAGACGTCT	0	24	RDR2-sensitive
ACGGATTTGGCACGGCATGAACTC	1	35	RDR2-sensitive
ACGGATTTTAGCCCAGGCCCGGTT	1	15	RDR2-sensitive
ACGGATTTTCTTTTATAGACGTCT	1	28	RDR2-sensitive
ACGGATTTTTGTTTCGTTGCTCGA	0	12	RDR2-sensitive
ACGGCAAAATAAGGGGGAGGACAG	0	10	RDR2-sensitive
ACGGCAAACGGTAACGGCTGACGG	0	9	RDR2-sensitive
ACGGCAAAGAAGCTGATTCCGGTA	0	12	RDR2-sensitive
ACGGCAAAGGAGCTGATTCCGGTA	2	77	RDR2-sensitive
ACGGCAAAGGAGCTGTGTCCGGTA	0	9	RDR2-sensitive
ACGGCAACCTATTTAGACGGACAT	0	10	RDR2-sensitive
ACGGCAACGGTCGGATCCAACGGT	2	23	RDR2-sensitive
ACGGCAAGAACTGAGGAGGGGCAC	0	9	RDR2-sensitive
ACGGCACATGACAATACCTGGCTG	0	13	RDR2-sensitive

ACGGCACCTGAACGAGGCAGACCA	0	14	RDR2-sensitive
ACGGCACCTGTAGTCTGTAGGCAC	0	22	RDR2-sensitive
ACGGCACGAAAAAGAACGGTCCAG	0	19	RDR2-sensitive
ACGGCACGAAAAAGCACGGTCCAG	0	20	RDR2-sensitive
ACGGCACGACACGAGTTGAACCGG	0	15	RDR2-sensitive
ACGGCACGACACTGGCACTAGCAC	1	19	RDR2-sensitive
ACGGCACGACAGGACACGGCACGG	0	12	RDR2-sensitive
ACGGCACGACAGGCCCGAGCACGC	0	9	RDR2-sensitive
ACGGCACGACTGTCGGCATGGCCT	0	21	RDR2-sensitive
ACGGCACGATTAGCTCCTAGGCAC	0	12	RDR2-sensitive
ACGGCACGATTAGGGTTTAGGCAC	0	14	RDR2-sensitive
ACGGCACGCTTCGCTAGCCGGGCC	0	33	RDR2-sensitive
ACGGCACGGCACAGCTGAGCGGAG	0	9	RDR2-sensitive
ACGGCACGGCACGGTTAAGCACTG	0	22	RDR2-sensitive
ACGGCACGGCACTGGCACTAGCAC	1	21	RDR2-sensitive
ACGGCACGGCTAAGCACTGGTAGT	0	9	RDR2-sensitive
ACGGCACGGCTAAGCAGGCTTAGT	0	9	RDR2-sensitive
ACGGCACGGCTATCGGATCGGGCT	0	12	RDR2-sensitive
ACGGCACGGGTAAGGCACGATCGT	0	14	RDR2-sensitive
ACGGCACGGTACGGATTAAAGCGG	0	13	RDR2-sensitive
ACGGCACGGTAGAATAAGCGGGCG	0	9	RDR2-sensitive
ACGGCACGTCCATGTCTAGATCGT	1	56	RDR2-sensitive
ACGGCACGTGCACCGGGTCGGGCT	1	44	RDR2-sensitive
ACGGCACGTTACGCGGGCTGGGCC	0	12	RDR2-sensitive
ACGGCACTAAGCACTATTAGGCAC	4	42	RDR2-sensitive
ACGGCACTAAGCACTATTAGGCAT	1	73	RDR2-sensitive
ACGGCACTACACAGGCTCATCCGT	0	43	RDR2-sensitive
ACGGCACTCTAGTTGAAGAAGGTT	580	120	RDR2-resistant
ACGGCACTGACGCACGGTACTGAC	0	21	RDR2-sensitive
ACGGCACTGCTCGACGCGTTCGTC	129	27	RDR2-resistant
ACGGCACTGGCACTAGCACGACCA	0	17	RDR2-sensitive
ACGGCACTTGCTGGCACTATACCA	301	11	RDR2-resistant
ACGGCAGAACACACTGATGGCAGA	0	12	RDR2-sensitive
ACGGCAGAACAGCAGACTACTCTT	0	39	RDR2-sensitive
ACGGCAGAACGACAGACTACTCTT	0	15	RDR2-sensitive
ACGGCAGAACGGCAGAACGCGTCA	0	26	RDR2-sensitive
ACGGCAGAACGGCAGAACGGCAGA	0	57	RDR2-sensitive
ACGGCAGAACGGCAGACTACCCTT	1	28	RDR2-sensitive
ACGGCAGAACGGCAGACTACTCTT	0	149	RDR2-sensitive
ACGGCAGGGTAGAGAAGCGACGGT	0	22	RDR2-sensitive
ACGGCAGTGTCTTATCTCCGACGG	0	16	RDR2-sensitive
ACGGCATAGAGAACGTTTCGTACT	0	11	RDR2-sensitive
ACGGCATCGTAGACAGCCGTCGGA	1	19	RDR2-sensitive
ACGGCATGGTTAGGACACGGCCCG	0	10	RDR2-sensitive
ACGGCATTAACTTATCTCCGACGG	0	18	RDR2-sensitive
ACGGCATTGCTACGTACCGTCGGC	33	5	RDR2-resistant
ACGGCATTTTGGGCTTTTTGGACC	0	9	RDR2-sensitive
ACGGCCAAAACCGTCGGACATAAG	0	16	RDR2-sensitive
ACGGCCAAAACCGTCGGACATTAG	0	14	RDR2-sensitive

ACGGCCAAAGCCGTCGGACATAAG	0	19	RDR2-sensitive
ACGGCCAGAGTAGAAGCACGACAG	0	27	RDR2-sensitive
ACGGCCAGCCGTCGGAAATAAGAC	0	15	RDR2-sensitive
ACGGCCAGCTAGCAAAGATGAGTG	0	10	RDR2-sensitive
ACGGCCAGGCAACGTTTCGGACGT	0	14	RDR2-sensitive
ACGGCCAGGTGTCGGACGGTCCGC	0	9	RDR2-sensitive
ACGGCCAGTCTGACAGCCGTCGGC	0	11	RDR2-sensitive
ACGGCCCCATAGGAAACCGTCGGA	0	10	RDR2-sensitive
ACGGCCCCATAGGCAACCGTCGGA	0	34	RDR2-sensitive
ACGGCCCCGTAGGCAACCGTCGGA	1	32	RDR2-sensitive
ACGGCCCGTAGTGCCTGGGCACGG	0	79	RDR2-sensitive
ACGGCCCGTCAGCCTGCTAGTCGT	1	21	RDR2-sensitive
ACGGCCCGTCAGCCTGGTAGTCGT	0	10	RDR2-sensitive
ACGGCCCGTGAGCCTGTTAGTCGT	14	119	RDR2-sensitive
ACGGCCCGTGAGTCTGTTAGTCGT	8	228	RDR2-sensitive
ACGGCCCTGGCTCTTTGATGGCTC	0	14	RDR2-sensitive
ACGGCCTAAGTTGGACGCGTGCGT	0	13	RDR2-sensitive
ACGGCCTCCTACAGGACCGTCGGA	3	16	RDR2-sensitive
ACGGCCTCGATGAACTGCCGGCGC	1	19	RDR2-sensitive
ACGGCCTCGTACAGATCCGTCGGA	0	11	RDR2-sensitive
ACGGCCTCGTACCGAGCCGTCGGA	1	12	RDR2-sensitive
ACGGCCTCGTACGTAGCCGTCGGA	0	14	RDR2-sensitive
ACGGCCTGGTGACGTGGCTGGCGC	3	17	RDR2-sensitive
ACGGCCTGTCAGCCTGTTAGTCGT	0	17	RDR2-sensitive
ACGGCCTTCAGCGACCTACGGTTG	1	16	RDR2-sensitive
ACGGCCTTGTAGAAAGCCGTCGGA	0	10	RDR2-sensitive
ACGGCCTTGTAGGAAGCCGTCGGA	0	16	RDR2-sensitive
ACGGCCTTTAGCGACCTACGGTTG	0	31	RDR2-sensitive
ACGGCGAACCGGAAGCAGCTCCAG	0	15	RDR2-sensitive
ACGGCGACTGGTAAAGGAGACGAC	0	11	RDR2-sensitive
ACGGCGCTTAACTCGGACGTCGGT	0	13	RDR2-sensitive
ACGGCGTAAGTACATTGGTGACGG	0	9	RDR2-sensitive
ACGGCGTAGATTCATGGAAGGCAG	0	14	RDR2-sensitive
ACGGCGTCGTTACCGAGAGCAGAA	0	12	RDR2-sensitive
ACGGCGTCTGTACACGGGAACGGT	100	0	RDR2-resistant
ACGGCTAACTCGGTCTTCGACGGT	0	12	RDR2-sensitive
ACGGCTACCGTCGGAACAGTCGGA	0	17	RDR2-sensitive
ACGGCTACCTACAAGACCGTCGGA	0	10	RDR2-sensitive
ACGGCTACCTGCGCCTAGGGCTGA	2	20	RDR2-sensitive
ACGGCTACCTTCGCCTAGGGCTGA	3	32	RDR2-sensitive
ACGGCTACGATCGCAGAACAGTAA	0	15	RDR2-sensitive
ACGGCTACGCTGACAGCCGTCGGA	0	9	RDR2-sensitive
ACGGCTACGGACCGGGCTCTCGGA	1	14	RDR2-sensitive
ACGGCTACTGTAGCCCACGGGCCG	0	16	RDR2-sensitive
ACGGCTACTTGCGCCTAGGGCTGA	0	25	RDR2-sensitive
ACGGCTAGCAGACTGACGGACCAT	0	10	RDR2-sensitive
ACGGCTAGGAACCAGACGGTCTGT	0	13	RDR2-sensitive
ACGGCTAGGCACGGTAGAAGATCC	0	11	RDR2-sensitive
ACGGCTATAGCTGAAGATGCTCAA	0	13	RDR2-sensitive

ACGGCTATCAGTCGGTCGTCTGAC	0	9	RDR2-sensitive
ACGGCTCAACATCCTGACGGACAG	0	24	RDR2-sensitive
ACGGCTCCTCCGCGAACGGCTCAA	1	12	RDR2-sensitive
ACGGCTCGTGAGCCTGTTAGTCGT	0	16	RDR2-sensitive
ACGGCTCTAGTGAAACACGTCATC	0	9	RDR2-sensitive
ACGGCTCTGAATCTGCAACGGTCG	1	18	RDR2-sensitive
ACGGCTCTGGCTCCTTGATGGCTC	0	34	RDR2-sensitive
ACGGCTCTGGCTCTTTGATGGCTC	2	111	RDR2-sensitive
ACGGCTCTGTCTCTTTGATGGCTC	1	32	RDR2-sensitive
ACGGCTGAGATCGCAGAACGGCAA	0	27	RDR2-sensitive
ACGGCTGAGATCGCAGAATGACAG	0	16	RDR2-sensitive
ACGGCTGAGCACGTGGGCCGGCAC	1	25	RDR2-sensitive
ACGGCTGAGCACGTGGGCTGGCAC	0	17	RDR2-sensitive
ACGGCTGCACGGTACTCCAAGTAC	0	14	RDR2-sensitive
ACGGCTGCCTAGGAGACCGTCGGA	0	56	RDR2-sensitive
ACGGCTGCGTGTACCCGTCGGAAA	0	11	RDR2-sensitive
ACGGCTGGAGTAGAGACCGGTGTG	0	10	RDR2-sensitive
ACGGCTGGCACTCGGCAAAGAGGC	2	17	RDR2-sensitive
ACGGCTGGCAGACTGACCGTCGGA	0	16	RDR2-sensitive
ACGGCTGGGCGGCAAAGGATTCGC	0	9	RDR2-sensitive
ACGGCTGGTCCAAGCTCGACACGG	0	16	RDR2-sensitive
ACGGCTTACTTAGAGACCGTCGGA	0	14	RDR2-sensitive
ACGGCTTAGATTCACCGAAGGCAG	0	10	RDR2-sensitive
ACGGCTTGTCTGACAGCAGTCGGA	0	17	RDR2-sensitive
ACGGCTTGTCTGATAGTCGTCGGT	0	11	RDR2-sensitive
ACGGCTTTGTAGGAAATTGTCGGC	0	11	RDR2-sensitive
ACGGCTTTTTGTAGCTTCGTCGTG	2	18	RDR2-sensitive
ACGGGAACGAGGAACACGGTACGT	0	20	RDR2-sensitive
ACGGGAACGAGGAACGCGGTACGT	0	9	RDR2-sensitive
ACGGGAATGTACCAACTGACGGTG	0	9	RDR2-sensitive
ACGGGACAACTAGGATAGGGACGT	0	17	RDR2-sensitive
ACGGGACCCGCCTTAAGCCGTCGG	0	15	RDR2-sensitive
ACGGGACCTTAGAATGCTTGGCAA	0	18	RDR2-sensitive
ACGGGACGACTGTGAAGACGACGA	1	104	RDR2-sensitive
ACGGGACGGATACATGTACTACCC	0	12	RDR2-sensitive
ACGGGACGGATACGGAACAGCCAA	0	13	RDR2-sensitive
ACGGGACGGATACGGATACAGAGA	0	10	RDR2-sensitive
ACGGGACGGATACGGATACAGGGA	0	9	RDR2-sensitive
ACGGGACGGTGAATGCTTCTCCAT	0	64	RDR2-sensitive
ACGGGACTAACGGGCACATAACAA	0	13	RDR2-sensitive
ACGGGACTCAGCTGAGGCAGCCAT	0	9	RDR2-sensitive
ACGGGACTGCTGAAGATTAGGGTT	0	15	RDR2-sensitive
ACGGGACTGTAAACGTAGAGAGGC	0	14	RDR2-sensitive
ACGGGACTGTCCGGTGGACTAGGC	0	12	RDR2-sensitive
ACGGGAGGGAAGGGAGCGGATCTG	0	11	RDR2-sensitive
ACGGGAGGGAGGACCATCACTACG	43	4	RDR2-resistant
ACGGGAGTACACTGGGATGAGCAC	0	14	RDR2-sensitive
ACGGGAGTACACTGGGATGAGCAT	0	19	RDR2-sensitive
ACGGGAGTACGCTAGGATGAGCAT	1	40	RDR2-sensitive

ACGGGAGTACGCTGAGATGAGCAT	0	12	RDR2-sensitive
ACGGGAGTACGCTGGGATGAGCAT	2	27	RDR2-sensitive
ACGGGATGGGACGGGACGATCCAT	0	10	RDR2-sensitive
ACGGGATTTTTCGGACTTCGGCCG	3	68	RDR2-sensitive
ACGGGCAAACTGAGCGCGTGACGG	0	11	RDR2-sensitive
ACGGGCACACAGAGCGGTAGGGGG	43	1	RDR2-resistant
ACGGGCACACAGAGCGGTAGGGGT	201	0	RDR2-resistant
ACGGGCAGTGGAAGATGATGTGCG	0	30	RDR2-sensitive
ACGGGCATAGTGTCGGGCTAGCAC	0	10	RDR2-sensitive
ACGGGCATCAACTCTGCAGGTAGA	0	18	RDR2-sensitive
ACGGGCATGCGTACTGTCGTGGAC	0	20	RDR2-sensitive
ACGGGCCAGAATCAACACGGTCGA	0	12	RDR2-sensitive
ACGGGCCATGAGCAGCAGGGTCGA	0	12	RDR2-sensitive
ACGGGCCCGGCACGAACTGATGGG	0	10	RDR2-sensitive
ACGGGCCCTTAGGGATTGTTCGGT	0	12	RDR2-sensitive
ACGGGCCGACAGCATGGACGAGCA	1	52	RDR2-sensitive
ACGGGCCGAGCTGACCAGGGACAG	0	14	RDR2-sensitive
ACGGGCCGATCCGATACTAGGCAT	0	12	RDR2-sensitive
ACGGGCCTTGCCTGGGTCGCGGCA	0	16	RDR2-sensitive
ACGGGCGACGGTAGAAGAGGAAGA	0	9	RDR2-sensitive
ACGGGCGCATGAACTACGGCGAGC	0	9	RDR2-sensitive
ACGGGCGCCGGCAGGCAACTTCTC	0	9	RDR2-sensitive
ACGGGCGGCCCGCACTGTTTGGAC	0	31	RDR2-sensitive
ACGGGCGGCCCGTACTGTTTGGAC	0	9	RDR2-sensitive
ACGGGCGGGCAGGCGTATGGAGGC	0	12	RDR2-sensitive
ACGGGCGTTTCTTAGAACAGTCGG	1	28	RDR2-sensitive
ACGGGCTAAACGGGCTAAACGGGC	0	12	RDR2-sensitive
ACGGGCTAGACGCACGCGGGCCAC	1	13	RDR2-sensitive
ACGGGCTAGATGCACACGGGCCAC	0	13	RDR2-sensitive
ACGGGCTAGATGCACGCGGGCCAC	0	80	RDR2-sensitive
ACGGGCTAGATGCATGCGGGCCAC	0	10	RDR2-sensitive
ACGGGCTAGGTTTACAACGTGCCG	0	15	RDR2-sensitive
ACGGGCTAGTTTGGTGTGTGGCGT	0	9	RDR2-sensitive
ACGGGCTATATGGGCTAAACGGGC	1	42	RDR2-sensitive
ACGGGCTCACTGCTGGACCGGTCC	0	14	RDR2-sensitive
ACGGGCTCGACAACACGCGAAGCG	0	14	RDR2-sensitive
ACGGGCTCGACGAGATGAAGCCGG	1	40	RDR2-sensitive
ACGGGCTGAGCTGGCATGACCCGA	0	18	RDR2-sensitive
ACGGGCTGCCGGCGTCCTTTACGG	0	10	RDR2-sensitive
ACGGGCTGCGGGTTTGATGGCCAT	0	9	RDR2-sensitive
ACGGGCTGCTGGACCAGCGTCGCA	0	11	RDR2-sensitive
ACGGGCTTAGAGGTAAACGGGCCG	0	33	RDR2-sensitive
ACGGGCTTCGTGCCGGGCTCACGG	0	30	RDR2-sensitive
ACGGGCTTCGTGCCGGGCTCGCGG	2	39	RDR2-sensitive
ACGGGCTTCGTGTCGGGCTCGCGG	0	10	RDR2-sensitive
ACGGGGAATCTGGCGGAAGAGCGA	0	9	RDR2-sensitive
ACGGGGAATTAGGACGAGACGCGG	0	9	RDR2-sensitive
ACGGGGACATGTAATATCACGGTA	0	10	RDR2-sensitive
ACGGGGACGGAGACAGAGAAGCAT	0	9	RDR2-sensitive

ACGGGGACGGGGATGGAGAAGCAT	0	18	RDR2-sensitive
ACGGGGACTGTTGGCAGACCGGTT	0	12	RDR2-sensitive
ACGGGGCATTGTAAGTGGCAGAGT	61	9	RDR2-resistant
ACGGGGCGCTGTGGTCCATATGGC	0	18	RDR2-sensitive
ACGGGGGCTTTGGCAGAACTGCAC	0	29	RDR2-sensitive
ACGGGTAATAGTGGAGGATGTCCT	0	11	RDR2-sensitive
ACGGGTACGGATTTGGAACGGCAA	0	11	RDR2-sensitive
ACGGGTACGGGTTTGGGACGGCAA	0	78	RDR2-sensitive
ACGGGTACGGGTTTGGGACGGTAA	0	16	RDR2-sensitive
ACGGGTACGGGTTTGGGATAGCAA	0	9	RDR2-sensitive
ACGGGTACGGGTTTGGGATGGCAA	0	9	RDR2-sensitive
ACGGGTAGGATACGGAATCACAAA	0	23	RDR2-sensitive
ACGGGTAGGATACGGAATCACTAA	0	78	RDR2-sensitive
ACGGGTAGGATACGGAATCGCTAA	0	16	RDR2-sensitive
ACGGGTAGGATACGTGATAGCTAA	0	16	RDR2-sensitive
ACGGGTAGGATAGGTGATAGCTAA	0	13	RDR2-sensitive
ACGGGTAGTTAAGAATCGGGACGG	0	11	RDR2-sensitive
ACGGGTAGTTAAGATTCGGGACGG	1	12	RDR2-sensitive
ACGGGTATTAGAACGTTCCGGCCT	0	10	RDR2-sensitive
ACGGGTATTAGAACGTTCCGGCTT	1	12	RDR2-sensitive
ACGGGTCATGCTGTGGTTCGGGTC	0	9	RDR2-sensitive
ACGGGTCCCTGTAGCGAGTCCGGT	0	11	RDR2-sensitive
ACGGGTCCTGTAGACGGCCAATGC	0	19	RDR2-sensitive
ACGGGTCGATAACATGGACGAGCA	0	11	RDR2-sensitive
ACGGGTCGATCGGTATACGCGTAT	0	13	RDR2-sensitive
ACGGGTCGGATACGGATACTACCC	0	47	RDR2-sensitive
ACGGGTCGGATACGGATAGTACCC	0	18	RDR2-sensitive
ACGGGTCGGTCTGAAACACGGCTC	2	63	RDR2-sensitive
ACGGGTCGGTGCGAGACAGGCGGA	0	16	RDR2-sensitive
ACGGGTCTCTGTGGAGAACGGGAT	0	40	RDR2-sensitive
ACGGGTCTGAGTCCGGCACGACAC	0	43	RDR2-sensitive
ACGGGTCTGCGGCAATGTATCATG	0	9	RDR2-sensitive
ACGGGTCTGTATGAGCACGGCCAA	0	9	RDR2-sensitive
ACGGGTGACGGATATGGATAGCAT	0	15	RDR2-sensitive
ACGGGTGCTCGGCTGAGACAGCGT	0	10	RDR2-sensitive
ACGGGTGGTAGAACGCGGCAGTGT	0	14	RDR2-sensitive
ACGGGTGTAGAGTGTAGACGCCTA	1	113	RDR2-sensitive
ACGGGTTACTGTTATTGATCGGCT	72	10	RDR2-resistant
ACGGGTTCGACGACTCGACGGTAA	0	11	RDR2-sensitive
ACGGGTTGGTCTGTTACGGTCATG	0	19	RDR2-sensitive
ACGGGTTTTGGATATCCGTCGGAT	0	9	RDR2-sensitive
ACGGTACACAATCTGATCTGACGG	40	0	RDR2-resistant
ACGGTACCCTAAGAACCGTCGGAA	0	10	RDR2-sensitive
ACGGTACCCTAGGAACCGTCGGAA	0	11	RDR2-sensitive
ACGGTACCTGTACGTTGCAACGGC	0	10	RDR2-sensitive
ACGGTACGAACAAGCGGGCGGGCT	0	14	RDR2-sensitive
ACGGTACGAATAAGCGGGCGGGCT	0	24	RDR2-sensitive
ACGGTACGACTGTTAAACGGCCCA	1	24	RDR2-sensitive
ACGGTACTATAGTCATGTCGTGCT	0	15	RDR2-sensitive

ACGGTACTGACGCAAAGAATGGCT	0	11	RDR2-sensitive
ACGGTAGAATAAACGGGCGGGCTT	0	74	RDR2-sensitive
ACGGTAGAATAAGCGGACGGACTT	0	64	RDR2-sensitive
ACGGTAGAATAAGCGGGCAGGTTT	0	9	RDR2-sensitive
ACGGTAGAATAAGCGGGCGGACTT	0	37	RDR2-sensitive
ACGGTAGAATAAGCGGGCGGGCTA	0	40	RDR2-sensitive
ACGGTAGAATAAGCGGGCGGGCTC	0	18	RDR2-sensitive
ACGGTAGAATAAGCGGGCGGGCTT	28	619	RDR2-sensitive
ACGGTAGAATAAGCGGGCGGGTTT	0	25	RDR2-sensitive
ACGGTAGAATAAGTGGACGGGCTT	1	27	RDR2-sensitive
ACGGTAGAATAAGTGGGCGGGCTT	0	21	RDR2-sensitive
ACGGTAGAATGGACGGCTGAGATC	0	18	RDR2-sensitive
ACGGTAGTGTAGACCAGGCACAAC	0	13	RDR2-sensitive
ACGGTATCGTAGACAGCCGTCGGA	0	9	RDR2-sensitive
ACGGTATGAAGACGCTGCACCCGT	0	23	RDR2-sensitive
ACGGTCAACGCACGGCATGAGCTT	0	19	RDR2-sensitive
ACGGTCAACTGAACGCGGTGGGCC	0	13	RDR2-sensitive
ACGGTCAAGCCTTATCTCCGACGG	0	9	RDR2-sensitive
ACGGTCAATTTATCTCCGACGGCT	0	9	RDR2-sensitive
ACGGTCACGTAGAAAGCATGTCTT	0	17	RDR2-sensitive
ACGGTCAGGCAGAGCAACAGGATG	1	15	RDR2-sensitive
ACGGTCATACGGCGAACAGAACGA	0	22	RDR2-sensitive
ACGGTCATCTATACTCTCCAGCAG	0	10	RDR2-sensitive
ACGGTCATGGCCTGACACGAGCAT	0	33	RDR2-sensitive
ACGGTCCAACGAAGACACGACATG	0	9	RDR2-sensitive
ACGGTCCAAGACAACCGTCGGACA	0	14	RDR2-sensitive
ACGGTCCAAGCACTGCACGACGCT	0	9	RDR2-sensitive
ACGGTCCATCTGTACGGCTGTACC	0	20	RDR2-sensitive
ACGGTCCCGTAGGCAACCGTCGGA	0	13	RDR2-sensitive
ACGGTCCGACGAAGACACGACGTG	0	10	RDR2-sensitive
ACGGTCCGCAGCTCTGGGCCGGAC	1	20	RDR2-sensitive
ACGGTCCGGATGTGTGCAGATCGG	0	9	RDR2-sensitive
ACGGTCCGTAGATACGCAGATGAT	0	12	RDR2-sensitive
ACGGTCCGTCGCTCTGGACCGGAC	0	11	RDR2-sensitive
ACGGTCCGTGAGCCTGTTAGTCGT	1	81	RDR2-sensitive
ACGGTCCGTGGCTCTGGACCGGAT	0	36	RDR2-sensitive
ACGGTCCTGTAGGAGGCCGTCGGA	0	33	RDR2-sensitive
ACGGTCGACTGCAACGCATTAAAT	2	36	RDR2-sensitive
ACGGTCGACTGCAACGCATTTAAT	1	34	RDR2-sensitive
ACGGTCGACTGCTACAGACGCCAA	1	24	RDR2-sensitive
ACGGTCGACTGCTACAGGCGCCAA	5	29	RDR2-sensitive
ACGGTCGCGACAGAACACGGTACT	0	16	RDR2-sensitive
ACGGTCGCTGTTACGGTAGTCCTT	0	10	RDR2-sensitive
ACGGTCGGCTGACGTGGCGTGCAC	4	35	RDR2-sensitive
ACGGTCGGTACCGGTGCATGGCCT	0	21	RDR2-sensitive
ACGGTCGGTCCGGCACAACCCGTT	0	12	RDR2-sensitive
ACGGTCGTGACCCGGCATGAGCAT	0	106	RDR2-sensitive
ACGGTCTACCCGTTTAAGTCTGAG	19	0	RDR2-resistant
ACGGTCTAGATTGGTGGAAGGCAG	0	18	RDR2-sensitive

ACGGTCTATTTAGTAGGGCGGTAA	0	64	RDR2-sensitive
ACGGTCTCCTAGGCAGCCGTCGGA	0	11	RDR2-sensitive
ACGGTCTCGTAGACAGCCGTCGGA	0	16	RDR2-sensitive
ACGGTCTGAAGACGGGTATAAGCC	0	10	RDR2-sensitive
ACGGTCTGCGCACGGTGCTGTCAT	0	13	RDR2-sensitive
ACGGTCTGCTGAAGATGGCCTGAC	0	13	RDR2-sensitive
ACGGTCTGCTGACGTGGCTGGCGC	4	48	RDR2-sensitive
ACGGTCTGTAACCGGAAGGTCCGG	0	18	RDR2-sensitive
ACGGTCTGTAACCTGGGCGCAGGA	1	25	RDR2-sensitive
ACGGTCTGTAGACGAGGGTCGGAC	2	18	RDR2-sensitive
ACGGTCTGTAGTAGGAATGTGCAT	1	38	RDR2-sensitive
ACGGTCTGTGCCGCTACGATGGAC	0	11	RDR2-sensitive
ACGGTCTGTGCTCTGGGCCGGATG	0	17	RDR2-sensitive
ACGGTCTGTGCTGCTACGGTGGAC	0	29	RDR2-sensitive
ACGGTCTGTGGCCAGAGGCTGGAT	0	15	RDR2-sensitive
ACGGTCTTAGGGCCCTGGCGACGG	0	12	RDR2-sensitive
ACGGTCTTGTAGGTAGCCGTCGGA	0	25	RDR2-sensitive
ACGGTGAAGAATAAGCTCGACGAG	77	1	RDR2-resistant
ACGGTGAGCTAACTCGGCATGACT	0	9	RDR2-sensitive
ACGGTGATCGGGCTGAACCGGCAT	0	9	RDR2-sensitive
ACGGTGATGGCTGTAAGCGCGCGG	0	9	RDR2-sensitive
ACGGTGCAATCCGGTCGAGTCGGA	0	9	RDR2-sensitive
ACGGTGCAGATTGGCTGAAGGCAG	1	14	RDR2-sensitive
ACGGTGCCGAGTGGAAGGGACGTC	0	12	RDR2-sensitive
ACGGTGCTGAGTTAAAGCGACGGT	0	15	RDR2-sensitive
ACGGTGGAAGAAGCTGGTGCCCAT	143	20	RDR2-resistant
ACGGTGGAAGAAGCTGGTGCCCCT	17	0	RDR2-resistant
ACGGTGGACTGTTCGTGATGGCGC	1	44	RDR2-sensitive
ACGGTGGAGACAGACAGAGGACGA	0	34	RDR2-sensitive
ACGGTGGCAGAACGGGAATGACAG	0	11	RDR2-sensitive
ACGGTGGGCTGCACGCTGGGACGG	0	10	RDR2-sensitive
ACGGTGTAGATTGGCTGAAGGCAG	0	11	RDR2-sensitive
ACGGTGTAGATTTACCAAAGGCAG	0	10	RDR2-sensitive
ACGGTGTATTATGAAGAGCGGCAA	0	27	RDR2-sensitive
ACGGTGTGGTAGCTGCACGGACGG	0	10	RDR2-sensitive
ACGGTGTGTAGACGTCCATATCGT	0	10	RDR2-sensitive
ACGGTTAATTGGTAGGCACGGTAA	0	75	RDR2-sensitive
ACGGTTAATTTGTAGGTACGGTAA	0	9	RDR2-sensitive
ACGGTTAGAAAACCGGGTCGGGCC	2	60	RDR2-sensitive
ACGGTTAGAAAATCGGGTCGGACC	0	13	RDR2-sensitive
ACGGTTAGACTGTTGCTGACGGCT	0	16	RDR2-sensitive
ACGGTTATAAAACCGGGTCGGGAC	0	10	RDR2-sensitive
ACGGTTCAGATCGGTTAGACGACA	0	11	RDR2-sensitive
ACGGTTCAGATCGTTAGATAACCT	0	13	RDR2-sensitive
ACGGTTCAGATCGTTGGATAACCC	0	52	RDR2-sensitive
ACGGTTCAGATCGTTGGATAATCT	0	9	RDR2-sensitive
ACGGTTCATCTCTGCAAGGACTGC	1	41	RDR2-sensitive
ACGGTTCGCTAGCCGGGTCTGACG	0	11	RDR2-sensitive
ACGGTTCGGATCGATAGAAGGCAG	0	9	RDR2-sensitive

ACGGTTCTCGGATTGGATCGGTAT	1	24	RDR2-sensitive
ACGGTTCTCTACGCTCTCCAGCAG	0	13	RDR2-sensitive
ACGGTTCTCTTGAGTAATTGACGC	0	13	RDR2-sensitive
ACGGTTCTGACCATATCCTTGGCC	15	0	RDR2-resistant
ACGGTTCTGTGGAGAGGGTATCTC	0	13	RDR2-sensitive
ACGGTTCTTGTTGAGTGTTTGGCA	0	10	RDR2-sensitive
ACGGTTCTTGTTGAGTGTTTGGCG	3	28	RDR2-sensitive
ACGGTTGCCTACGGGGCCGTCGGA	0	9	RDR2-sensitive
ACGGTTGCTCTGGGCCATCGGATA	0	10	RDR2-sensitive
ACGGTTGGACGATCGGGTGGGGGC	0	13	RDR2-sensitive
ACGGTTGGACTTAACGTTATCGGT	0	11	RDR2-sensitive
ACGGTTGGATCTTGAACTAACGGA	0	10	RDR2-sensitive
ACGGTTGGGTGACGTGGCTGGCGC	0	16	RDR2-sensitive
ACGGTTGTACTTATATCTGACGGC	1	13	RDR2-sensitive
ACGGTTGTCAACTGTAGATGGCTT	0	12	RDR2-sensitive
ACGGTTGTCTTGGACCGTCGGATA	0	9	RDR2-sensitive
ACGGTTGTTAGCTAGATTCGCGGA	4	18	RDR2-sensitive
ACGGTTTAGATTGTTGGATAACAC	0	17	RDR2-sensitive
ACGGTTTCTGCAGACGGGTTGGAT	0	16	RDR2-sensitive
ACGGTTTCTGTAAGAAGGGGGCGA	0	14	RDR2-sensitive
ACGGTTTGTAGAAGACATCGGCTC	0	33	RDR2-sensitive
ACGGTTTGTCAGATCGGATCGGCT	0	9	RDR2-sensitive
ACGGTTTGTGACCTGACAGCCGGA	0	12	RDR2-sensitive
ACGGTTTTAAGCCGTCGGACATAA	0	9	RDR2-sensitive
ACGTAAATTTACTTCGGTTTAGGG	0	9	RDR2-sensitive
ACGTAACTCGGGAGTGGACGGGAC	0	9	RDR2-sensitive
ACGTAAGCGGACTGACTATCGGAT	0	10	RDR2-sensitive
ACGTAATGGGCGCTGTAGGGGGCA	0	46	RDR2-sensitive
ACGTACATACGCGTAGAAGGACAA	0	12	RDR2-sensitive
ACGTACCGGAGACGTGAGCTGCAT	0	15	RDR2-sensitive
ACGTACCGGGCGTAGAAGATGCGG	0	38	RDR2-sensitive
ACGTACCTAGGAGGACTGCGCTAC	0	14	RDR2-sensitive
ACGTACGCTAGACTGTGACGCGCT	0	25	RDR2-sensitive
ACGTACGTAGAGAAGGAGCCGCTC	0	10	RDR2-sensitive
ACGTACGTAGGACAGGCCGGTCTC	0	16	RDR2-sensitive
ACGTACGTGCTGATGGAACCCGGC	0	13	RDR2-sensitive
ACGTACGTTGGCACTAATAGGCAT	1	74	RDR2-sensitive
ACGTACTGCACAGCCGAACGGTAA	0	14	RDR2-sensitive
ACGTACTGTTTGACGTTCGACGGG	0	23	RDR2-sensitive
ACGTAGAACCGAACCGTGTGGGAC	0	10	RDR2-sensitive
ACGTAGAACTGGGTCGTACCGGAC	0	59	RDR2-sensitive
ACGTAGAATATATCTCGGCGCCAT	1	19	RDR2-sensitive
ACGTAGAATCTTATATCCGACGGG	0	18	RDR2-sensitive
ACGTAGAATGAGGATAGGGAGCGC	0	12	RDR2-sensitive
ACGTAGACACTCGGTGAAGACCGA	0	10	RDR2-sensitive
ACGTAGACCGCCGAACGCGGACAT	0	10	RDR2-sensitive
ACGTAGACGGCAGAACGGCAGAAC	0	34	RDR2-sensitive
ACGTAGAGAGATTCTAGGACGGTT	0	15	RDR2-sensitive
ACGTAGAGCATGACCGGCTGGTGC	15	0	RDR2-resistant

ACGTAGAGCGGAGTAGGCTGGCGC	0	16	RDR2-sensitive
ACGTAGAGCGTATGTTAACGGCGT	0	30	RDR2-sensitive
ACGTAGAGGATGCTGTAGAGGGCA	0	19	RDR2-sensitive
ACGTAGAGGCACTATAGAGGGCAC	0	15	RDR2-sensitive
ACGTAGAGGGCGCTGTAGAGGCAC	1	130	RDR2-sensitive
ACGTAGAGGGCGCTGTAGGGGGCA	5	121	RDR2-sensitive
ACGTAGAGTGCATCTGGCGCCGGA	62	8	RDR2-resistant
ACGTAGATGGCAGAACACGTAGAC	0	9	RDR2-sensitive
ACGTAGCAGATCAGCGAGGACGAC	0	9	RDR2-sensitive
ACGTAGCCGACGGAGCTTTGCGAC	19	1	RDR2-resistant
ACGTAGGACTGTGCAGGCCCGGCC	0	132	RDR2-sensitive
ACGTAGGACTGTGCAGGCCCGGCT	1	39	RDR2-sensitive
ACGTAGGAGGCGCTGTAGGGGCAC	0	202	RDR2-sensitive
ACGTAGGAGGCGCTGTAGGGGGCA	3	52	RDR2-sensitive
ACGTAGGGACCGGCAACAAGACGA	0	11	RDR2-sensitive
ACGTAGGGACGCTGTAGGGGGCAC	0	51	RDR2-sensitive
ACGTAGGGACTGACATCAAGACGA	0	25	RDR2-sensitive
ACGTAGGGACTGGCATCAAGACGA	0	26	RDR2-sensitive
ACGTAGGGAGCGCTGTAGGGAGCA	0	9	RDR2-sensitive
ACGTAGGGAGCGCTGTAGGGGGCA	0	38	RDR2-sensitive
ACGTAGGGGACGCTGTAGAGGGCA	0	68	RDR2-sensitive
ACGTAGGGGACGCTGTAGGGGCAC	0	25	RDR2-sensitive
ACGTAGGGGACGCTGTAGGGGGCA	0	20	RDR2-sensitive
ACGTAGGGGCGCTGTAGAGGGCAC	3	599	RDR2-sensitive
ACGTAGGGGCGCTGTAGAGGGCCC	0	11	RDR2-sensitive
ACGTAGGGGCGCTGTAGGGGGCAC	3	139	RDR2-sensitive
ACGTAGGGGGCGCTGTAGAGGCAC	0	49	RDR2-sensitive
ACGTAGGGGGCGCTGTAGAGGGCA	0	27	RDR2-sensitive
ACGTAGGGGGCGCTGTAGGAGGCA	0	69	RDR2-sensitive
ACGTAGGGGGCGCTGTAGGGGCAC	6	269	RDR2-sensitive
ACGTAGGGGGCGCTGTAGGGGCAT	2	32	RDR2-sensitive
ACGTAGGGGGCGCTGTAGGGGCCC	0	9	RDR2-sensitive
ACGTAGGGGGCGCTGTAGGGGGCA	18	257	RDR2-sensitive
ACGTAGGGGGCGCTGTAGGGGGCC	0	12	RDR2-sensitive
ACGTAGGGGGCGCTGTAGGGGGGC	0	15	RDR2-sensitive
ACGTAGGGGGCGCTGTAGTGGGCA	0	20	RDR2-sensitive
ACGTAGGGGGGCGCTGTAGGAGGC	0	13	RDR2-sensitive
ACGTAGGGTGCGCTGTAGGAGGCA	0	12	RDR2-sensitive
ACGTAGGTCTCGGTTGAAGGGCAC	0	9	RDR2-sensitive
ACGTAGTCCGTAGACGATGGCTTA	0	14	RDR2-sensitive
ACGTAGTGGGCGCTGTAGGGGCAC	2	354	RDR2-sensitive
ACGTAGTGGGCGCTGTAGGGGCCC	0	15	RDR2-sensitive
ACGTAGTGGTCTGGGACAAAGCAA	0	9	RDR2-sensitive
ACGTATGACCGTATAGACAGGCAC	0	9	RDR2-sensitive
ACGTATGGATTGGACGGGATTGGA	0	16	RDR2-sensitive
ACGTATGGGGCGCTGTAGGGGGCA	0	22	RDR2-sensitive
ACGTATGGTCTGTTGGTGGCTGCA	0	11	RDR2-sensitive
ACGTATGTAGAACCTGTGGGGGCG	0	10	RDR2-sensitive
ACGTATTCGGACAAGTGCGGCTGG	0	12	RDR2-sensitive

ACGTCAAAATGTAGTTAGCGGAGT	124	9	RDR2-resistant
ACGTCAAGCGACTTTAGTCGGATA	19	0	RDR2-resistant
ACGTCACCGGTCTGCACATGCATC	16	0	RDR2-resistant
ACGTCACTGTAAATATGTTTAGGA	0	11	RDR2-sensitive
ACGTCAGATCCACGTAGACGTCGA	0	15	RDR2-sensitive
ACGTCAGCTGTAGACGGATGTTGA	0	13	RDR2-sensitive
ACGTCATACGTGCTGCAGAGCGGT	0	13	RDR2-sensitive
ACGTCATGCCGGCCTGCTAACTGT	0	9	RDR2-sensitive
ACGTCCATGTCTAGATCGTGCCGG	0	69	RDR2-sensitive
ACGTCCATGTCTAGATCGTGTCGG	0	14	RDR2-sensitive
ACGTCCGACGGCTGCCAGAGAAGC	0	14	RDR2-sensitive
ACGTCCGACGGCTGTCAGACAGGC	0	12	RDR2-sensitive
ACGTCCGATGGCTGTCAGACAGGC	0	9	RDR2-sensitive
ACGTCCTCGGTCCAAACACGGCTC	0	16	RDR2-sensitive
ACGTCCTGTGGAAGAAGCGGCGCC	0	10	RDR2-sensitive
ACGTCGCGGTGTGTGTGGAACGGC	1	15	RDR2-sensitive
ACGTCTACTGAGCGAACACGGCAA	0	15	RDR2-sensitive
ACGTCTGTATGCACTCAGGGGCGG	0	108	RDR2-sensitive
ACGTGAAGGACGCCGACGACTGCA	64	0	RDR2-resistant
ACGTGAATTGCTGATCCTGGCACG	0	9	RDR2-sensitive
ACGTGACAGTCAGGCAGCCTCGGG	0	12	RDR2-sensitive
ACGTGACAGTGGAGTCGGCGCCAA	2	21	RDR2-sensitive
ACGTGACCTGTAGATGAGGTCTAT	0	22	RDR2-sensitive
ACGTGACGGACTTATTTCGGGCGG	0	9	RDR2-sensitive
ACGTGACGGCCGTTGAAACTGGTG	0	11	RDR2-sensitive
ACGTGAGGACATTGCAGAACGGAT	0	24	RDR2-sensitive
ACGTGCACTGGGCCAGGCTGGCAC	0	15	RDR2-sensitive
ACGTGCACTGTATAAGGGGAGAGG	0	19	RDR2-sensitive
ACGTGCAGACCGGTGACGTAGAGT	35	0	RDR2-resistant
ACGTGCCACGTAGATCCAGGTCAC	1	21	RDR2-sensitive
ACGTGCCGGGCTCATTTCGGGCGG	0	21	RDR2-sensitive
ACGTGCCGGGCTTATTTCGGGCGG	0	9	RDR2-sensitive
ACGTGCGGACCGTCCGGCCCCAGG	0	10	RDR2-sensitive
ACGTGCGGAGGAAGATAAAGGTGT	0	11	RDR2-sensitive
ACGTGCGGGCGCGTGTGTCGCGGC	0	19	RDR2-sensitive
ACGTGCTGCGTTGGCACAAGTCGG	0	10	RDR2-sensitive
ACGTGCTGGACTTATTTCGGGCGG	1	16	RDR2-sensitive
ACGTGCTGTGCTGACACAACCGGA	0	13	RDR2-sensitive
ACGTGCTTCGTGCCGGACTAACGG	0	10	RDR2-sensitive
ACGTGGAACCGAGCTCGACGCCAC	0	9	RDR2-sensitive
ACGTGGAACCGAGCTCGGCGCCAC	0	14	RDR2-sensitive
ACGTGGAACCGAGCTCGGCGCCAT	2	22	RDR2-sensitive
ACGTGGACAATTCCGGAACGGCGA	0	15	RDR2-sensitive
ACGTGGACCAATCAGATCGCGACA	4	176	RDR2-sensitive
ACGTGGACCAATCAGATCGCGCCA	1	35	RDR2-sensitive
ACGTGGACCAGTCAGATCGCGCCA	1	94	RDR2-sensitive
ACGTGGACCAGTCAGATCGCGTCA	0	18	RDR2-sensitive
ACGTGGACCGTCCGACTTCGGCGC	0	12	RDR2-sensitive
ACGTGGACGCAGGACAGAAGCCGT	0	10	RDR2-sensitive

ACGTGGACGCATGTAGGAGCGGGA	0	13	RDR2-sensitive
ACGTGGAGTAGGCAAGTGTTGGAC	1	22	RDR2-sensitive
ACGTGGATGGAGAATCGGCTCTAC	0	13	RDR2-sensitive
ACGTGGATTTCTAGCAGAGAGCAC	0	18	RDR2-sensitive
ACGTGGGCCTCAGAGCAGGACGAG	0	75	RDR2-sensitive
ACGTGGGCCTCCATGTTGGACGAG	0	11	RDR2-sensitive
ACGTGGGCCTCTGAGCTGGACGAG	16	251	RDR2-sensitive
ACGTGGGCCTCTGAGCTGGACGTG	0	16	RDR2-sensitive
ACGTGGTTGGGCTGACGACGGAAA	0	13	RDR2-sensitive
ACGTGTAGGTACATGTCGGATCAC	0	11	RDR2-sensitive
ACGTGTATATGTAGCGCGGCCTAA	0	11	RDR2-sensitive
ACGTGTCCGGACCATACCATGTAC	1	12	RDR2-sensitive
ACGTGTCGGCAAGCGAGAGGACTG	0	10	RDR2-sensitive
ACGTGTCGGCACGCATGAGAGGCT	0	9	RDR2-sensitive
ACGTGTCGGGCGAAGCAGGCCGCG	0	9	RDR2-sensitive
ACGTGTCGGGCTCATTTCGGGCGG	1	21	RDR2-sensitive
ACGTGTCGGGCTTATTTCGGGCGG	0	11	RDR2-sensitive
ACGTGTCGTGCTGGTCCGGCTATT	0	16	RDR2-sensitive
ACGTGTGACGGGCCACCGGACTGT	0	9	RDR2-sensitive
ACGTGTGACTGTTATGAAGGTATT	0	25	RDR2-sensitive
ACGTGTGGACTACGTGGAGACGCA	0	9	RDR2-sensitive
ACGTGTGGACTACGTGGAGGCGCA	0	14	RDR2-sensitive
ACGTGTGGACTATGTAGAGGCGTA	0	42	RDR2-sensitive
ACGTGTGGACTATGTGGAAGCGCA	1	32	RDR2-sensitive
ACGTGTGGAGATGGCGCTGATGAC	0	24	RDR2-sensitive
ACGTGTGTGGGCTCTGACGCACGG	1	23	RDR2-sensitive
ACGTGTTAGTCTGTGAGGATGGTC	0	10	RDR2-sensitive
ACGTGTTCGTACGTGGATCGGTGA	3	30	RDR2-sensitive
ACGTGTTTGACGACTCGACGGCAA	4	20	RDR2-sensitive
ACGTGTTTTGTGGGCTGAGCCGGT	1	12	RDR2-sensitive
ACGTTAACTAGTGGCGTTTTGGAT	0	15	RDR2-sensitive
ACGTTACCTGGACGTGTGAAGCGC	0	17	RDR2-sensitive
ACGTTACGCGGGCTGGGCCGTGCT	0	19	RDR2-sensitive
ACGTTACGCGGGCTGGGTCGTGCT	0	14	RDR2-sensitive
ACGTTACGTGCGAGATCTCCGGAC	0	9	RDR2-sensitive
ACGTTACGTGCGAGATCTCCGGGC	0	23	RDR2-sensitive
ACGTTACTGCTGTAGTATGGATGG	0	13	RDR2-sensitive
ACGTTACTGGAGAGCGTAGAGGAC	0	16	RDR2-sensitive
ACGTTAGACGGGCTGGGTCGTGCT	1	13	RDR2-sensitive
ACGTTAGACGGGCTGTGCCGTGCT	0	12	RDR2-sensitive
ACGTTCCGGGCTTTAGAGGGTGTG	0	21	RDR2-sensitive
ACGTTCGCGGATTTGGTTTCGGCT	0	10	RDR2-sensitive
ACGTTCGGCTCGTCTGGCTGGGGC	0	9	RDR2-sensitive
ACGTTCTTAAGTTAAGTTCGTCGG	0	15	RDR2-sensitive
ACGTTGACACTGCGGACGGTGAGA	0	16	RDR2-sensitive
ACGTTGACTGTGACCAGACGCGTG	0	10	RDR2-sensitive
ACGTTGACTTTGTGGAAGCGTGTG	0	14	RDR2-sensitive
ACGTTGCGCGGACGCTAGGGGCAC	0	10	RDR2-sensitive
ACGTTGCTGGAGAGAGTAGAGGAC	0	10	RDR2-sensitive

ACGTTGCTGGAGAGCGTAGAAGAC	0	12	RDR2-sensitive
ACGTTGCTGGAGAGCGTAGAGGAC	0	54	RDR2-sensitive
ACGTTGCTGGAGAGTGTAGAAGAC	0	26	RDR2-sensitive
ACGTTGCTGGAGGGAGTAGAGGAC	6	87	RDR2-sensitive
ACGTTGCTGGATGGAGTAGAGGAC	0	21	RDR2-sensitive
ACGTTGCTGGGCGCGTTGAGGACG	0	9	RDR2-sensitive
ACGTTGCTGTAGCGAGTAGAGGAC	1	74	RDR2-sensitive
ACGTTGTACGGCGTGGTAGACGGA	0	9	RDR2-sensitive
ACGTTGTACGGCGTGGTAGACGGG	0	11	RDR2-sensitive
ACGTTGTGGATTTGGCGCAGGCTC	0	9	RDR2-sensitive
ACGTTGTTGAACGTGTAGAGGACA	0	10	RDR2-sensitive
ACGTTGTTGGAGGGGTAGAGGACG	0	26	RDR2-sensitive
ACGTTTATGGCTAGGCTCGTTGGC	0	16	RDR2-sensitive
ACGTTTATTATGTCGGTCCGGCGC	32	0	RDR2-resistant
ACGTTTCGACGCTCTGGGTGGCCG	0	13	RDR2-sensitive
ACGTTTGGACCCGAAGCTCGGCGC	1	18	RDR2-sensitive
ACGTTTGGTTCGTTGTATCTAGGA	0	20	RDR2-sensitive
ACGTTTTAGGGCTGGTTTGGTAGC	0	15	RDR2-sensitive
ACTAAAGCGGTCAGACGGTGGAGA	0	12	RDR2-sensitive
ACTAAATCGTTTGGATAGCGTCAC	0	10	RDR2-sensitive
ACTAAATTTTGTAGTGGCTTGGAT	153	1	RDR2-resistant
ACTAAATTTTTGTCGTGTCGGTAC	0	12	RDR2-sensitive
ACTAACAGGCACGGCACGGATCTT	0	16	RDR2-sensitive
ACTAACAGTTTAGATCGTTGGACA	0	16	RDR2-sensitive
ACTAACATCGGTACCTTTGGCATG	21	0	RDR2-resistant
ACTAACCGGACCGGGTCGTGCCTA	0	13	RDR2-sensitive
ACTAACCGGACCGGGTCGTGCCTG	0	10	RDR2-sensitive
ACTAACCTCTGGATAATGGGGGCA	22	2	RDR2-resistant
ACTAACGACGGCAGAACGGCAGAA	0	10	RDR2-sensitive
ACTAAGGACTGAACATCATTTATC	29	2	RDR2-resistant
ACTAAGGGACATGTAGCGTCGGAT	0	17	RDR2-sensitive
ACTAATAATTTGTTGTGTCGATCG	19	1	RDR2-resistant
ACTAATACTTTGATCAGAGGGTGA	21	1	RDR2-resistant
ACTAATTATTGGTCGTGGTATGTC	26	1	RDR2-resistant
ACTAATTGGATGTAAGCATGTTTT	32	0	RDR2-resistant
ACTAATTTAAAGGATCGTCTCATC	0	11	RDR2-sensitive
ACTAATTTGGAGACTGCTTGGTAT	47	0	RDR2-resistant
ACTACACAAAGGACGGAGCGGCTC	0	23	RDR2-sensitive
ACTACATTTATGTGATCTTAGGGT	0	9	RDR2-sensitive
ACTACCAGTGCAGATCGTCCGGAC	0	51	RDR2-sensitive
ACTACCATCCGATCTTTGGACATC	0	11	RDR2-sensitive
ACTACCATCTGATCTTTGGACATC	0	11	RDR2-sensitive
ACTACCCAGTCGGACGGTCCGCAT	0	9	RDR2-sensitive
ACTACCGGAATCGACGGCTTTGCC	0	14	RDR2-sensitive
ACTACCGGAATTCGGCTCTTTGCC	0	14	RDR2-sensitive
ACTACCGGCAAACTCACTCGGCAA	0	9	RDR2-sensitive
ACTACCTATCGGGCCGTGCCGGCA	0	15	RDR2-sensitive
ACTACGATCAGGACGAGATCGGAT	44	2	RDR2-resistant
ACTACGCTCTGGGCTGGACGGTCC	0	12	RDR2-sensitive

ACTACGGCTAGCAGACGGACTCAT	0	10	RDR2-sensitive
ACTACTACCGTACGCGATTGTCTC	0	9	RDR2-sensitive
ACTACTCGATTGTGGATCAACGGC	0	10	RDR2-sensitive
ACTACTCTGGGCCAGCAACAGCAC	1	39	RDR2-sensitive
ACTACTGGTTCTCTGCTCGGTGGC	0	14	RDR2-sensitive
ACTACTGTCGTCCGACGGCCCACG	71	7	RDR2-resistant
ACTACTGTTGATCCGGCGACGGTG	0	9	RDR2-sensitive
ACTACTGTTTTTTGGACGGCCGTC	0	23	RDR2-sensitive
ACTACTTAAGACTGTAGTGTAGGC	0	10	RDR2-sensitive
ACTACTTATTGCTGCTCGTCGACC	32	0	RDR2-resistant
ACTAGAACGAGATTGTGTATTGAA	17	0	RDR2-resistant
ACTAGAACGATGCTTGAGGCGATG	17	1	RDR2-resistant
ACTAGACCTGTAGCCTGACGGTGA	0	14	RDR2-sensitive
ACTAGACTAGTAGCAAGGCCTCAG	0	21	RDR2-sensitive
ACTAGACTCATGTCGTGTCGGCAC	0	11	RDR2-sensitive
ACTAGACTCGACAGATGTATGACC	16	0	RDR2-resistant
ACTAGAGCTCTCGGCACAGGGACT	1	15	RDR2-sensitive
ACTAGAGGACGACGAACGTGCAGA	0	30	RDR2-sensitive
ACTAGAGTAGATATCATCTTTAAT	41	0	RDR2-resistant
ACTAGAGTAGATATCATTTTTAAT	113	1	RDR2-resistant
ACTAGATCCGAGGGACTGACGGGT	13	37	RDR2-sensitive
ACTAGATCTGAGGGACTGACGGGT	0	30	RDR2-sensitive
ACTAGATCTGAGGGACTGACGGTT	27	0	RDR2-resistant
ACTAGCACACGATCGGACGCGCAT	0	10	RDR2-sensitive
ACTAGCACGGTGTATTCTTGGCAT	0	11	RDR2-sensitive
ACTAGCTGAGAGAACGTTGCGCGG	0	10	RDR2-sensitive
ACTAGGAACGCTGGGCCAGCACGG	0	11	RDR2-sensitive
ACTAGGACTGAACGGCTACATTCT	0	10	RDR2-sensitive
ACTAGGAGCCTAGGACGACGGTGA	0	9	RDR2-sensitive
ACTAGGGCTCTGAGCCATTAGAGG	1	32	RDR2-sensitive
ACTAGGGTCGGTTGTGTTATCATC	18	0	RDR2-resistant
ACTAGGGTTGTACAGTTTGACGGT	1	24	RDR2-sensitive
ACTAGGTGAGTGCCCGTGCGTTGC	0	10	RDR2-sensitive
ACTAGGTTGTTCGGGCCAATGGTA	0	118	RDR2-sensitive
ACTAGGTTTTAGGGATTGTTCGGT	0	9	RDR2-sensitive
ACTAGTCATCGGACCATGCCGGCA	0	13	RDR2-sensitive
ACTAGTCCAGGCTGAAGCTAGCAT	0	11	RDR2-sensitive
ACTAGTCGACTGATCGGTGGCAAT	0	10	RDR2-sensitive
ACTAGTCGGTTGCCCGTGCGTTGC	0	12	RDR2-sensitive
ACTAGTCGTTAGGATCCTCGGGTC	2	26	RDR2-sensitive
ACTAGTCTTTAGAGAAACTGCATG	231	30	RDR2-resistant
ACTAGTGCCTGCAGATTCGGTGAG	15	0	RDR2-resistant
ACTAGTTGTTCTGAAAGACGCATC	0	17	RDR2-sensitive
ACTATAATATCGTCCGTAGATGGC	0	9	RDR2-sensitive
ACTATACTAGTCGGGTCAGGTCAT	0	14	RDR2-sensitive
ACTATAGACGACTGCTGACGGCGC	0	11	RDR2-sensitive
ACTATAGACGGCTGCTGACGGCCC	0	15	RDR2-sensitive
ACTATAGACGGCTGCTGACGGTCC	1	29	RDR2-sensitive
ACTATAGCGTAGTGACTGAGTCGT	0	37	RDR2-sensitive

ACTATAGTCTGATTGTAGGTGGTA	1	14	RDR2-sensitive
ACTATATCAAGGACTATGGCGTCA	0	13	RDR2-sensitive
ACTATATCATGGACTGTGACGTCA	0	14	RDR2-sensitive
ACTATATCGTCGACTTATGGTGTG	0	10	RDR2-sensitive
ACTATATTAAGGCTGTAAGGGGTA	0	11	RDR2-sensitive
ACTATCGCGGACCGTCCGGCCTAA	1	14	RDR2-sensitive
ACTATCGCGTTATGGTCGTCGGTA	1	18	RDR2-sensitive
ACTATCGTCGCGCGGCTTCGCCAC	0	10	RDR2-sensitive
ACTATCGTTGTATGTAGAGATGGC	0	21	RDR2-sensitive
ACTATCGTTTGGGCCATATTTCGG	21	0	RDR2-resistant
ACTATCTGTACAGAGCGGCCTGAT	1	26	RDR2-sensitive
ACTATGATTGTGGAGGATCGTTGT	0	18	RDR2-sensitive
ACTATGCGACTGTCAGAAGGTGAC	0	12	RDR2-sensitive
ACTATGCTAGTAGCAAGGTCTCGG	0	16	RDR2-sensitive
ACTATGGCTGCAGAGGATCGTTGT	0	12	RDR2-sensitive
ACTATGGTTGCGGAGGATCGTTGT	1	18	RDR2-sensitive
ACTATGGTTGTAGAGGATCGTTGT	0	15	RDR2-sensitive
ACTATTAAACGGATCGTGCTTCGG	0	23	RDR2-sensitive
ACTATTACCTTTATTGTTGGACAA	21	0	RDR2-resistant
ACTATTGAAGACGGTCTAAGGCTG	0	9	RDR2-sensitive
ACTATTGCGGATCGTCTGGCCCAG	0	12	RDR2-sensitive
ACTATTGTTGGTCTGTGATGATGG	0	11	RDR2-sensitive
ACTATTTCTGTAGTGTTTTGGTCT	0	10	RDR2-sensitive
ACTATTTCTGTAGTGTTTTGTCTC	0	22	RDR2-sensitive
ACTATTTGGACCAGGCCGAATCGG	0	11	RDR2-sensitive
ACTATTTTCTGTTCGGATTGTCGG	0	10	RDR2-sensitive
ACTCAACACGCAAACCGTCCGGAC	0	19	RDR2-sensitive
ACTCAACGTTTAGACTCGCTCGGT	0	11	RDR2-sensitive
ACTCAAGACGCCTCTGAACGACGG	1	32	RDR2-sensitive
ACTCAATACTGGACTGGATTGGTT	0	12	RDR2-sensitive
ACTCACTCGCGAGTCTTGTCGGAT	0	14	RDR2-sensitive
ACTCAGAACTGGTACGGACAAGGG	19	1	RDR2-resistant
ACTCAGACAAGCAGAGTCGAACGC	0	14	RDR2-sensitive
ACTCAGACTGTCCGTGCACACCGA	0	10	RDR2-sensitive
ACTCAGAGGTGTAGAGAAGGCATG	0	17	RDR2-sensitive
ACTCAGAGGTGTAGAGACGACATG	0	10	RDR2-sensitive
ACTCAGCATAGACGGACGCTCGAC	0	9	RDR2-sensitive
ACTCAGCCTGTAGTGTCGGCCCAG	2	39	RDR2-sensitive
ACTCAGCGAACTGACTGCTCTCGG	0	10	RDR2-sensitive
ACTCAGCTCGGGCGAGACGGACAG	0	11	RDR2-sensitive
ACTCAGGACCACTGAACCGGACGT	0	9	RDR2-sensitive
ACTCAGGACCCTAGCAACAGGCAA	0	17	RDR2-sensitive
ACTCAGGATCTAGAAAGTGACGGA	0	17	RDR2-sensitive
ACTCAGTAGACGACTCGGATGAAC	0	9	RDR2-sensitive
ACTCATATTCACTGTAGCGGCATG	0	10	RDR2-sensitive
ACTCATCGGATACGGGACGGATAC	0	10	RDR2-sensitive
ACTCATCGGCTACAAATGGGCATT	0	30	RDR2-sensitive
ACTCATCGTTGAAGTGGGAGGGCT	0	22	RDR2-sensitive
ACTCATCTGGACAGGGGCACGCAG	0	20	RDR2-sensitive

ACTCATCTGGGCAGGGACACGCAA	0	19	RDR2-sensitive
ACTCATCTGGGCTGGGGCACGCGG	0	11	RDR2-sensitive
ACTCATGACCTATCGGACTAGGCC	0	12	RDR2-sensitive
ACTCATGTCGCATCGCACGGACAC	0	19	RDR2-sensitive
ACTCATGTCTGTTGGATGGTTTGA	19	0	RDR2-resistant
ACTCATTGGACTGGCACGACACGA	0	9	RDR2-sensitive
ACTCCAAAACTCTGGATGGAGCAG	0	10	RDR2-sensitive
ACTCCAAAACTCTGGGTGGAGCAG	0	10	RDR2-sensitive
ACTCCAACAATAGATTGATTCGGT	0	9	RDR2-sensitive
ACTCCACCGGACGAGGCGAGGCAA	1	63	RDR2-sensitive
ACTCCATGTAGTGCTCGTTAGAAG	0	18	RDR2-sensitive
ACTCCCGTCGCAACGCACAGGCAC	0	9	RDR2-sensitive
ACTCCCGTCGCAACGCACGGACAT	0	11	RDR2-sensitive
ACTCCGAGGCTGTCAGAGAGACGT	0	13	RDR2-sensitive
ACTCCGCACGGACTGTTCGGTGAT	0	15	RDR2-sensitive
ACTCCGCTATAATTCACCGGACTG	0	10	RDR2-sensitive
ACTCCGGACGAGAGACGCAGTCAC	0	22	RDR2-sensitive
ACTCCGGGTCCTGTTTTATAGACG	0	12	RDR2-sensitive
ACTCCGTCGGCTACGTTAGTCGGT	19	1	RDR2-resistant
ACTCCGTGTGGGCTTCTGACGGAT	0	23	RDR2-sensitive
ACTCCTCACGAGGATCAGGATCTG	0	9	RDR2-sensitive
ACTCCTCGGTATGTATGTGGGCTA	1	13	RDR2-sensitive
ACTCCTGGCAGAACCGTGCACCAT	0	26	RDR2-sensitive
ACTCCTTCGGACTCTGCCAACGGA	0	12	RDR2-sensitive
ACTCCTTGGACTACGGCGAACACG	0	24	RDR2-sensitive
ACTCGACGAGCTGGGCAGAGCAGA	0	9	RDR2-sensitive
ACTCGACGGACCAAGTATGCCGGT	1	20	RDR2-sensitive
ACTCGACTCGTGGATCCGATCGGC	1	26	RDR2-sensitive
ACTCGACTGGACACTCGCGGCCCA	0	9	RDR2-sensitive
ACTCGATCGTAGCAGCGTGACGTA	0	19	RDR2-sensitive
ACTCGATCGTGGATTTATGATGGT	0	20	RDR2-sensitive
ACTCGATCTGAAGACCCTGACGGT	0	9	RDR2-sensitive
ACTCGATTCGAATCGTCTGCTTCT	65	0	RDR2-resistant
ACTCGCAGCCTGCAGCAACGGACG	1	13	RDR2-sensitive
ACTCGCATTCGTCGGGTCGGGTAT	0	14	RDR2-sensitive
ACTCGCGACAGACGGAACAGATGC	0	26	RDR2-sensitive
ACTCGCGAGTCGGAACGAGTTGGC	0	11	RDR2-sensitive
ACTCGCGAGTCTTGTCGGATCGGA	0	11	RDR2-sensitive
ACTCGCGTAGGACAGTTCGGCTAG	0	18	RDR2-sensitive
ACTCGCGTGGATTGTGGTGGGTTG	0	9	RDR2-sensitive
ACTCGCTACCTACTCTGGGCCGGA	0	15	RDR2-sensitive
ACTCGCTTACGTTGGATCTGGCTA	0	15	RDR2-sensitive
ACTCGGAACCCGGACTGTTCGGCC	0	13	RDR2-sensitive
ACTCGGAAGCGACACACGTCTGGA	0	18	RDR2-sensitive
ACTCGGAAGGAGGCTAGTAGGAAC	0	13	RDR2-sensitive
ACTCGGACCACCTGCCACGTCGAC	1	18	RDR2-sensitive
ACTCGGACCAGAATACCACAGCAT	0	9	RDR2-sensitive
ACTCGGACCAGGCCCAAACAGCGG	0	12	RDR2-sensitive
ACTCGGAGCCACAAGCCATGACGT	0	29	RDR2-sensitive

ACTCGGAGCCATAGATCACGGCGT	0	9	RDR2-sensitive
ACTCGGATGGAAGGTCAACGACGG	0	16	RDR2-sensitive
ACTCGGCAAACTGTACATCGGCAA	2	17	RDR2-sensitive
ACTCGGCAAACTGTATATCGGTAA	0	9	RDR2-sensitive
ACTCGGCAAAGAACACTCGGCAAA	2	33	RDR2-sensitive
ACTCGGCAAAGAACCTAAGCCGGT	0	13	RDR2-sensitive
ACTCGGCAAAGAACCTATACCGGT	0	13	RDR2-sensitive
ACTCGGCAAAGAACGCGATTCCGG	0	40	RDR2-sensitive
ACTCGGCAAAGAACGCTCGGCGAA	2	28	RDR2-sensitive
ACTCGGCAAAGAAGGCACACCTGG	0	9	RDR2-sensitive
ACTCGGCAAAGAAGGCTCGGCAAA	2	26	RDR2-sensitive
ACTCGGCAAAGACGCTGTCTCCGT	1	21	RDR2-sensitive
ACTCGGCAAAGACTGACGGCCGTC	2	15	RDR2-sensitive
ACTCGGCAAAGATTTCATCGGCAA	9	40	RDR2-sensitive
ACTCGGCAAAGCCTCGGATTCCGG	0	13	RDR2-sensitive
ACTCGGCAAAGGCTATTTTACACT	5	24	RDR2-sensitive
ACTCGGCAAAGGGACTGGCAAAGG	0	9	RDR2-sensitive
ACTCGGCAAAGTCTCGGATTCCGG	2	27	RDR2-sensitive
ACTCGGCACAACCAAGCACTCGGC	0	16	RDR2-sensitive
ACTCGGCACAGAAACACACTCGGC	0	10	RDR2-sensitive
ACTCGGCACAGAAAGACACTCGGC	0	9	RDR2-sensitive
ACTCGGCACAGAACGACACTCGGC	1	23	RDR2-sensitive
ACTCGGCACAGAACGGCACTCGGC	3	50	RDR2-sensitive
ACTCGGCACAGAATGATCGGCGAA	0	17	RDR2-sensitive
ACTCGGCACAGAGAAACACTCGGC	0	12	RDR2-sensitive
ACTCGGCACAGCCAAGCACTCGGC	4	54	RDR2-sensitive
ACTCGGCACAGCGAAGCACTCGGC	0	12	RDR2-sensitive
ACTCGGCACAGCTTCAGACGTCAG	0	10	RDR2-sensitive
ACTCGGCACAGGAAAACCCTCGGC	0	9	RDR2-sensitive
ACTCGGCACAGGACAACACTCGGC	1	22	RDR2-sensitive
ACTCGGCACAGGACAGCACTCGGC	0	9	RDR2-sensitive
ACTCGGCACAGGACCACACTCGGC	4	20	RDR2-sensitive
ACTCGGCACAGGACCCCACTCGGC	4	31	RDR2-sensitive
ACTCGGCACAGGACGACACTCGGC	2	48	RDR2-sensitive
ACTCGGCACAGGACGGCACTCGGC	3	29	RDR2-sensitive
ACTCGGCACAGGGCGACACTCGGC	0	18	RDR2-sensitive
ACTCGGCACAGGGCGGCACTCGGC	0	14	RDR2-sensitive
ACTCGGCACAGGTAGGCACTCGGC	1	33	RDR2-sensitive
ACTCGGCACATCCAAGCACTCGGC	1	14	RDR2-sensitive
ACTCGGCACCGAACGGCACTCGGC	0	10	RDR2-sensitive
ACTCGGCACCGTAGATCATGGCGC	0	11	RDR2-sensitive
ACTCGGCACGGGCAGACTTCGGGT	0	10	RDR2-sensitive
ACTCGGCACTCATAGACGGATCTC	0	10	RDR2-sensitive
ACTCGGCACTCTCGGCAACCGCGC	3	20	RDR2-sensitive
ACTCGGCAGAGGACAGCACTCGGC	0	15	RDR2-sensitive
ACTCGGCAGCAGGAGGAGGGACTC	0	11	RDR2-sensitive
ACTCGGCAGCAGTAGGAGGAGCTC	0	11	RDR2-sensitive
ACTCGGCAGCGGTAGAAGGGGCTC	0	28	RDR2-sensitive
ACTCGGCATAGAAAGACGCTCGAC	0	9	RDR2-sensitive

ACTCGGCATAGACACACACTCGGC	0	9	RDR2-sensitive
ACTCGGCATAGGTAGACACTCGGC	1	17	RDR2-sensitive
ACTCGGCATGCTGCAGCCATCTGC	0	11	RDR2-sensitive
ACTCGGCCCAGTAATACAGACGTT	0	18	RDR2-sensitive
ACTCGGCGAACTATACATCGGCAA	0	11	RDR2-sensitive
ACTCGGCGAACTGTACATCGGCAA	3	28	RDR2-sensitive
ACTCGGCGACTGCACGGCGAACGC	0	22	RDR2-sensitive
ACTCGGCGTGTCGACCCTTCGGCT	0	43	RDR2-sensitive
ACTCGGCTACGACGGTGACTCGGC	0	13	RDR2-sensitive
ACTCGGCTATAATTCACCGGACTG	2	18	RDR2-sensitive
ACTCGGCTCGGCTCGAAGCGGCTC	70	1	RDR2-resistant
ACTCGGCTCGGCTCGGATCGGCTC	0	10	RDR2-sensitive
ACTCGGCTGGCTGACGAAGAGACA	1	60	RDR2-sensitive
ACTCGGGACAAGTTCGATGACCGC	0	12	RDR2-sensitive
ACTCGGTAAAGCTAGGCTCTCGGC	0	11	RDR2-sensitive
ACTCGGTACAGGTCCACACTCGGC	1	12	RDR2-sensitive
ACTCGGTCAGCTAGTGAGAGGTTC	0	29	RDR2-sensitive
ACTCGGTCAGTCGGTGCGGCGATT	1	17	RDR2-sensitive
ACTCGGTGAACTGTAAATCGGCAA	0	15	RDR2-sensitive
ACTCGTACAGTAGGAAGTGAGGCG	0	13	RDR2-sensitive
ACTCGTACGTGTTAGCCTTCGGTA	1	17	RDR2-sensitive
ACTCGTACTCGTAGATAGAGGCTT	227	47	RDR2-resistant
ACTCGTAGTACTATGGCACGACAT	0	27	RDR2-sensitive
ACTCGTATGGACTATTGGTGTCGT	0	10	RDR2-sensitive
ACTCGTCACTGTAACCCGATGGTG	0	10	RDR2-sensitive
ACTCGTCAGGCAAAAGTGTGGCAA	0	10	RDR2-sensitive
ACTCGTGGGTCTGTTATTGGGCAA	4	25	RDR2-sensitive
ACTCGTGGTCAGCTCGGCTCGGCT	0	10	RDR2-sensitive
ACTCGTGGTCAGTTCGGCTCGGCT	0	10	RDR2-sensitive
ACTCGTTAATGGATCAGGTCGGGC	0	10	RDR2-sensitive
ACTCGTTATAGCGTACTGTGGCAT	0	28	RDR2-sensitive
ACTCGTTTGGCACGGCTTCTCCAA	0	11	RDR2-sensitive
ACTCGTTTGGCACGGCTTCTCTAG	1	28	RDR2-sensitive
ACTCTAAAACTCTGGGTGGAGCAG	5	126	RDR2-sensitive
ACTCTAAATCGAGGGTAGACTGCT	0	18	RDR2-sensitive
ACTCTAAGACACAACTTAAGACAC	0	11	RDR2-sensitive
ACTCTAAGAGTTAGGACTGGGCAT	0	18	RDR2-sensitive
ACTCTAAGCTCGTCGGTTGGCATG	0	11	RDR2-sensitive
ACTCTACAGCAGGACTGCTCTACC	0	11	RDR2-sensitive
ACTCTACATCGGACCACAGAGAGG	0	10	RDR2-sensitive
ACTCTACCGGACAAGACAGTGGCG	1	94	RDR2-sensitive
ACTCTACGGGCTGATTTGGTGGTA	0	9	RDR2-sensitive
ACTCTAGAGCGTATTGGACGGTTG	0	63	RDR2-sensitive
ACTCTATACTACTAGGTTGTTCGG	0	16	RDR2-sensitive
ACTCTATCAGTCCGTCGGGCGGCA	0	23	RDR2-sensitive
ACTCTATCATCAGCTTGGGACAGC	29	1	RDR2-resistant
ACTCTATCCCGGTGTGGAACGGTT	0	15	RDR2-sensitive
ACTCTATCTCTGTAGCGTTCGGCA	0	41	RDR2-sensitive
ACTCTATGGGCTGTCTGATGGCAA	0	26	RDR2-sensitive

ACTCTATTGGATGGAAGATGTATG	0	27	RDR2-sensitive
ACTCTCAACTGTAGCGGTGTGGTA	0	14	RDR2-sensitive
ACTCTCAGCAGGTACGACGGACAT	0	110	RDR2-sensitive
ACTCTCAGCGAACTGTACATCGGC	0	48	RDR2-sensitive
ACTCTCCACATTTAGCAACTCGGT	0	13	RDR2-sensitive
ACTCTCCCGTTGCAGTGGACATGA	24	0	RDR2-resistant
ACTCTCCGCGCGCTGTAGAAGGCA	0	9	RDR2-sensitive
ACTCTCGGCAAAGGAACAGGCAAA	0	13	RDR2-sensitive
ACTCTCGGGCCATACACGACACAA	0	11	RDR2-sensitive
ACTCTCGTCATGGCGTACTGTCCC	0	9	RDR2-sensitive
ACTCTCGTGGATGACGCTGGTATT	0	9	RDR2-sensitive
ACTCTCTATGACAGCGCGGACGGT	0	11	RDR2-sensitive
ACTCTCTCTCTCGGACTCGGTCAA	0	25	RDR2-sensitive
ACTCTCTCTGCAGGATGACTCTCA	124	6	RDR2-resistant
ACTCTCTGCGCAGGGGCGGACTGT	0	22	RDR2-sensitive
ACTCTCTGCGCTATTGTAGACGGT	0	23	RDR2-sensitive
ACTCTCTGCTCAGAGACGGACGGT	0	10	RDR2-sensitive
ACTCTCTGGTCAGGCGCGGACGGT	0	17	RDR2-sensitive
ACTCTCTGTAGCGGTTCGGACGGT	1	19	RDR2-sensitive
ACTCTCTGTGGAGGCACGGACGGT	0	10	RDR2-sensitive
ACTCTCTGTGGCGGCACGAACGGT	0	11	RDR2-sensitive
ACTCTGAATTTCTGGACAAGAGGA	0	11	RDR2-sensitive
ACTCTGACCTGACGAACGACACGT	0	9	RDR2-sensitive
ACTCTGACGGAGACTGTCTACTGC	3	16	RDR2-sensitive
ACTCTGACTCAGATTATGCCGGTT	39	4	RDR2-resistant
ACTCTGACTCGGGCTAGACTGCTC	0	12	RDR2-sensitive
ACTCTGATCTTCGTATTTGTGCAT	0	9	RDR2-sensitive
ACTCTGCAGATCTCCCGAAGAGGC	1	15	RDR2-sensitive
ACTCTGCAGGTAGAGCTGTACGGG	0	44	RDR2-sensitive
ACTCTGCATGTAGAGCTGTACGGG	0	21	RDR2-sensitive
ACTCTGCCTGTTGACGCAACACTG	0	23	RDR2-sensitive
ACTCTGCGAGACGGACCGACTGAC	0	9	RDR2-sensitive
ACTCTGCTAGTGGACCCGTGGGAT	0	21	RDR2-sensitive
ACTCTGCTCGGACCGAGACGAGGC	1	54	RDR2-sensitive
ACTCTGGACCAGACTCTACAGGCG	0	66	RDR2-sensitive
ACTCTGGACCCTTCAGACGAACAT	0	9	RDR2-sensitive
ACTCTGGATTAGAGCTGCTTCACC	0	9	RDR2-sensitive
ACTCTGGTCACTGTTCACGGCACT	564	8	RDR2-resistant
ACTCTGGTCCTTGCTGACGAACGT	0	26	RDR2-sensitive
ACTCTGGTCGTGCGGGTTGGGCAG	0	16	RDR2-sensitive
ACTCTGGTGGTCGGGAGGAGGCGT	0	19	RDR2-sensitive
ACTCTGTAGCAAAGCACGGACATA	0	13	RDR2-sensitive
ACTCTGTAGCAACGCACGAGCATA	0	9	RDR2-sensitive
ACTCTGTAGCAACGCACGGGCATA	0	13	RDR2-sensitive
ACTCTGTAGCAACGCACGGGCATT	0	52	RDR2-sensitive
ACTCTGTAGCAACGCACGGGTATA	0	11	RDR2-sensitive
ACTCTGTAGCAACGTACGGGCATA	0	22	RDR2-sensitive
ACTCTGTAGGAAATGCATAGACAG	1	17	RDR2-sensitive
ACTCTGTATAGGTACGAGGACCGA	0	12	RDR2-sensitive

ACTCTGTATCTTAGAACGCGCACA	1	20	RDR2-sensitive
ACTCTGTGCGTCGGGTCGACAGGC	0	9	RDR2-sensitive
ACTCTGTTGGATGGAAGATGCATG	1	173	RDR2-sensitive
ACTCTGTTGGATGTAAGATGCATG	0	10	RDR2-sensitive
ACTCTGTTGGGTGGAAGATGCATG	0	12	RDR2-sensitive
ACTCTGTTTGGTTGTAGGGGCTAA	0	10	RDR2-sensitive
ACTCTTAGAGGTATCGTTGTGCTG	77	3	RDR2-resistant
ACTCTTGAACCCCTCGGATGGTTG	26	0	RDR2-resistant
ACTCTTGACGTGTAGAGGACGAGA	0	17	RDR2-sensitive
ACTCTTGGATGAGGCACTGATGGT	33	2	RDR2-resistant
ACTCTTGGCGTGACTCGGCGACCT	2	30	RDR2-sensitive
ACTCTTGTGGGATCTGACGTGCAC	0	9	RDR2-sensitive
ACTCTTTAACCGGACCGGACGCAT	1	19	RDR2-sensitive
ACTCTTTAGGGTGTATTGCTGGAG	0	15	RDR2-sensitive
ACTCTTTTAGTGGACCCGGGGATA	0	10	RDR2-sensitive
ACTCTTTTGGGTCAGGGCTGGGCT	0	9	RDR2-sensitive
ACTGAAATGTAGGATCGGACGGCT	0	11	RDR2-sensitive
ACTGAACAGTCCGCCGGGTGGCAT	0	10	RDR2-sensitive
ACTGAACCCTAGCACTCCGGATTT	0	12	RDR2-sensitive
ACTGAACCTGAACTAGGAGACCCT	0	9	RDR2-sensitive
ACTGAACCTGCAGCAGTAGCTTAG	0	13	RDR2-sensitive
ACTGAACCTGTCAGGAAGCTGCTG	0	11	RDR2-sensitive
ACTGAACGAAGGGAGCCGAGCCGT	1	70	RDR2-sensitive
ACTGAACGGATTTAGACGACGTAG	0	9	RDR2-sensitive
ACTGAACTAGAGCTCCGGCCGGAA	1	21	RDR2-sensitive
ACTGAAGACTGCAGCGGGCGACGC	0	19	RDR2-sensitive
ACTGAAGAGGATTGGAGAGGCTAG	0	15	RDR2-sensitive
ACTGAAGCCGGCGGAGGAGAGCAT	0	12	RDR2-sensitive
ACTGAAGGATTACGGGGAGGCTAC	25	2	RDR2-resistant
ACTGAAGGCTGCGCAGAAGTCAAA	3	22	RDR2-sensitive
ACTGAAGGGCAGCAAGGCACTGGC	0	9	RDR2-sensitive
ACTGAATCTTCGCACGCACGGACC	0	10	RDR2-sensitive
ACTGAATGTAGGGACGTTCTGCCC	0	13	RDR2-sensitive
ACTGACACAGGCGGAACTGACACG	0	11	RDR2-sensitive
ACTGACACGCGGGACTGAGCAGGA	0	9	RDR2-sensitive
ACTGACACGTAGGAGGCGCTGTAG	0	16	RDR2-sensitive
ACTGACACTGCAAGCAGCTGGATA	0	10	RDR2-sensitive
ACTGACACTGTATGAATCGTGCAC	0	27	RDR2-sensitive
ACTGACACTGTATGAATCGTGCCT	0	15	RDR2-sensitive
ACTGACAGGAGCCGAACGTGTCAA	0	47	RDR2-sensitive
ACTGACCAAGGAGCCGGAGCCTCT	0	11	RDR2-sensitive
ACTGACCGATTGCCAGTGCTGCAT	1	15	RDR2-sensitive
ACTGACCGGCCTGACAGAAGGACA	2	35	RDR2-sensitive
ACTGACCGTCTACAGTAGGGCATC	0	17	RDR2-sensitive
ACTGACCTCTAGAACCGATGGCTC	1	46	RDR2-sensitive
ACTGACCTGCTGAGATTTTATATT	14	0	RDR2-resistant
ACTGACGACTGTTGGCTCACCGGA	0	16	RDR2-sensitive
ACTGACGCGACCTGACATGGCATT	1	15	RDR2-sensitive
ACTGACGGATGACGTGGTCTAGCT	0	29	RDR2-sensitive

ACTGACGGCAAGATAACGGGCTCT	0	10	RDR2-sensitive
ACTGACGGCCGTCAGCTCTGGGAC	1	32	RDR2-sensitive
ACTGACGGCTGACGGCGAGTGGAC	1	27	RDR2-sensitive
ACTGACGTAGACTGTGACACGGAT	0	10	RDR2-sensitive
ACTGACGTGGCACGTAACTCGGCG	0	9	RDR2-sensitive
ACTGACGTTGTCGCAGTAGACGAG	5	37	RDR2-sensitive
ACTGACTAAGCTCGGCTCACCTAC	1	17	RDR2-sensitive
ACTGACTAGGGTAGAGACTGGTAT	0	12	RDR2-sensitive
ACTGACTAGTGACCCAAGGACATA	0	10	RDR2-sensitive
ACTGACTAGTGACCTAAGGACATA	0	9	RDR2-sensitive
ACTGACTCTGCTCATGCGTGGACC	0	15	RDR2-sensitive
ACTGACTGGCAGACTGACCTGCAG	1	12	RDR2-sensitive
ACTGACTGTATGTTTTCAGTACGG	0	12	RDR2-sensitive
ACTGACTTAGACGGATTTAGATAA	1	26	RDR2-sensitive
ACTGACTTCGTCTATGTGTGGCAA	0	14	RDR2-sensitive
ACTGACTTTATTTTGGCATCTCTT	40	2	RDR2-resistant
ACTGAGACACGTGGAAGACATCAT	0	12	RDR2-sensitive
ACTGAGACGGAGGACGAGAGTCAG	0	19	RDR2-sensitive
ACTGAGAGCTCTTTCTTGATTCTA	34	1	RDR2-resistant
ACTGAGCACGGCCTGACAGTCTGA	0	9	RDR2-sensitive
ACTGAGCCTGACACGACATAGTTA	0	14	RDR2-sensitive
ACTGAGCGGGCTTCATGACCGGTG	0	30	RDR2-sensitive
ACTGAGCGGTCGCATACCCTGCAT	0	20	RDR2-sensitive
ACTGAGCGTAGTACACTTGGCATA	1	27	RDR2-sensitive
ACTGAGGCTTTGATACCAGATGAT	0	26	RDR2-sensitive
ACTGAGGGTAGCACACTCGGCATA	0	18	RDR2-sensitive
ACTGATAAGCTGCTGCAGAAGCTG	0	10	RDR2-sensitive
ACTGATACGATTGGACACGGCCTA	0	10	RDR2-sensitive
ACTGATACGATTGGACACGGCCTG	0	16	RDR2-sensitive
ACTGATACGATTGGACACGGCCTT	0	11	RDR2-sensitive
ACTGATAGATGGCGTAGACCGGAA	1	14	RDR2-sensitive
ACTGATCACGGCTAGACGGTTCGC	0	11	RDR2-sensitive
ACTGATCGATGGCACGCGCCGGAA	0	18	RDR2-sensitive
ACTGATCGCTTGATAGATGGGCAG	0	9	RDR2-sensitive
ACTGATCGGACCAACAGACTGGCC	0	9	RDR2-sensitive
ACTGATCGGTCGGTCAGGTTGGTA	0	13	RDR2-sensitive
ACTGATGATTTAGTGAACGTTGAT	51	0	RDR2-resistant
ACTGATGATTTAGTGAATGTTGAC	55	0	RDR2-resistant
ACTGATGATTTTAGTAGATATGGG	31	3	RDR2-resistant
ACTGATGCGCAGAACGACGACGAG	0	9	RDR2-sensitive
ACTGATGGAGGGAGTAGAGGACGA	0	9	RDR2-sensitive
ACTGATGGGCCCCAAGGATCGCGG	0	11	RDR2-sensitive
ACTGATGGTTGCAATGTAGTGGTG	1	15	RDR2-sensitive
ACTGATGTTCTTGATTGGTCGGTC	107	1	RDR2-resistant
ACTGATTAGGGCAGGCTGAGACGC	0	13	RDR2-sensitive
ACTGATTCAGCGTCGGCTTGGCCA	22	0	RDR2-resistant
ACTGATTCTGGACACGCTTGACGG	0	9	RDR2-sensitive
ACTGATTGGATCGTAGGGGAGCGG	0	33	RDR2-sensitive
ACTGCAAATAGTACGGACGGTTGA	0	9	RDR2-sensitive

ACTGCAACGAACCACTTGACGGTG	0	24	RDR2-sensitive
ACTGCAAGACGCTATATCTGAGGT	0	13	RDR2-sensitive
ACTGCAAGCGATGACCGTTTTTGG	17	0	RDR2-resistant
ACTGCACCGGACCCAAGGCGAGCT	0	15	RDR2-sensitive
ACTGCACCGGATCGGAGGATGTCA	0	10	RDR2-sensitive
ACTGCACCGGGCCCAAGGCGAGCT	0	9	RDR2-sensitive
ACTGCACGGTCAACTGAACGCGGT	0	36	RDR2-sensitive
ACTGCAGACTGAGAAGGCGTCCCA	0	31	RDR2-sensitive
ACTGCAGAGGATTGTGGTATAGAG	0	10	RDR2-sensitive
ACTGCAGAGTAGAAGTGTCGACAG	0	10	RDR2-sensitive
ACTGCAGATCGAGGATTTCCGGCG	0	11	RDR2-sensitive
ACTGCAGCTGTAGTTGGGCGCTAT	0	14	RDR2-sensitive
ACTGCAGTCGGAGTGGCGCAGGCG	3	37	RDR2-sensitive
ACTGCATCGACTCTATACGGCTAT	0	29	RDR2-sensitive
ACTGCATGCGGAGTGGGATGGGGA	1	14	RDR2-sensitive
ACTGCATGGACGTGGAGATGGCAC	2	18	RDR2-sensitive
ACTGCCAAAGCAACTCGACGACGC	2	28	RDR2-sensitive
ACTGCCAGTTGATCACAGGACAAT	0	9	RDR2-sensitive
ACTGCCGAGATTAGAACCGACGGG	0	14	RDR2-sensitive
ACTGCCGCTGTAGACAGAAGATGG	0	22	RDR2-sensitive
ACTGCCTCATCGCAGCCGTTGGAT	0	39	RDR2-sensitive
ACTGCCTGGCCTGACTAGAAGCGT	0	12	RDR2-sensitive
ACTGCGACTGGACACGACGTGGCG	0	19	RDR2-sensitive
ACTGCGGCTCTGGTGAACGGCGGA	0	14	RDR2-sensitive
ACTGCGTACTGGAACGCGGCGTCT	0	12	RDR2-sensitive
ACTGCGTTGAGAACCTGCGGCGAT	0	21	RDR2-sensitive
ACTGCTAAGAGCCTAGGACGACGG	0	9	RDR2-sensitive
ACTGCTAGGAGCCTAGGACGACGG	0	26	RDR2-sensitive
ACTGCTAGGAGCTTAGGACGACGG	0	13	RDR2-sensitive
ACTGCTCCGGACCAACAGTACAGC	0	9	RDR2-sensitive
ACTGCTCGGCAGGGATCTGATCGG	0	25	RDR2-sensitive
ACTGCTCTACTGCATGGTCGTCGC	0	14	RDR2-sensitive
ACTGCTGACGGGAGGGAAGGGAGC	1	18	RDR2-sensitive
ACTGCTGACTGTACGGCTGTACCG	0	14	RDR2-sensitive
ACTGCTGGACTGATGAATAGTGTG	0	9	RDR2-sensitive
ACTGCTGGAGAGCATAGAGGACCA	0	44	RDR2-sensitive
ACTGCTGTAGACGGCCTTAGGAGC	0	17	RDR2-sensitive
ACTGCTGTAGAGGAGCACGAGCGA	0	14	RDR2-sensitive
ACTGCTGTAGGACTAATCAGTAGA	48	0	RDR2-resistant
ACTGCTGTCGCACCTGATGACGTA	0	10	RDR2-sensitive
ACTGCTGTCGGAGCGCATCTTCAT	0	14	RDR2-sensitive
ACTGGAAAACGAACTCCACGGTGC	0	14	RDR2-sensitive
ACTGGAAACGTAGAGGACGACCCT	0	9	RDR2-sensitive
ACTGGAAACTGCATGCACCGCGAC	0	12	RDR2-sensitive
ACTGGAAACTGCATGGACCGCGGC	0	11	RDR2-sensitive
ACTGGAACCTAACACTGACGGTTT	0	17	RDR2-sensitive
ACTGGAACCTGGCTCTGGAGATGC	0	16	RDR2-sensitive
ACTGGAACGGAACGGCTTCATACC	0	27	RDR2-sensitive
ACTGGAACGGAGCGGCTCTGTTTT	0	9	RDR2-sensitive

ACTGGAACTGGAGCCCGGCCTTGA	18	0	RDR2-resistant
ACTGGAACTTGGAACTGGATCCGG	0	19	RDR2-sensitive
ACTGGAAGGGCATAGCTGAGAAAA	1	49	RDR2-sensitive
ACTGGACAAGCACCTGACGGACAC	2	28	RDR2-sensitive
ACTGGACAAGCACTGCGCCGCATT	0	9	RDR2-sensitive
ACTGGACAGGTAAAACGACGGGCT	0	11	RDR2-sensitive
ACTGGACAGTCCGACGTGTGGCAC	0	11	RDR2-sensitive
ACTGGACAGTGGATACCCGACGGG	0	14	RDR2-sensitive
ACTGGACCAGAACGGCTCTTGGCT	1	17	RDR2-sensitive
ACTGGACCATGCCAGGCTCGGACC	1	21	RDR2-sensitive
ACTGGACCGCCTGTGATCTGTGTT	0	12	RDR2-sensitive
ACTGGACCGTGTGGTAACGACTAC	0	18	RDR2-sensitive
ACTGGACCTGTGGTAGACGGGCAA	0	17	RDR2-sensitive
ACTGGACGACGAGCGCACTCTAAA	0	14	RDR2-sensitive
ACTGGACGAGAGGGACATGGTCGA	0	10	RDR2-sensitive
ACTGGACGCCGTGGCGTTGACGTG	0	21	RDR2-sensitive
ACTGGACGCTCTTCTTCGACGGAT	0	9	RDR2-sensitive
ACTGGACGGATGGGTTGGATGCAT	0	16	RDR2-sensitive
ACTGGACGTGTAGAGGACGTATTT	0	22	RDR2-sensitive
ACTGGACTAGGACGCAAGCCTCAA	0	19	RDR2-sensitive
ACTGGACTGCTAATTAGGCCGTGT	0	16	RDR2-sensitive
ACTGGACTGGCTGAGTACATGCAG	0	16	RDR2-sensitive
ACTGGACTGGCTGAGTGCATGCAA	0	12	RDR2-sensitive
ACTGGACTGGCTGAGTGCATGCAG	0	99	RDR2-sensitive
ACTGGACTGTCCGGTACGCCCATC	0	13	RDR2-sensitive
ACTGGACTGTCCGGTGCGCCCATC	0	20	RDR2-sensitive
ACTGGACTGTCCGGTGTCACATGA	0	22	RDR2-sensitive
ACTGGAGAGCGTAGAGGACCGTTT	0	12	RDR2-sensitive
ACTGGAGGCAGAACGCAGAACAGG	0	28	RDR2-sensitive
ACTGGAGGCAGAACGGCAGAACGG	0	19	RDR2-sensitive
ACTGGAGTCGGTATGCCACTGGTC	0	14	RDR2-sensitive
ACTGGAGTGGGTAGGAGAGAGACG	0	10	RDR2-sensitive
ACTGGATACGAGATTATGATTGGT	0	14	RDR2-sensitive
ACTGGATGTGCGTGGGTTGACGTA	0	9	RDR2-sensitive
ACTGGATGTTGGCGAATGCTGGTT	17	0	RDR2-resistant
ACTGGATTAGACATGCACGCACAA	0	19	RDR2-sensitive
ACTGGATTCGGGACCGGATACGGA	0	16	RDR2-sensitive
ACTGGCAAGAGCATTTGACCACGA	0	11	RDR2-sensitive
ACTGGCACTATACTAGTCGGGTCA	0	11	RDR2-sensitive
ACTGGCAGCAAGATAATGAGCTAT	0	22	RDR2-sensitive
ACTGGCAGGAGCATTTGACCACGA	0	21	RDR2-sensitive
ACTGGCAGTAAGATAATGGGCTAT	0	12	RDR2-sensitive
ACTGGCATGGTTGTAGGACGGCAG	0	18	RDR2-sensitive
ACTGGCATGTAAGCAGTAGGACAT	1	119	RDR2-sensitive
ACTGGCCCACTGTACGCGAGCATG	0	11	RDR2-sensitive
ACTGGCCCATAGCACTGACGGTTC	0	9	RDR2-sensitive
ACTGGCCCGAACCAACAGTAGAAC	0	9	RDR2-sensitive
ACTGGCCCGTAGCACTGGTGGTTA	0	10	RDR2-sensitive
ACTGGCCTGCTTCAGACGACTCGA	1	48	RDR2-sensitive

ACTGGCCTGGAACAGCAAAGACGC	0	19	RDR2-sensitive
ACTGGCCTTTAACACTGACGGTTC	0	23	RDR2-sensitive
ACTGGCCTTTAGAACTAGCGGCAC	0	41	RDR2-sensitive
ACTGGCGAAGATCGGTGCTCTGGA	0	10	RDR2-sensitive
ACTGGCTAACGATTCTGACGGCGT	1	15	RDR2-sensitive
ACTGGCTACAACGGCTTAGGACAT	0	23	RDR2-sensitive
ACTGGCTACTGTAGTTCACGGGCC	0	61	RDR2-sensitive
ACTGGCTGGCTGTAACGGTAGCTC	0	18	RDR2-sensitive
ACTGGCTTTCTGAACAATTGGCTT	0	11	RDR2-sensitive
ACTGGGACCCTAGAACACGTGGTA	0	16	RDR2-sensitive
ACTGGGACGGTAGCGGACGACGAA	1	41	RDR2-sensitive
ACTGGGACGTTGCAGAGTGGACGG	0	12	RDR2-sensitive
ACTGGGACTGGACAGCAGCTATGC	0	19	RDR2-sensitive
ACTGGGATGGCAGTGCTTGATCAC	3	36	RDR2-sensitive
ACTGGGCAGGTTGTTGGGCGTCAT	0	12	RDR2-sensitive
ACTGGGCATGCTCGGACGGTCTAG	0	11	RDR2-sensitive
ACTGGGCCAAGCCAACTTAGAGCT	0	9	RDR2-sensitive
ACTGGGCCATGCCAACCTGAGGCT	1	15	RDR2-sensitive
ACTGGGCCCTAGTACCGACGGTTC	0	10	RDR2-sensitive
ACTGGGCCTGTAGTGACGTGCGTT	0	17	RDR2-sensitive
ACTGGGCGCGGCTGGACGGTACGG	0	10	RDR2-sensitive
ACTGGGCTGAGATTATTAGGTCAT	0	38	RDR2-sensitive
ACTGGGCTTCTGAAAATCGGTATC	1	28	RDR2-sensitive
ACTGGGGATAGAGCGCTGCGGGAT	0	9	RDR2-sensitive
ACTGGGGCACAGGAGAACGCGCGA	0	9	RDR2-sensitive
ACTGGGGCAGAACGCGGAATGGCC	0	9	RDR2-sensitive
ACTGGGTCAGACTGTGTAGAGGGA	1	15	RDR2-sensitive
ACTGGTACAGACTGAGGCTAGCAT	0	15	RDR2-sensitive
ACTGGTAGGCTCATTAAGAAGGGT	18	1	RDR2-resistant
ACTGGTATCTGGGCTAATGGACGG	0	16	RDR2-sensitive
ACTGGTCAGGCCCGTCGCATCAAC	0	16	RDR2-sensitive
ACTGGTCAGGCCTGTCGCATCAAC	171	0	RDR2-resistant
ACTGGTCCAGGCTGAGACTAGCAT	0	10	RDR2-sensitive
ACTGGTCCTGATGACTGGACGGTC	0	11	RDR2-sensitive
ACTGGTCGACTGGACTGGGAGGTA	0	12	RDR2-sensitive
ACTGGTCGCGGACCGTCCGGCCCA	0	15	RDR2-sensitive
ACTGGTCGCTTGATAGATGGGCAG	0	9	RDR2-sensitive
ACTGGTCGGTGGTGTATTGGGTTG	0	10	RDR2-sensitive
ACTGGTCGGTTGACGGTCCTCGCG	1	15	RDR2-sensitive
ACTGGTGACTGGTGAGCAGGCAGG	0	12	RDR2-sensitive
ACTGGTGTGCGGGATTGGACGGCT	0	13	RDR2-sensitive
ACTGGTGTTTCTTAGAATCGTCGG	0	14	RDR2-sensitive
ACTGGTGTTTCTTAGTACCGTCGG	0	10	RDR2-sensitive
ACTGGTTAGCCTGATCGATCTGGG	0	16	RDR2-sensitive
ACTGGTTCCTAACACTGACGGTTA	0	21	RDR2-sensitive
ACTGGTTCGCACTGGAGGAGGCCA	0	34	RDR2-sensitive
ACTGGTTCGGATGACGTCAGATAG	0	13	RDR2-sensitive
ACTGGTTCTAGGCCGGGTCGTCAA	0	71	RDR2-sensitive
ACTGGTTGGTCGTGAGCGGAGGAA	0	12	RDR2-sensitive

ACTGGTTTAGGTGCTGTCGGGAAG	0	14	RDR2-sensitive
ACTGGTTTAGTTGATCTCGGATAA	1	13	RDR2-sensitive
ACTGGTTTCGTCGATCTCGGATAA	2	21	RDR2-sensitive
ACTGGTTTGGTCGCTTGGAGGCTA	0	10	RDR2-sensitive
ACTGTAAAATGGACGGTTAGATGG	0	9	RDR2-sensitive
ACTGTAAAATGGACGGTTGGATGG	0	19	RDR2-sensitive
ACTGTAAACGTAGAGAGGCCCTGA	0	14	RDR2-sensitive
ACTGTAAAGGACGGATATAGAAGA	0	13	RDR2-sensitive
ACTGTAAAGGACGGATATAGAGGA	0	38	RDR2-sensitive
ACTGTAAATCGGCAACTACCTCTT	0	9	RDR2-sensitive
ACTGTAATATGGACGGTTGGATGG	0	37	RDR2-sensitive
ACTGTACACGAACGAAGTTGGCGC	0	9	RDR2-sensitive
ACTGTACATCGGCAACAACCTCTT	3	30	RDR2-sensitive
ACTGTACATCGGCAACAACTTCTT	0	9	RDR2-sensitive
ACTGTACATCGGCAACAGCCTCTT	2	139	RDR2-sensitive
ACTGTACATCGGCAACAGCTTCTT	4	25	RDR2-sensitive
ACTGTACATCGGCAACAGTCTCTT	0	11	RDR2-sensitive
ACTGTACATCGGCAACGACTTCTT	0	26	RDR2-sensitive
ACTGTACATCGGCAACGGCCTCTT	0	34	RDR2-sensitive
ACTGTACATCGGCAACGGCTTCTT	2	43	RDR2-sensitive
ACTGTACATCGGCAACTGCTTCTT	0	10	RDR2-sensitive
ACTGTACATCGGCAGCGGTCTCTT	0	17	RDR2-sensitive
ACTGTACTAGGGCACAAGACTGAA	0	13	RDR2-sensitive
ACTGTAGAAGGCTTTTTACTAGGA	35	7	RDR2-resistant
ACTGTAGAAGGCTTTTTACTTGGT	94	5	RDR2-resistant
ACTGTAGACTACACTGTTCGCAGA	0	25	RDR2-sensitive
ACTGTAGACTGTACTGTTCGCAGA	0	19	RDR2-sensitive
ACTGTAGAGTAGGGCTATAGTCTG	0	10	RDR2-sensitive
ACTGTAGATGTAGATCCACTCGGT	0	22	RDR2-sensitive
ACTGTAGCACATGTAGAGGGCACC	0	9	RDR2-sensitive
ACTGTAGCCCACGAGCCGGCCTGA	0	11	RDR2-sensitive
ACTGTAGCTGTAGGGGACGGAATT	0	18	RDR2-sensitive
ACTGTAGGGACACATATGTAGGAT	0	9	RDR2-sensitive
ACTGTAGGTCGGCTTGCCTGCCCA	0	13	RDR2-sensitive
ACTGTATAAATGGACGGTTGGATG	0	11	RDR2-sensitive
ACTGTATACTTGTCGTTCTCGGGC	0	9	RDR2-sensitive
ACTGTATAGAGCGAGATTGAGGCT	0	9	RDR2-sensitive
ACTGTATAGGATGGTATCTCGAAA	0	9	RDR2-sensitive
ACTGTATATCGGCAACGGCTTCTT	1	22	RDR2-sensitive
ACTGTATCAAGGACTATGACGTCA	0	10	RDR2-sensitive
ACTGTATCCGGACAGCCAGGATGG	0	9	RDR2-sensitive
ACTGTATGGAAGAATTTGGTGCAT	44	2	RDR2-resistant
ACTGTATTTTAGGACGGAGGGCGT	0	13	RDR2-sensitive
ACTGTCAGAGTAAGTATCGCTGGT	0	13	RDR2-sensitive
ACTGTCAGCACTCGGCAAAGATGT	12	128	RDR2-sensitive
ACTGTCCACTCTGCAACGGCCCAA	5	57	RDR2-sensitive
ACTGTCCACTCTGCAACGGCTCAA	0	35	RDR2-sensitive
ACTGTCCACTCTGGAACGGCCCAA	0	11	RDR2-sensitive
ACTGTCCCAATATGGCACGGCTGT	0	17	RDR2-sensitive

ACTGTCCCAATCTGGCGCGGCTGT	0	47	RDR2-sensitive
ACTGTCCCATAGCTTAGAACGGAT	0	10	RDR2-sensitive
ACTGTCCGACTGTCAGGCAGGAAA	0	11	RDR2-sensitive
ACTGTCCGCGACACCGTAGAGGGC	0	11	RDR2-sensitive
ACTGTCCGGTGAGCCAACGGTCGG	0	13	RDR2-sensitive
ACTGTCCTAATCTGACGCGGCTGT	0	19	RDR2-sensitive
ACTGTCCTGCGGCAACATACTCGG	7	41	RDR2-sensitive
ACTGTCCTGCGGCAACGTACTCGG	3	28	RDR2-sensitive
ACTGTCGAAGACTGTATTTCGGGT	0	10	RDR2-sensitive
ACTGTCGAGACTAAGGCTATCCAC	0	35	RDR2-sensitive
ACTGTCGAGTGTGGACTGGGTCGG	0	13	RDR2-sensitive
ACTGTCGCGTAGGACAATTCGGCT	0	18	RDR2-sensitive
ACTGTCGCGTAGGACAGCTCGGCT	0	16	RDR2-sensitive
ACTGTCGCGTAGGACAGTTCGGCT	3	71	RDR2-sensitive
ACTGTCGCTTGTACGAGATGGATT	0	39	RDR2-sensitive
ACTGTCGGTATGATCATGCGTGCT	0	13	RDR2-sensitive
ACTGTCGGTCTTCTGTTGGTTGGC	0	11	RDR2-sensitive
ACTGTCGTAATGTATGTAGCGGTC	0	12	RDR2-sensitive
ACTGTCTACTCTACAACGGCCCAA	0	9	RDR2-sensitive
ACTGTCTAGCGTGGTTGTTTGAAT	31	0	RDR2-resistant
ACTGTCTCAATCTGGCGCGGCTGT	0	9	RDR2-sensitive
ACTGTCTCAGGTCGCGGACGGTCC	0	21	RDR2-sensitive
ACTGTCTCCGACGAATGACGAGCT	0	10	RDR2-sensitive
ACTGTCTCGGCTCGTAGTGACGGC	0	30	RDR2-sensitive
ACTGTCTGAGACGATACGGACGCC	0	11	RDR2-sensitive
ACTGTCTGGACATGTATGCGGATG	0	33	RDR2-sensitive
ACTGTCTGGCCTGGGCTTCCAATA	0	17	RDR2-sensitive
ACTGTGAAACTGCGAATGGCTCAT	21	0	RDR2-resistant
ACTGTGAACGACGCAACGACGAAC	0	23	RDR2-sensitive
ACTGTGACCAGCAGTCTGACTCAT	0	21	RDR2-sensitive
ACTGTGACTGTGGCAGACTGGCTT	6	239	RDR2-sensitive
ACTGTGACTGTTGCAGACTGGCTT	1	69	RDR2-sensitive
ACTGTGAGTCTGTAGGGATGGTCT	0	11	RDR2-sensitive
ACTGTGCCGGATGGATATACATAC	0	10	RDR2-sensitive
ACTGTGCGAGCAGAGCGTGACTGT	0	9	RDR2-sensitive
ACTGTGCTAGCTAGGTCGGGTCGT	0	10	RDR2-sensitive
ACTGTGGACGCCTACGGTAGATAC	0	12	RDR2-sensitive
ACTGTGGCAGACTGGCTTGGGTAC	0	13	RDR2-sensitive
ACTGTGGGTGTGGCTTCGACGAAA	0	21	RDR2-sensitive
ACTGTGGTTATGGATGCTCGCAAA	43	3	RDR2-resistant
ACTGTGTACGGCTACATCGAACAT	0	13	RDR2-sensitive
ACTGTGTATCCCTGTAGAGTACGG	1	105	RDR2-sensitive
ACTGTGTATGACCGAATGACGCGA	0	12	RDR2-sensitive
ACTGTGTATGTGTAGATGATTGGT	0	16	RDR2-sensitive
ACTGTTAAGATGGAGGACGACGTT	0	14	RDR2-sensitive
ACTGTTAGACTGTCCACACGGGTT	1	12	RDR2-sensitive
ACTGTTAGAGCAGGGTGTTTTGTT	30	0	RDR2-resistant
ACTGTTAGGATTGATTTGGTGGCA	0	10	RDR2-sensitive
ACTGTTAGGTACTGGACGGATGGG	0	15	RDR2-sensitive

ACTGTTATATATTGCAGAGGACTA	26	1	RDR2-resistant
ACTGTTATCTGGACTAATGGGCGG	0	11	RDR2-sensitive
ACTGTTCACGGCACTTGCTGGCAC	121	6	RDR2-resistant
ACTGTTCACGTCGGCACCAGGCTG	33	1	RDR2-resistant
ACTGTTCACTCTGCAACGGCCCAA	0	21	RDR2-sensitive
ACTGTTCACTGTCCGGTGCGCCAC	0	9	RDR2-sensitive
ACTGTTCACTGTCCGGTGGCACAC	0	11	RDR2-sensitive
ACTGTTCAGAGACGGTACTTTGCT	37	0	RDR2-resistant
ACTGTTCCGCATTGTAGACTGCAT	0	14	RDR2-sensitive
ACTGTTCGATTGCGTAGAGGGTAA	0	13	RDR2-sensitive
ACTGTTCGGTACTGTTTACGGAGT	0	16	RDR2-sensitive
ACTGTTCGTTTGGCAGAGGGCAAT	0	9	RDR2-sensitive
ACTGTTCTCTAGATGCTCAAGACT	0	12	RDR2-sensitive
ACTGTTCTGCACTGTAGACTGCAT	0	19	RDR2-sensitive
ACTGTTCTGCGATCGTAGCCGTCC	0	9	RDR2-sensitive
ACTGTTGACGTGTAGAGGACGAGA	0	31	RDR2-sensitive
ACTGTTGACGTGTAGAGGACGTGA	0	22	RDR2-sensitive
ACTGTTGACTGTGCGAGCTTCGGA	0	10	RDR2-sensitive
ACTGTTGCAGCTGTTTGGATTGCT	0	10	RDR2-sensitive
ACTGTTGCGGGACTGCGGCAGCCT	0	11	RDR2-sensitive
ACTGTTGGACACGTCGATCGGGCT	0	16	RDR2-sensitive
ACTGTTGGACATGTCTTAAGGCTG	1	13	RDR2-sensitive
ACTGTTGGAGTAGCTTTTCCGGTG	1	72	RDR2-sensitive
ACTGTTGGAGTGAATGAAGATGGT	0	159	RDR2-sensitive
ACTGTTGGAGTTAATGAAGATGGT	1	21	RDR2-sensitive
ACTGTTGGATGTGGAGGATGTGGT	0	14	RDR2-sensitive
ACTGTTGGCACTCGGCAAAGACTG	0	27	RDR2-sensitive
ACTGTTGGCCTCTAGCACTGATGG	0	17	RDR2-sensitive
ACTGTTGGCGTGTAGAGGACGAGA	0	27	RDR2-sensitive
ACTGTTGGCTGTGCTGAATACGGA	0	45	RDR2-sensitive
ACTGTTGGGACCATGCTTCGTCGC	2	16	RDR2-sensitive
ACTGTTGGGGCGAAGGCAAAGACG	0	12	RDR2-sensitive
ACTGTTGGGGGTATGCTTCGTAGC	0	14	RDR2-sensitive
ACTGTTGGGGGTATGCTTCGTCGC	0	9	RDR2-sensitive
ACTGTTGGGTCTATGCTTCGTCGC	1	20	RDR2-sensitive
ACTGTTGGGTGTAGATGACGACGA	0	9	RDR2-sensitive
ACTGTTGTGGATGGTAGGCGTCAT	0	25	RDR2-sensitive
ACTGTTGTTGCGGACGCGAGCATG	0	17	RDR2-sensitive
ACTGTTTAGAGGACGCTGCTGGAG	1	42	RDR2-sensitive
ACTGTTTATACTCATTGGACTGGC	0	15	RDR2-sensitive
ACTGTTTCAATTCGATCGTGTCGG	0	18	RDR2-sensitive
ACTGTTTCCTGGAAGACGAGATCT	0	10	RDR2-sensitive
ACTGTTTCGCGTAGAGATGGCTTT	0	17	RDR2-sensitive
ACTGTTTCGTGTAGAGATGGCTTT	0	16	RDR2-sensitive
ACTGTTTCTACTCTTTGGACTGGC	1	17	RDR2-sensitive
ACTGTTTCTTCGGTCTGTGCAGGC	0	9	RDR2-sensitive
ACTGTTTGAACTGCTGCAGGCTAC	0	10	RDR2-sensitive
ACTGTTTGGCACTGTGGTGTGGGC	0	9	RDR2-sensitive
ACTGTTTTATACTCTGTAGACTGC	0	11	RDR2-sensitive

ACTGTTTTATATTGTAGAGTGCAT	0	14	RDR2-sensitive
ACTGTTTTGCACTGAAGTCTGCAT	0	14	RDR2-sensitive
ACTGTTTTTACACTTCGGGCTGGC	0	17	RDR2-sensitive
ACTGTTTTTACACTTTGGACTGGC	0	9	RDR2-sensitive
ACTGTTTTTTAGAACCGTCGGTAC	0	22	RDR2-sensitive
ACTTAAGACAGTAGTGTAGACGTC	0	12	RDR2-sensitive
ACTTAAGACAGTAGTGTAGACGTT	0	13	RDR2-sensitive
ACTTAAGACTATAGTGTAGGCGTT	0	29	RDR2-sensitive
ACTTAAGACTGTAGTGTAGGCGTT	0	32	RDR2-sensitive
ACTTAAGCCAGTAGTGTAGACGTC	0	81	RDR2-sensitive
ACTTAATTGTGGGTCTGGGCCGGC	1	13	RDR2-sensitive
ACTTACCGGATCAGGACGAGGGCA	0	16	RDR2-sensitive
ACTTACCGGCTCTAGAAGAGGTTT	0	15	RDR2-sensitive
ACTTAGATCGTTCTCATCTCCGGC	27	2	RDR2-resistant
ACTTAGGCACTTGTGTTGGACACT	18	86	RDR2-sensitive
ACTTAGGGCGGTGAGAAAGGCACA	0	10	RDR2-sensitive
ACTTATACGCCGGGACGGAATCGG	0	10	RDR2-sensitive
ACTTATCGAGCGGATCGACGTCAC	1	48	RDR2-sensitive
ACTTATCGTATTAGAAACGTCGGT	0	193	RDR2-sensitive
ACTTATCGTATTAGGAACGTGGGT	0	10	RDR2-sensitive
ACTTATCGTCTATTGTTGGAGGCA	0	110	RDR2-sensitive
ACTTATGTGATCTTAGGATATGCC	0	9	RDR2-sensitive
ACTTATGTGATCTTAGGGTGTGCC	1	17	RDR2-sensitive
ACTTATTAAGACTGTAAGGGTAGC	0	12	RDR2-sensitive
ACTTATTATCGACGGACGACGGTT	0	9	RDR2-sensitive
ACTTATTTAAAGGATCGTTCCATC	0	9	RDR2-sensitive
ACTTATTTGAGAGATCGTCCCATC	0	23	RDR2-sensitive
ACTTCAATCGAAGACTATCGTCGT	0	47	RDR2-sensitive
ACTTCAATCGAAGACTGTCGTCGT	2	17	RDR2-sensitive
ACTTCAATCGAAGATCGTCGTCGT	0	16	RDR2-sensitive
ACTTCAATCGAAGGTCATCGTCGT	0	14	RDR2-sensitive
ACTTCAATCGAAGGTCGTCGTCGT	9	92	RDR2-sensitive
ACTTCAATCGGAGACTGTCGTCGT	0	9	RDR2-sensitive
ACTTCAATTGAAGATTGTCGTCGT	0	9	RDR2-sensitive
ACTTCACTGACGTTGGATGGCTGC	24	3	RDR2-resistant
ACTTCATCGTATCGTGACGGCACT	0	12	RDR2-sensitive
ACTTCCATGCAGTTAGACTCGTTA	36	1	RDR2-resistant
ACTTCCCGTCAGCAGTAGAGACGT	0	14	RDR2-sensitive
ACTTCCGACGGCTTAAGACGGGTC	0	17	RDR2-sensitive
ACTTCCTCGGTCAACGGATGCCAC	0	17	RDR2-sensitive
ACTTCGAGCTGGTATGACACGGTC	0	55	RDR2-sensitive
ACTTCGAGCTGTAGTTAGTGCATG	0	12	RDR2-sensitive
ACTTCGCGACTGGACTCCGCGGCT	1	12	RDR2-sensitive
ACTTCGCTATCTGGACCTGACGGG	0	12	RDR2-sensitive
ACTTCGGACTAGCACGGCACGGCC	0	9	RDR2-sensitive
ACTTCGGACTGATACGGCACGACC	1	20	RDR2-sensitive
ACTTCGGACTGGAAGGGTAGGCAG	0	24	RDR2-sensitive
ACTTCGGACTGGCACGGCACGACC	1	19	RDR2-sensitive
ACTTCGGCAGACTCTTTCAGAAGC	0	12	RDR2-sensitive

ACTTCGGGCTGACACGACACGGCC	0	10	RDR2-sensitive
ACTTCGGGCTGACACGATACGGCC	0	9	RDR2-sensitive
ACTTCGGGCTGACACGGCACGGCC	0	10	RDR2-sensitive
ACTTCGGGCTGGCACGACACGGCC	1	25	RDR2-sensitive
ACTTCGGGCTGGCACGACACGGTC	3	52	RDR2-sensitive
ACTTCGGGCTGGCACGGCACGACC	0	15	RDR2-sensitive
ACTTCGGGCTGGCACGGCACGGCC	0	58	RDR2-sensitive
ACTTCGGGCTGGCACGGCACGGTC	0	12	RDR2-sensitive
ACTTCGGGCTGGCACGGCATGTCC	0	11	RDR2-sensitive
ACTTCGGGCTGGCACGGTATGACC	0	19	RDR2-sensitive
ACTTCGGGCTTGCATGGCACGGTT	0	9	RDR2-sensitive
ACTTCGGGGCTGGCACGACACGGT	0	18	RDR2-sensitive
ACTTCGTCGACCAGTTATTGGCTT	2	16	RDR2-sensitive
ACTTCGTCGAGTGTTTTCAGGCTT	0	12	RDR2-sensitive
ACTTCGTCGAGTGTTTTTCAGGCT	0	16	RDR2-sensitive
ACTTCTAGTTAGAACGTCGTTGCT	21	1	RDR2-resistant
ACTTCTCACGGACAGCGTCAGCAT	0	9	RDR2-sensitive
ACTTCTGACGTGTTGCTGAAATGG	0	19	RDR2-sensitive
ACTTCTGGCACTGTGGTTTGGCCC	0	10	RDR2-sensitive
ACTTCTGTGATCTGTTTGGAGCGC	0	10	RDR2-sensitive
ACTTCTGTGCGTAGATCGCGGGTC	1	13	RDR2-sensitive
ACTTCTTCATACTGTAGTAGGGGT	0	15	RDR2-sensitive
ACTTCTTGCTGCTAGTCGACGATC	16	0	RDR2-resistant
ACTTGAACATGGTCGACGGCAAAG	0	13	RDR2-sensitive
ACTTGAACATGGTCGACGGCGAGG	0	37	RDR2-sensitive
ACTTGAACCCGCGGAGACTCGAAG	0	13	RDR2-sensitive
ACTTGAACGACTGAGGGAGGCCGC	0	19	RDR2-sensitive
ACTTGAATCGTTGGTTTTGATGGT	0	16	RDR2-sensitive
ACTTGAATGGATCGAATCGGGTTT	0	20	RDR2-sensitive
ACTTGACACAGGACGAGAACGGGC	0	11	RDR2-sensitive
ACTTGACGGAGCCTCGGACGCTAG	36	6	RDR2-resistant
ACTTGACTGTGAATTGGGTTGGGT	0	30	RDR2-sensitive
ACTTGAGAAGTAGATCATGGTCGT	15	0	RDR2-resistant
ACTTGATCAGAGTGGACTGGGCAC	0	21	RDR2-sensitive
ACTTGATGGCTGATACGGACGGGA	0	9	RDR2-sensitive
ACTTGATGGCTGGTACGGACGGGA	0	14	RDR2-sensitive
ACTTGCACGCGGATAGCCAGGCTC	0	19	RDR2-sensitive
ACTTGCAGGACTAGCGGCGAAGAC	0	11	RDR2-sensitive
ACTTGCAGGATGATATAACAATGT	14	0	RDR2-resistant
ACTTGCGACTGAAAGACGACGGTT	0	23	RDR2-sensitive
ACTTGCTCTGTGATCTTCTCTATA	30	0	RDR2-resistant
ACTTGGACAGACAGACGGATCTTC	0	10	RDR2-sensitive
ACTTGGACCAGGCCTAAACAGCAG	0	20	RDR2-sensitive
ACTTGGACGAACCGTGCGAGGACG	0	17	RDR2-sensitive
ACTTGGACGAACGACTGGGCGGCG	0	11	RDR2-sensitive
ACTTGGACGGAACACCGGGTGGCG	0	12	RDR2-sensitive
ACTTGGACTTATTGAAGAGGACGA	122	6	RDR2-resistant
ACTTGGACTTCATGCGGTCGGCAC	0	12	RDR2-sensitive
ACTTGGACTTTATTGGACGCGCCG	2	15	RDR2-sensitive

ACTTGGATCATGTGGAAGACGACT	0	21	RDR2-sensitive
ACTTGGCGTAGACGAGGATGACGA	0	9	RDR2-sensitive
ACTTGGGAACTGGCTTACGTGAGC	0	11	RDR2-sensitive
ACTTGGGAAGGCGACGAACAGCAA	0	13	RDR2-sensitive
ACTTGGGGCTGTGTAAACAGGTAT	1	28	RDR2-sensitive
ACTTGGTCGGCTGCTACGATACGG	0	9	RDR2-sensitive
ACTTGGTCGGTCGGTGCGGCGATT	4	22	RDR2-sensitive
ACTTGTAAGCTGATAGAGACGCAT	0	12	RDR2-sensitive
ACTTGTAGAACACCTAGAGAAGAA	0	10	RDR2-sensitive
ACTTGTAGATCTATCAGATAACGT	0	9	RDR2-sensitive
ACTTGTATACTTTAGAGGCTAATT	16	0	RDR2-resistant
ACTTGTCAAGTAGTTCAGGGGCAC	6	24	RDR2-sensitive
ACTTGTCAGCGTAGTTAGCGGGTG	0	11	RDR2-sensitive
ACTTGTCCCATGCGGTCGACGGAC	0	28	RDR2-sensitive
ACTTGTCGCGTAGGACAGTTCGGC	0	15	RDR2-sensitive
ACTTGTGCTGATTGGATGACTTGA	2240	231	RDR2-resistant
ACTTGTGCTGATTGGATGACTTGC	22	2	RDR2-resistant
ACTTGTGCTGATTGGATGACTTGT	71	3	RDR2-resistant
ACTTGTTATGGATTTAAACCGGCT	0	53	RDR2-sensitive
ACTTGTTATGGATTTAAATCGGCT	0	9	RDR2-sensitive
ACTTGTTCGGGCTTGCTGCGGCTT	0	12	RDR2-sensitive
ACTTGTTCTGATCTGATCGCGGAT	0	14	RDR2-sensitive
ACTTGTTGCCTTGTTGGACATGCC	0	10	RDR2-sensitive
ACTTGTTGGTGGGCATGTTAGTAG	15	0	RDR2-resistant
ACTTGTTTGTGACTCAATGGCGTT	0	10	RDR2-sensitive
ACTTTAAGTGGGATATGGTGATGG	26	1	RDR2-resistant
ACTTTAATCAAAGGTCGTCGTCGT	0	11	RDR2-sensitive
ACTTTAATCGAAGGTCGTCGTCGT	1	12	RDR2-sensitive
ACTTTAATTGAAGGTCGTCGTCGA	0	11	RDR2-sensitive
ACTTTAGACGGACATTTTTGGCAT	0	13	RDR2-sensitive
ACTTTATACTGTAGTAGAGGGTAC	0	13	RDR2-sensitive
ACTTTCAGTGGGACATTCGACGTT	42	3	RDR2-resistant
ACTTTCAGTTACAGACGGATTGGT	0	16	RDR2-sensitive
ACTTTCATACTGTAGAAGAGGATA	0	50	RDR2-sensitive
ACTTTCGACGGCTGTGTCAGAGGC	0	21	RDR2-sensitive
ACTTTCGGATTCGGGTTTAGGTAT	0	119	RDR2-sensitive
ACTTTCGGTGGCCTCTGACACGGC	1	28	RDR2-sensitive
ACTTTCTAAGGCTGTTGAAAGACG	0	12	RDR2-sensitive
ACTTTCTATGGAAGCGCGGACGGT	0	10	RDR2-sensitive
ACTTTCTGCATCACTCTAGACGGT	1	22	RDR2-sensitive
ACTTTCTGCGGAGCCACGGACGGT	0	19	RDR2-sensitive
ACTTTGACGAAGGCCTGCGGCGAC	4	17	RDR2-sensitive
ACTTTGACTGTGGCAGATCGGCTT	0	9	RDR2-sensitive
ACTTTGATATTGTTGGCACTAAAA	48	6	RDR2-resistant
ACTTTGCGTTGCTAGATCGTTGTC	0	9	RDR2-sensitive
ACTTTGGACAGACGTTTTTGGCAT	1	18	RDR2-sensitive
ACTTTGGACGACGTGTTTTGGCAT	8	40	RDR2-sensitive
ACTTTGGACGATGTGTTTTGGCAT	0	10	RDR2-sensitive
ACTTTGGACGGACGTTCTTGGCAT	0	22	RDR2-sensitive

ACTTTGGACGTCCAGTTTTGGCAT	2	44	RDR2-sensitive
ACTTTGGACTGGCACGACACGGCT	0	23	RDR2-sensitive
ACTTTGGGCTAGCATGACACGGTT	0	35	RDR2-sensitive
ACTTTGGGCTGGTATGGTACGGTC	0	11	RDR2-sensitive
ACTTTGTATTCATCTCGGACCTCC	20	0	RDR2-resistant
ACTTTGTGCGGAGGAAGGATGTCC	1	18	RDR2-sensitive
ACTTTGTGGATATTGATCGTTGAG	0	11	RDR2-sensitive
ACTTTGTGTAGAGCGCGTGATCGT	0	9	RDR2-sensitive
ACTTTGTGTGTGAGACTGACGACA	0	14	RDR2-sensitive
ACTTTGTTAGGCTGTACCGGTGTA	0	13	RDR2-sensitive
ACTTTGTTTGGCTGATGAAGGTGC	50	5	RDR2-resistant
ACTTTTAAGACTGCATTAATGACA	46	0	RDR2-resistant
ACTTTTAGTATCGTTCGGAGCCAT	0	11	RDR2-sensitive
ACTTTTCGAGGATCTGACAGACGG	0	10	RDR2-sensitive
ACTTTTGGTGATAAATCGACGGGG	17	1	RDR2-resistant
ACTTTTTCGTGTCGTGACTAGGCT	0	23	RDR2-sensitive
ACTTTTTGTAGTGTTGATCTCGGA	0	12	RDR2-sensitive
AGAAAAACTTTGATGATCTGTCTT	18	1	RDR2-resistant
AGAAAACCGGAACTGTTTCGGCCA	0	15	RDR2-sensitive
AGAAAACCGGGTCGGACCGTCTGA	0	10	RDR2-sensitive
AGAAAACGGTACGAGTCGGGACGA	0	9	RDR2-sensitive
AGAAAACGGTACGAGTCGGGACGG	0	10	RDR2-sensitive
AGAAAACGGTACGGGTCGGAACGA	0	17	RDR2-sensitive
AGAAAACGGTACGGGTCGGGACGA	0	18	RDR2-sensitive
AGAAAACGGTACGGGTCGGGACGG	0	27	RDR2-sensitive
AGAAAACTGGACAGGGCTGGCTAA	0	12	RDR2-sensitive
AGAAAAGACGGCGTCGGTACCTGC	0	12	RDR2-sensitive
AGAAAAGACTGTAAATGACGGATA	1	27	RDR2-sensitive
AGAAAATAATGGGACTGAGGGCAT	0	11	RDR2-sensitive
AGAAAATCGGTTTAGGTCGTCTAA	0	14	RDR2-sensitive
AGAAACCGGATCGAAGATCGGCAT	0	14	RDR2-sensitive
AGAAACCGGCCCGTGGAGCAGGCG	0	12	RDR2-sensitive
AGAAACGGACCCAGGAACGTGCGG	3	53	RDR2-sensitive
AGAAACGGTGGAATTATCAATGCA	14	0	RDR2-resistant
AGAAAGACGCGCGACCAGAGACTT	0	16	RDR2-sensitive
AGAAAGACGGATGGACTCACCGCC	2	14	RDR2-sensitive
AGAAAGACTGCTATGCACGCTTAC	0	16	RDR2-sensitive
AGAAAGCTGACTGGGAGACGCCAC	0	24	RDR2-sensitive
AGAAAGGTCGCCGAGTCACGCCAA	1	13	RDR2-sensitive
AGAAAGTCGGGAACAGACGGACAC	0	12	RDR2-sensitive
AGAAATGGTGGCAGAACACGGAAC	0	28	RDR2-sensitive
AGAAATTGTGTGCTTTGGGCCGGC	2	50	RDR2-sensitive
AGAACACACCACTGTGGACGCCTA	0	9	RDR2-sensitive
AGAACACACGGCAAAGAAGCTGAT	1	12	RDR2-sensitive
AGAACACGAGGATGGCAGAACGGC	0	17	RDR2-sensitive
AGAACACGCGGCATGCAGACTCTC	0	18	RDR2-sensitive
AGAACACGGAATGGGCAGAACGCG	0	11	RDR2-sensitive
AGAACACGGCGCACATGTAGACAC	0	21	RDR2-sensitive
AGAACACGGCGCACATGTGAACGC	0	29	RDR2-sensitive

AGAACACGGGATCGACTGAGACGT	0	22	RDR2-sensitive
AGAACACGGGGAGGACAGAACGGC	0	24	RDR2-sensitive
AGAACACGGTGCACTTGTGGACGC	4	213	RDR2-sensitive
AGAACACGTAGACGGCAGAACGGC	0	17	RDR2-sensitive
AGAACACGTCAGAGGCAGACTACT	0	22	RDR2-sensitive
AGAACACGTCTGATTGGGTGGCGC	0	28	RDR2-sensitive
AGAACACTGTTTTGCATCGTGGAT	0	9	RDR2-sensitive
AGAACAGACGTGGAGCGGTGGCAC	0	14	RDR2-sensitive
AGAACAGACTCTGCTCGGATCGGT	0	14	RDR2-sensitive
AGAACAGAGCGGCTCCGTTCCTAG	0	17	RDR2-sensitive
AGAACAGAGGTGACAGGGGGCTAA	23	3	RDR2-resistant
AGAACAGCCGTTGGCAGAACACGT	0	53	RDR2-sensitive
AGAACAGCCGTTGGCAGAACGCGT	1	15	RDR2-sensitive
AGAACAGCCTGCAGCCACGGACAC	0	15	RDR2-sensitive
AGAACAGGAATGGTGGCAGAACAC	0	26	RDR2-sensitive
AGAACAGGACATAGGAGTAGGCGT	0	41	RDR2-sensitive
AGAACAGGGAATGACAGAACGCGG	0	9	RDR2-sensitive
AGAACAGGTACCGCAGGATGAGGC	1	12	RDR2-sensitive
AGAACAGTGGTGCTGTGGACGGCT	0	9	RDR2-sensitive
AGAACATCTGGTGTAGACGCCTAC	0	9	RDR2-sensitive
AGAACATGGTGTACTGTGGACGAC	0	31	RDR2-sensitive
AGAACCAAGCGACGGACGAAGCAA	0	17	RDR2-sensitive
AGAACCAGCGACGGTGCCCTCTGC	0	14	RDR2-sensitive
AGAACCAGGCTCAAGAGAACACGC	0	18	RDR2-sensitive
AGAACCGAGGAACCGAACCGAAGA	3	27	RDR2-sensitive
AGAACCGCGCATGCAGATCTCCGC	1	14	RDR2-sensitive
AGAACCGGAATGGTGGCAGAACAA	0	30	RDR2-sensitive
AGAACCGGCGACGGTGCTCTCTGC	4	19	RDR2-sensitive
AGAACCGGTTGGAATAGAACGGCT	0	12	RDR2-sensitive
AGAACCGTCGCAGAAGGAGGACAA	0	17	RDR2-sensitive
AGAACCGTCTGAGCTCAGGGCATC	1	16	RDR2-sensitive
AGAACCGTGCACCGTGGACGTCTA	0	11	RDR2-sensitive
AGAACCGTGCTGAATGTGGACGTC	1	43	RDR2-sensitive
AGAACCGTTTAGAGGACGCTGCTG	0	18	RDR2-sensitive
AGAACCTGTACGAGCCCGACACGG	1	27	RDR2-sensitive
AGAACGACCGCAACTGGGAGGCTA	0	11	RDR2-sensitive
AGAACGAGCCCGAGCTGAGACTGT	0	15	RDR2-sensitive
AGAACGAGCCTGAGCCGAGCCGAG	0	9	RDR2-sensitive
AGAACGCACCACTGTCGACGTCTA	0	9	RDR2-sensitive
AGAACGCGGAATGACCGACTACTA	0	11	RDR2-sensitive
AGAACGCGGAATGGCCGACTACTA	0	30	RDR2-sensitive
AGAACGCGGCAGTGTGGACGCCTA	0	14	RDR2-sensitive
AGAACGCGTCAGAGGCAGAACGGC	0	50	RDR2-sensitive
AGAACGGCAGAACACACTGATGGC	0	18	RDR2-sensitive
AGAACGGCAGAACAGCAGACTACT	0	40	RDR2-sensitive
AGAACGGCAGAACCAAGGAGACAT	0	10	RDR2-sensitive
AGAACGGCAGAACGCAGGAAGACA	0	9	RDR2-sensitive
AGAACGGCAGAACGCGTCAGAGGC	0	9	RDR2-sensitive
AGAACGGCAGAACGGCAGAACCAC	0	23	RDR2-sensitive

AGAACGGCAGAACGGCAGAACCGC	0	14	RDR2-sensitive
AGAACGGCAGAACGGCAGAACGAA	0	9	RDR2-sensitive
AGAACGGCAGAACGGCAGAACGCA	0	9	RDR2-sensitive
AGAACGGCAGAACGGCAGAACGCG	0	9	RDR2-sensitive
AGAACGGCAGAACGGCAGAACGGC	0	44	RDR2-sensitive
AGAACGGCAGAACGGCAGACTACC	0	27	RDR2-sensitive
AGAACGGCAGAACGGCAGACTACT	0	65	RDR2-sensitive
AGAACGGCAGACTACTCTTACCTT	1	19	RDR2-sensitive
AGAACGGCGTACGGTTGTGGACGC	0	22	RDR2-sensitive
AGAACGGGCAGAACCACGGTGCAC	0	10	RDR2-sensitive
AGAACGGTACGAGATATGACGATC	0	9	RDR2-sensitive
AGAACGGTAGAACCACGGTGGCAG	0	28	RDR2-sensitive
AGAACGGTAGAACCAGAGAGACAG	0	10	RDR2-sensitive
AGAACGGTAGAACGAGGGAGGCAG	0	10	RDR2-sensitive
AGAACGGTTTACAGACTCTTGTTG	1	14	RDR2-sensitive
AGAACGTGCGAGTGTGGACGCCTA	0	12	RDR2-sensitive
AGAACGTTGCTGTAGCGAGTAGAG	0	33	RDR2-sensitive
AGAACTAGTTGTCGGCATGGGCAA	0	10	RDR2-sensitive
AGAACTATCTGACCTGGGCTGACG	0	16	RDR2-sensitive
AGAACTCACGTGGAAGGATGACAT	1	37	RDR2-sensitive
AGAACTCCTGATCCATAGAGACAA	15	0	RDR2-resistant
AGAACTCGAGGAATTTCAGGCATT	1010	91	RDR2-resistant
AGAACTCTGCAGGTAGAGCTGTAC	1	22	RDR2-sensitive
AGAACTGACTGATGGCATCTCCTC	0	10	RDR2-sensitive
AGAACTGAGAGCCGCCATAGACGA	0	51	RDR2-sensitive
AGAACTGATCTGAGCCTGACACGG	0	31	RDR2-sensitive
AGAACTGGACCATGTCGGGCTCGG	0	9	RDR2-sensitive
AGAACTGTAGTCTTTATTGCAGAG	0	22	RDR2-sensitive
AGAACTGTGTTTCTTGGCATGCTC	0	12	RDR2-sensitive
AGAACTGTTAGTGCATTTTACGTT	48	3	RDR2-resistant
AGAACTGTTCGTTTTGATGTTGGC	0	10	RDR2-sensitive
AGAACTGTTTAGAGGACGCTGCTG	1	28	RDR2-sensitive
AGAACTGTTTTTAGACGATTGCAT	0	24	RDR2-sensitive
AGAACTTAGGGCGGGTGCACTCCG	0	14	RDR2-sensitive
AGAACTTCTTGGTCTGTGCCTCGG	0	13	RDR2-sensitive
AGAACTTGAACCAAACATGGCATG	14	0	RDR2-resistant
AGAACTTGGTCTGCTAGATGACGA	0	80	RDR2-sensitive
AGAAGAACTCACACGCACAGACAA	0	12	RDR2-sensitive
AGAAGAACTGAAGCTACTTGATCC	1	18	RDR2-sensitive
AGAAGAATCTTGCATCGTTGTGGC	1	47	RDR2-sensitive
AGAAGACAGAGCTCCAACGGTCGG	0	13	RDR2-sensitive
AGAAGACAGTGTAGACTGCCCCCG	0	9	RDR2-sensitive
AGAAGACCAGAGGAGCGACGACGC	0	13	RDR2-sensitive
AGAAGACCCTAGAACTGGAGGCGC	0	28	RDR2-sensitive
AGAAGACCCTGTTGAGCTTGACTC	154	17	RDR2-resistant
AGAAGACGACACTCTGCGCCATCA	0	15	RDR2-sensitive
AGAAGACGACGAACTCCGCCTCGC	2	32	RDR2-sensitive
AGAAGACGACGAACTCCGCTCCGC	2	47	RDR2-sensitive
AGAAGACGACGAACTCCGCTTCAC	0	16	RDR2-sensitive

AGAAGACGACGAACTCCGCTTCGC	12	130	RDR2-sensitive
AGAAGACGACTCTCTGCGCCATCG	1	17	RDR2-sensitive
AGAAGACGATGAACTAGATCATTC	0	10	RDR2-sensitive
AGAAGACGCCGGCTGTGAATGGGC	0	11	RDR2-sensitive
AGAAGACGCGCGAGAAGCTGGACG	0	40	RDR2-sensitive
AGAAGACGTAGAGTCAGACGACGT	0	30	RDR2-sensitive
AGAAGACGTAGTTGAACTCTTCAT	0	25	RDR2-sensitive
AGAAGACTCTGCGCCATTGTGAAC	0	20	RDR2-sensitive
AGAAGACTCTGGCGCACTGTCCAC	0	14	RDR2-sensitive
AGAAGACTGGAAAGAGGTGGCCGC	0	17	RDR2-sensitive
AGAAGACTGGTACACATGGTGCAC	0	16	RDR2-sensitive
AGAAGACTGGTGCACATGGTGCAC	0	21	RDR2-sensitive
AGAAGACTGTAAAAGACGGATATA	0	10	RDR2-sensitive
AGAAGACTGTAAAGGACGGATATA	0	49	RDR2-sensitive
AGAAGACTGTAAGCAGCCGTCGCA	0	12	RDR2-sensitive
AGAAGACTGTGGCCAGGGGCGGAC	1	17	RDR2-sensitive
AGAAGACTTTGTTGAGCGCCGCAC	1	12	RDR2-sensitive
AGAAGAGACTGTAAATGACGGATA	0	37	RDR2-sensitive
AGAAGAGGTCACCGTTGTAGGCAA	0	12	RDR2-sensitive
AGAAGATACTGTATCTCAGCTGGA	70	1	RDR2-resistant
AGAAGATCCGGCGAGGAAGGACAT	0	11	RDR2-sensitive
AGAAGATCCGTGCCGTGCCTGTTA	0	28	RDR2-sensitive
AGAAGATCTTGAAGGTGCTAACGT	38	0	RDR2-resistant
AGAAGATGTAGAGAACCCGTCTCC	2	17	RDR2-sensitive
AGAAGATGTTCCGACGGCTGCTAT	54	1	RDR2-resistant
AGAAGATGTTGTGCTGCTTCCTTT	120	4	RDR2-resistant
AGAAGCAATCCGGCTCGGAATCGT	0	21	RDR2-sensitive
AGAAGCACGGACGGTCCGTAACAC	0	9	RDR2-sensitive
AGAAGCAGGAGCTGGAGCTTTACC	0	9	RDR2-sensitive
AGAAGCAGGAGCTGGAGCTTTGCC	0	17	RDR2-sensitive
AGAAGCAGTAGAATGCACGGGCGC	0	10	RDR2-sensitive
AGAAGCATAGCTATTGGATCTGAT	43	0	RDR2-resistant
AGAAGCCAAGTAGAACGGAGTCGT	0	26	RDR2-sensitive
AGAAGCCCCTGAGCAGCAGTCGGT	0	25	RDR2-sensitive
AGAAGCCGAGAACACCAGTAGCGT	0	17	RDR2-sensitive
AGAAGCCGCAGCCGCAGCCGGATT	7	38	RDR2-sensitive
AGAAGCCGGTGGTTAGAATCTAGG	0	9	RDR2-sensitive
AGAAGCCTACGTTGATGATCGCGA	16	0	RDR2-resistant
AGAAGCGATGATGGTGTGGGAACT	1	22	RDR2-sensitive
AGAAGCGGAACAGACGACGCACAT	1	35	RDR2-sensitive
AGAAGCGGCTCCTCCTCTGCGTAT	0	13	RDR2-sensitive
AGAAGCGGCTCGAACTACCGTGAC	0	23	RDR2-sensitive
AGAAGCTCGGCGCCAATGATGACG	1	17	RDR2-sensitive
AGAAGCTGGAGCTGGTACTGACTC	2	15	RDR2-sensitive
AGAAGCTGGCTGAAAAGCTGACGC	0	14	RDR2-sensitive
AGAAGCTGGCTGAAAAGCTGACGT	2	60	RDR2-sensitive
AGAAGCTGGCTGCTGAGCGTTTGG	0	12	RDR2-sensitive
AGAAGCTGGTGCCCATGGGGTGAC	31	3	RDR2-resistant
AGAAGCTGTCGGCAAACGGCTCCA	2	20	RDR2-sensitive

AGAAGGAAACCTGGGAACCGGCAA	4	223	RDR2-sensitive
AGAAGGAAACCTGGGAACCGGTAA	0	89	RDR2-sensitive
AGAAGGAACAGGTAACGAGGAGAT	26	2	RDR2-resistant
AGAAGGACGACCGAAGACTGGAGC	0	10	RDR2-sensitive
AGAAGGACTTGCACATGGTTTTTA	21	1	RDR2-resistant
AGAAGGCAACCGAATGCGAACCAT	0	11	RDR2-sensitive
AGAAGGCAACCTGGGAACCGGTAA	0	33	RDR2-sensitive
AGAAGGCACCGCCACGGTTCTTGT	0	9	RDR2-sensitive
AGAAGGCCAGGTAGAGCGGTTGGT	0	50	RDR2-sensitive
AGAAGGCCGGCGCGTAGAGGACGC	0	17	RDR2-sensitive
AGAAGGCGTGAACGAAGACGGGAA	0	9	RDR2-sensitive
AGAAGGCTGCGATGAACCGGCGTC	1	15	RDR2-sensitive
AGAAGGCTGCTGAGCACGACACGC	0	11	RDR2-sensitive
AGAAGGCTGTAGTCAGGCCGTGAA	2	17	RDR2-sensitive
AGAAGGCTTTGCCGAGTGCCGCAC	0	30	RDR2-sensitive
AGAAGGGAACCTGGGAACCGGTAA	0	9	RDR2-sensitive
AGAAGGGAAGTCAGGAACGAGCAT	0	11	RDR2-sensitive
AGAAGGGCCACTGTCAGGTTTGAA	47	0	RDR2-resistant
AGAAGGGCTGCGTAGAGATCGGCG	0	11	RDR2-sensitive
AGAAGGGTCGGCTACAGTTTCTAT	0	9	RDR2-sensitive
AGAAGGTGCTGTATGCTGTTTTGA	39	4	RDR2-resistant
AGAAGGTGGGACGAGAATGGGCTG	0	11	RDR2-sensitive
AGAAGGTTGGCCGCGCGGGGACGA	2	15	RDR2-sensitive
AGAAGTCACGGACCAACCCGGCTC	0	12	RDR2-sensitive
AGAAGTCATTGTCGATGTACGGTT	0	11	RDR2-sensitive
AGAAGTCGCTACGGATGGACAGGC	0	14	RDR2-sensitive
AGAAGTCGCTACTGCAGAAGGCAG	0	9	RDR2-sensitive
AGAAGTCGCTGCTGTAGAAGGCGG	1	26	RDR2-sensitive
AGAAGTCTGCATGATACTGATGGT	0	10	RDR2-sensitive
AGAAGTGGCTCCTTCTCTGTGTAC	0	11	RDR2-sensitive
AGAAGTGTGGAATGATAGCTTTAG	24	1	RDR2-resistant
AGAAGTGTTGTATTGTATGTGGCT	0	10	RDR2-sensitive
AGAAGTTCCGTCGTACATCGTTAG	0	13	RDR2-sensitive
AGAAGTTGGTTCGATCTATTTGGG	24	1	RDR2-resistant
AGAAGTTGTATTGGTATTCTTCAG	46	0	RDR2-resistant
AGAAGTTTTAGGCATAGACCTCGT	1	12	RDR2-sensitive
AGAAGTTTTTATCACGTGCGTCGG	0	12	RDR2-sensitive
AGAATAAACTCTGATAGTCTGATT	16	0	RDR2-resistant
AGAATACTGTCCAAGCTTCGGCAT	55	4	RDR2-resistant
AGAATAGCAGGCTGGACGATGCAT	0	9	RDR2-sensitive
AGAATAGCGGTGTACTGTAGCCCC	0	9	RDR2-sensitive
AGAATAGTATTTTGATTGTGGGAC	61	7	RDR2-resistant
AGAATAGTCGGGCCAGCACGGCAC	1	17	RDR2-sensitive
AGAATAGTCGGGCCGGCACGGCAC	0	18	RDR2-sensitive
AGAATCAGGAGCTGAAGCTGTGCC	0	12	RDR2-sensitive
AGAATCAGGGATGGAACGACTCCG	0	10	RDR2-sensitive
AGAATCAGGGATGGAACGGCTCCG	0	39	RDR2-sensitive
AGAATCAGGGCGGCTGCACTCCAC	0	59	RDR2-sensitive
AGAATCCATGGTCGTCATCGTCGT	1	13	RDR2-sensitive

AGAATCGGACCTAAACAGCGGCTT	0	49	RDR2-sensitive
AGAATCGGGCGAAGACGAAGCCAG	0	9	RDR2-sensitive
AGAATCGGGTGAAGACGAAGCCAG	1	12	RDR2-sensitive
AGAATCGGGTGAAGACGAAGCTAG	2	28	RDR2-sensitive
AGAATCGGGTGAAGACGGAGCCAG	0	21	RDR2-sensitive
AGAATCTCGGTGATACTTATCTAC	0	10	RDR2-sensitive
AGAATCTTGCATCGTTGTGGCCTG	0	15	RDR2-sensitive
AGAATCTTTTAGAGAAGACTGTAA	0	28	RDR2-sensitive
AGAATCTTTTAGAGGAGACTGTAA	2	14	RDR2-sensitive
AGAATGACGGATTTTGATACTCAT	0	33	RDR2-sensitive
AGAATGGACGGCTGAGATCGCAGA	1	43	RDR2-sensitive
AGAATGGCAGAACGGCAGAACGGC	0	21	RDR2-sensitive
AGAATGGCAGAACGGCTGGCTACC	0	9	RDR2-sensitive
AGAATGTCTCGTCATAGTCAACTC	17	0	RDR2-resistant
AGAATGTCTGGACGCTGGAGATAG	0	15	RDR2-sensitive
AGAATGTTATATTCGTGTCGTCGC	0	10	RDR2-sensitive
AGAATGTTGCACAGGATTGTTGGC	80	10	RDR2-resistant
AGAATGTTGCATATGATTGTTGGC	66	0	RDR2-resistant
AGAATTGTATGACATGCTTAAATT	48	0	RDR2-resistant
AGAATTGTTTGGTATCTTAGGCAC	15	0	RDR2-resistant
AGAATTTGAGCTCAGATGCGCGTT	15	0	RDR2-resistant
AGAATTTGGGGATCGAGAGGCTAA	0	10	RDR2-sensitive
AGAATTTGTCAGCAGCTTTATGGA	102	1	RDR2-resistant
AGAATTTGTGCATATTGTTAGAAC	0	9	RDR2-sensitive
AGAATTTTCCGGCTACGTACTGAT	0	10	RDR2-sensitive
AGAATTTTTCGGCTACGTACTGAT	0	28	RDR2-sensitive
AGACAAAACGGGCAGGGACACCCT	0	10	RDR2-sensitive
AGACAACGAATTTCAGAGTGATGG	18	0	RDR2-resistant
AGACAAGACAACGGGCAGGGACAC	1	16	RDR2-sensitive
AGACAAGCCGTGTCGGGTCTGGAC	0	29	RDR2-sensitive
AGACAAGCTCTGCAACGGCGCACC	0	13	RDR2-sensitive
AGACAAGGAGATGGTGACCGCTGA	0	28	RDR2-sensitive
AGACAAGGATGTCGGGTGGAAGGC	0	10	RDR2-sensitive
AGACAAGGTAGATCGGTAAGCAAC	29	0	RDR2-resistant
AGACACACGCTAGGATCTCGGAGC	0	42	RDR2-sensitive
AGACACACGTAGCTCAGCACGGTC	0	11	RDR2-sensitive
AGACACAGGAGGAGGCAGACTCGG	0	15	RDR2-sensitive
AGACACCTGGACCTGATCCGGCTC	0	22	RDR2-sensitive
AGACACGATGGACGGTATGCACGG	0	12	RDR2-sensitive
AGACACGGAGCACTCTTCTGACGG	0	16	RDR2-sensitive
AGACACGGATCAAGCATGGACCAT	0	9	RDR2-sensitive
AGACACGGCGTAGACCAACTGGTA	0	11	RDR2-sensitive
AGACACGGGATGGCATAATAACAG	0	11	RDR2-sensitive
AGACACTACTGCACAGAAGCTCTA	23	2	RDR2-resistant
AGACACTACTTCGGCTACTTCGCG	40	5	RDR2-resistant
AGACACTCGGCAAAGAACGCGGTT	0	21	RDR2-sensitive
AGACACTCGGCACAGACTGGTTTG	7	27	RDR2-sensitive
AGACACTGACAGACATGAGGACGC	1	17	RDR2-sensitive
AGACAGAAGAGCATAGCTGAGCAA	1	16	RDR2-sensitive

AGACAGAAGCAGGACGTGACACAA	0	14	RDR2-sensitive
AGACAGACCGTAGGCACGAGGCAA	0	11	RDR2-sensitive
AGACAGACGTTATAATGGATTAGT	63	2	RDR2-resistant
AGACAGAGTGGACACCAGGGGCGA	1	14	RDR2-sensitive
AGACAGAGTGGATACCAGGGGCGA	0	11	RDR2-sensitive
AGACAGAGTTAGACGTGTGGTAGA	1	24	RDR2-sensitive
AGACAGCACGTAGGGACTGGCATC	0	39	RDR2-sensitive
AGACAGCCGGTACGACATGGAGCT	0	9	RDR2-sensitive
AGACAGCGGGGTAGATGCGAGCAG	0	24	RDR2-sensitive
AGACAGCTAGGATTGAGACGTCGG	0	9	RDR2-sensitive
AGACAGGCATATGTCGGACCTCAA	0	14	RDR2-sensitive
AGACAGGTCACGGAGCGGTATAAG	39	1	RDR2-resistant
AGACAGGTCTGTTAGGAACGGTAT	0	30	RDR2-sensitive
AGACAGTAGTGTAGACGTCCACAA	0	16	RDR2-sensitive
AGACAGTAGTGTAGACGTCCACAC	0	9	RDR2-sensitive
AGACAGTAGTGTAGACGTCCACAT	0	10	RDR2-sensitive
AGACAGTGCTGGAGAATGTGACGC	0	17	RDR2-sensitive
AGACAGTGGACTCTTCTGGCGGAT	0	25	RDR2-sensitive
AGACATAGTGTCGGCACGGTGCGA	0	26	RDR2-sensitive
AGACATCATGGTCGGGCTAGACGC	0	26	RDR2-sensitive
AGACATGAGTTACTGGGCCGTCGG	0	13	RDR2-sensitive
AGACATGCAGGGTGACGATGACAT	21	0	RDR2-resistant
AGACATGTATCTTGACGAAGACAC	0	20	RDR2-sensitive
AGACATGTTGGACTAGCGTGCGTG	1	19	RDR2-sensitive
AGACATTCTAACGGCTGGTCTCGG	57	5	RDR2-resistant
AGACATTGCTGGAGGATGCGACGC	0	10	RDR2-sensitive
AGACCAACAGTAGATCGGGCCCAT	0	21	RDR2-sensitive
AGACCAACTCCAGCAAGACTCCGT	0	15	RDR2-sensitive
AGACCAACTCCGGACGAGAGACGC	0	13	RDR2-sensitive
AGACCAACTGTAGAGACAGGTCAA	0	17	RDR2-sensitive
AGACCACAGAACGCGCGCACACAA	0	9	RDR2-sensitive
AGACCACCAACTCTCAGAAGACAA	0	44	RDR2-sensitive
AGACCACCAACTCTCAGCAGACAA	0	13	RDR2-sensitive
AGACCACCGGATCGGAGGAGTTGG	0	14	RDR2-sensitive
AGACCACTTGAACAGCGTCGGCAA	0	26	RDR2-sensitive
AGACCAGAGGACAGACCGGCAAAG	0	9	RDR2-sensitive
AGACCAGATCCGGACGGTCGGCGT	2	14	RDR2-sensitive
AGACCAGCGACTGCAGCGAAGACC	0	10	RDR2-sensitive
AGACCAGCGACTGCAGCGAAGGCC	3	43	RDR2-sensitive
AGACCAGCGGGAACGATCTTGTAA	0	9	RDR2-sensitive
AGACCAGGTCGAAGGGCAGGGGCA	0	46	RDR2-sensitive
AGACCATACGATCAGCGGGCGAGA	0	30	RDR2-sensitive
AGACCATCTGGACCTGACCTCGGA	0	14	RDR2-sensitive
AGACCCGGAAGACGACGAAACTCC	0	11	RDR2-sensitive
AGACCCGGAAGACGGCGAACTCCG	6	55	RDR2-sensitive
AGACCCGGCACGAATTAGGACCAT	1	19	RDR2-sensitive
AGACCGACACGTAGGAGGCGCTGT	0	17	RDR2-sensitive
AGACCGACACGTAGGGGACGCTGT	0	30	RDR2-sensitive
AGACCGACACGTAGGGGGCGCTGT	0	16	RDR2-sensitive

AGACCGACTGTTTGGTTACGCGGC	0	17	RDR2-sensitive
AGACCGAGGCTGTGCGACGCGGAG	0	9	RDR2-sensitive
AGACCGATCCGAGCAGAGTCTGTT	0	14	RDR2-sensitive
AGACCGATTCGGACTTTTCGGTGT	1	14	RDR2-sensitive
AGACCGCACTGCTCGAGGACATGG	0	31	RDR2-sensitive
AGACCGCAGTAGCAGCCCGTCGGC	0	9	RDR2-sensitive
AGACCGCGGACCGTTCGGCACCTG	0	38	RDR2-sensitive
AGACCGCGGATTGTCTGCGCCGGC	2	73	RDR2-sensitive
AGACCGCTGGACTGTCCGGTATGA	0	12	RDR2-sensitive
AGACCGGACGGCATGTTTGGACAT	0	27	RDR2-sensitive
AGACCGGATGACCGAGGCATGCAC	0	9	RDR2-sensitive
AGACCGGATGTAGAGACGTTCCAA	0	18	RDR2-sensitive
AGACCGGCAAAGACTAGAGGACAG	0	33	RDR2-sensitive
AGACCGGGCTGACACGTGCGCCAT	0	22	RDR2-sensitive
AGACCGGTAGACGCCAAGGGGCAA	0	11	RDR2-sensitive
AGACCGGTCAGTGGAGCGGCGAAG	4	35	RDR2-sensitive
AGACCGGTGGATAGACGCATCTGT	21	0	RDR2-resistant
AGACCGTAGAACGGTAGAACGGTA	0	9	RDR2-sensitive
AGACCGTAGGGACAGAGGAAGTAA	0	23	RDR2-sensitive
AGACCGTCCGGACGTACGCAGAGG	0	36	RDR2-sensitive
AGACCGTGTAGATGTTGACGTCGC	0	10	RDR2-sensitive
AGACCGTGTCTGGGCTGAAGGCGT	2	54	RDR2-sensitive
AGACCGTTGGGGTTCGACCGTTGG	0	12	RDR2-sensitive
AGACCTACGTAGCAGCCTCGGTGT	1	16	RDR2-sensitive
AGACCTAGACCTGCATAGAACCGG	0	10	RDR2-sensitive
AGACCTAGCTATGACACTGGCACC	0	10	RDR2-sensitive
AGACCTATTCAATCGTCGGGTCAG	0	27	RDR2-sensitive
AGACCTCCATTTCGTCGAAGCCAA	0	11	RDR2-sensitive
AGACCTGATCATGTTGGACGGGCC	0	26	RDR2-sensitive
AGACCTGCGGCAGGTACGAGGACT	0	10	RDR2-sensitive
AGACCTGGACAGAGATGAGCTGGA	0	15	RDR2-sensitive
AGACCTGGCCCATGACAGACTCGG	0	11	RDR2-sensitive
AGACCTGTAGGTGAGAGACCGCCG	0	51	RDR2-sensitive
AGACCTGTGGTTTGACGTGGAACA	0	10	RDR2-sensitive
AGACCTTAGGGACTTTGTCGTCGT	0	13	RDR2-sensitive
AGACCTTGTTCATTTGTGTCGGAT	15	0	RDR2-resistant
AGACCTTGTTCGTTTACGTCGGAT	6	29	RDR2-sensitive
AGACGAACACGGACGTGGCGAGGC	0	21	RDR2-sensitive
AGACGAACAGGGCAACAGACGAAC	0	17	RDR2-sensitive
AGACGAAGACCGGGACGAAGGCAT	0	21	RDR2-sensitive
AGACGAAGAGCGGACGGGATTCGA	0	10	RDR2-sensitive
AGACGAAGCGTCGGTTCGGCCTAG	0	11	RDR2-sensitive
AGACGAAGCTATGAATACTGCATT	0	11	RDR2-sensitive
AGACGAAGGGACGACAGTGCACAA	0	9	RDR2-sensitive
AGACGACAAGGCTGAAGAGTGCAA	0	15	RDR2-sensitive
AGACGACGAACTCGCAGAGGATGC	0	32	RDR2-sensitive
AGACGACGCCTACCGCTCTGACGG	0	31	RDR2-sensitive
AGACGACGCGACTGAGCATGGTGA	0	9	RDR2-sensitive
AGACGACGGACAGACTTACCCTTC	0	12	RDR2-sensitive

AGACGACGGCCTAGACCTCTCACG	0	10	RDR2-sensitive
AGACGACGGCCTAGACCTCTCCAA	0	11	RDR2-sensitive
AGACGACGGCTTGACAACTTGGAC	0	12	RDR2-sensitive
AGACGACGTAAACACGGGAGCTGC	0	12	RDR2-sensitive
AGACGACGTATTCGACGAGGACAG	0	27	RDR2-sensitive
AGACGACTCGAACTCGGGGTGGCT	0	10	RDR2-sensitive
AGACGACTCGTAGTGTTTCGGTAT	0	16	RDR2-sensitive
AGACGACTGGCAGACGTTACCCTT	0	10	RDR2-sensitive
AGACGACTGGCAGACTTACCCTTC	0	27	RDR2-sensitive
AGACGACTGGCAGATTTACCCTTC	1	26	RDR2-sensitive
AGACGACTGGGAGATTGCATGTTA	0	12	RDR2-sensitive
AGACGAGAGTGGAGGCGAGAGCTG	0	15	RDR2-sensitive
AGACGAGATGGTACCAGGACGTGC	0	11	RDR2-sensitive
AGACGAGCTCGTAGAGGAAGACGC	0	11	RDR2-sensitive
AGACGATAGTGTAGACCAGGCACA	0	10	RDR2-sensitive
AGACGATCCACGTCAGGTAGTCGG	0	12	RDR2-sensitive
AGACGATGCGTTGGATAGTCGGAT	0	9	RDR2-sensitive
AGACGATGGACTGACAGCGATGGC	0	9	RDR2-sensitive
AGACGCAGGCGACAGACAAACTCC	0	10	RDR2-sensitive
AGACGCAGGCTGCATGAGCGGATA	0	10	RDR2-sensitive
AGACGCATAGACGGTAGGGCAGGT	0	15	RDR2-sensitive
AGACGCATGGAACAGGCACCGGAC	0	10	RDR2-sensitive
AGACGCCGATGGGAACCGACGGAC	1	12	RDR2-sensitive
AGACGCCGATGGGAACTGACGGAC	0	10	RDR2-sensitive
AGACGCCTCTGAACGACGGAGATG	1	13	RDR2-sensitive
AGACGCCTCTGAACGACGTAGAAC	0	11	RDR2-sensitive
AGACGCGATGGTAGAACGTGGCGC	1	17	RDR2-sensitive
AGACGCGTTGAGATCGGACATGAC	0	12	RDR2-sensitive
AGACGCTTACAGAGCACGGGACGT	0	14	RDR2-sensitive
AGACGCTTCGGACTTGGTGCGGCT	1	13	RDR2-sensitive
AGACGGAAAACGGTAGACGGACGG	0	10	RDR2-sensitive
AGACGGAAATCCTCCGGGGTCGGC	0	13	RDR2-sensitive
AGACGGAAGTCTCTCGAGGTCGGC	2	18	RDR2-sensitive
AGACGGACAGAACAGTGATAGCGG	0	15	RDR2-sensitive
AGACGGACCGAGGCTACAACCTGC	0	12	RDR2-sensitive
AGACGGACCTAGGTCGGGCCTCAA	0	17	RDR2-sensitive
AGACGGACGCTGGACGCAGGAAGC	0	58	RDR2-sensitive
AGACGGACGGAACAGTGATAGCGG	0	12	RDR2-sensitive
AGACGGAGACAGAGTTAGACGTGT	0	24	RDR2-sensitive
AGACGGAGACTGAGTCAGACGAGT	0	11	RDR2-sensitive
AGACGGAGTGGTAGTGGAGGGGAC	5	22	RDR2-sensitive
AGACGGATACGGAGAGGAGAGAGA	1	14	RDR2-sensitive
AGACGGATACGGAGTCTGCTGGAC	0	9	RDR2-sensitive
AGACGGATCTAGACGACGTAGAGT	0	18	RDR2-sensitive
AGACGGATCTAGATCAGGCCTCAA	0	10	RDR2-sensitive
AGACGGATCTAGCTAGACGACGTA	0	33	RDR2-sensitive
AGACGGCAGAACGGCAGAACACGT	1	24	RDR2-sensitive
AGACGGCATAGAGAACGTTTCGTA	0	9	RDR2-sensitive
AGACGGCCTGAGTGGGACGGTTGG	1	13	RDR2-sensitive

AGACGGCTCTAAGATTATTTTGCA	0	11	RDR2-sensitive
AGACGGCTCTAAGATTTTTTTGCA	3	16	RDR2-sensitive
AGACGGCTGAGATCGCAGAATGGC	0	20	RDR2-sensitive
AGACGGCTGCTGACGGTCCTTTGC	12	35	RDR2-sensitive
AGACGGCTGTTGACGGTCCTTGGC	0	29	RDR2-sensitive
AGACGGGCAACAGATTATTGGCAT	0	13	RDR2-sensitive
AGACGGGCGTGAGAGAGGTGGAAA	0	16	RDR2-sensitive
AGACGGGCTCTCGGTGTTGTTGAA	14	0	RDR2-resistant
AGACGGGGAGGAGGAACAGAAGAA	19	0	RDR2-resistant
AGACGGGGGCTTTGGCAGAACTGC	0	23	RDR2-sensitive
AGACGGGTACGGGTTGGAACTACC	0	10	RDR2-sensitive
AGACGGGTGGTGGAATATAGGCTT	0	14	RDR2-sensitive
AGACGGTACAGAGCATTCATGCAC	3	20	RDR2-sensitive
AGACGGTAGACGGTTTTACTTTTA	0	21	RDR2-sensitive
AGACGGTAGTGTAGACCAGGCACA	0	20	RDR2-sensitive
AGACGGTATGGACCTAACTCAGGA	0	9	RDR2-sensitive
AGACGGTCACAAGATGCGACTGAA	0	15	RDR2-sensitive
AGACGGTCAGTGTCGTAGACGGTC	0	17	RDR2-sensitive
AGACGGTCTGTGCTGCTACGGTGG	0	11	RDR2-sensitive
AGACGGTCTGTGTCTGGTGATCGG	3	40	RDR2-sensitive
AGACGGTGGACTGTTCGTGATGGC	0	12	RDR2-sensitive
AGACGGTGGAGACAGACAGAGGAC	0	23	RDR2-sensitive
AGACGGTGTCATCGTAGGATCCGG	0	10	RDR2-sensitive
AGACGGTGTGGGACAAGGCAAGAA	0	9	RDR2-sensitive
AGACGGTTATTTGGACTGTTGTAG	0	9	RDR2-sensitive
AGACGGTTCATAGGTGCCCTGTTC	79	1	RDR2-resistant
AGACGGTTGGCTCGGACCTGGCAC	0	12	RDR2-sensitive
AGACGGTTTGTGACTTAGGGTCGG	0	11	RDR2-sensitive
AGACGGTTTTACTTTTATGACGCT	2	17	RDR2-sensitive
AGACGTACGCTAGTAGAGACGAAT	0	10	RDR2-sensitive
AGACGTACGGATGTAGTTCGGCTC	0	16	RDR2-sensitive
AGACGTACGTAGAGGAGAAGCTGC	0	15	RDR2-sensitive
AGACGTACTCTGCCTTGCTCGAGA	1	13	RDR2-sensitive
AGACGTAGACCACATGACCGGATG	0	9	RDR2-sensitive
AGACGTAGAGTCAGACGACGTTGC	0	10	RDR2-sensitive
AGACGTAGTTAATGCGATCGGACA	0	16	RDR2-sensitive
AGACGTATGTAGAGGAGGAGTCGC	0	13	RDR2-sensitive
AGACGTCCGAATGTGGTCCGGCTC	1	12	RDR2-sensitive
AGACGTCCGACGAAAACGATGGTA	0	14	RDR2-sensitive
AGACGTCCGGATGTGGTTCGGCTC	5	38	RDR2-sensitive
AGACGTCTAGACCTGATCCGGCTC	0	52	RDR2-sensitive
AGACGTCTGAACCTAGTCCGGCTC	1	23	RDR2-sensitive
AGACGTCTGAACCTGGTCCGGCTC	1	66	RDR2-sensitive
AGACGTCTGAATGTGGTCCGGCTC	2	17	RDR2-sensitive
AGACGTCTGGACATGATCCGGCTC	1	16	RDR2-sensitive
AGACGTCTGGACCTAGTTCGGCTC	0	9	RDR2-sensitive
AGACGTCTGGACCTGATCCGGCTC	0	11	RDR2-sensitive
AGACGTCTGGACCTGGTCCGGCTC	15	75	RDR2-sensitive
AGACGTCTGGACCTGGTTCGGCTC	0	15	RDR2-sensitive

AGACGTCTGGATCTGGTCCGGCTC	0	10	RDR2-sensitive
AGACGTCTGGTTGTGGTCCGGCTC	1	19	RDR2-sensitive
AGACGTGACTGCATATGTCTGACC	0	12	RDR2-sensitive
AGACGTGATAGGCCTGAGGTGCAT	1	24	RDR2-sensitive
AGACGTGGAATAGGAGCGAGCAAC	0	11	RDR2-sensitive
AGACGTGGTTGGGCTGACGACGGA	0	9	RDR2-sensitive
AGACGTGTGGATCTGATCCGGCTC	0	11	RDR2-sensitive
AGACGTGTTCTCGGCTGCTCGGCT	1	15	RDR2-sensitive
AGACGTTAGACCTGAGATGGATGG	0	12	RDR2-sensitive
AGACGTTGGGTTTGTTACCGGTAA	17	1	RDR2-resistant
AGACTAAAATTGAATCGAACGGTG	48	4	RDR2-resistant
AGACTACAGCGCTCAAGCCGGCAT	0	30	RDR2-sensitive
AGACTACAGTAGCGACGATCCGGC	0	22	RDR2-sensitive
AGACTACGAGAGATGAGACGACAC	0	9	RDR2-sensitive
AGACTACGAGTGGAATGAGCCGAC	0	11	RDR2-sensitive
AGACTACTGTCGTCCGACGGCCCA	69	3	RDR2-resistant
AGACTAGAACGATGGAAGTGGCAT	0	40	RDR2-sensitive
AGACTAGACTACCACGGCCTGAAA	0	12	RDR2-sensitive
AGACTAGATCTGAGGGACTGACGG	0	30	RDR2-sensitive
AGACTAGCAGACTTCGACAGAGCC	0	10	RDR2-sensitive
AGACTAGTATGTTCAATGCGGCTT	0	9	RDR2-sensitive
AGACTAGTCTATCAGCCTGACGTC	0	10	RDR2-sensitive
AGACTATAGTGTAGGCGTTCATGA	0	16	RDR2-sensitive
AGACTATCAACTGTAGACGGCTGC	0	30	RDR2-sensitive
AGACTATGAGAGATGAGACGACAC	0	14	RDR2-sensitive
AGACTATGGTGGCAGAACACGGAA	0	15	RDR2-sensitive
AGACTCAACTCTGTACTAATGGGC	0	10	RDR2-sensitive
AGACTCACCTGAACCTGGAGCCGT	0	22	RDR2-sensitive
AGACTCACTGGACCAGCTGGAAGA	2	37	RDR2-sensitive
AGACTCACTGGACCAGCTGGAGGA	0	10	RDR2-sensitive
AGACTCAGAGGTGTAGAGAAGGCA	0	10	RDR2-sensitive
AGACTCATACAAGCGCGGAACAGT	0	9	RDR2-sensitive
AGACTCATCGGCAGCAGACCAACA	0	27	RDR2-sensitive
AGACTCATGAGCTCTGAAGAGGCT	22	0	RDR2-resistant
AGACTCATGATCGAGCGGCTTGAA	0	16	RDR2-sensitive
AGACTCATGTTGGTCTGTGGGCAT	0	10	RDR2-sensitive
AGACTCCGATGGGAACCGACGGAC	1	22	RDR2-sensitive
AGACTCCGGACAGTGTGAGCGGTG	0	20	RDR2-sensitive
AGACTCGACTCGGCTCGATAACGG	0	11	RDR2-sensitive
AGACTCGCGTAGGACAGTTCGGCT	1	17	RDR2-sensitive
AGACTCGCTGCGGAAGGTCGTCAC	0	28	RDR2-sensitive
AGACTCGCTGGACCAGCTGGAAGA	0	22	RDR2-sensitive
AGACTCGGACAGGAAATTAGGCAC	0	13	RDR2-sensitive
AGACTCGGACGTGAGCGACGGAAT	0	14	RDR2-sensitive
AGACTCGGTGAACTGTAAATCGGC	0	25	RDR2-sensitive
AGACTCGTAGATGCAAGGACTGGA	0	16	RDR2-sensitive
AGACTCGTGTTGGTCTGTGCGGAT	1	21	RDR2-sensitive
AGACTCGTGTTGGTCTGTGGGGAC	0	18	RDR2-sensitive
AGACTCTAGAGCGTATTGGACGGT	2	101	RDR2-sensitive

AGACTCTAGAGCGTATTGGATGGT	0	20	RDR2-sensitive
AGACTCTAGGCCGTATTGGACGGT	0	9	RDR2-sensitive
AGACTCTCGGCAACGCGGCTCTTT	1	30	RDR2-sensitive
AGACTCTCTGGTAGACCTAGGCAT	0	28	RDR2-sensitive
AGACTCTGACTCGGGCTAGACTGC	1	19	RDR2-sensitive
AGACTCTGAGAATAGTTGCTGCTG	136	1	RDR2-resistant
AGACTCTGCTCGGATCGGTCTAAG	1	17	RDR2-sensitive
AGACTCTGTAGATATGAGCAGATT	0	9	RDR2-sensitive
AGACTCTGTCAACCTCGTAAAAGA	18	0	RDR2-resistant
AGACTCTGTGCAGCCAGGATCGAC	0	22	RDR2-sensitive
AGACTCTTGCGGCAACTCTCTTAG	0	13	RDR2-sensitive
AGACTCTTTAGACGCATTGACTAT	0	20	RDR2-sensitive
AGACTCTTTGGCATGTGAGGGCTG	0	11	RDR2-sensitive
AGACTGAACTGCTGCAGCGAGCAC	0	28	RDR2-sensitive
AGACTGACACGTAGGAGGCGCTGT	0	10	RDR2-sensitive
AGACTGACGGCCGTCAGCTCTGGG	0	23	RDR2-sensitive
AGACTGACGGTCGTCAGCTGTAGA	0	11	RDR2-sensitive
AGACTGACTCAGAGGGGCTGCTGC	0	16	RDR2-sensitive
AGACTGACTGCAACGCATGCCTCT	0	19	RDR2-sensitive
AGACTGACTGCAACGCTTGCCTCT	0	13	RDR2-sensitive
AGACTGACTGCATGCGTTGACCAA	0	11	RDR2-sensitive
AGACTGACTGGCAGACTGACCTGC	0	15	RDR2-sensitive
AGACTGACTTAGACGGATTTAGAT	0	23	RDR2-sensitive
AGACTGAGACGGAGGACGAGAGTC	0	15	RDR2-sensitive
AGACTGAGCGCGTGCATTGACCAA	0	10	RDR2-sensitive
AGACTGAGGCTATCTTGTTCCGGC	0	10	RDR2-sensitive
AGACTGATGTTGTAGCTTCATATG	50	2	RDR2-resistant
AGACTGATTCAGCGTCGGCTTGGC	44	0	RDR2-resistant
AGACTGCACTACTCGTAGAAGGTG	0	12	RDR2-sensitive
AGACTGCACTGCGTACATCTGCCT	2	20	RDR2-sensitive
AGACTGCAGAGTCGACATCGCCAC	0	9	RDR2-sensitive
AGACTGCATCGACTCTATACGGCT	1	48	RDR2-sensitive
AGACTGCATGCGGAGTGGGATGGG	1	17	RDR2-sensitive
AGACTGCCAGTGAACGGTCGGCGT	0	12	RDR2-sensitive
AGACTGCCTGACACTGAAGCGGTT	0	17	RDR2-sensitive
AGACTGCGAGACGGAACGACCGAT	0	10	RDR2-sensitive
AGACTGCGGACGTGACGGCGTGAC	0	12	RDR2-sensitive
AGACTGCGGACGTGACGGCTTGAC	0	10	RDR2-sensitive
AGACTGCTGACTGTACGGCTGTAC	0	23	RDR2-sensitive
AGACTGCTGAGGGTATTAAGAGTA	32	0	RDR2-resistant
AGACTGCTGGAGAGCGAAACGGTG	0	14	RDR2-sensitive
AGACTGCTGTAGACGGCCTTAGGA	0	17	RDR2-sensitive
AGACTGCTTGCAGCGATTGTCGTT	0	15	RDR2-sensitive
AGACTGGACCGGCCCGACACGAAT	0	9	RDR2-sensitive
AGACTGGACCTGAGCGCGGCTGAC	0	12	RDR2-sensitive
AGACTGGACTAGGCAACCAAACAC	1	13	RDR2-sensitive
AGACTGGACTGCTAATTAGGCCGT	0	11	RDR2-sensitive
AGACTGGACTGGCTGAGTGCATGC	0	22	RDR2-sensitive
AGACTGGAGGCGACGAAGGCTGTA	0	9	RDR2-sensitive

AGACTGGAGGCGACGACGGTTATA	0	15	RDR2-sensitive
AGACTGGAGTAGAGGCCGAAGGTA	0	12	RDR2-sensitive
AGACTGGCCGAGCCGATCGACGTT	3	40	RDR2-sensitive
AGACTGGGACCGGCTGCATGCAAG	0	9	RDR2-sensitive
AGACTGGGATCCATGGAAGGACGC	0	40	RDR2-sensitive
AGACTGGGCCAGGCAACCAAACAT	0	14	RDR2-sensitive
AGACTGGGCTGGATTCTAAGCCGG	0	15	RDR2-sensitive
AGACTGGGGCAGAACGCGGAATGG	0	25	RDR2-sensitive
AGACTGGTAGATGCAAGGACTGGA	0	21	RDR2-sensitive
AGACTGGTCAGTGGAGCGGTGGAG	2	16	RDR2-sensitive
AGACTGGTGGAACAAGGAAGCCGC	0	44	RDR2-sensitive
AGACTGGTGGGAAGCTACATCCTC	0	12	RDR2-sensitive
AGACTGGTTCGGATGACGTCAGAT	0	9	RDR2-sensitive
AGACTGTAAAAGACGGATATAGAG	1	16	RDR2-sensitive
AGACTGTAAAGAACGGATATAGAG	0	11	RDR2-sensitive
AGACTGTAAAGGACGGATATAGAG	1	128	RDR2-sensitive
AGACTGTAAAGTACGGATATAGAG	0	15	RDR2-sensitive
AGACTGTAAATGACGGATATAGAG	0	17	RDR2-sensitive
AGACTGTAAATTTTAGAGGACGTT	0	9	RDR2-sensitive
AGACTGTAAGGGGTAGGCTGCCTC	0	18	RDR2-sensitive
AGACTGTAATAAGAAGTATTGGCT	0	59	RDR2-sensitive
AGACTGTACAACGGCTCCCGCAGC	0	13	RDR2-sensitive
AGACTGTACACTAGACCATGGCAC	0	16	RDR2-sensitive
AGACTGTACGGTGACCTACCACGG	0	13	RDR2-sensitive
AGACTGTACTATTCGCATATTCAA	0	9	RDR2-sensitive
AGACTGTACTGAGGACGACGTCTC	0	10	RDR2-sensitive
AGACTGTACTGTTCGCATAGTGAA	1	22	RDR2-sensitive
AGACTGTACTGTTCGCGGAGTCGA	0	29	RDR2-sensitive
AGACTGTACTGTTCGTAGAGTGGA	2	17	RDR2-sensitive
AGACTGTAGCATGGTGTACTTTGT	0	38	RDR2-sensitive
AGACTGTAGCGGCCCACGTCACTC	0	25	RDR2-sensitive
AGACTGTAGGTTGGAGACGAGCAC	0	23	RDR2-sensitive
AGACTGTAGTGTTCACATAACGGA	0	13	RDR2-sensitive
AGACTGTATCTAGCATAGGGTGGG	0	12	RDR2-sensitive
AGACTGTATGGACCGACGCAGAGA	0	23	RDR2-sensitive
AGACTGTATTTCGGGTCCGAGCAG	0	39	RDR2-sensitive
AGACTGTCCATGTCTGGAGCTCGG	2	43	RDR2-sensitive
AGACTGTCCGGTGAGAAGCTGCAC	0	12	RDR2-sensitive
AGACTGTCGACGCACTACTACCGT	0	12	RDR2-sensitive
AGACTGTCGACGCACTGCTGGCAT	0	19	RDR2-sensitive
AGACTGTCTGCAGCGTTTGACGTT	0	18	RDR2-sensitive
AGACTGTCTTCAACGGATGACGCT	0	29	RDR2-sensitive
AGACTGTCTTGAGTAGACCGTGTA	0	17	RDR2-sensitive
AGACTGTGGACGCCTACGTTGTCT	0	9	RDR2-sensitive
AGACTGTGGAGGCTTACAACACTG	0	14	RDR2-sensitive
AGACTGTGGGAGCGGCGGGACCTT	0	9	RDR2-sensitive
AGACTGTGGGTCGAACATCGGCGT	0	21	RDR2-sensitive
AGACTGTGGTAAGCATGCAATGTC	47	2	RDR2-resistant
AGACTGTGGTGCACGGTTCTGCCT	1	18	RDR2-sensitive

AGACTGTGTAGAGGGAGCGGGAGC	0	9	RDR2-sensitive
AGACTGTGTAGCCGGAGCCGGTGA	0	9	RDR2-sensitive
AGACTGTGTATCTGGAGCAGCAGG	0	10	RDR2-sensitive
AGACTGTGTATGACCGAATGACGC	0	13	RDR2-sensitive
AGACTGTGTGCATGTGTTGGCTGC	0	15	RDR2-sensitive
AGACTGTGTGTAAGGCTATCTCCA	5	22	RDR2-sensitive
AGACTGTGTGTGTGTGTGGGCCGG	0	11	RDR2-sensitive
AGACTGTTACAGGAGGCTTCGGCT	0	17	RDR2-sensitive
AGACTGTTGCAGAGTGGACGGTTG	0	19	RDR2-sensitive
AGACTGTTGCATCTAATGACTGCA	0	27	RDR2-sensitive
AGACTGTTGCTGACGGCTCTTTGC	0	14	RDR2-sensitive
AGACTGTTGTAGATGGCAAGATAT	33	0	RDR2-resistant
AGACTGTTGTAGCTATCCGCAGAG	0	20	RDR2-sensitive
AGACTGTTGTCTTTATTGCAGAGT	0	11	RDR2-sensitive
AGACTGTTTGAATGTTTCGGCTAA	0	9	RDR2-sensitive
AGACTGTTTGTTTGCCTCTTAGGA	0	19	RDR2-sensitive
AGACTGTTTTACTTTTATGACGGT	0	90	RDR2-sensitive
AGACTTAACCGAACTATGTTGGCA	0	10	RDR2-sensitive
AGACTTAACTGTAGAGGAGGCCAA	0	11	RDR2-sensitive
AGACTTAATGGATCGTACGTCGGA	0	11	RDR2-sensitive
AGACTTACTGATTGTTGGCGCGCG	14	36	RDR2-sensitive
AGACTTAGGGCCCTGACGACGTGC	1	52	RDR2-sensitive
AGACTTATTTCCGACGGCTGGCCC	0	20	RDR2-sensitive
AGACTTATTTTGTGTTGGACGCCA	0	13	RDR2-sensitive
AGACTTCAATCGAAGATCGTCGTC	5	24	RDR2-sensitive
AGACTTCAATCGAAGATCGTCGTT	1	26	RDR2-sensitive
AGACTTCAATCGAAGGTCGTCGTC	13	42	RDR2-sensitive
AGACTTCAATTGAAGGTCGTCGTT	0	11	RDR2-sensitive
AGACTTCCTCTGCTGCGTCGGTAC	0	46	RDR2-sensitive
AGACTTCGGTCGGATTCGGGTCTG	0	16	RDR2-sensitive
AGACTTCTACCCACGGACGGACAT	0	21	RDR2-sensitive
AGACTTGCTCGGCGGGAGACATGA	0	13	RDR2-sensitive
AGACTTGGACAGAGCACAAGACAT	0	28	RDR2-sensitive
AGACTTGGACAGGAAGAAGAGCAG	0	11	RDR2-sensitive
AGACTTGGGACTTGATGGCCTTCC	9	38	RDR2-sensitive
AGACTTGTCGGTCTGATTGCCTGA	0	9	RDR2-sensitive
AGACTTGTTCTGAGCGTTGAGTCA	0	66	RDR2-sensitive
AGACTTTAGTCTCATATCGTCCAT	0	9	RDR2-sensitive
AGACTTTATTTGGACATTGTCGGT	0	15	RDR2-sensitive
AGACTTTCCTTTCGTCGGAGGCGA	0	11	RDR2-sensitive
AGACTTTGGTACACGGACGAGCAC	0	9	RDR2-sensitive
AGACTTTGTTTGATTACGTCGGAT	0	16	RDR2-sensitive
AGAGAAACTGAGGAAGTCTTGGCT	22	2	RDR2-resistant
AGAGAACACTGACGTGGCACGTGA	0	19	RDR2-sensitive
AGAGAACACTGTCGTCGGTACACC	0	15	RDR2-sensitive
AGAGAACCAGGCTCAAGAGAACAC	0	15	RDR2-sensitive
AGAGAACCGCGCGATCGTTGGTCA	2	19	RDR2-sensitive
AGAGAACCGTTTAGAGGACGCTGC	1	53	RDR2-sensitive
AGAGAACGACAGAGCAAGTGGCGC	0	9	RDR2-sensitive

AGAGAACGCGGAAATACTGTTTAT	25	0	RDR2-resistant
AGAGAACGCGGCACGTACTCTAGA	0	17	RDR2-sensitive
AGAGAACGCTGACGTGGCACGTGA	6	279	RDR2-sensitive
AGAGAACGCTGACGTGGCATGTGA	0	15	RDR2-sensitive
AGAGAACGCTGCTGGAGGACGTAG	0	58	RDR2-sensitive
AGAGAACGGCGTTGGCACTGCTGC	0	18	RDR2-sensitive
AGAGAACGTTGCTGGAGACAGCCT	1	14	RDR2-sensitive
AGAGAACGTTGCTGGAGATGGAGA	0	9	RDR2-sensitive
AGAGAACGTTGCTGTAGCGAGTAG	0	12	RDR2-sensitive
AGAGAACTCGGAATGGGAGGGCGT	0	11	RDR2-sensitive
AGAGAACTGAGTGGCGACGGACAG	1	12	RDR2-sensitive
AGAGAACTGTTTAGAGGACGCTAC	0	13	RDR2-sensitive
AGAGAAGACTGCCGCTGCTCTCTG	0	17	RDR2-sensitive
AGAGAAGACTGGCTCCTACCGCAG	1	12	RDR2-sensitive
AGAGAAGACTGTAAAAGACGGATA	1	58	RDR2-sensitive
AGAGAAGACTGTAAAGAACGGATA	0	33	RDR2-sensitive
AGAGAAGACTGTAAAGGACGGATA	4	445	RDR2-sensitive
AGAGAAGACTGTAAAGGACGGATT	0	9	RDR2-sensitive
AGAGAAGACTGTAAAGTACGGATA	1	31	RDR2-sensitive
AGAGAAGTAGAGGCTCATCGTCGC	0	9	RDR2-sensitive
AGAGAATCCAGCAGCGTCCTCTAA	0	19	RDR2-sensitive
AGAGAATCTCGCTGACCAGGATGA	0	13	RDR2-sensitive
AGAGACAGACGACCAGGAGACCTC	0	15	RDR2-sensitive
AGAGACAGAGGAAGGGAGTGGACA	0	9	RDR2-sensitive
AGAGACAGGTGCACATCTGACGGT	0	81	RDR2-sensitive
AGAGACAGTAGAGGACCGGCCCAG	0	11	RDR2-sensitive
AGAGACAGTCGCGTATGACAGCAA	0	11	RDR2-sensitive
AGAGACAGTTGTAGCATTTATCTT	1	18	RDR2-sensitive
AGAGACATCGGACCGTGATGTGCC	0	9	RDR2-sensitive
AGAGACCAGGAGCGACATCTGCAT	0	11	RDR2-sensitive
AGAGACCGAACCGGCAGAACACAA	0	35	RDR2-sensitive
AGAGACCGGTACGGATTATGTCAT	0	11	RDR2-sensitive
AGAGACCGGTATGCACACTCTCTC	0	13	RDR2-sensitive
AGAGACGAGAGTGGAGGCGAGAGC	0	19	RDR2-sensitive
AGAGACGCAGTAGTGCAAGATGAT	0	9	RDR2-sensitive
AGAGACGCGGATATGCTGGCGGGC	0	15	RDR2-sensitive
AGAGACGGACTCTTCGCGAAGAAC	0	15	RDR2-sensitive
AGAGACGGAGTATGCTTGCGACAT	0	13	RDR2-sensitive
AGAGACGGATCCAAGACGGTACTC	0	29	RDR2-sensitive
AGAGACGGCCGTAGGAAGTTAGCA	0	9	RDR2-sensitive
AGAGACGGGAGGACGGAGCATCGC	0	13	RDR2-sensitive
AGAGACGGGGAGGAGGAACAGAAG	19	1	RDR2-resistant
AGAGACGGGGTGGTAGGCGGGCGA	0	9	RDR2-sensitive
AGAGACGGTGTAGAGACGAACAGC	0	12	RDR2-sensitive
AGAGACGTACGTACAGAGCATCCA	0	32	RDR2-sensitive
AGAGACGTCATCAGATTTGTGGAG	0	10	RDR2-sensitive
AGAGACGTCTTAGGTGATCTGCCG	0	12	RDR2-sensitive
AGAGACGTCTTGGATGGCCTGCCG	0	9	RDR2-sensitive
AGAGACGTGATAGGCCTGAGGTGC	0	10	RDR2-sensitive

AGAGACGTGTAGCAGTCGTGCGCA	0	29	RDR2-sensitive
AGAGACGTTGAGCCGGACGAGCAT	0	11	RDR2-sensitive
AGAGACGTTTTCACTGGCGCTTTT	8	29	RDR2-sensitive
AGAGACTCAGGATCATCGGGTTCA	29	4	RDR2-resistant
AGAGACTGAGATGTTATATGTGCA	1	45	RDR2-sensitive
AGAGACTGAGTCAGACGGGTGGTA	2	14	RDR2-sensitive
AGAGACTGCAGGATGGTTGCCCAC	0	9	RDR2-sensitive
AGAGACTGCATGGTACAACCCTAA	0	11	RDR2-sensitive
AGAGACTGCTGAGCAGGAGCTCGT	1	24	RDR2-sensitive
AGAGACTGCTTAATACAATCCTGA	0	14	RDR2-sensitive
AGAGACTGGATTTAACAACGGTGT	0	20	RDR2-sensitive
AGAGACTGTTGGGAGCAAGGAGGA	34	1	RDR2-resistant
AGAGACTGTTGTCGATGTATGGTT	0	26	RDR2-sensitive
AGAGACTTGTGCATCCATTCCTGT	0	19	RDR2-sensitive
AGAGAGAACGCTGACGTGGCACGT	0	9	RDR2-sensitive
AGAGAGAAGAGGACCGACACGTAG	0	11	RDR2-sensitive
AGAGAGAAGCGTCGCCTGGAAGGC	0	10	RDR2-sensitive
AGAGAGAAGGGGACCGACACGTAG	2	21	RDR2-sensitive
AGAGAGATCACACTGCAAGGGCAT	0	15	RDR2-sensitive
AGAGAGATCGGCAGAACCGTGCAC	0	9	RDR2-sensitive
AGAGAGATTGCTGACGTGGCACGT	2	34	RDR2-sensitive
AGAGAGCACCGCCGCCGGTTCTTC	0	10	RDR2-sensitive
AGAGAGCATCGCCAGACGGTACAC	0	14	RDR2-sensitive
AGAGAGCATCGTCAGACGGTTCAT	0	9	RDR2-sensitive
AGAGAGCCGACGCGTAGGGGACGC	0	11	RDR2-sensitive
AGAGAGCCGGATTGGACTGGCATG	0	31	RDR2-sensitive
AGAGAGCCGTTGGACTCATGGCGC	4	44	RDR2-sensitive
AGAGAGCCTGCCGAGCGGAACACC	0	10	RDR2-sensitive
AGAGAGCGCTGTAGGGGGCACCGC	1	17	RDR2-sensitive
AGAGAGCTCTGCCAGACGGTCCTT	0	27	RDR2-sensitive
AGAGAGCTGAGACGCCGTGGACGA	1	14	RDR2-sensitive
AGAGAGGACGCCGATCGGAACAAC	1	40	RDR2-sensitive
AGAGAGGACGCGGCTCTAGTCGGA	15	0	RDR2-resistant
AGAGAGGATGTAGAAGGCGGTTGC	0	22	RDR2-sensitive
AGAGAGTCGTGTAGAAACGGGCTA	0	9	RDR2-sensitive
AGAGATCAGGCACAACGTCGGCGA	0	12	RDR2-sensitive
AGAGATCATATGTGCATTGCAGAT	20	0	RDR2-resistant
AGAGATCGCAGACGCTGGAGGCAA	0	9	RDR2-sensitive
AGAGATCTGGCATGTTGCTGCTGC	0	10	RDR2-sensitive
AGAGATCTGGCTACTGTCGTCAAA	0	9	RDR2-sensitive
AGAGATCTTGAGCGAAGTAGACGA	0	15	RDR2-sensitive
AGAGATGAGTATGTGGGCTGACGG	0	14	RDR2-sensitive
AGAGATGGGTACTCAGAACTGTCT	0	11	RDR2-sensitive
AGAGATGTAGTAGATGTGTTTCCT	2	32	RDR2-sensitive
AGAGATTGCAGATCGTTGGTATTA	0	11	RDR2-sensitive
AGAGATTGCTGACGTGGCACGTGA	0	82	RDR2-sensitive
AGAGATTGCTGCAGGTCGCCGGAG	1	15	RDR2-sensitive
AGAGATTGGTATAAGTTTGGATGG	0	10	RDR2-sensitive
AGAGATTGGTGACGTGGCACGTGA	0	12	RDR2-sensitive

AGAGATTGTAGATGGCAGCTCCAC	0	41	RDR2-sensitive
AGAGATTGTGAATGGTTTTTCGGT	0	9	RDR2-sensitive
AGAGATTTTGGCTGTGAGTTTAAA	41	0	RDR2-resistant
AGAGATTTTGGCTGTGATTTTAAA	81	0	RDR2-resistant
AGAGCAAAGGGGTAGAGATCACTC	0	10	RDR2-sensitive
AGAGCAAGCCTTCAAAATCGTCGT	0	14	RDR2-sensitive
AGAGCACAGACCTGGGCACCGGCT	1	13	RDR2-sensitive
AGAGCACGCGAGCTGTAGCGACGG	0	9	RDR2-sensitive
AGAGCACGGGCTGGCATAACACGA	0	35	RDR2-sensitive
AGAGCACTGTGCAAGAGGACGCTA	0	12	RDR2-sensitive
AGAGCAGAGTGTAGACGTCCACGG	0	12	RDR2-sensitive
AGAGCAGGACTGCAGGAGGGCGGA	0	22	RDR2-sensitive
AGAGCAGGACTTTGCCGAGTGCGG	2	17	RDR2-sensitive
AGAGCAGGCCTCCGAAATCGTCGT	2	37	RDR2-sensitive
AGAGCAGGGGAGTAGGACAAGCAT	0	16	RDR2-sensitive
AGAGCATCGAAGAGTCAGAGCCAT	0	10	RDR2-sensitive
AGAGCATGGACATGGAGTCGACGG	0	15	RDR2-sensitive
AGAGCCAAGCTCTGGACATGGCAG	0	10	RDR2-sensitive
AGAGCCAAGTAGAACGGAACGGTT	0	29	RDR2-sensitive
AGAGCCAAGTAGAACGGAGCCGTT	4	35	RDR2-sensitive
AGAGCCAGTAGATATTTTCGGCAT	0	9	RDR2-sensitive
AGAGCCCGAGGAACTCGCAGGAGA	0	13	RDR2-sensitive
AGAGCCCTGTGCCAGGTCGTGGAC	0	19	RDR2-sensitive
AGAGCCGACAGAAGATCCGGACGC	0	28	RDR2-sensitive
AGAGCCGGACGGCCTGGGGTCACC	69	0	RDR2-resistant
AGAGCCGTTGGACTCATGGCGCAC	8	37	RDR2-sensitive
AGAGCCTGGGCTGTTGGTTTCGGC	1	23	RDR2-sensitive
AGAGCCTGTAGATGGTGACGTCGT	0	11	RDR2-sensitive
AGAGCGACCAGTACGATCGGACGG	2	19	RDR2-sensitive
AGAGCGACTCAGACTGTTCTTCGG	1	15	RDR2-sensitive
AGAGCGAGCTGAGCCGCGGCGGAG	1	15	RDR2-sensitive
AGAGCGATGGCTTGCTTTGAGCAC	0	30	RDR2-sensitive
AGAGCGCACTGTATTGAGTCGGAA	0	20	RDR2-sensitive
AGAGCGCGCGTCAGGATAAGGTGT	1	12	RDR2-sensitive
AGAGCGCGTGACCAGAGACTGTAA	0	9	RDR2-sensitive
AGAGCGGCAACTTAGAGGACCCAT	0	18	RDR2-sensitive
AGAGCGGCTCCGTTCCTAGAATCA	3	33	RDR2-sensitive
AGAGCGGCTCTGTTCCTAGAATCA	1	13	RDR2-sensitive
AGAGCGGCTCTGTTCTGTCCGTCA	0	13	RDR2-sensitive
AGAGCGGGATTGGAACGGAGCGGC	1	19	RDR2-sensitive
AGAGCGGGATTGGTGTAAACGAAC	0	16	RDR2-sensitive
AGAGCGGGCTGGACAGCGGCTGGG	95	11	RDR2-resistant
AGAGCGGGTGGACAGAGTAGGAGA	0	16	RDR2-sensitive
AGAGCGGTGTCTCTGAACCGACAT	0	12	RDR2-sensitive
AGAGCGTGACTGTGAGAGCGGGAT	0	22	RDR2-sensitive
AGAGCGTGACTGTGCGAGCGGGCT	4	19	RDR2-sensitive
AGAGCGTTGTGCTGTCGTACGGCT	0	10	RDR2-sensitive
AGAGCTATATAGAACTAGAGCAGC	0	9	RDR2-sensitive
AGAGCTCGGCACCATAGGCTATGG	0	10	RDR2-sensitive

AGAGCTCTAGTAGACACTCGGCAA	0	20	RDR2-sensitive
AGAGCTCTAGTAGACACTCGGCAC	0	11	RDR2-sensitive
AGAGCTCTAGTAGGCACTCGGCAA	1	44	RDR2-sensitive
AGAGCTCTCGGCACAGGGACTGGC	5	75	RDR2-sensitive
AGAGCTCTCGTAGACACTCGGCAA	1	24	RDR2-sensitive
AGAGCTCTCGTAGGCACTCGGCAA	4	56	RDR2-sensitive
AGAGCTCTTCAAGTAGATTATTTG	16	0	RDR2-resistant
AGAGCTCTTTCTTGATTCTATGGT	18	0	RDR2-resistant
AGAGCTGACGGTGGAGGAACGAGA	0	9	RDR2-sensitive
AGAGCTGAGAGCCGGTGACGTGAA	19	0	RDR2-resistant
AGAGCTGAGGACAGTCGACGTCAT	2	18	RDR2-sensitive
AGAGCTGAGGATGTGAGGACGGAA	0	10	RDR2-sensitive
AGAGCTGCAGGTCTGGCGAGCTGA	0	13	RDR2-sensitive
AGAGCTGCGGACTGTCCGGCGTGA	0	10	RDR2-sensitive
AGAGCTGGACCTTTGAAAATGGCT	0	14	RDR2-sensitive
AGAGCTGGTAGATTACGACGGTGC	0	16	RDR2-sensitive
AGAGCTGGTCTCGGGCGAGTCGGA	0	10	RDR2-sensitive
AGAGCTGTACGGGCATCAACGCTA	1	27	RDR2-sensitive
AGAGCTTTCGGCAAAGGGACTGGC	0	9	RDR2-sensitive
AGAGGAAACACACGAGACACTGGA	0	10	RDR2-sensitive
AGAGGAAATCTGAAATTTAGCATA	44	8	RDR2-resistant
AGAGGAACCGGCTCCGATCAAGAC	0	26	RDR2-sensitive
AGAGGAACCTCCTCCGTTGGATTG	0	10	RDR2-sensitive
AGAGGAACTCTATGGTCGCAGCGG	0	27	RDR2-sensitive
AGAGGAAGCAGAATGGATGAGGAA	0	20	RDR2-sensitive
AGAGGAAGGAGAAGTCGTAACAAG	28	2	RDR2-resistant
AGAGGAATTCATGCTATTATTATA	23	0	RDR2-resistant
AGAGGACACCAGAAGACGTCTCTG	0	31	RDR2-sensitive
AGAGGACACCGAAGGGCACTCCAT	0	10	RDR2-sensitive
AGAGGACACCGCTAGACGGTACAC	0	9	RDR2-sensitive
AGAGGACACTGTCAGGCGTTACAC	0	16	RDR2-sensitive
AGAGGACAGAATCTTTTAGAGTGT	17	38	RDR2-sensitive
AGAGGACAGACCGGCAAAGACTAG	0	21	RDR2-sensitive
AGAGGACAGAGCCATATAGCACTC	0	9	RDR2-sensitive
AGAGGACAGCAGATCACCGTTGGT	0	12	RDR2-sensitive
AGAGGACAGCAGGGACAACAGCAT	0	16	RDR2-sensitive
AGAGGACAGGACCATATACCGCTC	1	102	RDR2-sensitive
AGAGGACAGGAGCATTCAATGCGC	0	9	RDR2-sensitive
AGAGGACCATTTAGAGGACGCTGC	5	57	RDR2-sensitive
AGAGGACCATTTAGAGGACGTTGC	0	10	RDR2-sensitive
AGAGGACCATTTAGAGGACGTTGT	2	18	RDR2-sensitive
AGAGGACCGAATTTAGGGGACGTT	1	18	RDR2-sensitive
AGAGGACCGACACGTAGAGGGCGC	0	139	RDR2-sensitive
AGAGGACCGACACGTAGGAGGCGC	3	38	RDR2-sensitive
AGAGGACCGACACGTAGGGAGCGC	1	29	RDR2-sensitive
AGAGGACCGACACGTAGGGGACGC	0	69	RDR2-sensitive
AGAGGACCGACACGTAGGGGGCGC	0	44	RDR2-sensitive
AGAGGACCGACGCGTAGAGGGCGC	0	18	RDR2-sensitive
AGAGGACCGACGCGTAGGAGGCGC	0	20	RDR2-sensitive

AGAGGACCGACGCGTAGGGGACGC	0	9	RDR2-sensitive
AGAGGACCGACGCGTAGGGGGCAC	0	28	RDR2-sensitive
AGAGGACCGAGATAGAGACGTTAC	0	15	RDR2-sensitive
AGAGGACCGTTCAGAGGACGCTGC	0	58	RDR2-sensitive
AGAGGACCGTTTAGAAGACGCTGC	0	13	RDR2-sensitive
AGAGGACCGTTTAGAGGACGACGC	0	22	RDR2-sensitive
AGAGGACCGTTTAGAGGACGCTGC	8	158	RDR2-sensitive
AGAGGACCGTTTAGAGGACGCTGT	4	21	RDR2-sensitive
AGAGGACCGTTTAGAGGACGTTGC	17	545	RDR2-sensitive
AGAGGACCGTTTAGAGGACGTTGT	4	87	RDR2-sensitive
AGAGGACCTTGCTGGGCGCCTAGA	0	51	RDR2-sensitive
AGAGGACCTTGTTGGAGACGTAGA	0	14	RDR2-sensitive
AGAGGACCTTGTTGGAGGCGTAGA	0	30	RDR2-sensitive
AGAGGACGAAGACGTACGCTACGT	0	24	RDR2-sensitive
AGAGGACGACCGCTCGCGCGCTAC	0	9	RDR2-sensitive
AGAGGACGACGAACGACGGCGAAC	0	16	RDR2-sensitive
AGAGGACGACGTCGTTATTGTCTC	0	22	RDR2-sensitive
AGAGGACGAGGACGTCATCTACTC	3	38	RDR2-sensitive
AGAGGACGAGGACGTCATTTACTC	0	10	RDR2-sensitive
AGAGGACGCACAATGAACCACCAC	0	13	RDR2-sensitive
AGAGGACGCACAGTGAACCACCAC	0	9	RDR2-sensitive
AGAGGACGCACAGTGAACCACTAC	0	21	RDR2-sensitive
AGAGGACGCATAGTAAACCACCAT	0	9	RDR2-sensitive
AGAGGACGCGCGGTAAGACTGAGC	0	39	RDR2-sensitive
AGAGGACGCGGCTCTAGTCGGATG	65	0	RDR2-resistant
AGAGGACGCTACAGTACTCCCCTT	0	60	RDR2-sensitive
AGAGGACGCTACTAGAGACGTAGA	0	10	RDR2-sensitive
AGAGGACGCTACTGAATTCTCTAT	0	28	RDR2-sensitive
AGAGGACGCTACTGGATTCTCTAT	1	30	RDR2-sensitive
AGAGGACGCTATAGAGGCCCTTGC	0	32	RDR2-sensitive
AGAGGACGCTCCTGAAGACGTAGA	0	9	RDR2-sensitive
AGAGGACGCTGAATATGGCCATGC	36	3	RDR2-resistant
AGAGGACGCTGCCTGAGACAGCCT	0	16	RDR2-sensitive
AGAGGACGCTGCTAGAGACCGTAG	0	10	RDR2-sensitive
AGAGGACGCTGCTGAAACGAGTAG	0	49	RDR2-sensitive
AGAGGACGCTGCTGAAGAGCATAG	0	11	RDR2-sensitive
AGAGGACGCTGCTGAAGATCATAG	0	16	RDR2-sensitive
AGAGGACGCTGCTGAATTCCTTAT	0	30	RDR2-sensitive
AGAGGACGCTGCTGGAGAGCATAG	0	43	RDR2-sensitive
AGAGGACGCTGCTGGAGAGCGTAG	0	29	RDR2-sensitive
AGAGGACGCTGCTGGAGAGTATAG	0	14	RDR2-sensitive
AGAGGACGCTGCTGGAGGACGTAG	0	27	RDR2-sensitive
AGAGGACGCTGCTGGAGGGAATAG	0	13	RDR2-sensitive
AGAGGACGCTGCTGGAGGGCGTAG	0	62	RDR2-sensitive
AGAGGACGCTGCTGGATACATAGA	0	11	RDR2-sensitive
AGAGGACGCTGCTGGATTCTCTAC	0	14	RDR2-sensitive
AGAGGACGCTGCTGGATTCTCTAT	164	464	RDR2-sensitive
AGAGGACGCTGCTGGATTTTCTAT	0	12	RDR2-sensitive
AGAGGACGCTGTAGGGGGCACCGC	0	29	RDR2-sensitive

AGAGGACGCTTTAGAGGACCTTGT	2	33	RDR2-sensitive
AGAGGACGCTTTAGAGTTTCTTGT	0	16	RDR2-sensitive
AGAGGACGGAATCTTTTAGAGTGT	18	50	RDR2-sensitive
AGAGGACGGAATGTACTGCTGCTG	0	9	RDR2-sensitive
AGAGGACGGTGCAGATTGGCTGAA	0	21	RDR2-sensitive
AGAGGACGGTGCTGAAGACATAAA	0	31	RDR2-sensitive
AGAGGACGGTGGCATCCCCGGCTT	0	10	RDR2-sensitive
AGAGGACGTACGGCGAACGCTAAA	0	70	RDR2-sensitive
AGAGGACGTACTTGCACACCCCAA	0	37	RDR2-sensitive
AGAGGACGTCAGCAAGGACCCAAC	3	22	RDR2-sensitive
AGAGGACGTCAGCAAGGACCCGAC	0	13	RDR2-sensitive
AGAGGACGTCAGCAAGGACTCAAC	3	73	RDR2-sensitive
AGAGGACGTCAGCAAGGACTCGAC	1	36	RDR2-sensitive
AGAGGACGTCGTAGTGTGTAGCGT	0	12	RDR2-sensitive
AGAGGACGTCGTTGCGGGTTCAAG	0	9	RDR2-sensitive
AGAGGACGTGATTTAGAGGACGTT	1	38	RDR2-sensitive
AGAGGACGTGGAACTGTAGATGAC	0	14	RDR2-sensitive
AGAGGACGTGTGGTACATTTCTAG	0	14	RDR2-sensitive
AGAGGACGTTGATGGAGACAGCCT	0	9	RDR2-sensitive
AGAGGACGTTGCTAGAGACAGCCT	0	18	RDR2-sensitive
AGAGGACGTTGCTGAAGACAGCCT	0	19	RDR2-sensitive
AGAGGACGTTGCTGAAGAGCGTAG	0	9	RDR2-sensitive
AGAGGACGTTGCTGAAGGAGGTAA	0	9	RDR2-sensitive
AGAGGACGTTGCTGAATTCTCTAT	2	19	RDR2-sensitive
AGAGGACGTTGCTGACAGTTCACC	0	10	RDR2-sensitive
AGAGGACGTTGCTGCAGGGAGTAG	0	9	RDR2-sensitive
AGAGGACGTTGCTGGAGACAACCT	0	14	RDR2-sensitive
AGAGGACGTTGCTGGAGACAGCCT	1	97	RDR2-sensitive
AGAGGACGTTGCTGGAGACAGTCT	5	55	RDR2-sensitive
AGAGGACGTTGCTGGAGAGAGTAG	1	33	RDR2-sensitive
AGAGGACGTTGCTGGAGAGCGTAG	5	93	RDR2-sensitive
AGAGGACGTTGCTGGAGAGTGTAG	0	15	RDR2-sensitive
AGAGGACGTTGCTGGAGATAGCCT	2	20	RDR2-sensitive
AGAGGACGTTGCTGGAGGGAGTAG	1	86	RDR2-sensitive
AGAGGACGTTGCTGGAGGGAGTCG	0	15	RDR2-sensitive
AGAGGACGTTGCTGGAGGGATCGT	0	41	RDR2-sensitive
AGAGGACGTTGCTGGAGGGGTATC	0	14	RDR2-sensitive
AGAGGACGTTGCTGGAGTTGGCGA	0	43	RDR2-sensitive
AGAGGACGTTGCTTTTGCTGGGAC	0	9	RDR2-sensitive
AGAGGACGTTGTAGCGGACCTTGC	0	13	RDR2-sensitive
AGAGGACGTTGTTGGAGACAGCCT	1	13	RDR2-sensitive
AGAGGACGTTTAAATGCAACTGTG	17	0	RDR2-resistant
AGAGGACTATCATATTGTGGCTGC	0	9	RDR2-sensitive
AGAGGACTCTGGCATGCAACACAC	0	9	RDR2-sensitive
AGAGGACTGAATGGAGGCGGCCGC	1	16	RDR2-sensitive
AGAGGACTGATGGGCGCAATTCAG	0	16	RDR2-sensitive
AGAGGACTGTCAGGTCCCCTACGG	0	10	RDR2-sensitive
AGAGGACTGTTTAGAGGACGCTGC	0	10	RDR2-sensitive
AGAGGACTTGATTTAGAGGACGTT	0	12	RDR2-sensitive

AGAGGACTTGTGGAGCATGATGGC	0	9	RDR2-sensitive
AGAGGAGACGCAAGAGCTGTCCGC	0	31	RDR2-sensitive
AGAGGAGACGCAAGAGCTGTTCGC	0	13	RDR2-sensitive
AGAGGAGACGGAGCTATGCCAAAC	0	11	RDR2-sensitive
AGAGGAGACTGTAAAGGACGGATA	2	625	RDR2-sensitive
AGAGGAGAGAGAATGTGTCGGCCG	0	12	RDR2-sensitive
AGAGGAGAGAGAATGTGTTGGCCG	1	20	RDR2-sensitive
AGAGGAGAGGGTACCGGTGAACAC	0	30	RDR2-sensitive
AGAGGAGAGTGTAGGCGTCCACAA	0	9	RDR2-sensitive
AGAGGAGAGTGTAGGCGTCCACAG	0	11	RDR2-sensitive
AGAGGAGGCAGTAGAGTGATGCGA	0	33	RDR2-sensitive
AGAGGAGTAGTAGAGAGGAGACAG	0	10	RDR2-sensitive
AGAGGATACCTGTAGTAGTGCGTC	0	10	RDR2-sensitive
AGAGGATACTGCTGGAGAGCATAG	0	10	RDR2-sensitive
AGAGGATAGCGGACGTTTCTTTCT	0	21	RDR2-sensitive
AGAGGATAGTGTAGGCGTCCACAC	1	13	RDR2-sensitive
AGAGGATATGAGGGCGGAAGAAAA	0	11	RDR2-sensitive
AGAGGATCAAACGGTGCTCTACGC	0	9	RDR2-sensitive
AGAGGATCAGACGGTCACAAGATG	0	12	RDR2-sensitive
AGAGGATCCCTGTAGTAGTGGCGA	0	9	RDR2-sensitive
AGAGGATCCGGCGAACAGGCTCAT	0	13	RDR2-sensitive
AGAGGATCGAATGGTATTCTACGT	0	67	RDR2-sensitive
AGAGGATCGGAACGTCGTCTACTC	0	43	RDR2-sensitive
AGAGGATCGGAGGAAGAAGGAGGA	0	15	RDR2-sensitive
AGAGGATCGGTACCTGCAGGCCAT	0	68	RDR2-sensitive
AGAGGATCGTGCAACAGGAGGCAT	41	2	RDR2-resistant
AGAGGATCTAACGGCGTCTCCTAA	0	15	RDR2-sensitive
AGAGGATCTGAAACTGGTCTCGGG	0	10	RDR2-sensitive
AGAGGATGAAGGCTATCATGACAT	176	38	RDR2-resistant
AGAGGATGACCAGTCTTGGCTGCA	20	0	RDR2-resistant
AGAGGATGACGGGTTCGACGACTC	1	39	RDR2-sensitive
AGAGGATGCTGCTAGAGGGATCGT	0	44	RDR2-sensitive
AGAGGATGCTGCTGGAGGACGTAG	0	38	RDR2-sensitive
AGAGGATGCTGTAGAGGGCACCGC	0	55	RDR2-sensitive
AGAGGATGGAATTTAGGGGACGTT	0	26	RDR2-sensitive
AGAGGATGGCAGTATGTCTCGTTC	0	11	RDR2-sensitive
AGAGGATGTACGGCGAACTCTAAA	0	15	RDR2-sensitive
AGAGGATGTGATTTAGAGGACGTT	1	15	RDR2-sensitive
AGAGGATGTGGCAGACACTCTAAA	0	19	RDR2-sensitive
AGAGGATGTTGCTGCTGGAGACAG	0	10	RDR2-sensitive
AGAGGATGTTGCTGGAGTTGACAA	0	12	RDR2-sensitive
AGAGGATTGCATGCAGACTGGGGC	0	13	RDR2-sensitive
AGAGGATTTTGACTTGTAGGGGAT	0	10	RDR2-sensitive
AGAGGATTTTGGCGATTGATAGAT	18	0	RDR2-resistant
AGAGGCACGGTGAAGTGTGGACGC	0	31	RDR2-sensitive
AGAGGCAGGTATCAGGGGAAACTA	14	0	RDR2-resistant
AGAGGCAGTGAGCCAGAGGTCGGA	1	32	RDR2-sensitive
AGAGGCATGTGGTCCATGGATGGT	34	2	RDR2-resistant
AGAGGCATTGGACGGTCCGGATCT	0	9	RDR2-sensitive

AGAGGCCAAGTAGAACGGAGTCGT	0	9	RDR2-sensitive
AGAGGCCAGTCCACGGTCGCCGGA	0	20	RDR2-sensitive
AGAGGCCGGAAGTTCTGTACCAAG	0	9	RDR2-sensitive
AGAGGCCGGAAGTTTGTGTACCAA	0	15	RDR2-sensitive
AGAGGCCGGTAGATTAACAGTCCC	1	21	RDR2-sensitive
AGAGGCGAGAGTGGAGGCGAGAGC	2	48	RDR2-sensitive
AGAGGCGCGGATATGTTGGTGGGC	0	13	RDR2-sensitive
AGAGGCGGCATGCAGGACAGACGG	1	22	RDR2-sensitive
AGAGGCGGCGCTGGCCAAGAGCTT	0	9	RDR2-sensitive
AGAGGCGTACTGTACGTTCGGTAA	1	15	RDR2-sensitive
AGAGGCTAAACTGTATGCGCGCAT	0	13	RDR2-sensitive
AGAGGCTACAGTTACAACCGTCGG	0	14	RDR2-sensitive
AGAGGCTACGGAACGCGCAGAAAA	0	27	RDR2-sensitive
AGAGGCTAGGGTTTCTGTCGACGA	0	12	RDR2-sensitive
AGAGGCTCGACACAAGAACGGCGT	0	12	RDR2-sensitive
AGAGGCTCGGCTCGGCTCGTTCGA	1	93	RDR2-sensitive
AGAGGCTCGGCTCGGTAACGGCTC	1	23	RDR2-sensitive
AGAGGCTCTGCACGGACGGGAAAA	0	9	RDR2-sensitive
AGAGGCTGCTATTGAGGATCTCCC	3	22	RDR2-sensitive
AGAGGCTGGAGCTCGAACCCGGTA	0	21	RDR2-sensitive
AGAGGCTGTAATTGAGGAGGGGCT	0	32	RDR2-sensitive
AGAGGGAAGCTGGATGCATGGATG	38	1	RDR2-resistant
AGAGGGACGGAACGAAGCGACCAC	0	13	RDR2-sensitive
AGAGGGACGGAACGAGGCGGCCAC	0	16	RDR2-sensitive
AGAGGGACGGAAGAGGACAACCAT	0	15	RDR2-sensitive
AGAGGGATCGACCAGGACAGACGG	0	9	RDR2-sensitive
AGAGGGATGGAACGAGACGGTCAC	2	24	RDR2-sensitive
AGAGGGATGGAATGAGGCGGCCAC	0	14	RDR2-sensitive
AGAGGGCAAATCGAAGACTCGCGT	0	14	RDR2-sensitive
AGAGGGCAACGTAGGGGAATTAGC	49	0	RDR2-resistant
AGAGGGCAATTACACTATGTTCAG	0	10	RDR2-sensitive
AGAGGGCACCGCCACGGTTCTTGT	0	30	RDR2-sensitive
AGAGGGCAGATATGTAGGCGTGGT	54	10	RDR2-resistant
AGAGGGCAGGGACTTTTCATTCGT	15	0	RDR2-resistant
AGAGGGCCTGGCAGGGAACGTCGC	0	10	RDR2-sensitive
AGAGGGCGCTGCTGAAGAACGTAA	0	10	RDR2-sensitive
AGAGGGCGCTGTAGAGGCACCACT	1	23	RDR2-sensitive
AGAGGGCGCTGTAGAGGGCACCGC	0	58	RDR2-sensitive
AGAGGGCGCTGTAGGAGGCACCGT	0	12	RDR2-sensitive
AGAGGGCGCTGTAGGGGACACCGC	0	10	RDR2-sensitive
AGAGGGCGCTGTAGGGGCACCGCT	0	11	RDR2-sensitive
AGAGGGCGCTGTAGGGGGCACCGC	7	156	RDR2-sensitive
AGAGGGCTAGAGCTAGTTTAGGCT	0	9	RDR2-sensitive
AGAGGGCTCGGCAACAGTACATCG	0	21	RDR2-sensitive
AGAGGGCTGCGCTGCGGGACGTAG	0	22	RDR2-sensitive
AGAGGGCTGCGGTGAAGGGTTTAG	0	11	RDR2-sensitive
AGAGGGCTGTGCGTATGGGAGCGG	0	10	RDR2-sensitive
AGAGGGGCATCCAGTGATCTGAAT	0	24	RDR2-sensitive
AGAGGGGTGGAATGAAGCGGTCAC	0	10	RDR2-sensitive

AGAGGGTCATGCGACAGTAGATGT	0	9	RDR2-sensitive
AGAGGGTCGATGCAACTTGACGGA	0	23	RDR2-sensitive
AGAGGGTCTTGTAGAGGACGTCGT	0	54	RDR2-sensitive
AGAGGGTGACGAGGATGATGCGGA	27	0	RDR2-resistant
AGAGGGTGGCATAGAACTGAGCTA	49	113	RDR2-sensitive
AGAGGGTTGCTGACGCTGACGGGA	0	9	RDR2-sensitive
AGAGGGTTGGCTATGCCCAGCTGT	0	17	RDR2-sensitive
AGAGGTAAATAGGTCGTGCCGGAC	0	11	RDR2-sensitive
AGAGGTAAGTGTGTGACTGATGGT	0	22	RDR2-sensitive
AGAGGTACGTGTGTGACTGATGGT	0	20	RDR2-sensitive
AGAGGTAGACCGCGCTGACGTTGT	1	25	RDR2-sensitive
AGAGGTAGCTGCAGACGGATGACC	0	9	RDR2-sensitive
AGAGGTATGGCCTGCTAGTAGTGT	0	16	RDR2-sensitive
AGAGGTCAGCAACATTGAACGTGT	8	102	RDR2-sensitive
AGAGGTCAGGACCCAATGATGCAT	0	16	RDR2-sensitive
AGAGGTCTAGGTCGTCGTCTCCCA	0	9	RDR2-sensitive
AGAGGTCTGGCCTGCTAGTAGTGT	0	9	RDR2-sensitive
AGAGGTGACGCAGAGGAGGACCGA	1	12	RDR2-sensitive
AGAGGTGACGTAGAGGAGGACCGA	0	11	RDR2-sensitive
AGAGGTGTCCGCTGTAGATGGCTG	0	13	RDR2-sensitive
AGAGGTGTGCTGTAGTAGTGGGCA	0	13	RDR2-sensitive
AGAGGTTCCCTGTAGTAGTGACGG	0	20	RDR2-sensitive
AGAGGTTGAAGACGCAGGAACCAA	0	15	RDR2-sensitive
AGAGGTTGCTGGAGACTGCCCATC	24	1	RDR2-resistant
AGAGTACGTCTGGTCTATGATGAT	0	9	RDR2-sensitive
AGAGTACTGCGACGAACACGGCAT	0	15	RDR2-sensitive
AGAGTAGAATGAGTGCGGATAGCC	0	10	RDR2-sensitive
AGAGTAGACAGTTGATCGGCCCGT	0	36	RDR2-sensitive
AGAGTAGACCGGAACCACGACAGC	0	18	RDR2-sensitive
AGAGTAGACGATAGAGCGGCTCGC	2	48	RDR2-sensitive
AGAGTAGACTGTAAAGGACGGATA	0	14	RDR2-sensitive
AGAGTAGAGACGAGGACGTAACAG	0	17	RDR2-sensitive
AGAGTAGAGCTGCTCTGGGGTGGA	0	16	RDR2-sensitive
AGAGTAGATCTCCGGCCGGGTGGC	0	10	RDR2-sensitive
AGAGTAGCAGGCTGGACGATGCAT	0	50	RDR2-sensitive
AGAGTAGGATACAGGATGAGCTAC	0	13	RDR2-sensitive
AGAGTAGTGGAAGGCGGGCTGGGC	162	22	RDR2-resistant
AGAGTATACGGCTGCAGCAGTTCA	0	13	RDR2-sensitive
AGAGTATATATCTTTTTGAGTCGG	0	9	RDR2-sensitive
AGAGTATGCTGTAGAGGATAGGAG	0	11	RDR2-sensitive
AGAGTCACGACTGTGGAGGATGGT	0	12	RDR2-sensitive
AGAGTCACGGGCGAGAAGAGGACG	0	13	RDR2-sensitive
AGAGTCAGTTATCGTCGTTTTGCC	19	0	RDR2-resistant
AGAGTCCTTGGTGGCCGTCGGACA	2	38	RDR2-sensitive
AGAGTCTGTTGGAACGCACGGTGC	0	16	RDR2-sensitive
AGAGTCTTAGTTCGTCGAGACTGG	0	10	RDR2-sensitive
AGAGTGAACGGAACAGAGCGTGAC	0	19	RDR2-sensitive
AGAGTGAACGGAACGGAGCGTGAC	12	108	RDR2-sensitive
AGAGTGAACTGGAACAGAGCGGCT	0	34	RDR2-sensitive

AGAGTGACTGTTACAGTTCGGTTA	0	23	RDR2-sensitive
AGAGTGCGCGCATCGGACGGCTGA	1	23	RDR2-sensitive
AGAGTGCTGTGTGAACGAGCCGGT	0	63	RDR2-sensitive
AGAGTGGACACCAGGGGCGAGTTC	1	14	RDR2-sensitive
AGAGTGGACAGTTGACATCGGCAT	0	11	RDR2-sensitive
AGAGTGGACAGTTGACGCCGGCCC	1	22	RDR2-sensitive
AGAGTGGACAGTTGACGCCGGCTC	2	15	RDR2-sensitive
AGAGTGGAGGCGAGAGCTGACGTG	0	26	RDR2-sensitive
AGAGTGGAGGGTAGACACATAGGT	0	9	RDR2-sensitive
AGAGTGGAGTTAGAACGGCCTTGG	0	10	RDR2-sensitive
AGAGTGGGAGGCAGAACCGTGCAC	0	12	RDR2-sensitive
AGAGTGTGCTGTAAAGGACGGAGA	0	12	RDR2-sensitive
AGAGTTAAGGCCACGTCGGTATCA	0	11	RDR2-sensitive
AGAGTTACTGTTTGATCCTTGCGG	1	15	RDR2-sensitive
AGAGTTCAAAGGCCTGATGTCTGA	40	3	RDR2-resistant
AGAGTTCCTTGTTGGACGGCGTAG	0	14	RDR2-sensitive
AGAGTTCTGGATGTGTGACTGCAA	0	22	RDR2-sensitive
AGAGTTGATCACCTCATCGTCCGG	0	10	RDR2-sensitive
AGAGTTGCTGCGTTGACCGTCGGG	21	0	RDR2-resistant
AGAGTTGGAGTCAGGAGATGTCGC	0	17	RDR2-sensitive
AGAGTTGGGAGCTGTAGACGGTGC	0	10	RDR2-sensitive
AGAGTTGGGGCGAGGAAGACGACG	1	15	RDR2-sensitive
AGAGTTGTCTAAGGTCCGTTGGAT	0	24	RDR2-sensitive
AGAGTTGTGGGCCGTTGGATGCAA	0	18	RDR2-sensitive
AGAGTTGTGGGCGGAGATGGGAAT	0	10	RDR2-sensitive
AGAGTTTATCTGTCGTCTGCGTCT	1	13	RDR2-sensitive
AGAGTTTCGTTGATTGTTGTCGAC	137	2	RDR2-resistant
AGATAAAGGAGGACGAGCGACCGA	0	9	RDR2-sensitive
AGATAACTCCACTCTGGGCGGCTT	33	1	RDR2-resistant
AGATAACTGTAATATGGACGGTTG	0	10	RDR2-sensitive
AGATAAGGCACTGACATTTGAAGT	20	0	RDR2-resistant
AGATAATTCGGGTTATACTTAGCT	52	3	RDR2-resistant
AGATACAGCTGCACGGTACTCCAA	0	42	RDR2-sensitive
AGATACAGGGCATATTCTCTCGCA	0	12	RDR2-sensitive
AGATACGAACGTGGACGAGCGGCT	2	14	RDR2-sensitive
AGATACGGCTGCACGGTACTCCAA	1	254	RDR2-sensitive
AGATACGGCTGCACGGTACTCCAT	0	10	RDR2-sensitive
AGATACGGCTGCACGGTACTCCCA	0	10	RDR2-sensitive
AGATACGTATGGTCTGTTGGTGGC	0	9	RDR2-sensitive
AGATACTCGGCAGAACCGTGCACC	0	17	RDR2-sensitive
AGATACTGACATCGAGGACGCCAT	1	15	RDR2-sensitive
AGATACTGCTGCACGGTACTCCAA	0	9	RDR2-sensitive
AGATACTGTATCTCAGCTGGAGGT	34	1	RDR2-resistant
AGATACTGTGGCGTAGACTTCGGC	1	25	RDR2-sensitive
AGATAGAACGGTTTACAGACTCTT	0	23	RDR2-sensitive
AGATAGAACTTCAGCGATCTGGTT	36	3	RDR2-resistant
AGATAGAAGTCATCTGAGGCGTCG	2	18	RDR2-sensitive
AGATAGAGACTGTCTGCTCGTCAG	0	12	RDR2-sensitive
AGATAGATGGGAGGCAGAACGGCA	0	9	RDR2-sensitive

AGATAGCATCGCCAGACGGTTCAT	0	12	RDR2-sensitive
AGATAGGACTGTCACGTTAGTCGA	0	10	RDR2-sensitive
AGATAGGTATCGTCATCGTCGACC	17	1	RDR2-resistant
AGATAGTCTGTTGTAGTGAGATGC	0	32	RDR2-sensitive
AGATAGTTTGAAAATGGTCGCTGT	45	10	RDR2-resistant
AGATAGTTTTCTGATTGCCAAGGA	16	0	RDR2-resistant
AGATATACATCAGGATTTCGTCGT	38	1	RDR2-resistant
AGATATACCTGACTGGGGCTTCCC	0	16	RDR2-sensitive
AGATATAGAGGACGTTGCTGGAGA	0	14	RDR2-sensitive
AGATATCATCATGTCGTCGAGGCG	0	22	RDR2-sensitive
AGATATCGGCTAGCTGGTTCTCGG	13	32	RDR2-sensitive
AGATATCGGCTATGTGATTTTCGG	32	3	RDR2-resistant
AGATATCGGGCAGGAGTAGTGGGC	2	62	RDR2-sensitive
AGATATCGTAGCAACGCACGGCAC	0	30	RDR2-sensitive
AGATATCGTCGGGGACTAGCGGTG	0	9	RDR2-sensitive
AGATATCTTTTCTGTGCGGAATAC	20	0	RDR2-resistant
AGATATGGACCCGGCTGAAAGACG	0	23	RDR2-sensitive
AGATATGTGCGGCAGTTTCTGTTT	0	13	RDR2-sensitive
AGATATGTTGGCTATTGCAAAGGT	21	0	RDR2-resistant
AGATATTGTTGGTCACTTCGTCAT	132	2	RDR2-resistant
AGATATTTTCGTCGGCCGGCCCAC	3	21	RDR2-sensitive
AGATATTTTCGTCGGTCGGCCCAC	0	10	RDR2-sensitive
AGATCACCGTTGGTTTGAAGATCG	0	34	RDR2-sensitive
AGATCACGGCGAGCTGGAGACTGC	0	18	RDR2-sensitive
AGATCACTCCTACGACCGGCGGCT	0	12	RDR2-sensitive
AGATCAGGTCGTAGGGATGGTCAG	0	18	RDR2-sensitive
AGATCATTCTATCTTTAAGTCGGT	0	16	RDR2-sensitive
AGATCATTTCATCTTTAAGTCGGT	0	15	RDR2-sensitive
AGATCCAGCACGGATTAGGATCGT	1	50	RDR2-sensitive
AGATCCGGACTGGGTGTTGGCCAT	13	31	RDR2-sensitive
AGATCGATATCAGGACTCGACGGC	0	17	RDR2-sensitive
AGATCGCTGAACGCTGACGGTGAC	5	30	RDR2-sensitive
AGATCGGAATGACCAATAGAGGCT	0	12	RDR2-sensitive
AGATCGGCCTGATATGAACCGGTA	0	9	RDR2-sensitive
AGATCGGGCATCGTGACGCATCAC	0	10	RDR2-sensitive
AGATCGGGCATCGTGACGCATCAG	0	18	RDR2-sensitive
AGATCGGGCATCGTGACGCATCAT	8	72	RDR2-sensitive
AGATCGGTGTTAGGATATGTCGGT	2	20	RDR2-sensitive
AGATCTACTGGACGACGAGCGCAC	0	51	RDR2-sensitive
AGATCTAGATCGGATGCACCAGAT	0	13	RDR2-sensitive
AGATCTAGATCGGATGCACCGGAT	0	11	RDR2-sensitive
AGATCTATGTGTCAGTCGCGGACC	0	10	RDR2-sensitive
AGATCTCAAAGCGTCGAATGTCCC	17	0	RDR2-resistant
AGATCTCGGGAACCATGAAGACGT	3	44	RDR2-sensitive
AGATCTGCAAGTCGTCGGCGTCAC	1	15	RDR2-sensitive
AGATCTGTAGATGCAGGCTTGCAG	0	25	RDR2-sensitive
AGATCTGTAGTGCCGAGCTAGGCG	0	10	RDR2-sensitive
AGATCTGTATGTACTGTTCGTCGA	4	24	RDR2-sensitive
AGATCTGTCGCATGCGTTGACTGC	0	9	RDR2-sensitive

AGATCTGTGGCGCCGAGCTAGGTG	0	10	RDR2-sensitive
AGATCTGTGTCTGAATAGGTGTGC	34	1	RDR2-resistant
AGATCTTGTATTTCTGTCTCGCCT	16	0	RDR2-resistant
AGATGAACGGACCTGGATGTGCAG	0	32	RDR2-sensitive
AGATGAACTCGCGAATGACGGCGC	0	10	RDR2-sensitive
AGATGAAGGACAAGCAGACTGCAA	0	24	RDR2-sensitive
AGATGAAGTCGGTATCAGATGCTT	28	3	RDR2-resistant
AGATGAATCCGTGACGGTAACCAA	0	15	RDR2-sensitive
AGATGACAGAGCAGCGGACAAGAT	0	9	RDR2-sensitive
AGATGACAGCAGCCTGTAGTGTAG	0	14	RDR2-sensitive
AGATGACAGTAGCCTGTAGTGTAG	0	10	RDR2-sensitive
AGATGACCCGGCGACCTTGAGACA	0	18	RDR2-sensitive
AGATGACCCTGGCTCACTTTGTCC	17	0	RDR2-resistant
AGATGACCGACATGTAGGGGGCGC	0	11	RDR2-sensitive
AGATGACCGTTGCTCCGCTGGCAC	0	10	RDR2-sensitive
AGATGACGGGTTAGACTTTATCGA	0	9	RDR2-sensitive
AGATGACGGGTTAGACTTTGTCGA	0	12	RDR2-sensitive
AGATGACGGGTTTGACGACTGGAC	4	17	RDR2-sensitive
AGATGACGTCAGCACTGGAGTCAC	0	9	RDR2-sensitive
AGATGACGTTGCTGGAGGGAGTAG	0	13	RDR2-sensitive
AGATGACTGGACACTAATAAACAA	0	15	RDR2-sensitive
AGATGACTTGATGAAATTAGGCTT	27	0	RDR2-resistant
AGATGAGGATGTGCCGGATTCGGC	15	0	RDR2-resistant
AGATGAGGATGTGCTAGATTCGGC	125	14	RDR2-resistant
AGATGAGTATGTGGGCTGACGGTC	0	12	RDR2-sensitive
AGATGATCCGCGACCTGACGGCAA	0	12	RDR2-sensitive
AGATGATGCGTACTGATCAGACAT	1	34	RDR2-sensitive
AGATGATGCGTACTGATTAGACAT	2	37	RDR2-sensitive
AGATGATGCGTACTGATTAGACTT	2	38	RDR2-sensitive
AGATGATGGACGTAGTGACCGCAT	0	27	RDR2-sensitive
AGATGATGGACTACCTCGTTGCTC	0	10	RDR2-sensitive
AGATGATGGACTAGCTCATTCCTC	0	30	RDR2-sensitive
AGATGCAACGGCGATCTGATAACT	21	0	RDR2-resistant
AGATGCACACGGACGGTCTAGATC	0	10	RDR2-sensitive
AGATGCACCTCTGCGGTCGGGACG	3	24	RDR2-sensitive
AGATGCAGAGCTGCTTGTCGGAAC	0	12	RDR2-sensitive
AGATGCAGGCTTGCAGCTCTTCAG	1	12	RDR2-sensitive
AGATGCGACAGGGTAGAAGGGGTA	0	12	RDR2-sensitive
AGATGCGACTGAACCAGCACAGAC	0	15	RDR2-sensitive
AGATGCGGAGAACATGGCAGATAG	0	12	RDR2-sensitive
AGATGCGGCGAGGCCTGAGGTTGG	74	3	RDR2-resistant
AGATGCGGGGAAGCACGACGACGT	0	10	RDR2-sensitive
AGATGCTGTAGGGAGGTGGGCCGG	0	10	RDR2-sensitive
AGATGGAACTGGAGGAGACGACGT	1	13	RDR2-sensitive
AGATGGAAGCGTACAGCACTCTAC	0	26	RDR2-sensitive
AGATGGAAGGCAGAACGGCAGAAC	0	13	RDR2-sensitive
AGATGGAATGTTACAGTCGTCGAG	0	13	RDR2-sensitive
AGATGGACGAACCTGGATGCACGG	0	16	RDR2-sensitive
AGATGGACGGCCAAGTCACTCCAG	0	10	RDR2-sensitive

AGATGGACGGTACAGATTGGCTAA	0	17	RDR2-sensitive
AGATGGACGGTGCAGATTGGCTGA	2	65	RDR2-sensitive
AGATGGACGGTGTAGATTCATGGA	0	9	RDR2-sensitive
AGATGGACGGTGTAGATTGGCTGA	1	56	RDR2-sensitive
AGATGGACGGTGTAGATTTGTGGA	0	19	RDR2-sensitive
AGATGGACTTTGTGTGGGGGCTAC	0	10	RDR2-sensitive
AGATGGAGGACAGTAGCGCGGTGG	68	14	RDR2-resistant
AGATGGAGGACTAAGGGGATGCGG	0	10	RDR2-sensitive
AGATGGATAGTTATCATACCTGCT	38	7	RDR2-resistant
AGATGGATCAGTTGTTGAGGACAT	24	3	RDR2-resistant
AGATGGATCATGGCACATGTACTT	48	2	RDR2-resistant
AGATGGATCGGCGCGAGACGGAGC	0	9	RDR2-sensitive
AGATGGATCTGTGACGGTAACTAA	0	23	RDR2-sensitive
AGATGGATGGACCTGGATGTGCGG	0	16	RDR2-sensitive
AGATGGCACAGTAGACGTCTCCAC	0	20	RDR2-sensitive
AGATGGCAGAACGGCAGAACACAC	0	51	RDR2-sensitive
AGATGGCAGAACGGCAGAACAGAG	0	22	RDR2-sensitive
AGATGGCAGAACGGCAGAACAGAT	0	62	RDR2-sensitive
AGATGGCAGTGTAGGCGTCCACAT	0	10	RDR2-sensitive
AGATGGCATAGTAGACGTCTCCAC	1	18	RDR2-sensitive
AGATGGCCTGGAGAGAGACAGTTG	0	9	RDR2-sensitive
AGATGGCGAGAATGACAGATGACA	63	6	RDR2-resistant
AGATGGGAGGCAGAACGGCAGAAC	0	23	RDR2-sensitive
AGATGGGCAGTCTCCGGCAACCTC	0	10	RDR2-sensitive
AGATGGGCGTGAGAGAGGTGGAAA	0	13	RDR2-sensitive
AGATGGGTCGGCACGAGACGGAGC	0	11	RDR2-sensitive
AGATGGGTCGGCGCGAGACGGAAC	11	90	RDR2-sensitive
AGATGGGTCGGCGCGAGACGGAGC	13	131	RDR2-sensitive
AGATGGGTTGGCGCAAGATGGAAC	0	18	RDR2-sensitive
AGATGGGTTGGCGCGAGACGGAAC	0	10	RDR2-sensitive
AGATGGGTTGGCGCGAGATGGAAC	5	40	RDR2-sensitive
AGATGGTAGTGTAGGCGTCCACAT	0	14	RDR2-sensitive
AGATGGTGAGTCGGCATATGTCTA	0	9	RDR2-sensitive
AGATGGTTTTGTTGACGTGTCATT	22	0	RDR2-resistant
AGATGTAGAGCACAAGGACGGTGA	0	18	RDR2-sensitive
AGATGTAGATGGTTTGCAAGAATT	26	2	RDR2-resistant
AGATGTCATCTCTCTGATGTTTGG	0	13	RDR2-sensitive
AGATGTCGGAGATATTTATGTGCT	2	29	RDR2-sensitive
AGATGTCGTTGGGGACTAGCGGTG	1	12	RDR2-sensitive
AGATGTCTGGGACCTAGTGTGGCT	0	10	RDR2-sensitive
AGATGTGAGACCTCTCAGGGGCAA	0	12	RDR2-sensitive
AGATGTGGAACAGACGACGCACAT	0	40	RDR2-sensitive
AGATGTGGAGACTTGGAGACGTGC	0	29	RDR2-sensitive
AGATGTGGCCTGACGACGGAGGTT	21	0	RDR2-resistant
AGATGTTAGAAGATGTTGTGCTGC	28	0	RDR2-resistant
AGATGTTAGGTTGGACGAGTCGGT	0	14	RDR2-sensitive
AGATGTTATCGCTCGATCATTATC	24	0	RDR2-resistant
AGATGTTCCGACGGCTGCTATACG	30	5	RDR2-resistant
AGATGTTTGGACCCGTCGGGCGGC	0	9	RDR2-sensitive

AGATGTTTGGTTTCTGGAACGGAT	0	12	RDR2-sensitive
AGATTAACAACGGTAAACTTTACT	62	1	RDR2-resistant
AGATTACAGGACGGCAGAACACAA	1	22	RDR2-sensitive
AGATTACCTCACTCGGATCGTCAT	0	24	RDR2-sensitive
AGATTAGGGGGACTGTAGTGAGGC	0	93	RDR2-sensitive
AGATTAGTAGGAGCCGCGTTCCCT	0	9	RDR2-sensitive
AGATTATAGGGATGTTTGGACTGA	0	11	RDR2-sensitive
AGATTATATCTTTGGTCTGGTTGA	17	1	RDR2-resistant
AGATTATTTCGTGTCGTGCTGGCC	0	9	RDR2-sensitive
AGATTCACCGAAGCGGACTGACGG	0	16	RDR2-sensitive
AGATTCACCGAAGGCAGAACACAA	0	11	RDR2-sensitive
AGATTCACCGACGGCAGAACCAAA	0	9	RDR2-sensitive
AGATTCAGACTGTCCGGTATGACA	0	11	RDR2-sensitive
AGATTCATCGACGGCAGAACCCGG	0	10	RDR2-sensitive
AGATTCATCGGTTGCGACGACGGT	0	13	RDR2-sensitive
AGATTCGAGGGACTGACGGGTTAT	0	9	RDR2-sensitive
AGATTCGCCATAGCAGCTCGTCGC	0	11	RDR2-sensitive
AGATTCGGGACGGATACAGGACGA	0	9	RDR2-sensitive
AGATTCGGGACGGATACAGGACGG	0	29	RDR2-sensitive
AGATTCTATTGTTTGGTCGTCTGT	0	15	RDR2-sensitive
AGATTCTCCGAAGCGGACTGACGG	0	15	RDR2-sensitive
AGATTCTCTGTCTGATATGTTCTC	148	6	RDR2-resistant
AGATTCTGACCTCTTGGCGCGCTT	42	1	RDR2-resistant
AGATTCTGATTGGTTGCTTGGATA	0	13	RDR2-sensitive
AGATTGAAGTGGATTGAGTGGTAT	0	11	RDR2-sensitive
AGATTGACCTAGTGCGAGCTGGAC	0	9	RDR2-sensitive
AGATTGAGTAGGATTGAATCGGTT	0	19	RDR2-sensitive
AGATTGCAGAACGGTAGGCTACCT	0	17	RDR2-sensitive
AGATTGCGGGCGAGGGACGCGGCG	23	3	RDR2-resistant
AGATTGCTGCTGCATCTCGGACAA	0	14	RDR2-sensitive
AGATTGGAGGACCAGGCACATCAC	0	11	RDR2-sensitive
AGATTGGATTGAGCAGGAGACTTT	0	10	RDR2-sensitive
AGATTGGCTGAAGGCAGAACCAGG	0	32	RDR2-sensitive
AGATTGGCTGAAGGCAGAACGCAA	0	53	RDR2-sensitive
AGATTGGTAGGAGCTACGTTCCCT	0	14	RDR2-sensitive
AGATTGTAGAGCTGTGCAGAGCGG	0	10	RDR2-sensitive
AGATTGTAGATGCATGTCGACGCT	0	13	RDR2-sensitive
AGATTGTAGATGGCAGCTCCGCTC	0	21	RDR2-sensitive
AGATTGTAGCGTTAGTACGGACTA	0	20	RDR2-sensitive
AGATTGTAGGGAGGGGATTTGGTG	0	19	RDR2-sensitive
AGATTGTGTGTCTCGATCGGTCGT	0	52	RDR2-sensitive
AGATTGTTAATGTAGGTATGTATA	15	0	RDR2-resistant
AGATTGTTCCAGGAGATGAGATGA	20	1	RDR2-resistant
AGATTGTTGAACAGATCGACGTCA	0	18	RDR2-sensitive
AGATTTACATGTAGTCTGACGTGA	0	13	RDR2-sensitive
AGATTTACTGGAGGCAGAACGCAG	0	21	RDR2-sensitive
AGATTTAGATATTGTTGGTCACTT	43	0	RDR2-resistant
AGATTTAGGGCTTTACTGTTGGAG	0	17	RDR2-sensitive
AGATTTAGGGTCTGTTTGGACAGA	0	10	RDR2-sensitive

AGATTTAGTGGAACTGGACGGTTG	0	11	RDR2-sensitive
AGATTTCTTGCTGACACGTGGAAC	0	10	RDR2-sensitive
AGATTTGATGTAGAGGGCAGATAT	28	1	RDR2-resistant
AGATTTGCACTGTGAGAAAGTCGG	0	9	RDR2-sensitive
AGATTTGGCATACTCTTTAAGCTC	17	0	RDR2-resistant
AGATTTGGGGCTTTACTGTTGGAG	2	17	RDR2-sensitive
AGATTTGTAGAGTGTACATCAAGG	0	9	RDR2-sensitive
AGATTTGTATCGTTTGACTCGGTA	0	10	RDR2-sensitive
AGATTTTATAGCGATCGGTTAGGA	3	22	RDR2-sensitive
AGATTTTATATTTTTTCGTCGGGT	149	9	RDR2-resistant
AGATTTTGACTTGTTTGGGATTGA	2	20	RDR2-sensitive
AGATTTTGAGTCTATTTCGTCGGC	0	20	RDR2-sensitive
AGATTTTGGCTGTGATTTTAAAAG	25	0	RDR2-resistant
AGCAAAACGGCACTGCATCTGCAT	0	10	RDR2-sensitive
AGCAAAACGGCTCTGGCTCCTTGA	0	16	RDR2-sensitive
AGCAAAACGGCTCTGGCTCTTTGA	0	16	RDR2-sensitive
AGCAAACGACGCGACGACGAGCTG	4	24	RDR2-sensitive
AGCAAACGGGCCGGAACGGTAAGC	0	9	RDR2-sensitive
AGCAAACGTGTGAGCACTCGGCAA	0	9	RDR2-sensitive
AGCAAACTAGAACGAGATTGTGTA	29	0	RDR2-resistant
AGCAAAGCGTCGAGCACTCGGCAA	0	12	RDR2-sensitive
AGCAACAAACAGGACGTAGGGTAT	0	17	RDR2-sensitive
AGCAACAGAAAGACGGATGGACTC	1	18	RDR2-sensitive
AGCAACAGAGCTCATGATCGTCGA	0	21	RDR2-sensitive
AGCAACAGTTTCAACGGTCGTCTC	0	10	RDR2-sensitive
AGCAACCGAGGACGTAGACGCGGC	2	36	RDR2-sensitive
AGCAACCGCTGTAGAGCGAGTCGG	0	14	RDR2-sensitive
AGCAACGACTGTAGAGCGAGTCGG	0	61	RDR2-sensitive
AGCAACGCACGGACATACACCTAG	0	27	RDR2-sensitive
AGCAACGCACGGACATATACCTAG	0	10	RDR2-sensitive
AGCAACGCAGGAGAGGCTGAACAA	0	11	RDR2-sensitive
AGCAACGGACGGTCGGTCGCTAAA	5	42	RDR2-sensitive
AGCAACGTCGAGGTAGAGCGTGCT	24	85	RDR2-sensitive
AGCAACGTTTGTTTGGTTGTTCGG	0	32	RDR2-sensitive
AGCAACTCCGAACCAGAGAGCTGT	0	12	RDR2-sensitive
AGCAAGACCGGCCCATTTGGACAT	0	22	RDR2-sensitive
AGCAAGACGGGACTGCCGAGCAGC	0	12	RDR2-sensitive
AGCAAGACTCTCGAGCAAGTCAGA	0	9	RDR2-sensitive
AGCAAGAGGACGTCAGCAAGGACT	2	18	RDR2-sensitive
AGCAAGGACCAGCAGACTGCAGGA	0	14	RDR2-sensitive
AGCAAGGACTGCGGCAGGCAGCAC	2	16	RDR2-sensitive
AGCAAGGCAGCGTAGACCCTCGGT	0	19	RDR2-sensitive
AGCAAGTACTTTTTGTTGGACACT	0	13	RDR2-sensitive
AGCAAGTCAGAACGCACCGGACAT	0	10	RDR2-sensitive
AGCAAGTTAAATGGCTACTCTGTT	0	14	RDR2-sensitive
AGCAAGTTGTTGATTGTGATGATG	72	7	RDR2-resistant
AGCAATCCGGCTCGGAATCGTTCC	0	10	RDR2-sensitive
AGCAATCCGGTTAGGAATCGTTCC	0	9	RDR2-sensitive
AGCAATGTAGGACATTGTTACGGT	136	19	RDR2-resistant

AGCACACAACATAAGGTCGCGGAC	0	13	RDR2-sensitive
AGCACACCACATATGATCGCCGAC	0	10	RDR2-sensitive
AGCACACGGCAAAGCGTCTGACAC	0	16	RDR2-sensitive
AGCACACGGCGCGACGAACGACAT	0	10	RDR2-sensitive
AGCACACGGTAGAGAAATCGGCAA	0	21	RDR2-sensitive
AGCACACTCACTCGTCGGCTTAGG	2	17	RDR2-sensitive
AGCACACTCGGCATAGTCAGACAC	0	16	RDR2-sensitive
AGCACAGACCTGGGCACCGGCTTA	0	10	RDR2-sensitive
AGCACAGATGTGGTATAACTTGTT	31	2	RDR2-resistant
AGCACAGTCCAGCTAGACTCGGAT	0	16	RDR2-sensitive
AGCACAGTGCTGTAGGGGGTGAAT	0	12	RDR2-sensitive
AGCACATGGACCTGATGGCTTTAA	16	0	RDR2-resistant
AGCACATGTAGAACATGTTCGGTT	0	10	RDR2-sensitive
AGCACATTTTGTTTGGTTGTTGGA	0	13	RDR2-sensitive
AGCACCAGTTTCAACGGTCGTCGT	0	133	RDR2-sensitive
AGCACCAGTTTTGATGGTCGTCGT	0	9	RDR2-sensitive
AGCACCCTGAACCCGGCTCTGCAG	0	11	RDR2-sensitive
AGCACCGCAGGGGACTTCGACAGG	53	0	RDR2-resistant
AGCACCGCGCGGACGAGGACGGCG	0	11	RDR2-sensitive
AGCACCGTTGGATATGGAGGGTGT	0	26	RDR2-sensitive
AGCACCGTTGGATGTAGAGGGTGT	0	133	RDR2-sensitive
AGCACCGTTGGATGTGAAAGGCGT	1	40	RDR2-sensitive
AGCACCGTTGGATGTGAAAGGTGT	1	15	RDR2-sensitive
AGCACCGTTGGATGTGAAGGGTGT	6	69	RDR2-sensitive
AGCACCGTTGGATGTGGAAGGTGT	4	251	RDR2-sensitive
AGCACCGTTGGATGTGGAGGATGT	0	14	RDR2-sensitive
AGCACCGTTGGATGTGGAGGGGGT	5	53	RDR2-sensitive
AGCACCGTTGGATGTGGAGGGTAT	0	72	RDR2-sensitive
AGCACCGTTGGATGTGGAGGGTGG	0	21	RDR2-sensitive
AGCACCGTTGGATGTGGAGGGTGT	172	2158	RDR2-sensitive
AGCACCGTTGGATGTGGTGGTGTG	0	19	RDR2-sensitive
AGCACCTGCAGAACATCGGCGACA	0	15	RDR2-sensitive
AGCACGAATTGCATTCGGGTCGGA	0	9	RDR2-sensitive
AGCACGACCCAGTAGAGCAGGCAG	0	41	RDR2-sensitive
AGCACGACCCTCTGTTCGACGTCA	0	13	RDR2-sensitive
AGCACGCCTTATATTGTGGGACAA	0	9	RDR2-sensitive
AGCACGCGCAGATGTAGGCAACCA	0	12	RDR2-sensitive
AGCACGCGGATGAGGGATGCGGAT	0	14	RDR2-sensitive
AGCACGGACTGTTCGGTGCGACGC	0	11	RDR2-sensitive
AGCACGGACTTATGCAGAGGAGCT	0	34	RDR2-sensitive
AGCACGGCACAGATCTGGCACGAC	0	15	RDR2-sensitive
AGCACGGCACGGAATTGAATCGGA	0	22	RDR2-sensitive
AGCACGGCCTGAAGACGGATCTAG	0	38	RDR2-sensitive
AGCACGGCGGGCTTTAACGGGCTG	0	14	RDR2-sensitive
AGCACGGCTAGGCCTGACACGGAT	0	15	RDR2-sensitive
AGCACGGGGGTCGCGGACTCGGCC	1	19	RDR2-sensitive
AGCACGGTCCAGGCACGACCCGGT	0	9	RDR2-sensitive
AGCACGGTCTGAAGGCGGGTCTGA	0	21	RDR2-sensitive
AGCACGGTTCAAAGGTAGGAGCGA	0	9	RDR2-sensitive

AGCACGTCACGGTTAGAGCACGGC	0	14	RDR2-sensitive
AGCACGTCTCGTTGGACATGCATG	0	18	RDR2-sensitive
AGCACGTGTGTCTAGTCATCGGTC	0	9	RDR2-sensitive
AGCACGTTAGACGGGCTGGGTCGT	0	34	RDR2-sensitive
AGCACTAGCGGCACTGGAAAACGC	0	12	RDR2-sensitive
AGCACTATAGAATTGACGTGGCGT	0	20	RDR2-sensitive
AGCACTCGACAAAGACTTCGGCAC	0	11	RDR2-sensitive
AGCACTCGGCAAAGACGTTGGCAC	0	42	RDR2-sensitive
AGCACTCGGCAAAGCCACCGGCAC	2	56	RDR2-sensitive
AGCACTCGGCAAAGCCGTCGGCAC	0	15	RDR2-sensitive
AGCACTCGGCAAAGCCTCCGGCAC	0	50	RDR2-sensitive
AGCACTCGGCAAAGCCTTCGGCAC	0	12	RDR2-sensitive
AGCACTCGGCAAAGCGGTCGACTC	0	11	RDR2-sensitive
AGCACTCGGCAAAGCTGTCGGCAC	0	17	RDR2-sensitive
AGCACTCGGCAAAGGAACTGGCAA	1	13	RDR2-sensitive
AGCACTCGGCAAAGTCTTCGGCAC	0	9	RDR2-sensitive
AGCACTCGGCAAATCGTCTGGCAC	0	27	RDR2-sensitive
AGCACTCGGCAAGGTAAGGAGCGA	0	9	RDR2-sensitive
AGCACTGGAAGGGCACAGCTGAGA	0	15	RDR2-sensitive
AGCACTGGAAGGGCATAGCTGAGA	0	16	RDR2-sensitive
AGCACTGGACAAGCACTGCGCCGC	0	16	RDR2-sensitive
AGCACTGGCGGCACTGAAAAACAC	1	44	RDR2-sensitive
AGCACTGGCGGCACTGAAAAACGC	4	117	RDR2-sensitive
AGCACTGGCGGCACTGAAAAACGT	0	20	RDR2-sensitive
AGCACTGGCGGCACTGAAAAGCGC	1	105	RDR2-sensitive
AGCACTGGCGGCACTGAAAAGCGT	0	23	RDR2-sensitive
AGCACTGGCGGCACTGAGAAACGC	3	17	RDR2-sensitive
AGCACTGTAGACACGGCTGCAGGT	0	14	RDR2-sensitive
AGCACTGTAGCAGATTTAGGGCGT	1	58	RDR2-sensitive
AGCACTGTAGCAGATTTAGGGGCT	0	12	RDR2-sensitive
AGCACTGTAGCAGCGCTAGGGGGC	1	14	RDR2-sensitive
AGCACTGTTAGATCCGGCACGGAT	0	13	RDR2-sensitive
AGCACTGTTAGGTACTGGACGGAT	0	30	RDR2-sensitive
AGCAGAACGCGGAATGACAGACTA	0	19	RDR2-sensitive
AGCAGAACGGTAGAACGGACGACT	1	14	RDR2-sensitive
AGCAGAACTCAAACCGGACGACAC	0	10	RDR2-sensitive
AGCAGAACTCTACGAGCCAGGCCC	0	13	RDR2-sensitive
AGCAGAAGGACTTGCACATGGTTT	25	0	RDR2-resistant
AGCAGACCACTGTCGATGCGGCGC	15	0	RDR2-resistant
AGCAGACCAGCAGAAGAACTCCAG	1	14	RDR2-sensitive
AGCAGACCCTAGCGACATGGATAT	0	9	RDR2-sensitive
AGCAGACGCGACAGTGGCACCCGG	0	11	RDR2-sensitive
AGCAGACTACAGCGCTCAAGCCGG	0	12	RDR2-sensitive
AGCAGACTAGTATATTTGTGCATA	34	1	RDR2-resistant
AGCAGACTGCTTCTCTCTCACGGC	0	16	RDR2-sensitive
AGCAGACTGTCCATATTTTGTAGC	31	0	RDR2-resistant
AGCAGACTGTGAAGTTGGACGGTA	0	24	RDR2-sensitive
AGCAGACTGTGTTGCCGGTATGGT	0	14	RDR2-sensitive
AGCAGACTGTTGCGCGACATCGAA	0	15	RDR2-sensitive

AGCAGACTGTTGTAAGGGTGGCAC	0	24	RDR2-sensitive
AGCAGACTTCGACAGAGCCTTGGT	0	9	RDR2-sensitive
AGCAGAGACTGCAGGATGGTTGCC	0	20	RDR2-sensitive
AGCAGAGAGAGAGCACGTGAACGC	0	10	RDR2-sensitive
AGCAGAGATGATTAGAGCTGATAA	0	10	RDR2-sensitive
AGCAGAGGACTAGATTTTTCTCTA	142	5	RDR2-resistant
AGCAGAGGCTACGGAACGCGCAGA	0	24	RDR2-sensitive
AGCAGAGTCGAACGCAGAGACCAC	0	36	RDR2-sensitive
AGCAGATAATGGGACAGAGACGAC	1	18	RDR2-sensitive
AGCAGATATCGATCGGGTACCCAT	0	15	RDR2-sensitive
AGCAGATCGACATGAGGCCGGATT	0	10	RDR2-sensitive
AGCAGATCGCGGTTGCAACGCCTT	1	23	RDR2-sensitive
AGCAGATCTGTTCACTGGTGGGTT	21	0	RDR2-resistant
AGCAGATGCAGGCTTGCAGCTCTT	1	55	RDR2-sensitive
AGCAGATGGATAAGCTTGTAGACA	59	3	RDR2-resistant
AGCAGATGGTAGAGTCATGTTCTC	0	12	RDR2-sensitive
AGCAGATGTAGGCCCACTCGCTCG	0	16	RDR2-sensitive
AGCAGATTAGGCGCGAGAAGTCAT	0	9	RDR2-sensitive
AGCAGATTAGGCGCGAGAAGTCGT	0	15	RDR2-sensitive
AGCAGATTGGTCGCATCTCCAGAG	0	25	RDR2-sensitive
AGCAGATTGGTCGCATCTCTAGAG	0	10	RDR2-sensitive
AGCAGCAACGTCGAGGAAGAGCAT	0	12	RDR2-sensitive
AGCAGCAATCAGCGGTGGAAACTC	0	9	RDR2-sensitive
AGCAGCAGAGCTCATGATCGTCGA	0	9	RDR2-sensitive
AGCAGCAGCTTTCTGTCGTCGGGT	9	36	RDR2-sensitive
AGCAGCAGTGCGGACAACTGACTT	0	10	RDR2-sensitive
AGCAGCGACCAAGAACGTGCCCAA	0	23	RDR2-sensitive
AGCAGCGACGTTGGTAGAGGGCGG	0	13	RDR2-sensitive
AGCAGCGGACAAGCAGCGAACAAT	0	15	RDR2-sensitive
AGCAGCGGATGCAACAAACTTGGT	0	21	RDR2-sensitive
AGCAGCGTCCTCTGAACGGTCCTC	0	10	RDR2-sensitive
AGCAGCTGACTGGTAGGCCTACAC	0	15	RDR2-sensitive
AGCAGCTTTCTGTCGTCGGGTGCA	3	18	RDR2-sensitive
AGCAGCTTTTGCAGTCGTCTGGTC	0	12	RDR2-sensitive
AGCAGGACGACGACGAAGCTCAGC	0	10	RDR2-sensitive
AGCAGGACGACGACGACCAGCAGC	0	10	RDR2-sensitive
AGCAGGACGACGACGATGCGGCGC	1	22	RDR2-sensitive
AGCAGGACGTGCAGATGATGGCGT	0	12	RDR2-sensitive
AGCAGGACTCTCGGCACAGAAATA	0	12	RDR2-sensitive
AGCAGGACTGCAGGAGGGCGGACG	0	20	RDR2-sensitive
AGCAGGACTGGATCCCACGTGCAG	0	15	RDR2-sensitive
AGCAGGAGAGTGTTGAACATCAGC	17	1	RDR2-resistant
AGCAGGAGCTGGAGCTTTGCCAAA	0	13	RDR2-sensitive
AGCAGGAGGACAACATGTACCCGC	0	12	RDR2-sensitive
AGCAGGAGGACAATATGTACCCGC	0	21	RDR2-sensitive
AGCAGGAGGCGAGAACGGGGCTAG	0	9	RDR2-sensitive
AGCAGGCCGCCGGTAGACGCTCAA	0	11	RDR2-sensitive
AGCAGGCTCCGCATCCCTCTCCGC	0	10	RDR2-sensitive
AGCAGGCTGAAGCATGTATGGCGT	0	25	RDR2-sensitive

AGCAGGCTGGACGATGCATGGCGC	0	17	RDR2-sensitive
AGCAGGCTGTAAGGTGTTCCACGT	0	17	RDR2-sensitive
AGCAGGGACGACGACGCTCAGCAG	0	12	RDR2-sensitive
AGCAGGGACTGTGGTGGCGGACTG	0	12	RDR2-sensitive
AGCAGGGCTGGCATGTTCATCACA	17	0	RDR2-resistant
AGCAGGGGCGAGAGTGGAGGCGAG	1	15	RDR2-sensitive
AGCAGGGGTGTCAGTACTATGGAA	0	13	RDR2-sensitive
AGCAGGTAACTGGGTGCTAGACTC	0	9	RDR2-sensitive
AGCAGGTAGGGAGCGGCTGGGCGA	0	20	RDR2-sensitive
AGCAGGTATCCTCGGACAAGTCGG	0	23	RDR2-sensitive
AGCAGGTCGCAGGATAGCGGATGA	0	11	RDR2-sensitive
AGCAGGTCGCGGTTGCAACGCCTT	2	16	RDR2-sensitive
AGCAGGTCGGCATGACGTGTCTGG	0	13	RDR2-sensitive
AGCAGGTTGTGACAGCTTCGACGC	0	9	RDR2-sensitive
AGCAGTAAGAAAATATGGACAATT	29	3	RDR2-resistant
AGCAGTAGAGACGCAAGAAGCTGT	1	27	RDR2-sensitive
AGCAGTAGAGGCGCAAGAAGATGT	0	16	RDR2-sensitive
AGCAGTAGAGGCGCAAGAAGCTGT	0	12	RDR2-sensitive
AGCAGTAGTTTGGACCGAGCGGCT	0	18	RDR2-sensitive
AGCAGTCGCGAGTACGCTCCCCAA	1	20	RDR2-sensitive
AGCAGTCGGCATATGCTACGGAGT	1	15	RDR2-sensitive
AGCAGTCGTCTGTAGTCGACGGCC	0	17	RDR2-sensitive
AGCAGTCTGGCACGTTCATCCTGT	0	10	RDR2-sensitive
AGCAGTTGAGAGTAGTGGAAGGCG	49	3	RDR2-resistant
AGCATAAGAGTGGAACGAGGGCGT	1	64	RDR2-sensitive
AGCATAATATCCGGTCTACTAGCA	22	1	RDR2-resistant
AGCATACAGAGTGGACGGTGGGTT	1	26	RDR2-sensitive
AGCATACCTCGGCGACAAGACCTG	0	10	RDR2-sensitive
AGCATACTCACCAGGACGTAGGGT	1	12	RDR2-sensitive
AGCATAGGACTGAGGAGAAGGCCT	0	19	RDR2-sensitive
AGCATATAGGTTTGTGGCTCGGTC	0	11	RDR2-sensitive
AGCATATGATGGCTTTATTACTCG	61	8	RDR2-resistant
AGCATATTTTCGTCGGCAGGCCGA	0	9	RDR2-sensitive
AGCATCACGAGATCAAGGACGCGC	2	20	RDR2-sensitive
AGCATCAGTTTCAACGGTCGTCAT	2	24	RDR2-sensitive
AGCATCAGTTTTAACGGTCGTCAT	0	11	RDR2-sensitive
AGCATCCAGACTACACTCGCGGAC	0	11	RDR2-sensitive
AGCATCGACTGTAGGCGCCGGCGC	0	10	RDR2-sensitive
AGCATCGGACTGTCCGGTGTGCAC	0	9	RDR2-sensitive
AGCATCGGATACGTGTAGTCGGCA	0	11	RDR2-sensitive
AGCATCGGTGTGGAAGACGACTGC	0	22	RDR2-sensitive
AGCATCTGCGTACGTGCGGACGGT	1	50	RDR2-sensitive
AGCATGACTGTACAAAGAGATGTT	39	1	RDR2-resistant
AGCATGAGTGCACGACCGACGGAC	0	13	RDR2-sensitive
AGCATGCAGGACACCGCAGGACAC	0	9	RDR2-sensitive
AGCATGCATCTAGACCGGCCTCTT	31	6	RDR2-resistant
AGCATGCCATGATCCGGACACCAA	0	9	RDR2-sensitive
AGCATGGACGAGCAGACACGACAC	2	104	RDR2-sensitive
AGCATGTAGACGCCACCGACATTA	0	12	RDR2-sensitive

AGCATGTCGCGTAGGACAGTTCGG	0	14	RDR2-sensitive
AGCATGTCGTGCTTGTCTTGGGCC	0	10	RDR2-sensitive
AGCATGTGATCTAGGGGTGGCAAC	31	0	RDR2-resistant
AGCATGTTTCTAGTCGGATAGGAT	0	10	RDR2-sensitive
AGCATTGATCAACTTCATTAAGTT	19	1	RDR2-resistant
AGCATTGATGGTGAAGTGTCTCTT	18	1	RDR2-resistant
AGCATTTGTACTGCATGACTTCAT	23	0	RDR2-resistant
AGCATTTTTGGCTGGTTTGGGCAT	14	0	RDR2-resistant
AGCCAAATCTCGGACACTCGGCAA	0	19	RDR2-sensitive
AGCCAACACTCGGCAAAGTCTCGG	0	17	RDR2-sensitive
AGCCAACGGCTGGCACTCGGCAAA	0	11	RDR2-sensitive
AGCCAACGGTCGGAAGGGGGCACC	2	14	RDR2-sensitive
AGCCAAGTAGAACGGAGCCGTTCC	1	14	RDR2-sensitive
AGCCAATACGTACGTAGGACAGGC	0	13	RDR2-sensitive
AGCCAATCCTCGGGCGAAGGCAGC	0	11	RDR2-sensitive
AGCCAATGTGGGACTAAGCCGGGA	0	14	RDR2-sensitive
AGCCACACGGCGGGATTCGTGGAG	0	11	RDR2-sensitive
AGCCACGGACGGTAAACACAACTC	0	12	RDR2-sensitive
AGCCACGGCGACTCTAACGGAGAC	0	11	RDR2-sensitive
AGCCACTGAACCTGAACTAGGAGA	0	17	RDR2-sensitive
AGCCACTGTTTAGAGACGACGTTC	0	9	RDR2-sensitive
AGCCAGAACTGTGTGAACCGGTAT	0	15	RDR2-sensitive
AGCCAGAAGGACGACCGAAGACTG	0	10	RDR2-sensitive
AGCCAGCTCGGGCTCGTAGCGGCT	0	15	RDR2-sensitive
AGCCAGCTCGTATGTAGACGGTTA	0	9	RDR2-sensitive
AGCCAGGCTGAAGAGGTTGATCAG	0	31	RDR2-sensitive
AGCCAGGCTGTGGAGAAACGGGTT	0	13	RDR2-sensitive
AGCCAGTAGTGTAGACGTCCACAT	0	45	RDR2-sensitive
AGCCATCTGTAGCGTGACGTGTGT	0	17	RDR2-sensitive
AGCCATGAATCCTGGATGAGACGT	0	13	RDR2-sensitive
AGCCATGACTGGACCATGTCGGGC	0	10	RDR2-sensitive
AGCCCAAACACGGCCTCGTTGGAT	0	16	RDR2-sensitive
AGCCCAACAGTGGTGTCGGCCCAC	0	9	RDR2-sensitive
AGCCCAACGCGTCGGGATGGCTGT	0	21	RDR2-sensitive
AGCCCAAGCACTGTTAGATCCGGC	0	14	RDR2-sensitive
AGCCCACCATCGCCAGCGTCGGAC	1	28	RDR2-sensitive
AGCCCACGAGTCGGTATGGCACAA	1	29	RDR2-sensitive
AGCCCACGGACTTAGATGGCCGAC	0	16	RDR2-sensitive
AGCCCAGATGGGTCGGCGCGAGAC	0	13	RDR2-sensitive
AGCCCAGTGTTGGGAGTATCACGG	0	9	RDR2-sensitive
AGCCCCAAGGCAGCTAGGAGCCGT	0	16	RDR2-sensitive
AGCCCCAGCCAGGAGGTCGATCGG	0	14	RDR2-sensitive
AGCCCCAGGCACGGACTGTCCGGA	0	11	RDR2-sensitive
AGCCCCCTGCGCAGCTGTGGACGG	0	12	RDR2-sensitive
AGCCCCGGAAGACGGCGAACTCCG	3	36	RDR2-sensitive
AGCCCCTGAGCAGCAGTCGGTTGG	1	31	RDR2-sensitive
AGCCCCTGCGCCAGGTCGCGGACC	2	19	RDR2-sensitive
AGCCCCTGCGTCAGGTCGCGGACC	0	14	RDR2-sensitive
AGCCCCTTCGCGGTGCTAGATCGT	3	30	RDR2-sensitive

AGCCCGACACGGTAGAATAAGCGG	0	15	RDR2-sensitive
AGCCCGAGACTCAGATGCTGCGGC	0	10	RDR2-sensitive
AGCCCGCGGACTTAGACGGTGTGA	0	9	RDR2-sensitive
AGCCCGCGGGCTAGCACAGCACGG	0	15	RDR2-sensitive
AGCCCGGACTAATAGGAAGGCCAT	2	40	RDR2-sensitive
AGCCCGGAGGACCAGAGCAGCAGA	1	15	RDR2-sensitive
AGCCCGGCACAGTAGAATAAGCGG	0	67	RDR2-sensitive
AGCCCGGCACGATAGAATAAGCAG	0	12	RDR2-sensitive
AGCCCGGCACGATAGAATAAGCGG	0	13	RDR2-sensitive
AGCCCGGCACGGTAGAATAAACGG	0	106	RDR2-sensitive
AGCCCGGCACGGTAGAATAAGCGG	14	634	RDR2-sensitive
AGCCCGGCACTGATACGATTGGAC	0	22	RDR2-sensitive
AGCCCGGCACTGTTTTTAGGACGG	0	21	RDR2-sensitive
AGCCCGGCCCTGCAGAACGGCAAG	0	9	RDR2-sensitive
AGCCCGTATTATTTCGTGTCGGAC	0	16	RDR2-sensitive
AGCCCGTGGACTTAGACGGCTCGG	0	10	RDR2-sensitive
AGCCCGTGTTATTTCGTGTCGGAC	0	15	RDR2-sensitive
AGCCCGTTAGGCCTCTGGCATGGC	0	9	RDR2-sensitive
AGCCCGTTTGACCCGTGATCCGGC	0	25	RDR2-sensitive
AGCCCTAGGACTATATTCGGTATT	1	29	RDR2-sensitive
AGCCCTAGGCGCGGACTGTCCGGA	0	9	RDR2-sensitive
AGCCCTAGTCAAACCGTCCGGACG	0	15	RDR2-sensitive
AGCCCTGATTGAGAGAACACGGAT	0	59	RDR2-sensitive
AGCCCTGGACGCAGCTAGCCGTTG	0	17	RDR2-sensitive
AGCCCTGTAGAAGCGCAGCTGGCG	0	11	RDR2-sensitive
AGCCCTGTAGTCAGCGTCGGCCCT	0	11	RDR2-sensitive
AGCCGAAATCGGACGGGAACCGGT	0	11	RDR2-sensitive
AGCCGACACTGTTGCAGCTGTTTG	0	44	RDR2-sensitive
AGCCGACATGTAGAGGGCGCTGTA	0	25	RDR2-sensitive
AGCCGACCGTTGGCTCACCGGACA	1	14	RDR2-sensitive
AGCCGACGAGTGAGTGTGCCGCGT	0	12	RDR2-sensitive
AGCCGACGCGTAGGGGACGCTGTA	0	35	RDR2-sensitive
AGCCGACGGCACACAGAAACTCAC	0	18	RDR2-sensitive
AGCCGACGGCGATTTTTGGACGAA	0	17	RDR2-sensitive
AGCCGACTCGGACTCGCAGTGGCT	0	12	RDR2-sensitive
AGCCGACTTATGGCGTGACTCGGA	0	16	RDR2-sensitive
AGCCGAGAGTGGACTGCACACGGA	0	11	RDR2-sensitive
AGCCGAGCACGGAGGTGTGGGCAT	0	9	RDR2-sensitive
AGCCGAGCCGAGCTGACTCTGTGG	0	10	RDR2-sensitive
AGCCGAGGACAGTGTTGAAGACGA	1	17	RDR2-sensitive
AGCCGAGTTGGTTAGATGGTCTGA	0	10	RDR2-sensitive
AGCCGATCCGAGCCGAGCCGAGCT	0	13	RDR2-sensitive
AGCCGATCTGGACCCGCATAGAAC	0	13	RDR2-sensitive
AGCCGATGTGGGACTAAAGCCGGA	0	14	RDR2-sensitive
AGCCGATGTGGGACTAAAGCCGGG	4	96	RDR2-sensitive
AGCCGCAGCCGGATTGCAGCCGCA	1	24	RDR2-sensitive
AGCCGCCAGGCACAACGTGCGGAC	0	19	RDR2-sensitive
AGCCGCCCTGGGAACGACGACGGC	0	11	RDR2-sensitive
AGCCGCCCTGGGGAACGACGACGG	0	55	RDR2-sensitive

AGCCGCGCATCGCCAGTATGGTAT	0	9	RDR2-sensitive
AGCCGCTCTGTTCTGTGTTTGGTA	1	19	RDR2-sensitive
AGCCGCTCTGTTGGAAGAAGGATT	0	18	RDR2-sensitive
AGCCGGAAACCTCTAGAGCATGGC	0	32	RDR2-sensitive
AGCCGGAACGAGGACTGAAACGGC	0	17	RDR2-sensitive
AGCCGGACCAGGTCCAGACGTCTA	0	9	RDR2-sensitive
AGCCGGAGTGATAGAAGAGGGTAC	0	16	RDR2-sensitive
AGCCGGAGTGATAGAGGAGGGTAC	0	11	RDR2-sensitive
AGCCGGATTGCAGCCGCAGCAAGC	2	31	RDR2-sensitive
AGCCGGATTGTAGCCGCAGCAAGC	0	15	RDR2-sensitive
AGCCGGATTGTATCGGTTGGCTGC	0	28	RDR2-sensitive
AGCCGGCACGAACGCAGAATACGA	0	11	RDR2-sensitive
AGCCGGCTGTACGTAGACGTCGAC	0	19	RDR2-sensitive
AGCCGGGTTGGACTGCACGACGGG	0	28	RDR2-sensitive
AGCCGGTAAGTGCTGAGCGCTCGT	1	17	RDR2-sensitive
AGCCGGTCGACGTGAATGAGCGGA	0	10	RDR2-sensitive
AGCCGGTGGGGTAGTCTGATGGCG	0	22	RDR2-sensitive
AGCCGGTGGTTAGAATCTAGGTGT	2	31	RDR2-sensitive
AGCCGGTTGTATGTTTGAGTTGGC	27	4	RDR2-resistant
AGCCGTATACTCTATCTGGGACGC	0	14	RDR2-sensitive
AGCCGTCACTGCTGATTGTTGCGA	0	12	RDR2-sensitive
AGCCGTCAGGACTGGGAACGCGGT	1	22	RDR2-sensitive
AGCCGTGGAGCAATGTAAGACGTC	0	10	RDR2-sensitive
AGCCGTGTCGGGTCTGGACTGGCT	0	47	RDR2-sensitive
AGCCGTTGATCTACAGGGGCGGAC	0	13	RDR2-sensitive
AGCCGTTGGAAGTGACCGTTGGCG	0	10	RDR2-sensitive
AGCCGTTGGACAGAAAGCAGCAGC	1	23	RDR2-sensitive
AGCCGTTGGTGTGGAGTTGCGCAT	0	10	RDR2-sensitive
AGCCTAATGTTATGGACTCGCATG	52	0	RDR2-resistant
AGCCTACAGGCTTAGACGGACCGG	0	11	RDR2-sensitive
AGCCTACGTCGTACTCCAGGTGTT	0	9	RDR2-sensitive
AGCCTACTTTGGTGATCGCGAGCA	43	0	RDR2-resistant
AGCCTAGACGGTAACTGATGGGAC	0	12	RDR2-sensitive
AGCCTAGCCTCGGACAAGGGCAGC	2	37	RDR2-sensitive
AGCCTAGTCGTCCTGGTCGTCCGA	0	10	RDR2-sensitive
AGCCTATCGCGTAGGACAGTTCGG	0	35	RDR2-sensitive
AGCCTCACGGACCGGACGGATCAC	0	18	RDR2-sensitive
AGCCTCAGTTGCCACGGTAGGATT	0	9	RDR2-sensitive
AGCCTCAGTTGCGGACCGTCTGGA	0	9	RDR2-sensitive
AGCCTCCACGGCGTCGATCTCGGT	0	12	RDR2-sensitive
AGCCTCCGCGTCGGAAACCGGCCC	0	21	RDR2-sensitive
AGCCTCCTGTAGCAGTCTATGGTA	0	16	RDR2-sensitive
AGCCTCGGACAAGGGCAGCCGACC	4	17	RDR2-sensitive
AGCCTCGGATTCCGGTAGTGATAC	0	11	RDR2-sensitive
AGCCTCGGATTCCGGTAGTGATGG	0	17	RDR2-sensitive
AGCCTCGGATTCCGGTAGTGGTAC	0	16	RDR2-sensitive
AGCCTCGGATTCCTGTAGTGATGG	1	13	RDR2-sensitive
AGCCTCGGCCTGTAGTGTTGGCTC	0	11	RDR2-sensitive
AGCCTCGGTCAGACGGGAGACGTT	0	10	RDR2-sensitive

AGCCTCTAAGGCAGGACTTAGGCA	0	11	RDR2-sensitive
AGCCTCTAAGGCGGGACTTAGGCA	1	20	RDR2-sensitive
AGCCTCTACGCCAGGTCGCGGACC	0	22	RDR2-sensitive
AGCCTCTGCGCCTGGCTTGCACGG	0	12	RDR2-sensitive
AGCCTCTGTGTCAGGTCGCGGACC	1	15	RDR2-sensitive
AGCCTCTTCGGACGGTCGGATGCT	0	17	RDR2-sensitive
AGCCTCTTCGGACGGTCGGATGGT	1	121	RDR2-sensitive
AGCCTGAACCAGACGAAGGTCGAG	2	36	RDR2-sensitive
AGCCTGACGCGTAGGACAGTTCGG	0	12	RDR2-sensitive
AGCCTGAGCGCGCTGTCGGGCGGC	148	0	RDR2-resistant
AGCCTGCAGACTTAGACGGCTTGG	0	14	RDR2-sensitive
AGCCTGCAGCAACGGACGGTACAC	0	9	RDR2-sensitive
AGCCTGCATCGGTTGAAACGGACG	0	12	RDR2-sensitive
AGCCTGCCCCCTCTGTCTTCGGAT	0	13	RDR2-sensitive
AGCCTGCTCAAGCCTTCGGACAAT	20	0	RDR2-resistant
AGCCTGCTGCAGCCTGTGCACTGG	0	17	RDR2-sensitive
AGCCTGCTGGAACTCAATTGGCAT	7	221	RDR2-sensitive
AGCCTGCTGGAGCTCAATTGGCAT	0	43	RDR2-sensitive
AGCCTGGACAGTGAGAAACGGAAT	0	18	RDR2-sensitive
AGCCTGGACTAGTTGAACGGGCCA	0	9	RDR2-sensitive
AGCCTGGCACGGTAGAATAAGCGG	0	26	RDR2-sensitive
AGCCTGGCTCAGACTGGACTGGCT	0	9	RDR2-sensitive
AGCCTGGCTGAGAGCATACAATAT	0	18	RDR2-sensitive
AGCCTGGCTGTAGGGGATGAGGCT	0	16	RDR2-sensitive
AGCCTGGCTGTGCATCGCGTGAGC	2	18	RDR2-sensitive
AGCCTGGGCAGTGAGAAACGGAAT	0	14	RDR2-sensitive
AGCCTGGGCTGTTGGTTTCGGCGG	0	13	RDR2-sensitive
AGCCTGGTCGGTAGAGCGACACGG	3	41	RDR2-sensitive
AGCCTGTAGGACATGGATGGGGGC	1	15	RDR2-sensitive
AGCCTGTAGGTGATGGAAAGCCAA	0	9	RDR2-sensitive
AGCCTGTAGGTGCGTATGTAGACA	0	10	RDR2-sensitive
AGCCTGTAGTTCGGCAAGGAACGC	0	9	RDR2-sensitive
AGCCTGTATAGTCGGGTCGAGGCA	0	15	RDR2-sensitive
AGCCTGTCGCGTAGGACAGTTCGG	2	117	RDR2-sensitive
AGCCTGTCGCGTAGGATAGTTCGG	1	22	RDR2-sensitive
AGCCTGTGAATGCTGGGAACACGG	0	12	RDR2-sensitive
AGCCTGTGCACTGGAGCAGCAGAG	0	25	RDR2-sensitive
AGCCTGTGCACTGGAGCAGCAGGG	0	27	RDR2-sensitive
AGCCTGTGTGGATGAAGATAGCGT	0	11	RDR2-sensitive
AGCCTGTTCGGGTAGAAACGCGGT	0	10	RDR2-sensitive
AGCCTGTTCTGCAAGCTCGTTGGA	0	12	RDR2-sensitive
AGCCTGTTGGAACTCAATTGGCAT	0	22	RDR2-sensitive
AGCCTTAATTTTTGGTATGTCGGG	0	12	RDR2-sensitive
AGCCTTAGACTGTGTTCAACGGAC	0	9	RDR2-sensitive
AGCCTTAGTCTGTGGAGCGGTAAG	0	12	RDR2-sensitive
AGCCTTATGTTCGATGACTGGCAT	0	9	RDR2-sensitive
AGCCTTATTTTCGGCGGCGTCTGA	0	9	RDR2-sensitive
AGCCTTCAGCTCGGGCTACTCGGC	0	13	RDR2-sensitive
AGCCTTCGTCGCTGTCGTTGCAAG	0	11	RDR2-sensitive

AGCCTTCGTCGGAGAAGGATGGCG	1	15	RDR2-sensitive
AGCCTTCTTTGCCGAGTGCCCGAC	0	12	RDR2-sensitive
AGCCTTGCTAGTGCTGTCGTCGAA	0	12	RDR2-sensitive
AGCCTTGCTGACTATAGGACCGAT	0	12	RDR2-sensitive
AGCCTTGCTTCTTGGTCGTCGCGG	0	11	RDR2-sensitive
AGCCTTGGCTGTATGGTATATGGA	0	14	RDR2-sensitive
AGCCTTGGGCTTTTGATTTGGCTT	16	0	RDR2-resistant
AGCCTTGTATCGGCCGGACACGGT	0	12	RDR2-sensitive
AGCCTTGTGTGATCTGACGTGCAT	0	17	RDR2-sensitive
AGCCTTTCGCGTAGGACAGTTCGG	0	13	RDR2-sensitive
AGCCTTTGCGTCAGGTCGCGGACC	0	22	RDR2-sensitive
AGCCTTTGCGTTAGGTCGCGGACC	0	15	RDR2-sensitive
AGCCTTTTAGGACTGGTTTGGCAG	0	10	RDR2-sensitive
AGCCTTTTCATCTTTAAGTCGGTT	40	63	RDR2-sensitive
AGCCTTTTCGGACTTGGGCCGGGC	0	10	RDR2-sensitive
AGCCTTTTGTCGGGCACTCGGCAA	0	26	RDR2-sensitive
AGCGAAAGTCTACGGCTTGTTACT	16	0	RDR2-resistant
AGCGAACGATGGAAGAACTGGCGC	0	10	RDR2-sensitive
AGCGAACTCGGAAAAGAGGCTCGG	0	26	RDR2-sensitive
AGCGAACTGTACATCGGCAACTGC	0	19	RDR2-sensitive
AGCGAAGACTGCATGGACAGACGC	0	21	RDR2-sensitive
AGCGAAGGCCTCGAGCGAAGGGTG	0	13	RDR2-sensitive
AGCGAAGTGAGCACACTGTACGAT	0	9	RDR2-sensitive
AGCGAAGTTGGACGCCAAGGCTGC	0	9	RDR2-sensitive
AGCGAATCGGGCTATGCCGGACGG	0	15	RDR2-sensitive
AGCGACAATCTCACTCGTCGGTCC	0	13	RDR2-sensitive
AGCGACAATTTACTCGTCGGTCCA	0	24	RDR2-sensitive
AGCGACACAGGAGGCCAAGGATAT	176	8	RDR2-resistant
AGCGACACTGTAGTGCGGACATAT	0	9	RDR2-sensitive
AGCGACAGAGCTCATAATCGTCGA	0	9	RDR2-sensitive
AGCGACAGAGCTCATGATCGTCGA	0	45	RDR2-sensitive
AGCGACAGATACGGAAGAGGACAG	0	13	RDR2-sensitive
AGCGACAGGCGTAGACACCCGGCA	0	10	RDR2-sensitive
AGCGACAGTAGAAGTTGGGAGGCG	0	9	RDR2-sensitive
AGCGACCAGCACGACAGACGAACT	1	23	RDR2-sensitive
AGCGACCAGCACGACGGACGAACT	0	10	RDR2-sensitive
AGCGACCAGCACGATGGACGAACT	0	11	RDR2-sensitive
AGCGACCAGTACGATCGGACGGAC	0	20	RDR2-sensitive
AGCGACCGAACTCCGGATAAGCAG	0	9	RDR2-sensitive
AGCGACCGACGGATGACACGGACG	0	11	RDR2-sensitive
AGCGACGACTTTGGGACACCGGCT	0	9	RDR2-sensitive
AGCGACGCGGTAGAAGCGAAGGAG	0	16	RDR2-sensitive
AGCGACGGCACTTACAACGAGCAT	0	12	RDR2-sensitive
AGCGACGGCACTTACAGCGACCAT	0	29	RDR2-sensitive
AGCGACGTAGGACGCCGACGACGG	0	13	RDR2-sensitive
AGCGACGTTGTGCTGAGCCGGCCT	0	9	RDR2-sensitive
AGCGACTCCTACTCTGTGGATGGT	0	26	RDR2-sensitive
AGCGACTCTGAGTGCACAGGACAG	0	22	RDR2-sensitive
AGCGACTGAAGAGTTGGAGGCCAA	0	19	RDR2-sensitive

AGCGACTGCAGCGAAAGGCGACGA	1	28	RDR2-sensitive
AGCGACTGCAGCGAAGGCCTCGAG	2	46	RDR2-sensitive
AGCGACTGCAGCGAGAGACGACGA	1	120	RDR2-sensitive
AGCGACTGCATCGCGCTGCCGATC	35	0	RDR2-resistant
AGCGACTGGAACAGGAACGAAGAC	0	20	RDR2-sensitive
AGCGACTGGTTGTAAGATGGCGTA	0	11	RDR2-sensitive
AGCGACTGTCGTGCCAGGCCGGGC	0	17	RDR2-sensitive
AGCGACTTCTTGAACGATAGGCTC	0	10	RDR2-sensitive
AGCGACTTGCGACTTCGACGGAGA	0	14	RDR2-sensitive
AGCGAGAAAGACGAAGGCTGGAGC	0	11	RDR2-sensitive
AGCGAGAACAACCCCTGACGTCGT	0	9	RDR2-sensitive
AGCGAGACATGGTTGATTTATGAC	21	0	RDR2-resistant
AGCGAGACCAGGCTTGTTGGACGG	0	10	RDR2-sensitive
AGCGAGACCGTCTGACGACGACAT	0	30	RDR2-sensitive
AGCGAGACGGATCATGTACAACTT	0	12	RDR2-sensitive
AGCGAGACTGCAAGAGACGCGGGT	0	12	RDR2-sensitive
AGCGAGAGACGACGAGCGAAGGAT	0	21	RDR2-sensitive
AGCGAGAGACGACGAGCGAAGGGT	3	32	RDR2-sensitive
AGCGAGAGTAGGACACTCGGCATA	0	15	RDR2-sensitive
AGCGAGATCTAGCTTGTCGAGCGG	0	13	RDR2-sensitive
AGCGAGCCAGCGAGCCACGAGCAT	0	31	RDR2-sensitive
AGCGAGCCAGGCTGAGACCGGTGA	0	10	RDR2-sensitive
AGCGAGCGGACGGTGCAGATTCAC	0	29	RDR2-sensitive
AGCGAGCGTTGGGCAGTGATGGAC	0	12	RDR2-sensitive
AGCGAGCTAGCGAGCCACGAGCAT	0	11	RDR2-sensitive
AGCGAGCTATAGAACAGGGGGCAT	0	67	RDR2-sensitive
AGCGAGCTGGGACTGGGAGAGGCA	0	15	RDR2-sensitive
AGCGAGGACGAACGTTGTTGCTCC	0	24	RDR2-sensitive
AGCGAGGACGACGTCGAATCTCAA	0	9	RDR2-sensitive
AGCGAGGATGGAATCGCAGTCGGA	0	10	RDR2-sensitive
AGCGAGGCCATAGACCAGAGGACA	0	24	RDR2-sensitive
AGCGAGGGAACTGAACGATGACGT	0	9	RDR2-sensitive
AGCGAGGGACTCGGACACTCGGCA	0	9	RDR2-sensitive
AGCGAGGGCGTCCACCAACCTGAC	0	12	RDR2-sensitive
AGCGAGTCATGAGTTAACGGGATC	17	0	RDR2-resistant
AGCGATAGAGGACTGAATGGAGGC	0	33	RDR2-sensitive
AGCGATAGCGGCAACTTTTTCATT	27	0	RDR2-resistant
AGCGATATGTAGAGTTGATTGCAT	0	11	RDR2-sensitive
AGCGATCGCGTGAAACGAGGCGTC	0	9	RDR2-sensitive
AGCGATCGGTACTGAACGGCGGAC	0	17	RDR2-sensitive
AGCGATCGGTTAGGATAAGCGTAA	0	21	RDR2-sensitive
AGCGATCTCAAGGACCCGGCCACC	0	19	RDR2-sensitive
AGCGATCTGGCACAGTAGCGGCTC	2	18	RDR2-sensitive
AGCGATCTGTAGAAGCGTGTCATT	0	13	RDR2-sensitive
AGCGATGACGGTCTGACTCAACGG	0	18	RDR2-sensitive
AGCGATGATCGCGCGTGACATCCT	0	21	RDR2-sensitive
AGCGATGCGGATGGACGCTCCAGA	0	12	RDR2-sensitive
AGCGATGGAGTGGAGCACGGTCTG	0	10	RDR2-sensitive
AGCGATGGGACTCACGTACGGTGC	1	26	RDR2-sensitive

AGCGATGGTAGGACCAGGCGACCA	0	9	RDR2-sensitive
AGCGATGTGGTAGTATGTGGGCAT	0	13	RDR2-sensitive
AGCGATGTGTATGACTGCGGCGTG	0	10	RDR2-sensitive
AGCGATTACATAGTTGGACATCAT	0	11	RDR2-sensitive
AGCGATTCGGACGGAGCAGGACGT	0	10	RDR2-sensitive
AGCGATTGGGACAACGACGTCATT	0	17	RDR2-sensitive
AGCGATTGTATGTAGAGACGTTCT	0	9	RDR2-sensitive
AGCGATTTGCGCGAACGACGGCTC	1	14	RDR2-sensitive
AGCGCACAAGAATTCATGGCCGGT	0	14	RDR2-sensitive
AGCGCACAGTAGAAAAACCGGCGA	0	41	RDR2-sensitive
AGCGCACCGCGATGGCAGAACCGC	0	9	RDR2-sensitive
AGCGCACCTGGTCGGTTTCTGGTC	0	11	RDR2-sensitive
AGCGCACGGACGGACGGTGTAGAT	0	10	RDR2-sensitive
AGCGCACTCGGCAAAGGGACTGGC	0	21	RDR2-sensitive
AGCGCAGAACTTTGCCGAGTGCGG	0	13	RDR2-sensitive
AGCGCAGCGACGACGACAACTCTC	0	14	RDR2-sensitive
AGCGCAGGACTCTGCCGAGAGCGG	0	31	RDR2-sensitive
AGCGCAGGACTTTGCCGAGTGCGG	3	45	RDR2-sensitive
AGCGCAGGTTGTATGGACAGACGC	0	31	RDR2-sensitive
AGCGCATCTGAGCATGTAGACGCC	1	215	RDR2-sensitive
AGCGCCGAGCTCTGACGTGGCGAC	0	17	RDR2-sensitive
AGCGCCGTAGATCTTGACAGACGT	0	42	RDR2-sensitive
AGCGCCTGCTCGGACGGAGGACGG	1	37	RDR2-sensitive
AGCGCGAACACAAGTCGGAGACAT	0	12	RDR2-sensitive
AGCGCGAACATAAGTCGGAGACAA	0	22	RDR2-sensitive
AGCGCGACACTAGCAAGACCCGAG	0	9	RDR2-sensitive
AGCGCGACAGCAGATACAAAGGCG	0	26	RDR2-sensitive
AGCGCGACCGAGAACTGGCGTGAC	0	12	RDR2-sensitive
AGCGCGACGGCAGAACCGTGCACT	0	15	RDR2-sensitive
AGCGCGATCACAACTCGGAGGCAT	0	23	RDR2-sensitive
AGCGCGATCGCAACTCGGAGGCAT	0	15	RDR2-sensitive
AGCGCGATGGCAGAACCAAACTAT	0	10	RDR2-sensitive
AGCGCGATTCCAACTCGGAGGCAT	0	17	RDR2-sensitive
AGCGCGCGATGGCAGAATTAAGGT	0	10	RDR2-sensitive
AGCGCGCTGTTGGAGCACGGTCAG	0	12	RDR2-sensitive
AGCGCGGACTTATCCAACGGCTGT	0	32	RDR2-sensitive
AGCGCGGCGTGTTGTAGTCCCGGC	0	23	RDR2-sensitive
AGCGCGGCTTGAAGACGGGTCTGG	0	13	RDR2-sensitive
AGCGCGGGCGAGAGCGAGGCGATA	0	25	RDR2-sensitive
AGCGCGGTGGTGGAGAAGTTGGCG	116	5	RDR2-resistant
AGCGCGTAGACCACTGAACACGGA	0	14	RDR2-sensitive
AGCGCTCAACTGTGGGACGGCTGC	0	22	RDR2-sensitive
AGCGCTCGCGAACTTTATGGCGTA	0	14	RDR2-sensitive
AGCGCTCGTGAACTTTATGGCGGA	0	57	RDR2-sensitive
AGCGCTCTGCAGTATGATGTCGTT	0	10	RDR2-sensitive
AGCGCTGACTCTTGGCATTCTCTT	291	35	RDR2-resistant
AGCGCTGACTGTATATAGCGAGGC	0	10	RDR2-sensitive
AGCGCTGCGGACACGGTGTTGCTC	0	9	RDR2-sensitive
AGCGCTGGTGCTGCAGTCAGGACA	0	9	RDR2-sensitive

AGCGCTGGTGCTGTAGTCAGGACA	0	12	RDR2-sensitive
AGCGCTGTGTGACGGTGAAGACGT	0	217	RDR2-sensitive
AGCGCTGTTGTGACTGGTCGTTGT	1	15	RDR2-sensitive
AGCGCTTCGTCTCGCAGTCGGCAT	3	40	RDR2-sensitive
AGCGGAACAACTACGGTCGGAAAC	0	9	RDR2-sensitive
AGCGGAAGACGTCTCGATGTAGAC	3	33	RDR2-sensitive
AGCGGAAGTAGAAGCAGCGGAGGC	5	24	RDR2-sensitive
AGCGGAATAGTCAGCAGCAGCAGT	0	10	RDR2-sensitive
AGCGGACAGAACAATTTACCGGTT	0	10	RDR2-sensitive
AGCGGACAGAACCATTTAGCGGTC	0	13	RDR2-sensitive
AGCGGACAGAACCATTTAGGGGTC	1	15	RDR2-sensitive
AGCGGACAGATGGTGCAGATTCAT	0	11	RDR2-sensitive
AGCGGACAGCATGGATTTCGGCCA	0	17	RDR2-sensitive
AGCGGACAGCGGCACAGGCAGCAG	0	9	RDR2-sensitive
AGCGGACAGGACGGTTTACAGACC	0	35	RDR2-sensitive
AGCGGACAGGGGAGGGCAGAACGG	0	16	RDR2-sensitive
AGCGGACATGAGCATTTAGCGGCT	0	12	RDR2-sensitive
AGCGGACCCTGTTAGACGGTGAGC	0	9	RDR2-sensitive
AGCGGACCTGAAGTAGAGAGACAC	0	25	RDR2-sensitive
AGCGGACCTTGCTGGAGAAGTAAA	0	16	RDR2-sensitive
AGCGGACCTTGTTGGAGATAGCCT	1	41	RDR2-sensitive
AGCGGACCTTTTAGCGGATGTTGT	0	10	RDR2-sensitive
AGCGGACGAAGACGTGCGGAACGC	0	17	RDR2-sensitive
AGCGGACGAAGCGAGCAGTCGCGA	0	18	RDR2-sensitive
AGCGGACGAGCGGCGACAACGCAC	1	33	RDR2-sensitive
AGCGGACGCGCGGCAGAACGCACC	0	26	RDR2-sensitive
AGCGGACGCTAACTGCGGGCGACT	0	19	RDR2-sensitive
AGCGGACGCTCAGCAAGGCGGGAG	0	9	RDR2-sensitive
AGCGGACGGACGACGGCAGCGCAC	0	12	RDR2-sensitive
AGCGGACGGATACAGAGTGTCACA	0	11	RDR2-sensitive
AGCGGACGGCATGGATTTCGGCCA	1	39	RDR2-sensitive
AGCGGACGGCCACTAGTGGACAGC	0	22	RDR2-sensitive
AGCGGACGGCCGTTACTTTACCGA	0	9	RDR2-sensitive
AGCGGACGGCTGTGTTTGACGCTA	1	26	RDR2-sensitive
AGCGGACGGCTGTGTTTGGCGCTA	0	20	RDR2-sensitive
AGCGGACGGTGCAGATTCACCGAA	0	31	RDR2-sensitive
AGCGGACGTTGTTGGGCGGAGACC	0	33	RDR2-sensitive
AGCGGACTGACGGTGTAGATTTAC	0	13	RDR2-sensitive
AGCGGACTGAGCTGAGGTATGGCG	0	10	RDR2-sensitive
AGCGGACTTCACCCAGGTCGACGC	0	12	RDR2-sensitive
AGCGGAGACTGCATGGAGTGCGCT	1	76	RDR2-sensitive
AGCGGAGACTGCATGGAGTGTGCT	0	20	RDR2-sensitive
AGCGGAGACTGTATGGAGTGCGCT	2	34	RDR2-sensitive
AGCGGAGAGGACGCTGAATATGGC	111	5	RDR2-resistant
AGCGGAGATGCAGAACACGTCGGC	0	9	RDR2-sensitive
AGCGGAGCACGTAGGGCAGAGGCA	0	16	RDR2-sensitive
AGCGGAGCGGCTCCTGAAGTCTAT	0	21	RDR2-sensitive
AGCGGAGCGGCTCCTGATGTCCAT	0	16	RDR2-sensitive
AGCGGAGCTCATGTAGGGTCCGGA	0	13	RDR2-sensitive

AGCGGAGCTCGTTCGTACCTCGGC	0	13	RDR2-sensitive
AGCGGAGCTGCTCACACGTCACAG	0	13	RDR2-sensitive
AGCGGAGGAGAGCTAGCGCCCTAA	1	19	RDR2-sensitive
AGCGGAGGCAGAACGGGTTGGTGT	0	24	RDR2-sensitive
AGCGGAGGCATAGCTCAACGCCAG	0	12	RDR2-sensitive
AGCGGAGGGACGGCAGAACCGTGC	0	17	RDR2-sensitive
AGCGGAGTAGAGCAGTTTGGTAGC	35	6	RDR2-resistant
AGCGGATACGGATACAAGGACCAA	0	11	RDR2-sensitive
AGCGGATCCTGGAAACGACGGAGT	4	32	RDR2-sensitive
AGCGGATCGTGCTGTGCCGGCCCA	0	12	RDR2-sensitive
AGCGGATCTCGAGACGACGTCGGC	0	9	RDR2-sensitive
AGCGGATCTGGTTTCGGCTAATTT	2	17	RDR2-sensitive
AGCGGATGACCCTACCAGCCACGC	0	14	RDR2-sensitive
AGCGGATGAGACGTATGCTATCTG	0	33	RDR2-sensitive
AGCGGATGGGGGCTGGCGACGCGC	21	1	RDR2-resistant
AGCGGATGGTACACTACACATGGT	0	89	RDR2-sensitive
AGCGGATTGACGTTGTGCGGCGAA	0	11	RDR2-sensitive
AGCGGATTTCAGTATACATGTCCA	0	14	RDR2-sensitive
AGCGGCACTGGAGTTTGGAAGGGC	0	11	RDR2-sensitive
AGCGGCAGAACCATGGAGTGTGCA	0	15	RDR2-sensitive
AGCGGCAGAGCTCATGATCGTCGA	2	88	RDR2-sensitive
AGCGGCAGAGCTCATGATTGTCGA	1	123	RDR2-sensitive
AGCGGCAGAGCTTATGATCGTCGA	0	28	RDR2-sensitive
AGCGGCAGCAGAACCATCGCGGGC	0	12	RDR2-sensitive
AGCGGCCTATTCAGACGGACACCT	0	23	RDR2-sensitive
AGCGGCCTGAAGTAGAGATGTCAA	0	10	RDR2-sensitive
AGCGGCGACGTAGATCAAGAGGGC	2	18	RDR2-sensitive
AGCGGCGATATCCTGGGTGGCACC	26	4	RDR2-resistant
AGCGGCGCGGAATCCGGACGTCGG	0	90	RDR2-sensitive
AGCGGCGGGACGTGGATGGTGTGG	32	0	RDR2-resistant
AGCGGCTCTGGCGACGTCTGTAAA	0	17	RDR2-sensitive
AGCGGCTCTGTTTTTTGTTTGGTT	3	26	RDR2-sensitive
AGCGGCTGAAGCAGAGACTCTAAA	0	12	RDR2-sensitive
AGCGGCTGCAGCGAAGACTTCGAG	0	15	RDR2-sensitive
AGCGGCTGCAGCGAAGGCCTCGAG	0	11	RDR2-sensitive
AGCGGCTGCAGCGAAGGCTTCGAA	1	12	RDR2-sensitive
AGCGGCTGCTCTGCATACGTTCAG	0	32	RDR2-sensitive
AGCGGCTGGTAGGCGGGGGTACAG	0	19	RDR2-sensitive
AGCGGCTGGTGATCGAACGGTTGA	0	11	RDR2-sensitive
AGCGGCTTCAATATGATCGTTGCA	0	20	RDR2-sensitive
AGCGGGAAGCGTAGGACAAGGCCT	0	9	RDR2-sensitive
AGCGGGACGAGCATCAGCTGACGC	1	17	RDR2-sensitive
AGCGGGAGACTGGTGAGCAACGGC	1	27	RDR2-sensitive
AGCGGGATCGATCGGAGAAGACGC	0	11	RDR2-sensitive
AGCGGGATGAGAACAGAGTGGCTC	0	13	RDR2-sensitive
AGCGGGATGGGAACAGAGCGGCTC	0	15	RDR2-sensitive
AGCGGGATGGGAACGGAACGGCTC	0	9	RDR2-sensitive
AGCGGGATGGGAACGGAGCGGCTC	2	72	RDR2-sensitive
AGCGGGATGGGAACGGAGCGGCTT	0	9	RDR2-sensitive

AGCGGGATTGGAACGGAGCGGCTC	9	75	RDR2-sensitive
AGCGGGATTGGAACGGAGCGGCTT	0	15	RDR2-sensitive
AGCGGGCAAGAAGAAGGAGCACAT	0	10	RDR2-sensitive
AGCGGGCACAACGCTAGGACGAGC	0	13	RDR2-sensitive
AGCGGGCAGAACCGTGCACCAGAT	0	9	RDR2-sensitive
AGCGGGCAGAACCGTGCGCTAGAT	0	12	RDR2-sensitive
AGCGGGCAGGGACACCCTTCGCAG	0	12	RDR2-sensitive
AGCGGGCAGTATAGTGCATCTTGC	0	14	RDR2-sensitive
AGCGGGCGGGAGCGACGAAGACGT	0	20	RDR2-sensitive
AGCGGGCGTATTTTTTCTTCGCGG	0	13	RDR2-sensitive
AGCGGGCTGCAGGCAGAACCGTGC	0	45	RDR2-sensitive
AGCGGGCTGGACAGCGGCTGGGAG	779	68	RDR2-resistant
AGCGGGCTGGACAGCGGCTGGGGG	20	0	RDR2-resistant
AGCGGGCTGGGAACAGAGCGGCTC	0	10	RDR2-sensitive
AGCGGGCTGGGAACGAAGCGGCTC	0	10	RDR2-sensitive
AGCGGGCTGGGAACGGAGCGGCTC	0	17	RDR2-sensitive
AGCGGGCTGGGAACGGAGCGGCTT	0	10	RDR2-sensitive
AGCGGGCTTGGGTCGGGCTAGCAC	0	16	RDR2-sensitive
AGCGGGGAAGGACAACAGACGCAT	0	16	RDR2-sensitive
AGCGGGGCAGTAGGAGCGACGTCT	0	10	RDR2-sensitive
AGCGGGTATGGGGCAACAGAGAAC	3	47	RDR2-sensitive
AGCGGGTCGGGTTGTAGTCGGGTA	2	21	RDR2-sensitive
AGCGGGTCGGGTTGTATTCGGATA	0	12	RDR2-sensitive
AGCGGGTCGGTCCGGCAGACACAC	0	16	RDR2-sensitive
AGCGGGTCTAAACGGGCATAGCAC	0	9	RDR2-sensitive
AGCGGGTTCAACGGATGACGACAT	0	10	RDR2-sensitive
AGCGGGTTGAGAACGAAGCGGCTC	0	11	RDR2-sensitive
AGCGGTAAGCATTTTAGTTGGTCA	40	4	RDR2-resistant
AGCGGTACCATCCTCTCTGGTCGT	0	14	RDR2-sensitive
AGCGGTACCCTCGGATTCGGCAGA	0	59	RDR2-sensitive
AGCGGTACTTCGGACAGACGCGAA	0	20	RDR2-sensitive
AGCGGTAGGGGTGGAGGGCGTTGG	30	0	RDR2-resistant
AGCGGTATGGGAACAGGGGCGGAG	15	0	RDR2-resistant
AGCGGTCAGACGGTGGAGACAGAC	0	36	RDR2-sensitive
AGCGGTCATTGTTGGAGACAGCCT	0	27	RDR2-sensitive
AGCGGTCCGCTACGAAGGGGGCAC	0	15	RDR2-sensitive
AGCGGTCGAAAACGGGCTATGCGG	0	20	RDR2-sensitive
AGCGGTCGACTCCGGTAGCGGCTC	1	36	RDR2-sensitive
AGCGGTCGGCGGCAACGAGGGCGT	0	18	RDR2-sensitive
AGCGGTCTATAGCTGACGGTCGTT	0	16	RDR2-sensitive
AGCGGTCTGGGTAGCGGATGCTGC	0	31	RDR2-sensitive
AGCGGTGCTGCAGAGCGGTATCTG	0	10	RDR2-sensitive
AGCGGTGGACGGTCACGGACAGAC	0	11	RDR2-sensitive
AGCGGTGGTATCTGACACGAGCTG	0	10	RDR2-sensitive
AGCGGTGTAGGAGGCATTCAACCG	0	10	RDR2-sensitive
AGCGGTTAAGGACTCACGCGGCGC	0	10	RDR2-sensitive
AGCGGTTACTGTATGCGGCTTGTC	0	19	RDR2-sensitive
AGCGGTTCACTGGAAAGACGACGC	0	24	RDR2-sensitive
AGCGGTTCTGTTTTTTGTTTGGTT	0	10	RDR2-sensitive

AGCGGTTGAGTCCTTGCTGACGTC	1	13	RDR2-sensitive
AGCGGTTGGGATTCCTGAAGACGC	0	19	RDR2-sensitive
AGCGGTTGGGATTCCTGGAGACGC	1	101	RDR2-sensitive
AGCGGTTTTTAGGCTGATACGGTA	0	19	RDR2-sensitive
AGCGGTTTTTGTAGGCTAAACGGT	0	17	RDR2-sensitive
AGCGTACACTGTGCTGACACTCGG	0	14	RDR2-sensitive
AGCGTACGCCGGCATGCAGGCTCA	0	9	RDR2-sensitive
AGCGTACTGGAGCTGCCGCTGGAA	0	9	RDR2-sensitive
AGCGTACTGGTTGAACGGATGCAA	0	13	RDR2-sensitive
AGCGTAGACTGTGTACAGCGCGGG	0	13	RDR2-sensitive
AGCGTAGAGCACCGTTTGATCCTC	0	12	RDR2-sensitive
AGCGTAGATACTCAGGAACGGCAA	1	119	RDR2-sensitive
AGCGTAGATGCTCAGGAACGGCAA	2	31	RDR2-sensitive
AGCGTAGATGCTCAGGAACGGCGA	0	13	RDR2-sensitive
AGCGTAGATGGTGGACTGTGCCGC	0	9	RDR2-sensitive
AGCGTAGCGGTATGGTAGGAAGGA	0	13	RDR2-sensitive
AGCGTAGCTGTGGAGTAGTAGGCT	1	37	RDR2-sensitive
AGCGTAGGACTCTGCCGAGTGCGG	0	13	RDR2-sensitive
AGCGTAGGACTTTACCGAGTGCGG	0	12	RDR2-sensitive
AGCGTAGGACTTTGCCGAGTGCGG	1	31	RDR2-sensitive
AGCGTAGGCTCGATGACGTGACGG	0	21	RDR2-sensitive
AGCGTAGGGCTGCAGGAACGGCAG	0	39	RDR2-sensitive
AGCGTAGTCTGTATGTGCGAGCAC	0	15	RDR2-sensitive
AGCGTAGTGACTGAGTCGTTGGTT	0	17	RDR2-sensitive
AGCGTATCGCGCTGTCTGATGGGA	0	13	RDR2-sensitive
AGCGTATGGGGCACTGTAGGAGGC	0	9	RDR2-sensitive
AGCGTCAACGATCGGCAGGGCGAC	1	15	RDR2-sensitive
AGCGTCCTTGAGTACCTCGTCGCC	15	0	RDR2-resistant
AGCGTCGATCTCTGACGTGGCGTC	0	18	RDR2-sensitive
AGCGTCTGTCTCTGGCGGCTCCAC	0	13	RDR2-sensitive
AGCGTGACCGACGAGCTAAGGCTG	1	23	RDR2-sensitive
AGCGTGACTCAAGCAGGACCCGTG	0	18	RDR2-sensitive
AGCGTGACTGCATGAGGAGGGTGA	0	10	RDR2-sensitive
AGCGTGACTGTGAGAGCGGGATGG	5	135	RDR2-sensitive
AGCGTGCACCGTTGAACGCATGGC	0	11	RDR2-sensitive
AGCGTGCGAGAGGGACGAAGACGA	0	11	RDR2-sensitive
AGCGTGCGCGCCTGGTCGGTACGG	0	17	RDR2-sensitive
AGCGTGCGGGAGCGACGAAGACGT	0	14	RDR2-sensitive
AGCGTGCGGGAGGGACGAAACCGG	0	11	RDR2-sensitive
AGCGTGGCTGGACGAAGCGTCATG	0	20	RDR2-sensitive
AGCGTGTCGGGCTGCTGTGCGGAG	0	13	RDR2-sensitive
AGCGTTATTTCCGTCGGTTGGCCG	0	10	RDR2-sensitive
AGCGTTCCATGTAGAGTAGACGAC	0	13	RDR2-sensitive
AGCGTTCGTGGGACGGGGACGGAG	0	10	RDR2-sensitive
AGCGTTCGTTTGGTTGGCCGTATA	0	17	RDR2-sensitive
AGCGTTCTGCGTGAGATCTCGGCG	0	9	RDR2-sensitive
AGCGTTCTGCTGCATCTCTCAGGC	0	25	RDR2-sensitive
AGCGTTGGACCAAGTCGACGGCTG	0	10	RDR2-sensitive
AGCGTTGGGCGATGCACCGTGCAT	0	9	RDR2-sensitive

AGCGTTGTACGGATGCAGTGCGCT	0	16	RDR2-sensitive
AGCGTTGTTGGACGCCTCGCGGAC	0	10	RDR2-sensitive
AGCGTTGTTGGACGCCTTGCGGAC	0	9	RDR2-sensitive
AGCTAAATTTTTAAGATCGTTGGT	0	17	RDR2-sensitive
AGCTAACGTGGCTGAACGCTGGAC	1	14	RDR2-sensitive
AGCTAAGAGACTGTATAAGGGCAT	0	10	RDR2-sensitive
AGCTAATTCAAATCGGACCGGCAC	0	11	RDR2-sensitive
AGCTAATTCATGTCGGGCCGGCAC	0	125	RDR2-sensitive
AGCTAATTCGGACCGCGCAGTCAC	0	18	RDR2-sensitive
AGCTAATTCGGGCTGGACCGGTAC	0	20	RDR2-sensitive
AGCTACAGGGACTGTCCGGTGCGC	0	10	RDR2-sensitive
AGCTACAGTCGGCGACACCGTCCT	1	17	RDR2-sensitive
AGCTACAGTCGGCGACATCGTCCT	2	16	RDR2-sensitive
AGCTACAGTGACTGTCCGGTGGTG	0	12	RDR2-sensitive
AGCTACCATCGTAGTTGATCACGG	0	9	RDR2-sensitive
AGCTACCATGCAGACCGTCAGACA	0	24	RDR2-sensitive
AGCTACGGACGGCTGCTGACGACG	0	9	RDR2-sensitive
AGCTACGTCAAGACAGAGGGCATC	0	9	RDR2-sensitive
AGCTACTCTCTTGGACGTTGTGAA	14	0	RDR2-resistant
AGCTACTGAGTACTGATCGGTCGG	0	9	RDR2-sensitive
AGCTACTGTGCTACTGGGCGGACA	0	26	RDR2-sensitive
AGCTACTGTGGAAACCGGCGTCGC	0	12	RDR2-sensitive
AGCTAGACGTGAACTGTGCAGGGG	0	9	RDR2-sensitive
AGCTAGAGGACGGTTTCGATGGAC	0	11	RDR2-sensitive
AGCTAGATCGAACGCGTGTGGCAC	0	39	RDR2-sensitive
AGCTAGATGGCAGAACGGCAGAAC	1	39	RDR2-sensitive
AGCTAGCAACCGGCGCCTCTGCTC	33	3	RDR2-resistant
AGCTAGCCGTTGGACTCATGGCAC	8	89	RDR2-sensitive
AGCTAGCCGTTGGACTCATGGCGC	2	52	RDR2-sensitive
AGCTAGCCGTTGGACTGATGGCAC	0	107	RDR2-sensitive
AGCTAGCCGTTGGGCTGATGGCAC	1	19	RDR2-sensitive
AGCTAGCCGTTGGTCTGATGGCAC	0	17	RDR2-sensitive
AGCTAGCTCGAAGGCTGACGAGGC	0	9	RDR2-sensitive
AGCTAGCTCTGACAGTAGACGTTG	0	28	RDR2-sensitive
AGCTAGGAAGCCTGGGAACGACGG	0	14	RDR2-sensitive
AGCTAGGACATAACCGCGCGGCAC	0	10	RDR2-sensitive
AGCTAGGACTACGTAGAAGAGGAA	0	15	RDR2-sensitive
AGCTAGGATTGAGACGTCGGATAA	0	14	RDR2-sensitive
AGCTAGGCTGTAGGGGGCACCCTA	0	24	RDR2-sensitive
AGCTAGGGGTAGGGTAATGATGGC	3	19	RDR2-sensitive
AGCTAGTCCACTGTTTCGAGCATA	0	11	RDR2-sensitive
AGCTAGTCGTTGGGCTGATGGCAC	0	10	RDR2-sensitive
AGCTAGTGCATGATTGTACCGGGC	0	10	RDR2-sensitive
AGCTATAGACGGCTGTTGACGGTC	0	13	RDR2-sensitive
AGCTATCCCGTCGGAAGTTTGGCT	1	14	RDR2-sensitive
AGCTATCGGTACGTGTAGAGATGG	0	10	RDR2-sensitive
AGCTATCGGTTCGCGGACTCGGCC	0	16	RDR2-sensitive
AGCTATCGTAGCAACGCACGGACA	0	24	RDR2-sensitive
AGCTATCTGACTCTGTAAATTGGC	0	11	RDR2-sensitive

AGCTATCTGAGATCGTTAATGGCA	0	15	RDR2-sensitive
AGCTATCTGGACCCGTCGGACAGC	0	9	RDR2-sensitive
AGCTATCTGGTCGCGGACTCGGCC	8	56	RDR2-sensitive
AGCTATCTGGTTGCGGACTCGGCC	0	13	RDR2-sensitive
AGCTATCTGTTCGCGGACTCGGCC	12	77	RDR2-sensitive
AGCTATCTGTTCGTAGACTCGGCC	0	10	RDR2-sensitive
AGCTATCTGTTCGTGGACTCGGCC	0	9	RDR2-sensitive
AGCTATGATGCGGGCTATGAGGCT	0	17	RDR2-sensitive
AGCTATTCGGACTACTGCAGATGT	0	9	RDR2-sensitive
AGCTATTCGGACTGTTAGAGGTGT	0	12	RDR2-sensitive
AGCTATTGGGCTACGCTCTCGGAC	0	13	RDR2-sensitive
AGCTATTGTCGACTGCTGACGGTG	0	15	RDR2-sensitive
AGCTATTTGGGCACGTCGGGCGGC	0	10	RDR2-sensitive
AGCTATTTTAGCTGCTCCGTTGGA	1	12	RDR2-sensitive
AGCTATTTTCGTCGGTCGGCCCAC	1	20	RDR2-sensitive
AGCTCAAGCGGTCTGTCGGTCGTC	16	0	RDR2-resistant
AGCTCAATCAAGTCGTCGTTGTAC	27	2	RDR2-resistant
AGCTCACGTCCCGCGGATCGGCTC	91	3	RDR2-resistant
AGCTCACTGTAAGATAAAAGGCAC	0	11	RDR2-sensitive
AGCTCAGATTATTTGGACTCGGCT	7	34	RDR2-sensitive
AGCTCAGCCGGATACGGGTAGGAT	0	12	RDR2-sensitive
AGCTCAGCCTGATCCGGATAGCGT	1	31	RDR2-sensitive
AGCTCAGCGAACTGTACATCGGCA	0	10	RDR2-sensitive
AGCTCAGCGGACAGAGGAGAAGCA	0	10	RDR2-sensitive
AGCTCAGGACCTTGACGACGGGCA	0	15	RDR2-sensitive
AGCTCAGGTTGCATGGACAGACGC	0	20	RDR2-sensitive
AGCTCATCCGGACCAGGCCGGCAC	0	21	RDR2-sensitive
AGCTCATCGGTTCGTAGACGTGGC	0	9	RDR2-sensitive
AGCTCATTGGACTGGCCCGGGCAC	0	46	RDR2-sensitive
AGCTCCAACAGCCAGGACGTCGGT	1	30	RDR2-sensitive
AGCTCCACCGGACGCCGAAGACGC	0	24	RDR2-sensitive
AGCTCCAGCCAGGAGATCGATCGG	0	25	RDR2-sensitive
AGCTCCCCGGATGGTAGCTTCGGC	1	18	RDR2-sensitive
AGCTCCCTGGATCGTAGCTTCGGC	0	11	RDR2-sensitive
AGCTCCCTGGATCGTAGCTTCGGT	0	11	RDR2-sensitive
AGCTCCCTGGATTGTAGCTTCGGC	0	24	RDR2-sensitive
AGCTCCCTGGATTGTAGCTTCGGT	1	48	RDR2-sensitive
AGCTCCGGACATATAGAGTCGGTA	0	17	RDR2-sensitive
AGCTCCGTCGGCTACGTATCACCA	18	0	RDR2-resistant
AGCTCCTGCACCAGGTCGCGGACC	5	46	RDR2-sensitive
AGCTCCTGCTTCCAGGTCGCGGAC	0	25	RDR2-sensitive
AGCTCCTGTGGTTGCATCGTCTGG	0	10	RDR2-sensitive
AGCTCCTTGGACTGTCTTGAGGTT	1	23	RDR2-sensitive
AGCTCGAAGGTGGATATGAGACAA	0	14	RDR2-sensitive
AGCTCGAAGGTGGATATGAGACAT	0	14	RDR2-sensitive
AGCTCGACGAACTGTACATCGGCA	4	34	RDR2-sensitive
AGCTCGACGAGAACTGTCAACGGC	0	10	RDR2-sensitive
AGCTCGACTCAGACTCAACTCGGC	0	16	RDR2-sensitive
AGCTCGACTCGGCTCGGACCGGCT	0	33	RDR2-sensitive

AGCTCGACTCGGCTTGGATCGGCT	0	20	RDR2-sensitive
AGCTCGACTCGGTTTAGAACGGCT	6	65	RDR2-sensitive
AGCTCGACTGAGGGTTGAAGACGT	1	12	RDR2-sensitive
AGCTCGAGCTCGACGGACCGGCTT	0	19	RDR2-sensitive
AGCTCGAGCTCGGAGCAACTCGGC	66	1	RDR2-resistant
AGCTCGCTAGGTGACTGAGCCGGT	0	10	RDR2-sensitive
AGCTCGGACAAGCTACAGGTGCAT	0	9	RDR2-sensitive
AGCTCGGATCGGTTTGTAGCGGCT	1	24	RDR2-sensitive
AGCTCGGCACTACAGATCACGGTG	0	50	RDR2-sensitive
AGCTCGGCGAACTGTACATCGGCA	1	14	RDR2-sensitive
AGCTCGGCGACTGTACATCGGCAA	0	9	RDR2-sensitive
AGCTCGGCTCGGATCGGATCGGCT	0	9	RDR2-sensitive
AGCTCGGCTCGGCTCGACTCGGCT	3	94	RDR2-sensitive
AGCTCGGCTCGGCTCGGATCGGCT	7	150	RDR2-sensitive
AGCTCGGCTCGGCTTGGATCGGTT	1	15	RDR2-sensitive
AGCTCGGCTGTATATTGGGTGACA	0	23	RDR2-sensitive
AGCTCGGCTTGACTCGGACCGGCT	0	21	RDR2-sensitive
AGCTCGGCTTGGGCTCGTTCGGAG	0	10	RDR2-sensitive
AGCTCGGGAGACGGATTGTAACGG	0	9	RDR2-sensitive
AGCTCGGGATACGGATTGTAACGG	0	15	RDR2-sensitive
AGCTCGGGGTAGGGTGATGATGGC	8	36	RDR2-sensitive
AGCTCGGTACCACGTCGGCGTCAC	0	9	RDR2-sensitive
AGCTCGTCAGAACTACAAGCCGAG	0	14	RDR2-sensitive
AGCTCGTCTCGACTTGAATCGGCT	0	15	RDR2-sensitive
AGCTCGTCTGTGGACTGCGGCTGC	0	14	RDR2-sensitive
AGCTCGTGGACGACAGCGCGGCAG	0	22	RDR2-sensitive
AGCTCGTTTCTGTGGAGCCAGGCT	1	41	RDR2-sensitive
AGCTCTACCCCAGGTTGTGGACTG	0	12	RDR2-sensitive
AGCTCTACTTAGATCGTTCTCATC	49	4	RDR2-resistant
AGCTCTAGACATGTCAGACTGCAC	0	18	RDR2-sensitive
AGCTCTAGATCCTGATCCTCGTGA	4	32	RDR2-sensitive
AGCTCTAGTATTTGTCAATCGGTT	15	0	RDR2-resistant
AGCTCTCCGGATGGTAGCTTCGGC	0	20	RDR2-sensitive
AGCTCTCGCTCGGGTAGACGGTTT	0	9	RDR2-sensitive
AGCTCTCGGGAACCTGAAGGCGCT	4	18	RDR2-sensitive
AGCTCTCGTGTTTGTGTCGTCTGG	0	11	RDR2-sensitive
AGCTCTCTCTGTATCATGGACGGT	1	12	RDR2-sensitive
AGCTCTCTGCAGGTACGACGGACG	0	9	RDR2-sensitive
AGCTCTCTGCGGCAGTGCGGACGG	0	18	RDR2-sensitive
AGCTCTGAATGTAGCGGTGGCGTC	0	11	RDR2-sensitive
AGCTCTGACAGTAGACGTTGCAGA	0	13	RDR2-sensitive
AGCTCTGACGCAGGCACAACACAC	0	14	RDR2-sensitive
AGCTCTGACGTGGCCGCAACGGCT	0	14	RDR2-sensitive
AGCTCTGCGGACGGTGATTTTAGA	0	27	RDR2-sensitive
AGCTCTGGACATGGCAGACTGCAC	0	38	RDR2-sensitive
AGCTCTGGACATGTCAGACTGCAC	2	104	RDR2-sensitive
AGCTCTGGGACGGCCGCTGACGGC	2	22	RDR2-sensitive
AGCTCTGGTTAAACTCTTGATGGA	0	9	RDR2-sensitive
AGCTCTGTAGAGGAAGGCCAACAT	0	9	RDR2-sensitive

AGCTCTGTAGGCGCTACGCGACAA	0	17	RDR2-sensitive
AGCTCTGTGGAATGCACGTCAATC	26	3	RDR2-resistant
AGCTCTGTGTTTGATTGTCTGCAT	1	38	RDR2-sensitive
AGCTCTTCAAGTAGATTATTTGTT	59	0	RDR2-resistant
AGCTCTTCGGATCTGGCATATTTT	28	0	RDR2-resistant
AGCTCTTCGGATTTGGCGTATTTT	0	9	RDR2-sensitive
AGCTCTTGTAGGTGTAGTCGTCTT	27	2	RDR2-resistant
AGCTCTTTCGGTTGTAGCTTCGGC	4	64	RDR2-sensitive
AGCTCTTTGGATCGTAGCTTCGGC	0	9	RDR2-sensitive
AGCTGAACGTATGGATGGCTGTGC	0	100	RDR2-sensitive
AGCTGAACTGATAAGCTGCTGCAG	6	62	RDR2-sensitive
AGCTGAAGATCTGCTACTGTATAT	17	1	RDR2-resistant
AGCTGAAGGAGAAGGGTCACTCGG	0	20	RDR2-sensitive
AGCTGAAGGATGGACGATCACGGT	43	0	RDR2-resistant
AGCTGAATTATGGAGACTCAGGCT	0	9	RDR2-sensitive
AGCTGACATCCTAGCTCGGTTCGT	7	43	RDR2-sensitive
AGCTGACATGAGGCAGACTGACAC	1	17	RDR2-sensitive
AGCTGACCATGAACCGAGCGAGAC	0	18	RDR2-sensitive
AGCTGACGACGTACTGCAGGCTGC	0	9	RDR2-sensitive
AGCTGACGAGAGGAGATGGACAAT	0	9	RDR2-sensitive
AGCTGACGCCTACGTGGATCTGAT	0	10	RDR2-sensitive
AGCTGACGGACTAGAGCAGTCGGG	0	14	RDR2-sensitive
AGCTGACGGCCGTTAGTCTTTGCC	11	65	RDR2-sensitive
AGCTGACGGCCGTTATCTTTGCTG	0	12	RDR2-sensitive
AGCTGACGGCTGTCAGTCTTTGCC	0	12	RDR2-sensitive
AGCTGACGGGCAGGATGAAGCAAA	0	21	RDR2-sensitive
AGCTGACGGGGCAATGAAGAACAA	0	12	RDR2-sensitive
AGCTGACGTGGACAAGAGAGAGGT	0	12	RDR2-sensitive
AGCTGACTCTGGCGAAATGACTGC	0	10	RDR2-sensitive
AGCTGACTGAAAAGCTGAGCGTTT	0	9	RDR2-sensitive
AGCTGACTGAAAAGCTGAGGCGTT	0	10	RDR2-sensitive
AGCTGACTGAGAGACGCCACCTAG	0	31	RDR2-sensitive
AGCTGACTGCATGCGCTCGCGGCT	1	38	RDR2-sensitive
AGCTGACTGGAAGACGCCACCTAG	0	13	RDR2-sensitive
AGCTGACTGGAAGACGCCATCTAG	0	9	RDR2-sensitive
AGCTGACTGGAAGATGACACCTAG	0	21	RDR2-sensitive
AGCTGACTGGGAGACGCCACCTAG	1	26	RDR2-sensitive
AGCTGACTGGGAGACGCCACTTAG	0	10	RDR2-sensitive
AGCTGACTGTGAGACGACACCAAG	0	11	RDR2-sensitive
AGCTGACTGTGCAGAGAAGCTGGT	0	9	RDR2-sensitive
AGCTGACTGTGGAGAAAAGTTGGC	0	9	RDR2-sensitive
AGCTGACTGTGGGGAAAAGCTGGC	0	11	RDR2-sensitive
AGCTGACTGTGGGGAAAAGGTGGC	0	18	RDR2-sensitive
AGCTGACTGTGGGGAGAAGCTGGT	1	119	RDR2-sensitive
AGCTGAGAATTAGGCGTCTTGAGG	0	10	RDR2-sensitive
AGCTGAGACTGGTCTGATGGCGTC	0	25	RDR2-sensitive
AGCTGAGAGCAGACTACCTGGCAC	0	10	RDR2-sensitive
AGCTGAGCGTTTGGCAGTCCGCAG	0	31	RDR2-sensitive
AGCTGAGGCGGCTTGTAGGACGGA	1	20	RDR2-sensitive

AGCTGATAGGAAGAGGTGGACAAT	0	23	RDR2-sensitive
AGCTGATAGGACTAGGCACACGGA	0	9	RDR2-sensitive
AGCTGATCGTTGGCTAGACGTGCG	0	16	RDR2-sensitive
AGCTGATGTGGCTTAGCTCGGCGC	1	12	RDR2-sensitive
AGCTGATGTGGGACGCCAGGCATT	0	23	RDR2-sensitive
AGCTGATTCCGGTAGTAGAGGGCC	0	9	RDR2-sensitive
AGCTGCAACAGTGTCGGCTTGCAG	0	10	RDR2-sensitive
AGCTGCACGGTACTCCAAGTATAA	2	21	RDR2-sensitive
AGCTGCAGAACTGCTTTCCAACGG	0	21	RDR2-sensitive
AGCTGCAGACTGGAACGATGTCGG	0	23	RDR2-sensitive
AGCTGCAGAGACTGATGGGCGCAA	0	50	RDR2-sensitive
AGCTGCAGCAGTCCGAACGGCTTT	1	34	RDR2-sensitive
AGCTGCCAGAAGCCGAAACAAACA	0	18	RDR2-sensitive
AGCTGCCAGGGCTGACTCATGGAC	0	9	RDR2-sensitive
AGCTGCCATCGAAGGACGGGCGAC	0	9	RDR2-sensitive
AGCTGCCTTGTAGAAGCTAGGCCC	0	26	RDR2-sensitive
AGCTGCGTCAGAATGTGTGGCATC	1	17	RDR2-sensitive
AGCTGCGTGGGACTTGGCGATAAC	0	17	RDR2-sensitive
AGCTGCGTTGAGACGGACAATCAC	0	11	RDR2-sensitive
AGCTGCTAGAGCCGAGGGAGACGT	0	29	RDR2-sensitive
AGCTGCTCCGTTGGATACGACGAA	0	10	RDR2-sensitive
AGCTGCTCGGAGGTAGTCGGCGCC	1	15	RDR2-sensitive
AGCTGCTCTGTTGGAAGAAGGATT	0	17	RDR2-sensitive
AGCTGCTGACGGACCAGAGGACGA	0	18	RDR2-sensitive
AGCTGCTGGCCATGGTGAGGATGA	0	9	RDR2-sensitive
AGCTGCTGTAGATCGGTCGGTGTA	2	15	RDR2-sensitive
AGCTGCTTGTCGGACGTCGGCCAC	0	10	RDR2-sensitive
AGCTGCTTTGTGCGGATCTGTTGG	1	33	RDR2-sensitive
AGCTGGAACGGAGCGGCTCTGTTT	0	10	RDR2-sensitive
AGCTGGACAGCAGGATATGATGCT	0	9	RDR2-sensitive
AGCTGGACAGTGTGATGGCGTCGT	0	9	RDR2-sensitive
AGCTGGACGCAGGGGAGATGGACC	0	11	RDR2-sensitive
AGCTGGACGGCCTGGTACATGTGT	0	12	RDR2-sensitive
AGCTGGAGACTGCACAGTCTGCGT	0	13	RDR2-sensitive
AGCTGGAGATGAACATCGACACAT	0	20	RDR2-sensitive
AGCTGGAGCAGAGTAGGACGAGGC	0	18	RDR2-sensitive
AGCTGGATCGTATGGCTGCTGGCT	15	0	RDR2-resistant
AGCTGGCAGAACGTTGGGTGTCGC	0	12	RDR2-sensitive
AGCTGGCGAATCTGACTGGCAGTA	21	0	RDR2-resistant
AGCTGGCTCGGGCTCGGATTGGCT	0	10	RDR2-sensitive
AGCTGGCTGCGAGCATACAGTTCA	0	16	RDR2-sensitive
AGCTGGCTGGTAGAGAGGACGCAC	0	20	RDR2-sensitive
AGCTGGCTGTAGAAAAAAGCTGGC	0	10	RDR2-sensitive
AGCTGGCTGTAGAGAAAAGCTGGC	0	18	RDR2-sensitive
AGCTGGCTGTAGGGAGAAGCTGGT	0	34	RDR2-sensitive
AGCTGGCTGTAGTGAGAAGCTGGT	0	17	RDR2-sensitive
AGCTGGCTGTGAGGAGAAGCTGGT	0	11	RDR2-sensitive
AGCTGGCTGTGCAGAGAAGCTGGT	0	19	RDR2-sensitive
AGCTGGCTGTGCAGAGAAGTTGGT	0	10	RDR2-sensitive

AGCTGGCTGTGGAGAGAAGCTGGT	0	17	RDR2-sensitive
AGCTGGCTGTGGGGAAAAGCTGGC	0	42	RDR2-sensitive
AGCTGGGAAAGGCGATCGTTCGGC	0	10	RDR2-sensitive
AGCTGGGACAGGCAGGACAAAGAC	0	17	RDR2-sensitive
AGCTGGGACTGGGAGAGGCAAAGC	0	24	RDR2-sensitive
AGCTGGGAGGAATGAACAGAGGAT	0	15	RDR2-sensitive
AGCTGGGCTGAGACACGGCACTAT	0	10	RDR2-sensitive
AGCTGGGGATGAGCATCGACGCAT	0	16	RDR2-sensitive
AGCTGGGGGAACGAAGACGGGCCT	0	9	RDR2-sensitive
AGCTGGGTCGACGATGTCAGAAGA	0	17	RDR2-sensitive
AGCTGGTGCTCGGCAAAATAGCTA	43	4	RDR2-resistant
AGCTGGTGGTTAGAATCTAGGTGT	0	13	RDR2-sensitive
AGCTGGTGGTTAGAATTTAGGTGT	18	75	RDR2-sensitive
AGCTGGTTGGAAACGGAGCGGCTC	0	9	RDR2-sensitive
AGCTGTAACGTTGGCTCTTGACGG	0	30	RDR2-sensitive
AGCTGTAAGAAGCTCGTCGTGGAC	0	11	RDR2-sensitive
AGCTGTACATCACGCTGGTCTAGG	0	9	RDR2-sensitive
AGCTGTACGGAGGCAGAACGCGGC	0	22	RDR2-sensitive
AGCTGTACGGGCATCAACGCTATA	0	13	RDR2-sensitive
AGCTGTACGTGAAGTCGGACACAG	0	11	RDR2-sensitive
AGCTGTAGACAGCCGCTAACGGAC	0	12	RDR2-sensitive
AGCTGTAGACGACCACTAACGGCC	0	10	RDR2-sensitive
AGCTGTAGACGGCCGCTAACGACC	3	22	RDR2-sensitive
AGCTGTAGACGGCTGCTAACGGCC	2	23	RDR2-sensitive
AGCTGTAGAGCACTCGGCAAAGAT	0	15	RDR2-sensitive
AGCTGTAGCGACGGAGAGGAGCAT	3	16	RDR2-sensitive
AGCTGTAGCTCATAGGCATGTAGC	0	18	RDR2-sensitive
AGCTGTAGGCATACCCTTTCGCAC	0	22	RDR2-sensitive
AGCTGTATCTCGACCGAACCCGGC	0	13	RDR2-sensitive
AGCTGTATGACTACAAGATGGTTG	4	47	RDR2-sensitive
AGCTGTCAACTAGCACTCGTCGTC	0	12	RDR2-sensitive
AGCTGTCGAGTGTGGGCTAGGCCG	0	10	RDR2-sensitive
AGCTGTCGATTGTGGGCTAGGCCG	0	15	RDR2-sensitive
AGCTGTCGCGTGGGTTAGTTGGGC	3	24	RDR2-sensitive
AGCTGTCGGCACTCGGCAAAGAGC	0	12	RDR2-sensitive
AGCTGTCGTGCAAGCGAGTTGGGC	0	9	RDR2-sensitive
AGCTGTCTGGATTGTGCCGGCCAA	0	18	RDR2-sensitive
AGCTGTCTGGATTGTGCCGGTCAA	0	18	RDR2-sensitive
AGCTGTCTGGGCACGTCGGGCGGC	2	54	RDR2-sensitive
AGCTGTCTGGGCACGTCGGGCGGG	0	15	RDR2-sensitive
AGCTGTGACTGTGGCAGATCGGCC	0	10	RDR2-sensitive
AGCTGTGATGAGGTCAGGACACAT	0	10	RDR2-sensitive
AGCTGTGCTGGATCTGAATGGCTA	2	197	RDR2-sensitive
AGCTGTGGACCGTGCCGTCGTCGG	0	19	RDR2-sensitive
AGCTGTGGACGACTACACTACCTT	0	11	RDR2-sensitive
AGCTGTGGACGCCTACTTTGCTAT	0	18	RDR2-sensitive
AGCTGTGGAGCACAACGACGGAGT	0	16	RDR2-sensitive
AGCTGTGGATGTAGATCTAGCGGC	0	16	RDR2-sensitive
AGCTGTGGGGCTCACCAGTCGACC	0	12	RDR2-sensitive

AGCTGTGTTACCTCGGCGCCACAG	0	9	RDR2-sensitive
AGCTGTTCAGACTGCTGCAGGTGT	0	12	RDR2-sensitive
AGCTGTTCGAACCTGGTTTGGCTG	0	12	RDR2-sensitive
AGCTGTTCGGATAACGGGTCACGG	0	13	RDR2-sensitive
AGCTGTTCTCTGAGTCGTTGGGAA	0	9	RDR2-sensitive
AGCTGTTGAACTACAAGACGGTGT	1	27	RDR2-sensitive
AGCTGTTGAGGCAGATCCGATCGG	1	18	RDR2-sensitive
AGCTGTTGGTTAGAATCTAGGTGT	1	12	RDR2-sensitive
AGCTGTTGTATTGTGGAAGGGCGT	0	50	RDR2-sensitive
AGCTGTTGTCGGCTACTGACGGTA	5	22	RDR2-sensitive
AGCTGTTTGGACTGCTGCAGCTGC	6	112	RDR2-sensitive
AGCTGTTTGGATTGCTGCAGCTGC	6	122	RDR2-sensitive
AGCTGTTTGGATTGCTGCGGCTGC	1	99	RDR2-sensitive
AGCTGTTTGGCAGAACTTCGGCTT	1	45	RDR2-sensitive
AGCTGTTTTTTCGATCGTTTCGAC	0	11	RDR2-sensitive
AGCTTATGTCCGACGGTTCCCTAG	0	9	RDR2-sensitive
AGCTTATGTTTGGTTGTCTGCACA	0	12	RDR2-sensitive
AGCTTATTTTCGGCGGACAAGCCC	0	10	RDR2-sensitive
AGCTTCAATTCTGCTAACTCGGTA	20	1	RDR2-resistant
AGCTTCAGAGCGGCACAGTAGCAT	0	11	RDR2-sensitive
AGCTTCAGGAGGCACCGGTCGGGC	2	16	RDR2-sensitive
AGCTTCAGTGCTGTTTGATTCCGG	21	0	RDR2-resistant
AGCTTCGCGGACTTTTAGATGGCC	0	9	RDR2-sensitive
AGCTTCTTAGAGGGACTATGGCCG	93	2	RDR2-resistant
AGCTTGACTCTAGTCCGACTTTGT	38	2	RDR2-resistant
AGCTTGAGGTTGACTGATGAGGAC	0	20	RDR2-sensitive
AGCTTGCACTGTGTCGAATGGATA	0	12	RDR2-sensitive
AGCTTGCGACAGAGCGATGATGGC	0	10	RDR2-sensitive
AGCTTGCGACTGAGCGATGATGGT	0	21	RDR2-sensitive
AGCTTGCGGAGTGGACTGCGCATG	0	18	RDR2-sensitive
AGCTTGGACTGCGGCGTAGGCCTA	1	62	RDR2-sensitive
AGCTTGGAGGGCGGTTGAGGACGG	2	14	RDR2-sensitive
AGCTTGGATTCTGACTTAGAGGCG	14	0	RDR2-resistant
AGCTTGGGGTAGGGTAATGATGGC	3	30	RDR2-sensitive
AGCTTGGGTTGCGGCACGTGCACT	20	0	RDR2-resistant
AGCTTGGTCGACTGTTGAAGTGCA	1	29	RDR2-sensitive
AGCTTGGTGCGACTCTGTTCTGGC	0	10	RDR2-sensitive
AGCTTGTAGACATAATAATTTAAA	86	1	RDR2-resistant
AGCTTGTCGACGGTTAGAGTGCAC	0	9	RDR2-sensitive
AGCTTGTCGAGCGGATGGACGTCA	8	51	RDR2-sensitive
AGCTTGTCGAGCGGATGTGACGTC	0	11	RDR2-sensitive
AGCTTGTCGAGCGGATTGACGTCA	3	34	RDR2-sensitive
AGCTTGTCGCGTAGGACAGTTCGG	1	24	RDR2-sensitive
AGCTTGTGATTGTACGTCCGGTGT	0	10	RDR2-sensitive
AGCTTGTTCTGAACATCAAGCCGG	0	11	RDR2-sensitive
AGCTTTACTGTTAGAGCAGGGTGT	101	0	RDR2-resistant
AGCTTTAGAACGACCTCTGATGGC	0	16	RDR2-sensitive
AGCTTTAGAACGGCCGCTGACGGT	0	13	RDR2-sensitive
AGCTTTAGGACGGCCGCTGACGGC	2	47	RDR2-sensitive

AGCTTTAGGCTTAATCGTGTCGGG	0	21	RDR2-sensitive
AGCTTTCGGCAAAGGGACTGGCAA	0	29	RDR2-sensitive
AGCTTTCGGCCCGTCGGACCGCGG	1	12	RDR2-sensitive
AGCTTTCTGGGTTGTAGCTTCGGC	0	9	RDR2-sensitive
AGCTTTGCATCGAGCGGAGACGTT	0	11	RDR2-sensitive
AGCTTTGGACGTTCATGCGCTCGT	0	20	RDR2-sensitive
AGCTTTGGGACGGCCGCTGACGGC	6	44	RDR2-sensitive
AGCTTTTGCAGTCGTCTGGTCACA	0	44	RDR2-sensitive
AGCTTTTTCTCGGGCACTCGGCAA	0	10	RDR2-sensitive
AGCTTTTTCTTCGATCTTCGGCAC	29	0	RDR2-resistant
AGGAAAAAGACTCGACGCGGGAGT	0	12	RDR2-sensitive
AGGAAACAATGGGACTGAGGGCAT	0	32	RDR2-sensitive
AGGAAACACTGTGTAAGGGGACGC	0	14	RDR2-sensitive
AGGAAACCTGGGAACCGGCAAAGA	3	92	RDR2-sensitive
AGGAAACCTGGGAACCGGCAAAGC	0	49	RDR2-sensitive
AGGAAACCTGGGAACCGGTAAAGA	1	14	RDR2-sensitive
AGGAAACCTGGGAACCGGTAAAGC	1	86	RDR2-sensitive
AGGAAACGTGGGAGAGGTGGCTGG	0	13	RDR2-sensitive
AGGAAACTAGGCACGGCGGGCTAG	2	27	RDR2-sensitive
AGGAAACTCTCTGGGTCGCCCGAC	1	32	RDR2-sensitive
AGGAAACTCTGCGCGGCTGGGGTT	0	9	RDR2-sensitive
AGGAAACTGTAGCCCCTATACGGT	0	18	RDR2-sensitive
AGGAAACTTATAACTTTTGATGGC	0	18	RDR2-sensitive
AGGAAAGCTAGTGGAGGACTACCT	0	19	RDR2-sensitive
AGGAAATATTCTCAACAACTGATC	32	0	RDR2-resistant
AGGAAATCGACGACTGGCACAAAA	0	9	RDR2-sensitive
AGGAAATCTGTGACGGCAGGGACT	62	0	RDR2-resistant
AGGAAATTAGGCACGGTGGGCTAG	0	33	RDR2-sensitive
AGGAACAAGCAGCTCCGACTCCGC	1	31	RDR2-sensitive
AGGAACAATGGACGTGGACGTCTT	0	11	RDR2-sensitive
AGGAACACGACGGTCAGTCGGCGA	0	13	RDR2-sensitive
AGGAACACGCTAGATGAAGGGCGC	0	15	RDR2-sensitive
AGGAACAGCGGACAGAACAGGCTA	0	10	RDR2-sensitive
AGGAACAGTAGACCAAGGCAGATT	0	34	RDR2-sensitive
AGGAACAGTAGACCCAAGTGGATT	0	9	RDR2-sensitive
AGGAACAGTAGACCCAGGCAGATT	0	14	RDR2-sensitive
AGGAACAGTAGACCCAGGCGGATT	5	93	RDR2-sensitive
AGGAACAGTAGACCCAGGTGGATT	3	21	RDR2-sensitive
AGGAACAGTGGTAGGAGGAGAGAG	0	16	RDR2-sensitive
AGGAACAGTGGTAGGGGGAGAGCG	0	22	RDR2-sensitive
AGGAACAGTGGTAGGGGGGAGAGC	0	12	RDR2-sensitive
AGGAACATGATGGTCAGTCGGCGA	0	14	RDR2-sensitive
AGGAACCACACGGAACGACGGAAC	0	10	RDR2-sensitive
AGGAACCAGGACCTAGCAGACCAT	0	23	RDR2-sensitive
AGGAACCAGGACCTAGCAGGCCAT	0	11	RDR2-sensitive
AGGAACCAGGATCCAGCAGGCCAT	0	17	RDR2-sensitive
AGGAACCAGGCTCGGCTCGGCTCA	0	10	RDR2-sensitive
AGGAACCCTAACTGATTCTGCGCA	7	23	RDR2-sensitive
AGGAACCCTGTGGTGCGGTCGGAT	0	19	RDR2-sensitive

AGGAACGCACCTGTCTGACGTCTG	0	11	RDR2-sensitive
AGGAACGCTGGAACACTGGGACGA	0	56	RDR2-sensitive
AGGAACGGCCCAGGACATGTGCGA	0	12	RDR2-sensitive
AGGAACGGCCCTGAATGCGGTCAA	0	14	RDR2-sensitive
AGGAACGGCCCTGCATGGATTCTC	0	30	RDR2-sensitive
AGGAACGGGAACGAGGAACACGGT	0	9	RDR2-sensitive
AGGAACGGGTAGAGGCAAATACAA	1	14	RDR2-sensitive
AGGAACGTCGACGACAGGAGCAGC	0	19	RDR2-sensitive
AGGAACGTGCCCGTGCGATGCCAC	0	12	RDR2-sensitive
AGGAACTAGAGTGCAGGGACATAA	1	45	RDR2-sensitive
AGGAACTCGCAGGAGAAGCCCCTG	2	26	RDR2-sensitive
AGGAACTCTAGAATGCTCGCGGTG	0	13	RDR2-sensitive
AGGAACTCTATCATCAGCTTGGGA	30	2	RDR2-resistant
AGGAACTGAGGACCTGCGGTACGG	0	14	RDR2-sensitive
AGGAACTGATGAAGTGATGGGCCG	0	9	RDR2-sensitive
AGGAACTGTAAATTTTTACGGCTC	1	80	RDR2-sensitive
AGGAACTGTAAATTTTTACGGCTT	0	16	RDR2-sensitive
AGGAACTGTTGCCTGGTTGGGTAT	14	0	RDR2-resistant
AGGAACTTTGGATGCAAACATGGA	37	0	RDR2-resistant
AGGAAGACGACTCTACACCATCGT	0	9	RDR2-sensitive
AGGAAGACGGGAGTAGAGGACCTC	0	29	RDR2-sensitive
AGGAAGACTGGCAGCGGCACTGAA	0	12	RDR2-sensitive
AGGAAGAGCACGCAGCAGCGCCAT	0	9	RDR2-sensitive
AGGAAGATGGAGCGGACACAACAC	0	12	RDR2-sensitive
AGGAAGATGGTAGACCAGCGCACT	2	14	RDR2-sensitive
AGGAAGCACCAGACACTATCATTT	63	4	RDR2-resistant
AGGAAGCAGACGAACAGAGAGCGC	0	12	RDR2-sensitive
AGGAAGGAGAAGTCGTAACAAGGT	24	1	RDR2-resistant
AGGAAGTCTGTAAGGTCAGGGGAA	0	11	RDR2-sensitive
AGGAAGTTAGTCAGCTGACGGCGT	0	13	RDR2-sensitive
AGGAATAAGCTCGGCGAGGATAGC	29	4	RDR2-resistant
AGGAATCGTTCCGGACTACGGTTT	1	27	RDR2-sensitive
AGGAATCTGGGCGGCTGCTGGCGC	0	15	RDR2-sensitive
AGGAATGAGGTGGTGATGGATCAA	0	10	RDR2-sensitive
AGGAATGATGGCAGAACACGGGAA	0	12	RDR2-sensitive
AGGAATGATGGCAGAACATGGAAT	0	9	RDR2-sensitive
AGGAATGGCAGTAGGTCGGATCCG	0	16	RDR2-sensitive
AGGAATGGCAGTGAGGCTTGACGG	0	12	RDR2-sensitive
AGGAATGGTAGCAGTATCGACGTT	0	19	RDR2-sensitive
AGGAATGGTGGCAGAACACGAGAA	0	14	RDR2-sensitive
AGGAATGGTGGCAGAACACGGAAT	0	25	RDR2-sensitive
AGGAATGTAGCGTTAGCACGGGTT	4	21	RDR2-sensitive
AGGAATGTAGGATAGCAACGGTTT	0	12	RDR2-sensitive
AGGAATGTCTAGTAAGCGCGAGTC	30	3	RDR2-resistant
AGGAATGTGTGCAACGGCAGCTGT	22	0	RDR2-resistant
AGGAATTACGTAATCCCCGTCGGC	0	11	RDR2-sensitive
AGGAATTCGTAGCTCGGCGCCAAG	4	22	RDR2-sensitive
AGGAATTGTCGAATCAAAACGGCC	0	11	RDR2-sensitive
AGGAATTTCAGGCATTTAATCATC	32	4	RDR2-resistant

AGGACAAAGCCGGGTGCATCTCAT	3	20	RDR2-sensitive
AGGACAAATCGACGCGACGAGCAG	0	18	RDR2-sensitive
AGGACAAATGTAGGTGGGCCTAAT	0	11	RDR2-sensitive
AGGACAACACGAAGGCCGCGGGTT	0	14	RDR2-sensitive
AGGACAACACGAGCCGCGGTTCCC	1	12	RDR2-sensitive
AGGACAACAGGACTGCAGAAGAAT	0	23	RDR2-sensitive
AGGACAACAGTAGCAGCACAACAG	0	23	RDR2-sensitive
AGGACAACGGACAGAAACGGTCGC	2	84	RDR2-sensitive
AGGACAACGTCAGCAGGGACTCGA	2	29	RDR2-sensitive
AGGACAACTGTAGGAGGGCCTAAT	0	20	RDR2-sensitive
AGGACAACTGTAGGTGGGCCTAAT	22	643	RDR2-sensitive
AGGACAACTGTAGGTGGGCCTACT	0	9	RDR2-sensitive
AGGACAACTGTAGGTGGGTCTAAT	2	33	RDR2-sensitive
AGGACAAGAATTCATGGGAGGCAA	0	15	RDR2-sensitive
AGGACAAGAATTTAGGGGGGCCAA	0	9	RDR2-sensitive
AGGACAAGCGCGCTACAACAACAC	0	12	RDR2-sensitive
AGGACAAGGCCTTGATCGGACGTT	0	10	RDR2-sensitive
AGGACAAGGGACGGAGGCGCTAGG	1	15	RDR2-sensitive
AGGACAATGAGACTCGGAGGCAGA	115	6	RDR2-resistant
AGGACACAACGAAGACATGGGCAT	0	13	RDR2-sensitive
AGGACACAACTCTGGCCCAGGCAC	0	14	RDR2-sensitive
AGGACACACTCAGGCGGCTCAGCT	0	18	RDR2-sensitive
AGGACACAGAGACGTCACGAACTC	0	9	RDR2-sensitive
AGGACACCAGAAGACGTCTCTGTC	1	41	RDR2-sensitive
AGGACACCTCGCTGGCGCTTTTGG	22	0	RDR2-resistant
AGGACACCTGGGAACCGGTAAAGC	0	12	RDR2-sensitive
AGGACACGAATCCAGTGAGGGCTC	0	11	RDR2-sensitive
AGGACACGACACCGAGGCGGGCAC	0	15	RDR2-sensitive
AGGACACGACGAAGACCTAGGCAT	0	17	RDR2-sensitive
AGGACACGACGAAGACCTGGGCAT	1	23	RDR2-sensitive
AGGACACGACGAAGACTCAGGCAT	1	12	RDR2-sensitive
AGGACACGAGAACAGTGAGGCTGC	0	15	RDR2-sensitive
AGGACACGCGGCGACAATTCACTC	0	11	RDR2-sensitive
AGGACACGGCACGGCTAGACACAG	0	18	RDR2-sensitive
AGGACACGGGTATGACGGATTTTG	0	31	RDR2-sensitive
AGGACACGTGTGGAGTATCGGCAC	0	17	RDR2-sensitive
AGGACACTACAGGCAGGTGAACAT	0	15	RDR2-sensitive
AGGACACTAGGAGACGTCCCTGTC	0	9	RDR2-sensitive
AGGACACTCCGCACAGTAAGACAC	0	20	RDR2-sensitive
AGGACACTCGACACATGAAGACAC	0	9	RDR2-sensitive
AGGACACTCGACACTGGAAGACAC	0	12	RDR2-sensitive
AGGACACTCGGCACAGAAACACAC	0	14	RDR2-sensitive
AGGACACTCGGCACAGAGGAACAC	1	21	RDR2-sensitive
AGGACACTCGGCACAGCGAAGAAC	0	10	RDR2-sensitive
AGGACACTCGGCACAGGAAAACCC	0	9	RDR2-sensitive
AGGACACTCGGCACAGGACCACAC	4	21	RDR2-sensitive
AGGACACTCGGCACAGGACGACAC	0	24	RDR2-sensitive
AGGACACTCGGCATAGATAGACAC	2	72	RDR2-sensitive
AGGACACTCGGCATAGGACCACAC	1	29	RDR2-sensitive

AGGACACTCGGTACAGGACGACAC	1	15	RDR2-sensitive
AGGACACTGGGATTTAGACAGGAT	0	9	RDR2-sensitive
AGGACAGAACGGCAGACTACTCTT	0	15	RDR2-sensitive
AGGACAGAACGGCTCCGTTCTACT	1	46	RDR2-sensitive
AGGACAGAACGGCTCCGTTTTACT	0	11	RDR2-sensitive
AGGACAGACATACTGCTGCTTGCA	0	15	RDR2-sensitive
AGGACAGACCCTCAGACGGACCTC	0	33	RDR2-sensitive
AGGACAGAGAAACGCTATAGCCAG	0	20	RDR2-sensitive
AGGACAGAGCATATTCCTTTAGGG	1	16	RDR2-sensitive
AGGACAGAGGAGAGGAGAGAGACG	0	10	RDR2-sensitive
AGGACAGATCGACGCGCAGGAGCC	0	19	RDR2-sensitive
AGGACAGCAGATCACCGTTGGTTT	0	10	RDR2-sensitive
AGGACAGCAGTAGCAGCACAACAG	0	17	RDR2-sensitive
AGGACAGCATAGGGGTGTTGACCA	0	20	RDR2-sensitive
AGGACAGCCAGGACAGGGACGTCT	0	14	RDR2-sensitive
AGGACAGGAACACAGTGGGCTGAC	1	17	RDR2-sensitive
AGGACAGGAGACCGCTCGTGACGA	0	10	RDR2-sensitive
AGGACAGGAGCATTCAATGTGCAT	0	26	RDR2-sensitive
AGGACAGGATATGGCTACATTGTT	19	0	RDR2-resistant
AGGACAGTACTATCCTGCGTTGTC	0	11	RDR2-sensitive
AGGACAGTAGGGGAGTAGACGCAT	0	16	RDR2-sensitive
AGGACAGTCCAGCAGGAACATCAG	0	9	RDR2-sensitive
AGGACAGTTCGGCTAGGCCGGTGA	0	9	RDR2-sensitive
AGGACAGTTGGTTAGGAAGGCTTC	0	11	RDR2-sensitive
AGGACATAAGTTGCACGCGCGCAT	0	9	RDR2-sensitive
AGGACATAATGGTCTTCGGACGAA	73	0	RDR2-resistant
AGGACATCAGGCCTGCGACGACGT	0	18	RDR2-sensitive
AGGACATCCCGACGACGACGCTAC	0	11	RDR2-sensitive
AGGACATCGCAACCCGAGAGCGGA	1	29	RDR2-sensitive
AGGACATCGTAACCCAAGAGCGGA	0	19	RDR2-sensitive
AGGACATCGTAACCCGAGAGCAGA	0	9	RDR2-sensitive
AGGACATCGTAACCCGAGAGCGGA	4	186	RDR2-sensitive
AGGACATCGTAACCTGAGAGCGGA	3	32	RDR2-sensitive
AGGACATCTGTAGGTAGGCCTAAT	0	17	RDR2-sensitive
AGGACATCTGTAGGTGGGCCTAAT	0	15	RDR2-sensitive
AGGACATCTTGTGATCGGTATGGC	61	4	RDR2-resistant
AGGACATGACCATCGCAGTGACGT	0	87	RDR2-sensitive
AGGACATGTACAGTGGAGAGACAC	0	10	RDR2-sensitive
AGGACATGTATAGTGGAGAGACAT	0	13	RDR2-sensitive
AGGACATGTGATCGGTATGGCTAT	0	16	RDR2-sensitive
AGGACATTCGGCATAGAGAGACAC	0	12	RDR2-sensitive
AGGACATTTCCTGAAAGCACAAAG	257	45	RDR2-resistant
AGGACCAACTGAAGCGCATCTCGC	1	13	RDR2-sensitive
AGGACCACACTCGGCAAAGACATG	0	9	RDR2-sensitive
AGGACCACACTCGGCAAAGACTTG	0	15	RDR2-sensitive
AGGACCACTGAACCGGACGTTGGC	3	54	RDR2-sensitive
AGGACCAGACTTAAGGGAGCCCGC	0	14	RDR2-sensitive
AGGACCAGAGCTCTCTTACAGAAG	1	19	RDR2-sensitive
AGGACCAGAGTGAGTGCAGTGAGT	0	9	RDR2-sensitive

AGGACCAGCAGACTGCAGGACCGC	0	9	RDR2-sensitive
AGGACCAGTAGAAGCTACAGCCGA	0	16	RDR2-sensitive
AGGACCATATTCTTGTGCTCTTGG	21	0	RDR2-resistant
AGGACCATCACTACGAAATTCGTT	22	1	RDR2-resistant
AGGACCATGAATCCCCTGGCAGAA	0	9	RDR2-sensitive
AGGACCATGCAAGTCTGATCCTGA	0	19	RDR2-sensitive
AGGACCATTTAGAGGACGCTGCTG	0	48	RDR2-sensitive
AGGACCATTTAGAGGACGTTGCTG	1	16	RDR2-sensitive
AGGACCATTTAGATGACGTTGCTG	0	16	RDR2-sensitive
AGGACCCAACCTGACGCTGTTGAC	0	16	RDR2-sensitive
AGGACCCACAGCAGAACTGCTAGT	0	14	RDR2-sensitive
AGGACCCACGCGAAGTCTAGGCAT	0	28	RDR2-sensitive
AGGACCCCACCTTGATCGACCCAA	0	10	RDR2-sensitive
AGGACCCCATCTCCTAGGCGTCGG	0	28	RDR2-sensitive
AGGACCCCCTCAAGCGACGGACAC	0	11	RDR2-sensitive
AGGACCCCCTCCAGCAACGGACAC	1	19	RDR2-sensitive
AGGACCCTGAGCTCGCTGGAGGCT	0	11	RDR2-sensitive
AGGACCCTTTGATCGATCCGGCAC	0	14	RDR2-sensitive
AGGACCGAACCGAGAAGGTGGCAC	0	10	RDR2-sensitive
AGGACCGAAGAGCAGGCGAGCAGC	0	10	RDR2-sensitive
AGGACCGACACGTAGAGGGCGCTG	0	48	RDR2-sensitive
AGGACCGACACGTAGGAGGCGCTG	0	17	RDR2-sensitive
AGGACCGACACGTAGGGGGCGCTG	0	25	RDR2-sensitive
AGGACCGACGATATTGCATAGCAC	0	20	RDR2-sensitive
AGGACCGACGCGTAGAGGGCGCTG	0	13	RDR2-sensitive
AGGACCGACGCGTAGGAGGCGCTG	0	14	RDR2-sensitive
AGGACCGAGAGATGACGATCGTGC	0	19	RDR2-sensitive
AGGACCGAGGACATCGACGGAGAT	3	23	RDR2-sensitive
AGGACCGCTGAAGACTAGCTCCGC	1	51	RDR2-sensitive
AGGACCGCTGACCTAGAGTAGCAT	0	12	RDR2-sensitive
AGGACCGGATGTAGGGACGTGCAG	0	31	RDR2-sensitive
AGGACCGTCTGAAGAAATGGGCAA	0	12	RDR2-sensitive
AGGACCGTTCAGAGGACGCTGCTG	0	44	RDR2-sensitive
AGGACCGTTTAGAAGACGCTGCTG	1	22	RDR2-sensitive
AGGACCGTTTAGAGGACGCTGCTA	0	18	RDR2-sensitive
AGGACCGTTTAGAGGACGCTGCTG	4	208	RDR2-sensitive
AGGACCGTTTAGAGGACGTTGCTG	16	560	RDR2-sensitive
AGGACCGTTTAGAGGACGTTGTTG	0	20	RDR2-sensitive
AGGACCGTTTAGATGACGCTGCTG	0	12	RDR2-sensitive
AGGACCGTTTAGATGACGTTGCTG	0	12	RDR2-sensitive
AGGACCTAGATGTGCTACTCGGAT	0	10	RDR2-sensitive
AGGACCTCGATGGGTGGAACAGGC	0	17	RDR2-sensitive
AGGACCTCGTCAGGCGTCAGTCGT	0	10	RDR2-sensitive
AGGACCTCGTTAGGCGTCAGTCGT	0	12	RDR2-sensitive
AGGACCTGACGACGACATGAGTAC	0	59	RDR2-sensitive
AGGACCTGAGAAGAGCAAGATCGA	0	16	RDR2-sensitive
AGGACCTGCCTGCAGCATGCTGGA	0	9	RDR2-sensitive
AGGACCTGTCCAAGAGGAGGACCA	0	10	RDR2-sensitive
AGGACCTGTCTGAGCAGAGGCTGA	0	33	RDR2-sensitive

AGGACCTGTTCGTTGGATGACCGC	2	16	RDR2-sensitive
AGGACCTGTTCTATGGATGACCGC	14	123	RDR2-sensitive
AGGACCTTCGGAAGACGAAGACCC	0	9	RDR2-sensitive
AGGACCTTCGGAAGAGGAAGACCC	0	11	RDR2-sensitive
AGGACCTTGTGACCCGATTTGCGG	28	5	RDR2-resistant
AGGACCTTGTTGGAGACGTAGAGG	0	11	RDR2-sensitive
AGGACCTTGTTGGAGGCGTAGAGG	2	18	RDR2-sensitive
AGGACCTTTGGAACCGGCCATTAT	0	9	RDR2-sensitive
AGGACGAAAATTCAGGGGGGCCAA	1	47	RDR2-sensitive
AGGACGAAATTGGGCAGCAGCTTT	0	9	RDR2-sensitive
AGGACGAACCACGCGAGGACGTGA	0	9	RDR2-sensitive
AGGACGAAGCCTGAACCAGACGAA	2	28	RDR2-sensitive
AGGACGAAGCTTGCAGAGAACCGG	0	15	RDR2-sensitive
AGGACGAAGGAAGCATAGGGCTTT	0	9	RDR2-sensitive
AGGACGACACTCGGCAAAGACAAG	0	10	RDR2-sensitive
AGGACGACACTCGGCAAAGACTAG	2	51	RDR2-sensitive
AGGACGACAGAGCAGAGAAGTGGC	0	10	RDR2-sensitive
AGGACGACCCAGTAGAAGGCCCGT	1	112	RDR2-sensitive
AGGACGACGAACGCCATGGCGGGT	0	11	RDR2-sensitive
AGGACGACGAACGTGCAGAGCTTC	2	25	RDR2-sensitive
AGGACGACGCGCTTTTACTCCGGA	0	9	RDR2-sensitive
AGGACGACGCTACTTCTTGCTGCT	47	13	RDR2-resistant
AGGACGACGCTGCGGACGGGCTGC	0	26	RDR2-sensitive
AGGACGACGGTAGGCGTGATCCGC	1	58	RDR2-sensitive
AGGACGACTCATCGGGAAGCCCGC	0	10	RDR2-sensitive
AGGACGACTCGGTAGAAGCCATGT	0	13	RDR2-sensitive
AGGACGAGAATCCACAGGGGCTAA	0	11	RDR2-sensitive
AGGACGAGATTCTTTGTAAGAGGG	78	5	RDR2-resistant
AGGACGAGGACAGGTGTAGGTGGT	0	9	RDR2-sensitive
AGGACGAGGACGACAATCGGGCGT	0	11	RDR2-sensitive
AGGACGAGGACGTCATCTACTCCC	0	16	RDR2-sensitive
AGGACGAGGAGTAGGTTGGTACGT	24	0	RDR2-resistant
AGGACGAGGATGACAATCAGGTGT	0	14	RDR2-sensitive
AGGACGAGGCGCTGGGCAGAGCAG	0	9	RDR2-sensitive
AGGACGATATGCAAGCAGACACAA	0	12	RDR2-sensitive
AGGACGATGAGCAGCAATGGCCAG	0	10	RDR2-sensitive
AGGACGATGCATGAACGGCTTGAT	0	17	RDR2-sensitive
AGGACGCAGACAACGACGACGCAA	0	14	RDR2-sensitive
AGGACGCAGACGCTAGGGTTCGGC	0	21	RDR2-sensitive
AGGACGCAGGACTAGCAGACTTCG	0	13	RDR2-sensitive
AGGACGCCGACGACTGCACGGCAC	55	2	RDR2-resistant
AGGACGCGAGCTGAAGCAGAGCGA	0	9	RDR2-sensitive
AGGACGCGCGAACGAGATAGGCAC	0	19	RDR2-sensitive
AGGACGCGCGGTAAGACTGAGCAT	0	39	RDR2-sensitive
AGGACGCGGCACCAGACGACGCAT	0	12	RDR2-sensitive
AGGACGCGGCTCTAGTCGGATGGA	56	0	RDR2-resistant
AGGACGCGGGTATGACGGATTTTG	0	12	RDR2-sensitive
AGGACGCTATAGAGGCCCTTGCTG	0	9	RDR2-sensitive
AGGACGCTGAATATGGCCATGCTT	17	0	RDR2-resistant

AGGACGCTGCTGAAACGAGTAGAG	0	21	RDR2-sensitive
AGGACGCTGCTGGAGACGGAGAAT	0	15	RDR2-sensitive
AGGACGCTGCTGGAGAGCATAGAG	0	9	RDR2-sensitive
AGGACGCTGCTGGAGGGCGTAGAG	0	22	RDR2-sensitive
AGGACGCTGTCGAGACCACCGGAT	0	55	RDR2-sensitive
AGGACGGAATGGGAATGGCAGAAC	1	27	RDR2-sensitive
AGGACGGACTGAGGGATGACCGGA	0	12	RDR2-sensitive
AGGACGGAGCGAAGACACGGCTGT	0	79	RDR2-sensitive
AGGACGGAGGATATGATGTGTAGT	61	0	RDR2-resistant
AGGACGGATATAGAGGACGTTGCT	0	18	RDR2-sensitive
AGGACGGATATAGAGGATGGATAT	0	17	RDR2-sensitive
AGGACGGCACTCGGCAAAGACTAG	0	10	RDR2-sensitive
AGGACGGCGATTAGGCCTGCTATG	25	2	RDR2-resistant
AGGACGGGACGACGTGGACAGCGG	0	9	RDR2-sensitive
AGGACGGGGAACAGGCTACCATAC	0	10	RDR2-sensitive
AGGACGGTAGAGCAGAGAAGCAGC	0	13	RDR2-sensitive
AGGACGGTATAGAGTGCCTTGCTG	0	33	RDR2-sensitive
AGGACGGTCTGACGTTGACTCGGC	0	15	RDR2-sensitive
AGGACGGTTCATAGCAGACCCTGC	0	9	RDR2-sensitive
AGGACGTAACTCGGGAGCGGGCAA	0	28	RDR2-sensitive
AGGACGTAACTCGGGAGTGGACGG	0	24	RDR2-sensitive
AGGACGTAACTCGTGAGGAGGCAA	0	17	RDR2-sensitive
AGGACGTAGCCCGGGAGCGGACGG	0	15	RDR2-sensitive
AGGACGTAGGCATTGTTCGGTCAT	0	9	RDR2-sensitive
AGGACGTAGGGTGTTACGCATCTC	4	41	RDR2-sensitive
AGGACGTCGACGACTGCACGGCAC	0	25	RDR2-sensitive
AGGACGTGAAGGACGCCGACGACT	28	1	RDR2-resistant
AGGACGTGACACAACAGGACGTAT	1	26	RDR2-sensitive
AGGACGTGATTTAGAGGACGTTGT	1	56	RDR2-sensitive
AGGACGTGCAGATGATGGCGTGAA	0	17	RDR2-sensitive
AGGACGTGCAGTACTGCGGTGCGG	0	9	RDR2-sensitive
AGGACGTGCATACTGGTCTCTCAT	0	9	RDR2-sensitive
AGGACGTGCTTTTCTAATATCTTA	18	1	RDR2-resistant
AGGACGTGGGACTAGCAGACTTCG	0	14	RDR2-sensitive
AGGACGTTGCTGGAGACAGCCTAA	0	13	RDR2-sensitive
AGGACGTTGCTGGAGAGAGTAGAG	0	9	RDR2-sensitive
AGGACGTTGTTGGAGAGGAAGGAG	0	12	RDR2-sensitive
AGGACGTTGTTTGGGACGGTGGAG	0	11	RDR2-sensitive
AGGACTAACGAAGGCAGAACGGCA	0	10	RDR2-sensitive
AGGACTAAGGGCATACTGCCTGAT	0	20	RDR2-sensitive
AGGACTACGGTGAGGTGCATGGAG	0	9	RDR2-sensitive
AGGACTACGTAGAAGAGGAAGGAA	0	15	RDR2-sensitive
AGGACTAGATTGATCATTGTCGGG	0	11	RDR2-sensitive
AGGACTAGGACCTCGTCAGGCGTC	1	72	RDR2-sensitive
AGGACTAGGACCTCGTTAGGCGTC	0	9	RDR2-sensitive
AGGACTAGGCCCATAGGCCGGCTC	0	11	RDR2-sensitive
AGGACTAGTTGGAACAGCACCGCA	0	9	RDR2-sensitive
AGGACTATCCTGGCCAAAACCGGT	51	3	RDR2-resistant
AGGACTATCCTGGCCAAAGCCGGT	0	13	RDR2-sensitive

AGGACTATGACGTCATCTTGGCAC	2	17	RDR2-sensitive
AGGACTATGGCGTCATCTTGGCAC	0	17	RDR2-sensitive
AGGACTATTTAGAGGACGCTGCTG	0	12	RDR2-sensitive
AGGACTCACATTTGCATCGATGAC	18	0	RDR2-resistant
AGGACTCACGTAGGAGGAATAGGT	1	38	RDR2-sensitive
AGGACTCACTACCGGAATTTGGCT	0	10	RDR2-sensitive
AGGACTCAGAGCCGTTCGGCCAGC	0	22	RDR2-sensitive
AGGACTCAGCACGGTTCGTCATGT	0	12	RDR2-sensitive
AGGACTCAGGAAGAGAGACGACCC	0	23	RDR2-sensitive
AGGACTCAGGACACACGACGACAC	0	31	RDR2-sensitive
AGGACTCAGGACACAGGACGACAC	0	26	RDR2-sensitive
AGGACTCCCTAGCTCGGCGTCAAG	0	17	RDR2-sensitive
AGGACTCCGCAACCAGACTCCGGT	0	10	RDR2-sensitive
AGGACTCGACAGCCCCGACAGCTA	2	25	RDR2-sensitive
AGGACTCGACAGCCCCGACAGCTT	0	16	RDR2-sensitive
AGGACTCGACGGCAGCGCAACGCC	0	32	RDR2-sensitive
AGGACTCGACTCTCCCCAAGGACG	15	0	RDR2-resistant
AGGACTCGCGGAGACGGGGGATTG	0	12	RDR2-sensitive
AGGACTCGCTCGCGCAGGGCTGCA	0	17	RDR2-sensitive
AGGACTCGGCAAGCGACGACGCGA	0	15	RDR2-sensitive
AGGACTCGGCACTTTGACGAAGGC	2	25	RDR2-sensitive
AGGACTCGGCGAGCAACGACGCGA	0	25	RDR2-sensitive
AGGACTCGGCGAGCAGCGACACGA	0	10	RDR2-sensitive
AGGACTCGGCGCTTTGACGAAGGC	1	17	RDR2-sensitive
AGGACTCTCGACACAAGGAGACAC	0	14	RDR2-sensitive
AGGACTCTCGACACACGTAGACAC	0	16	RDR2-sensitive
AGGACTCTCGGCACACAAAGACAC	0	30	RDR2-sensitive
AGGACTCTCGGCACACGAAGACAC	1	17	RDR2-sensitive
AGGACTCTCGGCACAGACTCGGCG	2	32	RDR2-sensitive
AGGACTCTCGGCACAGGAAGACAC	2	14	RDR2-sensitive
AGGACTCTCGGCATAGGAAGACAT	0	16	RDR2-sensitive
AGGACTCTCGGTACAGGAAGACAC	0	24	RDR2-sensitive
AGGACTCTCTGCGGAGGCGCGGAC	0	12	RDR2-sensitive
AGGACTCTGACTGAGCTCAGAGAT	0	10	RDR2-sensitive
AGGACTCTGCCGAGGGCGACACTC	0	11	RDR2-sensitive
AGGACTCTTGTCTGATCTTAAGGC	0	25	RDR2-sensitive
AGGACTGAACAGGCCGAATCGGCT	0	10	RDR2-sensitive
AGGACTGAACCGAGAGGATCCGGC	0	9	RDR2-sensitive
AGGACTGACAACAACGTCGGTTGC	35	1	RDR2-resistant
AGGACTGACACACGTGCGCGCCAC	0	9	RDR2-sensitive
AGGACTGACCGGCTGCATGAGGAT	45	0	RDR2-resistant
AGGACTGACGCGGCACCGACGGGC	0	10	RDR2-sensitive
AGGACTGAGCATATATGTAGGTCA	0	33	RDR2-sensitive
AGGACTGAGGATGGACGGTTACAC	0	9	RDR2-sensitive
AGGACTGAGTCGGCTCAAACCCAG	0	10	RDR2-sensitive
AGGACTGATGTCCACCTACGTCAT	0	10	RDR2-sensitive
AGGACTGCAGGAGGGCGGACGGCG	0	12	RDR2-sensitive
AGGACTGCAGGAGTAAGGACGCAT	0	10	RDR2-sensitive
AGGACTGCCACACGCGCGTGCCGC	0	11	RDR2-sensitive

AGGACTGCCCCTTCAGCAGGGACG	0	11	RDR2-sensitive
AGGACTGCTATACGTGCGCGACGG	0	17	RDR2-sensitive
AGGACTGGAGGGAGAGAGAAACGC	0	9	RDR2-sensitive
AGGACTGGATCCAGAAAGCAGCTT	0	9	RDR2-sensitive
AGGACTGGCGAGCGGCGACCGGCG	0	9	RDR2-sensitive
AGGACTGGGCATTTTTAACCCGAA	0	9	RDR2-sensitive
AGGACTGGGCCAGTAGGGCAGGCC	0	9	RDR2-sensitive
AGGACTGGGCCGCGTCAGCCAACC	0	19	RDR2-sensitive
AGGACTGGGCGTTCGGGTTTACCC	0	9	RDR2-sensitive
AGGACTGGGGGCACAAGGACGGAC	0	12	RDR2-sensitive
AGGACTGGGGGCATGGTGATGCTC	0	10	RDR2-sensitive
AGGACTGTACCGAGTGCGGCGCTC	0	18	RDR2-sensitive
AGGACTGTAGGAGCATAGGTTACA	0	9	RDR2-sensitive
AGGACTGTCACGTTAGTCGAGGAT	1	20	RDR2-sensitive
AGGACTGTCAGGTCCCCTACGGCT	1	14	RDR2-sensitive
AGGACTGTCCGGTGTGCCACCCTT	1	12	RDR2-sensitive
AGGACTGTGCAGGCACGACTCGGT	0	41	RDR2-sensitive
AGGACTGTGCAGGCCCGGCCCGGC	0	14	RDR2-sensitive
AGGACTGTGCGGACGGTGGTTGGT	0	15	RDR2-sensitive
AGGACTGTGTAGGCCCGGCCCGAC	0	22	RDR2-sensitive
AGGACTGTGTCGAGCGCGGCTCTC	0	11	RDR2-sensitive
AGGACTGTTCACAGTGTAGCGGCT	3	19	RDR2-sensitive
AGGACTGTTCCTTCAGCAGGGAGG	0	9	RDR2-sensitive
AGGACTGTTTAGAGGACGCTGCTG	1	24	RDR2-sensitive
AGGACTGTTTAGAGGACGTTGCTG	0	16	RDR2-sensitive
AGGACTTAGGGCGGGTACACTCCG	0	21	RDR2-sensitive
AGGACTTAGGGCGGGTGCACTCCA	0	52	RDR2-sensitive
AGGACTTAGGGCGGGTGCACTCCG	2	39	RDR2-sensitive
AGGACTTAGGGCGGGTGCACTCTG	0	17	RDR2-sensitive
AGGACTTCACCAAGTGCGGCTCTC	2	18	RDR2-sensitive
AGGACTTCAGCTGAAGGCCGTCGT	0	16	RDR2-sensitive
AGGACTTCAGCTGAAGGTCGTCGT	1	45	RDR2-sensitive
AGGACTTCTGGCCAGGAGAACGAG	0	9	RDR2-sensitive
AGGACTTGCTGCATGATGGCGCGA	0	9	RDR2-sensitive
AGGACTTGGAGAAGCCGTTCGGCC	0	10	RDR2-sensitive
AGGACTTGGAGGTGCTACTCGGAA	1	14	RDR2-sensitive
AGGACTTGGCGAGTAGCGACGCGA	0	9	RDR2-sensitive
AGGACTTGTAGAGCATGATGGCTT	0	32	RDR2-sensitive
AGGACTTGTGGAGCATGATGGCTT	0	32	RDR2-sensitive
AGGACTTTACCGAGTACGGCTCTC	0	13	RDR2-sensitive
AGGACTTTACCGAGTGCGGCTCTC	4	23	RDR2-sensitive
AGGACTTTGACTATTTTGCGGCCT	158	0	RDR2-resistant
AGGACTTTGCCAAGTGCGGCACTC	0	14	RDR2-sensitive
AGGACTTTGCCAAGTGCGGCGCTC	2	21	RDR2-sensitive
AGGACTTTGCCAAGTGCGGCTCTC	1	31	RDR2-sensitive
AGGACTTTGCCATGACAGCTGCAT	1	31	RDR2-sensitive
AGGACTTTGCCGAGTGCGGCACTC	16	51	RDR2-sensitive
AGGACTTTGCCGAGTGCGGCGCTC	52	106	RDR2-sensitive
AGGACTTTGCCGAGTGCGGCTCTC	24	57	RDR2-sensitive

AGGACTTTGTCGAGTGCGGCTCTC	9	50	RDR2-sensitive
AGGAGAAAGGTCGCCGAGTCACGC	0	9	RDR2-sensitive
AGGAGAACTGTAGGTGGGCCTAAT	1	17	RDR2-sensitive
AGGAGAAGGCACTAGAGCATGAGT	0	9	RDR2-sensitive
AGGAGAATCTGGCTGTGGGGAGAA	0	15	RDR2-sensitive
AGGAGAATCTGGCTGTGTAGAGAA	0	16	RDR2-sensitive
AGGAGAATGCTGAGGATGATGGGC	0	10	RDR2-sensitive
AGGAGACACGATGTAGGGTTGGAC	0	9	RDR2-sensitive
AGGAGACAGAACATGGCGCACAGA	0	9	RDR2-sensitive
AGGAGACAGCAGTGCAAGCCTCTG	0	12	RDR2-sensitive
AGGAGACATGACAGACCTAGTGTG	17	0	RDR2-resistant
AGGAGACCACCAACCAGAAGGACG	0	19	RDR2-sensitive
AGGAGACCCGTAACAGAACTAGTT	0	10	RDR2-sensitive
AGGAGACCGACACGTAGGAGGCGC	0	9	RDR2-sensitive
AGGAGACCGACACGTAGGGCGCTA	0	24	RDR2-sensitive
AGGAGACCGACACGTAGGGGACGC	0	37	RDR2-sensitive
AGGAGACCGACACGTAGGGGGCGC	1	13	RDR2-sensitive
AGGAGACCGTACTTGGGGCTGCGT	0	14	RDR2-sensitive
AGGAGACCGTAGCAGAGACTGTAA	0	17	RDR2-sensitive
AGGAGACCTACTGGGCTTGCTGAC	36	2	RDR2-resistant
AGGAGACCTAGCGACGGAGAACGG	0	15	RDR2-sensitive
AGGAGACCTCGAAGGGAGTAGGAG	0	19	RDR2-sensitive
AGGAGACGACAACCTAGACCTCTC	0	17	RDR2-sensitive
AGGAGACGAGAAGCTGCAACCTTT	1	14	RDR2-sensitive
AGGAGACGAGATCTGCGCGTATTA	0	9	RDR2-sensitive
AGGAGACGAGCTCTCTACATCTTC	0	12	RDR2-sensitive
AGGAGACGAGGCAGGGTAGCACTA	0	11	RDR2-sensitive
AGGAGACGCTGCTGAAGACTGAGA	0	10	RDR2-sensitive
AGGAGACGCTGTAGGGGACAGCAT	0	14	RDR2-sensitive
AGGAGACGGAGAGACCGAGGGCGA	0	9	RDR2-sensitive
AGGAGACGGGCACAGGAACATGAA	18	1	RDR2-resistant
AGGAGACGGGCAGCTGAAGGACAA	0	9	RDR2-sensitive
AGGAGACGGGCCTAGAACGCGTGT	0	12	RDR2-sensitive
AGGAGACGGTAGAAGCTTGTTTTC	0	11	RDR2-sensitive
AGGAGACGTAGAAGAGACGCGCGC	0	18	RDR2-sensitive
AGGAGACGTCAGCAAGGACCCGAC	0	26	RDR2-sensitive
AGGAGACGTCAGCAAGGACTCGAC	0	25	RDR2-sensitive
AGGAGACGTCAGCAAGGATCCGAC	0	11	RDR2-sensitive
AGGAGACGTCAGCAAGGATTCGAC	0	12	RDR2-sensitive
AGGAGACGTCTGACTTTTTTCCAT	54	0	RDR2-resistant
AGGAGACGTGGCTTGCATGAACAG	0	47	RDR2-sensitive
AGGAGACGTGTTTGACTCATGCAT	0	15	RDR2-sensitive
AGGAGACTAGGACGACAGTGACGA	0	13	RDR2-sensitive
AGGAGACTGAATTTAGGGCACGCT	0	13	RDR2-sensitive
AGGAGACTGACACGTAGGAGGCGC	1	40	RDR2-sensitive
AGGAGACTGATCCTGGAGTTGCAT	0	10	RDR2-sensitive
AGGAGACTGATTCCTGCCGTTGAT	0	11	RDR2-sensitive
AGGAGACTGCATGGAGTGCGCTAT	0	20	RDR2-sensitive
AGGAGACTGCTTGCTGCCACCCAA	0	14	RDR2-sensitive

AGGAGACTGGGTAACATAGACTTG	0	10	RDR2-sensitive
AGGAGACTGTAAACGATCCGGTAC	0	14	RDR2-sensitive
AGGAGACTGTAAAGGACGGATATA	0	32	RDR2-sensitive
AGGAGACTGTAGCGCCCGATAGGC	0	9	RDR2-sensitive
AGGAGACTGTCCACTGCTTCAAAC	38	0	RDR2-resistant
AGGAGACTTGGGGCTACTGTAAGG	0	15	RDR2-sensitive
AGGAGACTTTAGTACTGGTTCATG	0	9	RDR2-sensitive
AGGAGACTTTTGGGATTATAACAC	0	16	RDR2-sensitive
AGGAGAGACGTACTGGCACCCTGC	1	24	RDR2-sensitive
AGGAGAGAGAACGCTGACGTGGCA	0	14	RDR2-sensitive
AGGAGAGCACTGTGCAAGAGGACG	0	21	RDR2-sensitive
AGGAGAGCGGGCAGTCACACTCAC	0	11	RDR2-sensitive
AGGAGAGCTCTGTACCTTGGTCGG	0	13	RDR2-sensitive
AGGAGAGGAACAGAAGACAAGCGC	0	9	RDR2-sensitive
AGGAGAGGCAGAACGGCAGAACGG	0	16	RDR2-sensitive
AGGAGAGGGCAGCAGATCACCGTT	0	11	RDR2-sensitive
AGGAGATAGGCTGACGATCCTGAG	0	17	RDR2-sensitive
AGGAGATAGTTGAGCGGCTGGAGA	0	16	RDR2-sensitive
AGGAGATATCGAATTATGTCATGT	26	0	RDR2-resistant
AGGAGATGGCGTAGCAACACTAGC	0	11	RDR2-sensitive
AGGAGATGGTGACCGCTGATTGAT	0	15	RDR2-sensitive
AGGAGATGTAGTCACGCCTCGCAT	0	13	RDR2-sensitive
AGGAGATGTGGTCCAGCTAGACGG	0	43	RDR2-sensitive
AGGAGATTGCTCAGGCGTACTCAC	0	13	RDR2-sensitive
AGGAGATTGTTGTATTTACTGTGA	0	9	RDR2-sensitive
AGGAGCACCCCTGTCACTCGGTAC	1	31	RDR2-sensitive
AGGAGCACTAGAGCTTGGAAGGGC	1	13	RDR2-sensitive
AGGAGCAGGAGAACGCCGAAGCCC	0	12	RDR2-sensitive
AGGAGCAGTAGCAAACGTCGGCGC	0	9	RDR2-sensitive
AGGAGCATCTGGAAGGACGGTCTG	1	41	RDR2-sensitive
AGGAGCCAAGTAGAACGGAGCCGC	1	17	RDR2-sensitive
AGGAGCCAAGTAGAACGGAGCCGT	13	123	RDR2-sensitive
AGGAGCCAAGTAGAACGGAGTCAT	1	57	RDR2-sensitive
AGGAGCCAAGTAGAACGGAGTCGT	4	292	RDR2-sensitive
AGGAGCCAAGTAGAATGGAGTCGT	0	34	RDR2-sensitive
AGGAGCCCGTAGAAGACACGCTAT	0	19	RDR2-sensitive
AGGAGCCGGCGTACACTGTTAATG	0	10	RDR2-sensitive
AGGAGCCGGTAGAAGCTTGTTTTC	2	88	RDR2-sensitive
AGGAGCCGGTGTGAGAACAGGTTT	0	12	RDR2-sensitive
AGGAGCCTAGGACGACGGTGACGA	0	11	RDR2-sensitive
AGGAGCCTGCATGTACAAAGCCAA	0	13	RDR2-sensitive
AGGAGCGACTCTTCTCTGCACGGC	0	11	RDR2-sensitive
AGGAGCGCATCGCCGGCAGAAGGG	0	9	RDR2-sensitive
AGGAGCGCTCGTGAACTTTATGGC	0	9	RDR2-sensitive
AGGAGCGGCTCCTCCTCTGCATAC	0	10	RDR2-sensitive
AGGAGCGGCTCCTCCTCTGCGTAC	0	13	RDR2-sensitive
AGGAGCGGCTCCTTCTCTGCGTAC	0	16	RDR2-sensitive
AGGAGCGGCTCCTTCTCTGTACAC	0	13	RDR2-sensitive
AGGAGCGGCTCTTCTCTGCGTCAC	0	18	RDR2-sensitive

AGGAGCGGCTTCTCCTCTGCATAC	2	54	RDR2-sensitive
AGGAGCGGGCAGGATTCCACGGTA	0	24	RDR2-sensitive
AGGAGCGGTGCCTTCTCTGCGTAC	0	16	RDR2-sensitive
AGGAGCGGTTCCTTCTCTGCGTTT	0	10	RDR2-sensitive
AGGAGCGTAGGCGTGCACACCCGT	0	13	RDR2-sensitive
AGGAGCTAGAGCTGTGCCAAACGG	0	16	RDR2-sensitive
AGGAGCTAGGAACATGCAAGACAG	0	9	RDR2-sensitive
AGGAGCTGCAGTAGAAACCCGGAA	0	10	RDR2-sensitive
AGGAGCTGGAGCTGTGCCAAACAG	0	16	RDR2-sensitive
AGGAGCTGGAGCTGTGCCAAACGG	0	70	RDR2-sensitive
AGGAGCTGGAGCTTTACCAAACGG	0	16	RDR2-sensitive
AGGAGCTGGAGCTTTGCCAAACGG	1	22	RDR2-sensitive
AGGAGCTGGGAATGAAAACGGAGT	0	15	RDR2-sensitive
AGGAGCTGGTGTAGTGTACTGCAT	0	11	RDR2-sensitive
AGGAGCTGGTTTCCCTACGCTGAG	0	9	RDR2-sensitive
AGGAGCTGTTTTAAGGGAGGGTGG	0	15	RDR2-sensitive
AGGAGGAACCGCTGAAGACAGTCT	0	9	RDR2-sensitive
AGGAGGAAGGGCGACGTGATGTTC	24	0	RDR2-resistant
AGGAGGACGCAGGATATGACGGAT	0	84	RDR2-sensitive
AGGAGGACGCGGGATATGATGGAT	0	20	RDR2-sensitive
AGGAGGAGGGATGAATAAGGCTGC	0	22	RDR2-sensitive
AGGAGGATAGTGACCAGAGCCGGA	150	0	RDR2-resistant
AGGAGGATGAAGCATTCGACGACT	14	0	RDR2-resistant
AGGAGGCAGAACCGTGCACCACAG	0	10	RDR2-sensitive
AGGAGGCAGACTACTATTACACTC	0	12	RDR2-sensitive
AGGAGGCATCCTGTACGACGGCGG	18	1	RDR2-resistant
AGGAGGCCAAGGATATGAACAGAA	35	4	RDR2-resistant
AGGAGGCGAGATGTAGGTTTACTC	0	13	RDR2-sensitive
AGGAGGCGCTGTAGGGGCACCGCT	3	41	RDR2-sensitive
AGGAGGCGCTGTAGGGGGCACCGC	2	42	RDR2-sensitive
AGGAGGCGGCGATTCTGGAGGCAC	1	22	RDR2-sensitive
AGGAGGCGGCTGCAGCATCGACGT	0	10	RDR2-sensitive
AGGAGGCTAGTAGGAACGGTCCAA	1	47	RDR2-sensitive
AGGAGGGAAGGGAGCGGATCTGGT	0	10	RDR2-sensitive
AGGAGGGAGAAGACCAACGGACAG	0	20	RDR2-sensitive
AGGAGGGCAGAACGGCAGAACAGC	1	77	RDR2-sensitive
AGGAGGGCTGTATTCCACGACGCA	0	9	RDR2-sensitive
AGGAGGGGCACTAATTTTGACATT	15	0	RDR2-resistant
AGGAGGTAGCAACAGAAAGACGGA	0	10	RDR2-sensitive
AGGAGGTAGCGCGGGACGATGCGG	106	21	RDR2-resistant
AGGAGGTGCTGCGACGAGGACGAT	28	0	RDR2-resistant
AGGAGGTTGTAGGGGACGTTGCTG	0	16	RDR2-sensitive
AGGAGTACGGTACCGCTGGCTCAT	3	25	RDR2-sensitive
AGGAGTACGGTACTCATGTGCACA	0	9	RDR2-sensitive
AGGAGTAGCAATGCAGAACGCCGA	0	11	RDR2-sensitive
AGGAGTAGTAGAGATAGCGACCAG	0	13	RDR2-sensitive
AGGAGTCGCTAGAGCTTTCGTCGG	2	48	RDR2-sensitive
AGGAGTCGCTTGAGCTCTCGTCGG	4	18	RDR2-sensitive
AGGAGTCGGTAGAAGCTTGTTTTC	1	58	RDR2-sensitive

AGGAGTCTAGGTCGGCCTGCCTGA	0	10	RDR2-sensitive
AGGAGTGAGAAGACGATGGACTAG	0	12	RDR2-sensitive
AGGAGTGAGTCGAAGAGCGTCATG	41	0	RDR2-resistant
AGGAGTGTGACGGCTCGCAAGAGC	15	0	RDR2-resistant
AGGAGTGTTGACGGCCTGGTGCAC	1	20	RDR2-sensitive
AGGAGTTCAGGACTGTAGGGGCAG	0	11	RDR2-sensitive
AGGAGTTGGGAATGGGAATGGCAG	0	9	RDR2-sensitive
AGGAGTTGTGATGCATATGACTAT	27	3	RDR2-resistant
AGGATAAGACATTGGCGACACCGT	0	16	RDR2-sensitive
AGGATACAGGATGAGCTACTATCC	0	14	RDR2-sensitive
AGGATACCCGATGGACGAAGACGA	0	21	RDR2-sensitive
AGGATACCTGTAGTAGTGCGGCCT	0	32	RDR2-sensitive
AGGATACCTGTAGTAGTGCGTCAG	0	23	RDR2-sensitive
AGGATACGGGCAACACTTCTCACC	0	17	RDR2-sensitive
AGGATAGAACGAGCGGCTGAGATC	0	11	RDR2-sensitive
AGGATAGCGGTGTACTGTAGCCCC	0	24	RDR2-sensitive
AGGATAGGGCAGCCCCGGACGAGC	0	15	RDR2-sensitive
AGGATAGTCCTGGTTAATTCTGGT	63	1	RDR2-resistant
AGGATAGTGACCAGAGCCGGACGG	35	0	RDR2-resistant
AGGATATAAGGCGTAACCACCTCT	1	16	RDR2-sensitive
AGGATATCTGTCGCTGGAGGGCAC	0	22	RDR2-sensitive
AGGATATGATGTGTAGTCGGGAAA	16	0	RDR2-resistant
AGGATATGGAAGCGGCAGAACCGT	0	27	RDR2-sensitive
AGGATATGGATACACGTTCATTGC	0	12	RDR2-sensitive
AGGATCAAATCGGATTAGGATCGG	0	14	RDR2-sensitive
AGGATCAAGTCAAGTTAGGGCCGG	0	17	RDR2-sensitive
AGGATCACAGGAGACTTCGTGGCC	0	37	RDR2-sensitive
AGGATCACGTCGGCGCCCCGTCGG	0	9	RDR2-sensitive
AGGATCAGGATCCTCGGAGCGCAT	1	42	RDR2-sensitive
AGGATCAGGGGGAGCACATTTCTA	0	9	RDR2-sensitive
AGGATCCAGATCCTCGGAGCGCAT	0	12	RDR2-sensitive
AGGATCCCTGTAGTAGTGGCGATG	0	11	RDR2-sensitive
AGGATCCCTGTTGGAAGGATGTTT	0	15	RDR2-sensitive
AGGATCCTGACGAGTCGACGAGCT	0	19	RDR2-sensitive
AGGATCCTGCAGACAACGTTGATA	0	9	RDR2-sensitive
AGGATCGAAGCGGGTTAGGATCGG	0	20	RDR2-sensitive
AGGATCGGAACACATACGGATCAA	0	24	RDR2-sensitive
AGGATCGGAGATCTACGACGACTA	0	18	RDR2-sensitive
AGGATCGGATAACTCCTCACGGTG	1	25	RDR2-sensitive
AGGATCGGATCAAGTTAGGGCCGG	0	11	RDR2-sensitive
AGGATCGGATGAAGACGAAGCTAG	0	13	RDR2-sensitive
AGGATCGGGCTATATGGGTCGGCA	0	11	RDR2-sensitive
AGGATCGGTAGACCACCCAAGACG	0	11	RDR2-sensitive
AGGATCGTATGGTCGCGGGCGCAC	0	13	RDR2-sensitive
AGGATCGTTTAGAGGACGCTGCTG	0	41	RDR2-sensitive
AGGATCTAGAAAGTGACGGATTCC	6	33	RDR2-sensitive
AGGATCTAGAAGACGAAGGGTTCC	31	134	RDR2-sensitive
AGGATCTAGACGACTATTATAATA	70	1	RDR2-resistant
AGGATCTAGATCCTCGGAGCGCAT	0	17	RDR2-sensitive

AGGATCTCTGTGCCCGAAGACCGG	0	28	RDR2-sensitive
AGGATCTGAAGCGGTAGGGGGCTC	0	25	RDR2-sensitive
AGGATCTGCATGCATGCATCTGCG	0	22	RDR2-sensitive
AGGATCTGCGGTAGAAGCTGGAGC	0	10	RDR2-sensitive
AGGATCTGGACCCGAAGCTCGGCG	0	11	RDR2-sensitive
AGGATCTGGACTCGACGCTCGGCG	0	10	RDR2-sensitive
AGGATCTGGAGCTAAAGAAGCAGG	0	12	RDR2-sensitive
AGGATCTGGAGGAGCCGTGCCAAA	0	15	RDR2-sensitive
AGGATCTGGATCATCAGAGCGCAT	9	266	RDR2-sensitive
AGGATCTGGATCATCAGAGCGTAT	1	24	RDR2-sensitive
AGGATCTGGATCATTAGAGCGCAT	0	9	RDR2-sensitive
AGGATCTGGATCCTCAGAGCGCAT	0	9	RDR2-sensitive
AGGATCTGGCTGACAGCAAGACTG	0	10	RDR2-sensitive
AGGATCTGTCGAAGGTATTTTCTT	1	13	RDR2-sensitive
AGGATCTTATCTCGCGGGCGCGCT	0	15	RDR2-sensitive
AGGATCTTGTACGGCTGCGTCCGC	1	16	RDR2-sensitive
AGGATGAAATAGGAGTGAGGGCAC	0	34	RDR2-sensitive
AGGATGAAGGACTGACACGCGCAC	0	13	RDR2-sensitive
AGGATGAAGGACTGCCACACGCGC	3	28	RDR2-sensitive
AGGATGAAGGACTGCCACGCGCGC	0	9	RDR2-sensitive
AGGATGAAGGACTGCCACGCGCGT	0	19	RDR2-sensitive
AGGATGAAGGCGTGATAGGACCAG	0	11	RDR2-sensitive
AGGATGAATATAGAGGACGTTGCT	0	18	RDR2-sensitive
AGGATGACCAGTCTTGGCTGCATA	50	0	RDR2-resistant
AGGATGACGAACAAGGTGGCGACA	0	10	RDR2-sensitive
AGGATGACGAGCGACCGATCGAGC	0	9	RDR2-sensitive
AGGATGACGGGTTCGACGACTTGA	0	9	RDR2-sensitive
AGGATGACTGGATGTAGGAAGGTT	0	10	RDR2-sensitive
AGGATGACTGGTGACGCATGGTGC	0	27	RDR2-sensitive
AGGATGAGACGAGCCTGCTCACGC	0	60	RDR2-sensitive
AGGATGAGCTGAACCGTGTGCATT	0	10	RDR2-sensitive
AGGATGAGGAGTAGCATGTGAAGT	164	0	RDR2-resistant
AGGATGAGGATGACGGGTGACACA	0	15	RDR2-sensitive
AGGATGATGGTACCACTTGCAGTG	21	0	RDR2-resistant
AGGATGCTGTAGAGGGCACCGCTA	0	15	RDR2-sensitive
AGGATGGAACAGAGCCGAAGTTGT	0	12	RDR2-sensitive
AGGATGGAATCGCAATCGGACGGT	0	16	RDR2-sensitive
AGGATGGACGATCACGGTGGAGGA	15	0	RDR2-resistant
AGGATGGATATAGAGGACGTTGCT	0	11	RDR2-sensitive
AGGATGGATGTAATTCGTACCGGC	0	9	RDR2-sensitive
AGGATGGCAGAACGGCAGAACGGC	3	213	RDR2-sensitive
AGGATGGCAGAACGGCAGACTACT	0	13	RDR2-sensitive
AGGATGGGAATTTGGTGGGGGCGA	0	10	RDR2-sensitive
AGGATGGGCCGCAGGAAGAGAAAG	30	0	RDR2-resistant
AGGATGGGCTGAGCAACTGGCATG	0	12	RDR2-sensitive
AGGATGGGCTGTAGAATGGGCCGC	0	26	RDR2-sensitive
AGGATGGGTATGAGGATGGATCAA	0	12	RDR2-sensitive
AGGATGTAGAAAGTCACGACTGCA	47	0	RDR2-resistant
AGGATGTAGATGAGGCGCTGCTGT	0	11	RDR2-sensitive

AGGATGTAGATTTAGGCATTTGAT	0	31	RDR2-sensitive
AGGATGTAGCAAACCAGAAGGCAC	0	11	RDR2-sensitive
AGGATGTATATAGAGGACGTTGCT	1	57	RDR2-sensitive
AGGATGTCGGGCAGAACACGGTGC	0	125	RDR2-sensitive
AGGATGTCGGGTGGAAGGCAGAAC	0	73	RDR2-sensitive
AGGATGTGAGAACATGGGAGATGC	23	1	RDR2-resistant
AGGATGTGCTAGATTCGGCTTCTT	38	6	RDR2-resistant
AGGATGTGGACGCCTACACTACCA	0	12	RDR2-sensitive
AGGATGTGTTATTACGAAAACGGT	0	14	RDR2-sensitive
AGGATGTTGTACTAGATAGGCATG	47	0	RDR2-resistant
AGGATTACGTGCGGAGGAAGATAA	9	31	RDR2-sensitive
AGGATTACTACTTGCCGGACGCGC	1	17	RDR2-sensitive
AGGATTAGCAATTCTGGGAGACGT	0	11	RDR2-sensitive
AGGATTAGCAATTCTGGGGGACGT	0	13	RDR2-sensitive
AGGATTAGGACCTCGTTAGGCGTC	0	21	RDR2-sensitive
AGGATTAGGACGGGTGCACTCCGC	0	29	RDR2-sensitive
AGGATTCACCAGCAGAGCAATTTA	17	1	RDR2-resistant
AGGATTCAGTGAATCCGGTCGGTT	0	14	RDR2-sensitive
AGGATTCCACGGTACCGAGCAGGC	0	12	RDR2-sensitive
AGGATTCCTGTAGTGTTGGCACGG	1	67	RDR2-sensitive
AGGATTCGACAAGTAGGCGCTAGC	0	15	RDR2-sensitive
AGGATTCGACTTTTGACCCGGCCC	0	10	RDR2-sensitive
AGGATTCTCTACAGTGACTCGCAT	1	24	RDR2-sensitive
AGGATTGAAAGGGACTGAGGAGGA	0	10	RDR2-sensitive
AGGATTGACAGACATCGAGGGTAT	0	20	RDR2-sensitive
AGGATTGCAGGAAGGAAGCAAGAC	0	11	RDR2-sensitive
AGGATTGCATGCAGACTGGGCGGT	0	15	RDR2-sensitive
AGGATTGCATGCAGACTGGGGCCG	0	17	RDR2-sensitive
AGGATTGCATGTAGACCGGGCGGT	0	13	RDR2-sensitive
AGGATTGCCTAGCGACGACGTCTG	0	12	RDR2-sensitive
AGGATTGCGGGCTTACGGATGGAA	0	10	RDR2-sensitive
AGGATTGCTGCTTGATGGTTGGAT	0	37	RDR2-sensitive
AGGATTGCTGTACCATCGAGGCGA	0	13	RDR2-sensitive
AGGATTGCTTGCTACAGAAGTATC	17	1	RDR2-resistant
AGGATTGGAGGGGATTGAGGGGGA	0	13	RDR2-sensitive
AGGATTGGATTCGGAGGGGACGAC	0	20	RDR2-sensitive
AGGATTGGCTCTGAGGGTTGGGCT	30	5	RDR2-resistant
AGGATTGGGACAGTACTATCTAGT	0	11	RDR2-sensitive
AGGATTGGGAGTAGAAGCGAGGGC	0	9	RDR2-sensitive
AGGATTGGGCACCTAAGAAGGAAC	0	11	RDR2-sensitive
AGGATTGGGGACGCTTCACGCGAC	0	10	RDR2-sensitive
AGGATTGGGTGTGAAGTACGGATT	0	12	RDR2-sensitive
AGGATTGGTAGGAGCTACGTTCCC	1	72	RDR2-sensitive
AGGATTGGTAGGAGCTACGTTTCC	0	11	RDR2-sensitive
AGGATTGGTGGAGACGTAGACCGC	0	9	RDR2-sensitive
AGGATTGGTGGCAGAACACGGAAT	0	12	RDR2-sensitive
AGGATTGTAGCAAAACCAAGCGGC	42	4	RDR2-resistant
AGGATTGTGCGGCAGAACATGGCG	0	13	RDR2-sensitive
AGGATTGTGGTATAGAGTAAAGAA	0	9	RDR2-sensitive

AGGATTGTGGTATAGAGTAGATAA	1	15	RDR2-sensitive
AGGATTGTTGGCTAGTTTAATGGC	167	57	RDR2-resistant
AGGATTTAAGGCGGGTGCACTCCG	0	10	RDR2-sensitive
AGGATTTAGAAAGTGACGGATTCC	0	9	RDR2-sensitive
AGGATTTAGAGCGGGTGCGTTCCG	0	26	RDR2-sensitive
AGGATTTAGCGACTGACCGACGGT	1	13	RDR2-sensitive
AGGATTTAGCGACTGACCGTCGGT	1	76	RDR2-sensitive
AGGATTTAGGACGGGTGCACTCCG	0	9	RDR2-sensitive
AGGATTTAGGGCGGGTACATTCCG	0	9	RDR2-sensitive
AGGATTTAGGGCGGGTGCAATCCG	0	15	RDR2-sensitive
AGGATTTAGGGCGGGTGCACTCCG	5	149	RDR2-sensitive
AGGATTTAGGGCGGGTGCATTCCA	0	14	RDR2-sensitive
AGGATTTAGGGCGGGTGCATTCCG	1	53	RDR2-sensitive
AGGATTTCTAGACGACGCACGGAA	0	12	RDR2-sensitive
AGGATTTCTTGCTGACGTATGGAA	0	10	RDR2-sensitive
AGGATTTGAACATGCTGGATTCGG	2	29	RDR2-sensitive
AGGATTTGAACTCTCAGCTTGGCG	0	9	RDR2-sensitive
AGGATTTGGCATACTCTCTAAGCT	0	14	RDR2-sensitive
AGGATTTGGGGCTGGACGCGACGG	0	9	RDR2-sensitive
AGGATTTGTTTTACTGAGGTTCGG	0	10	RDR2-sensitive
AGGATTTTCCGACTCCGACGGTAA	0	22	RDR2-sensitive
AGGATTTTGACTTGTAGGGGATTT	0	16	RDR2-sensitive
AGGCAAAAGACCCTTGGACTTCCA	0	9	RDR2-sensitive
AGGCAAACCGACGTCTGACATCAT	0	20	RDR2-sensitive
AGGCAAACCTCGGGCGCGAGACGT	0	13	RDR2-sensitive
AGGCAAAGCGGGCAGGGACACCCT	0	10	RDR2-sensitive
AGGCAAAGTGTGGCAACTGAGGTA	3	48	RDR2-sensitive
AGGCAACAGAGAACATACCCACGC	0	17	RDR2-sensitive
AGGCAACAGTGTAGGCAGCTGGCT	0	10	RDR2-sensitive
AGGCAACCTGGGAACCGGTAAAGC	0	47	RDR2-sensitive
AGGCAACGGAACGTGTGATGGTTT	19	0	RDR2-resistant
AGGCAACTGGAACACATACGGTAA	0	12	RDR2-sensitive
AGGCAAGAGGTATGACGCGGACAC	0	34	RDR2-sensitive
AGGCAAGCGTTGGACTTGGCCGAA	0	12	RDR2-sensitive
AGGCAATCGGCAAAGACGGAACCT	0	9	RDR2-sensitive
AGGCAATTCTGAAATTTGGACATC	1	13	RDR2-sensitive
AGGCAATTCTGAGATTTGGACATC	0	18	RDR2-sensitive
AGGCACACAGGACTGTGCAGGCGC	0	20	RDR2-sensitive
AGGCACAGGCGTAGAGTATAACAG	0	12	RDR2-sensitive
AGGCACAGGCGTAGAGTATAGCAG	0	15	RDR2-sensitive
AGGCACATGATGCAATCTGCGCAA	0	9	RDR2-sensitive
AGGCACCAGTGGCTCCATTCACAC	2	14	RDR2-sensitive
AGGCACGACGTGGTTTATGGCTGA	0	9	RDR2-sensitive
AGGCACGATCGCAGTAGAAGATGA	0	13	RDR2-sensitive
AGGCACGCAGGACTGTGCAGGCAC	0	344	RDR2-sensitive
AGGCACGCAGGACTGTGCAGGCCC	0	19	RDR2-sensitive
AGGCACGCAGGACTGTGTAGGCCC	0	16	RDR2-sensitive
AGGCACGGACGCACGGTACCACTC	0	17	RDR2-sensitive
AGGCACGGCACGGTTAGGACACGG	0	13	RDR2-sensitive

AGGCACGGTAGAAAACCAGGTCGG	0	12	RDR2-sensitive
AGGCACGGTGAGCTAACTCGGCAT	2	48	RDR2-sensitive
AGGCACGTAGGACTGTGCAGGCCC	3	158	RDR2-sensitive
AGGCACGTGGGCTCTCTAGGCATG	0	30	RDR2-sensitive
AGGCACGTTAGAATGGACGGAGAC	0	12	RDR2-sensitive
AGGCACTACGTAGGATTCATCGGC	0	10	RDR2-sensitive
AGGCACTAGGGCTAGGGTTTTCAA	0	17	RDR2-sensitive
AGGCACTATTGCGCTCTCCAGCAG	0	30	RDR2-sensitive
AGGCACTCGACAAAGGGACTGGCA	0	11	RDR2-sensitive
AGGCACTCGAGTGATCGTCTGTTC	0	11	RDR2-sensitive
AGGCACTCGGCAAAGAATACAACA	0	13	RDR2-sensitive
AGGCACTCGGCAAAGAGACTGGCA	0	14	RDR2-sensitive
AGGCACTCGGCAAAGATACTGGCA	0	39	RDR2-sensitive
AGGCACTCGGCAAAGCCTCGGATT	0	12	RDR2-sensitive
AGGCACTCGGCAAAGCGTCCGGCA	0	9	RDR2-sensitive
AGGCACTCGGCAAAGGGACTGGCA	1	18	RDR2-sensitive
AGGCACTGAAGACACCGAGGAGGA	0	42	RDR2-sensitive
AGGCACTGAAGACTCGAGGAGGAA	0	10	RDR2-sensitive
AGGCACTGACACTATACTGACCGG	0	24	RDR2-sensitive
AGGCACTGAGGAGCCCCGACGGGT	0	12	RDR2-sensitive
AGGCACTGATGGTTGTTTTTGTAT	14	0	RDR2-resistant
AGGCACTGCATGTGGATCCTTGGA	54	8	RDR2-resistant
AGGCACTGGCACTATACTAACCGG	1	32	RDR2-sensitive
AGGCACTGGGAACTATGGAGGCAT	0	89	RDR2-sensitive
AGGCACTGTAGCACAATCCAGCAG	0	21	RDR2-sensitive
AGGCACTGTGACGCCCTAAGCCGG	0	13	RDR2-sensitive
AGGCACTGTGGGCTAGCCCGGCAC	2	21	RDR2-sensitive
AGGCACTGTTGCGCGCTCCAGCAG	0	9	RDR2-sensitive
AGGCACTGTTGGAGTGGTTTTTCC	0	9	RDR2-sensitive
AGGCACTGTTTCGCTCTCCAGCAG	0	52	RDR2-sensitive
AGGCACTTGTGTTGGACACTTAAT	0	14	RDR2-sensitive
AGGCAGAACAGCCGTTGGCAGAAC	0	13	RDR2-sensitive
AGGCAGAACCAGGGAGGCAGCCTA	0	10	RDR2-sensitive
AGGCAGAACCAGGGGAGGCAGCCT	0	16	RDR2-sensitive
AGGCAGAACGATAGACTACCCTTA	0	15	RDR2-sensitive
AGGCAGAACGATGGCACATGTGCA	0	20	RDR2-sensitive
AGGCAGAACGCAGAACAGGAGGGG	0	11	RDR2-sensitive
AGGCAGAACGGTAGAACCAGAGAG	0	11	RDR2-sensitive
AGGCAGAACGGTAGAACGGCAGAA	0	22	RDR2-sensitive
AGGCAGAACGGTAGACTACCCTTA	0	14	RDR2-sensitive
AGGCAGAAGGGGTAGAACAGGCCG	0	10	RDR2-sensitive
AGGCAGACGGGGGCTTTGGCAGAA	0	13	RDR2-sensitive
AGGCAGACTTCTCGTGAGCGACGC	0	15	RDR2-sensitive
AGGCAGATAGACGAAGCCTCTGAA	1	17	RDR2-sensitive
AGGCAGATCTCATAGGGACAGGGC	1	12	RDR2-sensitive
AGGCAGATCTGGGTCGGGCTTCAA	0	10	RDR2-sensitive
AGGCAGATGGACCGATTTGTTACC	19	0	RDR2-resistant
AGGCAGATTGGCCGCTCTCAACAG	3	40	RDR2-sensitive
AGGCAGATTGTGGCGACTTAGGTA	0	16	RDR2-sensitive

AGGCAGATTGTGGCGCCTTAGGTA	0	12	RDR2-sensitive
AGGCAGATTGTGGCGTCTTAGGTA	0	18	RDR2-sensitive
AGGCAGCAACGGCAATGTGGGCTC	0	18	RDR2-sensitive
AGGCAGCACCGGCGTAGAGCACTT	0	12	RDR2-sensitive
AGGCAGCAGCGGCAATGTAGGCTC	1	44	RDR2-sensitive
AGGCAGCAGCGGCAATGTGGGCTC	0	21	RDR2-sensitive
AGGCAGGACAGAGGACTACCGCTC	0	10	RDR2-sensitive
AGGCAGGACAGCCCCAGAACACGA	0	10	RDR2-sensitive
AGGCAGGACCGTACTTTAGACCAC	0	24	RDR2-sensitive
AGGCAGGATGTAGCGTATGACTGG	0	9	RDR2-sensitive
AGGCAGGCAGAGTAGGAGAGACGC	0	9	RDR2-sensitive
AGGCAGGCTCGGCTTGGCTCGCTC	0	10	RDR2-sensitive
AGGCAGGCTGTGGCACCTTCGGCA	1	41	RDR2-sensitive
AGGCAGGGAACAAGTTTGGGACGC	0	13	RDR2-sensitive
AGGCAGGGAGTAGACGACATTGCT	0	10	RDR2-sensitive
AGGCAGGGATGTGATCGATGGCAC	0	9	RDR2-sensitive
AGGCAGGTGAATATACGAACGCAC	0	10	RDR2-sensitive
AGGCAGTAAGGGGTAGGCTGTCAT	0	9	RDR2-sensitive
AGGCAGTAGAGAACATCCTCGTCC	0	10	RDR2-sensitive
AGGCAGTAGAGTGATGCGATGCAC	0	24	RDR2-sensitive
AGGCAGTTCGAGTCGGACGGTCAG	0	40	RDR2-sensitive
AGGCATAACTGGGGACGACACTGG	0	10	RDR2-sensitive
AGGCATCACGTAGGGACCGACATC	0	11	RDR2-sensitive
AGGCATCACGTAGGGACTGGCATC	0	11	RDR2-sensitive
AGGCATCACGTAGGGACTGGCGTC	0	110	RDR2-sensitive
AGGCATCAGTGGCTCCATTCACAC	0	10	RDR2-sensitive
AGGCATCTTCGGACTCAAGTCGGT	0	32	RDR2-sensitive
AGGCATGATGTAGCAAGTGTGGAC	0	26	RDR2-sensitive
AGGCATGCCCCATTTTCGTCGGTA	0	14	RDR2-sensitive
AGGCATGCTCGGCTACTCCAGTCC	41	0	RDR2-resistant
AGGCATGGACGGTCTACGGCTCAA	0	9	RDR2-sensitive
AGGCATGGGGTAGGCACTCGGCAA	0	9	RDR2-sensitive
AGGCATGTAGGACTGTGCAGGCCC	0	28	RDR2-sensitive
AGGCATTATGACGGAGCCGAACGC	0	11	RDR2-sensitive
AGGCATTCGTAGCTCGGCGCCAAG	2	34	RDR2-sensitive
AGGCATTGTCCAGTTCTCGTCGTG	0	12	RDR2-sensitive
AGGCATTTTGCTTGTTAGTCGGGA	0	9	RDR2-sensitive
AGGCCAACATCGGGCGGGAGACGC	0	18	RDR2-sensitive
AGGCCAACCTCGGACGGGAGACGC	0	47	RDR2-sensitive
AGGCCAACGGACAGTAGCGGTCAC	0	20	RDR2-sensitive
AGGCCAATTTCGGACACGAGACGC	0	9	RDR2-sensitive
AGGCCACACTCGGACGGGAGGCAC	0	15	RDR2-sensitive
AGGCCACAGGTAAAACCGGACGAC	0	21	RDR2-sensitive
AGGCCACTCTCGGGACTCGACGGC	0	10	RDR2-sensitive
AGGCCAGAAGATTCCTGCAGGTTT	17	0	RDR2-resistant
AGGCCAGGTAGAGCGGTTGGTTTT	0	82	RDR2-sensitive
AGGCCATAGACCAGAGGACAGACC	0	16	RDR2-sensitive
AGGCCATAGGATCGGACACCTAAG	0	12	RDR2-sensitive
AGGCCATAGTAGTGGCACGTCGGC	1	36	RDR2-sensitive

AGGCCATCCTCGGGCGGGAGACGC	0	10	RDR2-sensitive
AGGCCATCGTATTCTCGGACCCGG	0	10	RDR2-sensitive
AGGCCATGAACGAGACCCAGGCAC	0	11	RDR2-sensitive
AGGCCATGATGGAACTGACGTGGC	0	11	RDR2-sensitive
AGGCCATGCTCGGACGAGGCGGAG	0	9	RDR2-sensitive
AGGCCATGTCGGCGAGCAGAGCGA	0	14	RDR2-sensitive
AGGCCCAACGAACTCGAGCGGCAC	0	9	RDR2-sensitive
AGGCCCAAGAGGAATCTTTGCATC	0	9	RDR2-sensitive
AGGCCCGCAGGGGACTGGACTCGG	1	13	RDR2-sensitive
AGGCCCGCTGTATGCATTGACCGT	0	9	RDR2-sensitive
AGGCCCGGACAAGAAACTAGGCAC	0	9	RDR2-sensitive
AGGCCCGGATGACTCCAGAAGCAG	0	31	RDR2-sensitive
AGGCCCGTCGGACCATGGACCGGC	0	18	RDR2-sensitive
AGGCCCGTGGGCCGTCGGACGACA	340	0	RDR2-resistant
AGGCCGAAGCGACCATAAACTCTA	0	14	RDR2-sensitive
AGGCCGACACGTAGAGGGCGCTGT	0	20	RDR2-sensitive
AGGCCGACGGAGAATCCGGACTTC	0	9	RDR2-sensitive
AGGCCGACGGCGATTAAGTAACTC	0	10	RDR2-sensitive
AGGCCGACGGCTGTTAGACGGGCC	1	12	RDR2-sensitive
AGGCCGAGAAGACGAATGAGACGT	0	9	RDR2-sensitive
AGGCCGCTCTTGGGACTCGACGGC	0	9	RDR2-sensitive
AGGCCGGGAAGACGAACAGGACGT	1	13	RDR2-sensitive
AGGCCGGGAAGACGAACGGGACGT	2	15	RDR2-sensitive
AGGCCGGGCCTGTACGTGAAGCAT	0	11	RDR2-sensitive
AGGCCGGGCTAGGTAGAGCGAGGC	2	33	RDR2-sensitive
AGGCCGTCAACGAACGAGGACAGC	0	11	RDR2-sensitive
AGGCCGTCGACGTGTCATTGGCAA	0	11	RDR2-sensitive
AGGCCGTGCGTTATACGGAGTCGG	0	32	RDR2-sensitive
AGGCCGTGCTGCACTGTATGTCGT	0	10	RDR2-sensitive
AGGCCGTGGAGCAATGTAAGACGT	0	20	RDR2-sensitive
AGGCCGTTTTAGCAACCAACCGTA	0	11	RDR2-sensitive
AGGCCTATAGCAGGTCGGCTCGGA	0	12	RDR2-sensitive
AGGCCTATTTCTGTAGTCTATCGG	183	0	RDR2-resistant
AGGCCTCAAATTCTGACCCGTCGG	0	10	RDR2-sensitive
AGGCCTCCCGCGAAGACGTGACTG	0	12	RDR2-sensitive
AGGCCTCGAGCGAAGGGTGGCGTC	0	11	RDR2-sensitive
AGGCCTCGGGCGAGCCGGTGATGT	0	17	RDR2-sensitive
AGGCCTGAAGTGGACATATGGCAT	0	9	RDR2-sensitive
AGGCCTGATATTGCTCATGCGGTT	21	0	RDR2-resistant
AGGCCTGATCTCGCTCGTCGCGAC	0	19	RDR2-sensitive
AGGCCTGCAGGAAGCTTGAGACGA	0	27	RDR2-sensitive
AGGCCTGGACGGCTACTGTAGCCC	1	20	RDR2-sensitive
AGGCCTGGGTATTGAAGTCGGTGG	0	13	RDR2-sensitive
AGGCCTGTAGCTAGCTAGAAGTGG	1	27	RDR2-sensitive
AGGCCTGTGACGTGCCGGACCAAG	0	11	RDR2-sensitive
AGGCCTGTGGATTCGTTGCGGGAC	49	6	RDR2-resistant
AGGCCTGTGGCATGCACACTAGTT	110	7	RDR2-resistant
AGGCCTTAAATTCCGGCCCGTCGG	2	24	RDR2-sensitive
AGGCCTTAGACTATATTCAACGGT	0	12	RDR2-sensitive

AGGCCTTAGTTGACGGTAGGATCG	0	42	RDR2-sensitive
AGGCCTTGTTCGTTTACGTCGGAT	64	88	RDR2-sensitive
AGGCCTTGTTCGTTTCTGTCGGAT	111	43	RDR2-resistant
AGGCCTTGTTGGACGGGTTGGCGT	0	36	RDR2-sensitive
AGGCCTTGTTGGTTTACGTCGGAT	0	11	RDR2-sensitive
AGGCCTTTCGGACGGTCGGATGCT	0	18	RDR2-sensitive
AGGCGAACGGACAGTAACGGTCAC	1	13	RDR2-sensitive
AGGCGAAGGACAGTCAACGGACGA	0	13	RDR2-sensitive
AGGCGAATTTTGGGCGGAGAACGT	0	11	RDR2-sensitive
AGGCGACCTGGAATTGGGGGACGA	1	14	RDR2-sensitive
AGGCGAGACGAGCTTATTCTCTGG	1	12	RDR2-sensitive
AGGCGAGACGGAACTGCATCTCCT	0	13	RDR2-sensitive
AGGCGAGACGGAACTTCATCTCCT	0	19	RDR2-sensitive
AGGCGAGAGTGGAGGCGAGAGCTG	1	36	RDR2-sensitive
AGGCGAGTCTGTGGTTTTGATGGA	0	10	RDR2-sensitive
AGGCGATAGACTGTTCATGATGGT	1	18	RDR2-sensitive
AGGCGATCAGGATTGGTGACGGGC	0	12	RDR2-sensitive
AGGCGATTTCGGCGACGACGAACT	0	10	RDR2-sensitive
AGGCGCAAGATGCAACCCGGACAC	0	12	RDR2-sensitive
AGGCGCACGATAGAATACGGACGG	0	10	RDR2-sensitive
AGGCGCAGACTGTTCGGACCGCGG	0	11	RDR2-sensitive
AGGCGCATGTAGCGACGGAAGCAC	0	11	RDR2-sensitive
AGGCGCCAACGAACTCAGATGGTG	1	30	RDR2-sensitive
AGGCGCCGAGCTCTGACATGGCGT	7	79	RDR2-sensitive
AGGCGCGGACTGTCCGGACCGCGG	0	16	RDR2-sensitive
AGGCGCGGGACATGGAGAGGACGA	0	12	RDR2-sensitive
AGGCGCTGTAGGGGGCACCGCTGC	0	9	RDR2-sensitive
AGGCGCTTCGAACTACAACCGGTT	0	13	RDR2-sensitive
AGGCGGAACGACGGACCAGATCCC	0	9	RDR2-sensitive
AGGCGGAACTGACACAGGCGGAAC	0	19	RDR2-sensitive
AGGCGGAACTGCAGGAGCACGTGC	0	12	RDR2-sensitive
AGGCGGACGAGCACGGACGAGCAG	0	12	RDR2-sensitive
AGGCGGACTTGGATCGTGCCTCAA	0	15	RDR2-sensitive
AGGCGGATTAAGGCATCCTGGCCG	50	1	RDR2-resistant
AGGCGGCAGTCCTGAAAGTCTGGG	0	9	RDR2-sensitive
AGGCGGCATGCAGGACAGACGGCG	0	13	RDR2-sensitive
AGGCGGGACGGACGACCAACCTGC	0	30	RDR2-sensitive
AGGCGGGCACTCAGGAGTTCAGGC	18	1	RDR2-resistant
AGGCGGGCGCGGCAGAACGTGCGA	0	49	RDR2-sensitive
AGGCGGGCTGGGCAGCAGCTAGGA	283	29	RDR2-resistant
AGGCGGGCTTGGGACAGGCCTCAA	0	14	RDR2-sensitive
AGGCGGGTCTGAACCGGGCCTCAA	0	10	RDR2-sensitive
AGGCGGGTTGGAAATCGGACGGTT	0	9	RDR2-sensitive
AGGCGGGTTGGGCATTGACTGCAA	0	11	RDR2-sensitive
AGGCGTACAGGACCTGACCGTCCC	0	13	RDR2-sensitive
AGGCGTAGACGAGGGCTTCGACAT	0	10	RDR2-sensitive
AGGCGTAGGGGACCTGACCGTCTC	0	10	RDR2-sensitive
AGGCGTCTTGAGGGTACCCCTAAT	1	17	RDR2-sensitive
AGGCGTGAATTCTGTGGACGCGGA	0	10	RDR2-sensitive

AGGCGTGGAACAGGTATTGTAGTC	0	12	RDR2-sensitive
AGGCGTGGACTGTCCGGACCGCGG	1	22	RDR2-sensitive
AGGCGTGGATACGGAGGTGCGCAT	0	9	RDR2-sensitive
AGGCGTTAAGACTGAGATGGCTTT	0	13	RDR2-sensitive
AGGCTAAACTCGGGCGGGAGACGC	0	9	RDR2-sensitive
AGGCTAAACTGTTCGCGATGGCGT	0	29	RDR2-sensitive
AGGCTAACGGACTGGCATGGCAGA	0	19	RDR2-sensitive
AGGCTAATCCAACAGACTCGGTAA	0	17	RDR2-sensitive
AGGCTAATGTGGACGGGCAAGCTG	0	10	RDR2-sensitive
AGGCTACAGGGAACAAGACCGGCG	1	14	RDR2-sensitive
AGGCTACAGTTACAACCGTCGGAA	0	50	RDR2-sensitive
AGGCTACGAAGGAACAAGACCGGT	2	16	RDR2-sensitive
AGGCTACGATCTCGACGAGCGAAG	0	11	RDR2-sensitive
AGGCTACGGCTACCGTCGGAACAG	1	12	RDR2-sensitive
AGGCTACGGGGAACAAGACCGGCG	1	35	RDR2-sensitive
AGGCTAGCCTCGGGCAAAAGATGT	0	13	RDR2-sensitive
AGGCTAGCTTCGGGCGAGATGCAT	2	18	RDR2-sensitive
AGGCTAGGAGACTCGCTTGCACGC	0	9	RDR2-sensitive
AGGCTAGGGTTCCTGTCTGACGGC	0	14	RDR2-sensitive
AGGCTATATAGGATCTGAAGCGGT	0	13	RDR2-sensitive
AGGCTATCTGCAGTGACGAACGGT	0	11	RDR2-sensitive
AGGCTATGGTTCCTGTCTGACGGC	0	13	RDR2-sensitive
AGGCTCACATGAGTCAGGCGTCGG	0	13	RDR2-sensitive
AGGCTCACTAGATACGAGACAGGC	0	18	RDR2-sensitive
AGGCTCAGCCCCGGTCGTGGTCGT	0	16	RDR2-sensitive
AGGCTCAGCGGGTGCATTGACTAT	0	11	RDR2-sensitive
AGGCTCCAAAGTAGACGAACACGG	0	30	RDR2-sensitive
AGGCTCCAGTAGATCTTCACGGTA	0	19	RDR2-sensitive
AGGCTCCATAGGATCTGAAGCGGT	0	25	RDR2-sensitive
AGGCTCCCGGACTGGACCAGGCAA	3	213	RDR2-sensitive
AGGCTCCCGGACTGGACCAGGCAT	0	10	RDR2-sensitive
AGGCTCGAACTCCGGCGACGACGT	0	41	RDR2-sensitive
AGGCTCGAATCGGGCTCGTTCGGA	0	14	RDR2-sensitive
AGGCTCGACTCGGGCTCGTTCGGA	3	189	RDR2-sensitive
AGGCTCGATCGTGCTTTGGACTAA	0	12	RDR2-sensitive
AGGCTCGGACAAGAAACTAGGCAC	2	24	RDR2-sensitive
AGGCTCGGACAAGAAATTAGGCAC	0	12	RDR2-sensitive
AGGCTCGGACAGAAAACTAGGCAC	0	10	RDR2-sensitive
AGGCTCGGACAGGAAATTAGGCAC	1	60	RDR2-sensitive
AGGCTCGGACCGACCGCACCACAG	0	9	RDR2-sensitive
AGGCTCGGATGTAGGTTTGGATGG	0	12	RDR2-sensitive
AGGCTCGGCACATAGTACATCGGC	0	18	RDR2-sensitive
AGGCTCGGCACGGTTATTCTTACA	0	9	RDR2-sensitive
AGGCTCGGCCTGTCAAACACGGTT	0	11	RDR2-sensitive
AGGCTCGGCGAACTGTACATCGGC	4	17	RDR2-sensitive
AGGCTCGGCTATTGAATCGGACTG	0	10	RDR2-sensitive
AGGCTCGGCTCAGGCTCGTTCGGA	0	43	RDR2-sensitive
AGGCTCGGCTCGAGCTCGTTCGGA	0	16	RDR2-sensitive
AGGCTCGGCTCGGACTTGTTCGGA	0	12	RDR2-sensitive

AGGCTCGGCTCGGCTCAAAGGCGG	0	14	RDR2-sensitive
AGGCTCGGCTCGGGCTCGTTCGGA	11	69	RDR2-sensitive
AGGCTCGGCTCGGTAACGGCTCAC	0	12	RDR2-sensitive
AGGCTCGGCTCGTCTGGCTGGGGC	0	20	RDR2-sensitive
AGGCTCGGGCGAGGCGTGATCGAG	5	20	RDR2-sensitive
AGGCTCGGGCTGACTGGTTCGGCT	4	22	RDR2-sensitive
AGGCTCGGGCTGGTACGGCTACTA	0	11	RDR2-sensitive
AGGCTCGGTGGAATAGCAAGACGA	0	14	RDR2-sensitive
AGGCTCGTAGATGTGTCTTCCGGC	0	14	RDR2-sensitive
AGGCTCTAGTAGGACGTCGGCGAC	0	27	RDR2-sensitive
AGGCTCTAGTGTAGACTGCGGCGT	0	10	RDR2-sensitive
AGGCTCTATGGAATCCGAAGCGGT	0	11	RDR2-sensitive
AGGCTCTCCGGACTGGACCAGGCA	0	56	RDR2-sensitive
AGGCTCTCGGGAGATGGAGGACAG	125	4	RDR2-resistant
AGGCTCTGGAGATGCGACCAAAAT	1	16	RDR2-sensitive
AGGCTCTGTGAATAGTTGTTGACG	384	15	RDR2-resistant
AGGCTCTTAACCTTGTGGTCGTGG	2	18	RDR2-sensitive
AGGCTGAACCAGCGCCGAAGCTGC	0	17	RDR2-sensitive
AGGCTGAAGAGGCTGATCAGGCAA	0	13	RDR2-sensitive
AGGCTGACCTGAACCGACCTGACG	0	9	RDR2-sensitive
AGGCTGAGGACTGTGCGGACGGTG	0	10	RDR2-sensitive
AGGCTGATAGAGGAGGGAGGCCCG	0	10	RDR2-sensitive
AGGCTGATCGATGAAGACGTGCGC	0	10	RDR2-sensitive
AGGCTGATGCACGGGACTATCAAT	0	9	RDR2-sensitive
AGGCTGATGGTGAACGCAGGAGTA	0	11	RDR2-sensitive
AGGCTGCAAGAGTAATCGGTCATT	0	10	RDR2-sensitive
AGGCTGCAAGAGTAGTCGGTCATT	0	10	RDR2-sensitive
AGGCTGCAAGCATGAACAAGCATA	0	13	RDR2-sensitive
AGGCTGCACTAACGAGAGGACTGA	0	20	RDR2-sensitive
AGGCTGCAGCTGCCGAGGACGCAT	0	10	RDR2-sensitive
AGGCTGCAGGATGACTGGGAGGCT	0	14	RDR2-sensitive
AGGCTGCATCTCGAGGACGAGCTG	0	9	RDR2-sensitive
AGGCTGCATCTGTGGAGACAGGCC	0	9	RDR2-sensitive
AGGCTGCCTCGACCTGAAGACGCC	0	30	RDR2-sensitive
AGGCTGCCTCGACCTGAAGACGCT	0	17	RDR2-sensitive
AGGCTGCGATGAACCGGCGTCTTC	0	12	RDR2-sensitive
AGGCTGCGTGCGACGAAGAAGACG	0	22	RDR2-sensitive
AGGCTGCTCTCGGGACTTCGACGG	0	72	RDR2-sensitive
AGGCTGGACATTGGACACTCCGGC	115	6	RDR2-resistant
AGGCTGGACGATGCATGGCGCTGC	0	28	RDR2-sensitive
AGGCTGGACTTGGACCGAGCTAGA	0	47	RDR2-sensitive
AGGCTGGAGCAGAGACGTCGGCAA	0	11	RDR2-sensitive
AGGCTGGCGTGTAGAAGGCAGGCA	1	17	RDR2-sensitive
AGGCTGGCTCGGACGAGACGGACT	0	22	RDR2-sensitive
AGGCTGGTAGAAGGAGTAGGGCAT	0	9	RDR2-sensitive
AGGCTGGTCTGAACGGCAAATGTA	0	10	RDR2-sensitive
AGGCTGGTGGACACTCGGCGAAGA	0	10	RDR2-sensitive
AGGCTGTAAGAAGGGCGTCCACAC	0	19	RDR2-sensitive
AGGCTGTAAGGGGTAGGCTGCCTC	1	12	RDR2-sensitive

AGGCTGTAAGGTGTTCCACGTCAT	0	100	RDR2-sensitive
AGGCTGTAATTGAGGAGGGGCTGA	0	35	RDR2-sensitive
AGGCTGTACACTGGAGCAGTATGG	0	10	RDR2-sensitive
AGGCTGTAGCACGTAGGGAGCCGT	0	28	RDR2-sensitive
AGGCTGTAGCTGCCTAGCTTGGAT	0	26	RDR2-sensitive
AGGCTGTAGTGAGGCTTCATTGGA	1	17	RDR2-sensitive
AGGCTGTAGTTATGGTAGTGCCGT	0	10	RDR2-sensitive
AGGCTGTATTGGCGTAGAGGGCTT	0	15	RDR2-sensitive
AGGCTGTCATTGGGCAAAAACTTC	0	10	RDR2-sensitive
AGGCTGTCGCGTAGAACAGTTCGG	0	18	RDR2-sensitive
AGGCTGTCGCGTAGGACAGTTCGG	0	12	RDR2-sensitive
AGGCTGTCTGGACTCTAGGGGCAT	0	10	RDR2-sensitive
AGGCTGTGAGCCAGTGCTGCGGTG	0	23	RDR2-sensitive
AGGCTGTGCACTGTTCAGATACGG	0	12	RDR2-sensitive
AGGCTGTGGATGATCCGGTATGTT	0	25	RDR2-sensitive
AGGCTGTGGCACCTTCGGCACCAC	0	23	RDR2-sensitive
AGGCTGTGTGGAAACAAAGGCGTG	0	9	RDR2-sensitive
AGGCTGTTTCTGACCGGACCGTCT	0	9	RDR2-sensitive
AGGCTTAATCGTGTCGGGCTCAAA	0	18	RDR2-sensitive
AGGCTTACGGTACGGGAACAGGCT	0	10	RDR2-sensitive
AGGCTTACTGTCTGCTATCGTCGC	0	32	RDR2-sensitive
AGGCTTAGGGGACTGCTACACCGA	0	9	RDR2-sensitive
AGGCTTATGTCCGACGGCTTTGAC	0	11	RDR2-sensitive
AGGCTTCAAAGTGTCGGACCGGGC	0	14	RDR2-sensitive
AGGCTTCACCAGAAGCTGGTCGGC	0	12	RDR2-sensitive
AGGCTTCACTAGAACTGTGCCGGT	0	22	RDR2-sensitive
AGGCTTCAGGTGGATGATGAGCTA	0	28	RDR2-sensitive
AGGCTTCGCCTTGCAGGTCGTCGT	0	10	RDR2-sensitive
AGGCTTCGCGGACGGTTCGGCCAG	0	17	RDR2-sensitive
AGGCTTCGTGCCGGGCTCGTGGGC	0	11	RDR2-sensitive
AGGCTTCTGGGCAAGTCTGTCGGG	0	9	RDR2-sensitive
AGGCTTGATCTCGCTCGTCGTGAC	0	13	RDR2-sensitive
AGGCTTGCTTAGCAGGGACTTGGT	1	12	RDR2-sensitive
AGGCTTGTAACTCTATCAGACAGA	1	18	RDR2-sensitive
AGGCTTGTAGGACCATGGGACGGT	0	10	RDR2-sensitive
AGGCTTTGCCGAGTGCTTCGGACA	0	9	RDR2-sensitive
AGGCTTTGGAAAGTTGGACCGGTT	71	0	RDR2-resistant
AGGCTTTTAACCTTGTGGTCGTGG	0	9	RDR2-sensitive
AGGGAAAAATCGTGCCGGGCCGGC	0	11	RDR2-sensitive
AGGGAAAAATCGTGTCGGGCCGGC	0	11	RDR2-sensitive
AGGGAAAAATGTAGAGGGGACCGC	0	22	RDR2-sensitive
AGGGAAACTGCACGAACACAGCAT	0	9	RDR2-sensitive
AGGGAAACTGCGCGAACACAGCAT	0	28	RDR2-sensitive
AGGGAACAAGCAGGCTCAGCTCGA	0	13	RDR2-sensitive
AGGGAACCAGCAGAACAAGCACAT	0	15	RDR2-sensitive
AGGGAACCGACACGTAGGGGGCGT	0	12	RDR2-sensitive
AGGGAACCGGCAGAACAAGCACAT	0	10	RDR2-sensitive
AGGGAACCTGACAGCAGGCGTCGT	0	9	RDR2-sensitive
AGGGAACGCTACTGGAGACTGAGA	0	12	RDR2-sensitive

AGGGAACGGTAGACCAACGGCTAG	0	16	RDR2-sensitive
AGGGAACGTGTAGTACGGGTAGGC	0	10	RDR2-sensitive
AGGGAACGTTGTTGTAGACGGCCT	0	9	RDR2-sensitive
AGGGAACTAAAAGGCGGCTCAGCT	1	20	RDR2-sensitive
AGGGAACTCGAGCCCTGCGTCAGG	0	10	RDR2-sensitive
AGGGAACTCGTGTCCGCAAGACGT	0	11	RDR2-sensitive
AGGGAACTGAACGATGACGTGGAC	0	10	RDR2-sensitive
AGGGAACTGGATTACACGGCTTCA	0	28	RDR2-sensitive
AGGGAACTGTAGCCCAACGGCTAG	0	32	RDR2-sensitive
AGGGAAGAAGAGGACGCTGCCTGT	0	10	RDR2-sensitive
AGGGAAGACGAACGCTGCCCTCGG	0	9	RDR2-sensitive
AGGGAAGACGTCGCTCCTGCTCGC	0	12	RDR2-sensitive
AGGGAAGCGAAGAGTAGAGGACAC	0	13	RDR2-sensitive
AGGGAAGGCAGAACGGCAGAACAC	0	14	RDR2-sensitive
AGGGAAGGGAGCGGATCTGGTGAT	3	19	RDR2-sensitive
AGGGAAGGGCGTACTCGATCGTGT	0	25	RDR2-sensitive
AGGGAATACCCAGTCGGAAGTACC	1	22	RDR2-sensitive
AGGGAATATTCTCCTCAGATCCGG	31	126	RDR2-sensitive
AGGGAATGCAGAGCATACAGCGAA	0	14	RDR2-sensitive
AGGGAATGGGAATAGGCTAGGCGA	0	12	RDR2-sensitive
AGGGAATGGTGACAGAACACGGAA	0	9	RDR2-sensitive
AGGGAATGGTGATTTGGATTCGAA	24	2	RDR2-resistant
AGGGACAACAGAGAACGACAGAGC	0	14	RDR2-sensitive
AGGGACAACAGAGGTCAGAGGACG	0	9	RDR2-sensitive
AGGGACAACAGGGGACAACACTAG	1	21	RDR2-sensitive
AGGGACAACCAGAATCGTGCGCTA	0	34	RDR2-sensitive
AGGGACAACGACACGACATTGAAA	0	9	RDR2-sensitive
AGGGACAACGGTGGACAAAACTAG	0	11	RDR2-sensitive
AGGGACAACTTTGATCAGCCGCAT	0	13	RDR2-sensitive
AGGGACAAGAACGGGAAAGGCAGC	0	65	RDR2-sensitive
AGGGACAAGAGAGGACGTCAGGGA	0	13	RDR2-sensitive
AGGGACAAGCAGCTTCGACTCCGC	0	12	RDR2-sensitive
AGGGACAAGCATAACTATCATCGC	18	0	RDR2-resistant
AGGGACAAGGGACAGAAAGTCGGT	0	10	RDR2-sensitive
AGGGACACAAGGCGCGTGAAACGT	0	16	RDR2-sensitive
AGGGACACAATTCGGTTGTTGGAC	0	9	RDR2-sensitive
AGGGACACCCTTCGCAGGACTCGG	3	90	RDR2-sensitive
AGGGACACGAGCAGACGTAGGCTT	0	30	RDR2-sensitive
AGGGACACGCAGCAACAAATTCAC	2	16	RDR2-sensitive
AGGGACACTTACGAAGGCAAGCAT	0	13	RDR2-sensitive
AGGGACAGACGGACAACAGCCAGC	0	10	RDR2-sensitive
AGGGACAGACGGACAGCAGCCAGC	0	20	RDR2-sensitive
AGGGACAGAGACGGCTGAAAGCGA	0	10	RDR2-sensitive
AGGGACAGCTGTAGGAGCGGTCGA	0	24	RDR2-sensitive
AGGGACAGCTGTTGGAGCGGTCGA	7	57	RDR2-sensitive
AGGGACAGGCTGATGCTCGTGTAC	0	19	RDR2-sensitive
AGGGACAGGGCAGCAGAATATCTT	0	25	RDR2-sensitive
AGGGACAGTAGGAGGGCCGAGGAG	0	9	RDR2-sensitive
AGGGACAGTGAATGCAGTGTCGTT	0	40	RDR2-sensitive

AGGGACATAGGAAATTACGGCCAC	0	10	RDR2-sensitive
AGGGACATAGGTAGAAACGGCCCT	0	9	RDR2-sensitive
AGGGACATCCCCTGTCGTTCTCTC	0	16	RDR2-sensitive
AGGGACATGCGGACAACAGCCAGC	0	11	RDR2-sensitive
AGGGACATGGAACCACCAGACGGT	0	14	RDR2-sensitive
AGGGACATGTAGCGTCGGATATGA	0	32	RDR2-sensitive
AGGGACCAGTATGGCGAGACGAGC	0	14	RDR2-sensitive
AGGGACCCACAGAAGAGTGTGAAC	0	33	RDR2-sensitive
AGGGACCCCCCTGTCTCGAAACGC	0	37	RDR2-sensitive
AGGGACCCCCGGTCTCGGAACGCC	2	16	RDR2-sensitive
AGGGACCCCCTGTCTCGAAACGCC	2	18	RDR2-sensitive
AGGGACCCTGAAATCTGTTGGCGC	0	11	RDR2-sensitive
AGGGACCGACACATAGGGAGCGCT	0	12	RDR2-sensitive
AGGGACCGACACGTAGAGAGCGCT	0	11	RDR2-sensitive
AGGGACCGACACGTAGGGGGCGCT	0	11	RDR2-sensitive
AGGGACCGACATCGAGACGGGGTG	0	9	RDR2-sensitive
AGGGACCGAGCAAGGGATGAGCGC	0	10	RDR2-sensitive
AGGGACCGATACTCAAGGCACCAG	0	11	RDR2-sensitive
AGGGACCGATACTCAAGGCATCAG	0	15	RDR2-sensitive
AGGGACCGATGCTCAAGGCACCAG	0	50	RDR2-sensitive
AGGGACCTCAGCTTCGGCGTCATT	34	0	RDR2-resistant
AGGGACCTCATCCTGAACGAAGGG	1	17	RDR2-sensitive
AGGGACCTCCTCTACACTCAGGGC	0	20	RDR2-sensitive
AGGGACCTGAAAAGCTAGGCGTTT	0	16	RDR2-sensitive
AGGGACCTGCCGACTTGACGGAGA	1	15	RDR2-sensitive
AGGGACCTGTATGATGGACGAGCA	0	13	RDR2-sensitive
AGGGACCTTCCTAAGAATTGGCAC	11	31	RDR2-sensitive
AGGGACCTTCCTAGGAATTGGCAC	0	9	RDR2-sensitive
AGGGACCTTCGGCCATCTCTTGGA	2	17	RDR2-sensitive
AGGGACCTTTAAGCTACGAGCCAG	0	29	RDR2-sensitive
AGGGACGAACTCTACAGGTAGCGT	0	14	RDR2-sensitive
AGGGACGAAGACGAAGCGTCGGTT	1	19	RDR2-sensitive
AGGGACGAAGACGGACGAAGACGA	1	19	RDR2-sensitive
AGGGACGACAGAACAGAGAAGCGA	2	20	RDR2-sensitive
AGGGACGACGCGCGCAACTCGAAC	0	11	RDR2-sensitive
AGGGACGACTGACGTGAGGACCAT	1	13	RDR2-sensitive
AGGGACGAGAGAGGACGCCAGAAA	0	10	RDR2-sensitive
AGGGACGAGAGAGGACGTCAGGGG	0	10	RDR2-sensitive
AGGGACGAGAGTGAAGGCGAGAGC	0	9	RDR2-sensitive
AGGGACGAGAGTGGAGGCGAGAGC	1	47	RDR2-sensitive
AGGGACGCAGACACGATTCACCTA	2	16	RDR2-sensitive
AGGGACGCAGGGACGAAGCTTCAC	0	11	RDR2-sensitive
AGGGACGCTCGGTATACAACTTTG	0	9	RDR2-sensitive
AGGGACGCTGTAGGGGGCACCGCT	0	15	RDR2-sensitive
AGGGACGGAGCCAGAAAAAAGTTT	0	13	RDR2-sensitive
AGGGACGGCAGAACCGTGCGCCAC	0	32	RDR2-sensitive
AGGGACGGCTGACGTGAGGACCAT	2	34	RDR2-sensitive
AGGGACGGCTGGCGTGAGGACCAT	0	14	RDR2-sensitive
AGGGACGGGAACAGCCTTCGGGCT	14	0	RDR2-resistant

AGGGACGGGCCCTATTGAATTCAA	0	11	RDR2-sensitive
AGGGACGGTCTGTGATCAGGAGTC	0	9	RDR2-sensitive
AGGGACGGTGACGGACGCCAACGG	0	9	RDR2-sensitive
AGGGACGTCATCTATCGTTCTCTC	0	9	RDR2-sensitive
AGGGACGTGACCTACATAGACAGA	0	13	RDR2-sensitive
AGGGACGTGGGGCACGGTAACCAA	0	9	RDR2-sensitive
AGGGACGTGGTAATGTAGACGACA	0	10	RDR2-sensitive
AGGGACGTTCTGCTCGAATCGGTA	0	13	RDR2-sensitive
AGGGACGTTGTTGAGAATGGCCGC	0	10	RDR2-sensitive
AGGGACGTTTCGTCGGTCGGCAAT	0	10	RDR2-sensitive
AGGGACTACAGGCCAGCAAGGTAC	1	12	RDR2-sensitive
AGGGACTACTGACAAGCAAGGTAC	2	59	RDR2-sensitive
AGGGACTAGCTTGATCAGTTGCTC	0	17	RDR2-sensitive
AGGGACTAGGGTTGTGATGGGCAA	0	13	RDR2-sensitive
AGGGACTATACTGCGTACATCTGC	0	14	RDR2-sensitive
AGGGACTATACTGCGTACGACAAC	0	9	RDR2-sensitive
AGGGACTCAAAACCTTGCGTCTTT	0	12	RDR2-sensitive
AGGGACTCCACGCAAAGACGTCGG	0	13	RDR2-sensitive
AGGGACTCCGTACAAGCCAAAGGC	66	7	RDR2-resistant
AGGGACTCCTCACTCGAGGAAGAC	15	0	RDR2-resistant
AGGGACTCGGCTTGCATCGTTCCA	0	15	RDR2-sensitive
AGGGACTCGTATACCGAGCAGATA	0	13	RDR2-sensitive
AGGGACTCTCTACATACGCAACAA	0	12	RDR2-sensitive
AGGGACTGACGTGTTATTCCTATT	0	10	RDR2-sensitive
AGGGACTGATTTTGTAACGGTCAG	0	10	RDR2-sensitive
AGGGACTGCACAGCGTACATCTTC	0	42	RDR2-sensitive
AGGGACTGCACGACGTACATCTTC	0	15	RDR2-sensitive
AGGGACTGCACGGCGTACATCTTC	0	12	RDR2-sensitive
AGGGACTGCACTGCGTACACCTTC	0	20	RDR2-sensitive
AGGGACTGCACTGCGTACATCTGC	0	81	RDR2-sensitive
AGGGACTGCACTGCGTACATCTTC	1	27	RDR2-sensitive
AGGGACTGCAGGGCAGAGAAGCGG	1	14	RDR2-sensitive
AGGGACTGCCGGCCCAATAGCCCA	0	14	RDR2-sensitive
AGGGACTGCTGACGTGAGGACCAT	0	51	RDR2-sensitive
AGGGACTGGCAAAGGGGCCCACTG	0	12	RDR2-sensitive
AGGGACTGGCATCAAGACGAGATG	0	21	RDR2-sensitive
AGGGACTGGCGTCGAAGCGAGGCG	1	18	RDR2-sensitive
AGGGACTGGCGTCGAAGCGAGGGA	0	17	RDR2-sensitive
AGGGACTGGCTGCTCGCAACGGCG	0	24	RDR2-sensitive
AGGGACTGGTTGGCACGGCAGGGC	0	12	RDR2-sensitive
AGGGACTTGTGCCGCAGCAGCCTC	2	14	RDR2-sensitive
AGGGACTTTTCATTCGTCTGGCCA	69	0	RDR2-resistant
AGGGAGAACACTCGGCAAAGTTAT	0	15	RDR2-sensitive
AGGGAGAAGGACAACGGACAGAAA	0	15	RDR2-sensitive
AGGGAGAATCCGGCTGTGTAGAGA	0	11	RDR2-sensitive
AGGGAGACCTTCCTGGTCGGACGG	0	11	RDR2-sensitive
AGGGAGACGGATACTCTACAGCTT	0	16	RDR2-sensitive
AGGGAGACGGATGCGGAGACTGCT	0	9	RDR2-sensitive
AGGGAGACGGGTACTCTACAACTT	0	17	RDR2-sensitive

AGGGAGACGGGTACTCTACAGCTT	0	23	RDR2-sensitive
AGGGAGATGGCAGAACAATGGTGC	0	12	RDR2-sensitive
AGGGAGCCGACATGTAGAGGGCGC	0	23	RDR2-sensitive
AGGGAGCCGGCGTGTAGGGGCACC	0	10	RDR2-sensitive
AGGGAGCCGTAGCACCCTCGGCAT	0	15	RDR2-sensitive
AGGGAGCGCTGTAGGAGGCACCGC	1	109	RDR2-sensitive
AGGGAGCGCTGTAGGGAGCACCGC	0	40	RDR2-sensitive
AGGGAGCGCTGTAGGGGCACCACT	0	25	RDR2-sensitive
AGGGAGCGCTGTAGGGGGCACCGC	1	108	RDR2-sensitive
AGGGAGCGTGGGCGACGGTAGTGC	0	9	RDR2-sensitive
AGGGAGCTGGACCATGGCGAGCAA	0	24	RDR2-sensitive
AGGGAGCTGTAGCACCCTCGGCAT	0	16	RDR2-sensitive
AGGGAGGCGGGGGCATATGGACGG	0	9	RDR2-sensitive
AGGGAGGCTAGATGAGGAACGGTT	0	12	RDR2-sensitive
AGGGAGGTGCTGTAGCCGGCTAGG	0	39	RDR2-sensitive
AGGGAGTAGACGCACGACCTACGC	0	29	RDR2-sensitive
AGGGAGTAGCAGAACACGGAATGA	0	9	RDR2-sensitive
AGGGAGTCGGTAGAGCGCGGCGCG	0	9	RDR2-sensitive
AGGGATAAAAGAGGACGACCACAT	0	22	RDR2-sensitive
AGGGATAAAGGACCTGACCGTCCC	0	31	RDR2-sensitive
AGGGATAACCCTCTGGTCGTCTAT	0	12	RDR2-sensitive
AGGGATAACTGGGGACAACACGGG	0	11	RDR2-sensitive
AGGGATAAGAGAGAACGCCAGGAG	0	12	RDR2-sensitive
AGGGATACCCTTATTCCCGTCGGG	0	12	RDR2-sensitive
AGGGATAGAATAGGACGGACACAT	0	31	RDR2-sensitive
AGGGATAGATCTGAGCAAGACGTC	0	11	RDR2-sensitive
AGGGATAGTGGACGGCCGAAGCAG	1	20	RDR2-sensitive
AGGGATATTGCATGCTCTTAGTCA	0	10	RDR2-sensitive
AGGGATATTTTACTGTAAATGGCG	1	42	RDR2-sensitive
AGGGATCAGCTCGGGCGAGACGGA	0	9	RDR2-sensitive
AGGGATCAGTGAGTGGGCCGGCCC	0	10	RDR2-sensitive
AGGGATCGTTGCTGGAGATGGTAG	0	10	RDR2-sensitive
AGGGATCTGTATGGAAGAGCGGAC	0	18	RDR2-sensitive
AGGGATGACTCTGGGTACGTCCGA	4	22	RDR2-sensitive
AGGGATGATCGACCTGGGCTTCCT	0	9	RDR2-sensitive
AGGGATGCTGGACAGCTTTTGACG	1	14	RDR2-sensitive
AGGGATGCTGGGCTGCAGGGACGT	0	18	RDR2-sensitive
AGGGATGGAAGAATGTGGCCACAT	0	10	RDR2-sensitive
AGGGATGGAATGAGGCGGCCACGT	1	17	RDR2-sensitive
AGGGATGGCAGAACCGTGCACCAA	0	14	RDR2-sensitive
AGGGATGGTGGCAGAACACGGAAT	0	13	RDR2-sensitive
AGGGATTAAGTAATTCCCGTCGGC	0	9	RDR2-sensitive
AGGGATTGAGTGATATGGGAGGGA	0	26	RDR2-sensitive
AGGGATTGGGTGAAAGGCTTGGAT	0	33	RDR2-sensitive
AGGGATTGGTCTAGCCGAGGGCAG	2	27	RDR2-sensitive
AGGGATTGTGCTCGAAGGCGGCCA	0	9	RDR2-sensitive
AGGGATTGTGCTCGAAGGCGGCTT	0	14	RDR2-sensitive
AGGGATTTGACTGATTGGTATGGT	14	0	RDR2-resistant
AGGGATTTGATTGTGTGTCGTCGG	30	0	RDR2-resistant

AGGGATTTGCACCAACTGGGCCAT	0	9	RDR2-sensitive
AGGGCAAACTCGGCATATGTGCGT	0	11	RDR2-sensitive
AGGGCAAACTGAAGATGCCGGACA	0	9	RDR2-sensitive
AGGGCAAACTTCGAACCAGACCGC	0	17	RDR2-sensitive
AGGGCAACGGTATGAAGATAGCAA	0	10	RDR2-sensitive
AGGGCACAGTGGAACGGAGGGAAA	0	9	RDR2-sensitive
AGGGCACGGCACGGTAGAAGATCC	0	9	RDR2-sensitive
AGGGCACTACAAGACGAACTGCAC	0	13	RDR2-sensitive
AGGGCACTGATTAGATCTAGGCGA	0	21	RDR2-sensitive
AGGGCACTGTACGAGGGTATTTAT	0	20	RDR2-sensitive
AGGGCACTGTACGGGGGTATTTAT	2	23	RDR2-sensitive
AGGGCACTGTGCGGGGGTATTTAT	0	10	RDR2-sensitive
AGGGCACTGTGGCATAGATAACTA	0	12	RDR2-sensitive
AGGGCAGAACAGAGTGGCAGCCTA	0	13	RDR2-sensitive
AGGGCAGAACGACAGAACGGCCGA	0	20	RDR2-sensitive
AGGGCAGAACGGCAGAACAGCAGA	0	39	RDR2-sensitive
AGGGCAGAACGGTAGACTACTCTT	0	10	RDR2-sensitive
AGGGCAGAGTTGGATTGGGTCATT	0	11	RDR2-sensitive
AGGGCAGATCTGTAGATGCAGGCT	0	10	RDR2-sensitive
AGGGCAGCCTGGAGAAGATGACCA	0	17	RDR2-sensitive
AGGGCAGGGACGTAGTCAACGCGA	1	14	RDR2-sensitive
AGGGCAGGGTTGATGTTGGACAAC	16	0	RDR2-resistant
AGGGCAGTAGGGGAGTAGGCGTGT	0	9	RDR2-sensitive
AGGGCAGTAGGTAGAGAGCGCGCG	0	28	RDR2-sensitive
AGGGCAGTAGTCAAGAGCGTGCTG	0	19	RDR2-sensitive
AGGGCAGTGTCGGCGACAGCTCTT	1	16	RDR2-sensitive
AGGGCATAAGGCAGCAACCTCTGA	0	10	RDR2-sensitive
AGGGCATGATGCATCAGACAGCAC	0	10	RDR2-sensitive
AGGGCATGGCACTACATACACATT	5	87	RDR2-sensitive
AGGGCATGGCAGATGATGACGACA	0	31	RDR2-sensitive
AGGGCATGTACAACCCAGATACGG	0	41	RDR2-sensitive
AGGGCATGTACAGTAGAGAGACAC	0	13	RDR2-sensitive
AGGGCATGTACAGTGAAGAGACAT	0	9	RDR2-sensitive
AGGGCATGTACAGTGGAGAGACAC	11	139	RDR2-sensitive
AGGGCATGTATAGTGGAGAGACAT	3	23	RDR2-sensitive
AGGGCATGTGGATCGACACGGCAT	1	20	RDR2-sensitive
AGGGCCAAGCACTCGGCAAAGCCA	1	14	RDR2-sensitive
AGGGCCAGCAGAGAACGACAGAGC	0	16	RDR2-sensitive
AGGGCCATGGACGAAGACGCAGGC	0	21	RDR2-sensitive
AGGGCCCACGCGATCTGAGGCGGC	0	9	RDR2-sensitive
AGGGCCCAGAAGAATAGACGACCA	0	34	RDR2-sensitive
AGGGCCCCCTCAAGCAACGGACAC	3	41	RDR2-sensitive
AGGGCCCCTCTGACCACTGGACGT	0	20	RDR2-sensitive
AGGGCCCCTGTTGGAAGGATGTTT	0	11	RDR2-sensitive
AGGGCCGCTCGGAGCACTCGGCAA	0	11	RDR2-sensitive
AGGGCCGGTCCTGAGATCTCGGAT	0	23	RDR2-sensitive
AGGGCCGTGTCTGGACCGAAGGCG	0	9	RDR2-sensitive
AGGGCCGTTTAGAGGACGCTGCTG	0	15	RDR2-sensitive
AGGGCCTACGCTCCTACCGTCGGA	20	0	RDR2-resistant

AGGGCCTCGGATTTTGTCGGCACG	0	19	RDR2-sensitive
AGGGCCTGGCAGGGAACGTCGCTG	0	13	RDR2-sensitive
AGGGCCTGTTTGGAAATGCGGTTT	0	15	RDR2-sensitive
AGGGCCTGTTTGGTTTGTGGCTAA	2	15	RDR2-sensitive
AGGGCCTTAAGACCTGTACGTCAT	0	16	RDR2-sensitive
AGGGCCTTACATTATCCCCGTCGG	0	12	RDR2-sensitive
AGGGCCTTCTCATCGACCCGTCGG	40	6	RDR2-resistant
AGGGCCTTCTCATCGACTCGTCGG	41	0	RDR2-resistant
AGGGCGAAGGAGAACGGCGACCAC	0	12	RDR2-sensitive
AGGGCGACACTCGGCAAAGACTAG	1	17	RDR2-sensitive
AGGGCGACAGCCTGAGCGCGCTGT	19	1	RDR2-resistant
AGGGCGACGGTACGAAGATAACAA	0	26	RDR2-sensitive
AGGGCGACGGTATGAAGATAGCAA	5	100	RDR2-sensitive
AGGGCGACTGAGGGCGTAGAGGCT	0	20	RDR2-sensitive
AGGGCGACTGGACGGTTGGGCTAC	0	19	RDR2-sensitive
AGGGCGACTGTATGAAGATAGCAA	0	44	RDR2-sensitive
AGGGCGAGAGGACGATCAGGGACA	0	18	RDR2-sensitive
AGGGCGCTCCCTAGAATACGGATA	0	17	RDR2-sensitive
AGGGCGCTGCGCAGATCGACGTCT	28	0	RDR2-resistant
AGGGCGCTGGAAAGAGGCATCTGG	0	18	RDR2-sensitive
AGGGCGCTGGCTCCTTTCCGGCGT	21	0	RDR2-resistant
AGGGCGCTGTAGAGGCACCACTGC	0	16	RDR2-sensitive
AGGGCGCTGTAGAGGGCACCGCTG	0	26	RDR2-sensitive
AGGGCGCTGTAGCAGGCACCGCTG	1	38	RDR2-sensitive
AGGGCGCTGTAGGGGCACCGCTGC	0	15	RDR2-sensitive
AGGGCGCTGTAGGGGGCACCGCTG	1	22	RDR2-sensitive
AGGGCGGACTGTCCGCGGCCTGGG	0	13	RDR2-sensitive
AGGGCGGCACCACTGTTGGGCTAC	0	15	RDR2-sensitive
AGGGCGGCTGGGAGGAGGGCAGAC	58	1	RDR2-resistant
AGGGCGGGGTTGATGTTGGACAAC	52	15	RDR2-resistant
AGGGCGGGTTGTGGTTCGTGCAGC	0	10	RDR2-sensitive
AGGGCGTAACATAGAAGCGGACGG	0	10	RDR2-sensitive
AGGGCGTAGAGGCTCGGCGAGGCA	0	10	RDR2-sensitive
AGGGCGTAGCATATGGCTGGTTAT	2	43	RDR2-sensitive
AGGGCGTGGAGTAGATGCAGGCAA	0	9	RDR2-sensitive
AGGGCGTGTTCGGCTGGCTACAAG	0	16	RDR2-sensitive
AGGGCTAACCAGCAGAACACACGT	0	12	RDR2-sensitive
AGGGCTAACGACTAAGGATGGCAA	1	12	RDR2-sensitive
AGGGCTAAGGACACCATGTGGCAA	0	9	RDR2-sensitive
AGGGCTAAGGTCTCGTTCGTCTGC	0	15	RDR2-sensitive
AGGGCTACGGCGATTAACTCGGAA	0	10	RDR2-sensitive
AGGGCTAGAAACGATCCGAGACGA	0	11	RDR2-sensitive
AGGGCTAGACTCAGTGAGGCTCGG	1	35	RDR2-sensitive
AGGGCTAGATCTAGGAAGCAGCTT	0	15	RDR2-sensitive
AGGGCTAGGTCCTCTGCCTGACGG	0	16	RDR2-sensitive
AGGGCTAGTTTGGATCGGCGCGCC	0	19	RDR2-sensitive
AGGGCTATTGACGTCGACTTTATG	39	2	RDR2-resistant
AGGGCTATTGTGCAAAAATGACGT	8	47	RDR2-sensitive
AGGGCTCAAGCACTTCTCCGACGG	3	44	RDR2-sensitive

AGGGCTCAAGCGCTCCTCCGACGG	24	96	RDR2-sensitive
AGGGCTCAGACACTTCTCCGACGG	0	22	RDR2-sensitive
AGGGCTCAGGCACTCCTCCGACGG	0	36	RDR2-sensitive
AGGGCTCAGGCACTTCTCCGACGG	9	65	RDR2-sensitive
AGGGCTCAGGCGCTCCTCCGACGG	0	9	RDR2-sensitive
AGGGCTCCATGCATGGACTTGGCA	0	11	RDR2-sensitive
AGGGCTCGGCAACAGTACATCGGC	0	13	RDR2-sensitive
AGGGCTCGGCGAACAGTACATCGG	0	9	RDR2-sensitive
AGGGCTCGGGTTTCTGCCGGACGG	0	16	RDR2-sensitive
AGGGCTCTCGGCACAGGGACTGGC	3	43	RDR2-sensitive
AGGGCTCTGCTCCTCTGAAAGCGG	0	13	RDR2-sensitive
AGGGCTGAATCCAGGAAGCAGCTT	0	14	RDR2-sensitive
AGGGCTGACATGGCATTGACGCGG	0	9	RDR2-sensitive
AGGGCTGACCTGGCGCTGACGTGG	5	27	RDR2-sensitive
AGGGCTGACCTGGCTCTGACGTGG	3	16	RDR2-sensitive
AGGGCTGACGCGACGTCGACGTGA	0	14	RDR2-sensitive
AGGGCTGAGCAGAGACAGATCCAG	0	9	RDR2-sensitive
AGGGCTGATTTAGTGACCCGGCAT	0	12	RDR2-sensitive
AGGGCTGATTTGGTGGTAAGGGAT	0	21	RDR2-sensitive
AGGGCTGATTTGGTGGTTAGGGAT	44	4	RDR2-resistant
AGGGCTGCACCAGTGGAAACGCGC	0	15	RDR2-sensitive
AGGGCTGCAGGAACGGCAGATTGA	1	163	RDR2-sensitive
AGGGCTGCCGTCGGCTTCGTGCTC	0	11	RDR2-sensitive
AGGGCTGCGCGGAGGGGATAGACA	0	9	RDR2-sensitive
AGGGCTGCGGACACCGTTGAAGAC	0	14	RDR2-sensitive
AGGGCTGCGTAGAGATCGGCGAGC	0	20	RDR2-sensitive
AGGGCTGCTACCCTAGAACCAAGC	40	729	RDR2-sensitive
AGGGCTGCTGCACGCACGACGCGA	0	15	RDR2-sensitive
AGGGCTGCTGTGAAGACGATCCTC	1	30	RDR2-sensitive
AGGGCTGGACAAAATACTCGCGGC	0	17	RDR2-sensitive
AGGGCTGGACAAAATGCTCGCGGC	0	30	RDR2-sensitive
AGGGCTGGACGAAATACTCGTGGC	0	13	RDR2-sensitive
AGGGCTGGACGAAGCATGAGGGTT	0	65	RDR2-sensitive
AGGGCTGGACGGTCCGCGACCTGA	0	11	RDR2-sensitive
AGGGCTGGATCAAGGCAGCAACTT	0	10	RDR2-sensitive
AGGGCTGGATCCAGGAAGCAGCTA	0	13	RDR2-sensitive
AGGGCTGGATCCAGGAAGCAGCTT	19	184	RDR2-sensitive
AGGGCTGGATCCAGGAAGCAGTTT	0	9	RDR2-sensitive
AGGGCTGGATCTAGGAAGCAGCTT	3	46	RDR2-sensitive
AGGGCTGGATTCAGGAAGCAGCTT	0	12	RDR2-sensitive
AGGGCTGGATTTAGGAAGCAGCTT	0	12	RDR2-sensitive
AGGGCTGGCGGATAGGTCGGGGGC	0	9	RDR2-sensitive
AGGGCTGGCGTGGCAACGATGCAG	0	10	RDR2-sensitive
AGGGCTGGCGTTCCGTAGATATGC	0	11	RDR2-sensitive
AGGGCTGGGAAGGCTGTAGACAGG	0	9	RDR2-sensitive
AGGGCTGGGGTGTTACGGGACGGA	0	16	RDR2-sensitive
AGGGCTGGGTACCGTCGGAGATAA	0	10	RDR2-sensitive
AGGGCTGGGTGGTGTACGGACGGA	0	9	RDR2-sensitive
AGGGCTGGTGTTGGACGAATACGG	0	9	RDR2-sensitive

AGGGCTGTAGACAGGTCGTCAGGC	0	10	RDR2-sensitive
AGGGCTGTATCTGGTGGACCAGGC	0	12	RDR2-sensitive
AGGGCTGTCAGGAGTAAGTACCGC	0	12	RDR2-sensitive
AGGGCTGTGCGTATGGGAGCGGGT	0	17	RDR2-sensitive
AGGGCTGTGTATAGGGCTGTCTAT	0	10	RDR2-sensitive
AGGGCTGTTCTTGCTTACCGGTGC	0	11	RDR2-sensitive
AGGGCTTACCTGGCTCTGACGTGG	6	44	RDR2-sensitive
AGGGCTTATTTTTCAGCTTCGGCT	2	25	RDR2-sensitive
AGGGCTTCGCTGTTTTGGAGACGC	0	9	RDR2-sensitive
AGGGCTTCTCTGCCTGACGATCGG	2	36	RDR2-sensitive
AGGGCTTGTTCGGTTACACCAATC	4	51	RDR2-sensitive
AGGGCTTGTTCGGTTACACTAATC	0	18	RDR2-sensitive
AGGGCTTGTTCGGTTAGCTCTCAA	1	15	RDR2-sensitive
AGGGCTTGTTCGGTTATACCCCAA	0	9	RDR2-sensitive
AGGGCTTGTTCGGTTATTCCCAAT	12	65	RDR2-sensitive
AGGGCTTGTTCGGTTCTACCCCAA	3	35	RDR2-sensitive
AGGGCTTTCTGTTAAGACTTGTAT	0	18	RDR2-sensitive
AGGGCTTTTCGGGCTGACCCGGCC	0	14	RDR2-sensitive
AGGGGAACCGCTGAAGACAGTCTG	0	9	RDR2-sensitive
AGGGGAACCTGGCTCTGGAGATGC	1	20	RDR2-sensitive
AGGGGAACTCTGCTGCTACGGCTT	0	12	RDR2-sensitive
AGGGGAAGAGAACGCTGACGTGGC	0	10	RDR2-sensitive
AGGGGAAGATGCGGGACGATGCTT	16	0	RDR2-resistant
AGGGGAAGGGGGCGTACTGTAGCC	0	30	RDR2-sensitive
AGGGGAATCGCGCGATGGCAGAAC	0	11	RDR2-sensitive
AGGGGACAACACAGGATAGTACTG	1	20	RDR2-sensitive
AGGGGACACAGGCTAGGGTGCGAC	19	0	RDR2-resistant
AGGGGACAGAACCGTACATCATGA	0	18	RDR2-sensitive
AGGGGACAGTGAACAAGGCATCCC	0	18	RDR2-sensitive
AGGGGACATATGTTGAGCACCAAA	0	12	RDR2-sensitive
AGGGGACCCTCGGGCGAGACGTGA	0	14	RDR2-sensitive
AGGGGACCGAATTTAGGGGACGTT	8	139	RDR2-sensitive
AGGGGACCGACACATAGGGGGCGC	0	18	RDR2-sensitive
AGGGGACCGACACGTAATGGGCGC	0	50	RDR2-sensitive
AGGGGACCGACACGTAGAGAGCGC	0	19	RDR2-sensitive
AGGGGACCGACACGTAGAGGATGC	0	23	RDR2-sensitive
AGGGGACCGACACGTAGAGGCACT	0	9	RDR2-sensitive
AGGGGACCGACACGTAGAGGGCGC	2	45	RDR2-sensitive
AGGGGACCGACACGTAGGAGGCGC	2	30	RDR2-sensitive
AGGGGACCGACACGTAGGGACGCT	0	54	RDR2-sensitive
AGGGGACCGACACGTAGGGAGCGC	1	90	RDR2-sensitive
AGGGGACCGACACGTAGGGGACGC	3	139	RDR2-sensitive
AGGGGACCGACACGTAGGGGCGCT	0	37	RDR2-sensitive
AGGGGACCGACACGTAGGGGGCGC	4	247	RDR2-sensitive
AGGGGACCGACACGTAGGGGGCGT	0	13	RDR2-sensitive
AGGGGACCGACACGTAGTGGGCGC	0	17	RDR2-sensitive
AGGGGACCGACGCGTAGAGGGCGC	0	17	RDR2-sensitive
AGGGGACCGACGCGTAGGATGCGC	0	13	RDR2-sensitive
AGGGGACCGACGTGTAGGGGGCGC	0	10	RDR2-sensitive

AGGGGACCGATACGTAGGGGACGT	0	36	RDR2-sensitive
AGGGGACCGTGTTGCACTTATAAG	0	9	RDR2-sensitive
AGGGGACCTAGTGTGGAAGACCAT	0	9	RDR2-sensitive
AGGGGACCTGGTGTGGAAGACTAT	1	18	RDR2-sensitive
AGGGGACGAAATTTAGGGGACGCT	3	51	RDR2-sensitive
AGGGGACGAAATTTAGGGGACGTT	2	40	RDR2-sensitive
AGGGGACGACCAGAATTTGTTTTC	14	0	RDR2-resistant
AGGGGACGAGATCTAGATTCCAGC	1	18	RDR2-sensitive
AGGGGACGCTACTGTAGACTGAGA	0	17	RDR2-sensitive
AGGGGACGCTCTAGATGATCTTAT	0	9	RDR2-sensitive
AGGGGACGCTGCTGGAGACAACCG	1	12	RDR2-sensitive
AGGGGACGCTGCTGGAGACAGCCT	0	15	RDR2-sensitive
AGGGGACGCTGCTGGAGACAGTCT	0	16	RDR2-sensitive
AGGGGACGCTGCTGGAGACTGAGA	2	22	RDR2-sensitive
AGGGGACGCTGTAAGGGGCACTGC	0	19	RDR2-sensitive
AGGGGACGCTGTAGAGGGCACCGC	1	307	RDR2-sensitive
AGGGGACGCTGTAGGGGACACCGC	0	19	RDR2-sensitive
AGGGGACGCTGTAGGGGCACCGCT	0	83	RDR2-sensitive
AGGGGACGCTGTAGGGGGCACCGC	4	105	RDR2-sensitive
AGGGGACGCTTTAGAGGACCTTGT	0	43	RDR2-sensitive
AGGGGACGCTTTAGAGTATCTTGT	0	9	RDR2-sensitive
AGGGGACGGAATTTAGGGGACGCT	1	17	RDR2-sensitive
AGGGGACGGGTATGGGACGAATTT	0	20	RDR2-sensitive
AGGGGACGGGTATGGGACTAATTT	0	9	RDR2-sensitive
AGGGGACGTCGCTGAAGATTGAGA	0	11	RDR2-sensitive
AGGGGACGTGATTGGTTGCCGGAT	0	11	RDR2-sensitive
AGGGGACGTTGCTAGAGACAGCCT	2	14	RDR2-sensitive
AGGGGACGTTGCTGAAGATAGCCT	1	73	RDR2-sensitive
AGGGGACGTTGCTGGAGACACAGA	5	57	RDR2-sensitive
AGGGGACGTTGCTGGAGACAGCCT	2	81	RDR2-sensitive
AGGGGACGTTGCTGGAGACTGAGA	2	15	RDR2-sensitive
AGGGGACTCCAAGCTGAACTTCGC	0	10	RDR2-sensitive
AGGGGACTGACGCGTAGGGGGCGC	0	11	RDR2-sensitive
AGGGGACTGGGAGGGATGAGGCAT	0	23	RDR2-sensitive
AGGGGAGAGCGGGCTGGACAGCGG	67	9	RDR2-resistant
AGGGGAGATGTGGACGTCGACGGT	0	16	RDR2-sensitive
AGGGGAGGCGACAACGTTGAACAT	0	12	RDR2-sensitive
AGGGGAGGCTGTAGTGAGGCACTA	0	12	RDR2-sensitive
AGGGGAGGGAATCTTGCTGACGTG	0	17	RDR2-sensitive
AGGGGAGGGCAGAACAGAATAGCA	0	17	RDR2-sensitive
AGGGGAGGGCAGAACAGAGTGGCA	0	21	RDR2-sensitive
AGGGGAGTGGACTGCATGCCCGGC	0	16	RDR2-sensitive
AGGGGAGTGTAGAGAGCGACGTCT	0	14	RDR2-sensitive
AGGGGATACCCAGTCGGAAGTACC	0	10	RDR2-sensitive
AGGGGATAGAATTTAGGGGACGTT	2	26	RDR2-sensitive
AGGGGATAGAGCTGACACCTTAGG	0	13	RDR2-sensitive
AGGGGATGACAGCTTCGACGACGA	0	12	RDR2-sensitive
AGGGGATGACGGATTCGACAACTC	0	11	RDR2-sensitive
AGGGGATGACGGGTTCGACGACTC	1	18	RDR2-sensitive

AGGGGATGACGGGTTCGATGACTC	0	21	RDR2-sensitive
AGGGGATGGAATCTTGCTGACGTG	1	41	RDR2-sensitive
AGGGGATGTAGAGGGAGAGTAGAA	0	11	RDR2-sensitive
AGGGGCAACGGTACACTAACCTGA	36	2	RDR2-resistant
AGGGGCACATACTGGATGAGACAA	0	11	RDR2-sensitive
AGGGGCACGTAGATGGCAGAACCA	0	21	RDR2-sensitive
AGGGGCACGTAGGGCGTACCAAGA	0	18	RDR2-sensitive
AGGGGCACTGTAGGGGGCACCGCT	0	10	RDR2-sensitive
AGGGGCCGGTAGTCAGTGTCTTGG	0	12	RDR2-sensitive
AGGGGCGAGAGTGGAGACGAGAGC	2	21	RDR2-sensitive
AGGGGCGAGAGTGGAGGCGAGAGC	3	142	RDR2-sensitive
AGGGGCGAGATAGAACACCAGAGG	0	13	RDR2-sensitive
AGGGGCGCAATGTGGATTAGATGC	0	11	RDR2-sensitive
AGGGGCGCGCCCGTGTTCTTTGCC	17	1	RDR2-resistant
AGGGGCGCGGAACGAAGGCAGAAC	0	27	RDR2-sensitive
AGGGGCGCGGTTAGACGGTTAGGA	1	15	RDR2-sensitive
AGGGGCGCTGTAGAGGGCACCGCT	1	100	RDR2-sensitive
AGGGGCGCTGTAGGGACACCGCTG	0	9	RDR2-sensitive
AGGGGCGCTGTAGGGGGCACCGCT	0	11	RDR2-sensitive
AGGGGCGCTGTAGGGGTACCGCTG	0	29	RDR2-sensitive
AGGGGCGGACGGTCCGTGTAACAC	0	13	RDR2-sensitive
AGGGGCTACAACATATACTTCCTA	23	0	RDR2-resistant
AGGGGCTAGTTTGGATCGGCACGC	0	16	RDR2-sensitive
AGGGGCTAGTTTGGATCGGCGCGC	0	22	RDR2-sensitive
AGGGGCTATTTGGACAGACGCCTA	0	19	RDR2-sensitive
AGGGGCTCGGACTCGACCTCGGCC	1	13	RDR2-sensitive
AGGGGCTCTCGGAAATAAGACGTT	0	15	RDR2-sensitive
AGGGGCTCTCTGCATTGATGGAAG	0	11	RDR2-sensitive
AGGGGCTGAGGGCAGCAGTGGCAC	0	12	RDR2-sensitive
AGGGGCTGATGCTCAAGGCATAAG	0	12	RDR2-sensitive
AGGGGCTGCACGGCTTTACGTCAG	1	19	RDR2-sensitive
AGGGGCTGCGACCAGCGCGCTGGA	1	29	RDR2-sensitive
AGGGGCTGCTTCGGTCTGGGGCAC	0	10	RDR2-sensitive
AGGGGCTGGGTGTACGGACGGACG	0	12	RDR2-sensitive
AGGGGCTGTTTCGGTCTGGGGCAC	0	9	RDR2-sensitive
AGGGGCTGTTTGGTGTGTGGCCTA	0	11	RDR2-sensitive
AGGGGCTGTTTGGTTCGTGGCTAA	1	13	RDR2-sensitive
AGGGGCTTCAGAATGGACACGCGC	0	9	RDR2-sensitive
AGGGGCTTCTCTGGTCGCTCAGGT	0	27	RDR2-sensitive
AGGGGGAACCGCTGAAGACAGTCT	0	40	RDR2-sensitive
AGGGGGAACCGCTGCGGGAGATGA	1	16	RDR2-sensitive
AGGGGGACTGAGGGCGTAGAGGCT	0	11	RDR2-sensitive
AGGGGGACTGTAGTGAGGCTCCAA	0	9	RDR2-sensitive
AGGGGGACTGTAGTGAGGCTCCAT	0	45	RDR2-sensitive
AGGGGGCACGGTAGAGGGCTGCGC	0	21	RDR2-sensitive
AGGGGGCACGGTTGCGGACAGTCT	0	10	RDR2-sensitive
AGGGGGCACTGTAGGGGGCACCAC	0	13	RDR2-sensitive
AGGGGGCGCTGTAGAGGGCACCGC	0	65	RDR2-sensitive
AGGGGGCGCTGTAGGAGGCACCGC	0	44	RDR2-sensitive

AGGGGGCGCTGTAGGGAGCACCGC	0	13	RDR2-sensitive
AGGGGGCGCTGTAGGGGACACCGC	0	11	RDR2-sensitive
AGGGGGCGCTGTAGGGGCACCGCT	0	24	RDR2-sensitive
AGGGGGCGCTGTAGGGGGCACCGC	2	19	RDR2-sensitive
AGGGGGCGCTGTAGTGGGCACCGC	0	18	RDR2-sensitive
AGGGGGCTCAGCGACTGACGGGAA	34	6	RDR2-resistant
AGGGGGCTCTGCCAGCGATGACAC	0	12	RDR2-sensitive
AGGGGGCTGTAGCACCCTCGGCAT	0	16	RDR2-sensitive
AGGGGGTAGTGCTACATGTTTCAA	0	10	RDR2-sensitive
AGGGGTAGAACCGTGCACCGTGAA	0	11	RDR2-sensitive
AGGGGTAGATCTGAGCAAGACGTC	0	53	RDR2-sensitive
AGGGGTCGGCGTGTAGGGGGCACC	0	11	RDR2-sensitive
AGGGGTCGGTATGCATGCCCTGGG	0	16	RDR2-sensitive
AGGGGTGAACTCTAGGTGACGTGA	0	9	RDR2-sensitive
AGGGGTGGCCTGTAGCCGTCACGG	0	18	RDR2-sensitive
AGGGGTGTTGATCTCGGTCGTCGT	0	18	RDR2-sensitive
AGGGGTTACTCTGGGTCGGCAGGC	0	9	RDR2-sensitive
AGGGGTTGCATGCAGACTGGGACC	0	11	RDR2-sensitive
AGGGGTTGGACGTGGAGAAGATGG	0	15	RDR2-sensitive
AGGGGTTGGGCGTGAGGAAGATGG	0	15	RDR2-sensitive
AGGGGTTGTCTAGGCTCGGGCCGG	2	37	RDR2-sensitive
AGGGTAAACAGAATAACTAGGCTT	14	0	RDR2-resistant
AGGGTAACAGAAGCTGCGCGGCGT	0	9	RDR2-sensitive
AGGGTACAAGGCGCATCCTGGACA	0	11	RDR2-sensitive
AGGGTACCCCGTAGGAGATGGCAT	0	11	RDR2-sensitive
AGGGTACTGTATGCCGCGGTGACA	0	10	RDR2-sensitive
AGGGTACTGTTTACTGTCCGGTGC	0	10	RDR2-sensitive
AGGGTAGACAGAGGGAATAGACGA	0	10	RDR2-sensitive
AGGGTAGACAGTAGGAATAGACGA	0	26	RDR2-sensitive
AGGGTAGACCGGGGGAATAGACGA	0	9	RDR2-sensitive
AGGGTAGACTTAGACGGAGGGTAG	0	15	RDR2-sensitive
AGGGTAGAGCTTAGAGCAGGGCGG	0	10	RDR2-sensitive
AGGGTAGATCTGAGCAAGACGTCC	0	11	RDR2-sensitive
AGGGTAGATGCGACAGGGTAGAAG	0	13	RDR2-sensitive
AGGGTAGCAGATGGGCTTGTGGAG	0	9	RDR2-sensitive
AGGGTAGCCGTCGGAGATAAGGCG	1	27	RDR2-sensitive
AGGGTAGCGAAGATGTGGGGCTGA	0	10	RDR2-sensitive
AGGGTAGCTCTTGAGGTGCAGCGG	0	22	RDR2-sensitive
AGGGTAGGATACAAGATGAGCTAA	0	15	RDR2-sensitive
AGGGTAGGATACAGGATGAGCTAA	1	71	RDR2-sensitive
AGGGTAGGATACAGGATGAGCTAC	1	80	RDR2-sensitive
AGGGTAGGATACAGGATGGGCTAA	0	11	RDR2-sensitive
AGGGTAGGATACATGATGAGCTAA	0	16	RDR2-sensitive
AGGGTAGGATACGGAATGACCATG	0	10	RDR2-sensitive
AGGGTAGGATACGGGATGAGCTAA	0	21	RDR2-sensitive
AGGGTAGTACACTCGGCATATCCA	0	12	RDR2-sensitive
AGGGTATCCACTGCAAGACGCTAT	0	23	RDR2-sensitive
AGGGTATCGACCATTCGGATAGCA	0	14	RDR2-sensitive
AGGGTATTGTATATAGTAAGGCTT	17	0	RDR2-resistant

AGGGTCAAAGTAGGATGAGCCGGA	0	12	RDR2-sensitive
AGGGTCAACAGAGAACGACAGAGC	0	10	RDR2-sensitive
AGGGTCAAGTAGAGGCGAGTTGAA	0	13	RDR2-sensitive
AGGGTCACCCTAGGACTGGGCGTT	1	18	RDR2-sensitive
AGGGTCACGTTGCAACTCACGGCG	18	1	RDR2-resistant
AGGGTCAGCAGAGAACGACAGAAC	1	15	RDR2-sensitive
AGGGTCAGCAGAGAACGACAGAGC	1	190	RDR2-sensitive
AGGGTCAGCTGCAGGGAACAACGG	0	36	RDR2-sensitive
AGGGTCAGTAGCATACAAGGAATG	0	22	RDR2-sensitive
AGGGTCATTGTGTCCTGTCGGGCC	0	10	RDR2-sensitive
AGGGTCCAGCGGCCGGAAGAGCAC	27	2	RDR2-resistant
AGGGTCCAGTGGACGTCAGATCAT	0	9	RDR2-sensitive
AGGGTCCATGCTCTGCTCAGGCAC	173	4	RDR2-resistant
AGGGTCGACGATCTGTACGACGGT	0	9	RDR2-sensitive
AGGGTCGATGCAACTTGACGGATT	0	21	RDR2-sensitive
AGGGTCGTACCCTGTTGACTCGGG	0	13	RDR2-sensitive
AGGGTCGTTCCGAAGGGCTGGTTC	44	3	RDR2-resistant
AGGGTCTCCTGGATCAATGTTTGA	16	0	RDR2-resistant
AGGGTCTCGAACGGCAAGGGCTTC	3	24	RDR2-sensitive
AGGGTCTCGCATGCAGCTGACGAA	0	9	RDR2-sensitive
AGGGTCTGACGCAGAAGAACTGTC	0	14	RDR2-sensitive
AGGGTCTGATAAAATAGAGGACGT	0	16	RDR2-sensitive
AGGGTCTGGACGGTCCGCGATGGC	0	22	RDR2-sensitive
AGGGTCTGTTTACCTCTAGAAGAC	2	15	RDR2-sensitive
AGGGTCTGTTTGGCATGGCTTCAG	0	9	RDR2-sensitive
AGGGTCTGTTTGGTAGAGCTACGG	0	29	RDR2-sensitive
AGGGTGAATATGAACGCGGAGTAT	1	14	RDR2-sensitive
AGGGTGACAACTTATGAAGGCCGA	0	18	RDR2-sensitive
AGGGTGACACGCATAGGACATCGT	1	22	RDR2-sensitive
AGGGTGACGAGGATGATGCGGATA	21	0	RDR2-resistant
AGGGTGATGAACAGTGACACGCTA	4	93	RDR2-sensitive
AGGGTGATGAACAGTGACGCGCTA	0	25	RDR2-sensitive
AGGGTGCATCGCCGGTCGGACAGA	0	15	RDR2-sensitive
AGGGTGCATGCGGCTGATGAGCAT	52	11	RDR2-resistant
AGGGTGCCACCAATTGATGATCGT	21	1	RDR2-resistant
AGGGTGCGCGGATTTCAGCACGGA	23	1	RDR2-resistant
AGGGTGCTCCGAGATCGGCAGGAC	0	14	RDR2-sensitive
AGGGTGCTCTGAAGGTCGGTAGGC	0	10	RDR2-sensitive
AGGGTGGACTGCAGCAGCCGGCAT	0	40	RDR2-sensitive
AGGGTGGAGATTGACGGAAGGCAG	0	11	RDR2-sensitive
AGGGTGGAGGTAGATATGCGGAAA	0	11	RDR2-sensitive
AGGGTGGATCTGAGGCGAGTCGGT	0	10	RDR2-sensitive
AGGGTGGTAGACGCAACAATAGAC	0	9	RDR2-sensitive
AGGGTGTAGCGTATGGCTGGTTAT	0	9	RDR2-sensitive
AGGGTGTAGGCTTACGGTACAGAA	0	18	RDR2-sensitive
AGGGTGTTACACTGTTACTCGGCA	0	9	RDR2-sensitive
AGGGTGTTTGGCATGCTCTGGCCA	0	17	RDR2-sensitive
AGGGTTAGGATTTTTTTTGTCGGC	0	9	RDR2-sensitive
AGGGTTCGTTTCCTGGGAGCGCGG	0	12	RDR2-sensitive

AGGGTTCTCTCTGTAGGAGCACGG	0	14	RDR2-sensitive
AGGGTTCTCTGTAGGCCGGACGGT	0	24	RDR2-sensitive
AGGGTTCTGGAGCAGTTGACCGTT	1	20	RDR2-sensitive
AGGGTTCTTTTCTCTCGGACACAC	0	10	RDR2-sensitive
AGGGTTGAGCAGGGGATGACGGGT	0	11	RDR2-sensitive
AGGGTTGCAGAATGACGCCGCCGC	0	12	RDR2-sensitive
AGGGTTGCTAGAACTAGAGGCCGT	0	10	RDR2-sensitive
AGGGTTGCTGACGCTGACGGGACG	0	15	RDR2-sensitive
AGGGTTGGAGGTGGATATGCGGAA	0	9	RDR2-sensitive
AGGGTTGGATCCAGAAAGCAGCTT	0	9	RDR2-sensitive
AGGGTTGGGGGTCAGACGAGGCAT	0	9	RDR2-sensitive
AGGGTTGTCACAGAACCGTTGGAC	0	16	RDR2-sensitive
AGGGTTGTCCGGCAGAGGATCTGA	0	14	RDR2-sensitive
AGGGTTGTCTGAAGTCCGTTGGAC	4	18	RDR2-sensitive
AGGGTTGTCTGAGATCCGTTGGAC	1	102	RDR2-sensitive
AGGGTTGTCTGAGGTCCGTTGGAC	29	71	RDR2-sensitive
AGGGTTGTCTGAGGTCTGTTGGAC	11	30	RDR2-sensitive
AGGGTTGTCTGATGTCTGTTGGAC	7	77	RDR2-sensitive
AGGGTTGTGCATGAGCCAGGGCAT	0	13	RDR2-sensitive
AGGGTTGTGGGCCGTTGGATGCAA	0	9	RDR2-sensitive
AGGGTTGTGGGCCGTTGGATGCTA	0	14	RDR2-sensitive
AGGGTTGTTTTCTTTGATTCGGAT	0	14	RDR2-sensitive
AGGGTTTAGGACTTTAGGCGGCAC	0	18	RDR2-sensitive
AGGGTTTAGGGGTGTACTGTAGCC	0	17	RDR2-sensitive
AGGGTTTAGGGTTTAGGGTTTAGG	18	0	RDR2-resistant
AGGGTTTGTGCCCACGGACACGGG	0	10	RDR2-sensitive
AGGTAAACGGACCGTGTTAGGCTA	1	28	RDR2-sensitive
AGGTAAACTTTAGGTAGACTGCTA	0	12	RDR2-sensitive
AGGTAACACGACTCGACGCTACAG	0	17	RDR2-sensitive
AGGTAACAGAGGACGGGGGAATAA	0	9	RDR2-sensitive
AGGTAACATCGGGTCGGATTCGGA	0	26	RDR2-sensitive
AGGTAACTGAGGTACCGTCTGGTC	0	18	RDR2-sensitive
AGGTAACTGATGGACCACCCGGCC	0	11	RDR2-sensitive
AGGTAACTGTAGCCATGCGCGGAC	0	24	RDR2-sensitive
AGGTAACTTACCGATTCGGAGCAC	0	11	RDR2-sensitive
AGGTAAGTCTGTGGATCAAAAGGT	20	0	RDR2-resistant
AGGTAAGTGTGTGACTGATGGTGG	0	10	RDR2-sensitive
AGGTAATACGACCGACGGCTCTAA	0	12	RDR2-sensitive
AGGTAATTCTGGGTCGGACCGCAA	0	9	RDR2-sensitive
AGGTAATTGGTTCGATTGGAATGT	0	10	RDR2-sensitive
AGGTACAAGGCGCATCCTGGACAT	0	27	RDR2-sensitive
AGGTACACTAGAGTAGATATCATC	74	0	RDR2-resistant
AGGTACCACAGAATGAGGCGCATG	0	15	RDR2-sensitive
AGGTACCACAGGATGAGGCGCATG	2	25	RDR2-sensitive
AGGTACCCTCGGACTAAAGTCGGC	1	21	RDR2-sensitive
AGGTACCGCAGGATGAGGCGCATG	4	34	RDR2-sensitive
AGGTACCTGTAGTACCTATGCATG	97	5	RDR2-resistant
AGGTACCTTCGGACTCAAGTCGGT	0	9	RDR2-sensitive
AGGTACGACGGACATTCCGGCTAT	0	12	RDR2-sensitive

AGGTACGACTGTACGAGTGCGGAT	2	36	RDR2-sensitive
AGGTACGGAGTGAGAACAGAGGCG	0	10	RDR2-sensitive
AGGTACGGTACGACTACGAGCGGC	0	9	RDR2-sensitive
AGGTACGGTTAGAAACAGGGTCGG	0	10	RDR2-sensitive
AGGTACGTGATGCGTCGGCACAAC	0	9	RDR2-sensitive
AGGTACTACTACCGTGTCGGCACG	1	28	RDR2-sensitive
AGGTACTCGGAAAAGCCGTCGGTT	0	14	RDR2-sensitive
AGGTACTCTTAGGATGATTGGTTG	0	10	RDR2-sensitive
AGGTACTCTTATTGTCGGCGTTTC	2	16	RDR2-sensitive
AGGTACTCTTTAGGGCGACGCATT	1	34	RDR2-sensitive
AGGTACTGCAGGATGAGGCGCATG	0	14	RDR2-sensitive
AGGTACTGGACGGATGGGTTGAAT	0	17	RDR2-sensitive
AGGTACTGGAGACTCCTACAGAAG	0	50	RDR2-sensitive
AGGTACTGGTCCCTGGTCAACGAC	0	10	RDR2-sensitive
AGGTACTGTAAAATAGAGGACGTG	0	24	RDR2-sensitive
AGGTACTGTGAACTATGGCTGCAT	0	23	RDR2-sensitive
AGGTACTGTGCACTGTCCGGTGGC	1	24	RDR2-sensitive
AGGTACTGTGGATCGGCCTAGTAC	0	53	RDR2-sensitive
AGGTACTGTGTACGGACCATGCCC	0	16	RDR2-sensitive
AGGTACTGTTCACTGTCCGGTGGC	2	43	RDR2-sensitive
AGGTACTGTTTACTGTCCGGTGGC	2	20	RDR2-sensitive
AGGTACTGTTTGATGTCCGGTGTG	4	32	RDR2-sensitive
AGGTACTGTTTGCTGTCCGGTGTG	10	27	RDR2-sensitive
AGGTACTTATGAACTGTAATCGGC	0	11	RDR2-sensitive
AGGTACTTGAACTGGACCCGAGGC	0	27	RDR2-sensitive
AGGTACTTGTTTATCTTAGTCACG	20	0	RDR2-resistant
AGGTAGAACACAAGGGAGGCAGGC	0	9	RDR2-sensitive
AGGTAGAACATCTGGTGTAGACGC	0	9	RDR2-sensitive
AGGTAGAACCAGCGTGAGCCGTGT	0	12	RDR2-sensitive
AGGTAGAACCAGGGGAGGCAGCCT	0	19	RDR2-sensitive
AGGTAGAACCGCTGCGGGAGATAA	0	28	RDR2-sensitive
AGGTAGAACCTAGGCAGACCACGA	0	16	RDR2-sensitive
AGGTAGAACGGTAGAACCAGGCAG	0	14	RDR2-sensitive
AGGTAGAATGTTTCGTCGTACGTC	0	17	RDR2-sensitive
AGGTAGAATTGTTTTTGATGGGTA	0	32	RDR2-sensitive
AGGTAGACACTCGGCACAGACTGG	8	81	RDR2-sensitive
AGGTAGAGCTGCAGGCAGAGAGCA	1	16	RDR2-sensitive
AGGTAGAGCTGTACGGGCATCAAC	3	226	RDR2-sensitive
AGGTAGATCGGTAAGCAACTCCTT	16	0	RDR2-resistant
AGGTAGATTTGGACGGCGCAGGCG	0	9	RDR2-sensitive
AGGTAGCACTCTCAGCAAAGGCTC	0	32	RDR2-sensitive
AGGTAGCAGCGGATGCAACAAACT	0	18	RDR2-sensitive
AGGTAGCGCGGGACGATGCGGCCC	18	1	RDR2-resistant
AGGTAGCTCGGCGCCAAGATCTAT	0	15	RDR2-sensitive
AGGTAGGACCGAGGACGCGGGATG	0	9	RDR2-sensitive
AGGTAGGCACTCGGCAAAGACAAG	3	63	RDR2-sensitive
AGGTAGGCGGCATGTAGGGCAAGA	0	21	RDR2-sensitive
AGGTAGGGGACGGACGTAGAGGCG	1	48	RDR2-sensitive
AGGTAGGGGGCGGACGTAGAGGTG	0	21	RDR2-sensitive

AGGTAGTACACCTGGTCGTTGCAC	0	11	RDR2-sensitive
AGGTATCCTCGGACAAGTCGGCGT	2	45	RDR2-sensitive
AGGTATCGCGGCTGCAGAGGCAGA	0	14	RDR2-sensitive
AGGTATCGGATACCGGGCGGATAC	0	9	RDR2-sensitive
AGGTATCGGGCCAGCACGAAACGG	0	11	RDR2-sensitive
AGGTATCTTCGGACTCAAGTCGGC	3	25	RDR2-sensitive
AGGTATCTTCGGACTCAAGTCGGT	4	31	RDR2-sensitive
AGGTATCTTCGGACTCGAATCGGC	20	0	RDR2-resistant
AGGTATCTTCGGACTCGAGTCGGT	2	63	RDR2-sensitive
AGGTATCTTCGGGCTCGAGTCGGT	0	10	RDR2-sensitive
AGGTATGAATTGTTATGTTGACTG	0	10	RDR2-sensitive
AGGTATGCCCCATTTTCGTCGGTA	0	31	RDR2-sensitive
AGGTATGCTGTAGTAGTGCACCAA	0	9	RDR2-sensitive
AGGTATGGTAGACTGTCCGGCTAT	2	30	RDR2-sensitive
AGGTATTGGCCCGTCGGACGGCAC	2	22	RDR2-sensitive
AGGTCAAAGTAGGATGAGACGGGA	0	13	RDR2-sensitive
AGGTCAAAGTAGGATGAGTCGGAG	0	10	RDR2-sensitive
AGGTCAAAGTAGGATGAGTCGGGG	0	13	RDR2-sensitive
AGGTCAAGTTGGGTTTGTCGGGTC	0	20	RDR2-sensitive
AGGTCACACTAGGACGGATCTATA	0	9	RDR2-sensitive
AGGTCACTGTGGGAAACGGAGACG	0	11	RDR2-sensitive
AGGTCAGAACGGCAGACTACTCTT	0	16	RDR2-sensitive
AGGTCAGACGGTTCGCGATGGCAC	0	9	RDR2-sensitive
AGGTCAGCCCTGGACGCAGCTAGC	0	19	RDR2-sensitive
AGGTCATGTGGTTTTCGGCGAGGC	0	11	RDR2-sensitive
AGGTCATGTTAGTCGGGTCGGGTG	0	14	RDR2-sensitive
AGGTCATTCTATCTTTAAGTCGGT	15	82	RDR2-sensitive
AGGTCATTGAACTTGGTATGTTGT	0	11	RDR2-sensitive
AGGTCCCCCTGCTTGACGATGGAC	0	10	RDR2-sensitive
AGGTCCGAACTCCAGCAGGGTCCC	0	9	RDR2-sensitive
AGGTCCGAGGGAACAGAAGCGTCG	0	9	RDR2-sensitive
AGGTCCGGACGAACGAGAGGCCAC	0	9	RDR2-sensitive
AGGTCCGGATGACTCCAGAAGCAG	0	10	RDR2-sensitive
AGGTCCTACCGACTCCTGATACGG	0	18	RDR2-sensitive
AGGTCCTCGAACTTGGCATGTTGT	0	18	RDR2-sensitive
AGGTCCTGCAGAAAGAGACAGCTT	0	59	RDR2-sensitive
AGGTCCTGCAGGAAGAAACAGCTT	1	17	RDR2-sensitive
AGGTCCTGTAGAAAGAGACAGCTT	0	49	RDR2-sensitive
AGGTCCTGTAGCTGGCGGGCGCCG	38	1	RDR2-resistant
AGGTCGACCAACTTTGACGTGGAC	0	16	RDR2-sensitive
AGGTCGAGACGGCCGTCAGTCGGT	0	9	RDR2-sensitive
AGGTCGCAAAGCTCCGTCGGCTAC	18	0	RDR2-resistant
AGGTCGCAGCTCATGGACGGACAT	0	16	RDR2-sensitive
AGGTCGGCAGGCTACCTAAGACGT	3	18	RDR2-sensitive
AGGTCGGGCTATTTTCGTCGGCCG	0	10	RDR2-sensitive
AGGTCGGGTGAGAACGTGGGACGG	0	15	RDR2-sensitive
AGGTCGGTCAGTGGAGCGGCGAAG	1	25	RDR2-sensitive
AGGTCGGTGTAGTTGACGCGCCAA	0	40	RDR2-sensitive
AGGTCGTAAAGCTCCATCGGCTAC	17	1	RDR2-resistant

AGGTCGTAGTTGGATAAGGTCGGC	21	2	RDR2-resistant
AGGTCGTGACTGAGCTGCCGGTAG	0	54	RDR2-sensitive
AGGTCGTGCTTCGGCTGGCGGCTC	0	22	RDR2-sensitive
AGGTCTAAATGGACTGGATATCGG	0	12	RDR2-sensitive
AGGTCTAACTGAACAGAGGAAGAT	0	10	RDR2-sensitive
AGGTCTAATGTGGCGCTGACGGGA	3	25	RDR2-sensitive
AGGTCTATAGAACGGTTAGGGCAT	0	11	RDR2-sensitive
AGGTCTCATCTGACTTCTGACGGT	0	10	RDR2-sensitive
AGGTCTCCGGAACCCTTCGTCTTC	25	197	RDR2-sensitive
AGGTCTCGACTGATCGACTTCGGC	22	1	RDR2-resistant
AGGTCTCGGGCAGCACATACTAGG	0	12	RDR2-sensitive
AGGTCTCTGGCCTAGCCGAACTGT	0	10	RDR2-sensitive
AGGTCTGACGAGCTGACACGGTCA	0	40	RDR2-sensitive
AGGTCTGCAAGCTGATCCTTGGTG	42	8	RDR2-resistant
AGGTCTGCATGATATTGATGGTTG	3	28	RDR2-sensitive
AGGTCTGCTACGAAGAAGACGACT	0	9	RDR2-sensitive
AGGTCTGCTGTGAAGAAGACGATC	1	17	RDR2-sensitive
AGGTCTGGACCGTGCCGGCACGAC	0	11	RDR2-sensitive
AGGTCTGGCCGATGGAACAGGCGT	0	15	RDR2-sensitive
AGGTCTGGCGAGCTGACGCGGTCA	0	21	RDR2-sensitive
AGGTCTGGGACGAAATAGACTGGC	0	11	RDR2-sensitive
AGGTCTGGTCGCTGGGACAGGCGC	1	45	RDR2-sensitive
AGGTCTGGTCGTTGGGACAGGCGC	0	27	RDR2-sensitive
AGGTCTGTTAAGCGGGACTGGGTC	18	0	RDR2-resistant
AGGTCTGTTACGAAGAAGATGACT	0	15	RDR2-sensitive
AGGTCTGTTCCGAAGAATACGCCA	0	10	RDR2-sensitive
AGGTCTGTTTCCTGGGAGTCTGGC	0	9	RDR2-sensitive
AGGTCTGTTTGGTTTGATGGCTAA	0	10	RDR2-sensitive
AGGTCTTATTGGAGCGAAGGCGAA	0	11	RDR2-sensitive
AGGTCTTCGGAACCCTTCGTCTTC	0	11	RDR2-sensitive
AGGTCTTGTAGGAAGAAGCAGCTT	0	18	RDR2-sensitive
AGGTCTTGTAGGAAGAAGCGGCTT	1	14	RDR2-sensitive
AGGTCTTGTTGGTCTGGTCTAGTG	0	9	RDR2-sensitive
AGGTCTTTTACTCTGCTAGTGGAC	0	11	RDR2-sensitive
AGGTCTTTTTAAACTTGAGTCGGG	0	13	RDR2-sensitive
AGGTGAACGGACGGACGAACGGTG	0	59	RDR2-sensitive
AGGTGACATGAGATGGATCCACTC	0	12	RDR2-sensitive
AGGTGACCGGCATGTAGATGTGGC	6	26	RDR2-sensitive
AGGTGACGCAGAGGAGGACCGACA	0	10	RDR2-sensitive
AGGTGACGGCCAGAGTAGAAGCAC	3	32	RDR2-sensitive
AGGTGACGTACTGAAGTAGACGAC	0	24	RDR2-sensitive
AGGTGACTCGATTGTCCGGACACC	0	19	RDR2-sensitive
AGGTGACTGGTCCAGAGTTATGGC	0	9	RDR2-sensitive
AGGTGACTGTTGGAGGTTCGGTGC	0	14	RDR2-sensitive
AGGTGAGAGGGCAGAACATGGTGC	0	18	RDR2-sensitive
AGGTGAGCTCGGACGGTCTGCCGC	0	9	RDR2-sensitive
AGGTGATCGGCTGAAGACGCGCCA	0	48	RDR2-sensitive
AGGTGATGATGAGCTGGACGGTGT	0	9	RDR2-sensitive
AGGTGATGTAGACGGACTGACGGT	0	15	RDR2-sensitive

AGGTGATTGTAGCCATGCACGGAC	0	10	RDR2-sensitive
AGGTGCACTAGAGTAGATATCATT	127	7	RDR2-resistant
AGGTGCACTGGTAGAACAACGGAG	0	40	RDR2-sensitive
AGGTGCACTGGTGAGACAACGGAG	0	9	RDR2-sensitive
AGGTGCAGCGGTCCGTAGAGGTAA	0	9	RDR2-sensitive
AGGTGCATCTACAGGATTGTCCGG	1	33	RDR2-sensitive
AGGTGCATTTGTTGAACGGGCCGG	0	10	RDR2-sensitive
AGGTGCCGGCGAATCAGGTTTCTG	0	10	RDR2-sensitive
AGGTGCGGACAATTCTAGTGGCGT	10	60	RDR2-sensitive
AGGTGCGGGTGTGGTTTGATGGGT	0	11	RDR2-sensitive
AGGTGCTCTGACCGGCGCGCACGC	0	13	RDR2-sensitive
AGGTGCTGCAGTGACGACTTTGGA	1	13	RDR2-sensitive
AGGTGCTGTATGCTGTTTTGATGG	56	9	RDR2-resistant
AGGTGCTGTGTGAACGGGCCGGTT	1	18	RDR2-sensitive
AGGTGCTTTTAGGAGTAGGACGGT	2	16	RDR2-sensitive
AGGTGCTTTTGGGTAGGACGGTGT	3	20	RDR2-sensitive
AGGTGCTTTTTGGAGTAGGACGGT	1	27	RDR2-sensitive
AGGTGGAATGGTAGAGGAGGATAT	0	12	RDR2-sensitive
AGGTGGACGATGAGCTGGATGGCG	0	16	RDR2-sensitive
AGGTGGACGCTTGATCGACGACAA	0	9	RDR2-sensitive
AGGTGGACTGACTGAGGACGAGGC	0	28	RDR2-sensitive
AGGTGGACTGACTGAGGATGAGGC	0	11	RDR2-sensitive
AGGTGGACTGGACAACATGATGGA	2	14	RDR2-sensitive
AGGTGGACTGGACAGCATGACGGA	0	15	RDR2-sensitive
AGGTGGACTGGACAGCATGATGGA	1	32	RDR2-sensitive
AGGTGGACTGGCTGAGGACGAGGC	0	20	RDR2-sensitive
AGGTGGAGGTTTTAGGTATTCTTT	17	0	RDR2-resistant
AGGTGGAGTGGTAGAGAAGGGTAT	0	13	RDR2-sensitive
AGGTGGATAGCGGTGTCAGGGACT	21	1	RDR2-resistant
AGGTGGATATGCGGAAGGCTACAA	2	18	RDR2-sensitive
AGGTGGCACTGAACTGGTCGTCCG	0	19	RDR2-sensitive
AGGTGGCACTGTGGAGAGAAGACC	0	30	RDR2-sensitive
AGGTGGCACTGTGGAGAGACGACC	0	24	RDR2-sensitive
AGGTGGCCTGTAGTCCAGCACGGT	0	13	RDR2-sensitive
AGGTGGCGGCGAACTGAGAGGAAT	0	9	RDR2-sensitive
AGGTGGCTGTGTGAACGGGCCGGT	0	15	RDR2-sensitive
AGGTGGGCTTGGGGAGAGATGTAG	0	9	RDR2-sensitive
AGGTGGTCTGTGGACGAGGCGAGA	0	22	RDR2-sensitive
AGGTGGTGATCCAATCGTTGGCCA	16	0	RDR2-resistant
AGGTGGTGTGTAGAGATGGCACAG	0	9	RDR2-sensitive
AGGTGGTTGATGGATAAGTTGGAT	210	0	RDR2-resistant
AGGTGTAACCTTTGAACACGGTCT	0	18	RDR2-sensitive
AGGTGTACATTCGGGCTGCACGGC	0	19	RDR2-sensitive
AGGTGTAGACTGCAGAGAGGACAG	0	12	RDR2-sensitive
AGGTGTAGAGCACAGGAACGGTGG	0	12	RDR2-sensitive
AGGTGTAGCAACAAGGAGTCGGTG	0	17	RDR2-sensitive
AGGTGTCATGTAGCTGGTTTGTCA	95	2	RDR2-resistant
AGGTGTCGACCCTGTGCGCCTGGA	0	12	RDR2-sensitive
AGGTGTCGCAATACGGTAGGGCAT	0	48	RDR2-sensitive

AGGTGTCGGTGCTGGAGTGGGGGC	1	12	RDR2-sensitive
AGGTGTCTGGACAAGTAGCAGCAT	0	16	RDR2-sensitive
AGGTGTCTGTACTGTTGGTGTCGA	0	9	RDR2-sensitive
AGGTGTCTGTTAGAAAAGGTGTCT	1	40	RDR2-sensitive
AGGTGTGACTGTCGATTCGGCCGC	0	37	RDR2-sensitive
AGGTGTGATTGTAAGAAACAAGGC	20	0	RDR2-resistant
AGGTGTGATTGTATAGGGACGCTA	0	10	RDR2-sensitive
AGGTGTGCACTGTAGCGACGGTGG	0	11	RDR2-sensitive
AGGTGTGCAGTACATGGACGGTAA	0	9	RDR2-sensitive
AGGTGTGGACGCTGACGGTACTAT	0	15	RDR2-sensitive
AGGTGTGGGCATAAGAGGAAGGAG	1	17	RDR2-sensitive
AGGTGTGTCTCAAGGTCGCCGGGT	0	10	RDR2-sensitive
AGGTGTTAGCGACTCGGTGGAAGA	0	33	RDR2-sensitive
AGGTGTTAGTCTGTTAGGATGGAT	0	12	RDR2-sensitive
AGGTGTTCAGACTGCTACAGCCGG	0	10	RDR2-sensitive
AGGTGTTCTTTTTAGGATCTTCGG	0	22	RDR2-sensitive
AGGTGTTGATGGACTGTTTGGTAG	16	0	RDR2-resistant
AGGTGTTGCTAGTAGAACGGACGG	0	51	RDR2-sensitive
AGGTGTTGGAGGCTTATACCCTGA	39	0	RDR2-resistant
AGGTGTTGTTGAATGGTCATGAGA	295	0	RDR2-resistant
AGGTGTTTGGCATGCTCTAGCTAG	0	12	RDR2-sensitive
AGGTGTTTTAGGAGTAGGACGGTG	8	24	RDR2-sensitive
AGGTGTTTTTAGGAGTAGGACGGT	26	291	RDR2-sensitive
AGGTGTTTTTCGGACCTTCGGCTT	2	37	RDR2-sensitive
AGGTTAAACCATGTCGGGCCACGG	0	10	RDR2-sensitive
AGGTTAACGGGCCGTATCAGACTG	0	11	RDR2-sensitive
AGGTTAAGAAGGTATGATAACTAT	51	2	RDR2-resistant
AGGTTACAGAGTAGGCGTGCACAC	0	10	RDR2-sensitive
AGGTTATACATAGATCTGAATCGT	0	10	RDR2-sensitive
AGGTTATACCCCTCAGCCGTCGGT	0	14	RDR2-sensitive
AGGTTATCGTACTGGCCTCGGCGT	0	18	RDR2-sensitive
AGGTTCAAGATCATCGGAGCGCAT	0	9	RDR2-sensitive
AGGTTCAGATTGTGCGGATCCGGC	0	10	RDR2-sensitive
AGGTTCAGGATCATCGGAGCGCAT	0	10	RDR2-sensitive
AGGTTCCCTGTAGTAGTGACGGGC	0	14	RDR2-sensitive
AGGTTCCCTGTAGTAGTGAGGTAT	0	15	RDR2-sensitive
AGGTTCCTACAGGAGAGGATCGTG	31	0	RDR2-resistant
AGGTTCGGATGGCTCCAGGAGCGA	0	10	RDR2-sensitive
AGGTTCGGCTCGACTCGACGACGG	1	25	RDR2-sensitive
AGGTTCGGGCCGCTTAGAGAGGCG	0	9	RDR2-sensitive
AGGTTCGGGCGGCTATAAAAACAC	0	10	RDR2-sensitive
AGGTTCGGGCTGCTGCAAGGTGTA	0	12	RDR2-sensitive
AGGTTCGTGAGTCGGCTCGGCTGG	0	9	RDR2-sensitive
AGGTTCTCCCCTTTTTGTCGGGCC	0	70	RDR2-sensitive
AGGTTCTCTCCTAGGTCGTCCTAC	0	45	RDR2-sensitive
AGGTTCTCTGAAGACTACCTGCAT	0	13	RDR2-sensitive
AGGTTCTGAACACTGATAGTTGGC	0	35	RDR2-sensitive
AGGTTCTGCCACGCCTGATCTCGG	0	22	RDR2-sensitive
AGGTTCTGGAGACTTCTACAGAAG	0	9	RDR2-sensitive

AGGTTCTGTCCGGAGCGTGCGGGA	1	12	RDR2-sensitive
AGGTTCTGTCGGGAGCGTGCGGGA	0	15	RDR2-sensitive
AGGTTGAAGAGGCTGATCAGGCAA	0	15	RDR2-sensitive
AGGTTGAAGTAGATTACATCGGAT	71	0	RDR2-resistant
AGGTTGATGGAGGAGTGTGACGGC	54	2	RDR2-resistant
AGGTTGATTAATTTTGTACGGCTC	106	1	RDR2-resistant
AGGTTGCAGGATGGCAGAACGGCA	0	21	RDR2-sensitive
AGGTTGCCTCTGGGTCGAGTCGGG	0	10	RDR2-sensitive
AGGTTGCCTGTGAGGCGAGGTCGG	0	11	RDR2-sensitive
AGGTTGCTGGGAAGAGAGCGGACT	0	9	RDR2-sensitive
AGGTTGGAGACTGTTGGCGGCTAG	0	11	RDR2-sensitive
AGGTTGGAGCAACAGGATCGACGA	0	11	RDR2-sensitive
AGGTTGGATCGACGTAGAGGCAGT	1	27	RDR2-sensitive
AGGTTGGCACTGGCGGAAACGCGG	0	19	RDR2-sensitive
AGGTTGGGGTGGTGGATGTCGCGG	23	1	RDR2-resistant
AGGTTGGGTGAGGACGTGGGACGG	0	20	RDR2-sensitive
AGGTTGGTCGGCACTCGGCAGCCC	1	15	RDR2-sensitive
AGGTTGTACGAATACGGCGAACTG	0	10	RDR2-sensitive
AGGTTGTAGAGGTAATCGGCCTAG	0	21	RDR2-sensitive
AGGTTGTATGAGTGGCTAATGGTT	15	0	RDR2-resistant
AGGTTGTATGGCTTGGTCTCTCGG	0	11	RDR2-sensitive
AGGTTGTGGACTGTCCGGCCCTTG	0	13	RDR2-sensitive
AGGTTGTTGGGCGTCATAGTCTAT	0	10	RDR2-sensitive
AGGTTTACGAGAGCTGACGACATG	34	2	RDR2-resistant
AGGTTTATTAGACTGGCAGTACCT	15	0	RDR2-resistant
AGGTTTCGGATAACTGGAGTCGGT	0	18	RDR2-sensitive
AGGTTTCGGATAGCGGGTACCCAT	0	52	RDR2-sensitive
AGGTTTCGTAGAGCAGGAAGAGAA	0	10	RDR2-sensitive
AGGTTTCGTAGAGCAGGAGAGGAA	0	12	RDR2-sensitive
AGGTTTCGTAGAGCAGGAGGGGAA	1	49	RDR2-sensitive
AGGTTTGCGAGAACCGAACCTCGG	3	36	RDR2-sensitive
AGGTTTGCTGTAGTAGTGCGAGCA	0	25	RDR2-sensitive
AGGTTTGGAATTCGAATTGAGGCA	20	1	RDR2-resistant
AGGTTTGGACTGAGTTTGCGGCCT	0	13	RDR2-sensitive
AGGTTTTCCTGCCTGACGTCCGGA	4	25	RDR2-sensitive
AGGTTTTCGAGAGCTGACGTCATG	32	5	RDR2-resistant
AGGTTTTCTCCTGTTCCTCGGACA	20	2	RDR2-resistant
AGGTTTTGGAACTATGAACCGGTT	415	69	RDR2-resistant
AGGTTTTGTAACTATGAACCGGTT	205	0	RDR2-resistant
AGGTTTTGTAGAGCAGGAGGGGAA	0	15	RDR2-sensitive
AGGTTTTTCGAAGTGAGTCTGGGT	40	1	RDR2-resistant
AGGTTTTTTACTTTTTATCCATCA	14	0	RDR2-resistant
AGGTTTTTTGTAGACGAGCCGAAT	0	20	RDR2-sensitive
AGTAAACTTTGGATAGACTGCTGC	0	9	RDR2-sensitive
AGTAAAGGTAGTCGTTTAGGACAT	0	13	RDR2-sensitive
AGTAAATAAAGCATATCTCGTCGT	1	13	RDR2-sensitive
AGTAACGGACGTCTCTGATGGCGT	0	9	RDR2-sensitive
AGTAACTCTGCAAGGTGGGACCGA	1	13	RDR2-sensitive
AGTAACTCTGCAAGGTGGGACCGC	2	28	RDR2-sensitive

AGTAACTCTGCAAGGTGGGGCCGC	1	19	RDR2-sensitive
AGTAAGCCGACGGACCACGAGCAT	0	15	RDR2-sensitive
AGTAAGTCGAAAGGAGCATGGCAT	0	49	RDR2-sensitive
AGTAATGTTCTTGTAGTTTGATGG	24	1	RDR2-resistant
AGTAATTGGCGTCCGGATCGGTGT	0	9	RDR2-sensitive
AGTACAAGGGTCGGTGGAGCGGAC	0	31	RDR2-sensitive
AGTACACGCGGCAGAAGTCAGAGC	0	11	RDR2-sensitive
AGTACACTCGGCACAGGACGACAC	0	15	RDR2-sensitive
AGTACACTCGGCATATCCAGACAC	0	9	RDR2-sensitive
AGTACAGCTTCGGATTCGGGTCGG	3	23	RDR2-sensitive
AGTACAGGACTGTAGATAGGGCAT	0	9	RDR2-sensitive
AGTACATCAGACCTGCGAGGACGT	0	18	RDR2-sensitive
AGTACCCACTGTAGATGACAGCAA	0	17	RDR2-sensitive
AGTACCGAGCTTGGACTGCGGCGT	4	160	RDR2-sensitive
AGTACCGTTGGATGTAGATGGTGT	3	194	RDR2-sensitive
AGTACCTGAAAGAGCGCTAGGCAT	0	10	RDR2-sensitive
AGTACGATGGACTAGCACGAGCAT	0	12	RDR2-sensitive
AGTACGGACTCACAGTACGGATGT	0	20	RDR2-sensitive
AGTACTACTGTTTTTTGGACGGCC	1	177	RDR2-sensitive
AGTACTAGGCTGCAGATGGGGCAT	0	9	RDR2-sensitive
AGTACTAGTGATTGACGTCTGGAT	0	32	RDR2-sensitive
AGTACTAGTGGCAACTGCTCGAAG	43	2	RDR2-resistant
AGTACTATCTACCAGGTCGTGGAC	0	10	RDR2-sensitive
AGTACTCGGCGTGAGTAGAGCGGT	0	13	RDR2-sensitive
AGTACTCGGTTGCAGATGGAGCAT	0	10	RDR2-sensitive
AGTACTCTGGCTGCCTGACGACGC	0	20	RDR2-sensitive
AGTACTGGCCTGCTTCAGACGACT	0	17	RDR2-sensitive
AGTACTGGGATGTAGATGGGGCAT	0	20	RDR2-sensitive
AGTACTGGGCTGCAAATGGGGCAT	0	9	RDR2-sensitive
AGTACTGGGCTGCAGATGGGGCAT	0	27	RDR2-sensitive
AGTACTGTAGCACCGGTAGGAGCA	0	12	RDR2-sensitive
AGTACTGTAGCACCGGTAGGGGCA	0	27	RDR2-sensitive
AGTACTGTATCGAATTATTAGGCC	0	18	RDR2-sensitive
AGTACTGTGACGGAACCTCCCAAG	0	36	RDR2-sensitive
AGTACTGTGAGCAGGTGCAGACGC	41	0	RDR2-resistant
AGTACTGTGCCAGAATCGAAGCAT	2	54	RDR2-sensitive
AGTACTTAAGGTTGTCAGATGTTC	20	1	RDR2-resistant
AGTACTTAGAGTCTGATTGGTTGC	0	26	RDR2-sensitive
AGTACTTGAGCCCTGTAGAAGCGC	0	18	RDR2-sensitive
AGTACTTGTGCAGATTTGTGGATT	23	1	RDR2-resistant
AGTACTTTGGTTGTTGATCTCGGC	1	13	RDR2-sensitive
AGTACTTTTTATCGGACACTCGGC	1	37	RDR2-sensitive
AGTACTTTTTCTCGGGCACTCGGC	18	62	RDR2-sensitive
AGTACTTTTTGTCGGGCACTCGGC	15	87	RDR2-sensitive
AGTAGAAACCGGCACACATCTCTT	0	11	RDR2-sensitive
AGTAGAAACCTTGTCGGAAGGCGT	5	25	RDR2-sensitive
AGTAGAACACTCGGCATAGACCGT	0	10	RDR2-sensitive
AGTAGAACCGGACATTCGGCCACA	0	16	RDR2-sensitive
AGTAGAACGAGACCAGGACGACAC	0	19	RDR2-sensitive

AGTAGAACGAGGCGAGGACGACAC	0	21	RDR2-sensitive
AGTAGAACGCGTGACTGCTGGTCC	0	12	RDR2-sensitive
AGTAGAACGGACGGACGGTGCAGA	0	9	RDR2-sensitive
AGTAGAACGGTAGAACCACGGTGG	0	12	RDR2-sensitive
AGTAGAACGTGATCTCGACGCCAT	0	18	RDR2-sensitive
AGTAGAAGGGTTGGCTGGCTGCAT	0	169	RDR2-sensitive
AGTAGAAGGGTTGGCTGGCTGCCT	0	11	RDR2-sensitive
AGTAGACAAGCTCTGCAACGGCGC	1	34	RDR2-sensitive
AGTAGACACGGACGCGGAGGGCAT	1	13	RDR2-sensitive
AGTAGACCGCTGCGGACATTTGTT	0	11	RDR2-sensitive
AGTAGACGATAGAGCGGCTCGCTG	0	15	RDR2-sensitive
AGTAGACGCATAGCCGACGAGCAT	0	9	RDR2-sensitive
AGTAGACGGTACTGTCACCACTGC	0	12	RDR2-sensitive
AGTAGACTGAACTGCTGCAGCCGT	2	16	RDR2-sensitive
AGTAGACTGAATGGAGATGGCCGC	0	12	RDR2-sensitive
AGTAGACTGGCCTCTGAACGGGCA	2	15	RDR2-sensitive
AGTAGACTGGCCTCTGGACGGGCA	5	24	RDR2-sensitive
AGTAGAGACCGGTGTGTGCTCCAA	0	12	RDR2-sensitive
AGTAGAGACTTGTGCATCCATTCC	0	12	RDR2-sensitive
AGTAGAGCGCATGATTGGCTTCCT	0	11	RDR2-sensitive
AGTAGAGGGCGTATCACTCAGCAC	0	11	RDR2-sensitive
AGTAGAGTTCTCGGCTGCAGCGTT	0	17	RDR2-sensitive
AGTAGATATAGGAGATGGAATCTT	1	14	RDR2-sensitive
AGTAGATCACTGTGGTGCGTGCAC	0	18	RDR2-sensitive
AGTAGATGTAAATTCATTCGGCAT	14	0	RDR2-resistant
AGTAGCAAGGTCTCGGGCAGCACA	0	9	RDR2-sensitive
AGTAGCACGGAAGGGACGCTGCAT	1	16	RDR2-sensitive
AGTAGCACTGGACAAGCACTGCGC	1	15	RDR2-sensitive
AGTAGCCAAGTAGAACGGAGTCGT	0	19	RDR2-sensitive
AGTAGCCTTTGGACTACACTCGGT	0	14	RDR2-sensitive
AGTAGCGACTGTCCGAGGCGTGAT	0	9	RDR2-sensitive
AGTAGCTGAGAGCGTTGCGTGGAC	0	9	RDR2-sensitive
AGTAGCTGATAGAGCGTTGCGCGG	0	9	RDR2-sensitive
AGTAGCTGGCTGCACGGACGTCGG	0	10	RDR2-sensitive
AGTAGGAATGGACGTCATTCTGAT	0	10	RDR2-sensitive
AGTAGGACACTCGGCATAGATAGA	0	12	RDR2-sensitive
AGTAGGACGTCTGGAGCGGCGAAA	0	9	RDR2-sensitive
AGTAGGACTGGGCATAAAACCCGA	0	9	RDR2-sensitive
AGTAGGAGGATACGGTGCGACTGT	22	1	RDR2-resistant
AGTAGGATAAGTCGGGGGCGTCAC	0	9	RDR2-sensitive
AGTAGGATGAGACAGGGGCGCCAC	0	12	RDR2-sensitive
AGTAGGATGAGACGGAGATGACAT	0	9	RDR2-sensitive
AGTAGGATGAGGTGGAGGCGACAT	0	10	RDR2-sensitive
AGTAGGATGAGTCGGAGACGCCAC	0	48	RDR2-sensitive
AGTAGGATGAGTCGGGGACGCCAC	0	11	RDR2-sensitive
AGTAGGATGAGTCGGGGGCGCCAC	0	30	RDR2-sensitive
AGTAGGGATGAAAACGGGACGGAT	0	9	RDR2-sensitive
AGTAGGGCTCGTGCTGAGATCGCC	1	12	RDR2-sensitive
AGTAGGGTACGTAGCGGGAGTGTT	29	1	RDR2-resistant

AGTAGGGTGTTACGCTCCGGCGGC	0	16	RDR2-sensitive
AGTAGTCGGCATATGCTACGAAGT	0	9	RDR2-sensitive
AGTAGTCGTGCTGGGCAGAATCGC	0	15	RDR2-sensitive
AGTAGTGCGTCTGACATGTGAGGC	0	9	RDR2-sensitive
AGTAGTGCTCTGTGCAAAGAGGCG	0	34	RDR2-sensitive
AGTAGTGGAAGGCGGGCTGGGCAG	1593	261	RDR2-resistant
AGTAGTGGAAGGCGGGCTGGGCCG	71	11	RDR2-resistant
AGTAGTGGATTGGGCATGTTCGTT	3	19	RDR2-sensitive
AGTAGTGTAGACCATATGCCGTGT	47	1	RDR2-resistant
AGTAGTGTGAGCCGCTGAAGACTG	0	11	RDR2-sensitive
AGTAGTGTTTGTTTGGTTCGTTGT	1	13	RDR2-sensitive
AGTATAACGAGCCGAGCCGAGCTG	0	10	RDR2-sensitive
AGTATACGGCTGCAGCAGTTCAGT	0	13	RDR2-sensitive
AGTATAGACTGTACTGTTCGCGGA	0	12	RDR2-sensitive
AGTATAGTCTGATTGTAGGTGGTA	0	12	RDR2-sensitive
AGTATAGTGTCGTGTCTGGACGTC	0	11	RDR2-sensitive
AGTATATATCTTTTTGAGTCGGAC	0	10	RDR2-sensitive
AGTATATGGACTGAAATGTGCATG	34	2	RDR2-resistant
AGTATCACGACTGTATAGGATGGT	6	60	RDR2-sensitive
AGTATCAGATCGGATTGGTCCGCA	0	11	RDR2-sensitive
AGTATCGGTGTAACACGGCTCGAC	0	9	RDR2-sensitive
AGTATCTGATTACGGTTGGACTAA	0	35	RDR2-sensitive
AGTATCTGCACACGGCACGGTCTA	0	10	RDR2-sensitive
AGTATGAATCTACTGGTGCTCGTT	16	0	RDR2-resistant
AGTATGACTGTTATGTATGGCCAA	0	10	RDR2-sensitive
AGTATGGACTAGTAGCATACCGGT	0	28	RDR2-sensitive
AGTATGGGACAGCGACCAGCACGA	0	17	RDR2-sensitive
AGTATGGTAGGGCTGACGCGGTGC	1	39	RDR2-sensitive
AGTATTAGGGTAGAGGCCGGACAA	0	19	RDR2-sensitive
AGTATTAGTGGAGGGAGAGGGCTG	0	16	RDR2-sensitive
AGTATTCTGTATTGTAGATTGCAT	0	11	RDR2-sensitive
AGTATTGGGTTGTAGGTGGGGCAT	0	12	RDR2-sensitive
AGTATTTAGTCGGGTCGGGTTGGC	0	30	RDR2-sensitive
AGTATTTCCGGTCCGTCTGACAAC	0	18	RDR2-sensitive
AGTATTTTCGGTTGGTAGTGGCAA	76	7	RDR2-resistant
AGTATTTTGTTTGTCGGGACGGAT	0	14	RDR2-sensitive
AGTCAAACATTGATCTAGGAGACC	25	2	RDR2-resistant
AGTCAAACCCGGTCGTTGGATGAC	0	10	RDR2-sensitive
AGTCAAACGCTTGATGTGACCCGG	0	13	RDR2-sensitive
AGTCAAACGCTTGATGTGACTCGG	0	32	RDR2-sensitive
AGTCAAGACATGCGGAGGACATAG	0	9	RDR2-sensitive
AGTCAAGCGGAGCGAGGTGAACAC	0	22	RDR2-sensitive
AGTCACATGGATTTGTTGATGGAT	0	12	RDR2-sensitive
AGTCACCGGAGTTCGTATGACTGG	0	9	RDR2-sensitive
AGTCACCTGGTGCCGAAGACGTCG	3	19	RDR2-sensitive
AGTCACGGATACAGTTGACCGCTG	1	34	RDR2-sensitive
AGTCACGGTTGACTGGGAGACGAT	0	10	RDR2-sensitive
AGTCACTGTAGCTGTCCGGTGCGC	0	11	RDR2-sensitive
AGTCAGAACTCGTCGACTACGGTT	0	13	RDR2-sensitive

AGTCAGAGCTCCAACGGTCGGAAC	1	12	RDR2-sensitive
AGTCAGCCCGTCGGGCGGCACGGC	0	9	RDR2-sensitive
AGTCAGTAGGTCGTCATGACGCGT	0	10	RDR2-sensitive
AGTCATATAGCTAGTCTGTCGGAG	1	12	RDR2-sensitive
AGTCATCATTAGATCTAGATCGGA	1	25	RDR2-sensitive
AGTCATCGGACCATGCCGGCACGG	0	9	RDR2-sensitive
AGTCATGCGAGACAGACTGGAGGC	0	20	RDR2-sensitive
AGTCATTTTTTTCGTCGTAGGTGT	0	10	RDR2-sensitive
AGTCCACCGCTGCATCGTTTGTGA	0	9	RDR2-sensitive
AGTCCACTGTCGTCAGCGTCGGAC	0	24	RDR2-sensitive
AGTCCAGACTGTTGGCAGCGGCAA	0	13	RDR2-sensitive
AGTCCAGGACTGCAGGGACGGTGC	0	24	RDR2-sensitive
AGTCCATCTTGGATATGTGACGGT	0	14	RDR2-sensitive
AGTCCCTCGAACGGTCGCCGGGAA	8	47	RDR2-sensitive
AGTCCGATCGGCATGTCTAGGTAC	0	16	RDR2-sensitive
AGTCCGCAGCAGCTTTTGGTGGCC	0	10	RDR2-sensitive
AGTCCGCGACCAGACGTAGAGGCT	1	15	RDR2-sensitive
AGTCCGCGGACTTAGACGGCCCGA	0	15	RDR2-sensitive
AGTCCGGAACTCCCAAGAAGGCAT	0	12	RDR2-sensitive
AGTCCGGACCGTTTAGGCCTATAG	0	10	RDR2-sensitive
AGTCCGGATGTACACAAAGGAGGA	0	28	RDR2-sensitive
AGTCCGGATTAGAAAGCAGGTCGT	0	24	RDR2-sensitive
AGTCCGGCACGGTAGAATAAGCGG	0	14	RDR2-sensitive
AGTCCGGCTCTGCGATCTCACGGC	144	61	RDR2-resistant
AGTCCGGTGAGGTCGATCTCGGGC	1	12	RDR2-sensitive
AGTCCGTAGCAGCTTTTGGTGGCC	0	10	RDR2-sensitive
AGTCCGTAGCGTGACAGAAGTCGG	0	31	RDR2-sensitive
AGTCCTAGACTGTCTCGGCAACAC	0	16	RDR2-sensitive
AGTCCTAGATCGTGGACCGACGCC	0	11	RDR2-sensitive
AGTCCTCGACCACGTCGGCAACAC	0	9	RDR2-sensitive
AGTCCTGCGACGGCGCACAGACTC	19	0	RDR2-resistant
AGTCCTGTAGACTGTCCGGTGCGC	0	10	RDR2-sensitive
AGTCCTGTAGACTGTCCGGTGTGC	3	33	RDR2-sensitive
AGTCCTGTCGAGCACGGCACTGTA	1	35	RDR2-sensitive
AGTCCTTGATATAGTAGTCGTCGA	1	13	RDR2-sensitive
AGTCCTTGGTGGCCGTCGGACATA	3	24	RDR2-sensitive
AGTCCTTGTAGACTGTGCCGAAGA	37	4	RDR2-resistant
AGTCGACAGAGCCGAAGTGACCGC	5	33	RDR2-sensitive
AGTCGACATGGCCATACATCTGAT	0	13	RDR2-sensitive
AGTCGAGGTTGTCGACCGTCGGTG	0	9	RDR2-sensitive
AGTCGATCGTTGGTCACTTGGCAC	0	19	RDR2-sensitive
AGTCGATTGAATCGGTCTCGGCCC	0	13	RDR2-sensitive
AGTCGCGGGCGGATATTATCTATG	0	11	RDR2-sensitive
AGTCGCTTGAGCTTTCGTCGGAAT	51	9	RDR2-resistant
AGTCGTAGACGGCTGTCAGGAGTA	0	10	RDR2-sensitive
AGTCGTGCTCGGACCGGACTGGCA	0	42	RDR2-sensitive
AGTCGTGTCGCGACCTGGACGGCT	1	12	RDR2-sensitive
AGTCGTTTAGAACATGATTGTGCA	0	9	RDR2-sensitive
AGTCGTTTAGGACATGATTGTGCA	7	208	RDR2-sensitive

AGTCTAAGATGTAGAAGTGATGGT	0	10	RDR2-sensitive
AGTCTACGGCTCTGGGCTAGACGG	1	22	RDR2-sensitive
AGTCTAGACCACAGAACCTCCGGC	0	9	RDR2-sensitive
AGTCTAGTCGTGCTCGGACCGGAC	0	15	RDR2-sensitive
AGTCTAGTGCAGTTCTGAGCGTCG	0	10	RDR2-sensitive
AGTCTATAAATGTAACTGGTCGTA	44	5	RDR2-resistant
AGTCTATAGGTCTGTTTGGTTCGG	0	10	RDR2-sensitive
AGTCTATCCGTCTGATTGGTTGCA	0	11	RDR2-sensitive
AGTCTATCGCGTAGGACAGTTCGG	0	12	RDR2-sensitive
AGTCTATGACACTGAGCTGGACGG	0	15	RDR2-sensitive
AGTCTATGTTGATGATCGCGAGGA	24	0	RDR2-resistant
AGTCTCAACGGTCGATCGTCGTGC	34	2	RDR2-resistant
AGTCTCAGTACTAGAACAGAGCGG	0	12	RDR2-sensitive
AGTCTCATCGTTTGGAGCGGCTTC	0	13	RDR2-sensitive
AGTCTCGGACGCAGACGTGCCCCT	0	10	RDR2-sensitive
AGTCTCGGCGAACTGTACATCGGC	0	11	RDR2-sensitive
AGTCTCGGCGAACTGTATATCGGC	0	12	RDR2-sensitive
AGTCTCGGTAAACTGTACATCGGC	0	31	RDR2-sensitive
AGTCTCGGTGAACTGTACATCGGC	0	9	RDR2-sensitive
AGTCTCGTTGTATACTGTTGGCTG	0	25	RDR2-sensitive
AGTCTCTAGAACGCGGCAATTCTT	2	15	RDR2-sensitive
AGTCTCTGTAGAGTGGCAGATTCG	39	0	RDR2-resistant
AGTCTGAACAGCTGTAGCAGGTGT	0	14	RDR2-sensitive
AGTCTGAACCGCTGCAGCAGGTGT	1	22	RDR2-sensitive
AGTCTGAACGTATGCAGAGCAGCC	0	9	RDR2-sensitive
AGTCTGAACTGTAAGTTTGTTGGC	0	10	RDR2-sensitive
AGTCTGAAGACGGATGAAAACATA	60	0	RDR2-resistant
AGTCTGATTTTTGGACTTGTCGGT	0	14	RDR2-sensitive
AGTCTGCAGCAGCTTTTGATGGTC	0	11	RDR2-sensitive
AGTCTGCATGATACTGATGGTTGC	1	37	RDR2-sensitive
AGTCTGCATGATACTGATGGTTGT	1	18	RDR2-sensitive
AGTCTGCGGTGTAGTTTGGACGGT	0	20	RDR2-sensitive
AGTCTGGAGATGTAGCACCGGCGC	2	21	RDR2-sensitive
AGTCTGGCGGACACGGATTAGGAT	0	10	RDR2-sensitive
AGTCTGTAGCAGCTTTTGATGGCC	0	40	RDR2-sensitive
AGTCTGTAGCAGCTTTTGGTGGCC	0	77	RDR2-sensitive
AGTCTGTAGGCCGACATGACGCGC	0	12	RDR2-sensitive
AGTCTGTAGGGGTAAGACGGACGA	0	13	RDR2-sensitive
AGTCTGTATCTCGGATTTCGGGCA	0	36	RDR2-sensitive
AGTCTGTATGATACTGATGGTTGC	1	39	RDR2-sensitive
AGTCTGTATGATACTGATGGTTGT	2	41	RDR2-sensitive
AGTCTGTATGGACGGATTCAGAGA	0	19	RDR2-sensitive
AGTCTGTATTATTTTATGTCGGAT	1	57	RDR2-sensitive
AGTCTGTCGCGTAGGACAATTCGG	0	26	RDR2-sensitive
AGTCTGTCGCGTAGGACAGTTCGG	0	13	RDR2-sensitive
AGTCTGTGAACTCCGCTGCGACGG	0	15	RDR2-sensitive
AGTCTGTGAACTGCGCTGCGACGG	0	10	RDR2-sensitive
AGTCTGTGAACTTCGCTGCGACGG	0	30	RDR2-sensitive
AGTCTGTTGGAACGCACGGTGCAA	0	10	RDR2-sensitive

AGTCTGTTGGATTGTTATTCAGGC	173	30	RDR2-resistant
AGTCTTAGACAGATCGCAGCGGTA	0	10	RDR2-sensitive
AGTCTTCCACGTCGGTCACGTCAT	0	11	RDR2-sensitive
AGTCTTCGATCGAGCAGCTGACGA	0	9	RDR2-sensitive
AGTCTTCGGATTGACTGTCGCTAG	3	29	RDR2-sensitive
AGTCTTCGTCGATGACAGCACGGT	1	20	RDR2-sensitive
AGTCTTGCGTGAACTGTATATCGG	0	12	RDR2-sensitive
AGTCTTGGATATAGAGTTGTAGGC	27	3	RDR2-resistant
AGTCTTGGCTGCATAACGAAGAAA	16	0	RDR2-resistant
AGTCTTGGGTTGTACTGCAGAGGC	0	21	RDR2-sensitive
AGTCTTGGTCGGAGGCAGCGGCGC	0	13	RDR2-sensitive
AGTCTTTAGAGAAACTGCATGTAA	61	2	RDR2-resistant
AGTCTTTGTCAAGTGTCGTCTGGT	0	14	RDR2-sensitive
AGTCTTTTATCATTTAGGTCGGTT	14	0	RDR2-resistant
AGTCTTTTCATCTTTAAGTCGGTT	11	31	RDR2-sensitive
AGTCTTTTCGGGCTTGAGCCGGAC	0	15	RDR2-sensitive
AGTGAAACGGCACGGTTGGACGCT	0	24	RDR2-sensitive
AGTGAACAGGTGATCTGACGGCCA	373	11	RDR2-resistant
AGTGAACAGTAGACGCGCGTCCTC	0	14	RDR2-sensitive
AGTGAACAGTAGGAATGGGCGCGC	0	19	RDR2-sensitive
AGTGAACATGTAGGCGCGGCTGAT	0	14	RDR2-sensitive
AGTGAACCGGACCGAGACGTAGTG	1	12	RDR2-sensitive
AGTGAACGGAACGGAGCGTGACTG	1	22	RDR2-sensitive
AGTGAACGGCAGACTGGAACGTAC	0	17	RDR2-sensitive
AGTGAACTGGAACAGAGCGGCTCT	1	30	RDR2-sensitive
AGTGAACTGTAACAGAGCGGCTCC	0	10	RDR2-sensitive
AGTGAATATGATATTGATTGTGGC	24	3	RDR2-resistant
AGTGAATGTCGCCGCGTGCCCCAG	0	13	RDR2-sensitive
AGTGACACACTAGATCTGGAGGAA	0	10	RDR2-sensitive
AGTGACACACTAGATCTGGAGGGA	0	10	RDR2-sensitive
AGTGACACATGAACTAGCGGACGG	0	9	RDR2-sensitive
AGTGACACGCTAGATCTAGGAGGA	0	11	RDR2-sensitive
AGTGACACGCTAGATCTAGGGAGA	0	10	RDR2-sensitive
AGTGACACGCTAGATCTAGGGGGA	6	22	RDR2-sensitive
AGTGACACGCTAGATCTGAGGGAG	0	9	RDR2-sensitive
AGTGACACGCTAGATCTGAGGGGA	4	21	RDR2-sensitive
AGTGACACGCTAGATCTGGAGGAA	0	15	RDR2-sensitive
AGTGACACGCTAGATCTGGAGGGA	0	21	RDR2-sensitive
AGTGACACGCTAGATCTGGGAAGA	0	18	RDR2-sensitive
AGTGACACGCTAGATCTGGGGAGA	2	15	RDR2-sensitive
AGTGACACGCTAGATCTGGGGGAG	3	25	RDR2-sensitive
AGTGACACGCTAGATCTGGGGGGA	9	83	RDR2-sensitive
AGTGACACGCTAGATCTGTGGGAG	4	18	RDR2-sensitive
AGTGACACGCTAGATCTGTGGGGA	0	21	RDR2-sensitive
AGTGACAGACGATCTCCAGGGACG	0	11	RDR2-sensitive
AGTGACAGTGACAGGGGCGACGAC	20	0	RDR2-resistant
AGTGACCAGCACGACAGACGAACT	3	20	RDR2-sensitive
AGTGACGACGTACATCTCTTATGT	29	0	RDR2-resistant
AGTGACGACTTTGGACGACGTGTT	1	23	RDR2-sensitive

AGTGACGCGCATGAATGGATTAAC	18	1	RDR2-resistant
AGTGACGGACACTGTTTGGTTACC	0	13	RDR2-sensitive
AGTGACGGATTACTACTATTGCGA	7	49	RDR2-sensitive
AGTGACGGATTCCTACTATTACAA	0	20	RDR2-sensitive
AGTGACGGATTCCTACTATTGCAA	2	18	RDR2-sensitive
AGTGACGGTCCTAGAGCTATTTGC	1	15	RDR2-sensitive
AGTGACGGTCTGACTACAGTGGAT	0	17	RDR2-sensitive
AGTGACGGTGACGGATCCTTTGCC	1	14	RDR2-sensitive
AGTGACGGTTTGAACAGGGCGCAC	0	10	RDR2-sensitive
AGTGACGTAGGAGGTCTGACGGTT	0	12	RDR2-sensitive
AGTGACGTGGACACTGCAGATGGT	0	11	RDR2-sensitive
AGTGACTCAACCTTTTCGTTGGCA	37	4	RDR2-resistant
AGTGACTCTCGACACCGACGTGCT	0	9	RDR2-sensitive
AGTGACTCTGCAAGGTGGGACCGA	0	17	RDR2-sensitive
AGTGACTGAATATATGCAGAAAGA	14	0	RDR2-resistant
AGTGACTGACAAGACGGCGATCAC	0	10	RDR2-sensitive
AGTGACTGTGGGAATAGTCCGGAT	0	10	RDR2-sensitive
AGTGACTGTTCACTGTCCGGTGTG	0	15	RDR2-sensitive
AGTGACTTTTGTCGGGCACTCGGC	2	18	RDR2-sensitive
AGTGAGACGAGGCATGGACGACAC	0	17	RDR2-sensitive
AGTGAGACGGCTGTTGCTACTTGG	0	13	RDR2-sensitive
AGTGAGACGTCTGTTGCTACTTGG	3	25	RDR2-sensitive
AGTGAGAGAGTGTTGAACAGGCAT	0	11	RDR2-sensitive
AGTGAGATGAGACCAGGACGACAT	0	22	RDR2-sensitive
AGTGAGCCTCTGAGTTCTGGCGTT	0	11	RDR2-sensitive
AGTGAGCCTGGCTCAGACTGGACT	0	9	RDR2-sensitive
AGTGAGCGGGGCTGAGGTATGGTA	0	18	RDR2-sensitive
AGTGAGGACGGAATGGGAATGGCA	0	11	RDR2-sensitive
AGTGAGGAGTGAGAAGATGATGGA	0	13	RDR2-sensitive
AGTGAGGCGTCTGTTACTACTTGG	1	19	RDR2-sensitive
AGTGAGGGCTCTGGCGACGACGAA	0	9	RDR2-sensitive
AGTGAGTAGAGGACGTTCTGATCC	0	21	RDR2-sensitive
AGTGAGTTGCAGTAGCCGGAACAG	0	9	RDR2-sensitive
AGTGAGTTGCAGTAGCCGGGACAG	1	59	RDR2-sensitive
AGTGAGTTGCAGTAGGCGAGACAT	0	25	RDR2-sensitive
AGTGAGTTGCAGTAGTCGGGACAG	0	12	RDR2-sensitive
AGTGAGTTTGTATGAGTGAGGTTT	14	0	RDR2-resistant
AGTGATAGTGCAGTGTCGGACGAC	0	12	RDR2-sensitive
AGTGATCACCAAGTCGTCGACGTA	37	2	RDR2-resistant
AGTGATCGGCTTGCACGGATCGTA	0	12	RDR2-sensitive
AGTGATCTGACGACCTACACGGAT	0	9	RDR2-sensitive
AGTGATGGTATGAACGGTCCGCGT	0	13	RDR2-sensitive
AGTGATGTGGTTGTGTGGAGGAGG	17	1	RDR2-resistant
AGTGATTATAGCTCTGAGATGGTA	0	13	RDR2-sensitive
AGTGATTCCGGACTTGGGAGAGGA	0	13	RDR2-sensitive
AGTGATTCTCTAGATGTAGCGGCT	0	14	RDR2-sensitive
AGTGATTGACGTTCGGATCGGCGC	0	11	RDR2-sensitive
AGTGATTGATGTCCGGATCGGCGC	0	11	RDR2-sensitive
AGTGATTGCATAGGATATTTTCCC	0	11	RDR2-sensitive

AGTGATTGGCACGCAGATCGACAC	0	9	RDR2-sensitive
AGTGCACAGGCTGCAGCAGGCTAC	4	38	RDR2-sensitive
AGTGCACAGGCTGTAGCAGGCTAC	0	14	RDR2-sensitive
AGTGCACAGGCTGTAGCAGGCTAT	6	104	RDR2-sensitive
AGTGCACGGAGACGACGAACAAAG	0	13	RDR2-sensitive
AGTGCACGGTCAACTGAACGCGGT	0	11	RDR2-sensitive
AGTGCACTGTTAGATATGTTGCGG	0	14	RDR2-sensitive
AGTGCAGGACAACATATTCGCATC	20	0	RDR2-resistant
AGTGCAGTCTACGGTGCAGAACAT	0	9	RDR2-sensitive
AGTGCATGCGTGTAGAGGACTGCA	0	22	RDR2-sensitive
AGTGCATGGTCGCTGGAACGACGA	0	9	RDR2-sensitive
AGTGCATTTTGTCGGGCACTCGGC	1	22	RDR2-sensitive
AGTGCCCTAAGTACATGTCGTCGT	0	14	RDR2-sensitive
AGTGCCCTCTGACGGGAAGTCGGT	0	25	RDR2-sensitive
AGTGCCGAGGACGGGGAACGGCGA	0	9	RDR2-sensitive
AGTGCCGGGCTGGGCATGACACGA	1	52	RDR2-sensitive
AGTGCCTACGAGAGCTCTCGGCAT	0	19	RDR2-sensitive
AGTGCCTGAGCCCTGTAGAAGCAC	0	10	RDR2-sensitive
AGTGCCTGAGCCCTGTAGAAGCGC	0	9	RDR2-sensitive
AGTGCCTTTTGTCGGACACTCGGC	4	34	RDR2-sensitive
AGTGCGAGGAATGGCAGAACACGG	0	12	RDR2-sensitive
AGTGCGCATGTAGGACTGGGCATT	0	13	RDR2-sensitive
AGTGCGCGGACGGCTTCCAGCGGA	0	9	RDR2-sensitive
AGTGCGGCACTCGACAAAGACGTC	0	10	RDR2-sensitive
AGTGCGGCGGGACAATGAAGGCAT	0	25	RDR2-sensitive
AGTGCGGCTACGGATGAAGCGGTT	0	16	RDR2-sensitive
AGTGCGTGGGGCTTGCTGTGCGGC	0	10	RDR2-sensitive
AGTGCTACTCTGAGCTGCAGCGGG	0	25	RDR2-sensitive
AGTGCTAGCTGTAGAGCACTCGGC	1	36	RDR2-sensitive
AGTGCTATAGAACAGAAGGGCACA	0	9	RDR2-sensitive
AGTGCTATAGAACAGGAGGGCATA	0	9	RDR2-sensitive
AGTGCTATGGTCACTGTACTCGGT	1	20	RDR2-sensitive
AGTGCTCGCGGTCGGACATCCCGG	14	0	RDR2-resistant
AGTGCTCTCTGGTCAGGCGCGGAC	0	20	RDR2-sensitive
AGTGCTCTCTGTAGCAGCACGGAC	0	10	RDR2-sensitive
AGTGCTGACTTGCGAGATCGTCTT	0	13	RDR2-sensitive
AGTGCTGGTGGAATGGTTCGGCTT	0	10	RDR2-sensitive
AGTGCTGTGTGAACGAGCCGGTTT	0	34	RDR2-sensitive
AGTGCTTCGGACACTCGGCAAAGC	0	14	RDR2-sensitive
AGTGCTTCGGAGGACGAAGGGCAT	0	17	RDR2-sensitive
AGTGCTTCTGTTAGATGTTGGATA	0	14	RDR2-sensitive
AGTGCTTGAGCCCTGTAGAAGCGC	0	10	RDR2-sensitive
AGTGCTTGAGCCCTGTAGAAGCGT	7	122	RDR2-sensitive
AGTGCTTTCTGTCGGGCACTCGGC	0	19	RDR2-sensitive
AGTGCTTTTTATCGGACACTCGGC	4	56	RDR2-sensitive
AGTGCTTTTTGTCGGACACTCGGC	0	12	RDR2-sensitive
AGTGCTTTTTGTCGGGCACTCGGC	15	218	RDR2-sensitive
AGTGCTTTTTTTCGGACACTCGGC	6	43	RDR2-sensitive
AGTGGAAAATAGGCTGACGAGCAA	0	12	RDR2-sensitive

AGTGGAACCGGTAGCACGTACAAT	0	35	RDR2-sensitive
AGTGGAACGGAGGGAAAACGACAC	2	14	RDR2-sensitive
AGTGGAACTCCTAGCGGTGGGCAT	0	36	RDR2-sensitive
AGTGGAAGCAACACGGTCGTTGGT	0	13	RDR2-sensitive
AGTGGAAGGAGCTGCTGATGTCGT	0	16	RDR2-sensitive
AGTGGAAGGGCAAAACTGAGCCAA	0	13	RDR2-sensitive
AGTGGAAGGGGAGGAGACGGACGC	0	32	RDR2-sensitive
AGTGGAAGGTGAGGCTGATCTGAA	50	1	RDR2-resistant
AGTGGAATAGGCGAGCGACGACGT	0	13	RDR2-sensitive
AGTGGAATTTTGCAGTGGGCCCGC	1	12	RDR2-sensitive
AGTGGACAAGATTTACAGGTTCGG	0	11	RDR2-sensitive
AGTGGACACCAGGGGCGAGTTCAC	0	15	RDR2-sensitive
AGTGGACAGGAACAACAAGGTCGT	0	10	RDR2-sensitive
AGTGGACAGTCAGGCAACGCTCTC	0	10	RDR2-sensitive
AGTGGACAGTTGACGCCGGCCCGT	3	47	RDR2-sensitive
AGTGGACAGTTGACGCCGGCTCGT	0	10	RDR2-sensitive
AGTGGACCGTAAGCTCGAGAAGAC	0	14	RDR2-sensitive
AGTGGACCTGAAGTAGAGAGACAC	0	35	RDR2-sensitive
AGTGGACGAGGACGTGCGCAATGC	0	75	RDR2-sensitive
AGTGGACGCGTAGAACGGCGTCGA	0	30	RDR2-sensitive
AGTGGACGGTCTGCTGCTCATGCG	0	31	RDR2-sensitive
AGTGGACGTGCTGCTACTCCCCTC	0	9	RDR2-sensitive
AGTGGACTCCTCAACGGGGACTAG	0	17	RDR2-sensitive
AGTGGACTGTCAGCGGCCTAGTGC	0	10	RDR2-sensitive
AGTGGACTGTGTGCATGGAGACGA	0	61	RDR2-sensitive
AGTGGACTGTTCACTGTCCGGTGC	2	14	RDR2-sensitive
AGTGGAGACGAGAGCTGACGTGGC	0	17	RDR2-sensitive
AGTGGAGCGGACCAGCATCGGCGT	0	12	RDR2-sensitive
AGTGGAGGCGAGAACTGACGTGGC	0	10	RDR2-sensitive
AGTGGAGGCGAGAGCTGACGTGGC	0	48	RDR2-sensitive
AGTGGAGGGCCTAGGATAGCAGAT	17	0	RDR2-resistant
AGTGGATAGGCTCTATGTATCTAG	0	9	RDR2-sensitive
AGTGGATCTGGACGCACAAGCGGC	1	12	RDR2-sensitive
AGTGGATGGCTGCAGCGATTGACT	0	10	RDR2-sensitive
AGTGGATGGTTAACGTTGGCTCGT	0	25	RDR2-sensitive
AGTGGATGTAGCATATTTAAAGAT	0	9	RDR2-sensitive
AGTGGATGTGAGAGGTGGAGGGAC	0	9	RDR2-sensitive
AGTGGATTGTTACCTCAAAGATGG	0	48	RDR2-sensitive
AGTGGCACGGACAGTCTACGGCTC	0	26	RDR2-sensitive
AGTGGCACTAGACTCTTACGGCGT	0	40	RDR2-sensitive
AGTGGCAGAACCGCGCAACAACGT	0	16	RDR2-sensitive
AGTGGCATTGAACGGATCAATATA	17	0	RDR2-resistant
AGTGGCCTGGTAGTCATGTCGGAC	0	9	RDR2-sensitive
AGTGGCTGGTTTGGAGTTGTACGG	0	15	RDR2-sensitive
AGTGGCTTGACTCGGTTTGGACGG	0	21	RDR2-sensitive
AGTGGGAGAGGCTCTGCACGGACG	0	13	RDR2-sensitive
AGTGGGAGTAGAACTGCGAAGGGG	0	10	RDR2-sensitive
AGTGGGAGTGGAGCTTTTGCGGCC	0	9	RDR2-sensitive
AGTGGGCGCTGTAGGGGCACCGCT	0	20	RDR2-sensitive

AGTGGGCTAACGACGACGGATCAG	36	2	RDR2-resistant
AGTGGTAGCGACCCTGGACTCGGA	0	15	RDR2-sensitive
AGTGGTCACTAGTAGGGATGGCAA	0	31	RDR2-sensitive
AGTGGTCCATGTCCAGATCGTCCG	0	10	RDR2-sensitive
AGTGGTCGAAGCTGTAGGTCGGGC	0	10	RDR2-sensitive
AGTGGTCGAAGGAATAGGTCAGGC	0	11	RDR2-sensitive
AGTGGTGGACAAGACAGCGATCAC	0	13	RDR2-sensitive
AGTGGTGTGCTGTAGTGCGGGCCA	0	13	RDR2-sensitive
AGTGGTTATGGATGGTTTGGCGTA	18	0	RDR2-resistant
AGTGGTTCGGGTCGCTGGAGCATA	0	17	RDR2-sensitive
AGTGGTTGCACGACTGAGAACGGA	0	13	RDR2-sensitive
AGTGGTTGGACCGTCTGACGGCAT	0	10	RDR2-sensitive
AGTGGTTGGAGTGTAGTCAGCATC	17	0	RDR2-resistant
AGTGTAAAGTGATTGTATTGCATA	0	10	RDR2-sensitive
AGTGTAAGACGGAGACTGAGTCAG	0	58	RDR2-sensitive
AGTGTAATGATCTGTGGAATACAA	0	20	RDR2-sensitive
AGTGTACGACGGAGACTGAGCCAG	0	49	RDR2-sensitive
AGTGTACGACGGAGGCTGAGTCAG	0	10	RDR2-sensitive
AGTGTAGACCAGGCACAACCATGC	0	18	RDR2-sensitive
AGTGTAGACGTGGGCTTCGGCATT	0	9	RDR2-sensitive
AGTGTAGAGGTAAAGATGGACATA	0	18	RDR2-sensitive
AGTGTAGATTCTCCGAAGCGGACT	0	26	RDR2-sensitive
AGTGTAGCACGACCCGTTGGACAT	0	10	RDR2-sensitive
AGTGTAGCTCTGCGGACGGTGATT	0	65	RDR2-sensitive
AGTGTAGGACGGAGTTGAGGCCGA	0	51	RDR2-sensitive
AGTGTAGGATCTCGAAGGCGACAG	0	50	RDR2-sensitive
AGTGTAGTATCTGATTACGGTTGG	0	21	RDR2-sensitive
AGTGTATCTCTGCGGACGACGATT	0	34	RDR2-sensitive
AGTGTATCTCTGCGGTCGGTGATT	1	41	RDR2-sensitive
AGTGTATGACTGATGTCGGGTGCC	15	0	RDR2-resistant
AGTGTATTCTGAGAAGCAGCTGAA	5	53	RDR2-sensitive
AGTGTCACATCTGTAGACGACGGT	0	23	RDR2-sensitive
AGTGTCACTGATAATTGGAACATA	54	6	RDR2-resistant
AGTGTCACTTGTAGAGATGGCCAA	0	52	RDR2-sensitive
AGTGTCAGACCAACGGCTAGGCGC	0	10	RDR2-sensitive
AGTGTCAGACTCGGCAAAGACTTC	0	9	RDR2-sensitive
AGTGTCAGGCTGGCATACACTCGT	0	29	RDR2-sensitive
AGTGTCATACCACGGGTCGTCCGA	0	66	RDR2-sensitive
AGTGTCCGGACGGGCAGGCTTCAG	0	9	RDR2-sensitive
AGTGTCGAGTCAGGTAGGGTTGGC	0	20	RDR2-sensitive
AGTGTCGGATCGGCCCATGCTACC	0	11	RDR2-sensitive
AGTGTCGGCACAATACAACACAGT	0	14	RDR2-sensitive
AGTGTCGGGCTTGAGCTGCGGCGC	1	20	RDR2-sensitive
AGTGTCGTAGACGGTCCGATGCCC	0	11	RDR2-sensitive
AGTGTCGTGGCTTACGGTGGAAGC	0	14	RDR2-sensitive
AGTGTCTACTGTACCGGCACTCAG	0	12	RDR2-sensitive
AGTGTCTACTGTACTGACGCTCGG	0	9	RDR2-sensitive
AGTGTCTCTGTGTCTCGACGTCAT	0	16	RDR2-sensitive
AGTGTCTGGGCACTGGTTAGACAC	1	18	RDR2-sensitive

AGTGTCTTTTGTCGGACACTCGGC	0	27	RDR2-sensitive
AGTGTCTTTTGTCGGGCACTCGGC	18	159	RDR2-sensitive
AGTGTGACTAAGCTGAGTTGTGCG	14	0	RDR2-resistant
AGTGTGCTGTAAAGGACGGAGAAT	0	12	RDR2-sensitive
AGTGTGGAACCCGAATGATGGCAC	0	14	RDR2-sensitive
AGTGTGGACTCCAAGAAGACGATA	0	17	RDR2-sensitive
AGTGTGGCACTCGACAAAGACGTC	0	21	RDR2-sensitive
AGTGTGGCTCTGATACCATGTAGA	36	4	RDR2-resistant
AGTGTGGTATCTGATTACGGTTGG	0	18	RDR2-sensitive
AGTGTGTAGGCTTACGATACGGTA	0	9	RDR2-sensitive
AGTGTGTCGGTTTAGGCGTTGGCC	0	9	RDR2-sensitive
AGTGTGTGACTGATGGTGGATCGC	0	24	RDR2-sensitive
AGTGTGTGACTGATGGTGGGTCGC	0	10	RDR2-sensitive
AGTGTGTTTGGTTGTAGGTTGCAT	3	144	RDR2-sensitive
AGTGTGTTTTGTCGGGCACTCGGC	0	13	RDR2-sensitive
AGTGTTATCTGTTAGACTGTCGGT	0	9	RDR2-sensitive
AGTGTTATGTCGCTGGCACTCGGC	1	23	RDR2-sensitive
AGTGTTCTGCTGCATCTCTCGGGC	0	10	RDR2-sensitive
AGTGTTGAATCTAGGCCTGGGCAT	0	22	RDR2-sensitive
AGTGTTGAGTTGCTGGGACGTCGT	0	12	RDR2-sensitive
AGTGTTGATTGTAGTATTGTCCGG	0	17	RDR2-sensitive
AGTGTTGCTGGAAACGGAGACGGA	1	21	RDR2-sensitive
AGTGTTGGAACTGTATTAGGGTTT	0	48	RDR2-sensitive
AGTGTTGGACATGGTCTGTAGGCA	0	9	RDR2-sensitive
AGTGTTGGACGCCCGAAAAAGGCA	0	9	RDR2-sensitive
AGTGTTGGAGCCTGTACACGCGGC	1	14	RDR2-sensitive
AGTGTTGGCTGTAGAGATGGCCAA	0	13	RDR2-sensitive
AGTGTTGGGTCTTGACTGACGGAT	0	9	RDR2-sensitive
AGTGTTGTGTGTGGGGATGGTCTA	1	35	RDR2-sensitive
AGTGTTGTTCGATCGGACATCCGC	0	10	RDR2-sensitive
AGTGTTGTTGGACGGAAAAAGGTG	0	12	RDR2-sensitive
AGTGTTTAGGGTGATGAACAGTGA	8	73	RDR2-sensitive
AGTGTTTCGATACGGCACGGGCAT	0	9	RDR2-sensitive
AGTGTTTGGTTGAGAGTCAAGCGG	1	12	RDR2-sensitive
AGTGTTTGTATTCTGATAATTGGT	4	18	RDR2-sensitive
AGTGTTTTATTTCGGGCACTCGGC	6	50	RDR2-sensitive
AGTGTTTTTTGTCGGGCACTCGGC	13	57	RDR2-sensitive
AGTTAAAAAGCTCGTAGTTGGACC	65	5	RDR2-resistant
AGTTAAATGGCTACTCTGTTGGAG	0	11	RDR2-sensitive
AGTTAATTGTTTTGGTTGTCTGGC	0	14	RDR2-sensitive
AGTTACGCGGCTGGCGGCTGGAAG	0	12	RDR2-sensitive
AGTTACTGCGGTATTCAACATAAT	28	0	RDR2-resistant
AGTTACTGTTTGATCCTTGCGGCC	0	14	RDR2-sensitive
AGTTACTTCCGACGGCTTAAGACG	0	11	RDR2-sensitive
AGTTAGATGAGTCGGGGACGACAC	0	9	RDR2-sensitive
AGTTAGCCGTTGGACTCATGGCAC	1	26	RDR2-sensitive
AGTTAGCGGCTCGTTTGACCACAC	0	14	RDR2-sensitive
AGTTAGTTGCAGTAGCCGGGACAG	0	13	RDR2-sensitive
AGTTATAAGCTGTCGAACGTACGG	0	10	RDR2-sensitive

AGTTATATGAGTCGGACTAGCATG	0	11	RDR2-sensitive
AGTTATATTTTGGAGACTGTGTCG	0	9	RDR2-sensitive
AGTTATCACGTAGGGACTGGCGTC	0	10	RDR2-sensitive
AGTTATCGTTTAGGACAACGGCAC	0	11	RDR2-sensitive
AGTTATCTAGGATTTATCGTTGTG	26	0	RDR2-resistant
AGTTATGATCTGCTGACTGGTCGA	0	9	RDR2-sensitive
AGTTATGGACTGACGGTTGGTCGC	0	30	RDR2-sensitive
AGTTATTTCTGTAGCCTGTGCGGC	0	17	RDR2-sensitive
AGTTCAACTCTGGACGCTGGATGA	85	7	RDR2-resistant
AGTTCAAGACGGTATGCTGTGGGT	25	2	RDR2-resistant
AGTTCAGACTGCGGGGGGTGTTAG	0	13	RDR2-sensitive
AGTTCAGTTTGGCTCAGAGCGGTT	0	17	RDR2-sensitive
AGTTCATACGGATACTCATTAATA	15	0	RDR2-resistant
AGTTCATAGACGGTAACAGAGCTC	0	10	RDR2-sensitive
AGTTCATGTCGCTGTAGTGGCGTT	0	11	RDR2-sensitive
AGTTCCACTTAACTGTCGTGCTGG	17	0	RDR2-resistant
AGTTCCAGAGCTCTTCAAGTAGAT	55	1	RDR2-resistant
AGTTCGACGGCTTATTACATTAAG	0	14	RDR2-sensitive
AGTTCGCGGCAGAAGTCAGAGCAG	0	9	RDR2-sensitive
AGTTCGGCTAGGCCAGAGACCTGC	0	13	RDR2-sensitive
AGTTCGGCTAGGCCGGAGACCTGC	0	20	RDR2-sensitive
AGTTCGGCTCGGCTCAGATCGGCT	3	16	RDR2-sensitive
AGTTCGTCCCTGACAGCAGAACGC	0	11	RDR2-sensitive
AGTTCGTCGATCGGATCTTCGGTT	0	28	RDR2-sensitive
AGTTCGTCTGTACTGATAGTTGGG	0	11	RDR2-sensitive
AGTTCGTCTGTATATATAAGCATT	0	10	RDR2-sensitive
AGTTCGTGTGCGGCTGACCGTTCT	21	2	RDR2-resistant
AGTTCGTTGGGCTGTAGGCCGGCT	0	10	RDR2-sensitive
AGTTCTCGGGCTGGTATGTTGACG	0	11	RDR2-sensitive
AGTTCTCTCGGGCGAGTGAGCGGC	0	9	RDR2-sensitive
AGTTCTGGCTGATATGACACGGTT	0	68	RDR2-sensitive
AGTTCTGTAGGGTTTGGTCGGCAC	0	21	RDR2-sensitive
AGTTCTGTATTAAAGCGTAGACGA	21	0	RDR2-resistant
AGTTGAACCCTGCTGCGTAGACGT	0	11	RDR2-sensitive
AGTTGAACGGACCGAGCAGGCCAG	0	17	RDR2-sensitive
AGTTGAATTCTGCATCCGGAAGGC	0	9	RDR2-sensitive
AGTTGACCGCTGCAGTTGAAGAGT	0	23	RDR2-sensitive
AGTTGACGCCTACGTGGATCTGAT	0	9	RDR2-sensitive
AGTTGACGGGTTTGATGACATGAC	177	23	RDR2-resistant
AGTTGACGGTTTTGATGACATGAC	88	0	RDR2-resistant
AGTTGACTCGGAGACGATGGCCTA	0	10	RDR2-sensitive
AGTTGACTGGAAGACGACGGTCTA	0	11	RDR2-sensitive
AGTTGACTGGGAGACGACAGCCTA	0	9	RDR2-sensitive
AGTTGACTGGGAGACGACGGCCTA	4	109	RDR2-sensitive
AGTTGACTGGGAGACGATGGCCTA	0	9	RDR2-sensitive
AGTTGACTGGTAGACGACGGCGTA	0	9	RDR2-sensitive
AGTTGACTGTTTAGATTGATGCAT	0	9	RDR2-sensitive
AGTTGAGAGTAGTGGAAGGCGGGC	130	11	RDR2-resistant
AGTTGATGTGGTCTAGCTCGGCGC	0	9	RDR2-sensitive

AGTTGATTCTACAGTCGGCCCGTC	1	17	RDR2-sensitive
AGTTGCAACTGTAGCTGCAGAGTC	0	10	RDR2-sensitive
AGTTGCAGCTGGAGAAGCAGGCCA	0	9	RDR2-sensitive
AGTTGCCGTAGCAACGCACGGACA	0	11	RDR2-sensitive
AGTTGCCTGAACAACGACGGCCTT	0	11	RDR2-sensitive
AGTTGCCTGTAGTAGTTCAGCCGG	0	14	RDR2-sensitive
AGTTGCGGACGGTATGAGAGGCAC	0	20	RDR2-sensitive
AGTTGCTACATGTAGAAATGCATC	0	10	RDR2-sensitive
AGTTGCTCGGACAGCGTCAGCAAC	0	44	RDR2-sensitive
AGTTGCTGCGTTGACCGTCGGGAC	31	0	RDR2-resistant
AGTTGCTTAGACTGTACAACGGCT	0	25	RDR2-sensitive
AGTTGCTTATTGTCAAGGACTATC	24	2	RDR2-resistant
AGTTGGAATGAACAGTTTGACGTT	45	6	RDR2-resistant
AGTTGGACCTATCTCGTGACGGAC	0	13	RDR2-sensitive
AGTTGGATAACTGATGTCTTTCGA	26	1	RDR2-resistant
AGTTGGATCCAGACGACATGACAA	22	0	RDR2-resistant
AGTTGGCGAGCTCGAGAAGACCAG	0	14	RDR2-sensitive
AGTTGGCGGACTCACCACGGCCTG	0	14	RDR2-sensitive
AGTTGGCGGCAATAGTACTGACGT	0	9	RDR2-sensitive
AGTTGGCTGCGAGCATACAGTTCA	2	31	RDR2-sensitive
AGTTGGGAATGGGAATGGCAGAAC	0	11	RDR2-sensitive
AGTTGGGATAGATGGCAGAACCGT	0	12	RDR2-sensitive
AGTTGGGTTTGTCGGGTCGGGCGG	0	12	RDR2-sensitive
AGTTGTAGATCTTTATTGCAGAGA	0	10	RDR2-sensitive
AGTTGTAGCAGGCAGAGGACTTTC	0	13	RDR2-sensitive
AGTTGTATTGAAGGGCAAGCCACC	28	1	RDR2-resistant
AGTTGTATTGATTCGATTGTTGAT	0	14	RDR2-sensitive
AGTTGTCACTGTTGACGATCGCTC	17	1	RDR2-resistant
AGTTGTCGACTGAGAAGTAGGCTC	0	14	RDR2-sensitive
AGTTGTCGCTGCATACTACAAAAT	0	9	RDR2-sensitive
AGTTGTCGCTGGATAGTGCAAAAT	0	24	RDR2-sensitive
AGTTGTCTATAGCGTCGGGTTACC	37	1	RDR2-resistant
AGTTGTGGGACCGAATCGGTGGAT	0	16	RDR2-sensitive
AGTTGTGGTATTGTAAACTGCGGT	0	24	RDR2-sensitive
AGTTGTTCGGACTGCTGCGGCTGC	1	33	RDR2-sensitive
AGTTGTTGACTGGAACCAACGGCT	0	16	RDR2-sensitive
AGTTGTTTCCTGTTGGCATTTCTA	79	0	RDR2-resistant
AGTTTAAGATGTAGAATTGATGGT	0	10	RDR2-sensitive
AGTTTAATATCTGATATGTGGACC	45	1	RDR2-resistant
AGTTTAGATCGTGCCGACGGTGCT	37	3	RDR2-resistant
AGTTTAGGCAGACATACTGAAGTT	120	13	RDR2-resistant
AGTTTAGGGTCGACACAGCGGAAG	25	0	RDR2-resistant
AGTTTATGACTGTACAGCGGTGAT	17	0	RDR2-resistant
AGTTTATGGAGATGGATGATGATT	33	0	RDR2-resistant
AGTTTATTCATGGATCTGGTTCGG	28	1	RDR2-resistant
AGTTTCAAACTGATTGTATGAAGA	19	0	RDR2-resistant
AGTTTCAACGGTCGTCATACGTCA	0	16	RDR2-sensitive
AGTTTCAACGGTCGTCATGCGTCA	1	34	RDR2-sensitive
AGTTTCAACGGTCGTCTCGCGTCA	1	12	RDR2-sensitive

AGTTTCACGGATGTAGGTGCGGGT	0	12	RDR2-sensitive
AGTTTCCGACGGCCTACACTGTTT	1	15	RDR2-sensitive
AGTTTCTACTATCGTTGTGCGTCA	0	15	RDR2-sensitive
AGTTTCTGGCTGAATGTAACGGTA	0	10	RDR2-sensitive
AGTTTCTGGCTGGCTGTAACGGTA	11	75	RDR2-sensitive
AGTTTCTGGCTGGCTGTAACGGTT	0	10	RDR2-sensitive
AGTTTCTTCGGCAACCTTGGCGGC	19	0	RDR2-resistant
AGTTTGCGACTCGGACAGGCCTAG	0	21	RDR2-sensitive
AGTTTGCGGAGGCCGAGGATGACA	14	0	RDR2-resistant
AGTTTGGACTGGTGGAAGGGCTAT	0	23	RDR2-sensitive
AGTTTGGACTGGTGGGAGGGCTAT	2	53	RDR2-sensitive
AGTTTGGATAGGAACAGCCATGGC	0	14	RDR2-sensitive
AGTTTGGCCGGCTATATAACCATC	0	12	RDR2-sensitive
AGTTTGGCTGGGGCGGCACATCTG	27	2	RDR2-resistant
AGTTTGGCTGTCGCGTACGTCTCT	60	0	RDR2-resistant
AGTTTGTAGGACAGCATATTCACA	0	9	RDR2-sensitive
AGTTTGTCTAGTCGTTGGTGTCGT	0	16	RDR2-sensitive
AGTTTGTCTCGACCTCGGCTTCGT	0	12	RDR2-sensitive
AGTTTGTTCTCTGAATTCGGCTCA	0	13	RDR2-sensitive
AGTTTGTTTGATGGTACGTGCTAC	73	7	RDR2-resistant
AGTTTTACTGTAGTGATCGGCGCC	0	62	RDR2-sensitive
AGTTTTAGGACGGCCGCTGACGGC	0	14	RDR2-sensitive
AGTTTTAGGCGGCTCGCGGCTATG	19	0	RDR2-resistant
AGTTTTAGGTCTGGATCTAGGTTC	18	0	RDR2-resistant
AGTTTTCACTATCGTCGTGCATCA	0	10	RDR2-sensitive
AGTTTTCGATCGTTATCTGGTGCA	0	10	RDR2-sensitive
AGTTTTCGGACGAGGACGCAACGG	0	11	RDR2-sensitive
AGTTTTCGGGACTGTTTCGGGCAA	0	11	RDR2-sensitive
AGTTTTCGTGACTGTTTCGGGCAA	2	18	RDR2-sensitive
AGTTTTCTGTTAGAATTGGATGGA	0	13	RDR2-sensitive
AGTTTTGCACTGCAAGAAAGTCGG	0	10	RDR2-sensitive
AGTTTTGGACCCTTAGCTCGGCGC	3	30	RDR2-sensitive
AGTTTTGGACCCTTTGCTCGGCGC	3	35	RDR2-sensitive
AGTTTTGGACGTACGGACGGATGC	0	11	RDR2-sensitive
AGTTTTGGGCGTAAGGACGGATGC	0	11	RDR2-sensitive
AGTTTTTCTGATTAAATGGACAAC	0	13	RDR2-sensitive
AGTTTTTGCATCTATCGTTCGGCC	0	13	RDR2-sensitive
AGTTTTTTTATTTCTGTAGTCGCA	24	1	RDR2-resistant
ATAAAAATTCGTTCGGCTACGGTC	0	10	RDR2-sensitive
ATAAAAGGGTATTCATCGTCTGAC	50	1	RDR2-resistant
ATAAAATGTAAGGATCGGATCGGG	19	0	RDR2-resistant
ATAAACACCGTATATTCGTCGGCC	0	9	RDR2-sensitive
ATAAACACGGACTCTGTCGGTCAC	0	14	RDR2-sensitive
ATAAACCCGTTAGGTCTGTCGGAT	0	14	RDR2-sensitive
ATAAACTTTGATTAGCCGGAACGA	2	15	RDR2-sensitive
ATAAACTTTGATTAGCCGGAATAA	7	43	RDR2-sensitive
ATAAACTTTGATTAGCTGGAACGA	8	36	RDR2-sensitive
ATAAAGGAATGTAGCTTACGTGGC	32	0	RDR2-resistant
ATAAAGTTTGTAGCACGGGTGACA	15	0	RDR2-resistant

ATAAATGACGGATGTAGAGGACGT	0	20	RDR2-sensitive
ATAAATTCACTAGTCGGTCCGGTT	0	9	RDR2-sensitive
ATAAATTCGAAGGTTACTGGCATT	17	1	RDR2-resistant
ATAAATTTTTACTCGTCAGATACC	22	1	RDR2-resistant
ATAACAACTTGTGCTGATTGGATG	15	0	RDR2-resistant
ATAACACAACCGAGCTCGGCGCCA	0	9	RDR2-sensitive
ATAACAGCGTAGACGGTTCTGTCC	0	12	RDR2-sensitive
ATAACAGCTCAGGACCTTGACGAC	0	9	RDR2-sensitive
ATAACATCGTACGAGGCTTGGCAT	0	19	RDR2-sensitive
ATAACATGGAGCGGAAGTAGGTTC	31	1	RDR2-resistant
ATAACCTGTGGACGCCTACACTAA	0	16	RDR2-sensitive
ATAACGCGGTATTAAGTTGGAATG	28	3	RDR2-resistant
ATAACGGATGTAGAGACGACGTTA	0	11	RDR2-sensitive
ATAACGTCAAAATGTAGTTAGCGG	47	0	RDR2-resistant
ATAACTCCCGTCGGCTGGCGGCGC	0	10	RDR2-sensitive
ATAACTCTGGCCTAGGCACGTCAT	0	10	RDR2-sensitive
ATAACTGTACGGACGGTCCGGCCC	0	12	RDR2-sensitive
ATAACTTGACGGATCGCACGGCCT	45	3	RDR2-resistant
ATAACTTTCGTGACTGTTCCGGCC	0	9	RDR2-sensitive
ATAAGACAGGTCACGGAGCGGTAT	29	2	RDR2-resistant
ATAAGACAGTAGTGTAGACGTCCA	0	20	RDR2-sensitive
ATAAGACCTTCGGCGACGAAGCAT	0	9	RDR2-sensitive
ATAAGACTTATATTATCATTCATT	32	1	RDR2-resistant
ATAAGAGACTACGTTCGACGGCTG	0	9	RDR2-sensitive
ATAAGAGTAGAATCTTGACGACAT	0	9	RDR2-sensitive
ATAAGATCCTACGTCTGACGGTTG	0	15	RDR2-sensitive
ATAAGATTTAAGGATCAAGTCGGA	0	9	RDR2-sensitive
ATAAGATTTAATGATCGGATCGGA	0	10	RDR2-sensitive
ATAAGCGGGCGGGCTTGGACAGGA	0	11	RDR2-sensitive
ATAAGCGGGGGACGTACTCGACAG	0	11	RDR2-sensitive
ATAAGGACCAGAGTGTGCATGTTG	39	2	RDR2-resistant
ATAAGGATTGCATTTTTCATTATC	29	3	RDR2-resistant
ATAAGGCCTGTGGCATGCACACTA	33	0	RDR2-resistant
ATAAGGCGTGCTCTCTCTATGCAT	1	19	RDR2-sensitive
ATAAGGGCTAGTTTGGATCGGCGC	0	25	RDR2-sensitive
ATAAGTAATTTGGATGAATCGGAC	30	0	RDR2-resistant
ATAAGTACTCGTTGGAAAGGTATG	0	12	RDR2-sensitive
ATAAGTCAATTATTTTCGTCGGCC	2	14	RDR2-sensitive
ATAAGTCCGACTGCATACAATATA	0	12	RDR2-sensitive
ATAAGTCTTTGCCGTCGGAGATAA	2	17	RDR2-sensitive
ATAAGTGCCTGTAGATAGGGTGTA	1	17	RDR2-sensitive
ATAAGTGTTGGACTAGCACGGCCA	0	16	RDR2-sensitive
ATAAGTTTAGATCGTGCCGACGGT	33	3	RDR2-resistant
ATAATACTGATGAATTGGTCGGCC	0	59	RDR2-sensitive
ATAATATTTTAGCAAGTCGGCAAT	15	0	RDR2-resistant
ATAATGAGAACTTTATGATGTGGA	16	0	RDR2-resistant
ATAATGATCGAGCGATGACATTTA	41	2	RDR2-resistant
ATAATGTTGCATAGGATTGTTGGC	0	12	RDR2-sensitive
ATAATTCACCGGACTGTCTGGTGT	0	13	RDR2-sensitive

ATAATTCACTGGACTGTTTGGTGT	0	10	RDR2-sensitive
ATAATTCATCGGACTGTTCGGTGT	1	12	RDR2-sensitive
ATAATTTGGACTGGTGGGAGGGCT	1	25	RDR2-sensitive
ATAATTTGTAGATCTTTCCATGGC	73	2	RDR2-resistant
ATAATTTTTTGTCTGTTGGTTAGT	25	0	RDR2-resistant
ATACAAATTGTAAGTAATGCGTTC	28	0	RDR2-resistant
ATACAACACTCGGCAAAGCCTCGG	0	10	RDR2-sensitive
ATACAACCATACTGTCGGCGTTTC	18	1	RDR2-resistant
ATACAACGACGGACGGTCCGCATA	0	11	RDR2-sensitive
ATACAAGCGGTAGACTTTTTACAT	1	76	RDR2-sensitive
ATACAAGCGGTAGGATTTTTACAT	4	44	RDR2-sensitive
ATACAAGCGGTAGGCTTTTTACAT	1	20	RDR2-sensitive
ATACAAGGTGACTGAACCCCGACG	0	15	RDR2-sensitive
ATACAATCTGTAGTGTTATATGCA	0	15	RDR2-sensitive
ATACAATGTTATTTTCGTCGGCAA	2	27	RDR2-sensitive
ATACACAAATTTACTCGTCGGTGC	0	12	RDR2-sensitive
ATACACAAGTGTGACTCGTCGATT	0	11	RDR2-sensitive
ATACACACTCTGCCTGATTGGACG	1	13	RDR2-sensitive
ATACACCGGTACAACCTAGGCTTA	0	13	RDR2-sensitive
ATACACGGAGACGGAACACGGTGC	0	16	RDR2-sensitive
ATACACTACTGAGGGAGAGGACAG	0	9	RDR2-sensitive
ATACACTATGGGTCGATTTCGGAT	32	4	RDR2-resistant
ATACACTCGGTCTTTGTTGTACCT	24	0	RDR2-resistant
ATACACTGTGCTGATTTCTGTGCA	0	22	RDR2-sensitive
ATACAGATAGTAGAGTTACTCATT	23	2	RDR2-resistant
ATACAGATCGGACTAGCACGGTTC	0	41	RDR2-sensitive
ATACAGGCATGCTCGGCTACTCCA	20	0	RDR2-resistant
ATACAGGTAGAGTGTGTTGGGCAC	0	11	RDR2-sensitive
ATACAGTCTCCGCTGCAGAAGGTG	1	13	RDR2-sensitive
ATACATATTGGATCCGTAACCTGA	14	0	RDR2-resistant
ATACATCTAGTAGAGCGTGATAGC	0	10	RDR2-sensitive
ATACATGACTGGGTACTCATACCC	17	0	RDR2-resistant
ATACATGGCCAGTAGATAATGTCA	0	12	RDR2-sensitive
ATACATGGTCTGTTGGTTAGCATC	0	10	RDR2-sensitive
ATACATGTATTGTTTCTGCACGAG	19	0	RDR2-resistant
ATACATGTTCGGATATCCTGCGGC	0	10	RDR2-sensitive
ATACATTGTGGATCTAGGATTACA	15	0	RDR2-resistant
ATACATTTCTTCTATTCTGTGGTA	16	0	RDR2-resistant
ATACCAAACATCTCTGTAGTATGT	0	10	RDR2-sensitive
ATACCACTTGTAGGATCTAGGACA	20	2	RDR2-resistant
ATACCAGCCCGAGAACTTGGGACC	1	13	RDR2-sensitive
ATACCAGTTTTACACTCTGCAGAG	7	33	RDR2-sensitive
ATACCATGCTATTTGTCGGTCACA	39	0	RDR2-resistant
ATACCCAGGTCGCGGATCGTCCGG	0	14	RDR2-sensitive
ATACCCATCGGGCTGTGCCGGCAC	0	10	RDR2-sensitive
ATACCCGGATACTGTAGCTTCGGA	0	15	RDR2-sensitive
ATACCCTGCTGCGTCGGATGGCAT	0	17	RDR2-sensitive
ATACCGACGGATGCTGTGATGGTA	0	14	RDR2-sensitive
ATACCGGCTTTGACCTTGACGGGT	0	9	RDR2-sensitive

ATACCGGTCGTTGCTGGAGGCATC	0	19	RDR2-sensitive
ATACCGGTCGTTGCTGGAGGCGTC	0	10	RDR2-sensitive
ATACCTAATGTTAGGCTGTGGCGT	0	15	RDR2-sensitive
ATACCTACAGGTTTGTTGTCGGGA	72	2	RDR2-resistant
ATACCTGACTGGGGCTTCCCATAC	0	19	RDR2-sensitive
ATACCTGTACATGTGGGCCGGCCT	51	2	RDR2-resistant
ATACCTTTCTCGGCCTTTTGGCTA	73	3	RDR2-resistant
ATACGAAGCTGTAATTCTTGGCCT	81	8	RDR2-resistant
ATACGACAGGATCGGCTCGGCTCT	0	37	RDR2-sensitive
ATACGACATGTAGTAGCCCCGAAT	0	10	RDR2-sensitive
ATACGACATTGTAGATGACACTGT	0	12	RDR2-sensitive
ATACGACGACGACGGTTCATCTGT	0	9	RDR2-sensitive
ATACGACTACTTAGGAACATGGCT	0	84	RDR2-sensitive
ATACGAGACATGTGAAGCTTGCTT	79	10	RDR2-resistant
ATACGAGCATCCGAACATCCATGT	14	0	RDR2-resistant
ATACGATCAGCGGGCGAGACGCAC	0	9	RDR2-sensitive
ATACGATGGGACGCATGATTCTTG	0	12	RDR2-sensitive
ATACGATTGGACACGGCCTGACTC	0	12	RDR2-sensitive
ATACGATTGGTGAGTTGAGCGACT	0	18	RDR2-sensitive
ATACGCAGAGTAGAAGTCGCTCCT	0	25	RDR2-sensitive
ATACGCCGTTCTGGCCCGTCGGAA	0	14	RDR2-sensitive
ATACGCGGAAGGCGGTCTGAACTC	0	12	RDR2-sensitive
ATACGGAAAACGGGACGGAAACAG	0	12	RDR2-sensitive
ATACGGAAATTTTAGTGCGGATCA	31	0	RDR2-resistant
ATACGGAACTAGTCGGACACGGAT	0	17	RDR2-sensitive
ATACGGAAGGCACGATGAACGCTA	0	12	RDR2-sensitive
ATACGGATTGCACGCGAAAGGCAT	0	10	RDR2-sensitive
ATACGGCTCGAACTTCCAGTGTGC	0	9	RDR2-sensitive
ATACGGGACAGACGAGAACTGCAT	0	11	RDR2-sensitive
ATACGGGATTTTTCGGACTTCGGC	0	23	RDR2-sensitive
ATACGGGCTCTGTTGGAACGGTAA	0	9	RDR2-sensitive
ATACGGGGCTATAGGTAGCATTGT	0	9	RDR2-sensitive
ATACGGGTAGTTAAGATTCGGGAC	0	17	RDR2-sensitive
ATACGGTCCCTGCTGGAAACGCCT	0	30	RDR2-sensitive
ATACGGTGCTGTCAGCGACGCGAA	112	1	RDR2-resistant
ATACGGTGTGTATTTTCGTCGGTC	6	36	RDR2-sensitive
ATACGGTGTTTATTTTCGTCGGGC	0	10	RDR2-sensitive
ATACGGTGTTTATTTTCGTCGGTC	0	15	RDR2-sensitive
ATACGGTTAAGACTCTATAACTAA	34	2	RDR2-resistant
ATACGTCAGACGCCGGTGTGACCT	0	10	RDR2-sensitive
ATACGTCAGACGCCGGTGTGATCT	0	12	RDR2-sensitive
ATACGTCAGACGGCGGTGTGACCT	0	14	RDR2-sensitive
ATACGTCGGGTAGGGTACGGATAG	0	21	RDR2-sensitive
ATACGTCTTTGACAAGGGACCGGC	0	9	RDR2-sensitive
ATACGTGCTGCAGAGCGGTGCTGC	0	11	RDR2-sensitive
ATACGTGTCGTTGTGTGGTGTCGT	14	0	RDR2-resistant
ATACGTTAGGCAGGCAACTGGCAT	0	10	RDR2-sensitive
ATACGTTATTTTTTTCGTCGGTTC	0	10	RDR2-sensitive
ATACGTTGACTTTATAGTTGTCTG	1	16	RDR2-sensitive

ATACGTTGACTTTGTGGAAGCGTG	0	28	RDR2-sensitive
ATACGTTTAAACTCTGCGTAGAGG	18	0	RDR2-resistant
ATACTAAACTGTAGTATTGTAAAT	0	15	RDR2-sensitive
ATACTAACCGGACCGGGTCGTGCC	0	49	RDR2-sensitive
ATACTAATCGGACCGGGTCGTGCC	0	9	RDR2-sensitive
ATACTACTAGGTTGTTCGGGCCAA	0	12	RDR2-sensitive
ATACTACTCTATTAAGTCTGTAGC	0	10	RDR2-sensitive
ATACTATGAATTAAGAACAAGGCA	0	10	RDR2-sensitive
ATACTATGCACGGGTCGGATACAA	0	16	RDR2-sensitive
ATACTATGGAGGTAGGTCTAGCTC	23	1	RDR2-resistant
ATACTATTATTAGCAAGATGTCGG	18	40	RDR2-sensitive
ATACTCGTATGGACTATTGGTGTC	0	30	RDR2-sensitive
ATACTCTAGACTTATTTGACTGCA	0	14	RDR2-sensitive
ATACTCTGTAGACTGCACAAGCGT	0	18	RDR2-sensitive
ATACTCTGTGACGACGGCGGCTCT	7	33	RDR2-sensitive
ATACTGAAGTTACTATGAAGGATT	142	23	RDR2-resistant
ATACTGACGGATGACGTGGTCTAG	0	9	RDR2-sensitive
ATACTGCAGCTTTGATGTGCCATA	0	13	RDR2-sensitive
ATACTGCTGCTGTTCATCCTTCTG	16	0	RDR2-resistant
ATACTGCTGTGTGACTCGTCCTAC	0	9	RDR2-sensitive
ATACTGCTTATCTCTGACGGCTAT	0	9	RDR2-sensitive
ATACTGGAACGGAACGGCTTCATA	0	20	RDR2-sensitive
ATACTGGATTGCATGCAAAGAGCC	0	18	RDR2-sensitive
ATACTGGTTCAGAACGGAGCCCTA	0	13	RDR2-sensitive
ATACTGTAATTCGCGCGGACTAAA	1	13	RDR2-sensitive
ATACTGTACCTATTAGCTCGGATC	0	11	RDR2-sensitive
ATACTGTAGAAGACGCAGCCGCAG	0	20	RDR2-sensitive
ATACTGTAGAAGAGGGTACCCCTA	1	39	RDR2-sensitive
ATACTGTAGAAGAGGGTACCCCTG	1	60	RDR2-sensitive
ATACTGTAGAAGAGGGTACCCCTT	1	58	RDR2-sensitive
ATACTGTAGAAGCCGCAGCCGCAA	1	31	RDR2-sensitive
ATACTGTAGAAGCCGCAGCCGCAG	33	904	RDR2-sensitive
ATACTGTAGAAGCCGCAGCCGCAT	0	11	RDR2-sensitive
ATACTGTAGAAGCCGCAGCCGCCG	1	49	RDR2-sensitive
ATACTGTAGATGGCGTTGTTTGAC	0	9	RDR2-sensitive
ATACTGTAGCGCGTCATGACGGTT	0	9	RDR2-sensitive
ATACTGTAGCGGACTACTTATCGT	0	24	RDR2-sensitive
ATACTGTAGGAGAGGGTACCCCTG	0	12	RDR2-sensitive
ATACTGTAGTAACGGATACCCTGT	2	15	RDR2-sensitive
ATACTGTAGTAACGGGTACCCCAT	0	16	RDR2-sensitive
ATACTGTAGTAACGGGTACCCCTG	0	11	RDR2-sensitive
ATACTGTAGTAACGGGTACCCCTT	0	9	RDR2-sensitive
ATACTGTAGTAAGGGATACCCTGT	0	14	RDR2-sensitive
ATACTGTAGTAAGGGGTACCCTAG	0	19	RDR2-sensitive
ATACTGTAGTAGAGGGTACCCCTA	0	20	RDR2-sensitive
ATACTGTAGTAGAGGGTACCCCTG	2	44	RDR2-sensitive
ATACTGTAGTAGAGGGTACCCCTT	0	17	RDR2-sensitive
ATACTGTAGTATTGAGCAATACTA	0	9	RDR2-sensitive
ATACTGTAGTATTGAGCAATACTG	0	14	RDR2-sensitive

ATACTGTATCTCAGCTGGAGGTGC	39	10	RDR2-resistant
ATACTGTATTTATAGCGACGATTG	0	15	RDR2-sensitive
ATACTGTCCAAGCTTCGGCATCTT	26	3	RDR2-resistant
ATACTGTCGTAGCCCTGATGGCCG	0	15	RDR2-sensitive
ATACTGTGCTGCACTATAGCTTGT	19	0	RDR2-resistant
ATACTGTGGAAGAGGGTACCCCTG	0	9	RDR2-sensitive
ATACTGTGGAAGAGGGTACCCCTT	0	11	RDR2-sensitive
ATACTGTGGCGTAGACTTCGGCGT	0	33	RDR2-sensitive
ATACTGTGGCGTGCTGAGCGTCTA	0	27	RDR2-sensitive
ATACTGTTATGAGATGGAGGACGA	0	10	RDR2-sensitive
ATACTGTTCATGCTGTAGAGGCTA	0	9	RDR2-sensitive
ATACTGTTGTGGTATCTGGATCGT	0	9	RDR2-sensitive
ATACTGTTTTTAGACGATTGCATG	21	2	RDR2-resistant
ATACTTACCAGGTCGGACCGGCCC	0	14	RDR2-sensitive
ATACTTAGTAGATTTTGATAGAGC	20	2	RDR2-resistant
ATACTTATAGGTATGGATCTGGAC	0	12	RDR2-sensitive
ATACTTATTTTCGACTATGGCTAG	14	0	RDR2-resistant
ATACTTCGGGCTGACACGATACGG	0	26	RDR2-sensitive
ATACTTCGGGCTGACACGGTACGG	0	24	RDR2-sensitive
ATACTTCGGGCTGGCACGACACGG	0	9	RDR2-sensitive
ATACTTCGGGCTGGCACGGCACGG	0	12	RDR2-sensitive
ATACTTCGGGCTTGTACGACACGG	0	20	RDR2-sensitive
ATACTTGGTGACTTGCTGGTGCAT	17	0	RDR2-resistant
ATACTTGTGAATTTTGTTGCATAC	21	2	RDR2-resistant
ATAGAAATTTTATTGGTTGTCAGC	21	0	RDR2-resistant
ATAGAACAGTAGTAGCTTGTGCAT	49	3	RDR2-resistant
ATAGAACCGGACCGTGCCGGGCAT	0	13	RDR2-sensitive
ATAGAACCGGATCGTGCTAGGCTT	0	17	RDR2-sensitive
ATAGAACGACTGCTGACGGTTCTT	0	10	RDR2-sensitive
ATAGAACGGTAGAATGGACGGCTA	0	9	RDR2-sensitive
ATAGAACTGCCGTGGCGCTGACGT	1	66	RDR2-sensitive
ATAGAACTTATGATCAGTTACGGA	14	0	RDR2-resistant
ATAGAATCGGACTCGAATCGGGCC	0	9	RDR2-sensitive
ATAGAATGTATGAGGCTGGAAGAG	29	1	RDR2-resistant
ATAGACCAGAGGACAGACCGGCAA	0	16	RDR2-sensitive
ATAGACGACTGCTGACGGCGCTTT	0	12	RDR2-sensitive
ATAGACGACTGCTGACGGTCCTTT	0	17	RDR2-sensitive
ATAGACGCGATGGTAGAACGTGGC	0	16	RDR2-sensitive
ATAGACGGCTCTTTTAAATTTGTT	34	0	RDR2-resistant
ATAGACGGCTGCTGACGGACCTTT	0	10	RDR2-sensitive
ATAGACGGCTGCTGACGGCCCTTT	8	49	RDR2-sensitive
ATAGACGGCTGCTGACGGCTCTTT	0	10	RDR2-sensitive
ATAGACGGCTGCTGACGGTCCTTT	6	54	RDR2-sensitive
ATAGACGGCTGCTGATGGTCCTTT	0	14	RDR2-sensitive
ATAGACGGCTGTTGACGGTCCTTG	0	55	RDR2-sensitive
ATAGACGGGGACGAGAGAGATGGA	18	1	RDR2-resistant
ATAGACGTACGGATGTAGTTCGGC	0	10	RDR2-sensitive
ATAGACGTAGCACCGTTGGATGTG	0	16	RDR2-sensitive
ATAGACGTCTAGACCTGATCCGGC	0	10	RDR2-sensitive

ATAGACGTCTGAACCTGGTCCGGC	0	21	RDR2-sensitive
ATAGACGTGTGGATCTGATCCGGC	0	10	RDR2-sensitive
ATAGACTACTGTCGTCCGACGGCC	62	2	RDR2-resistant
ATAGACTGATTCAGCGTCGGCTTG	20	0	RDR2-resistant
ATAGACTGGACTGCTAATTAGGCC	0	11	RDR2-sensitive
ATAGACTGGGGCAGAACGCGGAAT	0	10	RDR2-sensitive
ATAGACTGTAACGGCGAGCGGTAT	0	12	RDR2-sensitive
ATAGACTGTAGTGTGGTGTCTAGG	0	10	RDR2-sensitive
ATAGACTTAGGGCCCTGGCGACGG	0	15	RDR2-sensitive
ATAGAGACGGGTGGATTAAGGCGT	0	9	RDR2-sensitive
ATAGAGCTGCAGGTCTGGCGAGCT	0	9	RDR2-sensitive
ATAGAGGACGAAGACGTACGCTAC	0	22	RDR2-sensitive
ATAGAGGACGGAATCTTTTAGAGT	2	23	RDR2-sensitive
ATAGAGGACGTTGCTGGAGACAGT	1	12	RDR2-sensitive
ATAGAGGATGAATATAGAGGACGT	1	13	RDR2-sensitive
ATAGAGGATGGATATAGAGGACGT	1	20	RDR2-sensitive
ATAGAGGATGTATATAGAGGACGT	1	27	RDR2-sensitive
ATAGAGTTTCAATGTAGACCGGCT	0	11	RDR2-sensitive
ATAGATAATTTTCGTCGGCAGGCC	0	11	RDR2-sensitive
ATAGATCCATATGCTCGTTGGACC	41	10	RDR2-resistant
ATAGATCTCTCCTGTACGTGGCTA	0	10	RDR2-sensitive
ATAGATCTGGCGAGCTGACGCAAG	0	11	RDR2-sensitive
ATAGATGGACGGTGTAGATTGGCT	0	28	RDR2-sensitive
ATAGATGGATCAGTTGTTGAGGAC	21	1	RDR2-resistant
ATAGATGGCATAGTAGACGTCTCC	0	10	RDR2-sensitive
ATAGATGGCTCTGGTGATCGTCGT	0	10	RDR2-sensitive
ATAGATGTCATAGCTTGATTATTA	23	1	RDR2-resistant
ATAGATGTGGAACAGACGACGCAC	0	9	RDR2-sensitive
ATAGATTGAGTTATGATTGTTTGT	15	0	RDR2-resistant
ATAGATTGTAGATGGCAGCTCCGC	0	11	RDR2-sensitive
ATAGATTGTAGCTGTAGTAGTCGG	0	18	RDR2-sensitive
ATAGATTTATTGACTGGGAGGGTT	99	35	RDR2-resistant
ATAGATTTTGACGAGCTGGAACGA	0	12	RDR2-sensitive
ATAGCACATGACGCTGAACTACGG	0	10	RDR2-sensitive
ATAGCACTGGAAGGGCACAGCTGA	0	11	RDR2-sensitive
ATAGCAGCAGACTACAGCGCTCAA	0	12	RDR2-sensitive
ATAGCATATTTTCGTCGGCAGGCC	0	12	RDR2-sensitive
ATAGCATTGATCAACTTCATTAAG	28	0	RDR2-resistant
ATAGCCAACGGACTGGTTCGGCGC	0	14	RDR2-sensitive
ATAGCCGACGGCTGTCAGACTGGC	0	13	RDR2-sensitive
ATAGCCTATTTTCGTCGGCAGGCC	0	13	RDR2-sensitive
ATAGCCTATTTTCGTCGGCAGGCT	0	9	RDR2-sensitive
ATAGCCTCGGGGTGGACAACGGTT	17	1	RDR2-resistant
ATAGCGAAGCTGACATGTATCATT	23	0	RDR2-resistant
ATAGCGACGATTGTCAGACGCCAA	0	10	RDR2-sensitive
ATAGCGCACCGGACTGTTAGGTGT	0	13	RDR2-sensitive
ATAGCGGATGTAGACGTTCGGTAC	0	17	RDR2-sensitive
ATAGCGGCGGGACGGAGATGGTGT	19	0	RDR2-resistant
ATAGCGGCGGGACGTGGATGGGGT	22	0	RDR2-resistant

ATAGCGGCGGGACGTGGATGGTGT	34	1	RDR2-resistant
ATAGCGTACTGTGGCATCCGTCAT	0	13	RDR2-sensitive
ATAGCGTAGTGACTGAGTCGTTGG	0	13	RDR2-sensitive
ATAGCGTTTCTGTGGTCTCGGTAA	2	60	RDR2-sensitive
ATAGCTACGATTCGTTGGTCGTTA	217	1	RDR2-resistant
ATAGCTACGATTCGTTGGTCGTTT	14	0	RDR2-resistant
ATAGCTAGTCTGTCGGAGATTTAT	0	9	RDR2-sensitive
ATAGCTATTGGATCTGATCCGGTT	17	1	RDR2-resistant
ATAGCTCGCGAACCGGCTCGGCTT	0	9	RDR2-sensitive
ATAGCTCTGAGATGGTATCTTCGT	0	11	RDR2-sensitive
ATAGGAAGTATTCTCTATCGTCGT	1	26	RDR2-sensitive
ATAGGACAAGTATGTGACTGGTAA	0	21	RDR2-sensitive
ATAGGACAGTTGTCACGGTTCTGT	0	12	RDR2-sensitive
ATAGGACCCTCATGACATTGGCGT	0	28	RDR2-sensitive
ATAGGACCCTGCTGGAGAGAGCAT	0	14	RDR2-sensitive
ATAGGACGACTCGGTAGAAGCCAT	0	82	RDR2-sensitive
ATAGGACGGAGGATATGATGTGTA	185	0	RDR2-resistant
ATAGGACTGGACGGTCCGTGACCT	0	9	RDR2-sensitive
ATAGGATTAGAAGTTGGATGGTCG	16	0	RDR2-resistant
ATAGGCCACTGTAGAGTAGGCGGT	0	10	RDR2-sensitive
ATAGGCTATGGCGCCGAGGTACGT	0	16	RDR2-sensitive
ATAGGCTCTGATACCACCTGCAGC	30	2	RDR2-resistant
ATAGGCTGGAATGAATACGCGCAC	0	27	RDR2-sensitive
ATAGGGACAACCAGAATCGTGCGC	1	18	RDR2-sensitive
ATAGGGAGCGCTGTAGGAGGCACC	0	15	RDR2-sensitive
ATAGGGGCGCTGTAGGGGTACCGC	0	24	RDR2-sensitive
ATAGGTAATCAGGTCGGTGGGCAT	0	14	RDR2-sensitive
ATAGGTACAGTATCGGACAGCGAC	0	9	RDR2-sensitive
ATAGGTACTGTGGATCGGCCTAGT	1	17	RDR2-sensitive
ATAGGTAGGACGGCGCGGCTGCTT	44	1	RDR2-resistant
ATAGGTAGGGGACGGACGTAGAGG	0	34	RDR2-sensitive
ATAGGTCTGCATGATATTGATGGT	1	75	RDR2-sensitive
ATAGGTCTGTTTGGTTCATGGCTA	0	14	RDR2-sensitive
ATAGGTGCGGGTGTGGTTTGATGG	0	9	RDR2-sensitive
ATAGGTTAGATTGTGCATAGGCTT	23	0	RDR2-resistant
ATAGTACTCGGATAGTGCGGCTTT	0	10	RDR2-sensitive
ATAGTAGAATGTCTTAGATGTCAT	30	0	RDR2-resistant
ATAGTAGAGATGAATCATGACACA	15	0	RDR2-resistant
ATAGTAGTCGTCGAACGCGCGCAA	0	10	RDR2-sensitive
ATAGTAGTCGTCGAACGCGCGCGA	1	13	RDR2-sensitive
ATAGTAGTGCTCTGTGCAAAGAGG	0	13	RDR2-sensitive
ATAGTATTGACTGAGAGAGGCATT	29	5	RDR2-resistant
ATAGTCAACTGTACAGGGCCTTAA	0	47	RDR2-sensitive
ATAGTCACGACTATAGATGGCCAG	0	18	RDR2-sensitive
ATAGTCACGGACGGATATTATCTA	0	23	RDR2-sensitive
ATAGTCCGGCCTAGCTAGAAGCGT	0	13	RDR2-sensitive
ATAGTCTAGTGTAGGTGCGGATGT	0	17	RDR2-sensitive
ATAGTCTGATTGTAGGTGGTAGTC	0	9	RDR2-sensitive
ATAGTGACCAGAGCCGGACGGCCT	382	0	RDR2-resistant

ATAGTGACTGTCTGCTTGGGCGTG	22	1	RDR2-resistant
ATAGTGCCGGGCTGGGCATGACAC	0	14	RDR2-sensitive
ATAGTGGACGCGTAGAACGGCGTC	0	14	RDR2-sensitive
ATAGTGGATTTTATGTTGTGCATT	29	0	RDR2-resistant
ATAGTGTCGTGTCTGGACGTCATC	0	31	RDR2-sensitive
ATAGTTGGACATAGATGGCAATGG	0	13	RDR2-sensitive
ATAGTTGGACCTATCTCGTGACGG	0	13	RDR2-sensitive
ATAGTTGGGGTAGATATGGGACGG	0	9	RDR2-sensitive
ATAGTTGGTGGACGGAGGCAGGAC	0	13	RDR2-sensitive
ATAGTTGTTGACTTAGATTTACGG	0	14	RDR2-sensitive
ATAGTTTATGTGTAGTTCGGTATA	37	1	RDR2-resistant
ATAGTTTGGACTGGTGGGAGGGCT	0	11	RDR2-sensitive
ATAGTTTGTTTGATGGTACGTGCT	42	4	RDR2-resistant
ATAGTTTTACACTGTAGATTGCAT	0	16	RDR2-sensitive
ATAGTTTTACTGTAGTGATCGGCG	0	21	RDR2-sensitive
ATATAAACTTTGATTAGCCGGAAC	5	50	RDR2-sensitive
ATATAAACTTTGATTAGCCGGAAT	4	17	RDR2-sensitive
ATATAAACTTTGATTAGTCGGAAC	0	11	RDR2-sensitive
ATATAAATTGTGGTCGCTCTTTTC	0	10	RDR2-sensitive
ATATAAGACAGTAGTGTAGACGTC	0	19	RDR2-sensitive
ATATAAGTGTAATGATCTGTGGAA	1	63	RDR2-sensitive
ATATAAGTGTTGGACTAGCACGGC	0	25	RDR2-sensitive
ATATAATTGATGAATTGGTCGGCC	18	0	RDR2-resistant
ATATACCAGGAATGTAGTAGGTAA	0	9	RDR2-sensitive
ATATACCTTCGGCATATACTTGTG	0	13	RDR2-sensitive
ATATACCTTGCTGTGAGACAGAAT	47	1	RDR2-resistant
ATATACGGTGTGTATTTTCGTCGG	0	14	RDR2-sensitive
ATATACTCGTATGGACTATTGGTG	0	12	RDR2-sensitive
ATATACTGTAGCGGACTACTTATC	0	12	RDR2-sensitive
ATATAGAGACGCGATAGCCCTCAG	0	23	RDR2-sensitive
ATATAGAGTTTCGTTGATTGTTGT	75	2	RDR2-resistant
ATATAGATCGGAGCGGCAATCCTT	0	12	RDR2-sensitive
ATATAGCGTTTCTGTGGTCTCGGT	0	10	RDR2-sensitive
ATATAGCGTTTGTGTGGTCTCGGT	0	9	RDR2-sensitive
ATATAGGACCGTCCAGTACGACAT	0	12	RDR2-sensitive
ATATAGGACGAGGATGTGCCTCAT	0	16	RDR2-sensitive
ATATAGGGCAGGTATAACGGGAGC	0	12	RDR2-sensitive
ATATAGTAGTCGTCGAACGCGCAC	1	12	RDR2-sensitive
ATATATAAATCTGAAATTCTAATT	15	0	RDR2-resistant
ATATATAAGTATGTTATTGCGTCG	15	0	RDR2-resistant
ATATATACTGGAACAGAACGGCTC	0	12	RDR2-sensitive
ATATATATATTTGCGTCGGATACC	58	1	RDR2-resistant
ATATATATGCAGAATTCATTTCCC	24	2	RDR2-resistant
ATATATCGAAGCAATTTCTAGTGG	23	3	RDR2-resistant
ATATATCGAAGTCTGCATGATACT	1	32	RDR2-sensitive
ATATATGAACATGCAGACTGTGGC	0	11	RDR2-sensitive
ATATATGGACACCATGATGGGGCG	16	0	RDR2-resistant
ATATATGTACATGAACCTTTGGAC	15	0	RDR2-resistant
ATATATTGCAGAGGACTACATGTT	62	0	RDR2-resistant

ATATATTGGACTATATCAGGCTAC	0	14	RDR2-sensitive
ATATATTGTCTTTATGTCGTCGTC	0	10	RDR2-sensitive
ATATATTTCTCGGTCTTTTGGTTA	26	0	RDR2-resistant
ATATATTTTCGTCGGCCGGCCCAC	0	13	RDR2-sensitive
ATATATTTTCGTCGGCCGGCCCAT	0	9	RDR2-sensitive
ATATCAAGGACTATGGCGTCATCT	0	10	RDR2-sensitive
ATATCAAGTTGGAAGTATGCTTTT	15	0	RDR2-resistant
ATATCATACTGATGTTTGTCGTTC	0	14	RDR2-sensitive
ATATCATGACACGTGGACAGGTTT	21	2	RDR2-resistant
ATATCCATCGGGCCGTATCTAGGC	0	11	RDR2-sensitive
ATATCCCTACGTTTGATTGTCTGT	0	11	RDR2-sensitive
ATATCCGACGGCTGTCAGAGGGGC	0	10	RDR2-sensitive
ATATCCGAGGGCTGGGAGGCGGAC	0	17	RDR2-sensitive
ATATCCTGCGGGCTAGTACGGCAC	0	11	RDR2-sensitive
ATATCGCCGAGCTCTGACGTGGCT	0	12	RDR2-sensitive
ATATCGCTGTTTGAATCCGGGTAT	1	14	RDR2-sensitive
ATATCGGACCGTGCCAGCCCGGCA	0	10	RDR2-sensitive
ATATCGGACGGTCTACGACATATC	0	11	RDR2-sensitive
ATATCGGCTAGTTGATTTTCGGTG	39	3	RDR2-resistant
ATATCGGCTATGTGATTTTCGGTG	53	3	RDR2-resistant
ATATCGGGCTAAACGGCGTAACTT	0	47	RDR2-sensitive
ATATCGGGCTGTGCTGTCCAACGG	0	9	RDR2-sensitive
ATATCGGGTCTGTTTGTTTCGGCT	1	12	RDR2-sensitive
ATATCGGTTAGTTGATTTTCGGTG	23	0	RDR2-resistant
ATATCTATATCTAGCTCCGTCGGG	0	9	RDR2-sensitive
ATATCTCACATGTGGGTCGTCCGG	0	9	RDR2-sensitive
ATATCTGATCTGTTAGATGGTCTT	15	0	RDR2-resistant
ATATCTGCCTCTGATAGTCGGAAT	0	9	RDR2-sensitive
ATATCTGTATGAATAATTTCTAAC	17	0	RDR2-resistant
ATATCTTAGAATTCTCTTAACGGC	55	11	RDR2-resistant
ATATCTTAGAATTCTTTTTGCGGC	53	1	RDR2-resistant
ATATGAAATTTGTGGAAGTGCTAT	33	0	RDR2-resistant
ATATGAACAAGTAGAACATTAAGA	43	3	RDR2-resistant
ATATGAACATGCAGACTGTGGCCA	0	9	RDR2-sensitive
ATATGAAGTGTTGTAGGCATGACA	0	11	RDR2-sensitive
ATATGAATCGTGATCGTGTCGTGC	0	12	RDR2-sensitive
ATATGACAATATTCAGTAGACTTA	134	6	RDR2-resistant
ATATGACAATTCTTAGAAATCTTA	14	0	RDR2-resistant
ATATGACACTGTGGACAGCGTTTA	0	16	RDR2-sensitive
ATATGACATTTTGCTGATAGGGCC	34	1	RDR2-resistant
ATATGACGCAGGACGACCGTTGAA	15	0	RDR2-resistant
ATATGACTGCGGAAGATTGTATGG	0	10	RDR2-sensitive
ATATGACTGGACGGAACCCGGCAT	0	13	RDR2-sensitive
ATATGAGTGTAGATGGTAGAAGAA	0	11	RDR2-sensitive
ATATGAGTTTATGCAGTTGGGATT	30	0	RDR2-resistant
ATATGATATCATGACACGTGGACA	35	6	RDR2-resistant
ATATGATCATCGATGACGAGCGTG	0	14	RDR2-sensitive
ATATGATCTTGTTTCTGTTGGTTT	28	0	RDR2-resistant
ATATGATGTGTAGTCGGGAAAGAA	14	0	RDR2-resistant

ATATGATTGTTTAAGTGATTTCAA	15	0	RDR2-resistant
ATATGATTTGTGGGTCTGACGGTA	0	14	RDR2-sensitive
ATATGCATTCTGTAGGAAGACAAC	32	3	RDR2-resistant
ATATGCTGCTGGATTCTCAAGACG	21	0	RDR2-resistant
ATATGCTGGAGCGGTATTACTCAA	59	3	RDR2-resistant
ATATGCTTGATAATTGAGGACTAA	21	0	RDR2-resistant
ATATGCTTTTTTGAACAGATGATG	17	1	RDR2-resistant
ATATGGACCCGGCTGAAAGACGTA	0	13	RDR2-sensitive
ATATGGACGGTCTACACTATTGAC	0	9	RDR2-sensitive
ATATGGACGGTGTACTTAGCATCA	0	13	RDR2-sensitive
ATATGGAGACTACAGGAGCCGCTC	0	10	RDR2-sensitive
ATATGGAGTGGTTAATAATGGAAA	17	0	RDR2-resistant
ATATGGATCGGACTACCATGGCCC	0	12	RDR2-sensitive
ATATGGCAACTGTCGACGAGCACG	31	0	RDR2-resistant
ATATGGGCAAAACGACGGTATCTG	43	0	RDR2-resistant
ATATGGGCTAAACGGGCTAAACGG	0	10	RDR2-sensitive
ATATGGGCTAAGTGGAACCGGTAG	0	17	RDR2-sensitive
ATATGGTGCACTGAATTTAAGCAT	23	0	RDR2-resistant
ATATGGTTGTATGTACAAATTTAA	20	2	RDR2-resistant
ATATGTAGACTCCAGAAGCCGTTC	0	10	RDR2-sensitive
ATATGTAGGACGGATTATAAGCAA	0	11	RDR2-sensitive
ATATGTAGTTCTAGTGAGAGGTGT	0	12	RDR2-sensitive
ATATGTATAATATAGATCTTGGAC	32	1	RDR2-resistant
ATATGTATTACTATTGAAGATGTT	22	0	RDR2-resistant
ATATGTATTGAGAATAATCGAATA	0	11	RDR2-sensitive
ATATGTATTTTCTGTGATCTTCGG	0	11	RDR2-sensitive
ATATGTCACTCTCTGTATCGTCGT	0	10	RDR2-sensitive
ATATGTCAGACTAAAGCGTCGGTT	0	18	RDR2-sensitive
ATATGTGAAGGTAGAAATAACAAC	40	0	RDR2-resistant
ATATGTGATCGAATTCGGATGCGC	0	10	RDR2-sensitive
ATATGTGCACTATGTAGAAGACCG	0	10	RDR2-sensitive
ATATGTGCTGCAGCGGTGGACTGT	0	14	RDR2-sensitive
ATATGTGGACACTGATATGTGCAT	0	10	RDR2-sensitive
ATATGTGGATACTCGGCTATAAGT	22	0	RDR2-resistant
ATATGTGTCGGGCTAGCACGGCCT	0	9	RDR2-sensitive
ATATGTTCGGACGCTCAGAAAGGC	41	8	RDR2-resistant
ATATGTTCTTAGTGTAGGCGTGCA	1	21	RDR2-sensitive
ATATGTTGCAGGTTCGTCGTTGTC	33	1	RDR2-resistant
ATATGTTTATGTTCTGTAGGAGAC	30	1	RDR2-resistant
ATATTAATGTAGACGAGATTAGAC	25	2	RDR2-resistant
ATATTACTGGACGGTTCGACCACA	0	10	RDR2-sensitive
ATATTAGATGATCATGTTGGGTGC	0	11	RDR2-sensitive
ATATTAGATGTGTCGGCGTGTGCA	29	1	RDR2-resistant
ATATTAGCAAGGGATGAGCTCACT	53	4	RDR2-resistant
ATATTATTTGATCGGATATCGGAT	8	50	RDR2-sensitive
ATATTCAAATGGGTCGTCGTGATC	0	11	RDR2-sensitive
ATATTCATGTCGTACTGGACGGTC	0	35	RDR2-sensitive
ATATTCCGGGAATGTAGCAGGTAA	0	10	RDR2-sensitive
ATATTCGACTTTAGTGCACGGTTC	0	9	RDR2-sensitive

ATATTCGGATTCGGACTATGCGGG	15	0	RDR2-resistant
ATATTCGGCATTTTCTATAGATAT	42	0	RDR2-resistant
ATATTCGGGACGGATACGGGTAAT	4	18	RDR2-sensitive
ATATTCGGGACGGATATGAGTAAT	0	9	RDR2-sensitive
ATATTGACTGTTGTCGACGGATAT	0	9	RDR2-sensitive
ATATTGATCAATTTCGCCGGCTAC	17	1	RDR2-resistant
ATATTGCAGAGGACTACATGTTTC	335	0	RDR2-resistant
ATATTGCCTGGTACTGTGATCGGT	0	16	RDR2-sensitive
ATATTGGAATGTCATGATAGTCTT	19	1	RDR2-resistant
ATATTGGACTGGGCCACGGCCTGA	0	12	RDR2-sensitive
ATATTGGCGCTTAGGGCTGGGCAA	0	18	RDR2-sensitive
ATATTGGTTGGTACTGTGACCGGC	0	11	RDR2-sensitive
ATATTGTAGAAGCCGCAGCCGCAG	1	16	RDR2-sensitive
ATATTGTCTTTATGTCGTCGTCGT	0	10	RDR2-sensitive
ATATTGTTATATATGAACCTTGAT	25	0	RDR2-resistant
ATATTGTTTGTCGTTTCGGGGTAC	28	4	RDR2-resistant
ATATTGTTTGTGCATCTGTCGGAT	159	7	RDR2-resistant
ATATTTAAATTTCACTCTGCGAAT	16	0	RDR2-resistant
ATATTTAGCAACTATGACGTCGGG	0	13	RDR2-sensitive
ATATTTAGCAACTGTGACTTCGGG	0	16	RDR2-sensitive
ATATTTATGTCTAGACTCGTTGGC	0	17	RDR2-sensitive
ATATTTCAGAGGCTGTGTCGTCGT	0	9	RDR2-sensitive
ATATTTCGATTTGAGGCATTAGTT	0	20	RDR2-sensitive
ATATTTCTCGGTCTTTTGGTTAAA	15	0	RDR2-resistant
ATATTTGTAGGGATAGGATGGATA	97	5	RDR2-resistant
ATATTTTACATGGTCGTCCAACAG	0	11	RDR2-sensitive
ATATTTTACTGTAAATGGCGATGC	0	10	RDR2-sensitive
ATATTTTAGCAAGTCGGCAATACC	114	12	RDR2-resistant
ATATTTTCTGCGGAATCGTCGTTT	88	1	RDR2-resistant
ATATTTTGGAGACTGTGACGTTGT	0	14	RDR2-sensitive
ATATTTTGGAGACTGTGTCGTTGT	0	9	RDR2-sensitive
ATATTTTGGGGTGTAGGTTCGGTT	0	10	RDR2-sensitive
ATATTTTTCTTCGCGGATACGGAT	0	16	RDR2-sensitive
ATATTTTTCTTTTCGTCGACGCTA	0	10	RDR2-sensitive
ATCAAATAATTTGTATGTCGTCGC	0	10	RDR2-sensitive
ATCAACAGTGTCTGACGTGAGGCT	101	8	RDR2-resistant
ATCAACCATCTAGGACAAGGGCAT	0	32	RDR2-sensitive
ATCAACCCATCTGGACAGAGGCAT	0	9	RDR2-sensitive
ATCAACGGAACGCGGACTCGGCGC	0	21	RDR2-sensitive
ATCAACTCATCTGGACAGGGGCAC	0	12	RDR2-sensitive
ATCAACTCATCTGGGCAGGGACAC	0	15	RDR2-sensitive
ATCAACTCGGAGACGACGACTGGC	0	34	RDR2-sensitive
ATCAACTCTCTCTGCAGGATGACT	59	3	RDR2-resistant
ATCAACTGTCCACTCTGCAACGGC	0	29	RDR2-sensitive
ATCAACTGTCTACTCTACAACGGC	1	19	RDR2-sensitive
ATCAACTGTGGGGCATATATGCAT	58	8	RDR2-resistant
ATCAAGACGGTGTGGGACAAGGCA	15	0	RDR2-resistant
ATCAAGCAGACTGTTGCGCGACAT	0	31	RDR2-sensitive
ATCAAGCAGACTGTTGCGCGACCT	0	12	RDR2-sensitive

ATCAAGCGGTCTGTCGGTCGTCAT	0	9	RDR2-sensitive
ATCAAGCTGACTGAGAGACGCCAC	0	11	RDR2-sensitive
ATCAAGCTGACTGGGAGACGCCAC	0	20	RDR2-sensitive
ATCAAGGTCGGTGTAGTTGACGCG	0	9	RDR2-sensitive
ATCAAGTTGGAAGTATGCTTTTAT	31	0	RDR2-resistant
ATCAATACTGCATGGACTACGGAT	0	15	RDR2-sensitive
ATCAATAGATTGATTCGGTTGCCC	0	9	RDR2-sensitive
ATCAATGTTTGACTGTCGCGTGCC	1717	88	RDR2-resistant
ATCAATTGATTGATTGTAGCGATA	46	3	RDR2-resistant
ATCACAAATGAACGATGACGGTCT	0	13	RDR2-sensitive
ATCACACATGTAGACAGTTGGCGC	1	15	RDR2-sensitive
ATCACACGTTGTGGATTTGGCGCA	0	10	RDR2-sensitive
ATCACACTGCAAGGCATGAACCGG	0	11	RDR2-sensitive
ATCACCCGCGGTAACTGACGGATA	0	16	RDR2-sensitive
ATCACCGTCTACTCTGCAACGGAC	0	12	RDR2-sensitive
ATCACCTACAGGCTGTATGGACGG	0	14	RDR2-sensitive
ATCACGACTGTATAGGATGGTATC	0	33	RDR2-sensitive
ATCACGATTGCGGTATTCAAACCT	28	0	RDR2-resistant
ATCACGCTGACTATAGATGGCCAA	0	10	RDR2-sensitive
ATCACGGTGTTGTCTCGGACGTGC	0	23	RDR2-sensitive
ATCACGTAGGGACTGGCGTCGAAG	0	17	RDR2-sensitive
ATCACTCGTACTGACTGATCGGTG	0	22	RDR2-sensitive
ATCACTGCGTGAGAACTTGCGGCG	0	9	RDR2-sensitive
ATCACTGCGTTGAGAACCTGCGGC	0	39	RDR2-sensitive
ATCACTGCTGTAGGATGACGGTTG	35	2	RDR2-resistant
ATCACTGTAGTTGTTTTGGCGGCT	0	16	RDR2-sensitive
ATCACTGTGAACTATCCGGCCTCA	0	18	RDR2-sensitive
ATCACTGTGACGGCTGCTCTTGAC	0	13	RDR2-sensitive
ATCACTTAGTATGATGGACCGGAG	0	9	RDR2-sensitive
ATCACTTATTCTCGCGAACGGCAT	0	10	RDR2-sensitive
ATCACTTGAACATGGTCGACGGCG	0	10	RDR2-sensitive
ATCAGAATTTACAAGGACGTGCTT	53	0	RDR2-resistant
ATCAGACGCGGACCGTCCGGCTTG	0	17	RDR2-sensitive
ATCAGACTATAGGAAAGTTCGGTT	25	4	RDR2-resistant
ATCAGATACGGACACACCGCGGAT	0	10	RDR2-sensitive
ATCAGATCGATAGAACAGACGACA	2	21	RDR2-sensitive
ATCAGATCGCAGGCGACAAGGCTT	0	22	RDR2-sensitive
ATCAGATCGTCACGGCATGATCGT	0	10	RDR2-sensitive
ATCAGATCTGGACTCTACGATCAA	0	11	RDR2-sensitive
ATCAGCAAGGTCGGTAACCACAGG	0	9	RDR2-sensitive
ATCAGCAGAGGACTGGTCACTGGC	0	29	RDR2-sensitive
ATCAGCATCGTTCGGGTCGACGTC	2	19	RDR2-sensitive
ATCAGCGAACTGGGAGGATGTCGG	0	9	RDR2-sensitive
ATCAGGACGTAGGGTGTTACGCAT	0	19	RDR2-sensitive
ATCAGGACTCGACGGCAGCGCAAC	0	32	RDR2-sensitive
ATCAGGCAAGACTAGGACAGGCGC	1	14	RDR2-sensitive
ATCAGGCAGATCTCATAGGGACAG	1	23	RDR2-sensitive
ATCAGGCCACTAGACGGGACTGTC	0	9	RDR2-sensitive
ATCAGGCCGAGGACTGGGGGCATG	0	31	RDR2-sensitive

ATCAGGCTACGTAGAGGGAGCGGC	0	12	RDR2-sensitive
ATCAGGGCATGGCACTACATACAC	0	32	RDR2-sensitive
ATCAGGTCGTAAAGCTCCATCGGC	23	2	RDR2-resistant
ATCAGGTCTGGTGATTGTAAGGTA	53	0	RDR2-resistant
ATCAGTCGGGTATCAGTCAGACGA	0	12	RDR2-sensitive
ATCAGTGACGTGGACACGGTTAGC	34	0	RDR2-resistant
ATCAGTTGGTATCAGAGCAAGGTT	0	9	RDR2-sensitive
ATCAGTTTACTGTAGTGAAGGATT	0	9	RDR2-sensitive
ATCATACTGATGTTTGTCGTTCTG	0	12	RDR2-sensitive
ATCATAGAGTTGTCTTGGCGTCGG	0	13	RDR2-sensitive
ATCATAGTGATATTTGTCGTTCTG	0	15	RDR2-sensitive
ATCATATCGGACGTACCTGGTGGC	0	10	RDR2-sensitive
ATCATATTTGTGAGGATCTTCGGA	0	20	RDR2-sensitive
ATCATCATCATCTGATCTGCGGTA	32	4	RDR2-resistant
ATCATCATGTCGTCGAGGCGAGCA	0	9	RDR2-sensitive
ATCATCCGGGTTGTAGGTGACGTC	0	15	RDR2-sensitive
ATCATCGGATAGTAGGTAGGTGGT	0	9	RDR2-sensitive
ATCATCTCGTATGGACCAGGCTAA	1	16	RDR2-sensitive
ATCATCTGTTTAGGTCGTTGAGTA	0	15	RDR2-sensitive
ATCATCTTCTAAATATGTCGGACA	0	9	RDR2-sensitive
ATCATGAGTTATCAGGTCGCCGTT	33	3	RDR2-resistant
ATCATGCCCTGTGACTGTCGTCAA	0	10	RDR2-sensitive
ATCATGTTTTTGAGGACCTTCGGA	15	34	RDR2-sensitive
ATCATGTTTTTGAGGATCTTCGGA	1	14	RDR2-sensitive
ATCATTAGACTGTCTTCAACGGAT	0	11	RDR2-sensitive
ATCATTAGATCTAGATCGGATACA	0	23	RDR2-sensitive
ATCATTAGATCTAGATCGGATGCA	2	113	RDR2-sensitive
ATCATTAGATCTATATCGGATGCA	0	19	RDR2-sensitive
ATCATTAGTGATTGTAGCGGCAAG	0	12	RDR2-sensitive
ATCATTATTGTAGGACACGTCATG	0	22	RDR2-sensitive
ATCATTCAATCAACTGTGGGGCAT	49	5	RDR2-resistant
ATCATTCGTTGGGTACATTTTAGT	30	0	RDR2-resistant
ATCATTGTCGTTTCTGTTCGGCTT	0	12	RDR2-sensitive
ATCATTGTTGGATCAAAGGGCATC	25	0	RDR2-resistant
ATCATTTCTATCCTTCGTCGGCCA	1	43	RDR2-sensitive
ATCATTTGGTTTGAATCGCGGAAC	0	11	RDR2-sensitive
ATCATTTGGTTTGAATCGCGGAAT	0	9	RDR2-sensitive
ATCATTTTTGTAGAGCTAAACGGT	0	9	RDR2-sensitive
ATCCAAAAAGGCTCTAGACGACGT	0	11	RDR2-sensitive
ATCCAAACTGAGAACCGTCGTCGT	0	10	RDR2-sensitive
ATCCAAGACTGTCTAGCGTGGTTG	28	0	RDR2-resistant
ATCCAATCTCGGAGGAATGGGCAC	0	13	RDR2-sensitive
ATCCACATGATTGGTTGCTCGGAT	0	41	RDR2-sensitive
ATCCACCGGGTCTGCTAGAAGGCG	0	12	RDR2-sensitive
ATCCACGACCTAGAACCGGACGGT	0	11	RDR2-sensitive
ATCCACGGGTCGGACCGTCCGGCT	0	10	RDR2-sensitive
ATCCACGTGATTGGTTGCTCGGAT	0	18	RDR2-sensitive
ATCCACTGCGAGAACATGGCGGGC	1	18	RDR2-sensitive
ATCCACTGTATGGCTTCGGCGGCA	62	1	RDR2-resistant

ATCCAGAACTCTGCAGGTAGAGCT	0	11	RDR2-sensitive
ATCCAGACTGGGATGACGCTGGAT	0	35	RDR2-sensitive
ATCCAGATTGTAGATGCATGTCGA	0	9	RDR2-sensitive
ATCCAGCAGATTGTGGACGCGGAT	0	14	RDR2-sensitive
ATCCAGCCGGACTGCTACGCGCGA	0	14	RDR2-sensitive
ATCCAGGGCTGCTGTGAAGACGAT	0	15	RDR2-sensitive
ATCCATAGTTGAACTGATGTGGTT	14	0	RDR2-resistant
ATCCATATCCTAATCCGTCGGGTC	0	63	RDR2-sensitive
ATCCATATCCTGATCCGTCGGTTC	0	10	RDR2-sensitive
ATCCATATCCTTATCCGTCGGGTC	1	37	RDR2-sensitive
ATCCATCCCGTCAGCAGTAGAGGC	0	11	RDR2-sensitive
ATCCATCGGGTATTAGGTCGCGGG	0	28	RDR2-sensitive
ATCCATGTCGGATTCCATGGCGCC	0	22	RDR2-sensitive
ATCCATGTGGATTGAGTGGGATTG	1	12	RDR2-sensitive
ATCCATTCCGGACCGGCTGATTCC	0	21	RDR2-sensitive
ATCCATTCTTGGATTCGTCACTGT	38	2	RDR2-resistant
ATCCATTTGGCTGGTCTGTTGGAC	1	33	RDR2-sensitive
ATCCATTTTGGAGTTTGTCTGACG	0	18	RDR2-sensitive
ATCCCAAGAATCGGGCGAAGACGA	0	23	RDR2-sensitive
ATCCCAAGAATCGGGTGAAGACGA	0	28	RDR2-sensitive
ATCCCAAGAGTCGGGTGAAGACGA	0	9	RDR2-sensitive
ATCCCAAGAGTCGGGTGAAGACGG	2	26	RDR2-sensitive
ATCCCAAGGATCGGATGAAGACGA	0	29	RDR2-sensitive
ATCCCAGATTGTCGTCCTAGGCAC	1	18	RDR2-sensitive
ATCCCAGGTTGTAGACCGTTCGGC	0	33	RDR2-sensitive
ATCCCCGGATCCACTAGAAGACGA	0	49	RDR2-sensitive
ATCCCGGACGTTAGGCAAAAATAA	0	9	RDR2-sensitive
ATCCCGGTGTGGAACGGTTTCTAA	0	9	RDR2-sensitive
ATCCCGGTTCGGACAAGAGAAGAC	0	16	RDR2-sensitive
ATCCCGTATCGGACTCGTTCGGTG	0	9	RDR2-sensitive
ATCCCGTCAGCAGTAGAGGCGCAA	0	9	RDR2-sensitive
ATCCCTCTGAATCGGACGGACAAT	0	9	RDR2-sensitive
ATCCCTGGACTTTTTTTCTTGGCA	22	0	RDR2-resistant
ATCCCTGGCCGGCAGAACACGATC	0	9	RDR2-sensitive
ATCCCTGGGCACGCGTCGGCACGC	0	40	RDR2-sensitive
ATCCCTGTAGAGTACGGAAATAAC	0	10	RDR2-sensitive
ATCCCTTACGGCCACAGTAGACGG	0	10	RDR2-sensitive
ATCCGAACCGAGTTGACCATGAAC	0	9	RDR2-sensitive
ATCCGAACGAGATGACTTGAGCTA	19	0	RDR2-resistant
ATCCGAAGCTGTAGTATCCGGGGC	1	12	RDR2-sensitive
ATCCGACACAAACGAACAAGGCCT	0	11	RDR2-sensitive
ATCCGACCCGATAGAACCCGCGGA	0	18	RDR2-sensitive
ATCCGACGAGTAGAAACCGGTCAC	0	10	RDR2-sensitive
ATCCGACGGAAACGAACAAGGCCT	1	22	RDR2-sensitive
ATCCGACTGCGGCTGCGGCTTCTA	3	84	RDR2-sensitive
ATCCGAGATTTGTTGAGAGAGCAT	0	26	RDR2-sensitive
ATCCGAGGTGTAGAAAGGGTCAAC	18	0	RDR2-resistant
ATCCGAGTCGCTGTATGGTTGGTG	0	15	RDR2-sensitive
ATCCGATTGGTCAGGACGACGTCA	0	36	RDR2-sensitive

ATCCGATTTCTCGGGATCGTTCGA	0	76	RDR2-sensitive
ATCCGCAGCTCTGAGCCAGACGGT	0	12	RDR2-sensitive
ATCCGCGATCTAGGGACGGACGGT	0	23	RDR2-sensitive
ATCCGCGCCTAGGGCTTGGACGGT	0	12	RDR2-sensitive
ATCCGCGGATACGGTTATGGATAT	0	14	RDR2-sensitive
ATCCGCGGTCGCTGTTACGGTAGT	0	11	RDR2-sensitive
ATCCGCGTCGTAGAGTTGCTCGGC	0	19	RDR2-sensitive
ATCCGGACAGGAAACTAAGCACGG	0	10	RDR2-sensitive
ATCCGGACGACGTGCATCTGGGAT	0	9	RDR2-sensitive
ATCCGGACGGGTAAGAAACGGATA	2	43	RDR2-sensitive
ATCCGGATCAGATTCAGATGGGGG	57	1	RDR2-resistant
ATCCGGATCAGGCTGAGCTCGGGC	0	10	RDR2-sensitive
ATCCGGATTGGGTATTGGCCATGC	17	0	RDR2-resistant
ATCCGGCACAAACGAACAAGGCCT	0	11	RDR2-sensitive
ATCCGGCAGAAACGAACAAGGCCT	0	20	RDR2-sensitive
ATCCGGGACGTGGACGGCTACAAT	0	15	RDR2-sensitive
ATCCGGGCGGGTAAGAAACGGATA	0	9	RDR2-sensitive
ATCCGGGCTGTGAGAGGAGGGGAA	0	9	RDR2-sensitive
ATCCGGGTTGTAGGTGACGTCGGA	1	78	RDR2-sensitive
ATCCGGTCGTTTTGGTGGAGCGGA	2	15	RDR2-sensitive
ATCCGGTCTGTGGATAGAGGAGAC	0	10	RDR2-sensitive
ATCCGGTGCGACGTAGAGAAGACG	0	18	RDR2-sensitive
ATCCGGTTAGGAATCGTTCGGATA	0	9	RDR2-sensitive
ATCCGTATCCCGGAGGCGTAGGAA	0	9	RDR2-sensitive
ATCCGTATCCCGGAGGCGTAGGAG	14	37	RDR2-sensitive
ATCCGTCATTGGTGGAGGATTCGG	1	29	RDR2-sensitive
ATCCGTCGACGGCTGGCACAGGCT	0	15	RDR2-sensitive
ATCCGTCTGATTGGTTGCATGTAT	0	9	RDR2-sensitive
ATCCGTCTGGGGTGGTATGTCGAC	0	10	RDR2-sensitive
ATCCGTCTTGTGTGGTCTGTCGAC	0	13	RDR2-sensitive
ATCCGTGTCGGGCCTAGCCGTGCT	0	11	RDR2-sensitive
ATCCGTGTCTAGGGCTTGGACGGT	0	11	RDR2-sensitive
ATCCGTTGGAATTGGATGGCCGAG	0	12	RDR2-sensitive
ATCCGTTGGACATGGCATGGCCTG	15	0	RDR2-resistant
ATCCTAATCCGTCGGGTCGGATAT	0	10	RDR2-sensitive
ATCCTACCTGTAGGCTCACGGCAT	0	14	RDR2-sensitive
ATCCTACGTCGTGAAGATCGGGCA	0	36	RDR2-sensitive
ATCCTCAGTTGACGCCGTAGTTTC	0	9	RDR2-sensitive
ATCCTCATTTACTCGTCGGGGAAA	0	11	RDR2-sensitive
ATCCTCCGGATCGGATCTTGGCAA	0	49	RDR2-sensitive
ATCCTCCGGTTGGATCGTTGGAGA	0	30	RDR2-sensitive
ATCCTCGACTCGGAGCTGGATGGT	0	10	RDR2-sensitive
ATCCTCGATTCAGGCTGCCGGTTA	0	17	RDR2-sensitive
ATCCTCGCGGACCGTCTGGGGTGC	0	9	RDR2-sensitive
ATCCTCGGCAGAACCGTGCACCAA	0	9	RDR2-sensitive
ATCCTCGGTAAAGGTTGTCGGTAC	0	15	RDR2-sensitive
ATCCTCGGTATGAACGGAAGGTGT	0	16	RDR2-sensitive
ATCCTCTTGGGATTCTTTTGTCGG	0	10	RDR2-sensitive
ATCCTGAGCGCGCTGTCGGGCGGC	0	10	RDR2-sensitive

ATCCTGGGCTGGCATCTCGACGGG	0	15	RDR2-sensitive
ATCCTGGGTCGTCGTTTGGCCATA	0	11	RDR2-sensitive
ATCCTGTACGACGGCTGTCTCAGC	0	10	RDR2-sensitive
ATCCTGTACGCAAAGGACGTCGGT	0	11	RDR2-sensitive
ATCCTGTAGGCAAAGGACGCCGGT	0	17	RDR2-sensitive
ATCCTTAAGACGGTAGTGTAGACC	0	9	RDR2-sensitive
ATCCTTAAGGACTGATTTGGTGAC	0	9	RDR2-sensitive
ATCCTTACAGTGAGGTTGTCGGTG	0	11	RDR2-sensitive
ATCCTTACTAGATACGTCCGGATA	0	14	RDR2-sensitive
ATCCTTGCCTTCTGTCGTCGGGCG	1	22	RDR2-sensitive
ATCCTTGGAAGGCTGTAGTAGGGC	0	56	RDR2-sensitive
ATCCTTTTGGATTGTTGGTTCGTG	49	1	RDR2-resistant
ATCGAACAGGTAGAGATTGTTGTC	0	11	RDR2-sensitive
ATCGAACCCAGGACTTGGAGGTGC	0	12	RDR2-sensitive
ATCGAACCTCTGCCTTAGACTGGG	0	27	RDR2-sensitive
ATCGAACTGGGACTAGAGGACGAC	0	20	RDR2-sensitive
ATCGAACTGTGACCGATTCGGTAG	0	17	RDR2-sensitive
ATCGAATATTTTGTAGACCGTCGC	0	10	RDR2-sensitive
ATCGAATCTAGAACACTTGCGGCG	0	17	RDR2-sensitive
ATCGAATTTGTACGGCTACATCGA	1	12	RDR2-sensitive
ATCGACACAAGGCTGTATCGGCAC	0	10	RDR2-sensitive
ATCGACAGAGAGCACGACCGACGC	0	37	RDR2-sensitive
ATCGACAGCGGATGCACAAACCAT	1	18	RDR2-sensitive
ATCGACCGAGGCGGCATCTGACAG	0	13	RDR2-sensitive
ATCGACGACTGACAATATGGCTGC	0	11	RDR2-sensitive
ATCGACGACTGGCACAAAAGGCAC	2	71	RDR2-sensitive
ATCGACGACTTGTACAAAAGGCAT	0	10	RDR2-sensitive
ATCGACGTAGAGCCGAAGAGAAGC	0	15	RDR2-sensitive
ATCGACGTAGAGCCGAAGAGAGAC	0	15	RDR2-sensitive
ATCGACGTCTGTCAAGATCTACGG	0	10	RDR2-sensitive
ATCGACTATGTACGGCTACATCGA	2	24	RDR2-sensitive
ATCGACTCGTGTAGGCAACCAAAC	0	17	RDR2-sensitive
ATCGACTGAGACCCCATCGGCCTG	0	11	RDR2-sensitive
ATCGACTGCATACGACTACATGGA	0	11	RDR2-sensitive
ATCGACTGCGTACGACTACATCGA	2	62	RDR2-sensitive
ATCGACTGCTCCATCGTGCGGCTG	0	9	RDR2-sensitive
ATCGACTGCTTGCGCTACCGGCAT	0	23	RDR2-sensitive
ATCGACTGTAGCTCGATGGTTCGT	3	38	RDR2-sensitive
ATCGACTGTCCGGAATACCGTAGA	0	31	RDR2-sensitive
ATCGACTGTGTCACCGTGAAGGCG	0	20	RDR2-sensitive
ATCGACTTCGTACGGCTACATCGA	3	16	RDR2-sensitive
ATCGACTTGATGTGCTTCGTTTGG	231	6	RDR2-resistant
ATCGAGAAGCTAGAGCCGGAGTCC	0	9	RDR2-sensitive
ATCGAGACGGGGTGACCGAACGAC	0	40	RDR2-sensitive
ATCGAGAGTAGGACATTCGGCATA	1	47	RDR2-sensitive
ATCGAGCATGCGGACTTAGACGGT	0	11	RDR2-sensitive
ATCGAGCCCGCGGACTTAGACGGT	0	17	RDR2-sensitive
ATCGAGCCTGTAGACTTAGACGGT	0	10	RDR2-sensitive
ATCGAGCGATGACATTTATTCTGA	16	0	RDR2-resistant

ATCGAGCTGGTGGATCAGGCCATG	49	0	RDR2-resistant
ATCGAGGTGTCGTAGACGGTCCGC	3	17	RDR2-sensitive
ATCGAGGTTCGGATGCAGGATCGG	0	13	RDR2-sensitive
ATCGAGTTGCGATTGTCGGCGCCT	0	30	RDR2-sensitive
ATCGATATCACGTCAGAGCTCGGC	1	36	RDR2-sensitive
ATCGATATCAGGACTCGACGGCAG	0	51	RDR2-sensitive
ATCGATATCGGGACTCGACGGCAG	2	24	RDR2-sensitive
ATCGATCAGACGACCTCAGACGGT	0	10	RDR2-sensitive
ATCGATCCGATGCTGTACAGACGC	0	14	RDR2-sensitive
ATCGATCGCGCGCGAGTGAGGCAT	0	13	RDR2-sensitive
ATCGATCGGTCACGGCAAGAAGGT	0	11	RDR2-sensitive
ATCGATCTGGCATTCTTTAGACGC	0	9	RDR2-sensitive
ATCGATCTGGTTGGGCTTGGTCGG	0	9	RDR2-sensitive
ATCGATGACTACATGTAGGGACGT	0	13	RDR2-sensitive
ATCGATGATCGTATGGTTTCGGAT	0	12	RDR2-sensitive
ATCGATGCCTGGATGTAGAGACGT	0	9	RDR2-sensitive
ATCGATGCTTGGAACATGGATGGT	0	26	RDR2-sensitive
ATCGATGGACAGGAACGTCGGCCA	0	9	RDR2-sensitive
ATCGATGGACAGGAATGTCGGCCC	0	9	RDR2-sensitive
ATCGATGGACGGCAGAACGGCAGA	0	26	RDR2-sensitive
ATCGATGGGCAGGAACGTCGGCCG	1	13	RDR2-sensitive
ATCGATGTACTGTGTGGCGAGCTC	0	12	RDR2-sensitive
ATCGATGTGTAGTGTGATGGTCTG	31	1	RDR2-resistant
ATCGATTCGGTCTTGTGTCGGCTC	0	11	RDR2-sensitive
ATCGATTCTGTACTGTAGCAGGTT	0	15	RDR2-sensitive
ATCGATTGTTAGCAGTATGTTGAT	77	7	RDR2-resistant
ATCGCAAACTCTGGGCGACGTGCG	0	9	RDR2-sensitive
ATCGCAATCGGACGGTGGAGATGG	0	9	RDR2-sensitive
ATCGCAATCGGACGGTGGGGATGG	1	52	RDR2-sensitive
ATCGCACGAGACAGGCTAGCACGG	0	17	RDR2-sensitive
ATCGCAGAAGGCCAGGTAGAGCGG	0	21	RDR2-sensitive
ATCGCAGGACTGACCGGCTGCATG	74	0	RDR2-resistant
ATCGCATCAGACGGTGGAGAGCAA	0	11	RDR2-sensitive
ATCGCATCTGTTTCGTCGGTCGCG	0	21	RDR2-sensitive
ATCGCATGGGCTCGACGTCGGCGT	0	9	RDR2-sensitive
ATCGCCATCGTAGATCGTCCGGAG	2	36	RDR2-sensitive
ATCGCCCCTGGACGGTCGCGGACC	45	3	RDR2-resistant
ATCGCGACGATAACACTACGGATG	0	10	RDR2-sensitive
ATCGCGCGACGTGAGACCACGGTT	0	15	RDR2-sensitive
ATCGCGCGATGGCAGAACAGGGCG	0	14	RDR2-sensitive
ATCGCGCGGAAGGCCAGGTAGAGC	1	20	RDR2-sensitive
ATCGCGCTGTTGGCTACGTCTTCA	5	77	RDR2-sensitive
ATCGCGGTGGGACTCACGGCGAAC	0	15	RDR2-sensitive
ATCGCGTAGGACAGTTCGGCAAGG	0	13	RDR2-sensitive
ATCGCGTAGGACAGTTCGGCGAGG	0	16	RDR2-sensitive
ATCGCGTAGGACAGTTCGGCTAGA	0	22	RDR2-sensitive
ATCGCGTAGGACAGTTCGGCTAGG	0	41	RDR2-sensitive
ATCGCTAGCTGAAGTAGGACGAGC	0	27	RDR2-sensitive
ATCGCTAGGGACTCAGAGAGAGGG	0	10	RDR2-sensitive

ATCGCTCAACGGCAGAACAGATGG	0	17	RDR2-sensitive
ATCGCTCGCTCGGAGGACGCGCAC	0	9	RDR2-sensitive
ATCGCTCTGAAGGGCCTGAAGGCG	0	9	RDR2-sensitive
ATCGCTGCCCTTGAGTGCCAGCTC	24	0	RDR2-resistant
ATCGCTTCGTCGGGGGCTTGCAAG	0	9	RDR2-sensitive
ATCGCTTGATTGCATGCTTGCACC	0	12	RDR2-sensitive
ATCGCTTGGTCGTCGTGCGTGCAT	4	29	RDR2-sensitive
ATCGCTTTGTCTAGATTGTCTAAA	24	0	RDR2-resistant
ATCGGAAAAGACGGGACACCACAC	0	15	RDR2-sensitive
ATCGGAAACAAAATCGAAAACGGT	0	12	RDR2-sensitive
ATCGGAACTGAGCGAGGATGAGTC	1	14	RDR2-sensitive
ATCGGAATGCAGCGGAGGAATGTT	21	0	RDR2-resistant
ATCGGACACACGGCAGAGATGCTG	0	10	RDR2-sensitive
ATCGGACACTCTAAGGGCATGTTC	0	9	RDR2-sensitive
ATCGGACAGCGACCAGCACGACAG	0	35	RDR2-sensitive
ATCGGACCATGCCGGCACGGACCC	0	15	RDR2-sensitive
ATCGGACCGTGCCGGCCCAGCACT	0	11	RDR2-sensitive
ATCGGACCTTGGCGATACAGTCGA	0	12	RDR2-sensitive
ATCGGACGTCAGCTGTAGACGGAT	0	19	RDR2-sensitive
ATCGGACTGTCCGGTGTGCACTGT	3	16	RDR2-sensitive
ATCGGACTTTTTTTCAATTGGTTG	14	0	RDR2-resistant
ATCGGAGCTGTTAGGTTGGCCGCA	0	15	RDR2-sensitive
ATCGGAGGAGGGTTATTTGATAAA	14	0	RDR2-resistant
ATCGGATACGCATGGAAACGGATT	0	10	RDR2-sensitive
ATCGGATAGTAGGTAGGTGGTCGT	0	17	RDR2-sensitive
ATCGGATATCGGATACTTGTCGGA	0	9	RDR2-sensitive
ATCGGATCCCAACGCAAGTAGCAT	0	9	RDR2-sensitive
ATCGGATCCCGAGGGCACGCACGC	0	12	RDR2-sensitive
ATCGGATCCGATCTGACGGACCAG	0	10	RDR2-sensitive
ATCGGATCCTCGTATACCCGACGG	0	10	RDR2-sensitive
ATCGGATCGAATACAGACGGATAT	0	27	RDR2-sensitive
ATCGGATCGAATACGGACGGATAT	1	113	RDR2-sensitive
ATCGGATCGGATACGGACGGATAT	0	14	RDR2-sensitive
ATCGGATCGGGCTGGCCCAGGCAC	0	18	RDR2-sensitive
ATCGGATCGTGATCACACGGCTGG	0	12	RDR2-sensitive
ATCGGATCGTGGATGTTTATCGGT	0	10	RDR2-sensitive
ATCGGATGCGAACGAGCGAGGACA	0	23	RDR2-sensitive
ATCGGATTCATCGGTAACGGTATT	0	13	RDR2-sensitive
ATCGGATTGCGACGCATTTACGGC	0	15	RDR2-sensitive
ATCGGATTGTGCCGGTATGACACT	1	81	RDR2-sensitive
ATCGGATTTCTATATATGCAGATG	17	0	RDR2-resistant
ATCGGATTTGTGCCGGTAGTTCTT	0	14	RDR2-sensitive
ATCGGCAAGCTGTTCGACGACTTC	0	9	RDR2-sensitive
ATCGGCAATACACGTGAACGGCGC	0	9	RDR2-sensitive
ATCGGCATGTACTTAGGGCACTAG	0	9	RDR2-sensitive
ATCGGCATGTACTTAGGGCGCTAG	0	66	RDR2-sensitive
ATCGGCATTGGTAGTTTGGTGGCT	0	21	RDR2-sensitive
ATCGGCATTGTACCGGCAGATCCG	1	17	RDR2-sensitive
ATCGGCCAGGCAGAACGGGTTCCT	0	37	RDR2-sensitive

ATCGGCCCGCTAGACACGGCTCAT	0	23	RDR2-sensitive
ATCGGCCGACTAGACAGACGCATG	0	10	RDR2-sensitive
ATCGGCTAGACGATAGGCTGACGG	0	11	RDR2-sensitive
ATCGGCTATCTTGTTCTCACGGCT	1	41	RDR2-sensitive
ATCGGCTCCGGCAGAAAAGCTGCT	0	132	RDR2-sensitive
ATCGGCTCCGGCAGAGAAGCTGCT	0	22	RDR2-sensitive
ATCGGCTCCTGAACGAGGCTGCAG	0	11	RDR2-sensitive
ATCGGCTCGGCTCGCTACGAAAAC	0	9	RDR2-sensitive
ATCGGCTCTAGCAGAGAAGCTGCT	1	26	RDR2-sensitive
ATCGGCTCTGTTTGTAGAAGTCGT	0	13	RDR2-sensitive
ATCGGCTGACTCCGACCAGGTCGA	55	10	RDR2-resistant
ATCGGCTGCCGCAGATTTCGCCAT	0	9	RDR2-sensitive
ATCGGCTGCGAGATTAGCGGTGTG	0	15	RDR2-sensitive
ATCGGCTGCGGCTGGACCTTGCAG	0	9	RDR2-sensitive
ATCGGCTGGACAGGGAAGAAGAGC	2	30	RDR2-sensitive
ATCGGCTGGGAAGCATGCCTTGTG	0	15	RDR2-sensitive
ATCGGCTTGGCAGATGAGAGACGA	0	19	RDR2-sensitive
ATCGGCTTTGACTGAGCGGACGGT	0	23	RDR2-sensitive
ATCGGGACGGATACAGGACGGGAC	0	14	RDR2-sensitive
ATCGGGACTCGACGACAGCGCAAC	0	17	RDR2-sensitive
ATCGGGACTCGACGGCAGCACAAC	0	11	RDR2-sensitive
ATCGGGACTCGACGGCAGCGCAAC	0	13	RDR2-sensitive
ATCGGGACTCGACGGCGAACGGCG	0	9	RDR2-sensitive
ATCGGGCAACCCGGACGGATTCAC	0	37	RDR2-sensitive
ATCGGGCACCTAAGCAGGAACACG	0	24	RDR2-sensitive
ATCGGGCACTCAGCGAAGACGTCT	0	9	RDR2-sensitive
ATCGGGCAGGAATGGGCAGACCAA	0	9	RDR2-sensitive
ATCGGGCAGGAGTAGTGGGCAGGC	0	9	RDR2-sensitive
ATCGGGCATCGTGACGCATCATCT	0	11	RDR2-sensitive
ATCGGGCATGCAGGATGGTCCCAG	0	9	RDR2-sensitive
ATCGGGCCAACAACGAGTCAGCGG	0	9	RDR2-sensitive
ATCGGGCCCACATGCGCGGACGGT	0	11	RDR2-sensitive
ATCGGGCCGAAACCATAAGCACGG	0	20	RDR2-sensitive
ATCGGGCCGAAATCATAAGCACGG	0	11	RDR2-sensitive
ATCGGGCCGACTCGCCAGACACGG	0	11	RDR2-sensitive
ATCGGGCGGGTAGTACACGGATAT	0	23	RDR2-sensitive
ATCGGGCTAAACGGCGTAACTTTT	0	10	RDR2-sensitive
ATCGGGCTGATTGGATGGGCGGAC	0	10	RDR2-sensitive
ATCGGGCTGGGACCAGAGGATGAT	0	13	RDR2-sensitive
ATCGGGCTGTGCTGTCCAACGGAT	0	21	RDR2-sensitive
ATCGGGCTTACTGGACAGGCCGAC	0	9	RDR2-sensitive
ATCGGGCTTGCAGGACGGGTCGGC	0	9	RDR2-sensitive
ATCGGGTACGGTGGGCGTGGGTAT	1	15	RDR2-sensitive
ATCGGGTACGTCATACGCTGGAAC	1	24	RDR2-sensitive
ATCGGGTCCTGTTTTATAGACGTC	0	12	RDR2-sensitive
ATCGGGTGAAGACGAAGCTAGCAG	2	83	RDR2-sensitive
ATCGGGTGAAGACGGAGCCAGCAA	0	38	RDR2-sensitive
ATCGGGTGTTCTGACCACCGGTAT	14	0	RDR2-resistant
ATCGGTACGACATGTAACAGGCTC	0	9	RDR2-sensitive

ATCGGTACGACTCATGACGGGCTC	0	52	RDR2-sensitive
ATCGGTACTCTAGATCACGTCATA	0	13	RDR2-sensitive
ATCGGTATGGCTTGTAGATCGGAT	0	9	RDR2-sensitive
ATCGGTATGGTAGAGAGTCGGTGC	0	9	RDR2-sensitive
ATCGGTATGGTAGAGAGTCGGTGG	0	10	RDR2-sensitive
ATCGGTCCGGTCTGTGATAGGTTG	0	10	RDR2-sensitive
ATCGGTCGTGTATTTAGTCGGTCC	0	18	RDR2-sensitive
ATCGGTCTATGGTCTGACAGGCGT	0	16	RDR2-sensitive
ATCGGTCTCCATAGTGGACGCGTA	0	10	RDR2-sensitive
ATCGGTGCTCTGTCTGCGCGCGTC	0	9	RDR2-sensitive
ATCGGTGGGGAGTACGGCTCTAGT	0	9	RDR2-sensitive
ATCGGTGTCCGATCTGACGGCTGA	0	19	RDR2-sensitive
ATCGGTGTGATCTTTAGAACCGGC	0	24	RDR2-sensitive
ATCGGTTCCAGTCTCTGACCGAGC	0	15	RDR2-sensitive
ATCGGTTGTGTTGCTGCACGGCGC	0	12	RDR2-sensitive
ATCGGTTGTTGAGCAAGTCGGTAA	0	9	RDR2-sensitive
ATCGTAACTCGGATGTATGTTGGT	75	2	RDR2-resistant
ATCGTAATCGGATACTGTCGGCGT	0	19	RDR2-sensitive
ATCGTACATCTTCTGCATCCGGTT	128	1	RDR2-resistant
ATCGTACCGGGCTGTGCCGAGGCA	0	57	RDR2-sensitive
ATCGTAGACAGCCGTCGGAGATAA	0	17	RDR2-sensitive
ATCGTATGATCTCTGCTGCGAAGA	0	27	RDR2-sensitive
ATCGTATTAGAAACGTCGGTACCC	1	52	RDR2-sensitive
ATCGTCACGTCGTTCGGATGGCTG	0	10	RDR2-sensitive
ATCGTCACTCGTTGGAAGCGTCGT	0	16	RDR2-sensitive
ATCGTCAGATCGGATCGGTCAGTT	0	11	RDR2-sensitive
ATCGTCCTCGGGCAAGACGCGCTG	0	10	RDR2-sensitive
ATCGTCGACCCTGCTGATTCCGGC	0	14	RDR2-sensitive
ATCGTCTATTGTTGGAGGCAGCCT	0	23	RDR2-sensitive
ATCGTCTCTTTGGACACGTGGCAG	0	9	RDR2-sensitive
ATCGTGACCTACTGATATGGCTGA	0	22	RDR2-sensitive
ATCGTGATATCGGAGGAGGGTTAT	18	0	RDR2-resistant
ATCGTGATGGGTAGGATCAAGCAT	0	10	RDR2-sensitive
ATCGTGATTAGTCGGGCTAGTCGG	0	12	RDR2-sensitive
ATCGTGCCTATGGACTAAAATGGA	0	16	RDR2-sensitive
ATCGTGGGCCGGGCAAAGACTACA	0	9	RDR2-sensitive
ATCGTGTACCGTAGGGAATGAGGC	0	22	RDR2-sensitive
ATCGTGTATCGTACTGTGCGTCGT	1	20	RDR2-sensitive
ATCGTGTCCGGACTAGCACGGTCC	0	10	RDR2-sensitive
ATCGTGTCGGATTGATTCGCGGCT	0	23	RDR2-sensitive
ATCGTGTCGGGATAGCGGCTCAAG	0	18	RDR2-sensitive
ATCGTGTGATTCCTAGGTCGTCGA	0	11	RDR2-sensitive
ATCGTGTTAGTATGATCTGAAGAC	1	25	RDR2-sensitive
ATCGTTATATTTGTAGAGTGGCCC	0	38	RDR2-sensitive
ATCGTTCAGATCGTTGGATAAATC	0	9	RDR2-sensitive
ATCGTTCGTCGGGTGGCACGGCAC	1	13	RDR2-sensitive
ATCGTTCGTGCCGACGTAGAGGGC	0	19	RDR2-sensitive
ATCGTTCTGTCTCGTCGGCGTGCA	0	9	RDR2-sensitive
ATCGTTGGACAATGGTTATGTGAC	50	8	RDR2-resistant

ATCGTTTCGAGTCGGGGTCGTCAA	0	14	RDR2-sensitive
ATCGTTTCTGTTCTGCACTGCGGT	0	10	RDR2-sensitive
ATCGTTTGAGATGGTTTGGACATA	3	19	RDR2-sensitive
ATCGTTTTAGTGGAGCGAGGGCAT	0	10	RDR2-sensitive
ATCTAAACTCTGGAACAGAGCTGC	0	10	RDR2-sensitive
ATCTAAACTCTGGAGCAGAGCTGC	3	119	RDR2-sensitive
ATCTAAACTCTGGAGTAGAGCTGC	0	23	RDR2-sensitive
ATCTAACTGTAGAAAGGTTTTGGA	0	9	RDR2-sensitive
ATCTAAGTATATGGTGGCTTTATT	65	0	RDR2-resistant
ATCTAATCCGGGCTGAACCGGCAT	0	10	RDR2-sensitive
ATCTACGGAGGACACGACGAGCGC	0	12	RDR2-sensitive
ATCTACTAACAATTAATCGTGGAC	0	9	RDR2-sensitive
ATCTACTAACAATTACTCGTGGAC	32	354	RDR2-sensitive
ATCTACTAACAATTACTCGTGGCC	4	44	RDR2-sensitive
ATCTACTAACAATTCATCGCGGGC	0	10	RDR2-sensitive
ATCTACTAATAATTACTCGTGGAC	3	24	RDR2-sensitive
ATCTACTCCATTGCAGCTGAACGT	0	29	RDR2-sensitive
ATCTACTGTAGGGCCACTAGGCAC	0	12	RDR2-sensitive
ATCTACTTTCTGTTCGTCGGTTCA	0	9	RDR2-sensitive
ATCTAGACTGCATGCATTGAGATG	0	21	RDR2-sensitive
ATCTAGCGGCTGCCAGAGACGACT	0	11	RDR2-sensitive
ATCTAGGACTCGGTGCATTGGCAG	14	0	RDR2-resistant
ATCTAGGAGGAGAGAGAAGGGGAC	0	11	RDR2-sensitive
ATCTAGGATCGTATGGTCGCGGGC	0	11	RDR2-sensitive
ATCTAGGCTGTGAGAGTAGGGAGA	0	11	RDR2-sensitive
ATCTAGTACTGTATAAAATCGGAG	0	13	RDR2-sensitive
ATCTAGTCGTCTAGTATCGTGGTT	0	22	RDR2-sensitive
ATCTAGTGAGCGGGGCTGAGGTAT	0	14	RDR2-sensitive
ATCTATACAGTCGTCGGGAGGCTG	0	28	RDR2-sensitive
ATCTATAGACTGCGGCTGCAACAG	0	11	RDR2-sensitive
ATCTATAGTTATGGTCTGTCTCGA	0	12	RDR2-sensitive
ATCTATATACCTTGCTGTGAGACA	56	3	RDR2-resistant
ATCTATATCCGGATTCGTCGAGTC	0	10	RDR2-sensitive
ATCTATATCTAGCTCCGTCGGGTC	0	14	RDR2-sensitive
ATCTATCACTTTTAAGACTGCATT	71	1	RDR2-resistant
ATCTATCTCGGATCTCATGGCGAC	26	0	RDR2-resistant
ATCTATGACCGTGGATATGACTGC	0	47	RDR2-sensitive
ATCTATTTAGTTTGACTAGCGGCT	0	9	RDR2-sensitive
ATCTATTTGATGTTGAATACGGTT	14	0	RDR2-resistant
ATCTCACATGTGGGTCGTCCGGAT	0	14	RDR2-sensitive
ATCTCACGGGATTGGAACAGGGCC	0	9	RDR2-sensitive
ATCTCAGACCGAGGGATCGCGGCC	0	10	RDR2-sensitive
ATCTCAGATGATGGTTCTAACAAT	1	20	RDR2-sensitive
ATCTCAGCTGAAGGTACACTAGAG	40	0	RDR2-resistant
ATCTCAGTGGATCGTGGCAGCAAG	31	0	RDR2-resistant
ATCTCCAACAGTTGGTCGGACACT	0	10	RDR2-sensitive
ATCTCCACGGATGAAAAGGGGACA	0	14	RDR2-sensitive
ATCTCCACGGATGAAAAGGGGACG	0	14	RDR2-sensitive
ATCTCCAGTAGAGATCGACTCGGA	0	10	RDR2-sensitive

ATCTCCATAAAGGGTGTCGCGGAC	0	14	RDR2-sensitive
ATCTCCCGTCAGCAGTAGAGGCAC	1	55	RDR2-sensitive
ATCTCCCGTCGGCTTCGTTATGGC	0	27	RDR2-sensitive
ATCTCCGACGGATCTGTACGAGGC	0	48	RDR2-sensitive
ATCTCCGACGGCACTCAGACTAGC	0	12	RDR2-sensitive
ATCTCCGACGGCCTCGTACACAGC	0	13	RDR2-sensitive
ATCTCCGACGGCCTCGTACAGAGC	2	17	RDR2-sensitive
ATCTCCGACGGCCTCGTAGGCGGC	1	28	RDR2-sensitive
ATCTCCGACGGCTAGAAGAGACGC	0	13	RDR2-sensitive
ATCTCCGACGGCTCTGTACGAGGC	43	237	RDR2-sensitive
ATCTCCGACGGCTCTGTATGAGGC	1	14	RDR2-sensitive
ATCTCCGACGGCTCTGTGCGAGGC	0	12	RDR2-sensitive
ATCTCCGACGGCTGTAATACGGGC	3	32	RDR2-sensitive
ATCTCCGACGGCTGTGTACGAGGC	4	171	RDR2-sensitive
ATCTCCGGACGGCTAAGCCCACAC	0	31	RDR2-sensitive
ATCTCCGGAGCCAGAATCCAACGG	0	9	RDR2-sensitive
ATCTCCGTTGGACCAACGACTGCA	0	11	RDR2-sensitive
ATCTCCTCGGCTGCATGCGCACAT	0	12	RDR2-sensitive
ATCTCGAACCGGCGACGATGGCGT	3	23	RDR2-sensitive
ATCTCGACGAACTGTACATCGGTA	0	16	RDR2-sensitive
ATCTCGACGTACTGTGACGGTGGG	0	12	RDR2-sensitive
ATCTCGATCGGATGACAAGAGAAG	0	12	RDR2-sensitive
ATCTCGCGAGATTGGAACAGGGCC	0	37	RDR2-sensitive
ATCTCGCGGATATGGATTGACGAA	0	33	RDR2-sensitive
ATCTCGGACAAATAGCAGAGTTGT	0	11	RDR2-sensitive
ATCTCGGACAAGCCATGATGCCAT	0	12	RDR2-sensitive
ATCTCGGACAGCCGCGCAGTAGAC	0	12	RDR2-sensitive
ATCTCGGACATACGATGGTGGCGT	0	80	RDR2-sensitive
ATCTCGGACATGCGACGATGGCGT	0	15	RDR2-sensitive
ATCTCGGACATGCGACGATGGTGT	1	14	RDR2-sensitive
ATCTCGGACCAGGCTTCAATCCCT	103	1	RDR2-resistant
ATCTCGGACCAGGCTTCATTCCCC	116	2	RDR2-resistant
ATCTCGGACCAGGCTTCATTCCCT	35	1	RDR2-resistant
ATCTCGGACCGGCGACGGTGGCAT	0	19	RDR2-sensitive
ATCTCGGACTCGCGACGGTGTCGT	0	20	RDR2-sensitive
ATCTCGGTGAACTGTACATTGGCA	0	31	RDR2-sensitive
ATCTCGTCGCGCGGCTTCGCCACT	1	13	RDR2-sensitive
ATCTCGTGACTCTGCGCGCACAGA	0	9	RDR2-sensitive
ATCTCGTGGCACTCGGCAAAGAGC	0	12	RDR2-sensitive
ATCTCGTTTCTGGGATCCCGTCGG	0	9	RDR2-sensitive
ATCTCGTTTCTGTGGAGCCAGGCT	0	12	RDR2-sensitive
ATCTCGTTTGGATGATTTTCGTGA	0	9	RDR2-sensitive
ATCTCTAATTTTGTCGGTTCGGAT	0	14	RDR2-sensitive
ATCTCTCAAAGACTTGGATGTGCA	21	0	RDR2-resistant
ATCTCTCTAATCTTGTCGGCTAAG	34	4	RDR2-resistant
ATCTCTCTGCTGTAGTTTTGGCAT	78	28	RDR2-resistant
ATCTCTGACGGCTCTGTACGAGGC	0	11	RDR2-sensitive
ATCTCTGACGGGCTGGTGCTGGCA	0	10	RDR2-sensitive
ATCTCTGACTGCGAAGATCGACAA	0	31	RDR2-sensitive

ATCTCTGACTGCGAAGATCGACAG	0	15	RDR2-sensitive
ATCTCTGCGGTCGGTGATTTCGGA	1	44	RDR2-sensitive
ATCTCTGGTCTGGACTGCGGACGG	1	16	RDR2-sensitive
ATCTCTGTAGCGTTCGGCATTGAG	1	22	RDR2-sensitive
ATCTCTGTCGGATGTCGGAGACGA	0	11	RDR2-sensitive
ATCTCTTGTAGTTTTTTGTTGTCT	29	0	RDR2-resistant
ATCTCTTGTGAACCAGACTGGCCA	0	62	RDR2-sensitive
ATCTCTTTACTAGTCGAGGACGTT	0	9	RDR2-sensitive
ATCTGAAATCTCTGTATACTAAAC	0	11	RDR2-sensitive
ATCTGAACACAATTTCTTATGGCC	19	0	RDR2-resistant
ATCTGAACGCGCGTGCAAGGGCAA	0	12	RDR2-sensitive
ATCTGAACTTTGGAAAGTGGTGGA	1	16	RDR2-sensitive
ATCTGAACTTTTAGCTGCATTAAA	22	1	RDR2-resistant
ATCTGAATTTTGTTGGACTTCGGC	46	6	RDR2-resistant
ATCTGACAGTGGTTCCGCGGTAAA	0	10	RDR2-sensitive
ATCTGACATATGAGGAGTGGACAT	0	10	RDR2-sensitive
ATCTGACATGTCGGGTACAGATTC	1	15	RDR2-sensitive
ATCTGACCGTGATGTGAAGTTTGT	2	17	RDR2-sensitive
ATCTGACCTGGGCTGACGACTGCC	0	9	RDR2-sensitive
ATCTGACCTGGGCTGACGACTGTC	0	10	RDR2-sensitive
ATCTGACGGTCGGTAACGTAACTA	0	10	RDR2-sensitive
ATCTGACGGTGACCTGCCTGACGG	0	18	RDR2-sensitive
ATCTGACTCTGACAGCAACGGTCG	1	15	RDR2-sensitive
ATCTGACTTGAAACTTGCTTGAAG	16	0	RDR2-resistant
ATCTGAGACGGTAGGTTATACTAG	0	15	RDR2-sensitive
ATCTGAGCACTGTGTGATGACGTC	4	33	RDR2-sensitive
ATCTGAGCATGTAGACGCCACCGA	0	36	RDR2-sensitive
ATCTGAGCGTCGGCAGGGGCAGTC	15	0	RDR2-resistant
ATCTGAGCGTGCAGGCTGATCTCG	0	10	RDR2-sensitive
ATCTGAGTATCGTAGAGATTGCAT	0	11	RDR2-sensitive
ATCTGAGTATTGTGTGGATTACGT	1	16	RDR2-sensitive
ATCTGAGTCGGCTCGACACGGTCC	0	15	RDR2-sensitive
ATCTGATACGTTGAGGAATGGATT	0	9	RDR2-sensitive
ATCTGATATTCGTGACGGTGTCAT	0	11	RDR2-sensitive
ATCTGATCTGATCCAATCGCGGAC	0	25	RDR2-sensitive
ATCTGATGAGTGTGACCGACGAGC	0	11	RDR2-sensitive
ATCTGATGGACCTGTATGTAGGAT	0	11	RDR2-sensitive
ATCTGATGTGGCATTTTTGGAAAC	0	13	RDR2-sensitive
ATCTGATTACGGTTGGACTAAAAC	0	25	RDR2-sensitive
ATCTGATTGTGAATGTAGTTGGAG	0	10	RDR2-sensitive
ATCTGCAGAGCTGATTTCCGTGTG	0	28	RDR2-sensitive
ATCTGCCAGTTGATCACAGGACAA	0	13	RDR2-sensitive
ATCTGCGACGCAGGATCGGACGTT	0	24	RDR2-sensitive
ATCTGCGCATCTGTGGAGCCAGGC	1	211	RDR2-sensitive
ATCTGCGTGATTGGTTGTCCTATA	0	11	RDR2-sensitive
ATCTGCTAGAGCGATCACAGACGA	0	9	RDR2-sensitive
ATCTGCTCAGGCTGTACGCGGACA	0	14	RDR2-sensitive
ATCTGCTCTGGTAGAGAAGCTGCT	0	20	RDR2-sensitive
ATCTGCTGCTTAGATCATCGTCAA	16	0	RDR2-resistant

ATCTGCTGGACCTGCAGGTGGGGT	0	16	RDR2-sensitive
ATCTGCTGGTGCGGACTAGGAGGC	0	15	RDR2-sensitive
ATCTGGAACGGAACGGCTCTATTT	0	9	RDR2-sensitive
ATCTGGAACGGAGCGGCTCTGTTT	2	97	RDR2-sensitive
ATCTGGAACTGAATTCATGGGGCA	0	10	RDR2-sensitive
ATCTGGACAAATAAGCTAGACTGT	0	12	RDR2-sensitive
ATCTGGACACCGGTATAGCTTCTT	1	13	RDR2-sensitive
ATCTGGACCCGCATAGAACCGGAC	0	18	RDR2-sensitive
ATCTGGACCGGTTCAGTAGCGCAC	0	10	RDR2-sensitive
ATCTGGACGCAGGAGCGACGCCTT	1	21	RDR2-sensitive
ATCTGGACTAACATGGACGGACAC	0	13	RDR2-sensitive
ATCTGGACTGGACGAGATGACGAG	0	16	RDR2-sensitive
ATCTGGACTGGGGCACGCGGCATA	0	9	RDR2-sensitive
ATCTGGACTGGGGCACGCGGCGAC	0	19	RDR2-sensitive
ATCTGGACTGGTTCGGCGAAGGGC	4	42	RDR2-sensitive
ATCTGGACTGTAGCCCCGGTCGGC	0	9	RDR2-sensitive
ATCTGGCAGATAGATCATCGGGAT	0	20	RDR2-sensitive
ATCTGGCCCGTCAGACAGAAACGT	0	11	RDR2-sensitive
ATCTGGCCTGCATGCATTGAGATG	0	20	RDR2-sensitive
ATCTGGCGCTTAATGAACTGGTAT	0	14	RDR2-sensitive
ATCTGGCGGCTGAAGCGAGGACGA	2	85	RDR2-sensitive
ATCTGGCGTGAGAGAGAAGAGCGT	0	10	RDR2-sensitive
ATCTGGCTCTGATACCACTGAAGG	19	1	RDR2-resistant
ATCTGGCTGTAGGGAGAATCCGGC	0	17	RDR2-sensitive
ATCTGGCTTATGAAACGGTGACCA	22	1	RDR2-resistant
ATCTGGGCTGGAGCACGCGGCGAC	0	19	RDR2-sensitive
ATCTGGGCTGGGACACGCAGCGAC	0	19	RDR2-sensitive
ATCTGGGCTGGGACACGCGGCGAC	2	85	RDR2-sensitive
ATCTGGGCTGGGGCACGCGGCGAC	7	38	RDR2-sensitive
ATCTGGGTTGAAATGAATGGTGTT	22	0	RDR2-resistant
ATCTGGTCGGATCTGATCGTTGTG	25	4	RDR2-resistant
ATCTGGTGCACATAGAAGACTGGT	3	37	RDR2-sensitive
ATCTGGTGGAACTGCAGGTGGCGT	0	18	RDR2-sensitive
ATCTGGTGGACCTGCAGGCGAGGT	0	12	RDR2-sensitive
ATCTGGTGGACCTGCAGGTGGGGC	3	111	RDR2-sensitive
ATCTGGTGGACCTGCAGGTGGGGT	0	20	RDR2-sensitive
ATCTGGTGGACCTGTAGATGGGGT	0	25	RDR2-sensitive
ATCTGGTGGACCTGTAGGTGGGGC	0	66	RDR2-sensitive
ATCTGGTGGATCTGCAGGTGGGGT	0	24	RDR2-sensitive
ATCTGGTGGCACTGTCGATCCGGT	0	9	RDR2-sensitive
ATCTGGTTCTATGTAGGTCTAGGC	0	19	RDR2-sensitive
ATCTGGTTGTGCTTGATCCGTGGC	14	0	RDR2-resistant
ATCTGGTTGTGGATGCGGCTTCTA	0	9	RDR2-sensitive
ATCTGTACAGAGCGGCCTGATGTA	0	17	RDR2-sensitive
ATCTGTACAGGATAGAGTTGTGAC	19	55	RDR2-sensitive
ATCTGTACAGTCGTCGGAAGGCTG	2	92	RDR2-sensitive
ATCTGTACAGTCGTCGGGAGGCCG	0	30	RDR2-sensitive
ATCTGTACAGTCGTCGGGAGGCTG	0	17	RDR2-sensitive
ATCTGTACAGTCGTCGTGAGGCCG	0	9	RDR2-sensitive

ATCTGTACGAGCTTAAATGGCGAT	0	19	RDR2-sensitive
ATCTGTAGAGCCAGGCTTCAGCGG	1	15	RDR2-sensitive
ATCTGTAGGACACATGTAGGTGGT	0	23	RDR2-sensitive
ATCTGTAGGAGCCTAGAGGTGCTT	0	9	RDR2-sensitive
ATCTGTAGTATAGAGTTGAAGCAT	0	10	RDR2-sensitive
ATCTGTAGTTTATAATCGGACGGT	2	16	RDR2-sensitive
ATCTGTAGTTTTACGACGTTTCAT	0	23	RDR2-sensitive
ATCTGTATCGACTCTTACCGGTGT	2	21	RDR2-sensitive
ATCTGTCCGAGTCAGAACCCGTGC	0	12	RDR2-sensitive
ATCTGTCGACTGTTGACGTGCTAC	1	17	RDR2-sensitive
ATCTGTCGGGTAGCAGATACGCAA	0	18	RDR2-sensitive
ATCTGTCGTGCTTATGGGAAGGGA	0	11	RDR2-sensitive
ATCTGTCTAGATGAGCTCGTAGGT	16	0	RDR2-resistant
ATCTGTCTCGATTGATGACGCGTG	0	9	RDR2-sensitive
ATCTGTCTCGTTTGGACTTCCGGA	0	10	RDR2-sensitive
ATCTGTGACGGCAGGGACTTGCGT	19	0	RDR2-resistant
ATCTGTGCGGACCGTGCCAGAGGC	0	11	RDR2-sensitive
ATCTGTGGAATAAGGGCATCCCGA	0	16	RDR2-sensitive
ATCTGTGGAATAGGGGCATCCCGG	0	9	RDR2-sensitive
ATCTGTGGAGCCAGGCTCACGCGA	1	17	RDR2-sensitive
ATCTGTGGCGCTCGTTTTGACGGA	0	9	RDR2-sensitive
ATCTGTGGGAGCGCATGCAACGTG	0	18	RDR2-sensitive
ATCTGTGTCCGACTTGTTCCGCAT	0	14	RDR2-sensitive
ATCTGTGTCGGGCTCAAACGGATC	0	19	RDR2-sensitive
ATCTGTGTCTATGATTTGTGGGAC	1	13	RDR2-sensitive
ATCTGTGTCTATGATTTGTGGGTC	1	50	RDR2-sensitive
ATCTGTGTTAGGATTGAAAACGGA	0	12	RDR2-sensitive
ATCTGTGTTGGATGATATTGTATT	73	3	RDR2-resistant
ATCTGTGTTTATGATTTGTAGGCC	0	10	RDR2-sensitive
ATCTGTTCCGGACCGGCATGACTT	0	12	RDR2-sensitive
ATCTGTTCGCGGACTCGGCCCTAT	0	9	RDR2-sensitive
ATCTGTTCGGGCTCAAACGGACAG	0	15	RDR2-sensitive
ATCTGTTCGGGCTTATTCGTGCTG	0	16	RDR2-sensitive
ATCTGTTCTATAGGATCGTACCGT	45	4	RDR2-resistant
ATCTGTTCTGTATCTAACGGTGTT	0	9	RDR2-sensitive
ATCTGTTGCTAGTCTGATCGTCGC	0	11	RDR2-sensitive
ATCTGTTGGACCTGCAGGTGGGGT	0	13	RDR2-sensitive
ATCTGTTTAGTGAGGATCGTCCGT	0	19	RDR2-sensitive
ATCTGTTTCTCGGTGTAGTTAGGC	23	2	RDR2-resistant
ATCTGTTTGAACTTGGTGTGAGGC	16	0	RDR2-resistant
ATCTGTTTGACGTGTTACATAACT	30	2	RDR2-resistant
ATCTGTTTGGCAGAACTTCGGCTT	8	128	RDR2-sensitive
ATCTGTTTTGCTCGGCAACACGGT	0	10	RDR2-sensitive
ATCTGTTTTTTCTGCGGCTGATGA	20	1	RDR2-resistant
ATCTGTTTTTTTTCTGATTTGGGC	26	0	RDR2-resistant
ATCTTAAGGATGGTGCGTCGTAGA	31	5	RDR2-resistant
ATCTTACAAGTGATCTTTGATGGC	46	1	RDR2-resistant
ATCTTACTCACTATGTGTTGGACA	0	9	RDR2-sensitive
ATCTTAGAATTCTTTTTGCGGCAT	16	0	RDR2-resistant

ATCTTAGAATTCTTTTTGCGGCCT	32	0	RDR2-resistant
ATCTTAGGACTTAGGGCGGGTGCA	0	19	RDR2-sensitive
ATCTTAGGACTTAGGGCGGGTGGA	0	11	RDR2-sensitive
ATCTTAGGATTAGGACGGGTGCAT	0	16	RDR2-sensitive
ATCTTAGGATTAGGGCGGGTGCAT	0	11	RDR2-sensitive
ATCTTAGGATTTAGGGCGGGTGCA	0	11	RDR2-sensitive
ATCTTATGGTCCGACTGTACCGGT	4	19	RDR2-sensitive
ATCTTATTTGAGAGATCGTCCTAT	0	10	RDR2-sensitive
ATCTTATTTGAGAGATCGTTTCAT	0	12	RDR2-sensitive
ATCTTCAACGCGGGCTGACGTCAT	8	113	RDR2-sensitive
ATCTTCATGCTGTAGAAGATGGTA	0	23	RDR2-sensitive
ATCTTCCGGTAAATTTAGAGGACG	0	12	RDR2-sensitive
ATCTTCCTGGGATTCTTTTGTCGG	0	11	RDR2-sensitive
ATCTTCGACGGCTGTAATAAGGGC	0	9	RDR2-sensitive
ATCTTCGACGGTTTACAGCTTATG	0	12	RDR2-sensitive
ATCTTCGGACTCAAGTCAGGCATC	2	28	RDR2-sensitive
ATCTTCGGACTCAAGTCGGTATCA	0	19	RDR2-sensitive
ATCTTCGTTGACGGATATTGAGAT	25	0	RDR2-resistant
ATCTTCTCTGTATCGTTCCAATTT	118	3	RDR2-resistant
ATCTTCTTAGGATGCTTTTGTCGG	0	12	RDR2-sensitive
ATCTTCTTGGGATGCTTTTGTCGG	1	12	RDR2-sensitive
ATCTTCTTGGGATTCTTTTGTCGG	0	42	RDR2-sensitive
ATCTTGAACTATTGAAGGCTCGTT	0	14	RDR2-sensitive
ATCTTGAAGATGGATGACTTTTGT	26	4	RDR2-resistant
ATCTTGACGACTGTACAGATTGCG	0	12	RDR2-sensitive
ATCTTGAGTTGTAGCTAGGGACGT	0	11	RDR2-sensitive
ATCTTGCAGAAACTGGACGAGCGC	0	9	RDR2-sensitive
ATCTTGCAGGAACTGGACGAGCGC	1	35	RDR2-sensitive
ATCTTGCATCGTTGTGGCCTGGCC	1	22	RDR2-sensitive
ATCTTGGATATGTGACGGTGGCTT	3	19	RDR2-sensitive
ATCTTGGCCGGCTGGAGATGGAGC	0	18	RDR2-sensitive
ATCTTGTTGGTCGGTAAAGGTATT	2	21	RDR2-sensitive
ATCTTGTTTTATAGACGTCCGGAC	0	10	RDR2-sensitive
ATCTTTAGGACTGATTTGATGGAT	0	12	RDR2-sensitive
ATCTTTATTGCAGAGATTCTGGTT	2	19	RDR2-sensitive
ATCTTTCGGATCGGCTGGCCACAC	0	19	RDR2-sensitive
ATCTTTGCCTTCTGTCGTCGGGCG	0	18	RDR2-sensitive
ATCTTTGGGGCTGATTGATGGGTA	0	11	RDR2-sensitive
ATCTTTGGTTGTGCGTGATTCGGT	19	0	RDR2-resistant
ATCTTTGTAAAGGCTACGTAGTGA	0	9	RDR2-sensitive
ATCTTTTAGAGAAGACTGTAAAAG	0	10	RDR2-sensitive
ATCTTTTAGAGAAGACTGTAAAGA	0	23	RDR2-sensitive
ATCTTTTAGAGAAGACTGTAAAGG	0	40	RDR2-sensitive
ATCTTTTAGAGGAGACTGTAAAGG	1	35	RDR2-sensitive
ATCTTTTCAGGATCAGACTTGCAT	0	20	RDR2-sensitive
ATGAAACACGACGGATGCGGCTTT	0	24	RDR2-sensitive
ATGAAATGTGGGTCTGATGGCAAT	0	16	RDR2-sensitive
ATGAACCACCGCAGCTAGGACCGA	0	14	RDR2-sensitive
ATGAACGTCCGACGCTGACGATGG	0	11	RDR2-sensitive

ATGAAGACGTCATGTGCACTCACT	19	0	RDR2-resistant
ATGAAGACTGTAAGGGGGTAGGCT	0	12	RDR2-sensitive
ATGAAGGCGCTGTGGAGAAGACGC	0	26	RDR2-sensitive
ATGAAGTGAATTGTTGGACACGGT	0	9	RDR2-sensitive
ATGAATCTTAGTGACTACCGTGGC	21	2	RDR2-resistant
ATGAATGCCTGAAACGTAATGAGA	23	1	RDR2-resistant
ATGAATTCTTGTGCGCTGGGACGA	1	14	RDR2-sensitive
ATGAATTGTGGACGTCTACGTTAG	0	11	RDR2-sensitive
ATGACAATATTCAGTAGACTTAAG	38	1	RDR2-resistant
ATGACACGGTCTGAAGACGGGTAT	0	14	RDR2-sensitive
ATGACACTAGGTACGATTCGGAGC	14	0	RDR2-resistant
ATGACACTCTGCAATAAAGACGAT	0	11	RDR2-sensitive
ATGACACTCTGCTCTGATCTGTCT	28	0	RDR2-resistant
ATGACACTGTACGAGGCTTGGCAT	38	0	RDR2-resistant
ATGACAGAGCTGGAGAAGATCGCC	24	1	RDR2-resistant
ATGACAGGACGGGATGGGACGGGA	0	10	RDR2-sensitive
ATGACAGGTTGTTGATATGGCATG	0	9	RDR2-sensitive
ATGACATGTAGGGTCAGTGGCATA	0	15	RDR2-sensitive
ATGACATGTGGGACCAGTGGCATA	0	14	RDR2-sensitive
ATGACATGTGGGATCTATGGTATA	0	15	RDR2-sensitive
ATGACATGTGGGGCCAGTGGCATA	1	32	RDR2-sensitive
ATGACCCAGACTCGGCACAGTTAT	1	13	RDR2-sensitive
ATGACCTGGACGCAAGAGCGACTC	0	10	RDR2-sensitive
ATGACGACATACATCTGTGCTTAT	1	22	RDR2-sensitive
ATGACGATGTAGCATTAGGGGTAT	0	9	RDR2-sensitive
ATGACGCGTGGCAGAGAACTGCAT	1	31	RDR2-sensitive
ATGACGGATGTAGAGGACGTTGCT	0	17	RDR2-sensitive
ATGACGGATTATATTCAGACTGCA	27	0	RDR2-resistant
ATGACGTAACTCGGGAGTGGACGG	0	13	RDR2-sensitive
ATGACGTGTTTGACGACTCGACGG	1	12	RDR2-sensitive
ATGACTAGGCTCGTTGGCTCGGCG	1	21	RDR2-sensitive
ATGACTAGTTGTTCTGAAAGACGC	0	14	RDR2-sensitive
ATGACTATCTTTATTCATGAGCAT	26	0	RDR2-resistant
ATGACTATGTGACTAATACGTGCT	23	1	RDR2-resistant
ATGACTCGCCAGAACGACGCGCCG	0	14	RDR2-sensitive
ATGACTCTCGGCACATGCAGGCAC	0	10	RDR2-sensitive
ATGACTCTGGTCACTGTTCACGGC	354	19	RDR2-resistant
ATGACTGCAGAGGATTGTGGTATA	2	64	RDR2-sensitive
ATGACTGCAGAGGATTGTGTTATA	0	10	RDR2-sensitive
ATGACTGCGGGCGGATATTTTCAA	0	41	RDR2-sensitive
ATGACTGCTTCATGATCTACGGTG	27	2	RDR2-resistant
ATGACTGGGCACACGTGCTGGCAC	0	27	RDR2-sensitive
ATGACTGGTCTCTGTGGAAGCGTA	0	29	RDR2-sensitive
ATGACTGTCATACGCATGCTCCGC	0	12	RDR2-sensitive
ATGACTGTGACGCTGGAAGACTGC	0	11	RDR2-sensitive
ATGACTGTGGTTATGGATGCTCGC	30	1	RDR2-resistant
ATGACTGTTCATTGCGCATTTGAG	28	3	RDR2-resistant
ATGACTGTTGACCGAACACGGTAT	0	10	RDR2-sensitive
ATGACTGTTGGGTATATAATCTTG	14	0	RDR2-resistant

ATGACTGTTTCTTCTAGACGGTTC	0	9	RDR2-sensitive
ATGACTGTTTCTTCTGGACGGTTC	0	26	RDR2-sensitive
ATGACTGTTTGGAAAATGTGGTTT	0	18	RDR2-sensitive
ATGACTTGCTTGGTTGTGGATCGT	0	10	RDR2-sensitive
ATGACTTTGCACTAGATACTGGCT	0	10	RDR2-sensitive
ATGACTTTGGACAGACGTTTTTGG	0	9	RDR2-sensitive
ATGAGAAGACTGTTGTAGATGGCA	118	1	RDR2-resistant
ATGAGACAGACGTTATAATGGATT	19	1	RDR2-resistant
ATGAGACATGTTGGACTAGCGTGC	0	10	RDR2-sensitive
ATGAGACGGTTCATAGGTGCCCTG	652	18	RDR2-resistant
ATGAGATGAGACTAGCTCGTTGGT	22	0	RDR2-resistant
ATGAGCCAAGTAGAACGGAGCCGT	2	15	RDR2-sensitive
ATGAGCCTGACTGAGATGAGCCAT	1	49	RDR2-sensitive
ATGAGCCTGATAGCATACTGACGA	0	22	RDR2-sensitive
ATGAGCTCTGCCATGTCTAGACAA	0	10	RDR2-sensitive
ATGAGCTGTATTTTGTGAGATCGT	0	13	RDR2-sensitive
ATGAGGAATGAGGTGGTGATGGAT	0	10	RDR2-sensitive
ATGAGGACAACATGCATTGCTTAT	32	2	RDR2-resistant
ATGAGGACGTGTATATGATACAGC	19	0	RDR2-resistant
ATGAGGAGGCTGGAGAGGTGGCTC	15	0	RDR2-resistant
ATGAGGATGTGCCGGATTCGGCTT	82	2	RDR2-resistant
ATGAGGATGTGCTAGATTCGGCTT	83	3	RDR2-resistant
ATGAGGTTGGTCTGTTGCCCCGGC	0	10	RDR2-sensitive
ATGAGTAGAGGACGTTCTGATCCT	0	14	RDR2-sensitive
ATGAGTCCGATGGCATACTGGCGA	0	27	RDR2-sensitive
ATGAGTCGGGCGTCCGGACGGTTT	0	9	RDR2-sensitive
ATGAGTCTGGTGTATGTTGACATG	34	4	RDR2-resistant
ATGAGTCTGGTGTATGTTGGCATG	102	0	RDR2-resistant
ATGAGTCTGTTAGGCCCGTCGGAC	0	22	RDR2-sensitive
ATGAGTGTGTGACTCGTGCGGTGC	24	0	RDR2-resistant
ATGAGTTGTAGAAAGAGGCTCGGC	0	9	RDR2-sensitive
ATGATAGCGGTGTAGTGAGACGAC	3	19	RDR2-sensitive
ATGATATTGATTGTGGCGATGTTT	18	0	RDR2-resistant
ATGATCGAGCGATGACATTTATTC	50	3	RDR2-resistant
ATGATCGGACGATTTATGTGCATT	0	16	RDR2-sensitive
ATGATCGGTTAACTGCATAGCATG	0	15	RDR2-sensitive
ATGATCTACCTGTAGAGATGGCAA	0	17	RDR2-sensitive
ATGATCTATTATGTTTGTTAGGTC	24	1	RDR2-resistant
ATGATCTGTCGTTTCCTGTTGGCA	38	6	RDR2-resistant
ATGATCTTGTTTCTGTTGGTTTTA	59	1	RDR2-resistant
ATGATGCCAGGATTGATGGTAATC	17	0	RDR2-resistant
ATGATGGACTCTTGGATGAGGCAC	44	9	RDR2-resistant
ATGATGTCTTCTTGAAAATTCGGT	14	0	RDR2-resistant
ATGATGTTCTTATGCGGATGATGA	14	0	RDR2-resistant
ATGATGTTGTATTGATGAGATGGT	17	1	RDR2-resistant
ATGATTGAGGATAGTGAAGTCGGT	18	0	RDR2-resistant
ATGATTTACTCTGTCATGGATATT	25	2	RDR2-resistant
ATGATTTAGTGAACGTTGATTTTA	19	0	RDR2-resistant
ATGATTTATGGATTCGATGGCATA	0	15	RDR2-sensitive

ATGATTTGTGGATCCGATGGCATA	0	17	RDR2-sensitive
ATGATTTGTGGGCCCGATGGCATA	0	21	RDR2-sensitive
ATGATTTGTGGGTCCGATGGCATA	7	668	RDR2-sensitive
ATGATTTGTGGGTCCGATGGTATA	0	15	RDR2-sensitive
ATGATTTGTGGGTCTGACGGTAAA	0	14	RDR2-sensitive
ATGATTTGTTATTATGGTCTGATC	44	3	RDR2-resistant
ATGATTTTAGTAGATATGGGTATG	57	3	RDR2-resistant
ATGCAAAATTTGTAGAATGGCATT	0	10	RDR2-sensitive
ATGCAACAGGCGGATATAGACCAT	0	19	RDR2-sensitive
ATGCAACGGACGGGTACGGGCTAT	1	23	RDR2-sensitive
ATGCACAAAGATCTGGAAATTTAT	15	0	RDR2-resistant
ATGCACACCTGCATGACTGTCTGA	0	9	RDR2-sensitive
ATGCACACGGACGGTCTAGATCGC	0	12	RDR2-sensitive
ATGCACACGTGGACCGTCCGGCTC	0	19	RDR2-sensitive
ATGCACATGCTCGTGTTGTCGGTA	0	21	RDR2-sensitive
ATGCACCTCTGCGGTCGGGACGAA	2	19	RDR2-sensitive
ATGCACGGGCGTAGAAGGAGGCAT	1	22	RDR2-sensitive
ATGCACTGTGGAAGATTGCAGGAA	0	16	RDR2-sensitive
ATGCAGCGATTTGGTTACTTTGGT	0	13	RDR2-sensitive
ATGCAGCGGAGGAATGTTTTTATA	54	6	RDR2-resistant
ATGCAGCTCGGCGTCATGAATCAT	0	14	RDR2-sensitive
ATGCAGGACTTAGAATGGGGGCAC	0	13	RDR2-sensitive
ATGCAGGATCGGATAACTCCTCAC	0	17	RDR2-sensitive
ATGCAGGATGGTGGGAGAAGTCAC	0	25	RDR2-sensitive
ATGCAGGGCGGTGGACGAAGGCAC	0	15	RDR2-sensitive
ATGCAGGGTGACGATGACATGAAC	46	1	RDR2-resistant
ATGCAGGTCGGTATCCTCGTATAC	0	10	RDR2-sensitive
ATGCAGTCGGCATTCAGTTTTGCA	0	10	RDR2-sensitive
ATGCAGTTGGGATTAGCGTGCATT	34	0	RDR2-resistant
ATGCATAAGTATCTGGATGACAAT	52	2	RDR2-resistant
ATGCATATACCCGTCGGATATTGC	0	10	RDR2-sensitive
ATGCATCTAGACCGGCCTCTTCTT	42	1	RDR2-resistant
ATGCATCTTCAAGTTGAATTCGGT	0	16	RDR2-sensitive
ATGCATGTTGACGGTGGTCCAGAA	17	0	RDR2-resistant
ATGCATGTTTGACTGAGTTAGGCA	0	18	RDR2-sensitive
ATGCATTAACTGTGAACAAGACGT	0	10	RDR2-sensitive
ATGCATTCGAGGATACTCCATAGG	147	3	RDR2-resistant
ATGCCAGATCGCGGGTACTCGGCA	0	9	RDR2-sensitive
ATGCCAGATTGGAGGACCAGGCAC	0	9	RDR2-sensitive
ATGCCAGGTCGCGGACCGTCCGGC	0	15	RDR2-sensitive
ATGCCAGTTGGTCTGTATGTGCGG	0	11	RDR2-sensitive
ATGCCCGGCAAATGTAGCGGCTGA	1	49	RDR2-sensitive
ATGCCCTACTGTAGACGGTCAGAC	0	10	RDR2-sensitive
ATGCCCTGATTGGACTGGACCCAA	0	38	RDR2-sensitive
ATGCCGACGGATGCTGTGATGGTA	0	18	RDR2-sensitive
ATGCCGCGGTGTAGGGATGGGCGA	0	14	RDR2-sensitive
ATGCCGTGACTGGACCGTGGCTGC	0	18	RDR2-sensitive
ATGCCTAGGCTCGTTGGCTCGGCG	1	25	RDR2-sensitive
ATGCCTGTAGTCCGGCATTTCCGG	0	9	RDR2-sensitive

ATGCCTTTGTAGATCCGAGCGGCC	4	37	RDR2-sensitive
ATGCGAAGACTGGTCTGCATGCGC	0	15	RDR2-sensitive
ATGCGAATTCGAACTATGTGGAAT	46	3	RDR2-resistant
ATGCGACCACGTAAGACTGCTTTT	0	10	RDR2-sensitive
ATGCGACGCGTAAGGGGCACTCTA	0	11	RDR2-sensitive
ATGCGACTGCCATGTTTTTCGGAT	3	21	RDR2-sensitive
ATGCGACTGCGGCTTCTACATTAT	0	9	RDR2-sensitive
ATGCGACTGGCGCGTGAATGGTTT	0	18	RDR2-sensitive
ATGCGAGACAGACTGGAGGCCAGA	0	17	RDR2-sensitive
ATGCGATTAAGCGGGCTAGGCTAG	0	10	RDR2-sensitive
ATGCGATTGTGGAGACCGTGCTGT	0	13	RDR2-sensitive
ATGCGATTTTCTGTAGGTTCGGGT	0	9	RDR2-sensitive
ATGCGCCTCTGCAGAGAACACCGC	0	11	RDR2-sensitive
ATGCGCGACGGGCTGAAGACGCGC	0	10	RDR2-sensitive
ATGCGCGGACCGTCCGGCCCACAG	0	9	RDR2-sensitive
ATGCGCTGCGGACGGTCTTAGTGT	0	10	RDR2-sensitive
ATGCGGAAGTTTGGCTACTGTATT	0	21	RDR2-sensitive
ATGCGGAGAGGAGAGAGAAGACGT	0	9	RDR2-sensitive
ATGCGGAGTGCAAATACGGAGTCT	0	16	RDR2-sensitive
ATGCGGAGTGCTGCTGGAGTTGGT	0	10	RDR2-sensitive
ATGCGGAGTGGACGGAGTAGGAGC	0	9	RDR2-sensitive
ATGCGGAGTGGTTAGTACAGTCAT	0	9	RDR2-sensitive
ATGCGGATCAGGCGTGCAGCTCAG	0	20	RDR2-sensitive
ATGCGGATCGGAGCCAGCCAACGG	0	27	RDR2-sensitive
ATGCGGCAGGTACCTCGGCGCCAA	0	9	RDR2-sensitive
ATGCGGCATGTAGGGAGCATTCTA	0	14	RDR2-sensitive
ATGCGGCGCGTAGGGGCACTTTAC	0	9	RDR2-sensitive
ATGCGGCTGCCATGTTTTTCGGAT	1	14	RDR2-sensitive
ATGCGGGAGTTTGGCTACTGTATT	0	30	RDR2-sensitive
ATGCGGGTTTGCGCTGAACGTCGC	0	14	RDR2-sensitive
ATGCGGTCAGCGCGGCACTGGTTC	18	1	RDR2-resistant
ATGCGGTCTGTAGGGGCACCCTAT	0	25	RDR2-sensitive
ATGCGTACTGTCGTGGACGGCCGG	0	12	RDR2-sensitive
ATGCGTCGCTGTGTTCGGCAAGCT	1	42	RDR2-sensitive
ATGCGTGCTGCAGAGCGGTATCTG	0	18	RDR2-sensitive
ATGCGTGTACGTGACAACTGCTGT	17	0	RDR2-resistant
ATGCGTGTGTAGAAGCGACGGAGT	0	17	RDR2-sensitive
ATGCGTTCGGTCTAGATGGATGTA	0	9	RDR2-sensitive
ATGCGTTGACTGAGCAAGTGCGTC	0	23	RDR2-sensitive
ATGCGTTGCGACGGCTCACAACAA	0	12	RDR2-sensitive
ATGCGTTGGGCTGAGACGCGCCGC	0	12	RDR2-sensitive
ATGCGTTTCCTGTAGTGTACGGCA	0	18	RDR2-sensitive
ATGCTAGTAGCAAGGTCTCGGGCA	0	48	RDR2-sensitive
ATGCTATGAATTAAGAACAAGGCA	1	16	RDR2-sensitive
ATGCTCACTTTAAATCGTATCGGA	0	20	RDR2-sensitive
ATGCTCGGCGCCAATGATGACGGC	0	22	RDR2-sensitive
ATGCTCGGCGCCAATGATGATGGT	0	9	RDR2-sensitive
ATGCTCTGCGTGACTCATGTGATC	14	0	RDR2-resistant
ATGCTCTGGATAGTTTGATTGCAC	29	170	RDR2-sensitive

ATGCTCTGTGAACTCGCGGCTCAT	17	0	RDR2-resistant
ATGCTGACCTCTGGCCTTCGCAGC	0	17	RDR2-sensitive
ATGCTGACGCGCGGAACGACGACG	0	11	RDR2-sensitive
ATGCTGACTGTAGGAGGCACCCCA	0	21	RDR2-sensitive
ATGCTGCACGTAAGGACTAGGCAC	0	10	RDR2-sensitive
ATGCTGCAGAACTGAAGGGGTGTT	0	15	RDR2-sensitive
ATGCTGCGGAAATCGGAACGGCGT	0	12	RDR2-sensitive
ATGCTGCTGCTCCCTGACGGCACT	521	45	RDR2-resistant
ATGCTGCTGGATTCTCAAGACGTG	68	0	RDR2-resistant
ATGCTGCTGGTGCTGGTCTCGTTA	135	2	RDR2-resistant
ATGCTGGAGCGGTATTACTCAAAG	39	3	RDR2-resistant
ATGCTGGATTTGCTGGTCGCTGGA	4	19	RDR2-sensitive
ATGCTGGTCTGCATACACTCATGC	0	9	RDR2-sensitive
ATGCTGTAGAAGAGGGTACCCCTA	0	39	RDR2-sensitive
ATGCTGTAGAAGAGGGTACCCCTG	0	21	RDR2-sensitive
ATGCTGTAGAAGAGGGTACCCCTT	0	23	RDR2-sensitive
ATGCTGTAGGAGAGGGTACCCCTA	1	15	RDR2-sensitive
ATGCTGTAGGAGAGGGTACCCCTG	1	29	RDR2-sensitive
ATGCTGTAGGAGAGGGTACCCCTT	0	10	RDR2-sensitive
ATGCTGTAGTAAGGGGTACCCTGG	0	14	RDR2-sensitive
ATGCTGTAGTAAGGTGTACCCCTG	0	13	RDR2-sensitive
ATGCTGTATCATTTCGTCGGCACA	0	23	RDR2-sensitive
ATGCTGTGGATCGATGGCTCGACC	0	36	RDR2-sensitive
ATGCTGTGTTCTTTCAATTCGGTG	0	9	RDR2-sensitive
ATGCTGTTGGCTTGAGCAGTAGGA	0	14	RDR2-sensitive
ATGCTTCGGACCGATCTATCGGAC	0	10	RDR2-sensitive
ATGCTTCGGGTCGATTCGTCAGGC	0	10	RDR2-sensitive
ATGCTTGGCCGTTCGATCGTTGGC	0	15	RDR2-sensitive
ATGCTTGTTTGCAGATGAACGGAC	0	35	RDR2-sensitive
ATGCTTGTTTGCAGATGGACGGAC	0	10	RDR2-sensitive
ATGCTTTAATGGATTCAAATGAAG	24	1	RDR2-resistant
ATGGAAAACGAAGATGGAATCATG	75	0	RDR2-resistant
ATGGAAAACTAGAGGGCAACCCAC	0	9	RDR2-sensitive
ATGGAAACTGAAAGGTCAGTCGGC	0	41	RDR2-sensitive
ATGGAAAGTTTGAGGCGTATCATT	25	0	RDR2-resistant
ATGGAACAAAGCACGGCCTGACAC	0	15	RDR2-sensitive
ATGGAACCCCATTTGTCGGTCGGA	0	28	RDR2-sensitive
ATGGAACCTGTACGACGCGGCCAC	0	24	RDR2-sensitive
ATGGAAGATGCATCTGTAGATCGA	16	0	RDR2-resistant
ATGGAAGGTGGAGGTTTTAGGTAT	27	0	RDR2-resistant
ATGGAATCTTGCTGACGTGGCTGC	0	10	RDR2-sensitive
ATGGAATCTTTTAGAGAAGACTGT	1	35	RDR2-sensitive
ATGGAATGGACAGCTTGCTTCGGC	26	1	RDR2-resistant
ATGGAATGTATAAATAATGGCTAA	26	0	RDR2-resistant
ATGGACAAGCTCATAGGACGACAT	0	9	RDR2-sensitive
ATGGACACAAGCTGACGACGACGG	0	84	RDR2-sensitive
ATGGACACAGAAGACATTCTGCAT	31	0	RDR2-resistant
ATGGACACGTGGAGTAGACGCCAT	0	12	RDR2-sensitive
ATGGACAGCCGAGCAAGACTAAGA	14	0	RDR2-resistant

ATGGACAGGACGGCTCGAGCACAG	0	15	RDR2-sensitive
ATGGACAGTACGGTCTGAAGGCGG	1	14	RDR2-sensitive
ATGGACAGTCGACTGAAGATGGCG	0	15	RDR2-sensitive
ATGGACAGTCTGAACGTATGCAGA	0	24	RDR2-sensitive
ATGGACCAATTACACGTAGCCGAG	0	9	RDR2-sensitive
ATGGACCAGATAGTTTGCTTGCAC	0	10	RDR2-sensitive
ATGGACCAGCACTAAGCACGGCAC	0	9	RDR2-sensitive
ATGGACCATGTCACTTGACGACAC	0	23	RDR2-sensitive
ATGGACCCGGCTGAAAGACGTACC	0	64	RDR2-sensitive
ATGGACCGAGCTTGGATCAGCATG	0	9	RDR2-sensitive
ATGGACCGATCTAGCATGGCTCGA	0	9	RDR2-sensitive
ATGGACCTCGCGGACGAACGGCTA	78	20	RDR2-resistant
ATGGACCTGATGGCTTTAATGGCA	133	24	RDR2-resistant
ATGGACGAACCAGAAGATCTCCAA	0	11	RDR2-sensitive
ATGGACGAACCAGAAGATCTCCAG	0	32	RDR2-sensitive
ATGGACGACAGAATCGATCGGTTG	0	12	RDR2-sensitive
ATGGACGAGCAGACACGACACAGA	0	14	RDR2-sensitive
ATGGACGAGCAGACACGACACGGA	0	16	RDR2-sensitive
ATGGACGAGCCGAGCCGAGCCGAG	0	16	RDR2-sensitive
ATGGACGATCACGGTGGAGGACGA	26	0	RDR2-resistant
ATGGACGGCGGCGCGTACGGGAAC	0	11	RDR2-sensitive
ATGGACGGCGGGGAGGGTCGCGCA	35	0	RDR2-resistant
ATGGACGGTGTACTTAGCATCAAC	1	30	RDR2-sensitive
ATGGACGTAGGATGGACGGTGGAG	0	18	RDR2-sensitive
ATGGACTAGTAGCATACCGGTGCG	0	10	RDR2-sensitive
ATGGACTATCAGCGAAGGGTACCT	0	26	RDR2-sensitive
ATGGACTCGCGACAGGAGGACCGA	0	11	RDR2-sensitive
ATGGACTCTCCTCGTCAGCTGGCT	24	0	RDR2-resistant
ATGGACTCTTGGATGAGGCACTGA	86	16	RDR2-resistant
ATGGACTGAACGTAGATGGAAGAT	0	12	RDR2-sensitive
ATGGACTGACACTAAGCACGGCAT	0	28	RDR2-sensitive
ATGGACTGCGGGATCGGACGGCTG	0	11	RDR2-sensitive
ATGGACTGCTGGCGCACATCATAC	0	16	RDR2-sensitive
ATGGACTGGTTAGTGACGGACCAC	0	9	RDR2-sensitive
ATGGACTGTTCGCGTTTAAAGGCA	0	20	RDR2-sensitive
ATGGACTTAGAGCCCTGGCGACGG	2	67	RDR2-sensitive
ATGGACTTAGGGCCCTAGCGACGG	2	17	RDR2-sensitive
ATGGACTTAGGGCCCTGACGACGG	0	82	RDR2-sensitive
ATGGACTTAGGGCCCTGGCGACGG	0	71	RDR2-sensitive
ATGGACTTAGGGCCTTAGCGACGG	0	28	RDR2-sensitive
ATGGACTTGTCGGCGTACTGCTGT	2	21	RDR2-sensitive
ATGGACTTGTTTGGCCTCATCGGA	18	0	RDR2-resistant
ATGGAGAGCAGAACGCAGGGACGA	0	9	RDR2-sensitive
ATGGAGAGTCGTGTAGAGACGGAC	0	20	RDR2-sensitive
ATGGAGCATCTGATGTCGGACGAC	0	13	RDR2-sensitive
ATGGAGGACAGTAGCGCGGTGGTG	71	14	RDR2-resistant
ATGGAGTATCTGATGTCGGACGAC	0	11	RDR2-sensitive
ATGGATAAAACTCTATTAGGGCGT	0	16	RDR2-sensitive
ATGGATACAAGCTGACGACGACGG	0	15	RDR2-sensitive

ATGGATAGAAGATCTTAAAGGTGT	39	2	RDR2-resistant
ATGGATATTGCTGTGAACCGTCGC	0	11	RDR2-sensitive
ATGGATATTTGTAGAAGTATCGGA	0	12	RDR2-sensitive
ATGGATCAGCGACTGCTCCGGCAT	0	10	RDR2-sensitive
ATGGATCCTATGTCTATATCGTCA	34	0	RDR2-resistant
ATGGATCGGAGGGGATTGATACGG	0	14	RDR2-sensitive
ATGGATCTGAACACGTGACGACGC	0	10	RDR2-sensitive
ATGGATCTGTCCGTGCTTGTGCGG	0	39	RDR2-sensitive
ATGGATGAACTGCTGGATACGGTC	0	10	RDR2-sensitive
ATGGATGGACCTGGATGTGCGGAT	0	10	RDR2-sensitive
ATGGATTAGTGATACTAAGGACTG	50	6	RDR2-resistant
ATGGATTCGATGGCATAATGGCGA	0	12	RDR2-sensitive
ATGGATTGAAGTGGATTGATACGG	0	11	RDR2-sensitive
ATGGATTGAGAGCCTATTCGGTTG	0	13	RDR2-sensitive
ATGGATTGAGTGGGATTGGATGGG	3	22	RDR2-sensitive
ATGGATTGCAGCAGTAGTACGATC	0	11	RDR2-sensitive
ATGGATTGCAGCTGCAGCAGTCCA	2	32	RDR2-sensitive
ATGGATTGGAGGGGATTGATACGG	1	23	RDR2-sensitive
ATGGATTGTTTGCAGAGCACCGCC	0	12	RDR2-sensitive
ATGGATTGTTTTATTTACCCACGG	0	10	RDR2-sensitive
ATGGATTTGCAGATTTGATGGTTG	0	14	RDR2-sensitive
ATGGATTTGTATAGGTATGGGTTT	0	11	RDR2-sensitive
ATGGATTTGTTGATGGATTTAATC	0	29	RDR2-sensitive
ATGGCAAACGTAGATGGCACCCTC	18	0	RDR2-resistant
ATGGCAATGTGGCTGGCATGACTG	14	0	RDR2-resistant
ATGGCACACGCGAGAACACGGGAT	0	12	RDR2-sensitive
ATGGCACGTACCTACCTGTCGGAT	0	28	RDR2-sensitive
ATGGCACTCTGACGGAGACTGTCT	42	9	RDR2-resistant
ATGGCAGAACACGGAATGAGACAG	0	10	RDR2-sensitive
ATGGCAGACCGTAAGCAGGGACAC	0	19	RDR2-sensitive
ATGGCATACTGGTGAAGCTCACAA	0	15	RDR2-sensitive
ATGGCATATGTGTAGATGACTTGA	22	0	RDR2-resistant
ATGGCCAGTAGATAATGTCAGGCC	0	16	RDR2-sensitive
ATGGCCAGTTCTCGATTTGTTGTA	77	2	RDR2-resistant
ATGGCCCGTGAACCTGTTAGTCGT	1	28	RDR2-sensitive
ATGGCCCGTGGGCCGTCGGACGAC	37	3	RDR2-resistant
ATGGCCTCCATTTCGTCGAATCCC	0	10	RDR2-sensitive
ATGGCGAGAATGACAGATGACAAG	55	5	RDR2-resistant
ATGGCGGAACTGACAGGAGCCGAA	0	11	RDR2-sensitive
ATGGCGTGAGAACTTCAGGAGGTA	18	0	RDR2-resistant
ATGGCGTGTCTGAGCGACGTAATC	21	0	RDR2-resistant
ATGGCGTTGAACCATCTCCGTCGG	0	9	RDR2-sensitive
ATGGCTAGGATCGTTGGCTCGGCG	0	9	RDR2-sensitive
ATGGCTAGGCTCGTTGGCTCGGCG	0	40	RDR2-sensitive
ATGGCTAGGTTCGTTGGCTCGGCG	0	22	RDR2-sensitive
ATGGCTATAGATATTTTTCACGGA	0	12	RDR2-sensitive
ATGGCTCCTGCTCTAGACGCTAAA	0	15	RDR2-sensitive
ATGGCTCGACATTGAAGAATGCTG	18	0	RDR2-resistant
ATGGCTCTGATACCAGTTGTTGGC	0	10	RDR2-sensitive

ATGGCTGCAGAGGATCGTTGTCGT	0	9	RDR2-sensitive
ATGGCTGTACGAACTAACGGCTAA	0	12	RDR2-sensitive
ATGGCTTAGTGTGACACAGGGGCG	0	9	RDR2-sensitive
ATGGCTTATACCCGCTCTCGTCGT	0	13	RDR2-sensitive
ATGGCTTCAGCTCTGGCAGTAGAC	2	18	RDR2-sensitive
ATGGCTTCCAAAATATCAGGACTG	0	12	RDR2-sensitive
ATGGCTTGCTTGGCTGTGGGTCGT	0	15	RDR2-sensitive
ATGGCTTGGGTCGATCTGACATGA	0	12	RDR2-sensitive
ATGGCTTGTAGTCGGACATGTCAG	2	26	RDR2-sensitive
ATGGCTTTCCTGGACACATGGCAC	0	9	RDR2-sensitive
ATGGGAATGGCAGAACACGGAATG	0	12	RDR2-sensitive
ATGGGACAGCGACCAGCACGACGG	0	15	RDR2-sensitive
ATGGGACATGCATGGGCACGGCAC	0	13	RDR2-sensitive
ATGGGACCAGTGGAAGCGGACGCA	14	0	RDR2-resistant
ATGGGACCGTAGTAAAGCCTCGGA	0	9	RDR2-sensitive
ATGGGACCTTCGACGACGAAGCAT	0	12	RDR2-sensitive
ATGGGACCTTCGGCGATGAAGCAG	0	9	RDR2-sensitive
ATGGGACTGAGGGCATACTTGCAA	0	9	RDR2-sensitive
ATGGGACTGGATATGCGGGCGCAG	0	10	RDR2-sensitive
ATGGGAGGCAGAACGGCAGAACGC	1	14	RDR2-sensitive
ATGGGAGGCTGTAGTGAGGCTTCA	0	15	RDR2-sensitive
ATGGGAGTGGCACGAGAGGATGGT	0	9	RDR2-sensitive
ATGGGATGTTGTTGAGTATGGTGT	31	2	RDR2-resistant
ATGGGATTGCGGCATTTTTCTCTA	59	0	RDR2-resistant
ATGGGCAACAGAGAACATACCCAC	0	12	RDR2-sensitive
ATGGGCAGAACGCGGAATGGCCGA	0	11	RDR2-sensitive
ATGGGCATAGATATTTTTCACGGG	0	21	RDR2-sensitive
ATGGGCCGGCACTAAGCACGACAC	0	15	RDR2-sensitive
ATGGGCGAACCCTCAGTGGACTTC	15	0	RDR2-resistant
ATGGGCGACCGAACTCTCCGGCTA	21	1	RDR2-resistant
ATGGGCTAAACGGGCTAAACGGGC	0	10	RDR2-sensitive
ATGGGCTACGATACCGCTGTGTAT	0	14	RDR2-sensitive
ATGGGCTGACCGGGCTACAGGCAC	0	30	RDR2-sensitive
ATGGGCTGATCGGGCTACAGGCAC	0	17	RDR2-sensitive
ATGGGCTGCGCGCGGTAGAATGAA	0	9	RDR2-sensitive
ATGGGCTGGATCTGGTTCGGGCTG	0	16	RDR2-sensitive
ATGGGCTGTCTCCAGTGGACCCAT	0	15	RDR2-sensitive
ATGGGCTGTCTGATAGGCTTCGGC	2	101	RDR2-sensitive
ATGGGGATGTAAGCGGAGACTCAT	0	10	RDR2-sensitive
ATGGGGCACTGTAGGAGGCACCGC	0	9	RDR2-sensitive
ATGGGGCGGCATGGACACATTCTC	29	0	RDR2-resistant
ATGGGGCTGTAGGGGGCACTCCAC	2	33	RDR2-sensitive
ATGGGGGCTGTAGGGGGCACTCCA	1	19	RDR2-sensitive
ATGGGTCCGATGGCATACTGACGA	0	20	RDR2-sensitive
ATGGGTCCGATGGCATACTGGCGA	0	31	RDR2-sensitive
ATGGGTCGGCGCGAGACGGAACAC	2	19	RDR2-sensitive
ATGGGTCGGCGCGAGACGGAGCAC	3	38	RDR2-sensitive
ATGGGTCGGCGCGAGACGGAGCGC	0	17	RDR2-sensitive
ATGGGTCGGCTCGAAGAACGGCTC	0	12	RDR2-sensitive

ATGGGTCGGGCTGGGACCAGAGGA	0	12	RDR2-sensitive
ATGGGTCGGGCTTAGACCGGCATA	0	13	RDR2-sensitive
ATGGGTCGTGTCGGATCGGAGGCA	0	13	RDR2-sensitive
ATGGGTCGTGTCTGGCGAGCCGGC	0	13	RDR2-sensitive
ATGGGTCTGCACCGGTTGAGACGC	0	11	RDR2-sensitive
ATGGGTGAAGGCGGAACGTTCTAA	0	11	RDR2-sensitive
ATGGGTTGGCTTTGGCATCGTCGT	0	12	RDR2-sensitive
ATGGGTTGTATATGGTTGACAGGT	23	2	RDR2-resistant
ATGGTAATGGGTTGTATATGGTTG	44	0	RDR2-resistant
ATGGTACACTACACATGGTCGGAC	0	15	RDR2-sensitive
ATGGTACGTGTAGAAGCATGTCGG	0	11	RDR2-sensitive
ATGGTACGTGTAGGAGCATGTCGG	0	16	RDR2-sensitive
ATGGTAGAATAAGCGGGCGGGCTT	0	14	RDR2-sensitive
ATGGTAGACGTGAGCAGCATGAGT	0	9	RDR2-sensitive
ATGGTAGACTGATTGATGATTGCA	83	1	RDR2-resistant
ATGGTAGACTGTACGGTGACCTAC	0	21	RDR2-sensitive
ATGGTAGAGGAGGCAGTAGAGTGA	0	11	RDR2-sensitive
ATGGTAGATTCAGATGAAGACTGT	44	1	RDR2-resistant
ATGGTAGGGCTGACGCGGTGCCGA	0	12	RDR2-sensitive
ATGGTCAAACGGACGATTCGGCAC	0	15	RDR2-sensitive
ATGGTCAAGTTACATGTCGGATAA	1	48	RDR2-sensitive
ATGGTCACAGGCAGGAACTTAGGC	0	15	RDR2-sensitive
ATGGTCAGGAGATATAGCAATTTT	15	0	RDR2-resistant
ATGGTCATAAAATGTGAAGTCGTG	0	11	RDR2-sensitive
ATGGTCCCGTTAGGCCCGTCGGGC	0	9	RDR2-sensitive
ATGGTCTGGCTGTAGCTTGCAGGA	0	21	RDR2-sensitive
ATGGTCTGTTTAGAGTGAGGCTAG	0	69	RDR2-sensitive
ATGGTCTTTTGTTTCAGGTGTGAC	15	0	RDR2-resistant
ATGGTGACCTCGACAAGCAGCTTG	49	2	RDR2-resistant
ATGGTGAGCTTTGGGCAGACTGGT	0	13	RDR2-sensitive
ATGGTGAGGATGACTGGTGACGCA	0	13	RDR2-sensitive
ATGGTGAGGATGAGGATGACGGGT	0	21	RDR2-sensitive
ATGGTGATTTGGATTCGAAATCGG	17	1	RDR2-resistant
ATGGTGCACGATATGGACGTCTAC	0	9	RDR2-sensitive
ATGGTGGAGCCGATCAGTGACGTG	18	0	RDR2-resistant
ATGGTGGCAGAACACGGGAATGAC	0	10	RDR2-sensitive
ATGGTGTCAAGCGGCGTATCCTAT	15	0	RDR2-resistant
ATGGTTAACTCGGAAGATGCTGCT	0	11	RDR2-sensitive
ATGGTTACTGTTTGATATCCACGG	4	46	RDR2-sensitive
ATGGTTAGGTGCAGTAGATGTGTA	82	7	RDR2-resistant
ATGGTTATGTGACTGATTATGGTG	416	40	RDR2-resistant
ATGGTTCGATGACATACTGACGAA	0	10	RDR2-sensitive
ATGGTTCGGCTGCTTCGATGGCAA	0	36	RDR2-sensitive
ATGGTTCGGTCAATGCAACGGATG	31	0	RDR2-resistant
ATGGTTCGTTCCGGTGATTGACGT	0	12	RDR2-sensitive
ATGGTTGACTTAGAATCGATAGGC	0	21	RDR2-sensitive
ATGGTTGAGGATCTAGACGACTAT	52	0	RDR2-resistant
ATGGTTGATGGTGTGACTTGGCCT	1	13	RDR2-sensitive
ATGGTTGCGGAGGATCGTTGTCGT	0	13	RDR2-sensitive

ATGGTTGGTCGCTATAGATGTCGT	0	18	RDR2-sensitive
ATGGTTGTAGGCCTGACCACGGAC	0	11	RDR2-sensitive
ATGGTTGTGGAGGCTACGCAGTAC	0	17	RDR2-sensitive
ATGGTTGTTGTTTCATTCGCGGTT	41	0	RDR2-resistant
ATGGTTTCGTGTTAGCTTCAGGCA	20	1	RDR2-resistant
ATGGTTTTGTAGATAGCATGGTAT	0	16	RDR2-sensitive
ATGTAAACTTTGTAGCAGAGCTGC	0	17	RDR2-sensitive
ATGTAAATCTTTCAACGGGACTGC	1	16	RDR2-sensitive
ATGTAAGTGAGATAGTAATGGTAT	0	12	RDR2-sensitive
ATGTACAATTTATGCTCTGTCGGT	0	12	RDR2-sensitive
ATGTACCGTAAAGGCACGCGTCGG	0	23	RDR2-sensitive
ATGTACCTGTAGTACTTGTGCAGA	37	5	RDR2-resistant
ATGTACGAGGGAACTTCTACCGGC	0	18	RDR2-sensitive
ATGTACGCGAGGAACGATGGCTGC	0	28	RDR2-sensitive
ATGTACGCTCTGCTGAATATGGTC	0	13	RDR2-sensitive
ATGTAGAACAGGACTGTCTGCGGC	0	17	RDR2-sensitive
ATGTAGAATAATGATGGATATTGG	3	16	RDR2-sensitive
ATGTAGAATGACAGAACGCGGAAT	0	9	RDR2-sensitive
ATGTAGAATTTGTATGCAAGCAAG	251	20	RDR2-resistant
ATGTAGACTCCAGAAGCCGTTCCG	0	10	RDR2-sensitive
ATGTAGACTCCTATGGCGTTGTCA	102	4	RDR2-resistant
ATGTAGAGCTGTACGGGCATCAAC	0	17	RDR2-sensitive
ATGTAGAGGAGTAGCACATCCGGT	0	12	RDR2-sensitive
ATGTAGAGGGCGCTGTATGGGTAC	0	9	RDR2-sensitive
ATGTAGAGTGCATCAGACGCCGGA	78	1	RDR2-resistant
ATGTAGCGGCTGAAGCAGAGACTC	1	29	RDR2-sensitive
ATGTAGCTGGTGGTGGATGGACAT	0	20	RDR2-sensitive
ATGTAGGACAACGTCGTTGATGAT	0	9	RDR2-sensitive
ATGTAGGACCGGATGTAGGGACGT	0	12	RDR2-sensitive
ATGTAGGACTGCTTCCAGAGGTCG	0	12	RDR2-sensitive
ATGTAGGACTGTGCAGGCCCGGCC	1	19	RDR2-sensitive
ATGTAGGCAGTGGCGGAGCTCCGT	0	10	RDR2-sensitive
ATGTAGGCCTCCGAGTTGGACGAG	0	11	RDR2-sensitive
ATGTAGTGGCTCAGGACAAGACGA	0	23	RDR2-sensitive
ATGTAGTTCTCCGGCGGCGTCTAG	0	9	RDR2-sensitive
ATGTAGTTGCACGGGCTTTTGAAC	14	0	RDR2-resistant
ATGTAGTTGGTAGACGGTTCTCAT	0	62	RDR2-sensitive
ATGTATCAACTCTGTCTTGAGCAT	0	10	RDR2-sensitive
ATGTATTGGGTGGGATTGAGCCGG	0	15	RDR2-sensitive
ATGTATTTTCTGTGATCTTCGGGC	0	9	RDR2-sensitive
ATGTCAAGTTACTGCGGTATTCAA	93	13	RDR2-resistant
ATGTCACTCTCTCGTATCGTCGTC	0	9	RDR2-sensitive
ATGTCAGACTGACCTAGATTGGCC	0	26	RDR2-sensitive
ATGTCAGAGGCAGAACGGCAGACT	0	11	RDR2-sensitive
ATGTCAGAGGTAGGCTACTATTGC	0	11	RDR2-sensitive
ATGTCAGTACTTGTGGTTGTTGGA	1	33	RDR2-sensitive
ATGTCATATTAGATGTGTCGGCGT	76	2	RDR2-resistant
ATGTCATCCGACGGCATGTCTACT	159	0	RDR2-resistant
ATGTCATGTTGAGCTTAGATGGTT	0	43	RDR2-sensitive

ATGTCCAACGACTGTTAGACGGGC	1	28	RDR2-sensitive
ATGTCCAATAGGTCGTGTCGGCAT	0	17	RDR2-sensitive
ATGTCCACTGTAGAGGCATACTGG	0	11	RDR2-sensitive
ATGTCCGACGGCATCGTAGACAGC	4	177	RDR2-sensitive
ATGTCCGACGGCATTAACCTATCT	1	12	RDR2-sensitive
ATGTCCGACGGCATTAACTTATCT	1	40	RDR2-sensitive
ATGTCCGACGGCCCTGTACGAGGC	1	18	RDR2-sensitive
ATGTCCGACGGCCTGCGCTTATGT	0	9	RDR2-sensitive
ATGTCCGACGGCTACGCTGACAGC	0	29	RDR2-sensitive
ATGTCCGACGGCTGAAGGCTGGTA	0	12	RDR2-sensitive
ATGTCCGACGGCTGCCTAGGAGAC	0	14	RDR2-sensitive
ATGTCCGACGGCTGTAATACGGGC	0	12	RDR2-sensitive
ATGTCCGACGGCTGTCAGACGGGC	0	13	RDR2-sensitive
ATGTCCGACGGCTGTCAGATGGGC	0	18	RDR2-sensitive
ATGTCCGACGGCTGTCTTGGGCCC	0	9	RDR2-sensitive
ATGTCCGACGGTCCTGTAGGAGGC	4	195	RDR2-sensitive
ATGTCCGACGGTTCTGTACGAGGC	0	17	RDR2-sensitive
ATGTCCGACGGTTGCCTATGGGGC	0	11	RDR2-sensitive
ATGTCCGATGGCTGTCAGACAGGC	0	13	RDR2-sensitive
ATGTCCGGAATGAGTGAAAGGGCT	0	23	RDR2-sensitive
ATGTCCTCCGCATGTCTTGACTGT	0	9	RDR2-sensitive
ATGTCGAGTCTGGGCTTTTCGGCA	0	13	RDR2-sensitive
ATGTCGCGTAGGACAGTTCGGCAA	4	61	RDR2-sensitive
ATGTCGCGTAGGACAGTTCGGCTA	1	39	RDR2-sensitive
ATGTCGCGTAGGACAGTTCGGCTT	0	34	RDR2-sensitive
ATGTCGGCTGTAGGGGCACCCTAT	0	14	RDR2-sensitive
ATGTCGGCTGTAGGGGCATCCTAT	0	14	RDR2-sensitive
ATGTCGGCTGTAGGGGGCACCCTA	0	22	RDR2-sensitive
ATGTCGTACGGGCACACAGAGCGG	16	0	RDR2-resistant
ATGTCGTACTGGACGGTCCTATAT	0	15	RDR2-sensitive
ATGTCGTGACCCATGGGCGGGCAT	0	16	RDR2-sensitive
ATGTCGTGAGTTTCTGGTGGTCAT	0	9	RDR2-sensitive
ATGTCGTGCGTAGTGTCGGCCCAT	0	26	RDR2-sensitive
ATGTCTACTGTACTGGCACTCGGC	1	23	RDR2-sensitive
ATGTCTAGACTGTTGGGATGGCAA	0	9	RDR2-sensitive
ATGTCTAGATCGTGCCGGACCGGC	0	10	RDR2-sensitive
ATGTCTAGATCGTGCCGGGCCGGC	0	22	RDR2-sensitive
ATGTCTAGATTGTATCGGTGCGTG	0	18	RDR2-sensitive
ATGTCTAGCATGTCGTCCTCGGAT	108	7	RDR2-resistant
ATGTCTATAGGCTCTCTAAGGCGC	25	4	RDR2-resistant
ATGTCTGACGGCTGTGAGACGGGC	0	12	RDR2-sensitive
ATGTCTGCTAGACGGTTGGCCCTG	1	18	RDR2-sensitive
ATGTCTGGACCAGGTTCGACGGAT	0	14	RDR2-sensitive
ATGTCTGGACCGATGTATAGGCAC	0	33	RDR2-sensitive
ATGTCTGGGCCTAGGGACCGGCAC	0	53	RDR2-sensitive
ATGTCTGTGTGCTTTGTACGGCTG	0	26	RDR2-sensitive
ATGTCTGTTGACCTGTTCTGACGG	0	21	RDR2-sensitive
ATGTCTGTTGGATGGTTTGATAGA	75	0	RDR2-resistant
ATGTCTGTTTATTTTGGTCTTCTT	37	0	RDR2-resistant

ATGTCTTCAGTTAGCGTGTCGGTA	48	0	RDR2-resistant
ATGTCTTGACGTGTGGGATGGCAC	0	34	RDR2-sensitive
ATGTCTTGTATGAATTCCACGGCC	72	13	RDR2-resistant
ATGTCTTTTACAAATCGTTTGGCA	0	15	RDR2-sensitive
ATGTGAACTACTGGACACGGTATA	1	14	RDR2-sensitive
ATGTGAACTCTTGGTTTTGATGGT	0	11	RDR2-sensitive
ATGTGACCTGGTCCAGGCTGACGC	0	20	RDR2-sensitive
ATGTGACGTAGATGTTGTGGCTAG	0	18	RDR2-sensitive
ATGTGACTGATTCTACAACAGAAG	22	0	RDR2-resistant
ATGTGAGCTAGGACCGCTGAAGAC	0	9	RDR2-sensitive
ATGTGATTTGGCATTCGACTCCTT	33	0	RDR2-resistant
ATGTGCACACGTGGATCGTCCGGC	0	11	RDR2-sensitive
ATGTGCACTATGTAGAAGACCGTA	0	9	RDR2-sensitive
ATGTGCGCCGGGCTGAGCAGGCAG	0	9	RDR2-sensitive
ATGTGCTCCGAATCGGCGTGGCTT	21	1	RDR2-resistant
ATGTGCTGGTTCTGACTCGGGCGG	0	14	RDR2-sensitive
ATGTGGAACCTTTGGAAGGAGCGC	1	29	RDR2-sensitive
ATGTGGAACTGGAGGAGGCGACGC	0	9	RDR2-sensitive
ATGTGGAAGCGTTATACTGTCTGT	0	9	RDR2-sensitive
ATGTGGACCTTTGGTGGCTGTTCT	0	16	RDR2-sensitive
ATGTGGACGCTATGATGCATGGAC	0	20	RDR2-sensitive
ATGTGGACGCTGAGGAGGTGGCTC	23	0	RDR2-resistant
ATGTGGACGTGCATGTGGACGTGC	0	9	RDR2-sensitive
ATGTGGAGACTTGGAGACGTGCGC	0	33	RDR2-sensitive
ATGTGGCACTCGGCAAAGACTGAC	0	10	RDR2-sensitive
ATGTGGCACTGTCGTTTGGGTCAT	33	2	RDR2-resistant
ATGTGGCGCTGACGGGACGTTCTA	0	13	RDR2-sensitive
ATGTGGCTGTACGGACGCGGATGG	0	9	RDR2-sensitive
ATGTGGGACCGTCTGACACGGCAC	0	13	RDR2-sensitive
ATGTGGGCCTGGCACTGAGGACAG	0	15	RDR2-sensitive
ATGTGGGCTGTACTTTAGGGGTGT	0	13	RDR2-sensitive
ATGTGGTTTATTATAGTTGGATAA	36	0	RDR2-resistant
ATGTGTACCACTCTGGGCCGGCGT	1	41	RDR2-sensitive
ATGTGTACGGTCTGGGCTATTAAC	0	9	RDR2-sensitive
ATGTGTAGTCCCTGAACTTGGCAT	0	71	RDR2-sensitive
ATGTGTAGTGTGATGATCTGGGTA	33	0	RDR2-resistant
ATGTGTAGTGTGATGATCTGGGTT	55	0	RDR2-resistant
ATGTGTAGTGTGATGGTCTGGGTA	43	7	RDR2-resistant
ATGTGTAGTGTGATGGTCTGGGTT	55	3	RDR2-resistant
ATGTGTCCAGGATGAAGATAGGTT	39	1	RDR2-resistant
ATGTGTCGTACTGTTATGAGATGG	0	21	RDR2-sensitive
ATGTGTCGTCGCAACGCACGGGCA	0	33	RDR2-sensitive
ATGTGTGGACGAGGTAGCGAAGGC	15	0	RDR2-resistant
ATGTGTTGACTAAGTGACGGACAC	0	29	RDR2-sensitive
ATGTGTTGGACCATCTAAAGGTGA	26	2	RDR2-resistant
ATGTGTTTCGTTGTGATGACTGCA	0	9	RDR2-sensitive
ATGTGTTTGACATGATCTGGGAAA	35	0	RDR2-resistant
ATGTGTTTGGTTGCTTGGATGTGA	0	13	RDR2-sensitive
ATGTGTTTGGTTTCGGAACGACGT	0	21	RDR2-sensitive

ATGTGTTTTTAGGAGTAGGACGGT	0	12	RDR2-sensitive
ATGTTAAAATAGAATTGTTTGGTA	23	0	RDR2-resistant
ATGTTACGTCGGCGCTACAGTTGT	0	12	RDR2-sensitive
ATGTTAGACTGCCTGCAACGGTGC	2	32	RDR2-sensitive
ATGTTCAGACAAGAGGATTGGATA	0	12	RDR2-sensitive
ATGTTCAGTAGATCGTGTGTATAA	0	17	RDR2-sensitive
ATGTTCCCAGGCTATTTTCGTCGG	0	9	RDR2-sensitive
ATGTTCCGGACTTCAAACGGTGTG	0	15	RDR2-sensitive
ATGTTCGACGACTTTCTTGACGGT	0	32	RDR2-sensitive
ATGTTCGAGACAGGAGACTAGCTG	1	24	RDR2-sensitive
ATGTTCGATGACTGGCATGTAAGC	0	11	RDR2-sensitive
ATGTTCGATGACTTATCTGACGGC	0	36	RDR2-sensitive
ATGTTCGGACGCTCAGAAAGGCTT	144	13	RDR2-resistant
ATGTTCGTCGCTGGAGGGGGGCCT	2	21	RDR2-sensitive
ATGTTCGTTCGGAGCATGAGGCTA	0	16	RDR2-sensitive
ATGTTCTGTGTGATATAATGGCAT	0	9	RDR2-sensitive
ATGTTCTTAGAACACATGTTGGTA	44	1	RDR2-resistant
ATGTTCTTGATTGGTCGGTCAAAC	46	0	RDR2-resistant
ATGTTGAAGCTCTGCATTGACGTC	219	0	RDR2-resistant
ATGTTGACATGGAAAGTTTGAGGC	44	0	RDR2-resistant
ATGTTGCGCTGACTGTTGGATGGA	0	10	RDR2-sensitive
ATGTTGCGTTGGATATGTGGGCAT	0	10	RDR2-sensitive
ATGTTGCTGGATTCTCAAGACGTG	40	3	RDR2-resistant
ATGTTGGACGGGCCGGCTCGGTAC	0	9	RDR2-sensitive
ATGTTGGACTAGCGTGCGTGCCGT	0	15	RDR2-sensitive
ATGTTGGACTTGCAGGGTTGGCCT	0	16	RDR2-sensitive
ATGTTGGATAAAATGGCGTGAGAA	90	5	RDR2-resistant
ATGTTGGCTAGAATGTAGAAGGCT	1	33	RDR2-sensitive
ATGTTGGGCTTTTTACCGGTTAGG	28	3	RDR2-resistant
ATGTTGGGGCGAAGGCAAAGACGC	0	15	RDR2-sensitive
ATGTTGTACTAGATAGGCATGTCC	19	0	RDR2-resistant
ATGTTGTCGGTGCGGAGAGGCAGA	0	10	RDR2-sensitive
ATGTTGTCTGGCTCGAGGTGCAGA	17	0	RDR2-resistant
ATGTTGTTTTACATCACAGTCGGT	2	20	RDR2-sensitive
ATGTTTAGAGCATTTTTTGTAGGA	14	0	RDR2-resistant
ATGTTTAGAGTTCGTCGTACGTCC	0	15	RDR2-sensitive
ATGTTTATGAACTGTAGGTGCATG	0	9	RDR2-sensitive
ATGTTTCTTTCGTACTGTCGGCGT	0	9	RDR2-sensitive
ATGTTTGAATGTATGTAATGGCAC	0	16	RDR2-sensitive
ATGTTTGACGATGGACAGCGCGGT	0	9	RDR2-sensitive
ATGTTTGACTGTCGCGTGCCTTTG	34	5	RDR2-resistant
ATGTTTGGACTGACACCTACGGCG	0	9	RDR2-sensitive
ATGTTTGGATGCTTGGATGGTCAC	0	16	RDR2-sensitive
ATGTTTGGGCTCAGACTTCGGCTC	0	14	RDR2-sensitive
ATGTTTGGGTCGACGGCCAGGCAC	0	34	RDR2-sensitive
ATGTTTGGTTGACTGTACATGGTA	0	26	RDR2-sensitive
ATGTTTGGTTGACTGTACGTGGTA	0	64	RDR2-sensitive
ATGTTTGGTTGACTGTATTTGGTT	0	44	RDR2-sensitive
ATGTTTGTGACGGACGAGTTGGCT	0	14	RDR2-sensitive

ATGTTTGTGCATTCTGTAGGCGGT	1	13	RDR2-sensitive
ATGTTTGTTCTGTAGATCCGTGCG	1	25	RDR2-sensitive
ATGTTTGTTGGCTAGATTTGCGGA	23	73	RDR2-sensitive
ATGTTTTAGGATGTCGGAAACGGT	0	11	RDR2-sensitive
ATGTTTTCGTTGGGCTGTGACGGT	0	9	RDR2-sensitive
ATGTTTTCTGAACAGGTCTGGCAC	0	85	RDR2-sensitive
ATGTTTTTTGACTGTATGTCGGGT	0	12	RDR2-sensitive
ATTAAACGGATCGTGCTTCGGGCC	0	35	RDR2-sensitive
ATTAAAGGTTTTTATTCGGACAAA	25	3	RDR2-resistant
ATTAAAGTTTATGGTCGCTTCGGC	0	21	RDR2-sensitive
ATTAAATAGGTCGTGGATTAGCAT	0	10	RDR2-sensitive
ATTAACGGCAGAACGGCAGAACGG	0	17	RDR2-sensitive
ATTAACGGGCTGGACCGACACGGC	0	9	RDR2-sensitive
ATTAACGGGCTTAGAGTTAAACGG	0	9	RDR2-sensitive
ATTAACTATGTCGGGCCGGCCCAT	0	10	RDR2-sensitive
ATTAACTGTCCACTCTGCAACGGC	0	10	RDR2-sensitive
ATTAAGACTGGGCATATTACTCGC	0	12	RDR2-sensitive
ATTAAGACTGTAAGGGGTAGGCTG	0	15	RDR2-sensitive
ATTAAGACTGTAAGGGTAGCCTAC	0	14	RDR2-sensitive
ATTAAGGATGGACTTGGCTTCAAG	20	2	RDR2-resistant
ATTAAGGCTGTAAGAGTAGTCGGC	0	17	RDR2-sensitive
ATTAAGGCTGTAAGGGGTAGCATG	0	10	RDR2-sensitive
ATTAAGGCTGTAAGGGGTAGGCTG	0	14	RDR2-sensitive
ATTAATAGGGACAGTCGGGGGCAT	28	1	RDR2-resistant
ATTAATTGGATATTCGGATTCGGA	23	0	RDR2-resistant
ATTACACTCTGTAGGAAATGCATA	0	10	RDR2-sensitive
ATTACATGTGGAACAGCACGTGCG	0	14	RDR2-sensitive
ATTACCTCGGCTGACTGACGGTTC	0	64	RDR2-sensitive
ATTACGCTGATAGAAAGATTGTCC	15	0	RDR2-resistant
ATTACTATGTGATGTGGACCTTAT	30	0	RDR2-resistant
ATTACTGTTGAAGACATATTTGCT	55	1	RDR2-resistant
ATTACTTGAACATGGTCGACGGCA	0	9	RDR2-sensitive
ATTACTTGGGCTGCATATGGAGAT	15	0	RDR2-resistant
ATTAGAATTTATGGTCGCTTCGGC	0	15	RDR2-sensitive
ATTAGAATTTATGGTCGTTTCGGC	0	14	RDR2-sensitive
ATTAGACATACTGATAGATGGCGC	0	13	RDR2-sensitive
ATTAGACGGAACCGGGCAGAACGC	0	10	RDR2-sensitive
ATTAGACGGATGGATTGGATGCAT	0	11	RDR2-sensitive
ATTAGACTTACTGATAGATGGCAC	2	18	RDR2-sensitive
ATTAGACTTACTGATCGATGGCAC	6	79	RDR2-sensitive
ATTAGACTTACTGATTGTTGGCGC	4	46	RDR2-sensitive
ATTAGAGTTTACGGTCGTTTCGGC	0	31	RDR2-sensitive
ATTAGAGTTTACGGTCGTTTCGGT	0	12	RDR2-sensitive
ATTAGAGTTTATGATCGCTTCGGC	0	26	RDR2-sensitive
ATTAGAGTTTATGATCGTTTCGGC	0	26	RDR2-sensitive
ATTAGAGTTTATGGTCGCTTCGGC	0	12	RDR2-sensitive
ATTAGAGTTTATGGTCGTTTCGGT	1	25	RDR2-sensitive
ATTAGCAACGGACGGTCGGTCGCT	5	33	RDR2-sensitive
ATTAGCACCTGCAGAACATCGGCG	1	13	RDR2-sensitive

ATTAGCATTGGTGGTAGTAAGTAT	23	1	RDR2-resistant
ATTAGCGTCAGGAGGTCGGCCCAC	21	0	RDR2-resistant
ATTAGCTATCGGACGTAACAGGCC	0	11	RDR2-sensitive
ATTAGCTATGATGTTTGGATGTTT	30	0	RDR2-resistant
ATTAGGACGCGCGAATTACAGTAT	0	13	RDR2-sensitive
ATTAGGATTCCTGTAGTGTTGGCA	0	13	RDR2-sensitive
ATTAGGGGACGGGTATGGGACTAA	0	13	RDR2-sensitive
ATTAGGGGGACTGTAGTGAGGCTC	0	13	RDR2-sensitive
ATTAGGGTAGAATACAGGATGAGC	0	10	RDR2-sensitive
ATTAGGTTGTCAGGTTGGATGGAT	0	9	RDR2-sensitive
ATTAGTAAAATTGGTGCGTCGTTC	15	0	RDR2-resistant
ATTAGTCTCTGTAGAGTGGCAGAT	46	0	RDR2-resistant
ATTAGTGACGCGCATGAATGGATT	42	1	RDR2-resistant
ATTAGTGGGATTCTACCTTGGCAT	0	12	RDR2-sensitive
ATTAGTTGTCGTTTTGGATAAGGT	4	20	RDR2-sensitive
ATTATAACAGTCGTCTGAATCCTC	15	0	RDR2-resistant
ATTATAGACTGTAGTGTGGTGTCT	0	12	RDR2-sensitive
ATTATAGATCCATGTAGTAGCATT	29	1	RDR2-resistant
ATTATATCGATTGTTAGCAGTATG	21	0	RDR2-resistant
ATTATATGCATTCTGTAGGAAGAC	25	2	RDR2-resistant
ATTATCGTGCGGAGGTATAGATGG	0	16	RDR2-sensitive
ATTATCGTTGAACTGTTAGTCGGT	0	15	RDR2-sensitive
ATTATCTATGATCGTGGATATGAC	0	11	RDR2-sensitive
ATTATGGTTTTGTCGACATTATGA	19	0	RDR2-resistant
ATTATGTTGCGACAGACTGGCTGT	0	12	RDR2-sensitive
ATTATTCAAGGACTGTTCAGGCAA	177	3	RDR2-resistant
ATTATTGTGGTCTGTAATGAGGTT	0	10	RDR2-sensitive
ATTATTTCAACATGGTTGTTCGGA	54	2	RDR2-resistant
ATTATTTGATCGGATATCGGATAC	3	48	RDR2-sensitive
ATTATTTTGTTGTAGATAAATGGC	49	4	RDR2-resistant
ATTCAAATTGGACTTTAAAAGCAT	265	24	RDR2-resistant
ATTCAACTGGAAGATGCATCTGCC	0	14	RDR2-sensitive
ATTCAACTTGAAGATGCATCTGCC	0	16	RDR2-sensitive
ATTCAAGGACTGTTCAGGCAAGGA	26	3	RDR2-resistant
ATTCAATATGTTATCTTGTCGGAT	55	6	RDR2-resistant
ATTCAATCGGTAGGAGCGACGGGC	3	37	RDR2-sensitive
ATTCACAAGTGGAGGACCCTAGAC	36	1	RDR2-resistant
ATTCACACAATTGGTCATCGCGCT	18	0	RDR2-resistant
ATTCACGCAGGGACGGTAGAGCAA	0	33	RDR2-sensitive
ATTCACGGACCGGGTACAAGCCAT	1	39	RDR2-sensitive
ATTCACGGACCGGGTACTATCCAT	0	12	RDR2-sensitive
ATTCACGTGACTGTATTTGAGGAT	0	9	RDR2-sensitive
ATTCACTGTAGCGGCATGCATCTT	0	34	RDR2-sensitive
ATTCAGACTGTCCGGTATGACACC	0	11	RDR2-sensitive
ATTCAGAGACGGTTAGATTTTGCT	20	1	RDR2-resistant
ATTCAGATATCTGTCGGATCGGAT	0	12	RDR2-sensitive
ATTCAGCCTGTTGATGATCTCGTT	1	38	RDR2-sensitive
ATTCAGCGTCGGCTTGGCCATCAC	14	0	RDR2-resistant
ATTCATATACTTATCCGTCGGATC	0	15	RDR2-sensitive

ATTCATATCGGGCCGAAACGGGTT	0	14	RDR2-sensitive
ATTCATCTGAGATTGATTGTAGCT	16	0	RDR2-resistant
ATTCATGCCGGGCTCGGATCAGGC	0	10	RDR2-sensitive
ATTCATGCTGTTAGATCGCGGACC	0	48	RDR2-sensitive
ATTCATGGACTGAACGTAGATGGA	0	67	RDR2-sensitive
ATTCATGTCGGGCCGGCACGATCC	0	9	RDR2-sensitive
ATTCATGTCGTACTGGACGGTCCT	0	36	RDR2-sensitive
ATTCATTTTTATCTTCTGACGCT	39	0	RDR2-resistant
ATTCCAGGTCTGTGGGCAAGACGG	0	9	RDR2-sensitive
ATTCCATCAGTTATCTTGTCGGAT	21	0	RDR2-resistant
ATTCCATCGGTGCTGCAGTGGCGT	1	22	RDR2-sensitive
ATTCCCGTCGGCATGTGTCAGGGC	0	9	RDR2-sensitive
ATTCCTCGGATGTAGTTGGTGGCC	0	13	RDR2-sensitive
ATTCCTGGGACTCGACAAGCTGGC	0	10	RDR2-sensitive
ATTCCTGTAGCAGCCATGGACGGA	0	9	RDR2-sensitive
ATTCCTGTAGTGTTGGCACGGCTT	0	12	RDR2-sensitive
ATTCGAAATCGGAATGCAGCGGAG	21	0	RDR2-resistant
ATTCGAACGACTAACCTTAGGCAA	0	141	RDR2-sensitive
ATTCGAACGACTAACTTTAGGCAA	1	13	RDR2-sensitive
ATTCGAACGACTAATCTTAGGCAA	0	11	RDR2-sensitive
ATTCGAACGACTAATTTTAGGCAA	0	10	RDR2-sensitive
ATTCGAAGGTTACTGGCATTGTGA	17	0	RDR2-resistant
ATTCGAATGTTATTTTCGTCGGCC	0	20	RDR2-sensitive
ATTCGACCTTTTTACTCTACGGCC	0	10	RDR2-sensitive
ATTCGAGAGAGTAGAATTGCGGAT	0	9	RDR2-sensitive
ATTCGAGGCGGATTAAGGCATCCT	15	0	RDR2-resistant
ATTCGAGTCAGCTGTTGAAACGGT	1	24	RDR2-sensitive
ATTCGATGGCTTGTTGACGGTTAT	1	14	RDR2-sensitive
ATTCGCAATAGGGCACTGTGGCAT	0	10	RDR2-sensitive
ATTCGCGGGTATAGTCACGGACGG	0	13	RDR2-sensitive
ATTCGCGTATAGCGTTGGACAGTC	0	11	RDR2-sensitive
ATTCGCTGTAGATGGGCCGCGACC	0	10	RDR2-sensitive
ATTCGCTTCGGATTAAAGCCGGTT	0	13	RDR2-sensitive
ATTCGGAACCCAGAACACGCGCAC	0	32	RDR2-sensitive
ATTCGGACCGGGCTCGGACCGGAC	0	15	RDR2-sensitive
ATTCGGACGACTAACCTTAGGCAA	2	27	RDR2-sensitive
ATTCGGACTATGCGGGGTGCAAAG	20	0	RDR2-resistant
ATTCGGAGTGTGGAAGATGGTTCT	0	10	RDR2-sensitive
ATTCGGATTAAATTTATGATGGCA	1	19	RDR2-sensitive
ATTCGGATTTCGGTTACCCATTGC	0	9	RDR2-sensitive
ATTCGGCAAGAACCCACGGGTCAT	0	15	RDR2-sensitive
ATTCGGCGAGAACCCACGGGTCAT	2	48	RDR2-sensitive
ATTCGGCTAATGTAGGTAGGGCGC	0	24	RDR2-sensitive
ATTCGGCTTGCTCTCTAAGCAGAA	0	10	RDR2-sensitive
ATTCGGGACGGATACGGGTAATAC	0	11	RDR2-sensitive
ATTCGGGCGGATAAGAGACGGATA	0	24	RDR2-sensitive
ATTCGGTCTATTAGGGTAATTCGG	0	45	RDR2-sensitive
ATTCGGTCTATTCGGGTTCGGGTT	0	10	RDR2-sensitive
ATTCGGTCTATTCGGGTTCGGTTC	0	26	RDR2-sensitive

ATTCGGTTCTGTTGCAACACACGG	0	23	RDR2-sensitive
ATTCGTACAGTAGGAAGTGAGGCG	0	15	RDR2-sensitive
ATTCGTACAGTGGGAAGTGAGGCG	0	27	RDR2-sensitive
ATTCGTACCGGACCGAAACGGGTT	0	30	RDR2-sensitive
ATTCGTACCGGGCCGAAACAGGTT	0	10	RDR2-sensitive
ATTCGTACCGGGCCGAAACGGGTT	0	55	RDR2-sensitive
ATTCGTACCGGGCTGAAACAGGTT	0	17	RDR2-sensitive
ATTCGTACTGGACCGAAACGGATT	0	9	RDR2-sensitive
ATTCGTACTGGGCTGAAACGGGTT	0	13	RDR2-sensitive
ATTCGTAGCGTGGGCTGTTGAGGC	0	13	RDR2-sensitive
ATTCGTATCGGGCTGAAACGGGTT	0	65	RDR2-sensitive
ATTCGTATCTCTGTAGCCAGGCTC	0	13	RDR2-sensitive
ATTCGTATGTAGGGATAAAAACGG	0	9	RDR2-sensitive
ATTCGTCAACAGATAGATGGGCTC	0	17	RDR2-sensitive
ATTCGTCCTGTAGATCTGTGCGGC	0	16	RDR2-sensitive
ATTCGTCGAGTGTTTTATAGGCTT	0	11	RDR2-sensitive
ATTCGTGCCGGGCTGGTCTAGGCC	0	10	RDR2-sensitive
ATTCGTGTCAGCTGTGGAAACGGT	6	55	RDR2-sensitive
ATTCGTGTCAGCTGTTGAAACGGT	8	66	RDR2-sensitive
ATTCGTGTCGGGCCAGTCCGGGCT	0	10	RDR2-sensitive
ATTCGTGTCGGGCCTAATCGTGCC	0	14	RDR2-sensitive
ATTCGTGTCTGCTGTGGAAACGGT	0	47	RDR2-sensitive
ATTCGTTCTGTATCCAACGGTGTT	0	10	RDR2-sensitive
ATTCGTTGAGCCAGATGATGACTG	33	2	RDR2-resistant
ATTCGTTGCTACTGTATAAAGACG	0	10	RDR2-sensitive
ATTCGTTGGGTAGACAGGTACTAG	0	11	RDR2-sensitive
ATTCGTTGGTCGTTAAAATTAATA	17	0	RDR2-resistant
ATTCGTTGGTTGTTGCATATTGGT	0	16	RDR2-sensitive
ATTCGTTGTGGCTTACTGCGGCTT	0	29	RDR2-sensitive
ATTCGTTTACTTGAATGGGGACGC	14	0	RDR2-resistant
ATTCGTTTCTGTACCATGAGTCAT	0	11	RDR2-sensitive
ATTCTAAACCGTCGTTGGATGATC	46	0	RDR2-resistant
ATTCTAACGGCTGGTCTCGGTAAC	58	1	RDR2-resistant
ATTCTAAGTCTGTGGGCAAGATGG	10	38	RDR2-sensitive
ATTCTACCGTGCCGGGCTCGGACC	0	14	RDR2-sensitive
ATTCTACCGTGCCGGGTTCGGACC	0	65	RDR2-sensitive
ATTCTATAAGACGGTAGTGTAGAC	0	26	RDR2-sensitive
ATTCTATCTTTAAGTCGGTTTGCA	1	13	RDR2-sensitive
ATTCTATCTTTAAGTCGGTTTGTA	0	10	RDR2-sensitive
ATTCTATGTTTGGCAAGCAGAACG	0	13	RDR2-sensitive
ATTCTATTTGGGTTATCGTGGACA	40	1	RDR2-resistant
ATTCTCACATGGGTCGTGTCGGAT	0	18	RDR2-sensitive
ATTCTCAGACAAATCTCGGCTCAC	0	14	RDR2-sensitive
ATTCTCCGAAGCGGACTGACGGTG	0	12	RDR2-sensitive
ATTCTCTGTATGTAGTTGGTAGCC	0	10	RDR2-sensitive
ATTCTGAAAAGCAGCTGAACTGAT	0	14	RDR2-sensitive
ATTCTGAAATAGAAAAGCTGACGT	0	12	RDR2-sensitive
ATTCTGAACCTCGTGCATTGTAGC	23	0	RDR2-resistant
ATTCTGACGAACTGTTGAGGACGA	0	18	RDR2-sensitive

ATTCTGACTGTCCGTCCATATGGC	1	12	RDR2-sensitive
ATTCTGACTTAGAGGCGTTCAGTC	18	0	RDR2-resistant
ATTCTGAGAAGCAGCTGAACTGAT	0	52	RDR2-sensitive
ATTCTGCTGGGCTCTACACGAAGA	17	0	RDR2-resistant
ATTCTGGAATTGCATATCTTGAAG	17	0	RDR2-resistant
ATTCTGGACAAAGAGTTAAGGTAC	25	0	RDR2-resistant
ATTCTGGACAGTGGATAGAGGTAG	0	9	RDR2-sensitive
ATTCTGGACGCTGATCTGGCGCAT	0	16	RDR2-sensitive
ATTCTGTAACGTGTGAATCGCCTG	0	12	RDR2-sensitive
ATTCTGTACGAATACGTCTGTCGG	0	13	RDR2-sensitive
ATTCTGTCGGGAGCGTGCGGGAGG	0	9	RDR2-sensitive
ATTCTGTCTGATTGGTTCGGTGTG	0	15	RDR2-sensitive
ATTCTGTGGTGTAGTGCTAAACGG	0	25	RDR2-sensitive
ATTCTGTTTGTGGTTCTTTCGGTT	91	7	RDR2-resistant
ATTCTTAAGACGGTAGTGTAGACC	0	15	RDR2-sensitive
ATTCTTATTGGACTTTGTGTGCGG	0	97	RDR2-sensitive
ATTCTTCGGATATAGTTGGTGGAC	0	10	RDR2-sensitive
ATTCTTGCTGCTTGGTCTATCTGG	14	0	RDR2-resistant
ATTCTTGGTTGTCGACTGCATGGC	0	16	RDR2-sensitive
ATTCTTGTGCGCTGGGACGACGGT	0	12	RDR2-sensitive
ATTCTTTTTAGGTGACCTGACAAC	25	3	RDR2-resistant
ATTGAAATCGGTTGAATTCGGTAT	19	0	RDR2-resistant
ATTGAACATTTTACTCGGCGTCAT	0	10	RDR2-sensitive
ATTGAACCTCGGATACGGGTAGGA	0	33	RDR2-sensitive
ATTGAACTCGGTTGAATTCGGTAT	26	1	RDR2-resistant
ATTGAACTCTCACGCATAGCGCAT	0	26	RDR2-sensitive
ATTGAATCGGACCGTGCCCGGCAC	0	35	RDR2-sensitive
ATTGAATCGGACTGTGCCGGATGG	0	13	RDR2-sensitive
ATTGAATCTACGCAGGACTTCGCC	19	0	RDR2-resistant
ATTGACCGGACTTAGATCGGTATG	0	9	RDR2-sensitive
ATTGACGCCTAGGTCTCGGATGCG	0	16	RDR2-sensitive
ATTGACGTCAACTGTATTTAGGGA	0	20	RDR2-sensitive
ATTGACTGAACCCATGGGATGAAC	25	0	RDR2-resistant
ATTGACTGGTGGAGACGAAGGCAT	0	16	RDR2-sensitive
ATTGACTTCGGTACTGACGTCATC	65	0	RDR2-resistant
ATTGAGATGTGACTGTTGGTGGCT	0	9	RDR2-sensitive
ATTGAGGTATTGTAAACTGCGGTA	0	11	RDR2-sensitive
ATTGAGTAAGAAGTGGAAAGATGT	37	1	RDR2-resistant
ATTGAGTTGCAGTAGATGAGATAT	0	10	RDR2-sensitive
ATTGAGTTGTAAGACTGTATGCAT	0	10	RDR2-sensitive
ATTGATAGGACGGTATCGCCGGTG	0	16	RDR2-sensitive
ATTGATCGTTGAGCAAGTCGGTGA	0	12	RDR2-sensitive
ATTGATGCTGCAGAACTAGGCATT	0	21	RDR2-sensitive
ATTGATGGGCGGTACGGTATGGAA	17	0	RDR2-resistant
ATTGATGGTAATCGCTTCTGGACT	25	2	RDR2-resistant
ATTGATTACATAACTGGACGTCAT	0	10	RDR2-sensitive
ATTGATTCTGTAGCAAAGCACGGT	0	22	RDR2-sensitive
ATTGATTGATGGGCTTATATGGTG	14	0	RDR2-resistant
ATTGATTGCATTGCGGGTACGGAC	46	0	RDR2-resistant

ATTGATTGTAGGCTGTGTTGGTTG	0	17	RDR2-sensitive
ATTGATTGTTGATCGATTGTTGGT	0	10	RDR2-sensitive
ATTGATTTTGATTCGATTGTTGGT	0	14	RDR2-sensitive
ATTGATTTTGTAGCAACGCACGGG	0	10	RDR2-sensitive
ATTGATTTTTTGGAGTGGGATGGT	0	12	RDR2-sensitive
ATTGCCACAGATGGTGAGTCGGCA	0	15	RDR2-sensitive
ATTGCCACCGCGGACTGTTCGGAT	0	15	RDR2-sensitive
ATTGCCGCTGTACGACGACGCCGG	0	19	RDR2-sensitive
ATTGCCTGATTGTGATTTTCGGTT	0	9	RDR2-sensitive
ATTGCCTGGTACTGTGATCGGTAG	0	61	RDR2-sensitive
ATTGCCTGTAGAGCGTGTCGGCGA	0	50	RDR2-sensitive
ATTGCGCGAGACAGTGTAAGATGG	0	9	RDR2-sensitive
ATTGCGCGGCGGACTAGCGGCTAG	0	14	RDR2-sensitive
ATTGCGCTGTTCGGAATACCTATT	0	10	RDR2-sensitive
ATTGCTACGGATTGTGAGTCGGCA	0	15	RDR2-sensitive
ATTGCTCTCGGACCGAACGGGCGT	0	40	RDR2-sensitive
ATTGCTGGAGAGCGTAGAAGACTG	0	19	RDR2-sensitive
ATTGCTGGAGGGAGTAGAGGACGT	0	9	RDR2-sensitive
ATTGCTGTAGAAGTGTGCAGGCCG	0	24	RDR2-sensitive
ATTGCTTGGTCGTCGTGCGTGCAT	0	13	RDR2-sensitive
ATTGGAACGATACAGAGAAGAGAC	17	0	RDR2-resistant
ATTGGAACGATACAGAGAAGATTA	56	2	RDR2-resistant
ATTGGAACTGCTGGACGATGGTGT	0	9	RDR2-sensitive
ATTGGAAGCCTGTATGTGGACGGT	0	10	RDR2-sensitive
ATTGGAATTCCAAGGGTCGTTGGC	0	9	RDR2-sensitive
ATTGGACAGAAGATAGCGGGGCGC	0	14	RDR2-sensitive
ATTGGACAGGAAAGCCTGAAACGG	0	16	RDR2-sensitive
ATTGGACCCTGAAACACACGAGAC	19	0	RDR2-resistant
ATTGGACGGATGGGTTGGATACAT	1	23	RDR2-sensitive
ATTGGACGGGATCGAGACGGATTT	0	9	RDR2-sensitive
ATTGGACGGTCCGGATCTATCCGA	0	24	RDR2-sensitive
ATTGGACGGTTGAGATAGTCGGAA	0	14	RDR2-sensitive
ATTGGACTAGTTGATGTTTTGGCA	15	0	RDR2-resistant
ATTGGACTGTCCGGTGTGCCCATC	1	19	RDR2-sensitive
ATTGGACTTTCGGTATTATAACAG	0	38	RDR2-sensitive
ATTGGACTTTGTGTGCGGTGGGCC	0	13	RDR2-sensitive
ATTGGACTTTGTGTGCGGTGGGGC	0	14	RDR2-sensitive
ATTGGACTTTGTGTGCGGTGGGGT	0	13	RDR2-sensitive
ATTGGAGGGACGAGAGAGGACGTC	0	9	RDR2-sensitive
ATTGGAGGGACTAGATCTGCGGTA	0	13	RDR2-sensitive
ATTGGATCGTGCCTGTACCCGGCT	0	9	RDR2-sensitive
ATTGGATGCCTGTATCGGACGCGC	0	12	RDR2-sensitive
ATTGGATGCTTGTTTGTAGATGGA	0	13	RDR2-sensitive
ATTGGATTGTGTGTTGGTGTGTGC	53	3	RDR2-resistant
ATTGGCATGTACTTAGGGCGCTAG	1	16	RDR2-sensitive
ATTGGCGCCGGCACACACATGCTC	0	19	RDR2-sensitive
ATTGGCTCCGGCAGAGAAGCTGCT	0	17	RDR2-sensitive
ATTGGCTCTAGGTATCGATGGCAC	0	19	RDR2-sensitive
ATTGGCTGAGTCTGTGGCAGGCAT	1	26	RDR2-sensitive

ATTGGGACACTATATACACGACGT	14	0	RDR2-resistant
ATTGGGCAGAGACTTCATGATCAT	28	3	RDR2-resistant
ATTGGGGTCGGGGTGATTCGGGTG	20	0	RDR2-resistant
ATTGGGTACTGTAGGACGGCTAAT	0	9	RDR2-sensitive
ATTGGGTCCGTGCTCGTCTGAGGC	17	1	RDR2-resistant
ATTGGTACTGTAGAAGACTAGGTT	0	21	RDR2-sensitive
ATTGGTAGTTTGGTGGCTCGTCGT	0	24	RDR2-sensitive
ATTGGTATTAGTGATGCATGTATG	22	0	RDR2-resistant
ATTGGTCAGTTCGGATCGGGTGGC	0	9	RDR2-sensitive
ATTGGTCATCGCGCTTGGTTGAAA	33	0	RDR2-resistant
ATTGGTCCAGGCTGAGGCTAGCAT	0	9	RDR2-sensitive
ATTGGTCCCCTTAGTATCGTCACA	0	12	RDR2-sensitive
ATTGGTCCGTAGCACCGACGGTTC	0	10	RDR2-sensitive
ATTGGTCGAACTTTGATGTTGCAT	55	0	RDR2-resistant
ATTGGTCGCTGTAGGGGTTACAAG	0	9	RDR2-sensitive
ATTGGTCGGGTCGAGTCAGGTCGG	0	13	RDR2-sensitive
ATTGGTCGGGTCGGGTTGGTCGGA	0	9	RDR2-sensitive
ATTGGTCGGGTTGAAGGAGAGGTG	0	11	RDR2-sensitive
ATTGGTCGTGGTATGTCTTAGGTT	34	0	RDR2-resistant
ATTGGTGAATGTATCAGTAGTCGG	0	9	RDR2-sensitive
ATTGGTGGTAGTAAGTATGGTTTA	22	2	RDR2-resistant
ATTGGTTCGGTAGACACGGTCTAT	0	22	RDR2-sensitive
ATTGGTTGGTACTGTGACCGGCAG	0	9	RDR2-sensitive
ATTGTAATGATCTGTGGAATACAA	0	11	RDR2-sensitive
ATTGTAGAACGGTAGAATGGACGG	0	12	RDR2-sensitive
ATTGTAGACTTAGTAGAAGCAGGT	0	9	RDR2-sensitive
ATTGTAGACTTGATCAACGGAGAC	0	11	RDR2-sensitive
ATTGTAGACTTTCATGAAGGGCAT	4	17	RDR2-sensitive
ATTGTAGAGTGGACGGTTGATACC	0	19	RDR2-sensitive
ATTGTAGAGTTCGTACGAACGGTT	0	11	RDR2-sensitive
ATTGTAGCATTCTGTCGGGAGCGT	0	39	RDR2-sensitive
ATTGTAGGCTGTGTTGGTTGGTTC	0	27	RDR2-sensitive
ATTGTAGGCTTCTCCAGTAGACCG	0	12	RDR2-sensitive
ATTGTAGTCGATTGTAAAGGGCAT	2	26	RDR2-sensitive
ATTGTAGTCTCAAGCGCACGACAT	0	31	RDR2-sensitive
ATTGTAGTCTCTGCACTAGAGTAG	0	24	RDR2-sensitive
ATTGTAGTTGGACGGCGACCGTGT	0	12	RDR2-sensitive
ATTGTCAAATGGGCTGAGCCGGTC	0	9	RDR2-sensitive
ATTGTCACATGTAAGTTTAACGGG	1	12	RDR2-sensitive
ATTGTCAGGCGCCAGAAATCGGTA	0	23	RDR2-sensitive
ATTGTCATGTGACTGTTGGTGGCT	0	12	RDR2-sensitive
ATTGTCCTCTGTTGACTGCGGCTT	0	17	RDR2-sensitive
ATTGTCCTTGTAACCTTGATGGCT	0	13	RDR2-sensitive
ATTGTCGACCCTGCTGATTTCGGT	0	32	RDR2-sensitive
ATTGTCGAGCTTGCTGATGTGGCC	14	0	RDR2-resistant
ATTGTCGCGGGCGGATATTTACCA	0	16	RDR2-sensitive
ATTGTCGCTGTAAGTAGATGTGCC	0	15	RDR2-sensitive
ATTGTCGGTCACTTTCTTCAGGCT	0	10	RDR2-sensitive
ATTGTCTGAATTACTTGCTGAAGA	35	1	RDR2-resistant

ATTGTCTGACGATGATTTCATGGC	35	1	RDR2-resistant
ATTGTCTGGAAATGTGGTTGCATA	14	0	RDR2-resistant
ATTGTCTTACACTGTAGACTGCAT	0	9	RDR2-sensitive
ATTGTCTTTTTCGCTCGGTCACGG	0	12	RDR2-sensitive
ATTGTGCACTGTGAACGCCTACGG	0	14	RDR2-sensitive
ATTGTGGCGAGTAGAAGAGCCCGT	0	9	RDR2-sensitive
ATTGTGGGCAGTGAAGATGGAGAT	42	0	RDR2-resistant
ATTGTGGTATTGTAAACTGCGGTA	0	11	RDR2-sensitive
ATTGTGTCACTGTTACATGATCGT	0	11	RDR2-sensitive
ATTGTGTCGGGCCATGCATTGAGC	0	18	RDR2-sensitive
ATTGTGTCGTTGTAGTTAAAAGCA	51	4	RDR2-resistant
ATTGTGTGTCGTCGGTTTTGCATC	18	0	RDR2-resistant
ATTGTTAGGACCATGCTTCGTCGT	0	19	RDR2-sensitive
ATTGTTAGTCGGTATACATTGCCA	0	12	RDR2-sensitive
ATTGTTAGTCTGTGGGATGTGGCT	0	18	RDR2-sensitive
ATTGTTCCAGGAGATGAGATGACA	14	0	RDR2-resistant
ATTGTTGAATTTTTTTCGCTTGGA	36	0	RDR2-resistant
ATTGTTGACTGTGAAGCCGGAGAT	32	3	RDR2-resistant
ATTGTTGATGTGGAACAATCGGGA	20	0	RDR2-resistant
ATTGTTGGCTAGTTTAATGGCGCT	57	7	RDR2-resistant
ATTGTTGGGACCATGCTTCGTCGC	0	13	RDR2-sensitive
ATTGTTGGGGGTATACTTCGTAGC	0	10	RDR2-sensitive
ATTGTTGGTCTGTGATGATGGTCT	0	16	RDR2-sensitive
ATTGTTGTCTGCTGTGGCTACGGT	54	7	RDR2-resistant
ATTGTTTATTTAATCGGGCATTTT	33	5	RDR2-resistant
ATTGTTTGAATTTCGGGCCGGCCT	0	11	RDR2-sensitive
ATTGTTTGGTATCTTAGGCACAAT	112	0	RDR2-resistant
ATTGTTTGGTTACCTGTACGCGGA	0	28	RDR2-sensitive
ATTGTTTTAACATGGTTGTTCGGA	52	1	RDR2-resistant
ATTGTTTTACATCACAGTCGGTGC	0	11	RDR2-sensitive
ATTGTTTTGAGAGTTTGGGAGCCC	25	0	RDR2-resistant
ATTTAAAGGACCGTTTAGAGGACG	1	14	RDR2-sensitive
ATTTAACAACTTATGGTCGGTCGC	0	9	RDR2-sensitive
ATTTAAGACTGAGACATACCGGGC	0	14	RDR2-sensitive
ATTTAATAGTGCCGGGCTGGGCAT	1	26	RDR2-sensitive
ATTTACAAGGACGTGCTTTTCTAA	30	0	RDR2-resistant
ATTTACGACGGCCGTTGGATGGAA	0	17	RDR2-sensitive
ATTTACGACGGCCGTTGGATGGGA	0	17	RDR2-sensitive
ATTTACGGAGGACACGGCGAGCGC	0	11	RDR2-sensitive
ATTTACTGTAGCATCATACATCTT	0	12	RDR2-sensitive
ATTTACTGTGACTGTAGCATCGGC	0	31	RDR2-sensitive
ATTTAGAACTGTGTTTCTTGGCAT	0	9	RDR2-sensitive
ATTTAGAGAACTGTTTAGAGGACG	1	14	RDR2-sensitive
ATTTAGAGGACCATTTAGAGGACG	1	15	RDR2-sensitive
ATTTAGAGGACCGTTTAGAGGACG	21	197	RDR2-sensitive
ATTTAGAGGACGCTGCTGGAGAGC	0	18	RDR2-sensitive
ATTTAGAGGACTATTTAGAGGACG	0	11	RDR2-sensitive
ATTTAGCAAAAAATGTGTAGTTGT	0	9	RDR2-sensitive
ATTTAGTGGACTTAGAAATCGGCC	0	13	RDR2-sensitive

ATTTAGTTTGGAAACGGAAGCATC	77	3	RDR2-resistant
ATTTATAGACTCGAGAAGCCGCTC	0	12	RDR2-sensitive
ATTTATCGGACAGTGCATTTTAGC	0	11	RDR2-sensitive
ATTTATGATGTAGGGGCTCGGCGT	0	11	RDR2-sensitive
ATTTATGCGCATGCATTGGATCGT	0	11	RDR2-sensitive
ATTTATGTGTAGCGTCGTATATCA	28	0	RDR2-resistant
ATTTATTAGTTTCGCATGTCGGTC	0	13	RDR2-sensitive
ATTTATTGTAGTGGTCTGGGTGGT	25	0	RDR2-resistant
ATTTATTTCTGGTCACTCGTCGGT	0	16	RDR2-sensitive
ATTTCAACATGGTTGTTCGGATTC	14	0	RDR2-resistant
ATTTCAACGGTCGTCATGCGTCAT	0	17	RDR2-sensitive
ATTTCAATCGGATTCGAGATCGGA	0	9	RDR2-sensitive
ATTTCACCCACTAGTCTGGACGCT	0	10	RDR2-sensitive
ATTTCAGGATGTAGTTTTGCCGGA	30	0	RDR2-resistant
ATTTCAGTTCGGTTCGGTACGGTT	0	26	RDR2-sensitive
ATTTCATACTGTGTCGGACCAAGC	0	9	RDR2-sensitive
ATTTCATGACTGTGAGACGTGAGC	0	9	RDR2-sensitive
ATTTCCTTTTATAGACGTCTGGAC	1	17	RDR2-sensitive
ATTTCGATTTGCTGTAGCGTTAGC	24	0	RDR2-resistant
ATTTCGCTGGTACTGTGGTGGGCA	0	30	RDR2-sensitive
ATTTCGGACCAGGCTTCAATCCCT	20	0	RDR2-resistant
ATTTCGGACCGGCGACGTTGGCAT	1	31	RDR2-sensitive
ATTTCGGCCACTGTTTTGCGTCAT	0	13	RDR2-sensitive
ATTTCGGCGCCAACGGCTACCTGC	0	11	RDR2-sensitive
ATTTCGGGCTGACACGTCACGGCC	0	9	RDR2-sensitive
ATTTCGGGCTTAATCGGGTAGCAA	0	14	RDR2-sensitive
ATTTCGGGTCCGAGCAGAACGACA	0	10	RDR2-sensitive
ATTTCGGTCTGATCGGTTTCGGTT	0	12	RDR2-sensitive
ATTTCGGTTCGGTACGGTTCGGTT	0	10	RDR2-sensitive
ATTTCGGTTCGGTCTATTCGGTCC	0	11	RDR2-sensitive
ATTTCGGTTCGGTCTATTCGGTTC	0	9	RDR2-sensitive
ATTTCGGTTCGGTTCGGTACGGTT	4	54	RDR2-sensitive
ATTTCGGTTTTTTGTAGTTCGGTT	0	9	RDR2-sensitive
ATTTCGTGGAGCTGGAGCGGTCCC	0	9	RDR2-sensitive
ATTTCTAACTTTCAGTGGGACATT	47	0	RDR2-resistant
ATTTCTAGTGGCATTGAACGGATC	24	0	RDR2-resistant
ATTTCTATCCTTCGTCGGCCAACC	0	64	RDR2-sensitive
ATTTCTCATCGGCACCCTATTTTC	14	0	RDR2-resistant
ATTTCTCGGTCTTTTGGTTAAAAT	63	1	RDR2-resistant
ATTTCTCGGTTCATCGGCAAAGTC	0	16	RDR2-sensitive
ATTTCTCTGTTGGCTAGGACGGTT	22	1	RDR2-resistant
ATTTCTCTTCATCGTATGGACATC	0	15	RDR2-sensitive
ATTTCTGCATATATGATTTTCGGT	15	0	RDR2-resistant
ATTTCTTAGTCTTTGAACCAGAGG	0	22	RDR2-sensitive
ATTTCTTGATCTTTGAACCAGAGG	0	9	RDR2-sensitive
ATTTCTTGGTCTTTGAACCAGAGG	0	12	RDR2-sensitive
ATTTCTTGTGAACTGTGTCGTTGT	0	11	RDR2-sensitive
ATTTCTTTGATGGTCGTGGATCCC	25	2	RDR2-resistant
ATTTCTTTTGAGTTGTCGTTGGAT	0	15	RDR2-sensitive

ATTTCTTTTTAGTTGTCGTTGGAT	3	18	RDR2-sensitive
ATTTGAATGATGCGTCGCCGGCAC	1	16	RDR2-sensitive
ATTTGACCCGAATCTGTGCGTGCT	17	1	RDR2-resistant
ATTTGACGACGTGGATGGCCTCAT	0	9	RDR2-sensitive
ATTTGACTGCGGTTTCTACGGTAT	0	10	RDR2-sensitive
ATTTGAGCACTGTGTGATGATGTC	3	22	RDR2-sensitive
ATTTGAGCATCGTTGGACAATGGT	50	15	RDR2-resistant
ATTTGAGGAATGGATACATGATGC	0	16	RDR2-sensitive
ATTTGAGTAGAGAGTTTAGAAGAT	19	0	RDR2-resistant
ATTTGATCTAGACTGTTTGGTGTA	0	10	RDR2-sensitive
ATTTGATGGATGTAGATGGACGGT	0	23	RDR2-sensitive
ATTTGATGGTGCGGGGATGGGCGT	15	0	RDR2-resistant
ATTTGATTGATGATTTGTTCTATT	28	2	RDR2-resistant
ATTTGATTTGATTGGTTATTCGGA	32	4	RDR2-resistant
ATTTGCAAACTGTAGCTGTATGGC	0	22	RDR2-sensitive
ATTTGCAACTTATGGTCTGTCGGT	1	55	RDR2-sensitive
ATTTGCATCTGTGGAGTGGTTATC	22	0	RDR2-resistant
ATTTGCCGACTGTTTTTAAGGCTT	0	36	RDR2-sensitive
ATTTGCTCTGTGTCGTTGGGAGAT	20	0	RDR2-resistant
ATTTGCTTGACTGTGGCGACGCCA	0	9	RDR2-sensitive
ATTTGCTTTGAAAGCACCGTCGGC	124	15	RDR2-resistant
ATTTGGACTGGTGGGAGGGCTATT	0	11	RDR2-sensitive
ATTTGGATCTGACCTGGCTCGGCT	0	9	RDR2-sensitive
ATTTGGATGGTGTGAGCAGTTCGG	50	1	RDR2-resistant
ATTTGGCCTGGCTGCAGAAGACTC	0	11	RDR2-sensitive
ATTTGGCGGCGTCACTTTATGTTT	0	22	RDR2-sensitive
ATTTGGCTGCGGAGAATGTGCATG	47	7	RDR2-resistant
ATTTGGGACATGCTAAAGACTGCG	15	0	RDR2-resistant
ATTTGGGATAACAAATGTGGATGG	14	0	RDR2-resistant
ATTTGGTACTGTAGTTGTGGGTAC	0	27	RDR2-sensitive
ATTTGGTAGAGCAGGTCATTAGGT	33	0	RDR2-resistant
ATTTGGTATTCGTAGATACGGTTT	0	9	RDR2-sensitive
ATTTGGTCGACAGAACTGCGGCAC	0	14	RDR2-sensitive
ATTTGGTGGACCTGTAGGTGGGGC	0	23	RDR2-sensitive
ATTTGTACAGTCGTCGGGAGGCTT	0	21	RDR2-sensitive
ATTTGTACCGGGCTGAAATGGGTT	0	33	RDR2-sensitive
ATTTGTACCTGAAGGACGCGGCGC	0	13	RDR2-sensitive
ATTTGTACGGCTACATCGAACATA	0	9	RDR2-sensitive
ATTTGTAGCCTGATCCGAGCACGG	1	35	RDR2-sensitive
ATTTGTAGGGATAGGATGGATAAT	26	0	RDR2-resistant
ATTTGTAGTCCTCTCACAGAAAGG	0	12	RDR2-sensitive
ATTTGTCGAATCAGGAATCACGGT	0	27	RDR2-sensitive
ATTTGTCGAGCGCTTGGCAGGTAC	1	15	RDR2-sensitive
ATTTGTCTCATGCACTGTCGGCCC	0	11	RDR2-sensitive
ATTTGTCTCGTCTGGACTTCCGGC	0	9	RDR2-sensitive
ATTTGTCTCGTCTGGACTTTCGGC	0	26	RDR2-sensitive
ATTTGTCTCGTTTGGACTTCCGGC	0	10	RDR2-sensitive
ATTTGTCTCGTTTGGACTTTCGGC	0	13	RDR2-sensitive
ATTTGTCTCTTGCACTGTCGGCCT	0	9	RDR2-sensitive

ATTTGTCTGATATACATTTGCATG	0	9	RDR2-sensitive
ATTTGTGACACAATTTCGTTGGAT	93	0	RDR2-resistant
ATTTGTGCAGGGCTGTGCCGGTAA	0	11	RDR2-sensitive
ATTTGTGCTACTGTGGATGGTGTT	1	21	RDR2-sensitive
ATTTGTGGCACTGAGCACGGTCGG	1	26	RDR2-sensitive
ATTTGTGGGCCTAGACTGTCACGG	0	10	RDR2-sensitive
ATTTGTGGTTCTGATCGCATGATA	30	0	RDR2-resistant
ATTTGTGTCGTACGAACTTGGCTA	0	9	RDR2-sensitive
ATTTGTGTCGTACGAACTTGGCTG	7	75	RDR2-sensitive
ATTTGTGTCGTTCGAACTTGGCTG	0	32	RDR2-sensitive
ATTTGTTAGACTATGTGTGTGGGC	0	13	RDR2-sensitive
ATTTGTTCGGTGGGCAGTCGGCTT	0	11	RDR2-sensitive
ATTTGTTGCAACTGTCATGGGCAT	0	10	RDR2-sensitive
ATTTGTTGGCTCTGTACATGCGGG	0	16	RDR2-sensitive
ATTTGTTGTATGAAGAGCATGATA	23	1	RDR2-resistant
ATTTTAAGTTGTGTCTTACGGTGT	0	11	RDR2-sensitive
ATTTTACAGGACGTGTTAGCGGTT	0	11	RDR2-sensitive
ATTTTACTGTAAATGGCGATGCAT	1	47	RDR2-sensitive
ATTTTAGAAGGAGTGAGGTCGGAA	17	0	RDR2-resistant
ATTTTAGCCGGCCTGTTTGGTATT	0	9	RDR2-sensitive
ATTTTAGCGGTCTGGGTAGCGGAT	0	9	RDR2-sensitive
ATTTTAGCTGCTCCGTTGGATACG	0	11	RDR2-sensitive
ATTTTATATTTTTTCGTCGGGTCT	27	0	RDR2-resistant
ATTTTATGATTGTAGTGAGGTATA	0	13	RDR2-sensitive
ATTTTATGGTTGTAGTATGGTATA	0	10	RDR2-sensitive
ATTTTATGTCGGATCATACATTGT	0	17	RDR2-sensitive
ATTTTCCGTTCGGATTGTCGGGAT	0	9	RDR2-sensitive
ATTTTCGACGACATCTGACACGGT	0	13	RDR2-sensitive
ATTTTCGACGGCTGTATCGGTAGC	0	12	RDR2-sensitive
ATTTTCGACGGTCTCTGGCACGGC	1	12	RDR2-sensitive
ATTTTCGGCGACTGTGTCAGACGC	0	19	RDR2-sensitive
ATTTTCGGCGGCTTCTGACACGGC	3	34	RDR2-sensitive
ATTTTCGGGTTTGATCTGGGCGGC	0	10	RDR2-sensitive
ATTTTCTTGGGATTCTTTTGTCGG	0	32	RDR2-sensitive
ATTTTCTTTTATAGACGTCTGGAC	0	46	RDR2-sensitive
ATTTTGAGAAGCAGCTGAACTGAT	0	11	RDR2-sensitive
ATTTTGCACGGATCGGCCAAGTTC	22	0	RDR2-resistant
ATTTTGCACGGTCTGAGGCACTAT	0	28	RDR2-sensitive
ATTTTGGATTGTAGCGTGATTGGA	0	9	RDR2-sensitive
ATTTTGGGGCATGGATTGAGGCAT	0	27	RDR2-sensitive
ATTTTGTAAATTTTGATCTGTCTT	0	10	RDR2-sensitive
ATTTTGTAGATAGATGGATCAGTT	625	84	RDR2-resistant
ATTTTGTAGTGGCTTGGATTTAAT	19	0	RDR2-resistant
ATTTTGTATCTATAGACTGCGGCT	0	10	RDR2-sensitive
ATTTTGTCTAACTGTTGAAAGGAT	109	16	RDR2-resistant
ATTTTGTTGCGCGGGACTAACGGA	0	17	RDR2-sensitive
ATTTTGTTGGACTTCGGCTTCAAG	23	0	RDR2-resistant
ATTTTGTTGTGGAGTGTAAGGCAC	46	8	RDR2-resistant
ATTTTTACAGACGGATTCGGTTAT	0	10	RDR2-sensitive

ATTTTTACTGTAGTAAGACGATAC	0	18	RDR2-sensitive
ATTTTTAGTAGAGAGTCTTGGATA	14	0	RDR2-resistant
ATTTTTATAGGTCACGTTGTTGGA	0	15	RDR2-sensitive
ATTTTTCTGAGGTAGTACGTATCC	14	0	RDR2-resistant
ATTTTTGCATTTCGGGCTGGCATG	0	15	RDR2-sensitive
ATTTTTGCTACAGTCGTCGACACC	5	44	RDR2-sensitive
ATTTTTGGACCCTCTGCTCGGCGC	1	19	RDR2-sensitive
ATTTTTGGACCCTTTGCTCGGCAT	0	10	RDR2-sensitive
ATTTTTGGACCCTTTGCTCGGCGC	2	46	RDR2-sensitive
ATTTTTGGACCCTTTGCTCGGCGT	1	24	RDR2-sensitive
ATTTTTGGTGATAAGAGGACATGA	14	0	RDR2-resistant
ATTTTTGTCACTGTGAATTGCGGC	1	13	RDR2-sensitive
ATTTTTGTGAGGGGCTGCAAACGG	0	16	RDR2-sensitive
ATTTTTGTTCGGTCGGAATCGGTA	0	16	RDR2-sensitive
ATTTTTTAGATTCGGGACGGATAC	0	10	RDR2-sensitive
ATTTTTTGTGGTGTTTGTAGGGAC	38	0	RDR2-resistant
ATTTTTTGTTCGGTCGGAATCGGT	1	64	RDR2-sensitive
CAAAAAATACTCGGGCCAGCACGG	0	9	RDR2-sensitive
CAAAAATTACTCGGGCCAGCACGG	0	10	RDR2-sensitive
CAAAACATGCTCGGGCTGGGTCGG	0	20	RDR2-sensitive
CAAAAGATTTTTGGACCGTTTCGT	0	16	RDR2-sensitive
CAAAAGCACGGCCCACAAAGGCAC	0	10	RDR2-sensitive
CAAAATAGGTTGTATCGGTAGACA	15	0	RDR2-resistant
CAAAATGAGCGGACGGTCCGGCCC	2	15	RDR2-sensitive
CAAACACAGAACAGAGCGGCTCCG	0	15	RDR2-sensitive
CAAACACAGAGCTCGCACGCGCAT	2	77	RDR2-sensitive
CAAACACCCCGTAGACTAGGCATG	0	13	RDR2-sensitive
CAAACACTGGACGGACAGCGACGG	0	22	RDR2-sensitive
CAAACAGCTGCAACAGTGTCGGCT	0	12	RDR2-sensitive
CAAACAGTGGTGGACGCAGGACAT	0	43	RDR2-sensitive
CAAACATTCGATGGGACAGGGGCT	0	9	RDR2-sensitive
CAAACCTGTTTTTGAGCTCGTTGG	0	14	RDR2-sensitive
CAAACGGACTAGACTGGCTCGAAA	0	9	RDR2-sensitive
CAAACGGCGTAGCATTGGCGACAT	0	16	RDR2-sensitive
CAAACTCTCGGCAAAACGCGACAT	0	9	RDR2-sensitive
CAAACTGCAGAGACTGATGGGCAC	2	27	RDR2-sensitive
CAAACTGCAGAGACTGATGGGCGC	0	18	RDR2-sensitive
CAAAGACAAAACGGGCAGGGACAC	0	31	RDR2-sensitive
CAAAGCAGATGTAGGCCCACTCGC	0	19	RDR2-sensitive
CAAAGCGACTGGAGGGCAGAGGAA	0	12	RDR2-sensitive
CAAAGGCTATTTTACACTCGGCAA	6	36	RDR2-sensitive
CAAAGGCTGTATCCCGAGAGGCAA	1	29	RDR2-sensitive
CAAAGGGCTGAATCCAGGAAGCAG	0	9	RDR2-sensitive
CAAAGGGCTGGATCCAGAAAGCAG	0	16	RDR2-sensitive
CAAAGGGCTGGATCCAGGAAGCAA	0	15	RDR2-sensitive
CAAAGGGCTGGATCCAGGAAGCAG	5	99	RDR2-sensitive
CAAAGGGCTGGATCTAGGAAGCAG	1	15	RDR2-sensitive
CAAAGTAACTAGAACGGAATGGCT	0	10	RDR2-sensitive
CAAAGTAGGACGGGGCAGGGACAA	1	23	RDR2-sensitive

CAAAGTAGGATGAGACAGGGGCGC	0	14	RDR2-sensitive
CAAAGTAGGATGAGTCGGGGACGC	0	9	RDR2-sensitive
CAAAGTAGGATGAGTCGGGGGCGC	0	49	RDR2-sensitive
CAAAGTATGATGAGTCGGGGGCGC	0	9	RDR2-sensitive
CAAAGTCTTCGTCACTGTAACGGT	0	9	RDR2-sensitive
CAAAGTGAGACGAGGCATGGACGA	0	15	RDR2-sensitive
CAAAGTTGACTGAGAGACGATGGC	0	9	RDR2-sensitive
CAAAGTTGACTGGGAGACGACGAC	0	19	RDR2-sensitive
CAAAGTTGACTGGGAGACGACGGC	4	37	RDR2-sensitive
CAAATAACTGTAGAATGGACGGTT	0	9	RDR2-sensitive
CAAATAAGCGGGTCGGGCTCGGAC	0	18	RDR2-sensitive
CAAATAGGACCACCATGCAGGCAT	0	9	RDR2-sensitive
CAAATATTCGGCGAAGGACGGCTC	0	15	RDR2-sensitive
CAAATCGTTCGTCTGACTTGGGTA	44	6	RDR2-resistant
CAAATGACGTGGGACTGGTTGCAT	0	12	RDR2-sensitive
CAAATGGGCTGGACTGGATTCGGC	0	21	RDR2-sensitive
CAAATGGTTTTGGGTCCTTTGGCA	15	0	RDR2-resistant
CAAATGTCTGATCGTTTGGAGGGA	26	2	RDR2-resistant
CAAATTGAGCACTGGACTGGACGG	0	17	RDR2-sensitive
CAAATTGCGTCGCGACCGAGCGGA	0	10	RDR2-sensitive
CAAATTTCGTGGAGCTGGAGCGGT	0	25	RDR2-sensitive
CAAATTTGAACTACTAGGCGACAA	0	9	RDR2-sensitive
CAAATTTGTACTAACGGACGACAT	0	14	RDR2-sensitive
CAACAAAACGTTGTATATGTGGAC	0	9	RDR2-sensitive
CAACAAAACTAGGACAGAACGGCT	0	11	RDR2-sensitive
CAACAAACTGTAGCGGACCAGCAG	0	12	RDR2-sensitive
CAACAATCCACTCTGCAACGGCCT	0	9	RDR2-sensitive
CAACACATTTTTTCGATTGTCGGA	17	0	RDR2-resistant
CAACACTGTGCTGTGGATCCACGA	0	15	RDR2-sensitive
CAACAGCAGTAGGGACGTGAGAAG	0	24	RDR2-sensitive
CAACAGCTCGACTCGGACCGGCAT	2	66	RDR2-sensitive
CAACAGTTGTGATTCGGTATGGCT	49	0	RDR2-resistant
CAACATGTGCGGACTGTCCGGCTT	0	12	RDR2-sensitive
CAACCAAACTTCACGCGTCGGGTC	0	11	RDR2-sensitive
CAACCAAAGGACTGGATCTAGGAA	0	9	RDR2-sensitive
CAACCAAAGGGCTGGATCCAGGAA	5	61	RDR2-sensitive
CAACCAAAGGGCTGGATTCAGGAA	1	21	RDR2-sensitive
CAACCAGAAGGACGACCGAAGACA	0	10	RDR2-sensitive
CAACCAGAAGGACGACCGAAGACT	0	40	RDR2-sensitive
CAACCATAGTTGACTGGGAGACGA	0	10	RDR2-sensitive
CAACCATCCATTCTGTAACGGCTC	0	9	RDR2-sensitive
CAACCCACACCTAGAATTCGTCGT	0	11	RDR2-sensitive
CAACCCATCTGGGCAGGGGCACGC	0	11	RDR2-sensitive
CAACCCGTTCGGCCGGACAGTCAC	0	16	RDR2-sensitive
CAACCCTGTAGAGCGCGGCGAGGT	0	9	RDR2-sensitive
CAACCGAAGGATAGAGCAGACGAG	0	17	RDR2-sensitive
CAACCGGAGGATGACGTACATGAC	0	11	RDR2-sensitive
CAACCGGGAACAGACTGAGATGGT	0	9	RDR2-sensitive
CAACCGTAGAGCCAGAGGCAAGGA	0	14	RDR2-sensitive

CAACCGTTAGGGTTCGACCGTTGG	0	9	RDR2-sensitive
CAACCTACTCCTTGGACTACGGCG	0	39	RDR2-sensitive
CAACCTTGCCAAGACTGTCGTCGT	0	18	RDR2-sensitive
CAACGAATTTTATAGAAGCTGGCT	1	24	RDR2-sensitive
CAACGACTGCAGCGAAGGCCTCGA	2	28	RDR2-sensitive
CAACGACTGTACAGATTGCGCCAC	0	17	RDR2-sensitive
CAACGAGAGATGGGACGGGATCAA	0	17	RDR2-sensitive
CAACGAGGGATGGGACGGGATCAA	0	16	RDR2-sensitive
CAACGATACTGCAGAATCGGGCAT	0	17	RDR2-sensitive
CAACGATATGTTGTCGTCGGTAGC	0	15	RDR2-sensitive
CAACGATGGATTCTGCAGGGACAG	0	9	RDR2-sensitive
CAACGCGACTCTGTCAGGAACGGA	0	24	RDR2-sensitive
CAACGCGTCTGAGACTGTTCGGTG	0	9	RDR2-sensitive
CAACGGAACGATGGGACGACGCAA	0	11	RDR2-sensitive
CAACGGAACGCGGACTCGGCGCAC	0	23	RDR2-sensitive
CAACGGAACGTGTGATGGTTTAAC	14	0	RDR2-resistant
CAACGGAAGGTAGATGACCGGCGC	0	17	RDR2-sensitive
CAACGGACCATTCACGAGACGCGT	0	11	RDR2-sensitive
CAACGGACGAGGGTTGCAATCAAC	3	19	RDR2-sensitive
CAACGGAGACAAGACAACGGGCAG	2	16	RDR2-sensitive
CAACGGAGACAAGACGACGGACAG	0	13	RDR2-sensitive
CAACGGAGACAAGACGACTGGCAG	1	16	RDR2-sensitive
CAACGGGGAGTAGGTTTGCAAGAA	3	21	RDR2-sensitive
CAACGGTCGGAAGGGGGCACCGGA	6	24	RDR2-sensitive
CAACGGTCGGCTGACGTGGCGTGC	1	29	RDR2-sensitive
CAACGGTCGGCTGGGCCAACGGTC	1	16	RDR2-sensitive
CAACGGTCGGTCGGGCCAACGGTC	1	14	RDR2-sensitive
CAACGGTCTGCTGACGTGGCTGGC	0	29	RDR2-sensitive
CAACGTAGAGCATGACCGGCTGGT	52	1	RDR2-resistant
CAACGTAGAGTTGTAGTTGCGGAC	35	1	RDR2-resistant
CAACGTCATCTCTGAGGTCGTCGT	0	12	RDR2-sensitive
CAACGTGACTGTACGGGACGGACA	0	12	RDR2-sensitive
CAACGTGTGGGGAGTGTGGACGAA	23	1	RDR2-resistant
CAACGTTAGGTTGTAGAGGGCGTT	0	10	RDR2-sensitive
CAACTAAAGGGCTGGATCCAGGAA	0	16	RDR2-sensitive
CAACTAAGAGACTCTATTGTACAA	0	14	RDR2-sensitive
CAACTACTCTTTTGGACGCTGTGA	82	10	RDR2-resistant
CAACTAGGGATGGAACGGGATCAA	0	12	RDR2-sensitive
CAACTATAGACGGCTGCTGACGGT	0	11	RDR2-sensitive
CAACTATTTATTGGACTGGGCAAT	18	1	RDR2-resistant
CAACTCAGCCTGTAGTGTCGGCCC	1	42	RDR2-sensitive
CAACTCATCTCGGACCATCGCATG	0	36	RDR2-sensitive
CAACTCATCTGGACAGGGGCACGC	0	14	RDR2-sensitive
CAACTCATCTGGGCAGGGACACGC	0	31	RDR2-sensitive
CAACTCGGACTCCGCAAGACGGAC	0	18	RDR2-sensitive
CAACTCGGAGACGACGACGGGCAC	0	14	RDR2-sensitive
CAACTCGGAGACGACGACTGGCAC	0	29	RDR2-sensitive
CAACTCGGAGGCATCACGTAGGGA	0	13	RDR2-sensitive
CAACTCGGCTCGGCTCGAAGCGGC	36	1	RDR2-resistant

CAACTCGGCTCGGCTCGGATCGGC	0	9	RDR2-sensitive
CAACTCGGGAAGGCGACGAACAGC	0	10	RDR2-sensitive
CAACTCTAGGTGGGACACGCGTAT	0	14	RDR2-sensitive
CAACTCTCTTGATCGTAACTCGGA	27	0	RDR2-resistant
CAACTCTTCAACCCGCAACGACGT	0	9	RDR2-sensitive
CAACTCTTCGTCAAGCCGGCAGGT	0	10	RDR2-sensitive
CAACTCTTCGTCACGTCGGCAGGT	0	12	RDR2-sensitive
CAACTCTTGAACCCGCAACGACGT	0	66	RDR2-sensitive
CAACTGCACGAGCAGACGCCGGGC	27	0	RDR2-resistant
CAACTGCCCACTCTGCAACGGTAT	0	16	RDR2-sensitive
CAACTGCTGTGCTGTATGACGGAT	0	10	RDR2-sensitive
CAACTGCTGTGGAAATGGCTGGCG	28	2	RDR2-resistant
CAACTGGAACTGGAGCCCGGCCTT	28	3	RDR2-resistant
CAACTGGCAGATACTCCCAAGGGA	0	10	RDR2-sensitive
CAACTGTAGACGGCTGCTGACGAC	0	21	RDR2-sensitive
CAACTGTCCACTCTGCAACGGCCC	2	176	RDR2-sensitive
CAACTGTCCACTCTGCAACGGCCT	1	16	RDR2-sensitive
CAACTGTCCACTCTGCAACGGCTC	1	23	RDR2-sensitive
CAACTGTCCACTCTGGAACGGCCC	0	36	RDR2-sensitive
CAACTGTCTACTCTACAACGGCCC	0	31	RDR2-sensitive
CAACTGTCTTTAATGTCGTCGGTT	0	10	RDR2-sensitive
CAACTGTGGGACGGCTGCAGGTGT	0	11	RDR2-sensitive
CAACTGTTCACTCTGCAACGGCCC	0	35	RDR2-sensitive
CAACTTAAGGGCTATTGACGTCGA	31	2	RDR2-resistant
CAACTTTCTTGTTTTATTCGGACC	19	0	RDR2-resistant
CAAGAACTCGAGGAATTTCAGGCA	36	4	RDR2-resistant
CAAGAAGCTGTTGTGGACTGGCAA	0	13	RDR2-sensitive
CAAGAATCGAACTCGGGTAGGCAT	0	15	RDR2-sensitive
CAAGAATCGGCTCGGAGACGACAA	0	39	RDR2-sensitive
CAAGAATGTGATCTATGGCAAGGA	21	2	RDR2-resistant
CAAGACACACGCTAGGATCTCGGA	0	9	RDR2-sensitive
CAAGACATGTATCTTGACGAAGAC	0	17	RDR2-sensitive
CAAGACATGTTACCTCGACGTCAT	1	43	RDR2-sensitive
CAAGACGGGACGGGATGAAACGAT	0	11	RDR2-sensitive
CAAGACGGGATGGGACAGGACGAT	0	17	RDR2-sensitive
CAAGACTGGACGGTCCATGAGCAG	0	29	RDR2-sensitive
CAAGACTGGAGGACCTGCCGGCGT	0	12	RDR2-sensitive
CAAGACTTCGGATCGGTTTTGGGC	30	2	RDR2-resistant
CAAGAGCGGAAGAAACTACGGCGT	0	15	RDR2-sensitive
CAAGAGCTGGCCGACTCATGGCAT	0	11	RDR2-sensitive
CAAGATCCAGGTACTGTTCGGCGA	0	11	RDR2-sensitive
CAAGATTCCGGTAGCACGAGACGT	0	14	RDR2-sensitive
CAAGCAACGGACACACCTTCGGCT	0	17	RDR2-sensitive
CAAGCAATCGGTCTTTCTTCTGCT	19	0	RDR2-resistant
CAAGCACGAGATTCTGATTGGTTG	0	12	RDR2-sensitive
CAAGCACGGCACTAGACTCGTGTC	1	19	RDR2-sensitive
CAAGCACGTGAGCTCTCGTATGGC	0	12	RDR2-sensitive
CAAGCACGTTGGGGTAGGCTAGAG	0	17	RDR2-sensitive
CAAGCACTCGGCAAACATGGCTCT	0	45	RDR2-sensitive

CAAGCAGACTGTTGCGCGACCTCA	0	21	RDR2-sensitive
CAAGCAGATCTGGGACACCCTCAA	7	66	RDR2-sensitive
CAAGCAGGCTGATCGAATTTGGAA	23	1	RDR2-resistant
CAAGCAGTCTCGGATGAAGTGGTA	57	6	RDR2-resistant
CAAGCATACGGATACGGAGGGCGT	0	16	RDR2-sensitive
CAAGCATCGTGAACTGTCCGGCAC	0	26	RDR2-sensitive
CAAGCATTAGGTCGTTTCTGAGAC	0	15	RDR2-sensitive
CAAGCCCGCCCGCTTATTCTACCG	0	15	RDR2-sensitive
CAAGCCCGCGGACTTAGACGGCCC	0	15	RDR2-sensitive
CAAGCCGACTGCAGACGCTATCAG	0	16	RDR2-sensitive
CAAGCCGAGGGACGAGTAGGCTAT	0	10	RDR2-sensitive
CAAGCCTACGTAGAACTGGGTCGT	0	41	RDR2-sensitive
CAAGCGAGACGAGGTGAACACCAG	0	11	RDR2-sensitive
CAAGCGAGGATCTAGAGGAGCCGT	0	10	RDR2-sensitive
CAAGCGCGCTGTTGGAGCACGGTC	0	9	RDR2-sensitive
CAAGCGGACCGCCTGATACGGCAC	0	9	RDR2-sensitive
CAAGCGGAGACGAGGTGAACACAA	1	50	RDR2-sensitive
CAAGCGGTAGACTTTTTACATCAC	0	23	RDR2-sensitive
CAAGCGGTAGGATTTTTACATCAC	0	13	RDR2-sensitive
CAAGCGTTGTACGGATGCAGTGCG	0	13	RDR2-sensitive
CAAGCTAACACAACTCGGCGTCAT	0	11	RDR2-sensitive
CAAGCTAAGCGGACCGGTAAGCAC	0	42	RDR2-sensitive
CAAGCTAGATCTCGCTCGTCGCAA	0	15	RDR2-sensitive
CAAGCTAGCAACCGGCGCCTCTGC	32	1	RDR2-resistant
CAAGCTCGGCACTACAGATCACGG	0	10	RDR2-sensitive
CAAGCTCTCGGCGAAACGCGACGC	0	10	RDR2-sensitive
CAAGCTCTGGACATGGCAGACTGC	1	50	RDR2-sensitive
CAAGCTCTGGACATGTCAGACTGC	0	12	RDR2-sensitive
CAAGCTCTTACGTGGCATCGCCGG	0	12	RDR2-sensitive
CAAGCTCTTGTAGGTGTAGTCGTC	26	3	RDR2-resistant
CAAGCTGCAGAGACTGATGGGCAC	2	73	RDR2-sensitive
CAAGCTGCAGAGACTGATGGGCAT	1	12	RDR2-sensitive
CAAGCTGCAGAGACTGATGGGCGC	1	116	RDR2-sensitive
CAAGCTGCAGAGACTGGTGGACAC	0	33	RDR2-sensitive
CAAGCTGCAGAGCCTGATGGGCGC	2	23	RDR2-sensitive
CAAGCTGCAGAGGCTGATGGACAC	0	14	RDR2-sensitive
CAAGCTGTGTTACCTCGACGTCAC	0	46	RDR2-sensitive
CAAGCTGTGTTACCTCGGCGCCAT	0	14	RDR2-sensitive
CAAGGAACAGTAGATGTCCGGTGT	0	19	RDR2-sensitive
CAAGGAACCCTGTGGTGCGGTCGG	0	12	RDR2-sensitive
CAAGGAACTAGAGTGCAGGGACAT	0	89	RDR2-sensitive
CAAGGAACTAGAGTGGAGGGACAT	0	10	RDR2-sensitive
CAAGGACAACTGTAGGTGGGCCTA	3	41	RDR2-sensitive
CAAGGACAACTGTAGGTGGGCCTT	0	11	RDR2-sensitive
CAAGGACCAACTGAAGCGCATCAC	0	10	RDR2-sensitive
CAAGGACGACGACACGGCGAGCAC	0	11	RDR2-sensitive
CAAGGACGCGAACTTGAAGGGCGG	0	11	RDR2-sensitive
CAAGGACTGTGGCGTCATCTCGGC	1	47	RDR2-sensitive
CAAGGACTGTGGCGTCATCTTGGC	1	14	RDR2-sensitive

CAAGGACTGTTCAGGCAAGGACAT	19	1	RDR2-resistant
CAAGGAGGAGAAGGAAGACTCGAC	51	0	RDR2-resistant
CAAGGAGGGGATGGATCGAAGAAC	15	0	RDR2-resistant
CAAGGATGGGGACGATGCTTTCAT	0	9	RDR2-sensitive
CAAGGATTGTGGCGTCATCTCGAC	0	14	RDR2-sensitive
CAAGGATTGTGGCGTCATCTCGGC	1	14	RDR2-sensitive
CAAGGCACGCAGGACTGTGCAGGC	1	25	RDR2-sensitive
CAAGGCACTATCGGAATTCGGCTC	0	9	RDR2-sensitive
CAAGGCATACGTAGCAGCTTCGGT	0	11	RDR2-sensitive
CAAGGCGTCGGTGTCTGGGCGGTA	24	3	RDR2-resistant
CAAGGCTAGTGAGCGAGGAAGGCA	19	0	RDR2-resistant
CAAGGCTCTTAACCTTAAGGTCGT	1	16	RDR2-sensitive
CAAGGCTCTTAACCTTGTGGTCGT	4	34	RDR2-sensitive
CAAGGCTGAGACGGGACCATCCCC	0	11	RDR2-sensitive
CAAGGCTGTCAGCGATGACGTGGT	0	10	RDR2-sensitive
CAAGGCTTCTAACCTTGTGGTCGT	2	26	RDR2-sensitive
CAAGGCTTTTAACCTTGTGGTCGT	0	9	RDR2-sensitive
CAAGGGAGTGGAACCAGAGGACGC	0	11	RDR2-sensitive
CAAGGGCAGTAGGGGAGTGGTCAG	1	15	RDR2-sensitive
CAAGGGCTGACGCGACGTCGACGT	0	10	RDR2-sensitive
CAAGGGGACTGTGACAAGTGGTAT	0	32	RDR2-sensitive
CAAGGGGGAGACCTCACGTCGGAT	2	15	RDR2-sensitive
CAAGGGGTAGAGCGAGGCGGACGA	0	12	RDR2-sensitive
CAAGGTAACACTGATCGACGCCAC	0	9	RDR2-sensitive
CAAGGTAGAGCGTGCTGATAACGT	0	10	RDR2-sensitive
CAAGGTCAGCTTGGCTCGGCTCGG	0	13	RDR2-sensitive
CAAGGTCGGTGTAGTTGACGCGCC	1	79	RDR2-sensitive
CAAGGTCTCGAACGGCAAGGGCTT	3	23	RDR2-sensitive
CAAGGTCTCGAACGGCACCGGCTT	1	28	RDR2-sensitive
CAAGGTCTGACGTGGCATCAACGG	0	9	RDR2-sensitive
CAAGGTGCAGAGACTGATGGGCAC	0	9	RDR2-sensitive
CAAGGTGGTGGAAGGAGGCGTGGA	20	1	RDR2-resistant
CAAGGTGGTGTGTAGAGATGGCAC	0	19	RDR2-sensitive
CAAGGTGTGTCTCAAGGTCGCCGG	0	18	RDR2-sensitive
CAAGGTTAGCCTCGGACGAGTCAT	0	22	RDR2-sensitive
CAAGGTTTCGTAGAGCAGGAGGGG	0	24	RDR2-sensitive
CAAGTACGACAGGCCGATGATCAT	0	11	RDR2-sensitive
CAAGTACTCGGTATGCAGGGGCGA	0	31	RDR2-sensitive
CAAGTAGAACGGAGCCGTTCCATC	0	16	RDR2-sensitive
CAAGTAGAACGGAGCCGTTCTGTC	0	10	RDR2-sensitive
CAAGTAGAGGCGAGTTGAACCCGA	0	36	RDR2-sensitive
CAAGTCAAGCTAGAGCCGGATCAT	0	13	RDR2-sensitive
CAAGTCAAGTTAGGGCCGGATCAT	0	24	RDR2-sensitive
CAAGTCAGCGTGAACAGTACGGGA	2	21	RDR2-sensitive
CAAGTCAGGACTCGCCACGGGCGG	38	0	RDR2-resistant
CAAGTCAGTTGTAGTAGTAGGCTC	0	9	RDR2-sensitive
CAAGTCCGCGGACTTAGACGGCCC	2	21	RDR2-sensitive
CAAGTCCTGTAGACTGTCCGGTGT	0	47	RDR2-sensitive
CAAGTCGTGCTCGGGCTGGGCCGG	0	10	RDR2-sensitive

CAAGTGAAATGTATGAACAGGTAT	0	11	RDR2-sensitive
CAAGTGGGAACTCCGCGTCGGCGC	0	59	RDR2-sensitive
CAAGTGTAGCTCTGCGGACGGTGA	0	11	RDR2-sensitive
CAAGTGTAGGATCTCGAAGGCGAC	0	17	RDR2-sensitive
CAAGTGTATCTCTGCGGACGACGA	0	11	RDR2-sensitive
CAAGTTACGGAGAGCTGACGTCAT	66	0	RDR2-resistant
CAAGTTAGCGCTGTGTGACGGTGA	0	23	RDR2-sensitive
CAAGTTCTACTGTAGTCATGGCAT	1	12	RDR2-sensitive
CAAGTTGCAGCACTAGTAGGCGGT	0	24	RDR2-sensitive
CAAGTTGGCGAGCGAAGCGGAGGC	2	34	RDR2-sensitive
CAAGTTGGGTTTGTCGGGTCGGGC	0	15	RDR2-sensitive
CAATACGTACGTAGGACAGGCCGG	0	13	RDR2-sensitive
CAATAGCTGGACGGCCTGGTACAT	0	20	RDR2-sensitive
CAATATAGTCGTTACTGGTATGGC	0	9	RDR2-sensitive
CAATATGTTATCTTGTCGGATGTT	19	1	RDR2-resistant
CAATATTATGTACGGCTATTGAAC	17	0	RDR2-resistant
CAATCACTGCGTGAGAACCGACGG	0	9	RDR2-sensitive
CAATCCATCCGGGCAGGGACACGC	0	11	RDR2-sensitive
CAATCGAACTGGGCTGGCACGGAC	0	12	RDR2-sensitive
CAATCGCGTCGTCGATCTCGTCGT	0	18	RDR2-sensitive
CAATCGGAAAGGCAGTAGTCGCAT	0	10	RDR2-sensitive
CAATCGGACGGGTAGTACACGGAT	0	13	RDR2-sensitive
CAATCGGGCGGGTAGAACGCGGAT	0	16	RDR2-sensitive
CAATCGGGCGGGTAGTACACGGAT	1	52	RDR2-sensitive
CAATCGGGTGGGTAGTACGCGGAT	0	36	RDR2-sensitive
CAATCGTATGAGTGGGCAGGGACG	0	19	RDR2-sensitive
CAATCGTGATATCGGAGGAGGGTT	55	1	RDR2-resistant
CAATCGTTGGGCCGCATACACCGC	0	14	RDR2-sensitive
CAATCTGGCTTATGAAACGGTGAC	153	9	RDR2-resistant
CAATCTTGTAGAACAGATGGCAGA	17	0	RDR2-resistant
CAATCTTTCGGATCGGCTGGCCAC	0	12	RDR2-sensitive
CAATCTTTTTGGAGAGCTCGACGG	20	1	RDR2-resistant
CAATGACAGGACGGGATGGGACGG	0	12	RDR2-sensitive
CAATGACATGTGGGGTCCATGGTA	0	10	RDR2-sensitive
CAATGCAGGCTATAGTGAGGACGA	0	11	RDR2-sensitive
CAATGCCAAGCAGATCTGGGACAC	0	9	RDR2-sensitive
CAATGGAAGTTGGCTGCGAGCATA	3	17	RDR2-sensitive
CAATGGACCAAGCTTCAGTAGACC	41	6	RDR2-resistant
CAATGGACTGGACAGGCACGCCGC	1	14	RDR2-sensitive
CAATGGACTTAGACGGTGCTACAA	0	14	RDR2-sensitive
CAATGGACTTCTCGCGACGTCGGG	104	40	RDR2-resistant
CAATGGAGTGGATGTACCGCGCAT	0	11	RDR2-sensitive
CAATGTAGGCTATAGCGAGGGCGA	0	10	RDR2-sensitive
CAATTCAGCCTGTAGTGTCGGCCC	0	22	RDR2-sensitive
CAATTCGGCGGGTAGTACACGGAT	0	14	RDR2-sensitive
CAATTCTCTGAACGGGCTCGGCAC	0	24	RDR2-sensitive
CAATTGATGATCGTTCTTTTTGGC	27	3	RDR2-resistant
CAATTGGATGCATGAGCGGGGACG	0	9	RDR2-sensitive
CAATTGTCTACTCTGTAACGGTCC	0	11	RDR2-sensitive

CAATTGTTGATGTGGAACAATCGG	70	13	RDR2-resistant
CAATTGTTTATTTAATCGGGCATT	23	1	RDR2-resistant
CAATTTTGTTGGATTAGATGTGGG	19	0	RDR2-resistant
CACAAAGCTGACTGGGAGACGCCA	0	22	RDR2-sensitive
CACAAATAAGCGGGTCGGGCTCGG	0	9	RDR2-sensitive
CACAAATAATTTGTGTGTCGTCGC	0	9	RDR2-sensitive
CACAACGTCAGCAACGGTCGTCGT	0	14	RDR2-sensitive
CACAACGTGGAAGTAGGCAAGTGT	0	31	RDR2-sensitive
CACAACTACAAGCCGGGTTAGCGT	0	10	RDR2-sensitive
CACAACTCCGTTGGACTACGGGTG	24	2	RDR2-resistant
CACAACTCTGAACCTCGACGACAC	0	9	RDR2-sensitive
CACAAGACGAAGGCCTGCGGTGAC	0	10	RDR2-sensitive
CACAAGACTGACTCAGAGGGGCTG	0	12	RDR2-sensitive
CACAAGTCCGGACTGGTGCGGCTT	4	32	RDR2-sensitive
CACAAGTGGAGGACCCTAGACTAA	36	2	RDR2-resistant
CACAATTGGTCATCGCGCTTGGTT	15	0	RDR2-resistant
CACACACTGCTGGACTGGACGCAT	0	94	RDR2-sensitive
CACACAGACTGGACCTGAGCGCGG	0	14	RDR2-sensitive
CACACATGCAGAGGACACGGGAAA	0	18	RDR2-sensitive
CACACATGTAGACAGTTGGCGCGT	0	15	RDR2-sensitive
CACACCGCGCACTGTAGACGACGG	0	25	RDR2-sensitive
CACACCGGACAAGGAACAGTGCAT	0	17	RDR2-sensitive
CACACGACGGACACCAAGCGGGGA	1	12	RDR2-sensitive
CACACGCTGGACGAACGAGATCGG	0	11	RDR2-sensitive
CACACGTAGACACTCGGTGAAGAC	0	15	RDR2-sensitive
CACACGTAGAGCCGCAGGGACACA	0	15	RDR2-sensitive
CACACGTTACGTGCGAGATCTCGG	0	11	RDR2-sensitive
CACACGTTGGACTGTTCGGTGCAT	0	18	RDR2-sensitive
CACACTCGGCAAAGAACGCTCGGC	0	25	RDR2-sensitive
CACACTGAGCGGTATGCAACTCCC	0	11	RDR2-sensitive
CACACTGCAGAGGCTTGGACGGAC	0	15	RDR2-sensitive
CACACTGTAGTAAGGGGTACCCAT	0	11	RDR2-sensitive
CACACTTATGTCTGATGGTTGGAC	15	0	RDR2-resistant
CACACTTTGCCTAAGGTTAGTCGT	1	12	RDR2-sensitive
CACAGACTCCGGTGAACAGGACGC	0	10	RDR2-sensitive
CACAGACTGGACCTGAGCGCGGCT	0	19	RDR2-sensitive
CACAGACTGTAGGTTGGAGACGAG	0	9	RDR2-sensitive
CACAGAGCGGTAGGGGTGGAGGGC	88	0	RDR2-resistant
CACAGCAGTTCGGCTCGGCTCGGA	1	23	RDR2-sensitive
CACAGCTCGAACGACGTGTGGCAT	3	147	RDR2-sensitive
CACAGGACGACACTCGGCAAAGAC	0	20	RDR2-sensitive
CACAGGACTCAGGAAGAGAGACGA	0	18	RDR2-sensitive
CACAGGAGCCTGAGCCGGGGGAAG	17	0	RDR2-resistant
CACAGGAGCGACATGAACACCGGA	0	12	RDR2-sensitive
CACAGGCACTGGTGGAGATGGCGA	0	9	RDR2-sensitive
CACAGGTAGGCACTCGGCAAAGAC	1	24	RDR2-sensitive
CACAGTAGAATAAGCGGACGGGCT	0	12	RDR2-sensitive
CACAGTAGAATAAGCGGGCGGGCT	0	10	RDR2-sensitive
CACAGTAGAGCGCATGATTGGCTT	0	38	RDR2-sensitive

CACAGTAGGACGAGCACCGGCGAT	0	12	RDR2-sensitive
CACAGTCAGAGAGACAGGGGACGC	0	12	RDR2-sensitive
CACAGTCGGTGCACCACAAGCGGT	1	33	RDR2-sensitive
CACAGTCGTGTGCTGACGCGGTCA	0	9	RDR2-sensitive
CACATAAAACCGGGCTGTGTCGGA	0	22	RDR2-sensitive
CACATACACGTTTGGACCACGCAT	0	11	RDR2-sensitive
CACATCGACGACTGTAGTGGGTGT	0	34	RDR2-sensitive
CACATCGACTGTTCAGAGGGCCGC	0	15	RDR2-sensitive
CACATCGGCATGTACTTAGGGCGC	3	18	RDR2-sensitive
CACATCGTCGATGGGTGAAGGGTA	21	2	RDR2-resistant
CACATCTAGGATGCGTGCGGCGTC	0	9	RDR2-sensitive
CACATCTTAGGACTTAGGGCGGGT	0	20	RDR2-sensitive
CACATGCGTCGCTGTGTTCGGCAA	1	22	RDR2-sensitive
CACATGTTCGGTGACTGCGGCTAG	0	12	RDR2-sensitive
CACCAAAATGAGCGGACGGTCCGG	1	28	RDR2-sensitive
CACCAAATTGAGCGGGCGGACGGT	1	23	RDR2-sensitive
CACCAATGACTGGAAGTAGGGACG	0	10	RDR2-sensitive
CACCACCACTTCGTCAGGACGCAT	0	10	RDR2-sensitive
CACCACGGACAGTCCGGACCGCGG	0	11	RDR2-sensitive
CACCACGTGACTGCTACGCCGGAA	0	25	RDR2-sensitive
CACCACTCGCTGGATTGGGTTCGT	0	13	RDR2-sensitive
CACCAGACTGTGCGAGCAGAGCGT	0	13	RDR2-sensitive
CACCAGCCCGGCTATAGCGCGCAT	0	9	RDR2-sensitive
CACCAGGACGTAGGGTGTTACGCA	0	9	RDR2-sensitive
CACCAGTTTCAACGGTCGTCGTGC	0	13	RDR2-sensitive
CACCATATTGAGCGGGCGGACGGT	0	11	RDR2-sensitive
CACCATTACTTGGAGATGGTCGGC	0	10	RDR2-sensitive
CACCATTTGTGGCACTGAGCACGG	0	18	RDR2-sensitive
CACCCAAGACGGATCTAGACGACG	5	44	RDR2-sensitive
CACCCACGTCTGGACCTGCGCGGC	0	10	RDR2-sensitive
CACCCCATGATCGAAGCGGAGGCA	0	37	RDR2-sensitive
CACCCCATTCTCGGCAGAGGGCGT	3	23	RDR2-sensitive
CACCCCGTCAGGGCTTGTTCGGTT	0	13	RDR2-sensitive
CACCCCTCCGGTCGTTGACGTCAT	0	20	RDR2-sensitive
CACCCCTCCGGTCGTTGACGTCGT	0	10	RDR2-sensitive
CACCCGACGGACTGTCCGGTGCAT	0	15	RDR2-sensitive
CACCCGGCACGAACTCGGCCCGAA	0	9	RDR2-sensitive
CACCCTGACTGTTCGGATACGGTT	0	9	RDR2-sensitive
CACCGAAGACTGGAAGTAGGGACG	0	10	RDR2-sensitive
CACCGAAGGCAGAACCAGGGGACA	0	9	RDR2-sensitive
CACCGAAGGCAGAACCAGGGGAGG	0	14	RDR2-sensitive
CACCGACCAGGTGCTCTGACCGGC	1	57	RDR2-sensitive
CACCGACTCGGACGTCATCGGCGC	0	10	RDR2-sensitive
CACCGACTCGGATGTCATCGGCGC	11	43	RDR2-sensitive
CACCGAGACTGCATGCGTGGACGT	0	39	RDR2-sensitive
CACCGATATCGTCGACAAGGTCGG	0	11	RDR2-sensitive
CACCGATCTCTCGGGACTGGCGTC	0	11	RDR2-sensitive
CACCGATGACTGGAAGTAGGGACG	1	15	RDR2-sensitive
CACCGCAGACGACTCACCGGACAG	0	10	RDR2-sensitive

CACCGCGACGTGGACAGCCAGCAC	0	9	RDR2-sensitive
CACCGCGGCTGGCATAGTGCACAC	0	34	RDR2-sensitive
CACCGCGTCGACGTAGGAACGGTG	0	19	RDR2-sensitive
CACCGGACAATCACTGTAGACGGT	0	10	RDR2-sensitive
CACCGGACACTGAACAGTAGATGT	0	21	RDR2-sensitive
CACCGGACACTGCACAGTAGATGT	0	21	RDR2-sensitive
CACCGGACAGAACTGAGGGGATGT	0	9	RDR2-sensitive
CACCGGACAGGTCCTGTAGACGGT	1	24	RDR2-sensitive
CACCGGACAGTCACTGTAGACGGT	2	48	RDR2-sensitive
CACCGGACAGTGAACAGTAACGGA	0	10	RDR2-sensitive
CACCGGACAGTGAACAGTGCGCGG	0	25	RDR2-sensitive
CACCGGACATCCAGGCGGGCCCAG	0	13	RDR2-sensitive
CACCGGACTGTCCGGCGTGCCCAT	0	9	RDR2-sensitive
CACCGGACTGTTCGGTGAGCCAAC	2	17	RDR2-sensitive
CACCGGAGGACCTGTAGAGACGGA	0	57	RDR2-sensitive
CACCGGATACCCGATACCCGACGG	0	11	RDR2-sensitive
CACCGGATCTGGCATGGCACACAC	0	9	RDR2-sensitive
CACCGGGCTGGACGGTCGAGTGGG	0	11	RDR2-sensitive
CACCGGTCGGTCTGGTGCACGGTT	0	17	RDR2-sensitive
CACCGGTGAGAACGCTGAGCCGGT	27	3	RDR2-resistant
CACCGTACGAGGCTTGGCATGGGC	25	1	RDR2-resistant
CACCGTCCATGTTCTCATCGGCAC	0	29	RDR2-sensitive
CACCGTCTGATGCGATCGAAGGTC	0	15	RDR2-sensitive
CACCGTCTGTTTAACCCACGGCAA	0	20	RDR2-sensitive
CACCGTGCGACCTGAGAACGGCCT	0	13	RDR2-sensitive
CACCGTGCGCTGGGGGAAGAGAGC	22	1	RDR2-resistant
CACCTACAGGCTGTATGGACGGAA	0	18	RDR2-sensitive
CACCTAGGACACTCGACACTCGGC	0	12	RDR2-sensitive
CACCTCAATCTGACTGGATGGCTG	0	22	RDR2-sensitive
CACCTCTCGGAAGGGATCTGTCGG	0	13	RDR2-sensitive
CACCTGGATCATGTGGAAGACGAC	0	28	RDR2-sensitive
CACCTGTAAGAGCCTTTGACGGGA	0	24	RDR2-sensitive
CACCTGTAAGAGCGTCGACGGAAC	0	13	RDR2-sensitive
CACCTGTAAGAGGCTTTTGACGGA	0	17	RDR2-sensitive
CACCTGTAGCCTGACGGTGACCTC	0	9	RDR2-sensitive
CACCTGTAGGGGCACGTAGATGGC	0	10	RDR2-sensitive
CACCTGTCTTATATCTATGGCATG	14	0	RDR2-resistant
CACCTGTGGAGCTGCGTTCCGGAT	0	10	RDR2-sensitive
CACCTTCTCTGGACACGTGGCGGC	0	21	RDR2-sensitive
CACCTTCTGCAGCGGAGACTGTAT	0	10	RDR2-sensitive
CACGAAACTAAACACGGCGGGCAA	0	10	RDR2-sensitive
CACGAAAGGCAATGTAGGGCGGTT	0	14	RDR2-sensitive
CACGAACACGGATAGCGGACGCAC	0	16	RDR2-sensitive
CACGAAGCTCTGACGTGACGACGT	2	70	RDR2-sensitive
CACGAATAAGCGGGACGGGCTCGG	0	22	RDR2-sensitive
CACGAATAAGCGGGTCGGGCTCGG	0	25	RDR2-sensitive
CACGAATAAGCGGGTTGGCTCGGA	0	12	RDR2-sensitive
CACGACACACTGGACGGAAGAGCA	0	12	RDR2-sensitive
CACGACAGATAGCACACGAGACAC	2	17	RDR2-sensitive

CACGACCATGTAGCTCGGCGTCAT	0	19	RDR2-sensitive
CACGACCCAACGAAGACACGACAT	0	12	RDR2-sensitive
CACGACGACGATGATCCGAGCGGC	0	15	RDR2-sensitive
CACGACGACTGTACAGATTACGCC	0	14	RDR2-sensitive
CACGACGGATGCGGCTTCGACGAA	0	11	RDR2-sensitive
CACGACGGCATGTACTTAGGGCAC	4	24	RDR2-sensitive
CACGACGGCATGTACTTAGGGCGC	6	32	RDR2-sensitive
CACGACTCTCGGCAACGGATATCT	2003	215	RDR2-resistant
CACGACTGTATAGGATGGTATCTC	0	9	RDR2-sensitive
CACGACTGTGAGCTACAACGGTGT	0	32	RDR2-sensitive
CACGACTGTGTGGTTCACCGGCAT	1	15	RDR2-sensitive
CACGACTTGGACACGGGAGCGGCT	0	20	RDR2-sensitive
CACGAGACAGTGTAAGACGGAGAC	0	34	RDR2-sensitive
CACGAGACGATGATCCGAGCGGCA	33	1	RDR2-resistant
CACGAGATGGAGGCAAACTGGCTA	0	20	RDR2-sensitive
CACGAGCACGGACTGGACCGGCCC	0	18	RDR2-sensitive
CACGAGCCCACGGACTTAGATGGC	0	21	RDR2-sensitive
CACGAGCTGACGCGGCGTCGACGT	0	11	RDR2-sensitive
CACGAGTGCGGACCACGAGTGCGG	0	22	RDR2-sensitive
CACGAGTGTGTGACTATCGCTGCC	61	1	RDR2-resistant
CACGATAGAATAAGCGGGCGGGCT	0	9	RDR2-sensitive
CACGATATAGGGCTGGACGGTTCA	0	14	RDR2-sensitive
CACGATCTTGTGGAGCGTGACACC	2	72	RDR2-sensitive
CACGATGAGACTGGAGTAGAGGCC	0	22	RDR2-sensitive
CACGATGCAGGCACGAGAACGCGT	0	10	RDR2-sensitive
CACGATGGCATGTACTTAGGGCGC	2	15	RDR2-sensitive
CACGATTAGTCAGGCTGGTCGGCT	20	0	RDR2-resistant
CACGATTCACGACGGAACGGCGAT	0	9	RDR2-sensitive
CACGCACTGAAGACATGGAGGCAC	4	27	RDR2-sensitive
CACGCAGCACACTCACTCGTCGGC	9	78	RDR2-sensitive
CACGCAGGACTGTGCAGGCACGGC	0	14	RDR2-sensitive
CACGCATCTGATCATGGAGGACGT	0	9	RDR2-sensitive
CACGCCAGACTGCTGACTGTACGG	1	12	RDR2-sensitive
CACGCCGTGCGACTGGGTAGGCAG	0	35	RDR2-sensitive
CACGCCTGCTAGACGAGTCGGACA	0	19	RDR2-sensitive
CACGCGAAGTAGCAGAATGAGCGC	0	15	RDR2-sensitive
CACGCGACAAATGGGACACACGGA	1	25	RDR2-sensitive
CACGCGACGAATGGGACACACGGA	0	17	RDR2-sensitive
CACGCGACTGTGGTTGTTGGGGAC	0	9	RDR2-sensitive
CACGCGGAACGACGATGAGATCAT	0	9	RDR2-sensitive
CACGCGGCACACTCACTCGTCGGC	5	27	RDR2-sensitive
CACGCGGGACTGCCACGTCAGGAA	0	17	RDR2-sensitive
CACGCGGTAGAACCACCAGAAGAT	0	14	RDR2-sensitive
CACGCGTGCGCGAGATCTGAAGGC	0	9	RDR2-sensitive
CACGCTACAGTAGAAGGGGACCAA	0	10	RDR2-sensitive
CACGCTATACTGCATGCACTCAGC	0	16	RDR2-sensitive
CACGCTGTGGGCTGGGGGCGACGG	0	9	RDR2-sensitive
CACGGAACTGTTGGCGTGCCAGGC	0	11	RDR2-sensitive
CACGGAAGACAGTGCTAGGGAACA	0	9	RDR2-sensitive

CACGGAAGCGAACGAACGGTGCAG	0	10	RDR2-sensitive
CACGGAAGCGAACGGATGGTACAG	0	10	RDR2-sensitive
CACGGAAGGGACGCTGCATATAGC	0	11	RDR2-sensitive
CACGGAATGAGCAGAACGCGGAAT	0	10	RDR2-sensitive
CACGGACACGACTGGGCCCGGCAC	0	9	RDR2-sensitive
CACGGACCCCTAGGACTACGGTGT	0	17	RDR2-sensitive
CACGGACCGTACTGAAGCGTGCAT	0	52	RDR2-sensitive
CACGGACCGTCCAAGCCCTAGGCG	0	15	RDR2-sensitive
CACGGACCGTTTGAAAGCGCGCAG	0	19	RDR2-sensitive
CACGGACGCGTCTGTTCACCGGTT	44	6	RDR2-resistant
CACGGAGAGACGGCTCTAAGATTT	0	19	RDR2-sensitive
CACGGAGCGGTATAAGGAGCAGAA	49	7	RDR2-resistant
CACGGAGCTCTGACATGACGACGT	1	35	RDR2-sensitive
CACGGAGGTGTGGGCATAAGAGGA	2	15	RDR2-sensitive
CACGGATCTGTTAGCGTGCCGGGC	0	9	RDR2-sensitive
CACGGATTAGGACTGTACCGGCCT	0	14	RDR2-sensitive
CACGGATTGAAGGCCTGGAACGGT	0	10	RDR2-sensitive
CACGGCAAAAATGGGTAGTGGGAT	16	0	RDR2-resistant
CACGGCAAGGTCGACGACGAGATA	0	9	RDR2-sensitive
CACGGCACAGGCCCACAAAAGCAC	0	18	RDR2-sensitive
CACGGCACGGTAGAATAAGCGGGC	0	17	RDR2-sensitive
CACGGCACTGGCACGGCACGGCAC	0	15	RDR2-sensitive
CACGGCACTGGCACGGCATGACAC	0	13	RDR2-sensitive
CACGGCATCCAGCTGGCACGGCAA	20	1	RDR2-resistant
CACGGCATGAACTCGACACGGCAT	0	19	RDR2-sensitive
CACGGCCAGGTGTCGGACGGTCCG	0	9	RDR2-sensitive
CACGGCCGCTGAACTGCTTGACGG	0	13	RDR2-sensitive
CACGGCCTAGTAGACGGTGTGCCC	0	31	RDR2-sensitive
CACGGCGACTGGTAAAGGAGACGA	0	9	RDR2-sensitive
CACGGCGTGGAATCTTTGGCGGCG	0	9	RDR2-sensitive
CACGGCTAGGCACGGTAGAAGATC	0	15	RDR2-sensitive
CACGGGACGGCGAAGCGGACGCTC	2	31	RDR2-sensitive
CACGGGACTGCTGAAGATTAGGGT	0	16	RDR2-sensitive
CACGGGAGCGAGATGTAGACGGAT	0	13	RDR2-sensitive
CACGGGAGGCAGAACATGGTGCAT	0	10	RDR2-sensitive
CACGGGCTGAGTTGGTGTCCCAAG	0	14	RDR2-sensitive
CACGGGCTGTATGAGCGGATGCAA	0	49	RDR2-sensitive
CACGGGTAAGGATACGGACAACAC	0	14	RDR2-sensitive
CACGGGTAAGGATACGGGCAACAC	0	14	RDR2-sensitive
CACGGGTACGGGTTTGGGACGGCA	0	20	RDR2-sensitive
CACGGGTGCACGGGCTGTCCGGTG	1	22	RDR2-sensitive
CACGGGTTGTAAGGGATACCACAT	0	22	RDR2-sensitive
CACGGTAAATTGGGCTGGCACGGC	0	9	RDR2-sensitive
CACGGTACCGAGCAGGCACGACAC	0	16	RDR2-sensitive
CACGGTAGAATAAACGGGCGGGCT	0	40	RDR2-sensitive
CACGGTAGAATAAGCGGACGGACT	0	23	RDR2-sensitive
CACGGTAGAATAAGCGGACGGGCT	0	25	RDR2-sensitive
CACGGTAGAATAAGCGGGCAGGCT	0	12	RDR2-sensitive
CACGGTAGAATAAGCGGGCAGGTT	0	17	RDR2-sensitive

CACGGTAGAATAAGCGGGCGGACT	0	18	RDR2-sensitive
CACGGTAGAATAAGCGGGCGGGCT	21	1328	RDR2-sensitive
CACGGTAGAATAAGCGGGCGGGGT	2	15	RDR2-sensitive
CACGGTAGAATAAGCGGGCGGGTT	2	85	RDR2-sensitive
CACGGTAGAATAAGTGGACGGGCT	0	15	RDR2-sensitive
CACGGTAGAGAGCACGGTGAGCAC	0	19	RDR2-sensitive
CACGGTCGCGTGCTGGAGCGGATA	0	9	RDR2-sensitive
CACGGTCGCTGTTACGGTAGTCCT	0	27	RDR2-sensitive
CACGGTGACTGGTAACGGAGACGT	1	17	RDR2-sensitive
CACGGTGCTTTTGGGTAGGACGGT	0	12	RDR2-sensitive
CACGGTGGAGGACGAGGAGTAGGT	68	1	RDR2-resistant
CACGGTGTTGTCTCGGACGTGCGC	0	10	RDR2-sensitive
CACGGTTAGAAAACCGGGTCGGGC	0	12	RDR2-sensitive
CACGGTTAGAAAATCGGGTCGGAC	0	11	RDR2-sensitive
CACGGTTAGGAAACCGGGTCGGGC	0	11	RDR2-sensitive
CACGGTTCTGACCATATCCTTGGC	16	0	RDR2-resistant
CACGGTTCTGTGACGGAGTGGTAT	0	31	RDR2-sensitive
CACGGTTCTTGTTGAGTGTTTGGC	2	76	RDR2-sensitive
CACGGTTGACTGGGAGACGATGGC	0	12	RDR2-sensitive
CACGTAATGGGCGCTGTAGGGGGC	0	11	RDR2-sensitive
CACGTACGGTCAGAGTAGCAGGCT	0	12	RDR2-sensitive
CACGTAGAGGGCGCTGTAGGGGGC	3	20	RDR2-sensitive
CACGTAGATCTGAATGTATGGCGA	0	11	RDR2-sensitive
CACGTAGATGAGCTGACAGGTGGT	0	12	RDR2-sensitive
CACGTAGGAGGCGCTGTAGGGGCA	0	20	RDR2-sensitive
CACGTAGGAGGCGCTGTAGGGGGC	1	16	RDR2-sensitive
CACGTAGGGACCGACATCAAGACG	0	27	RDR2-sensitive
CACGTAGGGACCGACATCGAGACG	0	14	RDR2-sensitive
CACGTAGGGAGCGCTGTAGGGGGC	0	11	RDR2-sensitive
CACGTAGGGCGCTGTAGCAGGCAC	0	13	RDR2-sensitive
CACGTAGGGGACGCTGTAGAGGGC	0	38	RDR2-sensitive
CACGTAGGGGCGCTGTAGAGGGCA	0	30	RDR2-sensitive
CACGTAGGGGGCGCTGTAGGAGGC	0	13	RDR2-sensitive
CACGTAGGGGGCGCTGTAGGGGCA	1	26	RDR2-sensitive
CACGTAGGGGGCGCTGTAGGGGGC	7	96	RDR2-sensitive
CACGTAGTGGGCGCTGTAGGGGCA	0	23	RDR2-sensitive
CACGTCACGAAGCTCGGCGCCATA	0	18	RDR2-sensitive
CACGTCACGGTTAGAGCACGGCCC	0	9	RDR2-sensitive
CACGTCAGAGCTCGGCGCCATAGG	0	9	RDR2-sensitive
CACGTCAGATACCAGGCATCTCTC	0	14	RDR2-sensitive
CACGTCAGGAAGCTCGGCGCCACA	0	10	RDR2-sensitive
CACGTCCCTCAGTAGACAAGGCAT	0	14	RDR2-sensitive
CACGTCGACTGTTCAGAGGGCCGC	0	18	RDR2-sensitive
CACGTCGTAATCTGCAGTGGGCAC	0	9	RDR2-sensitive
CACGTCGTGCTTTCGTTGGGTCGT	0	11	RDR2-sensitive
CACGTCGTGTCTTAGTTGGGTCGT	0	10	RDR2-sensitive
CACGTCTGTATGCACTCAGGGGCG	0	25	RDR2-sensitive
CACGTGACATGTAGGGGAAGGCAC	0	10	RDR2-sensitive
CACGTGGACCAATCAGATCGCGAC	0	53	RDR2-sensitive

CACGTGGACCAATCAGATCGCGCC	1	33	RDR2-sensitive
CACGTGGACCAGTCAGATCGCGCC	1	17	RDR2-sensitive
CACGTGGCATGGGAGATCCGCGGC	27	0	RDR2-resistant
CACGTGGCTGACAAGTAAGAGCGT	1	12	RDR2-sensitive
CACGTGGGCCTCAGAGCAGGACGA	0	15	RDR2-sensitive
CACGTGGGCCTCTGAGCTGGACGA	3	28	RDR2-sensitive
CACGTGGTTTGTGATGGACGTCAA	0	9	RDR2-sensitive
CACGTGTACAGCAACGACGGACGC	0	10	RDR2-sensitive
CACGTGTGCTGTAGCCTGAGGCTG	0	11	RDR2-sensitive
CACGTGTGGACTATGTAGAGGCGT	1	45	RDR2-sensitive
CACGTTCCGGGCTTTAGAGGGTGT	0	17	RDR2-sensitive
CACGTTGGCATAGGTAAAGCGCGG	0	10	RDR2-sensitive
CACGTTGGCATGTACACAGGGCTC	0	15	RDR2-sensitive
CACGTTGGCATGTACACAGGGCTT	1	28	RDR2-sensitive
CACGTTGGCTAGAGGGTGGAAGGC	3	16	RDR2-sensitive
CACGTTGTTGTGGGCCGTGCTCGT	0	10	RDR2-sensitive
CACGTTTCGACGCTCTGGGTGGCC	0	11	RDR2-sensitive
CACTAAAAAGCACGGCACTGGCAC	0	12	RDR2-sensitive
CACTACTTCGGCTACTTCGCGGCG	106	4	RDR2-resistant
CACTAGCTGAAGAAGGACGAGCGG	0	10	RDR2-sensitive
CACTAGCTGAAGGAGGATGAGCGA	0	9	RDR2-sensitive
CACTAGCTGAAGTAGGACGAGCGG	0	11	RDR2-sensitive
CACTAGGGTCGGTTGTGTTATCAT	20	2	RDR2-resistant
CACTAGGGTTGTACAGTTTGACGG	0	10	RDR2-sensitive
CACTAGTCGTTAGGATCCTCGGGT	0	18	RDR2-sensitive
CACTAGTGGACAGCAGCAGACGAC	0	9	RDR2-sensitive
CACTATATTGGACTGGGCCACGGC	0	9	RDR2-sensitive
CACTATCGGGCTGATTGGATGGGC	1	15	RDR2-sensitive
CACTATGGTGGGACTGGGATAGGC	0	10	RDR2-sensitive
CACTCCTGCAGCGAGGTCGCGGAC	0	19	RDR2-sensitive
CACTCGACAAAGAGGGCTCGGCAT	1	37	RDR2-sensitive
CACTCGACAGCTTCGGACGTTCAT	0	26	RDR2-sensitive
CACTCGACAGCTTCGGGCGTTCAT	0	14	RDR2-sensitive
CACTCGACAGCTTTGGGCGTTCAT	0	15	RDR2-sensitive
CACTCGACTGTCCAACCCGGTGGC	17	75	RDR2-sensitive
CACTCGAGACCAGCACAAGAGCGC	1	21	RDR2-sensitive
CACTCGAGACCAGCATAGGGACGT	0	16	RDR2-sensitive
CACTCGAGACTAGCACAGGAGCGC	0	11	RDR2-sensitive
CACTCGAGACTAGCACAGGGGCGC	0	17	RDR2-sensitive
CACTCGCTCCGACCAGATGACTGA	0	9	RDR2-sensitive
CACTCGGCAAAGAACACTCGGCAA	21	490	RDR2-sensitive
CACTCGGCAAAGAACACTCGGCAC	0	11	RDR2-sensitive
CACTCGGCAAAGAACGCTCGGCAA	1	29	RDR2-sensitive
CACTCGGCAAAGAACGCTCGGCGA	19	295	RDR2-sensitive
CACTCGGCAAAGAACGTTCGGCGA	0	15	RDR2-sensitive
CACTCGGCAAAGAAGACTCGGCAA	7	89	RDR2-sensitive
CACTCGGCAAAGAAGACTCGGCAT	0	13	RDR2-sensitive
CACTCGGCAAAGAAGGCTCGGCAA	17	283	RDR2-sensitive
CACTCGGCAAAGAAGGCTCGGCAT	2	16	RDR2-sensitive

CACTCGGCAAAGAAGGCTCGGCGA	1	12	RDR2-sensitive
CACTCGGCAAAGACGGCTCTCGGC	1	13	RDR2-sensitive
CACTCGGCAAAGACTGACGGTCGT	1	12	RDR2-sensitive
CACTCGGCAAAGATGGCTCTCGGC	0	9	RDR2-sensitive
CACTCGGCAAAGGCTATTTTACAC	1	38	RDR2-sensitive
CACTCGGCACAGCCAAGCACTCGG	1	23	RDR2-sensitive
CACTCGGCACAGGACGCACTCGGC	0	18	RDR2-sensitive
CACTCGGCACAGGACGGCACTCAT	0	12	RDR2-sensitive
CACTCGGCACAGGACGGCACTCGG	2	42	RDR2-sensitive
CACTCGGCACAGGACGGCGCTCAG	1	14	RDR2-sensitive
CACTCGGCACAGGGCGGCACTCGG	2	39	RDR2-sensitive
CACTCGGCAGAGGACAGCACTCGG	1	39	RDR2-sensitive
CACTCGGCTGGCTGACGAAGAGAC	6	24	RDR2-sensitive
CACTCGTCACTGTAACCCGATGGT	0	18	RDR2-sensitive
CACTCGTGACCCGTGGGCCGGCAT	0	14	RDR2-sensitive
CACTCTATATACGGGGCTGTCGGT	0	23	RDR2-sensitive
CACTCTCAACTGTAGCGGTGTGGT	0	13	RDR2-sensitive
CACTCTGAAGGCACATGACGACCT	0	15	RDR2-sensitive
CACTCTGCAACGACCTAGCCGGAG	0	12	RDR2-sensitive
CACTCTGGACCATGCCGTGCTGCT	0	36	RDR2-sensitive
CACTCTGGATGAAGCGAACGGTCT	0	11	RDR2-sensitive
CACTCTGGGCCAGCGAAACGACTG	2	14	RDR2-sensitive
CACTCTGTAGCAACGCACAGGCGT	0	17	RDR2-sensitive
CACTCTTGAACCCCTCGGATGGTT	45	0	RDR2-resistant
CACTGAACCGGACGTTGGCGCGCC	0	25	RDR2-sensitive
CACTGAACGGATTTAGACGACGTA	1	20	RDR2-sensitive
CACTGACAGAATGGATCGACGCAA	0	11	RDR2-sensitive
CACTGACCAGTGGTACCGGACAAC	0	9	RDR2-sensitive
CACTGACCGACCTGACAGAAGGAC	0	11	RDR2-sensitive
CACTGACCGGCCTGACAAGAGGAC	0	9	RDR2-sensitive
CACTGACCGGCCTGACAGAAAGAC	0	11	RDR2-sensitive
CACTGACCGGCCTGACAGAAGGAC	5	19	RDR2-sensitive
CACTGACCGGTCTGACAAGAGGAC	0	14	RDR2-sensitive
CACTGACCGTCTACAGTAGGGCAT	0	20	RDR2-sensitive
CACTGACTGGTCTGACAGAAGGAC	0	9	RDR2-sensitive
CACTGATACGATTGGACACGGCCT	0	100	RDR2-sensitive
CACTGATCTGACTTAATCCGGCAT	116	2	RDR2-resistant
CACTGATGTTCTTGATTGGTCGGT	26	2	RDR2-resistant
CACTGCAAATTAGAGCTCGTCGCC	16	0	RDR2-resistant
CACTGCAGTCGGAGTGGCGCAGGC	11	28	RDR2-sensitive
CACTGCGTGAGAACCGAACACGGT	0	11	RDR2-sensitive
CACTGCGTTGAGAACCTGCGGCGA	0	37	RDR2-sensitive
CACTGCTGAATTCTATCACGGATG	87	0	RDR2-resistant
CACTGGACGGACAGCGACGGTCGG	0	34	RDR2-sensitive
CACTGGACTGTCCGGTGAGCCAAC	0	17	RDR2-sensitive
CACTGGACTGTCCGGTGCGCCCAT	0	18	RDR2-sensitive
CACTGGACTGTGCGGTGCGCCCAT	0	9	RDR2-sensitive
CACTGGATCAAGATCGTACGGCGA	0	10	RDR2-sensitive
CACTGGGAATGTGGACGTCTACAC	0	12	RDR2-sensitive

CACTGTAACCTCGAAGCACGTCAT	0	11	RDR2-sensitive
CACTGTAGACGGTCCGGTGCGGAT	0	16	RDR2-sensitive
CACTGTAGAGGCATACTGGACCGA	0	22	RDR2-sensitive
CACTGTAGCAATGGTAGGGGGCAC	0	15	RDR2-sensitive
CACTGTAGCAGCGATAGGGGGCAC	0	9	RDR2-sensitive
CACTGTAGCAGCGCTAGGGGGCAA	1	13	RDR2-sensitive
CACTGTAGCAGCTTTAGCGGTCAC	0	15	RDR2-sensitive
CACTGTAGCAGTGGTAGGGGGCAC	1	21	RDR2-sensitive
CACTGTAGCATGTCCGGTGCGGAT	1	17	RDR2-sensitive
CACTGTAGCGATGTCAGGCGCCGT	0	10	RDR2-sensitive
CACTGTAGCGGCATGCATCTTCCA	0	17	RDR2-sensitive
CACTGTAGCTTTAGGGGACCGAAT	0	22	RDR2-sensitive
CACTGTCACCTCTGCCTCAAGCGG	0	9	RDR2-sensitive
CACTGTCAGCACTCGGCAAAGATG	0	21	RDR2-sensitive
CACTGTCGCTTGTACGAGATGGAT	0	18	RDR2-sensitive
CACTGTGACTCGAGCTGAATACGT	0	12	RDR2-sensitive
CACTGTGGCTTGGTAAGAACGGAT	0	15	RDR2-sensitive
CACTGTGTTGAGAACCTGCGACGG	0	10	RDR2-sensitive
CACTGTTAGTAGCAGCATCGTCGT	0	24	RDR2-sensitive
CACTGTTCACGGCACTTGCTGGCA	26	2	RDR2-resistant
CACTGTTCACGTCGGCACCAGGCT	30	3	RDR2-resistant
CACTGTTGACCAGTAGGACCGGAC	0	15	RDR2-sensitive
CACTGTTGGAGGGAAGGCGAAGAC	0	9	RDR2-sensitive
CACTGTTGGTACTTCAAGATTGGA	0	22	RDR2-sensitive
CACTGTTTTGATGTGTTAGGTCGG	0	13	RDR2-sensitive
CACTGTTTTTACACTTCGGGCTGG	0	22	RDR2-sensitive
CACTTAGGTCCTCGAACTTGGCAT	0	10	RDR2-sensitive
CACTTATTCGTGTCGGGCCGGCCC	0	15	RDR2-sensitive
CACTTCAACCTGGGATCGAGGGCA	0	9	RDR2-sensitive
CACTTCAACGGATCCACTCGGCAC	0	21	RDR2-sensitive
CACTTCACCATTGTCGGCACCAAC	0	13	RDR2-sensitive
CACTTGAACATGGTCGACGGCGAG	0	22	RDR2-sensitive
CACTTGAACATGGTCGACGGTGAG	0	9	RDR2-sensitive
CACTTGACAGAGTGAGGCTGACAG	0	10	RDR2-sensitive
CACTTGGACGAACGACTGGGCGGC	0	11	RDR2-sensitive
CACTTGTCGTGACTCGGTCATCAT	0	9	RDR2-sensitive
CACTTTATCTCGGATCGGATACGG	0	15	RDR2-sensitive
CACTTTCGGCAGGACGATATGCAA	0	11	RDR2-sensitive
CACTTTGTTAGGCTGTACCGGTGT	0	10	RDR2-sensitive
CACTTTTGGTGTTCAACACGTCGG	0	10	RDR2-sensitive
CACTTTTTTATTTGGTTTGTGGAA	0	13	RDR2-sensitive
CAGAAAAGGTGGACTCGGACGATC	0	12	RDR2-sensitive
CAGAAAGACGGATGGACTCACCGC	0	50	RDR2-sensitive
CAGAACAACCCGAGAGTCGTCGAC	0	14	RDR2-sensitive
CAGAACACGAGTGGGGCAGAACAA	0	9	RDR2-sensitive
CAGAACAGACAGAGCACCACACAG	0	17	RDR2-sensitive
CAGAACATGCACCACTGTGGACGC	0	9	RDR2-sensitive
CAGAACCAGAACCAGATCGAGCGC	0	31	RDR2-sensitive
CAGAACGACAGAACGGCAGAACGC	0	14	RDR2-sensitive

CAGAACGGCACTCGGCAAAGACAA	3	20	RDR2-sensitive
CAGAACGGCAGAACGGCAGAACGC	0	12	RDR2-sensitive
CAGAACTCACCTCACGGAGGACAT	0	16	RDR2-sensitive
CAGAACTCCCGCTCAGTAGAGACA	52	122	RDR2-sensitive
CAGAACTCCTGATCCATAGAGACA	64	0	RDR2-resistant
CAGAACTCTGCAGGTAGAGCTGTA	1	39	RDR2-sensitive
CAGAACTCTGCCCAGACACGTCAT	0	22	RDR2-sensitive
CAGAACTGCGTACGCTGCCGTCAA	0	15	RDR2-sensitive
CAGAAGACGCGCGAGAAGCTGGAC	0	13	RDR2-sensitive
CAGAAGGAAGGGCACTCAGATCGA	0	12	RDR2-sensitive
CAGAAGGAAGGGCACTCGGATCGA	0	11	RDR2-sensitive
CAGAAGTACGTAGTCGCTGTCCGA	0	9	RDR2-sensitive
CAGAAGTGTGAGGAGGCGTAGGAA	27	0	RDR2-resistant
CAGAATACTGTCCAAGCTTCGGCA	20	0	RDR2-resistant
CAGAATCGTGCCGGATCACGGGTC	0	20	RDR2-sensitive
CAGAATCTGCGCGACGAACCGGCT	0	9	RDR2-sensitive
CAGACAACGAATTTCAGAGTGATG	46	1	RDR2-resistant
CAGACATACTGGTAGACGACGCGA	0	12	RDR2-sensitive
CAGACCACTCTCGGGACTCGACGG	0	13	RDR2-sensitive
CAGACCAGCGACTGCAGCGAAGGC	0	25	RDR2-sensitive
CAGACCAGCGGCTGCAGCGAAGAC	0	9	RDR2-sensitive
CAGACCATCTGGACCTGACCTCGG	0	16	RDR2-sensitive
CAGACCGAGGCTGTGCGACGCGGA	1	15	RDR2-sensitive
CAGACCGCGGACCGTTCGGCACCT	0	17	RDR2-sensitive
CAGACCGGTCAGTGGAGCGGCGAA	6	39	RDR2-sensitive
CAGACGAACGACGGGGATGACGAC	27	0	RDR2-resistant
CAGACGAAGGCCTGCGGTGACGTC	3	20	RDR2-sensitive
CAGACGACTAGCAGAGGTGAGCGT	0	48	RDR2-sensitive
CAGACGACTCGAACGGCGCCTCGC	1	29	RDR2-sensitive
CAGACGAGTGGTAGACAGGGATGG	0	20	RDR2-sensitive
CAGACGGACGCTGGACGCAGGAAG	0	11	RDR2-sensitive
CAGACGGATGGTAGAAAGGGTTAG	0	19	RDR2-sensitive
CAGACGGATGGTAGACAAGGTTAG	0	17	RDR2-sensitive
CAGACGGCATGCAGGACAGACGAC	0	12	RDR2-sensitive
CAGACGGTGGAGACAGACAGAGGA	0	11	RDR2-sensitive
CAGACGTGGAATAAGAGCGAGCAA	0	53	RDR2-sensitive
CAGACGTGGAATAGGAGCGAGCAA	1	16	RDR2-sensitive
CAGACGTGGAGTAGAAGCGAGCAA	0	10	RDR2-sensitive
CAGACGTTGAGCTGGACAGGTCGC	0	11	RDR2-sensitive
CAGACTCCGGACAGTGTGAGCGGT	0	9	RDR2-sensitive
CAGACTCTCGAGCAAGCCAGACAA	0	9	RDR2-sensitive
CAGACTCTCGAGCAAGCTAAGCGA	0	11	RDR2-sensitive
CAGACTGAATCTGATAAGAATGGA	0	9	RDR2-sensitive
CAGACTGACCCTGACACTACGGCT	0	17	RDR2-sensitive
CAGACTGACGCGCAGACTGACCGC	0	13	RDR2-sensitive
CAGACTGAGCTGAACTTCGGCTGC	0	10	RDR2-sensitive
CAGACTGCTTCTCTCTCACGGCAA	0	11	RDR2-sensitive
CAGACTGGACTGGCTGAGTGCATG	0	24	RDR2-sensitive
CAGACTGGCCGAGCCGATCGACGT	2	15	RDR2-sensitive

CAGACTGGTAGATGCAAGGACTGG	0	10	RDR2-sensitive
CAGACTGGTCAGTGGAGCGGTGGA	3	16	RDR2-sensitive
CAGACTGGTTTAGGTGCTGTCGGG	0	9	RDR2-sensitive
CAGACTGTGACAGCTGGGTCCCGT	0	9	RDR2-sensitive
CAGACTGTGTTCAACGGGGGACGC	0	13	RDR2-sensitive
CAGACTTCAATCGAAGATCGTCGT	0	9	RDR2-sensitive
CAGACTTCAATCGAAGGTCGTCGT	2	61	RDR2-sensitive
CAGAGACAAAACAGGCAGGGACAC	0	12	RDR2-sensitive
CAGAGACAAAACGAGCAGGGACAC	0	12	RDR2-sensitive
CAGAGACAAAACGGGCAGGGACAC	0	17	RDR2-sensitive
CAGAGACAAAGCAGGCAGGGACAC	1	14	RDR2-sensitive
CAGAGACAGAACAGGCAGGGACAC	0	30	RDR2-sensitive
CAGAGACGTGTAGCAGTCGTGCGC	0	11	RDR2-sensitive
CAGAGATCGCGACAACCGGAGGTA	22	0	RDR2-resistant
CAGAGCAGACAAGTCGGACCTCAA	0	22	RDR2-sensitive
CAGAGCAGAGGCTACGGAACGCGC	0	14	RDR2-sensitive
CAGAGCCGGACGGCCTGGGGTCAC	34	0	RDR2-resistant
CAGAGCGGGATTGGTGTAAACGAA	0	12	RDR2-sensitive
CAGAGCGGTAGGGGTGGAGGGCGT	16	0	RDR2-resistant
CAGAGCGGTGCTGCAGAGCGGTAT	0	16	RDR2-sensitive
CAGAGGACAATACTGGATAGTCAT	0	28	RDR2-sensitive
CAGAGGACCTGAGCTGCTCGGCGT	0	12	RDR2-sensitive
CAGAGGACGCTGCTGAAGAGCATA	0	10	RDR2-sensitive
CAGAGGAGTGTACCACAGAGGATG	20	2	RDR2-resistant
CAGAGGCGTAGACAGTGGGTGGTC	0	9	RDR2-sensitive
CAGAGGCTACGGAACGCGCAGAAA	0	17	RDR2-sensitive
CAGAGGGTCTGTACGGCGTATCGG	1	13	RDR2-sensitive
CAGAGGGTGGCATAGAACTGAGCT	17	87	RDR2-sensitive
CAGAGTCAGCTCGGCTCGGCTCGT	0	11	RDR2-sensitive
CAGAGTGAACGGAACGGAGCGTGA	0	16	RDR2-sensitive
CAGAGTGGACAGTTGACGCCGGCC	0	10	RDR2-sensitive
CAGAGTGGACTGGGCACGCACGGT	0	13	RDR2-sensitive
CAGAGTGGGCTAACGACGACGGAT	70	3	RDR2-resistant
CAGAGTTAGACGTGTGGTAGACAT	0	10	RDR2-sensitive
CAGATAAGTTTAGATCGTGCCGAC	50	3	RDR2-resistant
CAGATACGGAAGGCGACTCCAAGT	20	2	RDR2-resistant
CAGATACGGCTGCACGGTACTCCA	0	81	RDR2-sensitive
CAGATACGGGTAGGATAGGTGATA	0	12	RDR2-sensitive
CAGATAGATGGGAGGCAGAACGGC	0	20	RDR2-sensitive
CAGATCACACGTGGGCCTTAGGAC	36	3	RDR2-resistant
CAGATCAGGCTACGTAGAGGGAGC	0	9	RDR2-sensitive
CAGATCCAAAGTCGGGCGGGGCAC	1	35	RDR2-sensitive
CAGATCGACGACCCTGGACGCGGT	0	16	RDR2-sensitive
CAGATCGATATCAGGACTCGACGG	1	26	RDR2-sensitive
CAGATCGATATCGGGACTCGACGG	1	12	RDR2-sensitive
CAGATCGATGGACGGCAGAACGGC	0	25	RDR2-sensitive
CAGATCGCTGAACGCTGACGGTGA	2	33	RDR2-sensitive
CAGATCGGGTTGGTCGAGTCGGAT	0	9	RDR2-sensitive
CAGATCGTAAACCAGGGCAGGCAT	0	9	RDR2-sensitive

CAGATCTACTGGACGACGAGCGCA	0	25	RDR2-sensitive
CAGATCTCTGCCGCCAGGACGGCG	24	0	RDR2-resistant
CAGATGAGGATGTGCCGGATTCGG	14	0	RDR2-resistant
CAGATGAGTCGGCGCGAGACGGAA	0	10	RDR2-sensitive
CAGATGATGCGTACTGATTAGACT	0	13	RDR2-sensitive
CAGATGCACCTCTGCGGTCGGGAC	0	28	RDR2-sensitive
CAGATGCATGTGGAGACCGGTGAT	23	0	RDR2-resistant
CAGATGGGTCGAGCGCGTGGGCGA	6	27	RDR2-sensitive
CAGATGGGTCGGCGCGAGACGGAA	3	23	RDR2-sensitive
CAGATGGGTCGGCGCGAGACGGAG	3	57	RDR2-sensitive
CAGATGTCGGGGTGGGTGAGCGGT	52	0	RDR2-resistant
CAGATTAGTAGGAGCCGCGTTCCC	0	18	RDR2-sensitive
CAGATTCACCGACGGCAGAACCAA	0	22	RDR2-sensitive
CAGATTGATGGGCGGTACGGTATG	14	0	RDR2-resistant
CAGATTGGTAGGAGCTACGTTCCC	0	14	RDR2-sensitive
CAGATTGTAGATGCATGTCGACGC	0	13	RDR2-sensitive
CAGATTGTTAGAGACAGACGTTGT	0	16	RDR2-sensitive
CAGCAACCGCGGCGGCGAACACGG	17	1	RDR2-resistant
CAGCAACGGACACACCTTCGGCTC	2	23	RDR2-sensitive
CAGCAACGTCGAGGTAGAGCGTGC	10	84	RDR2-sensitive
CAGCACAACTGCAGGTCGGCCGGT	0	11	RDR2-sensitive
CAGCACAGTAGGTATCTCGGCGTT	0	11	RDR2-sensitive
CAGCACAGTCCAGCTAGACTCGGA	0	13	RDR2-sensitive
CAGCACCACTGTTGGGCTGCCGGT	0	12	RDR2-sensitive
CAGCACCGCGCGGACGAGGACGGC	0	16	RDR2-sensitive
CAGCACGACACGGAACTGCATCAC	0	14	RDR2-sensitive
CAGCACGACGGAGCCGATGAGCAG	1	12	RDR2-sensitive
CAGCACGAGACGTAGTGAACCGGA	0	24	RDR2-sensitive
CAGCACGCGACTGTGGTTGTTGGG	0	13	RDR2-sensitive
CAGCACGGAAGGGAATAAGACGGG	0	18	RDR2-sensitive
CAGCACGTGCTAGCGATGGCGGCT	33	0	RDR2-resistant
CAGCACTACGGGCTGAGGCTACAG	0	13	RDR2-sensitive
CAGCAGACTACAGCCTCAGACTAT	0	18	RDR2-sensitive
CAGCAGATCGCGGTTGCAACGCCT	0	12	RDR2-sensitive
CAGCAGCGGCTCTAGTATCTTCGT	0	10	RDR2-sensitive
CAGCAGCTTCAAAAACATGGACGC	0	11	RDR2-sensitive
CAGCAGCTTTTGCAGTCGTCTGGT	1	16	RDR2-sensitive
CAGCAGGCTCCGCATCCCTCTCCG	0	12	RDR2-sensitive
CAGCAGGGACGAGAGTGAAGGCGA	1	17	RDR2-sensitive
CAGCAGGGTCGACGAGGTCAGCAG	0	9	RDR2-sensitive
CAGCAGGTGATCGGCTGAAGACGC	0	22	RDR2-sensitive
CAGCAGTAGAGGCGCAAGAAGCTG	0	9	RDR2-sensitive
CAGCATCTGTGGCGCGACGACGGA	0	11	RDR2-sensitive
CAGCATGCCGGGCTTGGGCGTCGT	0	23	RDR2-sensitive
CAGCATGTAGCGAGTGTAGGAGAT	19	1	RDR2-resistant
CAGCATGTGTTAGGTCGGCGCCAA	0	11	RDR2-sensitive
CAGCCAATAGTGCAACGGCCGGAC	32	3	RDR2-resistant
CAGCCACTGTAGACTGTCCGGTGT	0	11	RDR2-sensitive
CAGCCAGAAGGACGACCGAAGACT	0	11	RDR2-sensitive

CAGCCAGGAGATCGATCGGGTCGC	0	15	RDR2-sensitive
CAGCCATCTGTAGCGTGACGTGTG	0	9	RDR2-sensitive
CAGCCCACGAGTCGGTATGGCACA	0	9	RDR2-sensitive
CAGCCCCGCGTCGCACGGATTCGT	180	11	RDR2-resistant
CAGCCCTGATTGAGAGAACACGGA	0	15	RDR2-sensitive
CAGCCCTGGACGCAGCTAGCCGTT	0	10	RDR2-sensitive
CAGCCGCAGCCGGATTGCAGCCGC	2	16	RDR2-sensitive
CAGCCGGAGGTAGGGTCCAGCGGC	45	4	RDR2-resistant
CAGCCGGCTGTACGTAGACGTCGA	0	10	RDR2-sensitive
CAGCCTAAGCCTGCTACGTACTGT	0	10	RDR2-sensitive
CAGCCTGGACCAGTTGAACGGACC	0	21	RDR2-sensitive
CAGCCTGGACCAGTTGAACGGGCC	0	11	RDR2-sensitive
CAGCCTTAGTCTGTGGAGCGGTAA	0	9	RDR2-sensitive
CAGCGAACTGTACATCGGCAACAG	1	24	RDR2-sensitive
CAGCGAACTGTACATCGGCAACGG	0	19	RDR2-sensitive
CAGCGAACTGTACATCGGCAACTG	0	10	RDR2-sensitive
CAGCGACGACAGCGACTCGGCTAC	1	24	RDR2-sensitive
CAGCGACGGTAGCCTAAGAGGAGA	0	11	RDR2-sensitive
CAGCGACTGCAGCGAAAGGCGACG	0	40	RDR2-sensitive
CAGCGACTGCAGCGAAGACCTCGA	0	25	RDR2-sensitive
CAGCGACTGCAGCGAAGGCCTCGA	2	111	RDR2-sensitive
CAGCGACTGCAGCGAGAGACGACG	0	42	RDR2-sensitive
CAGCGACTGCGACGGCTGCATGCT	0	9	RDR2-sensitive
CAGCGACTGCGGCGAAGACTTCGA	0	32	RDR2-sensitive
CAGCGAGAACGACGACATCGACGT	0	9	RDR2-sensitive
CAGCGAGAGTGCAGACGAGGTGAT	16	0	RDR2-resistant
CAGCGAGCGTTGGGCAGTGATGGA	0	11	RDR2-sensitive
CAGCGAGGTAGGACGGACAACCAA	0	16	RDR2-sensitive
CAGCGCGGGCAATAGCGAGGCGAT	0	9	RDR2-sensitive
CAGCGCGGGCGAGAGCGAGGCGAT	0	10	RDR2-sensitive
CAGCGGACGCTAACTGCGGGCGAC	0	10	RDR2-sensitive
CAGCGGACGGCCACTAGTGGACAG	0	9	RDR2-sensitive
CAGCGGACTTAGCTTGACAGGCAA	0	10	RDR2-sensitive
CAGCGGAGACAAGACAACGGGCAG	0	13	RDR2-sensitive
CAGCGGAGACTGCATGGAGTGCGC	0	19	RDR2-sensitive
CAGCGGAGACTGTATGGAGTGCGC	0	45	RDR2-sensitive
CAGCGGCGATATCCTGGGTGGCAC	28	1	RDR2-resistant
CAGCGGCTGCAGCGAAGACTTCGA	0	21	RDR2-sensitive
CAGCGGCTGCAGCGAAGGCCTCGA	0	20	RDR2-sensitive
CAGCGGCTGCAGCGAAGGCTTCGA	0	9	RDR2-sensitive
CAGCGGCTGGGAGGAGGGCCGAGG	31	1	RDR2-resistant
CAGCGGCTTGGACTTGGACGGAAC	0	13	RDR2-sensitive
CAGCGGGAGACTGGCTCTAGTGAT	0	24	RDR2-sensitive
CAGCGGGAGACTGGTGAGCAACGG	0	21	RDR2-sensitive
CAGCGGGGTAGATGCGAGCAGCAC	0	15	RDR2-sensitive
CAGCGGTGTGCTCTGCAACCGGTT	0	10	RDR2-sensitive
CAGCGTAGCATGTATCTCGGCGTC	0	11	RDR2-sensitive
CAGCGTGATAGTGTCAGACGTCGG	0	22	RDR2-sensitive
CAGCGTGCCTGATGCTCGGCCCAG	0	17	RDR2-sensitive

CAGCGTGCGTAGGCATACACTCTG	0	10	RDR2-sensitive
CAGCGTTGGGTTGCGGCGAGCCCC	17	0	RDR2-resistant
CAGCTAGCCGTTGGACTGATGGCA	0	12	RDR2-sensitive
CAGCTAGGAGCAGGGGAGAGCGGG	156	11	RDR2-resistant
CAGCTATTGCACTGCTGGCCGGAC	28	1	RDR2-resistant
CAGCTATTGTCGACTGCTGACGGT	0	17	RDR2-sensitive
CAGCTCAGCTCGGCTCGACTCGGC	0	11	RDR2-sensitive
CAGCTCCAACAGCCAGGACGTCGG	0	17	RDR2-sensitive
CAGCTCCGCCCTGTTGGTCTCGGC	0	12	RDR2-sensitive
CAGCTCGAGGTAGACGACGGCCTG	3	53	RDR2-sensitive
CAGCTCGGCTCGGCTCAGATCGGC	0	9	RDR2-sensitive
CAGCTCGGCTCGGCTCGACTCGGC	2	67	RDR2-sensitive
CAGCTCGGCTCGGCTCGGATCGGC	0	72	RDR2-sensitive
CAGCTCGGCTCGGCTCGTTAAGGT	0	18	RDR2-sensitive
CAGCTCGGCTCGGCTCGTTTACGA	0	27	RDR2-sensitive
CAGCTCGGCTCGGCTCGTTTGCGA	0	9	RDR2-sensitive
CAGCTCGGGCGAGACGGACAGAGC	0	12	RDR2-sensitive
CAGCTCGGTGGCAAGGTAGAAGGC	0	33	RDR2-sensitive
CAGCTCGTAGCGACTCGACGGTAC	0	21	RDR2-sensitive
CAGCTCGTGGACGACAGCGCGGCA	0	34	RDR2-sensitive
CAGCTCGTTTGAATTTTGGGTCGT	0	30	RDR2-sensitive
CAGCTCTGAGGATGAACGATCCAC	0	11	RDR2-sensitive
CAGCTCTGGGACGGCCGCTGACGG	3	30	RDR2-sensitive
CAGCTGAAGACACGCCGAACGCGG	0	14	RDR2-sensitive
CAGCTGAAGGGTGACGCGTAGGAC	0	9	RDR2-sensitive
CAGCTGAGAATTAGGCGTCTTGAG	1	15	RDR2-sensitive
CAGCTGCAGAGCCAAGACGGGACC	26	2	RDR2-resistant
CAGCTGCAGCAGTCCGAACGGCTT	0	14	RDR2-sensitive
CAGCTGCTCGGAGGTAGTCGGCGC	2	14	RDR2-sensitive
CAGCTGCTGTAGATCGGTCGGTGT	0	16	RDR2-sensitive
CAGCTGGACCGGATACTACGCATA	0	9	RDR2-sensitive
CAGCTGGCTGGTAGAGAGGACGCA	0	9	RDR2-sensitive
CAGCTGTAGACGGATGTTGACGGC	0	10	RDR2-sensitive
CAGCTGTAGACGGCCGCTAACGAC	0	9	RDR2-sensitive
CAGCTGTAGACGGCCGCTAACGGC	1	66	RDR2-sensitive
CAGCTGTAGACGGCCGCTAATGGC	0	10	RDR2-sensitive
CAGCTGTAGACGGCTGCTAACGGC	0	9	RDR2-sensitive
CAGCTGTAGCAATGGGATGGTTAC	0	21	RDR2-sensitive
CAGCTGTAGCTAGGATGGCAACAG	1	23	RDR2-sensitive
CAGCTGTATGACTACAAGATGGTT	1	21	RDR2-sensitive
CAGCTGTGCAGAAAAGCTGTTCGT	1	16	RDR2-sensitive
CAGCTGTGGAAACGGTAAGCTCAT	0	11	RDR2-sensitive
CAGCTGTGGAAACGGTAAGCTGAT	7	124	RDR2-sensitive
CAGCTGTTGAAACGGTAAGCTGAT	0	18	RDR2-sensitive
CAGCTGTTGTCGGCTACTGACGGT	0	16	RDR2-sensitive
CAGCTTCGGGAAACTCCAACGGCT	1	48	RDR2-sensitive
CAGCTTTAGAACGGCCGCTGACGG	0	14	RDR2-sensitive
CAGCTTTAGGACGGCCACTGACGG	0	15	RDR2-sensitive
CAGCTTTAGGACGGCCGCTGACGG	1	17	RDR2-sensitive

CAGCTTTGGGACGGCCGCTGACGG	0	19	RDR2-sensitive
CAGCTTTTCTGAGAATCTGGCTGT	1	17	RDR2-sensitive
CAGGAAACGGTACTGTCGCTTGGC	0	13	RDR2-sensitive
CAGGAAACTAGGCACGATGGGCAA	3	62	RDR2-sensitive
CAGGAAACTAGGCACGGTGGACAA	0	12	RDR2-sensitive
CAGGAACAGTAGACCCAGGCGGAT	0	22	RDR2-sensitive
CAGGAACATCTGCTCGATGGACAT	0	9	RDR2-sensitive
CAGGAACATGGCCGCGCGACCCTC	32	4	RDR2-resistant
CAGGAACTCTCTGAGACAGCGGTT	0	9	RDR2-sensitive
CAGGAACTGTAAATTTTTACGGCT	0	34	RDR2-sensitive
CAGGAAGACTGGCAGCGGCACTGA	0	9	RDR2-sensitive
CAGGAAGGAGCAGTCTGCATCTAA	0	9	RDR2-sensitive
CAGGACAAAGGACGAAGGCGTCAG	0	11	RDR2-sensitive
CAGGACAAAGGACGGAGGCGTCAG	2	34	RDR2-sensitive
CAGGACAACACTCGGCAAAGACAT	0	22	RDR2-sensitive
CAGGACAAGTGCTGCGCAGGTCGG	0	24	RDR2-sensitive
CAGGACACCGTCTCTAGCTTGCGG	0	11	RDR2-sensitive
CAGGACAGACGAAGGACAGACGAA	0	9	RDR2-sensitive
CAGGACAGCACTCGGCAAAGACAA	1	14	RDR2-sensitive
CAGGACATCGTAACCCGAGAGCGG	2	34	RDR2-sensitive
CAGGACCACACTCGGCAAAGACAC	1	12	RDR2-sensitive
CAGGACCACACTCGGCAAAGACAT	1	42	RDR2-sensitive
CAGGACCATGTAGGAGGCGCCAAT	0	9	RDR2-sensitive
CAGGACCCCACTCAGCAAAGACAT	1	14	RDR2-sensitive
CAGGACCCCACTCGGCAAAGACAA	0	16	RDR2-sensitive
CAGGACCCCACTCGGCAAAGACAC	5	53	RDR2-sensitive
CAGGACCCTACTCGGCAAAGACAC	0	9	RDR2-sensitive
CAGGACCCTGACGACAAACGGCGT	0	59	RDR2-sensitive
CAGGACCCTGAGCTCGCTGGAGGC	0	17	RDR2-sensitive
CAGGACCGAAGAGCAGGCGAGCAG	0	15	RDR2-sensitive
CAGGACCTAAGGAGTGCTACTCAG	0	10	RDR2-sensitive
CAGGACCTGAGGAGTGCTACTCAA	0	11	RDR2-sensitive
CAGGACCTGCCAAACATCAGTCGC	2	34	RDR2-sensitive
CAGGACCTGCCAAACATCAGTCGT	0	34	RDR2-sensitive
CAGGACCTGCTGCGCAACACGGAT	0	9	RDR2-sensitive
CAGGACGAAGCCTGAACCAGACGA	3	210	RDR2-sensitive
CAGGACGAAGCCTGAACGAGACGA	0	21	RDR2-sensitive
CAGGACGAAGCCTGAGCAAGACGA	0	17	RDR2-sensitive
CAGGACGAAGCCTGAGCGAGACGA	3	27	RDR2-sensitive
CAGGACGAAGGAAGCATAGGGCTT	0	29	RDR2-sensitive
CAGGACGAAGGCTGAGCCAGACGA	0	24	RDR2-sensitive
CAGGACGACACGAGATGAGACGAT	0	9	RDR2-sensitive
CAGGACGACACTCGGCAAAGACAA	2	70	RDR2-sensitive
CAGGACGACACTCGGCAAAGACAG	0	21	RDR2-sensitive
CAGGACGACGACGATGCGGCGCAG	0	25	RDR2-sensitive
CAGGACGAGACGGGATGGAATGAT	0	13	RDR2-sensitive
CAGGACGAGCATGCATGCATTCAT	1	15	RDR2-sensitive
CAGGACGATAAGCGCGTAAGGCAA	0	30	RDR2-sensitive
CAGGACGCCAAACCAGATCGCGGT	1	25	RDR2-sensitive

CAGGACGCCAACCAGATCGCGGTC	2	33	RDR2-sensitive
CAGGACGCCAACCAGGTCGCGGTC	0	17	RDR2-sensitive
CAGGACGCGGAAGAAGGGAAGGAT	0	11	RDR2-sensitive
CAGGACGGCACTCGGCAAAGACAA	3	26	RDR2-sensitive
CAGGACGGGACGGGACGAGACGAT	0	12	RDR2-sensitive
CAGGACGGGATATGATAGGACGAT	0	17	RDR2-sensitive
CAGGACGGGATGGGATGGGACGAT	0	62	RDR2-sensitive
CAGGACGGGTTGGGTTGGGACGAT	0	9	RDR2-sensitive
CAGGACTAACGAAGGCAGAACGGC	0	10	RDR2-sensitive
CAGGACTAGCGGGGAGGAGCCCAA	1	13	RDR2-sensitive
CAGGACTATCCTGGCCAAAACCGG	29	0	RDR2-resistant
CAGGACTCAGGACACACGACGACA	0	20	RDR2-sensitive
CAGGACTCAGGACACAGGACGACA	0	14	RDR2-sensitive
CAGGACTCGAACACGGGCTGGTGA	0	9	RDR2-sensitive
CAGGACTCGACGGCAGCGCAACGC	0	48	RDR2-sensitive
CAGGACTCTCGGCACAGACTCCAT	0	23	RDR2-sensitive
CAGGACTGACCGGCTGCATGAGGA	125	1	RDR2-resistant
CAGGACTGCGAGGAGATGTGTCGC	0	12	RDR2-sensitive
CAGGACTGGGAACGCGGTCGACGG	1	25	RDR2-sensitive
CAGGACTGGGCCGCGTCAGCCAAC	0	36	RDR2-sensitive
CAGGACTTCTGGCCAGGAGAACGA	0	15	RDR2-sensitive
CAGGAGAGGATTTTGGCGATTGAT	24	0	RDR2-resistant
CAGGAGCGGGCAGGATTCCACGGT	0	29	RDR2-sensitive
CAGGAGGACAAGCCCATGGACGGT	0	10	RDR2-sensitive
CAGGAGGAGGATAGGACGGAGGAT	129	1	RDR2-resistant
CAGGAGGAGGATGGGGCTTTCGAC	18	1	RDR2-resistant
CAGGAGGCGAGAGCGGGACTGGAG	0	9	RDR2-sensitive
CAGGAGTCGTCTCAGATTGGGCAC	0	13	RDR2-sensitive
CAGGAGTTCAGGACTGTAGGGGCA	0	14	RDR2-sensitive
CAGGATAAGGACAGGAAGGAGCAT	0	25	RDR2-sensitive
CAGGATACCAAACATGCGGACCTT	0	10	RDR2-sensitive
CAGGATAGGGCAGCCCCGGACGAG	0	9	RDR2-sensitive
CAGGATCTAGAAAGTGACGGATTC	0	12	RDR2-sensitive
CAGGATCTAGAAGACGAAGGGTTC	0	62	RDR2-sensitive
CAGGATCTAGAAGGCGAAGGGTTC	0	15	RDR2-sensitive
CAGGATGGCAGAACGGCAGAACGG	0	10	RDR2-sensitive
CAGGATGTACCAGTTGGACCGGAC	0	9	RDR2-sensitive
CAGGATGTAGTTTTGCCGGATGAA	15	0	RDR2-resistant
CAGGATTCCTGTAGTGCGTTGGTC	0	17	RDR2-sensitive
CAGGATTGCAGAGGCGGAATGGAT	0	10	RDR2-sensitive
CAGGATTGGATTCGGAGGGGACGA	0	10	RDR2-sensitive
CAGGCAACGTAGCATAGCACTCTC	0	16	RDR2-sensitive
CAGGCAAGGCACAGATACACTCTG	0	10	RDR2-sensitive
CAGGCACGCAGGACTGTGCAGGCC	0	10	RDR2-sensitive
CAGGCACGGCACGACAGGACACGG	0	10	RDR2-sensitive
CAGGCACGTAGGACTGTGCAGGCC	0	47	RDR2-sensitive
CAGGCACGTAGGACTGTGCAGGCT	0	25	RDR2-sensitive
CAGGCACGTGGGCTCCCTAGGCAC	0	12	RDR2-sensitive
CAGGCACGTGGGCTCTCCAGGCAC	0	10	RDR2-sensitive

CAGGCACGTGGGCTCTCTAGGCAC	0	16	RDR2-sensitive
CAGGCACGTGGGCTCTCTAGGCAT	0	86	RDR2-sensitive
CAGGCACGTGGGCTGGCACGGCAC	0	15	RDR2-sensitive
CAGGCACTGGCACTATACTAACCA	0	11	RDR2-sensitive
CAGGCACTGGCACTATACTAACCG	3	131	RDR2-sensitive
CAGGCACTGTAGACTGTCCGGTGC	0	18	RDR2-sensitive
CAGGCACTGTAGACTGTCCGGTGT	0	10	RDR2-sensitive
CAGGCACTGTAGGCTGTCCGGTGC	0	13	RDR2-sensitive
CAGGCAGCACCGGCGTAGAGCACT	0	9	RDR2-sensitive
CAGGCAGCAGCGGCAATGTAGGCT	1	12	RDR2-sensitive
CAGGCAGTGACGACGTGGACGCAT	2	16	RDR2-sensitive
CAGGCAGTTGCTCAGGACCTGGCC	0	13	RDR2-sensitive
CAGGCATCTTCGGACTCAAGTCGG	0	26	RDR2-sensitive
CAGGCATGACCCGCTATAGCTCTG	0	14	RDR2-sensitive
CAGGCATGCTCGGCTACTCCAGTC	39	1	RDR2-resistant
CAGGCCACGACGGACAGAGATCAG	0	14	RDR2-sensitive
CAGGCCACGGTGGACAATAATCAG	1	14	RDR2-sensitive
CAGGCCACTCTCGGGACTCGACGG	0	9	RDR2-sensitive
CAGGCCAGACAGTGCAATCCGGTC	0	9	RDR2-sensitive
CAGGCCAGGCCTGATCATGTTGGA	0	13	RDR2-sensitive
CAGGCCGAGCTAGCACGTGGGCGC	1	23	RDR2-sensitive
CAGGCCTAGACGACGTAGAGTCAA	0	9	RDR2-sensitive
CAGGCCTGTGGATTCGTTGCGGGA	45	13	RDR2-resistant
CAGGCGAAGCTCTCTGAGGACGAC	12	33	RDR2-sensitive
CAGGCGACGCAAACACAAGAGCTG	0	10	RDR2-sensitive
CAGGCGACGCTTCTCTCTTGGCAT	0	9	RDR2-sensitive
CAGGCGAGACGGAACCCGAGGCGA	0	13	RDR2-sensitive
CAGGCGCCAACGAACTCAGATGGT	0	14	RDR2-sensitive
CAGGCGCTAACACTCGGCAAACAT	2	17	RDR2-sensitive
CAGGCGGCATGCAGGACAGACGAC	0	11	RDR2-sensitive
CAGGCGGGTTGGGCATTGACTGCA	0	13	RDR2-sensitive
CAGGCGTCAGAACTGTGAAGGCTA	0	10	RDR2-sensitive
CAGGCGTGAGTCTCTTCTCCTCGG	0	12	RDR2-sensitive
CAGGCTACAGCACACGTGGACCAA	0	19	RDR2-sensitive
CAGGCTACAGGGAACAAGACCGGC	4	18	RDR2-sensitive
CAGGCTACGAAGGAACAAGACCGG	1	14	RDR2-sensitive
CAGGCTACTCTGGGAAGACAACCA	0	9	RDR2-sensitive
CAGGCTAGCTGAACTGCAGAGGCA	0	14	RDR2-sensitive
CAGGCTATAGCACACGTGGACCAA	0	9	RDR2-sensitive
CAGGCTCCAGCACTCCTCCGACGG	0	13	RDR2-sensitive
CAGGCTCCAGCGCTCCTCCGACGG	1	16	RDR2-sensitive
CAGGCTCCCGGACTGGACCAGGCA	1	46	RDR2-sensitive
CAGGCTCCGGTAGGACGTCGCATC	0	11	RDR2-sensitive
CAGGCTCCGGTAGGACGTCGCGTC	10	28	RDR2-sensitive
CAGGCTCTAGTAGGACGTCGGCGA	0	10	RDR2-sensitive
CAGGCTCTGCAGACGATACCCATC	6	24	RDR2-sensitive
CAGGCTCTGGAGATGCGACCAAAA	0	15	RDR2-sensitive
CAGGCTGAACTCCGGTGAACAGGC	0	20	RDR2-sensitive
CAGGCTGACCGGCGACAAACTCGT	0	19	RDR2-sensitive

CAGGCTGAGACCGGTGATTTGCAT	0	27	RDR2-sensitive
CAGGCTGAGGACTGTGCGGACGGT	0	54	RDR2-sensitive
CAGGCTGATGAGAGGAGGGGGACT	0	9	RDR2-sensitive
CAGGCTGATGCACGGGACTATCAA	0	12	RDR2-sensitive
CAGGCTGATGGGAGGAGGGGGACT	0	10	RDR2-sensitive
CAGGCTGCCGCGGACCTGGCTCGC	0	11	RDR2-sensitive
CAGGCTGCCTCGACCTGAAGACGC	6	59	RDR2-sensitive
CAGGCTGCTCGACGGTGAACTGGC	0	15	RDR2-sensitive
CAGGCTGCTGGGCCATGACGGCAA	0	10	RDR2-sensitive
CAGGCTGGCCGACGGTGAACCGAC	1	17	RDR2-sensitive
CAGGCTGGCTCTGGCGAACTGGCT	2	22	RDR2-sensitive
CAGGCTGGTCGACGGTGAAGTGAC	0	9	RDR2-sensitive
CAGGCTGTACAGGAAACTACGCAT	0	19	RDR2-sensitive
CAGGCTGTACGCGTGCAAATTGAG	0	15	RDR2-sensitive
CAGGCTGTAGCAGGCTATAGTCTC	0	15	RDR2-sensitive
CAGGCTGTATGAGCGGATACTCGT	0	67	RDR2-sensitive
CAGGCTGTGCGCGTGCAAATTGAG	0	9	RDR2-sensitive
CAGGCTGTGGGCGTGCAAATCTAG	0	15	RDR2-sensitive
CAGGCTTGGACCGGTTCCTTTCGT	0	9	RDR2-sensitive
CAGGGAAAGATCTGTGGGGGCTGT	17	1	RDR2-resistant
CAGGGAACATGGAACTGGCGTCGC	0	13	RDR2-sensitive
CAGGGAACTCCAAGCTGAACTCAT	0	15	RDR2-sensitive
CAGGGACAAGAACGGGAAAGGCAG	0	10	RDR2-sensitive
CAGGGACACACAGCAGAACAACAT	0	32	RDR2-sensitive
CAGGGACAGTAGGAGGGCCGAGGA	4	18	RDR2-sensitive
CAGGGACGGACGATATCTTAGCGA	0	13	RDR2-sensitive
CAGGGAGCTTCGGCATGATGACAC	0	12	RDR2-sensitive
CAGGGAGGCTGAAGGCAGACTGGC	0	12	RDR2-sensitive
CAGGGCAATACGGCTGGAGCCACA	17	0	RDR2-resistant
CAGGGCACGGCACGGTAGAAGATC	0	11	RDR2-sensitive
CAGGGCAGCCTGTAGCTACGGACA	1	14	RDR2-sensitive
CAGGGCAGTATACGGATGAAGCAA	0	9	RDR2-sensitive
CAGGGCATGGCACTACATACACAT	0	39	RDR2-sensitive
CAGGGCTGACCTGGCGCTGACGTG	0	19	RDR2-sensitive
CAGGGCTGAGCAGACGGATAGCGC	0	67	RDR2-sensitive
CAGGGCTGAGCAGACGGATAGCGT	0	16	RDR2-sensitive
CAGGGCTGCTGTGAAGACGATCCT	0	14	RDR2-sensitive
CAGGGCTTGTTCGGTTATTCCCAA	0	12	RDR2-sensitive
CAGGGCTTTTCGGGCTGACCCGGC	1	18	RDR2-sensitive
CAGGGGACAACACTGGATAGTCTT	0	41	RDR2-sensitive
CAGGGGACGAGATCTAGATTCCAG	0	9	RDR2-sensitive
CAGGGGCGACGACAAGAAGGGAAG	19	0	RDR2-resistant
CAGGGGCGAGAGTGGAGGCGAGAG	2	27	RDR2-sensitive
CAGGGGCTCTCGGAAATAAGACGT	0	16	RDR2-sensitive
CAGGGGTTGTAGAACGACGGCCAC	0	26	RDR2-sensitive
CAGGGTATGGATAGACGACGACGG	0	9	RDR2-sensitive
CAGGGTCGTACCCTGTTGACTCGG	0	16	RDR2-sensitive
CAGGGTGCTTTAAGGATCGTGCGA	0	11	RDR2-sensitive
CAGGGTTACGTGCACGGGGGCAAT	0	9	RDR2-sensitive

CAGGGTTTCCTGACTGCTGGACGG	0	10	RDR2-sensitive
CAGGGTTTGGGGACGCGCGGTGAT	57	0	RDR2-resistant
CAGGTACCACAGGATGAGGCGCAT	2	22	RDR2-sensitive
CAGGTACCCTCGGACTAAAGTCGG	1	59	RDR2-sensitive
CAGGTACCGCAGGATGAGGCGCAT	2	21	RDR2-sensitive
CAGGTACGGTTCACTGTTCGGTGT	0	10	RDR2-sensitive
CAGGTACTCTCGGACTAAAGTCGG	1	19	RDR2-sensitive
CAGGTACTGTTTGCTGTCCGGTGT	0	26	RDR2-sensitive
CAGGTAGACACTCGGCAAAGACAA	0	23	RDR2-sensitive
CAGGTAGGCACTCGGCAAAGACAA	7	100	RDR2-sensitive
CAGGTAGGCACTCGGCAAAGACAT	0	10	RDR2-sensitive
CAGGTAGTAGGCTGAACTGGGCGT	0	29	RDR2-sensitive
CAGGTATAGTAGGGGGTAGGGAAC	0	13	RDR2-sensitive
CAGGTATCTTCGGACTCAAGTCGG	1	25	RDR2-sensitive
CAGGTATCTTCGGACTCAGGTCGG	0	11	RDR2-sensitive
CAGGTATCTTCGGACTCGAGTCGG	0	9	RDR2-sensitive
CAGGTATCTTCGGGCTCGAGTCGG	0	11	RDR2-sensitive
CAGGTCACGGACCGTCATCGGCTC	0	13	RDR2-sensitive
CAGGTCACGGACTGTCCGGCCCTC	0	21	RDR2-sensitive
CAGGTCCTGTAGACTGTCCGGTGC	1	66	RDR2-sensitive
CAGGTCCTGTAGACTGTCCGGTGT	0	11	RDR2-sensitive
CAGGTCCTGTAGCTTGTCCGGTGC	0	12	RDR2-sensitive
CAGGTCGCGGACTGTCCGGCCCAG	1	15	RDR2-sensitive
CAGGTCGCGGACTGTCCGGCCCCT	0	24	RDR2-sensitive
CAGGTCGGACCCGTGTTGAGTGGC	0	9	RDR2-sensitive
CAGGTCGGGTGCGCTTGCAGTAGG	6	67	RDR2-sensitive
CAGGTCGGTCAGTGGAGCGGCGAA	5	43	RDR2-sensitive
CAGGTCGTGACTGAGCTGCCGGTA	0	9	RDR2-sensitive
CAGGTCGTGGACTCTTCGGTACTG	0	22	RDR2-sensitive
CAGGTCTCCGGAACCCTTCGTCTT	3	17	RDR2-sensitive
CAGGTCTGCACTCGGCAAAGACAT	0	11	RDR2-sensitive
CAGGTGAACGGACGGACGAACGGT	0	20	RDR2-sensitive
CAGGTGCTCTGAGTAAGCTGGACA	16	0	RDR2-resistant
CAGGTGGACGATGAGCTGGACGGC	0	10	RDR2-sensitive
CAGGTGGATGATGAGCTAGACGGC	0	16	RDR2-sensitive
CAGGTGGCACTGTGGAGAGAAGAC	0	10	RDR2-sensitive
CAGGTGTTAGCGACTCGGTGGAAG	0	14	RDR2-sensitive
CAGGTTGAAGAGGCTGATCAGGCA	0	10	RDR2-sensitive
CAGGTTGGACTGGCCCACGTGCCT	0	9	RDR2-sensitive
CAGGTTGTAGAGGTAATCGGCCTA	0	43	RDR2-sensitive
CAGGTTGTGGACTGTCCGGCCCTT	0	22	RDR2-sensitive
CAGTACAGTGGACACTCGGCACAG	0	9	RDR2-sensitive
CAGTACTGTACTGAGTTTTGTCGT	0	41	RDR2-sensitive
CAGTAGCGGCGTGGATGAAGGGGC	15	0	RDR2-resistant
CAGTAGTGTAGACGTCACACCCAT	0	12	RDR2-sensitive
CAGTATCGTAGACTCAGCGGCCAG	0	9	RDR2-sensitive
CAGTATGCCATCGGACCCACAAAT	0	10	RDR2-sensitive
CAGTCACTGTAGACGGTCCGGTGC	0	27	RDR2-sensitive
CAGTCAGGCACTGAGGCACGGCGC	0	10	RDR2-sensitive

CAGTCCAGATACGAACGTTGGACA	29	0	RDR2-resistant
CAGTCCGTGTGTAGATCAGGTCAG	0	9	RDR2-sensitive
CAGTCCTGCTGTAGATGGTCCAAC	0	17	RDR2-sensitive
CAGTCGATGCAGGCAGATGTACGC	1	12	RDR2-sensitive
CAGTCGATGCGGAATAGGAGGCAA	1	13	RDR2-sensitive
CAGTCGATGTGGAATAGGAGGCAA	1	20	RDR2-sensitive
CAGTCGCACGGTCTGTTGTTGGCT	0	9	RDR2-sensitive
CAGTCGCTGGTCTGACAGAGACAA	3	69	RDR2-sensitive
CAGTCGGAAAGGCAGTAGTCGCAT	1	15	RDR2-sensitive
CAGTCGGAGTGGCGCAGGCGGCGC	11	51	RDR2-sensitive
CAGTCGGCTGAGAATGCACGTGCG	0	42	RDR2-sensitive
CAGTCGGCTGTGAAATGCAGGTGC	0	11	RDR2-sensitive
CAGTCGGCTGTGAATGCACGTGCG	0	17	RDR2-sensitive
CAGTCGTGCGGAACCAGCAAGCGG	0	10	RDR2-sensitive
CAGTCTCAACATCTGTAGTGGCGT	0	9	RDR2-sensitive
CAGTCTCGCATAGAACCGGATCGT	0	73	RDR2-sensitive
CAGTCTCGTCCTCACTGTCGTCGC	0	11	RDR2-sensitive
CAGTCTCGTTCTCACTGTCGGCGC	0	11	RDR2-sensitive
CAGTCTGCCTCGACCTGAAGACGC	2	34	RDR2-sensitive
CAGTGAACAGTGACTGTCCGGTGC	0	12	RDR2-sensitive
CAGTGAACTGTATATCGGCAACAA	0	9	RDR2-sensitive
CAGTGACGTGGACACGGTTAGCCC	43	0	RDR2-resistant
CAGTGACTATGGGCCGGACGGTTC	1	28	RDR2-sensitive
CAGTGACTGTACAACGCTAGGCAT	0	30	RDR2-sensitive
CAGTGACTGTTCACTGTCCGGTGT	0	13	RDR2-sensitive
CAGTGAGCAGTAGATCCAGAGCAT	0	9	RDR2-sensitive
CAGTGAGCGTAGGCATGCAACGGC	0	9	RDR2-sensitive
CAGTGAGGACGGAATGGGAATGAC	0	15	RDR2-sensitive
CAGTGATGGGACCAGTGGAAGCGG	14	0	RDR2-resistant
CAGTGCACAGGCTGCAGCAGGCTA	0	13	RDR2-sensitive
CAGTGCACAGGCTGTAGCAGGCTA	0	21	RDR2-sensitive
CAGTGCAGGCGAGAGCGAGGCGAC	0	9	RDR2-sensitive
CAGTGCCGTGCAGTCGTCGGCGTC	47	5	RDR2-resistant
CAGTGCGGGCTGCCCATTTGGCAC	0	18	RDR2-sensitive
CAGTGCTATAGAACAGAAGGGCAA	0	17	RDR2-sensitive
CAGTGCTATAGAACAGAAGGGCAC	0	11	RDR2-sensitive
CAGTGCTATAGAACAGAGGGTCAT	0	15	RDR2-sensitive
CAGTGCTATAGAACAGGAGGGCAT	1	95	RDR2-sensitive
CAGTGCTATAGAATAGAAGGGCAT	0	10	RDR2-sensitive
CAGTGGGCCGAACGGAACGCGCAT	0	11	RDR2-sensitive
CAGTGGTAGCGACCCTGGACTCGG	0	9	RDR2-sensitive
CAGTTAAAAAGCTCGTAGTTGGAC	48	0	RDR2-resistant
CAGTTCGACTGCTCGGTTGTCACC	0	10	RDR2-sensitive
CAGTTCGGTACGGCGACGTCGACT	29	1	RDR2-resistant
CAGTTCGTGTGGCTGGGACAGCAA	0	19	RDR2-sensitive
CAGTTCTACGTAGGCTTGGGTCGA	0	20	RDR2-sensitive
CAGTTGAAGATGAGGTAGGCTATA	20	0	RDR2-resistant
CAGTTGAGAGTAGTGGAAGGCGGG	22	0	RDR2-resistant
CAGTTGATATCGGCAACTCGGCAT	0	36	RDR2-sensitive

CAGTTGCACGTGGAACAGAGACGC	0	37	RDR2-sensitive
CAGTTGGACGTCCAGTTTGGGTAT	0	12	RDR2-sensitive
CAGTTGGGACACTCGGCAAAGAGC	4	34	RDR2-sensitive
CAGTTGGGTGCCTAGAGTTGCATA	22	1	RDR2-resistant
CAGTTGTTTCCTGTTGGCATTTCT	68	0	RDR2-resistant
CAGTTTACTGTAGTGGAGAGTGGT	0	15	RDR2-sensitive
CAGTTTAGGAGGGAGCTCGGCGGC	0	61	RDR2-sensitive
CAGTTTATTATTTGGTAGTCTGAA	0	9	RDR2-sensitive
CAGTTTCAACGATCGTCCCGCGTC	0	10	RDR2-sensitive
CAGTTTCAACGGTCGTCATGCGTC	0	12	RDR2-sensitive
CAGTTTCTGGCTGGCTGTAACGGT	2	74	RDR2-sensitive
CAGTTTGAAGCGGATCCAGACCTC	0	15	RDR2-sensitive
CAGTTTGTAGGACAGCATATTCGC	0	9	RDR2-sensitive
CAGTTTTTAATGTAGAATCAGGAC	31	2	RDR2-resistant
CATAAAGCTGACTGGGAGACGCCA	0	19	RDR2-sensitive
CATAAAGGCACTAGGGCTGGGCAA	0	13	RDR2-sensitive
CATAAAGTTTGTAGCACGGGTGAC	89	1	RDR2-resistant
CATAAGATCCTACGTCTGACGGTT	0	11	RDR2-sensitive
CATAAGTGCCTGTAGATAGGGTGT	0	12	RDR2-sensitive
CATAATACTGTGATTGGTCGCCGG	0	9	RDR2-sensitive
CATAATATCGGACCTAGATGCGGT	22	0	RDR2-resistant
CATACAACGACGGACGGTCCGCAT	0	18	RDR2-sensitive
CATACGATGGGACGCATGATTCTT	0	11	RDR2-sensitive
CATACGGTGCTGTCAGCGACGCGA	172	7	RDR2-resistant
CATACGTCCGACGGCTATTGGAAC	0	11	RDR2-sensitive
CATACTGTAGAAGAGGGTACCCCT	1	65	RDR2-sensitive
CATACTGTAGTAACGGGTACCCAT	1	13	RDR2-sensitive
CATACTGTAGTAACGGGTACCCCT	0	10	RDR2-sensitive
CATACTGTAGTAAGGGGTACCCAT	0	31	RDR2-sensitive
CATACTGTGGAAGAGGGTACCCCT	0	38	RDR2-sensitive
CATAGAATTCGGCCCGTCAGGTAT	0	14	RDR2-sensitive
CATAGACACAGACGGTCCCACCAG	0	14	RDR2-sensitive
CATAGACCAGAGGACAGACCGGCA	0	9	RDR2-sensitive
CATAGACCATACGATCAGCGGGCG	0	14	RDR2-sensitive
CATAGACTGCATGCGGAGTGGGAT	0	19	RDR2-sensitive
CATAGACTGTAACGGCGAGCGGTA	0	10	RDR2-sensitive
CATAGATGTGGCCTGACGACGGAG	31	1	RDR2-resistant
CATAGCACTCGGCAAAGAAGGCAC	0	9	RDR2-sensitive
CATAGCAGGACGTGCACAGGACTC	0	9	RDR2-sensitive
CATAGGACCAACGACTGCCAACGG	0	10	RDR2-sensitive
CATAGGCTATGGCGCCGAGGTCGT	1	14	RDR2-sensitive
CATAGGTACTGTGGATCGGCCTAG	0	10	RDR2-sensitive
CATAGTGACGTGGACTGTCCGCAC	0	9	RDR2-sensitive
CATAGTGGACGCGTAGAACGGCGT	2	98	RDR2-sensitive
CATAGTTGACTGGGAGACGACGGC	0	13	RDR2-sensitive
CATAGTTGACTGGGAGACGACGGT	0	12	RDR2-sensitive
CATATATGTCCTCTTAGCTTTGGC	14	0	RDR2-resistant
CATATCGGGTAAAACTCGTCGGGT	0	13	RDR2-sensitive
CATATCTAATTATGGGTCGTGCTT	33	0	RDR2-resistant

CATATCTGGCTGGCTAGGGTGGCG	0	9	RDR2-sensitive
CATATGGACGGTGGTCGATGGCGA	0	10	RDR2-sensitive
CATATGGATGCTGTAAGATGACAT	0	12	RDR2-sensitive
CATATGGCGGGACTGGGCGTGCAT	0	10	RDR2-sensitive
CATATGTATTGTGGACGTCTACAC	0	54	RDR2-sensitive
CATATGTGTGAGACTTGGTCCCAG	0	17	RDR2-sensitive
CATATTCACTGTAGCGGCATGCAT	1	12	RDR2-sensitive
CATCAACATCGGATGGATCTGACA	19	0	RDR2-resistant
CATCAAGCTGACTGGGAGACGCCA	1	17	RDR2-sensitive
CATCAAGTACGGGTTCGGGCACAA	0	9	RDR2-sensitive
CATCACCCACTTCGTCGGATGCAA	1	26	RDR2-sensitive
CATCAGATCGAGATCGGGCGGCAT	0	19	RDR2-sensitive
CATCAGCTAGAGCGTCGGCGGCGT	0	11	RDR2-sensitive
CATCAGTCGCATGCACTCTCGGAT	40	4	RDR2-resistant
CATCAGTTATCTTGTCGGATGTTT	17	0	RDR2-resistant
CATCAGTTTTAGGACGGCCGCTGA	0	10	RDR2-sensitive
CATCATTTCTATCCTTCGTCGGCC	0	11	RDR2-sensitive
CATCCAGGCGGGCCCAGAAGACAG	0	13	RDR2-sensitive
CATCCCTCTGAATCGGACGGACAA	0	17	RDR2-sensitive
CATCCGACCGTCCGAAGAGGCTAA	0	9	RDR2-sensitive
CATCCGCAGCGGGGCACTGCACAC	0	11	RDR2-sensitive
CATCCGGACAAACGCAGAGAAGGA	0	10	RDR2-sensitive
CATCCGGACATACGCAGAGGAGAC	0	21	RDR2-sensitive
CATCCGGACGTATGCAGAGGAGGA	0	21	RDR2-sensitive
CATCCGGGACGTGGACGGCTACAA	0	16	RDR2-sensitive
CATCCGGGTTGTAGGTGACGTCGG	0	53	RDR2-sensitive
CATCCGGTCTGTGGATAGAGGAGA	0	9	RDR2-sensitive
CATCCGTAGTGTCATCGTCGGGAT	0	14	RDR2-sensitive
CATCCGTGTGGACTGGGCCGCCGT	0	10	RDR2-sensitive
CATCCTCGGGCGGGAGACGCGGTC	0	14	RDR2-sensitive
CATCCTGGTTTGCAGTCGGGCTGT	0	10	RDR2-sensitive
CATCCTGTACGACGGCTGTCTCAG	0	9	RDR2-sensitive
CATCCTTACAGTGAGGTTGTCGGT	0	12	RDR2-sensitive
CATCCTTCTAGTGGACCCGGGGAT	0	13	RDR2-sensitive
CATCCTTGCCTTCTGTCGTCGGGC	4	20	RDR2-sensitive
CATCGAAGGCAGAACCAGGGAGGC	0	11	RDR2-sensitive
CATCGACCTGGAGGAATGACGCGA	0	14	RDR2-sensitive
CATCGACGGAGAGCAGATGGGGCG	29	2	RDR2-resistant
CATCGAGCTGGTGGATCAGGCCAT	157	1	RDR2-resistant
CATCGAGTTCTGTATTAAAGCGTA	63	6	RDR2-resistant
CATCGAGTTGCGATTGTCGGCGCC	0	9	RDR2-sensitive
CATCGATCCAGGATTCAGGTGCAC	0	10	RDR2-sensitive
CATCGATCTGGACGGTCCGCGCAT	0	35	RDR2-sensitive
CATCGATCTGTCATCGGACGACGC	0	28	RDR2-sensitive
CATCGCAGAGACGGACGGGACAAC	0	23	RDR2-sensitive
CATCGCAGGACTGACCGGCTGCAT	72	0	RDR2-resistant
CATCGCCATCGTAGATCGTCCGGA	0	12	RDR2-sensitive
CATCGCCCTGCTGTGCTTCGTCGG	0	11	RDR2-sensitive
CATCGCGCGGAAGGCCAGGTAGAG	1	14	RDR2-sensitive

CATCGCTGGCTGAGAGATAGGGCC	0	9	RDR2-sensitive
CATCGGAAATGGGAGCGGGAGCGA	0	11	RDR2-sensitive
CATCGGACGGTCCGCAGTAGAGAC	0	9	RDR2-sensitive
CATCGGATCAGCATCGGAGGGCGA	0	9	RDR2-sensitive
CATCGGATTTCGGACGGGTTTCGG	0	9	RDR2-sensitive
CATCGGGTAGCGGGTATGGGGCAA	2	54	RDR2-sensitive
CATCGGGTGACGGATATGGATAGC	0	19	RDR2-sensitive
CATCGGTTGTCCATGGATCTGCGC	0	21	RDR2-sensitive
CATCGTACTGAGCGAAGTTGGCAG	0	15	RDR2-sensitive
CATCGTCAAGCAGGGGAACCTCAT	0	14	RDR2-sensitive
CATCGTCAAGCAGGGGGACCTCAT	2	97	RDR2-sensitive
CATCGTCGACCCTGCTGATTCCGG	0	10	RDR2-sensitive
CATCGTGCGTGATGGTGACCTCGA	20	2	RDR2-resistant
CATCGTGTCGGGATGACGGTCCAA	0	9	RDR2-sensitive
CATCGTTATATTTGTAGAGTGGCC	0	10	RDR2-sensitive
CATCGTTGGGCCGCATACACCGCT	0	12	RDR2-sensitive
CATCTAGACTGGGGCACGCGGCGA	0	13	RDR2-sensitive
CATCTAGCGGGTGAATGATGCATT	29	3	RDR2-resistant
CATCTAGGCTGGGGCACGCGGCGA	0	12	RDR2-sensitive
CATCTATTTATAGGCACAGGACGC	1	13	RDR2-sensitive
CATCTCCAGTAGAGATCGACTCGG	0	12	RDR2-sensitive
CATCTCCGGACGGCTAAGCCCACA	0	10	RDR2-sensitive
CATCTCGGACATACGATGGTGGCG	0	12	RDR2-sensitive
CATCTCGGCGTAGGTGAGCAGGCC	0	9	RDR2-sensitive
CATCTCGTGCGACTGTCCGGTGAG	0	11	RDR2-sensitive
CATCTCTGGACCGGATCCGACGCC	0	22	RDR2-sensitive
CATCTGAACCAAGGGGCCAGCCGG	0	9	RDR2-sensitive
CATCTGCTCAGGCTGTACGCGGAC	0	12	RDR2-sensitive
CATCTGGACTGGGGCACGCGGCAT	1	18	RDR2-sensitive
CATCTGGACTGGGGCACGCGGCGA	0	39	RDR2-sensitive
CATCTGGATGGCACTGCAACGGCA	38	3	RDR2-resistant
CATCTGGGCAGGGACACGCAGCAA	0	21	RDR2-sensitive
CATCTGGGCTAGGACACGCGGCGA	0	20	RDR2-sensitive
CATCTGGGCTAGGGCACGCGGCGA	0	12	RDR2-sensitive
CATCTGGGCTGGAGCACGCGGCGA	1	23	RDR2-sensitive
CATCTGGGCTGGGACACGCGGCGA	3	121	RDR2-sensitive
CATCTGGGCTGGGGCACGCAGCAC	1	30	RDR2-sensitive
CATCTGGGCTGGGGCACGCGACGA	1	16	RDR2-sensitive
CATCTGGGCTGGGGCACGCGGCAC	3	46	RDR2-sensitive
CATCTGGGCTGGGGCACGCGGCGA	6	144	RDR2-sensitive
CATCTGGGCTGGGGCACGTGGCGA	0	10	RDR2-sensitive
CATCTGTACGGCTGTACCGGAGGC	0	10	RDR2-sensitive
CATCTGTAGGCGGCGCAGCAGCAA	1	12	RDR2-sensitive
CATCTGTATCTAGGGCTGGGCATT	0	11	RDR2-sensitive
CATCTGTGGGAGCGCATGCAACGT	0	11	RDR2-sensitive
CATCTGTGTCTATGATTTGTCGGT	0	9	RDR2-sensitive
CATCTGTGTTAGGATTGAAAACGG	0	25	RDR2-sensitive
CATCTTAGGACTTAGGGCGGGTGC	0	16	RDR2-sensitive
CATCTTAGGATTAGGGCGGGTGCA	0	10	RDR2-sensitive

CATCTTAGGATTTAGGGCGGGTGC	0	16	RDR2-sensitive
CATCTTCCACAGCTCGAACGACGT	0	10	RDR2-sensitive
CATCTTGACCTGTAGTGTCGGCAT	1	43	RDR2-sensitive
CATCTTGGATATGTGACGGTGGCT	0	23	RDR2-sensitive
CATCTTGTTGGTCGGTAAAGGTAT	0	14	RDR2-sensitive
CATCTTTGGATTAGGACAGGTGTG	0	20	RDR2-sensitive
CATGAACCGAGCGAACCGACGACC	0	11	RDR2-sensitive
CATGAACCTCGGACGGCTGTTGAA	0	9	RDR2-sensitive
CATGAACTCGGCAGCGGTAGAAGG	0	12	RDR2-sensitive
CATGAAGATCGGGCATCGTGACGC	0	9	RDR2-sensitive
CATGACACGTTAGACGGCCTGGAC	0	9	RDR2-sensitive
CATGACAGACCTGCTGCAACACGC	0	10	RDR2-sensitive
CATGACCGCAGAGGATTGTGGTAT	0	23	RDR2-sensitive
CATGACCTGCCGTAGAAGCGACTC	0	12	RDR2-sensitive
CATGACGAAGACCTGGGCATCTGG	0	10	RDR2-sensitive
CATGACGACGACGGCTCTCAGCCC	0	11	RDR2-sensitive
CATGACGGGCTATCGGCTAGACGA	0	9	RDR2-sensitive
CATGACTCGGTGTGGGAAGGATGG	0	10	RDR2-sensitive
CATGACTCTGGTCACTGTTCACGG	27	1	RDR2-resistant
CATGACTGCAGAGGATTGTGGTAT	0	76	RDR2-sensitive
CATGACTGGTCTCTGTGGAAGCGT	0	14	RDR2-sensitive
CATGAGCAGCTCAGGACTCTCTGA	0	15	RDR2-sensitive
CATGAGGACTACCAGATCGTGGCT	0	18	RDR2-sensitive
CATGAGGATGAGGAGAAGGAGGGT	16	0	RDR2-resistant
CATGAGTCGGCAGCTGAAGGAGAA	0	17	RDR2-sensitive
CATGAGTCTGGTGTATGTTGGCAT	60	0	RDR2-resistant
CATGATCGGTTAACTGCATAGCAT	0	20	RDR2-sensitive
CATGATCGGTTACCTGCATAGCAT	0	16	RDR2-sensitive
CATGCAGACTGTGACAGCTGGGTC	0	12	RDR2-sensitive
CATGCAGGGTGACGATGACATGAA	16	0	RDR2-resistant
CATGCAGTTGTAGGCTGACGCCAA	0	136	RDR2-sensitive
CATGCAGTTGTAGGCTGACGCCCA	0	9	RDR2-sensitive
CATGCCAGATTGGAGGACCAGGCA	0	13	RDR2-sensitive
CATGCCTTCCTTGGATTCTGGCGT	0	10	RDR2-sensitive
CATGCCTTTGTAGATCCGAGCGGC	0	12	RDR2-sensitive
CATGCGAACGGGAAGTAGCGCGCC	0	10	RDR2-sensitive
CATGCGACTGGCACAACGTCCGAT	35	4	RDR2-resistant
CATGCGAGACAGACTGGAGGCCAG	0	11	RDR2-sensitive
CATGCGAGCACCAGATGCTGACAT	0	17	RDR2-sensitive
CATGCGGAGGACATAGATGCGGAT	2	23	RDR2-sensitive
CATGCGGGAAGAGACGGAATTCAT	5	28	RDR2-sensitive
CATGCGGGGATGGTCGTTGGCAGC	79	2	RDR2-resistant
CATGCGTCAGCACACGAGAGGCAT	0	9	RDR2-sensitive
CATGCGTGTGTAGAAGCGACGGAG	0	20	RDR2-sensitive
CATGCTAATCTTCTCTGTATCGTT	93	2	RDR2-resistant
CATGCTCTGTTCCCATCTCGGCGT	2	15	RDR2-sensitive
CATGCTGCAGAACTGAAGGGGTGT	0	16	RDR2-sensitive
CATGCTGCTCCGGCTGAAGATCGT	0	11	RDR2-sensitive
CATGCTGCTTGGGACCGACGGAAA	0	10	RDR2-sensitive

CATGCTGTAGAAGAGGGTACCCCT	2	48	RDR2-sensitive
CATGCTGTAGAAGATGGTACCCCT	0	13	RDR2-sensitive
CATGCTGTAGGAGAGGGTACCCCT	0	12	RDR2-sensitive
CATGCTGTAGTAAGGTGTACCCCT	0	10	RDR2-sensitive
CATGCTGTCGGTACGTGATGTCAT	0	11	RDR2-sensitive
CATGCTGTCGTTGGTAAAGAGGTT	17	0	RDR2-resistant
CATGCTGTTAGATCGCGGACCATC	0	11	RDR2-sensitive
CATGCTTTGGATAGGAAATGGGCA	17	1	RDR2-resistant
CATGCTTTGGTCTGGGCTTGGTAT	0	10	RDR2-sensitive
CATGCTTTTGTCTGGGCTTGGTAT	0	13	RDR2-sensitive
CATGGAATACCCCTGGAACGACGA	18	0	RDR2-resistant
CATGGACAGAGATCTGAGGAGCGG	0	24	RDR2-sensitive
CATGGACCTCGCGGACGAACGGCT	56	14	RDR2-resistant
CATGGACCTGATGGCTTTAATGGC	22	1	RDR2-resistant
CATGGACGAAGACGCAGGCAACCG	0	9	RDR2-sensitive
CATGGACGACAGCATCAAGGACCT	0	9	RDR2-sensitive
CATGGACGACGTAGCATCAACGGT	0	14	RDR2-sensitive
CATGGACGAGCAGACACGACACAG	0	14	RDR2-sensitive
CATGGACGGCGGGGAGGGTCGCGC	38	3	RDR2-resistant
CATGGACTGCCGGACGTTGGTCGG	0	18	RDR2-sensitive
CATGGACTGTCCAGCCTTAGGCGC	0	23	RDR2-sensitive
CATGGACTGTGGCGTCATCTCGGC	0	23	RDR2-sensitive
CATGGACTTGACAGCGGTAGGAGA	1	16	RDR2-sensitive
CATGGATACGGATACGGATAGCAC	0	13	RDR2-sensitive
CATGGATACGGATACGGGTAGCAC	0	11	RDR2-sensitive
CATGGATGGGAGGAAGAAGATGAA	0	10	RDR2-sensitive
CATGGATGGTAGAGGTGCAGTGAG	0	11	RDR2-sensitive
CATGGCAATGTGGCTGGCATGACT	41	1	RDR2-resistant
CATGGCACTGAGGTAACACCGCTC	0	13	RDR2-sensitive
CATGGCAGATGTGGACGTCTACAC	0	25	RDR2-sensitive
CATGGCAGTGTAGGACGGAGTTGA	0	41	RDR2-sensitive
CATGGCCTTGTCGTTGGAGAGCAT	0	9	RDR2-sensitive
CATGGCTAAGAACCGTGTGATCGT	0	12	RDR2-sensitive
CATGGGACAGTAGCTCAACGGCTA	0	10	RDR2-sensitive
CATGGGCTGTTGGGTGCCGGTCGG	0	12	RDR2-sensitive
CATGGGGACTCAGAAGGAATCGGT	14	0	RDR2-resistant
CATGGGTCTGTAGAGGGCGGGCAT	0	18	RDR2-sensitive
CATGGTAGAATAAGCGGGCGGGCT	0	22	RDR2-sensitive
CATGGTCGTCACCGTCGTCTGGGC	1	20	RDR2-sensitive
CATGGTCGTCATCGTCGTCTGGGC	0	9	RDR2-sensitive
CATGGTGAGGATGACGAGTGACGC	0	10	RDR2-sensitive
CATGGTGAGGATGAGGATGACGGG	1	31	RDR2-sensitive
CATGGTTCGTGGGTCTGGGCCGGA	0	12	RDR2-sensitive
CATGTAACGCACGTTCGGACGCCT	0	54	RDR2-sensitive
CATGTACAGACGCTGGATCCTCGG	0	10	RDR2-sensitive
CATGTACTGTAGAGTGTCCGGTGC	0	42	RDR2-sensitive
CATGTAGCAGAACAGGACACGCAT	0	13	RDR2-sensitive
CATGTCAGAGCTCCGTGCCATGGA	0	13	RDR2-sensitive
CATGTCAGAGGCAGAACACGTCAG	0	9	RDR2-sensitive

CATGTCAGAGGCAGAACGGCAGAC	0	13	RDR2-sensitive
CATGTCAGCGATAGGAACCGGCCA	0	11	RDR2-sensitive
CATGTCATATTAGATGTGTCGGCG	34	0	RDR2-resistant
CATGTCATGTTGAGCTTAGATGGT	0	27	RDR2-sensitive
CATGTCCAGGACTGTGGAAGCGGT	0	14	RDR2-sensitive
CATGTCCCTGAACCAGCACACTGC	0	10	RDR2-sensitive
CATGTCGACATCACGTAGGGGCAG	2	18	RDR2-sensitive
CATGTCGCGTAGGACAGTTCGGCT	0	17	RDR2-sensitive
CATGTCGGGGCTGAGGTCGGACGG	0	10	RDR2-sensitive
CATGTCGTCCCCTGTTACGGTGTG	0	9	RDR2-sensitive
CATGTCGTGTCTTCGTTGGGTCGT	0	14	RDR2-sensitive
CATGTCTAGATCGTGCCGGGCCGG	0	13	RDR2-sensitive
CATGTCTGTGTACGTCAAACGGTT	0	9	RDR2-sensitive
CATGTCTTCAGTTAGCGTGTCGGT	19	0	RDR2-resistant
CATGTCTTCTCGGGCAGCGTCGGT	0	15	RDR2-sensitive
CATGTGGAAGACGACTGACATCAT	0	47	RDR2-sensitive
CATGTGGAAGACGGCTGGCATCAT	0	14	RDR2-sensitive
CATGTGGACCCAAACAGTGGACGC	0	35	RDR2-sensitive
CATGTGGACTATAGAGGGCTTCAC	0	9	RDR2-sensitive
CATGTGGAGTAGCTCAAGCGGCCT	4	21	RDR2-sensitive
CATGTTGAAGCTCTGCATTGACGT	19	0	RDR2-resistant
CATGTTGGACGACATACCGGCCTT	0	20	RDR2-sensitive
CATGTTGGATAAAATGGCGTGAGA	32	5	RDR2-resistant
CATGTTGGCTAGAATGTAGAAGGC	0	15	RDR2-sensitive
CATGTTGGCTAGAGGGTGGAAGGC	1	43	RDR2-sensitive
CATGTTGGTGACTGACGGCCGCGC	0	10	RDR2-sensitive
CATGTTGGTGAGCGGAGCGAAGGC	1	12	RDR2-sensitive
CATGTTTGACGATGGACAGCGCGG	0	11	RDR2-sensitive
CATGTTTGGTGGGCTGGTTGAGGA	0	11	RDR2-sensitive
CATGTTTGTCTTCTTCGTCGCGCA	17	0	RDR2-resistant
CATGTTTTTAATAACGTGTCGGGC	0	11	RDR2-sensitive
CATTAACGACATGAAGAACGTCAT	0	9	RDR2-sensitive
CATTAAGCGTGTCGGGCTTGGCAA	0	13	RDR2-sensitive
CATTAGATCTAGATCGGATGCACC	0	16	RDR2-sensitive
CATTAGGATTCCTGTAGTGTTGGC	0	9	RDR2-sensitive
CATTCATGCTGTTAGATCGCGGAC	0	24	RDR2-sensitive
CATTCGGAACCCAGAACACGCGCA	0	24	RDR2-sensitive
CATTCGGCTGGAGGACAAGGGCAA	1	14	RDR2-sensitive
CATTCTATCTTTAAGTCGGTTCGA	3	30	RDR2-sensitive
CATTCTGCTGAGCGCGGACGGTCC	0	11	RDR2-sensitive
CATTCTGTTTGTGGTTCTTTCGGT	65	4	RDR2-resistant
CATTCTTGCATCCAGGTCGCGGAC	2	15	RDR2-sensitive
CATTGACCAATGACCCAGGGACGC	0	11	RDR2-sensitive
CATTGACCGTATCTCTGATGGCAT	0	12	RDR2-sensitive
CATTGCCTGTAGAGCGTGTCGGCG	2	20	RDR2-sensitive
CATTGCGTCATGTGGACCGTTCGA	0	9	RDR2-sensitive
CATTGGACTGTCCGGTGTGCCCAT	0	33	RDR2-sensitive
CATTGGGTACTGTAGGACGGCTAA	0	10	RDR2-sensitive
CATTGGTGGATTGGTGGGAGGAAG	20	0	RDR2-resistant

CATTGTAACTCGAGCTGAATACGT	0	17	RDR2-sensitive
CATTGTAAGTACTGTGACGGAACC	0	11	RDR2-sensitive
CATTGTCAGCTCTAGATGTAGGTA	0	9	RDR2-sensitive
CATTGTCCTTGGGCAAGACGCGCT	20	0	RDR2-resistant
CATTGTTGACTGTGAAGCCGGAGA	41	1	RDR2-resistant
CATTTACGTGGGGCAGCAGGCTGC	1	16	RDR2-sensitive
CATTTAGAGGACGCTGCTGGAGAG	0	10	RDR2-sensitive
CATTTATTTCTGGTCACTCGTCGG	0	10	RDR2-sensitive
CATTTCGGGCTTAGACTGGACCGG	1	14	RDR2-sensitive
CATTTCGTTTGTAGATGGTTCGTA	14	0	RDR2-resistant
CATTTCTATCCTTCGTCGGCCAAC	0	16	RDR2-sensitive
CATTTGCTTGACTGTGGCGACGCC	0	13	RDR2-sensitive
CATTTGGAGGGTAGACGATGGCTC	0	15	RDR2-sensitive
CATTTGGGCTGGTAGGCGGTACAT	0	9	RDR2-sensitive
CATTTTACCTGGACAGGATGGCTC	0	14	RDR2-sensitive
CATTTTCCATCCAACGGTCGTCGC	0	18	RDR2-sensitive
CATTTTGCTACAGTCGTCGACACC	16	102	RDR2-sensitive
CATTTTGTAGATAGATGGATCAGT	51	10	RDR2-resistant
CATTTTGTTGTGGAGTGTAAGGCA	22	2	RDR2-resistant
CATTTTTGGACCCTTTGCTCGGCG	5	20	RDR2-sensitive
CCAAAACACTCAGTCGGGCTTCAC	0	13	RDR2-sensitive
CCAAAAGCTGCTGCGGACTGCCAA	0	10	RDR2-sensitive
CCAAAAGCTGTTGCGGACTGCCAA	0	9	RDR2-sensitive
CCAAAATGAGCGGACGGTCCGGCC	0	12	RDR2-sensitive
CCAAACACAGAACAGAGCGGCTCC	0	9	RDR2-sensitive
CCAAACAGCGGACTGGAACGGCTC	0	12	RDR2-sensitive
CCAAACAGCTGCAACAGTGTCGGC	1	47	RDR2-sensitive
CCAAACCTGCAGGACTAGGCCCAT	0	10	RDR2-sensitive
CCAAAGAAGAGGGCAACGTAGGGG	23	0	RDR2-resistant
CCAAAGACTGGCACATGACGAGGA	0	10	RDR2-sensitive
CCAAAGCGACTGGAGGGCAGAGGA	0	10	RDR2-sensitive
CCAAAGTTGACTGGGAGACGACGA	5	85	RDR2-sensitive
CCAAAGTTGACTGGGAGACGACGG	4	204	RDR2-sensitive
CCAAATTCTTGGTAGAATCGATGG	25	1	RDR2-resistant
CCAACACAGCTAGCAGGTCGGCAT	0	13	RDR2-sensitive
CCAACACTCTTAAGCACGGCTGGT	0	13	RDR2-sensitive
CCAACAGCAGTAGGGACGTGAGAA	2	35	RDR2-sensitive
CCAACCAAAGGGCTGGATCCAGGA	0	43	RDR2-sensitive
CCAACCAAAGGGCTGGATCTAGGA	0	22	RDR2-sensitive
CCAACCGAAACCGTAGAGAGGGCC	28	1	RDR2-resistant
CCAACCGAAGGATAGAGCAGACGA	0	31	RDR2-sensitive
CCAACCTAATTGTGGGACTGGGCC	0	14	RDR2-sensitive
CCAACCTGAACGACGTGGCGCCAA	2	18	RDR2-sensitive
CCAACGGACGAGGGTTGCAATCAA	1	30	RDR2-sensitive
CCAACGGAGTAGAGCAGAACGGTT	0	10	RDR2-sensitive
CCAACGGTCGGCCGGGCCAACGGT	45	106	RDR2-sensitive
CCAACGGTCGGCTGGGCCAACGGT	0	16	RDR2-sensitive
CCAACGGTGCTGTGTCAGGGGGTT	15	34	RDR2-sensitive
CCAACTCGAATTCCTGGCTGAGGC	0	9	RDR2-sensitive

CCAACTGGAACTGGAGCCCGGCCT	40	3	RDR2-resistant
CCAACTGTAGGTCGCTGAAGACGC	0	20	RDR2-sensitive
CCAAGAATCGGGTGAAGACGGAGC	0	9	RDR2-sensitive
CCAAGACACACGCTAGGATCTCGG	0	17	RDR2-sensitive
CCAAGATCTGTATAGCCTCGGCAT	1	21	RDR2-sensitive
CCAAGATCTGTGGCATCGAGTCGT	0	9	RDR2-sensitive
CCAAGGAACTGTAGCCCAACGGCT	0	10	RDR2-sensitive
CCAAGGAAGGTGAGCAAACTCCAT	0	12	RDR2-sensitive
CCAAGGAGGAGCTGGCCAGAGCAT	0	17	RDR2-sensitive
CCAAGGGATACGTAGCAGCTTCGG	0	13	RDR2-sensitive
CCAAGGTTTCGTAGAGCAGGAGAA	0	27	RDR2-sensitive
CCAAGGTTTCGTAGAGCAGGAGAG	0	18	RDR2-sensitive
CCAAGGTTTCGTAGAGCAGGAGGA	0	14	RDR2-sensitive
CCAAGGTTTCGTAGAGCAGGAGGG	0	27	RDR2-sensitive
CCAAGTAGAACGGAGCCGCTCCAT	0	18	RDR2-sensitive
CCAAGTAGAACGGAGCCGTTCCAT	1	86	RDR2-sensitive
CCAAGTAGAACGGAGCCGTTTCAT	0	20	RDR2-sensitive
CCAAGTAGAACGGAGCTGTTCCAT	0	9	RDR2-sensitive
CCAAGTAGAACGGAGTCGTTCCAT	0	23	RDR2-sensitive
CCAAGTCGCTAGGGCTGGTGGCAT	0	17	RDR2-sensitive
CCAAGTGTAGGATCTCGAAGGCGA	0	12	RDR2-sensitive
CCAATAGATCGTGCCGTGGGCCAA	0	10	RDR2-sensitive
CCAATAGCCTGATCGTTGGATTAA	0	11	RDR2-sensitive
CCAATCTAATTGTGGGTCTGGGCC	1	27	RDR2-sensitive
CCAATCTCTGCATCGCGTGGACGC	0	10	RDR2-sensitive
CCAATGACTGGAAGTAGGGACGTT	0	9	RDR2-sensitive
CCAATGATTGAGGATTCAGACGAC	19	1	RDR2-resistant
CCAATGCAACGGGCCTGTACGGAC	0	12	RDR2-sensitive
CCAATGGAAGTTGGCTGCGAGCAT	0	12	RDR2-sensitive
CCAATGGGTGTGGGCAAGGGACGG	2	20	RDR2-sensitive
CCAATGGTTGAGGATCTAGACGAC	32	0	RDR2-resistant
CCACAAGTCAGAGCTCCAACGGTC	2	15	RDR2-sensitive
CCACAATCTGGACTGTCCGGCTAT	0	25	RDR2-sensitive
CCACACGAACTGGAAGAAGTGCAA	0	23	RDR2-sensitive
CCACACGAACTGGAGAAAGTGCAA	0	21	RDR2-sensitive
CCACAGGCGCGGACCGTCCGGACC	0	16	RDR2-sensitive
CCACAGGCGCGGACTGTCCGGACC	0	14	RDR2-sensitive
CCACATCCAACGGTGCTGCGTCAG	0	9	RDR2-sensitive
CCACATGTCAGAAGCTCCAACGGT	0	13	RDR2-sensitive
CCACCAAAAGCTGCTGCGGACTGC	0	11	RDR2-sensitive
CCACCAGCGGTCTCGGTAGGCTAG	0	14	RDR2-sensitive
CCACCAGTGAACAGATCTGCTCGG	52	0	RDR2-resistant
CCACCATCACCAGCGTCGGGCGTT	0	12	RDR2-sensitive
CCACCCAAGACGGATCTAGACGAC	1	23	RDR2-sensitive
CCACCGCGTCGACGCAGGAACGGT	0	12	RDR2-sensitive
CCACCGCGTCGACGTAGGAACGGT	0	25	RDR2-sensitive
CCACCGCGTCGGTGTAGGAACGGT	0	20	RDR2-sensitive
CCACCGGACGTCCCTAGACCGCAT	0	16	RDR2-sensitive
CCACCGGAGAAGGCAACCAAACGC	0	14	RDR2-sensitive

CCACCGGAGTCCGACCTGACGGTT	0	10	RDR2-sensitive
CCACCTGTGGATTGTGGAGGTGTG	0	9	RDR2-sensitive
CCACGAAAGGAACAGACGGCTCAT	1	22	RDR2-sensitive
CCACGAACAGACATGGTACGGCTC	0	22	RDR2-sensitive
CCACGAACCGGACGGCACACCTGC	0	11	RDR2-sensitive
CCACGACCTAGAACCGGACGGTCC	0	12	RDR2-sensitive
CCACGACTTGGACACGGGAGCGGC	0	15	RDR2-sensitive
CCACGAGTCGGTATGGCACAACAC	0	21	RDR2-sensitive
CCACGATCTGGACACAGAAGCGGC	1	14	RDR2-sensitive
CCACGCGCCTGCAGAGAAGAGCAT	0	9	RDR2-sensitive
CCACGCTCCGGACTGAATGCGCAC	0	9	RDR2-sensitive
CCACGGATCTGAGGAAGAAGACGC	0	32	RDR2-sensitive
CCACGGCTAGGAACCAGACGGTCT	0	25	RDR2-sensitive
CCACGGCTGAAGCTCGGACGGTCC	0	12	RDR2-sensitive
CCACGGCTGCAGCGGTGTCCCTAC	0	14	RDR2-sensitive
CCACGGTACATGACTGGACGGTCC	0	9	RDR2-sensitive
CCACGGTCCGGACTGTCCGCGGTG	0	17	RDR2-sensitive
CCACGGTCGTCGCGAAGCCGCGCT	1	12	RDR2-sensitive
CCACGGTCTGAACGGTACGCGCGT	0	13	RDR2-sensitive
CCACGTATCCCGGCTGAGACGCGA	0	13	RDR2-sensitive
CCACGTCACGAAGCTCGGCGCCAC	0	17	RDR2-sensitive
CCACGTCACGAAGCTCGGCGCCAT	0	36	RDR2-sensitive
CCACGTCAGGAAGCTCGGCGCCAC	0	9	RDR2-sensitive
CCACGTCAGGAAGCTCGGCGCCAT	0	9	RDR2-sensitive
CCACGTCAGGCAGACAGGCGCCAA	0	11	RDR2-sensitive
CCACGTCATGAAGCTCGGCGCCAT	0	26	RDR2-sensitive
CCACGTGGTGTGAGCTGTGACGGC	73	12	RDR2-resistant
CCACGTTGGAAGAGCTGTTCGGTT	0	10	RDR2-sensitive
CCACGTTGGCATGTACACAGGGCT	7	48	RDR2-sensitive
CCACGTTGGCATGTACATAGGGCT	1	29	RDR2-sensitive
CCACTAGATTGTAGGGGTGGTCGG	0	10	RDR2-sensitive
CCACTCACGGACTCATCAGGGCAT	1	19	RDR2-sensitive
CCACTCGAGACTAGCACAGGGGCG	0	11	RDR2-sensitive
CCACTCTGACCTGACGAACGACAC	0	19	RDR2-sensitive
CCACTCTGGTCGACGGAAGGGCAA	0	13	RDR2-sensitive
CCACTGAACCGGACGTTGGCGCGC	0	33	RDR2-sensitive
CCACTGACCGGCCTGACAGAAGGA	5	23	RDR2-sensitive
CCACTGACCGGTCTGACAGAAGGA	2	20	RDR2-sensitive
CCACTGCGCTGTAGGGTCGGTCAG	0	9	RDR2-sensitive
CCACTGCGTTTGGTGTAGAGATGG	0	13	RDR2-sensitive
CCACTGGAGAAAGCCTGAGGCTGC	0	11	RDR2-sensitive
CCACTGGCCAGTCTGACAGAAGGA	1	13	RDR2-sensitive
CCACTGGGCTTTGACACACGACGG	0	13	RDR2-sensitive
CCACTGTACATGCCCTGAAGACGA	0	15	RDR2-sensitive
CCACTGTAGACCAGGCAACACCAC	0	11	RDR2-sensitive
CCACTGTAGCGCGTAGAGGATGAT	0	21	RDR2-sensitive
CCACTGTATGGCTTCGGCGGCAAG	35	1	RDR2-resistant
CCACTGTCAGTTCTCAATCGGCCT	0	12	RDR2-sensitive
CCACTGTTATGCGGGCTGAGCCGG	1	16	RDR2-sensitive

CCACTGTTTTTACACTTCGGGCTG	0	28	RDR2-sensitive
CCACTTATTCGTATCGTGCCGGGC	0	15	RDR2-sensitive
CCACTTCTGGCACTGTGGTTTGGC	0	13	RDR2-sensitive
CCACTTGTAGGAAACGGGTGACGC	0	9	RDR2-sensitive
CCACTTGTGCGGACAGGCATGGAC	0	10	RDR2-sensitive
CCACTTTGTGGACTTGCTCGACGG	0	10	RDR2-sensitive
CCAGAACCAGATCGAGCGCGCGTA	1	36	RDR2-sensitive
CCAGAACTCCCGCTCAGCAGAGAC	22	0	RDR2-resistant
CCAGAACTCCCGCTCAGTAGAGAC	97	36	RDR2-resistant
CCAGAACTCCTGATCCATAGAGAC	38	1	RDR2-resistant
CCAGAACTCTGCAGGTAGAGCTGT	2	161	RDR2-sensitive
CCAGAAGACAGAAGCTCCAACGGT	0	11	RDR2-sensitive
CCAGAAGACGACGAACTCCGCTTC	0	9	RDR2-sensitive
CCAGAAGCTACTGCGGACTGTCAA	0	9	RDR2-sensitive
CCAGAAGGACTTCGTGGACCCGGC	0	11	RDR2-sensitive
CCAGACATGTGTGGTTCCTTGGAG	34	0	RDR2-resistant
CCAGACCAGACGAGTAGACGACGC	0	13	RDR2-sensitive
CCAGACCCGCATAGAACCAGGCTC	0	9	RDR2-sensitive
CCAGACGGATGGTAGAAAGGGTTA	0	9	RDR2-sensitive
CCAGACGGTGTGTACGATAGACGT	0	12	RDR2-sensitive
CCAGACTGAGCAGATTCTTGGCTG	2	14	RDR2-sensitive
CCAGACTGCTGACTGTACGGCTGT	0	9	RDR2-sensitive
CCAGACTGTGCTCGATGACGGTGC	0	11	RDR2-sensitive
CCAGACTGTTCGGTGGCACATCGG	0	9	RDR2-sensitive
CCAGAGACTGTAAATGTGGCGGAT	0	18	RDR2-sensitive
CCAGAGCTCTTCAAGTAGATTATT	23	0	RDR2-resistant
CCAGAGCTGCGGACTGTCCGGCGT	0	9	RDR2-sensitive
CCAGATACGGAAGGCGACTCCAAG	31	1	RDR2-resistant
CCAGATACGGCTGCACGGTACTCC	0	52	RDR2-sensitive
CCAGATAGATGGGAGGCAGAACGG	0	15	RDR2-sensitive
CCAGATCGATGGACGGCAGAACGG	0	16	RDR2-sensitive
CCAGATCTCCTGGAGGCAGAACGC	2	16	RDR2-sensitive
CCAGATCTTGGCGAGGACGAGCAC	0	16	RDR2-sensitive
CCAGATGAGTCGGCGCGAGACGGA	0	12	RDR2-sensitive
CCAGATGGGTCGGCGCGAGACGGA	7	45	RDR2-sensitive
CCAGATTGAGCGAAGGCAGAACGG	0	27	RDR2-sensitive
CCAGCACGAATAAGCGGGTCGGGC	0	18	RDR2-sensitive
CCAGCACGACTAGCAGGCTGACGG	0	13	RDR2-sensitive
CCAGCACGGAAGGGAATAAGACGG	0	32	RDR2-sensitive
CCAGCACGTGTGCCCAGTCATCGG	0	11	RDR2-sensitive
CCAGCATCTGTGGCGCGACGACGG	2	27	RDR2-sensitive
CCAGCCTGGCGCACCGGACACTGT	0	15	RDR2-sensitive
CCAGCGACTGCAGCGAAAGGCGAC	1	18	RDR2-sensitive
CCAGCGACTGCAGCGAAGGCCTCG	0	14	RDR2-sensitive
CCAGCGACTGCAGCGAGAGACGAC	2	21	RDR2-sensitive
CCAGCGACTGCAGTGAAAGGCGAC	0	9	RDR2-sensitive
CCAGCTATTTTCGTCGGTTGGCCT	0	20	RDR2-sensitive
CCAGCTCGAGGTAGACGACGGCCT	0	37	RDR2-sensitive
CCAGCTCGGCTCGGCTCGACTCGG	0	9	RDR2-sensitive

CCAGCTGAGAATTAGGCGTCTTGA	0	15	RDR2-sensitive
CCAGCTGTAGCAATGGGATGGTTA	0	27	RDR2-sensitive
CCAGGAAACGCGGCTAGGGTTCGT	0	42	RDR2-sensitive
CCAGGACACAGGAAGGGAAGGCAA	0	37	RDR2-sensitive
CCAGGACCCTGACCTCGGTGACGG	0	9	RDR2-sensitive
CCAGGACGCCAACCAGATCGCGGT	0	16	RDR2-sensitive
CCAGGACGCCAACCAGGTCGCGGT	0	20	RDR2-sensitive
CCAGGACGTAGGGTGTTACGCATC	0	11	RDR2-sensitive
CCAGGACTTTGTTGCGGCCCGGAC	0	19	RDR2-sensitive
CCAGGATCCGAAGACAGAGGAGGC	1	18	RDR2-sensitive
CCAGGATTGCAGAGGCGGAATGGA	0	10	RDR2-sensitive
CCAGGCACGCAGGACTGTGCAGGC	0	12	RDR2-sensitive
CCAGGCACGCAGGACTGTGTAGGC	0	12	RDR2-sensitive
CCAGGCACGGCACGGTTAGAGCAC	0	22	RDR2-sensitive
CCAGGCACGGCACTAAGAACGGGC	0	20	RDR2-sensitive
CCAGGCACGGCATGGTTAGAGCAC	0	21	RDR2-sensitive
CCAGGCACGTAGGACTGTGCAGGC	0	101	RDR2-sensitive
CCAGGCACGTGGGCTCTCTAGGCA	0	9	RDR2-sensitive
CCAGGCATGTAGGACTGTGCAGGC	0	13	RDR2-sensitive
CCAGGCCTGTGGATTCGTTGCGGG	55	8	RDR2-resistant
CCAGGCTCCCGGACTGGACCAGGC	0	30	RDR2-sensitive
CCAGGCTGAGGACTGTGCGGACGG	0	13	RDR2-sensitive
CCAGGCTTCTGACAGCACTGACAA	0	16	RDR2-sensitive
CCAGGGAACAGTAGCACAACGGTT	0	9	RDR2-sensitive
CCAGGGAACTGTAGCCCAACGGCT	0	15	RDR2-sensitive
CCAGGGACCTGCCGACTTGACGGA	0	13	RDR2-sensitive
CCAGGTAGAGATGAGAGAGGACAT	0	9	RDR2-sensitive
CCAGGTCAGACAGTGCAATCCGGT	0	11	RDR2-sensitive
CCAGGTCGCGGACCGTTCGGCCCC	0	14	RDR2-sensitive
CCAGGTCGCGGACTGTCCGGCCCC	2	14	RDR2-sensitive
CCAGGTCGGGTGCGCTTGCAGTAG	12	129	RDR2-sensitive
CCAGGTCGTGACTGAGCTGCCGGT	0	26	RDR2-sensitive
CCAGGTCGTGGACTGTACGGCCAC	0	10	RDR2-sensitive
CCAGGTGCTCTGACCGGCGCGCAC	0	13	RDR2-sensitive
CCAGGTGCTCTTGACGATCGTCGT	0	9	RDR2-sensitive
CCAGGTTCGGCTCGGTCGTCTGGC	0	12	RDR2-sensitive
CCAGGTTGAAGAGGCTGATCAGGC	0	10	RDR2-sensitive
CCAGGTTGGCCTGAGGCTTGTCGT	0	82	RDR2-sensitive
CCAGGTTGTAGAGGTAATCGGCCT	0	47	RDR2-sensitive
CCAGTAGTAGTGCTGGAACGGCAT	0	9	RDR2-sensitive
CCAGTCAGGACGCGGTCAACCCAG	0	25	RDR2-sensitive
CCAGTCATAGACACGGGATGGCAT	1	47	RDR2-sensitive
CCAGTCATTCGTCGAGATCGGCTC	0	16	RDR2-sensitive
CCAGTCCACGGTCTGCTATGGCGC	0	9	RDR2-sensitive
CCAGTCGACAGAAGGCAAGATCAG	0	27	RDR2-sensitive
CCAGTCGAGAGTCGAGCGAGGCAG	0	10	RDR2-sensitive
CCAGTCGCTGCTAGAGGACGTGTG	0	88	RDR2-sensitive
CCAGTCTGTAGGATCTAGGACACC	0	9	RDR2-sensitive
CCAGTGAGCGTAGGCATGCAACGG	0	9	RDR2-sensitive

CCAGTGCACAGGCTGCAGCAGGCT	0	10	RDR2-sensitive
CCAGTGCACAGGCTGTAGCAGGCT	1	55	RDR2-sensitive
CCAGTGGAAGCGGACGCATCGATA	63	2	RDR2-resistant
CCAGTTGGCCCAATTGTAGTCGGA	0	11	RDR2-sensitive
CCAGTTGGGCCCGAATACGCGCAT	0	15	RDR2-sensitive
CCAGTTTCAACGGTCGTCATGCGT	0	24	RDR2-sensitive
CCATAAACTGTAGCGGGTTTTCGG	1	16	RDR2-sensitive
CCATAAAGTTTGTAGCACGGGTGA	20	0	RDR2-resistant
CCATACGATCAGCGGGCGAGACGC	0	18	RDR2-sensitive
CCATAGTGGACGCGTAGAACGGCG	0	18	RDR2-sensitive
CCATAGTTAACTGAGAGACGACGG	0	18	RDR2-sensitive
CCATAGTTGACTGGGAGACGACGA	0	18	RDR2-sensitive
CCATAGTTGACTGGGAGACGACGG	0	44	RDR2-sensitive
CCATATGGATTGAGGGGGATTGAG	0	9	RDR2-sensitive
CCATATTTGGACTGGACCGCGCAT	1	14	RDR2-sensitive
CCATCACCATCGGACTGCCCACGG	0	11	RDR2-sensitive
CCATCACCGCGGGCTTAGAAGGCT	0	14	RDR2-sensitive
CCATCACGGTCTGTACAGATGGAT	0	13	RDR2-sensitive
CCATCACTAACGGGCTGACGTCAA	1	18	RDR2-sensitive
CCATCAGACTGTGTTCAACGGTGT	0	52	RDR2-sensitive
CCATCAGCGTCGACTAGGGATGGC	0	31	RDR2-sensitive
CCATCAGTTATCTTGTCGGATGTT	18	0	RDR2-resistant
CCATCCCGTCAGCAGTAGAGGCGC	2	36	RDR2-sensitive
CCATCGACGGAGAGCAGATGGGGC	20	1	RDR2-resistant
CCATCGCAGAAGGCCAGGTAGAGC	0	41	RDR2-sensitive
CCATCGCCATCGTAGATCGTCCGG	0	14	RDR2-sensitive
CCATCGGGTGACGGATATGGATAG	0	20	RDR2-sensitive
CCATCGTCAAGCAGGGGACCTCAT	0	47	RDR2-sensitive
CCATCGTCAAGTAGGGGACCTCAT	0	39	RDR2-sensitive
CCATCTCACTGTCAGGATCGCCAT	25	3	RDR2-resistant
CCATCTCGCGGTCCTGTTTGGCAG	0	9	RDR2-sensitive
CCATCTCGGACATGCGACGATGGC	0	47	RDR2-sensitive
CCATCTCGGACTCGCGACGGTGTC	0	15	RDR2-sensitive
CCATCTCTGGACCGGATCCGACGC	0	9	RDR2-sensitive
CCATCTGAACTGGGTCTGGCGCAT	0	11	RDR2-sensitive
CCATCTGGATGGCACTGCAACGGC	47	2	RDR2-resistant
CCATCTGGGCTGGAGCACGCGGCG	0	13	RDR2-sensitive
CCATCTGGGCTGGGACACGCGGCG	9	53	RDR2-sensitive
CCATCTGGGCTGGGGCACGCAGCA	0	18	RDR2-sensitive
CCATCTGGGCTGGGGCACGCGGCA	8	55	RDR2-sensitive
CCATCTGGGCTGGGGCACGCGGCG	31	147	RDR2-sensitive
CCATCTTGGACCTGTGACGGTGGC	0	26	RDR2-sensitive
CCATCTTGGATATGTGACGGTGGC	0	45	RDR2-sensitive
CCATGAACATAGACCGGCCCCCAT	0	12	RDR2-sensitive
CCATGAACCGAGCGAGCCAGCGAG	0	9	RDR2-sensitive
CCATGAACTCGGCAGCGGTAGAAG	0	12	RDR2-sensitive
CCATGACTTTAGGTCGGACGGTTT	0	10	RDR2-sensitive
CCATGCGTGTGTAGAAGCGACGGA	2	34	RDR2-sensitive
CCATGGAACTCTCTTCCCTGACGG	1	19	RDR2-sensitive

CCATGGATCTTGGCGCTGAGGTGT	0	9	RDR2-sensitive
CCATGGATGTAGATGGGTTTGCAT	0	12	RDR2-sensitive
CCATGGATTGAGTGGGATTGGAAA	0	9	RDR2-sensitive
CCATGGATTGGATGGGATTGGAAA	0	22	RDR2-sensitive
CCATGGGTTGTAGAACGACGGCCA	0	9	RDR2-sensitive
CCATGGTAGTGGGGGCACTGAAGC	39	0	RDR2-resistant
CCATGTAACGCACGTTCGGACGCC	0	10	RDR2-sensitive
CCATGTAACGCACGTTCGGACGCT	0	9	RDR2-sensitive
CCATGTCGTCCCCTGTTACGGTGT	0	179	RDR2-sensitive
CCATGTCTGGAGCTCGGACGGCCT	0	37	RDR2-sensitive
CCATGTGGATTGAGCGGGATTGGA	0	13	RDR2-sensitive
CCATGTGGATTGAGTGGGATTGGA	2	23	RDR2-sensitive
CCATGTGGCAAACTAGCTCGGCAT	1	12	RDR2-sensitive
CCATGTTCGGGTCTCGGGTACGGT	0	14	RDR2-sensitive
CCATGTTTGTCACTGTTGAGGCAT	0	10	RDR2-sensitive
CCATTACATTGGTCGGATTGGGCT	0	10	RDR2-sensitive
CCATTAGTAGACATGCCGTCGGAT	35	2	RDR2-resistant
CCATTAGTGGATTGGTGGGAGGAA	62	0	RDR2-resistant
CCATTCAATCGGTAGGAGCGACGG	1	19	RDR2-sensitive
CCATTCATGCTGTTAGATCGCGGA	0	12	RDR2-sensitive
CCATTCCGTTGGCAGGCAGTACGC	0	11	RDR2-sensitive
CCATTCGAGGGACTCGATCACGCC	1	12	RDR2-sensitive
CCATTCTCGGCAGAGGGCGTAGGA	0	10	RDR2-sensitive
CCATTCTCTATTCGATCTCGTCGT	0	9	RDR2-sensitive
CCATTCTGCTGAGCGCGGACGGTC	0	9	RDR2-sensitive
CCATTGCCTGTAGAGCGTGTCGGC	1	28	RDR2-sensitive
CCATTGGTGGATTGGTGGGAGGAA	36	1	RDR2-resistant
CCATTTAGCTACAGTCGGCGACAC	0	15	RDR2-sensitive
CCATTTGCCACGGGGTTGATCGGA	0	15	RDR2-sensitive
CCATTTTGCTACAGTCGGCGACAC	2	14	RDR2-sensitive
CCATTTTGCTACAGTCGTCGACAC	2	43	RDR2-sensitive
CCCAAAAACCTGATCCGTCGGCAC	0	9	RDR2-sensitive
CCCAAAGAGGATTGGAGGGGCTAA	0	11	RDR2-sensitive
CCCAAAGTGTAGACACAACGGCTC	0	9	RDR2-sensitive
CCCAACGATCGGCTGACGTGGCAA	2	22	RDR2-sensitive
CCCAACGGGCGGCGTGCTGAATCC	57	5	RDR2-resistant
CCCAACGGTCGGCTGACGTGGCAT	0	9	RDR2-sensitive
CCCAAGCACGTTGGGGTAGGCTAG	0	11	RDR2-sensitive
CCCAAGCACTGTTAGATCCGGCAC	0	18	RDR2-sensitive
CCCAATCTTTTCGTCGTCGCGCAT	0	9	RDR2-sensitive
CCCACAATACTCGTGTCGGTGCAT	0	9	RDR2-sensitive
CCCACACGGGCCTTCAGAACGGTT	0	10	RDR2-sensitive
CCCACAGGCGCGGACCGTCCGGAC	3	21	RDR2-sensitive
CCCACAGGCGCGGACTGTCCGGAC	3	167	RDR2-sensitive
CCCACAGGCGCGGACTGTTCGGAC	0	18	RDR2-sensitive
CCCACAGGCGTGGACTGTCCGGAC	0	13	RDR2-sensitive
CCCACCTGTAGGTCCAGCAGATAA	0	9	RDR2-sensitive
CCCACGAACTCTTGCGAGAGACAT	0	16	RDR2-sensitive
CCCACGAACTCTTGCGGGGGGCAT	2	19	RDR2-sensitive

CCCACGCACTGTTCACGGCTCCAG	0	12	RDR2-sensitive
CCCACTCAGGACCACTGAACCGGA	0	33	RDR2-sensitive
CCCACTGACCAGTGGTACCGGACA	0	9	RDR2-sensitive
CCCACTGCTAGTCCCCGACGACAT	0	10	RDR2-sensitive
CCCAGAAGACAGAAGCTCCAACGG	0	15	RDR2-sensitive
CCCAGAAGACAGAGCTCCAACGGT	0	11	RDR2-sensitive
CCCAGATACGGCTGCACGGTACTC	0	14	RDR2-sensitive
CCCAGATGAGTCGGCGCGAGACGG	0	10	RDR2-sensitive
CCCAGATGGGTCGGCGCGAGACGG	1	38	RDR2-sensitive
CCCAGCCAGGAGGTCGATCGGGTC	0	12	RDR2-sensitive
CCCAGCTGTAGCAATGGGATGGTT	0	41	RDR2-sensitive
CCCAGGAATCTGCACGCACGTCAT	0	11	RDR2-sensitive
CCCAGGACTTTGTTGCGGCCCGGA	0	12	RDR2-sensitive
CCCAGGGCTGAGCAGACGGATAGC	0	10	RDR2-sensitive
CCCAGTAGTTGACGGGCTCAGGAT	0	10	RDR2-sensitive
CCCATAGGCAACCGTCGGACATAA	0	12	RDR2-sensitive
CCCATCGTACGGAGAGAGGCCCAG	0	19	RDR2-sensitive
CCCATCTGGACTGGGGCACGCGGC	0	15	RDR2-sensitive
CCCATCTGGGCTGGGACACGCGGC	7	81	RDR2-sensitive
CCCATCTGGGCTGGGGCACGCAGC	5	26	RDR2-sensitive
CCCATCTGGGCTGGGGCACGCGGC	29	67	RDR2-sensitive
CCCATGAGATTCCCGGCTGGACGG	0	16	RDR2-sensitive
CCCATGGCAGTGTAGGACGGAGTT	0	17	RDR2-sensitive
CCCATGTTTGGATGCTTGGATGGT	0	16	RDR2-sensitive
CCCATTCATGCTGTTAGATCGCGG	0	15	RDR2-sensitive
CCCATTTGCCACGGGGTTGATCGG	0	13	RDR2-sensitive
CCCATTTTGCGCGGTCTGAGGCAC	1	58	RDR2-sensitive
CCCCAACGGTCGGCTGACGTGGCA	0	16	RDR2-sensitive
CCCCAACGGTCTGCTGACGTGGCT	0	20	RDR2-sensitive
CCCCAGTCTAAGGCAGAGGTTCGA	0	16	RDR2-sensitive
CCCCATCACGGTCTGTACAGATGG	1	28	RDR2-sensitive
CCCCCAATGGACTCTAAGATGGTA	0	10	RDR2-sensitive
CCCCCAGATCTAGCGTGTCACTGT	3	37	RDR2-sensitive
CCCCCATCACTAACGGGTCGGCGT	0	9	RDR2-sensitive
CCCCCGACGGATAAACGAGGACGG	0	9	RDR2-sensitive
CCCCCGGGCATTGTATTAAGAAGA	0	10	RDR2-sensitive
CCCCCGTCAGCAGTAGAGACGCAA	0	14	RDR2-sensitive
CCCCCGTCAGCAGTAGAGGCGCAA	1	33	RDR2-sensitive
CCCCCGTGCAAGGTCTCGAACGGC	3	22	RDR2-sensitive
CCCCCTGACACAGCACCGTTGGAT	2	20	RDR2-sensitive
CCCCCTTGGTACTGTACGCATGGC	0	13	RDR2-sensitive
CCCCGAATCCCGGACGTTAGGCAA	0	13	RDR2-sensitive
CCCCGATGGCAAACGTAGATGGCA	25	3	RDR2-resistant
CCCCGCCCTGTTAACTTCCGACGG	0	14	RDR2-sensitive
CCCCGGAAGACGGCGAACTCCGCT	10	27	RDR2-sensitive
CCCCGGATCCACTAGAAGACGAAA	0	11	RDR2-sensitive
CCCCGGATCGTAGGCGCAGAGGCT	0	10	RDR2-sensitive
CCCCGGCTTGTAGTTGTGCTTAAA	0	9	RDR2-sensitive
CCCCGGGCTGCATGACGTAGGTGA	0	10	RDR2-sensitive

CCCCGGGTAGAATGGCGTACACAA	0	14	RDR2-sensitive
CCCCGGTCCGATGGCAGAACAGGC	0	9	RDR2-sensitive
CCCCGTCAGCAGTAGAGGCGCAAG	0	9	RDR2-sensitive
CCCCGTGGAGCAGGACATTGGCGT	0	16	RDR2-sensitive
CCCCTACAGTCGACGGACTTGGCA	0	14	RDR2-sensitive
CCCCTAGATCTAGCGTGTCACTGT	0	11	RDR2-sensitive
CCCCTAGCACTGGCGGCACTGAAA	2	19	RDR2-sensitive
CCCCTAGCCACTGAACGTTAGCAT	0	11	RDR2-sensitive
CCCCTCGAGGCTGTGGGAGCGCAT	0	17	RDR2-sensitive
CCCCTCGTGGAATGTAGCGTTGGC	0	12	RDR2-sensitive
CCCCTGAGCAGCAGTCGGTTGGTG	0	10	RDR2-sensitive
CCCCTGATCAGTAGGAGGGCTCGG	2	21	RDR2-sensitive
CCCCTGGAGCTGCTGCATATGGAC	20	2	RDR2-resistant
CCCCTGGATGAGAAATGAAGGCAC	0	21	RDR2-sensitive
CCCCTGTAGATCAACGGCTGAAAA	0	31	RDR2-sensitive
CCCCTGTAGATGGTATGGCTTGAG	1	22	RDR2-sensitive
CCCCTGTGCTTTGACGTGGGACGG	0	9	RDR2-sensitive
CCCCTTACGGAAGCAAGAGGACGT	1	20	RDR2-sensitive
CCCCTTCTTGTCGTCGCCTCGGTC	40	5	RDR2-resistant
CCCGAACAGCTTGAGAACGCCATC	0	35	RDR2-sensitive
CCCGAACCAACAGTAGAACGGTAT	0	29	RDR2-sensitive
CCCGAACCAACAGTAGAGCAGTAT	0	10	RDR2-sensitive
CCCGAACTCGCGAGCTGAGACTGT	0	30	RDR2-sensitive
CCCGAACTGTTGTGTTGGCTTCAT	1	21	RDR2-sensitive
CCCGAAGCTGACTGTGGATGCGGA	0	9	RDR2-sensitive
CCCGAATTGTAGTCTGGAGAGGCG	56	3	RDR2-resistant
CCCGACACGGCACGGTACTGGCAC	0	16	RDR2-sensitive
CCCGACACGGTAGAATAAGCGGGC	1	29	RDR2-sensitive
CCCGACCAAGACTCTCCGAAGACA	0	11	RDR2-sensitive
CCCGACCCAGGGCTCGGACTCGGC	0	9	RDR2-sensitive
CCCGACGACGACACTACGGACGGT	0	28	RDR2-sensitive
CCCGACGACTGTACAGATTACGCC	0	14	RDR2-sensitive
CCCGACGACTGTACAGATTACGTC	0	13	RDR2-sensitive
CCCGACGACTGTACAGATTGCGCC	0	17	RDR2-sensitive
CCCGACGACTGTATAGATTGCGTC	1	24	RDR2-sensitive
CCCGACGGACTGCGCGGGTCACGG	0	15	RDR2-sensitive
CCCGACTCGTATATTTGACCGCAT	0	11	RDR2-sensitive
CCCGACTCGTTGACGGCGCCTCGT	1	12	RDR2-sensitive
CCCGACTGAGACACGAGAGGCGAT	1	21	RDR2-sensitive
CCCGACTGTAGGCACCTTCGGCAC	0	9	RDR2-sensitive
CCCGACTTAGGAACCGCCAGTGGA	0	18	RDR2-sensitive
CCCGACTTGTAGGCGAGAAGTGGT	0	9	RDR2-sensitive
CCCGACTTTGACTCGACCTGACGG	0	33	RDR2-sensitive
CCCGAGATACGTAGGAATTGGCCG	0	17	RDR2-sensitive
CCCGAGGACGATGGGCAGATACGC	0	9	RDR2-sensitive
CCCGAGGCTTTAGAATAGGGCTGC	0	12	RDR2-sensitive
CCCGAGGTATCGAAGAGTCGGCAC	0	18	RDR2-sensitive
CCCGATATATCGGATTGTGCCGGT	0	10	RDR2-sensitive
CCCGATCCAGAGGACGAGCGGCGT	0	14	RDR2-sensitive

CCCGATGAGGCGAAGACTGGACGA	0	11	RDR2-sensitive
CCCGATGGCAAACGTAGATGGCAC	29	1	RDR2-resistant
CCCGATGTGGCTAGAAGAATGCAA	0	36	RDR2-sensitive
CCCGCACTGTTAGGAGCCGGCGTA	0	85	RDR2-sensitive
CCCGCCTCGTTGGATTGCTCGGCA	0	11	RDR2-sensitive
CCCGCGAGCGGGCTGCGAGGGCAT	55	0	RDR2-resistant
CCCGCGGACAGTACGAAGCGCGGA	0	9	RDR2-sensitive
CCCGCGGGTGCTGTTGGGAGACGC	0	11	RDR2-sensitive
CCCGCGTAGAACCAGACCGTGCTG	0	16	RDR2-sensitive
CCCGCTTATTCGTGTAGGGCCGGC	0	15	RDR2-sensitive
CCCGGAAGACGGCGAACTCCGCTA	0	10	RDR2-sensitive
CCCGGAAGACGGCGAACTCCGCTC	81	290	RDR2-sensitive
CCCGGAATACAGAGAAGGCTCCAA	0	12	RDR2-sensitive
CCCGGACACGGCACTAAAAAGCAC	0	21	RDR2-sensitive
CCCGGACACGGTCGGACACGGTAC	0	20	RDR2-sensitive
CCCGGACCGCTGGATACGCAGGAT	0	13	RDR2-sensitive
CCCGGACGCCGTGGCGTTGACGTG	1	37	RDR2-sensitive
CCCGGACGGGTGGCTACATGACCT	0	9	RDR2-sensitive
CCCGGACTGGACCAGGCAACCAAA	0	30	RDR2-sensitive
CCCGGAGAGGATTGGAGGGGCTAA	4	17	RDR2-sensitive
CCCGGAGGCAGAACCAGGGGAGGC	0	11	RDR2-sensitive
CCCGGATCCACTAGAAGACGAAAA	0	10	RDR2-sensitive
CCCGGATGCAGGACACACGGTCAC	0	26	RDR2-sensitive
CCCGGATTTAAGGGATAAAGTCGG	0	12	RDR2-sensitive
CCCGGCAAATGTAGCGGCTGAGGA	1	14	RDR2-sensitive
CCCGGCACAGTAGAATAAGCGGAC	2	190	RDR2-sensitive
CCCGGCACAGTAGAATAAGCGGGC	0	16	RDR2-sensitive
CCCGGCACGACTAGCAGGCTGACG	0	34	RDR2-sensitive
CCCGGCACGATAGAATAAGCAGGC	0	41	RDR2-sensitive
CCCGGCACGATAGAATAAGCGGGC	0	19	RDR2-sensitive
CCCGGCACGGCACGGCACTGACAC	0	18	RDR2-sensitive
CCCGGCACGGCAGAATAAGCGGGC	0	13	RDR2-sensitive
CCCGGCACGGGAGAATAAGCGGGC	2	15	RDR2-sensitive
CCCGGCACGGGTACGACTGGGCAC	0	9	RDR2-sensitive
CCCGGCACGGTACGAATAAGCGGG	2	20	RDR2-sensitive
CCCGGCACGGTAGAATAAACGGGC	6	346	RDR2-sensitive
CCCGGCACGGTAGAATAAGCGGAC	10	402	RDR2-sensitive
CCCGGCACGGTAGAATAAGCGGCC	0	13	RDR2-sensitive
CCCGGCACGGTAGAATAAGCGGGA	0	20	RDR2-sensitive
CCCGGCACGGTAGAATAAGCGGGC	85	4716	RDR2-sensitive
CCCGGCACGGTAGAATAAGCGGGG	2	27	RDR2-sensitive
CCCGGCACGGTAGAATAAGCGGGT	2	52	RDR2-sensitive
CCCGGCACGGTAGAATAAGTGGGC	1	19	RDR2-sensitive
CCCGGCACGGTTATTCTTACACGG	0	9	RDR2-sensitive
CCCGGCACTGTTTTTAGGACGGGC	0	32	RDR2-sensitive
CCCGGCATGGTAGAATAAGCGGGC	2	135	RDR2-sensitive
CCCGGCCAGGTTACGTAGACGAGC	0	14	RDR2-sensitive
CCCGGCCCTAAACCACGTCGTCGT	0	9	RDR2-sensitive
CCCGGCGCGGTAGAATAAGCGGGC	1	25	RDR2-sensitive

CCCGGCTATGGAGAATGCGACGGA	0	9	RDR2-sensitive
CCCGGCTGTATGAGTCAGGTCGGC	0	16	RDR2-sensitive
CCCGGCTGTGTCTGTGGTCGTCAA	1	13	RDR2-sensitive
CCCGGCTTTAGTCCCACATCGGCT	2	28	RDR2-sensitive
CCCGGGAGCAGGATGAACGAGCGC	55	10	RDR2-resistant
CCCGGGCACGGCACTAAAAAGCAC	0	20	RDR2-sensitive
CCCGGGCGCGAACATAATCACCAG	0	9	RDR2-sensitive
CCCGGGCTGTGAGAGAAGAGGAAA	0	23	RDR2-sensitive
CCCGGGCTGTGAGAGGAGGGGAAA	2	38	RDR2-sensitive
CCCGGGGAAATACCCGCTCGTCGT	0	11	RDR2-sensitive
CCCGGTACGGTAGAATAAGCGGGC	0	19	RDR2-sensitive
CCCGGTACGGTAGAATAAGTGGGC	0	14	RDR2-sensitive
CCCGGTCCAATGGACTTAGACGGT	0	9	RDR2-sensitive
CCCGGTCGGCAACACGGACGACGG	0	13	RDR2-sensitive
CCCGGTGACCCCAGGTCGTCCGAC	16	0	RDR2-resistant
CCCGGTGCAGGATCTAGAAGACGA	16	200	RDR2-sensitive
CCCGGTGTAGGATCTAGAAGACGA	0	18	RDR2-sensitive
CCCGGTTAGGCACGGCACGAACAG	1	20	RDR2-sensitive
CCCGGTTTTGGACGGTTTTACACG	15	0	RDR2-resistant
CCCGTACAAGGTCTCGAACGGCAA	0	10	RDR2-sensitive
CCCGTACCCATGAACATAGACCGG	0	10	RDR2-sensitive
CCCGTAGAACTCCAGTGGGACTGC	0	12	RDR2-sensitive
CCCGTAGACCAGGAAGAACGGCGT	1	24	RDR2-sensitive
CCCGTAGACTAGGAAGAACGGCGT	1	39	RDR2-sensitive
CCCGTAGATCTCGAGCAAGGCAGA	0	46	RDR2-sensitive
CCCGTATGGACTGTATCCCGGTAT	0	13	RDR2-sensitive
CCCGTCAGCAGTAGAGGCGCAAGA	3	59	RDR2-sensitive
CCCGTCAGCAGTAGAGGCGCGAGA	1	32	RDR2-sensitive
CCCGTCTGACCGAGGCTCGTTGGG	0	135	RDR2-sensitive
CCCGTCTTTCTGTAGGGTGGCCAA	1	43	RDR2-sensitive
CCCGTCTTTCTGTAGGGTGGCCAT	1	70	RDR2-sensitive
CCCGTGACTGGGACAGGCCATGGT	0	18	RDR2-sensitive
CCCGTGCAAGGTCTCGAACGGCAA	2	24	RDR2-sensitive
CCCGTGCAAGGTCTCGAACGGCAC	0	10	RDR2-sensitive
CCCGTGCCTAGCGTCGGATCGGTT	0	9	RDR2-sensitive
CCCGTGGCTATAAGGTCGGATGGT	0	14	RDR2-sensitive
CCCGTGGGCTTGACTTGAGGCATA	0	14	RDR2-sensitive
CCCGTGGGTATAGTCATGGACGGA	0	9	RDR2-sensitive
CCCGTGGGTCTGTTATTGGGCAAA	8	38	RDR2-sensitive
CCCGTGTCGGCACTGTAGGTCGGC	1	28	RDR2-sensitive
CCCGTGTGACTAGACGATCGGCCT	0	11	RDR2-sensitive
CCCGTTCTGTAGCGGGTGAGACGT	0	55	RDR2-sensitive
CCCGTTCTGTATGTCCTGATAGGC	0	12	RDR2-sensitive
CCCGTTGAGCAACTCTGTGAGGCA	0	9	RDR2-sensitive
CCCGTTGCACGTCTGTAGATGGCC	0	10	RDR2-sensitive
CCCGTTGGGCTAGCACGACACGGC	0	10	RDR2-sensitive
CCCGTTGGGCTAGCCCGAGCACGG	0	19	RDR2-sensitive
CCCGTTGGGCTGACCCAAGCACGG	0	11	RDR2-sensitive
CCCGTTGTATTGGTCGGATCGGAT	1	35	RDR2-sensitive

CCCGTTTAACTGTGGGCGTGCGGT	0	27	RDR2-sensitive
CCCGTTTGACCCGTGATCCGGCAC	1	54	RDR2-sensitive
CCCGTTTGGCACGAAGCACGATGG	0	34	RDR2-sensitive
CCCGTTTGGCCTGTGGCACGGCAC	0	26	RDR2-sensitive
CCCTAACTGTACAGGTGTGAGCGT	0	31	RDR2-sensitive
CCCTAATCTGAACGTGATGGCTTT	0	9	RDR2-sensitive
CCCTAATGAGTCGGGCGTCCGGAC	0	15	RDR2-sensitive
CCCTACAGACTAGACAGAGGACGA	0	12	RDR2-sensitive
CCCTACCGTCAGCAGTAGAGGCGC	0	11	RDR2-sensitive
CCCTAGCACTGGCGGAACTGAGAA	0	28	RDR2-sensitive
CCCTAGCACTGGCGGCACTGAAAA	4	32	RDR2-sensitive
CCCTAGTACTGGCGGCACTGAAAA	0	9	RDR2-sensitive
CCCTCAAAGGCGTAGGAAGAGCAT	0	10	RDR2-sensitive
CCCTCACGCGGCAGATGTAAACAG	0	12	RDR2-sensitive
CCCTCAGATCTAGCGTGTCACTGT	0	9	RDR2-sensitive
CCCTCCACGCGGCGGACTCGGTGT	0	12	RDR2-sensitive
CCCTCCCGTCAACAGTAGAGGCGC	1	25	RDR2-sensitive
CCCTCCCGTCAGCAGTAGAGACGC	6	162	RDR2-sensitive
CCCTCCCGTCAGCAGTAGAGGCAC	4	36	RDR2-sensitive
CCCTCCCGTCAGCAGTAGAGGCGA	2	16	RDR2-sensitive
CCCTCCCGTCAGCAGTAGAGGCGC	84	1166	RDR2-sensitive
CCCTCCCGTCAGCAGTAGAGGCGT	7	196	RDR2-sensitive
CCCTCCCGTCAGCAGTAGATGCGC	0	12	RDR2-sensitive
CCCTCCCGTCAGGAGTAGAGGCGT	0	14	RDR2-sensitive
CCCTCCCGTCAGTAGTAGAGGCGC	1	17	RDR2-sensitive
CCCTCCCGTTAGCAGTAGAGACGC	0	19	RDR2-sensitive
CCCTCGAACGGTCGCCGGGAAGAA	8	26	RDR2-sensitive
CCCTCGCTCGGCGTCTGAGGCGAC	1	14	RDR2-sensitive
CCCTCGGGTTGTGTGCGCGGCTAT	0	11	RDR2-sensitive
CCCTCGGGTTTCGTGCATGCTCAC	0	19	RDR2-sensitive
CCCTCGGTTTCTGTGCGCGGCTGT	0	17	RDR2-sensitive
CCCTCGTACATCGTCAGTCATCAG	0	9	RDR2-sensitive
CCCTCGTGCAAGGTCTCGAACGGC	3	25	RDR2-sensitive
CCCTCTAGCCTGGATTGTACGGAC	0	13	RDR2-sensitive
CCCTCTCGTCAGCAGTAGAGGCGC	2	29	RDR2-sensitive
CCCTCTCGTCAGCGGATTAGGCGT	0	10	RDR2-sensitive
CCCTCTCTGCGACGGTGTGGACGG	0	10	RDR2-sensitive
CCCTCTGAGGCTCTGACCGAGGAC	2	43	RDR2-sensitive
CCCTCTGCGACGGTGTGGACGGTC	0	11	RDR2-sensitive
CCCTCTGGTCAGGCGCGGACGGTC	0	13	RDR2-sensitive
CCCTCTGTAAGCTGAATGCGTCCA	0	9	RDR2-sensitive
CCCTCTGTTTGGAGACTGGCTGAT	0	12	RDR2-sensitive
CCCTGAACTCGGCTCGAACGGCGA	15	0	RDR2-resistant
CCCTGACATCGTCGGCGATGCGGT	0	15	RDR2-sensitive
CCCTGACCCGAGTAGAGAGGAGGA	18	0	RDR2-resistant
CCCTGACGATACTCTTGTTGAAGA	16	0	RDR2-resistant
CCCTGACGGCACTCTAGTTGAAGA	66	18	RDR2-resistant
CCCTGACTGTTTTGTCTCCGTCGG	0	14	RDR2-sensitive
CCCTGAGATCGGACGGTCCGCGGT	0	12	RDR2-sensitive

CCCTGAGCGGCGGGCTGTTCGCGG	0	9	RDR2-sensitive
CCCTGAGCTGAGCATGAGGATTGT	0	10	RDR2-sensitive
CCCTGAGGTCGGATGGTCCACGGT	1	23	RDR2-sensitive
CCCTGAGTCCGGACGGTCCGCGGT	0	9	RDR2-sensitive
CCCTGATCAGTAGGAGGGCTCGGG	0	10	RDR2-sensitive
CCCTGATTCTAGGAACGGAGCCGC	0	9	RDR2-sensitive
CCCTGATTGTAGAAACGGAGCCGC	0	14	RDR2-sensitive
CCCTGCGATACGGTCGGTCGTCGT	0	12	RDR2-sensitive
CCCTGCGATTCGGTCGGTTGGCGT	0	11	RDR2-sensitive
CCCTGCGATTTGGTCGGTCGGCGT	0	29	RDR2-sensitive
CCCTGCGATTTGGTCGGTTGGCGT	5	64	RDR2-sensitive
CCCTGCGGTCTGGAGCGGTGACAG	0	11	RDR2-sensitive
CCCTGCGTAGGATGAGACGAGCCT	0	10	RDR2-sensitive
CCCTGGACCGACGAGTGAGATTGT	0	11	RDR2-sensitive
CCCTGGACTTGTGGAGTACGCCGG	1	12	RDR2-sensitive
CCCTGGAGGATGAGGTCGAGGACT	19	0	RDR2-resistant
CCCTGGATGTAGTTGGTAGACGGT	1	34	RDR2-sensitive
CCCTGGTCTGGCGCACCGGACTGT	4	18	RDR2-sensitive
CCCTGTACCTAGCTGCTGCCCAGC	26	2	RDR2-resistant
CCCTGTACGTGTGGATACCTCGGA	12	58	RDR2-sensitive
CCCTGTAGAAGCACAGCTGGCGGT	4	27	RDR2-sensitive
CCCTGTAGAAGCACAGCTGTCGGT	0	18	RDR2-sensitive
CCCTGTAGAAGCATAGCTGGCGGT	1	14	RDR2-sensitive
CCCTGTAGAAGCGCAGCTGGCGGT	0	9	RDR2-sensitive
CCCTGTAGAAGCGTAGCTGGCGGT	2	41	RDR2-sensitive
CCCTGTAGAGTGAGGATTTGGGTG	0	10	RDR2-sensitive
CCCTGTAGATCAACGGCTGAAAAT	0	18	RDR2-sensitive
CCCTGTAGATCAACGGCTGAAGAT	0	9	RDR2-sensitive
CCCTGTATACTGGACGGTCCGCAG	1	17	RDR2-sensitive
CCCTGTCGGCTACTTTGATGGGCT	0	10	RDR2-sensitive
CCCTTACACTGGATTGTTCGGATA	0	10	RDR2-sensitive
CCCTTATTGGTTGGGCTAAGGTAT	0	24	RDR2-sensitive
CCCTTCCCTCCCGTCAGCAGTAGA	1	16	RDR2-sensitive
CCCTTCCGTCAACAGTAGAGGCGC	0	13	RDR2-sensitive
CCCTTCCGTCAGCAGTAGAGGCGC	10	178	RDR2-sensitive
CCCTTCCGTCAGCAGTAGAGGCGT	2	26	RDR2-sensitive
CCCTTCCGTCAGGAGTAGAGGCGC	0	10	RDR2-sensitive
CCCTTCGTTCTTAGGCTCAGCGGC	155	14	RDR2-resistant
CCCTTCTACTGTAGCTATGTGCAT	0	20	RDR2-sensitive
CCCTTGCATGTCGGCTGTAGGGGC	0	12	RDR2-sensitive
CCGAAACCGTAGAGAGGGCCACCG	19	1	RDR2-resistant
CCGAAATCGGACGGGAACCGGTAT	0	10	RDR2-sensitive
CCGAACAGCTCGGACGGAGTCCAA	0	9	RDR2-sensitive
CCGAACCGAACCCGAATAGACCGA	1	30	RDR2-sensitive
CCGAACCGAACGCAGCTGTAGCAT	0	19	RDR2-sensitive
CCGAACCGCCTGTGGAGACGACAC	0	12	RDR2-sensitive
CCGAACCGGAAGCACTGAGACGAA	0	11	RDR2-sensitive
CCGAACCGTATTGGACCTTGGCAT	0	86	RDR2-sensitive
CCGAACGGGCGTAGGAACAGCGGA	0	12	RDR2-sensitive

CCGAACGGTCTGCGGTAGTAACGC	0	9	RDR2-sensitive
CCGAACTCGGCACGGTCTGGTTAT	0	9	RDR2-sensitive
CCGAAGACTGGAAGTAGGGACGTT	0	10	RDR2-sensitive
CCGAAGACTGGAGCGAGCAGGGCA	0	16	RDR2-sensitive
CCGAAGATGTACGATGATGAGGAG	22	1	RDR2-resistant
CCGAAGCGGACTGACGGTGTAGAT	0	9	RDR2-sensitive
CCGAAGCTGTAGTATCCGGGGCAT	3	75	RDR2-sensitive
CCGAAGGACCTGCCGGCTTGACGG	0	38	RDR2-sensitive
CCGAAGGACCTGTGAGACGACCAG	0	11	RDR2-sensitive
CCGAAGTCTGTAGGCAGCGGCGAC	0	28	RDR2-sensitive
CCGAATAGCGGACGCTCGGAAGTC	0	12	RDR2-sensitive
CCGAATCCCTGGGCACGCGTCGGC	0	16	RDR2-sensitive
CCGAATGGAGCTGGCAGACGACGT	0	16	RDR2-sensitive
CCGAATGGGCTGTCTGATAGGCTT	1	50	RDR2-sensitive
CCGACAAAAGGCACTCGGCAAAGA	0	37	RDR2-sensitive
CCGACAAGCAGCTCTGCATCTCGT	0	21	RDR2-sensitive
CCGACACAACTAGCAGGCTGACAG	0	11	RDR2-sensitive
CCGACACGACTAGCAGGCTGATGG	0	18	RDR2-sensitive
CCGACACGGTCTGGTTTTATGCGG	0	9	RDR2-sensitive
CCGACACGGTTAATTGGTAGGCAC	0	11	RDR2-sensitive
CCGACACGTAGAGGGCGCTGTAGG	0	14	RDR2-sensitive
CCGACACGTAGGAGGCGCTGTAGG	0	28	RDR2-sensitive
CCGACAGCCTGGATGGACGCGCGT	0	14	RDR2-sensitive
CCGACATCGGGCGACGGATCCGGC	0	9	RDR2-sensitive
CCGACCAAGACTCTCCGAAGACAA	0	9	RDR2-sensitive
CCGACCAGGTGCTCTGACCGGCGC	0	38	RDR2-sensitive
CCGACCATGGACGAGCAGAACGCC	0	9	RDR2-sensitive
CCGACCCAGGGCTCGGACTCGGCC	0	12	RDR2-sensitive
CCGACCCAGGGGCTCGGACTCGGC	61	6	RDR2-resistant
CCGACCGGGCTGTCCGCACGGAGC	0	14	RDR2-sensitive
CCGACCGTTGCAGATCTGGCGCAC	1	13	RDR2-sensitive
CCGACCGTTGGCTCACCGGACAGT	2	18	RDR2-sensitive
CCGACCTAGACTCCTCCAATCGGC	0	10	RDR2-sensitive
CCGACGACGTGTGGTAGAAGGGGC	0	9	RDR2-sensitive
CCGACGACTGTACAGATTACGCCA	0	28	RDR2-sensitive
CCGACGACTGTACAGATTGCGCCA	0	18	RDR2-sensitive
CCGACGACTGTACAGATTGCGCTA	0	9	RDR2-sensitive
CCGACGACTGTACATATTACGCCA	0	13	RDR2-sensitive
CCGACGACTGTATAGATTGCGTCA	0	9	RDR2-sensitive
CCGACGACTGTTTAGGAAGCCGTC	0	15	RDR2-sensitive
CCGACGAGACTGGGTGCGGTTCAG	0	13	RDR2-sensitive
CCGACGCCTGGTGGACGGAACGGC	2	15	RDR2-sensitive
CCGACGCGACTGACGGATAAGCCA	0	12	RDR2-sensitive
CCGACGGAAACGAACAAGGCCTAA	0	9	RDR2-sensitive
CCGACGGATGACGGATACAAGCGT	0	23	RDR2-sensitive
CCGACGGCTGCAATTTTAGACGGT	0	9	RDR2-sensitive
CCGACGGCTTAAGACGGGTCCCGT	0	24	RDR2-sensitive
CCGACGGGCACTCGATAAAGATGG	0	10	RDR2-sensitive
CCGACGGGCTAACTTACGACGGTT	3	17	RDR2-sensitive

CCGACGGGCTGGAAATTGAGGCAT	0	9	RDR2-sensitive
CCGACGTGACAGCGCATATGACAA	0	14	RDR2-sensitive
CCGACGTGGAACCGAGCTCGGCGC	0	10	RDR2-sensitive
CCGACTAGTAGTGGTTAACGGCCA	0	9	RDR2-sensitive
CCGACTCAGAACTGGTACGGACAA	14	38	RDR2-sensitive
CCGACTCGGCCTGAAGCACGGTAG	0	19	RDR2-sensitive
CCGACTGAAACACGTGGGTCTCGT	0	21	RDR2-sensitive
CCGACTGCCGACGCTACACACTGA	0	9	RDR2-sensitive
CCGACTGCGGCTGCGGCTTCTACA	0	12	RDR2-sensitive
CCGACTGTTGATCCAAGGGCGCCT	0	16	RDR2-sensitive
CCGACTGTTTGGTTACGCGGCCGA	0	27	RDR2-sensitive
CCGACTTTGACTCGACCTGACGGT	0	13	RDR2-sensitive
CCGAGACGGCGTCAGATTCTCCTT	0	15	RDR2-sensitive
CCGAGACGGTAGGAAAACGGACTG	0	10	RDR2-sensitive
CCGAGACTGCATGCGTGGACGTGA	0	16	RDR2-sensitive
CCGAGAGCGCTGGAGAGAACGGAT	0	10	RDR2-sensitive
CCGAGAGCTTGGCCCTCGCGTCGG	0	10	RDR2-sensitive
CCGAGAGGACTAGGGTTTCAGCGG	0	19	RDR2-sensitive
CCGAGATACGTAGGAATTGGCCGT	0	20	RDR2-sensitive
CCGAGATACGTAGGAATTGGTCGT	1	44	RDR2-sensitive
CCGAGATCGGACGGCTTGGATGGC	25	0	RDR2-resistant
CCGAGATCTCAGGACCGGCCCTAG	0	12	RDR2-sensitive
CCGAGCAACTCTGCGAGACGGACT	0	9	RDR2-sensitive
CCGAGCACGGCACGGTAGAGGCCC	0	9	RDR2-sensitive
CCGAGCCGCTGTAGGGTGTAGCAT	0	9	RDR2-sensitive
CCGAGCCGCTGTAGGTTGTCGCGT	1	14	RDR2-sensitive
CCGAGCGCGTGGAACTGACGGCAC	1	14	RDR2-sensitive
CCGAGCTACCTGCCGCGCTGTCGT	0	10	RDR2-sensitive
CCGAGCTCCTGCACCAGGTCGCGG	0	28	RDR2-sensitive
CCGAGCTCGGTACCACGTCGGCGC	1	12	RDR2-sensitive
CCGAGCTCGGTGCCACGTCGACGC	0	14	RDR2-sensitive
CCGAGCTCGGTGCCACGTCGGCGC	0	9	RDR2-sensitive
CCGAGCTGACCATGAAACGAGCGA	0	11	RDR2-sensitive
CCGAGCTGACCATGAACCAAGCGA	0	9	RDR2-sensitive
CCGAGCTGACCCTGAAACGAGCGA	0	15	RDR2-sensitive
CCGAGCTTGGACTGCGGCGTAGGC	0	13	RDR2-sensitive
CCGAGGAATAACAGCGTAGACGGT	0	48	RDR2-sensitive
CCGAGGACGCGCTGCTGACGGTGT	0	13	RDR2-sensitive
CCGAGGATAGACTCAGGCAAGGCG	0	9	RDR2-sensitive
CCGAGGGACCGTTTGAGAGAGGGA	21	2	RDR2-resistant
CCGAGGGACCTGCCGGCTTGACGG	1	16	RDR2-sensitive
CCGAGGGACTCCACGCAAAGACGT	5	35	RDR2-sensitive
CCGAGGGACTCCACGCGAAGACGT	2	25	RDR2-sensitive
CCGAGGGACTGACGGGTTATTCCT	10	28	RDR2-sensitive
CCGAGGTACGTGCTACGCTGGCAC	4	21	RDR2-sensitive
CCGAGGTACGTGCTACGCTGGCGC	0	9	RDR2-sensitive
CCGAGGTACGTGCTGCGCTGGCAC	0	14	RDR2-sensitive
CCGAGGTACGTGCTGCGTTGGCAC	0	9	RDR2-sensitive
CCGAGTCGCTGTATGGTTGGTGGA	0	9	RDR2-sensitive

CCGAGTGGCTAGGACTGGGCGGAC	0	9	RDR2-sensitive
CCGATACCAGCTGTGACGGAACCT	0	20	RDR2-sensitive
CCGATCAGTGACGTGGACACGGTT	149	0	RDR2-resistant
CCGATCCGGGCGGAAGACATTGTC	29	5	RDR2-resistant
CCGATCCGGTGGTCTCGACAGCGT	0	21	RDR2-sensitive
CCGATCGAAGTTGTGGGCGAGGCT	0	19	RDR2-sensitive
CCGATCGGCAAGCGGGAGGACCAT	1	12	RDR2-sensitive
CCGATCTGAACCCGGTGAGCACAG	0	10	RDR2-sensitive
CCGATGACTGGAAGTAGGGACGTT	0	24	RDR2-sensitive
CCGATGACTGGGCACACGTGCTGA	0	10	RDR2-sensitive
CCGATGACTGGGCACACGTGCTGG	1	16	RDR2-sensitive
CCGATGATTTCGTGGAACGGGCAT	9	44	RDR2-sensitive
CCGATGCCCTACTGTAGACGGTCA	0	20	RDR2-sensitive
CCGATGCTGCGATCCTGAAGACGG	0	12	RDR2-sensitive
CCGATGGACACGGGTACAATTCCT	0	9	RDR2-sensitive
CCGATGGACCGCAGCGGGAGAGGA	23	0	RDR2-resistant
CCGATGGGACGCACGCTGGTACGG	0	12	RDR2-sensitive
CCGATGGGTACTCTGACGGGTCGT	0	24	RDR2-sensitive
CCGATGTGGGACTAAAGCCGGGGC	1	12	RDR2-sensitive
CCGATTATCCGTCGGGCCTAACGG	0	9	RDR2-sensitive
CCGATTATGTGTCGGGCCTGACGG	0	14	RDR2-sensitive
CCGATTCGGCTGTAGCGAAGATGC	0	9	RDR2-sensitive
CCGATTGATGAACCGGAAGCGCGG	0	9	RDR2-sensitive
CCGCAAGAGTGGCATGATCCTGAC	0	13	RDR2-sensitive
CCGCAATCTGGGCGCAAGAACGGC	0	10	RDR2-sensitive
CCGCACAAGCACGGACAGATCCAT	0	12	RDR2-sensitive
CCGCACCTGTGGTATGGACGGTCC	0	19	RDR2-sensitive
CCGCACTCGGCGACTGCACGGCGA	0	12	RDR2-sensitive
CCGCAGATGTAGACATTGTGGCAC	0	11	RDR2-sensitive
CCGCAGCCGGATTGCAGCCGCAGC	0	17	RDR2-sensitive
CCGCAGCGCACGGTGGTCTGGGTG	21	1	RDR2-resistant
CCGCAGCTCTGAGCCAGACGGTCC	0	18	RDR2-sensitive
CCGCAGTATACGGTAGGACGGTTT	0	11	RDR2-sensitive
CCGCATACCGGACAGATGACGCAG	0	11	RDR2-sensitive
CCGCATAGAACCGGACTGGGTCGG	0	9	RDR2-sensitive
CCGCATCATTGATTTTGTCGTCGT	0	9	RDR2-sensitive
CCGCATCTGAGGCTCGGACGGTCT	0	16	RDR2-sensitive
CCGCATGCGTCTGGCCTGTGACGG	0	16	RDR2-sensitive
CCGCATTTTGCACGGTCTGAGGCA	0	19	RDR2-sensitive
CCGCCACTGTGCAGAATTGCGGAC	0	12	RDR2-sensitive
CCGCCCCTGTAGATCAACGGCTGA	2	324	RDR2-sensitive
CCGCCCTGGGGAACGACGACGGTA	0	38	RDR2-sensitive
CCGCCGATGATGTAGGGACGTTGT	0	13	RDR2-sensitive
CCGCCGCGGCTTAGACACGACGTG	0	19	RDR2-sensitive
CCGCCTCGTTGGATTGCTCGGCAG	0	13	RDR2-sensitive
CCGCGACACAGAGCCGGACGGTCC	0	12	RDR2-sensitive
CCGCGACCTGGATGCAAGAATGGC	0	19	RDR2-sensitive
CCGCGACCTGGCGCAGAAGCTGAC	0	18	RDR2-sensitive
CCGCGACCTGGTACAGAGGCTGGC	0	9	RDR2-sensitive

CCGCGACCTGGTGCAGGAGCTCGG	3	92	RDR2-sensitive
CCGCGACTGCTTCATGATCTACGG	5	20	RDR2-sensitive
CCGCGACTGTGCAGAGACCACCGC	0	9	RDR2-sensitive
CCGCGAGACCAGCACGAAGCCCGC	0	9	RDR2-sensitive
CCGCGAGCGGGCTGCGAGGGCATA	24	0	RDR2-resistant
CCGCGCATGTGTAGGGACGACGAA	0	12	RDR2-sensitive
CCGCGCCTGACCAGAGAGCACCGC	1	42	RDR2-sensitive
CCGCGCCTGTAGATCAACGGCTGA	2	86	RDR2-sensitive
CCGCGCCTGTAGGCCGGACGGTCC	1	18	RDR2-sensitive
CCGCGCCTGTGGCTCAGACGGTAC	0	10	RDR2-sensitive
CCGCGCCTGTGGGCTGGACGGTCC	0	9	RDR2-sensitive
CCGCGCGCTGTAGAAGGCACCGTC	0	28	RDR2-sensitive
CCGCGCGGGCGTTTTTATAGGCAT	0	13	RDR2-sensitive
CCGCGCGTGAACAATGATGGCTGA	0	12	RDR2-sensitive
CCGCGCGTGCGTAGAGACGGTGAA	0	27	RDR2-sensitive
CCGCGCTCTGGAACTGACGGTCTG	0	17	RDR2-sensitive
CCGCGGACGGTAAAACACGGACGG	0	13	RDR2-sensitive
CCGCGGACGTTAGAGAATCGCGGC	1	15	RDR2-sensitive
CCGCGGAGACATCGGAACACGGCT	0	9	RDR2-sensitive
CCGCGGCACAGGGTCGGACGGTCC	0	19	RDR2-sensitive
CCGCGGCTCTAGGTCGGACGGTCC	0	14	RDR2-sensitive
CCGCGGCTGGAACCTGGAAGCGGA	0	37	RDR2-sensitive
CCGCGGTATAGGACCGGACGGTCT	0	24	RDR2-sensitive
CCGCGGTATAGGGCTGTACGGGGT	0	13	RDR2-sensitive
CCGCGGTCCGGACTGTCCACGGTG	0	9	RDR2-sensitive
CCGCGGTCTGAGTGCAGGAGCGAC	0	34	RDR2-sensitive
CCGCGTCGAAGAAGAAGTCCCGGA	1	12	RDR2-sensitive
CCGCGTCTGTGGGCCGGACGGTCC	0	9	RDR2-sensitive
CCGCGTGCATCTAGCCCGTGTCGG	0	19	RDR2-sensitive
CCGCGTGCTGGGCTTGGACGCCAT	0	31	RDR2-sensitive
CCGCGTGTAGATCATTGTGGCGTA	0	29	RDR2-sensitive
CCGCGTTGGACAAAGGGCTGCGGC	0	11	RDR2-sensitive
CCGCTAGATCGACGATGGACGGGT	24	0	RDR2-resistant
CCGCTAGTACTGTAGCACCGGTAG	0	19	RDR2-sensitive
CCGCTAGTCCTAGACTGTCTCGGC	1	15	RDR2-sensitive
CCGCTCCGCCTGTAGAGATGGCAA	0	23	RDR2-sensitive
CCGCTCGACTCGTGGAAACCGGCC	1	13	RDR2-sensitive
CCGCTCGGCTGAACGTGATGGCGT	0	28	RDR2-sensitive
CCGCTCTCGCCTGCCTATCGCCGG	0	11	RDR2-sensitive
CCGCTCTGACCGCCCAGATGGCAA	0	9	RDR2-sensitive
CCGCTCTGAGCCAAACCGAGACGC	0	13	RDR2-sensitive
CCGCTCTGGATTAGCATGGATTGG	0	22	RDR2-sensitive
CCGCTCTGGCCTCTGGACGCGGCT	0	18	RDR2-sensitive
CCGCTCTGTGTGCCCGTACGACAT	18	0	RDR2-resistant
CCGCTGAAGACTGAATGTAGGGAC	0	22	RDR2-sensitive
CCGCTGAGAACACGGGACAGACGC	1	28	RDR2-sensitive
CCGCTGAGTCTGTATACACGAGTG	0	11	RDR2-sensitive
CCGCTGCGACTAGTCTGAGGACGC	0	11	RDR2-sensitive
CCGCTGCTCTGCGCGTCTAGTGGT	36	2	RDR2-resistant

CCGCTGCTGGATCTCGACACGCGT	0	15	RDR2-sensitive
CCGCTGTAGACAGAAGATGGGCGA	0	16	RDR2-sensitive
CCGCTTATTCGTGCCGGGCTCGGA	0	11	RDR2-sensitive
CCGCTTATTCGTGTAGGGCCGGCC	0	43	RDR2-sensitive
CCGCTTATTCGTGTCGGGCCGGCC	2	27	RDR2-sensitive
CCGCTTATTCGTGTCGGGCCGGTC	0	20	RDR2-sensitive
CCGCTTTCGGACCGTGCTAGCATG	0	12	RDR2-sensitive
CCGGAACAAGGTCTGGAATGGGCT	0	15	RDR2-sensitive
CCGGAACCTTGCATGCGTATGCAT	0	12	RDR2-sensitive
CCGGAACGATGCAAAGCAGCACAG	0	9	RDR2-sensitive
CCGGAAGACGGCGAACTCCGCTCC	11	474	RDR2-sensitive
CCGGAAGGAGGTAGAGCTGTACGG	0	13	RDR2-sensitive
CCGGAATTGCTCGCTGAAGTTCGG	0	9	RDR2-sensitive
CCGGACACTTGAGGGCATAAACGT	0	17	RDR2-sensitive
CCGGACAGTCCGCGGTGGTGGCGC	2	18	RDR2-sensitive
CCGGACAGTCCGGTGAATTATAGC	2	19	RDR2-sensitive
CCGGACAGTCCGGTGCACCATCGG	0	14	RDR2-sensitive
CCGGACAGTCCGGTGCGCCAGCCA	0	13	RDR2-sensitive
CCGGACAGTGAACAGTAACGGATC	0	13	RDR2-sensitive
CCGGACATGAAACGAGGTACGGTT	0	11	RDR2-sensitive
CCGGACCAGCCTACTCAAGCACGG	0	24	RDR2-sensitive
CCGGACCCGCATAGAACCAGGCCG	0	16	RDR2-sensitive
CCGGACCCGCATAGAACCGGGCTG	0	11	RDR2-sensitive
CCGGACCCTGACAGAGAATTGCAC	32	4	RDR2-resistant
CCGGACCGAGCACGCAGAGACGAT	0	13	RDR2-sensitive
CCGGACGATCTACGATGGCGATGG	1	14	RDR2-sensitive
CCGGACGCAGTCTGCACGCGCGGA	0	12	RDR2-sensitive
CCGGACGGTATGCGAGGTAGGCGC	0	21	RDR2-sensitive
CCGGACGGTCTGCGGTGGTGGCAC	1	27	RDR2-sensitive
CCGGACGTACACAGGAGGAGCCGC	0	11	RDR2-sensitive
CCGGACGTGGGCGGCGGAATTGGT	2	20	RDR2-sensitive
CCGGACGTTGGGCACGATAGACGC	0	9	RDR2-sensitive
CCGGACTCTGTAGGGTGTGCACGG	0	20	RDR2-sensitive
CCGGACTGGACCAGGCAACCAAAC	0	9	RDR2-sensitive
CCGGACTGGGTGTTGGCCATGCAG	0	11	RDR2-sensitive
CCGGACTGTCCGCGGTGGTGGCGC	10	48	RDR2-sensitive
CCGGACTGTCCGGTGAGCCAACGG	3	20	RDR2-sensitive
CCGGACTGTCCGGTGCGCCATCGA	8	67	RDR2-sensitive
CCGGACTGTCCGGTGCGCCATCGC	3	28	RDR2-sensitive
CCGGACTGTCCGGTGGTGCACCAG	1	12	RDR2-sensitive
CCGGACTGTCCGGTGTGCCACCAG	1	15	RDR2-sensitive
CCGGACTGTCCGGTGTGCCACCGA	0	14	RDR2-sensitive
CCGGACTGTCCGGTGTGCCAGCGG	0	13	RDR2-sensitive
CCGGACTTCGGAGTGTGTACGCGG	0	10	RDR2-sensitive
CCGGAGCCTGTAGTTCGGCAAGGA	0	14	RDR2-sensitive
CCGGAGCTCGCACCCTCGTCGTGT	0	9	RDR2-sensitive
CCGGAGGACCTGCCGGCTTGACGG	1	33	RDR2-sensitive
CCGGAGGACCTGTAGAGACGGAGC	0	18	RDR2-sensitive
CCGGAGGATAGCGTGCATGACGTA	0	10	RDR2-sensitive

CCGGAGGCAGAACACGTCAGAGGC	0	11	RDR2-sensitive
CCGGAGGCAGAACCAGGGGAGGCA	0	9	RDR2-sensitive
CCGGAGGCTGATCGATGAAGACGT	0	19	RDR2-sensitive
CCGGAGGTAGGGTCCAGCGGCCGG	49	0	RDR2-resistant
CCGGAGTCATGTGGACGGAGTCGA	0	9	RDR2-sensitive
CCGGAGTGGACTGTGTAAGCGGAT	0	15	RDR2-sensitive
CCGGATAAGCAGAACCAGGCTCAA	5	200	RDR2-sensitive
CCGGATACGGATAGTTAGAAACGG	0	15	RDR2-sensitive
CCGGATACGGGTAGGATACGGGAT	0	11	RDR2-sensitive
CCGGATACGGGTAGGATACGTGAT	0	9	RDR2-sensitive
CCGGATAGATGGGAGGCAGAACGG	0	19	RDR2-sensitive
CCGGATAGGACACCCAGAGACCAT	0	10	RDR2-sensitive
CCGGATATAACTCCCGAGGACGAC	0	30	RDR2-sensitive
CCGGATATCGACGTAGCATGCCAC	0	13	RDR2-sensitive
CCGGATATGGCATGAACCGGCCGA	0	12	RDR2-sensitive
CCGGATATTGAACCTCGGATACGG	0	15	RDR2-sensitive
CCGGATATTGGCTCTCGGATACGG	0	15	RDR2-sensitive
CCGGATCAATTAACGGCAGAACGG	0	10	RDR2-sensitive
CCGGATCGAGCTCAGCCAGATACG	0	26	RDR2-sensitive
CCGGATCGGCCTGAGCTTCGCGGC	0	17	RDR2-sensitive
CCGGATCTGCTTGGAGGCGTCGTT	3	30	RDR2-sensitive
CCGGATGACTCCAGAAGCAGTCCA	0	17	RDR2-sensitive
CCGGATGAGAAATGAAGGCACCAG	0	9	RDR2-sensitive
CCGGATGAGGAGAGGAGACTGCTT	0	9	RDR2-sensitive
CCGGATGCTGTGGCCCACGGACGG	0	11	RDR2-sensitive
CCGGATGGCTATGTGCTTAGTGCG	18	1	RDR2-resistant
CCGGATGGTCCGCGACTTAGGCAC	0	9	RDR2-sensitive
CCGGATGTACGCAGAGGAGCCGCT	0	10	RDR2-sensitive
CCGGATGTTGTGCACAGAGGGCAC	1	25	RDR2-sensitive
CCGGATTGCAGCCGCAGCAAGCCG	0	16	RDR2-sensitive
CCGGATTGCTAACTGTGTCGGCCC	0	20	RDR2-sensitive
CCGGATTGCTTCGGAGACTGGCGT	0	14	RDR2-sensitive
CCGGATTGTAGCCGCAGCAAGCCG	0	16	RDR2-sensitive
CCGGATTGTATCGGTTGGCTGCAA	0	10	RDR2-sensitive
CCGGATTGTCCGCAGTAGTGATGT	0	16	RDR2-sensitive
CCGGCAAATGTAGCGGCTGAGGAA	0	29	RDR2-sensitive
CCGGCAACAGAAGGTGGACAACAA	0	12	RDR2-sensitive
CCGGCACAAACGAACAAGGCCTAA	0	11	RDR2-sensitive
CCGGCACAAATAAGCGGGTTGGAC	0	11	RDR2-sensitive
CCGGCACGAAGCCCGCTTTTTGGG	0	20	RDR2-sensitive
CCGGCACGACTAACAGACTCACGG	0	25	RDR2-sensitive
CCGGCACGACTAACAGGCTCACGG	0	19	RDR2-sensitive
CCGGCACGACTAGCAGGCTCACGG	0	15	RDR2-sensitive
CCGGCACGACTAGCAGGCTGACGG	0	84	RDR2-sensitive
CCGGCACGACTAGCAGGCTGGCAC	0	22	RDR2-sensitive
CCGGCACGAGAGGCACTAGTCCAA	0	10	RDR2-sensitive
CCGGCACGCACAGGGAAAAATCGT	3	49	RDR2-sensitive
CCGGCACGCTGAGGAACGGGAGAA	0	12	RDR2-sensitive
CCGGCACGGATATGACTGGACGGA	0	15	RDR2-sensitive

CCGGCACGGCACGGCACTGACACG	0	10	RDR2-sensitive
CCGGCACGGCACGGTACTCGACGG	0	17	RDR2-sensitive
CCGGCACGGCCTGGTTCTATGCGG	0	16	RDR2-sensitive
CCGGCACGGTACGAACAAGCGGGC	0	25	RDR2-sensitive
CCGGCACGGTACGAATAAGCGGGC	0	14	RDR2-sensitive
CCGGCACGGTAGAATAAACGGGCG	0	11	RDR2-sensitive
CCGGCACGGTAGAATAAGCGGACG	0	10	RDR2-sensitive
CCGGCACGGTAGAATAAGCGGGCA	1	67	RDR2-sensitive
CCGGCACGGTAGAATAAGCGGGCC	0	20	RDR2-sensitive
CCGGCACGGTAGAATAAGCGGGCG	9	287	RDR2-sensitive
CCGGCACGGTAGAATAAGCGGGCT	3	46	RDR2-sensitive
CCGGCACGGTATGAACAAGCGCGC	0	17	RDR2-sensitive
CCGGCACGGTATGAATAAGCGGGC	0	33	RDR2-sensitive
CCGGCACGTGAGCAGAGGATACAC	0	17	RDR2-sensitive
CCGGCACTATTTGGACCAGGCCGA	0	14	RDR2-sensitive
CCGGCACTGTTTTGGACGAGGGCT	0	11	RDR2-sensitive
CCGGCACTGTTTTTAGGACGGGCT	0	30	RDR2-sensitive
CCGGCACTTAGGCCTACGCCGCAG	0	15	RDR2-sensitive
CCGGCAGAAACGAACAAGGCCTAA	0	18	RDR2-sensitive
CCGGCAGTAGGCTCGTGACCCGGC	0	10	RDR2-sensitive
CCGGCAGTGGGCTTGTGATTCGGC	0	17	RDR2-sensitive
CCGGCATAGGCCTGACACACCGGC	0	11	RDR2-sensitive
CCGGCATGACTAGCAGGCTGACGG	0	10	RDR2-sensitive
CCGGCATGGTAGAATAAGCGGGCG	0	10	RDR2-sensitive
CCGGCCATGGTGTAGAAGCGACGG	1	66	RDR2-sensitive
CCGGCCCAGTTACTCGGACGCTGC	0	10	RDR2-sensitive
CCGGCCCTGTGGAAGTGACGCCGA	3	27	RDR2-sensitive
CCGGCCTGACAGAAGGACAGCGCC	0	12	RDR2-sensitive
CCGGCGACTCGACGATGGCTCCGC	0	10	RDR2-sensitive
CCGGCGATAGGCAGGCGAGAGCGG	0	10	RDR2-sensitive
CCGGCGCTCGGACGGAAGGCGCGT	0	12	RDR2-sensitive
CCGGCGGCAGGCTTGAGGCAGGAC	0	9	RDR2-sensitive
CCGGCGTAGGTTCGGCGTAGGCAA	0	10	RDR2-sensitive
CCGGCGTTGGAGCTTGGCCGTCGT	0	11	RDR2-sensitive
CCGGCTAATGTAGATGGGAGGTGA	0	10	RDR2-sensitive
CCGGCTACGCTCGTCGGGCGGCAC	1	20	RDR2-sensitive
CCGGCTAGACTCGACAGAGATTGG	0	18	RDR2-sensitive
CCGGCTATTCCTGATTTTTTTGGC	17	1	RDR2-resistant
CCGGCTCCGTAAAACGCACGGCCT	0	19	RDR2-sensitive
CCGGCTCCGTAAAACGCACGGCGT	3	98	RDR2-sensitive
CCGGCTCGGCTCGGCTCGTTAGTG	1	12	RDR2-sensitive
CCGGCTGAAAGACGTACCCATGGC	2	21	RDR2-sensitive
CCGGCTGCATGCGAAGGATAGCGT	0	16	RDR2-sensitive
CCGGCTGGAATGAATCAAGGGAAG	0	14	RDR2-sensitive
CCGGGAACAGGATCTGGATGTCAC	0	9	RDR2-sensitive
CCGGGAAGAAGAACAGCAGCACGC	0	18	RDR2-sensitive
CCGGGAAGTGGGCGCATGCATGCA	29	0	RDR2-resistant
CCGGGACGAGTAAGGCGGAGCCAA	1	13	RDR2-sensitive
CCGGGACGGATACAGGTACTACCC	0	17	RDR2-sensitive

CCGGGACGTGGACGGCTACAATAC	0	23	RDR2-sensitive
CCGGGACTTCTTCTTCGACGCGGT	0	10	RDR2-sensitive
CCGGGAGCAGGATGAACGAGCGCC	68	6	RDR2-resistant
CCGGGAGCGAGATCCAGGCGCCGC	1	16	RDR2-sensitive
CCGGGAGCGAGATCCAGGCGCCGT	0	15	RDR2-sensitive
CCGGGATACGGATACCTCACTCAT	1	16	RDR2-sensitive
CCGGGCAAAGATAACGGTGGAAAT	0	10	RDR2-sensitive
CCGGGCACTCGAGGGCATATACGT	0	14	RDR2-sensitive
CCGGGCACTCGGCAAACACTACTT	0	10	RDR2-sensitive
CCGGGCAGTCTCGGACGCAGACGT	0	41	RDR2-sensitive
CCGGGCAGTTTGCGTAGAGAAGAC	0	14	RDR2-sensitive
CCGGGCCATGACTGAGGACGTGAC	0	11	RDR2-sensitive
CCGGGCCGAATGCATGACGGGCTT	0	12	RDR2-sensitive
CCGGGCCGTATAATCACGCGGGCT	0	10	RDR2-sensitive
CCGGGCGATAGTGGTGTAGATGGC	1	21	RDR2-sensitive
CCGGGCGGAAGACATTGTCAGGTG	134	27	RDR2-resistant
CCGGGCGTAGGGTTCCTTACGGCT	0	11	RDR2-sensitive
CCGGGCTAAACTTTAGCAGGAGCA	0	18	RDR2-sensitive
CCGGGCTAAACTTTAGCAGGGGCA	0	9	RDR2-sensitive
CCGGGCTAGGATATCACAGTCGGG	0	9	RDR2-sensitive
CCGGGCTATGGACGTGGTCGTGGT	0	9	RDR2-sensitive
CCGGGCTCATCAGCGAGGTAGTAC	0	9	RDR2-sensitive
CCGGGCTCGGACAAGAAACTAGGC	1	19	RDR2-sensitive
CCGGGCTCGGACAGGAAACTAGGC	2	14	RDR2-sensitive
CCGGGCTCGGACAGGAAATTAGGC	2	14	RDR2-sensitive
CCGGGCTCTGATACCAGATGTCAG	1	20	RDR2-sensitive
CCGGGCTCTGTAGGGTGTGCGCGG	0	12	RDR2-sensitive
CCGGGCTGATTTGGTGACAAGGCG	0	9	RDR2-sensitive
CCGGGCTGGAGGCACGACAGGCCT	0	15	RDR2-sensitive
CCGGGCTGTCCGCGTGGAGCAGGC	0	12	RDR2-sensitive
CCGGGCTGTGACTGTCCTAGGCGC	2	14	RDR2-sensitive
CCGGGCTGTGAGAGAAGAGGAAAT	0	19	RDR2-sensitive
CCGGGCTGTGAGAGGAGGGGAAAT	3	34	RDR2-sensitive
CCGGGCTTAGGCTGCATCAGCCAG	0	12	RDR2-sensitive
CCGGGCTTTAAGGGACAAAGTCGG	0	10	RDR2-sensitive
CCGGGGACCTGCCGACTTGACGGA	1	25	RDR2-sensitive
CCGGGTAGTTCAGGACTGCAACGG	0	14	RDR2-sensitive
CCGGGTCGGATACGGGTACTACTC	0	9	RDR2-sensitive
CCGGGTCGGGCTAGCTAACGCGGT	0	13	RDR2-sensitive
CCGGGTGCGGCACATGGCTAAGAC	14	0	RDR2-resistant
CCGGGTGTAGAGATGGATTCGGAT	0	12	RDR2-sensitive
CCGGGTTGGACTGCACGACGGGCT	0	15	RDR2-sensitive
CCGGTACACCGTTGGATATTGGAC	0	19	RDR2-sensitive
CCGGTACGCTACGGACTTAAACGG	0	21	RDR2-sensitive
CCGGTAGACTGGGTGGATGACGCT	0	10	RDR2-sensitive
CCGGTAGATCTGACGGACGAGCTT	0	11	RDR2-sensitive
CCGGTAGTAGGCCCGTGACCCGGC	0	15	RDR2-sensitive
CCGGTATTGCTCGCCGAAGTTCGG	0	9	RDR2-sensitive
CCGGTCAGAGGAACGTGCACTGTC	17	1	RDR2-resistant

CCGGTCAGTGGAGCGGCGAAGTGA	12	85	RDR2-sensitive
CCGGTCATTTTGGTAGAGCAGGAG	0	17	RDR2-sensitive
CCGGTCATTTTTGTGGAGCGGGAG	0	9	RDR2-sensitive
CCGGTCCAGACCTGACACGGCTAA	0	15	RDR2-sensitive
CCGGTCCGAACCCGGCACGGTAGA	0	13	RDR2-sensitive
CCGGTCCGAACCTGGCACGGTAGA	0	17	RDR2-sensitive
CCGGTCCGGTTAAAGAGTAGGCAT	0	46	RDR2-sensitive
CCGGTCCGGTTAGTATAGTGCCAG	0	9	RDR2-sensitive
CCGGTCCTGAGTCTAGACGGTCTG	0	18	RDR2-sensitive
CCGGTCGACCTGGACACGGATGGC	0	22	RDR2-sensitive
CCGGTCGACGTGAATGAGCGGATC	0	15	RDR2-sensitive
CCGGTCGTTTTGGTGGAGCGGAAG	0	9	RDR2-sensitive
CCGGTCTGAATTCGGCATGGCACG	0	9	RDR2-sensitive
CCGGTCTGACAAGAGGACAGCGCC	0	9	RDR2-sensitive
CCGGTCTGGCACGAATAAACGGAC	0	11	RDR2-sensitive
CCGGTCTGTGATGGGAGAATGCAT	0	9	RDR2-sensitive
CCGGTCTGTGGATAGAGGAGACAT	1	25	RDR2-sensitive
CCGGTCTTCGTCTTCCTGGATCGA	0	9	RDR2-sensitive
CCGGTGAATTATAGCGGAGCGCCT	0	11	RDR2-sensitive
CCGGTGACGTAGAGAGCATCAGGC	42	10	RDR2-resistant
CCGGTGACTGTCCGGCCGAACGGG	0	32	RDR2-sensitive
CCGGTGATTGACGATCGGACGGTT	0	13	RDR2-sensitive
CCGGTGCAGGATCTAGAAGACGAA	8	85	RDR2-sensitive
CCGGTGCGACGTAGAGAAGACGCC	0	11	RDR2-sensitive
CCGGTGGACCTGCAGGTGGGGCAT	0	10	RDR2-sensitive
CCGGTGTAGGATCTCGAAGGCGAC	0	10	RDR2-sensitive
CCGGTTCGTTGAGGCTTTGATGGC	0	11	RDR2-sensitive
CCGGTTCTTGTTGAGTGATTGGCG	0	13	RDR2-sensitive
CCGGTTTTAGGCCAAGGTCGCGGT	0	30	RDR2-sensitive
CCGGTTTTCTGTTTTCTGGCGGCC	35	0	RDR2-resistant
CCGTAACCGATCGTGCGGGGCCGT	0	17	RDR2-sensitive
CCGTACCCACCGGGTATCCGTCGG	0	10	RDR2-sensitive
CCGTACCCATGAACATAGACCGGC	0	15	RDR2-sensitive
CCGTACCGGACTGCACACGCGCAT	0	38	RDR2-sensitive
CCGTACGAAGTCGGTGCAGGAAGA	0	11	RDR2-sensitive
CCGTACGACGCTGGAAGTAAGGTT	0	14	RDR2-sensitive
CCGTACTGTTCACGCGGACTTGGC	0	9	RDR2-sensitive
CCGTACTGTTCACGCTGACTTGGC	2	22	RDR2-sensitive
CCGTACTGTTCAGGCTGACTTGGC	0	15	RDR2-sensitive
CCGTAGACCTCACCCTCGCGCGTA	2	17	RDR2-sensitive
CCGTAGACCTGAACTCGACGGGTA	0	18	RDR2-sensitive
CCGTAGACGAGTTGGACGGTTCGT	0	44	RDR2-sensitive
CCGTAGATCTCGAGCAAGGCAGAG	0	24	RDR2-sensitive
CCGTAGCGTGACAGAAGTCGGTAT	0	9	RDR2-sensitive
CCGTAGCGTGACAGTAGTCGGCAT	0	12	RDR2-sensitive
CCGTAGGACGACCCAGTAGAAGGC	0	39	RDR2-sensitive
CCGTAGGATTGCTGCTTGATGGTT	0	36	RDR2-sensitive
CCGTATAGAACCTGGTCGTGCCGG	0	20	RDR2-sensitive
CCGTATGAGTGTAGAGGGTAGGAG	0	9	RDR2-sensitive

CCGTATTACTTCGTGTCGGATCGT	0	16	RDR2-sensitive
CCGTCAACAGTAGAGGCGCAAGAA	0	13	RDR2-sensitive
CCGTCACACTGTAGCTGCCGACGG	0	9	RDR2-sensitive
CCGTCAGCAGTAGAGACGCAAGAA	0	50	RDR2-sensitive
CCGTCAGCAGTAGAGGCACAAGAA	0	23	RDR2-sensitive
CCGTCAGCAGTAGAGGCGCAACAA	0	10	RDR2-sensitive
CCGTCAGCAGTAGAGGCGCAAGAA	5	287	RDR2-sensitive
CCGTCAGCAGTAGAGGCGCGAGAA	2	55	RDR2-sensitive
CCGTCAGCAGTAGAGGCGTAAGAA	0	19	RDR2-sensitive
CCGTCAGCTGTAGACGGCCGCTAA	1	25	RDR2-sensitive
CCGTCATCGCTTGGTCGTCGTCGT	0	10	RDR2-sensitive
CCGTCCACTCTGTAACAACGGTCC	0	15	RDR2-sensitive
CCGTCCATCGTCGATCTAGCGGAC	14	0	RDR2-resistant
CCGTCCGATCTCGGCTGAACGGCC	31	0	RDR2-resistant
CCGTCCGCGCCTGACCAGAGAGCA	1	37	RDR2-sensitive
CCGTCCGCGCCTGTGCAGAGAGCA	0	13	RDR2-sensitive
CCGTCGACGAAGCAGCGAAGTCAA	0	14	RDR2-sensitive
CCGTCGTCGGCGCGGACGGTATCC	16	0	RDR2-resistant
CCGTCTACAGTAGGGCATCGGACC	0	10	RDR2-sensitive
CCGTCTGGAAGGGTGATTGTCGTG	0	22	RDR2-sensitive
CCGTGAACTGTACATCGGCAACAG	0	18	RDR2-sensitive
CCGTGACCTCGGCGCAGGAGCTGC	0	43	RDR2-sensitive
CCGTGACTCTAAGCCAGACGGCCC	0	11	RDR2-sensitive
CCGTGACTGGAACCGAGAGGAGGA	1	20	RDR2-sensitive
CCGTGACTGGGACAGGCCATGGTC	0	23	RDR2-sensitive
CCGTGACTTGGACGTAGGAGTGGC	1	24	RDR2-sensitive
CCGTGAGCAGATAGAACCGTGCAC	0	12	RDR2-sensitive
CCGTGAGCATCAGCTGGTCGTCGC	0	22	RDR2-sensitive
CCGTGAGCCCGGCACGAAGCCCGC	1	14	RDR2-sensitive
CCGTGAGCCTGTTAGTCGTGCCGG	0	12	RDR2-sensitive
CCGTGAGGTTGGTGGAGGCCGGCG	143	19	RDR2-resistant
CCGTGAGTAGGACTAGGCATTCGG	0	12	RDR2-sensitive
CCGTGAGTCTGTTAGTCGTGCCGG	3	50	RDR2-sensitive
CCGTGATCTGGACGCAGGAGCGAC	0	51	RDR2-sensitive
CCGTGCCTAGCGTCGGATCGGTTT	0	10	RDR2-sensitive
CCGTGCCTGTAGCCCGGTCAGCCC	0	10	RDR2-sensitive
CCGTGCGCTGGGGGAAGAGAGCAA	24	1	RDR2-resistant
CCGTGCTATAGAACAGAAGGGCGC	0	9	RDR2-sensitive
CCGTGCTCTGGTTGGCGGCTCGAC	0	10	RDR2-sensitive
CCGTGCTGTCGAGCAGGAGCTGAT	0	28	RDR2-sensitive
CCGTGGAACGCCTAGGACAACGGT	0	25	RDR2-sensitive
CCGTGGAGCTTTGAGCGGACAGCT	0	10	RDR2-sensitive
CCGTGGATTTAGCTCGGGATACGG	0	14	RDR2-sensitive
CCGTGGCACAAGGTCGGACGGTCC	0	23	RDR2-sensitive
CCGTGGCTCTGGACCGGACGGTCT	0	14	RDR2-sensitive
CCGTGGCTCTGGACCGGATGGTTC	0	12	RDR2-sensitive
CCGTGGCTCTGGGCAGGATGGTCC	0	10	RDR2-sensitive
CCGTGGCTTTAGGACAGACGGTTT	0	21	RDR2-sensitive
CCGTGGGTCTGTTATTGGGCAAAA	1	26	RDR2-sensitive

CCGTGGTCCGGACAGTTCGCGGTG	0	11	RDR2-sensitive
CCGTGGTTCTGCCCTCGGCGTCGG	0	17	RDR2-sensitive
CCGTGTAGAGTGGACGGTTGACAT	0	11	RDR2-sensitive
CCGTGTAGTGGGCTGCGATGGCAC	0	11	RDR2-sensitive
CCGTGTCCACGTCACTGATCGGCT	24	0	RDR2-resistant
CCGTGTCGGCACTGTAGGTCGGCT	0	10	RDR2-sensitive
CCGTGTCTAGGACTCGGACGGTCC	0	26	RDR2-sensitive
CCGTGTCTGGACCGAAGGCGTGGC	0	15	RDR2-sensitive
CCGTGTCTGGGCTGAAGGCGTGGC	0	11	RDR2-sensitive
CCGTGTCTTCACGTCGGATAGCGG	0	9	RDR2-sensitive
CCGTGTTTGTACCGGGTCGGGCGG	0	10	RDR2-sensitive
CCGTTAGAATTCGGCGGAGGACGA	0	11	RDR2-sensitive
CCGTTAGCAACTCACTGGAGACAC	2	16	RDR2-sensitive
CCGTTCAAGGGACTGCACGGCGTA	0	10	RDR2-sensitive
CCGTTCGAGAGACTGCACGGCGTA	0	9	RDR2-sensitive
CCGTTCGGCTTGAGGAATGCGGGC	16	0	RDR2-resistant
CCGTTCGTTTAGCTTTGAACCGGA	0	10	RDR2-sensitive
CCGTTCTGGTAGAATAGCCCGGCT	0	10	RDR2-sensitive
CCGTTCTGTAGCGGGTGAGACGTT	0	29	RDR2-sensitive
CCGTTGACTGTTCATATAATTAAC	22	2	RDR2-resistant
CCGTTGAGCAACTCTGTGAGGCAG	0	17	RDR2-sensitive
CCGTTGGAAGTTGGCAGACGACGT	0	9	RDR2-sensitive
CCGTTGGACAGACGTGCACAGCGC	0	9	RDR2-sensitive
CCGTTGGACTCATGGCGCACCGGA	5	25	RDR2-sensitive
CCGTTGGAGGCTGCTGATGTGCAC	0	9	RDR2-sensitive
CCGTTGGATGTGGAAGGTGTGGTC	0	12	RDR2-sensitive
CCGTTGGATGTGGAGGATGTGGTC	0	9	RDR2-sensitive
CCGTTGGATGTGGAGGGTGTGGTC	3	60	RDR2-sensitive
CCGTTGGATTGGTGGTATAGACGA	1	18	RDR2-sensitive
CCGTTGGCAGAACGCGTCAGAGGC	1	22	RDR2-sensitive
CCGTTGGCTCTCGGCAAAGACTGA	0	41	RDR2-sensitive
CCGTTGGGAAGGGAGCTTCGAGGC	66	6	RDR2-resistant
CCGTTGGGCTGGCCCGAGCACGGC	0	16	RDR2-sensitive
CCGTTGTCATCGGGCTTTGTGCGT	0	25	RDR2-sensitive
CCGTTGTTATCGGGCTTTGTGCAT	1	51	RDR2-sensitive
CCGTTTAACTGTGGGCGTGCGGTG	0	42	RDR2-sensitive
CCGTTTACTGTAGTGCACGGCCTG	0	59	RDR2-sensitive
CCGTTTACTGTAGTGCACGGGCGT	0	9	RDR2-sensitive
CCGTTTACTGTAGTGTGAGTGCGT	0	33	RDR2-sensitive
CCGTTTATCTGTCGGGCCTAACGG	0	11	RDR2-sensitive
CCGTTTCGGACCGGCATACCTCAT	0	19	RDR2-sensitive
CCGTTTGTAGGGCATAGTGGTCAC	0	16	RDR2-sensitive
CCGTTTGTGACGACCGTAGATGGC	0	17	RDR2-sensitive
CCGTTTTCGGAATGAGTGACGTGC	0	11	RDR2-sensitive
CCGTTTTCGGAATGGGTGACGTGC	3	78	RDR2-sensitive
CCGTTTTGACTACGGCTCGTTGGT	0	10	RDR2-sensitive
CCGTTTTGTCTCCGTCGGACCGGC	0	21	RDR2-sensitive
CCGTTTTTGCCTCGGGTTGTACGG	0	19	RDR2-sensitive
CCTAAACTGAGCAGGACGGGCTGT	0	15	RDR2-sensitive

CCTAACCGTCCAGGCTTGTGGCAT	0	19	RDR2-sensitive
CCTAACGTAGACTGTGGTGCACGG	1	17	RDR2-sensitive
CCTAAGATCGGACGGTCCGCGGTC	0	13	RDR2-sensitive
CCTAATCTGTAGGAGCCTAGAGGT	0	10	RDR2-sensitive
CCTAATTTTCGGCGGCTGGCGCAT	0	10	RDR2-sensitive
CCTACAGGCGCGGACCGTCCGGAC	0	9	RDR2-sensitive
CCTACAGGCTGTATGGACGGAAAA	0	11	RDR2-sensitive
CCTACGTATAGCTCTGACAGGCGT	0	14	RDR2-sensitive
CCTACGTCGTGAAGATCGGGCATC	1	69	RDR2-sensitive
CCTACTATCGCTGCGACTCGGCCG	0	9	RDR2-sensitive
CCTACTCCTTGGACTACGGCGAAC	0	38	RDR2-sensitive
CCTACTCTCGGACGTCAATGCAGA	44	0	RDR2-resistant
CCTACTGTAGACGGTCAGTGTCGT	0	14	RDR2-sensitive
CCTAGAAACCTGATTCGCCGGCAC	0	9	RDR2-sensitive
CCTAGAATCAGGGATGGAACGGCT	0	13	RDR2-sensitive
CCTAGCACTGGCGGAACTGAGAAA	0	53	RDR2-sensitive
CCTAGCACTGGCGGCACTGAAAAA	3	28	RDR2-sensitive
CCTAGCACTGGCGGCACTGAAAAG	0	20	RDR2-sensitive
CCTAGCTGTAGCAGCCGAAGGCGT	0	36	RDR2-sensitive
CCTAGGACACTCGACACTCGGCAA	0	12	RDR2-sensitive
CCTAGGATCTCTCCTCCCCGACGG	1	16	RDR2-sensitive
CCTAGGCAGCTAGAACGGTAGAAT	0	9	RDR2-sensitive
CCTAGGCTGAGAGAGGAGGAAGGT	0	15	RDR2-sensitive
CCTAGGCTGAGAGAGGAGGGAGGC	1	16	RDR2-sensitive
CCTAGGCTGTACCGGTGCATGCAC	2	26	RDR2-sensitive
CCTAGTACGAGAGGAACCGTTGAT	74	7	RDR2-resistant
CCTAGTCGGTGTAGACGTGCGGTA	0	12	RDR2-sensitive
CCTATCAGACTATCTATCGGACAT	18	1	RDR2-resistant
CCTATCGCGTAGGACAGTTCGGCA	0	9	RDR2-sensitive
CCTATCGCGTAGGACAGTTCGGCT	0	17	RDR2-sensitive
CCTATCGGACGCTGTATCAGGCAT	0	10	RDR2-sensitive
CCTATGGTTGTGGAGGCTACGCAG	0	9	RDR2-sensitive
CCTATTAGGACGCGGGTATGACGG	0	15	RDR2-sensitive
CCTATTCGTACCGGGCCGAAACGG	2	93	RDR2-sensitive
CCTATTCGTACTGGGCTGAAACGG	0	15	RDR2-sensitive
CCTATTTCTGTAGTCTATCGGTTT	58	0	RDR2-resistant
CCTCACGTCGACTGTTCAGAGGGC	2	51	RDR2-sensitive
CCTCAGACTGTTATGCATGTGCAA	0	15	RDR2-sensitive
CCTCAGCCTGGACCAGTTGAACGG	0	37	RDR2-sensitive
CCTCAGCCTTAGCTCGTCGGTCAC	0	9	RDR2-sensitive
CCTCAGCGAACTGTACATTGGCAA	0	11	RDR2-sensitive
CCTCAGGCCATAGGATCGGACACC	0	9	RDR2-sensitive
CCTCAGTGGTTCTTGTTGTTTCGG	281	1	RDR2-resistant
CCTCAGTTGACGCCGTAGTTTCTT	0	12	RDR2-sensitive
CCTCATAGCGACACTGTAGTGCGG	0	11	RDR2-sensitive
CCTCATCTTCGGACCCGACGGCAT	0	12	RDR2-sensitive
CCTCCAAAGACTGGCACATGACGA	0	9	RDR2-sensitive
CCTCCACGCGGCGGACTCGGTGTC	0	9	RDR2-sensitive
CCTCCCGACGACTGTACAGATTGC	0	9	RDR2-sensitive

CCTCCCGTCAACAGTAGAGGCGCA	0	9	RDR2-sensitive
CCTCCCGTCAGCAGTAGAGACGCA	0	52	RDR2-sensitive
CCTCCCGTCAGCAGTAGAGGCGCA	13	163	RDR2-sensitive
CCTCCCGTCAGCAGTAGAGGCGCG	2	21	RDR2-sensitive
CCTCCCGTCAGCAGTAGAGGCGTA	1	21	RDR2-sensitive
CCTCCCTGTATCGAGCCGGACGGT	0	16	RDR2-sensitive
CCTCCGAATTGTGATCGCGCGGAC	0	22	RDR2-sensitive
CCTCCGGAATCGTAGCCACGGCGT	0	11	RDR2-sensitive
CCTCCTCATCTTCGGACCCGACGG	0	24	RDR2-sensitive
CCTCCTCCGTTGGATTGGTGGTAT	0	70	RDR2-sensitive
CCTCCTCTACACGCTTTCGGACGG	0	9	RDR2-sensitive
CCTCCTCTGCACACTTCCGGACGG	0	37	RDR2-sensitive
CCTCCTCTGCACGCTTCCGGACGG	0	18	RDR2-sensitive
CCTCCTCTGCACGCTTTCGGACGG	0	24	RDR2-sensitive
CCTCCTCTGGGTGACTCGGGCGGC	1	14	RDR2-sensitive
CCTCCTTGAGCGACAGATCGTCAT	0	9	RDR2-sensitive
CCTCGAAACCAAGATCTGTGGCAT	0	17	RDR2-sensitive
CCTCGAAACCTGGAGAGCGAGCAA	0	9	RDR2-sensitive
CCTCGAACGAGCCGAGCCGAGCCT	0	10	RDR2-sensitive
CCTCGACGACTGCGGCGACATGGC	0	11	RDR2-sensitive
CCTCGACGGTACAGCTTACGGTGT	0	13	RDR2-sensitive
CCTCGACTCGGAGCTGGATGGTCT	0	10	RDR2-sensitive
CCTCGACTCGTGGATCCGATCGGC	3	29	RDR2-sensitive
CCTCGACTGGATCCCGCGCAACAC	0	9	RDR2-sensitive
CCTCGACTTCAAGAACGCCGGCCT	0	10	RDR2-sensitive
CCTCGAGGACGACGCTGCGGACGG	0	29	RDR2-sensitive
CCTCGAGTTCGACTGCGCAGACGT	0	9	RDR2-sensitive
CCTCGATGGGCTAGCAATGACGCT	0	9	RDR2-sensitive
CCTCGCTTAGAATCAGGAGCTGGA	0	17	RDR2-sensitive
CCTCGGACAAGTCGGCGTTGCTGT	0	17	RDR2-sensitive
CCTCGGACTCAAGTCAGGCATCAC	0	10	RDR2-sensitive
CCTCGGATACGGGTAGGATACGGA	3	61	RDR2-sensitive
CCTCGGATTCCGCAGACGATGCAC	0	11	RDR2-sensitive
CCTCGGCGAACTGTACATCGGCAA	0	14	RDR2-sensitive
CCTCGGCGTCATAGACTGTAACGG	0	9	RDR2-sensitive
CCTCGGCTCCGGCAGCGGCTCTAG	1	20	RDR2-sensitive
CCTCGGGCCGCAACTAGGCTGCAT	0	10	RDR2-sensitive
CCTCGGGCGAGACGGAAACCCCTC	0	14	RDR2-sensitive
CCTCGGGCGAGTCGAAGGTGCGTC	115	10	RDR2-resistant
CCTCGGGCTCTTCAACCGGATCGT	1	12	RDR2-sensitive
CCTCGGTGAACCTAATGTCGGCAA	0	14	RDR2-sensitive
CCTCGGTTAGTCGGGCCTGGCTAG	0	11	RDR2-sensitive
CCTCGGTTCTTGTAGAAATTCGGT	3	18	RDR2-sensitive
CCTCGTCGTCGAGGCTGCGGCTAC	1	14	RDR2-sensitive
CCTCGTCTACTGCGACAACGTCAG	0	12	RDR2-sensitive
CCTCGTTGTAGCCTCCGCGTCGGA	0	10	RDR2-sensitive
CCTCTACACGCTTTCGGACGGTCT	0	10	RDR2-sensitive
CCTCTAGGGCTGTGTGTTTGCGGT	0	12	RDR2-sensitive
CCTCTCGTCGAGACGACAAGTCAT	0	10	RDR2-sensitive

CCTCTCTCGTTTGGACTTCCGGCC	0	13	RDR2-sensitive
CCTCTCTGCGACGGTGTGGACGGG	0	20	RDR2-sensitive
CCTCTGACCACTGGACGTTAGCAC	0	14	RDR2-sensitive
CCTCTGACGTACTACAGTGGACAG	0	14	RDR2-sensitive
CCTCTGACGTGGCAGTAACGACTC	0	13	RDR2-sensitive
CCTCTGAGGCTCTGACCGAGGACC	0	23	RDR2-sensitive
CCTCTGATCAGTAGGGAGGCTCGG	7	35	RDR2-sensitive
CCTCTGATCAGTAGGGGGGCTCGG	3	16	RDR2-sensitive
CCTCTGCACAGGGGCGGACGGTCC	2	27	RDR2-sensitive
CCTCTGCAGAGGCGCGGACGGTCC	0	11	RDR2-sensitive
CCTCTGCATCGAGGAGTGGCTCGG	0	14	RDR2-sensitive
CCTCTGCGACGGTGTGGACGGTCC	0	11	RDR2-sensitive
CCTCTGCGGCGGCGTGGACGGTCC	0	12	RDR2-sensitive
CCTCTGGGTTGTAGGTGACGTCGG	0	9	RDR2-sensitive
CCTCTGGTCAGGACTGCGGACGGT	0	31	RDR2-sensitive
CCTCTGGTCAGGCGCGGACGGTCC	0	33	RDR2-sensitive
CCTCTGTAGAGGCGCGGACGGTCC	2	35	RDR2-sensitive
CCTCTGTAGTTCAATTGGGGACGG	0	16	RDR2-sensitive
CCTCTGTATCTGTGGGAGCAGGCT	0	10	RDR2-sensitive
CCTCTGTCGTGCTTATGGGAAGGG	0	10	RDR2-sensitive
CCTCTGTTGACTGCGGCTTCACAT	0	11	RDR2-sensitive
CCTCTGTTGACTGTTGTTGCGGAC	0	24	RDR2-sensitive
CCTCTTCGGGACCGTAGCGTTGGG	1	19	RDR2-sensitive
CCTCTTGATCTTGGGGACACGCGC	0	10	RDR2-sensitive
CCTCTTTGCTGAGTGCCCGTTGGA	0	10	RDR2-sensitive
CCTCTTTGTTGGACGGAGAGAGGA	0	10	RDR2-sensitive
CCTGAACAGCTTCCTGAGGGTATT	139	0	RDR2-resistant
CCTGAACAGCTTTCTGAGGGTATT	60	0	RDR2-resistant
CCTGAACGAGGCTGCAGAAGTCGT	0	10	RDR2-sensitive
CCTGAAGACGCACGGAACAAGGAC	1	15	RDR2-sensitive
CCTGAAGACGTAGAGGACAGGAAT	0	21	RDR2-sensitive
CCTGACACCTTTATCCTGACGCGC	2	15	RDR2-sensitive
CCTGACACTCGGCAAAGAAGGCTC	0	19	RDR2-sensitive
CCTGACACTCGGCAAAGAGAGCAC	0	10	RDR2-sensitive
CCTGACACTTGGTGGGGACTTGGT	0	13	RDR2-sensitive
CCTGACAGAAGGACAGCGCCGCCT	0	10	RDR2-sensitive
CCTGACATTCGGCAAAGGCTCGGT	0	11	RDR2-sensitive
CCTGACCAGAGAGCACCGCCGCCG	0	9	RDR2-sensitive
CCTGACCTTACAGACTTCCTGCAT	0	12	RDR2-sensitive
CCTGACGAAGCAAGGGCTGTGACC	0	9	RDR2-sensitive
CCTGACGACGGACAGCGTGAGTAC	0	11	RDR2-sensitive
CCTGACGAGTCGACGAGCTGATGA	0	10	RDR2-sensitive
CCTGACGCAGCACCGTTGGATGTG	0	11	RDR2-sensitive
CCTGACGCGTAGGACAGTTCGGCT	0	25	RDR2-sensitive
CCTGACGCTGTTGACCCACGACGG	0	14	RDR2-sensitive
CCTGACGGACGGCATGTGGTTCAA	1	15	RDR2-sensitive
CCTGACGGACTGCGGAGGCGACAT	1	26	RDR2-sensitive
CCTGACGGTGGCGAGGATGACGAA	1	13	RDR2-sensitive
CCTGACGTACTGCGGAGGCGACAT	0	15	RDR2-sensitive

CCTGACGTGGACTGGAGCTACGGT	0	29	RDR2-sensitive
CCTGACTCTGACGCCCGGAAGCGC	0	13	RDR2-sensitive
CCTGACTCTTGGTAGACTGGCTGA	0	79	RDR2-sensitive
CCTGACTGTGTAGTGTGGATGGCC	0	9	RDR2-sensitive
CCTGACTGTTTTGTCTCCGTCGGA	0	31	RDR2-sensitive
CCTGACTTGGGCTGATTTGCGGTT	0	11	RDR2-sensitive
CCTGAGACCTGTAGCAAAGGGGCC	0	10	RDR2-sensitive
CCTGAGAGCCGGCAGCTGAGACGT	0	11	RDR2-sensitive
CCTGAGATGATGCTAGGCTCGGCA	14	0	RDR2-resistant
CCTGAGCTCTGTGGAATGCACGTC	182	67	RDR2-resistant
CCTGAGGAGCTAAGCATTCGACGA	0	15	RDR2-sensitive
CCTGAGGGTGGACTGTTCGCGGTC	0	9	RDR2-sensitive
CCTGAGTGTAGAGGAGGTCCCTGA	0	115	RDR2-sensitive
CCTGATAGCGTCTGCAGTCGGCTT	0	15	RDR2-sensitive
CCTGATCACGTACTGGAGCTAGGC	0	26	RDR2-sensitive
CCTGATCATCGTACATCTTCGGCA	45	2	RDR2-resistant
CCTGATCATGTTGGACGGGCCGAG	0	10	RDR2-sensitive
CCTGATCATGTTGGACGGGCCGGC	0	28	RDR2-sensitive
CCTGATCGACGAGGACGGCTAGGG	0	17	RDR2-sensitive
CCTGATCTCGGCAGCCTGGCGGTC	0	20	RDR2-sensitive
CCTGATCTGCGACTGACTGACGGC	0	9	RDR2-sensitive
CCTGATCTGCTGACGGGGAATCGA	2	41	RDR2-sensitive
CCTGATGCAAGATCTCGAAGGCGA	0	12	RDR2-sensitive
CCTGATGCTGTTCAGAGGTCGGAC	8	32	RDR2-sensitive
CCTGCACGTGGACTCTAAGGGCAT	0	53	RDR2-sensitive
CCTGCAGAACACTACAGAACGGGT	0	12	RDR2-sensitive
CCTGCAGTACGTCAGAGGAACCGG	0	15	RDR2-sensitive
CCTGCATCATCTGACTGATTGGCT	18	0	RDR2-resistant
CCTGCATCGGTTGAAACGGACGAT	0	10	RDR2-sensitive
CCTGCCCAGTCGGCTGCAGGAGAC	0	11	RDR2-sensitive
CCTGCCGGCGTGGTGGAGAGTCGT	0	16	RDR2-sensitive
CCTGCCGGGACTGAGGAGATGCAT	0	9	RDR2-sensitive
CCTGCGACGGCGCACAGACTCCTC	18	0	RDR2-resistant
CCTGCGAGACAGAGCGGACGACAG	0	10	RDR2-sensitive
CCTGCGATCGTGGAGCAAAGGCGT	0	10	RDR2-sensitive
CCTGCGCACGCATGGACTGTTCGG	0	12	RDR2-sensitive
CCTGCGCACGCGCGGACCGTCCGG	1	16	RDR2-sensitive
CCTGCGCGCGATCTGTGACCGGTT	0	9	RDR2-sensitive
CCTGCGCGCGTAGAAGCTGGGCTG	0	27	RDR2-sensitive
CCTGCGCTGAGCCGGACGGTCCAC	1	12	RDR2-sensitive
CCTGCGGAAGTCTGCGTGGACTGC	0	11	RDR2-sensitive
CCTGCGGGACTACGGTTACAAATT	17	0	RDR2-resistant
CCTGCGTCGAGACGGACGGTCCGC	0	21	RDR2-sensitive
CCTGCTAGAACTGCAAGCGAGCAT	0	12	RDR2-sensitive
CCTGCTGACGCGCAGAACGACGAC	0	9	RDR2-sensitive
CCTGCTGTGGACGTGTACACTACG	0	10	RDR2-sensitive
CCTGCTTCAGACGACTCGAACGGC	0	22	RDR2-sensitive
CCTGCTTGGACGGCTACGGACAAC	0	12	RDR2-sensitive
CCTGGACAACGTGAGCGGAGGCTT	0	14	RDR2-sensitive

CCTGGACACCAGGATGACGAGGAG	23	3	RDR2-resistant
CCTGGACAGCACCGGCCGTAGATC	0	11	RDR2-sensitive
CCTGGACAGTCAGCAAGCTGGCAT	0	18	RDR2-sensitive
CCTGGACCAATTGAACGGGCCGAG	0	24	RDR2-sensitive
CCTGGACCAGTTGAACCGGCCGAA	0	9	RDR2-sensitive
CCTGGACCAGTTGAACGGACCGAG	0	32	RDR2-sensitive
CCTGGACCAGTTGAACGGGCCGAA	0	10	RDR2-sensitive
CCTGGACCTGTTGAAGCACGTCAT	0	48	RDR2-sensitive
CCTGGACGCGGGACGAACACGAAG	0	10	RDR2-sensitive
CCTGGACTGAGCCCGGCTAGACTC	0	26	RDR2-sensitive
CCTGGACTGGGGAAGATGGTGCCT	0	20	RDR2-sensitive
CCTGGACTGTGGAAGATGGTGCTT	0	10	RDR2-sensitive
CCTGGACTTCTGCGAGGACCACCA	0	9	RDR2-sensitive
CCTGGACTTGGATACAGATCGGAT	0	9	RDR2-sensitive
CCTGGACTTGTCGAGTACGCCGGA	1	14	RDR2-sensitive
CCTGGACTTTGGGCTAGGATTGGC	0	31	RDR2-sensitive
CCTGGAGGCAGAACGCAAAAAGGC	0	14	RDR2-sensitive
CCTGGAGGCCCACGTGGGATTCGG	0	18	RDR2-sensitive
CCTGGATCATGTGGAAGACGACTG	0	28	RDR2-sensitive
CCTGGATCCACTGTAGTAGTGCAA	0	10	RDR2-sensitive
CCTGGATGTAGTTGGTAGACGGTT	0	40	RDR2-sensitive
CCTGGATTGGTTCGGTAGACACGG	0	13	RDR2-sensitive
CCTGGCACGGTAGAATAAGCGGGC	2	186	RDR2-sensitive
CCTGGCACTATTCGGACTGGGCTT	0	13	RDR2-sensitive
CCTGGCAGAAGGACCGTTGGACAT	0	17	RDR2-sensitive
CCTGGCCAGCAGCCTGATCGTTGG	0	15	RDR2-sensitive
CCTGGCGAAGTACTGAGCTGACGG	0	12	RDR2-sensitive
CCTGGCGGACTGCGGAGGCGACAT	1	15	RDR2-sensitive
CCTGGCGGCTAGGACTGACTTGGC	0	9	RDR2-sensitive
CCTGGCTCAGACTGGACTGGCTGA	0	21	RDR2-sensitive
CCTGGCTCGAGACTGACCCAGCAT	0	17	RDR2-sensitive
CCTGGCTCGCAAGAAAATCGGTGT	0	10	RDR2-sensitive
CCTGGCTCGCTAGAAACGGAAGAA	0	9	RDR2-sensitive
CCTGGCTCTGATACCACTGAAGGA	22	1	RDR2-resistant
CCTGGCTCTGGAGATGCGACCAAT	0	29	RDR2-sensitive
CCTGGCTGAGTAGAAACACGGGGT	0	36	RDR2-sensitive
CCTGGCTGTACGGCAACCAAACAC	0	17	RDR2-sensitive
CCTGGCTGTATCGAATGTGTGCAT	0	10	RDR2-sensitive
CCTGGCTGTTGACTGCATGGCTGC	0	37	RDR2-sensitive
CCTGGCTTCGAGGATGAACGGTAC	6	22	RDR2-sensitive
CCTGGGAACCGGCAAAGACATCTT	0	9	RDR2-sensitive
CCTGGGAACCGGCAAAGATGTCTT	0	14	RDR2-sensitive
CCTGGGACTCGACAAGCTGGCCAG	0	10	RDR2-sensitive
CCTGGGACTCGACAAGCTGGGCAG	0	11	RDR2-sensitive
CCTGGGCAGTGAGAAACGGAATAT	0	10	RDR2-sensitive
CCTGGGCGCAGGACAACACGAAGG	0	14	RDR2-sensitive
CCTGGGCGCAGGACGAACACGAAG	0	10	RDR2-sensitive
CCTGGGCGCGGGACGAACACGAAG	20	103	RDR2-sensitive
CCTGGGCTGCCGGCTACGCTCGTC	0	12	RDR2-sensitive

CCTGGGCTGGCATCTCGACGGGAT	0	14	RDR2-sensitive
CCTGGGCTGTAATGTGCAGACAAC	0	12	RDR2-sensitive
CCTGGGCTGTTGGTTTCGGCGGAG	0	9	RDR2-sensitive
CCTGGGCTTTTCGGGCTGGCACGG	0	15	RDR2-sensitive
CCTGGGGAGTAAGCGTTTGGAGGC	15	0	RDR2-resistant
CCTGGGGTCGGGCGAAGCGGAGTT	40	7	RDR2-resistant
CCTGGGGTGGAACATAGCGAGGAC	0	13	RDR2-sensitive
CCTGGGTCGAGTGTCGAACACGGT	0	9	RDR2-sensitive
CCTGGGTGTAGAAGAGGTCCCCGA	0	14	RDR2-sensitive
CCTGGGTTGTAACAGAGAAGGCAG	0	9	RDR2-sensitive
CCTGGTAGTGCCCTCTGCGACGGT	0	14	RDR2-sensitive
CCTGGTCGGTACGTGGTCGGGGCT	0	19	RDR2-sensitive
CCTGGTCGTCCGACTAATCGTCGA	3	33	RDR2-sensitive
CCTGGTGAGACTGCACGACGGCGC	0	10	RDR2-sensitive
CCTGGTGCAGGAGCTCGGGTTCCC	0	18	RDR2-sensitive
CCTGGTGCGGCTGAACCGGATGGC	0	10	RDR2-sensitive
CCTGGTGGTTGGCACAGTAGTCGG	0	10	RDR2-sensitive
CCTGTACCCGACAGGTCGGATATC	0	9	RDR2-sensitive
CCTGTACGTGTGGATACCTCGGAA	2	16	RDR2-sensitive
CCTGTACGTGTGGATACCTCGGAG	769	95	RDR2-resistant
CCTGTACGTGTGGATACCTCGGCG	32	2	RDR2-resistant
CCTGTAGACTGTCCGGTGCGCCAA	0	16	RDR2-sensitive
CCTGTAGATCTGTTGGCACCGGCC	0	9	RDR2-sensitive
CCTGTAGCGCTGACTCTTGGCATT	207	58	RDR2-resistant
CCTGTAGGCAAAGGACGCCGGTGC	0	11	RDR2-sensitive
CCTGTAGTACCTATGCATGTTGAC	38	0	RDR2-resistant
CCTGTCACGTGACCCGAACCGGCA	0	23	RDR2-sensitive
CCTGTCAGACCAGCGACTGCGGCG	1	14	RDR2-sensitive
CCTGTCGAGCACGGCACTGTACAG	0	20	RDR2-sensitive
CCTGTCGAGCAGGCCACCTGGCAT	0	13	RDR2-sensitive
CCTGTCGAGCTGGAACAAGAGCGG	0	11	RDR2-sensitive
CCTGTCGAGTGGCACTGGTACGGT	0	19	RDR2-sensitive
CCTGTCGCGGGCTGGGACAAGGCA	0	22	RDR2-sensitive
CCTGTCGCGTAGGACAGTTCGGCA	0	34	RDR2-sensitive
CCTGTCGCGTAGGACAGTTCGGCT	2	293	RDR2-sensitive
CCTGTCGCTTCTCTCGTTGTCGGA	29	0	RDR2-resistant
CCTGTCGGGACCCGAAAGATGGTG	26	4	RDR2-resistant
CCTGTCGGTATTCTCTGTGGAGGC	0	10	RDR2-sensitive
CCTGTCGTGTTGGACTTAGGTGGT	0	10	RDR2-sensitive
CCTGTCTGACTGAGGCATCGTCAC	0	9	RDR2-sensitive
CCTGTCTGGTCGGATACAGAGGTT	0	10	RDR2-sensitive
CCTGTCTTCTGGCTGTTAACGGAC	0	11	RDR2-sensitive
CCTGTGGAATGGACTGGTCGTCGC	0	11	RDR2-sensitive
CCTGTGGACCTCTTTTGTCGGCAC	0	21	RDR2-sensitive
CCTGTGGAGCAAGGAAGCGAACGC	0	17	RDR2-sensitive
CCTGTGTGACTCTGTAGTGTGCAT	0	9	RDR2-sensitive
CCTGTGTGTTCTGGAAGTCCGGAT	0	12	RDR2-sensitive
CCTGTGTTCAGCCTGTAGTAGCAT	0	11	RDR2-sensitive
CCTGTGTTGCAGCTGTAGGGGCAT	0	13	RDR2-sensitive

CCTGTTCGGTTGTTAGGGATTGGA	0	19	RDR2-sensitive
CCTGTTCGGTTTGTAGGGATTGGA	0	16	RDR2-sensitive
CCTGTTCTCTGACATCTGCAGAAT	0	9	RDR2-sensitive
CCTGTTGCCCTCGGCCTGGAACGG	0	10	RDR2-sensitive
CCTGTTGCGGGCTGAGCGCGCGGA	0	9	RDR2-sensitive
CCTGTTTGAACTGTGGCACGGCAT	1	34	RDR2-sensitive
CCTGTTTGACTGCGGCGTGTGGAC	0	35	RDR2-sensitive
CCTGTTTGGCACGAAGCACGATGG	0	9	RDR2-sensitive
CCTGTTTGTTTCGGCTTCTGGCAG	0	13	RDR2-sensitive
CCTGTTTTCGACGGTCTCTGACGG	0	10	RDR2-sensitive
CCTTAACACAAGACGCTGGGCACT	0	10	RDR2-sensitive
CCTTAACACTGGACGCTGGGCACT	1	12	RDR2-sensitive
CCTTAACGTTGACACTGCGGACGG	0	17	RDR2-sensitive
CCTTACATCGAGACGTTGGGCACT	0	10	RDR2-sensitive
CCTTACATCGGGACGTTGGGCACT	0	12	RDR2-sensitive
CCTTACCGAAGGGCAGTGGACACT	0	11	RDR2-sensitive
CCTTACGCGTCTAGCTGCATGGCT	0	9	RDR2-sensitive
CCTTACTGGACGCCTAGAGGACGC	0	14	RDR2-sensitive
CCTTACTTCCAGCGTCGTACGGAG	0	19	RDR2-sensitive
CCTTAGTCTGTGGAGCGGTAAGGC	0	9	RDR2-sensitive
CCTTAGTGGGCTGGGCTGGGCTAC	2	41	RDR2-sensitive
CCTTCATACTGTAGAAGAGGGTAC	0	9	RDR2-sensitive
CCTTCATCACCCACTTCGTCGGAT	0	9	RDR2-sensitive
CCTTCCAGACGGCTCGAATTACAT	0	17	RDR2-sensitive
CCTTCCCGTCAGCAGTAGAGACGC	0	20	RDR2-sensitive
CCTTCCCGTCAGCAGTAGAGGCGC	0	16	RDR2-sensitive
CCTTCCCTCCCGTCAGCAGTAGAG	0	10	RDR2-sensitive
CCTTCCGTCAGCAGTAGAGGCGCA	3	17	RDR2-sensitive
CCTTCGCCCCTGTAGCACGGATGG	32	3	RDR2-resistant
CCTTCGCTGTAGTCGCCGGTCCGA	0	15	RDR2-sensitive
CCTTCGGCTGCATGTGAAGATGAC	0	11	RDR2-sensitive
CCTTCGGGGCTGCGGACGACTGGC	14	0	RDR2-resistant
CCTTCGTCCCGACCGCAGAGGTGC	1	43	RDR2-sensitive
CCTTCGTTCTTAGGCTCAGCGGCA	50	6	RDR2-resistant
CCTTCTAGTGGACCCGGGGATATC	2	19	RDR2-sensitive
CCTTCTAGTGGACTCGGGGATATC	0	17	RDR2-sensitive
CCTTCTCGACTGCGGAACCGGTGC	0	9	RDR2-sensitive
CCTTCTCTCGGTTCGGCTGTACGG	0	9	RDR2-sensitive
CCTTCTCTGCACGCTTCCGGACGG	1	15	RDR2-sensitive
CCTTCTGTCGACTGGCGCACCGGA	1	16	RDR2-sensitive
CCTTCTTGACAAGCCGGACGGTAC	0	28	RDR2-sensitive
CCTTGAAATTGGGACTAATGAGAC	0	9	RDR2-sensitive
CCTTGATGATTAGTGGGTTGTCGC	0	11	RDR2-sensitive
CCTTGATGTATCGTGAGGTTGGCT	15	0	RDR2-resistant
CCTTGATTTGATGGGCGACGACAA	0	23	RDR2-sensitive
CCTTGCAAGGCGGAACGACTGATC	0	10	RDR2-sensitive
CCTTGCATGTCGGCTGTAGGAGGC	0	11	RDR2-sensitive
CCTTGCATGTCGGCTGTAGGGGCA	0	15	RDR2-sensitive
CCTTGCATGTCGGCTGTAGGGGGC	0	44	RDR2-sensitive

CCTTGCCGCTCGGATCATCGTCGT	0	79	RDR2-sensitive
CCTTGCCTTCTGTCGTCGGGCGCA	10	87	RDR2-sensitive
CCTTGCTATGTCTGGACCTGGTAA	0	15	RDR2-sensitive
CCTTGCTGGGCGTGTAGAGGACGT	0	39	RDR2-sensitive
CCTTGGAAGGCTGTAGTAGGGCCA	0	20	RDR2-sensitive
CCTTGGACTACGGCGAACACGGAC	0	27	RDR2-sensitive
CCTTGGCTAGTAGGAGTCGTGGTG	28	0	RDR2-resistant
CCTTGGGACTGAACGAGGACGCGC	1	74	RDR2-sensitive
CCTTGGGTCGCGCGGACATCCGGC	41	2	RDR2-resistant
CCTTGGTCTGCAGGAACATGACGG	0	11	RDR2-sensitive
CCTTGGTGTAGAGGGCATACTGAA	0	38	RDR2-sensitive
CCTTGTCCTAAGGACCTCGGACAA	0	10	RDR2-sensitive
CCTTGTCTCAAAGACCTCAGACGG	0	16	RDR2-sensitive
CCTTGTGCGGGCAAGCGACGACGT	0	10	RDR2-sensitive
CCTTGTGTCTCAAGGACTCCGGAT	0	9	RDR2-sensitive
CCTTGTTGGACGGAGAAAGGAGGT	0	17	RDR2-sensitive
CCTTGTTGGACGGAGAGAGGAGAT	0	27	RDR2-sensitive
CCTTGTTGGACGGGGAGAGGAGGT	0	10	RDR2-sensitive
CCTTTACACAGTAGGATTGTCGGC	0	9	RDR2-sensitive
CCTTTCCGTCAACAGTAGAGGCGC	0	16	RDR2-sensitive
CCTTTCTGTGCACTCGCGGACGGT	0	11	RDR2-sensitive
CCTTTGAGGGCATCTCGGATGTGT	14	0	RDR2-resistant
CCTTTGTTTGCTCGGTACTTCGGT	15	0	RDR2-resistant
CCTTTTCCCGTTGTAGTTAGGCTG	0	11	RDR2-sensitive
CCTTTTCGTCCTGTGGAGAGGTTA	2	22	RDR2-sensitive
CCTTTTCTCGGGCACTCGGCAAAG	0	11	RDR2-sensitive
CCTTTTGTCGGGCACTCGGCAAAG	0	10	RDR2-sensitive
CCTTTTTTTGGTGGTCGTCTTGGT	42	1	RDR2-resistant
CGAAAACATGTGGAGCAGAGGCAA	1	19	RDR2-sensitive
CGAAACAACGAGCACGGTGGACAG	0	10	RDR2-sensitive
CGAAACAGCGAGCACGGTGGACAG	0	14	RDR2-sensitive
CGAAACATTCTGGGCGACGTGGAC	0	10	RDR2-sensitive
CGAAAGCGTGTAGAGGAGGATCAG	0	14	RDR2-sensitive
CGAAAGGCACTGACCCAAGCTCGG	0	19	RDR2-sensitive
CGAAATACGTCGGGTAGGGTACGG	0	121	RDR2-sensitive
CGAAATTAGTCTCTGTAGAGTGGC	27	0	RDR2-resistant
CGAACAAGCCCTGACGGAACTCGT	0	12	RDR2-sensitive
CGAACAAGTTCTGAGCGGTGCCGT	0	13	RDR2-sensitive
CGAACAGACGTAGATGGACGACGG	0	18	RDR2-sensitive
CGAACAGAGAGCTCCCAGCAACGG	0	31	RDR2-sensitive
CGAACATGGCGGAACTGACAGGAG	0	19	RDR2-sensitive
CGAACCAAGAGTACCTGACTGCAA	0	29	RDR2-sensitive
CGAACCACTGCATGCAGACAGCAT	0	16	RDR2-sensitive
CGAACCAGCGACGGTGCTCTCTGC	0	18	RDR2-sensitive
CGAACCATGCTCGGACCAGGCTAA	0	12	RDR2-sensitive
CGAACCCAGGACCTGAGGAGTGCT	12	29	RDR2-sensitive
CGAACCCAGGACTTAGAGGTGCTA	0	9	RDR2-sensitive
CGAACCCAGGACTTGGAGGTGCTA	0	22	RDR2-sensitive
CGAACCCGTTTGGCACGAAGCACG	0	13	RDR2-sensitive

CGAACCGAACCCGAATAGACCGAA	0	9	RDR2-sensitive
CGAACCGAGCTGCAGCGAACCCAA	0	9	RDR2-sensitive
CGAACCGCGGAACAGGATTCGGCC	0	14	RDR2-sensitive
CGAACCGGAAGCACTGAGACGAAC	0	21	RDR2-sensitive
CGAACCGGACGGCACACCTGCCAA	0	19	RDR2-sensitive
CGAACGAACGGGCGGCGACTGCAC	0	16	RDR2-sensitive
CGAACGACTGGGCGGCGGCTGGAC	0	9	RDR2-sensitive
CGAACGGAACTGCGTGGAGACGAA	0	12	RDR2-sensitive
CGAACGGACAATGGCGACGTGCAA	0	11	RDR2-sensitive
CGAACGGACAGTAACGGTCGCCAT	0	9	RDR2-sensitive
CGAACGGATTGGCATCACCACGAC	0	11	RDR2-sensitive
CGAACGGCTAGGAACCCCTCCGGT	53	12	RDR2-resistant
CGAACGGGCGTAGGAACAGCGGAG	0	12	RDR2-sensitive
CGAACGTAGAAGCGCAGTGCAGAC	0	18	RDR2-sensitive
CGAACTCAGGACCTGGAGGTGCTA	0	10	RDR2-sensitive
CGAACTCAGGATCCGGCGAGGCAA	0	19	RDR2-sensitive
CGAACTCGGCACGGCATAGACCGA	0	28	RDR2-sensitive
CGAACTGGAAGAAGTGCAAGTCGT	0	20	RDR2-sensitive
CGAACTGGAGAAAGTGCAAGCCGT	0	12	RDR2-sensitive
CGAACTGGAGGACCTGCCTGCAGC	0	9	RDR2-sensitive
CGAACTGGGACTAGAGGACGACAA	0	13	RDR2-sensitive
CGAACTGGGACTAGAGGACGACGA	2	47	RDR2-sensitive
CGAACTGTACAGATGCGGAGCTAT	0	9	RDR2-sensitive
CGAACTGTACATCGACAACGGCTT	0	23	RDR2-sensitive
CGAACTGTACATCGGCAACAGCAT	8	186	RDR2-sensitive
CGAACTGTACATCGGCAACAGCCT	2	238	RDR2-sensitive
CGAACTGTACATCGGCAACAGCTA	0	9	RDR2-sensitive
CGAACTGTACATCGGCAACAGCTT	16	207	RDR2-sensitive
CGAACTGTACATCGGCAACGGCCT	0	22	RDR2-sensitive
CGAACTGTACATCGGCAACGGCTT	0	28	RDR2-sensitive
CGAACTGTATATCAGCAACGGCTT	0	16	RDR2-sensitive
CGAACTGTGAAACTGCGAATGGCT	57	9	RDR2-resistant
CGAACTGTGTTACATCGGCGTCAT	0	84	RDR2-sensitive
CGAACTGTGTTACCTCAGCGTCAT	0	43	RDR2-sensitive
CGAACTGTGTTACTTCGACGTCAT	0	90	RDR2-sensitive
CGAACTTACACATGTCGGCGTCAT	0	15	RDR2-sensitive
CGAAGACAAAACGGGCAGGGACAC	0	50	RDR2-sensitive
CGAAGACCTGGGCATCTGGAGGCG	0	9	RDR2-sensitive
CGAAGACGATGGCGACAGGAACAC	0	12	RDR2-sensitive
CGAAGACTGGAAGTAGGGACGTTC	0	10	RDR2-sensitive
CGAAGACTGGAGCGAGCAGGGCAA	0	11	RDR2-sensitive
CGAAGACTTGGACGGGCAGGGCAA	0	11	RDR2-sensitive
CGAAGACTTGGGCGGGCAGGGCAA	0	11	RDR2-sensitive
CGAAGAGACGTACGTACAGAGCAT	0	9	RDR2-sensitive
CGAAGAGTCAAGGACGGGAACGGC	0	15	RDR2-sensitive
CGAAGATGGTATGACTCGGCCGCC	35	3	RDR2-resistant
CGAAGCACGGAATAGACACGGCAC	0	10	RDR2-sensitive
CGAAGCGGACTGACGGTGTAGATT	0	22	RDR2-sensitive
CGAAGGACGAAGGGCATTAACCAT	0	14	RDR2-sensitive

CGAAGGACGACGTCATCACCACGC	0	9	RDR2-sensitive
CGAAGGAGGCGGCTGCAGCATCGA	0	10	RDR2-sensitive
CGAAGGAGGCTCGGCTCGGTTCGA	0	20	RDR2-sensitive
CGAAGGAGGTAGAGGCATCATCGG	0	18	RDR2-sensitive
CGAAGGCAGAACCAGGGGACACAG	0	12	RDR2-sensitive
CGAAGGCAGAACCAGGGGAGGCAG	0	27	RDR2-sensitive
CGAAGGCAGAACCGTGCACCACAC	0	9	RDR2-sensitive
CGAAGGCAGAACGGCAGAACGGCA	0	11	RDR2-sensitive
CGAAGGCCTGCGGTGACGTCTCCC	0	10	RDR2-sensitive
CGAAGGCGTGGCACGACGGGCTAG	0	14	RDR2-sensitive
CGAAGGGACTGTACCTGGCGGCGT	27	49	RDR2-sensitive
CGAAGGGGAATAGAACGGCTCCAT	0	16	RDR2-sensitive
CGAAGGGTCCCCGTCGAACGTCAA	0	14	RDR2-sensitive
CGAAGGTCTTGCGTGAAGAAGCAG	0	9	RDR2-sensitive
CGAAGTCGGTGCAGGAAGATGTAC	1	26	RDR2-sensitive
CGAAGTCTGCATGATACTGATGGT	7	23	RDR2-sensitive
CGAATAAGCGGGACGGGCTCGGAC	0	17	RDR2-sensitive
CGAATAAGCGGGATGGGCTCGGAC	0	20	RDR2-sensitive
CGAATAAGCGGGCCGGGCTCGGAC	3	42	RDR2-sensitive
CGAATAAGCGGGTCGGGCTCGGAC	0	13	RDR2-sensitive
CGAATAGAATTGTCGTGACATCAT	0	13	RDR2-sensitive
CGAATCCCTGGGCACGCGTCGGCA	0	17	RDR2-sensitive
CGAATCCTGGATGAGACGTGACAG	0	23	RDR2-sensitive
CGAATCGGACGCCACAACTGCGGC	0	9	RDR2-sensitive
CGAATCGGCTGCCACACCTACGGC	0	11	RDR2-sensitive
CGAATGGAAGGACACGGACAGCGG	1	18	RDR2-sensitive
CGAATGGGCTGTCTGATAGGCTTC	0	18	RDR2-sensitive
CGAATGTTTGAACCTCCGTTCGGG	1	88	RDR2-sensitive
CGAATTCGAACTATGTGGAATACG	20	0	RDR2-resistant
CGAATTCGTAGAGCCTAAAGGCGT	0	9	RDR2-sensitive
CGAATTCGTGGAGCCTGAAGGCGT	0	23	RDR2-sensitive
CGAATTGCATTCGGACCGGACTAA	0	9	RDR2-sensitive
CGAATTTTTTTTGGATCATGTCGT	0	10	RDR2-sensitive
CGACAAAAGGCACTCGGCAAAGAA	0	17	RDR2-sensitive
CGACAACCCTGTAGAGCGCGGCGA	0	31	RDR2-sensitive
CGACAACGAGCTCAGGGAAGGTGT	1	13	RDR2-sensitive
CGACAAGAACCCTGGCGACGTCAA	0	39	RDR2-sensitive
CGACAAGTTGTAGCTCGGCGCCAA	0	22	RDR2-sensitive
CGACACCACTGTTGGGCTGCCGGC	4	20	RDR2-sensitive
CGACACCACTGTTGGGCTGTCGGC	0	15	RDR2-sensitive
CGACACCACTGTTGGGCTGTCGGT	0	9	RDR2-sensitive
CGACACGACGGTACTGGTCTAGGC	0	14	RDR2-sensitive
CGACACGACTAGCAGGCTGATGGG	0	9	RDR2-sensitive
CGACACGACTCTCGGCAACGGATA	657	54	RDR2-resistant
CGACACGACTGTGCGACGCCGCAT	3	16	RDR2-sensitive
CGACACGATAGAATAAGCGGGCGG	0	9	RDR2-sensitive
CGACACGCTGTTGACTCGGGTCGT	1	282	RDR2-sensitive
CGACACGGCACGGTACTGGCACGG	0	9	RDR2-sensitive
CGACACGGCATGTAGTGAACGGGG	0	9	RDR2-sensitive

CGACACGGGCTAGATGCACGCGGG	1	15	RDR2-sensitive
CGACACGGTAGAATAAGCGGGCAG	0	44	RDR2-sensitive
CGACACGGTAGAATAAGCGGGCGG	1	24	RDR2-sensitive
CGACACGTAGAGGGCGCTGTAGGG	1	25	RDR2-sensitive
CGACACGTAGGAGGCGCTGTAGGG	0	14	RDR2-sensitive
CGACACGTAGGGGACGCTGTAGAG	0	10	RDR2-sensitive
CGACACGTAGGGGGCGCTGTAGGG	0	16	RDR2-sensitive
CGACACGTAGTGAACCGGGCTCGT	0	10	RDR2-sensitive
CGACACGTGGACCAATCAGATCGC	0	28	RDR2-sensitive
CGACACGTGGACCAGTCAGATCGC	2	15	RDR2-sensitive
CGACACTAAATTATTTCGGGCCGT	0	10	RDR2-sensitive
CGACACTGTGCTGTGGGTCCACGA	0	15	RDR2-sensitive
CGACACTGTTGCAGCTGTTTGGAC	0	17	RDR2-sensitive
CGACACTGTTGCAGCTGTTTGGAT	1	18	RDR2-sensitive
CGACAGAGAGCACACAAGACACAT	2	27	RDR2-sensitive
CGACAGAGGAAACACACGAGACAC	0	14	RDR2-sensitive
CGACAGATAGCACACGAGACACAT	0	11	RDR2-sensitive
CGACAGCACTGTAGACACGGCTGC	0	11	RDR2-sensitive
CGACAGCGCGGCAGGTAGCTCGGC	1	17	RDR2-sensitive
CGACAGCGTCGCCACTGGAGGCAT	0	23	RDR2-sensitive
CGACAGGCTAACAGAACGGTACAG	0	10	RDR2-sensitive
CGACAGTAGACACACAGACGTGGC	0	16	RDR2-sensitive
CGACAGTGCTCTCTGCACAGGCGT	0	27	RDR2-sensitive
CGACAGTTGGCACTCCAGGTAGAG	0	21	RDR2-sensitive
CGACAGTTTCTCTCCAAGGGACGT	0	10	RDR2-sensitive
CGACAGTTTCTGTTCATGCCGGTT	18	0	RDR2-resistant
CGACATCACATGTGGACCGTCCGG	0	16	RDR2-sensitive
CGACATCGAGACGGGGCGACTGAA	0	10	RDR2-sensitive
CGACATGGACTGTAGGAGCGGGGT	1	12	RDR2-sensitive
CGACATGTGGAACGTGGCGCGGTT	0	26	RDR2-sensitive
CGACATGTGTTACCTCGGCGTCAT	0	14	RDR2-sensitive
CGACCAAAGTATTGTAGACGGTCC	0	10	RDR2-sensitive
CGACCAAGACTCTCCGAAGACAAT	1	13	RDR2-sensitive
CGACCAAGGGACGTTCTGGGTGGC	0	10	RDR2-sensitive
CGACCAGACGCTCATACACCTCAC	2	19	RDR2-sensitive
CGACCAGAGACTCATACACCTCAC	0	14	RDR2-sensitive
CGACCAGCACGACGGACGAACTAA	2	16	RDR2-sensitive
CGACCATGGATTCTCAGGCGCCAA	0	23	RDR2-sensitive
CGACCATGGATTCTCGGGCGGCAA	1	38	RDR2-sensitive
CGACCCATCTGGACTGGGGCACGC	0	18	RDR2-sensitive
CGACCCATCTGGGCTGGGGCACGC	9	61	RDR2-sensitive
CGACCCCAGGGCTCGGACTCGGGC	87	36	RDR2-resistant
CGACCCGAACCGAACCATTAACGG	0	11	RDR2-sensitive
CGACCCGAACCTGCAGAAAATCGT	0	19	RDR2-sensitive
CGACCCGGCTTTGGCGGACTCGGC	0	9	RDR2-sensitive
CGACCCGTAGTGCTGGCACGGGCA	0	12	RDR2-sensitive
CGACCCGTGAGTAGGACTAGGCAT	1	35	RDR2-sensitive
CGACCCTAACTCTAATGGACCGGC	0	12	RDR2-sensitive
CGACCCTGTAGCAATGACGCGCAT	0	16	RDR2-sensitive

CGACCCTGTAGCAATGACGCGCGC	0	21	RDR2-sensitive
CGACCGACTGTTGGCTCACCGGAC	0	11	RDR2-sensitive
CGACCGATCGGAATCAGATGACGC	0	9	RDR2-sensitive
CGACCGCGCCTGTAGACCGGACGG	0	22	RDR2-sensitive
CGACCGTACACTCTTAAAGCGGCT	2	14	RDR2-sensitive
CGACCGTACTGGCATGACACGGCT	0	9	RDR2-sensitive
CGACCGTAGGACTGAAGACGTGTG	0	20	RDR2-sensitive
CGACCGTGAGAGCCGACGTGCGTG	0	14	RDR2-sensitive
CGACCGTTGGACAGGCGCGCGGCT	0	11	RDR2-sensitive
CGACCGTTGGCTCACCGGACAGTC	10	38	RDR2-sensitive
CGACCGTTGGCTGAGGCACGTGGC	0	35	RDR2-sensitive
CGACCTAGACTCCTCCAATCGGCG	0	9	RDR2-sensitive
CGACCTAGACTCCTCTAATCGGCG	0	9	RDR2-sensitive
CGACCTCGCTAGGACTGTCGTCAT	0	12	RDR2-sensitive
CGACCTCGGCTGCAGCGGCGGGGA	15	0	RDR2-resistant
CGACCTCTGGAAGCAGTACTACGT	0	13	RDR2-sensitive
CGACCTGGTGCAGGAGCTCGGGTT	1	15	RDR2-sensitive
CGACCTGTACAACGATGCTGCGGC	0	18	RDR2-sensitive
CGACGAACAAGTTCTGAGCGGTGC	0	20	RDR2-sensitive
CGACGAACAGCAAGCTGTGGACGA	0	15	RDR2-sensitive
CGACGAACTGTACATCATCGGCAA	0	14	RDR2-sensitive
CGACGAACTGTACATCGGCAACAA	0	30	RDR2-sensitive
CGACGAACTGTACATCGGCAACAG	0	96	RDR2-sensitive
CGACGAAGACCTGGGCATCTGGAG	0	14	RDR2-sensitive
CGACGACAGACGGCGTCTGAGCAC	0	18	RDR2-sensitive
CGACGACCTTCAGCTTGACGACGA	0	9	RDR2-sensitive
CGACGACGAGAAGAAGCTTGGCGA	0	12	RDR2-sensitive
CGACGACGCAGGAGCTCTCCGGCT	0	9	RDR2-sensitive
CGACGACGGACGGCGTAATTCCGG	0	18	RDR2-sensitive
CGACGACTCGACGGCAAATGACGA	0	16	RDR2-sensitive
CGACGACTCGACTGTGAACGGCGC	0	19	RDR2-sensitive
CGACGACTCTCGGGTTGTTCTGGA	0	12	RDR2-sensitive
CGACGACTCTCGGGTTGTTCTGGG	0	16	RDR2-sensitive
CGACGACTGCACGGCACTGCTCGA	35	78	RDR2-sensitive
CGACGACTGTACAGATTACGCCAC	0	48	RDR2-sensitive
CGACGACTGTACAGATTACGCCGC	0	32	RDR2-sensitive
CGACGACTGTACAGATTACGTCAC	0	24	RDR2-sensitive
CGACGACTGTACAGATTGCACCAC	0	20	RDR2-sensitive
CGACGACTGTACAGATTGCGCCAC	2	65	RDR2-sensitive
CGACGACTGTACAGATTGCGCCAT	1	26	RDR2-sensitive
CGACGACTGTACAGATTGCGTCAC	0	15	RDR2-sensitive
CGACGACTGTACATATTACGCCAC	0	17	RDR2-sensitive
CGACGACTGTATAGATTGCGTCAC	2	63	RDR2-sensitive
CGACGACTTCGTTGATCTTCCGGC	15	0	RDR2-resistant
CGACGAGAACACCCTGATAGACGC	0	10	RDR2-sensitive
CGACGAGCAGAAACAGGAGCAGGA	22	0	RDR2-resistant
CGACGAGCTAGAACTGCGGCGGAG	0	13	RDR2-sensitive
CGACGAGCTAGATCTGCGACGGAG	0	10	RDR2-sensitive
CGACGAGGACGCCGCCAACATCGC	6	23	RDR2-sensitive

CGACGATGCGGCGCAGCAGGACGA	0	10	RDR2-sensitive
CGACGCAACGACGAACGACGACGA	0	17	RDR2-sensitive
CGACGCACTGAAGTTCAACGACGG	0	10	RDR2-sensitive
CGACGCACTGGCACAGACGAAGAC	0	9	RDR2-sensitive
CGACGCCATGTCAGAGCTCGGCGC	0	12	RDR2-sensitive
CGACGCCTACCGCTCTGACGGCGA	1	24	RDR2-sensitive
CGACGCGACTGACGGATAAGCCAA	0	49	RDR2-sensitive
CGACGCGGAAACGAGCATGCTGGC	0	13	RDR2-sensitive
CGACGCGGCGTATGAGATGGACGG	0	12	RDR2-sensitive
CGACGCGTAGAGGGCGCTGTAGGG	0	11	RDR2-sensitive
CGACGCTGGAGCCAACGACGACAT	0	23	RDR2-sensitive
CGACGGAAGCACACTGAGAAGCAG	0	13	RDR2-sensitive
CGACGGAAGGTGTTGCTAGTAGAA	0	9	RDR2-sensitive
CGACGGACTGGACGAGTGCGACGG	0	13	RDR2-sensitive
CGACGGAGACGGATATGGATACAT	1	70	RDR2-sensitive
CGACGGAGACTGAGCCAGACGGAT	0	59	RDR2-sensitive
CGACGGAGCCGGATATGGATACAT	2	87	RDR2-sensitive
CGACGGAGCCGGGTATGAATACAT	0	17	RDR2-sensitive
CGACGGAGGCTGAGTCAGACGTGT	0	13	RDR2-sensitive
CGACGGAGGGGAAGGCAGGGCAGC	43	2	RDR2-resistant
CGACGGATGCGGCTTCGACGAAAT	0	23	RDR2-sensitive
CGACGGATGCTAGTAGAATGGCAT	0	33	RDR2-sensitive
CGACGGCAGTGTCTTATCTCCGAC	0	13	RDR2-sensitive
CGACGGCCACGACATCACATGAAC	6	22	RDR2-sensitive
CGACGGCCTCCTTATCTCCGACGG	0	10	RDR2-sensitive
CGACGGCCTTCAGCTTGACGACGA	0	9	RDR2-sensitive
CGACGGCGGCTTGGCGAGGGCGAG	23	0	RDR2-resistant
CGACGGCTGTCAGGGATGAGTCAT	0	9	RDR2-sensitive
CGACGGGAACGGCTACGAATACAG	0	12	RDR2-sensitive
CGACGGGAGCTACGAGCAGCAGAC	0	9	RDR2-sensitive
CGACGGGCTGCCTGCACACATGGC	0	13	RDR2-sensitive
CGACGGGGCATTGTAAGTGGCAGA	298	39	RDR2-resistant
CGACGGTAGCCGCAGCCTTGACGA	0	9	RDR2-sensitive
CGACGGTCTGCTCGTGGAAGTCGC	0	13	RDR2-sensitive
CGACGGTCTGTAACCTAGGCACAG	0	14	RDR2-sensitive
CGACGGTGCCGAGTGGAAGGGACG	0	14	RDR2-sensitive
CGACGGTGCTCTCTGCACAGACGC	0	21	RDR2-sensitive
CGACGGTGCTCTCTGCACAGGCGT	1	34	RDR2-sensitive
CGACGGTGCTCTCTGGTCAGGCGC	1	19	RDR2-sensitive
CGACGGTTTCTGCAGACGGGTTGG	0	13	RDR2-sensitive
CGACGGTTTTACTGAGAGCGGCAT	0	9	RDR2-sensitive
CGACGTAGACCAAGAGGGCTTCTC	1	23	RDR2-sensitive
CGACGTAGAGCCGAAGAGAAGCGA	0	26	RDR2-sensitive
CGACGTAGAGCCGAAGAGAGACGA	1	94	RDR2-sensitive
CGACGTAGAGCCGAAGAGAGGTGA	0	26	RDR2-sensitive
CGACGTAGCGCCCTTGACAACGGT	0	31	RDR2-sensitive
CGACGTAGGCTGAACCGGTATCAC	0	12	RDR2-sensitive
CGACGTCATGGGGTTGAAGATGAG	35	1	RDR2-resistant
CGACGTGCGGACTGCAGAGACGGT	0	14	RDR2-sensitive

CGACGTGGAACCGAGCTCGGCGCC	9	65	RDR2-sensitive
CGACGTGGACATCACGAACGGCGA	0	11	RDR2-sensitive
CGACGTGGACGCATATGGAAGCAG	0	9	RDR2-sensitive
CGACGTGGACGCATGTAGGAGCGG	0	29	RDR2-sensitive
CGACGTTGAACCGGCTGCTCTCAG	0	22	RDR2-sensitive
CGACGTTGACTGACTGCTCGGCAT	3	16	RDR2-sensitive
CGACGTTGAGCTGAGTGGGGGCAC	0	10	RDR2-sensitive
CGACGTTGGGCTGAGTGGAGGCAC	0	25	RDR2-sensitive
CGACGTTTTGAGATCTTTAGTGTC	22	1	RDR2-resistant
CGACTACTCTGGGCCAGCAACAGC	0	13	RDR2-sensitive
CGACTACTTAGGAACATGGCTCAC	0	9	RDR2-sensitive
CGACTAGAGACGTACTGCGCGGCC	0	13	RDR2-sensitive
CGACTATTTTGTCGATCCGAGGCA	39	3	RDR2-resistant
CGACTATTTTGTCGATCCGTGGCA	33	0	RDR2-resistant
CGACTCATGGCATAACTCGGCTAG	0	11	RDR2-sensitive
CGACTCCGGCAGCGGCTCTAGTAT	0	9	RDR2-sensitive
CGACTCCTCCAACGCGGTCGTCGA	1	26	RDR2-sensitive
CGACTCCTGGCAGAACCGTGCACC	0	9	RDR2-sensitive
CGACTCGACTCGAAGGTGGCTCGT	0	24	RDR2-sensitive
CGACTCGTAGTGCTTCGGCATGGC	0	10	RDR2-sensitive
CGACTCGTTCTAACTCGGCGGCAA	1	16	RDR2-sensitive
CGACTCTACGTCGTCTAGATCCGT	1	13	RDR2-sensitive
CGACTCTAGGCCACGGACCGTCTG	0	9	RDR2-sensitive
CGACTCTCGGCAACGGATATCTCG	22	2	RDR2-resistant
CGACTCTGACAACGGAGGACTCGC	0	9	RDR2-sensitive
CGACTCTGTCAGGAACGGAACTGG	0	9	RDR2-sensitive
CGACTCTTGGCGTGACTCGGCGAC	2	27	RDR2-sensitive
CGACTGAAGAGCGCGCGTCAGGAT	1	12	RDR2-sensitive
CGACTGAATCAGGACCATCGTCGT	0	13	RDR2-sensitive
CGACTGACACTCGGCAAAGAGGCT	1	17	RDR2-sensitive
CGACTGACCAAATTGCAGGGGCAT	1	18	RDR2-sensitive
CGACTGACCCTAGAAGAAGCACAG	0	26	RDR2-sensitive
CGACTGCAAATATACAGAGGGCAT	0	12	RDR2-sensitive
CGACTGCAGCGAGAGACGACGAGC	0	9	RDR2-sensitive
CGACTGCATCCTCATCAACGTCGG	0	10	RDR2-sensitive
CGACTGCATGGCTGACGCAAGCAC	0	15	RDR2-sensitive
CGACTGCCGACGCTACACACTGAC	0	15	RDR2-sensitive
CGACTGCGGCTGCGGCTTCTACAG	0	39	RDR2-sensitive
CGACTGCGTACGACTACATCGAAC	1	29	RDR2-sensitive
CGACTGCTAGGAGCCTAAGACGAT	0	13	RDR2-sensitive
CGACTGCTAGGAGCCTAGGACGAC	0	10	RDR2-sensitive
CGACTGGAACAGGAACGAAGACGT	0	68	RDR2-sensitive
CGACTGGAAGGTATACACACGCAC	51	1	RDR2-resistant
CGACTGGACGGAGACCTACCTGGT	0	9	RDR2-sensitive
CGACTGGACGTACCTGGAAGGCGC	0	46	RDR2-sensitive
CGACTGGAGACGGCGAGCGTCCAC	2	18	RDR2-sensitive
CGACTGGAGCAGGAGCACAACTAT	0	15	RDR2-sensitive
CGACTGGCTGAGGAGGGTGGAGAC	0	13	RDR2-sensitive
CGACTGTAATGGAGGGCAAGGCGC	0	28	RDR2-sensitive

CGACTGTACGAGTGCGGATTTGAT	0	10	RDR2-sensitive
CGACTGTAGCTAAATGGTTCGTGC	0	9	RDR2-sensitive
CGACTGTAGCTCGATGGTTCGTGC	0	14	RDR2-sensitive
CGACTGTATTTTAGCGTAGGCGAA	0	19	RDR2-sensitive
CGACTGTATTTTAGCGTAGGTGAA	0	11	RDR2-sensitive
CGACTGTCTAGACCGCTACGCCAG	0	13	RDR2-sensitive
CGACTGTGAACGACGCAACGACGA	0	17	RDR2-sensitive
CGACTGTGCACCTGGGCTACGGCC	0	15	RDR2-sensitive
CGACTGTGGCAGCCACAACGGCGT	0	17	RDR2-sensitive
CGACTGTGGGGGCAGATATCACTC	0	11	RDR2-sensitive
CGACTGTTCCGACGGAAGCCGCAG	0	11	RDR2-sensitive
CGACTGTTCCGACGGTAGCCGCAG	4	39	RDR2-sensitive
CGACTGTTCCTGACGGGCCTGGCT	0	12	RDR2-sensitive
CGACTGTTCGGCGTGCATAGACAT	0	19	RDR2-sensitive
CGACTGTTCTGACGGTAGCCGCAG	0	17	RDR2-sensitive
CGACTGTTGACTGGGCGCGCGGCT	0	9	RDR2-sensitive
CGACTGTTGGCACTCGGCAAAGAC	3	35	RDR2-sensitive
CGACTGTTTAGGAAGCCGTCGGAC	0	14	RDR2-sensitive
CGACTTAAAACGCGACGGGGCATT	61	15	RDR2-resistant
CGACTTATGGCGTGACTCGGCAAG	0	10	RDR2-sensitive
CGACTTATGGCGTGACTCGGCGAG	0	18	RDR2-sensitive
CGACTTCGTACGGCTACATCGAAC	1	125	RDR2-sensitive
CGACTTCGTGTGGGCAGGGCTGGC	41	1	RDR2-resistant
CGACTTCGTTGATCTTCCGGCGTC	24	0	RDR2-resistant
CGACTTCTATACGGATCGGAGGCC	58	0	RDR2-resistant
CGACTTCTTAGGACGGCAGTAGAG	0	9	RDR2-sensitive
CGACTTGTAGATCGGTGTCCGAAA	0	14	RDR2-sensitive
CGACTTGTGGGTAAAGATGCGCAA	0	14	RDR2-sensitive
CGACTTTAGACGCGGACCGTCCAG	2	17	RDR2-sensitive
CGACTTTGACTCGACCTGACGGTT	1	20	RDR2-sensitive
CGAGAAAACTTCCCGGTCGGTCAC	0	13	RDR2-sensitive
CGAGAACCGACCGGCTCGACCCTT	66	2	RDR2-resistant
CGAGAACTCTACTGCCGACTGCAC	0	80	RDR2-sensitive
CGAGAACTCTACTGTCGACTGCAC	0	20	RDR2-sensitive
CGAGAAGACCAGAGGAGCGACGAC	0	19	RDR2-sensitive
CGAGAATATGCATGGACACGACAT	0	9	RDR2-sensitive
CGAGACAACACAACTCGGCGTCAT	0	9	RDR2-sensitive
CGAGACACCACCTCTGAGCGTCGT	0	21	RDR2-sensitive
CGAGACAGTGTAAGACGGAGACAG	0	213	RDR2-sensitive
CGAGACAGTGTAAGATGGAGACAG	0	57	RDR2-sensitive
CGAGACATATTACCTCGGTGTCGT	0	11	RDR2-sensitive
CGAGACATGTTAGCTCGACGTCAC	0	16	RDR2-sensitive
CGAGACCAACAGGGCGGAGCTGGC	0	13	RDR2-sensitive
CGAGACCCGTGAGCCCGGCATGAA	0	11	RDR2-sensitive
CGAGACGATCTGAGCCGAGCCGAG	0	13	RDR2-sensitive
CGAGACGGACTGAGCTGAGGCGAC	0	16	RDR2-sensitive
CGAGACGGCGTCAGATTCTCCTTC	0	9	RDR2-sensitive
CGAGACGGGGTGACCGAACGACGA	0	16	RDR2-sensitive
CGAGACGGTTGCACGAGACAGTGT	0	10	RDR2-sensitive

CGAGACGTGCTAGGCTAAGATCGG	0	15	RDR2-sensitive
CGAGACGTGTAACCTCGGCGCCAA	0	12	RDR2-sensitive
CGAGACGTGTAACCTCGGCGTCAA	0	17	RDR2-sensitive
CGAGACTAGGACGGCTGCAGTTGC	0	9	RDR2-sensitive
CGAGACTCTGTCTTCGTAGGACGG	0	9	RDR2-sensitive
CGAGACTGATCTGCATGCACGTAC	0	16	RDR2-sensitive
CGAGACTGCATGCGTGGACGTGAC	0	35	RDR2-sensitive
CGAGACTGGACCGGCCCGACACGA	0	10	RDR2-sensitive
CGAGACTGGAGGCAGAACACGACG	0	10	RDR2-sensitive
CGAGACTGGGGTCCTTAAGCCGAC	0	10	RDR2-sensitive
CGAGACTGGTGAAGGACGCGGCAG	0	10	RDR2-sensitive
CGAGACTGTTGGGAGCAAGGAGGA	42	8	RDR2-resistant
CGAGACTTCGGATGGCCGCTAGGT	21	1	RDR2-resistant
CGAGACTTTGTCGAGTGTTGGCAT	2	19	RDR2-sensitive
CGAGAGACCTGACTGACGAGCGAC	0	17	RDR2-sensitive
CGAGAGAGGACGTCAGGGGACGAT	0	13	RDR2-sensitive
CGAGAGCTCTCGTAGACACTCGGC	1	19	RDR2-sensitive
CGAGAGCTGGCCGACTCATGGCAT	2	15	RDR2-sensitive
CGAGAGCTGTTTGGCGACGAGCAT	0	10	RDR2-sensitive
CGAGAGGAACCCGAAGATGAGCGT	0	12	RDR2-sensitive
CGAGAGGAGGCTAGGAACAGACAG	0	12	RDR2-sensitive
CGAGAGGGTGTAGGATGCGATGGT	25	3	RDR2-resistant
CGAGAGTCTGTCTCTGTCGTCTGT	0	12	RDR2-sensitive
CGAGATCGCGGTCCACGGACGGGC	0	20	RDR2-sensitive
CGAGATCGCTGCCCAGAATGTCGT	0	11	RDR2-sensitive
CGAGATCGCTGGAAGGCAGAACGG	0	12	RDR2-sensitive
CGAGATCGGACGGCTTGGATGGCC	20	2	RDR2-resistant
CGAGATCGGGCTTGCAGGACGGGT	0	10	RDR2-sensitive
CGAGATCTCCGAGCGGTTGCACGT	0	13	RDR2-sensitive
CGAGATCTCTGGGCAGTTGCACGT	0	30	RDR2-sensitive
CGAGATCTGGAACAGGCTCGACGA	0	17	RDR2-sensitive
CGAGATGAAGACGCCAAGAACCAG	0	11	RDR2-sensitive
CGAGATGAAGACGCCAGGAACCAG	1	32	RDR2-sensitive
CGAGATGCAGGGCCTGGCACCGAC	17	0	RDR2-resistant
CGAGATGCGCGAGGACGACAGCAC	0	10	RDR2-sensitive
CGAGATGTGCGACGAGCTGGGGCC	56	1	RDR2-resistant
CGAGATGTGTAAACTCGGCGCCAT	0	19	RDR2-sensitive
CGAGATGTGTAAACTCGGCGTCAT	0	9	RDR2-sensitive
CGAGATGTGTTACCTCGGCGCCAT	0	9	RDR2-sensitive
CGAGATGTGTTAGCTCGGCGTCAT	0	19	RDR2-sensitive
CGAGATGTGTTATCTTGACGTCGT	0	9	RDR2-sensitive
CGAGATTACTTAGGGCGGTGAGAA	0	9	RDR2-sensitive
CGAGCAAAGCGGACCGGTAAGCAT	0	27	RDR2-sensitive
CGAGCAACGGGCAACGGATGAGGT	45	5	RDR2-resistant
CGAGCAACTCTGCGAGACGGACTA	0	10	RDR2-sensitive
CGAGCACACGGACTTAGATGGCCC	0	11	RDR2-sensitive
CGAGCACGGACCGAAGTATGCTAC	17	0	RDR2-resistant
CGAGCACGGACTGGACCGGCCCGA	0	14	RDR2-sensitive
CGAGCAGACTGTGTTGCCGGTATG	0	9	RDR2-sensitive

CGAGCAGAGGGCGACTGAGGGCGT	0	17	RDR2-sensitive
CGAGCAGCAGACCTCGATGAACCT	0	16	RDR2-sensitive
CGAGCAGGGCTACGGCGTGAACAA	0	9	RDR2-sensitive
CGAGCATAGTGGCCTGATGAGCAT	0	12	RDR2-sensitive
CGAGCCAAGTAGAGCCGAGCTGAA	0	14	RDR2-sensitive
CGAGCCAGCTCGAACTCGGACGAG	0	14	RDR2-sensitive
CGAGCCCGCGGACTTAGACGGTGT	0	38	RDR2-sensitive
CGAGCCCGGCACGGTAGAATAAGC	0	11	RDR2-sensitive
CGAGCCGCTGTAGGTTGTCGCGTT	4	25	RDR2-sensitive
CGAGCCGGACCAGGTCCAGACGTC	4	35	RDR2-sensitive
CGAGCCGGAGGCGAGATGGACTAT	0	22	RDR2-sensitive
CGAGCCGTGTTAACTCGACGTCAT	0	19	RDR2-sensitive
CGAGCCTGATCGAGCCCGAGCCGG	0	22	RDR2-sensitive
CGAGCGCTGAACACTCGGCACAGA	0	13	RDR2-sensitive
CGAGCGGACGAAGCGAGCAGTCGC	0	11	RDR2-sensitive
CGAGCGGATGCAGTCAATTGGCCT	0	12	RDR2-sensitive
CGAGCGGATGCGCGAGTGAATGAG	0	9	RDR2-sensitive
CGAGCGGATGGACGTCAAACAGGA	0	11	RDR2-sensitive
CGAGCGGGCTGCGAGGGCATAGAG	28	0	RDR2-resistant
CGAGCGGGCTGGGAACGGAGCGGC	6	35	RDR2-sensitive
CGAGCGGGTGGACGACTTCGGGGC	17	1	RDR2-resistant
CGAGCTAACGGGCTGTCTCGGCAC	0	18	RDR2-sensitive
CGAGCTAAGCCGACTCGTGAGCCT	0	9	RDR2-sensitive
CGAGCTACAACGTGTCGGCGCCAA	0	14	RDR2-sensitive
CGAGCTAGACGGTCTGCGCCTGAT	0	11	RDR2-sensitive
CGAGCTAGCACAGCCCGAAGTCGG	0	13	RDR2-sensitive
CGAGCTAGCATGACTCGAAGTCGG	1	13	RDR2-sensitive
CGAGCTATGTAAACTCGGCGTCAT	0	16	RDR2-sensitive
CGAGCTCCTGCACCAGGTCGCGGA	0	11	RDR2-sensitive
CGAGCTCGACTCGGCTCTGCTCGG	0	9	RDR2-sensitive
CGAGCTCGGACACGTCGACGTCGT	0	16	RDR2-sensitive
CGAGCTCGGTACCACGTCGACGCC	0	9	RDR2-sensitive
CGAGCTCGGTGCCACGTCGACGCC	0	12	RDR2-sensitive
CGAGCTCTGACATGGCAGCGACGG	0	10	RDR2-sensitive
CGAGCTCTGACGTAGCAGCCACGT	1	17	RDR2-sensitive
CGAGCTCTGACGTGGCCGCAACGG	0	24	RDR2-sensitive
CGAGCTCTGACGTGGCGACGACAG	0	22	RDR2-sensitive
CGAGCTCTGACGTGGCGACGGGAG	0	11	RDR2-sensitive
CGAGCTGACCATGAAACGAGCGAG	0	20	RDR2-sensitive
CGAGCTGACCATGAACCAAGCGAG	0	31	RDR2-sensitive
CGAGCTGACCATGAACCGAGCGAG	0	49	RDR2-sensitive
CGAGCTGACCCTGAAACGAGCGAG	0	74	RDR2-sensitive
CGAGCTGACTATGAACCGAGCGAG	0	10	RDR2-sensitive
CGAGCTGAGCCGAGTAGAGCCGAG	0	10	RDR2-sensitive
CGAGCTGATGGACATCGCCACCAA	2	15	RDR2-sensitive
CGAGCTGGACCGAGCCCGACTCAT	0	10	RDR2-sensitive
CGAGCTGTATTACCTCGGCGCCGT	1	19	RDR2-sensitive
CGAGCTGTATTACCTCGGCGTCAT	0	58	RDR2-sensitive
CGAGCTGTCGGCACCATCTTTCCT	0	24	RDR2-sensitive

CGAGCTGTGGCATGGTATACTGAG	0	12	RDR2-sensitive
CGAGCTGTGTGACGTGGCAGTCTG	0	21	RDR2-sensitive
CGAGCTGTGTTAACTCGGCGCCAT	0	11	RDR2-sensitive
CGAGCTGTGTTAACTCGGCGTCAT	0	19	RDR2-sensitive
CGAGCTGTGTTACATCGACGCCAT	1	50	RDR2-sensitive
CGAGCTGTGTTACCTAGGCGCCAT	0	23	RDR2-sensitive
CGAGCTGTGTTACCTCGACGCCAT	1	26	RDR2-sensitive
CGAGCTGTGTTACCTCGACGTCAT	0	69	RDR2-sensitive
CGAGCTGTGTTACCTCGGCGCCAC	2	128	RDR2-sensitive
CGAGCTGTGTTACCTCGGCGCCAT	15	228	RDR2-sensitive
CGAGCTGTGTTACCTCGGCGCCCT	1	15	RDR2-sensitive
CGAGCTGTGTTACCTCGGCGCCGA	0	19	RDR2-sensitive
CGAGCTGTGTTACCTCGGCGCCGT	0	17	RDR2-sensitive
CGAGCTGTGTTACCTCGGCGTCAC	1	21	RDR2-sensitive
CGAGCTGTGTTACCTCGGCGTCAT	2	78	RDR2-sensitive
CGAGCTGTGTTACCTCGGCGTCGT	2	35	RDR2-sensitive
CGAGCTGTGTTACCTTGGCGCCAT	0	9	RDR2-sensitive
CGAGCTGTGTTACTTCGACGCCAT	0	10	RDR2-sensitive
CGAGCTGTGTTACTTCGGCGCCAT	3	41	RDR2-sensitive
CGAGCTGTGTTATCTTGGCGTCAT	0	19	RDR2-sensitive
CGAGCTTGGACTGCGGCGTAGGCC	0	17	RDR2-sensitive
CGAGGAACCGAACCGAACCGAAGA	0	14	RDR2-sensitive
CGAGGAACGAGGAACACGGTACGT	0	15	RDR2-sensitive
CGAGGAACTCGCAGGAGAAGCCCC	0	11	RDR2-sensitive
CGAGGAAGCTGCCGAGGACGTCGT	1	21	RDR2-sensitive
CGAGGAATCTGTCAAGGACGTCGG	0	12	RDR2-sensitive
CGAGGAATGACGTGGCAGCAACAG	0	9	RDR2-sensitive
CGAGGACAACGACAAGGACGAGCT	0	13	RDR2-sensitive
CGAGGACAGTGTTGAAGACGACGA	0	17	RDR2-sensitive
CGAGGACATAGCTGTGGGCTGGCT	0	19	RDR2-sensitive
CGAGGACCGAGGACATCGACGGAG	0	13	RDR2-sensitive
CGAGGACCGATCTGAACAGGGCAT	0	13	RDR2-sensitive
CGAGGACGACGCTGCGGACGGGCT	0	22	RDR2-sensitive
CGAGGACGCTGGCAAGGAACACGG	12	43	RDR2-sensitive
CGAGGACGGGGAACAGGCTACCAT	1	63	RDR2-sensitive
CGAGGACGTAAAGGACGCCGGCGT	0	9	RDR2-sensitive
CGAGGACGTGGACGGCACCGACGA	0	12	RDR2-sensitive
CGAGGACTAGACCAGTGGACTGCT	0	13	RDR2-sensitive
CGAGGACTAGGCCCATAGGCCGGC	0	9	RDR2-sensitive
CGAGGACTAGTGGAGAGTGCGGAC	0	11	RDR2-sensitive
CGAGGACTGAACCGAGTAGGACGA	0	30	RDR2-sensitive
CGAGGACTGGGGGCATGGTGATGC	0	10	RDR2-sensitive
CGAGGACTGTGGCGTCATCTTGGC	3	34	RDR2-sensitive
CGAGGAGACCGTAGCAGAGACTGT	0	18	RDR2-sensitive
CGAGGAGCTCTGTCCAGCTGCAGA	22	0	RDR2-resistant
CGAGGATAGACTCAGGCAAGGCGT	0	11	RDR2-sensitive
CGAGGATTGTGGCGTCATCTTGGC	0	28	RDR2-sensitive
CGAGGCACCGAACTCAGGATCCGG	0	14	RDR2-sensitive
CGAGGCAGAACTAGTGGAGGCATA	0	10	RDR2-sensitive

CGAGGCGGGAACCAGCAGAACGTG	0	10	RDR2-sensitive
CGAGGCTACGGCTACCGTCGGAAC	0	15	RDR2-sensitive
CGAGGCTAGGCTCGGGTGAAGCGT	7	26	RDR2-sensitive
CGAGGCTGCGGCTACCGTCGGAAC	5	26	RDR2-sensitive
CGAGGGAAGCATGGTATAGCTCAC	0	16	RDR2-sensitive
CGAGGGACCTGCCGGCTTGATGGA	0	16	RDR2-sensitive
CGAGGGACTGCACTCTGTCGGCAT	0	20	RDR2-sensitive
CGAGGGAGTTCTCGAGGACGTCGT	0	21	RDR2-sensitive
CGAGGGATGGGTGCTCGTTTGGTC	17	0	RDR2-resistant
CGAGGGCTAGACATGTGGGCCGGT	0	11	RDR2-sensitive
CGAGGGCTGACGCGGTGCCATCGT	0	13	RDR2-sensitive
CGAGGGGCGTAGAACGGGCGGCGT	0	15	RDR2-sensitive
CGAGGGGCTGGACGCGCTTCCCAG	4	18	RDR2-sensitive
CGAGGGTAATCTCGGACGAGTGAC	0	17	RDR2-sensitive
CGAGGGTAGATCTGAGCAAGACGT	0	11	RDR2-sensitive
CGAGGGTAGATTTGAGCAGAGCGT	0	9	RDR2-sensitive
CGAGGGTGCATGCGGCTGATGAGC	15	0	RDR2-resistant
CGAGGTAACACAGCTCGACGCCAT	2	53	RDR2-sensitive
CGAGGTAACACAGCTCGACGTCAT	0	30	RDR2-sensitive
CGAGGTAACACAGCTCGGCGCCAC	0	33	RDR2-sensitive
CGAGGTAACACAGCTCGGCGCCAT	2	86	RDR2-sensitive
CGAGGTAACACAGCTCGGCGTCAT	0	10	RDR2-sensitive
CGAGGTAACACAGCTCGGCGTCGT	0	21	RDR2-sensitive
CGAGGTAACACATGTCGGCGCCAT	0	17	RDR2-sensitive
CGAGGTAACACTGTTCGACGTCAC	1	19	RDR2-sensitive
CGAGGTACGGCTTGACGTCTGGGA	0	16	RDR2-sensitive
CGAGGTAGAGCGTGCTGATAACGT	0	32	RDR2-sensitive
CGAGGTCCTGTAGCTGGCGGGCGC	119	29	RDR2-resistant
CGAGGTCGCAGCTCATGGACGGAC	0	11	RDR2-sensitive
CGAGGTCTGACGTGGCAACGACGG	0	17	RDR2-sensitive
CGAGGTCTGACGTGGCAGCAACGG	1	32	RDR2-sensitive
CGAGGTGAGAGGGCAGAACATGGT	0	11	RDR2-sensitive
CGAGGTGCTTTTGGGTAGGACGGT	0	16	RDR2-sensitive
CGAGGTTGGTCTTGGACAAGGCGT	0	38	RDR2-sensitive
CGAGTAGAGCCGAGCTGAAATCGA	0	21	RDR2-sensitive
CGAGTATTGTTTTGGTGAGTCTGG	14	0	RDR2-resistant
CGAGTCAAGCCGAGCTACTAACGA	0	11	RDR2-sensitive
CGAGTCGAATCTACTGTTGTGGCT	0	18	RDR2-sensitive
CGAGTCGAGACTGCTTTTGTGGCT	0	9	RDR2-sensitive
CGAGTCGAGCCGAGCTACTAACGA	1	17	RDR2-sensitive
CGAGTCGGTATCACAGTAGGAGAA	0	21	RDR2-sensitive
CGAGTCGTGGACTGACCCAGGCGT	0	11	RDR2-sensitive
CGAGTCGTGGACTGACCGAGGCAT	0	9	RDR2-sensitive
CGAGTCTGTGTGTGCCGGCGCCAA	0	9	RDR2-sensitive
CGAGTGCAAGTAGATACTCGGCAA	0	12	RDR2-sensitive
CGAGTGCACTGCCATGGACGTCAT	0	16	RDR2-sensitive
CGAGTGCGGCACTCGGCAAAGCCT	0	10	RDR2-sensitive
CGAGTGGACGAAGAGTGATGGCCT	0	15	RDR2-sensitive
CGAGTGGATTATGTGAACAGGTAT	0	43	RDR2-sensitive

CGAGTGGGATGTATGTGACGACAT	0	16	RDR2-sensitive
CGAGTGGTAGACAGGGATGGATAA	0	9	RDR2-sensitive
CGAGTGGTAGACAGGGTTGGATAA	1	16	RDR2-sensitive
CGAGTGGTAGATAGGGTTGGATAA	0	21	RDR2-sensitive
CGAGTGTCAGGCAGTACTCGGCAA	0	10	RDR2-sensitive
CGAGTGTCAGTCGGTACTCGGCAA	0	10	RDR2-sensitive
CGAGTGTCAGTCGGTCCTCGGCAA	0	11	RDR2-sensitive
CGAGTGTGGGCTAGGGACGGATAG	1	13	RDR2-sensitive
CGAGTTAGGGTTAGAGAACGGCGT	0	21	RDR2-sensitive
CGAGTTGTAGAGCACGATGGCGTC	0	11	RDR2-sensitive
CGAGTTTCTTTCGGGTCGGGCTAA	0	32	RDR2-sensitive
CGATAAAAACGGGGCAAGGGACGG	0	13	RDR2-sensitive
CGATAAGTTCGACGGTTTTACCAC	0	9	RDR2-sensitive
CGATACCAGCTGTGACGGAACCTC	2	55	RDR2-sensitive
CGATACCTGGTGTGAATTGCAGAA	20	0	RDR2-resistant
CGATAGAACGGACGGTGCAGATGC	0	9	RDR2-sensitive
CGATAGATGGTAGAACGGCAGGCT	0	24	RDR2-sensitive
CGATAGGACTACTCGAGTCGGCTT	0	30	RDR2-sensitive
CGATAGGGCCCTGAGTCGTTGGAG	0	14	RDR2-sensitive
CGATATAGTAGTCGTCGAACGCAC	0	15	RDR2-sensitive
CGATATAGTAGTCGTCGAACGCGC	0	18	RDR2-sensitive
CGATATATTTTCGTCGGCCGGCCC	1	13	RDR2-sensitive
CGATATATTTTCGTCGGTCGGCCC	0	37	RDR2-sensitive
CGATATGCGGCATCTCTGGATCGT	0	15	RDR2-sensitive
CGATATGTGTTAACTCGGCGTCAT	0	18	RDR2-sensitive
CGATATTCCCCAAGCGCTCGACAA	1	14	RDR2-sensitive
CGATCACGGTGGAGGACGAGGAGT	25	0	RDR2-resistant
CGATCACGTCTGACGCAGCGGCAT	0	9	RDR2-sensitive
CGATCAGACTGCCGGACAGGGCAT	0	11	RDR2-sensitive
CGATCAGACTGTCCACATGGAGGC	0	10	RDR2-sensitive
CGATCAGACTTGGGCAGCGTACAA	0	9	RDR2-sensitive
CGATCAGGCTCGGCTCGGCTCGGT	5	77	RDR2-sensitive
CGATCAGTGTTACCTTGACGTCAT	0	9	RDR2-sensitive
CGATCATGCTCGGCTCGGCTCGGT	0	15	RDR2-sensitive
CGATCATGGCTAGACTGGAGGCGA	0	14	RDR2-sensitive
CGATCCATCTTGGACCTGTGACGG	0	12	RDR2-sensitive
CGATCCGATCTGACGATAGGCATG	0	21	RDR2-sensitive
CGATCCGCGGTCGCTGTTACGGTA	0	11	RDR2-sensitive
CGATCCGGATCTGCTTGGAGGCGT	2	19	RDR2-sensitive
CGATCGGACGATCCGCGATGACGC	0	13	RDR2-sensitive
CGATCGGATTAGCTAGCAGGTCGT	0	23	RDR2-sensitive
CGATCTAGTCATCGGATTGGTTGT	0	12	RDR2-sensitive
CGATCTATGGACTCGCGACAGGAG	0	9	RDR2-sensitive
CGATCTCGTCGTCAAGCTGAAGGC	0	9	RDR2-sensitive
CGATCTCGTCGTCGTTCCGTGCAT	0	16	RDR2-sensitive
CGATCTGAACAGGGCATGCCGCTT	0	9	RDR2-sensitive
CGATCTGAAGTCGGGCGGGCACGG	0	25	RDR2-sensitive
CGATCTGACGTTCTGGATTGGCGC	0	26	RDR2-sensitive
CGATCTGTAGTGGAACAGGCTCAT	0	9	RDR2-sensitive

CGATCTGTATCTGCCACGTGACGC	38	2	RDR2-resistant
CGATCTGTGGTTCCTAGACGGTAC	0	19	RDR2-sensitive
CGATGACAAGACACGTGCTGGCAA	0	11	RDR2-sensitive
CGATGACATACTGGCGAAGCCCAC	0	24	RDR2-sensitive
CGATGACGAACTCCAGGCGCGGAT	0	10	RDR2-sensitive
CGATGACTCACCAGAACGACGCGC	0	11	RDR2-sensitive
CGATGACTCGCCAGAACGACGCGC	0	49	RDR2-sensitive
CGATGACTGGAAGTAGGGACGTTC	1	45	RDR2-sensitive
CGATGACTGGATGTAGGGACGTTC	0	19	RDR2-sensitive
CGATGACTGGGCACACGTGCTGAC	0	22	RDR2-sensitive
CGATGACTGGGCACACGTGCTGGC	9	180	RDR2-sensitive
CGATGACTGGGCATACGTGCTGGC	0	11	RDR2-sensitive
CGATGACTGTGTCTCGTGAGGCAG	0	9	RDR2-sensitive
CGATGAGAACCACGACGTCGGCAC	0	10	RDR2-sensitive
CGATGAGACTAGAGTAGAGGCCGA	0	23	RDR2-sensitive
CGATGAGACTGAGGTACGACCACT	27	0	RDR2-resistant
CGATGAGACTGGAGTAGAAGCCGA	0	14	RDR2-sensitive
CGATGAGACTGGAGTAGAGGCCGA	8	156	RDR2-sensitive
CGATGAGACTGGAGTAGGAGCCGA	0	9	RDR2-sensitive
CGATGCAGTAGATCGGGACGGCAG	1	14	RDR2-sensitive
CGATGCCCTACTGTAGACGGTCAG	3	54	RDR2-sensitive
CGATGCCGCGGTGTAGGGATGGGC	1	33	RDR2-sensitive
CGATGCGACGGTGCACAGGAACAA	0	9	RDR2-sensitive
CGATGCGACTTGTGGGAAGAGGCT	0	9	RDR2-sensitive
CGATGCGAGGTAGAGGGTAGGGAA	15	0	RDR2-resistant
CGATGCGATTGTGGAGACCGTGCT	1	23	RDR2-sensitive
CGATGCGGCACAGCAGGACGACGA	1	45	RDR2-sensitive
CGATGCGGCGCAGCAGGACGACGA	0	23	RDR2-sensitive
CGATGCGTACAGAACAGAGGGCAT	0	23	RDR2-sensitive
CGATGCTCTCTGTGGACACGCGGA	0	9	RDR2-sensitive
CGATGGACGGCAGAACGGCAGAAC	1	30	RDR2-sensitive
CGATGGATCTGTGGGACAGCGGCT	0	11	RDR2-sensitive
CGATGGCATACTGGCGAAGCCCAC	0	30	RDR2-sensitive
CGATGGCATACTGGCGAAGCTCAT	0	31	RDR2-sensitive
CGATGGCATACTGGTGAAGCTCAC	1	85	RDR2-sensitive
CGATGGCGATTGTGGAACAGGCAC	0	18	RDR2-sensitive
CGATGGGAACATGGACGGCGGGGA	10	44	RDR2-sensitive
CGATGGGACTCACGTACGGTGCAT	0	13	RDR2-sensitive
CGATGTCGAACTCGCGACAGACGA	0	16	RDR2-sensitive
CGATGTCTTTCGCATCACCGTCGG	0	11	RDR2-sensitive
CGATGTGACGTAGATGTTGTGGCT	0	18	RDR2-sensitive
CGATGTGCGCCGGGCTGAGCAGGC	0	12	RDR2-sensitive
CGATGTGGAGGGCGCTGCGCAGAT	25	0	RDR2-resistant
CGATGTGGGACTAAAGCCGAGGCA	0	9	RDR2-sensitive
CGATGTGGGACTAAAGCCGGGACG	0	16	RDR2-sensitive
CGATGTGGGACTAAAGCCGGGGCG	0	17	RDR2-sensitive
CGATGTGTAGTGTGATGGTCTGGG	921	63	RDR2-resistant
CGATGTTATAGGACTTGACGTCAA	0	17	RDR2-sensitive
CGATGTTGGCCTCGAGTGAGTCGT	0	17	RDR2-sensitive

CGATTAGGCTCGGCTCGGCTCGGT	0	11	RDR2-sensitive
CGATTATCCGTCGGGCCTAACGGG	0	21	RDR2-sensitive
CGATTATGTAAACGGGCCTGGCGT	0	9	RDR2-sensitive
CGATTATTCAAGGACTGTTCAGGC	603	19	RDR2-resistant
CGATTCAATCGACTAATCGGGCTA	0	11	RDR2-sensitive
CGATTCCATCGGTGCTGCAGTGGC	0	9	RDR2-sensitive
CGATTCGATCAATGGACTGGGCGG	0	9	RDR2-sensitive
CGATTCGGTAGATTATAATGGCAA	0	26	RDR2-sensitive
CGATTGGAACCGCGAGCAGCAGAG	0	16	RDR2-sensitive
CGATTGGACCAGGCCTGACGGTTG	0	14	RDR2-sensitive
CGATTGGAGGAGTCTAGGTCGGCC	0	16	RDR2-sensitive
CGATTGTGACTGGTTCTGCGGCAC	0	11	RDR2-sensitive
CGATTGTGGAGACCGTGCTGTCAT	0	22	RDR2-sensitive
CGATTGTTACTTGGTACTTGCAGA	17	0	RDR2-resistant
CGATTTATTCGCGGGCTAGCACGG	0	10	RDR2-sensitive
CGATTTCAGCTCGGCTCTACTCGG	0	10	RDR2-sensitive
CGATTTGGTCGGTCGGTGCGGCGA	0	9	RDR2-sensitive
CGCAAAACTGTATCGAGATAGCGT	0	11	RDR2-sensitive
CGCAAACACGGACGAGCAGGGCAA	0	28	RDR2-sensitive
CGCAAAGAACGGCAGCGCTCTCAT	0	13	RDR2-sensitive
CGCAAAGACTGAGATGCACCGGTG	0	10	RDR2-sensitive
CGCAAAGGACTGGCGAGCGGCGAC	0	11	RDR2-sensitive
CGCAAAGGTAGAAGCAGCGAACAG	0	10	RDR2-sensitive
CGCAACAGCTCGACTCGGACCGGC	0	13	RDR2-sensitive
CGCAACCTAGGACTCAGCACGGTT	1	22	RDR2-sensitive
CGCAACTTGAATGGATCGAATCGG	0	11	RDR2-sensitive
CGCAAGCAGTAGATCACGGAACAG	1	46	RDR2-sensitive
CGCAAGTACGGATCACCGCAGGAC	17	0	RDR2-resistant
CGCAAGTTAGCGCTGTGTGACGGT	0	64	RDR2-sensitive
CGCAAGTTCGGATAGGGACGGCTG	0	11	RDR2-sensitive
CGCAATCGGACGGTGGAGATGGAG	0	10	RDR2-sensitive
CGCAATCGGACGGTGGGGATGGAC	0	14	RDR2-sensitive
CGCAATGATGGCTAGGAACAACAA	1	13	RDR2-sensitive
CGCACCCTAGGCTCTCGGGAGATG	39	1	RDR2-resistant
CGCACCGGACAAGGAACAGTGCAT	0	20	RDR2-sensitive
CGCACCGGACAGTGAACAGTGCAT	0	33	RDR2-sensitive
CGCACGCGTCGGTGCGTAGGCGGC	0	11	RDR2-sensitive
CGCACGGATTAGCTAGCAGGTCGT	0	93	RDR2-sensitive
CGCACGGCACGGCACTGGCACTAG	1	34	RDR2-sensitive
CGCACGTCGTCGCGTGTCGTTCGC	4	32	RDR2-sensitive
CGCACTCGACTGGACACTCGCGGC	0	20	RDR2-sensitive
CGCACTCGGCAAAGAAGGCTCGGC	3	27	RDR2-sensitive
CGCACTCGGCAAAGGGACTGGCAA	0	13	RDR2-sensitive
CGCACTGTGGAGCGGGAGTTGCAT	3	25	RDR2-sensitive
CGCACTGTGGGATGCCTTCGTCGC	0	15	RDR2-sensitive
CGCAGAAGGCCAGGTAGAGCGGTT	0	20	RDR2-sensitive
CGCAGACCATACGGCCCTAGGACC	0	10	RDR2-sensitive
CGCAGACTGTTCGCGTGTGGGCGG	0	9	RDR2-sensitive
CGCAGAGACGGACGGGACAACCGC	0	10	RDR2-sensitive

CGCAGCAGCTCGGCTCGGCTCGGA	0	11	RDR2-sensitive
CGCAGCATCTGTAGGCGGCGCCGC	0	19	RDR2-sensitive
CGCAGCCACGGACGGTAAACACAA	0	10	RDR2-sensitive
CGCAGCCGGATTGCAGCCGCAGCA	0	21	RDR2-sensitive
CGCAGCGACGCGCAGGACCTCGAC	0	9	RDR2-sensitive
CGCAGGACACTTGGACGAGCGGCT	0	10	RDR2-sensitive
CGCAGGACCAGGCCCATTTGGAAC	0	18	RDR2-sensitive
CGCAGGACGTAACATGAGAGCGGG	0	9	RDR2-sensitive
CGCAGGACTCTGCCGAGAGCGGCC	0	12	RDR2-sensitive
CGCAGGACTGACCGGCTGCATGAG	62	1	RDR2-resistant
CGCAGGACTGTGCAGGCACGGCAC	0	47	RDR2-sensitive
CGCAGGACTGTGCAGGCCCGGCCC	0	21	RDR2-sensitive
CGCAGGACTGTGCAGGCCCGGCCT	0	12	RDR2-sensitive
CGCAGGACTGTGTAGGCCCGGCCC	0	12	RDR2-sensitive
CGCAGGACTTTGCCGAGTGCGGCT	0	14	RDR2-sensitive
CGCAGGGGAAAGGAACAGGAGCAC	0	16	RDR2-sensitive
CGCAGGTTGTATGGACAGACGCAA	0	29	RDR2-sensitive
CGCAGGTTGTGGACAGTCCGTGAT	0	23	RDR2-sensitive
CGCAGTCTTCGCCGTGGAACAGGC	24	2	RDR2-resistant
CGCAGTGACAACGAACAGACGCGC	0	10	RDR2-sensitive
CGCAGTGATTGGCGCTCGGATCGG	0	9	RDR2-sensitive
CGCAGTTTTTCTAGGCTGGTCGGA	0	12	RDR2-sensitive
CGCATACCGGACAGATGACGCAGT	0	9	RDR2-sensitive
CGCATAGAACCAGGTCGTGTCGGG	0	11	RDR2-sensitive
CGCATCGGCCAGGCAGAACGGGTT	0	12	RDR2-sensitive
CGCATCTCGCCTGTGGAGCGTCGT	0	49	RDR2-sensitive
CGCATCTGAGCATGTAGACGCCAC	0	24	RDR2-sensitive
CGCATGACACGGGTCTGGACGCGT	0	9	RDR2-sensitive
CGCATGAGCCTGACTGAGATGAGC	0	10	RDR2-sensitive
CGCATGATTCGATCGGGAGCGGAT	0	9	RDR2-sensitive
CGCATGCGTCGCTGTGTTCGGCAA	0	18	RDR2-sensitive
CGCATGTGTTCGGTCTAGATGGAT	0	24	RDR2-sensitive
CGCATTGTCGGTCGTTTTAGGCAT	0	9	RDR2-sensitive
CGCATTTTGCACGGTCTGAGGCAC	0	65	RDR2-sensitive
CGCCAAAATTCGGACGACGTGCAT	0	26	RDR2-sensitive
CGCCAACCATGATGTCTTGGTGGC	26	2	RDR2-resistant
CGCCAAGACCTGGGACTTGATGGC	1	27	RDR2-sensitive
CGCCAAGACTTGGGACTTGATGGC	3	22	RDR2-sensitive
CGCCAATCACTGCGTGAGAACCGG	0	14	RDR2-sensitive
CGCCACGTTTGGAGAGCGTCGGAG	0	13	RDR2-sensitive
CGCCACTAGAAATAGCACAGTCGG	0	15	RDR2-sensitive
CGCCACTGTGCAGAATTGCGGACA	0	20	RDR2-sensitive
CGCCAGATCTGGGACGACGTGCGC	0	9	RDR2-sensitive
CGCCAGCCGTAGAGCAGAGTACGT	0	9	RDR2-sensitive
CGCCAGGTCGTGGACTCTTCGGTA	0	11	RDR2-sensitive
CGCCATAAGTCGGCAGCTGAAGGA	0	11	RDR2-sensitive
CGCCATCGCAGGACGGCAGACCAT	0	9	RDR2-sensitive
CGCCATGTGGACTATAGAGGGCTT	1	17	RDR2-sensitive
CGCCATTGCTGTAGAAGTGTGCAG	0	9	RDR2-sensitive

CGCCCACTGTGTGTTCGTTGCTTC	0	13	RDR2-sensitive
CGCCCATTTGGCCTGTGGCACGGC	0	15	RDR2-sensitive
CGCCCCTACAGCTAGGTCGCGGAC	0	10	RDR2-sensitive
CGCCCCTGCGTCGGAATTGGTGTA	30	1	RDR2-resistant
CGCCCCTGTAGATCAACGGCTGAA	8	133	RDR2-sensitive
CGCCCCTGTGCAGAGAGCACCGCC	0	11	RDR2-sensitive
CGCCCGCTTATTCTACCGTGCCGG	0	25	RDR2-sensitive
CGCCCGTAGCTCTGGCCTAGGCAT	0	14	RDR2-sensitive
CGCCCGTCTGACAGCCGTCGGCTA	0	13	RDR2-sensitive
CGCCCGTTTGGCCCGTGGCACGGC	0	9	RDR2-sensitive
CGCCCGTTTGGCCTGTGGCACGGC	0	41	RDR2-sensitive
CGCCCTATCGTCGTCGCCAGGAGA	22	0	RDR2-resistant
CGCCCTGGGGAACGACGACGGTAG	0	15	RDR2-sensitive
CGCCGACGGCTTGTCTGACAGCAT	0	9	RDR2-sensitive
CGCCGACTGCAGGGGACAAGTCAT	0	25	RDR2-sensitive
CGCCGACTGTAGGGGACAAGTCAT	0	14	RDR2-sensitive
CGCCGAGAGGCAGAACGATGGCAC	0	11	RDR2-sensitive
CGCCGAGCTCGGTACCACGTCGGC	2	23	RDR2-sensitive
CGCCGAGCTCGGTGCCACGTCGAC	0	12	RDR2-sensitive
CGCCGAGCTCGGTGCCACGTCGGC	2	30	RDR2-sensitive
CGCCGAGCTCTGACGTGGCCGCAA	1	14	RDR2-sensitive
CGCCGAGCTCTGACGTGGCGACGA	0	11	RDR2-sensitive
CGCCGAGCTCTGACGTGGCGACGG	0	11	RDR2-sensitive
CGCCGAGCTGTGACGTGGCCGCAA	0	20	RDR2-sensitive
CGCCGAGGTACGTGCTACGCTGGC	0	20	RDR2-sensitive
CGCCGAGGTACGTGCTGCGCTGGC	1	14	RDR2-sensitive
CGCCGAGGTCCTGTAGCTGGCGGG	58	3	RDR2-resistant
CGCCGATGACTGGAAGTAGGGACG	0	13	RDR2-sensitive
CGCCGCACGGAGACGCAGTAGGAC	0	9	RDR2-sensitive
CGCCGCGGCTTAGACACGACGTGG	0	13	RDR2-sensitive
CGCCGCTGCAGCTGTTAGGACGGC	0	11	RDR2-sensitive
CGCCGGAATCAGCAGGATCGACGA	0	10	RDR2-sensitive
CGCCGGAATCAGCAGGGTCGACGA	4	115	RDR2-sensitive
CGCCGGGAAGAAGAACAGCAGCAC	3	17	RDR2-sensitive
CGCCGGTGACGAACTCCAAGCGGC	0	48	RDR2-sensitive
CGCCGGTTCGTCGACAGCTAAGGC	0	22	RDR2-sensitive
CGCCGTCATGTCGTTGGATGGAAA	0	16	RDR2-sensitive
CGCCGTCGTGACAATTGAACGGGT	0	9	RDR2-sensitive
CGCCTAAGCTGAAGGATGGACGAT	47	0	RDR2-resistant
CGCCTAAGGGTGCAGCCGGACTAA	0	11	RDR2-sensitive
CGCCTACGCCTCCAAATCGTCGAA	0	26	RDR2-sensitive
CGCCTCAGCCTTAGCTCGTCGGTC	0	9	RDR2-sensitive
CGCCTCCAAATCGTCGAAGCCGCA	0	12	RDR2-sensitive
CGCCTCGTGCGGGCTTGGCGTGAA	17	1	RDR2-resistant
CGCCTCTGTGCTGTAGACGCGGTT	0	28	RDR2-sensitive
CGCCTGACCAGAGAGCACCGCCGC	1	26	RDR2-sensitive
CGCCTGCAACTGCGGTCTCGTCGT	0	15	RDR2-sensitive
CGCCTGCCCGGACCGAGGACAGAT	1	17	RDR2-sensitive
CGCCTGCTCGGACGGAGGACAGAT	9	41	RDR2-sensitive

CGCCTGCTCGGACGGAGGACGGAT	6	26	RDR2-sensitive
CGCCTGCTCGGACGGTGGACGGAT	2	18	RDR2-sensitive
CGCCTGGACTGGGGAAGATGGTGC	0	10	RDR2-sensitive
CGCCTGGCTCCCTGGAAACGGCAC	0	10	RDR2-sensitive
CGCCTGTAGATCAACGGCTGAAAA	0	9	RDR2-sensitive
CGCCTGTGGGTTGCCTGACGCGGT	0	54	RDR2-sensitive
CGCCTGTGTAGAGGCTAACCGGAC	0	16	RDR2-sensitive
CGCCTGTTTGAACTGTGGCACGGC	2	50	RDR2-sensitive
CGCCTTCTAGTGGACCTGAGGGAT	0	23	RDR2-sensitive
CGCCTTTTTTAGACGTGTCGTACT	17	0	RDR2-resistant
CGCGAAAAAGAACCCACGGCGCCC	3	24	RDR2-sensitive
CGCGAACTGTGGCAGGAGCGGGTT	0	10	RDR2-sensitive
CGCGAAGACGTGACTGCACCTGTA	0	9	RDR2-sensitive
CGCGACACAGAGCCGGACGGTCCG	0	11	RDR2-sensitive
CGCGACACAGCACGCTGACGACAC	0	9	RDR2-sensitive
CGCGACACCGTAGAGGGCACCGAC	0	18	RDR2-sensitive
CGCGACACGTGGACCAGTCAGATC	0	10	RDR2-sensitive
CGCGACAGGAAGCGATGACGACGC	0	24	RDR2-sensitive
CGCGACAGTGGAGGCATGCACGGA	0	19	RDR2-sensitive
CGCGACATAGAGCAATAGGGGGAG	25	1	RDR2-resistant
CGCGACCCACAAACAAGGACCGGC	0	10	RDR2-sensitive
CGCGACCCACAAACAGGACTGGCA	1	16	RDR2-sensitive
CGCGACCGAGATTCTGGAGGTGGC	0	10	RDR2-sensitive
CGCGACCGTGGTCGTGTTCGTCGA	0	13	RDR2-sensitive
CGCGACCTAGACGCTGGAGCGGCT	0	10	RDR2-sensitive
CGCGACCTGGGCACATGAGCGGCT	0	13	RDR2-sensitive
CGCGACCTGGTGCAGGAGCTCGGG	0	12	RDR2-sensitive
CGCGACGAATGGGACACACGGAGC	0	11	RDR2-sensitive
CGCGACGAATTCGGCAACCATCTC	0	10	RDR2-sensitive
CGCGACGAGTAGACGCAACGGCGC	0	14	RDR2-sensitive
CGCGACGCGTGTAGAAGGGCGGAC	0	9	RDR2-sensitive
CGCGACGGGGCATTGTAAGTGGCA	40	7	RDR2-resistant
CGCGACGGTAGAACCAACGCGCAT	0	9	RDR2-sensitive
CGCGACGGTGGAACCAACGCGCGT	0	14	RDR2-sensitive
CGCGACTCTGGTAGGATGGATGGA	42	0	RDR2-resistant
CGCGACTGATCAAGGCTGAGACGC	0	14	RDR2-sensitive
CGCGACTGTAGCACCGCAGTGCAT	0	11	RDR2-sensitive
CGCGACTGTGCTGGTAGACCACAC	0	9	RDR2-sensitive
CGCGACTGTGGTCGTGTTCATCGA	0	20	RDR2-sensitive
CGCGACTTAGACGTGGTGTGGCGC	0	14	RDR2-sensitive
CGCGACTTGCGACGGAGACGGGCG	16	0	RDR2-resistant
CGCGAGACACAGGCGCTGATGCAT	0	27	RDR2-sensitive
CGCGAGACAGTGTAAGATGGAGAC	1	201	RDR2-sensitive
CGCGAGAGAGTGGAGCCTAGGCAT	1	33	RDR2-sensitive
CGCGAGCATCGGTGTGGAAGACGA	0	15	RDR2-sensitive
CGCGAGCTAAACGAGCCAGCTCGA	0	14	RDR2-sensitive
CGCGAGGCGAAGGACAGTCAACGG	0	12	RDR2-sensitive
CGCGATAGTCGTCATACGTTGAGC	0	11	RDR2-sensitive
CGCGATCCGCGACGAATTCGGCAA	0	23	RDR2-sensitive

CGCGATCCGCGGACGACGAGCAC	1	12	RDR2-sensitive
CGCGCAACTCGAACACACGGTCAC	0	9	RDR2-sensitive
CGCGCAAGATGGCAATAGCTCTGT	0	15	RDR2-sensitive
CGCGCACCCTAGGCTCTCGGGAGA	165	6	RDR2-resistant
CGCGCACCGGACATGAACAGTGGC	0	10	RDR2-sensitive
CGCGCACTGTAGACGACGGCCCAA	0	20	RDR2-sensitive
CGCGCAGATTCTGGAACGTGCAGC	0	15	RDR2-sensitive
CGCGCAGCGCGTCGGCAGGGGCGC	28	2	RDR2-resistant
CGCGCATACGTACAGAGACTCCAT	0	19	RDR2-sensitive
CGCGCATGCAGAACGAGCACTCAA	0	14	RDR2-sensitive
CGCGCATGTGTAGGGACGACGAAA	0	9	RDR2-sensitive
CGCGCCGGAATCAGCAGGGTCGAC	0	41	RDR2-sensitive
CGCGCCTGTAGATCAACGGCTGAA	1	44	RDR2-sensitive
CGCGCGATGGCAGAACAGGGCGTT	0	154	RDR2-sensitive
CGCGCGATTGTGGGGCATGTTTAT	0	9	RDR2-sensitive
CGCGCGCTGTAGAAGGCACCGTCA	0	13	RDR2-sensitive
CGCGCGGAAGGCCAGGTAGAGCGG	1	76	RDR2-sensitive
CGCGCGGACTCGGCCGAACGGCGC	0	14	RDR2-sensitive
CGCGCGGATGGCAGAGGCGAACTC	0	9	RDR2-sensitive
CGCGCGGGAGAACGGGCTGACGGA	0	9	RDR2-sensitive
CGCGCGTCAGGATAAAGGTGTCAG	2	14	RDR2-sensitive
CGCGCGTGCGTAGAGACGGTGAAG	0	15	RDR2-sensitive
CGCGCTATGCTGAAGGTCGTCCGT	0	16	RDR2-sensitive
CGCGCTCACCTCAGTATCGTCGCA	14	0	RDR2-resistant
CGCGCTCTGAACCAGACGACCCGC	0	22	RDR2-sensitive
CGCGCTCTGTCATGCTCGACGGTT	0	14	RDR2-sensitive
CGCGCTGCTGTGGAGGACGAGCGC	0	11	RDR2-sensitive
CGCGCTGTACACTCCCGCGCCGGC	0	11	RDR2-sensitive
CGCGGAAACCCAGGTAGAGCGGTT	0	17	RDR2-sensitive
CGCGGAAAGCCTGCAGGACGTCGC	1	25	RDR2-sensitive
CGCGGAAAGGCTAGGTAGAGCGGC	0	12	RDR2-sensitive
CGCGGAACAGGATTCGGCCCACTA	0	14	RDR2-sensitive
CGCGGAAGGCCAGGTAGAGCGGTT	1	35	RDR2-sensitive
CGCGGACACCACTTTGCGTCGGAC	14	0	RDR2-resistant
CGCGGACAGAACTGCAGCAGACCG	0	25	RDR2-sensitive
CGCGGACAGTCCACGGCACAGGAT	0	9	RDR2-sensitive
CGCGGACATTGTCGCAGTAGACGA	0	11	RDR2-sensitive
CGCGGACCACGAAGCGAAGGTCGT	0	12	RDR2-sensitive
CGCGGACCTTGGGGCCGGCGCCCT	46	0	RDR2-resistant
CGCGGACCTTTCGGAAGTGTGCAG	0	10	RDR2-sensitive
CGCGGACGTTAGGGAAATCGCGGC	0	11	RDR2-sensitive
CGCGGACTCGCTGCGCACGGGCAA	0	9	RDR2-sensitive
CGCGGAGCCTCAGATGAAGAACGG	0	9	RDR2-sensitive
CGCGGAGCTGACGGTGTAGATTAT	0	9	RDR2-sensitive
CGCGGATAGTCCGGCACCAGACGC	0	9	RDR2-sensitive
CGCGGATGGGAATGGCAGAACCGG	0	19	RDR2-sensitive
CGCGGATTGATCGGGCGAGCGGTT	0	11	RDR2-sensitive
CGCGGCACAGGCAGAACCGTGCAC	0	12	RDR2-sensitive
CGCGGCACAGGGTCGGACGGTCCG	0	15	RDR2-sensitive

CGCGGCAGGGCGCTGGCTCCTTTC	292	8	RDR2-resistant
CGCGGCAGGTAGCTCGGCGCCAAG	0	19	RDR2-sensitive
CGCGGCAGTACGACATAGGCGCAT	0	15	RDR2-sensitive
CGCGGCCCGGACACGACAGACGAC	0	11	RDR2-sensitive
CGCGGCCCTCTGAACAGTCGACGT	0	26	RDR2-sensitive
CGCGGCCGGCAGCACATCACGGGC	0	9	RDR2-sensitive
CGCGGCCTCGTAGCTCGGCGCCAC	3	28	RDR2-sensitive
CGCGGCGCACTGGCGGAATGTGGC	0	15	RDR2-sensitive
CGCGGCGCGGTCATCAGTAGGGTA	48	3	RDR2-resistant
CGCGGCGGCGAACACGGACCATAG	14	0	RDR2-resistant
CGCGGCGTATGAGATGGACGGCAC	0	9	RDR2-sensitive
CGCGGCGTGTTGTAGTCCCGGCAC	0	19	RDR2-sensitive
CGCGGCTGGAACCTGGAAGCGGAA	0	11	RDR2-sensitive
CGCGGCTGGAACCTGGAAGCGGAG	1	30	RDR2-sensitive
CGCGGCTTAGACACGACGTGGCGT	1	187	RDR2-sensitive
CGCGGGAACGGCGTATCTGGGCGG	0	9	RDR2-sensitive
CGCGGGACCGAGGACGGGACAGGC	14	59	RDR2-sensitive
CGCGGGATTGCAGAAGCGGCGGCT	1	14	RDR2-sensitive
CGCGGGCGAGAGTGGAGGCGACAG	0	9	RDR2-sensitive
CGCGGGCGTAGAGAAATGCGCGGC	0	9	RDR2-sensitive
CGCGGGCTAGCTAACGCGGCACAG	0	10	RDR2-sensitive
CGCGGGCTGGCTAACGCGGTCCAG	0	32	RDR2-sensitive
CGCGGGCTGGCTGACGCGGTCCAG	0	11	RDR2-sensitive
CGCGGGCTGGGACAAGGCATGACA	0	35	RDR2-sensitive
CGCGGGGACGAGGACGAGACAGGC	21	49	RDR2-sensitive
CGCGGGGCGAGCATGCAGAACCGT	0	20	RDR2-sensitive
CGCGGTACCAACGGCGATACTCGA	1	32	RDR2-sensitive
CGCGGTCGCTCTGTGCCTCGGCAT	0	14	RDR2-sensitive
CGCGGTCGCTGTTACGGTAGTCCT	0	17	RDR2-sensitive
CGCGGTCTGAGTGCAGGAGCGACG	0	10	RDR2-sensitive
CGCGGTGACTTGTAGTGGCAGAGA	0	10	RDR2-sensitive
CGCGGTGCGATACTCACGACGGTT	0	11	RDR2-sensitive
CGCGGTGCTTTTGGATAGGACGGT	0	45	RDR2-sensitive
CGCGGTGCTTTTGGGTAGGACGGT	1	60	RDR2-sensitive
CGCGGTGGAAGGATGAGATGAGAT	0	17	RDR2-sensitive
CGCGGTGTAGGGATGGGCGAGCAC	0	26	RDR2-sensitive
CGCGGTGTGTGTGGAACGGCGGCG	1	33	RDR2-sensitive
CGCGGTTGGCACTGCGGTCTTTGG	15	0	RDR2-resistant
CGCGTAACTGCGTCGGCGCGTCGC	0	11	RDR2-sensitive
CGCGTAAGACTGTGTTCAGCGGTT	0	10	RDR2-sensitive
CGCGTACAACTCAGGGACAGGGCA	0	10	RDR2-sensitive
CGCGTACCCTAGGCTCTCGGGAGA	31	0	RDR2-resistant
CGCGTACCTGAAAGAAGCCGTCCA	1	14	RDR2-sensitive
CGCGTACGGCGAGCTCGGACGTGC	0	16	RDR2-sensitive
CGCGTAGAACGGCGTCGACACCAA	0	19	RDR2-sensitive
CGCGTAGAGGGCGCTGTAGGGGGC	0	11	RDR2-sensitive
CGCGTAGGACAATTCGGCTAGGCC	0	13	RDR2-sensitive
CGCGTAGGACAGTTCGGCAAGGCC	0	48	RDR2-sensitive
CGCGTAGGACAGTTCGGCTAGGCC	3	119	RDR2-sensitive

CGCGTAGGGGACGCTGTAGGGGCA	1	12	RDR2-sensitive
CGCGTAGGGGGCGCTGTAGGGGCA	0	17	RDR2-sensitive
CGCGTAGGGGGCGCTGTAGGGGGC	0	11	RDR2-sensitive
CGCGTATGGTGCGCTGTAGGAGGC	0	10	RDR2-sensitive
CGCGTCAGAGGCAGAACACGTCAG	0	37	RDR2-sensitive
CGCGTCAGAGGCAGAACGGCAGAC	0	17	RDR2-sensitive
CGCGTCGAGACGTGCATTGGTCGT	0	11	RDR2-sensitive
CGCGTCGAGCTGAGACAGTGCGGT	0	9	RDR2-sensitive
CGCGTCGAGGCGTGCATTGATCGT	2	25	RDR2-sensitive
CGCGTCGTTCGTGGAAGACCCGGT	0	9	RDR2-sensitive
CGCGTCTGGGCGAGAGGGAGGCGA	0	10	RDR2-sensitive
CGCGTGAGACTAAGGAGCGGCGGC	0	9	RDR2-sensitive
CGCGTGCATCTAGCCCGTGTCGGA	0	32	RDR2-sensitive
CGCGTGCATCTAGCCCGTGTCGGG	0	9	RDR2-sensitive
CGCGTGCCCCAGCCCAGATGGGTC	0	9	RDR2-sensitive
CGCGTGCGCAGAACAGATTAGGGT	4	53	RDR2-sensitive
CGCGTGCGTCGGTAGATATGGCAT	0	9	RDR2-sensitive
CGCGTGCTGGGCTTGGACGCCATA	0	14	RDR2-sensitive
CGCGTGGATACTGCGTGGAATCGT	0	21	RDR2-sensitive
CGCGTGGCCGCCTCTGTATTACGG	0	10	RDR2-sensitive
CGCGTGGGTGGACTGAACGACGGA	0	12	RDR2-sensitive
CGCGTGGTCTGTGTGCTTCGGTAT	0	15	RDR2-sensitive
CGCGTGTATGGACAGGCTAGGCGC	0	11	RDR2-sensitive
CGCGTGTGTGCAGGACGATGGCGT	0	34	RDR2-sensitive
CGCGTTCAGATCTGTGTGAGGCAC	1	44	RDR2-sensitive
CGCGTTCCATACCTCTGAAGCGGT	0	9	RDR2-sensitive
CGCGTTGGACATGTGTGGACGGGT	0	10	RDR2-sensitive
CGCGTTGGAGAACTAGCAGGCCGC	0	11	RDR2-sensitive
CGCGTTGGAGGAGTCGGCAGCCGC	0	13	RDR2-sensitive
CGCGTTTCGGACTCCAGAGGGTGT	0	33	RDR2-sensitive
CGCTAAAATACAGTCGGGCGTCAA	0	34	RDR2-sensitive
CGCTAAAATCCGGACGACGTGCAT	1	60	RDR2-sensitive
CGCTAAGGACACAACACTCGGCAT	0	10	RDR2-sensitive
CGCTAATCTGTAGGATAGTATGGT	0	10	RDR2-sensitive
CGCTAATGAATCTGGATGGGGCGT	0	11	RDR2-sensitive
CGCTAATGTCGGTGGCGTCTACAT	0	16	RDR2-sensitive
CGCTACAATCGACGACTGCGGCGC	0	14	RDR2-sensitive
CGCTACGGTTTCTGAGAAGGGCAA	0	10	RDR2-sensitive
CGCTAGATCTACCGGACGACGAGC	0	11	RDR2-sensitive
CGCTAGATCTACTGGACGACGAGC	0	17	RDR2-sensitive
CGCTAGCGACGAGGACTGACGCGG	0	11	RDR2-sensitive
CGCTAGCTGAAGTAGGACGAGCGA	0	21	RDR2-sensitive
CGCTAGGGTTGGACCGCGCGGCGC	0	14	RDR2-sensitive
CGCTAGTACTGTAGCACCGGTAGG	2	64	RDR2-sensitive
CGCTATACTGCATGCACTCAGCCA	0	9	RDR2-sensitive
CGCTATAGATATCGTCGGGGACTA	0	12	RDR2-sensitive
CGCTATATTTATCGGACAGTGCAT	1	18	RDR2-sensitive
CGCTATCGATATCGGATTGGTCGT	0	12	RDR2-sensitive
CGCTATCTGGCTGAGTCGTCGAGT	0	14	RDR2-sensitive

CGCTATGTGTAGAGGCTGTACGGC	0	11	RDR2-sensitive
CGCTCAACGGCAGAACAGATGGCT	0	10	RDR2-sensitive
CGCTCACGCGAGAACTCGTCCGGC	0	16	RDR2-sensitive
CGCTCAGGCTGTCAGGAGATGCAT	0	66	RDR2-sensitive
CGCTCATCCCGGACGTTGCGGCAA	0	9	RDR2-sensitive
CGCTCCACCAACTAAGAACGGCCA	69	3	RDR2-resistant
CGCTCCTGAAGACGTAGAGGACAG	0	20	RDR2-sensitive
CGCTCCTGCAACGAGGTCGCGGAC	0	10	RDR2-sensitive
CGCTCCTGCAGCGAGATTGCGGAC	0	14	RDR2-sensitive
CGCTCCTGCAGCGAGGTCGTGGAC	0	20	RDR2-sensitive
CGCTCCTGGGCTGCAACTAGGCGT	0	11	RDR2-sensitive
CGCTCCTGTAGCGAGGTCGTGGAC	0	11	RDR2-sensitive
CGCTCGACAAAGAAGGCTCGGCAA	0	18	RDR2-sensitive
CGCTCGACGAACTGTACATCGGCA	0	9	RDR2-sensitive
CGCTCGACTCGTGGCCAGCTCGGC	0	15	RDR2-sensitive
CGCTCGACTGCGTAGAAGAGGATT	0	12	RDR2-sensitive
CGCTCGCGCGAATACGAATTCGGA	0	10	RDR2-sensitive
CGCTCGCTCGGCTGAGATGGCGGC	39	5	RDR2-resistant
CGCTCGGCAAAGAACACTCGGCAA	1	15	RDR2-sensitive
CGCTCGGCGAACGACGGCGCTCAG	0	10	RDR2-sensitive
CGCTCGGCGAACTGTACATCGGCA	5	209	RDR2-sensitive
CGCTCGTCAGAAGGGGTCACGGAT	45	5	RDR2-resistant
CGCTCTCGGGAATTGAACGGCGGC	0	9	RDR2-sensitive
CGCTCTCGGGAATTTGCTGACGGC	5	35	RDR2-sensitive
CGCTCTGAACCAGACGACCCGCGT	0	11	RDR2-sensitive
CGCTCTGGCAGGTGGGTCTCACGC	0	10	RDR2-sensitive
CGCTCTGGGCTGTGCGTGTGCGGC	0	45	RDR2-sensitive
CGCTGAACACTCGGCACAGAACGG	1	33	RDR2-sensitive
CGCTGAATCAGTCTATGTGGCGTG	21	0	RDR2-resistant
CGCTGACATGTGGAGCCCGGCTGT	0	19	RDR2-sensitive
CGCTGACGTGTAGAGCCAGGCTAC	0	13	RDR2-sensitive
CGCTGACGTTGTCGCAGTAGACGA	8	50	RDR2-sensitive
CGCTGAGCCGGTGGTACCTGAGAT	29	0	RDR2-resistant
CGCTGAGCTCTGGCGTAAGGACGA	0	21	RDR2-sensitive
CGCTGAGTCTGTATACACGAGTGA	0	19	RDR2-sensitive
CGCTGCAGAATGGATGGTTGACAT	0	15	RDR2-sensitive
CGCTGCCGGAACCGAGGGAGACGT	1	26	RDR2-sensitive
CGCTGCCGGAGCCGAGGGAGACGT	0	19	RDR2-sensitive
CGCTGCGACTGTATAAGCAGAGGT	0	12	RDR2-sensitive
CGCTGCTGGAGAGCGTAGAGGACC	0	9	RDR2-sensitive
CGCTGCTGTACTGGGTCTTCGGAG	0	13	RDR2-sensitive
CGCTGCTGTGGACCCGGAGTAGGC	0	14	RDR2-sensitive
CGCTGCTGTGGAGGAGGGATGCAC	0	14	RDR2-sensitive
CGCTGCTTCAACACTGAGACGGGA	14	0	RDR2-resistant
CGCTGGACCTTTGCGCAGACTGCT	0	12	RDR2-sensitive
CGCTGGACGCAGGAAGCAGAACGG	0	43	RDR2-sensitive
CGCTGGAGCAGCTCGAGGACGCGC	0	13	RDR2-sensitive
CGCTGGAGCTGGAGCTGGAGCCGG	1	13	RDR2-sensitive
CGCTGGCGACGACAGAACCGTGCA	0	9	RDR2-sensitive

CGCTGGCGGCCCAGCCTGAACGGC	0	9	RDR2-sensitive
CGCTGGGACAGAGATCAGGCACAA	0	10	RDR2-sensitive
CGCTGTACACTCCCGCGCCGGCAG	0	16	RDR2-sensitive
CGCTGTAGAAAACCCGCTAGACGA	0	11	RDR2-sensitive
CGCTGTAGACAGAAGATGGGCGAG	0	25	RDR2-sensitive
CGCTGTAGAGGGCACCGCTGCGGC	0	24	RDR2-sensitive
CGCTGTAGGAGGCACCGCTGCGGC	0	14	RDR2-sensitive
CGCTGTAGGGGCACCGCTGCGGCC	0	10	RDR2-sensitive
CGCTGTAGGGGGCACCGCTGCGGC	5	49	RDR2-sensitive
CGCTGTCGGGCGGCCACCAAGACA	87	4	RDR2-resistant
CGCTGTCTTCTCTGTGAGCGTGCA	0	14	RDR2-sensitive
CGCTGTTCGTCGAGTGTTTGGCAC	0	13	RDR2-sensitive
CGCTGTTGACTCGGGCCGTGCCGG	0	15	RDR2-sensitive
CGCTGTTGACTCGGGTCGTGCCGG	0	15	RDR2-sensitive
CGCTGTTGCATCGGGCTGTGCCGG	0	11	RDR2-sensitive
CGCTTACGCCTCCAAATCGTCGAA	0	10	RDR2-sensitive
CGCTTAGAAGTTATGGACGGCGTG	20	1	RDR2-resistant
CGCTTATTCGTATCGGACCGGCCC	0	17	RDR2-sensitive
CGCTTATTCGTGTAGGGCCGGCCT	0	15	RDR2-sensitive
CGCTTATTCGTGTCGGGCCGGCCC	2	14	RDR2-sensitive
CGCTTATTCGTGTCGGGCCGGCCT	0	9	RDR2-sensitive
CGCTTCCCCTGTTGAGGGCGTCGG	0	11	RDR2-sensitive
CGCTTCCCTCACCCGCGTGGACGC	2	26	RDR2-sensitive
CGCTTCCTGCGGACTGTGGGCGGT	1	13	RDR2-sensitive
CGCTTCGCCTGGACGCAGGGACGC	0	17	RDR2-sensitive
CGCTTCTAGGGTTGGATCGCACGG	0	11	RDR2-sensitive
CGCTTCTCGGGCACCGTAGGCGGC	1	27	RDR2-sensitive
CGCTTCTCGGGCACCGTAGGCGGG	0	9	RDR2-sensitive
CGCTTCTGTCGCAAGCTCGTTGGT	0	9	RDR2-sensitive
CGCTTGCGATCTCAGACTTGGCAT	0	9	RDR2-sensitive
CGCTTGGCAAAGAGGAGGACTCAG	1	12	RDR2-sensitive
CGCTTGGCCTCGCTGACGTCGGTT	26	2	RDR2-resistant
CGCTTGTAGAGCCTGGATCAGGAT	0	16	RDR2-sensitive
CGCTTGTATCCGGCGGACTAGGCT	0	13	RDR2-sensitive
CGCTTTAGGGGAACTGTTGGAGAC	0	17	RDR2-sensitive
CGGAAAACACGTAGGGGCAGGTCC	26	0	RDR2-resistant
CGGAAAACGGTAGACGGACGGTGA	0	20	RDR2-sensitive
CGGAAAAGGACACCGCGAAGACAT	0	20	RDR2-sensitive
CGGAAACAGTAGTCCCGGACGGAT	1	14	RDR2-sensitive
CGGAAACAGTGTAGGCCGTCGGAA	3	78	RDR2-sensitive
CGGAAACCTCTAGAGCATGGCGAA	0	15	RDR2-sensitive
CGGAAACGGAAACAGGAACGGGAT	0	19	RDR2-sensitive
CGGAAACGGGAGCGGGATAAACGG	6	35	RDR2-sensitive
CGGAAACGGTAACTCCGGAAACGG	0	34	RDR2-sensitive
CGGAAAGACTACAGTAGCGACGAT	0	9	RDR2-sensitive
CGGAAAGGAGACAGCGCTTGGAAC	0	12	RDR2-sensitive
CGGAAATAAGTCCTGAACCGTCGG	0	11	RDR2-sensitive
CGGAAATAGAGCTATTTTCGACGG	1	33	RDR2-sensitive
CGGAAATAGCGTTATTTCCGTCGG	1	13	RDR2-sensitive

CGGAACAACTACGGTCGGAAACGG	0	28	RDR2-sensitive
CGGAACACGAACAGCGAGAGGAAC	0	9	RDR2-sensitive
CGGAACAGGATGGACGCGGTGGAC	14	0	RDR2-resistant
CGGAACAGTCGGAAAGGCAGTAGT	4	20	RDR2-sensitive
CGGAACCCAACGGCCTGGTGACGT	0	13	RDR2-sensitive
CGGAACCCGGACTGTTCGGCCCAT	0	20	RDR2-sensitive
CGGAACCGTAGGCGGCCTTAGCAG	0	11	RDR2-sensitive
CGGAACGAAGGCAGAACCGTGCAC	0	14	RDR2-sensitive
CGGAACGATTCCGAGCCGGATTGC	0	52	RDR2-sensitive
CGGAACGATTCCTGACCGGATTGC	0	9	RDR2-sensitive
CGGAACGATTTCTGACCGGATTGC	0	16	RDR2-sensitive
CGGAACTTGTTCGGCATAGATCCA	0	16	RDR2-sensitive
CGGAAGAACTCACCTTCGACGGTC	0	14	RDR2-sensitive
CGGAAGACACATCTACGAGCCTGC	0	11	RDR2-sensitive
CGGAAGACGACGAAACTCCGCCTC	3	22	RDR2-sensitive
CGGAAGACGGCGAACTCCGCTCCA	2	28	RDR2-sensitive
CGGAAGACGGCGAACTCCGCTCCC	0	10	RDR2-sensitive
CGGAAGACGGCGAACTCCGCTCCG	21	295	RDR2-sensitive
CGGAAGATGCAACGGCGATCTGAT	79	0	RDR2-resistant
CGGAAGCACGTAGAGACGAGCCGC	1	18	RDR2-sensitive
CGGAAGCACTCGGCGAAGCCTTAA	0	21	RDR2-sensitive
CGGAAGCAGCAGAGTCAACGACGT	0	13	RDR2-sensitive
CGGAAGCGCGGTGACGAAGCTCAA	0	13	RDR2-sensitive
CGGAAGGAGGCTAGTAGGAACGGT	0	17	RDR2-sensitive
CGGAAGGAGGTAGAGCTGTACGGG	0	27	RDR2-sensitive
CGGAAGGCAGAACAAGCAGAGACA	0	9	RDR2-sensitive
CGGAAGGCAGAACAAGCAGAGGCA	1	83	RDR2-sensitive
CGGAAGGCCAGGTAGAGCGGTTGG	0	18	RDR2-sensitive
CGGAAGGCTGTGGATGATCCGGTA	0	17	RDR2-sensitive
CGGAAGTACGCTGGGATGAGCATC	0	12	RDR2-sensitive
CGGAAGTATGGAAACGGTGGGCGG	0	20	RDR2-sensitive
CGGAAGTCTCTCGAGGTCGGCTGC	4	23	RDR2-sensitive
CGGAAGTGTGGCGCGGCGTGGGGA	0	11	RDR2-sensitive
CGGAAGTTAGACTGCGGACCGGGC	0	10	RDR2-sensitive
CGGAATAAGCAGGGTCGACGACGT	1	31	RDR2-sensitive
CGGAATAGTAGACGGGGATAGGAC	1	12	RDR2-sensitive
CGGAATAGTCGGGAACGGAAACGG	0	100	RDR2-sensitive
CGGAATCAGCAGAGTCGACGACGT	1	29	RDR2-sensitive
CGGAATCAGCAGGATCGACGACGT	0	13	RDR2-sensitive
CGGAATCAGCAGGCTCGACGACAT	0	15	RDR2-sensitive
CGGAATCAGCAGGGTCGACGACGT	3	76	RDR2-sensitive
CGGAATCAGCAGGGTCGACGAGGT	0	10	RDR2-sensitive
CGGAATCAGCAGGGTCGACGATGT	0	30	RDR2-sensitive
CGGAATCAGTAGGGTCGACGACGT	0	11	RDR2-sensitive
CGGAATCAGTCGGGGAGCCGGACA	0	9	RDR2-sensitive
CGGAATCGGGAGCTCCAAGTGGGC	31	0	RDR2-resistant
CGGAATCGGTATCGGAATCGGCGT	3	20	RDR2-sensitive
CGGAATGATTCCGAGCCGGATTGT	0	12	RDR2-sensitive
CGGAATGCTGTAACTCCACCGGCT	0	15	RDR2-sensitive

CGGAATGGACAGAACAGGGATGGC	0	9	RDR2-sensitive
CGGAATGGCAGAACGCGGAATGGC	0	9	RDR2-sensitive
CGGAATGGGAATGGCAGAACACGG	0	16	RDR2-sensitive
CGGAATTGGAGAGTGGAGGCGTGT	0	20	RDR2-sensitive
CGGACAACACGTGACTACGTCTCC	0	9	RDR2-sensitive
CGGACAACCTGAACCTGAGACCAT	0	13	RDR2-sensitive
CGGACAAGAAACTAGGCACGGTGG	0	12	RDR2-sensitive
CGGACAATGGCGACGTGCAAACAG	1	15	RDR2-sensitive
CGGACACAGAATCAAGCATGGCGT	2	36	RDR2-sensitive
CGGACACATCACCGGCTCGCCCAA	0	9	RDR2-sensitive
CGGACACATCTCCGGCTCGCCCGA	4	17	RDR2-sensitive
CGGACACCTGACTCCGGGCGGCTT	0	10	RDR2-sensitive
CGGACACTCGGCATAGGTAGACAC	1	13	RDR2-sensitive
CGGACACTGTCCGGTGTGCACCGG	1	18	RDR2-sensitive
CGGACAGAACAGTCAGCCCGTCGG	0	12	RDR2-sensitive
CGGACAGAACTGCAGCAGACCAGT	14	0	RDR2-resistant
CGGACAGAACTGCAGCAGACCGGT	1	23	RDR2-sensitive
CGGACAGAGAATCAAGCACAGCGT	0	10	RDR2-sensitive
CGGACAGGAAACTAAGCACGACAG	0	14	RDR2-sensitive
CGGACAGGAAACTAAGCCCGGCAC	0	9	RDR2-sensitive
CGGACAGGAAACTAGGCACGGTGG	0	19	RDR2-sensitive
CGGACAGGAAATTAGGCACGGTGG	0	10	RDR2-sensitive
CGGACAGGGCACCGGACGGTCCGG	24	1	RDR2-resistant
CGGACAGTCCGGTGCGCCAGCCAC	0	9	RDR2-sensitive
CGGACAGTGAACAGTGCGCGGGCA	0	9	RDR2-sensitive
CGGACATAACAGAAGTGGGCTCAG	0	10	RDR2-sensitive
CGGACATAACCTACCTCCGACGGT	0	10	RDR2-sensitive
CGGACATAACCTATCTCCGACGGT	0	22	RDR2-sensitive
CGGACATAAGCATAGGGCCGTCGG	0	10	RDR2-sensitive
CGGACATAAGCGCAGGCCGTCGGA	0	10	RDR2-sensitive
CGGACATAAGCTATCTCCGACGGC	0	11	RDR2-sensitive
CGGACATAAGGCTACCTCCGACGG	0	9	RDR2-sensitive
CGGACATAGAGTTATATCGGATGG	0	14	RDR2-sensitive
CGGACATAGCCTATCTCCGACGGC	0	9	RDR2-sensitive
CGGACATGTCCGGTGCGCCAACGG	2	15	RDR2-sensitive
CGGACCAAGAATCCACGATGTCGC	0	9	RDR2-sensitive
CGGACCAGCGACTGCAGCGAAGGC	1	40	RDR2-sensitive
CGGACCAGCGACTGCGACGAAGGC	0	18	RDR2-sensitive
CGGACCAGCGGCTGCAACGAAGGC	0	10	RDR2-sensitive
CGGACCAGCGGCTGCAGCGAAGGC	2	20	RDR2-sensitive
CGGACCAGCTGAAGGGTGACGCGT	0	21	RDR2-sensitive
CGGACCAGGTCCAGACGTCTATAA	0	28	RDR2-sensitive
CGGACCATGTCTGGCTTAGATCGG	0	15	RDR2-sensitive
CGGACCCAAAACACCGACAGACGC	0	12	RDR2-sensitive
CGGACCCAGGAACGTGCGGATCCC	0	10	RDR2-sensitive
CGGACCCCTGTACATGAAGAACGG	0	18	RDR2-sensitive
CGGACCCGGGTAAGGAGGAGGATA	17	0	RDR2-resistant
CGGACCGCGCTCTGAACCAGACGA	0	11	RDR2-sensitive
CGGACCGGACCCAAAAAGCGGGCT	1	15	RDR2-sensitive

CGGACCGGACTACCCGACGGACTC	0	12	RDR2-sensitive
CGGACCGTAGGCCTAGATACGGCC	0	11	RDR2-sensitive
CGGACCGTCCGAGCATCATCTCAG	0	11	RDR2-sensitive
CGGACCGTCTGACCTCAGGGCCGG	0	9	RDR2-sensitive
CGGACCGTGCCGGGCTCGAATCGG	0	10	RDR2-sensitive
CGGACCGTGCTGGCTCAAGACCGG	0	12	RDR2-sensitive
CGGACCTTGGATCTAACGGACGGC	0	11	RDR2-sensitive
CGGACCTTGGGGCCGGCGCCCTTC	18	0	RDR2-resistant
CGGACCTTGTTGGAGATAGCCTAA	0	13	RDR2-sensitive
CGGACGAACACCGCGGCACCGGGC	0	9	RDR2-sensitive
CGGACGAACGGGCCGTACCGGCAC	0	24	RDR2-sensitive
CGGACGAAGACGTACTACATGCGT	0	9	RDR2-sensitive
CGGACGAATACGGGGAGGAGACGC	0	9	RDR2-sensitive
CGGACGAATACGGGGAGGAGGCGC	0	9	RDR2-sensitive
CGGACGACCTCATGCAGGAGCAGC	0	13	RDR2-sensitive
CGGACGAGAGGAGGCTAGGAACAG	0	9	RDR2-sensitive
CGGACGAGATGGATCTGTGACGGT	0	15	RDR2-sensitive
CGGACGATCTACGATGGCGATGGA	0	10	RDR2-sensitive
CGGACGCACGCTCTTGGCGATGGC	0	18	RDR2-sensitive
CGGACGCGGATAGAAGGTAACCAG	0	9	RDR2-sensitive
CGGACGCTACAGGTAGAGGCACAA	0	16	RDR2-sensitive
CGGACGGAGGACAGATCGACGCGC	4	44	RDR2-sensitive
CGGACGGAGGTAGAGCTGTACGGG	0	10	RDR2-sensitive
CGGACGGCGGACTCGCAGAGTCGC	0	14	RDR2-sensitive
CGGACGGCTAAGCCCACACTGGAG	0	11	RDR2-sensitive
CGGACGGCTACGATCGCAGAACAG	1	37	RDR2-sensitive
CGGACGGGATTCGACGAAATGGAG	0	12	RDR2-sensitive
CGGACGGGCCTGACTGCGGATGAT	0	9	RDR2-sensitive
CGGACGGTAAAACACGGACGGGCT	1	65	RDR2-sensitive
CGGACGGTCCGCATACAACGACGG	0	11	RDR2-sensitive
CGGACGGTCCGCGCATGCGCAGAG	2	15	RDR2-sensitive
CGGACGGTCCGCGCGTGCGCAGAG	1	16	RDR2-sensitive
CGGACGGTCCGCTGTCTGGACGGT	0	9	RDR2-sensitive
CGGACGGTCTGACCTGTGGATGGT	0	9	RDR2-sensitive
CGGACGTACATAGAGAAGGAGCCA	0	10	RDR2-sensitive
CGGACGTACCTGAAGCCAGACGGT	1	12	RDR2-sensitive
CGGACGTTGTGCCAGTCGCATGGA	23	1	RDR2-resistant
CGGACGTTGTTGGGCGGAGACCGG	0	33	RDR2-sensitive
CGGACTACGCTCTGGGCTGGACGG	3	34	RDR2-sensitive
CGGACTAGCACGGCCCGAAGTCGA	0	10	RDR2-sensitive
CGGACTAGCATTGTTCGAAGGCGT	0	9	RDR2-sensitive
CGGACTATCTGGCGTAACGCGCAG	0	16	RDR2-sensitive
CGGACTATTTTCGTCGGCCGGCCC	2	34	RDR2-sensitive
CGGACTCATCAGGGCATCCTCCAC	0	31	RDR2-sensitive
CGGACTCCTCTGGCTGGCACGCAC	0	9	RDR2-sensitive
CGGACTCGGTCAAGGCCAGACGAC	0	9	RDR2-sensitive
CGGACTCGTTCTGAACGTCGAGCT	0	11	RDR2-sensitive
CGGACTCTCGCACTCGCACGGTAA	0	9	RDR2-sensitive
CGGACTCTGTAGGGTGTGCACGGT	0	18	RDR2-sensitive

CGGACTGACGGCGCGACGGCCAAA	59	11	RDR2-resistant
CGGACTGATCTATTAGAAGGGCCC	19	0	RDR2-resistant
CGGACTGGAAGCCCGGTACGGCCT	0	10	RDR2-sensitive
CGGACTGGCGACTACAACGAAGGC	0	9	RDR2-sensitive
CGGACTGTCCGGTGAGCCAACGGT	65	97	RDR2-sensitive
CGGACTGTCCGGTGCACCATCGAA	28	3	RDR2-resistant
CGGACTGTCCGGTGCACCCGACGA	3	17	RDR2-sensitive
CGGACTGTCCGGTGCGCCAGGCGA	4	21	RDR2-sensitive
CGGACTGTCCGGTGCGCCCATCGA	0	20	RDR2-sensitive
CGGACTGTCCGGTGCGCCCATCGC	2	17	RDR2-sensitive
CGGACTGTCCGGTGCGCCCGACGA	4	19	RDR2-sensitive
CGGACTGTCCGGTGCGCCCGTCGA	0	14	RDR2-sensitive
CGGACTGTCCGGTGTGCCACCGAA	4	20	RDR2-sensitive
CGGACTGTCCGGTGTGCCAGCGGA	13	38	RDR2-sensitive
CGGACTGTCCGGTGTGCCCATCGA	2	15	RDR2-sensitive
CGGACTGTTAGGTGTACCACCCGA	0	23	RDR2-sensitive
CGGACTGTTCGTCATAGCGTGCGT	0	10	RDR2-sensitive
CGGACTTAGGTCGGTATGGTACAA	0	13	RDR2-sensitive
CGGACTTCGGAGTGTGTACGCGGA	0	13	RDR2-sensitive
CGGAGAACAAGACCGGCGTCCCAT	2	17	RDR2-sensitive
CGGAGAAGACCAAGGACGAAACAG	0	17	RDR2-sensitive
CGGAGAAGACCAAGGATGAGGCAC	0	14	RDR2-sensitive
CGGAGAAGCCCTCTTGATCTACGT	1	12	RDR2-sensitive
CGGAGAAGGACACGACGAAGACAT	5	21	RDR2-sensitive
CGGAGAAGGACACGACGAAGACCT	0	14	RDR2-sensitive
CGGAGAAGGAGGTAGCAACAGAAA	0	9	RDR2-sensitive
CGGAGACAAAACGGGCAGGGACAC	5	28	RDR2-sensitive
CGGAGACAACATGTAGGGACCGGC	0	31	RDR2-sensitive
CGGAGACAGGACCGAGCACGACAG	0	13	RDR2-sensitive
CGGAGACAGTAGAGGCAGAACCGA	0	14	RDR2-sensitive
CGGAGACGGATACGGAGAGGAGAG	0	9	RDR2-sensitive
CGGAGACGGTGGGCCACCATGAGG	22	0	RDR2-resistant
CGGAGACGTCAAACACGGGAACGA	0	12	RDR2-sensitive
CGGAGACTGAGCCAGACGGATGGT	0	65	RDR2-sensitive
CGGAGACTGAGTCAGACGAGTGGT	0	25	RDR2-sensitive
CGGAGACTGCATGGAGTGCGCTAG	1	14	RDR2-sensitive
CGGAGACTGCATGGAGTGCGCTAT	1	48	RDR2-sensitive
CGGAGACTGCATGGAGTGTGCTAT	0	13	RDR2-sensitive
CGGAGACTGTATGGAGTGCGCTAT	0	72	RDR2-sensitive
CGGAGAGCGCACTGAGAGAGACGA	0	17	RDR2-sensitive
CGGAGAGGAGTAGAACGGTTCCAT	0	17	RDR2-sensitive
CGGAGATAAGACACTGCCGTCGGA	0	12	RDR2-sensitive
CGGAGATAAGGCTTTGCCGTCGGA	0	14	RDR2-sensitive
CGGAGATACTAGAGCCGCTGCCGG	5	32	RDR2-sensitive
CGGAGATAGGAAAGGCAGAACCAG	0	27	RDR2-sensitive
CGGAGATGGACTAGAGCGCGGTGA	0	9	RDR2-sensitive
CGGAGATGTCACGTAGGGACTGGC	0	9	RDR2-sensitive
CGGAGATTGCAGTCCACAGCCATT	15	0	RDR2-resistant
CGGAGCAGACGTAGTGCGCGGCGA	0	9	RDR2-sensitive

CGGAGCCAGCAACGTGATATTCAT	0	12	RDR2-sensitive
CGGAGCCTGACCGAGAAAGGACAC	0	38	RDR2-sensitive
CGGAGCCTGTAGTTCGGCAAGGAA	0	14	RDR2-sensitive
CGGAGCGGACTCTTGTCGGTGGGT	21	0	RDR2-resistant
CGGAGCGGCTGCAGGTTTCTCGCC	0	14	RDR2-sensitive
CGGAGCGTGACTGTGAGAGCGGGA	0	18	RDR2-sensitive
CGGAGCTCCGACACGGCAGTACGT	0	12	RDR2-sensitive
CGGAGCTCTCAGCCAGAAAATGGT	0	12	RDR2-sensitive
CGGAGCTGAGGACAAGTCGGCGTT	24	2	RDR2-resistant
CGGAGCTGTACACCGGCACGACAA	0	9	RDR2-sensitive
CGGAGCTGTTGACCAGGTGGGGCC	16	0	RDR2-resistant
CGGAGCTGTTTACCAGGACGTCGT	0	9	RDR2-sensitive
CGGAGCTTTGCGACCTGATAGCGG	31	2	RDR2-resistant
CGGAGGAATGTTTTTATAGCGGCG	18	0	RDR2-resistant
CGGAGGACCAGAGCAGCAGACAGC	0	9	RDR2-sensitive
CGGAGGACCTGCCGGCTTGACGAA	3	28	RDR2-sensitive
CGGAGGACCTGCCGGCTTGACGGA	6	77	RDR2-sensitive
CGGAGGACCTGTAGAGACGGAGCG	0	10	RDR2-sensitive
CGGAGGACTGGACAGAGGTGGATG	86	9	RDR2-resistant
CGGAGGACTTGCCGGCGTGATGGA	0	10	RDR2-sensitive
CGGAGGAGGACCTCAAGAAGTCGT	0	10	RDR2-sensitive
CGGAGGATATGATGTGTAGTCGGG	25	0	RDR2-resistant
CGGAGGCAAAGCGGGCAGGGACAC	6	21	RDR2-sensitive
CGGAGGCAGAACACAAAAAGGCAG	0	9	RDR2-sensitive
CGGAGGCAGAACAGCCGTTGGCAG	0	15	RDR2-sensitive
CGGAGGCAGAACGGCAGAACGGGT	2	17	RDR2-sensitive
CGGAGGCAGAACGGTCAGAGACAG	0	19	RDR2-sensitive
CGGAGGCAGACCAAAACGTAGGCA	22	0	RDR2-resistant
CGGAGGCATCACGTAGGGACTAGC	1	12	RDR2-sensitive
CGGAGGCTGAGTCAGACGTGTGGT	0	9	RDR2-sensitive
CGGAGGCTTGGTGGAAAAGTGCAC	0	10	RDR2-sensitive
CGGAGGGACGGCAGAACCGTGCGC	0	12	RDR2-sensitive
CGGAGGGACTCAAGAACGCCGGAT	31	3	RDR2-resistant
CGGAGGGCAGAACTGTACGCGCGC	0	53	RDR2-sensitive
CGGAGGGGACGACGCCACGATGGC	62	6	RDR2-resistant
CGGAGGTAGAACCACCGGCGCTGC	1	12	RDR2-sensitive
CGGAGGTGTGGGCATAAGAGGAAG	0	23	RDR2-sensitive
CGGAGTAGACTGGAAACGAGCCGA	0	29	RDR2-sensitive
CGGAGTAGCTGACGTGGACGGTGA	0	13	RDR2-sensitive
CGGAGTCAGATCTGGGAATGCGGG	0	14	RDR2-sensitive
CGGAGTCATCACGTAGGGACTGGC	0	13	RDR2-sensitive
CGGAGTCATGTGGACGGAGTCGAG	0	9	RDR2-sensitive
CGGAGTGACTGGAATCGCTGTGAC	0	11	RDR2-sensitive
CGGAGTGATTTTTAGAGACAGCGT	0	20	RDR2-sensitive
CGGAGTGATTTTTGGGGACAGCGT	0	9	RDR2-sensitive
CGGAGTGGACTGTGTAAGCGGATG	0	25	RDR2-sensitive
CGGAGTTAGACGAGTGGTGGATGT	0	12	RDR2-sensitive
CGGAGTTAGACGGGTGACAGACAA	0	40	RDR2-sensitive
CGGAGTTAGACGGGTGATAGACAA	0	38	RDR2-sensitive

CGGAGTTGGGAATGGGAATGGCAG	0	11	RDR2-sensitive
CGGAGTTGTCGCCGGAGTTGGAAC	0	27	RDR2-sensitive
CGGATAAACACTAGAGGCGCGGAT	0	9	RDR2-sensitive
CGGATAAGCAGAACCAGGCTCAAC	0	40	RDR2-sensitive
CGGATACAGGTAGGATACGGAATC	0	9	RDR2-sensitive
CGGATACGGATACAGGGACCGGAT	2	27	RDR2-sensitive
CGGATACGGATACTGATTCGGGAC	1	65	RDR2-sensitive
CGGATACGGGTACCCAGAATTCGG	0	9	RDR2-sensitive
CGGATACGGGTACGAAAACGGGAC	0	11	RDR2-sensitive
CGGATACGGGTACTGATTCGGGAC	1	37	RDR2-sensitive
CGGATACGGGTAGGATACGGAATC	0	69	RDR2-sensitive
CGGATACGGGTAGGATACGGGATG	0	19	RDR2-sensitive
CGGATACGGGTAGGATACGTGATA	0	9	RDR2-sensitive
CGGATACGGGTAGTTAAGAATCGG	0	9	RDR2-sensitive
CGGATACGGGTAGTTAAGATTCGG	0	10	RDR2-sensitive
CGGATACTTTTGGGGCACCTGGCA	17	1	RDR2-resistant
CGGATAGAGAATCAAGCACGGCGT	0	14	RDR2-sensitive
CGGATAGGAACTAAGCACGGTGAG	0	12	RDR2-sensitive
CGGATAGGGGCATTCGAGGCGGAT	362	14	RDR2-resistant
CGGATAGGGGCATTCGAGGCGGGT	24	0	RDR2-resistant
CGGATAGTATTTTGATGTAGTCGG	0	14	RDR2-sensitive
CGGATAGTTAGAAACGGGACGGAT	2	281	RDR2-sensitive
CGGATAGTTAGAAACGGGACGGCT	0	10	RDR2-sensitive
CGGATAGTTAGAAACGGGGCGGAT	0	17	RDR2-sensitive
CGGATAGTTGGAAACGGGACGGAT	0	11	RDR2-sensitive
CGGATATAACTCCCGAGGACGACC	0	45	RDR2-sensitive
CGGATATAGTTGGTGGACGGAGGC	0	9	RDR2-sensitive
CGGATATCTTATCTGAATAGTCGT	0	10	RDR2-sensitive
CGGATATGACTCGGCGAGCCCGGC	0	10	RDR2-sensitive
CGGATATGACTGGACGGAACCCGG	0	9	RDR2-sensitive
CGGATATTCGGGTCGGATACGGGT	2	21	RDR2-sensitive
CGGATCAAGCTCAGCCGGATACGG	0	9	RDR2-sensitive
CGGATCACTCTGGATGAAGCGAAC	0	9	RDR2-sensitive
CGGATCAGGTCTAGACGTCTATAA	0	9	RDR2-sensitive
CGGATCATCAGGCAGGAACACCAA	0	18	RDR2-sensitive
CGGATCATGGAGCGTTTAGCACGG	0	16	RDR2-sensitive
CGGATCCCTCTGGATAGATCGCGT	0	19	RDR2-sensitive
CGGATCCGCATAGAACCGTGTCGT	0	19	RDR2-sensitive
CGGATCCGGAGGGCAGTATACCAG	0	13	RDR2-sensitive
CGGATCGACTCCCCTGCAGGGCTG	20	0	RDR2-resistant
CGGATCGAGCTCAGCCAGATACGG	1	63	RDR2-sensitive
CGGATCGATAGAAGGCAGAACCGT	0	9	RDR2-sensitive
CGGATCGATATCGCCTAGGGACGC	0	10	RDR2-sensitive
CGGATCGGAGGATGTCACGCGCGA	0	10	RDR2-sensitive
CGGATCGGATACGCATCGAAACGG	0	39	RDR2-sensitive
CGGATCGGATACGCATGGAAACAG	0	13	RDR2-sensitive
CGGATCGGATACGCATGGAAACGG	0	58	RDR2-sensitive
CGGATCGGGCTTGCCGAGCGGCTC	2	17	RDR2-sensitive
CGGATCGTAGGCGCAGAGGCTGGT	0	9	RDR2-sensitive

CGGATCTAGACGACGTAGAGTCAA	1	13	RDR2-sensitive
CGGATCTGAGGATGCCGTCGACGG	0	11	RDR2-sensitive
CGGATCTGCGGCGCTCGGAGCCGG	1	14	RDR2-sensitive
CGGATCTGGGCCTCTGCGACACGG	0	10	RDR2-sensitive
CGGATCTGGGCTGTGGCGCAGAGA	0	15	RDR2-sensitive
CGGATCTGGGGAAGACGGGGGCGC	0	18	RDR2-sensitive
CGGATCTTGGCAACAACACGGACA	0	12	RDR2-sensitive
CGGATGAACGCACAGTAACGGGTT	0	10	RDR2-sensitive
CGGATGACCGAGGCATGCACTGAA	0	13	RDR2-sensitive
CGGATGACTCCAGAAGCAGTCCAA	0	20	RDR2-sensitive
CGGATGACTCTGGAAGCAGTCCAA	1	16	RDR2-sensitive
CGGATGCAGAGCCCTCCTCGGCGT	0	10	RDR2-sensitive
CGGATGCATGAGCCAGGTCGACGG	0	19	RDR2-sensitive
CGGATGCCGCAGGAGCTCGGCGAG	0	9	RDR2-sensitive
CGGATGGAACTAGCAGAATGGCAG	0	10	RDR2-sensitive
CGGATGGCATTGAAGGGGACTGGT	0	10	RDR2-sensitive
CGGATGGGGGCTGGCGACGCGCAC	41	3	RDR2-resistant
CGGATGGTCTGGCCCTGAGGACGG	0	9	RDR2-sensitive
CGGATGTACCGAGCCAACAAAGAC	0	21	RDR2-sensitive
CGGATGTACGGAGCCAACAAAGAC	0	18	RDR2-sensitive
CGGATGTAGAGACGACGTGAGTAT	23	2	RDR2-resistant
CGGATGTAGGGACGTGCAGCCCAA	0	12	RDR2-sensitive
CGGATGTCCTGCCGATCTCGGAGC	0	14	RDR2-sensitive
CGGATGTGAAGGCCTACACTACTA	0	9	RDR2-sensitive
CGGATGTTACTCAAGCGTCGACAC	0	9	RDR2-sensitive
CGGATGTTGTTGGACGCGTACCGA	0	21	RDR2-sensitive
CGGATTAGAACCGTGCCGGTCCGG	0	11	RDR2-sensitive
CGGATTAGCACGGCCTGAAGACAG	2	45	RDR2-sensitive
CGGATTAGTAGGCTAGATGGTCGT	0	17	RDR2-sensitive
CGGATTATGCTAGACTGGAGGCAC	3	112	RDR2-sensitive
CGGATTCATTGACTCGTCGTCGGT	31	3	RDR2-resistant
CGGATTCGGCGAGGGCCTCGGCGT	3	22	RDR2-sensitive
CGGATTCGTAGAGCCTAAAGGCGT	0	19	RDR2-sensitive
CGGATTCGTAGAGCCTGAAGACGT	0	10	RDR2-sensitive
CGGATTCGTAGAGCCTGAAGGCGT	1	29	RDR2-sensitive
CGGATTGACGGAGAGTTGATGCAG	2	24	RDR2-sensitive
CGGATTGAGACCATGCCGGTTCAG	0	10	RDR2-sensitive
CGGATTGCACCCGGAATCGTTCCA	0	14	RDR2-sensitive
CGGATTGCACTGTCTGGCCTGGGC	1	23	RDR2-sensitive
CGGATTGCAGCCGCAGCAAGCCGC	1	34	RDR2-sensitive
CGGATTGCGACGCATTTACGGCCC	0	12	RDR2-sensitive
CGGATTGGTGCAAACCCGGACGGC	4	22	RDR2-sensitive
CGGATTGGTGCAAACCCGGACGGT	0	10	RDR2-sensitive
CGGATTGTAGCCGCAGCAAGCCGC	2	51	RDR2-sensitive
CGGATTGTCCGCTCACAGGGCCGG	3	39	RDR2-sensitive
CGGATTGTGCGTACAGGGGTCCGG	0	9	RDR2-sensitive
CGGATTTCGGTAGCGGCTACCCAT	1	33	RDR2-sensitive
CGGATTTCGGTAGTAGTTGCGGGA	0	9	RDR2-sensitive
CGGATTTCTCCCTCGCAGTCGGTT	0	12	RDR2-sensitive

CGGATTTGACTGCGATGGCGGCTT	0	9	RDR2-sensitive
CGGATTTGCTCTCAACGGCTCCTA	0	9	RDR2-sensitive
CGGATTTGGCTTGTCGGGCGGCCC	0	16	RDR2-sensitive
CGGATTTGTAAGCACGAAGCCCGG	0	11	RDR2-sensitive
CGGATTTTACTGGCACGGCCCTGA	0	9	RDR2-sensitive
CGGCAAACTGTACATCGGCAACAG	0	9	RDR2-sensitive
CGGCAAAGAACACTCGGCAAAGAT	3	21	RDR2-sensitive
CGGCAAAGAACCAAGACCTGGGCA	0	10	RDR2-sensitive
CGGCAAAGAACGCGATTCCGGTAG	7	43	RDR2-sensitive
CGGCAAAGAAGACTCGGCAAACAG	0	14	RDR2-sensitive
CGGCAAAGAAGGCTCGACAAACAG	0	9	RDR2-sensitive
CGGCAAAGAAGGCTCGGCAAACAA	0	13	RDR2-sensitive
CGGCAAAGAAGGCTCGGCAAACAG	9	222	RDR2-sensitive
CGGCAAAGAAGGCTCGGCACACAG	1	16	RDR2-sensitive
CGGCAAAGAAGTCTCGGCACGCAG	0	11	RDR2-sensitive
CGGCAAAGAATCGATCGGTCGCGT	0	9	RDR2-sensitive
CGGCAAAGAGCACAGACCTGGGCA	1	24	RDR2-sensitive
CGGCAAAGAGGAACCTGACGGCGC	2	15	RDR2-sensitive
CGGCAAAGAGGACACACAATGCAT	0	29	RDR2-sensitive
CGGCAAAGAGGGCTCGACATACAG	0	15	RDR2-sensitive
CGGCAAAGCCTCGGATTCCGGTAG	0	9	RDR2-sensitive
CGGCAAAGGCTATTTTACACTCGG	2	32	RDR2-sensitive
CGGCAACGAGCCTCTTTACCGAGT	0	9	RDR2-sensitive
CGGCAACGAGCTCAGGGAAGGTGT	1	12	RDR2-sensitive
CGGCAATCGGACGGTTGAGATGGA	0	9	RDR2-sensitive
CGGCACAACTAGACTGGCAGGGGC	0	11	RDR2-sensitive
CGGCACACACAAGCTCGGCGCCAA	1	44	RDR2-sensitive
CGGCACACACAGGCTCGGCGCCAA	0	9	RDR2-sensitive
CGGCACACAGAAGCTCGGCGCCAA	2	138	RDR2-sensitive
CGGCACACAGAAGCTCGGCGCCCA	0	10	RDR2-sensitive
CGGCACAGAGAAGCTCGGCGCCAA	1	24	RDR2-sensitive
CGGCACAGATCTGGCACGACCCAA	0	19	RDR2-sensitive
CGGCACAGGACGACACTCGGCAAA	1	18	RDR2-sensitive
CGGCACAGTAGAATAAGCGGACGG	2	108	RDR2-sensitive
CGGCACAGTAGAATAAGCGGGCGG	0	10	RDR2-sensitive
CGGCACATGGAAGCTCGGCGCCAA	2	67	RDR2-sensitive
CGGCACATGGGTTAGAGAACGGCG	0	13	RDR2-sensitive
CGGCACCACTGTTGGGCTGTCGGC	0	9	RDR2-sensitive
CGGCACCGAGGAAAATGACAGCAG	0	10	RDR2-sensitive
CGGCACGAACTCGGCACGACACAT	0	9	RDR2-sensitive
CGGCACGACACGGGCACGATTCAT	0	15	RDR2-sensitive
CGGCACGACACGGGCATGATTCAT	0	9	RDR2-sensitive
CGGCACGACACTGTTAGGGCACGG	0	13	RDR2-sensitive
CGGCACGACGGCTAAGTGAACTAC	0	10	RDR2-sensitive
CGGCACGACTAACAGACTCACGGG	0	29	RDR2-sensitive
CGGCACGACTAACAGGCTCACGGA	0	19	RDR2-sensitive
CGGCACGACTAACAGGCTCACGGG	0	16	RDR2-sensitive
CGGCACGACTAGACGAAGGCACGA	0	17	RDR2-sensitive
CGGCACGACTAGCAAACTGACGGA	0	13	RDR2-sensitive

CGGCACGACTAGCAGGCTGACGGG	0	27	RDR2-sensitive
CGGCACGATAGAATAAGCGGGCAG	0	25	RDR2-sensitive
CGGCACGATAGAATAAGCGGGCGG	1	37	RDR2-sensitive
CGGCACGATTCTGGCCCGGTCCAA	0	15	RDR2-sensitive
CGGCACGCAGCACACAGAGGACGC	0	11	RDR2-sensitive
CGGCACGCCGACCCATCTGGGCAC	0	12	RDR2-sensitive
CGGCACGCTGTTGACTCGGGCCGT	0	41	RDR2-sensitive
CGGCACGCTGTTGACTCGGGTCGT	2	76	RDR2-sensitive
CGGCACGCTGTTGCATCGGGCTGT	0	23	RDR2-sensitive
CGGCACGGATATGACTGGACGGAA	0	13	RDR2-sensitive
CGGCACGGCACGGCACTGACACGA	0	23	RDR2-sensitive
CGGCACGGCACTGGCACTAGCACG	2	19	RDR2-sensitive
CGGCACGGCCTGGTTCTATGCGGG	0	13	RDR2-sensitive
CGGCACGGGTACGACTGGGCACGG	0	18	RDR2-sensitive
CGGCACGGTAAATTGGGCTGGCAC	0	9	RDR2-sensitive
CGGCACGGTACGAACAAGCGGGCG	0	12	RDR2-sensitive
CGGCACGGTAGAATAAACGGGCGG	0	127	RDR2-sensitive
CGGCACGGTAGAATAAGCGGACGG	5	246	RDR2-sensitive
CGGCACGGTAGAATAAGCGGGCAA	0	11	RDR2-sensitive
CGGCACGGTAGAATAAGCGGGCAG	2	127	RDR2-sensitive
CGGCACGGTAGAATAAGCGGGCCG	0	13	RDR2-sensitive
CGGCACGGTAGAATAAGCGGGCGA	0	50	RDR2-sensitive
CGGCACGGTAGAATAAGCGGGCGC	0	31	RDR2-sensitive
CGGCACGGTAGAATAAGCGGGCGG	12	1543	RDR2-sensitive
CGGCACGGTAGAATAAGCGGGCGT	0	64	RDR2-sensitive
CGGCACGGTAGAATAAGTGGACGG	1	291	RDR2-sensitive
CGGCACGGTAGAATAAGTGGGCGG	0	97	RDR2-sensitive
CGGCACGTAGTGAACCGGGTTCGT	0	57	RDR2-sensitive
CGGCACTAAAAAGCACGGCACTGG	0	14	RDR2-sensitive
CGGCACTATTAATTGTCGGGCCGT	0	18	RDR2-sensitive
CGGCACTATTTGGACCGGACCGAA	0	11	RDR2-sensitive
CGGCACTCTAGTTGAAGAAGGTTA	42	5	RDR2-resistant
CGGCACTGATAATTTGTTCGGCGT	0	26	RDR2-sensitive
CGGCACTGATACGATTGGACACGG	0	28	RDR2-sensitive
CGGCACTGCGGGCCGAGACAGCGG	1	14	RDR2-sensitive
CGGCACTGGCACGGCATGACACGT	0	22	RDR2-sensitive
CGGCACTGTAGGTCGGCTTGCCTA	0	19	RDR2-sensitive
CGGCACTGTAGGTCGGCTTGCCTG	1	25	RDR2-sensitive
CGGCACTGTAGGTCGGCTTGCCTT	0	28	RDR2-sensitive
CGGCACTGTGGGCCGAGACAGCGG	1	19	RDR2-sensitive
CGGCACTGTTTTTAGGACGGGCTA	0	26	RDR2-sensitive
CGGCAGAACGGCAGAACACGTCAG	0	13	RDR2-sensitive
CGGCAGAACGGCAGAACGCGTCAG	0	13	RDR2-sensitive
CGGCAGAACGGCAGAACGGCAGAC	0	10	RDR2-sensitive
CGGCAGAACGGCAGAACTTGTCAG	0	14	RDR2-sensitive
CGGCAGAACGGCAGACTACTCTTA	0	12	RDR2-sensitive
CGGCAGACACACGTAGCTCAGCAC	0	11	RDR2-sensitive
CGGCAGACGGGAATACTGAGTCGA	0	10	RDR2-sensitive
CGGCAGACTACGCTAGATACCTCT	0	21	RDR2-sensitive

CGGCAGACTCAGAGGTGTAGAGAA	0	13	RDR2-sensitive
CGGCAGCCTGAATCGAGGATTCGC	1	14	RDR2-sensitive
CGGCAGCGGCTCTAGTATCTCCGT	0	11	RDR2-sensitive
CGGCAGCTACGACATGATACTCCT	5	19	RDR2-sensitive
CGGCAGCTGAAGGAGAAGGGTCAC	0	11	RDR2-sensitive
CGGCAGGAGAGGTATTGAAGACAA	0	11	RDR2-sensitive
CGGCAGTAGAGTTCTCGGCTGCAG	0	9	RDR2-sensitive
CGGCAGTCTGGCTGTGCATCGCGT	0	13	RDR2-sensitive
CGGCAGTGCTCTCTGCACAGGCGC	0	16	RDR2-sensitive
CGGCAGTGCTCTCTGGTCAGGCGC	2	24	RDR2-sensitive
CGGCAGTGTCTTATCTCCGACGGT	1	13	RDR2-sensitive
CGGCATAGATCTAGCACGGCCCAA	0	9	RDR2-sensitive
CGGCATATCCAGACACTCGGCGAA	0	15	RDR2-sensitive
CGGCATATCCTGTGGTTGGCTCAG	0	9	RDR2-sensitive
CGGCATCGTAGACAGCCGTCGGAG	0	16	RDR2-sensitive
CGGCATGATACTGAACCGTGGCTT	0	9	RDR2-sensitive
CGGCATGCCGTGCCTGGACCACGA	0	18	RDR2-sensitive
CGGCATGGACACAAGCTGACGACG	0	15	RDR2-sensitive
CGGCATGGACTGCGGCAGTGCTCC	0	10	RDR2-sensitive
CGGCATGGTAGAATAAGCGGGCGG	0	30	RDR2-sensitive
CGGCATGGTGTGCCTAGATCACGG	0	12	RDR2-sensitive
CGGCATGGTTAAATGGGCTGGCAC	0	9	RDR2-sensitive
CGGCATGTAGTCGTACACGAGCAC	0	10	RDR2-sensitive
CGGCATGTAGTCGTACACGAGCAT	1	103	RDR2-sensitive
CGGCATTAACTCCAAACTAACGGT	27	1	RDR2-resistant
CGGCATTCTGACGACCTGCACGGC	0	11	RDR2-sensitive
CGGCCAGGAGTAGGACGCGAGGTA	14	0	RDR2-resistant
CGGCCATCCTGGACTCGACGTCAT	0	12	RDR2-sensitive
CGGCCATGGTGTAGAAGCGACGGC	1	16	RDR2-sensitive
CGGCCATGGTGTAGAAGCGACGGT	0	12	RDR2-sensitive
CGGCCATTGAGAGTGACGGAACAT	0	22	RDR2-sensitive
CGGCCCAACCTGAACCATTAACGG	0	15	RDR2-sensitive
CGGCCCACTTTTATACGGACTCGG	0	11	RDR2-sensitive
CGGCCCAGCGTGCCTGATGCTCGG	0	13	RDR2-sensitive
CGGCCCATGTAGAGTCGACGTGAG	0	18	RDR2-sensitive
CGGCCCGAGACCGCATAGAACCGG	0	9	RDR2-sensitive
CGGCCCGATCTGAGCCTGGCACGG	0	11	RDR2-sensitive
CGGCCCGCCAACCCTGGAATCGGT	15	0	RDR2-resistant
CGGCCCGGACACGACAGACGACAC	0	10	RDR2-sensitive
CGGCCCGGGACCGCATAGAATCAG	0	18	RDR2-sensitive
CGGCCCGGGCACGCCTCTAGTCGG	0	18	RDR2-sensitive
CGGCCCGTAGTGCCTGGGCACGGC	0	49	RDR2-sensitive
CGGCCCTGAGGGTGGACTGTTCGC	1	20	RDR2-sensitive
CGGCCGAACTCCAGATGTAGCGTC	0	9	RDR2-sensitive
CGGCCGACAGACTGAACCACCGGC	0	26	RDR2-sensitive
CGGCCGTCCACGACAGTACGCATG	0	9	RDR2-sensitive
CGGCCGTTTCTCTGGATGCAGGCT	0	21	RDR2-sensitive
CGGCCTAAAGCACGGACTGTTCGG	1	23	RDR2-sensitive
CGGCCTAGCCGTGGCCGGTCGGCA	145	0	RDR2-resistant

CGGCCTAGCCGTGGCCGGTCGGCC	19	0	RDR2-resistant
CGGCCTCGTAGCTCGGCGCCACAG	2	82	RDR2-sensitive
CGGCCTCGTAGGAAGCCGTCGGAG	0	12	RDR2-sensitive
CGGCCTGAGTGGGACGGTTGGCTC	0	12	RDR2-sensitive
CGGCCTGGCACGGCACAGACCCAT	0	9	RDR2-sensitive
CGGCCTGGGTGGGACGGTTGGCTC	4	49	RDR2-sensitive
CGGCCTGTGTGGGACGGTTGGCTC	0	10	RDR2-sensitive
CGGCCTGTTGACCGTGTCGGACAA	0	17	RDR2-sensitive
CGGCCTTGATCCCTATCTCGTCGT	0	16	RDR2-sensitive
CGGCCTTGTAGAAAGCCGTCGGAT	0	12	RDR2-sensitive
CGGCCTTGTAGGAAGCCGTCGGAG	0	9	RDR2-sensitive
CGGCGAACAGGACCCATGGAAGGC	0	20	RDR2-sensitive
CGGCGAACCGTGCAGTAGATTCAC	1	31	RDR2-sensitive
CGGCGAACTGAACATCGGCAACAT	0	14	RDR2-sensitive
CGGCGAACTGTACATCGACAACGG	0	35	RDR2-sensitive
CGGCGAACTGTACATCGGCAACAA	5	161	RDR2-sensitive
CGGCGAACTGTACATCGGCAACAG	66	1856	RDR2-sensitive
CGGCGAACTGTACATCGGCAACAT	1	34	RDR2-sensitive
CGGCGAACTGTACATCGGCAACCG	0	20	RDR2-sensitive
CGGCGAACTGTACATCGGCAACGG	0	20	RDR2-sensitive
CGGCGAACTGTACATCGGCAATAG	0	11	RDR2-sensitive
CGGCGAACTGTACATCGGCACCAG	0	9	RDR2-sensitive
CGGCGAACTGTACATCGGCCACAG	0	11	RDR2-sensitive
CGGCGAACTGTATATCGGCAACAG	0	27	RDR2-sensitive
CGGCGAAGGTCGAGGACGGAAGAC	0	10	RDR2-sensitive
CGGCGAATTCTGGGTCAGGCGGCT	0	11	RDR2-sensitive
CGGCGAATTCTGGGTGCGGCGGCT	0	10	RDR2-sensitive
CGGCGACACTCGGCAAAGACTGGT	2	18	RDR2-sensitive
CGGCGACGTACGGCTATAATTCAC	1	33	RDR2-sensitive
CGGCGACGTAGACCAAGAGGGCTT	10	33	RDR2-sensitive
CGGCGACGTAGATCAAGAGGGCTT	7	23	RDR2-sensitive
CGGCGACTCAGACCTGGAGAGCAT	0	21	RDR2-sensitive
CGGCGACTCTCGGGTTGTTCTGGG	1	25	RDR2-sensitive
CGGCGACTGCACGGCACTGCTCGA	1	43	RDR2-sensitive
CGGCGACTGCAGCGCGACGATGGT	0	9	RDR2-sensitive
CGGCGAGAACTGCTGCCTGGGCGG	80	2	RDR2-resistant
CGGCGAGGACGACGACGTCGGCGT	0	9	RDR2-sensitive
CGGCGAGTTGTAGAGCACGATGGC	0	12	RDR2-sensitive
CGGCGATGCTCTCTGCGTAGACGC	0	10	RDR2-sensitive
CGGCGATGTTGGCGGCGTCCTCGT	64	17	RDR2-resistant
CGGCGCAGGAACGTTCGACGACAT	0	11	RDR2-sensitive
CGGCGCGAGACGGAACACAAAGGG	0	11	RDR2-sensitive
CGGCGCGAGACGGAACACAAGGGG	0	10	RDR2-sensitive
CGGCGCGAGACGGAGCACGAAGGG	0	9	RDR2-sensitive
CGGCGCGAGAGGAGGATGGGTGAT	22	0	RDR2-resistant
CGGCGCGGCCTCGTAGCTCGGCGC	0	10	RDR2-sensitive
CGGCGCTCGGACGGAAGGCGCGTG	3	45	RDR2-sensitive
CGGCGCTCTGCTGGTGTGAGATGG	0	10	RDR2-sensitive
CGGCGCTGTCTGCACACACGGCAC	1	13	RDR2-sensitive

CGGCGGCAACTGGCTTATTTTCGG	2	21	RDR2-sensitive
CGGCGGCAGGCTTGAGGCAGGACC	0	26	RDR2-sensitive
CGGCGGCAGGGACAGGCTGATGCT	0	17	RDR2-sensitive
CGGCGGCGCGAGAGGAGGATGGGT	31	0	RDR2-resistant
CGGCGGCGTTCTGGAGACGAGCAA	0	9	RDR2-sensitive
CGGCGTAATCTGTACAGTCGTCGT	0	9	RDR2-sensitive
CGGCGTACACTGTTAATGGAGTCT	0	9	RDR2-sensitive
CGGCGTACGGAAGGCTGACGACGT	0	10	RDR2-sensitive
CGGCGTACGGCGGAACACGGGCGT	0	26	RDR2-sensitive
CGGCGTAGAGTCGAAGAGAGGCGA	0	9	RDR2-sensitive
CGGCGTCAGGCGTAGTTAATGCGG	0	13	RDR2-sensitive
CGGCGTCATAGACTGTAACGGCGA	0	14	RDR2-sensitive
CGGCGTCTGCGCACGGGAACGGTA	23	0	RDR2-resistant
CGGCGTCTGTACACGGGAACGGTA	124	0	RDR2-resistant
CGGCGTCTGTACACGGGAACGGTT	40	0	RDR2-resistant
CGGCGTGACTCGCGAGCGAGGATG	0	9	RDR2-sensitive
CGGCGTGACTCTAGCAGGAGCCAT	0	12	RDR2-sensitive
CGGCGTGAGTTTGAGGTCGTCGTT	0	10	RDR2-sensitive
CGGCGTGCCTGGACCATGACTGCG	0	9	RDR2-sensitive
CGGCGTGCGCTGTTAGGAGCCGGC	0	11	RDR2-sensitive
CGGCGTGCTGACACATTGGACGGA	0	9	RDR2-sensitive
CGGCGTGGAGCTCCAAGCGAGAAG	0	11	RDR2-sensitive
CGGCGTGGGAGGCAGAACCGTGCA	0	10	RDR2-sensitive
CGGCGTGTCTCTGGCGGAAGAGCT	0	17	RDR2-sensitive
CGGCGTGTGCCTGAACCGGGCCGG	1	12	RDR2-sensitive
CGGCTAATGTAGATGGGAGGTGAT	0	24	RDR2-sensitive
CGGCTAATTGTATCAGCCCGTCGG	0	10	RDR2-sensitive
CGGCTACGTACTGATTTGTTTCCT	0	12	RDR2-sensitive
CGGCTACTGTAGCCCACGAGCCGG	0	54	RDR2-sensitive
CGGCTACTGTAGCCCACGGGCCGG	0	12	RDR2-sensitive
CGGCTACTTACTCTGCATTGAGGT	0	10	RDR2-sensitive
CGGCTAGACGATAGGCTGACGGCA	0	12	RDR2-sensitive
CGGCTAGGGCTCCTGCCTGACGGC	0	12	RDR2-sensitive
CGGCTATCTGTAGTCGCGTGGTAA	0	10	RDR2-sensitive
CGGCTATTGAGAGTGACGGAACAT	0	29	RDR2-sensitive
CGGCTCCGACTCTGCTAGACGGGC	0	10	RDR2-sensitive
CGGCTCCGGCAGCGGCTCTAGTAT	0	9	RDR2-sensitive
CGGCTCCTCTGAAAACGGCTCCGA	0	9	RDR2-sensitive
CGGCTCGAACTCGTTCGGAGCGTC	0	10	RDR2-sensitive
CGGCTCGACACAGAGAAGACTCGC	0	9	RDR2-sensitive
CGGCTCGACGGCATGAATCATGGC	0	10	RDR2-sensitive
CGGCTCGACTCGAAGGTGGCTCGC	0	33	RDR2-sensitive
CGGCTCGACTTGGCTCGGAGCGGC	0	9	RDR2-sensitive
CGGCTCGATCCGGACCAAGTCTGA	0	10	RDR2-sensitive
CGGCTCGATTACTGACGGTAACAA	0	23	RDR2-sensitive
CGGCTCGCCCACGGCACTGGACAT	0	9	RDR2-sensitive
CGGCTCGGATTAGTTAGCAGGCCA	0	10	RDR2-sensitive
CGGCTCGGCTCAAAGGCGGCTCGC	0	35	RDR2-sensitive
CGGCTCGGCTCGAAGGTGGCTCGC	0	14	RDR2-sensitive

CGGCTCGGCTCGGCTCGCTGCGGC	0	15	RDR2-sensitive
CGGCTCGGCTCGGCTCGGAGCGGC	1	41	RDR2-sensitive
CGGCTCGGCTCGGCTCGGCTCGGT	0	10	RDR2-sensitive
CGGCTCGGCTCGGCTCGGTGCGGC	0	9	RDR2-sensitive
CGGCTCGGCTCGGCTCGTTAGTGT	0	13	RDR2-sensitive
CGGCTCGGCTCGGTGAGGCTCGGT	1	12	RDR2-sensitive
CGGCTCGGCTTGAAGGCGGCTCGC	0	10	RDR2-sensitive
CGGCTCGGGCTCGTTCGAAGCGTC	0	15	RDR2-sensitive
CGGCTCGTGTTTGGTAGACTGCAT	0	38	RDR2-sensitive
CGGCTCGTTAGGCAGAAGGATCGT	0	12	RDR2-sensitive
CGGCTCGTTAGTAGCTCGGCTCGA	2	32	RDR2-sensitive
CGGCTCTCGGCAAAGACTGACGGC	1	77	RDR2-sensitive
CGGCTCTCGGTAAAGACTGACGGC	0	9	RDR2-sensitive
CGGCTCTCTGTCTCGGCGACTCGG	0	12	RDR2-sensitive
CGGCTCTGAATCTGCAACGGTCGG	0	11	RDR2-sensitive
CGGCTCTGTGACGTGTGAGCGGCT	0	12	RDR2-sensitive
CGGCTGAAAGACGTACCCATGGCA	0	22	RDR2-sensitive
CGGCTGAATGGACCGAGGAGTCGT	0	19	RDR2-sensitive
CGGCTGACTCTATCAGTCCGTCGG	0	10	RDR2-sensitive
CGGCTGAGACTGTATGCGTCGAAT	0	19	RDR2-sensitive
CGGCTGAGATCGCAGAACGGCAAA	0	10	RDR2-sensitive
CGGCTGAGATCGCAGAACGGCAGA	0	11	RDR2-sensitive
CGGCTGAGCAACCCTACGAGGCAA	0	13	RDR2-sensitive
CGGCTGAGCGGACGACATCACAAC	0	11	RDR2-sensitive
CGGCTGAGCGTAGTCTGCATGCAC	0	9	RDR2-sensitive
CGGCTGCAAGAACTCGACAGACGC	0	10	RDR2-sensitive
CGGCTGCAGGAACTCGGCAGACGT	0	19	RDR2-sensitive
CGGCTGCATGAGCCAGGTCGACGG	0	12	RDR2-sensitive
CGGCTGCATGCAAGGACGGAGCGA	0	11	RDR2-sensitive
CGGCTGCCACTCTGGGCCAGCGAA	4	17	RDR2-sensitive
CGGCTGCCTAGACGACGGACGGAC	0	15	RDR2-sensitive
CGGCTGCCTTTGGATGCTGATGGA	0	15	RDR2-sensitive
CGGCTGCGGATTCACGGAAGACGT	1	20	RDR2-sensitive
CGGCTGCGGCTGCGGCTTCTACAG	0	24	RDR2-sensitive
CGGCTGCTCTGCATACGTTCAGAC	0	30	RDR2-sensitive
CGGCTGCTGTAGCGGGCGACGCAA	0	16	RDR2-sensitive
CGGCTGCTGTGGACAGAAGCTGGT	0	17	RDR2-sensitive
CGGCTGCTGTGGAGAGAAGCTGGT	0	14	RDR2-sensitive
CGGCTGGAATCACCATCGGGGCTA	36	2	RDR2-resistant
CGGCTGGACAGGGAAGAAGAGCAC	0	106	RDR2-sensitive
CGGCTGGACGACTGCATCTGTGCT	0	9	RDR2-sensitive
CGGCTGGAGAGAGAGTGCGGGCAG	0	17	RDR2-sensitive
CGGCTGGAGGGCGCTACGACCGCT	0	12	RDR2-sensitive
CGGCTGGCACTCGGCAAAGAGGCT	0	25	RDR2-sensitive
CGGCTGGCAGAACACACGGCTGGT	0	13	RDR2-sensitive
CGGCTGGGAGGAGGGCCGAGGGCG	136	3	RDR2-resistant
CGGCTGGGCGCTCGGCTTAGGCAT	41	7	RDR2-resistant
CGGCTGGGCTGTAGTGCGGCGCAC	0	11	RDR2-sensitive
CGGCTGTATAGGGGCATGTAGGCC	22	1	RDR2-resistant

CGGCTGTATGAGTCAGGTCGGCGC	0	110	RDR2-sensitive
CGGCTGTCTACGATGCCGTCGGAC	0	12	RDR2-sensitive
CGGCTGTCTAGAAGACCGTCGGAC	0	10	RDR2-sensitive
CGGCTTAAAACCGTCGGAGATAAG	0	9	RDR2-sensitive
CGGCTTAAGACGGGTCCCGTCGGA	0	10	RDR2-sensitive
CGGCTTAAGGTCGGATATGATCGT	0	13	RDR2-sensitive
CGGCTTATTAGTAGCTCGGCTCGA	1	18	RDR2-sensitive
CGGCTTATTTGTCAGCCGTCGGAC	0	9	RDR2-sensitive
CGGCTTCGTCGACGGCGCTCGCCC	0	10	RDR2-sensitive
CGGCTTGAAGACGAAGGCGTGCAT	0	14	RDR2-sensitive
CGGCTTGACTCGGACCGGCTTGAT	0	17	RDR2-sensitive
CGGCTTGCTGCCTCTGGATGGCGT	1	13	RDR2-sensitive
CGGCTTGCTGTCTCTGGATGGCGT	0	13	RDR2-sensitive
CGGCTTGGACGGAACACCGGGCGG	0	9	RDR2-sensitive
CGGCTTGGCGTAGAGAAGACTCGG	0	13	RDR2-sensitive
CGGCTTGTAGCCAGCCGAACACGC	2	38	RDR2-sensitive
CGGCTTTAGACGGTAGCTGACGAC	1	12	RDR2-sensitive
CGGGAAATAGGACAGAGACGAGTT	0	10	RDR2-sensitive
CGGGAACAGAAACGGGAGCGGGAT	0	23	RDR2-sensitive
CGGGAACAGGAACGGGAGCGGGAT	0	9	RDR2-sensitive
CGGGAACCTGAAGGCGCTCCTCAC	5	48	RDR2-sensitive
CGGGAACGAGAATGTTGGACGGAA	0	42	RDR2-sensitive
CGGGAACGGAAACGGGAGCGGGAT	2	46	RDR2-sensitive
CGGGAAGACGAACAGGACGTCCGA	0	13	RDR2-sensitive
CGGGAAGACGAACGGGACGTCCGA	0	11	RDR2-sensitive
CGGGAAGTGGGCGCATGCATGCAT	17	0	RDR2-resistant
CGGGAATAGAAACAGGAGCGAGAT	0	9	RDR2-sensitive
CGGGAATTCTGGAGCTGAGGGCGC	1	18	RDR2-sensitive
CGGGACAACTGATAAGCAAGGCCT	0	10	RDR2-sensitive
CGGGACACGTGGCCGAGCCAACGG	0	11	RDR2-sensitive
CGGGACACTGACAGATGGACCTAG	0	16	RDR2-sensitive
CGGGACAGACTGAGCTGAGGCGAC	0	10	RDR2-sensitive
CGGGACAGCCAGGACAGGGACGTC	0	26	RDR2-sensitive
CGGGACAGTCAGGACAGGGACGTC	1	29	RDR2-sensitive
CGGGACAGTTAGGACAGAGACGTC	0	10	RDR2-sensitive
CGGGACCAACTCCACTACTGGCAT	0	10	RDR2-sensitive
CGGGACCGAGGACGGGACAGGCGC	3	28	RDR2-sensitive
CGGGACCGCGTGTAGACGTGTATG	1	19	RDR2-sensitive
CGGGACCGGTTCTTACGCTTCTCC	36	4	RDR2-resistant
CGGGACGAACACGAAGGCCGCGGG	8	54	RDR2-sensitive
CGGGACGAAGGGAACTAAAAGGCG	0	15	RDR2-sensitive
CGGGACGACTGTGAAGACGACGAT	0	28	RDR2-sensitive
CGGGACGAGCCAGATGAGACGCCG	0	13	RDR2-sensitive
CGGGACGCAAGCTGTATGAGCGGA	0	9	RDR2-sensitive
CGGGACGCCTACAGAGCATGACGA	0	15	RDR2-sensitive
CGGGACGGAGATGGTGTGGGGGAC	29	0	RDR2-resistant
CGGGACGGATACAGCTAGATACAG	0	11	RDR2-sensitive
CGGGACGGCGAAGCGGACGCTCAG	1	13	RDR2-sensitive
CGGGACGGGGATGGTGTGGGGGAC	42	9	RDR2-resistant

CGGGACGTGGATGGTGTGGGGGAC	75	0	RDR2-resistant
CGGGACGTGGATGGTGTGGGGGCC	14	0	RDR2-resistant
CGGGACGTTCTAGCTCGACGCCAT	0	16	RDR2-sensitive
CGGGACTAACGACGGCAGAACGGC	0	18	RDR2-sensitive
CGGGACTCGACGGCAGCACAACAC	0	11	RDR2-sensitive
CGGGACTCGACGGCAGCGCAACAC	0	19	RDR2-sensitive
CGGGACTGATATCGGTAGGATACT	0	12	RDR2-sensitive
CGGGACTGGGCGTGCATACTGCAT	0	11	RDR2-sensitive
CGGGACTGTTGGAGCACTGGCGGC	0	16	RDR2-sensitive
CGGGAGACGCAGTCACGACTCGAC	0	9	RDR2-sensitive
CGGGAGACTGGTGAGCAACGGCAA	2	122	RDR2-sensitive
CGGGAGAGAGAACCGGTCGGCGTT	0	9	RDR2-sensitive
CGGGAGATGAGCAAGAGCTTCGGA	0	19	RDR2-sensitive
CGGGAGATTCATGCGGACCGCGGC	18	1	RDR2-resistant
CGGGAGCGGAACAACTACGGTCGG	0	34	RDR2-sensitive
CGGGAGGCTTGAACCGAAGCAGAG	0	11	RDR2-sensitive
CGGGAGTAGAAAGACGGATGACAT	0	9	RDR2-sensitive
CGGGAGTAGGGGGCTAAATTTTTT	57	0	RDR2-resistant
CGGGAGTGTACAGCGCGATGGCAG	0	9	RDR2-sensitive
CGGGAGTTACTTAATCCCCGTCGG	0	29	RDR2-sensitive
CGGGAGTTAGTTAATCCCCGTCGG	0	11	RDR2-sensitive
CGGGATCTCGGCGTAGTAGCGGAT	0	38	RDR2-sensitive
CGGGATCTGATTGACGGCTTTCCA	0	9	RDR2-sensitive
CGGGATCTGTAACCTTTTCACGGT	0	9	RDR2-sensitive
CGGGATCTGTTGAAGATGGCCTAA	0	10	RDR2-sensitive
CGGGATGAACACGAGAGGACACGA	0	10	RDR2-sensitive
CGGGATGGCAGAACCGTGCACCGT	0	9	RDR2-sensitive
CGGGATTGACCTCGAGCAAGCCGG	0	9	RDR2-sensitive
CGGGATTGACTGGCCGCGAGACAA	0	12	RDR2-sensitive
CGGGATTGCACTCGGGCGAGTCGT	1	13	RDR2-sensitive
CGGGATTTACTCTAAAACCGACGG	1	18	RDR2-sensitive
CGGGATTTGTAGTGGGCAGTAGTG	0	9	RDR2-sensitive
CGGGCAAAGGATGGCATGGCTGTA	0	10	RDR2-sensitive
CGGGCAACATCAGGAACGGGGCAT	0	9	RDR2-sensitive
CGGGCAACCCACCAAGGCGGACAG	0	25	RDR2-sensitive
CGGGCAATCTCTGCAGCAGATCGA	1	13	RDR2-sensitive
CGGGCACACAGAGCGGTAGGGGTG	80	0	RDR2-resistant
CGGGCACACGTGACGTACGCTGAT	0	16	RDR2-sensitive
CGGGCACCCTGGACCTGACGTCAT	1	17	RDR2-sensitive
CGGGCACCTAAGCAGGAACACGAC	1	14	RDR2-sensitive
CGGGCACGAATAAGTGGGTCGGAC	1	17	RDR2-sensitive
CGGGCACGACTGCAGCGACTGAAG	0	9	RDR2-sensitive
CGGGCACGCATGGAACCGGACCGT	0	10	RDR2-sensitive
CGGGCACGGCTATAGTCAATGCAC	0	25	RDR2-sensitive
CGGGCACGGGTACAACATTTTCAT	0	19	RDR2-sensitive
CGGGCACGTAGGGCGGCAAGACAT	0	11	RDR2-sensitive
CGGGCACTCCTGGCAGAACCGTGC	0	11	RDR2-sensitive
CGGGCACTCGACAAAGAAGGCTTC	0	10	RDR2-sensitive
CGGGCACTCGACAAAGAAGGCTTT	0	37	RDR2-sensitive

CGGGCACTCGGCAAACACTACTTT	0	21	RDR2-sensitive
CGGGCACTCGGCAAAGAACGGTTT	1	18	RDR2-sensitive
CGGGCACTCGGCAAAGAAGACTTT	1	25	RDR2-sensitive
CGGGCACTCGGCAAAGAAGGATTT	0	11	RDR2-sensitive
CGGGCACTCGGCAAAGAAGGCTTT	6	286	RDR2-sensitive
CGGGCACTCGGCAAAGACAGCTTT	0	21	RDR2-sensitive
CGGGCAGAACACGGTGCACTTGTG	0	19	RDR2-sensitive
CGGGCAGAACTCAACCGCAGGCGT	1	19	RDR2-sensitive
CGGGCAGAGAATCGCGTGATTGGT	0	12	RDR2-sensitive
CGGGCAGAGTCTGACGTAAGGCAT	0	10	RDR2-sensitive
CGGGCAGCCAGAACGTGACGACAA	0	9	RDR2-sensitive
CGGGCAGCGTTGGAAACGGGGCAT	0	13	RDR2-sensitive
CGGGCAGGAACAAACGATGCGGTT	0	9	RDR2-sensitive
CGGGCAGGAGCTCACGACCGGTGT	1	16	RDR2-sensitive
CGGGCAGTCTCGGACGCAGACGTG	0	9	RDR2-sensitive
CGGGCATCAACTCTGCAGGTAGAG	1	19	RDR2-sensitive
CGGGCATCGTGACGCATCATCTGG	1	54	RDR2-sensitive
CGGGCATGTTGGCACCGGCTGGCG	29	3	RDR2-resistant
CGGGCCACAACTCTGGCACGTCAT	0	22	RDR2-sensitive
CGGGCCAGAATCGTGTCGGGTCAC	0	9	RDR2-sensitive
CGGGCCAGGGACGGGGTAGAGGGC	1002	1	RDR2-resistant
CGGGCCAGGTTGGAACGGGCACGG	0	11	RDR2-sensitive
CGGGCCCAGAAGACAGAGCTCCAA	1	16	RDR2-sensitive
CGGGCCCGTAGACGATCGTGCCGG	0	10	RDR2-sensitive
CGGGCCGAATGCATGACGGGCTAT	0	47	RDR2-sensitive
CGGGCCGACAGCATGGACGAGCAG	0	11	RDR2-sensitive
CGGGCCGAGTATGGACAGATACGG	0	18	RDR2-sensitive
CGGGCCGATAGACAACGGACGGTT	0	9	RDR2-sensitive
CGGGCCGCAGAGAACAAGACCGGT	25	79	RDR2-sensitive
CGGGCCGCAGGGAACAAGACCGGT	3	28	RDR2-sensitive
CGGGCCGGGACAGCACGTCGTGAC	0	9	RDR2-sensitive
CGGGCCGGGTATGAGTAGATACGG	0	17	RDR2-sensitive
CGGGCCGGGTATGGACAGATACGG	3	27	RDR2-sensitive
CGGGCCTACATAGAACTGGGTCGT	0	21	RDR2-sensitive
CGGGCCTAGCTCCAGTCGTGTCAT	0	9	RDR2-sensitive
CGGGCCTCCAATTCTGGATGTCGG	0	9	RDR2-sensitive
CGGGCCTGTACGTGAAGCATGAGC	0	9	RDR2-sensitive
CGGGCCTTTGGCAGAACGCGTCAG	0	20	RDR2-sensitive
CGGGCGAAAGGACAATGTAGGCAC	0	15	RDR2-sensitive
CGGGCGAAGCAGGCTTCCTCTGGA	0	16	RDR2-sensitive
CGGGCGACGCACTGGCACAGACGA	0	22	RDR2-sensitive
CGGGCGACGGTAGAAGAGGAAGAC	0	13	RDR2-sensitive
CGGGCGACGTAGGGTAGGACGGGT	0	10	RDR2-sensitive
CGGGCGGAAAACTCCGAGGAGCGA	1	15	RDR2-sensitive
CGGGCGGACGGTTGTGGCGAGGGC	0	17	RDR2-sensitive
CGGGCGGATAAGTGACGGATACAG	0	14	RDR2-sensitive
CGGGCGGATACGGACGGATAGTAG	2	37	RDR2-sensitive
CGGGCGGATACGGACGGATATTAG	0	38	RDR2-sensitive
CGGGCGGATGATGATCAAGGAGGC	49	10	RDR2-resistant

CGGGCGGATTTCGGCATAAAACGT	1	18	RDR2-sensitive
CGGGCGGCCCGAATGTACACCTAT	2	14	RDR2-sensitive
CGGGCGGCGTGAGTACAATAGTGA	0	11	RDR2-sensitive
CGGGCGGCTGTTTGGCAAGTCGGT	0	9	RDR2-sensitive
CGGGCGGGTAGTACACGGATATAT	0	11	RDR2-sensitive
CGGGCGTAGAAGGAGGCATCTCGC	0	16	RDR2-sensitive
CGGGCGTGCAGACTGCAGACACGT	0	24	RDR2-sensitive
CGGGCGTGCAGACTGCATACACGT	0	62	RDR2-sensitive
CGGGCGTTCGGTAGAGCGGGGGCA	0	17	RDR2-sensitive
CGGGCGTTTCTTAGAACAGTCGGT	1	50	RDR2-sensitive
CGGGCTAAACGGCCGGTAAGCAC	1	14	RDR2-sensitive
CGGGCTAAACGGGTACGGGTTCGG	0	14	RDR2-sensitive
CGGGCTAAACTGCTGGTCCAAGCA	0	9	RDR2-sensitive
CGGGCTAAACTTTAGCAGGAGCAA	1	32	RDR2-sensitive
CGGGCTAACGGGCTGACTCGGCAC	0	18	RDR2-sensitive
CGGGCTAACGGGCTGGTTCGGCAC	0	12	RDR2-sensitive
CGGGCTACAGGCAGAACTGTGCAC	0	14	RDR2-sensitive
CGGGCTAGCACAGCACGAAGACAT	0	24	RDR2-sensitive
CGGGCTAGCACGACATGACACGGA	0	9	RDR2-sensitive
CGGGCTAGCACGACCTGAAGGCGG	0	9	RDR2-sensitive
CGGGCTAGCACGACTTAAATGCGG	0	10	RDR2-sensitive
CGGGCTAGCACGGCCTGAAAGCGG	1	12	RDR2-sensitive
CGGGCTAGCACGGCCTGAAGACGG	0	21	RDR2-sensitive
CGGGCTAGCACGGCCTGAAGTCGG	0	30	RDR2-sensitive
CGGGCTAGTACGGCACGACCTGTC	2	43	RDR2-sensitive
CGGGCTAGTTTGGCAGCAACGTGG	0	15	RDR2-sensitive
CGGGCTATACCTGACGGGCCGAAT	0	13	RDR2-sensitive
CGGGCTATATGGGCTAAACGGGCT	0	16	RDR2-sensitive
CGGGCTATGCGGACCGGTAAGCAC	0	14	RDR2-sensitive
CGGGCTATGCGGGACGGTAAGCAC	0	10	RDR2-sensitive
CGGGCTATTTTCGTCGGCCGACCC	0	23	RDR2-sensitive
CGGGCTATTTTCGTCGGCCGGCCC	2	25	RDR2-sensitive
CGGGCTCAGCCTCGGAAGACGACG	14	0	RDR2-resistant
CGGGCTCAGTCCAGGTCGGCCTAG	0	15	RDR2-sensitive
CGGGCTCGACAACACGCGAAGCGA	2	31	RDR2-sensitive
CGGGCTCGGACAAGAAACTAGGCA	0	54	RDR2-sensitive
CGGGCTCGGACAGAAAACTAGGCA	0	11	RDR2-sensitive
CGGGCTCGGACAGGAAACTAGGCA	1	32	RDR2-sensitive
CGGGCTCGGACAGGAAATTAGGCA	4	41	RDR2-sensitive
CGGGCTCTGTAGGGTGTGCGCGGT	0	10	RDR2-sensitive
CGGGCTGACGCTTGCCGACGGCGC	0	10	RDR2-sensitive
CGGGCTGACTCGGTAAGCACGGAA	0	11	RDR2-sensitive
CGGGCTGAGCCAATGGCACGGGCC	26	2	RDR2-resistant
CGGGCTGAGGCCGTGGTAGATCGG	0	14	RDR2-sensitive
CGGGCTGATTGGTTGGACGGACGC	0	10	RDR2-sensitive
CGGGCTGCATGACGTAGGTGACGA	4	33	RDR2-sensitive
CGGGCTGCGGGATACCAAAGAGGC	0	10	RDR2-sensitive
CGGGCTGCGTCCTGACGGAGGTAT	0	9	RDR2-sensitive
CGGGCTGGACAGCGGCTGGGAGGA	888	43	RDR2-resistant

CGGGCTGGACAGCGGCTGGGAGGC	23	2	RDR2-resistant
CGGGCTGGACAGCGGCTGGGAGGG	63	4	RDR2-resistant
CGGGCTGGACAGCGGCTGGGAGGT	16	0	RDR2-resistant
CGGGCTGGACCGGGCCGAATGCAT	0	9	RDR2-sensitive
CGGGCTGGCACGGGCACGAAACCA	0	11	RDR2-sensitive
CGGGCTGGCTCGGCACGATCTAGA	0	10	RDR2-sensitive
CGGGCTGGCTGACGCGGTCCAGAC	0	11	RDR2-sensitive
CGGGCTGGGACCAGAGGACGACGA	0	19	RDR2-sensitive
CGGGCTGGGACTAGAGAACGACGA	0	9	RDR2-sensitive
CGGGCTGTAATGACTGGTTGTCGT	0	15	RDR2-sensitive
CGGGCTGTAATTCGTGCGCGACGG	1	13	RDR2-sensitive
CGGGCTGTCCGCACGGAGCAGGCC	1	13	RDR2-sensitive
CGGGCTGTCCGCGTGGAGCAGGCC	0	16	RDR2-sensitive
CGGGCTGTTCTACCAGAGCGGCAA	1	24	RDR2-sensitive
CGGGCTTAAAACGGGCGACCTCAA	0	15	RDR2-sensitive
CGGGCTTAACGGGCTTAGAGGTAA	0	31	RDR2-sensitive
CGGGCTTACTGGACAGGCCGACGT	0	21	RDR2-sensitive
CGGGCTTAGAGATAAACGGGTCGT	0	28	RDR2-sensitive
CGGGCTTAGAGCTAAACGGGCCGT	3	41	RDR2-sensitive
CGGGCTTAGAGGTAAACGGGCCGT	5	166	RDR2-sensitive
CGGGCTTAGAGGTAAACGGGTCGT	3	68	RDR2-sensitive
CGGGCTTAGAGTTAAACGGGTCGT	0	19	RDR2-sensitive
CGGGCTTAGGCTGTGATCCAGGCA	0	10	RDR2-sensitive
CGGGCTTCACTTGATCCGTGTCGG	0	14	RDR2-sensitive
CGGGCTTCAGGAAAATGACCGGGA	0	9	RDR2-sensitive
CGGGCTTGAATCGGCATGGACCAA	0	15	RDR2-sensitive
CGGGCTTGACAGGAAACTAGGCAC	3	59	RDR2-sensitive
CGGGCTTGCAGGACGGGTCGGCGT	0	34	RDR2-sensitive
CGGGCTTGCTCGTCGTAGTCGGAT	0	28	RDR2-sensitive
CGGGCTTGGACAGGAAACTAGGCA	5	112	RDR2-sensitive
CGGGCTTGGACAGGGAACTAGGCA	0	11	RDR2-sensitive
CGGGCTTGGCCTCAGGTGAGTCGT	0	17	RDR2-sensitive
CGGGCTTGGCGGAATCAGCGGGGA	25	2	RDR2-resistant
CGGGCTTGTTCTGAGCGGCGAGCC	0	9	RDR2-sensitive
CGGGCTTTAGAGGGTGTGTGCGGT	0	12	RDR2-sensitive
CGGGGAACAAGACCGGCGTCCCAT	2	17	RDR2-sensitive
CGGGGAACGGGGCATCAGAGCAGA	0	9	RDR2-sensitive
CGGGGAATCCTCATGGAACGGCTT	0	9	RDR2-sensitive
CGGGGAATTAGGACGAGACGCGGT	0	9	RDR2-sensitive
CGGGGACACAGAGCAGAGCAACGC	0	30	RDR2-sensitive
CGGGGACCTGCCGACTTGACGGAG	0	36	RDR2-sensitive
CGGGGACGAGGAATAGGATACCAT	0	33	RDR2-sensitive
CGGGGACGAGGACGAGACAGGCGC	19	46	RDR2-sensitive
CGGGGACGGGGAATAGGATACCAT	0	37	RDR2-sensitive
CGGGGAGCTATCTGGACCCGTCGG	0	12	RDR2-sensitive
CGGGGAGGAGGAACAGAAGAAAAA	23	0	RDR2-resistant
CGGGGAGGGTCGCGCAGCCAGGTT	24	0	RDR2-resistant
CGGGGATAACTTAACTCCCGTCGG	0	10	RDR2-sensitive
CGGGGATAAGGTTATCCACGTCGG	0	9	RDR2-sensitive

CGGGGATCGGGAGGTAGAGGATGG	0	15	RDR2-sensitive
CGGGGATGGTCGTTGGCAGCCGGT	816	6	RDR2-resistant
CGGGGATTAAGTAACTCCCGTCGG	0	15	RDR2-sensitive
CGGGGATTCCTTAACTCTCGTCGG	0	17	RDR2-sensitive
CGGGGCAGAGGCACCTGAACGCAT	0	11	RDR2-sensitive
CGGGGCATCAACACGGGCATGAAG	52	1	RDR2-resistant
CGGGGCATTGTAAGTGGCAGAGTG	78	8	RDR2-resistant
CGGGGCCACGGCCGCGCCATGCGT	26	0	RDR2-resistant
CGGGGCCTTGGAGATAGATGACGT	0	11	RDR2-sensitive
CGGGGCTGACCTGATCGAGATCGC	0	16	RDR2-sensitive
CGGGGGCTCAAGCTGGATTATCGA	3	20	RDR2-sensitive
CGGGGGCTGATAGTGGCAGACTAG	0	10	RDR2-sensitive
CGGGGGGACGGTGCTCGCCTTCGT	18	0	RDR2-resistant
CGGGGGTAAATCTGAGCAAGACGT	0	27	RDR2-sensitive
CGGGGGTAGATCTGAGCAAGACGT	0	9	RDR2-sensitive
CGGGGGTTGAAGGGGAAGATGCGG	24	1	RDR2-resistant
CGGGGTCGGGTGCTGAACCGGCGC	0	12	RDR2-sensitive
CGGGGTGGGTGAGCGGTTGACGTC	15	0	RDR2-resistant
CGGGTAAGGAGGCGGAGGGTGGGA	15	0	RDR2-resistant
CGGGTACGAACCACCTGACGGTGT	0	14	RDR2-sensitive
CGGGTACGGGTTCGGATATCGGAT	0	9	RDR2-sensitive
CGGGTACTCGACAAAGAAGGCTTT	0	16	RDR2-sensitive
CGGGTAGAAACCAAGTTGGACCGG	0	68	RDR2-sensitive
CGGGTAGAATGGCGTACACAAGAT	0	14	RDR2-sensitive
CGGGTAGCTGTCTGGGCACGTCGG	0	15	RDR2-sensitive
CGGGTAGGATACGGAATCACTAAT	0	9	RDR2-sensitive
CGGGTAGGGATTGTGAACGGGTAT	0	9	RDR2-sensitive
CGGGTAGTTAGAAACGGGACGGAT	0	9	RDR2-sensitive
CGGGTAGTTCAGGACTGCAACGGC	0	19	RDR2-sensitive
CGGGTATGAGGGGAGGTGAGTCGT	0	9	RDR2-sensitive
CGGGTATGAGTAGATACGGCGGCC	0	10	RDR2-sensitive
CGGGTATTTCTCGTCGGAGGGTAT	0	10	RDR2-sensitive
CGGGTCAAAGTCGGGTCGTGGGTC	0	10	RDR2-sensitive
CGGGTCAAATGTAGAAAACGGTAC	0	17	RDR2-sensitive
CGGGTCACGGAGAACAAGACCGGC	21	46	RDR2-sensitive
CGGGTCACGGGCTAAACGGGCGGC	2	33	RDR2-sensitive
CGGGTCCGGAATAGTCGGGAACGG	2	20	RDR2-sensitive
CGGGTCCGTAGAGCCTGAAGGCAT	0	11	RDR2-sensitive
CGGGTCCTGTTTTATAGACGTCCG	0	10	RDR2-sensitive
CGGGTCCTGTTTTATAGACGTCTG	0	13	RDR2-sensitive
CGGGTCGTGCCTGGGCCGAAGGGT	0	12	RDR2-sensitive
CGGGTCTAGCTGTGCTTGGGTCGG	0	12	RDR2-sensitive
CGGGTCTCTGTGGAGAACGGGATG	0	9	RDR2-sensitive
CGGGTGAACGACAGTTGAACGGTT	0	9	RDR2-sensitive
CGGGTGAATGATGCATTTATTGAC	28	1	RDR2-resistant
CGGGTGACCAGAAGACGAAGGAGA	40	4	RDR2-resistant
CGGGTGCGCTTGCAGTAGGGGGTT	7	31	RDR2-sensitive
CGGGTGCGCTTGTAGTAGGGGGTT	0	10	RDR2-sensitive
CGGGTGCTGAGTAGGAGTCCGCAT	0	31	RDR2-sensitive

CGGGTGGACCATGGCATACTCTAT	0	18	RDR2-sensitive
CGGGTGGACTGGTGTATGTCACGG	0	13	RDR2-sensitive
CGGGTGGGTAGTACGCGGATATAT	0	9	RDR2-sensitive
CGGGTGGTAGAACGCGGCAGTGTG	0	9	RDR2-sensitive
CGGGTGTAGCTGTGCTCGGCCGG	0	9	RDR2-sensitive
CGGGTGTGTAGACGTGGGACGCGC	0	10	RDR2-sensitive
CGGGTTACCCATATAAGGTCGCGG	0	11	RDR2-sensitive
CGGGTTACTGTTATTGATCGGCTC	363	89	RDR2-resistant
CGGGTTATGCTACCTCACGTCGGT	0	14	RDR2-sensitive
CGGGTTCGGGCCAGCAAAGACCAA	0	35	RDR2-sensitive
CGGGTTCGGGTTCGGTTTTCGGTT	0	9	RDR2-sensitive
CGGGTTCGGGTTTCGGGTTACGGG	0	9	RDR2-sensitive
CGGGTTGAGACTGTAGCATGGTGT	0	10	RDR2-sensitive
CGGGTTGAGGCTCAGACACGGCAC	0	12	RDR2-sensitive
CGGGTTGCTCGGAACCGAAAACGG	0	9	RDR2-sensitive
CGGGTTGGACTGCACGACGGGCTC	1	21	RDR2-sensitive
CGGGTTTGGGCATGTCACTAGGCA	0	10	RDR2-sensitive
CGGGTTTTCGGGTTCGGGTTCGGT	0	12	RDR2-sensitive
CGGTAAAACTTGGTCGGCCGGTAA	0	11	RDR2-sensitive
CGGTAAAGGACTGTCAGCGGCCGT	0	18	RDR2-sensitive
CGGTAAGTCAGCTGCAGGAGGCCA	0	9	RDR2-sensitive
CGGTAATGAGGCTCGGCTCGGCTC	1	22	RDR2-sensitive
CGGTACACCCCCTGTAGACCCGGC	0	23	RDR2-sensitive
CGGTACCAACGGCGATACTCGAAA	0	11	RDR2-sensitive
CGGTACCCTAAGAACCGTCGGAAA	0	23	RDR2-sensitive
CGGTACCCTAGGAACCGTCGGAAC	0	20	RDR2-sensitive
CGGTACGAACAAGCAGGCCGGGCT	0	14	RDR2-sensitive
CGGTACGAACAAGCGGGCGGGCTT	0	9	RDR2-sensitive
CGGTACGAACACGGCACGACACAT	0	9	RDR2-sensitive
CGGTACGAATAAGCGGGCGGGCTT	0	12	RDR2-sensitive
CGGTACGACCTGTGGAGGAGCGGT	0	33	RDR2-sensitive
CGGTACGCGTCTGGGCTTGGACGG	0	10	RDR2-sensitive
CGGTACGGTAGAATAAGTGGGCGG	1	44	RDR2-sensitive
CGGTACTAAGCACGGTAGGACGGT	0	21	RDR2-sensitive
CGGTACTCTCTGGTCAGGCGCGGA	0	9	RDR2-sensitive
CGGTACTGGGAGAAGGAAAGGCAC	0	24	RDR2-sensitive
CGGTAGAACTGACGTGGCACGTAC	1	24	RDR2-sensitive
CGGTAGAATAAGCGGGCGGGCTTA	0	9	RDR2-sensitive
CGGTAGAATAAGCGGGCGGGCTTG	6	175	RDR2-sensitive
CGGTAGAATATTTGGTTTTATAGG	30	0	RDR2-resistant
CGGTAGAGCTCTTCGGTGCGGGCA	0	16	RDR2-sensitive
CGGTAGGACCAACAATGTCAGCAT	0	20	RDR2-sensitive
CGGTAGGAGACGCAGTCAGGACTC	0	22	RDR2-sensitive
CGGTAGGGATGGATATGGATGGCA	44	2	RDR2-resistant
CGGTAGTGCCCTCTCTGCGACGGT	0	13	RDR2-sensitive
CGGTAGTGGGCTCATGACTCGGCA	0	9	RDR2-sensitive
CGGTATGCTTTTTGATCAACGGAC	66	18	RDR2-resistant
CGGTATGGGAACAGGGGCGGAGGC	14	0	RDR2-resistant
CGGTCAACTGTATCCGTGACTGCA	0	16	RDR2-sensitive

CGGTCAAGCAGTAGGTGGTGGCTA	14	0	RDR2-resistant
CGGTCAATGTAGCCAGGCAGACGC	0	14	RDR2-sensitive
CGGTCAGACAAGGCAGAACGCGGT	1	22	RDR2-sensitive
CGGTCAGACGGGAGACGTTGGAGC	0	9	RDR2-sensitive
CGGTCAGAGGTAGAACGGTCAAAA	0	15	RDR2-sensitive
CGGTCAGGTAGACCTCCACGGTGT	2	28	RDR2-sensitive
CGGTCAGTAGAGCGGCGAAGTGAC	3	26	RDR2-sensitive
CGGTCAGTAGCGACTTACGTGGCA	2	26	RDR2-sensitive
CGGTCAGTCGGCTGGGCGAGCGTG	0	12	RDR2-sensitive
CGGTCAGTGGAGCAGCGGAGTGAC	2	17	RDR2-sensitive
CGGTCAGTGGAGCGGCGAAGTGAC	99	328	RDR2-sensitive
CGGTCAGTGGAGCGGTGAAGTGAC	2	16	RDR2-sensitive
CGGTCCACGGCCTGAACCGGACGG	0	9	RDR2-sensitive
CGGTCCACGGGCTCCAAGCGAGAG	0	11	RDR2-sensitive
CGGTCCAGTATGAATATCCGGCCA	0	10	RDR2-sensitive
CGGTCCCTGTAAATTTAGGTTCGG	0	14	RDR2-sensitive
CGGTCCGAACCCGGCACGGTAGAA	1	100	RDR2-sensitive
CGGTCCGAACCTGGCACGGTAGAA	0	10	RDR2-sensitive
CGGTCCGACTAGAGGCGTGTCCAG	0	15	RDR2-sensitive
CGGTCCGAGCCCGGCACGGTAGAA	0	26	RDR2-sensitive
CGGTCCGCCCCTGTAGATCAACGG	4	247	RDR2-sensitive
CGGTCCGCGCCTGTAGATCAACGG	0	88	RDR2-sensitive
CGGTCCGCGCTCTGAGCCAGTCGG	0	14	RDR2-sensitive
CGGTCCGCGCTCTGGAACTGACGG	0	14	RDR2-sensitive
CGGTCCGGACATAACGTAGGGGCT	0	42	RDR2-sensitive
CGGTCCGGACATGCGCAGATCGGC	0	9	RDR2-sensitive
CGGTCCGGACATGTGCAGATCGGC	0	9	RDR2-sensitive
CGGTCCGGGCTAGCTGGAGACTGG	0	15	RDR2-sensitive
CGGTCCGTCAGACACGGTTAGAAA	1	12	RDR2-sensitive
CGGTCCGTGACCTGAGCACAGGAG	0	13	RDR2-sensitive
CGGTCCGTGATGACGCATGATCGT	0	12	RDR2-sensitive
CGGTCCTGTAGGAGGCCGTCGGAG	1	16	RDR2-sensitive
CGGTCGAACCTGACAAGGGAGGTC	0	9	RDR2-sensitive
CGGTCGACCAACTCTGCGAGACGG	0	10	RDR2-sensitive
CGGTCGAGACTCGAGAGTGGGCAG	0	11	RDR2-sensitive
CGGTCGAGATTGACGGCAGAACGG	0	11	RDR2-sensitive
CGGTCGAGCAGCTCGAGAATGCAT	0	24	RDR2-sensitive
CGGTCGAGGAACAAAAGGACGGAC	0	10	RDR2-sensitive
CGGTCGCACCAAAGCTGTTCGGAT	32	0	RDR2-resistant
CGGTCGCGACACAGTTGATGACGA	0	12	RDR2-sensitive
CGGTCGCGCTCGGCTGCTCGTCGT	0	9	RDR2-sensitive
CGGTCGCGTACTCGTAGTGGGCCT	0	9	RDR2-sensitive
CGGTCGCTATAGAGGTACGTGTGT	0	9	RDR2-sensitive
CGGTCGCTGACATGTAGGGGCGTG	0	18	RDR2-sensitive
CGGTCGCTGTTACGGTAGTCCTTC	0	15	RDR2-sensitive
CGGTCGGAAACGGTATTTATTCGG	0	9	RDR2-sensitive
CGGTCGGCTGTGAATGCACGTGCG	0	32	RDR2-sensitive
CGGTCGGTGCGGCGATTTGGTCAG	0	9	RDR2-sensitive
CGGTCGTACCCTAGAGCCGGACGG	0	23	RDR2-sensitive

CGGTCGTGCTGACGGATACAGAAA	0	14	RDR2-sensitive
CGGTCGTGTTCTCTGCATAGGCGT	0	11	RDR2-sensitive
CGGTCGTTCTGCTTCCTGTCGGCG	0	10	RDR2-sensitive
CGGTCTACGGTTCGAACAGTTCGT	0	15	RDR2-sensitive
CGGTCTAGACTGGCATGCTGACCC	0	12	RDR2-sensitive
CGGTCTAGATCCAAGCGCGGGCGT	98	8	RDR2-resistant
CGGTCTAGCACAGCTCGAAGGCGG	1	28	RDR2-sensitive
CGGTCTATTCGGGTTCGGTTCGGG	0	28	RDR2-sensitive
CGGTCTCCTAGGCAGCCGTCGGAC	1	18	RDR2-sensitive
CGGTCTCTAGCCAGTGTTGACGTG	20	2	RDR2-resistant
CGGTCTCTAGCGAGCGTTGACGTG	0	14	RDR2-sensitive
CGGTCTGAATTCGGCATGGCACGT	0	11	RDR2-sensitive
CGGTCTGACAAGAGGACAGCGCCG	0	10	RDR2-sensitive
CGGTCTGACAGAAGGACAGCGCCG	0	26	RDR2-sensitive
CGGTCTGACAGTCTGGGTAGGCGC	0	16	RDR2-sensitive
CGGTCTGAGCCTGGCACGATCCGG	0	19	RDR2-sensitive
CGGTCTGAGTCCGGCACGGTAGAA	0	33	RDR2-sensitive
CGGTCTGCGTCTGGAACCGTACGG	0	21	RDR2-sensitive
CGGTCTGCTAGGCACGGTTAGAAA	0	11	RDR2-sensitive
CGGTCTGCTGCTAGGGGATGACGG	0	13	RDR2-sensitive
CGGTCTGGCCGATGGAACAGGCGC	0	16	RDR2-sensitive
CGGTCTGGCTCTGACCGGTCTGCT	38	0	RDR2-resistant
CGGTCTGGGCTAGCCGGAGACTGG	0	17	RDR2-sensitive
CGGTCTGGGTGAGACGGTTGGCTC	0	12	RDR2-sensitive
CGGTCTGTAAATCGGAGTGGGCGC	0	9	RDR2-sensitive
CGGTCTGTAACCTAGGCACAGCAC	0	24	RDR2-sensitive
CGGTCTGTAACCTGGGCGCAGGAG	0	20	RDR2-sensitive
CGGTCTGTTCGGGTTCGGTTCGGG	0	15	RDR2-sensitive
CGGTCTGTTGGGCCCCAGCATGGC	0	9	RDR2-sensitive
CGGTCTTAAGGTCGGACGGTCCGC	0	17	RDR2-sensitive
CGGTGAAAGGCTCGAAGGCGTGAT	48	1	RDR2-resistant
CGGTGAACGACGTGACGACGGATG	0	9	RDR2-sensitive
CGGTGAACTGTACATCGATAACGG	0	9	RDR2-sensitive
CGGTGAACTGTACATCGGCAACAA	0	40	RDR2-sensitive
CGGTGAACTGTACATCGGCAACAG	0	70	RDR2-sensitive
CGGTGAATTATAGCCGTACGTCGC	1	19	RDR2-sensitive
CGGTGAATTTTAGCCGTACGCCGT	0	9	RDR2-sensitive
CGGTGAATTTTAGCCGTACGTCGT	0	10	RDR2-sensitive
CGGTGACGAACTCCAAGCGGCCCA	0	55	RDR2-sensitive
CGGTGACGACTCCTGATCCGGAAG	0	21	RDR2-sensitive
CGGTGACTGTCCGGCCGAACGGGT	0	129	RDR2-sensitive
CGGTGAGATTTTAGAGGACGGGTT	23	1	RDR2-resistant
CGGTGAGTAGACGAGTCAAGCCGG	0	10	RDR2-sensitive
CGGTGAGTTCTGACAGACTGACGA	0	13	RDR2-sensitive
CGGTGATCCAAGACGAGACGGTGC	0	14	RDR2-sensitive
CGGTGATGGCTGTAAGCGCGCGGA	0	21	RDR2-sensitive
CGGTGCAGGATCTAGAAGACGAAG	0	30	RDR2-sensitive
CGGTGCAGGTGAACGGACGGACGA	0	21	RDR2-sensitive
CGGTGCAGTTGAACCCCTCGACGG	1	19	RDR2-sensitive

CGGTGCGACGTAGAGAAGACGCCG	0	16	RDR2-sensitive
CGGTGCGACTGCAGGATGGGGCAT	0	15	RDR2-sensitive
CGGTGCGGCTGGAGAGAGAGTGCG	0	10	RDR2-sensitive
CGGTGCGGCTTGAAGGTGAAACGG	0	16	RDR2-sensitive
CGGTGCGGTACTGGACTGTCCGGT	0	11	RDR2-sensitive
CGGTGCTACGAACCAGGGCAGGCT	0	15	RDR2-sensitive
CGGTGCTCGAGGGGCAAGTCGGCA	18	1	RDR2-resistant
CGGTGCTCGGAACTAGACGGTCTG	0	20	RDR2-sensitive
CGGTGCTCGGTGGGCTTCGATCGT	0	9	RDR2-sensitive
CGGTGCTCTCTGACCAGGCGCGGA	0	10	RDR2-sensitive
CGGTGCTCTCTGCACAGGGGCGGA	0	18	RDR2-sensitive
CGGTGCTCTCTGGTCAAGCGCGGA	0	9	RDR2-sensitive
CGGTGCTCTCTGGTCAGACGCGGA	1	17	RDR2-sensitive
CGGTGCTCTCTGGTCAGGCGCGGA	4	87	RDR2-sensitive
CGGTGCTTTTGGGTAGGACGGTGC	1	24	RDR2-sensitive
CGGTGCTTTTGGGTAGGACGGTGT	0	24	RDR2-sensitive
CGGTGGAATAGCGAGCAGAGCCAG	0	13	RDR2-sensitive
CGGTGGAATAGCGAGCCGAGCCAG	0	28	RDR2-sensitive
CGGTGGACATACTTCTCAAGGCGT	0	22	RDR2-sensitive
CGGTGGACATCAAGCTCGGGTCGG	0	10	RDR2-sensitive
CGGTGGACATGGATACGGGTTGAA	0	9	RDR2-sensitive
CGGTGGACTGGGACGAGCACGCAC	0	18	RDR2-sensitive
CGGTGGAGACTTGGGGGCTGGGGT	20	0	RDR2-resistant
CGGTGGAGCTCTTCGGATCTGGCA	27	0	RDR2-resistant
CGGTGGCACACTGGACAGTCCGAT	0	13	RDR2-sensitive
CGGTGGCACACTGGACAGTCCGGT	4	26	RDR2-sensitive
CGGTGGCAGAGACTGTAATTTCAT	0	10	RDR2-sensitive
CGGTGGCCGAAGCTCTGGCGGCAA	0	13	RDR2-sensitive
CGGTGGGCTCGACCGAGATGGACA	0	17	RDR2-sensitive
CGGTGGTGCACTGGACCGTGTGGT	0	13	RDR2-sensitive
CGGTGGTGCATCGGACTGTCCGGT	1	12	RDR2-sensitive
CGGTGGTGCTCTCTGGTCAGACGT	0	14	RDR2-sensitive
CGGTGGTGCTCTCTGGTCAGGCGC	0	9	RDR2-sensitive
CGGTGGTGGGTCTCGAAGTCGGTG	69	13	RDR2-resistant
CGGTGTAAATAGCTCTAGAGACGT	0	12	RDR2-sensitive
CGGTGTAACTGCGGGGCAACGGCT	1	35	RDR2-sensitive
CGGTGTACCATTGGACTGTCCGGT	0	19	RDR2-sensitive
CGGTGTACCTGTCGCTCAAGACGT	0	14	RDR2-sensitive
CGGTGTAGATTGGCTGAAGGCAGA	0	49	RDR2-sensitive
CGGTGTCAACGAGGGATGGGACGG	0	13	RDR2-sensitive
CGGTGTCGCCCCGGGTAGAATGGC	0	14	RDR2-sensitive
CGGTGTCTCTGAACCGACATTGGC	0	10	RDR2-sensitive
CGGTGTGACTGTCCAACGAGCGTG	0	12	RDR2-sensitive
CGGTGTGATCTTTAGAACCGGCCA	0	28	RDR2-sensitive
CGGTGTGGAAGACGACTGCGCAGC	0	12	RDR2-sensitive
CGGTGTGGACTCCGAGAAGACGAT	0	19	RDR2-sensitive
CGGTGTGTGCTCCAAGAAGACGAT	1	14	RDR2-sensitive
CGGTGTTAATATATTTCGTTGGAG	14	0	RDR2-resistant
CGGTGTTCGGACGGTATACACGGT	0	10	RDR2-sensitive

CGGTTACATCGGAACGAGACCCGG	0	16	RDR2-sensitive
CGGTTAGTCGGGCCTGGCTAGCAC	1	61	RDR2-sensitive
CGGTTCACTACGTGTCGTGTCGGG	0	10	RDR2-sensitive
CGGTTCAGAACTTATTTCCGACGG	1	32	RDR2-sensitive
CGGTTCAGATCGGTTAGACGACAT	0	13	RDR2-sensitive
CGGTTCAGGACTTATTTCCGACGG	1	51	RDR2-sensitive
CGGTTCAGGCTGTGTGGATCGGTT	0	22	RDR2-sensitive
CGGTTCATGGTCAACTCGGTTCGG	0	11	RDR2-sensitive
CGGTTCATGGTCAGCTCGGCTCAG	0	10	RDR2-sensitive
CGGTTCATGGTCAGCTCGGCTCGG	0	10	RDR2-sensitive
CGGTTCCAGTCTCTGACCGAGCAA	2	50	RDR2-sensitive
CGGTTCCATACGGGCTAGGACCAA	0	10	RDR2-sensitive
CGGTTCCTCTGCGTCGGTTCGGCT	0	9	RDR2-sensitive
CGGTTCGCGCTCTGGACCGGACGG	1	15	RDR2-sensitive
CGGTTCGGCTGTACGGGAAGCATG	0	11	RDR2-sensitive
CGGTTCGGCTGTGTGGATAGGTTG	0	14	RDR2-sensitive
CGGTTCGGCTGTGTGGATCGGTTG	0	12	RDR2-sensitive
CGGTTCGGTACGGTTCGGTTCGGT	2	66	RDR2-sensitive
CGGTTCGGTCTGGGTGGAGCGGTT	0	9	RDR2-sensitive
CGGTTCGGTTCGGTACGGTTCGGT	2	18	RDR2-sensitive
CGGTTCGTAGGCTGGACGGTCCGC	0	19	RDR2-sensitive
CGGTTCGTATTGAGTGATTGGCGC	0	13	RDR2-sensitive
CGGTTCGTCGACAGCTAAGGCCGC	1	231	RDR2-sensitive
CGGTTCGTGTTGAGTGATTGGCGC	0	18	RDR2-sensitive
CGGTTCGTTAACGAGCCAGGTCGG	0	12	RDR2-sensitive
CGGTTCTCCAAGCACAAGAGACAA	0	12	RDR2-sensitive
CGGTTCTCCAAGCACAGGAGACAA	0	16	RDR2-sensitive
CGGTTCTCCAAGCACAGTAGACAA	0	22	RDR2-sensitive
CGGTTCTCCAAGCACATGAGACAA	0	16	RDR2-sensitive
CGGTTCTCGGTTATTTCGGTTCGG	0	20	RDR2-sensitive
CGGTTCTCGGTTCTTCGGTTCGGT	0	16	RDR2-sensitive
CGGTTCTCTAAGCACAGGAGACAA	0	12	RDR2-sensitive
CGGTTCTGCCTCCTCACGACGCAT	0	11	RDR2-sensitive
CGGTTCTTAGGGTACCGTCGGAAA	0	9	RDR2-sensitive
CGGTTCTTCGGTTCGGTTCCTCGG	8	95	RDR2-sensitive
CGGTTCTTCGGTTCGGTTCGGTTC	0	21	RDR2-sensitive
CGGTTCTTCTTGAGTATTTGGCGC	2	18	RDR2-sensitive
CGGTTCTTCTTGAGTGATTGGCGC	0	10	RDR2-sensitive
CGGTTCTTGTTGAGTGATTGACGG	0	14	RDR2-sensitive
CGGTTCTTGTTGAGTGATTGGCGC	1	34	RDR2-sensitive
CGGTTCTTGTTGAGTGATTGGCGT	0	16	RDR2-sensitive
CGGTTCTTGTTGAGTGTTTGGCAC	0	11	RDR2-sensitive
CGGTTCTTGTTGAGTGTTTGGCGC	1	22	RDR2-sensitive
CGGTTGAACCGACCCAGGAGCTGG	0	9	RDR2-sensitive
CGGTTGAATTCGGTATCTTTGGTT	15	0	RDR2-resistant
CGGTTGAGATTGCAGGATGGCAGA	0	17	RDR2-sensitive
CGGTTGATGTGTGTGGGGACGGAG	43	5	RDR2-resistant
CGGTTGCGGCTGCGGCTTCTACAG	0	21	RDR2-sensitive
CGGTTGGACCGCTGCAGAATGGAT	0	9	RDR2-sensitive

CGGTTGGACCGTTACAGAGTGGAT	0	13	RDR2-sensitive
CGGTTGGACCGTTGCAGAGTGGAC	0	20	RDR2-sensitive
CGGTTGGGATATAGGTGGGGGCAT	0	9	RDR2-sensitive
CGGTTGGGATTCCTGGAGACGCGA	0	9	RDR2-sensitive
CGGTTGGGCATGTATTGACCGGTA	0	11	RDR2-sensitive
CGGTTGGGCCGTTGCAGAGTGGAC	5	23	RDR2-sensitive
CGGTTGTCAGAGCGGAGTAGACGA	0	10	RDR2-sensitive
CGGTTGTGGGAGTAGGAGGTCGAC	1	13	RDR2-sensitive
CGGTTTAAGGCTTATTCCCGTCGG	0	11	RDR2-sensitive
CGGTTTAGGGCTTATTCCCGTCGG	1	22	RDR2-sensitive
CGGTTTAGTGCTTATTCCCGTCGG	0	11	RDR2-sensitive
CGGTTTCGGTTGGGTGTCGGGGGT	24	0	RDR2-resistant
CGGTTTGGCTCAGAGCGGCTCCGT	0	19	RDR2-sensitive
CGGTTTGGGGCTTACTTCCGTCGG	0	9	RDR2-sensitive
CGGTTTTGTAGGATGGAAGCGGAT	0	9	RDR2-sensitive
CGGTTTTTCGGTTCGGTTCCTCGG	0	9	RDR2-sensitive
CGGTTTTTCTGCGGGTGATGAGAC	72	0	RDR2-resistant
CGTAAACGAGATGCAGAGCTGCTT	0	11	RDR2-sensitive
CGTAAACTCGATCCCCGTAGACGT	1	17	RDR2-sensitive
CGTAACAGGGGACGACATGGGACG	0	28	RDR2-sensitive
CGTAACTGCGTCGGCGCGTCGCGT	0	9	RDR2-sensitive
CGTAAGTCGCTACTGACCGGCCAT	2	18	RDR2-sensitive
CGTACATCGTCAGTCATCAGGCAC	3	31	RDR2-sensitive
CGTACCAGAACTCCAGAAGAGCAG	0	10	RDR2-sensitive
CGTACCAGGACTGCCGCGTGGCAC	2	21	RDR2-sensitive
CGTACCCCTGTAGATGGTATGGCT	0	19	RDR2-sensitive
CGTACCGCGGCACAGACACGGCAT	0	10	RDR2-sensitive
CGTACCTGGGGTGTAGAAGGCAGG	0	9	RDR2-sensitive
CGTACGAAGTCGGTGCAGGAAGAT	1	27	RDR2-sensitive
CGTACGAGCAGCTGGGTTGGAGGA	23	2	RDR2-resistant
CGTACGATGGGACGCATGATTCTT	0	10	RDR2-sensitive
CGTACGCAGTCGACACAGGTAGAT	0	10	RDR2-sensitive
CGTACGGCGAAGATGACGGGTCAT	0	11	RDR2-sensitive
CGTACGGCGTACGGCGGAACACGG	0	10	RDR2-sensitive
CGTACGGGCACACAGAGCGGTAGG	14	0	RDR2-resistant
CGTACGTATTGGCTCGATCGTCGA	0	14	RDR2-sensitive
CGTACTGGGACTAGAGGACGACGA	0	14	RDR2-sensitive
CGTACTGTTCACGCTGACTTGGCA	17	58	RDR2-sensitive
CGTAGAAACTCACACGCTCGGGGC	0	16	RDR2-sensitive
CGTAGAAAGTGACTGCGACGACAT	0	10	RDR2-sensitive
CGTAGAAATCCATCGGTTGTTGAA	24	1	RDR2-resistant
CGTAGAACATTTCTTTGCAAGAGG	14	0	RDR2-resistant
CGTAGAACTGCTTCCAGAGGTCGA	0	9	RDR2-sensitive
CGTAGAAGCTGTAGCCGCAGTCGG	0	11	RDR2-sensitive
CGTAGAATGGTAGAACGGCAGGCT	0	14	RDR2-sensitive
CGTAGACACCCACCAGCAGGACAT	25	4	RDR2-resistant
CGTAGACTCATGATCGAGCGGCTT	0	50	RDR2-sensitive
CGTAGACTGGATGAGCGGATGCAA	0	34	RDR2-sensitive
CGTAGAGAGCAGTAGAGCACCACC	0	10	RDR2-sensitive

CGTAGAGCCTGAAGGCGTGGAGAG	2	18	RDR2-sensitive
CGTAGAGGACAGCGAGGAGCAGAG	0	17	RDR2-sensitive
CGTAGAGGGACCTGTTGAGCCGGC	3	18	RDR2-sensitive
CGTAGAGGGCGCTGTAGGGGGCAC	2	29	RDR2-sensitive
CGTAGAGTGCATCTGGCGCCGGAG	20	2	RDR2-resistant
CGTAGATAACCGCGGCCTTCACAG	0	13	RDR2-sensitive
CGTAGATCAAGAGGGCTTCTCCGG	0	9	RDR2-sensitive
CGTAGATCTCGAGCAAGGCAGAGT	0	22	RDR2-sensitive
CGTAGATCTTGGCGCCGAGGTAGG	0	9	RDR2-sensitive
CGTAGATCTTTGGTGGACGGTTGT	0	18	RDR2-sensitive
CGTAGCAACATCAAGGTAGAGCGT	2	20	RDR2-sensitive
CGTAGCAACTTTTGGTGACGTCAT	0	10	RDR2-sensitive
CGTAGCACGAACGGTGGACGCCAA	0	10	RDR2-sensitive
CGTAGCACGAACGGTGGGCGCCAA	0	9	RDR2-sensitive
CGTAGCAGTTCGGCTCGGCTCGAC	0	9	RDR2-sensitive
CGTAGCCTAGGGTGTAGAAGGCAG	0	18	RDR2-sensitive
CGTAGCCTGAGCTAGCTAGGGGTA	1	17	RDR2-sensitive
CGTAGCCTGGGGTGTAGAAGGCAG	2	23	RDR2-sensitive
CGTAGCGGGAGTGTTTGACAGGTT	15	0	RDR2-resistant
CGTAGGAAATCGGGCTGAGCCGGA	0	17	RDR2-sensitive
CGTAGGAAGTTAGTCAGCTGACGG	0	13	RDR2-sensitive
CGTAGGACAGATCGGCTAGGCCAG	0	9	RDR2-sensitive
CGTAGGACAGTTCGGCAAGGCCAA	0	15	RDR2-sensitive
CGTAGGACAGTTCGGCAAGGCCGG	1	90	RDR2-sensitive
CGTAGGACAGTTCGGCTAGACCAG	0	9	RDR2-sensitive
CGTAGGACAGTTCGGCTAGACCGG	0	18	RDR2-sensitive
CGTAGGACAGTTCGGCTAGGCCAA	1	77	RDR2-sensitive
CGTAGGACAGTTCGGCTAGGCCAG	2	91	RDR2-sensitive
CGTAGGACAGTTCGGCTAGGCCGG	3	100	RDR2-sensitive
CGTAGGACAGTTCGGCTAGGTCAG	0	9	RDR2-sensitive
CGTAGGACGACCCAGTAGAAGGCC	0	11	RDR2-sensitive
CGTAGGACGCCACAGATCGATCGG	0	12	RDR2-sensitive
CGTAGGACTGCTTACAGAGGTCGA	0	9	RDR2-sensitive
CGTAGGACTGCTTCCAGAGGTCGA	0	53	RDR2-sensitive
CGTAGGACTGTGCAGGCCCGGCCC	0	25	RDR2-sensitive
CGTAGGAGGAATAGGTCTGGCGGC	0	20	RDR2-sensitive
CGTAGGAGGCAAGGTGCAGCTCGG	0	10	RDR2-sensitive
CGTAGGATTGCTGCTTGATGGTTG	0	12	RDR2-sensitive
CGTAGGCTGGCGTGTAGAAGGCAG	1	16	RDR2-sensitive
CGTAGGGAGCGCTGTAGGGGGCAC	1	15	RDR2-sensitive
CGTAGGGCTGCAGGAACGGCAGAT	0	33	RDR2-sensitive
CGTAGGGCTGTGCGTTGCGGACAG	0	30	RDR2-sensitive
CGTAGGGGACGCTGTAGGGGGCAC	0	9	RDR2-sensitive
CGTAGGGGGCGCTGTAGAGGGCAC	0	12	RDR2-sensitive
CGTAGGGGGCGCTGTAGGAGGCAC	0	12	RDR2-sensitive
CGTAGGGGGCGCTGTAGGGGGCAC	1	18	RDR2-sensitive
CGTAGGTGTGTGTGATGTCGGCAT	0	16	RDR2-sensitive
CGTAGTAGACTATAGCGTCGGCGT	0	9	RDR2-sensitive
CGTATAAGCTGTAGAAGCCGGTAT	0	21	RDR2-sensitive

CGTATACAGGTCGAACGGATGCAA	0	9	RDR2-sensitive
CGTATACTCTATCTGGGACGCTAT	0	9	RDR2-sensitive
CGTATCATAGCGGATTGGTCTGAC	0	12	RDR2-sensitive
CGTATGCGTTGGGCTGAGACGCGC	0	11	RDR2-sensitive
CGTATGTAGAACCTGTGGGGGCGC	0	14	RDR2-sensitive
CGTATGTCATGGGCTGCGGTGCGC	0	14	RDR2-sensitive
CGTATGTGCGGACGACCCGATGGA	0	11	RDR2-sensitive
CGTATTCCTGTACAAGATGAGCTT	0	11	RDR2-sensitive
CGTATTCGACGAGGACAGGATGGT	0	9	RDR2-sensitive
CGTATTGTTTGAATTTCGGGTCGT	0	9	RDR2-sensitive
CGTCAACGAGTCGGGTTGTTTGGG	4	25	RDR2-sensitive
CGTCAACGATCGGCAGGGCGACAC	0	16	RDR2-sensitive
CGTCAAGGACCAACTGAAGCGCAT	0	16	RDR2-sensitive
CGTCAATCTTGAGCAGGTCGTCGT	1	15	RDR2-sensitive
CGTCACACTGTAGCTGCCGACGGC	0	11	RDR2-sensitive
CGTCACCGCCCACTCTGCAACGGC	1	39	RDR2-sensitive
CGTCAGATCTACCGGACGACGAGC	0	9	RDR2-sensitive
CGTCAGATCTGGGACGGCCGCTGA	0	9	RDR2-sensitive
CGTCAGCAGTAGAGGCGCAAGAAG	2	29	RDR2-sensitive
CGTCAGCGGACAGACGTATAGCAT	0	13	RDR2-sensitive
CGTCAGCTCTGGGACGGCCGCTGA	0	22	RDR2-sensitive
CGTCAGCTTTGGGACGGCCGCTGA	0	14	RDR2-sensitive
CGTCATCCCCGTCGTTCGTCTGGC	28	0	RDR2-resistant
CGTCATCCGGGTTGTAGGTGACGT	0	12	RDR2-sensitive
CGTCATTCGAGCTGTGGAGGACGG	0	44	RDR2-sensitive
CGTCCACTCTGTAACAACGGTCCA	0	15	RDR2-sensitive
CGTCCAGGCGAGTCGAACGAGCAT	0	9	RDR2-sensitive
CGTCCATGCTGTCGGCCCGTACGG	0	14	RDR2-sensitive
CGTCCATGTCTAGATCGTGCCGGG	0	18	RDR2-sensitive
CGTCCATGTTTAGGCTGTATTGGC	0	19	RDR2-sensitive
CGTCCCGGACCTGTAGAATACCGG	0	25	RDR2-sensitive
CGTCCGACGGCTGTCAGACAGGCT	0	11	RDR2-sensitive
CGTCCGATCTCGGCTGAACGGCCC	22	0	RDR2-resistant
CGTCCGCACCTGACCAGAGAGCAC	0	11	RDR2-sensitive
CGTCCGCGACTGTGCAGAGACCAC	0	29	RDR2-sensitive
CGTCCGCGCCTGACCAGAGAGCAC	8	196	RDR2-sensitive
CGTCCGCGCCTGACCAGAGAGCAT	1	32	RDR2-sensitive
CGTCCGCGCCTGTGCAGAGAGCAT	0	13	RDR2-sensitive
CGTCCGCGGTCGGCAGAATGGAGC	1	13	RDR2-sensitive
CGTCCGCGTCTGACCAGAGGGCAC	0	23	RDR2-sensitive
CGTCCGGACATACACAGAGAAGAA	0	11	RDR2-sensitive
CGTCCGGACGTACACAGAGGAGAA	0	12	RDR2-sensitive
CGTCCGGACGTACGCAAAGGAGGA	0	15	RDR2-sensitive
CGTCCGGACGTACGCAGAGGAGGA	1	26	RDR2-sensitive
CGTCCGGACGTACGCAGATGAGGA	0	11	RDR2-sensitive
CGTCCGGACGTACGTAGAGAAGGA	0	27	RDR2-sensitive
CGTCCGGCTTGGCGTAGAGAAGAC	0	10	RDR2-sensitive
CGTCCGTAGAATCCATGTGCGCAT	0	19	RDR2-sensitive
CGTCCGTAGTGTCGTCGTCGGGAT	0	12	RDR2-sensitive

CGTCCGTTTGGCCCGTGGCACGGC	0	16	RDR2-sensitive
CGTCCTCGAGCTGCAGCATGACGG	0	15	RDR2-sensitive
CGTCCTGCTGAGCGGCGTCGTCGT	0	10	RDR2-sensitive
CGTCCTGTAGTCGGAGGAAGACGA	0	12	RDR2-sensitive
CGTCGACCCTGCTGATTCCGGCGC	0	11	RDR2-sensitive
CGTCGACGTGGCACCGAGCTCGGC	1	22	RDR2-sensitive
CGTCGATCGCGGAGCTGACGGTGT	0	16	RDR2-sensitive
CGTCGATCTCTGACGTGGCGTCGG	0	11	RDR2-sensitive
CGTCGATTGGATGAGGACCGGCAC	0	14	RDR2-sensitive
CGTCGTCAAGCTGAAGGCCGTCGT	0	36	RDR2-sensitive
CGTCGTCAAGCTGAAGGTCGTCGT	0	10	RDR2-sensitive
CGTCGTCGTTCCGTGCATCGTCAA	0	11	RDR2-sensitive
CGTCGTCGTTCTGCGCATCAGCAA	0	12	RDR2-sensitive
CGTCGTCTGGGCAGGGCGTAGCAT	0	13	RDR2-sensitive
CGTCGTCTGTCGGAGATGAGGCGT	0	14	RDR2-sensitive
CGTCTACAGTGACTGTTCGGTGGC	0	9	RDR2-sensitive
CGTCTAGACTTGAACCGGTAGTGT	0	12	RDR2-sensitive
CGTCTCCCATCCAACGGTCGTCGT	0	14	RDR2-sensitive
CGTCTCGGGCGTACCACGGCGCAA	0	16	RDR2-sensitive
CGTCTCGGTTTGGCTCAGAGCGGC	0	10	RDR2-sensitive
CGTCTGCAGAAACCGTCGGCCCAG	0	10	RDR2-sensitive
CGTCTGCAGAGCCTGAGCGGGCAG	3	21	RDR2-sensitive
CGTCTGCGGCTCTGGCGTTGGCGT	0	10	RDR2-sensitive
CGTCTGCGGTGGACTGGGACGAGC	0	17	RDR2-sensitive
CGTCTGCGTCGCCGTAGAGGGCAC	0	12	RDR2-sensitive
CGTCTGGAGACGTCTTGGATGGCC	0	9	RDR2-sensitive
CGTCTGGGACTCTGGGAGGACGTC	0	9	RDR2-sensitive
CGTCTGTATGCACTCAGGGGCGGA	0	59	RDR2-sensitive
CGTCTTCATGCTGTAGAAGAGGGT	0	15	RDR2-sensitive
CGTGAACAGACCACACGTCGGCAC	0	9	RDR2-sensitive
CGTGAACGGGACGACTGTGAAGAC	0	18	RDR2-sensitive
CGTGAACTTGTTGGGCGGAGACGA	0	9	RDR2-sensitive
CGTGAAGACTAAAGTGAAGATCGA	0	9	RDR2-sensitive
CGTGAAGACTGTGGCAGAGACTGT	0	17	RDR2-sensitive
CGTGAAGAGACGCCTCTGGCTCGT	0	12	RDR2-sensitive
CGTGAAGATCGGGCATCATGACGC	0	12	RDR2-sensitive
CGTGAAGATCGGGCATCGTGACAC	0	17	RDR2-sensitive
CGTGAAGATCGGGCATCGTGACGC	4	57	RDR2-sensitive
CGTGAATAGGACTAGGGATGGCAA	0	9	RDR2-sensitive
CGTGACGGACTTATTTCGGGCGGT	0	14	RDR2-sensitive
CGTGACTCGCAAGAACTAGCGGCG	0	9	RDR2-sensitive
CGTGACTGGAACCGAGAGGAGGAA	0	10	RDR2-sensitive
CGTGACTGGACCGTGGCTGCGGCA	0	18	RDR2-sensitive
CGTGACTGGGCCTCTGGCCAGGCA	0	17	RDR2-sensitive
CGTGACTGTGAGAGCGGGATGGAA	0	9	RDR2-sensitive
CGTGACTGTGAGAGCGGGATGGGA	4	41	RDR2-sensitive
CGTGACTGTGAGAGCGGGATTGGA	1	58	RDR2-sensitive
CGTGACTGTTTCGGGCAACACGGA	1	21	RDR2-sensitive
CGTGACTTGACGCAGGAGCGACTC	0	11	RDR2-sensitive

CGTGACTTGGACGTAGGAGTGGCT	0	17	RDR2-sensitive
CGTGAGAACTGGCACGTAGACCGC	0	19	RDR2-sensitive
CGTGAGCAGATAGAACCGTGCACC	0	31	RDR2-sensitive
CGTGAGCCTGGGCAGTTAGGACGC	1	15	RDR2-sensitive
CGTGAGGAGGCAGAACCGTGCACC	0	21	RDR2-sensitive
CGTGAGGCTATAGAGTAGAATGGC	0	17	RDR2-sensitive
CGTGAGGTTGGTGGAGGCCGGCGA	26	2	RDR2-resistant
CGTGAGTCTGTTAGTCGTGCCGGG	0	19	RDR2-sensitive
CGTGATATCGTCAGTGGATCCGGA	0	13	RDR2-sensitive
CGTGATCAACTCGGCTCGGCTGAC	0	13	RDR2-sensitive
CGTGATCAGGAACAGGAGCAGAGA	0	25	RDR2-sensitive
CGTGATGCCACAGATGATCGTCTT	0	10	RDR2-sensitive
CGTGATTGGTTGCCGGATAAGCAG	0	21	RDR2-sensitive
CGTGATTTAGAGGACGTTGTTGGA	0	9	RDR2-sensitive
CGTGCAACTCATCGCGCGATCGTC	0	13	RDR2-sensitive
CGTGCAGGTCCGGACGTGGGCGGC	5	38	RDR2-sensitive
CGTGCAGTCGTCGGCGTCCTTCAC	25	0	RDR2-resistant
CGTGCAGTGACTGTAGATGGGCTA	0	45	RDR2-sensitive
CGTGCCACAGGTCAAACGGACGAC	0	10	RDR2-sensitive
CGTGCCACGGTGGAAGAAGCTGGT	114	31	RDR2-resistant
CGTGCCGGGGACTCGACAAGTCGG	0	17	RDR2-sensitive
CGTGCCGGGTGGCCCGTTTGGACA	0	19	RDR2-sensitive
CGTGCCTGGACCGTGCTTTTTCGT	0	9	RDR2-sensitive
CGTGCCTTCGTTGGGCCGTGCCGG	0	12	RDR2-sensitive
CGTGCCTTTTGTGCCAGTCGTCGA	3	36	RDR2-sensitive
CGTGCCTTTTGTGCTAGTCGTCGA	0	12	RDR2-sensitive
CGTGCGAACTGATCGTGGTGGGCC	0	10	RDR2-sensitive
CGTGCGACGTGTAGAGTGACGTGC	0	14	RDR2-sensitive
CGTGCGACTGGGTAGGCAGCGCGA	0	11	RDR2-sensitive
CGTGCGAGAAGCCGAGAACACCAG	0	10	RDR2-sensitive
CGTGCGCTGTGTGGACACGACGTC	0	9	RDR2-sensitive
CGTGCGGAAGGCCAGGTAGAGCGG	0	21	RDR2-sensitive
CGTGCGGAGGTATAGATGGCCAAA	0	13	RDR2-sensitive
CGTGCGGATGTATAGATGGCCAAA	0	9	RDR2-sensitive
CGTGCGGCTGGATGGCATAGGCAA	0	12	RDR2-sensitive
CGTGCGTTGCTACGGATAGATCGT	0	9	RDR2-sensitive
CGTGCTGAATCCTTTGCAGACGAC	17	0	RDR2-resistant
CGTGCTGACTGTGCCGGACGAGCA	0	12	RDR2-sensitive
CGTGCTGCGGGCTGTCGCGTGCGC	0	21	RDR2-sensitive
CGTGCTGGACCAGGCTTCGTGCTA	0	12	RDR2-sensitive
CGTGCTGTCCGTGCGCGTCGTCGT	0	10	RDR2-sensitive
CGTGCTGTCGAGCAGGAGCTGATG	0	19	RDR2-sensitive
CGTGCTGTGCTGACACAACCGGAT	0	9	RDR2-sensitive
CGTGCTGTTCGACTGCAGGAACGG	0	10	RDR2-sensitive
CGTGCTTCCGGACTGGCCCGGCAT	0	12	RDR2-sensitive
CGTGCTTCGGGCCGGCCCATCAGG	0	9	RDR2-sensitive
CGTGCTTCGTGCCGGGCTAACGGG	0	12	RDR2-sensitive
CGTGCTTGAACTGACTAGGAGCAT	0	21	RDR2-sensitive
CGTGCTTGGACAGGTAGGCCGGCC	0	11	RDR2-sensitive

CGTGCTTGGGCAAACGGCTGGCAC	0	33	RDR2-sensitive
CGTGGAAATTGAGGGGGCTGGCCT	0	14	RDR2-sensitive
CGTGGAACAATGGGTTGCTGGCAC	0	22	RDR2-sensitive
CGTGGAACGCCTAGGACAACGGTG	0	29	RDR2-sensitive
CGTGGAACTGTTCAGCCGGTGCTC	0	9	RDR2-sensitive
CGTGGAAGGCCAGGTAGAGCGGTT	1	40	RDR2-sensitive
CGTGGAATGGACGAGCCGAGCCGA	1	125	RDR2-sensitive
CGTGGAATTAGGCTCGGCTCGGGC	0	13	RDR2-sensitive
CGTGGAATTGTAGGTGACACGGTT	1	15	RDR2-sensitive
CGTGGACACCTAGCTCGGCGCCAT	0	11	RDR2-sensitive
CGTGGACATGAAACTAGGCACGGT	0	17	RDR2-sensitive
CGTGGACCAATCAGAAGCCGCCAC	0	11	RDR2-sensitive
CGTGGACCAATCAGATCGCGACAC	2	157	RDR2-sensitive
CGTGGACCAATCAGATCGCGCCAC	0	40	RDR2-sensitive
CGTGGACCAATCAGATCGCGTCAC	0	9	RDR2-sensitive
CGTGGACCAGTCAGATCGCGCCAC	0	43	RDR2-sensitive
CGTGGACCAGTCAGATCGCGTCAC	0	33	RDR2-sensitive
CGTGGACCGTCCAGACGTACGCAT	0	11	RDR2-sensitive
CGTGGACCGTCTGACCTAGAGGTA	0	9	RDR2-sensitive
CGTGGACGCACGTGGAAGCCGGAT	0	16	RDR2-sensitive
CGTGGACGCATGTAGGAGCGGGAA	0	33	RDR2-sensitive
CGTGGACGTCACCCTCGGCTCCGG	1	22	RDR2-sensitive
CGTGGACGTCTCCCTCGGCTCCGG	8	64	RDR2-sensitive
CGTGGACTCCGTAGCAAAGCACGG	0	11	RDR2-sensitive
CGTGGACTGGCCTCTCCGGCGAAG	0	12	RDR2-sensitive
CGTGGAGAACTGAGTGACGCGCGT	0	9	RDR2-sensitive
CGTGGAGCAATGTAAGACGTCCAT	0	24	RDR2-sensitive
CGTGGAGTCTGTTAGGCAGTTCGT	0	9	RDR2-sensitive
CGTGGATAACGTAACTCCCGTCGG	0	28	RDR2-sensitive
CGTGGATATGACTGCGGGCGGATA	1	29	RDR2-sensitive
CGTGGATCATTACAGGGGCTTCGG	0	11	RDR2-sensitive
CGTGGATCTGACGTGGCAGTACCT	0	21	RDR2-sensitive
CGTGGATCTGAGCAGCCAAGCAGC	0	18	RDR2-sensitive
CGTGGATCTGTTGATGTACGGCTC	0	9	RDR2-sensitive
CGTGGATGTAGAAATAGGGGTGGT	0	9	RDR2-sensitive
CGTGGATTCGTGATTGGCGATGGG	27	0	RDR2-resistant
CGTGGCAATTGTAGGCGTGGCATC	0	15	RDR2-sensitive
CGTGGCCACAGGAACTGAGGGGTA	14	0	RDR2-resistant
CGTGGCCTGGCTGGATGGAGCGGT	0	9	RDR2-sensitive
CGTGGCTCGCTGGCTCGCTCGGAT	0	30	RDR2-sensitive
CGTGGCTCTGGACCGGACGGTCTG	0	19	RDR2-sensitive
CGTGGCTGCGGCATGTGGATCGAT	0	12	RDR2-sensitive
CGTGGCTTGTAGTGGGCTTTTCGC	10	56	RDR2-sensitive
CGTGGCTTTTGATCTCGGCGTCAT	1	21	RDR2-sensitive
CGTGGGAGCTCAGACGGATTTCAG	0	11	RDR2-sensitive
CGTGGGATTGTAGTGTTAGCACGG	0	27	RDR2-sensitive
CGTGGGCCTCTGAGCTGGACGAGG	1	17	RDR2-sensitive
CGTGGGCTGGTACAACACGGTCTG	0	21	RDR2-sensitive
CGTGGGGCTCACAGGGCACGACGC	0	16	RDR2-sensitive

CGTGGGTCCTGTTTGTTTCGGCTT	0	11	RDR2-sensitive
CGTGGGTGATAGAACGGACGGTGC	0	10	RDR2-sensitive
CGTGGTATTGTTTCGGCTCATGTC	16	0	RDR2-resistant
CGTGGTCAGCTCGGCTCGGCTCGG	0	26	RDR2-sensitive
CGTGGTGCTTTTAGGTAGGACGGT	0	10	RDR2-sensitive
CGTGGTGCTTTTGAGTAGGACGGT	0	27	RDR2-sensitive
CGTGGTGCTTTTGGGTAGGACGGT	0	35	RDR2-sensitive
CGTGGTGGATGAACAGCGGACGTT	16	0	RDR2-resistant
CGTGTACAGCAACGACGGACGCAA	0	10	RDR2-sensitive
CGTGTACTTGAGGGAACGAGGCTC	0	9	RDR2-sensitive
CGTGTAGAATAGAGGACGCGACAC	0	12	RDR2-sensitive
CGTGTAGATCTCCCGGCATGTCGT	0	15	RDR2-sensitive
CGTGTAGCGCTCTGCGAGAGGTAT	0	29	RDR2-sensitive
CGTGTAGTGGGCTGCGATGGCACC	0	14	RDR2-sensitive
CGTGTCACGGACCAAACGGGCGGC	0	9	RDR2-sensitive
CGTGTCAGCTGTGGAAACGGTAAG	1	18	RDR2-sensitive
CGTGTCCACGGAAACGGCTCTCAA	0	15	RDR2-sensitive
CGTGTCCACGTCACTGATCGGCTC	44	0	RDR2-resistant
CGTGTCCTAACCGTGTCTGATCGT	0	10	RDR2-sensitive
CGTGTCGAACCACTGGAAGGGCAC	0	22	RDR2-sensitive
CGTGTCGGCACTGTAGGTCGGCTT	0	76	RDR2-sensitive
CGTGTCGGGTCACGGGCCAAACGG	0	16	RDR2-sensitive
CGTGTCGTGCTCAGGAAGAGGTAT	0	9	RDR2-sensitive
CGTGTCTGCTGTGGAAACGGTAAG	2	15	RDR2-sensitive
CGTGTCTTCACGTCGGATAGCGGA	0	15	RDR2-sensitive
CGTGTCTTCGTTGGGTCGTGACGG	0	9	RDR2-sensitive
CGTGTCTTTCACTGTGGATAGGCG	0	22	RDR2-sensitive
CGTGTGCGTTATCGAGCTCGTCGT	0	17	RDR2-sensitive
CGTGTGGACTACGTGGAGGCGCAA	0	9	RDR2-sensitive
CGTGTGGACTATGTGGAAGCGCAG	0	10	RDR2-sensitive
CGTGTGGCATAAGTACGGCGTCGG	0	9	RDR2-sensitive
CGTGTGTGAATCTGAGCGTCGGCA	47	7	RDR2-resistant
CGTGTTACGGACCAAACGGACGGC	0	15	RDR2-sensitive
CGTGTTATCTCGGTGCCATGGCTT	0	13	RDR2-sensitive
CGTGTTATTTTCGTCGGCCGGCCC	0	11	RDR2-sensitive
CGTTAATTGATCCGGACCGTCCGT	0	16	RDR2-sensitive
CGTTACCTATGGATTGGACGGGAT	0	9	RDR2-sensitive
CGTTACGTATGGATTGGACGGGAT	0	13	RDR2-sensitive
CGTTACTTCTAGTTAGAACGTCGT	45	1	RDR2-resistant
CGTTAGGTTGACGGGCCGTGCCGG	0	10	RDR2-sensitive
CGTTAGTAACTCGGCTCGACTCGG	0	11	RDR2-sensitive
CGTTAGTAGCTCGGCTCGACTCGG	1	59	RDR2-sensitive
CGTTATAATGGATTAGTGATACTA	93	6	RDR2-resistant
CGTTATATTTGTAGAGTGGCCCAG	0	12	RDR2-sensitive
CGTTATCTCCGGACGGCTAAGCCC	0	10	RDR2-sensitive
CGTTATGACTGTGGGGGATGTCGG	0	26	RDR2-sensitive
CGTTCAATTGGACCAGGCTGAGGC	0	9	RDR2-sensitive
CGTTCACTCCACGGAACTCGTCGT	0	12	RDR2-sensitive
CGTTCAGGACGAGCATGCATGCAT	2	16	RDR2-sensitive

CGTTCCTGTAGCGAGCCGAGCCGA	0	15	RDR2-sensitive
CGTTCGAAGGCGGACTTGGATCGT	0	33	RDR2-sensitive
CGTTCGAGCTGTGGAAGATGGCCC	2	22	RDR2-sensitive
CGTTCGATCCGACGACAACGGCAA	0	9	RDR2-sensitive
CGTTCGGACGCCTGGCTCGATGGC	0	10	RDR2-sensitive
CGTTCGGAGCGATGTAGGGAAGGA	0	18	RDR2-sensitive
CGTTCGGCCGGACAGTCACCGGAT	0	17	RDR2-sensitive
CGTTCGGTTGTACGCAGGGGCGGA	0	13	RDR2-sensitive
CGTTCGTCTGACTTGGGTATAGGG	40	2	RDR2-resistant
CGTTCTTACAGCGAGGTCGCGGAT	0	25	RDR2-sensitive
CGTTGACACTGCGGACGGTGAGAG	0	18	RDR2-sensitive
CGTTGACGTGGCACCGAGCTCGGC	4	25	RDR2-sensitive
CGTTGACTGAGGGCGCGGTGGCGT	0	22	RDR2-sensitive
CGTTGATCCTGGAGTGGACGAGGT	0	12	RDR2-sensitive
CGTTGATGCTTCGGGCCAGGTCGG	0	14	RDR2-sensitive
CGTTGATGCTTCGGGCCGGGTCGG	0	37	RDR2-sensitive
CGTTGATGCTTCGGGCCGGGTCGT	0	9	RDR2-sensitive
CGTTGATGCTTCGGGCTGGGTCGG	0	11	RDR2-sensitive
CGTTGCAGAGTAGGCCATGACGGT	0	11	RDR2-sensitive
CGTTGCAGAGTGGACAGTTGACGC	0	159	RDR2-sensitive
CGTTGCAGAGTGGACAGTTGACGT	0	11	RDR2-sensitive
CGTTGCAGAGTGGACGGTTGACAC	0	11	RDR2-sensitive
CGTTGCAGAGTGGACGGTTGACGC	0	10	RDR2-sensitive
CGTTGCATCGGAAGAGGACGGACG	0	21	RDR2-sensitive
CGTTGCCACGGCAGAGCTCGGCAC	0	39	RDR2-sensitive
CGTTGCCGGAGAAGGACACGACGA	1	21	RDR2-sensitive
CGTTGCGACGGCTCACAACAACAC	0	9	RDR2-sensitive
CGTTGCTGAACACGTAGAGGACAA	0	23	RDR2-sensitive
CGTTGCTGGAGAGCGTAGAGGACC	0	16	RDR2-sensitive
CGTTGCTGGAGGGAGTAGAGGACA	0	9	RDR2-sensitive
CGTTGCTGGAGGGAGTAGAGGACG	4	31	RDR2-sensitive
CGTTGGAGCTCTGACTGGTGGGGC	1	14	RDR2-sensitive
CGTTGGAGTTGGACGCGACGCGGA	0	12	RDR2-sensitive
CGTTGGATGTGGAGGGTGTGGTCT	0	35	RDR2-sensitive
CGTTGGCGCACTGTTGGCACACAG	0	9	RDR2-sensitive
CGTTGGCTCTCGGCAAAGACTGAC	2	51	RDR2-sensitive
CGTTGGCTTCTCTGTTCTGGAGAC	0	16	RDR2-sensitive
CGTTGGTGGCTGACTGCAAGCAAA	30	0	RDR2-resistant
CGTTGTACGGCGTGGTAGACGGGC	0	9	RDR2-sensitive
CGTTGTATCGCACGAGACAGGCTA	0	32	RDR2-sensitive
CGTTGTTGGAGGGGTAGAGGACGT	2	45	RDR2-sensitive
CGTTTAATAGTGTCGGGCCGGGCC	0	10	RDR2-sensitive
CGTTTACTGTAGTGCACGGCCTGT	0	17	RDR2-sensitive
CGTTTAGAGGACGCTGCTGGAGAG	0	9	RDR2-sensitive
CGTTTAGGACAACGACACGGTCTC	0	20	RDR2-sensitive
CGTTTAGGGCAGTGGGTAACGGTT	0	11	RDR2-sensitive
CGTTTATTCGTACCGGGGCGGCTC	0	13	RDR2-sensitive
CGTTTCTGTGGAGCCAGGCTGAGA	0	36	RDR2-sensitive
CGTTTCTTTTTATTTGTCGCTGGA	9	32	RDR2-sensitive

CGTTTGAATTTCGGGCCGTGCCGG	0	11	RDR2-sensitive
CGTTTGAATTTTGGGTCGTATCGG	0	9	RDR2-sensitive
CGTTTGGCACGAAGCACGATGGGC	0	11	RDR2-sensitive
CGTTTGTAACTCAGCGAGCGTCAT	0	22	RDR2-sensitive
CGTTTGTGACGACCGTAGATGGCC	0	12	RDR2-sensitive
CGTTTGTTTGGTTGCTGGATACAT	0	20	RDR2-sensitive
CGTTTTAGAGTTTGTCGGTCGCCT	0	9	RDR2-sensitive
CGTTTTCGGAATGGGTGACGTGCG	2	20	RDR2-sensitive
CGTTTTGAGCCTGGCGGACGGTCC	0	9	RDR2-sensitive
CGTTTTTCTCGGGCGCAGTAACGG	0	12	RDR2-sensitive
CTAAAAAGAGCAGTGCGGACGGTC	0	16	RDR2-sensitive
CTAAAACTCTGGGTGGAGCAGCTC	3	24	RDR2-sensitive
CTAAACTCTGGAGCAGAGCTGCTC	1	177	RDR2-sensitive
CTAAATTATTTCGGATCGTGTCGT	0	9	RDR2-sensitive
CTAAATTGGACTGAGCAGGGGATA	0	9	RDR2-sensitive
CTAAATTTCGGTCCGTCGGCCCAT	0	15	RDR2-sensitive
CTAACAGGACGGCTGAAAGCACAC	0	28	RDR2-sensitive
CTAACATTCGGCGAACGCGGCGTT	0	12	RDR2-sensitive
CTAACCCGCCATGGCGTTCGTCGT	0	11	RDR2-sensitive
CTAACCGTCCAGGCTTGTGGCATG	0	10	RDR2-sensitive
CTAACCGTGACTGTCGGGCCAGGC	0	19	RDR2-sensitive
CTAACCGTGTCTGGTGGGTCGGGC	0	12	RDR2-sensitive
CTAACGGGCTGGCCTGAAGCACGG	0	10	RDR2-sensitive
CTAACGGGCTGGTTCGAAACACGG	0	13	RDR2-sensitive
CTAACGTGGGTCGGGGCGGGCGGC	32	1	RDR2-resistant
CTAACGTGTCGGGCTGGCACGGCT	0	13	RDR2-sensitive
CTAACTGACTCTGCGCACGCACGG	0	12	RDR2-sensitive
CTAACTGACTCTGCGCACGCGCGG	0	86	RDR2-sensitive
CTAACTGACTCTGCGCGCGCGCGG	1	24	RDR2-sensitive
CTAACTGACTCTGCGCGCGCGGAC	1	21	RDR2-sensitive
CTAACTGACTCTGTGCACGCGCGG	0	9	RDR2-sensitive
CTAACTTATCGAGCGGATCGACGT	0	27	RDR2-sensitive
CTAACTTATTGGACGGATCGGCGT	3	31	RDR2-sensitive
CTAACTTGTCGGACGGGCCGGTGT	1	16	RDR2-sensitive
CTAACTTTCAGTGGGACATTCGAC	41	4	RDR2-resistant
CTAAGACTTTAGGCCTTGTTCGGT	0	9	RDR2-sensitive
CTAAGATCTGTGGCGCCAAGGTAT	0	12	RDR2-sensitive
CTAAGCGACGCTGAGCAGAAGATT	0	11	RDR2-sensitive
CTAAGCGACGCTGAGCAGAGGACT	0	19	RDR2-sensitive
CTAAGCGTTTTGCCTGTTAGTCGG	0	9	RDR2-sensitive
CTAAGGCTCAGCCCCGATCGCGGT	0	9	RDR2-sensitive
CTAAGGTTAGGCAGATTGTGGCGC	0	13	RDR2-sensitive
CTAAGGTTAGGCAGATTGTGGCGT	1	24	RDR2-sensitive
CTAAGGTTAGTCGTTCGAATTGAA	0	12	RDR2-sensitive
CTAAGTCTGTGGGCAAGATGGCCT	0	12	RDR2-sensitive
CTAAGTGTAGATCGCTGAAGGCAT	0	10	RDR2-sensitive
CTAAGTTCGGGCCTAATCGGGTAT	0	11	RDR2-sensitive
CTAATACGATAAGTTCGACGGTTT	0	9	RDR2-sensitive
CTAATACTGTAAGTTCGACGGTTT	0	16	RDR2-sensitive

CTAATACTGTAAGTTTGACGGTTT	1	16	RDR2-sensitive
CTAATATATCGGGCTATGTCGGAC	0	11	RDR2-sensitive
CTAATCATGTTGGACGGGCCGGCT	0	10	RDR2-sensitive
CTAATCGGACTTATGCCGGGTCGG	0	15	RDR2-sensitive
CTAATCTCGGGATACGCAGACGGT	0	13	RDR2-sensitive
CTAATGAGTCGGGCGTCCGGACGG	0	13	RDR2-sensitive
CTAATGCGGGGGACAGGGAGGCAC	1	14	RDR2-sensitive
CTAATGGACGGGCACGAAGCACGA	0	33	RDR2-sensitive
CTAATGGGCGGGCACGAAGCACGA	0	11	RDR2-sensitive
CTAATGTATTTCTCTCGGATACGG	0	10	RDR2-sensitive
CTAATGTTATGGACTCGCATGTTG	20	0	RDR2-resistant
CTAATTGTGGGACTGGGCCGGCAC	3	84	RDR2-sensitive
CTAATTGTGGGCCTGGGCCGGCAC	1	14	RDR2-sensitive
CTAATTGTGGGCTGTGCCGTGGGC	21	0	RDR2-resistant
CTAATTGTGGGTCTGGGCCGGCAC	1	42	RDR2-sensitive
CTAATTGTGGGTCTGGGCCGGCAT	1	27	RDR2-sensitive
CTACACACGGGCTGCACGGAAAAG	0	11	RDR2-sensitive
CTACACATGTGTGGACTGTCCGGC	0	27	RDR2-sensitive
CTACACCTCTGAGTCTGCCGGCAC	0	28	RDR2-sensitive
CTACACTGTCGAGACTAAGGCTAT	0	9	RDR2-sensitive
CTACAGAAATAGGCCTCGATCGAC	17	0	RDR2-resistant
CTACAGAAATAGGCCTCGATCGAT	68	0	RDR2-resistant
CTACAGATAGCGGACGCATGTCAT	0	9	RDR2-sensitive
CTACAGATCGCTGAACGCTGACGG	0	12	RDR2-sensitive
CTACAGCGCCCCCTACGTGTCGGT	0	22	RDR2-sensitive
CTACATACATGTGGGTCGTCCGAG	0	10	RDR2-sensitive
CTACATGTAGCGGCTGAAGCAGAG	0	10	RDR2-sensitive
CTACATGTGGCACTGTCGTTTGGG	20	1	RDR2-resistant
CTACCAAGTGGGCTAGATGGGCTT	3	32	RDR2-sensitive
CTACCACCTGTAGCCTGACGGTGA	0	14	RDR2-sensitive
CTACCCACAGGTATAATCACGGAC	0	11	RDR2-sensitive
CTACCCACAGGTATAGTCGCGGAC	0	60	RDR2-sensitive
CTACCCACGGGTATAGTCGCGGGC	0	12	RDR2-sensitive
CTACCCGTGGGTCTGTTATTGGGC	3	43	RDR2-sensitive
CTACCCTCAGTAAAGGTTGTCGGT	0	9	RDR2-sensitive
CTACCGACGACTGTACAGATTGCA	0	9	RDR2-sensitive
CTACCGATTGCTGGACTGGATGGT	0	9	RDR2-sensitive
CTACCGTCAGCAGTAGAGGCGCAA	0	10	RDR2-sensitive
CTACCGTCTCGGACAGGCAGTGGA	0	11	RDR2-sensitive
CTACCTATCGGGCCGTGCCGGCAC	0	12	RDR2-sensitive
CTACCTCGTTGGGCTGCTCGGTAG	0	10	RDR2-sensitive
CTACCTGAAGGAGGACGAACGGTT	0	15	RDR2-sensitive
CTACCTGTAGCGCTGACTCTTGGC	21	2	RDR2-resistant
CTACCTGTCAGAGACCGAACCGGC	0	11	RDR2-sensitive
CTACCTTAGGGGTTCAGAGATCGT	0	12	RDR2-sensitive
CTACGACGAGCAAACCTGAGGCGC	0	9	RDR2-sensitive
CTACGACTAGTGTATTCATACGGT	16	0	RDR2-resistant
CTACGATCCAGGGAGCTTCGGCAT	0	21	RDR2-sensitive
CTACGCATCTGTGGAGCCAGGCTC	0	25	RDR2-sensitive

CTACGCCAAAGGATCTCTGACGTC	0	10	RDR2-sensitive
CTACGGACTCTTAGCACTGGCGGG	0	17	RDR2-sensitive
CTACGGCAACGCTTGGAACATCAA	1	22	RDR2-sensitive
CTACGGCTCTGGGCTAGACGGTCT	1	30	RDR2-sensitive
CTACGGTTTCGGTTGGGTGTCGGG	18	0	RDR2-resistant
CTACGTAGAACTGGGTCGTACCGG	0	14	RDR2-sensitive
CTACGTCCGTGCTGGCTGGCGGCT	0	9	RDR2-sensitive
CTACGTGGACATAGGTTTAGGCAA	0	11	RDR2-sensitive
CTACGTTGGCATGATACATTCGGC	0	18	RDR2-sensitive
CTACTAACAATTACTCGTGGACCC	0	17	RDR2-sensitive
CTACTAACTATTGTTGTCGGCACA	0	12	RDR2-sensitive
CTACTACAGCTGTGGATGTGGGCA	0	10	RDR2-sensitive
CTACTAGACTTTAGTACTGACGGT	0	9	RDR2-sensitive
CTACTAGAGCTCTCGGCACAGGGA	0	11	RDR2-sensitive
CTACTATATCAAGGACTATGGCGT	0	52	RDR2-sensitive
CTACTCCTTGGACTACGGCGAACA	1	38	RDR2-sensitive
CTACTCTGACAGCGGCGAGGCCGA	0	15	RDR2-sensitive
CTACTCTGCGCAGGCGTGGACCGT	0	11	RDR2-sensitive
CTACTCTGTAACTTATAAGACGAT	0	11	RDR2-sensitive
CTACTCTGTGGTTATACTATGGCA	27	0	RDR2-resistant
CTACTCTGTTGGATGGAAGATGCA	0	26	RDR2-sensitive
CTACTCTTTGGACTGGCACGACGC	0	9	RDR2-sensitive
CTACTGACCTCTAGAACCGATGGC	0	16	RDR2-sensitive
CTACTGCAGAAGGACTTGAAGCAT	0	110	RDR2-sensitive
CTACTGCGTCAAGGACTGCGGCGT	0	13	RDR2-sensitive
CTACTGCTGACGGGAGGGAAGGGA	0	23	RDR2-sensitive
CTACTGGACAATAGCGTGAGGTAT	0	21	RDR2-sensitive
CTACTGGCCCATAGCACTGACGGT	0	12	RDR2-sensitive
CTACTGGCCCCTAGCACTGACGGT	0	11	RDR2-sensitive
CTACTGGCCTTTAACACTGACGGT	0	9	RDR2-sensitive
CTACTGTACCTGTGGGCCCGTCTT	0	13	RDR2-sensitive
CTACTGTAGACGGTCAGTGTCGTA	0	11	RDR2-sensitive
CTACTGTAGCCCACGAGCCGGCCT	0	9	RDR2-sensitive
CTACTGTAGTTCACGGGCCGGCCC	0	12	RDR2-sensitive
CTACTGTCACGTAGGTACCGACGC	0	13	RDR2-sensitive
CTACTGTCACTCTCTGCACGCGGT	0	18	RDR2-sensitive
CTACTGTTTATTTTGTTTGGCATC	21	0	RDR2-resistant
CTACTTCCTTGGGCTGTCTTCGGT	0	20	RDR2-sensitive
CTACTTCGACTGGGCGGCGCTCAT	0	14	RDR2-sensitive
CTACTTGGGCCTCCTGACGCGGTG	0	12	RDR2-sensitive
CTACTTTTCGTTTGGTTCGTCTGT	0	17	RDR2-sensitive
CTAGAAACCTGATTCGCCGGCACC	0	12	RDR2-sensitive
CTAGAACCGAACAGCGAGAGGCGC	0	24	RDR2-sensitive
CTAGAACCGAGAGCGACGATGCGT	0	32	RDR2-sensitive
CTAGAACGACTGACGCCTGACGAG	0	31	RDR2-sensitive
CTAGAACGGGCTCGACGAGAGGAA	0	9	RDR2-sensitive
CTAGAAGACGAAGGGTTCCGGAGA	0	16	RDR2-sensitive
CTAGAAGGTTGGCTTGGACGTCGT	0	13	RDR2-sensitive
CTAGAATCAAGGATGGAACGGCTC	0	9	RDR2-sensitive

CTAGAATCAGGAATGGAACGGCTC	0	32	RDR2-sensitive
CTAGAATCAGGGATGGAACGGCTC	5	306	RDR2-sensitive
CTAGAATCAGGGATGGAACGGCTT	0	12	RDR2-sensitive
CTAGAATCAGGGATGGAGCGGCTC	0	20	RDR2-sensitive
CTAGAATCGGGGATGGAACGGCTC	0	9	RDR2-sensitive
CTAGAATCTGGGATGGAACGGCTC	0	27	RDR2-sensitive
CTAGACACGGACTGGTGACCGGGC	0	16	RDR2-sensitive
CTAGACACTCCGACGCTTAGGCGA	0	9	RDR2-sensitive
CTAGACCCTCTCTGAAGACGTCGC	0	15	RDR2-sensitive
CTAGACCGAAGGACCTGCCGGCTT	0	13	RDR2-sensitive
CTAGACTATCCGTCGGAGCGGCGC	0	10	RDR2-sensitive
CTAGACTCTACGCGTGATCGAGCT	0	9	RDR2-sensitive
CTAGACTGAGACGGAGGACGAGAG	0	17	RDR2-sensitive
CTAGACTGAGAGAGGAGGGAGGCC	0	13	RDR2-sensitive
CTAGACTGTATGGCGTAGCGGCGC	0	42	RDR2-sensitive
CTAGACTGTCACGGCACAAAGCGT	0	16	RDR2-sensitive
CTAGACTGTGGACGCGGTGGGAGA	0	11	RDR2-sensitive
CTAGACTTGAACCGGTAGTGTCGT	1	93	RDR2-sensitive
CTAGAGCTCTCGGCACAGGGACTG	0	13	RDR2-sensitive
CTAGAGGAGCTGTACCAAACGGGT	0	10	RDR2-sensitive
CTAGATCACGGACCGTTCAGGCAG	0	15	RDR2-sensitive
CTAGATCCTCGGAGCGCATGGCAG	1	13	RDR2-sensitive
CTAGATCCTGCACCGGGAGAGGGC	0	14	RDR2-sensitive
CTAGATCGGATGCACCAGATCGCT	0	13	RDR2-sensitive
CTAGATCTACTGGACGACGAGCGC	0	13	RDR2-sensitive
CTAGATCTGAGGGACTGACGGTTT	32	0	RDR2-resistant
CTAGATGGCAGAACGGCAGAACAG	0	26	RDR2-sensitive
CTAGATGTGGAGACTTGGAGACGT	0	31	RDR2-sensitive
CTAGATTCTGGCCATAGCTCGGTT	0	12	RDR2-sensitive
CTAGATTGGCTCGAGGCAGAACGG	0	21	RDR2-sensitive
CTAGCACACTAGAACGGAGCTCAA	0	9	RDR2-sensitive
CTAGCACGACTAGCAGGCTGACGG	0	17	RDR2-sensitive
CTAGCACTATAGAATTGACGTGGC	0	14	RDR2-sensitive
CTAGCACTGGCGGAACTGAGAAAC	0	123	RDR2-sensitive
CTAGCACTGGCGGCACTGAAAAAC	0	40	RDR2-sensitive
CTAGCACTGGCGGCACTGAAAAGC	1	45	RDR2-sensitive
CTAGCAGTCGGCGTAGTGTATGGC	0	9	RDR2-sensitive
CTAGCCCCTGTGCCAGGTCGCGGA	0	12	RDR2-sensitive
CTAGCCGCGCGTCGGAACCCGGTG	0	9	RDR2-sensitive
CTAGCGAGCAGTTGTAGGCCCCTG	1	19	RDR2-sensitive
CTAGCGCATCGGACTGTTCGGGTG	0	10	RDR2-sensitive
CTAGCGGACCTGAAGTAGAGAGAC	0	10	RDR2-sensitive
CTAGCGGACGGCTGTGTTTGACGC	0	10	RDR2-sensitive
CTAGCGGACGGCTGTGTTTGGCGC	0	20	RDR2-sensitive
CTAGCGTGACAACTGCCGTCGGAG	0	14	RDR2-sensitive
CTAGCTCTGATAGCATGTTCGGTT	0	10	RDR2-sensitive
CTAGCTGAACGTATGGATGGCTGT	0	9	RDR2-sensitive
CTAGCTGGAGACGAAGCTCGGAAA	0	9	RDR2-sensitive
CTAGCTGGGCCACGTAGACAGCAT	1	20	RDR2-sensitive

CTAGCTGTGCTTGGGTCGGGTCGG	0	17	RDR2-sensitive
CTAGCTTGTCGAGCGGACCGGTAT	0	27	RDR2-sensitive
CTAGCTTGTCGAGCGGATGGACGT	1	44	RDR2-sensitive
CTAGCTTGTCGAGCGGATTGACGT	1	31	RDR2-sensitive
CTAGGACGACGACGATGACGACGT	0	16	RDR2-sensitive
CTAGGACTCGGTGCATTGGCAGTT	16	0	RDR2-resistant
CTAGGAGAGGACGCTACTGGAGAG	0	9	RDR2-sensitive
CTAGGAGCCTAGGACGACGGTGAC	0	9	RDR2-sensitive
CTAGGAGTTCTGGACCTGGAGCGT	0	13	RDR2-sensitive
CTAGGAGTTGTCTAGGCTCGGGCA	3	32	RDR2-sensitive
CTAGGATCTCTACTCCCCGACGGG	0	11	RDR2-sensitive
CTAGGATCTCTCCTCCCCGACGGG	0	9	RDR2-sensitive
CTAGGATGAACCGCGCGAGGACGT	0	11	RDR2-sensitive
CTAGGATGCCGGACATGCACGCAT	0	9	RDR2-sensitive
CTAGGATGTGGCACGGTGGCCCAA	0	9	RDR2-sensitive
CTAGGATGTGGCACGGTGGCCCAG	0	9	RDR2-sensitive
CTAGGCACGGAGCTGGCACAACAC	0	17	RDR2-sensitive
CTAGGCACGGTAGAATAAGCGGGC	0	16	RDR2-sensitive
CTAGGCATTTTGCTTGTTAGTCGG	4	25	RDR2-sensitive
CTAGGCTCTCGGGAGATGGAGGAC	57	2	RDR2-resistant
CTAGGCTGAGAGAGGAGGGAGGCC	0	10	RDR2-sensitive
CTAGGCTGTAGCCCCGGTCACGGC	1	81	RDR2-sensitive
CTAGGCTGTGAGAGTAGGGAGACC	0	28	RDR2-sensitive
CTAGGCTGTGAGCCAGTGCTGCGG	0	22	RDR2-sensitive
CTAGGCTGTGCGCGTGCAAATCGA	0	10	RDR2-sensitive
CTAGGGAACTGGATTACACGGCTT	0	11	RDR2-sensitive
CTAGGGCATGGCAGATGATGACGA	0	11	RDR2-sensitive
CTAGGGCTCAGCACGGTTCGTCGT	0	14	RDR2-sensitive
CTAGGGCTGACCTGGCATCGACGT	0	22	RDR2-sensitive
CTAGGGGCCAGTGGAAATCAGCAT	0	11	RDR2-sensitive
CTAGGGGCCAGTGGAAATCGTCAT	0	11	RDR2-sensitive
CTAGGGGTCAGTGGAAATCAGCAT	3	79	RDR2-sensitive
CTAGGGGTTGTCTAGGCTCGGGCC	0	10	RDR2-sensitive
CTAGGGTGGACTGCAGCAGCCGGC	0	12	RDR2-sensitive
CTAGGTAGGCTTGGACATTGGCAC	0	11	RDR2-sensitive
CTAGGTCGTGGACTGTCCGGCCAA	1	34	RDR2-sensitive
CTAGGTCTGCTGTGAAGAAGACGA	0	11	RDR2-sensitive
CTAGGTGACCTACTGATCTCGGAG	0	28	RDR2-sensitive
CTAGTAACTGTTTTTTCCTTGGCT	140	8	RDR2-resistant
CTAGTATTGTAGAAGCTTCGGCTC	0	20	RDR2-sensitive
CTAGTCAGGACGCGGTCAACCCAG	0	26	RDR2-sensitive
CTAGTCATCGGACCATGCCGGCAC	0	9	RDR2-sensitive
CTAGTCATGGGCTGGGCCAGGCAA	0	15	RDR2-sensitive
CTAGTCCGGTATGTAGGGGTGGAT	0	9	RDR2-sensitive
CTAGTCGGCAGGAAGATCAGAGGC	0	11	RDR2-sensitive
CTAGTCGGTGTAGACGTGCGGTAG	0	14	RDR2-sensitive
CTAGTCGTTGGACCCAGACGGTCT	0	15	RDR2-sensitive
CTAGTCGTTTTGGTAGAGCGGTAA	0	34	RDR2-sensitive
CTAGTGGGCTGGGCTAGATTTGGC	0	46	RDR2-sensitive

CTAGTGTGGTATCTGATTACGGTT	0	14	RDR2-sensitive
CTAGTTAGGGTTATGTGGGCAAAT	0	9	RDR2-sensitive
CTAGTTCGGTTCTCGGTTCTTCGG	2	14	RDR2-sensitive
CTAGTTGAAGAAGGTTATGATGAT	80	6	RDR2-resistant
CTAGTTTGACTGGTTAGAATGGCT	0	9	RDR2-sensitive
CTAGTTTGTCGAACGGATTGGCAT	0	24	RDR2-sensitive
CTATAAGTCTGTAGTGTAGACGTC	0	21	RDR2-sensitive
CTATAATTTGTGGATCCGATGACA	0	19	RDR2-sensitive
CTATACACGTGTGGACCGTCTGAC	1	25	RDR2-sensitive
CTATACCACGGACTCACTTGGCAG	0	31	RDR2-sensitive
CTATACCAGCGTTTGATCGTCGTC	2	19	RDR2-sensitive
CTATACTACTAGGTTGTTCGGGCC	0	14	RDR2-sensitive
CTATAGACGGCTGCTGACGGTCCT	1	13	RDR2-sensitive
CTATAGCAGGTCGGCTCGGACAGT	0	11	RDR2-sensitive
CTATAGCTACTCTGATTTAGACGT	0	10	RDR2-sensitive
CTATAGGCTATAGCGCAGAGACGT	0	10	RDR2-sensitive
CTATAGGGCAGTAGCTGAGAGCAT	0	9	RDR2-sensitive
CTATATACTGGAACGGAACGGCTT	0	32	RDR2-sensitive
CTATATCAAGGACTATGACGTCAT	0	9	RDR2-sensitive
CTATATCAAGGACTATGGCGTCAT	2	27	RDR2-sensitive
CTATATCAAGGACTGTGGCGTCAT	0	61	RDR2-sensitive
CTATATCATGGACTGTGACGTCAT	1	56	RDR2-sensitive
CTATATCATGGACTGTGGCGTCAT	0	38	RDR2-sensitive
CTATATCGAGAACTGTGGCGTCAT	0	9	RDR2-sensitive
CTATATCGAGGACTGTGGCGTCAT	1	98	RDR2-sensitive
CTATATCGAGGATTGTGGCGTCAT	4	36	RDR2-sensitive
CTATATTCATGTCGTACTGGACGG	0	13	RDR2-sensitive
CTATATTGGACTGGGCCACGGCCT	0	12	RDR2-sensitive
CTATCATACGTGGGATCGTAGCGT	0	11	RDR2-sensitive
CTATCCCTGATTGTAGAAACGGAG	0	12	RDR2-sensitive
CTATCGAGCAACTCTGCGAGACGG	0	9	RDR2-sensitive
CTATCGCGTAGGACAGTTCGGCAA	5	121	RDR2-sensitive
CTATCGCGTAGGACAGTTCGGCTA	0	50	RDR2-sensitive
CTATCGCGTAGGACAGTTCGGCTT	0	13	RDR2-sensitive
CTATCGCGTAGGACAGTTTGGCAA	1	23	RDR2-sensitive
CTATCGGACGCTGTATCAGGCATA	0	11	RDR2-sensitive
CTATCGGACTAGGCCAGAGTGCAC	0	11	RDR2-sensitive
CTATCGGGCACTCAGCGAAGACGT	0	62	RDR2-sensitive
CTATCGGGCACTGTATGAGGCATA	0	12	RDR2-sensitive
CTATCGGGTGACGGATATGGATAG	0	10	RDR2-sensitive
CTATCGTCAGATCGGATCGGTCAG	0	11	RDR2-sensitive
CTATCGTGTAGGACAGCTCGGCAA	0	10	RDR2-sensitive
CTATCGTGTTGGGCTTAGGTGGTT	0	15	RDR2-sensitive
CTATCGTGTTGTGCTGGGATGGTT	0	14	RDR2-sensitive
CTATCTCCGACGACTGGTGAGGTT	0	12	RDR2-sensitive
CTATCTGACCTGGGCTGACGACTG	0	9	RDR2-sensitive
CTATCTGATCCCGGACCACAGCGG	0	19	RDR2-sensitive
CTATCTGTGAAGGGCTGAAGGCGT	0	21	RDR2-sensitive
CTATCTTACTGTAGTTAGACGCGT	0	9	RDR2-sensitive

CTATGACACTGAGCTGGACGGTCT	1	70	RDR2-sensitive
CTATGACATGTGGAGCCAGTGGCA	0	9	RDR2-sensitive
CTATGACATGTGGGACCAGTGGCA	0	28	RDR2-sensitive
CTATGACATGTGGGGCCAGTGGCA	0	64	RDR2-sensitive
CTATGACTGGTGGGACCGTCTGTG	0	46	RDR2-sensitive
CTATGAGCCCCCGGACTGCGGCGC	0	15	RDR2-sensitive
CTATGAGCCTGTAGGACCGCGACA	0	10	RDR2-sensitive
CTATGATTTATGGATTCGATGGCA	1	14	RDR2-sensitive
CTATGATTTGTGAACCGATGGCAT	0	16	RDR2-sensitive
CTATGATTTGTGGATCCGATGGCA	0	22	RDR2-sensitive
CTATGATTTGTGGGCCCGATGGCA	0	19	RDR2-sensitive
CTATGATTTGTGGGTCCGATGACA	0	38	RDR2-sensitive
CTATGATTTGTGGGTCCGATGGCA	0	248	RDR2-sensitive
CTATGCGAGACTGGACCGGCCCGA	0	14	RDR2-sensitive
CTATGCTGAAGCTCCCTGGATCGT	0	9	RDR2-sensitive
CTATGGAGCTGCCCACGTCGGCAA	0	11	RDR2-sensitive
CTATGGATGGCAGAACGGCAGAAC	0	13	RDR2-sensitive
CTATGGATGGTGGAGGAAGTGCAG	1	14	RDR2-sensitive
CTATGTAAGTCGGCATATGTCCAT	0	9	RDR2-sensitive
CTATGTCCGACGGCATCGTAGACA	0	11	RDR2-sensitive
CTATGTCCTAGATATTGTCGTCGT	0	12	RDR2-sensitive
CTATGTCGTGGGTCCGTGTCGGTC	0	10	RDR2-sensitive
CTATGTTAGAACTCCGGCGAGGAG	27	0	RDR2-resistant
CTATGTTTCGATCGGGCCAGGCCA	0	15	RDR2-sensitive
CTATGTTTGACGGCTGCCTTGGAG	0	10	RDR2-sensitive
CTATTAAACGGGCCGTGCTTCGGA	0	10	RDR2-sensitive
CTATTAAGACTGTAAGGGGTAGGC	0	9	RDR2-sensitive
CTATTCGCTGTAGATGGGCCGCGA	0	10	RDR2-sensitive
CTATTCGGGTTCGGTTCGGGTTCG	0	9	RDR2-sensitive
CTATTCGTACCGGGCCGAAACGGG	0	17	RDR2-sensitive
CTATTGAGCCTGGCCGTAGGAGGA	0	9	RDR2-sensitive
CTATTGCACTGTAATTGATGGCGA	0	15	RDR2-sensitive
CTATTGGGGGACGCATAGGAACAG	0	16	RDR2-sensitive
CTATTGTTGGAGGCAGCCTTAGAC	0	11	RDR2-sensitive
CTATTGTTGGTCTGTGATGATGGT	0	9	RDR2-sensitive
CTATTTGTGCAGGGCTGTGCCGGT	0	10	RDR2-sensitive
CTATTTTCGGCGGCTGTGTCAGGC	0	12	RDR2-sensitive
CTATTTTTTTGAGACGGTATGGAC	0	11	RDR2-sensitive
CTCAAAACCCGGGCTGTGAGAGGA	3	37	RDR2-sensitive
CTCAACGAAGAAGGCTCGGCACAC	0	11	RDR2-sensitive
CTCAACGAGCTGTAGAAAGAGGTT	0	12	RDR2-sensitive
CTCAACGTCGATCGGCTCGGCCAG	0	9	RDR2-sensitive
CTCAAGGGCACTGTACGAGGGTAT	0	26	RDR2-sensitive
CTCAAGGGCACTGTACGGAGGTAT	0	9	RDR2-sensitive
CTCAAGGGCACTGTACGGGGGTAT	0	10	RDR2-sensitive
CTCAATATTTGGTTGTAACGTCGT	17	1	RDR2-resistant
CTCAATGAATCAGGAATTGTCGGT	0	17	RDR2-sensitive
CTCAATGGTTCGTGCGGGACGCAC	0	13	RDR2-sensitive
CTCAATGGTTCGTGCTGGACGCAC	0	11	RDR2-sensitive

CTCAATTATAATTCTCGGACGCGC	0	15	RDR2-sensitive
CTCACACTGCAGAGGCTTGGACGG	0	14	RDR2-sensitive
CTCACATAGACCTGAACGAGCGGT	0	16	RDR2-sensitive
CTCACCGTATGACACTGGAGGCAT	0	18	RDR2-sensitive
CTCACGCTCGGGGATGGCTCAGAC	25	0	RDR2-resistant
CTCACGGGCACTGTGCGGGGGTAT	2	15	RDR2-sensitive
CTCACGGGTACTGTACTGGGGTAT	0	11	RDR2-sensitive
CTCACGTAGATCTGAATGTATGGC	0	14	RDR2-sensitive
CTCACGTCGACTGTTCAGAGGGCC	0	29	RDR2-sensitive
CTCACTAGTGTGTGGTCGGTCGAT	0	9	RDR2-sensitive
CTCACTGAGTCGGCTGTGGATGGC	0	15	RDR2-sensitive
CTCACTGGTCTGTGGATGCGGAGT	0	14	RDR2-sensitive
CTCACTTCTTCGGTCTTCGGTCAT	0	11	RDR2-sensitive
CTCAGACTGTATGACTAAGGGCAT	0	10	RDR2-sensitive
CTCAGAGTCGTTGGTGCTTGCATA	14	0	RDR2-resistant
CTCAGCAATCCACCTGGTCGTCGT	0	9	RDR2-sensitive
CTCAGCAGGCTGTAAGGTGTTCCA	0	10	RDR2-sensitive
CTCAGCCTGGACCAGTTGAACGGA	1	45	RDR2-sensitive
CTCAGCCTGGACCAGTTGAACGGG	0	9	RDR2-sensitive
CTCAGCCTGGCTCCACAGAAACGA	0	9	RDR2-sensitive
CTCAGCCTGGCTCGCTAGAAACGG	0	9	RDR2-sensitive
CTCAGCGAACTGTACATCGGCAAC	0	35	RDR2-sensitive
CTCAGCTGGACCGGATACTACGCA	0	10	RDR2-sensitive
CTCAGCTGGACGCAGGGGAGATGG	0	10	RDR2-sensitive
CTCAGGACCCTGACGACAAACGGC	0	12	RDR2-sensitive
CTCAGGATCTAGAAAGTGACGGAT	0	33	RDR2-sensitive
CTCAGGCCATAGGATCGGACACCT	0	23	RDR2-sensitive
CTCAGGCCATAGGATTGGGCACCT	0	9	RDR2-sensitive
CTCAGGCTAGACCAGTTGAACGGC	0	10	RDR2-sensitive
CTCAGGCTATGGACGCAGGGGCTT	0	13	RDR2-sensitive
CTCAGTACTAGAACAGAGCGGTTC	0	11	RDR2-sensitive
CTCAGTCGTGTAGAAGGAGGGCGT	0	31	RDR2-sensitive
CTCAGTGGATCGTGGCAGCAAGGC	46	1	RDR2-resistant
CTCATAATATTTTAGCAAGTCGGC	18	1	RDR2-resistant
CTCATCCCATGGACGACGTAGCAT	0	12	RDR2-sensitive
CTCATCCTTCCACCGCGGCCGGCA	0	10	RDR2-sensitive
CTCATCGTTTGGAGCGGCTTCTAG	0	11	RDR2-sensitive
CTCATCTCAGGACTTAGGGCGGGT	0	11	RDR2-sensitive
CTCATCTGTGTACGTCCGGACGGT	0	11	RDR2-sensitive
CTCATCTTAGGACTTAGGGCGGGT	0	33	RDR2-sensitive
CTCATCTTAGGATTTAGAGCGGGT	0	9	RDR2-sensitive
CTCATCTTAGGATTTAGGACGGGT	0	16	RDR2-sensitive
CTCATCTTAGGATTTAGGGCGGGT	1	23	RDR2-sensitive
CTCATGTCGCATCGCACGGACACT	0	22	RDR2-sensitive
CTCATGTCTGTTGGATGGTTTGAT	29	0	RDR2-resistant
CTCATTCGGAGGGTGAGGACAACA	0	11	RDR2-sensitive
CTCATTGTCAAGCTTGGAGGCGTC	29	4	RDR2-resistant
CTCATTGTCGAGCTTAGAGGCGTC	43	4	RDR2-resistant
CTCATTGTCGAGCTTGGAGGCGTC	111	24	RDR2-resistant

CTCCAAACCCTAGCGTCGGCGGCT	0	10	RDR2-sensitive
CTCCAAAGACTGGCACATGACGAG	0	15	RDR2-sensitive
CTCCAACGAGGACTAGGCAAGCAT	0	16	RDR2-sensitive
CTCCAACGGTCAGAACCCAACGGC	0	10	RDR2-sensitive
CTCCAAGAACCAAGCGACGGACGA	0	12	RDR2-sensitive
CTCCACATTTAGGGTCTCTGGCAC	0	11	RDR2-sensitive
CTCCACGGAGCGTGGTCGGTCGGA	0	9	RDR2-sensitive
CTCCACGGATGAAAAGGGGACGCA	2	56	RDR2-sensitive
CTCCACTCTGAAGGACGACGTCAT	1	12	RDR2-sensitive
CTCCAGCAGCGTCCTCTAAACGGT	0	13	RDR2-sensitive
CTCCAGCCAGGAGATCGATCGGGT	0	48	RDR2-sensitive
CTCCAGGACAGATCTAGACGACGT	0	9	RDR2-sensitive
CTCCAGGACTCGCCGCTGAAGAGG	16	0	RDR2-resistant
CTCCAGGAGGAGGATAGGACGGAG	16	0	RDR2-resistant
CTCCAGGTTGGACTGTGCGGTTGC	0	20	RDR2-sensitive
CTCCAGTAGTTCTCTAAGCAGTAG	0	13	RDR2-sensitive
CTCCATGACGACGACGGCTCTCAG	0	10	RDR2-sensitive
CTCCCCACAGCTAGATTCTCGGAA	0	16	RDR2-sensitive
CTCCCCACGTCAGAGGATCTCGTT	0	17	RDR2-sensitive
CTCCCCGTATTCGTCCGACACCAG	1	14	RDR2-sensitive
CTCCCGCGAGCGGGCTGCGAGGGC	38	0	RDR2-resistant
CTCCCGGGAGCGAGATCCAGGCGC	1	13	RDR2-sensitive
CTCCCGGTGCAGGATCTAGAAGAC	0	16	RDR2-sensitive
CTCCCGGTGTAGGATCTAGAAGAC	0	11	RDR2-sensitive
CTCCCGTCAACAGTAGAGGCGCAA	0	22	RDR2-sensitive
CTCCCGTCAGCAGTAGAGACGCAA	3	141	RDR2-sensitive
CTCCCGTCAGCAGTAGAGGCACAA	0	45	RDR2-sensitive
CTCCCGTCAGCAGTAGAGGCGAAA	0	9	RDR2-sensitive
CTCCCGTCAGCAGTAGAGGCGCAA	17	953	RDR2-sensitive
CTCCCGTCAGCAGTAGAGGCGCAC	0	22	RDR2-sensitive
CTCCCGTCAGCAGTAGAGGCGCAT	1	27	RDR2-sensitive
CTCCCGTCAGCAGTAGAGGCGCCA	0	17	RDR2-sensitive
CTCCCGTCAGCAGTAGAGGCGCGA	1	118	RDR2-sensitive
CTCCCGTCAGCAGTAGAGGCGTAA	0	24	RDR2-sensitive
CTCCCGTCAGCAGTAGAGGTGCAA	2	16	RDR2-sensitive
CTCCCGTCAGCAGTAGATGCGCAA	0	13	RDR2-sensitive
CTCCCGTCAGTAGTAGAGGCGCAA	1	20	RDR2-sensitive
CTCCCGTTAGCAGTAGAGGCGCAA	0	12	RDR2-sensitive
CTCCCTCATCGTCAGGGCGGCGTC	0	18	RDR2-sensitive
CTCCCTCGGCGTGACGACGGATGC	0	9	RDR2-sensitive
CTCCCTGACGGCACTCTAGTTGAA	76	24	RDR2-resistant
CTCCCTGGAAGGACTCAGTCGGGA	0	9	RDR2-sensitive
CTCCCTGGATTGTAGCTTCGGCAT	0	16	RDR2-sensitive
CTCCCTGGATTGTAGCTTCGGTAT	0	13	RDR2-sensitive
CTCCCTGTATCGAGCCGGACGGTT	0	17	RDR2-sensitive
CTCCCTTATGAAACAGCTGGGCAT	2	54	RDR2-sensitive
CTCCGACCTGTAGCGAGATGGGAC	0	27	RDR2-sensitive
CTCCGAGGCGACTGTTGGAGGCGA	0	15	RDR2-sensitive
CTCCGATATATAGAGGGTCGGCGT	0	14	RDR2-sensitive

CTCCGCACTCTGACACATCGGCAC	0	9	RDR2-sensitive
CTCCGCCTGGCTCCCTGGAAACGG	0	10	RDR2-sensitive
CTCCGCTATAATTCACCGGACTGT	0	34	RDR2-sensitive
CTCCGGACTGGACCAGGCAACCAA	1	21	RDR2-sensitive
CTCCGGGTCCTGTTTTATAGACGT	0	24	RDR2-sensitive
CTCCGGTGATCTCTGCGACGGCAC	0	27	RDR2-sensitive
CTCCGTAAGGACCTGTTCGTTGGA	0	10	RDR2-sensitive
CTCCGTCGTTTACTGTATCGTGCA	1	15	RDR2-sensitive
CTCCGTCTGAAGGTGTCCGGTCAT	0	10	RDR2-sensitive
CTCCGTCTTACACTGTCTCGTGCA	0	10	RDR2-sensitive
CTCCGTGCTCGGCTCGCCCACGGC	0	11	RDR2-sensitive
CTCCGTGTGGGCTTCTGACGGATA	0	14	RDR2-sensitive
CTCCGTTGGACCAACGACTACGGC	0	9	RDR2-sensitive
CTCCGTTGGATTGGTGGTATAGAC	0	19	RDR2-sensitive
CTCCGTTGGTGCAGCGAGACTGGC	0	9	RDR2-sensitive
CTCCGTTGTAGAATATTTGTCGGT	0	17	RDR2-sensitive
CTCCGTTTACTGTAGTGCACGGCC	0	30	RDR2-sensitive
CTCCGTTTACTGTAGTGGAGGGCA	0	9	RDR2-sensitive
CTCCGTTTTGGTCGTCCGTCGGAT	0	9	RDR2-sensitive
CTCCTACACTGTCGAGACTAAGGC	0	11	RDR2-sensitive
CTCCTCACAGCTAGATTCTCGGAA	0	15	RDR2-sensitive
CTCCTCATCGTAGGTGTCGTCGCC	72	6	RDR2-resistant
CTCCTCATCTTCGGACCCGACGGC	0	9	RDR2-sensitive
CTCCTCCCCGTATTCGTCCGACAC	0	11	RDR2-sensitive
CTCCTCCGTTGGATTGGTGGTATA	0	51	RDR2-sensitive
CTCCTCGGTATGTATGTGGGCTAA	0	12	RDR2-sensitive
CTCCTCGTCAGACGTAGGGAGGTG	0	12	RDR2-sensitive
CTCCTCTGCACACTTCCGGACGGT	1	23	RDR2-sensitive
CTCCTCTGCACACTTCTGGACGGT	0	13	RDR2-sensitive
CTCCTCTGCACGCTTCCGGACGGT	0	11	RDR2-sensitive
CTCCTCTGCACGCTTTCGGACGGT	2	18	RDR2-sensitive
CTCCTCTGCATACGTCCGGACGGT	0	9	RDR2-sensitive
CTCCTCTGCATACGTCCGGATGGT	0	14	RDR2-sensitive
CTCCTCTGCATACTTCCGGACGGT	0	25	RDR2-sensitive
CTCCTCTGCGTACATCCGGACGGT	0	17	RDR2-sensitive
CTCCTCTGCGTACGCTCGGACGGT	0	11	RDR2-sensitive
CTCCTCTGCGTACGTCCGGACGGT	1	51	RDR2-sensitive
CTCCTCTGCGTACTTCCGGACGGT	1	27	RDR2-sensitive
CTCCTCTGGGTGACTCGGGCGGCT	0	12	RDR2-sensitive
CTCCTCTGTGTACGTCCGGACGGT	0	11	RDR2-sensitive
CTCCTGATCTGTGGGAGACTCGGT	0	15	RDR2-sensitive
CTCCTGCACCAGGTCGCGGACCGT	0	17	RDR2-sensitive
CTCCTGGGCACGGATAGGGGCATT	41	11	RDR2-resistant
CTCCTGTCAGCAGTAGAGGCGCAA	0	14	RDR2-sensitive
CTCCTTCCCTTGGTCGCTTCGGAC	0	9	RDR2-sensitive
CTCCTTGCTGGTTGTTCTGACGGT	0	9	RDR2-sensitive
CTCCTTGGACTACGACGAACACGG	0	9	RDR2-sensitive
CTCCTTGGACTACGGCGAACACGG	0	54	RDR2-sensitive
CTCCTTTCTGTCGACTGCGGACGG	0	9	RDR2-sensitive

CTCCTTTGTGTACATCCGGACTGT	0	9	RDR2-sensitive
CTCGAAAAAGCGGCTCGGCTCGGC	0	14	RDR2-sensitive
CTCGAACGGTCGCTGGAAGACGAA	0	10	RDR2-sensitive
CTCGAACTCAGAGCAGCTCGGCTT	0	9	RDR2-sensitive
CTCGAAGCACGACGGGTAGATGAT	0	9	RDR2-sensitive
CTCGAAGGAGCAGGAGAACGCCGA	0	18	RDR2-sensitive
CTCGAATCGGCTCGAGAGCATCGT	0	14	RDR2-sensitive
CTCGACACAGTTGGACATTGGCAA	0	9	RDR2-sensitive
CTCGACAGCAGACGCCATGGACGC	0	14	RDR2-sensitive
CTCGACCCATCTGGGCTGGGGCAC	1	19	RDR2-sensitive
CTCGACCTCGGAAGACGGACTCCG	0	11	RDR2-sensitive
CTCGACCTCGGCCTCGGAAGACAG	0	11	RDR2-sensitive
CTCGACGAACTCTCTGTAGCGGTT	0	9	RDR2-sensitive
CTCGACGAACTGTACATCGGCAAC	1	32	RDR2-sensitive
CTCGACGACTGGCAGAAGGACCGT	0	60	RDR2-sensitive
CTCGACGACTGTACAGATTGCGTC	0	22	RDR2-sensitive
CTCGACGAGCTGGGCAGAGCAGAG	0	9	RDR2-sensitive
CTCGACGCAAGGGCTGAACCAGAA	0	14	RDR2-sensitive
CTCGACGCGTCTGTGGAAGGGCGA	0	12	RDR2-sensitive
CTCGACGTCGGCACAAAAAGGCAT	0	11	RDR2-sensitive
CTCGACTCGGCTCGATAACGGCTC	0	22	RDR2-sensitive
CTCGACTCGTGGAAACCGGCCGGT	0	13	RDR2-sensitive
CTCGACTCGTGGATCCGATCGGCA	0	30	RDR2-sensitive
CTCGACTGAGTGGGGCGGAGACGC	0	11	RDR2-sensitive
CTCGACTGTCCAACCCGGTGGCAA	0	66	RDR2-sensitive
CTCGACTTAGCGCACGGACGACGC	0	13	RDR2-sensitive
CTCGAGAAACTCTAAGAATGACGA	198	5	RDR2-resistant
CTCGAGATTTATGAATGACTAGGA	18	0	RDR2-resistant
CTCGAGCAACGGGCAACGGATGAG	20	1	RDR2-resistant
CTCGAGCAAGGCAGAGTACGTCTG	0	9	RDR2-sensitive
CTCGAGCCAGAACCAGAACCAGAA	0	10	RDR2-sensitive
CTCGAGCTCGTAGGAAGACTAGGT	0	9	RDR2-sensitive
CTCGAGGACGACGCTGCGGACGGG	0	13	RDR2-sensitive
CTCGAGTAGGAGAGTGGACATCAT	0	17	RDR2-sensitive
CTCGAGTGTGGACACAGAGACGTC	0	11	RDR2-sensitive
CTCGATAACTCGCTCGACTCGGCT	0	39	RDR2-sensitive
CTCGATAGGACTACTCGAGTCGGC	0	13	RDR2-sensitive
CTCGATATAGTAGTCGTCGAACGC	0	9	RDR2-sensitive
CTCGATATTTGGTTGTAACGTCGT	56	14	RDR2-resistant
CTCGATCGTGTCCTCTGCCCGGTA	0	9	RDR2-sensitive
CTCGATCTATGGAGACGTGGACGG	0	10	RDR2-sensitive
CTCGATGGACGCGTGCAGGAGGCT	0	9	RDR2-sensitive
CTCGATTAGGACACGGCACGACAG	0	23	RDR2-sensitive
CTCGATTTGGGCCGTGTAGTGGGC	0	9	RDR2-sensitive
CTCGCACAAAGGAACTATCGGCAA	0	100	RDR2-sensitive
CTCGCACAGTAGACCGTAGACGGC	0	10	RDR2-sensitive
CTCGCAGCCTGCAGCAACGGACGG	0	15	RDR2-sensitive
CTCGCAGGAGAAGCCCCTGAGCAG	1	14	RDR2-sensitive
CTCGCAGTTCCGGCTAATGTAGAT	0	13	RDR2-sensitive

CTCGCATCTGAGACGAAGACGAGC	0	9	RDR2-sensitive
CTCGCCGACTGTAATGGAGGGCAA	0	10	RDR2-sensitive
CTCGCCGCTCTCAGGACTCGACGG	0	14	RDR2-sensitive
CTCGCCGCTCTCGGGATTCGACGG	1	13	RDR2-sensitive
CTCGCCGGCTGCAATGGAGGGCAA	2	21	RDR2-sensitive
CTCGCCTCGTTGGGTTGCTCGGCA	0	11	RDR2-sensitive
CTCGCCTGACGTCGGGCTCGGTGC	0	11	RDR2-sensitive
CTCGCGCCCAGGAACTCGTCGGAG	0	11	RDR2-sensitive
CTCGCGCCTCTACTGCTGACGGGA	0	17	RDR2-sensitive
CTCGCGCGGGACGGTAGAGGGCCC	0	11	RDR2-sensitive
CTCGCGGAGAGCTTGCAGGACGTC	0	10	RDR2-sensitive
CTCGCGTGCGTAGGACGAGTTGGC	0	37	RDR2-sensitive
CTCGCTACCTACTCTGGGCCGGAC	0	13	RDR2-sensitive
CTCGCTCTGAGCTGCTGAGACGTT	0	19	RDR2-sensitive
CTCGCTGACGCACGGAATGACGAC	0	13	RDR2-sensitive
CTCGCTGGCGTGTATAGGACGGTC	0	15	RDR2-sensitive
CTCGCTGTAGGAGCGGCTCTTCTC	0	17	RDR2-sensitive
CTCGCTTAGAATCAGGAGCTGGAG	0	16	RDR2-sensitive
CTCGGAAAAACGGCTCGGCTCGGC	0	14	RDR2-sensitive
CTCGGAAAAGAGGCTCGGCTCGGC	0	12	RDR2-sensitive
CTCGGAACTAGCAGGTAGCAGAAT	0	9	RDR2-sensitive
CTCGGAAGACGGACTCCGCCTCGC	0	12	RDR2-sensitive
CTCGGAAGACGGCGAACTCCGCTC	1	17	RDR2-sensitive
CTCGGAAGACGGTCTCCGCCTCGC	1	18	RDR2-sensitive
CTCGGAAGTTAGACTGCGGACCGG	0	11	RDR2-sensitive
CTCGGACAAGAAACTAGGCACGGT	0	27	RDR2-sensitive
CTCGGACACTCGATACAGAGGCGT	0	10	RDR2-sensitive
CTCGGACACTCGGCAAAGAGGTTT	0	21	RDR2-sensitive
CTCGGACAGGAAACTAGGCACGGT	0	77	RDR2-sensitive
CTCGGACAGGAAATTAGGCACGGT	0	13	RDR2-sensitive
CTCGGACGGGCCTGACTGCGGATG	0	20	RDR2-sensitive
CTCGGACGGTAGACGCAGTCACGT	0	9	RDR2-sensitive
CTCGGACTAAAGTCGGCATTGCAG	0	9	RDR2-sensitive
CTCGGACTACGGCACACTCATGAC	0	44	RDR2-sensitive
CTCGGACTCGGCGAGGGCCTCGGC	36	8	RDR2-resistant
CTCGGAGAAGACTGTGCACACCTC	0	9	RDR2-sensitive
CTCGGAGGGCGTCGAACATGGTGT	20	0	RDR2-resistant
CTCGGAGTGACCGACAGAACGCGG	0	18	RDR2-sensitive
CTCGGATACAGGATCGGATACGGA	0	13	RDR2-sensitive
CTCGGATACAGGTAGGATACGGAA	1	20	RDR2-sensitive
CTCGGATACGGGTAGGATACGGAA	2	259	RDR2-sensitive
CTCGGATACGGGTAGGATACGGCA	0	9	RDR2-sensitive
CTCGGATCCTGCCCAGCTCGTCGT	0	18	RDR2-sensitive
CTCGGATCTGTGACGCACGGCCAT	0	14	RDR2-sensitive
CTCGGATTCTACCCAGCTCGTCGT	0	35	RDR2-sensitive
CTCGGATTTTACTGGCACGGCCCT	0	29	RDR2-sensitive
CTCGGATTTTGTCGGCACGGCCAC	0	18	RDR2-sensitive
CTCGGATTTTGTCGGCACGGCCCT	0	11	RDR2-sensitive
CTCGGCAAACAGTACATCGGCAAA	3	19	RDR2-sensitive

CTCGGCAAACGGGGCTCTCGGCAA	3	42	RDR2-sensitive
CTCGGCAAAGAACCTAAGCCGGTG	0	12	RDR2-sensitive
CTCGGCAAAGAACGCGATTCCGGT	3	96	RDR2-sensitive
CTCGGCAAAGAACGCTCGGCGAAC	3	131	RDR2-sensitive
CTCGGCAAAGAAGGCTCGGCAAAC	0	9	RDR2-sensitive
CTCGGCAAAGACACAAGCACTCGG	0	9	RDR2-sensitive
CTCGGCAAAGACGGCTCTCGGCAA	0	31	RDR2-sensitive
CTCGGCAAAGACTGACGGCCGTCA	0	17	RDR2-sensitive
CTCGGCAAAGATGGCGCTCGGCAA	1	17	RDR2-sensitive
CTCGGCAAAGATGGCTCTCGGCAA	0	13	RDR2-sensitive
CTCGGCAAAGCCTCGGATTCCGGT	1	29	RDR2-sensitive
CTCGGCAAAGCCTTGGATTCCGGT	0	17	RDR2-sensitive
CTCGGCAAAGGCTCGGATTCCGGT	0	10	RDR2-sensitive
CTCGGCAAAGGGACTGGCAAAGGG	0	13	RDR2-sensitive
CTCGGCAAAGGGGGCTCTCGGCAA	0	25	RDR2-sensitive
CTCGGCAAAGTCTCGGATTCCGGT	2	18	RDR2-sensitive
CTCGGCAAGCGTTCGGACGTTCAT	0	11	RDR2-sensitive
CTCGGCACACCATGAAAGCGACGG	0	12	RDR2-sensitive
CTCGGCACAGAACGGCACTCGGCA	2	15	RDR2-sensitive
CTCGGCACAGCATACACTCGGCAA	0	9	RDR2-sensitive
CTCGGCACAGCCAAGCACTCGGCA	0	19	RDR2-sensitive
CTCGGCACAGGAAAACACTCGGCA	1	18	RDR2-sensitive
CTCGGCACAGGAATACACTCGGCA	0	15	RDR2-sensitive
CTCGGCACAGGACAACACTCGGCA	0	9	RDR2-sensitive
CTCGGCACAGGACCACACTCGGCA	0	12	RDR2-sensitive
CTCGGCACAGGACGACACTCGGCA	0	20	RDR2-sensitive
CTCGGCACAGGACGCACTCGGCAA	2	24	RDR2-sensitive
CTCGGCACAGGTAGGCACTCGGCA	5	23	RDR2-sensitive
CTCGGCACGGTAGAATAAGCGGGC	0	10	RDR2-sensitive
CTCGGCAGCGGGCGTAGGAGCATA	0	16	RDR2-sensitive
CTCGGCATAGAACGACACTCGGCA	0	9	RDR2-sensitive
CTCGGCATAGGTAGACACTCGGCA	0	10	RDR2-sensitive
CTCGGCATAGTTAACGATCGGCGT	0	25	RDR2-sensitive
CTCGGCATATAAGGACACTCGGCA	0	15	RDR2-sensitive
CTCGGCATGTAGGTTGATCGGTAA	0	25	RDR2-sensitive
CTCGGCCACGGAAGACAGACTCGA	1	21	RDR2-sensitive
CTCGGCCAGGCTGACGTGGACTGC	0	15	RDR2-sensitive
CTCGGCCAGGTTACGTAGACGAGC	0	16	RDR2-sensitive
CTCGGCCCCGTAGACCAGGAAGAA	2	17	RDR2-sensitive
CTCGGCGAACTGTACATCGGCAAC	11	227	RDR2-sensitive
CTCGGCGAACTGTACATCGGCTAT	0	34	RDR2-sensitive
CTCGGCGAGCTGTTGAACACGGGC	0	10	RDR2-sensitive
CTCGGCGGCGAGGTACTCGAGGAC	104	27	RDR2-resistant
CTCGGCGTAGATGATGTTGAGGCT	13	96	RDR2-sensitive
CTCGGCGTAGTAGCGGATTTTCCA	2	26	RDR2-sensitive
CTCGGCGTCTCAACAGAAGAACAG	0	14	RDR2-sensitive
CTCGGCGTCTCAGCAGAAGAACAG	0	12	RDR2-sensitive
CTCGGCGTCTCAGCGGAAGAACAG	2	31	RDR2-sensitive
CTCGGCTCCGGCAGCGGCTCTAGT	1	16	RDR2-sensitive

CTCGGCTCGCCCACGGCACTGGAC	0	28	RDR2-sensitive
CTCGGCTCGGTGAGGCTCGGTGGA	0	10	RDR2-sensitive
CTCGGCTCGTTAGTAGATCGGCTC	0	17	RDR2-sensitive
CTCGGCTCGTTCGAGGCTCGCGAG	0	16	RDR2-sensitive
CTCGGCTCTCGCATCGATGAAGAA	35	2	RDR2-resistant
CTCGGCTCTGCTGAAGGAGGATGT	0	9	RDR2-sensitive
CTCGGCTGACGTAGAGACAGGCAA	0	19	RDR2-sensitive
CTCGGCTGCCTAGACGACGGACGG	0	10	RDR2-sensitive
CTCGGCTGCGACAATGGACAGAAG	0	15	RDR2-sensitive
CTCGGCTGCTACGACGGGTGTGCA	0	10	RDR2-sensitive
CTCGGCTGGCTGACGAAGAGACAG	0	37	RDR2-sensitive
CTCGGCTGGCTGACGAAGAGACAT	0	11	RDR2-sensitive
CTCGGCTTGCGGCGCACTTATGGC	0	13	RDR2-sensitive
CTCGGCTTTAGTCCCACATCGGCT	0	17	RDR2-sensitive
CTCGGGAAACAACATGTAGCGGAT	0	15	RDR2-sensitive
CTCGGGAAACAGTTGGAAACGGAT	0	10	RDR2-sensitive
CTCGGGCAAAAACAGTCGTCGCGG	0	9	RDR2-sensitive
CTCGGGCACTCGACAAAGAGGTTT	0	11	RDR2-sensitive
CTCGGGCACTCGGCAAAGAAAAGC	0	12	RDR2-sensitive
CTCGGGCACTCGGCAAAGAGGTTT	0	77	RDR2-sensitive
CTCGGGCACTCGGCAAAGTGGTTT	0	9	RDR2-sensitive
CTCGGGCCGACGAGTGAGTGTCGC	0	9	RDR2-sensitive
CTCGGGCGAGCCGAAGGTGTGTCC	0	9	RDR2-sensitive
CTCGGGCGAGCCGGAGATGTGTCC	0	24	RDR2-sensitive
CTCGGGCGAGCCGGTGATGTGTCC	0	12	RDR2-sensitive
CTCGGGCTCCTGTTTGGATGAGGC	0	17	RDR2-sensitive
CTCGGGCTCGCAGCAGGTCGGTTC	0	9	RDR2-sensitive
CTCGGGCTGGCTCGCCAGAAACGA	0	13	RDR2-sensitive
CTCGGGCTTGCTCGTCGTAGTCGG	1	80	RDR2-sensitive
CTCGGGGAGGGTAGGGAATGACGG	0	11	RDR2-sensitive
CTCGGGGCCTTGGAGATAGATGAC	0	10	RDR2-sensitive
CTCGGGGTAGGGTGATGATGGCGC	14	48	RDR2-sensitive
CTCGGGTAGCTAGATGCAAGACGT	0	11	RDR2-sensitive
CTCGGGTCCACTAGAAGGCGACAT	0	11	RDR2-sensitive
CTCGGGTCTGAGCTGCAGGGCACG	0	10	RDR2-sensitive
CTCGGGTCTTGCTAGTGTCGCGCT	0	14	RDR2-sensitive
CTCGGGTTCCCTGCCTGACGGCCG	0	13	RDR2-sensitive
CTCGGGTTGAGAACAACAGGGGAC	0	12	RDR2-sensitive
CTCGGGTTGTTCTGGGCACCGCAT	0	9	RDR2-sensitive
CTCGGTAAAGAGGGCTCGGCACAC	0	10	RDR2-sensitive
CTCGGTAACGTGTGGAGCGGTCAC	0	12	RDR2-sensitive
CTCGGTAATGAGGCTCGGCTCGGC	3	35	RDR2-sensitive
CTCGGTACAGGGAAACACTCGGCA	0	15	RDR2-sensitive
CTCGGTACAGGTCCACACTCGGCA	0	9	RDR2-sensitive
CTCGGTACGGATAATGGATCGGCT	0	9	RDR2-sensitive
CTCGGTAGGACCTGGTCAGCCGGT	0	14	RDR2-sensitive
CTCGGTATAGAATGACACTCGGTT	0	12	RDR2-sensitive
CTCGGTATGAACGGAAGGTGTCGT	0	9	RDR2-sensitive
CTCGGTCAGACGGGAGACGTTGGA	0	9	RDR2-sensitive

CTCGGTCAGGAGAGAAGCCGTGCG	0	10	RDR2-sensitive
CTCGGTCTAGATCCAAGCGCGGGC	89	10	RDR2-resistant
CTCGGTCTCGCTGTGGGTCAGCAT	0	9	RDR2-sensitive
CTCGGTCTGCGGCGCACTTATGGC	0	11	RDR2-sensitive
CTCGGTCTGGCGGATGCAACAGGC	0	15	RDR2-sensitive
CTCGGTCTTCGACGGTCATCTCAA	0	9	RDR2-sensitive
CTCGGTGAACTGTACATTGGCAAC	0	14	RDR2-sensitive
CTCGGTGACCGTAGGCGGGTGGCT	0	9	RDR2-sensitive
CTCGGTGAGGCTCGGTGGAATAGC	0	10	RDR2-sensitive
CTCGGTGATGACTTCAAGCAGCAT	18	0	RDR2-resistant
CTCGGTGCCTGAACGAGCTCGGCT	0	11	RDR2-sensitive
CTCGGTGGTTGATGGCTCCGGCAT	1	22	RDR2-sensitive
CTCGGTGTAGCTCACGAGCTGGCT	0	9	RDR2-sensitive
CTCGGTTCGGTCCTAGACGCGCGT	0	15	RDR2-sensitive
CTCGGTTCTGCGGGAGACGATCGG	0	9	RDR2-sensitive
CTCGGTTCTTGTAGAAATTCGGTT	1	88	RDR2-sensitive
CTCGGTTGGGGACAGAACGGTCGG	0	10	RDR2-sensitive
CTCGGTTTCTGTGCGCGGCTGTGT	0	14	RDR2-sensitive
CTCGTACACAGCCGTCGGAGATAA	0	9	RDR2-sensitive
CTCGTACAGAGCCGTCGGAGATAA	1	27	RDR2-sensitive
CTCGTACAGTAGGAAGTGAGGCGT	0	15	RDR2-sensitive
CTCGTACGTGTTAGCCTTCGGTAT	4	18	RDR2-sensitive
CTCGTAGTACTATGGCACGACATA	0	9	RDR2-sensitive
CTCGTAGTCCTCTAGGCAGATCGA	0	12	RDR2-sensitive
CTCGTAGTGGCAGAACCGCGCAAC	0	9	RDR2-sensitive
CTCGTATCGTGCGTTGTTGTGGCG	67	2	RDR2-resistant
CTCGTATTGCTGTTGCTCATCGGC	0	9	RDR2-sensitive
CTCGTCAAAGCGCCGAGGACTCGG	0	9	RDR2-sensitive
CTCGTCCATGCTGTCGGCCCGTAC	0	12	RDR2-sensitive
CTCGTCGAAGGGCAGAGGCACCGA	0	9	RDR2-sensitive
CTCGTCGACCCTGCTGATTCCGGC	2	16	RDR2-sensitive
CTCGTCGTGGTGTATGATAGGGCT	0	13	RDR2-sensitive
CTCGTGAGCCAGAGCCAAAGCCGT	0	14	RDR2-sensitive
CTCGTGAGCCAGAGCTAAAGCCGT	0	16	RDR2-sensitive
CTCGTGCAAGGTCTCGAACGGCAA	1	31	RDR2-sensitive
CTCGTGCGACTGTCCGGTGAGGCC	0	9	RDR2-sensitive
CTCGTGCTTGGACGAGGACACGGC	19	0	RDR2-resistant
CTCGTGGACGACAGCGCGGCAGGT	0	12	RDR2-sensitive
CTCGTGGCTCGCTGGCTCGCTCGG	0	30	RDR2-sensitive
CTCGTGTATACAGACTCAGCGGTG	0	14	RDR2-sensitive
CTCGTGTCGGGCCAAGCCGGCTTG	0	9	RDR2-sensitive
CTCGTTGACACTTTGGACGCCGGC	0	21	RDR2-sensitive
CTCGTTGATCTGGAACCCAGGCAT	0	9	RDR2-sensitive
CTCGTTGTGGGCTGCAAGCGGTCT	0	20	RDR2-sensitive
CTCGTTTCTGTAGAGCCAGGCTGA	0	11	RDR2-sensitive
CTCGTTTCTGTGGAGCCAGGCTGA	0	43	RDR2-sensitive
CTCGTTTCTGTGGAGTCAGGCTGA	0	10	RDR2-sensitive
CTCGTTTTGGGCTCCGGACGGTTT	0	10	RDR2-sensitive
CTCGTTTTTGTGGAGCCAGGCTGA	0	15	RDR2-sensitive

CTCTAAAACTCTGGGTGGAGCAGC	0	30	RDR2-sensitive
CTCTAACCTTGTCGTGGACGCTGT	0	17	RDR2-sensitive
CTCTACATCGTTTGGAGACGTCTT	0	10	RDR2-sensitive
CTCTACATGTAGCGGCTGAAGCAG	0	20	RDR2-sensitive
CTCTACGTCGATTAGAGGAGTCTA	2	23	RDR2-sensitive
CTCTACTAACTATTGTTGTCGGCA	0	13	RDR2-sensitive
CTCTACTCCCTCCAGCAACGTCAT	0	9	RDR2-sensitive
CTCTACTGCTGACGGGAGGGAAGG	1	32	RDR2-sensitive
CTCTAGAACCGCCACTATAGAGGC	0	9	RDR2-sensitive
CTCTAGAGCGTATTGGACGGTTGA	0	25	RDR2-sensitive
CTCTAGGGCTGTGTGTTTGCGGTA	0	23	RDR2-sensitive
CTCTATACACGTGTGGACCGTCTG	0	14	RDR2-sensitive
CTCTATAGATTGCAGCTCCGGCAG	0	9	RDR2-sensitive
CTCTATCAGTCCGTCGGGCGGCAA	0	11	RDR2-sensitive
CTCTATCGTACACTGTTTCGCGCA	0	10	RDR2-sensitive
CTCTATCGTCTACTCTAAAGCAGA	0	9	RDR2-sensitive
CTCTATCTCTGTAGCGTTCGGCAT	0	41	RDR2-sensitive
CTCTATGGATTGCAGATGCAGCAG	0	10	RDR2-sensitive
CTCTATGGATTGCAGCTGCAGCAG	0	33	RDR2-sensitive
CTCTATGTCAGATGGAGCGCTCGG	0	9	RDR2-sensitive
CTCTATTGGGCGGGACCGAGCATG	0	9	RDR2-sensitive
CTCTCAGCAGGTACGACGGACATT	0	36	RDR2-sensitive
CTCTCCCGTCAGCAGTAGAGACGC	0	12	RDR2-sensitive
CTCTCCCGTCAGCAGTAGAGGCGC	1	70	RDR2-sensitive
CTCTCCCGTCAGCAGTAGAGGCGT	1	12	RDR2-sensitive
CTCTCCCGTCAGTAGTAGAGGCGC	0	11	RDR2-sensitive
CTCTCCGCGCGCTGTAGAAGGCAC	3	62	RDR2-sensitive
CTCTCGAGGTCGGCTGCCCTTGGC	1	18	RDR2-sensitive
CTCTCGAGGTCGGCTGCCTTCGGC	12	32	RDR2-sensitive
CTCTCGGACTACGGCACACTCATG	0	16	RDR2-sensitive
CTCTCGGACTCGGTCAAGGCCAGA	0	10	RDR2-sensitive
CTCTCGGCAAAGACTGACGGTCGT	0	24	RDR2-sensitive
CTCTCGGCAACGGATATCTCGGCT	44	10	RDR2-resistant
CTCTCGGCCAGCAGAACGATCGAT	0	16	RDR2-sensitive
CTCTCGGGAATTTGCTGACGGCGT	0	14	RDR2-sensitive
CTCTCGGTTTGGAGCTGCTCCGGC	2	17	RDR2-sensitive
CTCTCGTCAGCAGTAGAGGCGCAA	0	32	RDR2-sensitive
CTCTCGTCAGCAGTAGAGGCGTAA	0	9	RDR2-sensitive
CTCTCGTTCGCATGACTGGGCATG	0	10	RDR2-sensitive
CTCTCGTTTGGACTTCCGGCCGGG	0	14	RDR2-sensitive
CTCTCTAACCGCCTGAATCGTCGT	0	9	RDR2-sensitive
CTCTCTACCGGACACGTTAGCATA	0	10	RDR2-sensitive
CTCTCTACTAGAGCGTGGACGGAT	0	9	RDR2-sensitive
CTCTCTATGGATTGCAGCTGCAGC	0	46	RDR2-sensitive
CTCTCTCGGACTCGGTCAAGGCCA	0	22	RDR2-sensitive
CTCTCTCGTCAGCAGTAGAGGCGT	1	75	RDR2-sensitive
CTCTCTCGTCAGCAGTAGAGGTGT	2	31	RDR2-sensitive
CTCTCTCGTCGACCTCTTGCAGAA	0	14	RDR2-sensitive
CTCTCTCGTTTGGACTTCCGGCCG	0	44	RDR2-sensitive

CTCTCTCTCGGACTCGGTCAAGGC	0	34	RDR2-sensitive
CTCTCTCTGCAGGATGACTCTCAT	49	2	RDR2-resistant
CTCTCTGATCAGGCACGGACGGTC	0	12	RDR2-sensitive
CTCTCTGCACAGGCGCGGACGGTC	1	35	RDR2-sensitive
CTCTCTGCACAGGCGTGGACGGTC	0	11	RDR2-sensitive
CTCTCTGCACAGGGGCGGACGGTC	4	41	RDR2-sensitive
CTCTCTGCCGGACACGTTAGCATA	2	33	RDR2-sensitive
CTCTCTGCCGGATACGTTAGCATA	0	11	RDR2-sensitive
CTCTCTGCGACGGTGTGGACGGGT	0	14	RDR2-sensitive
CTCTCTGCGCAGACTGTCCGGTGC	0	9	RDR2-sensitive
CTCTCTGCGCTATTGTAGACGGTT	0	15	RDR2-sensitive
CTCTCTGCGGAGCCGATGACGGTC	0	12	RDR2-sensitive
CTCTCTGCGGAGGCGTAGACGGTC	0	11	RDR2-sensitive
CTCTCTGGGCCGTGCTTGGACACA	0	20	RDR2-sensitive
CTCTCTGGTCAAGCGCGGACGGTC	1	18	RDR2-sensitive
CTCTCTGGTCAGACGCGGACGGTC	3	62	RDR2-sensitive
CTCTCTGGTCAGACGTGGACGGTC	0	17	RDR2-sensitive
CTCTCTGGTCAGGACTATGGACGG	0	14	RDR2-sensitive
CTCTCTGGTCAGGCACGGACGGTC	0	9	RDR2-sensitive
CTCTCTGGTCAGGCGCGGACGGTC	15	271	RDR2-sensitive
CTCTCTGGTCAGGCGTGGACGGTC	1	20	RDR2-sensitive
CTCTCTGTAGAGGCGTGGACGGTC	0	17	RDR2-sensitive
CTCTCTGTAGCGGTTCGGACGGTT	0	14	RDR2-sensitive
CTCTCTGTGTTGGGTTGCTGGGAA	0	16	RDR2-sensitive
CTCTGACCATCGCGCATCTGACGC	0	9	RDR2-sensitive
CTCTGACCCAGGACTTTGTTGCGG	0	10	RDR2-sensitive
CTCTGACCTAGCCGAACTGTCCTA	0	21	RDR2-sensitive
CTCTGACGGGCTGGTGCTGGCACG	0	16	RDR2-sensitive
CTCTGACTGTAGCGCGCGACCGGT	0	23	RDR2-sensitive
CTCTGAGGACGACGGGACGTCGGT	0	18	RDR2-sensitive
CTCTGATCAGTAGGAGGGCTCGGG	1	15	RDR2-sensitive
CTCTGATCAGTAGGGAGGCTCGGA	5	20	RDR2-sensitive
CTCTGATCAGTAGGGGGGCTCGGG	1	13	RDR2-sensitive
CTCTGCACGCTTCCGGACGGTCTG	0	9	RDR2-sensitive
CTCTGCAGAGTGTAAAACTGGTAT	2	30	RDR2-sensitive
CTCTGCAGATCTCCCGAAGAGGCT	0	9	RDR2-sensitive
CTCTGCAGATTGTAAAACCGGTAT	0	16	RDR2-sensitive
CTCTGCAGCTGTAGGTGGCTTCTT	0	13	RDR2-sensitive
CTCTGCAGGTAGAGCTGTACGGGC	0	19	RDR2-sensitive
CTCTGCATGTAGAGCTGTACGGGC	0	11	RDR2-sensitive
CTCTGCCACGTCAGCTAGCTCGGT	0	12	RDR2-sensitive
CTCTGCCGGACACGTTAGCGCATA	0	19	RDR2-sensitive
CTCTGCCTCGCAGGGTTGGACGGT	0	10	RDR2-sensitive
CTCTGCCTGACGGCCGGACGGTCC	0	11	RDR2-sensitive
CTCTGCGAAGACTGGGCCTCGGGC	1	18	RDR2-sensitive
CTCTGCGCAGACTGTCCGGTGCCT	0	9	RDR2-sensitive
CTCTGCGCGTGCATGTAGAAGTGG	0	19	RDR2-sensitive
CTCTGCGGACGGCGATTTCGGAGG	0	12	RDR2-sensitive
CTCTGCGGCAGAGGCTGAACGGGC	0	10	RDR2-sensitive

CTCTGCTAGTGGACCCGTGGGATA	0	13	RDR2-sensitive
CTCTGCTCAGCCAGAACTCGGCAA	0	11	RDR2-sensitive
CTCTGCTCGGACCGAGACGAGGCC	0	12	RDR2-sensitive
CTCTGCTCGGCTCGATGGTACAGC	0	9	RDR2-sensitive
CTCTGCTCGGCTGGACGACTGCAT	1	132	RDR2-sensitive
CTCTGCTCGTAGTGGACCAGCATG	0	9	RDR2-sensitive
CTCTGCTGAGCGGGAGTTCTGGAC	21	0	RDR2-resistant
CTCTGCTGAGCGGGAGTTCTGGAT	14	0	RDR2-resistant
CTCTGCTGCTCCAGTGCACAGGCT	0	10	RDR2-sensitive
CTCTGGACATGGCAGACTGCACCA	0	12	RDR2-sensitive
CTCTGGACCAGACTCTACAGGCGC	0	19	RDR2-sensitive
CTCTGGACCTGCTTTTAGCGGCTT	2	20	RDR2-sensitive
CTCTGGACCTGCTTTTAGTGGCTT	0	15	RDR2-sensitive
CTCTGGAGTTAGGGTGTTTGGCAT	3	34	RDR2-sensitive
CTCTGGATAATGGGGGCATGAGCA	46	12	RDR2-resistant
CTCTGGCCAGGCATGTAGGACTGT	0	10	RDR2-sensitive
CTCTGGCCTAGCCGAACTGTCCTA	1	33	RDR2-sensitive
CTCTGGCGTTGGCTGAGCAGTCGG	0	19	RDR2-sensitive
CTCTGGCTCTGTGACGGTGACCAT	0	24	RDR2-sensitive
CTCTGGGCATCTGAGGCATCTGAG	1	15	RDR2-sensitive
CTCTGGGCTAGACTTATATGGCAT	0	13	RDR2-sensitive
CTCTGGTACCGTAGCAAGATGATT	0	16	RDR2-sensitive
CTCTGGTCACTGTTCACGGCACTT	21	0	RDR2-resistant
CTCTGGTCAGACGCGGACGGTCCG	3	29	RDR2-sensitive
CTCTGGTCAGGACTACGGACGGTC	0	23	RDR2-sensitive
CTCTGGTCAGGACTATGGACGGTC	0	19	RDR2-sensitive
CTCTGGTCAGGACTGCGGACGGTC	12	111	RDR2-sensitive
CTCTGGTCAGGCGCGGACGGTCCG	5	62	RDR2-sensitive
CTCTGGTCAGGCGCGGACGGTCCT	0	9	RDR2-sensitive
CTCTGGTCATGACTGCGGACGGTC	1	32	RDR2-sensitive
CTCTGGTCTGGACTGCGGACGGTC	0	20	RDR2-sensitive
CTCTGTAGAGTGTAAAACTGGTAT	0	32	RDR2-sensitive
CTCTGTAGCAGCACGGACGGTCCG	0	11	RDR2-sensitive
CTCTGTAGCGGGCTTAGACGACCC	0	12	RDR2-sensitive
CTCTGTAGCGTTCGGCATTGAGTC	0	45	RDR2-sensitive
CTCTGTAGGCCGGACGGTCCGCGC	0	12	RDR2-sensitive
CTCTGTAGGGCTGGGCATTCGGTT	0	9	RDR2-sensitive
CTCTGTATCTGTGGGAGCAGGCTC	0	19	RDR2-sensitive
CTCTGTATGGTGGAATTATGTCAT	1	31	RDR2-sensitive
CTCTGTCAGACCAGCGACTGCAGC	0	24	RDR2-sensitive
CTCTGTCAGACCAGCGGCTGCAGC	0	15	RDR2-sensitive
CTCTGTCGACGAGCACACCGGACA	0	19	RDR2-sensitive
CTCTGTCGACTGAAGGGCGCGCGT	1	20	RDR2-sensitive
CTCTGTCGAGATGCGCAAGGACGA	0	14	RDR2-sensitive
CTCTGTCGAGATGCGCGAGGACGA	0	23	RDR2-sensitive
CTCTGTCGGGTCTGGTGGGGACGG	0	15	RDR2-sensitive
CTCTGTCGTGCTTATGGGAAGGGA	6	122	RDR2-sensitive
CTCTGTCGTGCTTCTGGGAAGGGA	0	12	RDR2-sensitive
CTCTGTGCGTCACGGACGGTCCGA	0	9	RDR2-sensitive

CTCTGTGCTCGTAGAAGGTGGGAC	0	15	RDR2-sensitive
CTCTGTGCTGTAGACGCGGTTTCT	0	10	RDR2-sensitive
CTCTGTGGATTTACTCGCCGGCAC	0	21	RDR2-sensitive
CTCTGTGTCGAGCCGGACGGTCGA	1	19	RDR2-sensitive
CTCTGTTCACTGTAAGACATTGGC	1	19	RDR2-sensitive
CTCTGTTCTGGAGACGCAGGCGAC	0	12	RDR2-sensitive
CTCTGTTTTGGAGACGCATCTGAC	0	11	RDR2-sensitive
CTCTGTTTTGGAGACGTAGGCGAC	0	15	RDR2-sensitive
CTCTTAATTGCAGGTTGTTGGACA	31	1	RDR2-resistant
CTCTTCACGGAGAAACGGCTCTAA	0	13	RDR2-sensitive
CTCTTCACGGAGAGACGACTCTAA	0	11	RDR2-sensitive
CTCTTCACGGAGAGACGGCTCTAA	6	183	RDR2-sensitive
CTCTTCACGGAGAGACGGCTCTAT	0	11	RDR2-sensitive
CTCTTCAGGCTGCATGCGTGAGTA	0	9	RDR2-sensitive
CTCTTCAGGCTGTATACGCGGGTA	0	72	RDR2-sensitive
CTCTTCCGTCAGCAGTAGAGGCGC	0	46	RDR2-sensitive
CTCTTCGCGGAGAGACGGCTCTAA	1	91	RDR2-sensitive
CTCTTCGGGACCGTAGCGTTGGGC	0	14	RDR2-sensitive
CTCTTCGTCAAGCCGGCAGGTCTC	0	9	RDR2-sensitive
CTCTTCTTTTGTTTCATGTTGGAT	53	1	RDR2-resistant
CTCTTGACGTGTAGAGGACGAGAA	0	16	RDR2-sensitive
CTCTTGTAAGGCACGCAGGACTGT	0	12	RDR2-sensitive
CTCTTGTTCCATTGTCGTCGTGAC	0	12	RDR2-sensitive
CTCTTTCTGCAGGACCTTCGGCGA	0	10	RDR2-sensitive
CTCTTTGACTGACTGTGGCGTGGC	0	10	RDR2-sensitive
CTCTTTGCTGAGTGCCCGTTGGAC	0	29	RDR2-sensitive
CTCTTTGTTGGACGGAGAGAGGAG	0	15	RDR2-sensitive
CTGAAAATCTGGCTGTGGGGAGAA	0	12	RDR2-sensitive
CTGAAACCTGAATATTTGACGGAT	0	9	RDR2-sensitive
CTGAAAGGCTAGAACTGATCCGGC	0	17	RDR2-sensitive
CTGAACAACGACGGCCTTCAGCTT	0	11	RDR2-sensitive
CTGAACACTCGGCACAGAACGGCA	0	12	RDR2-sensitive
CTGAACACTCGGCACAGGACGCAC	0	13	RDR2-sensitive
CTGAACACTCGGCACAGGGGGCTC	1	13	RDR2-sensitive
CTGAACACTCGGCACAGGTAGGCA	2	19	RDR2-sensitive
CTGAACACTGACCAACGATCGCGC	0	16	RDR2-sensitive
CTGAACAGTCGACGTGAGGACCGT	0	9	RDR2-sensitive
CTGAACCAGACGAAGGACGGGCTG	0	14	RDR2-sensitive
CTGAACCCGTCGACTCGCACGCAT	5	26	RDR2-sensitive
CTGAACGTCGAGCCAGGCCGAGCT	0	12	RDR2-sensitive
CTGAACTCGACGGGTACTCGACAC	0	31	RDR2-sensitive
CTGAACTGATAAGCTGCTGCAGAA	0	13	RDR2-sensitive
CTGAACTGCCTGAGTCTGCATCAG	0	12	RDR2-sensitive
CTGAACTGTTGATGTTGATGACGT	0	14	RDR2-sensitive
CTGAAGACTGAATGTAGGGACGTT	0	9	RDR2-sensitive
CTGAAGACTGCTGCTTGATCCGCT	24	3	RDR2-resistant
CTGAAGATGCAGAGGCCAGAACAT	0	9	RDR2-sensitive
CTGAAGCTTCGGTAGAAGGCTGGT	14	0	RDR2-resistant
CTGAAGGATGGACGATCACGGTGG	53	0	RDR2-resistant

CTGAAGGGTTTGAGGGGACGACGC	1	20	RDR2-sensitive
CTGAATATCTGAGCTGGGCGGCAT	0	9	RDR2-sensitive
CTGAATCCTGATCGTCTGATGCAG	0	10	RDR2-sensitive
CTGAATCGTTGGTTTTGATGGCAT	0	15	RDR2-sensitive
CTGAATCTGATCCGGATCTTCGCT	25	3	RDR2-resistant
CTGAATTTTGTTCAGGGATTTCGG	0	10	RDR2-sensitive
CTGACAAACTGTGGAGGAGGAGAC	0	23	RDR2-sensitive
CTGACAGAAGACGACGGGGAACGT	0	11	RDR2-sensitive
CTGACAGAAGGACAGCGCCGCCTG	1	28	RDR2-sensitive
CTGACAGTGCTCTCTACAGGACGG	0	38	RDR2-sensitive
CTGACAGTGGCACTCGGCAAAGAA	1	14	RDR2-sensitive
CTGACATAGCAGCTCCGACTTCAC	0	13	RDR2-sensitive
CTGACATCCACGGGCTAGCAGGCG	0	15	RDR2-sensitive
CTGACATCGATGTAGCGGCGACAC	0	19	RDR2-sensitive
CTGACATCTGCAGAACGCAACCAC	0	13	RDR2-sensitive
CTGACATGTATGCTTAGTTGACGA	24	2	RDR2-resistant
CTGACATGTCGGGTACAGATTCGG	2	28	RDR2-sensitive
CTGACCACGTCGTGTCTTCGTCGG	0	9	RDR2-sensitive
CTGACCAGAGAGCACCGCCACGGT	0	9	RDR2-sensitive
CTGACCAGAGAGCACCGCCGCCGG	1	34	RDR2-sensitive
CTGACCAGTCTGACAGAAGGACAG	0	12	RDR2-sensitive
CTGACCATGAACCGAGCGAGCCAG	1	17	RDR2-sensitive
CTGACCCCCTTGTAAAGCTGGCCT	19	0	RDR2-resistant
CTGACCCGTCGGGTTTGGGTTCGG	0	22	RDR2-sensitive
CTGACCGACCTGACAGAAGGACAG	0	10	RDR2-sensitive
CTGACCGAGCCGAGGGACTCCTAC	4	18	RDR2-sensitive
CTGACCGAGGCTCGTTGGGAAGAC	0	10	RDR2-sensitive
CTGACCGGCCTGACAAGAGGACAG	0	23	RDR2-sensitive
CTGACCGGCCTGACAGAAAGACAG	1	17	RDR2-sensitive
CTGACCGGCCTGACAGAAGGACAG	2	49	RDR2-sensitive
CTGACCTGGCTCTGACGTGGTGGC	0	12	RDR2-sensitive
CTGACGAACTGTTGAGGACGACAC	0	70	RDR2-sensitive
CTGACGAGTCGACGAGCTGATGAG	0	10	RDR2-sensitive
CTGACGCAGCACCGTTGGATGTGG	0	29	RDR2-sensitive
CTGACGCGCGGAACGACGACGAGA	1	14	RDR2-sensitive
CTGACGCGTAGGACAGTTCGGCTA	0	14	RDR2-sensitive
CTGACGCGTAGGACAGTTCGGCTT	0	14	RDR2-sensitive
CTGACGCTGCTAGATGGTGATCGT	1	43	RDR2-sensitive
CTGACGGCACTCTAGTTGAAGAAG	94	8	RDR2-resistant
CTGACGGCCGATGAGCAACAGCAA	0	14	RDR2-sensitive
CTGACGGGAGGGAAGGGAGCGGAT	2	19	RDR2-sensitive
CTGACGGGCTGACGGACTCACGGC	0	9	RDR2-sensitive
CTGACGTAGCAGCTTCGACTTCAT	0	17	RDR2-sensitive
CTGACGTGGACTGGAGCTACGGTG	0	17	RDR2-sensitive
CTGACTAGACACATGCAACTTCTT	0	12	RDR2-sensitive
CTGACTATAGTCTGATCGTGACGT	0	9	RDR2-sensitive
CTGACTGAAGAGGACGCAATCCGA	0	10	RDR2-sensitive
CTGACTGAGAGACGCCACCTAGAT	0	22	RDR2-sensitive
CTGACTGAGATGAGCCATAAGCGA	0	10	RDR2-sensitive

CTGACTGCGGGACGGGCGGACGGC	0	25	RDR2-sensitive
CTGACTGCTTCCTCTGTAGCAGGA	0	19	RDR2-sensitive
CTGACTGGCTGATGGAGCAAGGCA	17	1	RDR2-resistant
CTGACTGGGAGACGCCACCTAGAT	1	21	RDR2-sensitive
CTGACTGTAGCGCGCGACCGGTTC	0	10	RDR2-sensitive
CTGACTGTCGTATGGTGCACCGGA	0	40	RDR2-sensitive
CTGACTGTTCTGTCCGAGCACGGC	0	14	RDR2-sensitive
CTGACTTAGCGTAGCCTGCATCAG	0	10	RDR2-sensitive
CTGACTTGAAACTTGCTTGAAGAA	32	2	RDR2-resistant
CTGAGAAACGTGGACGGCTGATGC	0	16	RDR2-sensitive
CTGAGAATCCATGGTCGTCACCGT	0	9	RDR2-sensitive
CTGAGAATCTGGCTGTGAGGAGAA	0	18	RDR2-sensitive
CTGAGAGTGCCCTTTCGGTCGGAC	0	22	RDR2-sensitive
CTGAGATTGATTGTAGCTATTGGC	826	101	RDR2-resistant
CTGAGCAAGACGGCGACCTCTGGG	0	10	RDR2-sensitive
CTGAGCACTGTGTGATGACGTCCA	0	11	RDR2-sensitive
CTGAGCAGCAGTCGGTTGGTGGAG	1	16	RDR2-sensitive
CTGAGCATGTAGACGCCACCGACA	0	34	RDR2-sensitive
CTGAGCCCTGTAGAAGCACAGCTG	2	48	RDR2-sensitive
CTGAGCCGAGTAGAGCCGAGCTGA	1	52	RDR2-sensitive
CTGAGCGGGCTTCATGACCGGTGT	0	32	RDR2-sensitive
CTGAGCTCTGACGTGGCGTCCTAG	0	9	RDR2-sensitive
CTGAGCTCTGTGGAATGCACGTCA	232	83	RDR2-resistant
CTGAGGAACCAGGCTCGGCTCGGC	1	22	RDR2-sensitive
CTGAGGACGAGGCACTAGGGCGGA	0	15	RDR2-sensitive
CTGAGGAGCCAGAACCTGAGACGT	0	10	RDR2-sensitive
CTGAGGCACTGTTGCGCGCTCCAG	0	31	RDR2-sensitive
CTGAGGCATGGTATGGATCGACGC	0	11	RDR2-sensitive
CTGAGGCTAGACGGTCCGGACGGT	0	10	RDR2-sensitive
CTGAGGGTTGGGCTCGGGGGTCCC	14	0	RDR2-resistant
CTGAGGTATATGGCGGCGGAGGAC	14	0	RDR2-resistant
CTGAGGTGTCCTGCTGAGCGTCGT	0	10	RDR2-sensitive
CTGAGTCCTGTAGGAGCACAGCTG	1	19	RDR2-sensitive
CTGAGTGTAGAGGAGGTCCCTGAG	1	17	RDR2-sensitive
CTGATATGAGCTGTGGAATGCGGA	0	38	RDR2-sensitive
CTGATCATATCCAGTAGTAGTGCG	43	1	RDR2-resistant
CTGATCATGTTGGACGGGCCGGCT	0	52	RDR2-sensitive
CTGATGATGGTAGAGAATTGTTTC	142	15	RDR2-resistant
CTGATGGACTGGCCCGAAACACGG	0	18	RDR2-sensitive
CTGATGTTCTTGATTGGTCGGTCA	44	0	RDR2-resistant
CTGATTACGGTTGGACTAAAACAA	0	14	RDR2-sensitive
CTGATTAGACTTACTGATAGATGG	0	9	RDR2-sensitive
CTGATTCCGTCTGGACCGAGGCAA	0	13	RDR2-sensitive
CTGATTGCAGAGCTCGCCAGACGA	0	18	RDR2-sensitive
CTGATTGGATGGCTGTGTAGGCAC	0	10	RDR2-sensitive
CTGATTGGTACAATGAATATGGTA	0	13	RDR2-sensitive
CTGATTGGTCGGTCCGAAGCACGG	0	63	RDR2-sensitive
CTGATTGGTTGACTGCATAATCTT	0	13	RDR2-sensitive
CTGATTTCGCTTGGTTCGTCTGTC	0	14	RDR2-sensitive

CTGATTTGTAGTGCTTTGCACGGC	0	18	RDR2-sensitive
CTGATTTTTTCGGACGGATCGGAT	0	16	RDR2-sensitive
CTGCAACCTGGTCAGTCGGCGCGG	1	12	RDR2-sensitive
CTGCAACGGACCAGCTGAAGGGTG	0	12	RDR2-sensitive
CTGCACACGCGTGGAACGTCCGGC	0	10	RDR2-sensitive
CTGCACATTGAAGACGTGGGCTGC	0	16	RDR2-sensitive
CTGCACCCCGCGGATTTGCTCGAG	0	12	RDR2-sensitive
CTGCACCTGAACAGCATAGGGCCT	0	19	RDR2-sensitive
CTGCACGGACGGGAAAACGAAGAA	0	12	RDR2-sensitive
CTGCACGGATGGTTTGGTGGACGG	0	10	RDR2-sensitive
CTGCACGTGGTAGATACAAGGCGC	0	14	RDR2-sensitive
CTGCACTGTAGCCAACATAGGCAA	0	20	RDR2-sensitive
CTGCAGAACACTACAGAACGGGTC	0	9	RDR2-sensitive
CTGCAGACGGTACAGAGCATTCAT	0	10	RDR2-sensitive
CTGCAGACTGAGAAGGCGTCCCAA	0	14	RDR2-sensitive
CTGCAGACTGTGCACTAGAGCAGC	0	12	RDR2-sensitive
CTGCAGAGGATTGTGGTATAGAGT	0	19	RDR2-sensitive
CTGCAGCCTGCAACTCGTCGAGAC	0	23	RDR2-sensitive
CTGCAGCCTGTGCACTGGAGCAGC	0	12	RDR2-sensitive
CTGCAGCGACGTACTCGGCACCGA	0	10	RDR2-sensitive
CTGCAGCGGGAGACTGGTGAGCAA	0	15	RDR2-sensitive
CTGCAGGATCGAAAGGAGGCAAGG	21	0	RDR2-resistant
CTGCAGGCTGCAGCCACGGACACA	0	13	RDR2-sensitive
CTGCAGGCTTGGACAGGAAAACGA	0	16	RDR2-sensitive
CTGCAGGTAGAGCTGTACGGGCAT	1	108	RDR2-sensitive
CTGCAGTACGTCAGAGGAACCGGC	1	14	RDR2-sensitive
CTGCAGTTCGGTATTGGAAGGCAA	0	26	RDR2-sensitive
CTGCATAGTCGGGTGGCACACCAG	0	10	RDR2-sensitive
CTGCATCGCGGTCTTGCTCGGCAT	0	9	RDR2-sensitive
CTGCATCGGTTGAAACGGACGATT	0	12	RDR2-sensitive
CTGCATGACGTAGGTGACGAGCGT	0	19	RDR2-sensitive
CTGCATGGGACCTCTGCTCGACGG	0	9	RDR2-sensitive
CTGCATGTAGAGCTGTACGGGCAT	0	40	RDR2-sensitive
CTGCCACGTCACGAAGCTCGGCGC	0	20	RDR2-sensitive
CTGCCACGTTGGTAAGCTCGGCGT	0	9	RDR2-sensitive
CTGCCCATCGGACCTTCAGTACGG	0	12	RDR2-sensitive
CTGCCCTGTTTTCTGTAGAGGTAT	0	10	RDR2-sensitive
CTGCCGACTTATGGCGTGACTCGG	0	14	RDR2-sensitive
CTGCCGCTGTAGACAGAAGATGGG	0	9	RDR2-sensitive
CTGCCGGATTGATGGAGAGTCGGT	0	11	RDR2-sensitive
CTGCCGGCGTGGTGGAGAGTCGTT	0	15	RDR2-sensitive
CTGCCGGCTTGACGAAGAGGTGAT	0	11	RDR2-sensitive
CTGCCGGGACTGAGGAGATGCATT	0	15	RDR2-sensitive
CTGCCTAGAGGACTACGAGGACGG	0	11	RDR2-sensitive
CTGCCTCGACTGCATGAACGGCGC	0	9	RDR2-sensitive
CTGCCTGACGGTCGGACGGTCCGC	1	12	RDR2-sensitive
CTGCCTTCGTTCAATCTGGATCGT	0	20	RDR2-sensitive
CTGCGACACGTCAGAGCTCGGCAC	0	12	RDR2-sensitive
CTGCGACAGGGTAGATGCGACAGG	0	21	RDR2-sensitive

CTGCGACATGGACTGTAGGAGCGG	1	47	RDR2-sensitive
CTGCGACTGTGCAGAGAGCACCGC	0	24	RDR2-sensitive
CTGCGACTTCAGTAGACGCGGCCT	0	17	RDR2-sensitive
CTGCGATGCAGAACTGATTGGCGA	0	10	RDR2-sensitive
CTGCGATTCGGTCGGTCGGTGCGG	1	12	RDR2-sensitive
CTGCGATTTGGTCGGTCGGTGCGG	2	18	RDR2-sensitive
CTGCGCACACGTAGACGGTCTGTG	1	36	RDR2-sensitive
CTGCGCACATGTGGACCGTCCGAT	0	9	RDR2-sensitive
CTGCGCACGCGCGGACTGTCCGGA	0	10	RDR2-sensitive
CTGCGCACGCGCGGACTGTCCGGC	0	10	RDR2-sensitive
CTGCGCACGCGTGGATTGTCCGGA	0	10	RDR2-sensitive
CTGCGCACGTGCGGACTGTCCGGC	0	11	RDR2-sensitive
CTGCGCAGTTGAACTCGAGGTGGC	0	21	RDR2-sensitive
CTGCGCATCTGTGGAGCCAGGCTC	4	821	RDR2-sensitive
CTGCGCATCTGTGGAGTCAGGCTC	0	19	RDR2-sensitive
CTGCGCATGTAGTCTTGAGGGCGT	0	22	RDR2-sensitive
CTGCGCCGGATCGGAGGATGTCAT	1	13	RDR2-sensitive
CTGCGCCTAGGGCTGACAAGACGC	0	9	RDR2-sensitive
CTGCGCCTAGGGCTGACATGGCAT	1	19	RDR2-sensitive
CTGCGCCTAGGGCTGACCTGGCAT	0	9	RDR2-sensitive
CTGCGCGCGCAGAAGTCAGAGCAG	0	11	RDR2-sensitive
CTGCGCGCGGAACGAGGAGGGCAG	0	13	RDR2-sensitive
CTGCGCGCGTAGAAGCTGGGCTGC	0	14	RDR2-sensitive
CTGCGCGGACAGAACTGCAGCAGA	9	26	RDR2-sensitive
CTGCGCGGGGATGGAATTGGACGG	0	18	RDR2-sensitive
CTGCGCGTGCATGTAGAAGTGGTG	0	25	RDR2-sensitive
CTGCGCGTGCATGTGGAAGCGGTG	0	21	RDR2-sensitive
CTGCGCTATTGTAGACGGTTCGAG	0	42	RDR2-sensitive
CTGCGCTGGAAGACTGTCCGCATG	0	10	RDR2-sensitive
CTGCGGACGGTGATTTTAGAGGCT	0	13	RDR2-sensitive
CTGCGGACTGTGGGCGGTCGGCTT	0	10	RDR2-sensitive
CTGCGGATTAGCAGTAGGGACCGC	0	10	RDR2-sensitive
CTGCGGCAATATACTCGGCTTCGG	4	21	RDR2-sensitive
CTGCGGCAATGTACTCGGCTTCGG	14	77	RDR2-sensitive
CTGCGGCGTAGGCCTAAGTGACGG	0	14	RDR2-sensitive
CTGCGGCTGCGGCTTCTACAGTAT	0	24	RDR2-sensitive
CTGCGGGCTGGTTGACGCGGTCTA	0	35	RDR2-sensitive
CTGCGGGCTGTACGGCGCGGAGTA	9	25	RDR2-sensitive
CTGCGTACGCGTGGACGGTCTGCA	0	10	RDR2-sensitive
CTGCGTCAGACACGGACCGTCCGA	0	15	RDR2-sensitive
CTGCGTCATCGTAGATCGTCCGGC	0	64	RDR2-sensitive
CTGCGTCCGAGACTGCCCGGGCTG	0	9	RDR2-sensitive
CTGCGTGAGGACTGAGGAGCCGGC	0	9	RDR2-sensitive
CTGCGTGCAGGACCGGATGACGCA	0	11	RDR2-sensitive
CTGCGTGCTGTTTGGACGGTCCGT	0	10	RDR2-sensitive
CTGCGTGGCCTGTGGCGAAGTCGG	0	10	RDR2-sensitive
CTGCGTTTGGTGTAGAGATGGCAA	0	11	RDR2-sensitive
CTGCTAACTCTTACTCGGACGGTG	0	9	RDR2-sensitive
CTGCTAGGAGCCTAGGACGACGGT	1	12	RDR2-sensitive

CTGCTAGTCGTGTCGGGCCGGCCC	1	12	RDR2-sensitive
CTGCTATCCAGATTGTAGATGCAT	0	14	RDR2-sensitive
CTGCTATTGTAGGGCTTGTTCGGT	0	21	RDR2-sensitive
CTGCTCACTCTACGCGGATGACGC	0	10	RDR2-sensitive
CTGCTCAGGCTGTACGCGGACAGA	0	10	RDR2-sensitive
CTGCTCCTGTTTCTGCTCGTCGTA	33	1	RDR2-resistant
CTGCTCGTTGTATTGGTCGGTCAG	0	14	RDR2-sensitive
CTGCTCTCCGAACTCTGATGGCAC	0	10	RDR2-sensitive
CTGCTCTCTGCGGCACACCGGACA	0	11	RDR2-sensitive
CTGCTGAAGGCTTTATACCACTGT	0	10	RDR2-sensitive
CTGCTGACGCGCAGAACGACGACG	0	13	RDR2-sensitive
CTGCTGACTCTGTCGGGCCGGCCC	0	9	RDR2-sensitive
CTGCTGACTGTACGGCTGTACCGT	0	15	RDR2-sensitive
CTGCTGACTGTGCCAGACTGGCTC	0	11	RDR2-sensitive
CTGCTGATAGACCTGGCTACTAGA	0	28	RDR2-sensitive
CTGCTGATCAGATCATCGACGGTA	0	12	RDR2-sensitive
CTGCTGCCTGATCCTGAAGACGTT	0	29	RDR2-sensitive
CTGCTGCGGGCTGAGCGCGCGGAA	1	15	RDR2-sensitive
CTGCTGGAAGGGCCCGAACGGTAT	0	13	RDR2-sensitive
CTGCTGGAGAGCGTAGAGGACCAT	0	14	RDR2-sensitive
CTGCTGGGGACGGATGGAACACAC	0	13	RDR2-sensitive
CTGCTGGTGCGGACTAGGAGGCAA	1	68	RDR2-sensitive
CTGCTGTAGTGACATGGACGACAT	1	15	RDR2-sensitive
CTGCTGTTTGCTGTGGACGGATGT	0	10	RDR2-sensitive
CTGCTTCAGACGACTCGAACGGCG	0	21	RDR2-sensitive
CTGCTTGGACTGAGGCACGTAGAA	0	13	RDR2-sensitive
CTGCTTTGTCTCTGTCGGACCAGC	0	10	RDR2-sensitive
CTGCTTTTGGCTGGATTCCTGGGC	55	4	RDR2-resistant
CTGGAACCGGTGTATTTTGAAGAG	0	13	RDR2-sensitive
CTGGAACCGTCTGTGTAGAGGCTA	0	15	RDR2-sensitive
CTGGAACCTGGAAGCGGAGCTCGG	0	13	RDR2-sensitive
CTGGAACGAGCCGTCTGAAGGTGC	0	11	RDR2-sensitive
CTGGAACGGGCTCGACGAGATGAA	0	26	RDR2-sensitive
CTGGAACTGAAGGCGCGCCTGGGC	0	17	RDR2-sensitive
CTGGAAGACGGCGAACTCCGCTCC	0	12	RDR2-sensitive
CTGGAATGTGGAAGCATACAGCGC	0	11	RDR2-sensitive
CTGGACAAAATGCTCGCGGCTCGT	0	15	RDR2-sensitive
CTGGACAACGTGAGCGGAGGCTTC	0	33	RDR2-sensitive
CTGGACACTCGGCAAAGACCCCAT	0	14	RDR2-sensitive
CTGGACACTCGGCACAGGACCCCA	0	11	RDR2-sensitive
CTGGACACTCGGCACAGGACGACA	0	18	RDR2-sensitive
CTGGACAGAGATGAGCTGGAGCGG	0	9	RDR2-sensitive
CTGGACAGCGGCTGGGAGGAGGGC	2091	105	RDR2-resistant
CTGGACAGCGGCTGGGAGGAGGGG	59	2	RDR2-resistant
CTGGACAGGGATACGCAGCAACAA	0	13	RDR2-sensitive
CTGGACAGGGGCACGCAACAACAA	0	11	RDR2-sensitive
CTGGACAGTAGAAGTTGCAGGTAT	0	9	RDR2-sensitive
CTGGACAGTGGATAGAGGTAGGCT	0	14	RDR2-sensitive
CTGGACAGTTCAACCTGCGGACGG	0	13	RDR2-sensitive

CTGGACATGGCAGACTGCACCAGC	0	39	RDR2-sensitive
CTGGACATGTCAGACTGCACCAGC	0	49	RDR2-sensitive
CTGGACCACTGCGTGCATTGACTC	1	20	RDR2-sensitive
CTGGACCAGCTTACATGACGCGTG	0	9	RDR2-sensitive
CTGGACCAGTTAGACAGACCGAGC	0	10	RDR2-sensitive
CTGGACCAGTTGAACGGACCGAGC	0	13	RDR2-sensitive
CTGGACCAGTTGAACGGGCCGAAC	0	9	RDR2-sensitive
CTGGACCCGGCACGACTTTGGCGT	0	94	RDR2-sensitive
CTGGACCCGGCACGACTTTGGTGT	0	22	RDR2-sensitive
CTGGACCGACGAAAATGAGGCGTA	0	9	RDR2-sensitive
CTGGACCGCATCCAACGACGACAT	0	9	RDR2-sensitive
CTGGACCGGACGGCGTTAACCCTT	0	10	RDR2-sensitive
CTGGACCGTTTGCGCAGAGAGACC	0	10	RDR2-sensitive
CTGGACCTAGACCGCGTCAGCCAG	0	11	RDR2-sensitive
CTGGACCTGAGCGCGGCTGACCAG	0	10	RDR2-sensitive
CTGGACCTGCCACCTGACGAGCAA	0	11	RDR2-sensitive
CTGGACCTGGACTCAAAGCTCGGC	0	28	RDR2-sensitive
CTGGACGAACAGACAGACAGGCAT	0	11	RDR2-sensitive
CTGGACGAGGCCGTGAATTTGCAC	0	9	RDR2-sensitive
CTGGACGATGCATGGCGCTGCTGC	0	13	RDR2-sensitive
CTGGACGCGGGACGAACACGAAGG	0	15	RDR2-sensitive
CTGGACGCTCCTCATGAAGATCAA	0	9	RDR2-sensitive
CTGGACGGCAGTAGAAAACGACGT	0	11	RDR2-sensitive
CTGGACGGCTACTGTAGCCCACGG	0	15	RDR2-sensitive
CTGGACGGGGCTGGTGGACAGGTA	1	16	RDR2-sensitive
CTGGACGGTATGCACGGTGGGCAA	0	19	RDR2-sensitive
CTGGACGGTGTGCACGGTGGGCAT	1	17	RDR2-sensitive
CTGGACGGTTTCTAGTAGCGGCGT	0	10	RDR2-sensitive
CTGGACGTAGGGCCTACAAGGCAG	0	16	RDR2-sensitive
CTGGACGTGGACGACGGAATTGGT	0	10	RDR2-sensitive
CTGGACTCTCGACTCTAGGGGCAT	0	16	RDR2-sensitive
CTGGACTCTGAGCCGTTGGAGGTG	0	35	RDR2-sensitive
CTGGACTCTGTCCGAGCTGTTCGG	0	9	RDR2-sensitive
CTGGACTGGACGAGATGACGAGTG	0	11	RDR2-sensitive
CTGGACTGGGACACGCAGCGACAA	1	14	RDR2-sensitive
CTGGACTGGGGAAGATGGTGCCTG	1	27	RDR2-sensitive
CTGGACTGGGGCACGCGACGACAT	0	10	RDR2-sensitive
CTGGACTGGGGCACGCGGCGACAA	0	12	RDR2-sensitive
CTGGACTGGGGCACGCGGCGACAC	0	10	RDR2-sensitive
CTGGACTGGGGCACGCGGCGACAT	0	13	RDR2-sensitive
CTGGACTGGTTCGGCGAAGGGCTG	3	67	RDR2-sensitive
CTGGACTGTCCAGTGGAGCACCGG	0	116	RDR2-sensitive
CTGGACTGTCCATCGTAGCGGCAC	0	18	RDR2-sensitive
CTGGACTGTCCGCGATGACGCAGA	0	9	RDR2-sensitive
CTGGACTGTCCGCGGTGGTGGCGC	0	20	RDR2-sensitive
CTGGACTGTCTACCGTAGCGGCAC	3	20	RDR2-sensitive
CTGGACTGTGCTGAAGAGCTGCAA	0	24	RDR2-sensitive
CTGGACTGTGGAAGATGGTGCTTG	0	13	RDR2-sensitive
CTGGACTTATTTCGGGCGGCCCGA	0	10	RDR2-sensitive

CTGGAGACAGAGAACGAGCAACAG	0	11	RDR2-sensitive
CTGGAGACGTAGCACGTGTACAGC	0	10	RDR2-sensitive
CTGGAGGACCTGTCGGTTTGACGG	0	12	RDR2-sensitive
CTGGAGTAGCCGAGCATGCCTGTA	49	0	RDR2-resistant
CTGGAGTGGGTAGGAGAGAGACGA	0	14	RDR2-sensitive
CTGGAGTGTGGCGCGGTGTGGCGT	0	9	RDR2-sensitive
CTGGATAGACAGCGGGGTAGATGC	0	14	RDR2-sensitive
CTGGATAGAGGACGCACAGTGAAC	0	12	RDR2-sensitive
CTGGATCAGGTCAGACTCGTGCAA	0	9	RDR2-sensitive
CTGGATCATGTGGAAGACGACTGA	0	39	RDR2-sensitive
CTGGATCATTAGAGCGCATGGGAG	0	12	RDR2-sensitive
CTGGATCTCGCAGGGGGTTGGGGT	14	0	RDR2-resistant
CTGGATGACGTACAGAACGACGAC	0	13	RDR2-sensitive
CTGGATGCGGCAGAACCGTGCACC	1	31	RDR2-sensitive
CTGGATGGACTAGCTCGAGCGGTG	0	12	RDR2-sensitive
CTGGATGGTAAAGAGGACGGACCT	0	13	RDR2-sensitive
CTGGATGTAGGGACGTTCCACCGG	0	10	RDR2-sensitive
CTGGATGTAGTTGGTAGACGGTTC	0	11	RDR2-sensitive
CTGGATGTGGCAGAACCGTGCACC	0	17	RDR2-sensitive
CTGGATGTTTGAGACTCAGACGGT	0	11	RDR2-sensitive
CTGGATTCGGGACCGGATACGGAT	2	96	RDR2-sensitive
CTGGATTGGTTCGGTAGACACGGT	0	32	RDR2-sensitive
CTGGATTGTTTCGGAGGGTCGGAC	0	12	RDR2-sensitive
CTGGATTTGTACGGTACGGGGCGC	0	9	RDR2-sensitive
CTGGCAAGAGACTCGATCCGGCAT	0	12	RDR2-sensitive
CTGGCAATCGGACGGTCGAGATGG	0	10	RDR2-sensitive
CTGGCACAACCTGAACGACGTGGC	0	9	RDR2-sensitive
CTGGCACAATCAGCAGGTCGGCAT	0	9	RDR2-sensitive
CTGGCACCTTCGCCCCGTCGTCGT	0	9	RDR2-sensitive
CTGGCACGACACAGATCTGGCATG	0	11	RDR2-sensitive
CTGGCACGAGACCCGAACCGGCAT	0	9	RDR2-sensitive
CTGGCACGGCACGACAGATGACTC	0	29	RDR2-sensitive
CTGGCACGGTAGAATAAGCGGGCG	0	16	RDR2-sensitive
CTGGCACGTGACCCGAACCGGCAC	1	15	RDR2-sensitive
CTGGCACTATTCGGACCGGGCTAA	0	10	RDR2-sensitive
CTGGCATGAACCCGGCACTGACAT	0	19	RDR2-sensitive
CTGGCATGTAAGCAGTAGGACATA	0	18	RDR2-sensitive
CTGGCATTCAAGGGCGGCGACAGC	18	0	RDR2-resistant
CTGGCCAGCAGCCTGATCGTTGGA	0	27	RDR2-sensitive
CTGGCCCAAGCATGACACTGACAA	0	21	RDR2-sensitive
CTGGCCTAGAGAACCTGACGGCGT	0	9	RDR2-sensitive
CTGGCCTGGAAATGTAGAGGACGT	0	14	RDR2-sensitive
CTGGCCTGGATGGACTCACGGCGT	2	22	RDR2-sensitive
CTGGCCTGGCTACGAAGCGTGCTG	0	10	RDR2-sensitive
CTGGCGAAGGAGGATGGAGGGGCA	16	0	RDR2-resistant
CTGGCGCGACTGCTAGCACGGCAC	0	11	RDR2-sensitive
CTGGCGGAACATTGCGACAACCAT	0	10	RDR2-sensitive
CTGGCGGCGGACAGCAGAGAGCGG	0	9	RDR2-sensitive
CTGGCGTAGACTTGCAGATCTGGC	1	27	RDR2-sensitive

CTGGCGTAGGAGCGGCTCCTCTGC	0	13	RDR2-sensitive
CTGGCGTGGGATGTAGGTGGGCGC	0	30	RDR2-sensitive
CTGGCGTTGGCTGAGCAGTCGGAT	0	9	RDR2-sensitive
CTGGCTACTGTAGTTCACGGGCCG	0	27	RDR2-sensitive
CTGGCTCCTCTAAGCCCTCGTCGT	0	10	RDR2-sensitive
CTGGCTCGAGCGGATTGCAGCGTA	0	9	RDR2-sensitive
CTGGCTCGCTCGGATCATGGTCAG	0	18	RDR2-sensitive
CTGGCTCGGCTCGGCTCGTTAGAA	0	18	RDR2-sensitive
CTGGCTCGGCTTGGCTCGTTAGAA	0	9	RDR2-sensitive
CTGGCTCTCGGCAAAGACTGACGG	0	16	RDR2-sensitive
CTGGCTCTGACCGGTCTGCTGCAG	14	0	RDR2-resistant
CTGGCTCTGACGTGGTGGCGGCGC	0	13	RDR2-sensitive
CTGGCTCTGATACCACTGAAGGAT	17	1	RDR2-resistant
CTGGCTGAGAAACGATTTTTGCAT	0	16	RDR2-sensitive
CTGGCTGAGTAGAAACACGGGGTT	0	29	RDR2-sensitive
CTGGCTGCTCTCGGGTTTCGTCGG	1	14	RDR2-sensitive
CTGGCTGGCACGGACACGGTATGT	0	12	RDR2-sensitive
CTGGCTGGCTGTAACGGTAGCTCT	0	9	RDR2-sensitive
CTGGCTGTAGGGAGAATCCGGCTT	0	9	RDR2-sensitive
CTGGCTTAGAGTCACGGACCGTCC	0	12	RDR2-sensitive
CTGGCTTCCCTGGCTCTTTGACGG	0	16	RDR2-sensitive
CTGGCTTCTCTGGCTCTGTGACGG	0	16	RDR2-sensitive
CTGGGAAGAGGTGGACGACGACGG	0	16	RDR2-sensitive
CTGGGAAGCATCGGACTGTCCGGT	1	18	RDR2-sensitive
CTGGGAAGCTTTGGGATCTCGGGC	27	1	RDR2-resistant
CTGGGACAGGCAGGACAAAGACGT	0	9	RDR2-sensitive
CTGGGACATGTAGACGGTCGAGAC	1	12	RDR2-sensitive
CTGGGACCTAGATGTGCTACTCGG	0	9	RDR2-sensitive
CTGGGACGGTAGCGGACGACGAAA	0	9	RDR2-sensitive
CTGGGACGTTGCAGAGTGGACGGT	0	9	RDR2-sensitive
CTGGGACTCTGGGAGGACGTCGCC	0	12	RDR2-sensitive
CTGGGAGACGACGGCCTAGACCTC	2	14	RDR2-sensitive
CTGGGAGCGTGAAGATGATCTGAT	0	22	RDR2-sensitive
CTGGGAGGAGGGCCGAGGGCGGGG	17	1	RDR2-resistant
CTGGGAGGAGGGCCGAGGGCGGGT	137	3	RDR2-resistant
CTGGGCAAAATACTCGTGGCTCGT	0	13	RDR2-sensitive
CTGGGCAAAATACTCGTGGGTCGT	0	11	RDR2-sensitive
CTGGGCACGACACAGTACGGCTGA	0	15	RDR2-sensitive
CTGGGCACGCGTCGGCACGCACGC	0	19	RDR2-sensitive
CTGGGCACGGCACGGCACGGCTAG	0	10	RDR2-sensitive
CTGGGCAGCAGCTAGGAGCAGGGG	47	4	RDR2-resistant
CTGGGCAGGAGGAGGTGCTGGGGA	59	0	RDR2-resistant
CTGGGCAGGGACACGCAACAACAA	1	16	RDR2-sensitive
CTGGGCAGGGACACGCAGCAACAA	2	37	RDR2-sensitive
CTGGGCATAACGCTGAGGCAGCAC	0	10	RDR2-sensitive
CTGGGCATGAGGCGCATAGGACAC	0	19	RDR2-sensitive
CTGGGCGACGTGCGAGAGGAACAG	0	11	RDR2-sensitive
CTGGGCGATTGGTAGGAGAGGGCG	0	11	RDR2-sensitive
CTGGGCGCAGGACAACACGAAGGC	17	77	RDR2-sensitive

CTGGGCGCGGGACGAACACGAAGG	24	178	RDR2-sensitive
CTGGGCGTAGGACAACACGAAGGC	0	9	RDR2-sensitive
CTGGGCGTGACAGTTAGACAGCGA	0	10	RDR2-sensitive
CTGGGCGTGTAGAGGACGTGTCTG	0	14	RDR2-sensitive
CTGGGCTACGGGCTGAAGACCAGT	0	13	RDR2-sensitive
CTGGGCTAGGACACGCGGCGACAA	0	35	RDR2-sensitive
CTGGGCTAGGGCGTGCAACATCAT	0	11	RDR2-sensitive
CTGGGCTCTGAATGGTGTGCACGG	0	10	RDR2-sensitive
CTGGGCTGAAAACGAGGGGTTCGC	0	9	RDR2-sensitive
CTGGGCTGAAAACGAGGTGTTCGC	0	9	RDR2-sensitive
CTGGGCTGCAGCCCCGATCGCGGC	0	11	RDR2-sensitive
CTGGGCTGCTGACGTAGGTGACGA	0	14	RDR2-sensitive
CTGGGCTGGAGCACGCGGCGACAA	0	12	RDR2-sensitive
CTGGGCTGGGACACGCAACGACAA	0	9	RDR2-sensitive
CTGGGCTGGGACACGCAGCGACAA	1	32	RDR2-sensitive
CTGGGCTGGGACACGCGACGACAA	0	10	RDR2-sensitive
CTGGGCTGGGACACGCGGCGACAA	1	119	RDR2-sensitive
CTGGGCTGGGACACGCGGCGACAT	0	14	RDR2-sensitive
CTGGGCTGGGACGCGCAGCGACAA	0	12	RDR2-sensitive
CTGGGCTGGGGCACGCAGCACACT	0	19	RDR2-sensitive
CTGGGCTGGGGCACGCGACGACAA	0	20	RDR2-sensitive
CTGGGCTGGGGCACGCGACGACAT	0	71	RDR2-sensitive
CTGGGCTGTAGCCCCGGTCACGGC	0	26	RDR2-sensitive
CTGGGCTTCATATAGGTGCGCGGA	0	10	RDR2-sensitive
CTGGGCTTGTGTGAGATGGTGCAT	0	26	RDR2-sensitive
CTGGGCTTTTCGGGCTGGCACGGC	1	31	RDR2-sensitive
CTGGGGCTGTAGCAAGTACCGGGG	0	14	RDR2-sensitive
CTGGGGCTTGCACTCGAACACGGA	0	9	RDR2-sensitive
CTGGGGCTTGGCAGGGGATGACGA	0	27	RDR2-sensitive
CTGGGTAGCACTGAACAATCCGGT	0	15	RDR2-sensitive
CTGGGTCTCATGGACCTCGCGGAC	21	0	RDR2-resistant
CTGGGTGAGACGGTTGGCTCGGAC	0	9	RDR2-sensitive
CTGGGTGTAGAAGAGGTCCCCGAG	0	9	RDR2-sensitive
CTGGTAACGCGGCATGACATACCT	0	11	RDR2-sensitive
CTGGTACCATGTAGAAGTGAGGAA	0	21	RDR2-sensitive
CTGGTACTGTGACCGGCAACTAAA	0	11	RDR2-sensitive
CTGGTAGAGACTGCTGAGCAGGAG	0	9	RDR2-sensitive
CTGGTAGGACTCTCTGCGGAGGCG	0	25	RDR2-sensitive
CTGGTATGACTAGCAGGCTGACGG	0	55	RDR2-sensitive
CTGGTCAACAGGACGGTGTGACAA	0	13	RDR2-sensitive
CTGGTCAACTGGCTGGGCGCGGAC	0	9	RDR2-sensitive
CTGGTCACTGTTCACGGCACTTGC	407	16	RDR2-resistant
CTGGTCAGATGCGGTTGAACCGGC	14	0	RDR2-resistant
CTGGTCAGGACCGATTGAACCGGC	1	19	RDR2-sensitive
CTGGTCGAGCTGAAGGAAGTGCGG	0	12	RDR2-sensitive
CTGGTCGCTCGCTCTGCCTGACGG	0	15	RDR2-sensitive
CTGGTCTAGAACTGTTTCCGACGT	0	9	RDR2-sensitive
CTGGTGAGTCTGGACGTTGCTGGC	0	33	RDR2-sensitive
CTGGTGATGGTGGGCTTTGGCGGA	0	19	RDR2-sensitive

CTGGTGCACTCATGGACAGAAGGC	0	11	RDR2-sensitive
CTGGTGGACCTGCAGGTGGGGCCT	0	50	RDR2-sensitive
CTGGTGGACCTGTAGGTGGGGCCT	2	33	RDR2-sensitive
CTGGTGGACTTACAGGTGAGACGT	0	9	RDR2-sensitive
CTGGTGTGCTAATGTCGTACGGGC	16	0	RDR2-resistant
CTGGTTCGGACTGCAGGAGTGGGT	76	3	RDR2-resistant
CTGGTTCTGCCCATCCTAGTCGGT	0	9	RDR2-sensitive
CTGGTTGGACCACGTGGAGGACGT	0	11	RDR2-sensitive
CTGGTTGGACCACTGACGAGATGG	1	12	RDR2-sensitive
CTGGTTGTCGGCGAAGTTGAAGGC	0	10	RDR2-sensitive
CTGTAAACGTAGAGAGGCCCTGAG	0	9	RDR2-sensitive
CTGTAACCGTCTGTGTAGAGGCTA	0	18	RDR2-sensitive
CTGTAACGTTGGCTCTTGACGGAC	0	22	RDR2-sensitive
CTGTAAGCTCTCCAACAAGGACAC	0	15	RDR2-sensitive
CTGTACAAGATGAGCTCGGCCCAG	0	9	RDR2-sensitive
CTGTACAGGATAGAGTTGTGACTG	0	14	RDR2-sensitive
CTGTACGAATACGTCTGTCGGCAC	0	19	RDR2-sensitive
CTGTACGAGCTCCGGCACCGGCGT	0	14	RDR2-sensitive
CTGTACGGGCTGCTGGAGATAGAC	0	9	RDR2-sensitive
CTGTACTGTGTTGGTCGGCGGCTG	0	15	RDR2-sensitive
CTGTAGAACACGCCTGCCAGACGA	0	28	RDR2-sensitive
CTGTAGAACACGCCTGCTAGACGA	0	12	RDR2-sensitive
CTGTAGACTGGAGCAGCAGGAACG	0	21	RDR2-sensitive
CTGTAGATCAACGGCTGAAAACGC	0	9	RDR2-sensitive
CTGTAGATCAACGGCTGAAAATGC	0	9	RDR2-sensitive
CTGTAGATCTGTTGGCACCGGCCC	0	17	RDR2-sensitive
CTGTAGCAATGACGCGCGCGGCCT	0	9	RDR2-sensitive
CTGTAGCAGTCGACCGTTGGATCC	0	10	RDR2-sensitive
CTGTAGCTACTCTGATTTAGACGT	0	10	RDR2-sensitive
CTGTAGGACCTGGGGGCAGATCGT	0	12	RDR2-sensitive
CTGTAGGCTGATCGGTCGGAAGGA	0	11	RDR2-sensitive
CTGTAGGTAGGACGTGGACACGGA	15	0	RDR2-resistant
CTGTAGTACCTATGCATGTTGACG	195	0	RDR2-resistant
CTGTATACGTAGGGACGGATCCAG	2	15	RDR2-sensitive
CTGTATATTGAAATACAGTCGGAC	0	10	RDR2-sensitive
CTGTATCAAGGACTATGACGTCAT	0	14	RDR2-sensitive
CTGTATCGCGGACTCCAAACGGAT	0	11	RDR2-sensitive
CTGTATCGGTTGAAACGGACGATT	0	10	RDR2-sensitive
CTGTATCTATCGGCTTATTTACAT	24	1	RDR2-resistant
CTGTATCTCGGATTTCGGGCACGG	0	23	RDR2-sensitive
CTGTATCTGGACGCGCAGGCTCGG	0	9	RDR2-sensitive
CTGTATGACGGACAGACAGGACAA	0	13	RDR2-sensitive
CTGTATGAGTAGGGACGCAGGGAC	0	10	RDR2-sensitive
CTGTATGCACTCAGGGGCGGAGAG	0	10	RDR2-sensitive
CTGTATGCACTCAGGGGCGGAGGG	0	35	RDR2-sensitive
CTGTATGCACTCGGTCGTCTTGAC	0	11	RDR2-sensitive
CTGTATGCCTCCAACAAGACACGC	0	9	RDR2-sensitive
CTGTATTGGCGTAGAGGGCTTCAC	0	12	RDR2-sensitive
CTGTATTTTCGTCGGCAGGCCCAC	0	9	RDR2-sensitive

CTGTCACGTAGGACAGTTCGGCAA	1	24	RDR2-sensitive
CTGTCACGTGACCCGAACCGGCAC	0	36	RDR2-sensitive
CTGTCAGACCAGCGGCTGCAGCGA	0	16	RDR2-sensitive
CTGTCAGACTGGCCAGTGGAGCGG	5	59	RDR2-sensitive
CTGTCAGACTGGTCAGTGGAGCGG	0	34	RDR2-sensitive
CTGTCAGCAGTAGAGGCGCAAGAA	2	43	RDR2-sensitive
CTGTCAGCAGTAGAGGCGCGAGAA	0	20	RDR2-sensitive
CTGTCAGCAGTAGAGGCGTAAGAA	0	10	RDR2-sensitive
CTGTCAGGCTACAAGGGTAGACAG	0	10	RDR2-sensitive
CTGTCAGGTCCCCTACGGCTCCAG	0	9	RDR2-sensitive
CTGTCAGGTCGGTCAGTGGAGCGG	0	11	RDR2-sensitive
CTGTCAGTTGTAGACGGCTGCTGA	0	10	RDR2-sensitive
CTGTCCAAGCAGAACTGTCCACAC	0	13	RDR2-sensitive
CTGTCCACTCTGCAACGGCCCAAC	6	29	RDR2-sensitive
CTGTCCCAGCGACCAGACCTCGGA	0	14	RDR2-sensitive
CTGTCCGAGACGGTAGGAAAACGG	0	20	RDR2-sensitive
CTGTCCGGTGAACCAGCAGAGCAA	2	19	RDR2-sensitive
CTGTCGACTGAAGGGCGCGCGTCA	0	14	RDR2-sensitive
CTGTCGACTGGAGGACGTGCGTCA	0	12	RDR2-sensitive
CTGTCGAGCTGAACGAACACGGGT	0	9	RDR2-sensitive
CTGTCGAGCTTTGGAGGTGGTGGC	0	21	RDR2-sensitive
CTGTCGAGTGGCACTGGTACGGTC	0	12	RDR2-sensitive
CTGTCGCAAGCTCGTTGGTCACAG	0	18	RDR2-sensitive
CTGTCGCGGGCTGGGACAAGGCAT	0	68	RDR2-sensitive
CTGTCGCGTAGAACAGTTCGGCAA	0	20	RDR2-sensitive
CTGTCGCGTAGGACAATTCGGCTA	0	27	RDR2-sensitive
CTGTCGCGTAGGACAATTCGGCTT	0	21	RDR2-sensitive
CTGTCGCGTAGGACAGTTCGGCAA	7	547	RDR2-sensitive
CTGTCGCGTAGGACAGTTCGGCAC	0	13	RDR2-sensitive
CTGTCGCGTAGGACAGTTCGGCAT	2	27	RDR2-sensitive
CTGTCGCGTAGGACAGTTCGGCCA	0	15	RDR2-sensitive
CTGTCGCGTAGGACAGTTCGGCGA	0	12	RDR2-sensitive
CTGTCGCGTAGGACAGTTCGGCTA	9	501	RDR2-sensitive
CTGTCGCGTAGGACAGTTCGGCTC	1	28	RDR2-sensitive
CTGTCGCGTAGGACAGTTCGGCTT	3	352	RDR2-sensitive
CTGTCGCGTAGGATAGTTCGGCAA	0	36	RDR2-sensitive
CTGTCGGGCGGCCACCAAGACAGC	53	8	RDR2-resistant
CTGTCGTATTGGACTTGATGGCTT	0	9	RDR2-sensitive
CTGTCGTATTGTACTTGGATGGTT	0	11	RDR2-sensitive
CTGTCGTGACTTTCCCAACCGGAT	0	11	RDR2-sensitive
CTGTCGTGCTTATGGGAAGGGAGA	3	70	RDR2-sensitive
CTGTCGTGTTGGACTTAGGTGGTT	0	14	RDR2-sensitive
CTGTCTATAGCACTGGAAGGGCAC	0	11	RDR2-sensitive
CTGTCTGACCAGACGAACGGACGG	0	16	RDR2-sensitive
CTGTCTGACTGAGGCATCGTCACT	0	11	RDR2-sensitive
CTGTCTGAGCAGAGGCTGAAGCAG	1	40	RDR2-sensitive
CTGTCTGAGCCAGAACCCGCGCAC	0	9	RDR2-sensitive
CTGTCTGTCGGAGATGGGCCGCAT	0	14	RDR2-sensitive
CTGTCTGTCGGGAGATGAGCAGAA	0	11	RDR2-sensitive

CTGTCTTCGGGCTGTGCTAGTCGG	0	15	RDR2-sensitive
CTGTCTTGCGGATTTACTCGACGG	0	12	RDR2-sensitive
CTGTCTTGTAGAAGGGGTATGAAA	0	11	RDR2-sensitive
CTGTGACTAGCAGAGCAGCCCCAT	0	9	RDR2-sensitive
CTGTGACTCGGCGAGCTGGGCAGA	0	9	RDR2-sensitive
CTGTGAGAACGACACGAAGCCTGC	0	16	RDR2-sensitive
CTGTGAGAGCGGGATTGGAACGGA	0	16	RDR2-sensitive
CTGTGATTAGATCGGATCAGGTAT	0	23	RDR2-sensitive
CTGTGCACTGGAGCAGCAGGGACG	1	14	RDR2-sensitive
CTGTGCACTGGAGCAGCAGGGGCG	0	23	RDR2-sensitive
CTGTGCAGAGAGCACCGCCGCCGG	0	15	RDR2-sensitive
CTGTGCATCCTATAGCGGCTACAG	0	9	RDR2-sensitive
CTGTGCGGACGATTGCTTGCAGGC	0	9	RDR2-sensitive
CTGTGCTCGCAGAAGGTGGGACGG	1	19	RDR2-sensitive
CTGTGCTCGTAGAAGGTGGGACGG	0	18	RDR2-sensitive
CTGTGGAAAAAGCTGCTGTAGGCT	0	24	RDR2-sensitive
CTGTGGAGCAAGGAAGCGAACGCA	0	15	RDR2-sensitive
CTGTGGAGCCAGGCTCTGGAGATG	0	14	RDR2-sensitive
CTGTGGATATGGACCGGAAGGTAC	0	18	RDR2-sensitive
CTGTGGCACTGAACCTGAGATCGA	0	37	RDR2-sensitive
CTGTGGCTGAAAAGTGTGGCGCGG	0	10	RDR2-sensitive
CTGTGGCTTAGGGTCGGGCAGTCT	0	11	RDR2-sensitive
CTGTGGGCCGGACTGTCCGCGCGT	0	12	RDR2-sensitive
CTGTGGGTCTATACCGTGTCGGGC	0	16	RDR2-sensitive
CTGTGGGTCTTGTCGAGTCGGATA	0	11	RDR2-sensitive
CTGTGGTCTAGACTGTATGGCGTA	0	12	RDR2-sensitive
CTGTGGTGTAGTGCTAAACGGGCT	0	17	RDR2-sensitive
CTGTGTAGAGGCTAACCAGACCGC	0	13	RDR2-sensitive
CTGTGTAGAGGCTAACCGGACCGC	0	48	RDR2-sensitive
CTGTGTAGCGGAGAACAAGATGTC	21	2	RDR2-resistant
CTGTGTAGGGATAATCTGGGACGG	0	14	RDR2-sensitive
CTGTGTATCCCTGTAGAGTACGGA	0	12	RDR2-sensitive
CTGTGTGTTGACTGTATTTAGGTT	0	15	RDR2-sensitive
CTGTGTTCTCTGACTGAACGACAT	0	15	RDR2-sensitive
CTGTGTTTGGTAAGCTGGAACGGA	1	14	RDR2-sensitive
CTGTGTTTGGTAATCTGGAACGGA	2	128	RDR2-sensitive
CTGTTAGTCGTGCCGGGCCGGCCC	1	26	RDR2-sensitive
CTGTTATGAGATGGAGGACGACAT	1	19	RDR2-sensitive
CTGTTCACCGGAGGCTGGCCTGGC	0	16	RDR2-sensitive
CTGTTCACGGCACTTGCTGGCACT	97	5	RDR2-resistant
CTGTTCACGTCGGCACCAGGCTGC	16	0	RDR2-resistant
CTGTTCACTGTAAGACATTGGCAT	0	9	RDR2-sensitive
CTGTTCATGCTGTAGAGGCTACAG	0	13	RDR2-sensitive
CTGTTCATGTTGTAGAGGCTACAG	0	9	RDR2-sensitive
CTGTTCCACGACGAAGACTGCGCC	60	2	RDR2-resistant
CTGTTCCATGTAGCGGCGCTCTGC	0	10	RDR2-sensitive
CTGTTCCTCGTCAAGCTCGGGCAT	0	23	RDR2-sensitive
CTGTTCGAACCAGAATTGAGCGGC	0	9	RDR2-sensitive
CTGTTCGGTTGACTGTGTGATGGA	0	10	RDR2-sensitive

CTGTTCGTTGCCGGAGAAGGACAC	0	38	RDR2-sensitive
CTGTTCGTTGGTGGAACGACGGGC	175	7	RDR2-resistant
CTGTTCTGGAGACGCAGGCGACAG	0	16	RDR2-sensitive
CTGTTCTTCTTCTGTAGGGGGTGT	0	10	RDR2-sensitive
CTGTTGACTCGGGCCGTGCCGGTT	0	25	RDR2-sensitive
CTGTTGCAACGTACGGGTACTCAC	0	9	RDR2-sensitive
CTGTTGCATCGGGCTGTGCCGGTT	3	28	RDR2-sensitive
CTGTTGCCCTCGGCCTGGAACGGT	0	16	RDR2-sensitive
CTGTTGGAGAGCGTAGAAGACCAT	0	10	RDR2-sensitive
CTGTTGGAGCGAAGGCAAAGACGC	0	23	RDR2-sensitive
CTGTTGGAGGGAAGGCGAAGACGC	0	12	RDR2-sensitive
CTGTTGGAGTGAATGAAGATGGTG	0	10	RDR2-sensitive
CTGTTGGATTTCATCTGACGGCGC	0	10	RDR2-sensitive
CTGTTGGCGTGTAGAGGACGAGAA	0	10	RDR2-sensitive
CTGTTGGCTGTGCTGAATACGGAT	0	9	RDR2-sensitive
CTGTTGGGCTGCTCCATACGGTAA	0	22	RDR2-sensitive
CTGTTGGGCTGCTCTATACGGTAA	0	34	RDR2-sensitive
CTGTTGGGGCGAAGGCAAAGACGC	3	34	RDR2-sensitive
CTGTTGGGGCGAAGGCAAAGACGT	0	14	RDR2-sensitive
CTGTTGGGGCGAAGGCGAAGACGC	0	17	RDR2-sensitive
CTGTTGGGGCGAAGGCGAAGACGT	0	9	RDR2-sensitive
CTGTTGGTTCGGGCCAGTGGTCGG	0	9	RDR2-sensitive
CTGTTGTGTTCTGTATGCGTTGGC	163	5	RDR2-resistant
CTGTTTAGTCAGGCTGATGCACGG	0	35	RDR2-sensitive
CTGTTTATCGGTTGGGCTGGCGGG	17	1	RDR2-resistant
CTGTTTGAACTGTGGCACGGCATG	0	15	RDR2-sensitive
CTGTTTGACTACTCTATTGGATGG	0	15	RDR2-sensitive
CTGTTTGAGCCTGACCTGCAACGG	0	10	RDR2-sensitive
CTGTTTGCGACTGAGAGCCTAGGC	1	22	RDR2-sensitive
CTGTTTGGACGCGGGCGCCGGCGC	0	9	RDR2-sensitive
CTGTTTGGCACGGCTCCTCTAGAT	0	11	RDR2-sensitive
CTGTTTGGCTACTCTGTTGGATGG	0	28	RDR2-sensitive
CTGTTTTCGACGGTCTCTGACGGC	0	33	RDR2-sensitive
CTGTTTTGGAGACGCATCTGACAG	0	14	RDR2-sensitive
CTGTTTTGTTTAAGGGTCACGGCA	24	0	RDR2-resistant
CTTAAAAACACTCGGCAAAGGCTT	0	9	RDR2-sensitive
CTTAAAACCTTTTTAAGGACTGGA	20	2	RDR2-resistant
CTTAAAGTCCGGGCTGTGAGAGGA	0	9	RDR2-sensitive
CTTAAAGTTGGGTTTGTTTTGGAC	24	2	RDR2-resistant
CTTAACGGGCTTAGAGGTAAACGG	0	17	RDR2-sensitive
CTTAACGTTGACACTGCGGACGGT	0	60	RDR2-sensitive
CTTAACTCGTTAAGTTGGACGGTT	0	15	RDR2-sensitive
CTTAACTGAGTAGAACTGACGGTC	0	16	RDR2-sensitive
CTTAATAGTTGTAGAGATGACGAC	0	13	RDR2-sensitive
CTTAATCGACACGACTCTCGGCAA	3	50	RDR2-sensitive
CTTACACTGTTGGAGGCTCGTCAA	0	16	RDR2-sensitive
CTTACATTGTAGACCGTACGGTTC	0	10	RDR2-sensitive
CTTACCACGGTGTCGACTCGGTAT	0	15	RDR2-sensitive
CTTACCGGAGTAGAGCTTGGCGGT	0	9	RDR2-sensitive

CTTACCTTCTAGTGGACTCGGGGA	0	30	RDR2-sensitive
CTTACGGAAGCAAGAGGACGTCAG	1	31	RDR2-sensitive
CTTACGGAAGCAGGACAACGTCAG	1	17	RDR2-sensitive
CTTACGGAAGCAGGACGACGTCAG	0	13	RDR2-sensitive
CTTACTCCGATTATTCAAGGACTG	14	0	RDR2-resistant
CTTACTGACGCGCGGAACGACGAC	0	9	RDR2-sensitive
CTTACTGATAGACTGTCATCTAAA	34	0	RDR2-resistant
CTTACTGGACGCCTAGAGGACGCG	0	31	RDR2-sensitive
CTTACTGGACGTGTAGAGGACGTA	0	59	RDR2-sensitive
CTTACTTTGATATTGTTGGCACTG	14	0	RDR2-resistant
CTTAGGACGGCAGTAGAGATGGCA	1	26	RDR2-sensitive
CTTAGGATTTAGGGCGGGTGCATT	1	13	RDR2-sensitive
CTTAGGCACTTGTGTTGGACACTA	1	22	RDR2-sensitive
CTTAGGCACTTGTGTTGGACACTT	0	13	RDR2-sensitive
CTTAGGCCAGGCTCTGCAGACGCG	0	14	RDR2-sensitive
CTTAGGTACTACTACCGTGTCGGC	0	11	RDR2-sensitive
CTTAGGTGGACTGGACAGCATGAT	0	10	RDR2-sensitive
CTTATATGTCGGCTGTAGGGGCAT	2	25	RDR2-sensitive
CTTATCGTATTAGAAACGTCGGTA	0	27	RDR2-sensitive
CTTATCGTATTAGGAACGTGGGTA	0	15	RDR2-sensitive
CTTATCTCCGACGGCTACCCTGAC	1	18	RDR2-sensitive
CTTATGGTCCGACTGTACCGGTAC	1	53	RDR2-sensitive
CTTATGTAAGCTGGGGATGAGCAT	0	21	RDR2-sensitive
CTTATGTACGTTGGGGATGAGCAT	1	27	RDR2-sensitive
CTTATTCTTCACCGTCGTCGTTAC	0	10	RDR2-sensitive
CTTATTTGGTCTCTGTATGATGGA	0	23	RDR2-sensitive
CTTATTTTCGGCGGCTATTTTCGT	0	16	RDR2-sensitive
CTTCAAGTCGCGTCCTTAGGGCAT	0	9	RDR2-sensitive
CTTCAAGTTGAATTCGGTCTCGGT	2	19	RDR2-sensitive
CTTCACCAAGGGGCTATCGTCGAG	0	9	RDR2-sensitive
CTTCACGGAGAGACGGCTCTAAGA	0	14	RDR2-sensitive
CTTCACGGGAGGGAGGACCATCAC	102	19	RDR2-resistant
CTTCACTGTAGCGATGTCAGGCGC	0	11	RDR2-sensitive
CTTCACTTGGATCATGTGGAAGAC	0	10	RDR2-sensitive
CTTCAGACGACTCGAACGGCGCCT	0	13	RDR2-sensitive
CTTCAGACGTAGGGCGGCTATCCC	0	29	RDR2-sensitive
CTTCAGCTGGCTGTAGCGGGGGCT	0	14	RDR2-sensitive
CTTCAGGACACTCGGCATCACCAC	0	9	RDR2-sensitive
CTTCAGGCGGGTCGGATACGGATA	0	24	RDR2-sensitive
CTTCAGGCGGGTCGGATACGGATC	0	10	RDR2-sensitive
CTTCAGGCTCCACGAATTCGGCAA	0	9	RDR2-sensitive
CTTCAGGCTCTACGAATCCGGCAA	0	17	RDR2-sensitive
CTTCAGTCGTGACAACTTGGCGGA	0	12	RDR2-sensitive
CTTCATACTGTAGAAGAGGGTACC	0	83	RDR2-sensitive
CTTCATACTGTAGTAAGGGGTACC	1	34	RDR2-sensitive
CTTCATCCATCGGGCTTCGTGCGT	0	11	RDR2-sensitive
CTTCATCGCAGGGACGGGAATGAG	0	9	RDR2-sensitive
CTTCATCTTCATCGTCTGCAGGCA	17	0	RDR2-resistant
CTTCATGCTGTAGAAGAGGGCACC	0	11	RDR2-sensitive

CTTCATGCTGTAGAAGAGGGTACC	0	21	RDR2-sensitive
CTTCATGCTGTAGAAGATGGTACC	0	26	RDR2-sensitive
CTTCATGCTGTAGGAGAGGGTACC	0	11	RDR2-sensitive
CTTCATTGGCTGTATTATAGTGGA	17	1	RDR2-resistant
CTTCCAAGGTTTCGTAGAGCAGGA	0	10	RDR2-sensitive
CTTCCATCAGAACTCCATCCGGCT	0	10	RDR2-sensitive
CTTCCCAACGGGCGGCGTGCTGAA	79	8	RDR2-resistant
CTTCCCGTCAGCAGTAGAGACGCA	0	11	RDR2-sensitive
CTTCCCTCCCGTCAGCAGTAGAGG	0	16	RDR2-sensitive
CTTCCGGAGGGTTGTTGGATGCCG	0	15	RDR2-sensitive
CTTCCGGATTTGCTCTCAACGGCT	33	3	RDR2-resistant
CTTCCGGCAGGTGTGGGACGTGCT	0	15	RDR2-sensitive
CTTCCGTCAGCAGTAGAGGCGCAA	1	115	RDR2-sensitive
CTTCCTCTAGACGGCTCTTGGCAA	0	9	RDR2-sensitive
CTTCCTCTGGACCTGTTGATGGAT	0	14	RDR2-sensitive
CTTCCTCTGGTATCACGATTGCGG	15	0	RDR2-resistant
CTTCCTGCAGGACCTTCGGCGACG	2	28	RDR2-sensitive
CTTCCTGTGGCTAATTGTGACGGT	0	9	RDR2-sensitive
CTTCCTGTTCTTGAGCGTCGGCAC	0	16	RDR2-sensitive
CTTCGACGAGACGAACGCAGCGGT	1	22	RDR2-sensitive
CTTCGACGATTTGGAGGCGTAGGC	0	13	RDR2-sensitive
CTTCGACGTTTTGGAGGCGTAGGC	0	10	RDR2-sensitive
CTTCGATCGAGCAGCTGACGAGTA	1	41	RDR2-sensitive
CTTCGCACGTGTGGACCGTTCGAC	1	16	RDR2-sensitive
CTTCGCCGCTCCACTGACCGGCCT	0	18	RDR2-sensitive
CTTCGCGGGCGAGAGTGGAGGCGA	1	15	RDR2-sensitive
CTTCGCGTGACCTGGAGACGTCGT	0	9	RDR2-sensitive
CTTCGCTACTGTAGCAGACGGTTT	1	22	RDR2-sensitive
CTTCGCTAGTTGGGCCTGACGGAC	0	12	RDR2-sensitive
CTTCGCTATCGCGGACTGTTCGGC	0	10	RDR2-sensitive
CTTCGCTATCTGGACCTGACGGGC	0	24	RDR2-sensitive
CTTCGGACGGTCGGATGGTCGGAT	0	11	RDR2-sensitive
CTTCGGACTCAAGTCAGGCATCAC	1	12	RDR2-sensitive
CTTCGGACTGGAAGGGTAGGCAGC	0	16	RDR2-sensitive
CTTCGGAGTTTGAAGCTAGAGGTG	14	0	RDR2-resistant
CTTCGGATTCGGGTCGGATACGGA	0	61	RDR2-sensitive
CTTCGGCACTCGGCAAAGGCACGG	0	13	RDR2-sensitive
CTTCGGCGACGAAGCATAGACCCA	0	10	RDR2-sensitive
CTTCGGCTACAGCAGGCACCCCGC	0	9	RDR2-sensitive
CTTCGGCTACGAAGCATACCCCCA	0	9	RDR2-sensitive
CTTCGGGACCGTAGCGTTGGGCTA	0	18	RDR2-sensitive
CTTCGGGCAAAGGATGGCATGGCT	0	13	RDR2-sensitive
CTTCGGGCTGGCACGGTATGACCC	0	10	RDR2-sensitive
CTTCGGGTAGACGAAGTCTTGGCT	0	9	RDR2-sensitive
CTTCGGTAAGTAGGGGATCGACAT	0	12	RDR2-sensitive
CTTCGGTCCTGAGAGAAGGTAGAA	0	11	RDR2-sensitive
CTTCGTACGAGCAGCTGGGTTGGA	22	0	RDR2-resistant
CTTCGTCAAGCCGGCAGGTCCTCC	0	14	RDR2-sensitive
CTTCGTCAGGACACACATGGAGGC	0	22	RDR2-sensitive

CTTCGTCCATCGGGCTTCGCGCGT	0	22	RDR2-sensitive
CTTCGTCCCGACCGCAGAGGTGCA	0	37	RDR2-sensitive
CTTCGTCCCGATCGTAGAGGTGCA	0	14	RDR2-sensitive
CTTCGTCGATGACAGCACGGTCTC	0	21	RDR2-sensitive
CTTCGTCGTGTCCTTCTCCGGCAA	0	34	RDR2-sensitive
CTTCGTCTTGTAGTGTAGAGGACG	0	9	RDR2-sensitive
CTTCGTGTAGGACCTTCGGCGCAA	0	12	RDR2-sensitive
CTTCGTTCTTAGGCTCAGCGGCAA	24	3	RDR2-resistant
CTTCTAGGTAGGGCTGGACGTGGC	0	12	RDR2-sensitive
CTTCTAGTAACAATTGTAGTCGGG	1	17	RDR2-sensitive
CTTCTAGTTAGAACGTCGTTGCTC	29	5	RDR2-resistant
CTTCTATGTTTAGAGTTCGTCGTA	0	30	RDR2-sensitive
CTTCTCAAGATCGACAGCCGGCAC	0	12	RDR2-sensitive
CTTCTCAGTGCTGAAGCCGGACGG	1	14	RDR2-sensitive
CTTCTCCGTCGAGGCCGAAGACAC	0	12	RDR2-sensitive
CTTCTCGTTGTGGGCTGCAAGCGG	0	21	RDR2-sensitive
CTTCTCTCAGGACCGAAGGTACAT	0	13	RDR2-sensitive
CTTCTCTGCACGCTTCCGGACGGT	0	22	RDR2-sensitive
CTTCTCTGCACGGCGCTGGACGGT	0	9	RDR2-sensitive
CTTCTCTGCGTACATCCGGACGGT	1	13	RDR2-sensitive
CTTCTCTGCGTATGTCTGGACGGT	0	17	RDR2-sensitive
CTTCTCTGGACACGTGGCGGCTTC	0	13	RDR2-sensitive
CTTCTCTGTGTATGTCCGGACGGT	0	9	RDR2-sensitive
CTTCTGACATGTGGGCCACCGGAC	0	12	RDR2-sensitive
CTTCTGACGTGTTGCTGAAATGGA	0	16	RDR2-sensitive
CTTCTGCCGGAAAGAGGAGGGCGC	0	11	RDR2-sensitive
CTTCTGCTCTGAGCCGGTTTGCAT	0	9	RDR2-sensitive
CTTCTGTAGCTGTTGGGAGGCCGG	0	16	RDR2-sensitive
CTTCTGTCAGACCGGTCAGTGGAG	0	10	RDR2-sensitive
CTTCTGTCAGACTGGCCAGTGGAG	1	17	RDR2-sensitive
CTTCTGTCGACTGGCGCACCGGAC	0	13	RDR2-sensitive
CTTCTGTGGCACTCGGCAAAGAGG	1	16	RDR2-sensitive
CTTCTGTTCGGAATAATCTACGGT	0	9	RDR2-sensitive
CTTCTGTTGACTGTTGTTGCGGAC	0	21	RDR2-sensitive
CTTCTTACCTTCTAGTGGACTCGG	0	14	RDR2-sensitive
CTTGAACATGGTCGACGGCGAGGC	1	32	RDR2-sensitive
CTTGAACATGGTCGACGGCGAGGT	1	26	RDR2-sensitive
CTTGAACTGGGCTACTTCGGACAA	0	11	RDR2-sensitive
CTTGACGAACTGTATATTGGCAAC	0	9	RDR2-sensitive
CTTGACGACACGCGGAACGACGAC	1	28	RDR2-sensitive
CTTGACGACGCACAGAATGACGAT	0	16	RDR2-sensitive
CTTGACGACGCACGGAATGACGAC	0	14	RDR2-sensitive
CTTGACGACTGTACAGATTGCGTC	0	34	RDR2-sensitive
CTTGACGATGCACGGAACGACGAC	0	17	RDR2-sensitive
CTTGACGTGTAGAGGACGAGAATC	0	9	RDR2-sensitive
CTTGACTATTGGACTAGGCGAGAC	19	0	RDR2-resistant
CTTGACTGGGACGGCGATCACGAC	0	10	RDR2-sensitive
CTTGACTTGAGGGCCGGACACGAC	0	13	RDR2-sensitive
CTTGAGAGCCTGGCTGAGATGAGC	0	11	RDR2-sensitive

CTTGAGCCCTGTAGAAGCACAGCT	0	27	RDR2-sensitive
CTTGAGCCCTGTAGAAGCGTAGCT	0	11	RDR2-sensitive
CTTGATCCGGACACTGAAGAGGAG	0	13	RDR2-sensitive
CTTGATCTGGGGAGCGGCGTGGCA	0	10	RDR2-sensitive
CTTGATCTGTCAAGGCTGTCACGG	0	10	RDR2-sensitive
CTTGATGACACGCGGAACGACGAT	0	28	RDR2-sensitive
CTTGATGACGCACGGAACGACGAC	1	141	RDR2-sensitive
CTTGATGACGCACGGAATGACGAC	2	16	RDR2-sensitive
CTTGATGACGCACGGATCGACGAC	1	17	RDR2-sensitive
CTTGATGACGCGCGGAACGACGAC	0	34	RDR2-sensitive
CTTGATGACGCGCGGAATGACGAC	1	20	RDR2-sensitive
CTTGATGACGTACGGAACGACGAC	1	32	RDR2-sensitive
CTTGATGACGTGCGGAACGACGAC	0	11	RDR2-sensitive
CTTGATGACTCGCAGAACGACGAC	0	13	RDR2-sensitive
CTTGATGATGCACGGAACGACGAC	0	12	RDR2-sensitive
CTTGCACAGAAGGACTGCTTGCAT	0	29	RDR2-sensitive
CTTGCAGGAGCTAGGAACATGCAA	1	22	RDR2-sensitive
CTTGCAGTAGGGGGTTACAAGCGT	0	21	RDR2-sensitive
CTTGCATCGTTGTGGCCTGGCCCA	0	24	RDR2-sensitive
CTTGCATGCTGACTGTAGGAGGCA	0	12	RDR2-sensitive
CTTGCATGTCGGCTGTAGGAGGCA	0	10	RDR2-sensitive
CTTGCATGTCGGCTGTAGGGGCAC	0	81	RDR2-sensitive
CTTGCATGTCGGCTGTAGGGGCCC	0	13	RDR2-sensitive
CTTGCATGTCGGCTGTAGGGGGCA	0	32	RDR2-sensitive
CTTGCCTTCTGTCGTCGGGCGCAC	7	103	RDR2-sensitive
CTTGCCTTCTGTCGTCGGGGGCAC	0	12	RDR2-sensitive
CTTGCCTTCTGTCGTCGGGTGCAC	5	24	RDR2-sensitive
CTTGCGATCCGACTACGACGAGCA	1	21	RDR2-sensitive
CTTGCGCCTCTACTGCTGACGGGA	1	34	RDR2-sensitive
CTTGCGTCTCTACTGCTGACGGGA	0	30	RDR2-sensitive
CTTGCTCGACGAGGACTTGGTCGT	0	10	RDR2-sensitive
CTTGCTCGAGCTGTGGGTCGTGAC	3	27	RDR2-sensitive
CTTGCTCGGACTCGCACGCAGGCT	0	12	RDR2-sensitive
CTTGCTCGGCGAGGACTTGGTCGT	0	16	RDR2-sensitive
CTTGCTGACACGCGGAACGACGAC	0	28	RDR2-sensitive
CTTGCTGACACGCGGAACGACGAT	0	54	RDR2-sensitive
CTTGCTGACGCGCAGAACGACGAC	0	11	RDR2-sensitive
CTTGCTGACGCGCGGAACGACGAC	5	290	RDR2-sensitive
CTTGCTGACGCGCGGAATGACGAC	0	21	RDR2-sensitive
CTTGCTGACGTATGGAACGACAAC	1	35	RDR2-sensitive
CTTGCTGACGTGACTGCATAGTAA	0	16	RDR2-sensitive
CTTGCTGACGTGGCTGCATAGTAA	0	19	RDR2-sensitive
CTTGCTGAGGCGCGGAACGACGAC	0	17	RDR2-sensitive
CTTGCTGATGCGCAGAACGACGAC	1	27	RDR2-sensitive
CTTGCTGCTGCTGTAGGAGACGTG	0	10	RDR2-sensitive
CTTGCTGGAGGCGAAAAGGAGCTC	0	14	RDR2-sensitive
CTTGCTGGGCGTGTAGAGGACGTG	0	194	RDR2-sensitive
CTTGCTTGGCGGGGACTTGGTCGG	0	12	RDR2-sensitive
CTTGCTTGGCGGGGACTTGGTCGT	0	10	RDR2-sensitive

CTTGGAAGGCTGTAGTAGGGCCAT	1	32	RDR2-sensitive
CTTGGACAGGAAACTAGGCACGGC	6	67	RDR2-sensitive
CTTGGACAGGGAACTAGGCACGGT	0	10	RDR2-sensitive
CTTGGACGAACCGTGCGAGGACGT	0	10	RDR2-sensitive
CTTGGACTACGGCGAACACGGACA	0	27	RDR2-sensitive
CTTGGACTATCTCTCAGATGGATG	25	1	RDR2-resistant
CTTGGACTTGGACTGGGAGGAGGA	0	17	RDR2-sensitive
CTTGGAGCCACTGTAGAAGTGCAA	0	18	RDR2-sensitive
CTTGGAGCCGGTGGAGGATGACAG	0	9	RDR2-sensitive
CTTGGATCCACCACTGTATGCATG	0	10	RDR2-sensitive
CTTGGATCCTGCCCAGCTCGTCGT	0	9	RDR2-sensitive
CTTGGATTCCGGTAGTGACGTCAT	0	23	RDR2-sensitive
CTTGGATTCTGACTTAGAGGCGTT	40	2	RDR2-resistant
CTTGGATTGGTTCGGTAGACACGG	0	14	RDR2-sensitive
CTTGGCAGATGGGTAACATGACTT	0	16	RDR2-sensitive
CTTGGCGAACTGTACATCGGCAAC	0	20	RDR2-sensitive
CTTGGCGAAGACTGGGCCTCGGGC	4	37	RDR2-sensitive
CTTGGCGTAGACGAGGATGACGAT	1	29	RDR2-sensitive
CTTGGCGTAGAGAAGACTCGGCTC	0	35	RDR2-sensitive
CTTGGCGTAGGATTTCGCGGTGTC	0	14	RDR2-sensitive
CTTGGGACTGAACGAGGACGCGCC	2	68	RDR2-sensitive
CTTGGGATGCTTTTGTCGGCGCGA	0	9	RDR2-sensitive
CTTGGGATTCTTTTGTCGGCGTGA	0	9	RDR2-sensitive
CTTGGGCAGTAGGAAGGTATGTAT	0	11	RDR2-sensitive
CTTGGGCCAGGCTCACCAGATACG	0	11	RDR2-sensitive
CTTGGGCTCCCTGCTGCGCGGTGT	0	22	RDR2-sensitive
CTTGGGCTCCCTTCTGCACGGTGT	0	10	RDR2-sensitive
CTTGGGGATACACGCACGACGACG	0	9	RDR2-sensitive
CTTGGGGATGCACGAACGACGACA	0	10	RDR2-sensitive
CTTGGGGTAGGGTAATGATGGCGC	1	13	RDR2-sensitive
CTTGGTCTTAGCTCGATATCGGCA	22	0	RDR2-resistant
CTTGGTGAAGACTGGGCCTCGGGC	1	19	RDR2-sensitive
CTTGGTGTAGAGGGCATACTGAAT	0	9	RDR2-sensitive
CTTGTACGCAGCCTCTGTGGACGG	0	9	RDR2-sensitive
CTTGTCATGCTGAGGGCTAAAGGC	2	57	RDR2-sensitive
CTTGTCCCATGCGGTCGACGGACA	0	17	RDR2-sensitive
CTTGTCCGAGTCCTGTAGCGGCAT	0	13	RDR2-sensitive
CTTGTCCGGTAGAGTATTCGGTGA	0	9	RDR2-sensitive
CTTGTCCTAAGGACCTCGGACAAC	0	13	RDR2-sensitive
CTTGTCCTGGCTGTACGGCAACCA	0	13	RDR2-sensitive
CTTGTCGACATACTGTTGTGGTAC	0	17	RDR2-sensitive
CTTGTCGAGCGGATTGACGTCATG	0	11	RDR2-sensitive
CTTGTCGCGTAGGACAGTTCGGCT	0	35	RDR2-sensitive
CTTGTCGGCGTACTGTTGTGGTAC	0	9	RDR2-sensitive
CTTGTCGTATTGAGATTGATGGCT	0	14	RDR2-sensitive
CTTGTCTCCAGATATATCGTCGTC	26	2	RDR2-resistant
CTTGTCTGACGACTGGACGGTCCG	0	10	RDR2-sensitive
CTTGTCTGCTGAGAGTTGGTGGTC	0	27	RDR2-sensitive
CTTGTCTGGTATTGTGGCACGGTT	20	1	RDR2-resistant

CTTGTGAACCAGACTGGCCACATA	0	9	RDR2-sensitive
CTTGTGCACACATGGACTGTCCGT	0	16	RDR2-sensitive
CTTGTGGACCTTGATGAAGAGCGT	0	10	RDR2-sensitive
CTTGTGTTGGCTGTACGCATTACC	0	14	RDR2-sensitive
CTTGTTAGCTGGCTCTGAGGACGA	0	13	RDR2-sensitive
CTTGTTCAGTCTGTAATCCGTGCA	0	19	RDR2-sensitive
CTTGTTCGTTTGTGCTGGATTGGT	0	9	RDR2-sensitive
CTTGTTGGACGGAGAAAGGAGGTC	0	9	RDR2-sensitive
CTTGTTGGACGGGGAGAGGAGGTC	0	14	RDR2-sensitive
CTTGTTGGGACCATGCTTCGTCGC	0	9	RDR2-sensitive
CTTGTTGGTTCTTTGGCATTTGGA	18	0	RDR2-resistant
CTTGTTTCTGTGGAGCCAGGCTGA	0	14	RDR2-sensitive
CTTGTTTGGCTGGACAGATTGGCT	0	16	RDR2-sensitive
CTTTACCGAGCGCAGGACGTTGCC	0	9	RDR2-sensitive
CTTTAGAGGGTCTTGTTGGAGCCA	1	15	RDR2-sensitive
CTTTAGCGACGGCACTTACAGCGA	0	29	RDR2-sensitive
CTTTAGGCTCTACGAATTCGGCAA	0	13	RDR2-sensitive
CTTTATACTGTAGTAGAGGGTACC	0	9	RDR2-sensitive
CTTTATGGATCGGGAGTTATGGAC	150	0	RDR2-resistant
CTTTATTTGCTCTGTGTCGTTGGG	21	0	RDR2-resistant
CTTTATTTGTTGGGTAGACGGGTA	0	9	RDR2-sensitive
CTTTCACTGTGGATAGGCGACGGA	0	10	RDR2-sensitive
CTTTCATGGGACTGCACAGGAGAC	0	9	RDR2-sensitive
CTTTCCCACAGGATGTAGACGGAA	0	36	RDR2-sensitive
CTTTCCCAGACTGTCGGGCCTTAG	0	9	RDR2-sensitive
CTTTCCGACGGGCCAGAACGGCGT	0	24	RDR2-sensitive
CTTTCCGTTTTGGCGTTTGGCTGT	0	12	RDR2-sensitive
CTTTCCTGTGGAAGCCGTCGTCGT	0	9	RDR2-sensitive
CTTTCGCCGAGCACTTGGGGCATC	0	9	RDR2-sensitive
CTTTCGCGTAGGACAGTTCGGCAA	0	104	RDR2-sensitive
CTTTCGCGTAGGACAGTTCGGCAT	0	9	RDR2-sensitive
CTTTCGCTTGTGGAAACTGGCATG	25	0	RDR2-resistant
CTTTCGGGCACTCGGCAAAGACGT	0	48	RDR2-sensitive
CTTTCGGTGGCCCTCTCTACGGTT	33	1	RDR2-resistant
CTTTCGTCAGCTCGTAGAATGGGC	0	15	RDR2-sensitive
CTTTCGTCGCAACGCACGGGCATA	0	10	RDR2-sensitive
CTTTCTAGCTCATATGGTCGGCAT	15	0	RDR2-resistant
CTTTCTGCAGGACCTTCGGCGACG	0	17	RDR2-sensitive
CTTTCTGTCGTCGGGTGCACCGGA	4	17	RDR2-sensitive
CTTTGACAGGATGTAGCAGGGAGA	0	9	RDR2-sensitive
CTTTGACTGACTGTGGCGTGGCTA	0	38	RDR2-sensitive
CTTTGAGAGTGTGGTTGGGCGAAT	2	28	RDR2-sensitive
CTTTGCACGCGCAGAACCGAGCTC	1	34	RDR2-sensitive
CTTTGCATGAGTAGAGCCGGGATG	0	42	RDR2-sensitive
CTTTGCCGAGTGCCTTTTGTCGGG	0	17	RDR2-sensitive
CTTTGCCGAGTGTTTTTAAGGCTT	0	9	RDR2-sensitive
CTTTGCCTTCTGTCGTCGGGCGCA	5	58	RDR2-sensitive
CTTTGGAAGGCTGTAGTCGGGCCG	0	11	RDR2-sensitive
CTTTGGACTGGCACGACACGGCTC	0	23	RDR2-sensitive

CTTTGGACTGGCACGACGCGGCTC	0	14	RDR2-sensitive
CTTTGGCCTGGAACAAACACGGAC	0	11	RDR2-sensitive
CTTTGGGTAGACTGCTGCAGACGG	0	9	RDR2-sensitive
CTTTGGGTGAACTGCTGGAGTCGG	0	14	RDR2-sensitive
CTTTGGTTTGGATGTTTTTGGGGT	15	0	RDR2-resistant
CTTTGTCGACTGTTTTTCAGGCTA	0	11	RDR2-sensitive
CTTTGTCGAGTAACAGGTGACGTT	0	10	RDR2-sensitive
CTTTGTCGAGTGTTTTTACGGCTT	0	11	RDR2-sensitive
CTTTGTCGAGTGTTTTTCAGGCTT	0	9	RDR2-sensitive
CTTTGTCTCTGTCGGACCAGCGGC	2	20	RDR2-sensitive
CTTTGTGCCGGCAGACTCAGAGGT	1	17	RDR2-sensitive
CTTTGTGCGGAGGAAGGATGTCCA	0	9	RDR2-sensitive
CTTTGTTAATAATTAGAGCCGGAC	0	11	RDR2-sensitive
CTTTGTTCGGCGGCTCGGCACCAC	0	12	RDR2-sensitive
CTTTGTTGGACGGAGAGAGGAGGT	0	13	RDR2-sensitive
CTTTGTTGGGCTCTCCGCATGAGA	46	1	RDR2-resistant
CTTTGTTTGCTCGGTACTTCGGTT	131	0	RDR2-resistant
CTTTGTTTGGTTGCTTGTACGGAC	0	12	RDR2-sensitive
CTTTTAAACCGGACCGCCTGTGAT	0	14	RDR2-sensitive
CTTTTAGAGAAGACTGTAAAAGAC	0	11	RDR2-sensitive
CTTTTAGAGAAGACTGTAAAGGAC	0	44	RDR2-sensitive
CTTTTAGAGCGATGTTGGATGGCA	19	0	RDR2-resistant
CTTTTCATCTTTAAGTCGGTTCCC	1	14	RDR2-sensitive
CTTTTCGGACTTGAACCGGGCGGC	0	15	RDR2-sensitive
CTTTTCGGGCTTAGGCCAGGCCGG	0	9	RDR2-sensitive
CTTTTCGGGCTTGGGCCGGGCCGG	1	32	RDR2-sensitive
CTTTTCGGGCTTGGGTCGGACGGC	0	24	RDR2-sensitive
CTTTTCGGTTCTGTTTTTGTGGTT	0	9	RDR2-sensitive
CTTTTCGTCCTGTGGAGAGGTTAG	1	61	RDR2-sensitive
CTTTTCTCGGGCACTCGGCAAAGA	0	26	RDR2-sensitive
CTTTTGGAACTGCGCTGACGTCAT	0	53	RDR2-sensitive
CTTTTGTCGCTGTAGGCACATGGC	1	37	RDR2-sensitive
CTTTTGTCGGGCACTCGGCAAAGA	0	93	RDR2-sensitive
CTTTTGTTTAAGGGTCACGGCAAT	15	0	RDR2-resistant
CTTTTTAGTTGTCGCTGGATAGTT	2	46	RDR2-sensitive
CTTTTTAGTTGTCGTTGGATAGTT	0	12	RDR2-sensitive
CTTTTTATAGGACCTTCGGCGACG	1	13	RDR2-sensitive
CTTTTTATCGGGCACTCGGCAAAG	1	13	RDR2-sensitive
CTTTTTCGCTCGGTCACGGTCACC	0	45	RDR2-sensitive
CTTTTTGATCCTTCGATGTCGGCT	77	7	RDR2-resistant
CTTTTTTCGTGCCGGACCATGCAT	0	19	RDR2-sensitive
CTTTTTTTGGTGGTCGTCTTGGTT	18	0	RDR2-resistant
GAAAAACGTCACTGTAAATAGGTT	0	17	RDR2-sensitive
GAAAACGGACGGAAACGGACGGAA	0	12	RDR2-sensitive
GAAAACGGGCACGTGGAAGGGCAC	1	16	RDR2-sensitive
GAAAACGGGCGAATACGGACGGAT	0	9	RDR2-sensitive
GAAAACGGGCGGATACGGACGGAT	4	122	RDR2-sensitive
GAAAACTCTGAACGAGCCTGGCAC	0	11	RDR2-sensitive
GAAAATACAGATTATTTTCGTCGG	0	9	RDR2-sensitive

GAAAATAGGCTATTTTCGTCGGTT	1	18	RDR2-sensitive
GAAAATATGCTATTTTCGTCGGTT	1	15	RDR2-sensitive
GAAAATGTGGCTGTACGGACGCGG	0	17	RDR2-sensitive
GAAAATTATCTATTTTCGTCGGTT	0	54	RDR2-sensitive
GAAACACCTTCGGAAGACTGGCAC	0	21	RDR2-sensitive
GAAACACGACGGATGCGGCTTCGA	0	13	RDR2-sensitive
GAAACACGACGGGTACGGCTTTGA	0	9	RDR2-sensitive
GAAACAGTCTGATTGTCCGGCATT	0	9	RDR2-sensitive
GAAACAGTGTAGGCCGTCGGAAAC	0	10	RDR2-sensitive
GAAACATTCTGGGCGACGTGGACA	0	10	RDR2-sensitive
GAAACCGCATCTGAGGACATCCAT	0	10	RDR2-sensitive
GAAACCGTTGGATCTAGATTGGAT	2	31	RDR2-sensitive
GAAACGACTGTTGGGCTTCGGCGT	2	25	RDR2-sensitive
GAAACGGACAAGGGCGTGCCACAC	0	31	RDR2-sensitive
GAAACGGACCCAGGAACGTGCGGA	0	30	RDR2-sensitive
GAAACGGGACGGATACGGATACAG	0	29	RDR2-sensitive
GAAACTCGAGCTCGTGAGCCGGCT	0	9	RDR2-sensitive
GAAACTCGGATCTGTGACGCACGG	1	21	RDR2-sensitive
GAAACTGCAGCTGAAGGACGACGA	0	9	RDR2-sensitive
GAAACTGGGTCAGGTCGGGCGGCC	0	11	RDR2-sensitive
GAAACTGTAGCAATGCATGTGCAT	0	9	RDR2-sensitive
GAAACTGTAGCCCCTATACGGTGA	0	27	RDR2-sensitive
GAAAGACTGTGCAGGGAGGGGGAA	0	9	RDR2-sensitive
GAAAGCCGTTGGACATAAGGGCAT	0	25	RDR2-sensitive
GAAAGCGGGCTTGGGTCGGGCTAG	0	12	RDR2-sensitive
GAAAGCTCGGGCTAAGGACACGGG	0	10	RDR2-sensitive
GAAAGGCTAGAACTGATCCGGCTC	1	16	RDR2-sensitive
GAAAGTCGCCTAGAGGGGGGTGAA	0	11	RDR2-sensitive
GAAAGTCTAGCTGTGCGCGTGCAA	0	13	RDR2-sensitive
GAAAGTCTGACGGCCGATGAGCAA	0	9	RDR2-sensitive
GAAAGTTGGGGGCTCGAAGACGAT	2	15	RDR2-sensitive
GAAATACGTCGGGTAGGGTACGGA	1	34	RDR2-sensitive
GAAATAGCAGTGGGCTGTGGTCAT	0	22	RDR2-sensitive
GAAATCCAGGCACTGTCGGCGAAC	0	9	RDR2-sensitive
GAAATCGACGACCGGCTAAGACGC	0	12	RDR2-sensitive
GAAATCTTTTAGAGAAGACTGTAA	0	13	RDR2-sensitive
GAAATGACTGTAGAGTAGGGCTAT	0	23	RDR2-sensitive
GAAATGGACAAGCCGAGCCGAGCT	0	9	RDR2-sensitive
GAAATGGACAAGGGCGTGCCACAC	7	95	RDR2-sensitive
GAAATGGGAGCGGGAGCGGAACAA	0	16	RDR2-sensitive
GAAATTAGTCTCTGTAGAGTGGCA	198	1	RDR2-resistant
GAAATTCTCTGAGGACGACGGGAC	0	10	RDR2-sensitive
GAAATTTAGGGGACGCTGCTGGAG	0	11	RDR2-sensitive
GAAATTTAGGGGACGTTGCTGGAG	1	14	RDR2-sensitive
GAAATTTTATTTGCACGTTTGGAC	0	24	RDR2-sensitive
GAACAACAGCGATGCGGATGGACG	0	10	RDR2-sensitive
GAACAACAGGTGACTGCAGAGAGG	0	16	RDR2-sensitive
GAACAACAGTCTGATGGCGCTCTC	0	17	RDR2-sensitive
GAACAACGCAGTGACGGACGACAT	0	10	RDR2-sensitive

GAACAACTCGGACTCCGCAAGACG	0	11	RDR2-sensitive
GAACAAGATCGTATCTGGCCCGGC	0	11	RDR2-sensitive
GAACAAGATGGACCATAGGGCGAC	0	12	RDR2-sensitive
GAACACATAGTCGGTCGAGTCGTC	0	9	RDR2-sensitive
GAACACCGCGCAGCAGGGAGCCCA	0	10	RDR2-sensitive
GAACACGCTTGTTCGGACAACGCA	0	43	RDR2-sensitive
GAACACGCTTGTTCGGACGACGCA	0	16	RDR2-sensitive
GAACACGGAATGGTGGCAGAACAC	0	19	RDR2-sensitive
GAACACTCGGCAAAGTCTCGGTAA	0	9	RDR2-sensitive
GAACACTCGGCACAGAACGGCACT	9	193	RDR2-sensitive
GAACACTCGGCACAGGACAGCACT	1	13	RDR2-sensitive
GAACACTCGGCACAGGACGACACT	4	42	RDR2-sensitive
GAACACTCGGCACAGGACGCACTC	1	13	RDR2-sensitive
GAACACTCGGCACAGGACGGCACT	1	17	RDR2-sensitive
GAACACTCGGCACCGAACGGCACT	1	20	RDR2-sensitive
GAACACTCGGCATAGAACGGCACT	0	11	RDR2-sensitive
GAACACTGATAGGCGCAAGGACGT	0	30	RDR2-sensitive
GAACAGAAGGGCACAACTGAGCAA	1	29	RDR2-sensitive
GAACAGAAGGGCACAGCTGAGCAA	0	18	RDR2-sensitive
GAACAGACCCTTAGTCGGATCGGA	0	13	RDR2-sensitive
GAACAGATCTGGGCTAGGAGGCAC	2	23	RDR2-sensitive
GAACAGCAAGCTGTGGACGATCAC	0	17	RDR2-sensitive
GAACAGCATGCTGTCCGCGTCGGC	1	12	RDR2-sensitive
GAACAGCCGTTGGCAGAACACGTC	0	20	RDR2-sensitive
GAACAGCCGTTGGCAGAACGCGTC	1	18	RDR2-sensitive
GAACAGCGGACTGTGTTGTGGCAC	0	12	RDR2-sensitive
GAACAGGGCAACAGACGAACAGGC	0	17	RDR2-sensitive
GAACAGGTACCTTCGGACTCGGAT	0	30	RDR2-sensitive
GAACATACGTCCGACGGCTGTCAG	0	15	RDR2-sensitive
GAACATATTGATCGTATCTGCGGA	0	24	RDR2-sensitive
GAACATGAAGTGCGGTCAGGACGA	1	31	RDR2-sensitive
GAACATGCAGGACGCGGACCTCAC	1	17	RDR2-sensitive
GAACATGTGAGGATTGTGACGCAT	0	18	RDR2-sensitive
GAACATTCGCGGGGACGGGGACGA	0	11	RDR2-sensitive
GAACCAAGCGACGGACGAAGCAAC	0	17	RDR2-sensitive
GAACCAATCAGGCGGAAGGGACGA	0	12	RDR2-sensitive
GAACCACCGTCGTCATGGACGCAC	0	14	RDR2-sensitive
GAACCACGACGAGCTCGAGCTGGC	0	10	RDR2-sensitive
GAACCACGTCGCCTGTTTGGGCAT	0	14	RDR2-sensitive
GAACCACTTGACGGTGCTCTCTGC	0	24	RDR2-sensitive
GAACCAGAACCAGATCGAGCGCGC	0	15	RDR2-sensitive
GAACCAGACGAAGGACGGGCTGAT	0	26	RDR2-sensitive
GAACCAGATTGTAGGACTGGTCGG	0	13	RDR2-sensitive
GAACCAGCACACGATCGGACGCGC	0	18	RDR2-sensitive
GAACCAGTTTGAAGGGGTGGTCGG	0	33	RDR2-sensitive
GAACCAGTTTGCAGGGGTGGTCGG	2	40	RDR2-sensitive
GAACCAGTTTGTAGGGGTGGTCGG	0	32	RDR2-sensitive
GAACCATCGCGGGCTGTACGACGG	1	22	RDR2-sensitive
GAACCATCGGACTACAGTCGGTAA	0	15	RDR2-sensitive

GAACCATTTGGACTGACTTGGCAT	0	16	RDR2-sensitive
GAACCCACGGGCTTAGACGGTCTG	0	28	RDR2-sensitive
GAACCCAGGACCTGAGGAGTGCTA	0	14	RDR2-sensitive
GAACCCATGCATATACCCGTCGGA	2	18	RDR2-sensitive
GAACCCGGGACTCGTGAGAACGGC	1	12	RDR2-sensitive
GAACCGAACGGACGGTGCAGATTC	0	11	RDR2-sensitive
GAACCGAATCACGGCGTCGTCGGT	2	19	RDR2-sensitive
GAACCGACTGGCTCAGAGCGCGGA	0	18	RDR2-sensitive
GAACCGACTGTTGGTCACGACATA	0	10	RDR2-sensitive
GAACCGAGGAACCGAACCGAAGAA	10	36	RDR2-sensitive
GAACCGCCTAGCTGTGGACGACTA	0	13	RDR2-sensitive
GAACCGCGGAACAGGATTCGGCCC	0	69	RDR2-sensitive
GAACCGGACAGGAGGGTTGGGCAT	0	29	RDR2-sensitive
GAACCGGTCTCAGCTGAACGACGC	0	9	RDR2-sensitive
GAACCGTAGACCGTCCGGCCTCAG	1	21	RDR2-sensitive
GAACCGTCAGGCTGTGTGCGCGGA	0	13	RDR2-sensitive
GAACCGTCGGAACTAGTGGAGGCC	0	13	RDR2-sensitive
GAACCGTCTCTGTAGATGCGGGCT	1	37	RDR2-sensitive
GAACCGTGCGAGGACGTGAAGGAC	1	18	RDR2-sensitive
GAACCGTTTAGAGGACGCTGCTGG	0	13	RDR2-sensitive
GAACCTGGAAGCGGAGCTCGGCGC	0	18	RDR2-sensitive
GAACCTGGCCGACGACATGAACTA	0	10	RDR2-sensitive
GAACCTTGGATCTATTGAGCAGAA	33	2	RDR2-resistant
GAACCTTTGTTCGGCGGCTCGGCA	0	19	RDR2-sensitive
GAACGAACCGGCCAGTAGAGACGA	0	13	RDR2-sensitive
GAACGAACGGACGAACGATCGGAC	0	11	RDR2-sensitive
GAACGAAGACGGGAACCGGTGAAA	0	11	RDR2-sensitive
GAACGAAGGCAGAACGGTAGGCTA	0	33	RDR2-sensitive
GAACGACACAAGGACGAACGACGA	0	9	RDR2-sensitive
GAACGACCGCAACTGGGAGGCTAA	0	11	RDR2-sensitive
GAACGACGCAACGACGAACGACGA	2	67	RDR2-sensitive
GAACGACGCAGAGGACGCAGGCTC	0	18	RDR2-sensitive
GAACGACGTGACGACGAACGACGG	1	20	RDR2-sensitive
GAACGACGTGACGACGGATGAGCT	0	41	RDR2-sensitive
GAACGACTCTGACGATTTGGCGGC	0	14	RDR2-sensitive
GAACGACTGACGAAGAAGGGCGAT	0	15	RDR2-sensitive
GAACGACTGGGCGGCGGCTGGACA	0	14	RDR2-sensitive
GAACGAGGACTGAAACGGCCGGAT	0	11	RDR2-sensitive
GAACGAGTTCTAGGGGGTAGACGG	0	11	RDR2-sensitive
GAACGATCGGAATTTCGGCAGCAT	3	49	RDR2-sensitive
GAACGATGAGATTGGTCGGCGGAC	0	21	RDR2-sensitive
GAACGATGCGGAGGCCTGTCGCGG	0	9	RDR2-sensitive
GAACGATGTCGGCAGCCCATGCAC	0	13	RDR2-sensitive
GAACGCACGAACGAACAATGGCGA	0	18	RDR2-sensitive
GAACGCACGAACGGACAATGGCGA	0	17	RDR2-sensitive
GAACGCATCGGTAATCGGGACGGA	0	9	RDR2-sensitive
GAACGCCCAGATCGATCAGGCTAA	0	10	RDR2-sensitive
GAACGCCTAGATCGGAGGAGAGAA	0	9	RDR2-sensitive
GAACGCGACAGGGACGGTGACGGA	0	9	RDR2-sensitive

GAACGCGGAAGTACAAGACGACAT	0	9	RDR2-sensitive
GAACGCGGAATGGCCGACTACTAT	3	21	RDR2-sensitive
GAACGCGGCAACACACGGCTCAAC	0	9	RDR2-sensitive
GAACGCGTCAGAGGCAGAACACGT	0	14	RDR2-sensitive
GAACGCGTCAGAGGCAGAACGGCA	0	12	RDR2-sensitive
GAACGCGTGGAAGAGGCACTGTAG	0	25	RDR2-sensitive
GAACGCTGGAACACTGGGACGAGC	0	9	RDR2-sensitive
GAACGGAACAGAGCGTGACTGTGA	0	10	RDR2-sensitive
GAACGGAACGGAGCATGACTGCGC	0	19	RDR2-sensitive
GAACGGAACGTTGAGGTCGGCGTA	0	10	RDR2-sensitive
GAACGGACAATGGCGACGTGCAAA	1	12	RDR2-sensitive
GAACGGACCTGGATGTGCAGAGAT	1	28	RDR2-sensitive
GAACGGACGAGACGCGACGCCATA	0	12	RDR2-sensitive
GAACGGACGGCTTAGATTGGCTGA	0	26	RDR2-sensitive
GAACGGACGGTCTAGATTGGTGGA	0	12	RDR2-sensitive
GAACGGACGGTTGACATTGCAGAA	0	14	RDR2-sensitive
GAACGGACGTGGGATGTTGGCGGA	1	12	RDR2-sensitive
GAACGGAGATGGCTGGAACAGCGT	0	12	RDR2-sensitive
GAACGGAGTTGCTGAGCCGTCGGA	0	13	RDR2-sensitive
GAACGGCAGAACAGCAGACTACTC	0	37	RDR2-sensitive
GAACGGCAGAACGAGTGAAGGCAT	0	10	RDR2-sensitive
GAACGGCAGAACGCAGGAAGACAG	0	22	RDR2-sensitive
GAACGGCAGAACGGCAGAACGCAG	0	18	RDR2-sensitive
GAACGGCAGAACGGCAGAACGCGT	0	27	RDR2-sensitive
GAACGGCAGAACGGCAGACTACTC	0	54	RDR2-sensitive
GAACGGCATGCTGCTCGCGTCGGT	0	9	RDR2-sensitive
GAACGGCTCCTCCGCGAACGGCTC	0	12	RDR2-sensitive
GAACGGGACGACTGTGAAGACGAC	6	173	RDR2-sensitive
GAACGGGAGTGAGCTCATCCGGTT	0	9	RDR2-sensitive
GAACGGGCGTAGGAACAGCGGAGC	1	15	RDR2-sensitive
GAACGGTAGAACCACGGTGGCAGA	0	10	RDR2-sensitive
GAACGGTAGAATGGACGACTGAGA	0	9	RDR2-sensitive
GAACGGTAGAATGGACGGCTGAGA	0	17	RDR2-sensitive
GAACGGTCAGAGCCTAGGGACGAA	0	11	RDR2-sensitive
GAACGGTCGAACGGAACAGCAGAC	0	11	RDR2-sensitive
GAACGGTCGCCAGAAGAGGAACAG	0	9	RDR2-sensitive
GAACGGTCGCCGGGAAGAAGACGA	1	21	RDR2-sensitive
GAACGGTCTGCGGCATAGGGTCGA	1	13	RDR2-sensitive
GAACGGTCTGTAGACGAGGGTCGG	2	40	RDR2-sensitive
GAACGGTCTGTGCTCTGGGCCGGA	1	47	RDR2-sensitive
GAACGGTGGCAGAACGGGAATGAC	0	9	RDR2-sensitive
GAACGGTTCGCGCTCTGGACCGGA	3	49	RDR2-sensitive
GAACGTCCGACGCTGACGATGGTG	0	21	RDR2-sensitive
GAACGTTCGCGGGGATGGAGACGG	2	15	RDR2-sensitive
GAACGTTGCTGTAGCGAGTAGAGG	0	9	RDR2-sensitive
GAACGTTTTTTTGGATTGATGGTA	81	2	RDR2-resistant
GAACTACCTGACTTGGGCTGACGA	0	12	RDR2-sensitive
GAACTATCTGACCTGGGCTGACGA	1	37	RDR2-sensitive
GAACTCGCGACAGACGGAACAGAT	0	9	RDR2-sensitive

GAACTCGGAAAAGAGGCTCGGCTC	0	22	RDR2-sensitive
GAACTCGGTCATTCAGGGACAGTG	0	9	RDR2-sensitive
GAACTCTATCATCAGCTTGGGACA	41	2	RDR2-resistant
GAACTGAACCTGACGAGGCCGCAC	0	9	RDR2-sensitive
GAACTGCGCCGGATCGGAGGATGT	0	10	RDR2-sensitive
GAACTGCGGCTCTGGTGAACGGCG	0	34	RDR2-sensitive
GAACTGGACGAACACAACCACGGT	0	19	RDR2-sensitive
GAACTGGAGGACCTGCCTGCAGCA	1	38	RDR2-sensitive
GAACTGGATGAACCGGCTCGGCTA	0	14	RDR2-sensitive
GAACTGGGACTAGAGGACGACGAA	0	44	RDR2-sensitive
GAACTGGGCATCTGTAGGCGACAT	0	29	RDR2-sensitive
GAACTGTACATCGACAACGGCTTT	0	14	RDR2-sensitive
GAACTGTACATCGGCAACAGCCTA	1	12	RDR2-sensitive
GAACTGTACATCGGCAACAGCCTC	2	16	RDR2-sensitive
GAACTGTAGCCTTTATTGCAGAGT	0	9	RDR2-sensitive
GAACTGTGGCACGGCATGATTTTA	0	12	RDR2-sensitive
GAACTGTTTGATCGGCTCTTCATA	18	0	RDR2-resistant
GAACTTAGGATGCTCTGAGGTCGG	0	9	RDR2-sensitive
GAACTTCCAGGCGACGGCTGTAAT	0	9	RDR2-sensitive
GAACTTGATGGCTGGTACGGACGG	0	11	RDR2-sensitive
GAACTTGGTCTGCTAGATGACGAA	0	11	RDR2-sensitive
GAACTTGTGGACCAGAATATGCAT	0	10	RDR2-sensitive
GAACTTGTTGGGCGGAGACGACCG	0	9	RDR2-sensitive
GAACTTTGTTCGTCGATGGACGCT	0	23	RDR2-sensitive
GAAGAAAACAGGCAGAATTCTACA	42	6	RDR2-resistant
GAAGAACAGCGGTGTGCTCTGCAA	0	28	RDR2-sensitive
GAAGAACCCGGACCAGCACGGCAC	0	12	RDR2-sensitive
GAAGAACTCGAACCAGATGCCCGT	0	16	RDR2-sensitive
GAAGAACTCGCAGGAGAAGCCCCT	0	11	RDR2-sensitive
GAAGAACTTGGCGTAGACGACGAA	0	12	RDR2-sensitive
GAAGAAGAACCTGAGCGGACGTGC	0	14	RDR2-sensitive
GAAGAAGACCGGCGCGTAGGAGAC	0	14	RDR2-sensitive
GAAGAAGACGACTCTCTGCGCCAT	0	9	RDR2-sensitive
GAAGAAGGCGGCCAAGTTGGCGAG	0	10	RDR2-sensitive
GAAGACAAAACGGGCAGGGACACC	0	10	RDR2-sensitive
GAAGACAACGACGGACGTTGGGCA	0	9	RDR2-sensitive
GAAGACACTGGCAGCACGAAGACT	0	10	RDR2-sensitive
GAAGACAGGCACTACCGGACTCAC	0	9	RDR2-sensitive
GAAGACATGGACATCTGGAGGCGA	0	33	RDR2-sensitive
GAAGACCGCTGGACTGTCCGGTAT	0	11	RDR2-sensitive
GAAGACCTGAGCATCTGAAGGCGA	0	25	RDR2-sensitive
GAAGACCTGAGCATCTGGAGGCGA	8	26	RDR2-sensitive
GAAGACCTGGACATCTGGAGACGA	0	10	RDR2-sensitive
GAAGACCTGGACATCTGGAGGCGA	2	40	RDR2-sensitive
GAAGACCTGGACATCTGTAGGCGA	0	16	RDR2-sensitive
GAAGACCTGGGCATCTGAAGGCGA	3	19	RDR2-sensitive
GAAGACCTGGGCATCTGGAGACGA	2	15	RDR2-sensitive
GAAGACCTGGGCATCTGGAGGCGA	1	30	RDR2-sensitive
GAAGACCTGGGTATCTGGAGGCGA	0	12	RDR2-sensitive

GAAGACGAAGCGTCGGTTCGGCCT	1	20	RDR2-sensitive
GAAGACGACAGTAGAGGCGAGATA	0	21	RDR2-sensitive
GAAGACGACGAACTCGCAGAGGAT	0	19	RDR2-sensitive
GAAGACGACGGCTGCTCTATGCTT	0	12	RDR2-sensitive
GAAGACGACGTAGAGGACGCACAG	0	13	RDR2-sensitive
GAAGACGACTCTCTGCGCCATCGC	0	10	RDR2-sensitive
GAAGACGACTGATGTGGGAGTCCA	66	2	RDR2-resistant
GAAGACGCAGGCAACCGAACACGC	0	11	RDR2-sensitive
GAAGACGCCGCATCTGTACAACAC	0	25	RDR2-sensitive
GAAGACGCGCGAGAAGCTGGACGT	0	15	RDR2-sensitive
GAAGACGGCCTGAGTGGGACGGTT	5	22	RDR2-sensitive
GAAGACGGCTGTGACGGAGGGCGA	0	9	RDR2-sensitive
GAAGACGGGAGTAGAGGACCTCAG	0	18	RDR2-sensitive
GAAGACGTAGAGTCAGACGACGTT	0	15	RDR2-sensitive
GAAGACGTCATGTGCACTCACTAT	35	0	RDR2-resistant
GAAGACGTGACTGCATATGTCTGA	0	12	RDR2-sensitive
GAAGACTCGCGTAGGACAGTTCGG	0	20	RDR2-sensitive
GAAGACTGGAAGTAGGGACGTTCT	0	13	RDR2-sensitive
GAAGACTGGGCCTCGGGCGAGCCG	0	13	RDR2-sensitive
GAAGACTGTAAAGGACGGATATAG	2	14	RDR2-sensitive
GAAGACTGTAAGCAGCCACGGCGT	0	12	RDR2-sensitive
GAAGACTGTAAGCAGCCGTCGCAT	0	14	RDR2-sensitive
GAAGACTGTAAGGGGGTAGGCTGC	1	62	RDR2-sensitive
GAAGACTGTTGTAGATGGCAAGAT	23	0	RDR2-resistant
GAAGAGACAGTAGAGGACCGGCCC	0	11	RDR2-sensitive
GAAGAGACGGACATACTCGGAGAA	0	12	RDR2-sensitive
GAAGAGACGTACGTACAGAGCATC	0	24	RDR2-sensitive
GAAGAGAGCGCTGTAGTGCACGGG	0	10	RDR2-sensitive
GAAGAGAGCTATCTGGTCGCGGAC	0	9	RDR2-sensitive
GAAGAGAGTGACGTAGGGACGACA	0	18	RDR2-sensitive
GAAGAGCCAAGTAGAACGGAGCCA	0	10	RDR2-sensitive
GAAGAGCCAAGTAGAACGGAGCCG	2	45	RDR2-sensitive
GAAGAGGACCGACACGTAGAGGGC	0	10	RDR2-sensitive
GAAGAGGATGGCAGTATGTCTCGT	0	33	RDR2-sensitive
GAAGAGTAGAGGATGATGATAAGT	24	1	RDR2-resistant
GAAGATAGCTATCTGGTCGCGGAC	0	16	RDR2-sensitive
GAAGATAGCTATCTGTTCGCGGAC	2	17	RDR2-sensitive
GAAGATCCGGCAGTGACGAAGCAG	0	17	RDR2-sensitive
GAAGATCCTGACAGTAGCCGCCAT	0	9	RDR2-sensitive
GAAGATCGGCAGCAGCGGAGCTAT	0	12	RDR2-sensitive
GAAGATCGGGCATCATGACGCATC	0	16	RDR2-sensitive
GAAGATCGGGCATCGTGACACATC	0	14	RDR2-sensitive
GAAGATCGGGCATCGTGACGCATC	4	230	RDR2-sensitive
GAAGATCTGTGGAATAGGGGCATC	1	17	RDR2-sensitive
GAAGCAACCTTGGCCAGATCGTCT	0	11	RDR2-sensitive
GAAGCAAGGGCGGACGGTGGAGAG	0	9	RDR2-sensitive
GAAGCAGCCTCGGCTCGGTCTCGT	0	14	RDR2-sensitive
GAAGCAGGAGCTGGAGCTTTGCCA	1	24	RDR2-sensitive
GAAGCAGTAGAAGTCGGTACGGAT	0	11	RDR2-sensitive

GAAGCATAGCACCAGATCGCGGAC	0	9	RDR2-sensitive
GAAGCCCCTGAGCAGCAGTCGGTT	5	93	RDR2-sensitive
GAAGCCCTTTGGTGAAGACGTCGG	1	13	RDR2-sensitive
GAAGCCTCGGACGACAGACGACAG	0	9	RDR2-sensitive
GAAGCCTCGGACGACGGACGACAT	1	20	RDR2-sensitive
GAAGCCTCTGAAGAAGTTCGTCAT	4	36	RDR2-sensitive
GAAGCCTGAACCAGACGAAGGACG	1	20	RDR2-sensitive
GAAGCCTGAACCAGACGAAGGTCG	0	9	RDR2-sensitive
GAAGCGACGTAGGATGAGCGGTGT	1	18	RDR2-sensitive
GAAGCGCACGGACGGACGGTGTAG	0	10	RDR2-sensitive
GAAGCGCGTCTGAACGGAGGGTTT	0	24	RDR2-sensitive
GAAGCGGACTGACGGTGTAGATTT	0	18	RDR2-sensitive
GAAGCGGACTGGATCAGGCAGTGT	0	11	RDR2-sensitive
GAAGCGGAGGCAGAACGGGTTGGT	0	29	RDR2-sensitive
GAAGCGGTCGGTTGGTGAGGCGGG	0	10	RDR2-sensitive
GAAGCGTCTAGACTTGAACCGGTA	0	19	RDR2-sensitive
GAAGCTAGACTCTCGGCAACGCGG	0	25	RDR2-sensitive
GAAGCTCGGCGCCAATGATGACGG	3	20	RDR2-sensitive
GAAGCTCTCTGAGGACGACGGGCC	3	21	RDR2-sensitive
GAAGCTCTGAACGGATTCGGCCAC	0	11	RDR2-sensitive
GAAGCTCTGGTCAACGGATTGCGG	2	16	RDR2-sensitive
GAAGCTGACTGAATAGGTGGACGT	0	12	RDR2-sensitive
GAAGCTGCTCGTACGTGACGCCAA	0	9	RDR2-sensitive
GAAGCTGGGCTGGCGAAACGGTGA	1	19	RDR2-sensitive
GAAGCTGTAGAAGCCGGTACGGAT	0	9	RDR2-sensitive
GAAGCTGTGGACGCCTACTTTGCT	3	26	RDR2-sensitive
GAAGCTTGACTGGGACGGCGATCA	0	9	RDR2-sensitive
GAAGCTTTTCGTCGGCAGGCCCAC	0	10	RDR2-sensitive
GAAGGAAACCTGGGAACCGGCAAA	3	56	RDR2-sensitive
GAAGGAAACCTGGGAACCGGTAAA	0	29	RDR2-sensitive
GAAGGAAGGGTAGGGTCGTTGGAG	0	9	RDR2-sensitive
GAAGGACAAGCAGACTGCAACTTT	0	15	RDR2-sensitive
GAAGGACACGACGAAGACATGGAC	0	9	RDR2-sensitive
GAAGGACACGAGAACAGTGAGGCT	0	12	RDR2-sensitive
GAAGGACACGGACAGCGGAAGCGA	0	9	RDR2-sensitive
GAAGGACCATGTGGAAGAAGCCGC	0	30	RDR2-sensitive
GAAGGACCCAGAAACGACCCCTAG	0	14	RDR2-sensitive
GAAGGACCGGTAAGATGACCACAG	0	9	RDR2-sensitive
GAAGGACCTGTGAGACGACCAGAG	0	15	RDR2-sensitive
GAAGGACGCCGACGACTGCACGGC	100	3	RDR2-resistant
GAAGGACGCTGTCGAGACCACCGG	2	45	RDR2-sensitive
GAAGGACGGCTCTCGGCAAAGGAC	1	17	RDR2-sensitive
GAAGGACGTCGACGACTGCACGGC	0	17	RDR2-sensitive
GAAGGACTCGAGCGAAGGGTGGCG	0	9	RDR2-sensitive
GAAGGACTCGATACGTGACGCTAT	0	36	RDR2-sensitive
GAAGGACTCGGCAAGCGACGACGC	0	12	RDR2-sensitive
GAAGGACTCGGCGAGCAGCGACAC	0	11	RDR2-sensitive
GAAGGACTGACACACGCGCGCGTC	0	9	RDR2-sensitive
GAAGGACTGCCACACGCGCGCGCG	5	21	RDR2-sensitive

GAAGGACTGCCACGCGCGTGCCGC	0	13	RDR2-sensitive
GAAGGACTGCTATACGTGCGCGAC	0	21	RDR2-sensitive
GAAGGACTGTCACGCGCGCGCTGC	0	9	RDR2-sensitive
GAAGGACTTAGGGGAAGCAGCATA	22	0	RDR2-resistant
GAAGGACTTGGCGAGTAGCGACGC	0	18	RDR2-sensitive
GAAGGACTTTGCCATGACAGCTGC	0	17	RDR2-sensitive
GAAGGAGACGTAGAAGAGACGCGC	1	57	RDR2-sensitive
GAAGGAGCAGGAGAACGCCGAAGC	0	19	RDR2-sensitive
GAAGGAGCTGTAGCTAGGAAGAGG	0	10	RDR2-sensitive
GAAGGAGGACGCAGGATATGACGG	2	21	RDR2-sensitive
GAAGGATAGAGCAGACGAGACGGT	0	9	RDR2-sensitive
GAAGGATGTCGGGCAGAACACGGT	0	17	RDR2-sensitive
GAAGGATTTATAGTAGATACGGAT	0	12	RDR2-sensitive
GAAGGCACGGACGCACGGTACCAC	1	18	RDR2-sensitive
GAAGGCATCACGTAGGGACTGGCG	0	15	RDR2-sensitive
GAAGGCCAGGTAGAGCGGTTAGTT	0	19	RDR2-sensitive
GAAGGCCAGGTAGAGCGGTTGGTT	0	138	RDR2-sensitive
GAAGGCCGAAGATACTGCACTGTC	0	16	RDR2-sensitive
GAAGGCCGTCGGAAATAAGGCTAT	0	11	RDR2-sensitive
GAAGGCCTCGAGCGAAGGGTGGCG	0	15	RDR2-sensitive
GAAGGCGATCAGGATTGGTGACGG	0	13	RDR2-sensitive
GAAGGCGCTGTGGAGAAGACGCCG	1	20	RDR2-sensitive
GAAGGCGGGCTGGGCAGCAGCTAG	19	0	RDR2-resistant
GAAGGCTCTTAACCTTGTGGTCGT	0	15	RDR2-sensitive
GAAGGCTGGCAGAGTGGAGGACGC	0	10	RDR2-sensitive
GAAGGGAACTAAAAGGCGGCTCAG	2	35	RDR2-sensitive
GAAGGGAATAAGACGGGCCAGCAC	0	9	RDR2-sensitive
GAAGGGACAGTAGCAAACACCGGA	2	23	RDR2-sensitive
GAAGGGACCTGAAAAGCTAGGCGT	0	17	RDR2-sensitive
GAAGGGACGAGAGAGGACGCCAGA	0	9	RDR2-sensitive
GAAGGGAGTGTGGGTGTATCGGCA	0	10	RDR2-sensitive
GAAGGGATAGAATAGGACGGACAC	0	24	RDR2-sensitive
GAAGGGCGTGGAGTAGATGCAGGC	0	11	RDR2-sensitive
GAAGGGCTACGGCGATTAACTCGG	1	17	RDR2-sensitive
GAAGGGCTGCGTAGAGATCGGCGA	0	9	RDR2-sensitive
GAAGGGCTGGACGAAGCATGAGGG	0	14	RDR2-sensitive
GAAGGGCTTAAGCGAGGAGCTGGT	0	14	RDR2-sensitive
GAAGGGGAAGGGGGTGTACTGTAG	0	9	RDR2-sensitive
GAAGGGGACCGACACGTAGGGGGC	1	21	RDR2-sensitive
GAAGGGGACTGACACGTAGGGCGC	0	12	RDR2-sensitive
GAAGGGGGCACGGTAGAGGGCTGC	0	76	RDR2-sensitive
GAAGGGTTTTGTGAAGATATCGGC	51	0	RDR2-resistant
GAAGGTAATACTGTAGCGCGTCAT	1	16	RDR2-sensitive
GAAGGTAGCTATCTGGTCGCGGAC	0	27	RDR2-sensitive
GAAGGTCCTGCAGAAAGAGACAGC	0	19	RDR2-sensitive
GAAGGTCGGCGACAATGATGGCCC	0	12	RDR2-sensitive
GAAGTACCTGTAGGTCGCGGCGGC	0	9	RDR2-sensitive
GAAGTAGAACGCTCGCATCCGGTA	0	10	RDR2-sensitive
GAAGTCATTGTGGTGCGCGGACGG	0	19	RDR2-sensitive

GAAGTCGACGCAGAGGATCCCACT	0	10	RDR2-sensitive
GAAGTCGCGCCTGTGCATACCAGC	0	15	RDR2-sensitive
GAAGTCGGAGCTGCTACGTTAGAG	0	10	RDR2-sensitive
GAAGTCGTAGTAGCACCAGACGTC	0	12	RDR2-sensitive
GAAGTCTGCGTGGTATTGATGGTT	22	0	RDR2-resistant
GAAGTCTGGAGCGAGCTGGTGAGG	0	13	RDR2-sensitive
GAAGTCTGGCTGTGAACACGGCAA	0	9	RDR2-sensitive
GAAGTGAGGGAGGTGGGGACAGTC	0	10	RDR2-sensitive
GAAGTGAGTAGAGGACGTTCTGAT	0	10	RDR2-sensitive
GAAGTGCCTGAGCCCTGTAGAAGC	0	19	RDR2-sensitive
GAAGTGCGGCGGGCTGTGGCGGCT	0	12	RDR2-sensitive
GAAGTGCTTGAGCCCTGTAGAAGC	0	13	RDR2-sensitive
GAAGTGGACTAGTAAAAATTGGCA	42	3	RDR2-resistant
GAAGTGGAGAACCGAAGACGACGC	0	16	RDR2-sensitive
GAAGTTCTTCAAGCTCTGGATGGC	0	13	RDR2-sensitive
GAAGTTGATGTGGTCTAGCTCGGC	0	9	RDR2-sensitive
GAAGTTGATTGTAGCTCGATGGTT	0	11	RDR2-sensitive
GAAGTTGGGCCGGATTGTTTGGGC	0	9	RDR2-sensitive
GAAGTTTGCGACTCGGACAGGCCT	2	26	RDR2-sensitive
GAAGTTTTATTGTTTTATCCGGTG	44	1	RDR2-resistant
GAATAACATAACGCCCGTCGGCCA	0	10	RDR2-sensitive
GAATAACGGACAGAGCCGGGTCGG	0	11	RDR2-sensitive
GAATAAGACCGTAGGGACAGAGGA	0	10	RDR2-sensitive
GAATAAGCAGGCCGGACTTGACAG	0	14	RDR2-sensitive
GAATACAGCACGGATCGGTTGGAT	0	9	RDR2-sensitive
GAATACTCTGTCGTATCGGGTCGG	0	12	RDR2-sensitive
GAATAGAGCGGACGGAGCCACGGA	0	10	RDR2-sensitive
GAATAGGGTCGTGTCTGGTCCGGC	0	11	RDR2-sensitive
GAATAGGTCGGTTTAGAGGTGGTT	39	0	RDR2-resistant
GAATAGTTGGAGGTAGGAGGGCCT	0	9	RDR2-sensitive
GAATATAGAGGACGTTGCTGGAGA	0	15	RDR2-sensitive
GAATATTCCGGATCTGAGCGGCTC	0	21	RDR2-sensitive
GAATATTTTCGTCGGCCGGCCCAC	0	9	RDR2-sensitive
GAATCAAGCGAGCTGACGAACCAC	0	31	RDR2-sensitive
GAATCAGGAATGGAACGGCTCCGT	0	17	RDR2-sensitive
GAATCAGGGATGGAACGGCTCCGT	1	12	RDR2-sensitive
GAATCCATGGTCGTCATCGTCGTC	0	11	RDR2-sensitive
GAATCCCTGGGCACGCGTCGGCAC	0	38	RDR2-sensitive
GAATCGAGCGAGCTGACGAGCCAT	0	20	RDR2-sensitive
GAATCGGACCTAAACAGCGGCTTC	0	14	RDR2-sensitive
GAATCGGCCTGATGGGACTAACGG	0	9	RDR2-sensitive
GAATCGGGTGAAGACGGAGCCAGC	0	11	RDR2-sensitive
GAATCGGTCAGGAACTGGAGGCAT	4	99	RDR2-sensitive
GAATCTATCTCGGATCTCATGGCG	24	0	RDR2-resistant
GAATCTCGACGTACTGTGACGGTG	3	41	RDR2-sensitive
GAATCTCGCAGGAGACCACTCTGA	0	9	RDR2-sensitive
GAATCTCGTTTGGATAATCGTCGT	0	17	RDR2-sensitive
GAATCTCTCGGACTACGGCACACT	0	15	RDR2-sensitive
GAATCTGAGCGTCGGCAGGGGCAG	43	9	RDR2-resistant

GAATCTTGCTGACGTGGCTGCATA	0	9	RDR2-sensitive
GAATGAATGTAGAACGGCAGGCTA	0	12	RDR2-sensitive
GAATGACTCTGCGATACGGCTGAT	14	0	RDR2-resistant
GAATGAGGACGACGGTAGATGTAT	0	11	RDR2-sensitive
GAATGAGTGGGCTGCTAATGGTAA	24	0	RDR2-resistant
GAATGATAATGATCGAGCGATGAC	29	1	RDR2-resistant
GAATGATCGCGGCACGGAATCGGA	1	13	RDR2-sensitive
GAATGCAACACGAGGACTTCCCAG	87	6	RDR2-resistant
GAATGCACGAACGGACAATGGCGA	0	12	RDR2-sensitive
GAATGCACGGATCTGGATATGCGG	0	9	RDR2-sensitive
GAATGCTCCTGCGATACGGTCGGT	2	14	RDR2-sensitive
GAATGCTGGTCTGCATACACTCAT	0	17	RDR2-sensitive
GAATGCTTTTACAGGTCGTTTGGA	0	10	RDR2-sensitive
GAATGGACGACTGACCGCGGGACG	0	20	RDR2-sensitive
GAATGGACGACTGAGACAGCAGAA	0	14	RDR2-sensitive
GAATGGACGGCTGAGATCGCAGAA	1	74	RDR2-sensitive
GAATGGATTGGGAAGGGACGGAAC	0	16	RDR2-sensitive
GAATGGGAATGGCAGAACACGGAA	1	63	RDR2-sensitive
GAATGGGCTTTGCAGTGAGGACAG	0	9	RDR2-sensitive
GAATGGGCTTTGCAGTGAGGACGG	3	23	RDR2-sensitive
GAATGGGGACGCAGATGAGGGATT	167	0	RDR2-resistant
GAATGGTCTTTGGACGAAGGTCAT	29	2	RDR2-resistant
GAATGGTGGCAGAACACGGAATGA	0	35	RDR2-sensitive
GAATGGTGTGATCGGTGGACGCAA	0	21	RDR2-sensitive
GAATGGTTCGGCTGCTTCGATGGC	0	16	RDR2-sensitive
GAATGTACCTGGTAGACACGTCGA	0	10	RDR2-sensitive
GAATGTACTGTATACGGATTGCAT	0	35	RDR2-sensitive
GAATGTAGGGCGAGTAGAGACGCG	0	18	RDR2-sensitive
GAATGTATTTCACGTCGGAAACAG	0	18	RDR2-sensitive
GAATGTTCTGACGCTAGTTCGATC	17	1	RDR2-resistant
GAATGTTGGCTGGCTCGAGGCATC	14	0	RDR2-resistant
GAATTAGTCGACGGAGTTGGGCAA	0	13	RDR2-sensitive
GAATTATAGCCGGACGGTCCGCAC	0	10	RDR2-sensitive
GAATTATAGCGGAGTGGCTCCCAG	0	10	RDR2-sensitive
GAATTCATGGCCGGTCAGTAGCGA	0	10	RDR2-sensitive
GAATTCCGGTAGTGATGGCTCATG	0	24	RDR2-sensitive
GAATTCTGTGGACGCGGACGCGGT	3	70	RDR2-sensitive
GAATTGACGGTGACTTGTAACAAT	16	0	RDR2-resistant
GAATTGTGGAGCGGGCAGAACCGT	0	19	RDR2-sensitive
GAATTTAGGGGACGTTGCTGGAGA	3	36	RDR2-sensitive
GAATTTGTGGTTCTGATCGCATGA	15	0	RDR2-resistant
GACAAAATGCTCGCGGCTCGTCGG	0	15	RDR2-sensitive
GACAAACCGGACACTTGAGGGCAT	3	27	RDR2-sensitive
GACAAACTGTGGAGGAGGAGACGA	0	22	RDR2-sensitive
GACAAAGACTTCGGCACTCGGCAA	1	43	RDR2-sensitive
GACAACAGGGGACAACACTGGATA	0	9	RDR2-sensitive
GACAACCCTGTAGAGCGCGGCGAG	0	109	RDR2-sensitive
GACAACTGCCGTCGGCTTGGCTAA	4	37	RDR2-sensitive
GACAACTGTAGACGGCCCCCGCCC	0	10	RDR2-sensitive

GACAACTGTAGCAAAATGGTTCGT	0	10	RDR2-sensitive
GACAAGCTGGAACAGGGACGCGTC	0	9	RDR2-sensitive
GACAAGGAGATGGTGACCGCTGAT	0	21	RDR2-sensitive
GACAAGGTAGATCGGTAAGCAACT	23	0	RDR2-resistant
GACAAGGTTCTCGGATCAGGCTGC	0	12	RDR2-sensitive
GACAATCCACTGTATGGCTTCGGC	47	6	RDR2-resistant
GACAATCGAACTGGGCTGGCACGG	0	16	RDR2-sensitive
GACAATCGGCCAGGTCGACGACGC	2	19	RDR2-sensitive
GACAATCGGGCGGGTACAACACGG	0	15	RDR2-sensitive
GACAATCGGGCGGGTAGAACGCGG	0	10	RDR2-sensitive
GACAATCGGGCGGGTAGTACACGG	0	15	RDR2-sensitive
GACAATCGGGCGTAGCGAGACGAC	1	57	RDR2-sensitive
GACAATCGGGTGGGTAGTACGCGG	0	27	RDR2-sensitive
GACAATTCACTCGTCGGTCCAGGG	0	45	RDR2-sensitive
GACAATTCGGCGGGTAGTACACGG	0	9	RDR2-sensitive
GACAATTTACTCGTCGGTCCAGGG	20	188	RDR2-sensitive
GACAATTTACTCGTCGGTCTAGGG	3	22	RDR2-sensitive
GACAATTTTACTCGTCGGTCCAGG	3	18	RDR2-sensitive
GACACAAAGTCAGTTATCGTCGTT	22	0	RDR2-resistant
GACACAACTCGGACAACGTGGCGT	0	19	RDR2-sensitive
GACACAACTCTGGCCCAGGCACGT	0	9	RDR2-sensitive
GACACAATCTGTACAGTCGTCGGG	0	15	RDR2-sensitive
GACACAGATTAGGATCGTGCCGGT	0	9	RDR2-sensitive
GACACATTAGACGGGCTGGGTCGT	0	11	RDR2-sensitive
GACACCACTGTTGAGCTACCGGCT	0	22	RDR2-sensitive
GACACCACTGTTGGGCTGTCGGAC	0	12	RDR2-sensitive
GACACCACTGTTGGGCTGTCGGCC	0	29	RDR2-sensitive
GACACCCGATCCAGGAAGAGGCTA	0	15	RDR2-sensitive
GACACCCGTGGTCGTAGGATAGCC	0	18	RDR2-sensitive
GACACCCTTCGCAGGACTCGGCAC	2	38	RDR2-sensitive
GACACCGCGGCTGGCATAGTGCAC	0	10	RDR2-sensitive
GACACCGTCTGTTGGGCCGTTGGG	4	23	RDR2-sensitive
GACACCTGGACTGGAGAAGATGGT	1	12	RDR2-sensitive
GACACGAATAAGCAGGCCGGACTT	0	13	RDR2-sensitive
GACACGACTCTCGGCAACGGATAT	772	50	RDR2-resistant
GACACGCGGAACGACGACGAGATC	2	31	RDR2-sensitive
GACACGCGGAACGACGATGAGATC	0	13	RDR2-sensitive
GACACGCTTCTACAGATCGCTCGA	0	12	RDR2-sensitive
GACACGGACCGTACTGAAGCGTGC	0	9	RDR2-sensitive
GACACGGATCTGTTAGCGTGCCGG	0	9	RDR2-sensitive
GACACGGATTAGGACCATGCCGGT	0	20	RDR2-sensitive
GACACGGATTAGGACTGTACCGGC	0	9	RDR2-sensitive
GACACGGCACGATTGGAACCAGGC	0	19	RDR2-sensitive
GACACGGGCTAGATGCACGCGGGC	1	15	RDR2-sensitive
GACACGGTAGAATAAGCGGGCGGG	0	14	RDR2-sensitive
GACACGGTATCTGTCGGGCCTTTT	0	11	RDR2-sensitive
GACACGGTCCGTAGTGCTTCGGCA	0	11	RDR2-sensitive
GACACGGTCTGAAGACGGGTATAA	0	14	RDR2-sensitive
GACACGTAGGGCGCTGTAGCAGGC	0	66	RDR2-sensitive

GACACGTCGACAGAAACCAACGGT	0	18	RDR2-sensitive
GACACTACGGGCTGAATTGGTGGC	1	14	RDR2-sensitive
GACACTACTGTTGGACTGCCGGCT	0	29	RDR2-sensitive
GACACTATAGTAGCGAGTCGGATC	0	9	RDR2-sensitive
GACACTCGGCAAAGAAGGCTCCAG	0	10	RDR2-sensitive
GACACTCGGCAAAGAAGGCTCGGC	6	47	RDR2-sensitive
GACACTCGGCAAAGACTGCGGCAC	0	18	RDR2-sensitive
GACACTCGGCAAAGACTGGTCCAG	1	12	RDR2-sensitive
GACACTCGGCAAAGAGACTGGCAA	1	12	RDR2-sensitive
GACACTCGGCAAAGTCTGACGGAC	0	17	RDR2-sensitive
GACACTCGGCTCAGACACGGCACT	0	11	RDR2-sensitive
GACACTGAGCTGGACGGTCTATAA	0	9	RDR2-sensitive
GACACTGCGGACGGTGAGAGGCGC	0	32	RDR2-sensitive
GACACTGGATATAGGATTGGGCAT	0	10	RDR2-sensitive
GACACTGTAGTACACGACCCGGTC	0	12	RDR2-sensitive
GACACTGTCTGGTACTGTCGGCGT	0	17	RDR2-sensitive
GACACTGTGCTAGCTAGGTCGGGT	0	9	RDR2-sensitive
GACACTGTGGACAGCGTTTACGAC	0	16	RDR2-sensitive
GACACTGTGGGTCGAGAAGTCGAT	0	12	RDR2-sensitive
GACAGAACACGGAATGAGGACGGA	0	10	RDR2-sensitive
GACAGAACACGGAATGGCTGACTA	0	10	RDR2-sensitive
GACAGAACACGGAATGGGGCAGCC	0	12	RDR2-sensitive
GACAGAACGACAGAACGGCAGAAC	0	11	RDR2-sensitive
GACAGAACGGAACGAGATGGACAA	0	72	RDR2-sensitive
GACAGACACAATGACGGACCTCAA	0	10	RDR2-sensitive
GACAGACATGGGAATGGGAACGGT	0	15	RDR2-sensitive
GACAGACTGATGTGGCACCAACAT	1	15	RDR2-sensitive
GACAGACTGTAGTGGTGGAAGATG	0	11	RDR2-sensitive
GACAGAGACGGACCCACGAATCAT	0	10	RDR2-sensitive
GACAGAGACTGAGTCAGACGAGCT	0	12	RDR2-sensitive
GACAGAGACTGAGTTAGACGAGCT	0	11	RDR2-sensitive
GACAGAGCAGAGGCTACGGAACGC	0	13	RDR2-sensitive
GACAGAGCTCATGATCGTCGAGAC	0	19	RDR2-sensitive
GACAGAGTGGGCTAACGACGACGG	47	4	RDR2-resistant
GACAGAGTTAGACGTGTGGTAGAC	1	32	RDR2-sensitive
GACAGAGTTAGACGTGTGGTAGAT	0	15	RDR2-sensitive
GACAGCCGTCGACGGGAGCGGGAT	0	10	RDR2-sensitive
GACAGCGCGGCAGGTAGCTCGGCG	0	16	RDR2-sensitive
GACAGCTAGGATTGAGACGTCGGA	0	27	RDR2-sensitive
GACAGCTCTGAGGAGATTCTGGTG	0	10	RDR2-sensitive
GACAGCTGTAGAATCGCGGGCGTC	0	10	RDR2-sensitive
GACAGGAAACTAGGCACGGCGGGC	3	28	RDR2-sensitive
GACAGGAAGCTGATCGACGTCGAG	0	10	RDR2-sensitive
GACAGGACGGCAAGCGGTGAGCAG	0	20	RDR2-sensitive
GACAGGACTAAGAAACGGCGGAAC	0	13	RDR2-sensitive
GACAGGACTGACTGGCAGACTGGC	4	22	RDR2-sensitive
GACAGGATTTGTAGCGCATGCCAG	2	17	RDR2-sensitive
GACAGGCACTGTAGGCTATCCGGT	0	10	RDR2-sensitive
GACAGGCACTGTAGGCTGTCCGGT	0	10	RDR2-sensitive

GACAGGCCTGTCGTACTGGGTCGG	0	16	RDR2-sensitive
GACAGGGATTGTGCTCGAAGGCGG	0	20	RDR2-sensitive
GACAGGTATGAAGTGGGCTGACTT	1	12	RDR2-sensitive
GACAGGTCACGGAGCGGTATAAGG	19	1	RDR2-resistant
GACAGGTTCGACGACTTGACGGTA	0	9	RDR2-sensitive
GACAGGTTCGTCGTGCCTCCGGCC	0	13	RDR2-sensitive
GACAGTAATGGACGGTCATCACTC	0	15	RDR2-sensitive
GACAGTAGATCGGACGGTCCGCAA	0	19	RDR2-sensitive
GACAGTCACATATAGCCGTCGGAC	0	14	RDR2-sensitive
GACAGTCACTGTAGACGGTCCGGT	2	30	RDR2-sensitive
GACAGTCACTGTAGATGGTCCGGT	0	11	RDR2-sensitive
GACAGTCAGTCGGCGAGCTGGCAA	0	9	RDR2-sensitive
GACAGTCGAGCAGGGGATGATGGG	0	19	RDR2-sensitive
GACAGTCGATGTAGCAAGGCCCAG	0	27	RDR2-sensitive
GACAGTCGGGATACAGGTGGGGGC	0	13	RDR2-sensitive
GACAGTCTGGTATAGTCGGTGCGC	0	12	RDR2-sensitive
GACAGTGACAGGGGCGACGACAAG	94	20	RDR2-resistant
GACAGTGACGAAGACAATGGCGGC	0	9	RDR2-sensitive
GACAGTGCCACTGGAGATCGACGA	0	10	RDR2-sensitive
GACAGTTGGCACTCCAGGTAGAGG	1	12	RDR2-sensitive
GACAGTTTACTCGTCGGTCCAGGG	1	25	RDR2-sensitive
GACAGTTTCCTAGGCCGTCGTCGG	0	10	RDR2-sensitive
GACATAATGGGTCAGAGGACACTT	0	14	RDR2-sensitive
GACATAGGGTCGGACGGTCCACGG	0	11	RDR2-sensitive
GACATAGGTACTGTGGATCGGCCT	0	91	RDR2-sensitive
GACATATATTATTTCGTGTCGGAC	0	9	RDR2-sensitive
GACATCACCCTTCAGGTCGTCTGG	0	26	RDR2-sensitive
GACATCGACGTAGAGGAAGCAGAC	0	9	RDR2-sensitive
GACATCGGGCGACGGATCCGGCTT	2	30	RDR2-sensitive
GACATCTAGGACCACGACGGACAC	0	15	RDR2-sensitive
GACATCTGGGAAGGCTGGACGGTC	1	13	RDR2-sensitive
GACATGAGGACGACGACGAGCGAC	0	14	RDR2-sensitive
GACATGCGGTGTGGACGAAGATGC	0	11	RDR2-sensitive
GACATGGACATCTGGAGGCGACAT	1	17	RDR2-sensitive
GACATGGACTGTAGGAGCGGGGTT	1	40	RDR2-sensitive
GACATGGCACGTAGTGAACCGGAC	0	9	RDR2-sensitive
GACATGTACTGTAGAGTGTCCGGT	19	56	RDR2-sensitive
GACATGTCCCCGACGATCGACGGA	0	11	RDR2-sensitive
GACATGTCGTGCTCGTGACGGACC	0	10	RDR2-sensitive
GACATGTGGGACTGAGAGGTGGGC	0	17	RDR2-sensitive
GACATTCGTGGCAACGCACGGGCA	0	9	RDR2-sensitive
GACATTCTAACGGCTGGTCTCGGT	33	3	RDR2-resistant
GACATTTCACTCGTCGGCCCAGGG	1	23	RDR2-sensitive
GACATTTCACTCGTCGGTCCAGGG	0	16	RDR2-sensitive
GACCAAACAGCGGACTGGAACGGC	0	75	RDR2-sensitive
GACCAACCGTAGATCGTTGAGGGC	0	11	RDR2-sensitive
GACCAACGACGACAGTGAGGACGC	0	9	RDR2-sensitive
GACCAAGGCTCTGTCGAAGTCTGC	0	12	RDR2-sensitive
GACCACAGTCGTGTGCTGACGCGG	0	9	RDR2-sensitive

GACCACCAGCATGGCGTCGGCGGC	1	12	RDR2-sensitive
GACCACCCCATGATCGAAGCGGAG	0	40	RDR2-sensitive
GACCACGTCGACAGAAGCGGAACA	0	46	RDR2-sensitive
GACCACGTTCGGCTCGTCTGGCTG	1	16	RDR2-sensitive
GACCACTGAACCGGACGTTGGCGC	2	52	RDR2-sensitive
GACCAGACCGTAGAGGAAGGCGTT	0	26	RDR2-sensitive
GACCAGAGACTGTAAATGTGGCGA	0	21	RDR2-sensitive
GACCAGAGAGCACCGCCGCCGGTT	0	16	RDR2-sensitive
GACCAGATCGACGGCAACACACTT	0	9	RDR2-sensitive
GACCAGCACGACAGACGAACTAAG	0	51	RDR2-sensitive
GACCAGCACGACGGACGAACTAAG	2	106	RDR2-sensitive
GACCAGCGACTGCAGCGAAGGCCT	1	53	RDR2-sensitive
GACCAGCGGCTGCAGCGAAGGCCT	0	9	RDR2-sensitive
GACCAGGACGACATATATGGACGA	0	10	RDR2-sensitive
GACCAGGGGTTGTAGAACGACGGC	0	10	RDR2-sensitive
GACCAGGTCGAAGGGCAGGGGCAC	0	19	RDR2-sensitive
GACCAGTCTGACAGAAGGACAGCG	3	16	RDR2-sensitive
GACCATAATTAGGGGTACCCTCAA	0	9	RDR2-sensitive
GACCATCTGGGCTAGGACACGCGG	0	11	RDR2-sensitive
GACCATGAACCGAGCGAGCCAGCG	0	12	RDR2-sensitive
GACCATGGACGAGCAGAACGCCGG	0	10	RDR2-sensitive
GACCATGTTCAAGTGATTGCTCGG	2	24	RDR2-sensitive
GACCATGTTCAAGTGATTGTTCGG	1	14	RDR2-sensitive
GACCCAATCTTGGTCGTTCGTTGG	0	11	RDR2-sensitive
GACCCAATTGTAGTCGGATGGCGA	1	13	RDR2-sensitive
GACCCAGGGACGCAGCCTCACATA	0	9	RDR2-sensitive
GACCCATATTGTTTCGTGTCGGGC	0	20	RDR2-sensitive
GACCCATCTGGGCTGGGACACGCA	0	14	RDR2-sensitive
GACCCATCTGGGCTGGGGCACGCA	0	9	RDR2-sensitive
GACCCCTGGCTGCAGATCGTTCGC	1	113	RDR2-sensitive
GACCCGAATGGTCGGTTGTATCGT	0	10	RDR2-sensitive
GACCCGCGGGCTGGCTGACATGGC	0	9	RDR2-sensitive
GACCCGGAAGACGGCGAACTCCGC	25	211	RDR2-sensitive
GACCCGGACCCGCATAGAATCGGA	0	15	RDR2-sensitive
GACCCGGACTAAACTTTAGCAGGA	0	9	RDR2-sensitive
GACCCGGAGTAGAGCTGCGGCTGC	0	9	RDR2-sensitive
GACCCGGCACGGATATGACTCGGC	0	9	RDR2-sensitive
GACCCGGCTGAAAGACGTACCCAT	0	20	RDR2-sensitive
GACCCGGGCTAAACTTTAGCAGAA	0	13	RDR2-sensitive
GACCCGGGCTAAACTTTAGCAGAG	0	9	RDR2-sensitive
GACCCGGGCTAAACTTTAGCAGGA	1	59	RDR2-sensitive
GACCCGTGAGTAGGACTAGGCATT	0	23	RDR2-sensitive
GACCCGTTGTCGGAGATCTCGGGC	0	12	RDR2-sensitive
GACCCGTTTAACTGTGGGCGTGCG	0	11	RDR2-sensitive
GACCCTAAGCCACAGATCGTTCGT	0	58	RDR2-sensitive
GACCCTGTATACTGGACGGTCCGC	0	14	RDR2-sensitive
GACCCTGTCGCCAGGTCGCGGACC	0	13	RDR2-sensitive
GACCGAAGACTGGAGCGAGCAGGG	0	9	RDR2-sensitive
GACCGACACGTAGAGGATGCTGTA	0	16	RDR2-sensitive

GACCGACACGTAGAGGGCGCTGTA	1	168	RDR2-sensitive
GACCGACACGTAGGAGGCGCTGTA	2	94	RDR2-sensitive
GACCGACACGTAGGGGACGCTGTA	2	126	RDR2-sensitive
GACCGACACGTAGGGGGCGCTGTA	10	148	RDR2-sensitive
GACCGACATGGACCGATGAGGCAC	0	10	RDR2-sensitive
GACCGACATGTAGGGGGCGCTGTA	1	50	RDR2-sensitive
GACCGACGCGTAGAGGGCGCTGTA	0	34	RDR2-sensitive
GACCGACGCGTAGGAGGCGCTGTA	0	11	RDR2-sensitive
GACCGACTACTGTAGCTCACGGGC	0	17	RDR2-sensitive
GACCGACTGTAAAATTTTATCGGT	0	13	RDR2-sensitive
GACCGAGGCAATAGGACGCAGGCT	0	14	RDR2-sensitive
GACCGAGGCTCGTTGGGAAGACTT	0	26	RDR2-sensitive
GACCGAGTGACAGGTACCGTCGGA	0	9	RDR2-sensitive
GACCGATACGTAGGGGACGTGGTA	0	10	RDR2-sensitive
GACCGATTCGGACTTTTCGGTGTC	0	9	RDR2-sensitive
GACCGCACTGCTCGAGGACATGGA	0	9	RDR2-sensitive
GACCGCAGTGCCGGACTCGGACGG	0	10	RDR2-sensitive
GACCGCATGCGTCGCTGTGTTCGG	0	15	RDR2-sensitive
GACCGCGCCTGTAGACCGGACGGT	1	30	RDR2-sensitive
GACCGCGGACGGTAAAACACGGAC	0	13	RDR2-sensitive
GACCGCGGAGACATCGGAACACGG	0	14	RDR2-sensitive
GACCGCGGAGTAGGGAGACAGACG	0	10	RDR2-sensitive
GACCGCGGGCTGGGCACGTGTGGC	0	18	RDR2-sensitive
GACCGCGTTGGAGGAGTCGGCAGC	0	10	RDR2-sensitive
GACCGCTCGAGGACTATTGGCGTG	0	13	RDR2-sensitive
GACCGGACACAGACGACGATGCAG	0	9	RDR2-sensitive
GACCGGACACCTTCAGACGGAGAC	0	21	RDR2-sensitive
GACCGGACAGTCTAGAACGGGCAC	0	14	RDR2-sensitive
GACCGGACTTAGATCGGTATGGTC	0	12	RDR2-sensitive
GACCGGATGGAAAACGGCCCTGCG	0	13	RDR2-sensitive
GACCGGATGTCAGAGGCACAAGAA	0	10	RDR2-sensitive
GACCGGATTAGTAGGCTAGATGGT	2	16	RDR2-sensitive
GACCGGCATGTAGATGTGGCCCAG	3	61	RDR2-sensitive
GACCGGCCTGACAAGAGGACAGCG	1	18	RDR2-sensitive
GACCGGCCTGACAGAAAGACAGCG	2	33	RDR2-sensitive
GACCGGCCTGACAGAAGGACAGCG	10	141	RDR2-sensitive
GACCGGCGACGACAGTGAGGACGA	0	10	RDR2-sensitive
GACCGGCGCGTAGGAGACGCTGTA	0	10	RDR2-sensitive
GACCGGCGTCGGTGTCGTTCGGAT	0	9	RDR2-sensitive
GACCGGGCGTGGACGGAATTCGGT	9	43	RDR2-sensitive
GACCGGTCAGTGGAGCGGCGAAGT	0	13	RDR2-sensitive
GACCGGTCGGAAAGTCTTGGGCAC	0	16	RDR2-sensitive
GACCGGTCGGAGAACGTGGATGGT	0	15	RDR2-sensitive
GACCGGTCTGACAAGAGGACAGCG	1	50	RDR2-sensitive
GACCGGTCTGACAGAAGGACAGCG	14	115	RDR2-sensitive
GACCGGTGGATAGACGCATCTGTG	29	0	RDR2-resistant
GACCGGTTTGGTGGGATCGTTGAG	0	15	RDR2-sensitive
GACCGTATCGGGGGCTCGACGGAC	0	28	RDR2-sensitive
GACCGTATGTGACTGGTAACGGAC	0	18	RDR2-sensitive

GACCGTCACTGTTAGTAGCAGCAT	0	14	RDR2-sensitive
GACCGTCCGGACCACAGACGCGGA	0	17	RDR2-sensitive
GACCGTCCGGTCTGTAGGCGCGGA	0	43	RDR2-sensitive
GACCGTCGACGGCATCCTCAGATC	0	15	RDR2-sensitive
GACCGTCGATCTAGGACCACGGAT	0	11	RDR2-sensitive
GACCGTCGGTGAACGAAACGTCAT	0	37	RDR2-sensitive
GACCGTCTGGCCTGTAGGCGCGGA	0	14	RDR2-sensitive
GACCGTGCCGGGCCGTGTTTGGAC	1	18	RDR2-sensitive
GACCGTGCTATGCAATATCGTCGG	0	10	RDR2-sensitive
GACCGTGGATATGATTGTGGGCGG	0	9	RDR2-sensitive
GACCGTGTAGAATAGAGGACGCGA	0	19	RDR2-sensitive
GACCGTTAGGGTTCGACCGTTGGA	2	17	RDR2-sensitive
GACCGTTGGATGGAAAATGGACGG	0	25	RDR2-sensitive
GACCGTTTAGAGGACGCTGCTGAA	0	12	RDR2-sensitive
GACCGTTTAGAGGACGCTGCTGGA	0	20	RDR2-sensitive
GACCGTTTAGAGGACGTTGCTGGA	0	28	RDR2-sensitive
GACCTAACACTAGGCACACGTCGG	0	13	RDR2-sensitive
GACCTAATATATGTCGTGTCGGAC	0	9	RDR2-sensitive
GACCTACTCCTCGGACTGCGGCAC	0	27	RDR2-sensitive
GACCTAGAACCGGACGGTCCACGC	0	9	RDR2-sensitive
GACCTAGACCTGCATAGAACCGGA	0	13	RDR2-sensitive
GACCTAGCTGTAGGGGCGGCTCCT	0	9	RDR2-sensitive
GACCTAGGCGTAGAAGCGGCTCCT	0	9	RDR2-sensitive
GACCTAGTACTTGTAGAAGGGCAC	0	9	RDR2-sensitive
GACCTATGGTAGACTGTACGGTGA	0	38	RDR2-sensitive
GACCTATTTTCTATCGTACCGGGC	0	49	RDR2-sensitive
GACCTCAGCTGTAGACGGCCGCTA	2	18	RDR2-sensitive
GACCTCGCTGTAGGAGCGGCTCTT	0	28	RDR2-sensitive
GACCTCGGAAGACGGACTCCGCCT	1	17	RDR2-sensitive
GACCTCGGACGACGGTCTCCGCCT	0	9	RDR2-sensitive
GACCTCTCAGGACAAAGTCGGTAT	0	18	RDR2-sensitive
GACCTCTCGGTGGTTGTCTCGGTC	33	1	RDR2-resistant
GACCTGAAGGCGGATCTAGACCAG	0	13	RDR2-sensitive
GACCTGATCATGTTGGACGGGCCG	0	18	RDR2-sensitive
GACCTGCAACGGGCCGAACTCGGA	1	16	RDR2-sensitive
GACCTGGACATCTGGAGACGACAT	0	9	RDR2-sensitive
GACCTGGACGCGGGACGAACACGA	0	10	RDR2-sensitive
GACCTGGGCGCGGGACGAACACGA	2	36	RDR2-sensitive
GACCTGGGCGTAGGAGCGGCTCCT	0	18	RDR2-sensitive
GACCTGGGCGTAGGAGCGGTGTCT	0	10	RDR2-sensitive
GACCTGGTGCTTCGGCTGCGGCAA	0	10	RDR2-sensitive
GACCTGGTTGGACCGCATTGGCAC	0	18	RDR2-sensitive
GACCTGTAGATTGAACTAGGTCGG	0	10	RDR2-sensitive
GACCTGTAGGCGCGGATCGTCCGG	2	17	RDR2-sensitive
GACCTGTGACTGGCAAACAACATC	0	9	RDR2-sensitive
GACCTGTGGACCTCTTTTGTCGGC	0	13	RDR2-sensitive
GACCTGTTCTGACGGTCATCGGAC	0	20	RDR2-sensitive
GACCTTCAGACGTAGGGCGGCTAT	1	32	RDR2-sensitive
GACCTTCCCAAGGTTTGATCGCGG	0	12	RDR2-sensitive

GACCTTCTGGCCGCGGATCGTCTG	0	13	RDR2-sensitive
GACCTTGAAGCCGGCGCGGTAGAG	0	9	RDR2-sensitive
GACCTTGTTGGAGGCGTAGAGGAC	0	25	RDR2-sensitive
GACCTTTCGGCACTGGGCAGGCGT	0	20	RDR2-sensitive
GACCTTTTCCCGGTCGTTGCACTG	19	1	RDR2-resistant
GACGAAACTGTCGAGTCACTCGGT	78	9	RDR2-resistant
GACGAAATACTCGTGGCTCGCTGG	0	9	RDR2-sensitive
GACGAAATGCTCGTGGCTCGCTGG	1	12	RDR2-sensitive
GACGAACACGAAGGCCGCGGGATT	2	19	RDR2-sensitive
GACGAACAGACGTAGATGGACGAC	0	9	RDR2-sensitive
GACGAACAGAGACGAGAGAGCCAG	2	16	RDR2-sensitive
GACGAACAGCAAGCTGTGAACGAA	0	10	RDR2-sensitive
GACGAACAGCAAGCTGTGGACGAT	1	31	RDR2-sensitive
GACGAACAGGGCAACAGACGAACA	0	32	RDR2-sensitive
GACGAACAGTAGGAGGAGGAGCAT	0	18	RDR2-sensitive
GACGAACCCGGGACCTGGAGATGC	0	15	RDR2-sensitive
GACGAACCGAACGCGCTGCGACGA	1	12	RDR2-sensitive
GACGAACCGCGGAACAGGATTCGG	0	18	RDR2-sensitive
GACGAACCGCGGAAGCCGGAGCTC	0	19	RDR2-sensitive
GACGAACCGGCTGGAACAGTAGAA	0	13	RDR2-sensitive
GACGAACGGACGGCTTAGATTGGC	1	34	RDR2-sensitive
GACGAACGGACGGTCTAGATTGGT	0	17	RDR2-sensitive
GACGAACGTAGAAGCGCAGTGCAG	1	29	RDR2-sensitive
GACGAACGTTGGCGCGACAGACAC	0	9	RDR2-sensitive
GACGAACTCCAAGCGGCCCAGCCA	0	17	RDR2-sensitive
GACGAACTCGTCGCTCTCGGGAAA	0	11	RDR2-sensitive
GACGAACTCTAACGTGGCTTCGGC	0	12	RDR2-sensitive
GACGAACTGGCGTGCGAAGACGAC	0	11	RDR2-sensitive
GACGAAGACCGGATGTAGAGACGT	0	9	RDR2-sensitive
GACGAAGACCGGCGACGAAGGCAT	3	18	RDR2-sensitive
GACGAAGACCTAGGCATCTGGAGG	0	10	RDR2-sensitive
GACGAAGACGACCAGAGCTGGCAT	0	50	RDR2-sensitive
GACGAAGACGGACGAAGACGACCT	0	17	RDR2-sensitive
GACGAAGACGTGCGGGAGGGACGA	2	41	RDR2-sensitive
GACGAAGCAGAGACTTGAGGACGA	0	15	RDR2-sensitive
GACGAAGGACAGACGAACAGACGT	0	12	RDR2-sensitive
GACGAAGGCTGGAGCGAAGGGTAG	0	9	RDR2-sensitive
GACGAATCGGGACCGCATTGGCAC	0	13	RDR2-sensitive
GACGAATCTGTACCGTCGACTCGC	0	16	RDR2-sensitive
GACGACAACGGTGGAGGCTGCATG	0	12	RDR2-sensitive
GACGACAAGACTCATCGGCAGCAG	1	30	RDR2-sensitive
GACGACAAGGACAGCAACCTCAAG	0	16	RDR2-sensitive
GACGACACAATGAGGTGAACACGG	0	9	RDR2-sensitive
GACGACACGCGGAACGACGACGAG	0	67	RDR2-sensitive
GACGACACTACGGACGGTGAAGAT	2	19	RDR2-sensitive
GACGACACTCTGCGCCATCACGGA	0	17	RDR2-sensitive
GACGACACTGGTGGCTCGACGGAC	0	9	RDR2-sensitive
GACGACACTGTAGGTGCGGCGACA	1	26	RDR2-sensitive
GACGACAGACAGACAGTTGAGCAT	0	13	RDR2-sensitive

GACGACAGACGGCGTCTGAGCACG	2	25	RDR2-sensitive
GACGACAGGAACGTGCTGGCGGAT	0	11	RDR2-sensitive
GACGACAGGGGAATATCGAGACGG	0	10	RDR2-sensitive
GACGACAGTAGACACACAGACGTG	1	35	RDR2-sensitive
GACGACAGTAGACACAGAGACGTG	0	34	RDR2-sensitive
GACGACAGTGAGGACGAGACTGGC	0	18	RDR2-sensitive
GACGACATAGGTCGTGTGGGCTGT	0	9	RDR2-sensitive
GACGACATCGTTATCTCCGGACGG	0	18	RDR2-sensitive
GACGACCATGGATTCTCGGGCGGC	4	79	RDR2-sensitive
GACGACCCAGTAGAAGGCCCGTGA	0	10	RDR2-sensitive
GACGACCCTGGACCTGGAGTGGTG	0	10	RDR2-sensitive
GACGACCGAAGACTTGGGCGGGCA	0	14	RDR2-sensitive
GACGACCTCAGCTGTAGACGGCCG	1	13	RDR2-sensitive
GACGACGAACTTGGACGATGGCAA	0	14	RDR2-sensitive
GACGACGAAGAACCAGATCGGTGT	0	13	RDR2-sensitive
GACGACGACGACGACGAAGGCGGT	23	1	RDR2-resistant
GACGACGAGAAGAAGCTTGGCGAC	0	12	RDR2-sensitive
GACGACGAGCTGGGCAGGATCCGA	0	15	RDR2-sensitive
GACGACGAGGACGCCGCCAACATC	47	8	RDR2-resistant
GACGACGAGGACTGGCGCGTGGTC	0	17	RDR2-sensitive
GACGACGATATAGGAGGGTGGCAT	0	9	RDR2-sensitive
GACGACGATGAATCTGAGGCGGCG	0	9	RDR2-sensitive
GACGACGCACAGAATGACGATGAT	0	11	RDR2-sensitive
GACGACGCACGGAATGACGACGGA	0	16	RDR2-sensitive
GACGACGCAGGACAGAGTGTGGAT	0	9	RDR2-sensitive
GACGACGCAGGAGCTCTCCGGCAC	0	10	RDR2-sensitive
GACGACGCGGCACGACAAGCTGGA	0	10	RDR2-sensitive
GACGACGCTGTAGTGAGGTTGCAA	0	21	RDR2-sensitive
GACGACGGCGACGACGACGACGGT	29	0	RDR2-resistant
GACGACGTAGAGCAGAAGAGAGAT	0	14	RDR2-sensitive
GACGACGTAGAGCCGAAGAGAGGT	0	11	RDR2-sensitive
GACGACGTGGACAGCGGACGCCAA	0	9	RDR2-sensitive
GACGACTCGACGACGAGCTGGGCA	0	18	RDR2-sensitive
GACGACTCGACGGTAAACACGAAC	0	11	RDR2-sensitive
GACGACTCGACGGTAAACGCGAAC	2	42	RDR2-sensitive
GACGACTCGACTGTGAACGGCGCA	0	15	RDR2-sensitive
GACGACTCGTCCGAGCTTAGACGA	0	9	RDR2-sensitive
GACGACTGAGATGCAGAACACGGT	0	15	RDR2-sensitive
GACGACTGATTCGGAACGGAGGCG	0	19	RDR2-sensitive
GACGACTGCAACGGCAAACTCTAC	0	30	RDR2-sensitive
GACGACTGCACGGCACTGCTCGAC	113	44	RDR2-resistant
GACGACTGCTTTGACCGAGAGGAA	0	13	RDR2-sensitive
GACGACTGGGACTCTGGGAGTGGC	0	10	RDR2-sensitive
GACGACTGGGAGATTGCATGTTAT	0	11	RDR2-sensitive
GACGACTGGTAACAGAGACGACGC	0	14	RDR2-sensitive
GACGACTGTACAGATTACGCCACA	0	12	RDR2-sensitive
GACGACTGTACAGATTACGCCGCA	0	57	RDR2-sensitive
GACGACTGTACAGATTGCGCCACA	0	24	RDR2-sensitive
GACGACTGTAGATGGTGACGGGGC	0	11	RDR2-sensitive

GACGACTGTAGCAAAATGGTTCGT	7	85	RDR2-sensitive
GACGACTGTAGGAAGCCGTCGGAT	4	64	RDR2-sensitive
GACGACTGTATAGATTGCGTTGCA	0	14	RDR2-sensitive
GACGACTGTGCGGTCTGGGGCATC	0	11	RDR2-sensitive
GACGACTGTGTACGGCTACATCGA	0	56	RDR2-sensitive
GACGACTGTTGGGGCGAAGGCGAA	0	10	RDR2-sensitive
GACGAGACGAGGCAAACGACTGAC	0	17	RDR2-sensitive
GACGAGACGGTAACATCACGACGA	0	13	RDR2-sensitive
GACGAGAGCGGTACTTCGGACAGA	0	15	RDR2-sensitive
GACGAGAGGCAGAACAAACGGAAC	0	10	RDR2-sensitive
GACGAGATGGATCTGTGACGGTAA	0	12	RDR2-sensitive
GACGAGATTGTGTCTGGAGCGAGC	0	22	RDR2-sensitive
GACGAGCATGTGGAGGAGGACAGT	0	14	RDR2-sensitive
GACGAGCCTGTAGCATGCAACCGG	0	18	RDR2-sensitive
GACGAGCGAAGGGTGGCGTCTCTG	0	15	RDR2-sensitive
GACGAGCGGGCAGGATATGGAAGC	0	18	RDR2-sensitive
GACGAGCGTCACACATGCGAACAG	0	9	RDR2-sensitive
GACGAGCGTCGTGGCGTTGTCGGC	3	31	RDR2-sensitive
GACGAGCTGCAGTTGGACATCCAT	0	13	RDR2-sensitive
GACGAGGACCCGCAGCTGGACGTC	0	12	RDR2-sensitive
GACGAGGACTGACGCGGAGGCCGA	4	58	RDR2-sensitive
GACGAGGACTGGCGCGTGGTCAAC	0	9	RDR2-sensitive
GACGAGGAGAAGCAGGCTTCTGAA	0	12	RDR2-sensitive
GACGAGGAGAGGTAGACGTAACTA	27	62	RDR2-sensitive
GACGAGGGTAGACACGAGCAGCAT	0	43	RDR2-sensitive
GACGAGTTGACGACGGATCGCTGT	0	9	RDR2-sensitive
GACGATAGTGTAGACCAGGCACAA	0	19	RDR2-sensitive
GACGATCACGGTGGAGGACGAGGA	72	0	RDR2-resistant
GACGATCAGGATTCAGGGGCAGGC	0	11	RDR2-sensitive
GACGATCCACGTCAGGTAGTCGGA	0	9	RDR2-sensitive
GACGATCCTGAACATGTCCGGCAG	0	30	RDR2-sensitive
GACGATCGGGCAAGACAACGGATT	0	13	RDR2-sensitive
GACGATCTGCAACCCTGGACCGGA	0	10	RDR2-sensitive
GACGATCTGGTACTGCATCGGCGT	0	10	RDR2-sensitive
GACGATCTTGTACTGCATCGGCGC	0	28	RDR2-sensitive
GACGATGACCAACAGTAGGACGAC	0	12	RDR2-sensitive
GACGATGACTGAAAGTAGGGACGT	0	17	RDR2-sensitive
GACGATGACTGGAAGTAGGGACGT	1	30	RDR2-sensitive
GACGATGAGGACATGGAGAGACGA	0	10	RDR2-sensitive
GACGATGATCGGGAGCGGCTCCGT	0	13	RDR2-sensitive
GACGATGCGACGGTAGATGGCGAC	0	42	RDR2-sensitive
GACGATGCGGCGCAGCAGGACGAC	1	31	RDR2-sensitive
GACGATGGCGCCAAGAAGACTGAT	1	45	RDR2-sensitive
GACGATGTCGGCGTAGAGGAAGCA	0	9	RDR2-sensitive
GACGATTGGACTCAGGACGGACGA	2	15	RDR2-sensitive
GACGATTTGGAGGCGTAGGCGAGC	0	10	RDR2-sensitive
GACGATTTGTCGATTTATCTGGCA	0	10	RDR2-sensitive
GACGATTTTGGCCGTCGGAAGTCA	0	11	RDR2-sensitive
GACGCAACGACGAACGACGACGAC	0	18	RDR2-sensitive

GACGCAACGACGAACGACGACGAT	0	14	RDR2-sensitive
GACGCAAGCTGTATGAGCGGATGT	0	9	RDR2-sensitive
GACGCAAGGAATGAGGACGAGCAA	0	11	RDR2-sensitive
GACGCAAGGACGACAGAGCAGAGA	0	9	RDR2-sensitive
GACGCACGACGACCGTTGAAACTG	0	14	RDR2-sensitive
GACGCACGGAACGACGACGAGATC	0	9	RDR2-sensitive
GACGCACGTCGGCTAGAATAAGGC	0	11	RDR2-sensitive
GACGCACTGTAAATTTAGCGGCAA	0	9	RDR2-sensitive
GACGCAGACCAGCAAGAACGACGA	0	14	RDR2-sensitive
GACGCAGAGCGGTTCATGTAGAAC	0	9	RDR2-sensitive
GACGCAGAGGAGGACCGACAACAA	2	30	RDR2-sensitive
GACGCAGGCGACAGACAAACTCCA	0	11	RDR2-sensitive
GACGCATGACGACCGTTGAAACTG	1	19	RDR2-sensitive
GACGCATGCGGGAGGTGGGCTGGC	0	11	RDR2-sensitive
GACGCATGGTACGACGACGAGCTC	0	12	RDR2-sensitive
GACGCCCGACTGTATTTTAGCGTA	0	18	RDR2-sensitive
GACGCCCGGCTGTAGGGATGGTGC	0	18	RDR2-sensitive
GACGCCCGGGCTGGATTCAGGCAG	0	11	RDR2-sensitive
GACGCCTAGGTCTCGGATGCGGTC	2	43	RDR2-sensitive
GACGCCTCTGAACGACGGAGATGT	0	10	RDR2-sensitive
GACGCGAAGAACTTGGCGTAGACG	0	22	RDR2-sensitive
GACGCGACTGACGGATAAGCCAAC	0	9	RDR2-sensitive
GACGCGACTGTAGGCGTTGATGGC	5	122	RDR2-sensitive
GACGCGAGGTAGAACAGGATCGGT	0	9	RDR2-sensitive
GACGCGCAGGGCAGTGGAAGACGT	0	12	RDR2-sensitive
GACGCGCGAGGCAGAACACGGACT	0	10	RDR2-sensitive
GACGCGCGGAACGACGACAAGATC	0	10	RDR2-sensitive
GACGCGCGGAACGACGACAGTCAA	0	9	RDR2-sensitive
GACGCGCGGAACGACGACGAGATC	0	84	RDR2-sensitive
GACGCGCGGAATGACGACGAGATC	0	14	RDR2-sensitive
GACGCGCTGTAGGTTCGGTGGCGT	0	9	RDR2-sensitive
GACGCGGAAACGAGCATGCTGGCA	0	13	RDR2-sensitive
GACGCGGAGGCAGAACATGGTGTA	0	9	RDR2-sensitive
GACGCGGATAGGACAACAGCGGCT	3	16	RDR2-sensitive
GACGCGGATAGGACAACGATGGTT	0	40	RDR2-sensitive
GACGCGGATAGGACAACGGTGGTT	0	39	RDR2-sensitive
GACGCGTAGGACGTAACCCGGTAG	0	16	RDR2-sensitive
GACGCGTGTAGAAGGGCGGACCAG	0	9	RDR2-sensitive
GACGCTACTGAAGACGACGTTCCC	14	0	RDR2-resistant
GACGCTATAGACAACTCCCCGGCG	29	1	RDR2-resistant
GACGCTATCTGTACAGTCGTCGGA	0	41	RDR2-sensitive
GACGCTCGGGACTAGTGCGGCTGA	0	13	RDR2-sensitive
GACGCTCTGGATGCGGCAGAACCG	0	10	RDR2-sensitive
GACGCTGACGACGAGGACTGGCGC	0	13	RDR2-sensitive
GACGCTGCTGGAGACGGAGAATAT	1	20	RDR2-sensitive
GACGCTGGACGCAGGAAGCAGAAC	0	21	RDR2-sensitive
GACGCTGGCAAGGAACACGGGCGC	24	3	RDR2-resistant
GACGCTGGCACGGTCGGACGGCTT	0	11	RDR2-sensitive
GACGCTGTAGAGATAGACCCGCAG	0	9	RDR2-sensitive

GACGCTGTAGTGGCTCAGGCACGG	0	11	RDR2-sensitive
GACGCTGTGTAGAGCATGGGTGGT	0	40	RDR2-sensitive
GACGCTGTTATAGAAGGATGGCTT	0	12	RDR2-sensitive
GACGCTGTTGTAGAATGTAGAGGA	0	10	RDR2-sensitive
GACGCTTCTTCGTGCTGTCCGGCG	0	9	RDR2-sensitive
GACGCTTGTAGGAATATGGGGCTA	0	10	RDR2-sensitive
GACGGAAAACGGTAGACGGACGGT	0	9	RDR2-sensitive
GACGGAAACGGCGTGGGATGGCAA	0	10	RDR2-sensitive
GACGGAAAGTAAGGGTACAGGCGT	0	17	RDR2-sensitive
GACGGAACGTGTAACGACGACGGT	0	12	RDR2-sensitive
GACGGAAGGGAAGGGAGCGGATCT	2	29	RDR2-sensitive
GACGGAAGTCTCTCGAGGTCGGCT	19	45	RDR2-sensitive
GACGGAATCACTGCTCGCGGACGG	0	9	RDR2-sensitive
GACGGACACCAAGCGGGGAGGCTC	0	17	RDR2-sensitive
GACGGACATGCGATCGAACGCGGA	0	11	RDR2-sensitive
GACGGACCACCCAACAAGACGCAT	0	13	RDR2-sensitive
GACGGACCATGTCTGGTAGAAGAA	0	48	RDR2-sensitive
GACGGACGAACTAAGTCGCGAGGT	0	12	RDR2-sensitive
GACGGACGAACTAAGTCGCGGCGA	1	14	RDR2-sensitive
GACGGACGAACTAAGTCGTGACGA	1	12	RDR2-sensitive
GACGGACGAACTAAGTTACGGCGA	0	13	RDR2-sensitive
GACGGACGCTAGCGGACGCGCGCT	0	12	RDR2-sensitive
GACGGACGGCGTAATTCCGGCGAG	0	23	RDR2-sensitive
GACGGACGGCGTAATTCCGGTGAG	0	9	RDR2-sensitive
GACGGACGGTAGATAGCAGGCGGA	0	15	RDR2-sensitive
GACGGACTGCCTGCAGGACAGCAG	0	16	RDR2-sensitive
GACGGACTGGACGAGTGCGACGGC	1	26	RDR2-sensitive
GACGGAGACAAGAACGTCAGACGG	0	11	RDR2-sensitive
GACGGAGACAAGAATACGACGCCA	0	12	RDR2-sensitive
GACGGAGACAGAACGGGCAGTGAT	0	9	RDR2-sensitive
GACGGAGACAGAGTTAGACGTGTG	0	28	RDR2-sensitive
GACGGAGACGGAGTCTTTGCCGAG	0	22	RDR2-sensitive
GACGGAGACTGAGCCAGACGGATG	0	28	RDR2-sensitive
GACGGAGACTGAGTCAGACGAGTG	0	12	RDR2-sensitive
GACGGAGAGAAGAATACGACGCCA	0	14	RDR2-sensitive
GACGGAGAGTTGATGCAGATCTGA	1	14	RDR2-sensitive
GACGGAGATCAGCAGAACGAGCAT	0	18	RDR2-sensitive
GACGGAGATGGAGAACGCGACGGG	0	10	RDR2-sensitive
GACGGAGCGGCAGAACACGATTGT	0	18	RDR2-sensitive
GACGGAGCTGATGCGATGAAGGCG	0	18	RDR2-sensitive
GACGGAGGACGAGAGTCAGGAGAA	0	9	RDR2-sensitive
GACGGAGGACTCTGACAGGGGCGT	0	15	RDR2-sensitive
GACGGAGGCTGAGTCAGACGTGTG	0	46	RDR2-sensitive
GACGGAGTTAGACGAGTGGTGGAT	0	9	RDR2-sensitive
GACGGAGTTAGACGGGTGACAGAC	0	19	RDR2-sensitive
GACGGATACGGAGAGGAGAGAGAA	0	34	RDR2-sensitive
GACGGATACGGATACAGAGACCGA	0	12	RDR2-sensitive
GACGGATACGGATACAGGGACCGG	1	39	RDR2-sensitive
GACGGATACGGGTATGGATACATA	0	14	RDR2-sensitive

GACGGATCCAGAACAAGGGGGCTA	0	13	RDR2-sensitive
GACGGATCGACGCGTACCTTCTCC	0	9	RDR2-sensitive
GACGGATCTAGCTAGACGACGTAG	0	12	RDR2-sensitive
GACGGATGACGGATACAAGCGTTT	0	12	RDR2-sensitive
GACGGATGACGTGGTCTAGCTCGG	0	35	RDR2-sensitive
GACGGATGACGTGTCAGACCTTAG	0	9	RDR2-sensitive
GACGGATGAGCTCAACGACGTCAT	1	174	RDR2-sensitive
GACGGATGATGTCGAAGACGCCCT	0	10	RDR2-sensitive
GACGGATGCTAGTAGAATGGCATG	0	10	RDR2-sensitive
GACGGATGCTGTGATGGTATTATG	0	9	RDR2-sensitive
GACGGATGGCAGAACCACGGGGTG	0	10	RDR2-sensitive
GACGGATGTAGAGGACGTTGCTGG	0	19	RDR2-sensitive
GACGGATTCGACAACTCGACGGCA	2	28	RDR2-sensitive
GACGGATTTCTCCTCGGCTCCGGC	3	19	RDR2-sensitive
GACGGCAAACGACGAGAACGACGA	0	19	RDR2-sensitive
GACGGCAACAAGTGGATATGACGA	0	11	RDR2-sensitive
GACGGCAATATCAATCTGGCGATG	0	12	RDR2-sensitive
GACGGCACGAACACCTGTGCAGAA	0	9	RDR2-sensitive
GACGGCACTAGACAAACAGACGTG	0	21	RDR2-sensitive
GACGGCACTAGACATACAGACGTG	0	16	RDR2-sensitive
GACGGCACTCTAGTTGAAGAAGGT	23	1	RDR2-resistant
GACGGCACTGCATAACGCGTCCAG	0	9	RDR2-sensitive
GACGGCACTGGCGACTCGGCGGAC	1	12	RDR2-sensitive
GACGGCACTGTACGGCAATAATAG	0	19	RDR2-sensitive
GACGGCACTGTGGACGAAGCGGAA	0	10	RDR2-sensitive
GACGGCACTTACAGCGACCATCCT	5	31	RDR2-sensitive
GACGGCAGAACGGCAGAACAAGAG	0	9	RDR2-sensitive
GACGGCAGCCTGAGAAGTCAGCAG	0	16	RDR2-sensitive
GACGGCAGCGACTTGGGCGAGCAC	0	10	RDR2-sensitive
GACGGCAGCGTACGCAGTTCTGAA	1	58	RDR2-sensitive
GACGGCAGCGTGACTCTAGCGAGA	1	25	RDR2-sensitive
GACGGCAGTAGAGATGGCAATGGG	0	12	RDR2-sensitive
GACGGCATGCAGGACAGACGACGT	3	44	RDR2-sensitive
GACGGCATGCAGGGCAGACGACGT	0	17	RDR2-sensitive
GACGGCATGTACTTAGGGCACTAG	3	17	RDR2-sensitive
GACGGCATGTACTTAGGGCGCTAG	0	34	RDR2-sensitive
GACGGCATGTCTACTAATGGGCAA	20	0	RDR2-resistant
GACGGCCAAAGCTATCGGACACAA	0	27	RDR2-sensitive
GACGGCCACCTTATCTCTGACGGC	0	10	RDR2-sensitive
GACGGCCAGAAAACGACATGAGCG	0	20	RDR2-sensitive
GACGGCCAGAGTAGAAGCACGACA	0	38	RDR2-sensitive
GACGGCCCCTAGAAGATTGTCGGA	0	10	RDR2-sensitive
GACGGCCGTTGGATGGAAAATGGA	1	22	RDR2-sensitive
GACGGCCGTTGGATGGGAGACGGA	0	31	RDR2-sensitive
GACGGCCTACACTGTTTCCGACGG	1	12	RDR2-sensitive
GACGGCCTAGGAAACTGTCGGACA	0	10	RDR2-sensitive
GACGGCCTCGTCGGCGACAGCATG	0	9	RDR2-sensitive
GACGGCCTGAGTAGGACGGTTGGC	0	9	RDR2-sensitive
GACGGCCTGCACTAGTTCCGACGG	0	9	RDR2-sensitive

GACGGCCTGGGTAAGACGTGTCGT	0	15	RDR2-sensitive
GACGGCCTTCAACTTGACGACGAG	0	9	RDR2-sensitive
GACGGCCTTCAGCTTGACGACGAG	0	40	RDR2-sensitive
GACGGCGAACGACATGACGACGAA	3	27	RDR2-sensitive
GACGGCGAACGACGTGACGACGAA	0	10	RDR2-sensitive
GACGGCGAAGTAACTACACCTCTA	0	9	RDR2-sensitive
GACGGCGACTGTGGCACGCGGCAC	0	18	RDR2-sensitive
GACGGCGCTGACGGCGTGAACCTA	0	17	RDR2-sensitive
GACGGCGTCTGTACACGGGAACGG	29	0	RDR2-resistant
GACGGCGTGCATGGACTCGGGTCA	0	9	RDR2-sensitive
GACGGCGTTGTGGTGAATAGGAAA	3	66	RDR2-sensitive
GACGGCTACAACTATGGCACCATG	125	0	RDR2-resistant
GACGGCTACGACCTGGGCGAGCGC	0	13	RDR2-sensitive
GACGGCTACTGTAGCCCACGGGCC	0	22	RDR2-sensitive
GACGGCTCCTCGGACGACGGCTGT	0	15	RDR2-sensitive
GACGGCTCGCGTGCTTTGGCCCGG	0	11	RDR2-sensitive
GACGGCTCGCTGTGCGTGAAACGT	0	10	RDR2-sensitive
GACGGCTCTAAGATTTTTTTGCAA	3	19	RDR2-sensitive
GACGGCTGACCTGCTCGAGGACGG	0	21	RDR2-sensitive
GACGGCTGACGGCACTTTACCGAT	0	10	RDR2-sensitive
GACGGCTGACGGCGAGTGGACGGA	0	17	RDR2-sensitive
GACGGCTGAGATCGCAGAACGGCA	1	23	RDR2-sensitive
GACGGCTGAGATCGCAGAATGGCA	3	19	RDR2-sensitive
GACGGCTGGCAGAAGCTTGTCTGC	0	9	RDR2-sensitive
GACGGCTGGGGACGCAAGTAGGGA	0	15	RDR2-sensitive
GACGGCTGTGACGGAGGGCGACCT	0	9	RDR2-sensitive
GACGGCTGTGAGGAAAGAGAAGAA	0	9	RDR2-sensitive
GACGGCTTACAACAATACCCACGT	0	11	RDR2-sensitive
GACGGGAATTAACTGGGCCGACGG	1	12	RDR2-sensitive
GACGGGACGGATACATGTACTACC	0	15	RDR2-sensitive
GACGGGAGGCAGAACATGGTGCAC	0	30	RDR2-sensitive
GACGGGAGGGAAGGGAGCGGATCT	2	51	RDR2-sensitive
GACGGGAGGGAATGAAGCGGATCT	0	10	RDR2-sensitive
GACGGGAGTGTAGAGGACGGGGCC	0	11	RDR2-sensitive
GACGGGCACTCCTGGCAGAACCGT	2	57	RDR2-sensitive
GACGGGCAGGATGAAGCAAAAGGA	0	13	RDR2-sensitive
GACGGGCAGGTAGAGAGCGCGGAC	0	11	RDR2-sensitive
GACGGGCCAAGAGAAGGACATGGC	0	17	RDR2-sensitive
GACGGGCGCATGAACTACGGCGAG	0	17	RDR2-sensitive
GACGGGCGGCGTGAGTACAATAGT	0	12	RDR2-sensitive
GACGGGCGTAGATGCGTAGACGTG	0	9	RDR2-sensitive
GACGGGCGTCAGCTTTAGAACGGC	0	12	RDR2-sensitive
GACGGGCGTGAGAGAGGTGGAAAT	5	19	RDR2-sensitive
GACGGGCTCGACAATGATACGGAA	0	19	RDR2-sensitive
GACGGGCTCGATGACGATACGGGA	0	9	RDR2-sensitive
GACGGGCTGAAATTTAAAGCACGG	0	16	RDR2-sensitive
GACGGGCTGACATTTAGAGCACGG	1	57	RDR2-sensitive
GACGGGCTGACGGACTCACGGCTC	0	18	RDR2-sensitive
GACGGGCTGCGGGATACCAAAGAG	0	16	RDR2-sensitive

GACGGGCTGCTGGACCAGCGTCGC	0	12	RDR2-sensitive
GACGGGCTTCTAGGTAAACGGATC	0	12	RDR2-sensitive
GACGGGCTTGCACGAGAGGGCTTT	0	20	RDR2-sensitive
GACGGGGACTGCAGGCGGATTCGG	0	12	RDR2-sensitive
GACGGGGCATTGTAAGTGGCAGAG	75	8	RDR2-resistant
GACGGGGGCTTTGGCAGAACTGCA	0	20	RDR2-sensitive
GACGGGGTGGAGAACGACCACGGC	0	12	RDR2-sensitive
GACGGGGTGGAGAACGACCGCGGC	1	27	RDR2-sensitive
GACGGGTACGGGTAGATATCCATA	0	9	RDR2-sensitive
GACGGGTAGTCTCTGACAGGTAGA	0	10	RDR2-sensitive
GACGGGTCACCGGATCGCCTGGCA	0	11	RDR2-sensitive
GACGGGTCGCTGATGAAGACAGGA	0	17	RDR2-sensitive
GACGGGTCGGTCTGAAACACGGCT	1	30	RDR2-sensitive
GACGGGTTCGACGACTCGACGGTA	0	48	RDR2-sensitive
GACGGGTTCGACGACTTGACGGTA	2	28	RDR2-sensitive
GACGGGTTCGATGACTCGACGGCA	2	18	RDR2-sensitive
GACGGTAAACGACACGACGACGAG	0	10	RDR2-sensitive
GACGGTAAACGACGCGACGACGAG	0	13	RDR2-sensitive
GACGGTAAGCTTATGTCTGACGGT	0	16	RDR2-sensitive
GACGGTAAGGTGGAACATCGGCGT	1	26	RDR2-sensitive
GACGGTACAGAACCAGGGAGGCAG	0	12	RDR2-sensitive
GACGGTACGGTCTGGGAGCGACGG	1	27	RDR2-sensitive
GACGGTACTCTCTGCGGGAGCGTG	0	15	RDR2-sensitive
GACGGTAGCGACTTGGGCGAGCAT	0	12	RDR2-sensitive
GACGGTAGTGTAGACCAGGCACAA	0	21	RDR2-sensitive
GACGGTATCTGACTTTGTGCCAGT	23	0	RDR2-resistant
GACGGTATGGACCTAACTCAGGAG	0	26	RDR2-sensitive
GACGGTCAATGTATCTCCGACGGC	0	10	RDR2-sensitive
GACGGTCAGGTAGGTAGAGGACAT	0	18	RDR2-sensitive
GACGGTCAGTCGGCGAGCTGGCAA	0	15	RDR2-sensitive
GACGGTCCACGCCTGATGGTCGGA	0	14	RDR2-sensitive
GACGGTCCCACCAGTCATAGACAC	0	10	RDR2-sensitive
GACGGTCCGCAACGGGAAACTGGA	0	14	RDR2-sensitive
GACGGTCCGCGACCTGGCGCAGAG	0	9	RDR2-sensitive
GACGGTCCGGACACGTAGCAGCAT	1	16	RDR2-sensitive
GACGGTCCGTAGATACGCAGATGA	0	10	RDR2-sensitive
GACGGTCCGTGGCCCTGAGCCGGA	0	11	RDR2-sensitive
GACGGTCCGTGGCTCTGGACCGGA	0	17	RDR2-sensitive
GACGGTCCGTGGCTCTGGGCCGGA	0	11	RDR2-sensitive
GACGGTCGCGACAGAACACGGTAC	0	40	RDR2-sensitive
GACGGTCGCGTAGGACAGTTCGGC	2	17	RDR2-sensitive
GACGGTCGTGCCCGTGCCACGGTG	44	8	RDR2-resistant
GACGGTCGTTGGATGGGAGACGGA	0	26	RDR2-sensitive
GACGGTCTAGATTGGTGGAAGGCA	0	10	RDR2-sensitive
GACGGTCTCCTGTAGTGTAGTGCC	0	12	RDR2-sensitive
GACGGTCTGCTCGTGGAAGTCGCA	0	15	RDR2-sensitive
GACGGTCTGTCGCTGCATCAGTGA	0	9	RDR2-sensitive
GACGGTCTGTGACCTGGCGCAGGA	0	19	RDR2-sensitive
GACGGTCTGTGATCAGGAGTCGGA	0	20	RDR2-sensitive

GACGGTCTGTGCCGCTACGATGGA	1	12	RDR2-sensitive
GACGGTCTGTGCTGCTACGGTGGA	1	19	RDR2-sensitive
GACGGTCTGTGGCACAGGGTTGGA	1	14	RDR2-sensitive
GACGGTGAAGGACGCCTGGGCGGC	1	33	RDR2-sensitive
GACGGTGACGGCTCGTGCTCCCAT	0	13	RDR2-sensitive
GACGGTGAGGGTAGACAAGGCGCT	0	11	RDR2-sensitive
GACGGTGCAGATTGGCTGAAGGCA	0	10	RDR2-sensitive
GACGGTGCCGAGTGGAAGGGACGT	0	37	RDR2-sensitive
GACGGTGCGGCATGACAAACTGAA	0	50	RDR2-sensitive
GACGGTGCGGCATGGCAAACTGAA	0	13	RDR2-sensitive
GACGGTGCTACGGGATTGACTGGC	1	27	RDR2-sensitive
GACGGTGCTGAACGTCGACGTCGT	0	10	RDR2-sensitive
GACGGTGGACCTGCAGGACGTGCT	0	10	RDR2-sensitive
GACGGTGGGATGATAGCACGGATT	0	13	RDR2-sensitive
GACGGTGTAGAGTAGAGATGCCTG	0	13	RDR2-sensitive
GACGGTGTAGATTCACGGAAGCGT	0	17	RDR2-sensitive
GACGGTGTAGATTTACCAAAGGCA	0	12	RDR2-sensitive
GACGGTGTGGGACAAGGCAAGAAG	0	13	RDR2-sensitive
GACGGTGTTCGTAGGATCGTGCGT	1	25	RDR2-sensitive
GACGGTTACTGCTTGCACCATGGC	0	22	RDR2-sensitive
GACGGTTCGGACATCCTTCGGTGT	0	52	RDR2-sensitive
GACGGTTCGGCGTTGACGGAGAAT	0	9	RDR2-sensitive
GACGGTTGAGATTGCAGAACGGTA	0	16	RDR2-sensitive
GACGGTTGATCGATCAGACCTCAG	0	9	RDR2-sensitive
GACGGTTGTAGATATGTTCGACGG	0	12	RDR2-sensitive
GACGGTTGTCAGACGGACCGGAAA	0	10	RDR2-sensitive
GACGGTTTATTTAGAGATCGCTGG	0	9	RDR2-sensitive
GACGGTTTCTGCAGACGGGTTGGA	0	24	RDR2-sensitive
GACGGTTTGTGACCTGACAGCCGG	0	14	RDR2-sensitive
GACGGTTTTGGCGTCGGACGTTGT	0	13	RDR2-sensitive
GACGTAACTCGGGAGTGGACGGGA	0	13	RDR2-sensitive
GACGTACGGAACGACGACGAGCTC	0	9	RDR2-sensitive
GACGTACGTAGAGAAGGAGCCGCT	0	29	RDR2-sensitive
GACGTACTGTGACGGTGGGGATGA	0	34	RDR2-sensitive
GACGTACTGTTTGACGTTCGACGG	0	100	RDR2-sensitive
GACGTACTTTAGAGTAGAGGCTGA	0	42	RDR2-sensitive
GACGTAGACCAAGAGGGCTTCTCC	7	48	RDR2-sensitive
GACGTAGACCGCGAACGGCGACAT	0	16	RDR2-sensitive
GACGTAGACTGGATGAGCGGATGC	0	17	RDR2-sensitive
GACGTAGAGCCGAAGAGAGGTGAA	0	12	RDR2-sensitive
GACGTAGATCAAGAGGGCTTCTCC	1	18	RDR2-sensitive
GACGTAGATCCGAGCGGGAAAGGA	0	10	RDR2-sensitive
GACGTAGCAGATCAGCGAGGACGA	0	9	RDR2-sensitive
GACGTAGCAGATCGGCGAGGACGA	0	14	RDR2-sensitive
GACGTCCCGGCGGATCGGCTCAAG	2	17	RDR2-sensitive
GACGTCTTCGCACGGATCGACCGG	0	10	RDR2-sensitive
GACGTGAAGGACGCCGACGACTGC	169	1	RDR2-resistant
GACGTGAATCCTCCGGGGTCGGCT	3	20	RDR2-sensitive
GACGTGACACTAACGGCTCCCTGC	0	9	RDR2-sensitive

GACGTGACGACGAACGACGGCGAA	0	10	RDR2-sensitive
GACGTGACGGCACAAACTCTTCAT	0	18	RDR2-sensitive
GACGTGAGCGTGATGACTGGGCAG	1	16	RDR2-sensitive
GACGTGAGCTGAAGCCGGGTGAGC	0	23	RDR2-sensitive
GACGTGAGGAACAACTGGATCGGC	0	10	RDR2-sensitive
GACGTGCGGACTGCAGAGACGGTG	0	11	RDR2-sensitive
GACGTGCGGGAGGGACGAAGACGG	2	15	RDR2-sensitive
GACGTGCTCGGGCAGAGAAAGCAG	0	9	RDR2-sensitive
GACGTGGAACCGAGCTCGACGCCA	0	11	RDR2-sensitive
GACGTGGAACCGAGCTCGGCGCCA	1	14	RDR2-sensitive
GACGTGGACGCATCTGAAAGCAGA	0	9	RDR2-sensitive
GACGTGGACGCATGTAGGAGCGGG	0	20	RDR2-sensitive
GACGTGGACGCTTGTGGGAGCCGG	0	9	RDR2-sensitive
GACGTGGACGGCACCGACGACAGC	0	10	RDR2-sensitive
GACGTGGACTGGAGCTACGGTGAG	1	13	RDR2-sensitive
GACGTGGACTGTCCGCGGCACTGG	0	9	RDR2-sensitive
GACGTGGCTATAGCCTGAGGCTGT	1	12	RDR2-sensitive
GACGTGGCTGGCGCACCGGACTGT	0	10	RDR2-sensitive
GACGTGGGACGTAGGTGGACGCGC	0	10	RDR2-sensitive
GACGTGGTACATGAAAGCTCGGTA	30	0	RDR2-resistant
GACGTGGTTGGGCTGACGACGGAA	0	15	RDR2-sensitive
GACGTGGTTTAGGGCTAGACTGGA	0	10	RDR2-sensitive
GACGTGTCGGGCTCGTGCAGGGTT	0	18	RDR2-sensitive
GACGTGTTGAGCTGTAGTGGTGGA	0	10	RDR2-sensitive
GACGTGTTTGACAACTCGACGGCA	0	23	RDR2-sensitive
GACGTGTTTGACGACTCGACGGCA	8	119	RDR2-sensitive
GACGTGTTTGACGACTCGACGGTA	2	41	RDR2-sensitive
GACGTGTTTGATGACTCGACGGTA	0	20	RDR2-sensitive
GACGTTACTGGAGAGCGTAGAGGA	0	11	RDR2-sensitive
GACGTTCGAAGGAGGTAGAGGCAT	0	26	RDR2-sensitive
GACGTTGACTCGGCTGAACTCGGC	0	10	RDR2-sensitive
GACGTTGACTGTCCGGCAAAGCAC	0	10	RDR2-sensitive
GACGTTGCTGCGGATCTGATCGGC	0	9	RDR2-sensitive
GACGTTGCTGGACGCCATAGCGGA	0	9	RDR2-sensitive
GACGTTGCTGGAGACACAGAAGAT	0	24	RDR2-sensitive
GACGTTGCTGGAGAGCATAGAGGC	0	12	RDR2-sensitive
GACGTTGCTGGAGAGCGTAGAGGA	0	27	RDR2-sensitive
GACGTTGCTGGAGAGTGTAGAAGA	0	11	RDR2-sensitive
GACGTTGCTGGAGGGAGTAGAGGA	5	97	RDR2-sensitive
GACGTTGCTGGATGGAGTAGAGGA	0	11	RDR2-sensitive
GACGTTGCTGTGGATCCGATCGGC	0	24	RDR2-sensitive
GACGTTGGAGCTCTTGATGGGCAG	0	19	RDR2-sensitive
GACGTTGTCGCAGTAGACGAGGGT	7	47	RDR2-sensitive
GACGTTGTTGGAGGGGTAGAGGAC	0	32	RDR2-sensitive
GACTAAAGCGGTCAGACGGTGGAG	2	52	RDR2-sensitive
GACTAAGGGACATGTAGCGTCGGA	0	61	RDR2-sensitive
GACTACACAGAACTTGGTGTCGGC	0	18	RDR2-sensitive
GACTACAGAGTGGACAGTTGACGC	0	19	RDR2-sensitive
GACTACGACGAGCAAGCCTGACGC	0	9	RDR2-sensitive

GACTACGCTCTGGGCTGGACGGTC	1	12	RDR2-sensitive
GACTACGGACGTACTAGAAGACGA	0	29	RDR2-sensitive
GACTACTATATCAAGGACTATGGC	1	13	RDR2-sensitive
GACTACTGCGTCAAGGACTGCGGC	0	20	RDR2-sensitive
GACTACTGTCGTCCGACGGCCCAC	58	3	RDR2-resistant
GACTACTTATCGTCTATTGTTGGA	0	11	RDR2-sensitive
GACTAGACTGAGACGGAGGACGAG	0	27	RDR2-sensitive
GACTAGATCTGAGGGACTGACGGG	0	20	RDR2-sensitive
GACTAGATGTAGAGACGTTTTGCT	0	17	RDR2-sensitive
GACTAGATTTAGGACGGTCCGCGA	0	10	RDR2-sensitive
GACTAGCGGGCTTGACAGATCGGA	6	43	RDR2-sensitive
GACTAGTCGACTGATCGGTGGCAA	0	42	RDR2-sensitive
GACTAGTTGTTCTGAAAGACGCAT	0	20	RDR2-sensitive
GACTATCGTGTAGGACAGCTCGGC	0	14	RDR2-sensitive
GACTATGCGTCGGCTGTATTGGTA	21	1	RDR2-resistant
GACTATGCGTCGGTTGTATTGGTA	54	0	RDR2-resistant
GACTATTAGCACCAGGATCGTCGC	0	9	RDR2-sensitive
GACTATTTTCGTCGGCCGGCCCAC	0	21	RDR2-sensitive
GACTCAAAAGTAGGACTAAGTCGG	0	18	RDR2-sensitive
GACTCAACGTTTAGACTCGCTCGG	1	13	RDR2-sensitive
GACTCACAAGATCGTTCCGGTGGC	0	21	RDR2-sensitive
GACTCACTAGGACTCGAGCGGTAG	0	11	RDR2-sensitive
GACTCAGAGGTGTAGAGAAGGCAT	0	15	RDR2-sensitive
GACTCAGAGGTGTAGAGACGACAT	2	16	RDR2-sensitive
GACTCAGTGACGTAGTGACGTGTG	1	12	RDR2-sensitive
GACTCATCGGGCTCCCGACACGGC	0	16	RDR2-sensitive
GACTCCACGGATGGCGAACGGCGT	0	9	RDR2-sensitive
GACTCCACGTCGTCTGGGGACGCC	0	10	RDR2-sensitive
GACTCCCTGTTTGACGTGTCGGAT	0	13	RDR2-sensitive
GACTCCGATGGGAACCGACGGACA	0	11	RDR2-sensitive
GACTCCTCCAACGCGGTCGTCGAC	0	11	RDR2-sensitive
GACTCCTCTGCGTACGTTCGGACA	0	21	RDR2-sensitive
GACTCGACGAGACGGTAACATCAC	0	10	RDR2-sensitive
GACTCGACGGCAAACGATGAGGTC	0	12	RDR2-sensitive
GACTCGACTGTCCGGACACGGTGC	5	57	RDR2-sensitive
GACTCGCCAGAACGACGCGCCGAT	1	14	RDR2-sensitive
GACTCGCGTAGGACAGTTCGGCTA	0	15	RDR2-sensitive
GACTCGCTCTAGGGGTCGTCGGAT	1	15	RDR2-sensitive
GACTCGGACCACGACCTAGATGGC	0	15	RDR2-sensitive
GACTCGGACCGGCTTGATGTTCAG	0	16	RDR2-sensitive
GACTCGGACGGTCCACGATGGCAT	1	17	RDR2-sensitive
GACTCGGACTAAACTTTAGCAGGA	0	17	RDR2-sensitive
GACTCGGATTAGCTAGCAGGTCGT	0	11	RDR2-sensitive
GACTCGGCTACGACGGTGACTCGG	0	11	RDR2-sensitive
GACTCGGCTCGTTAGTAGCTCGGC	0	9	RDR2-sensitive
GACTCGGGCGATTGATTCCGGCTA	0	9	RDR2-sensitive
GACTCGGTGATTCGCAGACTCGGC	0	15	RDR2-sensitive
GACTCGGTGGACTGGACTGATGGG	0	9	RDR2-sensitive
GACTCGTAGATGCAAGGACTGGAT	0	13	RDR2-sensitive

GACTCTAGAGCGTATTGGACGGTT	0	13	RDR2-sensitive
GACTCTCCAAGACGGACAGAACAT	1	63	RDR2-sensitive
GACTCTCGGCAACGGATATCTCGG	62	11	RDR2-resistant
GACTCTCGGGCTCTCGGCAACAAG	0	13	RDR2-sensitive
GACTCTCTGGTAGACCTAGGCATT	0	12	RDR2-sensitive
GACTCTCTGTTTGACGTGTCGGAT	0	11	RDR2-sensitive
GACTCTGGACTGTTCAGGCGCTAC	0	14	RDR2-sensitive
GACTCTGTAGCAAAGCACGGACAT	0	40	RDR2-sensitive
GACTCTGTAGCAACGCACGAGCAT	0	12	RDR2-sensitive
GACTCTGTAGCAACGCACGGGCAT	0	33	RDR2-sensitive
GACTCTGTAGCAACGCACGGGTAT	0	37	RDR2-sensitive
GACTCTGTAGCAACGTACGGGCAT	0	46	RDR2-sensitive
GACTCTGTCAGGAACGGAACTGGG	0	20	RDR2-sensitive
GACTCTGTGGAACCGACGTAGCAC	0	14	RDR2-sensitive
GACTCTGTTGAATTCGGTGTGTCA	0	9	RDR2-sensitive
GACTCTTGGATGAGGCACTGATGG	18	1	RDR2-resistant
GACTCTTGGCATCGACGGCCTTTA	0	16	RDR2-sensitive
GACTCTTGGCGTGACTCGGCGACC	8	56	RDR2-sensitive
GACTCTTGGTATCGACGGCCTTTA	0	71	RDR2-sensitive
GACTGAAAAGCTGAGCGTTTGGCA	2	14	RDR2-sensitive
GACTGAAACGTAGGATCGGACGGC	0	12	RDR2-sensitive
GACTGAACCAGCACAGACGGACAC	0	36	RDR2-sensitive
GACTGAAGGGGAGTAGACGTGGCA	0	25	RDR2-sensitive
GACTGACAACAGGAGCGAGAGCAG	0	11	RDR2-sensitive
GACTGACACGTAGGAGGCGCTGTA	0	14	RDR2-sensitive
GACTGACGAAGAGCGTTGATGCCA	0	12	RDR2-sensitive
GACTGACGCGACCTGACATGGCAT	1	21	RDR2-sensitive
GACTGACGGTGTAGATTTACCAAA	0	10	RDR2-sensitive
GACTGACTAGCGGAGCAACGACAT	0	11	RDR2-sensitive
GACTGACTGAGGACGAGGCACTAG	0	10	RDR2-sensitive
GACTGACTGGCAGAGGAACAACAT	0	17	RDR2-sensitive
GACTGACTGTATGGCGGGGGGCGC	0	13	RDR2-sensitive
GACTGACTGTGGCGTGGCTAGCCA	0	27	RDR2-sensitive
GACTGAGAACGGAGCTGAGGATGG	0	9	RDR2-sensitive
GACTGAGATCGCAGAATGGCAGAA	0	21	RDR2-sensitive
GACTGAGATTGCAGAACGGTAGGA	0	14	RDR2-sensitive
GACTGAGCCAGACGGATGGTAGAA	0	80	RDR2-sensitive
GACTGAGCCGGCCTAGACAGACAT	0	13	RDR2-sensitive
GACTGAGCGGACGGTCTAGACTAT	0	16	RDR2-sensitive
GACTGAGGCACGTAGAACTGGCGT	0	12	RDR2-sensitive
GACTGAGGGACAGATACCGTCGGA	0	11	RDR2-sensitive
GACTGAGTCAGACAAGTGGTAGAC	0	13	RDR2-sensitive
GACTGATATCCGTCGGATTTCGGA	0	19	RDR2-sensitive
GACTGATCAAGTAGAGTCATGGCC	19	1	RDR2-resistant
GACTGATTCGGAACGGAGGCGGCT	0	9	RDR2-sensitive
GACTGATTCGGACGAAGGAGGGTT	2	18	RDR2-sensitive
GACTGATTGATAGGCTCTCGGCAA	0	9	RDR2-sensitive
GACTGATTGGGACGGAGGAGGCTT	0	16	RDR2-sensitive
GACTGCAACGGCAAACTCTACGTA	0	11	RDR2-sensitive

GACTGCAAGGACTGCGGCAGACAG	4	23	RDR2-sensitive
GACTGCACGGAGCCAACAAAACGG	0	11	RDR2-sensitive
GACTGCAGAGGATTGTGGTATAGA	0	11	RDR2-sensitive
GACTGCCACGTCAGGGAGCTCGGC	2	19	RDR2-sensitive
GACTGCCCATCTAGAGCTTGACGG	0	15	RDR2-sensitive
GACTGCCGGACCGGAGAACGACGG	0	17	RDR2-sensitive
GACTGCGGAGAGAAAAGAGGACGT	15	0	RDR2-resistant
GACTGCGGCGAAGACTTCGAGCGG	0	17	RDR2-sensitive
GACTGCGGCGGCGTTGTGGACGGC	0	25	RDR2-sensitive
GACTGCGGCTGCGGCTTCTACAGT	7	84	RDR2-sensitive
GACTGCGGGCAGTAGCGGCCTTTA	0	11	RDR2-sensitive
GACTGCGGGCTACAGAAGACTGAA	0	12	RDR2-sensitive
GACTGCTAGGAGCCTAGGACGACG	0	10	RDR2-sensitive
GACTGGAACAGGAACGAAGACGTT	0	35	RDR2-sensitive
GACTGGAAGTAGGGACGTTCTGCC	1	42	RDR2-sensitive
GACTGGACAGGAGAGAGAGAGCAG	0	10	RDR2-sensitive
GACTGGACCAGGCAACCAAACATA	0	14	RDR2-sensitive
GACTGGACCGAGAACTTGGCGGAG	0	11	RDR2-sensitive
GACTGGACGGAACCCGGCATGCCA	0	17	RDR2-sensitive
GACTGGACGGTCCGTGACCTCGGC	0	16	RDR2-sensitive
GACTGGACGGTGATGCAAGCTCGT	0	10	RDR2-sensitive
GACTGGACGGTGGACGCACCCGAC	0	11	RDR2-sensitive
GACTGGACGTACCTGGAAGGCGCG	0	13	RDR2-sensitive
GACTGGACGTAGGGACATTCGGCT	0	20	RDR2-sensitive
GACTGGACTGTTCGGGAGTCGGAA	0	29	RDR2-sensitive
GACTGGATCGGCGAGATGGCGAAC	0	10	RDR2-sensitive
GACTGGATGTAGGGACGTTCTCGC	0	17	RDR2-sensitive
GACTGGATTGTTTCGGAGGGTCGG	0	24	RDR2-sensitive
GACTGGCATGTAGATGTGGCCCAG	0	9	RDR2-sensitive
GACTGGCCCACTGTACGCGAGCAT	0	26	RDR2-sensitive
GACTGGCCTGTCTGACTGAGGCAT	1	35	RDR2-sensitive
GACTGGCGCATCTGATGGCTCTAC	0	15	RDR2-sensitive
GACTGGCTACTGTAGTTCACGGGC	1	53	RDR2-sensitive
GACTGGCTCATGCGGCACTGACTT	0	26	RDR2-sensitive
GACTGGCTGATGGAGCAAGGCAAC	15	0	RDR2-resistant
GACTGGGACGAGTAGGAGTACGAC	0	13	RDR2-sensitive
GACTGGGACGGTGTAACACTCCAA	0	32	RDR2-sensitive
GACTGGGAGTAGAGCAGGGCACAG	0	11	RDR2-sensitive
GACTGGGCCCAGTTGACTCGGTGT	0	16	RDR2-sensitive
GACTGGTACGGGCTCGCTCGGCAA	0	15	RDR2-sensitive
GACTGGTAGATGCAAGGACTGGAT	0	9	RDR2-sensitive
GACTGTACTGTGGAAGGAGCCCGC	0	17	RDR2-sensitive
GACTGTCAGGTCCCCTACGGCTCC	0	22	RDR2-sensitive
GACTGTCCATGTCTGGAGCTCGGA	0	11	RDR2-sensitive
GACTGTCGGAGATGCGAGCAGCAG	0	25	RDR2-sensitive
GACTGTCGTCGCTGTTGCGTGCCT	1	14	RDR2-sensitive
GACTGTCTACTGTACGTACTGGCA	1	15	RDR2-sensitive
GACTGTCTCCAGTAGAGCGCGTAA	0	9	RDR2-sensitive
GACTGTCTGACGTAGAGATGGCAA	0	16	RDR2-sensitive

GACTGTGAACGACGCAACGACGAA	0	9	RDR2-sensitive
GACTGTGCTGTCGGGAAGATCTAG	0	12	RDR2-sensitive
GACTGTGGCAGACTGGCTTGGGTA	0	10	RDR2-sensitive
GACTGTGGGGGATGTCGGCTCGAC	0	29	RDR2-sensitive
GACTGTGTCTGTGCGACTGTGCGG	1	12	RDR2-sensitive
GACTGTTCGACGGAGAGAGCCGGA	0	17	RDR2-sensitive
GACTGTTCGGAGAAGGAGGCGCTC	0	18	RDR2-sensitive
GACTGTTGGATGGAGATACGACGG	0	24	RDR2-sensitive
GACTGTTGGATTTCATCTGACGGC	0	12	RDR2-sensitive
GACTGTTGTTGCGGACGCGAGCAT	1	45	RDR2-sensitive
GACTGTTTGGACTGCTGCGGCTGT	0	14	RDR2-sensitive
GACTTACTGATTGTTGGCGCGCGC	3	29	RDR2-sensitive
GACTTATCCTTTGCGGTCGGCGGA	0	15	RDR2-sensitive
GACTTATGGTCGGTCGCTATAGAG	0	12	RDR2-sensitive
GACTTCGTACGGCTACATCGAACA	3	22	RDR2-sensitive
GACTTCGTAGCAACGCACGAGCAT	0	13	RDR2-sensitive
GACTTCTTAGGACGGCAGTAGAGA	0	11	RDR2-sensitive
GACTTGAGCGACTGAGGGAGACCG	0	10	RDR2-sensitive
GACTTGATCAGAGTGGACTGGGCA	0	20	RDR2-sensitive
GACTTGATCTAGGTGGCGTCTCCC	1	17	RDR2-sensitive
GACTTGATTCGGACCGGGCCGGGC	0	16	RDR2-sensitive
GACTTGATTTGATCGATTGTTGGG	0	14	RDR2-sensitive
GACTTGATTTGTTCGATTGTTGGG	4	17	RDR2-sensitive
GACTTGGACGGAACACCGGGTGGC	0	9	RDR2-sensitive
GACTTGGACGTAGGAGTGGCTCCT	0	12	RDR2-sensitive
GACTTGGACTGGGAGGAGGAGGAT	0	13	RDR2-sensitive
GACTTGGTACTGTACTGTGGTGTG	0	9	RDR2-sensitive
GACTTGTCCCATGCGGTCGACGGA	0	61	RDR2-sensitive
GACTTTAAATTGAACTAACGGCAT	21	0	RDR2-resistant
GACTTTCTGCAGGACCTTCGGCGA	0	14	RDR2-sensitive
GACTTTGTAGGTAGCCGTCGGAGA	0	10	RDR2-sensitive
GACTTTTCATTCGTCTGGCCAAGG	108	0	RDR2-resistant
GACTTTTCTGGCAAGTCGGGCGAC	0	17	RDR2-sensitive
GACTTTTGGTGATAAATCGACGGG	14	0	RDR2-resistant
GAGAAACTCCGGTCTGTGACGCAT	0	19	RDR2-sensitive
GAGAAACTCGGGCCTGTGACGCAC	1	15	RDR2-sensitive
GAGAAACTCGGGCCTGTGACGCAT	1	30	RDR2-sensitive
GAGAACAGCGGACTGTGTTGTGGC	0	13	RDR2-sensitive
GAGAACCGTTTAGAGGACGCTGCT	1	15	RDR2-sensitive
GAGAACGCTATGTCCCTCGTCGGG	0	13	RDR2-sensitive
GAGAACGCTGACGTGGCACGTGAG	4	48	RDR2-sensitive
GAGAACGTTGCTGTAGCGAGTAGA	0	9	RDR2-sensitive
GAGAACTGTAGTAGTGACCCGGTG	0	11	RDR2-sensitive
GAGAACTGTGGACGGGTGCAGCAA	1	25	RDR2-sensitive
GAGAAGACGGAACTGTTCAGGCGC	0	9	RDR2-sensitive
GAGAAGACTGTAAAGGACGGATAT	0	85	RDR2-sensitive
GAGAAGCACTGTGGCTCAGATGGA	0	14	RDR2-sensitive
GAGAAGCCCCTGAGCAGCAGTCGG	1	14	RDR2-sensitive
GAGAAGCGGCGCGTTTGGCTCCAG	1	15	RDR2-sensitive

GAGAAGGCTGTGTGAAAGACGGTT	0	15	RDR2-sensitive
GAGAATGGACTGTGCACGGTGAGG	0	12	RDR2-sensitive
GAGAATGGTGGACTCGAACCGGAA	0	11	RDR2-sensitive
GAGAATTAGGCGTCTTGAGGGTAC	0	11	RDR2-sensitive
GAGACAACTGTAGACGGCCCCCGC	1	37	RDR2-sensitive
GAGACAAGGACAGGTGCATGTGGG	1	14	RDR2-sensitive
GAGACACGCCGGACTGTGTAGGCT	0	14	RDR2-sensitive
GAGACACGGGCTGTATGAGCGGAT	0	16	RDR2-sensitive
GAGACACTCGGCATAGACATACAC	1	12	RDR2-sensitive
GAGACAGACGGTAGGCCAGAAAGT	0	11	RDR2-sensitive
GAGACAGAGTTAGACGTGTGGTAG	1	214	RDR2-sensitive
GAGACAGCGGAACGCAGGAGAACC	0	10	RDR2-sensitive
GAGACAGGCTAGCACGGAACTTAA	0	27	RDR2-sensitive
GAGACAGTGTAAGACGGAGACAGA	0	10	RDR2-sensitive
GAGACAGTGTAGGACAGAGACTGA	0	37	RDR2-sensitive
GAGACATGTCGTGCTCGTGACGGA	0	13	RDR2-sensitive
GAGACATGTTGGACTAGCGTGCGT	1	132	RDR2-sensitive
GAGACCCCTCAGGGGCAAAGTCGG	0	41	RDR2-sensitive
GAGACCGTAGAACGGTAGAACGGT	0	18	RDR2-sensitive
GAGACCGTAGGAAGTTAGTGGCTC	1	12	RDR2-sensitive
GAGACCGTGATAGCACCAGACGCC	0	10	RDR2-sensitive
GAGACCTCTCAGGACAAAGTCGGT	0	9	RDR2-sensitive
GAGACGACCCTGGACCTGGAGTGG	0	11	RDR2-sensitive
GAGACGACGGCCTAGACCACTCAC	1	13	RDR2-sensitive
GAGACGATTGGACTCAGGACGGAC	0	32	RDR2-sensitive
GAGACGCAAGCTGTATGAGCGGAT	0	22	RDR2-sensitive
GAGACGCAGGCTGTATGAGCGGAT	1	56	RDR2-sensitive
GAGACGCCCGGCTGTAGGGATGGT	3	16	RDR2-sensitive
GAGACGGAAATCCTCCGGGGTCGG	3	35	RDR2-sensitive
GAGACGGAACACAAGGGAGAACAA	0	10	RDR2-sensitive
GAGACGGAAGTCCCTCGAGGTCGG	0	14	RDR2-sensitive
GAGACGGAGTTAGACGGGTGACAG	0	67	RDR2-sensitive
GAGACGGCCGTAGGAAGTTAGCAT	0	12	RDR2-sensitive
GAGACGGGCATGCACCACGCTGAT	0	15	RDR2-sensitive
GAGACGGGGAGGAGGAACAGAAGA	28	1	RDR2-resistant
GAGACGGTGAGAACACGAGGGCAA	1	18	RDR2-sensitive
GAGACGGTGTAAGACGGAGACTGA	0	16	RDR2-sensitive
GAGACGGTTCATAGGTGCCCTGTT	27	1	RDR2-resistant
GAGACGTAGACTGGATGAGCGGAT	2	183	RDR2-sensitive
GAGACTAGGACGGTATCTGATCGT	0	12	RDR2-sensitive
GAGACTATAGCCTGCTGCAGTTGG	1	17	RDR2-sensitive
GAGACTCAGGATCATCGGGTTCAT	34	7	RDR2-resistant
GAGACTCGACTTTGCGCCGGTGGA	23	2	RDR2-resistant
GAGACTCGAGAGTGGGCAGAACGG	0	9	RDR2-sensitive
GAGACTCGGCATCATATAGGGCGT	0	11	RDR2-sensitive
GAGACTCTAGAGCGTATTGGACGG	1	55	RDR2-sensitive
GAGACTCTAGAGCGTGTTGGACGG	0	15	RDR2-sensitive
GAGACTGACAGCAGGGGACTTCGC	0	13	RDR2-sensitive
GAGACTGAGACCCACTTGTCGGCT	0	27	RDR2-sensitive

GAGACTGAGCCAGACGGATGGTAG	0	44	RDR2-sensitive
GAGACTGAGTCAGACGAGTGGTAG	0	22	RDR2-sensitive
GAGACTGAGTCAGACGGGTGGTAG	0	9	RDR2-sensitive
GAGACTGATCTGCATGCACGTACC	0	15	RDR2-sensitive
GAGACTGCCTGACACTGAAGCGGT	0	18	RDR2-sensitive
GAGACTGCTGGAGAGCGAAACGGT	0	13	RDR2-sensitive
GAGACTGGACCGGCCCGACACGAA	0	10	RDR2-sensitive
GAGACTGGAGGCAGAACACGACGC	0	19	RDR2-sensitive
GAGACTGGAGTAGAGGCCGAAGGT	6	65	RDR2-sensitive
GAGACTGGCCAGCACCTGCACGGC	0	14	RDR2-sensitive
GAGACTGGTCTGATGGCGTCGCTC	0	17	RDR2-sensitive
GAGACTGGTTCTGTGCAGCGGTGT	0	10	RDR2-sensitive
GAGACTGTATCTAGCATAGGGTGG	0	45	RDR2-sensitive
GAGACTGTCTTCAACGGATGACGC	0	34	RDR2-sensitive
GAGACTGTGACTATGGGACGACAT	0	12	RDR2-sensitive
GAGACTGTGAGAGCGTTACCGGAA	0	15	RDR2-sensitive
GAGACTGTGGATGCGGTACGCTAA	0	9	RDR2-sensitive
GAGACTGTTTGGAGCAAGGAGGAG	11	48	RDR2-sensitive
GAGAGAAGGGGACCGACACGTAGG	2	15	RDR2-sensitive
GAGAGAATCTGGCTGTGAGGAGAA	0	11	RDR2-sensitive
GAGAGACTGTTGGGAGCAAGGAGG	250	0	RDR2-resistant
GAGAGAGAAGGGGACCGACACGTA	2	14	RDR2-sensitive
GAGAGAGACTATAGAGTTGGGCGG	1	15	RDR2-sensitive
GAGAGAGGGCGACGGCACTCACGC	0	15	RDR2-sensitive
GAGAGATCACACTGCAAGGGCATA	0	16	RDR2-sensitive
GAGAGATGGAACGGAACATGGCGT	1	23	RDR2-sensitive
GAGAGCAGACAAGTCGGACCTCAA	0	26	RDR2-sensitive
GAGAGCAGGGCTGGAGGTGGGCGA	0	11	RDR2-sensitive
GAGAGCCAGGCTGTACGCGTGCAA	0	19	RDR2-sensitive
GAGAGCCAGGCTGTGCGCGTGCAA	0	15	RDR2-sensitive
GAGAGCCATCGGACTGTTCGGTGA	0	10	RDR2-sensitive
GAGAGCCCCTGTTGGCCGTCGGAC	4	20	RDR2-sensitive
GAGAGCCGGAGTACGTAACTGCAT	0	13	RDR2-sensitive
GAGAGCGAGAGCCTTATGCGTCGG	0	11	RDR2-sensitive
GAGAGCGGAAGTAGAAGCAGCGGA	9	38	RDR2-sensitive
GAGAGCGGGATTGGAACGGAGCGG	0	34	RDR2-sensitive
GAGAGCGGGCTGGACAGCGGCTGG	190	34	RDR2-resistant
GAGAGCTCTAGTAGACACTCGGCA	0	10	RDR2-sensitive
GAGAGCTCTCGGCACAGGGACTGG	1	32	RDR2-sensitive
GAGAGCTCTCGTAGACACTCGGCA	0	9	RDR2-sensitive
GAGAGCTCTCTGTGCGTCACGGAC	0	12	RDR2-sensitive
GAGAGCTGACGTGACAGGGACGAG	0	9	RDR2-sensitive
GAGAGCTGACGTGGCAGGGGCGAG	1	12	RDR2-sensitive
GAGAGCTGAGGATGTGAGGACGGA	0	11	RDR2-sensitive
GAGAGGACGCGGCTCTAGTCGGAT	80	0	RDR2-resistant
GAGAGGAGGAGGAGCACGCTGGGA	0	9	RDR2-sensitive
GAGAGGAGGATGGGTGATGTGGAC	31	0	RDR2-resistant
GAGAGGATCGTGCAACAGGAGGCA	27	0	RDR2-resistant
GAGAGGCAGAACGATGGCACATGT	0	15	RDR2-sensitive

GAGAGGCTCTGCACGGACGGGAAA	0	12	RDR2-sensitive
GAGAGGGAGTCGGTAGAGCGCGGC	1	24	RDR2-sensitive
GAGAGGGGCATCCAGTGATCTGAA	0	19	RDR2-sensitive
GAGAGGTAGGGCTGGTTGGCGAGC	0	17	RDR2-sensitive
GAGAGGTCATGTGGTTTTCGGCGA	0	15	RDR2-sensitive
GAGAGTAGTGGAAGGCGGGCTGGG	59	2	RDR2-resistant
GAGAGTCACGTAGAAGCGAGGTAA	0	13	RDR2-sensitive
GAGAGTCACGTAGGAGCGAGGTAA	0	20	RDR2-sensitive
GAGAGTCACGTAGGGACGAAGTAA	0	9	RDR2-sensitive
GAGAGTCCTTGGTGGCCGTCGGAC	1	13	RDR2-sensitive
GAGAGTCGTGTAGAAACGGGCTAT	0	11	RDR2-sensitive
GAGAGTCTGACTGTGCACGTGCAA	0	23	RDR2-sensitive
GAGAGTCTGGCTGTCCGCGTGCAA	0	11	RDR2-sensitive
GAGAGTGAACAGTAGCGACGACAA	0	20	RDR2-sensitive
GAGAGTGACGTAGGAGCGCGGTAA	0	21	RDR2-sensitive
GAGAGTGGAGGCGAGAGCTGACGT	0	18	RDR2-sensitive
GAGAGTGGGAGGCAGAACCGTGCA	1	32	RDR2-sensitive
GAGATAAGGCTTGACCGTCGGAAA	0	9	RDR2-sensitive
GAGATCAGGCCTTGTTGGACGGGT	0	9	RDR2-sensitive
GAGATCGAACATGTAGCCAGGCAC	1	17	RDR2-sensitive
GAGATCGATTGTAGATCGATGGTT	0	10	RDR2-sensitive
GAGATCGCAGAACGGCAGAACGGC	1	106	RDR2-sensitive
GAGATCGCAGAACGGCAGAACGGT	0	24	RDR2-sensitive
GAGATCGGGCTGTCGCGGACGCGG	0	9	RDR2-sensitive
GAGATCGGGCTTGCAGGACGGGTC	0	9	RDR2-sensitive
GAGATCGTAGAATGGTAGAACGGC	0	12	RDR2-sensitive
GAGATCGTGTCGTGTCGGACCAGA	0	12	RDR2-sensitive
GAGATCTCGTGAAGGGCAGGACAG	0	12	RDR2-sensitive
GAGATCTGGAACAGGCTCGACGAG	0	16	RDR2-sensitive
GAGATGACGCGCTGCAGTTCACGA	0	9	RDR2-sensitive
GAGATGAGACTGGTGTTAGGGCAT	0	9	RDR2-sensitive
GAGATGAGTATGTGGGCTGACGGT	0	21	RDR2-sensitive
GAGATGCAGAGCTGCTTGTCGGAA	0	20	RDR2-sensitive
GAGATGGCGAGAATGACAGATGAC	23	1	RDR2-resistant
GAGATGTAGACTGAATAAGCGGAT	0	9	RDR2-sensitive
GAGATTAAGTCAGACGGATGGTAG	0	18	RDR2-sensitive
GAGATTACCCTCGGGCGACGGAAC	0	45	RDR2-sensitive
GAGATTCCAGTAGACGGTTCCTGA	0	9	RDR2-sensitive
GAGATTGAGGGACGGCAGAACGGC	0	18	RDR2-sensitive
GAGATTGATTGTAGCTATTGGCTC	131	12	RDR2-resistant
GAGATTGCAGAACGGTAGGCTACC	1	31	RDR2-sensitive
GAGATTGCAGGATGGCAGAACGAC	0	13	RDR2-sensitive
GAGATTGCAGGATGGCAGAACGGC	0	16	RDR2-sensitive
GAGATTGCCAGGACGGGACGGGAT	0	10	RDR2-sensitive
GAGATTGTAAACATACAGGTGCTA	101	2	RDR2-resistant
GAGATTGTACAACGGTAGAATGGA	0	14	RDR2-sensitive
GAGATTGTAGAACGGTAGAATGGA	2	39	RDR2-sensitive
GAGATTGTATGATTGAGGCTAGTC	14	0	RDR2-resistant
GAGATTGTCTAACGGCAGAACGGT	0	11	RDR2-sensitive

GAGATTGTGTCTGGAGCGAGCTGG	1	12	RDR2-sensitive
GAGATTTTATATTTTTTCGTCGGG	149	6	RDR2-resistant
GAGCAAAGACAGGGCGTGGACTGC	1	14	RDR2-sensitive
GAGCAAAGCTGGCAGAACGTTGGG	1	16	RDR2-sensitive
GAGCAAAGTAGGCAGGACACTGGA	0	11	RDR2-sensitive
GAGCAAATTGTTCGCATCTCTGGA	0	46	RDR2-sensitive
GAGCAACAGGTAGGACGACACGCA	0	9	RDR2-sensitive
GAGCAACTCTGCGAGACGGACTAG	0	11	RDR2-sensitive
GAGCAACTCTGTGAGGTAGGACGA	0	13	RDR2-sensitive
GAGCAAGCAGGACGCCTGAATCAT	0	28	RDR2-sensitive
GAGCAAGGCTGTGAGGCGGGGCAA	0	10	RDR2-sensitive
GAGCAAGTCTGTGAGACGTGACAG	0	11	RDR2-sensitive
GAGCACACCGCTGTTCTTCGGGCA	0	12	RDR2-sensitive
GAGCACACGTGAAGCCCGTTGGAC	0	17	RDR2-sensitive
GAGCACAGACTGTAGGTTGGAGAC	0	14	RDR2-sensitive
GAGCACAGCGTCGATCTGAACGGC	1	16	RDR2-sensitive
GAGCACAGGACTGTCAGGTGGCAC	2	16	RDR2-sensitive
GAGCACCAGGGACAGTAGGAGGGC	0	31	RDR2-sensitive
GAGCACCGGACTGTCCGATGGCAC	0	33	RDR2-sensitive
GAGCACCTGGTTGTCGGCGAAGTT	0	9	RDR2-sensitive
GAGCACGGACGAGCAGAGAGACAG	0	9	RDR2-sensitive
GAGCACGGACTGGACCGGCCCGAC	1	16	RDR2-sensitive
GAGCAGAACTGGGCCTGATGTTGT	0	36	RDR2-sensitive
GAGCAGAGGCTACGGAACGCGCAG	0	14	RDR2-sensitive
GAGCAGATGTAGTACAACGTGCGC	1	24	RDR2-sensitive
GAGCAGATTGGTCGCATCTCCAGA	0	16	RDR2-sensitive
GAGCAGCAACGTCGGCTACACTGG	0	10	RDR2-sensitive
GAGCAGCAGTCGGTTGGTGGAGGC	0	53	RDR2-sensitive
GAGCAGCGACGCGAGATCTCCGAT	0	9	RDR2-sensitive
GAGCAGCGGGCAGACGAACAGGGC	0	24	RDR2-sensitive
GAGCAGCTGGGTTGGAGGAATCAA	23	3	RDR2-resistant
GAGCAGGAACGTCGACGACAGGAG	0	14	RDR2-sensitive
GAGCAGGACATTGGCGTTCTCGAT	0	45	RDR2-sensitive
GAGCAGGACGGGAGGGAACGACGT	0	9	RDR2-sensitive
GAGCAGGACGTGCATGGATCTCAA	0	9	RDR2-sensitive
GAGCAGGACTCTCGGCACAGAAAT	0	10	RDR2-sensitive
GAGCAGGACTCTCGGCACAGACTC	0	10	RDR2-sensitive
GAGCAGGACTGGATCCCACGTGCA	0	51	RDR2-sensitive
GAGCAGGACTGGGTCCCACGTGCA	1	14	RDR2-sensitive
GAGCAGGCTGGGAACGGAGCGGCT	0	9	RDR2-sensitive
GAGCAGGCTGTGAAGAGGATCATC	11	29	RDR2-sensitive
GAGCAGGGGAGAGCGGGCTGGACA	37	7	RDR2-resistant
GAGCAGTATTATCTCGGCACCTGT	0	46	RDR2-sensitive
GAGCAGTCGCGATACTCGGATCGG	0	10	RDR2-sensitive
GAGCAGTCTATCTCTGAGCGACGC	1	15	RDR2-sensitive
GAGCATAGTGTAGACCATTTTCGT	0	15	RDR2-sensitive
GAGCATATTTAGAAGTATCTGGTT	26	0	RDR2-resistant
GAGCATCGGTGTGGAAGACGACTG	0	67	RDR2-sensitive
GAGCATCGGTGTGGAGGACGACTG	0	18	RDR2-sensitive

GAGCATCGTTGGACAATGGTTATG	30	2	RDR2-resistant
GAGCATGTAGACGCCACCGACATT	0	25	RDR2-sensitive
GAGCCAACGGATGACACTCGGCAA	0	12	RDR2-sensitive
GAGCCAACGGTCGGAAGGAAGCAC	1	18	RDR2-sensitive
GAGCCAACGGTCGGAAGGGGGCAC	4	38	RDR2-sensitive
GAGCCAACTGGTAGATAGATGCGC	0	14	RDR2-sensitive
GAGCCACAGCCAGTAGAAGACTCT	0	9	RDR2-sensitive
GAGCCACTCCTGTGTCTAGATCGT	0	10	RDR2-sensitive
GAGCCATACCGTGACTGAGACGTG	0	10	RDR2-sensitive
GAGCCCACGGACTTAGATGGCCGA	0	9	RDR2-sensitive
GAGCCCCCCGCGGTACGGATCGTT	0	9	RDR2-sensitive
GAGCCCGCGGACTTAGACGGTGTG	1	44	RDR2-sensitive
GAGCCCGGTCTGCTTATTCGTCGA	0	11	RDR2-sensitive
GAGCCCGTAGCAGTCGGACGGTGT	1	32	RDR2-sensitive
GAGCCCTGTAGAAGCGTAGCTGGC	1	40	RDR2-sensitive
GAGCCGACTGACTGAGCCGATCGA	0	10	RDR2-sensitive
GAGCCGAGCTGAGATGGTTCTCTG	0	27	RDR2-sensitive
GAGCCGGACCAGGTCCAGACGTCT	1	21	RDR2-sensitive
GAGCCGGACGGTCCACGTGTGCAT	0	14	RDR2-sensitive
GAGCCGGCTCGAGCTCGGAGCGGC	0	13	RDR2-sensitive
GAGCCGGTAAGCTGACGAGGACGC	0	9	RDR2-sensitive
GAGCCTCGGTCAGACGGGAGACGT	0	15	RDR2-sensitive
GAGCCTGACTGAGATGAGCCATAA	0	11	RDR2-sensitive
GAGCCTGCACTGAGCCGAGCCGAG	0	12	RDR2-sensitive
GAGCCTGGACAGTGAGAAACGGAA	0	23	RDR2-sensitive
GAGCCTGGCTGACGACGTGACCGC	3	21	RDR2-sensitive
GAGCCTGGGCAGTTAGGACGCGGC	0	11	RDR2-sensitive
GAGCCTGGGCTGTTGGTTTCGGCG	1	17	RDR2-sensitive
GAGCCTTGCTAGTGCTATCGTCGA	0	15	RDR2-sensitive
GAGCCTTGCTAGTGCTGTCGTCGA	3	134	RDR2-sensitive
GAGCCTTGCTTCCTGGTCGTCGCG	0	9	RDR2-sensitive
GAGCCTTGCTTCTTGGTCGTCGCG	2	24	RDR2-sensitive
GAGCCTTGTCAGACCTGTGGCGTA	0	13	RDR2-sensitive
GAGCGACACAGGAGGCCAAGGATA	23	0	RDR2-resistant
GAGCGACGACGAGGACACGGGCAT	1	21	RDR2-sensitive
GAGCGACGGATAGAGTTGGTGGAC	0	10	RDR2-sensitive
GAGCGAGAGGACGGTCAAGGACAG	0	10	RDR2-sensitive
GAGCGAGATCCAGGCGCCGTCGGC	0	14	RDR2-sensitive
GAGCGAGATCCAGGCGCCGTCGGT	0	9	RDR2-sensitive
GAGCGAGGGACTCGGACACTCGGC	0	25	RDR2-sensitive
GAGCGAGTTAGCTGATCGGTTCGG	0	13	RDR2-sensitive
GAGCGATCTGTAGAAGCGTGTCAT	1	37	RDR2-sensitive
GAGCGATGTAGGGAGGATGGGCGT	0	25	RDR2-sensitive
GAGCGCAGCGCGGCAGAACATGGC	0	12	RDR2-sensitive
GAGCGCAGGACTCTGCCGAGGGCG	0	11	RDR2-sensitive
GAGCGCATGAACGTCCGACGCTGG	0	28	RDR2-sensitive
GAGCGCCATATTGAGCGGTCGGAC	0	10	RDR2-sensitive
GAGCGCGCTGTCGGGCGGCCACCA	25	4	RDR2-resistant
GAGCGCGGAGGCGAGGATGTCGGG	0	10	RDR2-sensitive

GAGCGCTGTAGGGGGCACCGCTGC	0	10	RDR2-sensitive
GAGCGGAACAACTACGGTCGGAAA	0	30	RDR2-sensitive
GAGCGGAACGACGGTAGAACACAC	0	9	RDR2-sensitive
GAGCGGAGCACGTAGGGCAGAGGC	0	9	RDR2-sensitive
GAGCGGAGCTGAGGACAAGTCGGC	15	0	RDR2-resistant
GAGCGGATGATGGTGCGTGATCGT	0	9	RDR2-sensitive
GAGCGGATGGACGTCAAACAGGAC	0	15	RDR2-sensitive
GAGCGGGATGAGAACGGAGCGGCT	0	31	RDR2-sensitive
GAGCGGGATGGGAACAGAGCGGCT	0	15	RDR2-sensitive
GAGCGGGATGGGAACGGAGCGGCT	4	93	RDR2-sensitive
GAGCGGGATGTTGTCGGTGCGGAG	0	13	RDR2-sensitive
GAGCGGGATTGGAACGGAGCGGCT	3	117	RDR2-sensitive
GAGCGGGCTAGGAACAGAGCGGCT	0	15	RDR2-sensitive
GAGCGGGCTGAGAACGGAGCGGCT	2	16	RDR2-sensitive
GAGCGGGCTGAGAATGGAGCGGCT	0	9	RDR2-sensitive
GAGCGGGCTGGACAGCGGCTGGGA	1469	245	RDR2-resistant
GAGCGGGCTGGACAGCGGCTGGGG	145	31	RDR2-resistant
GAGCGGGCTGGACAGCGGCTGGGT	24	1	RDR2-resistant
GAGCGGGCTGGGAACGAAGCGGCT	0	16	RDR2-sensitive
GAGCGGGCTGGGAACGGAGCGGCT	5	130	RDR2-sensitive
GAGCGGGCTGGGAACGGAGTGGCT	1	20	RDR2-sensitive
GAGCGGGCTGGGCAGCGACTGGGA	39	0	RDR2-resistant
GAGCGGGCTGTTTGGGCGCACAAC	0	13	RDR2-sensitive
GAGCGGGTTGAGAACGAAGCGGCT	0	35	RDR2-sensitive
GAGCGGGTTGGGAACGGAGCGGCT	0	9	RDR2-sensitive
GAGCGGTACTTCGGACAGACGCGA	0	15	RDR2-sensitive
GAGCGGTGATTGAGCTCACGACGG	0	9	RDR2-sensitive
GAGCGTACGCCGGCATGCAGGCTC	0	9	RDR2-sensitive
GAGCGTAGTCTGAATGTACGAGCA	0	10	RDR2-sensitive
GAGCGTCCGACGGCTCAGCAACTC	0	11	RDR2-sensitive
GAGCGTCGACGGCGTGCTGTTCAC	1	13	RDR2-sensitive
GAGCGTGACCGACGAGCTAAGGCT	0	11	RDR2-sensitive
GAGCGTGACGACTGGGCAGTGGAC	0	11	RDR2-sensitive
GAGCGTGACTGCATGAGGAGGGTG	0	9	RDR2-sensitive
GAGCGTGACTGTGCGAGCGGGCTG	0	11	RDR2-sensitive
GAGCGTGGATCAAGCACTGGACGA	0	9	RDR2-sensitive
GAGCGTTCGCGGGGACGAGGACGG	0	15	RDR2-sensitive
GAGCGTTCGCGGGGACGGGGACGG	0	17	RDR2-sensitive
GAGCGTTGGGTAGAGAGCGAGGTA	0	61	RDR2-sensitive
GAGCTACAGTTACGGTCTGCAGGA	0	9	RDR2-sensitive
GAGCTAGACGCCACGTAGACACTA	0	19	RDR2-sensitive
GAGCTAGCTCTGACAGTAGACGTT	0	17	RDR2-sensitive
GAGCTAGCTTGGATCGTCGACACA	0	15	RDR2-sensitive
GAGCTCAGTCCTGCTGTAGATGGT	0	20	RDR2-sensitive
GAGCTCATTGGACTGGCCCGGGCA	1	40	RDR2-sensitive
GAGCTCCCACGGTCGTCGCGAAGC	0	12	RDR2-sensitive
GAGCTCCTCGTTGGACATGGCGTC	0	21	RDR2-sensitive
GAGCTCCTGCACCAGGTCGCGGAC	8	271	RDR2-sensitive
GAGCTCGACGTCAGGATTTATGGC	0	17	RDR2-sensitive

GAGCTCGGACAGGAAAACGTGCCT	0	10	RDR2-sensitive
GAGCTCGGACAGGAAACTAGGCAT	0	17	RDR2-sensitive
GAGCTCGGGTTCCCTGCCTGACGG	4	19	RDR2-sensitive
GAGCTCGTTTCTGTGGAGCCAGGC	1	47	RDR2-sensitive
GAGCTCTCGGCACAGGGACTGGCG	0	12	RDR2-sensitive
GAGCTCTCTGCGGCAGTGCGGACG	0	19	RDR2-sensitive
GAGCTCTGGGTTGTGCGGCTGTGT	0	9	RDR2-sensitive
GAGCTCTGTTGGAACGGTAAACAA	0	20	RDR2-sensitive
GAGCTGACCACGAACCGAGCGAGC	0	10	RDR2-sensitive
GAGCTGACCATGAAACGAGCGAGC	0	20	RDR2-sensitive
GAGCTGACCATGAACCAAGCGAGC	0	16	RDR2-sensitive
GAGCTGACCATGAACCGAGCGAGA	0	13	RDR2-sensitive
GAGCTGACCATGAACCGAGCGAGC	4	94	RDR2-sensitive
GAGCTGACCCTGAAACGAGCGAGC	0	14	RDR2-sensitive
GAGCTGACGCGGCGTCGACGTGGC	0	10	RDR2-sensitive
GAGCTGACTATGAACCGAGCGAGC	0	23	RDR2-sensitive
GAGCTGACTTGGACTCGCAGCAGA	0	21	RDR2-sensitive
GAGCTGAGAGTTGGACGAATCGGA	0	9	RDR2-sensitive
GAGCTGAGGATGTGAGGACGGAAG	0	19	RDR2-sensitive
GAGCTGAGTCCTGCTGTAGATGGT	0	19	RDR2-sensitive
GAGCTGAGTCTTGGCTGTACACAG	0	22	RDR2-sensitive
GAGCTGGACGGCGTTGTTGACTGC	0	22	RDR2-sensitive
GAGCTGGAGACGCGCTGGAGCTGC	0	9	RDR2-sensitive
GAGCTGGAGCTCTGGACATGGCAG	0	13	RDR2-sensitive
GAGCTGGCGAATCTGACTGGCAGT	17	0	RDR2-resistant
GAGCTGGCTCAAACTCGAAGCGGT	0	12	RDR2-sensitive
GAGCTGGGCAGAGCAGAGGGACAA	1	14	RDR2-sensitive
GAGCTGGTCGACCGTTGGAGGCTT	0	9	RDR2-sensitive
GAGCTGTACGGGCATCAACGCTAT	0	14	RDR2-sensitive
GAGCTGTGCTTGGACCTGAGTCGG	0	25	RDR2-sensitive
GAGCTGTGGAATGCGGATACTACC	0	45	RDR2-sensitive
GAGCTGTGTCTCAGAACCGACGTC	0	10	RDR2-sensitive
GAGCTGTGTGACGTGGCAGTCTGC	0	11	RDR2-sensitive
GAGCTGTGTTACATCGACGCCATA	0	9	RDR2-sensitive
GAGCTGTGTTACCTCGGCGCCATA	0	9	RDR2-sensitive
GAGCTTAGCCCACTGGACACGGAG	0	12	RDR2-sensitive
GAGCTTATCGGAGGACTGACCGGT	23	0	RDR2-resistant
GAGCTTGACTCTAGTCCGACTTTG	58	2	RDR2-resistant
GAGCTTGGACTGCGGCGTAGGCCT	5	66	RDR2-sensitive
GAGCTTGGGTCCTCTGCCTGACGG	0	18	RDR2-sensitive
GAGGAAACCGTAGCTCGGGGACGG	0	11	RDR2-sensitive
GAGGAACACGGTACGTCTGTTAAA	0	11	RDR2-sensitive
GAGGAACACTGAACGCCGCGACGA	0	12	RDR2-sensitive
GAGGAACAGAAGACAAGCGCTCAA	0	39	RDR2-sensitive
GAGGAACCACAGTGTAGACATCAT	0	12	RDR2-sensitive
GAGGAACCAGGCTCGGCTCGGCTC	2	14	RDR2-sensitive
GAGGAACCGAACCGAACCGAAGAA	0	26	RDR2-sensitive
GAGGAACCGGAAGCGGAACGTGAA	0	9	RDR2-sensitive
GAGGAACGGGAACGAGGAACACGG	1	26	RDR2-sensitive

GAGGAACGGGAACGAGGAACGCGG	0	11	RDR2-sensitive
GAGGAACGGGAACGTGGTACGCGG	0	21	RDR2-sensitive
GAGGAACGGTGGCTGCATGAGTGG	0	10	RDR2-sensitive
GAGGAACGGTGTCTGCATGAGTTG	0	21	RDR2-sensitive
GAGGAACTCGCAGGAGAAGCCCCT	0	12	RDR2-sensitive
GAGGAACTCTAGAATGCTCGCGGT	0	10	RDR2-sensitive
GAGGAACTCTATGGTCGCAGCGGT	0	25	RDR2-sensitive
GAGGAACTGGCAGAGACAACGGGT	0	10	RDR2-sensitive
GAGGAAGGACGAAGGAGAAGGAGC	0	9	RDR2-sensitive
GAGGAAGTTGGGTTCGACGGTGAA	0	12	RDR2-sensitive
GAGGAATAACAGCGTAGACGGTTC	0	17	RDR2-sensitive
GAGGAATGGATTGGGAAGGGACGG	0	11	RDR2-sensitive
GAGGAATGGATTGGGGAGGGATGG	0	13	RDR2-sensitive
GAGGAATGGATTGTGGAGGGATGG	0	12	RDR2-sensitive
GAGGACAACAGTGACACACAGAAG	0	13	RDR2-sensitive
GAGGACAACAGTGGCACACAGAAG	0	19	RDR2-sensitive
GAGGACAACAGTGGCATACAGAAG	0	22	RDR2-sensitive
GAGGACAACGACAAGGACGAGCTG	0	13	RDR2-sensitive
GAGGACACCACCTAGAGACGACAA	0	11	RDR2-sensitive
GAGGACACCAGAAGACGTCTCTGT	1	15	RDR2-sensitive
GAGGACACCAGGGGACGTCTCTGT	0	10	RDR2-sensitive
GAGGACAGACACAGTAGGCGCCCT	0	15	RDR2-sensitive
GAGGACAGACTCCGGGAGACCCGT	0	23	RDR2-sensitive
GAGGACAGGGAGACACTCGGCGGC	1	13	RDR2-sensitive
GAGGACAGGGAGGCGCTCGACGGC	5	27	RDR2-sensitive
GAGGACAGGGAGGCGCTCGGCGGC	8	45	RDR2-sensitive
GAGGACAGTAGAAGACGACAGCTC	0	9	RDR2-sensitive
GAGGACAGTGAGGACTGAGGAGGC	0	18	RDR2-sensitive
GAGGACATAGCTGTGGGCTGGCTT	0	13	RDR2-sensitive
GAGGACATGACAGAGCAGGCCGTG	0	12	RDR2-sensitive
GAGGACATGGACGCGACCATTCAA	0	14	RDR2-sensitive
GAGGACATGGAGTAGCAGAGTGTG	0	9	RDR2-sensitive
GAGGACCAGAGCAGCAGACAGCCC	0	9	RDR2-sensitive
GAGGACCATCATTATCGTGCGGAG	3	92	RDR2-sensitive
GAGGACCATCATTATCGTGCGGAT	0	12	RDR2-sensitive
GAGGACCATTTAGAGGACGTTGCT	0	11	RDR2-sensitive
GAGGACCCCCTCCAGCAACGGACA	0	9	RDR2-sensitive
GAGGACCCGCAGCTGGACGTCCGG	0	9	RDR2-sensitive
GAGGACCCTAGCGACGGGCGACGT	0	9	RDR2-sensitive
GAGGACCGACACGTAGAGGGCGCT	0	37	RDR2-sensitive
GAGGACCGACACGTAGGAGGCGCT	0	11	RDR2-sensitive
GAGGACCGACACGTAGGGGACGCT	0	10	RDR2-sensitive
GAGGACCGACACGTAGGGGGCGCT	2	16	RDR2-sensitive
GAGGACCGACGCGTAGGGGGCACC	0	16	RDR2-sensitive
GAGGACCGAGATAAGACAGACTAG	0	10	RDR2-sensitive
GAGGACCGAGGACATCGACGGAGA	1	31	RDR2-sensitive
GAGGACCGATCTGAACAGGGCATG	0	18	RDR2-sensitive
GAGGACCGTTCAGAGGACGCTGCT	0	14	RDR2-sensitive
GAGGACCGTTTAGAGGACGCTGCT	5	26	RDR2-sensitive

GAGGACCGTTTAGAGGACGTTGCT	24	268	RDR2-sensitive
GAGGACCGTTTAGAGGACGTTGTT	6	46	RDR2-sensitive
GAGGACCTCTGGTGACCGTCGGAT	0	10	RDR2-sensitive
GAGGACCTGCCGACTTGACGGAGA	0	9	RDR2-sensitive
GAGGACCTGCCGGCTTGACGGAGA	0	12	RDR2-sensitive
GAGGACCTTCGGAAGCCGAAGACC	0	9	RDR2-sensitive
GAGGACCTTCGGAGGACGAAGACC	0	9	RDR2-sensitive
GAGGACCTTGTTGGAGGCGTACAG	0	14	RDR2-sensitive
GAGGACCTTGTTGGAGGCGTAGAG	0	32	RDR2-sensitive
GAGGACGACGACGACGAAGACGAT	0	13	RDR2-sensitive
GAGGACGACGCCATACTCTTGATT	0	11	RDR2-sensitive
GAGGACGACGCTGCGGACGGGCTG	0	25	RDR2-sensitive
GAGGACGACTAGAAACGGATCCAG	0	23	RDR2-sensitive
GAGGACGAGGACGTCATCTACTCC	0	11	RDR2-sensitive
GAGGACGAGGCACTAGGGCGGAGC	0	15	RDR2-sensitive
GAGGACGAGGCGTTGGGACGGAGC	0	9	RDR2-sensitive
GAGGACGAGTAGGGATGGCAATGG	0	10	RDR2-sensitive
GAGGACGCAGGATATGACGGATGC	3	107	RDR2-sensitive
GAGGACGCGGCTCTAGTCGGATGG	17	0	RDR2-resistant
GAGGACGCTCCTGAAGACGTAGAG	0	9	RDR2-sensitive
GAGGACGGAATGAGAATGGCAGAA	0	13	RDR2-sensitive
GAGGACGGAATGGGAATGGCAGAA	1	37	RDR2-sensitive
GAGGACGGAGCCAGGCAACGACGC	0	11	RDR2-sensitive
GAGGACGGCACACAGATCTCCGAT	0	10	RDR2-sensitive
GAGGACGGGCTGACGAACAGGCTC	2	21	RDR2-sensitive
GAGGACGGTATAGAGTGCCTTGCT	0	9	RDR2-sensitive
GAGGACGGTGCACGGTTCTGCCGT	0	12	RDR2-sensitive
GAGGACGGTGGAAGGTGGACGGGC	0	10	RDR2-sensitive
GAGGACGGTTTCGATGGACCAGAG	0	10	RDR2-sensitive
GAGGACGTAAAGGACGCCGGCGTC	0	9	RDR2-sensitive
GAGGACGTCATCTACTCACTCCAG	1	21	RDR2-sensitive
GAGGACGTCATCTACTCCCTCCAG	0	17	RDR2-sensitive
GAGGACGTGAAGGACGCCGACGAC	31	0	RDR2-resistant
GAGGACGTGAAGGACGCCGGCGAC	1	13	RDR2-sensitive
GAGGACGTGACGCTGATGCCGGAC	0	9	RDR2-sensitive
GAGGACGTGCAGTACTGCGGTGCG	0	12	RDR2-sensitive
GAGGACGTTGTTGGAGAGGAAGGA	0	10	RDR2-sensitive
GAGGACTACAGAGCGAGAGATCGG	0	19	RDR2-sensitive
GAGGACTAGACCAGTGGACTGCTC	0	21	RDR2-sensitive
GAGGACTAGACTGAGACGGAGGAC	1	26	RDR2-sensitive
GAGGACTAGGCCCATAGGCCGGCT	2	27	RDR2-sensitive
GAGGACTATGACAATGAGGACCGA	1	18	RDR2-sensitive
GAGGACTCCGCAACCAGACTCCGG	0	14	RDR2-sensitive
GAGGACTCGAATTTGAAGGGCGGT	0	10	RDR2-sensitive
GAGGACTGAACCGAGTAGGACGAG	0	12	RDR2-sensitive
GAGGACTGACACGGTGTTAACGTA	0	12	RDR2-sensitive
GAGGACTGACGCGGAGGCCGACGT	0	21	RDR2-sensitive
GAGGACTGCGGCCCAAGTCGTGAA	2	19	RDR2-sensitive
GAGGACTGGACAGAGGTGGATGCC	36	2	RDR2-resistant

GAGGACTGGCGCGTGGTCAACCAC	0	16	RDR2-sensitive
GAGGACTGTCCGGCCTATAGGCGT	0	19	RDR2-sensitive
GAGGACTGTGCGGACGGTGGTTGG	2	58	RDR2-sensitive
GAGGACTTGGATTTGAAGGGCGGT	0	9	RDR2-sensitive
GAGGACTTTAGCGTCGGCCGCTGC	0	11	RDR2-sensitive
GAGGAGAAATCCGTCGATTGCCGG	0	53	RDR2-sensitive
GAGGAGAAGAGACTCATGCCCGAC	81	10	RDR2-resistant
GAGGAGAAGCAGGCTTCTGAAGCT	0	13	RDR2-sensitive
GAGGAGAATCTGGCTGTACGGAAA	0	9	RDR2-sensitive
GAGGAGACCGTAGCAGAGACTGTA	0	12	RDR2-sensitive
GAGGAGACGAAGACATCGAGCGAA	0	13	RDR2-sensitive
GAGGAGACTGTAAAGGACGGATAT	3	194	RDR2-sensitive
GAGGAGACTGTGGATGCAGGTTAT	0	10	RDR2-sensitive
GAGGAGAGAGAACGCTGACGTGGC	0	28	RDR2-sensitive
GAGGAGAGAGAATGTGTCGGCCGC	0	43	RDR2-sensitive
GAGGAGAGATGATGACGACGCCAC	0	9	RDR2-sensitive
GAGGAGATGGTAGACTAATGTTCC	38	2	RDR2-resistant
GAGGAGCAAGTAGAACGGAGTCGT	0	9	RDR2-sensitive
GAGGAGCAGGAACGTCGACGACAG	3	30	RDR2-sensitive
GAGGAGCCAAGTAGAACGGAGCCG	0	23	RDR2-sensitive
GAGGAGCCAAGTAGAACGGAGTCG	0	23	RDR2-sensitive
GAGGAGCCCGTAGAAGACACGCTA	0	9	RDR2-sensitive
GAGGAGCGAGAAGATGATGGACTA	0	21	RDR2-sensitive
GAGGAGCTGACCAGAGCATGCCAA	1	12	RDR2-sensitive
GAGGAGCTGTTTTAAGGGAGGGTG	0	32	RDR2-sensitive
GAGGAGGACCTGTCTGAGCAGAGG	0	12	RDR2-sensitive
GAGGAGGATAGTGACCAGAGCCGG	43	0	RDR2-resistant
GAGGAGGATGAAGCATTCGACGAC	15	0	RDR2-resistant
GAGGAGGCAGAACCGTGCACCACA	0	10	RDR2-sensitive
GAGGAGGCGGTACGTCAACCCTGA	0	28	RDR2-sensitive
GAGGAGGCTGTAGTCGAGCGGCAT	0	10	RDR2-sensitive
GAGGAGGCTGTAGTCGAGTGGCGT	0	14	RDR2-sensitive
GAGGAGGGATGAATAAGGCTGCAG	0	10	RDR2-sensitive
GAGGAGGGCAGAACGGCAGAACAG	0	65	RDR2-sensitive
GAGGAGGGCGGGGTTGATGTTGGA	27	4	RDR2-resistant
GAGGAGGTGGTGGCTATGTTTCTA	26	1	RDR2-resistant
GAGGAGTAAGAAGATGATGGACTA	0	18	RDR2-sensitive
GAGGAGTCAAGTAGAACGGGCTGT	0	9	RDR2-sensitive
GAGGAGTCTAGCTGGAACGGATAT	0	12	RDR2-sensitive
GAGGAGTGAGAAGACGATGGACTA	0	10	RDR2-sensitive
GAGGAGTGAGAAGATGATGGACTA	0	15	RDR2-sensitive
GAGGAGTGAGGAGAATTTGGCTGT	0	37	RDR2-sensitive
GAGGAGTGGTAGAAGGATAGCTAC	0	13	RDR2-sensitive
GAGGAGTGTGACGGCTCGCAAGAG	37	1	RDR2-resistant
GAGGATAACCGACGCGAGCAGCAT	0	23	RDR2-sensitive
GAGGATACCCGATGGACGAAGACG	0	9	RDR2-sensitive
GAGGATACTGTACGGGAAGGTCGG	0	12	RDR2-sensitive
GAGGATAGCGGTGTACTGTAGCCC	0	11	RDR2-sensitive
GAGGATAGTCGACGCAAGCAGCAT	0	20	RDR2-sensitive

GAGGATAGTGACCAGAGCCGGACG	54	0	RDR2-resistant
GAGGATCGATTTGGCACTCGGCAA	0	12	RDR2-sensitive
GAGGATCGTGCAACAGGAGGCATC	133	11	RDR2-resistant
GAGGATCTAGAGGAGCCGTGCCAA	0	14	RDR2-sensitive
GAGGATCTAGAGGAGTCGTGCCAA	0	16	RDR2-sensitive
GAGGATCTCCACTCTGAAGGACGA	1	24	RDR2-sensitive
GAGGATCTGAAGGAGCCGTGCCAA	0	10	RDR2-sensitive
GAGGATCTGGAGGAGCCGTGCCAA	1	14	RDR2-sensitive
GAGGATGAAGACCGAGGTGGGCGT	1	42	RDR2-sensitive
GAGGATGACGGCCTGTCATGACAA	0	13	RDR2-sensitive
GAGGATGAGGATGACGGGTGACAC	0	121	RDR2-sensitive
GAGGATGCTGAACTCCAGCACGGC	0	22	RDR2-sensitive
GAGGATGGAATCGCAATCGGACGG	0	14	RDR2-sensitive
GAGGATGGAGAACCGGAGCGAGGC	0	10	RDR2-sensitive
GAGGATGGCAGAACGGCAGACTAC	0	35	RDR2-sensitive
GAGGATGGCAGAATACGGTGCAAT	0	9	RDR2-sensitive
GAGGATGGCATTGCTGGACCCCGG	0	9	RDR2-sensitive
GAGGATGGGATGGGTATGAGACGC	0	18	RDR2-sensitive
GAGGATGGGTATAGGGATGGATCA	0	9	RDR2-sensitive
GAGGATGGTGGCAGAACCGTGCAC	0	12	RDR2-sensitive
GAGGATGTGCTAGATTCGGCTTCT	22	1	RDR2-resistant
GAGGATTACGCGGCAAAACCTGGC	0	10	RDR2-sensitive
GAGGATTCTCTGGATAGAGGGCGC	0	13	RDR2-sensitive
GAGGATTCTTGCAGCTGACGACGC	0	12	RDR2-sensitive
GAGGATTGTGCGGCAGAACATGGC	0	20	RDR2-sensitive
GAGGATTGTGCGGCAGAACATGGT	0	9	RDR2-sensitive
GAGGATTGTTTAGAGGACGTTGTT	1	12	RDR2-sensitive
GAGGATTTTCCGACTCCGACGGTA	0	17	RDR2-sensitive
GAGGATTTTGACTTGTAGGGGATT	0	11	RDR2-sensitive
GAGGCAAGCAGACATACATGACAC	0	11	RDR2-sensitive
GAGGCACGTAGGACTGTGCAGGCC	0	28	RDR2-sensitive
GAGGCACTAGTTTGAGGCATTGTT	0	12	RDR2-sensitive
GAGGCACTATAGAGGGCACCGCTG	0	11	RDR2-sensitive
GAGGCACTGTTGGAGTGGTTTTTC	0	22	RDR2-sensitive
GAGGCAGAACGATGGCACATGTGC	0	13	RDR2-sensitive
GAGGCAGAATGGTTGCGTGAACTC	0	10	RDR2-sensitive
GAGGCAGAGCTTTCGGCCCGTCGG	1	13	RDR2-sensitive
GAGGCAGAGGACCCAACCTGACGC	0	12	RDR2-sensitive
GAGGCAGTTCGAGTCGGACGGTCA	0	17	RDR2-sensitive
GAGGCATATATAGGACGAGGATGT	0	9	RDR2-sensitive
GAGGCATCACGTAGAGACCGGCAT	0	9	RDR2-sensitive
GAGGCATGTCATAGGTAGAGCGGT	0	10	RDR2-sensitive
GAGGCATGTGGTCCATGGATGGTG	18	1	RDR2-resistant
GAGGCCAGAAGATTCCTGCAGGTT	22	0	RDR2-resistant
GAGGCCATAGACCAGAGGACAGAC	1	22	RDR2-sensitive
GAGGCCCAAACATACTGTCGTCGT	0	13	RDR2-sensitive
GAGGCCCAAGAGAACAAGAGGCAA	0	11	RDR2-sensitive
GAGGCCCGTGAGCTCGGCACGAAG	0	11	RDR2-sensitive
GAGGCGAAGACTGGACGATCGAGT	0	9	RDR2-sensitive

GAGGCGACTGTTGAACCCTTCGGA	2	16	RDR2-sensitive
GAGGCGAGGACTCTCGGACGGAAT	0	15	RDR2-sensitive
GAGGCGCTGTAGGGGCACCGCTGC	1	49	RDR2-sensitive
GAGGCGGCATGCAGGACAGACGGC	2	17	RDR2-sensitive
GAGGCGTTAAGACTGAGATGGCTT	0	20	RDR2-sensitive
GAGGCTACAGTTACAACCGTCGGA	0	14	RDR2-sensitive
GAGGCTAGGCTCGGACGAGGCGTG	1	17	RDR2-sensitive
GAGGCTAGGGTTCCTGTCTGACGG	0	9	RDR2-sensitive
GAGGCTCGGTGGAATAGCAAGACG	0	16	RDR2-sensitive
GAGGCTCTGTGAATAGTTGTTGAC	47	0	RDR2-resistant
GAGGCTGAAACATCGGAGCGACGA	0	11	RDR2-sensitive
GAGGCTGAACTCGGGGGAGGTCGT	0	10	RDR2-sensitive
GAGGCTGAGTCAGACGTGTGGTAG	0	17	RDR2-sensitive
GAGGCTGGACGGTCTGCACTTAGT	0	11	RDR2-sensitive
GAGGCTGGACTCGGGTAAGATCGC	0	9	RDR2-sensitive
GAGGCTGGAGCTCGAACCCGGTAC	0	11	RDR2-sensitive
GAGGCTGGGCTCGGATGAGGTCGC	0	17	RDR2-sensitive
GAGGCTGTAATTGAGGAGGGGCTT	0	13	RDR2-sensitive
GAGGCTGTCAGATCCGACGCGCAT	0	15	RDR2-sensitive
GAGGCTGTGCTGGACGAGGGACGC	0	10	RDR2-sensitive
GAGGCTGTGGATGCGAGACGCTAT	0	11	RDR2-sensitive
GAGGCTGTGGATGCGGGACGCTAA	0	11	RDR2-sensitive
GAGGCTTTGGAAAGTTGGACCGGT	22	0	RDR2-resistant
GAGGGAAGAGTAGAGGATGATGAT	37	2	RDR2-resistant
GAGGGAAGCATGGTACAACTCACC	0	14	RDR2-sensitive
GAGGGAAGGGAGCGGATCTGGTGA	4	20	RDR2-sensitive
GAGGGAATCGGAAAGGCAGAACAC	0	12	RDR2-sensitive
GAGGGACAACAGAGGACAACACTA	1	21	RDR2-sensitive
GAGGGACAACAGAGGACAACGCAG	1	17	RDR2-sensitive
GAGGGACAACAGGGGACAACACAG	18	67	RDR2-sensitive
GAGGGACAACAGGGGACAACACTA	6	24	RDR2-sensitive
GAGGGACAACAGGGGACAACACTG	7	39	RDR2-sensitive
GAGGGACAGACGGACAGCAGCCAG	0	9	RDR2-sensitive
GAGGGACCGTTTGAGAGAGGGAAG	3	16	RDR2-sensitive
GAGGGACGGATCGGGATGGCAGAA	0	11	RDR2-sensitive
GAGGGACTAGCGACATGGTCACAG	0	10	RDR2-sensitive
GAGGGACTATACTGCGTACATCTG	0	33	RDR2-sensitive
GAGGGACTCGGACACTCGGCAAAG	0	12	RDR2-sensitive
GAGGGACTGACGGGTTATTCCGAT	1	15	RDR2-sensitive
GAGGGACTGCACAGCGTACATCTT	15	92	RDR2-sensitive
GAGGGACTGCACGACGTACATCTT	1	12	RDR2-sensitive
GAGGGACTGCACTGCGTACACCTT	2	20	RDR2-sensitive
GAGGGACTGCACTGCGTACATCTA	3	16	RDR2-sensitive
GAGGGACTGCACTGCGTACATCTG	8	60	RDR2-sensitive
GAGGGACTGCACTGCGTACATCTT	9	60	RDR2-sensitive
GAGGGACTGTACTGCGTACACCTG	0	9	RDR2-sensitive
GAGGGACTTTTGTGCAGTGACTAA	20	0	RDR2-resistant
GAGGGAGATGGCAGAACAATGGTG	0	11	RDR2-sensitive
GAGGGATAACTGGGGACAACACGG	2	41	RDR2-sensitive

GAGGGATCTTCAGTGACCGTCGGA	0	35	RDR2-sensitive
GAGGGATGAATAAGGCTGCAGCAT	0	29	RDR2-sensitive
GAGGGCAAGTGAACAGGAGACCAA	0	10	RDR2-sensitive
GAGGGCAGAACGGCAGAACAGCAG	0	17	RDR2-sensitive
GAGGGCAGAACTGTACGCGCGCAC	0	27	RDR2-sensitive
GAGGGCAGCAGTGGCACACAGAAG	0	9	RDR2-sensitive
GAGGGCCCAGAAGAATAGACGACC	2	23	RDR2-sensitive
GAGGGCGCTGCGCAGATCGACGTC	15	0	RDR2-resistant
GAGGGCGCTGTAGGGGGCACCGCT	0	12	RDR2-sensitive
GAGGGCTATTTCCGAGAGCCGGTT	0	11	RDR2-sensitive
GAGGGCTCGGCAACAGTACATCGG	0	24	RDR2-sensitive
GAGGGCTCTCGACGCGAAGCCGTT	0	12	RDR2-sensitive
GAGGGCTCTGGCGATGACGAAGTT	0	13	RDR2-sensitive
GAGGGCTTCCAGACGCGGAGACGC	0	13	RDR2-sensitive
GAGGGGAGTGTAGAGAGCGACGTC	0	19	RDR2-sensitive
GAGGGGCAGCGGCAGCAGAACCAT	0	10	RDR2-sensitive
GAGGGGCTGTAAAAGGTAGGGTTT	1	19	RDR2-sensitive
GAGGGGCTGTAAAAGGTGGGGTTT	0	10	RDR2-sensitive
GAGGGGTCTGCAGGGCGTACCAGA	0	11	RDR2-sensitive
GAGGGTACCCCTAATTATGGTCCC	4	22	RDR2-sensitive
GAGGGTAGACAAGGCGCTGACGTA	0	9	RDR2-sensitive
GAGGGTAGATCTGAGCAAGACGTC	0	16	RDR2-sensitive
GAGGGTAGATTTGAGCAGAGCGTC	0	35	RDR2-sensitive
GAGGGTCAAGTAGAGGCGAGTTGA	0	11	RDR2-sensitive
GAGGGTCATCCTTGTGTCGCGGAC	0	11	RDR2-sensitive
GAGGGTCGACGATCTGTACGACGG	0	11	RDR2-sensitive
GAGGGTCGATGACGGGCGAGCGGT	0	9	RDR2-sensitive
GAGGGTGAGGATGGGTAGAGAGGC	0	9	RDR2-sensitive
GAGGGTGATGCTCGACGAACGGAC	1	46	RDR2-sensitive
GAGGGTGCTGTACATATACGACGT	0	20	RDR2-sensitive
GAGGGTTGCTGACGCTGACGGGAC	0	9	RDR2-sensitive
GAGGGTTGGCGATCTGTACGACGG	0	9	RDR2-sensitive
GAGGTAAACGGGCCGTGTCGGGCT	0	13	RDR2-sensitive
GAGGTAAGTGTGTGACTGATGGTG	0	9	RDR2-sensitive
GAGGTACCCGTCTGAATAGGCTGC	0	12	RDR2-sensitive
GAGGTACGTGATGCGTCGGCACAA	0	27	RDR2-sensitive
GAGGTACTGGACTATAGATGGCAA	0	14	RDR2-sensitive
GAGGTACTGTAAAAGATGGTGTTT	0	10	RDR2-sensitive
GAGGTAGAGCAGTGCTGATGACGT	10	98	RDR2-sensitive
GAGGTAGAGCTAGAGAGGGGACAG	0	11	RDR2-sensitive
GAGGTAGAGCTGTACGGGCATCAA	0	22	RDR2-sensitive
GAGGTAGGGCTGGTTGGCGAGCGG	0	9	RDR2-sensitive
GAGGTAGGTGTAGTTGGGGACGAC	3	31	RDR2-sensitive
GAGGTAGTACACCTGGTCGTTGCA	0	50	RDR2-sensitive
GAGGTAGTCGAACTGCGCGAGCAT	0	19	RDR2-sensitive
GAGGTATCACTTCGGACATGACGG	0	17	RDR2-sensitive
GAGGTATTCGCCGGAGGACGTCGT	1	12	RDR2-sensitive
GAGGTATTGTTCGTTTCTGTCGGA	42	0	RDR2-resistant
GAGGTCAAAGTAGGATGAGGCGGG	0	11	RDR2-sensitive

GAGGTCAAAGTAGGATGAGTCGGA	1	30	RDR2-sensitive
GAGGTCAAAGTAGGATGAGTCGGG	0	23	RDR2-sensitive
GAGGTCAAAGTAGGATGAGTCGGT	0	35	RDR2-sensitive
GAGGTCAAGTTGGGTTTGTCGGGT	0	13	RDR2-sensitive
GAGGTCCACCCATCACCCGTCGGT	0	10	RDR2-sensitive
GAGGTCCTGTAGCTGGCGGGCGCC	219	18	RDR2-resistant
GAGGTCGACGGTTCGAATACTAAC	63	11	RDR2-resistant
GAGGTCGAGGTAGATGTGGGCTAT	0	14	RDR2-sensitive
GAGGTCGCAGCTCATGGACGGACA	0	12	RDR2-sensitive
GAGGTCGGCTTCGGGCAAGGCGTA	0	11	RDR2-sensitive
GAGGTCGGGGCCGAGATTAGACGT	0	10	RDR2-sensitive
GAGGTCGGGTGCTGAGCAGGCGTA	0	10	RDR2-sensitive
GAGGTCGGGTGTTGGGCAGGCGTA	0	9	RDR2-sensitive
GAGGTCGTTGTAGATCCGAGGCTT	0	10	RDR2-sensitive
GAGGTCTATGTAGGATGAGGCGTG	0	17	RDR2-sensitive
GAGGTCTGACTCTTCTACGTGCAC	0	11	RDR2-sensitive
GAGGTCTGGTCGCTGGGACAGGCG	0	13	RDR2-sensitive
GAGGTCTTATTGGAGCGAAGGCGA	0	12	RDR2-sensitive
GAGGTGACGTAGAGGAGGACCGAC	0	9	RDR2-sensitive
GAGGTGACTCTGTGGCTATGAAAG	0	10	RDR2-sensitive
GAGGTGCACTAGAGTAGATATCAT	24	1	RDR2-resistant
GAGGTGCCCGTCTGAATAGGCTGC	1	23	RDR2-sensitive
GAGGTGGCTCTGGACGCGGGACGC	0	29	RDR2-sensitive
GAGGTGTTACAAGGTCGTCGCGTT	0	12	RDR2-sensitive
GAGGTGTTCGCCTGGATCGGAGGC	0	34	RDR2-sensitive
GAGGTTAATGTAGACCACAGGCGT	0	86	RDR2-sensitive
GAGGTTACTGGAGGCTGACGGCGT	0	12	RDR2-sensitive
GAGGTTCGGATGCAGGATCGGATA	0	19	RDR2-sensitive
GAGGTTCGGCAAGCTCGACGACGA	0	66	RDR2-sensitive
GAGGTTCTGTAGCTTGGTGGTGGA	0	9	RDR2-sensitive
GAGGTTGACTGATGAGGACGTCGG	0	22	RDR2-sensitive
GAGGTTGCAGGATGGCAGAACGGC	0	80	RDR2-sensitive
GAGGTTGGTCTGTTGCCCCGGCAT	0	15	RDR2-sensitive
GAGGTTGGTCTTGGACAAGGCGTG	0	85	RDR2-sensitive
GAGGTTGGTGGAGGCCGGCGAGCT	853	59	RDR2-resistant
GAGGTTGTGTAGCAGTGACGACAT	4	87	RDR2-sensitive
GAGGTTGTGTAGTAGTGACGACAT	0	56	RDR2-sensitive
GAGGTTGTTCTCGGACGAGTCGTG	0	12	RDR2-sensitive
GAGGTTTGGAATTCGAATTGAGGC	16	0	RDR2-resistant
GAGGTTTTGGAACTATGAACCGGT	51	12	RDR2-resistant
GAGGTTTTGTAACTATGAACCGGT	22	0	RDR2-resistant
GAGTAAACTTTGGATAGACTGCTG	0	40	RDR2-sensitive
GAGTACTCGGCAGACGACAGCGCT	0	9	RDR2-sensitive
GAGTACTGAGTGACACTCGGCATG	0	9	RDR2-sensitive
GAGTACTGATCGGTCGGTCAGGTT	0	9	RDR2-sensitive
GAGTACTGTGTGAATTACGTGTGG	0	9	RDR2-sensitive
GAGTACTTTTGTCGGATACTCGGC	0	14	RDR2-sensitive
GAGTAGAATGAGTGCGGATAGCCT	0	10	RDR2-sensitive
GAGTAGAGCGACGACGCTGCGGCG	14	0	RDR2-resistant

GAGTAGATCACTGTGGTGCGTGCA	0	9	RDR2-sensitive
GAGTAGGACGGTGCTGTTGACTGT	0	12	RDR2-sensitive
GAGTAGGACGGTGTCAACGACTGT	3	38	RDR2-sensitive
GAGTAGGACGGTGTCATAGATCAT	0	36	RDR2-sensitive
GAGTAGGACGGTGTCGACAACTGT	0	9	RDR2-sensitive
GAGTAGGACGGTGTCGACGACTGT	0	38	RDR2-sensitive
GAGTAGGACGGTGTCGCCGACTGT	2	16	RDR2-sensitive
GAGTAGGACTTTGCCGAGCGCGGC	1	13	RDR2-sensitive
GAGTAGGCTCGGGCCCAAGCGGCT	0	16	RDR2-sensitive
GAGTAGGGCTCGTGCTGAGATCGC	7	39	RDR2-sensitive
GAGTAGTAGGGACTAGCAGATGAT	0	9	RDR2-sensitive
GAGTAGTGGAAGGCGGGCTGGGCA	283	47	RDR2-resistant
GAGTATCGTGTGGATTGAGTGCGG	0	10	RDR2-sensitive
GAGTATGTTGATGGGCAGGAACCT	1	19	RDR2-sensitive
GAGTATTTCTGATCGTTGGATCAA	0	9	RDR2-sensitive
GAGTATTTTTTGGTCGTGCTGGAC	0	15	RDR2-sensitive
GAGTCAAGACGGGATGGGACAGGA	0	21	RDR2-sensitive
GAGTCAAGGCTGAGACGGGACCAT	0	17	RDR2-sensitive
GAGTCAATCTGATCGCAGGGGCGC	0	11	RDR2-sensitive
GAGTCACGGGCGAGAAGAGGACGA	0	10	RDR2-sensitive
GAGTCCGATGGCATACTGGCGAAG	0	12	RDR2-sensitive
GAGTCCGCGTGCTTAGACGGTCTG	0	12	RDR2-sensitive
GAGTCCTGTAGGAGCACAGCTGGC	0	13	RDR2-sensitive
GAGTCGGCTTGAGCTCGGAGCGGC	0	13	RDR2-sensitive
GAGTCGGTAGAGCGCGGCGCGCAC	0	9	RDR2-sensitive
GAGTCGTAGATCTTTGGTGGACGG	0	9	RDR2-sensitive
GAGTCTGTATCTCGGATTTCGGGC	0	27	RDR2-sensitive
GAGTCTTAAATTCCGGCTCGTCGG	0	69	RDR2-sensitive
GAGTCTTCTTCACTACATCGTCGC	0	11	RDR2-sensitive
GAGTCTTTTCGGGCTTGAGCCGGA	0	14	RDR2-sensitive
GAGTGAACTCACGAGACGCACCAA	0	10	RDR2-sensitive
GAGTGAACTGGAACAGAGCGGCTC	5	36	RDR2-sensitive
GAGTGAACTGGAACAGAGCGGCTT	0	14	RDR2-sensitive
GAGTGAAGTAGAACGGATCGGCTC	0	11	RDR2-sensitive
GAGTGACTGAACCAGTGGCAGCTC	0	14	RDR2-sensitive
GAGTGACTGAGTGTGAGTGGGCTG	0	10	RDR2-sensitive
GAGTGAGGACAAAGTGCGCACAAA	2	35	RDR2-sensitive
GAGTGAGTGTGCTGCGTGCCCCAG	0	19	RDR2-sensitive
GAGTGATGGTTTGCCGGACAACAG	0	14	RDR2-sensitive
GAGTGCAACCCTCGTTCGTTGGAA	0	12	RDR2-sensitive
GAGTGCAGGACTTTGCCGAGGACG	0	11	RDR2-sensitive
GAGTGCCCTCTGACGGGAAGTCGG	0	15	RDR2-sensitive
GAGTGCGCGGACTGTCCGGCCATG	0	9	RDR2-sensitive
GAGTGCGGCACTCGGCAAAGCCTT	0	11	RDR2-sensitive
GAGTGCTGGGGTACAACAGACGAC	0	9	RDR2-sensitive
GAGTGCTGTGAATGGAACGGACAT	0	9	RDR2-sensitive
GAGTGCTGTGTGAACGAGCCGGTT	0	11	RDR2-sensitive
GAGTGGAAGACTGATTGACCGGGC	0	9	RDR2-sensitive
GAGTGGACGAAGAGTGATGGCCTG	0	9	RDR2-sensitive

GAGTGGACGGTCTGCTGCTCATGC	0	9	RDR2-sensitive
GAGTGGACGGTGCAGATTGGCTGA	1	12	RDR2-sensitive
GAGTGGAGACTGGGACACACGGAT	0	13	RDR2-sensitive
GAGTGGAGTGATAGAGGACGCTAC	0	9	RDR2-sensitive
GAGTGGAGTTAGAACGGCCTTGGA	0	24	RDR2-sensitive
GAGTGGATGATGGACGAGATGTGC	0	23	RDR2-sensitive
GAGTGGATTGAGGTGGTATAAGGT	0	9	RDR2-sensitive
GAGTGGGAGAGGCTCTGCACGGAC	1	17	RDR2-sensitive
GAGTGGGAGATGCTCTGCACGGAC	0	10	RDR2-sensitive
GAGTGGGCAGAACGGCAGAACGGC	0	11	RDR2-sensitive
GAGTGGTCTGTGGTCCGAGAGGCT	0	16	RDR2-sensitive
GAGTGTAGCACTCGGCAAAGAGAG	0	11	RDR2-sensitive
GAGTGTCAGACGGTCCGAGCCATT	0	10	RDR2-sensitive
GAGTGTCGTACAGGTATTCGGCCA	0	9	RDR2-sensitive
GAGTGTCTTAGACCTGGACCCGGC	0	9	RDR2-sensitive
GAGTGTGACTCTCGGGCTCTCGGC	0	13	RDR2-sensitive
GAGTGTGAGATTGGGCCATCTGTG	34	0	RDR2-resistant
GAGTGTGCTGCGTGCCCCAGCCCA	0	11	RDR2-sensitive
GAGTGTGGAACTCGGCAAAGACTG	0	9	RDR2-sensitive
GAGTGTGGCACTCGACAAAGACGT	0	28	RDR2-sensitive
GAGTGTTGGGTCTTGACTGACGGA	2	14	RDR2-sensitive
GAGTTAATCTGATCGGAGGGGCGC	0	17	RDR2-sensitive
GAGTTACGGACAAGCGGATGCTGC	0	13	RDR2-sensitive
GAGTTAGGGTTAGAGAACGGCGTC	0	12	RDR2-sensitive
GAGTTATCCTGGACTTGTCGGCGT	0	12	RDR2-sensitive
GAGTTATCGTGGACTTGTCGGCGT	0	17	RDR2-sensitive
GAGTTCTAGTCGACTGGACGGGTT	0	15	RDR2-sensitive
GAGTTCTGATGGGATCCGGTGCTT	155	27	RDR2-resistant
GAGTTGATCTGATCGGGAGGCCGC	0	13	RDR2-sensitive
GAGTTGCATACGCCAGGTCGTCAG	0	9	RDR2-sensitive
GAGTTGCGGATGGTCGGATGGCGT	0	10	RDR2-sensitive
GAGTTGGATCTGTTGGTGTGGTGT	0	14	RDR2-sensitive
GAGTTGGGACTGTCGCAACGGCGC	0	19	RDR2-sensitive
GAGTTGGGTGAAGTCGGAGGCTAA	0	10	RDR2-sensitive
GAGTTGTGGGCGATGATTGGCAGC	14	0	RDR2-resistant
GAGTTTCCCCGTCGGTCTGGGCAT	0	10	RDR2-sensitive
GAGTTTCGTTGATTGTTGTCGACA	36	1	RDR2-resistant
GAGTTTGTGAGGTTGATATCGGAT	59	0	RDR2-resistant
GATAAAATCGGGTCGGGTACGGGT	1	12	RDR2-sensitive
GATAAACTGGGCTGTAAGGCGGAC	0	9	RDR2-sensitive
GATAAATGGGTCGGCTCGACGGCG	0	70	RDR2-sensitive
GATAAATTCGGGTCGGGTACGGGT	0	9	RDR2-sensitive
GATAACAGACGTGGGACAGGTGGC	0	29	RDR2-sensitive
GATAACCGGTGGAATCGACGACGC	0	17	RDR2-sensitive
GATAACTCGGACGCGAGAGCGCAT	0	9	RDR2-sensitive
GATAAGAATGGGACTGGGCCATAG	0	9	RDR2-sensitive
GATAAGACACTGCCGTCGGACATA	0	18	RDR2-sensitive
GATAAGACTGGAGTAGAGGCCGAA	0	15	RDR2-sensitive
GATAAGTTTAGATCGTGCCGACGG	48	5	RDR2-resistant

GATAATAACGGACAGGGGAGGGCA	0	17	RDR2-sensitive
GATAATCTGGAACGGAGCGGCTCT	0	17	RDR2-sensitive
GATAATGGCATACTGGTGGGACCG	0	20	RDR2-sensitive
GATACAACGAGACTGCGGACGAGC	0	14	RDR2-sensitive
GATACACAGGGACAAGAACGGGAA	0	9	RDR2-sensitive
GATACAGATTGTTTTAACATGGCT	17	0	RDR2-resistant
GATACCAGCTGTGACGGAACCTCC	0	51	RDR2-sensitive
GATACGACGGACGTATGGATGGCC	0	9	RDR2-sensitive
GATACGCAGCAGACGACACTCTAG	0	11	RDR2-sensitive
GATACGGACAGTAGCGATGACGGG	2	40	RDR2-sensitive
GATACGGACCATTGGGGCGGCTGG	0	9	RDR2-sensitive
GATACGGACGGTACCGATTGACGG	0	13	RDR2-sensitive
GATACGGGTAGGATACGGAATCAC	0	13	RDR2-sensitive
GATACGGTCATACGGCGAACAGAA	0	10	RDR2-sensitive
GATACGGTCGGTTGGAGAGGCGAT	0	11	RDR2-sensitive
GATACGGTCGGTTGGCGAGGCGAT	0	12	RDR2-sensitive
GATACGGTCGGTTGGTAAGGCGAT	2	34	RDR2-sensitive
GATACGGTCGGTTGGTGAGGCGAT	1	18	RDR2-sensitive
GATACGTATGGTCTGTTGGTGGCT	0	20	RDR2-sensitive
GATACGTCCGTAGTTCGTCGTCGA	0	9	RDR2-sensitive
GATACTATGGGCTGAGCTGGTGGC	1	16	RDR2-sensitive
GATACTGAACCGTGGCTTGGACGA	0	58	RDR2-sensitive
GATACTGTAGCCCCTAAACGGTGA	0	13	RDR2-sensitive
GATAGAATGGACGGCTGAGATCGC	1	26	RDR2-sensitive
GATAGACATGTCGGAACAGGTCGG	0	11	RDR2-sensitive
GATAGACCGGACAGGCCCTTGGGC	19	0	RDR2-resistant
GATAGACGGGGACGAGAGAGATGG	73	1	RDR2-resistant
GATAGACTGAGGAGAGGAGGATGG	0	9	RDR2-sensitive
GATAGACTGTAACCTCGCTGACGG	0	10	RDR2-sensitive
GATAGAGGACTGAATGGAGGCGAC	0	16	RDR2-sensitive
GATAGAGGACTGGAGGGAGGCGGG	0	10	RDR2-sensitive
GATAGAGGACTGGATGGAGACGAC	0	9	RDR2-sensitive
GATAGAGGATGGCGAGCAGGACGT	0	17	RDR2-sensitive
GATAGATCATCGGGATCGACGGGC	0	32	RDR2-sensitive
GATAGATGGTAGAACGGCAGGCTA	0	69	RDR2-sensitive
GATAGCAGGACTCTCGGCACAGAA	0	10	RDR2-sensitive
GATAGCGGTGTAGTGAGACGACGT	14	258	RDR2-sensitive
GATAGCTCGGACAGGGAGGCGAGG	0	10	RDR2-sensitive
GATAGCTTTGGCCGTCGGAAGTTA	0	11	RDR2-sensitive
GATAGGACACCTGATCACGGTCAC	0	30	RDR2-sensitive
GATAGGACGGGACGAGATGAGACG	0	15	RDR2-sensitive
GATAGGACGGTATCGCCGGTGCTC	0	28	RDR2-sensitive
GATAGGCAGGCGAGAGCGGGGCGA	0	13	RDR2-sensitive
GATAGGGGCATTCGAGGCGGATTA	44	0	RDR2-resistant
GATAGGGTACTGCCGACGGACATT	0	12	RDR2-sensitive
GATAGGTCTGGACCGTGCCGGCAC	0	33	RDR2-sensitive
GATAGGTTAATGCCGTCGGACATA	1	22	RDR2-sensitive
GATAGTCGTAGAGTAGCGTCGGCG	122	0	RDR2-resistant
GATAGTGAAGACGACGTGGAGCAT	0	28	RDR2-sensitive

GATAGTGACCAGAGCCGGACGGCC	89	0	RDR2-resistant
GATAGTTGTATGGACGGTGTCGGA	0	11	RDR2-sensitive
GATAGTTGTGGGACCGAATCGGTG	1	15	RDR2-sensitive
GATATAGAGGACGTTGCTGGAGAC	0	25	RDR2-sensitive
GATATAGGGCTGGACGGTTCACGA	0	9	RDR2-sensitive
GATATAGTTGGTGGACGGAGGCAG	0	9	RDR2-sensitive
GATATATGAGTCGGCACGACGGTG	0	37	RDR2-sensitive
GATATATGGGTCGGCACGACGGTA	0	15	RDR2-sensitive
GATATATGGGTTGGCACGACGGCG	0	9	RDR2-sensitive
GATATCAGGACTCGACGGCAGCGC	0	10	RDR2-sensitive
GATATCCAGTCGGCCAGGGCGCGC	0	13	RDR2-sensitive
GATATCCTCGTAGGACGGGACGCC	0	10	RDR2-sensitive
GATATCGGAGGAGAGGGCATTCAC	0	11	RDR2-sensitive
GATATCGGGCAGGAGTAGTGGGCA	0	21	RDR2-sensitive
GATATCTGATTACAGTTGGACTAA	0	9	RDR2-sensitive
GATATCTGATTACGATTGGACTAA	0	9	RDR2-sensitive
GATATGAAGGCGGGTATGGAACGA	0	14	RDR2-sensitive
GATATGACACGGCGATATATGGAT	15	0	RDR2-resistant
GATATGACACGGCGATGTATGGAT	14	0	RDR2-resistant
GATATGAGCTGTGGAATGCGGATA	0	51	RDR2-sensitive
GATATGCAGCCTGACTGAGCGGAT	0	9	RDR2-sensitive
GATATGGACCCGGCTGAAAGACGT	5	85	RDR2-sensitive
GATATGGCATCGGACGGCTGGCTT	0	9	RDR2-sensitive
GATATGGGCAATAGGAACGGACGG	0	9	RDR2-sensitive
GATATGGTTGAAGCTCGACGGTAT	0	16	RDR2-sensitive
GATATTATTTGATCGGATATCGGA	2	37	RDR2-sensitive
GATATTCGGGTCGGATACGGATAC	0	9	RDR2-sensitive
GATATTGCATAGCACGGTCGGTAG	0	9	RDR2-sensitive
GATATTGTTTGTGGGTTATTGGCT	14	0	RDR2-resistant
GATATTGTTTTACATCACAGTCGG	0	25	RDR2-sensitive
GATATTTTCTGCGGAATCGTCGTT	64	0	RDR2-resistant
GATATTTTTCGGATTATGTTTGGA	21	0	RDR2-resistant
GATCAATGTTTGACTGTCGCGTGC	81	3	RDR2-resistant
GATCAATTGATTGATTGTAGCGAT	175	8	RDR2-resistant
GATCACATCGGTTCTCTTAGGCAT	0	13	RDR2-sensitive
GATCACATCTTGGCCGGTCGTCGA	0	19	RDR2-sensitive
GATCACTATCATTGTCGGGACAGT	14	0	RDR2-resistant
GATCACTCTGGACCATGCCGTGCT	0	14	RDR2-sensitive
GATCACTTGGGTCGTACGACGTAT	0	10	RDR2-sensitive
GATCAGACCAGGACGCGATATCAG	0	9	RDR2-sensitive
GATCAGACTGTCCACATGGAGGCG	1	15	RDR2-sensitive
GATCAGAGTGGACTGGGCACGCAC	0	13	RDR2-sensitive
GATCAGATTAACTCGGACGATCGT	0	9	RDR2-sensitive
GATCAGCATTGGCGTCGTGACGGT	21	1	RDR2-resistant
GATCAGGCTACGTAGAGGGAGCGG	0	12	RDR2-sensitive
GATCAGGGGCGCGGTTAGACGGTT	2	15	RDR2-sensitive
GATCAGGTTTCTGGAGAGCGTCGT	0	16	RDR2-sensitive
GATCAGTGACGTGGACACGGTTAG	20	1	RDR2-resistant
GATCATGGCTAGACTGGAGGCGAC	2	36	RDR2-sensitive

GATCATGTGGAAGACGACTGACAT	1	36	RDR2-sensitive
GATCATGTTGGACGGGCCGAGCGG	0	13	RDR2-sensitive
GATCCAAAGTCGGGCGGGGCACGG	0	9	RDR2-sensitive
GATCCAGCAGCAACGTCGAGGTAG	0	9	RDR2-sensitive
GATCCAGTTCTTCGTCGTCGGTGC	0	14	RDR2-sensitive
GATCCATATTATTTCGTGTCGGGC	0	14	RDR2-sensitive
GATCCATCTTGGACCTGTGACGGT	0	9	RDR2-sensitive
GATCCCGAGCCACTGTAGCAGCAT	0	14	RDR2-sensitive
GATCCCTTGGGTCGGTAGCTTGCA	22	0	RDR2-resistant
GATCCGACCTGAGGACCGGACGGT	0	11	RDR2-sensitive
GATCCGCGACGAATTCGGCAACCA	0	12	RDR2-sensitive
GATCCGCGATCTAGGGACGGACGG	0	19	RDR2-sensitive
GATCCGCGGCATAGGACTGGACGG	0	18	RDR2-sensitive
GATCCGCGGCTCAGGGATGGACGG	0	11	RDR2-sensitive
GATCCGCGGGAGGTAGGGGCGAGC	0	16	RDR2-sensitive
GATCCGGACCAAGTCTGAAAAGGC	0	9	RDR2-sensitive
GATCCGGACCCGTATAGAATCGGA	0	12	RDR2-sensitive
GATCCGGACTTGCTTGGAGGCGTC	0	10	RDR2-sensitive
GATCCGGAGGATAGAAGACTGGGA	0	10	RDR2-sensitive
GATCCGGAGTCATGTGGACGGAGT	0	12	RDR2-sensitive
GATCCGGATCTGCTTGGAGGCGTC	4	57	RDR2-sensitive
GATCCGGCACGGATTAGAACCGGC	0	13	RDR2-sensitive
GATCCGGGCGGAAGACATTGTCAG	6	25	RDR2-sensitive
GATCCGGTCGGCTGGCGAGGCGAT	1	17	RDR2-sensitive
GATCCGTCGACGGCTGGCACAGGC	0	17	RDR2-sensitive
GATCCGTGTCTAGGGCTTGGACGG	0	17	RDR2-sensitive
GATCCGTTGGACATGGCATGGCCT	48	0	RDR2-resistant
GATCCTACGGACGAGGTCTCGGAT	0	19	RDR2-sensitive
GATCCTACGTCGTGAAGATCGGGC	0	33	RDR2-sensitive
GATCCTCTGCGTCAGACACGGACC	3	55	RDR2-sensitive
GATCCTGACGGAAGACGACACCAT	0	15	RDR2-sensitive
GATCCTGCGATTTGGTCGGTCGGT	2	18	RDR2-sensitive
GATCCTGTTAGTGGCCTCGTCGGA	1	13	RDR2-sensitive
GATCCTTTTGGATTGTTGGTTCGT	67	1	RDR2-resistant
GATCGAACGATCGGACGAACGGAC	0	19	RDR2-sensitive
GATCGAACTGGGACTAGAGGACGA	0	25	RDR2-sensitive
GATCGAAGCGGAGGCAGAACGGGT	0	12	RDR2-sensitive
GATCGAATAGTAGAATAGAGCGGA	0	25	RDR2-sensitive
GATCGACTGCTCTGTCCAAGACGT	0	12	RDR2-sensitive
GATCGAGTCACTCGTGTGGACTGA	0	14	RDR2-sensitive
GATCGAGTCGCTCGAATGGACTGA	0	17	RDR2-sensitive
GATCGAGTCGCTCGTATGGACTGA	1	18	RDR2-sensitive
GATCGATATCAGGACTCGACGGCA	1	28	RDR2-sensitive
GATCGATATCGGGACTCGACGGCA	0	12	RDR2-sensitive
GATCGATGACTGGACACACGGCAC	0	13	RDR2-sensitive
GATCGATGCCCTGAGCTCAGACGG	0	25	RDR2-sensitive
GATCGCCGTCGGATATAAGACGAC	1	17	RDR2-sensitive
GATCGCGTCTTAGCCGGTCGTCGA	0	22	RDR2-sensitive
GATCGGAAACACAGGTGTCGGCGT	0	10	RDR2-sensitive

GATCGGAATGAGGGGATGACGGAT	0	13	RDR2-sensitive
GATCGGAATTCGACCTAGGACGAT	0	11	RDR2-sensitive
GATCGGACAGTACTGCAAGACGCT	0	9	RDR2-sensitive
GATCGGACGGTCCACGATGGCGTA	0	15	RDR2-sensitive
GATCGGATACGCATGGAAACGGAT	0	24	RDR2-sensitive
GATCGGATCAAACCAGGCTACCAA	0	12	RDR2-sensitive
GATCGGATCAGTCGGCAACGGTAT	0	13	RDR2-sensitive
GATCGGATGCACGGCTACCTGCGC	0	37	RDR2-sensitive
GATCGGATTCACCGGTAACGGTAT	3	48	RDR2-sensitive
GATCGGATTCATCGGTAACGGTAT	5	58	RDR2-sensitive
GATCGGCACATGTAGGGATGGCAA	0	9	RDR2-sensitive
GATCGGCACCAGACGAAGACCGGC	3	21	RDR2-sensitive
GATCGGGATCTGTTGTTCGTCGGG	0	11	RDR2-sensitive
GATCGGGCGAGCGGTTGTTTGGCG	0	23	RDR2-sensitive
GATCGGGCTACAGGCACGGCACGT	0	13	RDR2-sensitive
GATCGGGCTGGCCCACGTGCCTAG	0	11	RDR2-sensitive
GATCGGGTCTGAACTGGAGCCGTC	1	25	RDR2-sensitive
GATCGGGTGCTGGACACAGGGCAT	0	18	RDR2-sensitive
GATCGGGTTGTGCCAGCGCAGCAT	0	15	RDR2-sensitive
GATCGGTAGGCTCCAGCATGATGC	0	15	RDR2-sensitive
GATCGGTCTGTGGTTGTATGGTAC	0	18	RDR2-sensitive
GATCGGTTACGGGCTCGGGCCGGC	0	14	RDR2-sensitive
GATCGTAGATACACGTCGGCGCTT	0	9	RDR2-sensitive
GATCGTCTTATCCTTCGTCGTGAT	0	11	RDR2-sensitive
GATCGTTGTCGGGCCTAGGCCGGC	0	39	RDR2-sensitive
GATCTAACCGTTGATCTGTCAGAA	0	9	RDR2-sensitive
GATCTAACTTGTCGGACGGGCCGG	0	13	RDR2-sensitive
GATCTAATCTCGGGACACGCGGAC	1	23	RDR2-sensitive
GATCTACGGGCGGGGAATCGCGGC	0	11	RDR2-sensitive
GATCTACTGGACGACGAGCGCACT	0	12	RDR2-sensitive
GATCTAGAACGGGCTCGACGAGAG	0	9	RDR2-sensitive
GATCTAGCTTGTCGGGCGGAGGTT	0	9	RDR2-sensitive
GATCTATATACCTTGCTGTGAGAC	32	1	RDR2-resistant
GATCTATGACGTCGAGACGAGCGC	0	9	RDR2-sensitive
GATCTCCGGTGATCTCTGCGACGG	0	39	RDR2-sensitive
GATCTCGTCGTCAAGCTGAAGGCC	0	12	RDR2-sensitive
GATCTCTCGACGGCTACGTGACAC	1	12	RDR2-sensitive
GATCTCTGACGGGCTGGTGCTGGC	3	64	RDR2-sensitive
GATCTCTGCGACGGCACGGACGGT	0	16	RDR2-sensitive
GATCTCTGCGAGGACGAGAACGGT	0	19	RDR2-sensitive
GATCTCTGTATTATGAGGGGGTGT	0	11	RDR2-sensitive
GATCTGAACGCATGGATGTGTGTA	0	9	RDR2-sensitive
GATCTGAAGCCGGGTCTGAGCCAG	0	9	RDR2-sensitive
GATCTGAAGTCGGGCGGGCACGGA	0	14	RDR2-sensitive
GATCTGACCTTGCTCGGGCGGTAT	0	19	RDR2-sensitive
GATCTGATCCACTGTATCGTGCTA	1	12	RDR2-sensitive
GATCTGATCTAGGTGGCGTCTCCC	0	10	RDR2-sensitive
GATCTGATCTAGGTGGCGTTTCTC	2	42	RDR2-sensitive
GATCTGATCTATTCGCGGACCATC	0	10	RDR2-sensitive

GATCTGATCTCGCAGCAACGTCGA	0	19	RDR2-sensitive
GATCTGCGACGCAGGATCGGACGT	0	12	RDR2-sensitive
GATCTGGAACAGGCTCGACGAGAG	0	12	RDR2-sensitive
GATCTGGACCCGAAGCTCGGCGTC	0	11	RDR2-sensitive
GATCTGGACCCGCATAGAACCGGA	0	48	RDR2-sensitive
GATCTGGACGCAGGAGCGACGCCT	0	24	RDR2-sensitive
GATCTGGATTGACGTGGCACGTAC	1	19	RDR2-sensitive
GATCTGGCAATCGGACGGTCGAGA	0	11	RDR2-sensitive
GATCTGGCACAGTAGCGGCTCCTC	0	15	RDR2-sensitive
GATCTGGCATCGGACGGCTGGCTT	0	15	RDR2-sensitive
GATCTGGTCGGCGGGTCTCGCGGC	0	12	RDR2-sensitive
GATCTGGTTGGGCTTGGTCGGTAA	0	10	RDR2-sensitive
GATCTGTAGCGCCGAGCTAGACGC	0	9	RDR2-sensitive
GATCTGTAGTTTATAATCGGACGG	0	12	RDR2-sensitive
GATCTGTCATCGGACGACGCACAG	0	25	RDR2-sensitive
GATCTGTCGCCTCTGGGCCGGTAA	0	9	RDR2-sensitive
GATCTGTCGTTTCCTGTTGGCATT	39	6	RDR2-resistant
GATCTGTCTTGTGCGGTCTGTCGA	0	12	RDR2-sensitive
GATCTGTGGAATACAAACAACGGT	0	11	RDR2-sensitive
GATCTGTGGAATACAAATAACGGT	0	12	RDR2-sensitive
GATCTGTGTCTGAATAGGTGTGCT	60	11	RDR2-resistant
GATCTGTTACGTCAATCTGCTGGT	15	0	RDR2-resistant
GATCTTAGGTGGACTGGACAGCAT	0	16	RDR2-sensitive
GATCTTCGTCGGTGTAGCGAGGCG	0	18	RDR2-sensitive
GATCTTCTGACATCGTCGACCCAG	3	25	RDR2-sensitive
GATCTTCTTTGTGGGTCGTGCGGA	1	31	RDR2-sensitive
GATCTTGCATGACTGTTGGGTATA	15	0	RDR2-resistant
GATCTTGCCTTCTGTCGTCGGGCG	0	11	RDR2-sensitive
GATCTTTGTTCGGCTGGCTGGCTG	0	10	RDR2-sensitive
GATGAAAACGGTCGGAAACGGTAT	0	17	RDR2-sensitive
GATGAACCACGTGACGGTACTCTC	0	11	RDR2-sensitive
GATGAACGCGTGGAAGAGGCACTG	0	12	RDR2-sensitive
GATGAACGGTGCAGATTGGCTGAA	0	13	RDR2-sensitive
GATGAACTGGCACGAGCACGACAC	0	17	RDR2-sensitive
GATGAACTGTAGTGTTTGTTGGCT	1	21	RDR2-sensitive
GATGAACTGTTCGAACTGCGGATC	0	9	RDR2-sensitive
GATGAAGACCGGCGACGAAGGCAT	0	12	RDR2-sensitive
GATGAAGACTGACACGACGCCGAC	0	13	RDR2-sensitive
GATGAAGCGGTCGCTGTAGACGGT	1	13	RDR2-sensitive
GATGAAGGACTGCCACACGCGCGC	8	218	RDR2-sensitive
GATGAAGGACTGCCACACGCGCGT	0	52	RDR2-sensitive
GATGAAGGACTGCCACACGTGCGT	0	9	RDR2-sensitive
GATGAAGGACTGCCACATGCGGAC	0	11	RDR2-sensitive
GATGAAGGACTGCCACGCGCGCGC	0	12	RDR2-sensitive
GATGAAGGCGCTGTGGAGAAGACG	0	10	RDR2-sensitive
GATGAATAGTGTGCTAGACTCCGC	0	12	RDR2-sensitive
GATGACAACGGCAGCGACTCGGAC	1	12	RDR2-sensitive
GATGACACACGGAATGACGACGAG	0	12	RDR2-sensitive
GATGACACGCGGAACGACGATGAG	1	28	RDR2-sensitive

GATGACAGACTGATGTGGCACCAA	0	38	RDR2-sensitive
GATGACAGAGCAACGTTGGACGGA	0	12	RDR2-sensitive
GATGACAGTGAGGCTCGACGGCGA	0	9	RDR2-sensitive
GATGACATGGTAGAACACGTCGGA	0	13	RDR2-sensitive
GATGACCAGCTGTAGAGGACACAC	1	21	RDR2-sensitive
GATGACCAGGGAACTCAGCACGGA	0	42	RDR2-sensitive
GATGACCGACATGTAGGGGGCGCT	0	10	RDR2-sensitive
GATGACCGTTTGAAGCCGTCGGAT	0	15	RDR2-sensitive
GATGACCTGTGACTGGCAAACAAC	0	14	RDR2-sensitive
GATGACGAACTTGGACGATGGCAA	0	9	RDR2-sensitive
GATGACGCACGGAACGACGACGAC	0	43	RDR2-sensitive
GATGACGCACGGAACGACGACGAG	1	24	RDR2-sensitive
GATGACGCACGGAATGACGACGAG	1	20	RDR2-sensitive
GATGACGCACGGATCGACGACGAC	2	25	RDR2-sensitive
GATGACGCATGGTACGACGACGAG	0	10	RDR2-sensitive
GATGACGCGCGGAACGACGACGAC	0	12	RDR2-sensitive
GATGACGCGCGGAACGACGACGAG	0	11	RDR2-sensitive
GATGACGCGCGGAATGACGACAAG	1	20	RDR2-sensitive
GATGACGGGCTGCGGGATACCAAA	0	11	RDR2-sensitive
GATGACGTCATATTAGACATCTGT	181	36	RDR2-resistant
GATGACGTGCTGCGGGATACCAAA	0	10	RDR2-sensitive
GATGACTCACCAGAACGACGCGCT	0	14	RDR2-sensitive
GATGACTCGACGGTAAACGCGAAC	1	14	RDR2-sensitive
GATGACTCGCAGAACGACGACGAG	0	9	RDR2-sensitive
GATGACTCGCCAGAACGACACGTT	1	23	RDR2-sensitive
GATGACTCGCCAGAACGACGCGCC	3	92	RDR2-sensitive
GATGACTCGCCAGAACGACGCGCT	0	19	RDR2-sensitive
GATGACTCGCTAGAACGACGCGCC	0	9	RDR2-sensitive
GATGACTCTCCAGAACGACGCGCC	1	27	RDR2-sensitive
GATGACTCTTGGTATCGACGGCCT	0	13	RDR2-sensitive
GATGACTGAGCACACGCGCTGGCA	4	32	RDR2-sensitive
GATGACTGAGCACACGTGCTGGCA	1	12	RDR2-sensitive
GATGACTGCAGATAGGGAGAGACA	0	10	RDR2-sensitive
GATGACTGCATACTTATCCATCGT	1	12	RDR2-sensitive
GATGACTGCGGCGGCGTTGTGGAC	0	11	RDR2-sensitive
GATGACTGGAAGTAGGGACGCTAT	0	13	RDR2-sensitive
GATGACTGGAAGTAGGGACGTTCT	0	29	RDR2-sensitive
GATGACTGGCTCATGCGGCACTGA	1	17	RDR2-sensitive
GATGACTGGGCACACGTGCTGGCA	2	139	RDR2-sensitive
GATGACTGGGCATACGTGCTGGCA	3	26	RDR2-sensitive
GATGACTGGTGACGCATGGTGCAC	0	35	RDR2-sensitive
GATGACTGTCGGAGATGCGAGCAG	0	12	RDR2-sensitive
GATGAGAAGACTGTTGTAGATGGC	21	0	RDR2-resistant
GATGAGACTAGAGTAGAGGCCGAA	1	18	RDR2-sensitive
GATGAGACTGGAGTAGAAGCCGAA	1	23	RDR2-sensitive
GATGAGACTGGAGTAGAGGCCGAA	12	197	RDR2-sensitive
GATGAGACTGGAGTAGAGGCCGCA	0	9	RDR2-sensitive
GATGAGACTGGAGTAGAGGTCGAA	1	12	RDR2-sensitive
GATGAGACTGGAGTAGGAGCCGAC	0	17	RDR2-sensitive

GATGAGCACGTACTCTCCGTCGGC	1	24	RDR2-sensitive
GATGAGCCTTTGCAGAATCTGATG	26	1	RDR2-resistant
GATGAGCTCGTATCGGCCTAGGCA	0	14	RDR2-sensitive
GATGAGCTCTGCCAGCAATCGCAA	0	11	RDR2-sensitive
GATGAGCTGACTGTTGGATAGCGG	0	11	RDR2-sensitive
GATGAGCTGGCACGAGCACGACAC	0	19	RDR2-sensitive
GATGAGGACAACAGTGGCACACAG	0	22	RDR2-sensitive
GATGAGGATGGATATGGGATGCTA	0	12	RDR2-sensitive
GATGAGGATGTGCCGGATTCGGCT	20	0	RDR2-resistant
GATGAGGATGTGCTAGATTCGGCT	58	6	RDR2-resistant
GATGAGTATGTGGGCTGACGGTCT	0	24	RDR2-sensitive
GATGAGTATTTCTGATCGTTGGAT	0	19	RDR2-sensitive
GATGAGTTTGTCGTCGTTCGTACC	24	1	RDR2-resistant
GATGATAGCGGTGTAGTGAGACGA	3	91	RDR2-sensitive
GATGATAGGATGTAATTCGGCTGC	0	11	RDR2-sensitive
GATGATCACGGCAGACAGACAGTG	0	10	RDR2-sensitive
GATGATCAGAGACACAAGGGCAGC	0	21	RDR2-sensitive
GATGATCAGGAAGGAGCGTGGCGT	0	13	RDR2-sensitive
GATGATCATGACTTGAAACGGTGT	0	14	RDR2-sensitive
GATGATCGTTCTTTTTGGCCAACT	22	0	RDR2-resistant
GATGATTTGGCGGCGTCACTTTAT	0	19	RDR2-sensitive
GATGCACAGTAGGACCTTCGGCGA	0	19	RDR2-sensitive
GATGCACCTCTGCGGTCGGGACGA	3	28	RDR2-sensitive
GATGCACTACCGGAATCGACGGCT	0	10	RDR2-sensitive
GATGCACTGATGGTAGAAGAACGT	0	9	RDR2-sensitive
GATGCAGATCCCGGGAGAAGACGT	2	49	RDR2-sensitive
GATGCAGGACGACCGTTGAAACAG	0	20	RDR2-sensitive
GATGCAGTACAAGATCGTCCCGGA	0	9	RDR2-sensitive
GATGCAGTCGGAGATGGACGCGGT	38	7	RDR2-resistant
GATGCATCTTCAAGTTGAATTCGG	4	20	RDR2-sensitive
GATGCCCTACTGTAGACGGTCAGA	0	20	RDR2-sensitive
GATGCCCTACTGTAGACGGTCAGT	0	11	RDR2-sensitive
GATGCCGCGGTGTAGGGATGGGCG	0	24	RDR2-sensitive
GATGCGACAGGGCCTCTGCGGCAT	1	29	RDR2-sensitive
GATGCGACAGGGTAGAAGGGGTAT	0	25	RDR2-sensitive
GATGCGACGCAGCAGGACGACGAC	0	12	RDR2-sensitive
GATGCGACGGTAGATGGCGACGAC	0	9	RDR2-sensitive
GATGCGACTGAACCAGCACAGACG	0	20	RDR2-sensitive
GATGCGATTGTGGAGACCGTGCTG	3	29	RDR2-sensitive
GATGCGCGGGGTAGACAAACGGAC	0	10	RDR2-sensitive
GATGCGCGGGGTAGACAAACGGGC	0	55	RDR2-sensitive
GATGCGGAAGTTTGGCTACTGTAT	0	22	RDR2-sensitive
GATGCGGAGCCTGCTGGAGAGCGC	0	11	RDR2-sensitive
GATGCGGCACAGCAGGACGACGAC	0	26	RDR2-sensitive
GATGCGGCGAGGCCTGAGGTTGGA	18	1	RDR2-resistant
GATGCGGCGCAGCAGGACGACGAC	3	48	RDR2-sensitive
GATGCGGCGTCGGATCCGGTCCAG	1	13	RDR2-sensitive
GATGCGGCTGAACTGGATGACGTC	0	27	RDR2-sensitive
GATGCGGGAGCTGCTGGAGACGTC	0	56	RDR2-sensitive

GATGCGGGAGTTTGGCTACTGTAT	0	21	RDR2-sensitive
GATGCGGGCTGTCAGGAGACGTAA	0	10	RDR2-sensitive
GATGCTCGACGAACGGACGGCTTA	0	15	RDR2-sensitive
GATGCTCGGCTGGGAATCGTGGCT	0	11	RDR2-sensitive
GATGCTCGTAGGACAGAGACCGGC	0	14	RDR2-sensitive
GATGCTCTCTGTGGACACGCGGAC	0	14	RDR2-sensitive
GATGCTCTGGATAGTTTGATTGCA	7	210	RDR2-sensitive
GATGCTGCAGAACTAGGCATTGGG	0	48	RDR2-sensitive
GATGCTTCGAGCCAGGTTGGACTG	0	9	RDR2-sensitive
GATGCTTCGGACTTGGTGCGGCTA	0	10	RDR2-sensitive
GATGCTTTTGGACAGGACGACGTT	0	9	RDR2-sensitive
GATGCTTTTGGGTAGGACGACGTC	0	11	RDR2-sensitive
GATGCTTTTGTGAGTCGGCAACAT	0	9	RDR2-sensitive
GATGGAAAGCTAGAGCAGAGTCAT	0	9	RDR2-sensitive
GATGGAACACAAGAGGTGGGACAA	1	16	RDR2-sensitive
GATGGAACTGTGGGACGCGGTGGA	1	13	RDR2-sensitive
GATGGAAGGCAGAACGGCAGGCTA	0	31	RDR2-sensitive
GATGGAAGTAGAGTCCGGCCAGGT	0	14	RDR2-sensitive
GATGGAATCTGGATGACGTGACGT	0	10	RDR2-sensitive
GATGGACACTGCACAGAAGGACAC	0	14	RDR2-sensitive
GATGGACAGAAGAGGCAGCTCGAC	1	17	RDR2-sensitive
GATGGACAGCGAGAGGAGGAGGAG	0	11	RDR2-sensitive
GATGGACATCTATAACGCCGGCAC	0	10	RDR2-sensitive
GATGGACCAACGGCTGCACATGCA	0	17	RDR2-sensitive
GATGGACCAAGAGCGGTGAAGGAC	0	19	RDR2-sensitive
GATGGACCAATTACACGTAGCCGA	3	72	RDR2-sensitive
GATGGACCACTCACGAACAGAGGC	0	15	RDR2-sensitive
GATGGACCAGCACGGAGGATTCAT	0	15	RDR2-sensitive
GATGGACCAGGTGGGATGGAACAG	0	9	RDR2-sensitive
GATGGACCATGCACCCATGGACGT	0	10	RDR2-sensitive
GATGGACCGGTCTGAAGCATGGAA	0	10	RDR2-sensitive
GATGGACGAACTAAGTCGCGACGA	0	16	RDR2-sensitive
GATGGACGAACTATGTCGCGGCGA	1	12	RDR2-sensitive
GATGGACGAAGAGCGAACGCACGG	0	21	RDR2-sensitive
GATGGACGAGGAAGCAAGCCTGGC	0	9	RDR2-sensitive
GATGGACGATGCAGATTGGCTGAA	1	18	RDR2-sensitive
GATGGACGCAATCGGACAGTCGGG	0	10	RDR2-sensitive
GATGGACGCAATCGGACGGTCGAG	0	23	RDR2-sensitive
GATGGACGCGTCCGGCTCCGGCAT	0	9	RDR2-sensitive
GATGGACGCTCCAGATTGAACGAA	0	21	RDR2-sensitive
GATGGACGGAGAAGCATTCCCCAT	0	9	RDR2-sensitive
GATGGACGGCAGAACGGCAGAACA	0	41	RDR2-sensitive
GATGGACGGTGCAGATTGGCTGAA	0	28	RDR2-sensitive
GATGGACGGTGTGGAGGCGAAGAG	1	15	RDR2-sensitive
GATGGACTAGCACGAGTACGACAT	0	26	RDR2-sensitive
GATGGACTGACCTGTAGAAGGCAA	2	31	RDR2-sensitive
GATGGACTGACGTGGCGTCGACGT	1	20	RDR2-sensitive
GATGGACTGAGACATGGGGACCGA	0	12	RDR2-sensitive
GATGGACTGCGAGCTGTCCCGGTT	0	28	RDR2-sensitive

GATGGACTGCGGGATCGGACGGCT	0	19	RDR2-sensitive
GATGGACTGGCATGAGTACGACAT	0	12	RDR2-sensitive
GATGGACTGGCCCGAAACACGGAA	0	18	RDR2-sensitive
GATGGACTGTAAGGTACGCACCAG	0	12	RDR2-sensitive
GATGGACTTAGGTTATGCCGGCTC	0	13	RDR2-sensitive
GATGGAGAACGATGACGACGGTTC	0	13	RDR2-sensitive
GATGGAGCATGTGACACGAGGCAG	0	13	RDR2-sensitive
GATGGAGGACAGTAGCGCGGTGGT	68	16	RDR2-resistant
GATGGAGGTAGGCGCGTGCACGGA	2	21	RDR2-sensitive
GATGGAGTTGGACGGTGTCATCAG	0	9	RDR2-sensitive
GATGGATAAGTTAGGAGCACGATC	24	0	RDR2-resistant
GATGGATAGTTATCATACCTGCTT	18	1	RDR2-resistant
GATGGATCACGTGGAGCTGCCGGC	0	9	RDR2-sensitive
GATGGATCAGTTGTTGAGGACATT	105	39	RDR2-resistant
GATGGATGTAGATGGACGGTGTAG	0	14	RDR2-sensitive
GATGGATTCGGATCGGATACGCAT	2	25	RDR2-sensitive
GATGGATTCTGCAGGGACAGGCGC	0	10	RDR2-sensitive
GATGGCAAGGAAGATCCGCTCGGA	1	14	RDR2-sensitive
GATGGCAGAACGGCAGAACACACT	0	15	RDR2-sensitive
GATGGCAGAACGGCAGAACAGATA	0	16	RDR2-sensitive
GATGGCAGCGTAGAGGACGACATC	0	15	RDR2-sensitive
GATGGCAGTACGACTCGTTCCAGA	0	12	RDR2-sensitive
GATGGCAGTACGACTCGTTCGAGA	1	39	RDR2-sensitive
GATGGCAGTACGACTCGTTCGAGT	0	14	RDR2-sensitive
GATGGCAGTTAGGCTATGCGACTC	0	10	RDR2-sensitive
GATGGCATGTACTTAGGGCGCTAG	1	25	RDR2-sensitive
GATGGCCAAGCCGACGCTGAATCA	32	0	RDR2-resistant
GATGGCCAGGAACAACAACGACGG	0	13	RDR2-sensitive
GATGGCCCATGTCGGATCGGTCCA	0	9	RDR2-sensitive
GATGGCGACAAGTGACGAAGACAA	0	18	RDR2-sensitive
GATGGCGAGAATGACAGATGACAA	27	3	RDR2-resistant
GATGGCGATTGTGGAACAGGCACA	0	10	RDR2-sensitive
GATGGCGCAGACTGTGGGAGCGGC	2	15	RDR2-sensitive
GATGGCGGGCGGGCGTAGGTAGGC	0	9	RDR2-sensitive
GATGGCTAGGAACAACAACGACGG	1	127	RDR2-sensitive
GATGGCTAGGAACAACGACGACGG	0	14	RDR2-sensitive
GATGGCTAGGTCTGTTAAGAGGTT	0	45	RDR2-sensitive
GATGGCTGCAGCGATTGACTGGAT	0	18	RDR2-sensitive
GATGGCTGGTACGGACGGGAGGAG	0	10	RDR2-sensitive
GATGGCTTTTGCTCTAGACGCCGT	0	14	RDR2-sensitive
GATGGGAATGGCAGAACACGGAAA	0	10	RDR2-sensitive
GATGGGACACACGATACTACGGAC	0	10	RDR2-sensitive
GATGGGACACGCTTGACGAGACGC	0	16	RDR2-sensitive
GATGGGACCAGTGGAAGCGGACGC	18	0	RDR2-resistant
GATGGGACGAGTGGAGGACATCAC	0	11	RDR2-sensitive
GATGGGACGCACGCTGGTACGGAA	0	16	RDR2-sensitive
GATGGGAGCGGCTGGGAGCAGAGG	14	0	RDR2-resistant
GATGGGAGGCAGAACGGCAGAACG	0	10	RDR2-sensitive
GATGGGCAAGGTGGATGGCGGCGC	0	15	RDR2-sensitive

GATGGGCCATGCACACACGGACGC	0	33	RDR2-sensitive
GATGGGCGCGAAGTAGGCCGGCAT	1	23	RDR2-sensitive
GATGGGCGCGAAGTAGGGCGGCAC	0	13	RDR2-sensitive
GATGGGCGTGAAGTAGGCCGGCAC	0	19	RDR2-sensitive
GATGGGCTGCGACACACGCGGATG	0	9	RDR2-sensitive
GATGGGCTGTAAAAGAGGGTGTTT	1	14	RDR2-sensitive
GATGGGCTGTAGAATGGGCCGCAC	0	12	RDR2-sensitive
GATGGGCTGTCACGAACACGGCAT	0	17	RDR2-sensitive
GATGGGCTTGGAATGGTGCTCGGT	0	12	RDR2-sensitive
GATGGGGAGTGTTGCTGGAAACGG	1	61	RDR2-sensitive
GATGGGGTAGACAAAGGATAGCAT	0	19	RDR2-sensitive
GATGGGGTGACCGAACGACGACGG	0	10	RDR2-sensitive
GATGGGGTTGGTAGCGGACGGTGT	0	9	RDR2-sensitive
GATGGGTACGGGTATGGAATGGTA	1	67	RDR2-sensitive
GATGGGTCGAGCGCGAGGGCGAGC	0	9	RDR2-sensitive
GATGGGTCGAGCGCGTGGGCGAGC	9	34	RDR2-sensitive
GATGGGTCGGCCCGAATCACGGAA	0	12	RDR2-sensitive
GATGGGTCGGCGCGAGACGGAACA	1	12	RDR2-sensitive
GATGGGTCGGCGCGAGACGGAGCA	0	32	RDR2-sensitive
GATGGGTCGGCGCGAGACGGAGCG	1	24	RDR2-sensitive
GATGGGTCGTCAGATCACGTCGGA	0	11	RDR2-sensitive
GATGGGTCTGGACCGTGCCGGTAT	0	25	RDR2-sensitive
GATGGGTTCGACGACTCGACGGTA	0	14	RDR2-sensitive
GATGGTACACTACACATGGTCGGA	0	18	RDR2-sensitive
GATGGTAGGACCAGGCGACCACGT	0	15	RDR2-sensitive
GATGGTAGGCTTTGGACGGACGGA	0	10	RDR2-sensitive
GATGGTATTGCGCCAGGTCGCGGA	0	14	RDR2-sensitive
GATGGTCAGTCGGCGAGCTGGCAA	0	14	RDR2-sensitive
GATGGTCCGTGGCTCTGGACCGGA	0	13	RDR2-sensitive
GATGGTCCTTTAACGAGCGTCGGT	0	15	RDR2-sensitive
GATGGTCGTGGATCCCTTGATGGC	88	5	RDR2-resistant
GATGGTCTGGACACGTAGCGGCGC	0	24	RDR2-sensitive
GATGGTCTGTATCGCGTACCGGTT	0	13	RDR2-sensitive
GATGGTCTGTGGACACGGATCCAT	4	26	RDR2-sensitive
GATGGTCTGTGGCTCTAGGCCGGA	0	21	RDR2-sensitive
GATGGTGAACTATGCCTGAGCGGG	15	0	RDR2-resistant
GATGGTGACCTCGACAAGCAGCTT	27	0	RDR2-resistant
GATGGTGACTGTGCGGCCAACTGC	0	13	RDR2-sensitive
GATGGTGAGCTGTGTTACCTCGGC	0	12	RDR2-sensitive
GATGGTGAGCTTTGGGCAGACTGG	0	29	RDR2-sensitive
GATGGTGAGTCGGCATATGTCCAT	0	11	RDR2-sensitive
GATGGTGAGTCGGCATATGTCTAT	0	12	RDR2-sensitive
GATGGTGCGGTAGAAGCCAGGCAT	0	17	RDR2-sensitive
GATGGTGGAGGAGACGAGATCTGC	0	9	RDR2-sensitive
GATGGTGGGCTTTAGACGGACTGG	0	9	RDR2-sensitive
GATGGTGGGCTTTGGACGAAGGCA	0	10	RDR2-sensitive
GATGGTGGGCTTTGGCGGACTGGC	0	73	RDR2-sensitive
GATGGTGTCGGGTGGATAAGGCAT	0	14	RDR2-sensitive
GATGGTTAAGACTGTGTTCAACGG	0	11	RDR2-sensitive

GATGGTTCCGGATCTGAGGAGAAT	0	11	RDR2-sensitive
GATGGTTCTGCAGAAGGAGTTGTC	32	2	RDR2-resistant
GATGGTTGAGATCGTAGAACGGCA	0	12	RDR2-sensitive
GATGGTTTGCCGGACAGCGACGGA	0	13	RDR2-sensitive
GATGGTTTGTCGGACAACAGCGGC	0	12	RDR2-sensitive
GATGTACACGGGACTAGATGGAGC	0	11	RDR2-sensitive
GATGTACGTCGAGCGCAGGAACGG	0	47	RDR2-sensitive
GATGTACTTCTGGATACGGGTCGG	0	10	RDR2-sensitive
GATGTAGACTCTTATGTCCGACGG	0	19	RDR2-sensitive
GATGTAGAGCACAAGGACGGTGAT	0	12	RDR2-sensitive
GATGTAGAGCCGAAGAGAGGTGAA	1	12	RDR2-sensitive
GATGTAGAGCCGACACAAGACCGA	0	27	RDR2-sensitive
GATGTAGAGGTCGGCTTCCCGGAA	2	20	RDR2-sensitive
GATGTAGAGTGCATCAGACGCCGG	56	0	RDR2-resistant
GATGTAGAGTGGACGGTGATGGCC	0	13	RDR2-sensitive
GATGTAGTCTCTCTGCTCGGTCGT	0	41	RDR2-sensitive
GATGTAGTCTGTAGCGGAGAGTAT	0	19	RDR2-sensitive
GATGTATCTTCAAGTTGAATTCGG	1	12	RDR2-sensitive
GATGTATGTAGAGGAGTAGCGCAT	0	27	RDR2-sensitive
GATGTCAAGTTACTGCGGTATTCA	14	0	RDR2-resistant
GATGTCACGTACTGGAGACGAGCT	0	15	RDR2-sensitive
GATGTCAGGCCACAGAGAGACGCG	0	10	RDR2-sensitive
GATGTCATCGACGTAGAGGAGGCA	0	9	RDR2-sensitive
GATGTCCAAACAGGTCGCACGGTC	0	9	RDR2-sensitive
GATGTCCACCTACGTCATGGACTG	0	14	RDR2-sensitive
GATGTCCTGTAGCTCAACGGTGAT	0	11	RDR2-sensitive
GATGTCGACGGAGCGCTGATGGCG	0	19	RDR2-sensitive
GATGTCGGCGTAGAGGAAGCAGAC	0	10	RDR2-sensitive
GATGTCGGGCAGAACACGGTGCAC	0	42	RDR2-sensitive
GATGTCGTCGTGTCTCTGGCGGCA	0	13	RDR2-sensitive
GATGTCGTTGTGCGAACCAGGCAT	0	12	RDR2-sensitive
GATGTCTAGCATGTCGTCCTCGGA	42	4	RDR2-resistant
GATGTCTAGTCGGTCTCATCGCAT	0	9	RDR2-sensitive
GATGTCTATGTGCTTTGTGCGGCT	25	2	RDR2-resistant
GATGTCTCACTGCAACGTCGGGCA	0	10	RDR2-sensitive
GATGTCTCTGAACAGGCTGAGCAT	0	23	RDR2-sensitive
GATGTCTGGATTGGAGCAGGCCAT	1	15	RDR2-sensitive
GATGTCTGGATTGGAGCAGGCCGT	0	12	RDR2-sensitive
GATGTCTTCGACCGACGAACCGGC	1	14	RDR2-sensitive
GATGTGAGGACTAGACTGAGACGG	0	14	RDR2-sensitive
GATGTGCAGTGTGATGGTCTGGGT	15	0	RDR2-resistant
GATGTGGACATATAGGACGGCTGC	0	10	RDR2-sensitive
GATGTGGACTGACACGAAGGCCGA	0	67	RDR2-sensitive
GATGTGGCCAGACTCTGCAGACGC	0	29	RDR2-sensitive
GATGTGGCTCAGCAACGATGGCAT	0	9	RDR2-sensitive
GATGTGGCTCGACAACGATGACGT	1	23	RDR2-sensitive
GATGTGGCTCGGCAACGACGACGT	1	20	RDR2-sensitive
GATGTGGGACGCCAGGCATTGACT	0	44	RDR2-sensitive
GATGTGGGACTAAAGCCAGGACGC	0	12	RDR2-sensitive

GATGTGGGACTAAAGCCGAGGCAC	1	22	RDR2-sensitive
GATGTGGGACTAAAGCCGGGACAC	0	9	RDR2-sensitive
GATGTGGGACTAAAGCCGGGACGC	3	48	RDR2-sensitive
GATGTGGGACTAAAGCCGGGGCGC	3	86	RDR2-sensitive
GATGTGGGCAGCAGAACCAATCGG	0	24	RDR2-sensitive
GATGTGGTACTATGTGGGAGCGTT	1	12	RDR2-sensitive
GATGTGGTTGTGTGGAGGAGGTGC	52	6	RDR2-resistant
GATGTGTAGTGCGATGGTCTGGGT	14	0	RDR2-resistant
GATGTGTAGTGTGATGATCTGGGT	74	0	RDR2-resistant
GATGTGTAGTGTGATGGCCTGGGT	20	0	RDR2-resistant
GATGTGTAGTGTGATGGGCTGGGT	21	0	RDR2-resistant
GATGTGTAGTGTGATGGTCTGGGA	26	0	RDR2-resistant
GATGTGTAGTGTGATGGTCTGGGC	43	1	RDR2-resistant
GATGTGTAGTGTGATGGTCTGGGG	60	0	RDR2-resistant
GATGTGTAGTGTGATGGTCTGGGT	13261	261	RDR2-resistant
GATGTTACGGCACCTGAACGAGGC	1	32	RDR2-sensitive
GATGTTCTGTAATTGTTGTCGGTA	0	9	RDR2-sensitive
GATGTTCTTGATTGGTCGGTCAAA	17	0	RDR2-resistant
GATGTTGATCCAAGGGAACGCGCA	0	15	RDR2-sensitive
GATGTTGATCCAAGGGGACGTGCG	0	10	RDR2-sensitive
GATGTTGCTGGACGCCGTAGCGGA	0	32	RDR2-sensitive
GATGTTGGAGGCAGGGTCGACGAG	0	11	RDR2-sensitive
GATGTTGGCCAACTCGAACGTCGT	0	15	RDR2-sensitive
GATGTTGGGCAGTCGCGAAGACGC	0	10	RDR2-sensitive
GATGTTGTGACTGAGCCGGCCTAG	0	11	RDR2-sensitive
GATGTTGTTTTACATCACAGTCGG	1	17	RDR2-sensitive
GATGTTTAGTGGACTGTGTGCATG	0	28	RDR2-sensitive
GATGTTTCTATACTGTAGCGGTTT	0	10	RDR2-sensitive
GATGTTTCTTCGTCAGGACACACA	1	24	RDR2-sensitive
GATGTTTGGACTGACACCTACGGC	0	12	RDR2-sensitive
GATGTTTTTTCGGATCTTTGGCTA	14	0	RDR2-resistant
GATGTTTTTTGACTGTATGTCGGG	0	20	RDR2-sensitive
GATTAATTATCGGGGCTGGGACGG	0	9	RDR2-sensitive
GATTAATTTTTGGTCGTGCTGGGC	0	10	RDR2-sensitive
GATTACATCGGTTCTCTTAGGCAT	3	25	RDR2-sensitive
GATTAGACTTACTGATCGATGGCA	11	30	RDR2-sensitive
GATTAGACTTACTGATTGTTGGCG	35	70	RDR2-sensitive
GATTAGATGGTATCGTTCGTCGGT	0	11	RDR2-sensitive
GATTAGCTAGCGGGCTGTGCCGGT	0	9	RDR2-sensitive
GATTAGCTGGGGGAACGAAGACGG	0	12	RDR2-sensitive
GATTAGGGATTTAGCGGTGGGCGT	0	13	RDR2-sensitive
GATTAGGGGAGGCTGTAGTGGGGC	0	28	RDR2-sensitive
GATTAGGGGGACTGTAGTGAGGCT	0	40	RDR2-sensitive
GATTAGTCGGGACCTTGCGCGGTT	3	31	RDR2-sensitive
GATTATAGGGCTCGATCTCTCGGG	0	10	RDR2-sensitive
GATTATCTGTTCGGGCTCAAACGG	1	45	RDR2-sensitive
GATTATGGGAGGCTGTAGTGAGGC	0	10	RDR2-sensitive
GATTATGTTCGTCAGGCTGGGCAC	0	32	RDR2-sensitive
GATTATTCAAGGACTGTTCAGGCA	1091	43	RDR2-resistant

GATTATTCAAGGACTGTTCAGGCT	36	4	RDR2-resistant
GATTCAATATGTTATCTTGTCGGA	45	4	RDR2-resistant
GATTCAATCGACTAATCGGGCTAT	0	11	RDR2-sensitive
GATTCACGCAGGGACGGTAGAGCA	0	39	RDR2-sensitive
GATTCCAGGACGGAGCCGTATGAG	0	11	RDR2-sensitive
GATTCCGGTAGTGTAGTGGGCGAC	0	24	RDR2-sensitive
GATTCGAAATCGGAATGCAGCGGA	42	4	RDR2-resistant
GATTCGAAGATTCGGCGGAGACAG	0	10	RDR2-sensitive
GATTCGAAGGCTGTCGCGTAGGAC	0	10	RDR2-sensitive
GATTCGCGGCGTGGATGTTGAGGC	0	10	RDR2-sensitive
GATTCGCGGGCGGGAGGAAGACGC	0	42	RDR2-sensitive
GATTCGCGGGCGTGAGGAAGACGC	0	14	RDR2-sensitive
GATTCGGCTCTGCAAGTTGGACGC	0	10	RDR2-sensitive
GATTCGGGGTAGGACGATGATGGC	3	40	RDR2-sensitive
GATTCGGGGTAGGATGATGATGGC	8	55	RDR2-sensitive
GATTCGGGGTGGGATGATGATGGC	1	14	RDR2-sensitive
GATTCGGTCGGTCGGTGCGGCGAT	5	44	RDR2-sensitive
GATTCGGTGTAGATACAACCGGTT	47	13	RDR2-resistant
GATTCGTTTCGGTATCTGGGCGGC	0	22	RDR2-sensitive
GATTCTACAGTCGGCCCGTCGATC	0	16	RDR2-sensitive
GATTCTCCGAAGCGGACTGACGGT	0	13	RDR2-sensitive
GATTCTGTAGCAACGCACGGGCAT	0	29	RDR2-sensitive
GATTGAACGAAGGCAGAACGGCAG	0	9	RDR2-sensitive
GATTGAAGTCTGGAGCGAGCTGGT	2	14	RDR2-sensitive
GATTGACGTTCGGATCGGCGCCAA	0	10	RDR2-sensitive
GATTGACTGGATGGCGTACTCTAT	0	39	RDR2-sensitive
GATTGAGTGGGATTGGATGGGTTT	2	27	RDR2-sensitive
GATTGATCCAATTGCTGAGGCGTA	14	0	RDR2-resistant
GATTGATGGTAATCGCTTCTGGAC	217	11	RDR2-resistant
GATTGATTGGACGTTTGACGGACG	23	3	RDR2-resistant
GATTGATTGTAGGCTGTGTTGGTT	9	33	RDR2-sensitive
GATTGCACCCTGATCGAGGACGCT	0	12	RDR2-sensitive
GATTGCAGAACGGTAGGCTACCTT	0	12	RDR2-sensitive
GATTGCAGAGCTCGCCAGACGACC	0	26	RDR2-sensitive
GATTGCAGGATGGCAGAACGACAG	0	20	RDR2-sensitive
GATTGCGGGATAGAGGACGAACAG	0	9	RDR2-sensitive
GATTGCGTCTGGCACTGGCGGCCC	0	11	RDR2-sensitive
GATTGCGTCTGGCACTGGCGGCCT	0	22	RDR2-sensitive
GATTGCTGGTCGTGCTGGTCGGAG	0	14	RDR2-sensitive
GATTGGAAGCCTGTATGTGGACGG	0	25	RDR2-sensitive
GATTGGACCAGGCCTGACGGTTGC	1	21	RDR2-sensitive
GATTGGACCGCAGGTAGATTTGGA	1	33	RDR2-sensitive
GATTGGACTCAGGACGGACGACAC	0	25	RDR2-sensitive
GATTGGAGACTCGGACGGTGACAG	0	9	RDR2-sensitive
GATTGGATTCGGAGGGGACGACGC	0	17	RDR2-sensitive
GATTGGATTGAGGAGCAGGGTCAT	0	9	RDR2-sensitive
GATTGGATTGGGACATCAACACGG	0	9	RDR2-sensitive
GATTGGCAGGAAGCAGAACGACAG	0	19	RDR2-sensitive
GATTGGCATATCCAAGTCGGTCTG	0	10	RDR2-sensitive

GATTGGCCGGCTGGACAGGGCGTA	0	17	RDR2-sensitive
GATTGGCTGAAGGCAGAACAGGGG	0	10	RDR2-sensitive
GATTGGCTGAAGGCAGAACCAGGG	1	20	RDR2-sensitive
GATTGGCTGAAGGCAGAACGAGGT	0	47	RDR2-sensitive
GATTGGCTGAAGGCAGAACGCAAG	0	15	RDR2-sensitive
GATTGGGAGTAGAAGCGAGGGCGA	0	20	RDR2-sensitive
GATTGGGTCGTATTGTGAGGCGTT	0	104	RDR2-sensitive
GATTGGTCGCATCTCCAGAGCCAG	0	10	RDR2-sensitive
GATTGGTCGGTCCGAAGCACGGAA	0	22	RDR2-sensitive
GATTGGTTCGGTAGACACGGTCTA	0	12	RDR2-sensitive
GATTGGTTGCTTGGATACGACGAC	0	32	RDR2-sensitive
GATTGTATCGACTATGTACGGCTA	0	21	RDR2-sensitive
GATTGTATCTGGAGCGAGCTGGCG	0	9	RDR2-sensitive
GATTGTATTGCACGGAGATGGGCT	0	10	RDR2-sensitive
GATTGTCAAATGGGCTGAGCCGGT	0	16	RDR2-sensitive
GATTGTCATCGAACTGTTAGTCGG	0	10	RDR2-sensitive
GATTGTCGGCACTACAACAGCGTA	0	9	RDR2-sensitive
GATTGTCTACAGTAGATTGTGCAT	0	14	RDR2-sensitive
GATTGTGGACGCCTACAGTAGATA	0	13	RDR2-sensitive
GATTGTGGGGTAGTGGAGCGTGCA	0	19	RDR2-sensitive
GATTGTGTCTGGAGCGAGCTGGCG	0	11	RDR2-sensitive
GATTGTGTGTCGTCGGTTTTGCAT	22	0	RDR2-resistant
GATTGTTACGACCAGACGGAGCAT	0	16	RDR2-sensitive
GATTGTTGGGGCGAAGGCGAAGAC	2	16	RDR2-sensitive
GATTGTTGGTTTTGGCTACTCAGG	81	4	RDR2-resistant
GATTGTTTTAACATGGTTGTTCGG	66	0	RDR2-resistant
GATTTAAAGGCGTAGACGAGGGTT	0	23	RDR2-sensitive
GATTTAATCGGGCCAAGGCACAAC	0	9	RDR2-sensitive
GATTTAATTAAATGGATCGACGGG	0	17	RDR2-sensitive
GATTTAGGGCTAGTTTGGATCGGC	3	20	RDR2-sensitive
GATTTATATTGTATGCAGTCGGAC	0	13	RDR2-sensitive
GATTTCCGGAGCGTGGGAACGGCG	0	14	RDR2-sensitive
GATTTCGCGGACGTGTGGAGGCCA	0	9	RDR2-sensitive
GATTTCGGTTAGTTCGGTTTCGGT	0	9	RDR2-sensitive
GATTTCTCGGGATCGTTCGACGAC	2	35	RDR2-sensitive
GATTTGAGGATCGTGCGAGGCTGT	0	13	RDR2-sensitive
GATTTGATCTATTCGCGGACGTCT	0	10	RDR2-sensitive
GATTTGATGGATATAGATGGACGG	0	10	RDR2-sensitive
GATTTGATGGATGTAGATGGACGG	0	11	RDR2-sensitive
GATTTGATGTAGAGGGCAGATATG	19	0	RDR2-resistant
GATTTGATTGGTTATTCGGATGCA	14	0	RDR2-resistant
GATTTGGCCTGAGAGATGCAGCAG	0	16	RDR2-sensitive
GATTTGGCGGCGTCACTTTATGTT	0	33	RDR2-sensitive
GATTTGGGCAGCGACATACTCGGG	21	0	RDR2-resistant
GATTTGGTCGGTCGGCGCGGCGAT	4	21	RDR2-sensitive
GATTTGGTCGGTCGGTGCGGCGAT	15	90	RDR2-sensitive
GATTTGTAGCGCTCCACGGATCGG	0	11	RDR2-sensitive
GATTTGTAGTGTGATGGTCTGGGT	16	0	RDR2-resistant
GATTTGTCTCGTTTGGACTTTCGG	0	10	RDR2-sensitive

GATTTGTCTCTTGCACTGTCGGCC	0	15	RDR2-sensitive
GATTTGTCTCTTGTACTGTCGGCC	0	21	RDR2-sensitive
GATTTGTGCTACTGTGGATGGTGT	1	31	RDR2-sensitive
GATTTGTTGGTCTTGTGTTGGCGT	0	10	RDR2-sensitive
GATTTTATAGCGATCGGTTAGGAT	17	51	RDR2-sensitive
GATTTTATATTTTTTCGTCGGGTC	221	5	RDR2-resistant
GATTTTCGTGACTGTTTCGGGCAA	1	13	RDR2-sensitive
GATTTTCGTTGGAGCAGCAGACGC	0	15	RDR2-sensitive
GATTTTCTCTTCAATCGTCACCAT	31	0	RDR2-resistant
GATTTTGACTTGTAGGGGATTTAA	0	9	RDR2-sensitive
GATTTTGGACCCTTAGCTCGGCAT	0	17	RDR2-sensitive
GATTTTGTAGCAACGCACGGGCAT	0	12	RDR2-sensitive
GATTTTGTCGTTTCTGGAACCGGC	0	12	RDR2-sensitive
GATTTTTCGGGATTGGGCTGGGCC	0	10	RDR2-sensitive
GATTTTTCGGGCCAGTAGAGGGCA	0	13	RDR2-sensitive
GATTTTTCGGGCCAGTGGAGGGCA	21	41	RDR2-sensitive
GATTTTTCGGGCTAGTGGAGGGCA	0	16	RDR2-sensitive
GATTTTTTGGACCAGCACGAGCAT	0	10	RDR2-sensitive
GCAAAATGCGGATGTGGAGTCGGA	0	9	RDR2-sensitive
GCAAACATTCGGACGGAAAGGCGT	0	10	RDR2-sensitive
GCAAACGGATGGATCGCGAGCGGC	0	9	RDR2-sensitive
GCAAAGACGCCACCCTTCGCTCGA	0	9	RDR2-sensitive
GCAAATAACGTGCGGACCGGGCGC	0	10	RDR2-sensitive
GCAAATCGTTTGGCAAATCGGCTC	0	11	RDR2-sensitive
GCAAATGACGGACACAGGGGAGAA	0	16	RDR2-sensitive
GCAACACGAGGACTTCCCAGGAGG	21	2	RDR2-resistant
GCAACCTAGGACTCAGCACGGTTC	0	10	RDR2-sensitive
GCAACGACAAATTTACTCGTCGGT	0	10	RDR2-sensitive
GCAACGACAATTTACTCGTCGGTC	3	21	RDR2-sensitive
GCAACGACTGTAGAGCGAGTCGGC	0	30	RDR2-sensitive
GCAACGCGTCTGAGACTGTTCGGT	1	15	RDR2-sensitive
GCAACGGAAGTACGCTGGGATGAG	0	16	RDR2-sensitive
GCAACGGACACACCTTCGGCTCGC	0	10	RDR2-sensitive
GCAACGGACGGGTACGGGCTATAA	0	23	RDR2-sensitive
GCAACGGATGGCACTTGTACACGC	0	12	RDR2-sensitive
GCAACGGGAGTACGCTAGGATGAG	0	9	RDR2-sensitive
GCAACGGTCAGTAGTAACGGCTAT	1	18	RDR2-sensitive
GCAACGGTCCAGACTCGGATAGAA	0	13	RDR2-sensitive
GCAACGGTCGACTGCAACGCATTA	0	17	RDR2-sensitive
GCAACGGTCGACTGCAACGCATTT	0	12	RDR2-sensitive
GCAACGTAGGCTCGACAAAGAGGA	0	13	RDR2-sensitive
GCAACGTCCCAGTCGGAGGGTGGC	0	9	RDR2-sensitive
GCAACGTCGAGGTAGAGCGTGCTG	0	13	RDR2-sensitive
GCAACTCCGGGCTGGTTGGCGCAC	0	9	RDR2-sensitive
GCAACTCGGGTGTCGTCTGATGGC	0	15	RDR2-sensitive
GCAACTTGTATACTTTAGAGGCTA	36	0	RDR2-resistant
GCAAGAAGGTGCTCGTCGTCGGTT	14	0	RDR2-resistant
GCAAGACGGTGTCGATCTGAGCGG	0	9	RDR2-sensitive
GCAAGAGGACGTCAGCAAGGACTC	2	38	RDR2-sensitive

GCAAGAGGATTTGTTGTATGAAGA	43	0	RDR2-resistant
GCAAGATCTCGAAGGCGACGGGTT	0	15	RDR2-sensitive
GCAAGATCTTGCGGACGACGGGTT	0	9	RDR2-sensitive
GCAAGATGATCGATTCGGAGGCGA	0	18	RDR2-sensitive
GCAAGCTTTATGGTTCGATTGGAA	16	0	RDR2-resistant
GCAAGGAATGAGGACGAGCAAGAA	0	12	RDR2-sensitive
GCAAGGACGGAGCGAAGACACGGC	0	13	RDR2-sensitive
GCAAGGAGACGTCAGCAAGGACTC	4	25	RDR2-sensitive
GCAAGGAGGCGGCGGACAAGTACT	0	9	RDR2-sensitive
GCAAGGCCCGTGGGCCGTCGGACG	15	0	RDR2-resistant
GCAAGGGACGGTGTAGTGGATCAC	0	10	RDR2-sensitive
GCAAGGGAGTGTAGAGATGGCCAA	0	10	RDR2-sensitive
GCAAGGTAGGTCGCGGACACGCAC	0	9	RDR2-sensitive
GCAAGGTCTCGAACGGCAAGGGCT	0	10	RDR2-sensitive
GCAAGGTCTCGAACGGCACCGGCT	0	9	RDR2-sensitive
GCAAGTACCGGATGACTGAGCGGA	0	10	RDR2-sensitive
GCAAGTCGCGGATTGTCCGGCCCA	0	16	RDR2-sensitive
GCAAGTGAACATGTAGGCGCGGCT	0	10	RDR2-sensitive
GCAAGTGATCCTGTTGACCGTTGT	15	0	RDR2-resistant
GCAAGTGCGGACTGTCCGCGGCCT	0	11	RDR2-sensitive
GCAAGTGTAGCTCTGCGGACGGCG	0	9	RDR2-sensitive
GCAAGTGTAGCTCTGCGGACGGTG	0	28	RDR2-sensitive
GCAAGTTAGCGCTGTGTGACGGTG	0	17	RDR2-sensitive
GCAAGTTCGGATAGGGACGGCTGA	1	13	RDR2-sensitive
GCAATCCGGCTGCGGCTGCGGCTT	0	12	RDR2-sensitive
GCAATCCGGCTGCGGCTTCTACAG	0	14	RDR2-sensitive
GCAATCCGGCTGTGGCTGCGGCTT	3	19	RDR2-sensitive
GCAATCGGGCGGGTAGTACACGGA	0	12	RDR2-sensitive
GCAATGACAGGACGGGACGGGATG	0	17	RDR2-sensitive
GCAATGACGGACGCTATCACCTGC	0	10	RDR2-sensitive
GCAATGCGGACTGCAGGCACGCAT	0	26	RDR2-sensitive
GCAATGTAGGACATTGTTACGGTG	33	1	RDR2-resistant
GCAATTTGAGCATCGTTGGACAAT	36	7	RDR2-resistant
GCACAACACTGTAGCCGGCTAACC	0	9	RDR2-sensitive
GCACAACCGTAGAGCCAGAGGCAA	0	22	RDR2-sensitive
GCACAAGACTGCATGCGCTCGCGG	0	33	RDR2-sensitive
GCACAAGTCGGGCTGGTGCGGCAC	0	11	RDR2-sensitive
GCACACAGGACTGTGCAGGCGCAG	0	18	RDR2-sensitive
GCACACATTTGACGCGTTGGACTG	0	10	RDR2-sensitive
GCACACCTTGGCACTAAGGACTGT	0	11	RDR2-sensitive
GCACACCTTGGTACTAAGGACTGT	0	16	RDR2-sensitive
GCACAGACCGTTCGGCTCGACGTA	0	9	RDR2-sensitive
GCACAGACTGTAGGTTGGAGACGA	0	39	RDR2-sensitive
GCACAGAGACGGGAGGACGGAGCA	0	23	RDR2-sensitive
GCACAGCCGGCTGTACGTAGACGT	0	17	RDR2-sensitive
GCACAGGACATTGTCGAACGGCTT	1	23	RDR2-sensitive
GCACAGGACGTAGAGGAGTAGAAG	0	9	RDR2-sensitive
GCACAGTAGAATAAGCGGACGGGC	0	13	RDR2-sensitive
GCACATGGACCTGATGGCTTTAAT	24	2	RDR2-resistant

GCACATGGGCTGTTGGGTGCCGGT	0	17	RDR2-sensitive
GCACATGTAGATCGGTATCCTCGC	0	9	RDR2-sensitive
GCACCAAATTGAGCGGGCGGACGG	0	10	RDR2-sensitive
GCACCAACGAGGATTAGTCGGAAC	0	13	RDR2-sensitive
GCACCAACGAGGATTAGTCGGGAC	4	29	RDR2-sensitive
GCACCAACTTCGGATTGACGGCGT	0	20	RDR2-sensitive
GCACCAGGTCGCGGACCGTCCGGC	32	169	RDR2-sensitive
GCACCAGTTTCAACGGTCGTCGTG	1	13	RDR2-sensitive
GCACCATCGCAGGACTGACCGGCT	99	0	RDR2-resistant
GCACCCCCGGACAGAAGGCAACTA	0	10	RDR2-sensitive
GCACCGACGTCGAGCAGGAAGCGT	1	15	RDR2-sensitive
GCACCGGACACAGCTACTATTCAC	0	19	RDR2-sensitive
GCACCGGACAGTGAACAGTAACGG	1	56	RDR2-sensitive
GCACCGGACAGTGAACAGTGCATG	2	27	RDR2-sensitive
GCACCGGACTGTCCGATGGCACAC	0	9	RDR2-sensitive
GCACCGGACTGTCCGATGGCGCAC	0	11	RDR2-sensitive
GCACCGGACTGTTAGGTGTACCAC	0	11	RDR2-sensitive
GCACCGGACTGTTCGGTGAGCCAA	1	15	RDR2-sensitive
GCACCGTCATCGCTTGGTCGTCGT	1	101	RDR2-sensitive
GCACCGTCATTGCTTGGTCGTCGT	0	111	RDR2-sensitive
GCACCGTTGGATGTAGAGGGTGTG	0	25	RDR2-sensitive
GCACCGTTGGATGTGGAAGGTGTG	6	61	RDR2-sensitive
GCACCGTTGGATGTGGAGGGTGTG	47	412	RDR2-sensitive
GCACCTCGGCGACGAACAGCACGG	0	11	RDR2-sensitive
GCACCTCGGGCGTGGAAGTGGCAC	0	10	RDR2-sensitive
GCACCTGAACAGCATAGGGCCTGA	0	9	RDR2-sensitive
GCACCTGCTGATAGACCTGGCTAC	0	10	RDR2-sensitive
GCACCTGGACATATCGAAGAGCAC	0	9	RDR2-sensitive
GCACCTGGTCGGTACGTGGTCGGG	0	15	RDR2-sensitive
GCACCTGGTCGGTGGGTGGTCGGT	7	28	RDR2-sensitive
GCACCTGTGGTATGGACGGTCCGT	1	35	RDR2-sensitive
GCACGACACGACTGTTAAACGGAT	1	14	RDR2-sensitive
GCACGACACTGTTAGGGCACGGCC	0	12	RDR2-sensitive
GCACGACATGATTAGGACACGGCC	0	13	RDR2-sensitive
GCACGACCCAGTAGAGCAGGCAGT	0	12	RDR2-sensitive
GCACGACCCTCTGTTCGACGTCAA	0	10	RDR2-sensitive
GCACGACGGCAATACCAATCTGAC	0	9	RDR2-sensitive
GCACGACGGGCTGGGCGTAGACGA	0	12	RDR2-sensitive
GCACGACTAGCACACTGACGGGCC	0	10	RDR2-sensitive
GCACGACTCGTAGTGCTTCGGCAT	0	38	RDR2-sensitive
GCACGACTCTACGGACGACGTCAA	0	9	RDR2-sensitive
GCACGACTGTGGACCAGGCCGCGT	0	12	RDR2-sensitive
GCACGACTGTGTACATGGCGACAT	0	10	RDR2-sensitive
GCACGAGACAGGCTAGCACGGAAC	0	11	RDR2-sensitive
GCACGATAGGACACCTGATCACGG	0	10	RDR2-sensitive
GCACGATCTGGGCACAGGAGCGAC	0	9	RDR2-sensitive
GCACGATGGGCTTGAGCCGGACGG	0	16	RDR2-sensitive
GCACGCAGCACACTCACTCGTCGG	8	73	RDR2-sensitive
GCACGCAGGACTGTGCAGGCACGG	1	32	RDR2-sensitive

GCACGCCGTGCGACTGGGTAGGCA	1	14	RDR2-sensitive
GCACGCGGCACACTCACTCGTCGG	3	17	RDR2-sensitive
GCACGCGGGCGGGTGTAGAGAGGC	0	13	RDR2-sensitive
GCACGCGTAGAAGGATACATACAT	0	11	RDR2-sensitive
GCACGCGTCGGTGCGTAGGCGGCG	0	10	RDR2-sensitive
GCACGCTGGGCTGTGGAGCGCGGG	0	15	RDR2-sensitive
GCACGGAAAAAAAGCACGGCCCAG	0	13	RDR2-sensitive
GCACGGAAGGGACGCTGCATATAG	0	19	RDR2-sensitive
GCACGGACCGCTCGGCTTATCGGC	0	14	RDR2-sensitive
GCACGGACTGTGAACGCCTACGGT	0	21	RDR2-sensitive
GCACGGACTTATGCAGAGGAGCTA	0	10	RDR2-sensitive
GCACGGATCGACGACTACGTACAC	0	17	RDR2-sensitive
GCACGGCACGACAGGACACGGCAC	0	45	RDR2-sensitive
GCACGGCACGACTAAGGACCCTTA	0	9	RDR2-sensitive
GCACGGCACGGAATTGAATCGGAC	0	14	RDR2-sensitive
GCACGGCACGGTAGAATAAGCGGG	0	14	RDR2-sensitive
GCACGGCACGGTTAGGACACGGGC	0	25	RDR2-sensitive
GCACGGCGGGCTCACTATCGGCAC	0	15	RDR2-sensitive
GCACGGCTAGGCCTGACACGGATT	0	10	RDR2-sensitive
GCACGGGACCGAGACGAGACGCGT	0	27	RDR2-sensitive
GCACGGGACTATCAATTGTCGGGC	0	13	RDR2-sensitive
GCACGGGGCTGTTATGACCGGCAT	0	9	RDR2-sensitive
GCACGGTAGAATAAACGGGCGGGC	0	32	RDR2-sensitive
GCACGGTAGAATAAGCGGACGGAC	0	65	RDR2-sensitive
GCACGGTAGAATAAGCGGACGGGC	0	13	RDR2-sensitive
GCACGGTAGAATAAGCGGGCAGGC	0	10	RDR2-sensitive
GCACGGTAGAATAAGCGGGCGGAC	1	22	RDR2-sensitive
GCACGGTAGAATAAGCGGGCGGGC	3	346	RDR2-sensitive
GCACGGTAGAATAAGCGGGCGGGG	1	15	RDR2-sensitive
GCACGGTAGAATAAGCGGGCGGGT	0	38	RDR2-sensitive
GCACGGTAGAATAAGTGGACGGGC	0	27	RDR2-sensitive
GCACGGTAGAATAAGTGGGCGGGC	0	11	RDR2-sensitive
GCACGGTAGATGAATGGACGGTAT	0	14	RDR2-sensitive
GCACGGTCGGTTACGGGCCCGGGC	0	12	RDR2-sensitive
GCACGGTCGTGACCCGGCATGAGC	0	9	RDR2-sensitive
GCACGGTTAATTGGTAGGCACGGT	0	10	RDR2-sensitive
GCACGTAGAGAGATTCTAGGACGG	0	12	RDR2-sensitive
GCACGTAGATGGCAGAACCAGAGC	0	15	RDR2-sensitive
GCACGTAGGACTGTGCAGGCCCGG	0	59	RDR2-sensitive
GCACGTATCCGCATCGGACCATAT	0	18	RDR2-sensitive
GCACGTATTTTCGTCGGCCACCAC	0	9	RDR2-sensitive
GCACGTCGAGAAGGTGACAACGGA	0	11	RDR2-sensitive
GCACGTCGTCCGGATTTTAGCGGA	0	32	RDR2-sensitive
GCACGTGCGGACTGTCCGGCCCCC	0	10	RDR2-sensitive
GCACGTGGGAATGAGAATAGGCTT	1	20	RDR2-sensitive
GCACGTGGGAATGGGAATAGGCTT	2	31	RDR2-sensitive
GCACGTGGGCCTCTGAGCTGGACG	0	25	RDR2-sensitive
GCACGTGGGCTCTCTAGGCATGGC	0	14	RDR2-sensitive
GCACGTGTAGAACAGATTAGGGTT	0	11	RDR2-sensitive

GCACGTTTGGACATGTATAGGCGC	0	12	RDR2-sensitive
GCACGTTTGGACCCGAAGCTCGGC	7	111	RDR2-sensitive
GCACGTTTGGACCCGATGCTCGGC	1	41	RDR2-sensitive
GCACTAACGGATCGTGCTTCGGAC	0	10	RDR2-sensitive
GCACTACACTGGACTAGAGCGCAA	0	16	RDR2-sensitive
GCACTACGAGAAAGTCGGCTTTTA	3	23	RDR2-sensitive
GCACTATATTGGACTGGGCCACGG	0	15	RDR2-sensitive
GCACTCAGCCAGTCCAGTCTGAGC	0	15	RDR2-sensitive
GCACTCGACTGGACACTCGCGGCC	0	13	RDR2-sensitive
GCACTCGCGTTAGGACGTGGCGTA	2	21	RDR2-sensitive
GCACTCTAGCCGGGCTGGTGTCGG	0	24	RDR2-sensitive
GCACTCTCGGGCCATACACGACAC	0	10	RDR2-sensitive
GCACTGCATGGACGTGGAGATGGC	1	14	RDR2-sensitive
GCACTGCATGTGGATCCTTGGACA	106	18	RDR2-resistant
GCACTGGAAGGGCATAGCTGAGAA	0	10	RDR2-sensitive
GCACTGGAGCAGCAGGGGCGAGAG	0	15	RDR2-sensitive
GCACTGTACTCGGCAAAGACCTGC	0	14	RDR2-sensitive
GCACTGTAGACGACGGCCCAACAT	0	18	RDR2-sensitive
GCACTGTAGATTACACCAGGGGCG	1	17	RDR2-sensitive
GCACTGTAGCACAATCCAGCAGAT	0	10	RDR2-sensitive
GCACTGTAGCAGCGCTAGGGGGCA	0	13	RDR2-sensitive
GCACTGTAGCGCATTGAATGCGGT	3	21	RDR2-sensitive
GCACTGTAGGGATAGAAGATGCCT	1	17	RDR2-sensitive
GCACTGTAGGGGGCAACGGGACTA	0	10	RDR2-sensitive
GCACTGTAGGTCGGCTTGCCTGCC	0	10	RDR2-sensitive
GCACTGTGGAAGATTGCAGGAACG	0	12	RDR2-sensitive
GCACTGTGGAGCGGGAGTTGCATA	0	34	RDR2-sensitive
GCACTGTTAGGTACTGGACGGATG	0	32	RDR2-sensitive
GCACTGTTCCGCTCTGTAGACTGC	0	18	RDR2-sensitive
GCACTTCGGGCTGGCACGGCACGG	0	9	RDR2-sensitive
GCACTTCGGGCTGGCACGGTACGG	0	33	RDR2-sensitive
GCACTTTGGACTGGTACGGCACAG	0	12	RDR2-sensitive
GCACTTTGGGCTAGCATGACACGG	0	21	RDR2-sensitive
GCAGAACACGGAATGAGACAGCCT	0	14	RDR2-sensitive
GCAGAACACGGCGCACATGTAGAC	0	10	RDR2-sensitive
GCAGAACACGTCAGAGGCAGACTA	0	24	RDR2-sensitive
GCAGAACAGCAGTTGGCAGAACAC	0	9	RDR2-sensitive
GCAGAACATGTCAGAGGTAGGCTA	0	10	RDR2-sensitive
GCAGAACCGTGCTGAATGTGGACG	0	86	RDR2-sensitive
GCAGAACCTGACAGGGGAGGCTAG	0	13	RDR2-sensitive
GCAGAACGAGTGGGCTGTTTTCAG	0	15	RDR2-sensitive
GCAGAACGGCAGAACACACTGATG	0	15	RDR2-sensitive
GCAGAACGGCAGAACGGCAGAACC	0	11	RDR2-sensitive
GCAGAACGGCAGAACGGCAGACTA	0	9	RDR2-sensitive
GCAGAACGGCAGACTACTCTTACC	0	13	RDR2-sensitive
GCAGAACGGTAGAACGGACGACTG	0	26	RDR2-sensitive
GCAGAACGGTAGGCTACCTTTGGA	0	48	RDR2-sensitive
GCAGAACTTTGCCAAGTGCGGCTT	0	9	RDR2-sensitive
GCAGAACTTTGCCGAGGGCGGTAC	0	9	RDR2-sensitive

GCAGAAGACAGGGAACGTGACCAT	0	10	RDR2-sensitive
GCAGAAGGGCTACTCGTGCATCAT	1	20	RDR2-sensitive
GCAGAAGGGCTGCGTAGAGATCGG	0	14	RDR2-sensitive
GCAGAAGGGGTAGAACAGGCCGAG	0	9	RDR2-sensitive
GCAGAATACTGTCCAAGCTTCGGC	19	1	RDR2-resistant
GCAGAATGCAGACAAGGGACGCAG	0	10	RDR2-sensitive
GCAGACAGTAGAGGGCGTGATCAG	0	11	RDR2-sensitive
GCAGACCAGCAAGAACGACGACGT	0	12	RDR2-sensitive
GCAGACCGGTGACGTAGAGTGCAT	84	25	RDR2-resistant
GCAGACCGTCTGGACCACCGGTGC	0	11	RDR2-sensitive
GCAGACCGTTTGAGCCTACGCATG	0	14	RDR2-sensitive
GCAGACCTCTGTACCAGGCGTAGT	0	15	RDR2-sensitive
GCAGACGACGTACTCAGGGACGCA	0	9	RDR2-sensitive
GCAGACGAGTAGATGGCGGACAAC	0	11	RDR2-sensitive
GCAGACGCAAGGACGACAGAGCAG	0	12	RDR2-sensitive
GCAGACGCAGATATGAACGCGCAA	0	23	RDR2-sensitive
GCAGACGCTGGTATAGACATGGAT	1	16	RDR2-sensitive
GCAGACGGCTGCAAACTAGAATGT	0	10	RDR2-sensitive
GCAGACTCAGAACGCTGTGCAGAA	0	36	RDR2-sensitive
GCAGACTGCAGACAAGCAAAGCAA	0	10	RDR2-sensitive
GCAGACTGCATACACGTCCACACG	0	13	RDR2-sensitive
GCAGACTGGAACGATGTCGGCGTC	0	10	RDR2-sensitive
GCAGACTGGGACCGGCTACATGCG	0	79	RDR2-sensitive
GCAGACTGGGACCGGCTGCATGCA	0	9	RDR2-sensitive
GCAGACTGTCTGTACCTATGGGGC	0	14	RDR2-sensitive
GCAGACTGTGCAAGCGGAGCGTGA	0	9	RDR2-sensitive
GCAGACTTGCTCGGCGGGAGACAT	0	9	RDR2-sensitive
GCAGAGAAGGAGGTAGCAACAGAA	0	10	RDR2-sensitive
GCAGAGACGACGGAGTTCGCTGAA	0	11	RDR2-sensitive
GCAGAGACGTGTAGCAGTCGTGCG	0	34	RDR2-sensitive
GCAGAGACTGATGGGCGCAATTCA	0	18	RDR2-sensitive
GCAGAGAGCATCGCCAGACGGTAC	0	13	RDR2-sensitive
GCAGAGAGGACGCCGATCGGAACA	0	12	RDR2-sensitive
GCAGAGAGGATTGGAGCTGGCGGC	0	10	RDR2-sensitive
GCAGAGCGCCGATACATGGAACAG	0	12	RDR2-sensitive
GCAGAGCGCCGCTACATGGAACAG	0	9	RDR2-sensitive
GCAGAGCGGCAAGTTAGAGCGACC	0	16	RDR2-sensitive
GCAGAGCTCGGCACCATAGGCTAT	0	19	RDR2-sensitive
GCAGAGGACATATCTGGTGCACAT	0	12	RDR2-sensitive
GCAGAGGACCTGGTCGAGCGCTAC	0	21	RDR2-sensitive
GCAGAGGACTCTGACAGCGAGCGT	0	10	RDR2-sensitive
GCAGAGGAGGACCGACAACAATGA	0	22	RDR2-sensitive
GCAGAGGATATGAGGGCGGAAGAA	0	29	RDR2-sensitive
GCAGAGGATGACGGGTTCGACGAC	0	11	RDR2-sensitive
GCAGAGGCCGGAAGTGACTTTCAA	0	12	RDR2-sensitive
GCAGAGGCTACGGAACGCGCAGAA	0	26	RDR2-sensitive
GCAGAGGGTTGCTGACGCTGACGG	0	19	RDR2-sensitive
GCAGAGTCGAACGCAGAGACCACA	1	12	RDR2-sensitive
GCAGAGTGAACGGAACAGAGCGTG	0	10	RDR2-sensitive

GCAGAGTGAACGGAACGGAGCGTG	5	21	RDR2-sensitive
GCAGAGTGAACTGGAACAGAGCGG	2	31	RDR2-sensitive
GCAGAGTGGACAGTTGAACCGGTC	0	11	RDR2-sensitive
GCAGAGTGGACAGTTGACGCCGAC	0	12	RDR2-sensitive
GCAGAGTGGACAGTTGACGCCGGC	4	58	RDR2-sensitive
GCAGAGTGTGGACCTGGGCACGGT	0	10	RDR2-sensitive
GCAGAGTTCTGGATGTGTGACTGC	0	27	RDR2-sensitive
GCAGATAGGCCGTGACCGACTGAA	0	9	RDR2-sensitive
GCAGATATCGGGCCTGTTTGGTGT	0	41	RDR2-sensitive
GCAGATCCCGGGAGAAGACGTCCG	0	13	RDR2-sensitive
GCAGATCGAGCATAGAACCGGCAC	1	24	RDR2-sensitive
GCAGATCGATAGAACAGACGACAA	0	9	RDR2-sensitive
GCAGATCTGAACTCGCAGCAACGT	0	12	RDR2-sensitive
GCAGATCTGATCCAGCAGCAACGT	0	9	RDR2-sensitive
GCAGATCTGATCCCGCAGCAACGT	0	26	RDR2-sensitive
GCAGATCTGATCTCGCAGCAACGT	1	17	RDR2-sensitive
GCAGATCTGGGAGCCGTTGGCGCA	4	29	RDR2-sensitive
GCAGATGAACGGACCTGGATGTGC	1	90	RDR2-sensitive
GCAGATGACTAGCTACCTGACGAC	0	14	RDR2-sensitive
GCAGATGACTGGCGGCTTCTCCGG	0	9	RDR2-sensitive
GCAGATGAGGGATTTGATTGTGTG	16	0	RDR2-resistant
GCAGATGATGTTGACGAGTACTGA	18	0	RDR2-resistant
GCAGATGGACGGACCTGGATGCGT	0	68	RDR2-sensitive
GCAGATGGACGGTGTAGATTGGCT	0	22	RDR2-sensitive
GCAGATGGATGGACCTGGATGTGC	0	20	RDR2-sensitive
GCAGATGGCGGACATTGCAGACGG	0	13	RDR2-sensitive
GCAGATGTAGAGCACAAGGACGGT	0	45	RDR2-sensitive
GCAGATGTAGTACAACGTGCGCAA	0	17	RDR2-sensitive
GCAGATGTGGAGCATAAGGACGGT	0	17	RDR2-sensitive
GCAGATTCACCGAAGGCAGAACCA	0	23	RDR2-sensitive
GCAGATTCGTAGACGACATCCCGT	0	11	RDR2-sensitive
GCAGATTGGCAGGAAGCAGAACGA	0	9	RDR2-sensitive
GCAGATTGGCTGAAGGCAGAACCA	0	12	RDR2-sensitive
GCAGATTGGCTGAAGGCAGAACGA	0	52	RDR2-sensitive
GCAGATTGGTCGCATCTCCAGAGC	0	20	RDR2-sensitive
GCAGATTTACTGGAGGCAGAACGC	0	18	RDR2-sensitive
GCAGCAACGTCGAGGTAGAGCGTG	0	11	RDR2-sensitive
GCAGCACACTCACTCGTCGGCTTA	2	48	RDR2-sensitive
GCAGCACCGTTGGATGTGGAGGGT	0	42	RDR2-sensitive
GCAGCAGAGCTCATGATCGTCGAG	0	10	RDR2-sensitive
GCAGCAGCTCGGCTCGGCTCGGAC	0	33	RDR2-sensitive
GCAGCAGTCGGTTGGTGGAGGCAA	2	24	RDR2-sensitive
GCAGCAGTCGTTTGGTGGAGGCAA	2	14	RDR2-sensitive
GCAGCATCCAAGGACAGCCGGCGT	1	17	RDR2-sensitive
GCAGCCCAGACAGCCTCGGACGCA	0	11	RDR2-sensitive
GCAGCCGACGGCACACAGAAACTC	0	10	RDR2-sensitive
GCAGCCGGACGAGTGAGAGACTGC	0	27	RDR2-sensitive
GCAGCCGGATTGCAGCCGCAGCAA	1	85	RDR2-sensitive
GCAGCCGGATTGTAGCCGCAGCAA	0	10	RDR2-sensitive

GCAGCCTGATAGTCGGATCGGCCT	0	24	RDR2-sensitive
GCAGCCTGCAGCAACGGACGGTAC	0	16	RDR2-sensitive
GCAGCCTGGGGAACAGCCGACGTC	0	12	RDR2-sensitive
GCAGCCTGTAGATTTGGTCGGCAA	0	11	RDR2-sensitive
GCAGCGAAGGCTTCGAACGAAGGG	0	9	RDR2-sensitive
GCAGCGACAATCTCACTCGTCGGT	0	26	RDR2-sensitive
GCAGCGACAATTTACTCGTCGGTC	13	148	RDR2-sensitive
GCAGCGACAATTTACTCGTCGGTT	0	10	RDR2-sensitive
GCAGCGACGTACTCGGCACCGAAC	0	10	RDR2-sensitive
GCAGCGATCTCAAGGACCCGGCCA	0	10	RDR2-sensitive
GCAGCGATGGTAGGACCAGGCGAC	0	42	RDR2-sensitive
GCAGCGCGACTGTTTTATAGGCCT	0	9	RDR2-sensitive
GCAGCGCGGCAGAACATGGCACAC	0	16	RDR2-sensitive
GCAGCGGCAATTTACTCGTCGGTC	0	9	RDR2-sensitive
GCAGCGGCGCGGAATCCGGACGTC	1	63	RDR2-sensitive
GCAGCGGCGTACTCTATACCACAC	0	13	RDR2-sensitive
GCAGCGGCTTGGACTTGGACGGAA	0	26	RDR2-sensitive
GCAGCGGGCAGACGAACAGGGCAA	0	10	RDR2-sensitive
GCAGCGGGGCAGTAGGAGCGACGT	0	13	RDR2-sensitive
GCAGCGGTGTAGAAGGCCTTCAAC	0	37	RDR2-sensitive
GCAGCTAGACGCGTAAGGACCATT	2	26	RDR2-sensitive
GCAGCTAGGAGCAGGGGAGAGCGG	26	0	RDR2-resistant
GCAGCTCTGACGCAGGCACAACAC	0	18	RDR2-sensitive
GCAGCTGTAGGAAGTTAGCGGCTC	0	15	RDR2-sensitive
GCAGCTGTATGACTACAAGATGGT	0	22	RDR2-sensitive
GCAGCTTTCTGTCGTCGGGTGCAC	8	38	RDR2-sensitive
GCAGCTTTTGCAGTCGTCTGGTCA	0	17	RDR2-sensitive
GCAGGAACAGTAGACCAAGGCAGA	3	20	RDR2-sensitive
GCAGGAACAGTAGACCCAGGCGGA	1	43	RDR2-sensitive
GCAGGAACAGTAGACTCAGGCGGA	0	12	RDR2-sensitive
GCAGGAACGGTAGATCGAGCAGAG	0	32	RDR2-sensitive
GCAGGAAGATGTACGCTGTGCAGT	1	32	RDR2-sensitive
GCAGGAATTGTCGAATCAAAACGG	0	14	RDR2-sensitive
GCAGGACAACACGAAGGCCGCGGG	0	11	RDR2-sensitive
GCAGGACAACAGGACTGCAGAAGA	0	22	RDR2-sensitive
GCAGGACAACAGTAGCAGCACAAC	0	11	RDR2-sensitive
GCAGGACAACCAGGACAGGGACGT	0	15	RDR2-sensitive
GCAGGACAACGAGCGGCTGGGCGG	0	10	RDR2-sensitive
GCAGGACAACGTCAGCAGGGACTC	1	16	RDR2-sensitive
GCAGGACACATGTAGGTGGTCGTA	0	10	RDR2-sensitive
GCAGGACACTCGGCACAGGAACAC	0	9	RDR2-sensitive
GCAGGACACTCGGCACAGGACCAC	2	15	RDR2-sensitive
GCAGGACAGCAGTAGCAGCACAGC	1	12	RDR2-sensitive
GCAGGACCGCGTCGAAGAAGAAGT	0	10	RDR2-sensitive
GCAGGACCTGATAGAGTGTAACGG	0	9	RDR2-sensitive
GCAGGACCTTCAGCGACGAAGCAT	3	32	RDR2-sensitive
GCAGGACCTTCGACGACGAAGCAT	0	53	RDR2-sensitive
GCAGGACCTTCGGCGACGAAGCAT	0	31	RDR2-sensitive
GCAGGACCTTTAGCGACGAAGCAT	0	10	RDR2-sensitive

GCAGGACCTTTGGCGACGAAGCAT	0	14	RDR2-sensitive
GCAGGACGAAAATTCAGGGGGCAA	0	12	RDR2-sensitive
GCAGGACGACGACGACGACGACGC	0	10	RDR2-sensitive
GCAGGACGATGGTAGAGACGACAA	0	23	RDR2-sensitive
GCAGGACGGCTGACGGACGACGCA	1	16	RDR2-sensitive
GCAGGACGTAAACCGGGAGCGGAC	0	10	RDR2-sensitive
GCAGGACGTAACATGAGAGCGGGC	0	9	RDR2-sensitive
GCAGGACGTAGCCCGGGAGCGGAC	0	13	RDR2-sensitive
GCAGGACTAACGAAGGCAGAACGG	0	30	RDR2-sensitive
GCAGGACTCTGCCGAGAGCGGCCC	6	35	RDR2-sensitive
GCAGGACTCTGCCGAGGGCGACAC	0	19	RDR2-sensitive
GCAGGACTGACCGGCTGCATGAGG	88	0	RDR2-resistant
GCAGGACTGCAAGGAGGACAGCGT	0	21	RDR2-sensitive
GCAGGACTGCACTGTAGGGGGCAA	0	13	RDR2-sensitive
GCAGGACTGCAGGAGGGCGGACGG	0	22	RDR2-sensitive
GCAGGACTGCAGGCGACCAGGCGT	0	9	RDR2-sensitive
GCAGGACTGTACCGAGTGCGGCGC	0	11	RDR2-sensitive
GCAGGACTGTGCAGGCACGGCACG	0	29	RDR2-sensitive
GCAGGACTGTGCAGGCACGGCCCG	0	22	RDR2-sensitive
GCAGGACTGTGCAGGCCCGGCCCG	1	19	RDR2-sensitive
GCAGGACTGTGTCGAGCGCGGCTC	0	11	RDR2-sensitive
GCAGGACTTGAGTGCGCATATCTT	1	17	RDR2-sensitive
GCAGGACTTTACCGAGGACGACAC	0	28	RDR2-sensitive
GCAGGACTTTACCGAGTGCGGCTC	4	21	RDR2-sensitive
GCAGGACTTTGACGAATGCGGCTC	0	11	RDR2-sensitive
GCAGGACTTTGCCAAGTGCGGCTC	0	14	RDR2-sensitive
GCAGGACTTTGTCGAGGGCGACAC	0	9	RDR2-sensitive
GCAGGACTTTGTCGAGTGCGGCGC	1	15	RDR2-sensitive
GCAGGACTTTGTCGAGTGCGGCTC	9	61	RDR2-sensitive
GCAGGAGATGAATGATGTGATGTT	17	1	RDR2-resistant
GCAGGAGCTAGGAACATGCAAGAC	0	14	RDR2-sensitive
GCAGGAGCTGTGCCGAGGACGAAG	0	10	RDR2-sensitive
GCAGGAGGACGTCAGCAAGGACTC	1	22	RDR2-sensitive
GCAGGAGGCGAGAGCGGGACTGGA	0	9	RDR2-sensitive
GCAGGATAGACGCAAGGACGGTAG	0	11	RDR2-sensitive
GCAGGATATGACGGATGCGATCAT	0	16	RDR2-sensitive
GCAGGATATGGAAGCGGCAGAACC	0	9	RDR2-sensitive
GCAGGATCGGATAACTCCTCACGG	0	10	RDR2-sensitive
GCAGGATCTAGAAGACGAAGGGTT	6	100	RDR2-sensitive
GCAGGATGAACGCAGGAAGACTGG	0	9	RDR2-sensitive
GCAGGATGGCAGAACGGCAGAACG	0	13	RDR2-sensitive
GCAGGATTTTGCCGAGGACAGCAC	0	9	RDR2-sensitive
GCAGGCACGACCTAGGACTGGCAT	1	14	RDR2-sensitive
GCAGGCAGAGTCGATTGTTAGGCA	0	11	RDR2-sensitive
GCAGGCAGCGTGGAACACGTGCGT	0	14	RDR2-sensitive
GCAGGCATGCAGGACAGACGGCGC	2	21	RDR2-sensitive
GCAGGCCCGATAGCATACTGACGA	0	16	RDR2-sensitive
GCAGGCCGGTGATGTAGAGTGCAT	20	1	RDR2-resistant
GCAGGCCTGGGTATTGAAGTCGGT	0	12	RDR2-sensitive

GCAGGCGCAGAACAGATTAGGGTT	0	11	RDR2-sensitive
GCAGGCTCGGGCTGGTACGGCTAC	1	17	RDR2-sensitive
GCAGGCTGTGACGCATAATGCCCA	0	13	RDR2-sensitive
GCAGGCTGTGTACGTGCTGTCGGA	0	9	RDR2-sensitive
GCAGGGAAGGGCGTACTCGATCGT	1	19	RDR2-sensitive
GCAGGGACAAGAGAGGACACCAAA	0	10	RDR2-sensitive
GCAGGGACTTTTCATTCGTCTGGC	144	0	RDR2-resistant
GCAGGGAGGGGGAATGAGAAAGAC	5	21	RDR2-sensitive
GCAGGGATTGGGATCGCGCCGCGC	29	0	RDR2-resistant
GCAGGGCAACACAGCGAAGGACAA	0	9	RDR2-sensitive
GCAGGGCCATGGACGAAGACGCAG	0	28	RDR2-sensitive
GCAGGGCGTAGCATATGGCTGGTT	0	9	RDR2-sensitive
GCAGGGCTGTAGACAGGTCGTCAG	0	14	RDR2-sensitive
GCAGGGGAACCTCATGGTCGTCGC	0	10	RDR2-sensitive
GCAGGGGAGAGCGGGCTGGACAGC	230	37	RDR2-resistant
GCAGGGGCATCTAAGCGACGCTGA	0	11	RDR2-sensitive
GCAGGGGCGCGGAACGAAGGCAGA	0	9	RDR2-sensitive
GCAGGGGCGGATATTAGGCCTGAG	0	11	RDR2-sensitive
GCAGGGGCGTTAGCGCGGACTCAC	0	13	RDR2-sensitive
GCAGGGTGGCATCAGATTCTGGCT	0	10	RDR2-sensitive
GCAGGTAACCGATCATGGACTCAA	0	23	RDR2-sensitive
GCAGGTACGACGGACATTCCGGCT	0	9	RDR2-sensitive
GCAGGTACGCTACAGAACATGGTT	0	16	RDR2-sensitive
GCAGGTATGGTAGACTGTCCGGCT	3	16	RDR2-sensitive
GCAGGTCACGGCATGCAAACTCTG	0	9	RDR2-sensitive
GCAGGTCGCTCTGACGGACGCCAA	0	14	RDR2-sensitive
GCAGGTCGGCACTCGGCAGTGATG	0	13	RDR2-sensitive
GCAGGTCGGTATCGTTACATACAA	0	26	RDR2-sensitive
GCAGGTGAACGGACGGACGAACGG	0	10	RDR2-sensitive
GCAGGTGATCGGCTGAAGACGCGC	0	17	RDR2-sensitive
GCAGGTGTAGATCACAGGGACGAT	0	9	RDR2-sensitive
GCAGGTGTAGGAGTCTAAAGGCAT	0	17	RDR2-sensitive
GCAGGTGTGGAACACAAGTACGGT	0	13	RDR2-sensitive
GCAGGTGTGGAGCACAATAACGGT	0	9	RDR2-sensitive
GCAGGTGTGGAGCACAGGAACGGT	0	17	RDR2-sensitive
GCAGGTGTGGAGCACTGGGACGGT	0	13	RDR2-sensitive
GCAGGTGTGGAGCATAGGGACGGT	0	14	RDR2-sensitive
GCAGGTGTGGAGCATATGGACGGT	1	15	RDR2-sensitive
GCAGGTTATAGTCGGGTTAGGCGA	0	17	RDR2-sensitive
GCAGGTTGGAGACTGTTGGCGGCT	0	12	RDR2-sensitive
GCAGTAAAGGTAGTCGTTTAGGAC	0	11	RDR2-sensitive
GCAGTACAAGATCGTCCCGGAGAC	0	11	RDR2-sensitive
GCAGTACGTCAGAGGAACCGGCTC	1	47	RDR2-sensitive
GCAGTAGAAGTCGGTACGGATCAA	0	17	RDR2-sensitive
GCAGTAGATCGGGACGGCAGACAA	1	17	RDR2-sensitive
GCAGTAGATTGTGTTATAACTTGT	0	9	RDR2-sensitive
GCAGTAGTTTGGACCGAGCGGCTC	0	24	RDR2-sensitive
GCAGTATGACTGGATCACACGGAC	0	21	RDR2-sensitive
GCAGTATGCCACCGATATCGTCGA	0	9	RDR2-sensitive

GCAGTCCCTCGAACGGTCGCCGGG	0	18	RDR2-sensitive
GCAGTCCCTTGAACGGTCGCCGGA	0	11	RDR2-sensitive
GCAGTCGGCAGTAGAGTTCTCGGC	0	29	RDR2-sensitive
GCAGTCTATAATGTAGAACGGTGT	0	19	RDR2-sensitive
GCAGTCTCTCGAACGGTCGTCATA	1	15	RDR2-sensitive
GCAGTCTGGCTGAGCGGATGCAAA	0	15	RDR2-sensitive
GCAGTGACGGCTCTCTGGCGTCGA	0	11	RDR2-sensitive
GCAGTGACTGTACAACGCTAGGCA	0	18	RDR2-sensitive
GCAGTGAGGACGGAACGGGAATGA	0	11	RDR2-sensitive
GCAGTGATTGGCGCTCGGATCGGC	0	21	RDR2-sensitive
GCAGTGCACGGAGACGACGAACAA	0	11	RDR2-sensitive
GCAGTGCATGCGGAACTTGTTCGG	1	45	RDR2-sensitive
GCAGTGTAGGACGGAGTTGAGGCC	0	12	RDR2-sensitive
GCAGTGTGTTTGGACGGCTGACCA	0	15	RDR2-sensitive
GCAGTTACTGGGACTAGGACGCAT	0	16	RDR2-sensitive
GCAGTTTTGGACCCTTAGCTCGGC	3	53	RDR2-sensitive
GCAGTTTTGGACCCTTTGCTCGGC	3	41	RDR2-sensitive
GCAGTTTTTCTAGGCTGGTCGGAC	3	65	RDR2-sensitive
GCATAAATTTTAGTATCGCCGGGG	16	0	RDR2-resistant
GCATAAGAGTGGAACGAGGGCGTC	0	10	RDR2-sensitive
GCATAATTTCGGGCTGGTCCGGTC	0	12	RDR2-sensitive
GCATACAACCGGACTGTGTCGGAC	0	17	RDR2-sensitive
GCATACACAAGTGTGACTCGTCGA	0	14	RDR2-sensitive
GCATACACGCGGGTCGTCTGGTTC	0	10	RDR2-sensitive
GCATACAGAGTGGACGGTGGGTTA	0	9	RDR2-sensitive
GCATACCATGGCGATCTGTTCGGG	0	11	RDR2-sensitive
GCATACGATGGCTCAGGACTACAT	0	15	RDR2-sensitive
GCATACGGAAGGCACGATGAACGC	0	57	RDR2-sensitive
GCATACGTGTGATCTAGACTGCAT	0	21	RDR2-sensitive
GCATAGAACACCCCTGGACGGCAG	0	12	RDR2-sensitive
GCATAGAACCAGGTCGTGTCGGGC	0	12	RDR2-sensitive
GCATAGATTGTAGATGGCAGCTCC	0	9	RDR2-sensitive
GCATAGCCCTCCCTCTGAAATGGA	0	12	RDR2-sensitive
GCATAGGACTGGGGAAAAACGGCG	3	21	RDR2-sensitive
GCATAGTAGGACACTCGGCATAGA	0	17	RDR2-sensitive
GCATATGATGGCTTTATTACTCGC	290	13	RDR2-resistant
GCATATGGATGACTGTGCCTGCAT	0	15	RDR2-sensitive
GCATATTTACGACGGCCGTTGGAT	0	13	RDR2-sensitive
GCATCAAGCAGAGGATCGTGGGCT	0	9	RDR2-sensitive
GCATCAGACGGTGGAGAGCAACAC	0	9	RDR2-sensitive
GCATCCACGGTAGAAGAGGACGAC	0	10	RDR2-sensitive
GCATCCATGTCGGATTCCATGGCG	1	17	RDR2-sensitive
GCATCCGGACTCCGATTGCGACGG	0	16	RDR2-sensitive
GCATCCTGTACGACGGCGGTGGCC	28	0	RDR2-resistant
GCATCCTTCGGTTGTCTGGCACAC	0	10	RDR2-sensitive
GCATCGACTGCGTACGACTACATC	2	18	RDR2-sensitive
GCATCGGATCGTGATCACACGGCT	0	10	RDR2-sensitive
GCATCGGGCTGTGCCGGTTCGGGT	0	10	RDR2-sensitive
GCATCTCCCGTCAGGACTCGGCCT	3	41	RDR2-sensitive

GCATCTGACTGCATCGACTGCATA	3	47	RDR2-sensitive
GCATCTGAGCATGTAGACGCCACC	0	13	RDR2-sensitive
GCATCTGTGGAGCCAGGCTCACGC	0	30	RDR2-sensitive
GCATCTTCGGACTCAAGTCGGTAT	0	31	RDR2-sensitive
GCATCTTGGACGTACACGGCGAAT	0	17	RDR2-sensitive
GCATCTTGGACGTATACGCCGAAT	0	13	RDR2-sensitive
GCATGACACGTTAGACGGCCTGGA	0	11	RDR2-sensitive
GCATGACGTACTGAACCGGCCGGC	0	11	RDR2-sensitive
GCATGAGCCAGAGCCGAAGCTGAA	0	16	RDR2-sensitive
GCATGAGCCTGACTGAGATGAGCC	0	11	RDR2-sensitive
GCATGAGGACGTGCAGTACTGCGG	0	16	RDR2-sensitive
GCATGAGGCAGGAACTGCATTACT	38	2	RDR2-resistant
GCATGATAGACAGGCTCTCGACAA	0	36	RDR2-sensitive
GCATGATTCACGCAGGGACGGTAG	0	14	RDR2-sensitive
GCATGCAAGGACGGAGCGAAGACA	0	17	RDR2-sensitive
GCATGCAAGGTAGGTCGCGGACAC	0	9	RDR2-sensitive
GCATGCAGATGTAGAGCGAGGCTT	0	9	RDR2-sensitive
GCATGCGGCAGAACGGCAGAACGG	0	13	RDR2-sensitive
GCATGCGGCTGACATGAATATGGC	0	27	RDR2-sensitive
GCATGCGGGCACGTAGCGTTGCAT	0	9	RDR2-sensitive
GCATGCGTAGAGACACGAGAACGG	0	10	RDR2-sensitive
GCATGCGTAGAGACGTGGGGACGA	0	10	RDR2-sensitive
GCATGCGTCGAGCAGGAACGACAC	0	11	RDR2-sensitive
GCATGCGTCGCTGTGTTCGGCAAG	0	9	RDR2-sensitive
GCATGGAACCTGTACGACGCGGCC	0	12	RDR2-sensitive
GCATGGACACAAGCTGACGACGAC	0	15	RDR2-sensitive
GCATGGACGAGCAGACACGACACA	0	38	RDR2-sensitive
GCATGGACGTTAGTCACGGACGGT	0	13	RDR2-sensitive
GCATGGACTATCAGCGAAGGGTAC	0	40	RDR2-sensitive
GCATGGACTGCGGCAGTGCTCCGG	0	9	RDR2-sensitive
GCATGGGCTGTGCTACAGAGGCAT	0	18	RDR2-sensitive
GCATGGTAGAGGAGGCAGTAGAGT	0	22	RDR2-sensitive
GCATGGTGTAGCAAGTGTGGACGC	0	9	RDR2-sensitive
GCATGGTTCGTGGGTCTGGGCCGG	0	15	RDR2-sensitive
GCATGTACAGTGGAGAGACACCAA	0	9	RDR2-sensitive
GCATGTACGCGAGGAACGATGGCT	0	12	RDR2-sensitive
GCATGTAGTCTTGAGGGCGTCGGG	0	9	RDR2-sensitive
GCATGTCCGGCATCCTAGATACAA	0	47	RDR2-sensitive
GCATGTCCTGTAGGCACGGTTCGT	0	10	RDR2-sensitive
GCATGTCGCACTGGCGTTCCGGAG	0	12	RDR2-sensitive
GCATGTCGCGTAGGACAGTTCGGC	2	27	RDR2-sensitive
GCATGTCGTGCGCCCGCGCCCTTC	0	12	RDR2-sensitive
GCATGTGAAGTAGAGCAACGGGAA	0	12	RDR2-sensitive
GCATGTGTTGGTCGCGGTATTCGC	0	9	RDR2-sensitive
GCATGTTGACGGTGGTCCAGAACT	36	0	RDR2-resistant
GCATGTTGGTACAGGACGTGGCTA	0	9	RDR2-sensitive
GCATGTTTGGACAGGCGTCTACAA	0	9	RDR2-sensitive
GCATTAGACGAGCTGAGTAGGGCT	0	9	RDR2-sensitive
GCATTCCGGGCTCTAGACGGTGTG	1	21	RDR2-sensitive

GCATTCGAGGCGGATTAAGGCATC	33	4	RDR2-resistant
GCATTCGTACTGTAGTAACGGGTA	0	10	RDR2-sensitive
GCATTCGTACTGTCTTGGGCCAAG	0	11	RDR2-sensitive
GCATTCGTTGGACCGTGCCGGCCC	0	17	RDR2-sensitive
GCATTGGACGAACGTTTTTGGCAT	2	14	RDR2-sensitive
GCATTGTAGTTTTGGACGAGCGGT	50	0	RDR2-resistant
GCATTTAGGACCTGACGACGACAT	0	47	RDR2-sensitive
GCATTTATGTTGATTTGGACGGTT	0	10	RDR2-sensitive
GCCAAAACTGGATGTAGGGACGTT	0	9	RDR2-sensitive
GCCAAACAGGAGGGCTAGAGCCAG	1	53	RDR2-sensitive
GCCAAACGCGGAATAGAACGGCTC	1	82	RDR2-sensitive
GCCAACACGGACGAGAACGGGCGT	0	18	RDR2-sensitive
GCCAACATGTGCGGACTGTCCGGC	0	11	RDR2-sensitive
GCCAACCGACTGGAGCAGGAGCAC	0	11	RDR2-sensitive
GCCAACCTCGGACGGGAGACGCAG	0	18	RDR2-sensitive
GCCAACCTCGGACGGGAGACGTAG	0	27	RDR2-sensitive
GCCAACGGTCGGCCGGGCCAACGG	5	21	RDR2-sensitive
GCCAACGGTCGGTCGGGCCAACGG	3	16	RDR2-sensitive
GCCAAGAGAGAAGCGTCGCCTGGA	0	24	RDR2-sensitive
GCCAAGCGGAGATAGGGTGAACAC	0	9	RDR2-sensitive
GCCAAGTCAGCGTGAACAGTACGG	1	34	RDR2-sensitive
GCCAATAGATCTCGGACTCGAGGC	0	10	RDR2-sensitive
GCCAATCACGGACGACAAGGATGG	0	28	RDR2-sensitive
GCCAATTTAGTCGGGCTAAGGCAC	0	9	RDR2-sensitive
GCCACAAAGCTGACTGGGAGACGC	0	23	RDR2-sensitive
GCCACAACTCTGGCCCAGACACGT	0	9	RDR2-sensitive
GCCACACAAGACGGATATAGACGA	0	14	RDR2-sensitive
GCCACACGCTGGACTGTCCGGTAC	0	11	RDR2-sensitive
GCCACACGTAGAGCCGCAGGGACA	0	9	RDR2-sensitive
GCCACACGTTGGACTGTCCGGTGC	0	10	RDR2-sensitive
GCCACACGTTGGACTGTTCGGTGC	0	32	RDR2-sensitive
GCCACAGGACTGAGGATGGACGGT	0	11	RDR2-sensitive
GCCACCACCGCGGACAGTCCGGAC	6	34	RDR2-sensitive
GCCACCACCGCGGACAGTTCGGAC	1	34	RDR2-sensitive
GCCACCACCGTGGACTGTCCGGAC	1	13	RDR2-sensitive
GCCACCAGTGTGGACTGTCCGGAC	0	63	RDR2-sensitive
GCCACCCAAGACGGATCTAGACGA	0	25	RDR2-sensitive
GCCACCCGTGCAGGTCGTTAGGTC	0	10	RDR2-sensitive
GCCACCTCGTCGACTGGTCGTCGT	0	15	RDR2-sensitive
GCCACCTTCCTTGGATCGCGCGGA	0	11	RDR2-sensitive
GCCACGGCAGAGCTCGGCACCATA	0	15	RDR2-sensitive
GCCACGGGTCGGTGCGAGACAGGC	1	14	RDR2-sensitive
GCCACGTGGAGCTCGAAGGTGGAT	0	9	RDR2-sensitive
GCCACTAGAAATAGCACAGTCGGT	0	12	RDR2-sensitive
GCCACTCTGACCTGATGGACGATT	0	17	RDR2-sensitive
GCCACTGAACTCGAAGACGAACGT	0	16	RDR2-sensitive
GCCACTGTAGACGAGAGGAAGCCG	0	9	RDR2-sensitive
GCCACTGTATGGAAGAATTTGGTG	42	1	RDR2-resistant
GCCACTTCTGTGCGTAGATCGCGG	0	19	RDR2-sensitive

GCCACTTGTTCGAACGTGGGTCGG	0	9	RDR2-sensitive
GCCAGAAAGCTGACTGGGAGACGC	0	14	RDR2-sensitive
GCCAGACCGCGGACCGTTCGGCAC	0	23	RDR2-sensitive
GCCAGACGGATGGTAGAAAGGGTT	0	38	RDR2-sensitive
GCCAGACTCTGCAGACGCAAGTAA	0	12	RDR2-sensitive
GCCAGACTGCTGACTGTACGGCTG	0	21	RDR2-sensitive
GCCAGACTGCTGGACCATGGCGGC	0	10	RDR2-sensitive
GCCAGACTGTGTGCATGGCTGCAT	0	10	RDR2-sensitive
GCCAGAGCTCTGCAGCAATGCCGC	0	17	RDR2-sensitive
GCCAGATACGGAAGGCGACTCCAA	19	0	RDR2-resistant
GCCAGATCACGGACCATTCGGCTC	0	9	RDR2-sensitive
GCCAGGACAAAGGATGGTCGTCAA	0	9	RDR2-sensitive
GCCAGGACGTAGAGTGTTACGCAT	0	26	RDR2-sensitive
GCCAGGACGTAGGATGTTACGCAT	1	16	RDR2-sensitive
GCCAGGACGTAGGGTGTTACGCAT	2	143	RDR2-sensitive
GCCAGGACTGGAGTTCGTCGGCAC	0	10	RDR2-sensitive
GCCAGGCACTGACACTATACTGAC	1	12	RDR2-sensitive
GCCAGGCACTGTTAGAAAACCGGT	0	19	RDR2-sensitive
GCCAGGCCTGGACGGCTACTGTAG	0	9	RDR2-sensitive
GCCAGGCTAGAGAATGCGTCACGG	0	17	RDR2-sensitive
GCCAGGGACCTGCCGACTTGACGG	0	17	RDR2-sensitive
GCCAGGTCGCGGACGGTCCGGCCA	46	11	RDR2-resistant
GCCAGGTCGTGGACTCTTCGGTAC	0	60	RDR2-sensitive
GCCAGGTGTTAGCGACTCGGTGGA	0	20	RDR2-sensitive
GCCAGGTTCGGATCTGGATCGGAC	0	10	RDR2-sensitive
GCCAGGTTGAAGAGGCTGATCAGG	0	19	RDR2-sensitive
GCCAGTACAGTAGACATTCGGCAA	0	41	RDR2-sensitive
GCCAGTATGACATCGGACCCACAA	0	12	RDR2-sensitive
GCCAGTATGCCATCGGACACACAA	0	27	RDR2-sensitive
GCCAGTATGCCATCGGACCCACAA	0	49	RDR2-sensitive
GCCAGTATGCCATCGGACTCATAA	0	15	RDR2-sensitive
GCCAGTATGCTATCGGACTCACAA	0	17	RDR2-sensitive
GCCAGTCGGTACTCGGCAAAGAAA	3	52	RDR2-sensitive
GCCATAAAGCTGACTGGGAGACGC	0	10	RDR2-sensitive
GCCATAAGTCGGCCAGCTGAAGGA	0	12	RDR2-sensitive
GCCATACCGTGACTGAGACGTGAT	0	21	RDR2-sensitive
GCCATCAAGCTGACTGGGAGACGC	0	9	RDR2-sensitive
GCCATCAGGTGCGGACTGTCCGGC	0	9	RDR2-sensitive
GCCATCGCAGGTCGTTTGTTCGTA	0	10	RDR2-sensitive
GCCATCGGGCTGCATTCAGGCGAA	0	15	RDR2-sensitive
GCCATGACGGACGCAGAGGATCGA	0	14	RDR2-sensitive
GCCATGAGGAGATGAGCATCTGAC	79	0	RDR2-resistant
GCCATGAGTCGGCCAGCTGAAGGA	0	20	RDR2-sensitive
GCCATGCTCGGACGAGGCGGAGAT	0	14	RDR2-sensitive
GCCCAAACAGGTTGTCGGGTGCGC	0	10	RDR2-sensitive
GCCCACAGACTTAGAATACGGTTC	0	34	RDR2-sensitive
GCCCACAGGCGCGGACTGTCCGGA	1	23	RDR2-sensitive
GCCCACCCGTTGGTCGAAGTCTGA	0	10	RDR2-sensitive
GCCCACCTGTAAGAGCGTCGACGG	0	14	RDR2-sensitive

GCCCACGGCTGCGAGCAGAACGAC	0	17	RDR2-sensitive
GCCCACTCAGGACCACTGAACCGG	0	35	RDR2-sensitive
GCCCACTGACCAGCAGGACTCGGC	0	15	RDR2-sensitive
GCCCAGACGGCACGGTAGGCCTGT	0	12	RDR2-sensitive
GCCCAGCTGTAGCAATGGGATGGT	0	43	RDR2-sensitive
GCCCAGTCGGCGGCATCCTCCCAC	30	2	RDR2-resistant
GCCCATGGGGTGACGCCGGAAAGG	338	0	RDR2-resistant
GCCCCAGAAGACGACGAACTCCGC	0	9	RDR2-sensitive
GCCCCCGGATGCAGGACACACGGT	0	11	RDR2-sensitive
GCCCCGCACGACTGTGGACCAGGC	0	11	RDR2-sensitive
GCCCCGCTGTAGATCTGGTCGGCA	0	19	RDR2-sensitive
GCCCCGGAAGACGGCGAACTCCGA	0	20	RDR2-sensitive
GCCCCGGAAGACGGCGAACTCCGC	22	130	RDR2-sensitive
GCCCCGGACGTATGCAGTAGGCGT	0	14	RDR2-sensitive
GCCCCGGACGTATGCAGTAGGTGT	1	21	RDR2-sensitive
GCCCCGGGACTGTGTCCGAGCGGT	2	36	RDR2-sensitive
GCCCCTACAGCTAGGTCGCGGACC	0	16	RDR2-sensitive
GCCCCTGAATCCTGATCGTCTGAT	0	15	RDR2-sensitive
GCCCCTGTAGATCAACGGCTGAAA	1	90	RDR2-sensitive
GCCCCTGTTTGGATGGTTGGTCGG	0	13	RDR2-sensitive
GCCCCTTAGGGAGAGATCTTAGGA	0	9	RDR2-sensitive
GCCCGAACCAACAGTAGAACGGTA	0	11	RDR2-sensitive
GCCCGAATTGTAGTCTGGAGAGGC	35	72	RDR2-sensitive
GCCCGACCTAGAGCACGACGCCAT	0	10	RDR2-sensitive
GCCCGACTGCCGAAAATACGACAT	0	15	RDR2-sensitive
GCCCGAGATGTAGGTCGACGACGG	0	10	RDR2-sensitive
GCCCGAGTACCTGTAGAGACGCAT	0	11	RDR2-sensitive
GCCCGCACTGTTAGGAGCCGGCGT	0	10	RDR2-sensitive
GCCCGCGACAGTAGATCGGACGGT	0	14	RDR2-sensitive
GCCCGCTTATTCTACCGTGCCGGG	0	28	RDR2-sensitive
GCCCGGACTGCGTGCTGCATGGCT	0	9	RDR2-sensitive
GCCCGGACTGCGTGTTGCATGGCT	0	9	RDR2-sensitive
GCCCGGCACAGTAGAATAAGCGGA	2	119	RDR2-sensitive
GCCCGGCACGATCTAGACATGGAC	1	21	RDR2-sensitive
GCCCGGCACGGTAGAATAAACGGG	1	68	RDR2-sensitive
GCCCGGCACGGTAGAATAAGCGGA	0	40	RDR2-sensitive
GCCCGGCACGGTAGAATAAGCGGG	11	469	RDR2-sensitive
GCCCGGCACGGTAGAATAAGCGGT	0	10	RDR2-sensitive
GCCCGGCACGGTAGAATAAGTGGA	0	10	RDR2-sensitive
GCCCGGCTATGGAGAATGCGACGG	0	9	RDR2-sensitive
GCCCGGCTGTAGGGATGGTGCCGC	0	19	RDR2-sensitive
GCCCGGCTGTATGAGTCAGGTCGG	0	9	RDR2-sensitive
GCCCGGTCCAATGGACTTAGACGG	0	11	RDR2-sensitive
GCCCGTAGATCTCGAGCAAGGCAG	1	17	RDR2-sensitive
GCCCGTGGCGTAGACGCGAGACGC	0	30	RDR2-sensitive
GCCCGTGTCGGCACTGTAGGTCGG	0	46	RDR2-sensitive
GCCCGTTTGGCCTGTGGCACGGCA	0	14	RDR2-sensitive
GCCCTAATCGGACTTATGCCGGGT	0	11	RDR2-sensitive
GCCCTACGGACGAGCTCCGGCATG	0	10	RDR2-sensitive

GCCCTACTGTAGACGGTCAGACAC	0	9	RDR2-sensitive
GCCCTACTGTAGACGGTCAGTGTC	1	30	RDR2-sensitive
GCCCTCAGCTCGGATCGCGCGGAT	0	9	RDR2-sensitive
GCCCTCCAATTGAGTCGTCGGAGC	0	10	RDR2-sensitive
GCCCTCTCGGAACCGGATGACTGC	0	18	RDR2-sensitive
GCCCTCTGGTCAGGCGCGGACGGT	0	10	RDR2-sensitive
GCCCTCTGTAAGCTGAATGCGTCC	0	10	RDR2-sensitive
GCCCTCTGTAAGCTGAATGCGTCT	0	9	RDR2-sensitive
GCCCTGAGGGTGGACTGTTCGCGG	0	16	RDR2-sensitive
GCCCTGAGGTCGGACGGTCCGCGG	0	15	RDR2-sensitive
GCCCTGGACATGCAAGTGAACGAA	0	9	RDR2-sensitive
GCCCTGGCTGATCTGATACGGCAT	0	14	RDR2-sensitive
GCCCTGGGGAACGACGACGGTAGC	0	10	RDR2-sensitive
GCCCTGTAGAAGCACAGCTGTCGG	1	56	RDR2-sensitive
GCCCTGTAGAAGCGCAGCTGGCGT	0	25	RDR2-sensitive
GCCCTGTAGAAGCGTAGCTGACGG	0	14	RDR2-sensitive
GCCCTGTGTCGTCGACGTTGGTGC	0	10	RDR2-sensitive
GCCCTTAAAGGCGTGACTCGGGCA	1	40	RDR2-sensitive
GCCCTTAGCCCCAAGAGCGTCGGT	110	26	RDR2-resistant
GCCCTTGGCGTAGGATTTCGCGGT	0	36	RDR2-sensitive
GCCGAAATCGGACGGGAACCGGTA	0	29	RDR2-sensitive
GCCGAACCGGGAGCAGAACGCCCT	0	12	RDR2-sensitive
GCCGAAGAAGGACACGACGAAGAC	0	15	RDR2-sensitive
GCCGAAGACGTCTCTGCATGTTGT	0	10	RDR2-sensitive
GCCGAAGACTGAAAGTAGGGACGT	0	47	RDR2-sensitive
GCCGAAGACTGGATGTAGAGACGT	0	16	RDR2-sensitive
GCCGAAGACTGGATGTAGGGACGT	0	13	RDR2-sensitive
GCCGAAGATGTACGATGATGAGGA	47	1	RDR2-resistant
GCCGAATACTGGATGTAGGGACGT	3	26	RDR2-sensitive
GCCGAATTGGTTCTGTTGGGCTGC	0	9	RDR2-sensitive
GCCGACACGTAGGGGGCGCTGTAG	0	15	RDR2-sensitive
GCCGACACGTCGGTGTTCTCGGAC	0	18	RDR2-sensitive
GCCGACCAACCCTCTGGCAAGCAG	0	12	RDR2-sensitive
GCCGACCGTTGCAGATCTGGCGCA	0	10	RDR2-sensitive
GCCGACCGTTGGCTCACCGGACAG	1	12	RDR2-sensitive
GCCGACGACTGCACGGCACTGCTC	39	8	RDR2-resistant
GCCGACGATCTGACGACCTGGTGC	0	29	RDR2-sensitive
GCCGACGTGCTGACTGTGCCGGAC	0	12	RDR2-sensitive
GCCGACGTGTGGTCTGTTCACGGA	0	20	RDR2-sensitive
GCCGACTGAGCATACGATGGAGAC	0	12	RDR2-sensitive
GCCGACTGTAGCAAAATGGTTCGT	0	16	RDR2-sensitive
GCCGACTGTAGCTAAATGGTTCGT	0	25	RDR2-sensitive
GCCGAGAACTCTACTGCCGACTGC	0	57	RDR2-sensitive
GCCGAGAACTCTACTGTCGACTGC	0	17	RDR2-sensitive
GCCGAGAGGCAGAACGATGGCACA	0	28	RDR2-sensitive
GCCGAGATCTCAGGACTATGGTTA	0	10	RDR2-sensitive
GCCGAGCAGACTGCAGACAAACAA	0	10	RDR2-sensitive
GCCGAGCGTAGTCTGTATGTGCGA	0	10	RDR2-sensitive
GCCGAGCTCGGACACGTCGACGTC	0	10	RDR2-sensitive

GCCGAGCTCTGACGTGGCCGCAAC	0	12	RDR2-sensitive
GCCGAGCTCTGACGTGGCGACGAC	3	19	RDR2-sensitive
GCCGAGCTCTGACGTGGCGACGGG	0	17	RDR2-sensitive
GCCGAGCTCTGACGTGGTTTTGGC	0	12	RDR2-sensitive
GCCGAGCTCTGATATGGCCAAGAC	0	11	RDR2-sensitive
GCCGAGGACAGTGTTGAAGACGAC	0	12	RDR2-sensitive
GCCGAGGACTGGGGGCATGGTGAT	0	12	RDR2-sensitive
GCCGAGGTCCTGTAGCTGGCGGGC	138	20	RDR2-resistant
GCCGAGTCGCAGCGATAGTAGGAA	0	10	RDR2-sensitive
GCCGAGTGTGGGCTAGGGACGGAT	0	14	RDR2-sensitive
GCCGATCAGTGACGTGGACACGGT	90	3	RDR2-resistant
GCCGATCCATCTGGGCTGGGGCAC	1	31	RDR2-sensitive
GCCGATCCGAGCCGAGCCGAGCTG	0	9	RDR2-sensitive
GCCGATCCGGACTTGCTTGGAGGC	2	17	RDR2-sensitive
GCCGATCCGGATCTGCTTGGAGGC	8	83	RDR2-sensitive
GCCGATCCGGCGGACAGAGACGAC	0	9	RDR2-sensitive
GCCGATCCGGGCGGAAGACATTGT	39	2	RDR2-resistant
GCCGATCGGGGACGGTTATTTCGG	1	37	RDR2-sensitive
GCCGATGACTAAAGAACAGGCCGG	0	12	RDR2-sensitive
GCCGATGACTGAAAGTAGGGACGT	0	14	RDR2-sensitive
GCCGATGACTGCAAGTAGTGACGT	0	25	RDR2-sensitive
GCCGATGACTGGAAGTAGGGACGC	0	10	RDR2-sensitive
GCCGATGACTGGAAGTAGGGACGT	0	68	RDR2-sensitive
GCCGATGACTGGATGTAGGGACGT	1	26	RDR2-sensitive
GCCGATGACTGGGCACACGTGCTG	1	20	RDR2-sensitive
GCCGATGGGAACATGGACGGCGGG	44	86	RDR2-sensitive
GCCGATGTACAGTTCGCCGAGCGT	0	9	RDR2-sensitive
GCCGATGTGGGACTAAAGCCGGGG	1	14	RDR2-sensitive
GCCGCACGGGATCTTAAGACACGA	0	9	RDR2-sensitive
GCCGCACGTCGTCAGGGCCCTAAG	0	12	RDR2-sensitive
GCCGCAGCCGCAGCCGGATTGCAG	0	11	RDR2-sensitive
GCCGCCACTGAGAACCGTTCGGAC	0	21	RDR2-sensitive
GCCGCCCTGGGGAACGACGACGGT	0	95	RDR2-sensitive
GCCGCCGCGGCTTAGACACGACGT	0	15	RDR2-sensitive
GCCGCCTGGGTAAGGATCTGTCGG	0	12	RDR2-sensitive
GCCGCCTTGGGCTGTAATGTTGGC	0	28	RDR2-sensitive
GCCGCGACTTGCAACTTCGACGGA	0	11	RDR2-sensitive
GCCGCGACTTGCGACTTCGACGGA	20	52	RDR2-sensitive
GCCGCGACTTGTGACTTCGACGGA	0	13	RDR2-sensitive
GCCGCGGCTGGAACCTGGAAGCGG	0	66	RDR2-sensitive
GCCGCGGTCGTCCCTGATGTTGGC	0	10	RDR2-sensitive
GCCGCGGTGTAGGGATGGGCGAGC	1	23	RDR2-sensitive
GCCGCTACGGTGGATAGTCGGAAC	0	11	RDR2-sensitive
GCCGCTAGTACTGTAGCACCGGTA	1	127	RDR2-sensitive
GCCGCTCTGAGCCAAACCGAGACG	0	13	RDR2-sensitive
GCCGCTCTGAGCCGAGCCGAGCCG	0	9	RDR2-sensitive
GCCGCTCTGTTCTGTATTTGGTAA	0	16	RDR2-sensitive
GCCGCTCTGTTCTGTGTTTGGTAA	1	32	RDR2-sensitive
GCCGCTCTTATAGCGAGGTCGCGG	0	9	RDR2-sensitive

GCCGCTTTAAGGGATCTGTTGGAG	0	22	RDR2-sensitive
GCCGGAACCGAGGGAGACGTCCAC	1	28	RDR2-sensitive
GCCGGAATCAGCAGAGTCGACGAC	0	9	RDR2-sensitive
GCCGGAATCAGCAGGATCGACGAC	0	17	RDR2-sensitive
GCCGGAATCAGCAGGGTCGACGAC	0	22	RDR2-sensitive
GCCGGAATCAGCAGGGTCGACGAT	2	59	RDR2-sensitive
GCCGGACATGTTCAGGATCGTCGT	2	24	RDR2-sensitive
GCCGGACCAGGTCCAGACGTCTAT	2	62	RDR2-sensitive
GCCGGACCAGGTTCAGACGTCTAT	0	10	RDR2-sensitive
GCCGGACCGTAGGCCTAGATACGG	0	10	RDR2-sensitive
GCCGGACTTAGAGCCCAAGCACGG	0	13	RDR2-sensitive
GCCGGAGAAGACAGCTATCCCCAT	0	15	RDR2-sensitive
GCCGGAGAAGGACACGACGAAGAC	2	115	RDR2-sensitive
GCCGGAGACGAGGCAGAGCTGCAA	0	10	RDR2-sensitive
GCCGGAGACGGTACTATAGATCCA	1	15	RDR2-sensitive
GCCGGAGCCTGTACACGCGGCTGT	0	35	RDR2-sensitive
GCCGGAGGATAGCACGCAAGACGT	0	9	RDR2-sensitive
GCCGGAGGATAGCGTGCATGACGT	0	24	RDR2-sensitive
GCCGGAGTAGCTGACGTGGACGGT	1	19	RDR2-sensitive
GCCGGATAAGCAGAACCAGGCTCA	0	91	RDR2-sensitive
GCCGGATATTGTGGAGAAGGGCGT	0	10	RDR2-sensitive
GCCGGATCACGGGTCAAACGGGCT	0	10	RDR2-sensitive
GCCGGATCAGAGGATGTCACGCGC	0	16	RDR2-sensitive
GCCGGATCAGGTCTAGACGTCTAT	0	9	RDR2-sensitive
GCCGGATCGGAGGATGTCACGCGC	1	16	RDR2-sensitive
GCCGGATCTGAGGATGAGAAGGGC	0	10	RDR2-sensitive
GCCGGATTATCTCTATGCAGGCAA	0	9	RDR2-sensitive
GCCGGATTGATGAAGAGTTGGCGT	0	9	RDR2-sensitive
GCCGGATTTGACTGCGATGGCGGC	0	37	RDR2-sensitive
GCCGGCACGCAGATCGCAGACGCA	0	9	RDR2-sensitive
GCCGGCCTCGAGCAGATAGGACAT	1	13	RDR2-sensitive
GCCGGCGATGTAGGAGAGCGGGAC	0	11	RDR2-sensitive
GCCGGCGTGTAGATCAAGAGTGGA	0	9	RDR2-sensitive
GCCGGCTAAGCAGAACAGATCCAT	0	13	RDR2-sensitive
GCCGGCTAGACGACGCAAACACAT	0	14	RDR2-sensitive
GCCGGCTTGAGCTCGGTGCGGCTC	0	9	RDR2-sensitive
GCCGGGATGGCATATCTGCACGAA	0	9	RDR2-sensitive
GCCGGGCTAATTGGACCGTCGGGC	0	12	RDR2-sensitive
GCCGGGCTACTTGGGCCGTCGGGC	0	10	RDR2-sensitive
GCCGGGCTAGGTAGAGCGAGGCAA	0	21	RDR2-sensitive
GCCGGGCTATTTGGGTCGTTGGGC	1	15	RDR2-sensitive
GCCGGGCTGACTCGGTAAGCACGG	0	17	RDR2-sensitive
GCCGGGCTGGCCTAGAAAGTACGG	0	11	RDR2-sensitive
GCCGGGCTGGGCATGACACGACAG	0	14	RDR2-sensitive
GCCGGGGACCTGCCGACTTGACGG	0	39	RDR2-sensitive
GCCGGGTCACGGGCTAAACGGGCG	0	10	RDR2-sensitive
GCCGGGTTACGTAGTGCAGTGCAT	0	15	RDR2-sensitive
GCCGGGTTGGACTGCACGACGGGC	1	25	RDR2-sensitive
GCCGGTATGGTCGAGGACTGGCTA	0	16	RDR2-sensitive

GCCGGTCGACGTGAATGAGCGGAT	0	13	RDR2-sensitive
GCCGGTCGGCTATGCACGGCTCAC	0	19	RDR2-sensitive
GCCGGTCTCTAGCGAGCGTTGACG	0	38	RDR2-sensitive
GCCGGTCTGGACCAGTTGAACGGG	0	20	RDR2-sensitive
GCCGGTGACCTGCCGACTTGACGG	0	18	RDR2-sensitive
GCCGGTGACGAACTCCAAGCGGCC	0	84	RDR2-sensitive
GCCGGTGATGTAGAGTGCATCAGA	19	1	RDR2-resistant
GCCGGTGCAGGTGAACGGACGGAC	0	16	RDR2-sensitive
GCCGGTGGGGTAGTCTGATGGCGC	0	11	RDR2-sensitive
GCCGGTGGTAGACTGGAGCTCGGC	0	10	RDR2-sensitive
GCCGGTGGTCAGACGTTGGATGAA	0	10	RDR2-sensitive
GCCGGTGTTTGTAGTTATCGACGT	0	10	RDR2-sensitive
GCCGGTTCGTCGACAGCTAAGGCC	0	10	RDR2-sensitive
GCCGGTTCTTGTTGAGTGTTTGGC	3	37	RDR2-sensitive
GCCGTAGGATTGCTGCTTGATGGT	1	46	RDR2-sensitive
GCCGTAGGATTGCTGTTTGATGGT	0	14	RDR2-sensitive
GCCGTAGTCGTTGGTCCAACGGAG	0	13	RDR2-sensitive
GCCGTCATGTCGTTGGATGGAAAA	0	106	RDR2-sensitive
GCCGTCTGGAAGGGTGATTGTCGT	0	100	RDR2-sensitive
GCCGTGACGGTGACTTTTCTTTGC	0	15	RDR2-sensitive
GCCGTGAGCAGATAGAACCGTGCA	0	18	RDR2-sensitive
GCCGTGCCGGACTTGGATCAGGCT	0	19	RDR2-sensitive
GCCGTGCCGGGCCAAGAACGACGG	1	15	RDR2-sensitive
GCCGTGCGACGTGTAGAGTGACGT	1	12	RDR2-sensitive
GCCGTGCGTTATACGGAGTCGGAT	0	57	RDR2-sensitive
GCCGTGCTGTCGAGCAGGAGCTGA	0	29	RDR2-sensitive
GCCGTGGCTGAGGATTCTCGCGGC	0	12	RDR2-sensitive
GCCGTGGGCCGATGACTGGGCATA	0	12	RDR2-sensitive
GCCGTGTAGAACAAAAGAGGCCAC	0	14	RDR2-sensitive
GCCGTGTATGGTTGTCGGATACGG	0	9	RDR2-sensitive
GCCGTGTCGGGCTCGTGCCGTGCT	0	14	RDR2-sensitive
GCCGTGTCGGGTCTGGACTGGCTA	1	13	RDR2-sensitive
GCCGTGTCGTGCCTGTAGCCCGGT	0	16	RDR2-sensitive
GCCGTTAGGGTTCGACCGTTGGAG	3	17	RDR2-sensitive
GCCGTTGGTCGTCGCTCTTCCGGT	0	9	RDR2-sensitive
GCCGTTGTCGTCGGATCGAACGGC	0	10	RDR2-sensitive
GCCTAAACTGGACTTGAGCTACGG	0	12	RDR2-sensitive
GCCTAAAGCACGGACTGTTCGGTG	0	10	RDR2-sensitive
GCCTAAATGGTTGTTCGGATAGAA	0	10	RDR2-sensitive
GCCTAACAGGACGGCTGAAAGCAC	0	31	RDR2-sensitive
GCCTACAGGTCGGACGGTCCGCGC	0	9	RDR2-sensitive
GCCTACCAATTAACCGTGTCGGAC	0	15	RDR2-sensitive
GCCTACCATCGGACTGTTCGGTGC	0	13	RDR2-sensitive
GCCTACCGAGTGGGACTGCGTCGT	0	13	RDR2-sensitive
GCCTACGCCTCCAAATCGTCGAAG	0	23	RDR2-sensitive
GCCTAGCCGAACTGTCCTACGCGA	3	35	RDR2-sensitive
GCCTAGCGGGCTGTCTTGAGGTAC	0	30	RDR2-sensitive
GCCTAGGTACGGCACGAATACTCT	0	12	RDR2-sensitive
GCCTATAGCAGGTCGGCTCGGACA	0	24	RDR2-sensitive

GCCTATCCTTGCATCGCTCGGCAC	0	10	RDR2-sensitive
GCCTATCGCGTAGGACAGTTCGGC	0	14	RDR2-sensitive
GCCTATCGGATCGGAGAAGCCCGC	7	48	RDR2-sensitive
GCCTATTGGGTCGTGCGTTGTCGG	0	16	RDR2-sensitive
GCCTATTTCTGTAGTCTATCGGTT	143	0	RDR2-resistant
GCCTCACGCCAGGTCGCGGACCGT	0	19	RDR2-sensitive
GCCTCAGCCTTAGCTCGTCGGTCA	1	25	RDR2-sensitive
GCCTCCAAATCGTCGAAGCCGCAA	0	17	RDR2-sensitive
GCCTCCAAATCGTCGAAGCCGCAC	1	47	RDR2-sensitive
GCCTCCAAATCGTCGAAGCCGCAT	0	100	RDR2-sensitive
GCCTCCAACAGCGTATCGTCGGTG	0	16	RDR2-sensitive
GCCTCCGAATTGTGATCGCGCGGA	0	12	RDR2-sensitive
GCCTCGCGCGGGACGGTAGAGGGC	0	18	RDR2-sensitive
GCCTCGCGCGTCTGTATGAAGCAT	0	11	RDR2-sensitive
GCCTCGCGCGTCTGTATGGAGCAT	0	15	RDR2-sensitive
GCCTCGCGTGTCTGTATGGAGCAT	0	17	RDR2-sensitive
GCCTCGGTTAGTCGGGCCTGGCTA	0	14	RDR2-sensitive
GCCTCTACTGCTGACGGGAGGGAA	0	15	RDR2-sensitive
GCCTCTCGGAAGGGACTTCGTCGA	0	16	RDR2-sensitive
GCCTCTGTCGGCTCTGTGAGGTGC	0	9	RDR2-sensitive
GCCTCTTCGGACGGTCGGATGGTC	0	19	RDR2-sensitive
GCCTGAAAGGTGACGTAGGACGAC	0	13	RDR2-sensitive
GCCTGAACGCTTGGATGCCGGCCT	0	22	RDR2-sensitive
GCCTGACCAGAGAGCACCGCCGCC	8	47	RDR2-sensitive
GCCTGACCAGAGAGCACCGCCGCT	1	24	RDR2-sensitive
GCCTGACCAGAGAGCACCGCTGCC	1	13	RDR2-sensitive
GCCTGACGACGGTGAACGCGCGAA	0	10	RDR2-sensitive
GCCTGACGCGTAGGACAGTTCGGC	0	40	RDR2-sensitive
GCCTGACTCTAAACCATGCGGTGT	0	12	RDR2-sensitive
GCCTGAGACTGTACGTGACGACGT	0	22	RDR2-sensitive
GCCTGAGAGAACCACAGAAACGGT	0	11	RDR2-sensitive
GCCTGAGCAGAGCATGGACCCTTC	42	1	RDR2-resistant
GCCTGAGCGCGCTGTCGGGCGGCC	17	0	RDR2-resistant
GCCTGATAGAAGGGCTCTCGGCAT	0	9	RDR2-sensitive
GCCTGATCATGTTGGACGGGCCGG	1	43	RDR2-sensitive
GCCTGATCCTGAAGACGTTGGCCG	1	12	RDR2-sensitive
GCCTGATTGGTCGGATATGGGCGT	0	18	RDR2-sensitive
GCCTGCAACTCGTCGAGACGCCGA	0	10	RDR2-sensitive
GCCTGCACGGATTGTTGTTACGGT	1	12	RDR2-sensitive
GCCTGCGCTGTAGATGCCGATGGT	0	13	RDR2-sensitive
GCCTGCGTTGGACACTTGGACGGC	0	11	RDR2-sensitive
GCCTGCTAGACGAGTCGGACACAG	0	33	RDR2-sensitive
GCCTGCTCGGACGGAGGACGGATC	4	22	RDR2-sensitive
GCCTGCTGTGGAAACCAAGACTGT	0	14	RDR2-sensitive
GCCTGCTTCAGACGACTCGAACGG	0	21	RDR2-sensitive
GCCTGCTTGGACGAACCGCGGAAC	0	11	RDR2-sensitive
GCCTGGACCAATTGAACGGGCCGA	0	11	RDR2-sensitive
GCCTGGACCAGTTGAACCGGCCGA	0	13	RDR2-sensitive
GCCTGGACCAGTTGAACGGACCGA	0	24	RDR2-sensitive

GCCTGGACCAGTTGAACGGGCCGA	0	15	RDR2-sensitive
GCCTGGACCATGACTGCGACACGC	0	56	RDR2-sensitive
GCCTGGACCGTGCTTTTTCGTGCC	0	18	RDR2-sensitive
GCCTGGACGGAGCAGCTGATGCGC	0	10	RDR2-sensitive
GCCTGGACGGCTACTGTAGCCCAC	0	26	RDR2-sensitive
GCCTGGACTGGGGAAGATGGTGCC	1	40	RDR2-sensitive
GCCTGGACTGTGGAAGATGGTGCT	1	28	RDR2-sensitive
GCCTGGCACGGTAGAATAAGCGGG	0	32	RDR2-sensitive
GCCTGGCTCCAGAGATGCGAACAA	0	20	RDR2-sensitive
GCCTGGCTGGCCAGATACTGGCAT	0	16	RDR2-sensitive
GCCTGGCTGTAAGCACGCATCCAA	0	9	RDR2-sensitive
GCCTGGCTTCCTCTGGACGATGGT	2	16	RDR2-sensitive
GCCTGGGCAGTTAGGACGCGGCAA	0	16	RDR2-sensitive
GCCTGGGCTGCCGGCTACGCTCGT	1	20	RDR2-sensitive
GCCTGGGCTGTAATGTGCAGACAA	0	67	RDR2-sensitive
GCCTGGGCTGTTGGATGGTAAGAT	0	12	RDR2-sensitive
GCCTGGGCTGTTGGTTTCGGCGGA	7	49	RDR2-sensitive
GCCTGGGTGGGACGGTTGGCTCGG	0	12	RDR2-sensitive
GCCTGGTAGTGCCCTCTGCGACGG	1	69	RDR2-sensitive
GCCTGGTAGTGTCCTCTGCGACGG	0	14	RDR2-sensitive
GCCTGGTCGGTACGGTGCGGTCAG	0	46	RDR2-sensitive
GCCTGGTCGGTAGAGCGACACGGA	0	14	RDR2-sensitive
GCCTGGTCGTCGATCTCCAGTGGC	0	9	RDR2-sensitive
GCCTGTACCTCTGCGCTTTGACGG	0	13	RDR2-sensitive
GCCTGTAGACCGGACGGTCCGCGC	0	16	RDR2-sensitive
GCCTGTAGATAGGGTGTACCTGCG	0	12	RDR2-sensitive
GCCTGTAGATCAACGGCTGAAAAC	1	19	RDR2-sensitive
GCCTGTAGCACCTGGCAAAGCAGC	0	11	RDR2-sensitive
GCCTGTAGCACTAGCGGCACTGAA	0	11	RDR2-sensitive
GCCTGTAGGCCGGACGGTCCGCGC	0	13	RDR2-sensitive
GCCTGTAGTGCCTGGGCACGACAC	0	10	RDR2-sensitive
GCCTGTAGTGTAGTTGGACATCAA	0	15	RDR2-sensitive
GCCTGTAGTTAGTATTTGCGCGGA	21	1	RDR2-resistant
GCCTGTATAGAGCCGAGACGTGCT	1	12	RDR2-sensitive
GCCTGTATTGTCGTATCGTCTGAC	0	10	RDR2-sensitive
GCCTGTCAACTGGGTGGAACGGAC	0	9	RDR2-sensitive
GCCTGTCCGGTGGCATGATAGAAC	34	4	RDR2-resistant
GCCTGTCGAGCTTTGGAGGTGGTG	0	15	RDR2-sensitive
GCCTGTCGCGGGCTGGGACAAGGC	0	47	RDR2-sensitive
GCCTGTCGCGTAGGACAGTTCGGC	8	225	RDR2-sensitive
GCCTGTCGCGTAGGATAGTTCGGC	1	18	RDR2-sensitive
GCCTGTCGGTGCCCTCTACGGTGT	0	13	RDR2-sensitive
GCCTGTGACTGTGACATCGGCTGA	0	13	RDR2-sensitive
GCCTGTGGACTGCGCGATGGCGGT	0	18	RDR2-sensitive
GCCTGTGGCGCCGAGCTATGACGT	0	15	RDR2-sensitive
GCCTGTGGGATTTGTCGAAGAGGT	0	9	RDR2-sensitive
GCCTGTGGTTCGGACGGTCCGCGC	0	12	RDR2-sensitive
GCCTGTGTGAACCTCCAGCAGCAC	15	0	RDR2-resistant
GCCTGTTAGCCAGAATCGTCGAGC	0	11	RDR2-sensitive

GCCTGTTCGGCTGTTCCTGGCGGC	1	32	RDR2-sensitive
GCCTGTTCTCTGACATCTGCAGAA	8	41	RDR2-sensitive
GCCTGTTTGAACTGTGGCACGGCA	1	24	RDR2-sensitive
GCCTTAACGGGCTGTGGGCCGGCC	0	10	RDR2-sensitive
GCCTTACGCGCTTATCGTCCTGGA	2	15	RDR2-sensitive
GCCTTAGAACTCTCCCCTCGACGG	0	9	RDR2-sensitive
GCCTTAGTTCGTCGAGAGTGGCAC	1	12	RDR2-sensitive
GCCTTATGGGTCGGTGGCGTCGGA	0	9	RDR2-sensitive
GCCTTCAAATCGTCGAAGCCGCAT	0	17	RDR2-sensitive
GCCTTCAAATTGTGATCGCGCGGA	0	10	RDR2-sensitive
GCCTTCACACTGTAGTAAGGGGTA	1	14	RDR2-sensitive
GCCTTCACGTCGAACTGCTTGGCA	0	14	RDR2-sensitive
GCCTTCAGGCTCTACGAATTCGGC	1	14	RDR2-sensitive
GCCTTCATACTGTAGTAACGGGTA	0	11	RDR2-sensitive
GCCTTCGGACTCGTAGCCACAGGC	0	15	RDR2-sensitive
GCCTTCGTTGGACCGTGTCGGCCC	2	16	RDR2-sensitive
GCCTTCGTTGGATCGTGCCGGCTC	0	15	RDR2-sensitive
GCCTTCGTTGGATCGTGTCGGCCC	0	14	RDR2-sensitive
GCCTTCTAGGATCGGATCGCGCGG	1	12	RDR2-sensitive
GCCTTCTCTGTAGGAGACGACATA	0	11	RDR2-sensitive
GCCTTCTGTCGTCGGGCGCACCGG	6	44	RDR2-sensitive
GCCTTCTGTCGTCGGGGGCACCGG	0	11	RDR2-sensitive
GCCTTGGGACTGAACGAGGACGCG	1	14	RDR2-sensitive
GCCTTGGGCTGTAATGTTGGCTGT	0	11	RDR2-sensitive
GCCTTGTATCGGCCGGACACGGTT	1	15	RDR2-sensitive
GCCTTGTCGTGCTGTTTATCGGTT	178	34	RDR2-resistant
GCCTTGTGTTGGATTGTACGGTGT	0	9	RDR2-sensitive
GCCTTTAAATCGTCGAAGCCGCAC	0	18	RDR2-sensitive
GCCTTTAACGTACTGAACCGGTGT	0	17	RDR2-sensitive
GCCTTTATGTGATGATTTGACGGT	0	11	RDR2-sensitive
GCCTTTATTGTATCGTCGGGACGG	0	9	RDR2-sensitive
GCCTTTCCGACTGTTCTGACGGTA	0	29	RDR2-sensitive
GCCTTTGACGGAGGCTGAGTCGGA	0	13	RDR2-sensitive
GCCTTTGTAGATCCGAGCGGCCAG	0	55	RDR2-sensitive
GCGAAACACATGTGGATGGGGCAT	0	10	RDR2-sensitive
GCGAAACCTGATGGATGAGAGCGG	0	13	RDR2-sensitive
GCGAAACGACTGTTGGGCTTCGGC	0	9	RDR2-sensitive
GCGAACACCAGTCGGAGACGTCAC	0	22	RDR2-sensitive
GCGAACCAGTAACGAGATGAGCAC	1	13	RDR2-sensitive
GCGAACCGCGGAACAGGATTCGGC	1	31	RDR2-sensitive
GCGAACGCGGCAGAACAAAGCAAA	0	14	RDR2-sensitive
GCGAACGGAACTGCGTGGAGACGA	0	10	RDR2-sensitive
GCGAAGAAGACGACACTCTGCGCC	0	11	RDR2-sensitive
GCGAAGACGTAAGAGCCAGGCGCT	0	19	RDR2-sensitive
GCGAAGACGTGACTGCATCTGTAC	0	12	RDR2-sensitive
GCGAAGACTGGACGATCGAGTCAT	0	10	RDR2-sensitive
GCGAAGAGAATTGGACCAAGGCAA	0	11	RDR2-sensitive
GCGACAATTTACTCGTCGGTCCAG	1	18	RDR2-sensitive
GCGACAGAGCTCATGATCGTCGAG	0	9	RDR2-sensitive

GCGACAGGCGTAGACACCCGGCAA	2	25	RDR2-sensitive
GCGACAGGTGTAGGCTCTAGCGGC	0	10	RDR2-sensitive
GCGACAGTAGATCGGACGGTCCGC	0	14	RDR2-sensitive
GCGACCCACAAACAGGACTGGCAT	0	34	RDR2-sensitive
GCGACCCCAGGTCAGTCGGGACTA	650	94	RDR2-resistant
GCGACCGACCGTTGGCTGAGGCAC	0	11	RDR2-sensitive
GCGACCTGGCGCAGAAGCTGACGT	1	22	RDR2-sensitive
GCGACGACACTGGTGGCTCGACGG	0	9	RDR2-sensitive
GCGACGACTGTAGATGGTGACGGG	0	19	RDR2-sensitive
GCGACGACTGTGTACGGCTACATC	0	11	RDR2-sensitive
GCGACGAGGACGATTGGCGCCAAC	22	0	RDR2-resistant
GCGACGCTCAGAACTGCACTAGAC	0	10	RDR2-sensitive
GCGACGCTGAGCAGAGGACTTCAT	0	16	RDR2-sensitive
GCGACGGCAGAACCACGGCAGAAC	0	16	RDR2-sensitive
GCGACGGCTAAAACGACGGAACAA	0	18	RDR2-sensitive
GCGACGGCTTACAACAATACCCAC	0	20	RDR2-sensitive
GCGACGTAATCGGACTTTATTGGA	21	2	RDR2-resistant
GCGACTAGGGAAGAAGAGGACGCT	0	12	RDR2-sensitive
GCGACTCGAACTCGGATGGCGGTC	0	9	RDR2-sensitive
GCGACTCGGCTACGACGGTGACTC	0	11	RDR2-sensitive
GCGACTCTGGTAGGATGGATGGAG	16	0	RDR2-resistant
GCGACTGAACCAGATGGCACCTTT	0	14	RDR2-sensitive
GCGACTGAACCAGCACAGACGGAC	0	27	RDR2-sensitive
GCGACTGAGAGCCTAGACAGATGG	0	47	RDR2-sensitive
GCGACTGAGAGCCTAGGCAGATAG	0	18	RDR2-sensitive
GCGACTGAGAGCCTAGGCAGATGG	2	49	RDR2-sensitive
GCGACTGCGGATCGGCTAAATCAC	0	9	RDR2-sensitive
GCGACTGGACGTACCTGGAAGGCG	0	26	RDR2-sensitive
GCGACTGGATCGACAGCGAGCGGC	0	13	RDR2-sensitive
GCGACTGTCGACGGAAAGCTACAA	0	17	RDR2-sensitive
GCGACTGTGGTCGTGTTCATCGAA	6	32	RDR2-sensitive
GCGACTGTTGAAGGTGCACAAGAA	0	10	RDR2-sensitive
GCGACTTACACTCTGTCACGGTGT	1	15	RDR2-sensitive
GCGACTTCAGTAGACGCGGCCTCT	0	12	RDR2-sensitive
GCGACTTCCATAGAGCAGGGACGA	0	26	RDR2-sensitive
GCGAGAACGGAACCGAGACGACTG	0	10	RDR2-sensitive
GCGAGACACAGGCACTGATGCATA	0	11	RDR2-sensitive
GCGAGACACAGGCGCTGATGCATA	1	65	RDR2-sensitive
GCGAGACAGTGTAAGACGGAGACT	0	11	RDR2-sensitive
GCGAGACAGTGTAAGATGGAGACA	0	28	RDR2-sensitive
GCGAGACTGAAACGTAGGATCGGA	0	13	RDR2-sensitive
GCGAGACTGTTGGGAGCAAGGAGG	50	6	RDR2-resistant
GCGAGATCTAGACGCTGTCGGCAA	0	58	RDR2-sensitive
GCGAGATTAGCGGGACGAAGACGC	0	11	RDR2-sensitive
GCGAGCAGTGGACGAGCGGAACGG	0	13	RDR2-sensitive
GCGAGCAGTTGTAGGCCCCTGCAC	0	14	RDR2-sensitive
GCGAGCATACAGTTCACTGTCGGG	0	14	RDR2-sensitive
GCGAGCCAGGCTGAAGAGGTTGAT	0	15	RDR2-sensitive
GCGAGCGGGCTAGGAACAGAGCGG	1	17	RDR2-sensitive

GCGAGCTGGGACTGGGAGAGGCAA	1	15	RDR2-sensitive
GCGAGCTGTAGCGACGGAGAGGAG	0	16	RDR2-sensitive
GCGAGCTTAGCCCACTGGACACGG	0	9	RDR2-sensitive
GCGAGGACCGATCTGAACAGGGCA	0	10	RDR2-sensitive
GCGAGGACGTGAAGGACGCCGGCG	0	9	RDR2-sensitive
GCGAGGACTAGACCAAAGAGGATG	0	9	RDR2-sensitive
GCGAGGCCATAGACCAGAGGACAG	0	11	RDR2-sensitive
GCGAGGCCTGAGGTTGGAGGTGGC	31	1	RDR2-resistant
GCGAGGGACTCGGACACTCGGCAA	0	14	RDR2-sensitive
GCGAGTAGCAACTCCGGATGACGT	0	10	RDR2-sensitive
GCGAGTGGAAGACTGATTGACCGG	0	18	RDR2-sensitive
GCGAGTTGTAGAGCACGATGGCGT	0	50	RDR2-sensitive
GCGATAATGGTTGAGGCATGTGAT	17	0	RDR2-resistant
GCGATACGGTCGGTTGGTAAGGCG	0	23	RDR2-sensitive
GCGATACGGTCGGTTGGTGAGGCG	1	14	RDR2-sensitive
GCGATTGATTGGACGTTTGACGGA	19	1	RDR2-resistant
GCGATTGGAGAACAGCGGGGTTAT	0	14	RDR2-sensitive
GCGATTGGGAATTAAAGCCGAGCG	0	14	RDR2-sensitive
GCGATTGTGGACGCCTACAGTAGA	0	11	RDR2-sensitive
GCGATTTGGTCGGTCGGTGCGGCG	1	18	RDR2-sensitive
GCGCAACGGTCGACTGCAACGCAT	2	19	RDR2-sensitive
GCGCAACTGTCTCTTTCGCTCGGC	0	10	RDR2-sensitive
GCGCAAGATGGCAATAGCTCTGTT	0	9	RDR2-sensitive
GCGCAAGGGCGGACTGTCCGCGGC	11	31	RDR2-sensitive
GCGCAATCTGTACAGTCGTCGGGA	0	15	RDR2-sensitive
GCGCACACTGGTGGACGCCCTCGT	0	9	RDR2-sensitive
GCGCACAGACGTAGGACGGGTGGC	0	14	RDR2-sensitive
GCGCACAGGACGAAGAGCGGCGAC	0	11	RDR2-sensitive
GCGCACCCAGTGCAGTGTCGCCGG	0	9	RDR2-sensitive
GCGCACTGTGGAGCGGGAGTTGCA	0	26	RDR2-sensitive
GCGCAGAACAGATTAGGGTTCCGA	1	15	RDR2-sensitive
GCGCAGAACCGAGCTCTAAAGCAA	0	15	RDR2-sensitive
GCGCAGAACGACGACGACTTTCGA	1	12	RDR2-sensitive
GCGCAGAACTTTGCCGAGGGCGGT	1	13	RDR2-sensitive
GCGCAGAACTTTGCCGAGTGCGGC	0	15	RDR2-sensitive
GCGCAGACCTCTGTACCAGGCGTA	0	18	RDR2-sensitive
GCGCAGATCGACGACTAGCAAGGC	0	9	RDR2-sensitive
GCGCAGATGTAGGCAACCAAACAC	0	10	RDR2-sensitive
GCGCAGATTAGGCTGTATGGTCAT	0	11	RDR2-sensitive
GCGCAGCACGTACCTCGGCGCCAT	0	10	RDR2-sensitive
GCGCAGCAGGACTTGCAGAACGGT	0	9	RDR2-sensitive
GCGCAGCGCGGCAGAACATGGCAC	0	19	RDR2-sensitive
GCGCAGGACACTCGGCACATCTAA	0	9	RDR2-sensitive
GCGCAGGACACTTGGACGAGCGGC	0	9	RDR2-sensitive
GCGCAGGACGTAACCCGGGAGCGG	2	41	RDR2-sensitive
GCGCAGGACTAACGAAGGCAGAAC	0	51	RDR2-sensitive
GCGCAGGACTTTGCCGAGTGCGGC	2	14	RDR2-sensitive
GCGCAGGAGGCAGAACATGGGCGT	1	45	RDR2-sensitive
GCGCAGGTTGTATGGACAGACGCA	0	9	RDR2-sensitive

GCGCAGTAGGGCTTACAGAACGGC	0	54	RDR2-sensitive
GCGCAGTCGACTCAGCACCCGCAT	0	10	RDR2-sensitive
GCGCAGTGTGTTTGGACGGCTGAC	0	10	RDR2-sensitive
GCGCAGTTGAACTCGAGGTGGCGT	0	10	RDR2-sensitive
GCGCAGTTTTTCTAGGCTGGTCGG	0	46	RDR2-sensitive
GCGCATAACTCGGTCTCGGACGTC	0	10	RDR2-sensitive
GCGCATCTGAGCATGTAGACGCCA	0	15	RDR2-sensitive
GCGCATCTGTGGAGCCAGGCTCAC	0	51	RDR2-sensitive
GCGCCAGACCGCGGACCGTTCGGC	0	12	RDR2-sensitive
GCGCCAGGTCGCGGACCGTCCGGC	11	36	RDR2-sensitive
GCGCCAGGTCGTGGACTCTTCGGT	0	27	RDR2-sensitive
GCGCCATATTGAGCGGTCGGACGG	0	9	RDR2-sensitive
GCGCCATCGCGGATAGTCCGGCAC	0	17	RDR2-sensitive
GCGCCATGTGGACTATAGAGGGCT	0	44	RDR2-sensitive
GCGCCCGGCACAACTAGACTGGCA	0	14	RDR2-sensitive
GCGCCCTTAGGTCGGACGGTCCGC	21	0	RDR2-resistant
GCGCCGAGCTCGGTACCACGTCGG	0	10	RDR2-sensitive
GCGCCGAGCTCTGACGTGGCCGCA	0	10	RDR2-sensitive
GCGCCGTCATGTCGTTGGATGGAA	0	42	RDR2-sensitive
GCGCCGTTGGATGGACTGACCGGC	2	83	RDR2-sensitive
GCGCCTGGACTGGGGAAGATGGTG	0	10	RDR2-sensitive
GCGCCTGTAGACCGGACGGTCCGC	0	31	RDR2-sensitive
GCGCCTGTAGATCAACGGCTGAAA	0	28	RDR2-sensitive
GCGCCTGTAGGCCGGACGGTCCGC	4	76	RDR2-sensitive
GCGCCTGTGGGTCGGACGGTCCGC	0	10	RDR2-sensitive
GCGCGACATGCGGATCGGAGCCAG	0	9	RDR2-sensitive
GCGCGACGGACGGAGTTCGGCGAC	0	24	RDR2-sensitive
GCGCGACGGCAGAACCACGGCAGA	0	50	RDR2-sensitive
GCGCGAGACAGTGTAAGATGGAGA	0	18	RDR2-sensitive
GCGCGAGGGTAGGACTGGGCAGAA	0	11	RDR2-sensitive
GCGCGAGGGTGAGGTCGACGGTTC	0	11	RDR2-sensitive
GCGCGAGGTGTAGAGTGGTGGCAA	0	25	RDR2-sensitive
GCGCGAGTGGAAGACTGATTGACC	0	20	RDR2-sensitive
GCGCGCCAGGACGAACAGACGAGC	3	30	RDR2-sensitive
GCGCGCCGGAATCAGCAGGGTCGA	0	34	RDR2-sensitive
GCGCGCGAGCTGTAGCGACGGAGA	0	12	RDR2-sensitive
GCGCGCGGGCCGAAAACAACGGTT	0	9	RDR2-sensitive
GCGCGCGTGCTGTTCGGAAGACGT	0	12	RDR2-sensitive
GCGCGCTACTGTAGACCCCCGGCC	0	11	RDR2-sensitive
GCGCGCTGTAGAAGGCACCGTCAA	1	71	RDR2-sensitive
GCGCGCTGTCGGGCGGCCACCAAG	19	0	RDR2-resistant
GCGCGGAACGACGACGACTTTCAG	3	23	RDR2-sensitive
GCGCGGAAGGCCAGGTAGAGCGGT	0	57	RDR2-sensitive
GCGCGGAGTAGAAACCAAGTTGGA	0	10	RDR2-sensitive
GCGCGGCAGGCAGAACAGAGCGGC	1	13	RDR2-sensitive
GCGCGGCAGGCAGAACAGAGCGTG	0	22	RDR2-sensitive
GCGCGGCAGGTATCTCGGCACCAG	0	9	RDR2-sensitive
GCGCGGCATGTATAGGACAGGCGT	0	23	RDR2-sensitive
GCGCGGCGGGAACAGATTCGGTAC	0	9	RDR2-sensitive

GCGCGGCTGTGGACGTACGGCGAG	0	12	RDR2-sensitive
GCGCGGGACGAACACGAAGGCCGC	8	107	RDR2-sensitive
GCGCGGGACTAACGACGGCAGAAC	0	18	RDR2-sensitive
GCGCGGGACTAACGGAGGCAGAAC	3	41	RDR2-sensitive
GCGCGGGCGCGTAAATTTGACGGA	0	27	RDR2-sensitive
GCGCGGGCGTTCGGTAGAGCGGGG	0	11	RDR2-sensitive
GCGCGGGGATGGAATTGGACGGTG	0	9	RDR2-sensitive
GCGCGGGGTAGAAACCAAGTTGGA	0	9	RDR2-sensitive
GCGCGGGTTGGTGCTGACAGGCAT	0	10	RDR2-sensitive
GCGCGGTACTTGGGGCAGAAGCAT	0	9	RDR2-sensitive
GCGCGTATTGGATGGTAGACGCAT	0	11	RDR2-sensitive
GCGCGTCAGGATAAAGGTGTCAGG	3	35	RDR2-sensitive
GCGCGTCAGGATAAGGTGTCAGGC	1	23	RDR2-sensitive
GCGCGTGACATCCTCTGATCCGGC	1	18	RDR2-sensitive
GCGCGTGACGCAAGACGGTGGAAC	0	10	RDR2-sensitive
GCGCGTGCATGTAGAAGTGGTGGT	0	38	RDR2-sensitive
GCGCGTGGGACTGGGTGTGCAGGC	0	13	RDR2-sensitive
GCGCGTGGTGAACTCGAGCGCGGC	1	19	RDR2-sensitive
GCGCTACAGAACCGTCCAGAAGAA	0	9	RDR2-sensitive
GCGCTATGTGTAGAGGCTGTACGG	0	9	RDR2-sensitive
GCGCTCCCGGACGACGAGAACGGC	0	9	RDR2-sensitive
GCGCTCGTGAACTTTATGGCGGAA	1	39	RDR2-sensitive
GCGCTCTCGGGCAACCGTCGTCGT	0	11	RDR2-sensitive
GCGCTGAACTCCGGAACGGATCGG	0	20	RDR2-sensitive
GCGCTGTAGAGGGCACCGCTGCGG	0	14	RDR2-sensitive
GCGCTGTAGGAGCACCGCTGCGGC	1	12	RDR2-sensitive
GCGCTGTAGGAGGCACCGCTGCGG	0	34	RDR2-sensitive
GCGCTGTAGGCCACGTGTCAGCAT	0	10	RDR2-sensitive
GCGCTGTAGGCTAGGGGATGCGCA	0	9	RDR2-sensitive
GCGCTGTAGGGACACCGCTGCGGC	0	16	RDR2-sensitive
GCGCTGTAGGGACACTGCTGCGGC	1	24	RDR2-sensitive
GCGCTGTAGGGGCACCGCTGCGGC	0	152	RDR2-sensitive
GCGCTGTAGGGGCACTGCTGCGGC	0	9	RDR2-sensitive
GCGCTGTAGGGGGCACCGCTGCGG	0	31	RDR2-sensitive
GCGCTGTAGGGGTACCGCTGCGGC	0	11	RDR2-sensitive
GCGCTGTCCTTCTGTCAGACCGGT	17	100	RDR2-sensitive
GCGCTGTCGGCGCTGTTGAGGCCA	0	17	RDR2-sensitive
GCGCTGTCGGGCGGCCACCAAGAC	232	22	RDR2-resistant
GCGCTGTCTTTCTGTCAGACCGGT	0	9	RDR2-sensitive
GCGCTTTCCGGACTTCAGAGGGTG	0	14	RDR2-sensitive
GCGGAAACAGTAGTCCCGGACGGA	0	17	RDR2-sensitive
GCGGAAACGAGCATGCTGGCACAA	0	45	RDR2-sensitive
GCGGAAAGGCTAGGTAGAGCGGCT	0	10	RDR2-sensitive
GCGGAACAGGATTCGGCCCACTAT	0	36	RDR2-sensitive
GCGGAACCGCGGACCACGAAGCGA	0	13	RDR2-sensitive
GCGGAACGAAGGCAGAACCGTGCA	0	18	RDR2-sensitive
GCGGAACGACGACGACTTTCAGCT	0	13	RDR2-sensitive
GCGGAACGACGACGAGATCATGTC	0	13	RDR2-sensitive
GCGGAACGACGACGAGATCGTGTC	0	18	RDR2-sensitive

GCGGAACGAGGAGGGCAGAACGGC	0	16	RDR2-sensitive
GCGGAACGGCTGCTCCTGATCCGG	0	9	RDR2-sensitive
GCGGAACGTCGTCACTTGGATGGT	0	13	RDR2-sensitive
GCGGAACTCATGGGCCTGCCGGCG	0	10	RDR2-sensitive
GCGGAACTGACACGCGAAGGTTGA	0	12	RDR2-sensitive
GCGGAACTGCAGGAGCACGTGCGC	0	21	RDR2-sensitive
GCGGAACTGGGCAGAGGACTCGCG	0	12	RDR2-sensitive
GCGGAACTTGTTCGGCATAGATCC	0	21	RDR2-sensitive
GCGGAAGAACCGGTCAGAGAGCAT	0	13	RDR2-sensitive
GCGGAAGACGACGCGACTGAGCAT	0	17	RDR2-sensitive
GCGGAAGATATGCGCACTCAAGTC	0	20	RDR2-sensitive
GCGGAAGATCGCCGACGGCGCCTG	0	16	RDR2-sensitive
GCGGAAGCTGCACTTGCTGATCAG	0	21	RDR2-sensitive
GCGGAAGCTGGTTACACGGGCGCT	0	9	RDR2-sensitive
GCGGAAGGCCAGGTAGAGCGGTTG	1	16	RDR2-sensitive
GCGGAAGGGCATAACTGAGCACAA	1	14	RDR2-sensitive
GCGGAATGGGAGAGGGCAGAACGG	0	11	RDR2-sensitive
GCGGAATGGTTCCAGATCTGAGGA	0	11	RDR2-sensitive
GCGGAATGGTTCCGGATCTGAGGA	3	91	RDR2-sensitive
GCGGACAATTCTAGTGGCGTCGGC	4	20	RDR2-sensitive
GCGGACACGCTGTGGCAGAACCGT	0	17	RDR2-sensitive
GCGGACACTCGGCAAACGACTGAT	0	9	RDR2-sensitive
GCGGACAGCACGCGAACGACGCAT	0	20	RDR2-sensitive
GCGGACAGTCTGAAACGTTGCGGA	0	15	RDR2-sensitive
GCGGACAGTCTGATCCTTAGGCAT	0	13	RDR2-sensitive
GCGGACATGAAACATGATGGGCAA	1	28	RDR2-sensitive
GCGGACATTGTCGCAGTAGACGAG	0	18	RDR2-sensitive
GCGGACCAGAGACAGAGTGGGCAC	0	16	RDR2-sensitive
GCGGACCATCGAGCTACAGTCGGT	0	30	RDR2-sensitive
GCGGACCATCTAACTCGTCGGGCA	0	15	RDR2-sensitive
GCGGACCATCTGACCGGGGTCGGC	0	22	RDR2-sensitive
GCGGACCATCTGGACGTACGCAGA	0	26	RDR2-sensitive
GCGGACCATTCACGCGCATGTCGT	2	37	RDR2-sensitive
GCGGACCGACACAGAGAAGACACC	0	9	RDR2-sensitive
GCGGACCGACACTAGGAAGACGTC	0	12	RDR2-sensitive
GCGGACCGAGCCAGCATCGACGTA	0	31	RDR2-sensitive
GCGGACCGCGCACGCGAACGGCAA	0	13	RDR2-sensitive
GCGGACCGTCCATACCACAGGTAC	0	9	RDR2-sensitive
GCGGACCGTCCGACCGTCAGGCAG	0	9	RDR2-sensitive
GCGGACCGTCCGGACGTACGCAGA	0	12	RDR2-sensitive
GCGGACCGTCCGGACGTCAGGCAG	0	16	RDR2-sensitive
GCGGACCGTCCGGATCATCAGGCA	0	19	RDR2-sensitive
GCGGACCGTCCGGCATATAGGCAC	0	9	RDR2-sensitive
GCGGACCGTCCGGCCCACAGACGC	0	9	RDR2-sensitive
GCGGACCGTCCGGCCTAAGACGTG	0	11	RDR2-sensitive
GCGGACCGTCCGGCTATAATTCAC	0	9	RDR2-sensitive
GCGGACCGTCTGGACGTACGCAGA	0	10	RDR2-sensitive
GCGGACCTGGTACGATAAGACGTC	0	9	RDR2-sensitive
GCGGACGACTGCAGGCAGAACCGT	0	12	RDR2-sensitive

GCGGACGAGAATTGCATGCGCGTA	0	9	RDR2-sensitive
GCGGACGCGGTAGGACGTGGCGTA	1	18	RDR2-sensitive
GCGGACGGGATTCGACGAAATGGA	0	11	RDR2-sensitive
GCGGACGGTGTAGATTTACGGAAG	1	13	RDR2-sensitive
GCGGACGTGGCTTTCTAGCTCGGC	0	10	RDR2-sensitive
GCGGACGTTGTTGGAGGCTTCCAG	0	10	RDR2-sensitive
GCGGACTAGGAGGCAAAATATCAA	3	16	RDR2-sensitive
GCGGACTATCTGTGCCACCGGCGC	0	27	RDR2-sensitive
GCGGACTTGGCATTTGCTTTTGCG	0	9	RDR2-sensitive
GCGGAGAACGTGCTACGTCTGGAC	0	9	RDR2-sensitive
GCGGAGACATCGGAACACGGCTAC	0	12	RDR2-sensitive
GCGGAGACGAACAGAGCAGCTCGA	0	10	RDR2-sensitive
GCGGAGACGGATGCAGAGGCTGCT	0	34	RDR2-sensitive
GCGGAGACGGATGCGGAGGCTGCT	0	11	RDR2-sensitive
GCGGAGACTGCATGGAGTGCGCTA	0	20	RDR2-sensitive
GCGGAGACTGCTGGACTGATGAAT	0	18	RDR2-sensitive
GCGGAGACTGCTGGAGAGCGAAAC	0	15	RDR2-sensitive
GCGGAGAGGCAGATGAATTCGGTC	0	17	RDR2-sensitive
GCGGAGCACGCAATCTGTCTGAGC	0	13	RDR2-sensitive
GCGGAGCAGGGACCAAAAGCGCAT	0	20	RDR2-sensitive
GCGGAGCCTGACAAGGGAGGACAG	0	13	RDR2-sensitive
GCGGAGCCTGATAAGGGAGAACGG	0	22	RDR2-sensitive
GCGGAGCCTGCTGGAGAGCGCAAT	1	22	RDR2-sensitive
GCGGAGCTGTACGACCGGAGGCAG	0	13	RDR2-sensitive
GCGGAGGAAGGATGTCCAGTGCCG	0	14	RDR2-sensitive
GCGGAGGACTGACGCGGCACCGAC	0	17	RDR2-sensitive
GCGGAGGATCTGGCCAAGCGCGTC	0	10	RDR2-sensitive
GCGGAGGATTGCGGTATAGAGTAA	0	13	RDR2-sensitive
GCGGAGGATTGTAGTATAGGGTAG	0	31	RDR2-sensitive
GCGGAGGGACGAGCAAACGAACAG	0	9	RDR2-sensitive
GCGGAGGGAGCGTACGCTGGACGG	0	12	RDR2-sensitive
GCGGAGGTAGAACACAGGGGTGTG	42	4	RDR2-resistant
GCGGAGTAACAGGTGGCAGGACAA	0	11	RDR2-sensitive
GCGGAGTACTGTAGACGCACGGAG	0	41	RDR2-sensitive
GCGGAGTCCGAACGGGTTGACGGT	0	9	RDR2-sensitive
GCGGAGTCTGCTGGAGCGCGCAAC	0	13	RDR2-sensitive
GCGGAGTGCAAATACGGAGTCTGC	0	10	RDR2-sensitive
GCGGAGTGGGATGGGGAGTGTTGC	2	16	RDR2-sensitive
GCGGATAAGTCTATCGGACAGCAT	0	14	RDR2-sensitive
GCGGATACGGACGGATATTAGCTC	0	67	RDR2-sensitive
GCGGATACGTAAGGGCATGTACAG	0	15	RDR2-sensitive
GCGGATACGTTATAGGCTGAGGGA	19	1	RDR2-resistant
GCGGATACTCTAGTGCAGATGGAA	0	9	RDR2-sensitive
GCGGATAGCGACAGAATTGGGCAG	0	13	RDR2-sensitive
GCGGATAGGACAACAGCGGCTGAT	0	17	RDR2-sensitive
GCGGATAGTCTGAAGCAGTGGAGC	0	10	RDR2-sensitive
GCGGATAGTCTGAGAGAGAAGAGT	0	19	RDR2-sensitive
GCGGATCACGACTGCGGTTGGCGT	0	10	RDR2-sensitive
GCGGATCAGGCGTGCAGCTCAGAG	0	17	RDR2-sensitive

GCGGATCCTGGAAACGACGGAGTA	2	18	RDR2-sensitive
GCGGATCGACTCCCCTGCAGGGCT	80	0	RDR2-resistant
GCGGATCGCGACAGTGGAGGCATG	0	36	RDR2-sensitive
GCGGATCGGCAACACTGCGAAGTC	0	13	RDR2-sensitive
GCGGATCTCACGCTCGGGGATGGC	22	0	RDR2-resistant
GCGGATCTCTGATGCAGTGGCTGT	0	9	RDR2-sensitive
GCGGATCTGGTCGAGATGTGCGAC	73	7	RDR2-resistant
GCGGATGACAGGCTGCCTCGGCGT	0	23	RDR2-sensitive
GCGGATGATTGTGGTATAGAGTAA	0	12	RDR2-sensitive
GCGGATGCTGTTGGACGCATTCAG	0	48	RDR2-sensitive
GCGGATGGACGTCAAACAGGACAA	2	98	RDR2-sensitive
GCGGATGGGAATGGCAGAACCGGA	0	15	RDR2-sensitive
GCGGATGTCCAAGGAGTGCTCGGC	1	13	RDR2-sensitive
GCGGATGTGGCTTCGACGAAACGG	0	11	RDR2-sensitive
GCGGATGTGGTTAACGGGCCGGCG	0	9	RDR2-sensitive
GCGGATGTTGTTGGACGCGGGTAC	0	11	RDR2-sensitive
GCGGATTCCGGTAGTGAGGACGTC	0	17	RDR2-sensitive
GCGGATTCGGACCAGATGGATTGC	0	9	RDR2-sensitive
GCGGATTGATCGGGCGGGGGCGGG	0	15	RDR2-sensitive
GCGGATTGATCGGGCGGGGGCGGT	0	24	RDR2-sensitive
GCGGATTGCGGCGACGGCGTGGAC	0	10	RDR2-sensitive
GCGGATTGGCGTTGTGCGGCGAAG	0	23	RDR2-sensitive
GCGGCAACCGGCAGAACCGTGCAC	0	12	RDR2-sensitive
GCGGCAACTGAAGCGGGTGGTGTC	0	10	RDR2-sensitive
GCGGCAAGAACACGTACTCGGCAC	0	24	RDR2-sensitive
GCGGCACACTCACTCGTCGGCTTA	0	18	RDR2-sensitive
GCGGCACACTGGCCCATCTGGCAC	1	18	RDR2-sensitive
GCGGCACGGAATCGGACGATACAT	0	19	RDR2-sensitive
GCGGCACGTGATGGATGACGACGG	0	14	RDR2-sensitive
GCGGCACGTGGACCCATCCGGCAC	0	12	RDR2-sensitive
GCGGCACGTGGACCGATCGGGCAC	0	9	RDR2-sensitive
GCGGCACTCACTCGTCGGCCTGAG	0	9	RDR2-sensitive
GCGGCACTCGACAAAGACGTCTTT	0	31	RDR2-sensitive
GCGGCAGAGCTCATGATCGTCGAG	0	16	RDR2-sensitive
GCGGCAGAGCTTATGATCGTCGAG	0	10	RDR2-sensitive
GCGGCAGCGGCAGAACCATGGAGT	1	16	RDR2-sensitive
GCGGCAGGGCGCTGGCTCCTTTCC	222	8	RDR2-resistant
GCGGCATCACGGCGCATGAACCGA	1	43	RDR2-sensitive
GCGGCATCCTGACCTGACGACTGC	29	0	RDR2-resistant
GCGGCATGTGGATCGATCGGGCAT	0	11	RDR2-sensitive
GCGGCATTGGTGGAACTGGGCGGC	1	26	RDR2-sensitive
GCGGCCAAGTAGGACTGCTTCCAG	0	10	RDR2-sensitive
GCGGCCACGGCAGAACAACAGGGT	0	11	RDR2-sensitive
GCGGCCCAGAACATCTAAGGGCAT	4	33	RDR2-sensitive
GCGGCCGGTTGCAGAAGTAGGATG	0	10	RDR2-sensitive
GCGGCCTATGGATAGACACGAGCA	0	11	RDR2-sensitive
GCGGCCTGTCCAAGCAGAACTGTC	0	14	RDR2-sensitive
GCGGCGACAATTTACTCGTCGGTC	1	21	RDR2-sensitive
GCGGCGACCTTGCGGAGCGACTGG	0	10	RDR2-sensitive

GCGGCGACGAAGAACACTCACGGC	0	11	RDR2-sensitive
GCGGCGACGTAGACCAAGAGGGCT	4	21	RDR2-sensitive
GCGGCGACGTAGATCAAGAGGGCT	1	16	RDR2-sensitive
GCGGCGAGTGTAGAGAAGGACGGT	0	10	RDR2-sensitive
GCGGCGAGTTGTAGAGCACGATGG	0	20	RDR2-sensitive
GCGGCGCAGCAGGACGACGACGAC	0	13	RDR2-sensitive
GCGGCGCCGCTGTGGTGAGACTGC	0	11	RDR2-sensitive
GCGGCGCGGAAGCAGACTGGCGAT	0	9	RDR2-sensitive
GCGGCGTAGGCCTAAGTGACGGCA	0	11	RDR2-sensitive
GCGGCGTAGGCTTAAGTGCCGGCA	0	10	RDR2-sensitive
GCGGCGTGGGAACAACGGCGATAA	0	56	RDR2-sensitive
GCGGCGTGGGAACCGGAGCGGTGA	0	9	RDR2-sensitive
GCGGCTACAGGAACTCCGGGAGGC	0	17	RDR2-sensitive
GCGGCTACCGTCGGAACAGTCGGA	3	74	RDR2-sensitive
GCGGCTACGTAGATCAAGAGGGCT	2	16	RDR2-sensitive
GCGGCTAGGCCGATCGGGGACGGT	0	10	RDR2-sensitive
GCGGCTAGGGCTCCTGCCTGACGG	1	25	RDR2-sensitive
GCGGCTCAGCTCGGATCGGATCGG	0	9	RDR2-sensitive
GCGGCTCTGGTCAGACTGTGGCAT	0	12	RDR2-sensitive
GCGGCTGAACCGGAACGTGGCGTT	0	19	RDR2-sensitive
GCGGCTGAACTGCGGCGATGGCGC	0	13	RDR2-sensitive
GCGGCTGACATGAATATGGCGAGC	1	42	RDR2-sensitive
GCGGCTGCGGCTTCTACAGTATTT	10	142	RDR2-sensitive
GCGGCTGGAGTAGAGACCGGTGTG	0	14	RDR2-sensitive
GCGGCTGGGACTTCTGCCTGACGG	2	57	RDR2-sensitive
GCGGCTTAGACACGACGTGGCGTC	0	64	RDR2-sensitive
GCGGGAACCAGCAGAACGTGCACC	0	11	RDR2-sensitive
GCGGGAAGCCTACGGCACTGAGTA	0	15	RDR2-sensitive
GCGGGAAGCCTACGGCACTGGATA	1	53	RDR2-sensitive
GCGGGAAGTGGACATACGGTACGA	0	14	RDR2-sensitive
GCGGGACAACTAAGACAGGGACGT	0	53	RDR2-sensitive
GCGGGACAACTAGGATAGAGACGT	0	28	RDR2-sensitive
GCGGGACAAGAACACGAAGGCCGC	0	16	RDR2-sensitive
GCGGGACAATCAAGATAGGGACGT	0	11	RDR2-sensitive
GCGGGACACCCAGGACAGGGACGT	0	15	RDR2-sensitive
GCGGGACAGACAGGACAGAGACGT	0	9	RDR2-sensitive
GCGGGACAGACAGGATAGAGACGT	0	16	RDR2-sensitive
GCGGGACAGCCAGGACAGAGACGT	1	29	RDR2-sensitive
GCGGGACAGCCAGGACAGGGACGT	1	26	RDR2-sensitive
GCGGGACAGCTAGGACAGGGACGT	0	14	RDR2-sensitive
GCGGGACAGTCAGGACAGAGACGT	0	28	RDR2-sensitive
GCGGGACAGTCAGGACAGGGACGT	0	27	RDR2-sensitive
GCGGGACAGTTAGGACAGAGACGT	2	37	RDR2-sensitive
GCGGGACAGTTAGGATAGAGACGT	0	14	RDR2-sensitive
GCGGGACCGAGGACGGGACAGGCG	10	49	RDR2-sensitive
GCGGGACGAAAACGAAAACGGGAT	0	13	RDR2-sensitive
GCGGGACGAACACGAAGGCCACGG	0	12	RDR2-sensitive
GCGGGACGAGTGAATATAGGGGAT	5	22	RDR2-sensitive
GCGGGACGGCTGACGGACGACGCA	0	24	RDR2-sensitive

GCGGGACTGCATGATGCGTACGGC	0	20	RDR2-sensitive
GCGGGACTGCCACGTCAGGAAGCT	0	9	RDR2-sensitive
GCGGGACTGCGGCAGCCTCACGGA	0	24	RDR2-sensitive
GCGGGACTGCGTGCTACGTACGGC	0	10	RDR2-sensitive
GCGGGACTGTACGACCGGAGGCAG	0	14	RDR2-sensitive
GCGGGAGAACCAGTCAGAGAGCGT	0	9	RDR2-sensitive
GCGGGAGACTGGCTCTAGTGATGT	0	20	RDR2-sensitive
GCGGGAGAGAGAACCGGTCGGCGT	0	10	RDR2-sensitive
GCGGGAGCCTGACTCTAGAGATGC	0	11	RDR2-sensitive
GCGGGAGCTAAGTGAATGTCGGAG	0	10	RDR2-sensitive
GCGGGAGCTGAGAGATGACGCGAC	1	12	RDR2-sensitive
GCGGGAGCTGCAGCGGTAAACTGT	0	14	RDR2-sensitive
GCGGGAGCTGCTGGAGACGTCTAC	0	56	RDR2-sensitive
GCGGGAGGCAGAACGTGCACCACC	0	9	RDR2-sensitive
GCGGGAGGGACGAAGACGAAGCGT	0	47	RDR2-sensitive
GCGGGAGGGACGAAGACGGACGAA	0	27	RDR2-sensitive
GCGGGAGTGTACAGCGCGATGGCA	0	20	RDR2-sensitive
GCGGGAGTTTGGCTACTGTATTTT	0	15	RDR2-sensitive
GCGGGATAGCCAGGACAGAGACGT	0	19	RDR2-sensitive
GCGGGATCTGGATTCGCGGCGAAT	0	14	RDR2-sensitive
GCGGGATGAGAACGGAGCGGCTCC	0	11	RDR2-sensitive
GCGGGATGGACTGCGGGATCGGAC	2	48	RDR2-sensitive
GCGGGATGGGAACGGAGCGGCTCC	1	22	RDR2-sensitive
GCGGGATGGTTCCGGATCTGAGGA	0	15	RDR2-sensitive
GCGGGATTGGAACGGAGCGGCTCC	0	59	RDR2-sensitive
GCGGGATTGGACGGCTGAGATTGT	0	9	RDR2-sensitive
GCGGGCAGGGGCAGCGTAGAGAGG	0	12	RDR2-sensitive
GCGGGCCCACAAGTCAGAAGCTCC	0	32	RDR2-sensitive
GCGGGCCCAGAATTTGGAGGATGG	21	0	RDR2-resistant
GCGGGCCCTGAGTGTAGAGGAGGT	0	11	RDR2-sensitive
GCGGGCGAGTCTCTGGCGCGTGCA	0	10	RDR2-sensitive
GCGGGCGCGGCAGAACGTGCGAGT	0	27	RDR2-sensitive
GCGGGCGCGGCAGAATAGAGGAGA	1	17	RDR2-sensitive
GCGGGCGGGATGCGGCAGAACAGT	0	18	RDR2-sensitive
GCGGGCGTTCGGTAGAGCGGGGGC	0	61	RDR2-sensitive
GCGGGCGTTTGGTAGAGCGGGGGC	0	30	RDR2-sensitive
GCGGGCTACTGTAGCTTGGATGGC	0	13	RDR2-sensitive
GCGGGCTAGAACGAGGCAGCTCGG	0	18	RDR2-sensitive
GCGGGCTAGCACGGCATGGCACGG	0	9	RDR2-sensitive
GCGGGCTAGTACGGCACGACCTGT	0	64	RDR2-sensitive
GCGGGCTCTGACTCAGGCATCCGC	0	15	RDR2-sensitive
GCGGGCTGACAACGCGCGGGGGCA	0	13	RDR2-sensitive
GCGGGCTGACGTCATCCGCGACAC	0	10	RDR2-sensitive
GCGGGCTGCAGGCAGAACCGTGCA	0	11	RDR2-sensitive
GCGGGCTGCGTCCTGACGGAGGTA	0	10	RDR2-sensitive
GCGGGCTGGATCGGCGCAGTAGCT	0	11	RDR2-sensitive
GCGGGCTGGGAACGGAGCGGCTCC	0	14	RDR2-sensitive
GCGGGCTGGGACAAGGCATGACAC	0	93	RDR2-sensitive
GCGGGCTGGTTGACGCGGTCTAAG	0	14	RDR2-sensitive

GCGGGCTGTGCGATGCGCGGGATG	0	14	RDR2-sensitive
GCGGGCTTATAGGACTGAGTCGGC	0	12	RDR2-sensitive
GCGGGCTTGGACAGGAAACTAGGC	6	121	RDR2-sensitive
GCGGGGACGAGGACGAGACAGGCG	7	29	RDR2-sensitive
GCGGGGACGATGCAGGAACGACGC	0	10	RDR2-sensitive
GCGGGGATGGTCGTTGGCAGCCGG	45	1	RDR2-resistant
GCGGGGGCAGTCTACGGTACTCTC	0	12	RDR2-sensitive
GCGGGGTAGATCTGAGCAAGACGT	0	16	RDR2-sensitive
GCGGGGTTGATGTTGGACAACACA	78	7	RDR2-resistant
GCGGGTAACCAATCGGACGGTATA	0	10	RDR2-sensitive
GCGGGTAGGAAGCGAGGACAGTCT	0	12	RDR2-sensitive
GCGGGTCGGGTTTTGTTCGGGTAT	0	9	RDR2-sensitive
GCGGGTGAGGGAAGCGAGCGGCCC	4	18	RDR2-sensitive
GCGGGTGAGTGAGCGGTAGAAGAT	92	0	RDR2-resistant
GCGGGTTGAGAACGAAGCGGCTCC	0	20	RDR2-sensitive
GCGGGTTTGGACAGGAAACTAGGC	0	11	RDR2-sensitive
GCGGGTTTTCTGAAGCTAGTGGAA	13	31	RDR2-sensitive
GCGGTAAACCCTAGCGTCGGCGGC	1	20	RDR2-sensitive
GCGGTACACCGTTGGATATTGGAT	0	9	RDR2-sensitive
GCGGTACATGTGGAGGGTTGGCTC	0	12	RDR2-sensitive
GCGGTACCAACGGCGATACTCGAA	0	32	RDR2-sensitive
GCGGTACCCTCGGATTCGGCAGAC	0	27	RDR2-sensitive
GCGGTACGGTATGAGACGAGCTAC	0	10	RDR2-sensitive
GCGGTACTGGACTGTCCGGTGTCA	0	12	RDR2-sensitive
GCGGTAGAACCACCAGAAGATCAA	0	33	RDR2-sensitive
GCGGTAGACGACAGGGACGGACAT	0	13	RDR2-sensitive
GCGGTAGGGGTGGAGGGCGTTGGG	14	0	RDR2-resistant
GCGGTCAAACAGAAACAGGGACAT	0	16	RDR2-sensitive
GCGGTCCGGACTGTCCGCGGTGGG	0	10	RDR2-sensitive
GCGGTCCGGACTGTCCGCGGTGGT	6	43	RDR2-sensitive
GCGGTCGCTGTAGACGGTGCGCAA	0	9	RDR2-sensitive
GCGGTCGCTTCTTTTGTGGACGGT	0	10	RDR2-sensitive
GCGGTCGGGAGCTGTCGCGTGGGT	0	10	RDR2-sensitive
GCGGTCTATTCGCTGTAGATGGGC	0	36	RDR2-sensitive
GCGGTCTGACTCAACAGGAGCGAT	0	10	RDR2-sensitive
GCGGTCTGACTCAACGGGAGCGAT	1	17	RDR2-sensitive
GCGGTCTGAGTGCAGGAGCGACGC	0	17	RDR2-sensitive
GCGGTCTGTACCGAAGGAGATGTG	0	10	RDR2-sensitive
GCGGTCTTTGGTGTACATGTTGAA	16	0	RDR2-resistant
GCGGTGAGGCCTGGACGCGAACGG	2	20	RDR2-sensitive
GCGGTGCGATACTCACGACGGTTT	0	38	RDR2-sensitive
GCGGTGCTCTCTGGTCAGGCGCGG	2	21	RDR2-sensitive
GCGGTGCTGCAGAGCGGTATCTGT	0	32	RDR2-sensitive
GCGGTGCTTTTGGGTAGGACGGTG	2	21	RDR2-sensitive
GCGGTGGGCCTGAACACGAGCGAC	0	18	RDR2-sensitive
GCGGTGTATTATGAAGAGCGGCAA	0	63	RDR2-sensitive
GCGGTGTGGTAGTTACGTGGACGG	0	21	RDR2-sensitive
GCGGTGTGTGTGGAACGGCGGCGG	0	56	RDR2-sensitive
GCGGTGTGTGTGGAACGGCGGCGT	0	60	RDR2-sensitive

GCGGTTAAGGCTCTCTGCTTCGGC	72	8	RDR2-resistant
GCGGTTATCGCGGAAGGAAGGCGT	0	16	RDR2-sensitive
GCGGTTCTTGTGTAGTGATTGGCG	0	15	RDR2-sensitive
GCGGTTGGCACTGCGGTCTTTGGT	26	0	RDR2-resistant
GCGTACAACTCAGGGACAGGGCAG	0	37	RDR2-sensitive
GCGTACACAAGTGTCACCCGTCGG	0	10	RDR2-sensitive
GCGTACACAAGTGTGACTCGTCGA	0	21	RDR2-sensitive
GCGTACAGATGTAGAGTCAGGCTC	0	14	RDR2-sensitive
GCGTACAGGAGAGCCGAGGGCGTG	0	18	RDR2-sensitive
GCGTACAGGAGGGCCGAGGACGTG	1	21	RDR2-sensitive
GCGTACAGGAGGGCCGAGGGCGTG	1	22	RDR2-sensitive
GCGTACCCGAGGACGAAGACGCCG	0	13	RDR2-sensitive
GCGTACGAAGCGAACACACTGGCG	0	11	RDR2-sensitive
GCGTACGAGGATGGGCAGAACCGT	0	15	RDR2-sensitive
GCGTAGAACGGCGTCGACACCAAT	0	17	RDR2-sensitive
GCGTAGACTGCGTATAGGCTTACT	0	12	RDR2-sensitive
GCGTAGACTGTTGATAGGGCACAA	1	20	RDR2-sensitive
GCGTAGAGAGCGCTGTAGGGGCAC	0	11	RDR2-sensitive
GCGTAGAGGGCGCTGTAGAGGGCA	0	19	RDR2-sensitive
GCGTAGAGGGCGCTGTAGGAGGCA	0	12	RDR2-sensitive
GCGTAGAGGGCGCTGTAGGGGGCA	0	31	RDR2-sensitive
GCGTAGAGGGCGCTGTAGGGTGTC	0	13	RDR2-sensitive
GCGTAGATATACCTGACTGGGGCT	5	101	RDR2-sensitive
GCGTAGATGGCAGAACCAGAGCGG	0	19	RDR2-sensitive
GCGTAGATTCTCCATCGAAGCCGT	0	9	RDR2-sensitive
GCGTAGGACAACAGGTTGATTCGT	0	9	RDR2-sensitive
GCGTAGGACAGTTCGGCAAGGCCG	0	25	RDR2-sensitive
GCGTAGGACAGTTCGGCTAGGCCA	7	80	RDR2-sensitive
GCGTAGGACAGTTCGGCTAGGCCC	0	9	RDR2-sensitive
GCGTAGGACAGTTCGGCTAGGCCG	0	28	RDR2-sensitive
GCGTAGGACGTAACCCGGAAGCGG	0	16	RDR2-sensitive
GCGTAGGACGTAACCCGGTAGCGG	0	10	RDR2-sensitive
GCGTAGGACGTAACTCGGGAGCGG	0	9	RDR2-sensitive
GCGTAGGACTCTTCGGATGTTCGG	0	12	RDR2-sensitive
GCGTAGGACTTTGCCGAGTGCGGC	0	32	RDR2-sensitive
GCGTAGGAGGCGCTGTAGGGGCAC	0	54	RDR2-sensitive
GCGTAGGCTCGAACTCCGACGGTG	0	55	RDR2-sensitive
GCGTAGGGCTCATCTCAGCCAGGC	0	9	RDR2-sensitive
GCGTAGGGGACGCTGTAGGGGCAC	0	75	RDR2-sensitive
GCGTAGGGGGCGCTGTAGGGGCAC	0	44	RDR2-sensitive
GCGTAGGGTCGCTGTAGGGGGCAC	0	9	RDR2-sensitive
GCGTAGGTTCGGCCTGGGTGCATT	2	27	RDR2-sensitive
GCGTAGTAGCTGACTGCGGGGACG	0	21	RDR2-sensitive
GCGTAGTCGAACTCGAGGTGGCAC	0	9	RDR2-sensitive
GCGTAGTGGGCGCTGTAGGGGCAC	1	62	RDR2-sensitive
GCGTAGTTGGGTTGACTCGACGGT	0	9	RDR2-sensitive
GCGTATAGCGTTGGACAGTCCGGA	0	15	RDR2-sensitive
GCGTATGGTGCGCTGTAGGAGGCA	0	13	RDR2-sensitive
GCGTCATCGTAGATCGTCCGGCAC	3	32	RDR2-sensitive

GCGTCTCGAGACATACTGACGGAT	0	11	RDR2-sensitive
GCGTCTGATCGTGGAGGACGTGCA	0	10	RDR2-sensitive
GCGTCTGTCGAACCTGGCTGACGG	0	11	RDR2-sensitive
GCGTCTTTGGAAGGCTGTAGTCGG	7	30	RDR2-sensitive
GCGTGAACTTGTTGGGCGGAGACG	0	13	RDR2-sensitive
GCGTGAAGCTTGATCCTGTCGGAA	0	10	RDR2-sensitive
GCGTGACAAGGACTGTGCGGGCGT	0	16	RDR2-sensitive
GCGTGACAGTTAGAACAGAGACGT	0	9	RDR2-sensitive
GCGTGACATCCTCTGATCCGGCGC	1	43	RDR2-sensitive
GCGTGACGACTAGGCAGTGGGCAA	3	21	RDR2-sensitive
GCGTGACGGACGACGGTGCCACAA	0	9	RDR2-sensitive
GCGTGACTGACGAGCTAAGGCTGA	1	16	RDR2-sensitive
GCGTGACTGTGAGAGCGGGATGGG	1	52	RDR2-sensitive
GCGTGAGATGGCAAATAGCTCTGT	0	9	RDR2-sensitive
GCGTGAGATGGCAAGTAGCTCTAT	0	12	RDR2-sensitive
GCGTGATCGAGTCGCTCGTATGGA	0	15	RDR2-sensitive
GCGTGATGACTGGGCAGTGGGCAA	1	12	RDR2-sensitive
GCGTGATTTACGGACGGACGGTGC	0	10	RDR2-sensitive
GCGTGCAGATGGATGGTAGAATAT	0	10	RDR2-sensitive
GCGTGCATCTAGCCCGTGTCGGAC	4	38	RDR2-sensitive
GCGTGCATGTAGAAGTGGTGGTAA	0	21	RDR2-sensitive
GCGTGCCACCGGCTAGGTCGTCGT	0	17	RDR2-sensitive
GCGTGCGATGTAGTGGGACAGGGC	0	9	RDR2-sensitive
GCGTGCGCAGAACAGATTAGGGTT	5	28	RDR2-sensitive
GCGTGCGCGGACTGTCCGGCCTAG	0	16	RDR2-sensitive
GCGTGCGGGCTGTTCGTGGCGGGC	0	9	RDR2-sensitive
GCGTGCGGGTAGGAAGCGAGGACA	0	19	RDR2-sensitive
GCGTGCTGGGCTGCTGAGGCGGGG	0	10	RDR2-sensitive
GCGTGCTGTAGAAGCGTTGTGGCT	0	13	RDR2-sensitive
GCGTGGACACGGACGGCAAGACTC	0	12	RDR2-sensitive
GCGTGGACTGTCTATGCATTTCCT	0	9	RDR2-sensitive
GCGTGGAGGGGCTTGTAGGGGGCT	0	9	RDR2-sensitive
GCGTGGATCTGAGCAGCCAAGCAG	0	18	RDR2-sensitive
GCGTGGATTGTTGGTTTTGGCTAC	15	0	RDR2-resistant
GCGTGGCCCTGTAGCTATCGGTAA	0	17	RDR2-sensitive
GCGTGGCTGTAGGTGGGACCGCAC	0	17	RDR2-sensitive
GCGTGGGTGTGGAAGCAACAACGG	0	9	RDR2-sensitive
GCGTGTAACTCGAGCTCGACGACA	0	9	RDR2-sensitive
GCGTGTAGACGGTGGACTGTTCGT	0	35	RDR2-sensitive
GCGTGTAGTGAAGCCGCCGGTCGT	0	10	RDR2-sensitive
GCGTGTATCTAGCCCGTGTCGGAC	0	9	RDR2-sensitive
GCGTGTCAGGATAAGGTGTCAGGC	1	44	RDR2-sensitive
GCGTGTGGAGACGGTGGCAGAACC	0	13	RDR2-sensitive
GCGTGTGTAGAAGCGACGGAGTTC	1	18	RDR2-sensitive
GCGTTAACTCGCGACTGTGGTCGT	0	24	RDR2-sensitive
GCGTTAACTGTGTGTGAGTCGGTG	0	9	RDR2-sensitive
GCGTTATACGGAGTCGGATGCGGA	0	20	RDR2-sensitive
GCGTTCGAACTCTGGAAGTAGCGT	0	10	RDR2-sensitive
GCGTTCGGTAGAGCGGGGGCAGTC	0	13	RDR2-sensitive

GCGTTCGGTGCGGACCGACGGCTC	0	10	RDR2-sensitive
GCGTTGACGGCAGAACCGTGCACC	0	11	RDR2-sensitive
GCGTTGCTCGGATCTAGACGGTTG	0	16	RDR2-sensitive
GCGTTGGCACAAACGGACGTCGTG	0	15	RDR2-sensitive
GCGTTGTAGACAAGGATGTCGGGT	0	10	RDR2-sensitive
GCGTTTACCGTCGAGTCGTCGAAC	0	12	RDR2-sensitive
GCGTTTCCTGTAGTGTACGGCATA	0	60	RDR2-sensitive
GCGTTTTCTGTAGTGCAAGGCGGT	0	10	RDR2-sensitive
GCTAAAGGACTACAGAGGACAGTC	1	18	RDR2-sensitive
GCTAAAGGCTTTAGCGACCGACAT	0	11	RDR2-sensitive
GCTAAATTTGGCGGACAGGACGGT	0	9	RDR2-sensitive
GCTAACACCGGACAAGCAGCGCAT	0	9	RDR2-sensitive
GCTAACAGACGTGGAACAGGTGGC	1	60	RDR2-sensitive
GCTAACAGACGTGGGACAGATGGC	0	11	RDR2-sensitive
GCTAACAGACGTGGGACAGGTGGC	11	53	RDR2-sensitive
GCTAACAGACGTGGGGCAGGTGGC	13	42	RDR2-sensitive
GCTAACAGTCGGCGTGTCGTGCAT	0	29	RDR2-sensitive
GCTAACCCGGCTTGTAGTTGTGCT	2	15	RDR2-sensitive
GCTAACCGGCGGAATCGACGACAC	1	39	RDR2-sensitive
GCTAACCGGCGGAATCGACGACGC	0	16	RDR2-sensitive
GCTAACCGGTGGAATCGACGACAC	0	23	RDR2-sensitive
GCTAACGCGGTCCAGGCTCGGCAC	0	10	RDR2-sensitive
GCTAACGCGGTTTAGGCTCGGCAC	0	21	RDR2-sensitive
GCTAACGGACTGGCATGGCAGAGT	0	10	RDR2-sensitive
GCTAACTGTTGGGGCGAAGGCATA	0	10	RDR2-sensitive
GCTAAGTCGGGTAGCGCGGGCATG	1	47	RDR2-sensitive
GCTAATCTGTAGGATAGTATGGTC	1	15	RDR2-sensitive
GCTAATGAGCCGGACGGTCCACAC	0	11	RDR2-sensitive
GCTAATGTCGTACGGGCACACAGA	70	0	RDR2-resistant
GCTAATTCATGTCGGGCCGGCACG	0	10	RDR2-sensitive
GCTACAACGAGACTGCGGACGAGC	10	28	RDR2-sensitive
GCTACACAAGTGTGGAACTTCGGC	45	0	RDR2-resistant
GCTACACCTGATTGTCATCGTCGT	0	14	RDR2-sensitive
GCTACAGGTGTGGTAAGTCTGGTC	60	0	RDR2-resistant
GCTACAGTCGGCGACACCGTCCTA	0	25	RDR2-sensitive
GCTACAGTTACGGTCTGCAGGAGA	0	9	RDR2-sensitive
GCTACATATGTAGGGTCGGCTCGG	0	14	RDR2-sensitive
GCTACATTTGGCGTCGGTGCGGCT	0	15	RDR2-sensitive
GCTACCCGACGGACTGTTCGGTGC	3	26	RDR2-sensitive
GCTACCGGACCGGCTCGACACGGC	0	9	RDR2-sensitive
GCTACCTGCCGCGCTGTCGTCCAC	0	9	RDR2-sensitive
GCTACGAACTGGGGAACTGCCGGA	0	10	RDR2-sensitive
GCTACGAGCGAGATCTTGTCGGTG	0	9	RDR2-sensitive
GCTACGGACTTAAACGGGTCGGAT	0	11	RDR2-sensitive
GCTACGGGCTGAAGACCAGTTGGC	0	9	RDR2-sensitive
GCTACGGTTGTTAGCTATATTCGC	0	11	RDR2-sensitive
GCTACGTCTGCAGCAAGAACCGGT	0	20	RDR2-sensitive
GCTACTAAAGAAGCTATTGTCGGT	19	0	RDR2-resistant
GCTACTCTGAGCTGCAGCGGGCGA	0	49	RDR2-sensitive

GCTACTCTGTTGGATGGAAGATGC	2	55	RDR2-sensitive
GCTACTGAGTACTGATCGGTCGGT	0	12	RDR2-sensitive
GCTACTGGAGAACGTAGAGGACCG	0	10	RDR2-sensitive
GCTACTGTAGATCGACGAAGGCAA	0	28	RDR2-sensitive
GCTACTGTAGTTCACGGCCGGCC	1	31	RDR2-sensitive
GCTACTGTTGCAGTTTGGACGGAC	0	13	RDR2-sensitive
GCTACTGTTGTATGGACCCGGCGC	0	11	RDR2-sensitive
GCTAGAACAGAGCGGTCGCGACGA	0	10	RDR2-sensitive
GCTAGAACTGATCCGGCTCTGGAA	0	17	RDR2-sensitive
GCTAGAAGACAAGGTAGATCGGTA	26	1	RDR2-resistant
GCTAGACCTGGACGTAGAAAGGAG	0	10	RDR2-sensitive
GCTAGACGACATGGAGGAAGCTGC	0	9	RDR2-sensitive
GCTAGACGTAGTTACTGCGACCGA	0	14	RDR2-sensitive
GCTAGACTCGTACCTCGACGCCAT	0	32	RDR2-sensitive
GCTAGAGGACGGTTTCGATGGACC	0	12	RDR2-sensitive
GCTAGATCAGGTCGTAGGGATGGT	0	12	RDR2-sensitive
GCTAGATCCGGACTGGGTGTTGGC	31	65	RDR2-sensitive
GCTAGATCGAGACGAGTAAGGCAA	0	16	RDR2-sensitive
GCTAGATCTACTGGACGACGAGCG	0	9	RDR2-sensitive
GCTAGATGGCAGAACGGCAGAACA	0	32	RDR2-sensitive
GCTAGATGGTGATCGTGCGGCCAA	0	10	RDR2-sensitive
GCTAGCAGTAGACGAAGAACGCAC	0	22	RDR2-sensitive
GCTAGCCGCTGAAGACTGGATGTA	0	23	RDR2-sensitive
GCTAGCCGTTGGACTCATGGCACA	0	9	RDR2-sensitive
GCTAGCCTCGGACGAGCCGGCCTC	0	38	RDR2-sensitive
GCTAGCGACGAGGACTGACGCGGA	0	24	RDR2-sensitive
GCTAGCTGAAGAAGGACGAGCAAT	0	9	RDR2-sensitive
GCTAGCTGAAGTAGGACGAGCGAC	0	18	RDR2-sensitive
GCTAGCTGGAGACGAAGCTCGGAA	0	15	RDR2-sensitive
GCTAGGACGTAGGGTGTTACGCAT	0	19	RDR2-sensitive
GCTAGGAGCCTAGGACAACGGTGA	0	11	RDR2-sensitive
GCTAGGAGCCTAGGACGACGGTGA	1	47	RDR2-sensitive
GCTAGGAGCTTAGAACGACGGTGA	0	12	RDR2-sensitive
GCTAGGAGCTTAGGACGACGGTGA	0	28	RDR2-sensitive
GCTAGGATCCTAGGACGACGGTGA	0	22	RDR2-sensitive
GCTAGGCAGACAGATACTCGGCAA	2	20	RDR2-sensitive
GCTAGGCTGTAGGGGGCACCCTAT	0	36	RDR2-sensitive
GCTAGGGAACTGGATTACACGGCT	0	22	RDR2-sensitive
GCTAGGGGTTCGGACATATGGCAC	0	19	RDR2-sensitive
GCTAGGGTGGACTGCAGCAGCCGG	0	9	RDR2-sensitive
GCTAGGTAGGCGGCATGTAGGGCA	0	10	RDR2-sensitive
GCTAGGTCGCGGACCGTCCGGCCC	0	10	RDR2-sensitive
GCTAGGTCTAAGGGTAGGGGTGGA	15	0	RDR2-resistant
GCTAGTAACTGTTTTTTCCTTGGC	22	0	RDR2-resistant
GCTAGTACTGTAGCACCGGTAGGA	0	60	RDR2-sensitive
GCTAGTACTGTAGCACCGGTAGGG	0	12	RDR2-sensitive
GCTAGTAGAACGGACGGACGGTGC	2	36	RDR2-sensitive
GCTAGTAGCAAGGTCTCGGGCAGC	0	38	RDR2-sensitive
GCTAGTATGCCATCGGACCCACAA	0	13	RDR2-sensitive

GCTAGTCATATGGGCTTTGACGGA	0	11	RDR2-sensitive
GCTAGTCATGTGGGATTTGATGGA	0	11	RDR2-sensitive
GCTAGTCATGTGGGATTTGGCGGA	0	19	RDR2-sensitive
GCTAGTCCTAGACTGTCTCGGCAA	2	38	RDR2-sensitive
GCTAGTGGGATTGGGATAGGAGGA	0	9	RDR2-sensitive
GCTAGTTCGTCGTTGTTTCGTCAT	0	9	RDR2-sensitive
GCTATACTGCATGCACTCAGCCAG	0	11	RDR2-sensitive
GCTATACTGGACTAGGGGCGTAAG	0	11	RDR2-sensitive
GCTATAGACGGCTACTGACGACAC	0	10	RDR2-sensitive
GCTATAGACGGCTGTTGACGGTCC	0	30	RDR2-sensitive
GCTATATTTGGCGGACAGGAGGCT	0	11	RDR2-sensitive
GCTATCACTGTGAACTATCCGGCC	3	27	RDR2-sensitive
GCTATCCTTGTCGGCATGTCGGAT	0	9	RDR2-sensitive
GCTATCCTTTTTTTGGTGGTCGTC	42	2	RDR2-resistant
GCTATCTCATCGTGGGCTGGGCAA	0	9	RDR2-sensitive
GCTATCTGGCTGAGTCGTCGAGTA	0	18	RDR2-sensitive
GCTATCTGGTCAGGCGCGGACGGT	0	14	RDR2-sensitive
GCTATCTGTGGAGATGTGGATGGT	3	104	RDR2-sensitive
GCTATCTGTTCGCGGACTCGGCCC	0	9	RDR2-sensitive
GCTATGCACTGTGGAAGATTGCAG	0	12	RDR2-sensitive
GCTATGTACGACAAGCTCCGGCGC	2	29	RDR2-sensitive
GCTATGTCCGACGGCATCGTAGAC	2	21	RDR2-sensitive
GCTATTGACGCCTAGGTCTCGGAT	0	24	RDR2-sensitive
GCTATTGACGTCGACTTTATGGAT	32	2	RDR2-resistant
GCTATTGACTGTGCGAGCTTCGGA	6	47	RDR2-sensitive
GCTATTGTCGACTGCTGACGGTGT	0	18	RDR2-sensitive
GCTCAAATGTCTGTCGGGCTCGGC	2	25	RDR2-sensitive
GCTCAACCAGGTTGTCGGGTGCGC	0	10	RDR2-sensitive
GCTCAACGAACTGTACATCGGCAA	1	14	RDR2-sensitive
GCTCAACGCGGGCAGCGAACGGAA	0	9	RDR2-sensitive
GCTCAAGATGTCGGGCCCCAACGG	3	23	RDR2-sensitive
GCTCACCGGAACTACGAGGACGAT	0	14	RDR2-sensitive
GCTCACGGTATGCGGTCGTAGTGT	0	19	RDR2-sensitive
GCTCACGTCGTCGAAGTTGCGCAT	1	19	RDR2-sensitive
GCTCACGTGCGGACGGTCCGGGCA	2	17	RDR2-sensitive
GCTCACTTTAAATCGTATCGGACA	0	9	RDR2-sensitive
GCTCAGAACTGCACTAGACTTCAT	0	15	RDR2-sensitive
GCTCAGACCCTTGACGACGAACGG	0	9	RDR2-sensitive
GCTCAGACTGGAACAGGCAACCAA	0	9	RDR2-sensitive
GCTCAGCGAACTGTACATCGGCAA	6	86	RDR2-sensitive
GCTCAGCGGACAGAGGAGAAGCAT	1	36	RDR2-sensitive
GCTCAGGAAGATCTTCGGCAACAC	0	12	RDR2-sensitive
GCTCAGGACCCTGACGACAAACGG	0	16	RDR2-sensitive
GCTCAGGACCTTGACGACGGGCAA	2	92	RDR2-sensitive
GCTCAGGATCTAGAAAGTGACGGA	4	30	RDR2-sensitive
GCTCAGTCAGGCTGCATATCCCAC	0	11	RDR2-sensitive
GCTCAGTCGTCACTGTGCCGGCCT	0	22	RDR2-sensitive
GCTCATCGCGGACGAGCACGCCGG	0	15	RDR2-sensitive
GCTCATTCGTCGAAGAAGGCAGGA	0	9	RDR2-sensitive

GCTCCAAATCGTCAACGGTCGGCT	1	16	RDR2-sensitive
GCTCCAACAGCCAGGACGTCGGTC	0	10	RDR2-sensitive
GCTCCAACGGTCGGAACCCAACGG	2	30	RDR2-sensitive
GCTCCAGATTGAACGAAGGCAGAA	0	17	RDR2-sensitive
GCTCCAGCCAGGAGATCGATCGGG	0	11	RDR2-sensitive
GCTCCATATAGTGATCGTGCGGGC	0	12	RDR2-sensitive
GCTCCATCACTAACGGGCTGGCAT	0	9	RDR2-sensitive
GCTCCCATCGTCCAGCGAGAACGG	0	13	RDR2-sensitive
GCTCCCGGACGACGAGAACGGCGT	0	24	RDR2-sensitive
GCTCCCGGACTGGACCAGGCAACC	0	10	RDR2-sensitive
GCTCCCTGACGGCACTCTAGTTGA	44	10	RDR2-resistant
GCTCCCTGGATTGTAGCTTCGGTA	0	68	RDR2-sensitive
GCTCCCTGGTCAGACGCGGACGGT	1	24	RDR2-sensitive
GCTCCGCTATAATTCACCGGACTG	2	14	RDR2-sensitive
GCTCCGGACCAACAGTACAGCATA	0	9	RDR2-sensitive
GCTCCGGACGATCTACGATGGCGA	0	16	RDR2-sensitive
GCTCCGGATGTACGCAGCAGGCCT	0	12	RDR2-sensitive
GCTCCGGCTTGAACTCGTCGGCGT	0	14	RDR2-sensitive
GCTCCGGTAGGACGTCGCGTCGGC	1	13	RDR2-sensitive
GCTCCGGTCGTGTGGCGCTGCGGC	2	14	RDR2-sensitive
GCTCCGTGCGGACTGTCCGGTGAT	0	9	RDR2-sensitive
GCTCCTACAGCAGGGTCGCGGACC	0	9	RDR2-sensitive
GCTCCTACCTTGTGTCGATCGGAT	0	13	RDR2-sensitive
GCTCCTCTCGGCCAACAGAACGGT	0	9	RDR2-sensitive
GCTCCTGCAACCTGGTCAGTCGGC	2	17	RDR2-sensitive
GCTCCTGCAACTCGGTCAGTCGGT	8	42	RDR2-sensitive
GCTCCTGCAACTTGGTCAGTCGGT	8	48	RDR2-sensitive
GCTCCTGCGATACGGTCGGTCGGC	2	17	RDR2-sensitive
GCTCCTGCGATTTGGTCGGTCGGC	5	21	RDR2-sensitive
GCTCCTGCGATTTGGTCGGTCGGT	7	24	RDR2-sensitive
GCTCCTGGACGCGTAGGTTCGGCC	0	14	RDR2-sensitive
GCTCCTGGGCTGCAACTAGGCGTG	0	18	RDR2-sensitive
GCTCCTTGGACTGTCTTGAGGTTA	0	11	RDR2-sensitive
GCTCCTTGTTGGACCGAGAGAGGA	0	14	RDR2-sensitive
GCTCCTTGTTGGACGGAGAAAGGA	0	13	RDR2-sensitive
GCTCCTTGTTGGACGGAGAGAGGA	0	38	RDR2-sensitive
GCTCGAAAAAGCGGCTCGGCTCGG	0	9	RDR2-sensitive
GCTCGAACTCACAACCTAGTGCAT	0	13	RDR2-sensitive
GCTCGACAAGGACTTGGCAGGCCG	0	12	RDR2-sensitive
GCTCGACAAGTAGTGCATCGGCAA	0	10	RDR2-sensitive
GCTCGACACGTAGAGGACGAGAAT	0	9	RDR2-sensitive
GCTCGACACTCGGCTCAGACACGG	0	10	RDR2-sensitive
GCTCGACGAACGGACGGCTTAGAT	0	14	RDR2-sensitive
GCTCGACGAACTGTACATCGGCAA	1	70	RDR2-sensitive
GCTCGACGAACTGTACATTGGCAA	0	12	RDR2-sensitive
GCTCGACGAACTGTATATCGGCAA	0	9	RDR2-sensitive
GCTCGACGAGAACTGTCAACGGCG	0	12	RDR2-sensitive
GCTCGACGAGATGAAGCCGGACAT	3	39	RDR2-sensitive
GCTCGACGGGCTGGTTGGTCGGCA	0	10	RDR2-sensitive

GCTCGACGTTGTGACTTATGGCAT	1	22	RDR2-sensitive
GCTCGACTCGGACCGGCATAACGT	0	12	RDR2-sensitive
GCTCGACTCGGCTCGGACCGGCTC	0	13	RDR2-sensitive
GCTCGACTCGGCTTGGATCGGCTC	0	31	RDR2-sensitive
GCTCGACTCGGTTTAGAACGGCTC	0	28	RDR2-sensitive
GCTCGACTCGTGGAAACCGGCCGG	0	19	RDR2-sensitive
GCTCGACTGCGTAGAAGAGGATTC	0	11	RDR2-sensitive
GCTCGAGAGCCAGGCTGTGCGCGT	1	12	RDR2-sensitive
GCTCGAGAGTCTTGCTGTGCGCGT	0	9	RDR2-sensitive
GCTCGAGCCAGAACCAGAACCAGA	0	10	RDR2-sensitive
GCTCGAGCTCGGAGCAACTCGGCT	77	0	RDR2-resistant
GCTCGATAACTCGCTCGACTCGGC	0	18	RDR2-sensitive
GCTCGATCGTGGCGTTGTATGCGG	0	10	RDR2-sensitive
GCTCGATGTCCGGATTGAAGGCAA	0	12	RDR2-sensitive
GCTCGCAAGTTCGGATAGGGACGG	0	16	RDR2-sensitive
GCTCGCCTGTAGGACGTACGTGCT	0	19	RDR2-sensitive
GCTCGCGGACGAAGTGCAGGTCGA	0	20	RDR2-sensitive
GCTCGCGTGGGACTGGGTATGCAG	0	10	RDR2-sensitive
GCTCGCTCTGGACTCGCAAGCCGG	0	21	RDR2-sensitive
GCTCGCTTCAAGGTCGTGGATATT	45	0	RDR2-resistant
GCTCGCTTTTGACTGGGACGGGAT	0	9	RDR2-sensitive
GCTCGGACAAGAAACTAGGCACGG	0	14	RDR2-sensitive
GCTCGGACAGGAAACTAGGCACGG	2	17	RDR2-sensitive
GCTCGGACAGGAAATTAGGCACGG	0	14	RDR2-sensitive
GCTCGGACCGGACCCAAAAAGCGG	0	10	RDR2-sensitive
GCTCGGACCGGACCGACACGGTAT	0	9	RDR2-sensitive
GCTCGGAGGTACTGGCATGTGTAG	0	12	RDR2-sensitive
GCTCGGATGGGACTTGGTCGGCCG	0	17	RDR2-sensitive
GCTCGGATGTAGGTTTGGATGGCT	0	13	RDR2-sensitive
GCTCGGATGTATAACACACAGCAT	0	13	RDR2-sensitive
GCTCGGATTAGCTCAGACCGGTTT	0	9	RDR2-sensitive
GCTCGGCAAACAGTACATCGGCAA	10	184	RDR2-sensitive
GCTCGGCAAACAGTGCATCGGCAA	1	15	RDR2-sensitive
GCTCGGCAAACTGTACATCGGCAA	1	40	RDR2-sensitive
GCTCGGCAAAGAAAGGTCGTCGTA	0	11	RDR2-sensitive
GCTCGGCAAATAGTACATCGGCAA	0	19	RDR2-sensitive
GCTCGGCACATAGTACATCGGCAA	0	37	RDR2-sensitive
GCTCGGCACTACAGATCACGGTGT	0	31	RDR2-sensitive
GCTCGGCATGGTACGAATAAGCGG	1	17	RDR2-sensitive
GCTCGGCATGTAGGTTGATCGGTA	4	64	RDR2-sensitive
GCTCGGCCCGAGACCGCATAGAAC	0	9	RDR2-sensitive
GCTCGGCCTGTCAAACACGGTTAG	0	10	RDR2-sensitive
GCTCGGCGAACAGTACATCGGCAA	8	40	RDR2-sensitive
GCTCGGCGAACTGTACATCGGCAA	40	513	RDR2-sensitive
GCTCGGCGAACTGTACATCGGCAC	2	20	RDR2-sensitive
GCTCGGCGAACTGTACATCGGCCA	0	20	RDR2-sensitive
GCTCGGCGAACTGTATATCGGCAA	2	14	RDR2-sensitive
GCTCGGCGGCGGCAGGATAGAAAC	0	13	RDR2-sensitive
GCTCGGCGTCAAGATCTGCGGTGC	0	10	RDR2-sensitive

GCTCGGCTCGACTCGAAGGTGGCT	0	12	RDR2-sensitive
GCTCGGCTCGAGCTCGGAGCGGCT	0	9	RDR2-sensitive
GCTCGGCTCGGATCGGATCGGCTC	0	25	RDR2-sensitive
GCTCGGCTCGGATCGGCTCGATAG	0	9	RDR2-sensitive
GCTCGGCTCGGCTCGACTCGGCTC	3	61	RDR2-sensitive
GCTCGGCTCGGCTCGGAGCGGCTC	0	12	RDR2-sensitive
GCTCGGCTCGGCTCGGATCGGCTC	15	155	RDR2-sensitive
GCTCGGCTCGGCTCGGCATTTCAC	0	14	RDR2-sensitive
GCTCGGCTGGACGACTGCATCTGT	0	17	RDR2-sensitive
GCTCGGCTTGACTCGGACCGGCTT	0	16	RDR2-sensitive
GCTCGGGAACCAGGCTGTGGGCGT	0	24	RDR2-sensitive
GCTCGGGTGAACCAGGCTGAAGTT	0	10	RDR2-sensitive
GCTCGGTAAACTGTACATCGGCAA	0	11	RDR2-sensitive
GCTCGGTAAAGGACTGTCAGCGGC	0	10	RDR2-sensitive
GCTCGGTACGGATAATGGATCGGC	0	15	RDR2-sensitive
GCTCGGTAGCCTGGCTGAGACGAG	0	10	RDR2-sensitive
GCTCGGTAGGACCTGGTCAGCCGG	0	14	RDR2-sensitive
GCTCGGTAGGGACTTGGTCGGCCT	0	9	RDR2-sensitive
GCTCGGTGCCTGAACGAGCTCGGC	2	15	RDR2-sensitive
GCTCGTCGAGCCAAGCTGAGACCG	0	10	RDR2-sensitive
GCTCGTCGTCGTACCATGCGTCAT	0	12	RDR2-sensitive
GCTCGTCGTCGTTCCGTACGTCAT	0	11	RDR2-sensitive
GCTCGTCTCGACTTGAATCGGCTT	0	9	RDR2-sensitive
GCTCGTGCATGTGAGACAGGGGAC	15	0	RDR2-resistant
GCTCGTGGACCAGATTGAGGGGCT	0	9	RDR2-sensitive
GCTCGTGGCTCGTGGTAGATCGGA	1	12	RDR2-sensitive
GCTCGTGTCGTGTAGTGTAAGGCT	0	9	RDR2-sensitive
GCTCGTGTTGTCGGTACATGCATT	0	22	RDR2-sensitive
GCTCGTTCGGAGCGTCGAGCCGGT	0	11	RDR2-sensitive
GCTCGTTTCTGTGGAGCCAGGCTG	2	99	RDR2-sensitive
GCTCTAACTTTCGGTCTGTCGTGC	0	20	RDR2-sensitive
GCTCTACCTCGTCGGATTTGGCGT	0	9	RDR2-sensitive
GCTCTAGTTGCGGACTCAGGCGTT	0	12	RDR2-sensitive
GCTCTATCGTCTACTCTAAAGCAG	1	16	RDR2-sensitive
GCTCTATCTCGACGGCAACTTCAC	0	15	RDR2-sensitive
GCTCTCAACTGTAGCGGTGTGGTA	0	12	RDR2-sensitive
GCTCTCCAGGTTGGACTGTGCGGT	0	82	RDR2-sensitive
GCTCTCCGGACTGGACCAGGCAAC	0	36	RDR2-sensitive
GCTCTCCGGATGGTAGCTTCGGCT	1	19	RDR2-sensitive
GCTCTCGGAACTAGTCAACGCATG	0	22	RDR2-sensitive
GCTCTCGGCACAGGGACTGGCGGG	1	12	RDR2-sensitive
GCTCTCGGCACAGGGACTGGCTGT	0	11	RDR2-sensitive
GCTCTCGGGACTTCGACGGCGGTG	0	10	RDR2-sensitive
GCTCTCTAGTAGAACGGTTCGGTG	0	23	RDR2-sensitive
GCTCTCTAGTCAGGCGCGGACGGT	1	15	RDR2-sensitive
GCTCTCTATGATAGCGCGGACGGT	0	11	RDR2-sensitive
GCTCTCTGATCAGGCGCGGACGGT	0	10	RDR2-sensitive
GCTCTCTGCACAGACGCGGACGGT	0	9	RDR2-sensitive
GCTCTCTGCACAGGCGCGGACGGT	0	42	RDR2-sensitive

GCTCTCTGCACAGGGGCGGACGGT	2	37	RDR2-sensitive
GCTCTCTGCATAGACGTGGACGGT	0	14	RDR2-sensitive
GCTCTCTGCATAGGCGTGGACGGT	0	9	RDR2-sensitive
GCTCTCTGCGGACACATGGATGGT	0	28	RDR2-sensitive
GCTCTCTGCGGAGCCGATGACGGT	0	11	RDR2-sensitive
GCTCTCTGCGGAGGAGTAGACGGT	1	21	RDR2-sensitive
GCTCTCTGCGGAGGCGTAGACGGT	1	26	RDR2-sensitive
GCTCTCTGCGGCGGCGTAGACGGG	0	12	RDR2-sensitive
GCTCTCTGCGGCGGCGTAGACGGT	1	12	RDR2-sensitive
GCTCTCTGCGGCGGTACGGACGGT	0	9	RDR2-sensitive
GCTCTCTGGTCAAGCGCGGACGGT	0	32	RDR2-sensitive
GCTCTCTGGTCAGACGAGGACGGT	4	26	RDR2-sensitive
GCTCTCTGGTCAGACGCGGACGGT	2	118	RDR2-sensitive
GCTCTCTGGTCAGACGTGGACGGT	2	41	RDR2-sensitive
GCTCTCTGGTCAGGCACGGACGGT	0	18	RDR2-sensitive
GCTCTCTGGTCAGGCGCGGACGGG	2	14	RDR2-sensitive
GCTCTCTGGTCAGGCGCGGACGGT	20	433	RDR2-sensitive
GCTCTCTGGTCAGGCGTAGACGGT	1	31	RDR2-sensitive
GCTCTCTGGTCAGGCGTGGACGGT	4	53	RDR2-sensitive
GCTCTCTGGTCAGGTGCGGACGGT	0	9	RDR2-sensitive
GCTCTCTGGTCGGACGCGGACGGT	0	16	RDR2-sensitive
GCTCTCTGGTTAGGCGTGGACGGT	0	13	RDR2-sensitive
GCTCTCTGTAGAAGCACGGACGGT	0	29	RDR2-sensitive
GCTCTCTGTAGAGACGCGGACGGT	0	29	RDR2-sensitive
GCTCTCTGTAGAGGCGCGGACGGT	0	13	RDR2-sensitive
GCTCTCTGTAGAGGCGTGGACGGT	1	112	RDR2-sensitive
GCTCTCTGTAGCAGCACGGACGGT	0	41	RDR2-sensitive
GCTCTCTGTGATAGCATGGACGGT	0	14	RDR2-sensitive
GCTCTCTGTGGAACCACGGACGGT	0	21	RDR2-sensitive
GCTCTCTGTGGAAGCGAGGACGAC	0	17	RDR2-sensitive
GCTCTCTGTGGAGACGCGGACGGT	1	20	RDR2-sensitive
GCTCTCTGTGGAGGCACGAACGGT	0	39	RDR2-sensitive
GCTCTCTGTGGAGGCACGGACGGT	0	14	RDR2-sensitive
GCTCTCTGTGGAGGCGCGAACGGT	0	11	RDR2-sensitive
GCTCTCTGTGGAGGCGCGGACGGT	1	28	RDR2-sensitive
GCTCTCTGTGGAGGCGCTGACGGT	0	36	RDR2-sensitive
GCTCTCTGTGGAGGCGTGGACGGT	1	13	RDR2-sensitive
GCTCTCTGTGGAGTCGTGGACGGT	0	46	RDR2-sensitive
GCTCTCTGTGGATGCTTAGACGGT	0	11	RDR2-sensitive
GCTCTCTTCGGCTGCACTGACGGT	0	17	RDR2-sensitive
GCTCTCTTCTACAGCGCGGACGGT	0	12	RDR2-sensitive
GCTCTGACAGTAGACGTTGCAGAT	0	9	RDR2-sensitive
GCTCTGACTGAGACGGTGTATTGC	1	18	RDR2-sensitive
GCTCTGCACGGACGGGAAAACGAA	0	24	RDR2-sensitive
GCTCTGCAGATCTCCCGAAGAGGC	1	17	RDR2-sensitive
GCTCTGCCACGTCAGCTAGCTCGG	0	9	RDR2-sensitive
GCTCTGCCTCGCAGGATTGCTCGG	0	18	RDR2-sensitive
GCTCTGCGAACTGTACATCGGTAA	0	9	RDR2-sensitive
GCTCTGCGGACGGTGATTTTAGAG	0	10	RDR2-sensitive

GCTCTGCTCGGCTCGATGGTACAG	0	10	RDR2-sensitive
GCTCTGGACATGGCAGACTGCACC	2	31	RDR2-sensitive
GCTCTGGACATGTCAGGAAGCTGC	0	20	RDR2-sensitive
GCTCTGGACGACAGCCTGCACGAA	0	12	RDR2-sensitive
GCTCTGGACTGTGTACATGATGGA	0	10	RDR2-sensitive
GCTCTGGATAGTTTGATTGCACGC	1	20	RDR2-sensitive
GCTCTGGATGCGGCAGAACCGTGC	0	12	RDR2-sensitive
GCTCTGGATGTGGCAGAACCGTGC	0	22	RDR2-sensitive
GCTCTGGCCTCTGGACGCGGCTGC	0	11	RDR2-sensitive
GCTCTGTAGCCTCCAAATTCGGTA	0	23	RDR2-sensitive
GCTCTGTAGGCGCTACGCGACAAG	0	9	RDR2-sensitive
GCTCTGTGAATAGTTGTTGACGCT	44	1	RDR2-resistant
GCTCTGTGACGACGGCGGCTCTCA	1	25	RDR2-sensitive
GCTCTGTGACGACGGCGGCTCTCC	0	9	RDR2-sensitive
GCTCTGTGGAATGCACGTCAATCC	17	0	RDR2-resistant
GCTCTGTGGAGGCGTGGACGGTCC	0	18	RDR2-sensitive
GCTCTTAACCTTGTGGTCGTGGGT	0	9	RDR2-sensitive
GCTCTTATGAACGGATCGTCCGGT	0	11	RDR2-sensitive
GCTCTTCATGATAGATCGTTTCGA	0	9	RDR2-sensitive
GCTCTTCGGCTATAACTTCGGCGT	0	11	RDR2-sensitive
GCTCTTGAAGCGGACGGACGACGG	2	43	RDR2-sensitive
GCTCTTGCTGTAGACCGAGGTGGC	2	15	RDR2-sensitive
GCTCTTTAGACGGCTCTAGCTCAG	0	12	RDR2-sensitive
GCTCTTTCGGTTGTAGCTTCGGCT	1	30	RDR2-sensitive
GCTCTTTGAGTTGGATCTGTTGGT	0	36	RDR2-sensitive
GCTCTTTGCGACAGCGTGGACGGT	0	11	RDR2-sensitive
GCTCTTTGGTCAGGCGCGGACGGT	2	23	RDR2-sensitive
GCTGAACACTCGGCACAGAACGAC	2	19	RDR2-sensitive
GCTGAACACTCGGCACAGAACGGC	1	47	RDR2-sensitive
GCTGAACACTCGGCACAGGACGAC	3	19	RDR2-sensitive
GCTGAACACTCGGCACAGGTAGGC	5	24	RDR2-sensitive
GCTGAACACTCGGTACAGGACGAC	1	17	RDR2-sensitive
GCTGAACCAGGTTCAGGTCGGACC	0	36	RDR2-sensitive
GCTGAACCGGATGCACTGTTGGAC	0	12	RDR2-sensitive
GCTGAACCTTATGGATGACTGGCT	0	10	RDR2-sensitive
GCTGAACGTATGGATGACTGTGCT	0	27	RDR2-sensitive
GCTGAACGTATGGATGGCTGTGCT	0	23	RDR2-sensitive
GCTGAACTGAAGAAGGCCTGTCAC	0	9	RDR2-sensitive
GCTGAACTGATAAGCTGCTGCAGA	1	17	RDR2-sensitive
GCTGAAGAAGAACGCGGAACAGGA	0	12	RDR2-sensitive
GCTGAAGACTAGCTCCGCAGATAC	0	27	RDR2-sensitive
GCTGAAGACTGAATGTAGGGACGT	0	110	RDR2-sensitive
GCTGAAGACTGGCTGCGCACTGTG	0	17	RDR2-sensitive
GCTGAAGATTGGCTGCGCACTGTG	2	16	RDR2-sensitive
GCTGAAGGATGGACGATCACGGTG	41	0	RDR2-resistant
GCTGAAGGATGGTGCGCAGGACGT	0	9	RDR2-sensitive
GCTGAAGGCTGGTAGGCGGGCCGT	0	9	RDR2-sensitive
GCTGAAGTAGGACGAGTCACACAG	0	10	RDR2-sensitive
GCTGAATCGTTTTTGGGCCAGGCT	0	17	RDR2-sensitive

GCTGAATTTGGTGGGCTGGTGGCT	0	28	RDR2-sensitive
GCTGACAATGCAGTATCTTCGGCC	18	0	RDR2-resistant
GCTGACACACGGAACAACGACGAC	0	16	RDR2-sensitive
GCTGACACATTGGACGGAAGAGCA	0	24	RDR2-sensitive
GCTGACACGCGGAACGACGATGAG	1	42	RDR2-sensitive
GCTGACACGTGGAACGACGACGAC	0	49	RDR2-sensitive
GCTGACATCTAGGACCACGACGGA	0	15	RDR2-sensitive
GCTGACATGACCCAGACTCGGCAC	0	10	RDR2-sensitive
GCTGACATGTATGCTTAGTTGACG	87	2	RDR2-resistant
GCTGACATTGTCGCAGTAGACGAG	1	12	RDR2-sensitive
GCTGACCAGCGGAATCGATGACGC	1	50	RDR2-sensitive
GCTGACCATGAACCAAGCGAGCAA	0	16	RDR2-sensitive
GCTGACCATGAACCGAGCGAGCAA	0	19	RDR2-sensitive
GCTGACCATGAACCGAGCGAGCCA	0	25	RDR2-sensitive
GCTGACCATGAACCGAGCGAGCTA	0	21	RDR2-sensitive
GCTGACCATGGCACTCGGCAAAGA	1	31	RDR2-sensitive
GCTGACCATGGCACTCGGCAAGAA	0	27	RDR2-sensitive
GCTGACCGAGCCGAGGGACTCCTA	4	41	RDR2-sensitive
GCTGACCGGGCCAAGGACACGACA	0	10	RDR2-sensitive
GCTGACCGGGCTACAGGCACGACA	0	13	RDR2-sensitive
GCTGACCTCGGACGGCTCCCTCGA	1	15	RDR2-sensitive
GCTGACCTGAACCGACCTGACGCC	1	13	RDR2-sensitive
GCTGACCTGATAGGCTGATACGGT	0	66	RDR2-sensitive
GCTGACCTTGAACCGAGCGAGCCA	0	14	RDR2-sensitive
GCTGACGAAGTAGACCGGCCTCTG	0	10	RDR2-sensitive
GCTGACGACGTCAACTCTAGCGGC	0	18	RDR2-sensitive
GCTGACGAGTATAGAACGGAGCTT	0	14	RDR2-sensitive
GCTGACGCACGGAATGACGACGAG	0	30	RDR2-sensitive
GCTGACGCACGGATTGACGACGAC	1	19	RDR2-sensitive
GCTGACGCAGATAGGACAACAGTG	0	19	RDR2-sensitive
GCTGACGCGCAGAACGACGACGAC	0	23	RDR2-sensitive
GCTGACGCGCGGAACAACGACGAC	0	11	RDR2-sensitive
GCTGACGCGCGGAACGACGACAAG	1	20	RDR2-sensitive
GCTGACGCGCGGAACGACGACGAC	1	21	RDR2-sensitive
GCTGACGCGCGGAACGACGACGAG	5	278	RDR2-sensitive
GCTGACGCGCGGAACGACGACGCG	0	9	RDR2-sensitive
GCTGACGCGCGGAACGACGGCGAC	0	14	RDR2-sensitive
GCTGACGCGCGGAATGACGACGAG	0	30	RDR2-sensitive
GCTGACGCGCGGAATGATGACGAC	0	20	RDR2-sensitive
GCTGACGCGGATAGGACAACGGTG	0	48	RDR2-sensitive
GCTGACGCGTCGGCTACCGTGGCA	0	9	RDR2-sensitive
GCTGACGCTGCTGACGCCTGACGG	0	12	RDR2-sensitive
GCTGACGGACGACACGGCTAGGCT	3	21	RDR2-sensitive
GCTGACGGAGACCGCGACCTCTAC	35	0	RDR2-resistant
GCTGACGGGAGATAAGGGAACGGA	0	18	RDR2-sensitive
GCTGACGGTGTAGATTCACGGAAG	0	10	RDR2-sensitive
GCTGACGTCCGACGGCTCACTCGA	0	17	RDR2-sensitive
GCTGACGTGACTGCATAGTAACAT	0	105	RDR2-sensitive
GCTGACGTGGACGCATGTGGGAGC	0	12	RDR2-sensitive

GCTGACGTGGATAGGACAACGGCA	0	21	RDR2-sensitive
GCTGACGTGGCTGCATAGTAACAT	7	342	RDR2-sensitive
GCTGACGTGGCTGCATAGTAAGAT	2	61	RDR2-sensitive
GCTGACGTGGCTGCATAGTGACAT	0	10	RDR2-sensitive
GCTGACGTTGTCGCAGTAGACGAG	44	175	RDR2-sensitive
GCTGACTCAGGCTACGGGGAACAA	7	28	RDR2-sensitive
GCTGACTCGATGACCTGACGGCAA	0	13	RDR2-sensitive
GCTGACTCGGACGGGTAAGCACGG	0	14	RDR2-sensitive
GCTGACTCTGCACTGCACGTGACG	0	9	RDR2-sensitive
GCTGACTGAGTTGTGGCTACCTGT	0	9	RDR2-sensitive
GCTGACTGTACGGCTGTACCGTTC	0	12	RDR2-sensitive
GCTGACTGTCGTATGGTGCACCGG	2	23	RDR2-sensitive
GCTGACTGTGAGATACACCAGGCT	0	14	RDR2-sensitive
GCTGACTGTGGGGAGAAGCTGGTG	0	13	RDR2-sensitive
GCTGACTGTTCTGTCCGAGCACGG	1	19	RDR2-sensitive
GCTGACTTCGGACGGCTCCCTCGA	0	18	RDR2-sensitive
GCTGACTTCGTAGCAACGCACGGA	1	76	RDR2-sensitive
GCTGAGAACCCGAACGTCACCGGC	3	18	RDR2-sensitive
GCTGAGACAGAAGAGCAACCTCAA	0	15	RDR2-sensitive
GCTGAGACTGTCGAGGTGGTGCTA	0	15	RDR2-sensitive
GCTGAGAGGCGGCATGCAGGACAG	2	22	RDR2-sensitive
GCTGAGATCGCAGAACGGCAGAAC	0	48	RDR2-sensitive
GCTGAGATCGCAGAATGGCAGAAC	0	25	RDR2-sensitive
GCTGAGCCGAGTAGAGCCGAGCTG	0	18	RDR2-sensitive
GCTGAGCCTAGCTGTAGACCAGAC	0	13	RDR2-sensitive
GCTGAGCGTGACTGCATGAGGAGG	0	13	RDR2-sensitive
GCTGAGCGTTTGGCAGTCTGCAGC	4	22	RDR2-sensitive
GCTGAGGACTGTGCGGACGGTGGT	0	19	RDR2-sensitive
GCTGAGGATGTGAGGACGGAAGAA	0	32	RDR2-sensitive
GCTGAGGCGCGGAACGACGACGAG	1	16	RDR2-sensitive
GCTGAGGCGGCTTGTAGGACGGAG	1	13	RDR2-sensitive
GCTGAGGCTTGGCAACGATGGCGC	3	19	RDR2-sensitive
GCTGAGTCGCGACAGCACGACGGA	19	1	RDR2-resistant
GCTGAGTCTGTATACACGAGTGAA	0	27	RDR2-sensitive
GCTGAGTGCCCGTTGGACTAACGC	0	10	RDR2-sensitive
GCTGATAGACCTGGCTACTAGAGA	0	13	RDR2-sensitive
GCTGATCACGAGTCGAGCGAGCTC	0	11	RDR2-sensitive
GCTGATCCCAGGTAGATAAGACAA	0	22	RDR2-sensitive
GCTGATCCGGATCTGCTTGGAGGC	2	28	RDR2-sensitive
GCTGATCCGGTTGGCTGTGACGGA	1	15	RDR2-sensitive
GCTGATCGATGAAGACGTGCGCTG	0	10	RDR2-sensitive
GCTGATCGGGCTACAGGCACGGCA	0	14	RDR2-sensitive
GCTGATCGGGTAAGAGTTACGCAT	0	9	RDR2-sensitive
GCTGATCGTAGCTCGATGGTTCGT	0	16	RDR2-sensitive
GCTGATGATGGCACTGTTACAACT	91	1	RDR2-resistant
GCTGATGCACGGAACGACGACGAC	0	10	RDR2-sensitive
GCTGATGCACTGTGTGACGAGCCC	1	13	RDR2-sensitive
GCTGATGCGCAGAACGACGACGAG	0	36	RDR2-sensitive
GCTGATGGACAGTTGGTCGGCGGC	0	10	RDR2-sensitive

GCTGATGGAGGATAGACTGGTGCT	53	3	RDR2-resistant
GCTGATGGAGTTGGACGATGTCAT	0	9	RDR2-sensitive
GCTGATGGGGGTTACCAGCTGAGA	0	13	RDR2-sensitive
GCTGATTTAATCGGGCCAAGGCAC	2	79	RDR2-sensitive
GCTGATTTCTCTGTCGATGGGCAC	0	13	RDR2-sensitive
GCTGCAACAGTGTCGGCTTGTAGC	0	23	RDR2-sensitive
GCTGCAAGAACTCGACAGACGCAG	0	15	RDR2-sensitive
GCTGCAAGTCGCGGATTGTCCGGC	0	13	RDR2-sensitive
GCTGCAGACCGAGCTTGGACAGAA	0	13	RDR2-sensitive
GCTGCAGACTGTGCACTAGAGCAG	0	21	RDR2-sensitive
GCTGCAGCGGCGTACTCTATACCA	0	14	RDR2-sensitive
GCTGCAGCGGGAGACTGGTGAGCA	2	43	RDR2-sensitive
GCTGCAGCTGAGACAGAAGAGCAA	0	10	RDR2-sensitive
GCTGCATCCGGATGGCACAGGCAA	0	9	RDR2-sensitive
GCTGCATCTTGGACGTACACGGCG	0	10	RDR2-sensitive
GCTGCATGAGTCAGGTCGGCGGGA	0	13	RDR2-sensitive
GCTGCCACTCTGACCTGATGGACG	0	9	RDR2-sensitive
GCTGCCGGAACCGAGGGAGACGTC	0	13	RDR2-sensitive
GCTGCCGGAGCCGAGGGAGACGTC	0	10	RDR2-sensitive
GCTGCGAAGAAGACGACCCTCTGC	0	15	RDR2-sensitive
GCTGCGACCAGCGCGCTGGACGTT	0	11	RDR2-sensitive
GCTGCGACGAGGACGATTGGCGCC	100	0	RDR2-resistant
GCTGCGACGTGTAGCCTGCAGGGG	0	15	RDR2-sensitive
GCTGCGATGCAGAACTGATTGGCG	0	13	RDR2-sensitive
GCTGCGCTGGGCTGGCGGCTGAAA	0	9	RDR2-sensitive
GCTGCGGAAATGGGCCAAGACGAT	0	52	RDR2-sensitive
GCTGCGGGCTAGGCTCAGGCAGAA	0	43	RDR2-sensitive
GCTGCGGGCTGAGATTAGACAGAA	0	14	RDR2-sensitive
GCTGCGTGATTTTATTGGACGGTA	0	11	RDR2-sensitive
GCTGCGTTGGCACAAACGGACGTC	0	35	RDR2-sensitive
GCTGCTACACGACAAGAGAACCGG	0	14	RDR2-sensitive
GCTGCTAGAGACACTCATGACGGC	0	26	RDR2-sensitive
GCTGCTCCAGTGCACAGGCTGCAG	0	17	RDR2-sensitive
GCTGCTCCGGCTGAAGATCGTCGT	1	16	RDR2-sensitive
GCTGCTCGCGTCGGCGATTCTCGT	0	15	RDR2-sensitive
GCTGCTCGGACTCTGACCACGGAG	0	34	RDR2-sensitive
GCTGCTCGGATTGTGGGCAGGTGT	2	19	RDR2-sensitive
GCTGCTGACATTAGGACGATGCAT	0	10	RDR2-sensitive
GCTGCTGCTGTAGGAGACGTGGTG	0	10	RDR2-sensitive
GCTGCTGGAAGAAGTAGAGGACGA	0	14	RDR2-sensitive
GCTGCTGGAGAACGTAGAAGACCA	0	20	RDR2-sensitive
GCTGCTGGAGAGCATAGAAGACCA	0	30	RDR2-sensitive
GCTGCTGGAGAGCGTAGAGGACCA	1	40	RDR2-sensitive
GCTGCTGGAGGGAATAGAGGACGT	3	28	RDR2-sensitive
GCTGCTGGAGGGAGTAGACGACGA	0	18	RDR2-sensitive
GCTGCTGGAGGGCGTAGAGGACCA	0	20	RDR2-sensitive
GCTGCTGGAGGGGTAGAGGACGTC	0	14	RDR2-sensitive
GCTGCTGGAGTGAGTAGAGGACGA	0	13	RDR2-sensitive
GCTGCTGGATTCTCAAGACGTGGT	15	0	RDR2-resistant

GCTGCTGGCAGAATTTACGGGCAA	0	19	RDR2-sensitive
GCTGCTGGCCTGTGGATAGCACGC	0	18	RDR2-sensitive
GCTGCTGGGACTTCTGCCTGACGG	10	56	RDR2-sensitive
GCTGCTGGGCTGCGGCTGAAGACC	0	23	RDR2-sensitive
GCTGCTGTAGAAGGCGGGCGCTGA	0	22	RDR2-sensitive
GCTGCTGTAGTCTGTAGACGGGAG	0	10	RDR2-sensitive
GCTGCTGTCTGGATAGAGGACGCA	0	13	RDR2-sensitive
GCTGCTGTGCCGCTCTGAAGCCGG	0	9	RDR2-sensitive
GCTGCTGTGGACCCGGAGTAGGCG	1	36	RDR2-sensitive
GCTGCTGTGGGCTTCAAGGTGGTG	0	11	RDR2-sensitive
GCTGCTGTTAGATATGAGATGGAT	0	19	RDR2-sensitive
GCTGCTTAGAGACGAGGACGCGAG	0	25	RDR2-sensitive
GCTGCTTCGGACTGGGGCACCGGA	1	23	RDR2-sensitive
GCTGCTTCGGGCTGGGGCACCGGA	1	13	RDR2-sensitive
GCTGCTTGGGCTCCTCCGTCGGAT	0	10	RDR2-sensitive
GCTGCTTTAAGGGATCTGTTGGAG	0	9	RDR2-sensitive
GCTGCTTTGTGCGGATCTGTTGGG	0	20	RDR2-sensitive
GCTGGAACACTGGGACGAGCGCGG	0	11	RDR2-sensitive
GCTGGAACCTGGAAGCGGAGCTCG	0	12	RDR2-sensitive
GCTGGAACTCAATTGGCATTACCC	0	16	RDR2-sensitive
GCTGGAAGAAGTAGAGGACGACCT	0	24	RDR2-sensitive
GCTGGAAGGAGTAGAGGACGTCAT	0	15	RDR2-sensitive
GCTGGAATTATTTGCATCTGTGGA	17	0	RDR2-resistant
GCTGGACAAACAGATCACGCGAGC	0	9	RDR2-sensitive
GCTGGACAACTTGGACGCAGCGGC	1	22	RDR2-sensitive
GCTGGACAACTTGGACGCAGTGGC	0	26	RDR2-sensitive
GCTGGACACAAAAAGGAGGCTGTA	0	11	RDR2-sensitive
GCTGGACACCTGTAGGGGCACGTA	0	16	RDR2-sensitive
GCTGGACACTCGGCACAGGACCAC	0	12	RDR2-sensitive
GCTGGACACTCGGCACAGGACCCC	0	31	RDR2-sensitive
GCTGGACACTCGGCACAGGACGAC	6	46	RDR2-sensitive
GCTGGACACTGCAGGGGAACTGAG	0	9	RDR2-sensitive
GCTGGACAGCGGCTGGGAGGAGGG	25	2	RDR2-resistant
GCTGGACATAGAAATCACGCGCAC	0	37	RDR2-sensitive
GCTGGACATTAGAAGAAGACTTGT	0	10	RDR2-sensitive
GCTGGACCAATGGAGACAAGACAA	0	14	RDR2-sensitive
GCTGGACCACAGAATTCGGCCCGT	0	10	RDR2-sensitive
GCTGGACCAGTACGGGAGCCTCGA	0	19	RDR2-sensitive
GCTGGACCCGGCACGACTTTGGCG	0	12	RDR2-sensitive
GCTGGACCCTTTACACGACTGGAT	0	13	RDR2-sensitive
GCTGGACCGCTGGAGCAGAGACGT	0	14	RDR2-sensitive
GCTGGACCGGGCCGAATGCATGAC	0	21	RDR2-sensitive
GCTGGACCTATAGAGGAGATAGTT	0	13	RDR2-sensitive
GCTGGACCTCAGCAGACTCGTGTC	0	14	RDR2-sensitive
GCTGGACCTGCAGGTGGAGTCTAT	1	19	RDR2-sensitive
GCTGGACCTGGACGGAACGGCTGG	0	28	RDR2-sensitive
GCTGGACCTTTGCGCAGACTGCTT	0	14	RDR2-sensitive
GCTGGACGACGGCTTAACAACTTG	0	9	RDR2-sensitive
GCTGGACGATGCATGGCGCTGCTG	0	17	RDR2-sensitive

GCTGGACGCAGGAAGCAGAACGGC	0	79	RDR2-sensitive
GCTGGACGCAGGAAGTAGAACGGC	0	21	RDR2-sensitive
GCTGGACGCAGGGGAGATGGACCC	1	33	RDR2-sensitive
GCTGGACGGCGTTGTTGACTGCGA	0	9	RDR2-sensitive
GCTGGACGTAGGGCCTACAAGGCA	0	12	RDR2-sensitive
GCTGGACGTTGCTGGGGGCCGCCT	0	10	RDR2-sensitive
GCTGGACTAACAGGCAACAATCGA	0	11	RDR2-sensitive
GCTGGACTAGCTCTCGGCAAAGAC	0	15	RDR2-sensitive
GCTGGACTCGAGGGAGACGGAGCT	0	10	RDR2-sensitive
GCTGGACTCGGTGCAGAACGGTTG	0	15	RDR2-sensitive
GCTGGACTCGTCACCAGGAGACTG	0	45	RDR2-sensitive
GCTGGACTCTCGCGATACGTGAAT	0	9	RDR2-sensitive
GCTGGACTGAGATACGGATTGCAT	0	10	RDR2-sensitive
GCTGGACTGATGAATAGTGCGCGG	0	21	RDR2-sensitive
GCTGGACTGATGAATAGTGCGCTA	0	18	RDR2-sensitive
GCTGGACTGATGAATAGTGTGCTA	0	167	RDR2-sensitive
GCTGGACTGATGAATAGTGTGCTC	0	35	RDR2-sensitive
GCTGGACTGATGAATAGTGTGCTT	0	16	RDR2-sensitive
GCTGGACTGCTGGAAATGACGCGG	0	10	RDR2-sensitive
GCTGGACTGGACGCATATACCTAG	0	12	RDR2-sensitive
GCTGGACTGGCCCGGTGGCACTAT	0	17	RDR2-sensitive
GCTGGACTGGTGAATAGTGTGCTA	0	9	RDR2-sensitive
GCTGGACTGTGTGCGGCTTTCTAT	0	16	RDR2-sensitive
GCTGGACTTCTATTGGTTTGACGT	2	22	RDR2-sensitive
GCTGGAGAAGGACACGACGAAGAC	1	16	RDR2-sensitive
GCTGGAGAAGGCTAGACTCGCCAT	68	6	RDR2-resistant
GCTGGAGACATAAAGGAGGACTGC	0	18	RDR2-sensitive
GCTGGAGACGAAGCTCGGAGTCAT	1	13	RDR2-sensitive
GCTGGAGACTGGCGCTGCGCACCC	1	29	RDR2-sensitive
GCTGGAGAGAGTAGAGGACGTCCT	0	37	RDR2-sensitive
GCTGGAGAGCGTAGAAGACCGTTT	0	19	RDR2-sensitive
GCTGGAGAGCGTAGAAGACTGTTT	0	13	RDR2-sensitive
GCTGGAGAGTGTAGAAGACCGTTT	0	92	RDR2-sensitive
GCTGGAGCACTGTAGCGGGCCGGT	0	14	RDR2-sensitive
GCTGGAGCAGAGTAGGACGAGGCT	0	29	RDR2-sensitive
GCTGGAGCGAGTAGAGGACGTCCT	0	10	RDR2-sensitive
GCTGGAGCGTGCTACTCAAGACAA	0	18	RDR2-sensitive
GCTGGAGCTGGAGCTGGAGCCGGA	0	9	RDR2-sensitive
GCTGGAGGAAGTAGAGGACGACAC	0	15	RDR2-sensitive
GCTGGAGGAGACCCTGACGGAGAC	0	10	RDR2-sensitive
GCTGGAGGGAGTAGACGACGAGAG	0	19	RDR2-sensitive
GCTGGAGGGAGTAGAGGACGTCCT	2	104	RDR2-sensitive
GCTGGAGGGAGTAGAGGACGTTCT	0	24	RDR2-sensitive
GCTGGAGGGAGTAGAGGGCGTCCT	0	15	RDR2-sensitive
GCTGGAGGGAGTAGATGACGTCAT	2	27	RDR2-sensitive
GCTGGAGGGAGTAGATGACGTCCT	1	69	RDR2-sensitive
GCTGGAGGGAGTAGATGATGTCCT	0	11	RDR2-sensitive
GCTGGAGGGCTAGAGGACGTCGAG	0	9	RDR2-sensitive
GCTGGAGGGCTTGCGTACACTCAA	0	9	RDR2-sensitive

GCTGGAGTGAGTAGAGGACGATCT	0	17	RDR2-sensitive
GCTGGAGTGAGTAGATGACGTCCT	0	35	RDR2-sensitive
GCTGGAGTTCGGACCTACCCGGAG	0	10	RDR2-sensitive
GCTGGATACACTGTTGGACACGGT	0	10	RDR2-sensitive
GCTGGATAGATGCAGGAGCTCACA	0	35	RDR2-sensitive
GCTGGATCAGATCTGCATCAACTC	1	13	RDR2-sensitive
GCTGGATCTTGAGTGGAGCGACGG	0	22	RDR2-sensitive
GCTGGATGCAGTAGAGGACGTCAT	2	15	RDR2-sensitive
GCTGGATGGTGACTGTGCGGCCAA	1	26	RDR2-sensitive
GCTGGATTGCGGGTGTGCAGGCGT	0	12	RDR2-sensitive
GCTGGCAACGGTGATTTCGCGGAC	0	54	RDR2-sensitive
GCTGGCAAGGAACACGGGCGCGCC	40	4	RDR2-resistant
GCTGGCAAGTTCGGATAGGGACGG	0	33	RDR2-sensitive
GCTGGCAAGTTTGGATAGGGACGG	0	10	RDR2-sensitive
GCTGGCAATTGTGGAAACGCAGAA	2	21	RDR2-sensitive
GCTGGCACAAACGGACGTCGTGGC	1	32	RDR2-sensitive
GCTGGCACACTGGACGGAAGAGCA	0	9	RDR2-sensitive
GCTGGCACGGACTAGACGACAGGC	0	27	RDR2-sensitive
GCTGGCACTGCAGACTGAGAAGGC	0	33	RDR2-sensitive
GCTGGCCATGGTGAGGATGACGAG	0	10	RDR2-sensitive
GCTGGCCTAGGACCGCATAGAACC	0	11	RDR2-sensitive
GCTGGCCTCTAGCACTGACGACAC	0	10	RDR2-sensitive
GCTGGCCTGTGGATAGCACGCGCG	1	29	RDR2-sensitive
GCTGGCGAAAGAGGCTCGGCTCGG	0	14	RDR2-sensitive
GCTGGCGAGTGAGGGCTGACACGG	0	9	RDR2-sensitive
GCTGGCGTCCTTAGATCCGTCGGA	0	11	RDR2-sensitive
GCTGGCGTGTATAGGACGGTCTGA	0	20	RDR2-sensitive
GCTGGCTCTCGGCAAAGACTGACG	2	99	RDR2-sensitive
GCTGGCTGATAGACCACGACGGAC	0	9	RDR2-sensitive
GCTGGCTGCGAGCATACAGTTCAC	0	9	RDR2-sensitive
GCTGGCTGCGTGAACAAGATACGG	0	21	RDR2-sensitive
GCTGGGAAGCTTTGGGATCTCGGG	55	5	RDR2-resistant
GCTGGGACAAGGCATGACACAGAT	0	25	RDR2-sensitive
GCTGGGACAGGCAGGACAGGACGT	0	26	RDR2-sensitive
GCTGGGACTTCTGCCTGACGGCCG	4	48	RDR2-sensitive
GCTGGGACTTGACGAGCTAGGCAG	0	10	RDR2-sensitive
GCTGGGAGCTCTCTGTTCGATGGC	2	21	RDR2-sensitive
GCTGGGCAAGCAATACGATGACAA	0	11	RDR2-sensitive
GCTGGGCAGGAACTGAGCAGGCAT	0	21	RDR2-sensitive
GCTGGGCAGTGGGAGGCACGACCT	0	13	RDR2-sensitive
GCTGGGCAGTTACAGAATGGACGA	0	11	RDR2-sensitive
GCTGGGCCGTGCTCTGATTGACGG	0	19	RDR2-sensitive
GCTGGGCCGTGCTCTGGTTGGCGG	0	16	RDR2-sensitive
GCTGGGCCGTGCTTATGATTTCGG	0	12	RDR2-sensitive
GCTGGGCGTGTAGAGGACGTGTCT	0	106	RDR2-sensitive
GCTGGGCTATCTGGATAAGTAGAA	0	11	RDR2-sensitive
GCTGGGCTGCGGCTGAAGACCTGC	1	56	RDR2-sensitive
GCTGGGGCGCTGGGCTGTAGTGGC	0	11	RDR2-sensitive
GCTGGGGTCGGGAGCAGACGTGAG	0	14	RDR2-sensitive

GCTGGGTAGACGCAGTAGCTGCAT	0	12	RDR2-sensitive
GCTGGGTCGACGATGTCAGAAGAT	0	11	RDR2-sensitive
GCTGGGTCGGGCTAGAATGGGCTT	0	23	RDR2-sensitive
GCTGGGTCGTTACAGAGTGGGCGT	0	15	RDR2-sensitive
GCTGGGTTGTCTGTTCGATGGCGC	2	16	RDR2-sensitive
GCTGGGTTTGCCCTGATCGCCGGC	2	46	RDR2-sensitive
GCTGGTAATCCGGATGATAGGCGT	0	9	RDR2-sensitive
GCTGGTACTGTATCGGCTATCCAC	0	20	RDR2-sensitive
GCTGGTACTGTGGTGGGCAGCTAT	1	18	RDR2-sensitive
GCTGGTCAGTCGGAGGAGATAGGT	0	12	RDR2-sensitive
GCTGGTCAGTGGAGCGGCGAAGTG	0	15	RDR2-sensitive
GCTGGTCATGTGGGTTTTGGCGGT	80	0	RDR2-resistant
GCTGGTCATGTGGTCTTTGACGGA	0	9	RDR2-sensitive
GCTGGTCGAGCGGACGAAGCGAGC	0	10	RDR2-sensitive
GCTGGTCGGCAGACACTCGGCAAA	1	14	RDR2-sensitive
GCTGGTCGTGCTGGTCGGAGGGGC	1	17	RDR2-sensitive
GCTGGTCTCGAGTGGATAAGGCAT	0	10	RDR2-sensitive
GCTGGTCTGACAGAGACAAAACGG	1	13	RDR2-sensitive
GCTGGTCTGGCTCGAGCACGGCTA	0	14	RDR2-sensitive
GCTGGTGACGTCGTAGATGAGCAT	0	10	RDR2-sensitive
GCTGGTGCTGCAGTCAGGACATCC	0	11	RDR2-sensitive
GCTGGTGGACGAAGAGCGCGGATA	0	12	RDR2-sensitive
GCTGGTGTTGGACGAATACGGGGA	0	10	RDR2-sensitive
GCTGGTGTTGTAGGTGTAGTCGTG	0	13	RDR2-sensitive
GCTGGTTGGAAACGGAGCGGCTCC	0	12	RDR2-sensitive
GCTGGTTGGTGGCCTCGTTGGACG	0	9	RDR2-sensitive
GCTGGTTTGGTCGTTTGGAGGCTA	0	11	RDR2-sensitive
GCTGTAACGTTGGCTCTTGACGGA	0	11	RDR2-sensitive
GCTGTAAGGAGGCAGAACCGTGCA	0	42	RDR2-sensitive
GCTGTACGACATGGTAGACGGGCT	0	18	RDR2-sensitive
GCTGTACGACTGGAGGCAGAACGG	0	24	RDR2-sensitive
GCTGTACGGAGGCAGAACGCGGCT	0	24	RDR2-sensitive
GCTGTACGGCGTGGTAGACAGGCT	0	13	RDR2-sensitive
GCTGTACGGCGTGGTAGACGGGCT	2	63	RDR2-sensitive
GCTGTAGCGAGTAGAGGACGTCCT	0	10	RDR2-sensitive
GCTGTAGCGGGATCGGGATCGGAC	0	10	RDR2-sensitive
GCTGTAGGACTAATCAGTAGATGT	15	0	RDR2-resistant
GCTGTATCGCTGGAGAAACGACGG	0	30	RDR2-sensitive
GCTGTCAGCTGGAGAAGACAAGGA	0	21	RDR2-sensitive
GCTGTCAGGCAGACACTCGGCAAA	3	16	RDR2-sensitive
GCTGTCCCTGGCTGAGAAGATGAC	0	9	RDR2-sensitive
GCTGTCCGAACAGAACGATGCGCC	1	13	RDR2-sensitive
GCTGTCCTTCTGTCAGACCGGTCA	4	21	RDR2-sensitive
GCTGTCGAGACCACCGGATCGGAG	0	84	RDR2-sensitive
GCTGTCGAGCAGGAGCTGATGCGG	0	16	RDR2-sensitive
GCTGTCGAGTGTGGGCTGGGCCGG	0	32	RDR2-sensitive
GCTGTCGATTGTGGGCTAGGCCGG	0	10	RDR2-sensitive
GCTGTCGCAGAGAGGACGCCGATC	0	16	RDR2-sensitive
GCTGTCGCGTAGGACAGATCGGCT	0	13	RDR2-sensitive

GCTGTCGCGTAGGACAGTTCGGCT	3	111	RDR2-sensitive
GCTGTCGCTCGCCCTCTGGATCGG	0	14	RDR2-sensitive
GCTGTCGGGCCGGCACGACACGGT	0	9	RDR2-sensitive
GCTGTCGTCCACGAGCTGACGCGG	0	12	RDR2-sensitive
GCTGTCGTGCGTAGAGAAGAGGAG	0	25	RDR2-sensitive
GCTGTCGTGCTGGGCTTGGTGGCC	3	18	RDR2-sensitive
GCTGTCGTGTACGACTCGTCTGGC	0	26	RDR2-sensitive
GCTGTCTCGGGCGGATTTCGGCAT	7	28	RDR2-sensitive
GCTGTCTCTAAGGCGTGACTCGGG	0	12	RDR2-sensitive
GCTGTCTCTGCAACTCGGCCCTAG	0	12	RDR2-sensitive
GCTGTCTGGATAGAGGACGCACAG	0	13	RDR2-sensitive
GCTGTCTGGGCACGTCGGGCGGGC	0	9	RDR2-sensitive
GCTGTCTGTAGAGGTGCGGACGGT	0	20	RDR2-sensitive
GCTGTCTTGGATGGTGTAGAGGCT	0	12	RDR2-sensitive
GCTGTGAAAGCTGATCTGGATGAC	15	0	RDR2-resistant
GCTGTGACGACAAGACTCATCGGC	0	41	RDR2-sensitive
GCTGTGCTGGATCTGAATGGCTAC	0	16	RDR2-sensitive
GCTGTGGAAAAAGCTGCTGTAGGC	0	19	RDR2-sensitive
GCTGTGGACTGCAATCTCCGGCTT	19	0	RDR2-resistant
GCTGTGGACTGTGAGCGGTGGAAA	0	9	RDR2-sensitive
GCTGTGGGACGACGGCGCCTGAGC	0	10	RDR2-sensitive
GCTGTGGGCCAGGCTTAGACAGAG	0	14	RDR2-sensitive
GCTGTGGGCTGTGGCACCAGAGAC	0	11	RDR2-sensitive
GCTGTGTACGACAAGCTCCGACGC	1	16	RDR2-sensitive
GCTGTGTACTGTAGCTGTCAGGCA	2	16	RDR2-sensitive
GCTGTGTAGAGCATGGGTGGTGAG	0	11	RDR2-sensitive
GCTGTGTAGTGTGATGGTCTGGGT	15	0	RDR2-resistant
GCTGTGTATGACAAGCTCCGACGC	5	20	RDR2-sensitive
GCTGTTCAAGTCGGTGCATGGAAA	0	9	RDR2-sensitive
GCTGTTCGGATGATCGCATGCAAT	41	2	RDR2-resistant
GCTGTTCTTCGGGCAAAGGATGGC	0	9	RDR2-sensitive
GCTGTTGACTCGGGCCGTGCCGGT	0	13	RDR2-sensitive
GCTGTTGACTGTGCGAGCTTCGGA	1	18	RDR2-sensitive
GCTGTTGCATCGGGCTGTGCCGGT	1	21	RDR2-sensitive
GCTGTTGCGACGACGACTTCGGAC	0	12	RDR2-sensitive
GCTGTTGGAGCAGAAGCAATGCAA	0	44	RDR2-sensitive
GCTGTTGGAGGAACTCAACCAGGC	0	13	RDR2-sensitive
GCTGTTGGGCTAGCGGCTGGTGCT	0	23	RDR2-sensitive
GCTGTTGGGGCGAAGGCAAAGACG	0	10	RDR2-sensitive
GCTGTTGTATTGTGGAAGGGCGTT	0	21	RDR2-sensitive
GCTGTTGTCGGCTACTGACGGTAC	1	23	RDR2-sensitive
GCTGTTGTGTTCTGTATGCGTTGG	17	0	RDR2-resistant
GCTGTTTCTCCCTGCAGGACCTTC	0	9	RDR2-sensitive
GCTGTTTGGACTGCTGCAGCTGCA	1	18	RDR2-sensitive
GCTGTTTTCCTGTCAGGTCGGTCA	1	17	RDR2-sensitive
GCTTAACACTCGGCACAGGACGCA	0	12	RDR2-sensitive
GCTTAACCCGGTCTGAAGCTTCAA	0	13	RDR2-sensitive
GCTTACCTGGACCTTTGGAGCGGC	0	11	RDR2-sensitive
GCTTACGGTACAGAACTAGGCTAA	0	21	RDR2-sensitive

GCTTAGACTGTACAACGGCTCCCG	0	33	RDR2-sensitive
GCTTAGCGAACTGTACATCGGCAA	0	9	RDR2-sensitive
GCTTAGCGGATTGTTTGGTAGGCT	0	9	RDR2-sensitive
GCTTAGCTGTGGTGTTCGTGTGGA	1	13	RDR2-sensitive
GCTTAGGTTCGGCTTAGAGAGGCT	0	9	RDR2-sensitive
GCTTAGTCGGAGGTGTGTGTGGTC	18	0	RDR2-resistant
GCTTAGTGCGACTGTGTTCAACGG	58	3	RDR2-resistant
GCTTATAGGACTGAGTCGGCTCAA	0	9	RDR2-sensitive
GCTTATTCGTGTAGGGCCGGCCTG	0	9	RDR2-sensitive
GCTTCAACATAGTGGATCGTTCAT	0	10	RDR2-sensitive
GCTTCAATATGATCGTTGCAGGAC	0	14	RDR2-sensitive
GCTTCACCAGGAGAGATGTGGACA	0	14	RDR2-sensitive
GCTTCACGACGTCGAGCTGGGCAC	0	11	RDR2-sensitive
GCTTCAGACGACTCGAACGGCGCC	0	38	RDR2-sensitive
GCTTCAGATTCGGGTCGGATACGG	0	10	RDR2-sensitive
GCTTCAGCGGACGAAGGGCATTAA	0	10	RDR2-sensitive
GCTTCCTACTGTAGCATGATGGCG	0	18	RDR2-sensitive
GCTTCCTGCGGACTGTGGGCGGTC	1	83	RDR2-sensitive
GCTTCGCCTGGACGCAGGGACGCC	0	9	RDR2-sensitive
GCTTCGCGAGGACCGTGGGCGGGC	0	15	RDR2-sensitive
GCTTCGCTCGGATGGATAGCGGAT	0	11	RDR2-sensitive
GCTTCGGACACTCGGCAAAGCAGT	0	13	RDR2-sensitive
GCTTCGGACCGGCCCATCAGACCC	0	17	RDR2-sensitive
GCTTCGGACGGTCCATGATGGCGT	0	14	RDR2-sensitive
GCTTCGGACTGGCACGACACGGCC	2	17	RDR2-sensitive
GCTTCGGATTCGGGTCGGATACAG	0	19	RDR2-sensitive
GCTTCGGATTTGAGATAAAGATGT	0	12	RDR2-sensitive
GCTTCGGCTGAGGGCCCGACGGCC	0	12	RDR2-sensitive
GCTTCGGGCTTCAAAGTGTCGGGC	1	25	RDR2-sensitive
GCTTCGGTTCAAGCCTCCCGACGG	0	9	RDR2-sensitive
GCTTCGTCTGGTCACGGTTGTTCT	0	9	RDR2-sensitive
GCTTCGTGTCGGGCTAACGGGTCA	0	12	RDR2-sensitive
GCTTCGTTGGATAGCTCGGCGGAC	0	9	RDR2-sensitive
GCTTCTAGGGCTATAGACGGTCGC	0	10	RDR2-sensitive
GCTTCTAGGGTTGGATCGCACGGA	0	13	RDR2-sensitive
GCTTCTAGTCGGGCCGGTCCAGTC	0	11	RDR2-sensitive
GCTTCTCGGATCTTCGGACGCGGC	0	14	RDR2-sensitive
GCTTCTCGGCGTTCGGTAGGCGGA	0	11	RDR2-sensitive
GCTTCTCGGGCACCGTAGGCGGCT	0	27	RDR2-sensitive
GCTTCTCGGGCACCGTAGGCGGGT	0	13	RDR2-sensitive
GCTTCTCGGTGACCGTAGGCGGGT	0	9	RDR2-sensitive
GCTTCTCTGGCTCTGTGACGGTGA	0	10	RDR2-sensitive
GCTTCTCTGTAGGGTTATCGCGCT	0	9	RDR2-sensitive
GCTTCTGTTAGATGTTGGATAGAC	0	14	RDR2-sensitive
GCTTCTGTTAGATGTTGGATGGAC	0	9	RDR2-sensitive
GCTTCTTCGTGTAGGACCTTCGGC	1	21	RDR2-sensitive
GCTTCTTTCTGTAAGACCTTCGGC	2	28	RDR2-sensitive
GCTTGACGAACTGTACATTGGCAA	0	11	RDR2-sensitive
GCTTGACGAACTGTATATTGGCAA	0	16	RDR2-sensitive

GCTTGAGATCTACGGACCCGAGGC	0	25	RDR2-sensitive
GCTTGAGCCCTGTAGAAGCACAGC	1	22	RDR2-sensitive
GCTTGATCGGCACCAGACGAAGAC	0	17	RDR2-sensitive
GCTTGATCGGCTGGTTGGGCAGGC	0	15	RDR2-sensitive
GCTTGATGGAAGTAGAGTCCGGCC	0	10	RDR2-sensitive
GCTTGCAAGAGCCAAGAACGTCGT	0	25	RDR2-sensitive
GCTTGCAGAGACGGACGGGAACGG	0	9	RDR2-sensitive
GCTTGCAGGTTGTCGGGCCGGCAC	1	76	RDR2-sensitive
GCTTGCATCGCGGGATACAATGAG	0	9	RDR2-sensitive
GCTTGCATTGCTGCACCGTTGGAT	0	13	RDR2-sensitive
GCTTGCGGGCTGTCGGACCGGCAC	0	19	RDR2-sensitive
GCTTGCGGTCGTAGCACGGGGCGT	1	13	RDR2-sensitive
GCTTGCTCGTCGTAGTCGGATCGC	2	25	RDR2-sensitive
GCTTGGACAGGAAACTAGGCACGG	5	127	RDR2-sensitive
GCTTGGACAGTATTCCGTGATGGT	36	0	RDR2-resistant
GCTTGGACCAGCAGTTTAGCCCGT	0	10	RDR2-sensitive
GCTTGGACCGGGCCAGAATCGTGC	0	23	RDR2-sensitive
GCTTGGACGGAACACCGGGCGGCG	0	11	RDR2-sensitive
GCTTGGACGGACGCCTAGACGGAC	0	17	RDR2-sensitive
GCTTGGACTGCGGCGTAGGCCTAA	3	39	RDR2-sensitive
GCTTGGACTGTCCATGGTGTTCAT	0	12	RDR2-sensitive
GCTTGGACTGTGGCAGCGAGACGA	0	15	RDR2-sensitive
GCTTGGATTCTGACTTAGAGGCGT	38	3	RDR2-resistant
GCTTGGCACACCGGACTGTGCGGT	0	9	RDR2-sensitive
GCTTGGCCCTGAAGACTGGACGGT	0	9	RDR2-sensitive
GCTTGGCGAACTGTACATCGGCAA	4	107	RDR2-sensitive
GCTTGGCGTAGACGAGGATGACGA	1	25	RDR2-sensitive
GCTTGGCGTAGAGAAGACTCGGCT	0	52	RDR2-sensitive
GCTTGGCTAGAACGTCGACGACAT	0	34	RDR2-sensitive
GCTTGGCTCTGCTCGGATCGGCTC	1	39	RDR2-sensitive
GCTTGGCTGTAACAGAGGAGATTA	0	9	RDR2-sensitive
GCTTGGCTTCTGAGCGACGGTCAT	0	13	RDR2-sensitive
GCTTGGGCGAACCGCGGAACAGGA	0	9	RDR2-sensitive
GCTTGGGCTCCCTCTGCGCGGTGT	0	12	RDR2-sensitive
GCTTGGGCTCTCTGCTGCGCGGTA	0	11	RDR2-sensitive
GCTTGGGCTGCGGCGTAGGCTTAA	0	10	RDR2-sensitive
GCTTGGTCGACTGTTGAAGTGCAA	0	44	RDR2-sensitive
GCTTGGTCTTAGCTCGATATCGGC	19	0	RDR2-resistant
GCTTGGTTGGTCTCTGGCAGACGG	0	19	RDR2-sensitive
GCTTGTACGCGAGGAACGATGGCT	0	20	RDR2-sensitive
GCTTGTACGGACTTGGTCGGTCTA	0	11	RDR2-sensitive
GCTTGTCGAGCGGACCGGTATCAC	1	23	RDR2-sensitive
GCTTGTCGAGCGGATGGACGTCAA	2	52	RDR2-sensitive
GCTTGTCGAGCGGATGGACGTCAC	2	23	RDR2-sensitive
GCTTGTCGAGCGGATTGACGTCAC	0	9	RDR2-sensitive
GCTTGTCGAGCGGATTGACGTCAT	9	161	RDR2-sensitive
GCTTGTCGCGTAGGACAGTTCGGC	0	31	RDR2-sensitive
GCTTGTCGGAACTCCGTATGGTGC	0	27	RDR2-sensitive
GCTTGTTCGGACAACGCATACAAC	3	22	RDR2-sensitive

GCTTGTTTCTGTGGAGCCAGGCTG	0	9	RDR2-sensitive
GCTTGTTTGCAGATGGACGGACCT	0	9	RDR2-sensitive
GCTTTACTGTTAGAGCAGGGTGTT	131	0	RDR2-resistant
GCTTTAGCGGTCTGCTGGACACAG	0	13	RDR2-sensitive
GCTTTAGGACGGCCGCTGACGGCT	0	9	RDR2-sensitive
GCTTTATAGTTAGGACTGGGCGTT	0	10	RDR2-sensitive
GCTTTCAATTGCCACGATCGCGGT	0	16	RDR2-sensitive
GCTTTCAGGAGTAGGACGGTGTTA	0	22	RDR2-sensitive
GCTTTCCGGACTTCAGAGGGTGTG	0	21	RDR2-sensitive
GCTTTCCTCGGCGACGGCTCTAGC	38	1	RDR2-resistant
GCTTTCGCTGATCGTATGGAGTAA	0	22	RDR2-sensitive
GCTTTCTACGACAGTATGGACGGT	0	22	RDR2-sensitive
GCTTTCTGCAGATGCGTGGACGGT	2	32	RDR2-sensitive
GCTTTCTGGTCAGGCGCGGACGGT	7	33	RDR2-sensitive
GCTTTGAAGTAGGGCAGGAAACGA	0	11	RDR2-sensitive
GCTTTGCAGTGAGGACGGAATGAG	0	10	RDR2-sensitive
GCTTTGCAGTGAGGACGGAATGGG	1	15	RDR2-sensitive
GCTTTGCGACCTGATAGCGGCATA	18	0	RDR2-resistant
GCTTTGGACGGACGGACTGGGCGT	0	58	RDR2-sensitive
GCTTTGGCGTGGGACAGACGAGAG	1	18	RDR2-sensitive
GCTTTGGCGTGGGACAGACGAGGA	0	10	RDR2-sensitive
GCTTTGGGACGGCCTGTTAGGCAC	0	9	RDR2-sensitive
GCTTTGGTACATTTAGGCATGACT	17	0	RDR2-resistant
GCTTTGTCTCTGTCGGACCAGCGG	0	18	RDR2-sensitive
GCTTTGTGCGGATCTGTTGGGAGC	0	19	RDR2-sensitive
GCTTTTGGGTAGGACGACGTCGTT	1	25	RDR2-sensitive
GCTTTTGGGTAGGACGGTGCTGTT	0	9	RDR2-sensitive
GCTTTTGGGTAGGATGACGTCGTT	0	9	RDR2-sensitive
GCTTTTGTAGGCCTGTGACGTGCC	0	172	RDR2-sensitive
GCTTTTGTGCGACACGTAGATCGG	0	30	RDR2-sensitive
GCTTTTGTGGGCCTGTGCCGTGCC	1	15	RDR2-sensitive
GCTTTTTAGTAGACCGCTGCGGAC	0	11	RDR2-sensitive
GCTTTTTCGGAGTAGGACGGTGTT	1	13	RDR2-sensitive
GCTTTTTGGAGTAGGACGGTGTTA	0	14	RDR2-sensitive
GCTTTTTGGTGTAGGACGGTGTTA	0	16	RDR2-sensitive
GCTTTTTGTTGGACACTCGGCATA	0	36	RDR2-sensitive
GCTTTTTTATCGGACACTCGGCAA	1	57	RDR2-sensitive
GCTTTTTTCTAAATCGTTTGGCAA	0	11	RDR2-sensitive
GGAAACAGTGTAGGCCGTCGGAAA	1	51	RDR2-sensitive
GGAAACTGAGCACAGGATGGACGC	3	21	RDR2-sensitive
GGAAACTGTAGCCCCTATACGGTG	1	22	RDR2-sensitive
GGAAATAGAGCTATTTTCGACGGT	0	11	RDR2-sensitive
GGAAATGGGCCAAGACGATGTCGA	0	9	RDR2-sensitive
GGAACAACAAGGTCGTCCGACATC	2	18	RDR2-sensitive
GGAACACGATGGTCAGTCGGCGAG	0	10	RDR2-sensitive
GGAACACGGGATCGACGAGGACGC	0	12	RDR2-sensitive
GGAACACTCGGCACAGAACGACAC	0	16	RDR2-sensitive
GGAACACTCGGCATAGACAGACAC	0	10	RDR2-sensitive
GGAACAGTCGGAAAGGCAGTAGTC	0	23	RDR2-sensitive

GGAACAGTCGGAGAGGCAGTAGTC	4	20	RDR2-sensitive
GGAACCAGGCTCGGCTCGGCTCAA	0	16	RDR2-sensitive
GGAACCCGGACTGTTCGGCCCATA	0	30	RDR2-sensitive
GGAACCGACTGTGTAGAGGCTAAC	0	11	RDR2-sensitive
GGAACCGGACTGCCGACACACGGC	1	20	RDR2-sensitive
GGAACCGTCGCAGAAGGAGGACAA	0	10	RDR2-sensitive
GGAACCGTCGGAACTAGTGGAGGC	0	22	RDR2-sensitive
GGAACCGTTGGAACTAGTGGAGGC	0	12	RDR2-sensitive
GGAACGAAGGCAGAACCGTGCACC	0	9	RDR2-sensitive
GGAACGCGGCGCATCCGAATTCGA	0	9	RDR2-sensitive
GGAACGGAAACGGGAGCGGGATAA	0	10	RDR2-sensitive
GGAACGGACAGTAACGGTAGCCAT	0	12	RDR2-sensitive
GGAACGGGCTCGACGAGATGAAGC	0	9	RDR2-sensitive
GGAACGGTCGAACGGAACAGCAGA	0	14	RDR2-sensitive
GGAACGTTGTACGGCGTGGTAGAC	0	16	RDR2-sensitive
GGAACTCTATGGTCGCAGCGGTGT	0	10	RDR2-sensitive
GGAACTGACACAGGCGGAACTGAC	0	10	RDR2-sensitive
GGAACTGGGCAGAGGACTCGCGGA	0	18	RDR2-sensitive
GGAACTTGTTCGGCATAGATCCAC	1	14	RDR2-sensitive
GGAAGAAATCGCCGGCGACGTCAG	1	30	RDR2-sensitive
GGAAGAATCTGGCTGTGTAGAGAA	1	12	RDR2-sensitive
GGAAGACGACGAACTCCGCCTCGC	0	15	RDR2-sensitive
GGAAGACGACGAACTCCGCTTCGC	4	28	RDR2-sensitive
GGAAGACGGCGAACTCCGCTCCGC	14	84	RDR2-sensitive
GGAAGACGGCGAACTCCGCTCTGC	0	9	RDR2-sensitive
GGAAGACGTCATGTAGGAGCCAGC	0	9	RDR2-sensitive
GGAAGAGGTCACCGTTGTAGGCAA	0	15	RDR2-sensitive
GGAAGATCGCCGACGGCGCCTGGA	2	15	RDR2-sensitive
GGAAGATCTGTGGAATAAGGGCAT	0	11	RDR2-sensitive
GGAAGATCTGTGGAATAGGGGCAT	0	18	RDR2-sensitive
GGAAGCGGCGTTGACTCGGTTTGT	0	9	RDR2-sensitive
GGAAGCGTTGCACTGTATGCGGCT	0	20	RDR2-sensitive
GGAAGCTCGGCGCCAATGATGACG	0	12	RDR2-sensitive
GGAAGGAAATCGGGCACCGGACAG	0	13	RDR2-sensitive
GGAAGGACTGTGCACTGCATTCAT	0	16	RDR2-sensitive
GGAAGGAGGCTAGTAGGAACGGTC	1	17	RDR2-sensitive
GGAAGGAGGTAGAGCTGTACGGGC	0	11	RDR2-sensitive
GGAAGGATGTCGGGCAGAACACGG	1	14	RDR2-sensitive
GGAAGGATTCGGACCACAATGGGA	15	0	RDR2-resistant
GGAAGGCCAGGTAGAGCGGTTGGT	2	56	RDR2-sensitive
GGAAGGCGGGCTGGGCAGCAGCTA	46	10	RDR2-resistant
GGAAGGCGGTCTGAACTCTAGGAG	0	10	RDR2-sensitive
GGAAGGCTGTAGACAGGTCGTCAG	1	60	RDR2-sensitive
GGAAGGCTGTAGTAGGGCCATGAA	0	12	RDR2-sensitive
GGAAGGCTGTAGTCGGGCCGTGAA	8	29	RDR2-sensitive
GGAAGGCTGTGGATGATCCGGTAT	0	26	RDR2-sensitive
GGAAGGGAGACGGGTTCTCTACAT	1	16	RDR2-sensitive
GGAAGGGCTCGCCGGAAGAAACAA	0	11	RDR2-sensitive
GGAAGGGGACAGTGAACAAGGCAT	1	15	RDR2-sensitive

GGAAGGGGCAGTACACAAGAACAC	0	33	RDR2-sensitive
GGAAGGTAACAGAGCGCGGGGCAT	0	11	RDR2-sensitive
GGAAGGTCGTATGACGGACGGCGT	0	26	RDR2-sensitive
GGAAGGTCTGCGTACTCAGGTCAA	0	33	RDR2-sensitive
GGAAGTCAAAACTGAGAGCGGCAA	0	31	RDR2-sensitive
GGAAGTCTTGGACGTTGACAGCAC	0	15	RDR2-sensitive
GGAATAGACTGGGGCAGAACGCGG	0	10	RDR2-sensitive
GGAATAGTCGGGAACGGAAACGGG	1	24	RDR2-sensitive
GGAATCTTGCTGACGTGACTGCAT	0	12	RDR2-sensitive
GGAATCTTGCTGACGTGGCTGCAT	1	13	RDR2-sensitive
GGAATCTTTTAGAGAAGACTGTAA	1	33	RDR2-sensitive
GGAATGGACGAGCCGAGCCGAGCC	0	10	RDR2-sensitive
GGAATGGACGAGCCGAGCCGAGCT	0	12	RDR2-sensitive
GGAATGGATTGGGGAGGGATGGAA	0	23	RDR2-sensitive
GGAATGGGAATGGCAGAACACGGA	2	40	RDR2-sensitive
GGAATGGGAATGGCAGAACATGGA	0	10	RDR2-sensitive
GGAATGGGAATGGCAGAACCAGGA	0	18	RDR2-sensitive
GGAATGGGCAGAACGCAGAATGGC	0	17	RDR2-sensitive
GGAATGGGCAGAACGCGGAATGAC	0	19	RDR2-sensitive
GGAATGGGCAGAACGCGGAATGGC	10	32	RDR2-sensitive
GGAATGGTTCCGGATCTGAGGAGA	6	64	RDR2-sensitive
GGAATGTCACGGCATAGGCACTGA	0	11	RDR2-sensitive
GGAATTATGGGTTCGGATTCGGTT	0	10	RDR2-sensitive
GGAATTCCTCTTCCGGTCGTGCGC	0	14	RDR2-sensitive
GGAATTGCGGGACTGCAGGACGGC	0	9	RDR2-sensitive
GGAATTTTAGATCGGAATCGGTAT	0	21	RDR2-sensitive
GGACAACTTTGGCTGTTGGGCTGC	0	13	RDR2-sensitive
GGACAAGAAGGACTGTGCGGCCAT	0	9	RDR2-sensitive
GGACACACCGCGGATACTTCTGGA	0	13	RDR2-sensitive
GGACACGACTGAGGAGTGGACGTG	0	9	RDR2-sensitive
GGACAGAACAGTCAGCCCGTCGGG	0	16	RDR2-sensitive
GGACAGATCGACGCGCAGGAGCCT	0	14	RDR2-sensitive
GGACAGGGCTTGTAGAGAGGTTGT	0	18	RDR2-sensitive
GGACAGTCCGCCGGCTCATTTCTG	0	11	RDR2-sensitive
GGACAGTCGGGACGCATTGGACGG	0	18	RDR2-sensitive
GGACAGTCTGAACGTATGCAGAGC	0	10	RDR2-sensitive
GGACATCTTGTGATCGGTATGGCT	20	1	RDR2-resistant
GGACCAGGGGTTGTAGAACGACGG	0	15	RDR2-sensitive
GGACCAGTTGAACGGACCGAGCAG	0	16	RDR2-sensitive
GGACCCGACGCGACTGACGGATAA	0	10	RDR2-sensitive
GGACCCGGCTGAAAGACGTACCCA	2	168	RDR2-sensitive
GGACCGACACGTAGAGGGCGCTGT	0	13	RDR2-sensitive
GGACCGACGCGTAGAGGGCGCTGT	0	9	RDR2-sensitive
GGACCGACGGTACTTACTCCTGGC	3	25	RDR2-sensitive
GGACCGATGGACTAACGGGCTGGC	0	11	RDR2-sensitive
GGACCGCAGGTAGATTTGGACGGC	1	12	RDR2-sensitive
GGACCGCGGACCGTTCGGCCTCAG	4	33	RDR2-sensitive
GGACCGGACTAGCACGAATTGCAT	0	32	RDR2-sensitive
GGACCGGATTGTCCGCGATGGCGT	0	13	RDR2-sensitive

GGACCGGGCTAAATTCGTGGCGTG	0	9	RDR2-sensitive
GGACCGGGCTATATGGGACGACAT	1	46	RDR2-sensitive
GGACCGTCCGCGCCTGACCAGAGA	1	17	RDR2-sensitive
GGACCTGGACTCAAAGCTCGGCGC	1	32	RDR2-sensitive
GGACGACAGCGCGGCAGGTAGCTC	5	57	RDR2-sensitive
GGACGACGACTGATTCGGAACGGA	1	14	RDR2-sensitive
GGACGACTGAGATCGCAGAATGGC	0	10	RDR2-sensitive
GGACGACTGTGAAGACGACGATGT	0	10	RDR2-sensitive
GGACGCAATCGGACGGTCGAGATA	0	14	RDR2-sensitive
GGACGCGACTCGAACTCGGATGGC	0	20	RDR2-sensitive
GGACGGAAACGGACGGAAAAACCC	0	13	RDR2-sensitive
GGACGGACGGTCCACGACCTGCGA	2	14	RDR2-sensitive
GGACGGCGTAGAGAACGTTTCGTA	0	15	RDR2-sensitive
GGACGGCTGAGATCGCAGAACGGC	1	42	RDR2-sensitive
GGACGGCTGAGATCGCAGAATGAC	2	15	RDR2-sensitive
GGACGGCTGAGATCGCAGAATGGC	0	22	RDR2-sensitive
GGACGGTCCAGATCGATGGACGGC	0	15	RDR2-sensitive
GGACGGTCCGCGGCGTAAGGCTAC	0	10	RDR2-sensitive
GGACGGTCCGCGTCTGAGACTCGG	0	10	RDR2-sensitive
GGACGGTCGCGACAGAACACGGTA	0	12	RDR2-sensitive
GGACGGTCGTACCCTAGAGCCGGA	0	55	RDR2-sensitive
GGACGGTTGAGATTGCAGAACGGT	0	23	RDR2-sensitive
GGACGGTTGGCTCGGACCTGGCAC	2	16	RDR2-sensitive
GGACGTACCTGGAACTGAAGGCGC	0	10	RDR2-sensitive
GGACGTGGAACTGTAGATGACTGT	0	11	RDR2-sensitive
GGACGTGGGACTAGCAGACTTCGT	0	21	RDR2-sensitive
GGACGTTGTTGGGCGGAGACCGGA	0	16	RDR2-sensitive
GGACTAAAGTCGGCATTGCAGCAG	0	9	RDR2-sensitive
GGACTAGACTGAGACGGAGGACGA	0	54	RDR2-sensitive
GGACTCAAGTCGGTATCACAGCAG	0	9	RDR2-sensitive
GGACTCAGCCTAAGATCGACGGTG	0	16	RDR2-sensitive
GGACTCCGTCGGCTGGACAACGGC	0	10	RDR2-sensitive
GGACTCGTTCTGAACGTCGAGCTG	0	22	RDR2-sensitive
GGACTGACCGGCTGCATGAGGATT	14	0	RDR2-resistant
GGACTGGACTGTCCGCGATGACGC	1	23	RDR2-sensitive
GGACTGGCTACTGTAGTTCACGGG	0	12	RDR2-sensitive
GGACTGGCTGAGTGCATGCAGTAT	0	10	RDR2-sensitive
GGACTGTTCAGGCAAGGACATGGT	17	1	RDR2-resistant
GGACTGTTGTAGGAGTGTAGGCGC	1	12	RDR2-sensitive
GGACTTGCTCGGCGTTGACTTGAT	0	22	RDR2-sensitive
GGACTTGGACAGGAAACTAGGCAC	0	15	RDR2-sensitive
GGACTTGGACTGGAAACTAGGCAC	0	17	RDR2-sensitive
GGAGAAAAGACGGCGTCGGTACCT	0	11	RDR2-sensitive
GGAGAAAGTCGACGAGCACTGCTT	0	13	RDR2-sensitive
GGAGAACGGACAAAGGACGGACGG	0	16	RDR2-sensitive
GGAGAACGTAGCAGCATGAGCGGA	0	21	RDR2-sensitive
GGAGAACTCGAGGCCAGATCAGGC	2	19	RDR2-sensitive
GGAGAACTCTCCCGTTGCAGTGGA	49	0	RDR2-resistant
GGAGAAGAATAGTCGGGCCGGCAC	0	10	RDR2-sensitive

GGAGAAGACGGAACTGTTCAGGCG	0	9	RDR2-sensitive
GGAGAAGGACAACTGTAGGAGGGC	0	9	RDR2-sensitive
GGAGAATCATGTAGAGATGGGCTC	0	14	RDR2-sensitive
GGAGAATCGTGTAGAGACGGGCTC	0	9	RDR2-sensitive
GGAGAATCTGACTGTGGAGAGGAT	1	28	RDR2-sensitive
GGAGACAGAGTTAGACGTGTGGTA	1	26	RDR2-sensitive
GGAGACCGGACGGAAGAAAACGCT	0	14	RDR2-sensitive
GGAGACGACGGCCTAGACCACTCA	0	15	RDR2-sensitive
GGAGACGATGAACCCCAAGAGCAT	1	31	RDR2-sensitive
GGAGACGATGAACCCCAGGAGCAT	2	15	RDR2-sensitive
GGAGACGCAGGCTGCATGAGCGGA	0	12	RDR2-sensitive
GGAGACGCGCGGGCTGTTCGGCGT	1	12	RDR2-sensitive
GGAGACGGATGCAGAGGCTGCTGG	0	13	RDR2-sensitive
GGAGACGGATGCGGAGACTGCTGG	1	31	RDR2-sensitive
GGAGACGGATGCGGAGGCTGCTGG	1	20	RDR2-sensitive
GGAGACGGATTGTAACGGGTCCAA	0	14	RDR2-sensitive
GGAGACGGGCGTGAGAGAGGTGGA	0	9	RDR2-sensitive
GGAGACGTAGACCACGAACGGCGA	0	10	RDR2-sensitive
GGAGACGTAGACCGCGAACGGCGA	0	30	RDR2-sensitive
GGAGACGTAGAGGATCTGACCGTC	0	11	RDR2-sensitive
GGAGACGTCGTATATGTACAGCAC	0	9	RDR2-sensitive
GGAGACGTGGAGAAGATCGGCAGC	0	9	RDR2-sensitive
GGAGACTCACGTGGGATTCGGCCT	1	58	RDR2-sensitive
GGAGACTCGGCGAGACGGTAACAT	1	12	RDR2-sensitive
GGAGACTGAAGACGGTAGGGTCGT	0	11	RDR2-sensitive
GGAGACTGAGTCAGACAAGTGGTA	0	10	RDR2-sensitive
GGAGACTGATCAATCGGACACCGC	4	138	RDR2-sensitive
GGAGACTGCTGAGCAGGAGCTCGT	0	29	RDR2-sensitive
GGAGACTGCTGGAGAGCGAAACGG	1	37	RDR2-sensitive
GGAGACTGGATCTAACAGCGGCGT	0	13	RDR2-sensitive
GGAGACTGTAAAGGACGGATATAG	0	9	RDR2-sensitive
GGAGACTGTGAGATCCGGTAGCCA	0	30	RDR2-sensitive
GGAGACTGTGTAAAGGCACCGAAC	0	28	RDR2-sensitive
GGAGACTGTGTAGCCGGAGCCGGT	0	31	RDR2-sensitive
GGAGAGAATCTGACTGTGGGAAGA	0	13	RDR2-sensitive
GGAGAGAATCTGGCTGTGAGGAGA	0	15	RDR2-sensitive
GGAGAGAATCTGGCTGTGGCGAAA	0	17	RDR2-sensitive
GGAGAGAATCTGGCTGTGGGAAGA	2	16	RDR2-sensitive
GGAGAGACATTGGCACTTGGTCGA	0	9	RDR2-sensitive
GGAGAGAGAACGCTGACGTGGCAC	0	10	RDR2-sensitive
GGAGAGCAGGGTGACGGAGGACTC	0	9	RDR2-sensitive
GGAGAGGACGACGTGCAGCCTGAT	0	13	RDR2-sensitive
GGAGAGGAGAGAGAATGTGTCGGC	3	25	RDR2-sensitive
GGAGAGGATCGTGCAACAGGAGGC	71	1	RDR2-resistant
GGAGAGGCTCTGCACGGACGGGAA	1	23	RDR2-sensitive
GGAGATAAGACACTGCCGTCGGAC	0	10	RDR2-sensitive
GGAGATAAGGCTTGACCGTCGGAA	0	21	RDR2-sensitive
GGAGATAAGGCTTTGCCGTCGGAA	1	13	RDR2-sensitive
GGAGATACTAGAACCGCTGACGGA	0	19	RDR2-sensitive

GGAGATACTAGAGCCGCTGACGGA	1	12	RDR2-sensitive
GGAGATACTAGAGCCGCTGCCGGA	4	21	RDR2-sensitive
GGAGATACTAGAGCTGCTGCCGGA	0	9	RDR2-sensitive
GGAGATACTCGGCAGAACCGTGCA	0	12	RDR2-sensitive
GGAGATAGGTTAATGCCGTCGGAC	0	9	RDR2-sensitive
GGAGATCCGGCGACTGGGGGACGG	0	10	RDR2-sensitive
GGAGATCCTCGGCAGAACCGTGCA	0	12	RDR2-sensitive
GGAGATCCTGGACGGGAGCACGGT	0	14	RDR2-sensitive
GGAGATCGGGCTGTCGGGGTTGGC	0	18	RDR2-sensitive
GGAGATCGGTAGAACGAGCAGAGT	0	16	RDR2-sensitive
GGAGATGGGTCTGGTCAGGACGAC	1	13	RDR2-sensitive
GGAGATTGAGGGACGGCAGAACGG	0	10	RDR2-sensitive
GGAGATTTGTTACCTGGACAGCGG	0	14	RDR2-sensitive
GGAGCAAAAGACTGTTGACGGCAT	0	27	RDR2-sensitive
GGAGCAACTCGGCTCGGCTCGAAG	16	0	RDR2-resistant
GGAGCACCGGACTGTCCGATGGCA	0	20	RDR2-sensitive
GGAGCACTCGGCATGTGGAGACCA	0	15	RDR2-sensitive
GGAGCATCGCTGTAGGTACAGGCT	0	21	RDR2-sensitive
GGAGCATCTGATGTCGGACGACAC	0	26	RDR2-sensitive
GGAGCATCTGGAAGGACGGTCTGG	0	16	RDR2-sensitive
GGAGCCGGATATGGATACATGTTT	0	11	RDR2-sensitive
GGAGCCGTAGGGAACCTGACGGAC	0	9	RDR2-sensitive
GGAGCGACGGGCGGTGTGTACAAA	71	3	RDR2-resistant
GGAGCGACTGAATCGGATGACATC	0	15	RDR2-sensitive
GGAGCGAGATCCAGGCGCCGTCGG	0	15	RDR2-sensitive
GGAGCGAGCTGTAGACGGGTTCAA	2	107	RDR2-sensitive
GGAGCGAGTTGTAGACGGGTTCAA	0	9	RDR2-sensitive
GGAGCGATGTAGGGAGGATGGGCG	0	14	RDR2-sensitive
GGAGCGCGCGAGCTGTAGCGACGG	1	21	RDR2-sensitive
GGAGCGGAGGGACGGCAGAACCGT	0	10	RDR2-sensitive
GGAGCGTCGTAGCGACCGCGGCTC	0	10	RDR2-sensitive
GGAGCGTGACTGTACGAGCAGGCT	0	9	RDR2-sensitive
GGAGCGTGACTGTGAGAGCGGGAT	6	72	RDR2-sensitive
GGAGCGTTTGAGCCCTGTAGAAGC	1	12	RDR2-sensitive
GGAGCTCAGTCCTGCTGTAGATGG	3	39	RDR2-sensitive
GGAGCTCGGACGGCCTGCTATGGC	0	9	RDR2-sensitive
GGAGCTGATCCGGATCTGCTTGGA	0	11	RDR2-sensitive
GGAGCTGGACCATGCACTAGACGA	0	12	RDR2-sensitive
GGAGCTGGGCTGTGGCGCAGAGAA	0	9	RDR2-sensitive
GGAGCTGTGGAGTAGTATACGGAC	0	10	RDR2-sensitive
GGAGGAAACTGGAGCGGGGCGCGA	0	11	RDR2-sensitive
GGAGGAAGACGAGCGAAGGGACGA	0	16	RDR2-sensitive
GGAGGACAAGCCCATGGACGGTGA	0	12	RDR2-sensitive
GGAGGACAGAATCTAGGGTTGCAT	0	9	RDR2-sensitive
GGAGGACAGTAGCGCGGTGGTGGA	129	51	RDR2-resistant
GGAGGACATAGATGCGGATAGCAG	0	16	RDR2-sensitive
GGAGGACATGAGCGCGGATAACAA	0	10	RDR2-sensitive
GGAGGACATGGAAGACCGTTTGAA	0	24	RDR2-sensitive
GGAGGACCTGCCGGCTTGACGGAG	0	10	RDR2-sensitive

GGAGGACCTGTAGAGACGGAGCGA	0	12	RDR2-sensitive
GGAGGACCTGTCTGAGCAGAGGCT	0	12	RDR2-sensitive
GGAGGACCTTGCAGTACCGGCAAA	16	0	RDR2-resistant
GGAGGACGAGGAGTAGGTTGGTAC	56	0	RDR2-resistant
GGAGGACGCAGGATATGACGGATG	5	58	RDR2-sensitive
GGAGGACGCGCTGCACAGCTTGAG	0	9	RDR2-sensitive
GGAGGACGCGGGATATGATGGATG	1	17	RDR2-sensitive
GGAGGACGGGCTGACGAACAGGCT	1	14	RDR2-sensitive
GGAGGACTGACGCGGCACCGACGG	0	19	RDR2-sensitive
GGAGGACTGGACAGAGGTGGATGC	50	7	RDR2-resistant
GGAGGACTTCATGACTTCACGGCT	0	9	RDR2-sensitive
GGAGGACTTGTAGAGCATGATGGC	1	24	RDR2-sensitive
GGAGGACTTTGACTATTTTGCGGC	37	0	RDR2-resistant
GGAGGAGACTGTAAAGGACGGATA	0	17	RDR2-sensitive
GGAGGAGCCCGTAGAAGACACGCT	0	9	RDR2-sensitive
GGAGGAGCGCTGGAGCCTGGCGGA	1	12	RDR2-sensitive
GGAGGATAGTGACCAGAGCCGGAC	101	0	RDR2-resistant
GGAGGATCACGCAGTAGAGGGCTC	0	10	RDR2-sensitive
GGAGGATCATACGACATGGACGAC	0	9	RDR2-sensitive
GGAGGATCCGGAGTCTGAAGCGGT	0	10	RDR2-sensitive
GGAGGATGACGTGCAGGACATCGT	0	15	RDR2-sensitive
GGAGGATTGGGAGTAGAAGCGAGG	0	13	RDR2-sensitive
GGAGGATTGTAGTATAGGGTAGAG	0	14	RDR2-sensitive
GGAGGATTGTGGTATAGAGTGCTG	0	12	RDR2-sensitive
GGAGGCATCATAGGGACTGGCGTC	0	9	RDR2-sensitive
GGAGGCATCCTGTACGACGGCGGG	88	4	RDR2-resistant
GGAGGCATCCTGTACGACGGCGGT	582	34	RDR2-resistant
GGAGGCATGCAGGACAGACGACGT	0	13	RDR2-sensitive
GGAGGCGAGATGGACTATTAGTGT	0	10	RDR2-sensitive
GGAGGCGGAGGTAGACAGGGCGGG	0	11	RDR2-sensitive
GGAGGCGTCAGACGTAGGACGGCT	2	28	RDR2-sensitive
GGAGGCTCGAAGCGATACTGACGT	51	83	RDR2-sensitive
GGAGGCTGGCATAAACAGAACACA	0	10	RDR2-sensitive
GGAGGCTGGGTAGGAGAGAGACGA	0	25	RDR2-sensitive
GGAGGCTGTCTGTTCGATGGCGCA	2	24	RDR2-sensitive
GGAGGGACGAAGACGGACGAAGAC	1	18	RDR2-sensitive
GGAGGGACGAGAGAGAACGACAGG	0	26	RDR2-sensitive
GGAGGGACGAGAGAGGACACCAGG	0	22	RDR2-sensitive
GGAGGGACGAGAGAGGACGCCAGG	2	30	RDR2-sensitive
GGAGGGACGAGAGAGGACGTCAGG	0	10	RDR2-sensitive
GGAGGGACGGCAGAACCGTGCGCC	0	13	RDR2-sensitive
GGAGGGACTCAGCACTAGACCTGA	0	11	RDR2-sensitive
GGAGGGATCCGTTGGACATGGCAT	21	0	RDR2-resistant
GGAGGGCAGAACGGCAGAACAGCA	0	28	RDR2-sensitive
GGAGGGCCTTGTAGAGGACGCTGT	0	24	RDR2-sensitive
GGAGGGCGCTGCGCAGATCGACGT	18	0	RDR2-resistant
GGAGGGCGTTGGGGCAGCGTGGCC	31	0	RDR2-resistant
GGAGGGCTGTATTCCACGACGCAG	0	9	RDR2-sensitive
GGAGGGGCTGACAGAGGGCTGCGT	0	9	RDR2-sensitive

GGAGGGTCTTGTAGAGACGAGCTC	0	10	RDR2-sensitive
GGAGGGTGTAGGCTTACGGTACAG	0	17	RDR2-sensitive
GGAGGTGACGTAGAGGAAGACCGA	0	9	RDR2-sensitive
GGAGGTGCTGCGACGAGGACGATT	16	0	RDR2-resistant
GGAGGTGGATATGTGGAAGACTAC	3	54	RDR2-sensitive
GGAGGTGGATATGTGGAAGGCTAC	1	19	RDR2-sensitive
GGAGGTTGACGGAGATACTAGAGC	1	28	RDR2-sensitive
GGAGGTTGACGGAGATACTGGAGC	1	20	RDR2-sensitive
GGAGGTTGGTTGTGCGGAGCGGAC	0	11	RDR2-sensitive
GGAGTAAGCGTTTGGAGGCCAGAA	16	0	RDR2-resistant
GGAGTACTGTAGACGCACGGAGAA	0	22	RDR2-sensitive
GGAGTAGACGTAGCAGAACGACGA	0	11	RDR2-sensitive
GGAGTAGACTGGAAACGAGCCGAG	0	18	RDR2-sensitive
GGAGTAGGACGGTGTCAACGACTG	9	29	RDR2-sensitive
GGAGTAGGACGGTGTCGACGACTG	0	16	RDR2-sensitive
GGAGTATCTGATGTCGGACGACAC	0	29	RDR2-sensitive
GGAGTCAGATCTGGGAATGCGGGC	0	25	RDR2-sensitive
GGAGTCCAGGACTGCAGGGGCGGT	1	16	RDR2-sensitive
GGAGTCGAACCCAGGACCTGAGGA	0	12	RDR2-sensitive
GGAGTCGAACTCAGGACCTGAGGA	2	16	RDR2-sensitive
GGAGTCGAGTGCTGGACAGACGCA	0	10	RDR2-sensitive
GGAGTCGAGTGCTGGACAGACGTA	0	11	RDR2-sensitive
GGAGTCTGCTGGAAACGGGGACGG	0	28	RDR2-sensitive
GGAGTCTGCTGGAACGCACGGTGC	0	11	RDR2-sensitive
GGAGTCTGTAGACTGGAGCAGCAG	0	10	RDR2-sensitive
GGAGTGAAGTAGAACGGATCGGCT	0	18	RDR2-sensitive
GGAGTGACTCTGAGGACGACGATG	0	10	RDR2-sensitive
GGAGTGAGCTGTAGACGGGTTCAA	0	13	RDR2-sensitive
GGAGTGAGTAGAGGACGATCTGAT	1	48	RDR2-sensitive
GGAGTGAGTAGAGGACGTTCTGAT	1	37	RDR2-sensitive
GGAGTGGACTGTCAGCGGCCTAGT	0	13	RDR2-sensitive
GGAGTGGATTGAGGGGGATTGGAG	16	52	RDR2-sensitive
GGAGTGGGAGGCAGAACCGTGCAC	0	27	RDR2-sensitive
GGAGTTCAGGACTGTAGGGGCAGT	0	10	RDR2-sensitive
GGAGTTCGGACCTACCCGGAGGCT	0	13	RDR2-sensitive
GGAGTTGACTTGAGACCTAGGCAC	0	17	RDR2-sensitive
GGAGTTGCTGAGCCGTCGGACGCT	0	9	RDR2-sensitive
GGAGTTGGGAATGGGAATGGCAGA	0	13	RDR2-sensitive
GGAGTTGGGCTGTCCGCGTGCGTA	0	13	RDR2-sensitive
GGAGTTTCTTCTTCGTCGGATACT	26	0	RDR2-resistant
GGATAACTCTTCGTCGACGAGCAA	0	16	RDR2-sensitive
GGATACAGACGGATAGTAGGTCAT	0	11	RDR2-sensitive
GGATACCCGATGGACGAAGACGAT	0	9	RDR2-sensitive
GGATACGACGGACGTATGGATGGC	0	12	RDR2-sensitive
GGATACGAGACGGATACGGGACGA	0	11	RDR2-sensitive
GGATACGGAATCGAGTGGCTCAGA	0	9	RDR2-sensitive
GGATACGGACGGATACTAGGTCAT	0	18	RDR2-sensitive
GGATACGGACGGATATTAGCTCAT	0	83	RDR2-sensitive
GGATACGGATACAGGGACCGGATA	3	24	RDR2-sensitive

GGATACGGATAGTTAGAAACGGGA	1	13	RDR2-sensitive
GGATACGGGACGGATACGGAACAG	0	9	RDR2-sensitive
GGATACGGGCTCTGTTGGAACGGT	0	35	RDR2-sensitive
GGATACTTCGCGGATACTTTTGGA	0	9	RDR2-sensitive
GGATAGAAGGAGCCGGTAGAAGCT	0	10	RDR2-sensitive
GGATAGAGTCGGATACATGGTCAT	0	17	RDR2-sensitive
GGATAGGACAACGACAGCTGACGA	0	15	RDR2-sensitive
GGATAGGACAATGGCAGCTGACGA	0	10	RDR2-sensitive
GGATAGGGGCATTCGAGGCGGATT	14	0	RDR2-resistant
GGATAGGTACTGTGCGAAGTCCGG	0	13	RDR2-sensitive
GGATAGTCCGGACTCAAGACGGGA	0	9	RDR2-sensitive
GGATAGTGACCAGAGCCGGACGGC	79	0	RDR2-resistant
GGATAGTTAGAAACGGGACGGATA	0	17	RDR2-sensitive
GGATATAGAGGACGTTGCTGGAGA	0	22	RDR2-sensitive
GGATATGACTGCGGGCGGATATTT	0	12	RDR2-sensitive
GGATATGACTGGACGGAACCCGGC	1	71	RDR2-sensitive
GGATATGCGGAAGGCTACAACTAG	0	10	RDR2-sensitive
GGATATTAAATGTAGTCGGATTAT	0	17	RDR2-sensitive
GGATATTATTTGATCGGATATCGG	5	21	RDR2-sensitive
GGATCAATGTTTGACTGTCGCGTG	42	2	RDR2-resistant
GGATCCAAACACGTAGAGGGCTAG	0	13	RDR2-sensitive
GGATCCATCTCTTCGGGCGGTTAG	0	14	RDR2-sensitive
GGATCCGATCGGCATCGACTCTCC	0	10	RDR2-sensitive
GGATCCGGATTGAGTGATGGCCAT	2	24	RDR2-sensitive
GGATCCGTTGGACATGGCATGGCC	40	0	RDR2-resistant
GGATCGAACTGGGACTAGAGGACG	0	17	RDR2-sensitive
GGATCGACGATTCGGGTAGGGACG	0	10	RDR2-sensitive
GGATCGACTTGACTCACGAGGCAT	0	15	RDR2-sensitive
GGATCGAGTACGGACGGATATCAC	0	9	RDR2-sensitive
GGATCGGACAACTCCTCACGGTGT	0	9	RDR2-sensitive
GGATCGGACCGAGTCGGTAAGCAC	0	11	RDR2-sensitive
GGATCGGATACGCATGGAAACGGA	0	17	RDR2-sensitive
GGATCGGATGTGGATCACCCACGG	0	15	RDR2-sensitive
GGATCGGCGCGAGACGGAACACAA	0	14	RDR2-sensitive
GGATCGGGATGGCAGAACCGTGCA	0	11	RDR2-sensitive
GGATCGGGCTGGGACCAGAGGACC	0	11	RDR2-sensitive
GGATCGGGCTGGGACCAGAGGATG	0	26	RDR2-sensitive
GGATCGGTACCTGCAGGCCATGGC	0	10	RDR2-sensitive
GGATCTGCGTCCGAGGCTGTCTGG	0	11	RDR2-sensitive
GGATCTGGACTCGACGCTCGGCGC	0	11	RDR2-sensitive
GGATCTTAATCTTATCGTCAGAGA	23	2	RDR2-resistant
GGATCTTATTTTCGTCGTCGGAAT	0	14	RDR2-sensitive
GGATCTTCCGCTTCCGGTCGTTGA	0	10	RDR2-sensitive
GGATGAATCAACCCGGCTTAGAAT	0	9	RDR2-sensitive
GGATGACACGTCACGAAGCTCGGC	0	12	RDR2-sensitive
GGATGACTCTATAGGTGCTGACGG	0	13	RDR2-sensitive
GGATGAGAGGAGAGAGTGTGGAGA	4	24	RDR2-sensitive
GGATGAGAGTCACGTAGGGACGAA	0	10	RDR2-sensitive
GGATGAGGAGTAGCATGTGAAGTT	22	0	RDR2-resistant

GGATGATCCGTCGCATACATGTGT	0	9	RDR2-sensitive
GGATGCAGACGGATAGTAGGTCAT	0	10	RDR2-sensitive
GGATGCGAAGACTGCAGAGTGGAA	0	18	RDR2-sensitive
GGATGCTCTGGATAGTTTGATTGC	0	95	RDR2-sensitive
GGATGGAATCGCAATCGGACGGTG	0	50	RDR2-sensitive
GGATGGACCTGGATGTGCGGATGC	0	9	RDR2-sensitive
GGATGGACGATCACGGTGGAGGAC	53	0	RDR2-resistant
GGATGGACGGTTGAGATCGTTGGA	0	10	RDR2-sensitive
GGATGGACTAGCTCGAGCGGTGTC	0	11	RDR2-sensitive
GGATGGACTGACCGGCCCGTCGGT	0	17	RDR2-sensitive
GGATGGACTGCGGGATCGGACGGC	2	19	RDR2-sensitive
GGATGGATATAGAGGACGTTGCTG	0	23	RDR2-sensitive
GGATGGATGGATGAGGAAGGATGA	0	10	RDR2-sensitive
GGATGGCGTGGACAGAACCGTGCA	0	22	RDR2-sensitive
GGATGGCTAGAGGATAGGTGGAAC	0	16	RDR2-sensitive
GGATGGCTGCAGCGATTGACTGGA	1	12	RDR2-sensitive
GGATGGCTGTATCCGGACGCACAG	0	14	RDR2-sensitive
GGATGGGAATGGCAGAACCGGAAT	0	14	RDR2-sensitive
GGATGGGACGGGACGATCACTCAA	1	13	RDR2-sensitive
GGATGGTCAGTGACTATGGGCCGG	0	9	RDR2-sensitive
GGATGGTCGTTGGCAGCCGGTGAT	20	0	RDR2-resistant
GGATGGTCTGTGGCTCTAGGCCGG	3	26	RDR2-sensitive
GGATGTAATTCGGCTGCAGGGACC	0	16	RDR2-sensitive
GGATGTAGATGGACGGTGTAGATT	0	12	RDR2-sensitive
GGATGTAGTTGGTAGACGGTTCTC	0	12	RDR2-sensitive
GGATGTCCTGACTGCAGCACCAGC	0	23	RDR2-sensitive
GGATGTCGACGAGTTGGAGACTTG	2	14	RDR2-sensitive
GGATGTCGACGAGTTGGAGGCCTC	1	16	RDR2-sensitive
GGATGTCGACGATGGCAGAACCGT	1	16	RDR2-sensitive
GGATGTCGGCGTGCGTGTGGAGAC	0	9	RDR2-sensitive
GGATGTCGGGCAGAACACGGTGCA	0	25	RDR2-sensitive
GGATGTCTGGACCAGGTTCGACGG	1	34	RDR2-sensitive
GGATGTCTGGCTGGCCATGACGTG	0	18	RDR2-sensitive
GGATGTTGGGCTTAGAAACCGGCC	0	14	RDR2-sensitive
GGATGTTGTCCAGCATGTAGACGT	0	21	RDR2-sensitive
GGATGTTGTTGGACGCGTACCGAA	1	38	RDR2-sensitive
GGATGTTTGAGACTCAGACGGTCC	0	13	RDR2-sensitive
GGATGTTTTTTGACTGTATGTCGG	1	18	RDR2-sensitive
GGATTACGTGCGGAGGAAGATGAA	1	17	RDR2-sensitive
GGATTAGGATCGTGCCGGCCCGAC	0	43	RDR2-sensitive
GGATTCCTGGCTGTTGACTGCATG	2	14	RDR2-sensitive
GGATTCGAGATCGGATACGGATAG	0	21	RDR2-sensitive
GGATTCGGATCGGATACGCATGGA	0	10	RDR2-sensitive
GGATTCGGGATCGGATACGGATAG	9	181	RDR2-sensitive
GGATTCGGGTCGGATACGGAGCGA	1	18	RDR2-sensitive
GGATTCTTGGCTGTCGACTGCATG	1	12	RDR2-sensitive
GGATTGAACTGCTCCGGTCGTCGC	0	11	RDR2-sensitive
GGATTGAATCCTCGGACGACGGAC	0	9	RDR2-sensitive
GGATTGAGTGGGATTGGATGGGTT	19	77	RDR2-sensitive

GGATTGATACGGATTGGAGGGGAT	4	42	RDR2-sensitive
GGATTGCACTGTCTGGCCTGGGCT	0	11	RDR2-sensitive
GGATTGCGCGACGGATGACTCCGA	1	17	RDR2-sensitive
GGATTGGAAGGGATTGGAGAGGAT	0	12	RDR2-sensitive
GGATTGGGCTGAGCGAGTGGGCAT	0	13	RDR2-sensitive
GGATTGGTCTTGCGGATCTGGTCG	25	3	RDR2-resistant
GGATTGGTTCGGTAGACACGGTCT	0	22	RDR2-sensitive
GGATTGTCGGCACTACAACAGCGT	0	13	RDR2-sensitive
GGATTGTGATCGTGTCGGCCCGGC	0	9	RDR2-sensitive
GGATTGTTGTCTGGCTCGGTGTCT	23	0	RDR2-resistant
GGATTGTTTTAGAGGGTCGGGCAT	1	17	RDR2-sensitive
GGATTTACTCGCCGGCACAGACAG	0	9	RDR2-sensitive
GGATTTAGGACGGGTGCACTCCGC	0	19	RDR2-sensitive
GGATTTAGGGCGGGTGCACTCTGC	0	13	RDR2-sensitive
GGATTTGAGGGGAAGGGGACGACC	24	1	RDR2-resistant
GGATTTGATTGTGTGTCGTCGGTT	129	0	RDR2-resistant
GGATTTGGACCAGACATGACGGAC	0	41	RDR2-sensitive
GGATTTGGACCCTTAACTCGGCGC	0	10	RDR2-sensitive
GGATTTGGGATCGGATACGGATAG	0	9	RDR2-sensitive
GGATTTGGTCCGGCACGAACCCAG	0	24	RDR2-sensitive
GGATTTTATCTGATATGTAGACGA	0	14	RDR2-sensitive
GGCAAACCCAGCCTGATCGACGAG	0	9	RDR2-sensitive
GGCAAACGACGAGATCAACGACGC	0	19	RDR2-sensitive
GGCAAACGACGAGATCGACGACGC	1	12	RDR2-sensitive
GGCAAACGACGAGATCGATGACGC	0	21	RDR2-sensitive
GGCAAACGACGCGACGACGAGCTG	0	19	RDR2-sensitive
GGCAAACGACGTGACGACGAGCTG	0	9	RDR2-sensitive
GGCAAACTGTGGCAACTGAGGCAA	0	10	RDR2-sensitive
GGCAAAGAACGCTCGGCGAACTGT	7	48	RDR2-sensitive
GGCAAAGAAGGCTCGGCGAACTGT	0	12	RDR2-sensitive
GGCAAAGACGTTGGCACTTGGCAA	0	15	RDR2-sensitive
GGCAAAGAGGCTGGCACTCGGCAA	0	15	RDR2-sensitive
GGCAAAGCCTCGGATTCCGGTAGT	0	12	RDR2-sensitive
GGCAAAGCGTCCGGCACTCGGCAA	0	17	RDR2-sensitive
GGCAAATCGTCCGGCACTCGGCAA	0	9	RDR2-sensitive
GGCAAATGATTGAGGGGACAGCTT	0	24	RDR2-sensitive
GGCAACCCCGGGTGTGCGGTCGGA	0	12	RDR2-sensitive
GGCAACCCGGACGGATTCACACAT	0	10	RDR2-sensitive
GGCAACGGATATCTCGGCTCTCGC	26	3	RDR2-resistant
GGCAAGAACAGTAGTCCCGGACGG	0	14	RDR2-sensitive
GGCAAGTTCGGATAGGGACGGCTG	1	50	RDR2-sensitive
GGCAATCGGACGGTCGAGATGGAG	0	27	RDR2-sensitive
GGCAATCGGGCGGGTAGAACACGG	0	12	RDR2-sensitive
GGCAATCGGGCGGGTAGAACGCGG	0	17	RDR2-sensitive
GGCAATCGGGCGGGTAGTACACGG	3	23	RDR2-sensitive
GGCAATGTGGCTGGCATGACTGAC	34	0	RDR2-resistant
GGCAATTGTAGGCGTGGCATCCAA	0	32	RDR2-sensitive
GGCACACAGGACTGTGCAGGCGCA	0	10	RDR2-sensitive
GGCACACCGGACAGTCTGATGGCA	0	9	RDR2-sensitive

GGCACACTCACTCGTCGGCTCAGG	0	11	RDR2-sensitive
GGCACAGAGTCAGTTATCGTCGTT	56	1	RDR2-resistant
GGCACAGCAGTCGGAGCAGGGCAG	2	16	RDR2-sensitive
GGCACAGGACATTGTCGAACGGCT	1	16	RDR2-sensitive
GGCACAGTAGAATAAGCGGACGGG	0	9	RDR2-sensitive
GGCACCAACGAGGATTAGTCGGAA	2	14	RDR2-sensitive
GGCACCAACGAGGATTAGTCGGGA	1	17	RDR2-sensitive
GGCACCGGACAGTGAACAGTGCAT	1	30	RDR2-sensitive
GGCACCGGACCTCTAGCATGGCGT	0	15	RDR2-sensitive
GGCACCGGACTGTCCGATGTGCAC	4	18	RDR2-sensitive
GGCACCGTTGGATGTGGAGGGTGT	0	25	RDR2-sensitive
GGCACGAATTGCGGGCTGGATCGT	2	40	RDR2-sensitive
GGCACGACAGGCTAGGCTCGGCAC	0	11	RDR2-sensitive
GGCACGACGTGCCTTGGTCGCGGC	0	9	RDR2-sensitive
GGCACGACTAACAGACTCACGGGC	0	60	RDR2-sensitive
GGCACGATCTACACTTATCTGTAA	14	0	RDR2-resistant
GGCACGATCTGTACAATGATGGCG	0	10	RDR2-sensitive
GGCACGATGGACTGACACGAGCAC	0	12	RDR2-sensitive
GGCACGATGGGCTGGCACGAGCAC	0	20	RDR2-sensitive
GGCACGCACGGACAGCAGGGACGC	0	20	RDR2-sensitive
GGCACGCAGCGACAATTTACTCGT	0	13	RDR2-sensitive
GGCACGCAGGACTGTGCAGGCACG	0	31	RDR2-sensitive
GGCACGCAGTGACGGTCTGACTAC	1	12	RDR2-sensitive
GGCACGCCGCCTGTAGGATGGCCT	0	10	RDR2-sensitive
GGCACGCGGCGACAATTTACTCGT	1	38	RDR2-sensitive
GGCACGCGGCGACATTTCACTCGT	0	12	RDR2-sensitive
GGCACGGACACAGCACCATGACGG	0	16	RDR2-sensitive
GGCACGGACCCGATGAATGACGGC	0	11	RDR2-sensitive
GGCACGGAGCTCTGACATGACGAC	0	23	RDR2-sensitive
GGCACGGATTAGCTAGCGGGTCAT	0	11	RDR2-sensitive
GGCACGGATTGAAGCAGCCCCGGA	0	11	RDR2-sensitive
GGCACGGCACGACAGATGACTCGG	0	14	RDR2-sensitive
GGCACGGCATGAACTCGACACGGC	0	12	RDR2-sensitive
GGCACGGTAGAATAAACGGGCGGG	0	22	RDR2-sensitive
GGCACGGTAGAATAAGCGGACGGA	0	21	RDR2-sensitive
GGCACGGTAGAATAAGCGGGCGGA	0	29	RDR2-sensitive
GGCACGGTAGAATAAGCGGGCGGG	2	242	RDR2-sensitive
GGCACGGTAGAATAAGTGGACGGG	0	27	RDR2-sensitive
GGCACGGTAGAATAAGTGGGCGGG	0	13	RDR2-sensitive
GGCACGTTACGCGGGCTGGGCCGT	0	10	RDR2-sensitive
GGCACGTTACGCGGGCTGGGTCGT	2	25	RDR2-sensitive
GGCACGTTAGACGGGCTGGGTCGT	0	16	RDR2-sensitive
GGCACTATACTAACCGGGTCGGAT	0	13	RDR2-sensitive
GGCACTATACTAATCGGACCGGGT	0	11	RDR2-sensitive
GGCACTCGACAAAGGGACTGGCAA	0	12	RDR2-sensitive
GGCACTCGACAAATCGTTCGGCAC	0	10	RDR2-sensitive
GGCACTCGGCAAAGAAGGAAACCT	1	25	RDR2-sensitive
GGCACTCGGCAAAGAAGGCTTTGC	2	22	RDR2-sensitive
GGCACTCGGCAAAGAATATGGCAC	1	39	RDR2-sensitive

GGCACTCGGCAAAGACGACCTCTT	0	18	RDR2-sensitive
GGCACTCGGCAAAGATACTGGCAA	1	26	RDR2-sensitive
GGCACTCGGCAAAGGGACTGGCAA	0	31	RDR2-sensitive
GGCACTGATACGATTGGACACGGC	0	20	RDR2-sensitive
GGCACTGTAGGTCGGCTTGCCTGC	0	41	RDR2-sensitive
GGCACTTGTGGCCGACGTTGGGGA	34	0	RDR2-resistant
GGCAGAACAACAGGGTGTGGACGC	0	11	RDR2-sensitive
GGCAGAACACGGAACGATAGACTA	0	14	RDR2-sensitive
GGCAGAACACGGAATGACAGACTA	0	9	RDR2-sensitive
GGCAGAACAGGTCAAAGGCAGAAC	0	13	RDR2-sensitive
GGCAGAACGCGGAATGGCCGACTA	0	12	RDR2-sensitive
GGCAGAACGCGTCAGAGGCAGAAC	2	46	RDR2-sensitive
GGCAGAACGGCAGAACACACTGAT	1	98	RDR2-sensitive
GGCAGAACGGCAGAACAGATAGAG	0	30	RDR2-sensitive
GGCAGAACGGCAGAACAGCAGACT	0	13	RDR2-sensitive
GGCAGAACGGCAGAACGACAGAAC	0	9	RDR2-sensitive
GGCAGAACGGCAGAACGCGTCAGA	0	10	RDR2-sensitive
GGCAGAACGGCAGAACGGCAGAAC	0	41	RDR2-sensitive
GGCAGAACGGCAGAACTTGTCAGA	0	9	RDR2-sensitive
GGCAGAACGGCAGACTACTCTTAC	2	81	RDR2-sensitive
GGCAGAACGGCAGACTACTCTTAG	0	17	RDR2-sensitive
GGCAGAACGGCAGACTACTCTTAT	0	9	RDR2-sensitive
GGCAGAACGGGAATGACAGAACAG	0	17	RDR2-sensitive
GGCAGAACGGGAATGGCAGAACAG	0	15	RDR2-sensitive
GGCAGAACGTGCGAGTGTGGACGC	0	78	RDR2-sensitive
GGCAGAAGACAAAGGAACGCACAC	0	9	RDR2-sensitive
GGCAGACCGTAAGCAGGGACACGC	0	9	RDR2-sensitive
GGCAGACGACTTAGGTACGCTGAC	3	18	RDR2-sensitive
GGCAGACGGGGGCTTTGGCAGAAC	0	10	RDR2-sensitive
GGCAGACTGCGGACGTGACGGCGT	0	15	RDR2-sensitive
GGCAGACTGGCACTGACACTGGTA	1	16	RDR2-sensitive
GGCAGACTGGCTTGGGTACATCAA	0	11	RDR2-sensitive
GGCAGAGACTGCAGTGCCTCTCGT	0	13	RDR2-sensitive
GGCAGAGCGACTTTAGACGGACAT	0	19	RDR2-sensitive
GGCAGAGCTCATGATCGTCGAGAC	0	17	RDR2-sensitive
GGCAGAGCTTATGATCGTCGAGAC	0	14	RDR2-sensitive
GGCAGATAGACGAAGCCTCTGAAG	0	10	RDR2-sensitive
GGCAGATAGGTACGTGCACTCTGC	2	18	RDR2-sensitive
GGCAGATGACTAGCTACCTGACGA	0	30	RDR2-sensitive
GGCAGATTTGATGTAGAGGGCAGA	24	2	RDR2-resistant
GGCAGCACTTGACCGTCGGACATA	0	9	RDR2-sensitive
GGCAGCGCGACTGGAGGAGCGCAT	1	13	RDR2-sensitive
GGCAGCGTCTGTCTCTGGCGGCTC	0	17	RDR2-sensitive
GGCAGCTGACGGTCTTGACCTGAA	2	14	RDR2-sensitive
GGCAGCTGTAGCCCAAGTAGCATA	15	0	RDR2-resistant
GGCAGCTGTTGGATTGCGATCGGA	0	11	RDR2-sensitive
GGCAGGAACAGTAGACCAAGGCAG	1	20	RDR2-sensitive
GGCAGGAACTGCATTACTTGCAAG	83	3	RDR2-resistant
GGCAGGACGGGACGACGTGGACAG	0	18	RDR2-sensitive

GGCAGGCTGTGGCACCTTCGGCAC	0	83	RDR2-sensitive
GGCAGGGACTTTTCATTCGTCTGG	40	0	RDR2-resistant
GGCAGGGCAGAGAAGCGACGACAT	2	17	RDR2-sensitive
GGCAGGGCGTAGCATATGACTGAT	0	13	RDR2-sensitive
GGCAGGTCGGCACTCGGCAGTGAT	1	12	RDR2-sensitive
GGCAGGTGACTGAGGGGGTGGCAA	0	9	RDR2-sensitive
GGCAGTCCGCAGCAGCTTTTGGTG	0	13	RDR2-sensitive
GGCAGTCGCGTAGACGCTCCCCAA	0	10	RDR2-sensitive
GGCAGTCTGCAGCAGCTTTTGGTG	1	15	RDR2-sensitive
GGCAGTGACGATGACAGTGAACGG	0	14	RDR2-sensitive
GGCAGTGCGGATACGTAAGGGCAT	0	10	RDR2-sensitive
GGCAGTGGACCCGTGACTCGGCAC	0	11	RDR2-sensitive
GGCAGTGGGCCTGTGATCCGGCAC	0	11	RDR2-sensitive
GGCAGTGGGCTTGTGATTCGGCAC	0	21	RDR2-sensitive
GGCAGTGTAGGACGGAGTTGAGGC	0	12	RDR2-sensitive
GGCATACATAGAGCACAAGGACGT	0	9	RDR2-sensitive
GGCATAGGGCTCGACGGTCTGTAA	0	10	RDR2-sensitive
GGCATATGACGACAGTAGACACAC	0	26	RDR2-sensitive
GGCATCGTGACGCATCATCTGGTA	0	10	RDR2-sensitive
GGCATGACACGACTGGAGCTAGGC	0	13	RDR2-sensitive
GGCATGACTGGCACTGTACCCAAG	24	1	RDR2-resistant
GGCATGACTGTTGAGGCGGGGGCA	0	11	RDR2-sensitive
GGCATGATCCTGACGGAAGACGAC	0	9	RDR2-sensitive
GGCATGCTGTAACTGAAACCGGTC	0	9	RDR2-sensitive
GGCATGGACACAAGCTGACGACGA	0	67	RDR2-sensitive
GGCATGGATACAAGCTGACGACGA	0	16	RDR2-sensitive
GGCATGGTTTGTCGGTCATAAGAA	24	0	RDR2-resistant
GGCATGTATGGACAGGCTAGACGC	0	18	RDR2-sensitive
GGCATGTGGATCGATCGGGCATGA	0	11	RDR2-sensitive
GGCATTAGAACACGGGCTGAAGAC	0	10	RDR2-sensitive
GGCATTTGCTGGGCTGTTGTGGGC	66	0	RDR2-resistant
GGCCAACCTCGGACGGGAGACGCA	0	11	RDR2-sensitive
GGCCAACTGAAGGAGAACGAGCAA	0	10	RDR2-sensitive
GGCCACCTAGGGAACCGTCGGACA	0	13	RDR2-sensitive
GGCCACTCGGAAACATTCTGCGGC	2	14	RDR2-sensitive
GGCCAGGACTGGAGTTCGTCGGCA	0	9	RDR2-sensitive
GGCCAGGAGGAGGTAGCGCGGGAC	25	1	RDR2-resistant
GGCCAGGGACGGGGTAGAGGGCAG	44	0	RDR2-resistant
GGCCAGGTTCGGTACGAGAGTCGT	0	22	RDR2-sensitive
GGCCAGTCTGACAGAAGGACAGCG	5	34	RDR2-sensitive
GGCCATGAGTTGGAGACGGAACAA	0	11	RDR2-sensitive
GGCCCAATTGTAGTCGGACGGCGA	1	33	RDR2-sensitive
GGCCCACAGGCGCGGACTGTCCGG	2	66	RDR2-sensitive
GGCCCAGGATGATGAGATAGACGT	0	13	RDR2-sensitive
GGCCCAGTTGGTCTCTATCGTTGG	2	15	RDR2-sensitive
GGCCCATAGGCGCGGACTGTCCGG	0	15	RDR2-sensitive
GGCCCATGTAACGCACGTTCGGAC	0	13	RDR2-sensitive
GGCCCGAACCAACAGTAGAACGGT	1	64	RDR2-sensitive
GGCCCGACGGTCTGAAAATTGGAG	0	10	RDR2-sensitive

GGCCCGAGACCGCATAGAACCGGA	0	13	RDR2-sensitive
GGCCCGCAGCTGGTCTAAACGGAC	0	20	RDR2-sensitive
GGCCCGCGACAGTAGATCGGACGG	0	25	RDR2-sensitive
GGCCCGGACCAAGCTGAAGAACAG	0	28	RDR2-sensitive
GGCCCGGACTGCGTGCTGCATGGC	0	17	RDR2-sensitive
GGCCCGGCCTGTCAGATACGGTTA	0	9	RDR2-sensitive
GGCCCGTGGGCCGTCGGACGACAG	37	1	RDR2-resistant
GGCCCGTTGGACCCCTATCACGGC	1	16	RDR2-sensitive
GGCCCTATGCGTGGATCGTCCGGC	0	9	RDR2-sensitive
GGCCCTGTGTTGCGGATCGTTCGT	0	24	RDR2-sensitive
GGCCGAAGCCGGCTTCCTCTGGAC	0	9	RDR2-sensitive
GGCCGAATTGGTTCTGTTGGGCTG	0	15	RDR2-sensitive
GGCCGACACGTAGAGGGCGCTGTA	0	15	RDR2-sensitive
GGCCGACCGTTGGACAGGCGCGCG	0	10	RDR2-sensitive
GGCCGACCGTTGGCTCACCGGACA	19	73	RDR2-sensitive
GGCCGACCTGGACTGAGCCCGGCT	0	10	RDR2-sensitive
GGCCGAGACGAACTTCAGAAGCGA	1	21	RDR2-sensitive
GGCCGCAATTTGAGCATCGTTGGA	27	3	RDR2-resistant
GGCCGCACTGTGGGATGACGTCGT	0	10	RDR2-sensitive
GGCCGCCAAGGCCATCGATCGGCA	18	0	RDR2-resistant
GGCCGCCTAGGGAACTGTCGGACA	0	10	RDR2-sensitive
GGCCGCTCGGATCTACAAAGGCAT	0	16	RDR2-sensitive
GGCCGGTCTGGACCAGTTGAACGG	0	21	RDR2-sensitive
GGCCGTTGGATGGAAAATGGACGG	0	33	RDR2-sensitive
GGCCTAAAGCACGGACTGTTCGGT	0	16	RDR2-sensitive
GGCCTACAGGCGCGGACTGTCCGG	0	9	RDR2-sensitive
GGCCTAGACTTGTGAGAGGACTGA	0	9	RDR2-sensitive
GGCCTAGAGTCGGACGATCCACGA	0	13	RDR2-sensitive
GGCCTAGGACCGCATAGAACCGGA	0	14	RDR2-sensitive
GGCCTATTCAGACGGACACCTCAC	0	9	RDR2-sensitive
GGCCTATTTCTGTAGTCTATCGGT	28	0	RDR2-resistant
GGCCTCCGGACGCGATGAGGACGT	0	15	RDR2-sensitive
GGCCTCGGATTTTACTGGCACGGC	0	20	RDR2-sensitive
GGCCTCGGGCGAGACGGAAATCCT	0	21	RDR2-sensitive
GGCCTCGGGCGAGACGGAGATCCT	0	13	RDR2-sensitive
GGCCTCGTACAGAGCCGTCGGAGA	1	14	RDR2-sensitive
GGCCTCGTACAGATCCGTCGGAGA	0	11	RDR2-sensitive
GGCCTGCATCTGGTCGGACGGTCC	0	9	RDR2-sensitive
GGCCTGCCGGGACTGAGGAGATGC	2	26	RDR2-sensitive
GGCCTGGGTGGGACGGTTGGCTCG	1	12	RDR2-sensitive
GGCCTGGTTGTGGACTGAGGGCAT	2	21	RDR2-sensitive
GGCCTTAACGGGCTGTGGGCCGGC	3	28	RDR2-sensitive
GGCCTTAGTTGACGGTAGGATCGA	0	50	RDR2-sensitive
GGCCTTGGGACTGAACGAGGACGC	4	81	RDR2-sensitive
GGCGAACAATGACACTCGGCACAG	0	10	RDR2-sensitive
GGCGAACCGCGGAACAGGATTCGG	0	47	RDR2-sensitive
GGCGAACCTGGATCGAGCGGACGC	0	13	RDR2-sensitive
GGCGAACTGAAGATTGGGAGCGAC	0	11	RDR2-sensitive
GGCGAACTGTACATCGACAACGGC	0	12	RDR2-sensitive

GGCGAACTGTACATCGGCAACAGC	6	73	RDR2-sensitive
GGCGAACTGTACATCGGCAACGGC	0	11	RDR2-sensitive
GGCGAAGCCGCGACATAGAGCAAT	21	2	RDR2-resistant
GGCGAATCTGGTAGTAGACAGCGG	0	9	RDR2-sensitive
GGCGACACGGATGGTACGCGGCTC	0	9	RDR2-sensitive
GGCGACATACAACACTGACGGCGA	0	10	RDR2-sensitive
GGCGACATGATGGAGCAGAGCGTG	0	14	RDR2-sensitive
GGCGACATTCACTCGTCGGCTCAG	0	30	RDR2-sensitive
GGCGACCGACGAACCGCAAGGACG	0	9	RDR2-sensitive
GGCGACCGAGAACGACATAGACGC	0	15	RDR2-sensitive
GGCGACCGAGCTCTGGCCACAGAA	0	9	RDR2-sensitive
GGCGACCGTAGAAAGTTAGCGGCT	0	23	RDR2-sensitive
GGCGACCTTGCGGAGCGACTGGCT	2	36	RDR2-sensitive
GGCGACGAATGGAAGGACACGGAC	0	10	RDR2-sensitive
GGCGACGACAGTAGAGGAAGACGC	0	11	RDR2-sensitive
GGCGACGACGGACCGTGGCTACGA	0	22	RDR2-sensitive
GGCGACGAGGAAGAAGAAGGACAC	0	18	RDR2-sensitive
GGCGACGAGTAAGTAGAAGGACAC	0	34	RDR2-sensitive
GGCGACGATGGGAACAGGGACCGC	0	12	RDR2-sensitive
GGCGACGCGGAGAAACTTCGGCAC	0	13	RDR2-sensitive
GGCGACGCGGCCTCGTAGCTCGGC	1	22	RDR2-sensitive
GGCGACGCTGGACGCGGGAGCTGA	0	11	RDR2-sensitive
GGCGACGGATACGGAAGAGGACAG	0	10	RDR2-sensitive
GGCGACGGTAGACGGCAGAACCAA	0	34	RDR2-sensitive
GGCGACGTACGGCTATAATTCACC	6	26	RDR2-sensitive
GGCGACGTAGACCAAGAGGGCTTC	6	70	RDR2-sensitive
GGCGACGTAGATCAAGAGGGCTTC	9	46	RDR2-sensitive
GGCGACGTGGACGAGTCGGACGCT	0	9	RDR2-sensitive
GGCGACGTTTGAGCAAGAGGACAC	0	16	RDR2-sensitive
GGCGACTGCACGGCACTGCTCGAC	4	30	RDR2-sensitive
GGCGACTGGTAAAGGAGACGACGC	0	13	RDR2-sensitive
GGCGACTGTGCGGTCTGCGGCGTC	0	9	RDR2-sensitive
GGCGAGAACTGCTGCCTGGGCGGT	24	2	RDR2-resistant
GGCGAGACCGTACCCATAATGCAA	0	11	RDR2-sensitive
GGCGAGACGAGCAGAGAACAGTGA	0	11	RDR2-sensitive
GGCGAGACGGTAACATCACGACGA	0	14	RDR2-sensitive
GGCGAGACGTGAATCCTCCGGGGT	0	9	RDR2-sensitive
GGCGAGAGCGGGACTGGAGGCGGG	0	11	RDR2-sensitive
GGCGAGAGTGGAGACGAGAGCTGA	0	10	RDR2-sensitive
GGCGAGAGTGGAGGCGAGAGCTGA	0	13	RDR2-sensitive
GGCGAGATGGCGAACGACGATCGC	0	12	RDR2-sensitive
GGCGAGATGGCGAACGACGATCGT	0	15	RDR2-sensitive
GGCGAGATGGCGAACGACGATTAC	0	11	RDR2-sensitive
GGCGAGCAACAGGTAGGACGACAC	1	24	RDR2-sensitive
GGCGAGCAGGAACTAAACGGACAT	0	23	RDR2-sensitive
GGCGAGCATGCAGAACCGTGCTGA	0	17	RDR2-sensitive
GGCGAGCGGCAGAGCAACGACGAA	0	13	RDR2-sensitive
GGCGAGGACGAGTAGGGATGGCAA	0	9	RDR2-sensitive
GGCGAGGATCATGGTCGAGGACGA	0	9	RDR2-sensitive

GGCGAGGGCTGACACGGCGCTCAC	0	14	RDR2-sensitive
GGCGAGTGTAGAGAAGGACGGTAC	0	13	RDR2-sensitive
GGCGATAACGGCGAGCTAGAGCAT	0	9	RDR2-sensitive
GGCGATAACGGCGAGCTGGAGCGT	1	15	RDR2-sensitive
GGCGATCCAAGTCGGAGACAGCTC	0	10	RDR2-sensitive
GGCGATCCACGACCTAGAACCGGA	0	10	RDR2-sensitive
GGCGATCCGACATGGGCTAGACGC	0	14	RDR2-sensitive
GGCGATCGAGTCGAACTAGTACGG	2	47	RDR2-sensitive
GGCGATGGAACTGTGGGACGCGGT	0	19	RDR2-sensitive
GGCGATGGTGGGCTTTAGACGGAC	0	24	RDR2-sensitive
GGCGATGTAGTTCTCCGGCGGCGT	2	20	RDR2-sensitive
GGCGCAATCTGTACAATCGTCGTG	0	10	RDR2-sensitive
GGCGCAATCTGTACAGTCGTCGGG	5	26	RDR2-sensitive
GGCGCACCGGAATCGATCGAGACA	17	0	RDR2-resistant
GGCGCACTGTAGGGATAGAAGATG	0	22	RDR2-sensitive
GGCGCAGAAGCGGATTCTTCTCTG	0	9	RDR2-sensitive
GGCGCAGACTGTCCGGACCGCGGA	0	10	RDR2-sensitive
GGCGCATGTTCGTTCTGTCTGGAC	2	14	RDR2-sensitive
GGCGCATTGGCGGAATGTGGCGGC	0	9	RDR2-sensitive
GGCGCCGAGCTCTGACGTGGCGAC	0	11	RDR2-sensitive
GGCGCGAACACCAGTCGGAGACGT	0	12	RDR2-sensitive
GGCGCGACGGAACCGAACAGGCGT	1	14	RDR2-sensitive
GGCGCGACGTGACGACTTAGGCAT	0	42	RDR2-sensitive
GGCGCGACTCGCGAGCGAGGATGG	0	12	RDR2-sensitive
GGCGCGACTGCAAGAGCCACGGCT	0	12	RDR2-sensitive
GGCGCGATATGGCAGAACCGTGCG	0	10	RDR2-sensitive
GGCGCGCGGATTGATCGGGCGGGG	0	11	RDR2-sensitive
GGCGCGGCGTGGGAACAACGGCGA	0	15	RDR2-sensitive
GGCGCGTTGACGGCAGAACCGTGC	0	11	RDR2-sensitive
GGCGCGTTGAGGACGTGTCTGAAC	0	52	RDR2-sensitive
GGCGCGTTTGGCTGGTCTGTTGGA	0	17	RDR2-sensitive
GGCGCTCGATCGGAGGAAGGCTAC	0	9	RDR2-sensitive
GGCGCTCGGCACGGGCTAGACGCA	0	9	RDR2-sensitive
GGCGCTCGGTCGGCTGAAGGCGTT	0	10	RDR2-sensitive
GGCGCTCGTTACTGTACTTGGCAT	0	9	RDR2-sensitive
GGCGCTCTGGCTGGCTCGACTGCT	64	11	RDR2-resistant
GGCGCTGTTTTCCTGTCAGGTCGG	0	9	RDR2-sensitive
GGCGGAAACAGTAGTCCCGGACGG	0	10	RDR2-sensitive
GGCGGAACAATCGCAGAACGAGGA	0	19	RDR2-sensitive
GGCGGAACGACGGACCAGATCCCT	0	10	RDR2-sensitive
GGCGGAAGAAGGCGAACTCGGCTC	0	9	RDR2-sensitive
GGCGGACAGAGTAGAAGAGAGGAC	0	23	RDR2-sensitive
GGCGGACCATTCACGCGCATGTCG	1	16	RDR2-sensitive
GGCGGACGCGACAGATCAATGCTG	1	22	RDR2-sensitive
GGCGGAGATACTCGGCAGAACCGT	0	18	RDR2-sensitive
GGCGGAGCCTGACAGAGGGACAGC	15	0	RDR2-resistant
GGCGGAGCTCGGCGTAAAGATCTG	0	9	RDR2-sensitive
GGCGGAGTCTGATAGGAGGGTCGA	0	41	RDR2-sensitive
GGCGGATCAGTAGAGGATGTCAGA	0	9	RDR2-sensitive

GGCGGATCTGACGGTGTGCCTCAC	0	23	RDR2-sensitive
GGCGGATTCCTGTGGCGCGACGGC	1	13	RDR2-sensitive
GGCGGATTGCGCGAGACAGTGTAA	0	15	RDR2-sensitive
GGCGGCAGCAAGGACGGTGACGGC	15	0	RDR2-resistant
GGCGGCAGGATAGAAACGCGCGGT	0	13	RDR2-sensitive
GGCGGCAGGGACAGGCTGATGCTC	0	14	RDR2-sensitive
GGCGGCATGCAGGACAGACGACGC	0	27	RDR2-sensitive
GGCGGCATGCAGGACAGACGGCGC	2	62	RDR2-sensitive
GGCGGCGTGTGGAAAGGGACTGGC	0	9	RDR2-sensitive
GGCGGCTGAGATGAACGCGCGGCT	0	12	RDR2-sensitive
GGCGGGAGCTGAGAGATGACGCGA	0	13	RDR2-sensitive
GGCGGGCCTAGAATACAGACAGGG	0	27	RDR2-sensitive
GGCGGGCCTGGAGAAGAATAGGGT	1	16	RDR2-sensitive
GGCGGGCGCGGCAGAACGTGCGAG	0	9	RDR2-sensitive
GGCGGGTTCCGTAGATCCATCGGC	0	9	RDR2-sensitive
GGCGGTACGGTATGAGACGAGCTA	0	21	RDR2-sensitive
GGCGTACACTGTTAATGGAGTCTT	0	9	RDR2-sensitive
GGCGTAGACGAGGATGACGATCGA	1	12	RDR2-sensitive
GGCGTAGCGGGCCCGCAGAACCGG	0	10	RDR2-sensitive
GGCGTAGGGTGTAGGTGGACGTGC	0	11	RDR2-sensitive
GGCGTAGTAGCTGGTTGCACGGAC	0	17	RDR2-sensitive
GGCGTCGCTGTGCGTTACCTCGGC	0	16	RDR2-sensitive
GGCGTCTCGAGACATACTGACGGA	0	34	RDR2-sensitive
GGCGTCTGTACACGGGAACGGTAA	29	0	RDR2-resistant
GGCGTGAATTCTGTGGACGCGGAC	0	13	RDR2-sensitive
GGCGTGACTCGGCAAGAGCTGGCC	1	37	RDR2-sensitive
GGCGTGACTCGGCGAGAGCTGGCC	6	63	RDR2-sensitive
GGCGTGCCGGGCTACTTGGGCCGT	0	14	RDR2-sensitive
GGCGTGCGCTGTTAGGAGCCGGCG	0	10	RDR2-sensitive
GGCGTGCGTCAGGATAAAGGTGTC	0	9	RDR2-sensitive
GGCGTGGACTGTCCGGACCGCGGA	0	14	RDR2-sensitive
GGCGTGTAGAGGACGTGTCTGAAC	0	16	RDR2-sensitive
GGCGTGTATAGCTGTTCTGTTGGA	0	13	RDR2-sensitive
GGCGTGTCGGGCTGCACGACGGGT	0	9	RDR2-sensitive
GGCGTGTCTCTGGCGGAAGAGCTC	1	25	RDR2-sensitive
GGCGTTCGGTAGAGCGGGGGCAGT	0	18	RDR2-sensitive
GGCGTTGTCGGCAGCATAGGCATG	0	19	RDR2-sensitive
GGCGTTTCGCGCGGGCGTTCGGTA	0	15	RDR2-sensitive
GGCGTTTCTTAGAACAGTCGGTGC	2	15	RDR2-sensitive
GGCGTTTCTTCGGTTCGAGGACGG	0	9	RDR2-sensitive
GGCGTTTTAAGTCGGACGGTCCGC	0	11	RDR2-sensitive
GGCTAACCGGACCGCCTGTACTGA	0	11	RDR2-sensitive
GGCTAATGGACTGTATCGGGGCAG	0	18	RDR2-sensitive
GGCTACACGACGGCTCGGAACCAA	0	9	RDR2-sensitive
GGCTACCCTGACAGCCGTCGGACA	0	9	RDR2-sensitive
GGCTACCGTCGGAACAGTCGGAAA	0	16	RDR2-sensitive
GGCTACCGTCGGAACAGTCGGAGA	2	26	RDR2-sensitive
GGCTACGGGTGGTAGAACGCGGCA	0	13	RDR2-sensitive
GGCTACGTAGATCAAGAGGGCTTC	1	13	RDR2-sensitive

GGCTACTCTGTTGGATGGAAGATG	1	41	RDR2-sensitive
GGCTACTGTAGTTCACGGGCCGGC	1	32	RDR2-sensitive
GGCTAGAACTGATCCGGCTCTGGA	0	23	RDR2-sensitive
GGCTAGAAGATGGCACCGGACTGT	0	14	RDR2-sensitive
GGCTAGACACAGTAGAAGATCCGT	0	30	RDR2-sensitive
GGCTAGACTGGAGACGACAACAGA	0	12	RDR2-sensitive
GGCTAGACTGGAGGCGACGACGGA	1	46	RDR2-sensitive
GGCTAGACTGGAGGCGACGACGGT	1	40	RDR2-sensitive
GGCTAGAGCGTTGGTTGAAGGCAT	0	14	RDR2-sensitive
GGCTAGGAACAACAACGACGGTAT	0	11	RDR2-sensitive
GGCTAGGGCTTCTCTGCCTGACGG	3	32	RDR2-sensitive
GGCTAGGGTTTAGAATGAGGGCGG	0	10	RDR2-sensitive
GGCTAGGTTCGTTGGCTCGGCGAA	4	24	RDR2-sensitive
GGCTATATTCGTCGGTAAACCCAC	0	37	RDR2-sensitive
GGCTATATTCGTCGGTACACCCAC	0	17	RDR2-sensitive
GGCTATCTTTGCTCTATTTGTCGG	1	50	RDR2-sensitive
GGCTATTGACGTCGACTTTATGGA	72	3	RDR2-resistant
GGCTATTTTCGTCGGCCGGCCCAC	3	25	RDR2-sensitive
GGCTCAATTGTAGTCGGACGGTGA	0	15	RDR2-sensitive
GGCTCAATTGTAGTTGGACGGCGA	0	9	RDR2-sensitive
GGCTCAGATCGGACTGGTCCGGCC	0	20	RDR2-sensitive
GGCTCAGGATCTAGAAAGTGACGG	3	20	RDR2-sensitive
GGCTCCACGGCTGTGCGTCTGGCA	0	11	RDR2-sensitive
GGCTCCATAGGATCTGAAGCGGTA	0	10	RDR2-sensitive
GGCTCCCGGACTGGACCAGGCAAC	0	103	RDR2-sensitive
GGCTCCTCGGACGACGGCTGTGCT	0	9	RDR2-sensitive
GGCTCGCGAGGCATACCCTGATAT	0	9	RDR2-sensitive
GGCTCGGATAAGGTAGAGGGACGT	0	13	RDR2-sensitive
GGCTCGGATAGGAACTAAGCACGG	0	15	RDR2-sensitive
GGCTCGGATGACGTGTTTCACTAG	0	12	RDR2-sensitive
GGCTCGGCTCGGACTCGTTCTGAA	0	11	RDR2-sensitive
GGCTCGGCTCGGCTCGGAGCGGCT	0	10	RDR2-sensitive
GGCTCGGGGTAGAGTGATGATGGC	0	9	RDR2-sensitive
GGCTCGTGGCTCGTGGTAGATCGG	0	10	RDR2-sensitive
GGCTCGTGGGCTACAGTAGCCGGT	0	10	RDR2-sensitive
GGCTCGTTGGATCACCGAAGCGCA	0	13	RDR2-sensitive
GGCTCTACGTTGTACATGTACTAA	21	1	RDR2-resistant
GGCTCTATCGTCTTTTCGTCTGTC	0	15	RDR2-sensitive
GGCTCTCCGGACTGGACCAGGCAA	0	27	RDR2-sensitive
GGCTCTCGGCAAAGACTGACGACC	1	20	RDR2-sensitive
GGCTCTCGGCAAAGACTGACGGAC	0	14	RDR2-sensitive
GGCTCTCGGCAAAGACTGACGGCC	2	17	RDR2-sensitive
GGCTCTCGGCAAAGACTGACGGTC	8	34	RDR2-sensitive
GGCTCTCGGGAGATGGAGGACAGT	180	10	RDR2-resistant
GGCTCTCGGTAAAGTCGGCTTTGC	0	11	RDR2-sensitive
GGCTCTGGACTGCAGACAGGCGAC	1	19	RDR2-sensitive
GGCTCTGTCGACTGAAGGGCGCGC	5	26	RDR2-sensitive
GGCTCTGTCGACTGGAGGACGTGC	1	13	RDR2-sensitive
GGCTCTGTCGACTGGAGGGCGCGC	0	9	RDR2-sensitive

GGCTCTGTCGACTGGAGGGCGTGC	0	29	RDR2-sensitive
GGCTCTTGATCGCAAAGGACACAC	0	9	RDR2-sensitive
GGCTCTTGCAAGCATTAGATCGTT	1	23	RDR2-sensitive
GGCTCTTGCAAGCATTAGGTCGTT	6	51	RDR2-sensitive
GGCTCTTTGAGTTGGATCTGTTGG	0	18	RDR2-sensitive
GGCTCTTTGGACCGGTAGAAACGA	0	10	RDR2-sensitive
GGCTGAAAAGCTGAGCGTTTGGCA	3	37	RDR2-sensitive
GGCTGAAAAGCTGATTGTTTGGCA	0	11	RDR2-sensitive
GGCTGAACTGTGCTGAAGAGCTGC	0	10	RDR2-sensitive
GGCTGACAGGACTGTCCGGTGTGC	0	9	RDR2-sensitive
GGCTGACATCTAGGACCACGACGG	1	53	RDR2-sensitive
GGCTGACCTGAACCGACCTGACGC	0	18	RDR2-sensitive
GGCTGACCTGATAGGCTGATACGG	0	37	RDR2-sensitive
GGCTGACGGCGAGTGGACGGACGA	0	13	RDR2-sensitive
GGCTGAGAAGGAGGCTGTAGCGGC	1	16	RDR2-sensitive
GGCTGAGATCGCAGAACGGCAGAA	0	13	RDR2-sensitive
GGCTGAGATCGCAGAATGGCAGAA	1	34	RDR2-sensitive
GGCTGAGATCGTAGAATGGCAGAA	0	12	RDR2-sensitive
GGCTGAGATTGTAGAACGGTAGAA	1	29	RDR2-sensitive
GGCTGAGCGTTGGTCAAATGCATG	26	1	RDR2-resistant
GGCTGAGTCAGACGTGTGGTAGAA	0	21	RDR2-sensitive
GGCTGATCAGAGGATGGCGGAGAT	0	29	RDR2-sensitive
GGCTGATCGATGAAGACGTGCGCT	0	11	RDR2-sensitive
GGCTGATGAAGGCGCTGTGGAGAA	1	32	RDR2-sensitive
GGCTGCATGAGCTAGATCGGCGGA	0	9	RDR2-sensitive
GGCTGCATGAGTCAGGTCGGCGGG	0	29	RDR2-sensitive
GGCTGCCACGTCAGCATAGTCGGC	0	13	RDR2-sensitive
GGCTGCCTAGGAGACCGTCGGACA	0	11	RDR2-sensitive
GGCTGCGGACACCGTTGAAGACAG	0	21	RDR2-sensitive
GGCTGCGGACTGCGGTGCGGCTCC	0	37	RDR2-sensitive
GGCTGCGGCATGTGGATCGATCGG	0	23	RDR2-sensitive
GGCTGGAACCTGGAAGCGGAGCTC	0	9	RDR2-sensitive
GGCTGGACAGTAGCACAAGCGCAC	0	13	RDR2-sensitive
GGCTGGACATCGGAACACAGACGG	0	11	RDR2-sensitive
GGCTGGACCCGGCACGACTTTGGC	0	60	RDR2-sensitive
GGCTGGACCCGGCACGACTTTGGT	0	24	RDR2-sensitive
GGCTGGACGACGGCTTAACAACTT	1	13	RDR2-sensitive
GGCTGGACTTGGACCGAGCTAGAC	0	9	RDR2-sensitive
GGCTGGAGCTGGGCTGTCGCACGG	0	9	RDR2-sensitive
GGCTGGAGTAGAAATGGACGTCAT	1	15	RDR2-sensitive
GGCTGGAGTAGGAATGGACGTCAT	2	29	RDR2-sensitive
GGCTGGATCGGCGAGATGGCGAAC	0	19	RDR2-sensitive
GGCTGGATCTCAGTGGATCGTGGC	53	17	RDR2-resistant
GGCTGGCGGCTGGAAGCTGGAGGC	0	58	RDR2-sensitive
GGCTGGCGTGTAGAAGGCAGGCAC	0	9	RDR2-sensitive
GGCTGGGAGGAGGGCCGAGGGCGG	25	0	RDR2-resistant
GGCTGGGCGCTCGGCTTAGGCATC	22	0	RDR2-resistant
GGCTGGGCTGTCTGTTCGATGGCG	4	32	RDR2-sensitive
GGCTGGTCTGAACGGCAAATGTAG	0	16	RDR2-sensitive

GGCTGTCCGTCGATGGCAGAACGG	0	14	RDR2-sensitive
GGCTGTGAGCTGTGGAAAAAGCAG	0	20	RDR2-sensitive
GGCTGTGCTGTTGGAAAGATGTAG	0	13	RDR2-sensitive
GGCTGTGGACTGCAATCTCCGGCT	236	14	RDR2-resistant
GGCTTATTTTCGGCGGCTTCTGGC	0	9	RDR2-sensitive
GGCTTCAATATGATCGTTGCAGGA	0	36	RDR2-sensitive
GGCTTCAGCTCTGGCAGTAGACCT	0	15	RDR2-sensitive
GGCTTCGGACCAGGCTTCATTCCC	78	2	RDR2-resistant
GGCTTCGTCTGAGAGGCGTCGGAG	1	13	RDR2-sensitive
GGCTTCTCTGCCTGACGATCGGAC	1	13	RDR2-sensitive
GGCTTCTTCGTCATCGTCTGCAGA	23	0	RDR2-resistant
GGCTTGAACTGCCTGAGTCTGCAT	1	16	RDR2-sensitive
GGCTTGAGCTGTGTCGGATCGGAC	0	11	RDR2-sensitive
GGCTTGGACCAGATCGGACCGGAC	0	18	RDR2-sensitive
GGCTTGGACGGAACACCGGGCGGC	0	9	RDR2-sensitive
GGCTTGGACGGAACACGGGGCGGC	0	15	RDR2-sensitive
GGCTTGGACTATGATCAGGACGAG	0	12	RDR2-sensitive
GGCTTGGACTTGGACCGACACGGT	0	9	RDR2-sensitive
GGCTTGGCCCTGAAGACTGGACGG	0	20	RDR2-sensitive
GGCTTGGCGTAGAGAAGACTCGGC	0	28	RDR2-sensitive
GGCTTGGGCGTAGACCGGCACTGC	0	18	RDR2-sensitive
GGCTTTAGAGCATCGGATCGACGG	0	10	RDR2-sensitive
GGCTTTATCGTGCCTATGGACTAA	0	15	RDR2-sensitive
GGCTTTGTGCGGAAGCGACCTGAG	0	18	RDR2-sensitive
GGCTTTTCGGACTAGCACGAGCAT	0	77	RDR2-sensitive
GGCTTTTCGGGCTAGCACGAGCAC	0	24	RDR2-sensitive
GGCTTTTGTCACTGTAAGGGGGAG	17	0	RDR2-resistant
GGCTTTTTATCGGACACTCGGCAA	3	20	RDR2-sensitive
GGGAAAAATGTAGAGGGGACCGCT	0	11	RDR2-sensitive
GGGAAACTGTAGCCCCTATACGGT	0	24	RDR2-sensitive
GGGAAACTGTTGGCGAATGGCGTC	0	9	RDR2-sensitive
GGGAAAGAGAACGCTGACGTGGCA	0	15	RDR2-sensitive
GGGAACACTGGCAGAACACGGAAT	0	28	RDR2-sensitive
GGGAACCTGGAGGACTGGATCGGC	0	15	RDR2-sensitive
GGGAACCTGTCTGTTCGATGGCGC	5	42	RDR2-sensitive
GGGAACGACAGAACACGGAATGAC	0	10	RDR2-sensitive
GGGAACGAGAATGTTGGACGGAAA	0	23	RDR2-sensitive
GGGAACGAGGAACGAGGAACACGG	1	31	RDR2-sensitive
GGGAACGAGGAACGGGAACGAGGA	0	13	RDR2-sensitive
GGGAACTGTAGACGATACGGTGAC	0	15	RDR2-sensitive
GGGAACTGTGGACGCCTACGGTAG	0	37	RDR2-sensitive
GGGAACTTGAGGAGCTGATGCGGC	47	3	RDR2-resistant
GGGAAGAAGAACAGCAGCACGCGT	3	29	RDR2-sensitive
GGGAAGACGGCAGAACCGTGCACC	0	20	RDR2-sensitive
GGGAAGAGAACGCTGACGTGGCAC	0	16	RDR2-sensitive
GGGAAGAGGCGGACATACCTGTTC	0	9	RDR2-sensitive
GGGAAGATCTGTGGAATAGGGGCA	0	17	RDR2-sensitive
GGGAAGCTGTTAGAGCAGAAGCAA	0	51	RDR2-sensitive
GGGAAGCTGTTGGAGCAGAAGCAA	0	25	RDR2-sensitive

GGGAAGGCTGTAGACAGGTCGTCA	0	13	RDR2-sensitive
GGGAATCGGAAAGGCAGAACACGG	0	21	RDR2-sensitive
GGGAATGACAGAACACGGAATGGC	0	11	RDR2-sensitive
GGGAATGACAGAACGCGGAATGGC	0	24	RDR2-sensitive
GGGAATGAGAATGGCAGAACCAGG	0	11	RDR2-sensitive
GGGAATGGACTGTACGCAGGGAGG	0	12	RDR2-sensitive
GGGAATGGCAGAACAACACGGAAT	0	12	RDR2-sensitive
GGGAATGGCAGAACACGGAATGAC	1	28	RDR2-sensitive
GGGAATGGCAGAACACGGAATGAG	0	22	RDR2-sensitive
GGGAATGGCAGAACACGGAATGGC	1	83	RDR2-sensitive
GGGAATGGCAGAACACGGAATGGG	0	28	RDR2-sensitive
GGGAATGGCAGAACAGAACACGGA	0	17	RDR2-sensitive
GGGAATGGCAGAACATGGAATGGC	0	23	RDR2-sensitive
GGGAATGGGAATGACAGAACACGG	1	25	RDR2-sensitive
GGGAATGGGAATGGCAGAACACGG	0	13	RDR2-sensitive
GGGAATTGGACTGGGTCGAGACGG	0	11	RDR2-sensitive
GGGACAAAGTCGGGTACATCTCAT	0	25	RDR2-sensitive
GGGACAACAGAGGTCAGAGGACGA	0	12	RDR2-sensitive
GGGACAACAGGGGACAACACTAGA	1	14	RDR2-sensitive
GGGACAACAGGGGACAACACTGGA	0	16	RDR2-sensitive
GGGACAACTTAGGACAGACAGGAC	0	16	RDR2-sensitive
GGGACAATCAAGATAGGGACGTCA	0	9	RDR2-sensitive
GGGACAATCTGTACAGTCGTCGGA	0	11	RDR2-sensitive
GGGACACACATAATGAGACGCTAT	0	13	RDR2-sensitive
GGGACACACGATACTACGGACGGT	0	16	RDR2-sensitive
GGGACACATGTAGATACGGGGAAG	0	9	RDR2-sensitive
GGGACACCAATAGAGTAGAGACGA	0	10	RDR2-sensitive
GGGACACCCTTCGCAGGACTCGGC	11	306	RDR2-sensitive
GGGACACGAGCAGACGTAGGCTTT	0	14	RDR2-sensitive
GGGACACGCAGCGACAATTTACTC	1	18	RDR2-sensitive
GGGACACTCGACACAGAGGAACAC	0	12	RDR2-sensitive
GGGACACTCGGCACACAGGAACAC	0	10	RDR2-sensitive
GGGACACTGTTCACTCCTAGAGGC	0	10	RDR2-sensitive
GGGACAGAAGGAGTACGCGACAAT	44	0	RDR2-resistant
GGGACAGCACGATCTGACGGAAGC	0	9	RDR2-sensitive
GGGACAGCCAGGACAGGGACGTCT	0	13	RDR2-sensitive
GGGACAGCGACCAGCACGACGGAC	0	10	RDR2-sensitive
GGGACAGCGACCAGCACGATGGAC	0	29	RDR2-sensitive
GGGACAGCGTGGACATATTAACGG	0	10	RDR2-sensitive
GGGACAGCTGAAGACTGACAGGAT	0	9	RDR2-sensitive
GGGACAGTCAGGACAGGACGTCAT	0	16	RDR2-sensitive
GGGACATATCATCAGAGTGGGGCT	1	12	RDR2-sensitive
GGGACATGGAGAGGACGACGGGCA	0	12	RDR2-sensitive
GGGACATGTATAACGAGAGGCTAC	0	11	RDR2-sensitive
GGGACATGTGTATCTCACGGCAAG	0	13	RDR2-sensitive
GGGACCAATGTGAAGACGGACAGG	0	16	RDR2-sensitive
GGGACCAGTGGCATACAAGGAATG	0	41	RDR2-sensitive
GGGACCCCCTAGATCGACGTGGAA	0	9	RDR2-sensitive
GGGACCCGCCTTAAGCCGTCGGAA	0	16	RDR2-sensitive

GGGACCCGTCTTAAGCCGTCGGAA	0	9	RDR2-sensitive
GGGACCGACACGTAGAGGGCGCTG	2	16	RDR2-sensitive
GGGACCGACACGTAGGAGGCGCTG	0	13	RDR2-sensitive
GGGACCGACACGTAGGGGACGCTG	1	20	RDR2-sensitive
GGGACCGACACGTAGGGGGCGCTG	5	24	RDR2-sensitive
GGGACCGCAGTGCCGGACTCGGAC	0	11	RDR2-sensitive
GGGACCGGATACGGATAGTTAGAA	1	22	RDR2-sensitive
GGGACCGTAGCTCCATGCAGTCTG	0	16	RDR2-sensitive
GGGACCTAGATGTGCTACTCGGAA	0	22	RDR2-sensitive
GGGACCTTCGGCGATGAAGCAGGC	0	11	RDR2-sensitive
GGGACGAAATAGACTGGCTTCGAA	0	9	RDR2-sensitive
GGGACGAACACGAAGGCCGCGGGA	12	54	RDR2-sensitive
GGGACGAACGAGCGAGTGCGGTAG	0	28	RDR2-sensitive
GGGACGAAGACGGACGAAGACGAC	0	53	RDR2-sensitive
GGGACGAAGGGAACTAAAAGGCGA	0	18	RDR2-sensitive
GGGACGAAGGGAACTAAAAGGCGG	3	78	RDR2-sensitive
GGGACGACAGAACAGAGAAGCGAC	0	9	RDR2-sensitive
GGGACGAGGAATAGGATACCATAC	1	17	RDR2-sensitive
GGGACGAGGCATCTGAAGCAGGAG	0	10	RDR2-sensitive
GGGACGAGTGAATATAGGGGATGA	0	17	RDR2-sensitive
GGGACGAGTGAATATAGGGGATGG	0	47	RDR2-sensitive
GGGACGATGCAGGAACGACGCTGC	0	9	RDR2-sensitive
GGGACGCAAGCTGTATGAGCGGAT	0	9	RDR2-sensitive
GGGACGCAGATGAGGGATTTGATT	20	0	RDR2-resistant
GGGACGCATGCTGGTACGGGACGT	1	15	RDR2-sensitive
GGGACGCCTACAGAGCATGACGAC	1	20	RDR2-sensitive
GGGACGCGCGGTGATGGCTCGAAT	17	0	RDR2-resistant
GGGACGCGTAATACGACAGGACGC	2	24	RDR2-sensitive
GGGACGCTGTAGAGGGCACCGCTG	0	11	RDR2-sensitive
GGGACGCTGTAGGGGCACCGCTGC	0	30	RDR2-sensitive
GGGACGCTTGGGAATGGGAAACGG	0	12	RDR2-sensitive
GGGACGGATACAGAACGGGACCGG	0	9	RDR2-sensitive
GGGACGGATACGGACGGATACTAG	0	15	RDR2-sensitive
GGGACGGTTGGCTCGGACCTGGCA	4	29	RDR2-sensitive
GGGACGTGAGCCTGGCTGACGACG	3	25	RDR2-sensitive
GGGACGTGAGCTTGGCTGACGACG	0	9	RDR2-sensitive
GGGACGTGATTGGTTGCCGGATAA	0	14	RDR2-sensitive
GGGACGTGGTAATGTAGACGACAC	0	11	RDR2-sensitive
GGGACGTGTAATACGACAGGACGC	0	15	RDR2-sensitive
GGGACGTTACTGAGTGGACGGTTG	0	9	RDR2-sensitive
GGGACGTTCTGCCTGAAGTAGCAT	1	18	RDR2-sensitive
GGGACGTTCTGCTCGAATCGGTAT	0	24	RDR2-sensitive
GGGACGTTCTGTACGAACCGGTAT	0	16	RDR2-sensitive
GGGACTACATCCGGCGATTCTCGA	2	14	RDR2-sensitive
GGGACTAGACCTGAGTGCGTAGAC	1	16	RDR2-sensitive
GGGACTAGCAGACTTCGACGGAGC	1	18	RDR2-sensitive
GGGACTCAGCTGAGGCAGCCATGC	3	21	RDR2-sensitive
GGGACTCGACGACAGCGCAACACC	0	10	RDR2-sensitive
GGGACTCGGACACTCGGCAAAGGC	0	9	RDR2-sensitive

GGGACTCGGATCTAAGCACGATGC	0	12	RDR2-sensitive
GGGACTGAACGAGGACGCGCCGTG	0	11	RDR2-sensitive
GGGACTGATTGCATACTGGCGAAG	0	13	RDR2-sensitive
GGGACTGCACGACGTACATCTTCC	0	12	RDR2-sensitive
GGGACTGCACTCTAGAGGAGCTGG	0	11	RDR2-sensitive
GGGACTGCACTGCGTACACCTTCC	1	13	RDR2-sensitive
GGGACTGCACTGCGTACATCTGCC	4	22	RDR2-sensitive
GGGACTGCACTGCGTACATCTTCC	7	26	RDR2-sensitive
GGGACTGCACTGCGTACCCCTTCC	0	16	RDR2-sensitive
GGGACTGCGAGTGCGAGACGAGAG	0	13	RDR2-sensitive
GGGACTGCGGCAGCCTCACGGACG	0	10	RDR2-sensitive
GGGACTGGGCGTGCATACTGCATA	0	11	RDR2-sensitive
GGGACTGTAAACGTAGAGAGGCCC	0	13	RDR2-sensitive
GGGACTGTAGTATATATAGATGTA	21	2	RDR2-resistant
GGGACTTCGTGCCCGGTCAGAGGA	16	0	RDR2-resistant
GGGACTTGGCGATAACGACGAGCT	0	22	RDR2-sensitive
GGGACTTGGCGATAACGGCGAGCT	1	19	RDR2-sensitive
GGGACTTGGCGTAGACGAGGATGG	2	17	RDR2-sensitive
GGGACTTGTTTGGGACAGCTCCAA	0	10	RDR2-sensitive
GGGAGAATCTGACTGTGAGGAGAA	0	27	RDR2-sensitive
GGGAGAATCTGACTGTGTAGAGAA	0	10	RDR2-sensitive
GGGAGAATCTGGCTGTGAGAAGAA	0	17	RDR2-sensitive
GGGAGAATCTGGCTGTGTAGAGAA	9	33	RDR2-sensitive
GGGAGACGACGACCTAGACCACTC	0	9	RDR2-sensitive
GGGAGACGGATGCGGAGACTGCTG	1	24	RDR2-sensitive
GGGAGACGGGTACTCTACAACTTC	0	20	RDR2-sensitive
GGGAGACGGGTACTCTACAGCTTC	2	25	RDR2-sensitive
GGGAGACGGGTTCTCTACATCTTC	0	12	RDR2-sensitive
GGGAGACTGGTGAGCAACGGCAAA	0	18	RDR2-sensitive
GGGAGACTGTCTGTTCGATGGCGC	0	10	RDR2-sensitive
GGGAGAGCGGGCTGGACAGCGGCT	102	15	RDR2-resistant
GGGAGAGGCTCTGCACGGACGGGA	0	17	RDR2-sensitive
GGGAGATGGCAGAACAATGGTGCA	0	11	RDR2-sensitive
GGGAGCAAGTGGAATGAAGGGTAT	1	50	RDR2-sensitive
GGGAGCCCACTCTGCGACGCCGGT	0	11	RDR2-sensitive
GGGAGCGAGATGTAGACGGATTAA	0	37	RDR2-sensitive
GGGAGCTCATAGTAGATGCCTGGC	0	16	RDR2-sensitive
GGGAGCTCTCTGTTCGATGGCGCA	0	9	RDR2-sensitive
GGGAGCTGCTGGAGACGTCTACAG	0	23	RDR2-sensitive
GGGAGGACGGGCTGACGAACAGGC	0	16	RDR2-sensitive
GGGAGGACTGGGGATGACTGCGGC	1	15	RDR2-sensitive
GGGAGGAGGGCCGAGGGCGGGTGA	73	4	RDR2-resistant
GGGAGGCAGAACGGCAGAACGACA	0	9	RDR2-sensitive
GGGAGGCAGAACGGCAGAACGCAG	0	36	RDR2-sensitive
GGGAGGCAGAACGGCAGAACGGCA	0	16	RDR2-sensitive
GGGAGGCTCTGGTACCATGTAGAA	0	13	RDR2-sensitive
GGGAGGCTGTAGTGAGGCTTCATT	0	32	RDR2-sensitive
GGGAGGCTGTCTGCTCGATGGCGC	0	17	RDR2-sensitive
GGGAGGCTGTCTGTTCGATGGCGC	0	30	RDR2-sensitive

GGGAGGCTGTGCACTGGACTCGGC	0	10	RDR2-sensitive
GGGAGGTTACTGGAGGCTGACGGC	0	9	RDR2-sensitive
GGGAGGTTGACGGAGATACTAGAG	6	32	RDR2-sensitive
GGGAGTAGAGGACGACGAATGTGC	1	15	RDR2-sensitive
GGGAGTCTGGCTGTGCGCGTGCAA	0	10	RDR2-sensitive
GGGAGTGGAGGACGAGTCGATTGC	0	9	RDR2-sensitive
GGGAGTGGGAGGCAGAACCGTGCA	0	22	RDR2-sensitive
GGGAGTGTACAGCGCGATGGCAGA	0	9	RDR2-sensitive
GGGAGTGTGGGACCACCTCGGCAT	0	18	RDR2-sensitive
GGGAGTGTGGGACCACCTCGGCGT	1	19	RDR2-sensitive
GGGAGTTACGTTATCCACGTCGGC	0	14	RDR2-sensitive
GGGATAATTCTGAGGCTCACACGG	0	31	RDR2-sensitive
GGGATACGGAGAGGGAGTCTGCTG	0	14	RDR2-sensitive
GGGATACGGATTGTAACGGGTCAG	0	10	RDR2-sensitive
GGGATACTGAGACCTGGACACGGA	0	11	RDR2-sensitive
GGGATAGACTTAGACGGAGGGTAG	0	18	RDR2-sensitive
GGGATAGATGGCAGAACCGTGCAC	0	23	RDR2-sensitive
GGGATATACGGAAAACGGGACGGA	1	13	RDR2-sensitive
GGGATATGCAGCCTGACTGAGCGG	0	40	RDR2-sensitive
GGGATCCGTTGGACATGGCATGGC	30	0	RDR2-resistant
GGGATCGGACAACTGGTGGGCGGA	0	11	RDR2-sensitive
GGGATCGGATACGGATAGTTAGAA	2	16	RDR2-sensitive
GGGATCTCGGCGTAGTAGCGGATT	0	12	RDR2-sensitive
GGGATGAAAACGGTCGGGAACGGT	0	10	RDR2-sensitive
GGGATGAACGCAATCGGACGGTTG	0	9	RDR2-sensitive
GGGATGAATCAACCCGGCTTAGAA	0	12	RDR2-sensitive
GGGATGACACGTCACGAAGCTCGG	1	17	RDR2-sensitive
GGGATGATAGAATGGACGGCTGAA	0	9	RDR2-sensitive
GGGATGCATTTGTCTCGTCTGGAC	0	10	RDR2-sensitive
GGGATGCGGAGTCTGCTGGAGCGC	0	9	RDR2-sensitive
GGGATGGACTGCGGGATCGGACGG	1	59	RDR2-sensitive
GGGATGGATTTGTCTCGTCTGGAC	1	19	RDR2-sensitive
GGGATGGATTTGTCTCGTTTGGAC	0	21	RDR2-sensitive
GGGATGGTCGTTGGCAGCCGGTGA	45	0	RDR2-resistant
GGGATGGTTCCGGATCTGAGGAGA	0	22	RDR2-sensitive
GGGATGTGAGAGACCGGTATGCAC	1	19	RDR2-sensitive
GGGATGTTGTGACTGAGCCGGCCT	0	13	RDR2-sensitive
GGGATTAGTGGAGAGGTAGACGGT	0	22	RDR2-sensitive
GGGATTCCTGGAGACGCGATGGAG	0	10	RDR2-sensitive
GGGATTCTGTGGTTCGTGGTGGTC	45	0	RDR2-resistant
GGGATTGCGGCATTTTTCTCTATG	17	0	RDR2-resistant
GGGATTGGACGGCTGAGATTGTAG	0	11	RDR2-sensitive
GGGATTGTAGCGTTAGCACGAGCT	0	18	RDR2-sensitive
GGGATTGTATCTAGAGCAAGCCGG	0	14	RDR2-sensitive
GGGATTGTGCGTAGGAGCGGTGTT	23	0	RDR2-resistant
GGGATTTCAAGGAACACGACGGTC	0	9	RDR2-sensitive
GGGATTTGATTGTGTGTCGTCGGT	105	0	RDR2-resistant
GGGCAAGACTGCATGAGACTCCAA	1	66	RDR2-sensitive
GGGCACAGAGTAGTCACAAGGCAT	0	10	RDR2-sensitive

GGGCACGACTGCAGCGACTGAAGA	0	13	RDR2-sensitive
GGGCACGCGGCACTCACTCGTCGG	0	9	RDR2-sensitive
GGGCACGCGGCGACATTCACTCGT	1	13	RDR2-sensitive
GGGCACGTAGATGGCAGAACAGGT	0	15	RDR2-sensitive
GGGCACTCGGCAAAGAAGGCTTTG	10	28	RDR2-sensitive
GGGCAGAACCTGACGAGAAGTCGT	0	60	RDR2-sensitive
GGGCAGAACGCGGCTACTGTGGAC	1	17	RDR2-sensitive
GGGCAGAACTGTACGCGCGCACAC	0	9	RDR2-sensitive
GGGCAGATCTCCAACTGGGACGAC	31	4	RDR2-resistant
GGGCAGCTGGGACAGGCAGGACAA	0	14	RDR2-sensitive
GGGCAGGAACAGTAGACCAAGGCA	1	12	RDR2-sensitive
GGGCAGGATGGCAGAACAGTGCAC	0	9	RDR2-sensitive
GGGCAGGCTGCATCTGTGGAGACA	0	11	RDR2-sensitive
GGGCAGGGCGTAGCGTATGTCTGG	0	10	RDR2-sensitive
GGGCAGTCTCGGACGCAGACGTGC	0	42	RDR2-sensitive
GGGCAGTCTGTGACCTGGCAGCAG	1	27	RDR2-sensitive
GGGCAGTGTCGGCGACAGCTCTTC	0	12	RDR2-sensitive
GGGCATACTTGTAAGGATGGGCAC	0	10	RDR2-sensitive
GGGCATAGTGTCGGGCTAGCACGG	0	10	RDR2-sensitive
GGGCATCTACCAGGGGTAGGACGG	0	16	RDR2-sensitive
GGGCATCTGGACTGGTTCGGCGAA	0	10	RDR2-sensitive
GGGCATCTTAGACGAAGGACGGTC	0	15	RDR2-sensitive
GGGCATGCGTACTGTCGTGGACGG	0	16	RDR2-sensitive
GGGCATGGATTGAGGCATGGATTT	0	20	RDR2-sensitive
GGGCATGGCAGATGATGACGACAT	0	9	RDR2-sensitive
GGGCATTTGGTCTAGTGGTATGAT	17	0	RDR2-resistant
GGGCCACGCGTCGACTGAGCGGAG	0	25	RDR2-sensitive
GGGCCACGGTAGAACTGATGTGGC	0	12	RDR2-sensitive
GGGCCAGACTGTGCGCGATGGCGT	0	28	RDR2-sensitive
GGGCCAGTGGCATACAAGGAACGA	0	12	RDR2-sensitive
GGGCCATGACTGAGGACGTGACAC	0	12	RDR2-sensitive
GGGCCATTGTAGAGTGGACGGTTG	0	24	RDR2-sensitive
GGGCCCATGTAACGCACGTTCGGA	0	20	RDR2-sensitive
GGGCCCGGACAGGAAATTAGGCAC	0	9	RDR2-sensitive
GGGCCCGGCCTGTCAGATACGGTT	0	9	RDR2-sensitive
GGGCCGGGCAAAGACTACAGTTGA	0	14	RDR2-sensitive
GGGCCGGGCGTGCAGACTGCAGAC	0	13	RDR2-sensitive
GGGCCGGGCTGTTCGACAGGCTAA	0	26	RDR2-sensitive
GGGCCTCAAATTCCGACCCGTCGG	4	43	RDR2-sensitive
GGGCCTCAATTTCCGTCCCGTCGG	0	10	RDR2-sensitive
GGGCCTCCCTGTATCGAGCCGGAC	0	15	RDR2-sensitive
GGGCCTCGGATTTTACTGGCACGG	0	17	RDR2-sensitive
GGGCCTCGGGCGAGACGGAAGTCT	0	10	RDR2-sensitive
GGGCCTCGGGCGAGTCGAAGGTGC	3	16	RDR2-sensitive
GGGCCTGCATCTGGTCGGACGGTC	1	21	RDR2-sensitive
GGGCCTGGCTGAGTAGAAACACGG	0	14	RDR2-sensitive
GGGCCTTAAATTCCAGCTCGTCGT	0	9	RDR2-sensitive
GGGCCTTGGGACTGAACGAGGACG	5	48	RDR2-sensitive
GGGCGACGACTTCGTACGGCGGAC	27	4	RDR2-resistant

GGGCGACGGTAGACGGCAGAACCA	0	9	RDR2-sensitive
GGGCGACGGTATGAAGATAGCAAC	1	15	RDR2-sensitive
GGGCGACTCGACCGCGGACGGTAA	2	20	RDR2-sensitive
GGGCGACTGTATGAAGATAGCAAC	0	10	RDR2-sensitive
GGGCGACTTACACTCTGTCACGGT	0	9	RDR2-sensitive
GGGCGAGTCTGAAGAGCGGATGAG	0	20	RDR2-sensitive
GGGCGCCTAGAGGAGGCTTCTGAA	0	10	RDR2-sensitive
GGGCGCTCTGCAGGCAGCTGACGA	0	10	RDR2-sensitive
GGGCGCTGTAGAGGGCACCGCTGC	0	47	RDR2-sensitive
GGGCGCTGTAGGAGGCACCGCTGC	0	16	RDR2-sensitive
GGGCGCTGTAGGGGGCACCGCTGC	3	22	RDR2-sensitive
GGGCGGACGGTTGTGGCGAGGGCA	0	15	RDR2-sensitive
GGGCGGATACGGACGGATAGTAGC	1	29	RDR2-sensitive
GGGCGGATACGGACGGATATTAGC	0	10	RDR2-sensitive
GGGCGTGTAGAGGACGTGTCTGAA	0	93	RDR2-sensitive
GGGCGTTATGTTATTCTCGTCGGC	1	26	RDR2-sensitive
GGGCGTTCGGTAGAGCGGGGGCAG	0	26	RDR2-sensitive
GGGCGTTGTCGGCAGCATAGGCAT	0	45	RDR2-sensitive
GGGCGTTTCTTAGAACAGTCGGTG	1	12	RDR2-sensitive
GGGCGTTTGGTAGAGCGGGGGCAG	1	13	RDR2-sensitive
GGGCTACGATTGATCGGTAAGGCT	0	12	RDR2-sensitive
GGGCTAGACGGTCTGTAACCTGGG	0	9	RDR2-sensitive
GGGCTCAGACAGTAAACTAGGCAC	0	11	RDR2-sensitive
GGGCTCAGACTTCGGCTCACGGAC	0	9	RDR2-sensitive
GGGCTCCATGGGATCTGAAGCGGT	0	10	RDR2-sensitive
GGGCTCCTGCACCAGGTCGCGGAC	7	76	RDR2-sensitive
GGGCTCCTGTACCAGGTCGCGGAC	2	20	RDR2-sensitive
GGGCTCGGACAAGAAACTAGGCAC	6	165	RDR2-sensitive
GGGCTCGGACAAGAAATTAGGCAC	1	29	RDR2-sensitive
GGGCTCGGACAAGATATTAGGCAC	0	15	RDR2-sensitive
GGGCTCGGACAGAAAACTAGGCAA	0	10	RDR2-sensitive
GGGCTCGGACAGAAAACTAGGCAC	3	20	RDR2-sensitive
GGGCTCGGACAGAAAACTAGGCAT	4	25	RDR2-sensitive
GGGCTCGGACAGGAAACTAGGCAC	7	148	RDR2-sensitive
GGGCTCGGACAGGAAATTAGGCAC	11	201	RDR2-sensitive
GGGCTCGGACATGAAACTAGGCAT	0	32	RDR2-sensitive
GGGCTCGGATAGGAAATTAGGCAC	4	17	RDR2-sensitive
GGGCTCGGCCTGTCAAACACGGTT	0	10	RDR2-sensitive
GGGCTCGGGCGAGACGGAAACTGT	0	23	RDR2-sensitive
GGGCTCTCGGAAGTTAGACTGCGG	1	15	RDR2-sensitive
GGGCTGATCGGGCTACAGGCACGG	0	12	RDR2-sensitive
GGGCTGCAGGAACGGCAGATTGAG	0	41	RDR2-sensitive
GGGCTGCGGCGTAGGCTTAAGTGC	0	20	RDR2-sensitive
GGGCTGCGGCTGAAGACCTGCTCC	1	19	RDR2-sensitive
GGGCTGGACTGCTGCGCCTGCGGC	2	16	RDR2-sensitive
GGGCTGGACTTGGGCTGGGACGAC	0	10	RDR2-sensitive
GGGCTGGATCGGCGCAGTAGCTAT	0	13	RDR2-sensitive
GGGCTGGGACTAGAGAACGACGAA	0	14	RDR2-sensitive
GGGCTGGGCGTAGACGAACAGCAC	0	25	RDR2-sensitive

GGGCTGGTCCGGCACTGTTTTGGA	0	16	RDR2-sensitive
GGGCTGTATGTATGGAGACGAGCT	0	14	RDR2-sensitive
GGGCTTCCTGTAGTTAGAGAGAGC	0	15	RDR2-sensitive
GGGCTTCGCCAGTATGCCATCGGA	0	24	RDR2-sensitive
GGGCTTCGCCAGTATGTCATCGGA	1	24	RDR2-sensitive
GGGCTTCGTGCCGGACTCGCGGGC	0	16	RDR2-sensitive
GGGCTTCGTGCTGGTCTCGCGGGC	0	9	RDR2-sensitive
GGGCTTGACTTGAGACACGGACAC	0	19	RDR2-sensitive
GGGCTTGAGCTGTGTCGGATCGGA	0	15	RDR2-sensitive
GGGCTTGGACAAGAAACTAGGCAC	4	69	RDR2-sensitive
GGGCTTGGACAGAAAACTAGGCAC	0	22	RDR2-sensitive
GGGCTTGGACAGGAAACTAGGCAA	1	37	RDR2-sensitive
GGGCTTGGACAGGAAACTAGGCAC	48	936	RDR2-sensitive
GGGCTTGGACAGGAAACTAGGCAT	0	42	RDR2-sensitive
GGGCTTGGACAGGAAATTAGGCAC	2	14	RDR2-sensitive
GGGCTTGGACAGGGAACTAGGCAC	2	53	RDR2-sensitive
GGGCTTGGACATGAAACTAGGCAC	0	19	RDR2-sensitive
GGGCTTTACTGTTGGAGCGGAGTG	0	9	RDR2-sensitive
GGGCTTTCTCTCGGTTCGTCTGAC	0	11	RDR2-sensitive
GGGGAAAAACAGGAACGAGACGGA	0	9	RDR2-sensitive
GGGGAAACTGTAGCCCCTATACGG	0	10	RDR2-sensitive
GGGGAAACTGTTGGCGAATGGCGT	0	21	RDR2-sensitive
GGGGAAAGAGAACGCTGACGTGGC	1	16	RDR2-sensitive
GGGGAAAGGTAGGATAGAGACGTC	0	25	RDR2-sensitive
GGGGAACAGAACGCTGACGTGGCA	0	9	RDR2-sensitive
GGGGAACCGCGGAACAGGATTCGG	1	32	RDR2-sensitive
GGGGAACCGCTGAAGACAGTCTAA	0	9	RDR2-sensitive
GGGGAACCGCTGAAGACAGTCTGA	0	31	RDR2-sensitive
GGGGAACCGCTGAAGACAGTCTTA	0	15	RDR2-sensitive
GGGGAACTCTGCTGTCGACGGGCG	1	15	RDR2-sensitive
GGGGAACTGTAGACGATACGGTGA	0	21	RDR2-sensitive
GGGGAAGACGACAGAACCGTGCAC	1	24	RDR2-sensitive
GGGGAAGAGAACGCTGACGTGGCA	1	14	RDR2-sensitive
GGGGAAGCTGTTAGAGCAGAAGCA	0	10	RDR2-sensitive
GGGGAAGCTGTTGGAGCAGAAGCA	0	9	RDR2-sensitive
GGGGAATCGATGGGGACGGTTGAT	32	4	RDR2-resistant
GGGGAATTAGGACGAGACGCGGTG	0	15	RDR2-sensitive
GGGGACAACAAGCGCGAGCTCGGC	37	0	RDR2-resistant
GGGGACAAGGGACACAGTATGAGG	1	21	RDR2-sensitive
GGGGACACATCTGACGGCCTTCCT	0	9	RDR2-sensitive
GGGGACACGAGAAAGGGAAGACAG	0	14	RDR2-sensitive
GGGGACAGCCTGAACACATAGAGG	0	10	RDR2-sensitive
GGGGACAGCTCAAACATGATGGGG	0	15	RDR2-sensitive
GGGGACAGGTAGGATAGAGACGTC	0	17	RDR2-sensitive
GGGGACAGGTGAGAGGCAAGATGT	0	13	RDR2-sensitive
GGGGACAGTGAACAAGGCATCCCG	0	9	RDR2-sensitive
GGGGACAGTGAACAGTTGGGTCGG	0	12	RDR2-sensitive
GGGGACCGAACGCACCAAAGATGT	0	18	RDR2-sensitive
GGGGACCGAATTTAGGGGACGTTG	0	9	RDR2-sensitive

GGGGACCGACACGTAGAGGGCGCT	0	10	RDR2-sensitive
GGGGACCGACACGTAGGAGGCGCT	0	12	RDR2-sensitive
GGGGACCGACACGTAGGGACGCTG	0	9	RDR2-sensitive
GGGGACCGACACGTAGGGAGCGCT	0	17	RDR2-sensitive
GGGGACCGACACGTAGGGGACGCT	0	22	RDR2-sensitive
GGGGACCGACACGTAGGGGCGCTG	0	17	RDR2-sensitive
GGGGACCGACACGTAGGGGGCGCT	4	29	RDR2-sensitive
GGGGACCGATCCGCAGGACGTGAG	23	0	RDR2-resistant
GGGGACCTGCCGACTTGACGGAGA	1	17	RDR2-sensitive
GGGGACGAGGACGAGACAGACGCT	0	12	RDR2-sensitive
GGGGACGAGGACGAGACAGGCGCT	22	48	RDR2-sensitive
GGGGACGCAGATGAGGGATTTGAT	49	0	RDR2-resistant
GGGGACGCATAGGAACAGGTCCAG	0	9	RDR2-sensitive
GGGGACGCTGAGAACGCGGACGTC	0	9	RDR2-sensitive
GGGGACGCTGTAGAGGGCACCGCT	1	13	RDR2-sensitive
GGGGACGCTGTAGGGGCACCGCTG	1	16	RDR2-sensitive
GGGGACGGCCATGAACTTCGCGTA	13	42	RDR2-sensitive
GGGGACGGCCATGAACTTGGCGTA	4	27	RDR2-sensitive
GGGGACGGGAACAGGCTACCATAC	0	10	RDR2-sensitive
GGGGACGGTAAACGGTAACAGGAC	0	9	RDR2-sensitive
GGGGACGGTCTACACTGCTAACTT	0	44	RDR2-sensitive
GGGGACGTAGCTCAAATGGTAGAG	33	6	RDR2-resistant
GGGGACGTAGCTCATATGGTAGAG	86	5	RDR2-resistant
GGGGACGTCAGACTCATACGGCAA	0	10	RDR2-sensitive
GGGGACTAGACCGATGAGGAGGAC	0	13	RDR2-sensitive
GGGGACTGCGAGTGCGAGACGAGA	0	36	RDR2-sensitive
GGGGACTGGTGCGCTGACGTGCTG	0	9	RDR2-sensitive
GGGGACTGTGACAAGTGGTATCAG	0	12	RDR2-sensitive
GGGGAGAAGGGGGATACTGTAGCC	0	9	RDR2-sensitive
GGGGAGACGGACACGGAAGGCAGG	0	12	RDR2-sensitive
GGGGAGACTGTAGTGGGGCTCCAT	0	13	RDR2-sensitive
GGGGAGAGCGGGCTGGACAGCGGC	217	27	RDR2-resistant
GGGGAGGCGACTGAGAATGGCATA	0	9	RDR2-sensitive
GGGGAGGCTGTAGTGAGGCACTAT	0	29	RDR2-sensitive
GGGGAGGCTGTAGTGGGGCTCCAT	0	12	RDR2-sensitive
GGGGAGGGCAGAACGGCAGAACAC	1	22	RDR2-sensitive
GGGGAGTGTAGAGAGCGACGTCTA	0	17	RDR2-sensitive
GGGGATACACGCACGACGACGACC	0	17	RDR2-sensitive
GGGGATATGTAGGATAGGGACGTC	0	12	RDR2-sensitive
GGGGATCCCTGGACCGACGAGTAA	0	23	RDR2-sensitive
GGGGATCCGCGAGGACAAAGATGG	0	12	RDR2-sensitive
GGGGATCCGCGGGCGTCAATGGCG	15	0	RDR2-resistant
GGGGATCCTCGGCAGAACCGTGCA	0	16	RDR2-sensitive
GGGGATCGAGCCGGGGATGACGTG	0	26	RDR2-sensitive
GGGGATCGAGGACGGGGAACAGGC	0	13	RDR2-sensitive
GGGGATCGGGAGGTAGAGGATGGC	1	16	RDR2-sensitive
GGGGATGCACGAACGACGACGGGA	1	18	RDR2-sensitive
GGGGATGGACGCAATCGGACAGTC	0	10	RDR2-sensitive
GGGGATGGACGCAATCGGACGGTC	1	24	RDR2-sensitive

GGGGATGGGTATGAAGATGGATCA	0	11	RDR2-sensitive
GGGGATGGGTATGGGGATGGATCA	0	14	RDR2-sensitive
GGGGATGGTCGTTGGCAGCCGGTG	65	0	RDR2-resistant
GGGGATGTAGCTCAAATGGTAGAG	246	28	RDR2-resistant
GGGGATGTAGCTCAGATGGTAGAG	298	43	RDR2-resistant
GGGGATGTAGTTCAGATGGTAGAA	176	37	RDR2-resistant
GGGGATGTTAGACTGTGTGTGGGC	0	24	RDR2-sensitive
GGGGATTATGTTAACTCCCGTCGG	1	23	RDR2-sensitive
GGGGATTCCTTAACTCTCGTCGGC	1	22	RDR2-sensitive
GGGGATTGTTTGGCCACCAGGCGT	1	13	RDR2-sensitive
GGGGATTTGGGGAGGCTAGACGGT	0	16	RDR2-sensitive
GGGGCAACGGTACACTAACCTGAA	56	1	RDR2-resistant
GGGGCACCCCTTCAGATCGATCGA	1	28	RDR2-sensitive
GGGGCACGCGGCACACTCACTCGT	3	24	RDR2-sensitive
GGGGCACGCGGCGACAATTTACTC	3	24	RDR2-sensitive
GGGGCACGCGGCGACATTCACTCG	0	16	RDR2-sensitive
GGGGCACGCGGCGACATTTCACTC	0	11	RDR2-sensitive
GGGGCACGTAGATGGCAGAACCAG	0	69	RDR2-sensitive
GGGGCACTGTAGGAGGCACCGCTG	0	9	RDR2-sensitive
GGGGCAGAGGCAGAACAATGTGCA	0	16	RDR2-sensitive
GGGGCATCGTCACTTGAATCGGAT	65	4	RDR2-resistant
GGGGCATGGATTGAGGCATGGATT	0	15	RDR2-sensitive
GGGGCCAGTGGCATACAAGGAACG	0	90	RDR2-sensitive
GGGGCCAGTGGCATACAAGGAATG	1	21	RDR2-sensitive
GGGGCCTTCGGTCTTGGTCGTCCT	1	14	RDR2-sensitive
GGGGCGCTGTAGAGGCACCGCTGC	0	9	RDR2-sensitive
GGGGCGCTGTAGAGGGCACCGCTG	1	80	RDR2-sensitive
GGGGCGCTGTAGGAGGCACCGCTG	1	26	RDR2-sensitive
GGGGCGCTGTAGGGGCACCGCTGC	0	49	RDR2-sensitive
GGGGCGCTGTAGGGGCATCGCTGC	0	9	RDR2-sensitive
GGGGCTATGGTAGAGGCAAGTCAA	0	19	RDR2-sensitive
GGGGCTATGTGGAAGTACAGATGG	0	11	RDR2-sensitive
GGGGCTCCATAGAATCCGAAGCGG	0	14	RDR2-sensitive
GGGGCTCCATGAACTCGGCAGCGG	0	21	RDR2-sensitive
GGGGCTCCATGGACTCGGCAGCGG	0	15	RDR2-sensitive
GGGGCTGACTGTCGTATGGTGCAC	3	31	RDR2-sensitive
GGGGCTGAGAACCACCGACGTCAT	0	46	RDR2-sensitive
GGGGCTGAGAACCACCGTCGTCAT	3	62	RDR2-sensitive
GGGGCTGAGAACCGCCGTCGTCAT	8	26	RDR2-sensitive
GGGGCTGAGTCCTCTGTCTGACGG	0	9	RDR2-sensitive
GGGGCTGAGTTACAAAGACAGCAT	0	33	RDR2-sensitive
GGGGCTGATTGATGGGTACCACAA	0	14	RDR2-sensitive
GGGGCTGATTGCAACGTTCGTCCA	0	33	RDR2-sensitive
GGGGCTGCTACCCTAGAACCAAGC	0	11	RDR2-sensitive
GGGGCTGGGGGTTGAACAGAAGGC	0	10	RDR2-sensitive
GGGGCTGGTGTCGGACGAATACGG	6	46	RDR2-sensitive
GGGGCTGTAGCAAGTACCGGGGCA	0	11	RDR2-sensitive
GGGGCTGTAGGGGGCACTCCACGG	1	18	RDR2-sensitive
GGGGCTTAAATACTTCGGACCGGA	1	29	RDR2-sensitive

GGGGCTTCAGAACTATGTGCGGCC	0	9	RDR2-sensitive
GGGGCTTCAGAATGGACACGCGCA	0	20	RDR2-sensitive
GGGGCTTCCTGTAGTTAGAGAGAG	1	12	RDR2-sensitive
GGGGCTTTCGACGACTTCATCGAC	24	1	RDR2-resistant
GGGGGAAACTGTATAACGGCGCCT	0	14	RDR2-sensitive
GGGGGAACAGAACGCTGATGTGGC	1	17	RDR2-sensitive
GGGGGAACCGCTGAAGACAGTCTC	0	13	RDR2-sensitive
GGGGGAACCGCTGAAGACAGTCTG	0	24	RDR2-sensitive
GGGGGACACATACAAGGATACTGA	0	10	RDR2-sensitive
GGGGGACCGATCCGCAGGACGTGA	39	1	RDR2-resistant
GGGGGACTGTAGTGAGGCTCCATA	0	13	RDR2-sensitive
GGGGGACTTGCTGCTGCCTGCGGC	30	5	RDR2-resistant
GGGGGAGGAGTAGGAGCGCACATA	17	0	RDR2-resistant
GGGGGATGGTGTTGCTGACGGTAA	0	10	RDR2-sensitive
GGGGGATTGGAGGAGATTGATGGA	0	10	RDR2-sensitive
GGGGGCACGTAGATGGCAGAACAG	0	40	RDR2-sensitive
GGGGGCACGTAGATGGCAGAACCA	0	28	RDR2-sensitive
GGGGGCACTGTAGGGGGCACCACT	0	9	RDR2-sensitive
GGGGGCAGTCTACGGTACTCTCTT	0	16	RDR2-sensitive
GGGGGCATCAGACGGAACACCTAG	0	14	RDR2-sensitive
GGGGGCGACTGTAGTGGCACGGCG	0	13	RDR2-sensitive
GGGGGCGCTGTAGAGGGCACCGCT	0	15	RDR2-sensitive
GGGGGCGCTGTAGGAGGCACCGCT	1	13	RDR2-sensitive
GGGGGCGCTGTGGAGCAGAGGGGA	60	3	RDR2-resistant
GGGGGCTGCATACCCAGATCTCGA	2	42	RDR2-sensitive
GGGGGCTGCTCTCGCAAAAACCGG	4	24	RDR2-sensitive
GGGGGGACCAGATCTGAGACCGGC	1	19	RDR2-sensitive
GGGGGGACTGCCTCATCGTTGGGA	0	10	RDR2-sensitive
GGGGGTAGATCTGAGCAAGACGTC	0	18	RDR2-sensitive
GGGGGTCCCTGAACCGACGAGTAA	1	12	RDR2-sensitive
GGGGGTCTCTGGACCGACGAGTAA	0	12	RDR2-sensitive
GGGGGTGAACAATGGCGACAACAG	2	18	RDR2-sensitive
GGGGGTGGTAGCTTGCATGGAAGA	36	0	RDR2-resistant
GGGGGTGTAGCTCATATGGTAGAG	132	7	RDR2-resistant
GGGGTAACATCTCGGTTGTAGGTC	0	12	RDR2-sensitive
GGGGTACGTAGATGGCAGAACCGG	0	75	RDR2-sensitive
GGGGTAGAACCGAACAAGACCGTA	0	10	RDR2-sensitive
GGGGTAGAACCGGGCGCATAACAT	0	15	RDR2-sensitive
GGGGTAGAACCGTGCACCGTGAAC	0	9	RDR2-sensitive
GGGGTAGATCTGAGCAAGACGTCC	0	25	RDR2-sensitive
GGGGTCACTGTAGAAGCTTCGGTA	0	31	RDR2-sensitive
GGGGTCAGGGGAGGCAGCTGTAGC	0	9	RDR2-sensitive
GGGGTCATGCTGTGGATCGATGGC	1	70	RDR2-sensitive
GGGGTCGAACTAGGACTAGAGGAC	2	15	RDR2-sensitive
GGGGTCGAACTGGGACTAGAGGAC	1	16	RDR2-sensitive
GGGGTCGGGTGCTGAACCGGCGCA	1	12	RDR2-sensitive
GGGGTCTATCCTTGCGTTCGTCGT	0	14	RDR2-sensitive
GGGGTCTGTAGTGGTTAAGATGGA	0	21	RDR2-sensitive
GGGGTGACAGTGGATAACTACGGA	0	9	RDR2-sensitive

GGGGTGACTGGAGCCTCACGCGGT	2	19	RDR2-sensitive
GGGGTGGAACATAGCGAGGACGAA	1	21	RDR2-sensitive
GGGGTGGATAGGCGGAAACTGAAA	1	12	RDR2-sensitive
GGGGTGGCCTGTAGCCGTCACGGA	1	12	RDR2-sensitive
GGGGTGGGCTGTGGAGGAGGACGG	0	14	RDR2-sensitive
GGGGTGGTACCCGATTGAAGGAAC	113	2	RDR2-resistant
GGGGTGTTAGGTGCTCTGGTCGGA	0	10	RDR2-sensitive
GGGGTTACCAGCTGAGAATTAGGC	0	10	RDR2-sensitive
GGGGTTAGGGCACTAGAAGATCGC	14	0	RDR2-resistant
GGGGTTGGAATGATGATGGAGCAG	0	15	RDR2-sensitive
GGGGTTGGACGTGGAGAAGATGGT	1	25	RDR2-sensitive
GGGGTTGGGCGTGAGGAAGATGGT	0	13	RDR2-sensitive
GGGGTTGGGCTGATCAACGGTGCA	0	13	RDR2-sensitive
GGGGTTGTACGAGGTCCGTTGGAC	0	12	RDR2-sensitive
GGGGTTGTAGAACGACGGCCACGC	0	10	RDR2-sensitive
GGGTAAACACCAGAGACGCGGATA	0	18	RDR2-sensitive
GGGTAAACTGCTGAAGACGACCAA	0	17	RDR2-sensitive
GGGTAACCTGTTGAACACGGTATC	0	11	RDR2-sensitive
GGGTAACTGAGAACCGCCAGTGGA	0	10	RDR2-sensitive
GGGTAAGCAAAGCAGGTAGGACGC	0	13	RDR2-sensitive
GGGTACACTGTAGCCGCTATAGGA	3	24	RDR2-sensitive
GGGTACGTAGATGGCAGAACCGGT	0	26	RDR2-sensitive
GGGTACGTAGCGGGAGTGTTTGAC	14	0	RDR2-resistant
GGGTACTCGTGCAGGACGAGCTGT	0	22	RDR2-sensitive
GGGTACTCTAGGAATGAGACGGAT	0	10	RDR2-sensitive
GGGTACTGTAGCATATATATAGGC	14	0	RDR2-resistant
GGGTACTTCAGATTCGGGACGGAT	0	9	RDR2-sensitive
GGGTAGAAACCAAGTTGGACCGGC	0	31	RDR2-sensitive
GGGTAGAACAGTGAATGGCAGAAC	0	12	RDR2-sensitive
GGGTAGAACCCTAGCGAAGTGCGC	0	17	RDR2-sensitive
GGGTAGAACGCGGATACATGGAAC	0	10	RDR2-sensitive
GGGTAGAACGCGGATATATGGTTC	0	13	RDR2-sensitive
GGGTAGAATCTTTTTTGACGGGTA	0	19	RDR2-sensitive
GGGTAGAATTCGGGTACCCGGTAT	0	13	RDR2-sensitive
GGGTAGAATTTTTTTTGATGGGTA	0	10	RDR2-sensitive
GGGTAGACACGAGCAACACGACAT	0	14	RDR2-sensitive
GGGTAGACAGTAGGAATAGACGAT	0	38	RDR2-sensitive
GGGTAGACATGAGCAGCACGACGT	0	10	RDR2-sensitive
GGGTAGAGGGCAGGGACTTTTCAT	18	0	RDR2-resistant
GGGTAGATCTGAGCAAGACGTCCA	2	17	RDR2-sensitive
GGGTAGATCTGAGCAAGACGTCGA	0	17	RDR2-sensitive
GGGTAGCACACTCGGCATAGTCAG	0	12	RDR2-sensitive
GGGTAGCACTGAACAATCCGGTGA	0	15	RDR2-sensitive
GGGTAGCGGATCTTGGTCTCGGAC	9	35	RDR2-sensitive
GGGTAGGACATGCGTGCAAACTAG	0	9	RDR2-sensitive
GGGTAGGACGGTGCTGTCGACTGT	0	43	RDR2-sensitive
GGGTAGGACGGTGCTGTTGACTGT	1	27	RDR2-sensitive
GGGTAGTACACTCGGCATATCCAG	0	20	RDR2-sensitive
GGGTAGTGCGGGCTTACTCGGCGT	0	20	RDR2-sensitive

GGGTAGTTAAGATTCGGGACGGAT	1	45	RDR2-sensitive
GGGTATGGGGATGGGTATGAAGAT	0	10	RDR2-sensitive
GGGTATTAAATGTAGTCGGATTAT	3	71	RDR2-sensitive
GGGTCAAACTGGGACTAGAGGACG	1	14	RDR2-sensitive
GGGTCACTGTAGAGGATCCGGCGC	0	14	RDR2-sensitive
GGGTCAGCAGAGAACGACAGAGCA	1	18	RDR2-sensitive
GGGTCAGGCTGTATTTTCGTCGGC	0	17	RDR2-sensitive
GGGTCAGTCTGTTAGCTCGTCGGC	0	33	RDR2-sensitive
GGGTCATCTGTCGGGCTATGGCAT	0	9	RDR2-sensitive
GGGTCATGCTGTGGATCGATGGCT	0	29	RDR2-sensitive
GGGTCATGTCGGGCTCGAGTCGGA	0	10	RDR2-sensitive
GGGTCCGATGGCATACTGACGAAG	0	12	RDR2-sensitive
GGGTCCGATGGCATACTGGCGAAG	0	71	RDR2-sensitive
GGGTCCGGAGTAGTCACATGGCAT	0	10	RDR2-sensitive
GGGTCCGGAGTGGTTCCATGGCAT	0	11	RDR2-sensitive
GGGTCCGTAGATCTCAAGCAAGGC	0	14	RDR2-sensitive
GGGTCCTGTAGATGACTAGTGCAT	1	17	RDR2-sensitive
GGGTCGAAGGGTCGGACGAGGCGA	0	38	RDR2-sensitive
GGGTCGATTTCGGATATCCAACGG	19	1	RDR2-resistant
GGGTCGCGGTAGCGGCACGTCGGC	2	15	RDR2-sensitive
GGGTCGGAATTGGAGAGTGGAGGC	0	19	RDR2-sensitive
GGGTCGGCGCGAGACGGAACACAA	2	39	RDR2-sensitive
GGGTCGGGCTGGGACCAGAGGACG	0	11	RDR2-sensitive
GGGTCGGGCTGTCACGTGGGCTAT	0	14	RDR2-sensitive
GGGTCGTGACTCTGGACTAGACGG	0	10	RDR2-sensitive
GGGTCGTGCTGTGCTCCAGGCCGG	0	71	RDR2-sensitive
GGGTCGTGTGGAACTAACGGCGAT	0	11	RDR2-sensitive
GGGTCTCAAATTTCGGCTCGTCGG	0	13	RDR2-sensitive
GGGTCTCTGTGGAGAACGGGATGG	0	13	RDR2-sensitive
GGGTCTGGACTGTTCGACGGAGAG	1	17	RDR2-sensitive
GGGTCTGGCTGTGGTGTAGGCTTG	0	13	RDR2-sensitive
GGGTCTGTGAACCAGCAGGGAGTC	39	4	RDR2-resistant
GGGTCTGTTGAACGGTCGATCCGG	1	24	RDR2-sensitive
GGGTCTTAAATTCCGGTCCGTCGA	0	11	RDR2-sensitive
GGGTGAACTGCTGAACACGGTCTT	0	16	RDR2-sensitive
GGGTGAACTGCTGGACACGGTGTT	0	12	RDR2-sensitive
GGGTGAACTGCTGTAGTCGGCCTT	0	14	RDR2-sensitive
GGGTGAAGACGGAACCAACAACAT	0	19	RDR2-sensitive
GGGTGAATCTGGATCTGGACGGAT	0	9	RDR2-sensitive
GGGTGACAAACACCAGCTCGTCTG	0	9	RDR2-sensitive
GGGTGACGAGGATGATGCGGATAG	18	0	RDR2-resistant
GGGTGACTGGAGCCTCACGCGGTG	1	39	RDR2-sensitive
GGGTGACTGGGTCCTGCTGCGGCT	0	9	RDR2-sensitive
GGGTGAGACTGTGAGAGAGACTGC	0	19	RDR2-sensitive
GGGTGATGTGGACGCTGAGGAGGT	29	0	RDR2-resistant
GGGTGATTGGTAGGCTACGCTCAG	0	19	RDR2-sensitive
GGGTGCCGATGGGAACATGGACGG	10	30	RDR2-sensitive
GGGTGGAACATAGCGAGGACGAAC	0	26	RDR2-sensitive
GGGTGGACAGAGTAGGAGAGAGCA	0	27	RDR2-sensitive

GGGTGGACCATGGCATACTCTATG	0	14	RDR2-sensitive
GGGTGGACTGAACGACGGACGGTT	0	16	RDR2-sensitive
GGGTGGACTGTGTAGATTCACGGA	0	9	RDR2-sensitive
GGGTGGATCTCTGATACAGTGGCT	0	11	RDR2-sensitive
GGGTGGGATTCGTAGGCGGGGGCA	0	9	RDR2-sensitive
GGGTGGTACCCGATTGAAGGAACA	84	0	RDR2-resistant
GGGTGTAGATCTGAGCAAGGCGTC	0	10	RDR2-sensitive
GGGTGTCGGGTGCATACAAAGCAC	0	18	RDR2-sensitive
GGGTGTCGGGTGCATACAAAGCAT	0	16	RDR2-sensitive
GGGTGTCTGATGTCTGTTGGACAA	2	18	RDR2-sensitive
GGGTGTGGACGCCTACAGTAGACA	49	2	RDR2-resistant
GGGTGTTCAGCTGAAGGTCGTCGT	0	18	RDR2-sensitive
GGGTGTTTGGTCTAGTGGTATGAT	14	0	RDR2-resistant
GGGTTACTGTTATTGATCGGCTCC	65	6	RDR2-resistant
GGGTTATCTGTGCTCTGTTGTCGG	3	17	RDR2-sensitive
GGGTTCATCCCGGCAATGGAACCA	23	0	RDR2-resistant
GGGTTCGGCACTGGAGCTGGCGGA	0	11	RDR2-sensitive
GGGTTCTCTGTAGGCCGGACGGTC	0	14	RDR2-sensitive
GGGTTGAAAGTCAAGCGGGAACGA	0	9	RDR2-sensitive
GGGTTGAACCAGGTTGTAGAGGTA	0	24	RDR2-sensitive
GGGTTGACGCAAGACGGAACACAA	0	18	RDR2-sensitive
GGGTTGAGACTGTAGCATGGTGTA	0	100	RDR2-sensitive
GGGTTGCATGCAGACTGGGACCGG	0	13	RDR2-sensitive
GGGTTGCTGGGAAGGGAGCGGGCT	0	10	RDR2-sensitive
GGGTTGGCGCGAGACGGAACACAA	2	20	RDR2-sensitive
GGGTTGTCGCCAGAGAGAACGGAA	0	41	RDR2-sensitive
GGGTTTAGGACGGCAAAACTCAGC	0	17	RDR2-sensitive
GGGTTTCCAACCTATCGGCCGGAC	0	15	RDR2-sensitive
GGGTTTCGCTATATATCATCGGAC	0	11	RDR2-sensitive
GGGTTTCGGTCTAGGGCTCGGTTT	0	10	RDR2-sensitive
GGGTTTGGACAGGAAACTAGGCAC	0	90	RDR2-sensitive
GGGTTTGGACATGTCCCTAGGCAC	0	9	RDR2-sensitive
GGGTTTGGCCTGACACGAACACGG	0	19	RDR2-sensitive
GGGTTTGGTTCGGCACGAACTCGG	0	9	RDR2-sensitive
GGTAAAATCTCGGGCACTCGGCAA	0	22	RDR2-sensitive
GGTAAACGACGCGACGACGAGCTG	0	15	RDR2-sensitive
GGTAACGACAGACTGACGGGACAT	0	10	RDR2-sensitive
GGTAACGCGGCATGACATACCTGG	1	30	RDR2-sensitive
GGTAAGACGGGAACACTAGCGCAA	0	24	RDR2-sensitive
GGTAAGTTCGACGGCCTATTACAT	0	10	RDR2-sensitive
GGTAAGTTGGAACGGAGCGGCTCT	0	42	RDR2-sensitive
GGTAATCGGCTGAGTTATGGCATT	3	34	RDR2-sensitive
GGTAATCTAGAACGGAGCGGCTCT	0	13	RDR2-sensitive
GGTAATCTGGAACGGAACGGCTCT	1	20	RDR2-sensitive
GGTAATCTGGAACGGAGCGGCTCT	12	215	RDR2-sensitive
GGTAATCTGGAACGGAGTGGCTCT	1	12	RDR2-sensitive
GGTAATCTGGAATGGAGCGGCTCT	1	31	RDR2-sensitive
GGTAATGGGTTGTATATGGTTGAC	32	0	RDR2-resistant
GGTACACCCCCTGTAGACCCGGCT	0	12	RDR2-sensitive

GGTACACCGTTGGATATTGGATGG	0	12	RDR2-sensitive
GGTACAGATTCGTCGAAGGCAGAA	0	9	RDR2-sensitive
GGTACCACGTCGGCGCCACGTCGT	0	9	RDR2-sensitive
GGTACCCTAAGAACCGTCGGAAAC	1	21	RDR2-sensitive
GGTACCCTAGGAACCGTCGGAAAC	0	11	RDR2-sensitive
GGTACCCTCGGACTAAAGTCGGCA	0	11	RDR2-sensitive
GGTACCCTCGGATTCCGCAGACGA	0	12	RDR2-sensitive
GGTACCGGATACCGCTGCAGACGT	0	9	RDR2-sensitive
GGTACCGGTAGAACTGCCACGGAC	2	19	RDR2-sensitive
GGTACCTTCGCCGGCGACGAGCAT	0	11	RDR2-sensitive
GGTACGAATGATCGCGGCACGGAA	0	12	RDR2-sensitive
GGTACGACGGGCTAGGTTCGGCAT	3	39	RDR2-sensitive
GGTACGACTCATGACGGGCTCGAG	0	10	RDR2-sensitive
GGTACGACTGTTAAACGGCCCAAC	0	24	RDR2-sensitive
GGTACGCGACTGCATCGACGACAT	0	9	RDR2-sensitive
GGTACGCTACTGAAGAACGAGCAG	0	12	RDR2-sensitive
GGTACGGATTAGGACTGTGTCGGT	0	21	RDR2-sensitive
GGTACGGATTGGGATCGTGTCGGT	0	10	RDR2-sensitive
GGTACGGGACCAGACTGAGCGGCT	0	21	RDR2-sensitive
GGTACGTGTAGAAGCATGTCGGAT	0	10	RDR2-sensitive
GGTACGTGTAGGTACATGTCGGAT	0	17	RDR2-sensitive
GGTACGTTGTACGGCGTGGTAGAC	0	20	RDR2-sensitive
GGTACTATTGTTGGCATCGATCGT	1	31	RDR2-sensitive
GGTACTCGGGCTGCGTCGAGGCGT	2	14	RDR2-sensitive
GGTACTTCGGCTGCGACAAGACGC	1	14	RDR2-sensitive
GGTACTTGAGCCCTGTAGAAGCGT	0	10	RDR2-sensitive
GGTAGAACGCGGCAGTGTGAACGC	0	23	RDR2-sensitive
GGTAGAACGGATGGTTGAGATCGT	0	17	RDR2-sensitive
GGTAGAACGTGGCTAGTAGAGGTG	0	11	RDR2-sensitive
GGTAGAAGTGTGGAATGATAGCTT	14	0	RDR2-resistant
GGTAGAATAAGCGGACGGACTTGG	0	17	RDR2-sensitive
GGTAGAATAAGCGGGCGGGCTTGG	0	47	RDR2-sensitive
GGTAGAATGGACGGCTGAGATCGC	1	119	RDR2-sensitive
GGTAGACACTCGGCACAGACTGGT	2	31	RDR2-sensitive
GGTAGACCTCTGCACGATGACGAT	26	1	RDR2-resistant
GGTAGACGGGCTACTCTCCCAAGA	0	9	RDR2-sensitive
GGTAGACGGGCTGCTCTCCCAAGA	0	25	RDR2-sensitive
GGTAGACGGTTTTACTTTTATGAC	0	10	RDR2-sensitive
GGTAGACTGGCTGAGAGCATATAA	0	18	RDR2-sensitive
GGTAGAGAGGAAGAACAGGACGCT	5	20	RDR2-sensitive
GGTAGAGGAGGCAGTAGAGTGATG	0	9	RDR2-sensitive
GGTAGAGGGCTGCGCTGCGGGACG	0	63	RDR2-sensitive
GGTAGATCGAGCAGAGCACTGCAT	0	10	RDR2-sensitive
GGTAGATCTGAGCAAGACGTCCAG	1	20	RDR2-sensitive
GGTAGCAGGAGGAGTTGGTAGAGG	0	12	RDR2-sensitive
GGTAGCCTGTCTGAACGGACGCAT	2	37	RDR2-sensitive
GGTAGCTCTGATGACGACTGTTAC	0	11	RDR2-sensitive
GGTAGCTCTGATGGCGACTGTTAC	0	9	RDR2-sensitive
GGTAGCTGTAGGTAGGACGTGGAC	21	0	RDR2-resistant

GGTAGGACTCTCTGCGGAGGCGCG	0	11	RDR2-sensitive
GGTAGGATGGGCTGTAGAATGGGC	0	17	RDR2-sensitive
GGTAGGCCGACCGTAGGATATCCA	0	9	RDR2-sensitive
GGTAGGCGGCATGTAGGGCAAGAG	0	11	RDR2-sensitive
GGTAGGCGGCTGGTACCTTGCAGT	0	11	RDR2-sensitive
GGTAGGCTCGGACGAGACGGACAT	0	13	RDR2-sensitive
GGTAGGGCAGGTAAGCGACGACAT	0	21	RDR2-sensitive
GGTAGGGGGCTGCACTGCAGGACG	0	10	RDR2-sensitive
GGTAGGGGTGGAGGGCGTTGGGGC	94	1	RDR2-resistant
GGTAGTAGGCCCTGGGCGCGACGG	0	9	RDR2-sensitive
GGTAGTCGATGTAGTCATGGGCGG	0	50	RDR2-sensitive
GGTAGTCGGCCCTGTAGTCGCGGT	0	14	RDR2-sensitive
GGTAGTCGGGGATGGCAGATGCAA	0	31	RDR2-sensitive
GGTAGTCGTTTAGGACATGATTGT	0	30	RDR2-sensitive
GGTAGTGGGATATAGGGATGGAAA	0	11	RDR2-sensitive
GGTAGTGGGCTCATGACTCGGCAC	0	23	RDR2-sensitive
GGTAGTGTTTGGATGCAGGGACGA	0	10	RDR2-sensitive
GGTAGTTCAGGACTGCAACGGCAC	0	13	RDR2-sensitive
GGTAGTTGCCTGAACAACGACGGC	0	12	RDR2-sensitive
GGTAGTTGCCTGAACGACGACGAC	0	13	RDR2-sensitive
GGTAGTTGCCTGAACGACGACGGC	0	9	RDR2-sensitive
GGTAGTTGGGAAGTGGAACGACGG	0	9	RDR2-sensitive
GGTATAACCTCGCCGGAGAAGACA	0	11	RDR2-sensitive
GGTATAACTCGTAGCGCTTCGGCA	0	19	RDR2-sensitive
GGTATAGATCGGGCTAGCACGGCT	0	13	RDR2-sensitive
GGTATCAGGCTAACGACTAGGCGC	1	12	RDR2-sensitive
GGTATCTGATTACGGTGGGACTAA	0	23	RDR2-sensitive
GGTATCTGATTACGGTTGGACTAA	3	176	RDR2-sensitive
GGTATGAACTGCTGTGGGGGACAG	0	9	RDR2-sensitive
GGTATGACTAGCAGGCTGACGGGC	0	26	RDR2-sensitive
GGTATGGACCTAACTCAGGAGCGG	0	54	RDR2-sensitive
GGTATGGCTCGTAGTGCTTCGGCA	1	56	RDR2-sensitive
GGTATGGCTCTAGAGCAGAATCGC	2	39	RDR2-sensitive
GGTATGTATGTAGGGGTGGCGGGC	0	9	RDR2-sensitive
GGTATTGTAGAACGGAGCGGTTCT	0	15	RDR2-sensitive
GGTATTTTAGACACGGATTGGTAT	0	11	RDR2-sensitive
GGTCAAACTGGGACTAGAGGACGA	0	16	RDR2-sensitive
GGTCAAAGTAGGATGAGTCGGAGA	0	9	RDR2-sensitive
GGTCAATCAGGACGGACTTGGCAT	0	22	RDR2-sensitive
GGTCACAGGCAGGAACTTAGGCTC	0	21	RDR2-sensitive
GGTCACTCGGCTGACGTACGCGGT	1	15	RDR2-sensitive
GGTCACTTATAGCCGTCGGACATA	0	13	RDR2-sensitive
GGTCAGACGGTGGAGACAGACAGA	0	17	RDR2-sensitive
GGTCAGCAGAGAACGACAGAGCAA	0	9	RDR2-sensitive
GGTCAGCTCGGCTCGGATCGGCTC	0	17	RDR2-sensitive
GGTCAGGACTGTGGATGGTCCGGC	0	11	RDR2-sensitive
GGTCAGGCCTGTCGCATCAACGAA	15	0	RDR2-resistant
GGTCAGGCTCTAGTAGGACGTCGG	0	11	RDR2-sensitive
GGTCAGTGACTATGGGCCGGACGG	0	24	RDR2-sensitive

GGTCAGTTAGGACGCGGCAACCAA	0	10	RDR2-sensitive
GGTCATATCGTCGATCGTTTTGCA	0	12	RDR2-sensitive
GGTCCAAACCCGGCACGGTAGAAT	0	12	RDR2-sensitive
GGTCCAAGTTCGGCATGATCTGGT	0	18	RDR2-sensitive
GGTCCACGGCTAGGAACCAGACGG	0	17	RDR2-sensitive
GGTCCACGGTGACGGCACGAACAC	0	9	RDR2-sensitive
GGTCCAGCGCGGCTGATCGTTCGC	0	14	RDR2-sensitive
GGTCCATCTCGGACATACGATGGT	1	17	RDR2-sensitive
GGTCCATCTCGGACTCGCGACGGT	0	10	RDR2-sensitive
GGTCCATGGTATAGGACTGGACGG	0	9	RDR2-sensitive
GGTCCCAGTAGGACTGTAGGAGGC	0	19	RDR2-sensitive
GGTCCGAACCCGGCACGGTAGAAT	4	287	RDR2-sensitive
GGTCCGAACCCGGCATGGTAGAAT	0	48	RDR2-sensitive
GGTCCGAACCTGGCACGGTAGAAT	0	12	RDR2-sensitive
GGTCCGAAGTCGGGTCTAAGACGA	0	13	RDR2-sensitive
GGTCCGACGGACTAATGGGCCGGC	0	10	RDR2-sensitive
GGTCCGACTCGGCCTGAAGCACGG	0	10	RDR2-sensitive
GGTCCGAGCCCGGCACGATAGAAT	0	9	RDR2-sensitive
GGTCCGAGCCCGGCACGGTAGAAT	0	38	RDR2-sensitive
GGTCCGCAGACTGGTTGACGCGAC	1	30	RDR2-sensitive
GGTCCGCCCCTGTAGATCAACGGC	4	55	RDR2-sensitive
GGTCCGCCTTGACTGTGACAGCAT	0	13	RDR2-sensitive
GGTCCGCGCCTGTAGATCAACGGC	5	64	RDR2-sensitive
GGTCCGCGGCTCTAGAACGGACGG	0	15	RDR2-sensitive
GGTCCGCGGCTCTGGTCTGGACGG	2	21	RDR2-sensitive
GGTCCGCGGTATAGGGCTGTACGG	0	9	RDR2-sensitive
GGTCCGGAATAGTCGGGAACGGAA	1	26	RDR2-sensitive
GGTCCGGACTGTCCGCGGTACTGT	1	14	RDR2-sensitive
GGTCCGGACTGTCCGCGGTGGTGA	1	12	RDR2-sensitive
GGTCCGGGCATGTGCAGATCGGCT	1	18	RDR2-sensitive
GGTCCGGGCTAGCTGGAGACTGGC	0	22	RDR2-sensitive
GGTCCTAGACTGTAGATGGCGAGC	0	9	RDR2-sensitive
GGTCCTAGATCGACGGTCCGCGAC	2	14	RDR2-sensitive
GGTCCTATCGGGTCGGATACCTAA	0	9	RDR2-sensitive
GGTCCTGTAGACTGTCCGGTGCGC	12	30	RDR2-sensitive
GGTCGAAGGGTCGGACGAGGCGAA	0	17	RDR2-sensitive
GGTCGACTGTTGAAGTGCAAGTGA	0	9	RDR2-sensitive
GGTCGAGATTGACGGCAGAACGGA	0	10	RDR2-sensitive
GGTCGATATCGGTCGTGGCCTCAA	0	11	RDR2-sensitive
GGTCGATATGGTAGTAGAGGACGA	0	10	RDR2-sensitive
GGTCGATCTCTCCTGTACGTGGCT	1	13	RDR2-sensitive
GGTCGCATCTCTGGAGCTAGGCTT	0	10	RDR2-sensitive
GGTCGCTATAGATATCGTCGGGGA	0	76	RDR2-sensitive
GGTCGCTATAGATGTCGTCGGGAA	0	9	RDR2-sensitive
GGTCGCTATAGATGTCGTCGGGGA	0	20	RDR2-sensitive
GGTCGGCCCACTGTACGCAAGCAT	0	9	RDR2-sensitive
GGTCGGCCCACTGTACGCGAGCAT	0	19	RDR2-sensitive
GGTCGTACCCTAGAGCCGGACGGT	0	26	RDR2-sensitive
GGTCTATCGCTCAGGACCAGACGG	0	23	RDR2-sensitive

GGTCTATGTCGTCGTTGGCTCCAG	0	10	RDR2-sensitive
GGTCTCATAGGCAATTGTCGGACA	0	21	RDR2-sensitive
GGTCTCCTAGGCAGCCGTCGGACA	1	79	RDR2-sensitive
GGTCTCTATCGTTGGCCCGTGCAT	1	39	RDR2-sensitive
GGTCTCTGTGGAAGCGTAGACGGT	0	10	RDR2-sensitive
GGTCTGACCTGAACCCGACACGGC	0	10	RDR2-sensitive
GGTCTGAGTCCGGCACGGTAGAAT	0	21	RDR2-sensitive
GGTCTGCATGGCTCCAAGGGACGT	1	37	RDR2-sensitive
GGTCTGCTTCTGAGGACTGGACGG	2	15	RDR2-sensitive
GGTCTGGCCATAGATACGGACGGT	0	11	RDR2-sensitive
GGTCTGTAGCAGTCGACTGTTGGA	0	9	RDR2-sensitive
GGTCTGTGGCACAGGGTTGGACGG	0	14	RDR2-sensitive
GGTCTGTTTGGTTGGGCTGTGGCT	0	12	RDR2-sensitive
GGTCTTAGGTGGACTGGACAGCAT	3	42	RDR2-sensitive
GGTCTTCGCATTGAACGTCGGTCA	0	11	RDR2-sensitive
GGTCTTCGTGCCGGGCTCGCGGAC	0	10	RDR2-sensitive
GGTCTTCTCGTCGGATCGGTTAAG	0	9	RDR2-sensitive
GGTCTTGTTCTGCTGGCTGATGGA	18	1	RDR2-resistant
GGTCTTTAGGCTGATCAATCGGAC	0	10	RDR2-sensitive
GGTGAAAGTGCGGCCTGTAGCGGC	0	17	RDR2-sensitive
GGTGAACCTGCGGAAGGATCATTG	40	3	RDR2-resistant
GGTGAACGACGTGACGACGGATGA	1	16	RDR2-sensitive
GGTGAACGCGACAGGGACGGTGAC	0	11	RDR2-sensitive
GGTGAACGGACGGACGAACGGTGC	0	17	RDR2-sensitive
GGTGAAGATGTCGGCATACTGTGA	0	15	RDR2-sensitive
GGTGAAGTCGGAGGCTAACAGCAT	0	63	RDR2-sensitive
GGTGAAGTTCGGCCCGCTAGACAC	0	9	RDR2-sensitive
GGTGAATACTCTGCAGTCGGATGA	0	9	RDR2-sensitive
GGTGAATCTGCACCGTCCGTTCGG	0	30	RDR2-sensitive
GGTGACCAGAAACTGCCAGAAGCC	0	10	RDR2-sensitive
GGTGACCTACTGATCTCGGAGCAC	0	9	RDR2-sensitive
GGTGACGAGGAAGCAGAAGGACAC	0	11	RDR2-sensitive
GGTGACGCGCAGAACGTAACTCGA	0	11	RDR2-sensitive
GGTGACGGGCTGACAGCTGACTGC	0	10	RDR2-sensitive
GGTGACTACTCGCTGGGAGAACGG	0	10	RDR2-sensitive
GGTGACTGACGACCGAGAAGCGGT	0	11	RDR2-sensitive
GGTGACTGCTAGATAGGATGGTCG	0	11	RDR2-sensitive
GGTGACTGGATAGGAGGCTTCGGA	0	9	RDR2-sensitive
GGTGACTGGCGCATCTGATGGCTC	0	27	RDR2-sensitive
GGTGACTGTCCGGCCGAACGGGTT	0	68	RDR2-sensitive
GGTGACTGTGTGGCATTCGGCGAG	2	15	RDR2-sensitive
GGTGAGACGGTAGTGGGCGCAGAA	0	14	RDR2-sensitive
GGTGAGACTGTCACCGGAGCTGGC	16	0	RDR2-resistant
GGTGAGAGATGTAGTAGATGTGTT	3	16	RDR2-sensitive
GGTGAGCTTTGGGCAGACTGGTCC	0	19	RDR2-sensitive
GGTGAGGAATTGTGGACGGATGAA	0	9	RDR2-sensitive
GGTGAGGACAGACACAGTAGGCGC	0	14	RDR2-sensitive
GGTGAGGATGACGAGTGACGCATG	0	28	RDR2-sensitive
GGTGAGGATGACTGGTGACGCATG	0	27	RDR2-sensitive

GGTGAGGATGAGGATGACGGGTGA	0	43	RDR2-sensitive
GGTGATCGATCAAGTTTCTGCCGA	0	10	RDR2-sensitive
GGTGATCGGACGGTCCACGATGGC	0	9	RDR2-sensitive
GGTGATCGGCAGCAGATCCAGACG	0	9	RDR2-sensitive
GGTGATCGGCTGAAGACGCGCCAA	0	14	RDR2-sensitive
GGTGATCGGGCTGAACCGGCATGA	0	9	RDR2-sensitive
GGTGATGCGGACGATACAGCCAGC	0	27	RDR2-sensitive
GGTGATGGACAGGGCTGACGTGGC	0	18	RDR2-sensitive
GGTGATGGACGTAATTGGACGGTT	0	17	RDR2-sensitive
GGTGATGGCTGTAAGCGCGCGGAA	0	45	RDR2-sensitive
GGTGCAATCTGTACAGTCGTCGGT	0	13	RDR2-sensitive
GGTGCACCCCCGGACAGAAGGCAA	0	14	RDR2-sensitive
GGTGCACCGGACTGTACGGTGGTG	0	9	RDR2-sensitive
GGTGCACTGGTAGAACAACGGAGC	0	18	RDR2-sensitive
GGTGCAGATCGATAGATGGCAGAA	0	9	RDR2-sensitive
GGTGCAGGATCTAGAAGACGAAGG	0	9	RDR2-sensitive
GGTGCATGCGTCGAGCAGGAACGA	0	14	RDR2-sensitive
GGTGCATGTGCGCGGTACTCGGAC	0	29	RDR2-sensitive
GGTGCCCATGGGGTGACGCCGGAA	17	0	RDR2-resistant
GGTGCCGATGGGAACATGGACGGC	5	66	RDR2-sensitive
GGTGCCTCTGCCATGAATCGTCGC	0	10	RDR2-sensitive
GGTGCGACTCTGTTCTGGCGCGCG	3	56	RDR2-sensitive
GGTGCGACTGAACCAGATGACACC	1	54	RDR2-sensitive
GGTGCGAGCAGTGCTGACATCGGT	23	0	RDR2-resistant
GGTGCGCCTGATCAACCGGACGAA	0	13	RDR2-sensitive
GGTGCGCGGACGAACAACGACGGA	0	14	RDR2-sensitive
GGTGCGGATTCTTTGCGATGGCTT	44	70	RDR2-sensitive
GGTGCGGCCACGGCAGAACAACAG	0	10	RDR2-sensitive
GGTGCGGCTGAACCGGAACGTGGC	0	15	RDR2-sensitive
GGTGCGGGCTGCACGAGTAGATGT	0	9	RDR2-sensitive
GGTGCGTCGTGGAACTGAGAAGAC	0	10	RDR2-sensitive
GGTGCGTGTGGGACGGATTCAGAG	0	14	RDR2-sensitive
GGTGCTCGGAACTAGACGGTCTGC	3	19	RDR2-sensitive
GGTGCTCGGCAAAATAGCTAGCTT	35	3	RDR2-resistant
GGTGCTCGGCAATGTCTTCGGCTT	21	0	RDR2-resistant
GGTGCTCTCTGAACAGGCGCGGAT	0	11	RDR2-sensitive
GGTGCTCTCTGACCAGGCGCGGAC	3	25	RDR2-sensitive
GGTGCTCTCTGATCAGGCACGGAC	0	16	RDR2-sensitive
GGTGCTCTCTGCACAGACGCGGAC	0	20	RDR2-sensitive
GGTGCTCTCTGCACAGGCGCGGAC	6	56	RDR2-sensitive
GGTGCTCTCTGGTCAAGCGCGGAC	0	22	RDR2-sensitive
GGTGCTCTCTGGTCAGACGCGGAC	0	19	RDR2-sensitive
GGTGCTCTCTGGTCAGGCGCGGAC	14	298	RDR2-sensitive
GGTGCTCTCTGTACAGGCGCGGAC	0	9	RDR2-sensitive
GGTGCTCTGTGGAGGCGTGGACGG	0	13	RDR2-sensitive
GGTGCTGTAGCCGTCGGCAATCAA	0	10	RDR2-sensitive
GGTGCTGTATGCTGTTTTGATGGC	20	1	RDR2-resistant
GGTGCTTTCTGCACAGGCGCGGAC	0	9	RDR2-sensitive
GGTGCTTTTTCGGAGTAGGACGGT	0	9	RDR2-sensitive

GGTGGAAACGCGGGCGTTGACGGC	0	9	RDR2-sensitive
GGTGGAACCTGACAGGGATGCCAG	0	27	RDR2-sensitive
GGTGGAATTGTGGGCAGAGAAGCC	0	10	RDR2-sensitive
GGTGGACACAGTCAGACAGACAAG	0	9	RDR2-sensitive
GGTGGACAGAAGTCAACAGATCGC	1	16	RDR2-sensitive
GGTGGACAGAAGTCAACAGATCGT	0	9	RDR2-sensitive
GGTGGACAGAGTAGGAGAGAGCAT	0	35	RDR2-sensitive
GGTGGACAGGTACGAGCAGATGAT	0	53	RDR2-sensitive
GGTGGACATGGAATTGGAGAGGAT	0	12	RDR2-sensitive
GGTGGACATGGATACGGGTTTGAA	2	16	RDR2-sensitive
GGTGGACATGTAGGGCTTATTCGG	0	9	RDR2-sensitive
GGTGGACCTGCAGGTGGGGCCTAT	3	27	RDR2-sensitive
GGTGGACCTGTAGGTGGGGCCTAT	1	38	RDR2-sensitive
GGTGGACCTTAGACCGTTCGGCAC	0	17	RDR2-sensitive
GGTGGACGATGAGCTAGACGACGT	0	19	RDR2-sensitive
GGTGGACGATGAGCTGGACGGCGT	1	56	RDR2-sensitive
GGTGGACGATGAGCTGGATGGCGT	1	44	RDR2-sensitive
GGTGGACTATGAACAGCTAGACGA	0	58	RDR2-sensitive
GGTGGACTCGTACCTCCGTGTCGT	0	15	RDR2-sensitive
GGTGGACTCTCTTGCCTAGGCCGG	0	14	RDR2-sensitive
GGTGGACTGACTGAGGACGAGGCA	0	13	RDR2-sensitive
GGTGGACTGGACAGCATGACGGAT	0	9	RDR2-sensitive
GGTGGACTGGACAGCATGATGGAT	2	20	RDR2-sensitive
GGTGGACTGGCACTCGGCAAAGAA	0	16	RDR2-sensitive
GGTGGACTGGGACGAGCACGCACC	0	13	RDR2-sensitive
GGTGGACTGTGCCTTATGGGTCGG	2	17	RDR2-sensitive
GGTGGAGACAGACAGAGGACGACT	0	11	RDR2-sensitive
GGTGGAGAGGTAGACGGCGCTGAC	6	37	RDR2-sensitive
GGTGGAGATGGCGAACGACGAGCA	0	16	RDR2-sensitive
GGTGGAGCGATTTGTCTGGTTAAT	18	1	RDR2-resistant
GGTGGAGGGAGTAGCAGCGACGAC	0	12	RDR2-sensitive
GGTGGAGTGGACTGTCAGCGGCCT	0	15	RDR2-sensitive
GGTGGAGTTGGTGGAGCAGGTCAT	0	10	RDR2-sensitive
GGTGGATAGAGATGACGACGCTAG	0	9	RDR2-sensitive
GGTGGATCTGAGACGGTAGGTTAT	1	29	RDR2-sensitive
GGTGGATGATGAGCTAGACGGCGT	0	12	RDR2-sensitive
GGTGGATGATGAGCTGGACGGCGT	4	25	RDR2-sensitive
GGTGGATGATGAGCTGGACGGTGT	2	22	RDR2-sensitive
GGTGGATGGTGGAAGTCAGTCGGC	0	9	RDR2-sensitive
GGTGGATTCGGATCGGATACGCAT	0	18	RDR2-sensitive
GGTGGCACTGTGGAGAGAAGACCA	0	10	RDR2-sensitive
GGTGGCTCTGGACGCGGGACGCGA	0	9	RDR2-sensitive
GGTGGCTGACAGCTAGGACACGCA	0	14	RDR2-sensitive
GGTGGCTGTAGTTTAGTGGTAAGA	58	13	RDR2-resistant
GGTGGCTGTAGTTTAGTGGTGAGA	142	24	RDR2-resistant
GGTGGCTGTAGTTTAGTGGTTAGA	86	29	RDR2-resistant
GGTGGCTTAGGTACTGTGTACGGA	0	22	RDR2-sensitive
GGTGGGACATAGAACGGACGGTTT	0	10	RDR2-sensitive
GGTGGGACGATGATCGGGAGCGGC	0	9	RDR2-sensitive

GGTGGGACTGGGATAGGCCGAGGC	1	19	RDR2-sensitive
GGTGGGAGGAGACAGAACATGGCG	0	16	RDR2-sensitive
GGTGGGCCGTATGTATGAAGACGA	0	14	RDR2-sensitive
GGTGGGCTCGACCGAGATGGACAT	0	13	RDR2-sensitive
GGTGGGCTGGCACGAAGCACGCAA	0	13	RDR2-sensitive
GGTGGGCTGGTTGAGGAGTTGGGC	0	16	RDR2-sensitive
GGTGGGCTGTATGCATGGAGACGA	0	13	RDR2-sensitive
GGTGGGCTGTATGTATGGAGACGA	1	28	RDR2-sensitive
GGTGGGCTGTGCCTTATGGGTCGG	0	12	RDR2-sensitive
GGTGGGGAACTGTAGACGATACGG	0	11	RDR2-sensitive
GGTGGGGACATGGGCAGTGGGACG	0	10	RDR2-sensitive
GGTGGGGACGCTGAGAACGCGGAC	0	12	RDR2-sensitive
GGTGGGGCTGACTGTCGTATGGTG	1	14	RDR2-sensitive
GGTGGGTCTGGTCGGACGACGCGC	6	58	RDR2-sensitive
GGTGGTACTCTCTGCGCAGGGGCG	2	17	RDR2-sensitive
GGTGGTCACTGTCAGGGAGGGCGT	0	10	RDR2-sensitive
GGTGGTCTGGGCTGCCTCGGCTGC	0	21	RDR2-sensitive
GGTGGTCTGTGGCATGTGGGACGG	1	19	RDR2-sensitive
GGTGGTGAAGACTCCGAAGATCGT	0	9	RDR2-sensitive
GGTGGTGACAGTATGACACGACGG	0	12	RDR2-sensitive
GGTGGTTGATGGATAAGTTGGATC	53	0	RDR2-resistant
GGTGTAAATAGCTCTAGAGACGTC	0	10	RDR2-sensitive
GGTGTACATTCGGGCTGCACGGCT	0	10	RDR2-sensitive
GGTGTACCATTGGACTGTCCGGTG	1	14	RDR2-sensitive
GGTGTACTCTAATGAGCTGGACGC	173	27	RDR2-resistant
GGTGTAGACCACGAGCTCCGACGT	7	53	RDR2-sensitive
GGTGTAGAGGGCATACTGAATGAA	0	10	RDR2-sensitive
GGTGTAGATCGATGGATGGCAGAA	0	9	RDR2-sensitive
GGTGTAGATTGGCTGAAGGCAGAA	0	40	RDR2-sensitive
GGTGTAGCGGAGTAGTAGACCGGC	0	15	RDR2-sensitive
GGTGTAGGAGTAGATCACAGGCGT	1	14	RDR2-sensitive
GGTGTAGGATCTCGAAGGCGACAG	0	9	RDR2-sensitive
GGTGTAGGATGCGATGGTAGAGGC	170	2	RDR2-resistant
GGTGTAGTCTCTCCAAGTGGCTGG	0	20	RDR2-sensitive
GGTGTATGAGCCTCTGGTCGACGA	0	12	RDR2-sensitive
GGTGTATGCGGCTCGCTCTGCCAA	3	34	RDR2-sensitive
GGTGTCACTGAACTCAGAGAGGTC	0	15	RDR2-sensitive
GGTGTCACTGTAAACCTACGGCAA	0	9	RDR2-sensitive
GGTGTCAGACCAACGGCTAGGCGC	0	13	RDR2-sensitive
GGTGTCAGATCGACGGCAACATAC	0	10	RDR2-sensitive
GGTGTCGAACGTAGTTAATGCGGC	1	67	RDR2-sensitive
GGTGTCGACCTCGGGCGAGACGGA	0	10	RDR2-sensitive
GGTGTCGACTATGCCAGCCGCGGT	1	12	RDR2-sensitive
GGTGTCGACTTCGGGCGTGGCGGA	0	14	RDR2-sensitive
GGTGTCGGCATCGGCATAGCCCGA	0	14	RDR2-sensitive
GGTGTCGGGGTGCATACAAAGCAC	2	17	RDR2-sensitive
GGTGTCGGGGTGCATACAAAGCAT	0	14	RDR2-sensitive
GGTGTCGTCGTGTAGTTGGTTATC	42	2	RDR2-resistant
GGTGTCGTGGTGTAGTTGGTTATC	385	32	RDR2-resistant

GGTGTCGTTGAATGCAAGATGGAT	3	22	RDR2-sensitive
GGTGTCGTTGTGTAGTTGGTTATC	60	1	RDR2-resistant
GGTGTCTCCGTTGGGCCGACGTCT	0	10	RDR2-sensitive
GGTGTCTGTAGCACGGGTTGGTAT	0	10	RDR2-sensitive
GGTGTGAGCTGTGACGGCTGAGTT	72	7	RDR2-resistant
GGTGTGATCTCAGGCAGGACAGGC	0	9	RDR2-sensitive
GGTGTGATCTGGATTGACGTGGCA	0	9	RDR2-sensitive
GGTGTGATCTTTAGAACCGGCCAT	1	48	RDR2-sensitive
GGTGTGCACTGTAGCGACGGTGGT	0	25	RDR2-sensitive
GGTGTGCGGGATTGGACGGCTGAG	1	29	RDR2-sensitive
GGTGTGCTGTAGTAGTGGGCACGG	0	15	RDR2-sensitive
GGTGTGGAAGCGACAGAACATGGC	0	21	RDR2-sensitive
GGTGTGGAGCGGTAGAATGGACGG	0	14	RDR2-sensitive
GGTGTGTACAAAGGGCAGGGACGT	75	24	RDR2-resistant
GGTGTGTAGAGCAAGGGTGAGTAC	0	9	RDR2-sensitive
GGTGTGTCTCAAGGTCGCCGGGTC	0	9	RDR2-sensitive
GGTGTGTTTGGTTGGAGAGCGGAT	0	17	RDR2-sensitive
GGTGTTCTGGAACGGGCTCGACGA	0	12	RDR2-sensitive
GGTGTTGCTAGTAGAACGGACGGA	0	24	RDR2-sensitive
GGTGTTGGACGAATACGGGGAGGA	0	9	RDR2-sensitive
GGTGTTGGGTGCTGGGTAGGCGTA	0	9	RDR2-sensitive
GGTGTTTCTAGGAGTAGGACGGTG	19	1	RDR2-resistant
GGTGTTTCTTAGAACTGTCGGCAC	0	10	RDR2-sensitive
GGTGTTTTAGGAGTAGGACGGTAT	0	19	RDR2-sensitive
GGTGTTTTAGGAGTAGGACGGTGT	6	203	RDR2-sensitive
GGTGTTTTTTCGGACCTTCGGCAT	0	9	RDR2-sensitive
GGTGTTTTTTCGGACCTTCGGCGT	1	15	RDR2-sensitive
GGTTAATAATCGTGTCGGGCCGGC	0	10	RDR2-sensitive
GGTTAATCGTTGGCGTGTCCCGGT	0	31	RDR2-sensitive
GGTTACCAGCTGAGAATTAGGCGT	10	53	RDR2-sensitive
GGTTACCTGCAAACGGTCGTCTGA	0	10	RDR2-sensitive
GGTTACCTGGACGGAGATTTGAGC	0	12	RDR2-sensitive
GGTTACTGGATCAAGGAGGACGTT	0	18	RDR2-sensitive
GGTTACTGTTATTGATCGGCTCCA	21	1	RDR2-resistant
GGTTAGATTCGTGTCCGGACCGGC	0	13	RDR2-sensitive
GGTTAGTTCGGTTCCGGTTCTCGG	0	30	RDR2-sensitive
GGTTATCACACAGGGTCGTCTCGT	0	10	RDR2-sensitive
GGTTATCGGGCTGAACCATGCCGG	0	30	RDR2-sensitive
GGTTATGTGACTGATTATGGTGGT	49	2	RDR2-resistant
GGTTCAACGTGAGGACGGTACGGA	0	9	RDR2-sensitive
GGTTCAGGACTTATTTCCGACGGT	7	64	RDR2-sensitive
GGTTCAGTATCGTGTCGGGCCGGC	0	17	RDR2-sensitive
GGTTCAGTCTGTCGGCCGGACACA	0	10	RDR2-sensitive
GGTTCCACGTCGGCAACACGTCAT	0	12	RDR2-sensitive
GGTTCCCATGGCAGTGTAGGACGG	0	34	RDR2-sensitive
GGTTCCTACAGGAGAGGATCGTGC	24	0	RDR2-resistant
GGTTCGAAGGTGGGACTAGGTCGG	0	9	RDR2-sensitive
GGTTCGACGGATCTGATTGGGGGC	0	9	RDR2-sensitive
GGTTCGCAACTCTGGATCGGACGG	0	16	RDR2-sensitive

GGTTCGCGACTCTAGGCTAGACGA	0	11	RDR2-sensitive
GGTTCGCGGCTCTGGACTGGACGG	1	20	RDR2-sensitive
GGTTCGGAATAGTCGGGAACGGAT	0	9	RDR2-sensitive
GGTTCGGACGCTACAGGTAGAGGC	0	12	RDR2-sensitive
GGTTCGGCACCACAGATCTTGGCA	2	20	RDR2-sensitive
GGTTCGGTACGGTTCGGTTCGGTT	0	9	RDR2-sensitive
GGTTCGGTCTGGGTGAAGCGTCAC	0	16	RDR2-sensitive
GGTTCGGTCTTGGATTGGTTCGGT	0	18	RDR2-sensitive
GGTTCTCGGATTGGATCGGTATGG	0	14	RDR2-sensitive
GGTTCTCGGTTATTTCGGTTCGGT	0	9	RDR2-sensitive
GGTTCTGGCTCGAGCGGATTGCAG	0	10	RDR2-sensitive
GGTTCTGGTTCTGGCTCGAGCGGA	1	25	RDR2-sensitive
GGTTCTGGTTCTGGCTCGGGCGGA	0	9	RDR2-sensitive
GGTTCTTCGGTTCGGTTCCTCGGT	0	14	RDR2-sensitive
GGTTGAAGAGCCAAGTAGAACGGA	2	40	RDR2-sensitive
GGTTGAATTTCAGGCTGACTCGGT	0	16	RDR2-sensitive
GGTTGACACGGAATGGAAGATCGG	0	10	RDR2-sensitive
GGTTGACGGAGATACTAGAGCCAC	1	15	RDR2-sensitive
GGTTGACGGAGATACTAGAGCCGC	3	79	RDR2-sensitive
GGTTGACTGTACATGGTAGACGCA	0	35	RDR2-sensitive
GGTTGACTGTACGCAAGGAACGGT	0	36	RDR2-sensitive
GGTTGACTGTACGCGAGAAACGGT	0	24	RDR2-sensitive
GGTTGACTGTACGTGATAGACGCA	0	65	RDR2-sensitive
GGTTGACTGTACGTGGTAGATGCA	0	16	RDR2-sensitive
GGTTGACTGTACGTGTTAGACGCA	0	13	RDR2-sensitive
GGTTGACTTAGAATCGATAGGCAT	0	9	RDR2-sensitive
GGTTGAGACTAGGACGGTATCTGA	2	29	RDR2-sensitive
GGTTGAGATCGTAGAACGGCAGAA	0	16	RDR2-sensitive
GGTTGAGATTGCAGAACGGTAGGC	0	14	RDR2-sensitive
GGTTGAGATTGCAGGATGGCAGAA	0	24	RDR2-sensitive
GGTTGAGGAGCCAAGTAGAACGAA	0	24	RDR2-sensitive
GGTTGAGGAGCCAAGTAGAACGGA	8	687	RDR2-sensitive
GGTTGAGGAGCCAAGTAGAACGGT	0	9	RDR2-sensitive
GGTTGAGGAGCCAAGTAGAATGGA	1	19	RDR2-sensitive
GGTTGAGGAGCCGAGTAGAACGGA	0	11	RDR2-sensitive
GGTTGAGGAGTCAAGTAGAACGGA	0	9	RDR2-sensitive
GGTTGAGGATCTAGACGACTATTA	21	0	RDR2-resistant
GGTTGATTGTATGGGTAAGACGAG	0	12	RDR2-sensitive
GGTTGCAATGACAGGACGAGACGG	0	11	RDR2-sensitive
GGTTGCAATGACAGGACGGGACGG	0	34	RDR2-sensitive
GGTTGCAATGACAGGACGGGATGG	0	21	RDR2-sensitive
GGTTGCAATGATAGGACGGGACGG	1	21	RDR2-sensitive
GGTTGCAGAGTGAACGGAACAGAG	1	20	RDR2-sensitive
GGTTGCAGAGTGAACGGAACGGAA	1	13	RDR2-sensitive
GGTTGCAGAGTGAACGGAACGGAG	11	84	RDR2-sensitive
GGTTGCAGAGTGAATGGAACGGAG	0	10	RDR2-sensitive
GGTTGCAGATTGAACGGAACGGAG	0	11	RDR2-sensitive
GGTTGCATTGTAGTAGATCGACGG	1	24	RDR2-sensitive
GGTTGCCGGATAAGCAGAACCAGG	0	11	RDR2-sensitive

GGTTGCCGTAGACTGTGATGGCGC	0	9	RDR2-sensitive
GGTTGCCTATGGGGCCGTCGGACA	1	13	RDR2-sensitive
GGTTGCCTGCTTGTGGATGGACGT	0	18	RDR2-sensitive
GGTTGCCTGTACGTGAGGAACGGT	0	10	RDR2-sensitive
GGTTGCGGGACAAGATAGGGACGT	0	19	RDR2-sensitive
GGTTGCGGGAGGCAGAACGTGCAC	0	9	RDR2-sensitive
GGTTGCGGGTCAGGACGAGATGGA	0	11	RDR2-sensitive
GGTTGCTCGGATAGCTACACCCAG	0	9	RDR2-sensitive
GGTTGCTTGTACGGACTTGGTCGG	0	10	RDR2-sensitive
GGTTGGACGGACGAACATAGGTAT	0	9	RDR2-sensitive
GGTTGGAGAGCCAAGTAGAACGGA	0	27	RDR2-sensitive
GGTTGGATGGTCGAGGTGGGATGG	0	11	RDR2-sensitive
GGTTGGATTGGGACATCAACACGG	0	9	RDR2-sensitive
GGTTGGCTCGGACCTGGCACATAG	0	12	RDR2-sensitive
GGTTGGCTGCAGAGCAGGAGGACA	0	15	RDR2-sensitive
GGTTGGGTGTGGGATGGATATCAA	0	17	RDR2-sensitive
GGTTGGTCAGGAGTCGAGACGTCG	45	0	RDR2-resistant
GGTTGGTCGTACGAAGAGGGACGG	0	15	RDR2-sensitive
GGTTGGTCTTGGACAAGGCGTGGT	0	11	RDR2-sensitive
GGTTGTAAACGGGCTGTGTCGGTC	0	14	RDR2-sensitive
GGTTGTAATGACAGAACGGGACGA	0	11	RDR2-sensitive
GGTTGTAGGACTGCAGAAAGACGT	1	54	RDR2-sensitive
GGTTGTATGACGCCATGTAGACGC	0	17	RDR2-sensitive
GGTTGTCAGACGGACCGGAAATAC	0	15	RDR2-sensitive
GGTTGTCGAGCGAACGAAGTGGTT	0	10	RDR2-sensitive
GGTTGTCGGCGAAGTTGAAGGCGC	0	21	RDR2-sensitive
GGTTGTCTGCATCTGGAAGACGTG	0	13	RDR2-sensitive
GGTTGTCTGTGTGTAGATGCGGAG	0	10	RDR2-sensitive
GGTTGTCTTGGACCGTCGGATATA	0	10	RDR2-sensitive
GGTTGTGAGTCAAGGCTGAGACGG	0	51	RDR2-sensitive
GGTTGTGGGACAGCTAGGACGGGA	1	47	RDR2-sensitive
GGTTGTGTGGAGGAGGTGCTGGTT	84	6	RDR2-resistant
GGTTGTGTGGTGTGGATGGCTCAT	0	9	RDR2-sensitive
GGTTGTTACGGATTGATGGGTCGG	0	13	RDR2-sensitive
GGTTGTTCTCGGACGAGTCGTGGC	0	13	RDR2-sensitive
GGTTGTTGGAGCGGTAGCGGATGT	0	9	RDR2-sensitive
GGTTGTTGTACTGTGGACCGGCAT	0	9	RDR2-sensitive
GGTTGTTGTGTGGCTATAGACGGT	71	4	RDR2-resistant
GGTTGTTTGCCGGACGGCTAGATC	1	12	RDR2-sensitive
GGTTGTTTGGTTGACTGCACGTAA	0	13	RDR2-sensitive
GGTTTACTGGTCTGGTCGAACTAC	0	9	RDR2-sensitive
GGTTTAGGGCTTATTCCCGTCGGT	0	9	RDR2-sensitive
GGTTTATGGACATGTATCTTCGGA	0	12	RDR2-sensitive
GGTTTATTATAGTTGGATAACTGA	81	0	RDR2-resistant
GGTTTCCTATGGGGCCGTCGGACA	1	16	RDR2-sensitive
GGTTTCGCTTCGGTTCGCTCGGAC	0	12	RDR2-sensitive
GGTTTCGTCGTGTAGTTGGTTATC	14	0	RDR2-resistant
GGTTTCGTGGTGTAGTTGGTTATC	33	1	RDR2-resistant
GGTTTGGACTTCCAGCAGCTCGGC	0	13	RDR2-sensitive

GGTTTGGTCGTAGCACGAACGGTG	0	12	RDR2-sensitive
GGTTTGTGGCCTAGGACCAGATGA	0	11	RDR2-sensitive
GGTTTTATCGTGCCTATGGACTAA	1	52	RDR2-sensitive
GGTTTTCTTAGTGGGCTGGGTCGG	1	30	RDR2-sensitive
GGTTTTGTGAAGATATCGGCTAGT	83	4	RDR2-resistant
GTAAAAGACATCGGCTCCGGCAGA	0	10	RDR2-sensitive
GTAAAAGACGGATATAGAGGATGT	0	12	RDR2-sensitive
GTAAAATGGACGGTTGGATGGGTC	0	10	RDR2-sensitive
GTAAACGGACTAACAGGACGGGGT	0	18	RDR2-sensitive
GTAAACTCTTTTTAGATTGTCGGT	0	10	RDR2-sensitive
GTAAAGGACGGATATAGAGGATGT	2	26	RDR2-sensitive
GTAAAGTGGGAGGTGATCGTCTGT	0	10	RDR2-sensitive
GTAAATATGACTGCGGAAGATTGT	0	9	RDR2-sensitive
GTAAATTGTCGCCGCGTGCCCCAG	0	13	RDR2-sensitive
GTAAATTGTCGTCGCGTGCCCCAG	0	9	RDR2-sensitive
GTAACAACGACTGCGGATCGGCTA	34	0	RDR2-resistant
GTAACAATCTCGTCGCGCGGCTTC	0	13	RDR2-sensitive
GTAACACTCGGCAAAGAAGGCTCG	0	10	RDR2-sensitive
GTAACACTCGGCAAAGAGGCTCTT	0	15	RDR2-sensitive
GTAACACTCTCGTCGCGCGGCTTC	0	10	RDR2-sensitive
GTAACAGGCCGGCGAGGACGACGT	0	14	RDR2-sensitive
GTAACATCGGGTCGGATTCGGATA	0	10	RDR2-sensitive
GTAACATTCGCCGGATGCATGTGC	14	0	RDR2-resistant
GTAACATTGACTGTAGATGTGCAT	0	11	RDR2-sensitive
GTAACCCCTTGGTGAAGATGTCGG	0	11	RDR2-sensitive
GTAACCCTGTGATGCGTAGTCGGA	0	13	RDR2-sensitive
GTAACCCTTGTCTCTGACGGCTGA	0	9	RDR2-sensitive
GTAACGCGGCATGACATACCTGGA	0	10	RDR2-sensitive
GTAACGGGCTTCGGGCTAAACGGG	0	9	RDR2-sensitive
GTAACGTTGGCTCTTGACGGACAT	0	185	RDR2-sensitive
GTAACGTTGGCTGTTGACGGACAT	0	9	RDR2-sensitive
GTAACTAGAACGGAGTGGCTCCAA	1	24	RDR2-sensitive
GTAACTCGGATGTATGTTGGTTAC	28	1	RDR2-resistant
GTAACTCGGGAGTGGACGGGGCAC	0	11	RDR2-sensitive
GTAAGACATGTGGGGCCAGTGGCA	0	10	RDR2-sensitive
GTAAGACTGGCATGTAGGCGACAT	0	9	RDR2-sensitive
GTAAGAGGCTGCAAACCACTCGGT	1	17	RDR2-sensitive
GTAAGGTGTGTTGGGCCGAAACGG	1	31	RDR2-sensitive
GTAAGTACTGTGACGGAACCTCCC	0	12	RDR2-sensitive
GTAAGTCCGGCATACACTCGTCAG	0	14	RDR2-sensitive
GTAAGTCGTCGCAACGCACGGGCA	1	22	RDR2-sensitive
GTAATACGACAGGACGCGGACCAA	0	10	RDR2-sensitive
GTAATCGGAGGAGACATAGGGCAT	0	23	RDR2-sensitive
GTAATCGGATCGAATACGGACGGA	0	9	RDR2-sensitive
GTAATCTGGAACGGAGCGGCTCTG	0	19	RDR2-sensitive
GTAATGGATCTGTCTCGTTTGGAC	0	9	RDR2-sensitive
GTAATGGGCGCTGTAGGGGGCACC	0	19	RDR2-sensitive
GTAATGGGTTGTATATGGTTGACA	28	0	RDR2-resistant
GTAATTGGACTTTCGGTATTATAA	0	11	RDR2-sensitive

GTAATTGTGTGTCTGGGCCGGTAC	0	11	RDR2-sensitive
GTACAACGGTAGAATGGACGACTG	0	9	RDR2-sensitive
GTACAATCGACTGTTGATGGGCAT	0	12	RDR2-sensitive
GTACACAGCCGTCGGAGATAAGGC	0	22	RDR2-sensitive
GTACACAGTTGGAACACTCGGCAA	0	9	RDR2-sensitive
GTACACCGTTGGATATTGGATGGA	0	12	RDR2-sensitive
GTACACGCGGCTGTAGGTAAGGCT	1	14	RDR2-sensitive
GTACACTACACATGGTCGGACGGT	0	10	RDR2-sensitive
GTACAGACGCTGGATCCTCGGTAT	0	11	RDR2-sensitive
GTACAGCGCGATGGCAGAACCAAA	0	9	RDR2-sensitive
GTACAGCGGCAATAGAGCAAGGCA	0	11	RDR2-sensitive
GTACAGGAGGGCTGAGGGCGTGCT	0	9	RDR2-sensitive
GTACAGGCGAGTAGCGTGCGGACA	0	9	RDR2-sensitive
GTACAGGGCCTCGGATTTTGTCGG	0	11	RDR2-sensitive
GTACAGTAGGAAGTGAGGCGTCTG	3	21	RDR2-sensitive
GTACAGTGGGAAGTGAGGCGTCTG	4	18	RDR2-sensitive
GTACAGTTGGACCATCTACAGCAG	0	11	RDR2-sensitive
GTACATGTAGATGATTGGTAGTAT	0	10	RDR2-sensitive
GTACATGTAGGACCGATAAGGCGA	3	18	RDR2-sensitive
GTACCAGGTCGCGGACCGTCCGGC	5	33	RDR2-sensitive
GTACCAGGTCGCGGACTGTCCGGC	0	22	RDR2-sensitive
GTACCATCGAGTTGCGATTGTCGG	1	32	RDR2-sensitive
GTACCATTGGACTGTCCGGTGTGC	0	24	RDR2-sensitive
GTACCCCTGTAGATGGTATGGCTT	0	14	RDR2-sensitive
GTACCCGATAGATCGCAGCCGGAT	0	13	RDR2-sensitive
GTACCCTCGGACTAAAGTCGGCAT	4	45	RDR2-sensitive
GTACCGAGCTTGGACTGCGGCGTA	0	34	RDR2-sensitive
GTACCGGAATGAGGCAGGATTCAT	0	15	RDR2-sensitive
GTACCGGGCGTAGAAGATGCGGCC	0	9	RDR2-sensitive
GTACCGTTGGATGTAGATGGTGTG	0	63	RDR2-sensitive
GTACCTACCTGTCGGATTGGTCGG	0	44	RDR2-sensitive
GTACCTATCGTTGTATTGGTCGGG	0	9	RDR2-sensitive
GTACCTATTTCTGATCGTTATGGT	0	9	RDR2-sensitive
GTACCTCCTTGCTCTTGTCGTCGT	0	16	RDR2-sensitive
GTACCTCGAGACGACGACATGAAT	0	15	RDR2-sensitive
GTACCTGGGGTGTAGAAGGCAGGC	0	23	RDR2-sensitive
GTACCTGGTAGACACGTCGACGAA	0	66	RDR2-sensitive
GTACCTGTAGTACTTGTGCAGATT	31	3	RDR2-resistant
GTACCTGTGACTGTAGCTTCGGCT	0	11	RDR2-sensitive
GTACCTTCATACTGTAGTAACGGA	0	14	RDR2-sensitive
GTACCTTCGGACTAAAGTTGGCAT	5	50	RDR2-sensitive
GTACCTTCGGACTCAAGTCGGTAT	0	37	RDR2-sensitive
GTACCTTCGGACTCAAGTTGGTAT	0	12	RDR2-sensitive
GTACGAAGTCGGTGCAGGAAGATG	4	44	RDR2-sensitive
GTACGAATAAGCGGGCCGGATCTC	0	14	RDR2-sensitive
GTACGACACTGGTAGCGAAGACAT	0	10	RDR2-sensitive
GTACGACGTAGGCTGAACCGGTAT	0	12	RDR2-sensitive
GTACGACGTGGTAGACGGGCTGCT	0	18	RDR2-sensitive
GTACGAGCAGATGATGGAGGACAA	0	12	RDR2-sensitive

GTACGAGCAGCTGGGTTGGAGGAA	17	1	RDR2-resistant
GTACGAGCGAGAACCTGTGCGGTA	0	12	RDR2-sensitive
GTACGAGGACGCAACAGATCAGAA	0	14	RDR2-sensitive
GTACGATTTTATGCTTGAACACGG	2	14	RDR2-sensitive
GTACGCACGGACTATTCGCGGCTC	1	42	RDR2-sensitive
GTACGCAGACAGAACATGACGCAT	0	26	RDR2-sensitive
GTACGCATCGAGGACTGCGGCCAG	0	10	RDR2-sensitive
GTACGCGAGGAACGATGGCTGCAC	0	12	RDR2-sensitive
GTACGCGCACAGCAAGACTCTCGA	0	9	RDR2-sensitive
GTACGCGGCTCTAGACCGGACGGT	0	21	RDR2-sensitive
GTACGCGGTGGGAGAGATGAGGGC	21	0	RDR2-resistant
GTACGCTGTTAGAACGCCGGTGTG	0	9	RDR2-sensitive
GTACGGAGGCAGAACGCGGCTCGG	0	21	RDR2-sensitive
GTACGGAGTGAGAACAGAGGCGGG	0	9	RDR2-sensitive
GTACGGAGTGTAGGCGTCCAACGG	0	9	RDR2-sensitive
GTACGGATCAGCCGGCAGCGACAG	0	9	RDR2-sensitive
GTACGGATCGTTTTAGGGTTTCAC	0	18	RDR2-sensitive
GTACGGCTATAATTCACCGGACTG	6	37	RDR2-sensitive
GTACGGGCAACTTCAGAGACACAT	1	13	RDR2-sensitive
GTACGGGCCGACAGCATGGACGAG	0	23	RDR2-sensitive
GTACGGGTGTAGAGTGTAGACGCC	0	12	RDR2-sensitive
GTACGGTACACGCAGGGCCTCGGT	0	9	RDR2-sensitive
GTACGGTAGACGAAGGGACGACAG	0	14	RDR2-sensitive
GTACGGTCAGAGTAGCAGGCTGGA	0	26	RDR2-sensitive
GTACGTAGCGGGAGTGTTTGACAG	23	3	RDR2-resistant
GTACGTCAGAGGAACCGGCTCCGA	0	18	RDR2-sensitive
GTACGTCCTGTGGAAGAAGCGGCG	0	12	RDR2-sensitive
GTACGTCGAGCGCAGGAACGGGAA	0	17	RDR2-sensitive
GTACGTCGTGTGTAGAGATGGCAA	1	26	RDR2-sensitive
GTACGTGAATTGCTGATCCTGGCA	0	18	RDR2-sensitive
GTACGTGAGGAACGGTGTCTGCAT	0	13	RDR2-sensitive
GTACGTGAGGACATTGCAGAACGG	0	11	RDR2-sensitive
GTACGTGAGTTCTGATCCTGGCAC	0	22	RDR2-sensitive
GTACGTGCACATGTAGATCGGTAT	0	77	RDR2-sensitive
GTACGTGCTGATGGAACCCGGCAT	0	9	RDR2-sensitive
GTACGTGGCTAGGGATTGAGGGCA	2	30	RDR2-sensitive
GTACGTGGGCTTTCTGTACGGCAT	52	12	RDR2-resistant
GTACGTGTCGGCAAGCGAGAGGAA	0	9	RDR2-sensitive
GTACGTGTCGGCAAGCGAGAGGAC	0	10	RDR2-sensitive
GTACGTTTGGACCTGATGCTCGGC	0	10	RDR2-sensitive
GTACTACGACGGCAGCCAAAACTC	0	9	RDR2-sensitive
GTACTACTGTTTTTTGGACGGCCG	0	17	RDR2-sensitive
GTACTAGTGGACGTGCTCGTCGGC	0	13	RDR2-sensitive
GTACTCCCTTAGCGCATTGTCGGT	0	9	RDR2-sensitive
GTACTCGGGCTGCGTCGAGGCGTC	1	22	RDR2-sensitive
GTACTCTAATGAGCTGGACGCACT	31	3	RDR2-resistant
GTACTCTCGGACTAAAGTCGGCAT	0	38	RDR2-sensitive
GTACTCTGCCTGTTGACGCAACAC	0	10	RDR2-sensitive
GTACTCTGGTCGTCTAACAAGGTA	0	25	RDR2-sensitive

GTACTGAACCGCCTGGGACGTCAC	0	21	RDR2-sensitive
GTACTGAGGCATGAGCGTGATGGC	28	0	RDR2-resistant
GTACTGGAGACTCCTACAGAAGAT	0	10	RDR2-sensitive
GTACTGGCCTGCTTCAGACGACTC	0	20	RDR2-sensitive
GTACTGGGACTAGAGGACGACGAA	0	11	RDR2-sensitive
GTACTGTACTGTTTAGACGATGGA	0	16	RDR2-sensitive
GTACTGTAGACTGTTGTCGGCGTT	0	17	RDR2-sensitive
GTACTGTAGAGTGTCCGGTGCGCC	1	38	RDR2-sensitive
GTACTGTAGATAATAGGCTGTTTA	0	12	RDR2-sensitive
GTACTGTAGCACCGGTAGGAGCAA	0	10	RDR2-sensitive
GTACTGTAGCACCGGTAGGGGCAA	0	10	RDR2-sensitive
GTACTGTAGCGTCCCTGTCGGCGT	0	34	RDR2-sensitive
GTACTGTATCGAATTATTAGGCCA	0	35	RDR2-sensitive
GTACTGTGGACGCCTACACTGCGT	0	12	RDR2-sensitive
GTACTGTTCACGCGGACTTGGCAT	2	34	RDR2-sensitive
GTACTGTTCACGCTGACTTGGCAT	41	378	RDR2-sensitive
GTACTGTTGGAGTGAATGAAGATG	0	25	RDR2-sensitive
GTACTGTTTGGTTTTGTCGGCGTT	0	9	RDR2-sensitive
GTACTTAACTTAAGTTCGTCGGTA	0	9	RDR2-sensitive
GTACTTCTGGATACGGGTCGGATA	0	9	RDR2-sensitive
GTACTTTCTGAACGCTAGAGTGGC	0	11	RDR2-sensitive
GTACTTTTTCTCGGGCACTCGGCA	10	32	RDR2-sensitive
GTACTTTTTGTCGGGCACTCGGCA	1	20	RDR2-sensitive
GTAGAAAACGGTACGGGTCGGAAC	1	13	RDR2-sensitive
GTAGAAATCCATCGGTTGTTGAAT	193	23	RDR2-resistant
GTAGAACACCGCAGGAGTAGACGT	0	14	RDR2-sensitive
GTAGAACAGGACTGTCTGCGGCAC	0	109	RDR2-sensitive
GTAGAACCGAACCGTGTGGGACTC	0	9	RDR2-sensitive
GTAGAACGGTAGAACCACGGTGGC	0	21	RDR2-sensitive
GTAGAACGGTAGAATGGACGACTG	0	21	RDR2-sensitive
GTAGAACGGTAGAATGGACGGTAA	0	9	RDR2-sensitive
GTAGAACGTGGCTAGTAGAGGTGT	0	18	RDR2-sensitive
GTAGAACTGGACCATGTCGGGCTC	0	53	RDR2-sensitive
GTAGAACTGGGAGAACGACGACGC	0	42	RDR2-sensitive
GTAGAAGCCGCAGCCGCAGCCGGA	0	13	RDR2-sensitive
GTAGAAGCCGCAGCCGGATTGCAG	0	19	RDR2-sensitive
GTAGAAGCTTCGAGCCTTGGACGT	0	13	RDR2-sensitive
GTAGAAGGGAGCACTCGGACGCGC	0	30	RDR2-sensitive
GTAGAAGGGCGGACCAGAATGGTT	1	13	RDR2-sensitive
GTAGAATAAACGGGCGGGCTTGGA	0	45	RDR2-sensitive
GTAGAATAAGCGGACGGGCTTGGA	2	23	RDR2-sensitive
GTAGAATAAGCGGGCGGACTTGGA	0	14	RDR2-sensitive
GTAGAATAAGCGGGCGGGCTTGGA	2	435	RDR2-sensitive
GTAGAATAAGCGGGCGGGCTTGGC	0	11	RDR2-sensitive
GTAGAATAAGCGGGCGGGCTTGGG	0	11	RDR2-sensitive
GTAGAATAAGCGGGCGGGCTTGGT	0	10	RDR2-sensitive
GTAGAATGGACGGTTGGATGGGAC	0	25	RDR2-sensitive
GTAGAATGGGAAGTACTCGAGCAT	2	20	RDR2-sensitive
GTAGAATGGTAGAACGGCAGGCTA	1	19	RDR2-sensitive

GTAGACACTCGGCACAGACTGGTT	0	12	RDR2-sensitive
GTAGACACTCGGCACGGACTGGTT	0	12	RDR2-sensitive
GTAGACCACGAGCTCCGACGTCGG	3	40	RDR2-sensitive
GTAGACCAGCGAGCACGACGACGT	0	14	RDR2-sensitive
GTAGACCTATTCAATCGTCGGGTC	0	14	RDR2-sensitive
GTAGACGAGAAGCACGAGGGACAG	0	13	RDR2-sensitive
GTAGACGAGTTGGACGGTTCGTGT	0	25	RDR2-sensitive
GTAGACGATGTTGGGCCAGAAGAT	0	14	RDR2-sensitive
GTAGACGGCAGAACGGCAGAACAC	1	84	RDR2-sensitive
GTAGACGGGTGGTGGAATATAGGC	0	17	RDR2-sensitive
GTAGACGGTCTGTGCTGCTACGGT	0	17	RDR2-sensitive
GTAGACGTAGTAGACCGGCGAGGA	0	9	RDR2-sensitive
GTAGACTCATGATCGAGCGGCTTG	0	20	RDR2-sensitive
GTAGACTCGGACGTAGCTTCGGAT	0	9	RDR2-sensitive
GTAGACTGACGGGACACAGATAAC	0	16	RDR2-sensitive
GTAGACTGCTGTAGACGGCCTTAG	0	14	RDR2-sensitive
GTAGACTGGAAACGAGCCGAGTCA	0	10	RDR2-sensitive
GTAGACTGGAGCAGCAGGAACGGG	0	34	RDR2-sensitive
GTAGACTGGATGAGCGGATGCAAA	0	14	RDR2-sensitive
GTAGACTGTACGGTGACCTACCAC	0	9	RDR2-sensitive
GTAGACTGTATTAGATCCGGTTGA	0	10	RDR2-sensitive
GTAGACTGTGCCGAAGATGCCACC	50	2	RDR2-resistant
GTAGACTTGCAGATCTGGCCTCCA	17	38	RDR2-sensitive
GTAGACTTGCAGATCTGGCCTCTG	0	15	RDR2-sensitive
GTAGAGAGCATGTAGAACTGGCGT	0	10	RDR2-sensitive
GTAGAGAGCGCTGTAGGGGGCACC	1	38	RDR2-sensitive
GTAGAGATGGCAATGGGGACCCGA	0	9	RDR2-sensitive
GTAGAGCAGCGTCGGGGTCGGTGT	0	11	RDR2-sensitive
GTAGAGCGCAGGAGTGAATTCGAC	0	19	RDR2-sensitive
GTAGAGCGCGGCGCTGTATGTGAC	0	10	RDR2-sensitive
GTAGAGCGGGCGAAGACATGACCT	0	12	RDR2-sensitive
GTAGAGCGTAGAGGACCGTTTGAA	0	11	RDR2-sensitive
GTAGAGCTGTACGGGCATCAACGC	0	109	RDR2-sensitive
GTAGAGGACGCTGTAGGGGGCACC	0	17	RDR2-sensitive
GTAGAGGAGGCAGTAGAGTGATGC	0	12	RDR2-sensitive
GTAGAGGATGCTGTAGAGGGCACC	0	20	RDR2-sensitive
GTAGAGGCCGAAGGTAGGAGTCGA	0	10	RDR2-sensitive
GTAGAGGCGCAAGAAGATGTAGAG	0	14	RDR2-sensitive
GTAGAGGCTCGACACAAGAACGGC	0	10	RDR2-sensitive
GTAGAGGGCGCTGTAGAGGGCACC	0	24	RDR2-sensitive
GTAGAGGGCGCTGTAGGAGGCACC	0	11	RDR2-sensitive
GTAGAGGGCGCTGTAGGGGACACC	0	11	RDR2-sensitive
GTAGAGGGCGCTGTAGGGGGCACC	2	85	RDR2-sensitive
GTAGAGGGCTGCGCTGCGGGACGT	1	38	RDR2-sensitive
GTAGAGGTCGGCTTCCCGGAACAG	3	23	RDR2-sensitive
GTAGAGTCGAATAGGGGTGGAGGT	4	19	RDR2-sensitive
GTAGAGTGACGTGTAGGGGGCAGC	0	15	RDR2-sensitive
GTAGAGTGGACGGTTGACATCGGC	0	10	RDR2-sensitive
GTAGAGTGTGGTCTCTGCGTTCGA	2	19	RDR2-sensitive

GTAGAGTTAGGCAGACTAGGCTCA	0	12	RDR2-sensitive
GTAGATAGCAGAACGGCAGAACAT	0	9	RDR2-sensitive
GTAGATATACCTGACTGGGGCTTC	0	17	RDR2-sensitive
GTAGATATAGGAGATGGAATCTTT	0	17	RDR2-sensitive
GTAGATATTGGTGCACAGGACTGC	0	24	RDR2-sensitive
GTAGATCAACGGCTGAAAATGCAA	0	10	RDR2-sensitive
GTAGATCAAGAGGGCTTCTCCGGC	17	86	RDR2-sensitive
GTAGATCAATGCGGTCGACTGGAG	0	10	RDR2-sensitive
GTAGATCACGGCACTACCATGACT	0	15	RDR2-sensitive
GTAGATCATGGGCAGCAGATACGG	0	11	RDR2-sensitive
GTAGATCTTGAAGATGGATGACTT	17	1	RDR2-resistant
GTAGATGGCAGAACGGCAGAACAC	0	51	RDR2-sensitive
GTAGATGTTGTGGCTAGAGCGGAA	0	30	RDR2-sensitive
GTAGATTCTCCGAAGCGGACTGAC	1	22	RDR2-sensitive
GTAGATTGGCTGAAGGCAGAACAG	0	36	RDR2-sensitive
GTAGATTGGCTGAAGGCAGAACCA	0	37	RDR2-sensitive
GTAGCAACGCACGGACATACACCT	0	18	RDR2-sensitive
GTAGCAACGGTCGGTTGGTCGCTA	0	12	RDR2-sensitive
GTAGCAACGTACGGGCATATACCT	0	12	RDR2-sensitive
GTAGCACACAGTGGAATTGAGCAT	0	14	RDR2-sensitive
GTAGCACTCGGCAAAGGAACTGGC	1	18	RDR2-sensitive
GTAGCAGACCGGCGAAGAGAACGA	0	9	RDR2-sensitive
GTAGCAGATCAGCGAGGACGACGA	0	18	RDR2-sensitive
GTAGCAGCTCGGCTCGGCGCTCAG	0	10	RDR2-sensitive
GTAGCAGCTCGGCTTGACTCGGAC	0	13	RDR2-sensitive
GTAGCAGTCGACCGTTGGATCCGA	2	25	RDR2-sensitive
GTAGCATCGCACGGGTACGTACCT	0	14	RDR2-sensitive
GTAGCATTCTGTCGGGAGCGTGCG	2	14	RDR2-sensitive
GTAGCCGGAACAAGATAGCCTCAG	0	11	RDR2-sensitive
GTAGCCTCGGCCTGTAGTGTTGGC	0	9	RDR2-sensitive
GTAGCCTGGACAACGTGAGCGGAG	0	9	RDR2-sensitive
GTAGCCTGGACGTGGAGTACAAGA	20	1	RDR2-resistant
GTAGCCTGGACGTGGAGTATAAGA	33	0	RDR2-resistant
GTAGCCTGGCTGAGCGACGACGAA	0	11	RDR2-sensitive
GTAGCCTGTAGGGTTTGTGGCGGA	0	14	RDR2-sensitive
GTAGCGAAGACTGCATGGACAGAC	0	15	RDR2-sensitive
GTAGCGACAATTTACTCGTCGGTC	2	43	RDR2-sensitive
GTAGCGACTCGACGGTACGAACGA	0	31	RDR2-sensitive
GTAGCGCGCGATGGCAGAACCAAA	0	14	RDR2-sensitive
GTAGCGCTGACTCTTGGCATTCTC	1248	87	RDR2-resistant
GTAGCGGAGTAGTAGACCGGCGAA	0	12	RDR2-sensitive
GTAGCGGCTGAAGCAGAGACTCTA	0	9	RDR2-sensitive
GTAGCGGTGTAGAAGGCCTTCAAC	0	10	RDR2-sensitive
GTAGCGTAGCTCTACTTAGATCGT	24	0	RDR2-resistant
GTAGCGTCTGATTGTACGGTGGTT	0	18	RDR2-sensitive
GTAGCTAACTGGACCTAAGAGCAT	0	9	RDR2-sensitive
GTAGCTAGTATAGACATGGGCGGA	0	13	RDR2-sensitive
GTAGCTCGGCGCCAAGATCTGCGG	1	16	RDR2-sensitive
GTAGCTCGGCGCCAAGATCTGTGG	1	14	RDR2-sensitive

GTAGCTCGGCGCCACAGATCTTGG	2	29	RDR2-sensitive
GTAGCTCGGCTGTATATTGGGTGA	0	63	RDR2-sensitive
GTAGCTCTCGCGGATGCTGGGCAC	0	20	RDR2-sensitive
GTAGCTCTGCGGACGGTGATTTTA	0	18	RDR2-sensitive
GTAGCTCTGCGGACGGTGATTTTG	1	24	RDR2-sensitive
GTAGCTCTTGTATTGTAGTGACGT	0	42	RDR2-sensitive
GTAGCTGCAGACGGATGACCACAT	0	13	RDR2-sensitive
GTAGCTGGAGAACGTAGCAGCATG	0	31	RDR2-sensitive
GTAGCTGTAGGGGACGGAATTTAG	0	9	RDR2-sensitive
GTAGCTGTGGAGTAGTAGGCTGGT	0	9	RDR2-sensitive
GTAGCTTACGTGGCTGCAATCTGA	23	0	RDR2-resistant
GTAGCTTATACGTGCTGGGTCGGA	0	13	RDR2-sensitive
GTAGCTTCGGATTCGGGTCGGATA	0	13	RDR2-sensitive
GTAGGAAGTTAGCGGCTCGTTTGA	0	23	RDR2-sensitive
GTAGGAATGTGTGCAACGGCAGCT	30	1	RDR2-resistant
GTAGGAATTGTCGAATCAAAACGG	0	11	RDR2-sensitive
GTAGGACAACAGGACTGCAGAAGG	0	9	RDR2-sensitive
GTAGGACACATGTAGGTGGTCGTA	0	25	RDR2-sensitive
GTAGGACACCGCAGGACACTGGGC	0	9	RDR2-sensitive
GTAGGACACTCGGCACAGAACGAC	0	12	RDR2-sensitive
GTAGGACACTCGGCACAGGACGAC	0	12	RDR2-sensitive
GTAGGACACTCGGTACAGGTCCAC	0	10	RDR2-sensitive
GTAGGACAGACAGGACAGGGACGT	0	26	RDR2-sensitive
GTAGGACAGTCAGGACATAGACGT	0	13	RDR2-sensitive
GTAGGACAGTTCGGCTAGGCCAAG	0	11	RDR2-sensitive
GTAGGACAGTTCGGCTAGGCCAGA	1	15	RDR2-sensitive
GTAGGACAGTTCGGCTAGGCCAGG	0	10	RDR2-sensitive
GTAGGACAGTTCGGCTAGGCCGGA	0	10	RDR2-sensitive
GTAGGACAGTTCGGCTAGGCCGGT	0	11	RDR2-sensitive
GTAGGACCAGTGGCACACAAGTAA	0	15	RDR2-sensitive
GTAGGACCCTAGGAAGTCGACCAT	0	10	RDR2-sensitive
GTAGGACCGCTAACCTAGAGTAGA	0	9	RDR2-sensitive
GTAGGACCGCTGACCTAGAGTAGC	3	20	RDR2-sensitive
GTAGGACCGGATGTAGGGACGTGC	0	97	RDR2-sensitive
GTAGGACCTAGTAGCAGCAGTCAT	0	65	RDR2-sensitive
GTAGGACGACCCAGTAGAAGGCCC	0	12	RDR2-sensitive
GTAGGACGAGCAGGATAGACCTAG	0	10	RDR2-sensitive
GTAGGACGCCACAGATCGATCGGA	0	20	RDR2-sensitive
GTAGGACGGAGTTGAGGCCGAGAG	0	11	RDR2-sensitive
GTAGGACGTAACTCGGGAGCGGGC	0	18	RDR2-sensitive
GTAGGACTACTTCCAGAGGTCTGG	0	24	RDR2-sensitive
GTAGGACTAGGACCTCGTCAGGCG	1	36	RDR2-sensitive
GTAGGACTGCAGGGAGGACAGCGT	0	30	RDR2-sensitive
GTAGGACTGCTTACAGAGGTCGAA	0	11	RDR2-sensitive
GTAGGACTGCTTCCAGAGGTCGAA	4	64	RDR2-sensitive
GTAGGACTGTGCAGGCCCGGCCCG	1	27	RDR2-sensitive
GTAGGACTGTTTCTAGAGGTCGAA	0	9	RDR2-sensitive
GTAGGAGACGTGGCTTGCATGAAC	0	24	RDR2-sensitive
GTAGGAGATCGCGGTGTGGATGGC	0	9	RDR2-sensitive

GTAGGAGCCAGGAGCTAAGACGTT	0	11	RDR2-sensitive
GTAGGAGCCGGTAGAAACCACGTT	0	9	RDR2-sensitive
GTAGGAGGAGCCCGTAGAAGACAC	0	12	RDR2-sensitive
GTAGGAGGATACGGTGCGACTGTC	26	1	RDR2-resistant
GTAGGAGGCACCGCTGCGGCCATA	0	14	RDR2-sensitive
GTAGGAGGCGCTGTAGGGGGCACC	2	45	RDR2-sensitive
GTAGGAGGCGCTGTAGGGGGCATC	0	31	RDR2-sensitive
GTAGGAGGCGTTGTAGGGGGCACC	0	9	RDR2-sensitive
GTAGGAGTCCCTCGGCTCGGTCGG	2	29	RDR2-sensitive
GTAGGATACGGATACGGAGCCAGC	0	18	RDR2-sensitive
GTAGGATAGCTCGGAAGATGACAC	0	22	RDR2-sensitive
GTAGGATAGGGATCGACAACGGTT	0	9	RDR2-sensitive
GTAGGATCTAGGACACCGGCTAGA	0	16	RDR2-sensitive
GTAGGATGAGACGAGCCTGCTCAC	0	10	RDR2-sensitive
GTAGGATGAGGCGTGGACGACATC	0	23	RDR2-sensitive
GTAGGATGCGATGGTAGAGGCACA	15	0	RDR2-resistant
GTAGGATGGTACAGACGCTGTATA	0	17	RDR2-sensitive
GTAGGCAACCGTCGGACATACGGT	0	13	RDR2-sensitive
GTAGGCACTCGGCAAAGACAAGTT	0	13	RDR2-sensitive
GTAGGCCCAAACACTCAGAGACAA	0	33	RDR2-sensitive
GTAGGCCCGGATGACTCCAGAAGC	0	9	RDR2-sensitive
GTAGGCCTGCAGTAGATAGGATAT	0	10	RDR2-sensitive
GTAGGCCTGGGCAGAAAACTCGAA	0	16	RDR2-sensitive
GTAGGCCTGTGACGTGCCGGACCA	0	18	RDR2-sensitive
GTAGGCGACTGTAAGGCGGAGTGA	0	9	RDR2-sensitive
GTAGGCGATGACTGTCGGAGATGC	0	24	RDR2-sensitive
GTAGGCGTCGAAGGGGAGAAGGAT	44	4	RDR2-resistant
GTAGGCTCGAACTCCGACGGTGAC	0	16	RDR2-sensitive
GTAGGCTCGGACGTAGGCCCGGAT	1	18	RDR2-sensitive
GTAGGCTCGGATACAGGTTCGGAT	0	12	RDR2-sensitive
GTAGGCTGACGGCTCTCGGTTCAC	0	11	RDR2-sensitive
GTAGGCTGTAGCGTAGATTGGCCT	0	13	RDR2-sensitive
GTAGGCTGTAGTACCGCACGTCGT	0	41	RDR2-sensitive
GTAGGCTGTAGTAGTAGGACTGGG	0	9	RDR2-sensitive
GTAGGGACAGAGGAAGTAAGTCGA	0	10	RDR2-sensitive
GTAGGGACCGGCAACAAGACGAGA	0	9	RDR2-sensitive
GTAGGGACGACCCAGCAGACTCGC	0	15	RDR2-sensitive
GTAGGGACGACCTTGCAGACTCGC	0	10	RDR2-sensitive
GTAGGGACGGTCTGTGATCAGGAG	0	9	RDR2-sensitive
GTAGGGACTGACATCAAGACGAGA	0	18	RDR2-sensitive
GTAGGGACTGGCATAAGACGAGAC	0	9	RDR2-sensitive
GTAGGGACTGGCATCAAGACGAGA	0	73	RDR2-sensitive
GTAGGGACTGGCGTCGAAGCGAGG	0	12	RDR2-sensitive
GTAGGGAGCGCTGTAGGGGGCACC	2	86	RDR2-sensitive
GTAGGGATGAACACGAGAACACGC	0	14	RDR2-sensitive
GTAGGGCCGGCCTGGACACGCATA	0	41	RDR2-sensitive
GTAGGGCGAGTAGAGACGCGCAAA	0	12	RDR2-sensitive
GTAGGGCGCTGTAGCAGGCACCGC	0	119	RDR2-sensitive
GTAGGGCGGCAAGACATGACCACG	0	13	RDR2-sensitive

GTAGGGCGGGCTGACGAGTGAGGC	0	9	RDR2-sensitive
GTAGGGCTGACGCGGTGCCGACAT	0	23	RDR2-sensitive
GTAGGGCTGCAGGAACGGCAGATT	2	103	RDR2-sensitive
GTAGGGCTGGACGTGGCTTGTCAG	0	18	RDR2-sensitive
GTAGGGGACACCGCTGCGGCCATA	0	11	RDR2-sensitive
GTAGGGGACGCTGTAGAGGGCACC	2	212	RDR2-sensitive
GTAGGGGACGCTGTAGAGGGCAGC	2	28	RDR2-sensitive
GTAGGGGACGCTGTAGGGGCACCG	0	12	RDR2-sensitive
GTAGGGGACGCTGTAGGGGGCACC	2	63	RDR2-sensitive
GTAGGGGCACGTAGATGGCAGAAC	1	19	RDR2-sensitive
GTAGGGGCGCTGTAGAGGGCACCG	1	21	RDR2-sensitive
GTAGGGGCTGGAGGTGGAGGGGAC	41	0	RDR2-resistant
GTAGGGGCTGGAGGTGGAGGGGGC	21	0	RDR2-resistant
GTAGGGGCTGTAGGGGGCACCCTA	0	14	RDR2-sensitive
GTAGGGGGCGCTGTAGAGGGCACC	0	27	RDR2-sensitive
GTAGGGGGCGCTGTAGGAGGCACC	0	48	RDR2-sensitive
GTAGGGGGCGCTGTAGGGGACACC	0	11	RDR2-sensitive
GTAGGGGGCGCTGTAGGGGGCACC	1	75	RDR2-sensitive
GTAGGGGGCGCTGTAGGGGGCATC	0	9	RDR2-sensitive
GTAGGGGGCGCTGTAGTGGGCATC	0	10	RDR2-sensitive
GTAGGGGGCGTTGTAGAGGGCATC	0	10	RDR2-sensitive
GTAGGGGGCTGCACTGCAGGACGA	0	15	RDR2-sensitive
GTAGGGGGGCGCTGTAGGAGGCAC	0	25	RDR2-sensitive
GTAGGGGTAGATCTGAGCAAGACG	0	12	RDR2-sensitive
GTAGGGTAGGACGGGTGTTGGAGC	0	32	RDR2-sensitive
GTAGGGTCAGACTGCTAGGTCGGT	0	9	RDR2-sensitive
GTAGGGTCAGTAGCATACAAGGAA	0	37	RDR2-sensitive
GTAGGGTGGATTGGAGAGGACGTG	0	10	RDR2-sensitive
GTAGGGTTGAGACGCGTCCTCTAG	0	9	RDR2-sensitive
GTAGGTCTCGGTTGAAGGGCACGG	1	13	RDR2-sensitive
GTAGGTCTGTCAAGCTTGAGTCAT	0	12	RDR2-sensitive
GTAGGTGTAGAGCACAGGAACGGT	0	16	RDR2-sensitive
GTAGGTGTAGGAGTCTAATGGCAT	0	20	RDR2-sensitive
GTAGGTGTGCAGTACATGGACGGT	0	9	RDR2-sensitive
GTAGGTGTGGAGCACAGGGACTGT	0	9	RDR2-sensitive
GTAGGTTGATTAATTTTGTACGGC	15	0	RDR2-resistant
GTAGTACGGGTAGGCATGTAAACA	0	11	RDR2-sensitive
GTAGTACGTCAGAGGAACCGGCTC	2	20	RDR2-sensitive
GTAGTACTTGTGCAGATTTGTGGA	32	1	RDR2-resistant
GTAGTAGACCGGCGAGGACGACGA	0	31	RDR2-sensitive
GTAGTAGACTATAGCGTCGGCGTA	0	10	RDR2-sensitive
GTAGTAGCACTGGACAAGCACTGC	0	12	RDR2-sensitive
GTAGTAGCGCAGGATCTTGGGCAT	0	10	RDR2-sensitive
GTAGTAGCTGACTGCGGGGACGCT	0	10	RDR2-sensitive
GTAGTAGCTGTAGCTCATAGGCAT	1	133	RDR2-sensitive
GTAGTATTTTCGGTTGGTAGTGGC	92	5	RDR2-resistant
GTAGTCATATTTTGGTCCTAGGAG	33	0	RDR2-resistant
GTAGTCATGAAGGCGGCTGTAGAC	3	18	RDR2-sensitive
GTAGTCATGCTATAGATATGAGAC	14	0	RDR2-resistant

GTAGTCGATGTAGTCATGGGCGGA	0	106	RDR2-sensitive
GTAGTCGATGTAGTCATGGGCGGG	0	9	RDR2-sensitive
GTAGTCGTACACGAGCATCAGCCT	0	12	RDR2-sensitive
GTAGTCTGACGTGAGGTAGGTGTG	1	39	RDR2-sensitive
GTAGTCTGAGCTGGCAGCTGACGT	0	21	RDR2-sensitive
GTAGTCTTAGTTGGTCATGGCTGT	25	0	RDR2-resistant
GTAGTGAACCGGACCGAGACGTAG	1	43	RDR2-sensitive
GTAGTGAAGCCGCCGGTCGTAGGA	0	11	RDR2-sensitive
GTAGTGAATCGGGTCTGTGCCGGC	0	16	RDR2-sensitive
GTAGTGAGCGGAACGACGGTAGAA	0	12	RDR2-sensitive
GTAGTGCCTTGCGGAAGATATGCG	0	11	RDR2-sensitive
GTAGTGCTGGCACGGACATGACAT	1	22	RDR2-sensitive
GTAGTGGAAGGCGGGCTGGGCAGC	1021	110	RDR2-resistant
GTAGTGGACCGTGAAACAAGGCTC	0	9	RDR2-sensitive
GTAGTGGGAACATGCAGGACGCGG	0	10	RDR2-sensitive
GTAGTGTGCTGGAGACGGTTTGAG	18	1	RDR2-resistant
GTAGTGTTTGGATGCAGGGACGAG	0	9	RDR2-sensitive
GTAGTTCCCCTCTGGCAGAGTCGT	0	10	RDR2-sensitive
GTAGTTCGTGGACACGGAGGATGA	0	9	RDR2-sensitive
GTAGTTGGCGTAGACGACCTCGGC	0	10	RDR2-sensitive
GTAGTTGGGAAGTGGAACGACGGA	0	10	RDR2-sensitive
GTAGTTGGGGACGGCCATGAACTT	3	50	RDR2-sensitive
GTAGTTGGTAGACGGTTCTCATAG	0	12	RDR2-sensitive
GTAGTTGGTGTGTAGGATCGGTAA	0	9	RDR2-sensitive
GTAGTTTTGGACGTACGGACGGAT	0	17	RDR2-sensitive
GTATAAATGGACGGTTGGATGGGC	0	10	RDR2-sensitive
GTATAACCTCGCCGGAGAAGACAA	0	9	RDR2-sensitive
GTATAACCTCGCCGGAGAAGACAG	0	45	RDR2-sensitive
GTATAACTCGTAGCGCTTCGGCAT	0	21	RDR2-sensitive
GTATAACTGTGACGTAGATGACTT	35	6	RDR2-resistant
GTATACTCGGACCGACCCGTTGGA	0	12	RDR2-sensitive
GTATAGAACCTGGTCGTGCCGGAC	0	18	RDR2-sensitive
GTATAGAACGGAGCTCGGCGCCAT	0	9	RDR2-sensitive
GTATAGACGCTGGATCCTCGGTAT	0	10	RDR2-sensitive
GTATAGATCCATATGCTCGTTGGA	21	2	RDR2-resistant
GTATAGATCGGGCTAGCACGGCTC	0	11	RDR2-sensitive
GTATAGATGGATTTGTATAGGTAT	0	25	RDR2-sensitive
GTATAGCGTTGGACAGTCCGGATA	0	20	RDR2-sensitive
GTATAGGTTGGGCTGCACGTACGG	1	51	RDR2-sensitive
GTATAGTGTGAGAAAATTTGGTAT	255	4	RDR2-resistant
GTATATAGAGGACGTTGCTGGAGA	1	22	RDR2-sensitive
GTATCAAATTATTGTTCGGTCGAG	0	15	RDR2-sensitive
GTATCACGACTGTATAGGATGGTA	1	14	RDR2-sensitive
GTATCACTTCGGACATGACGGCAT	0	18	RDR2-sensitive
GTATCAGACTGTTCTTAGGCCGAG	0	11	RDR2-sensitive
GTATCCCCTGTAGCGTAGTCGGTC	4	36	RDR2-sensitive
GTATCCCGGCTGAGACGCGACATG	0	16	RDR2-sensitive
GTATCCCTGTAGAGTACGGAAATA	0	46	RDR2-sensitive
GTATCCGGTGGACTGAGGGGCGGT	0	10	RDR2-sensitive

GTATCGATGGGCAGGAACGTCGGC	0	20	RDR2-sensitive
GTATCGGGCTGAAACGGGTTCGGA	0	33	RDR2-sensitive
GTATCTAGAAGGCCGGACGCGCAC	0	9	RDR2-sensitive
GTATCTAGGATGCCGGACATGCAC	0	14	RDR2-sensitive
GTATCTCGGATTTCGGGCACGGGT	0	24	RDR2-sensitive
GTATCTGATTACGGTTGGACTAAA	0	17	RDR2-sensitive
GTATCTGGCTGGTCAGGACGGCGC	0	9	RDR2-sensitive
GTATCTGGTGGGCTGAGGGGCGGT	0	33	RDR2-sensitive
GTATCTTCGGACACAAGTCGGCAT	1	22	RDR2-sensitive
GTATCTTCGGACTCAAGTCGGCAC	0	10	RDR2-sensitive
GTATCTTCGGACTCAAGTCGGCAT	1	66	RDR2-sensitive
GTATCTTCGGACTCAAGTCGGTAC	0	9	RDR2-sensitive
GTATCTTCGGACTCAAGTCGGTAT	1	56	RDR2-sensitive
GTATCTTCGGACTCAGGTCGGCAT	2	18	RDR2-sensitive
GTATCTTCGGACTCGAGTCGGTAT	1	37	RDR2-sensitive
GTATCTTCGGATTCAAGTCGGCAT	1	23	RDR2-sensitive
GTATCTTCGGGCTCGAGTCGGTAT	0	18	RDR2-sensitive
GTATCTTCGGTCTCAAGTCGGCAT	0	18	RDR2-sensitive
GTATCTTTATTGGATCGTGTCGGT	0	11	RDR2-sensitive
GTATGAAATCTCCAGACGGACTGC	0	11	RDR2-sensitive
GTATGACATGTGGAGCCAGTGGCA	0	9	RDR2-sensitive
GTATGACGGCGGCAGGGATGGGGC	0	12	RDR2-sensitive
GTATGAGACGAGCTACGTGGATAA	0	10	RDR2-sensitive
GTATGAGTAGGGACGCAGGGACGA	0	61	RDR2-sensitive
GTATGAGTGTAGAGGTGTGGAGGA	0	10	RDR2-sensitive
GTATGATGGACTCTAGGATGAGGC	32	1	RDR2-resistant
GTATGATGGACTCTTGGATGAGGC	31	0	RDR2-resistant
GTATGCGGATTACTGTACGGGCAG	0	14	RDR2-sensitive
GTATGCTGTTGAGCCAGGGGACGT	1	25	RDR2-sensitive
GTATGCTTTTATGTCTGGAGGAAC	14	0	RDR2-resistant
GTATGGAATGGTACTACCCGACGG	0	17	RDR2-sensitive
GTATGGACCAACAGTGGAGTATGT	0	9	RDR2-sensitive
GTATGGACCTAACTCAGGAGCGGA	0	34	RDR2-sensitive
GTATGGACTAGTAGCATACCGGTG	0	26	RDR2-sensitive
GTATGGACTCTATGGCGTTGTTAT	20	1	RDR2-resistant
GTATGGACTGAGGACATCACGGTG	0	21	RDR2-sensitive
GTATGGAGAGTCGTGTAGAGACGG	0	11	RDR2-sensitive
GTATGGATTGGACGGGATTGGAAA	0	10	RDR2-sensitive
GTATGGCATAGTCGAGGACCTCTG	0	9	RDR2-sensitive
GTATGGCTTCGGCGGCAAGGGCAC	57	14	RDR2-resistant
GTATGGGACGACGACGATGATGGT	0	9	RDR2-sensitive
GTATGGGCAGTAGTCAAGCGGTAT	0	14	RDR2-sensitive
GTATGGGGCGCTGTAGGGGGCACC	0	22	RDR2-sensitive
GTATGGTGCTGTTGTCCAAGGTTA	0	10	RDR2-sensitive
GTATGTATATGGACCGATCTGGCA	25	1	RDR2-resistant
GTATGTCGAGCTTTGGAGGTGGTG	0	16	RDR2-sensitive
GTATGTCGCGTAGGACAGTTCGGC	0	27	RDR2-sensitive
GTATTAAATGTAGTCGGATTATAA	1	26	RDR2-sensitive
GTATTACATGGTCGTTATCTAGGA	14	0	RDR2-resistant

GTATTAGGGTAGAGGCCGGACAAT	0	9	RDR2-sensitive
GTATTATAAATGTGATCGAGGAAT	15	0	RDR2-resistant
GTATTATTTTATGTCGGATCATAC	0	13	RDR2-sensitive
GTATTCGCTATATGTTCGGACGCT	0	12	RDR2-sensitive
GTATTCTGAGCGAGTAGTCGGCTC	1	46	RDR2-sensitive
GTATTCTGAGTGAGTAGTCGGCTC	0	15	RDR2-sensitive
GTATTGACCGGTATCACGGGCTGT	0	9	RDR2-sensitive
GTATTGACTGAGATCGAGTCGGAT	0	13	RDR2-sensitive
GTATTGAGTGGGATTGAGCCGGTT	1	13	RDR2-sensitive
GTATTGCGGGCGGGACGATCGGGC	0	10	RDR2-sensitive
GTATTGGACGGGATCGAGACGGAT	2	14	RDR2-sensitive
GTATTGGGCCTGCGAACACGACGG	0	25	RDR2-sensitive
GTATTGGGCTTGCGAACACGACGG	1	113	RDR2-sensitive
GTATTGGTCTGTGGAGATGGTCTA	0	13	RDR2-sensitive
GTATTGGTCTTATGATACTTCGGT	0	12	RDR2-sensitive
GTATTGTAGGCGTGCACACCCATC	0	13	RDR2-sensitive
GTATTGTTTGAATTTCGGGCCGGT	0	9	RDR2-sensitive
GTATTTCGGGTCCGAGCAGAACGA	0	71	RDR2-sensitive
GTATTTGCTATATGTTCGGACGCT	44	0	RDR2-resistant
GTATTTGGACTGACACGGCACAAT	0	10	RDR2-sensitive
GTATTTGGGCTGTGAATAAAAGGC	0	16	RDR2-sensitive
GTCAAACACTCGGCAAAGGGGGCT	1	19	RDR2-sensitive
GTCAAACGCGGAATAGAACGGCTC	0	46	RDR2-sensitive
GTCAAACTCTCGGCGAACGACGAC	0	12	RDR2-sensitive
GTCAAACTGGGACTAGAGGACGAC	0	10	RDR2-sensitive
GTCAACAGATCGTGCGCTGGGCGT	0	34	RDR2-sensitive
GTCAACCATCGGCTTTGGGGCGGT	0	10	RDR2-sensitive
GTCAACCTCGTCGTTCGTCGTCAC	0	10	RDR2-sensitive
GTCAACGACTGTAGCAAAATGGTT	9	27	RDR2-sensitive
GTCAACGGAACGATGGGACGACGC	0	10	RDR2-sensitive
GTCAACTGTCCACTCTGCAACGGC	1	27	RDR2-sensitive
GTCAAGATTGGGTCGTGGACGGTT	0	10	RDR2-sensitive
GTCAAGCGGAGACGAGGTGAACAC	0	28	RDR2-sensitive
GTCAAGCGGAGGCGAGGTGAACAC	1	27	RDR2-sensitive
GTCAAGCGGGGACGAGGTGAACAC	0	12	RDR2-sensitive
GTCAAGCGGGGGCGAGGTGAACAC	1	14	RDR2-sensitive
GTCAAGCGTCGGCGCAGCATCCAG	0	12	RDR2-sensitive
GTCAAGGCTGCACAGATCGACGGC	0	10	RDR2-sensitive
GTCAAGTAGGATCGGGTCGTCGTT	81	24	RDR2-resistant
GTCAAGTCGGCGTTTCTTAACGGC	41	8	RDR2-resistant
GTCAAGTTACATGTCGGATAAGAT	0	9	RDR2-sensitive
GTCACACCCGGTTTTAGAAGGCAA	0	10	RDR2-sensitive
GTCACACCGGACAGCAGATCGCAT	0	15	RDR2-sensitive
GTCACCAGGGCTAGGGCGGCTGAC	0	9	RDR2-sensitive
GTCACCATATCGTGGAGTAGGCAT	0	13	RDR2-sensitive
GTCACCATTGTGATCGGACGCAAC	22	0	RDR2-resistant
GTCACCCTAGGTTGATTGTGGGAC	0	11	RDR2-sensitive
GTCACCGGACGAATGGGATAGGCT	0	10	RDR2-sensitive
GTCACCGTCGTCTGGGCAGGGCAT	4	20	RDR2-sensitive

GTCACCGTCGTCTGGGCAGGGCGT	1	21	RDR2-sensitive
GTCACCGTTGGATGTGGAGGGTGT	0	24	RDR2-sensitive
GTCACCTTTGTAGTATTTTCGGTT	40	1	RDR2-resistant
GTCACGTAGGGACTGGCATAAGAC	0	10	RDR2-sensitive
GTCACGTTGGCTAGAAGGTGGAAG	0	9	RDR2-sensitive
GTCACGTTGGCTAGAGGGTGGAAG	0	15	RDR2-sensitive
GTCACTCGGCTGACGTACGCGGTT	4	39	RDR2-sensitive
GTCACTCGGTACTCGGCAAAGAAA	1	48	RDR2-sensitive
GTCACTCGGTCCTCGAACTTGGCA	0	10	RDR2-sensitive
GTCACTCTGGATGGTCTGGACGGT	1	17	RDR2-sensitive
GTCAGAACTGCGAGCCGAGCCGAG	0	9	RDR2-sensitive
GTCAGACCGGTCAGTGGAGCGGCG	2	26	RDR2-sensitive
GTCAGATCCAACGACTAGACGCAT	0	19	RDR2-sensitive
GTCAGATCCAACGGCTAGGCGCAC	0	9	RDR2-sensitive
GTCAGATGCGGACGGCTGGACGGT	1	15	RDR2-sensitive
GTCAGATGGACTGCGCCAAGACGG	0	17	RDR2-sensitive
GTCAGATGGTGGACGACAGAGCGG	0	12	RDR2-sensitive
GTCAGCGCGAACACAAGTCGGAGA	0	9	RDR2-sensitive
GTCAGCTCGTGGACGACAGCGCGG	2	47	RDR2-sensitive
GTCAGGAGACTGAAGACGCCAGAG	0	9	RDR2-sensitive
GTCAGGGAGCACTCGGCAAAGAAA	0	10	RDR2-sensitive
GTCAGGTCGGTCAGTGGAAGGGCG	1	12	RDR2-sensitive
GTCAGGTCGGTCAGTGGAGCGGCG	3	40	RDR2-sensitive
GTCAGGTCGGTCAGTGGAGCGGTG	0	16	RDR2-sensitive
GTCAGTAGTCCCTGTATTCGGTTT	0	11	RDR2-sensitive
GTCAGTCGGCTGAGAATGCACGTG	0	11	RDR2-sensitive
GTCAGTCGGCTGTGAATGCAGGTG	0	9	RDR2-sensitive
GTCAGTGACTATGGGCCGGACGGT	0	14	RDR2-sensitive
GTCAGTGGAGTAGACAGGGCGCAT	0	11	RDR2-sensitive
GTCATACAGATCGGACTAGCACGG	0	19	RDR2-sensitive
GTCATATGCGCTGTCACGTCGGTA	0	16	RDR2-sensitive
GTCATATGGGTCGGGCTAGCACGG	0	10	RDR2-sensitive
GTCATCAAGCAGACTGTTGCGCGA	0	11	RDR2-sensitive
GTCATCAGATTGTCGACGACGGTT	0	11	RDR2-sensitive
GTCATCATTAGATCTAGATCGGAT	0	14	RDR2-sensitive
GTCATCCGGGTTGTAGGTGACGTC	2	91	RDR2-sensitive
GTCATCTATGTGGCGTAGCTCGGC	0	15	RDR2-sensitive
GTCATGGGCTACGATTGATCGGTA	0	12	RDR2-sensitive
GTCATGTTAGTCGGGTCGGGTGGC	0	25	RDR2-sensitive
GTCATGTTCGTGACGGGCTAAAAC	0	11	RDR2-sensitive
GTCATTAAGACTGATCGTGTTGGT	1	12	RDR2-sensitive
GTCATTCGAGCTGTGGAGGACGGC	0	21	RDR2-sensitive
GTCATTCGGCTGCAGCTTCGGCAT	0	14	RDR2-sensitive
GTCATTGTACGTTCGCACTGGACA	0	9	RDR2-sensitive
GTCATTTTCGACGGTCTCTGGCAC	0	12	RDR2-sensitive
GTCCAAACAGTATGGGTCGTCCGG	0	26	RDR2-sensitive
GTCCAAATGGGTGGATCGTTCCGG	0	14	RDR2-sensitive
GTCCAAGACACCTCTGATCGGCAT	1	12	RDR2-sensitive
GTCCAATCGAAGGGTGACACGCAT	0	12	RDR2-sensitive

GTCCACGGTCCGGACTGTCCGCGG	0	28	RDR2-sensitive
GTCCACGGTTGGGAGGCAGAACGG	0	14	RDR2-sensitive
GTCCACGTCGGGGGCTCGGACGGT	0	10	RDR2-sensitive
GTCCACTCTGTAACAACGGTCCAA	0	18	RDR2-sensitive
GTCCACTGTAGAGGCATACTGGAC	0	9	RDR2-sensitive
GTCCACTGTAGAGGCATACTGGGT	0	14	RDR2-sensitive
GTCCAGGACGTGGGGAACGCGCAT	0	11	RDR2-sensitive
GTCCATCGGACGTGAGGCAGCGGC	15	0	RDR2-resistant
GTCCATGACTCGGGACTGGATGGT	0	9	RDR2-sensitive
GTCCATGGTATAGGACTGGACGGT	0	11	RDR2-sensitive
GTCCATGTCTAGATCGTGCCGGAT	0	29	RDR2-sensitive
GTCCATGTCTAGATCGTGCCGGGC	0	125	RDR2-sensitive
GTCCATGTCTGGAGCTCGGACGGC	0	16	RDR2-sensitive
GTCCATTGATGGCGTCGGCGTTTT	0	9	RDR2-sensitive
GTCCCAATCTGGCGCGGCTGTGAG	0	9	RDR2-sensitive
GTCCCAGTAGGACTGTAGGAGGCT	0	11	RDR2-sensitive
GTCCCAGTCGGAGGGTGGCGCGCA	0	10	RDR2-sensitive
GTCCCAGTTATGGACCATCTGGAC	0	18	RDR2-sensitive
GTCCCATCGTACGGAGAGAGGCCC	0	11	RDR2-sensitive
GTCCCATCGTATGGAGAGAGGCAC	0	12	RDR2-sensitive
GTCCCATGCGGTCGACGGACATGA	0	25	RDR2-sensitive
GTCCCGGAAGACGGCGAACTCCGC	16	80	RDR2-sensitive
GTCCCGGACCTGTAGAATACCGGA	0	39	RDR2-sensitive
GTCCCGGGTTGCAATTCGATGGCG	15	0	RDR2-resistant
GTCCCTAGTTGGAGTGTTTGGCGC	0	13	RDR2-sensitive
GTCCCTCGAACGGTCGCCGGGAAG	0	9	RDR2-sensitive
GTCCCTGTTTCTGTTTGACCGCAT	0	15	RDR2-sensitive
GTCCCTTAGGGATGTGTTCGTCGA	0	55	RDR2-sensitive
GTCCCTTGGGATATGTTCGTCGAG	1	27	RDR2-sensitive
GTCCCTTGGGATGTGTTCGTCGAG	1	33	RDR2-sensitive
GTCCCTTGGGGATATGTTCGTCGA	1	110	RDR2-sensitive
GTCCCTTGGGGATGTGTTCGTCGA	3	185	RDR2-sensitive
GTCCCTTGGGTCGAGCTCGGCAAC	1	16	RDR2-sensitive
GTCCCTTTGATAAAGCGTCGGCAC	0	12	RDR2-sensitive
GTCCGACACGACGGTACTGGTCTA	0	13	RDR2-sensitive
GTCCGACGGTCCTGTAGGAGGCCA	3	17	RDR2-sensitive
GTCCGAGACCAATGGCAGAACGTC	0	9	RDR2-sensitive
GTCCGAGCTCTAGAGTGGAACCGT	0	22	RDR2-sensitive
GTCCGATGCCCTACTGTAGACGGT	0	25	RDR2-sensitive
GTCCGATTCGGTGAACAGTAGGTT	0	10	RDR2-sensitive
GTCCGCCCCTGTAGATCAACGGCT	0	50	RDR2-sensitive
GTCCGCGACCTGGAGCAGGGGCAA	0	9	RDR2-sensitive
GTCCGCGACTGTGGGCCGGACGGT	0	12	RDR2-sensitive
GTCCGCGCCTGTAGATCAACGGCT	5	28	RDR2-sensitive
GTCCGCGGACTTAGACGGCCCGAA	0	14	RDR2-sensitive
GTCCGCGGCTAGGGACCGAACGGC	0	41	RDR2-sensitive
GTCCGCGGTCCGGACTGTCCACGG	1	12	RDR2-sensitive
GTCCGGAAGAGTAGGTACGCGGCA	0	10	RDR2-sensitive
GTCCGGAATAGTCGGAAACGGAAA	0	9	RDR2-sensitive

GTCCGGAATAGTCGGGAACGGAAA	0	18	RDR2-sensitive
GTCCGGACATAACGTAGGGGCTAG	0	10	RDR2-sensitive
GTCCGGACGTACATAGAGAAGGAG	0	10	RDR2-sensitive
GTCCGGACGTACGCAAAGGAGGAG	0	11	RDR2-sensitive
GTCCGGACGTACGCAGAGGAGGAG	1	13	RDR2-sensitive
GTCCGGACGTACGCAGATGAGGAG	0	10	RDR2-sensitive
GTCCGGACGTACGTAGAGAAGGAG	2	19	RDR2-sensitive
GTCCGGACGTCAGGCAGGGAACGC	0	10	RDR2-sensitive
GTCCGGACTGTCCGCGGTGGTGGC	5	26	RDR2-sensitive
GTCCGGAGGCAGAACACGTCAGAG	0	9	RDR2-sensitive
GTCCGGATATAACTCCCGAGGACG	2	17	RDR2-sensitive
GTCCGGATTGAAGGCAAAGCCAGC	0	9	RDR2-sensitive
GTCCGGCACGGTAGAATAAGCGGG	0	15	RDR2-sensitive
GTCCGGGCTCTGCGAGCGTAGCAT	0	11	RDR2-sensitive
GTCCGGGCTGTGAGAGGAGGGGAA	0	10	RDR2-sensitive
GTCCGGTCTGTATATGCAGGGGTC	0	11	RDR2-sensitive
GTCCGGTGACGTAGAGAGCATCAG	2	19	RDR2-sensitive
GTCCGGTGCGCCAGACTGACTCGG	1	12	RDR2-sensitive
GTCCGGTGCGCCAGGCTGACTCGG	0	17	RDR2-sensitive
GTCCGTCCGGCATACACTCGTCAG	0	9	RDR2-sensitive
GTCCGTGATGCCACAGATGATCGT	0	10	RDR2-sensitive
GTCCGTGGCTCTGGGCAGGATGGT	0	14	RDR2-sensitive
GTCCGTTGGTGGCTGATCGAACGG	0	11	RDR2-sensitive
GTCCTAATCTGACGCGGCTGTGAG	0	9	RDR2-sensitive
GTCCTCCGGGTTGTAGGTGACGTC	0	15	RDR2-sensitive
GTCCTCCTGGCAGCACTCGGCAAA	0	9	RDR2-sensitive
GTCCTCTGTAGATTGCGTACCAAG	0	9	RDR2-sensitive
GTCCTGATCTGAACGCCGAGCGTA	0	10	RDR2-sensitive
GTCCTGCAGAACACTACAGAACGG	0	10	RDR2-sensitive
GTCCTGCTCGGGATCGTTGGGTAA	0	15	RDR2-sensitive
GTCCTGGACCTGTAGAATACCGGA	0	21	RDR2-sensitive
GTCCTGTCGAGCACGGCACTGTAC	0	10	RDR2-sensitive
GTCCTTACTGGTTTGGTCGTTTGG	0	10	RDR2-sensitive
GTCCTTCGGCGGAACCGTGGCATG	0	12	RDR2-sensitive
GTCCTTGCTGGTTTGGTCGTTTGG	0	32	RDR2-sensitive
GTCCTTGTCGGCGGGCAGCACTAG	0	20	RDR2-sensitive
GTCCTTGTCTGCGGACTGCGGCGC	0	12	RDR2-sensitive
GTCGAACCATATCGCTTCGTCGGG	0	23	RDR2-sensitive
GTCGAACTACTTGGACCGTCGGGC	0	10	RDR2-sensitive
GTCGAAGACTGGATGTAGAGACGT	0	12	RDR2-sensitive
GTCGAAGCCGCATCTGAGGACATC	0	12	RDR2-sensitive
GTCGAATACTGGATGTAGGGACGT	0	9	RDR2-sensitive
GTCGAATCGGCGCAGAGGCTGCTT	5	19	RDR2-sensitive
GTCGACAACGGCCTGCAGCATCGC	0	10	RDR2-sensitive
GTCGACGACGGACGGCGTAATTCC	0	12	RDR2-sensitive
GTCGACGACTGTAGCAAAATGGTT	2	23	RDR2-sensitive
GTCGACGCTCGGGGTGAAGAAGAC	0	10	RDR2-sensitive
GTCGACGTGGAACCGAGCTCGGCG	1	24	RDR2-sensitive
GTCGACGTGGCACCGAGCTCGGCG	2	21	RDR2-sensitive

GTCGACGTGGCACTGAGCTCGGCG	0	9	RDR2-sensitive
GTCGAGACCACCGGATCGGAGGAG	0	35	RDR2-sensitive
GTCGAGATTGACGGCAGAACGGAT	0	14	RDR2-sensitive
GTCGAGCACGGCACTGTACAGGCT	0	9	RDR2-sensitive
GTCGAGCCGGACCAGGTCCAGACG	0	10	RDR2-sensitive
GTCGAGCCGGACGGTCCACATGTG	0	9	RDR2-sensitive
GTCGAGCGGATTGACGTCATGTAA	0	11	RDR2-sensitive
GTCGAGCTTAGTGCTACGTCGGCG	0	9	RDR2-sensitive
GTCGAGTCGTGGACTGACCCAGGC	0	9	RDR2-sensitive
GTCGAGTCGTGGACTGACCGAGGC	0	9	RDR2-sensitive
GTCGAGTGTCGGCTGACAGTTGGC	1	13	RDR2-sensitive
GTCGATCATGGCTAGACTGGAGGC	1	24	RDR2-sensitive
GTCGATCCATCTGGGCTGGGGCAC	0	11	RDR2-sensitive
GTCGATCCGCGGTCGCTGTTACGG	0	9	RDR2-sensitive
GTCGATCGTTGGTCACTTGGCACA	0	9	RDR2-sensitive
GTCGATGCGGCACAGCAGGACGAC	0	14	RDR2-sensitive
GTCGATTAAGGGCGTGTTTGGCAT	0	14	RDR2-sensitive
GTCGATTAGAGGAGTCTAGGTCGG	2	42	RDR2-sensitive
GTCGATTGAGAGCAGCGTAGGCGT	0	12	RDR2-sensitive
GTCGCACTCGGTAAAGATAGGCTC	1	18	RDR2-sensitive
GTCGCATGTCGGTCGTTTGTCGGA	0	10	RDR2-sensitive
GTCGCCAACGTCGACGACTCTCGG	3	18	RDR2-sensitive
GTCGCGTAGGACAGTTCGGCTAGA	0	9	RDR2-sensitive
GTCGCGTAGGACAGTTCGGCTAGG	8	74	RDR2-sensitive
GTCGCGTTGCGATTAGATCGCGGT	0	9	RDR2-sensitive
GTCGCTATAGATATCGTCGGGGAC	0	41	RDR2-sensitive
GTCGCTATAGATGTCGTCGGGGAC	0	9	RDR2-sensitive
GTCGCTCAAGTCTGATCGCTCGTC	20	1	RDR2-resistant
GTCGCTTCGTTGGGCTTCATGGTA	0	15	RDR2-sensitive
GTCGGAACGATTCCGAGCCGGATT	0	10	RDR2-sensitive
GTCGGTCGGCTTGTAGTGTGCGGG	0	14	RDR2-sensitive
GTCGTAAAGATCGGGCATCGTGAC	0	9	RDR2-sensitive
GTCGTACAGCGGCAATAGAGCAAG	0	10	RDR2-sensitive
GTCGTAGAACATGACCACGGCCAC	0	9	RDR2-sensitive
GTCGTAGACTCATGATCGAGCGGC	0	36	RDR2-sensitive
GTCGTAGATCTTTGGTGGACGGTT	0	16	RDR2-sensitive
GTCGTGAAGATCGGGCATCGTGAC	9	108	RDR2-sensitive
GTCGTGAAGATCGGGCATCGTGCC	0	15	RDR2-sensitive
GTCGTGCTCGGACAGAAGAGTCAG	0	11	RDR2-sensitive
GTCGTGCTCGGACCGGACTGGCAC	0	10	RDR2-sensitive
GTCGTGTCGCGACCTGGACGGCTC	4	27	RDR2-sensitive
GTCGTGTCTCTGGCGGCAGAGCTC	0	10	RDR2-sensitive
GTCGTTCGTCAGGTCAGAGTGGCA	0	11	RDR2-sensitive
GTCGTTCTTACAGCGAGGTCGCGG	0	26	RDR2-sensitive
GTCGTTTAAAACCGTCGGAGGCAA	0	20	RDR2-sensitive
GTCGTTTAGGACATGATTGTGCAT	0	15	RDR2-sensitive
GTCTAAATGGACTGGATATCGGAT	0	15	RDR2-sensitive
GTCTAAGACACTCGGCAAAGGCTA	1	18	RDR2-sensitive
GTCTAAGCTCGGACGAGTCGTCAT	0	11	RDR2-sensitive

GTCTACCACCTGTAGCCTGACGGT	0	10	RDR2-sensitive
GTCTACCGTAGCGGCACGAACCAT	0	12	RDR2-sensitive
GTCTAGAAGCTCTGGATGACGCAT	0	11	RDR2-sensitive
GTCTAGATCGTGCCGGACCGGCCC	0	10	RDR2-sensitive
GTCTAGATCGTGCCGGGCCGGCCC	0	56	RDR2-sensitive
GTCTAGATTGTATCGGTGCGTGCA	0	17	RDR2-sensitive
GTCTAGCGGACCAGTCTAAGGCAT	0	10	RDR2-sensitive
GTCTAGCGGGCTGGTCCGAGGCAC	1	15	RDR2-sensitive
GTCTAGCTCAGTCGGTAGAGTGCA	0	13	RDR2-sensitive
GTCTAGTCGTTTTGGTAGAGCGGT	0	20	RDR2-sensitive
GTCTAGTGGACTGGCACTCGGCAA	0	9	RDR2-sensitive
GTCTAGTGTCGGATCGGCCCATGC	0	21	RDR2-sensitive
GTCTATACCGTGTCGGGCTAGGCT	0	26	RDR2-sensitive
GTCTATAGCGTCGGGTTACCAGAA	15	0	RDR2-resistant
GTCTATCGCGTAGGACAGTTCGGC	1	13	RDR2-sensitive
GTCTATCGCTCAGGACCAGACGGT	0	13	RDR2-sensitive
GTCTATCGGAGTAGAAGACGCCAT	0	9	RDR2-sensitive
GTCTATCGTACACACCGTCTGGAG	0	10	RDR2-sensitive
GTCTATGATTTGTGGGTCCGATGG	1	31	RDR2-sensitive
GTCTATGTTGATGATCGCGAGGAG	53	0	RDR2-resistant
GTCTATTCGCTGTAGATGGGCCGC	1	42	RDR2-sensitive
GTCTATTGTCGGGTCGGGTTGGCT	0	9	RDR2-sensitive
GTCTCAGATTGTGGATGGACAGCA	0	14	RDR2-sensitive
GTCTCAGCGGAAGAACAGCTACAG	0	13	RDR2-sensitive
GTCTCAGTACTAGAACAGAGCGGT	0	10	RDR2-sensitive
GTCTCATCTGACTTCTGACGGTAG	0	11	RDR2-sensitive
GTCTCATGGACCTCGCGGACGAAC	23	2	RDR2-resistant
GTCTCCTGATTGTGGTCCAGCGGT	0	14	RDR2-sensitive
GTCTCGAAGCGGACTGGATCAGGC	0	9	RDR2-sensitive
GTCTCGACGAGCTGTACATCGGCA	1	12	RDR2-sensitive
GTCTCGGAAACTGTACATCGGCAA	0	10	RDR2-sensitive
GTCTCGGACGGGCCTGACTGCGGA	0	10	RDR2-sensitive
GTCTCGGCAAACTGTATATTGGCA	0	14	RDR2-sensitive
GTCTCGGTTTGGCTCAGAGCGGCT	0	19	RDR2-sensitive
GTCTCGTCTTTTAATCTTCGGCTG	21	53	RDR2-sensitive
GTCTCGTTGGAAGACGCTTGATGT	0	17	RDR2-sensitive
GTCTCGTTGGACATGCATGAGGAG	0	13	RDR2-sensitive
GTCTCGTTTGGACTTCCGGACGGG	0	9	RDR2-sensitive
GTCTCGTTTGGACTTCCGGCCGGG	1	13	RDR2-sensitive
GTCTCGTTTGGACTTTCGGCCGGG	0	13	RDR2-sensitive
GTCTCTATCGTTGGCCCGTGCATG	0	17	RDR2-sensitive
GTCTCTATTGTCGGCTGGGGCATA	0	14	RDR2-sensitive
GTCTCTCGGCTATAGTTTTGGCAT	43	9	RDR2-resistant
GTCTCTCTGGATTGCAGTTGCAGC	0	10	RDR2-sensitive
GTCTCTGTAGAGTGGCAGATTCGA	28	0	RDR2-resistant
GTCTCTGTAGCTAGATCGCGGACA	0	9	RDR2-sensitive
GTCTCTGTCAGACCAGCGACTGCA	2	46	RDR2-sensitive
GTCTCTGTCGGACCAGCGGCTGCA	6	69	RDR2-sensitive
GTCTCTTCGGACTCAAGTCGGCAT	0	9	RDR2-sensitive

GTCTCTTTCGCTCGGCCAAGACGC	0	9	RDR2-sensitive
GTCTCTTTCGCTCGGCCAGGCCAT	0	9	RDR2-sensitive
GTCTCTTTCTGCAGGACCTTCGGC	0	21	RDR2-sensitive
GTCTGAACCCCAGACACGGACCGC	0	9	RDR2-sensitive
GTCTGAAGAGCTGCAAGCCTGCAT	0	10	RDR2-sensitive
GTCTGAATCTGGTGCGGCGGAACC	0	10	RDR2-sensitive
GTCTGACCGTCTACAGTAGGGCAT	5	46	RDR2-sensitive
GTCTGACGGACCGAACCCAACGGA	0	41	RDR2-sensitive
GTCTGACGGGTCGGCCTGAGGCAT	0	14	RDR2-sensitive
GTCTGAGACTCGGACGGTCCGTGT	0	15	RDR2-sensitive
GTCTGATCGTGCCTGGGACGTGCC	0	10	RDR2-sensitive
GTCTGATGGGTCGGCCCGAAGCAC	0	9	RDR2-sensitive
GTCTGATGGGTCGGCCCGAATCAC	0	10	RDR2-sensitive
GTCTGATTAGTTGGATAAACGGAC	0	9	RDR2-sensitive
GTCTGATTGGTCGGTCCGAAGCAC	0	9	RDR2-sensitive
GTCTGCACCGGTTGAGACGCTCAT	0	12	RDR2-sensitive
GTCTGCACGACTGGGCCAGTGACG	16	0	RDR2-resistant
GTCTGCGAACTTTGAGGTCGGCAA	4	19	RDR2-sensitive
GTCTGCGACGACTGTGTACGGCTA	0	12	RDR2-sensitive
GTCTGCGGCTCTGGCGTTGGCGTC	0	16	RDR2-sensitive
GTCTGCGGCTCTGGGCCGAACGGT	0	11	RDR2-sensitive
GTCTGCGGTGGACTGGGACGAGCA	0	11	RDR2-sensitive
GTCTGCTCGATGGCGCACCGGACA	0	10	RDR2-sensitive
GTCTGCTCGTCCATGCTGTCGGCC	0	9	RDR2-sensitive
GTCTGCTGGCCTGACACTCGGCAA	0	10	RDR2-sensitive
GTCTGGAAGGGTGATTGTCGTGGG	1	35	RDR2-sensitive
GTCTGGACTCTGGACCACTCAGGA	0	11	RDR2-sensitive
GTCTGGATCAACGCGTAGCTTCAT	0	15	RDR2-sensitive
GTCTGGCGAGCTGACGCAAGTGAC	0	9	RDR2-sensitive
GTCTGGCGGAAGTGCGGAGTCTGG	0	14	RDR2-sensitive
GTCTGGCGGGCTGATCCGGGGCAC	0	12	RDR2-sensitive
GTCTGGCGGGCTGGACCAAAACGG	0	27	RDR2-sensitive
GTCTGGCGGGCTGGTCCGAGGCAC	1	18	RDR2-sensitive
GTCTGGCTCTGACCGGTCTGCTGC	37	0	RDR2-resistant
GTCTGGGACAGCACGATCTGACGG	0	10	RDR2-sensitive
GTCTGGGTGGTGTAGTTGGTTATC	418	45	RDR2-resistant
GTCTGGGTTGTGTAGTTGGTTATC	34	2	RDR2-resistant
GTCTGGTATGGCTCTAGAGCAGAA	0	45	RDR2-sensitive
GTCTGGTCGCTGGGACAGGCGCGC	1	20	RDR2-sensitive
GTCTGGTCGGAAGCATGGCGCGAT	0	14	RDR2-sensitive
GTCTGGTGGACTGGCACTCGGCAA	0	39	RDR2-sensitive
GTCTGTAGACTGGAGCAGCAGGAA	0	9	RDR2-sensitive
GTCTGTAGATTATGTCCGACGGCT	0	23	RDR2-sensitive
GTCTGTAGCAACGCGAATAGCGGA	1	12	RDR2-sensitive
GTCTGTAGCAGCTTTTGATGGCCA	0	12	RDR2-sensitive
GTCTGTAGCAGCTTTTGGTGGCCA	1	27	RDR2-sensitive
GTCTGTAGGATCTAGGACACCGGC	0	18	RDR2-sensitive
GTCTGTATCTCGGATTTCGGGCAC	0	114	RDR2-sensitive
GTCTGTATGCACTCAGGGGCGGAG	2	414	RDR2-sensitive

GTCTGTATGCACTCAGGGGCGGCG	1	19	RDR2-sensitive
GTCTGTATGCACTCAGGGGCGGGG	0	15	RDR2-sensitive
GTCTGTATGGACGGATTCAGAGAC	0	11	RDR2-sensitive
GTCTGTCAAGCGTTGGAAGTGGTG	0	13	RDR2-sensitive
GTCTGTCAGGTGGGCCTCGGGCGG	1	20	RDR2-sensitive
GTCTGTCCGTGAGCCGAAGTCTGA	0	18	RDR2-sensitive
GTCTGTCCTGGATTGGTTCGGTAG	0	11	RDR2-sensitive
GTCTGTCGAGCTTTAGAGGTGGTG	0	13	RDR2-sensitive
GTCTGTCGAGCTTTGGAGGTGGTG	0	22	RDR2-sensitive
GTCTGTCGCGTAGGACAATTCGGC	1	33	RDR2-sensitive
GTCTGTCGCGTAGGACAGTTCGGC	0	20	RDR2-sensitive
GTCTGTCGCTCATGTGTGGACGGT	0	34	RDR2-sensitive
GTCTGTCTGGCCTCAGAAGAGCTG	0	15	RDR2-sensitive
GTCTGTGAACTGCGCTGCGACGGA	0	14	RDR2-sensitive
GTCTGTGACTCTGGGCCAGACGGT	2	25	RDR2-sensitive
GTCTGTGATTCTGACCGTCGACGT	0	13	RDR2-sensitive
GTCTGTGGATATGGACCGGAAGGT	0	11	RDR2-sensitive
GTCTGTGGCACAGGGTTGGACGGT	0	14	RDR2-sensitive
GTCTGTGGGCTGGTACGACACGAC	0	9	RDR2-sensitive
GTCTGTGTCTATGATTTGTGGGTC	4	18	RDR2-sensitive
GTCTGTTAGCTCGTCGGCCCATTT	0	9	RDR2-sensitive
GTCTGTTCGTTGTCACTGCGGCGC	0	11	RDR2-sensitive
GTCTGTTGGATTGTTATTCAGGCT	215	29	RDR2-resistant
GTCTGTTGGTCGGATGGTCTTCGC	0	14	RDR2-sensitive
GTCTGTTGTGTACTGTAATGGCTA	0	11	RDR2-sensitive
GTCTGTTGTTGGAGAGGTAGCGGA	0	21	RDR2-sensitive
GTCTGTTTGGCAGAACTTCGGCTT	4	60	RDR2-sensitive
GTCTTAACTACGGACTTAGACGGT	142	24	RDR2-resistant
GTCTTAGGTGGACTGGACAGCATG	3	23	RDR2-sensitive
GTCTTAGTTCGTCGAGACTGGCAC	0	10	RDR2-sensitive
GTCTTATGGACTGTAGTTGCAGCA	2	14	RDR2-sensitive
GTCTTATTATCTGACGACTGAGGA	0	14	RDR2-sensitive
GTCTTCAATTTCGTCGACTTCGGA	39	3	RDR2-resistant
GTCTTCACGCTGTAGAAGAGGGTA	0	15	RDR2-sensitive
GTCTTCACGTCGGATAGCGGAGCA	0	13	RDR2-sensitive
GTCTTCATACTGTAGAAGAGGGTA	0	39	RDR2-sensitive
GTCTTCATACTGTAGTAAGAGGTA	0	17	RDR2-sensitive
GTCTTCATACTGTAGTAAGGGGTA	2	65	RDR2-sensitive
GTCTTCATGCTGTAGAAGAGGGTA	2	35	RDR2-sensitive
GTCTTCATGCTGTAGGAGAGGGTA	0	20	RDR2-sensitive
GTCTTCATGCTGTAGTAAGGGGTA	0	16	RDR2-sensitive
GTCTTCATGCTGTAGTAAGGTGTA	0	36	RDR2-sensitive
GTCTTCGTCACTGTCATCCGCGGC	0	41	RDR2-sensitive
GTCTTCGTCGTGTCCTTCTCCGGC	2	19	RDR2-sensitive
GTCTTCGTTGGACCGTGTCGGCCC	0	46	RDR2-sensitive
GTCTTGCGGATTTACTCGACGGTG	0	22	RDR2-sensitive
GTCTTGCTATCATCGTTCGGTCAC	0	9	RDR2-sensitive
GTCTTGGATATAGAGTTGTAGGCT	48	2	RDR2-resistant
GTCTTGGTCGTCGATGCCACGCAA	2	21	RDR2-sensitive

GTCTTGTCGGCTTATGGTTGGTGT	1	26	RDR2-sensitive
GTCTTGTGAATTGGGAACGGGCAT	1	12	RDR2-sensitive
GTCTTTACGGTCTGTTTTGAGGAT	0	11	RDR2-sensitive
GTCTTTATAGATTGTAGCCGCGGC	0	12	RDR2-sensitive
GTCTTTATGCTGTAGTAAGGGGTA	1	28	RDR2-sensitive
GTCTTTGGAAGGCTGTAGTCGGGC	13	55	RDR2-sensitive
GTCTTTGGACTGTGCTAGCCCGGC	0	13	RDR2-sensitive
GTCTTTGTCGTCTTGGATCAGGCT	0	9	RDR2-sensitive
GTCTTTTGTCGGGCATTCGAAGGC	3	16	RDR2-sensitive
GTCTTTTTAAGGGTTTAAGTCGGA	0	12	RDR2-sensitive
GTCTTTTTCGCTCGGTCACGGTCA	0	35	RDR2-sensitive
GTGAAAAAAGTTGCTGTGGGCTGT	1	43	RDR2-sensitive
GTGAACAGGCATCTTCGGACTCAA	0	15	RDR2-sensitive
GTGAACAGGTATCCTCGGACTCAA	0	18	RDR2-sensitive
GTGAACAGGTATCTTCGGACTCAA	1	24	RDR2-sensitive
GTGAACGACGCAACGACGAACGAC	0	24	RDR2-sensitive
GTGAACGGAACGGAGCATGACTGC	0	12	RDR2-sensitive
GTGAACGGAACGGAGCGTGACTGT	3	19	RDR2-sensitive
GTGAACGGGACGACTGTGAAGACG	0	24	RDR2-sensitive
GTGAACTACTCGTCTGAAGGCTGG	0	11	RDR2-sensitive
GTGAACTTGTTGGGCGGAGACGAC	0	22	RDR2-sensitive
GTGAAGACGACAGTAGAGGCGAGA	0	14	RDR2-sensitive
GTGAAGATCGGGCATCGTGACGCA	7	44	RDR2-sensitive
GTGAAGATGTCGGCATACTGTGAT	0	10	RDR2-sensitive
GTGAAGGAGACGTAGAAGAGACGC	0	10	RDR2-sensitive
GTGAAGGCTGCAGTCGACGGGCGC	0	9	RDR2-sensitive
GTGAAGGGCTACGGCGATTAACTC	0	9	RDR2-sensitive
GTGAAGGTAGACCCTGTTGATGGC	28	4	RDR2-resistant
GTGAAGTCCGGACTGTCCGGTGTG	52	10	RDR2-resistant
GTGAATATGACTGCGGAAGATTGT	0	9	RDR2-sensitive
GTGAATCTGAGCGTCGGCAGGGGC	36	6	RDR2-resistant
GTGAATGCTGGGAACACGGGAGAA	1	14	RDR2-sensitive
GTGAATTGCTGATCCTGGCACGTA	2	38	RDR2-sensitive
GTGACAAGTTCTCTGAAAAGGCGA	33	7	RDR2-resistant
GTGACAGGTGTCTTAGTTCGTCGA	0	9	RDR2-sensitive
GTGACAGTGACAGGGGCGACGACA	42	9	RDR2-resistant
GTGACCAGACTGACTCGAAGGCGC	0	14	RDR2-sensitive
GTGACCAGAGACTGTAAATGTGGC	1	16	RDR2-sensitive
GTGACCGACTGTGGGGGCAGATAT	0	32	RDR2-sensitive
GTGACCTAGATCTGGCGAAGGCGT	0	35	RDR2-sensitive
GTGACCTCGGCGCAGGAGCTGCAC	0	11	RDR2-sensitive
GTGACCTGGGATCTACGTGGCATG	0	11	RDR2-sensitive
GTGACGACAAGACTCATCGGCAGC	0	13	RDR2-sensitive
GTGACGACGAGGAACACGAGCGGA	0	10	RDR2-sensitive
GTGACGACTTAGGCATGCTACCGA	0	9	RDR2-sensitive
GTGACGATTGACGACGCGAGCAGA	0	15	RDR2-sensitive
GTGACGCGTAGGACGTAACCCGGT	0	46	RDR2-sensitive
GTGACGGACTTATTTCGGGCGGTC	0	23	RDR2-sensitive
GTGACGGATTATATTCAGACTGCA	38	4	RDR2-resistant

GTGACGGCGTCTGTACACGGGAAC	15	0	RDR2-resistant
GTGACTAACTGTGGTGGATGACAT	0	15	RDR2-sensitive
GTGACTAGGGAACGTCGTACGGGC	0	20	RDR2-sensitive
GTGACTCGACTGACCGGACACGGT	0	11	RDR2-sensitive
GTGACTCGATTGTCCGGACACCGT	0	9	RDR2-sensitive
GTGACTCTGGACTAGACGGTTCAC	1	49	RDR2-sensitive
GTGACTCTGTACCGAGCCGGCCCG	0	17	RDR2-sensitive
GTGACTGACGACCGAGAAGCGGTT	0	16	RDR2-sensitive
GTGACTGAGCCGGCCTAGACAGAC	0	9	RDR2-sensitive
GTGACTGATGTAGATATAGGGCCT	0	12	RDR2-sensitive
GTGACTGGACCGTGGCTGCGGCAT	0	15	RDR2-sensitive
GTGACTGTCCAACGAGCGTGGGTG	0	9	RDR2-sensitive
GTGACTGTGAGAGCGGGATGGGAA	3	69	RDR2-sensitive
GTGACTGTGAGAGCGGGATTGGAA	2	51	RDR2-sensitive
GTGACTGTGGCAGACTGGCTTGGG	8	95	RDR2-sensitive
GTGACTGTGTCCAGCATACTGTGA	0	10	RDR2-sensitive
GTGACTGTTGCAGACTGGCTTGGG	0	13	RDR2-sensitive
GTGACTGTTGCTTCAGCAGAACGA	0	20	RDR2-sensitive
GTGACTGTTTCGGGCAACACGGAA	0	11	RDR2-sensitive
GTGACTTAGGGTCGGACGGTCCAT	0	10	RDR2-sensitive
GTGACTTGGACGTAGGAGTGGCTC	0	24	RDR2-sensitive
GTGAGAAAAGGGCTGTAGAGGGCT	0	23	RDR2-sensitive
GTGAGAACGACCGCGACTGAGGCA	0	12	RDR2-sensitive
GTGAGACAGACGGTAGGCCAGAAA	0	10	RDR2-sensitive
GTGAGACCAGGAGAGCAGGACCGC	0	9	RDR2-sensitive
GTGAGACTGAGACCCACTTGTCGG	0	24	RDR2-sensitive
GTGAGACTGGTGGATGTGAGGGGC	0	21	RDR2-sensitive
GTGAGAGCGGGATTGGAACGGAGC	0	14	RDR2-sensitive
GTGAGAGGTCTAGGTCGTCGTCTC	3	27	RDR2-sensitive
GTGAGAGTGTAGAAGGGACGAGAG	0	15	RDR2-sensitive
GTGAGCAACGGGAACGAGAATCGG	0	9	RDR2-sensitive
GTGAGCAGGTACCTTCGGACTCAA	1	16	RDR2-sensitive
GTGAGCAGGTATCTTCGGACTCAA	0	33	RDR2-sensitive
GTGAGCCAGGACCGCTGAAGGCTG	0	9	RDR2-sensitive
GTGAGCCAGGCTGTGGAGAAACGG	0	12	RDR2-sensitive
GTGAGCCGCTGAAGACTGGATGTA	0	17	RDR2-sensitive
GTGAGCCTGGACGGGTAGAAACGC	0	25	RDR2-sensitive
GTGAGCCTGGCTCCACAGATGCGC	0	9	RDR2-sensitive
GTGAGCGGGGCTGAGGTATGGTAC	1	16	RDR2-sensitive
GTGAGCTAGGACCGCTGAAGACTA	0	15	RDR2-sensitive
GTGAGCTGAAGCCGGGTGAGCCAC	0	9	RDR2-sensitive
GTGAGCTTCGCGTAGACTGGGCTA	0	10	RDR2-sensitive
GTGAGGACACACTTGTGAGGACGA	0	20	RDR2-sensitive
GTGAGGAGTGAGAAGATGATGGAC	0	17	RDR2-sensitive
GTGAGGATTGTGACGCATCAGTAT	0	25	RDR2-sensitive
GTGAGGCGAGGACTCTCGGACGGA	0	37	RDR2-sensitive
GTGAGGCGGAACGACGGACCAGAT	0	16	RDR2-sensitive
GTGAGGGCTGTGTGGAGACGACAT	0	10	RDR2-sensitive
GTGAGGGGCTGCAAACGGCCTGGT	0	12	RDR2-sensitive

GTGAGTAGAGGACGTTCTGATCCT	0	15	RDR2-sensitive
GTGAGTCAAGACGGGATGGGACAG	0	44	RDR2-sensitive
GTGAGTCCGATAGCATACTGGCGA	1	24	RDR2-sensitive
GTGAGTCTGTTAGTCGTGCCGGGC	0	10	RDR2-sensitive
GTGAGTTCTGATCCTGGCACGTAC	0	16	RDR2-sensitive
GTGAGTTGCAGTAGCCGGGAACAG	0	9	RDR2-sensitive
GTGAGTTGGTATGTTTTATGGTTG	34	2	RDR2-resistant
GTGAGTTTCTGATCCTGGCACGTA	1	16	RDR2-sensitive
GTGATCGGTCTGAACTGACAGCAG	0	12	RDR2-sensitive
GTGATCTGGACGCAGGAGCGACGC	0	36	RDR2-sensitive
GTGATGATCTGGACACGACGACGT	0	10	RDR2-sensitive
GTGATGGCTGTAAGCGCGCGGAAT	0	12	RDR2-sensitive
GTGATGGGAACATGTGGACGTCGA	0	10	RDR2-sensitive
GTGATGGGACCAGTGGAAGCGGAC	18	0	RDR2-resistant
GTGATGGGCTGTAAAAGAGGGTGT	0	18	RDR2-sensitive
GTGATGTGACTGTGGATTGGGCTG	0	11	RDR2-sensitive
GTGATGTGGACCAGGCGATAGCAA	0	11	RDR2-sensitive
GTGATGTGGTTGTGTGGAGGAGGT	23	2	RDR2-resistant
GTGATTATAGCTCTGAGATGGTAT	0	12	RDR2-sensitive
GTGATTCAGGACAAAGGAATGCGG	0	13	RDR2-sensitive
GTGATTCTCTAGATGTAGCGGCTC	0	19	RDR2-sensitive
GTGATTGGTTGCCGGATAAGCAGA	0	12	RDR2-sensitive
GTGATTGTATTCAAAGGAGCTGCA	14	0	RDR2-resistant
GTGCAAACGGGCAGTGGAAGATGA	0	13	RDR2-sensitive
GTGCAACGATTGACTGGATGGCGT	0	10	RDR2-sensitive
GTGCAAGTGTAGCTCTGCGGACGG	2	52	RDR2-sensitive
GTGCAATTGTAGCTCTGCGGACGG	0	9	RDR2-sensitive
GTGCACAGGACTCACGTAGGAGGA	0	16	RDR2-sensitive
GTGCACATGTAGATCGGTATCCTC	0	21	RDR2-sensitive
GTGCACCAAACGATTGTGGACGTC	0	11	RDR2-sensitive
GTGCACCAGGGGACTCCAAGCTGA	0	11	RDR2-sensitive
GTGCACCGGGTCGGGCTGGTACGG	2	82	RDR2-sensitive
GTGCACTGGAGCAACAGAGACGAG	0	43	RDR2-sensitive
GTGCACTGGAGCAGCAGGGGCGAG	0	18	RDR2-sensitive
GTGCACTGTAGCGACGGTGGTCGA	0	31	RDR2-sensitive
GTGCACTGTGAACGCCTACGGTAC	0	61	RDR2-sensitive
GTGCAGATGGGAATGGCAGAACAC	0	10	RDR2-sensitive
GTGCAGATTCCTGGGACTTGACGG	0	12	RDR2-sensitive
GTGCAGCGATCTCAAGGACCCGGC	0	9	RDR2-sensitive
GTGCAGCGCAGTAGAAGATGATGA	0	9	RDR2-sensitive
GTGCAGCGGCTTGGACTTGGACGG	0	21	RDR2-sensitive
GTGCAGGAGCTGTGCCGAGGACGA	0	12	RDR2-sensitive
GTGCAGGGCCATGGACGAAGACGC	0	14	RDR2-sensitive
GTGCAGGTCCGGACGTGGGCGGCG	1	13	RDR2-sensitive
GTGCAGTCCTTGATCGGTCGTCGG	0	9	RDR2-sensitive
GTGCAGTGACTGTAGATGGGCTAA	0	15	RDR2-sensitive
GTGCATCCATGCGATTGTGGGACA	0	26	RDR2-sensitive
GTGCATCGAGGTATCGCGGACGTT	0	9	RDR2-sensitive
GTGCATCGAGGTGTCGCGGACGGT	0	10	RDR2-sensitive

GTGCATCGAGGTGTCGTAGACGGT	0	17	RDR2-sensitive
GTGCATGGATTCTAGCTTCGACGG	0	9	RDR2-sensitive
GTGCATTCGTTGGACCGTGCCGGC	0	32	RDR2-sensitive
GTGCATTGGGTACTGTAGGACGGC	2	42	RDR2-sensitive
GTGCCAACAGTCGGCTCTCGGCAA	2	26	RDR2-sensitive
GTGCCAACCGTCGGCTCTCGGCAA	0	19	RDR2-sensitive
GTGCCAACGCAGCACGTACCTCGG	1	42	RDR2-sensitive
GTGCCACGCGGACTGCCACGTCAC	1	53	RDR2-sensitive
GTGCCACGCGGGCTGCCACGTCAT	3	29	RDR2-sensitive
GTGCCAGCCGTCGGCTCTCGGCAA	0	13	RDR2-sensitive
GTGCCAGCGCAGCACGTACCTCGG	0	23	RDR2-sensitive
GTGCCAGGTCGCGGACCGTCCGGC	1	38	RDR2-sensitive
GTGCCCCAGTTGGACACTCGGCAA	0	12	RDR2-sensitive
GTGCCGATGGACTGGAAACGGTAA	0	9	RDR2-sensitive
GTGCCGATGGGAACATGGACGGCG	3	24	RDR2-sensitive
GTGCCGCGGTGCTGTTTCACGCAT	1	17	RDR2-sensitive
GTGCCGCTGTAGATTAGACGGTGA	0	25	RDR2-sensitive
GTGCCGGCCTGGCTCGGTCATCGG	0	11	RDR2-sensitive
GTGCCGGGCCTAGGAAGCGTGCTT	0	11	RDR2-sensitive
GTGCCGTGGGCCGATGACTGGGCA	2	57	RDR2-sensitive
GTGCCTATGGACTAGAATGAAGGC	0	16	RDR2-sensitive
GTGCCTGGACCACGACTGCGACAT	0	18	RDR2-sensitive
GTGCCTGGTCTACACTATCGTCTT	0	12	RDR2-sensitive
GTGCCTGTTAGCCAGAATCGTCGA	0	14	RDR2-sensitive
GTGCCTTCGTTGGACTGGTGGCAG	0	25	RDR2-sensitive
GTGCCTTCGTTGGGCCGTGCCGGC	8	46	RDR2-sensitive
GTGCCTTCGTTGGGCTGTGGCGGC	0	15	RDR2-sensitive
GTGCCTTCTTTGGACTGTGCCGGC	0	19	RDR2-sensitive
GTGCCTTGAGCCCTGTAGAAGCAT	0	13	RDR2-sensitive
GTGCCTTGTCGTGCTGTTTATCGG	31	5	RDR2-resistant
GTGCCTTTTGTGCCAGTCGTCGAT	0	12	RDR2-sensitive
GTGCGACTCCGGAGCAACGGCTAT	1	12	RDR2-sensitive
GTGCGAGACCAACAGACACTCGGC	0	12	RDR2-sensitive
GTGCGAGATCTCGGGTGGTTGCAT	0	14	RDR2-sensitive
GTGCGATTGTCTAGATAACATGGC	0	11	RDR2-sensitive
GTGCGCCGGGCTGAGCAGGCAGCA	0	22	RDR2-sensitive
GTGCGCGGATTACTGTATGGGCAG	0	14	RDR2-sensitive
GTGCGCTGTAGGAGGCACCGCTGC	0	9	RDR2-sensitive
GTGCGGAAGGAATCGAGTGCTGGC	0	9	RDR2-sensitive
GTGCGGAAGGCCAGGTAGAGCGGT	0	33	RDR2-sensitive
GTGCGGACTATCTGTGCCACCGGC	0	36	RDR2-sensitive
GTGCGGAGAGGCAGATGAATTCGG	0	26	RDR2-sensitive
GTGCGGAGGATTGCGGTATAGAGT	0	14	RDR2-sensitive
GTGCGGATGCAAGGCGGCTCAGTC	0	9	RDR2-sensitive
GTGCGGCATTATGACGAGAGCGGT	0	14	RDR2-sensitive
GTGCGGCCTGTCCAAGCAGAACTG	0	14	RDR2-sensitive
GTGCGGCGAACTGTACAACGGCAA	0	9	RDR2-sensitive
GTGCGGCTGGATGGCATAGGCAAT	0	10	RDR2-sensitive
GTGCGGGAGGGACGAAGACGGCCT	0	14	RDR2-sensitive

GTGCGGGAGGGACGAAGACGTGCG	0	9	RDR2-sensitive
GTGCGGGCTTACTCGGCGTGTCGA	0	13	RDR2-sensitive
GTGCGGGTAACCAATCGGACGGTA	0	12	RDR2-sensitive
GTGCGGGTGGTACGATATCGGTAT	0	10	RDR2-sensitive
GTGCGGTGTAGTAGCTACGTGGGC	0	12	RDR2-sensitive
GTGCGTACGGCAGCCTCGGACGAT	0	9	RDR2-sensitive
GTGCGTAGACTGTTGATAGGGCAC	1	27	RDR2-sensitive
GTGCGTAGCTGTAGTGTGGGTCTA	0	9	RDR2-sensitive
GTGCGTAGGCTCGAACTCCGACGG	2	83	RDR2-sensitive
GTGCGTATCGAAGGCGGCTCGCTC	0	9	RDR2-sensitive
GTGCGTCCGCGCGATTGTGGGGCA	0	20	RDR2-sensitive
GTGCGTCCGTGCGATTGTGGGGCA	0	10	RDR2-sensitive
GTGCGTGACTGTGTGGCATCTCAA	0	58	RDR2-sensitive
GTGCGTGCAGATGGATGGTAGAAT	0	10	RDR2-sensitive
GTGCGTGGCTAGGGATTGAGGGCA	0	11	RDR2-sensitive
GTGCGTTATCGAGCTCGTCGTCGT	0	10	RDR2-sensitive
GTGCGTTCCTGTAGAACCAGGCTG	0	24	RDR2-sensitive
GTGCGTTGCGACGGCTCACAACAA	2	72	RDR2-sensitive
GTGCGTTGCGACGGCTTACAACAA	5	374	RDR2-sensitive
GTGCGTTGCGACGGCTTACAACAG	1	12	RDR2-sensitive
GTGCGTTGCGACGGCTTACAACAT	0	17	RDR2-sensitive
GTGCGTTGCGACGGTTTACAACAA	0	26	RDR2-sensitive
GTGCGTTGCTCGGATCTAGACGGT	1	13	RDR2-sensitive
GTGCGTTTTGGAGTAGGATGGTGT	1	13	RDR2-sensitive
GTGCTACTCTGAGCTGCAGCGGGC	0	16	RDR2-sensitive
GTGCTAGACTCTACGCGTGATCGA	0	17	RDR2-sensitive
GTGCTAGAGCCCTGTGTACGCGGT	2	15	RDR2-sensitive
GTGCTAGCACAACCCGGTCGTCGT	0	17	RDR2-sensitive
GTGCTAGCTGTAGAGCACTCGGCA	2	49	RDR2-sensitive
GTGCTATAGAACAGAAGGGCGCAG	0	10	RDR2-sensitive
GTGCTCAAATATGCGGGTCGTCGT	0	10	RDR2-sensitive
GTGCTCACGTCGGGGTGGAGACGC	0	12	RDR2-sensitive
GTGCTCGGACCGGACTGGCACGGA	1	65	RDR2-sensitive
GTGCTCGGATCAGGCTACAAATAT	0	13	RDR2-sensitive
GTGCTCGGTCTGAACTGATAGCAG	0	12	RDR2-sensitive
GTGCTCTAACTTTCGGTCTGTCGT	0	13	RDR2-sensitive
GTGCTCTCTGCGCAAGGGCGGACT	1	19	RDR2-sensitive
GTGCTCTCTGGTCAAGCGCGGACG	1	14	RDR2-sensitive
GTGCTCTCTGGTCAGGCGCGGACG	12	91	RDR2-sensitive
GTGCTCTCTGTCAGGCGCGGACGG	0	10	RDR2-sensitive
GTGCTCTCTGTGGAAGCGAGGACG	0	10	RDR2-sensitive
GTGCTCTGAATGTCAAAGTGAAGA	56	2	RDR2-resistant
GTGCTCTGACCGGCGCGCACGCTT	0	12	RDR2-sensitive
GTGCTCTGACGACGCCTCGGCATA	0	12	RDR2-sensitive
GTGCTCTGGTTGGCGGCCTGACGG	0	12	RDR2-sensitive
GTGCTCTGGTTGGCGGCTCGACGG	2	32	RDR2-sensitive
GTGCTCTGTGGAGGCGTGGACGGT	1	17	RDR2-sensitive
GTGCTGAATCCTTTGCAGACGACT	15	0	RDR2-resistant
GTGCTGACTGCGGGCTACAGAAGA	0	9	RDR2-sensitive

GTGCTGACTGTGCCGGACGAGCAC	2	25	RDR2-sensitive
GTGCTGATGGACCAGGACCGGTAA	0	9	RDR2-sensitive
GTGCTGATTCTCTTGCCATAGACG	16	0	RDR2-resistant
GTGCTGCAAGCTTTATGGTTCGAT	14	0	RDR2-resistant
GTGCTGCATCTTGGACGTACACGG	0	14	RDR2-sensitive
GTGCTGCGTTGGCACAACTCGGAC	0	14	RDR2-sensitive
GTGCTGGACTGGCCTGATTGGCAC	0	19	RDR2-sensitive
GTGCTGGACTGTCACTGACGCGCT	0	11	RDR2-sensitive
GTGCTGGATCTGAATGGCTACTGT	0	11	RDR2-sensitive
GTGCTGGGACTAAATTTTTGGCAT	0	14	RDR2-sensitive
GTGCTGGGCTCTTTGGACCGGTAG	0	9	RDR2-sensitive
GTGCTGGTCGGCAGACACTCGGCA	0	9	RDR2-sensitive
GTGCTGTAAGATATGATTGGTGTG	202	21	RDR2-resistant
GTGCTGTAGAAGCGTTGTGGCTGC	0	9	RDR2-sensitive
GTGCTGTAGCCACCAGTTGAAGGG	0	14	RDR2-sensitive
GTGCTGTAGTAGTGGGCACGGCAC	0	12	RDR2-sensitive
GTGCTGTCCGTGCGCGTCGTCGTT	0	9	RDR2-sensitive
GTGCTGTCGAGCAGGAGCTGATGC	0	20	RDR2-sensitive
GTGCTGTGGACGCGACGCAAAGAC	0	9	RDR2-sensitive
GTGCTGTGGGCCGATGACTAGGCA	2	30	RDR2-sensitive
GTGCTGTTCGGAAGACGTGGGGCA	2	22	RDR2-sensitive
GTGCTGTTGGAAAGATGTAGCGTA	1	84	RDR2-sensitive
GTGCTGTTTTCCTGTCAGAACGGT	1	14	RDR2-sensitive
GTGCTTAAGTCGTCGTCTCGGCAC	0	11	RDR2-sensitive
GTGCTTATAGGACTGAGTCGGCTC	0	23	RDR2-sensitive
GTGCTTCGACTGGTGGCCCGACGG	0	39	RDR2-sensitive
GTGCTTCGGCTGAGGGCCCGACGG	0	10	RDR2-sensitive
GTGCTTCGGGCCTCAGTTAGTCGG	0	14	RDR2-sensitive
GTGCTTCGGGCCTCGGTTAGTCGG	0	16	RDR2-sensitive
GTGCTTCGGGCTGGTCCATCAGGC	0	18	RDR2-sensitive
GTGCTTCGTGCCGGACTAACGGGC	0	27	RDR2-sensitive
GTGCTTCGTGCCGGGCTAACGGGC	1	26	RDR2-sensitive
GTGCTTCGTGTCGGGCTAACGGGT	0	13	RDR2-sensitive
GTGCTTCTTCTAGGGTCAGTCGGC	0	11	RDR2-sensitive
GTGCTTGAGCCCTGTAGAAGCGTA	1	22	RDR2-sensitive
GTGCTTGCGGGCTGTCGGACCGGC	0	15	RDR2-sensitive
GTGCTTGGACAGGTAGGCCGGCCC	0	12	RDR2-sensitive
GTGCTTGGACCGGACCAGAATCGT	0	24	RDR2-sensitive
GTGCTTGGACCGGGCCAGAATCGT	0	12	RDR2-sensitive
GTGCTTGGGCCTAGGATGTGGCAC	0	13	RDR2-sensitive
GTGCTTGGGCTCCCTGCTGCGCGG	0	9	RDR2-sensitive
GTGCTTGGGCTGGGGATGTGGCAC	0	29	RDR2-sensitive
GTGCTTGGGTCTGGGATGTGGCAA	0	11	RDR2-sensitive
GTGCTTGGTCTGTAGCTTCGGCCT	0	10	RDR2-sensitive
GTGCTTGTTCGTTGGTCTGGCATG	0	28	RDR2-sensitive
GTGCTTGTTGGCGATAGACGGACC	24	2	RDR2-resistant
GTGCTTTCAGGAGTAGGACGGTGT	2	28	RDR2-sensitive
GTGCTTTGGCGTGGAACAGACGAG	0	9	RDR2-sensitive
GTGCTTTGGCGTGGGACAGACGAG	8	48	RDR2-sensitive

GTGCTTTGGGACGGCCTGTTAGGC	0	9	RDR2-sensitive
GTGCTTTTAGGAGTAGGACGGTGT	4	18	RDR2-sensitive
GTGCTTTTATCGGACACTCGGCAC	3	69	RDR2-sensitive
GTGCTTTTTATCGGACACTCGGCA	0	15	RDR2-sensitive
GTGCTTTTTATCGGGCACTCGGCA	0	11	RDR2-sensitive
GTGCTTTTTGCAAATCGTTTGGCA	0	40	RDR2-sensitive
GTGCTTTTTGGAGTAGGACGGTGT	4	32	RDR2-sensitive
GTGCTTTTTGTCGGGCACTCGGCA	11	102	RDR2-sensitive
GTGCTTTTTGTTGGACACTCGGCA	0	17	RDR2-sensitive
GTGCTTTTTTATCGGACACTCGGC	0	15	RDR2-sensitive
GTGCTTTTTTCGGACACTCGGCAA	5	32	RDR2-sensitive
GTGCTTTTTTGTCGGGCACTCGGC	0	15	RDR2-sensitive
GTGCTTTTTTTCGGACACTCGGCA	2	34	RDR2-sensitive
GTGGAAAAAGCAGCTGTAGGCTAT	0	16	RDR2-sensitive
GTGGAAAAAGCTGCTGTAGGCTGT	1	31	RDR2-sensitive
GTGGAAAAAGTTGCTGTGGGCTGT	0	14	RDR2-sensitive
GTGGAAAAGACAGACTGTCGGCTC	0	12	RDR2-sensitive
GTGGAAAGGCAGAACCGTGCACCA	0	11	RDR2-sensitive
GTGGAAATGGGGAGCGCAGGGACA	2	17	RDR2-sensitive
GTGGAAATGGGGAGTGCAGGGACA	0	9	RDR2-sensitive
GTGGAACATAGCGAGGACGAACGT	1	86	RDR2-sensitive
GTGGAACCAGACCAGAACGAGCAT	0	31	RDR2-sensitive
GTGGAACCAGTGGCACACAAGTAA	0	10	RDR2-sensitive
GTGGAACCTGAGGCTCAAGAGCAT	0	15	RDR2-sensitive
GTGGAACTCTAGCGAGGGGCGGAG	0	23	RDR2-sensitive
GTGGAACTGCAGTGGAGCGGACCA	0	13	RDR2-sensitive
GTGGAACTGCATGACGATGGGCAG	0	13	RDR2-sensitive
GTGGAAGACATCATGCACTCCGGA	0	12	RDR2-sensitive
GTGGAAGACTGGAATGTGCGGCGA	0	15	RDR2-sensitive
GTGGAAGAGGGGAGCAAAGACCAA	0	9	RDR2-sensitive
GTGGAAGCGTTGCACTGTATGCGG	0	12	RDR2-sensitive
GTGGAAGGCCAGGTAGAGCGGTTA	0	28	RDR2-sensitive
GTGGAAGGCTAGGCATTCTGCTCA	0	11	RDR2-sensitive
GTGGAAGGTGTAGGCTTACGGTAC	0	9	RDR2-sensitive
GTGGAATGCGGATACTACCCAGAT	0	10	RDR2-sensitive
GTGGAATGGACGAGCCGAGCCGAG	1	110	RDR2-sensitive
GTGGAATGGACGAGCCGAGCTGAG	0	10	RDR2-sensitive
GTGGAATGTTGTGGAGCAGGCGTC	0	14	RDR2-sensitive
GTGGAATTGTAGGTGACACGGTTT	0	40	RDR2-sensitive
GTGGAATTTGATGAGACTAATGGC	15	0	RDR2-resistant
GTGGACACAACATGGATCGACGAC	0	9	RDR2-sensitive
GTGGACACAACGTGGATTGACGAC	0	12	RDR2-sensitive
GTGGACACCACGAGAGAGCTCGGT	0	33	RDR2-sensitive
GTGGACACCTGATGCTATCACGGC	0	46	RDR2-sensitive
GTGGACACGGACAGCAAGACCCTC	0	11	RDR2-sensitive
GTGGACAGACGGTAAGCAGGGACA	0	27	RDR2-sensitive
GTGGACAGAGCATGGGCATGGATT	0	9	RDR2-sensitive
GTGGACAGAGTAGGAGAGAGCATG	0	45	RDR2-sensitive
GTGGACAGATGAGTTGATCGGCCT	0	14	RDR2-sensitive

GTGGACAGCGTTTACGACTGCATA	0	23	RDR2-sensitive
GTGGACAGGAACCGCCGCACGGCT	3	26	RDR2-sensitive
GTGGACAGTAGGGTTACGCGTGAG	0	22	RDR2-sensitive
GTGGACAGTCGGGACGCATTGGAC	0	9	RDR2-sensitive
GTGGACATCGACGGTGGACATCGA	0	9	RDR2-sensitive
GTGGACATGAAACTAGGCACGGTG	0	18	RDR2-sensitive
GTGGACATGGATACGGGTTTGAAA	0	20	RDR2-sensitive
GTGGACCAATCAGATCGCGACACG	2	81	RDR2-sensitive
GTGGACCAGAAGGAGTAAGAGCAT	0	9	RDR2-sensitive
GTGGACCATCCGGACAAACGCAGA	0	15	RDR2-sensitive
GTGGACCATCTGAGACCTTGGCGT	0	10	RDR2-sensitive
GTGGACCATCTGGACATACGCAGA	0	20	RDR2-sensitive
GTGGACCATCTGGCATAGGACCGC	2	53	RDR2-sensitive
GTGGACCCACAGAACAGTGCCGGA	0	11	RDR2-sensitive
GTGGACCCACAGCACAGTGTCGTA	0	15	RDR2-sensitive
GTGGACCCAGAGCAGAATGAATGC	1	16	RDR2-sensitive
GTGGACCGACAGTGCAAGAGACAA	0	9	RDR2-sensitive
GTGGACCGTCCAAACCTGCGGCGC	0	10	RDR2-sensitive
GTGGACCGTCCAGACGTACGCATA	0	27	RDR2-sensitive
GTGGACCGTCTGAACGTACGAAGA	0	11	RDR2-sensitive
GTGGACCGTCTGACCTAGAGGTAC	0	17	RDR2-sensitive
GTGGACCGTCTGGCTACTAGGCAT	0	16	RDR2-sensitive
GTGGACCGTTCGACCACGAGACGC	2	35	RDR2-sensitive
GTGGACCTGCATACATATATCTGA	19	1	RDR2-resistant
GTGGACCTTGATGAAGAGCGTCTA	0	82	RDR2-sensitive
GTGGACGAAGAGCACGGATAGCGC	0	9	RDR2-sensitive
GTGGACGACAGCGCGGCAGGTAGC	1	16	RDR2-sensitive
GTGGACGACGGAACGACGGGTTCA	0	10	RDR2-sensitive
GTGGACGAGGACGTGCGCAATGCG	0	13	RDR2-sensitive
GTGGACGCACGTGGAAGCCGGATT	0	28	RDR2-sensitive
GTGGACGCATGTAGGAGCGGGAAT	0	61	RDR2-sensitive
GTGGACGCCCACAGTAGAGACATA	0	11	RDR2-sensitive
GTGGACGCCTACCAGATCTCGGTC	0	14	RDR2-sensitive
GTGGACGCGTAGTAGCATGTGTGC	0	9	RDR2-sensitive
GTGGACGCTGACGCTACAGACTTA	0	10	RDR2-sensitive
GTGGACGCTGACGGTACTATCTTA	0	16	RDR2-sensitive
GTGGACGCTGAGGAGGTGGCTCAT	47	0	RDR2-resistant
GTGGACGGCGACAGAGTGGACGGA	1	32	RDR2-sensitive
GTGGACGGTCCGGTCTTAGGCATG	0	9	RDR2-sensitive
GTGGACGTGCATGTGGACGTGCAG	0	10	RDR2-sensitive
GTGGACTAGCTCGGCATGGCTCAT	0	9	RDR2-sensitive
GTGGACTATAGAGGGCTTCACCGA	0	33	RDR2-sensitive
GTGGACTATGTAGAGGCGTAGTTG	0	20	RDR2-sensitive
GTGGACTATGTGGAAGCGCAGCTG	0	15	RDR2-sensitive
GTGGACTCATCTGGCACGACACGG	2	16	RDR2-sensitive
GTGGACTCCGTAGCAAAGCACGGG	0	12	RDR2-sensitive
GTGGACTCCTTAACGAGGACGTAA	0	10	RDR2-sensitive
GTGGACTCTCTCGGCTGAGCCGGC	1	16	RDR2-sensitive
GTGGACTGACACGAAGGCCGACGT	0	19	RDR2-sensitive

GTGGACTGACTGAGGACGAGGCAC	0	11	RDR2-sensitive
GTGGACTGACTGAGGATGAGGCAC	0	10	RDR2-sensitive
GTGGACTGAGCAGTGAAACGTCGA	1	13	RDR2-sensitive
GTGGACTGAGCAGTGAAGCGTCGA	0	9	RDR2-sensitive
GTGGACTGGCTGGTCGATGGCGTC	0	10	RDR2-sensitive
GTGGACTGGTGTATGTCACGGCCT	0	11	RDR2-sensitive
GTGGACTGTCCGGCCAAGAGACAC	0	24	RDR2-sensitive
GTGGACTGTCTATGCATTTCCTAC	0	21	RDR2-sensitive
GTGGACTGTCTGCACCGTGCAGAG	3	22	RDR2-sensitive
GTGGACTGTGCAGAGGAATGAGAA	0	9	RDR2-sensitive
GTGGACTTAGACGGCTCGGCCCAA	0	12	RDR2-sensitive
GTGGACTTAGGGCCCTAGCGACGG	0	12	RDR2-sensitive
GTGGACTTCAGTGCTGAAGATGTT	18	0	RDR2-resistant
GTGGACTTGTCGGCGTACTGCTAT	0	22	RDR2-sensitive
GTGGACTTGTCGGCGTACTGCTGT	0	23	RDR2-sensitive
GTGGACTTGTCGGCGTACTGTTGT	0	23	RDR2-sensitive
GTGGACTTGTCGGCGTGCTGCTGT	0	21	RDR2-sensitive
GTGGACTTGTCGGCGTGCTGTCGT	0	16	RDR2-sensitive
GTGGACTTGTGCAAACAGGCGTGC	0	13	RDR2-sensitive
GTGGAGAACGGACAAAGGACGGAC	0	19	RDR2-sensitive
GTGGAGAACTTGGACGGTGATCAC	1	20	RDR2-sensitive
GTGGAGACGAGAGCTGACGTGGCA	0	12	RDR2-sensitive
GTGGAGACTTGGAGACGTGCGCTA	2	22	RDR2-sensitive
GTGGAGATCGCATTCGGTGAGCAT	0	11	RDR2-sensitive
GTGGAGATGGCGAACGACGAGCAA	0	9	RDR2-sensitive
GTGGAGCAACTGCGAGAAGAACTT	0	12	RDR2-sensitive
GTGGAGCAAGGAAGCGAACGCAAG	0	16	RDR2-sensitive
GTGGAGCACAGGGACGATGGTCGA	1	14	RDR2-sensitive
GTGGAGCATGTTGGTGGCTATGAC	0	19	RDR2-sensitive
GTGGAGCCCTCTGGCAGACCGCGC	0	11	RDR2-sensitive
GTGGAGCGCAGCGTGGATAATGAC	83	4	RDR2-resistant
GTGGAGCGGTAGAATGGACGGTGC	0	21	RDR2-sensitive
GTGGAGCGGTGGCACAGTTGGACG	0	12	RDR2-sensitive
GTGGAGCTAGAGCTGTAAGACTTT	0	17	RDR2-sensitive
GTGGAGCTCGAAGGTGGATCTGAG	0	13	RDR2-sensitive
GTGGAGCTCTTCGGATCTGGCATA	45	8	RDR2-resistant
GTGGAGGACTCTAACTGCAGGCGT	0	14	RDR2-sensitive
GTGGAGGCGAGAGCTGACGTGGCA	0	32	RDR2-sensitive
GTGGAGGCGTCAGACGTAGGACGG	0	12	RDR2-sensitive
GTGGAGGGCCTTGTAGAGGACGCT	0	10	RDR2-sensitive
GTGGAGGGTGTAGGCTTACGGTAC	0	55	RDR2-sensitive
GTGGAGTACGGTATTACGCTCTGT	0	10	RDR2-sensitive
GTGGAGTAGGGCTTGCAGTCAGAC	0	14	RDR2-sensitive
GTGGAGTCTGACCATGTAGCTGGA	0	23	RDR2-sensitive
GTGGAGTGTATTGAGGCTTGGCAT	0	22	RDR2-sensitive
GTGGAGTGTTGCGCTGCGAGCGGC	0	67	RDR2-sensitive
GTGGATAACGTAACTCCCGTCGGC	0	10	RDR2-sensitive
GTGGATAACTCAGAGGTGCTGCAT	1	20	RDR2-sensitive
GTGGATACCTCGGAAGTGCTGCAT	1	49	RDR2-sensitive

GTGGATACCTCGGAGATGCTGCAT	2	14	RDR2-sensitive
GTGGATAGAGGACGCACAATGAAC	0	26	RDR2-sensitive
GTGGATAGGACAACGGCAGCTGAT	0	10	RDR2-sensitive
GTGGATAGGCTAGAAAGCGACATT	0	35	RDR2-sensitive
GTGGATATCTTGGAGGTGCTGCAT	0	10	RDR2-sensitive
GTGGATATGACTGCGGGCGGATAT	0	16	RDR2-sensitive
GTGGATCATCCCGGCGTGTAAAGT	0	10	RDR2-sensitive
GTGGATCCCGTTGGCTGACAGCAT	0	13	RDR2-sensitive
GTGGATCCGATGACATACTGGCGA	0	61	RDR2-sensitive
GTGGATCCGATGGCATACTGGCGA	1	27	RDR2-sensitive
GTGGATCGATGACTGGACACACGG	0	9	RDR2-sensitive
GTGGATCTAAACGCACAACGACAT	0	23	RDR2-sensitive
GTGGATCTTCGAGCTCCGACGGTG	0	12	RDR2-sensitive
GTGGATGAGGATGGATATAGCTTT	0	9	RDR2-sensitive
GTGGATGATGGACGAGATGTGCCT	0	31	RDR2-sensitive
GTGGATGCTGGGAACTGCGGATGG	0	23	RDR2-sensitive
GTGGATGCTGTAGGGACACCGCTG	0	9	RDR2-sensitive
GTGGATGTAGCAATTAGGGGGCGT	1	15	RDR2-sensitive
GTGGATTCACAGAAGCGAACGGAT	1	12	RDR2-sensitive
GTGGATTCACGGAAGCGAACGAAC	0	13	RDR2-sensitive
GTGGATTCACGGAAGCGAACGGAC	0	18	RDR2-sensitive
GTGGATTCACGGAAGCGAACGGAT	0	44	RDR2-sensitive
GTGGATTCCTGTAGTGAGGCCCAA	0	10	RDR2-sensitive
GTGGATTCGTGATTGGCGATGGGG	49	0	RDR2-resistant
GTGGATTGAGCGGGATTGGATGGG	5	44	RDR2-sensitive
GTGGATTGAGTGGGATTGGATGGG	4	152	RDR2-sensitive
GTGGATTGATTAATTTTGTACGGC	0	11	RDR2-sensitive
GTGGATTGGAGTGTATTGGACGGG	0	12	RDR2-sensitive
GTGGATTGGAGTGTATTGGGCGGG	0	9	RDR2-sensitive
GTGGATTGGGAACACCGAGGACGC	0	9	RDR2-sensitive
GTGGATTGGGTAGGATTGGATGGA	0	12	RDR2-sensitive
GTGGATTGGGTGGGATTGGATGGG	0	36	RDR2-sensitive
GTGGATTGTTACCTCAAAGATGGA	0	10	RDR2-sensitive
GTGGATTTGAACGCGAAGTGGCAC	0	13	RDR2-sensitive
GTGGATTTGTTTCCTCGTAGTTGT	0	20	RDR2-sensitive
GTGGCAAGTTAGGCAGCGAACCAA	1	27	RDR2-sensitive
GTGGCACCGAGGTAGATGACATGT	0	10	RDR2-sensitive
GTGGCACCGGACGATCTGCAGGCA	0	13	RDR2-sensitive
GTGGCACCGTGAAACTGAGACTGC	0	23	RDR2-sensitive
GTGGCACTAGACTCTTACGGCGTG	0	12	RDR2-sensitive
GTGGCACTCGACAAATCGTTCGGC	0	10	RDR2-sensitive
GTGGCACTCGGCAAAGAAGACTCT	0	10	RDR2-sensitive
GTGGCACTCGGCAAAGAATATGGC	0	9	RDR2-sensitive
GTGGCACTCGGCAAAGACTGACGG	0	9	RDR2-sensitive
GTGGCACTGGGCGCGGCTGGACGG	0	9	RDR2-sensitive
GTGGCACTGGTGGAGAGAAGGCAC	0	10	RDR2-sensitive
GTGGCACTGTGACAGCGTGGACGG	0	9	RDR2-sensitive
GTGGCACTGTGACAGCGTGGACGT	0	19	RDR2-sensitive
GTGGCACTGTGGAGAGAAGACCAC	0	20	RDR2-sensitive

GTGGCACTGTGGAGAGAAGACCGC	0	20	RDR2-sensitive
GTGGCACTGTGGAGAGACGACCGC	0	9	RDR2-sensitive
GTGGCATATGACGACAGTAGACAC	0	10	RDR2-sensitive
GTGGCATTGAGCATGTCGGACGGA	0	13	RDR2-sensitive
GTGGCCAACTGAAGGAGAACGAGC	0	11	RDR2-sensitive
GTGGCCAAGGAGGACTGAGGCGGT	0	13	RDR2-sensitive
GTGGCCAGACTCTGCAGACGCAAG	0	21	RDR2-sensitive
GTGGCCGTAGGATGCACGAGACGC	0	9	RDR2-sensitive
GTGGCCTAGAGTCGGACGATCCAC	2	17	RDR2-sensitive
GTGGCCTAGGACCAGATGATCCGC	0	9	RDR2-sensitive
GTGGCCTCGGGAGGCAGAACCGCA	0	9	RDR2-sensitive
GTGGCCTCTGGCAACGACGGACAT	0	12	RDR2-sensitive
GTGGCCTCTGTACAGCATTGAGCA	0	33	RDR2-sensitive
GTGGCCTGTAGGCCTGTGGGACGG	0	53	RDR2-sensitive
GTGGCCTGTAGTCCAGCACGGTTT	0	9	RDR2-sensitive
GTGGCCTGTCGACCCCAAGACGAC	0	12	RDR2-sensitive
GTGGCCTGTTGGCTCGACCGTCTG	19	0	RDR2-resistant
GTGGCCTTGTGCGGACCGTCTGGC	0	9	RDR2-sensitive
GTGGCGAAAACTGCAGATGGGCAT	0	14	RDR2-sensitive
GTGGCGAGTTGTAGAGCACGATGG	0	19	RDR2-sensitive
GTGGCGCGACAGTAGTAGGACGTC	0	12	RDR2-sensitive
GTGGCGCTGAGCTAGCTGACGCGG	0	13	RDR2-sensitive
GTGGCGCTGTAGTGGGATATAGAG	0	10	RDR2-sensitive
GTGGCGTCGAGCTTAGGGACACGG	0	11	RDR2-sensitive
GTGGCGTTGGTCTCCTGGGCACGG	289	60	RDR2-resistant
GTGGCTACAGGAGCTCCCACGGCT	0	9	RDR2-sensitive
GTGGCTAGAATCTGTAGTAGCAGG	20	1	RDR2-resistant
GTGGCTAGGGATTGAGGGCAGGAC	4	30	RDR2-sensitive
GTGGCTCACCCGGCTTCAGCTCAC	0	9	RDR2-sensitive
GTGGCTCGACAACGATGACGTGGG	0	10	RDR2-sensitive
GTGGCTCGGACGGTCCGCGATGGC	1	17	RDR2-sensitive
GTGGCTCTGGACCGGATGGTTCGT	0	10	RDR2-sensitive
GTGGCTGACAGTCGTCGAACGCAT	0	15	RDR2-sensitive
GTGGCTGACTGTCGTATGGTGCAC	0	11	RDR2-sensitive
GTGGCTGCAGAGAAGAGGAAACAA	0	11	RDR2-sensitive
GTGGCTGCGGCATGTGGATCGATC	0	9	RDR2-sensitive
GTGGCTGCGTCGAGCTGTGGCATG	0	11	RDR2-sensitive
GTGGCTGCTGTAGCGGGATCGGAT	0	19	RDR2-sensitive
GTGGCTGGGCGGGTAGAAGGGCTT	0	11	RDR2-sensitive
GTGGCTGTACGGACGCGGATGGAG	0	13	RDR2-sensitive
GTGGCTTAGGTACTGTGTACGGAC	0	17	RDR2-sensitive
GTGGCTTGTAGTGGGCTTTTCGCC	18	1	RDR2-resistant
GTGGCTTGTTTTCTGATATCGGAT	14	0	RDR2-resistant
GTGGGAACTCGACACGCTGTGACG	0	21	RDR2-sensitive
GTGGGAATCTTGATGATGCTGCAT	430	92	RDR2-resistant
GTGGGACAACGACGATGACGCTAC	0	9	RDR2-sensitive
GTGGGACACTGTAGGAGCGGTCGG	0	11	RDR2-sensitive
GTGGGACAGCCAGGACAGGGACGT	0	12	RDR2-sensitive
GTGGGACAGCTAGGACGGGATCGT	0	27	RDR2-sensitive

GTGGGACAGCTATGACAGAGACGT	0	13	RDR2-sensitive
GTGGGACAGTCAGAACAGAGACGT	0	26	RDR2-sensitive
GTGGGACCAGTAGCATACAAGGAA	0	47	RDR2-sensitive
GTGGGACCAGTGGCATACAAGGAA	0	18	RDR2-sensitive
GTGGGACCCTAGATAGATGGCAAG	0	11	RDR2-sensitive
GTGGGACCCTTACGTTGGACAGCA	0	9	RDR2-sensitive
GTGGGACGCCAGGCATTGACTACC	0	19	RDR2-sensitive
GTGGGACTAAAGCCGAGGCACTGA	1	65	RDR2-sensitive
GTGGGACTAAAGCCGGGGCGCTGA	1	36	RDR2-sensitive
GTGGGACTAGACCGACGAGGACGT	0	22	RDR2-sensitive
GTGGGACTGATTGCATACTGGCGA	0	10	RDR2-sensitive
GTGGGACTGTTGATGACGTAGTAA	0	10	RDR2-sensitive
GTGGGAGAGGCTCTGCACGGACGG	2	40	RDR2-sensitive
GTGGGAGATGCTCTGCACGGACGG	0	13	RDR2-sensitive
GTGGGAGTGTGGGACCACCTCGGC	0	10	RDR2-sensitive
GTGGGATGCTGTTGCTGAGGCTAC	0	14	RDR2-sensitive
GTGGGATGGTTCCGGATCTGAGGA	0	21	RDR2-sensitive
GTGGGATTCGGATCAGATACGCAT	0	11	RDR2-sensitive
GTGGGATTCGGCCTACTGTAACAG	1	23	RDR2-sensitive
GTGGGATTGGGCTGTGCAGGGTGT	17	0	RDR2-resistant
GTGGGCAAGCTGAGGATATTGGTG	0	10	RDR2-sensitive
GTGGGCACTGGACAGTTGATCGGC	0	11	RDR2-sensitive
GTGGGCATAAGAGGAAGGAGGACG	3	48	RDR2-sensitive
GTGGGCATCTTAGACGAAGGACGG	1	53	RDR2-sensitive
GTGGGCCAAAGCCTTCGTCGGAGA	0	9	RDR2-sensitive
GTGGGCCACGGACGAGAGGGCGCA	0	14	RDR2-sensitive
GTGGGCCAGGCTTAGACAGAGAAT	1	15	RDR2-sensitive
GTGGGCCATTGGATGCAAAGATAG	2	14	RDR2-sensitive
GTGGGCCCAAGCACTTAGGAACGG	0	11	RDR2-sensitive
GTGGGCCCAGCTATTTTCGTCGGT	0	9	RDR2-sensitive
GTGGGCCCAGGCAACACAAGACAC	0	10	RDR2-sensitive
GTGGGCCCGATGACATACTGGCGA	0	11	RDR2-sensitive
GTGGGCCGATAGAACGGACGGTGC	0	10	RDR2-sensitive
GTGGGCCTGGACTGAGAGGGGCCG	0	11	RDR2-sensitive
GTGGGCGCTGTAGGGGCACCGCTG	1	24	RDR2-sensitive
GTGGGCTAACGACGACGGATCAGA	112	15	RDR2-resistant
GTGGGCTCGTCCGACATGACACGG	0	13	RDR2-sensitive
GTGGGCTCTCTAGGCACGGCTCGT	1	16	RDR2-sensitive
GTGGGCTCTCTTGGCTGGACCGGT	0	9	RDR2-sensitive
GTGGGCTGCGACGCTACGTAGAAG	0	18	RDR2-sensitive
GTGGGCTGGCACGGCATGACCCGC	0	21	RDR2-sensitive
GTGGGCTGGTAGACAAAAGGCGGT	0	9	RDR2-sensitive
GTGGGCTGGTTACGCGCGGGTTGT	0	15	RDR2-sensitive
GTGGGCTGTGACGGCTTAGGCCAT	0	11	RDR2-sensitive
GTGGGCTTCTGACGGATACGGCCA	0	11	RDR2-sensitive
GTGGGGAACTGTAGACGATACGGT	0	13	RDR2-sensitive
GTGGGGAATGTTAGGAGTGGCTGC	0	24	RDR2-sensitive
GTGGGGAATTCTGTGGTCGGCTGG	2	14	RDR2-sensitive
GTGGGGCAGAACATGATGCACGGT	0	24	RDR2-sensitive

GTGGGGCGATAACGTAGAAGACAA	0	19	RDR2-sensitive
GTGGGGCGATAACGTAGGAGACAA	0	20	RDR2-sensitive
GTGGGGCTCGTTGCTGAAGGCTAG	0	15	RDR2-sensitive
GTGGGGGCACGTAGATGGCAGAAC	0	17	RDR2-sensitive
GTGGGGGTAGATGTGAGCAAGACG	0	9	RDR2-sensitive
GTGGGGTACTGAGTCGCCTGCGAA	0	11	RDR2-sensitive
GTGGGGTAGCTGTGCGGAAATGGC	16	0	RDR2-resistant
GTGGGTAAAGATGCGCAACCTCTG	1	14	RDR2-sensitive
GTGGGTCACTGTAGAGGATCCGGC	0	20	RDR2-sensitive
GTGGGTCCACATGAGTCAATGACA	0	23	RDR2-sensitive
GTGGGTCCGATGGCATACTGACGA	0	10	RDR2-sensitive
GTGGGTCCGATGGCATACTGGCGA	6	323	RDR2-sensitive
GTGGGTCCGATGGCATACTGGCGC	0	14	RDR2-sensitive
GTGGGTCCGATGGCATACTGGCGT	0	9	RDR2-sensitive
GTGGGTCCGATGTCATACTGGCGA	0	12	RDR2-sensitive
GTGGGTCGTCCGAGCACCAGACAC	2	14	RDR2-sensitive
GTGGGTCTCTCTGCGCAAACGGTC	1	17	RDR2-sensitive
GTGGGTCTCTGTGCTGGACGAGGC	0	12	RDR2-sensitive
GTGGGTCTGGACTGTTCGACGGAG	0	12	RDR2-sensitive
GTGGGTCTGGGCCGGACAGTGCAT	0	12	RDR2-sensitive
GTGGGTCTGGTCGGACGACGCGCA	0	18	RDR2-sensitive
GTGGGTGGGGTGTGTGACGAGCAT	0	10	RDR2-sensitive
GTGGGTTCTGGAACATGTTGAGGT	0	12	RDR2-sensitive
GTGGGTTGTCGCCAGAGAGAACGG	0	13	RDR2-sensitive
GTGGGTTGTGGAAGGGCGCGCGTG	0	12	RDR2-sensitive
GTGGTACAAGGACGAACGACAGGA	0	13	RDR2-sensitive
GTGGTACCGGTAGAACTGCCACGG	0	23	RDR2-sensitive
GTGGTACTATGTGGGAGCGTTGCA	0	11	RDR2-sensitive
GTGGTACTGTAGCAGCACGAAGTC	0	10	RDR2-sensitive
GTGGTAGACGGGCTGCTCTCCCAA	0	9	RDR2-sensitive
GTGGTAGAGAGGAAGAACAGGACG	2	22	RDR2-sensitive
GTGGTATAGAGTGCTGATCAGGAC	0	10	RDR2-sensitive
GTGGTATAGATCGGGCTAGCACGG	0	72	RDR2-sensitive
GTGGTCAACGCACGGCATGAGCTT	2	26	RDR2-sensitive
GTGGTCAGGTTGGCTCGACTCGGA	0	10	RDR2-sensitive
GTGGTCCGATGTAGAGTGGACGGT	0	95	RDR2-sensitive
GTGGTCGAGATTACTTAGGGCGGT	1	23	RDR2-sensitive
GTGGTCGGTCATGGTATGTGAGGC	0	14	RDR2-sensitive
GTGGTCTAGATCATGTGCTCGGTG	0	11	RDR2-sensitive
GTGGTCTGCTGTAGAGATGGCCAA	1	27	RDR2-sensitive
GTGGTCTGGTGGAAAGGGAGGGCT	1	32	RDR2-sensitive
GTGGTCTGTGGAGGGAGCAGCAGC	0	13	RDR2-sensitive
GTGGTGAAAGCCGAGGCCGTGGGG	15	0	RDR2-resistant
GTGGTGTACTCTAATGAGCTGGAC	19	0	RDR2-resistant
GTGGTGTAGTGCTAAACGGGCTAA	0	9	RDR2-sensitive
GTGGTGTAGTGCTAAACGGGCTAT	0	12	RDR2-sensitive
GTGGTTCCCGGGCTGAAACGACGA	0	9	RDR2-sensitive
GTGGTTGGCTCTGGTGGTCGGCAT	0	9	RDR2-sensitive
GTGGTTGTTGTAGTCGGATATCAC	0	11	RDR2-sensitive

GTGGTTTATTATAGTTGGATAACT	56	1	RDR2-resistant
GTGGTTTCGCTTCGGTTCGCTCGG	1	37	RDR2-sensitive
GTGTAAAAGTTGTATTGAAGGGCA	20	0	RDR2-resistant
GTGTAACACTCGGCAAAGAAGGCT	3	20	RDR2-sensitive
GTGTAACACTCGGCAAAGAGGCTC	1	16	RDR2-sensitive
GTGTAACTCGAGCTCGACGACATA	0	16	RDR2-sensitive
GTGTACATTCGGGCTGCACGGCTT	0	17	RDR2-sensitive
GTGTACCACCGGACTGTTCGACGG	0	15	RDR2-sensitive
GTGTACCACTCTGGGCCGGCGTAA	0	47	RDR2-sensitive
GTGTACCCGTCGGAAACAGTGCAA	0	19	RDR2-sensitive
GTGTACGACGGAGACTGAGCCAGA	0	9	RDR2-sensitive
GTGTACGAGCCTCTGGTCGATGGT	18	0	RDR2-resistant
GTGTACGTCCGGACGGTCCGCGCA	0	11	RDR2-sensitive
GTGTACTCTAATGAGCTGGACGCA	828	116	RDR2-resistant
GTGTACTCTAGGGCGATTGTCGAA	0	9	RDR2-sensitive
GTGTAGAACACTCGGCACATCCAA	0	30	RDR2-sensitive
GTGTAGAAGCGACGGAGTTCACCA	0	13	RDR2-sensitive
GTGTAGACGTGTATGCAGTATGCA	0	10	RDR2-sensitive
GTGTAGATCTGAGCAAGGCGTCGA	1	18	RDR2-sensitive
GTGTAGCAGGCTGCACCGAAGACT	33	0	RDR2-resistant
GTGTAGCGAAGACTGCATGGACAG	0	12	RDR2-sensitive
GTGTAGCGGAGTAGTAGACCGGCG	0	10	RDR2-sensitive
GTGTAGCTCTGCGGACGGTGATTT	0	25	RDR2-sensitive
GTGTAGCTTTCTTCACGTTGGGAT	18	0	RDR2-resistant
GTGTAGGACACTCGGCACAGCCAA	1	16	RDR2-sensitive
GTGTAGGACACTCGGCACAGTCAA	0	11	RDR2-sensitive
GTGTAGGATCTAGGACACCGGCTA	1	14	RDR2-sensitive
GTGTAGGATCTTAGGGCACGCAAA	17	0	RDR2-resistant
GTGTAGTACTCGTAGGGATGGCAA	0	12	RDR2-sensitive
GTGTAGTCTGAGCTGGCAGCTGAC	0	37	RDR2-sensitive
GTGTAGTGCTGACAGTAGTACTGC	0	9	RDR2-sensitive
GTGTATAACTGTGACGTAGATGAC	108	6	RDR2-resistant
GTGTATACATCGTGGACAGAGCAT	0	20	RDR2-sensitive
GTGTATCATGGGATTGGAAGGCAT	15	0	RDR2-resistant
GTGTATCCCTGTAGAGTACGGAAA	0	20	RDR2-sensitive
GTGTATCCTCTGACGTCGACTGCA	0	10	RDR2-sensitive
GTGTATCTCTGCGGACGACGATTT	0	11	RDR2-sensitive
GTGTATTCTGAGAAGCAGCTGAAC	1	36	RDR2-sensitive
GTGTCACATCGGACGGTCCGCAAC	0	10	RDR2-sensitive
GTGTCACATCTGTAGACGACGGTT	0	15	RDR2-sensitive
GTGTCAGACCATGGATCGAGGGCT	0	14	RDR2-sensitive
GTGTCAGACTCATAGAGGACGCAC	0	11	RDR2-sensitive
GTGTCAGACTTAGAACGACACGGT	1	39	RDR2-sensitive
GTGTCAGGATAAGGTGTCAGGCGA	0	13	RDR2-sensitive
GTGTCAGGCTGTGGGTTAAACGGG	0	11	RDR2-sensitive
GTGTCAGTACGTGGATCCGACGGT	1	12	RDR2-sensitive
GTGTCATCGTGGACTTGTTGGCGT	0	10	RDR2-sensitive
GTGTCATCTCTGGACACTCGGCAA	3	22	RDR2-sensitive
GTGTCCCTTGGGGATGTGTTCGTC	0	11	RDR2-sensitive

GTGTCCTATGTAGACACTCGGCAA	0	27	RDR2-sensitive
GTGTCCTCTATAGATCGGATCGGA	0	9	RDR2-sensitive
GTGTCCTCTGTAGACACTCGGCAA	1	18	RDR2-sensitive
GTGTCGCGGCACGACGATCTGGAT	0	15	RDR2-sensitive
GTGTCGGCACTGTAGGTCGGCTTG	0	20	RDR2-sensitive
GTGTCGTCATGGACTTGTCGGCGT	0	14	RDR2-sensitive
GTGTCGTGACAGATGGGCCGACGG	0	13	RDR2-sensitive
GTGTCGTGCCTGTAGCCCGGTCAG	0	18	RDR2-sensitive
GTGTCGTGCTGTGCTAGCCCGCGG	0	10	RDR2-sensitive
GTGTCGTGGATATGTGTATGGGTA	0	9	RDR2-sensitive
GTGTCGTTGTGGCTTTATCGGCGT	1	15	RDR2-sensitive
GTGTCTACTGTACTGACGCTCGGC	0	14	RDR2-sensitive
GTGTCTAGTGTCGGATCGGCCCAT	0	29	RDR2-sensitive
GTGTCTATCGGATTTCGGACGGGT	0	18	RDR2-sensitive
GTGTCTGGACCGAAGGCGTGGCAC	1	28	RDR2-sensitive
GTGTCTGGATGTTTGGATTGGCAT	0	9	RDR2-sensitive
GTGTCTGGCAGACTGGTCCGAGGC	0	12	RDR2-sensitive
GTGTCTGGGCTGAAGGCGTGGCAC	0	20	RDR2-sensitive
GTGTCTGGTCGTGGAGGACGCGCA	0	11	RDR2-sensitive
GTGTCTGTGCGACTGTGCGGTGGC	0	11	RDR2-sensitive
GTGTCTTCACGAATTTCTGGGGCA	15	0	RDR2-resistant
GTGTCTTCACGTCGGATAGCGGAG	0	25	RDR2-sensitive
GTGTCTTCGTTGGACATTCGGCAA	0	26	RDR2-sensitive
GTGTCTTCGTTGGACCGTGTCGGC	1	37	RDR2-sensitive
GTGTCTTCTGTGGACACTCGGCAA	0	14	RDR2-sensitive
GTGTCTTGTGTGTTACTCGGTCGT	0	10	RDR2-sensitive
GTGTCTTTCACTGTGGATAGGCGA	4	27	RDR2-sensitive
GTGTCTTTTACTGTGGATAGGCGA	0	9	RDR2-sensitive
GTGTCTTTTGTCGGACACTCGGCA	0	15	RDR2-sensitive
GTGTCTTTTGTCGGGCACTCGGCA	7	57	RDR2-sensitive
GTGTGAAAGCGGGTTGAGAAGGCT	0	10	RDR2-sensitive
GTGTGAACCTGCACCTGGGCGCAT	0	32	RDR2-sensitive
GTGTGAACTGTTGGAGATGGTCGT	0	32	RDR2-sensitive
GTGTGAAGCGCAGACTGCATGGAC	0	15	RDR2-sensitive
GTGTGAAGCGCAGGTTGTATGGAC	0	16	RDR2-sensitive
GTGTGAAGGCGGACAGATCTTCAT	0	10	RDR2-sensitive
GTGTGAATCTGAGCGTCGGCAGGG	39	8	RDR2-resistant
GTGTGAATTGCAGAATCCCGCGAA	17	1	RDR2-resistant
GTGTGACACTCGGCAAAGAAGGCT	0	12	RDR2-sensitive
GTGTGACCACGAGCAAATGACGCG	0	10	RDR2-sensitive
GTGTGACTCTCTGTTCGGCTGCAT	0	27	RDR2-sensitive
GTGTGACTGAGGGAAGCACGTGTT	0	21	RDR2-sensitive
GTGTGACTGATGGTGGGTCGCTAT	0	10	RDR2-sensitive
GTGTGACTGCATGTGTATAGCGTA	0	12	RDR2-sensitive
GTGTGAGACAGACGGTAGGCCAGA	0	27	RDR2-sensitive
GTGTGAGGCGGAACGACGGACCAG	0	12	RDR2-sensitive
GTGTGAGGGGGAGATTGTTGGGAA	1	28	RDR2-sensitive
GTGTGAGTGGGAGAGTAGGTGCAT	0	13	RDR2-sensitive
GTGTGAGTGTGTGTGGGCCGGCAC	0	9	RDR2-sensitive

GTGTGATCTCAGGCAGGACAGGCT	0	15	RDR2-sensitive
GTGTGATCTGGATTGACGTGGCAC	1	17	RDR2-sensitive
GTGTGATCTTTAGAACCGGCCATT	0	15	RDR2-sensitive
GTGTGCACTGTAGCGACGGTGGTC	2	26	RDR2-sensitive
GTGTGCAGGCAGCCCGTCGGACAC	0	11	RDR2-sensitive
GTGTGCCCTCTGAGTATCGGACGT	0	12	RDR2-sensitive
GTGTGCGCGCGTCGGCTATGGCAC	0	10	RDR2-sensitive
GTGTGCGGGCGACTCGACGGGCAC	0	10	RDR2-sensitive
GTGTGCGTCGTGCTTGGGTTGGCA	0	11	RDR2-sensitive
GTGTGCGTGGTTGTACTCCGGCAT	0	9	RDR2-sensitive
GTGTGCGTTGGCTGTGTGCGCGGC	7	117	RDR2-sensitive
GTGTGCTCTGAAGTGCTCTGCGGC	0	10	RDR2-sensitive
GTGTGCTGGAGACGGTTTGAGTGA	33	3	RDR2-resistant
GTGTGCTGTAGTAGTGGGCACGGC	0	10	RDR2-sensitive
GTGTGCTGTAGTTGGATGGCCATC	0	11	RDR2-sensitive
GTGTGCTGTTTCTCTGCAGGGCAA	0	13	RDR2-sensitive
GTGTGGAACGGCGGCGTCGGCGAA	0	10	RDR2-sensitive
GTGTGGAACGTACAACGGCTGAGC	0	25	RDR2-sensitive
GTGTGGAATCTGGTAGTAGGCTAT	0	16	RDR2-sensitive
GTGTGGACTATGTGGAAGCGCAGC	0	21	RDR2-sensitive
GTGTGGACTGGACAAGAAGGAGGA	0	14	RDR2-sensitive
GTGTGGATTCACGGAAGCGAACGG	0	12	RDR2-sensitive
GTGTGGCTATAGACGGTACATGAA	23	3	RDR2-resistant
GTGTGGGCAGAACCAGGATGAGAA	0	9	RDR2-sensitive
GTGTGGGCAGTGATGGACAAGGAT	0	11	RDR2-sensitive
GTGTGGGCATAAGAGGAAGGAGGA	0	9	RDR2-sensitive
GTGTGGGCCTCTGGTGAGACACGC	0	15	RDR2-sensitive
GTGTGGGCTCTGACGCACGGGCAA	1	24	RDR2-sensitive
GTGTGGGCTGGTTACGCGCGGGTT	0	16	RDR2-sensitive
GTGTGGGTCGGCACCATTGTTGGG	0	11	RDR2-sensitive
GTGTGTAGACTGATAGTGGCGCAA	0	14	RDR2-sensitive
GTGTGTAGGATCTAGGACACCGGC	0	9	RDR2-sensitive
GTGTGTAGGCTTACGATACGGTAC	0	39	RDR2-sensitive
GTGTGTCAGACTCATAGAGGACGC	0	15	RDR2-sensitive
GTGTGTCGTCGTAATACATGGGCA	42	1	RDR2-resistant
GTGTGTCTGCACGTGTCGGGCGAA	0	9	RDR2-sensitive
GTGTGTCTGCGATTGTCTAGCCAC	0	13	RDR2-sensitive
GTGTGTGACTGAGGGAAGCACGTG	0	12	RDR2-sensitive
GTGTGTGAGTGTGTGGACCGGCAC	0	32	RDR2-sensitive
GTGTGTGCGGACAGCCCCGACGGA	0	12	RDR2-sensitive
GTGTGTGCGTAGAGATGACGACAT	0	9	RDR2-sensitive
GTGTGTGGACGAGGTAGCGAAGGC	43	5	RDR2-resistant
GTGTGTGTCGGCAACAAAAGCATG	40	0	RDR2-resistant
GTGTGTGTGAGTGTGTGGACCGGC	0	15	RDR2-sensitive
GTGTGTGTGAGTGTGTGGGCCGGC	0	9	RDR2-sensitive
GTGTGTTCGCGGGGACGGGGACGG	0	17	RDR2-sensitive
GTGTGTTGACTGTATTTAGGTTTA	0	14	RDR2-sensitive
GTGTGTTGCGACGGCTTACAACAA	0	11	RDR2-sensitive
GTGTGTTTCGGACGTGTCGCGGCA	0	14	RDR2-sensitive

GTGTGTTTGGTTGCTTGGACGTGT	0	24	RDR2-sensitive
GTGTGTTTGGTTGTAGGTTGCATC	0	29	RDR2-sensitive
GTGTTAATATATTTCGTTGGAGAC	16	0	RDR2-resistant
GTGTTACCTCGGCGCCACAGGCTA	0	9	RDR2-sensitive
GTGTTAGACTGGTATACAATCCTC	0	10	RDR2-sensitive
GTGTTCCATGGACGACTCGTCGGG	0	9	RDR2-sensitive
GTGTTCTGTGGCATCGTACGGACA	0	9	RDR2-sensitive
GTGTTGAACTTGGCCGAACCACGG	0	9	RDR2-sensitive
GTGTTGAAGGCAGATACTCGGCAA	0	18	RDR2-sensitive
GTGTTGAATTCTGATCGACTGCAT	0	38	RDR2-sensitive
GTGTTGACGCGGATAGGACAACGG	0	15	RDR2-sensitive
GTGTTGACTAAGTGACGGACACTG	0	9	RDR2-sensitive
GTGTTGACTTTGACTGATCGTCTG	0	9	RDR2-sensitive
GTGTTGATTGTAGTATTGTCCGGA	0	10	RDR2-sensitive
GTGTTGCTAGTAGAACGGACGGAC	0	29	RDR2-sensitive
GTGTTGCTGGAAACGGAGACGGAT	1	25	RDR2-sensitive
GTGTTGGACATGGTCTGTAGGCAT	0	33	RDR2-sensitive
GTGTTGGGGCGAAGGCAAAGACGC	0	15	RDR2-sensitive
GTGTTGGGGCGAAGGCGAAGACGC	0	14	RDR2-sensitive
GTGTTGGTCTGTGGAGACGGTCTA	0	25	RDR2-sensitive
GTGTTGGTCTGTGGGGACGATATA	0	19	RDR2-sensitive
GTGTTGTAGGACTGCAGGGAGGAC	1	14	RDR2-sensitive
GTGTTGTTCTGTGGGGATGGTCTA	0	11	RDR2-sensitive
GTGTTTAACGTGGGGCTGAGAGGC	2	24	RDR2-sensitive
GTGTTTAGACTGTGTTCACCGCAT	0	10	RDR2-sensitive
GTGTTTCGCTGGGACAGTGGACGG	0	24	RDR2-sensitive
GTGTTTCTAGGAGTAGGACGGTGC	18	0	RDR2-resistant
GTGTTTCTGTTGGGCGGCGGGCTT	4	18	RDR2-sensitive
GTGTTTGCGTCACCTGGCCGGCTC	0	11	RDR2-sensitive
GTGTTTGGACCAGGCTAAATTCGT	0	13	RDR2-sensitive
GTGTTTGGACCGGGCTAAATTCGT	0	72	RDR2-sensitive
GTGTTTGGTAATCTGGAACGGAGC	1	13	RDR2-sensitive
GTGTTTGGTATTGTAGAACGGAGC	0	9	RDR2-sensitive
GTGTTTGGTTGTGAGTCAAGACGG	0	12	RDR2-sensitive
GTGTTTGTTGACTAGATTCTCGGA	0	9	RDR2-sensitive
GTGTTTTTAGGAGTAGGACGGTGT	70	327	RDR2-sensitive
GTGTTTTTTATCGTCACTCGGCAA	0	14	RDR2-sensitive
GTGTTTTTTTCGGGCACTCGGCAA	3	17	RDR2-sensitive
GTTAAAAAGCTCGTAGTTGGACCT	62	5	RDR2-resistant
GTTAACACTGTAGATATAAGGCTG	0	12	RDR2-sensitive
GTTAACACTGTAGCTTTAGGGGAC	23	50	RDR2-sensitive
GTTAACTGAGAGACGACGGCCTAG	0	10	RDR2-sensitive
GTTAAGCGGACCGAGTTTGGGCGG	0	9	RDR2-sensitive
GTTAAGTGCGCGGACGGCTTCCAG	6	100	RDR2-sensitive
GTTAAGTGGGTGTAGATAGGCTAT	0	9	RDR2-sensitive
GTTAATCGTTGGCGTGTCCCGGTA	0	10	RDR2-sensitive
GTTACAGGCCTGAGACAGACGCGC	0	9	RDR2-sensitive
GTTACATCGGAACGAGACCCGGAA	0	16	RDR2-sensitive
GTTACCAACTGAGAATTAGGCGTC	0	31	RDR2-sensitive

GTTACCAGCTGAGAATTAGGCGTC	12	145	RDR2-sensitive
GTTACCAGTTGGAACTGTTCGGGT	0	10	RDR2-sensitive
GTTACCGACTGTAGCTCGATGGTT	8	139	RDR2-sensitive
GTTACCGACTGTAGTCCGATGGTT	0	10	RDR2-sensitive
GTTACCTATGGATTGGACGGGATT	1	12	RDR2-sensitive
GTTACCTGTAGTGCACCGACGCGC	0	10	RDR2-sensitive
GTTACGAAATGCGAGGCTGAGGTA	53	2	RDR2-resistant
GTTACGCTGGCACAAACGGACGTC	0	10	RDR2-sensitive
GTTACGGAGAGCTGACGTCATGTC	39	0	RDR2-resistant
GTTACGTATGGATTGGACGGGATT	0	10	RDR2-sensitive
GTTACTCTTAGTAGGACTGGGCGT	0	9	RDR2-sensitive
GTTACTGGAGAGCGTAGAGGACCG	0	10	RDR2-sensitive
GTTACTGTCCATTGATGGCGTCGG	0	13	RDR2-sensitive
GTTACTGTGTTTCTTGGCGTGCTC	0	9	RDR2-sensitive
GTTACTGTTATTGATCGGCTCCAT	53	10	RDR2-resistant
GTTACTTCTAGTTAGAACGTCGTT	42	9	RDR2-resistant
GTTAGAATGGACGGAGACGACGAC	0	9	RDR2-sensitive
GTTAGACGTGTGGTAGATATGGTT	0	21	RDR2-sensitive
GTTAGACTGTGTGTGGGTCAGGGC	0	10	RDR2-sensitive
GTTAGACTGTGTGTGTGGGCCGGC	0	11	RDR2-sensitive
GTTAGAGCTCGTCCTTATAGGCAT	102	0	RDR2-resistant
GTTAGAGCTCGTTCTTATAGGCAT	127	3	RDR2-resistant
GTTAGATAGCACGGACACGGTAGC	0	14	RDR2-sensitive
GTTAGATCCAGGTGCTGTTGGCAA	0	10	RDR2-sensitive
GTTAGATGGGAGACGGACGACCAG	0	11	RDR2-sensitive
GTTAGCTGGCTGTGGGCCGTCGGA	0	19	RDR2-sensitive
GTTAGGAGCAGTAGAAAGCGGGTT	0	14	RDR2-sensitive
GTTAGGAGCCGGCGTACACTGTTA	0	31	RDR2-sensitive
GTTAGGCACGGCACTGTTAGGCAC	0	9	RDR2-sensitive
GTTAGGCTCTGCAGACACGAGCAA	0	9	RDR2-sensitive
GTTAGGCTGTGGCGTCCTAGGCAT	1	50	RDR2-sensitive
GTTAGGCTGTGGCGTTCTAGGCAT	0	32	RDR2-sensitive
GTTAGGGTTCCTAGTTTCTCGCGG	0	12	RDR2-sensitive
GTTAGGTACTGGACGGATGGGTTG	0	14	RDR2-sensitive
GTTAGGTGGGAACTCTAGCGGCTC	0	57	RDR2-sensitive
GTTAGGTTGCTGGGAAGAGAGCGG	0	15	RDR2-sensitive
GTTAGTAGATCGGCTCGACTCGGC	0	9	RDR2-sensitive
GTTAGTAGCTCGGCTCGACTCGGC	2	21	RDR2-sensitive
GTTAGTAGGCTGGCATGACGTGCT	3	22	RDR2-sensitive
GTTAGTATGGTAGGGCTGACGCGG	0	28	RDR2-sensitive
GTTAGTCAGCTGGAGGAGATAGGT	0	9	RDR2-sensitive
GTTAGTCAGGCGAGACAATGGCAT	1	13	RDR2-sensitive
GTTAGTCTGTGGGATGTGGCTGGC	0	12	RDR2-sensitive
GTTAGTGACTTGGCTCGATTCGGC	0	10	RDR2-sensitive
GTTAGTTGCCACTGTATGGAAGAA	21	1	RDR2-resistant
GTTAGTTGCCTGAACGACGACGAC	0	9	RDR2-sensitive
GTTAGTTGGGCCGCGATTGGTGTT	31	0	RDR2-resistant
GTTATATATTGCAGAGGACTACAT	14	0	RDR2-resistant
GTTATATCGTATGAGTATGGCGTG	18	0	RDR2-resistant

GTTATCGACTGTAGCTCGATGGTT	6	36	RDR2-sensitive
GTTATCGGGCTGAACCATGCCGGA	0	17	RDR2-sensitive
GTTATCTCCGACGGCTGCCTATGC	0	10	RDR2-sensitive
GTTATCTGGAACAGTGGACGACGG	0	12	RDR2-sensitive
GTTATCTGGACTAATGGGCGGCAC	3	29	RDR2-sensitive
GTTATCTGGTGGACCTGCAGGTGG	0	36	RDR2-sensitive
GTTATGACCCTGACACTCGGCAAA	0	12	RDR2-sensitive
GTTATGACTGTGGGGGATGTCGGC	0	16	RDR2-sensitive
GTTATGGACTGGCATGGTGGCGAG	0	19	RDR2-sensitive
GTTATGGATTTAAATCGGCTCAAT	0	10	RDR2-sensitive
GTTATGTAGACTGACGGGACACAG	0	9	RDR2-sensitive
GTTATGTCCGACGGCTACTGACAA	3	19	RDR2-sensitive
GTTATGTCGTGGCGGCGAAGATGT	0	9	RDR2-sensitive
GTTATGTCTGGTCGGAAGCATGGC	0	9	RDR2-sensitive
GTTATGTGACTGATTATGGTGGTA	159	12	RDR2-resistant
GTTATTGGTCTGCAGACGAGGCAT	0	42	RDR2-sensitive
GTTATTTCGGCCCGGGACTGGCAC	0	11	RDR2-sensitive
GTTATTTCTGTAGCCTGTGCGGCT	0	9	RDR2-sensitive
GTTATTTTCGTTGGGCCACGCCAT	0	9	RDR2-sensitive
GTTCAACGTGAGGACGGTACGGAT	1	20	RDR2-sensitive
GTTCAATTGGACCAGGCTGAGGCT	0	19	RDR2-sensitive
GTTCAATTGGTCCAGGCTGAGGCT	0	10	RDR2-sensitive
GTTCACAAGGTAACCATCGTCGTA	0	17	RDR2-sensitive
GTTCACCGGACTAAGCGTTGCATA	0	14	RDR2-sensitive
GTTCACGCGGACTTGGCATTTGCT	1	23	RDR2-sensitive
GTTCACTCTGTAGCAACGCACGGG	0	12	RDR2-sensitive
GTTCACTTTTTGACTGACTGGCAT	14	0	RDR2-resistant
GTTCAGAACTGCTGCGGCTTATAG	0	9	RDR2-sensitive
GTTCAGACCGTAGCAAGTGGTGAC	0	17	RDR2-sensitive
GTTCAGGACTTATTTCCGACGGTT	0	17	RDR2-sensitive
GTTCAGGATTGGGGCAGGAATGGT	0	11	RDR2-sensitive
GTTCAGGATTGGGGGCAGGAACGG	0	9	RDR2-sensitive
GTTCATCGGCGATCTCTTGGTGAC	0	11	RDR2-sensitive
GTTCATCGTCACGACGTTCGCCGT	0	9	RDR2-sensitive
GTTCATGACTGGTGGCCGGACGGT	0	38	RDR2-sensitive
GTTCATGGCTCTGGACTGGACGAT	1	12	RDR2-sensitive
GTTCATGTTGACTTTAGACGGTTT	0	9	RDR2-sensitive
GTTCCAATTCGTGTCGGGCCTCGG	0	10	RDR2-sensitive
GTTCCAGAGCTCTTCAAGTAGATT	20	0	RDR2-resistant
GTTCCAGTCGCTGCTAGAGGACGT	0	19	RDR2-sensitive
GTTCCATGGACTCGGGAGCGGTAT	0	12	RDR2-sensitive
GTTCCCATCAGATCGTCACGCGGT	0	10	RDR2-sensitive
GTTCCCATGGCAGTGTAGGACGGA	0	53	RDR2-sensitive
GTTCCCGACCTAGAACGACGAGGA	0	9	RDR2-sensitive
GTTCCCTGCCTGACGGTCGGACGG	0	18	RDR2-sensitive
GTTCCCTGTAGTAGTGACGGGCAC	0	14	RDR2-sensitive
GTTCCGACGGCTGCTATACGGACC	26	4	RDR2-resistant
GTTCCGCGGAGTGCAGAGAGGCGT	0	9	RDR2-sensitive
GTTCCGTTTACTGTAGTGACGACC	0	11	RDR2-sensitive

GTTCCTATACCCAGGTCGCGGATC	0	10	RDR2-sensitive
GTTCCTGGCAGCAAGATACCTGAT	0	10	RDR2-sensitive
GTTCCTGTAGCGAGCCGAGCCGAG	1	34	RDR2-sensitive
GTTCCTGTCGAACCAATCACGCAT	0	10	RDR2-sensitive
GTTCCTGTCTGACGGCCGAACGGT	0	10	RDR2-sensitive
GTTCCTTATTGGACGATGTAGAGA	0	10	RDR2-sensitive
GTTCCTTCAGGATGGTGAGACGTA	0	13	RDR2-sensitive
GTTCCTTCGGTTCGGTTAGTTCGG	8	62	RDR2-sensitive
GTTCCTTGTTGGACGGCGTAGAGA	0	25	RDR2-sensitive
GTTCGAACCGTAGACCGTCCGGCC	1	129	RDR2-sensitive
GTTCGACACCTGGCAGAAGGACCG	0	9	RDR2-sensitive
GTTCGACGGACCATTCACGCGCAT	0	9	RDR2-sensitive
GTTCGACGTGGTGCTCTACGGCAC	1	22	RDR2-sensitive
GTTCGACGTGGTGCTCTGCGGCAC	0	11	RDR2-sensitive
GTTCGAGCTGTGGAAGATGGCCCT	0	16	RDR2-sensitive
GTTCGATCTGACAGGAGTAGGGAT	0	10	RDR2-sensitive
GTTCGATGACTGGCATGTAAGCAG	0	12	RDR2-sensitive
GTTCGATGACTTATCTGACGGCCG	2	17	RDR2-sensitive
GTTCGCAACTCTGGATCGGACGGT	0	12	RDR2-sensitive
GTTCGCATCTCTAGAGCCAGGCTT	0	10	RDR2-sensitive
GTTCGCCGCGGACGACTCACCGGA	0	9	RDR2-sensitive
GTTCGCGACCTGGAGCAGGGCTAG	0	9	RDR2-sensitive
GTTCGCGCGTGTGGACCGGACGGT	0	9	RDR2-sensitive
GTTCGCGGCTAAGGGTCGGACGGT	0	15	RDR2-sensitive
GTTCGCGGGGACGAGGACGGAGAA	0	33	RDR2-sensitive
GTTCGCTCTGTTCCCTGCGTCGGT	0	9	RDR2-sensitive
GTTCGGAACCGGGTCGGGAACGGC	0	21	RDR2-sensitive
GTTCGGACCGGGCCCAAAAAGCGG	0	19	RDR2-sensitive
GTTCGGAGCGATGTAGGGAAGGAG	0	17	RDR2-sensitive
GTTCGGATATCGGATATAAGTCGT	0	13	RDR2-sensitive
GTTCGGATCTGGTAGTTTAGCGGC	61	0	RDR2-resistant
GTTCGGATGATCGCATGCAATGAT	106	5	RDR2-resistant
GTTCGGATGGCAGCTAGACTGTAC	0	16	RDR2-sensitive
GTTCGGATTGTCCGCCGTAGCGGC	0	13	RDR2-sensitive
GTTCGGCAAGCTCGACGACGACAC	0	16	RDR2-sensitive
GTTCGGCAAGCTCGACGACGACAT	0	20	RDR2-sensitive
GTTCGGCAAGGCTAGAGAATCGGT	0	9	RDR2-sensitive
GTTCGGCACCAGCAGTAGCAGCTA	0	9	RDR2-sensitive
GTTCGGCCCTGTGCTGCGGATCGT	0	11	RDR2-sensitive
GTTCGGCGAACAGTACATCGGCAA	0	9	RDR2-sensitive
GTTCGGCGAACTGTACATCGGCAA	0	17	RDR2-sensitive
GTTCGGCGACTCCGACGGATCTAA	0	9	RDR2-sensitive
GTTCGGCTAGACCGGAGGACCTGC	0	14	RDR2-sensitive
GTTCGGCTAGACCGGGAGACCTGC	0	9	RDR2-sensitive
GTTCGGCTCGACTCGGATCGGCTC	0	11	RDR2-sensitive
GTTCGGCTCGGCTCGGCTCGGCAT	0	12	RDR2-sensitive
GTTCGGCTGCTTCGATGGCAACAA	1	41	RDR2-sensitive
GTTCGGGCCGCTTAGAAGTGCGTA	2	31	RDR2-sensitive
GTTCGGGCCGCTTAGAGATGCGTA	3	46	RDR2-sensitive

GTTCGGGGTATTAAATGTAGTCGG	1	17	RDR2-sensitive
GTTCGGGTCCGGGCGAAAACACCA	31	2	RDR2-resistant
GTTCGGGTCCGGGCGAAGCCACCA	17	0	RDR2-resistant
GTTCGGGTCCGGGCGACGCCACCA	14	0	RDR2-resistant
GTTCGGGTGTCGGAGGCAAGAGGC	0	9	RDR2-sensitive
GTTCGGGTTGTGCCAGCGTAGCAT	0	10	RDR2-sensitive
GTTCGGTAGAGCGGGGGCAGTCTA	0	29	RDR2-sensitive
GTTCGGTCAATGCAACGGATGGTA	22	0	RDR2-resistant
GTTCGGTCCCTGTAAATTTAGGTT	0	10	RDR2-sensitive
GTTCGGTCTATTCGGGTTTGTGAA	0	20	RDR2-sensitive
GTTCGGTCTATTCGGTTTTGTGAA	0	29	RDR2-sensitive
GTTCGGTGAACTGTATATCGGCAA	0	15	RDR2-sensitive
GTTCGGTGCGGACCGACGGCTCTC	0	22	RDR2-sensitive
GTTCGGTGGTTCTCAATGGGCGGT	0	16	RDR2-sensitive
GTTCGGTGTGGATGCAAACGCAGA	0	12	RDR2-sensitive
GTTCGGTTAGTAGTGGATTGACGG	0	11	RDR2-sensitive
GTTCGGTTCGGGTTCGGGTTCGGT	0	15	RDR2-sensitive
GTTCGGTTGCTCAGGAACGGTGGC	0	11	RDR2-sensitive
GTTCGTAGGCTGGACGGTCCGCAA	0	13	RDR2-sensitive
GTTCGTGACTTGACGCAGGAGCGA	1	33	RDR2-sensitive
GTTCGTGTCCGGGCGAAAACACCA	35	2	RDR2-resistant
GTTCGTTGGGGGTCGGCTAGGGGC	1	12	RDR2-sensitive
GTTCGTTGGTCGACGGCGAGCTTC	17	0	RDR2-resistant
GTTCGTTTCACGTCGGGTTCACCA	36	2	RDR2-resistant
GTTCTAACTCGGACCAGGGGCGGA	0	9	RDR2-sensitive
GTTCTAGACTGTAGGCGCATCCAT	0	10	RDR2-sensitive
GTTCTAGATTGTAGGTTTTAGGCA	0	11	RDR2-sensitive
GTTCTATTTCTTTAGGATCTTCGG	0	12	RDR2-sensitive
GTTCTCATTCTTTAAGATCTTCGG	0	15	RDR2-sensitive
GTTCTCCGTTGACTGTCTACGGCT	18	0	RDR2-resistant
GTTCTCGGGCTGGTATGTTGACGC	6	33	RDR2-sensitive
GTTCTCGGTGTAGATGTGGGCGCC	0	9	RDR2-sensitive
GTTCTCGTGACTTGAGCGACGGAG	0	9	RDR2-sensitive
GTTCTCTAGGAATCTCGTTAGGAT	14	0	RDR2-resistant
GTTCTCTGAATTCGGCTCACTTAT	0	9	RDR2-sensitive
GTTCTCTGCGGAAGCCTGGACGGT	0	15	RDR2-sensitive
GTTCTCTGCGGGAGCGCTGACGGA	1	114	RDR2-sensitive
GTTCTCTGGTCAGGCGCGGACGGT	0	14	RDR2-sensitive
GTTCTCTGTGTGAGTGTGGACGGT	0	17	RDR2-sensitive
GTTCTCTTTCTTTAGGATCTTCGG	6	85	RDR2-sensitive
GTTCTGAACATCAAGCCGGTCCGA	0	13	RDR2-sensitive
GTTCTGAATTGTATGAGAGACGTT	1	26	RDR2-sensitive
GTTCTGAGCGTCGAGCCGGTCCGA	1	13	RDR2-sensitive
GTTCTGATCTCTGTCTGTCGTGGC	0	17	RDR2-sensitive
GTTCTGGAACGGGCTCGACGAGAT	0	11	RDR2-sensitive
GTTCTGGACTCCTGGGAAGATGAA	1	21	RDR2-sensitive
GTTCTGGCTGATATGACACGGTTC	0	11	RDR2-sensitive
GTTCTGGGGCTATGGTAGAGGCAA	0	25	RDR2-sensitive
GTTCTGGTTCTGGCTCGAGCGGAT	2	14	RDR2-sensitive

GTTCTGGTTCTGGCTCGGGCGGAT	1	16	RDR2-sensitive
GTTCTGTAGATCCGTGCGGCGGGG	0	18	RDR2-sensitive
GTTCTGTAGCGGGTGAGACGTTGA	0	9	RDR2-sensitive
GTTCTGTAGGGTTTGGTCGGCACA	0	9	RDR2-sensitive
GTTCTGTATTAAAGCGTAGACGAG	22	1	RDR2-resistant
GTTCTGTGGCACTCGGCAAAGAGG	0	13	RDR2-sensitive
GTTCTGTTTCGGGAGGTCGCCGGA	0	11	RDR2-sensitive
GTTCTTAAGTTAAGTTCGTCCGGC	0	14	RDR2-sensitive
GTTCTTACAGCGAGGTCGCGGATC	0	9	RDR2-sensitive
GTTCTTACGGTAAGTTCGACGGCT	0	12	RDR2-sensitive
GTTCTTCGGGCAAAGGATGGCATG	0	85	RDR2-sensitive
GTTCTTGTGACTGAGGGAGGCTAC	2	14	RDR2-sensitive
GTTCTTTAGGTTCGTGCTGGGCAT	88	1	RDR2-resistant
GTTCTTTTTAGGATCTTCGGCTAC	0	10	RDR2-sensitive
GTTCTTTTTTATGGTCTAGTCGGA	0	12	RDR2-sensitive
GTTGAAACGAACAGCTGAGGGCCG	0	13	RDR2-sensitive
GTTGAAACGACGGGCAGAACCGGT	0	12	RDR2-sensitive
GTTGAACACATGGAGGACGAGAAT	0	10	RDR2-sensitive
GTTGAACACTCGGCACAGGACTGC	0	10	RDR2-sensitive
GTTGAACATGAACTGTGTCGGCGT	0	11	RDR2-sensitive
GTTGAACCAGGCTGCAGGGACGAT	0	14	RDR2-sensitive
GTTGAACGACTGACGATTTGACGG	0	45	RDR2-sensitive
GTTGAACGGGTCGAGCGGTACAGA	0	16	RDR2-sensitive
GTTGAACTGGTCCAGGCTGAGGCT	0	14	RDR2-sensitive
GTTGAACTTCGGAATTTCGGTATC	0	9	RDR2-sensitive
GTTGAAGACATATTTGCTGCAGAC	18	1	RDR2-resistant
GTTGAAGAGCCAAGTAGAACGGAA	0	11	RDR2-sensitive
GTTGAAGAGCCAAGTAGAACGGAG	0	17	RDR2-sensitive
GTTGAAGAGCCAAGTGGAACGGAG	0	9	RDR2-sensitive
GTTGACACTGTCTTGGACGATGGC	2	14	RDR2-sensitive
GTTGACACTTTGGACGCCGGCACA	0	12	RDR2-sensitive
GTTGACCGCTGCAGTTGAAGAGTA	0	30	RDR2-sensitive
GTTGACCTTCTGACCAGTCGGCAC	5	28	RDR2-sensitive
GTTGACGAAGACCGCTATATTATT	50	2	RDR2-resistant
GTTGACGCGGATAGGACAACGGTG	0	63	RDR2-sensitive
GTTGACGCGTAGGACGTAATCCGG	0	19	RDR2-sensitive
GTTGACGTGGCACCGAGCTCGGCG	1	30	RDR2-sensitive
GTTGACTAAGTGACGGACACTGTT	0	9	RDR2-sensitive
GTTGACTCTGGAGCTGTCATGACG	2	18	RDR2-sensitive
GTTGACTCTGTAGCAACGCACGGG	0	51	RDR2-sensitive
GTTGACTCTGTAGCAACGTACGGG	0	80	RDR2-sensitive
GTTGACTGAGCACCGTTGGATGGA	0	11	RDR2-sensitive
GTTGACTGGGAGACGACGACCTAG	0	9	RDR2-sensitive
GTTGACTGGGAGACGACGGCCTAA	0	10	RDR2-sensitive
GTTGACTGGGAGACGACGGCCTAG	0	36	RDR2-sensitive
GTTGACTGGGAGACGATGGTCTAG	0	10	RDR2-sensitive
GTTGACTGTAATAGTGTGGCCCAG	0	20	RDR2-sensitive
GTTGACTGTACATGGTAGACGCAG	0	15	RDR2-sensitive
GTTGACTGTACGCAAGGAACGGTA	0	26	RDR2-sensitive

GTTGACTGTACGCGAGAAACGGTG	0	11	RDR2-sensitive
GTTGACTGTACGCGAGGAACGGTG	0	16	RDR2-sensitive
GTTGACTGTAGCTCGATGGTTCGT	0	11	RDR2-sensitive
GTTGACTGTATTTAGGTTTATGAA	0	16	RDR2-sensitive
GTTGACTGTGACCAGACGCGTGAT	1	22	RDR2-sensitive
GTTGACTGTTGTTGCGGACGCGAG	0	12	RDR2-sensitive
GTTGACTTTGTGGAAGCGTGTGTT	0	37	RDR2-sensitive
GTTGAGAAATCATCGATCTGTGCA	16	0	RDR2-resistant
GTTGAGAACTACGTTACCACGGAT	1	17	RDR2-sensitive
GTTGAGAGTAGTGGAAGGCGGGCT	46	2	RDR2-resistant
GTTGAGCAGAGGACCTAATGGTGA	0	10	RDR2-sensitive
GTTGAGCCCTGGCAGAACCGTGCA	0	11	RDR2-sensitive
GTTGAGGAGCCAAGTAGAACGGAA	0	11	RDR2-sensitive
GTTGAGGAGCCAAGTAGAACGGAG	0	99	RDR2-sensitive
GTTGAGGATTGCGCGACGGATGAC	0	10	RDR2-sensitive
GTTGAGGCCTGGCAACGATGGCGC	0	11	RDR2-sensitive
GTTGAGGCTTGGCAACGACGGCGC	0	10	RDR2-sensitive
GTTGAGGTTGCGGAAGCAGAGCGG	0	9	RDR2-sensitive
GTTGAGTGGACAAGTAGAGCCGGC	0	13	RDR2-sensitive
GTTGATACGGACCATTGGGGCGGC	0	16	RDR2-sensitive
GTTGATAGGGCACAAGACGACGAG	0	16	RDR2-sensitive
GTTGATCAAGGGACGCAGGGGCGT	0	13	RDR2-sensitive
GTTGATCCAAGGGAACGCGCAGAG	1	12	RDR2-sensitive
GTTGATCCAAGGGACACAGGGCGT	0	19	RDR2-sensitive
GTTGATCGTGGGCGCGGGACCGGC	21	1	RDR2-resistant
GTTGATCTACAGGGGCGGACCGTC	3	24	RDR2-sensitive
GTTGATGCTGCAGAACTAGGCATT	0	100	RDR2-sensitive
GTTGATGCTTCGGGCCGGGTCGGG	0	21	RDR2-sensitive
GTTGATGGACTGTTTGGTAGGGTT	40	1	RDR2-resistant
GTTGATTAATTTTGTACGGCTCGC	15	0	RDR2-resistant
GTTGATTGGAAGCCGGATCGTCTG	0	15	RDR2-sensitive
GTTGATTTGATGGATATAGATGGA	0	17	RDR2-sensitive
GTTGCAACGCACGGGCATATACCT	0	10	RDR2-sensitive
GTTGCAACGCACGGGTACTCACCT	0	10	RDR2-sensitive
GTTGCAACGCACGGGTACTGACCT	0	13	RDR2-sensitive
GTTGCAACTGTAGCTGCAGAGTCA	0	13	RDR2-sensitive
GTTGCACGGACAACATCGGCTGGT	0	9	RDR2-sensitive
GTTGCACGGATAGAAGGTTGGCGC	0	12	RDR2-sensitive
GTTGCAGAGTGAACGGAACGGAGC	0	23	RDR2-sensitive
GTTGCAGAGTGGACAGTTGACGCC	4	41	RDR2-sensitive
GTTGCAGCTGTTTGGACTGCTGCA	4	31	RDR2-sensitive
GTTGCAGGATGGCAGAACGGCAGA	1	21	RDR2-sensitive
GTTGCATACGCGGATACGCTCGGA	0	13	RDR2-sensitive
GTTGCATCCTTCGGTTGTCTGGCA	0	20	RDR2-sensitive
GTTGCATCGACGGCAGCGCGACAC	1	49	RDR2-sensitive
GTTGCATCGACGGCAGCGTGACTC	0	14	RDR2-sensitive
GTTGCATCGGAAGAGGACGGACGA	0	49	RDR2-sensitive
GTTGCATCTGTGACTGTGAGTGCA	0	14	RDR2-sensitive
GTTGCATGCAGACTGGGACCGGCT	0	10	RDR2-sensitive

GTTGCATTAGTCGGATCAGGTCGG	0	10	RDR2-sensitive
GTTGCCACGGCAGAGCTCGGCACC	0	14	RDR2-sensitive
GTTGCCACTGGTGAAGCTGACGGA	0	12	RDR2-sensitive
GTTGCCCTCGGCCTGGAACGGTGC	0	11	RDR2-sensitive
GTTGCCGACTGTAGCTCGATGGTT	1	13	RDR2-sensitive
GTTGCCGATGTACAGTTCGCCGAG	5	25	RDR2-sensitive
GTTGCCGATTCGACTGTCGTCGCT	2	15	RDR2-sensitive
GTTGCCGCACGTAGAGGACAGCGA	0	12	RDR2-sensitive
GTTGCCGGAATTGGACGGGGACGG	0	27	RDR2-sensitive
GTTGCCGGAGAAGGACACGACGAA	1	22	RDR2-sensitive
GTTGCCGGAGAAGGATACGACGAA	0	11	RDR2-sensitive
GTTGCCGGATAAGCAGAACCAGGC	1	26	RDR2-sensitive
GTTGCGACGGCTCACAACAATACC	0	10	RDR2-sensitive
GTTGCGACGGCTTACAACAATACC	1	14	RDR2-sensitive
GTTGCGACTGGAGTAGAGACCGGT	0	13	RDR2-sensitive
GTTGCGATGGCTGAATGACGGAAT	0	10	RDR2-sensitive
GTTGCGCAGGACTAACGAAGGCAG	2	104	RDR2-sensitive
GTTGCGCGGGACTAACGACGGCAG	1	17	RDR2-sensitive
GTTGCGCGGGACTAACGGAGGCAG	0	31	RDR2-sensitive
GTTGCGGAAGCAGAGCGGATCGGA	0	12	RDR2-sensitive
GTTGCGGAGGATCGTTGTCGTCAT	0	9	RDR2-sensitive
GTTGCGGATCAACGGCCTCGTCGC	0	9	RDR2-sensitive
GTTGCGGATTGCGCGAGACAGTGT	0	18	RDR2-sensitive
GTTGCGGCGACGGCATGAAGACAA	0	10	RDR2-sensitive
GTTGCGGCTGGAGTAGAGACCGGG	0	10	RDR2-sensitive
GTTGCGGCTGGAGTAGAGACCGGT	1	33	RDR2-sensitive
GTTGCGGGACTGCGGCAGCCTCAC	0	50	RDR2-sensitive
GTTGCGGGAGATAGAGGACGACTG	0	9	RDR2-sensitive
GTTGCGGGTCAGGACGAGATGGAA	0	10	RDR2-sensitive
GTTGCTACAACTAGGACTGGGCGT	0	35	RDR2-sensitive
GTTGCTACTGCGTACCGTCCGGAC	0	9	RDR2-sensitive
GTTGCTAGGAGCTTCGACGTCTGA	0	15	RDR2-sensitive
GTTGCTAGTAGAACGGACGGACGG	2	82	RDR2-sensitive
GTTGCTCCTGCTAGCTAGTCGGTG	0	9	RDR2-sensitive
GTTGCTCGGACAGCGTCGGCAACC	26	1	RDR2-resistant
GTTGCTCGGACTCTGACCACGGAG	0	52	RDR2-sensitive
GTTGCTCGGATCTAGACGGTTGTC	0	11	RDR2-sensitive
GTTGCTCGGCAGTATTTATTCGGC	0	9	RDR2-sensitive
GTTGCTGACTGTAGCTCGATGGTT	0	9	RDR2-sensitive
GTTGCTGATCGTAGCTCGATGGTT	0	10	RDR2-sensitive
GTTGCTGCAGGGAGTAGATGACGT	0	12	RDR2-sensitive
GTTGCTGGAAGGAGTAGAGGACGT	1	29	RDR2-sensitive
GTTGCTGGACGCCGTAGCGGACGT	0	22	RDR2-sensitive
GTTGCTGGAGAGAGTAGAGGACGT	0	172	RDR2-sensitive
GTTGCTGGAGAGAGTAGATGACGT	0	11	RDR2-sensitive
GTTGCTGGAGAGCGTAGAAGACCG	0	45	RDR2-sensitive
GTTGCTGGAGAGCGTAGAGGACCA	0	11	RDR2-sensitive
GTTGCTGGAGAGCGTAGAGGACCG	2	68	RDR2-sensitive
GTTGCTGGAGAGCGTAGAGGACTG	0	11	RDR2-sensitive

GTTGCTGGAGAGCGTAGATGACAG	0	9	RDR2-sensitive
GTTGCTGGAGAGTGTAGAAGACCG	0	41	RDR2-sensitive
GTTGCTGGAGCGAGTAGAGGACGT	0	23	RDR2-sensitive
GTTGCTGGAGCGCTACCGGACGGT	0	23	RDR2-sensitive
GTTGCTGGAGGAAATAGAGGACGT	0	28	RDR2-sensitive
GTTGCTGGAGGAAGTAGAGGACGT	3	22	RDR2-sensitive
GTTGCTGGAGGACATGGAAGACCG	0	28	RDR2-sensitive
GTTGCTGGAGGCATAGAAGAGCAT	0	9	RDR2-sensitive
GTTGCTGGAGGGAATAGAGGACGT	0	15	RDR2-sensitive
GTTGCTGGAGGGAGAAGATGACGT	0	20	RDR2-sensitive
GTTGCTGGAGGGAGTAGAGGACAT	4	47	RDR2-sensitive
GTTGCTGGAGGGAGTAGAGGACGC	0	14	RDR2-sensitive
GTTGCTGGAGGGAGTAGAGGACGG	0	24	RDR2-sensitive
GTTGCTGGAGGGAGTAGAGGACGT	27	686	RDR2-sensitive
GTTGCTGGAGGGAGTAGAGGGCGT	1	21	RDR2-sensitive
GTTGCTGGAGGGAGTAGATGACGT	1	74	RDR2-sensitive
GTTGCTGGAGGGCTAGAGGACGTC	0	16	RDR2-sensitive
GTTGCTGGAGGTAGTAGAGGACGT	0	15	RDR2-sensitive
GTTGCTGGAGTGAGTAGAGAACGT	0	10	RDR2-sensitive
GTTGCTGGAGTGAGTAGAGGACGT	4	21	RDR2-sensitive
GTTGCTGGAGTGAGTAGATGACGT	1	40	RDR2-sensitive
GTTGCTGGATAGAGTAGAGGACGT	0	27	RDR2-sensitive
GTTGCTGGATGCAGTAGAGGACGT	0	60	RDR2-sensitive
GTTGCTGGATGGAGTAGAGGACAT	0	40	RDR2-sensitive
GTTGCTGGCGCGTTGAGGACGTG	3	141	RDR2-sensitive
GTTGCTGGCGCGTTGGGGACGTG	2	18	RDR2-sensitive
GTTGCTGGCGTGTTGAGGACGTG	0	11	RDR2-sensitive
GTTGCTGGGGCTGTACCGGGCGTC	0	9	RDR2-sensitive
GTTGCTGTAGAAGTGAGGTAGCCA	0	17	RDR2-sensitive
GTTGCTGTAGCGAGTAGAGGACGT	2	193	RDR2-sensitive
GTTGCTGTGGATCCGATCGGCACC	0	22	RDR2-sensitive
GTTGCTGTTGGAGCCATACCGGAC	0	9	RDR2-sensitive
GTTGCTTAGACTGTACAACGGCTC	0	16	RDR2-sensitive
GTTGCTTCGGTTCCTGTCAGGCAT	1	29	RDR2-sensitive
GTTGCTTGGATACGACGACGCTGG	0	9	RDR2-sensitive
GTTGCTTGTACGGACTTGGTCGGT	0	13	RDR2-sensitive
GTTGGAACCGCTCCGAACTTCGGC	0	10	RDR2-sensitive
GTTGGAACTGACCGGGAAGACCGC	0	14	RDR2-sensitive
GTTGGAAGGCTGCAGTCGCATGGC	0	25	RDR2-sensitive
GTTGGAATTGGCTGGTATGATGGC	0	13	RDR2-sensitive
GTTGGAATTGTAGTAGGCGGGCGT	0	13	RDR2-sensitive
GTTGGACACTCGGCATAATCCTGC	0	16	RDR2-sensitive
GTTGGACCGGCGTACTATCTCGCG	1	16	RDR2-sensitive
GTTGGACGGAGAGAGGAGATCGTC	0	14	RDR2-sensitive
GTTGGACGGGCCGGCTCGGTACAG	0	32	RDR2-sensitive
GTTGGAGCTCTGACAGGTGGGGCC	0	12	RDR2-sensitive
GTTGGAGGACATCGGAGAACGGAT	0	9	RDR2-sensitive
GTTGGAGGACGACTAGAAACGGAT	0	10	RDR2-sensitive
GTTGGAGGCTTCCAGGACGGTGAG	0	15	RDR2-sensitive

GTTGGAGGCTTTCAGGACGGTGAG	0	31	RDR2-sensitive
GTTGGAGGGAGTAGAGGACGTCCT	0	9	RDR2-sensitive
GTTGGAGGGGGTAGAGGACGTCCT	0	11	RDR2-sensitive
GTTGGAGGGGTAGAGGACGTCCTC	1	17	RDR2-sensitive
GTTGGAGTTGGACGCGACGCGGAC	0	27	RDR2-sensitive
GTTGGAGTTGTTCGGGTAACGGGT	0	9	RDR2-sensitive
GTTGGATATTGGATGGATGGGCTG	0	10	RDR2-sensitive
GTTGGATCTTATTTTCGTCGTCGG	0	10	RDR2-sensitive
GTTGGATGCACGGTATCGGCTCAG	0	11	RDR2-sensitive
GTTGGATGCGGGTTGGACGGTCTG	0	10	RDR2-sensitive
GTTGGATGGAAAATGGACGGCCAG	2	21	RDR2-sensitive
GTTGGATGGACCAGGTGGGATGGA	0	26	RDR2-sensitive
GTTGGATGGGAGACGGACAACCAG	0	18	RDR2-sensitive
GTTGGATGGGAGACGGACGACCAG	0	16	RDR2-sensitive
GTTGGATGGTCGAGGTGGGATGGA	0	10	RDR2-sensitive
GTTGGATGTGGAGGGTGTGGTCTG	4	18	RDR2-sensitive
GTTGGATTCCGACCGTTGGAGCTT	0	9	RDR2-sensitive
GTTGGATTGAAGGGCAGGCTCCAA	0	10	RDR2-sensitive
GTTGGCACAAACGGACGTCGTGGC	1	30	RDR2-sensitive
GTTGGCACTCGGCAAAGACTGACG	1	40	RDR2-sensitive
GTTGGCACTCGGCAAAGACTTCAC	0	15	RDR2-sensitive
GTTGGCACTGGCGGAAACGCGGAG	0	13	RDR2-sensitive
GTTGGCCCAATTGTAGTCGGACGG	0	10	RDR2-sensitive
GTTGGCCTGTTGGACCCCCGGCAC	0	11	RDR2-sensitive
GTTGGCTAGGTAGGTGTGAGACGT	0	16	RDR2-sensitive
GTTGGCTCGGACCCTCTACGGCTC	0	14	RDR2-sensitive
GTTGGCTCTCGGCAAAGACTGACG	37	261	RDR2-sensitive
GTTGGCTTGGGCAGTAGGAAGGTA	0	106	RDR2-sensitive
GTTGGGACCATGCTTCGTCGCCGA	1	31	RDR2-sensitive
GTTGGGACTATGCTTCGTCGCCGA	0	12	RDR2-sensitive
GTTGGGAGGCTGTCTGTTCGATGG	0	15	RDR2-sensitive
GTTGGGATTGTGCGTAGGAGCGGT	59	0	RDR2-resistant
GTTGGGCCGTTGCAGAGTGGACAG	0	10	RDR2-sensitive
GTTGGGCGGAGACCGGACGGAAGA	0	12	RDR2-sensitive
GTTGGGCTGCTCCATACGGTAAAG	0	19	RDR2-sensitive
GTTGGGGACGGCCATGAACTTCGC	2	19	RDR2-sensitive
GTTGGGGCGAAGGCGAAGACGCTA	2	45	RDR2-sensitive
GTTGGGGCTGCGCAGATCCGGCGA	0	13	RDR2-sensitive
GTTGGGGCTGCGTAGATCCGGCGA	0	13	RDR2-sensitive
GTTGGGGCTTGTACTGTTAGGCAT	0	12	RDR2-sensitive
GTTGGGTCTTGACTGACGGATCAG	4	32	RDR2-sensitive
GTTGGGTGGATACAGGAGATCGCT	0	13	RDR2-sensitive
GTTGGGTTCGACGGTGAAGGAGAA	0	14	RDR2-sensitive
GTTGGGTTGCTGGGAAGGGAGCGG	1	21	RDR2-sensitive
GTTGGGTTGGTCGGATTGAGTGGC	0	12	RDR2-sensitive
GTTGGGTTTGTCGGGTCGGGCGGC	0	14	RDR2-sensitive
GTTGGTAATCGGCTGAGTTATGGC	0	10	RDR2-sensitive
GTTGGTACGGGCAGAACGAGCGTA	0	33	RDR2-sensitive
GTTGGTCGCTATAGATATCGTCGG	0	13	RDR2-sensitive

GTTGGTCTCCTGGGCACGGATAGG	31	6	RDR2-resistant
GTTGGTCTCGAGTGGATAAGGCAT	0	14	RDR2-sensitive
GTTGGTCTGTTGTAGAGTGGGCGG	0	12	RDR2-sensitive
GTTGGTCTTGGACAAGGCGTGGTT	0	20	RDR2-sensitive
GTTGGTGGAGCGATTTGTCTGGTT	160	6	RDR2-resistant
GTTGGTGGAGGCCGGCGAGCTGGT	32	2	RDR2-resistant
GTTGGTGGCGGCTTCGTGTGAATG	66	2	RDR2-resistant
GTTGGTTGTACTTGAGTTGATGTA	4	20	RDR2-sensitive
GTTGGTTGTGTGGTGTGGATGGCT	0	28	RDR2-sensitive
GTTGGTTTTGGCTACTCAGGCATC	22	0	RDR2-resistant
GTTGTACGGCGTGGTAGACGGACT	0	16	RDR2-sensitive
GTTGTACGGCGTGGTAGACGGGCT	3	77	RDR2-sensitive
GTTGTAGAACTTAAATCTTGCAAA	17	1	RDR2-resistant
GTTGTAGACCTCCGCAAACACGGT	0	13	RDR2-sensitive
GTTGTAGGACTGCAGGGAGGACAG	0	17	RDR2-sensitive
GTTGTAGGATCTCGAAGACGACAG	0	16	RDR2-sensitive
GTTGTAGGCCCCTGCACTCGGTAC	0	17	RDR2-sensitive
GTTGTAGGGAGTAGAGGACAACAT	0	10	RDR2-sensitive
GTTGTAGTGGGAAGCGGAGGCCTG	0	17	RDR2-sensitive
GTTGTATACTACTGAACTGACGTT	0	13	RDR2-sensitive
GTTGTATCATTTCGTCGGCACACT	0	10	RDR2-sensitive
GTTGTATCCTGAGTAGAGCGACGG	0	13	RDR2-sensitive
GTTGTATCGCACGAGACAGGCTAG	0	71	RDR2-sensitive
GTTGTATCTAGGATGCCGGACATG	0	9	RDR2-sensitive
GTTGTCAACTGTAGATGGCTTGGC	0	18	RDR2-sensitive
GTTGTCACTGTTGACGATCGCTCC	22	2	RDR2-resistant
GTTGTCAGGCTGTGCTAGCACGGG	0	14	RDR2-sensitive
GTTGTCCAGCATGTAGACGTGCAG	0	33	RDR2-sensitive
GTTGTCGACCCTGCTGATTCCGGC	2	24	RDR2-sensitive
GTTGTCGACCCTGTTGATTCCGGC	0	22	RDR2-sensitive
GTTGTCGACCTGGCCGATTGTCGT	0	13	RDR2-sensitive
GTTGTCGACGGTAGACGCACGGTG	1	12	RDR2-sensitive
GTTGTCGACTGGGAGTAGAGATGG	0	13	RDR2-sensitive
GTTGTCGACTGTATGTTCGGCTAA	0	14	RDR2-sensitive
GTTGTCGATGGAAGACCAGACGGT	0	12	RDR2-sensitive
GTTGTCGATGTACAGTTCGCCGAG	0	13	RDR2-sensitive
GTTGTCGATTCGACTGTCGTCGCT	0	12	RDR2-sensitive
GTTGTCGCAGTAGACGAGGGGGGC	1	16	RDR2-sensitive
GTTGTCGCAGTAGACTAGGGTGGC	0	13	RDR2-sensitive
GTTGTCGCGTAGGACAGTTCGGCT	0	20	RDR2-sensitive
GTTGTCGGGCTTGCTGGACGGCCT	0	21	RDR2-sensitive
GTTGTCGGTCTGGGCCGAGGTCGG	1	12	RDR2-sensitive
GTTGTCGTTACCAGAAGCAGCCTC	0	9	RDR2-sensitive
GTTGTCTGAATTACTTGCTGAAGA	21	0	RDR2-resistant
GTTGTCTGCGGAAGCTTGGACGGT	0	18	RDR2-sensitive
GTTGTCTGGACGTGTGAAGCGCAC	0	9	RDR2-sensitive
GTTGTCTGGGCTGTTTTGTGCATT	1	12	RDR2-sensitive
GTTGTCTGTCTTGTAGAAGGGGTA	2	19	RDR2-sensitive
GTTGTGAGTCAAGGCTGAGACGGG	0	9	RDR2-sensitive

GTTGTGCCGCTGTAGATTAGACGG	0	17	RDR2-sensitive
GTTGTGGAACCACGGCGATGACAT	0	16	RDR2-sensitive
GTTGTGGAACTGCAGTGGAGCGGA	0	17	RDR2-sensitive
GTTGTGGAAGGTAGAGGGCGACTG	0	9	RDR2-sensitive
GTTGTGGACGGCGACAGAGTGGAC	0	15	RDR2-sensitive
GTTGTGGGACAGCTAGGACGGGAT	2	83	RDR2-sensitive
GTTGTGGGGCTATGGTAGAGGCAA	0	21	RDR2-sensitive
GTTGTGTCTGTGCTCTCATCGGCT	0	12	RDR2-sensitive
GTTGTGTGTTTGGCGATCTTGGTA	21	0	RDR2-resistant
GTTGTGTTCTGTATGCGTTGGCAT	378	20	RDR2-resistant
GTTGTGTTGGGCTTAGATGGTTTC	0	13	RDR2-sensitive
GTTGTTATCGTGACAGGACGGCAA	0	9	RDR2-sensitive
GTTGTTATGGACTGTTCGTCAGGC	1	86	RDR2-sensitive
GTTGTTCAATCTGGATCGTCCATC	3	26	RDR2-sensitive
GTTGTTCCGCGGGCGTAGGGAGGC	0	10	RDR2-sensitive
GTTGTTCGGATGATCGCATGCAAT	43	1	RDR2-resistant
GTTGTTCGGGCCAATGGTACGGCT	1	63	RDR2-sensitive
GTTGTTGAAGTGAGTAGAGGACGT	0	10	RDR2-sensitive
GTTGTTGACTGTAGCTCGATGGTT	0	16	RDR2-sensitive
GTTGTTGAGCTCTTCCTGCATGGC	21	2	RDR2-resistant
GTTGTTGATGTAGTGAACCGGCTT	1	22	RDR2-sensitive
GTTGTTGATTGTAGCTTGATGGTT	0	13	RDR2-sensitive
GTTGTTGCTGGTACACGAGGGCGT	0	9	RDR2-sensitive
GTTGTTGGAATGAGTAGAGGACGT	0	54	RDR2-sensitive
GTTGTTGGAGAGGTAGCGGATGTC	0	10	RDR2-sensitive
GTTGTTGGAGGCTTCCAGAACGGT	0	9	RDR2-sensitive
GTTGTTGGAGGCTTCCAGGACGGT	0	37	RDR2-sensitive
GTTGTTGGAGGCTTTCAGGACGGT	0	31	RDR2-sensitive
GTTGTTGGAGGGAGTAGAGGACGT	0	50	RDR2-sensitive
GTTGTTGGAGGGATAGAGGACGTC	0	39	RDR2-sensitive
GTTGTTGGAGGGGGTAGAGGACGT	0	42	RDR2-sensitive
GTTGTTGGAGGGGTAGAGGACGTC	7	405	RDR2-sensitive
GTTGTTGGAGTGAGTAGACGACGT	0	32	RDR2-sensitive
GTTGTTGGAGTGAGTAGAGGACGT	0	48	RDR2-sensitive
GTTGTTGGCTATAGATGTACACGG	0	15	RDR2-sensitive
GTTGTTGGGACCATGCTTCGTCGC	0	11	RDR2-sensitive
GTTGTTGGGACTAGGGGTGGGCGT	0	9	RDR2-sensitive
GTTGTTGGGCGGAGACCGGACGGA	0	134	RDR2-sensitive
GTTGTTGGGCTCTCACAGATGGGG	0	9	RDR2-sensitive
GTTGTTGGGTACCTGGGAACACGG	0	19	RDR2-sensitive
GTTGTTGTAGGCTCTGGTGGTCGG	0	10	RDR2-sensitive
GTTGTTGTGTGGCTATAGACGGTA	46	2	RDR2-resistant
GTTGTTGTTGGACGCATTGCGCAC	0	17	RDR2-sensitive
GTTGTTTCTCTGCAGGACGATGGT	1	59	RDR2-sensitive
GTTGTTTGGAACCTTAGTTGCATG	16	0	RDR2-resistant
GTTGTTTGGTTACCTGGACGGAGA	1	30	RDR2-sensitive
GTTGTTTTTGTTTGATTTGTTGGT	20	0	RDR2-resistant
GTTTAAGACTGTTATGTTGACGGG	0	14	RDR2-sensitive
GTTTAATCTTAAGGATGGTGCGTC	21	0	RDR2-resistant

GTTTACTGAGAGCAGGACGACGCT	0	15	RDR2-sensitive
GTTTACTGTAGTGCACGGCCTGTC	1	14	RDR2-sensitive
GTTTAGAGGACGCTGCTGGAGAGA	0	14	RDR2-sensitive
GTTTAGAGGACGCTGCTGGAGAGC	1	33	RDR2-sensitive
GTTTAGAGGACGCTGCTGGAGGAC	0	32	RDR2-sensitive
GTTTAGAGGACGCTGCTGGAGGGC	7	31	RDR2-sensitive
GTTTAGAGGACGTTGCTGGAGGGA	10	61	RDR2-sensitive
GTTTAGAGGACGTTGCTGGAGGGG	0	12	RDR2-sensitive
GTTTAGATCGTGCCGACGGTGCTT	42	2	RDR2-resistant
GTTTAGGAGGGAGCTCGGCGGCAC	0	27	RDR2-sensitive
GTTTAGGCTAATGTGGACGGGCAA	0	14	RDR2-sensitive
GTTTAGGGCGTGTTGTTGGACGGA	0	12	RDR2-sensitive
GTTTAGGTAGAATGATTGTGGCAA	0	12	RDR2-sensitive
GTTTAGGTTCGCTGTGGGACAGGC	0	9	RDR2-sensitive
GTTTAGTCAGGCTGATGCACGGGA	0	23	RDR2-sensitive
GTTTATAGCTACGATTCGTTGGTC	51	0	RDR2-resistant
GTTTATCGGGACGGATACAGGTAT	0	13	RDR2-sensitive
GTTTATGGGAGGCTGTAGTGAGGC	0	24	RDR2-sensitive
GTTTATGGTCGATTTAGGGGAGGA	15	0	RDR2-resistant
GTTTCAACGGTCGTCATACGTCAT	0	10	RDR2-sensitive
GTTTCAACGGTCGTCATGCGTCAT	3	58	RDR2-sensitive
GTTTCCGGGCTGTCGGGCCGGCAC	0	10	RDR2-sensitive
GTTTCCTGGATTGTTGTTTGGGTT	20	2	RDR2-resistant
GTTTCGACGCTCTGGGTGGCCGGT	2	34	RDR2-sensitive
GTTTCGCGCGGGCGTTCGGTAGAG	0	13	RDR2-sensitive
GTTTCGCTGGGACAGTGGACGGCC	0	13	RDR2-sensitive
GTTTCGGACCAGGCTTCATTCCCC	25	1	RDR2-resistant
GTTTCGGACGTACGTGTGGATGCA	0	15	RDR2-sensitive
GTTTCGGGCAACTCGGAAACGGAA	0	12	RDR2-sensitive
GTTTCGGGCCAGTAGAGCACGGAA	0	15	RDR2-sensitive
GTTTCGGGTGTAGACTGGGGATGG	0	10	RDR2-sensitive
GTTTCGGTTCTGCGGTGTTGGCAT	0	12	RDR2-sensitive
GTTTCGTAATGTGATCGTTGAGAT	0	13	RDR2-sensitive
GTTTCGTCACTCTCAATGGCCGGT	0	12	RDR2-sensitive
GTTTCGTTGATTGTTGTCGACATT	22	0	RDR2-resistant
GTTTCTACCTGTAGGACCTTCGGC	0	30	RDR2-sensitive
GTTTCTAGTTGGGCGCATCAGACG	16	0	RDR2-resistant
GTTTCTCCCTGCAGGACCTTCGGC	4	35	RDR2-sensitive
GTTTCTCTGCGGACGGCGATTTCG	0	11	RDR2-sensitive
GTTTCTGCCGGATTGGTGGGTCGG	2	16	RDR2-sensitive
GTTTCTGCTGGATTGGTGGGTCGG	0	17	RDR2-sensitive
GTTTCTGGCTGGCTGTAACGGTAG	1	26	RDR2-sensitive
GTTTCTGTCGGATTGGTGGGTCGG	8	61	RDR2-sensitive
GTTTCTGTGGAGCCAGGCTGAAAG	0	9	RDR2-sensitive
GTTTCTGTGGAGCCAGGCTGAGAG	0	64	RDR2-sensitive
GTTTCTTACTGGGACGGTAGCGGA	1	24	RDR2-sensitive
GTTTCTTCCTGCAGGACCTTCGGC	6	118	RDR2-sensitive
GTTTCTTCCTGTAGGACCTTCGGC	0	20	RDR2-sensitive
GTTTCTTCCTGTGGGACCTTCGGC	1	65	RDR2-sensitive

GTTTCTTCTTACAGGATCTTCGGC	3	44	RDR2-sensitive
GTTTCTTCTTGTAGGACCTTCGGC	2	34	RDR2-sensitive
GTTTCTTTCTGCAGGAACTTCGGC	0	11	RDR2-sensitive
GTTTCTTTCTGCAGGACCTTCGGC	4	68	RDR2-sensitive
GTTTCTTTCTTTAGGATCTTCGGC	0	19	RDR2-sensitive
GTTTCTTTTTGTTAGTCGCTGGAT	68	2	RDR2-resistant
GTTTGAACTGTGGCACGGCATGAT	0	11	RDR2-sensitive
GTTTGAATCGCGGAACGATGTCAT	0	11	RDR2-sensitive
GTTTGAATCGCGGAACGATGTCGT	0	25	RDR2-sensitive
GTTTGAATTTCGGGCCGGCCACAG	0	12	RDR2-sensitive
GTTTGAATTTCGGGTCGTATCGGA	0	16	RDR2-sensitive
GTTTGACGATGGACAGCGCGGTGC	0	9	RDR2-sensitive
GTTTGACTGCCTCAGCATCGTCGT	0	11	RDR2-sensitive
GTTTGAGCGGATGCAGGCTGACGG	0	11	RDR2-sensitive
GTTTGAGTGGGTAGAGGTTCGGAA	0	14	RDR2-sensitive
GTTTGATGGTACGTGCTACTCGGA	27	2	RDR2-resistant
GTTTGCAACCGCGGATGTGATCGT	0	9	RDR2-sensitive
GTTTGCACGGACTGCTGAAGATGA	0	15	RDR2-sensitive
GTTTGCAGATGAACGGACCTGGAT	0	64	RDR2-sensitive
GTTTGCAGATGGACGGACCTGGAT	0	36	RDR2-sensitive
GTTTGCATAGTAGGACACTCGGCA	0	17	RDR2-sensitive
GTTTGCCGACTGCAGGAGACAAGT	1	12	RDR2-sensitive
GTTTGCGACGACCGTTGGATGGAA	0	15	RDR2-sensitive
GTTTGCGACTCGGACAGGCCTAGG	0	10	RDR2-sensitive
GTTTGCGACTGAGAGCCTAGGCAG	2	90	RDR2-sensitive
GTTTGCGGTTGGAGAGGAAGGATC	21	2	RDR2-resistant
GTTTGCTCGACAAGGACTTGGCAG	0	12	RDR2-sensitive
GTTTGGAATCTCTGACGGGCTGGT	1	18	RDR2-sensitive
GTTTGGACCGGGCTAAATTCGTGC	0	51	RDR2-sensitive
GTTTGGACCGGGCTAGATTCGTGC	0	18	RDR2-sensitive
GTTTGGACTGACACCTACGGCGCC	2	113	RDR2-sensitive
GTTTGGACTGAGGTTGCGGCCCAT	0	17	RDR2-sensitive
GTTTGGACTTCCAGCAGCTCGGCT	0	12	RDR2-sensitive
GTTTGGACTTTCGGCCGGGGGTGC	0	10	RDR2-sensitive
GTTTGGAGAGCGTCGGAGCCGGCT	0	14	RDR2-sensitive
GTTTGGATGTCTGGCTGTCACCGG	0	19	RDR2-sensitive
GTTTGGCAAGCAGAACGGAACCGC	2	18	RDR2-sensitive
GTTTGGCAATGGACTGGACAGGCA	0	11	RDR2-sensitive
GTTTGGCAGTTGGCTGTGGTCGGC	1	29	RDR2-sensitive
GTTTGGCTACTCTGTTGGATGGAA	0	11	RDR2-sensitive
GTTTGGGACTGTTAATCTACCGGC	1	15	RDR2-sensitive
GTTTGGGATCTCTGACGGGCTGGT	8	31	RDR2-sensitive
GTTTGGGCCTGAGACGCGGCACGG	0	16	RDR2-sensitive
GTTTGGGCGAAAGCTAGGCGGCGA	52	3	RDR2-resistant
GTTTGGGTCGACGGCCAGGCACGT	0	67	RDR2-sensitive
GTTTGGTAAGCTGGAACGGAGCGG	3	22	RDR2-sensitive
GTTTGGTAAGTTGGAACGGAGCGG	0	9	RDR2-sensitive
GTTTGGTAATCTGGAACGGAGCGG	9	96	RDR2-sensitive
GTTTGGTACTGTTAATCTACCGGC	0	13	RDR2-sensitive

GTTTGGTATTGTAGAACGGAGCGG	0	15	RDR2-sensitive
GTTTGGTCGGAGGGATGAGATGGT	1	12	RDR2-sensitive
GTTTGGTCTTGTTTTCGTCGTCGG	0	10	RDR2-sensitive
GTTTGGTGGCTCTGTACATACGGG	0	20	RDR2-sensitive
GTTTGGTGTGTAGAGCAAGGGTGA	0	13	RDR2-sensitive
GTTTGGTTAGAGGCATTGGACGGT	0	12	RDR2-sensitive
GTTTGGTTGCAATGACAGGACGGG	5	26	RDR2-sensitive
GTTTGGTTGCAGAGTGAACGGAAC	14	117	RDR2-sensitive
GTTTGGTTGCAGAGTGAACGGAAT	0	9	RDR2-sensitive
GTTTGGTTGCTAGCAGTCTCGGCG	2	33	RDR2-sensitive
GTTTGGTTGCTGGAAAGGGAGCGG	0	25	RDR2-sensitive
GTTTGGTTGGACGTACAAGCACGG	0	10	RDR2-sensitive
GTTTGGTTGGCTGCAGAGCAGGAG	1	44	RDR2-sensitive
GTTTGGTTGGCTGCATGCATATTT	0	10	RDR2-sensitive
GTTTGGTTTATAGAATGTGGTAGG	22	0	RDR2-resistant
GTTTGTAACGGTTGTGTTGAAGGA	53	2	RDR2-resistant
GTTTGTAGGACAGCATATTCACAT	0	13	RDR2-sensitive
GTTTGTATTGTGGCGAGTAGAAGA	0	12	RDR2-sensitive
GTTTGTCGAGTGTTTTTAAGGCTT	1	13	RDR2-sensitive
GTTTGTCGGGACGGATACGGGTAT	0	11	RDR2-sensitive
GTTTGTCTGCTGTGCACGTGCGTT	0	9	RDR2-sensitive
GTTTGTGACTCTGTACCGAGCCGG	1	71	RDR2-sensitive
GTTTGTGCCGGATTGGTGGGTCGG	3	36	RDR2-sensitive
GTTTGTGCTGGACGTGCGAGCGGT	0	11	RDR2-sensitive
GTTTGTGTCGGATTGGTGGGTCGG	8	118	RDR2-sensitive
GTTTGTTCTGTAGATCCGTGCGGC	2	30	RDR2-sensitive
GTTTGTTGGCTAGATTTGCGGAAT	30	136	RDR2-sensitive
GTTTGTTGGGCTGGATCGAGGTAT	0	28	RDR2-sensitive
GTTTGTTTAATGTAAGAACTAGGC	15	0	RDR2-resistant
GTTTGTTTAGCTGGAGAGGAGGGT	0	9	RDR2-sensitive
GTTTGTTTGATGGTACGTGCTACT	88	3	RDR2-resistant
GTTTGTTTGGGCCTGAGACGCGGC	0	37	RDR2-sensitive
GTTTGTTTTTGACAGGAACGTCGT	0	15	RDR2-sensitive
GTTTTAACGTGGGGCTGAGAGGCG	0	9	RDR2-sensitive
GTTTTACACTTCGGGCTGGCACGG	0	25	RDR2-sensitive
GTTTTACATCACAGTCGGTGCACC	1	18	RDR2-sensitive
GTTTTAGGAGTAGGACGGTGTCGC	0	38	RDR2-sensitive
GTTTTAGTTGTCGCTGGATAGTGC	2	16	RDR2-sensitive
GTTTTAGTTGTCGTTGGATAGTGC	2	16	RDR2-sensitive
GTTTTATTTCGGGCACTCGGCAAA	0	9	RDR2-sensitive
GTTTTCGACGGTCTCTGACGGCCA	1	40	RDR2-sensitive
GTTTTCGGAATGGGTGACGTGCGA	0	42	RDR2-sensitive
GTTTTCGGAATGGGTGACGTGCGG	5	77	RDR2-sensitive
GTTTTCGTGGTGTAGTTGGTTATC	32	3	RDR2-resistant
GTTTTCTGTTAGAATTGGATGGAC	0	9	RDR2-sensitive
GTTTTCTTGGGCTGTGTCCGGGCA	0	15	RDR2-sensitive
GTTTTGCACTGTAGTAGACTGCAT	1	100	RDR2-sensitive
GTTTTGGAGACGCATCTGACAGAC	0	9	RDR2-sensitive
GTTTTGGGACTGGTCCGTTAGGCA	0	13	RDR2-sensitive

GTTTTGGTCGACAGAGCATGGCAT	0	12	RDR2-sensitive
GTTTTGGTCGTGCTGGGTCAGGCT	1	16	RDR2-sensitive
GTTTTGTACTGTAGATAATAGGCT	0	41	RDR2-sensitive
GTTTTGTCGACTGTAAAAAGGGCT	0	10	RDR2-sensitive
GTTTTGTCTGACTGTATACACGGT	0	14	RDR2-sensitive
GTTTTGTGGGCTCGACGCAGGCAT	0	12	RDR2-sensitive
GTTTTTACACTTCGGGCTGGCATG	0	9	RDR2-sensitive
GTTTTTATGCTGTAGTAAGGGGTA	0	23	RDR2-sensitive
GTTTTTATTTGATCAGCTCGTCGT	0	15	RDR2-sensitive
GTTTTTCGGGCCAGTGGAGGGCAC	2	19	RDR2-sensitive
GTTTTTCTAGGCTGGTCGGACCAA	0	11	RDR2-sensitive
GTTTTTGAGGACCTTCGGACGCCG	0	12	RDR2-sensitive
GTTTTTGAGGACCTTCGGAGGACG	2	24	RDR2-sensitive
GTTTTTGCGCCTATCGGATCGGAG	0	15	RDR2-sensitive
GTTTTTGTGTCGTGCTGTACCGGC	0	15	RDR2-sensitive
GTTTTTTGACTGTATGTCGGGTAG	0	19	RDR2-sensitive
TAAAAAGCTCGTAGTTGGACCTTG	45	5	RDR2-resistant
TAAAAATTCGTTCGGCTACGGTCT	1	36	RDR2-sensitive
TAAAACATACTGTAAATTTGTTAC	14	0	RDR2-resistant
TAAAACGCGACGGGGCATTGTAAG	20	2	RDR2-resistant
TAAAACGCTGATGGAGGATAGACT	17	0	RDR2-resistant
TAAAACTGATTTAGGTATTGGGAC	20	0	RDR2-resistant
TAAAACTGTAGAAGGCTTTTTACT	147	13	RDR2-resistant
TAAAAGCTGCTAGTTTGTTCGGAT	29	2	RDR2-resistant
TAAAATAGTAAGTATTCGGATGTT	15	0	RDR2-resistant
TAAAATGTAAGGATCGGATCGGGT	40	5	RDR2-resistant
TAAAATTTAGAAGACGTAGTTGAA	0	12	RDR2-sensitive
TAAACACAGCACTCAGTGGGCATT	14	0	RDR2-resistant
TAAACCTAGGCTGTACCGGTGCAT	0	13	RDR2-sensitive
TAAACTTTAGAGTGCGCTCGTCGT	0	11	RDR2-sensitive
TAAACTTTGATTAGCCGGAACGAT	0	11	RDR2-sensitive
TAAACTTTGATTAGCTGGAACGAT	4	25	RDR2-sensitive
TAAAGAAGCAGGAGCTGGAGCTTT	0	11	RDR2-sensitive
TAAAGACTTATCTCCGACGGCTTT	1	12	RDR2-sensitive
TAAAGATTTTCTCTTCAATCGTCA	14	0	RDR2-resistant
TAAAGCCAGGGACGTGCAAGCTCT	0	13	RDR2-sensitive
TAAAGCTATTTGCACTGTCGTTGT	0	9	RDR2-sensitive
TAAAGGAATGTAGCTTACGTGGCT	16	0	RDR2-resistant
TAAAGGACCTTAGCATTAGGCGGA	37	1	RDR2-resistant
TAAAGGACTACAGAGGACAGTCAA	1	25	RDR2-sensitive
TAAAGGGCTGGATCCAGGAAGCAG	0	11	RDR2-sensitive
TAAAGGTAGTCGTTTAGGACATGA	0	28	RDR2-sensitive
TAAAGGTTAGGGACCGACGGTCAG	0	9	RDR2-sensitive
TAAAGTAGGATGAGGTGGAGGCGA	0	9	RDR2-sensitive
TAAAGTTGGTTCTTTAATTTCGGT	19	0	RDR2-resistant
TAAAGTTTCCTTTGTTGAGTCGGC	14	0	RDR2-resistant
TAAAGTTTGTAGCACGGGTGACAA	69	0	RDR2-resistant
TAAATATTCTACCCGTGGGTCTGT	2	59	RDR2-sensitive
TAAATATTCTATCCGTGGGTCTGT	1	14	RDR2-sensitive

TAAATGATCTACCTGTAGAGATGG	0	12	RDR2-sensitive
TAAATGGGTCGGCACTAAGCACGG	0	22	RDR2-sensitive
TAAATTATAGTGTAGAAATAGGTT	45	0	RDR2-resistant
TAAATTTTTACTCGTCAGATACCC	58	1	RDR2-resistant
TAAATTTTTGGACTGTGACGTGCC	0	12	RDR2-sensitive
TAAATTTTTTTTGGACCGTATCGT	0	9	RDR2-sensitive
TAACAACGACTTAGTATATGGCTC	44	2	RDR2-resistant
TAACAACTTAAGGGCTATTGACGT	31	2	RDR2-resistant
TAACAAGTTTGAATAGGTCGGTAT	71	3	RDR2-resistant
TAACAGACGTGGGGCAGGTGGCGC	2	38	RDR2-sensitive
TAACAGGTCTCGGGCTAAACGGGT	0	15	RDR2-sensitive
TAACAGTGCTCTCTGCGAAGACGT	0	9	RDR2-sensitive
TAACATCCGAGGGCTAGCAGGCGT	0	9	RDR2-sensitive
TAACATTCGCCGGATGCATGTGCA	27	1	RDR2-resistant
TAACCCACACCTAGAACTCGTCGT	5	40	RDR2-sensitive
TAACCCATGCCTAGAACTCGTCGT	0	13	RDR2-sensitive
TAACCGAATAAGACCGTAGGGACA	0	11	RDR2-sensitive
TAACCGAGCAAGTTCCGTAGACGT	0	10	RDR2-sensitive
TAACCGGGCTTGGACTGAGTCGGC	0	18	RDR2-sensitive
TAACCGTGGATTCGTGATTGGCGA	17	0	RDR2-resistant
TAACCTGGCTCGAGTCGATGACGT	0	20	RDR2-sensitive
TAACGAGCCAGCTCGAACTCGGAC	0	15	RDR2-sensitive
TAACGGACTTCGGGCTAAACGGGT	0	27	RDR2-sensitive
TAACGGGCCTTTGGCAGAACGCGT	0	18	RDR2-sensitive
TAACGGGCTGGCCCGAAGCACGGT	0	10	RDR2-sensitive
TAACGGGCTGGTTCGAAACACGGC	0	16	RDR2-sensitive
TAACGGGCTTAGAGCTAAACGGGC	0	16	RDR2-sensitive
TAACGGGCTTAGAGGTAAACGGGC	0	75	RDR2-sensitive
TAACGGGCTTAGAGGTAAACGGGT	0	24	RDR2-sensitive
TAACGGGCTTCGGGCTAAACGGGT	0	17	RDR2-sensitive
TAACGTAGAGCCTATGTCCGACGG	0	12	RDR2-sensitive
TAACGTCAAAATGTAGTTAGCGGA	53	3	RDR2-resistant
TAACGTCTAACTGTCCGTCGGCAT	0	18	RDR2-sensitive
TAACGTGGGGCTGAGAGGCGGCAT	0	13	RDR2-sensitive
TAACGTTCGCCGGATGCACGTGCA	29	0	RDR2-resistant
TAACGTTGACACTGCGGACGGTGA	1	80	RDR2-sensitive
TAACGTTGGGCTGAGAGCAGGCAT	2	32	RDR2-sensitive
TAACGTTGTCGTTTGTTTGGCGTT	25	1	RDR2-resistant
TAACTAATTGATGTCGAGCGTGAT	16	0	RDR2-resistant
TAACTACGGACTTAGACGGTACCC	43	2	RDR2-resistant
TAACTAGAACTTAGGTCAATCGCA	34	5	RDR2-resistant
TAACTCGACCTGCAGATTTGCTAT	0	11	RDR2-sensitive
TAACTCTGTGCCAGCAACCATGGC	0	12	RDR2-sensitive
TAACTGGTCGGCACGGTTAGGCAC	1	14	RDR2-sensitive
TAACTGTAAAATGGACGGTTGGAT	0	9	RDR2-sensitive
TAACTGTAATATGGACGGTTGGAT	0	12	RDR2-sensitive
TAACTGTCCACTCTGCAACGGCCT	0	9	RDR2-sensitive
TAACTGTCCCAATCTGGCGCGGCT	2	14	RDR2-sensitive
TAACTGTGACTGTGGCAGACTGGC	2	62	RDR2-sensitive

TAACTTATCGAGCGGATCGACGTC	0	13	RDR2-sensitive
TAACTTCTGATTGGTTGACGTCAT	0	43	RDR2-sensitive
TAACTTTCAGTGGGACATTCGACG	38	2	RDR2-resistant
TAACTTTGTGCTGTTGTTGATGGT	15	0	RDR2-resistant
TAACTTTTACATATGAAATCGGAT	4	22	RDR2-sensitive
TAACTTTTATCGGACACTCGGCAA	0	31	RDR2-sensitive
TAAGAAGCTAATGTAGACGTTCAC	0	9	RDR2-sensitive
TAAGACACAACTTAAGACACCCAC	0	9	RDR2-sensitive
TAAGACAGGTCACGGAGCGGTATA	27	3	RDR2-resistant
TAAGACAGTAGTGTAGACGTCCAC	0	9	RDR2-sensitive
TAAGACAGTAGTGTAGGCGTCCAC	0	11	RDR2-sensitive
TAAGACTCTTAACTCGGCTGGTAA	0	9	RDR2-sensitive
TAAGACTGAGATGGCTTTGGTTAC	0	14	RDR2-sensitive
TAAGACTGTAGAAGGCTTTTTACT	52	10	RDR2-resistant
TAAGACTTAGCCTCCCAGTTGCGG	0	22	RDR2-sensitive
TAAGAGACGACTATGACAGTTTAC	6	21	RDR2-sensitive
TAAGAGATCATATGTGCATTGCAG	55	4	RDR2-resistant
TAAGATATGATTGGTGTGCTTGCC	267	8	RDR2-resistant
TAAGATGTCTTCTTGAGGATTCGG	25	4	RDR2-resistant
TAAGATTCGGGACGGATACAGGAC	0	21	RDR2-sensitive
TAAGATTGTAACCTTCATTGGCAT	0	11	RDR2-sensitive
TAAGCACACAGACCGGCCTGACAC	0	9	RDR2-sensitive
TAAGCAGACTGTCATGATAATGCT	73	2	RDR2-resistant
TAAGCCCTGCTTTAGGACGTGGAC	0	10	RDR2-sensitive
TAAGCGGGCGGGCTTGGACAGGAA	0	17	RDR2-sensitive
TAAGCGTGTCGGGCTTGGCAACAC	0	13	RDR2-sensitive
TAAGCGTTTTGCTTGTTAGTTGGA	0	30	RDR2-sensitive
TAAGCTAGCTGCGAGGAGTCCGAC	17	0	RDR2-resistant
TAAGCTCGGGCTCGTTCAGGCATG	0	10	RDR2-sensitive
TAAGCTCTGCGCGGGATCGGCTAC	0	15	RDR2-sensitive
TAAGCTGGACTGGGCACAGCACGG	0	10	RDR2-sensitive
TAAGCTTATGTCCGACGGCCTCGT	0	12	RDR2-sensitive
TAAGGAAACGAGCTGGCTCGGCTT	0	12	RDR2-sensitive
TAAGGAACCGAGCCAGAGAACCAG	0	11	RDR2-sensitive
TAAGGACCAGAGTGTGCATGTTGA	21	1	RDR2-resistant
TAAGGATTGTGGTGTAAGATTGCA	0	12	RDR2-sensitive
TAAGGCACGCAGGACTGTGCAGGC	0	24	RDR2-sensitive
TAAGGCCTGTGGCATGCACACTAG	34	2	RDR2-resistant
TAAGGCGGGTCCCGTCGGAAGTAA	0	11	RDR2-sensitive
TAAGGCTGTAGAAGGCTTTTTACT	65	5	RDR2-resistant
TAAGGGCATGTACAGTGGAGAGAC	2	19	RDR2-sensitive
TAAGGGTCTGTTTGGTTCGGCTTT	0	9	RDR2-sensitive
TAAGGGTCTGTTTGGTTGGGCTGT	0	24	RDR2-sensitive
TAAGGTTATTCTTAGATCACTCGT	18	0	RDR2-resistant
TAAGTACATTGTAAAGCGTCGGGA	29	3	RDR2-resistant
TAAGTATATGGTGGCTTTATTACT	52	0	RDR2-resistant
TAAGTATTTGCTATATGTTCGGAC	103	0	RDR2-resistant
TAAGTCTGTAGCGTCAGCGTCCAC	0	13	RDR2-sensitive
TAAGTGTTGGACTAGCACGGCCAG	0	9	RDR2-sensitive

TAAGTTAGGAGCACGATCATTGTC	23	0	RDR2-resistant
TAAGTTCGTCGACTGGGACGGTGT	0	11	RDR2-sensitive
TAAGTTGGAATAATTCGGACGGTT	22	2	RDR2-resistant
TAAGTTGTATGTTTGGATGTGCAT	14	0	RDR2-resistant
TAAGTTGTGTCTTAGAGTGTGTCT	0	15	RDR2-sensitive
TAAGTTTAGATCGTGCCGACGGTG	30	2	RDR2-resistant
TAAGTTTCTGTTAGGGTCCGGCCA	23	0	RDR2-resistant
TAAGTTTGATTCTTGGATGGCATT	38	0	RDR2-resistant
TAAGTTTTGGAGAGCTGACGTCAT	21	0	RDR2-resistant
TAATAAAGATGTTGGTCTCGTCGT	0	9	RDR2-sensitive
TAATAACACAACCGAGCTCGGCGC	0	9	RDR2-sensitive
TAATACGATAAGTTCGACGGTTTT	0	9	RDR2-sensitive
TAATACTTTGATCAGAGGGTGATG	18	0	RDR2-resistant
TAATATTTTAGCAAGTCGGCAATA	54	2	RDR2-resistant
TAATCAACTCCTGAACGAAGCTGC	44	0	RDR2-resistant
TAATCGGATCGAATACGGACGGAT	3	20	RDR2-sensitive
TAATCGGATCGGATACGGACGGAT	0	12	RDR2-sensitive
TAATCGTGTCGGGCTGGCCCACGG	0	10	RDR2-sensitive
TAATCTGATCGGAGGGGCGCGGAT	0	16	RDR2-sensitive
TAATCTGGAACGGAGCGGCTCTGT	1	37	RDR2-sensitive
TAATCTTAAGGATGGTGCGTCGTA	26	1	RDR2-resistant
TAATGAGTCGGGCGTCCGGACGGT	0	17	RDR2-sensitive
TAATGAGTTTCGGACGAAGGTCAT	3	22	RDR2-sensitive
TAATGATCGAGCGATGACATTTAT	120	16	RDR2-resistant
TAATGGACCACCTTAGATGATCAT	86	10	RDR2-resistant
TAATGGACCAGAAGGCTTGTTTGT	72	4	RDR2-resistant
TAATGGACTGTATCGGGGCAGCAC	0	14	RDR2-sensitive
TAATGGCTAAGACTTGCATGGGCT	16	0	RDR2-resistant
TAATGGGTTGTATATGGTTGACAG	22	0	RDR2-resistant
TAATGGTAATGGGTTGTATATGGT	17	0	RDR2-resistant
TAATGTGAACTCATCTATTGGCAT	39	4	RDR2-resistant
TAATGTGTGAACAGGTATCTTCGG	0	52	RDR2-sensitive
TAATGTGTTTGACATGATCTGGGA	198	3	RDR2-resistant
TAATGTTATGGACTCGCATGTTGC	21	0	RDR2-resistant
TAATGTTGCATAGGATTGTTGGCT	0	19	RDR2-sensitive
TAATGTTTGTGTACTATTGGCATT	88	0	RDR2-resistant
TAATTAATTTGCACCGTTGGTCTG	1184	0	RDR2-resistant
TAATTCGGGCTAGTTTGGCAGCAA	0	10	RDR2-sensitive
TAATTCGGTTCGGTCTATTCGGGT	2	14	RDR2-sensitive
TAATTGCAGGTTGTTGGACAAAGA	19	0	RDR2-resistant
TAATTGTCCCTGTAGCATTAAGGT	0	9	RDR2-sensitive
TAATTGTGGTGGATGAATAGGCAC	134	1	RDR2-resistant
TAATTTGGACTGGTGGGAGGGCTA	1	20	RDR2-sensitive
TAATTTGTAGATCTTTCCATGGCC	43	1	RDR2-resistant
TAATTTGTAGGTCCGATGATATAT	0	17	RDR2-sensitive
TAATTTTCGACGGCCGTGACGCAT	0	11	RDR2-sensitive
TAATTTTGTTAAGAAGATACTGTA	24	2	RDR2-resistant
TAATTTTTATCGGGCACTCGGCAA	0	9	RDR2-sensitive
TAATTTTTGATCTACAGGTTTACC	31	6	RDR2-resistant

TACACAATCTGATCTGACGGAGTC	16	0	RDR2-resistant
TACACAATTTCGGACGGTTGGCGT	0	18	RDR2-sensitive
TACACATGTGTGGACTGTCCGGCT	0	17	RDR2-sensitive
TACACCGTTGGATATTGGACGGAT	0	11	RDR2-sensitive
TACACGGATCGGGCTGGACCGGGC	1	39	RDR2-sensitive
TACACGTTAGATGGGCTAGATCGT	0	13	RDR2-sensitive
TACACTACACATGGTCGGACGGTC	0	9	RDR2-sensitive
TACACTATGTAGATCTGTATGTAC	0	10	RDR2-sensitive
TACACTCGGCAAAGAACGCTCGGC	0	30	RDR2-sensitive
TACACTCGGCAAAGAAGGCTCGGC	0	11	RDR2-sensitive
TACACTGGGATGAGCACGATACAT	0	9	RDR2-sensitive
TACACTGTAGATTGCACGGTTCGT	0	11	RDR2-sensitive
TACACTGTGGTTGTGGATGTCGAC	52	5	RDR2-resistant
TACAGAAACGGACGGCACTGGCAC	0	57	RDR2-sensitive
TACAGAATTCTTGCGTACAGTGAT	0	15	RDR2-sensitive
TACAGACTCGGACGTGAGCGACGG	0	19	RDR2-sensitive
TACAGATCGCAGGAGATTTACAAT	0	10	RDR2-sensitive
TACAGATTGAAGGATATGGACGGC	18	1	RDR2-resistant
TACAGCTCTGCTAGCGTGATGGAT	17	0	RDR2-resistant
TACAGCTGTAGCTAGGATGGCAAC	0	9	RDR2-sensitive
TACAGGACATGTGTAGGTAGTCGT	0	21	RDR2-sensitive
TACAGGCATGCTCGGCTACTCCAG	27	3	RDR2-resistant
TACAGTCTCCGGTATGATAGGCAT	19	0	RDR2-resistant
TACAGTTGTTGGCTAGATTCGCGG	0	10	RDR2-sensitive
TACATATTGATCAATTTCGCCGGC	45	3	RDR2-resistant
TACATATTTGCTTGCAACTTGACA	15	0	RDR2-resistant
TACATCGGTAATAGCGGCTTCTTT	0	12	RDR2-sensitive
TACATCTGAACTTTTAGCTGCATT	72	2	RDR2-resistant
TACATGCCTTTAGTGACCGACGCC	14	0	RDR2-resistant
TACATGCTGCTGCTCCCTGACGGC	33	7	RDR2-resistant
TACATGGAACTGTAGTATGGTATT	14	0	RDR2-resistant
TACATGTGGCACTGTCGTTTGGGT	20	0	RDR2-resistant
TACATTTAAAACATTAGTGTCGGA	14	0	RDR2-resistant
TACATTTGGCGTCGGTGCGGCTGG	0	9	RDR2-sensitive
TACCAAGAGCAGAACTCTCGGCAC	0	11	RDR2-sensitive
TACCAAGAGCAGGACTCTCGGCAC	0	29	RDR2-sensitive
TACCAAGCGTAGTACACTCGGCAT	0	23	RDR2-sensitive
TACCACTCTGGGCCGGCGTAACGC	0	10	RDR2-sensitive
TACCACTGTAGCAGCTTTAGCGGT	0	9	RDR2-sensitive
TACCAGCAGACGGTCGAGCCAACA	24	0	RDR2-resistant
TACCAGCTGAGAATTAGGCGTCTT	1	36	RDR2-sensitive
TACCATCTTGTTCGGTGTCGGTAT	0	11	RDR2-sensitive
TACCATTTTGTAGATAGATGGATC	38	2	RDR2-resistant
TACCCATCGGGTATCGGGTCGCGG	2	22	RDR2-sensitive
TACCCCTTGTTGGTTCTTTGGCAT	22	0	RDR2-resistant
TACCCGACGGACTGTTCGGTGCAC	0	10	RDR2-sensitive
TACCCGATTCTCGGCACAGGGCGT	0	9	RDR2-sensitive
TACCCGTCATGCCGGACTGCCGGT	0	10	RDR2-sensitive
TACCCGTGCGTTGCGACGGCTCAC	0	9	RDR2-sensitive

TACCCGTGGGTCTGTTATTGGCAA	0	43	RDR2-sensitive
TACCCGTGGGTCTGTTATTGGGCA	5	76	RDR2-sensitive
TACCCGTGGTCTGTTATTGGGCAA	0	10	RDR2-sensitive
TACCCTAATGTATTTCTCTCGGAT	4	19	RDR2-sensitive
TACCCTCGGGCTATAAAAGGGCAA	0	13	RDR2-sensitive
TACCCTCTGCGACGGTGTGGACGG	0	15	RDR2-sensitive
TACCCTGGAGGATGAGGTCGAGGA	30	0	RDR2-resistant
TACCGAAAGCAGAACTCTCGGCAC	0	21	RDR2-sensitive
TACCGAGAGTAGAACACTCGGCAC	0	33	RDR2-sensitive
TACCGAGAGTAGGACACTCGGCAT	1	19	RDR2-sensitive
TACCGAGCGCAGGACGTTGCCGAG	0	12	RDR2-sensitive
TACCGATAGCAGGACTCTCGGCAC	3	44	RDR2-sensitive
TACCGATATCAGGACACTCGGCAT	0	39	RDR2-sensitive
TACCGCAGAGCGGATGAGGATGAT	21	1	RDR2-resistant
TACCGCATCGGACGGAGCGGCTGC	0	9	RDR2-sensitive
TACCGCGTAGGACAGTTCGGCTAG	0	13	RDR2-sensitive
TACCGGAATCGACGGCTTTGCCAA	1	19	RDR2-sensitive
TACCGGATACGGGTACTGATTCAA	0	16	RDR2-sensitive
TACCGGATTGGTGGGTCGGAACGA	0	13	RDR2-sensitive
TACCGGGCACTCGGCAAACACTAC	0	9	RDR2-sensitive
TACCGGGCTCATTTCGGACGGGAT	0	13	RDR2-sensitive
TACCTAATGTTAGGCTGTGGCGTC	0	9	RDR2-sensitive
TACCTACAGGTTTGTTGTCGGGAT	90	0	RDR2-resistant
TACCTATGGTTTGTAGATCTTGGT	53	3	RDR2-resistant
TACCTCGATGATGAGTAGATCTAT	0	10	RDR2-sensitive
TACCTCGGCGCCAATGATGACGGC	0	10	RDR2-sensitive
TACCTCGTAGGACCACATTCGCAT	0	21	RDR2-sensitive
TACCTGAGTGAACCTGGCAGTGGA	18	0	RDR2-resistant
TACCTGCTCGTTGTATTGGTCGGT	0	9	RDR2-sensitive
TACCTGGTTTTTGTCGGCTACGGT	32	1	RDR2-resistant
TACCTGTACTTATTATTACTGCTA	17	0	RDR2-resistant
TACCTGTAGCGCTGACTCTTGGCA	440	36	RDR2-resistant
TACCTGTAGTGCACCGACGCGCCC	0	9	RDR2-sensitive
TACCTGTTAGATTGCACTATGGAT	32	4	RDR2-resistant
TACCTTCGGACTAAAGTTGGCATT	0	14	RDR2-sensitive
TACCTTGTCAGTGTGGCTACTTAA	16	0	RDR2-resistant
TACCTTGTTGGACGGCTAGAGTAC	0	11	RDR2-sensitive
TACCTTTTGATCTACGGATTTACC	17	1	RDR2-resistant
TACCTTTTGCAGGTTCGACAGACA	17	0	RDR2-resistant
TACGAACTGCAGAAAGGGTTGGTT	69	3	RDR2-resistant
TACGAAGAAGCGGGGATTGTTGGT	16	0	RDR2-resistant
TACGAAGAAGCGTGGATTGTTGGT	22	0	RDR2-resistant
TACGAAGTCGGTGCAGGAAGATGT	3	26	RDR2-sensitive
TACGAATAGAGTCGGGCCAGGCAC	0	15	RDR2-sensitive
TACGAATAGGGTCGGGCCAGGCAC	0	30	RDR2-sensitive
TACGAATCTCTCGGACTACGGCAC	1	44	RDR2-sensitive
TACGACGAGCAAGCCTGACGCGCC	0	11	RDR2-sensitive
TACGACGGAGACTGAGCCAGACGG	0	13	RDR2-sensitive
TACGACGGAGGCTGAGTCAGACGT	0	13	RDR2-sensitive

TACGACGTAGAGCCGAAGAGAGGT	0	10	RDR2-sensitive
TACGACTAGTGTATTCATACGGTT	84	0	RDR2-resistant
TACGAGAACGGTAGAGGCATCCAT	0	10	RDR2-sensitive
TACGAGCAGCTGGGTTGGAGGAAT	88	8	RDR2-resistant
TACGATAATTCAGATTGCAGTCAT	18	0	RDR2-resistant
TACGATTACGGATTCAGATCGGAT	0	10	RDR2-sensitive
TACGATTTGTACTTCCTGATGGTG	27	1	RDR2-resistant
TACGCAGGGACGGCGGAAGATCGC	0	18	RDR2-sensitive
TACGCAGTTGGGCTAGTCGTGCGT	0	44	RDR2-sensitive
TACGCCCGCGCTAGCTCAGATCGT	0	16	RDR2-sensitive
TACGCGACTCGACGATGACGGTCT	0	9	RDR2-sensitive
TACGCTCCGGCGGCCCGAACCACT	0	9	RDR2-sensitive
TACGCTGGAACCGTGAACAGGCAT	1	47	RDR2-sensitive
TACGCTGGCACAAACGGACGTCGT	1	22	RDR2-sensitive
TACGCTGGGATGAGCACGATACAT	0	22	RDR2-sensitive
TACGGAAGCACGCGTTGGACGGAT	91	14	RDR2-resistant
TACGGACTTAGACGGTACCCTATT	25	4	RDR2-resistant
TACGGAGAGCTCGAAGGTGGATAT	0	11	RDR2-sensitive
TACGGAGCATAGACTGCACGCACT	0	12	RDR2-sensitive
TACGGATAGTTAGAAACGGGACGG	1	39	RDR2-sensitive
TACGGATCACCGCAGGACTTGGTA	41	1	RDR2-resistant
TACGGATGCTGCAGGAGGGTTCAT	0	17	RDR2-sensitive
TACGGATTCAGGTATATAGTGCAT	0	9	RDR2-sensitive
TACGGCGACACTCGGCAAAGACTG	0	9	RDR2-sensitive
TACGGCTAAAATTCACCGGACTGT	3	26	RDR2-sensitive
TACGGCTATAATTCACCGGACTGT	8	46	RDR2-sensitive
TACGGCTCCAGCAAGGCACTCTAT	0	19	RDR2-sensitive
TACGGCTGGAGTAGAGACCGGTGT	0	13	RDR2-sensitive
TACGGGACCAGACTGAGCGGCTAC	0	9	RDR2-sensitive
TACGGGCTGAAGACCAGTTGGCAT	1	31	RDR2-sensitive
TACGGGTAAGGATACGGACAACAC	1	12	RDR2-sensitive
TACGGGTACACAGAGGACTACCAT	0	20	RDR2-sensitive
TACGGGTCGGGCTTGGGTCGGCAC	0	9	RDR2-sensitive
TACGGTAGAATTTGGTATCCGCGA	0	13	RDR2-sensitive
TACGGTGTAGTATATTTGAGGCAC	6	28	RDR2-sensitive
TACGGTGTGTCTAGGGGGGTGAAT	0	10	RDR2-sensitive
TACGGTTTCGGTTGGGTGTCGGGG	20	1	RDR2-resistant
TACGTAGATGGCAGAACCGGTCAG	0	15	RDR2-sensitive
TACGTATCCGGACAGTCCGCGCAT	0	10	RDR2-sensitive
TACGTATGGTCTGTTGGTGGCTGC	0	9	RDR2-sensitive
TACGTCAAGCGACTTTAGTCGGAT	36	0	RDR2-resistant
TACGTCCGGCGCCTCAGACAGCAT	0	9	RDR2-sensitive
TACGTCCTGTGGAAGAAGCGGCGC	2	21	RDR2-sensitive
TACGTCTGCAGCAAGAACCGGTAA	0	17	RDR2-sensitive
TACGTGACAAAGATCGGGGCGCAA	0	21	RDR2-sensitive
TACGTGACAAAGATGGGGGCGCAA	0	9	RDR2-sensitive
TACGTGCAGCGCGTAGAAATCGGT	0	9	RDR2-sensitive
TACGTGCGGGCGCGTGTGTCGCGG	0	12	RDR2-sensitive
TACGTGGACCAGTCAGATCGCGTC	0	13	RDR2-sensitive

TACGTGGATCTGACGTGGCAGCAC	0	20	RDR2-sensitive
TACGTGGATCTGACGTGGCAGTAC	3	19	RDR2-sensitive
TACGTGGATCTGATATGGCAGCAC	0	9	RDR2-sensitive
TACGTGGATCTGATGTGGCAGCAC	0	35	RDR2-sensitive
TACGTGGCTAGGGATTGAGGGCAG	0	31	RDR2-sensitive
TACGTTGACTGTGACCAGACGCGT	0	16	RDR2-sensitive
TACGTTGTACGGCGTGGTAGACGG	0	29	RDR2-sensitive
TACTAATACTTTGATCAGAGGGTG	38	0	RDR2-resistant
TACTACAGCTGTGGATGTGGGCAC	0	13	RDR2-sensitive
TACTACTAGAACGATGCTTGAGGC	26	0	RDR2-resistant
TACTAGACCGAGAGATAGCTGCTT	15	0	RDR2-resistant
TACTAGAGCTCTCGGCACAGGGAC	0	10	RDR2-sensitive
TACTAGGACATGTATATAGGCTAA	0	13	RDR2-sensitive
TACTAGTTTGAGGATGCTTAGATA	41	0	RDR2-resistant
TACTATATCAAGGACTATGGCGTC	1	13	RDR2-sensitive
TACTATGGTTTGGTCTCGACGATG	14	0	RDR2-resistant
TACTATTATTTTCAGTTGGCATG	20	1	RDR2-resistant
TACTCAGTAATCGGCTCAGGGCAT	0	11	RDR2-sensitive
TACTCATATTCACTGTAGCGGCAT	0	50	RDR2-sensitive
TACTCCGTGTCTTTATCGTCGGAA	119	9	RDR2-resistant
TACTCCTTGGACTACGGCGAACAC	0	23	RDR2-sensitive
TACTCGACCTAGCATACTAGTATT	24	0	RDR2-resistant
TACTCGAGGTGCTCTGGGGAGGCA	16	0	RDR2-resistant
TACTCGTAACTCGTCCGACGACGG	0	19	RDR2-sensitive
TACTCGTACTCGTAGATAGAGGCT	272	31	RDR2-resistant
TACTCGTGCGTTGCGACGGCTCAC	0	20	RDR2-sensitive
TACTCTACCGGACAAGACAGTGGC	0	13	RDR2-sensitive
TACTCTCGGACGTCAATGCAGAGC	15	0	RDR2-resistant
TACTCTCTGGTCAGGCGCGGACGG	1	17	RDR2-sensitive
TACTCTGACTCAGATTATGCCGGT	91	24	RDR2-resistant
TACTCTGATTCAGACTATGCCGGT	129	30	RDR2-resistant
TACTCTGATTCAGACTATGCTGGT	18	1	RDR2-resistant
TACTCTGTTGGATGGAAGATGCAT	1	57	RDR2-sensitive
TACTGAACACCAGCGAGAGGCTGC	0	10	RDR2-sensitive
TACTGAACGCCAACGAGAGGCTGC	0	11	RDR2-sensitive
TACTGAACGCCAGTGAGAGGCTGC	0	11	RDR2-sensitive
TACTGAAGGCTGCGCAGAAGTCAA	0	15	RDR2-sensitive
TACTGACTCTGGGAGCTCGGCTGA	0	22	RDR2-sensitive
TACTGAGCGTAGTACACTTGGCAT	1	22	RDR2-sensitive
TACTGAGGGTAGCACACTCGGCAT	0	16	RDR2-sensitive
TACTGAGGTGAGCGCGGGCGGACG	85	0	RDR2-resistant
TACTGATAGGGACGAGACTTGCAT	17	1	RDR2-resistant
TACTGATCTGGATATGAATCGGTT	30	3	RDR2-resistant
TACTGATGATGGTAGAGAATTGTT	16	0	RDR2-resistant
TACTGATTTGGGATATATGGTGTG	33	0	RDR2-resistant
TACTGCAACTCTGACAAGATAAGA	26	0	RDR2-resistant
TACTGCAGCGGGAGACTCTATCAG	0	18	RDR2-sensitive
TACTGCTGACGGGAGGGAAGGGAG	2	21	RDR2-sensitive
TACTGCTGATACTGCTGACGGTGC	0	17	RDR2-sensitive

TACTGGGACGGTAGCGGACGACGA	0	17	RDR2-sensitive
TACTGTAGAAGACGCAGCCGCAGC	0	10	RDR2-sensitive
TACTGTAGAAGAGGGTACCCCTGA	0	22	RDR2-sensitive
TACTGTAGAAGCCGCAGCCGCAGC	11	362	RDR2-sensitive
TACTGTAGAAGCCGCAGCCGCAGT	0	16	RDR2-sensitive
TACTGTAGCACATGTAGGGGGCAC	0	12	RDR2-sensitive
TACTGTAGCCCACGAGCCGGCCTG	0	12	RDR2-sensitive
TACTGTAGCGCGTCATGACGGTTT	0	32	RDR2-sensitive
TACTGTAGGATTCACCAGCAGAGC	24	0	RDR2-resistant
TACTGTAGTGATCGGCGCCGGCGC	0	14	RDR2-sensitive
TACTGTCTAGTGATTGTTGATGAC	106	12	RDR2-resistant
TACTGTTAGAGCAGGGTGTTTTGT	37	0	RDR2-resistant
TACTGTTCACGCTGACTTGGCATT	28	51	RDR2-sensitive
TACTGTTCAGGCTGACTTGGCATT	6	36	RDR2-sensitive
TACTGTTCATGTTGTAGAGGCTAC	0	31	RDR2-sensitive
TACTGTTCTTGTGCGATTATGGTT	60	0	RDR2-resistant
TACTGTTTCTATATTTCGGAGGAT	82	2	RDR2-resistant
TACTTAGATAAATCTGATGATGGA	21	2	RDR2-resistant
TACTTATACATGTTAGCGGTCGTC	19	1	RDR2-resistant
TACTTATCGTTTCGGTCCGATGGC	0	12	RDR2-sensitive
TACTTATGTGCTAGTAGACCGGAT	60	2	RDR2-resistant
TACTTCCGACGGCTTAAGACGGGT	0	9	RDR2-sensitive
TACTTCCGGTTAGTTCATCGTTGT	20	0	RDR2-resistant
TACTTCGGACAGTCGGTAAAACGC	0	9	RDR2-sensitive
TACTTCTAGTTAGAACGTCGTTGC	27	0	RDR2-resistant
TACTTCTTGCTGCTCGTCGACGAT	59	6	RDR2-resistant
TACTTGAGATCTAGTGACTAGGAA	15	0	RDR2-resistant
TACTTGTGTTTGTACTGTATAGGC	34	1	RDR2-resistant
TACTTTCGGGTCAGGTCTTATCGT	0	9	RDR2-sensitive
TACTTTCTATCGGGCACTCGGCAA	4	19	RDR2-sensitive
TACTTTTTATCGGACACTCGGCAA	2	45	RDR2-sensitive
TACTTTTTATCGGGCACTCGGCAA	8	102	RDR2-sensitive
TACTTTTTCTCGGACACTCGGCAA	7	25	RDR2-sensitive
TACTTTTTCTCGGACACTCGGCAT	1	14	RDR2-sensitive
TACTTTTTCTCGGACAGTCGGTAA	1	22	RDR2-sensitive
TACTTTTTCTCGGGCACTCGGCAA	60	175	RDR2-sensitive
TACTTTTTCTCGGGCACTTGGCAA	0	10	RDR2-sensitive
TACTTTTTGTCGGGCACTCGGCAA	5	25	RDR2-sensitive
TACTTTTTTTCGGACACTCGGCAA	0	11	RDR2-sensitive
TACTTTTTTTCGGGCACTCGGCAA	4	38	RDR2-sensitive
TAGAAACCAAGTTGGACCGGCCGT	0	20	RDR2-sensitive
TAGAAACGGGACGGATACGGATAC	0	32	RDR2-sensitive
TAGAAATTTAACTAGAACTTAGGT	30	4	RDR2-resistant
TAGAAATTTAACTAGAACTTCGGT	81	0	RDR2-resistant
TAGAAATTTTGGATGGAGACCGGT	17	0	RDR2-resistant
TAGAACACTCGGCAAAGACGCTGT	0	11	RDR2-sensitive
TAGAACAGTAGTAGCTTGTGCATT	83	4	RDR2-resistant
TAGAACCGTCGATGTTAGGGGCAA	0	24	RDR2-sensitive
TAGAACTGTACGGTCTAATTCCAT	1	19	RDR2-sensitive

TAGAACTTTAGATTTAGACTGCTG	20	2	RDR2-resistant
TAGAAGACGAAGGGTTCCGGAGAC	0	20	RDR2-sensitive
TAGAATAAGCGGGCGGGCTTGGAC	1	25	RDR2-sensitive
TAGAATGGACGGCTGAGATCGCAG	1	29	RDR2-sensitive
TAGAATGTTGCACAGGATTGTTGG	17	0	RDR2-resistant
TAGAATTGTTGCTCTCTACGGTAA	100	7	RDR2-resistant
TAGAATTGTTTGGTATCTTAGGCA	40	0	RDR2-resistant
TAGAATTTTGGAAATAGGCTGGCT	19	0	RDR2-resistant
TAGACCAAGTCGTATTTTCGTGCT	25	2	RDR2-resistant
TAGACCAGTACCGTCGTGTCGGAC	0	10	RDR2-sensitive
TAGACCATAGCACCACTGACGGCT	48	0	RDR2-resistant
TAGACCGATCCGAGCAGAGTCTGT	0	16	RDR2-sensitive
TAGACGAAGACGACCAGAGCTGGC	0	10	RDR2-sensitive
TAGACGAATCTGCAGGCTGGATGG	0	12	RDR2-sensitive
TAGACGGAACCGGGCAGAACGCAG	0	27	RDR2-sensitive
TAGACGGCTTAAGATCGGCTCATC	42	10	RDR2-resistant
TAGACGGTCTGTGCTGCTACGGTG	0	15	RDR2-sensitive
TAGACGTGGAATAGGAGCGAGCAA	0	15	RDR2-sensitive
TAGACGTGTGGTAGATATGGTTAG	0	29	RDR2-sensitive
TAGACTAAAATTGAATCGAACGGT	31	0	RDR2-resistant
TAGACTACTGTCGTCCGACGGCCC	264	16	RDR2-resistant
TAGACTATAGAGTTGCATACAGAT	29	1	RDR2-resistant
TAGACTCAGCTAGTGCTTTTGGCA	22	1	RDR2-resistant
TAGACTCTCGGCAACGCGGCTCTT	0	11	RDR2-sensitive
TAGACTGCATGCGGAGTGGGATGG	0	13	RDR2-sensitive
TAGACTGCTGAGGGTATTAAGAGT	141	2	RDR2-resistant
TAGACTGGAAACGAGTGGAGTCGA	0	14	RDR2-sensitive
TAGACTGTAGCGTCGAGTCGTGTT	0	9	RDR2-sensitive
TAGACTGTGTGTGCGGACTGGCAC	0	9	RDR2-sensitive
TAGACTGTGTGTGTGGGCCGGCAC	0	10	RDR2-sensitive
TAGACTGTGTGTGTGGGCTGGCAC	0	12	RDR2-sensitive
TAGACTGTGTGTGTGGGTCGGCAC	0	13	RDR2-sensitive
TAGACTTACTGATTGTTGGCGCGC	1	27	RDR2-sensitive
TAGACTTGCTGTAGATGGCCTTAT	0	9	RDR2-sensitive
TAGAGAAGACTGTAAAGGACGGAT	0	23	RDR2-sensitive
TAGAGACATGTACAAACCCGGCAC	0	23	RDR2-sensitive
TAGAGACCAGAAGGACGAGGTCAT	0	12	RDR2-sensitive
TAGAGACGGCACTGGGATAGTCAT	3	38	RDR2-sensitive
TAGAGACGTTTTCACTGGCGCCAT	0	13	RDR2-sensitive
TAGAGATAGTCGCACAGGCACCAT	0	9	RDR2-sensitive
TAGAGCGTATTGGACGGTTGAGAT	0	36	RDR2-sensitive
TAGAGCTCTCGGCACAGGGACTGG	2	21	RDR2-sensitive
TAGAGCTGTAAGACTTTAGAAGAC	0	24	RDR2-sensitive
TAGAGCTGTTTGCACTGGCGTTTT	0	9	RDR2-sensitive
TAGAGGACCTTGTTGGAGACGTAG	0	10	RDR2-sensitive
TAGAGGACTGCCAGGAGTAAGCAC	0	17	RDR2-sensitive
TAGAGGAGACTGTAAAGGACGGAT	0	31	RDR2-sensitive
TAGAGGAGTCTAGGTCGGCCGGCC	2	19	RDR2-sensitive
TAGAGGATGAATATAGAGGACGTT	0	14	RDR2-sensitive

TAGAGGATGGATATAGAGGACGTT	0	13	RDR2-sensitive
TAGAGGATGTATATAGAGGACGTT	1	13	RDR2-sensitive
TAGAGGATGTGGCAGACACTCTAA	0	9	RDR2-sensitive
TAGAGGCATGTGGTCCATGGATGG	16	0	RDR2-resistant
TAGAGTTTCGTTGATTGTTGTCGA	14	0	RDR2-resistant
TAGATACCTCGGCGTTATAGGCAA	0	14	RDR2-sensitive
TAGATAGAACTTCAGCGATCTGGT	33	2	RDR2-resistant
TAGATATGTTAGTGACGACGTCGT	69	3	RDR2-resistant
TAGATCACTAGTTTCATGGACATC	34	3	RDR2-resistant
TAGATCCGGACTGGGTGTTGGCCA	53	8	RDR2-resistant
TAGATCGACCTGGGATATGGCTAC	0	12	RDR2-sensitive
TAGATCGGCACCCATGAACACGGT	0	15	RDR2-sensitive
TAGATCGTTTCGAGTCGGGGTCGT	0	15	RDR2-sensitive
TAGATGACTTGATGAAATTAGGCT	26	0	RDR2-resistant
TAGATGGCAGAACGGCAGAACAGA	0	10	RDR2-sensitive
TAGATGTAGATGGTTTGCAAGAAT	17	0	RDR2-resistant
TAGATTCGGGACGGATACGGGTAA	0	10	RDR2-sensitive
TAGATTCTGACCTCTTGGCGCGCT	99	4	RDR2-resistant
TAGATTGCAGCTGTAGCGGTTCAA	0	9	RDR2-sensitive
TAGATTTAGATATTGTTGGTCACT	15	0	RDR2-resistant
TAGATTTCTTTGATGGTCGTGGAT	39	0	RDR2-resistant
TAGATTTTGACCTCTTGGCGCGCT	14	0	RDR2-resistant
TAGCAAAGGGCGGATGACAGAGAC	31	0	RDR2-resistant
TAGCAACGGACGGTCGGTCGCTAA	3	53	RDR2-sensitive
TAGCACGACCCAGTAGAGCAGGCA	0	10	RDR2-sensitive
TAGCACGGACACGAGCTAGCACGG	0	9	RDR2-sensitive
TAGCAGAACGGTAGAACGGACGAC	0	17	RDR2-sensitive
TAGCAGACTAGTATATTTGTGCAT	24	1	RDR2-resistant
TAGCAGATGTGGGACTAAAGCCGG	0	12	RDR2-sensitive
TAGCAGGAGTAGATGAAGATGGTG	15	0	RDR2-resistant
TAGCAGGTCGCAAAGCTCCGTCGG	15	0	RDR2-resistant
TAGCATACCTCGGCGACAAGACCT	0	9	RDR2-sensitive
TAGCATAGTAGAACTGGTGTGGAT	21	1	RDR2-resistant
TAGCATGTAGACTCCTATGGCGTT	15	0	RDR2-resistant
TAGCATGTCTGTTTATTTTGGTCT	21	0	RDR2-resistant
TAGCATGTGATCTAGGGGTGGCAA	19	0	RDR2-resistant
TAGCATTTGATCGTCTGGTGGGTA	27	0	RDR2-resistant
TAGCCAACTGTATTTTAGCGTCGT	0	10	RDR2-sensitive
TAGCCCCTGCGCCAGGTCGCGGAC	0	11	RDR2-sensitive
TAGCCCCTGTGCCAGGTCGCGGAC	0	16	RDR2-sensitive
TAGCCCGGCTGTATGAGACAGGCC	0	23	RDR2-sensitive
TAGCCCTAGTCAAACCGTCCGGAC	0	13	RDR2-sensitive
TAGCCGATGTGGGACTAAAGCCGG	5	247	RDR2-sensitive
TAGCCGATGTGGGACTAATGCCGG	0	11	RDR2-sensitive
TAGCCGCGCGTCGGAACCCGGTGC	0	9	RDR2-sensitive
TAGCCGCTGCTCTGACAGAGACAA	0	32	RDR2-sensitive
TAGCCGGATAGCTGGACGGTCCAT	0	19	RDR2-sensitive
TAGCCGTTGGACAGAAAGCAGCAG	2	24	RDR2-sensitive
TAGCCTCGGGGTGGACAACGGTTC	63	3	RDR2-resistant

TAGCCTGGACCTGTTGAAGCACGT	0	13	RDR2-sensitive
TAGCCTTTGATCGTCTGGTGGGTA	83	0	RDR2-resistant
TAGCGAACACGGATGTCTCGGCGT	0	15	RDR2-sensitive
TAGCGAACTGTACATCGGCAATAT	0	9	RDR2-sensitive
TAGCGACGGACGGCGTGAACGCAA	0	9	RDR2-sensitive
TAGCGAGCAGTTGTAGGCCCCTGC	0	10	RDR2-sensitive
TAGCGCATCTGAGCATGTAGACGC	0	30	RDR2-sensitive
TAGCGCGGTGGTGGAGAAGTTGGC	27	0	RDR2-resistant
TAGCGCGTGCGCTGTAGCCGGCAT	3	32	RDR2-sensitive
TAGCGCTGACTCTTGGCATTCTCT	206	13	RDR2-resistant
TAGCGGACAGGACGGTTTACAGAC	0	10	RDR2-sensitive
TAGCGGACGGATACAGAGTGTCAC	0	21	RDR2-sensitive
TAGCGGCGGGACGGAGATGGTGTG	27	0	RDR2-resistant
TAGCGGGTATGGGGCAACAGAGAA	0	13	RDR2-sensitive
TAGCGGTGCCAGGCCTGGACGGCT	0	9	RDR2-sensitive
TAGCGTCGAGCGGTGTTACCTCAG	0	16	RDR2-sensitive
TAGCGTCGATCTCTGACGTGGCGT	0	19	RDR2-sensitive
TAGCGTTCCATGTAGAGTAGACGA	0	9	RDR2-sensitive
TAGCTATCTGGTCGCGGACTCGGC	0	11	RDR2-sensitive
TAGCTCAAGTCATCTCGTTCGGAT	119	0	RDR2-resistant
TAGCTCAGATTATTTGGACTCGGC	0	12	RDR2-sensitive
TAGCTCAGCTTGCTAGTTCGTCGT	3	52	RDR2-sensitive
TAGCTCCTGCACCAGGTCGCGGAC	0	17	RDR2-sensitive
TAGCTCGGCGCCAAGATCTGTGGC	0	11	RDR2-sensitive
TAGCTCGGCGCCACAGATCTTGGC	1	26	RDR2-sensitive
TAGCTCGGCGCTAAGATCTGTGGC	0	11	RDR2-sensitive
TAGCTCGGCGTCACAGATCTTGGC	0	11	RDR2-sensitive
TAGCTCGGCTCGGCTCGGATCGGC	1	16	RDR2-sensitive
TAGCTCGTTCGTAGTAGCCGGTAT	0	9	RDR2-sensitive
TAGCTCTGATAGCATGTTCGGTTA	0	13	RDR2-sensitive
TAGCTGAAGCATATGGTACTGACT	27	0	RDR2-resistant
TAGCTGAGACTGGTCTGATGGCGT	0	13	RDR2-sensitive
TAGCTGAGAGAACGTTGCGCGGAC	0	13	RDR2-sensitive
TAGCTGATGTGGGACGCCAGGCAT	0	30	RDR2-sensitive
TAGCTGCATGACTGTGGTTATGGA	50	6	RDR2-resistant
TAGCTGTAAGCACTCTCTCGGTAT	0	31	RDR2-sensitive
TAGCTGTAGCAGCCGAAGGCGTGT	0	14	RDR2-sensitive
TAGCTGTCAGAACTAGCCGTTGGA	2	18	RDR2-sensitive
TAGCTGTCCACAACTCAACGGTGC	2	77	RDR2-sensitive
TAGCTGTGGGGAGAATCTGGCTGT	0	19	RDR2-sensitive
TAGCTGTGTTGGACCGGCCCGTAA	0	10	RDR2-sensitive
TAGCTGTTACTCTACTGGCGGCAT	14	0	RDR2-resistant
TAGCTTCTTAGAGGGACTATGGCC	42	2	RDR2-resistant
TAGCTTGGATTCTGACTTAGAGGC	95	0	RDR2-resistant
TAGCTTGGTTGTTTTGATCGTTGT	42	0	RDR2-resistant
TAGCTTGTCGAGCGGATGGACGTC	0	22	RDR2-sensitive
TAGCTTGTCGAGCGGATTGACGTC	4	47	RDR2-sensitive
TAGGAACAGACAGAAGCAGGACGT	0	18	RDR2-sensitive
TAGGAACCGAAGTTTTTGACGTAT	0	9	RDR2-sensitive

TAGGAAGATGGTAGACCAGCGCAC	0	21	RDR2-sensitive
TAGGACACCTGATCACGGTCACAT	0	10	RDR2-sensitive
TAGGACAGTTCGGCTAGGCCAGAG	0	9	RDR2-sensitive
TAGGACAGTTCGGCTAGGCCGGTG	0	10	RDR2-sensitive
TAGGACATAATGGTCTTCGGACGA	355	9	RDR2-resistant
TAGGACATCGCAACCCGAGAGCGG	0	9	RDR2-sensitive
TAGGACATGATTGTGCATACCAAG	0	17	RDR2-sensitive
TAGGACATTGTTACGGTGGCGGCG	33	2	RDR2-resistant
TAGGACGATTGCTAGATGGGCCAG	20	0	RDR2-resistant
TAGGACGGCCCACAGTGCTGGCAC	0	12	RDR2-sensitive
TAGGACTACTTCCAGAGGTCTGGC	3	21	RDR2-sensitive
TAGGACTAGGACCTCGTCAGGCGT	0	39	RDR2-sensitive
TAGGACTAGGACCTCGTTAGGCGT	0	10	RDR2-sensitive
TAGGACTCAATATGGAATGTGCAT	0	10	RDR2-sensitive
TAGGACTCCCGATGCTTGACGCCA	25	4	RDR2-resistant
TAGGACTCTCTGCGGAGGCGCGGA	0	17	RDR2-sensitive
TAGGAGATATTTGGTTTTGATGAT	30	0	RDR2-resistant
TAGGAGCATCTGGAAGGACGGTCT	0	9	RDR2-sensitive
TAGGAGGAATAGGTCTGGCGGCGC	0	15	RDR2-sensitive
TAGGAGTAGATGTAGCATAGTTCT	1	27	RDR2-sensitive
TAGGAGTAGGACGGTGCCGACGAC	27	0	RDR2-resistant
TAGGAGTAGGACGGTGTCGCCGAC	2	18	RDR2-sensitive
TAGGATACATGAATCTTAGTGACT	17	1	RDR2-resistant
TAGGATATGTCTGACGGTCCACAG	0	14	RDR2-sensitive
TAGGATCAATCACAGAGAAGACAC	0	15	RDR2-sensitive
TAGGATCTCGAAGGCGACAGGTTT	0	12	RDR2-sensitive
TAGGATCTGAAGCGGTAGGGAGGC	0	11	RDR2-sensitive
TAGGATGTAACTCGGGAGCAGCAG	0	11	RDR2-sensitive
TAGGATGTCTAACCAAGTGCTGAT	37	0	RDR2-resistant
TAGGATTCGACAAGTAGGCGCTAG	0	17	RDR2-sensitive
TAGGATTTCTTCATAAGTAGTGGA	20	0	RDR2-resistant
TAGGCAAAGTGTGGCAACTGAGGT	0	14	RDR2-sensitive
TAGGCAACGGAACGTGTGATGGTT	183	0	RDR2-resistant
TAGGCACTGTCTGTCTGTCGTGAT	0	10	RDR2-sensitive
TAGGCAGGCTGTGGCACCTTCGGC	0	19	RDR2-sensitive
TAGGCATTTTGCTTGTTAGTCGGG	0	11	RDR2-sensitive
TAGGCCACGGAAGTTTGAGGCAAT	34	6	RDR2-resistant
TAGGCCAGCATGAACACGACCCAG	0	14	RDR2-sensitive
TAGGCCGGTCTGTAAGCACGGAAA	0	9	RDR2-sensitive
TAGGCGACTGAGCAGTAACCGGGC	0	10	RDR2-sensitive
TAGGCGCACAACCAACGTCTGGAC	0	10	RDR2-sensitive
TAGGCGCCCTGTTGGATTGTTGGG	15	0	RDR2-resistant
TAGGCGGAGTGGTAGTGGAGGGGA	23	1	RDR2-resistant
TAGGCGGGCTGCAGGAGATGTCAC	0	9	RDR2-sensitive
TAGGCTCGGGCGAGGCATGATCGA	1	22	RDR2-sensitive
TAGGCTCGGGCGAGGCGTGATCGA	6	49	RDR2-sensitive
TAGGCTCTCGGGAGATGGAGGACA	59	3	RDR2-resistant
TAGGCTGAGGGATGACACAGTCAA	20	0	RDR2-resistant
TAGGCTGTGAGAGTAGGGAGACCT	0	9	RDR2-sensitive

TAGGCTGTGAGCCAGTGCTGCGGT	0	27	RDR2-sensitive
TAGGCTTCTTCGTCATCGTCTGCA	26	0	RDR2-resistant
TAGGCTTGCAAAGGACGTCGCTGC	0	11	RDR2-sensitive
TAGGGAAGGATCAGCAGTAGACAT	1	12	RDR2-sensitive
TAGGGAGACGGATGCGGAGACTGC	0	30	RDR2-sensitive
TAGGGATTTAGCGGTGGGCGTCAT	0	9	RDR2-sensitive
TAGGGCAACAGGCATGAAGGGCAC	0	9	RDR2-sensitive
TAGGGCATGGCAGATGATGACGAC	0	10	RDR2-sensitive
TAGGGCCCTGACGACGGGTGGCGT	0	9	RDR2-sensitive
TAGGGCCCTGACGACGTGCGGCGT	1	70	RDR2-sensitive
TAGGGCCTACGCTCCTACCGTCGG	50	0	RDR2-resistant
TAGGGCGCATGACTGTGACGTCAT	0	11	RDR2-sensitive
TAGGGCTAGTTTGGATCGGCGCGC	0	10	RDR2-sensitive
TAGGGCTCTCGGCACAGGGACTGG	0	37	RDR2-sensitive
TAGGGCTCTGGCGACGGACGGCGT	0	12	RDR2-sensitive
TAGGGCTGGATATCGAGACGGTTC	0	12	RDR2-sensitive
TAGGGCTTCTCTGCCTGACGGCCG	1	17	RDR2-sensitive
TAGGGCTTGTTCGGTTATTCCCAA	0	28	RDR2-sensitive
TAGGGCTTGTTCGGTTATTTTCAA	0	22	RDR2-sensitive
TAGGGGATGGGGATGGGGAAGTTT	0	10	RDR2-sensitive
TAGGGGCGCTGTAGAGGGCACCGC	0	15	RDR2-sensitive
TAGGGGCTGGAGGTGGAGGGGACC	120	0	RDR2-resistant
TAGGGGGAACCGCTGAAGACAGTC	1	21	RDR2-sensitive
TAGGGGTAGATCTGAGCAAGACGT	0	49	RDR2-sensitive
TAGGGTAGGATACAGGATGAGCTA	1	34	RDR2-sensitive
TAGGGTCCTCTGCCTGACGGTCAG	0	9	RDR2-sensitive
TAGGGTCGACACAGCGGAAGAAAT	15	0	RDR2-resistant
TAGGGTTCGTTGCTGTTTGGTCAT	0	9	RDR2-sensitive
TAGGGTTCTCTGTAGGCCGGACGG	0	10	RDR2-sensitive
TAGGGTTGCGCGGCACGACACTGC	0	9	RDR2-sensitive
TAGGGTTGGAGAGTTGTTCCTAGA	20	0	RDR2-resistant
TAGGGTTGTGTGGCCATTGATCGT	4	17	RDR2-sensitive
TAGGGTTTGTGGAGTGCGAGAGGT	123	4	RDR2-resistant
TAGGGTTTTAGATTTCGTCTCGAT	282	6	RDR2-resistant
TAGGTACGATGAGCTAGTCGGCAC	2	21	RDR2-sensitive
TAGGTACTACTACCGTGTCGGCAC	0	13	RDR2-sensitive
TAGGTAGAGCGAGGCAATGGGCGC	0	9	RDR2-sensitive
TAGGTAGTGCCGTGCCTGGGCCGA	0	13	RDR2-sensitive
TAGGTAGTGTCGTGACTAGGCCGA	0	12	RDR2-sensitive
TAGGTCAGGACGGCACGACCCGAA	0	11	RDR2-sensitive
TAGGTCATGGATTGTTGCAGGTAA	0	9	RDR2-sensitive
TAGGTCCGAACTCCAGCAGGGTCC	0	13	RDR2-sensitive
TAGGTCGTAGAAAGTTAGCGGCTC	0	10	RDR2-sensitive
TAGGTCTGCATGATATTGATGGTT	0	10	RDR2-sensitive
TAGGTCTGCTGTGAAGAAGACGAT	0	19	RDR2-sensitive
TAGGTCTGTTAAGCGGGACTGGGT	25	1	RDR2-resistant
TAGGTGACCTACTGATCTCGGAGC	0	10	RDR2-sensitive
TAGGTGCAGTAGATGTGTATGGTA	61	6	RDR2-resistant
TAGGTGTAGCAACAAGGAGTCGGT	0	16	RDR2-sensitive

TAGGTGTTGTTGAATGGTCATGAG	22	0	RDR2-resistant
TAGGTTCGTGCTGGGCATTTGGTG	20	2	RDR2-resistant
TAGGTTGACGGGCCGTGCCGGGCT	0	9	RDR2-sensitive
TAGGTTGATTAATTTTGTACGGCT	124	5	RDR2-resistant
TAGGTTGCTGGGAAGAGAGCGGAC	0	9	RDR2-sensitive
TAGGTTGGTGTATTGGCAGTTTTT	14	0	RDR2-resistant
TAGGTTTTCCTGCCTGACGTCCGG	0	15	RDR2-sensitive
TAGTAAACAATATTATGTACGGCT	26	0	RDR2-resistant
TAGTAAAGAGAGTTGGATCGGCTT	19	1	RDR2-resistant
TAGTACCTGCCAAGCGCTCGACAA	2	29	RDR2-sensitive
TAGTACGGTATCTGATTACGGTTG	0	12	RDR2-sensitive
TAGTACTGTAGCACCGGTAGGAGC	0	24	RDR2-sensitive
TAGTACTGTGAGCAGGTGCAGACG	15	0	RDR2-resistant
TAGTACTTGTGCAGATTTGTGGAT	67	3	RDR2-resistant
TAGTAGAACGGACGGACGGTGCAG	0	11	RDR2-sensitive
TAGTAGAATGTCTTAGATGTCATT	27	0	RDR2-resistant
TAGTAGACTGTAGCGTCGAGTCGT	0	38	RDR2-sensitive
TAGTATGCTGTAGTGAGATGCTAT	0	9	RDR2-sensitive
TAGTATTTTCGGTTGGTAGTGGCA	171	6	RDR2-resistant
TAGTCAGAGTAGGGACGGAGCTAG	0	9	RDR2-sensitive
TAGTCAGCTGGAGGAGTTAGGTAA	0	11	RDR2-sensitive
TAGTCCACAGTAGGTCATCGGAAC	0	9	RDR2-sensitive
TAGTCGATGTAGTCATGGGCGGAC	0	31	RDR2-sensitive
TAGTCGGAGGTGTGTGTGGTCATA	16	0	RDR2-resistant
TAGTCGTAGAGTAGCGTCGGCGTC	20	0	RDR2-resistant
TAGTCGTGCCGGGCTTGGGATGGC	0	40	RDR2-sensitive
TAGTCGTGCTCGGACCGGACTGGC	0	14	RDR2-sensitive
TAGTCGTTGCTGTGTAGAGGGTAC	0	10	RDR2-sensitive
TAGTCTCGCACCGTGAGTCGGCAT	0	9	RDR2-sensitive
TAGTCTCGCACTGTGAGTCGGCAT	0	23	RDR2-sensitive
TAGTCTCTGTAGAGTGGCAGATTC	24	0	RDR2-resistant
TAGTCTGTGGAGCGGTAAGGCAGC	0	9	RDR2-sensitive
TAGTCTTCGCGGTAGACTTCGGTT	44	3	RDR2-resistant
TAGTCTTTTATCATTTAGGTCGGT	23	0	RDR2-resistant
TAGTCTTTTATCCTTTAGGTCGGT	15	0	RDR2-resistant
TAGTGAATAGGTCGGTTTAGAGGT	22	0	RDR2-resistant
TAGTGACCAGAGCCGGACGGCCTG	22	0	RDR2-resistant
TAGTGACTCTCTTCCCTGGCAGAT	42	0	RDR2-resistant
TAGTGCGCTTTGGACGAGTTGAGA	26	4	RDR2-resistant
TAGTGCGGCTGAACCAGATGACAC	1	77	RDR2-sensitive
TAGTGCGGCTGAACCGGATGACAC	0	10	RDR2-sensitive
TAGTGCTAGGCGTAGAAGGTGCAG	0	9	RDR2-sensitive
TAGTGCTGAACAGTAAGTCGGTAT	0	11	RDR2-sensitive
TAGTGGAAGGCGGGCTGGGCAGCA	293	25	RDR2-resistant
TAGTGGAGGAACATGCTTTGACCA	14	0	RDR2-resistant
TAGTGGCGCTCGGCGAGTCGACAT	0	10	RDR2-sensitive
TAGTGTATTCTGAGAAGCAGCTGA	4	20	RDR2-sensitive
TAGTGTCGGATCGGCCCATGCTAC	0	13	RDR2-sensitive
TAGTGTCGTGACTAGGCCGAGCAT	0	10	RDR2-sensitive

TAGTGTCGTGGACCGATCCGGCAT	0	25	RDR2-sensitive
TAGTGTCGTGTCGGGTCGAGACGG	0	10	RDR2-sensitive
TAGTGTCGTGTCTGGGCCGAGCAT	0	13	RDR2-sensitive
TAGTGTGGACTCCAAGAAGACGAT	0	9	RDR2-sensitive
TAGTGTGGTATCTGATTACGGTTG	0	11	RDR2-sensitive
TAGTTAACTGAGAGACGACGGCCT	0	13	RDR2-sensitive
TAGTTCAAGACGGTATGCTGTGGG	27	4	RDR2-resistant
TAGTTCGGTCCTAAAGCACGTCAT	0	12	RDR2-sensitive
TAGTTGAAGAAGGTTATGATGATT	96	4	RDR2-resistant
TAGTTGACTGAGAGACGACAGCAT	0	11	RDR2-sensitive
TAGTTGATGTCAAGTTACTGCGGT	43	1	RDR2-resistant
TAGTTGTGGATGCGGGAGTTTGGC	0	12	RDR2-sensitive
TAGTTTCTGATTGACTGTAACGGT	0	13	RDR2-sensitive
TAGTTTGAGGATGCTTAGATAGGT	24	0	RDR2-resistant
TAGTTTGTCGAACGGATTGGCATC	0	10	RDR2-sensitive
TAGTTTGTTTGATGGTACGTGCTA	85	4	RDR2-resistant
TAGTTTTACTGTAGTGATCGGCGC	0	12	RDR2-sensitive
TAGTTTTAGGTCTGGATCTAGGTT	113	1	RDR2-resistant
TAGTTTTCCAAATGTCTGATCGTT	23	1	RDR2-resistant
TAGTTTTTCGCTAGCGTAGCCTCC	17	0	RDR2-resistant
TATAAATTTTTACTCGTCAGATAC	19	0	RDR2-resistant
TATAACATGGAGCGGAAGTAGGTT	24	0	RDR2-resistant
TATAACGGACCGTGCCAGCCCGGC	1	15	RDR2-sensitive
TATAACTGTAGCGCGTAGGGGGCT	0	15	RDR2-sensitive
TATAAGTAATTTGGATGAATCGGA	24	0	RDR2-resistant
TATAAGTGTACATTTAAGTCGGAT	0	9	RDR2-sensitive
TATAATATTGATGAATTGGTCGGC	91	0	RDR2-resistant
TATACAAATTATCTCATCGTCGTA	20	0	RDR2-resistant
TATACCGGTCGTTGCTGGAGGCAT	0	17	RDR2-sensitive
TATACCTGTACATGTGGGCCGGCC	75	1	RDR2-resistant
TATACCTTGCTGTGAGACAGAATT	36	2	RDR2-resistant
TATACGGAAATTTTAGTGCGGATC	17	0	RDR2-resistant
TATACGGCGAAACAATGATGTCAT	0	10	RDR2-sensitive
TATACTCCAATGCATCCTGCCTAT	269	29	RDR2-resistant
TATAGAATCAGGAGTAGTGATGAT	18	0	RDR2-resistant
TATAGAATGTATGAGGCTGGAAGA	22	0	RDR2-resistant
TATAGACTACTGTCGTCCGACGGC	25	0	RDR2-resistant
TATAGACTGTAGTGTGGTGTCTAG	0	12	RDR2-sensitive
TATAGCTACGATTCGTTGGTCGTT	27	0	RDR2-resistant
TATAGGACGACTCGGTAGAAGCCA	0	19	RDR2-sensitive
TATAGGAGGGTATGCGCGAGAGAG	68	2	RDR2-resistant
TATAGGCGTAAGGAGAGGATCCAG	4	17	RDR2-sensitive
TATAGTCGTGTAGAGACGGGCTAT	0	9	RDR2-sensitive
TATAGTTTGTTTGATGGTACGTGC	41	1	RDR2-resistant
TATATAAGTATGTTATTGCGTCGT	39	1	RDR2-resistant
TATATACCTTGCTGTGAGACAGAA	27	1	RDR2-resistant
TATATATTGCAGAGGACTACATGT	55	0	RDR2-resistant
TATATATTTCTCGGTCTTTTGGTT	91	1	RDR2-resistant
TATATCAAGGACTGTGGCGTCATC	0	9	RDR2-sensitive

TATATCATGGACTGTGGCGTCATC	0	13	RDR2-sensitive
TATATCGGACCGTGCCAGCCCGGC	2	23	RDR2-sensitive
TATATGACAATATTCAGTAGACTT	41	5	RDR2-resistant
TATATGCATTCTGTAGGAAGACAA	43	3	RDR2-resistant
TATATGGTAGATTCAGATGAAGAC	18	0	RDR2-resistant
TATATTCGGGACGGATACGGGTAA	4	31	RDR2-sensitive
TATATTGCAGAGGACTACATGTTT	23	0	RDR2-resistant
TATATTGGTTGTTGTTGGACGCAT	0	9	RDR2-sensitive
TATATTGTTTGTGCATCTGTCGGA	84	3	RDR2-resistant
TATATTTGTAGGGATAGGATGGAT	136	2	RDR2-resistant
TATCAAAAGTAAGACACTCGGCAT	0	9	RDR2-sensitive
TATCAACCAACCGTAGGTCGCTGA	0	12	RDR2-sensitive
TATCACGACTGTATAGGATGGTAT	0	36	RDR2-sensitive
TATCACGATTGCGGTATTCAAACC	17	0	RDR2-resistant
TATCACTGCTGTAGGATGACGGTT	50	0	RDR2-resistant
TATCACTTTTAAGACTGCATTAAT	17	0	RDR2-resistant
TATCAGTCGGCTGCTGTTACGGTC	41	0	RDR2-resistant
TATCATTATTGTAGGACACGTCAT	0	23	RDR2-sensitive
TATCATTTTGTGGTTAGGAATAAT	39	1	RDR2-resistant
TATCCACGACCTTGAAGCGAGCTT	16	0	RDR2-resistant
TATCCAGAGGGTGGCATAGAACTG	10	27	RDR2-sensitive
TATCCCGGCTGCGTCTGTGACTGT	0	14	RDR2-sensitive
TATCCTCGGACTCAAGTCAGGCAT	0	20	RDR2-sensitive
TATCCTGTGTGGCTCAATAGAGGA	26	0	RDR2-resistant
TATCCTTTTTTTGGTGGTCGTCTT	30	2	RDR2-resistant
TATCGAAGAACAAGAGTGAGCATT	21	1	RDR2-resistant
TATCGAGAGCAGGACATTCGGCAC	0	13	RDR2-sensitive
TATCGAGAGTAGGACATTCGGCAT	3	58	RDR2-sensitive
TATCGAGCACTCGGCAGAACACGC	0	9	RDR2-sensitive
TATCGATCCTTTAGACCTTCGGAG	42	10	RDR2-resistant
TATCGCGGTGGGACTCACGGCGAA	0	11	RDR2-sensitive
TATCGCGTAGGACAGTTCGGCAAG	0	9	RDR2-sensitive
TATCGCGTAGGACAGTTCGGCTAG	2	135	RDR2-sensitive
TATCGCTTTGTCTAGATTGTCTAA	35	0	RDR2-resistant
TATCGGACACAAGCCTTAAGCCGT	0	21	RDR2-sensitive
TATCGGACGTCAGCTGTAGACGGA	0	9	RDR2-sensitive
TATCGGACTTTTTTTCAATTGGTT	24	3	RDR2-resistant
TATCGGCCGACTAGACAGACGCAT	0	37	RDR2-sensitive
TATCGGCGTCATGATCTGAGGCAT	0	11	RDR2-sensitive
TATCGGGCACTCGGCAAAGACTAC	0	10	RDR2-sensitive
TATCGGGCACTCGGCAAAGCAGAC	2	15	RDR2-sensitive
TATCGGGCACTCGGCAAAGTCGAC	0	9	RDR2-sensitive
TATCGGTCTTTGGGCTAAACGGGT	1	35	RDR2-sensitive
TATCGGTTTTCTCAGGAGCACGCA	23	0	RDR2-resistant
TATCGTACCGGGCTGTGCCGAGGC	0	17	RDR2-sensitive
TATCGTCAGTATAGGGAACGCGGC	0	12	RDR2-sensitive
TATCGTCGCGCGGCTTCGCCACTT	0	9	RDR2-sensitive
TATCGTGATTGTGACATTAGGTAT	0	13	RDR2-sensitive
TATCGTGTTGGGCTTAGGTGGTTT	0	10	RDR2-sensitive

TATCGTTCGGTAGAAAGCATTAAT	91	24	RDR2-resistant
TATCGTTGAACTGTTAGTCAGCAT	1	16	RDR2-sensitive
TATCGTTTAGGACAACGACACGGT	0	26	RDR2-sensitive
TATCTATATCAAGTTGGAAGTATG	19	0	RDR2-resistant
TATCTATCGGACATGAATGTGCAG	58	0	RDR2-resistant
TATCTATTGAGGATGGAGTCTGCC	31	0	RDR2-resistant
TATCTATTTGGTTGTCTGTTACAG	19	1	RDR2-resistant
TATCTCGACCCGTAGTGCTGGCAC	0	15	RDR2-sensitive
TATCTCGGCTCTCGCATCGATGAA	21	0	RDR2-resistant
TATCTCGGTGTTATAGATCATGGC	0	19	RDR2-sensitive
TATCTCTCCAGAAATTTGAGCATG	31	0	RDR2-resistant
TATCTCTTGTAGTTTTTTGTTGTC	14	0	RDR2-resistant
TATCTGAACACAATTTCTTATGGC	350	20	RDR2-resistant
TATCTGACCTGGGCTGACGACTGC	0	33	RDR2-sensitive
TATCTGACTAGTGGATGCACGCGC	0	9	RDR2-sensitive
TATCTGATTCGTCCGGTCCGGTAT	0	19	RDR2-sensitive
TATCTGGACTGGACGAGACGACGA	0	14	RDR2-sensitive
TATCTGGTGGACCTGCAGGTGGGG	0	11	RDR2-sensitive
TATCTGTAATATCTACACACTGGC	17	0	RDR2-resistant
TATCTGTATCTCTCGAAGTAGACC	19	1	RDR2-resistant
TATCTGTTTGGCAGAACTTCGGCT	0	11	RDR2-sensitive
TATCTTAGAATTCTCTTAACGGCT	55	5	RDR2-resistant
TATCTTAGAATTCTTTTTGCGGCA	23	0	RDR2-resistant
TATCTTAGAATTCTTTTTGCGGCC	35	3	RDR2-resistant
TATCTTCGGACTCAAGTCAGGCAT	0	22	RDR2-sensitive
TATCTTCGGATTCAAGTCAGGCAT	0	13	RDR2-sensitive
TATGAAAAACTCTAGTTTTGGTCC	16	0	RDR2-resistant
TATGAAACACGACGGATGCGGCTT	2	55	RDR2-sensitive
TATGAAAGTCGTCATGCAATTAGT	146	0	RDR2-resistant
TATGAAATGTGGGCCTGATGGCAA	0	16	RDR2-sensitive
TATGAACAAGTAGAACATTAAGAA	23	1	RDR2-resistant
TATGAACAGAACTCATTAAAGGCA	0	10	RDR2-sensitive
TATGAACTGCTGTGGGGGACAGAT	0	13	RDR2-sensitive
TATGAATGTATTGTAGGCGTGCAC	0	25	RDR2-sensitive
TATGAATTTATTGTAGACGTCCAT	0	24	RDR2-sensitive
TATGACAATATTCAGTAGACTTAA	28	4	RDR2-resistant
TATGACACTGTGGACAGCGTTTAC	0	11	RDR2-sensitive
TATGACATGTGGAGCCAGTGGCAT	0	16	RDR2-sensitive
TATGACATGTGGGACCAGTGGCAT	0	29	RDR2-sensitive
TATGACATGTGGGGCCAGTGGCAC	0	10	RDR2-sensitive
TATGACATGTGGGGCCAGTGGCAT	6	164	RDR2-sensitive
TATGACATGTGGGGCTAGTGGCAT	0	10	RDR2-sensitive
TATGACATTTTGCTGATAGGGCCG	68	2	RDR2-resistant
TATGACGGATAAAACTATATGTGT	0	11	RDR2-sensitive
TATGACTGAAGAGATTAGTTCGTC	55	1	RDR2-resistant
TATGACTGCAGAGGATTGTGGTAT	0	22	RDR2-sensitive
TATGACTGCGGAGAATTATGGTAT	0	10	RDR2-sensitive
TATGACTGCGGAGGATTATGGTAT	0	11	RDR2-sensitive
TATGAGACATAGATTATTTTTGGT	23	0	RDR2-resistant

TATGAGCAATTGATGGTACTCACT	21	0	RDR2-resistant
TATGAGCGATCGACAGCGTGACGT	37	0	RDR2-resistant
TATGAGTCTGTTAGGCCCGTCGGA	0	10	RDR2-sensitive
TATGAGTGCGGAGGATTGCGGTAT	1	23	RDR2-sensitive
TATGAGTGTAGAGGAGGAGAGCGC	0	9	RDR2-sensitive
TATGATCAGATTCGTTTAACAAAG	27	1	RDR2-resistant
TATGATCGGACGATTTATGTGCAT	1	30	RDR2-sensitive
TATGATCTATCGGGCATCCGTCAT	0	9	RDR2-sensitive
TATGATGACGGATTATATTCAGAC	17	0	RDR2-resistant
TATGATGGACTCTTGGATGAGGCA	33	3	RDR2-resistant
TATGATTTGTAGGCCCGATGGTAT	0	61	RDR2-sensitive
TATGATTTGTGGATCCGATGGCAT	3	58	RDR2-sensitive
TATGATTTGTGGGACTGATTGCAT	0	11	RDR2-sensitive
TATGATTTGTGGGCCTGATGGCAT	0	29	RDR2-sensitive
TATGATTTGTGGGTCCGATGGCAT	22	687	RDR2-sensitive
TATGCAAGCTTTGAAGATCGAGAA	14	0	RDR2-resistant
TATGCACTGTAGCGTACGAGTGAA	0	49	RDR2-sensitive
TATGCAGTTGGGATTAGCGTGCAT	73	7	RDR2-resistant
TATGCATTCTGTAGGAAGACAACA	24	3	RDR2-resistant
TATGCGAGACTGGACCGGCCCGAC	0	23	RDR2-sensitive
TATGCGCCTGAGGTGCTCGTCGGT	0	12	RDR2-sensitive
TATGCGTTGACTGAGCAAGTGCGT	0	10	RDR2-sensitive
TATGCTAGACTGGAGGCACGACGA	0	65	RDR2-sensitive
TATGCTAGCACGGCTCGAAGACAA	0	10	RDR2-sensitive
TATGCTCGACGGGATTGTCGGCCA	40	10	RDR2-resistant
TATGCTGCTGGATTCTCAAGACGT	23	0	RDR2-resistant
TATGCTGGAGCGGTATTACTCAAA	42	0	RDR2-resistant
TATGCTGGATGTAAGGTCGATAGG	18	1	RDR2-resistant
TATGGAACTTTGTTGAGATGTGTT	40	0	RDR2-resistant
TATGGAAGAATTTGGTGCATTCAT	18	0	RDR2-resistant
TATGGACATCAGGAGCCGCTCCGC	0	18	RDR2-sensitive
TATGGACCCGGCTGAAAGACGTAC	0	9	RDR2-sensitive
TATGGACCTCTCTAGAAGCAGCGT	0	14	RDR2-sensitive
TATGGACTAGTAGCATACCGGTGC	0	130	RDR2-sensitive
TATGGAGTGTTTATAGAACGTCGT	0	9	RDR2-sensitive
TATGGATTAAACATGCTGATGAGG	32	1	RDR2-resistant
TATGGATTGAACTGCTTACCCAAT	38	3	RDR2-resistant
TATGGATTGCAGCAGTAGTACGAT	0	13	RDR2-sensitive
TATGGATTTTGTTTCGGAAATGCA	20	0	RDR2-resistant
TATGGCGCCGAGCAAAGGGTCCAA	0	18	RDR2-sensitive
TATGGCGCCGAGCTGTGACGTGGC	1	15	RDR2-sensitive
TATGGCGTCGAGCTCTGACGCGGC	0	12	RDR2-sensitive
TATGGCTCCTGCTCTAGACGCTAA	0	13	RDR2-sensitive
TATGGCTTCGGCGGCAAGGGCACC	25	0	RDR2-resistant
TATGGCTTGATAGTATGACGACGG	0	9	RDR2-sensitive
TATGGGCTGATTAAAGGTACATTC	14	0	RDR2-resistant
TATGGGTACGGTAGAATTTGGTAT	0	21	RDR2-sensitive
TATGGGTGAACTGCTGAACACGGT	0	9	RDR2-sensitive
TATGGTAGACTGTACGGTGACCTA	0	9	RDR2-sensitive

TATGGTAGATTCAGATGAAGACTG	17	0	RDR2-resistant
TATGGTGGGGGCTCTGCAAGTGGA	25	1	RDR2-resistant
TATGGTTGTTGTTTCATTCGCGGT	48	0	RDR2-resistant
TATGGTTTGGTCTCGACGATGGAT	17	0	RDR2-resistant
TATGGTTTGTAGATCTTGGTCAAG	31	1	RDR2-resistant
TATGTAACTGGGCGGATAGGTGGA	0	10	RDR2-sensitive
TATGTACGACAAGCTCCGGCGCAG	1	16	RDR2-sensitive
TATGTAGAAATGTGCATAAATCAT	9	108	RDR2-sensitive
TATGTAGAATTTGTATGCAAGCAA	47	4	RDR2-resistant
TATGTAGCTATTGTAGGCGTGCAC	0	9	RDR2-sensitive
TATGTAGGAATGGCAACTGTACAT	0	13	RDR2-sensitive
TATGTAGGCTTGATGATCGAGGAC	24	2	RDR2-resistant
TATGTATAATGACAGATGTTGGAT	16	0	RDR2-resistant
TATGTATGTATTGTAGGCGTGCAC	1	22	RDR2-sensitive
TATGTATTGATGTTGGTAGGCTAC	0	16	RDR2-sensitive
TATGTCCGACGGCATCGTAGACAG	4	76	RDR2-sensitive
TATGTCGCGTAGGACAGTTCGGCT	0	22	RDR2-sensitive
TATGTCTAGATTGTATCGGTGCGT	0	11	RDR2-sensitive
TATGTCTGGTCGGAAGCATGGCGC	0	18	RDR2-sensitive
TATGTCTTGTATGAATTCCACGGC	51	3	RDR2-resistant
TATGTGACTGATTATGGTGGTATT	98	3	RDR2-resistant
TATGTGATTGTTGAAGGAGCGCAT	0	12	RDR2-sensitive
TATGTGCACTATGTAGAAGACCGT	0	17	RDR2-sensitive
TATGTGCAGTCGGACTCTTGGCGT	0	10	RDR2-sensitive
TATGTGGAGCTCGAAGGTGGATAT	0	13	RDR2-sensitive
TATGTGTTGACTAAGTGACGGACA	0	9	RDR2-sensitive
TATGTGTTTGGACATTCGTTTGCA	20	0	RDR2-resistant
TATGTTCGGACGCTCAGAAAGGCT	80	11	RDR2-resistant
TATGTTGCTGGATTCTCAAGACGT	20	0	RDR2-resistant
TATGTTGGAAGGGCAAAAGGGCAA	0	17	RDR2-sensitive
TATGTTGGCTATTGCAAAGGTGGT	49	2	RDR2-resistant
TATGTTGTCGTCGGTAGCGGCTAA	0	12	RDR2-sensitive
TATGTTGTTTACTGTTGTGATGGA	0	11	RDR2-sensitive
TATGTTTGTATCGTAGGCGTCCAT	0	34	RDR2-sensitive
TATGTTTGTTGGCTAGATTTGCGG	52	78	RDR2-sensitive
TATTAAACGGATCGTGCTTCGGGC	0	14	RDR2-sensitive
TATTAAACGGGCCGTGCTTCGGAC	0	23	RDR2-sensitive
TATTAAACGGGTCGTGCTTCGGGC	1	14	RDR2-sensitive
TATTAAATGGGTCGTGCCTCGGGC	0	11	RDR2-sensitive
TATTAAGAAATCATAATCGTTGGT	21	0	RDR2-resistant
TATTAAGGCTGTAAGAGTAGTCGG	0	26	RDR2-sensitive
TATTAAGGGCTTAGCGTAGACTGC	0	9	RDR2-sensitive
TATTACAGATAAGTTTAGATCGTG	36	0	RDR2-resistant
TATTACATGGTCGTTATCTAGGAT	26	0	RDR2-resistant
TATTAGCTATGATGTTTGGATGTT	46	0	RDR2-resistant
TATTAGGACGCGGGTATGACGGAT	0	28	RDR2-sensitive
TATTATAACAGTCGTCTGAATCCT	16	0	RDR2-resistant
TATTATAGACTGTATCTAGGCATA	56	3	RDR2-resistant
TATTATATCGATTGTTAGCAGTAT	29	2	RDR2-resistant

TATTCAAGGACTGTTCAGGCAAGG	65	4	RDR2-resistant
TATTCATAGAGACTCAGGATCATC	30	4	RDR2-resistant
TATTCGACTTTAGTGCACGGTTCT	0	18	RDR2-sensitive
TATTCGCTATATGTTCGGACGCTC	0	12	RDR2-sensitive
TATTCGGATTCGGACTATGCGGGG	14	0	RDR2-resistant
TATTCGGGTTTTCGGGTTCGGGTT	0	16	RDR2-sensitive
TATTCGGTGATGGGAATCAAGGCA	17	1	RDR2-resistant
TATTCGTACCGGGCCGAAACGGGT	0	23	RDR2-sensitive
TATTCGTGCTGGGCTCAAACGGGC	0	13	RDR2-sensitive
TATTCTACAGGGCTAGTTTGGCAA	15	0	RDR2-resistant
TATTCTGGGCTTGTGTGAGATGGT	0	10	RDR2-sensitive
TATTCTGTCTGATTGGTTCGGTGT	0	9	RDR2-sensitive
TATTGAAAATGGTCGTTCATGGCT	32	5	RDR2-resistant
TATTGAACTGTGGGCGACGTGCGC	0	11	RDR2-sensitive
TATTGACTTCGGTACTGACGTCAT	24	0	RDR2-resistant
TATTGCAGAGGACTACATGTTTCT	53	0	RDR2-resistant
TATTGGACTAGGCGAGACATTACA	19	0	RDR2-resistant
TATTGTAGACATAATCGAGTGGCT	0	12	RDR2-sensitive
TATTGTGGGACAATGATTGGAAGA	74	5	RDR2-resistant
TATTGTTTAGAACATCTACACGGT	0	19	RDR2-sensitive
TATTGTTTGTCGTTTCGGGGTACC	37	2	RDR2-resistant
TATTGTTTTATCCGGTGAGACTGA	38	1	RDR2-resistant
TATTGTTTTGGTGAGTCTGGAGAA	16	0	RDR2-resistant
TATTGTTTTTACTCTGCTGGACGA	0	11	RDR2-sensitive
TATTTACAAATGTAGATGTGTCTG	53	2	RDR2-resistant
TATTTACATGTGTTGTTTGGACTG	25	0	RDR2-resistant
TATTTAGTTTGGCATAGAAAGGTA	42	3	RDR2-resistant
TATTTATGGATACAGTTAGACTTC	38	1	RDR2-resistant
TATTTATGTGCATCTCGACCGGAC	51	4	RDR2-resistant
TATTTATGTGCTAGTAGACCGGAC	82	5	RDR2-resistant
TATTTATGTGCTAGTAGACCGGAT	83	11	RDR2-resistant
TATTTATGTGTAGCGTCGTATATC	57	0	RDR2-resistant
TATTTATTGGACTGGGCAATTACT	31	3	RDR2-resistant
TATTTCGGGCACTCGGCAAAGAGC	1	34	RDR2-sensitive
TATTTCTCGGTCTTTTGGTTAAAA	54	1	RDR2-resistant
TATTTGACTACTAGATATGTTGGC	20	0	RDR2-resistant
TATTTGACTGGAAGTTTAATGGCA	20	1	RDR2-resistant
TATTTGATCGGATATCGGATACTT	1	17	RDR2-sensitive
TATTTGCGCGGACAGTTTGGTGTA	34	0	RDR2-resistant
TATTTGCTATATGTTCGGACGCTC	101	0	RDR2-resistant
TATTTGCTCTGTGTCGTTGGGAGA	68	0	RDR2-resistant
TATTTGGTCTGTGGCTAAGGGCAT	0	13	RDR2-sensitive
TATTTGTCAATCGGTTTTCGGTCC	17	0	RDR2-resistant
TATTTGTGACACAATTTCGTTGGA	16	0	RDR2-resistant
TATTTTAGATATGTTAGTGACGAC	55	0	RDR2-resistant
TATTTTAGCAAGTCGGCAATACCA	55	2	RDR2-resistant
TATTTTATGGTTGTAGTGAGGTAT	0	20	RDR2-sensitive
TATTTTGTCTAACTGTTGAAAGGA	23	0	RDR2-resistant
TATTTTGTTGTAGATAAATGGCAC	25	4	RDR2-resistant

TATTTTGTTTGTCGGGACGGATAC	0	42	RDR2-sensitive
TATTTTTCATTATGGTTTTGTCGA	20	0	RDR2-resistant
TCAAAAACTGAACCCTGACGGTCT	0	11	RDR2-sensitive
TCAAAACACTGTCGGGCACGGCAC	0	17	RDR2-sensitive
TCAAAACCCGGGCTGTGAGAGGAG	0	14	RDR2-sensitive
TCAAAATGAGTTCGGCTTAAGGAT	32	3	RDR2-resistant
TCAAACAGATGAGGATGTGCTAGA	19	0	RDR2-resistant
TCAAACATTGATCTAGGAGACCAT	39	8	RDR2-resistant
TCAAACGGACAGGCTGGAATGGGC	0	11	RDR2-sensitive
TCAAACTGATTGTATGAAGATGCC	143	7	RDR2-resistant
TCAAACTGGTCTTAGGGTCGTCTG	15	0	RDR2-resistant
TCAAAGACCTGCTGATTTACTGCA	20	2	RDR2-resistant
TCAAATATAATTGTGTGTCGTCGT	141	2	RDR2-resistant
TCAAATATCTGTCGGACCCGGCAC	0	15	RDR2-sensitive
TCAAATATGATTGTGTGTCGTCGC	0	9	RDR2-sensitive
TCAAATGTCTGTCGAGCCCGGCAC	0	19	RDR2-sensitive
TCAAATGTCTGTCGGGCTCGGCAC	0	14	RDR2-sensitive
TCAAATGTCTGTCGGGTCCGGCAC	0	16	RDR2-sensitive
TCAACACATGTTGGATAAAATGGC	17	0	RDR2-resistant
TCAACCCATGGACTAGTTTGGCAT	1	14	RDR2-sensitive
TCAACCTTTTCGTTGGGTCATGTC	15	0	RDR2-resistant
TCAACGCGACTCTGTCAGGAACGG	0	62	RDR2-sensitive
TCAACGTGACTGTACGGGACGGAC	0	10	RDR2-sensitive
TCAACGTGAGGACGGTACGGATAT	1	50	RDR2-sensitive
TCAACTAATCTTCTTGAGCTCGGC	58	0	RDR2-resistant
TCAACTCATCTCGGACCATCGCAT	0	15	RDR2-sensitive
TCAACTCTCTCTGCAGGATGACTC	35	1	RDR2-resistant
TCAACTGCTGAATCATGGACGGAT	0	10	RDR2-sensitive
TCAACTGTAGTAGGAGGAGGGCAT	0	9	RDR2-sensitive
TCAACTGTCCACTCTGCAACGGCC	5	24	RDR2-sensitive
TCAACTGTCTACTCTACAACGGCC	2	15	RDR2-sensitive
TCAACTGTGGGGCATATATGCATT	74	3	RDR2-resistant
TCAACTGTTATGCATCCATCTAGC	34	1	RDR2-resistant
TCAAGAAACTGGTTACGTCTAGCT	47	2	RDR2-resistant
TCAAGAACTCGAGGAATTTCAGGC	77	9	RDR2-resistant
TCAAGAACTCTCAAATTGAAGGCT	44	6	RDR2-resistant
TCAAGACTAGGTGGGCAAGCTGCA	22	1	RDR2-resistant
TCAAGCCTGCCGCCGGTTCGTCGA	1	34	RDR2-sensitive
TCAAGGACTGTTCAGGCAAGGACA	25	4	RDR2-resistant
TCAAGGCACAGTGTCGGACGGTCC	0	14	RDR2-sensitive
TCAAGGGACTGCACGGCGTATCGT	0	9	RDR2-sensitive
TCAAGGTCGGTGTAGTTGACGCGC	0	17	RDR2-sensitive
TCAAGGTTTACGAGAGCTGACGAC	24	0	RDR2-resistant
TCAAGTCAGCTGTTGAAACGGTAA	0	16	RDR2-sensitive
TCAAGTCGGCGTTTCTTAACGGCG	92	5	RDR2-resistant
TCAAGTCGTCAGGCTACCCGGTGC	0	11	RDR2-sensitive
TCAAGTGGCCTTGAATCTGAGGAT	52	4	RDR2-resistant
TCAATAAATCTATGTCGGACGACT	19	38	RDR2-sensitive
TCAATAGAAATTTTGAATGGAGAC	17	0	RDR2-resistant

TCAATCGATGTGTAGTGTGATGGT	113	5	RDR2-resistant
TCAATGAAGACCGTTGGTGGGCAT	32	1	RDR2-resistant
TCAATGACATGTGGGGTCCATGGT	0	15	RDR2-sensitive
TCAATGATTGTGTAATGTGTTGGC	17	0	RDR2-resistant
TCAATGGTATATCTGGAGAAGCAT	0	10	RDR2-sensitive
TCAATGTGATAGGATGTAGAGGAG	15	0	RDR2-resistant
TCAATGTTTGACTGTCGCGTGCCT	639	35	RDR2-resistant
TCAATTGGACCAGGCTGAGGCTAG	0	78	RDR2-sensitive
TCAATTGGTCCAGGCTGAGGCTAG	1	18	RDR2-sensitive
TCAATTTCCAGATCTTTGTGCATA	179	3	RDR2-resistant
TCACAAGTGGAGGACCCTAGACTA	51	4	RDR2-resistant
TCACACTGCAGAGGCTTGGACGGA	0	9	RDR2-sensitive
TCACACTGTAGCTGCCGACGGCAC	0	11	RDR2-sensitive
TCACAGCAGTTCGGCTCGGCTCGG	0	39	RDR2-sensitive
TCACAGGCTGCAGCAGATGATGAT	0	9	RDR2-sensitive
TCACATCAAGCGTTTGACTTTCAA	0	9	RDR2-sensitive
TCACATCAGCTGGCTGTGGACGGC	0	12	RDR2-sensitive
TCACATGCATTGACCAGACCTCAT	0	9	RDR2-sensitive
TCACATGGATGTCAAGTCGGCGTT	27	0	RDR2-resistant
TCACATTTATTGTAGTGGTCTGGG	41	0	RDR2-resistant
TCACATTTGTGCAAAAATGGTCGG	0	9	RDR2-sensitive
TCACCAAGAGAGCTTACTTTGATA	26	0	RDR2-resistant
TCACCACTGTAGCGCGTAGAGGAT	0	30	RDR2-sensitive
TCACCAGATCGCGCCGGACACGGT	0	9	RDR2-sensitive
TCACCAGGACGTAGGGTGTTACGC	0	9	RDR2-sensitive
TCACCCATCGTGCCGGGCTGGGCT	0	10	RDR2-sensitive
TCACCCCCCTCTAGGCGACTTTCA	0	9	RDR2-sensitive
TCACCCGCGGTAACTGACGGATAT	0	25	RDR2-sensitive
TCACCCGGCTTCAGCTCACGTCAT	4	28	RDR2-sensitive
TCACCGAAGGCAGAACCAGGGGAG	2	18	RDR2-sensitive
TCACCGACTCGGATGTCATCGGCG	53	16	RDR2-resistant
TCACCGAGCCGAGCCGAGCCTGAT	0	9	RDR2-sensitive
TCACCGAGTTGGAGTAGGCAGGTC	20	1	RDR2-resistant
TCACCGGAAGGGCTCGGACGGTCC	0	9	RDR2-sensitive
TCACCGGACCAGGCTGGACGGCCC	0	9	RDR2-sensitive
TCACCGGACTGTCCAGTGGTGCAC	0	13	RDR2-sensitive
TCACCGGACTGTCCGATGGTGCAC	0	11	RDR2-sensitive
TCACCGGAGGACCTGTAGAGACGG	0	10	RDR2-sensitive
TCACCGGGCCATGACTGAGGACGT	0	12	RDR2-sensitive
TCACCGTACCCCTGTAGATGGTAT	0	76	RDR2-sensitive
TCACCGTCTACTCTGCAACGGCCC	0	10	RDR2-sensitive
TCACCTAGGTCGTGAGCTCTCGGC	49	6	RDR2-resistant
TCACCTCACCATCGTCGGAGGCAA	1	13	RDR2-sensitive
TCACCTGGATCATGTGGAAGACGA	1	73	RDR2-sensitive
TCACGACATGAGCTTGGGCGGGAC	0	12	RDR2-sensitive
TCACGACGCGTAGGATGGGCGGAC	0	13	RDR2-sensitive
TCACGAGCTGAGCTGACATCCTAG	0	30	RDR2-sensitive
TCACGAGCTGAGCTGACATTCTAG	0	11	RDR2-sensitive
TCACGATTAGTCAGGCTGGTCGGC	32	0	RDR2-resistant

TCACGCAGGAGGAGTCTGGCCGAA	0	9	RDR2-sensitive
TCACGCCATGAGTCGGCAGCTGAA	0	9	RDR2-sensitive
TCACGCGGACTTGGCATTTGCTTT	0	27	RDR2-sensitive
TCACGGACCAGGCCGAGCTAGCAC	0	12	RDR2-sensitive
TCACGGAGAGACGGCTCTAAGATT	0	33	RDR2-sensitive
TCACGGCTGGTATGAGGAAGACGA	1	16	RDR2-sensitive
TCACGGCTTGTGGGTAAAGTGCAC	1	15	RDR2-sensitive
TCACGGGACCTCGCAGGTAGACGT	0	9	RDR2-sensitive
TCACGGGACTGTCCGGTGGACTAG	0	12	RDR2-sensitive
TCACGGGAGGGAGGACCATCACTA	39	5	RDR2-resistant
TCACGGGCCACGATGACATGCATC	20	0	RDR2-resistant
TCACGGGCTCACTGCTGGACCGGT	0	14	RDR2-sensitive
TCACGGGTACGGGTTTGGGACGGC	0	17	RDR2-sensitive
TCACGTAACACACGGAGTCGGAGC	0	20	RDR2-sensitive
TCACGTCCCGCGGATCGGCTCCTC	101	14	RDR2-resistant
TCACGTTTATCGTCGAGTCGTCGG	1	35	RDR2-sensitive
TCACGTTTGCTGTCGAGTCGTCGA	1	16	RDR2-sensitive
TCACTAGTCGGTCCGGTTGAGGGT	0	12	RDR2-sensitive
TCACTAGTCTTTAGAGAAACTGCA	91	8	RDR2-resistant
TCACTATTACCTTTATTGTTGGAC	18	0	RDR2-resistant
TCACTCATATCGTGCTGTCGTCGT	0	12	RDR2-sensitive
TCACTCGGCTGACGTACGCGGTTA	0	16	RDR2-sensitive
TCACTCGGGCAGGGCTGAAGTGGA	0	9	RDR2-sensitive
TCACTCGGTTGATGGTCAGCTCAG	0	9	RDR2-sensitive
TCACTCTGGTCCTTGCTGACGGAC	0	9	RDR2-sensitive
TCACTCTTTGATCGGTTGATGGTG	34	3	RDR2-resistant
TCACTGAAGACTGCAGCGGGCGAC	0	9	RDR2-sensitive
TCACTGCATCGATCTGGACGGTCC	0	13	RDR2-sensitive
TCACTGCGAGAGATGGAACGGAAC	0	11	RDR2-sensitive
TCACTGCTGAATTCTATCACGGAT	28	0	RDR2-resistant
TCACTGGACTGTCCGGTGGTGCAC	1	12	RDR2-sensitive
TCACTGTGGATAGGCGACGGAGAA	0	10	RDR2-sensitive
TCACTGTTCACGGCACTTGCTGGC	17	1	RDR2-resistant
TCACTTGAACATGGTCGACGGCGA	0	14	RDR2-sensitive
TCACTTGAACTCCCTTTGAGGCAT	0	11	RDR2-sensitive
TCACTTGGATCATGTGGAAGACGA	0	11	RDR2-sensitive
TCAGAACGAGCCCGAACCGAGCAT	0	29	RDR2-sensitive
TCAGAACTCACCTCACGGAGGACA	0	9	RDR2-sensitive
TCAGAAGTGGATTGATGATGTCAT	0	15	RDR2-sensitive
TCAGAATTTACAAGGACGTGCTTT	38	0	RDR2-resistant
TCAGAATTTTGGGCCCTGCGCGGT	0	10	RDR2-sensitive
TCAGACACCCGCGGGCTTAGACGG	0	9	RDR2-sensitive
TCAGACAGATTGCGTGCTCCGCGT	0	22	RDR2-sensitive
TCAGACCGGTCAGTAGAGCGGCGA	0	11	RDR2-sensitive
TCAGACCGGTCAGTGGAGCGGCGA	4	29	RDR2-sensitive
TCAGACGGACGCTGGACGCAGGAA	0	23	RDR2-sensitive
TCAGACTGGACTGGCTGAGTACAT	0	26	RDR2-sensitive
TCAGACTGGACTGGCTGAGTGCAT	2	821	RDR2-sensitive
TCAGACTGGACTGGCTGAGTGCCT	0	29	RDR2-sensitive

TCAGACTGTTTCTTCGAGAGGTGT	0	9	RDR2-sensitive
TCAGACTTAGCTAGAAGTGGTCAT	20	0	RDR2-resistant
TCAGACTTAGCTAGAAGTGGTTAT	36	0	RDR2-resistant
TCAGAGACCGAACCGGCAGAACAC	0	11	RDR2-sensitive
TCAGAGACGGTTAGATTTTGCTCT	33	0	RDR2-resistant
TCAGAGCACCAACGTCGACGACAC	0	22	RDR2-sensitive
TCAGAGGACGCTGCTGAAGAGCAT	0	13	RDR2-sensitive
TCAGAGTCCAGACGATGTGAGCGC	0	25	RDR2-sensitive
TCAGAGTTTGGGCCTGTCAGACGG	0	10	RDR2-sensitive
TCAGATAAGGATAGTTGTATGGAC	26	0	RDR2-resistant
TCAGATATTTTGTGGCTACATAGT	23	0	RDR2-resistant
TCAGATCCGGGCAAGAAGCGACAG	0	13	RDR2-sensitive
TCAGATCTGATCAGTGGGGCGCGT	0	9	RDR2-sensitive
TCAGATGATGCGTACTGATTAGAC	1	43	RDR2-sensitive
TCAGATTAGAAGTTTGCACGGTTG	0	15	RDR2-sensitive
TCAGATTAGATTGCGTACCGTGTT	33	0	RDR2-resistant
TCAGATTTGGATCTGCAGAAGCAT	0	10	RDR2-sensitive
TCAGCACAAGCTGCAGAGACTGAT	1	87	RDR2-sensitive
TCAGCAGAATTCACGATTTATGTC	14	0	RDR2-resistant
TCAGCAGAGACTGTTAAGTTGAAC	1	12	RDR2-sensitive
TCAGCAGGCTGTAAGGTGTTCCAC	0	26	RDR2-sensitive
TCAGCAGTAGAGGCGCAAGAAGAT	0	16	RDR2-sensitive
TCAGCATAGACGGACGCTCGACAA	0	19	RDR2-sensitive
TCAGCATCGAGGACACATGGCTAA	0	9	RDR2-sensitive
TCAGCCTGGACCAGTTGAACGGAC	0	61	RDR2-sensitive
TCAGCCTGTTTGAGCCCAGCACGG	0	10	RDR2-sensitive
TCAGCCTTGATCAGTCGCGGCCGC	0	17	RDR2-sensitive
TCAGCCTTGATCTGTCGCGGCCGC	0	11	RDR2-sensitive
TCAGCTCGGCTGCCTAGACGACGG	0	12	RDR2-sensitive
TCAGCTCGTAGCGACTCGACGGTA	0	9	RDR2-sensitive
TCAGCTCGTGGACGACAGCGCGGC	0	11	RDR2-sensitive
TCAGCTGGACCGGATACTACGCAT	0	29	RDR2-sensitive
TCAGCTGGACGCAGGGGAGATGGA	0	11	RDR2-sensitive
TCAGCTGTAGACGGATGTTGACGG	2	15	RDR2-sensitive
TCAGCTGTAGACGGCCGCTAACGG	1	16	RDR2-sensitive
TCAGCTGTAGACGGCTGCTAACGG	0	14	RDR2-sensitive
TCAGCTGTTGGAACGGTAAGCTAA	0	15	RDR2-sensitive
TCAGCTTTTTCTTCGATCTTCGGC	15	0	RDR2-resistant
TCAGGAACTGACCAGAAACGGCAC	0	10	RDR2-sensitive
TCAGGACGAAGGAAGCATAGGGCT	0	9	RDR2-sensitive
TCAGGACGCCAACCAGATCGCGGT	0	11	RDR2-sensitive
TCAGGACTGAATCCAGGCTAGGAA	0	22	RDR2-sensitive
TCAGGACTTATTTCCGACGGTTTA	0	15	RDR2-sensitive
TCAGGAGATAAGTAGTGCCGGAGT	0	18	RDR2-sensitive
TCAGGAGATGAGCTGACAGCTACT	45	6	RDR2-resistant
TCAGGAGCGGGCAGGATTCCACGG	0	14	RDR2-sensitive
TCAGGAGCTGTGAATATAGACTGC	0	9	RDR2-sensitive
TCAGGAGTAGGACGGTGTTATCGA	4	22	RDR2-sensitive
TCAGGATCTAGAAAGTGACGGATT	0	66	RDR2-sensitive

TCAGGATGTAGAAAGTGACGGTTT	0	9	RDR2-sensitive
TCAGGATGTAGTTTTGCCGGATGA	48	0	RDR2-resistant
TCAGGCCATGAGGAGATGAGCATC	81	1	RDR2-resistant
TCAGGCGAAGCTCTCTGAGGACGA	2	18	RDR2-sensitive
TCAGGCTCTGCAGACGATACCCAT	11	62	RDR2-sensitive
TCAGGCTGTACGCGGACAGATGGA	0	12	RDR2-sensitive
TCAGGGCAGCCTGTAGCTACGGAC	0	19	RDR2-sensitive
TCAGGGCAGTAGGCCAGTAGATGT	0	16	RDR2-sensitive
TCAGGTAGGTGTAGTTGGGGACGG	7	23	RDR2-sensitive
TCAGGTCGGTCAGTGGAGCGGCGA	0	43	RDR2-sensitive
TCAGGTCGTAAAGCTCCATCGGCT	34	2	RDR2-resistant
TCAGGTGCGGCTTCGACGATTTAA	0	12	RDR2-sensitive
TCAGGTGCTCTGAGTAAGCTGGAC	36	1	RDR2-resistant
TCAGGTGGATGATGAGCTAGACGG	0	15	RDR2-sensitive
TCAGGTTTGGATCGGATCGAGCAA	0	9	RDR2-sensitive
TCAGTAGACCGACATGACGTGCAT	0	10	RDR2-sensitive
TCAGTAGGACCTTGGTGGAGGTAT	0	17	RDR2-sensitive
TCAGTATCATGCAGACTTCGATAT	0	17	RDR2-sensitive
TCAGTATGGATTTTATCATCGGAC	15	0	RDR2-resistant
TCAGTATGTCTGATCAGTACGCAT	0	21	RDR2-sensitive
TCAGTCAGGCTGCATATCCCACGT	0	23	RDR2-sensitive
TCAGTCATGTCAGTAGGTCGAGTC	18	0	RDR2-resistant
TCAGTCGTGTAGAAGGAGGGCGTG	19	0	RDR2-resistant
TCAGTCGTTGCTGTCGATGGGCAC	0	20	RDR2-sensitive
TCAGTCTCAACGGTCGATCGTCGT	78	2	RDR2-resistant
TCAGTGACGTGGACACGGTTAGCC	21	0	RDR2-resistant
TCAGTGGATCGTGGCAGCAAGGCC	17	1	RDR2-resistant
TCAGTGTGGAAGGTGTTGATGGAC	25	0	RDR2-resistant
TCAGTTATAGTTTGTTTGATGGTA	23	0	RDR2-resistant
TCAGTTATCGTCGTTTTGCCCATA	58	16	RDR2-resistant
TCAGTTGGGTGCCTAGAGTTGCAT	49	2	RDR2-resistant
TCAGTTGTGAATGGATCGACGCTC	0	9	RDR2-sensitive
TCAGTTTACTGTAGTGTGAGGTGT	0	9	RDR2-sensitive
TCAGTTTCAACGGTCGTCATGCGT	0	10	RDR2-sensitive
TCATAATATTTTAGCAAGTCGGCA	41	1	RDR2-resistant
TCATAATTCGGGCTAGTTTGGCAG	0	29	RDR2-sensitive
TCATAGAGACTCAGGATCATCGGG	79	8	RDR2-resistant
TCATAGCGACACTGTAGTGCGGAC	0	12	RDR2-sensitive
TCATAGTGGATTTTGTGTTGTGCA	39	1	RDR2-resistant
TCATATATCGTACCGGGCCGGGCC	0	9	RDR2-sensitive
TCATATATTCAATGAAAGCGGACA	19	0	RDR2-resistant
TCATATCGGGTAAAACTCGTCGGG	0	13	RDR2-sensitive
TCATATTAGATGTGTCGGCGTGTG	15	0	RDR2-resistant
TCATCAACAACGGGATATGGTAGC	0	13	RDR2-sensitive
TCATCACATGGACGTCAAGTCGGC	17	0	RDR2-resistant
TCATCAGATGTGTTGACTGGGCAT	0	12	RDR2-sensitive
TCATCAGTCCAGCAGGCTCCGCAT	0	36	RDR2-sensitive
TCATCAGTCCAGCAGTCTCCGCAT	0	14	RDR2-sensitive
TCATCATCGGACAGTGTGGATGGA	16	0	RDR2-resistant

TCATCATCGTACATCTTCGGCATC	1	26	RDR2-sensitive
TCATCCCGTCAACAGTAGAGGCGC	0	9	RDR2-sensitive
TCATCCGGACTCCAAACAAGGCAA	0	18	RDR2-sensitive
TCATCGACCGGTATACCTTTTGAT	86	34	RDR2-resistant
TCATCGAGCCAGGCGTCCGAACGT	0	14	RDR2-sensitive
TCATCGAGCTGGTGGATCAGGCCA	55	0	RDR2-resistant
TCATCGCAGAAGGCCAGGTAGAAC	0	9	RDR2-sensitive
TCATCGGACTGTCCGGCGAGTCAT	0	21	RDR2-sensitive
TCATCGGAGTGTTGGTCAGTCGGT	0	10	RDR2-sensitive
TCATCGGGTGACGGATATGGATAG	0	12	RDR2-sensitive
TCATCTGTGTACGTCCGGACGGTC	0	11	RDR2-sensitive
TCATCTTCATCGTCTGCAGGCATA	70	4	RDR2-resistant
TCATCTTCTACCACTGTTGGCATC	33	2	RDR2-resistant
TCATCTTTTGAGTTCTGACGGTGT	0	18	RDR2-sensitive
TCATGACCTGGACGCAAGAGCGAC	0	11	RDR2-sensitive
TCATGACGAGTACGACGAGCAGAA	25	0	RDR2-resistant
TCATGACTAGATGTAGAGACGTTT	0	9	RDR2-sensitive
TCATGACTGGTGGCCGGACGGTCC	0	17	RDR2-sensitive
TCATGACTTGGTCGATGACGAGCT	42	6	RDR2-resistant
TCATGATCCAGTGAAGCGTGAACT	21	0	RDR2-resistant
TCATGATGAATTTTGACCTTGCAT	21	1	RDR2-resistant
TCATGCCTTTGTAGATCCGAGCGG	0	20	RDR2-sensitive
TCATGCGTGCAACGGCATGGTCGG	0	9	RDR2-sensitive
TCATGGACCTCGCGGACGAACGGC	74	23	RDR2-resistant
TCATGGACTGTTCTAGAAGAAGGT	71	0	RDR2-resistant
TCATGGCTAGACTGGAGACGACAA	0	9	RDR2-sensitive
TCATGGCTAGACTGGAGGCGACGA	2	29	RDR2-sensitive
TCATGTCAGCTGTGGAAACGGTAA	3	54	RDR2-sensitive
TCATGTCAGCTGTTGAAACGGTAA	0	10	RDR2-sensitive
TCATGTCATATTAGATGTGTCGGC	158	0	RDR2-resistant
TCATGTCGCATCGCACGGACACTC	0	10	RDR2-sensitive
TCATGTCTCTATGGATCGGGAGTT	17	0	RDR2-resistant
TCATGTGTACCACTCTGGGCCGGC	0	30	RDR2-sensitive
TCATGTTGGCTGTGGAAACGGTAA	0	27	RDR2-sensitive
TCATGTTGTAGAGGCTACAGTTAT	0	10	RDR2-sensitive
TCATTAGAGCATATCGGTGGAAGA	17	0	RDR2-resistant
TCATTAGATCTAGATCGGATACAC	0	12	RDR2-sensitive
TCATTAGATCTAGATCGGATGCAC	0	45	RDR2-sensitive
TCATTAGGATTACGTGCGGAGGAA	0	13	RDR2-sensitive
TCATTCAATCAACTGTGGGGCATA	23	2	RDR2-resistant
TCATTCTGTTTGTGGTTCTTTCGG	44	1	RDR2-resistant
TCATTGAATATGGGTACGGTAGAA	0	15	RDR2-sensitive
TCATTGACCGGTATGCTTTTTGAT	33	7	RDR2-resistant
TCATTGACGACGGTGCTGATGGCT	85	3	RDR2-resistant
TCATTGATTGTGACTTGATGGTAC	0	17	RDR2-sensitive
TCATTGGACTGGCCCGGGCACGAC	0	9	RDR2-sensitive
TCATTGGGCAGAGACTTCATGATC	34	1	RDR2-resistant
TCATTGTCAAGCTTGGAGGCGTCG	23	1	RDR2-resistant
TCATTGTTGAATGGGTTGAAGGAC	0	13	RDR2-sensitive

TCATTTAGAGGAAGGAGAAGTCGT	64	6	RDR2-resistant
TCATTTGCCGGTACTGTGAGGCCC	15	0	RDR2-resistant
TCATTTTAGTTATTTGGTCCGGGC	15	0	RDR2-resistant
TCATTTTCTAAGTTGTGCTCGGCA	22	2	RDR2-resistant
TCATTTTGTCAGCAGCTGGACGTG	18	0	RDR2-resistant
TCATTTTTCTGAGGTAGTACGTAT	36	0	RDR2-resistant
TCATTTTTGGACCCTTTGCTCGGC	2	16	RDR2-sensitive
TCCAAAACTCTGGATGGAGCAGCT	0	10	RDR2-sensitive
TCCAAACACGTAGAGGGCTAGAGC	0	25	RDR2-sensitive
TCCAAACAGCTGCAACAGTGTCGG	0	21	RDR2-sensitive
TCCAAACAGTATGGGTCGTCCGGT	0	9	RDR2-sensitive
TCCAAACGGAGGTAGCAGTAGCGT	0	10	RDR2-sensitive
TCCAAAGTCGGGCGGGGCACGGAT	0	22	RDR2-sensitive
TCCAACCCAAACGGCGAAACTCAC	0	16	RDR2-sensitive
TCCAACGCGGTCGTCGACGGCGGT	0	12	RDR2-sensitive
TCCAACGGAGTAGAGCAGAACGGT	0	27	RDR2-sensitive
TCCAACGGAGTAGAGCGAAGTGGC	0	10	RDR2-sensitive
TCCAACGGAGTAGAGCGGAGTGGC	0	22	RDR2-sensitive
TCCAACGGCACTGATGACGTGGCA	0	14	RDR2-sensitive
TCCAACGGGCGACTGACGTGGCAC	0	14	RDR2-sensitive
TCCAACGGTCGAACCCTAACGGTT	0	10	RDR2-sensitive
TCCAACGGTGCTGTGTCAGGGGGT	101	2	RDR2-resistant
TCCAACTGCTGTGCTGTATGACGG	0	10	RDR2-sensitive
TCCAACTGGTCTTGTAGCGGTCGA	0	11	RDR2-sensitive
TCCAACTTGGGGGCTTCAGACATT	16	0	RDR2-resistant
TCCAAGAATGTGATCTATGGCAAG	19	0	RDR2-resistant
TCCAAGACCAACCTCGGACGGTAG	0	9	RDR2-sensitive
TCCAAGCGCGGGCGTGGATCTGGC	25	4	RDR2-resistant
TCCAAGGTCGGATACGGAAGGCAA	0	9	RDR2-sensitive
TCCAAGGTTTCGTAGAGCAGGAGA	0	14	RDR2-sensitive
TCCAAGGTTTCGTAGAGCAGGAGG	1	12	RDR2-sensitive
TCCAAGTGGATTATGTGAACAGGT	0	10	RDR2-sensitive
TCCAAGTGGGAACTCCGCGTCGGC	0	12	RDR2-sensitive
TCCAAGTGGGCAGTGAACGAGCGT	0	14	RDR2-sensitive
TCCAATCGGTTGGGGCATGTACAA	0	13	RDR2-sensitive
TCCAATGGACTTAGACGGTGCTAC	0	12	RDR2-sensitive
TCCACAATCTGAGACGTGCGGCAC	0	14	RDR2-sensitive
TCCACACGATACTGAGGACGGTGC	0	9	RDR2-sensitive
TCCACAGGAACGCTGCGAATCCGA	0	12	RDR2-sensitive
TCCACAGTTCGGACTGTTCGTCAT	1	70	RDR2-sensitive
TCCACATCTTAGGACTTAGGGCGG	0	15	RDR2-sensitive
TCCACATGTAGGTGAACGAGACGA	0	12	RDR2-sensitive
TCCACATGTCGGAGACGGTCTCGT	0	14	RDR2-sensitive
TCCACCACGCGGGGAAGACGTCGT	0	22	RDR2-sensitive
TCCACCACGCGGGGTAGACGTCGT	0	12	RDR2-sensitive
TCCACCTGGGCCAGTGACTCGGAC	0	14	RDR2-sensitive
TCCACGAACGTTTGCAGGGAACAT	0	18	RDR2-sensitive
TCCACGACACAAGGCTGGACAGGT	0	10	RDR2-sensitive
TCCACGACCAAGGGCTGGACGGTC	0	10	RDR2-sensitive

TCCACGACTTGGACACGGGAGCGG	0	12	RDR2-sensitive
TCCACGAGAGCTGTTGGAGAACGC	0	9	RDR2-sensitive
TCCACGCGGACCGTCTGGAGGCAC	0	15	RDR2-sensitive
TCCACGCTGACTGAGACAAGACGT	0	9	RDR2-sensitive
TCCACGGCACAAGATCGGACGGTC	0	11	RDR2-sensitive
TCCACGGCTAGGAACCAGACGGTC	0	14	RDR2-sensitive
TCCACGGCTGAAGCTCGGACGGTC	0	12	RDR2-sensitive
TCCACGGTCCGGACTGTCCGCGGT	0	27	RDR2-sensitive
TCCACGTACGAGGATCGAACGGTC	0	9	RDR2-sensitive
TCCACTGTATGGCTTCGGCGGCAA	106	9	RDR2-resistant
TCCAGAACCTGAACCAGTGGGTAC	0	22	RDR2-sensitive
TCCAGAACTATTGGGGACCTTCGG	0	9	RDR2-sensitive
TCCAGAACTCCTGATCCATAGAGA	28	0	RDR2-resistant
TCCAGAACTCTGCAGGTAGAGCTG	0	16	RDR2-sensitive
TCCAGACTAGAGCACCAGGAAGAC	24	0	RDR2-resistant
TCCAGAGGGTGGCATAGAACTGAG	114	198	RDR2-sensitive
TCCAGATACGAACGTTGGACAAAT	15	0	RDR2-resistant
TCCAGATGGGTCGGCGCGAGACGG	1	16	RDR2-sensitive
TCCAGCAAGGGCCTGTAAACGGTT	0	19	RDR2-sensitive
TCCAGCAGCGTCCTCTAAACGGTT	3	16	RDR2-sensitive
TCCAGCGGGGTACAGTGTCGAGCA	14	0	RDR2-resistant
TCCAGGACGATAAGCGCGTAAGGC	0	9	RDR2-sensitive
TCCAGGACGATAAGCGTAAGACAA	0	13	RDR2-sensitive
TCCAGGACGTAGGGTGTTACGCAT	0	10	RDR2-sensitive
TCCAGGATAAGACGATGCGAGCAA	0	11	RDR2-sensitive
TCCAGGGCTGTACCCGGACGGATC	1	13	RDR2-sensitive
TCCAGGTCACGCGGAGAACAGCAT	0	31	RDR2-sensitive
TCCAGTACATGCAGAGCCTTGGCG	1	30	RDR2-sensitive
TCCAGTCATCGGCCTACGACACAT	0	9	RDR2-sensitive
TCCAGTCGACAGAGCCGAAGTGAC	0	9	RDR2-sensitive
TCCAGTCGCTGCTAGAGGACGTGT	0	56	RDR2-sensitive
TCCAGTGCACAGGCTGCAGCAGGC	0	11	RDR2-sensitive
TCCAGTGCACAGGCTGTAGCAGGC	5	48	RDR2-sensitive
TCCAGTGGAGCACCGGACAACCAA	0	21	RDR2-sensitive
TCCATAGTGGACGCGTAGAACGGC	2	50	RDR2-sensitive
TCCATATCCTGCGGGCTAGTACGG	1	16	RDR2-sensitive
TCCATATTATTTGTGTCGGATCGT	0	33	RDR2-sensitive
TCCATCAGACTGTGTTCAACGGTG	0	21	RDR2-sensitive
TCCATCCCGGATTCTAGGAACGGA	0	15	RDR2-sensitive
TCCATCTACTTATGGGCATCGGAC	25	0	RDR2-resistant
TCCATCTCGGACTCGCGACGGTGT	1	49	RDR2-sensitive
TCCATCTCGTGACTCTGCGCGCAC	1	32	RDR2-sensitive
TCCATCTTGGATATGTGACGGTGG	0	12	RDR2-sensitive
TCCATGACGACGACGGCTCTCAGC	1	15	RDR2-sensitive
TCCATGCGTGTGTAGAAGCGACGG	0	27	RDR2-sensitive
TCCATGGGATTGCGGCATTTTTCT	40	0	RDR2-resistant
TCCATGGTATAGGACTGGACGGTC	0	22	RDR2-sensitive
TCCATGTTTAGGCTGTATTGGCGT	1	145	RDR2-sensitive
TCCATTAGTGGATTGGTGGGAGGA	37	0	RDR2-resistant

TCCATTCTGCTGAGCGCGGACGGT	0	15	RDR2-sensitive
TCCATTGCCTGTAGAGCGTGTCGG	0	12	RDR2-sensitive
TCCATTGTCGTCTAGTCCGGTTAG	33	2	RDR2-resistant
TCCCAACAGGAGCTGTGAACACGG	0	13	RDR2-sensitive
TCCCAACGGGCGGCGTGCTGAATC	126	20	RDR2-resistant
TCCCAAGAATCGGGCGAAGACGAA	0	26	RDR2-sensitive
TCCCAAGAATCGGGTGAAGACGAA	3	46	RDR2-sensitive
TCCCAAGAGTCGGGTGAAGACGGA	2	17	RDR2-sensitive
TCCCAAGGATCGGATGAAGACGAA	0	30	RDR2-sensitive
TCCCAGCACTAGTGGCACCGGACA	0	12	RDR2-sensitive
TCCCAGGAACTCTTGCAGGGGCAT	0	36	RDR2-sensitive
TCCCAGGACACCCACCCAGGACGC	0	9	RDR2-sensitive
TCCCAGTCGGAGGGTGGCGCGCAT	0	16	RDR2-sensitive
TCCCAGTTTCGCGGATGACAGGCT	0	10	RDR2-sensitive
TCCCATCCGGAACGGCGAAGGCAC	0	13	RDR2-sensitive
TCCCATGCGGTCGACGGACATGAC	0	15	RDR2-sensitive
TCCCATGCGGTCGACGGACATGGC	0	14	RDR2-sensitive
TCCCATGGCAGTGTAGGACGGAGG	0	17	RDR2-sensitive
TCCCATGGCAGTGTAGGACGGAGT	0	30	RDR2-sensitive
TCCCATGTCGTCCCCTGTTACGGT	0	48	RDR2-sensitive
TCCCCAATCCGGCTGTATACGCAT	0	15	RDR2-sensitive
TCCCCACACGTTGCGGCGTCTGAG	39	0	RDR2-resistant
TCCCCACAGCTAGATTCTCGGAAA	0	21	RDR2-sensitive
TCCCCCCGTCAGCAGTAGAGACGC	0	34	RDR2-sensitive
TCCCCCCGTCAGCAGTAGAGGCAC	3	35	RDR2-sensitive
TCCCCCCGTCAGCAGTAGAGGCGC	6	71	RDR2-sensitive
TCCCCGACGAGTAAATGAGGATGC	0	14	RDR2-sensitive
TCCCCGATCCAGAGGACGAGCGGC	0	10	RDR2-sensitive
TCCCCGGATCCACTAGAAGACGAA	1	27	RDR2-sensitive
TCCCCTAAATGTAGCGAACGGTGA	0	14	RDR2-sensitive
TCCCCTATCTGAGGTGACCCGGAC	50	3	RDR2-resistant
TCCCCTGCGTAGGATGAGACGAGC	0	19	RDR2-sensitive
TCCCCTGTGTACTGTAATGGGCTA	0	9	RDR2-sensitive
TCCCGAAACAGAACTGACTCGCAT	0	16	RDR2-sensitive
TCCCGAACAGCTTGAGAACGCCAT	0	17	RDR2-sensitive
TCCCGACGACTGTACAGATTACGC	0	10	RDR2-sensitive
TCCCGACGACTGTACAGATTGCGC	0	13	RDR2-sensitive
TCCCGACGACTGTATAGATTGCGT	0	25	RDR2-sensitive
TCCCGACTTTTGCAAATTTGGCAT	0	19	RDR2-sensitive
TCCCGAGACACTGAGACGACGTAA	0	14	RDR2-sensitive
TCCCGAGCCGGCTAGACGACGCAA	0	16	RDR2-sensitive
TCCCGAGGACTGATCGGTTACCAT	0	18	RDR2-sensitive
TCCCGATAACGTCTCGGTCGTCGT	0	23	RDR2-sensitive
TCCCGATCTCAGGTCTGTTGTCGG	1	15	RDR2-sensitive
TCCCGCGAGCGGGCTGCGAGGGCA	84	0	RDR2-resistant
TCCCGCGCTCGGTGGACCTTCGGT	494	70	RDR2-resistant
TCCCGGAAGACGGCGAACTCCGCT	1	22	RDR2-sensitive
TCCCGGACCTGTAGAATACCGGAT	0	10	RDR2-sensitive
TCCCGGACTGGACCAGGCAACCAA	0	32	RDR2-sensitive

TCCCGGAGAGTGCAGGATCGTCGA	0	9	RDR2-sensitive
TCCCGGATGAGACGTGACAGGTGA	1	14	RDR2-sensitive
TCCCGGGCTGAAACGACGAGGTTA	0	16	RDR2-sensitive
TCCCGGTACTCACTGACAGACGAT	0	9	RDR2-sensitive
TCCCGGTGCAGGATCTAGAAGACG	5	32	RDR2-sensitive
TCCCGTCAACAGTAGAGGCGCAAG	0	10	RDR2-sensitive
TCCCGTCAGCAGTAGAGACGCAAG	1	40	RDR2-sensitive
TCCCGTCAGCAGTAGAGACGCGAG	0	10	RDR2-sensitive
TCCCGTCAGCAGTAGAGGCGCAAG	6	248	RDR2-sensitive
TCCCGTCAGCAGTAGAGGCGCGAG	1	29	RDR2-sensitive
TCCCGTCGGCTTGGTCAGGTGGCC	16	0	RDR2-resistant
TCCCGTGATGGATTCGAACGTCGT	0	14	RDR2-sensitive
TCCCTACGCTGAGCCGGACGGTCC	0	9	RDR2-sensitive
TCCCTAGGACCGTAGCGTTAGCAT	0	13	RDR2-sensitive
TCCCTAGTCATCGGTACTCGGCAT	0	10	RDR2-sensitive
TCCCTCACCCTACCAGTAGACTGT	0	12	RDR2-sensitive
TCCCTCCAGGTGGGAGCGGTCGTC	15	0	RDR2-resistant
TCCCTCCCGTCAGCAGTAGAGACG	0	15	RDR2-sensitive
TCCCTCCCGTCAGCAGTAGAGGCA	0	21	RDR2-sensitive
TCCCTCCCGTCAGCAGTAGAGGCG	2	171	RDR2-sensitive
TCCCTCGAGGACGACGCTGCGGAC	0	39	RDR2-sensitive
TCCCTGAACTCGGCTCGAACGGCG	15	0	RDR2-resistant
TCCCTGACGGACTGCGGAGGCGAC	0	19	RDR2-sensitive
TCCCTGCCTGACGGTCGGACGGTC	0	13	RDR2-sensitive
TCCCTGCGACGGGTTCGGACGGTC	0	24	RDR2-sensitive
TCCCTGGACCAGCGGTACTTACTC	0	10	RDR2-sensitive
TCCCTGGACCGACGAGTAAATTGT	0	10	RDR2-sensitive
TCCCTGGATCTAGCGGACGGCTGT	0	16	RDR2-sensitive
TCCCTGGATGTAGTTGGTAGACGG	0	90	RDR2-sensitive
TCCCTGGATTGTAGCTTCGGTATA	0	31	RDR2-sensitive
TCCCTGGCGGACTGCGGAGGCGAC	1	13	RDR2-sensitive
TCCCTGGTCAGACGCGGACGGTCC	0	10	RDR2-sensitive
TCCCTGTAGTAGTGACGGGCACTC	0	14	RDR2-sensitive
TCCCTGTCTGACGAGCTGGACGGT	0	15	RDR2-sensitive
TCCCTTATGAAACAGCTGGGCATA	0	13	RDR2-sensitive
TCCCTTCCCTCCCGTCAGCAGTAG	0	10	RDR2-sensitive
TCCCTTCCGTCAGCAGTAGAGGCG	0	24	RDR2-sensitive
TCCGAAACGGTAGGAGGGGCTCGA	0	13	RDR2-sensitive
TCCGAACCCGGCACGGTAGAATAA	3	392	RDR2-sensitive
TCCGAACCCGGCATGGTAGAATAA	0	18	RDR2-sensitive
TCCGAACCGAAGTCAGATGGACAC	0	9	RDR2-sensitive
TCCGAACCGCCTGTGGAGACGACA	0	12	RDR2-sensitive
TCCGAACCGCGAACGTGGACCGTC	0	11	RDR2-sensitive
TCCGAACCTGGCACGGTAGAATAA	0	22	RDR2-sensitive
TCCGAACGAGCCTGAGCCGAGCCT	0	23	RDR2-sensitive
TCCGAACGGTCACGTAGAAAGCAT	0	34	RDR2-sensitive
TCCGAACTCGAACCGGCTCGTGAG	0	9	RDR2-sensitive
TCCGAAGCAGAATCGGGTCGGCCT	0	16	RDR2-sensitive
TCCGAAGCTGTACTATCCGGGTAT	2	22	RDR2-sensitive

TCCGAAGCTGTATTATCCGGGTAT	0	10	RDR2-sensitive
TCCGAAGGACGACGTCAGCACCAC	0	11	RDR2-sensitive
TCCGAAGGTAGAAGTTTGCCGGAA	40	5	RDR2-resistant
TCCGAAGTCGGGCAGGCATGGATT	59	4	RDR2-resistant
TCCGAATGGGCTGTCTGATAGGCT	0	94	RDR2-sensitive
TCCGAATTGTGATCGCGCGGACAA	0	38	RDR2-sensitive
TCCGACAGACTAGCTATATGACTC	0	11	RDR2-sensitive
TCCGACATCGGGCGACGGATCCGG	0	16	RDR2-sensitive
TCCGACCACTTCGCACGTAGACAG	0	22	RDR2-sensitive
TCCGACCGTCGACGGCATCCTCAG	0	17	RDR2-sensitive
TCCGACCTGTAGCGAGATGGGACA	0	17	RDR2-sensitive
TCCGACGACGAGGACGCCGCCAAC	77	19	RDR2-resistant
TCCGACGAGGACGCCACCAACATC	19	1	RDR2-resistant
TCCGACGCTGAGAGGACACCACAG	0	9	RDR2-sensitive
TCCGACGGATAAACGAGGACGGAT	0	11	RDR2-sensitive
TCCGACGGCCTACACTGTTTCCAA	0	9	RDR2-sensitive
TCCGACGGGCTAACTTACGACGGT	1	31	RDR2-sensitive
TCCGACGGGTAGACGAGAAAGCAT	0	10	RDR2-sensitive
TCCGACGGTAGGAGCGTAGGCCCT	24	0	RDR2-resistant
TCCGACTACGACGAGCAAGCCCGA	1	12	RDR2-sensitive
TCCGACTACGACGAGCAAGCCTGA	0	9	RDR2-sensitive
TCCGACTACTGCATATGGCTCGAA	0	11	RDR2-sensitive
TCCGACTCTGTAGCCAAACGGTGT	0	10	RDR2-sensitive
TCCGACTGCGGCTGCGGCTTCTAC	0	31	RDR2-sensitive
TCCGACTGCTGTGCCACTCGACGG	0	18	RDR2-sensitive
TCCGACTGGTGTTCGCGGCGACAA	0	11	RDR2-sensitive
TCCGACTGTTCCGACGGAAGCCGC	3	29	RDR2-sensitive
TCCGACTGTTCCGACGGTAGCCGC	14	70	RDR2-sensitive
TCCGACTGTTCCGACGGTAGCCGT	3	16	RDR2-sensitive
TCCGACTGTTCCGATGGAAGCCGC	4	17	RDR2-sensitive
TCCGACTTCGAGCGCGATGGATAG	0	21	RDR2-sensitive
TCCGAGACCAACCCTGGACGGAAA	0	9	RDR2-sensitive
TCCGAGACCAGCGGGAACGATCTT	0	17	RDR2-sensitive
TCCGAGACCAGCGGGAGCGATCTT	0	10	RDR2-sensitive
TCCGAGCACGGCTAGACCCGACAC	0	10	RDR2-sensitive
TCCGAGCCCGGCACGATAGAATAA	0	13	RDR2-sensitive
TCCGAGCCCGGCACGGTAGAATAA	0	127	RDR2-sensitive
TCCGAGCCGATTCTTGGTCGTCGT	0	13	RDR2-sensitive
TCCGAGCTCGGCGCCATAGATCTT	0	10	RDR2-sensitive
TCCGAGCTTGGACAGTACATCGGC	14	0	RDR2-resistant
TCCGAGGAATAACAGCGTAGACGG	0	21	RDR2-sensitive
TCCGAGGACCGAGGACATCGACGG	0	17	RDR2-sensitive
TCCGAGGCTTTTGCCGATTGTCGG	0	9	RDR2-sensitive
TCCGAGTATACGGGCTGAACGGGC	1	64	RDR2-sensitive
TCCGAGTTGGAAGTATGCAGGAGC	0	15	RDR2-sensitive
TCCGATACCAGCTGTGACGGAACC	2	14	RDR2-sensitive
TCCGATACCAGCTGTGGCAGAACC	4	20	RDR2-sensitive
TCCGATACGTTGAGGATGGAACAT	0	10	RDR2-sensitive
TCCGATCCAGGCAATTTGTCGAGC	0	11	RDR2-sensitive

TCCGATCCGAGATAAAGTGGGCAT	0	70	RDR2-sensitive
TCCGATCGAAGTTGTGGGCGAGGC	1	23	RDR2-sensitive
TCCGATCTGGGCGGCGTATCCTGC	0	12	RDR2-sensitive
TCCGATCTTCCCGGGATGTCGAGT	0	26	RDR2-sensitive
TCCGATGAAGTTGGATTAAAGGTT	46	12	RDR2-resistant
TCCGATGCCCTACTGTAGACGGTC	0	25	RDR2-sensitive
TCCGATGGGAACCGACGGACACAC	4	25	RDR2-sensitive
TCCGATTGAGGTGTTCTTTGTGGC	42	0	RDR2-resistant
TCCGCAAAATGGGCTGACAGGCAA	0	12	RDR2-sensitive
TCCGCAACGGGAAACTGGACGGTC	0	13	RDR2-sensitive
TCCGCAACTGTGGGCCGGACGGTC	0	23	RDR2-sensitive
TCCGCAATCTGGGCGCAAGAACGG	0	14	RDR2-sensitive
TCCGCACCTGTGGGCCGGACGGTC	0	13	RDR2-sensitive
TCCGCAGCTCTGGGCCGGACGGTC	0	27	RDR2-sensitive
TCCGCAGTCTAGAACCGGACGGTC	0	16	RDR2-sensitive
TCCGCATGCAGACTGTGACAGCTT	0	9	RDR2-sensitive
TCCGCATTTTGCACGGTCTGAGGC	0	12	RDR2-sensitive
TCCGCCATCTAGCGACGTCTTCGG	0	11	RDR2-sensitive
TCCGCCCCCTGCGGTACAGATCGT	0	9	RDR2-sensitive
TCCGCCCCTGTAGATCAACGGCTG	2	22	RDR2-sensitive
TCCGCCCCTGTAGATCAACGGCTT	1	52	RDR2-sensitive
TCCGCCTGGCTCCCTGGAAACGGC	0	16	RDR2-sensitive
TCCGCGACACAGGGTCTGACGGTC	0	9	RDR2-sensitive
TCCGCGACCTGACGACGAAAGCAC	0	15	RDR2-sensitive
TCCGCGACCTGACGCAGGGGCTAG	0	10	RDR2-sensitive
TCCGCGACCTGGCGCAGAAGCTGA	0	18	RDR2-sensitive
TCCGCGACGAATTCGGCAACCATC	0	12	RDR2-sensitive
TCCGCGACTGAGGAGAAGCGACGG	0	15	RDR2-sensitive
TCCGCGACTGTAAGCGATGCGCAT	0	11	RDR2-sensitive
TCCGCGACTGTGGGCCGGACGGTC	0	11	RDR2-sensitive
TCCGCGACTTAGGCACAGAAGCAG	1	18	RDR2-sensitive
TCCGCGAGGTGGACTGTTCATGGC	0	9	RDR2-sensitive
TCCGCGATATGGTTGATCGGCGAA	16	0	RDR2-resistant
TCCGCGATCTAGGGACGGACGGTC	1	17	RDR2-sensitive
TCCGCGCATGAGAACAGCAGGACA	0	11	RDR2-sensitive
TCCGCGCATGTGTAGGGACGACGA	0	23	RDR2-sensitive
TCCGCGCCTAGGGCTTGGACGGTC	0	12	RDR2-sensitive
TCCGCGCCTGGGGCTTAAACGGTT	0	10	RDR2-sensitive
TCCGCGCCTGTGGACCGGACGGTC	0	9	RDR2-sensitive
TCCGCGCCTGTGGGCCAGACGGTC	0	11	RDR2-sensitive
TCCGCGCGCTGTAGAAGGCACCGT	1	107	RDR2-sensitive
TCCGCGCTCTGGAACTGACGGTCT	0	25	RDR2-sensitive
TCCGCGCTCTGGATCGGACGGTCC	0	10	RDR2-sensitive
TCCGCGCTCTGGGACTGGACGGTC	0	14	RDR2-sensitive
TCCGCGGAATCCGGCGCTGAAGAT	3	21	RDR2-sensitive
TCCGCGGACCCAAGATAAGGAGGT	0	9	RDR2-sensitive
TCCGCGGATCACGCCTATCGTCGT	0	32	RDR2-sensitive
TCCGCGGCACAGGGTCGGACGGTC	0	42	RDR2-sensitive
TCCGCGGGATTGTAATGTCAGCAT	0	9	RDR2-sensitive

TCCGCGGTCCGGACTGTCCACGGT	0	15	RDR2-sensitive
TCCGCGGTCTGAGTGCAGGAGCGA	0	29	RDR2-sensitive
TCCGCGTCTGTGGGCCGGACGGTC	1	42	RDR2-sensitive
TCCGCGTGCAGGGTCTGGACGGTC	0	17	RDR2-sensitive
TCCGCGTGTGTGCAGGACGATGGC	0	13	RDR2-sensitive
TCCGCGTTTACGTAGGCAGAGCGT	0	11	RDR2-sensitive
TCCGCTGACGGACGACACGGCTAG	1	14	RDR2-sensitive
TCCGCTGCTGTACTGGGTCTTCGG	0	23	RDR2-sensitive
TCCGGAACCTGAACCAGTGGGTAC	0	16	RDR2-sensitive
TCCGGAAGAGTAGGTACGCGGCAC	0	11	RDR2-sensitive
TCCGGAATAGTCGGGAACGGAAAC	0	14	RDR2-sensitive
TCCGGACAAACGCAGAGAAGGAAC	0	23	RDR2-sensitive
TCCGGACAAGTAGCGCATTTCCGA	0	11	RDR2-sensitive
TCCGGACAGGAAACTAAGCACGGC	0	15	RDR2-sensitive
TCCGGACAGTCTGCGCAGAGCCGA	0	11	RDR2-sensitive
TCCGGACCGAGCCACCAGTGACGC	0	14	RDR2-sensitive
TCCGGACGCAGCCTGCACGCGCGG	0	14	RDR2-sensitive
TCCGGACGGCTAAGCCCACACTGG	1	16	RDR2-sensitive
TCCGGACGGGTAAGAAACGGATAA	0	18	RDR2-sensitive
TCCGGACGGTCCGCGCCTGTGGGC	1	12	RDR2-sensitive
TCCGGACGGTCTGCAGGAGTAGTC	0	9	RDR2-sensitive
TCCGGACGGTTTGACTAGGGCTAG	0	12	RDR2-sensitive
TCCGGACGTACATAGAGAAGGAGC	0	9	RDR2-sensitive
TCCGGACTAGCCCGATAAACACGG	0	10	RDR2-sensitive
TCCGGACTCAAGACGGGACGGTCT	0	9	RDR2-sensitive
TCCGGATATAACTCCCGAGGACGA	0	30	RDR2-sensitive
TCCGGATCTGATGGGTGCCGCGGC	0	11	RDR2-sensitive
TCCGGATCTGCTTGGAGGCGTCGC	5	24	RDR2-sensitive
TCCGGATCTGCTTGGAGGCGTCGT	1	21	RDR2-sensitive
TCCGGATCTGGATTCGAAGATGGT	0	10	RDR2-sensitive
TCCGGATGCAGCCTGCACGCGCGA	0	25	RDR2-sensitive
TCCGGATGTACGCAGCAGGCCTGG	0	18	RDR2-sensitive
TCCGGATGTTACTCAAGCGTCGAC	0	17	RDR2-sensitive
TCCGGATTTGAAAATAGGCTGGTG	0	13	RDR2-sensitive
TCCGGCACGGCCTGTATTCGGCAC	0	32	RDR2-sensitive
TCCGGCACGGTAGAATAAGCGGGC	2	145	RDR2-sensitive
TCCGGCAGAAACGAACAAGGCCTT	0	12	RDR2-sensitive
TCCGGCAGCGGCTCTAGTATCTCC	0	28	RDR2-sensitive
TCCGGCATGTTTGGGAGTTAGCTC	0	10	RDR2-sensitive
TCCGGCCCTGAGGACTAGACGGTC	0	10	RDR2-sensitive
TCCGGCGTACGGCGTCTCCGACAA	0	25	RDR2-sensitive
TCCGGCTAGTAGGGGTGAAAACGG	0	45	RDR2-sensitive
TCCGGCTGCGGCTGCGGCTTCTAC	0	34	RDR2-sensitive
TCCGGGAATGTAGCAGGTAACCGG	0	12	RDR2-sensitive
TCCGGGACGGATACAGGACGGGAT	0	38	RDR2-sensitive
TCCGGGAGCCTGGCTGACGCGAGC	0	22	RDR2-sensitive
TCCGGGCAAGAAACGACGGATCAA	0	12	RDR2-sensitive
TCCGGGCAGGTAAGAAACGGATAA	0	9	RDR2-sensitive
TCCGGGCGGAAGACATTGTCAGGT	71	23	RDR2-resistant

TCCGGGCGGATAAGTGACGGATAC	1	20	RDR2-sensitive
TCCGGGCTAGCACGATACAGTGGA	0	9	RDR2-sensitive
TCCGGGCTCTCGGACGGACGGTGT	0	37	RDR2-sensitive
TCCGGGCTGATTTGGTGACAAGGC	0	19	RDR2-sensitive
TCCGGGCTGTGAGAGGAGGGGAAA	0	11	RDR2-sensitive
TCCGGGGCTGAGTTGCAGAGACAG	0	11	RDR2-sensitive
TCCGGGTCCTGTTTTATAGACGTC	13	52	RDR2-sensitive
TCCGGGTTCTGTTTTATAGACGTC	0	10	RDR2-sensitive
TCCGGTGACAACAGTAGTGCGCGT	0	9	RDR2-sensitive
TCCGGTGACGACAGCATGACACAG	0	9	RDR2-sensitive
TCCGGTGACTGTCCGGCCGAACGG	2	49	RDR2-sensitive
TCCGGTGCGACGTAGAGAAGACGC	0	15	RDR2-sensitive
TCCGGTTAGGACTGCAGGAGTGGT	0	10	RDR2-sensitive
TCCGGTTCGACGCAGGGAAGACAT	0	13	RDR2-sensitive
TCCGGTTCTATGCGGTCTCGGGCC	0	9	RDR2-sensitive
TCCGTAACACAGGGTCGGACGGTC	0	22	RDR2-sensitive
TCCGTAACAGACGACTGGGATCGC	0	12	RDR2-sensitive
TCCGTACGATGGGACGCATGATTC	0	9	RDR2-sensitive
TCCGTACGGGCCGACAGCATGGAC	0	10	RDR2-sensitive
TCCGTCAAGTCGGCAGGTCTTTGG	0	9	RDR2-sensitive
TCCGTCAGTAGCTGTAGCCCGGCC	0	17	RDR2-sensitive
TCCGTCGAGCTGAGCCGAGTAGAG	0	13	RDR2-sensitive
TCCGTCGTTTACTGTATCGTGCAA	0	14	RDR2-sensitive
TCCGTCTTTGTTGCCGGTCGTCGT	0	13	RDR2-sensitive
TCCGTGACATAGGGTCGGACGGTC	0	10	RDR2-sensitive
TCCGTGATCTGGACGCAGGAGCGA	0	29	RDR2-sensitive
TCCGTGATCTGGTGTAGGGGCTAT	0	10	RDR2-sensitive
TCCGTGCACGGACTGATCCGGCCT	0	27	RDR2-sensitive
TCCGTGGAGAACTGAGTGACGCGC	0	20	RDR2-sensitive
TCCGTGGATTTGGGCCGGACGGTC	0	9	RDR2-sensitive
TCCGTGGCACAAGGTCGGACGGTC	0	13	RDR2-sensitive
TCCGTGGCCTAGAGTCGGACGATC	0	10	RDR2-sensitive
TCCGTGGCTCTGGACCGGATGGTT	0	15	RDR2-sensitive
TCCGTGTCTAGGACTCGGACGGTC	0	40	RDR2-sensitive
TCCGTGTCTAGGGCTTGGACGGTC	0	10	RDR2-sensitive
TCCGTGTCTGAAACTGATGTACGT	0	15	RDR2-sensitive
TCCGTGTCTGACGCAGAGGATCAT	0	76	RDR2-sensitive
TCCGTTCGAGCGGACTCGATGGCT	0	16	RDR2-sensitive
TCCGTTCTGGTAGAATAGCCCGGC	0	10	RDR2-sensitive
TCCGTTGCATTGGTCGGGTCGGAT	0	18	RDR2-sensitive
TCCGTTGTAGTCTAGTTGGTCAGG	49	2	RDR2-resistant
TCCGTTTACTGTAGTGCACGGCCT	0	28	RDR2-sensitive
TCCGTTTACTGTAGTGGAGGCTAA	0	12	RDR2-sensitive
TCCGTTTACTGTAGTGGAGGGCAG	0	10	RDR2-sensitive
TCCTAAACACGGCTGGTAACCACC	0	9	RDR2-sensitive
TCCTAACATCGCACCGGACGGTCC	0	10	RDR2-sensitive
TCCTAACCATGCCTGGCGGGTCGG	0	9	RDR2-sensitive
TCCTACACCTAGGTCGTGGACTGT	0	12	RDR2-sensitive
TCCTACGTCGTGAAGATCGGGCAT	4	84	RDR2-sensitive

TCCTACTATCGCTGCGACTCGGCC	1	12	RDR2-sensitive
TCCTACTGGGCTGATTTGGTGGAC	0	9	RDR2-sensitive
TCCTAGAACTGTCTGTATAGAGGC	0	9	RDR2-sensitive
TCCTAGAATCAGGGATGGAACGGC	1	14	RDR2-sensitive
TCCTAGAATCGGGGATGGAACGGC	0	9	RDR2-sensitive
TCCTAGACTGTAGATGGCGAGCAA	0	9	RDR2-sensitive
TCCTAGACTGTCTCGGCAACACAG	0	10	RDR2-sensitive
TCCTAGCACTGGCGGAACTGAGAA	0	9	RDR2-sensitive
TCCTAGGCTCCTAGCAGTCGGCGT	0	10	RDR2-sensitive
TCCTAGGGTCGTCTTGGGATCGGC	1	19	RDR2-sensitive
TCCTAGGGTGTGCCTTGGCGTCGG	0	20	RDR2-sensitive
TCCTAGGGTGTGTCTTGGCGTCGG	1	12	RDR2-sensitive
TCCTAGGTGTTGCCTAGGGTCGGC	0	9	RDR2-sensitive
TCCTAGTCGGTGTAGACGTGCGGT	0	44	RDR2-sensitive
TCCTATATCGCATGGATTGGATGG	0	11	RDR2-sensitive
TCCTATTAGTTGTCGTTTAGGACA	0	11	RDR2-sensitive
TCCTCAGACTGTCAGCAATGGCAT	0	37	RDR2-sensitive
TCCTCATCCATCGGGTTTCGGCGT	0	9	RDR2-sensitive
TCCTCATCTTAGGATTTAGGGCGG	0	15	RDR2-sensitive
TCCTCATGCTGAGTTGGACACCGT	0	11	RDR2-sensitive
TCCTCCAACGCGGTCGTCGACGGC	0	13	RDR2-sensitive
TCCTCCCGTCAGCAGTAGAGACGC	0	26	RDR2-sensitive
TCCTCCCGTCAGCAGTAGAGGCGC	11	102	RDR2-sensitive
TCCTCCCGTCAGCAGTAGAGGCGT	1	13	RDR2-sensitive
TCCTCGACTGTTTCTCCGGTTACC	21	2	RDR2-resistant
TCCTCGAGCGGCTCTGTTAGGCAT	0	16	RDR2-sensitive
TCCTCGATCCGGAGTAGTGGCGGC	0	28	RDR2-sensitive
TCCTCGGATGAGGAGGACTCGGAC	20	0	RDR2-resistant
TCCTCGGCCTGGCAGGAGCAGGTT	0	9	RDR2-sensitive
TCCTCGGTCATGAATCCTTGCAGA	23	0	RDR2-resistant
TCCTCGTCCTCCACCGTGATCGTC	18	0	RDR2-resistant
TCCTCTATCGTTCGGTAGGCTGGC	0	12	RDR2-sensitive
TCCTCTGAATTTGTAGAGCAGGTT	18	0	RDR2-resistant
TCCTCTGACGTGGGGGGTGGGCAC	0	10	RDR2-sensitive
TCCTCTGGTCAGGACTGCGGACGG	0	11	RDR2-sensitive
TCCTCTGTGGTTATATGTGGCAGC	29	1	RDR2-resistant
TCCTGACGAAGCAAGGGCTGTGAC	0	15	RDR2-sensitive
TCCTGACGACTGTACAGATTGCGT	0	11	RDR2-sensitive
TCCTGACGCGTAGGTTCGGCCTGG	0	43	RDR2-sensitive
TCCTGAGATCGCCGAGATGAGCAT	0	9	RDR2-sensitive
TCCTGAGATGGCTGAGATCGACGA	0	11	RDR2-sensitive
TCCTGAGCGCGCTGTCGGGCGGCC	0	18	RDR2-sensitive
TCCTGATAAGGCTCATGATGTGTT	376	0	RDR2-resistant
TCCTGATCATCGTACATCTTCGGC	51	16	RDR2-resistant
TCCTGATCCTCGTGAGGAGCTGAT	0	9	RDR2-sensitive
TCCTGATCTCGACAGCCTGGCGGT	0	22	RDR2-sensitive
TCCTGATCTCGGCAGCCTGGCGGT	0	31	RDR2-sensitive
TCCTGATGATGGTAGAGAATTGTT	18	0	RDR2-resistant
TCCTGCACCAGGTCGCGGACCGTC	0	10	RDR2-sensitive

TCCTGCACGTCCGGATACAGGCAT	0	9	RDR2-sensitive
TCCTGCCTGACGTCCGGACGGTCC	0	9	RDR2-sensitive
TCCTGCGATACGGTCGGTCGGCGT	4	43	RDR2-sensitive
TCCTGCGATACGGTCGGTCGTCGT	0	9	RDR2-sensitive
TCCTGCGATACGGTCGGTTGGCGA	0	15	RDR2-sensitive
TCCTGCGATACGGTCGGTTGGCGT	5	63	RDR2-sensitive
TCCTGCGATATGGTCGGTTGGCGT	0	9	RDR2-sensitive
TCCTGCGATTTGGTCGGTCGGCGC	1	26	RDR2-sensitive
TCCTGCGATTTGGTCGGTCGGTGC	2	29	RDR2-sensitive
TCCTGCGCGTCCGGATACAGGCAT	0	11	RDR2-sensitive
TCCTGCTAGCTGAACGAGGATGAG	0	9	RDR2-sensitive
TCCTGCTCGGATAGTCGGATTCCT	0	9	RDR2-sensitive
TCCTGCTGTAGGAAACCGCGTGCC	21	0	RDR2-resistant
TCCTGGACACCAGGATGACGAGGA	23	2	RDR2-resistant
TCCTGGACCTGTAGAATACCGGAT	0	12	RDR2-sensitive
TCCTGGACGCGTAGGTTCGGCCTG	0	11	RDR2-sensitive
TCCTGGACTTTGCCGAGTGCCGGA	0	17	RDR2-sensitive
TCCTGGATGAGACGTGACAGACAA	3	24	RDR2-sensitive
TCCTGGCAGAACCGTGCACCATAT	0	22	RDR2-sensitive
TCCTGGCTCTAGAGATGCGACCAA	0	30	RDR2-sensitive
TCCTGGGACCTCCGAAGCTGCCGG	0	13	RDR2-sensitive
TCCTGGGACCTTCGAAGCTGCCGG	1	13	RDR2-sensitive
TCCTGGGCTGCAACTAGGCGTGTT	1	21	RDR2-sensitive
TCCTGGGCTGGCATCTCGACGGGA	0	14	RDR2-sensitive
TCCTGGTTAACAAGATTCTCAGAC	15	0	RDR2-resistant
TCCTGTAATAGAGCGACGTTCAAC	30	2	RDR2-resistant
TCCTGTACAAGATGAGCTCGGCCC	0	10	RDR2-sensitive
TCCTGTAGATCTGTTGGCACCGGC	1	12	RDR2-sensitive
TCCTGTAGCCACTAAAGCCGACGG	0	21	RDR2-sensitive
TCCTGTAGCGAGCCGAGCCGAGCC	0	10	RDR2-sensitive
TCCTGTAGCGGGACGGACACGCAT	0	10	RDR2-sensitive
TCCTGTAGTGACTAACAGTGAGGC	44	10	RDR2-resistant
TCCTGTAGTGTTGGCACGGCTTGT	0	11	RDR2-sensitive
TCCTGTCAGCAGTAGAGGCGCAAG	0	12	RDR2-sensitive
TCCTGTCCTCGTCGAATACGTCGT	0	18	RDR2-sensitive
TCCTGTCGACAAGTCGGACGGTTT	0	28	RDR2-sensitive
TCCTGTCTGACGGCCGGACGGTCC	0	10	RDR2-sensitive
TCCTGTGATACGGTCGGTTGGCGT	1	12	RDR2-sensitive
TCCTGTTTGACTGCGGCGTGTGGA	0	9	RDR2-sensitive
TCCTTAACACAAGACGCTGGGCAC	1	16	RDR2-sensitive
TCCTTAAGATGGCAGTGTAGGCGT	0	13	RDR2-sensitive
TCCTTACGCCGGCTGTAGGGGCAC	0	11	RDR2-sensitive
TCCTTAGATTGAAGGCTCGGTCGT	0	10	RDR2-sensitive
TCCTTAGTGGGCTGGGCTGGGCTA	0	13	RDR2-sensitive
TCCTTATTTGTGAGATCGAGGACA	24	2	RDR2-resistant
TCCTTCGCGGGATTGCAGAAGCGG	0	16	RDR2-sensitive
TCCTTCGGTCGTAGCGCGAGATGG	1	16	RDR2-sensitive
TCCTTCGGTTCGGTTAGTTCGGTT	17	98	RDR2-sensitive
TCCTTCGTGGGATTGCAGAAGCGG	0	10	RDR2-sensitive

TCCTTGGACTACGACGAACACGGA	0	16	RDR2-sensitive
TCCTTGGACTACGGCGAACACGGA	0	46	RDR2-sensitive
TCCTTGGCTAGTAGGAGTCGTGGT	57	1	RDR2-resistant
TCCTTGGTACAGAACTTCCGGCCT	0	10	RDR2-sensitive
TCCTTGGTAGAAGTGTGGAATGAT	77	6	RDR2-resistant
TCCTTGTTGGACGGAGAAAGGAGG	0	11	RDR2-sensitive
TCCTTGTTGGACGGAGAGAGGAGA	0	11	RDR2-sensitive
TCCTTGTTGGACGGATAAAGGAGC	0	23	RDR2-sensitive
TCCTTGTTGGACGGCGTAGAGAAC	1	25	RDR2-sensitive
TCCTTGTTGGAGGTATACAAGAGC	0	14	RDR2-sensitive
TCCTTTACACAGTAGGATTGTCGG	0	18	RDR2-sensitive
TCCTTTCACCAAGTTGCTCGGACA	23	3	RDR2-resistant
TCCTTTGCAGACGACTTAAAACGC	15	0	RDR2-resistant
TCCTTTGGAGGAGTTAGAGCCGGA	0	11	RDR2-sensitive
TCCTTTTTTGCATCGCGAGACACA	0	9	RDR2-sensitive
TCCTTTTTTTGGTGGTCGTCTTGG	15	0	RDR2-resistant
TCGAAAAAGCGGCTCGGCTCGGCT	0	14	RDR2-sensitive
TCGAAAACCTGACTCGACTCGGCT	43	0	RDR2-resistant
TCGAAACACTAGGGACCTCGGCGC	0	10	RDR2-sensitive
TCGAACACAACTGTGTCAGGCTAC	14	0	RDR2-resistant
TCGAACATAGAGCTAAGACACTGT	0	10	RDR2-sensitive
TCGAACCCAGGACCTGAGGAGTGC	3	25	RDR2-sensitive
TCGAACCGGCTCGACGCTCAGAAT	1	17	RDR2-sensitive
TCGAACCGTAGACCGTCCGGCCTC	0	15	RDR2-sensitive
TCGAACGAGTCGTACTGCCATCAT	0	11	RDR2-sensitive
TCGAACGGCAAGGGCTCCGGAGGC	0	9	RDR2-sensitive
TCGAACGGCAAGGGCTTCGGAGGC	0	16	RDR2-sensitive
TCGAACTAGCGTTAGGACATTCTA	70	10	RDR2-resistant
TCGAACTCGAGTAGGGGAAGGCGC	0	11	RDR2-sensitive
TCGAACTCTATTGGACATGGCAAG	21	2	RDR2-resistant
TCGAAGACGAACGTAGAAGCGCAG	0	11	RDR2-sensitive
TCGAAGCAGCGGAGCCGATGACGT	0	11	RDR2-sensitive
TCGAATAAACTGACTGGCTGATGG	24	1	RDR2-resistant
TCGAATTCTTGAGAAATTGGGGTG	18	0	RDR2-resistant
TCGACACAGGACCACACTCGGCAA	1	16	RDR2-sensitive
TCGACACAGGACGGCACTCGGCAA	0	9	RDR2-sensitive
TCGACACATCTGATCGGATATTAT	0	11	RDR2-sensitive
TCGACACGACTAGCAGGCTGACGT	0	31	RDR2-sensitive
TCGACACGACTCTCGGCAACGGAT	126	30	RDR2-resistant
TCGACAGAGTGAGGCTGACAGCAG	3	45	RDR2-sensitive
TCGACATAAAACGGCTCTCGGCAA	0	9	RDR2-sensitive
TCGACATCAGAGCCCAATGACGAC	0	10	RDR2-sensitive
TCGACATCGACAGACACTCGGCAA	0	16	RDR2-sensitive
TCGACCACGTTCGGCTCGTCTGGC	0	51	RDR2-sensitive
TCGACCCGATGAGGATCGTCGGCG	20	0	RDR2-resistant
TCGACCGCGGACGGTAAAACACGG	0	16	RDR2-sensitive
TCGACGAAATAGAGGCTCAGGCGA	1	25	RDR2-sensitive
TCGACGAAATAGAGGCTCAGGCGG	0	11	RDR2-sensitive
TCGACGAAATGGAGGCGATGACGT	0	24	RDR2-sensitive

TCGACGAACTAAGACTTTCGGCAT	0	26	RDR2-sensitive
TCGACGACTGGCAGAAGGACCGTC	0	12	RDR2-sensitive
TCGACGACTGTACAGATTGCGCCA	1	13	RDR2-sensitive
TCGACGACTGTAGCAAAATGGTTC	0	23	RDR2-sensitive
TCGACGAGCAGACTGGGCGAGCAC	0	9	RDR2-sensitive
TCGACGAGGAGCCAACGAAGGATC	27	0	RDR2-resistant
TCGACGATTTAAAGGCGTAGACGA	0	9	RDR2-sensitive
TCGACGCTCTGGGTGGCCGGTATG	0	14	RDR2-sensitive
TCGACGGGACTTATTTTCGATGGC	0	9	RDR2-sensitive
TCGACGGGCTCTGCATGGGGGTGT	0	10	RDR2-sensitive
TCGACGGGGCTCGGGGGAGGATGC	87	2	RDR2-resistant
TCGACGGTTTTATCACGGGCCCAC	0	10	RDR2-sensitive
TCGACGTACAGAGACACTCGGCAT	0	20	RDR2-sensitive
TCGACGTACTGTGACGGTGGGGAT	0	15	RDR2-sensitive
TCGACGTAGAGCCGAAGAGAGGTG	0	19	RDR2-sensitive
TCGACGTGGAACCGAGCTCGGCGC	10	94	RDR2-sensitive
TCGACGTGGAAGACTCGGTGGCAA	0	10	RDR2-sensitive
TCGACGTTTTGTAGGCGTAGGCGA	0	18	RDR2-sensitive
TCGACTCAGACGGAGACACTACAC	8	29	RDR2-sensitive
TCGACTCGGTTTAGAACGGCTCGC	0	10	RDR2-sensitive
TCGACTCGTGGAAACCGGCCGGTT	0	14	RDR2-sensitive
TCGACTCGTGGATCCGATCGGCAA	0	39	RDR2-sensitive
TCGACTCGTGGATCCGATCGGCAT	0	17	RDR2-sensitive
TCGACTCGTGGATCTGATTGGCAA	0	10	RDR2-sensitive
TCGACTCTCCCCAAGGACGTTCAT	22	2	RDR2-resistant
TCGACTGCTGTGGACAGAAGCTGG	0	9	RDR2-sensitive
TCGACTGTCCGGAATACCGTAGAT	2	22	RDR2-sensitive
TCGACTTGATGTGCTTCGTTTGGT	132	1	RDR2-resistant
TCGAGAAACGGTATCTGTTTACAC	18	0	RDR2-resistant
TCGAGACAAGGACGAGGATGACAA	0	11	RDR2-sensitive
TCGAGACGTCGAGACGGTATGGGC	0	20	RDR2-sensitive
TCGAGACGTGCATCACATGAGCAG	0	36	RDR2-sensitive
TCGAGACTTCGGATGGCCGCTAGG	30	1	RDR2-resistant
TCGAGAGACCTGACTGACGAGCGA	0	9	RDR2-sensitive
TCGAGAGCCAGGCTGTGCGCGTGC	0	10	RDR2-sensitive
TCGAGATCTACGGGCCCGAGGCAC	0	44	RDR2-sensitive
TCGAGATCTACGGGCCCGAGGCAT	0	28	RDR2-sensitive
TCGAGATGTGCGACGAGCTGGGGC	16	0	RDR2-resistant
TCGAGCAACGGGCAACGGATGAGG	47	1	RDR2-resistant
TCGAGCAGACTGTGTTGCCGGTAT	0	10	RDR2-sensitive
TCGAGCCCGGCACGGTATGAACAA	0	17	RDR2-sensitive
TCGAGCCGAGCCAAGTAGAGCCGA	0	66	RDR2-sensitive
TCGAGCCGGACCAGGTCCAGACGT	1	33	RDR2-sensitive
TCGAGCGACTAAGGTTAGGCAGAT	0	9	RDR2-sensitive
TCGAGCGGACGGTGTCGCCAATGT	0	9	RDR2-sensitive
TCGAGCGGGCGGCTGCACATGCAT	0	10	RDR2-sensitive
TCGAGCTACAGTCGGTAACACCAT	0	9	RDR2-sensitive
TCGAGCTGAGCCGAGTAGAGCCGA	0	14	RDR2-sensitive
TCGAGCTTCTTCTTCACCGTCGTC	29	0	RDR2-resistant

TCGAGGAACGGAACACGGGCGCGC	0	11	RDR2-sensitive
TCGAGGAAGACGTGATGATGACCT	14	0	RDR2-resistant
TCGAGGAATTTCAGGCATTTAATC	15	0	RDR2-resistant
TCGAGGACTCGCAGGCACAACTAA	0	10	RDR2-sensitive
TCGAGGACTGTGGCGTCATCTTGG	0	9	RDR2-sensitive
TCGAGGACTTCAGCTGAAGGCTGT	0	27	RDR2-sensitive
TCGAGGACTTCAGCTGAAGGTCGT	0	24	RDR2-sensitive
TCGAGGCGGATTAAGGCATCCTGG	21	2	RDR2-resistant
TCGAGGGACCTGACTGACGAGCGG	0	17	RDR2-sensitive
TCGAGTAGGGCCGGCTGACTCCGT	0	11	RDR2-sensitive
TCGAGTAGTGCTCAGGGTCGTCGA	0	32	RDR2-sensitive
TCGAGTCAGCTGCTGAAACGGTAA	0	43	RDR2-sensitive
TCGAGTCAGCTGTTGAAACGGTAA	2	20	RDR2-sensitive
TCGAGTCAGCTGTTGGAACGGTAA	0	17	RDR2-sensitive
TCGAGTGTAGCCGAAGCTGTGCGC	0	13	RDR2-sensitive
TCGATAACGTTAGGCACTCGGCAA	0	17	RDR2-sensitive
TCGATAACTCGCTCGACTCGGCTA	0	10	RDR2-sensitive
TCGATAACTCGCTCGACTCGGCTC	4	56	RDR2-sensitive
TCGATAACTCGCTCGACTCGGCTT	1	26	RDR2-sensitive
TCGATAGACTGAGGAGAGGAGGAT	0	11	RDR2-sensitive
TCGATCACTTAGCGAGCTCGGTAA	0	46	RDR2-sensitive
TCGATCCACGTGGAACATCCCGGT	0	12	RDR2-sensitive
TCGATCCTTTAGACCTTCGGAGTT	21	1	RDR2-resistant
TCGATCGAATAGGACGACGAGCAT	0	11	RDR2-sensitive
TCGATCTACTTTACAACCGGCATA	36	1	RDR2-resistant
TCGATCTCAGATTCGTCGGTCGCA	26	3	RDR2-resistant
TCGATCTCTGAGTAGCTACGGTTT	0	12	RDR2-sensitive
TCGATCTGTCATCGGACGACGCAC	0	20	RDR2-sensitive
TCGATGACTCGCCAGAACGACGCG	0	9	RDR2-sensitive
TCGATGATGAGGCTCGGCTCGGCT	0	9	RDR2-sensitive
TCGATGCAACTTGACGGATTCGAT	0	9	RDR2-sensitive
TCGATGCGATTGTGGAGACCGTGC	0	10	RDR2-sensitive
TCGATGGACGGCAGAACGGCAGAA	0	17	RDR2-sensitive
TCGATGGCATACTGGTGAAGCTCA	0	11	RDR2-sensitive
TCGATGTCGGTCCCTACGTGATGC	0	18	RDR2-sensitive
TCGATGTGACGTAGATGTTGTGGC	0	20	RDR2-sensitive
TCGATGTGGAGGGCGCTGCGCAGA	15	0	RDR2-resistant
TCGATGTGTAGTGTGATGGTCTGG	27	2	RDR2-resistant
TCGATTACATGGCTGAGCGTTGGT	21	0	RDR2-resistant
TCGATTGGTTGTCTACTTGGTGTA	116	4	RDR2-resistant
TCGATTGTTAGCAGTATGTTGATT	15	0	RDR2-resistant
TCGATTTGGGCCGTGTAGTGGGCT	1	14	RDR2-sensitive
TCGCAAACTCTGGGCGACGTGCGA	0	9	RDR2-sensitive
TCGCAACCTAGGACTCAGCACGGT	0	10	RDR2-sensitive
TCGCAACTCTGGATCGGACGGTCC	0	15	RDR2-sensitive
TCGCAAGAACTGCGAAGTCGGCGA	0	18	RDR2-sensitive
TCGCAATACGGTAGGGCATCAGAC	0	12	RDR2-sensitive
TCGCAGAAAGTGGGACGGTGATGT	0	13	RDR2-sensitive
TCGCAGAAGATGGGACGGGAATGT	1	16	RDR2-sensitive

TCGCAGAAGGCCAGGTAGAGCGGT	0	21	RDR2-sensitive
TCGCAGACGCATGGAACAGGCACC	0	9	RDR2-sensitive
TCGCAGATGCACCTCTGCGGTCGG	0	77	RDR2-sensitive
TCGCAGCAACTCGGCTCGGAGCAA	0	11	RDR2-sensitive
TCGCAGCAGCTCGGCTCGGCTCGA	0	11	RDR2-sensitive
TCGCAGCAGCTCGGCTCGGCTCGG	0	47	RDR2-sensitive
TCGCAGCCTGCAGCAACGGACGGT	0	19	RDR2-sensitive
TCGCAGGACTGACCGGCTGCATGA	59	1	RDR2-resistant
TCGCAGGGACTGCTGGTACCGCGC	0	12	RDR2-sensitive
TCGCAGTAGCTCGGCTCGGCTCGG	0	14	RDR2-sensitive
TCGCATGGACCAAGCTAAGCTCAT	0	17	RDR2-sensitive
TCGCATTCGTACGCGTGTCGGTAC	1	14	RDR2-sensitive
TCGCCAAACAGCTCTCGGACGCGC	0	11	RDR2-sensitive
TCGCCAACGTCAGCGACTCTCGGA	0	11	RDR2-sensitive
TCGCCAATATGTCATCGGACCCAC	0	45	RDR2-sensitive
TCGCCAGAAGCTCCAGGACGCCAT	0	17	RDR2-sensitive
TCGCCAGGTCGCGGACCGTCCGGC	1	20	RDR2-sensitive
TCGCCAGTATGACATCGGACCCAC	1	44	RDR2-sensitive
TCGCCAGTATGCCATCGGACCCAC	3	127	RDR2-sensitive
TCGCCAGTATGCCATCGGACCCAT	0	17	RDR2-sensitive
TCGCCAGTATGCCATCGGACTCAT	0	31	RDR2-sensitive
TCGCCAGTATGCTATCGGACTCAC	0	29	RDR2-sensitive
TCGCCAGTATGGCATCGGACCCAC	0	51	RDR2-sensitive
TCGCCAGTATGTCATCGGACCCAC	0	17	RDR2-sensitive
TCGCCAGTGACGGGACAACAGCGC	0	12	RDR2-sensitive
TCGCCCATCTGACGAGCTCGGGAA	0	22	RDR2-sensitive
TCGCCCTGTTCGGCTGTCCGGCGC	0	9	RDR2-sensitive
TCGCCGACTGTAGCTAAATGGTTC	0	9	RDR2-sensitive
TCGCCGATTGTCGTCGTTCGTCGT	0	9	RDR2-sensitive
TCGCCGGCTTTGAGTAGAGAGGAA	0	17	RDR2-sensitive
TCGCCGGGCAGTCGGAAGAGACGT	0	9	RDR2-sensitive
TCGCCGTTGCGACTAGGAAGATGC	0	13	RDR2-sensitive
TCGCCTACGCCTCCAAATCGTCGA	1	33	RDR2-sensitive
TCGCCTATCTAGCGAACTCGGAAA	0	39	RDR2-sensitive
TCGCCTGTCGGCCTCTGACACGGC	0	12	RDR2-sensitive
TCGCCTTTCGCTGCAGTCGCTGGT	0	11	RDR2-sensitive
TCGCGACAGACGGAACAGATGCGA	0	9	RDR2-sensitive
TCGCGACAGGAAGCGATGACGACG	1	14	RDR2-sensitive
TCGCGACCCACAAACAAGGACCGG	0	12	RDR2-sensitive
TCGCGACCCACAAACAGGACTGGC	1	14	RDR2-sensitive
TCGCGACCTACGGCAGGAGCTGAG	0	12	RDR2-sensitive
TCGCGAGCCTGGAACGAGCCGAGC	0	14	RDR2-sensitive
TCGCGAGTCTTGTCGGATCGGATA	0	10	RDR2-sensitive
TCGCGATCCGCGACGAATTCGGCA	0	23	RDR2-sensitive
TCGCGCAGATTCTGGAACGTGCAG	0	28	RDR2-sensitive
TCGCGCATGTAGACGAGCGAGCTA	0	9	RDR2-sensitive
TCGCGCGATGGCAGAACAGGGCGT	0	55	RDR2-sensitive
TCGCGCGGAGACGCTAGACGACAC	0	9	RDR2-sensitive
TCGCGCGGCGTTGATGGGGGCATG	19	0	RDR2-resistant

TCGCGCGGGCGTAGCCGGTTAGAG	0	9	RDR2-sensitive
TCGCGCGGGCGTTCGGTAGAGCGG	0	28	RDR2-sensitive
TCGCGCTCGAAGTCGGAAGATCAG	0	10	RDR2-sensitive
TCGCGCTCTGGACCGGACGGTCAA	0	17	RDR2-sensitive
TCGCGCTTCGGTGCCGGACGGTAC	2	39	RDR2-sensitive
TCGCGGAAACCCAGGTAGAGCGGT	0	22	RDR2-sensitive
TCGCGGCTAAGGGTCGGACGGTCC	0	11	RDR2-sensitive
TCGCGGGATTGCAGAAGCGGCGGC	0	24	RDR2-sensitive
TCGCGGGCTGGGACAAGGCATGAC	0	24	RDR2-sensitive
TCGCGGTGCTTTTGGGTAGGACGG	0	21	RDR2-sensitive
TCGCGGTGTGTGTGGAACGGCGGC	0	25	RDR2-sensitive
TCGCGTAGGACAGTTCGGCAAGGC	1	62	RDR2-sensitive
TCGCGTAGGACAGTTCGGCTAGAC	0	24	RDR2-sensitive
TCGCGTAGGACAGTTCGGCTAGGC	2	243	RDR2-sensitive
TCGCGTCCAGAAGAGTACTCACAT	0	10	RDR2-sensitive
TCGCGTCCGCCGCCAAATCGTCAG	0	11	RDR2-sensitive
TCGCGTCCGCCGCTAAATCGTCAG	0	16	RDR2-sensitive
TCGCGTCGCGTGGAAAGGCAGAAC	0	23	RDR2-sensitive
TCGCGTGAGACTGAATGTACGGGC	0	9	RDR2-sensitive
TCGCGTGGGACTGAATGTACGGAC	0	20	RDR2-sensitive
TCGCGTGGTATAGCTGGGACGGAT	0	10	RDR2-sensitive
TCGCGTTTACCGTCAAGTCGTCGA	1	56	RDR2-sensitive
TCGCGTTTACCGTCGAGTCGTCGA	3	36	RDR2-sensitive
TCGCGTTTGCCGTCGAGTCGTCGA	0	9	RDR2-sensitive
TCGCTAGTATGCCATCGGACCCAC	0	14	RDR2-sensitive
TCGCTATCTGGCTGAGTCGTCGAG	0	31	RDR2-sensitive
TCGCTCCAGACACAATCTCGTCGT	0	10	RDR2-sensitive
TCGCTCCAGACTCGATCTCGTCGT	0	37	RDR2-sensitive
TCGCTCGACTCGGCTCGTTAGTAG	0	11	RDR2-sensitive
TCGCTCGGCTGAGATGGCGGCTTT	21	1	RDR2-resistant
TCGCTCGTTTCGTGGTCAGCTCGG	1	15	RDR2-sensitive
TCGCTGAAGGTCTGGTAGGAAGAA	0	18	RDR2-sensitive
TCGCTGCTAAACTCAATCACGGAA	33	3	RDR2-resistant
TCGCTGGATTGTGCAGGACGACGT	0	24	RDR2-sensitive
TCGCTGGCAATGGCAGGCTGACGT	0	14	RDR2-sensitive
TCGCTGGCCAGTGAGATCTGACGT	0	22	RDR2-sensitive
TCGCTGGCGCTTTTGGTCTCGGAT	35	6	RDR2-resistant
TCGCTGGCGTGTATAGGACGGTCT	0	14	RDR2-sensitive
TCGCTGGCTCGCTCGGATCATGGT	0	13	RDR2-sensitive
TCGCTGTACCATCGAGCCGAGCAG	0	37	RDR2-sensitive
TCGCTGTAGCCAGGAAGTTGAGCG	0	9	RDR2-sensitive
TCGCTGTAGTCGCCGGTCCGACGG	1	15	RDR2-sensitive
TCGCTTAGACTGTCTTCAACGCAT	0	10	RDR2-sensitive
TCGCTTCAAACACGATCTCGTCGT	0	9	RDR2-sensitive
TCGCTTGGACGGACGCCTAGACGG	0	10	RDR2-sensitive
TCGGAAAAGAGGCTCGGCTCGGCT	0	9	RDR2-sensitive
TCGGAAAAGCGGCTCGGCTCGGCT	0	10	RDR2-sensitive
TCGGAAAAGCGGCTTGACTCGGCT	0	11	RDR2-sensitive
TCGGAAACGGTATTTATTCGGTAA	5	100	RDR2-sensitive

TCGGAACACTCTAAGCCTCGGCAT	0	24	RDR2-sensitive
TCGGAACAGAGGTGTTGGCGAGAG	0	10	RDR2-sensitive
TCGGAACCACTCGGGACGGAAGAA	0	10	RDR2-sensitive
TCGGAACCCAGAACACGCGCACAC	0	10	RDR2-sensitive
TCGGAACCGGGTCGGGAACGGCGC	21	74	RDR2-sensitive
TCGGAACCGGGTCGGGAATGGCGC	3	20	RDR2-sensitive
TCGGAACTGAAGTCGGGTACCCGC	0	9	RDR2-sensitive
TCGGAAGAACCAGTCGGAGCGCGT	0	12	RDR2-sensitive
TCGGAAGGAGGCTAGTAGGAACGG	0	18	RDR2-sensitive
TCGGAAGTTAAGCCCTCCCGTCGG	0	9	RDR2-sensitive
TCGGAATTGAACTGGATTTGGTAT	0	21	RDR2-sensitive
TCGGAATTGGAGAGTGGAGGCGTG	0	9	RDR2-sensitive
TCGGAATTTTAGATCGGAATCGGT	0	10	RDR2-sensitive
TCGGAATTTTCTATCGGAATCGGT	1	13	RDR2-sensitive
TCGGACAAGAAACTAGGCACGGTG	0	19	RDR2-sensitive
TCGGACACTCGGCAAAGACTTTGC	0	23	RDR2-sensitive
TCGGACAGAAAACTAGCCACGGTT	0	13	RDR2-sensitive
TCGGACAGGAAACTAGGCACGGTG	1	36	RDR2-sensitive
TCGGACAGGAAATTAGGCACGGTG	0	17	RDR2-sensitive
TCGGACATAAGGCCTACGTCTGAC	0	12	RDR2-sensitive
TCGGACATACGATGGTGGCGTCCC	0	9	RDR2-sensitive
TCGGACCAGGCTTCATTCCTTTTT	16	0	RDR2-resistant
TCGGACCAGGCTTCATTCTTTTTT	17	0	RDR2-resistant
TCGGACCAGGCTTCATTTTTTTTT	15	0	RDR2-resistant
TCGGACCCTCTGGAGGATCGCGGA	1	20	RDR2-sensitive
TCGGACCGACATGAATAAGCGGAC	0	12	RDR2-sensitive
TCGGACCGGACTAGCACGAATTGC	0	12	RDR2-sensitive
TCGGACCTCGGTTGACGACGAGAC	0	13	RDR2-sensitive
TCGGACGAAGGTCGTGAAAGACGT	2	28	RDR2-sensitive
TCGGACGATCCGCACTGGTGGCGT	0	22	RDR2-sensitive
TCGGACGGTACTCCGAAGCGGCAA	0	9	RDR2-sensitive
TCGGACTATCCGTCGTAGCGGCGT	0	21	RDR2-sensitive
TCGGACTCATTTCGGACGGCCCAA	0	15	RDR2-sensitive
TCGGACTTTTTTTCAATTGGTTGC	17	0	RDR2-resistant
TCGGAGACGACGACTGGCACGCAC	0	9	RDR2-sensitive
TCGGAGCCCGTGGACTGGCACGAC	0	10	RDR2-sensitive
TCGGAGCGCATGGGAGGGTATGAT	0	9	RDR2-sensitive
TCGGAGCTGTCGACGGAAGACCAA	0	15	RDR2-sensitive
TCGGAGGAAGGCAGATTGTAGCAC	0	9	RDR2-sensitive
TCGGAGGATGTCATGCGCGATCGT	1	13	RDR2-sensitive
TCGGAGGCACGATAGGCTTGTCAT	0	12	RDR2-sensitive
TCGGAGGCAGACCAAAACGTAGGC	61	5	RDR2-resistant
TCGGAGGCATCACGTAGAGACCGG	0	10	RDR2-sensitive
TCGGAGTGTGTACGCGGAGGGCGC	0	47	RDR2-sensitive
TCGGAGTTTGAAGCTAGAGGTGTC	51	1	RDR2-resistant
TCGGATACAAGATCGGATACGGTT	0	12	RDR2-sensitive
TCGGATACAGGATCGGATACGGAT	0	11	RDR2-sensitive
TCGGATACGGATAGTTAGAAACGG	0	54	RDR2-sensitive
TCGGATACGGGACCAGATACGGGT	0	22	RDR2-sensitive

TCGGATACGGGTACCAAAACGGGA	0	12	RDR2-sensitive
TCGGATACGGGTAGGATACAGGAT	0	15	RDR2-sensitive
TCGGATACGGGTAGGATACGGAAT	3	123	RDR2-sensitive
TCGGATACTTGTCGGATAATGCAT	1	20	RDR2-sensitive
TCGGATAGAACGGTGGCAGAACAG	1	17	RDR2-sensitive
TCGGATAGGAACTAAGCACGGTGA	0	9	RDR2-sensitive
TCGGATATCGGATACTTGTCGGAT	0	14	RDR2-sensitive
TCGGATATCTGATCCGACGGATAA	0	17	RDR2-sensitive
TCGGATCATGAGCATACAGAGCAT	0	15	RDR2-sensitive
TCGGATCCAACGGTCGACTGCTAC	0	10	RDR2-sensitive
TCGGATCCCTGACAGTAGGAGCAT	1	14	RDR2-sensitive
TCGGATCCGGGCAAGAAACGACAT	0	14	RDR2-sensitive
TCGGATCCGGGCAAGAAACGACGG	1	15	RDR2-sensitive
TCGGATCCTGAGAGAAGAGGTCGC	0	9	RDR2-sensitive
TCGGATCCTGCCCCGGCTCGTCGT	0	9	RDR2-sensitive
TCGGATCGCGGCGACGGGGGCGGT	46	3	RDR2-resistant
TCGGATCGCTCCCGTTGAGTCGGA	0	10	RDR2-sensitive
TCGGATCTGGCATATTTTTCAAAT	32	3	RDR2-resistant
TCGGATCTTGGCAACAACACGGAC	0	18	RDR2-sensitive
TCGGATCTTGGCAACGACGCGCAC	2	30	RDR2-sensitive
TCGGATGACTCAGAACAGCCGGGT	1	15	RDR2-sensitive
TCGGATGATGAGCAGGTGGGGGTC	0	12	RDR2-sensitive
TCGGATGCAAGACCTGCGGCAGGT	0	16	RDR2-sensitive
TCGGATGCGAACGAGCGAGGACAA	0	9	RDR2-sensitive
TCGGATGTCATCGGCGCGTTCCTC	18	55	RDR2-sensitive
TCGGATTCCTGTAGTGGCCAGAGA	0	13	RDR2-sensitive
TCGGATTCGAATTCGAATACGGAT	0	11	RDR2-sensitive
TCGGATTCGGGTCAGATACGGAAC	0	10	RDR2-sensitive
TCGGATTCGGGTCGGATACGGAAC	0	17	RDR2-sensitive
TCGGATTCGGGTCGGATACGGAGC	2	59	RDR2-sensitive
TCGGATTGTCCGCCGTAGCGGCGC	0	22	RDR2-sensitive
TCGGATTGTGTCTCTACACGGTGT	0	9	RDR2-sensitive
TCGGATTTCGGACGGGTTTCGGAT	0	24	RDR2-sensitive
TCGGATTTCTATATATGCAGATGA	26	0	RDR2-resistant
TCGGATTTGGACCAGACATGACGG	0	9	RDR2-sensitive
TCGGCAAAGAACCTAAGCCGGTGC	0	16	RDR2-sensitive
TCGGCAAAGAACCTATACCGGTGC	0	9	RDR2-sensitive
TCGGCAAAGAACGCGATTCCGGTA	2	106	RDR2-sensitive
TCGGCAAAGAACGCGATTCCGGTT	0	18	RDR2-sensitive
TCGGCAAAGAATATGACACTCGGC	0	11	RDR2-sensitive
TCGGCAAAGACTCGGATTCCGGTA	0	12	RDR2-sensitive
TCGGCAAAGACTCGGATTCGGCCT	0	13	RDR2-sensitive
TCGGCAAAGACTGGTCTAGTGGAC	0	10	RDR2-sensitive
TCGGCAAAGAGCACAGACCTGGGC	0	11	RDR2-sensitive
TCGGCAAAGAGCCTGTTTCCGGTA	0	12	RDR2-sensitive
TCGGCAAAGATTGCAACACTCGGC	0	10	RDR2-sensitive
TCGGCAAAGCCTCGGATTCCGGTA	2	55	RDR2-sensitive
TCGGCAAAGCCTGCGATTCCGGTA	0	11	RDR2-sensitive
TCGGCAAAGCCTTGGATTCCGGTA	0	19	RDR2-sensitive

TCGGCAAAGCGTACTGCACTCGGC	0	15	RDR2-sensitive
TCGGCAAAGGAGCTGTGTCCGGTA	0	13	RDR2-sensitive
TCGGCAAAGGCGCGAATTCCGGTA	1	13	RDR2-sensitive
TCGGCAAAGGCTCGGATTCCGGTA	0	32	RDR2-sensitive
TCGGCAAAGGCTCTGATTCCGGTA	3	32	RDR2-sensitive
TCGGCAAAGTCTCGGATTCCGGTA	0	23	RDR2-sensitive
TCGGCAACGTCCTGCGCTCGGTAA	0	9	RDR2-sensitive
TCGGCAAGGAACGCTCAACACGGA	0	18	RDR2-sensitive
TCGGCACAACCAAGCACTCGGCAA	1	21	RDR2-sensitive
TCGGCACAACGCAGCATTCGACAA	0	12	RDR2-sensitive
TCGGCACAAGACACCACTCGGCAA	1	12	RDR2-sensitive
TCGGCACAGAAACACACTCGGCAA	2	16	RDR2-sensitive
TCGGCACAGAAAGACACTAGGCAA	0	9	RDR2-sensitive
TCGGCACAGAAAGACACTCGGCAA	0	10	RDR2-sensitive
TCGGCACAGAAATACACTCGGCAA	6	52	RDR2-sensitive
TCGGCACAGAACGACACTCGGCAA	3	47	RDR2-sensitive
TCGGCACAGAACGGCACTCGGCAA	4	77	RDR2-sensitive
TCGGCACAGAACGGCACTTGGCAA	0	16	RDR2-sensitive
TCGGCACAGAACTACACTCGGCAA	0	12	RDR2-sensitive
TCGGCACAGAACTCCACTCGGCAA	0	9	RDR2-sensitive
TCGGCACAGACAAGCACTCGGCAA	1	22	RDR2-sensitive
TCGGCACAGAGAAACACTCGGCAA	2	44	RDR2-sensitive
TCGGCACAGAGGAACACTCGGCAA	0	19	RDR2-sensitive
TCGGCACAGCCAAGCACTCGGCAA	35	225	RDR2-sensitive
TCGGCACAGCCCAGCACTCGGCAA	2	24	RDR2-sensitive
TCGGCACAGCGAAGCACTCGGCAA	2	22	RDR2-sensitive
TCGGCACAGGAATACACTCGGCAA	0	44	RDR2-sensitive
TCGGCACAGGACAACACTCGGCAA	7	43	RDR2-sensitive
TCGGCACAGGACAGCACTCGGCAA	4	40	RDR2-sensitive
TCGGCACAGGACCACACTCGGCAA	4	47	RDR2-sensitive
TCGGCACAGGACCCCACTCGGCAA	8	122	RDR2-sensitive
TCGGCACAGGACCCCACTCGGTAA	0	10	RDR2-sensitive
TCGGCACAGGACGACACTCGGCAA	9	139	RDR2-sensitive
TCGGCACAGGACGACACTCGGTAA	0	9	RDR2-sensitive
TCGGCACAGGACGACACTTGGCAA	0	11	RDR2-sensitive
TCGGCACAGGACGGCACTCGGCAA	10	74	RDR2-sensitive
TCGGCACAGGACGGCGCTCAGTAA	0	12	RDR2-sensitive
TCGGCACAGGACTCCACTCGGCAA	0	10	RDR2-sensitive
TCGGCACAGGATCCCACTCGGCAA	0	9	RDR2-sensitive
TCGGCACAGGGCGACACTCGGCAA	1	18	RDR2-sensitive
TCGGCACAGGGCGGCACTCGGCAA	2	23	RDR2-sensitive
TCGGCACAGGTAGGCACTCGGCAA	8	87	RDR2-sensitive
TCGGCACAGTCAAGCACTCGGCAA	0	16	RDR2-sensitive
TCGGCACATCCAAGCACTCGGCAA	3	41	RDR2-sensitive
TCGGCACCAGACGAAGACCGGGAC	0	10	RDR2-sensitive
TCGGCACGCGAGCTCGACGCGCAC	0	13	RDR2-sensitive
TCGGCACGGCACGACACAACTCAC	0	11	RDR2-sensitive
TCGGCACTGATAATTTGTTCGGCG	0	15	RDR2-sensitive
TCGGCACTGTAGGTCGGCTTGCCT	1	94	RDR2-sensitive

TCGGCAGAGGACAGCACTCGGCAA	11	155	RDR2-sensitive
TCGGCAGAGTGACTGGTTGGATAG	0	17	RDR2-sensitive
TCGGCAGGTCACACTAGGACGGAT	0	9	RDR2-sensitive
TCGGCAGTGGATCCGTGACCCGGC	0	12	RDR2-sensitive
TCGGCATACGAAGACACTCGGTAA	0	15	RDR2-sensitive
TCGGCATAGAACGACACTCGGCAA	2	20	RDR2-sensitive
TCGGCATAGAACGACGCTCGGCAA	0	9	RDR2-sensitive
TCGGCATAGACACACACTCGGCAA	0	13	RDR2-sensitive
TCGGCATAGACAGACACTCGGCAA	0	18	RDR2-sensitive
TCGGCATAGAGAAACACTCGGCAA	0	11	RDR2-sensitive
TCGGCATAGAGAAGCACTCGGCAA	0	19	RDR2-sensitive
TCGGCATAGCGAAGCACTCGGCAA	0	9	RDR2-sensitive
TCGGCATAGGACCACACTCGGCAA	1	19	RDR2-sensitive
TCGGCATAGGACCCCACTCGGCAA	3	19	RDR2-sensitive
TCGGCATAGGTAGACACTCGGCAC	6	31	RDR2-sensitive
TCGGCATAGTTAACGATCGGCGTT	0	11	RDR2-sensitive
TCGGCATATAAGGACACTCGGCAA	0	11	RDR2-sensitive
TCGGCATATACTGACGCTCGGCAA	1	20	RDR2-sensitive
TCGGCATATCCAGACACTCGGCGA	0	29	RDR2-sensitive
TCGGCATCTGTTTTTTCTGCGGCT	155	9	RDR2-resistant
TCGGCATGGACACAAGCTGACGAC	0	60	RDR2-sensitive
TCGGCATGGATACAAGCTGACGAC	1	14	RDR2-sensitive
TCGGCATGTGGAGACCAAGCTCGG	0	10	RDR2-sensitive
TCGGCCACGGAAGACAGACTCGAC	24	2	RDR2-resistant
TCGGCCACGTCAGTCTGTCGTCGT	0	20	RDR2-sensitive
TCGGCCAGCCGTCGGAAATAAGTC	0	11	RDR2-sensitive
TCGGCCCCGGAAGACGACGAACTC	2	18	RDR2-sensitive
TCGGCCTCGCAGCTGGACATGGCC	47	6	RDR2-resistant
TCGGCGAAATGTGACCCTCGGCAT	0	15	RDR2-sensitive
TCGGCGAACAAAGACACTCGGCAC	0	11	RDR2-sensitive
TCGGCGAACACAGACGAACTGCAA	0	11	RDR2-sensitive
TCGGCGAACGACAACACTCGGTAT	0	21	RDR2-sensitive
TCGGCGAACTGTACATCGGCAACA	0	14	RDR2-sensitive
TCGGCGAAGGACGGCTCTCGGCAA	1	25	RDR2-sensitive
TCGGCGAAGGGCTGGACGAAGCAT	0	9	RDR2-sensitive
TCGGCGAATGACGGTCCTCGGCAA	0	11	RDR2-sensitive
TCGGCGACGACGAACTTTACGGTG	0	9	RDR2-sensitive
TCGGCGCATAATGACACTCGGCAT	1	22	RDR2-sensitive
TCGGCGCGACGAACCTAGCGGTGC	0	14	RDR2-sensitive
TCGGCGCGAGACGGAACACAAGGA	0	12	RDR2-sensitive
TCGGCGCGAGACGGAACACAAGGG	3	16	RDR2-sensitive
TCGGCGCTAGAACAGAGCGGTCGC	0	33	RDR2-sensitive
TCGGCGTAGCCTGCACACGCGGAT	0	11	RDR2-sensitive
TCGGCGTAGTAGCGGATTTTCCAG	0	12	RDR2-sensitive
TCGGCTAACCGACGGAACGGACGG	0	10	RDR2-sensitive
TCGGCTAACGGGCTGACCCGGCAC	0	9	RDR2-sensitive
TCGGCTAAGCATGTTTGAATGGCT	18	1	RDR2-resistant
TCGGCTAGACGATAGGCTGACGGC	0	9	RDR2-sensitive
TCGGCTCGAACCGGCTCGACGCTC	0	9	RDR2-sensitive

TCGGCTCGACGGCCAGGATCTCGG	0	12	RDR2-sensitive
TCGGCTCGACTCGGCTCGTTTCAA	0	33	RDR2-sensitive
TCGGCTCGCGAGGCATACCCTGAT	1	12	RDR2-sensitive
TCGGCTCGCTCTGGGCTCGCGGCC	0	10	RDR2-sensitive
TCGGCTCGGATCGGCTCGATAGAA	0	39	RDR2-sensitive
TCGGCTCGGATCGGCTCGTTTGAA	2	77	RDR2-sensitive
TCGGCTCGGCTCGGACCGGCTCGA	0	15	RDR2-sensitive
TCGGCTCGGCTCGGAGCGGCTCGC	1	16	RDR2-sensitive
TCGGCTCGGCTCGGATCGGCTCGA	0	9	RDR2-sensitive
TCGGCTCGGCTCGGATCGGCTCGT	1	27	RDR2-sensitive
TCGGCTCGGCTTGGATCGGTTCGT	0	38	RDR2-sensitive
TCGGCTCGGGCTCGTTCGAAGCGT	0	9	RDR2-sensitive
TCGGCTCTCGGCAAAGACTGACGG	1	29	RDR2-sensitive
TCGGCTCTGTCGACTGAAGGGCGC	4	39	RDR2-sensitive
TCGGCTCTGTCGACTGGAGGGCGC	0	12	RDR2-sensitive
TCGGCTCTGTCGACTGGAGGGCGT	6	31	RDR2-sensitive
TCGGCTCTGTGACGTGTGAGCGGC	1	21	RDR2-sensitive
TCGGCTGACTGACGAAGAGACAGG	0	11	RDR2-sensitive
TCGGCTGAGATGGCGACTTTAGGT	50	3	RDR2-resistant
TCGGCTGCTCGTGGCGTAAACCAG	0	11	RDR2-sensitive
TCGGCTGCTGTGGACAGAAGCTGG	0	12	RDR2-sensitive
TCGGCTGGGTGGAACGGTTGGCTC	0	9	RDR2-sensitive
TCGGCTTAGAAAGACACTCGGCAT	0	25	RDR2-sensitive
TCGGCTTGGATCGGCTCGTTTGAA	0	9	RDR2-sensitive
TCGGCTTGTAGGGTCGGCCTAGGC	0	9	RDR2-sensitive
TCGGCTTTCGCTCTGGCCGACGGG	0	9	RDR2-sensitive
TCGGGAAACTCCAACGGCTAGTCC	1	24	RDR2-sensitive
TCGGGAACACAAGACTAGGCCTAT	15	0	RDR2-resistant
TCGGGAACAGAAACGGGAGCGGGA	0	10	RDR2-sensitive
TCGGGAACCAGGCTGTGGGCGTGC	0	29	RDR2-sensitive
TCGGGAACGGAAACGGGAGCGGGA	0	16	RDR2-sensitive
TCGGGACCGGATACGGATAGTTAG	0	22	RDR2-sensitive
TCGGGACCGGTTCTTACGCTTCTC	47	1	RDR2-resistant
TCGGGACCTGGACGCAGGAGCGAC	0	20	RDR2-sensitive
TCGGGACGCATTGGACGGGCCTAG	0	11	RDR2-sensitive
TCGGGACGGATACAGGACGGGACC	0	22	RDR2-sensitive
TCGGGACGGATACGGGTAATACCC	4	76	RDR2-sensitive
TCGGGACGGATACGGGTAATACCT	0	9	RDR2-sensitive
TCGGGACGGATACGGGTAGTACCT	0	25	RDR2-sensitive
TCGGGACGGATACGGGTATTACCC	0	9	RDR2-sensitive
TCGGGAGCCAGGCTGTGCACGTGC	1	21	RDR2-sensitive
TCGGGATCGGATACGGATAGTTAG	0	14	RDR2-sensitive
TCGGGATCTCGGCGTAGTAGCGGA	0	21	RDR2-sensitive
TCGGGCACCTAAGCAGGAACACGA	0	9	RDR2-sensitive
TCGGGCACTCGGCAAAGAACGGTT	0	10	RDR2-sensitive
TCGGGCACTCGGCAAAGAAGGCTT	41	138	RDR2-sensitive
TCGGGCACTCGGCAAAGACAGCTT	0	10	RDR2-sensitive
TCGGGCACTCGGCAAAGACGTCTT	0	11	RDR2-sensitive
TCGGGCACTCGGCAAAGAGGTCGG	0	9	RDR2-sensitive

TCGGGCCACGGGGAACAAGACCGG	0	12	RDR2-sensitive
TCGGGCCGCAGAGAACAAGACCGG	5	51	RDR2-sensitive
TCGGGCCGCAGGGAACAAGACCGG	1	20	RDR2-sensitive
TCGGGCCGCTTAGAGATGCGTAAC	5	23	RDR2-sensitive
TCGGGCCGGCTAAGCAGAACAGAT	0	12	RDR2-sensitive
TCGGGCCTAGACCAGTACCGTCGT	0	10	RDR2-sensitive
TCGGGCCTAGTCCAGCACTGACGG	3	21	RDR2-sensitive
TCGGGCGAGACGGAAATCCTCCGG	0	12	RDR2-sensitive
TCGGGCGAGCCGGAGATGTGTCCG	0	15	RDR2-sensitive
TCGGGCGAGGCGGAGTTTCGTCGT	0	11	RDR2-sensitive
TCGGGCGGCCACCAAGACAGCATG	135	13	RDR2-resistant
TCGGGCGTGCTCTGTAGACGTGAT	1	20	RDR2-sensitive
TCGGGCTCCGGACGGTGTGTGCGG	0	10	RDR2-sensitive
TCGGGCTCCTGCACCAGGTCGCGG	1	18	RDR2-sensitive
TCGGGCTCCTGTTTGATAAGGCAC	1	15	RDR2-sensitive
TCGGGCTCGTTCTGAGCGTCGAGC	0	10	RDR2-sensitive
TCGGGCTCTCGGCAACAAGGTCTC	0	10	RDR2-sensitive
TCGGGCTCTCGGCGTATACCGCGT	0	21	RDR2-sensitive
TCGGGCTGACACAGCACGGCTCAA	0	18	RDR2-sensitive
TCGGGCTGCTGCAAGGTGTAACAC	0	10	RDR2-sensitive
TCGGGCTGGCACAGCATAACACAA	0	9	RDR2-sensitive
TCGGGCTGGCACGGCACGACCCAA	0	11	RDR2-sensitive
TCGGGCTGGCACGGCATGGCTCAA	0	11	RDR2-sensitive
TCGGGCTGGCACGGTACGACCCAA	0	14	RDR2-sensitive
TCGGGCTGGCTCGCCAGAAACGAC	0	15	RDR2-sensitive
TCGGGCTGGTGCCGTGGACGTCAC	0	13	RDR2-sensitive
TCGGGCTGTCACGTGGGCTATCAA	0	34	RDR2-sensitive
TCGGGCTTAGGCCAGGCCGGACGG	0	14	RDR2-sensitive
TCGGGCTTATTACAGGCGTCCCAA	0	14	RDR2-sensitive
TCGGGCTTCAAAGTGTCGGGCCGG	0	15	RDR2-sensitive
TCGGGCTTGCTCGTCGTAGTCGGA	2	25	RDR2-sensitive
TCGGGGACGGGAACAGGCTACCAT	0	9	RDR2-sensitive
TCGGGGAGCGCTCTTGGTTGTGGC	16	0	RDR2-resistant
TCGGGGCTTGTGAGGATGGCCAGC	22	2	RDR2-resistant
TCGGGGTATTAAATGTAGTCGGAT	0	11	RDR2-sensitive
TCGGGTCACGGAGAACAAGACCGG	5	54	RDR2-sensitive
TCGGGTCATGTAGGTCGAGGTCGC	0	11	RDR2-sensitive
TCGGGTCCGAGCAGAACGACACAC	0	23	RDR2-sensitive
TCGGGTCGCAGGGCAAACGGACGG	0	12	RDR2-sensitive
TCGGGTCGGATACGGGTACCCGGA	0	9	RDR2-sensitive
TCGGGTCTTGCTAGTGTCGCGCTG	0	9	RDR2-sensitive
TCGGGTGAGTAGCAAAGCGAAGAG	0	23	RDR2-sensitive
TCGGGTGGACGGCGGTAAGAGCGA	0	11	RDR2-sensitive
TCGGGTGGTTACAGATCGCAGGAG	0	14	RDR2-sensitive
TCGGGTGGTTACAGATCGTAGGAG	0	13	RDR2-sensitive
TCGGGTGTGGAGGAGGTGCCTTGC	17	1	RDR2-resistant
TCGGGTTCGGATCGGGTATGGTAA	0	10	RDR2-sensitive
TCGGGTTTTCGGGTTCGGGTTCGG	0	40	RDR2-sensitive
TCGGTAAAACAACGGTCGGTCGGT	0	15	RDR2-sensitive

TCGGTAAAGCTAGACTCTCGGCAA	0	27	RDR2-sensitive
TCGGTAAAGCTAGGCTCTCGGCAA	3	24	RDR2-sensitive
TCGGTAAAGCTCGGCTCTCGGCAA	0	11	RDR2-sensitive
TCGGTAAAGTCAGGCTCTCGGCAA	0	12	RDR2-sensitive
TCGGTAAAGTTAGGAACCCGGCAA	1	15	RDR2-sensitive
TCGGTAAAGTTAGGCACTCGGCAA	0	9	RDR2-sensitive
TCGGTAAAGTTAGGCGCTCGGCAA	4	29	RDR2-sensitive
TCGGTAAAGTTAGGCTCTCGGCAA	8	78	RDR2-sensitive
TCGGTAACGAACTCAATCCGGCGA	0	17	RDR2-sensitive
TCGGTAACGAGGCTCGGCTCGGCT	0	27	RDR2-sensitive
TCGGTAATCCTAGCGGACGAGCTT	1	19	RDR2-sensitive
TCGGTAATGAGGCTCGGCTCGGCT	1	42	RDR2-sensitive
TCGGTACAATGGTTCATACTTATA	18	0	RDR2-resistant
TCGGTACAGGAAACCACTCGGCAA	0	10	RDR2-sensitive
TCGGTACAGGACGACACTCGGCAA	1	24	RDR2-sensitive
TCGGTACAGGGAAACACTCGGCAA	0	13	RDR2-sensitive
TCGGTACAGGTCCACACTCGGCAA	1	23	RDR2-sensitive
TCGGTACAGTTAGGCACTCGGCAA	0	10	RDR2-sensitive
TCGGTACCACGTCGGCGCCACGAC	0	11	RDR2-sensitive
TCGGTACGGATAATGGATCGGCTC	2	88	RDR2-sensitive
TCGGTACGGATAATGGATCGGCTT	0	29	RDR2-sensitive
TCGGTAGAACGAGCAGAGTAGCAA	0	23	RDR2-sensitive
TCGGTAGACACTCGGCAAAGACGT	0	22	RDR2-sensitive
TCGGTAGATTATAATGGCAATGGT	2	19	RDR2-sensitive
TCGGTAGGACCTGGTCAGCCGGTC	0	11	RDR2-sensitive
TCGGTAGGCAGGTTGCTCGGCAGC	0	11	RDR2-sensitive
TCGGTAGTGCCCTCTCTGCGACGG	0	28	RDR2-sensitive
TCGGTATCGACTGACATTCGGCAA	1	21	RDR2-sensitive
TCGGTCACGCGCAGGATGACTTCT	0	13	RDR2-sensitive
TCGGTCACTTACCGAGCTCGGTAA	1	12	RDR2-sensitive
TCGGTCAGACAAGGCAGAACGCGG	0	12	RDR2-sensitive
TCGGTCAGCTAGTGAGAGGTTCGG	0	15	RDR2-sensitive
TCGGTCAGTCGGCTGGGCGAGCGT	0	12	RDR2-sensitive
TCGGTCAGTGGAGCGGCGAAGTGA	3	21	RDR2-sensitive
TCGGTCATGTCGATCAGACTGCGT	0	10	RDR2-sensitive
TCGGTCCACGGACAGATACGACAT	2	30	RDR2-sensitive
TCGGTCCAGTATGAATATCCGGCC	0	13	RDR2-sensitive
TCGGTCCATAAGTGAGCACGGCAC	0	14	RDR2-sensitive
TCGGTCCATGCGAGTTTGTAGGTC	18	0	RDR2-resistant
TCGGTCCTAGACTGTAGATGGCGA	0	12	RDR2-sensitive
TCGGTCGTGCTGACGGATACAGAA	0	13	RDR2-sensitive
TCGGTCTAGATCCAAGCGCGGGCG	51	3	RDR2-resistant
TCGGTCTAGATGGATGTGTGCAGC	0	10	RDR2-sensitive
TCGGTCTATTAGGATAATTCGGTT	0	12	RDR2-sensitive
TCGGTCTATTAGGGTAATTCGGTT	1	84	RDR2-sensitive
TCGGTCTATTCGAGTTCGGTTCGG	0	10	RDR2-sensitive
TCGGTCTATTCGGGTTCGGTTCGA	0	15	RDR2-sensitive
TCGGTCTATTCGGGTTCGGTTCGG	0	175	RDR2-sensitive
TCGGTCTATTCGGGTTCGGTTCGT	0	10	RDR2-sensitive

TCGGTCTCCGCCGCCGCCGTCGGC	34	0	RDR2-resistant
TCGGTCTGAGCCTGGCAATGGCAT	0	13	RDR2-sensitive
TCGGTCTGTTCGGGTTCGGTTCGG	1	20	RDR2-sensitive
TCGGTCTTTGGGCTAAACGGGTAT	1	14	RDR2-sensitive
TCGGTGAACGGGTTGGTCGGCAGC	0	14	RDR2-sensitive
TCGGTGAAGCTAGACTCTCGGCAA	0	20	RDR2-sensitive
TCGGTGAGTAGACGAGCCGAGCAG	0	9	RDR2-sensitive
TCGGTGATTCGCAGACTCGGCCAG	0	22	RDR2-sensitive
TCGGTGCAGGAAGATGTACGCTGT	1	31	RDR2-sensitive
TCGGTGCCGGACGGTACGTGACCT	0	23	RDR2-sensitive
TCGGTGCCTGAACGAGCTCGGCTA	0	9	RDR2-sensitive
TCGGTGCCTGAACGAGCTCGGCTC	0	12	RDR2-sensitive
TCGGTGCTCGGAACTAGACGGTCT	0	9	RDR2-sensitive
TCGGTGGATTATAATGGCAATGGT	1	12	RDR2-sensitive
TCGGTGGCACAACCCGGACGTCGT	0	9	RDR2-sensitive
TCGGTGGTGGGTCTCGAAGTCGGT	25	4	RDR2-resistant
TCGGTGTCTGAACGAGCTCGGCTC	0	14	RDR2-sensitive
TCGGTGTCTGGTACCTAGAAACGG	0	22	RDR2-sensitive
TCGGTGTGGAGCGCAGCGTGGATA	15	0	RDR2-resistant
TCGGTTACTACGGAATGGAGGCCT	0	10	RDR2-sensitive
TCGGTTAGGCTCCGGTAGGACGTC	0	10	RDR2-sensitive
TCGGTTAGGTCATGTTAGTCGGGT	0	18	RDR2-sensitive
TCGGTTCGGCTGTACGGGAAGCAT	3	55	RDR2-sensitive
TCGGTTCGGGTTCGGGTTCGGTTT	0	12	RDR2-sensitive
TCGGTTCGGTACGGTTCGGTTCGG	0	40	RDR2-sensitive
TCGGTTCGGTCTATTCGGGTTTAT	0	9	RDR2-sensitive
TCGGTTCGGTCTATTCGGGTTTGT	1	49	RDR2-sensitive
TCGGTTCGGTCTATTCGGTCCACC	0	12	RDR2-sensitive
TCGGTTCGGTCTATTCGGTTTTGT	0	16	RDR2-sensitive
TCGGTTCGGTTCGGTACGGTTCGG	0	21	RDR2-sensitive
TCGGTTGGTTCTAGGACTGGGCGT	1	12	RDR2-sensitive
TCGGTTGTGTAGGGCTAGGATGGT	0	10	RDR2-sensitive
TCGGTTTTCTCAGGAGCACGCACA	19	0	RDR2-resistant
TCGGTTTTTTCGGTAGTTCGGTTC	0	10	RDR2-sensitive
TCGTAAAAACGGGTCGCTCAAGTC	15	0	RDR2-resistant
TCGTAACTCGGATGTATGTTGGTT	355	15	RDR2-resistant
TCGTAACTCGTCCGACGACGGCAC	1	77	RDR2-sensitive
TCGTACACGGCTGGCAAAGTGGTT	21	0	RDR2-resistant
TCGTACAGTAGGAAGTGAGACGTC	0	12	RDR2-sensitive
TCGTACAGTAGGAAGTGAGGCGTC	2	42	RDR2-sensitive
TCGTACAGTGGGAAGTGAGGCGTC	2	29	RDR2-sensitive
TCGTACATCTTCTGCATCCGGTTT	32	0	RDR2-resistant
TCGTACGAGGACGCAACAGATCAG	0	21	RDR2-sensitive
TCGTACGTCGTGTGTAGAGATGGC	0	21	RDR2-sensitive
TCGTACTGCGCGGATGAGGGCGTT	65	15	RDR2-resistant
TCGTACTTATTTCGGACGGCTCAA	1	13	RDR2-sensitive
TCGTAGAAACGGAGGCGTAGGCGA	0	12	RDR2-sensitive
TCGTAGAGCAGGAGGGGAAGGGAA	0	16	RDR2-sensitive
TCGTAGATCATGAACGCTATGCGG	0	16	RDR2-sensitive

TCGTAGATTTGGCAACCGGAGCAC	0	18	RDR2-sensitive
TCGTAGATTTTGGCATACTGACGT	0	10	RDR2-sensitive
TCGTAGCACGAACGGTGGGCGCCA	0	9	RDR2-sensitive
TCGTATGGAGCAGGTAGTCGAGAC	0	10	RDR2-sensitive
TCGTATTGTGGAGCTGCATGGCAT	0	26	RDR2-sensitive
TCGTCACTCCGGCGTACTCGACAA	0	9	RDR2-sensitive
TCGTCCCGTTGGACAGACGTGCAC	0	12	RDR2-sensitive
TCGTCCTCGAGATTTATGAATGAC	28	3	RDR2-resistant
TCGTCGAATCGGCGCAGAGGCTGC	10	46	RDR2-sensitive
TCGTCTCGTCGAGGGCTTCGTCGC	0	13	RDR2-sensitive
TCGTCTCGTCTTTTAATCTTCGGC	0	17	RDR2-sensitive
TCGTCTCTCTCCTGGTCGTGGATC	17	1	RDR2-resistant
TCGTCTGAGTCCTGACGTGTCGGT	0	9	RDR2-sensitive
TCGTGAACCAGATCGAGCTCAGAG	0	12	RDR2-sensitive
TCGTGACAATTGAACGGGTGGCAC	2	354	RDR2-sensitive
TCGTGACAATTGAACGGGTGGCCC	0	14	RDR2-sensitive
TCGTGACTCGCATGATCAGCTCGG	0	12	RDR2-sensitive
TCGTGACTCTGGACTAGACGGTTC	0	40	RDR2-sensitive
TCGTGACTGAGCTGCCGGTAGCGT	0	10	RDR2-sensitive
TCGTGACTGTTTCGGGCAACACAA	0	14	RDR2-sensitive
TCGTGACTGTTTCGGGCAACACGG	2	61	RDR2-sensitive
TCGTGACTGTTTCGGGTAACACGG	0	19	RDR2-sensitive
TCGTGAGAGGTCTAGGTCGTCGTC	0	11	RDR2-sensitive
TCGTGAGCCTTGTCGAGCCGAGCT	0	11	RDR2-sensitive
TCGTGATATCGGAGGAGGGTTATT	26	1	RDR2-resistant
TCGTGATCAACTCGGCTCGGCTGA	0	12	RDR2-sensitive
TCGTGATCTCTGCGAGGACGAGAA	0	14	RDR2-sensitive
TCGTGATCTTCTGACATCGTCGAC	3	29	RDR2-sensitive
TCGTGATCTTCTGACCTCGTCGAC	0	16	RDR2-sensitive
TCGTGCCCGGACACGGCTGACTGC	0	21	RDR2-sensitive
TCGTGCCGGGCTTGGGATGGCCGT	0	19	RDR2-sensitive
TCGTGCCGGGTCGGGCTTAGGCAC	0	9	RDR2-sensitive
TCGTGCGACTGTCCGGTGAGGCCT	0	13	RDR2-sensitive
TCGTGCGGAGGTATAGATGGCCAA	0	44	RDR2-sensitive
TCGTGCGGAGGTATAGATGGTCAA	0	26	RDR2-sensitive
TCGTGCGGATGTATAGATGGCCAA	0	19	RDR2-sensitive
TCGTGCGTGGGAGCTCAGGCGGAT	0	10	RDR2-sensitive
TCGTGCTAAACGGACCGGCTCGGC	0	13	RDR2-sensitive
TCGTGCTAACGCTACGATCATCGG	0	10	RDR2-sensitive
TCGTGCTGATGAACCAGGAGCGGT	0	20	RDR2-sensitive
TCGTGCTGATGGACCGGGAGCGGT	1	18	RDR2-sensitive
TCGTGCTGATGGACTGGGAGCGGT	0	10	RDR2-sensitive
TCGTGCTGCCACACTAGTTCTGTC	23	0	RDR2-resistant
TCGTGCTTCGTGCCGGGCTAACGG	0	9	RDR2-sensitive
TCGTGCTTGGACAGGTAGGCCGGC	0	13	RDR2-sensitive
TCGTGCTTGGACGAGGACACGGCG	14	0	RDR2-resistant
TCGTGGAAGGCCAGGTAGAGCGGT	0	25	RDR2-sensitive
TCGTGGACAGAGCATGGGCATGGA	0	15	RDR2-sensitive
TCGTGGAGCCCTCTGGCAGACCGC	0	13	RDR2-sensitive

TCGTGGCAATTGAAATCGTGGCAT	0	21	RDR2-sensitive
TCGTGGCAATTGAACGGGTGGCAC	3	241	RDR2-sensitive
TCGTGGCAATTGAACGGGTGGCCC	0	32	RDR2-sensitive
TCGTGGCAATTGTAGGCGTGGCAT	0	251	RDR2-sensitive
TCGTGGCAATTGTAGGCGTGGCCT	0	14	RDR2-sensitive
TCGTGGGCCGGGCAAAGACTACAG	0	10	RDR2-sensitive
TCGTGGGCTCTCGGTCGCGTGCGT	0	16	RDR2-sensitive
TCGTGGGCTTCCATGGGTCGTCAC	0	13	RDR2-sensitive
TCGTGGTGATCTGTTGGAGGCCAG	0	14	RDR2-sensitive
TCGTGGTGCTTTTGGGTAGGACGG	0	15	RDR2-sensitive
TCGTGTATACAGACTCAGCGGTGC	0	11	RDR2-sensitive
TCGTGTCAGCTATGGAAACGGTAA	0	13	RDR2-sensitive
TCGTGTCAGCTGTGGAAACGGTAA	3	182	RDR2-sensitive
TCGTGTCAGCTGTGGAATCGGTAA	0	10	RDR2-sensitive
TCGTGTCAGCTGTGGATGCGGTAA	0	10	RDR2-sensitive
TCGTGTCAGCTGTTGAAACGGTAA	0	39	RDR2-sensitive
TCGTGTCGACTCTAGATAGGGCAG	0	18	RDR2-sensitive
TCGTGTCGGGCCTCGGCAGGCTTA	1	12	RDR2-sensitive
TCGTGTCTGCTGTGGAAACGGTAA	0	57	RDR2-sensitive
TCGTGTGGCTGGGACAGCAAGGTT	0	9	RDR2-sensitive
TCGTGTGTGAATCTGAGCGTCGGC	305	19	RDR2-resistant
TCGTGTGTGATGCAGGACGATCGT	0	14	RDR2-sensitive
TCGTTAAGGAACCGAGCCAGAGAA	0	13	RDR2-sensitive
TCGTTATCATCGGGCTTTGTGCGT	0	10	RDR2-sensitive
TCGTTATGTTAACAAGCTGAAGAT	0	10	RDR2-sensitive
TCGTTATGTTGTAGCTTCAGTGGC	163	8	RDR2-resistant
TCGTTCGTCCGGATCTTTCGGTAA	0	9	RDR2-sensitive
TCGTTCTTAGGCTCAGCGGCAATC	23	1	RDR2-resistant
TCGTTGGCTCACTCGGTTGATGGT	0	10	RDR2-sensitive
TCGTTGTAGCAGGAGCTGGAGAGT	35	6	RDR2-resistant
TCGTTGTAGCCTCCGCGTCGGAAA	0	10	RDR2-sensitive
TCGTTGTAGGATCTCGAAGACGAC	0	23	RDR2-sensitive
TCGTTGTATCGCACGAGACAGGCT	0	31	RDR2-sensitive
TCGTTGTATCTAGGATGCCGGACA	0	9	RDR2-sensitive
TCGTTGTCTGGGCTGTTTGTGCAT	0	11	RDR2-sensitive
TCGTTTACTGTAGTGTATGACGGA	0	39	RDR2-sensitive
TCGTTTAGGACAACGACACGGTCT	0	13	RDR2-sensitive
TCGTTTAGGACATGATTGTGCATA	2	47	RDR2-sensitive
TCGTTTAGTTAATGATCGGTTCGG	0	58	RDR2-sensitive
TCGTTTCTCTGTCCGAGCCCGGCT	0	11	RDR2-sensitive
TCGTTTCTGTGGAGCCAGGCTGAG	0	9	RDR2-sensitive
TCGTTTCTTTCGGAGTGGAGGCAT	0	20	RDR2-sensitive
TCGTTTTAGTTGTCGCTGGATAGT	4	29	RDR2-sensitive
TCGTTTTAGTTGTCGCTGGATGGT	0	9	RDR2-sensitive
TCGTTTTTTTCGGACACTCGGCAA	0	22	RDR2-sensitive
TCTAAAACTCTGGGTGGAGCAGCT	0	28	RDR2-sensitive
TCTAAACGACGTAGAGCCGGAGAG	0	16	RDR2-sensitive
TCTAAACTCTGGAGCAGAGCTGCT	3	89	RDR2-sensitive
TCTAAACTCTGGAGTAGAGCTGCT	0	18	RDR2-sensitive

TCTAAACTGCTAGATTCGCGACGG	0	9	RDR2-sensitive
TCTAAACTTTAAAGTAGAGCTGCT	0	11	RDR2-sensitive
TCTAAATATAGAATGTATGAGGCT	26	0	RDR2-resistant
TCTAAATGTGGAGGGCTCCGGCAG	0	36	RDR2-sensitive
TCTAAATTTAGAGGACGCTGCTGG	1	13	RDR2-sensitive
TCTAACCATGTCTGGCGGATCTGC	0	9	RDR2-sensitive
TCTAACCTAAAGTCGGGCGGGCAC	0	10	RDR2-sensitive
TCTAACTGTAGCTTGGAGAGGCAT	0	17	RDR2-sensitive
TCTAACTTGGCTGACATGGTGCTC	43	2	RDR2-resistant
TCTAAGAATGACGATCACTAAACT	32	1	RDR2-resistant
TCTAAGACACAACTTAAGACACCC	0	10	RDR2-sensitive
TCTAAGTGGGAGAGTGGACGTCAT	0	15	RDR2-sensitive
TCTAAGTTTTGGAGAGCTGACGTC	25	0	RDR2-resistant
TCTAATACTGTAAGTTCGACGGTT	0	36	RDR2-sensitive
TCTAATACTGTAAGTTTGACGGTT	0	20	RDR2-sensitive
TCTAATATGACATGATGACGACGT	25	0	RDR2-resistant
TCTAATGGACCAGATAGTTTGCTT	0	9	RDR2-sensitive
TCTAATTGTGGTGGATGAATAGGC	28	0	RDR2-resistant
TCTACAAGTATTGGGTGTGCTGAT	56	0	RDR2-resistant
TCTACACTTATGCAAGGACTGCTC	0	16	RDR2-sensitive
TCTACAGGCGCGGACCGTCCGGAC	0	12	RDR2-sensitive
TCTACCCGTGGGTCTGTTATTGGA	0	9	RDR2-sensitive
TCTACCCGTGGGTCTGTTATTGGC	2	17	RDR2-sensitive
TCTACCGGATGGATGGGTCGGAAC	0	9	RDR2-sensitive
TCTACCGTCCACCGCTCTGGAGGC	16	0	RDR2-resistant
TCTACGACAGTATGGACGGTCCAC	0	19	RDR2-sensitive
TCTACGACGGGTAGGGTAGGGCAT	0	9	RDR2-sensitive
TCTACGACTTGAGCGACGACGCTG	0	10	RDR2-sensitive
TCTACGCGAGACAGACGCACCTGC	0	14	RDR2-sensitive
TCTACGCGGACCACAATGGCGCAT	0	10	RDR2-sensitive
TCTACGGAAGCGCGGACGGTCCAC	0	10	RDR2-sensitive
TCTACGGACTCCTCTGGCTGGCAC	0	14	RDR2-sensitive
TCTACGGTTTCGGTTGGGTGTCGG	23	1	RDR2-resistant
TCTACTGCTGACGGGAGGGAAGGG	0	31	RDR2-sensitive
TCTACTGGAGAAGCCTACAATCAT	0	10	RDR2-sensitive
TCTACTGTATGAAGCTAGCATGGC	0	16	RDR2-sensitive
TCTACTGTTTATTTTGTTTGGCAT	17	0	RDR2-resistant
TCTACTTGGCTCGGCTCGACGGAT	0	9	RDR2-sensitive
TCTAGAACATTCGGCTGAGCACAC	0	9	RDR2-sensitive
TCTAGAACCGTCACTATAGAGGCT	2	14	RDR2-sensitive
TCTAGAACGGACGGTCCGTGACCT	0	9	RDR2-sensitive
TCTAGAACGGGCTCGACGAGAGGA	0	28	RDR2-sensitive
TCTAGAACTCGCCCCTCGAGATGG	0	12	RDR2-sensitive
TCTAGAAGACGAAGGGTTCCGGAG	0	19	RDR2-sensitive
TCTAGAATGTTGCACAGGATTGTT	111	4	RDR2-resistant
TCTAGACATGTCAGACTGCACCAG	0	21	RDR2-sensitive
TCTAGACCGGCCTCTTCTTTTGCA	14	0	RDR2-resistant
TCTAGACTGCTGGGGCGTAATGGC	0	10	RDR2-sensitive
TCTAGAGCGTATTGGACGGTTGAG	1	43	RDR2-sensitive

TCTAGAGCTGCGGTGAGGATTCCC	0	13	RDR2-sensitive
TCTAGAGCTGTTTGCACTGGCGTT	2	65	RDR2-sensitive
TCTAGATCCTGCACCGGGAGAGGG	2	17	RDR2-sensitive
TCTAGATTGTATCGGTGCGTGCAC	0	10	RDR2-sensitive
TCTAGCACATGATAGGTCGGCCAT	0	11	RDR2-sensitive
TCTAGCACGGCACAGATCTGGCAC	1	192	RDR2-sensitive
TCTAGCAGTGGATGCAATAGCTCT	25	0	RDR2-resistant
TCTAGCGACGTTGTATTTGATGAG	36	5	RDR2-resistant
TCTAGCTGTGGGGAGAATCTGGCT	0	9	RDR2-sensitive
TCTAGCTTGTCGAGCGGATGGACG	0	20	RDR2-sensitive
TCTAGGAACTCCGTCGGATCGCGG	0	10	RDR2-sensitive
TCTAGGCTGTGAGAGTAGGGAGAC	0	21	RDR2-sensitive
TCTAGGGCTGTGTGTTTGCGGTAC	0	10	RDR2-sensitive
TCTAGTCAGCAATGAAGTCTGCTA	15	0	RDR2-resistant
TCTAGTCAGGCGCGAACGGTCCGC	0	10	RDR2-sensitive
TCTAGTCGATGATCAATGGCCACA	20	1	RDR2-resistant
TCTAGTGGCATTGAACGGATCAAT	16	0	RDR2-resistant
TCTAGTTGGGCGCATCAGGCGGTA	274	26	RDR2-resistant
TCTATAAGACAATAGTGTAGACGT	0	10	RDR2-sensitive
TCTATAAGACAGTAGTGTAGACGT	0	28	RDR2-sensitive
TCTATAAGGACCGAGTGTGGGCGT	0	9	RDR2-sensitive
TCTATAAGGCTGTAAGAAGGGCGT	0	9	RDR2-sensitive
TCTATAATTTGTAGGTCCGATGAT	0	23	RDR2-sensitive
TCTATACGTGGATCGTTGATCGGA	35	1	RDR2-resistant
TCTATACTACTAGGTTGTTCGGGC	0	38	RDR2-sensitive
TCTATAGCGGTACGGACGGTCTGT	0	30	RDR2-sensitive
TCTATATCAAGTTGGAAGTATGCT	42	1	RDR2-resistant
TCTATATGTTAGCAGTGTAGACGT	0	10	RDR2-sensitive
TCTATATTCGAGACAGGAGACTAT	2	18	RDR2-sensitive
TCTATCATGCCACCGGACAGGCTT	54	0	RDR2-resistant
TCTATCGCGTAGGACAGTTCGGCT	0	30	RDR2-sensitive
TCTATCGTGTTGTGCTGGGATGGT	0	13	RDR2-sensitive
TCTATCTCGGATCTCATGGCGACT	32	0	RDR2-resistant
TCTATCTCTGTAGCGTTCGGCATT	3	37	RDR2-sensitive
TCTATGAAATGTGGGTCTGATGGC	0	21	RDR2-sensitive
TCTATGACACTGAGCTGGACGGTC	0	9	RDR2-sensitive
TCTATGACATGTGGGGCCAGTGGC	0	24	RDR2-sensitive
TCTATGATATTAGAGATCTGACAG	0	10	RDR2-sensitive
TCTATGATTTGTAGGCCCGATGGT	0	11	RDR2-sensitive
TCTATGATTTGTGGATCCGATGGC	0	12	RDR2-sensitive
TCTATGATTTGTGGGTCCGATGGC	1	86	RDR2-sensitive
TCTATGCGGCGACGCGGACGGTTT	0	12	RDR2-sensitive
TCTATGGATTGCAGCTGCAGCAAT	0	9	RDR2-sensitive
TCTATGGATTGCAGCTGCAGCAGT	0	21	RDR2-sensitive
TCTATGTCGGACGACTGGTGTTTC	14	0	RDR2-resistant
TCTATGTTAGAACTCCGGCGAGGA	21	0	RDR2-resistant
TCTATGTTCGAGACAGAAGACTAG	0	12	RDR2-sensitive
TCTATGTTCGAGACAGGAGACTAG	0	40	RDR2-sensitive
TCTATGTTCGAGACAGGAGACTAT	0	49	RDR2-sensitive

TCTATGTTCGAGACAGGAGGCTAG	0	30	RDR2-sensitive
TCTATTATGTTGGACTTGGATGGT	0	12	RDR2-sensitive
TCTATTCAGGGCAACATACTGGCT	0	9	RDR2-sensitive
TCTCAAATTCATTGGGCAGAGACT	17	0	RDR2-resistant
TCTCAACTCTGCCTTAACGACATG	33	7	RDR2-resistant
TCTCACCCAATCGCCGGCGCGCAC	0	9	RDR2-sensitive
TCTCACTTCCAGAAGGGACTCTAT	0	26	RDR2-sensitive
TCTCAGACACTCGGCAAAGGCTAT	0	15	RDR2-sensitive
TCTCAGCAGGTACGACGGACATTC	0	11	RDR2-sensitive
TCTCAGCAGTCAGACACTCAGCAA	0	9	RDR2-sensitive
TCTCAGCCTGGACCAGTTGAACGG	0	14	RDR2-sensitive
TCTCAGCGAACTGTACATCGGCAA	0	42	RDR2-sensitive
TCTCAGCTGAAGGTACACTAGAGT	51	0	RDR2-resistant
TCTCAGCTGGACCGGATACTACGC	0	10	RDR2-sensitive
TCTCAGGAAACGGTGGAGCAACGC	0	11	RDR2-sensitive
TCTCAGGCGGCCTATGGAGTGCAT	0	10	RDR2-sensitive
TCTCAGGCTATGGACGCAGGGGCT	0	16	RDR2-sensitive
TCTCAGGTACCACCGGCTCAGCGT	16	0	RDR2-resistant
TCTCAGTCATGAAGTAGGAGCCAG	50	2	RDR2-resistant
TCTCAGTGCTGAAGCCGGACGGTC	0	10	RDR2-sensitive
TCTCAGTGGATCGTGGCAGCAAGG	31	3	RDR2-resistant
TCTCATAATATTTTAGCAAGTCGG	16	0	RDR2-resistant
TCTCATCCATCGGGCTTCGCGCAT	0	9	RDR2-sensitive
TCTCCACACATGCAGGCTGGACAC	0	65	RDR2-sensitive
TCTCCACATATGCAGGCTGGACAC	0	17	RDR2-sensitive
TCTCCACCACGCCGGCAGGTCCTC	1	12	RDR2-sensitive
TCTCCACGGATGCAACGAACGGAT	0	11	RDR2-sensitive
TCTCCAGCAGCGTCCTCTAAACGG	0	9	RDR2-sensitive
TCTCCAGGTTGGACTGTGCGGTTG	0	15	RDR2-sensitive
TCTCCCCAAGGACGTTCATTTGAT	59	3	RDR2-resistant
TCTCCCCACAGCTAGATTCTCGGA	0	12	RDR2-sensitive
TCTCCCGTCAGCAGTAGAGGCGCA	1	13	RDR2-sensitive
TCTCCCTGCAGGACCTTCGGCGAC	0	9	RDR2-sensitive
TCTCCGAAGCGGACTGACGGTGTA	0	11	RDR2-sensitive
TCTCCGACAGGTAGACGAGGACAG	0	9	RDR2-sensitive
TCTCCGACGGATCTGTACGAGGCC	0	9	RDR2-sensitive
TCTCCGACGGCTCTGTACGAGGCC	1	19	RDR2-sensitive
TCTCCGACGGCTGTGTACGAGGCC	1	13	RDR2-sensitive
TCTCCGACTGTTCCGACGGTAGCC	20	1	RDR2-resistant
TCTCCGCGCGCTGTAGAAGGCACC	1	13	RDR2-sensitive
TCTCCGGATGCAGGCTGGCGGCGT	4	39	RDR2-sensitive
TCTCCGTCGATGGAAGGACGCCAT	22	1	RDR2-resistant
TCTCCGTGAAGAGACGTCTCTGGC	0	9	RDR2-sensitive
TCTCCGTTGACTGTCTACGGCTGC	61	0	RDR2-resistant
TCTCCGTTTGGCAAGCAGAACTGA	0	11	RDR2-sensitive
TCTCCTAGTTGGGCTGAAACCGGC	0	15	RDR2-sensitive
TCTCCTCGAAGGGTCGGACACGGA	0	9	RDR2-sensitive
TCTCCTCGGGAGAGACACTGGAGC	18	0	RDR2-resistant
TCTCCTCTGCACGCTTTCGGACGG	0	13	RDR2-sensitive

TCTCCTGATCGCAGATGCACGTCT	0	9	RDR2-sensitive
TCTCCTGGGCACGGATAGGGGCAT	23	0	RDR2-resistant
TCTCCTTTGTAGAGCCACGTGCGT	0	10	RDR2-sensitive
TCTCGAACATAGAGCTAAGACACT	0	17	RDR2-sensitive
TCTCGACAGAGGGCGTAGGAGCAT	2	52	RDR2-sensitive
TCTCGACAGAGGGCGTATGAGCAT	0	17	RDR2-sensitive
TCTCGACGAACTGTACATCGGTAA	0	12	RDR2-sensitive
TCTCGACGACTGTACAGATTGCGT	0	9	RDR2-sensitive
TCTCGACGAGCTGTACATCGGCAA	0	25	RDR2-sensitive
TCTCGACGTGCAAGCTACTGGTAC	0	11	RDR2-sensitive
TCTCGAGACTGGCTTCGCCCCTAA	0	14	RDR2-sensitive
TCTCGAGGATTTGTAGTTACGGAT	0	9	RDR2-sensitive
TCTCGCGCCTAATCTGCTGACGGG	0	18	RDR2-sensitive
TCTCGCGTAGGAGAGTTCGGCTAG	0	10	RDR2-sensitive
TCTCGCTGCAGTCGCTGGTTTGAC	0	13	RDR2-sensitive
TCTCGGAACCACTCGGGACGGAAG	0	11	RDR2-sensitive
TCTCGGAACTTGGAGCAGAGTCAT	0	15	RDR2-sensitive
TCTCGGACACTCGGCATAGACTTT	0	9	RDR2-sensitive
TCTCGGACAGTCCGGTGGCTTCAC	0	19	RDR2-sensitive
TCTCGGACATAAGGCTACCTCCAA	0	9	RDR2-sensitive
TCTCGGACTCGGTCAAGGCCAGAC	1	13	RDR2-sensitive
TCTCGGAGCAGATGTAGAAGCGGC	0	9	RDR2-sensitive
TCTCGGATACGGGACCAGATACGG	1	20	RDR2-sensitive
TCTCGGATACGGGTAGGATACGGA	0	10	RDR2-sensitive
TCTCGGCAAAAGGACGGCGGCTGT	0	9	RDR2-sensitive
TCTCGGCAAAGACTGACGACCGTC	0	10	RDR2-sensitive
TCTCGGCAAAGACTGACGGCCGTC	3	53	RDR2-sensitive
TCTCGGCAAAGGGCGTGGGAGCAG	0	10	RDR2-sensitive
TCTCGGCACAGACAGACACTCGGC	0	10	RDR2-sensitive
TCTCGGCACAGGAAAACACTCGGC	0	12	RDR2-sensitive
TCTCGGCAGAGGACGTAGGAGCAT	0	30	RDR2-sensitive
TCTCGGCAGAGGGCGTAGGAGCAG	0	39	RDR2-sensitive
TCTCGGCAGAGGGCGTAGGAGCAT	9	88	RDR2-sensitive
TCTCGGCAGCGGGCGTAGGAGCAT	0	32	RDR2-sensitive
TCTCGGCGAACAGTATATCGGCAA	0	10	RDR2-sensitive
TCTCGGCGAACTGTACATCGGCAA	1	16	RDR2-sensitive
TCTCGGCGAACTGTATATCGGCAA	0	12	RDR2-sensitive
TCTCGGGAGCGAATTTTCAGGCAT	0	9	RDR2-sensitive
TCTCGGGCACTCGACAAAGAGGTT	2	19	RDR2-sensitive
TCTCGGGCACTCGGCAAAGAGGTT	8	83	RDR2-sensitive
TCTCGGGCACTCGGCAAAGATGTT	0	9	RDR2-sensitive
TCTCGGGCTCTCGGCGTATACCGC	0	9	RDR2-sensitive
TCTCGGGTGGAGCATATACGTCGT	2	37	RDR2-sensitive
TCTCGGTAAACTGTACATCGGCAA	0	18	RDR2-sensitive
TCTCGGTAACCTTGAGCATCTTGG	26	3	RDR2-resistant
TCTCGGTACAGGACAGATCGGCAC	0	13	RDR2-sensitive
TCTCGGTCTGAGCTTCCCAGAGAC	17	0	RDR2-resistant
TCTCGGTGGAACGCGACGCTCGAT	0	18	RDR2-sensitive
TCTCGGTTGTATTTCGTTTCGGTG	35	2	RDR2-resistant

TCTCGGTTTGGCTCAGAGCGGCTC	0	9	RDR2-sensitive
TCTCGTAGCACGAACGGTGGGCGC	0	14	RDR2-sensitive
TCTCGTATCGTGCGTTGTTGTGGC	230	7	RDR2-resistant
TCTCGTCGCGCGGCTTCGCCACTT	0	11	RDR2-sensitive
TCTCGTCGTCAAGCTGAAGGCCGT	0	40	RDR2-sensitive
TCTCGTCGTCAAGCTGAAGGTCGT	1	16	RDR2-sensitive
TCTCGTCGTCGTTCCGTACGTCAT	0	14	RDR2-sensitive
TCTCGTCGTCGTTCCGTGCGTCAT	1	28	RDR2-sensitive
TCTCGTGACTTGAGCGACGGAGGA	2	18	RDR2-sensitive
TCTCGTGCGTCGAAGGTTAGCGGT	53	1	RDR2-resistant
TCTCGTTTGGACTTCCGGCCGGGT	0	10	RDR2-sensitive
TCTCTAATTCGTGGATTTATGCAT	15	0	RDR2-resistant
TCTCTACAGGGACTCCGTACAAGC	44	7	RDR2-resistant
TCTCTACCTGGTTGCATCGCGGCT	0	11	RDR2-sensitive
TCTCTACGCCAAGCCGGACGGTCC	0	12	RDR2-sensitive
TCTCTACGCTGAGCCGGACGGTCC	0	10	RDR2-sensitive
TCTCTAGTCAGGCGCGAACGGTCC	0	9	RDR2-sensitive
TCTCTAGTCAGGCGCGGACGGTCC	0	18	RDR2-sensitive
TCTCTATAGAAGCACGGACGGTCC	0	13	RDR2-sensitive
TCTCTATGACAGCGCGGACGGTAC	0	33	RDR2-sensitive
TCTCTATGGATTGCAGCTGCAGCA	0	38	RDR2-sensitive
TCTCTCACCTACGGGTCTTCGGCT	15	0	RDR2-resistant
TCTCTCCCCCCAGATCTAGCGTGT	0	9	RDR2-sensitive
TCTCTCCCGTCAGCAGTAGAGGCG	0	14	RDR2-sensitive
TCTCTCGCACGCATACTCTCTACC	27	0	RDR2-resistant
TCTCTCGGACTACGGCACACTCAT	0	128	RDR2-sensitive
TCTCTCGGACTCGGTCAAGGCCAG	0	15	RDR2-sensitive
TCTCTCGGATCACCGGATCGACGA	0	10	RDR2-sensitive
TCTCTCGTTGTCGGAGTTTGGTGT	22	0	RDR2-resistant
TCTCTCGTTTGGACTTCCGGCCGG	0	26	RDR2-sensitive
TCTCTCTCCCCCCAGATCTAGCGT	0	18	RDR2-sensitive
TCTCTCTGCAGGATGACTCTCATC	64	6	RDR2-resistant
TCTCTCTGCTGTAGTTTTGGCATA	71	22	RDR2-resistant
TCTCTGACGAGTGTGTAGGACGGC	0	12	RDR2-sensitive
TCTCTGACGGGCTGGTGCTGGCAC	0	10	RDR2-sensitive
TCTCTGACTGACGTGCCGGACGGT	1	13	RDR2-sensitive
TCTCTGACTGTAGCGCGCGACCGG	0	14	RDR2-sensitive
TCTCTGATCAGGCACGGACGGTCC	0	39	RDR2-sensitive
TCTCTGATCAGGCGCGGACGGTCC	0	13	RDR2-sensitive
TCTCTGCACAGGCGCGGACGGTCC	0	26	RDR2-sensitive
TCTCTGCACAGGGGCGGACGGTCC	4	17	RDR2-sensitive
TCTCTGCCTGACGGCCGGACGGTC	1	14	RDR2-sensitive
TCTCTGCGACGACGGGCTGGACGG	0	10	RDR2-sensitive
TCTCTGCGACGGCACGGACGGTCT	0	14	RDR2-sensitive
TCTCTGCGCTATTGTAGACGGTTC	0	12	RDR2-sensitive
TCTCTGCGGAGCCGATGACGGTCC	0	18	RDR2-sensitive
TCTCTGCGGAGGAGTAGACGGTCC	0	13	RDR2-sensitive
TCTCTGCGGAGGCGTAGACGGTCC	0	15	RDR2-sensitive
TCTCTGCGGGAGCGCTGACGGAGC	0	10	RDR2-sensitive

TCTCTGCGTAGAGTTGCGGACGGT	0	9	RDR2-sensitive
TCTCTGCTGAGCGGGAGTTCTGGA	17	0	RDR2-resistant
TCTCTGGAATTGTAGCGTTAGCAT	0	13	RDR2-sensitive
TCTCTGGCGGACTGCGGAGGCGTC	0	13	RDR2-sensitive
TCTCTGGGCCGTGCTTGGACACAT	1	16	RDR2-sensitive
TCTCTGGTCAAACGCGGACGGTCC	0	14	RDR2-sensitive
TCTCTGGTCAAGCGCGGACGGTCC	0	56	RDR2-sensitive
TCTCTGGTCAGACGCAGACGGTAC	0	11	RDR2-sensitive
TCTCTGGTCAGACGCGGACGGTCC	2	173	RDR2-sensitive
TCTCTGGTCAGACGTGGACGGTCC	0	54	RDR2-sensitive
TCTCTGGTCAGGCACGGACGGTCC	0	22	RDR2-sensitive
TCTCTGGTCAGGCGCGAACGGTCC	0	20	RDR2-sensitive
TCTCTGGTCAGGCGCGGACGATCC	0	12	RDR2-sensitive
TCTCTGGTCAGGCGCGGACGGTCC	9	689	RDR2-sensitive
TCTCTGGTCAGGCGCGGACGGTCT	0	44	RDR2-sensitive
TCTCTGGTCAGGCGTGGACGGTCC	2	31	RDR2-sensitive
TCTCTGGTCAGGTGCGGACGGTCC	1	12	RDR2-sensitive
TCTCTGGTCGGACGCGGACGGTCC	0	12	RDR2-sensitive
TCTCTGTAGAAGCACGGACGGTCC	0	25	RDR2-sensitive
TCTCTGTAGAGGCGTGGACGGTCC	0	21	RDR2-sensitive
TCTCTGTAGAGTGGCAGATTCGAC	26	0	RDR2-resistant
TCTCTGTAGCAAGGTGGTTCTCCC	35	0	RDR2-resistant
TCTCTGTAGCAGCACGGACGGTCC	2	22	RDR2-sensitive
TCTCTGTAGCCGCATCACGCGCAT	0	10	RDR2-sensitive
TCTCTGTGGAACCACGGACGGTCC	0	9	RDR2-sensitive
TCTCTGTGGAGGCACGAACGGTCC	0	27	RDR2-sensitive
TCTCTGTGGAGGCACGGACGGTCC	0	9	RDR2-sensitive
TCTCTGTGGAGGCGCTGACGGTCC	0	18	RDR2-sensitive
TCTCTGTGGATAGATGGCAGGTTT	38	3	RDR2-resistant
TCTCTGTTTGTAGACACGTACGGG	44	5	RDR2-resistant
TCTCTGTTTGTCTACTGTCGCGGC	21	0	RDR2-resistant
TCTCTTAAACTGGAATACATGGCT	22	0	RDR2-resistant
TCTCTTACGGAAGCAGGACAACGT	0	19	RDR2-sensitive
TCTCTTACGGAAGCAGGACGACGT	1	54	RDR2-sensitive
TCTCTTACGGAAGCAGGAGGACGT	1	13	RDR2-sensitive
TCTCTTGGACTCTATTGTGCGCAT	0	10	RDR2-sensitive
TCTCTTGTAGCAGCTTAAGAAGCC	96	4	RDR2-resistant
TCTCTTGTCGTGTAGCAGCAGCAT	0	17	RDR2-sensitive
TCTCTTTAAGTCGGTATCGTCCTT	14	0	RDR2-resistant
TCTGAAAAGCTGACTGTGGGGAAA	0	13	RDR2-sensitive
TCTGAAAAGCTGGCTGTGGGGAAA	3	34	RDR2-sensitive
TCTGAAAGATATCGGTCTAAATAA	127	0	RDR2-resistant
TCTGAAAGTGACTGAATATATGCA	93	6	RDR2-resistant
TCTGAACGAGCCCGAGTCGAGCCT	0	13	RDR2-sensitive
TCTGAACGTCGAGCTGGTCCGAGC	0	23	RDR2-sensitive
TCTGAAGATGGAGAATGTCGTCAT	0	15	RDR2-sensitive
TCTGAAGCTGGAGAATGTCGTCAT	0	10	RDR2-sensitive
TCTGAAGCTGTGAAGGTCCGATCT	14	0	RDR2-resistant
TCTGAAGGCTGCAGACAGAGTCAT	0	11	RDR2-sensitive

TCTGAATGATTTTAGCGACTTGGA	18	0	RDR2-resistant
TCTGAATGGAAGGTGGAATGGTCA	27	0	RDR2-resistant
TCTGAATTGTATGAGAGACGTTGT	0	16	RDR2-sensitive
TCTGAATTTTGTTGGACTTCGGCT	43	0	RDR2-resistant
TCTGACATAGGTACTGTGGATCGG	0	13	RDR2-sensitive
TCTGACATCCACGGGCTAGCAGGC	0	11	RDR2-sensitive
TCTGACATGTCCAGAGCTTGGCTC	0	16	RDR2-sensitive
TCTGACCACTGGACGTTAGCACTT	0	11	RDR2-sensitive
TCTGACCCAGGACTTTGTTGCGGC	0	11	RDR2-sensitive
TCTGACCCCATGAATTCTCGGCCT	0	9	RDR2-sensitive
TCTGACCTGAACCCGACACGGCAC	0	27	RDR2-sensitive
TCTGACCTGGCTGCCTCGCTGCAT	0	9	RDR2-sensitive
TCTGACGACCAGAAGTTGCTGCAG	0	33	RDR2-sensitive
TCTGACGCGGATTGTGCGATGGTT	0	19	RDR2-sensitive
TCTGACGTGGCAGTACCTCGGCGC	0	12	RDR2-sensitive
TCTGACGTTCTGGATTGGCGCGCC	0	9	RDR2-sensitive
TCTGACGTTGGGCCAGATACGGAT	0	18	RDR2-sensitive
TCTGACTCGGACAGATTTTAGCAT	0	11	RDR2-sensitive
TCTGACTCGGACAGATTTTAGCGT	1	35	RDR2-sensitive
TCTGACTCGGGCGGATTTCAGCGT	0	34	RDR2-sensitive
TCTGACTCTGGCGCGCACTGTAGC	2	22	RDR2-sensitive
TCTGACTGATTGGCTGACTGACTG	15	0	RDR2-resistant
TCTGACTGCGAAGATCGACAATGT	0	11	RDR2-sensitive
TCTGACTGCTCGCGGTGGACCTGC	0	13	RDR2-sensitive
TCTGACTTGAAACTTGCTTGAAGA	103	6	RDR2-resistant
TCTGACTTGGACAGATTTTAGCGT	1	56	RDR2-sensitive
TCTGACTTTGCACATTCTGATGGT	12	32	RDR2-sensitive
TCTGAGAAGCAGCTGAACTGATAA	1	36	RDR2-sensitive
TCTGAGAATGCACTGGGCCAACCT	0	10	RDR2-sensitive
TCTGAGACCTTGGCGTAGACTGTC	0	10	RDR2-sensitive
TCTGAGACTGACAGGGCCCGGTTC	0	10	RDR2-sensitive
TCTGAGCCCGGCACGGTAGAATAA	0	10	RDR2-sensitive
TCTGAGCCTGGCACGGTAGAATAA	0	55	RDR2-sensitive
TCTGAGCGAGTAGTCGACTCTGTG	0	22	RDR2-sensitive
TCTGAGCGTGCAGGCTGATCTCAT	1	54	RDR2-sensitive
TCTGAGCGTGCAGGCTGATCTCGT	1	13	RDR2-sensitive
TCTGAGCGTGCAGGCTGGTCTCGT	0	9	RDR2-sensitive
TCTGAGCTGGAAACTAGGTGCGGT	22	0	RDR2-resistant
TCTGAGCTGGACGAGGCCGCACTG	0	9	RDR2-sensitive
TCTGAGGCTCTGACCGAGGACCAG	0	9	RDR2-sensitive
TCTGAGGCTGAGCATGCATGTCAT	19	0	RDR2-resistant
TCTGAGGGGCTGCAACGTTTGGAG	0	9	RDR2-sensitive
TCTGAGGGTCACGCACTGCTGCAT	0	10	RDR2-sensitive
TCTGAGGTGATTGAGGACATCATA	42	7	RDR2-resistant
TCTGAGTCAGATTTAGCCGGCGCT	14	0	RDR2-resistant
TCTGAGTCTACAGAACTTATGCAT	0	11	RDR2-sensitive
TCTGATAACTTCTGAGGTCTTGGC	74	0	RDR2-resistant
TCTGATACCAATTGTTGGAACCGG	1	18	RDR2-sensitive
TCTGATAGGATCTGCCGAAGGTGT	0	13	RDR2-sensitive

TCTGATCAAGTCTCGGACCGGTCT	0	10	RDR2-sensitive
TCTGATCAGTGCGGCCACGGACAT	0	12	RDR2-sensitive
TCTGATCCCACAGCATGGACTGGA	70	10	RDR2-resistant
TCTGATCCTGGCACGTACATGGTT	1	17	RDR2-sensitive
TCTGATCGGAGGGGCGCGGATCAT	0	12	RDR2-sensitive
TCTGATCTCGCGAGCAGTGCTGAT	0	13	RDR2-sensitive
TCTGATCTCGTAGACCGTGGTAAT	0	9	RDR2-sensitive
TCTGATCTGACGGAGTCTGTGGCT	17	0	RDR2-resistant
TCTGATCTTGTATGTGGATGGCAT	25	0	RDR2-resistant
TCTGATGAAGCTGCTGCATTAACT	29	0	RDR2-resistant
TCTGATGAAGTCGAAGCTGTTCGT	0	42	RDR2-sensitive
TCTGATGAGTTAGACATGAGTGCT	0	9	RDR2-sensitive
TCTGATGCCGGATCCTGAAGACGC	0	24	RDR2-sensitive
TCTGATTGGATGACTGCGTGTCCG	0	12	RDR2-sensitive
TCTGATTGGTACAATGAATATGGT	4	83	RDR2-sensitive
TCTGCAACGGACCAGCTGAAGGGT	1	14	RDR2-sensitive
TCTGCAATCAGGCTCGGCTCGGCT	0	13	RDR2-sensitive
TCTGCACAACAAGCAGCTGGACAC	14	0	RDR2-resistant
TCTGCACACTTCTGGACGGTCCGT	0	16	RDR2-sensitive
TCTGCACAGCTGGCGAACCAAACA	1	39	RDR2-sensitive
TCTGCACCGTAGACTGCACTGTTC	0	14	RDR2-sensitive
TCTGCACCTGACGTCGCCGGCGAT	0	12	RDR2-sensitive
TCTGCACGCGCGGACCGTTCGGAC	1	16	RDR2-sensitive
TCTGCACGCTTTCGGACGGTCCGC	0	12	RDR2-sensitive
TCTGCACGGACGGGAAAACGAAGA	0	16	RDR2-sensitive
TCTGCACTATTCAGCTGTAGGCAC	0	41	RDR2-sensitive
TCTGCACTGTAGACTGCATGGTTC	0	20	RDR2-sensitive
TCTGCACTGTAGACTGCATTGTTC	0	13	RDR2-sensitive
TCTGCAGGACGATGGTAGAGACGA	1	12	RDR2-sensitive
TCTGCAGGTAGAGCTGTACGGGCA	0	23	RDR2-sensitive
TCTGCATCACTCTAGACGGTCCGC	0	9	RDR2-sensitive
TCTGCATCCACCGGTACGCTCGAA	0	11	RDR2-sensitive
TCTGCATCTTTAGACGACTTCTGC	0	16	RDR2-sensitive
TCTGCATGTAGAGCTGTACGGGCA	0	12	RDR2-sensitive
TCTGCCAACGCTTGGGACTGGATC	0	10	RDR2-sensitive
TCTGCCACGTCAGCTAGCTCGGTG	0	23	RDR2-sensitive
TCTGCCAGCCATAGGACGTAGGAT	0	11	RDR2-sensitive
TCTGCCCAACAAGTCGGACGGTCT	0	9	RDR2-sensitive
TCTGCCCAAGTTTGACTGGTCCAC	0	13	RDR2-sensitive
TCTGCCTCTCGGACTCTCCGGCAT	0	20	RDR2-sensitive
TCTGCCTCTGACGCGTTCTGCCAA	1	33	RDR2-sensitive
TCTGCCTGTCAGGCGTTGGACGTC	63	3	RDR2-resistant
TCTGCCTGTTGACGCAACACTGCA	2	15	RDR2-sensitive
TCTGCGAACTGAGCAGGACCTCGA	0	11	RDR2-sensitive
TCTGCGAAGGCGCGGACGATCTGC	0	9	RDR2-sensitive
TCTGCGACAGCGCGGACAGTCTGC	0	10	RDR2-sensitive
TCTGCGACGGCACGGACGGTCTGT	0	13	RDR2-sensitive
TCTGCGACGGTGTGGACGGTCCGC	0	10	RDR2-sensitive
TCTGCGAGCTTTCGGATGGTCTGT	0	11	RDR2-sensitive

TCTGCGCACACGTAGACGGTCTGT	0	26	RDR2-sensitive
TCTGCGCATCTGTGGAGCCAGGCT	3	272	RDR2-sensitive
TCTGCGCCAAGTCGGAAAGTCCAG	0	20	RDR2-sensitive
TCTGCGCCTGTAGGCCGGACGGTC	0	9	RDR2-sensitive
TCTGCGCGTACAAGATGAGCTCAT	0	33	RDR2-sensitive
TCTGCGCGTACAAGCTGAGCTCAT	3	262	RDR2-sensitive
TCTGCGCGTACAAGCTGAGCTCGT	0	11	RDR2-sensitive
TCTGCGCGTGCAAAAGTGGCTCTT	0	11	RDR2-sensitive
TCTGCGCGTGCAGGCTGAGAGACA	0	9	RDR2-sensitive
TCTGCGCGTGCATGTAGAAGTGGT	0	42	RDR2-sensitive
TCTGCGCTATTGTAGACGGTTCGA	0	12	RDR2-sensitive
TCTGCGGAAGTGGCCTGAGGACAG	0	9	RDR2-sensitive
TCTGCGGCAGTACGAACGGTCCAC	0	31	RDR2-sensitive
TCTGCGGCGGCACAGAGAATCCGT	0	11	RDR2-sensitive
TCTGCGGGCAGGTTCACTCAGGTA	24	1	RDR2-resistant
TCTGCGGGTGATGAGACTTCTAGG	22	0	RDR2-resistant
TCTGCGGTAGGAGCTGGAGCTGGT	24	0	RDR2-resistant
TCTGCGGTCGGTGATTTCGGAGGC	1	14	RDR2-sensitive
TCTGCGGTGGACTGGGACGAGCAC	0	22	RDR2-sensitive
TCTGCGTAATCGGTCTGGTGGCAC	0	48	RDR2-sensitive
TCTGCGTACGCGTGGACGGTCTGC	0	10	RDR2-sensitive
TCTGCGTACGTCCGGACGGTCCGA	0	13	RDR2-sensitive
TCTGCGTACGTCCGGACGGTCCGC	0	9	RDR2-sensitive
TCTGCGTACGTCCGGACGGTCTGT	0	16	RDR2-sensitive
TCTGCGTAGGCGCGAACGGTTCGC	0	15	RDR2-sensitive
TCTGCGTATGTCTGGACGGTCCGT	1	17	RDR2-sensitive
TCTGCGTGCATTGACTTTGATTAA	0	10	RDR2-sensitive
TCTGCTATCATGACGACTCGACAA	0	9	RDR2-sensitive
TCTGCTATTGTAGGGCTTGTTCGG	0	9	RDR2-sensitive
TCTGCTCAGACTGTCCGGTTGCAC	0	18	RDR2-sensitive
TCTGCTCGAGAGAAAGGTGGAGCT	0	9	RDR2-sensitive
TCTGCTCGATGGCGCACCGGACAG	0	16	RDR2-sensitive
TCTGCTCGGCTCGATGGTACAGCG	0	9	RDR2-sensitive
TCTGCTCGGCTGGACGACTGCATC	0	19	RDR2-sensitive
TCTGCTCGGGCGGATTCCAGCGTA	0	13	RDR2-sensitive
TCTGCTCGTCCATGCTGTCGGCCC	0	27	RDR2-sensitive
TCTGCTCTGGACAGAGAAGGCTGC	0	9	RDR2-sensitive
TCTGCTGCAGTTCTGTCCGCGCAT	21	0	RDR2-resistant
TCTGCTGCCTGATCCTGAAGACGT	0	16	RDR2-sensitive
TCTGCTGGAAACGGGGACGGATAC	0	10	RDR2-sensitive
TCTGCTTCTGTCGACGACAACACC	35	4	RDR2-resistant
TCTGCTTGGTTGGACTGCGGCTGT	0	22	RDR2-sensitive
TCTGCTTTCGGACCGATGGCCCAT	0	13	RDR2-sensitive
TCTGCTTTCTGTATGCTGGCTCCT	20	0	RDR2-resistant
TCTGCTTTGATCTGGATCGTCCGT	0	27	RDR2-sensitive
TCTGCTTTTGCTAGTAGACGCTGA	0	9	RDR2-sensitive
TCTGGAACAGGCTCGACGAGAGGA	1	15	RDR2-sensitive
TCTGGAACGGAGCGGCTCTGTTTT	1	23	RDR2-sensitive
TCTGGAACGGGCTCGACGAGATGA	1	30	RDR2-sensitive

TCTGGAATTGCTTGAGCCTCGGTT	120	21	RDR2-resistant
TCTGGACAAATAAGCTAGACTGTT	0	13	RDR2-sensitive
TCTGGACACCGCATCCGACGACGA	0	9	RDR2-sensitive
TCTGGACACCGCATCTGACGACGA	0	9	RDR2-sensitive
TCTGGACAGTGGATAGAGGTAGGC	1	15	RDR2-sensitive
TCTGGACATGGCAGACTACACCAG	0	11	RDR2-sensitive
TCTGGACATGGCAGACTGCACCAG	0	101	RDR2-sensitive
TCTGGACATGTCAGACTGCAACAG	0	17	RDR2-sensitive
TCTGGACATGTCAGACTGCACCAA	0	15	RDR2-sensitive
TCTGGACATGTCAGACTGCACCAG	0	92	RDR2-sensitive
TCTGGACATGTCAGGAAGCTGCAG	0	22	RDR2-sensitive
TCTGGACATGTCAGGTGGCAGTGA	1	13	RDR2-sensitive
TCTGGACCAGACTCTACAGGCGCG	0	12	RDR2-sensitive
TCTGGACCCGCATAGAACCGGACC	0	13	RDR2-sensitive
TCTGGACCGACGAAAATGTGGCGT	0	11	RDR2-sensitive
TCTGGACCGGATCCGACGCCGCAT	0	12	RDR2-sensitive
TCTGGACCGGCCTAGAGCGAGCGT	0	13	RDR2-sensitive
TCTGGACGCACTCGTAGGACGCCA	0	19	RDR2-sensitive
TCTGGACGCAGGAGCGACGCCTTC	0	9	RDR2-sensitive
TCTGGACGCGGCTGCTAGACGCAC	0	19	RDR2-sensitive
TCTGGACGGACAAATCAGCGACAT	1	17	RDR2-sensitive
TCTGGACGGGGCTGGTGGACAGGT	0	16	RDR2-sensitive
TCTGGACTCATGGGCACCCGGCTC	0	12	RDR2-sensitive
TCTGGACTCGACGCTCGGCGCCAT	0	9	RDR2-sensitive
TCTGGACTGGTTCGGCGAAGGGCT	0	21	RDR2-sensitive
TCTGGAGACAGCGGCAGAGCTCAT	0	10	RDR2-sensitive
TCTGGAGACGTAGGCGACAGCCAA	0	9	RDR2-sensitive
TCTGGAGATTGCGGTTCTTGATGC	41	2	RDR2-resistant
TCTGGAGCGCGCTGGCATGACTGC	0	10	RDR2-sensitive
TCTGGAGCTGTAGCAGGTGATCGA	0	10	RDR2-sensitive
TCTGGATAAACCCGAGGCACTGAG	0	9	RDR2-sensitive
TCTGGATAATGGGGGCATGAGCAG	30	3	RDR2-resistant
TCTGGATAGTTTGATTGCACGCTT	2	71	RDR2-sensitive
TCTGGATGCGGCAGAACCGTGCAC	0	38	RDR2-sensitive
TCTGGATGTGGCAGAACCGTGCAC	0	38	RDR2-sensitive
TCTGGATTAGCATGGATTGGAATT	0	13	RDR2-sensitive
TCTGGATTCAGCTTTAGACCGGCC	38	2	RDR2-resistant
TCTGGCAACCAGGTAGTAACTCGC	0	15	RDR2-sensitive
TCTGGCACAATCCATATCTGGCAT	0	26	RDR2-sensitive
TCTGGCACAGTTGTAGACAGACAA	0	25	RDR2-sensitive
TCTGGCACGACACAGATCTGGCAT	0	242	RDR2-sensitive
TCTGGCACGACATATATCTGGTAC	0	9	RDR2-sensitive
TCTGGCACGGCCCATATCTGACAC	0	39	RDR2-sensitive
TCTGGCACGGCCCATATCTGGCAC	1	27	RDR2-sensitive
TCTGGCACGGCCCATATCTGGCAT	0	10	RDR2-sensitive
TCTGGCACGGTCCATATCTGGCAC	0	9	RDR2-sensitive
TCTGGCACTGAAGCAGGGACCCAT	0	11	RDR2-sensitive
TCTGGCCACCAAAAGCTGCTGCGG	0	9	RDR2-sensitive
TCTGGCCAGGCACGCAGAACTGTG	0	11	RDR2-sensitive

TCTGGCCAGGCACGCAGGACTGTG	0	9	RDR2-sensitive
TCTGGCCAGGCACGTAGGACTGTG	0	20	RDR2-sensitive
TCTGGCCCCAAAAGAACTGCTCGC	0	13	RDR2-sensitive
TCTGGCCGCCAGAAGCTGAAGCTC	0	16	RDR2-sensitive
TCTGGCCGCCAGAAGCTGAAGCTG	0	13	RDR2-sensitive
TCTGGCCGTTGGGCTCGACGCCAA	0	13	RDR2-sensitive
TCTGGCGACGGACGGCGTAAGTAC	0	17	RDR2-sensitive
TCTGGCGATCGGCGAGCTTAGCAA	0	13	RDR2-sensitive
TCTGGCGGCAGAACGGCAGAACAC	0	13	RDR2-sensitive
TCTGGCGTCAAGACAAGTATGGTT	26	2	RDR2-resistant
TCTGGCTCGAGCGGATTGCAGCGT	1	50	RDR2-sensitive
TCTGGCTCGAGCGGATTTAAGTGT	1	17	RDR2-sensitive
TCTGGCTCGGGCGGATCTAAGCGT	0	13	RDR2-sensitive
TCTGGCTCGGGCGGATTCCAGCGT	5	51	RDR2-sensitive
TCTGGCTCGTCTGGCATCCGGCAC	0	12	RDR2-sensitive
TCTGGCTCTAGTGTAGACGATGGT	0	10	RDR2-sensitive
TCTGGCTCTGACCGGTCTGCTGCA	226	0	RDR2-resistant
TCTGGCTCTGATACCACTGAAGGA	44	6	RDR2-resistant
TCTGGCTCTTTGATGGCTCTCTGT	0	12	RDR2-sensitive
TCTGGCTGAGCTCGATCCGGGTAT	0	17	RDR2-sensitive
TCTGGCTGATATGACACGGTTCAA	0	9	RDR2-sensitive
TCTGGCTGATCAGGACGACGTCAA	0	9	RDR2-sensitive
TCTGGCTGGCTGTAACGGTAGCTC	3	43	RDR2-sensitive
TCTGGCTGTAGGGAGAATCCGGCT	0	32	RDR2-sensitive
TCTGGCTGTGCTCGCGGGATGCAA	0	10	RDR2-sensitive
TCTGGCTTCTCCAAAAACGGCTTT	0	9	RDR2-sensitive
TCTGGGAATGTAGCAGGTAACCGA	0	9	RDR2-sensitive
TCTGGGACGTAGTGAGCACCGGAG	0	9	RDR2-sensitive
TCTGGGACTGAACTGGTATTTCTT	0	10	RDR2-sensitive
TCTGGGAGTGAACCGGTGCTGCAT	0	20	RDR2-sensitive
TCTGGGCACAACTGGACACAGCAC	0	10	RDR2-sensitive
TCTGGGCCAGCTTCTGACGGTCGC	0	11	RDR2-sensitive
TCTGGGCCGACGGTTTCTGCAGAC	0	14	RDR2-sensitive
TCTGGGCCGGACAGTGCATGACCT	0	16	RDR2-sensitive
TCTGGGCCGGACGATCCGCGACAT	0	13	RDR2-sensitive
TCTGGGCCGGACGGTTCGGGACCT	0	27	RDR2-sensitive
TCTGGGCTAGACGGTCTGTAACCT	0	35	RDR2-sensitive
TCTGGGCTCGGCTGCTCGTGGCAT	1	32	RDR2-sensitive
TCTGGGCTGGGACACGCGGCGACA	1	22	RDR2-sensitive
TCTGGGCTGGGGCACGCAGCACAC	0	12	RDR2-sensitive
TCTGGGCTGTGCGTGTGCGGCTGT	0	18	RDR2-sensitive
TCTGGGGACTCTATCGGGCGGCAT	14	0	RDR2-resistant
TCTGGGGCTGAGTTGCAGAGACAG	0	11	RDR2-sensitive
TCTGGGGCTGTAGCAAGTACCGGG	0	14	RDR2-sensitive
TCTGGGGGCAGCGGCAGAGCTCAT	0	10	RDR2-sensitive
TCTGGGTTGGGTTCTGGACGGTGT	0	16	RDR2-sensitive
TCTGGTACCTCGGCGCTGAGACGT	0	16	RDR2-sensitive
TCTGGTAGACGCTGCGAAGTTCAT	0	30	RDR2-sensitive
TCTGGTATCAAAGGTCTCGACGAT	0	9	RDR2-sensitive

TCTGGTCACGGTCTGCATGAGAAG	0	10	RDR2-sensitive
TCTGGTCACTGTTCACGGCACTTG	102	2	RDR2-resistant
TCTGGTCAGACGCAGACGGTACAC	0	21	RDR2-sensitive
TCTGGTCAGACGCGGACGGTCCAC	0	22	RDR2-sensitive
TCTGGTCAGACGCGGACGGTCCGC	0	12	RDR2-sensitive
TCTGGTCAGACGCGGACGGTCCGT	0	33	RDR2-sensitive
TCTGGTCAGACGTGGACGGTCCGC	0	13	RDR2-sensitive
TCTGGTCAGGCGCGGACGGTCCAC	0	23	RDR2-sensitive
TCTGGTCAGGCGCGGACGGTCCGC	6	71	RDR2-sensitive
TCTGGTCAGGCGCGGACGGTCCGT	0	25	RDR2-sensitive
TCTGGTCAGGCGTGGACGGTCCGC	1	27	RDR2-sensitive
TCTGGTCCGAGCTCGAGCAGGTGA	0	12	RDR2-sensitive
TCTGGTGGACCTGCAGGTGGGGCC	0	9	RDR2-sensitive
TCTGGTTCGGGCAGATCAGAGCGT	0	19	RDR2-sensitive
TCTGTAAGGCGTGAGAGACTCCAA	0	17	RDR2-sensitive
TCTGTAATGACGGTCTGTAACCCT	4	26	RDR2-sensitive
TCTGTACACGGGCGATGACCTCAC	0	22	RDR2-sensitive
TCTGTACAGAACGGAGCTCGGCGC	0	11	RDR2-sensitive
TCTGTACAGAATGGAGCTCGGCGC	0	15	RDR2-sensitive
TCTGTACAGTCGTCGGGAGGCCGT	0	31	RDR2-sensitive
TCTGTACAGTCGTCGGGAGGCTGT	0	9	RDR2-sensitive
TCTGTACAGTTGTCGGGAGGTTGT	0	9	RDR2-sensitive
TCTGTACCGTAGACCGCACTGTTC	0	18	RDR2-sensitive
TCTGTACGACACAGGGGCTGCTGA	0	9	RDR2-sensitive
TCTGTACTCTGCCCAGCATGTCGA	0	13	RDR2-sensitive
TCTGTAGATAGTTTGAAAATGGTC	31	5	RDR2-resistant
TCTGTAGCAGCTTTTGGTGGCCAG	0	12	RDR2-sensitive
TCTGTAGCGAACTGAGAATAAGGC	0	9	RDR2-sensitive
TCTGTAGCGGGATAGGACACGCAT	0	12	RDR2-sensitive
TCTGTAGCGGTTCGGACGGTTCGC	0	9	RDR2-sensitive
TCTGTAGGAAACGGATGACGCCTA	0	9	RDR2-sensitive
TCTGTAGGATCTAGGACACCGGCT	0	37	RDR2-sensitive
TCTGTAGTGGAACAGGCTCATGAG	0	9	RDR2-sensitive
TCTGTATCCATAGACCATACTCGT	0	12	RDR2-sensitive
TCTGTATCTATCGGCTTATTTACA	20	2	RDR2-resistant
TCTGTATCTTAGAACGCGCACAAT	0	10	RDR2-sensitive
TCTGTATGTACTCATCGACCGCAT	0	13	RDR2-sensitive
TCTGTATTGGTCTTCGACATGGCT	19	0	RDR2-resistant
TCTGTATTTGGTAATCTGGAACGG	0	42	RDR2-sensitive
TCTGTCAACTGGAGGACGCGCGTC	0	10	RDR2-sensitive
TCTGTCAAGTGGCACTGCAGTCGG	4	20	RDR2-sensitive
TCTGTCAGCAGTCGAACTAAACAT	0	12	RDR2-sensitive
TCTGTCAGGTGGGCCTCGGGCGGT	0	9	RDR2-sensitive
TCTGTCATCGGACGACGCACAGAA	0	14	RDR2-sensitive
TCTGTCATGTTAGGCTTCGGTGGT	0	12	RDR2-sensitive
TCTGTCATGTTGCACTTGGACGGT	177	19	RDR2-resistant
TCTGTCCAAGACGTCGAAGAGCAC	0	9	RDR2-sensitive
TCTGTCCAGCTGCAGAAGGTCCAA	35	0	RDR2-resistant
TCTGTCCGAGAGTGGAACGTTCAT	0	11	RDR2-sensitive

TCTGTCCGTGAGCCGAAGTCTGAG	0	11	RDR2-sensitive
TCTGTCCTTTACAGAACACTCTAA	0	28	RDR2-sensitive
TCTGTCGACGAGCACACCGGACAT	0	9	RDR2-sensitive
TCTGTCGACTGAAGGGCGCGCGTC	2	72	RDR2-sensitive
TCTGTCGACTGGAGGACGTGCGTC	5	25	RDR2-sensitive
TCTGTCGACTGGAGGGCGTGCGTC	0	17	RDR2-sensitive
TCTGTCGAGATGCGCAAGGACGAC	0	18	RDR2-sensitive
TCTGTCGAGATGCGCGAGGACGAC	0	11	RDR2-sensitive
TCTGTCGAGTGGCACAGCAGTCGG	0	9	RDR2-sensitive
TCTGTCGCGTAGGACAATTCGGCT	2	45	RDR2-sensitive
TCTGTCGCGTAGGACAGTTCGGCA	0	9	RDR2-sensitive
TCTGTCGCGTAGGACAGTTCGGCT	2	102	RDR2-sensitive
TCTGTCGGGAGCGTGCGGGAGGGA	0	10	RDR2-sensitive
TCTGTCGTACACTGGGTTGCGCAA	0	13	RDR2-sensitive
TCTGTCGTGCTTATGGGAAGGGAG	4	55	RDR2-sensitive
TCTGTCGTTGCTAGCGAAGGACGC	0	12	RDR2-sensitive
TCTGTCTCGCAGACTTGCTCGGCT	0	12	RDR2-sensitive
TCTGTCTCGCGGCGTCGGCGACTC	0	9	RDR2-sensitive
TCTGTCTCTGTCTGACCAGCGGTT	4	25	RDR2-sensitive
TCTGTCTGACATCTGACGACGCAT	10	31	RDR2-sensitive
TCTGTCTGACATCTGACGTCGCAT	0	10	RDR2-sensitive
TCTGTCTTATCTCGGTCCTCGGAC	1	23	RDR2-sensitive
TCTGTCTTGTAGAAGGGGTATGAA	2	29	RDR2-sensitive
TCTGTGACGCGGGACGTCGAGCAA	0	24	RDR2-sensitive
TCTGTGACGGCAGGGACTTGCGTT	59	0	RDR2-resistant
TCTGTGATCGTGCAGCAAGAGCGC	0	12	RDR2-sensitive
TCTGTGCACCATCGCAGGACTGAC	15	0	RDR2-resistant
TCTGTGCACTCGCGGACGGTCGTA	0	12	RDR2-sensitive
TCTGTGCATGAGTGGACGGTCTGT	0	10	RDR2-sensitive
TCTGTGCGTGCAGGTAGAGAGCCA	0	9	RDR2-sensitive
TCTGTGCGTTGCGACGGTATCTGT	0	11	RDR2-sensitive
TCTGTGCTCGTAGAAGGTGGGACG	0	9	RDR2-sensitive
TCTGTGCTGAGTGCTGACTGTCAT	0	26	RDR2-sensitive
TCTGTGCTTACAGACCGTCCGGAC	0	17	RDR2-sensitive
TCTGTGCTTGACTCCGGCTGGTGA	17	0	RDR2-resistant
TCTGTGCTTTGGACATATGCTGAA	14	0	RDR2-resistant
TCTGTGGAGCCAGGCTGAGAGGCT	1	28	RDR2-sensitive
TCTGTGGAGGCACGAACGGTCCGT	0	15	RDR2-sensitive
TCTGTGGCTCGGACGGTCCATGAT	1	13	RDR2-sensitive
TCTGTGGTCTAGACTGTATGGCGT	0	22	RDR2-sensitive
TCTGTGTATGTCCGGACGGTCCGC	0	21	RDR2-sensitive
TCTGTGTCGCATTGAACGGTCCGC	0	14	RDR2-sensitive
TCTGTGTCGTGCTTTAGTGGGCAT	0	26	RDR2-sensitive
TCTGTGTTAGGATTGAAAACGGAC	0	18	RDR2-sensitive
TCTGTGTTTGATAAGCTGGAACGG	1	12	RDR2-sensitive
TCTGTGTTTGATAATCTGGAACGG	0	11	RDR2-sensitive
TCTGTGTTTGGCAAGCAGAACTGA	0	26	RDR2-sensitive
TCTGTGTTTGGTAAGCTGGAACGG	2	15	RDR2-sensitive
TCTGTGTTTGGTAAGTTGGAACGG	1	21	RDR2-sensitive

TCTGTGTTTGGTAATCTAGAACGG	2	21	RDR2-sensitive
TCTGTGTTTGGTAATCTGGAACGG	31	295	RDR2-sensitive
TCTGTTAGATGTTGGATAGACGTT	0	50	RDR2-sensitive
TCTGTTAGATGTTGGATGGACGTT	0	9	RDR2-sensitive
TCTGTTCATTCAGTCAATCGTTGT	0	17	RDR2-sensitive
TCTGTTCCATGTAGCGGCGCTCTG	0	47	RDR2-sensitive
TCTGTTCCATGTATCGGCGCTCTG	0	31	RDR2-sensitive
TCTGTTCCCTGCGTCGGTCTCAGC	0	10	RDR2-sensitive
TCTGTTCGATGGCGCACCGGACAG	0	11	RDR2-sensitive
TCTGTTCGGTGAAGCAGCGAGCTC	0	42	RDR2-sensitive
TCTGTTCTAGTACTGAGACTGTTT	0	17	RDR2-sensitive
TCTGTTCTATAGCACTGGATGCAT	0	97	RDR2-sensitive
TCTGTTCTTAGAATCTGGAACGGA	0	11	RDR2-sensitive
TCTGTTGACCGTAGCCTAAGGCAT	0	14	RDR2-sensitive
TCTGTTGAGTATATGCATAGCGGT	19	0	RDR2-resistant
TCTGTTGCAGCGTGAACGGTCCAC	0	9	RDR2-sensitive
TCTGTTGCCGCACGTAGAGGACAG	0	16	RDR2-sensitive
TCTGTTGGATGGAAGATGCATGGT	0	13	RDR2-sensitive
TCTGTTGGATTGTTATTCAGGCTA	45	13	RDR2-resistant
TCTGTTGGGCTGCTCCATACGGTA	0	146	RDR2-sensitive
TCTGTTGGGCTGCTCTATACGGTA	0	27	RDR2-sensitive
TCTGTTGGTACGGGCAGAACGAGC	0	14	RDR2-sensitive
TCTGTTGTCAATCAGGTGGACTGC	0	17	RDR2-sensitive
TCTGTTGTCAATCAGGTGGGCTGC	0	10	RDR2-sensitive
TCTGTTGTTGGAGAGGTAGCGGAT	0	71	RDR2-sensitive
TCTGTTTCGGGAGGCGATCTTCGG	0	11	RDR2-sensitive
TCTGTTTCTCGGTGTAGTTAGGCC	43	1	RDR2-resistant
TCTGTTTGACCGCATAGCCGCCTT	0	9	RDR2-sensitive
TCTGTTTGACGTGTTACATAACTT	90	4	RDR2-resistant
TCTGTTTGAGTAAAGGGCACTGCT	24	3	RDR2-resistant
TCTGTTTGGAGACTGGCTGATCGC	0	20	RDR2-sensitive
TCTGTTTGGTTAAGCTGTGGCTAT	0	10	RDR2-sensitive
TCTGTTTGGTTGGGCTATGGCTGT	1	20	RDR2-sensitive
TCTGTTTGGTTGGGCTGTGGCTGT	1	21	RDR2-sensitive
TCTGTTTGGTTGGTGGGTAGGTGT	0	11	RDR2-sensitive
TCTGTTTGTAACGGTTGTGTTGAA	27	2	RDR2-resistant
TCTGTTTTTCTGCGGCTGATGAG	211	10	RDR2-resistant
TCTGTTTTTTTTCTGATTTGGGCA	35	0	RDR2-resistant
TCTTAAAGTTGGGTTTGTTTTGGA	54	2	RDR2-resistant
TCTTAACACATGATAGGGTTCGCA	33	0	RDR2-resistant
TCTTAACATGAAGTGGTTCTCTCA	67	4	RDR2-resistant
TCTTAACTACGGACTTAGACGGTA	67	4	RDR2-resistant
TCTTAACTCGTTAAGTTGGACGGT	0	13	RDR2-sensitive
TCTTAAGCGGTTGCAGTAAATCAG	25	1	RDR2-resistant
TCTTAATAGTTGTAGAGATGACGA	0	18	RDR2-sensitive
TCTTACGATGTGTATGCGCTCACT	22	0	RDR2-resistant
TCTTACTGGGACGGTAGCGGACGA	0	12	RDR2-sensitive
TCTTAGCTAGTAGTGATGAGGATC	40	0	RDR2-resistant
TCTTAGGACGGCAGTAGAGATGGC	0	9	RDR2-sensitive

TCTTAGGACTTAGGGCGGGTGCAC	1	16	RDR2-sensitive
TCTTAGGGTGCTCTGGGTCGGTAG	0	12	RDR2-sensitive
TCTTAGTACTAGAACAGAGCGGTT	0	13	RDR2-sensitive
TCTTAGTCACGATGGATATCATTT	22	0	RDR2-resistant
TCTTAGTTGGTGGAGCGATTTGTC	40	0	RDR2-resistant
TCTTATATCTATGGCATGACTAAA	20	1	RDR2-resistant
TCTTATTGGACTTTGTGTGCGGTG	2	29	RDR2-sensitive
TCTTATTTGACATCTTGACGTGCT	40	1	RDR2-resistant
TCTTCAACTTTGGGATTATTCGGA	15	0	RDR2-resistant
TCTTCACATGTAGATTCGTTAGTA	90	30	RDR2-resistant
TCTTCACGCTGTAGAAGAGGGTAC	0	12	RDR2-sensitive
TCTTCACGGAGAGACGGCTCTAAG	6	60	RDR2-sensitive
TCTTCAGTTAGCGTGTCGGTAGTG	17	0	RDR2-resistant
TCTTCATACTGTAGAAGAGGGTAA	0	10	RDR2-sensitive
TCTTCATACTGTAGAAGAGGGTAC	0	41	RDR2-sensitive
TCTTCATACTGTAGTAAGAGGTAA	0	12	RDR2-sensitive
TCTTCATACTGTAGTAAGGGGTAC	2	68	RDR2-sensitive
TCTTCATCAAATGGAAATCGTCGC	0	22	RDR2-sensitive
TCTTCATGCTGTAGAAGAAGGTAC	0	10	RDR2-sensitive
TCTTCATGCTGTAGAAGAGGGTAC	0	22	RDR2-sensitive
TCTTCATGCTGTAGAAGATGGTAC	0	11	RDR2-sensitive
TCTTCATGCTGTAGGAGAGGGTAC	0	11	RDR2-sensitive
TCTTCATGTGCGGCATCCTGACCT	14	0	RDR2-resistant
TCTTCATGTGTAGAAGATCTTGAA	16	0	RDR2-resistant
TCTTCATTTGATAATAGGCGAACT	49	0	RDR2-resistant
TCTTCCAAGTACCGTAGCTCGGCT	25	0	RDR2-resistant
TCTTCCACAGGACGTACACGGGAT	0	13	RDR2-sensitive
TCTTCCTGCAGGACCTTCGGCGAC	19	61	RDR2-sensitive
TCTTCGACCACGGCTTCGGCGCTC	29	1	RDR2-resistant
TCTTCGACGTTGACGGTGCGCTGC	0	10	RDR2-sensitive
TCTTCGCGGAGAGACGGCTCTAAG	0	9	RDR2-sensitive
TCTTCGGACTCAAGTCGGTATCAC	0	13	RDR2-sensitive
TCTTCGGACTCAAGTCGGTATGGC	1	14	RDR2-sensitive
TCTTCGGACTCGAGTCGGTATCAC	1	36	RDR2-sensitive
TCTTCGGGCTCGAGTCGGTATCAC	0	12	RDR2-sensitive
TCTTCGTCAAGCCGGCAGGTCTCT	0	18	RDR2-sensitive
TCTTCGTCATGTGGGAATCGTCGT	0	10	RDR2-sensitive
TCTTCGTGTAGCTGAGTTCGTCGT	0	9	RDR2-sensitive
TCTTCGTTGGTGTAGCGAGGCGAC	1	12	RDR2-sensitive
TCTTCTACCACTGTTGGCATCCTT	20	2	RDR2-resistant
TCTTCTAGTAACAATTGTAGTCGG	0	13	RDR2-sensitive
TCTTCTATGTTTAGAGTTCGTCGT	0	15	RDR2-sensitive
TCTTCTCAGCGATTGTCGGTGCCA	166	10	RDR2-resistant
TCTTCTCAGCGATTGTCGGTGCCT	14	0	RDR2-resistant
TCTTCTCTTTTGGGCTGTTCCGGA	39	0	RDR2-resistant
TCTTCTGCTGCAGTAATGATCGCT	35	0	RDR2-resistant
TCTTCTTACAGGATCTTCGGCGAC	0	20	RDR2-sensitive
TCTTCTTTTGTTTCATGTTGGATT	34	0	RDR2-resistant
TCTTGAACCCCTCGGATGGTTGTA	22	0	RDR2-resistant

TCTTGACCTTCGTCCGATGAGCAT	0	10	RDR2-sensitive
TCTTGACGACACGCGGAACGACGA	0	21	RDR2-sensitive
TCTTGACGACGCACGGAACGACGA	0	49	RDR2-sensitive
TCTTGACGATGCACGGAACGACGA	0	15	RDR2-sensitive
TCTTGACGTGTAGAGGACGAGAAT	0	12	RDR2-sensitive
TCTTGAGCCGGTTGTAAGTTTGGG	27	0	RDR2-resistant
TCTTGAGTTGTAGCTAGGGACGTC	0	9	RDR2-sensitive
TCTTGATCAAGTGAATGATGAGGA	36	1	RDR2-resistant
TCTTGATCTTGGGGACACGCGCAC	0	11	RDR2-sensitive
TCTTGATGACGCACGGAACGACGA	2	105	RDR2-sensitive
TCTTGATGACGCGCGGAACGACGA	0	28	RDR2-sensitive
TCTTGATGATGTAGGTGATGAGCC	18	1	RDR2-resistant
TCTTGATTAGGTCGGCTAAAACGG	0	10	RDR2-sensitive
TCTTGCAGCGATCGACGACGGAGC	30	0	RDR2-resistant
TCTTGCATTCTGAAGTTATGTCGT	0	10	RDR2-sensitive
TCTTGCGCCTCTACTGCTGACGGG	0	14	RDR2-sensitive
TCTTGCGTCTCTACTGCTGACGGG	0	17	RDR2-sensitive
TCTTGCTGACACGCGGAACGACGA	1	24	RDR2-sensitive
TCTTGCTGACGCGCAGAACGACGA	0	10	RDR2-sensitive
TCTTGCTGACGCGCGGAACGACGA	4	118	RDR2-sensitive
TCTTGCTGGAGGCGTAGAGAACGT	0	11	RDR2-sensitive
TCTTGCTGTAGACCGAGGTGGCCT	1	18	RDR2-sensitive
TCTTGGACTATCTCTCAGATGGAT	34	2	RDR2-resistant
TCTTGGATATGTGACGGTGGCTTC	0	14	RDR2-sensitive
TCTTGGCGAAGACTGGGCCTCGGG	2	21	RDR2-sensitive
TCTTGGCGTAGACGAGGATGACGA	0	13	RDR2-sensitive
TCTTGGCGTGACTCGGCGACCTTT	1	14	RDR2-sensitive
TCTTGGGATTTCTTGCTGACGCGC	1	17	RDR2-sensitive
TCTTGGGGATGCACGAACGACGAC	0	27	RDR2-sensitive
TCTTGGGGCTGCGTAAACAGGTAT	56	0	RDR2-resistant
TCTTGGGTTGTACTGCAGAGGCAC	0	9	RDR2-sensitive
TCTTGGTAACCTGGCTGAGAGCAT	0	13	RDR2-sensitive
TCTTGGTAGACTGGCTGAGAGCAT	0	18	RDR2-sensitive
TCTTGGTGAACTGTATATCGGTAA	0	10	RDR2-sensitive
TCTTGGTGGACTGGTGATCCGGTG	0	11	RDR2-sensitive
TCTTGTAATTACGGCTTGTAACCC	0	11	RDR2-sensitive
TCTTGTAGAAATTCGGTTCTTCAA	0	42	RDR2-sensitive
TCTTGTCTGACGACTGGACGGTCC	0	14	RDR2-sensitive
TCTTGTCTGCTGAGAGTTGGTGGT	1	12	RDR2-sensitive
TCTTGTCTGGCATTTGCAACCAAT	19	1	RDR2-resistant
TCTTGTGCGCTGGAATGACGACCA	0	13	RDR2-sensitive
TCTTGTGGGAGCATGGACTATCAG	0	10	RDR2-sensitive
TCTTGTTAGACTGGTGATCCGGTG	0	11	RDR2-sensitive
TCTTTAACCGGCAGCCTGAATCGA	0	25	RDR2-sensitive
TCTTTACCGAGCGCAGGACTTTGC	0	14	RDR2-sensitive
TCTTTACTTTAGGGGGATTGGAAC	14	0	RDR2-resistant
TCTTTAGATGGCTCAACACATGTT	146	1	RDR2-resistant
TCTTTATACTGTAGTAGAGGGTAC	0	15	RDR2-sensitive
TCTTTATATGCGGATGATGTGGTT	54	4	RDR2-resistant

TCTTTATGCTGTAGTAAGGGGTAC	0	11	RDR2-sensitive
TCTTTATGGATCGGGAGTTATGGA	16	0	RDR2-resistant
TCTTTATTCTTGGACAGGCAGCAG	32	1	RDR2-resistant
TCTTTCAGACCTACGATGCAAATT	81	0	RDR2-resistant
TCTTTCATTACTGTTGAAGACATA	18	0	RDR2-resistant
TCTTTCCCACAGGATGTAGACGGA	0	40	RDR2-sensitive
TCTTTCTGCAGGACCTTCGGCGAC	0	45	RDR2-sensitive
TCTTTGACTGACTGTGGCGTGGCT	0	14	RDR2-sensitive
TCTTTGAGAGTGTGGTTGGGCGAA	12	36	RDR2-sensitive
TCTTTGCATAGGCGCGGACGGTCC	0	9	RDR2-sensitive
TCTTTGCATGAGTAGAGCCGGGAT	0	14	RDR2-sensitive
TCTTTGCCGAGTGCCTTTTGTCGG	0	19	RDR2-sensitive
TCTTTGGAAATATGCTGGAGCGGT	22	2	RDR2-resistant
TCTTTGGACAACTGAGGACAACAT	0	12	RDR2-sensitive
TCTTTGGACCGCTTGAGAGGGGTT	0	10	RDR2-sensitive
TCTTTGGCGACGTGACGATGGCAT	1	17	RDR2-sensitive
TCTTTGGTAGATTGTCAACATAGG	21	0	RDR2-resistant
TCTTTGTTGAATGGAAATCGTCGT	0	13	RDR2-sensitive
TCTTTTACTGTAGTGGGAGACTCT	0	15	RDR2-sensitive
TCTTTTAGAGAAGACTGTAAAGGA	0	20	RDR2-sensitive
TCTTTTCATCCTGGACTTTTGGTA	20	2	RDR2-resistant
TCTTTTCGGTTCTGTTTTGTGGTT	1	12	RDR2-sensitive
TCTTTTGTTTCATGTTGGATTACC	101	0	RDR2-resistant
TCTTTTTAGTTGTCGCTGGATAGT	16	42	RDR2-sensitive
TCTTTTTCGCTCGGTCACGGTCAC	0	10	RDR2-sensitive
TCTTTTTCGTGCTGGGGGATGGAT	0	22	RDR2-sensitive
TGAAAAAGGTGAAAGACATCAGTT	23	1	RDR2-resistant
TGAAACAAGGAGACCTACTGGGCT	19	1	RDR2-resistant
TGAAACCTAGGAGACTGTCCACTG	14	0	RDR2-resistant
TGAAACGACGTAGCTTTTGCCGGA	19	1	RDR2-resistant
TGAAACTTGCTTGAAGAATGAGAT	19	0	RDR2-resistant
TGAAAGAAGTCGCGAGCTGGTCGG	5	29	RDR2-sensitive
TGAAAGACATGTACCGAGAGGCGT	0	11	RDR2-sensitive
TGAAAGCACCGTCGGCACGATCTA	35	8	RDR2-resistant
TGAAAGGCTAGAACTGATCCGGCT	0	21	RDR2-sensitive
TGAAAGTCGACTAGAGGAGGGTGA	0	10	RDR2-sensitive
TGAAAGTGACTGAATATATGCAGA	80	1	RDR2-resistant
TGAAATGAATGGTGTTGTGGTCTA	109	5	RDR2-resistant
TGAAATGTAGAAACAGCAAGAAAC	14	0	RDR2-resistant
TGAAATGTCTGTCGAGCCCGGCAC	0	9	RDR2-sensitive
TGAAATGTTGGGCTTTTTACCGGT	42	2	RDR2-resistant
TGAAATTATTTGGTTGCGTTGGCT	181	3	RDR2-resistant
TGAACACACTGATTTTCTGGGCAT	34	0	RDR2-resistant
TGAACACGCTGATTTTCTGGGCAT	39	4	RDR2-resistant
TGAACACTCGGCACACGTAGGCAC	0	9	RDR2-sensitive
TGAACACTCGGCACAGAACGACAC	2	38	RDR2-sensitive
TGAACACTCGGCACAGAACGGCAC	3	42	RDR2-sensitive
TGAACACTCGGCACAGGACGACAC	6	110	RDR2-sensitive
TGAACACTCGGCACAGGTAGGCAC	5	99	RDR2-sensitive

TGAACACTCGGCACCGAACGGCAC	0	9	RDR2-sensitive
TGAACACTCGGTACAGGACGACAC	1	31	RDR2-sensitive
TGAACAGACTTGACTCGATGCGCT	40	3	RDR2-resistant
TGAACAGATCTGCTCGGTTTGCCA	26	0	RDR2-resistant
TGAACAGTTCGGGCAGGAGCGAAT	0	13	RDR2-sensitive
TGAACAGTTTGACGTTAGTATAAT	19	0	RDR2-resistant
TGAACATGGTCGACGGCGAGGCCT	0	10	RDR2-sensitive
TGAACCAGACGAAGGACGGGCTGA	0	11	RDR2-sensitive
TGAACCCACGGGCTTAGACGGTCT	0	10	RDR2-sensitive
TGAACCGAGTAGGACGAGAGTCAA	0	11	RDR2-sensitive
TGAACCGCTGTAGATTGTATTCAT	0	9	RDR2-sensitive
TGAACCGGACCGAGACGTAGTGAA	0	9	RDR2-sensitive
TGAACCTTTGAGGGCATCTCGGAT	16	0	RDR2-resistant
TGAACGACGACTATAGATGGCCAA	0	10	RDR2-sensitive
TGAACGATGACGGTCTGTCGCTGC	0	37	RDR2-sensitive
TGAACGCAATCGGACGGTTGGGAT	0	11	RDR2-sensitive
TGAACGGACCTGGATGTGCAGAGA	0	12	RDR2-sensitive
TGAACGGATTGCTCGGTTGCGCAT	0	9	RDR2-sensitive
TGAACGGGACGACTGTGAAGACGA	1	57	RDR2-sensitive
TGAACGGGCACAAGAGAACGACAC	0	28	RDR2-sensitive
TGAACGGGTGGCACTGGAATCTCC	0	18	RDR2-sensitive
TGAACGTAACTGCAGAGGAACCAT	0	13	RDR2-sensitive
TGAACGTATGGATGGCTGTGCTGT	0	28	RDR2-sensitive
TGAACGTCATGCTGCATAGGAGGC	0	19	RDR2-sensitive
TGAACGTCCGACGCTGGCGATGGT	1	19	RDR2-sensitive
TGAACGTTTTTTTGGATTGATGGT	53	1	RDR2-resistant
TGAACTAAACAGAAGTTATGGATT	18	0	RDR2-resistant
TGAACTAAGACTTGGCTTTGTGGA	36	0	RDR2-resistant
TGAACTACTGCAGGCGTTACTCAT	0	11	RDR2-sensitive
TGAACTGGAAGTGGAACGAGCCAA	0	9	RDR2-sensitive
TGAACTGTAAATCGGCAACTACCT	0	13	RDR2-sensitive
TGAACTGTACATCGATAACGGCTT	0	15	RDR2-sensitive
TGAACTGTATCAGATCTATGTCGT	0	10	RDR2-sensitive
TGAACTTTCACTCTGCAAGCAGCT	25	3	RDR2-resistant
TGAACTTTTCTGGCTTGAGCGCAT	6	26	RDR2-sensitive
TGAAGAACACGAGCGGACGTACAT	0	13	RDR2-sensitive
TGAAGACGACTTAAAGTAATTGAT	14	0	RDR2-resistant
TGAAGACGGAGCCAGCAACGTGAT	1	44	RDR2-sensitive
TGAAGACGTAGCCAACAACGCGAT	0	11	RDR2-sensitive
TGAAGACTGAATGTAGGGACGTTC	0	10	RDR2-sensitive
TGAAGACTGTAAGGGGTAGGCTGC	1	19	RDR2-sensitive
TGAAGAGCCAAGTAGAACGGAGCC	0	11	RDR2-sensitive
TGAAGAGGCATCTGTGAACCTCAA	0	10	RDR2-sensitive
TGAAGATCGGGCATCGTGACGCAT	2	124	RDR2-sensitive
TGAAGATTTGAACTTGGAAGGCAC	14	0	RDR2-resistant
TGAAGCCTTGACGGACTCCAGCAT	23	0	RDR2-resistant
TGAAGCGGGTGAGTAGCAGTAGGC	40	5	RDR2-resistant
TGAAGCGGTCGCTGTAGACGGTGC	0	10	RDR2-sensitive
TGAAGCTCTGGTCACGAGGTGCAT	0	9	RDR2-sensitive

TGAAGGAACTAGGGCAGACGCTGT	0	10	RDR2-sensitive
TGAAGGACGCCGACGACTGCACGG	39	2	RDR2-resistant
TGAAGGACTGCCACACGCGCGCGC	0	65	RDR2-sensitive
TGAAGGACTGTCACACGCGCGCAC	0	19	RDR2-sensitive
TGAAGGACTTGGCCATCGTGGCGT	15	0	RDR2-resistant
TGAAGGATGGACGATCACGGTGGA	27	0	RDR2-resistant
TGAAGGCCGTCGTTGTTCAGGCAA	0	9	RDR2-sensitive
TGAAGGGCTGAAGGCGTGGATGGT	0	16	RDR2-sensitive
TGAAGGTAGACCCTGTTGATGGCG	72	15	RDR2-resistant
TGAAGTAGAACGGATCGGCTCCAT	0	12	RDR2-sensitive
TGAAGTTCGTCTAGGCTACGTCAT	0	44	RDR2-sensitive
TGAAGTTCGTCTCGGACACGTCAT	0	60	RDR2-sensitive
TGAAGTTTATGACTGTACAGCGGT	25	0	RDR2-resistant
TGAATACCGCAGTAACTTGACATC	25	0	RDR2-resistant
TGAATAGGTCGGTTTAGAGGTGGT	87	6	RDR2-resistant
TGAATAGTTGTTGACGCTGTGTTC	63	6	RDR2-resistant
TGAATATTTGTAGAAGTCTCGGAT	0	17	RDR2-sensitive
TGAATCAAGTTAGGACCGGATCAT	0	14	RDR2-sensitive
TGAATCATCGCTCGAACTTGGGTT	37	4	RDR2-resistant
TGAATCGGGTTGGACCGTGCCGGC	0	14	RDR2-sensitive
TGAATCTGAGCGTCGGCAGGGGCA	28	4	RDR2-resistant
TGAATCTGGTGAGCCTGGCTTCGT	0	10	RDR2-sensitive
TGAATGATTGTATGTAGATGGCTT	25	1	RDR2-resistant
TGAATGGCACATCAAACTCCTGTT	18	0	RDR2-resistant
TGAATGGGGACGCAGATGAGGGAT	14	0	RDR2-resistant
TGAATGTAAAAAATCTGAAGACAT	0	9	RDR2-sensitive
TGAATGTAGTTGTTTTGGCTTATT	17	0	RDR2-resistant
TGAATGTCGAAGCTAGTTCCGAAA	27	0	RDR2-resistant
TGAATTATAGCGGAGCGCCTCTGG	0	11	RDR2-sensitive
TGAATTCTGCACCCGGAAGGCGAT	0	13	RDR2-sensitive
TGAATTTGTGGTTCTGATCGCATG	117	0	RDR2-resistant
TGACAAAGACTAGAGCTGGAGTGG	16	0	RDR2-resistant
TGACAACCCGATGGATGATGGCTC	0	9	RDR2-sensitive
TGACAAGTCGATCGTTCCCACCAC	54	8	RDR2-resistant
TGACAAGTTCTCTGAAAAGGCGAA	136	6	RDR2-resistant
TGACAATATTCAGTAGACTTAAGA	93	2	RDR2-resistant
TGACACCGTACGAGGCTTGGCATG	66	5	RDR2-resistant
TGACACCTTTATCCTGACGCGCGC	0	9	RDR2-sensitive
TGACACGACTGGAGCTAGGCCCGT	0	40	RDR2-sensitive
TGACACGCCAATACCGTCGATCGT	0	9	RDR2-sensitive
TGACACGTGGACAGGTTTTATGAT	48	1	RDR2-resistant
TGACACTCGGCAAAGACTGACGGC	0	9	RDR2-sensitive
TGACACTGATAGGCGTGAGGACAT	0	9	RDR2-sensitive
TGACACTGCGGACGGTGAGAGGCG	0	12	RDR2-sensitive
TGACACTGTACGAGGCTTGGCATG	61	3	RDR2-resistant
TGACAGAAGGACAGCGCCGCCTGC	3	31	RDR2-sensitive
TGACAGCGGCCTCTAGCAAGACAT	1	13	RDR2-sensitive
TGACAGGACGGGACGATACCTCAA	0	11	RDR2-sensitive
TGACAGGACGGGACGGGATGGAAC	0	10	RDR2-sensitive

TGACAGGACGGGATGGGACGGGAC	2	41	RDR2-sensitive
TGACAGGACGGGATGGGATGGGAC	0	51	RDR2-sensitive
TGACAGGACTGTCCGGTGTGTCAG	0	14	RDR2-sensitive
TGACAGTAGTACTGCTTGGGGCGT	0	10	RDR2-sensitive
TGACAGTATGACACGACGGAGCAC	0	13	RDR2-sensitive
TGACAGTGCTCTCTACAGGACGGT	0	15	RDR2-sensitive
TGACAGTGCTCTCTGTAGAGGCGT	0	11	RDR2-sensitive
TGACAGTGTTCTCTGCACAGACGC	0	68	RDR2-sensitive
TGACATCATAAGGCCTGTGGCATG	43	9	RDR2-resistant
TGACATCGCTCAACGGCAGAACAG	0	15	RDR2-sensitive
TGACATGACGGGATGGGACGGGAC	0	14	RDR2-sensitive
TGACATGTAGGGTAAGTGGCACAT	0	11	RDR2-sensitive
TGACATGTATGCTTAGTTGACGAA	24	0	RDR2-resistant
TGACATTTTGCTGATAGGGCCGCA	76	3	RDR2-resistant
TGACCACCATAAGTGACTGATCGT	0	22	RDR2-sensitive
TGACCAGAGAGCACCGCCGCCGGT	0	11	RDR2-sensitive
TGACCAGCGCGGCATGTACAACCT	0	9	RDR2-sensitive
TGACCAGTAGAGTTCGATCCGCAT	19	0	RDR2-resistant
TGACCAGTCTGACAGAAGGACAGC	4	19	RDR2-sensitive
TGACCAGTCTTGGCTGCATAACGA	18	0	RDR2-resistant
TGACCCATTTTCGTGTCGGATCGT	0	10	RDR2-sensitive
TGACCCTGGCTGACCTGATACGGC	0	23	RDR2-sensitive
TGACCCTGGCTGACCTGATAGGCT	0	24	RDR2-sensitive
TGACCGAGGCTGTGAAGACCACAA	0	17	RDR2-sensitive
TGACCGCCGGTGGCAGAAGAACAC	0	9	RDR2-sensitive
TGACCGGCCTGACAAGAGGACAGC	1	17	RDR2-sensitive
TGACCGGCCTGACAGAAGGACAGC	17	94	RDR2-sensitive
TGACCGGCTGCATGAGGATTTTCT	33	0	RDR2-resistant
TGACCGGTATGCTTTTTGATCAAC	34	7	RDR2-resistant
TGACCGGTCTGACAAGAGGACAGC	0	23	RDR2-sensitive
TGACCGGTCTGACAGAAGGACAGC	6	61	RDR2-sensitive
TGACCGTAGGGACGTGAAGGTCAT	25	4	RDR2-resistant
TGACCGTCAGGCAGAGGACCCTAG	0	10	RDR2-sensitive
TGACCGTCTACAGTAGGGCATCGG	0	45	RDR2-sensitive
TGACCGTGGCTGGGCTGTCGGCGC	1	23	RDR2-sensitive
TGACCGTTATGCCTCTGACCGGTT	0	9	RDR2-sensitive
TGACCGTTCTGCCTCTGACCGGTT	1	16	RDR2-sensitive
TGACCGTTGGCGCGCATACCTTTT	17	0	RDR2-resistant
TGACCTACTCCTCGGACTGCGGCA	0	13	RDR2-sensitive
TGACCTCTCGGTGGTTGTCTCGGT	65	1	RDR2-resistant
TGACCTGATAGGCTGATACGGTAA	1	63	RDR2-sensitive
TGACCTGCCTGACGGTGCTCTCAC	1	17	RDR2-sensitive
TGACCTGTATCTCTCTGATGGCGT	0	32	RDR2-sensitive
TGACCTGTGACTGGCAAACAACAT	0	17	RDR2-sensitive
TGACCTGTGGGGCAGAGATGACAG	0	9	RDR2-sensitive
TGACCTTGTCGAAGCTGTATTGCA	16	0	RDR2-resistant
TGACGAAGAGACAGGGTCACTCGG	2	17	RDR2-sensitive
TGACGAAGTACGGCTGCGGAGTAA	0	9	RDR2-sensitive
TGACGACACACGGAATGACGACAG	0	12	RDR2-sensitive

TGACGACACGCGGAACGACGACGA	1	12	RDR2-sensitive
TGACGACACTAGACACACAGACAT	0	9	RDR2-sensitive
TGACGACACTAGACACACAGACGT	3	27	RDR2-sensitive
TGACGACAGTAGACACACAGACGT	0	23	RDR2-sensitive
TGACGACAGTAGACACAGAGACGT	0	9	RDR2-sensitive
TGACGACCGTTCGAGAGACTGCAT	0	23	RDR2-sensitive
TGACGACGACGGAGACTTTGCCGA	0	9	RDR2-sensitive
TGACGACGAGGAACACGAGCGGAC	0	9	RDR2-sensitive
TGACGACGGACAGCGTGAGTACAA	0	12	RDR2-sensitive
TGACGACGTGCGGCGTGAGTACAA	1	38	RDR2-sensitive
TGACGACTGGGCAGTGGACAACGC	0	15	RDR2-sensitive
TGACGAGCCGGACGGTTCGCTCGT	0	11	RDR2-sensitive
TGACGATGATGGAGCATGTGACAC	0	28	RDR2-sensitive
TGACGATGGCGCCAAGAAGACTGA	0	13	RDR2-sensitive
TGACGATTTGACGGCCACAGACGC	0	11	RDR2-sensitive
TGACGCAATGCGGACTGCAGGCAC	0	10	RDR2-sensitive
TGACGCACGGAACGACGACGAGAT	3	18	RDR2-sensitive
TGACGCACGGAATGACGACGAGAT	0	16	RDR2-sensitive
TGACGCAGGACGACCGTTGAAACT	49	1	RDR2-resistant
TGACGCAGTAGTCGCCGAACGCAT	0	29	RDR2-sensitive
TGACGCGATGCAGACTGCGAGCAC	0	10	RDR2-sensitive
TGACGCGCATGAATGGATTAACGA	21	2	RDR2-resistant
TGACGCGCGGAACGACGACAAGAT	0	9	RDR2-sensitive
TGACGCGCGGAACGACGACGAGAT	4	73	RDR2-sensitive
TGACGCGTAGGACAGTTCGGCTAG	0	36	RDR2-sensitive
TGACGCTGAGCTCTGACGTGGCGT	0	10	RDR2-sensitive
TGACGCTGTGTTCTGATTTCAGAA	39	0	RDR2-resistant
TGACGCTTTTAAGTAGGACGACAT	0	41	RDR2-sensitive
TGACGGACCAGACACAAAACGGGC	0	9	RDR2-sensitive
TGACGGACCAGAGGACGAACGCGT	0	15	RDR2-sensitive
TGACGGACGACACGGCTAGGCTCC	0	12	RDR2-sensitive
TGACGGAGAGTTGATGCAGATCTG	0	13	RDR2-sensitive
TGACGGCACTAGACATACAGACGT	0	10	RDR2-sensitive
TGACGGCACTCTAGTTGAAGAAGG	56	2	RDR2-resistant
TGACGGGAGGGAAGGGAGCGGATC	0	15	RDR2-sensitive
TGACGGGCGTGAGTCGGCGATCAT	0	14	RDR2-sensitive
TGACGGGCTTAGAGGTAAACGGGC	0	11	RDR2-sensitive
TGACGGGCTTAGAGGTAAACGGGT	0	14	RDR2-sensitive
TGACGGGGAATCGATGGGGACGGT	24	3	RDR2-resistant
TGACGGGTCGGTCTGAAACACGGC	0	16	RDR2-sensitive
TGACGGTACTCTCTGCGGAGGCGT	0	10	RDR2-sensitive
TGACGGTACTCTCTGCGGGAGCGT	0	11	RDR2-sensitive
TGACGGTCACTGTGGTGTAGGCAC	3	21	RDR2-sensitive
TGACGGTCGTCAGCTGTAGACGGC	1	22	RDR2-sensitive
TGACGGTCTGGCCATAGATACGGA	0	9	RDR2-sensitive
TGACGGTGACCTGCCTGACGGTGC	0	21	RDR2-sensitive
TGACGGTGCTCTCTGCAACGGCGC	0	20	RDR2-sensitive
TGACGGTGCTCTCTGCGGAAGCGT	0	14	RDR2-sensitive
TGACGGTGCTCTCTGGTCAGACGC	0	20	RDR2-sensitive

TGACGGTTGTCAGACGGACCGGAA	0	16	RDR2-sensitive
TGACGTACGATGACGGTGGAACAG	0	13	RDR2-sensitive
TGACGTAGAGCACTCCGGTGGCGT	0	13	RDR2-sensitive
TGACGTGAATTGCATTGGAACGGT	0	16	RDR2-sensitive
TGACGTGAGCTGAAGCCGGGTGAG	0	17	RDR2-sensitive
TGACGTGGACTGGAGCTACGGTGA	0	9	RDR2-sensitive
TGACGTTATCTATACAGTCGTCGG	0	10	RDR2-sensitive
TGACGTTGTCGCAGTAGACGAGGG	128	49	RDR2-resistant
TGACTAAACCGGACTAAAGGGCAT	0	10	RDR2-sensitive
TGACTAAATAAATGAGATCGTCAC	25	3	RDR2-resistant
TGACTACGCTTAGCTGAACGACGA	0	11	RDR2-sensitive
TGACTAGATAGGTTTGTATCGGAT	21	0	RDR2-resistant
TGACTATCGCTGCCCTTGAGTGCC	26	0	RDR2-resistant
TGACTCAAGAAACCTCAAGGGCAT	0	19	RDR2-sensitive
TGACTCGAGCGACTGAGAGAGGCT	0	14	RDR2-sensitive
TGACTCGTCCGAGCTCGAGCTGGC	0	10	RDR2-sensitive
TGACTCTAGATCTCCAAACCGGAT	0	9	RDR2-sensitive
TGACTCTGACCCCCCGCCGGTGTT	14	0	RDR2-resistant
TGACTCTGACGCCCGGAAGCGCGG	0	10	RDR2-sensitive
TGACTCTGCGATACGGCTGATCGG	29	5	RDR2-resistant
TGACTCTGGTCACTGTTCACGGCA	56	2	RDR2-resistant
TGACTCTGTAGCAACGTACGGGCA	0	9	RDR2-sensitive
TGACTCTTGACAAGATTTATGGCC	23	3	RDR2-resistant
TGACTGAAGAGATTAGTTCGTCCT	17	0	RDR2-resistant
TGACTGACGCGACCTGACATGGCA	3	22	RDR2-sensitive
TGACTGCTTCCTCTGTAGCAGGAA	0	10	RDR2-sensitive
TGACTGGAAGCGCGATAACCCTAC	0	16	RDR2-sensitive
TGACTGGAAGTAGGGACGTTCTGC	0	18	RDR2-sensitive
TGACTGGCTGATGGAGCAAGGCAA	44	4	RDR2-resistant
TGACTGGGAGACGACGACCTAGAC	20	43	RDR2-sensitive
TGACTGGGAGACGACGGCCTAGAT	1	16	RDR2-sensitive
TGACTGGTAGACAGAGGTCAGCAC	0	9	RDR2-sensitive
TGACTGTACGCAAGGAACGGTAAC	0	9	RDR2-sensitive
TGACTGTACGTTGCGATGGCTCAC	0	11	RDR2-sensitive
TGACTGTAGCGTTAGCACGGACGT	0	13	RDR2-sensitive
TGACTGTATCTGGCCAGCTAGGCT	0	30	RDR2-sensitive
TGACTGTATGTTGCGACGGCTCAC	0	16	RDR2-sensitive
TGACTGTCGTATGGTGCACCGGAC	4	32	RDR2-sensitive
TGACTGTCTGCTTGGGCGTGGCCT	22	1	RDR2-resistant
TGACTGTGACGCTGGAAGACTGCT	0	9	RDR2-sensitive
TGACTGTGAGAGCGAGATGGGAAC	0	19	RDR2-sensitive
TGACTGTGAGAGCGGGATAGGAAC	0	10	RDR2-sensitive
TGACTGTGAGAGCGGGATGGGAAC	0	80	RDR2-sensitive
TGACTGTGAGAGCGGGATTGGAAC	3	66	RDR2-sensitive
TGACTGTGCGAGCGGGTTGAGAAC	0	11	RDR2-sensitive
TGACTGTGGAGAAAAGTTGGCTGT	0	10	RDR2-sensitive
TGACTGTGGCATCGGCTTAGAGAA	0	13	RDR2-sensitive
TGACTGTGGGGAAAAGGTGGCTGT	0	36	RDR2-sensitive
TGACTGTGTCTCGTGAGGCAGCGG	0	30	RDR2-sensitive

TGACTGTTATCTGCATATGATGGT	0	12	RDR2-sensitive
TGACTGTTATGAAGGTATTGTTAT	0	15	RDR2-sensitive
TGACTGTTCATTGCGCATTTGAGG	19	0	RDR2-resistant
TGACTTGAACTGGGAGGAGGATGT	0	13	RDR2-sensitive
TGACTTGGACTCGCAGCAGATCGA	3	73	RDR2-sensitive
TGAGAAAGATGAATGAGTCTGCAT	0	9	RDR2-sensitive
TGAGAACCGAGATCGTGGACACGC	0	16	RDR2-sensitive
TGAGAAGACTGTTGTAGATGGCAA	26	0	RDR2-resistant
TGAGACAGCAAGAGTGAGCTCACA	0	17	RDR2-sensitive
TGAGACCGACGCAGGGAACAGAGC	1	13	RDR2-sensitive
TGAGACCGGGCGGGTGACTTGCAC	13	31	RDR2-sensitive
TGAGACGAGAGCGCAGATGACCAC	1	15	RDR2-sensitive
TGAGACGAGGAGCACAGATCTGGC	0	42	RDR2-sensitive
TGAGACGGAGGACGAGAGTCAGGA	0	21	RDR2-sensitive
TGAGACTAATGGCTCTCCAAGAGA	30	1	RDR2-resistant
TGAGACTCGGAGGCAGACCAAAAC	30	1	RDR2-resistant
TGAGACTCTGAGAATAGTTGCTGC	16	0	RDR2-resistant
TGAGACTCTTGAAGCTGCGCGCAT	0	12	RDR2-sensitive
TGAGACTGAGACCCACTTGTCGGC	0	18	RDR2-sensitive
TGAGACTGGAGTAGGAGCCGACGG	0	12	RDR2-sensitive
TGAGAGCAAGCGGATGAGGGACAA	0	14	RDR2-sensitive
TGAGAGCTCTTTCTTGATTCTATG	24	1	RDR2-resistant
TGAGAGCTGAGGATGTGAGGACGG	0	10	RDR2-sensitive
TGAGAGGACCAGGCTCAAGAGAGC	0	23	RDR2-sensitive
TGAGAGGCGGCATGCAGGACAGAC	1	21	RDR2-sensitive
TGAGAGTAGTGGAAGGCGGGCTGG	227	24	RDR2-resistant
TGAGAGTCTTTTCTTTAGTCGGGT	32	6	RDR2-resistant
TGAGAGTGGGAGGCAGAACCGTGC	0	10	RDR2-sensitive
TGAGATATCTGGACCGTCTGCGGT	0	9	RDR2-sensitive
TGAGATCGCAGAACGGCAGAACGG	0	44	RDR2-sensitive
TGAGATCGCAGAATGGCAGAACAC	0	19	RDR2-sensitive
TGAGATCGGGCTTGCTAGACAGGC	0	11	RDR2-sensitive
TGAGATCTAGAACGGGCTCGACGA	0	17	RDR2-sensitive
TGAGATCTCGACAGTCGACGCCAT	0	16	RDR2-sensitive
TGAGATCTCGGCAGTCACGGATGT	0	9	RDR2-sensitive
TGAGATCTCTCGGCAGGGAAACAA	0	9	RDR2-sensitive
TGAGATGATGCTAGGCTCGGCAAC	33	0	RDR2-resistant
TGAGATTGATTGTAGCTATTGGCT	716	54	RDR2-resistant
TGAGATTGCAGAACGGTAGGCTAC	0	34	RDR2-sensitive
TGAGATTGTAAACATACAGGTGCT	37	1	RDR2-resistant
TGAGATTGTCTAACGGCAGAACGG	0	10	RDR2-sensitive
TGAGATTGTTCCAGGAGATGAGAT	71	1	RDR2-resistant
TGAGATTTTATATTTTTTCGTCGG	58	1	RDR2-resistant
TGAGCAACTCTGTGAGGCAGACAG	0	15	RDR2-sensitive
TGAGCACGTACTCTCCGTCGGCAA	0	14	RDR2-sensitive
TGAGCACTGTGTGATGATGTCCAT	7	104	RDR2-sensitive
TGAGCAGAGCGCCGGTGACCTCGC	0	15	RDR2-sensitive
TGAGCAGATAGAACCGTGCACCAC	0	19	RDR2-sensitive
TGAGCATGTAGACGCCACCGACAT	0	29	RDR2-sensitive

TGAGCCAACGGTCGGCCGGGCCAA	9	28	RDR2-sensitive
TGAGCCATATCAGACTTAGACCGT	0	9	RDR2-sensitive
TGAGCCATTGTAGAGTGGACGGTT	0	10	RDR2-sensitive
TGAGCCCTGTAGAAGCACAACTGT	1	12	RDR2-sensitive
TGAGCCCTGTAGAAGCACAGCTGG	0	12	RDR2-sensitive
TGAGCCCTGTAGAAGCACAGCTGT	2	133	RDR2-sensitive
TGAGCCCTGTAGAAGCATAGCTGG	0	17	RDR2-sensitive
TGAGCCCTGTAGAAGCATAGCTGT	0	19	RDR2-sensitive
TGAGCCCTGTAGAAGCGTAGCTGG	0	29	RDR2-sensitive
TGAGCCCTGTGGAAGTACAGCTGT	0	12	RDR2-sensitive
TGAGCCGAGCTTGAGTGAACCCAA	0	14	RDR2-sensitive
TGAGCCGAGTAGAGCCGAGCTGAA	0	39	RDR2-sensitive
TGAGCCGATGTAGAGTGTGGCGCC	36	5	RDR2-resistant
TGAGCCGGGCTGGGGCACAGTCAG	0	40	RDR2-sensitive
TGAGCCTGACTGAGATGAGCCATA	0	11	RDR2-sensitive
TGAGCCTGAGTCTGATTCTGGCAT	0	15	RDR2-sensitive
TGAGCCTGGCACGGTAGAATAAGC	0	10	RDR2-sensitive
TGAGCCTGGCTTAGACGAACTGGT	0	23	RDR2-sensitive
TGAGCCTGGTTCAGATGATCTGAT	0	12	RDR2-sensitive
TGAGCCTTGTCAGACCTGTGGCGT	0	14	RDR2-sensitive
TGAGCCTTTGCAGAATCTGATGCA	78	2	RDR2-resistant
TGAGCGGATGCAGGCTGACGGCAT	0	13	RDR2-sensitive
TGAGCGGGATTGGATGGGTTTGAA	1	25	RDR2-sensitive
TGAGCGTTTGGCAGTCTGCAGCAG	1	17	RDR2-sensitive
TGAGCTAGCATGACCTGAAGGCAC	0	19	RDR2-sensitive
TGAGCTATATGTGCCTGACATCGG	0	20	RDR2-sensitive
TGAGCTCGGAGATGTGAGGCGGGA	18	0	RDR2-resistant
TGAGCTCTGTGCCACGTGGACTGC	0	13	RDR2-sensitive
TGAGCTCTGTGGAATGCACGTCAA	645	85	RDR2-resistant
TGAGCTCTGTGGAATGCACGTCAT	28	3	RDR2-resistant
TGAGCTGAAGCTCTCGGTAGTGTC	16	0	RDR2-resistant
TGAGCTGACAGCTACTCTCTTGGA	47	2	RDR2-resistant
TGAGCTGAGCTGCTAGATCGAGTT	16	0	RDR2-resistant
TGAGCTGGACGCACTTACGCCATC	30	5	RDR2-resistant
TGAGCTGGCATGACCCGAAGGCAT	0	15	RDR2-sensitive
TGAGCTGGTTTGAAATTCGGTCGT	0	11	RDR2-sensitive
TGAGCTGTGCAGGACCGAGTCATA	0	9	RDR2-sensitive
TGAGCTGTGTTATCTCGACGTCGT	1	17	RDR2-sensitive
TGAGCTTCGAACTGATGAAGAGCA	20	0	RDR2-resistant
TGAGCTTGACTCTAGTCCGACTTT	37	1	RDR2-resistant
TGAGGAACCAGGCTCGGCTCGGCT	0	13	RDR2-sensitive
TGAGGAAGCTGTAGGGCGGCCCGC	0	17	RDR2-sensitive
TGAGGAAGGATAAAGATGGTGGCT	21	2	RDR2-resistant
TGAGGACAACAGTGGCACACAGAA	1	22	RDR2-sensitive
TGAGGACATAGCAGAGCAGAAGGT	0	12	RDR2-sensitive
TGAGGACATGACAGAGCGGGCCAT	0	11	RDR2-sensitive
TGAGGACATTGCAGAACGGATCAT	0	16	RDR2-sensitive
TGAGGACGACACTGGGATGGTCAT	0	44	RDR2-sensitive
TGAGGACGCGCGTCTACTGTTCAC	0	16	RDR2-sensitive

TGAGGACGTGACACGGCAGGCTAG	0	14	RDR2-sensitive
TGAGGACGTGTATATGATACAGCC	38	1	RDR2-resistant
TGAGGACTAGACTGAGACGGAGGA	0	13	RDR2-sensitive
TGAGGACTGAACAGGCCGAATCGG	1	20	RDR2-sensitive
TGAGGAGAACTGGCTGACGACGT	0	9	RDR2-sensitive
TGAGGAGAGGTAGAGGAAGACCGA	26	4	RDR2-resistant
TGAGGAGCCAAGTAGAACGGAGCC	1	22	RDR2-sensitive
TGAGGAGCCAAGTAGAACGGAGTC	0	18	RDR2-sensitive
TGAGGAGGCAGAACCGTGCACCAC	0	36	RDR2-sensitive
TGAGGAGGCAGAACCGTGCACCGC	0	12	RDR2-sensitive
TGAGGAGGCGGCGATTCTGGAGGC	0	11	RDR2-sensitive
TGAGGATCTAGACGACTATTATAA	56	0	RDR2-resistant
TGAGGATGTGCTAGATTCGGCTTC	130	16	RDR2-resistant
TGAGGCAACAGAGAACATACCCAC	0	9	RDR2-sensitive
TGAGGCACGTAGGACTGTGCAGGC	0	11	RDR2-sensitive
TGAGGCATCGGACGAGACGAGCGC	0	12	RDR2-sensitive
TGAGGCGAGGACCATCATTATCGT	0	10	RDR2-sensitive
TGAGGCGATGGTAGCAGGGGAACA	35	0	RDR2-resistant
TGAGGCGCTGTGCAGGAAGGGAAT	18	0	RDR2-resistant
TGAGGCGGCTTGTAGGACGGAGGC	4	23	RDR2-sensitive
TGAGGCTCTGATACCAGATGATGC	2	20	RDR2-sensitive
TGAGGCTGAGCTGGTGGACACGGT	0	20	RDR2-sensitive
TGAGGCTGATCTGAATTGGCGCTT	17	0	RDR2-resistant
TGAGGCTTTGATACCAGATGATGC	1	41	RDR2-sensitive
TGAGGCTTTGATACCAGATGATGT	2	27	RDR2-sensitive
TGAGGGACAACAGGGGACAACACT	4	34	RDR2-sensitive
TGAGGGACCTTTGTTTGCTCGGTA	105	1	RDR2-resistant
TGAGGGCAGCAGTGGCACACAGAA	0	12	RDR2-sensitive
TGAGGGCATGTGGAGGCGGGGTTT	0	11	RDR2-sensitive
TGAGGGCCCGGAGACGCTTTAGTA	14	0	RDR2-resistant
TGAGGGCTTGTTCGGTTACACCAA	0	10	RDR2-sensitive
TGAGGGGCAACGGTACACTAACCT	18	0	RDR2-resistant
TGAGGTAACACAGCTCGGCGCCAT	0	13	RDR2-sensitive
TGAGGTAACTGGTGTAGACGACAA	0	9	RDR2-sensitive
TGAGGTCAAAGTAGGACGGGGCAG	1	29	RDR2-sensitive
TGAGGTCAAAGTAGGATGAGTCGG	0	12	RDR2-sensitive
TGAGGTCGACGGTTCGAATACTAA	2	19	RDR2-sensitive
TGAGGTCTATGTAGGATGAGGCGT	0	11	RDR2-sensitive
TGAGGTCTCTGTGACTGGAGTCGT	0	31	RDR2-sensitive
TGAGGTGAGGAATTGTGGACGGAT	0	11	RDR2-sensitive
TGAGGTGGTGATGGATCAACTCAT	0	9	RDR2-sensitive
TGAGGTTCTGTTACCGGTACTCAC	0	20	RDR2-sensitive
TGAGGTTGCAGGATGGCAGAACGG	0	10	RDR2-sensitive
TGAGGTTGCTGTCCTTGTCGTCGC	0	17	RDR2-sensitive
TGAGTAGCAGGACGCGGAACCTAG	0	11	RDR2-sensitive
TGAGTAGTGATCGCCGGGCGGCAG	0	16	RDR2-sensitive
TGAGTATCTGTAATACCAGAACGA	225	0	RDR2-resistant
TGAGTCTGGAGAAGGTGGACCCGG	34	3	RDR2-resistant
TGAGTCTGTTAGTCGTGCCGGGCC	0	10	RDR2-sensitive

TGAGTGAACTGCTAGACACGGCCT	0	14	RDR2-sensitive
TGAGTGACTGTGCGTTGCAACGGA	0	10	RDR2-sensitive
TGAGTGAGGACAAAGTGCGCACAA	3	30	RDR2-sensitive
TGAGTGGATGTTTTTGACGACGGC	18	1	RDR2-resistant
TGAGTGTAGAAAACTGGGAGACGC	0	25	RDR2-sensitive
TGAGTGTTGACTGCCTGCTGCCAT	0	16	RDR2-sensitive
TGAGTTATACTGTTAAGATGAGCC	24	1	RDR2-resistant
TGAGTTCTTGTTCTGTCTTGGGAT	16	0	RDR2-resistant
TGAGTTGGGCCGACCACGACGTGC	0	9	RDR2-sensitive
TGAGTTGGTATGTTTTATGGTTGC	20	0	RDR2-resistant
TGAGTTGTAGCTAGGGACGTCCAC	0	10	RDR2-sensitive
TGAGTTTCAAATTAGATCTCGGTG	124	1	RDR2-resistant
TGAGTTTGTGAGGTTGATATCGGA	35	0	RDR2-resistant
TGAGTTTGTGCGTAGGTGCGGCGT	0	17	RDR2-sensitive
TGATAAATCGATTCGGATCGCCGT	0	16	RDR2-sensitive
TGATAACTTGACGGATCGCACGGC	25	3	RDR2-resistant
TGATAATAGGCGAACTCCACTAAA	42	0	RDR2-resistant
TGATACCAACTGTGACGGAACCGC	0	11	RDR2-sensitive
TGATACCTCGTTAGGCTGCACGGT	14	0	RDR2-resistant
TGATACTGTTAGAAAGTTAGGCAT	0	9	RDR2-sensitive
TGATAGGGTTCGCAAGCTACTGAG	27	0	RDR2-resistant
TGATAGTCGTAGAGTAGCGTCGGC	135	0	RDR2-resistant
TGATAGTGACTGTCTGCTTGGGCG	16	0	RDR2-resistant
TGATATAGTAGTCGTCGAACGCGC	1	21	RDR2-sensitive
TGATATCTGGCTTTGCACGAAGGC	17	0	RDR2-resistant
TGATATTATCGGAACTTACATGGC	19	0	RDR2-resistant
TGATCAACGTCGCAGTAGCAGCAA	0	48	RDR2-sensitive
TGATCACCCTGGCAGAACCTACGC	0	9	RDR2-sensitive
TGATCAGTTGTTTCCTGTTGGCAT	21	0	RDR2-resistant
TGATCATCGACGGAGGGCAGCTGT	0	16	RDR2-sensitive
TGATCATGTTGGACGGGCCGGCTC	0	23	RDR2-sensitive
TGATCCAAGACTCCGAGCGAACGG	0	9	RDR2-sensitive
TGATCCACGCGCGGTGCACTGACC	141	2	RDR2-resistant
TGATCCTCTAGAACCTCGATGGTG	24	0	RDR2-resistant
TGATCCTCTGCGTCAGACACGGAC	0	12	RDR2-sensitive
TGATCCTGACATCGTTGGCGACGC	0	12	RDR2-sensitive
TGATCCTTCGATGTCGGCTCTTCC	61	6	RDR2-resistant
TGATCGACCAGTGACGGAGGACAC	0	10	RDR2-sensitive
TGATCGAGCGATGACATTTATTCT	22	1	RDR2-resistant
TGATCGGACGGTCCACGATGGCGT	0	54	RDR2-sensitive
TGATCGGATACTCTGCACCGGCCT	0	12	RDR2-sensitive
TGATCGGCACCAGACGAAGACCGG	0	18	RDR2-sensitive
TGATCTAGGTTGGACGGTCCGCAT	0	16	RDR2-sensitive
TGATCTCTGCGAGGACGAGAACGG	0	12	RDR2-sensitive
TGATCTGCGGTTCCTGGACGGTAC	0	10	RDR2-sensitive
TGATGACATGTTGATTACAGGCGA	24	0	RDR2-resistant
TGATGACATTGGAGTCTTGGCGTG	32	4	RDR2-resistant
TGATGACCTGTACCGGCCGAGCGT	0	13	RDR2-sensitive
TGATGACGCACGGAACGACGACGA	0	11	RDR2-sensitive

TGATGACGCACGGAATGACGACGA	0	16	RDR2-sensitive
TGATGACGCGCGGAACGACGACGA	0	9	RDR2-sensitive
TGATGACGCGCGGAATGACGACAA	0	15	RDR2-sensitive
TGATGACGGATATCAATGTGCTTG	14	0	RDR2-resistant
TGATGACTAGGCAGTGGACAACGC	0	9	RDR2-sensitive
TGATGACTTATGGAACAGAAACGA	0	11	RDR2-sensitive
TGATGATCGTTCTTTTTGGCCAAC	113	28	RDR2-resistant
TGATGATGGATGTACAATAGGAAC	106	10	RDR2-resistant
TGATGATGGTAGAGAATTGTTTCA	37	2	RDR2-resistant
TGATGATTTAGTAGATATGGGTA	171	4	RDR2-resistant
TGATGCAAGTAAACGAGACATTAG	18	0	RDR2-resistant
TGATGCGGCTGAACCGGATGACAC	0	22	RDR2-sensitive
TGATGCGGCTGAACTGGATGACGT	0	21	RDR2-sensitive
TGATGCTCGACGAACGGACGGCTT	0	9	RDR2-sensitive
TGATGCTTTTGAGTAGGACGACGT	0	9	RDR2-sensitive
TGATGCTTTTGGACAGGACGACGC	0	17	RDR2-sensitive
TGATGCTTTTGGGTAGGACGACGT	0	23	RDR2-sensitive
TGATGCTTTTGGGTAGGACGGCGT	0	9	RDR2-sensitive
TGATGCTTTTGGGTAGGATGACGT	0	12	RDR2-sensitive
TGATGCTTTTGTGTAGGACGACAT	1	47	RDR2-sensitive
TGATGGAAGAAGGGCGAGAGACAC	0	22	RDR2-sensitive
TGATGGAAGAAGGGCGGGAGACAC	1	18	RDR2-sensitive
TGATGGACACGACCGGACGTGCGT	0	16	RDR2-sensitive
TGATGGACCGGTCTGAAGCATGGA	0	16	RDR2-sensitive
TGATGGACTGGCCCGAAACACGGA	0	21	RDR2-sensitive
TGATGGACTGTTTGGTAGGGTTGA	26	2	RDR2-resistant
TGATGGATAAGTTAGGAGCACGAT	23	2	RDR2-resistant
TGATGGCAGAGCGACTTTGGGCGT	0	14	RDR2-sensitive
TGATGGCTATAAAACAGAACTGGC	14	0	RDR2-resistant
TGATGGGAACATGTGGACGTCGAT	0	19	RDR2-sensitive
TGATGGGCGGTACGGTATGGAAAT	18	0	RDR2-resistant
TGATGGGCTGACCCGAAGCACGGC	0	11	RDR2-sensitive
TGATGGGTCGGCCCGAATCACGGA	0	26	RDR2-sensitive
TGATGGTACTCTGCCTGTTGACGC	4	53	RDR2-sensitive
TGATGGTCGTAGAGTAGCGTCGGC	0	9	RDR2-sensitive
TGATGGTGACCTCGACAAGCAGCT	79	3	RDR2-resistant
TGATGGTTTGCCGGACAACAGCGG	0	13	RDR2-sensitive
TGATGTAATAAGTTAGTTCTCGAG	26	0	RDR2-resistant
TGATGTCAAGTTACTGCGGTATTC	54	4	RDR2-resistant
TGATGTCGGGGAGGGTCGAAGGAG	15	0	RDR2-resistant
TGATGTGGAACTCGCGATAGACGA	0	11	RDR2-sensitive
TGATGTGGGACGCCAGGCATTGAC	0	15	RDR2-sensitive
TGATGTGGTTGTGTGGAGGAGGTG	34	5	RDR2-resistant
TGATGTGGTTTATTATAGTTGGAT	21	2	RDR2-resistant
TGATGTTAACTTTGTGATGTGGAC	24	2	RDR2-resistant
TGATGTTCTTATGCGGATGATGAA	21	1	RDR2-resistant
TGATGTTCTTGTGTGGATGATGGA	14	0	RDR2-resistant
TGATGTTCTTGTGTGGATGATGGG	15	0	RDR2-resistant
TGATGTTGACGAGTACTGAGTCTA	136	19	RDR2-resistant

TGATGTTGTATTGATGAGATGGTG	25	4	RDR2-resistant
TGATTAGACTTACTGATTGTTGGC	4	21	RDR2-sensitive
TGATTCACGCAGGGACGGTAGAGC	1	19	RDR2-sensitive
TGATTCAGCGTCGGCTTGGCCATC	96	0	RDR2-resistant
TGATTCGGCTGAACCGGATGGCAC	0	10	RDR2-sensitive
TGATTCGGTACGGCTCGTTTACAT	0	11	RDR2-sensitive
TGATTGAGGATAGTGAAGTCGGTT	29	0	RDR2-resistant
TGATTGCAGAGCTCGCCAGACGAC	0	17	RDR2-sensitive
TGATTGGACTTTCATGCTGGGACA	16	0	RDR2-resistant
TGATTGGATCAAGAAATGCATGGC	21	0	RDR2-resistant
TGATTGGATCGTAGGGGAGCGGTT	0	11	RDR2-sensitive
TGATTGGGATGAAGCAGAGCACGC	0	9	RDR2-sensitive
TGATTGGGTCGTATTGTGAGGCGT	1	102	RDR2-sensitive
TGATTGGTCGGTCCGAAGCACGGA	0	18	RDR2-sensitive
TGATTGGTCTGCACGCTCCGGTAT	0	15	RDR2-sensitive
TGATTGGTTGCCGGATAAGCAGAA	0	75	RDR2-sensitive
TGATTGTATCGACTATGTACGGCT	0	17	RDR2-sensitive
TGATTGTATGTATCTTGTGGCATA	17	0	RDR2-resistant
TGATTGTGTGTCGTCGGTTTTGCA	111	0	RDR2-resistant
TGATTGTTTTGGCTTGCTTCAGAT	45	0	RDR2-resistant
TGATTTAATCGGGCCAAGGCACAA	0	13	RDR2-sensitive
TGATTTAATTAAATGGATCGACGG	3	23	RDR2-sensitive
TGATTTCTTTCGGGCACTCGGCAA	0	31	RDR2-sensitive
TGATTTGCGGTTCCTGGACGGTAT	0	27	RDR2-sensitive
TGATTTTTTTAGAAACAGGACGCT	20	0	RDR2-resistant
TGCAAACTGTAGCTGTATGGCCGC	0	14	RDR2-sensitive
TGCAACCAAAGACTCGTGAGGCGT	0	13	RDR2-sensitive
TGCAACGGAAGTACGCTGGGATGA	1	25	RDR2-sensitive
TGCAACGGACCAGCTGAAGGGTGA	0	13	RDR2-sensitive
TGCAACTCTGACAAGATAAGAGGA	26	0	RDR2-resistant
TGCAACTTGTATACTTTAGAGGCT	257	1	RDR2-resistant
TGCAAGACATGAACCGGACGTCAA	0	15	RDR2-sensitive
TGCAAGCAAGTAGTGTTGAATAAC	14	0	RDR2-resistant
TGCAAGCATTAGGTCGTTTCTGAG	0	11	RDR2-sensitive
TGCAAGCTTTATGGTTCGATTGGA	94	0	RDR2-resistant
TGCAAGGCATGAACCGGACGTCAA	0	13	RDR2-sensitive
TGCAAGGCTCACGAGCTGGAACGA	0	12	RDR2-sensitive
TGCAAGGCTGTACAGGCAAGCTGC	0	23	RDR2-sensitive
TGCAAGTGTAGCTCTGCGGACGGT	0	23	RDR2-sensitive
TGCAAGTTGTACGTAGACATGGTA	18	1	RDR2-resistant
TGCAATCCGGCTGCGGCTGCGGCT	0	18	RDR2-sensitive
TGCAATGACAGGACGGGACGGGAC	0	24	RDR2-sensitive
TGCAATGACAGGACGGGACGGGAT	0	33	RDR2-sensitive
TGCAATGACAGGACGGGATGAGAT	0	10	RDR2-sensitive
TGCAATGACAGGACGGGATGGGAC	0	32	RDR2-sensitive
TGCAATGACAGGACGGGATGGGAT	1	70	RDR2-sensitive
TGCAATGATAGGACGGGACGGGAT	0	12	RDR2-sensitive
TGCAATTCTCTGTCAGGGTCCGGA	32	4	RDR2-resistant
TGCAATTTATCGGGCACTCGGCAA	0	30	RDR2-sensitive

TGCACAGCGGAGCACACTATTCAT	0	9	RDR2-sensitive
TGCACAGGACTCACGTAGGAGGAA	0	16	RDR2-sensitive
TGCACAGGCTGCAGCAGGCTACAG	3	31	RDR2-sensitive
TGCACAGGCTGTAGCAGGCTATAG	0	33	RDR2-sensitive
TGCACCAGCCGGTCATGCTCTACG	21	0	RDR2-resistant
TGCACCAGGGAACTCCAAGCTGAA	0	13	RDR2-sensitive
TGCACCAGGGAACTCGAAGCTGAA	0	10	RDR2-sensitive
TGCACCAGGGGACCTCGCGCAGAA	0	24	RDR2-sensitive
TGCACCAGGGGACTCCAAGCTGAA	0	17	RDR2-sensitive
TGCACCAGGGGACTTCACGCTGAA	0	13	RDR2-sensitive
TGCACCATCGCAGGACTGACCGGC	312	0	RDR2-resistant
TGCACCATCTCGGACCGTCCGGAC	0	9	RDR2-sensitive
TGCACCCACGGACAGAAAGGCTAC	0	12	RDR2-sensitive
TGCACCGTCGCTGAGGCATGGCAA	0	29	RDR2-sensitive
TGCACCGTCGTCGAGGCATGGCAA	0	14	RDR2-sensitive
TGCACCTCTGCGGTCGGGACGAAG	2	49	RDR2-sensitive
TGCACCTGAACAGCATAGGGCCTT	0	10	RDR2-sensitive
TGCACCTGTAGAGATGGAAACCAA	0	35	RDR2-sensitive
TGCACGACTAACAGACTCACGGGC	0	9	RDR2-sensitive
TGCACGAGACGAGTCTGCATCTAT	0	15	RDR2-sensitive
TGCACGAGAGTGGGTAGACGACGT	2	26	RDR2-sensitive
TGCACGATCTGGGCACAGGAGCGA	0	11	RDR2-sensitive
TGCACGCACAGAACCGAGCTCCAA	0	17	RDR2-sensitive
TGCACGCACGTCGGCTCTCACGGT	0	11	RDR2-sensitive
TGCACGGCAGGGTAGAGAAGCGAC	0	10	RDR2-sensitive
TGCACTAGAGTAGATATCATTTTT	30	4	RDR2-resistant
TGCACTAGATGTAGAGATGTTGAT	17	1	RDR2-resistant
TGCACTGACCCACACTGACGGACT	28	5	RDR2-resistant
TGCACTGGAAGATACATGCACCGT	0	9	RDR2-sensitive
TGCACTGGACTCAGCATGAGACGT	0	12	RDR2-sensitive
TGCACTGTAGCGACGGTGGTCGAT	0	9	RDR2-sensitive
TGCACTGTGGAAGATTGCAGGAAC	0	11	RDR2-sensitive
TGCACTTCGGGCTGTCACGGCATG	0	12	RDR2-sensitive
TGCACTTGGACGGTGTCAGTACCT	74	0	RDR2-resistant
TGCAGAAATGTTCGGATGCAAGCT	22	0	RDR2-resistant
TGCAGAAGGCTGCGATGAACCGGC	0	16	RDR2-sensitive
TGCAGACGAGTAGATGGCGGACAA	0	15	RDR2-sensitive
TGCAGACTAGTGGACACTCGGCAA	0	9	RDR2-sensitive
TGCAGACTGCAGACACGTCCACAC	1	42	RDR2-sensitive
TGCAGACTGCATACACGTCCACAC	0	15	RDR2-sensitive
TGCAGAGACGGACGGGAACGGCAA	0	11	RDR2-sensitive
TGCAGAGCGGCAAGCTAGAGCGAC	156	56	RDR2-resistant
TGCAGAGCGGCAAGTTAGAGCGAC	0	14	RDR2-sensitive
TGCAGAGCGGTATCTGTGCAGCAC	0	16	RDR2-sensitive
TGCAGAGGACTACATGTTTCTCAG	21	0	RDR2-resistant
TGCAGAGTGAACGGAACAGAGCGT	1	23	RDR2-sensitive
TGCAGAGTGAACGGAACGGAGCGT	11	80	RDR2-sensitive
TGCAGAGTGGACAGTTGACGCCGG	1	49	RDR2-sensitive
TGCAGATCGCGACAGTGGAGGCAT	0	15	RDR2-sensitive

TGCAGATGTAGAGCACAAGGACGG	0	24	RDR2-sensitive
TGCAGCCTGTACACTGGAGCAGCA	0	18	RDR2-sensitive
TGCAGCCTGTGCACTGGAGCAGCA	0	55	RDR2-sensitive
TGCAGCCTGTGTACTGGAGCAGCA	0	10	RDR2-sensitive
TGCAGCGACGTACTCGGCACCGAA	0	14	RDR2-sensitive
TGCAGCGGCAAGCCCTACGGACAA	0	12	RDR2-sensitive
TGCAGCGGCTTGGACTTGGACGGA	0	14	RDR2-sensitive
TGCAGCGGTTCACTGGAAAGACGA	0	10	RDR2-sensitive
TGCAGCTAGACGCGTAAGGACCAT	1	17	RDR2-sensitive
TGCAGCTGTGGGCGCGCGATGAAT	0	9	RDR2-sensitive
TGCAGGAAGATGTACGCTGTGCAG	0	15	RDR2-sensitive
TGCAGGACAATACGGGGACGACAG	0	12	RDR2-sensitive
TGCAGGACACATGTAGGTGGTCGT	0	35	RDR2-sensitive
TGCAGGACATATGTAGGTGGTCGT	0	11	RDR2-sensitive
TGCAGGATCCTGACGAGTCGACGA	0	15	RDR2-sensitive
TGCAGGATCTCGAAGGCAACGGAT	0	9	RDR2-sensitive
TGCAGGATGGCAGAACGGCAGAAC	0	16	RDR2-sensitive
TGCAGGATTTGCTGGTCGCTGGAT	0	15	RDR2-sensitive
TGCAGGCCGGTGATGTAGAGTGCA	17	0	RDR2-resistant
TGCAGGCTTGCAGCTCTTCAGCAC	0	13	RDR2-sensitive
TGCAGGTGTGCACTGTAGCGACGG	0	17	RDR2-sensitive
TGCAGGTTCGGACTGGACCGGCAT	0	24	RDR2-sensitive
TGCAGTACGTCAGAGGAACCGGCT	0	34	RDR2-sensitive
TGCAGTCCCTCGAACGGTCGCCGG	5	33	RDR2-sensitive
TGCAGTCGACAGCCAAGAATCCAG	0	15	RDR2-sensitive
TGCAGTCGTAAACGGTGTACACAT	0	13	RDR2-sensitive
TGCAGTCTAATGTAGAAAGGGCTC	0	12	RDR2-sensitive
TGCAGTCTCTCGAACGGTCGTCAT	0	27	RDR2-sensitive
TGCAGTGACTAAGTATATTGAATT	54	1	RDR2-resistant
TGCAGTTCAGGAGATGTGGGGCAC	0	10	RDR2-sensitive
TGCAGTTGGGCATCCATGTGTAGT	0	14	RDR2-sensitive
TGCATAAATTTTAGTATCGCCGGG	24	1	RDR2-resistant
TGCATAACTTTCTCTTGTAGCAGC	17	1	RDR2-resistant
TGCATAGAATCGGACTGTGCCGGA	0	14	RDR2-sensitive
TGCATATCTGTGCTTGACTCCGGC	39	0	RDR2-resistant
TGCATATGTTGCAGGTTCGTCGTT	37	0	RDR2-resistant
TGCATATTTCACTCGATCTGTCGT	0	12	RDR2-sensitive
TGCATCGACTGCGTACGACTACAT	3	35	RDR2-sensitive
TGCATCGGACTGTCCGGTATGCAC	0	9	RDR2-sensitive
TGCATCGGGCTGTGCCGGTTCGGG	0	11	RDR2-sensitive
TGCATGAGTAGAGCCGGGATGCAT	0	12	RDR2-sensitive
TGCATGATTGTACCGGGCCGGCAT	0	18	RDR2-sensitive
TGCATGCACGGACGCAGCCTAGTT	0	11	RDR2-sensitive
TGCATGCAGACTGGGACCGGCTAC	0	14	RDR2-sensitive
TGCATGCATAAGTATCTGGATGAC	25	0	RDR2-resistant
TGCATGCATAGAGTCTGGCCACAT	1	17	RDR2-sensitive
TGCATGCGACGGCTGGTTGAGGCT	0	18	RDR2-sensitive
TGCATGCGGAGAGAAGCGAGACGC	0	10	RDR2-sensitive
TGCATGCGGAGTGGGATGGGGAGT	0	16	RDR2-sensitive

TGCATGCGGCTGATGAGCATGATC	44	6	RDR2-resistant
TGCATGCGTCGCTGTGTTCGGCAA	0	13	RDR2-sensitive
TGCATGGACCCACGAAGGACGCGC	0	11	RDR2-sensitive
TGCATGGTAGGGAGGCGGGGCAAA	16	0	RDR2-resistant
TGCATGGTGCTGGACGGTTGACAT	0	18	RDR2-sensitive
TGCATGTCGTGACCAGGGCAATAC	15	0	RDR2-resistant
TGCATGTCTAGAGGTAGAGGAGTA	65	15	RDR2-resistant
TGCATGTTGACGGTGGTCCAGAAC	124	0	RDR2-resistant
TGCATGTTTGACTGAGTTAGGCAT	0	9	RDR2-sensitive
TGCATTGACTGTGTACGACTACAT	0	11	RDR2-sensitive
TGCATTGATCGGGCCGGGACGGAC	0	18	RDR2-sensitive
TGCATTGGGTACTGTAGGACGGCT	0	17	RDR2-sensitive
TGCATTGGTGTTGAATTCGGCGTT	25	1	RDR2-resistant
TGCATTGTAGACCGCGGTGTTCGC	0	12	RDR2-sensitive
TGCATTGTAGTTTTGGACGAGCGG	15	0	RDR2-resistant
TGCATTTTATCGGGCACTCGGCAA	0	15	RDR2-sensitive
TGCCAAAACTGGATGTAGGGACGT	1	85	RDR2-sensitive
TGCCAACTGTGGTAGAAGATGAAC	1	16	RDR2-sensitive
TGCCAAGACTGACGCGTTTCTCGA	0	11	RDR2-sensitive
TGCCAATGTCAGACTATTCGGTGT	0	10	RDR2-sensitive
TGCCACACCTTGGCTTGTGCGGAC	101	14	RDR2-resistant
TGCCACATAGTAGACACTCGGCAA	1	40	RDR2-sensitive
TGCCACGCGGGACTGCCACGTCAC	0	9	RDR2-sensitive
TGCCACGGCAGAGCTCGGCACCAT	0	15	RDR2-sensitive
TGCCACGGTGGAAGAAGCTGGTGC	14	0	RDR2-resistant
TGCCACGTCACGAAGCTCGGCGCC	0	9	RDR2-sensitive
TGCCACGTCAGGAAGCTCGGCGCC	1	22	RDR2-sensitive
TGCCACTCATTGATCTTCGGGGGA	31	0	RDR2-resistant
TGCCACTCTGGGCCAGCGAAACGA	0	12	RDR2-sensitive
TGCCACTGTATGGAAGAATTTGGT	21	0	RDR2-resistant
TGCCAGACCGCATACTCGAAGCAG	0	10	RDR2-sensitive
TGCCAGATCGCGGGCACTCGGCAA	3	26	RDR2-sensitive
TGCCAGATCGCGGGTACTCGGCAA	0	12	RDR2-sensitive
TGCCAGATTGGTGGGTCGGAACGA	0	12	RDR2-sensitive
TGCCAGCACGACACGGAACTGCAT	0	10	RDR2-sensitive
TGCCAGCGCAGTAGATATCTCGGT	0	18	RDR2-sensitive
TGCCAGCGCTGTAGGTACCTCGGC	0	20	RDR2-sensitive
TGCCAGCTAGACTCGTACCTCGAC	0	10	RDR2-sensitive
TGCCAGTCGGTACTCGGCAAAGAA	0	26	RDR2-sensitive
TGCCATATGTTAGCTGGACGTCGC	0	15	RDR2-sensitive
TGCCATCGTGCAGGACAAGGGCAG	0	11	RDR2-sensitive
TGCCCACCGTAGATCATGAAGCAG	0	10	RDR2-sensitive
TGCCCAGTCATCGGCCCACGGCAC	0	22	RDR2-sensitive
TGCCCATGGGGTGACGCCGGAAAG	119	0	RDR2-resistant
TGCCCCACAATCGCACGGACGCAC	0	18	RDR2-sensitive
TGCCCCACAATCGCGCGGACGCAC	0	12	RDR2-sensitive
TGCCCCGTTGGCCTGTTTGGGTAA	0	22	RDR2-sensitive
TGCCCCTCTGATGGAGTATCGGGA	34	1	RDR2-resistant
TGCCCGACAAAAAGCACTCGGCAA	1	20	RDR2-sensitive

TGCCCGACAAAAAGTACTCGGCAA	0	14	RDR2-sensitive
TGCCCGACAAAAGACACTCGGCAA	1	24	RDR2-sensitive
TGCCCGACAAAAGGCACTCGGCAA	2	38	RDR2-sensitive
TGCCCGCGATCTGGCACTCGGCAA	3	16	RDR2-sensitive
TGCCCTACTGTAGACGGTCAGTGT	0	24	RDR2-sensitive
TGCCCTATGGACGAGCTCCGGCAT	0	13	RDR2-sensitive
TGCCCTCCCCATGAATCGTCGAAT	58	0	RDR2-resistant
TGCCCTCTACGACGGCACGGACGG	0	18	RDR2-sensitive
TGCCCTCTGACGGGAAGTCGGTAA	0	54	RDR2-sensitive
TGCCCTCTGAGTATCGGACGTGTG	0	9	RDR2-sensitive
TGCCCTCTGCACAGGGACGGACGG	0	47	RDR2-sensitive
TGCCCTCTGCACAGGGGCGGACGG	4	36	RDR2-sensitive
TGCCCTCTGGTCAGGCACGGACGG	3	16	RDR2-sensitive
TGCCCTCTGGTCAGGCGCGGACGG	0	21	RDR2-sensitive
TGCCCTCTGTAAGCTGAATGCGTC	0	51	RDR2-sensitive
TGCCCTTAAAGGCGTGACTCGGGC	0	21	RDR2-sensitive
TGCCGAACAACCCGGTGAGGTCGG	0	18	RDR2-sensitive
TGCCGACACGTCGGTGTTCTCGGA	0	9	RDR2-sensitive
TGCCGACACTAGACACACAGACGT	0	11	RDR2-sensitive
TGCCGACCGTTGCAGATCTGGCGC	3	30	RDR2-sensitive
TGCCGACGAGAGCCAGGACGGTGT	0	10	RDR2-sensitive
TGCCGACTGCAGGAGACAAGTCAT	1	16	RDR2-sensitive
TGCCGAGTACTTCGGACAGTCGGT	0	22	RDR2-sensitive
TGCCGATTGTCCGACAAAATGCAT	0	15	RDR2-sensitive
TGCCGCGACTTGCAACTTCGACGG	0	10	RDR2-sensitive
TGCCGCGACTTGCACTGACGGTTT	0	9	RDR2-sensitive
TGCCGCGACTTGCGACTTCGACGG	9	27	RDR2-sensitive
TGCCGCGGACCTGGCTCGCTCGGC	1	23	RDR2-sensitive
TGCCGCGGATGAAAGGTTGCGCAT	0	23	RDR2-sensitive
TGCCGCTCGGATCATCGTCGTCGT	0	10	RDR2-sensitive
TGCCGCTGTAGACAGAAGATGGGC	0	9	RDR2-sensitive
TGCCGCTGTAGATTAGACGGTGAT	0	28	RDR2-sensitive
TGCCGGAACGAGCTCGACTCGGCT	0	10	RDR2-sensitive
TGCCGGAACGCCTAGATCGGAGGA	0	10	RDR2-sensitive
TGCCGGAATCGGAAACGGGAACGA	0	10	RDR2-sensitive
TGCCGGAATTGGACGGGGACGGAC	0	12	RDR2-sensitive
TGCCGGACCGGAGAACGACGGCGT	0	9	RDR2-sensitive
TGCCGGATAAGCAGAACCAGGCTA	0	34	RDR2-sensitive
TGCCGGATAAGCAGAACCAGGCTC	1	157	RDR2-sensitive
TGCCGGATAAGCAGAACCAGGCTT	0	53	RDR2-sensitive
TGCCGGATTGGTGGGTCGAAACGA	0	10	RDR2-sensitive
TGCCGGGCTGGAGGCACGACAGGC	0	25	RDR2-sensitive
TGCCGGGCTGGAGGCATGGCGGGC	0	9	RDR2-sensitive
TGCCGGGTCACGGGCTAAACGGGC	0	9	RDR2-sensitive
TGCCGTACCGGACTGCACACGCGC	0	15	RDR2-sensitive
TGCCGTCGCGGCTGGGAAGGCGAA	0	9	RDR2-sensitive
TGCCGTGTCGGCACTAGGCGTCGT	0	9	RDR2-sensitive
TGCCTAACTGTGACTGTGGCAGAC	0	22	RDR2-sensitive
TGCCTAGGCTGTAGCCCCGGTCAC	0	24	RDR2-sensitive

TGCCTATCGTCAGATCGGATCGGT	0	12	RDR2-sensitive
TGCCTATGCGTTGCGACGGCTCAC	0	16	RDR2-sensitive
TGCCTCATTAGCTGGCAGTCGGTC	71	6	RDR2-resistant
TGCCTCCAAGGCGTGACTCGGGCA	0	9	RDR2-sensitive
TGCCTCCGAATTGTGATCGCGCGG	1	17	RDR2-sensitive
TGCCTCGGGCTGTCGGCGTTTCGA	0	9	RDR2-sensitive
TGCCTCTACTGTAAGAAGACAGGT	1	17	RDR2-sensitive
TGCCTCTTCAGGTCTTCGTTGGAT	36	5	RDR2-resistant
TGCCTGAAGCACTCGGCAAAGCCT	0	10	RDR2-sensitive
TGCCTGATGGGCTCCCGACGGTTT	0	12	RDR2-sensitive
TGCCTGCTTGGACGAACCGCGGAA	0	15	RDR2-sensitive
TGCCTGGCCTGGCACTATTCGGAC	0	11	RDR2-sensitive
TGCCTGTAGAGCGTGTCGGCGAGC	0	16	RDR2-sensitive
TGCCTGTGGGAGCACGAGTCTGGC	0	9	RDR2-sensitive
TGCCTTACGAGCCGGACGGTCCAC	0	13	RDR2-sensitive
TGCCTTCAAATTGTGATCGCGCGG	0	20	RDR2-sensitive
TGCCTTCGCCCCTGTAGCACGGAT	41	5	RDR2-resistant
TGCCTTCTCGGTTCCTGTGGCGGC	0	11	RDR2-sensitive
TGCCTTCTGCACAGGGGCGGACGG	5	109	RDR2-sensitive
TGCCTTGCTGTTCGTCGAGACGAC	18	0	RDR2-resistant
TGCCTTGTCGTGCTGTTTATCGGT	101	15	RDR2-resistant
TGCCTTGTCGTTGGTGGATCCGGT	0	9	RDR2-sensitive
TGCCTTTCTAGCTCATATGGTCGG	43	5	RDR2-resistant
TGCCTTTGTAGATCCGAGCGGCCA	0	14	RDR2-sensitive
TGCCTTTTATCGGACACTCGGCAA	0	19	RDR2-sensitive
TGCCTTTTCCACGGCGAAGACTGC	0	9	RDR2-sensitive
TGCCTTTTCTCGGGCACTCGGCAA	2	33	RDR2-sensitive
TGCCTTTTGTCGGACACTCGGCAA	10	56	RDR2-sensitive
TGCCTTTTGTCGGGCACTCGGCAA	110	1649	RDR2-sensitive
TGCCTTTTGTCGGGCACTCGGCAC	8	105	RDR2-sensitive
TGCCTTTTGTCGGGCACTCGGCAT	6	73	RDR2-sensitive
TGCCTTTTGTCGGGCACTCGGCCA	6	60	RDR2-sensitive
TGCCTTTTGTCGGGCACTCGGTAA	0	34	RDR2-sensitive
TGCCTTTTGTTGGACACTCGGCAA	4	75	RDR2-sensitive
TGCGAACCATGTACGTGCCAGGAT	2	20	RDR2-sensitive
TGCGAACTCGGCTGCAAAAGACAA	0	9	RDR2-sensitive
TGCGAAGAAGACGACACTCTGCGC	0	11	RDR2-sensitive
TGCGAAGAAGACGACCCTCTGCAC	0	9	RDR2-sensitive
TGCGAAGAAGACGACTCTCTGCAC	0	11	RDR2-sensitive
TGCGAAGACGTAAGAGCCAGGCGC	0	9	RDR2-sensitive
TGCGAAGACTGCAGAGTGGAAGCA	0	12	RDR2-sensitive
TGCGACACACGCGGATGAACGCAC	0	18	RDR2-sensitive
TGCGACACGGGCTCCGACGGGAAC	0	10	RDR2-sensitive
TGCGACATACTGTTTTTAGACGAT	33	0	RDR2-resistant
TGCGACCAGGAGGACACTCGGCAA	0	19	RDR2-sensitive
TGCGACCCTGTAGCAATGACGCGC	0	15	RDR2-sensitive
TGCGACGACGGCGTGCATGGACTC	0	9	RDR2-sensitive
TGCGACGAGGACGATTGGCGCCAA	42	0	RDR2-resistant
TGCGACGGCTCACAACAATACCCA	0	18	RDR2-sensitive

TGCGACTAGGAAGATGCTGCCCGC	0	15	RDR2-sensitive
TGCGACTGAACCAGATGACACCGT	0	15	RDR2-sensitive
TGCGACTGAGTCTGCGACGGTGAT	0	9	RDR2-sensitive
TGCGACTGGAGTAGAGACCGGTGT	0	18	RDR2-sensitive
TGCGACTGGATCGACAGCGAGCGG	0	23	RDR2-sensitive
TGCGACTGGCAGGACGTTGGGCTG	34	1	RDR2-resistant
TGCGACTGTTCAGTGACCGCGGTT	57	13	RDR2-resistant
TGCGACTTCAGTAGACGCGGCCTC	0	9	RDR2-sensitive
TGCGACTTGGGCACTTAAGCTGGA	30	5	RDR2-resistant
TGCGAGACAGTGTACAACGGAGAT	0	15	RDR2-sensitive
TGCGAGACTGGACCGGCCCGACAC	1	67	RDR2-sensitive
TGCGAGATGTGGACACTGCGCGGC	0	10	RDR2-sensitive
TGCGAGCACGACGAGGGTAGACAC	0	48	RDR2-sensitive
TGCGAGGACGTGAAGGACGCCGGC	0	10	RDR2-sensitive
TGCGAGGACTAGACCAAAGAGGAT	0	25	RDR2-sensitive
TGCGATGCAGAACTGATTGGCGAC	0	16	RDR2-sensitive
TGCGATGCTGCTGTGGATCGGGAT	1	14	RDR2-sensitive
TGCGATGCTTTTGTGTAGGACGGT	0	13	RDR2-sensitive
TGCGATGTAGAACAGCAGAGGCAG	0	10	RDR2-sensitive
TGCGCAACACGTAGAAGGCGACGT	0	22	RDR2-sensitive
TGCGCAAGGGCGGACTGTCCGCGG	0	18	RDR2-sensitive
TGCGCACCCTAGGCTCTCGGGAGA	87	0	RDR2-resistant
TGCGCACGCATGGACTGTTCGGCT	0	13	RDR2-sensitive
TGCGCACGCGCGGACTGTCCGGCC	0	9	RDR2-sensitive
TGCGCACGCGTGGATTGTCCGGAC	0	9	RDR2-sensitive
TGCGCAGACTGTCCGGTGCCTCAC	1	26	RDR2-sensitive
TGCGCAGAGACAGTTAGGGTTCCT	0	9	RDR2-sensitive
TGCGCAGCACGTAGAAGACGACGT	0	11	RDR2-sensitive
TGCGCAGGACTAACGAAGGCAGAA	0	35	RDR2-sensitive
TGCGCAGGGGCGGCGGAAGATCGC	0	14	RDR2-sensitive
TGCGCGACTTGTCCCTACCGGCTG	25	0	RDR2-resistant
TGCGCGAGACAGTGTAAGATGGAG	1	64	RDR2-sensitive
TGCGCGATGAGAGGACGAAGTCGT	0	12	RDR2-sensitive
TGCGCGCTCCAGCAGACTCCGCAT	2	58	RDR2-sensitive
TGCGCGCTCCAGCAGGCTCCGCAT	0	15	RDR2-sensitive
TGCGCGGGACTAACGGAGGCAGAA	0	13	RDR2-sensitive
TGCGCGGGGATGGAATTGGACGGT	0	9	RDR2-sensitive
TGCGCTATCCGGGCGAGTCGTCGG	0	32	RDR2-sensitive
TGCGCTCGGCTGCAGAAAGGCTAA	0	9	RDR2-sensitive
TGCGCTCTCCAGCAGGCTCCGCAT	0	10	RDR2-sensitive
TGCGCTGAGCCGGACGGTCCACGT	0	11	RDR2-sensitive
TGCGCTGGCACAAACGGACGTCGT	1	25	RDR2-sensitive
TGCGCTGGCATGAACGGACGTCGT	3	42	RDR2-sensitive
TGCGCTGGCCCAAACGGACGACGT	0	10	RDR2-sensitive
TGCGCTGTTGTGCCAAGAGTCGGA	0	9	RDR2-sensitive
TGCGCTTGCAGTAGGGGGTTACAA	0	15	RDR2-sensitive
TGCGGAAATGGGCCAAGACGATGT	0	80	RDR2-sensitive
TGCGGAACTGGGCAGAGGACTCGC	1	13	RDR2-sensitive
TGCGGAAGGCCAGGTAGAGCGGTT	0	9	RDR2-sensitive

TGCGGAAGTCTGCGTGGACTGCAC	0	46	RDR2-sensitive
TGCGGAAGTTTGGCTACTGTATTT	0	10	RDR2-sensitive
TGCGGACAATTCTAGTGGCGTCAG	1	13	RDR2-sensitive
TGCGGACAATTCTAGTGGCGTCGG	1	19	RDR2-sensitive
TGCGGACAGTCCTACGCAGGGCAT	0	11	RDR2-sensitive
TGCGGACAGTCTGAAACGTTGCGG	0	17	RDR2-sensitive
TGCGGACAGTTCTGCCAAAGTGGC	2	21	RDR2-sensitive
TGCGGACCATGTAGGGAAGGATGC	0	10	RDR2-sensitive
TGCGGACCGAGCCAGCATCGACGT	0	41	RDR2-sensitive
TGCGGACCGCGGCTGACGTTCGCC	28	3	RDR2-resistant
TGCGGAGAATGTGCATGCAACGAT	45	7	RDR2-resistant
TGCGGAGACGGATGCGGAAGCTGC	0	11	RDR2-sensitive
TGCGGAGACGGATGCGGAGCCTGC	0	19	RDR2-sensitive
TGCGGAGACGGATGCGGAGGCTGC	3	53	RDR2-sensitive
TGCGGAGAGGCAGATGAATTCGGT	0	18	RDR2-sensitive
TGCGGAGCAGGACGCCTTTCTGAT	0	9	RDR2-sensitive
TGCGGAGCCTGCTGGAGAGCGCAA	1	90	RDR2-sensitive
TGCGGAGCCTGCTGGAGCGCGCAA	0	28	RDR2-sensitive
TGCGGAGGGCAGAACTGTACGCGC	1	38	RDR2-sensitive
TGCGGAGTCTAGCATACTATTCAT	0	9	RDR2-sensitive
TGCGGAGTCTGCTGGAGCGCGCAA	0	45	RDR2-sensitive
TGCGGAGTGACGATGATGGAGCAT	1	22	RDR2-sensitive
TGCGGATATGATAACAACGTGCGT	0	9	RDR2-sensitive
TGCGGATCGCGACAGTGGAGGCAT	1	52	RDR2-sensitive
TGCGGATCTGGTCGAGATGTGCGA	33	2	RDR2-resistant
TGCGGATGCGGAGTGGGATGGGGA	0	16	RDR2-sensitive
TGCGGATGTTGTTGGACGCGGGTA	0	16	RDR2-sensitive
TGCGGATTACTGTACGGGCAGCAC	0	12	RDR2-sensitive
TGCGGATTCGATGAAATAGAGGCT	0	12	RDR2-sensitive
TGCGGCAACTGAAGCGGGTGGTGT	1	14	RDR2-sensitive
TGCGGCACAGCAGGACGACGACGA	0	19	RDR2-sensitive
TGCGGCACTCGACAAAGACGTCTT	0	11	RDR2-sensitive
TGCGGCAGTGGATCCTAGGAGCAA	0	12	RDR2-sensitive
TGCGGCATGTGGATCGATCGGGCA	0	13	RDR2-sensitive
TGCGGCCACGGCAGAACAACAGGG	0	10	RDR2-sensitive
TGCGGCCACGTCACAGCTCGGCGC	3	37	RDR2-sensitive
TGCGGCCTCCTAGCTCGGCGTCAT	1	25	RDR2-sensitive
TGCGGCGCAGCAGGACGACGACGA	0	12	RDR2-sensitive
TGCGGCGTAGGCCTAAGTGACGGC	0	9	RDR2-sensitive
TGCGGCTGAACCAGATGACACCAT	0	17	RDR2-sensitive
TGCGGCTGAACCGGAACGTGGCGT	0	13	RDR2-sensitive
TGCGGCTGAACCGGTTGACAACAT	0	9	RDR2-sensitive
TGCGGCTGAACCGGTTGGCAACAT	0	10	RDR2-sensitive
TGCGGCTGAACTGGATGACGTCGT	1	125	RDR2-sensitive
TGCGGCTGACAAAGAGACGGGCAA	0	9	RDR2-sensitive
TGCGGCTGCGGCTTCTACAGTATT	13	245	RDR2-sensitive
TGCGGCTGGAGAGAGAGTGCGGGC	0	11	RDR2-sensitive
TGCGGCTGGAGCAGAGACCGGTGT	0	9	RDR2-sensitive
TGCGGCTGGAGTAGAGACCGGTGT	2	54	RDR2-sensitive

TGCGGGACAGCCAGGACAGGGACA	0	15	RDR2-sensitive
TGCGGGACAGTCAGGACAGAGGTT	0	10	RDR2-sensitive
TGCGGGAGTTTGGCTACTGTATTT	0	18	RDR2-sensitive
TGCGGGCAAACGACGGTGACACAT	0	11	RDR2-sensitive
TGCGGGCTGACAACGCGCGGGGGC	0	14	RDR2-sensitive
TGCGGGCTGGTTGACGCGGTCTAA	0	24	RDR2-sensitive
TGCGGGCTTACTCGGCGTGTCGAC	0	41	RDR2-sensitive
TGCGGGTGAATTTTGGCGACAGCA	36	6	RDR2-resistant
TGCGGTATTGTAAACTGCGGTATT	0	16	RDR2-sensitive
TGCGGTCTGTACCGAAGGAGATGT	0	12	RDR2-sensitive
TGCGGTGATGTCATCCCTGTCGGT	0	9	RDR2-sensitive
TGCGGTGCTCTCTGGAAGATCGGT	0	28	RDR2-sensitive
TGCGGTGCTTTTGAGTAGGACGGT	0	9	RDR2-sensitive
TGCGGTGCTTTTGGGTAGGACGGT	2	18	RDR2-sensitive
TGCGGTGTAGTATATTTGGGGCAT	0	15	RDR2-sensitive
TGCGGTTCTTGTGTAGTGATTGGC	0	9	RDR2-sensitive
TGCGTACTGTCGTGGACGGCCGGC	0	10	RDR2-sensitive
TGCGTCCCGGGCTTCAGATGGTTT	0	9	RDR2-sensitive
TGCGTCGACTGCGTACGACTACAT	0	11	RDR2-sensitive
TGCGTCGAGATCCCCTGTTGACGG	0	9	RDR2-sensitive
TGCGTCTGCGAACTTTGAGGTCGG	0	10	RDR2-sensitive
TGCGTGCTGAAGAACGGAGATGGC	0	9	RDR2-sensitive
TGCGTGCTGTAGAAGCGTTGTGGC	0	13	RDR2-sensitive
TGCGTGGAACTGTGGTGTCGGCGA	0	10	RDR2-sensitive
TGCGTGGCTAGGGATTGAGGGCAG	2	20	RDR2-sensitive
TGCGTGGTGCGGTGGATGCGGAGC	17	0	RDR2-resistant
TGCGTTGCGACGGCTTACAACAAT	0	52	RDR2-sensitive
TGCGTTGCTCGGATCTAGACGGTT	0	10	RDR2-sensitive
TGCGTTGGCACAAACGGACGTCGT	2	123	RDR2-sensitive
TGCGTTTCCTGTAGTGTACGGCAT	0	12	RDR2-sensitive
TGCGTTTCGGGCTCCGGACGGTGT	0	9	RDR2-sensitive
TGCGTTTGGTGTAGAGATGGCAAT	0	12	RDR2-sensitive
TGCTAAAACGGCCTTCAGCGACCT	0	10	RDR2-sensitive
TGCTAAAGCGACTACAGAGGACAG	0	12	RDR2-sensitive
TGCTAAAGGTTAGGGACCGACGGT	0	23	RDR2-sensitive
TGCTAAAGTTTAGTCGGGTCACAT	0	9	RDR2-sensitive
TGCTAACGCGCGGAACGACGACAC	1	17	RDR2-sensitive
TGCTAACTGGACAGCACTCGGCAA	5	103	RDR2-sensitive
TGCTAATGTCGTACGGGCACACAG	74	0	RDR2-resistant
TGCTACACGACAAGAGAACCGGCT	0	11	RDR2-sensitive
TGCTACAGGACAGGTTCGGTACAC	0	9	RDR2-sensitive
TGCTACGAGCGAGATCTTGTCGGT	0	11	RDR2-sensitive
TGCTACGTACTGTTTTCGTCGACC	61	3	RDR2-resistant
TGCTAGATCTTGGGCACTCGGCAA	0	12	RDR2-sensitive
TGCTAGATGTGGGACACTCGGCAA	0	25	RDR2-sensitive
TGCTAGCGACATAACACTCGGCAA	0	19	RDR2-sensitive
TGCTAGCGATGGCGGCTGGAGAGC	17	0	RDR2-resistant
TGCTAGCTGAAGAAGGACGAGCAA	0	10	RDR2-sensitive
TGCTAGCTGGACAGCACTCGGCAA	1	12	RDR2-sensitive

TGCTAGCTGTAGAGCACTCGGCAA	1	59	RDR2-sensitive
TGCTAGGACCATAACACTCGGCAA	0	11	RDR2-sensitive
TGCTAGGACCATAGCACTAGGCAA	0	11	RDR2-sensitive
TGCTAGGAGCCTAGGACGACGGTG	0	15	RDR2-sensitive
TGCTAGGGGTCAGTGGAAATCAGC	0	10	RDR2-sensitive
TGCTAGGTTTCGGACGGTCCGGCC	22	1	RDR2-resistant
TGCTATAGATATGAGACAGACGTT	22	0	RDR2-resistant
TGCTATGAATTAAGAACAAGGCAA	5	68	RDR2-sensitive
TGCTATGACCATGGCACTCGGCAA	4	22	RDR2-sensitive
TGCTATGACCTTGACACTCGGCAA	0	11	RDR2-sensitive
TGCTATGGTTGTTGCACTCGGCAA	0	9	RDR2-sensitive
TGCTCAACACCGCCGAATCGTCGC	0	9	RDR2-sensitive
TGCTCACATGAGCACGGACGTCGC	0	13	RDR2-sensitive
TGCTCACATGAGCACGGACGTCGT	0	16	RDR2-sensitive
TGCTCAGACTGTCCGGTTGCACAC	0	12	RDR2-sensitive
TGCTCAGTCGTCACTGTGCCGGCC	0	9	RDR2-sensitive
TGCTCCCGTGGCTTGTAGTGGGCT	49	7	RDR2-resistant
TGCTCCGGACCAACAGTACAGCAT	0	24	RDR2-sensitive
TGCTCCGGTAGACGCTGCGTCCAT	0	15	RDR2-sensitive
TGCTCCTGCATGAGGTCGTCCGAT	0	15	RDR2-sensitive
TGCTCCTGTTTCTGCTCGTCGTAC	18	0	RDR2-resistant
TGCTCGATGGCGCACCGGACAGTC	3	19	RDR2-sensitive
TGCTCGGCATGTAGGTTGATCGGT	1	24	RDR2-sensitive
TGCTCGGGCTGGCACACGCGGCGA	0	9	RDR2-sensitive
TGCTCGTACGTCTAGAACCGGCGT	0	11	RDR2-sensitive
TGCTCGTAGGACAGAGACCGGCCT	0	32	RDR2-sensitive
TGCTCGTCCATGCTGTCGGCCCGT	0	9	RDR2-sensitive
TGCTCTAGTTGCGGACTCAGGCGT	0	10	RDR2-sensitive
TGCTCTCGAGAAGAACGGTAACAT	0	29	RDR2-sensitive
TGCTCTCGCAAACTCGGCTCCGGC	17	0	RDR2-resistant
TGCTCTCGGAACTAGTCAACGCAT	0	9	RDR2-sensitive
TGCTCTCGGGACTTCGACGACGGT	0	9	RDR2-sensitive
TGCTCTCTAGTCAGGCGCGAACGG	0	9	RDR2-sensitive
TGCTCTCTATAGCGGTACGGACGG	0	11	RDR2-sensitive
TGCTCTCTGATCAGGCACGGACGG	0	37	RDR2-sensitive
TGCTCTCTGCACAGGCACGGACGG	0	44	RDR2-sensitive
TGCTCTCTGCACAGGCGCGGACGG	6	108	RDR2-sensitive
TGCTCTCTGCACAGGCGTGGACGA	0	29	RDR2-sensitive
TGCTCTCTGCACAGGCGTGGACGG	0	15	RDR2-sensitive
TGCTCTCTGCACAGGGCCGGACGG	0	19	RDR2-sensitive
TGCTCTCTGCACAGGGGCGGACGA	0	12	RDR2-sensitive
TGCTCTCTGCACAGGGGCGGACGG	5	156	RDR2-sensitive
TGCTCTCTGCGGCACGCGGACGGT	0	29	RDR2-sensitive
TGCTCTCTGCGGCGCACGGACGGT	0	11	RDR2-sensitive
TGCTCTCTGGTCAAGCGCGGACGG	2	35	RDR2-sensitive
TGCTCTCTGGTCAGACGTGGACGG	0	23	RDR2-sensitive
TGCTCTCTGGTCAGCCGCGGACGG	0	13	RDR2-sensitive
TGCTCTCTGGTCAGGCACGGACGG	6	30	RDR2-sensitive
TGCTCTCTGGTCAGGCGCGAACGG	0	11	RDR2-sensitive

TGCTCTCTGGTCAGGCGCGGACGA	0	14	RDR2-sensitive
TGCTCTCTGGTCAGGCGCGGACGG	15	512	RDR2-sensitive
TGCTCTCTGGTCAGGCGTGGACGG	1	33	RDR2-sensitive
TGCTCTCTGGTCAGGGGCGGACGG	0	9	RDR2-sensitive
TGCTCTCTGTACAGGGGCGGACGG	0	10	RDR2-sensitive
TGCTCTCTGTAGAAGCACGGACGG	0	11	RDR2-sensitive
TGCTCTCTGTAGAGGCGTGGACGG	1	25	RDR2-sensitive
TGCTCTCTGTCAGGCGCGGACGGT	0	9	RDR2-sensitive
TGCTCTCTGTGGAGGCACGAACGG	0	10	RDR2-sensitive
TGCTCTCTGTGGAGGCACGGACGG	0	10	RDR2-sensitive
TGCTCTCTGTGGAGGCGCGGACGG	0	22	RDR2-sensitive
TGCTCTCTGTGGAGTCGTGGACGG	0	10	RDR2-sensitive
TGCTCTGAATGTCAAAGTGAAGAA	26	4	RDR2-resistant
TGCTCTGGTTGGCGGCCTGACGGC	0	12	RDR2-sensitive
TGCTCTGTGACGACGGCGGCTCTC	3	16	RDR2-sensitive
TGCTGAACACTCGGCACAGGACGA	1	22	RDR2-sensitive
TGCTGAACACTCGGCACAGGACGC	0	18	RDR2-sensitive
TGCTGAACACTCGGCACAGGACGG	0	10	RDR2-sensitive
TGCTGAACACTCGGTACAGGACGA	0	11	RDR2-sensitive
TGCTGAACGACAGTGGGACATCAG	0	14	RDR2-sensitive
TGCTGAAGAGCTGCAAGCCTGCAT	0	36	RDR2-sensitive
TGCTGACAATGCAGTATCTTCGGC	24	2	RDR2-resistant
TGCTGACACGCGGAACGACGACGA	0	16	RDR2-sensitive
TGCTGACGCGCAGAACGACGACGA	0	11	RDR2-sensitive
TGCTGACGCGCGGAACGACGACAA	0	19	RDR2-sensitive
TGCTGACGCGCGGAACGACGACGA	2	101	RDR2-sensitive
TGCTGACTGTAGGAGGCACCCCAC	0	60	RDR2-sensitive
TGCTGACTTCGTAGCAACGCACGG	0	10	RDR2-sensitive
TGCTGATAGCAGGACTCTCGGCAC	0	20	RDR2-sensitive
TGCTGATAGGGCCGCAATTTGAGC	22	1	RDR2-resistant
TGCTGATCAATCTGGTAGAGGATA	42	2	RDR2-resistant
TGCTGATGCGCAGAACGACGACGA	0	13	RDR2-sensitive
TGCTGATTCTCTTGCCATAGACGT	37	0	RDR2-resistant
TGCTGCAGAACTAGGCATTGGGTT	0	27	RDR2-sensitive
TGCTGCAGACTGCCAAACGCTCAG	0	20	RDR2-sensitive
TGCTGCAGCGAAGACTTCGAGCGA	0	9	RDR2-sensitive
TGCTGCAGTAGCCGGCGCGTCGGT	0	10	RDR2-sensitive
TGCTGCATATGGACCTCTTCGGAC	66	16	RDR2-resistant
TGCTGCCAGGAGGACACTCGGCAA	0	10	RDR2-sensitive
TGCTGCGACGAGGACGATTGGCGC	34	0	RDR2-resistant
TGCTGCGCTGGCACAAACGGACGT	0	10	RDR2-sensitive
TGCTGCGTTGGCACAAACGGACGT	0	14	RDR2-sensitive
TGCTGCTCCAGTGCACAGGCTGCA	0	12	RDR2-sensitive
TGCTGCTTAGAGACGAGGACGCGA	0	21	RDR2-sensitive
TGCTGCTTTTGAGCTGGACGGCGT	0	17	RDR2-sensitive
TGCTGCTTTTGGCGGGTGGTAGGT	14	0	RDR2-resistant
TGCTGGAAACGGGGACGGATGCAC	1	18	RDR2-sensitive
TGCTGGAATGTAGATAGATGTCAT	0	11	RDR2-sensitive
TGCTGGACACAAAAAGGAGGCTGT	0	11	RDR2-sensitive

TGCTGGACACTCGGCACAGGACCC	0	14	RDR2-sensitive
TGCTGGACACTCGGCACAGGACGA	2	23	RDR2-sensitive
TGCTGGACGCCATAGCGGACGTAT	0	15	RDR2-sensitive
TGCTGGACGCCGTAGCGGACGTAT	0	28	RDR2-sensitive
TGCTGGACTGATGAATAGTGTGCT	0	13	RDR2-sensitive
TGCTGGAGAGCATAGAGGACCATT	0	10	RDR2-sensitive
TGCTGGAGAGCATAGAGGACCGTT	0	17	RDR2-sensitive
TGCTGGAGAGCGTAGAAGACCGTT	0	9	RDR2-sensitive
TGCTGGAGAGCGTAGAGGACCATT	0	27	RDR2-sensitive
TGCTGGAGAGCGTAGAGGACCGTT	0	24	RDR2-sensitive
TGCTGGAGAGTGTAGAAGACCGTT	1	25	RDR2-sensitive
TGCTGGAGCGCTACCGGACGGTAA	0	19	RDR2-sensitive
TGCTGGAGCGGTATTACTCAAAGC	25	0	RDR2-resistant
TGCTGGAGGGAGTAGACGACGAGA	0	11	RDR2-sensitive
TGCTGGAGGGAGTAGAGGACGTCC	2	34	RDR2-sensitive
TGCTGGAGGGCGTAGAGGACCATT	0	26	RDR2-sensitive
TGCTGGAGGGCTAGAGGACGTCGA	0	9	RDR2-sensitive
TGCTGGAGGGGTAGAGGACGTCTT	0	10	RDR2-sensitive
TGCTGGATCTCGGGCACTCGGCAA	4	25	RDR2-sensitive
TGCTGGATCTTGAGTGGAGCGACG	0	10	RDR2-sensitive
TGCTGGATTGCTGACAGGTTGGCT	107	3	RDR2-resistant
TGCTGGATTTGCTGGTCGCTGGAT	2	20	RDR2-sensitive
TGCTGGCCTGTGGATAGCACGCGC	0	30	RDR2-sensitive
TGCTGGGAGTCCTTTGCATTCGGT	17	0	RDR2-resistant
TGCTGGTCTGTGGAGATAGGCTAT	0	17	RDR2-sensitive
TGCTGGTTGACTCACGGGGAGGTT	22	1	RDR2-resistant
TGCTGTAAGATATGATTGGTGTGC	58	5	RDR2-resistant
TGCTGTAGACGGCCTTAGGAGCGT	0	9	RDR2-sensitive
TGCTGTAGAGCGTAGAGGACCGTT	0	12	RDR2-sensitive
TGCTGTAGCACTTGGACAGTCGGA	0	9	RDR2-sensitive
TGCTGTAGTGGCGAACATATAGAA	43	1	RDR2-resistant
TGCTGTATCATTTCGTCGGCACAC	0	15	RDR2-sensitive
TGCTGTCAGGCAGACACTCGGCAA	0	9	RDR2-sensitive
TGCTGTCTGAACCATGGAAGATGC	0	13	RDR2-sensitive
TGCTGTCTGTAGAGGTGCGGACGG	0	11	RDR2-sensitive
TGCTGTGAGCCAGGCTATGAGCGT	0	14	RDR2-sensitive
TGCTGTGAGCCAGGCTATGCGCGT	0	11	RDR2-sensitive
TGCTGTGAGTCAGACTATACGCGT	0	9	RDR2-sensitive
TGCTGTGCCGCTCTGAAGCCGGTT	0	13	RDR2-sensitive
TGCTGTGGAAGTCTGGCAGGGGTT	0	10	RDR2-sensitive
TGCTGTGGACCCGGAGTAGGCGCC	0	14	RDR2-sensitive
TGCTGTGGACTGTGAGCGGTGGAA	0	13	RDR2-sensitive
TGCTGTGGGCTGTGAGATGTGTAA	0	14	RDR2-sensitive
TGCTGTGGGTCGATGACTGGGCAC	0	10	RDR2-sensitive
TGCTGTTAGATGTAAGATGGATGC	0	11	RDR2-sensitive
TGCTGTTAGCGGGTGGTAGGGAGC	30	0	RDR2-resistant
TGCTGTTCTGATGGAAGTTGGCGC	0	11	RDR2-sensitive
TGCTGTTGGATACGTAGAAGACGA	0	15	RDR2-sensitive
TGCTTAGTGTCGGGTCGGGTCGGT	0	13	RDR2-sensitive

TGCTTAGTTCTTGAGTGTTGGACC	62	2	RDR2-resistant
TGCTTCAGACGACTCGAACGGCGC	1	109	RDR2-sensitive
TGCTTCCAGGAGGACACTCGGCAA	0	14	RDR2-sensitive
TGCTTCCTACTGTAGCATGATGGC	0	16	RDR2-sensitive
TGCTTCGACTGGTGGCCCGACGGC	0	14	RDR2-sensitive
TGCTTCGGACACTCGGCAAAGCAC	0	13	RDR2-sensitive
TGCTTCGGACACTCGGCAAAGCAG	0	25	RDR2-sensitive
TGCTTCGGCTGAGGGCCCGACGGC	0	20	RDR2-sensitive
TGCTTCGGGCACTCGGCAAAGCGC	1	23	RDR2-sensitive
TGCTTCTAGTCGACGTAGAGACGA	0	21	RDR2-sensitive
TGCTTCTCGGCGAAGACTGGGCCT	7	24	RDR2-sensitive
TGCTTCTCTGCAGGACAATGGTAT	0	13	RDR2-sensitive
TGCTTCTCTGCAGGACGACGACAT	0	27	RDR2-sensitive
TGCTTCTCTGCAGGACGACGATAT	0	11	RDR2-sensitive
TGCTTCTCTGCAGGACGATGGTAT	0	15	RDR2-sensitive
TGCTTCTCTGCAGGGCGACGGTAT	0	9	RDR2-sensitive
TGCTTCTGCGATTGATTGGACGTT	14	0	RDR2-resistant
TGCTTCTTCGATCGTTCGGACGTC	14	0	RDR2-resistant
TGCTTCTTCTAGGGTCAGTCGGCC	0	12	RDR2-sensitive
TGCTTGACGTAGTGGCAGAACCGG	0	11	RDR2-sensitive
TGCTTGGACGGCTACGGACAACTC	0	10	RDR2-sensitive
TGCTTGGGCTCCCTGCTGCGCGGT	0	32	RDR2-sensitive
TGCTTGTACGCGAGGAACGATGGC	0	9	RDR2-sensitive
TGCTTTAACTCGGGCACTCGGCAA	0	20	RDR2-sensitive
TGCTTTAATGATCTGTAGGTGCAG	0	19	RDR2-sensitive
TGCTTTATTTCGGGCACTCGGCAA	2	18	RDR2-sensitive
TGCTTTCAGGAGTAGGACGGTGTT	1	12	RDR2-sensitive
TGCTTTCGGACCGATGGCCCATAT	0	11	RDR2-sensitive
TGCTTTCTATCGGGCACTCGGCAA	5	19	RDR2-sensitive
TGCTTTCTGCACAGGCGCGGACGG	0	10	RDR2-sensitive
TGCTTTCTGGTCAGGCGCGGACGG	0	9	RDR2-sensitive
TGCTTTCTGTCGGGCACTCGGCAA	0	24	RDR2-sensitive
TGCTTTCTGTCGGGTACTCGGCAA	0	11	RDR2-sensitive
TGCTTTCTTATACAAGCAGTCGGT	0	23	RDR2-sensitive
TGCTTTGTGTCGGACACTCGGCAA	0	13	RDR2-sensitive
TGCTTTTATGTAGGACAACGTCGT	0	23	RDR2-sensitive
TGCTTTTCATCGGACACTCGGCAA	0	14	RDR2-sensitive
TGCTTTTCATCGGGCACTAGGCAA	0	19	RDR2-sensitive
TGCTTTTGAGTAGGACGGTGTCAT	0	9	RDR2-sensitive
TGCTTTTGGCGGGTGGTAGGTAGT	20	0	RDR2-resistant
TGCTTTTGGGTAGGACGACGTCGT	4	48	RDR2-sensitive
TGCTTTTGGGTAGGACGGTGCTGT	0	11	RDR2-sensitive
TGCTTTTGGGTAGGATGACGTCGT	0	9	RDR2-sensitive
TGCTTTTGTGTAGGACGGTGTTGT	0	10	RDR2-sensitive
TGCTTTTGTTGTACTAGTCCGGCT	23	0	RDR2-resistant
TGCTTTTTATCGGACACTCGGCAA	18	306	RDR2-sensitive
TGCTTTTTATCGGACACTCGGCCA	0	13	RDR2-sensitive
TGCTTTTTATCGGACACTTGGCAA	0	45	RDR2-sensitive
TGCTTTTTATCGGGCACTCGGCAA	106	761	RDR2-sensitive

TGCTTTTTATCGGGCACTCGGCAC	4	25	RDR2-sensitive
TGCTTTTTATCGGGCACTCGGCAT	2	35	RDR2-sensitive
TGCTTTTTATCGGGCACTCGGCCA	5	24	RDR2-sensitive
TGCTTTTTATCGGGTACTCGGCAA	0	10	RDR2-sensitive
TGCTTTTTCTCGGACAGTCGGCAA	3	50	RDR2-sensitive
TGCTTTTTCTCGGGCACTCGGCAA	19	132	RDR2-sensitive
TGCTTTTTGATCAACGGACTTACC	26	2	RDR2-resistant
TGCTTTTTGCAAATCGTTTGGCAC	0	24	RDR2-sensitive
TGCTTTTTGGTGTAGGACGGTGTT	0	9	RDR2-sensitive
TGCTTTTTGTCGGACACTTGGTAA	0	11	RDR2-sensitive
TGCTTTTTGTCGGGCACTCGGCAA	1	82	RDR2-sensitive
TGCTTTTTGTCGGGCACTCGGCAC	0	11	RDR2-sensitive
TGCTTTTTGTCGGGCACTCGGCAT	0	11	RDR2-sensitive
TGCTTTTTGTCGGGCACTTGGCAA	0	37	RDR2-sensitive
TGCTTTTTGTTGGACACTCGGCAA	0	36	RDR2-sensitive
TGCTTTTTGTTGGACACTCGGCAT	0	130	RDR2-sensitive
TGCTTTTTTATCGGACACTCGGCA	0	14	RDR2-sensitive
TGCTTTTTTTCGGACACTCGGCAA	23	147	RDR2-sensitive
TGCTTTTTTTTGGACACTCGGCAA	0	11	RDR2-sensitive
TGGAAAACACTCGGCGAAGGCTTT	0	18	RDR2-sensitive
TGGAAAACGAACTCCACGGTGCAG	0	34	RDR2-sensitive
TGGAAAAGGCAGTAGAACCGGCGC	0	9	RDR2-sensitive
TGGAAACGGACAGGGGGCAGGCGT	0	10	RDR2-sensitive
TGGAAAGGCAGAACCGTGCACCAA	1	14	RDR2-sensitive
TGGAAATTCTGGGATACACTTATA	15	0	RDR2-resistant
TGGAACAAAGCACGGCCTGACACG	0	10	RDR2-sensitive
TGGAACAGACCCTGTGTGGACGCC	0	11	RDR2-sensitive
TGGAACATAGCGAGGACGAACGTT	0	9	RDR2-sensitive
TGGAACCAGATTATGACCGGTTGC	25	0	RDR2-resistant
TGGAACCGAGCTCGGCGCCACAGA	1	15	RDR2-sensitive
TGGAACCTGGAAGCGGAGCTCGGC	0	17	RDR2-sensitive
TGGAACGGACGACGACAACCTCAT	6	51	RDR2-sensitive
TGGAACTTGAATTATGGTGCTGGA	32	0	RDR2-resistant
TGGAACTTGGAACTGGATCCGGCT	0	22	RDR2-sensitive
TGGAACTTTTCTGACTGTTGAGGC	37	0	RDR2-resistant
TGGAAGACAGAGCGTGTTTGGCAT	0	11	RDR2-sensitive
TGGAAGATGCATCTGTAGATCGAC	18	0	RDR2-resistant
TGGAAGCACGTAGAGAAGGAGCAG	1	17	RDR2-sensitive
TGGAAGCTGTGAACCGTCATGCAA	0	17	RDR2-sensitive
TGGAAGGATCGACAGGCGCGAGAC	70	16	RDR2-resistant
TGGAAGGCCAGGTAGAGCGGTTAG	0	17	RDR2-sensitive
TGGAAGGCTAGGCATTCTGCTCAA	0	16	RDR2-sensitive
TGGAAGGCTGCAGTCGCATGGCGC	3	19	RDR2-sensitive
TGGAAGGCTGTAGTCGGGCCGTGA	0	9	RDR2-sensitive
TGGAAGTAGGGACGTTCTGCCCGA	0	9	RDR2-sensitive
TGGAAGTATGAGGACGCTCACCAA	0	16	RDR2-sensitive
TGGAAGTATGCTTTTATGTCTGGA	45	0	RDR2-resistant
TGGAATAAGGATTAACGTGCGGAT	17	0	RDR2-resistant
TGGAATCGAACGGCTCAGCTGCAT	0	11	RDR2-sensitive

TGGAATCTCTCGAGGGTAGACCAG	0	9	RDR2-sensitive
TGGAATCTGGATGACGTGACGTAA	0	16	RDR2-sensitive
TGGAATGAACAGTTTGACGTTAGT	40	3	RDR2-resistant
TGGAATGGACAGCTTGCTTCGGCA	16	0	RDR2-resistant
TGGAATGGCAGAACAGCGGGGCAT	0	9	RDR2-sensitive
TGGAATTGGCTGGTATGATGGCGT	0	16	RDR2-sensitive
TGGACAAACAGATCACGCGAGCAG	0	11	RDR2-sensitive
TGGACAAAGTCGGGTGCATCTCAT	0	13	RDR2-sensitive
TGGACAACAGACTGCACACGGCGA	0	10	RDR2-sensitive
TGGACAACCCTGTAGTAGATGCAC	0	47	RDR2-sensitive
TGGACAACCTGAACCTGAGACCAT	0	44	RDR2-sensitive
TGGACAACGAGATGACGGACACAT	1	15	RDR2-sensitive
TGGACAAGAGCGTAGCGATGCCAA	0	18	RDR2-sensitive
TGGACACAAGCTGACGACGACGGC	0	13	RDR2-sensitive
TGGACACACACTCGGCAAAGACAG	0	10	RDR2-sensitive
TGGACACCAAAATGAGCGGACGGT	3	75	RDR2-sensitive
TGGACACCAATAGCGCAATGGCAT	0	16	RDR2-sensitive
TGGACACCACAAGCCGATCGACAT	0	11	RDR2-sensitive
TGGACACCTGCGTGTACGGCCTGT	0	10	RDR2-sensitive
TGGACACGAAGAGAACGACTCCAT	0	31	RDR2-sensitive
TGGACACGACTGAGGAGTGGACGT	0	25	RDR2-sensitive
TGGACACGTTTACAGATACGACAT	0	9	RDR2-sensitive
TGGACACTCGGCACAGAAAGACAC	0	13	RDR2-sensitive
TGGACACTCGGCACAGGACGACAC	6	43	RDR2-sensitive
TGGACAGAACTGCAGCAGACCGGT	15	0	RDR2-resistant
TGGACAGAAGTCAACAGATCGCGC	0	32	RDR2-sensitive
TGGACAGACACATAATTACTCTTA	16	0	RDR2-resistant
TGGACAGACGGTAAGCCGGGACAC	0	10	RDR2-sensitive
TGGACAGAGCAGAGGCTACGGAAC	0	14	RDR2-sensitive
TGGACAGAGCATGGGCATGGATTT	0	11	RDR2-sensitive
TGGACAGATACGGCGACGGGCGAC	0	9	RDR2-sensitive
TGGACAGCGGCTGGGAGGAGGGCC	360	19	RDR2-resistant
TGGACAGGAAACTAGGCACGACAG	0	14	RDR2-sensitive
TGGACAGGAAACTAGGCACGACGC	2	134	RDR2-sensitive
TGGACAGGAAACTAGGCACGACGG	0	43	RDR2-sensitive
TGGACAGGAAACTAGGCACGGCGG	2	21	RDR2-sensitive
TGGACAGGAAACTAGGCACGGCGT	0	11	RDR2-sensitive
TGGACAGGAAACTAGGCACGGTAG	0	21	RDR2-sensitive
TGGACAGGAAAGCCTGAAACGGAG	0	12	RDR2-sensitive
TGGACAGGGAAGAAGAGCACGGAT	0	25	RDR2-sensitive
TGGACAGGTTTTATGATTTTCTGA	16	0	RDR2-resistant
TGGACAGTCTGAACGTATGCAGAG	0	9	RDR2-sensitive
TGGACAGTGCTGGCAGAACACGAG	0	18	RDR2-sensitive
TGGACAGTGTAAGCGGATGGGTAT	0	23	RDR2-sensitive
TGGACAGTTTGCAGTAGGAGTCGC	0	13	RDR2-sensitive
TGGACATAACTTTAGCTGACGGCA	19	0	RDR2-resistant
TGGACATAATGTTATATTCGACGG	0	10	RDR2-sensitive
TGGACATCGGAACACAGACGGCGT	0	10	RDR2-sensitive
TGGACATGCGCACGACCTTGGGCT	21	0	RDR2-resistant

TGGACATGCGTGGATGCGCTCTGT	0	14	RDR2-sensitive
TGGACATGGCACCCTGACGGAGCT	38	0	RDR2-resistant
TGGACATGTATACCCAGGACGGAC	0	9	RDR2-sensitive
TGGACATGTATACGTACGCCTCAG	0	10	RDR2-sensitive
TGGACATGTATAGCAGGAAGGTAT	1	12	RDR2-sensitive
TGGACATGTATAGCCGGATGTCGT	0	11	RDR2-sensitive
TGGACATGTATAGTCTGGGGGCAT	0	12	RDR2-sensitive
TGGACCAAAAGCGACGAAGGACGA	0	41	RDR2-sensitive
TGGACCAAACCGGGCTCATTTCGC	0	10	RDR2-sensitive
TGGACCAAATGACTAGGCACTCGT	0	11	RDR2-sensitive
TGGACCAAGAGCGGTGAAGGACGA	0	48	RDR2-sensitive
TGGACCAAGCACAGACTTCTGATG	25	0	RDR2-resistant
TGGACCAAGCAGCTCCGACTTCAT	0	18	RDR2-sensitive
TGGACCAAGTGGGCACCCATCCCT	21	0	RDR2-resistant
TGGACCAATCAGATCGCGACACGT	1	14	RDR2-sensitive
TGGACCAATCAGATCGCGCCACGT	0	10	RDR2-sensitive
TGGACCAATTACACGTAGCCGAGC	4	35	RDR2-sensitive
TGGACCAATTGAACGGGCCGAGCT	0	9	RDR2-sensitive
TGGACCAGATTGAGGGGCTCTCAA	1	20	RDR2-sensitive
TGGACCAGTCAGATCGCGCCACGT	0	15	RDR2-sensitive
TGGACCATGCAAGACTTCCACTGC	0	12	RDR2-sensitive
TGGACCATGCAGCTGCTTTGTCAC	2	18	RDR2-sensitive
TGGACCATGCATTAGACGAGCTGA	0	10	RDR2-sensitive
TGGACCATGCGAGACTTCCACTGC	0	11	RDR2-sensitive
TGGACCCCGGACTATTTGGCTAAA	15	0	RDR2-resistant
TGGACCCGAAGCTCGGCGCCACAG	0	13	RDR2-sensitive
TGGACCCGAAGGCAGGTAGGACAG	0	10	RDR2-sensitive
TGGACCCGAATGCAGATAGGACAG	0	16	RDR2-sensitive
TGGACCCGGAGACCCTCAACCGAC	70	12	RDR2-resistant
TGGACCCGGCACGACTTTGGCGTG	0	10	RDR2-sensitive
TGGACCCGTGACTGCTTGGAGGAC	35	6	RDR2-resistant
TGGACCCTTAGCTCGGCGCCACAG	0	18	RDR2-sensitive
TGGACCCTTAGCTCGGCGCCATAG	1	17	RDR2-sensitive
TGGACCCTTCAGGCGAACATTCTC	0	11	RDR2-sensitive
TGGACCCTTGTAGCCGGCTGCCAA	0	10	RDR2-sensitive
TGGACCCTTTGCTCGGCGCCATAG	0	26	RDR2-sensitive
TGGACCCTTTGCTCGGGACGCCAT	0	9	RDR2-sensitive
TGGACCGAAGGTGTGACACGACAG	0	13	RDR2-sensitive
TGGACCGACATGGACCGATGAGGC	0	18	RDR2-sensitive
TGGACCGAGAGCGGTGAAGGACGA	0	14	RDR2-sensitive
TGGACCGAGCCTTGACTTAATCGC	0	16	RDR2-sensitive
TGGACCGATCTGACGAGGAAACAT	0	21	RDR2-sensitive
TGGACCGATCTGGCACATTAAGGC	27	1	RDR2-resistant
TGGACCGCAGGTAGATTTGGACGG	2	23	RDR2-sensitive
TGGACCGCATGCTGATAGCCTCGA	122	15	RDR2-resistant
TGGACCGCTGTCGTGGCACGCCGT	0	14	RDR2-sensitive
TGGACCGGAAGCAGTGAAGGACGA	0	14	RDR2-sensitive
TGGACCGGCTTCTGCGCGACGCGC	0	21	RDR2-sensitive
TGGACCGGGAGCAGTGAAAGACGA	0	21	RDR2-sensitive

TGGACCGGGCCGAATGCATGACGG	0	15	RDR2-sensitive
TGGACCGGGCTAAATTCGTGCCGT	0	15	RDR2-sensitive
TGGACCGGGCTAAATTCGTGTCGT	0	13	RDR2-sensitive
TGGACCGTATCGGGGGCTCGACGG	0	17	RDR2-sensitive
TGGACCGTTCGACCACGAGACGCG	0	9	RDR2-sensitive
TGGACCGTTGCAGAGTGGACAGTT	0	9	RDR2-sensitive
TGGACCTAACTCAGGAGCGGACGT	0	38	RDR2-sensitive
TGGACCTCCTGGAGAAGCTGGCGT	0	11	RDR2-sensitive
TGGACCTGAGTAGCTATTTTACAT	0	12	RDR2-sensitive
TGGACCTGAGTCGGGGCACACCGT	0	19	RDR2-sensitive
TGGACCTGATGGCTTTAATGGCAA	49	4	RDR2-resistant
TGGACCTGCATACATATATCTGAT	22	0	RDR2-resistant
TGGACCTGGTCCCAGCGGTAGGCA	51	5	RDR2-resistant
TGGACGAACTCGCCCGGACCCGGC	4	36	RDR2-sensitive
TGGACGAAGCAGGGTCGACGACGT	0	18	RDR2-sensitive
TGGACGAAGGGTGGAGACTGCCAG	0	12	RDR2-sensitive
TGGACGAAGGTGGACCAGCGTCGT	0	10	RDR2-sensitive
TGGACGAAGTAGGGGCGGGCCCAA	0	9	RDR2-sensitive
TGGACGAAGTTATGTGCTGTCTGA	0	18	RDR2-sensitive
TGGACGACAGAACGGTGGGTTCAT	0	9	RDR2-sensitive
TGGACGACAGCGCGGCAGGTAGCT	0	10	RDR2-sensitive
TGGACGACGATCAAACGCTGGTAT	0	14	RDR2-sensitive
TGGACGACGGAACGACGGGTTCAT	0	15	RDR2-sensitive
TGGACGACGTAGCATCAACGGTAG	0	11	RDR2-sensitive
TGGACGAGAGTCAAGTAGGGACGA	0	14	RDR2-sensitive
TGGACGAGATGTGCCTGTGATGGT	0	11	RDR2-sensitive
TGGACGAGCACGAGCACGAACCGA	0	12	RDR2-sensitive
TGGACGAGCGAACGTGGACGCGGC	0	30	RDR2-sensitive
TGGACGAGGACTCCGCAGCCAGAC	0	15	RDR2-sensitive
TGGACGCAATCGGACGGTCGAGAT	0	9	RDR2-sensitive
TGGACGCAATCGGACGGTCGGGAT	1	12	RDR2-sensitive
TGGACGCAGGGGAGATGGACCCGC	0	78	RDR2-sensitive
TGGACGCCCACGAGGCAGAACCGT	0	11	RDR2-sensitive
TGGACGCTAAGGCCCATCACCCAT	0	16	RDR2-sensitive
TGGACGCTACATCTGGAGTTCGGC	0	16	RDR2-sensitive
TGGACGCTACTCAGTACCGGCGCC	60	4	RDR2-resistant
TGGACGCTGAGGAGGTGGCTCATC	21	0	RDR2-resistant
TGGACGGAACACCGGGCGGCGGAT	0	18	RDR2-sensitive
TGGACGGAACACCGGGTGCCGGAT	1	27	RDR2-sensitive
TGGACGGAACACCGGGTGGCGGAT	0	29	RDR2-sensitive
TGGACGGAATAGATGAGCGTGCAG	0	9	RDR2-sensitive
TGGACGGACAGCGACGGTCGGCAA	0	18	RDR2-sensitive
TGGACGGACCATCCAAGACCGGAC	0	10	RDR2-sensitive
TGGACGGACCTGGATGCGTGGATG	0	9	RDR2-sensitive
TGGACGGAGAGAGGAGATAGTCAT	0	11	RDR2-sensitive
TGGACGGAGAGAGGAGATCGTCGT	0	15	RDR2-sensitive
TGGACGGCGGGGAGGGTCGCGCAG	46	2	RDR2-resistant
TGGACGGCGTAGAGAACGTTTCGT	1	53	RDR2-sensitive
TGGACGGCGTAGAGAATGTTTCGT	0	19	RDR2-sensitive

TGGACGGCTAGAGGAGGCTAACAT	0	12	RDR2-sensitive
TGGACGGCTGAGATCGCAGAACGG	0	23	RDR2-sensitive
TGGACGGGCCTAGAATGTCAGCAT	0	11	RDR2-sensitive
TGGACGGGGTGTTACAAGTGGTAT	0	10	RDR2-sensitive
TGGACGGTCACGGACAGACGCTAT	0	34	RDR2-sensitive
TGGACGGTCCATGAGCAGGAACGT	0	11	RDR2-sensitive
TGGACGGTCCGCGTCTAAAGTCGG	0	41	RDR2-sensitive
TGGACGGTCTCATACGGCACTGAT	0	21	RDR2-sensitive
TGGACGGTCTGGATGCGGCAGAAC	0	10	RDR2-sensitive
TGGACGGTGCAGATGTGTGGACGT	0	24	RDR2-sensitive
TGGACGGTGTAGAGAACGTTTCGT	0	11	RDR2-sensitive
TGGACGTAAATGGACGGTTGGGAT	0	15	RDR2-sensitive
TGGACGTAATTGGACGGTTGGGAT	0	11	RDR2-sensitive
TGGACGTACCTGGAACTGAAGGCG	1	29	RDR2-sensitive
TGGACGTACGGCGAGATCTATCGG	0	16	RDR2-sensitive
TGGACGTAGGGTTGACGAGCGCAT	0	9	RDR2-sensitive
TGGACGTATATGCTGACATGGCAC	1	114	RDR2-sensitive
TGGACGTCCCTAGCTACAACTCAA	0	15	RDR2-sensitive
TGGACGTCCGCACTCTGGACCGGA	0	14	RDR2-sensitive
TGGACGTCCTGGGCCAGATGACGC	0	10	RDR2-sensitive
TGGACGTCCTGGGCCTGATGACGC	0	11	RDR2-sensitive
TGGACGTCCTGGGGTAGATGACGC	0	12	RDR2-sensitive
TGGACGTCGAAGGGGGTAGACGGG	0	9	RDR2-sensitive
TGGACGTCTACTGAGCGAACACGG	0	36	RDR2-sensitive
TGGACGTCTCTGGCTGACTGCGGG	26	4	RDR2-resistant
TGGACTAAAATGGAGGCCCAGCAC	0	13	RDR2-sensitive
TGGACTAAAGTACCCCTACGGCGC	0	13	RDR2-sensitive
TGGACTAACATGGACGGACACTAT	0	11	RDR2-sensitive
TGGACTAACCTCCCCACATGACGA	0	11	RDR2-sensitive
TGGACTAACCTCTCCACAGGACGA	2	172	RDR2-sensitive
TGGACTAACCTCTCCACATGACGA	0	11	RDR2-sensitive
TGGACTAACTGAGCAGTGAGTCAT	0	12	RDR2-sensitive
TGGACTAGAATGAAGGCCCAGCAC	0	22	RDR2-sensitive
TGGACTAGCGCTCGGCAAAGGGAG	0	9	RDR2-sensitive
TGGACTAGCTCTCGGCAAAGACAG	0	31	RDR2-sensitive
TGGACTAGTAGCATACCGGTGCGT	1	47	RDR2-sensitive
TGGACTAGTGATCCGGTGGGCCGG	0	12	RDR2-sensitive
TGGACTAGTTGATGTTTTGGCATA	18	1	RDR2-resistant
TGGACTATAAAAGGGAGGCCACAC	0	15	RDR2-sensitive
TGGACTATAAGCGGTGAAGGACGA	0	15	RDR2-sensitive
TGGACTATAGAGGGCTTCACCGAC	2	41	RDR2-sensitive
TGGACTATCAGCGAAGGGTACCTG	0	9	RDR2-sensitive
TGGACTATCTCTCAGATGGATGTC	54	6	RDR2-resistant
TGGACTATGTAGAGGCGTAGTTGC	0	19	RDR2-sensitive
TGGACTCAAGCACGATCAACACAT	0	14	RDR2-sensitive
TGGACTCAAGCATGATCAACGCAT	0	10	RDR2-sensitive
TGGACTCTCTCGGCTGAGCCGGCC	0	10	RDR2-sensitive
TGGACTCTTGGATGAGGCACTGAT	102	30	RDR2-resistant
TGGACTGACTTGGACGACAGGCGC	0	23	RDR2-sensitive

TGGACTGAGAGGGGCCGGATTGAT	0	9	RDR2-sensitive
TGGACTGCAAGCGGTGAAGGACGA	0	14	RDR2-sensitive
TGGACTGCGGGATCGGACGGCTGA	1	15	RDR2-sensitive
TGGACTGCTCAAGCAAGAAAGAAC	23	2	RDR2-resistant
TGGACTGCTCTCGGGTGCTGGCGT	2	15	RDR2-sensitive
TGGACTGGACAAGAAGGAGGACAA	0	14	RDR2-sensitive
TGGACTGGATTCGGCCCAAAACAC	0	115	RDR2-sensitive
TGGACTGGCTACTGTAGTTCACGG	0	16	RDR2-sensitive
TGGACTGGCTGAGTGCATGCAGTA	0	13	RDR2-sensitive
TGGACTGGGACGAGCACGCACCAG	0	10	RDR2-sensitive
TGGACTGGTCGTGATGACGCAGAT	0	11	RDR2-sensitive
TGGACTGTCCAACGCAGGATACGC	15	0	RDR2-resistant
TGGACTGTCCAGTGGAGCACCGGA	0	82	RDR2-sensitive
TGGACTGTCCGCGACTACGCAGAG	0	11	RDR2-sensitive
TGGACTGTCCGGCCACGGGACGGC	1	13	RDR2-sensitive
TGGACTGTCCGTTCTGAGGCCGGA	0	17	RDR2-sensitive
TGGACTGTTTGAATTTCCGGTCAT	0	13	RDR2-sensitive
TGGACTTACCAGTATTGAAGGAAA	36	2	RDR2-resistant
TGGACTTAGACGGTGCTACAACAG	0	32	RDR2-sensitive
TGGACTTAGAGCCCTGGCGACGGG	1	43	RDR2-sensitive
TGGACTTAGGACCGTGGCGACGGG	0	10	RDR2-sensitive
TGGACTTAGGGCCCTAGCGACGGT	0	12	RDR2-sensitive
TGGACTTAGGGCCCTGACGACGGG	0	13	RDR2-sensitive
TGGACTTAGGGCCCTGGCGACGGG	1	24	RDR2-sensitive
TGGACTTAGGGCTCTGGCGACGGA	0	10	RDR2-sensitive
TGGACTTCCGGACGGGTTCGTTAT	1	13	RDR2-sensitive
TGGACTTCCGGATCGTACCTAGGG	17	0	RDR2-resistant
TGGACTTCCGGATCGTTCTTAGGG	27	0	RDR2-resistant
TGGACTTGACTGCCTCGGCCGGCT	17	1	RDR2-resistant
TGGACTTGTTTGGCCTCATCGGAC	125	16	RDR2-resistant
TGGACTTTGGGCTAGGATTGGCCT	0	22	RDR2-sensitive
TGGAGAACGTAGCAGCATGAGCGG	0	12	RDR2-sensitive
TGGAGAAGACTCAGCCCGTGACGG	0	10	RDR2-sensitive
TGGAGAAGTTGGCGTTTGGGCGAA	26	0	RDR2-resistant
TGGAGACCTTGGGGCAGATGACGT	2	16	RDR2-sensitive
TGGAGACGAGAAGGATGAGGTCAT	0	20	RDR2-sensitive
TGGAGACGTAGGCGACAGACAAAC	0	9	RDR2-sensitive
TGGAGACGTTGGCGATGGCTTCTC	1	16	RDR2-sensitive
TGGAGACTTGGAGACGTGCGCTAT	0	15	RDR2-sensitive
TGGAGAGCAGAACGCAGGGACGAC	0	12	RDR2-sensitive
TGGAGAGCGGAGAGTTGGTGGCAT	21	0	RDR2-resistant
TGGAGAGTCATGTAGAGACGGGCT	0	12	RDR2-sensitive
TGGAGAGTGGAGGCGTGTGTAGAG	0	12	RDR2-sensitive
TGGAGAGTTTGGCGCTGGAGTCGG	17	0	RDR2-resistant
TGGAGATAAGGCACTGACATTTGA	27	1	RDR2-resistant
TGGAGATCGACGACCAGGCTGCAG	0	13	RDR2-sensitive
TGGAGATGCGACCAATCTGCTCGT	0	11	RDR2-sensitive
TGGAGCACTGTAGCGGGCCGGTTT	0	15	RDR2-sensitive
TGGAGCGACGGGAGCTACGAGCAG	0	10	RDR2-sensitive

TGGAGCGACTGAATCGGATGACAT	0	12	RDR2-sensitive
TGGAGCGCAGCGTGGATAATGACC	17	0	RDR2-resistant
TGGAGCGGATCATACAGTCGACAT	0	9	RDR2-sensitive
TGGAGCTCGGTTCTGTGCGTGCAA	0	19	RDR2-sensitive
TGGAGCTCTGACAAGTGGGGCCAC	1	34	RDR2-sensitive
TGGAGCTCTGACAGCTAGGGCCAC	0	10	RDR2-sensitive
TGGAGCTGAAAGATCGGGAGTCGT	0	10	RDR2-sensitive
TGGAGCTGAACTCTAGGAAGTCGT	0	19	RDR2-sensitive
TGGAGCTTTACTGTTAGAGCAGGG	270	0	RDR2-resistant
TGGAGGAAGTAGAGGACGACACAT	1	21	RDR2-sensitive
TGGAGGACAGTAGCGCGGTGGTGG	356	112	RDR2-resistant
TGGAGGACGAGGAGTAGGTTGGTA	24	0	RDR2-resistant
TGGAGGCAGAACGCAGAACCAGGA	0	15	RDR2-sensitive
TGGAGGCATACAGGAGGACATGGT	0	12	RDR2-sensitive
TGGAGGCCAGAAGATTCCTGCAGG	17	0	RDR2-resistant
TGGAGGCGAGAGCTGACGTGGCAG	0	12	RDR2-sensitive
TGGAGGCTCCTCCGAGATCGAGGT	17	0	RDR2-resistant
TGGAGGCTTTCAGGACGGTGAGTT	0	12	RDR2-sensitive
TGGAGGGACAAGAGAGGACGCCAG	0	11	RDR2-sensitive
TGGAGGGACGAGAGAGGACACCAG	2	17	RDR2-sensitive
TGGAGGGACGAGAGAGGACATCAG	0	10	RDR2-sensitive
TGGAGGGACGAGAGAGGACGACAG	0	26	RDR2-sensitive
TGGAGGGACGAGAGAGGACGCCAG	2	34	RDR2-sensitive
TGGAGGGACGAGAGAGGACGTCAG	0	12	RDR2-sensitive
TGGAGGGATACCGGATACTGTCGT	0	9	RDR2-sensitive
TGGAGGGATCCGTTGGACATGGCA	24	0	RDR2-resistant
TGGAGGGTAGACGATGGCTCACTA	0	16	RDR2-sensitive
TGGAGGTGAGCTCACTAGGCTGAA	30	0	RDR2-resistant
TGGAGTAGCCGAGCATGCCTGTAT	27	1	RDR2-resistant
TGGAGTCCCTGAACTCGGCTCGAA	20	0	RDR2-resistant
TGGAGTCGGCGCCAAGGTCAGCGG	0	9	RDR2-sensitive
TGGAGTCTGTTTTGATGATGCATG	82	1	RDR2-resistant
TGGAGTGAGTAGACGACGTTCCGA	0	23	RDR2-sensitive
TGGAGTGGACCGTGTTGCCGGCGT	0	15	RDR2-sensitive
TGGAGTGGATTGAGGGGGATTGGA	4	19	RDR2-sensitive
TGGAGTGGGTAGGAGAGAGACGAA	0	11	RDR2-sensitive
TGGAGTTAGACGGGTGGTGGACGT	0	41	RDR2-sensitive
TGGAGTTCGGACCTACCCGGAGGC	0	14	RDR2-sensitive
TGGAGTTGCATCGTCCCGACGGTC	15	0	RDR2-resistant
TGGAGTTTGGCTGTCGCGTGCGTC	20	0	RDR2-resistant
TGGATAAGCTTGTAGACATAATAA	25	4	RDR2-resistant
TGGATAAGTTAGGAGCACGATCAT	150	17	RDR2-resistant
TGGATACAACACTGAGGTAGTCAT	1	25	RDR2-sensitive
TGGATACATGCTAGGATGGACCAA	0	14	RDR2-sensitive
TGGATACATGTGCTCCGAATCGGC	29	5	RDR2-resistant
TGGATACGGAATCGAGTGGCTCAG	0	38	RDR2-sensitive
TGGATACGTAGATGCAGCAGATGT	0	11	RDR2-sensitive
TGGATACTCTCAGGACGGACGCAT	0	41	RDR2-sensitive
TGGATATAGAGGACGTTGCTGGAG	0	11	RDR2-sensitive

TGGATATCAGCATCGAGGACACAT	0	16	RDR2-sensitive
TGGATATGATCAGATTCGTTTAAC	68	3	RDR2-resistant
TGGATATGATCAGATTCGTTTAGC	58	7	RDR2-resistant
TGGATATGCGGAAGGCTACAACTA	0	9	RDR2-sensitive
TGGATATGGGAGGGCAGATTGGCA	19	0	RDR2-resistant
TGGATCAAGAAAGTAGATGAGGAA	28	0	RDR2-resistant
TGGATCAGGAACGGTGAAGGACGA	0	15	RDR2-sensitive
TGGATCATGTGGAAGACGACTGAC	0	20	RDR2-sensitive
TGGATCCAAACACGTAGAGGGCTA	0	27	RDR2-sensitive
TGGATCCACCAAGAACCACGGCAC	14	0	RDR2-resistant
TGGATCCGATGACATACTGGCGAA	0	26	RDR2-sensitive
TGGATCCGGAGGGCACTAGACCAG	0	9	RDR2-sensitive
TGGATCCTATGTCTATATCGTCAT	19	0	RDR2-resistant
TGGATCGAACTGCCCCGAGCACGG	1	43	RDR2-sensitive
TGGATCGACGTAGAGGCAGTGAGC	0	30	RDR2-sensitive
TGGATCGAGAGCGGTGAAGGACGA	0	15	RDR2-sensitive
TGGATCGGAAGCGGTAAAGGACAA	0	10	RDR2-sensitive
TGGATCTAGACTGATACGACCTAA	0	14	RDR2-sensitive
TGGATCTAGGTTCATGAGATGGAT	38	0	RDR2-resistant
TGGATCTGATCCGGTTTTGTCGTG	112	3	RDR2-resistant
TGGATCTGCAGAAGCACGCCGCAA	0	11	RDR2-sensitive
TGGATCTGCAGAAGCATGCCGCAA	0	54	RDR2-sensitive
TGGATCTGGACGAGGAACACACGG	0	9	RDR2-sensitive
TGGATCTTAATCTTATCGTCAGAG	52	4	RDR2-resistant
TGGATCTTCTCTGCAACAAGGCAA	0	9	RDR2-sensitive
TGGATCTTGCATGACTGTTGGGTA	23	0	RDR2-resistant
TGGATCTTTCTGGTGGAAGAACAT	0	9	RDR2-sensitive
TGGATCTTTGCTGAAGTGCTGGCT	32	1	RDR2-resistant
TGGATGAACTGCTGGATACGGTCT	0	9	RDR2-sensitive
TGGATGACAACTCAGATTTCAGCA	20	0	RDR2-resistant
TGGATGAGAGGAGAGAGTGTGGAG	0	13	RDR2-sensitive
TGGATGAGAGTCACGTAGGGACGA	0	19	RDR2-sensitive
TGGATGAGGCAAACCCTGCGGGAC	19	1	RDR2-resistant
TGGATGCAGAGACGTTCTGACCGA	0	12	RDR2-sensitive
TGGATGCATGTGTAGATGACGGAG	0	17	RDR2-sensitive
TGGATGCCTGTATCGGACGCGCAT	0	13	RDR2-sensitive
TGGATGCGCACGGGACTTGGGCGG	0	11	RDR2-sensitive
TGGATGCGTGGATGCGGGATAGAC	0	16	RDR2-sensitive
TGGATGCTGGGAACTGCGGATGGA	0	12	RDR2-sensitive
TGGATGGAAAATGGACGGCCAGAT	0	29	RDR2-sensitive
TGGATGGACGGACGAGCAACGCAT	0	17	RDR2-sensitive
TGGATGGACTAGCTCGAGCGGTGT	0	9	RDR2-sensitive
TGGATGGACTGCCGACGCCAAGAC	0	11	RDR2-sensitive
TGGATGGAGAAGAGGGAGGAGCAT	1	21	RDR2-sensitive
TGGATGGCAAAGGGATAGGTGGAA	0	12	RDR2-sensitive
TGGATGGCTCGGCCGTAGCGGCTA	50	0	RDR2-resistant
TGGATGGTGTGAGCAGTTCGGAGA	17	0	RDR2-resistant
TGGATGTAGATGGACGGTGTAGAT	0	27	RDR2-sensitive
TGGATGTCAAGTCGGCGTTTCTTA	20	0	RDR2-resistant

TGGATGTCTGGCTGTCACCGGCCT	0	16	RDR2-sensitive
TGGATGTGAGTGTTGACAAGCAGG	25	0	RDR2-resistant
TGGATGTGAGTTGGGCCAGGCCAG	0	9	RDR2-sensitive
TGGATGTGATAGCTCAATCCAGGA	27	2	RDR2-resistant
TGGATGTGCGGATGCGAGATAGAT	0	12	RDR2-sensitive
TGGATGTGGAGGATGTGGTCTGGA	0	14	RDR2-sensitive
TGGATGTTCATGAGATGGGACCAA	15	0	RDR2-resistant
TGGATGTTTGAGACTCAGACGGTC	0	9	RDR2-sensitive
TGGATGTTTTTGACGACGGCAGCC	39	0	RDR2-resistant
TGGATTAGTTGGAGGGCGATCAAT	19	0	RDR2-resistant
TGGATTATACACTCTGCAGAGGTT	16	0	RDR2-resistant
TGGATTCATCCGAAGATTTGTCGG	47	5	RDR2-resistant
TGGATTCCTGGCTGTTGACTGCAT	0	39	RDR2-sensitive
TGGATTCCTGGCTGTTGACTGCCT	0	10	RDR2-sensitive
TGGATTCGGATAGGAAACAGGCAC	2	31	RDR2-sensitive
TGGATTCGGGACCGGATACGGATA	0	23	RDR2-sensitive
TGGATTCGTGATTGGCGATGGGGA	22	0	RDR2-resistant
TGGATTCTGACTTAGAGGCGTTCA	18	0	RDR2-resistant
TGGATTCTGGTAGTTCAAGACGGT	83	7	RDR2-resistant
TGGATTCTTGGCTGTCGACTGCAT	0	18	RDR2-sensitive
TGGATTGAAGCCTCGGACGACGGA	0	10	RDR2-sensitive
TGGATTGAGTGGGATTGGATGGGT	3	41	RDR2-sensitive
TGGATTGCTGACAGGTTGGCTAAA	90	7	RDR2-resistant
TGGATTGGAGGGGATTGATACGGA	0	28	RDR2-sensitive
TGGATTGGAGTGGATTGAGATGGT	0	11	RDR2-sensitive
TGGATTGGAGTGGATTGAGGTGGT	1	42	RDR2-sensitive
TGGATTGGCGAGATGGCGAACGAC	0	10	RDR2-sensitive
TGGATTGGGACATCAACACGGCAC	3	31	RDR2-sensitive
TGGATTGGTGGGAGGAAGAAGGAA	81	0	RDR2-resistant
TGGATTGGTTCGGTAGACACGGTC	0	21	RDR2-sensitive
TGGATTGTGGCCGGGCTACAACAA	0	12	RDR2-sensitive
TGGATTGTTATTCAGGCTATCATC	187	58	RDR2-resistant
TGGATTGTTTCTACATGGTTTAGC	15	0	RDR2-resistant
TGGATTTGAGGGGAAGGGGACGAC	180	13	RDR2-resistant
TGGATTTTGATTGGATGCACTGCA	44	0	RDR2-resistant
TGGCAAAGACTATTTTATACTCGT	0	9	RDR2-sensitive
TGGCAAAGGGATAGGTGGAACCAA	0	14	RDR2-sensitive
TGGCAAAGTGGACGGAAACGGCGT	0	12	RDR2-sensitive
TGGCAAGGACAGCACCAAGGACGC	0	17	RDR2-sensitive
TGGCAAGTTCGGATAGGGACGGCT	0	13	RDR2-sensitive
TGGCAATGTGGCTGGCATGACTGA	14	0	RDR2-resistant
TGGCAATTGTAGGCGTGGCATCCA	0	9	RDR2-sensitive
TGGCACAAAATCAGTTATCGTCGT	15	0	RDR2-resistant
TGGCACAAACGGACGTCGTGGCAC	0	14	RDR2-sensitive
TGGCACAAAGTCAGTTATCGTCGT	77	105	RDR2-sensitive
TGGCACAATCTGTACAGTCGTCGA	0	12	RDR2-sensitive
TGGCACAATCTGTACAGTCGTCGC	0	17	RDR2-sensitive
TGGCACACGAACTTCGCCTGGCAT	0	9	RDR2-sensitive
TGGCACCTACACGACGGACGACGT	0	10	RDR2-sensitive

TGGCACGCGGACACGGTGAGGCAC	0	16	RDR2-sensitive
TGGCACGGACCCGATGAATGACGG	0	9	RDR2-sensitive
TGGCACGGTAGAATAAGCGGGCGG	0	48	RDR2-sensitive
TGGCACTATTGTTGGGCTGCCGGC	0	32	RDR2-sensitive
TGGCACTCGGCAAAGACTGACGGC	2	21	RDR2-sensitive
TGGCACTGCAGACTGAGAAGGCGT	0	12	RDR2-sensitive
TGGCACTGCAGTCGGAGTGGCGCA	12	47	RDR2-sensitive
TGGCAGAACACGGAATGACAGCCT	1	16	RDR2-sensitive
TGGCAGAACACTGGCAGAACACGG	0	14	RDR2-sensitive
TGGCAGATCTGCGACCGGGGCTGC	0	16	RDR2-sensitive
TGGCAGCTGTTGGATTGCGATCGG	0	11	RDR2-sensitive
TGGCAGGATGCATTCGAGGATACT	252	2	RDR2-resistant
TGGCAGGGATAGACTGGCTGGCAC	0	9	RDR2-sensitive
TGGCAGTCCGTAGCAGCTTTTGGT	0	9	RDR2-sensitive
TGGCAGTCTGCAGCAGCTTTTGGT	0	21	RDR2-sensitive
TGGCAGTGCGACAGCGACGAACGC	17	1	RDR2-resistant
TGGCATAACTCGGCTAGAGCTGGC	1	22	RDR2-sensitive
TGGCATATGTGTAGATGACTTGAT	15	0	RDR2-resistant
TGGCATCGATCGTGCCGGGCCTGC	0	9	RDR2-sensitive
TGGCATGACTGGCACTGTACCCAA	41	11	RDR2-resistant
TGGCATGATCCTGACGGAAGACGA	0	11	RDR2-sensitive
TGGCATGGACCCAGTGGACAACGG	0	19	RDR2-sensitive
TGGCATGGTTTGTCGGTCATAAGA	40	0	RDR2-resistant
TGGCATGTTTGTCGTCTTCGTCGC	89	0	RDR2-resistant
TGGCATGTTTGTCTTCTTCGTCGC	169	20	RDR2-resistant
TGGCATTGAAGGGGACTGGTCGGT	0	15	RDR2-sensitive
TGGCATTGCAGTAGACCGGAATAA	0	13	RDR2-sensitive
TGGCCAACGGTCGGAAGGAGGCAC	1	13	RDR2-sensitive
TGGCCAACTGAAGGAGAACGAGCA	0	11	RDR2-sensitive
TGGCCAAGCCGACGCTGAATCAGT	101	0	RDR2-resistant
TGGCCACCAAAAGCTGCTGCGGAC	1	14	RDR2-sensitive
TGGCCACCAAAAGCTGTTGCGGAC	0	16	RDR2-sensitive
TGGCCACGAGTCGAGCGAGCTCAC	0	9	RDR2-sensitive
TGGCCAGGAAGGACCCTGAGGCAT	0	17	RDR2-sensitive
TGGCCAGGAGGAGGTAGCGCGGGA	20	2	RDR2-resistant
TGGCCAGTCTGACAGAAGGACAGC	3	34	RDR2-sensitive
TGGCCATGCATGAACATCACCGAA	0	9	RDR2-sensitive
TGGCCCAAACGGACGACGTGGCGC	0	11	RDR2-sensitive
TGGCCCAAGACAGTACGAATGCAT	0	16	RDR2-sensitive
TGGCCCACATGTCAGAAGCTCCAA	0	16	RDR2-sensitive
TGGCCCGAACCAACAGTAGAACGG	0	48	RDR2-sensitive
TGGCCCGAACCAACAGTAGAGCAG	0	20	RDR2-sensitive
TGGCCCGTGGGCCGTCGGACGACA	34	5	RDR2-resistant
TGGCCCTGGCTGACTCGATACGGC	0	14	RDR2-sensitive
TGGCCCTGGCTGATCTGATACGGC	0	12	RDR2-sensitive
TGGCCCTTACTGAAACCGGATCGA	20	0	RDR2-resistant
TGGCCGAACTGTCTGCGATGGCAC	0	43	RDR2-sensitive
TGGCCGACTCATGGCATAACTCGG	0	10	RDR2-sensitive
TGGCCGACTCTTGGCGTGACTCGG	1	14	RDR2-sensitive

TGGCCGCTCTCGGGAATTCGTCGG	0	10	RDR2-sensitive
TGGCCGGAAGGACGGAGAGACCAT	0	11	RDR2-sensitive
TGGCCGGACGGTCTGCGCCTACGC	0	15	RDR2-sensitive
TGGCCTATGAACCGGATCTTCGTG	18	0	RDR2-resistant
TGGCCTCTGTACAGCATTGAGCAT	0	15	RDR2-sensitive
TGGCCTGACACGGATACATGACAT	0	14	RDR2-sensitive
TGGCCTGCTTCAGACGACTCGAAC	0	21	RDR2-sensitive
TGGCCTGGCTACGAAGCGTGCTGT	0	13	RDR2-sensitive
TGGCCTGTTGGCTCGACCGTCTGC	93	3	RDR2-resistant
TGGCCTTAAATTCTAGCCTGTCGG	0	12	RDR2-sensitive
TGGCCTTAATCCCTATCTCGTCGT	0	30	RDR2-sensitive
TGGCGAAAACTGCAGATGGGCATT	1	13	RDR2-sensitive
TGGCGAAAGAGACTCGGCTCGGCT	0	9	RDR2-sensitive
TGGCGAAAGAGGCTCGGCTCGGCT	1	20	RDR2-sensitive
TGGCGAAGACTGGGCCTCGGGCGA	0	19	RDR2-sensitive
TGGCGACGGACGGCGTAAGTACAA	0	28	RDR2-sensitive
TGGCGACGGGCGGCGTGAGTACAA	0	12	RDR2-sensitive
TGGCGAGATAGAAAGGTTCGGCTA	0	10	RDR2-sensitive
TGGCGAGATCGAGCGGTAGGCCAG	0	9	RDR2-sensitive
TGGCGAGCGAAGCGGAGGCCCGTC	0	13	RDR2-sensitive
TGGCGAGCGTGACCGACGAGCTAA	2	19	RDR2-sensitive
TGGCGAGTTGTAGAGCACGATGGC	0	17	RDR2-sensitive
TGGCGATCTGCAGCAGCTTTTGGT	0	18	RDR2-sensitive
TGGCGATGAACGGCGTAAGTACAA	0	9	RDR2-sensitive
TGGCGATGATGGAGCAGGTGACAC	1	20	RDR2-sensitive
TGGCGATGGTGGGCTTTAGACGGA	0	10	RDR2-sensitive
TGGCGATTGTGGGCTTTAGGCGGA	0	16	RDR2-sensitive
TGGCGCAATCTGTACAATCGTCGT	0	22	RDR2-sensitive
TGGCGCAATCTGTACAGTCGTCGG	0	14	RDR2-sensitive
TGGCGCAATCTGTAGAGTCGTCGG	0	14	RDR2-sensitive
TGGCGCAGAGCTAGATTGTCACGT	0	18	RDR2-sensitive
TGGCGCCTCGGGCTTGCTCGTCGT	0	10	RDR2-sensitive
TGGCGCCTGAGAATCCATGGTCGT	0	13	RDR2-sensitive
TGGCGCCTGTAGCAGTCGACCGTT	12	29	RDR2-sensitive
TGGCGCTCGGCAAAGACTGACGGC	0	14	RDR2-sensitive
TGGCGGAACTAGACAGGGGACCAG	0	16	RDR2-sensitive
TGGCGGAGCCAGCTCGAAATCAGA	20	0	RDR2-resistant
TGGCGGATCAGTAGAGGATGTCAG	0	26	RDR2-sensitive
TGGCGGATGATACAGAAGAGACGA	0	16	RDR2-sensitive
TGGCGGATTGCGCGAGACAGTGTA	0	10	RDR2-sensitive
TGGCGGCAGAACGGCAGAACACAT	0	15	RDR2-sensitive
TGGCGGCGTCCTCGTCGGAGGAGT	61	3	RDR2-resistant
TGGCGGCTGAAGCGAGGACGACGT	0	10	RDR2-sensitive
TGGCGGGCTGGGTCCAAAACGGGT	0	11	RDR2-sensitive
TGGCGGGTGGTAGGTAGTAGTGGT	18	0	RDR2-resistant
TGGCGGTACCCTCTGCGACGGTGT	0	46	RDR2-sensitive
TGGCGGTGCTCTCTGGTCAGACGC	1	16	RDR2-sensitive
TGGCGTAATATGTACAGTCGTCGG	0	28	RDR2-sensitive
TGGCGTAATCTGTACAGTCGTCGG	0	29	RDR2-sensitive

TGGCGTAGGACATGACGAGGCCAG	1	15	RDR2-sensitive
TGGCGTATGTGCGTAGATTGTCGT	0	9	RDR2-sensitive
TGGCGTCAAAGTCGGCTTCCCTAG	14	0	RDR2-resistant
TGGCGTCACTGTAGGATGGATGGA	0	12	RDR2-sensitive
TGGCGTCTCGAGACATACTGACGG	0	20	RDR2-sensitive
TGGCGTGACTCGGCAAGAGCTGGC	0	26	RDR2-sensitive
TGGCGTGACTCGGCGAGAGCTGGC	1	18	RDR2-sensitive
TGGCGTGAGTAGAGATGGGAACAC	0	27	RDR2-sensitive
TGGCGTGCTGTAACTGAAACCGGC	0	10	RDR2-sensitive
TGGCGTGGGTGTGTAGGGACGCGC	0	9	RDR2-sensitive
TGGCGTGTTTCTGTTGGACGACGG	0	9	RDR2-sensitive
TGGCGTTGGTCTCCTGGGCACGGA	504	76	RDR2-resistant
TGGCGTTTCTGTAGATGGGCCCGC	0	71	RDR2-sensitive
TGGCGTTTCTTGTAGATGTAGCAT	59	6	RDR2-resistant
TGGCTACGCGGAGGGCTCGAAGGC	38	2	RDR2-resistant
TGGCTACTGTAGCCCACGGGCCGG	0	16	RDR2-sensitive
[truncated: 140,912 more chars]
