# Supplementary material for: Loss of RNA–Dependent RNA Polymerase 2 (RDR2) Function Causes Widespread and Unexpected Changes in the Expression of Transposons, Genes, and 24-nt Small RNAs
Source: PLoS Genet. 2009 Nov 20;5(11):e1000737. doi: 10.1371/journal.pgen.1000737 (PMC2774947; doi:10.1371/journal.pgen.1000737)
Supplement: Table S8 — Primer sequences used for qRT-PCR experiment. (0.01 MB PDF) [file pgen.1000737.s013.pdf]

Table S8. Primer sequences used for qRT-PCR experiment

**Gene**[a]	**Sequence for forward primer**	**Sequence for reverse primer**
hAT	AATGCTATGGAGGTGGACGA	AGCAATCCATGCTTGTTTGA
ago4a	GCCCACCCACTATCATGTTC	TCTCGTCGGCATTAAAACCT
ago4b	TCGAAGTTCCTTGGATGACA	TCATTCCTGGAAAGCCAGAT
ago4c	CGCACCCAATCACCTAAAAT	CCGTGGCTTAAACAATGAGTC
ddm1	GCCCCTGCAGAAGTAGCTTT	TGGGTACCGTACGGAGAGTC
liguleless3	GATCTGGCATGTGGAGTGAC	CAACCTCAGCTCAGCAGATT
met1	TGCACCATGAGAAGTTTTCC	CTTAGCTTCATGGGTCATGG
MULE	AGTGCAAGTGTGGTGGTCAA	CCATGTTGTGATGCTAAAACG

[a] Details are provided in the legend of Figure 2.
